# Causes of death among children aged 5–14 years in the WHO European Region: a systematic analysis for the Global Burden of Disease Study 2016

**DOI:** 10.1016/S2352-4642(18)30095-6

**Published:** 2018-05

**Authors:** Hmwe H Kyu, Claudia E Stein, Cynthia Boschi Pinto, Ivo Rakovac, Martin W Weber, Tina Dannemann Purnat, Joseph E Amuah, Scott D Glenn, Kelly Cercy, Stan Biryukov, Audra L Gold, Adrienne Chew, Meghan D Mooney, Kevin F O'Rourke, Amber Sligar, Christopher J L Murray, Ali H Mokdad, Mohsen Naghavi

**Affiliations:** a Institute for Health Metrics and Evaluation, University of Washington, Seattle, WA, USA; b World Health Organization, Division of Information, Evidence, Research and Innovation, Copenhagen, Denmark; c Universidad Federal Fluminense, Niterói, Brazil; d Division of Noncommunicable Diseases and Promoting Health through the Life-Course, World Health Organization European Office for the Prevention and Control of Noncommunicable Diseases (NCD Office), Moscow, Russia

## Abstract

**Background:**

The mortality burden in children aged 5–14 years in the WHO European Region has not been comprehensively studied. We assessed the distribution and trends of the main causes of death among children aged 5–9 years and 10–14 years from 1990 to 2016, for 51 countries in the WHO European Region.

**Methods:**

We used data from vital registration systems, cancer registries, and police records from 1980 to 2016 to estimate cause-specific mortality using the Cause of Death Ensemble model.

**Findings:**

For children aged 5–9 years, all-cause mortality rates (per 100 000 population) were estimated to be 46·3 (95% uncertainty interval [UI] 45·1–47·5) in 1990 and 19·5 (18·1–20·9) in 2016, reflecting a 58·0% (54·7–61·1) decline. For children aged 10–14 years, all-cause mortality rates (per 100 000 population) were 37·9 (37·3–38·6) in 1990 and 20·1 (18·8–21·3) in 2016, reflecting a 47·1% (43·8–50·4) decline. In 2016, we estimated 10 740 deaths (95% UI 9970–11 542) in children aged 5–9 years and 10 279 deaths (9652–10 897) in those aged 10–14 years in the WHO European Region. Injuries (road injuries, drowning, and other injuries) caused 4163 deaths (3820–4540; 38·7% of total deaths) in children aged 5–9 years and 4468 deaths (4162–4812; 43·5% of total) in those aged 10–14 years in 2016. Neoplasms caused 2161 deaths (1872–2406; 20·1% of total deaths) in children aged 5–9 years and 1943 deaths (1749–2101; 18·9% of total deaths) in those aged 10–14 years in 2016. Notable differences existed in cause-specific mortality rates between the European subregions, from a two-times difference for leukaemia to a 20-times difference for lower respiratory infections between the Commonwealth of Independent States (CIS) and EU15 (the 15 member states that had joined the European Union before May, 2004).

**Interpretation:**

Marked progress has been made in reducing the mortality burden in children aged 5–14 years over the past 26 years in the WHO European Region. More deaths could be prevented, especially in CIS countries, through intervention and prevention efforts focusing on the leading causes of death, which are road injuries, drowning, and lower respiratory infections. The findings of our study could be used as a baseline to assess the effect of implementation of programmes and policies on child mortality burden.

**Funding:**

WHO and Bill & Melinda Gates Foundation.

## Introduction

Mortality rates in children younger than 5 years and the variations between countries in the WHO European Region are well studied and documented.^1^, ^2^, ^3^, ^4^ However, the mortality burden in older children (aged 5–14 years) is less well known. Increasing attention is being paid to older children,^5^, ^6^, ^7^, ^8^ but no studies thus far have provided a comprehensive assessment of the mortality burden in the 5–14 years age group in the European Region.

Substantial diversity exists between countries in the WHO European Region in terms of socioeconomic and political conditions and health risks.^2^, ^9^ For example, the highest country-specific under-5 mortality rates are 25 times higher than the lowest rates.^2^ Little information is available about variations in mortality rates for older children across countries in the European Region. In 2014, the 53 member states of the WHO European Region adopted a new strategy that aims to reduce the burden of avoidable disease and mortality in children of all ages,^10^ and the 5–14 years age group has been identified as one of its topmost priorities. For the planning of intervention and prevention efforts, information on region-specific and country-specific leading causes of death in these children is essential. In this Article, we aim to identify the main causes of death in children aged 5–9 years and 10–14 years in the WHO European Region, and summarise their distributions and trends from 1990 to 2016 for 51 of the 53 countries in the Region (the Global Burden of Disease [GBD] study produces estimates only for locations with a population greater than 50 000; therefore, Monaco and San Marino are not included in this analysis).

**Research in context**

**Evidence before this study**

Mortality rates in children aged 5–14 years have been estimated by the Global Burden of Diseases, Injuries, and Risk Factors Study 2013 and 2016, and the United Nations Inter-Agency Group for Child Mortality Estimation. However, the burden in this age group across countries in the WHO European Region has not been comprehensively assessed. We did a PubMed search on Nov 10, 2017, using the following search terms: “child mortality[MeSH] AND (trend OR trends) AND Europe[MeSH]”, which yielded 82 results. We identified 12 studies that reported trends in mortality due to a single cause in this age group. An additional five studies reported trends in all-cause mortality and cause-specific mortality, but these studies focused on a single country in Europe and the most recent period of estimation was 2011. We found no studies that reported comparable all-cause and cause-specific mortality estimates over time across countries in the WHO European Region.

**Added value of this study**

This analysis provides a comprehensive assessment of the distribution and trends of the main causes of death among children aged 5–9 years and 10–14 years for 51 countries in the WHO European Region from 1990 to 2016. To our knowledge, this is the first study to show comparable age-specific and sex-specific trends in cause-specific mortality rates for these age groups across countries, using all available data. Our findings will help countries to identify priority areas for interventions and will also serve as a baseline for analysing the effectiveness of programmes and policies over time.

**Implications of all the available evidence**

Although the mortality burden in children aged 5–14 years has been reduced substantially between 1990 and 2016 in the WHO European Region, marked difference in levels and causes of death still exists between countries. More efforts are needed to reduce mortality from the leading causes of death that are highly preventable or amenable to health care, particularly road injuries, drowning, lower respiratory infections, self-harm, and congenital birth defects. Concerted efforts are needed to reduce the massive inequalities in mortality between countries in the region.

## Methods

### Overview

Details of the design and methods of the GBD have been reported previously.^11^, ^12^, ^13^, ^14^, ^15^ Briefly, GBD 2016 included 264 causes of death; the International Classification of Diseases (ICD) codes for the GBD 2016 cause list are shown in the appendix. Cause-specific mortality in the WHO European Region was estimated using data from vital registration systems, cancer registries, verbal autopsy data (for Turkey only), and police records (for road injuries and homicide only) from 1980 to 2016. The quality and comparability of the cause-of-death data were assessed and enhanced through multiple steps, which included adjustment of data from vital registration systems for incompleteness; conversion of causes found in the original data to the GBD 2016 cause list; identification of garbage codes (ie, deaths assigned to causes that were not underlying causes of death) and redistribution to underlying causes; age–sex splitting of deaths that were reported in aggregated categories; and smoothing random fluctuations. The detailed methods for each step are available in the appendix of a previous GBD paper.^16^ Country-year-age-sex-specific raw data versus enhanced data and model estimates are shown in the online data visualisation of the cause-of-death database. We assessed the overall data quality for each country based on completeness, garbage coding, cause-list detail, and time periods covered, and assigned a quality score ranging from 0 stars (poorest) to 5 stars (best). Causes of death data used, the years for which data are available, vital registration completeness, data quality rating, and the percentage of garbage codes by country in the WHO European Region are shown in the appendix.

The Cause of Death Ensemble model (CODEm)^11^, ^14^, ^15^, ^17^, ^18^ was used to estimate cause-specific mortality for most causes. The CODEm strategy explored a diverse set of plausible models that apply different functional forms, including mixed-effects models and spatiotemporal Gaussian process regression for mortality rates and cause fractions, with varying combinations of covariates (appendix). All models for each cause of death were assessed using out-of-sample predictive validity tests and combined into an ensemble of models that perform best. For a few causes with a very small number of deaths or no deaths (eg, upper respiratory infection and diphtheria), we used negative binomial regression to deal with the overdispersion in the data.

### Grouping of countries

**Figure 1:**
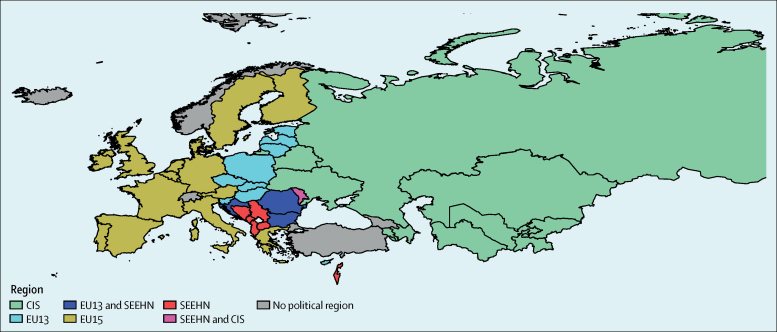
Countries in the WHO European Region and subregions Andorra, Georgia, Iceland, Norway, Switzerland, and Turkey are not included in the subregion groups. Bulgaria, Croatia, Romania, and Republic of Moldova belong to more than one subregion. CIS=commonwealth of independent states. EU13=countries that joined the European Union after May, 2004. EU15=countries that joined the European Union before May, 2004. SEEHN=South Eastern Europe Health Network.

In this study, we classified European countries into four subregions based on the official groupings formally agreed by member states of the WHO European Region, which are socioeconomically and politically diverse (figure 1). These subregions are EU15, the 15 Member States that had joined the European Union before May, 2004 (Austria, Belgium, Denmark, Finland, France, Germany, Greece, Ireland, Italy, Luxembourg, the Netherlands, Portugal, Spain, Sweden, and the UK); EU13, Member States that have joined the European Union since May, 2004 (Bulgaria, Croatia, Cyprus, Czechia, Estonia, Hungary, Latvia, Lithuania, Malta, Poland, Romania, Slovakia, and Slovenia); the South Eastern Europe Health Network (SEEHN; Albania, Bosnia and Herzegovina, Bulgaria, Croatia, Israel, Macedonia, Montenegro, Romania, Republic of Moldova, and Serbia); and the Commonwealth of Independent States (CIS; Armenia, Azerbaijan, Belarus, Kazakhstan, Kyrgyzstan, Republic of Moldova, Russian Federation, Tajikistan, Turkmenistan, Ukraine, and Uzbekistan). Andorra, Georgia, Iceland, Norway, Switzerland, and Turkey are not included in the subregion groups. This study complies with the Guidelines for Accurate and Transparent Health Estimates Reporting (GATHER) recommendations.

### Role of the funding source

Some authors are employed by WHO and had a role in all aspects of the study. The GBD enterprise is supported by funding from the Bill & Melinda Gates Foundation, which had no role in study design, data collection and analysis, interpretation of data, decision to publish, or preparation of the manuscript. The corresponding author had full access to all data in the study and had final responsibility for the decision to submit for publication.

## Results

In 2016, we estimated 10 740 deaths (95% uncertainty interval [UI] 9970–11 542) in children aged 5–9 years and 10 279 deaths (9652–10 897) in those aged 10–14 years in the WHO European Region. In 1990, the numbers of deaths were 29 994 (29 239–30 788) and 24 133 (23 722–24 562), reflecting a 64·2% (95% UI 61·4–66·8) decline in the number of all-cause deaths in children aged 5–9 years and a 57·4% (54·7–60·1) decrease in children aged 10–14 years (Table 1, Table 2; appendix). In 2016, mortality rates (per 100 000 people) were 19·5 (95% UI 18·1–20·9) in children aged 5–9 years and 20·1 (18·8–21·3) in those aged 10–14 years. In 1990, the death rates were 46·3 (45·1–47·5) and 37·9 (37·3–38·6), reflecting a 58·0% (95% UI 54·7–61·1) decline in all-cause mortality rates in children aged 5–9 years and a 47·1% (43·8–50·4) decline in children aged 10–14 years (Table 1, Table 2; appendix). The decrease in all-cause death rate was about four times greater in the 5–9 years age group than in the 10–14 years age group during the years 1990–2000 (appendix). The decreases in death rates were similar between the two age groups during the years 2000–16 (50·8% [46·6–54·5] in ages 5–9 years *vs* 45·2% [41·8–48·5] in ages 10–14 years; appendix).

**Table 1:** Number of deaths and death rates in 1990 and 2016 in the WHO European region, ages 5–9 years, both sexes

			**1990**		**2016**		**Percentage change from 1990 to 2016**	
			Number of deaths	Rate (per 100 000 people)	Number of deaths	Rate (per 100 000 people)	Number of deaths	Rate (per 100 000 people)
**All causes**			**29 994 (29 239 to 30 788)**	**46·3 (45·1 to 47·5)**	**10 740 (9970 to 11 542)**	**19·5 (18·1 to 20·9)**	**−64·2 (−66·8 to −61·4)**	**−58·0 (−61·1 to −54·7)**
**Injuries**			**14 596 (14 071 to 15 227)**	**22·5 (21·7 to 23·5)**	**4163 (3820 to 4540)**	**7·5 (6·9 to 8·2)**	**−71·5 (−73·9 to −68·7)**	**−66·5 (−69·4 to −63·2)**
	Transport injuries		5693 (5329 to 6154)	8·8 (8·2 to 9·5)	1589 (1412 to 1802)	2·9 (2·6 to 3·3)	−72·1 (−76·0 to −67·8)	−67·2 (−71·8 to −62·3)
		Road injuries	5463 (5112 to 5899)	8·4 (7·9 to 9·1)	1500 (1330 to 1696)	2·7 (2·4 to 3·1)	−72·5 (−76·4 to −68·4)	−67·7 (−72·3 to −62·9)
		Other transport injuries	229 (189 to 278)	0·4 (0·3 to 0·4)	88 (72 to 113)	0·2 (0·1 to 0·2)	−61·2 (−70·5 to −47·5)	−54·4 (−65·4 to −38·4)
	Unintentional injuries		8378 (7950 to 8802)	12·9 (12·3 to 13·6)	2272 (2082 to 2496)	4·1 (3·8 to 4·5)	−72·9 (−75·6 to −69·8)	−68·2 (−71·3 to −64·5)
		Falls	907 (582 to 1103)	1·4 (0·9 to 1·7)	225 (189 to 295)	0·4 (0·3 to 0·5)	−74·3 (−81·0 to −56·3)	−69·9 (−77·7 to −48·7)
		Drowning	4208 (3926 to 4507)	6·5 (6·1 to 7·0)	995 (887 to 1120)	1·8 (1·6 to 2·0)	−76·3 (−79·3 to −72·8)	−72·2 (−75·7 to −68·0)
		Fire, heat, and hot substances	746 (669 to 898)	1·2 (1·0 to 1·4)	235 (204 to 287)	0·4 (0·4 to 0·5)	−68·4 (−73·1 to −62·5)	−63·0 (−68·4 to −56·0)
		Poisonings	485 (326 to 568)	0·7 (0·5 to 0·9)	146 (106 to 183)	0·3 (0·2 to 0·3)	−69·6 (−76·0 to −60·1)	−64·3 (−71·9 to −53·2)
		Exposure to mechanical forces	741 (626 to 1040)	1·1 (1·0 to 1·6)	242 (207 to 310)	0·4 0·4 to 0·6)	−67·1 (−73·7 to −59·2)	−61·4 (−69·1 to −52·1)
		Adverse effects of medical treatment	178 (131 to 209)	0·3 (0·2 to 0·3)	77 (60 to 100)	0·1 (0·1 to 0·2)	−56·0 (−64·2 to −39·7)	−48·3 (−58·0 to −29·2)
		Animal contact	78 (56 to 101)	0·1 (0·1 to 0·2)	32 (26 to 42)	0·1 (0·0 to 0·1)	−58·5 (−70·5 to −40·9)	−51·3 (−65·4 to −30·6)
		Foreign body	360 (282 to 482)	0·6 (0·4 to 0·7)	192 (163 to 249)	0·3 (0·3 to 0·5)	−45·6 (−60·3 to −23·9)	−36·2 (−53·4 to −10·7)
		Environmental heat and cold exposure	93 (59 to 119)	0·1 (0·1 to 0·2)	29 (19 to 39)	0·1 (0·0 to 0·1)	−68·7 (−75·7 to −58·3)	−63·3 (−71·5 to −51·0)
		Other unintentional injuries	582 (469 to 721)	0·9 (0·7 to 1·1)	98 (84 to 115)	0·2 (0·2 to 0·2)	−82·9 (−87·0 to −77·9)	−79·9 (−84·7 to −74·1)
	Self-harm and interpersonal violence		456 (375 to 659)	0·7 (0·6 to 1·0)	198 (151 to 270)	0·4 (0·3 to 0·5)	−56·0 (−67·4 to −44·1)	−48·3 (−61·8 to −34·4)
		Interpersonal violence	456 (375 to 659)	0·7 (0·6 to 1·0)	198 (151 to 270)	0·4 (0·3 to 0·5)	−56·0 (−67·4 to −44·1)	−48·3 (−61·8 to −34·4)
**Non-communicable diseases**			**9804 (9298 to 10 389)**	**15·1 (14·3 to 16·0)**	**4742 (4332 to 5164)**	**8·6 (7·8 to 9·4)**	**−51·6 (−57·0 to −46·4)**	**−43·2 (−49·5 to −37·1)**
	Neoplasms		4391 (4133 to 4704)	6·8 (6·4 to 7·3)	2161 (1872 to 2406)	3·9 (3·4 to 4·4)	−50·7 (−59·2 to −44·0)	−42·1 (−52·1 to −34·2)
		Nasopharynx cancer	22 (18 to 28)	0·0 (0·0 to 0·0)	13 (11 to 15)	0·0 (0·0 to 0·0)	−42·9 (−57·4 to −27·5)	−33·0 (−50·0 to −14·9)
		Liver cancer	54 (47 to 63)	0·1 (0·1 to 0·1)	28 (24 to 32)	0·1 (0·0 to 0·1)	−48·1 (−59·5 to −35·6)	−39·0 (−52·5 to −24·4)
		Kidney cancer	133 (115 to 161)	0·2 (0·2 to 0·2)	84 (72 to 97)	0·2 (0·1 to 0·2)	−35·9 (−52·8 to −21·0)	−24·7 (−44·6 to −7·2)
		Brain and nervous system cancer	1117 (966 to 1260)	1·7 (1·5 to 1·9)	697 (574 to 808)	1·3 (1·0 to 1·5)	−37·3 (−52·3 to −22·2)	−26·3 (−44·0 to −8·7)
		Hodgkin's lymphoma	69 (45 to 88)	0·1 (0·1 to 0·1)	19 (14 to 24)	0·0 (0·0 to 0·0)	−72·0 (−79·9 to −62·3)	−67·1 (−76·5 to −55·8)
		Non-Hodgkin lymphoma	350 (253 to 406)	0·5 (0·4 to 0·6)	160 (132 to 189)	0·3 (0·2 to 0·3)	−53·5 (−64·6 to −32·5)	−45·5 (−58·5 to −20·8)
		Leukaemia	1983 (1814 to 2203)	3·1 (2·8 to 3·4)	743 (612 to 866)	1·3 (1·1 to 1·6)	−62·4 (−69·2 to −54·9)	−55·9 (−63·9 to −47·0)
		Other neoplasms	664 (592 to 803)	1·0 (0·9 to 1·2)	417 (349 to 475)	0·8 (0·6 to 0·9)	−36·7 (−53·9 to −23·7)	−25·7 (−45·8 to −10·4)
	Cardiovascular diseases		638 (589 to 700)	1·0 (0·9 to 1·1)	294 (256 to 337)	0·5 (0·5 to 0·6)	−53·9 (−60·8 to −46·2)	−45·8 (−54·0 to −36·9)
		Rheumatic heart disease	94 (83 to 109)	0·1 (0·1 to 0·2)	31 (26 to 38)	0·1 (0·0 to 0·1)	−66·4 (−73·9 to −59·0)	−60·6 (−69·3 to −51·9)
		Ischaemic heart disease	21 (19 to 24)	0·0 (0·0 to 0·0)	13 (10 to 15)	0·0 (0·0 to 0·0)	−40·9 (−53·3 to −26·2)	−30·6 (−45·2 to −13·4)
		Cerebrovascular disease	259 (226 to 300)	0·4 (0·3 to 0·5)	86 (70 to 109)	0·2 (0·1 to 0·2)	−66·4 (−75·1 to −56·7)	−60·6 (−70·7 to −49·2)
		Hypertensive heart disease	6 (5 to 7)	0·0 (0·0 to 0·0)	5 (4 to 6)	0·0 (0·0 to 0·0)	−16·0 (−32·3 to 1·0)	−1·4 (−20·5 to 18·5)
		Cardiomyopathy and myocarditis	157 (132 to 189)	0·2 (0·2 to 0·3)	86 (74 to 103)	0·2 (0·1 to 0·2)	−44·5 (−55·6 to −28·7)	−34·9 (−47·9 to −16·3)
		Endocarditis	19 (14 to 24)	0·0 (0·0 to 0·0)	17 (13 to 22)	0·0 (0·0 to 0·0)	−10·4 (−37·6 to 18·8)	5·1 (−26·8 to 39·4)
		Other cardiovascular and circulatory diseases	81 (67 to 102)	0·1 (0·1 to 0·2)	55 (44 to 69)	0·1 (0·1 to 0·1)	−31·6 (−48·9 to −11·1)	−19·7 (−40·0 to 4·4)
	Chronic respiratory diseases		296 (254 to 344)	0·5 (0·4 to 0·5)	82 (72 to 96)	0·1 (0·1 to 0·2)	−72·0 (−77·3 to −66·1)	−67·1 (−73·3 to −60·2)
		Chronic obstructive pulmonary disease	73 (60 to 96)	0·1 (0·1 to 0·1)	19 (16 to 24)	0·0 (0·0 to 0·0)	−73·5 (−79·9 to −67·0)	−68·9 (−76·4 to −61·3)
		Asthma	114 (82 to 158)	0·2 (0·1 to 0·2)	21 (17 to 27)	0·0 (0·0 to 0·0)	−80·9 (−86·5 to −73·7)	−77·6 (−84·2 to −69·1)
		Interstitial lung disease and pulmonary sarcoidosis	21 (16 to 28)	0·0 (0·0 to 0·0)	9 (6 to 10)	0·0 (0·0 to 0·0)	−58·0 (−73·0 to −43·5)	−50·8 (−68·3 to −33·6)
		Other chronic respiratory diseases	88 (50 to 133)	0·1 (0·1 to 0·2)	33 (26 to 43)	0·1 (0·0 to 0·1)	−59·6 (−76·2 to −34·9)	−52·6 (−72·0 to −23·5)
	Cirrhosis and other chronic liver diseases		222 (195 to 250)	0·3 (0·3 to 0·4)	125 (108 to 145)	0·2 (0·2 to 0·3)	−43·4 (−53·8 to −32·2)	−33·6 (−45·8 to −20·4)
		Cirrhosis and other chronic liver diseases due to hepatitis B	4 (3 to 5)	0·0 (0·0 to 0·0)	2 (2 to 3)	0·0 (0·0 to 0·0)	−32·3 (−46·2 to −16·4)	−20·5 (−36·8 to −1·9)
		Cirrhosis and other chronic liver diseases due to hepatitis C	3 (2 to 4)	0·0 (0·0 to 0·0)	2 (1 to 2)	0·0 (0·0 to 0·0)	−44·0 (−53·2 to −33·5)	−34·3 (−45·1 to −21·9)
		Cirrhosis and other chronic liver diseases due to other causes	216 (189 to 243)	0·3 (0·3 to 0·4)	121 (105 to 140)	0·2 (0·2 to 0·3)	−43·6 (−53·9 to −32·4)	−33·8 (−45·9 to −20·6)
	Digestive diseases		339 (286 to 390)	0·5 (0·4 to 0·6)	121 (102 to 153)	0·2 (0·2 to 0·3)	−64·1 (−71·3 to −51·4)	−57·8 (−66·3 to −43·0)
		Peptic ulcer disease	42 31 to 62)	0·1 (0·0 to 0·1)	11 (9 to 14)	0·0 (0·0 to 0·0)	−72·5 (−82·3 to −60·9)	−67·7 (−79·3 to −54·1)
		Gastritis and duodenitis	3 (2 to 5)	0·0 (0·0 to 0·0)	2 (1 to 2)	0·0 (0·0 to 0·0)	−51·0 (−72·3 to −26·3)	−42·5 (−67·4 to −13·5)
		Appendicitis	95 66 to 111)	0·1 (0·1 to 0·2)	23 (17 to 38)	0·0 (0·0 to 0·1)	−74·8 (−82·3 to −47·4)	−70·4 (−79·2 to −38·3)
		Paralytic ileus and intestinal obstruction	76 (57 to 93)	0·1 (0·1 to 0·1)	39 (31 to 52)	0·1 (0·1 to 0·1)	−47·7 (−59·9 to −24·2)	−38·6 (−52·9 to −11·0)
		Inguinal, femoral, and abdominal hernia	14 (9 to 19)	0·0 (0·0 to 0·0)	4 (3 to 6)	0·0 (0·0 to 0·0)	−68·5 (−79·6 to −53·4)	−63·0 (−76·1 to −45·2)
		Inflammatory bowel disease	33 (23 to 49)	0·1 (0·0 to 0·1)	14 (10 to 18)	0·0 (0·0 to 0·0)	−55·9 (−74·4 to −33·3)	−48·2 (−69·9 to −21·7)
		Vascular intestinal disorders	5 (3 to 7)	0·0 (0·0 to 0·0)	2 (1 to 3)	0·0 (0·0 to 0·0)	−56·1 (−74·8 to −24·4)	−48·4 (−70·5 to −11·3)
		Gallbladder and biliary diseases	8 (6 to 10)	0·0 (0·0 to 0·0)	2 (2 to 3)	0·0 (0·0 to 0·0)	−69·0 (−78·3 to −57·4)	−63·6 (−74·6 to −50·0)
		Pancreatitis	7 (5 to 9)	0·0 (0·0 to 0·0)	3 (1 to 4)	0·0 (0·0 to 0·0)	−62·1 (−75·1 to −44·2)	−55·5 (−70·8 to −34·5)
		Other digestive diseases	57 (40 to 76)	0·1 (0·1 to 0·1)	20 (16 to 26)	0·0 (0·0 to 0·0)	−63·1 (−74·9 to −45·0)	−56·7 (−70·6 to −35·5)
	Neurological disorders		737 (634 to 884)	1·1 (1·0 to 1·4)	426 373 to 492)	0·8 (0·7 to 0·9)	−41·6 (−54·4 to −24·8)	−31·5 (−46·4 to −11·7)
		Epilepsy	529 (444 to 636)	0·8 (0·7 to 1·0)	297 (253 to 361)	0·5 (0·5 to 0·7)	−43·1 (−56·4 to −20·5)	−33·2 (−48·8 to −6·7)
		Motor neuron disease	13 (11 to 17)	0·0 (0·0 to 0·0)	8 (6 to 10)	0·0 (0·0 to 0·0)	−40·4 (−55·0 to −22·2)	−30·0 (−47·2 to −8·6)
		Other neurological disorders	194 (164 to 248)	0·3 (0·3 to 0·4)	120 (104 to 139)	0·2 (0·2 to 0·3)	−37·0 (−56·0 to −20·8)	−26·1 (−48·4 to −7·1)
	Mental and substance use disorders		1 (0 to 1)	0·0 (0·0 to 0·0)	0 (0 to 1)	0·0 (0·0 to 0·0)	−35·4 (−63·8 to 10·6)	−24·2 (−57·5 to 29·8)
		Eating disorders	1 (0 to 1)	0·0 (0·0 to 0·0)	0 (0 to 1)	0·0 (0·0 to 0·0)	−35·4 (−63·8 to 10·6)	−24·2 (−57·5 to 29·8)
	Diabetes, urogenital, blood, and endocrine diseases		1115 (995 to 1324)	1·7 (1·5 to 2·0)	569 (500 to 646)	1·0 (0·9 to 1·2)	−48·8 (−58·2 to −40·5)	−39·9 (−50·9 to −30·2)
		Diabetes mellitus	89 (76 to 107)	0·1 (0·1 to 0·2)	36 (31 to 43)	0·1 (0·1 to 0·1)	−58·9 (−67·4 to −49·4)	−51·7 (−61·8 to −40·6)
		Acute glomerulonephritis	28 (18 to 49)	0·0 (0·0 to 0·1)	11 (8 to 17)	0·0 (0·0 to 0·0)	−57·4 (−79·2 to −25·8)	−50·0 (−75·5 to −12·9)
		Chronic kidney disease	252 (222 to 297)	0·4 (0·3 to 0·5)	127 (109 to 147)	0·2 (0·2 to 0·3)	−49·4 (−60·0 to −38·8)	−40·6 (−53·0 to −28·2)
		Urinary diseases and male infertility	78 (68 to 90)	0·1 (0·1 to 0·1)	41 (34 to 49)	0·1 (0·1 to 0·1)	−47·5 (−60·1 to −36·0)	−38·4 (−53·2 to −24·8)
		Haemoglobinopathies and haemolytic anaemias	255 (203 to 360)	0·4 (0·3 to 0·6)	92 (74 to 128)	0·2 (0·1 to 0·2)	−63·5 (−71·8 to −52·6)	−57·1 (−66·9 to −44·3)
		Endocrine, metabolic, blood, and immune disorders	412 (348 to 535)	0·6 (0·5 to 0·8)	261 (203 to 313)	0·5 (0·4 to 0·6)	−36·0 (−53·6 to −18·4)	−24·9 (−45·6 to −4·3)
	Musculoskeletal disorders		64 (47 to 112)	0·1 (0·1 to 0·2)	31 (24 to 52)	0·1 (0·0 to 0·1)	−51·4 (−63·0 to −34·6)	−43·0 (−56·6 to −23·2)
		Rheumatoid arthritis	16 (12 to 21)	0·0 (0·0 to 0·0)	5 (4 to 8)	0·0 (0·0 to 0·0)	−65·5 (−75·1 to −50·7)	−59·5 (−70·8 to −42·1)
		Other musculoskeletal disorders	49 (34 to 91)	0·1 (0·1 to 0·1)	25 (20 to 45)	0·0 (0·0 to 0·1)	−46·4 (−61·2 to −26·2)	−37·1 (−54·4 to −13·4)
	Other non-communicable diseases		2001 (1605 to 2224)	3·1 (2·5 to 3·4)	933 (788 to 1122)	1·7 (1·4 to 2·0)	−53·1 (−61·2 to −37·0)	−44·9 (−54·5 to −26·1)
		Skin and subcutaneous diseases	12 (9 to 16)	0·0 (0·0 to 0·0)	10 (7 to 13)	0·0 (0·0 to 0·0)	−12·8 (−31·0 to 4·4)	2·3 (−19·0 to 22·5)
**Communicable, maternal, neonatal, and nutritional diseases**			**5595 (4969 to 6197)**	**8·6 (7·7 to 9·6)**	**1835 (1635 to 2051)**	**3·3 (3·0 to 3·7)**	**−67·1 (−72·1 to −61·9)**	**−61·4 (−67·2 to −55·3)**
	HIV/AIDS and tuberculosis		291 (222 to 377)	0·4 (0·3 to 0·6)	72 (66 to 80)	0·1 (0·1 to 0·1)	−74·8 (−81·0 to −66·5)	−70·4 (−77·7 to −60·6)
	Diarrhoea, lower respiratory, and other common infectious diseases		4810 (4202 to 5429)	7·4 (6·5 to 8·4)	1608 (1422 to 1820)	2·9 (2·6 to 3·3)	−66·4 (−72·3 to −60·2)	−60·6 (−67·5 to −53·2)
		Diarrhoeal diseases	236 (171 to 329)	0·4 (0·3 to 0·5)	75 (50 to 114)	0·1 (0·1 to 0·2)	−67·8 (−77·8 to −54·4)	−62·2 (−73·9 to −46·5)
		Intestinal infectious diseases	134 (61 to 262)	0·2 (0·1 to 0·4)	62 (28 to 119)	0·1 (0·1 to 0·2)	−53·3 (−68·4 to −36·0)	−45·2 (−62·9 to −24·9)
		Lower respiratory infections	2523 (2210 to 2782)	3·9 (3·4 to 4·3)	1041 (894 to 1196)	1·9 (1·6 to 2·2)	−58·6 (−65·1 to −50·8)	−51·4 (−59·0 to −42·2)
		Upper respiratory infections	1 (1 to 2)	0·0 (0·0 to 0·0)	0 (0 to 1)	0·0 (0·0 to 0·0)	−64·7 (−87·4 to −19·3)	−58·6 (−85·2 to −5·2)
		Otitis media	8 (3 to 28)	0·0 (0·0 to 0·0)	0 (0 to 0)	0·0 (0·0 to 0·0)	−96·9 (−99·4 to −93·8)	−96·3 (−99·2 to −92·8)
		Meningitis	819 (498 to 1075)	1·3 (0·8 to 1·7)	170 (141 to 211)	0·3 (0·3 to 0·4)	−78·4 (−85·2 to −67·0)	−74·6 (−82·6 to −61·3)
		Encephalitis	343 (242 to 423)	0·5 (0·4 to 0·7)	229 (179 to 348)	0·4 (0·3 to 0·6)	−32·4 (−50·4 to −7·3)	−20·7 (−41·8 to 8·8)
		Diphtheria	11 (6 to 20)	0·0 (0·0 to 0·0)	1 (1 to 1)	0·0 (0·0 to 0·0)	−91·8 (−96·5 to −83·0)	−90·4 (−95·9 to −80·0)
		Whooping cough	86 (24 to 206)	0·1 (0·0 to 0·3)	16 (4 to 38)	0·0 (0·0 to 0·1)	−76·2 (−96·2 to −20·1)	−72·1 (−95·5 to −6·2)
		Tetanus	5 (3 to 9)	0·0 (0·0 to 0·0)	1 (1 to 1)	0·0 (0·0 to 0·0)	−74·3 (−88·3 to −58·3)	−69·9 (−86·3 to −51·0)
		Measles	614 (264 to 1266)	0·9 (0·4 to 2·0)	4 (2 to 9)	0·0 (0·0 to 0·0)	−99·3 (−99·6 to −98·8)	−99·2 (−99·5 to −98·6)
		Varicella and herpes zoster	28 (17 to 47)	0·0 (0·0 to 0·1)	8 (5 to 11)	0·0 (0·0 to 0·0)	−69·8 (−84·6 to −50·5)	−64·6 (−82·0 to −41·9)
	Neglected tropical diseases and malaria		36 (25 to 52)	0·1 (0·0 to 0·1)	19 (11 to 45)	0·0 (0·0 to 0·1)	−43·5 (−73·2 to 35·4)	−33·6 (−68·6 to 58·9)
	Nutritional deficiencies		90 (71 to 119)	0·1 (0·1 to 0·2)	29 (24 to 36)	0·1 (0·0 to 0·1)	−67·1 (−77·6 to −55·2)	−61·4 (−73·7 to −47·4)
	Other communicable, maternal, neonatal, and nutritional diseases		368 (319 to 431)	0·6 (0·5 to 0·7)	106 (89 to 126)	0·2 (0·2 to 0·2)	−71·0 (−76·5 to −64·8)	−66·0 (−72·4 to −58·7)

**Table 2:** Number of deaths and death rates in 1990 and 2016 in the WHO European Region, ages 10–14 years, both sexes

			**1990**		**2016**		Percentage change from 1990 to 2016	
			Number of deaths	Rate (per 100 000 people)	Number of deaths	Rate (per 100 000 people)	Number of deaths	Rate (per 100 000 people)
**All causes**			**24 133 (23 722 to 24 562)**	**37·9 (37·3 to 38·6)**	**10 279 (9652 to 10 897)**	**20·1 (18·8 to 21·3)**	**−57·4 (−60·1 to −54·7)**	**−47·1 (−50·4 to −43·8)**
**Injuries**			**12 335 (12 003 to 12 706)**	**19·4 (18·9 to 20·0)**	**4468 (4162 to 4812)**	**8·7 (8·1 to 9·4)**	**−63·8 (−66·4 to −60·9)**	**−55·0 (−58·2 to −51·4)**
	Transport injuries		4505 (4270 to 4771)	7·1 (6·7 to 7·5)	1495 (1337 to 1656)	2·9 (2·6 to 3·2)	−66·8 (−70·8 to −62·9)	−58·7 (−63·8 to −53·9)
		Road injuries	4216 (3995 to 4463)	6·6 (6·3 to 7·0)	1371 (1223 to 1516)	2·7 (2·4 to 3·0)	−67·5 (−71·5 to −63·7)	−59·6 (−64·6 to −54·9)
		Other transport injuries	289 (250 to 335)	0·5 (0·4 to 0·5)	125 (105 to 150)	0·2 (0·2 to 0·3)	−56·6 (−65·7 to −45·6)	−46·1 (−57·3 to −32·4)
	Unintentional injuries		6199 (5928 to 6483)	9·7 (9·3 to 10·2)	1986 (1829 to 2186)	3·9 (3·6 to 4·3)	−67·9 (−70·7 to −64·8)	−60·2 (−63·6 to −56·2)
		Falls	706 (499 to 819)	1·1 (0·8 to 1·3)	223 (193 to 286)	0·4 (0·4 to 0·6)	−67·7 (−74·8 to −48·2)	−59·9 (−68·7 to −35·7)
		Drowning	2857 (2658 to 3060)	4·5 (4·2 to 4·8)	817 (737 to 905)	1·6 (1·4 to 1·8)	−71·4 (−74·9 to −67·1)	−64·4 (−68·8 to −59·1)
		Fire, heat, and hot substances	400 (358 to 450)	0·6 (0·6 to 0·7)	141 (121 to 163)	0·3 (0·2 to 0·3)	−64·8 (−69·7 to −59·3)	−56·3 (−62·3 to −49·4)
		Poisonings	361 (258 to 421)	0·6 (0·4 to 0·7)	124 (87 to 152)	0·2 (0·2 to 0·3)	−65·6 (−72·5 to −55·8)	−57·2 (−65·8 to −45·1)
		Exposure to mechanical forces	689 (597 to 925)	1·1 (0·9 to 1·5)	267 (228 to 337)	0·5 (0·4 to 0·7)	−60·9 (−68·6 to −53·4)	−51·5 (−61·0 to −42·2)
		Adverse effects of medical treatment	136 (108 to 163)	0·2 (0·2 to 0·3)	66 (54 to 87)	0·1 (0·1 to 0·2)	−51·2 (−58·7 to −38·7)	−39·4 (−48·7 to −23·9)
		Animal contact	49 (33 to 62)	0·1 (0·1 to 0·1)	20 (16 to 25)	0·0 (0·0 to 0·0)	−58·9 (−69·2 to −42·4)	−48·9 (−61·7 to −28·4)
		Foreign body	288 (243 to 381)	0·5 (0·4 to 0·6)	155 (129 to 214)	0·3 (0·3 to 0·4)	−45·9 (−56·8 to −26·4)	−32·8 (−46·3 to −8·6)
		Environmental heat and cold exposure	82 (47 to 107)	0·1 (0·1 to 0·2)	28 (18 to 37)	0·1 (0·0 to 0·1)	−65·1 (−72·4 to −56·6)	−56·6 (−65·7 to −46·1)
		Other unintentional injuries	631 (533 to 739)	1·0 (0·8 to 1·2)	145 (124 to 170)	0·3 (0·2 to 0·3)	−76·8 (−81·5 to −71·2)	−71·2 (−77·0 to −64·2)
	Self-harm and interpersonal violence		1558 (1374 to 1775)	2·4 (2·2 to 2·8)	878 (713 to 1014)	1·7 (1·4 to 2·0)	−43·5 (−53·5 to −33·7)	−29·8 (−42·2 to −17·7)
		Self-harm	1023 (829 to 1145)	1·6 (1·3 to 1·8)	610 (479 to 726)	1·2 (0·9 to 1·4)	−40·3 (−51·2 to −28·9)	−25·8 (−39·4 to −11·7)
		Interpersonal violence	535 (429 to 747)	0·8 (0·7 to 1·2)	269 (198 to 346)	0·5 (0·4 to 0·7)	−49·4 (−59·8 to −37·1)	−37·1 (−50·0 to −21·9)
**Non-communicable diseases**			**8747 (8448 to 9129)**	**13·7 (13·3 to 14·3)**	**4564 (4272 to 4866)**	**8·9 (8·3 to 9·5)**	**−47·8 (−51·8 to −43·8)**	**−35·2 (−40·1 to −30·2)**
	Neoplasms		3697 (3523 to 3887)	5·8 (5·5 to 6·1)	1943 (1749 to 2101)	3·8 (3·4 to 4·1)	−47·4 (−53·8 to −41·9)	−34·7 (−42·6 to −27·9)
		Nasopharynx cancer	32 (26 to 40)	0·0 (0·0 to 0·1)	19 (17 to 23)	0·0 (0·0 to 0·0)	−38·7 (−54·0 to −20·1)	−23·8 (−42·9 to −0·7)
		Liver cancer	49 (44 to 55)	0·1 (0·1 to 0·1)	31 (27 to 36)	0·1 (0·1 to 0·1)	−36·5 (−47·2 to −24·7)	−21·1 (−34·3 to −6·4)
		Kidney cancer	46 (41 to 53)	0·1 (0·1 to 0·1)	34 (30 to 38)	0·1 (0·1 to 0·1)	−26·3 (−39·8 to −12·3)	−8·4 (−25·3 to 9·0)
		Brain and nervous system cancer	836 (761 to 924)	1·3 (1·2 to 1·5)	538 (458 to 619)	1·0 (0·9 to 1·2)	−35·5 (−46·7 to −23·9)	−19·9 (−33·8 to −5·5)
		Thyroid cancer	9 (8 to 10)	0·0 (0·0 to 0·0)	6 (5 to 7)	0·0 (0·0 to 0·0)	−33·7 (−40·8 to −26·0)	−17·7 (−26·5 to −8·1)
		Hodgkin's lymphoma	102 (84 to 120)	0·2 (0·1 to 0·2)	30 (26 to 35)	0·1 (0·0 to 0·1)	−70·3 (−76·1 to −64·1)	−63·1 (−70·3 to −55·4)
		Non-Hodgkin lymphoma	302 (228 to 342)	0·5 (0·4 to 0·5)	156 (133 to 181)	0·3 (0·3 to 0·4)	−47·9 (−57·7 to −31·5)	−35·3 (−47·4 to −14·9)
		Leukaemia	1597 (1468 to 1745)	2·5 (2·3 to 2·7)	678 (584 to 765)	1·3 (1·1 to 1·5)	−57·4 (−63·6 to −50·6)	−47·1 (−54·7 to −38·6)
		Other neoplasms	725 (655 to 832)	1·1 (1·0 to 1·3)	451 (392 to 504)	0·9 (0·8 to 1·0)	−37·6 (−49·8 to −27·3)	−22·5 (−37·7 to −9·6)
	Cardiovascular diseases		908 (854 to 975)	1·4 (1·3 to 1·5)	417 (376 to 460)	0·8 (0·7 to 0·9)	−54·0 (−59·2 to −48·5)	−42·8 (−49·3 to −36·1)
		Rheumatic heart disease	163 (148 to 179)	0·3 (0·2 to 0·3)	59 (52 to 67)	0·1 (0·1 to 0·1)	−63·7 (−69·4 to −57·3)	−54·9 (−61·9 to −47·0)
		Ischaemic heart disease	34 (30 to 39)	0·1 (0·0 to 0·1)	19 (16 to 23)	0·0 (0·0 to 0·0)	−43·4 (−55·1 to −30·4)	−29·7 (−44·2 to −13·5)
		Cerebrovascular disease	372 (336 to 421)	0·6 (0·5 to 0·7)	121 (103 to 141)	0·2 (0·2 to 0·3)	−67·3 (−73·4 to −61·1)	−59·4 (−66·9 to −51·6)
		Hypertensive heart disease	7 (6 to 7)	0·0 (0·0 to 0·0)	5 (4 to 6)	0·0 (0·0 to 0·0)	−21·2 (−33·1 to −9·0)	−2·1 (−16·9 to 13·1)
		Cardiomyopathy and myocarditis	209 (180 to 252)	0·3 (0·3 to 0·4)	127 (111 to 149)	0·2 (0·2 to 0·3)	−38·9 (−49·6 to −26·3)	−24·2 (−37·4 to −8·5)
		Endocarditis	22 (17 to 27)	0·0 (0·0 to 0·0)	19 (15 to 25)	0·0 (0·0 to 0·0)	−13·7 (−34·3 to 8·3)	7·2 (−18·4 to 34·5)
		Other cardiovascular and circulatory diseases	102 (84 to 124)	0·2 (0·1 to 0·2)	67 (56 to 80)	0·1 (0·1 to 0·2)	−33·2 (−47·9 to −14·9)	−17·0 (−35·3 to 5·7)
	Chronic respir atory diseases		336 (304 to 377)	0·5 (0·5 to 0·6)	103 (92 to 115)	0·2 (0·2 to 0·2)	−69·4 (−73·7 to −64·9)	−62·0 (−67·3 to −56·3)
		Chronic obstructive pulmonary disease	79 (66 to 104)	0·1 (0·1 to 0·2)	28 (24 to 34)	0·1 (0·0 to 0·1)	−64·3 (−72·5 to −56·1)	−55·7 (−65·8 to −45·5)
		Asthma	180 (153 to 214)	0·3 (0·2 to 0·3)	38 (33 to 45)	0·1 (0·1 to 0·1)	−79·0 (−83·1 to −73·8)	−73·9 (−79·0 to −67·5)
		Interstitial lung disease and pulmonary sarcoidosis	16 (11 to 20)	0·0 (0·0 to 0·0)	9 (5 to 11)	0·0 (0·0 to 0·0)	−44·1 (−65·9 to −26·4)	−30·5 (−57·7 to −8·6)
		Other chronic respiratory diseases	61 (37 to 89)	0·1 (0·1 to 0·1)	28 (21 to 38)	0·1 (0·0 to 0·1)	−51·2 (−70·0 to −27·0)	−39·4 (−62·7 to −9·3)
	Cirrhosis and other chronic liver diseases		245 (222 to 272)	0·4 (0·3 to 0·4)	167 (145 to 186)	0·3 (0·3 to 0·4)	−31·8 (−42·5 to −21·1)	−15·2 (−28·6 to −2·0)
		Cirrhosis and other chronic liver diseases due to hepatitis B	8 (6 to 10)	0·0 (0·0 to 0·0)	7 (5 to 9)	0·0 (0·0 to 0·0)	−15·2 (−30·2 to 1·0)	5·3 (−13·3 to 25·5)
		Cirrhosis and other chronic liver diseases due to hepatitis C	7 (5 to 9)	0·0 (0·0 to 0·0)	4 (3 to 6)	0·0 (0·0 to 0·0)	−33·7 (−44·0 to −21·8)	−17·6 (−30·5 to −2·8)
		Cirrhosis and other chronic liver diseases due to other causes	230 (208 to 257)	0·4 (0·3 to 0·4)	155 (135 to 174)	0·3 (0·3 to 0·3)	−32·3 (−43·0 to −21·6)	−15·9 (−29·2 to −2·6)
	Digestive diseases		289 (256 to 321)	0·5 (0·4 to 0·5)	115 (100 to 147)	0·2 (0·2 to 0·3)	−60·0 (−66·3 to −47·8)	−50·3 (−58·1 to −35·2)
		Peptic ulcer disease	36 (29 to 49)	0·1 (0·0 to 0·1)	12 (10 to 15)	0·0 (0·0 to 0·0)	−65·7 (−76·6 to −54·9)	−57·4 (−71·0 to −44·0)
		Gastritis and duodenitis	2 (2 to 3)	0·0 (0·0 to 0·0)	1 (1 to 2)	0·0 (0·0 to 0·0)	−48·3 (−62·7 to −32·2)	−35·7 (−53·6 to −15·8)
		Appendicitis	92 (65 to 106)	0·1 (0·1 to 0·2)	26 (21 to 38)	0·1 (0·0 to 0·1)	−70·9 (−78·1 to −45·1)	−63·8 (−72·9 to −31·7)
		Paralytic ileus and intestinal obstruction	60 (49 to 75)	0·1 (0·1 to 0·1)	32 (26 to 46)	0·1 (0·1 to 0·1)	−46·7 (−56·8 to −28·5)	−33·8 (−46·4 to −11·2)
		Inguinal, femoral, and abdominal hernia	12 (9 to 17)	0·0 (0·0 to 0·0)	5 (3 to 7)	0·0 (0·0 to 0·0)	−60·6 (−72·8 to −43·8)	−51·1 (−66·3 to −30·2)
		Inflammatory bowel disease	26 (19 to 39)	0·0 (0·0 to 0·1)	13 (10 to 16)	0·0 (0·0 to 0·0)	−47·8 (−68·3 to −24·1)	−35·1 (−60·6 to −5·7)
		Vascular intestinal disorders	6 (5 to 8)	0·0 (0·0 to 0·0)	3 (2 to 4)	0·0 (0·0 to 0·0)	−54·9 (−68·4 to −37·4)	−44·0 (−60·8 to −22·3)
		Gallbladder and biliary diseases	7 (6 to 10)	0·0 (0·0 to 0·0)	2 (2 to 4)	0·0 (0·0 to 0·0)	−64·4 (−71·9 to −55·9)	−55·8 (−65·0 to −45·3)
		Pancreatitis	11 (7 to 15)	0·0 (0·0 to 0·0)	5 (2 to 7)	0·0 (0·0 to 0·0)	−57·1 (−70·1 to −35·8)	−46·7 (−62·9 to −20·3)
		Other digestive diseases	37 (29 to 50)	0·1 (0·0 to 0·1)	17 (13 to 20)	0·0 (0·0 to 0·0)	−55·0 (−68·5 to −40·1)	−44·2 (−60·8 to −25·6)
	Neurological disorders		833 (739 to 962)	1·3 (1·2 to 1·5)	507 (460 to 562)	1·0 (0·9 to 1·1)	−38·9 (−48·5 to −26·4)	−24·0 (−36·1 to −8·6)
		Epilepsy	546 (467 to 639)	0·9 (0·7 to 1·0)	319 (280 to 371)	0·6 (0·5 to 0·7)	−41·0 (−52·2 to −24·5)	−26·8 (−40·6 to −6·2)
		Motor neuron disease	25 (22 to 29)	0·0 (0·0 to 0·0)	17 (14 to 19)	0·0 (0·0 to 0·0)	−32·8 (−45·7 to −19·9)	−16·5 (−32·6 to −0·4)
		Other neurological disorders	262 (229 to 317)	0·4 (0·4 to 0·5)	170 (150 to 192)	0·3 (0·3 to 0·4)	−34·4 (−49·5 to −21·5)	−18·5 (−37·3 to −2·5)
	Mental and substance use disorders		1 (1 to 1)	0·0 (0·0 to 0·0)	1 (1 to 1)	0·0 (0·0 to 0·0)	−17·9 (−44·1 to 13·3)	2·0 (−30·6 to 40·7)
		Eating disorders	1 (1 to 1)	0·0 (0·0 to 0·0)	1 (1 to 1)	0·0 (0·0 to 0·0)	−17·9 (−44·1 to 13·3)	2·0 (−30·6 to 40·7)
	Diabetes, urogenital, blood, and endocrine diseases		1036 (965 to 1164)	1·6 (1·5 to 1·8)	596 (543 to 665)	1·2 (1·1 to 1·3)	−42·4 (−48·4 to −36·3)	−28·4 (−35·9 to −20·9)
		Diabetes mellitus	115 (102 to 131)	0·2 (0·2 to 0·2)	63 (55 to 71)	0·1 (0·1 to 0·1)	−45·2 (−54·2 to −34·9)	−31·9 (−43·1 to −19·1)
		Acute glomerulonephritis	27 (17 to 44)	0·0 (0·0 to 0·1)	10 (8 to 15)	0·0 (0·0 to 0·0)	−58·8 (−79·3 to −30·7)	−48·8 (−74·3 to −14·0)
		Chronic kidney disease	251 (228 to 280)	0·4 (0·4 to 0·4)	154 (137 to 173)	0·3 (0·3 to 0·3)	−38·6 (−47·7 to −29·6)	−23·7 (−35·0 to −12·5)
		Urinary diseases and male infertility	82 (75 to 89)	0·1 (0·1 to 0·1)	50 (43 to 57)	0·1 (0·1 to 0·1)	−38·9 (−48·4 to −28·8)	−24·0 (−35·9 to −11·6)
		Haemoglobinopathies and haemolytic anaemias	191 (160 to 233)	0·3 (0·3 to 0·4)	84 (70 to 102)	0·2 (0·1 to 0·2)	−56·1 (−63·7 to −47·5)	−45·5 (−54·9 to −34·8)
		Endocrine, metabolic, blood, and immune disorders	371 (322 to 472)	0·6 (0·5 to 0·7)	236 (198 to 282)	0·5 (0·4 to 0·6)	−36·1 (−47·9 to −24·6)	−20·6 (−35·3 to −6·4)
	Musculoskeletal disorders		87 (69 to 134)	0·1 (0·1 to 0·2)	48 (39 to 72)	0·1 (0·1 to 0·1)	−44·7 (−55·3 to −30·2)	−31·3 (−44·5 to −13·3)
		Rheumatoid arthritis	18 (13 to 23)	0·0 (0·0 to 0·0)	5 (4 to 8)	0·0 (0·0 to 0·0)	−69·9 (−77·8 to −53·8)	−62·6 (−72·5 to −42·6)
		Other musculoskeletal disorders	69 (52 to 115)	0·1 (0·1 to 0·2)	42 (34 to 65)	0·1 (0·1 to 0·1)	−37·7 (−51·7 to −19·2)	−22·6 (−40·0 to 0·4)
	Other non-communicable diseases		1315 (1063 to 1457)	2·1 (1·7 to 2·3)	668 (588 to 793)	1·3 (1·1 to 1·5)	−48·9 (−56·1 to −35·1)	−36·5 (−45·4 to −19·4)
		Skin and subcutaneous diseases	12 (9 to 16)	0·0 (0·0 to 0·0)	10 (7 to 13)	0·0 (0·0 to 0·0)	−10·3 (−26·7 to 9·8)	11·5 (−9·0 to 36·4)
**Communicable, maternal, neonatal, and nutritional diseases**			**3051 (2748 to 3329)**	**4·8 (4·3 to 5·2)**	**1247 (1132 to 1378)**	**2·4 (2·2 to 2·7)**	**−59·1 (−63·6 to −54·1)**	**−49·1 (−54·8 to −42·9)**
HIV/AIDS and tuberculosis			169 (134 to 216)	0·3 (0·2 to 0·3)	62 (57 to 68)	0·1 (0·1 to 0·1)	−62·8 (−71·5 to −51·7)	−53·8 (−64·6 to −39·9)
Diarrhoea, lower respiratory, and other common infectious diseases			2515 (2230 to 2778)	4·0 (3·5 to 4·4)	1042 (939 to 1168)	2·0 (1·8 to 2·3)	−58·5 (−63·9 to −52·4)	−48·4 (−55·2 to −40·9)
		Diarrhoeal diseases	104 (75 to 141)	0·2 (0·1 to 0·2)	37 (27 to 53)	0·1 (0·1 to 0·1)	−63·5 (−73·5 to −51·4)	−54·7 (−67·1 to −39·7)
		Intestinal infectious diseases	145 (68 to 271)	0·2 (0·1 to 0·4)	64 (28 to 122)	0·1 (0·1 to 0·2)	−56·1 (−68·1 to −45·6)	−45·4 (−60·4 to −32·4)
		Lower respiratory infections	1368 (1238 to 1487)	2·1 (1·9 to 2·3)	662 (589 to 744)	1·3 (1·2 to 1·5)	−51·5 (−58·1 to −44·6)	−39·8 (−48·0 to −31·2)
		Upper respiratory infections	1 (0 to 2)	0·0 (0·0 to 0·0)	0 (0 to 1)	0·0 (0·0 to 0·0)	−59·3 (−84·8 to −9·0)	−49·5 (−81·1 to 13·0)
		Otitis media	6 (3 to 14)	0·0 (0·0 to 0·0)	0 (0 to 0)	0·0 (0·0 to 0·0)	−96·9 (−99·0 to −94·8)	−96·2 (−98·8 to −93·5)
		Meningitis	471 (347 to 585)	0·7 (0·5 to 0·9)	131 (111 to 163)	0·3 (0·2 to 0·3)	−71·6 (−78·8 to −60·9)	−64·7 (−73·6 to −51·4)
		Encephalitis	210 (141 to 252)	0·3 (0·2 to 0·4)	138 (111 to 193)	0·3 (0·2 to 0·4)	−33·6 (−49·1 to −14·0)	−17·5 (−36·8 to 6·8)
		Diphtheria	6 (3 to 10)	0·0 (0·0 to 0·0)	1 (0 to 1)	0·0 (0·0 to 0·0)	−89·7 (−95·5 to −79·2)	−87·2 (−94·5 to −74·2)
		Whooping cough	17 (5 to 43)	0·0 (0·0 to 0·1)	3 (1 to 8)	0·0 (0·0 to 0·0)	−74·1 (−95·6 to −14·3)	−67·8 (−94·5 to 6·5)
		Tetanus	2 (2 to 3)	0·0 (0·0 to 0·0)	1 (1 to 1)	0·0 (0·0 to 0·0)	−53·8 (−68·6 to −31·7)	−42·6 (−61·0 to −15·1)
		Measles	176 (76 to 366)	0·3 (0·1 to 0·6)	2 (1 to 3)	0·0 (0·0 to 0·0)	−99·0 (−99·4 to −98·3)	−98·7 (−99·3 to −97·9)
		Varicella and herpes zoster	9 (5 to 15)	0·0 (0·0 to 0·0)	3 (1 to 4)	0·0 (0·0 to 0·0)	−67·9 (−83·9 to −49·0)	−60·1 (−80·0 to −36·6)
Neglected tropical diseases and malaria			24 (18 to 34)	0·0 (0·0 to 0·1)	15 (9 to 30)	0·0 (0·0 to 0·1)	−34·4 (−63·1 to 32·5)	−18·4 (−54·2 to 64·6)
Nutritional deficiencies			66 (54 to 84)	0·1 (0·1 to 0·1)	24 (20 to 29)	0·0 (0·0 to 0·1)	−64·1 (−73·1 to −52·5)	−55·4 (−66·6 to −41·0)
Other communicable, maternal, neonatal, and nutritional diseases			256 (220 to 296)	0·4 (0·3 to 0·5)	100 (71 to 128)	0·2 (0·1 to 0·3)	−60·9 (−70·0 to −49·6)	−51·4 (−62·7 to −37·4)

**Figure 2:**
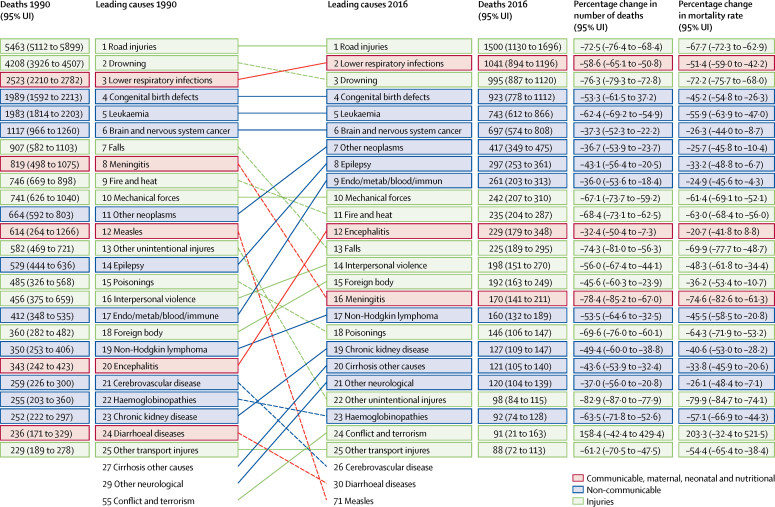
Top 25 causes of death in the WHO European Region, age 5–9 years, both sexes, 1990 and 2016

**Figure 3:**
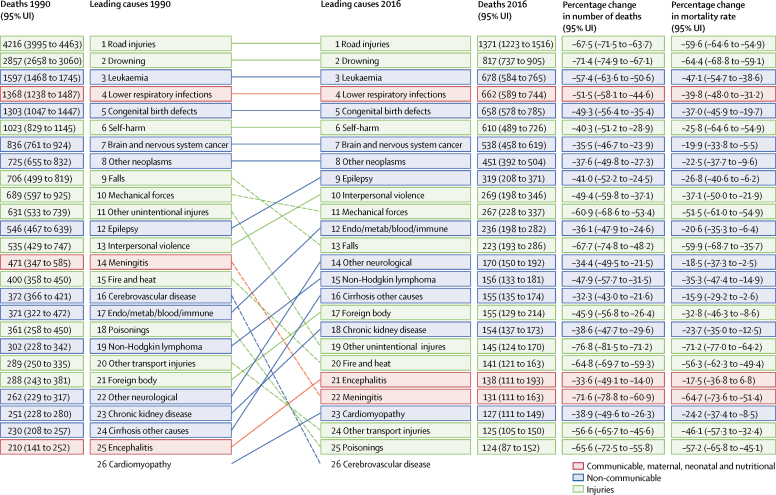
Top 25 causes of death in the WHO European Region, age 10–14 years, both sexes, 1990 and 2016

The leading causes of death were similar between the two age groups (Table 1, Table 2). Injuries caused 4163 deaths (95% UI 3820–4540; 38·7% of total deaths) in children aged 5–9 years and 4468 deaths (4162–4812; 43·5% of total) in those aged 10–14 years in 2016 (Table 1, Table 2). Injury mortality rates were almost twice as high in boys as in girls in both age groups (appendix). Road injuries remained a major contributor to injury mortality rates in both age groups in 2016 (Table 1, Table 2), despite a large decline of approximately 60% between 1990 and 2016 (Figure 2, Figure 3). The decrease in deaths due to road injuries from 1990 to 2016 varies throughout the European Region from a 79·1% (95% UI 76·9–81·0) decrease in EU15 to a 56·7% (51·1–62·0) decrease in CIS (appendix); in 2016, road injury mortality rates were four times higher in CIS than in EU15 (table 3; appendix). Even larger variations exist across individual countries: for example, road injury mortality rates (per 100 000 population) varied from 0·5 (95% UI 0·4–0·7) in Malta to 6·1 (4·4–8·3) in Kazakhstan (nearly a 12-times difference) in children 5–9 years old (appendix).

**Table 3:** Mortality rates for the top ten leading causes of death in 2016 globally and in the WHO European Region (51 countries and four subregions), ages 5–14 years, both sexes

		**Road injuries**	**Drowning**	**Lower respiratory infections**	**Congenital birth defects**	**Leukaemia**	**Brain and nervous system cancer**	**Other neoplasms**	**Epilepsy**	**Self-harm**	**Exposure to mechanical forces**
**Global**		**5·0 (4·6 to 5·4)**	**3·9 (3·6 to 4·3)**	**4·1 (3·3 to 4·7)**	**2·2 (2·0 to 2·5)**	**1·4 (1·3 to 1·6)**	**0·9 (0·8 to 1·0)**	**0·9 (0·8 to 1·0)**	**0·7 (0·6 to 0·8)**	**0·6 (0·6 to 0·7)**	**0·8 (0·7 to 0·8)**
**European Region**		**2·7 (2·4 to 3·0)**	**1·7 (1·6 to 1·9)**	**1·6 (1·4 to 1·8)**	**1·5 (1·3 to 1·8)**	**1·3 (1·1 to 1·5)**	**1·2 (1·0 to 1·3)**	**0·8 (0·7 to 0·9)**	**0·6 (0·5 to 0·7)**	**0·6 (0·5 to 0·7)**	**0·5 (0·4 to 0·6)**
	Albania	2·2 (1·5 to 3·1)	1·4 (0·9 to 2·2)	2·0 (1·3 to 2·8)	2·5 (1·6 to 3·7)	2·3 (1·5 to 3·2)	2·4 (1·5 to 3·5)	1·6 (1·0 to 2·3)	1·4 (0·9 to 2·0)	0·7 (0·4 to 1·2)	1·0 (0·6 to 1·9)
	Andorra	1·3 (0·9 to 1·6)	0·2 (0·1 to 0·3)	0·3 (0·1 to 0·4)	0·8 (0·6 to 1·0)	0·8 (0·5 to 1·1)	1·0 (0·8 to 1·3)	0·7 (0·5 to 1·0)	0·2 (0·2 to 0·3)	0·1 (0·1 to 0·2)	0·2 (0·1 to 0·3)
	Armenia	2·2 (1·7 to 2·9)	1·4 (1·1 to 1·9)	2·1 (1·6 to 2·7)	2·4 (1·7 to 3·4)	1·8 (1·4 to 2·5)	1·8 (1·3 to 2·5)	0·8 (0·6 to 1·0)	0·3 (0·2 to 0·4)	0·4 (0·2 to 0·5)	0·6 (0·4 to 0·8)
	Austria	1·0 (0·8 to 1·3)	0·3 (0·2 to 0·4)	0·1 (0·1 to 0·1)	1·1 (0·7 to 1·3)	0·6 (0·5 to 0·8)	0·8 (0·6 to 1·1)	0·5 (0·4 to 0·7)	0·2 (0·2 to 0·3)	0·3 (0·2 to 0·4)	0·3 (0·2 to 0·3)
	Azerbaijan	3·7 (2·4 to 5·2)	4·3 (2·9 to 7·1)	10·6 (7·6 to 14·6)	2·8 (1·5 to 4·7)	5·9 (3·6 to 8·6)	2·2 (1·5 to 3·1)	2·3 (1·3 to 3·2)	2·8 (1·9 to 4·2)	0·4 (0·3 to 0·6)	1·5 (0·9 to 2·1)
	Belarus	2·9 (2·2 to 3·7)	3·5 (2·7 to 4·5)	0·4 (0·3 to 0·6)	2·0 (1·5 to 3·1)	1·2 (0·9 to 1·6)	1·2 (0·8 to 1·6)	0·7 (0·5 to 0·9)	0·4 (0·2 to 0·6)	0·7 (0·5 to 1·0)	0·5 (0·4 to 0·7)
	Belgium	1·5 (1·3 to 1·8)	0·3 (0·2 to 0·3)	0·2 (0·2 to 0·2)	0·6 (0·5 to 0·8)	0·7 (0·6 to 0·9)	0·8 (0·6 to 1·2)	0·5 (0·4 to 0·6)	0·2 (0·2 to 0·3)	0·3 (0·2 to 0·4)	0·2 (0·2 to 0·3)
	Bosnia and Herzegovina	1·2 (1·0 to 1·5)	0·4 (0·3 to 0·6)	0·5 (0·4 to 0·7)	1·1 (0·8 to 1·3)	1·3 (0·8 to 1·7)	1·4 (1·0 to 1·9)	0·5 (0·4 to 0·7)	0·8 (0·6 to 1·0)	0·5 (0·3 to 0·7)	0·3 (0·2 to 0·4)
	Bulgaria	2·3 (1·6 to 3·1)	1·8 (1·3 to 2·4)	1·6 (1·2 to 2·3)	2·3 (1·6 to 3·1)	1·7 (1·2 to 2·3)	1·6 (1·1 to 2·1)	0·8 (0·6 to 1·1)	0·5 (0·4 to 0·7)	0·6 (0·4 to 0·8)	0·7 (0·5 to 1·0)
	Croatia	1·9 (1·6 to 2·3)	0·7 (0·5 to 0·8)	0·2 (0·2 to 0·3)	1·0 (0·8 to 1·3)	0·7 (0·6 to 1·0)	1·1 (0·8 to 1·5)	0·6 (0·4 to 0·7)	0·5 (0·4 to 0·6)	0·5 (0·3 to 0·7)	0·2 (0·2 to 0·3)
	Cyprus	2·1 (1·8 to 2·6)	0·9 (0·7 to 1·1)	0·2 (0·1 to 0·2)	0·6 (0·5 to 0·8)	0·9 (0·6 to 1·2)	0·8 (0·6 to 1·0)	0·5 (0·4 to 0·7)	0·1 (0·1 to 0·2)	0·1 (0·1 to 0·1)	0·3 (0·3 to 0·4)
	Czechia	1·3 (1·0 to 1·6)	0·6 (0·5 to 0·7)	0·4 (0·3 to 0·5)	0·7 (0·6 to 0·9)	0·6 (0·5 to 0·8)	1·0 (0·7 to 1·3)	0·5 (0·4 to 0·6)	0·3 (0·2 to 0·4)	0·3 (0·2 to 0·5)	0·3 (0·2 to 0·4)
	Denmark	1·1 (0·9 to 1·4)	0·2 (0·2 to 0·3)	0·1 (0·1 to 0·1)	0·7 (0·5 to 1·0)	0·6 (0·5 to 0·8)	0·8 (0·6 to 1·2)	0·4 (0·3 to 0·5)	0·1 (0·1 to 0·2)	0·1 (0·1 to 0·2)	0·2 (0·1 to 0·2)
	Estonia	1·3 (1·0 to 1·7)	1·6 (1·2 to 2·0)	0·4 (0·3 to 0·6)	1·4 (1·1 to 1·9)	1·1 (0·7 to 1·4)	1·1 (0·6 to 1·4)	0·5 (0·3 to 0·7)	0·3 (0·2 to 0·4)	0·5 (0·4 to 0·8)	0·4 (0·2 to 0·5)
	Finland	1·1 (0·9 to 1·4)	0·4 (0·3 to 0·5)	0·1 (0·1 to 0·1)	0·9 (0·7 to 1·1)	0·7 (0·5 to 0·9)	0·8 (0·6 to 1·1)	0·4 (0·3 to 0·6)	0·2 (0·1 to 0·2)	0·3 (0·2 to 0·4)	0·1 (0·1 to 0·2)
	France	1·1 (0·9 to 1·4)	0·4 (0·3 to 0·5)	0·1 (0·1 to 0·2)	0·6 (0·5 to 0·8)	0·8 (0·6 to 1·0)	0·8 (0·5 to 1·0)	0·6 (0·5 to 0·8)	0·2 (0·1 to 0·2)	0·3 (0·2 to 0·3)	0·2 (0·2 to 0·3)
	Georgia	3·6 (2·7 to 4·8)	2·0 (1·5 to 2·6)	2·8 (2·0 to 3·7)	1·7 (0·9 to 2·4)	3·1 (2·3 to 4·2)	1·2 (0·9 to 1·7)	1·0 (0·7 to 1·3)	0·5 (0·4 to 0·8)	0·3 (0·2 to 0·6)	1·0 (0·7 to 1·3)
	Germany	1·0 (0·8 to 1·2)	0·3 (0·2 to 0·3)	0·2 (0·1 to 0·2)	0·9 (0·6 to 1·1)	0·7 (0·6 to 0·9)	0·9 (0·7 to 1·2)	0·5 (0·4 to 0·6)	0·3 (0·3 to 0·4)	0·2 (0·1 to 0·3)	0·2 (0·1 to 0·2)
	Greece	2·1 (1·8 to 2·5)	0·5 (0·4 to 0·7)	0·4 (0·3 to 0·5)	1·6 (0·6 to 2·0)	1·1 (0·9 to 1·3)	1·3 (1·1 to 1·8)	0·4 (0·3 to 0·5)	0·1 (0·1 to 0·2)	0·1 (0·1 to 0·1)	0·2 (0·1 to 0·2)
	Hungary	1·5 (1·2 to 1·8)	0·8 (0·6 to 1·0)	0·3 (0·2 to 0·4)	1·4 (1·1 to 1·9)	1·0 (0·8 to 1·3)	1·2 (0·9 to 1·7)	0·6 (0·5 to 0·8)	0·4 (0·3 to 0·5)	0·4 (0·3 to 0·9)	0·2 (0·1 to 0·2)
	Iceland	1·0 (0·8 to 1·2)	0·4 (0·3 to 0·5)	0·3 (0·2 to 0·3)	0·9 (0·7 to 1·1)	0·8 (0·6 to 1·0)	1·5 (1·2 to 1·8)	0·5 (0·4 to 0·6)	0·2 (0·1 to 0·2)	0·2 (0·1 to 0·3)	0·1 (0·1 to 0·2)
	Ireland	1·2 (0·9 to 1·4)	0·3 (0·3 to 0·4)	0·2 (0·2 to 0·2)	1·0 (0·8 to 1·3)	0·9 (0·7 to 1·1)	0·9 (0·7 to 1·3)	0·6 (0·4 to 0·7)	0·2 (0·2 to 0·3)	0·3 (0·1 to 0·4)	0·2 (0·1 to 0·3)
	Israel	1·6 (1·3 to 1·9)	0·4 (0·3 to 0·5)	0·3 (0·3 to 0·4)	0·8 (0·6 to 1·0)	0·7 (0·6 to 0·9)	1·1 (0·9 to 1·4)	0·7 (0·5 to 0·8)	0·2 (0·2 to 0·3)	0·2 (0·1 to 0·3)	0·1 (0·1 to 0·2)
	Italy	1·2 (0·9 to 1·5)	0·2 (0·2 to 0·3)	0·2 (0·1 to 0·2)	0·7 (0·5 to 1·0)	1·1 (0·8 to 1·3)	0·7 (0·5 to 1·0)	0·7 (0·5 to 0·9)	0·1 (0·1 to 0·1)	0·1 (0·1 to 0·1)	0·2 (0·2 to 0·2)
	Kazakhstan	5·5 (4·2 to 7·1)	4·0 (3·1 to 5·1)	1·6 (1·2 to 2·2)	2·4 (1·7 to 3·9)	1·6 (1·2 to 2·2)	1·2 (0·8 to 1·6)	0·8 (0·5 to 1·1)	0·7 (0·5 to 1·3)	1·7 (1·1 to 2·3)	0·6 (0·4 to 0·8)
	Kyrgyzstan	5·3 (4·4 to 6·5)	3·8 (3·2 to 4·7)	4·3 (3·5 to 5·3)	2·2 (1·7 to 3·0)	1·7 (1·3 to 2·2)	0·9 (0·6 to 1·2)	0·9 (0·7 to 1·2)	1·5 (1·2 to 1·9)	1·4 (0·9 to 1·9)	0·7 (0·5 to 0·9)
	Latvia	1·9 (1·4 to 2·6)	2·7 (2·0 to 3·7)	0·4 (0·3 to 0·6)	1·9 (1·3 to 3·1)	1·2 (0·7 to 1·7)	1·2 (0·7 to 1·7)	0·7 (0·4 to 1·0)	0·2 (0·2 to 0·4)	0·5 (0·3 to 0·9)	0·5 (0·3 to 0·7)
	Lithuania	2·5 (2·0 to 3·0)	2·7 (2·2 to 3·2)	0·9 (0·7 to 1·1)	1·8 (1·4 to 2·6)	1·0 (0·8 to 1·3)	1·2 (0·9 to 1·6)	0·7 (0·5 to 0·9)	0·3 (0·2 to 0·3)	0·9 (0·7 to 1·3)	0·5 (0·4 to 0·6)
	Luxembourg	1·1 (0·9 to 1·4)	0·2 (0·1 to 0·2)	0·1 (0·1 to 0·2)	0·4 (0·3 to 0·5)	0·7 (0·5 to 0·9)	0·8 (0·6 to 1·2)	0·4 (0·3 to 0·5)	0·2 (0·2 to 0·3)	0·2 (0·1 to 0·2)	0·2 (0·2 to 0·3)
	Macedonia	2·2 (1·6 to 3·3)	1·0 (0·6 to 1·5)	0·7 (0·3 to 1·1)	0·9 (0·6 to 1·3)	1·3 (0·8 to 2·0)	1·5 (1·0 to 2·2)	0·7 (0·5 to 1·1)	0·4 (0·3 to 0·6)	0·4 (0·3 to 0·6)	0·3 (0·2 to 0·4)
	Malta	0·7 (0·6 to 0·9)	0·4 (0·3 to 0·4)	0·3 (0·3 to 0·4)	1·2 (1·0 to 1·5)	0·8 (0·6 to 1·0)	0·9 (0·7 to 1·1)	0·7 (0·5 to 0·8)	0·2 (0·1 to 0·2)	0·1 (0·1 to 0·2)	0·3 (0·2 to 0·3)
	Republic of Moldova	3·3 (2·4 to 4·3)	4·0 (2·9 to 5·2)	1·5 (1·1 to 2·1)	2·0 (1·4 to 2·8)	1·8 (1·2 to 2·4)	1·3 (0·9 to 1·8)	1·1 (0·8 to 1·5)	0·4 (0·3 to 0·6)	0·6 (0·4 to 0·9)	0·6 (0·4 to 0·8)
	Montenegro	1·9 (1·5 to 2·4)	0·5 (0·4 to 0·7)	0·5 (0·3 to 0·7)	0·5 (0·4 to 0·7)	1·2 (0·8 to 1·6)	1·6 (1·1 to 2·1)	0·3 (0·2 to 0·4)	0·2 (0·2 to 0·3)	0·5 (0·3 to 0·8)	0·6 (0·4 to 0·7)
	Netherlands	1·2 (1·0 to 1·4)	0·3 (0·2 to 0·3)	0·2 (0·2 to 0·3)	0·8 (0·6 to 1·2)	0·7 (0·5 to 0·8)	0·9 (0·6 to 1·1)	0·7 (0·6 to 0·8)	0·3 (0·2 to 0·3)	0·2 (0·1 to 0·3)	0·2 (0·1 to 0·2)
	Norway	0·9 (0·7 to 1·1)	0·3 (0·3 to 0·4)	0·1 (0·1 to 0·1)	0·8 (0·6 to 1·1)	0·6 (0·5 to 0·7)	0·9 (0·7 to 1·2)	0·4 (0·3 to 0·5)	0·2 (0·2 to 0·3)	0·3 (0·2 to 0·4)	0·2 (0·1 to 0·2)
	Poland	1·9 (1·4 to 2·6)	0·7 (0·5 to 0·9)	0·6 (0·4 to 0·8)	1·2 (0·8 to 1·6)	0·8 (0·5 to 1·1)	1·1 (0·7 to 1·6)	0·6 (0·4 to 0·8)	0·2 (0·2 to 0·3)	0·5 (0·3 to 0·7)	0·2 (0·2 to 0·4)
	Portugal	1·7 (1·4 to 2·0)	0·5 (0·4 to 0·6)	0·4 (0·4 to 0·5)	0·8 (0·6 to 1·0)	1·0 (0·8 to 1·2)	1·0 (0·8 to 1·2)	0·7 (0·6 to 0·9)	0·2 (0·2 to 0·3)	0·1 (0·1 to 0·2)	0·2 (0·2 to 0·3)
	Romania	3·0 (2·5 to 3·6)	2·3 (1·9 to 2·8)	2·3 (1·9 to 2·7)	1·0 (0·8 to 1·5)	1·2 (1·0 to 1·6)	1·3 (1·0 to 1·7)	0·8 (0·6 to 1·0)	0·5 (0·4 to 0·6)	0·6 (0·4 to 0·8)	0·3 (0·3 to 0·4)
	Russian Federation	4·2 (3·5 to 4·9)	3·5 (3·0 to 4·1)	1·1 (0·9 to 1·4)	2·0 (1·7 to 2·6)	1·2 (0·9 to 1·4)	1·2 (0·9 to 1·6)	0·9 (0·7 to 1·1)	0·2 (0·2 to 0·3)	1·3 (0·9 to 1·7)	0·8 (0·6 to 1·3)
	Serbia	2·3 (1·9 to 2·9)	1·1 (0·9 to 1·4)	0·6 (0·4 to 0·7)	1·2 (1·0 to 1·5)	1·1 (0·8 to 1·3)	1·6 (1·2 to 2·0)	0·9 (0·7 to 1·1)	0·7 (0·5 to 0·8)	0·4 (0·3 to 0·7)	0·4 (0·3 to 0·5)
	Slovakia	2·1 (1·6 to 2·7)	1·1 (0·8 to 1·4)	1·1 (0·8 to 1·4)	1·3 (0·9 to 1·6)	1·0 (0·7 to 1·3)	1·2 (0·9 to 1·6)	0·6 (0·5 to 0·9)	0·4 (0·3 to 0·5)	0·3 (0·2 to 0·5)	0·4 (0·3 to 0·6)
	Slovenia	1·2 (1·0 to 1·5)	0·3 (0·2 to 0·4)	0·1 (0·1 to 0·2)	0·8 (0·7 to 1·2)	0·6 (0·5 to 0·8)	0·9 (0·6 to 1·1)	0·4 (0·3 to 0·5)	0·1 (0·1 to 0·2)	0·5 (0·3 to 0·8)	0·2 (0·1 to 0·3)
	Spain	1·0 (0·8 to 1·2)	0·3 (0·2 to 0·4)	0·2 (0·1 to 0·2)	0·8 (0·6 to 1·0)	1·0 (0·8 to 1·2)	0·8 (0·6 to 1·0)	1·0 (0·8 to 1·2)	0·1 (0·1 to 0·2)	0·1 (0·1 to 0·2)	0·1 (0·1 to 0·2)
	Sweden	0·8 (0·7 to 1·0)	0·3 (0·2 to 0·4)	0·1 (0·1 to 0·1)	0·8 (0·6 to 1·4)	0·7 (0·5 to 0·8)	0·9 (0·7 to 1·1)	0·6 (0·4 to 0·7)	0·2 (0·1 to 0·2)	0·3 (0·2 to 0·4)	0·2 (0·2 to 0·3)
	Switzerland	1·0 (0·8 to 1·3)	0·3 (0·2 to 0·4)	0·1 (0·1 to 0·1)	0·9 (0·7 to 1·2)	0·6 (0·5 to 0·8)	0·6 (0·5 to 0·9)	0·4 (0·3 to 0·5)	0·2 (0·2 to 0·3)	0·3 (0·2 to 0·3)	0·2 (0·1 to 0·3)
	Tajikistan	3·7 (2·6 to 5·1)	9·0 (6·3 to 12·3)	16·3 (11·7 to 22·8)	2·3 (1·5 to 3·3)	2·6 (1·8 to 3·7)	1·3 (0·9 to 1·8)	1·1 (0·7 to 1·5)	2·8 (2·0 to 4·1)	0·5 (0·3 to 0·8)	1·0 (0·6 to 1·5)
	Turkey	4·9 (3·4 to 6·5)	1·0 (0·7 to 1·4)	1·2 (0·7 to 1·7)	2·5 (1·8 to 3·4)	2·3 (1·5 to 3·3)	1·7 (1·2 to 2·4)	1·3 (0·8 to 1·8)	1·1 (0·7 to 1·4)	0·4 (0·3 to 0·7)	1·0 (0·7 to 1·3)
	Turkmenistan	3·5 (2·5 to 4·8)	4·3 (3·1 to 5·9)	7·7 (5·4 to 10·2)	2·9 (1·6 to 4·3)	3·1 (2·2 to 4·2)	1·8 (1·2 to 2·6)	2·1 (1·4 to 2·9)	1·6 (1·0 to 2·5)	1·0 (0·6 to 1·3)	0·9 (0·6 to 1·2)
	Ukraine	2·8 (2·0 to 4·0)	3·6 (2·5 to 5·0)	0·7 (0·5 to 1·1)	2·8 (1·8 to 4·1)	1·5 (1·0 to 2·2)	1·4 (0·9 to 1·9)	1·0 (0·7 to 1·4)	0·4 (0·3 to 0·8)	0·7 (0·5 to 1·1)	0·6 (0·4 to 0·9)
	UK	0·9 (0·9 to 1·0)	0·2 (0·2 to 0·2)	0·3 (0·3 to 0·3)	0·9 (0·8 to 1·0)	0·7 (0·7 to 0·8)	0·9 (0·7 to 1·0)	0·7 (0·7 to 0·8)	0·3 (0·3 to 0·3)	0·1 (0·1 to 0·1)	0·2 (0·1 to 0·2)
	Uzbekistan	4·8 (3·5 to 6·5)	4·3 (3·2 to 5·8)	9·5 (7·1 to 12·2)	1·0 (0·7 to 1·4)	2·4 (1·7 to 3·2)	1·5 (1·1 to 2·0)	0·6 (0·5 to 0·9)	2·4 (1·8 to 3·2)	1·5 (0·9 to 2·2)	0·5 (0·4 to 0·6)
**EU13**		**2·1 (1·8 to 2·3)**	**1·2 (1·0 to 1·3)**	**0·9 (0·8 to 1·1)**	**1·2 (1·0 to 1·5)**	**1·0 (0·8 to 1·1)**	**1·2 (0·9 to 1·4)**	**0·6 (0·5 to 0·7)**	**0·3 (0·3 to 0·4)**	**0·5 (0·4 to 0·6)**	**0·3 (0·3 to 0·4)**
**EU15**		**1·1 (1·0 to 1·2)**	**0·3 (0·3 to 0·3)**	**0·2 (0·2 to 0·2)**	**0·8 (0·7 to 0·9)**	**0·8 (0·7 to 0·9)**	**0·9 (0·7 to 1·0)**	**0·6 (0·6 to 0·7)**	**0·2 (0·2 to 0·2)**	**0·2 (0·1 to 0·2)**	**0·2 (0·2 to 0·2)**
**SEEHN**		**2·3 (2·1 to 2·6)**	**1·5 (1·3 to 1·7)**	**1·2 (1·1 to 1·4)**	**1·2 (1·1 to 1·4)**	**1·2 (1·0 to 1·4)**	**1·4 (1·2 to 1·6)**	**0·8 (0·7 to 0·9)**	**0·5 (0·5 to 0·6)**	**0·5 (0·4 to 0·5)**	**0·4 (0·3 to 0·4)**
**CIS**		**4·1 (3·7 to 4·6)**	**4·0 (3·6 to 4·5)**	**3·8 (3·3 to 4·4)**	**2·1 (1·7 to 2·7)**	**1·8 (1·5 to 2·0)**	**1·3 (1·1 to 1·5)**	**0·9 (0·8 to 1·1)**	**0·9 (0·8 to 1·2)**	**1·2 (0·9 to 1·4)**	**0·7 (0·6 to 1·0)**

Andorra, Georgia, Iceland, Norway, Switzerland, and Turkey are not included in the subregion groups. Bulgaria, Croatia, Romania, and Republic of Moldova belong to more than one subregion. EU13=countries that joined the European Union after May, 2004. EU15=countries that joined the European Union before May, 2004. SEEHN=South Eastern Europe Health Network. CIS=commonwealth of independent states.

Mortality rates due to drowning, another notable contributor to injury mortality rates in children, also showed large decreases between 1990 and 2016, with a 72·2% (95% UI 68·0–75·7) decrease in children aged 5–9 years and 64·4% (59·1–68·8) decrease in the those aged 10–14 years (Figure 2, Figure 3). The decline in drowning mortality rates varied across subregions from 73·6% (95% UI 70·2–76·7) in EU13 to 63·3% (58·4–67·6) in CIS in children aged 5–14 years (appendix). In 2016, the mortality rate in children aged 5–14 years for drowning was 14 times higher in CIS than EU15 (table 3), with even greater variation at the country level: a nearly 45-times difference between Luxembourg (0·2 per 100 000 children) and Tajikistan (9·0 per 100 000 children; table 3).

In children aged 10–14 years, the percentage decrease in mortality rate for self-harm between 1990 and 2016 (25·8%; 95% UI 11·7–39·4) was much smaller than those for road injuries (59·6%; 54·9–64·6) and drowning (64·4%; 59·1–68·8; figure 3). At the subregion level, the decline in self-harm mortality rates was much smaller in CIS (12·5%) than the remaining three subregions, in which the decreases were all greater than 40% (appendix). In 2016, the mortality rate due to self-harm was more than six times higher in CIS than EU15 in children aged 10–14 years old. Self-harm mortality rates for children aged 10–14 years in 2016 varied greatly between countries, with a 20-times difference between the lowest (Greece; 0·2 per 100 000) and the highest (Kazakhstan; 4·0 per 100 000; appendix).

In 2016, neoplasms caused 2161 deaths (95% UI 1872–2406; 20·1% of total deaths) in children aged 5–9 years and 1943 deaths (1749–2101; 18·9% of total deaths) in those aged 10–14 years (Table 1, Table 2). Neoplasm mortality rates were 29·4% higher in boys than girls in the 5–9 years age group and 30·3% higher in boys than girls in the 10–14 years age group (appendix). Leukaemia was the leading cause of deaths due to neoplasms, followed by brain and nervous system cancer in both age groups. Between 1990 and 2016, leukaemia mortality rates decreased by 55·9% (95% UI 47·0–63·9) in the 5–9 years age group and 47·1% (38·6–54·7) in the 10–14 years age group (Figure 2, Figure 3), while the percentage decrease in mortality rates for brain and nervous system cancer was much smaller in both age groups (26·3% [95% UI 8·7–44·0] in children aged 5–9 years and 19·9% [5·5–33·8] in those aged 10–14 years; Figure 2, Figure 3). The decreases in mortality rates were not markedly different across subregions for leukaemia and brain and nervous system cancer. Leukaemia death rates were 45·4% higher in boys than girls aged 5–9 years and 60·0% higher in boys than girls aged 10–14 years (appendix). Brain and nervous system cancer mortality rates were not markedly different between boys and girls. The mortality rate for leukaemia was about two times higher in CIS than EU15 in 2016 (table 3).

Mortality rates from other causes of death also show varying degrees of differences within the European Region (table 3). In 2016, mortality rates for lower respiratory infections, the most common cause of communicable disease death, were about 20 times higher in CIS than EU15 countries. Encephalitis mortality rates in CIS countries were eight times greater than in EU15 countries (appendix). The mortality rate for congenital birth defects was 2·6 times higher and that for epilepsy was about 4·5 times higher in CIS than in EU15 countries.

**Figure 4:**
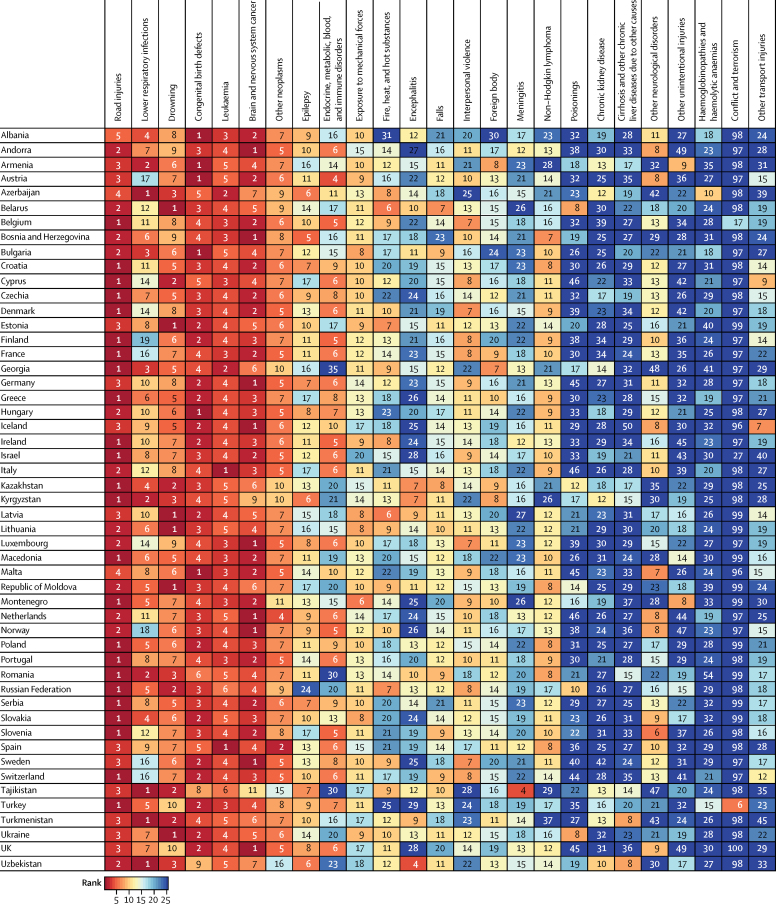
Rankings of top causes of death in WHO European Region, ages 5–9 years, both sexes, 2016

**Figure 5:**
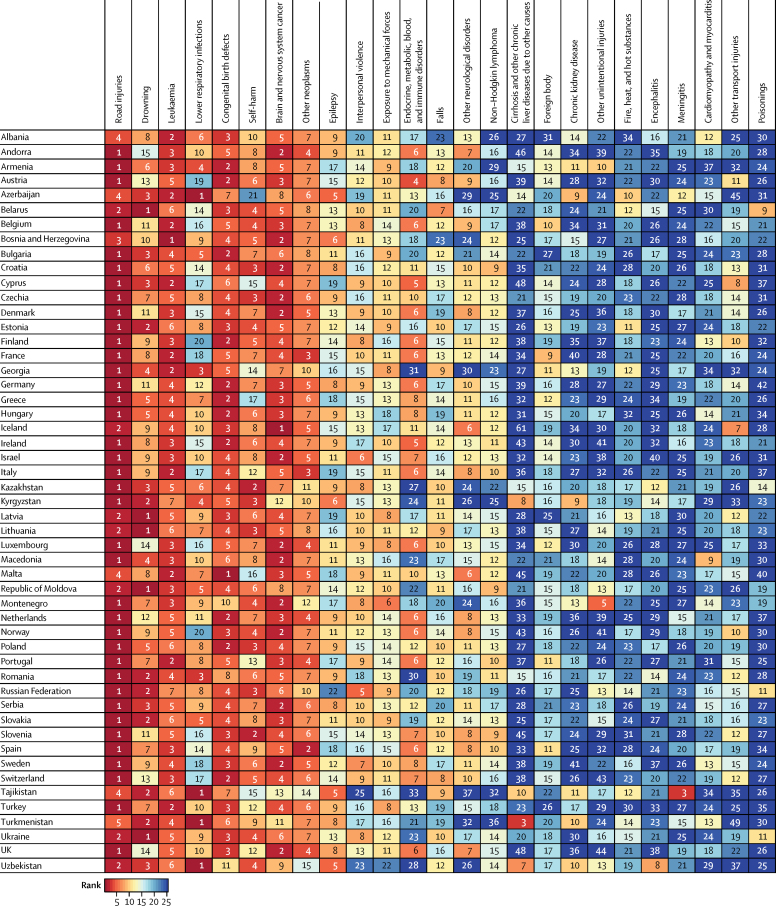
Rankings of top causes of death in WHO European Region, ages 10–14 years, both sexes, 2016

The rankings of the leading causes of death in each country in the WHO European Region in 2016 are shown on a dashboard for each age group (Figure 4, Figure 5). For children aged 5–9 years, the leading cause of death was road injuries in 24 countries, brain and nervous system cancer in nine countries, congenital birth defects in six countries, drowning in six countries, lower respiratory infections in four countries, and leukaemia in two countries (figure 4). For the 10–14 years age group, the leading cause of death was road injuries in 38 countries, drowning in five countries, lower respiratory infections in four countries, leukaemia in one country, brain and nervous system cancer in one country, and congenital birth defects in one country (figure 5). Lower respiratory infections were the first or second leading cause of death in several CIS countries in both age groups, whereas this cause was much lower in the ranking for the remaining groups of countries (Figure 4, Figure 5).

## Discussion

Substantial progress has been made in reducing mortality in children aged 5–14 years in the WHO European Region over the past 26 years. Despite the decline, deaths from causes that are preventable or amenable to high-quality health care (eg, injuries, congenital anomalies, leukaemia, and lower respiratory infections)^19^ remain large contributors to mortality among these children in 2016. Large differences in cause-specific mortality rates between EU15 and CIS countries are notable, ranging from a two-times difference in leukaemia to a 20-times difference in lower respiratory infections.

Road injuries were the main contributors to injury death rates for children aged 5–14 years, with death rates in CIS four times higher than EU15 in 2016. In CIS countries, child injury mortality rates increased substantially in the early 1990s as a consequence of political and economic transitions.^20^, ^21^, ^22^ Progress has been made in reducing the injury death rates over time but the decline could be accelerated further by concerted efforts. For example, according to a 2015 report by the WHO European Region,^23^ road safety laws addressing the key behavioural risk factors (speed, drunk driving, and not using helmets, seatbelts, and child car restraints) can reduce road injury deaths, but many countries have laws addressing only a subset of these risk factors. Having comprehensive laws addressing all five risk factors is therefore crucial to speed up the decline in road injury mortality rates. Furthermore, adherence to international vehicle safety standards and safer road infrastructure designs are also important to overcome premature deaths from road crashes. Many countries, especially those in the CIS group, have had rapid increases between 2010 and 2013, in motor vehicle ownership,^23^ and thus more organised efforts of society through legislation, enforcement, and social marketing are necessary to improve safety on the roads.

Drowning was a notable contributor to injury mortality rates in both age groups. Mortality rates from drowning were especially high in Tajikistan, Kazakhstan, Uzbekistan, Azerbaijan, Kyrgyzstan, and Turkmenistan. The high risk of drowning was not specific to children aged 5–14 years, but was also common in children younger than 5 years. Widespread irrigation practices, exposure to large expanses of unprotected waterways, the inability to swim, and scarce supervision might contribute to high drowning rates among children in these countries.^20^, ^24^ Intersectoral action is needed to implement effective interventions such as fencing, supervision, flotation devices, and water skills training.^25^

Self-harm was one of the top five causes of death among children aged 10–14 years in many countries in the European Region in 2016, with the highest mortality rate in Kazakhstan. A study by UNICEF in Kazakhstan reported that mental health conditions (especially depression) were key risk factors for youth suicides, and that specialised mental health services were not available in the country.^26^ Kyrgyzstan, Russian Federation, and Uzbekistan were also among the countries with the highest self-harm mortality rates in 2016. Self-poisoning with medication is a widely used method of suicide in many European countries,^27^ and prohibition of access to the medications commonly used in suicide has been shown to effectively prevent suicide.^28^ National suicide prevention strategies include multiple components such as restricting access to common lethal means and promoting access to mental health and other services, but such strategies are not present in most countries.^28^

Other leading causes of death in children aged 5–14 years are also highly preventable or amenable to high-quality health care. For example, lower respiratory infections, the leading cause of death mostly in CIS countries, are preventable through vaccination and reduction of exposure to risk factors,^29^, ^30^ and amenable to timely antimicrobial treatment.^29^ Certain congenital birth defects are preventable (eg, by folic acid supplementation for preventing neural tube defects)^31^ or amenable to surgical care (eg, congenital heart anomalies and neural tube defects).^32^, ^33^ About half of the deaths from congenital anomalies in children aged 5–14 years in 2016 were from congenital heart defects. Mortality rates from congenital anomalies vary widely across countries in the European Region. This might be partly explained by differences in prenatal screening policies and laws concerning termination of pregnancy for fetal anomaly in different countries.^34^

Despite a general decline in mortality in both age groups over the past 26 years, the rate of decline varies vastly across countries. For instance, leukaemia mortality rates show a stagnant trend or a slow decline in some countries (eg, Azerbaijan and Turkmenistan) but a sharp decline in others (eg, Russian Federation and Ukraine). The fast decline in the Russian Federation, for example, could be tracked to the successful implementation of treatment protocols tailored to the local conditions of the Russian health-care system.^35^

The challenges and limitations of the GBD approach in estimating all-cause and cause-specific mortality have been described extensively elsewhere,^11^, ^12^, ^15^ and we provide a brief summary of some of them here. First, although our systematic approach to redistributing garbage codes enhances the comparability of the cause-of-death data, this approach can cause our results to differ from countries' official statistics (even from those with complete vital registration systems). Second, we did not include any intermediate causes of death (eg, heart failure) in the cause list. They were treated as garbage codes and were reassigned to the possible underlying cause. Although the idea was to assign each cause uniquely to an underlying cause, this approach could mask intermediate causes that are important to note for purposes of health service delivery. In future research, we aim to report mortality estimates for intermediate causes as supplemental information. Third, for a very small number of locations and years with few or no data, we used covariates, borrowing strength across space and time to generate the mortality estimates; the scarcity of data in a particular location is reflected in the wide uncertainty intervals. Finally, time trends for some causes of death such as cancer might be influenced by changes in diagnostic technology; they were probably underdiagnosed in the past when diagnostic tests were done less frequently. Despite these limitations, our study used all available data and robust methods to produce comparable all-cause and cause-specific mortality estimates for children aged 5–9 years and 10–14 years over time across countries in the WHO European Region. A separate analysis of the GBD data focusing on the mortality burden in older adolescents and young adults could be complementary to this Article, and could provide a better picture of the health of children and young adults in the European Region.

In conclusion, our findings show large variations in trends in cause-specific mortality rates in children aged 5–14 years between 1990 and 2016 and across different countries in the WHO European Region. Differences between highest and lowest mortality rates ranged from a two-times difference to a 20-times difference for the leading causes of death across countries. Many causes of death are preventable or amenable to health care; although progress has been made in reducing mortality over time, the decline could be accelerated further through coordinated efforts between governments and stakeholders, such as legislators, local authorities, health-care professionals, and community members. Understanding the trends in causes of death in children allows governments and public health officials to identify priorities. Moreover, these findings could be used as a baseline to establish whether programmes and policies are effective in reducing the mortality burden in children aged 5–14 years in future.
