# Supplementary material for: Causes of death among children aged 5–14 years in the WHO European Region: a systematic analysis for the Global Burden of Disease Study 2016
Source: Lancet Child Adolesc Health. 2018 May;2(5):321–37. doi: 10.1016/S2352-4642(18)30095-6 (PMC5928398; doi:10.1016/S2352-4642(18)30095-6)
Supplement: Supplementary appendix [file mmc1.pdf]

THE LANCET
Child & Adolescent Health

Supplementary appendix

This appendix formed part of the original submission and has been peer reviewed. We post it as supplied by the authors.

Supplement to: Kyu HH, Stein CE, Pinto Boschi C, et al. Causes of death among children aged 5–14 years in the WHO European Region: a systematic analysis for the Global Burden of Disease Study 2016. *Lancet Child Adolesc Health* 2018; published online March 19. http://dx.doi.org/10.1016/S2352-4642(18)30095-6.

Appendix to “Levels and trends of the main causes of death among children aged 5–14 years in the WHO European Region: findings from the Global Burden of Diseases, Injuries, and Risk Factors (GBD) 2016 Study”

This appendix provides supplemental results for “Levels and trends of the main causes of death among children aged 5–14 years in the WHO European Region: findings from the Global Burden of Diseases, Injuries, and Risk Factors (GBD) 2016 Study.”

List of Appendix Figures and Tables

eTable 1: List of International Classification of Diseases (ICD) codes mapped to the Global Burden of Disease cause list for causes of death

eTable 2: Underlying indicators for percent well-certified for data source with maximum percent well certified in each 5-year time interval for WHO European region countries, 1980–2016

eTable 3: Number of deaths and rates (per 100,000) with percent change between 1990 and 2000 and between 2000 and 2016 in the WHO Euro region, ages 5 to 9, males

eTable 4: Number of deaths and rates (per 100,000) with percent change between 1990 and 2000 and between 2000 and 2016 in the WHO Euro region, ages 5 to 9, females

eTable 5: Number of deaths and rates (per 100,000) with percent change between 1990 and 2000 and between 2000 and 2016 in the WHO Euro region, ages 10 to 14, males

eTable 6: Number of deaths and rates (per 100,000) with percent change between 1990 and 2000 and between 2000 and 2016 in the WHO Euro region, ages 10 to 14, females

eTable 7: Mortality rates for the 11th to 20th leading causes of death in 2016 globally and in the WHO European Region (51 countries and four subregions), ages 5–14 years, both sexes

eTable 8: All-cause and cause-specific mortality (counts and rates) at the global level and by country in the WHO European Region for ages 5 to 9 and 10 to 14 for 1990, 2000, and 2016

eTable 9. Percent change in mortality rates for the leading causes of death in WHO European region between 1990 and 2016, ages 5-14, both sexes combined

eTable 10: Top 20 ranking sub-models for road injuries, males

eTable 1: List of International Classification of Diseases (ICD) codes mapped to the Global Burden of Disease cause list for causes of death		
**Cause**	**ICD10**	**ICD9**
Communicable, maternal, neonatal, and nutritional diseases	A00-A00.9, A01.0-A14, A15-A28.9, A30-A30.9, A32-A39.9, A48.1-A48.2, A48.4-A48.5, A49.1, A50-A58, A60-A60.9, A63-A63.8, A65-A65.0, A68-A70, A74, A74.8-A75.9, A77-A96.9, A98-A98.8, B00-B06.9, B10-B10.8, B15-B17.9, B19-B27.9, B29.4, B33-B33.1, B33.3-B33.8, B47-B48.8, B50-B53.8, B55.0, B56-B57.5, B60-B60.8, B63, B65-B67.9, B69-B72.0, B74.3-B75, B77-B77.9, B83-B83.8, B90-B92, B94.1-B94.2, B95-B95.5, B97.4-B97.6, D50.1-D50.8, D51-D52.0, D52.8-D53.9, D64.3, E00-E02, E40-E46.9, E51-E61.9, E63-E64.0, E64.2-E64.9, F07.1, G00.0-G00.8, G03-G03.8, G04-G05.8, G14-G14.6, H70-H70.9, I00, I02, I02.9, I98.0-I98.1, J01-J01.9, J02.0, J03.0, J04.0, J05-J05.0, J09-J15.8, J16-J16.9, J20-J21.9, J36-J36.0, K67.0-K67.8, K71.2, K71.6, K74.7-K74.8, K75.3, K76.3, K77.0, K93.0-K93.1, M03.1, M12.1, M49.0-M49.1, M73.0-M73.1, M89.6, N74.1-N74.2, N96, N98-N98.9, O00-O07.9, O09-O16.9, O20-O26.9, O28-O36.9, O40-O48.1, O60-O77.9, O80-O92.7, O96-O98.6, O98.8-P04.2, P04.5-P05.9, P07-P15.9, P19-P22.9, P23.0-P23.4, P24-P29.9, P35-P37.2, P37.5-P39.9, P50-P61.9, P70, P70.3-P72.9, P74-P78.9, P80-P81.9, P83-P84, P90-P94.9, P96, P96.3-P96.4, P96.8, R19.7, U04-U04.9, U06-U06.9, U82-U89, Z16-Z16.3	001-001.9, 002.0-030.9, 032-034.9, 036-036.3, 036.5-037.9, 040, 040.1-041.0, 042-066.9, 070-074.1, 074.3-075.9, 078.3-078.7, 079-079.7, 080-083.9, 084.0-084.5, 084.7-084.9, 085.0, 086-088, 088.8-088.9, 090-101.6, 104-104.9, 120-124.9, 125.4-125.9, 127-127.1, 128-129.0, 136-136.2, 137-139.0, 244.2, 260-263.9, 265-269.9, 280.1-280.8, 281.0-281.9, 320.0-320.8, 321-323.9, 381-383.9, 390-390.9, 392, 392.9, 425.6, 461-461.9, 464.0, 464.2, 464.4, 464.8-464.9, 466-469, 470.0, 475-475.9, 476.9, 480-482.8, 483.0-483.9, 484.0-484.7, 487-489, 630-636.9, 638-638.9, 640-679.1, 716.0, 730.4-730.6, 760-760.6, 760.8-768, 768.2-770, 770.1-775, 775.4-779.3, 779.6-779.8, V09-V09.9
HIV/AIDS and tuberculosis	A10-A14, A15-A19.9, B20-B24.9, B90-B90.9, K67.3, K93.0, M49.0, N74.1-N74.2, P37.0, U84.3	010-019.9, 042-044.9, 137-137.9, 138.0-138.9, 730.4-730.6
Tuberculosis	A10-A14, A15-A19.9, B90-B90.9, K67.3, K93.0, M49.0, N74.1-N74.2, P37.0, U84.3	010-019.9, 137-137.9, 138.0-138.9, 730.4-730.6
Drug-susceptible tuberculosis	A10-A14, A15-A19.9, B90-B90.9, K67.3, K93.0, M49.0, P37.0	010-019.9
Multidrug-resistant tuberculosis without extensive drug resistance	U84.3	
Extensively drug-resistant tuberculosis		
HIV/AIDS	B20-B24.9	042-044.9
Drug-susceptible HIV/AIDS - Tuberculosis	B20.0	
Multidrug-resistant HIV/AIDS - Tuberculosis without extensive drug resistance		
Extensively drug-resistant HIV/AIDS - Tuberculosis		
HIV/AIDS resulting in other diseases	B20.1-B23.9, B24.0	042-044.9
Diarrhea, lower respiratory, and other common infectious diseases	A00-A00.9, A01.0-A09.9, A33-A37.9, A39-A39.9, A48.1, A70, A83-A87.9, B01-B02.9, B05-B05.9, B94.1, B97.4-B97.6, F07.1, G00.0-G00.8, G03-G03.8, G04-G05.8, H70-H70.9, J01-J01.9, J04.0, J05-J05.0, J09-J15.8, J16-J16.9, J20-J21.9, J36-J36.0, P23.0-P23.4, P35.8, R19.7, U04-U04.9	001-001.9, 002.0-009.9, 032-033.9, 036-036.3, 036.5-037.9, 047-049.9, 052-053.9, 055-055.9, 062-064.9, 079.6, 139.0, 320.0-320.8, 321-323, 323.4-323.9, 381-383.9, 461-461.9, 464.0, 464.2, 464.4, 464.8-464.9, 466-469, 470.0, 475-475.9, 476.9, 480-482.8, 483.0-483.9, 484.0-484.3, 484.6-484.7, 487-489, 771.3
Diarrheal diseases	A00-A00.9, A02-A04.1, A04.3, A04.5-A07, A07.2-A07.4, A08-A09.9, R19.7	001-001.9, 003-006.9, 007.4-007.8, 008.2-009.9
Intestinal infectious diseases	A01.0-A01.4, A04.2, A04.4, A07.0-A07.1, A07.8-A07.9	002.0-002.9, 007-007.3, 007.9-008.1
Typhoid fever	A01.0	002.0
Paratyphoid fever	A01.1-A01.4	002.1-002.9
Other intestinal infectious diseases	A04.2, A04.4, A07.0-A07.1, A07.8-A07.9	007-007.3, 007.9-008.1
Lower respiratory infections	A48.1, A70, B97.4-B97.6, J09-J15.8, J16-J16.9, J20-J21.9, P23.0-P23.4, U04-U04.9	079.6, 466-469, 470.0, 480-482.8, 483.0-483.9, 484.1-484.2, 484.6-484.7, 487-489
Upper respiratory infections	J01-J01.9, J04.0, J05-J05.0, J36-J36.0	461-461.9, 464.0, 464.2, 464.4, 464.8-464.9, 475-475.9, 476.9
Otitis media	H70-H70.9	381-383.9
Meningitis	A39-A39.9, A87-A87.9, G00.0-G00.8, G03-G03.8	036-036.3, 036.5-036.9, 047-049.9, 320.0-320.8, 321-322.9

eTable 1: List of International Classification of Diseases (ICD) codes mapped to the Global Burden of Disease cause list for causes of death		
**Cause**	**ICD10**	**ICD9**
Pneumococcal meningitis	G00.1	320.1
H influenzae type B meningitis	G00.0	320.0
Meningococcal meningitis	A39-A39.9	036-036.3, 036.5-036.9
Other meningitis	A87-A87.9, G00.2-G00.8, G03-G03.8	047-049.9, 320.2-320.8, 321-322.9
Encephalitis	A83-A86.4, B94.1, F07.1, G04-G05.8	062-064.9, 139.0, 323, 323.4-323.9
Diphtheria	A36-A36.9	032-032.9
Whooping cough	A37-A37.9	033-033.9, 484.3
Tetanus	A33-A35.0	037-037.9, 771.3
Measles	B05-B05.9	055-055.9, 484.0
Varicella and herpes zoster	B01-B02.9, P35.8	052-053.9
Neglected tropical diseases and malaria	A30-A30.9, A68-A68.9, A69.2-A69.9, A75-A75.9, A77-A79.9, A82-A82.9, A90-A96.9, A98-A98.8, B33.0-B33.1, B50-B53.8, B55.0, B56-B57.5, B60-B60.8, B65-B67.9, B69-B72.0, B74.3-B75, B77-B77.9, B83-B83.8, B92, K93.1, P37.1, U06-U06.9	030-030.9, 060-061.8, 065-066.9, 071-071.9, 080-083.9, 084.0-084.5, 084.7-084.9, 085.0, 086-088, 088.8-088.9, 120-124.9, 125.4-125.9, 127-127.1, 128-129.0, 425.6
Malaria	B50-B53.8	084.0-084.5, 084.7-084.9
Leprosy	A30-A30.9	030-030.9
Chagas disease	B57-B57.5, K93.1	086-086.2, 086.9, 425.6
Leishmaniasis	B55.0	085.0
Visceral leishmaniasis	B55.0	085.0
African trypanosomiasis	B56-B56.9	086.3-086.5
Schistosomiasis	B65-B65.9	120-120.9
Cysticercosis	B69-B69.9	123.1
Cystic echinococcosis	B67-B67.4, B67.8-B67.9	122-122.4, 122.8-122.9
Dengue	A90-A91.9	061-061.8
Yellow fever	A95-A95.9	060-060.9
Rabies	A82-A82.9	071-071.9
Intestinal nematode infections	B77-B77.9	127.0
Ascariasis	B77-B77.9	127.0
Ebola	A98.4	
Zika virus	U06-U06.9	
Other neglected tropical diseases	A68-A68.9, A69.2-A69.9, A75-A75.9, A77-A79.9, A92-A94.0, A96-A96.9, A98-A98.3, A98.5-A98.8, B33.0-B33.1, B60-B60.8, B67.5-B67.7, B70-B71.9, B74.3-B75, B83-B83.8, P37.1	065-066.9, 080-083.9, 087-088, 088.8-088.9, 122.5-122.7, 123-123.0, 123.2-124.9, 125.4-125.6, 125.9, 127, 127.1, 128-129.0
Maternal disorders	N96, N98-N98.9, O00-O07.9, O09-O16.9, O20-O26.9, O28-O36.9, O40-O48.1, O60-O77.9, O80-O92.7, O96-O98.6, O98.8-O99.9	630-636.9, 638-638.9, 640-679.1
Maternal hemorrhage	O20-O20.9, O43.2, O44-O46.9, O62-O62.9, O67-O67.9, O70, O72-O72.3	640-641.9, 661-661.9, 665, 666-666.9
Maternal sepsis and other maternal infections	O23-O23.9, O85-O86.8, O91-O91.2	659.3, 670-670.9
Maternal hypertensive disorders	O10-O16.9	642-642.9
Maternal obstructed labor and uterine rupture	O32-O33.9, O64-O66.9, O71-O71.9	652-653.9, 660-660.9, 665.0-665.3
Maternal abortion, miscarriage, and ectopic pregnancy	N96, O00-O07.9	630-636.9, 638-638.9, 646.3
Indirect maternal deaths	O24-O25.3, O98-O98.6, O98.8-O99.9	646-646.2, 646.4-649.9
Late maternal deaths	O96-O97.9	
Maternal deaths aggravated by HIV/AIDS		
Other maternal disorders	N98-N98.9, O09-O09.9, O21-O22.9, O26-O26.9, O28-O31.8, O34-O36.9, O40-O43.1, O43.8-O43.9, O47-O48.1, O60-O61.9, O63-O63.9, O68-O69.9, O70.0-O70.9, O73-O77.9, O80-O84.9, O87-O90.9, O92-O92.7	643-645.2, 650-651.9, 654-659.2, 659.4-659.9, 662-664.9, 665.4-665.9, 667-669.9, 671-679.1
Neonatal disorders	P00-P04.2, P04.5-P05.9, P07-P15.9, P19-P22.9, P24-P29.9, P36-P36.9, P38-P39.9, P50-P61.9, P70, P70.3-P72.9, P74-P78.9, P80-P81.9, P83-P84, P90-P94.9, P96, P96.3-P96.4, P96.8	760-760.6, 760.8-768, 768.2-770, 770.1-771, 771.4-775, 775.4-779.3, 779.6-779.8
Neonatal preterm birth complications	P01.0-P01.1, P07-P07.3, P22-P22.9, P25-P28.9, P61.2, P77-P77.9	761.0-761.1, 765-765.9, 769-769.9, 770.2-770.9, 776.6, 777.5-777.6
Neonatal encephalopathy due to birth asphyxia and trauma	P01.7, P02-P03.9, P10-P15.9, P20-P21.9, P24-P24.9, P90-P91.9	761.7-763.9, 767-768, 768.2-768.9, 770.1, 772.1-772.9, 779.0-779.2
Neonatal sepsis and other neonatal infections	P36-P36.9, P38-P39.9	771.4-771.9
Hemolytic disease and other neonatal jaundice	P55-P59.9	773-774.9
Other neonatal disorders	P00-P01, P01.2-P01.6, P01.8-P01.9, P04-P04.2, P04.5-P05.9, P08-P09, P19-P19.9, P29-P29.9, P50-P54.9, P60-P61.1, P61.3-P61.9, P70, P70.3-P72.9, P74-P76.9, P78-P78.9, P80-P81.9, P83-P84, P92-P94.9, P96, P96.3-P96.4, P96.8	760-760.6, 760.8-761, 761.2-761.6, 764-764.9, 766-766.9, 770, 771, 772-772.0, 775, 775.4-776.5, 776.7-777.4, 777.7-779, 779.3, 779.6-779.8
Nutritional deficiencies	D50.1-D50.8, D51-D52.0, D52.8-D53.9, D64.3, E00-E02, E40-E46.9, E51-E61.9, E63-E64.0, E64.2-E64.9, M12.1	244.2, 260-263.9, 265-269.9, 280.1-280.8, 281.0-281.9, 716.0
Protein-energy malnutrition	E40-E46.9, E64.0	260-263.9

eTable 1: List of International Classification of Diseases (ICD) codes mapped to the Global Burden of Disease cause list for causes of death		
**Cause**	**ICD10**	**ICD9**
Iodine deficiency	E00-E02	244.2
Iron-deficiency anemia	D50.1-D50.8	280.1-280.8
Other nutritional deficiencies	D51-D52.0, D52.8-D53.9, D64.3, E51-E61.9, E63-E64, E64.2-E64.3, M12.1	265-269.9, 281.0-281.9, 716.0
Other communicable, maternal, neonatal, and nutritional diseases	A20-A28.9, A32-A32.9, A38-A38.9, A48.2, A48.4-A48.5, A49.1, A50-A58, A60-A60.9, A63-A63.8, A65-A65.0, A69-A69.1, A74, A74.8-A74.9, A80-A81.9, A88-A89.9, B00-B00.9, B03-B04, B06-B06.9, B10-B10.8, B15-B17.9, B19-B19.9, B25-B27.9, B29.4, B33, B33.3-B33.8, B47-B48.8, B63, B91, B94.2, B95-B95.5, G14-G14.6, I00, I02, I02.9, I98.0-I98.1, J02.0, J03.0, K67.0-K67.2, K67.8, K71.2, K71.6, K74.7-K74.8, K75.3, K76.3, K77.0, M03.1, M49.1, M73.0-M73.1, M89.6, P35-P35.3, P35.9, P37, P37.2, P37.5-P37.9, U82-U84, U85-U89, Z16-Z16.3	020-029, 034-034.9, 040, 040.1-041.0, 045-046.9, 050-051.9, 054-054.9, 056-059.9, 070-070.9, 072-074.1, 074.3-075.9, 078.3-078.7, 079-079.5, 079.7, 090-101.6, 104-104.9, 136-136.2, 138, 139, 323.0-323.3, 390-390.9, 392, 392.9, 484.4-484.5, 771.0-771.2, V09-V09.9
Sexually transmitted diseases excluding HIV	A50-A58, A60-A60.9, A63-A63.8, B63, I98.0, K67.0-K67.2, M03.1, M73.0-M73.1	054.1, 090-099.9
Syphilis	A50-A53.9, I98.0, K67.2, M03.1, M73.1	090-097.9
Chlamydial infection	A55-A56.8, K67.0	099
Gonococcal infection	A54-A54.9, K67.1, M73.0	098-098.9
Other sexually transmitted diseases	A57-A58, A63-A63.8	099.0-099.9
Hepatitis	B15-B17.9, B19-B19.9, B94.2, K71.2, K71.6, P35.3	070-070.9
Acute hepatitis A	B15-B15.9	070.0-070.1
Hepatitis B	B16-B16.9, B17.0, B19.1, P35.3	070.2-070.3
Hepatitis C	B17.1, B19.2	070.7
Acute hepatitis E	B17.2	
Other infectious diseases	A20-A28.9, A32-A32.9, A38-A38.9, A48.2, A48.4-A48.5, A49.1, A65-A65.0, A69-A69.1, A74, A74.8-A74.9, A80-A81.9, A88-A89.9, B00-B00.9, B03-B04, B06-B06.9, B10-B10.8, B25-B27.9, B29.4, B33, B33.3-B33.8, B47-B48.8, B91, B95-B95.5, G14-G14.6, I00, I02, I02.9, I98.1, J02.0, J03.0, K67.8, K74.7-K74.8, K75.3, K76.3, K77.0, M49.1, M89.6, P35-P35.2, P35.9, P37, P37.2, P37.5-P37.9, U82-U84, U85-U89, Z16-Z16.3	020-029, 034-034.9, 040, 040.1-041.0, 045-046.9, 050-051.9, 054-054.0, 054.2-054.9, 056-059.9, 072-074.1, 074.3-075.9, 078.3-078.7, 079-079.5, 079.7, 100-101.6, 104-104.9, 136-136.2, 138, 139, 323.0-323.3, 390-390.9, 392, 392.9, 484.4-484.5, 771.0-771.2, V09-V09.9
Non-communicable diseases	A46-A46.0, A66-A67.9, B18-B18.9, B33.2, B86, C00-C13.9, C15-C25.9, C30-C34.9, C37-C38.8, C40-C41.9, C43-C45.9, C47-C54.9, C56-C57.8, C58-C58.0, C60-C63.8, C64-C67.9, C68.0-C68.8, C69-C75.8, C81-C86.6, C88-C96.9, D00.1-D00.2, D01.0-D01.3, D02.0-D02.3, D03-D06.9, D07.0-D07.2, D07.4-D07.5, D09.0, D09.2-D09.3, D09.8, D10.0-D10.7, D11-D12.9, D13.0-D13.7, D14.0-D14.3, D15-D16.9, D22-D27.9, D28.0-D28.7, D29.0-D29.8, D30.0-D30.8, D31-D36, D36.1-D36.7, D37.1-D37.5, D38.0-D38.5, D39.1-D39.2, D39.8, D40.0-D40.8, D41.0-D41.8, D42-D43.9, D44.0-D44.8, D45-D47.0, D47.2-D47.9, D48.0-D48.6, D49.2-D49.4, D49.6, D52.1, D55-D58.9, D59.0-D59.3, D59.5-D59.6, D60-D61.9, D63.1, D64.0, D64.4, D66-D67, D68.0-D69.8, D70-D75.8, D76-D78.8, D86-D86.9, D89-D89.3, E03-E07.1, E09-E14.9, E15.0, E16.0-E16.9, E20-E34.8, E36-E36.8, E65-E68, E70-E85.2, E88-E89.9, F00-F03.9, F10-F16.9, F18-F19.9, F24, F50.0-F50.5, G10-G13.8, G20-G26.0, G30-G31.9, G35-G37.9, G40-G41.9, G45-G46.8, G47.3, G61-G61.9, G70-G73.7, G90-G90.9, G93.7, G95-G95.9, G97-G97.9, H05.0-H05.1, I01-I01.9, I02.0, I05-I09.9, I11-I13.9, I20-I25.9, I27.1, I28-I28.8, I30-I31.1, I31.8-I41.9, I42.1-I42.8, I43-I43.9, I47-I48.9, I51.0-I51.4, I60-I63.9, I65-I66.9, I67.0-I67.3, I67.5-I67.7, I68.0-I68.2, I69.0-I69.3, I70.2-I70.8, I71-I73.9, I77-I89.9, I95.2-I95.3, I97-I98, I98.2, I98.9, J30-J35.9, J37-J47.9, J60-J63.8, J65-J68.9, J70-J70.9, J82, J84-J84.9, J91-J92.9, J95-J95.9, K20-K29.9, K31-K31.8, K35-K38.9, K40-K46.9, K50-K52.9, K55-K62.9, K63.5, K64-K64.9, K66.8, K67, K68-K68.9, K70-K70.9, K71.3-K71.5, K71.7, K72.1-K74.6, K74.9, K75.1-K75.2, K75.4-K76.2, K76.4-K77, K77.8, K80-K83.9, K85-K86.9, K90-K91.9, K92.8, K93.8-K95.8, L00-L05.9, L08-L08.9, L10-L14.0, L51-L51.9, L88-L89.9, L93-L93.2, L97-L98.4, M00-M03.0, M03.2-M03.6, M05-M09.8, M30-M36.8, M40-M43.1, M65-M65.0, M71.0-M71.1, M72.5-M72.6, M80-M82.8, M86.3-M86.4, M87-M87.1, M88-M89.0, M89.5, M89.7-M89.9, N00-N08.8, N10-N12.9, N14-N16.8, N18-N18.9, N20-N23.0, N25-N28.1, N29-N32.0, N32.3-N32.4, N34-N34.3, N36-N36.9, N39-N39.2, N41-N41.9, N44-N44.0, N45-N45.9, N49-N49.9, N60-N60.9, N65-N65.1, N72-N72.0, N75-N77.8, N80-N81.9, N83-N83.9, N84.0-N84.1, N87-N87.9, N99-N99.9, P04.3-P04.4, P70.0-P70.2, P96.0-P96.2, P96.5, Q00-Q07.9, Q10.4-Q18.9, Q20-Q28.9, Q30-Q36, Q37-Q45.9, Q50-Q87.8, Q89-Q89.8, Q90-Q93.9, Q95-Q99.8, R50.2, R73-R73.9, R78.0-R78.5, R95-R95.9, X45-X45.9, X65-X65.9, Y15-Y15.9	035-035.9, 036.4, 074.2, 102-103.9, 133-133.6, 135-135.9, 136.6, 140-148.9, 150-158.9, 160-164.9, 170-175.9, 180-183.8, 184.0-184.4, 184.8, 185-186.9, 187.1-187.8, 188-188.9, 189.0-189.8, 190-194.8, 200-208.9, 209.0-209.1, 209.4-209.5, 210.0-210.9, 211.0-211.8, 212.0-212.8, 213-213.9, 217-220.9, 221.0-221.8, 222.0-222.8, 223.0-223.8, 224-228.9, 229.0, 229.8, 230.1-230.8, 231.0-231.2, 232-232.9, 233.0-233.2, 233.4-233.5, 233.7, 234.0-234.8, 235.0, 235.4, 235.6-235.8, 236.0-236.2, 236.4-236.5, 236.7, 237-237.3, 237.5-237.9, 238.0-238.9, 239.2-239.4, 239.6, 240-243.9, 244.0-244.1, 244.3-244.8, 245-246.9, 250-259.9, 270-273.9, 275-276, 277-277.2, 277.4-277.9, 278.0-278.8, 282-284.9, 286-286.5, 286.7-289.7, 290-292.9, 294.1-294.9, 303-303.9, 304.0-304.8, 305-305.9, 307.1, 327.2-327.8, 330-331.2, 331.5-337.9, 340-341.9, 345-345.9, 349, 349.2-349.8, 353.6-353.9, 356-356.9, 357.0-357.7, 358-359.9, 376.0-376.1, 391-391.9, 392.0, 393-398.9, 402-404.9, 410-414.9, 416.1, 417-417.9, 420-423, 423.1-424.9, 425.0-425.3, 425.5, 425.7-425.8, 427-427.3, 427.6-427.8, 429.0, 430-435.9, 437.0-437.2, 437.4-437.8, 440.2, 440.4, 441-443.9, 446-457, 457.1-457.9, 459, 459.1-459.3, 470, 470.9-474.9, 476-476.1, 477-479, 490-504.9, 506-506.9, 508-509, 515, 516-517.8, 518.6-518.7, 518.9, 519.0-519.4, 530-536.1, 536.4, 537-537.6, 537.8, 538-543.9, 550-553.6, 555-558.9, 560-560.3, 560.8-560.9, 562-562.1, 564-564.7, 565-566.9, 569.0-569.7, 571-571.9, 572.2-577.9, 579-583.9, 585-585.9, 588-590.9, 592-593.8, 594-599.6, 599.8, 601-602.9, 604-604.9, 608.2, 610-610.9, 617-618.9, 620-620.9, 621.4-621.9, 622.3-622.7, 629-629.8, 680-689, 694-695.5, 707-707.9, 710-711.9, 714-714.3, 714.8-714.9, 730.1, 732-732.9, 733.0-733.1, 740-749.0, 749.2-758.9, 759.0-759.8, 760.7, 775.0-775.3, 779.4-779.5, 787.1, 788.0, 790.2-790.3, 798-798.0, E850-E854, E860

eTable 1: List of International Classification of Diseases (ICD) codes mapped to the Global Burden of Disease cause list for causes of death		
**Cause**	**ICD10**	**ICD9**
Neoplasms	C00-C13.9, C15-C25.9, C30-C34.9, C37-C38.8, C40-C41.9, C43-C45.9, C47-C54.9, C56-C57.8, C58-C58.0, C60-C63.8, C64-C67.9, C68.0-C68.8, C69-C75.8, C81-C86.6, C88-C96.9, D00.1-D00.2, D01.0-D01.3, D02.0-D02.3, D03-D06.9, D07.0-D07.2, D07.4-D07.5, D09.0, D09.2-D09.3, D09.8, D10.0-D10.7, D11-D12.9, D13.0-D13.7, D14.0-D14.3, D15-D16.9, D22-D24.9, D26.0, D27-D27.9, D28.0-D28.1, D28.7, D29.0-D29.8, D30.0-D30.8, D31-D36, D36.1-D36.7, D37.1-D37.5, D38.0-D38.5, D39.1-D39.2, D39.8, D40.0-D40.8, D41.0-D41.8, D42-D43.9, D44.0-D44.8, D45-D47.0, D47.2-D47.9, D48.0-D48.6, D49.2-D49.4, D49.6, K31.7, K62.0-K62.1, K63.5, N60-N60.9, N84.0-N84.1, N87-N87.9	140-148.9, 150-158.9, 160-164.9, 170-175.9, 180-183.8, 184.0-184.4, 184.8, 185-186.9, 187.1-187.8, 188-188.9, 189.0-189.8, 190-194.8, 200-208.9, 209.0-209.1, 209.4-209.5, 210.0-210.9, 211.0-211.8, 212.0-212.8, 213-213.9, 217-217.8, 219.0, 220-220.9, 221.0-221.8, 222.0-222.8, 223.0-223.8, 224-228.9, 229.0, 229.8, 230.1-230.8, 231.0-231.2, 232-232.9, 233.0-233.2, 233.4-233.5, 233.7, 234.0-234.8, 235.0, 235.4, 235.6-235.8, 236.1-236.2, 236.4-236.5, 236.7, 237-237.3, 237.5-237.9, 238.0-238.9, 239.2-239.4, 239.6, 569.0, 610-610.9
Lip and oral cavity cancer	C00-C08.9, D10.0-D10.5, D11-D11.9	140-145.9, 210.0-210.6, 235.0
Nasopharynx cancer	C11-C11.9, D10.6	147-147.9, 210.7-210.9
Other pharynx cancer	C09-C10.9, C12-C13.9, D10.7	146-146.9, 148-148.9
Esophageal cancer	C15-C15.9, D00.1, D13.0	150-150.9, 211.0, 230.1
Stomach cancer	C16-C16.9, D00.2, D13.1, D37.1	151-151.9, 211.1, 230.2
Colon and rectum cancer	C18-C21.9, D01.0-D01.3, D12-D12.9, D37.3-D37.5	153-154.9, 209.1, 209.5, 211.3-211.4, 230.3-230.6
Liver cancer	C22-C22.9, D13.4	155-155.9, 211.5
Liver cancer due to hepatitis B		
Liver cancer due to hepatitis C		
Liver cancer due to alcohol use		
Liver cancer due to other causes		
Gallbladder and biliary tract cancer	C23-C24.9, D13.5	156-156.9
Pancreatic cancer	C25-C25.9, D13.6-D13.7	157-157.9, 211.6-211.7
Larynx cancer	C32-C32.9, D02.0, D14.1, D38.0	161-161.9, 212.1, 231.0, 235.6
Tracheal, bronchus, and lung cancer	C33-C34.9, D02.1-D02.3, D14.2-D14.3, D38.1	162-162.9, 212.2-212.3, 231.1-231.2, 235.7
Malignant skin melanoma	C43-C43.9, D03-D03.9, D22-D23.9, D48.5	172-172.9
Non-melanoma skin cancer	C44-C44.9, D04-D04.9, D49.2	173-173.9, 222.4, 232-232.9, 238.2
Non-melanoma skin cancer (squamous-cell carcinoma)	C44-C44.9, D04-D04.9, D49.2	173-173.9, 222.4, 232-232.9, 238.2
Breast cancer	C50-C50.9, D05-D05.9, D24-D24.9, D48.6, D49.3, N60-N60.9	174-175.9, 217-217.8, 233.0, 238.3, 239.3, 610-610.9
Cervical cancer	C53-C53.9, D06-D06.9, D26.0	180-180.9, 219.0, 233.1
Uterine cancer	C54-C54.9, D07.0-D07.2, N87-N87.9	182-182.8, 233.2
Ovarian cancer	C56-C56.9, D27-D27.9, D39.1	183-183.0, 220-220.9, 236.2
Prostate cancer	C61-C61.9, D07.5, D29.1, D40.0	185-185.9, 222.2, 236.5
Testicular cancer	C62-C62.9, D29.2-D29.8, D40.1-D40.8	186-186.9, 222.0, 222.3, 236.4
Kidney cancer	C64-C65.9, D30.0-D30.1, D41.0-D41.1	189.0-189.1, 189.5-189.6, 223.0-223.1
Bladder cancer	C67-C67.9, D09.0, D30.3, D41.4-D41.8, D49.4	188-188.9, 223.3, 233.7, 236.7, 239.4
Brain and nervous system cancer	C70-C72.9	191-192.9
Thyroid cancer	C73-C73.9, D09.3, D09.8, D34-D34.9, D44.0	193-193.9, 226-226.9
Mesothelioma	C45-C45.9	
Hodgkin lymphoma	C81-C81.9	201-201.9
Non-Hodgkin lymphoma	C82-C86.6, C96-C96.9	200-200.9, 202-202.9
Multiple myeloma	C88-C90.9	203-203.9
Leukemia	C91-C95.9	204-208.9
Acute lymphoid leukemia	C91.0	204.0
Chronic lymphoid leukemia	C91.1	204.1
Acute myeloid leukemia	C92.0, C92.3-C92.6, C93.0, C94.0, C94.2, C94.4-C94.5	205.0, 205.3, 206.0, 207.0
Chronic myeloid leukemia	C92.1	205.1, 206.1, 207.1
Other leukemia	C91.2-C91.9, C92.2, C92.7-C92.9, C93.1-C93.9, C94.1, C94.3, C94.6-C95.9	204.2-204.9, 205.2, 205.8-205.9, 206.2-207, 207.2-208.9
Other neoplasms	C17-C17.9, C30-C31.9, C37-C38.8, C40-C41.9, C47-C4A, C51-C52.9, C57-C57.8, C58-C58.0, C60-C60.9, C63-C63.8, C66-C66.9, C68.0-C68.8, C69-C69.9, C74-C75.8, D07.4, D09.2, D13.2-D13.3, D14.0, D15-D16.9, D28.0-D28.1, D28.7, D29.0, D30.2, D30.4-D30.8, D31-D33.9, D35-D36, D36.1-D36.7, D37.2, D38.2-D38.5, D39.2, D39.8, D41.2-D41.3, D42-D43.9, D44.1-D44.8, D45-D47.0, D47.2-D47.9, D48.0-D48.4, D49.6, K31.7, K62.0-K62.1, K63.5, N84.0-N84.1	152-152.9, 158-158.9, 160-160.9, 163-164.9, 170-171.9, 181-181.9, 182.9, 183.2-183.8, 184.0-184.4, 184.8, 187.1-187.8, 189.2-189.4, 189.8, 190-190.9, 194-194.8, 209.0, 209.4, 211.2, 211.8, 212.0, 212.4-212.8, 213-213.9, 221.0-221.8, 222.1, 222.8, 223.2, 223.8, 224-225.9, 227-228.9, 229.0, 229.8, 230.7-230.8, 233.4-233.5, 234.0-234.8, 235.4, 235.8, 236.1, 237-237.3, 237.5-237.9, 238.0-238.1, 238.4-238.9, 239.2, 239.6, 569.0
Cardiovascular diseases	B33.2, G45-G46.8, I01-I01.9, I02.0, I05-I09.9, I11-I11.9, I20-I25.9, I28-I28.8, I30-I31.1, I31.8-I41.9, I42.1-I42.8, I43-I43.9, I47-I48.9, I51.0-I51.4, I60-I63.9, I65-I66.9, I67.0-I67.3, I67.5-I67.6, I68.0-I68.2, I69.0-I69.3, I70.2-I70.8, I71-I73.9, I77-I83.9, I86-I89.0, I89.9, I98, K75.1	036.4, 074.2, 391-391.9, 392.0, 393-398.9, 402-402.9, 410-414.9, 417-417.9, 420-423, 423.1-424.9, 425.0-425.3, 425.5, 425.7-425.8, 427-427.3, 427.6-427.8, 429.0, 430-435.9, 437.0-437.2, 437.5-437.8, 440.2, 440.4, 441-443.9, 447-454.9, 456, 456.3-457, 457.1, 457.8-457.9, 459, 459.1-459.3

eTable 1: List of International Classification of Diseases (ICD) codes mapped to the Global Burden of Disease cause list for causes of death		
**Cause**	**ICD10**	**ICD9**
Rheumatic heart disease	I01-I01.9, I02.0, I05-I09.9	391-391.9, 392.0, 393-398.9
Ischemic heart disease	I20-I25.9	410-414.9
Cerebrovascular disease	G45-G46.8, I60-I63.9, I65-I66.9, I67.0-I67.3, I67.5-I67.6, I68.1-I68.2, I69.0-I69.3	430-435.9, 437.0-437.2, 437.5-437.8
Ischemic stroke	G45-G46.8, I63-I63.9, I65-I66.9, I67.2-I67.3, I67.5-I67.6, I69.3	433-435.9, 437.0-437.1, 437.5-437.8
Hemorrhagic stroke	I60-I62.9, I67.0-I67.1, I68.1-I68.2, I69.0-I69.2	430-432.9, 437.2
Hypertensive heart disease	I11-I11.9	402-402.9
Cardiomyopathy and myocarditis	B33.2, I40-I41.9, I42.1-I42.8, I43-I43.9, I51.4	422-422.9, 425.0-425.3, 425.5, 425.7-425.8, 429.0
Myocarditis	B33.2, I40-I41.9, I51.4	422-422.9
Alcoholic cardiomyopathy	I42.6	425.5
Other cardiomyopathy	I42.1-I42.5, I42.7-I42.8, I43-I43.9	425.0-425.3, 425.7-425.8, 429.0
Atrial fibrillation and flutter	I48-I48.9	427.3
Aortic aneurysm	I71-I71.9	441-441.9
Peripheral artery disease	I70.2-I70.8, I73-I73.9	440.2, 440.4, 443.0-443.9
Endocarditis	I33-I33.9, I38-I39.9	421-421.9, 424.9
Other cardiovascular and circulatory diseases	I28-I28.8, I30-I31.1, I31.8-I32.8, I34-I37.9, I47-I47.9, I51.0-I51.3, I68.0, I72-I72.9, I77-I83.9, I86-I89.0, I89.9, I98, K75.1	036.4, 074.2, 417-417.9, 420-420.9, 423, 423.1-424.8, 427-427.2, 427.6-427.8, 442-443, 447-454.9, 456, 456.3-457, 457.1, 457.8-457.9, 459, 459.1-459.3
Chronic respiratory diseases	D86-D86.2, D86.9, G47.3, J30-J35.9, J37-J47.9, J60-J63.8, J65-J68.9, J70-J70.1, J70.8-J70.9, J82, J84-J84.9, J91-J92.9	135-135.9, 136.6, 327.2-327.8, 470, 470.9-474.9, 476-476.1, 477-479, 490-504.9, 506-506.9, 508-509, 515, 516-517.8, 518.6, 518.9, 519.1-519.4
Chronic obstructive pulmonary disease	J40-J44.9, J47-J47.9	490-492.9, 494-494.9, 496-499
Pneumoconiosis	J60-J63.8, J65-J65.0, J92.0	500-504.9
Silicosis	J62-J62.9	502-502.9, 503.0, 503.9
Asbestosis	J61-J61.0, J92.0	501
Coal workers pneumoconiosis	J60-J60.0	500-500.9, 501.0-501.9
Other pneumoconiosis	J63-J63.8, J65-J65.0	503, 503.1, 504-504.9
Asthma	J45-J46.9	493-493.9
Interstitial lung disease and pulmonary sarcoidosis	D86-D86.2, D86.9, J84-J84.9	135-135.9, 136.6, 515, 516-516.9
Other chronic respiratory diseases	G47.3, J30-J35.9, J37-J39.9, J66-J68.9, J70-J70.1, J70.8-J70.9, J82, J91-J92, J92.9	327.2-327.8, 470, 470.9-474.9, 476-476.1, 477-479, 495-495.9, 506-506.9, 508-509, 517-517.8, 518.6, 518.9, 519.1-519.4
Cirrhosis and other chronic liver diseases	B18-B18.9, I85-I85.9, I98.2, K70-K70.9, K71.3-K71.5, K71.7, K72.1-K74.6, K74.9, K75.2, K75.4-K76.2, K76.4-K76.9, K77.8	456.0-456.2, 571-571.9, 572.2-573.9
Cirrhosis and other chronic liver diseases due to hepatitis B		
Cirrhosis and other chronic liver diseases due to hepatitis C		
Cirrhosis and other chronic liver diseases due to alcohol use		
Cirrhosis and other chronic liver diseases due to other causes		
Digestive diseases	I84-I84.9, K20-K29.9, K31-K31.6, K31.8, K35-K38.9, K40-K42.9, K44-K46.9, K50-K52.9, K55-K62, K62.2-K62.6, K62.8-K62.9, K64-K64.9, K66.8, K67, K68-K68.9, K77, K80-K83.9, K85-K86.9, K90-K90.9, K92.8, K93.8, M09.1	455-455.9, 530-536.1, 537-537.6, 537.8, 538, 540-543.9, 550-551.1, 551.3-552.1, 552.3-553.6, 555-558.9, 560-560.3, 560.8-560.9, 562-562.1, 564-564.1, 564.5-564.7, 565-566.9, 569.1-569.5, 569.7, 574-577.9, 579-579.2, 579.4-579.9, 787.1
Peptic ulcer disease	K25-K28.9, K31, K31.1-K31.6, K31.8	531-534.9
Gastritis and duodenitis	K29-K29.9	535-535.9
Appendicitis	K35-K37.9, K38.3-K38.9	540-542.9
Paralytic ileus and intestinal obstruction	K56-K56.9	560-560.3, 560.8-560.9
Inguinal, femoral, and abdominal hernia	K40-K42.9, K44-K46.9	550-551.1, 551.3-552.1, 552.3-553.0, 553.6
Inflammatory bowel disease	K50-K52.9, M09.1	555-556.9, 558-558.9, 569.5
Vascular intestinal disorders	K55-K55.9	557-557.9
Gallbladder and biliary diseases	K80-K83.9	574-576.9
Pancreatitis	K85-K86.9	577-577.9, 579.4
Other digestive diseases	I84-I84.9, K20-K24, K31.0, K38-K38.2, K57-K62, K62.2-K62.6, K62.8-K62.9, K64-K64.9, K66.8, K67, K68-K68.9, K77, K90-K90.9, K92.8, K93.8	455-455.9, 530-530.9, 536-536.1, 537-537.6, 537.8, 538, 543-543.9, 553.1-553.3, 562-562.1, 564-564.1, 564.5-564.7, 565-566.9, 569.1-569.4, 569.7, 579-579.2, 579.8-579.9, 787.1
Neurological disorders	F00-F03.9, G10-G13.8, G20-G21.0, G21.2-G24, G24.1-G25.0, G25.2-G25.3, G25.5, G25.8-G26.0, G30-G31.1, G31.8-G31.9, G35-G37.9, G40-G41.9, G61-G61.9, G70-G72, G72.2-G73.7, G90-G90.9, G95-G95.9, M33-M33.9	290-290.9, 294.1-294.9, 330-331.2, 331.5-337.9, 340-341.9, 345-345.9, 349, 349.2-349.8, 353.6-353.9, 356-356.9, 357.0-357.1, 357.3-357.4, 357.7, 358-359.9, 775.2
Alzheimer disease and other dementias	F00-F03.9, G30-G31.1, G31.8-G31.9	290-290.9, 294.1-294.9, 331-331.2

eTable 1: List of International Classification of Diseases (ICD) codes mapped to the Global Burden of Disease cause list for causes of death		
**Cause**	**ICD10**	**ICD9**
Parkinson disease	G20-G21.0, G21.2-G22.0	332-332.9
Epilepsy	G40-G41.9	345-345.9
Multiple sclerosis	G35-G35.9	340-340.9
Motor neuron disease	G12.2-G12.9	335-335.2, 335.8-335.9
Other neurological disorders	G10-G12.1, G13-G13.8, G23-G24, G24.1-G25.0, G25.2-G25.3, G25.5, G25.8-G26.0, G36-G37.9, G61-G61.9, G70-G72, G72.2-G73.7, G90-G90.9, G95-G95.9, M33-M33.9	330-330.9, 331.5-331.9, 333-334.9, 335.3, 336-337.9, 341-341.9, 349, 349.2-349.8, 353.6-353.9, 356-356.9, 357.0-357.1, 357.3-357.4, 357.7, 358-359.9, 775.2
Mental and substance use disorders	F10-F16.9, F18-F19.9, F24, F50.0-F50.5, G31.2, G72.1, P04.3-P04.4, P96.1, Q86.0, R78.0-R78.5, X45-X45.9, X65-X65.9, Y15-Y15.9	291-292.9, 303-303.9, 304.0-304.8, 305-305.9, 307.1, 357.5, 760.7, 790.3, E850-E854, E860
Alcohol use disorders	F10-F10.9, G31.2, G72.1, P04.3, Q86.0, R78.0, X45-X45.9, X65-X65.9, Y15-Y15.9	291-291.9, 303-303.9, 305.0, 357.5, 790.3, E860
Drug use disorders	F11-F16.9, F18-F19.9, P04.4, P96.1, R78.1-R78.5	292-292.9, 304.0-304.8, 305, 305.1-305.9, 760.7, E850-E854
Opioid use disorders	F11-F11.9, P96.1, R78.1	304.0, 305.5
Cocaine use disorders	F14-F14.9, R78.2	304.2, 305.6
Amphetamine use disorders	F15-F15.9	304.4, 305.7
Other drug use disorders	F13-F13.9, F16-F16.9, F18-F19.9, R78.3-R78.5	292-292.9, 304.1, 304.5-304.8, 305, 305.1, 305.3-305.4, 305.8-305.9
Eating disorders	F50.0-F50.5	307.1
Anorexia nervosa	F50.0-F50.1	307.1
Bulimia nervosa	F50.2-F50.5	
Diabetes, urogenital, blood, and endocrine diseases	D25-D26, D26.1-D26.9, D28.2, D52.1, D55-D58.9, D59.0-D59.3, D59.5-D59.6, D60-D61.9, D63.1, D64.0, D64.4, D66-D67, D68.0-D69.8, D70-D75.8, D76-D78.8, D86.8, D89-D89.3, E03-E07.1, E09-E14.9, E15.0, E16.0-E16.9, E20-E34.8, E36-E36.8, E65-E68, E70-E85.2, E88-E89.9, G21.1, G24.0, G25.1, G25.4, G25.6-G25.7, G72.0, G93.7, G97-G97.9, I12-I13.9, I95.2-I95.3, I97-I97.9, I98.9, J70.2-J70.5, J95-J95.9, K43-K43.9, K62.7, K91-K91.9, K94-K95.8, M87.1, N00-N08.8, N10-N12.9, N14-N16.8, N18-N18.9, N20-N23.0, N25-N28.1, N29-N32.0, N32.3-N32.4, N34-N34.3, N36-N36.9, N39-N39.2, N41-N41.9, N44-N44.0, N45-N45.9, N49-N49.9, N65-N65.1, N72-N72.0, N75-N77.8, N80-N81.9, N83-N83.9, N99-N99.9, P70.0-P70.2, P96.2, P96.5, Q61-Q62.8, R50.2, R73-R73.9	218-219, 219.1-219.9, 236.0, 240-243.9, 244.0-244.1, 244.3-244.8, 245-246.9, 250-259.9, 270-273.9, 275-276, 277-277.2, 277.4-277.9, 278.0-278.8, 282-284.9, 286-286.5, 286.7-289.7, 357.2, 357.6, 403-404.9, 518.7, 519.0, 536.4, 539-539.9, 551.2, 552.2, 564.2-564.4, 569.6, 579.3, 580-583.9, 585-585.9, 588-590.9, 592-593.8, 594-599.6, 599.8, 601-602.9, 604-604.9, 608.2, 617-618.9, 620-620.9, 621.4-621.9, 622.3-622.7, 629-629.8, 753-753.3, 775.0-775.1, 775.3, 779.4-779.5, 788.0, 790.2
Diabetes mellitus	E10-E10.1, E10.3-E11.1, E11.3-E12.1, E12.3-E13.1, E13.3-E14.1, E14.3-E14.9, P70.0-P70.2, R73-R73.9	250-250.3, 250.5-250.9, 357.2, 775.0-775.1, 790.2
Acute glomerulonephritis	N00-N01.9	580-580.9
Chronic kidney disease	D63.1, E10.2, E11.2, E12.2, E13.2, E14.2, I12-I13.9, N02-N08.8, N15.0, N18-N18.9, Q61-Q62.8	250.4, 403-404.9, 581-583.9, 585-585.9, 589-589.9, 753-753.3
Chronic kidney disease due to diabetes mellitus	E10.2, E11.2, E12.2, E13.2, E14.2	250.4
Chronic kidney disease due to hypertension	I12-I13.9	403-404.9
Chronic kidney disease due to glomerulonephritis	N03-N06.9	581-583.9
Chronic kidney disease due to other causes	N02-N02.9, N07-N08.8, N15.0, Q61-Q62.8	589-589.9, 753-753.3
Urinary diseases and male infertility	N10-N12.9, N15, N15.1-N16.8, N20-N23.0, N25-N28.1, N29-N32.0, N32.3-N32.4, N34-N34.3, N36-N36.9, N39-N39.2, N41-N41.9, N44-N44.0, N45-N45.9, N49-N49.9	588-588.9, 590-590.9, 592-593.8, 594-598.1, 598.8-599.6, 599.8, 601-602.9, 604-604.9, 608.2, 788.0
Interstitial nephritis and urinary tract infections	N10-N12.9, N15, N15.1-N16.8, N30-N30.9, N34-N34.3, N39.0-N39.2	590-590.9, 595-595.9, 597-597.9, 599.0
Urolithiasis	N20-N23.0	592-592.9, 594-594.9, 788.0
Other urinary diseases	N25-N28.1, N29-N29.8, N31-N32.0, N32.3-N32.4, N36-N36.9, N39, N41-N41.9, N44-N44.0, N45-N45.9, N49-N49.9	588-588.9, 593-593.8, 596-596.9, 598-598.1, 598.8-599, 599.1-599.6, 599.8, 601-602.9, 604-604.9, 608.2
Gynecological diseases	D25-D26, D26.1-D26.9, D28.2, E28.2, N72-N72.0, N75-N77.8, N80-N81.9, N83-N83.9	218-219, 219.1-219.9, 236.0, 256.4, 617-618.9, 620-620.9, 621.4-621.9, 622.3-622.7, 629-629.8
Uterine fibroids	D25-D26, D26.1-D26.9, D28.2	218-219, 219.1-219.9, 236.0
Polycystic ovarian syndrome	E28.2	256.4
Endometriosis	N80-N80.9	617-617.9
Genital prolapse	N81-N81.9	618-618.9
Other gynecological diseases	N72-N72.0, N75-N77.8, N83-N83.9	620-620.9, 621.4-621.9, 622.3-622.7, 629-629.8
Hemoglobinopathies and hemolytic anemias	D55-D58.9, D59.1, D59.3, D59.5, D60-D61.9, D64.0, D64.4	282-284.9
Thalassemias	D56-D56.9	282.4
Sickle cell disorders	D57-D57.2, D57.4-D57.8	282.6
G6PD deficiency	D55-D55.2	282.2-282.3
Other hemoglobinopathies and hemolytic anemias	D55.3-D55.9, D58-D58.9, D59.1, D59.3, D59.5, D60-D61.9, D64.0, D64.4	282-282.1, 282.7-284.9

eTable 1: List of International Classification of Diseases (ICD) codes mapped to the Global Burden of Disease cause list for causes of death		
**Cause**	**ICD10**	**ICD9**
Endocrine, metabolic, blood, and immune disorders	D52.1, D59.0, D59.2, D59.6, D66-D67, D68.0-D69.8, D70-D75.8, D76-D78.8, D86.8, D89-D89.3, E03-E07.1, E09-E09.9, E15.0, E16.0-E16.9, E20-E28.1, E28.3-E34.8, E36-E36.8, E65-E68, E70-E85.2, E88-E89.9, G21.1, G24.0, G25.1, G25.4, G25.6-G25.7, G72.0, G93.7, G97-G97.9, I95.2-I95.3, I97-I97.9, I98.9, J70.2-J70.5, J95-J95.9, K43-K43.9, K62.7, K91-K91.9, K94-K95.8, M87.1, N14-N14.4, N65-N65.1, N99-N99.9, P96.2, P96.5, R50.2	240-243.9, 244.0-244.1, 244.3-244.8, 245-246.9, 251-256.3, 256.8-259.9, 270-273.9, 275-276, 277-277.2, 277.4-277.9, 278.0-278.8, 286-286.5, 286.7-289.7, 357.6, 518.7, 519.0, 536.4, 539-539.9, 551.2, 552.2, 564.2-564.4, 569.6, 579.3, 598.2, 775.3, 779.4-779.5
Musculoskeletal disorders	I27.1, I67.7, L93-L93.2, M00-M03.0, M03.2-M03.6, M05-M09.0, M09.2-M09.8, M30-M32.9, M34-M36.8, M40-M43.1, M65-M65.0, M71.0-M71.1, M80-M82.8, M86.3-M86.4, M87-M87.0, M88-M89.0, M89.5, M89.7-M89.9	416.1, 437.4, 446-446.9, 695.4-695.5, 710-711.9, 714-714.3, 714.8-714.9, 730.1, 732-732.9, 733.0-733.1
Rheumatoid arthritis	M05-M06.9, M08.0-M08.8	714-714.3, 714.8-714.9
Other musculoskeletal disorders	I27.1, I67.7, L93-L93.2, M00-M03.0, M03.2-M03.6, M07-M08, M08.9-M09.0, M09.2-M09.8, M30-M32.9, M34-M36.8, M40-M43.1, M65-M65.0, M71.0-M71.1, M80-M82.8, M86.3-M86.4, M87-M87.0, M88-M89.0, M89.5, M89.7-M89.9	416.1, 437.4, 446-446.9, 695.4-695.5, 710-711.9, 730.1, 732-732.9, 733.0-733.1
Other non-communicable diseases	A46-A46.0, A66-A67.9, B86, D86.3, H05.0-H05.1, I89.1-I89.8, L00-L05.9, L08-L08.9, L10-L14.0, L51-L51.9, L88-L89.9, L97-L98.4, M72.5-M72.6, P96.0, Q00-Q07.9, Q10.4-Q18.9, Q20-Q28.9, Q30-Q36, Q37-Q45.9, Q50-Q60.6, Q63-Q86, Q86.1-Q87.8, Q89-Q89.8, Q90-Q93.9, Q95-Q99.8, R95-R95.9	035-035.9, 102-103.9, 133-133.6, 376.0-376.1, 457.2-457.3, 680-689, 694-695.3, 707-707.9, 740-749.0, 749.2-752.9, 753.4-758.9, 759.0-759.8, 798-798.0
Congenital birth defects	P96.0, Q00-Q07.9, Q10.4-Q18.9, Q20-Q28.9, Q30-Q36, Q37-Q45.9, Q50-Q60.6, Q63-Q86, Q86.1-Q87.8, Q89-Q89.8, Q90-Q93.9, Q95-Q99.8	740-749.0, 749.2-752.9, 753.4-758.9, 759.0-759.8
Neural tube defects	Q00-Q01.9, Q05-Q05.9	740-741.9, 742.0
Congenital heart anomalies	Q20-Q28.9	745-747.9
Orofacial clefts	Q35-Q36, Q37-Q37.9	749-749.0, 749.2-749.9
Down syndrome	Q90-Q90.9	758.0
Other chromosomal abnormalities	Q87-Q87.8, Q91-Q93.9, Q95-Q95.9, Q97-Q97.9, Q99-Q99.8	758, 758.1-758.6, 758.8-758.9
Congenital musculoskeletal and limb anomalies	Q65-Q79, Q79.6-Q79.9	742.5, 754-756.5, 756.8-756.9
Urogenital congenital anomalies	P96.0, Q50-Q56.4, Q60-Q60.6, Q63-Q64.9	752-752.9, 753.4-753.9
Digestive congenital anomalies	Q38-Q45.9, Q79.0-Q79.5	750-751.9, 756.6-756.7
Other congenital birth defects	Q02-Q04.9, Q06-Q07.9, Q10.4-Q18.9, Q30-Q34.9, Q57, Q80-Q86, Q86.1-Q86.8, Q89-Q89.8	742, 742.1-742.4, 742.8-744.9, 748-748.9, 757-757.9, 759.0-759.8
Skin and subcutaneous diseases	A46-A46.0, A66-A67.9, B86, D86.3, I89.1-I89.8, L00-L05.9, L08-L08.9, L10-L14.0, L51-L51.9, L88-L89.9, L97-L98.4, M72.5-M72.6	035-035.9, 102-103.9, 133-133.6, 457.2-457.3, 680-689, 694-695.3, 707-707.9
Cellulitis	L03-L03.9, M72.5-M72.6	681-682.9
Pyoderma	A46-A46.0, A66-A67.9, I89.1-I89.8, L00-L02.9, L04-L05.9, L08-L08.9, L88, L97-L98.4	035-035.9, 102-103.9, 457.2-457.3, 680-680.9, 683-689
Decubitus ulcer	L89-L89.9	707-707.9
Other skin and subcutaneous diseases	D86.3, L10-L14.0, L51-L51.9	694-695.3
Sudden infant death syndrome	R95-R95.9	798-798.0
Injuries	L55-L55.9, L56.3, L56.8-L56.9, L58-L58.9, U00-U03, V00-V86.9, V87.2-V87.3, V88.2-V88.3, V90-V98.8, W00-W46.2, W49-W62.9, W64-W70.9, W73-W75.9, W77-W81.9, W83-W94.9, W97.9, W99-X06.9, X08-X39.9, X46-X47, X47.1-X47.8, X48-X48.9, X50-X54.9, X57-X58.9, X60-X64.9, X66-Y08.9, Y35-Y84.9, Y87.0-Y87.1, Y88-Y88.3, Y89.0-Y89.1	349.0-349.1, 457.0, E800-E807, E830-E838, E840-E849, E856-E857, E861-E869, E870-E876, E878-E879, E880-E886, E888-E928, E930-E979, E990-E999
Transport injuries	V00-V86.9, V87.2-V87.3, V88.2-V88.3, V90-V98.8	E800-E807, E830-E838, E840-E849
Road injuries	V01-V04.9, V06-V80.9, V82-V82.9, V87.2-V87.3	
Pedestrian road injuries	V01-V04.9, V06-V09.9	
Cyclist road injuries	V10-V19.9	
Motorcyclist road injuries	V20-V29.9	
Motor vehicle road injuries	V30-V79.9, V87.2-V87.3	
Other road injuries	V80-V80.9, V82-V82.9	
Other transport injuries	V00-V00.8, V05-V05.9, V81-V81.9, V83-V86.9, V88.2-V88.3, V90-V98.8	E800-E807, E830-E838, E840-E849
Unintentional injuries	L55-L55.9, L56.3, L56.8-L56.9, L58-L58.9, W00-W46.2, W49-W62.9, W64-W70.9, W73-W75.9, W77-W81.9, W83-W94.9, W97.9, W99-X06.9, X08-X32.9, X39-X39.9, X46-X47, X47.1-X47.8, X48-X48.9, X50-X54.9, X57-X58.9, Y40-Y84.9, Y88-Y88.3	349.0-349.1, 457.0, E856-E857, E861-E869, E870-E876, E878-E879, E880-E886, E888-E906, E910-E928, E930-E949
Falls	W00-W19.9	E880-E886, E888
Drowning	W65-W70.9, W73-W74.9	E910
Fire, heat, and hot substances	X00-X06.9, X08-X19.9	E890-E899, E924
Poisonings	X46-X47, X47.1-X47.8, X48-X48.9	E856-E857, E861-E869
Exposure to mechanical forces	W20-W38.9, W40-W43.9, W45.0-W45.2, W46-W46.2, W49-W52, W75-W75.9	E913, E916-E922
Unintentional firearm injuries	W32-W34.9	E922
Unintentional suffocation	W75-W75.9	E913
Other exposure to mechanical forces	W20-W31.9, W35-W38.9, W40-W43.9, W45.0-W45.2, W46-W46.2, W49-W52	E916-E921

eTable 1: List of International Classification of Diseases (ICD) codes mapped to the Global Burden of Disease cause list for causes of death		
**Cause**	**ICD10**	**ICD9**
Adverse effects of medical treatment	Y40-Y84.9, Y88-Y88.3	349.0-349.1, 457.0, E870-E876, E878-E879, E930-E949
Animal contact	W52.0-W62.9, W64-W64.9, X20-X29.9	E905-E906
Venomous animal contact	X20-X29.9	E905
Non-venomous animal contact	W52.0-W62.9, W64-W64.9	E906
Foreign body	W44-W45, W45.3-W45.9, W78-W80.9, W83-W84.9	E911-E912, E914-E915
Pulmonary aspiration and foreign body in airway	W78-W80.9, W83-W84.9	E911-E912
Foreign body in other body part	W44-W45, W45.3-W45.9	E914-E915
Environmental heat and cold exposure	L55-L55.9, L56.3, L56.8-L56.9, L58-L58.9, W88-W94.9, W97.9, W99-W99.9, X30-X32.9, X39-X39.9	E900-E902, E926
Other unintentional injuries	W39-W39.9, W77-W77.9, W81-W81.9, W85-W87.9, X50-X54.9, X57-X58.9	E903-E904, E923, E925, E927-E928
Self-harm and interpersonal violence	X60-X64.9, X66-Y08.9, Y87.0-Y87.1	E950-E969
Self-harm	X60-X64.9, X66-X84.9, Y87.0	E950-E959
Self-harm by firearm	X72-X74.9	E955
Self-harm by other specified means	X60-X64.9, X66-X71.9, X75-X84.9, Y87.0	E950-E954, E956-E959
Interpersonal violence	X85-Y08.9, Y87.1	E960-E969
Physical violence by firearm	X93-X95.9	E965
Physical violence by sharp object	X99-X99.9	E966
Physical violence by other means	X85-X92.9, X96-X98.9, Y00-Y04.9, Y06-Y08.9, Y87.1	E961-E964, E967-E969
Forces of nature, conflict and terrorism, and executions and police conflict	U00-U03, X33-X38.9, Y35-Y38.9, Y89.0-Y89.1	E907-E909, E970-E979, E990-E999
Exposure to forces of nature	X33-X38.9	E907-E909
Conflict and terrorism	U00-U03, Y36-Y38.9, Y89.1	E979, E990-E999
Executions and police conflict	Y35-Y35.9, Y89.0	E970-E978
Still Born	P95-P95.9	768.0-768.1
Garbage Code (GBD Level 1)	A40-A41.9, A48.0, A48.3, A49.0, A59-A59.9, A71-A71.9, A74.0, B07-B07.9, B30-B30.9, B35-B36.9, B85-B85.4, B87-B88.9, B94.0, D50-D50.0, D50.9, D62-D63.0, D63.8-D64, D64.1-D64.2, D64.8-D65.9, D68, D69.9, E15, E16, E50-E50.9, E64.1, E85.3-E87.6, E87.8-E87.9, F06.2-F06.4, F07.2, F09-F09.9, F17-F17.9, F20-F23.9, F25-F49, F51-F99.0, G06-G08.0, G32-G32.8, G43-G44.2, G44.4-G44.8, G47-G47.2, G47.4-G47.9, G50-G60.9, G62-G65.2, G80-G83.9, G89-G89.4, G91-G91.2, G91.4-G92.9, G93.1-G93.2, G93.4-G93.6, G99-H05, H05.2-H69.9, H71-H99, I26-I26.9, I31.2-I31.4, I46-I46.9, I50-I50.9, I51.7, I67.4, I76, I95-I95.1, I95.8-I95.9, J69-J69.9, J80-J80.9, J85-J85.3, J86-J86.9, J93-J93.1, J93.8-J93.9, J94.2, J96-J96.9, J98.1-J98.3, K00-K19, K30, K65-K66.1, K66.9, K71-K71.1, K71.8-K72.0, K75.0, L20-L30.9, L40-L50.9, L52-L54.0, L56-L56.2, L56.4-L56.5, L57-L57.9, L59-L68.9, L70-L76.8, L80-L87.9, L90-L92.9, L94-L96, L98.5-L99.8, M04, M10-M12.0, M12.2-M29, M37-M39, M43.2-M49, M49.2-M64, M65.1-M71, M71.2-M72.4, M72.8-M73, M73.8-M79.9, M83-M86.2, M86.5-M86.9, M87.2-M87.9, M89.1-M89.4, M90-M99.9, N17-N17.9, N19-N19.9, N32.1-N32.2, N32.8-N33.8, N35-N35.9, N37-N37.8, N39.3-N39.8, N42-N43.4, N44.1-N44.8, N46-N48.9, N50-N53.9, N61-N64.9, N82-N82.9, N91-N91.5, N95, N95.1-N95.9, N97-N97.9, R02-R02.9, R03.1, R07.0, R08-R09, R09.3, R11-R12.0, R14-R15.9, R19-R19.6, R19.8-R23, R23.1-R30.9, R32-R50.1, R50.8-R57.9, R58.0-R72.9, R74-R78, R78.6-R94.8, R96-R99.9, U05, U07-U81, U90-U99, X40-X44.9, X47.0, X47.9, X49-X49.9, Y10-Y19.9, Z00-Z15.8, Z17	038-038.9, 040.0, 041.1, 076-078.2, 110-111.9, 125-125.3, 126-126.9, 127.2-127.9, 131-132.9, 133.8-134.9, 139.1, 139.9, 176-176.9, 247-248, 264-264.9, 274-274.9, 276.0-276.5, 276.7-276.9, 277.3, 280-280.0, 280.9-281, 285-285.9, 286.6, 293, 294-294.0, 295-302.9, 306-307.0, 307.2-307.4, 307.6-319.9, 324-327.1, 328-329, 338-339.1, 339.3-339.8, 342-344.9, 346-347.9, 350-353.5, 354-355.9, 360-376, 376.2-380.9, 384-389.9, 415-415.9, 423.0, 427.5, 427.9-428.9, 429.3, 437.3, 458-458.9, 459.0, 507-507.9, 510-510.9, 512-513.9, 518.0-518.2, 518.5, 520-529.9, 536.3, 536.8-536.9, 537.7, 537.9, 564.8-564.9, 567-568.9, 570-570.9, 572-572.1, 584-584.9, 586-587.9, 600-600.9, 603-603.9, 605-608.1, 608.3-609, 611-612.1, 615-616.9, 619-619.9, 621-621.3, 622-622.2, 622.8-623.6, 623.8-624.5, 624.8-628.9, 629.9, 690-693.9, 695.8-706.9, 708-709.9, 712-713.8, 715-716, 716.2-721.6, 721.8-730.0, 730.2-730.3, 730.7-731.9, 733, 733.2-734.2, 737-738, 738.2-739.9, 780-782.3, 782.6-784.6, 784.9, 785.4-786, 786.6, 786.8, 787, 787.3-788, 788.1-789, 790-790.1, 790.4-796.1, 796.3-797.9, 798.1-799, 799.2-799.9, E855, E858, E980-E982, V01-V08, V10
Garbage Code (GBD Level 2)	A14.9, A29, A45-A45.9, A47-A48, A48.8-A49, A49.3-A49.9, A61-A62, A72-A73, A76, A97, B08-B09, B11-B14, B28-B29, B31-B32.4, B34-B34.9, B61-B62, B68-B68.9, B73-B74.2, B76-B76.9, B78-B81.8, B84, B93-B94, B94.8-B94.9, B95.6-B97.3, B97.7-B99.9, D59, D59.4, D59.8-D59.9, G44.3, G91.3, G93.0, G93.3, I10-I10.9, I15-I15.9, I27-I27.0, I27.2-I27.9, I28.9, I70-I70.1, I70.9, I74-I75.8, J81-J81.1, J90-J90.0, J94-J94.1, J94.8-J94.9, K92.0-K92.2, N70-N71.9, N73-N74.0, N74.3-N74.8, R03-R03.0, R04-R06.9, R09.0-R09.2, R09.8-R10.9, R13-R13.9, R16-R18.9, R23.0, R58, S00, W47-W48, W63, W71-W72, W76-W76.9, W82, W95-W97, W98, X07, X55-X56, X59-X59.9, Y20-Y34.9, Y86-Y87, Y87.2, Y89, Y89.9-Y99.9	000-000.9, 041.2-041.9, 067-069, 078.8-078.9, 079.8-079.9, 089-089.9, 105-109.9, 119, 136.8-136.9, 139.8, 279-279.9, 304, 304.9, 307.5, 339.2, 401-401.9, 405-405.9, 416-416.0, 416.2-416.9, 440-440.1, 440.3, 440.8-440.9, 444-445.8, 511-511.9, 514-514.9, 515.0-515.9, 518, 518.3-518.4, 518.8, 536.2, 578-578.9, 599.7, 613-614.9, 714.4, 716.1, 721.7, 735-736.9, 738.0-738.1, 782.4, 784.7-784.8, 786.3, 787.0, 787.2, 789.0-789.9, 796.2, 799.0-799.1, 800-E80, E83, E839, E85, E859, E87, E877, E88, E887, E929, E983-E985, E988-E989

eTable 1: List of International Classification of Diseases (ICD) codes mapped to the Global Burden of Disease cause list for causes of death		
Cause	ICD10	ICD9
Garbage Code (GBD Level 3)	A01, A31-A31.9, A42-A44.9, A49.2, A64-A64.0, A99-A99.0, B37-B46.9, B49-B49.9, B55.1-B55.2, B58-B59.9, B89, C14-C14.9, C26-C29, C35-C36, C39-C39.9, C42, C46-C46.9, C55-C55.9, C57.9, C59, C63.9, C68, C68.9, C75.9-C80.9, C87, C97-D00.0, D01, D01.4-D02, D02.4-D02.9, D07, D07.3, D07.6-D09, D09.1, D09.7, D09.9-D10, D10.9, D13, D13.9-D14, D14.4, D17-D21.9, D28, D28.9-D29, D29.9-D30, D30.9, D36.0, D36.9-D37.0, D37.6-D38, D38.6-D39.0, D39.7, D39.9-D40, D40.9-D41, D41.9, D44, D44.9, D48, D48.7-D49.1, D49.5, D49.7-D49.9, D54, D75.9, D79-D85, D87-D88, D89.8-D99, E07.8-E08.9, E17-E19, E34.9-E35.8, E37-E39, E47-E49, E62, E69, E87.7, E90-E998, F04-F06.1, F06.5-F07.0, F07.8-F08, F50, F50.8-F50.9, G09-G09.9, G15-G19, G27-G29, G33-G34, G38-G39, G42, G48-G49, G66-G69, G74-G79, G84-G88, G93, G93.8-G94.8, G96-G96.9, G98-G98.9, I00.0, I03-I04, I14, I16-I19, I29-I29.9, I44-I45.9, I49-I49.9, I51, I51.6, I51.8-I59, I90-I94, I96-I96.9, I98.4-I98.8, I99-J00.0, J02, J02.8-J03, J03.8-J04, J04.1-J04.3, J05.1-J06.9, J48-J59, J71-J79, J81.9, J83, J85.9, J87-J89, J90.9, J93.6, J97-J98.0, J98.4-J99.8, K31.9-K34, K39, K47-K49, K53-K54, K63-K63.4, K63.8-K63.9, K69, K75, K78-K79, K84, K87-K89, K92, K92.9-K93, K96-K99, L06-L07, L09, L15-L19, L31-L39, L69, L77-L79, N09, N13-N13.9, N24, N28.8-N28.9, N38, N39.9-N40.9, N54-N59, N66-N69, N78-N79, N84, N84.2-N86, N88-N90.9, N92-N94.9, N95.0, O08-O08.9, O17-O19, O27, O37-O39, O49-O59, O78-O79, O93-O95.9, P06, P16-P18, P30-P34.2, P40-P49, P62-P69, P73, P79, P82, P85-P89, P96.9-P99.9, Q08-Q10.3, Q19, Q29, Q36.0-Q36.9, Q46-Q49, Q88, Q89.9, Q94, Q99.9-R01.2, R07, R07.1-R07.9, R31-R31.9	002, 031-031.9, 039-039.9, 085, 085.1-085.9, 088.0-088.7, 112-118.9, 130-130.9, 136.3-136.5, 149-149.9, 159-159.9, 165-169, 177-179.9, 183.9-184, 184.5, 184.9, 187, 187.9, 189, 189.9, 194.9-199.9, 209, 209.2-209.3, 209.6-210, 211, 211.9-212, 212.9, 214-216.9, 221, 221.9-222, 222.9-223, 223.9, 229, 229.1, 229.9-230.0, 230.9-231, 231.8-231.9, 233, 233.3, 233.6, 233.9-234, 234.9-235, 235.1-235.3, 235.5, 235.9-236, 236.3, 236.6, 236.9, 237.4, 239-239.1, 239.5, 239.7-239.9, 249-249.9, 276.6, 278, 293.0-293.9, 331.3-331.4, 348-348.9, 349.9, 357, 357.8-357.9, 399-400.0, 406-409.4, 418-419.9, 426-426.9, 427.4, 429, 429.2, 429.4-429.9, 459.5-460.9, 462-464, 464.1, 464.3, 464.5, 465-465.9, 505-505.9, 519, 519.8-519.9, 544-549, 553.8-553.9, 559-559.0, 560.4-560.7, 561, 562.2-563, 569, 569.8-569.9, 591-591.9, 593.9, 599.9, 623.7, 624.6, 637-637.9, 639-639.9, 749.1, 759, 759.9, 779.9, 782.5, 785-785.3, 786.0-786.2, 786.4-786.5, 786.7, 786.9, E986-E987
Garbage Code (GBD Level 4)	B54-B55, B55.9, B64, B82-B82.9, B83.9, D47.1, G00, G00.9-G02.8, G03.9, I42-I42.0, I42.9, I51.5, I64-I64.9, I67, I67.8-I68, I68.8-I69, I69.4-I69.9, J07-J08, J15.9, J17-J19.6, J22-J29, J64-J64.9, P23, P23.5-P23.9, P37.3-P37.4, V87-V87.1, V87.4-V88.1, V88.4-V89.9, V99-V99.0, Y09-Y09.9, Y85-Y85.9	084, 084.6, 238, 244, 244.9, 289.8-289.9, 320, 320.9, 425, 425.4, 425.9, 429.1, 436-437, 437.9-439.6, 482.9-483, 484, 484.8-486.9, 770.0, E808-E829

eTable 2: Underlying indicators for percent well-certified for data source with maximum percent well certified in each 5-year time interval for WHO European region countries, 1980-2016								
**Location**	**Time Window**	**Stars**	**Percent Well-Certified [PWC] (%)**	**Max PWC Data Year**	**Max PWC Data Source**	**Completeness (%)**	**Percent Major Garbage (%)**	**Verbal Autopsy Adjustment (None for VR) (%)**
Albania	1980-1984	0						
Albania	1985-1989	4	65.9	1989	Vital Registration	100.0	34.1	
Albania	1990-1994	4	67.0	1993	Vital Registration	97.7	31.4	
Albania	1995-1999	4	71.3	1997	Vital Registration	95.4	25.3	
Albania	2000-2004	4	65.8	2003	Vital Registration	91.8	28.4	
Albania	2005-2009	3	56.8	2008	Vital Registration	76.8	26.1	
Albania	2010-2016	3	45.0	2010	Vital Registration	56.8	20.8	
Andorra	1980-1984	0						
Andorra	1985-1989	0						
Andorra	1990-1994	0						
Andorra	1995-1999	0						
Andorra	2000-2004	0						
Andorra	2005-2009	0						
Andorra	2010-2016	0						
Armenia	1980-1984	4	69.9	1982	Vital Registration	81.6	14.3	
Armenia	1985-1989	4	76.4	1987	Vital Registration	88.4	13.6	
Armenia	1990-1994	4	82.1	1993	Vital Registration	99.1	17.2	
Armenia	1995-1999	4	81.8	1999	Vital Registration	93.2	12.2	
Armenia	2000-2004	5	87.4	2002	Vital Registration	100.0	12.6	
Armenia	2005-2009	5	90.8	2008	Vital Registration	99.3	8.6	
Armenia	2010-2016	5	91.9	2015	Vital Registration	100.0	8.1	
Austria	1980-1984	5	89.5	1981	Vital Registration	100.0	10.5	
Austria	1985-1989	5	90.6	1986	Vital Registration	100.0	9.4	
Austria	1990-1994	5	89.3	1990	Vital Registration	99.4	10.2	
Austria	1995-1999	5	88.6	1996	Vital Registration	100.0	11.4	
Austria	2000-2004	5	91.9	2003	Vital Registration	100.0	8.1	
Austria	2005-2009	5	90.8	2005	Vital Registration	100.0	9.2	
Austria	2010-2016	5	89.2	2013	Vital Registration	100.0	10.8	
Azerbaijan	1980-1984	4	71.7	1981	Vital Registration	83.8	14.4	
Azerbaijan	1985-1989	4	74.0	1988	Vital Registration	85.8	13.8	
Azerbaijan	1990-1994	4	79.7	1993	Vital Registration	91.0	12.4	
Azerbaijan	1995-1999	4	74.3	1995	Vital Registration	87.8	15.4	
Azerbaijan	2000-2004	4	73.2	2002	Vital Registration	80.7	9.2	
Azerbaijan	2005-2009	3	42.9	2007	Vital Registration	75.1	42.9	
Azerbaijan	2010-2016	0						
Belarus	1980-1984	4	81.4	1981	Vital Registration	97.5	16.4	
Belarus	1985-1989	5	86.6	1987	Vital Registration	99.7	13.1	
Belarus	1990-1994	4	77.1	1990	Vital Registration	99.2	22.3	
Belarus	1995-1999	4	79.9	1999	Vital Registration	100.0	20.1	
Belarus	2000-2004	4	83.0	2002	Vital Registration	100.0	17.0	
Belarus	2005-2009	4	82.7	2009	Vital Registration	99.6	17.0	
Belarus	2010-2016	4	82.6	2010	Vital Registration	100.0	17.4	
Belgium	1980-1984	4	77.0	1981	Vital Registration	99.0	22.2	
Belgium	1985-1989	4	77.2	1987	Vital Registration	99.6	22.4	
Belgium	1990-1994	4	81.1	1993	Vital Registration	100.0	18.9	
Belgium	1995-1999	4	84.1	1998	Vital Registration	100.0	15.9	
Belgium	2000-2004	4	83.1	2000	Vital Registration	100.0	16.9	
Belgium	2005-2009	4	83.0	2008	Vital Registration	100.0	17.0	
Belgium	2010-2016	4	80.2	2010	Vital Registration	99.8	19.6	
Bosnia and Herzegovina	1980-1984	0						
Bosnia and Herzegovina	1985-1989	3	64.4	1989	Vital Registration	94.8	32.0	
Bosnia and Herzegovina	1990-1994	3	64.5	1991	Vital Registration	93.5	31.0	
Bosnia and Herzegovina	1995-1999	0						
Bosnia and Herzegovina	2000-2004	0						
Bosnia and Herzegovina	2005-2009	0						
Bosnia and Herzegovina	2010-2016	4	68.8	2014	Vital Registration	95.6	28.0	
Bulgaria	1980-1984	4	80.4	1980	Vital Registration	100.0	19.6	
Bulgaria	1985-1989	4	80.7	1985	Vital Registration	100.0	19.3	
Bulgaria	1990-1994	4	79.7	1993	Vital Registration	99.5	19.9	
Bulgaria	1995-1999	4	76.0	1995	Vital Registration	99.2	23.3	
Bulgaria	2000-2004	4	71.8	2002	Vital Registration	100.0	28.2	
Bulgaria	2005-2009	4	73.5	2005	Vital Registration	100.0	26.5	
Bulgaria	2010-2016	4	70.3	2012	Vital Registration	100.0	29.7	
Croatia	1980-1984	0						
Croatia	1985-1989	4	82.7	1989	Vital Registration	98.2	15.7	
Croatia	1990-1994	4	83.7	1990	Vital Registration	98.4	14.9	
Croatia	1995-1999	4	80.7	1999	Vital Registration	97.7	17.5	
Croatia	2000-2004	4	84.1	2003	Vital Registration	100.0	15.9	
Croatia	2005-2009	5	86.5	2009	Vital Registration	98.0	11.7	
Croatia	2010-2016	5	87.9	2010	Vital Registration	97.2	9.5	
Cyprus	1980-1984	0						
Cyprus	1985-1989	0						
Cyprus	1990-1994	0						
Cyprus	1995-1999	2	28.7	1999	Vital Registration	81.9	65.0	
Cyprus	2000-2004	3	58.3	2004	Vital Registration	83.8	30.5	
Cyprus	2005-2009	4	66.7	2007	Vital Registration	86.5	22.8	
Cyprus	2010-2016	4	66.5	2014	Vital Registration	81.0	17.9	

Location	Time Window	Stars	Percent Well-Certified [PWC] (%)	Max PWC Data Year	Max PWC Data Source	Completeness (%)	Percent Major Garbage (%)	Verbal Autopsy Adjustment (None for VR) (%)
Czechia	1980-1984	0						
Czechia	1985-1989	5	90.3	1986	Vital Registration	100.0	9.7	
Czechia	1990-1994	5	89.4	1990	Vital Registration	100.0	10.6	
Czechia	1995-1999	4	84.8	1995	Vital Registration	100.0	15.2	
Czechia	2000-2004	5	85.1	2000	Vital Registration	100.0	14.9	
Czechia	2005-2009	4	84.8	2007	Vital Registration	98.7	14.2	
Czechia	2010-2016	5	87.8	2013	Vital Registration	100.0	12.2	
Denmark	1980-1984	4	80.6	1980	Vital Registration	100.0	19.4	
Denmark	1985-1989	4	78.8	1985	Vital Registration	100.0	21.2	
Denmark	1990-1994	4	84.0	1994	Vital Registration	100.0	16.0	
Denmark	1995-1999	5	86.7	1999	Vital Registration	100.0	13.2	
Denmark	2000-2004	5	85.3	2001	Vital Registration	100.0	14.7	
Denmark	2005-2009	4	84.1	2006	Vital Registration	100.0	15.9	
Denmark	2010-2016	4	84.6	2012	Vital Registration	100.0	15.4	
Estonia	1980-1984	5	89.0	1981	Vital Registration	99.0	10.1	
Estonia	1985-1989	5	90.9	1986	Vital Registration	100.0	9.1	
Estonia	1990-1994	5	93.7	1994	Vital Registration	100.0	6.3	
Estonia	1995-1999	5	93.0	1996	Vital Registration	99.8	6.8	
Estonia	2000-2004	5	92.0	2003	Vital Registration	100.0	8.0	
Estonia	2005-2009	5	93.8	2009	Vital Registration	99.6	5.8	
Estonia	2010-2016	5	93.8	2010	Vital Registration	100.0	6.2	
Finland	1980-1984	4	81.1	1980	Vital Registration	100.0	18.9	
Finland	1985-1989	5	90.5	1987	Vital Registration	99.4	8.9	
Finland	1990-1994	5	91.6	1993	Vital Registration	100.0	8.4	
Finland	1995-1999	5	95.7	1999	Vital Registration	99.9	4.2	
Finland	2000-2004	5	95.7	2000	Vital Registration	100.0	4.3	
Finland	2005-2009	5	94.5	2009	Vital Registration	99.4	4.9	
Finland	2010-2016	5	95.6	2013	Vital Registration	100.0	4.4	
France	1980-1984	4	76.2	1984	Vital Registration	100.0	23.8	
France	1985-1989	4	78.0	1988	Vital Registration	100.0	22.0	
France	1990-1994	4	78.1	1993	Vital Registration	100.0	21.9	
France	1995-1999	4	78.7	1998	Vital Registration	100.0	21.3	
France	2000-2004	4	79.1	2000	Vital Registration	100.0	20.9	
France	2005-2009	4	79.4	2005	Vital Registration	100.0	20.6	
France	2010-2016	4	77.9	2011	Vital Registration	99.6	21.8	
Georgia	1980-1984	5	85.9	1981	Vital Registration	95.8	10.4	
Georgia	1985-1989	4	83.2	1985	Vital Registration	93.7	11.2	
Georgia	1990-1994	4	78.0	1990	Vital Registration	84.9	8.1	
Georgia	1995-1999	4	74.2	1999	Vital Registration	85.8	13.5	
Georgia	2000-2004	4	77.6	2000	Vital Registration	87.6	11.5	
Georgia	2005-2009	3	51.2	2005	Vital Registration	80.5	36.4	
Georgia	2010-2016	3	58.7	2014	Vital Registration	99.4	40.9	
Germany	1980-1984	4	77.5	1983	Vital Registration	100.0	22.5	
Germany	1985-1989	4	78.2	1989	Vital Registration	98.3	20.5	
Germany	1990-1994	4	83.1	1994	Vital Registration	100.0	16.9	
Germany	1995-1999	4	83.9	1998	Vital Registration	100.0	16.1	
Germany	2000-2004	4	83.2	2004	Vital Registration	99.6	16.5	
Germany	2005-2009	4	83.6	2005	Vital Registration	100.0	16.4	
Germany	2010-2016	4	84.0	2013	Vital Registration	100.0	16.0	
Greece	1980-1984	4	79.7	1984	Vital Registration	98.8	19.4	
Greece	1985-1989	4	81.1	1985	Vital Registration	100.0	18.9	
Greece	1990-1994	4	71.3	1994	Vital Registration	99.5	28.4	
Greece	1995-1999	4	71.9	1995	Vital Registration	100.0	28.1	
Greece	2000-2004	4	72.2	2001	Vital Registration	99.6	27.5	
Greece	2005-2009	4	76.5	2007	Vital Registration	100.0	23.5	
Greece	2010-2016	4	74.1	2010	Vital Registration	98.6	24.9	
Hungary	1980-1984	5	90.6	1980	Vital Registration	100.0	9.4	
Hungary	1985-1989	5	89.3	1989	Vital Registration	100.0	10.7	
Hungary	1990-1994	5	89.9	1994	Vital Registration	100.0	10.1	
Hungary	1995-1999	5	90.8	1998	Vital Registration	100.0	9.2	
Hungary	2000-2004	5	92.6	2004	Vital Registration	100.0	7.4	
Hungary	2005-2009	5	93.3	2009	Vital Registration	100.0	6.7	
Hungary	2010-2016	5	93.6	2011	Vital Registration	100.0	6.4	
Iceland	1980-1984	5	91.3	1982	Vital Registration	99.6	8.4	
Iceland	1985-1989	5	92.8	1989	Vital Registration	100.0	7.2	
Iceland	1990-1994	5	94.0	1991	Vital Registration	100.0	6.0	
Iceland	1995-1999	5	94.1	1999	Vital Registration	100.0	5.9	
Iceland	2000-2004	5	93.5	2004	Vital Registration	100.0	6.5	
Iceland	2005-2009	5	92.8	2005	Vital Registration	99.9	7.1	
Iceland	2010-2016	5	91.4	2010	Vital Registration	100.0	8.6	
Ireland	1980-1984	5	90.1	1983	Vital Registration	100.0	9.9	
Ireland	1985-1989	5	91.1	1989	Vital Registration	100.0	8.9	
Ireland	1990-1994	5	91.5	1993	Vital Registration	100.0	8.5	
Ireland	1995-1999	5	90.7	1995	Vital Registration	100.0	9.3	
Ireland	2000-2004	5	90.6	2000	Vital Registration	100.0	9.4	
Ireland	2005-2009	5	92.5	2007	Vital Registration	100.0	7.5	
Ireland	2010-2016	5	92.4	2012	Vital Registration	100.0	7.6	
Israel	1980-1984	4	80.9	1983	Vital Registration	100.0	19.1	

Location	Time Window	Stars	Percent Well-Certified [PWC] (%)	Max PWC Data Year	Max PWC Data Source	Completeness (%)	Percent Major Garbage (%)	Verbal Autopsy Adjustment (None for VR) (%)
Israel	1985-1989	4	81.7	1989	Vital Registration	100.0	18.3	
Israel	1990-1994	4	82.8	1994	Vital Registration	100.0	17.2	
Israel	1995-1999	4	83.3	1996	Vital Registration	100.0	16.7	
Israel	2000-2004	4	81.8	2002	Vital Registration	100.0	18.2	
Israel	2005-2009	4	80.2	2005	Vital Registration	100.0	19.8	
Israel	2010-2016	4	79.0	2010	Vital Registration	97.9	19.2	
Italy	1980-1984	5	88.5	1980	Vital Registration	100.0	11.5	
Italy	1985-1989	5	87.8	1988	Vital Registration	99.9	12.1	
Italy	1990-1994	5	87.7	1991	Vital Registration	100.0	12.3	
Italy	1995-1999	5	87.3	1998	Vital Registration	100.0	12.7	
Italy	2000-2004	5	88.2	2003	Vital Registration	100.0	11.8	
Italy	2005-2009	5	88.7	2008	Vital Registration	100.0	11.3	
Italy	2010-2016	5	87.7	2012	Vital Registration	100.0	12.3	
Kazakhstan	1980-1984	4	76.3	1982	Vital Registration	95.2	19.8	
Kazakhstan	1985-1989	4	81.5	1988	Vital Registration	99.2	17.8	
Kazakhstan	1990-1994	5	89.5	1993	Vital Registration	100.0	10.5	
Kazakhstan	1995-1999	5	89.0	1995	Vital Registration	100.0	11.0	
Kazakhstan	2000-2004	4	82.2	2003	Vital Registration	95.8	14.2	
Kazakhstan	2005-2009	4	77.8	2008	Vital Registration	97.3	20.0	
Kazakhstan	2010-2016	5	86.1	2015	Vital Registration	98.3	12.5	
Kyrgyzstan	1980-1984	4	71.0	1981	Vital Registration	87.5	18.9	
Kyrgyzstan	1985-1989	4	76.4	1988	Vital Registration	89.4	14.6	
Kyrgyzstan	1990-1994	4	71.0	1994	Vital Registration	93.6	24.2	
Kyrgyzstan	1995-1999	4	73.0	1998	Vital Registration	92.2	20.8	
Kyrgyzstan	2000-2004	5	85.9	2002	Vital Registration	93.4	8.1	
Kyrgyzstan	2005-2009	5	87.7	2006	Vital Registration	94.0	6.7	
Kyrgyzstan	2010-2016	5	90.9	2014	Vital Registration	97.4	6.7	
Latvia	1980-1984	5	90.6	1984	Vital Registration	99.7	9.1	
Latvia	1985-1989	5	91.4	1985	Vital Registration	100.0	8.6	
Latvia	1990-1994	5	87.9	1990	Vital Registration	100.0	12.1	
Latvia	1995-1999	5	92.0	1999	Vital Registration	100.0	8.0	
Latvia	2000-2004	5	91.1	2000	Vital Registration	100.0	8.9	
Latvia	2005-2009	5	89.2	2007	Vital Registration	100.0	10.8	
Latvia	2010-2016	5	93.8	2013	Vital Registration	100.0	6.2	
Lithuania	1980-1984	5	87.6	1981	Vital Registration	99.2	11.7	
Lithuania	1985-1989	5	92.2	1988	Vital Registration	100.0	7.8	
Lithuania	1990-1994	5	91.7	1990	Vital Registration	100.0	8.3	
Lithuania	1995-1999	5	94.7	1997	Vital Registration	100.0	5.3	
Lithuania	2000-2004	5	92.6	2004	Vital Registration	100.0	7.4	
Lithuania	2005-2009	5	93.1	2009	Vital Registration	100.0	6.9	
Lithuania	2010-2016	5	94.4	2013	Vital Registration	100.0	5.6	
Luxembourg	1980-1984	5	86.4	1984	Vital Registration	100.0	13.6	
Luxembourg	1985-1989	5	86.7	1985	Vital Registration	100.0	13.3	
Luxembourg	1990-1994	5	85.3	1992	Vital Registration	100.0	14.7	
Luxembourg	1995-1999	4	84.9	1995	Vital Registration	99.3	14.6	
Luxembourg	2000-2004	4	82.2	2000	Vital Registration	100.0	17.8	
Luxembourg	2005-2009	4	78.2	2005	Vital Registration	100.0	21.8	
Luxembourg	2010-2016	4	82.0	2013	Vital Registration	99.2	17.3	
Macedonia	1980-1984	0						
Macedonia	1985-1989	0						
Macedonia	1990-1994	4	80.1	1994	Vital Registration	97.1	17.6	
Macedonia	1995-1999	4	81.5	1997	Vital Registration	98.2	17.0	
Macedonia	2000-2004	4	81.6	2003	Vital Registration	96.3	15.3	
Macedonia	2005-2009	4	78.9	2007	Vital Registration	98.2	19.7	
Macedonia	2010-2016	4	74.6	2012	Vital Registration	94.6	21.1	
Malta	1980-1984	4	81.0	1982	Vital Registration	99.9	18.9	
Malta	1985-1989	4	84.5	1989	Vital Registration	96.6	12.6	
Malta	1990-1994	5	88.4	1992	Vital Registration	100.0	11.6	
Malta	1995-1999	5	90.0	1997	Vital Registration	100.0	10.0	
Malta	2000-2004	5	89.0	2002	Vital Registration	99.5	10.5	
Malta	2005-2009	5	93.0	2009	Vital Registration	100.0	7.0	
Malta	2010-2016	5	90.9	2014	Vital Registration	98.5	7.6	
Republic of Moldova	1980-1984	4	83.9	1981	Vital Registration	96.3	12.9	
Republic of Moldova	1985-1989	5	87.1	1985	Vital Registration	97.7	10.8	
Republic of Moldova	1990-1994	4	77.2	1991	Vital Registration	94.3	18.1	
Republic of Moldova	1995-1999	4	84.8	1996	Vital Registration	94.9	10.7	
Republic of Moldova	2000-2004	5	90.0	2003	Vital Registration	93.2	3.4	
Republic of Moldova	2005-2009	5	89.6	2005	Vital Registration	92.5	3.1	
Republic of Moldova	2010-2016	5	90.3	2014	Vital Registration	93.1	3.0	
Montenegro	1980-1984	0						
Montenegro	1985-1989	0						
Montenegro	1990-1994	0						
Montenegro	1995-1999	0						
Montenegro	2000-2004	4	70.6	2004	Vital Registration	100.0	29.4	
Montenegro	2005-2009	4	72.9	2006	Vital Registration	100.0	27.1	
Montenegro	2010-2016	0						
Netherlands	1980-1984	5	88.2	1981	Vital Registration	99.9	11.7	
Netherlands	1985-1989	5	85.8	1987	Vital Registration	98.6	13.0	

Location	Time Window	Stars	Percent Well-Certified [PWC] (%)	Max PWC Data Year	Max PWC Data Source	Completeness (%)	Percent Major Garbage (%)	Verbal Autopsy Adjustment (None for VR) (%)
Netherlands	1990-1994	4	84.9	1990	Vital Registration	100.0	15.1	
Netherlands	1995-1999	4	84.0	1996	Vital Registration	100.0	16.0	
Netherlands	2000-2004	4	82.3	2003	Vital Registration	100.0	17.7	
Netherlands	2005-2009	4	83.3	2006	Vital Registration	100.0	16.7	
Netherlands	2010-2016	4	83.3	2012	Vital Registration	100.0	16.7	
Norway	1980-1984	4	78.6	1981	Vital Registration	100.0	21.4	
Norway	1985-1989	5	89.2	1988	Vital Registration	100.0	10.8	
Norway	1990-1994	5	88.4	1990	Vital Registration	100.0	11.6	
Norway	1995-1999	5	88.3	1997	Vital Registration	100.0	11.7	
Norway	2000-2004	5	86.4	2000	Vital Registration	100.0	13.6	
Norway	2005-2009	4	84.2	2006	Vital Registration	100.0	15.8	
Norway	2010-2016	4	83.0	2011	Vital Registration	100.0	17.0	
Poland	1980-1984	3	62.5	1980	Vital Registration	100.0	37.5	
Poland	1985-1989	3	60.3	1986	Vital Registration	100.0	39.7	
Poland	1990-1994	3	60.4	1994	Vital Registration	99.8	39.5	
Poland	1995-1999	4	71.6	1999	Vital Registration	100.0	28.4	
Poland	2000-2004	4	74.2	2002	Vital Registration	100.0	25.8	
Poland	2005-2009	4	73.6	2005	Vital Registration	100.0	26.4	
Poland	2010-2016	4	71.9	2011	Vital Registration	98.8	27.2	
Portugal	1980-1984	4	76.8	1984	Vital Registration	100.0	23.2	
Portugal	1985-1989	4	77.1	1985	Vital Registration	100.0	22.9	
Portugal	1990-1994	4	76.1	1991	Vital Registration	100.0	23.9	
Portugal	1995-1999	4	74.2	1996	Vital Registration	99.7	25.6	
Portugal	2000-2004	4	78.8	2002	Vital Registration	99.5	20.8	
Portugal	2005-2009	4	77.5	2009	Vital Registration	99.5	22.2	
Portugal	2010-2016	4	79.8	2014	Vital Registration	97.5	18.1	
Romania	1980-1984	4	77.4	1980	Vital Registration	100.0	22.6	
Romania	1985-1989	4	78.5	1989	Vital Registration	100.0	21.5	
Romania	1990-1994	4	83.3	1994	Vital Registration	99.6	16.4	
Romania	1995-1999	4	84.8	1999	Vital Registration	100.0	15.2	
Romania	2000-2004	5	85.5	2004	Vital Registration	99.4	14.0	
Romania	2005-2009	5	86.2	2006	Vital Registration	100.0	13.8	
Romania	2010-2016	5	85.5	2010	Vital Registration	100.0	14.5	
Russian Federation	1980-1984	4	81.6	1981	Vital Registration	99.4	18.0	
Russian Federation	1985-1989	5	88.4	1989	Vital Registration	98.2	10.0	
Russian Federation	1990-1994	5	87.8	1990	Vital Registration	99.3	11.6	
Russian Federation	1995-1999	4	84.6	1999	Vital Registration	99.9	15.3	
Russian Federation	2000-2004	5	87.6	2003	Vital Registration	100.0	12.4	
Russian Federation	2005-2009	5	88.9	2009	Vital Registration	100.0	11.1	
Russian Federation	2010-2016	5	88.4	2010	Vital Registration	100.0	11.6	
Serbia	1980-1984	0						
Serbia	1985-1989	0						
Serbia	1990-1994	0						
Serbia	1995-1999	4	73.1	1998	Vital Registration	92.4	20.9	
Serbia	2000-2004	4	75.1	2003	Vital Registration	94.2	20.4	
Serbia	2005-2009	4	79.7	2005	Vital Registration	96.5	17.4	
Serbia	2010-2016	4	77.9	2010	Vital Registration	93.5	16.7	
Slovakia	1980-1984	0						
Slovakia	1985-1989	0						
Slovakia	1990-1994	4	82.4	1994	Vital Registration	99.3	17.1	
Slovakia	1995-1999	4	82.7	1999	Vital Registration	100.0	17.3	
Slovakia	2000-2004	5	85.2	2003	Vital Registration	100.0	14.8	
Slovakia	2005-2009	5	90.3	2008	Vital Registration	99.9	9.6	
Slovakia	2010-2016	5	92.9	2012	Vital Registration	100.0	7.1	
Slovenia	1980-1984	0						
Slovenia	1985-1989	5	89.4	1985	Vital Registration	97.7	8.5	
Slovenia	1990-1994	5	91.1	1992	Vital Registration	98.6	7.6	
Slovenia	1995-1999	5	88.8	1997	Vital Registration	96.7	8.3	
Slovenia	2000-2004	5	88.3	2002	Vital Registration	100.0	11.7	
Slovenia	2005-2009	5	87.4	2009	Vital Registration	99.0	11.8	
Slovenia	2010-2016	5	87.3	2012	Vital Registration	99.3	12.1	
Spain	1980-1984	4	76.7	1984	Vital Registration	98.8	22.4	
Spain	1985-1989	4	78.9	1989	Vital Registration	100.0	21.1	
Spain	1990-1994	4	80.1	1993	Vital Registration	99.9	19.8	
Spain	1995-1999	4	83.3	1998	Vital Registration	100.0	16.7	
Spain	2000-2004	4	83.2	2002	Vital Registration	100.0	16.8	
Spain	2005-2009	4	84.0	2009	Vital Registration	100.0	16.0	
Spain	2010-2016	5	85.4	2014	Vital Registration	99.9	14.5	
Sweden	1980-1984	5	87.6	1980	Vital Registration	99.3	11.8	
Sweden	1985-1989	5	88.4	1988	Vital Registration	100.0	11.6	
Sweden	1990-1994	5	88.0	1990	Vital Registration	100.0	12.0	
Sweden	1995-1999	5	87.0	1997	Vital Registration	100.0	13.0	
Sweden	2000-2004	5	85.9	2001	Vital Registration	99.8	13.9	
Sweden	2005-2009	5	85.4	2006	Vital Registration	99.2	14.0	
Sweden	2010-2016	4	84.8	2014	Vital Registration	99.8	15.1	
Switzerland	1980-1984	4	69.3	1981	Vital Registration	100.0	30.7	
Switzerland	1985-1989	4	69.2	1986	Vital Registration	100.0	30.8	
Switzerland	1990-1994	4	68.3	1990	Vital Registration	100.0	31.7	

Location	Time Window	Stars	Percent Well-Certified [PWC] (%)	Max PWC Data Year	Max PWC Data Source	Completeness (%)	Percent Major Garbage (%)	Verbal Autopsy Adjustment (None for VR) (%)
Switzerland	1995-1999	4	84.6	1998	Vital Registration	100.0	15.4	
Switzerland	2000-2004	4	84.4	2003	Vital Registration	100.0	15.6	
Switzerland	2005-2009	5	86.6	2008	Vital Registration	99.8	13.3	
Switzerland	2010-2016	5	86.1	2010	Vital Registration	100.0	13.9	
Tajikistan	1980-1984	4	67.1	1981	Vital Registration	80.5	16.7	
Tajikistan	1985-1989	3	61.0	1988	Vital Registration	80.2	23.9	
Tajikistan	1990-1994	4	68.8	1993	Vital Registration	100.0	31.2	
Tajikistan	1995-1999	3	53.7	1995	Vital Registration	76.6	29.9	
Tajikistan	2000-2004	3	46.4	2002	Vital Registration	67.8	31.6	
Tajikistan	2005-2009	3	47.7	2005	Vital Registration	72.1	33.9	
Tajikistan	2010-2016	0						
Turkey	1980-1984	2	16.9	1984	Vital Registration	38.0	55.6	
Turkey	1985-1989	2	20.7	1989	Vital Registration	45.2	54.2	
Turkey	1990-1994	2	22.1	1993	Vital Registration	48.1	54.1	
Turkey	1995-1999	2	24.9	1998	Vital Registration	55.1	54.9	
Turkey	2000-2004	3	37.4	2002	Turkey Verbal Autopsy Survey 2003		41.5	64
Turkey	2005-2009	4	72.8	2009	Vital Registration	90.9	19.9	
Turkey	2010-2016	4	84.4	2013	Vital Registration	99.7	15.4	
Turkmenistan	1980-1984	4	83.9	1981	Vital Registration	95.6	12.2	
Turkmenistan	1985-1989	5	86.0	1986	Vital Registration	98.5	12.6	
Turkmenistan	1990-1994	4	79.7	1993	Vital Registration	95.4	16.5	
Turkmenistan	1995-1999	4	74.1	1995	Vital Registration	92.4	19.8	
Turkmenistan	2000-2004	4	65.5	2003	Vital Registration	77.1	15.1	
Turkmenistan	2005-2009	4	66.8	2005	Vital Registration	80.3	16.9	
Turkmenistan	2010-2016	4	70.6	2014	Vital Registration	93.0	24.1	
Ukraine	1980-1984	4	84.7	1981	Vital Registration	98.2	13.7	
Ukraine	1985-1989	5	87.8	1985	Vital Registration	100.0	12.2	
Ukraine	1990-1994	4	81.0	1994	Vital Registration	100.0	19.0	
Ukraine	1995-1999	4	83.5	1999	Vital Registration	100.0	16.5	
Ukraine	2000-2004	4	83.8	2000	Vital Registration	100.0	16.2	
Ukraine	2005-2009	5	89.0	2009	Vital Registration	100.0	11.0	
Ukraine	2010-2016	5	90.4	2012	Vital Registration	100.0	9.6	
United Kingdom	1980-1984	5	93.1	1983	Vital Registration	99.5	6.4	
United Kingdom	1985-1989	5	93.9	1985	Vital Registration	100.0	6.1	
United Kingdom	1990-1994	5	93.9	1991	Vital Registration	99.9	6.1	
United Kingdom	1995-1999	5	91.9	1995	Vital Registration	100.0	8.1	
United Kingdom	2000-2004	5	91.4	2002	Vital Registration	100.0	8.6	
United Kingdom	2005-2009	5	91.4	2007	Vital Registration	100.0	8.6	
United Kingdom	2010-2016	5	91.3	2010	Vital Registration	99.9	8.6	
Uzbekistan	1980-1984	4	82.6	1982	Vital Registration	97.8	15.5	
Uzbekistan	1985-1989	5	85.2	1985	Vital Registration	99.7	14.5	
Uzbekistan	1990-1994	4	80.0	1992	Vital Registration	95.0	15.7	
Uzbekistan	1995-1999	4	72.1	1995	Vital Registration	87.3	17.5	
Uzbekistan	2000-2004	3	61.1	2000	Vital Registration	75.7	19.4	
Uzbekistan	2005-2009	3	63.0	2005	Vital Registration	70.9	11.2	
Uzbekistan	2010-2016	4	65.3	2011	Vital Registration	72.2	9.6	

eTable 3: Number of deaths and rates (per 100,000) with percent change between 1990 and 2000 and between 2000 and 2016 in the WHO Euro region, ages 5 to 9, males

	1990		2000		2016		Change from 1990 to 2000		Change from 2000 to 2016	
	Count	Rate	Count	Rate	Count	Rate	Count	Rate	Count	Rate
**All causes**	**18,268 (17,759 to 18,783)**	**55.3 (53.7 to 56.8)**	**13,248 (12,804 to 13,764)**	**45.9 (44.4 to 47.7)**	**6,332 (5,864 to 6,838)**	**22.4 (20.7 to 24.2)**	**-27.5 (-30.7 to -24.0)**	**-16.9 (-20.6 to -13.0)**	**-52.2 (-56.2 to -47.9)**	**-51.3 (-55.3 to -46.9)**
**Injuries**	**9,900 (9,507 to 10,334)**	**30.0 (28.8 to 31.3)**	**6,578 (6,281 to 6,871)**	**22.8 (21.8 to 23.8)**	**2,667 (2,426 to 2,935)**	**9.4 (8.6 to 10.4)**	**-33.5 (-37.1 to -29.7)**	**-23.9 (-28.0 to -19.6)**	**-59.4 (-63.3 to -55.0)**	**-58.7 (-62.6 to -54.1)**
Transport injuries	3,715 (3,427 to 4,059)	11.2 (10.4 to 12.3)	2,197 (2,021 to 2,413)	7.6 (7.0 to 8.4)	982 (846 to 1,143)	3.5 (3.0 to 4.0)	-40.8 (-46.3 to -33.7)	-32.2 (-38.5 to -24.1)	-55.2 (-62.3 to -46.9)	-54.4 (-61.6 to -45.9)
Road injuries	3,559 (3,279 to 3,901)	10.8 (9.9 to 11.8)	2,075 (1,906 to 2,275)	7.2 (6.6 to 7.9)	925 (792 to 1,081)	3.3 (2.8 to 3.8)	-41.6 (-47.1 to -34.6)	-33.2 (-39.4 to -25.2)	-55.4 (-62.4 to -46.9)	-54.5 (-61.7 to -45.8)
Other transport injuries	156 (118 to 202)	0.5 (0.4 to 0.6)	123 (100 to 151)	0.4 (0.3 to 0.5)	58 (44 to 81)	0.2 (0.2 to 0.3)	-20.5 (-40.0 to 4.0)	-8.9 (-31.3 to 19.0)	-52.3 (-65.6 to -29.9)	-51.4 (-65.0 to -28.6)
Unintentional injuries	5,901 (5,560 to 6,245)	17.9 (16.8 to 18.9)	4,063 (3,822 to 4,328)	14.1 (13.2 to 15.0)	1,531 (1,393 to 1,695)	5.4 (4.9 to 6.0)	-31.1 (-35.7 to -26.0)	-21.1 (-26.3 to -15.3)	-62.3 (-66.2 to -57.9)	-61.6 (-65.6 to -57.1)
Falls	615 (327 to 776)	1.9 (1.0 to 2.3)	421 (263 to 523)	1.5 (0.9 to 1.8)	149 (121 to 204)	0.5 (0.4 to 0.7)	-30.4 (-44.9 to -14.2)	-20.3 (-36.9 to -1.7)	-63.5 (-74.6 to -32.3)	-62.8 (-74.2 to -31.0)
Drowning	3,239 (2,991 to 3,514)	9.8 (9.1 to 10.6)	2,158 (1,995 to 2,354)	7.5 (6.9 to 8.2)	739 (644 to 843)	2.6 (2.3 to 3.0)	-33.3 (-39.7 to -26.4)	-23.6 (-30.9 to -15.7)	-65.7 (-70.8 to -60.0)	-65.0 (-70.2 to -59.2)
Fire, heat, and hot substances	405 (352 to 498)	1.2 (1.1 to 1.5)	318 (272 to 360)	1.1 (0.9 to 1.2)	127 (107 to 155)	0.5 (0.4 to 0.5)	-21.1 (-34.7 to -8.9)	-9.7 (-25.2 to 4.3)	-59.8 (-66.9 to -50.4)	-59.0 (-66.3 to -49.5)
Poisonings	290 (181 to 355)	0.9 (0.5 to 1.1)	205 (143 to 252)	0.7 (0.5 to 0.9)	84 (56 to 113)	0.3 (0.2 to 0.4)	-28.7 (-42.4 to -11.4)	-18.3 (-34.1 to 1.5)	-58.7 (-70.7 to -39.7)	-57.9 (-70.1 to -38.6)
Exposure to mechanical forces	527 (431 to 767)	1.6 (1.3 to 2.3)	362 (295 to 495)	1.3 (1.0 to 1.7)	161 (134 to 212)	0.6 (0.5 to 0.7)	-31.1 (-39.9 to -21.1)	-21.1 (-31.2 to -9.6)	-55.1 (-64.0 to -42.0)	-54.2 (-63.3 to -40.9)
Adverse effects of medical treatment	105 (69 to 128)	0.3 (0.2 to 0.4)	84 (58 to 104)	0.3 (0.2 to 0.4)	49 (34 to 70)	0.2 (0.1 to 0.2)	-19.7 (-33.5 to -5.9)	-8.1 (-23.8 to 7.7)	-40.1 (-55.4 to -12.8)	-38.9 (-54.5 to -11.2)
Animal contact	48 (31 to 67)	0.1 (0.1 to 0.2)	39 (27 to 50)	0.1 (0.1 to 0.2)	21 (16 to 30)	0.1 (0.1 to 0.1)	-17.7 (-36.4 to 4.8)	-5.8 (-27.2 to 20.0)	-45.8 (-61.3 to -19.8)	-44.8 (-60.6 to -18.3)
Foreign body	220 (165 to 310)	0.7 (0.5 to 0.9)	175 (135 to 236)	0.6 (0.5 to 0.8)	112 (90 to 162)	0.4 (0.3 to 0.6)	-20.0 (-33.2 to -6.7)	-8.4 (-23.5 to 6.8)	-35.2 (-52.6 to -11.2)	-33.9 (-51.7 to -9.5)
Environmental heat and cold exposure	53 (27 to 74)	0.2 (0.1 to 0.2)	43 (23 to 58)	0.1 (0.1 to 0.2)	19 (11 to 26)	0.1 (0.0 to 0.1)	-17.9 (-36.4 to 4.1)	-6.0 (-27.2 to 19.2)	-56.0 (-66.7 to -40.4)	-55.1 (-66.1 to -39.2)
Other unintentional injuries	399 (311 to 511)	1.2 (0.9 to 1.5)	259 (213 to 309)	0.9 (0.7 to 1.1)	71 (59 to 86)	0.3 (0.2 to 0.3)	-34.3 (-48.5 to -17.9)	-24.8 (-41.0 to -6.0)	-72.4 (-78.9 to -63.8)	-71.9 (-78.4 to -63.1)
Self-harm and interpersonal violence	249 (171 to 422)	0.8 (0.5 to 1.3)	214 (123 to 284)	0.7 (0.4 to 1.0)	102 (61 to 150)	0.4 (0.2 to 0.5)	-12.0 (-39.0 to 15.2)	0.8 (-30.2 to 32.0)	-52.0 (-62.0 to -41.9)	-51.0 (-61.2 to -40.8)
Interpersonal violence	249 (171 to 422)	0.8 (0.5 to 1.3)	214 (123 to 284)	0.7 (0.4 to 1.0)	102 (61 to 150)	0.4 (0.2 to 0.5)	-12.0 (-39.0 to 15.2)	0.8 (-30.2 to 32.0)	-52.0 (-62.0 to -41.9)	-51.0 (-61.2 to -40.8)
Non-communicable diseases	5,460 (5,139 to 5,840)	16.5 (15.6 to 17.7)	4,295 (4,039 to 4,620)	14.9 (14.0 to 16.0)	2,656 (2,386 to 2,935)	9.4 (8.4 to 10.4)	-21.3 (-26.3 to -15.6)	-9.9 (-15.6 to -3.4)	-38.1 (-46.4 to -30.0)	-36.9 (-45.4 to -28.7)
Neoplasms	2,557 (2,370 to 2,834)	7.7 (7.2 to 8.6)	1,870 (1,734 to 2,053)	6.5 (6.0 to 7.1)	1,253 (1,062 to 1,417)	4.4 (3.8 to 5.0)	-26.8 (-32.5 to -20.6)	-16.2 (-22.7 to -9.1)	-32.8 (-44.7 to -21.9)	-31.6 (-43.7 to -20.4)
Nasopharynx cancer	17 (13 to 22)	0.0 (0.0 to 0.1)	13 (10 to 17)	0.0 (0.0 to 0.1)	9 (7 to 11)	0.0 (0.0 to 0.0)	-18.1 (-37.3 to 3.0)	-6.2 (-28.2 to 18.0)	-32.6 (-53.6 to -5.7)	-31.3 (-52.7 to -3.9)
Liver cancer	34 (28 to 43)	0.1 (0.1 to 0.1)	23 (20 to 27)	0.1 (0.1 to 0.1)	17 (14 to 21)	0.1 (0.1 to 0.1)	-32.7 (-46.0 to -19.0)	-22.9 (-38.1 to -7.2)	-23.4 (-42.8 to -2.4)	-21.9 (-41.7 to -0.5)
**Kidney cancer**	**66 (55 to 82)**	**0.2 (0.2 to 0.2)**	**55 (46 to 67)**	**0.2 (0.2 to 0.2)**	**43 (34 to 53)**	**0.2 (0.1 to 0.2)**	**-16.8 (-29.5 to -2.5)**	**-4.8 (-19.3 to 11.7)**	**-20.8 (-42.8 to 1.3)**	**-19.3 (-41.7 to 3.2)**
Brain and nervous system cancer	624 (510 to 724)	1.9 (1.5 to 2.2)	498 (434 to 576)	1.7 (1.5 to 2.0)	378 (284 to 456)	1.3 (1.0 to 1.6)	-20.0 (-30.4 to -7.4)	-8.4 (-20.3 to 6.0)	-23.5 (-44.2 to -4.1)	-22.0 (-43.1 to -2.3)
Hodgkin lymphoma	56 (34 to 74)	0.2 (0.1 to 0.2)	36 (26 to 48)	0.1 (0.1 to 0.2)	14 (10 to 19)	0.0 (0.0 to 0.1)	-35.1 (-54.4 to -12.9)	-25.7 (-47.8 to -0.2)	-60.3 (-72.9 to -42.7)	-59.5 (-72.4 to -41.6)
Non-Hodgkin lymphoma	253 (156 to 303)	0.8 (0.5 to 0.9)	169 (134 to 200)	0.6 (0.5 to 0.7)	110 (87 to 137)	0.4 (0.3 to 0.5)	-32.2 (-44.6 to -11.2)	-22.4 (-36.5 to 1.6)	-34.6 (-51.1 to -11.5)	-33.3 (-50.1 to -9.8)
Leukemia	1,141 (1,015 to 1,320)	3.5 (3.1 to 4.0)	773 (682 to 898)	2.7 (2.4 to 3.1)	452 (352 to 545)	1.6 (1.2 to 1.9)	-32.1 (-40.3 to -22.4)	-22.2 (-31.6 to -11.1)	-41.2 (-55.5 to -25.9)	-40.0 (-54.6 to -24.4)
Other neoplasms	366 (315 to 453)	1.1 (1.0 to 1.4)	303 (266 to 377)	1.1 (0.9 to 1.3)	229 (183 to 272)	0.8 (0.6 to 1.0)	-16.8 (-27.9 to -5.1)	-4.7 (-17.5 to 8.6)	-23.7 (-46.0 to -4.3)	-22.2 (-45.0 to -2.4)
Cardiovascular diseases	339 (303 to 388)	1.0 (0.9 to 1.2)	298 (268 to 336)	1.0 (0.9 to 1.2)	157 (133 to 186)	0.6 (0.5 to 0.7)	-11.9 (-23.3 to 0.5)	0.9 (-12.1 to 15.0)	-47.2 (-56.9 to -36.1)	-46.2 (-56.0 to -34.9)
Rheumatic heart disease	52 (43 to 65)	0.2 (0.1 to 0.2)	50 (42 to 61)	0.2 (0.1 to 0.2)	16 (13 to 21)	0.1 (0.0 to 0.1)	-4.2 (-22.5 to 14.7)	9.7 (-11.2 to 31.3)	-67.1 (-75.7 to -58.2)	-66.5 (-75.2 to -57.4)
Ischemic heart disease	12 (10 to 14)	0.0 (0.0 to 0.0)	14 (11 to 18)	0.0 (0.0 to 0.1)	7 (5 to 9)	0.0 (0.0 to 0.0)	20.8 (-3.5 to 48.4)	38.3 (10.5 to 70.0)	-49.6 (-63.9 to -31.6)	-48.6 (-63.2 to -30.3)
Cerebrovascular disease	133 (110 to 165)	0.4 (0.3 to 0.5)	100 (83 to 123)	0.3 (0.3 to 0.4)	44 (34 to 60)	0.2 (0.1 to 0.2)	-24.6 (-39.3 to -8.6)	-13.7 (-30.6 to 4.7)	-55.2 (-68.9 to -38.8)	-54.3 (-68.3 to -37.6)
Hypertensive heart disease	3 (3 to 3)	0.0 (0.0 to 0.0)	3 (3 to 3)	0.0 (0.0 to 0.0)	3 (2 to 3)	0.0 (0.0 to 0.0)	-5.5 (-15.4 to 5.6)	8.3 (-3.2 to 21.0)	-12.0 (-28.3 to 4.3)	-10.3 (-27.0 to 6.3)
Cardiomyopathy and myocarditis	82 (65 to 101)	0.2 (0.2 to 0.3)	78 (61 to 91)	0.3 (0.2 to 0.3)	48 (40 to 59)	0.2 (0.1 to 0.2)	-4.6 (-21.8 to 16.2)	9.3 (-10.4 to 33.1)	-37.6 (-51.0 to -19.1)	-36.4 (-50.0 to -17.5)
Endocarditis	10 (6 to 13)	0.0 (0.0 to 0.0)	9 (6 to 13)	0.0 (0.0 to 0.0)	9 (6 to 13)	0.0 (0.0 to 0.0)	-2.2 (-22.1 to 18.9)	11.9 (-10.8 to 36.2)	-0.3 (-35.9 to 36.7)	1.6 (-34.6 to 39.3)
Other cardiovascular and circulatory diseases	48 (37 to 65)	0.1 (0.1 to 0.2)	45 (35 to 58)	0.2 (0.1 to 0.2)	30 (22 to 39)	0.1 (0.1 to 0.1)	-4.9 (-26.6 to 19.2)	8.9 (-16.0 to 36.5)	-33.6 (-51.6 to -7.5)	-32.3 (-50.7 to -5.7)
Chronic respiratory diseases	176 (142 to 220)	0.5 (0.4 to 0.7)	128 (107 to 159)	0.4 (0.4 to 0.6)	44 (36 to 54)	0.2 (0.1 to 0.2)	-26.5 (-42.8 to -7.1)	-15.8 (-34.5 to 6.4)	-65.4 (-73.9 to -55.5)	-64.7 (-73.4 to -54.6)
Chronic obstructive pulmonary disease	42 (31 to 61)	0.1 (0.1 to 0.2)	35 (26 to 48)	0.1 (0.1 to 0.2)	10 (8 to 14)	0.0 (0.0 to 0.0)	-16.2 (-37.7 to 7.9)	-4.1 (-28.7 to 23.5)	-70.3 (-79.8 to -59.3)	-69.7 (-79.4 to -58.5)
Asthma	67 (43 to 104)	0.2 (0.1 to 0.3)	39 (26 to 58)	0.1 (0.1 to 0.2)	11 (8 to 15)	0.0 (0.0 to 0.1)	-40.8 (-60.1 to -18.1)	-32.2 (-54.3 to -6.2)	-71.2 (-82.2 to -56.6)	-70.7 (-81.9 to -55.8)
Interstitial lung disease and pulmonary sarcoidosis	10 (6 to 15)	0.0 (0.0 to 0.0)	7 (5 to 9)	0.0 (0.0 to 0.0)	4 (2 to 5)	0.0 (0.0 to 0.0)	-23.5 (-45.0 to -5.5)	-12.4 (-37.0 to 8.2)	-48.7 (-70.0 to -25.5)	-47.7 (-69.4 to -24.1)
Other chronic respiratory diseases	57 (27 to 94)	0.2 (0.1 to 0.3)	47 (27 to 70)	0.2 (0.1 to 0.2)	19 (14 to 27)	0.1 (0.0 to 0.1)	-13.2 (-45.7 to 34.3)	-0.6 (-37.9 to 53.7)	-56.6 (-75.3 to -28.4)	-55.8 (-74.8 to -27.0)
Cirrhosis and other chronic liver diseases	129 (103 to 153)	0.4 (0.3 to 0.5)	136 (113 to 159)	0.5 (0.4 to 0.6)	73 (61 to 87)	0.3 (0.2 to 0.3)	6.5 (-11.4 to 24.9)	22.0 (1.5 to 43.1)	-45.8 (-57.1 to -33.3)	-44.8 (-56.3 to -32.0)
Cirrhosis and other chronic liver diseases due to hepatitis B	3 (2 to 4)	0.0 (0.0 to 0.0)	4 (3 to 5)	0.0 (0.0 to 0.0)	2 (2 to 3)	0.0 (0.0 to 0.0)	23.1 (1.3 to 45.2)	40.9 (15.9 to 66.3)	-44.2 (-56.1 to -30.6)	-43.1 (-55.2 to -29.3)
Cirrhosis and other chronic liver diseases due to hepatitis C	2 (2 to 3)	0.0 (0.0 to 0.0)	2 (2 to 3)	0.0 (0.0 to 0.0)	1 (1 to 2)	0.0 (0.0 to 0.0)	-0.1 (-14.2 to 14.5)	14.4 (-1.8 to 31.1)	-43.1 (-52.7 to -31.8)	-42.0 (-51.8 to -30.4)
Cirrhosis and other chronic liver diseases due to other causes	123 (99 to 147)	0.4 (0.3 to 0.4)	130 (108 to 152)	0.5 (0.4 to 0.5)	70 (58 to 84)	0.2 (0.2 to 0.3)	6.3 (-11.7 to 24.8)	21.7 (1.2 to 42.9)	-45.9 (-57.3 to -33.4)	-44.9 (-56.4 to -32.1)
Digestive diseases	178 (139 to 210)	0.5 (0.4 to 0.6)	137 (115 to 161)	0.5 (0.4 to 0.6)	68 (54 to 98)	0.2 (0.2 to 0.3)	-22.7 (-35.4 to -6.8)	-11.5 (-26.1 to 6.7)	-50.2 (-62.7 to -29.8)	-49.2 (-62.0 to -28.5)
Peptic ulcer disease	22 (15 to 35)	0.1 (0.0 to 0.1)	16 (12 to 23)	0.1 (0.0 to 0.1)	6 (4 to 9)	0.0 (0.0 to 0.0)	-25.4 (-52.2 to 5.4)	-14.6 (-45.3 to 20.6)	-62.7 (-76.4 to -44.0)	-61.9 (-76.0 to -42.9)
Gastritis and duodenitis	1 (1 to 2)	0.0 (0.0 to 0.0)	1 (1 to 2)	0.0 (0.0 to 0.0)	1 (0 to 1)	0.0 (0.0 to 0.0)	-8.3 (-45.8 to 42.2)	5.0 (-37.9 to 62.8)	-32.8 (-69.5 to 19.2)	-31.5 (-68.9 to 21.5)
Appendicitis	50 (34 to 61)	0.2 (0.1 to 0.2)	30 (24 to 42)	0.1 (0.1 to 0.1)	12 (8 to 22)	0.0 (0.0 to 0.1)	-40.2 (-53.6 to -0.7)	-31.6 (-46.8 to 13.7)	-59.3 (-73.2 to -33.4)	-58.5 (-72.7 to -32.1)
Paralytic ileus and intestinal obstruction	45 (30 to 58)	0.1 (0.1 to 0.2)	40 (29 to 51)	0.1 (0.1 to 0.2)	25 (19 to 38)	0.1 (0.1 to 0.1)	-9.4 (-26.5 to 9.9)	3.8 (-15.9 to 25.8)	-36.7 (-54.5 to 2.5)	-35.5 (-53.7 to 4.5)
Inguinal, femoral, and abdominal hernia	8 (5 to 14)	0.0 (0.0 to 0.0)	7 (4 to 10)	0.0 (0.0 to 0.0)	3 (1 to 5)	0.0 (0.0 to 0.0)	-18.9 (-50.3 to 16.7)	-7.1 (-43.0 to 33.6)	-56.7 (-76.2 to -23.7)	-55.9 (-75.7 to -22.3)
Inflammatory bowel disease	16 (8 to 26)	0.0 (0.0 to 0.1)	14 (8 to 22)	0.0 (0.0 to 0.1)	7 (4 to 11)	0.0 (0.0 to 0.0)	-9.1 (-35.5 to 24.7)	4.1 (-26.2 to 42.8)	-44.7 (-72.7 to -3.3)	-43.6 (-72.2 to -1.4)
Vascular intestinal disorders	3 (1 to 5)	0.0 (0.0 to 0.0)	3 (1 to 4)	0.0 (0.0 to 0.0)	2 (1 to 3)	0.0 (0.0 to 0.0)	-15.0 (-38.1 to 13.7)	-2.7 (-29.2 to 30.2)	-44.5 (-69.8 to 2.7)	-43.4 (-69.2 to 4.7)
Gallbladder and biliary diseases	4 (3 to 6)	0.0 (0.0 to 0.0)	4 (3 to 5)	0.0 (0.0 to 0.0)	2 (1 to 2)	0.0 (0.0 to 0.0)	-18.4 (-44.4 to 12.3)	-6.6 (-36.4 to 28.6)	-54.0 (-71.5 to -24.5)	-53.1 (-71.0 to -23.0)
Pancreatitis	3 (1 to 4)	0.0 (0.0 to 0.0)	3 (1 to 4)	0.0 (0.0 to 0.0)	1 (1 to 2)	0.0 (0.0 to 0.0)	-14.0 (-32.6 to 8.2)	-1.5 (-22.9 to 23.9)	-42.8 (-65.5 to -11.3)	-41.7 (-64.8 to -9.6)
Other digestive diseases	24 (16 to 38)	0.1 (0.0 to 0.1)	20 (14 to 31)	0.1 (0.0 to 0.1)	9 (7 to 13)	0.0 (0.0 to 0.0)	-15.3 (-39.9 to 14.5)	-3.1 (-31.1 to 31.1)	-51.3 (-72.3 to -20.1)	-50.4 (-71.8 to -18.5)
Neurological disorders	413 (339 to 514)	1.3 (1.0 to 1.6)	373 (317 to 439)	1.3 (1.1 to 1.5)	234 (202 to 277)	0.8 (0.7 to 1.0)	-9.1 (-23.2 to 6.2)	4.0 (-12.1 to 21.6)	-36.6 (-51.1 to -17.4)	-35.4 (-50.2 to -15.9)
Epilepsy	307 (242 to 387)	0.9 (0.7 to 1.2)	289 (239 to 347)	1.0 (0.8 to 1.2)	168 (140 to 210)	0.6 (0.5 to 0.7)	-5.1 (-22.0 to 13.8)	8.7 (-10.7 to 30.3)	-41.1 (-56.9 to -16.4)	-40.0 (-56.1 to -14.8)
Motor neuron disease	5 (4 to 6)	0.0 (0.0 to 0.0)	4 (3 to 5)	0.0 (0.0 to 0.0)	2 (2 to 3)	0.0 (0.0 to 0.0)	-17.8 (-40.3 to 9.8)	-5.9 (-31.7 to 25.7)	-39.0 (-59.4 to -10.2)	-37.9 (-58.6 to -8.4)
Other neurological disorders	101 (82 to 139)	0.3 (0.2 to 0.4)	80 (68 to 100)	0.3 (0.2 to 0.3)	64 (52 to 78)	0.2 (0.2 to 0.3)	-20.0 (-36.2 to -4.7)	-8.4 (-27.0 to 9.2)	-19.1 (-43.7 to 5.7)	-17.6 (-42.7 to 7.7)
Mental and substance use disorders	0 (0 to 1)	0.0 (0.0 to 0.0)	0 (0 to 1)	0.0 (0.0 to 0.0)	0 (0 to 0)	0.0 (0.0 to 0.0)	-4.8 (-51.6 to 68.7)	9.0 (-44.6 to 93.1)	-37.8 (-70.9 to 18.2)	-36.6 (-70.3 to 20.5)
Eating disorders	0 (0 to 1)	0.0 (0.0 to 0.0)	0 (0 to 1)	0.0 (0.0 to 0.0)	0 (0 to 0)	0.0 (0.0 to 0.0)	-4.8 (-51.6 to 68.7)	9.0 (-44.6 to 93.1)	-37.8 (-70.9 to 18.2)	-36.6 (-70.3 to 20.5)
Diabetes, urogenital, blood, and endocrine diseases	569 (504 to 685)	1.7 (1.5 to 2.1)	530 (476 to 615)	1.8 (1.6 to 2.1)	300 (257 to 350)	1.1 (0.9 to 1.2)	-6.6 (-18.3 to 5.2)	6.9 (-6.5 to 20.4)	-43.3 (-52.9 to -33.3)	-42.2 (-52.0 to -32.0)
Diabetes mellitus	39 (32 to 50)	0.1 (0.1 to 0.2)	38 (31 to 47)	0.1 (0.1 to 0.2)	19 (16 to 24)	0.1 (0.1 to 0.1)	-2.9 (-26.4 to 23.6)	11.2 (-15.8 to 41.5)	-48.2 (-62.1 to -31.7)	-47.2 (-61.3 to -30.4)
Acute glomerulonephritis	16 (8 to 33)	0.0 (0.0 to 0.1)	16 (9 to 34)	0.1 (0.0 to 0.1)	6 (4 to 10)	0.0 (0.0 to 0.0)	13.2 (-53.8 to 151.5)	29.6 (-47.2 to 188.0)	-60.1 (-85.8 to -18.5)	-59.3 (-85.6 to -16.9)
Chronic kidney disease	135 (114 to 165)	0.4 (0.3 to 0.5)	131 (108 to 152)	0.5 (0.4 to 0.5)	66 (55 to 79)	0.2 (0.2 to 0.3)	-2.7 (-20.6 to 16.3)	11.4 (-9.1 to 33.2)	-49.0 (-59.7 to -37.8)	-48.0 (-58.9 to -36.6)
Urinary diseases and male infertility	40 (33 to 50)	0.1 (0.1 to 0.2)	41 (34 to 51)	0.1 (0.1 to 0.2)	21 (16 to 26)	0.1 (0.1 to 0.1)	4.5 (-20.1 to 34.0)	19.7 (-8.5 to 53.5)	-49.6 (-64.4 to -31.6)	-48.6 (-63.7 to -30.3)
Hemoglobinopathies and hemolytic anemias	135 (107 to 187)	0.4 (0.3 to 0.6)	118 (94 to 169)	0.4 (0.3 to 0.6)	48 (38 to 67)	0.2 (0.1 to 0.2)	-12.1 (-30.4 to 7.5)	0.7 (-20.3 to 23.1)	-58.6 (-68.7 to -46.7)	-57.8 (-68.1 to -45.7)
Endocrine, metabolic, blood, and immune disorders	204 (170 to 288)	0.6 (0.5 to 0.9)	185 (150 to 232)	0.6 (0.5 to 0.8)	139 (103 to 182)	0.5 (0.4 to 0.6)	-8.3 (-26.0 to 11.3)	5.0 (-15.2 to 27.5)	-24.5 (-42.7 to -2.4)	-23.0 (-41.6 to -0.5)

eTable 3: Number of deaths and rates (per 100,000) with percent change between 1990 and 2000 and between 2000 and 2016 in the WHO Euro region, ages 5 to 9, males

	1990		2000		2016		Change from 1990 to 2000		Change from 2000 to 2016	
	Count	Rate	Count	Rate	Count	Rate	Count	Rate	Count	Rate
Musculoskeletal disorders	34<br>(22 to 62)	0.1<br>(0.1 to 0.2)	27<br>(18 to 50)	0.1<br>(0.1 to 0.2)	15<br>(11 to 29)	0.1<br>(0.0 to 0.1)	-20.9<br>(-36.2 to -3.6)	-9.5<br>(-27.0 to 10.4)	-42.3<br>(-59.0 to -17.3)	-41.2<br>(-58.2 to -15.7)
Rheumatoid arthritis	7<br>(5 to 10)	0.0<br>(0.0 to 0.0)	4<br>(3 to 7)	0.0<br>(0.0 to 0.0)	3<br>(2 to 4)	0.0<br>(0.0 to 0.0)	-39.2<br>(-57.3 to -10.5)	-30.3<br>(-51.1 to 2.5)	-35.3<br>(-59.4 to -5.0)	-34.1<br>(-58.6 to -3.1)
Other musculoskeletal disorders	27<br>(17 to 54)	0.1<br>(0.1 to 0.2)	22<br>(15 to 45)	0.1<br>(0.1 to 0.2)	12<br>(9 to 25)	0.0<br>(0.0 to 0.1)	-15.2<br>(-33.4 to 6.0)	-3.0<br>(-23.7 to 21.4)	-43.4<br>(-60.8 to -16.9)	-42.3<br>(-60.0 to -15.3)
Other non-communicable diseases	1,064<br>(768 to 1,239)	3.2<br>(2.3 to 3.7)	795<br>(665 to 918)	2.8<br>(2.3 to 3.2)	512<br>(412 to 642)	1.8<br>(1.5 to 2.3)	-24.9<br>(-35.2 to -10.9)	-14.0<br>(-25.8 to 2.0)	-35.3<br>(-49.3 to -15.3)	-34.0<br>(-48.3 to -13.7)
Skin and subcutaneous diseases	6<br>(4 to 9)	0.0<br>(0.0 to 0.0)	6<br>(4 to 8)	0.0<br>(0.0 to 0.0)	5<br>(4 to 8)	0.0<br>(0.0 to 0.0)	-5.3<br>(-24.8 to 15.5)	8.4<br>(-13.8 to 32.2)	-6.9<br>(-31.1 to 20.0)	-5.1<br>(-29.8 to 22.4)
Communicable, maternal, neonatal, and nutritional diseases	2,908<br>(2,550 to 3,282)	8.8<br>(7.7 to 9.9)	2,376<br>(2,115 to 2,602)	8.2<br>(7.3 to 9.0)	1,009<br>(887 to 1,169)	3.6<br>(3.1 to 4.1)	-18.1<br>(-27.4 to -9.3)	-6.3<br>(-16.8 to 3.8)	-57.4<br>(-64.3 to -49.3)	-56.6<br>(-63.7 to -48.3)
HIV/AIDS and tuberculosis	206<br>(146 to 284)	0.6<br>(0.4 to 0.9)	127<br>(109 to 149)	0.4<br>(0.4 to 0.5)	34<br>(31 to 38)	0.1<br>(0.1 to 0.1)	-36.9<br>(-54.8 to -15.0)	-27.7<br>(-48.2 to -2.7)	-73.0<br>(-77.9 to -67.6)	-72.4<br>(-77.4 to -67.0)
Diarrhea, lower respiratory, and other common infectious diseases	2,444<br>(2,095 to 2,822)	7.4<br>(6.3 to 8.5)	2,042<br>(1,781 to 2,267)	7.1<br>(6.2 to 7.9)	888<br>(771 to 1,041)	3.1<br>(2.7 to 3.7)	-16.2<br>(-26.7 to -5.9)	-4.1<br>(-16.1 to 7.7)	-56.3<br>(-63.8 to -47.4)	-55.5<br>(-63.1 to -46.4)
Diarrheal diseases	119<br>(82 to 172)	0.4<br>(0.2 to 0.5)	103<br>(63 to 154)	0.4<br>(0.2 to 0.5)	40<br>(24 to 69)	0.1<br>(0.1 to 0.2)	-13.6<br>(-31.4 to 17.6)	-1.1<br>(-21.5 to 34.7)	-59.7<br>(-75.8 to -37.9)	-59.0<br>(-75.4 to -36.7)
Intestinal infectious diseases	74<br>(33 to 141)	0.2<br>(0.1 to 0.4)	53<br>(24 to 100)	0.2<br>(0.1 to 0.3)	34<br>(15 to 64)	0.1<br>(0.1 to 0.2)	-28.3<br>(-45.0 to -9.8)	-17.9<br>(-37.0 to 3.3)	-35.1<br>(-57.7 to -8.4)	-33.9<br>(-56.9 to -6.7)
Lower respiratory infections	1,325<br>(1,113 to 1,519)	4.0<br>(3.4 to 4.6)	1,341<br>(1,142 to 1,511)	4.6<br>(4.0 to 5.2)	559<br>(468 to 687)	2.0<br>(1.7 to 2.4)	1.5<br>(-11.2 to 15.2)	16.3<br>(1.6 to 31.9)	-58.2<br>(-66.3 to -47.5)	-57.4<br>(-65.7 to -46.5)
**Upper respiratory infections**	**1**<br>**(0 to 2)**	**0.0**<br>**(0.0 to 0.0)**	**1**<br>**(0 to 2)**	**0.0**<br>**(0.0 to 0.0)**	**0**<br>**(0 to 1)**	**0.0**<br>**(0.0 to 0.0)**	**20.9**<br>**(-69.6 to 216.1)**	**38.5**<br>**(-65.2 to 261.9)**	**-61.8**<br>**(-91.4 to 7.4)**	**-61.1**<br>**(-91.2 to 9.5)**
Otitis media	4<br>(2 to 12)	0.0<br>(0.0 to 0.0)	1<br>(0 to 2)	0.0<br>(0.0 to 0.0)	0<br>(0 to 0)	0.0<br>(0.0 to 0.0)	-79.7<br>(-93.3 to -59.2)	-76.8<br>(-92.4 to -53.3)	-77.9<br>(-93.1 to -61.2)	-77.5<br>(-93.0 to -60.5)
Meningitis	345<br>(232 to 484)	1.0<br>(0.7 to 1.5)	278<br>(176 to 375)	1.0<br>(0.6 to 1.3)	99<br>(74 to 136)	0.3<br>(0.3 to 0.5)	-19.1<br>(-35.6 to -0.7)	-7.4<br>(-26.2 to 13.7)	-62.9<br>(-75.7 to -44.9)	-62.2<br>(-75.2 to -43.8)
Encephalitis	203<br>(128 to 296)	0.6<br>(0.4 to 0.9)	192<br>(128 to 280)	0.7<br>(0.4 to 1.0)	141<br>(99 to 251)	0.5<br>(0.3 to 0.9)	-5.5<br>(-20.9 to 10.7)	8.2<br>(-9.4 to 26.8)	-26.3<br>(-49.1 to 0.1)	-24.9<br>(-48.1 to 2.0)
Diphtheria	7<br>(3 to 14)	0.0<br>(0.0 to 0.0)	3<br>(1 to 9)	0.0<br>(0.0 to 0.0)	1<br>(0 to 1)	0.0<br>(0.0 to 0.0)	-41.1<br>(-88.0 to 84.8)	-32.5<br>(-86.3 to 111.6)	-80.3<br>(-95.2 to -45.7)	-80.0<br>(-95.1 to -44.7)
Whooping cough	38<br>(10 to 92)	0.1<br>(0.0 to 0.3)	32<br>(10 to 71)	0.1<br>(0.0 to 0.2)	7<br>(2 to 17)	0.0<br>(0.0 to 0.1)	9.7<br>(-78.6 to 231.8)	25.6<br>(-75.5 to 280.0)	-73.5<br>(-95.3 to -17.2)	-73.0<br>(-95.2 to -15.6)
Tetanus	3<br>(1 to 6)	0.0<br>(0.0 to 0.0)	1<br>(1 to 4)	0.0<br>(0.0 to 0.0)	1<br>(0 to 1)	0.0<br>(0.0 to 0.0)	-45.6<br>(-78.2 to 33.3)	-37.8<br>(-75.0 to 52.6)	-48.7<br>(-84.9 to -9.7)	-47.7<br>(-84.7 to -8.0)
Measles	312<br>(132 to 640)	0.9<br>(0.4 to 1.9)	28<br>(11 to 62)	0.1<br>(0.0 to 0.2)	2<br>(1 to 5)	0.0<br>(0.0 to 0.0)	-91.1<br>(-93.7 to -87.9)	-89.8<br>(-92.8 to -86.1)	-91.9<br>(-95.0 to -87.6)	-91.7<br>(-94.9 to -87.3)
Varicella and herpes zoster	15<br>(7 to 28)	0.0<br>(0.0 to 0.1)	9<br>(5 to 15)	0.0<br>(0.0 to 0.1)	5<br>(2 to 7)	0.0<br>(0.0 to 0.0)	-33.2<br>(-71.9 to 39.7)	-23.5<br>(-67.8 to 60.0)	-46.5<br>(-74.2 to -11.2)	-45.5<br>(-73.7 to -9.5)
Neglected tropical diseases and malaria	22<br>(14 to 36)	0.1<br>(0.0 to 0.1)	24<br>(14 to 44)	0.1<br>(0.0 to 0.2)	12<br>(6 to 30)	0.0<br>(0.0 to 0.1)	13.0<br>(-39.4 to 100.1)	29.4<br>(-30.6 to 129.2)	-43.7<br>(-78.0 to 35.4)	-42.6<br>(-77.6 to 38.0)
Nutritional deficiencies	46<br>(33 to 70)	0.1<br>(0.1 to 0.2)	41<br>(31 to 55)	0.1<br>(0.1 to 0.2)	17<br>(13 to 23)	0.1<br>(0.0 to 0.1)	-9.4<br>(-39.9 to 29.0)	3.7<br>(-31.2 to 47.7)	-57.9<br>(-73.5 to -36.7)	-57.1<br>(-73.0 to -35.5)
Other communicable, maternal, neonatal, and nutritional diseases	191<br>(161 to 224)	0.6<br>(0.5 to 0.7)	142<br>(119 to 172)	0.5<br>(0.4 to 0.6)	58<br>(48 to 70)	0.2<br>(0.2 to 0.2)	-25.0<br>(-38.0 to -8.6)	-14.1<br>(-29.0 to 4.7)	-59.0<br>(-68.6 to -47.0)	-58.2<br>(-68.0 to -46.0)

eTable 4: Number of deaths and rates (per 100,000) with percent change between 1990 and 2000 and between 2000 and 2016 in the WHO Euro region, ages 5 to 9, females

	1990		2000		2016		Change from 1990 to 2000		Change from 2000 to 2016	
	Count	Rate	Count	Rate	Count	Rate	Count	Rate	Count	Rate
**All causes**	**11,726 (11,462 to 12,018)**	**36.9 (36.1 to 37.8)**	**9,038 (8,760 to 9,324)**	**32.8 (31.8 to 33.9)**	**4,408 (4,105 to 4,731)**	**16.4 (15.3 to 17.6)**	**-22.9 (-26.2 to -19.8)**	**-11.0 (-14.8 to -7.4)**	**-51.2 (-54.7 to -47.4)**	**-50.1 (-53.7 to -46.1)**
**Injuries**	**4,696 (4,455 to 4,996)**	**14.8 (14.0 to 15.7)**	**3,410 (3,244 to 3,624)**	**12.4 (11.8 to 13.2)**	**1,496 (1,374 to 1,643)**	**5.6 (5.1 to 6.1)**	**-27.3 (-31.4 to -22.7)**	**-16.1 (-20.8 to -10.8)**	**-56.1 (-60.5 to -51.5)**	**-55.1 (-59.6 to -50.3)**
Transport injuries	1,978 (1,799 to 2,194)	6.2 (5.7 to 6.9)	1,244 (1,114 to 1,393)	4.5 (4.0 to 5.1)	606 (527 to 698)	2.3 (2.0 to 2.6)	-37.0 (-44.4 to -29.5)	-27.3 (-35.8 to -18.6)	-51.1 (-59.1 to -41.4)	-50.0 (-58.2 to -40.0)
Road injuries	1,904 (1,733 to 2,107)	6.0 (5.5 to 6.6)	1,184 (1,061 to 1,329)	4.3 (3.9 to 4.8)	576 (500 to 664)	2.1 (1.9 to 2.5)	-37.8 (-45.2 to -30.4)	-28.2 (-36.7 to -19.7)	-51.2 (-59.3 to -41.3)	-50.1 (-58.3 to -40.0)
Other transport injuries	73 (60 to 91)	0.2 (0.2 to 0.3)	60 (50 to 74)	0.2 (0.2 to 0.3)	30 (23 to 40)	0.1 (0.1 to 0.1)	-17.1 (-34.4 to 1.6)	-4.4 (-24.3 to 17.3)	-48.9 (-63.4 to -31.4)	-47.7 (-62.6 to -29.8)
Unintentional injuries	2,477 (2,299 to 2,642)	7.8 (7.2 to 8.3)	1,876 (1,757 to 2,003)	6.8 (6.4 to 7.3)	741 (671 to 833)	2.8 (2.5 to 3.1)	-24.2 (-29.8 to -18.2)	-12.5 (-19.0 to -5.6)	-60.5 (-64.8 to -55.1)	-59.6 (-63.9 to -54.1)
Falls	293 (201 to 370)	0.9 (0.6 to 1.2)	217 (158 to 274)	0.8 (0.6 to 1.0)	77 (63 to 97)	0.3 (0.2 to 0.4)	-25.1 (-39.9 to -7.7)	-13.5 (-30.6 to 6.6)	-64.1 (-74.0 to -50.7)	-63.3 (-73.4 to -49.6)
Drowning	969 (873 to 1,073)	3.1 (2.7 to 3.4)	741 (666 to 819)	2.7 (2.4 to 3.0)	256 (217 to 303)	1.0 (0.8 to 1.1)	-23.4 (-32.0 to -14.0)	-11.6 (-21.5 to -0.7)	-65.4 (-71.6 to -57.5)	-64.6 (-70.9 to -56.6)
Fire, heat, and hot substances	341 (292 to 427)	1.1 (0.9 to 1.3)	262 (224 to 326)	1.0 (0.8 to 1.2)	107 (86 to 158)	0.4 (0.3 to 0.6)	-23.0 (-32.3 to -12.0)	-11.1 (-21.9 to 1.6)	-59.0 (-67.4 to -46.3)	-58.0 (-66.7 to -45.1)
Poisonings	195 (116 to 238)	0.6 (0.4 to 0.7)	143 (88 to 174)	0.5 (0.3 to 0.6)	63 (40 to 83)	0.2 (0.1 to 0.3)	-26.4 (-39.1 to -11.4)	-15.1 (-29.7 to 2.3)	-55.7 (-66.1 to -38.3)	-54.7 (-65.3 to -36.9)
Exposure to mechanical forces	213 (175 to 284)	0.7 (0.5 to 0.9)	172 (142 to 218)	0.6 (0.5 to 0.8)	81 (66 to 117)	0.3 (0.2 to 0.4)	-19.3 (-31.0 to -6.7)	-6.8 (-20.3 to 7.7)	-52.6 (-61.9 to -33.9)	-51.5 (-61.0 to -32.4)
Adverse effects of medical treatment	73 (54 to 93)	0.2 (0.2 to 0.3)	54 (40 to 73)	0.2 (0.1 to 0.3)	28 (22 to 37)	0.1 (0.1 to 0.1)	-25.4 (-38.0 to -13.8)	-13.9 (-28.4 to -0.5)	-48.1 (-57.3 to -37.9)	-46.9 (-56.3 to -36.5)
Animal contact	30 (20 to 45)	0.1 (0.1 to 0.1)	23 (15 to 31)	0.1 (0.1 to 0.1)	11 (9 to 13)	0.0 (0.0 to 0.0)	-23.1 (-45.5 to 2.7)	-11.3 (-37.1 to 18.6)	-50.2 (-65.8 to -31.0)	-49.0 (-65.0 to -29.4)
Foreign body	140 (105 to 185)	0.4 (0.3 to 0.6)	124 (96 to 162)	0.4 (0.3 to 0.6)	80 (67 to 104)	0.3 (0.2 to 0.4)	-11.3 (-22.7 to 0.6)	2.3 (-10.8 to 16.1)	-34.3 (-49.3 to -16.3)	-32.8 (-48.2 to -14.4)
Environmental heat and cold exposure	40 (22 to 57)	0.1 (0.1 to 0.2)	27 (16 to 39)	0.1 (0.1 to 0.1)	10 (7 to 16)	0.0 (0.0 to 0.1)	-31.5 (-47.1 to -15.8)	-21.0 (-38.9 to -2.8)	-62.6 (-72.3 to -51.8)	-61.8 (-71.6 to -50.7)
Other unintentional injuries	183 (126 to 264)	0.6 (0.4 to 0.8)	113 (82 to 151)	0.4 (0.3 to 0.5)	27 (23 to 33)	0.1 (0.1 to 0.1)	-37.1 (-55.6 to -16.6)	-27.4 (-48.8 to -3.7)	-75.2 (-82.7 to -65.0)	-74.6 (-82.3 to -64.2)
Self-harm and interpersonal violence	207 (154 to 308)	0.7 (0.5 to 1.0)	186 (124 to 223)	0.7 (0.5 to 0.8)	96 (66 to 147)	0.4 (0.2 to 0.5)	-9.1 (-31.7 to 10.2)	4.9 (-21.1 to 27.2)	-47.7 (-61.0 to -8.0)	-46.5 (-60.1 to -5.9)
Interpersonal violence	207 (154 to 308)	0.7 (0.5 to 1.0)	186 (124 to 223)	0.7 (0.5 to 0.8)	96 (66 to 147)	0.4 (0.2 to 0.5)	-9.1 (-31.7 to 10.2)	4.9 (-21.1 to 27.2)	-47.7 (-61.0 to -8.0)	-46.5 (-60.1 to -5.9)
Non-communicable diseases	4,344 (4,086 to 4,681)	13.7 (12.9 to 14.7)	3,460 (3,272 to 3,724)	12.6 (11.9 to 13.5)	2,086 (1,930 to 2,268)	7.8 (7.2 to 8.4)	-20.3 (-25.0 to -15.2)	-8.0 (-13.4 to -2.1)	-39.7 (-45.9 to -32.9)	-38.3 (-44.6 to -31.3)
Neoplasms	1,833 (1,692 to 2,002)	5.8 (5.3 to 6.3)	1,390 (1,284 to 1,517)	5.1 (4.7 to 5.5)	908 (787 to 1,010)	3.4 (2.9 to 3.8)	-24.1 (-30.0 to -17.6)	-12.4 (-19.1 to -4.8)	-34.6 (-43.9 to -25.3)	-33.1 (-42.6 to -23.6)
Nasopharynx cancer	6 (5 to 7)	0.0 (0.0 to 0.0)	5 (4 to 6)	0.0 (0.0 to 0.0)	4 (3 to 4)	0.0 (0.0 to 0.0)	-13.3 (-25.2 to -0.6)	0.0 (-13.6 to 14.8)	-25.3 (-37.6 to -9.9)	-23.6 (-36.2 to -7.8)
Liver cancer	20 (16 to 25)	0.1 (0.1 to 0.1)	16 (13 to 20)	0.1 (0.0 to 0.1)	10 (9 to 13)	0.0 (0.0 to 0.0)	-16.7 (-30.5 to -0.1)	-3.8 (-19.7 to 15.3)	-35.3 (-51.9 to -13.6)	-33.8 (-50.8 to -11.6)
**Kidney cancer**	**67 (55 to 83)**	**0.2 (0.2 to 0.3)**	**53 (45 to 64)**	**0.2 (0.2 to 0.2)**	**41 (35 to 49)**	**0.2 (0.1 to 0.2)**	**-20.6 (-33.4 to -5.8)**	**-8.3 (-23.1 to 8.7)**	**-20.6 (-40.5 to 1.6)**	**-18.8 (-39.1 to 3.9)**
Brain and nervous system cancer	492 (429 to 571)	1.5 (1.4 to 1.8)	411 (357 to 478)	1.5 (1.3 to 1.7)	318 (253 to 379)	1.2 (0.9 to 1.4)	-16.1 (-27.8 to -5.3)	-3.2 (-16.6 to 9.4)	-22.4 (-43.3 to -6.7)	-20.6 (-42.0 to -4.6)
Hodgkin lymphoma	13 (9 to 18)	0.0 (0.0 to 0.1)	10 (7 to 14)	0.0 (0.0 to 0.1)	5 (4 to 7)	0.0 (0.0 to 0.0)	-21.7 (-44.2 to 12.1)	-9.6 (-35.6 to 29.4)	-47.9 (-66.2 to -25.1)	-46.7 (-65.4 to -23.3)
Non-Hodgkin lymphoma	96 (79 to 117)	0.3 (0.2 to 0.4)	68 (56 to 83)	0.2 (0.2 to 0.3)	51 (40 to 62)	0.2 (0.2 to 0.2)	-29.1 (-42.4 to -13.1)	-18.2 (-33.5 to 0.3)	-24.5 (-45.1 to -1.3)	-22.8 (-43.8 to 1.0)
Leukemia	842 (733 to 972)	2.6 (2.3 to 3.1)	582 (488 to 674)	2.1 (1.8 to 2.4)	291 (242 to 351)	1.1 (0.9 to 1.3)	-30.7 (-39.6 to -20.3)	-20.0 (-30.3 to -8.0)	-49.7 (-59.1 to -36.3)	-48.6 (-58.2 to -34.8)
Other neoplasms	298 (258 to 373)	0.9 (0.8 to 1.2)	245 (215 to 298)	0.9 (0.8 to 1.1)	187 (156 to 220)	0.7 (0.6 to 0.8)	-17.8 (-28.5 to -6.6)	-5.1 (-17.5 to 7.8)	-22.7 (-43.3 to -4.2)	-20.9 (-42.0 to -2.0)
Cardiovascular diseases	298 (275 to 331)	0.9 (0.9 to 1.0)	249 (228 to 275)	0.9 (0.8 to 1.0)	137 (118 to 158)	0.5 (0.4 to 0.6)	-16.3 (-23.8 to -8.4)	-3.4 (-12.1 to 5.7)	-45.0 (-54.1 to -35.1)	-43.7 (-53.0 to -33.6)
Rheumatic heart disease	42 (37 to 48)	0.1 (0.1 to 0.2)	41 (35 to 48)	0.1 (0.1 to 0.2)	15 (12 to 19)	0.1 (0.0 to 0.1)	-3.0 (-16.2 to 12.9)	12.0 (-3.2 to 30.4)	-62.4 (-70.4 to -52.6)	-61.5 (-69.7 to -51.5)
Ischemic heart disease	10 (9 to 11)	0.0 (0.0 to 0.0)	11 (9 to 14)	0.0 (0.0 to 0.1)	6 (5 to 7)	0.0 (0.0 to 0.0)	14.2 (-4.1 to 35.2)	31.8 (10.7 to 56.0)	-48.9 (-62.2 to -34.0)	-47.7 (-61.3 to -32.4)
Cerebrovascular disease	126 (109 to 149)	0.4 (0.3 to 0.5)	89 (76 to 104)	0.3 (0.3 to 0.4)	42 (34 to 54)	0.2 (0.1 to 0.2)	-29.4 (-38.4 to -20.0)	-18.5 (-28.8 to -7.6)	-52.3 (-64.7 to -37.9)	-51.2 (-63.9 to -36.5)
Hypertensive heart disease	3 (2 to 4)	0.0 (0.0 to 0.0)	3 (2 to 3)	0.0 (0.0 to 0.0)	3 (2 to 3)	0.0 (0.0 to 0.0)	-9.6 (-20.2 to 3.2)	4.3 (-7.9 to 19.1)	-6.0 (-25.8 to 19.2)	-3.8 (-24.1 to 22.0)
Cardiomyopathy and myocarditis	75 (62 to 95)	0.2 (0.2 to 0.3)	67 (58 to 79)	0.2 (0.2 to 0.3)	38 (32 to 50)	0.1 (0.1 to 0.2)	-10.2 (-24.2 to 4.8)	3.6 (-12.4 to 21.0)	-42.7 (-54.2 to -28.4)	-41.4 (-53.1 to -26.8)
Endocarditis	9 (6 to 13)	0.0 (0.0 to 0.0)	8 (6 to 12)	0.0 (0.0 to 0.0)	8 (5 to 11)	0.0 (0.0 to 0.0)	-10.0 (-28.9 to 15.9)	3.8 (-17.9 to 33.8)	-7.5 (-37.6 to 27.9)	-5.3 (-36.1 to 30.9)
Other cardiovascular and circulatory diseases	33 (26 to 44)	0.1 (0.1 to 0.1)	31 (25 to 39)	0.1 (0.1 to 0.1)	26 (19 to 34)	0.1 (0.1 to 0.1)	-6.8 (-24.8 to 12.7)	7.6 (-13.2 to 30.1)	-16.6 (-39.2 to 12.8)	-14.7 (-37.8 to 15.4)
Chronic respiratory diseases	119 (99 to 144)	0.4 (0.3 to 0.5)	94 (79 to 113)	0.3 (0.3 to 0.4)	38 (33 to 46)	0.1 (0.1 to 0.2)	-20.6 (-34.6 to -1.9)	-8.4 (-24.5 to 13.2)	-59.0 (-67.9 to -48.4)	-58.0 (-67.1 to -47.2)
Chronic obstructive pulmonary disease	31 (24 to 41)	0.1 (0.1 to 0.1)	25 (19 to 32)	0.1 (0.1 to 0.1)	9 (7 to 11)	0.0 (0.0 to 0.0)	-19.5 (-36.4 to 0.8)	-7.1 (-26.6 to 16.3)	-63.2 (-72.8 to -50.6)	-62.3 (-72.1 to -49.5)
Asthma	47 (32 to 65)	0.1 (0.1 to 0.2)	32 (23 to 44)	0.1 (0.1 to 0.2)	11 (8 to 14)	0.0 (0.0 to 0.1)	-30.1 (-48.0 to -6.2)	-19.3 (-40.0 to 8.3)	-66.9 (-77.3 to -52.8)	-66.2 (-76.8 to -51.7)
Interstitial lung disease and pulmonary sarcoidosis	11 (8 to 16)	0.0 (0.0 to 0.1)	11 (7 to 15)	0.0 (0.0 to 0.1)	5 (3 to 6)	0.0 (0.0 to 0.0)	-2.9 (-24.8 to 26.3)	12.0 (-13.1 to 45.8)	-52.9 (-69.3 to -31.1)	-51.8 (-68.6 to -29.5)
Other chronic respiratory diseases	31 (17 to 49)	0.1 (0.1 to 0.2)	26 (15 to 42)	0.1 (0.1 to 0.2)	14 (9 to 20)	0.1 (0.0 to 0.1)	-11.6 (-42.6 to 32.9)	2.0 (-33.8 to 53.4)	-44.5 (-68.2 to -20.3)	-43.3 (-67.4 to -18.4)
Cirrhosis and other chronic liver diseases	94 (82 to 110)	0.3 (0.3 to 0.3)	101 (89 to 115)	0.4 (0.3 to 0.4)	52 (44 to 61)	0.2 (0.2 to 0.2)	8.0 (-8.1 to 24.3)	24.7 (6.1 to 43.5)	-48.3 (-59.3 to -36.2)	-47.1 (-58.4 to -34.8)
Cirrhosis and other chronic liver diseases due to hepatitis B	1 (1 to 1)	0.0 (0.0 to 0.0)	1 (1 to 1)	0.0 (0.0 to 0.0)	0 (0 to 1)	0.0 (0.0 to 0.0)	25.3 (4.3 to 45.7)	44.6 (20.4 to 68.2)	-47.6 (-59.1 to -34.9)	-46.4 (-58.1 to -33.4)
Cirrhosis and other chronic liver diseases due to hepatitis C	1 (1 to 1)	0.0 (0.0 to 0.0)	1 (1 to 1)	0.0 (0.0 to 0.0)	0 (0 to 1)	0.0 (0.0 to 0.0)	-1.2 (-14.8 to 12.8)	14.1 (-1.6 to 30.2)	-45.5 (-55.2 to -34.6)	-44.3 (-54.2 to -33.1)
Cirrhosis and other chronic liver diseases due to other causes	92 (80 to 108)	0.3 (0.3 to 0.3)	99 (88 to 113)	0.4 (0.3 to 0.4)	51 (43 to 60)	0.2 (0.2 to 0.2)	8.0 (-8.2 to 24.3)	24.6 (6.0 to 43.5)	-48.4 (-59.4 to -36.3)	-47.2 (-58.5 to -34.8)
Digestive diseases	161 (134 to 200)	0.5 (0.4 to 0.6)	113 (97 to 133)	0.4 (0.4 to 0.5)	53 (45 to 64)	0.2 (0.2 to 0.2)	-29.4 (-44.0 to -15.1)	-18.5 (-35.4 to -2.0)	-52.6 (-61.6 to -39.4)	-51.5 (-60.7 to -38.0)
Peptic ulcer disease	19 (13 to 32)	0.1 (0.0 to 0.1)	14 (11 to 19)	0.1 (0.0 to 0.1)	5 (4 to 7)	0.0 (0.0 to 0.0)	-26.0 (-54.1 to 0.6)	-14.6 (-47.0 to 16.1)	-61.3 (-73.3 to -45.7)	-60.4 (-72.7 to -44.4)
Gastritis and duodenitis	2 (1 to 3)	0.0 (0.0 to 0.0)	2 (1 to 3)	0.0 (0.0 to 0.0)	1 (1 to 1)	0.0 (0.0 to 0.0)	-17.9 (-46.9 to 20.5)	-5.3 (-38.7 to 39.1)	-45.5 (-69.3 to -14.5)	-44.2 (-68.6 to -12.5)
Appendicitis	45 (27 to 56)	0.1 (0.1 to 0.2)	27 (21 to 37)	0.1 (0.1 to 0.1)	11 (8 to 17)	0.0 (0.0 to 0.1)	-38.3 (-55.7 to -5.3)	-28.8 (-48.9 to 9.3)	-57.3 (-68.2 to -43.9)	-56.4 (-67.5 to -42.6)
Paralytic ileus and intestinal obstruction	31 (22 to 40)	0.1 (0.1 to 0.1)	23 (17 to 29)	0.1 (0.1 to 0.1)	14 (10 to 18)	0.1 (0.0 to 0.1)	-23.6 (-37.9 to -6.7)	-11.8 (-28.3 to 7.7)	-39.9 (-53.3 to -22.0)	-38.5 (-52.2 to -20.2)
Inguinal, femoral, and abdominal hernia	5 (3 to 8)	0.0 (0.0 to 0.0)	3 (2 to 5)	0.0 (0.0 to 0.0)	1 (1 to 2)	0.0 (0.0 to 0.0)	-36.0 (-57.6 to -10.5)	-26.1 (-51.0 to 3.3)	-55.6 (-73.8 to -31.8)	-54.6 (-73.2 to -30.3)
Inflammatory bowel disease	17 (12 to 26)	0.1 (0.0 to 0.1)	14 (10 to 19)	0.0 (0.0 to 0.1)	7 (5 to 9)	0.0 (0.0 to 0.0)	-21.3 (-39.3 to 2.2)	-9.1 (-30.0 to 18.0)	-49.1 (-64.9 to -30.2)	-47.9 (-64.1 to -28.6)
Vascular intestinal disorders	2 (1 to 3)	0.0 (0.0 to 0.0)	1 (1 to 1)	0.0 (0.0 to 0.0)	1 (0 to 1)	0.0 (0.0 to 0.0)	-31.5 (-55.5 to 0.8)	-20.9 (-48.6 to 16.4)	-42.0 (-63.9 to -7.8)	-40.7 (-63.1 to -5.7)
Gallbladder and biliary diseases	3 (2 to 4)	0.0 (0.0 to 0.0)	2 (2 to 3)	0.0 (0.0 to 0.0)	1 (1 to 1)	0.0 (0.0 to 0.0)	-36.1 (-50.5 to -18.2)	-26.3 (-42.8 to -5.6)	-63.3 (-73.7 to -49.3)	-62.5 (-73.1 to -48.2)
Pancreatitis	4 (3 to 6)	0.0 (0.0 to 0.0)	3 (2 to 4)	0.0 (0.0 to 0.0)	1 (1 to 2)	0.0 (0.0 to 0.0)	-29.3 (-48.6 to -4.4)	-18.4 (-40.7 to 10.4)	-56.7 (-71.4 to -33.9)	-55.7 (-70.8 to -32.4)
Other digestive diseases	33 (21 to 48)	0.1 (0.1 to 0.2)	25 (17 to 34)	0.1 (0.1 to 0.1)	11 (9 to 14)	0.0 (0.0 to 0.1)	-23.3 (-51.3 to 4.1)	-11.5 (-43.8 to 20.2)	-53.4 (-69.4 to -28.2)	-52.3 (-68.7 to -26.5)
Neurological disorders	324 (281 to 400)	1.0 (0.9 to 1.3)	296 (256 to 345)	1.1 (0.9 to 1.3)	191 (165 to 228)	0.7 (0.6 to 0.8)	-8.4 (-20.0 to 6.3)	5.8 (-7.6 to 22.7)	-34.8 (-47.8 to -16.0)	-33.3 (-46.6 to -14.0)
Epilepsy	222 (187 to 284)	0.7 (0.6 to 0.9)	214 (174 to 256)	0.8 (0.6 to 0.9)	130 (106 to 162)	0.5 (0.4 to 0.6)	-3.3 (-19.3 to 16.2)	11.6 (-6.8 to 34.1)	-38.9 (-53.3 to -17.1)	-37.5 (-52.2 to -15.2)
Motor neuron disease	9 (7 to 12)	0.0 (0.0 to 0.0)	7 (6 to 10)	0.0 (0.0 to 0.0)	6 (4 to 7)	0.0 (0.0 to 0.0)	-16.5 (-36.2 to 7.7)	-3.6 (-26.4 to 24.3)	-20.7 (-44.3 to 6.8)	-18.8 (-43.0 to 9.3)
Other neurological disorders	93 (76 to 115)	0.3 (0.2 to 0.4)	75 (62 to 90)	0.3 (0.2 to 0.3)	56 (48 to 66)	0.2 (0.2 to 0.2)	-19.2 (-31.3 to -5.3)	-6.8 (-20.7 to 9.3)	-23.7 (-42.4 to -2.2)	-21.9 (-41.1 to 0.0)
Mental and substance use disorders	0 (0 to 1)	0.0 (0.0 to 0.0)	0 (0 to 1)	0.0 (0.0 to 0.0)	0 (0 to 0)	0.0 (0.0 to 0.0)	10.6 (-42.6 to 78.8)	27.6 (-33.8 to 106.4)	-26.3 (-61.3 to 26.5)	-24.6 (-60.4 to 29.5)
Eating disorders	0 (0 to 1)	0.0 (0.0 to 0.0)	0 (0 to 1)	0.0 (0.0 to 0.0)	0 (0 to 0)	0.0 (0.0 to 0.0)	10.6 (-42.6 to 78.8)	27.6 (-33.8 to 106.4)	-26.3 (-61.3 to 26.5)	-24.6 (-60.4 to 29.5)
Diabetes, urogenital, blood, and endocrine diseases	546 (471 to 659)	1.7 (1.5 to 2.1)	493 (434 to 579)	1.8 (1.6 to 2.1)	269 (234 to 316)	1.0 (0.9 to 1.2)	-9.5 (-21.0 to 4.2)	4.5 (-8.8 to 20.3)	-45.1 (-54.0 to -34.0)	-43.9 (-52.9 to -32.4)
Diabetes mellitus	50 (40 to 62)	0.2 (0.1 to 0.2)	43 (37 to 51)	0.2 (0.1 to 0.2)	17 (14 to 20)	0.1 (0.1 to 0.1)	-13.5 (-30.8 to 5.4)	-0.1 (-20.1 to 21.6)	-59.9 (-68.5 to -50.1)	-59.0 (-67.8 to -48.9)
Acute glomerulonephritis	12 (6 to 26)	0.0 (0.0 to 0.1)	13 (7 to 27)	0.0 (0.0 to 0.1)	5 (3 to 10)	0.0 (0.0 to 0.0)	13.7 (-57.1 to 140.4)	31.2 (-50.5 to 177.5)	-52.4 (-81.5 to 8.0)	-51.3 (-81.1 to 10.5)
Chronic kidney disease	117 (98 to 145)	0.4 (0.3 to 0.5)	118 (100 to 138)	0.4 (0.4 to 0.5)	61 (51 to 72)	0.2 (0.2 to 0.3)	0.9 (-17.7 to 21.9)	16.5 (-5.0 to 40.7)	-48.1 (-59.4 to -35.6)	-46.9 (-58.5 to -34.1)
Urinary diseases and male infertility	38 (33 to 45)	0.1 (0.1 to 0.1)	36 (31 to 42)	0.1 (0.1 to 0.2)	20 (17 to 24)	0.1 (0.1 to 0.1)	-4.9 (-20.0 to 12.2)	9.7 (-7.7 to 29.6)	-43.9 (-57.2 to -29.5)	-42.6 (-56.2 to -27.9)
Hemoglobinopathies and hemolytic anemias	120 (80 to 187)	0.4 (0.3 to 0.6)	103 (71 to 158)	0.4 (0.3 to 0.6)	44 (33 to 71)	0.2 (0.1 to 0.3)	-13.0 (-32.1 to 10.2)	0.5 (-21.6 to 27.2)	-56.8 (-69.2 to -37.3)	-55.8 (-68.5 to -35.9)
Endocrine, metabolic, blood, and immune disorders	208 (152 to 280)	0.7 (0.5 to 0.9)	180 (125 to 237)	0.7 (0.5 to 0.9)	122 (89 to 153)	0.5 (0.3 to 0.6)	-12.9 (-30.6 to 10.7)	0.6 (-19.9 to 27.8)	-31.0 (-48.4 to -6.4)	-29.4 (-47.2 to -4.2)

eTable 4: Number of deaths and rates (per 100,000) with percent change between 1990 and 2000 and between 2000 and 2016 in the WHO Euro region, ages 5 to 9, females

	1990		2000		2016		Change from 1990 to 2000		Change from 2000 to 2016	
	Count	Rate	Count	Rate	Count	Rate	Count	Rate	Count	Rate
Musculoskeletal disorders	31 (23 to 51)	0.1 (0.1 to 0.2)	26 (20 to 44)	0.1 (0.1 to 0.2)	16 (13 to 25)	0.1 (0.0 to 0.1)	-15.1 (-28.9 to 1.0)	-2.0 (-17.9 to 16.6)	-38.4 (-53.3 to -20.1)	-37.0 (-52.2 to -18.3)
Rheumatoid arthritis	9 (6 to 13)	0.0 (0.0 to 0.0)	5 (4 to 9)	0.0 (0.0 to 0.0)	3 (2 to 4)	0.0 (0.0 to 0.0)	-44.0 (-57.2 to -21.0)	-35.4 (-50.6 to -8.9)	-43.1 (-61.7 to -20.3)	-41.8 (-60.8 to -18.4)
Other musculoskeletal disorders	22 (16 to 40)	0.1 (0.0 to 0.1)	21 (16 to 37)	0.1 (0.1 to 0.1)	13 (10 to 22)	0.0 (0.0 to 0.1)	-2.9 (-20.8 to 16.9)	12.1 (-8.6 to 34.9)	-37.1 (-53.5 to -18.2)	-35.6 (-52.5 to -16.3)
Other non-communicable diseases	936 (704 to 1,103)	2.9 (2.2 to 3.5)	698 (602 to 845)	2.5 (2.2 to 3.1)	421 (352 to 520)	1.6 (1.3 to 1.9)	-25.2 (-34.0 to -12.0)	-13.6 (-23.8 to 1.6)	-39.5 (-50.5 to -25.4)	-38.1 (-49.4 to -23.7)
Skin and subcutaneous diseases	6 (4 to 9)	0.0 (0.0 to 0.0)	6 (3 to 8)	0.0 (0.0 to 0.0)	5 (3 to 7)	0.0 (0.0 to 0.0)	-4.3 (-18.8 to 21.8)	10.4 (-6.3 to 40.6)	-8.6 (-31.8 to 13.4)	-6.5 (-30.2 to 16.0)
Communicable, maternal, neonatal, and nutritional diseases	2,687 (2,334 to 2,982)	8.5 (7.3 to 9.4)	2,167 (1,886 to 2,373)	7.9 (6.9 to 8.6)	825 (734 to 927)	3.1 (2.7 to 3.4)	-19.2 (-27.3 to -10.8)	-6.7 (-16.1 to 3.0)	-61.8 (-67.0 to -56.0)	-60.9 (-66.2 to -55.0)
HIV/AIDS and tuberculosis	86 (63 to 123)	0.3 (0.2 to 0.4)	92 (80 to 109)	0.3 (0.3 to 0.4)	38 (33 to 44)	0.1 (0.1 to 0.2)	9.8 (-25.7 to 43.8)	26.7 (-14.2 to 66.0)	-58.2 (-66.7 to -48.7)	-57.3 (-66.0 to -47.5)
Diarrhea, lower respiratory, and other common infectious diseases	2,366 (2,027 to 2,662)	7.4 (6.4 to 8.4)	1,885 (1,618 to 2,082)	6.8 (5.9 to 7.6)	720 (634 to 817)	2.7 (2.4 to 3.0)	-20.2 (-29.3 to -11.5)	-7.8 (-18.4 to 2.2)	-61.7 (-67.5 to -54.8)	-60.8 (-66.7 to -53.7)
Diarrheal diseases	117 (78 to 183)	0.4 (0.2 to 0.6)	102 (61 to 160)	0.4 (0.2 to 0.6)	35 (20 to 61)	0.1 (0.1 to 0.2)	-13.3 (-28.8 to 14.6)	0.1 (-17.8 to 32.3)	-65.1 (-79.2 to -45.8)	-64.3 (-78.7 to -44.5)
Intestinal infectious diseases	61 (27 to 118)	0.2 (0.1 to 0.4)	43 (20 to 86)	0.2 (0.1 to 0.3)	28 (13 to 54)	0.1 (0.0 to 0.2)	-27.7 (-42.7 to -8.7)	-16.5 (-33.8 to 5.4)	-33.7 (-55.3 to -5.0)	-32.2 (-54.2 to -2.8)
Lower respiratory infections	1,198 (1,053 to 1,333)	3.8 (3.3 to 4.2)	1,171 (1,035 to 1,296)	4.3 (3.8 to 4.7)	482 (411 to 560)	1.8 (1.5 to 2.1)	-2.1 (-13.9 to 10.0)	13.0 (-0.6 to 26.9)	-58.7 (-65.9 to -50.4)	-57.8 (-65.1 to -49.2)
**Upper respiratory infections**	**0 (0 to 1)**	**0.0 (0.0 to 0.0)**	**1 (0 to 1)**	**0.0 (0.0 to 0.0)**	**0 (0 to 0)**	**0.0 (0.0 to 0.0)**	**27.7 (-69.9 to 249.3)**	**47.4 (-65.3 to 303.3)**	**-62.6 (-91.6 to 11.2)**	**-61.8 (-91.4 to 13.7)**
Otitis media	5 (1 to 23)	0.0 (0.0 to 0.1)	1 (0 to 2)	0.0 (0.0 to 0.0)	0 (0 to 0)	0.0 (0.0 to 0.0)	-71.3 (-96.6 to -16.7)	-66.9 (-96.1 to -3.9)	-88.6 (-97.6 to -73.9)	-88.3 (-97.5 to -73.3)
Meningitis	474 (232 to 653)	1.5 (0.7 to 2.1)	351 (169 to 464)	1.3 (0.6 to 1.7)	71 (59 to 87)	0.3 (0.2 to 0.3)	-25.1 (-42.7 to -2.9)	-13.5 (-33.8 to 12.0)	-78.3 (-85.6 to -61.3)	-77.7 (-85.3 to -60.4)
Encephalitis	140 (86 to 176)	0.4 (0.3 to 0.6)	135 (81 to 172)	0.5 (0.3 to 0.6)	88 (65 to 119)	0.3 (0.2 to 0.4)	-3.1 (-15.5 to 13.2)	11.8 (-2.5 to 30.7)	-33.1 (-52.0 to -4.6)	-31.5 (-50.9 to -2.4)
Diphtheria	4 (2 to 9)	0.0 (0.0 to 0.0)	2 (1 to 6)	0.0 (0.0 to 0.0)	0 (0 to 1)	0.0 (0.0 to 0.0)	-34.6 (-86.4 to 108.1)	-24.5 (-84.2 to 140.3)	-81.4 (-95.3 to -50.8)	-81.0 (-95.2 to -49.7)
Whooping cough	48 (14 to 114)	0.2 (0.0 to 0.4)	42 (13 to 95)	0.2 (0.0 to 0.3)	9 (2 to 22)	0.0 (0.0 to 0.1)	12.5 (-77.5 to 226.2)	29.9 (-74.0 to 276.6)	-74.6 (-95.6 to -17.4)	-74.0 (-95.5 to -15.4)
Tetanus	2 (1 to 4)	0.0 (0.0 to 0.0)	1 (1 to 2)	0.0 (0.0 to 0.0)	1 (0 to 1)	0.0 (0.0 to 0.0)	-45.8 (-78.3 to 27.9)	-37.5 (-75.0 to 47.7)	-38.2 (-76.4 to -11.5)	-36.8 (-75.8 to -9.4)
Measles	303 (130 to 627)	1.0 (0.4 to 2.0)	28 (10 to 62)	0.1 (0.0 to 0.2)	2 (1 to 5)	0.0 (0.0 to 0.0)	-91.0 (-93.5 to -87.5)	-89.6 (-92.5 to -85.6)	-91.9 (-95.1 to -87.6)	-91.7 (-95.0 to -87.3)
Varicella and herpes zoster	13 (6 to 26)	0.0 (0.0 to 0.1)	8 (4 to 14)	0.0 (0.0 to 0.0)	3 (2 to 6)	0.0 (0.0 to 0.0)	-34.6 (-75.4 to 37.7)	-24.5 (-71.6 to 59.0)	-52.1 (-79.5 to -11.5)	-51.0 (-79.0 to -9.4)
Neglected tropical diseases and malaria	14 (9 to 25)	0.0 (0.0 to 0.1)	16 (10 to 26)	0.1 (0.0 to 0.1)	7 (4 to 21)	0.0 (0.0 to 0.1)	20.3 (-37.6 to 112.3)	38.8 (-28.0 to 145.1)	-53.0 (-80.4 to 29.9)	-51.9 (-79.9 to 32.9)
Nutritional deficiencies	44 (34 to 60)	0.1 (0.1 to 0.2)	34 (27 to 44)	0.1 (0.1 to 0.2)	12 (10 to 16)	0.0 (0.0 to 0.1)	-20.6 (-42.7 to 6.6)	-8.4 (-33.8 to 23.0)	-63.6 (-74.4 to -50.4)	-62.8 (-73.8 to -49.2)
Other communicable, maternal, neonatal, and nutritional diseases	177 (146 to 214)	0.6 (0.5 to 0.7)	140 (112 to 178)	0.5 (0.4 to 0.6)	48 (37 to 61)	0.2 (0.1 to 0.2)	-20.6 (-35.4 to -2.5)	-8.3 (-25.4 to 12.6)	-65.3 (-74.1 to -54.5)	-64.5 (-73.5 to -53.4)

eTable 5: Number of deaths and rates (per 100,000) with percent change between 1990 and 2000 and between 2000 and 2016 in the WHO Euro region, ages 10 to 14, males

	1990		2000		2016		Change from 1990 to 2000		Change from 2000 to 2016	
	Count	Rate	Count	Rate	Count	Rate	Count	Rate	Count	Rate
**All causes**	**15,307 (15,000 to 15,647)**	**47.2 (46.3 to 48.3)**	**14,778 (14,386 to 15,164)**	**44.9 (43.7 to 46.1)**	**6,342 (5,902 to 6,791)**	**24.1 (22.5 to 25.9)**	**-3.4 (-6.7 to -0.4)**	**-4.9 (-8.1 to -1.8)**	**-57.1 (-60.2 to -53.9)**	**-46.2 (-50.2 to -42.2)**
**Injuries**	**8,800 (8,523 to 9,107)**	**27.1 (26.3 to 28.1)**	**8,208 (7,862 to 8,517)**	**24.9 (23.9 to 25.9)**	**3,053 (2,810 to 3,333)**	**11.6 (10.7 to 12.7)**	**-6.7 (-11.2 to -2.6)**	**-8.1 (-12.5 to -4.0)**	**-62.8 (-66.1 to -59.1)**	**-53.4 (-57.6 to -48.8)**
Transport injuries	3,060 (2,847 to 3,291)	9.4 (8.8 to 10.2)	2,412 (2,206 to 2,634)	7.3 (6.7 to 8.0)	988 (867 to 1,126)	3.8 (3.3 to 4.3)	-21.1 (-28.5 to -13.9)	-22.3 (-29.5 to -15.1)	-59.0 (-65.1 to -52.5)	-48.6 (-56.3 to -40.5)
Road injuries	2,849 (2,651 to 3,069)	8.8 (8.2 to 9.5)	2,199 (2,017 to 2,403)	6.7 (6.1 to 7.3)	901 (786 to 1,025)	3.4 (3.0 to 3.9)	-22.8 (-29.7 to -15.5)	-23.9 (-30.8 to -16.8)	-59.0 (-65.2 to -52.4)	-48.6 (-56.4 to -40.3)
Other transport injuries	210 (175 to 256)	0.6 (0.5 to 0.8)	213 (175 to 257)	0.6 (0.5 to 0.8)	87 (70 to 111)	0.3 (0.3 to 0.4)	1.8 (-18.4 to 26.2)	0.3 (-19.6 to 24.4)	-58.6 (-68.9 to -44.3)	-48.2 (-61.0 to -30.2)
Unintentional injuries	4,568 (4,338 to 4,840)	14.1 (13.4 to 14.9)	4,233 (4,009 to 4,505)	12.9 (12.2 to 13.7)	1,415 (1,284 to 1,573)	5.4 (4.9 to 6.0)	-7.3 (-13.2 to -1.3)	-8.7 (-14.4 to -2.8)	-66.6 (-69.9 to -62.8)	-58.1 (-62.3 to -53.3)
Falls	550 (365 to 657)	1.7 (1.1 to 2.0)	493 (365 to 575)	1.5 (1.1 to 1.7)	167 (140 to 229)	0.6 (0.5 to 0.9)	-9.7 (-23.5 to 7.8)	-11.0 (-24.6 to 6.2)	-65.5 (-73.5 to -45.5)	-56.7 (-66.8 to -31.7)
Drowning	2,111 (1,926 to 2,302)	6.5 (5.9 to 7.1)	1,950 (1,780 to 2,134)	5.9 (5.4 to 6.5)	585 (515 to 665)	2.2 (2.0 to 2.5)	-7.5 (-16.8 to 2.7)	-8.9 (-18.0 to 1.1)	-69.9 (-74.3 to -64.8)	-62.3 (-67.7 to -55.9)
Fire, heat, and hot substances	266 (227 to 305)	0.8 (0.7 to 0.9)	274 (229 to 310)	0.8 (0.7 to 0.9)	90 (75 to 109)	0.3 (0.3 to 0.4)	3.5 (-10.2 to 18.8)	2.0 (-11.5 to 17.1)	-67.2 (-73.5 to -60.4)	-58.9 (-66.7 to -50.3)
Poisonings	232 (155 to 280)	0.7 (0.5 to 0.9)	210 (156 to 255)	0.6 (0.5 to 0.8)	76 (51 to 101)	0.3 (0.2 to 0.4)	-8.9 (-28.5 to 13.8)	-10.2 (-29.6 to 12.1)	-63.4 (-73.5 to -49.8)	-54.1 (-66.8 to -37.2)
Exposure to mechanical forces	555 (472 to 767)	1.7 (1.5 to 2.4)	501 (421 to 688)	1.5 (1.3 to 2.1)	202 (167 to 263)	0.8 (0.6 to 1.0)	-9.5 (-20.5 to 2.4)	-10.8 (-21.7 to 0.8)	-59.4 (-67.2 to -50.5)	-49.1 (-58.9 to -38.0)
Adverse effects of medical treatment	87 (62 to 108)	0.3 (0.2 to 0.3)	84 (61 to 106)	0.3 (0.2 to 0.3)	44 (33 to 63)	0.2 (0.1 to 0.2)	-2.9 (-17.6 to 9.1)	-4.4 (-18.8 to 7.5)	-47.1 (-57.6 to -29.4)	-33.7 (-46.9 to -11.5)
Animal contact	29 (19 to 38)	0.1 (0.1 to 0.1)	29 (19 to 38)	0.1 (0.1 to 0.1)	13 (9 to 18)	0.0 (0.0 to 0.1)	1.0 (-16.7 to 20.0)	-0.5 (-18.0 to 18.2)	-55.7 (-66.2 to -38.3)	-44.6 (-57.6 to -22.7)
Foreign body	197 (157 to 278)	0.6 (0.5 to 0.9)	184 (145 to 265)	0.6 (0.4 to 0.8)	100 (78 to 153)	0.4 (0.3 to 0.6)	-6.8 (-21.7 to 7.1)	-8.2 (-22.9 to 5.5)	-45.2 (-56.8 to -27.9)	-31.3 (-45.9 to -9.6)
Environmental heat and cold exposure	56 (26 to 77)	0.2 (0.1 to 0.2)	60 (28 to 83)	0.2 (0.1 to 0.3)	20 (11 to 27)	0.1 (0.0 to 0.1)	9.0 (-10.8 to 34.1)	7.4 (-12.1 to 32.1)	-65.9 (-74.4 to -54.6)	-57.3 (-67.9 to -43.1)
Other unintentional injuries	485 (400 to 586)	1.5 (1.2 to 1.8)	449 (378 to 523)	1.4 (1.1 to 1.6)	117 (98 to 141)	0.4 (0.4 to 0.5)	-6.9 (-22.7 to 12.1)	-8.3 (-23.8 to 10.5)	-73.8 (-79.1 to -67.2)	-67.1 (-73.9 to -58.9)
Self-harm and interpersonal violence	1,131 (906 to 1,316)	3.5 (2.8 to 4.1)	1,400 (946 to 1,609)	4.3 (2.9 to 4.9)	593 (431 to 714)	2.3 (1.6 to 2.7)	23.9 (-1.7 to 44.7)	22.1 (-3.1 to 42.6)	-57.3 (-65.0 to -44.1)	-46.5 (-56.1 to -29.9)
Self-harm	790 (594 to 908)	2.4 (1.8 to 2.8)	992 (646 to 1,149)	3.0 (2.0 to 3.5)	432 (301 to 532)	1.6 (1.1 to 2.0)	25.5 (3.8 to 46.9)	23.6 (2.3 to 44.7)	-56.1 (-64.8 to -40.3)	-45.0 (-55.8 to -25.1)
Interpersonal violence	341 (226 to 541)	1.1 (0.7 to 1.7)	408 (217 to 531)	1.2 (0.7 to 1.6)	161 (99 to 229)	0.6 (0.4 to 0.9)	21.7 (-12.2 to 65.2)	19.9 (-13.5 to 62.7)	-59.9 (-69.9 to -49.1)	-49.8 (-62.2 to -36.2)
Non-communicable diseases	4,895 (4,675 to 5,144)	15.1 (14.4 to 15.9)	4,769 (4,544 to 5,082)	14.5 (13.8 to 15.4)	2,594 (2,383 to 2,800)	9.9 (9.1 to 10.7)	-2.5 (-7.4 to 3.1)	-4.0 (-8.8 to 1.5)	-45.6 (-50.9 to -40.1)	-31.8 (-38.5 to -24.9)
Neoplasms	2,124 (1,981 to 2,288)	6.6 (6.1 to 7.1)	1,963 (1,831 to 2,145)	6.0 (5.6 to 6.5)	1,130 (989 to 1,241)	4.3 (3.8 to 4.7)	-7.5 (-13.8 to -0.5)	-8.9 (-15.1 to -2.0)	-42.3 (-50.8 to -34.6)	-27.7 (-38.3 to -18.1)
Nasopharynx cancer	25 (20 to 33)	0.1 (0.1 to 0.1)	25 (21 to 32)	0.1 (0.1 to 0.1)	15 (13 to 19)	0.1 (0.0 to 0.1)	1.5 (-18.5 to 23.4)	0.0 (-19.7 to 21.5)	-40.2 (-56.0 to -18.4)	-25.0 (-44.9 to 2.3)
**Liver cancer**	**32 (28 to 38)**	**0.1 (0.1 to 0.1)**	**31 (27 to 36)**	**0.1 (0.1 to 0.1)**	**21 (17 to 24)**	**0.1 (0.1 to 0.1)**	**-2.0 (-17.1 to 16.2)**	**-3.4 (-18.3 to 14.4)**	**-34.1 (-48.1 to -18.9)**	**-17.4 (-35.0 to 1.7)**
Kidney cancer	21 (18 to 25)	0.1 (0.1 to 0.1)	22 (19 to 26)	0.1 (0.1 to 0.1)	16 (13 to 19)	0.1 (0.1 to 0.1)	4.5 (-10.3 to 20.0)	3.0 (-11.7 to 18.3)	-26.5 (-43.3 to -9.4)	-7.9 (-28.9 to 13.6)
Brain and nervous system cancer	467 (400 to 531)	1.4 (1.2 to 1.6)	463 (408 to 526)	1.4 (1.2 to 1.6)	298 (245 to 355)	1.1 (0.9 to 1.4)	-0.7 (-12.8 to 11.3)	-2.1 (-14.1 to 9.7)	-35.3 (-48.8 to -21.7)	-18.9 (-35.9 to -1.9)
Thyroid cancer	5 (5 to 6)	0.0 (0.0 to 0.0)	5 (5 to 6)	0.0 (0.0 to 0.0)	3 (3 to 4)	0.0 (0.0 to 0.0)	-2.3 (-15.7 to 11.0)	-3.7 (-17.0 to 9.3)	-35.3 (-44.5 to -25.5)	-18.9 (-30.5 to -6.7)
Hodgkin lymphoma	68 (55 to 81)	0.2 (0.2 to 0.2)	57 (49 to 69)	0.2 (0.1 to 0.2)	21 (17 to 25)	0.1 (0.1 to 0.1)	-15.2 (-30.5 to 0.8)	-16.4 (-31.6 to -0.7)	-63.7 (-70.7 to -55.3)	-54.5 (-63.3 to -44.0)
Non-Hodgkin lymphoma	213 (142 to 252)	0.7 (0.4 to 0.8)	184 (148 to 213)	0.6 (0.4 to 0.6)	103 (83 to 127)	0.4 (0.3 to 0.5)	-12.9 (-27.7 to 6.7)	-14.2 (-28.8 to 5.1)	-43.8 (-55.4 to -28.9)	-29.6 (-44.1 to -10.9)
Leukemia	911 (821 to 1,033)	2.8 (2.5 to 3.2)	800 (717 to 916)	2.4 (2.2 to 2.8)	417 (347 to 489)	1.6 (1.3 to 1.9)	-12.1 (-21.8 to -1.4)	-13.4 (-23.0 to -2.9)	-47.6 (-58.0 to -36.0)	-34.4 (-47.4 to -19.9)
Other neoplasms	382 (335 to 449)	1.2 (1.0 to 1.4)	376 (331 to 448)	1.1 (1.0 to 1.4)	236 (199 to 274)	0.9 (0.8 to 1.0)	-1.4 (-14.2 to 11.2)	-2.9 (-15.5 to 9.5)	-36.7 (-51.5 to -22.3)	-20.7 (-39.2 to -2.6)
Cardiovascular diseases	491 (452 to 537)	1.5 (1.4 to 1.7)	470 (436 to 512)	1.4 (1.3 to 1.6)	234 (205 to 265)	0.9 (0.8 to 1.0)	-4.0 (-12.7 to 6.2)	-5.5 (-14.0 to 4.6)	-50.3 (-57.4 to -42.9)	-37.7 (-46.6 to -28.4)
Rheumatic heart disease	82 (71 to 96)	0.3 (0.2 to 0.3)	87 (77 to 98)	0.3 (0.2 to 0.3)	28 (23 to 33)	0.1 (0.1 to 0.1)	5.9 (-7.8 to 20.1)	4.3 (-9.2 to 18.3)	-68.0 (-74.4 to -61.3)	-59.9 (-67.9 to -51.5)
Ischemic heart disease	20 (17 to 25)	0.1 (0.1 to 0.1)	20 (17 to 25)	0.1 (0.1 to 0.1)	11 (8 to 14)	0.0 (0.0 to 0.1)	0.3 (-18.5 to 21.8)	-1.2 (-19.7 to 20.0)	-46.8 (-61.3 to -28.4)	-33.4 (-51.5 to -10.3)
Cerebrovascular disease	196 (173 to 227)	0.6 (0.5 to 0.7)	155 (135 to 184)	0.5 (0.4 to 0.6)	68 (56 to 84)	0.3 (0.2 to 0.3)	-20.5 (-30.8 to -9.0)	-21.7 (-31.9 to -10.3)	-55.8 (-66.1 to -44.5)	-44.6 (-57.5 to -30.5)
Hypertensive heart disease	3 (3 to 4)	0.0 (0.0 to 0.0)	3 (3 to 4)	0.0 (0.0 to 0.0)	3 (2 to 3)	0.0 (0.0 to 0.0)	7.3 (-5.5 to 23.9)	5.7 (-6.9 to 22.1)	-23.4 (-37.6 to -7.8)	-4.0 (-21.8 to 15.5)
Cardiomyopathy and myocarditis	120 (100 to 147)	0.4 (0.3 to 0.5)	129 (108 to 154)	0.4 (0.3 to 0.5)	77 (64 to 92)	0.3 (0.2 to 0.4)	8.2 (-6.0 to 24.8)	6.6 (-7.4 to 23.0)	-40.3 (-52.8 to -27.8)	-25.2 (-40.8 to -9.5)
Endocarditis	12 (8 to 15)	0.0 (0.0 to 0.0)	13 (9 to 17)	0.0 (0.0 to 0.1)	11 (8 to 15)	0.0 (0.0 to 0.1)	12.5 (-6.7 to 32.0)	10.8 (-8.1 to 30.0)	-16.2 (-37.1 to 6.7)	5.0 (-21.2 to 33.6)
Other cardiovascular and circulatory diseases	58 (45 to 75)	0.2 (0.1 to 0.2)	62 (49 to 77)	0.2 (0.2 to 0.2)	37 (28 to 46)	0.1 (0.1 to 0.2)	9.2 (-11.3 to 35.0)	7.6 (-12.6 to 33.0)	-40.8 (-55.4 to -23.4)	-25.9 (-44.1 to -4.0)
Chronic respiratory diseases	205 (179 to 241)	0.6 (0.6 to 0.7)	166 (147 to 192)	0.5 (0.4 to 0.6)	58 (50 to 68)	0.2 (0.2 to 0.3)	-18.5 (-31.4 to -4.0)	-19.7 (-32.4 to -5.4)	-65.2 (-71.8 to -57.6)	-56.4 (-64.6 to -46.8)
Chronic obstructive pulmonary disease	46 (35 to 67)	0.1 (0.1 to 0.2)	45 (36 to 61)	0.1 (0.1 to 0.2)	15 (12 to 19)	0.1 (0.0 to 0.1)	-1.0 (-23.7 to 23.7)	-2.5 (-24.8 to 21.8)	-65.5 (-75.5 to -54.6)	-56.8 (-69.4 to -43.1)
Asthma	116 (93 to 146)	0.4 (0.3 to 0.4)	82 (67 to 100)	0.2 (0.2 to 0.3)	22 (18 to 29)	0.1 (0.1 to 0.1)	-29.0 (-43.1 to -14.5)	-30.0 (-43.9 to -15.7)	-73.2 (-79.7 to -64.8)	-66.4 (-74.5 to -55.9)
Interstitial lung disease and pulmonary sarcoidosis	7 (3 to 10)	0.0 (0.0 to 0.0)	7 (4 to 9)	0.0 (0.0 to 0.0)	4 (2 to 5)	0.0 (0.0 to 0.0)	-2.9 (-24.7 to 24.0)	-4.3 (-25.8 to 22.2)	-41.7 (-66.5 to -13.7)	-27.0 (-58.0 to 8.1)
Other chronic respiratory diseases	37 (18 to 62)	0.1 (0.1 to 0.2)	33 (19 to 51)	0.1 (0.1 to 0.2)	17 (11 to 24)	0.1 (0.0 to 0.1)	-4.9 (-43.8 to 39.9)	-6.3 (-44.6 to 37.8)	-46.3 (-69.8 to -12.9)	-32.7 (-62.1 to 9.1)
Cirrhosis and other chronic liver diseases	132 (112 to 154)	0.4 (0.3 to 0.5)	214 (187 to 243)	0.6 (0.6 to 0.7)	95 (80 to 110)	0.4 (0.3 to 0.4)	63.0 (38.5 to 88.6)	60.6 (36.4 to 85.8)	-55.3 (-63.2 to -46.1)	-44.0 (-53.9 to -32.4)
Cirrhosis and other chronic liver diseases due to hepatitis B	6 (5 to 8)	0.0 (0.0 to 0.0)	12 (9 to 15)	0.0 (0.0 to 0.0)	5 (4 to 7)	0.0 (0.0 to 0.0)	92.1 (61.2 to 124.9)	89.2 (58.8 to 121.5)	-54.4 (-63.0 to -44.0)	-42.9 (-53.6 to -29.8)
Cirrhosis and other chronic liver diseases due to hepatitis C	5 (3 to 6)	0.0 (0.0 to 0.0)	7 (5 to 10)	0.0 (0.0 to 0.0)	3 (2 to 4)	0.0 (0.0 to 0.0)	52.5 (30.6 to 77.8)	50.2 (28.7 to 75.2)	-54.9 (-62.7 to -46.2)	-43.5 (-53.3 to -32.6)
Cirrhosis and other chronic liver diseases due to other causes	121 (102 to 142)	0.4 (0.3 to 0.4)	194 (170 to 222)	0.6 (0.5 to 0.7)	86 (72 to 101)	0.3 (0.3 to 0.4)	62.0 (37.3 to 87.8)	59.6 (35.3 to 85.0)	-55.3 (-63.3 to -46.1)	-44.0 (-54.1 to -32.5)
Digestive diseases	157 (129 to 180)	0.5 (0.4 to 0.6)	144 (125 to 174)	0.4 (0.4 to 0.5)	65 (54 to 97)	0.2 (0.2 to 0.4)	-7.9 (-20.7 to 10.4)	-9.3 (-21.9 to 8.8)	-54.7 (-63.6 to -39.9)	-43.3 (-54.4 to -24.7)
Peptic ulcer disease	18 (14 to 26)	0.1 (0.0 to 0.1)	19 (15 to 24)	0.1 (0.0 to 0.1)	6 (5 to 9)	0.0 (0.0 to 0.0)	6.2 (-25.6 to 38.7)	4.7 (-26.7 to 36.7)	-67.4 (-76.7 to -53.1)	-59.1 (-70.8 to -41.2)
Gastritis and duodenitis	1 (1 to 1)	0.0 (0.0 to 0.0)	1 (1 to 1)	0.0 (0.0 to 0.0)	1 (0 to 1)	0.0 (0.0 to 0.0)	-11.2 (-24.0 to 3.5)	-12.5 (-25.1 to 1.9)	-41.1 (-53.5 to -26.9)	-26.2 (-41.8 to -8.4)
Appendicitis	48 (31 to 57)	0.1 (0.1 to 0.2)	35 (29 to 50)	0.1 (0.1 to 0.2)	14 (10 to 23)	0.1 (0.0 to 0.1)	-25.8 (-41.7 to 18.9)	-26.9 (-42.6 to 17.1)	-61.4 (-72.2 to -41.1)	-51.6 (-65.2 to -26.2)
Paralytic ileus and intestinal obstruction	34 (24 to 44)	0.1 (0.1 to 0.1)	34 (26 to 46)	0.1 (0.1 to 0.1)	19 (15 to 32)	0.1 (0.1 to 0.1)	0.7 (-17.1 to 20.0)	-0.8 (-18.3 to 18.3)	-44.1 (-57.3 to -15.8)	-29.9 (-46.5 to 5.4)
Inguinal, femoral, and abdominal hernia	7 (4 to 12)	0.0 (0.0 to 0.0)	7 (3 to 11)	0.0 (0.0 to 0.0)	3 (1 to 5)	0.0 (0.0 to 0.0)	6.7 (-30.1 to 52.2)	5.1 (-31.1 to 49.9)	-57.6 (-73.1 to -32.1)	-46.9 (-66.3 to -14.9)
Inflammatory bowel disease	15 (10 to 24)	0.0 (0.0 to 0.1)	15 (11 to 22)	0.0 (0.0 to 0.1)	8 (6 to 11)	0.0 (0.0 to 0.0)	0.4 (-26.2 to 29.1)	-1.1 (-27.3 to 27.2)	-46.1 (-67.8 to -17.0)	-32.5 (-59.7 to 4.0)
Vascular intestinal disorders	4 (2 to 5)	0.0 (0.0 to 0.0)	4 (2 to 4)	0.0 (0.0 to 0.0)	2 (1 to 3)	0.0 (0.0 to 0.0)	-14.2 (-30.1 to 3.9)	-15.5 (-31.1 to 2.4)	-45.0 (-59.3 to -23.0)	-31.1 (-49.1 to -3.5)
Gallbladder and biliary diseases	4 (3 to 5)	0.0 (0.0 to 0.0)	3 (3 to 5)	0.0 (0.0 to 0.0)	2 (1 to 3)	0.0 (0.0 to 0.0)	-12.6 (-35.0 to 11.8)	-13.9 (-35.9 to 10.1)	-53.7 (-66.8 to -36.3)	-41.9 (-58.4 to -20.2)
Pancreatitis	4 (2 to 6)	0.0 (0.0 to 0.0)	4 (2 to 6)	0.0 (0.0 to 0.0)	2 (1 to 4)	0.0 (0.0 to 0.0)	5.1 (-17.3 to 33.5)	3.6 (-18.5 to 31.5)	-49.9 (-66.8 to -24.3)	-37.2 (-58.4 to -5.2)
Other digestive diseases	21 (16 to 31)	0.1 (0.0 to 0.1)	21 (16 to 31)	0.1 (0.0 to 0.1)	9 (7 to 13)	0.0 (0.0 to 0.0)	1.8 (-20.5 to 27.1)	0.3 (-21.7 to 25.2)	-55.6 (-70.7 to -38.3)	-44.4 (-63.2 to -22.7)
Neurological disorders	510 (442 to 603)	1.6 (1.4 to 1.9)	550 (479 to 630)	1.7 (1.5 to 1.9)	310 (276 to 351)	1.2 (1.1 to 1.3)	8.1 (-4.4 to 21.2)	6.5 (-5.9 to 19.4)	-43.4 (-52.9 to -31.6)	-29.0 (-41.0 to -14.3)
Epilepsy	316 (259 to 384)	1.0 (0.8 to 1.2)	365 (309 to 427)	1.1 (0.9 to 1.3)	184 (157 to 220)	0.7 (0.6 to 0.8)	16.3 (-2.4 to 37.0)	14.6 (-3.9 to 34.9)	-49.2 (-59.6 to -34.5)	-36.3 (-49.4 to -17.9)
Motor neuron disease	15 (13 to 18)	0.0 (0.0 to 0.1)	13 (11 to 16)	0.0 (0.0 to 0.0)	10 (8 to 12)	0.0 (0.0 to 0.0)	-13.7 (-29.5 to 4.2)	-15.0 (-30.6 to 2.6)	-25.5 (-43.0 to -4.4)	-6.6 (-28.5 to 19.8)
Other neurological disorders	178 (153 to 226)	0.6 (0.5 to 0.7)	171 (147 to 205)	0.5 (0.4 to 0.6)	116 (99 to 134)	0.4 (0.4 to 0.5)	-3.8 (-16.7 to 10.9)	-5.2 (-17.9 to 9.2)	-31.7 (-48.1 to -15.5)	-14.4 (-35.0 to 5.9)
Mental and substance use disorders	0 (0 to 1)	0.0 (0.0 to 0.0)	0 (0 to 1)	0.0 (0.0 to 0.0)	0 (0 to 0)	0.0 (0.0 to 0.0)	-1.1 (-46.5 to 75.1)	-2.6 (-47.3 to 72.5)	-30.6 (-67.5 to 31.2)	-13.0 (-59.2 to 64.3)
Eating disorders	0 (0 to 1)	0.0 (0.0 to 0.0)	0 (0 to 1)	0.0 (0.0 to 0.0)	0 (0 to 0)	0.0 (0.0 to 0.0)	-1.1 (-46.5 to 75.1)	-2.6 (-47.3 to 72.5)	-30.6 (-67.5 to 31.2)	-13.0 (-59.2 to 64.3)
Diabetes, urogenital, blood, and endocrine diseases	545 (491 to 632)	1.7 (1.5 to 2.0)	569 (517 to 641)	1.7 (1.6 to 1.9)	317 (276 to 369)	1.2 (1.0 to 1.4)	4.7 (-3.8 to 14.1)	3.1 (-5.2 to 12.4)	-44.3 (-51.2 to -37.0)	-30.2 (-38.9 to -21.1)
Diabetes mellitus	45 (39 to 53)	0.1 (0.1 to 0.2)	55 (48 to 63)	0.2 (0.1 to 0.2)	28 (24 to 32)	0.1 (0.1 to 0.1)	23.2 (1.9 to 44.8)	21.4 (0.3 to 42.6)	-49.6 (-59.2 to -38.4)	-36.9 (-48.9 to -22.8)
Acute glomerulonephritis	12 (7 to 22)	0.0 (0.0 to 0.1)	12 (7 to 20)	0.0 (0.0 to 0.1)	5 (3 to 8)	0.0 (0.0 to 0.0)	4.2 (-53.0 to 101.7)	2.7 (-53.7 to 98.7)	-54.8 (-79.0 to -17.2)	-43.3 (-73.7 to 3.7)
Chronic kidney disease	129 (113 to 150)	0.4 (0.3 to 0.5)	147 (130 to 165)	0.4 (0.4 to 0.5)	77 (66 to 89)	0.3 (0.3 to 0.3)	14.4 (-1.2 to 32.7)	12.7 (-2.6 to 30.8)	-47.6 (-56.8 to -38.2)	-34.3 (-45.8 to -22.5)
Urinary diseases and male infertility	41 (37 to 47)	0.1 (0.1 to 0.1)	50 (45 to 58)	0.2 (0.1 to 0.2)	26 (22 to 31)	0.1 (0.1 to 0.1)	22.0 (4.7 to 41.5)	20.2 (3.2 to 39.4)	-48.1 (-58.1 to -36.5)	-35.0 (-47.5 to -20.4)

eTable 5: Number of deaths and rates (per 100,000) with percent change between 1990 and 2000 and between 2000 and 2016 in the WHO Euro region, ages 10 to 14, males

	1990		2000		2016		Change from 1990 to 2000		Change from 2000 to 2016	
	Count	Rate	Count	Rate	Count	Rate	Count	Rate	Count	Rate
Hemoglobinopathies and hemolytic anemias	107<br>(86 to 135)	0.3<br>(0.3 to 0.4)	102<br>(79 to 131)	0.3<br>(0.2 to 0.4)	45<br>(34 to 57)	0.2<br>(0.1 to 0.2)	-4.4<br>(-21.3 to 15.6)	-5.8<br>(-22.4 to 13.8)	-55.8<br>(-64.4 to -43.9)	-44.6<br>(-55.5 to -29.7)
Endocrine, metabolic, blood, and immune disorders	210<br>(170 to 279)	0.6<br>(0.5 to 0.9)	203<br>(167 to 265)	0.6<br>(0.5 to 0.8)	137<br>(107 to 176)	0.5<br>(0.4 to 0.7)	-3.1<br>(-16.5 to 13.1)	-4.5<br>(-17.7 to 11.4)	-32.4<br>(-44.4 to -17.1)	-15.3<br>(-30.3 to 3.9)
Musculoskeletal disorders	36<br>(26 to 58)	0.1<br>(0.1 to 0.2)	35<br>(26 to 56)	0.1<br>(0.1 to 0.2)	19<br>(14 to 32)	0.1<br>(0.1 to 0.1)	-2.9<br>(-20.2 to 16.4)	-4.3<br>(-21.4 to 14.6)	-45.0<br>(-60.0 to -26.5)	-31.1<br>(-49.9 to -7.9)
Rheumatoid arthritis	7<br>(5 to 10)	0.0<br>(0.0 to 0.0)	5<br>(3 to 8)	0.0<br>(0.0 to 0.0)	2<br>(2 to 4)	0.0<br>(0.0 to 0.0)	-30.9<br>(-51.8 to -0.7)	-31.9<br>(-52.5 to -2.2)	-47.3<br>(-65.9 to -21.6)	-34.0<br>(-57.3 to -1.7)
Other musculoskeletal disorders	29<br>(20 to 50)	0.1<br>(0.1 to 0.2)	30<br>(22 to 49)	0.1<br>(0.1 to 0.1)	17<br>(12 to 28)	0.1<br>(0.0 to 0.1)	4.5<br>(-15.3 to 28.2)	2.9<br>(-16.6 to 26.3)	-44.5<br>(-60.4 to -25.8)	-30.5<br>(-50.4 to -7.1)
Other non-communicable diseases	695<br>(518 to 820)	2.1<br>(1.6 to 2.5)	657<br>(563 to 767)	2.0<br>(1.7 to 2.3)	366<br>(306 to 442)	1.4<br>(1.2 to 1.7)	-5.0<br>(-16.9 to 9.4)	-6.4<br>(-18.1 to 7.8)	-44.0<br>(-54.6 to -32.0)	-29.8<br>(-43.1 to -14.8)
Skin and subcutaneous diseases	6<br>(4 to 8)	0.0<br>(0.0 to 0.0)	7<br>(4 to 9)	0.0<br>(0.0 to 0.0)	5<br>(3 to 7)	0.0<br>(0.0 to 0.0)	22.3<br>(-5.1 to 50.5)	20.5<br>(-6.6 to 48.3)	-24.3<br>(-40.3 to -7.9)	-5.2<br>(-25.3 to 15.4)
Communicable, maternal, neonatal, and nutritional diseases	1,612<br>(1,439 to 1,784)	5.0<br>(4.4 to 5.5)	1,800<br>(1,653 to 1,940)	5.5<br>(5.0 to 5.9)	695<br>(618 to 785)	2.6<br>(2.4 to 3.0)	11.9<br>(2.5 to 21.2)	10.2<br>(1.0 to 19.4)	-61.4<br>(-66.0 to -56.2)	-51.6<br>(-57.4 to -45.2)
HIV/AIDS and tuberculosis	93<br>(72 to 123)	0.3<br>(0.2 to 0.4)	228<br>(204 to 258)	0.7<br>(0.6 to 0.8)	27<br>(24 to 31)	0.1<br>(0.1 to 0.1)	150.6<br>(84.6 to 218.6)	146.9<br>(81.9 to 213.9)	-88.1<br>(-89.9 to -86.0)	-85.0<br>(-87.3 to -82.5)
Diarrhea, lower respiratory, and other common infectious diseases	1,325<br>(1,156 to 1,489)	4.1<br>(3.6 to 4.6)	1,385<br>(1,234 to 1,524)	4.2<br>(3.8 to 4.6)	585<br>(514 to 676)	2.2<br>(2.0 to 2.6)	4.7<br>(-5.8 to 14.9)	3.1<br>(-7.2 to 13.2)	-57.7<br>(-63.9 to -50.8)	-47.0<br>(-54.7 to -38.4)
Diarrheal diseases	56<br>(39 to 79)	0.2<br>(0.1 to 0.2)	55<br>(35 to 81)	0.2<br>(0.1 to 0.2)	21<br>(13 to 33)	0.1<br>(0.0 to 0.1)	-2.7<br>(-22.6 to 24.8)	-4.1<br>(-23.8 to 23.0)	-61.1<br>(-74.2 to -44.4)	-51.3<br>(-67.7 to -30.3)
**Intestinal infectious diseases**	**77**<br>**(35 to 143)**	**0.2**<br>**(0.1 to 0.4)**	**55**<br>**(25 to 104)**	**0.2**<br>**(0.1 to 0.3)**	**35**<br>**(15 to 67)**	**0.1**<br>**(0.1 to 0.3)**	**-27.6**<br>**(-40.0 to -12.5)**	**-28.7**<br>**(-40.9 to -13.8)**	**-36.7**<br>**(-56.6 to -15.7)**	**-20.7**<br>**(-45.6 to 5.6)**
Lower respiratory infections	734<br>(645 to 823)	2.3<br>(2.0 to 2.5)	883<br>(782 to 986)	2.7<br>(2.4 to 3.0)	366<br>(314 to 431)	1.4<br>(1.2 to 1.6)	20.5<br>(6.3 to 34.9)	18.8<br>(4.7 to 32.9)	-58.5<br>(-65.3 to -50.7)	-48.0<br>(-56.6 to -38.3)
Upper respiratory infections	1<br>(0 to 1)	0.0<br>(0.0 to 0.0)	1<br>(0 to 2)	0.0<br>(0.0 to 0.0)	0<br>(0 to 1)	0.0<br>(0.0 to 0.0)	53.4<br>(-66.5 to 345.4)	51.1<br>(-67.0 to 338.8)	-65.2<br>(-91.9 to -0.7)	-56.4<br>(-89.8 to 24.4)
Otitis media	2<br>(1 to 6)	0.0<br>(0.0 to 0.0)	0<br>(0 to 1)	0.0<br>(0.0 to 0.0)	0<br>(0 to 0)	0.0<br>(0.0 to 0.0)	-81.6<br>(-91.8 to -66.0)	-81.8<br>(-91.9 to -66.5)	-75.8<br>(-91.3 to -60.1)	-69.6<br>(-89.1 to -50.0)
Meningitis	234<br>(162 to 318)	0.7<br>(0.5 to 1.0)	234<br>(157 to 304)	0.7<br>(0.5 to 0.9)	76<br>(57 to 104)	0.3<br>(0.2 to 0.4)	0.6<br>(-17.9 to 18.8)	-0.9<br>(-19.1 to 17.0)	-66.7<br>(-76.7 to -55.3)	-58.3<br>(-70.8 to -44.0)
Encephalitis	120<br>(77 to 169)	0.4<br>(0.2 to 0.5)	134<br>(93 to 199)	0.4<br>(0.3 to 0.6)	82<br>(61 to 135)	0.3<br>(0.2 to 0.5)	12.5<br>(-6.2 to 27.6)	10.9<br>(-7.6 to 25.7)	-38.7<br>(-52.8 to -25.1)	-23.1<br>(-40.8 to -6.2)
Diphtheria	4<br>(1 to 7)	0.0<br>(0.0 to 0.0)	2<br>(1 to 4)	0.0<br>(0.0 to 0.0)	0<br>(0 to 1)	0.0<br>(0.0 to 0.0)	-34.5<br>(-82.1 to 83.0)	-35.5<br>(-82.4 to 80.3)	-79.5<br>(-94.0 to -49.7)	-74.3<br>(-92.5 to -37.0)
Whooping cough	8<br>(2 to 19)	0.0<br>(0.0 to 0.1)	7<br>(2 to 16)	0.0<br>(0.0 to 0.0)	2<br>(1 to 4)	0.0<br>(0.0 to 0.0)	20.9<br>(-75.9 to 260.6)	19.1<br>(-76.3 to 255.2)	-72.8<br>(-94.3 to -21.9)	-65.9<br>(-92.8 to -2.1)
Tetanus	1<br>(1 to 2)	0.0<br>(0.0 to 0.0)	1<br>(1 to 1)	0.0<br>(0.0 to 0.0)	1<br>(0 to 1)	0.0<br>(0.0 to 0.0)	-36.1<br>(-57.9 to -1.8)	-37.0<br>(-58.5 to -3.2)	-36.1<br>(-54.2 to -11.3)	-20.0<br>(-42.6 to 11.1)
Measles	84<br>(36 to 178)	0.3<br>(0.1 to 0.6)	8<br>(4 to 17)	0.0<br>(0.0 to 0.1)	1<br>(1 to 2)	0.0<br>(0.0 to 0.0)	-90.1<br>(-92.6 to -87.0)	-90.2<br>(-92.7 to -87.2)	-88.1<br>(-92.8 to -80.1)	-85.1<br>(-91.0 to -75.0)
Varicella and herpes zoster	4<br>(2 to 9)	0.0<br>(0.0 to 0.0)	3<br>(2 to 5)	0.0<br>(0.0 to 0.0)	1<br>(1 to 2)	0.0<br>(0.0 to 0.0)	-23.2<br>(-70.4 to 50.4)	-24.3<br>(-70.8 to 48.1)	-56.1<br>(-79.7 to -25.5)	-45.0<br>(-74.6 to -6.6)
Neglected tropical diseases and malaria	15<br>(10 to 23)	0.0<br>(0.0 to 0.1)	20<br>(11 to 37)	0.1<br>(0.0 to 0.1)	10<br>(5 to 23)	0.0<br>(0.0 to 0.1)	35.6<br>(-20.8 to 146.2)	33.6<br>(-21.9 to 142.6)	-47.6<br>(-78.4 to 19.6)	-34.4<br>(-73.0 to 49.8)
Nutritional deficiencies	32<br>(25 to 44)	0.1<br>(0.1 to 0.1)	35<br>(28 to 44)	0.1<br>(0.1 to 0.1)	13<br>(10 to 16)	0.0<br>(0.0 to 0.1)	9.5<br>(-17.2 to 39.8)	7.9<br>(-18.4 to 37.7)	-63.2<br>(-74.1 to -50.5)	-53.9<br>(-67.5 to -38.0)
Other communicable, maternal, neonatal, and nutritional diseases	147<br>(121 to 181)	0.5<br>(0.4 to 0.6)	133<br>(105 to 169)	0.4<br>(0.3 to 0.5)	61<br>(42 to 85)	0.2<br>(0.2 to 0.3)	-9.0<br>(-28.8 to 14.4)	-10.4<br>(-29.8 to 12.7)	-54.0<br>(-67.4 to -32.2)	-42.3<br>(-59.2 to -15.1)

eTable 6: Number of deaths and rates (per 100,000) with percent change between 1990 and 2000 and between 2000 and 2016 in the WHO Euro region, ages 10 to 14, females

	1990		2000		2016		Change from 1990 to 2000		Change from 2000 to 2016	
	Count	Rate	Count	Rate	Count	Rate	Count	Rate	Count	Rate
**All causes**	**8,826 (8,662 to 9,010)**	**28.3 (27.7 to 28.9)**	**8,811 (8,617 to 8,998)**	**27.9 (27.3 to 28.5)**	**3,937 (3,685 to 4,220)**	**15.8 (14.8 to 16.9)**	**-0.2 (-3.4 to 2.9)**	**-1.2 (-4.4 to 1.9)**	**-55.3 (-58.3 to -51.9)**	**-43.5 (-47.3 to -39.2)**
**Injuries**	**3,535 (3,374 to 3,710)**	**11.3 (10.8 to 11.9)**	**3,533 (3,351 to 3,688)**	**11.2 (10.6 to 11.7)**	**1,415 (1,279 to 1,566)**	**5.7 (5.1 to 6.3)**	**-0.0 (-5.7 to 5.8)**	**-1.0 (-6.7 to 4.7)**	**-59.9 (-64.1 to -55.2)**	**-49.4 (-54.6 to -43.4)**
Transport injuries	1,445 (1,336 to 1,568)	4.6 (4.3 to 5.0)	1,207 (1,107 to 1,312)	3.8 (3.5 to 4.2)	507 (435 to 576)	2.0 (1.7 to 2.3)	-16.4 (-24.7 to -7.4)	-17.2 (-25.5 to -8.4)	-57.9 (-64.3 to -50.8)	-46.8 (-54.9 to -37.8)
Road injuries	1,367 (1,264 to 1,481)	4.4 (4.0 to 4.7)	1,124 (1,030 to 1,229)	3.6 (3.3 to 3.9)	470 (403 to 535)	1.9 (1.6 to 2.1)	-17.7 (-25.9 to -9.0)	-18.5 (-26.7 to -9.9)	-58.1 (-64.4 to -50.9)	-47.0 (-55.0 to -37.9)
Other transport injuries	78 (65 to 96)	0.3 (0.2 to 0.3)	83 (70 to 101)	0.3 (0.2 to 0.3)	37 (29 to 47)	0.1 (0.1 to 0.2)	7.1 (-14.5 to 33.2)	6.1 (-15.3 to 31.8)	-54.9 (-67.2 to -39.8)	-43.0 (-58.6 to -23.9)
Unintentional injuries	1,630 (1,527 to 1,730)	5.2 (4.9 to 5.5)	1,633 (1,527 to 1,732)	5.2 (4.8 to 5.5)	572 (508 to 646)	2.3 (2.0 to 2.6)	0.2 (-7.4 to 8.0)	-0.8 (-8.4 to 6.9)	-65.0 (-69.0 to -59.8)	-55.7 (-60.9 to -49.2)
Falls	156 (111 to 188)	0.5 (0.4 to 0.6)	146 (109 to 174)	0.5 (0.3 to 0.6)	56 (43 to 69)	0.2 (0.2 to 0.3)	-5.7 (-20.5 to 10.2)	-6.7 (-21.3 to 9.1)	-61.4 (-69.6 to -52.3)	-51.2 (-61.6 to -39.8)
Drowning	745 (676 to 815)	2.4 (2.2 to 2.6)	780 (702 to 857)	2.5 (2.2 to 2.7)	232 (199 to 276)	0.9 (0.8 to 1.1)	4.9 (-7.5 to 18.0)	3.8 (-8.5 to 16.8)	-70.1 (-75.3 to -62.3)	-62.3 (-68.8 to -52.4)
Fire, heat, and hot substances	135 (115 to 166)	0.4 (0.4 to 0.5)	139 (110 to 164)	0.4 (0.3 to 0.5)	51 (40 to 65)	0.2 (0.2 to 0.3)	3.4 (-10.3 to 19.6)	2.3 (-11.2 to 18.4)	-63.3 (-70.8 to -53.5)	-53.7 (-63.2 to -41.2)
Poisonings	129 (83 to 155)	0.4 (0.3 to 0.5)	127 (78 to 156)	0.4 (0.2 to 0.5)	47 (29 to 63)	0.2 (0.1 to 0.3)	-1.2 (-16.4 to 18.6)	-2.2 (-17.3 to 17.4)	-62.3 (-72.3 to -49.5)	-52.4 (-65.1 to -36.2)
Exposure to mechanical forces	134 (116 to 176)	0.4 (0.4 to 0.6)	141 (118 to 185)	0.4 (0.4 to 0.6)	65 (53 to 86)	0.3 (0.2 to 0.3)	5.4 (-9.8 to 20.2)	4.3 (-10.8 to 19.0)	-53.6 (-63.5 to -42.2)	-41.3 (-53.9 to -26.9)
Adverse effects of medical treatment	49 (38 to 65)	0.2 (0.1 to 0.2)	43 (35 to 61)	0.1 (0.1 to 0.2)	22 (17 to 30)	0.1 (0.1 to 0.1)	-11.7 (-23.7 to -2.0)	-12.6 (-24.5 to -3.0)	-49.4 (-55.7 to -41.6)	-36.0 (-44.0 to -26.2)
Animal contact	20 (11 to 30)	0.1 (0.0 to 0.1)	18 (11 to 24)	0.1 (0.0 to 0.1)	7 (6 to 9)	0.0 (0.0 to 0.0)	-8.9 (-38.3 to 18.9)	-9.8 (-38.9 to 17.7)	-58.8 (-71.7 to -39.6)	-47.9 (-64.3 to -23.7)
Foreign body	91 (72 to 112)	0.3 (0.2 to 0.4)	95 (78 to 124)	0.3 (0.2 to 0.4)	55 (46 to 71)	0.2 (0.2 to 0.3)	5.2 (-9.2 to 18.3)	4.1 (-10.1 to 17.1)	-42.2 (-52.3 to -30.1)	-27.0 (-39.8 to -11.7)
Environmental heat and cold exposure	27 (14 to 37)	0.1 (0.0 to 0.1)	25 (14 to 35)	0.1 (0.0 to 0.1)	8 (5 to 12)	0.0 (0.0 to 0.0)	-6.7 (-28.4 to 16.4)	-7.7 (-29.1 to 15.2)	-66.3 (-75.3 to -57.1)	-57.4 (-68.8 to -45.8)
Other unintentional injuries	146 (108 to 200)	0.5 (0.3 to 0.6)	120 (94 to 148)	0.4 (0.3 to 0.5)	28 (23 to 35)	0.1 (0.1 to 0.1)	-16.8 (-39.5 to 6.3)	-17.7 (-40.1 to 5.2)	-76.0 (-82.5 to -67.3)	-69.6 (-77.9 to -58.7)
Self-harm and interpersonal violence	427 (382 to 524)	1.4 (1.2 to 1.7)	555 (451 to 621)	1.8 (1.4 to 2.0)	285 (240 to 345)	1.1 (1.0 to 1.4)	30.5 (6.2 to 50.0)	29.2 (5.1 to 48.5)	-48.3 (-58.1 to -35.3)	-34.7 (-47.0 to -18.3)
Self-harm	233 (211 to 261)	0.7 (0.7 to 0.8)	295 (259 to 335)	0.9 (0.8 to 1.1)	178 (148 to 213)	0.7 (0.6 to 0.9)	26.9 (9.3 to 45.9)	25.6 (8.2 to 44.4)	-39.4 (-51.5 to -25.3)	-23.4 (-38.8 to -5.7)
Interpersonal violence	194 (154 to 287)	0.6 (0.5 to 0.9)	260 (162 to 317)	0.8 (0.5 to 1.0)	107 (75 to 155)	0.4 (0.3 to 0.6)	35.7 (-4.3 to 72.2)	34.3 (-5.2 to 70.4)	-58.1 (-69.3 to -32.6)	-47.0 (-61.2 to -14.9)
Non-communicable diseases	3,852 (3,694 to 4,063)	12.3 (11.8 to 13.0)	3,745 (3,597 to 3,941)	11.9 (11.4 to 12.5)	1,970 (1,840 to 2,120)	7.9 (7.4 to 8.5)	-2.8 (-7.1 to 1.5)	-3.7 (-8.0 to 0.5)	-47.4 (-51.4 to -42.9)	-33.5 (-38.6 to -27.9)
Neoplasms	1,573 (1,468 to 1,685)	5.0 (4.7 to 5.4)	1,471 (1,384 to 1,578)	4.7 (4.4 to 5.0)	813 (722 to 890)	3.3 (2.9 to 3.6)	-6.4 (-13.3 to -0.2)	-7.4 (-14.1 to -1.2)	-44.7 (-51.3 to -38.2)	-30.1 (-38.4 to -21.9)
Nasopharynx cancer	6 (6 to 7)	0.0 (0.0 to 0.0)	7 (6 to 8)	0.0 (0.0 to 0.0)	4 (4 to 5)	0.0 (0.0 to 0.0)	9.1 (-3.3 to 22.6)	8.0 (-4.3 to 21.4)	-40.1 (-48.6 to -29.4)	-24.3 (-35.1 to -10.8)
**Liver cancer**	**17 (14 to 21)**	**0.1 (0.0 to 0.1)**	**18 (15 to 22)**	**0.1 (0.0 to 0.1)**	**11 (9 to 13)**	**0.0 (0.0 to 0.1)**	**4.6 (-14.8 to 24.0)**	**3.5 (-15.7 to 22.7)**	**-40.0 (-53.3 to -23.3)**	**-24.2 (-41.0 to -3.1)**
Kidney cancer	25 (22 to 29)	0.1 (0.1 to 0.1)	25 (22 to 28)	0.1 (0.1 to 0.1)	18 (15 to 21)	0.1 (0.1 to 0.1)	-0.7 (-14.1 to 13.9)	-1.7 (-15.0 to 12.7)	-27.8 (-42.6 to -12.2)	-8.8 (-27.5 to 10.9)
Brain and nervous system cancer	369 (326 to 423)	1.2 (1.0 to 1.4)	374 (331 to 423)	1.2 (1.1 to 1.3)	239 (197 to 283)	1.0 (0.8 to 1.1)	1.6 (-10.2 to 14.2)	0.5 (-11.1 to 13.1)	-35.9 (-49.3 to -24.7)	-19.0 (-36.0 to -4.9)
Thyroid cancer	4 (4 to 4)	0.0 (0.0 to 0.0)	4 (4 to 4)	0.0 (0.0 to 0.0)	3 (3 to 3)	0.0 (0.0 to 0.0)	1.6 (-7.9 to 13.4)	0.6 (-8.8 to 12.2)	-30.4 (-38.9 to -22.0)	-12.1 (-22.8 to -1.4)
Hodgkin lymphoma	34 (24 to 45)	0.1 (0.1 to 0.1)	26 (20 to 35)	0.1 (0.1 to 0.1)	9 (7 to 12)	0.0 (0.0 to 0.0)	-21.0 (-42.4 to 10.6)	-21.8 (-43.0 to 9.5)	-63.9 (-74.9 to -49.1)	-54.4 (-68.3 to -35.7)
Non-Hodgkin lymphoma	89 (72 to 106)	0.3 (0.2 to 0.3)	87 (72 to 103)	0.3 (0.2 to 0.3)	53 (42 to 64)	0.2 (0.2 to 0.3)	-2.2 (-20.0 to 17.0)	-3.2 (-20.8 to 15.8)	-38.0 (-53.2 to -22.3)	-21.7 (-40.8 to -1.9)
Leukemia	686 (597 to 786)	2.2 (1.9 to 2.5)	592 (523 to 667)	1.9 (1.7 to 2.1)	261 (222 to 302)	1.0 (0.9 to 1.2)	-13.4 (-24.5 to -1.2)	-14.3 (-25.2 to -2.2)	-55.7 (-63.3 to -46.1)	-44.1 (-53.7 to -31.9)
Other neoplasms	343 (300 to 404)	1.1 (1.0 to 1.3)	338 (297 to 398)	1.1 (0.9 to 1.3)	214 (182 to 249)	0.9 (0.7 to 1.0)	-1.1 (-14.0 to 12.7)	-2.1 (-14.9 to 11.5)	-36.3 (-50.7 to -22.2)	-19.5 (-37.7 to -1.7)
Cardiovascular diseases	417 (383 to 459)	1.3 (1.2 to 1.5)	381 (352 to 412)	1.2 (1.1 to 1.3)	184 (166 to 207)	0.7 (0.7 to 0.8)	-8.6 (-16.5 to 0.1)	-9.6 (-17.3 to -0.9)	-51.6 (-57.8 to -44.7)	-38.9 (-46.7 to -30.1)
Rheumatic heart disease	81 (72 to 91)	0.3 (0.2 to 0.3)	87 (78 to 98)	0.3 (0.2 to 0.3)	31 (27 to 36)	0.1 (0.1 to 0.1)	8.4 (-4.9 to 22.4)	7.3 (-5.8 to 21.2)	-64.0 (-70.6 to -56.6)	-54.5 (-62.8 to -45.2)
Ischemic heart disease	13 (11 to 16)	0.0 (0.0 to 0.1)	15 (13 to 18)	0.0 (0.0 to 0.1)	8 (7 to 10)	0.0 (0.0 to 0.0)	16.4 (-3.5 to 37.1)	15.3 (-4.5 to 35.8)	-46.4 (-58.1 to -32.4)	-32.3 (-47.0 to -14.6)
Cerebrovascular disease	176 (154 to 204)	0.6 (0.5 to 0.7)	129 (113 to 148)	0.4 (0.4 to 0.5)	53 (45 to 62)	0.2 (0.2 to 0.2)	-26.6 (-35.0 to -16.9)	-27.3 (-35.6 to -17.7)	-58.9 (-67.0 to -50.0)	-48.0 (-58.3 to -36.8)
Hypertensive heart disease	3 (3 to 4)	0.0 (0.0 to 0.0)	3 (3 to 4)	0.0 (0.0 to 0.0)	3 (2 to 3)	0.0 (0.0 to 0.0)	-0.3 (-13.5 to 12.8)	-1.3 (-14.4 to 11.7)	-23.8 (-35.6 to -9.5)	-3.7 (-18.7 to 14.3)
Cardiomyopathy and myocarditis	89 (75 to 112)	0.3 (0.2 to 0.4)	89 (77 to 111)	0.3 (0.2 to 0.4)	50 (41 to 66)	0.2 (0.2 to 0.3)	0.8 (-11.8 to 14.5)	-0.2 (-12.7 to 13.4)	-43.8 (-54.4 to -31.4)	-29.0 (-42.4 to -13.3)
Endocarditis	10 (7 to 15)	0.0 (0.0 to 0.0)	10 (7 to 15)	0.0 (0.0 to 0.0)	8 (6 to 12)	0.0 (0.0 to 0.0)	-1.2 (-22.8 to 21.7)	-2.2 (-23.6 to 20.4)	-20.3 (-41.3 to 4.5)	0.7 (-25.8 to 32.1)
Other cardiovascular and circulatory diseases	44 (34 to 60)	0.1 (0.1 to 0.2)	46 (37 to 57)	0.1 (0.1 to 0.2)	31 (24 to 39)	0.1 (0.1 to 0.2)	5.1 (-17.5 to 27.7)	4.0 (-18.3 to 26.4)	-32.4 (-48.4 to -13.0)	-14.5 (-34.8 to 9.9)
Chronic respiratory diseases	131 (115 to 150)	0.4 (0.4 to 0.5)	109 (97 to 124)	0.3 (0.3 to 0.4)	45 (40 to 50)	0.2 (0.2 to 0.2)	-16.3 (-27.6 to -3.3)	-17.2 (-28.3 to -4.3)	-58.7 (-65.3 to -51.8)	-47.8 (-56.2 to -39.1)
Chronic obstructive pulmonary disease	34 (27 to 44)	0.1 (0.1 to 0.1)	32 (26 to 39)	0.1 (0.1 to 0.1)	13 (11 to 16)	0.1 (0.0 to 0.1)	-5.2 (-23.3 to 14.7)	-6.2 (-24.1 to 13.5)	-59.2 (-68.0 to -50.1)	-48.4 (-59.5 to -37.0)
Asthma	64 (54 to 76)	0.2 (0.2 to 0.2)	49 (41 to 57)	0.2 (0.1 to 0.2)	16 (13 to 19)	0.1 (0.1 to 0.1)	-24.2 (-35.5 to -11.7)	-24.9 (-36.2 to -12.6)	-67.2 (-73.0 to -60.6)	-58.6 (-65.9 to -50.2)
Interstitial lung disease and pulmonary sarcoidosis	9 (6 to 12)	0.0 (0.0 to 0.0)	9 (6 to 11)	0.0 (0.0 to 0.0)	5 (3 to 6)	0.0 (0.0 to 0.0)	0.6 (-23.7 to 26.5)	-0.4 (-24.5 to 25.2)	-43.2 (-60.6 to -23.7)	-28.3 (-50.2 to -3.6)
Other chronic respiratory diseases	24 (14 to 36)	0.1 (0.0 to 0.1)	20 (13 to 30)	0.1 (0.0 to 0.1)	11 (8 to 17)	0.0 (0.0 to 0.1)	-14.8 (-42.3 to 18.8)	-15.7 (-42.9 to 17.6)	-42.1 (-64.7 to -18.9)	-26.8 (-55.3 to 2.4)
Cirrhosis and other chronic liver diseases	113 (98 to 130)	0.4 (0.3 to 0.4)	161 (142 to 181)	0.5 (0.5 to 0.6)	71 (61 to 83)	0.3 (0.2 to 0.3)	42.8 (23.2 to 65.9)	41.4 (21.9 to 64.2)	-55.5 (-63.5 to -46.4)	-43.8 (-53.9 to -32.2)
Cirrhosis and other chronic liver diseases due to hepatitis B	2 (1 to 2)	0.0 (0.0 to 0.0)	3 (2 to 4)	0.0 (0.0 to 0.0)	1 (1 to 2)	0.0 (0.0 to 0.0)	69.8 (44.2 to 103.0)	68.1 (42.8 to 101.0)	-54.8 (-63.9 to -44.5)	-42.9 (-54.3 to -29.8)
Cirrhosis and other chronic liver diseases due to hepatitis C	2 (1 to 2)	0.0 (0.0 to 0.0)	2 (2 to 3)	0.0 (0.0 to 0.0)	1 (1 to 1)	0.0 (0.0 to 0.0)	29.8 (12.3 to 50.8)	28.5 (11.1 to 49.2)	-53.7 (-61.4 to -45.2)	-41.5 (-51.2 to -30.8)
Cirrhosis and other chronic liver diseases due to other causes	110 (95 to 126)	0.4 (0.3 to 0.4)	156 (138 to 175)	0.5 (0.4 to 0.6)	69 (59 to 80)	0.3 (0.2 to 0.3)	42.6 (22.9 to 65.8)	41.2 (21.7 to 64.1)	-55.5 (-63.6 to -46.4)	-43.8 (-54.0 to -32.3)
Digestive diseases	132 (115 to 151)	0.4 (0.4 to 0.5)	116 (102 to 132)	0.4 (0.3 to 0.4)	50 (43 to 59)	0.2 (0.2 to 0.2)	-11.4 (-23.9 to 0.9)	-12.3 (-24.7 to -0.1)	-56.7 (-63.1 to -46.8)	-45.3 (-53.4 to -32.8)
Peptic ulcer disease	17 (13 to 25)	0.1 (0.0 to 0.1)	18 (14 to 22)	0.1 (0.0 to 0.1)	6 (5 to 7)	0.0 (0.0 to 0.0)	4.4 (-28.5 to 37.9)	3.4 (-29.2 to 36.5)	-66.6 (-75.1 to -55.1)	-57.8 (-68.6 to -43.2)
Gastritis and duodenitis	1 (1 to 2)	0.0 (0.0 to 0.0)	1 (1 to 1)	0.0 (0.0 to 0.0)	1 (0 to 1)	0.0 (0.0 to 0.0)	-14.3 (-38.4 to 13.6)	-15.1 (-39.1 to 12.4)	-39.3 (-56.7 to -12.8)	-23.3 (-45.3 to 10.2)
Appendicitis	43 (27 to 54)	0.1 (0.1 to 0.2)	33 (26 to 43)	0.1 (0.1 to 0.1)	13 (10 to 18)	0.1 (0.0 to 0.1)	-22.8 (-41.1 to 10.3)	-23.6 (-41.7 to 9.2)	-61.6 (-69.6 to -51.0)	-51.4 (-61.6 to -38.0)
Paralytic ileus and intestinal obstruction	25 (20 to 34)	0.1 (0.1 to 0.1)	24 (19 to 32)	0.1 (0.1 to 0.1)	13 (10 to 17)	0.1 (0.0 to 0.1)	-2.5 (-20.7 to 17.5)	-3.5 (-21.6 to 16.3)	-48.6 (-58.2 to -34.9)	-35.1 (-47.2 to -17.7)
Inguinal, femoral, and abdominal hernia	5 (4 to 9)	0.0 (0.0 to 0.0)	5 (3 to 8)	0.0 (0.0 to 0.0)	2 (1 to 3)	0.0 (0.0 to 0.0)	-10.6 (-30.7 to 10.9)	-11.5 (-31.4 to 9.8)	-62.7 (-71.6 to -51.5)	-52.8 (-64.1 to -38.7)
Inflammatory bowel disease	11 (8 to 16)	0.0 (0.0 to 0.1)	10 (8 to 14)	0.0 (0.0 to 0.0)	5 (4 to 7)	0.0 (0.0 to 0.0)	-3.0 (-25.7 to 28.5)	-4.0 (-26.4 to 27.2)	-47.4 (-63.3 to -26.8)	-33.6 (-53.7 to -7.5)
Vascular intestinal disorders	2 (2 to 3)	0.0 (0.0 to 0.0)	2 (1 to 2)	0.0 (0.0 to 0.0)	1 (1 to 1)	0.0 (0.0 to 0.0)	-21.3 (-42.7 to 5.7)	-22.1 (-43.3 to 4.7)	-47.0 (-62.9 to -23.0)	-33.0 (-53.1 to -2.7)
Gallbladder and biliary diseases	3 (2 to 5)	0.0 (0.0 to 0.0)	2 (2 to 4)	0.0 (0.0 to 0.0)	1 (1 to 1)	0.0 (0.0 to 0.0)	-20.3 (-34.6 to -3.6)	-21.1 (-35.2 to -4.5)	-61.8 (-70.2 to -50.8)	-51.7 (-62.4 to -37.9)
Pancreatitis	6 (4 to 10)	0.0 (0.0 to 0.0)	5 (3 to 8)	0.0 (0.0 to 0.0)	2 (1 to 4)	0.0 (0.0 to 0.0)	-15.1 (-41.5 to 21.9)	-16.0 (-42.1 to 20.7)	-54.8 (-69.7 to -28.9)	-42.9 (-61.7 to -10.1)
Other digestive diseases	17 (12 to 23)	0.1 (0.0 to 0.1)	16 (12 to 21)	0.1 (0.0 to 0.1)	7 (6 to 10)	0.0 (0.0 to 0.0)	-2.6 (-29.5 to 25.4)	-3.6 (-30.2 to 24.1)	-52.6 (-67.8 to -32.6)	-40.1 (-59.3 to -14.8)
Neurological disorders	323 (282 to 385)	1.0 (0.9 to 1.2)	353 (312 to 398)	1.1 (1.0 to 1.3)	197 (174 to 224)	0.8 (0.7 to 0.9)	9.4 (-3.4 to 23.5)	8.3 (-4.4 to 22.3)	-44.0 (-52.9 to -31.9)	-29.2 (-40.4 to -14.0)
Epilepsy	230 (193 to 286)	0.7 (0.6 to 0.9)	261 (223 to 301)	0.8 (0.7 to 1.0)	135 (115 to 161)	0.5 (0.5 to 0.6)	14.0 (-3.6 to 34.1)	12.9 (-4.5 to 32.7)	-47.9 (-58.4 to -34.1)	-34.2 (-47.5 to -16.7)
Motor neuron disease	10 (8 to 12)	0.0 (0.0 to 0.0)	9 (7 to 11)	0.0 (0.0 to 0.0)	7 (6 to 8)	0.0 (0.0 to 0.0)	-5.7 (-23.1 to 15.1)	-6.7 (-23.9 to 14.0)	-22.3 (-40.1 to -2.6)	-1.8 (-24.3 to 23.0)
Other neurological disorders	83 (72 to 97)	0.3 (0.2 to 0.3)	82 (71 to 97)	0.3 (0.2 to 0.3)	55 (47 to 63)	0.2 (0.2 to 0.3)	-0.8 (-14.8 to 16.9)	-1.8 (-15.7 to 15.7)	-33.1 (-46.3 to -15.5)	-15.5 (-32.1 to 6.7)
Mental and substance use disorders	1 (0 to 1)	0.0 (0.0 to 0.0)	1 (1 to 1)	0.0 (0.0 to 0.0)	1 (0 to 1)	0.0 (0.0 to 0.0)	-1.4 (-28.6 to 30.1)	-2.4 (-29.4 to 28.8)	-6.4 (-32.2 to 25.4)	18.3 (-14.4 to 58.5)
Eating disorders	1 (0 to 1)	0.0 (0.0 to 0.0)	1 (1 to 1)	0.0 (0.0 to 0.0)	1 (0 to 1)	0.0 (0.0 to 0.0)	-1.4 (-28.6 to 30.1)	-2.4 (-29.4 to 28.8)	-6.4 (-32.2 to 25.4)	18.3 (-14.4 to 58.5)
Diabetes, urogenital, blood, and endocrine diseases	492 (448 to 559)	1.6 (1.4 to 1.8)	525 (483 to 580)	1.7 (1.5 to 1.8)	279 (253 to 312)	1.1 (1.0 to 1.3)	6.9 (-2.1 to 16.6)	5.8 (-3.1 to 15.4)	-46.7 (-52.3 to -40.7)	-32.7 (-39.8 to -25.1)
Diabetes mellitus	70 (60 to 85)	0.2 (0.2 to 0.3)	88 (78 to 101)	0.3 (0.2 to 0.3)	35 (30 to 41)	0.1 (0.1 to 0.2)	27.2 (4.1 to 50.7)	25.9 (3.1 to 49.2)	-60.2 (-67.4 to -52.3)	-49.7 (-58.8 to -39.7)
Acute glomerulonephritis	15 (8 to 28)	0.0 (0.0 to 0.1)	13 (8 to 22)	0.0 (0.0 to 0.1)	5 (4 to 9)	0.0 (0.0 to 0.0)	-4.9 (-59.8 to 83.9)	-5.9 (-60.2 to 82.0)	-53.3 (-78.9 to -10.3)	-41.0 (-73.3 to 13.3)
Chronic kidney disease	122 (107 to 141)	0.4 (0.3 to 0.5)	146 (130 to 165)	0.5 (0.4 to 0.5)	77 (67 to 88)	0.3 (0.3 to 0.4)	20.1 (4.1 to 37.3)	18.9 (3.1 to 35.9)	-47.1 (-56.0 to -37.7)	-33.2 (-44.4 to -21.3)
Urinary diseases and male infertility	40 (36 to 45)	0.1 (0.1 to 0.1)	49 (43 to 55)	0.2 (0.1 to 0.2)	24 (20 to 28)	0.1 (0.1 to 0.1)	20.8 (3.7 to 39.9)	19.5 (2.6 to 38.5)	-50.7 (-60.3 to -40.4)	-37.7 (-49.8 to -24.7)

eTable 6: Number of deaths and rates (per 100,000) with percent change between 1990 and 2000 and between 2000 and 2016 in the WHO Euro region, ages 10 to 14, females

	1990		2000		2016		Change from 1990 to 2000		Change from 2000 to 2016	
	**Count**	**Rate**	**Count**	**Rate**	**Count**	**Rate**	**Count**	**Rate**	**Count**	**Rate**
Hemoglobinopathies and hemolytic anemias	84<br>(61 to 110)	0.3<br>(0.2 to 0.4)	79<br>(57 to 104)	0.3<br>(0.2 to 0.3)	39<br>(31 to 55)	0.2<br>(0.1 to 0.2)	-5.3<br>(-22.7 to 15.5)	-6.2<br>(-23.5 to 14.3)	-50.9<br>(-62.0 to -38.9)	-38.0<br>(-52.0 to -22.8)
Endocrine, metabolic, blood, and immune disorders	161<br>(127 to 214)	0.5<br>(0.4 to 0.7)	150<br>(121 to 186)	0.5<br>(0.4 to 0.6)	99<br>(78 to 122)	0.4<br>(0.3 to 0.5)	-6.2<br>(-20.8 to 10.7)	-7.1<br>(-21.6 to 9.6)	-33.2<br>(-46.2 to -16.1)	-15.6<br>(-32.1 to 6.0)
Musculoskeletal disorders	51<br>(40 to 79)	0.2<br>(0.1 to 0.3)	51<br>(40 to 76)	0.2<br>(0.1 to 0.2)	29<br>(23 to 42)	0.1<br>(0.1 to 0.2)	0.7<br>(-15.8 to 20.1)	-0.3<br>(-16.7 to 18.9)	-43.0<br>(-55.8 to -28.4)	-28.0<br>(-44.2 to -9.5)
Rheumatoid arthritis	11<br>(7 to 15)	0.0<br>(0.0 to 0.0)	6<br>(5 to 11)	0.0<br>(0.0 to 0.0)	3<br>(2 to 5)	0.0<br>(0.0 to 0.0)	-40.2<br>(-57.1 to -6.9)	-40.8<br>(-57.5 to -7.8)	-54.6<br>(-68.3 to -36.2)	-42.7<br>(-59.9 to -19.4)
Other musculoskeletal disorders	40<br>(30 to 67)	0.1<br>(0.1 to 0.2)	44<br>(35 to 68)	0.1<br>(0.1 to 0.2)	26<br>(21 to 39)	0.1<br>(0.1 to 0.2)	13.0<br>(-8.6 to 38.2)	11.9<br>(-9.5 to 36.8)	-41.3<br>(-54.6 to -25.7)	-25.8<br>(-42.7 to -6.1)
Other non-communicable diseases	620<br>(476 to 721)	2.0<br>(1.5 to 2.3)	579<br>(479 to 702)	1.8<br>(1.5 to 2.2)	302<br>(260 to 384)	1.2<br>(1.0 to 1.5)	-6.5<br>(-16.3 to 5.2)	-7.4<br>(-17.1 to 4.1)	-47.6<br>(-55.7 to -37.4)	-33.9<br>(-44.0 to -20.9)
Skin and subcutaneous diseases	6<br>(4 to 9)	0.0<br>(0.0 to 0.0)	7<br>(4 to 10)	0.0<br>(0.0 to 0.0)	5<br>(3 to 8)	0.0<br>(0.0 to 0.0)	14.9<br>(-1.4 to 48.2)	13.7<br>(-2.4 to 46.7)	-23.3<br>(-38.6 to -6.3)	-3.1<br>(-22.5 to 18.3)
Communicable, maternal, neonatal, and nutritional diseases	1,439<br>(1,282 to 1,586)	4.6<br>(4.1 to 5.1)	1,533<br>(1,421 to 1,642)	4.9<br>(4.5 to 5.2)	552<br>(497 to 613)	2.2<br>(2.0 to 2.5)	6.7<br>(-2.1 to 15.4)	5.6<br>(-3.1 to 14.3)	-64.0<br>(-67.9 to -59.6)	-54.5<br>(-59.4 to -49.0)
HIV/AIDS and tuberculosis	76<br>(56 to 109)	0.2<br>(0.2 to 0.3)	192<br>(174 to 214)	0.6<br>(0.6 to 0.7)	35<br>(31 to 39)	0.1<br>(0.1 to 0.2)	158.3<br>(76.9 to 240.0)	155.6<br>(75.1 to 236.6)	-81.9<br>(-84.8 to -78.6)	-77.1<br>(-80.8 to -72.9)
Diarrhea, lower respiratory, and other common infectious diseases	1,190<br>(1,053 to 1,327)	3.8<br>(3.4 to 4.2)	1,195<br>(1,092 to 1,294)	3.8<br>(3.5 to 4.1)	457<br>(408 to 514)	1.8<br>(1.6 to 2.1)	0.6<br>(-9.3 to 10.0)	-0.4<br>(-10.2 to 8.9)	-61.7<br>(-66.5 to -56.1)	-51.6<br>(-57.7 to -44.6)
Diarrheal diseases	47<br>(31 to 74)	0.2<br>(0.1 to 0.2)	50<br>(32 to 76)	0.2<br>(0.1 to 0.2)	17<br>(11 to 28)	0.1<br>(0.0 to 0.1)	6.8<br>(-13.7 to 30.6)	5.7<br>(-14.6 to 29.2)	-66.6<br>(-78.3 to -52.7)	-57.8<br>(-72.6 to -40.3)
**Intestinal infectious diseases**	**69**<br>**(31 to 126)**	**0.2**<br>**(0.1 to 0.4)**	**48**<br>**(22 to 92)**	**0.2**<br>**(0.1 to 0.3)**	**29**<br>**(13 to 54)**	**0.1**<br>**(0.1 to 0.2)**	**-29.1**<br>**(-41.2 to -15.1)**	**-29.8**<br>**(-41.8 to -16.0)**	**-40.0**<br>**(-56.0 to -21.5)**	**-24.2**<br>**(-44.4 to -0.8)**
Lower respiratory infections	634<br>(565 to 709)	2.0<br>(1.8 to 2.3)	760<br>(685 to 836)	2.4<br>(2.2 to 2.6)	297<br>(256 to 341)	1.2<br>(1.0 to 1.4)	20.2<br>(6.1 to 34.6)	19.0<br>(5.1 to 33.2)	-60.9<br>(-67.0 to -53.9)	-50.6<br>(-58.3 to -41.7)
Upper respiratory infections	0<br>(0 to 1)	0.0<br>(0.0 to 0.0)	0<br>(0 to 1)	0.0<br>(0.0 to 0.0)	0<br>(0 to 0)	0.0<br>(0.0 to 0.0)	52.2<br>(-59.1 to 299.1)	50.6<br>(-59.6 to 295.1)	-63.3<br>(-90.7 to 2.7)	-53.7<br>(-88.3 to 29.8)
Otitis media	3<br>(1 to 10)	0.0<br>(0.0 to 0.0)	1<br>(0 to 1)	0.0<br>(0.0 to 0.0)	0<br>(0 to 0)	0.0<br>(0.0 to 0.0)	-79.6<br>(-95.0 to -48.9)	-79.8<br>(-95.0 to -49.5)	-85.5<br>(-95.4 to -72.0)	-81.7<br>(-94.1 to -64.6)
Meningitis	237<br>(169 to 299)	0.8<br>(0.5 to 1.0)	212<br>(154 to 255)	0.7<br>(0.5 to 0.8)	55<br>(47 to 65)	0.2<br>(0.2 to 0.3)	-9.9<br>(-27.7 to 7.7)	-10.8<br>(-28.4 to 6.6)	-73.5<br>(-79.5 to -64.4)	-66.5<br>(-74.1 to -55.1)
Encephalitis	90<br>(54 to 114)	0.3<br>(0.2 to 0.4)	101<br>(62 to 127)	0.3<br>(0.2 to 0.4)	55<br>(40 to 71)	0.2<br>(0.2 to 0.3)	12.3<br>(-3.3 to 28.1)	11.2<br>(-4.2 to 26.8)	-44.1<br>(-57.6 to -27.5)	-29.3<br>(-46.4 to -8.4)
Diphtheria	2<br>(1 to 4)	0.0<br>(0.0 to 0.0)	1<br>(0 to 3)	0.0<br>(0.0 to 0.0)	0<br>(0 to 0)	0.0<br>(0.0 to 0.0)	-31.9<br>(-84.1 to 90.5)	-32.6<br>(-84.3 to 88.6)	-79.3<br>(-94.0 to -49.2)	-73.9<br>(-92.4 to -35.9)
Whooping cough	9<br>(3 to 24)	0.0<br>(0.0 to 0.1)	9<br>(3 to 19)	0.0<br>(0.0 to 0.1)	2<br>(1 to 4)	0.0<br>(0.0 to 0.0)	18.1<br>(-77.5 to 263.0)	16.9<br>(-77.7 to 259.4)	-75.0<br>(-95.2 to -23.6)	-68.4<br>(-93.9 to -3.5)
Tetanus	1<br>(1 to 1)	0.0<br>(0.0 to 0.0)	1<br>(1 to 1)	0.0<br>(0.0 to 0.0)	0<br>(0 to 1)	0.0<br>(0.0 to 0.0)	-18.0<br>(-46.5 to -0.9)	-18.8<br>(-47.0 to -1.9)	-30.1<br>(-43.5 to -14.5)	-11.7<br>(-28.6 to 8.0)
Measles	92<br>(39 to 194)	0.3<br>(0.1 to 0.6)	9<br>(3 to 19)	0.0<br>(0.0 to 0.1)	1<br>(0 to 1)	0.0<br>(0.0 to 0.0)	-90.6<br>(-92.8 to -87.7)	-90.7<br>(-92.9 to -87.9)	-90.1<br>(-94.0 to -83.8)	-87.5<br>(-92.4 to -79.5)
Varicella and herpes zoster	5<br>(2 to 9)	0.0<br>(0.0 to 0.0)	4<br>(2 to 6)	0.0<br>(0.0 to 0.0)	2<br>(1 to 3)	0.0<br>(0.0 to 0.0)	-20.2<br>(-65.3 to 45.7)	-21.0<br>(-65.7 to 44.2)	-54.9<br>(-76.8 to -29.0)	-43.1<br>(-70.7 to -10.3)
Neglected tropical diseases and malaria	9<br>(6 to 16)	0.0<br>(0.0 to 0.1)	13<br>(8 to 21)	0.0<br>(0.0 to 0.1)	6<br>(3 to 12)	0.0<br>(0.0 to 0.0)	50.3<br>(-15.3 to 164.9)	48.8<br>(-16.2 to 162.2)	-54.5<br>(-77.1 to 7.3)	-42.6<br>(-71.0 to 35.6)
Nutritional deficiencies	34<br>(25 to 48)	0.1<br>(0.1 to 0.2)	30<br>(24 to 40)	0.1<br>(0.1 to 0.1)	11<br>(9 to 14)	0.0<br>(0.0 to 0.1)	-9.3<br>(-36.5 to 23.1)	-10.2<br>(-37.1 to 21.8)	-63.6<br>(-75.0 to -49.0)	-54.0<br>(-68.4 to -35.6)
Other communicable, maternal, neonatal, and nutritional diseases	109<br>(89 to 134)	0.4<br>(0.3 to 0.4)	92<br>(72 to 112)	0.3<br>(0.2 to 0.4)	39<br>(28 to 51)	0.2<br>(0.1 to 0.2)	-15.8<br>(-31.9 to 3.1)	-16.7<br>(-32.6 to 2.1)	-56.8<br>(-67.2 to -43.8)	-45.4<br>(-58.6 to -29.1)

	Endocrine, metabolic, blood, and immune disorders	Interpersonal violence	Falls	Fire, heat, and hot substances	Encephalitis	Foreign body	Non-Hodgkin lymphoma	Meningitis	Other neurological disorders	Chronic kidney disease
**Global**	**0·3 (0·3 to 0·4)**	**0·9 (0·8 to 1·0)**	**1·1 (1·0 to 1·2)**	**0·6 (0·5 to 0·6)**	**0·8 (0·5 to 1·1)**	**0·4 (0·4 to 0·5)**	**0·6 (0·5 to 0·7)**	**2·4 (1·8 to 3·3)**	**0·2 (0·2 to 0·2)**	**0·5 (0·5 to 0·6)**
**European Region**	**0·5 (0·4 to 0·5)**	**0·4 (0·3 to 0·6)**	**0·4 (0·4 to 0·5)**	**0·4 (0·3 to 0·4)**	**0·3 (0·3 to 0·5)**	**0·3 (0·3 to 0·4)**	**0·3 (0·3 to 0·3)**	**0·3 (0·2 to 0·4)**	**0·3 (0·2 to 0·3)**	**0·3 (0·2 to 0·3)**
Albania	0·7 (0·4 to 1·6)	0·4 (0·3 to 0·8)	0·4 (0·2 to 0·6)	0·2 (0·1 to 0·3)	0·8 (0·5 to 1·2)	0·2 (0·1 to 0·3)	0·3 (0·2 to 0·5)	0·5 (0·3 to 0·9)	0·9 (0·5 to 1·3)	0·7 (0·5 to 1·0)
Andorra	0·4 (0·3 to 0·6)	0·2 (0·1 to 0·3)	0·2 (0·1 to 0·2)	0·1 (0·1 to 0·2)	0·0 (0·0 to 0·1)	0·2 (0·1 to 0·3)	0·2 (0·1 to 0·2)	0·2 (0·1 to 0·3)	0·3 (0·2 to 0·4)	0·0 (0·0 to 0·1)
Armenia	0·3 (0·2 to 0·4)	0·3 (0·2 to 0·5)	0·5 (0·3 to 1·3)	0·4 (0·3 to 0·6)	0·3 (0·2 to 0·5)	0·5 (0·4 to 0·8)	0·1 (0·1 to 0·3)	0·2 (0·2 to 0·3)	0·2 (0·2 to 0·3)	0·5 (0·4 to 0·6)
Austria	0·7 (0·4 to 0·9)	0·2 (0·2 to 0·4)	0·3 (0·2 to 0·4)	0·1 (0·1 to 0·1)	0·1 (0·0 to 0·1)	0·2 (0·1 to 0·3)	0·1 (0·1 to 0·2)	0·1 (0·1 to 0·1)	0·3 (0·2 to 0·4)	0·1 (0·0 to 0·1)
Azerbaijan	1·5 (0·8 to 2·2)	0·7 (0·4 to 1·1)	1·0 (0·6 to 1·7)	1·9 (1·3 to 2·7)	1·2 (0·7 to 2·1)	1·1 (0·5 to 1·6)	0·7 (0·4 to 1·0)	1·4 (0·8 to 2·4)	0·3 (0·1 to 0·5)	1·9 (1·3 to 2·5)
Belarus	0·2 (0·1 to 0·3)	0·4 (0·3 to 0·6)	0·7 (0·4 to 1·0)	0·7 (0·5 to 0·9)	0·5 (0·3 to 0·7)	0·3 (0·2 to 0·4)	0·3 (0·2 to 0·4)	0·1 (0·1 to 0·2)	0·2 (0·2 to 0·3)	0·1 (0·1 to 0·1)
Belgium	0·5 (0·4 to 0·7)	0·4 (0·3 to 0·5)	0·2 (0·2 to 0·3)	0·2 (0·2 to 0·3)	0·1 (0·1 to 0·1)	0·2 (0·2 to 0·3)	0·2 (0·1 to 0·2)	0·1 (0·1 to 0·1)	0·2 (0·2 to 0·3)	0·0 (0·0 to 0·0)
Bosnia and Herzegovina	0·2 (0·1 to 0·3)	0·3 (0·2 to 0·5)	0·1 (0·1 to 0·2)	0·1 (0·1 to 0·2)	0·1 (0·0 to 0·3)	0·2 (0·1 to 0·3)	0·4 (0·3 to 0·6)	0·1 (0·1 to 0·2)	0·1 (0·1 to 0·1)	0·2 (0·1 to 0·2)
Bulgaria	0·3 (0·2 to 0·5)	0·4 (0·2 to 0·7)	0·6 (0·3 to 0·8)	0·3 (0·2 to 0·4)	0·4 (0·2 to 0·7)	0·2 (0·1 to 0·3)	0·5 (0·3 to 0·7)	0·2 (0·2 to 0·3)	0·3 (0·2 to 0·4)	0·3 (0·2 to 0·4)
Croatia	0·3 (0·2 to 0·4)	0·2 (0·1 to 0·3)	0·2 (0·1 to 0·3)	0·1 (0·1 to 0·1)	0·1 (0·1 to 0·2)	0·1 (0·1 to 0·2)	0·3 (0·2 to 0·4)	0·1 (0·1 to 0·1)	0·2 (0·2 to 0·3)	0·1 (0·1 to 0·1)
Cyprus	0·7 (0·5 to 1·0)	0·4 (0·3 to 0·5)	0·2 (0·1 to 0·2)	0·2 (0·1 to 0·2)	0·1 (0·1 to 0·1)	0·2 (0·1 to 0·2)	0·2 (0·2 to 0·3)	0·1 (0·1 to 0·2)	0·2 (0·2 to 0·3)	0·1 (0·1 to 0·1)
Czechia	0·4 (0·2 to 0·5)	0·2 (0·1 to 0·3)	0·2 (0·1 to 0·3)	0·1 (0·1 to 0·1)	0·1 (0·1 to 0·1)	0·2 (0·2 to 0·3)	0·2 (0·2 to 0·4)	0·1 (0·1 to 0·1)	0·2 (0·2 to 0·3)	0·1 (0·1 to 0·1)
Denmark	0·3 (0·2 to 0·4)	0·2 (0·1 to 0·3)	0·1 (0·0 to 0·2)	0·1 (0·1 to 0·2)	0·0 (0·0 to 0·1)	0·1 (0·1 to 0·2)	0·2 (0·1 to 0·2)	0·1 (0·1 to 0·2)	0·2 (0·1 to 0·2)	0·0 (0·0 to 0·1)
Estonia	0·2 (0·1 to 0·3)	0·3 (0·2 to 0·5)	0·3 (0·2 to 0·7)	0·4 (0·3 to 0·6)	0·2 (0·1 to 0·3)	0·3 (0·2 to 0·4)	0·2 (0·2 to 0·4)	0·1 (0·1 to 0·1)	0·2 (0·1 to 0·3)	0·1 (0·1 to 0·1)
Finland	0·5 (0·2 to 0·7)	0·3 (0·2 to 0·5)	0·1 (0·1 to 0·3)	0·1 (0·1 to 0·2)	0·1 (0·0 to 0·1)	0·1 (0·0 to 0·2)	0·2 (0·1 to 0·2)	0·1 (0·0 to 0·1)	0·2 (0·1 to 0·2)	0·0 (0·0 to 0·0)
France	0·5 (0·4 to 0·8)	0·3 (0·2 to 0·4)	0·2 (0·1 to 0·3)	0·1 (0·1 to 0·2)	0·1 (0·0 to 0·1)	0·3 (0·1 to 0·6)	0·2 (0·1 to 0·3)	0·1 (0·1 to 0·1)	0·2 (0·1 to 0·2)	0·0 (0·0 to 0·0)
Georgia	0·2 (0·1 to 0·3)	0·4 (0·2 to 0·6)	0·9 (0·7 to 1·4)	1·0 (0·7 to 1·5)	0·4 (0·2 to 0·8)	1·0 (0·5 to 1·7)	0·4 (0·2 to 0·5)	0·6 (0·4 to 0·8)	0·2 (0·1 to 0·2)	0·7 (0·5 to 0·9)
Germany	0·4 (0·3 to 0·7)	0·2 (0·2 to 0·3)	0·1 (0·1 to 0·2)	0·1 (0·1 to 0·2)	0·1 (0·0 to 0·1)	0·1 (0·1 to 0·2)	0·2 (0·1 to 0·2)	0·1 (0·1 to 0·1)	0·2 (0·2 to 0·3)	0·1 (0·0 to 0·1)
Greece	0·3 (0·2 to 0·4)	0·2 (0·1 to 0·3)	0·2 (0·1 to 0·2)	0·1 (0·1 to 0·2)	0·0 (0·0 to 0·1)	0·2 (0·1 to 0·3)	0·2 (0·1 to 0·3)	0·1 (0·1 to 0·2)	0·2 (0·1 to 0·2)	0·1 (0·1 to 0·1)
Hungary	0·5 (0·3 to 0·7)	0·2 (0·2 to 0·4)	0·1 (0·1 to 0·4)	0·1 (0·1 to 0·1)	0·1 (0·1 to 0·2)	0·2 (0·1 to 0·3)	0·3 (0·2 to 0·4)	0·1 (0·1 to 0·1)	0·2 (0·2 to 0·3)	0·1 (0·1 to 0·2)
Iceland	0·2 (0·2 to 0·4)	0·2 (0·1 to 0·3)	0·2 (0·1 to 0·2)	0·1 (0·1 to 0·1)	0·0 (0·0 to 0·1)	0·1 (0·1 to 0·1)	0·2 (0·1 to 0·3)	0·1 (0·1 to 0·2)	0·4 (0·3 to 0·5)	0·0 (0·0 to 0·0)
Ireland	0·7 (0·5 to 0·9)	0·2 (0·1 to 0·3)	0·2 (0·1 to 0·2)	0·2 (0·1 to 0·2)	0·0 (0·0 to 0·1)	0·2 (0·1 to 0·2)	0·2 (0·1 to 0·3)	0·2 (0·1 to 0·2)	0·2 (0·1 to 0·2)	0·0 (0·0 to 0·0)
Israel	0·5 (0·4 to 0·9)	0·4 (0·3 to 0·6)	0·2 (0·1 to 0·2)	0·1 (0·1 to 0·2)	0·1 (0·0 to 0·1)	0·2 (0·1 to 0·2)	0·2 (0·2 to 0·3)	0·1 (0·1 to 0·2)	0·2 (0·2 to 0·3)	0·1 (0·1 to 0·1)
Italy	0·4 (0·3 to 0·5)	0·1 (0·1 to 0·3)	0·1 (0·1 to 0·2)	0·1 (0·1 to 0·1)	0·1 (0·1 to 0·1)	0·1 (0·1 to 0·2)	0·2 (0·2 to 0·3)	0·1 (0·1 to 0·1)	0·2 (0·2 to 0·3)	0·1 (0·0 to 0·1)
Kazakhstan	0·3 (0·2 to 0·4)	0·8 (0·5 to 1·4)	0·9 (0·6 to 1·4)	0·6 (0·4 to 0·8)	1·0 (0·7 to 1·5)	0·7 (0·4 to 1·5)	0·3 (0·2 to 0·4)	0·4 (0·3 to 0·5)	0·2 (0·1 to 0·2)	0·3 (0·2 to 0·4)
Kyrgyzstan	0·4 (0·2 to 0·5)	0·5 (0·4 to 0·9)	0·8 (0·6 to 1·5)	0·5 (0·4 to 0·8)	1·0 (0·7 to 1·6)	0·8 (0·6 to 1·1)	0·3 (0·2 to 0·3)	0·6 (0·4 to 0·7)	0·2 (0·2 to 0·3)	0·9 (0·7 to 1·0)
Latvia	0·2 (0·1 to 0·3)	0·4 (0·2 to 0·6)	0·4 (0·2 to 0·8)	0·6 (0·4 to 0·8)	0·3 (0·2 to 0·6)	0·1 (0·1 to 0·2)	0·3 (0·2 to 0·5)	0·1 (0·0 to 0·1)	0·2 (0·1 to 0·3)	0·1 (0·1 to 0·2)
Lithuania	0·3 (0·2 to 0·4)	0·4 (0·3 to 0·6)	0·4 (0·3 to 0·8)	0·3 (0·2 to 0·4)	0·2 (0·1 to 0·4)	0·3 (0·2 to 0·4)	0·3 (0·2 to 0·5)	0·1 (0·1 to 0·1)	0·2 (0·2 to 0·3)	0·1 (0·1 to 0·1)

(Table 7 continues on next page)

	Endocrine, metabolic, blood, and immune disorders	Interpersonal violence	Falls	Fire, heat, and hot substances	Encephalitis	Foreign body	Non-Hodgkin lymphoma	Meningitis	Other neurological disorders	Chronic kidney disease
(Continued from previous page)										
Luxembourg	0·3 (0·2 to 0·6)	0·2 (0·2 to 0·4)	0·2 (0·1 to 0·3)	0·1 (0·0 to 0·1)	0·1 (0·0 to 0·1)	0·2 (0·1 to 0·4)	0·2 (0·1 to 0·2)	0·1 (0·0 to 0·1)	0·1 (0·1 to 0·2)	0·0 (0·0 to 0·0)
Macedonia	0·2 (0·1 to 0·3)	0·3 (0·2 to 0·4)	0·3 (0·2 to 0·4)	0·1 (0·1 to 0·2)	0·2 (0·1 to 0·3)	0·2 (0·1 to 0·3)	0·3 (0·2 to 0·5)	0·1 (0·1 to 0·2)	0·2 (0·1 to 0·3)	0·2 (0·1 to 0·2)
Malta	0·3 (0·2 to 0·4)	0·3 (0·2 to 0·5)	0·2 (0·2 to 0·3)	0·1 (0·1 to 0·1)	0·1 (0·1 to 0·1)	0·1 (0·1 to 0·2)	0·2 (0·2 to 0·3)	0·1 (0·1 to 0·1)	0·4 (0·3 to 0·5)	0·1 (0·1 to 0·1)
Republic of Moldova	0·3 (0·2 to 0·4)	0·4 (0·3 to 0·9)	0·5 (0·3 to 0·7)	0·5 (0·3 to 0·7)	0·4 (0·2 to 0·6)	0·4 (0·3 to 0·6)	0·7 (0·4 to 1·0)	0·2 (0·2 to 0·3)	0·2 (0·2 to 0·3)	0·2 (0·2 to 0·3)
Montenegro	0·2 (0·1 to 0·3)	0·5 (0·3 to 0·7)	0·2 (0·1 to 0·2)	0·2 (0·1 to 0·2)	0·1 (0·1 to 0·2)	0·3 (0·2 to 0·4)	0·3 (0·2 to 0·4)	0·1 (0·0 to 0·1)	0·1 (0·1 to 0·1)	0·2 (0·2 to 0·3)
Netherlands	0·6 (0·5 to 0·7)	0·2 (0·2 to 0·4)	0·1 (0·1 to 0·2)	0·1 (0·1 to 0·1)	0·1 (0·0 to 0·1)	0·1 (0·1 to 0·2)	0·2 (0·1 to 0·2)	0·2 (0·1 to 0·2)	0·3 (0·3 to 0·4)	0·0 (0·0 to 0·0)
Norway	0·4 (0·3 to 0·6)	0·2 (0·2 to 0·3)	0·2 (0·1 to 0·2)	0·2 (0·2 to 0·2)	0·0 (0·0 to 0·1)	0·1 (0·1 to 0·2)	0·2 (0·1 to 0·2)	0·1 (0·1 to 0·2)	0·3 (0·2 to 0·4)	0·1 (0·0 to 0·1)
Poland	0·2 (0·2 to 0·3)	0·2 (0·1 to 0·3)	0·2 (0·2 to 0·4)	0·1 (0·1 to 0·2)	0·2 (0·1 to 0·3)	0·2 (0·1 to 0·2)	0·2 (0·2 to 0·4)	0·1 (0·1 to 0·1)	0·2 (0·1 to 0·3)	0·1 (0·1 to 0·1)
Portugal	0·5 (0·4 to 0·7)	0·4 (0·3 to 0·5)	0·3 (0·2 to 0·3)	0·1 (0·1 to 0·2)	0·1 (0·1 to 0·1)	0·3 (0·2 to 0·4)	0·3 (0·2 to 0·4)	0·1 (0·1 to 0·2)	0·2 (0·2 to 0·2)	0·1 (0·1 to 0·1)
Romania	0·1 (0·1 to 0·2)	0·2 (0·1 to 0·5)	0·4 (0·3 to 0·6)	0·3 (0·2 to 0·3)	0·4 (0·3 to 0·5)	0·3 (0·3 to 0·4)	0·4 (0·3 to 0·6)	0·2 (0·1 to 0·2)	0·2 (0·2 to 0·2)	0·2 (0·2 to 0·2)
Russian Federation	0·3 (0·2 to 0·3)	1·2 (0·7 to 1·5)	0·6 (0·5 to 0·9)	0·8 (0·6 to 1·0)	0·4 (0·3 to 0·6)	0·4 (0·3 to 0·5)	0·3 (0·2 to 0·4)	0·2 (0·2 to 0·3)	0·3 (0·3 to 0·4)	0·2 (0·1 to 0·2)
Serbia	0·4 (0·3 to 0·6)	0·4 (0·3 to 0·5)	0·2 (0·1 to 0·2)	0·1 (0·1 to 0·2)	0·3 (0·2 to 0·4)	0·2 (0·1 to 0·3)	0·3 (0·2 to 0·4)	0·1 (0·1 to 0·2)	0·4 (0·3 to 0·5)	0·1 (0·1 to 0·2)
Slovakia	0·3 (0·2 to 0·4)	0·4 (0·2 to 0·5)	0·3 (0·2 to 0·5)	0·1 (0·1 to 0·2)	0·1 (0·1 to 0·1)	0·3 (0·2 to 0·4)	0·3 (0·2 to 0·5)	0·2 (0·1 to 0·2)	0·3 (0·2 to 0·5)	0·1 (0·1 to 0·1)
Slovenia	0·5 (0·3 to 0·6)	0·1 (0·1 to 0·2)	0·2 (0·2 to 0·3)	0·0 (0·0 to 0·1)	0·1 (0·0 to 0·1)	0·1 (0·1 to 0·2)	0·3 (0·2 to 0·3)	0·1 (0·0 to 0·1)	0·4 (0·3 to 0·5)	0·0 (0·0 to 0·0)
Spain	0·5 (0·3 to 0·6)	0·1 (0·1 to 0·3)	0·2 (0·1 to 0·2)	0·1 (0·1 to 0·1)	0·1 (0·1 to 0·1)	0·2 (0·1 to 0·3)	0·2 (0·2 to 0·3)	0·1 (0·1 to 0·2)	0·2 (0·2 to 0·3)	0·1 (0·1 to 0·1)
Sweden	0·2 (0·2 to 0·5)	0·3 (0·2 to 0·4)	0·1 (0·1 to 0·2)	0·2 (0·1 to 0·2)	0·0 (0·0 to 0·1)	0·1 (0·1 to 0·2)	0·2 (0·1 to 0·2)	0·1 (0·0 to 0·1)	0·2 (0·1 to 0·2)	0·0 (0·0 to 0·0)
Switzerland	0·4 (0·3 to 0·5)	0·3 (0·2 to 0·4)	0·3 (0·2 to 0·4)	0·1 (0·1 to 0·1)	0·1 (0·0 to 0·1)	0·1 (0·1 to 0·2)	0·1 (0·1 to 0·2)	0·1 (0·0 to 0·1)	0·2 (0·2 to 0·3)	0·0 (0·0 to 0·0)
Tajikistan	0·3 (0·2 to 0·4)	0·4 (0·3 to 0·7)	1·5 (1·1 to 2·1)	1·4 (0·9 to 2·0)	1·0 (0·6 to 1·5)	0·8 (0·5 to 1·3)	0·3 (0·2 to 0·4)	3·6 (2·3 to 5·9)	0·2 (0·1 to 0·2)	1·2 (0·9 to 1·7)
Turkey	1·0 (0·7 to 1·5)	0·4 (0·3 to 0·6)	0·6 (0·4 to 0·8)	0·3 (0·1 to 0·4)	0·2 (0·1 to 0·3)	0·4 (0·3 to 0·7)	0·5 (0·3 to 0·7)	0·4 (0·2 to 0·6)	0·5 (0·3 to 0·7)	0·5 (0·4 to 0·7)
Turkmenistan	0·8 (0·4 to 1·2)	0·7 (0·5 to 1·2)	0·8 (0·5 to 1·3)	1·2 (0·8 to 1·7)	1·0 (0·4 to 1·8)	1·1 (0·6 to 1·7)	0·3 (0·2 to 0·4)	1·1 (0·8 to 1·5)	0·3 (0·2 to 0·5)	1·5 (1·1 to 2·0)
Ukraine	0·2 (0·1 to 0·3)	0·5 (0·3 to 0·9)	0·6 (0·4 to 0·9)	0·5 (0·3 to 0·7)	0·3 (0·2 to 0·5)	0·3 (0·2 to 0·5)	0·3 (0·2 to 0·4)	0·2 (0·1 to 0·3)	0·2 (0·1 to 0·3)	0·1 (0·1 to 0·1)
UK	0·6 (0·5 to 0·7)	0·2 (0·1 to 0·2)	0·1 (0·1 to 0·2)	0·1 (0·1 to 0·2)	0·0 (0·0 to 0·1)	0·1 (0·1 to 0·2)	0·2 (0·2 to 0·2)	0·2 (0·1 to 0·2)	0·3 (0·3 to 0·3)	0·0 (0·0 to 0·0)
Uzbekistan	0·2 (0·2 to 0·4)	0·3 (0·2 to 0·6)	0·9 (0·6 to 1·3)	0·7 (0·5 to 1·0)	2·2 (1·3 to 4·0)	0·7 (0·5 to 1·0)	0·7 (0·4 to 1·0)	0·6 (0·4 to 1·0)	0·2 (0·2 to 0·3)	1·1 (0·8 to 1·4)
**EU13**	**0·3 (0·2 to 0·3)**	**0·2 (0·2 to 0·4)**	**0·3 (0·2 to 0·4)**	**0·2 (0·1 to 0·2)**	**0·2 (0·2 to 0·3)**	**0·2 (0·2 to 0·3)**	**0·3 (0·2 to 0·4)**	**0·1 (0·1 to 0·1)**	**0·2 (0·2 to 0·3)**	**0·1 (0·1 to 0·1)**
**EU15**	**0·5 (0·4 to 0·6)**	**0·2 (0·2 to 0·3)**	**0·2 (0·1 to 0·2)**	**0·1 (0·1 to 0·2)**	**0·1 (0·0 to 0·1)**	**0·2 (0·1 to 0·3)**	**0·2 (0·2 to 0·2)**	**0·1 (0·1 to 0·1)**	**0·2 (0·2 to 0·3)**	**0·0 (0·0 to 0·1)**
**SEEHN**	**0·3 (0·3 to 0·5)**	**0·3 (0·3 to 0·5)**	**0·3 (0·2 to 0·4)**	**0·2 (0·2 to 0·2)**	**0·3 (0·2 to 0·3)**	**0·2 (0·2 to 0·3)**	**0·4 (0·3 to 0·4)**	**0·2 (0·1 to 0·2)**	**0·3 (0·2 to 0·3)**	**0·2 (0·2 to 0·2)**
**CIS**	**0·3 (0·2 to 0·4)**	**0·8 (0·5 to 1·0)**	**0·8 (0·6 to 1·0)**	**0·8 (0·7 to 0·9)**	**0·8 (0·6 to 1·2)**	**0·5 (0·5 to 0·7)**	**0·4 (0·3 to 0·4)**	**0·6 (0·5 to 0·7)**	**0·3 (0·2 to 0·3)**	**0·5 (0·4 to 0·6)**

Andorra, Georgia, Iceland, Norway, Switzerland, and Turkey are not included in the subregion groups. Bulgaria, Croatia, Romania, and Republic of Moldova belong to more than one subregion. EU13=countries that joined the European Union after May, 2004. EU15=countries that joined the European Union before May, 2004. SEEHN=South Eastern Europe Health Network. CIS=commonwealth of independent states.

Table 7. **Mortality rates for the 11th to 20th leading causes of death in 2016 globally and in the WHO European Region (51 countries and four subregions), ages 5–14 years, both sexes**

eTable 8. All-cause and cause-specific mortality (counts and rates) at the global level and by country in the WHO European Region for ages 5 to 9 and 10 to 14 for 1990, 2000, and 2016

Location	Cause	Age	Sex	1990		2000		2016		Percent change 1990 to 2016	
				Count	Rate	Count	Rate	Count	Rate	Count	Rate
Global	All causes	5 to 9	Both	784744 (777308 to 792168)	136.5 (135.2 to 137.7)	684139 (677822 to 691529)	111.4 (110.3 to 112.6)	419099 (409064 to 429633)	65.3 (63.7 to 66.9)	-46.6 (-48.0 to -45.2)	-52.2 (-53.4 to -50.9)
Global	All causes	5 to 9	Female	363761 (359650 to 367810)	129.6 (128.1 to 131.0)	312915 (308955 to 316816)	105.1 (103.8 to 106.4)	184940 (180662 to 189422)	59.6 (58.2 to 61.1)	-49.2 (-50.5 to -47.8)	-54.0 (-55.2 to -52.7)
Global	All causes	5 to 9	Male	420983 (416466 to 425417)	143.0 (141.5 to 144.5)	371224 (367384 to 375340)	117.2 (116.0 to 118.5)	234159 (228476 to 240215)	70.5 (68.8 to 72.4)	-44.4 (-45.9 to -42.8)	-50.7 (-52.1 to -49.3)
Global	All causes	10 to 14	Both	503707 (498353 to 509502)	95.4 (94.3 to 96.5)	502688 (498139 to 507566)	80.2 (79.5 to 81.0)	357146 (351555 to 363134)	58.4 (57.5 to 59.4)	-29.1 (-30.4 to -27.7)	-38.7 (-39.8 to -37.5)
Global	All causes	10 to 14	Female	224812 (221837 to 227795)	87.1 (85.9 to 88.2)	217329 (214494 to 220314)	71.2 (70.3 to 72.2)	151918 (149087 to 154927)	51.5 (50.5 to 52.5)	-32.4 (-34.0 to -30.8)	-40.9 (-42.3 to -39.5)
Global	All causes	10 to 14	Male	278895 (275636 to 282331)	103.3 (102.1 to 104.5)	285359 (282765 to 288083)	88.7 (87.9 to 89.5)	205228 (201907 to 208660)	64.9 (63.9 to 66.0)	-26.4 (-27.8 to -24.9)	-37.1 (-38.3 to -35.8)
Global	Tuberculosis	5 to 9	Both	17872 (14528 to 21365)	3.1 (2.5 to 3.7)	13090 (10911 to 15518)	2.1 (1.8 to 2.5)	5848 (5037 to 6735)	0.9 (0.8 to 1.0)	-67.3 (-73.1 to -59.0)	-70.7 (-75.9 to -63.3)
Global	Tuberculosis	5 to 9	Female	9713 (7462 to 12326)	3.5 (2.7 to 4.4)	7235 (5749 to 9033)	2.4 (1.9 to 3.0)	3176 (2492 to 3957)	1.0 (0.8 to 1.3)	-67.3 (-75.8 to -53.0)	-70.4 (-78.1 to -57.5)
Global	Tuberculosis	5 to 9	Male	8159 (6700 to 9628)	2.8 (2.3 to 3.3)	5854 (4884 to 6756)	1.8 (1.5 to 2.1)	2672 (2293 to 3112)	0.8 (0.7 to 0.9)	-67.2 (-73.1 to -59.9)	-71.0 (-76.2 to -64.5)
Global	Tuberculosis	10 to 14	Both	17742 (15472 to 19996)	3.4 (2.9 to 3.8)	15135 (13447 to 16835)	2.4 (2.1 to 2.7)	7467 (6633 to 8337)	1.2 (1.1 to 1.4)	-57.9 (-62.5 to -51.6)	-63.6 (-67.5 to -58.1)
Global	Tuberculosis	10 to 14	Female	9584 (8056 to 11142)	3.7 (3.1 to 4.3)	8133 (6911 to 9370)	2.7 (2.3 to 3.1)	3932 (3325 to 4586)	1.3 (1.1 to 1.6)	-59.0 (-65.4 to -50.0)	-64.1 (-69.7 to -56.3)
Global	Tuberculosis	10 to 14	Male	8157 (7011 to 9294)	3.0 (2.6 to 3.4)	7002 (6058 to 7903)	2.2 (1.9 to 2.5)	3535 (3073 to 4064)	1.1 (1.0 to 1.3)	-56.7 (-62.6 to -49.1)	-63.0 (-68.1 to -56.5)
Global	HIV/AIDS	5 to 9	Both	1371 (1159 to 1668)	0.2 (0.2 to 0.3)	22017 (20299 to 24010)	3.6 (3.3 to 3.9)	21420 (20268 to 22612)	3.3 (3.2 to 3.5)	1462.8 (1193.0 to 1758.6)	1299.6 (1058.0 to 1564.6)
Global	HIV/AIDS	5 to 9	Female	652 (547 to 800)	0.2 (0.2 to 0.3)	10904 (10063 to 11893)	3.7 (3.4 to 4.0)	10568 (9994 to 11171)	3.4 (3.2 to 3.6)	1522.0 (1230.2 to 1844.4)	1368.1 (1104.0 to 1659.9)
Global	HIV/AIDS	5 to 9	Male	719 (613 to 869)	0.2 (0.2 to 0.3)	11113 (10235 to 12117)	3.5 (3.2 to 3.8)	10851 (10274 to 11458)	3.3 (3.1 to 3.5)	1409.1 (1159.4 to 1685.7)	1238.2 (1016.8 to 1483.4)
Global	HIV/AIDS	10 to 14	Both	459 (399 to 543)	0.1 (0.1 to 0.1)	11208 (10066 to 12669)	1.8 (1.6 to 2.0)	30865 (29293 to 32605)	5.1 (4.8 to 5.3)	6628.4 (5561.0 to 7721.5)	5716.6 (4793.8 to 6661.5)
Global	HIV/AIDS	10 to 14	Female	195 (165 to 239)	0.1 (0.1 to 0.1)	5540 (4983 to 6255)	1.8 (1.6 to 2.1)	15339 (14562 to 16188)	5.2 (4.9 to 5.5)	7752.9 (6314.8 to 9295.0)	6771.8 (5513.4 to 8121.2)
Global	HIV/AIDS	10 to 14	Male	263 (231 to 304)	0.1 (0.1 to 0.1)	5667 (5086 to 6426)	1.8 (1.6 to 2.0)	15526 (14722 to 16422)	4.9 (4.7 to 5.2)	5794.5 (4953.3 to 6719.4)	4937.4 (4218.5 to 5727.8)
Global	Diarrheal diseases	5 to 9	Both	63826 (35363 to 105793)	11.1 (6.1 to 18.4)	53326 (31248 to 87673)	8.7 (5.1 to 14.3)	33206 (21029 to 53325)	5.2 (3.3 to 8.3)	-48.0 (-67.1 to -19.3)	-53.4 (-70.6 to -27.7)
Global	Diarrheal diseases	5 to 9	Female	32893 (13215 to 64213)	11.7 (4.7 to 22.9)	27428 (12054 to 54197)	9.2 (4.0 to 18.2)	15677 (7591 to 30320)	5.1 (2.4 to 9.8)	-52.3 (-73.2 to -18.5)	-56.9 (-75.8 to -26.3)
Global	Diarrheal diseases	5 to 9	Male	30933 (14928 to 56339)	10.5 (5.1 to 19.1)	25898 (13246 to 48463)	8.2 (4.2 to 15.3)	17530 (9450 to 31416)	5.3 (2.8 to 9.5)	-43.3 (-64.4 to -15.7)	-49.8 (-68.5 to -25.3)
Global	Diarrheal diseases	10 to 14	Both	30868 (17579 to 52747)	5.8 (3.3 to 10.0)	28598 (17279 to 47556)	4.6 (2.8 to 7.6)	18317 (11587 to 29800)	3.0 (1.9 to 4.9)	-40.7 (-58.8 to -14.4)	-48.7 (-64.4 to -26.0)
Global	Diarrheal diseases	10 to 14	Female	16464 (6677 to 32499)	6.4 (2.6 to 12.6)	15218 (6741 to 30721)	5.0 (2.2 to 10.1)	9135 (4256 to 17971)	3.1 (1.4 to 6.1)	-44.5 (-64.7 to -11.6)	-51.4 (-69.1 to -22.6)
Global	Diarrheal diseases	10 to 14	Male	14404 (7205 to 27310)	5.3 (2.7 to 10.1)	13380 (6875 to 25739)	4.2 (2.1 to 8.0)	9182 (4855 to 17040)	2.9 (1.5 to 5.4)	-36.3 (-55.6 to -12.5)	-45.5 (-62.1 to -25.2)
Global	Intestinal infectious diseases	5 to 9	Both	69468 (37997 to 115548)	12.1 (6.6 to 20.1)	58959 (32785 to 97412)	9.6 (5.3 to 15.9)	37410 (21382 to 59903)	5.8 (3.3 to 9.3)	-46.1 (-56.8 to -33.5)	-51.8 (-61.3 to -40.5)
Global	Intestinal infectious diseases	5 to 9	Female	33633 (17902 to 56996)	12.0 (6.4 to 20.3)	28310 (15639 to 46797)	9.5 (5.3 to 15.7)	16761 (9404 to 27212)	5.4 (3.0 to 8.8)	-50.2 (-61.3 to -35.5)	-54.9 (-65.0 to -41.6)
Global	Intestinal infectious diseases	5 to 9	Male	35835 (19476 to 59730)	12.2 (6.6 to 20.3)	30649 (17144 to 50166)	9.7 (5.4 to 15.8)	20649 (11722 to 33545)	6.2 (3.5 to 10.1)	-42.4 (-53.1 to -30.2)	-48.9 (-58.4 to -38.1)
Global	Intestinal infectious diseases	10 to 14	Both	40741 (21497 to 64290)	7.7 (4.1 to 12.2)	37045 (19630 to 58549)	5.9 (3.1 to 9.3)	25742 (13596 to 40579)	4.2 (2.2 to 6.6)	-36.8 (-45.3 to -28.6)	-45.4 (-52.7 to -38.3)

eTable 8. All-cause and cause-specific mortality (counts and rates) at the global level and by country in the WHO European Region for ages 5 to 9 and 10 to 14 for 1990, 2000, and 2016

Global	Intestinal infectious diseases	10 to 14	Female	19076 (10037 to 30570)	7.4 (3.9 to 11.8)	17136 (9037 to 27211)	5.6 (3.0 to 8.9)	11407 (6103 to 18157)	3.9 (2.1 to 6.2)	-40.2 (-50.1 to -29.9)	-47.7 (-56.3 to -38.6)
Global	Intestinal infectious diseases	10 to 14	Male	21666 (11661 to 34156)	8.0 (4.3 to 12.6)	19909 (10810 to 31467)	6.2 (3.4 to 9.8)	14335 (7726 to 22899)	4.5 (2.4 to 7.2)	-33.8 (-41.8 to -25.9)	-43.5 (-50.3 to -36.7)
Global	Lower respiratory infections	5 to 9	Both	54674 (44362 to 64754)	9.5 (7.7 to 11.3)	46806 (38791 to 53994)	7.6 (6.3 to 8.8)	32874 (26213 to 38071)	5.1 (4.1 to 5.9)	-39.9 (-50.3 to -24.8)	-46.1 (-55.5 to -32.7)
Global	Lower respiratory infections	5 to 9	Female	27115 (20628 to 33877)	9.7 (7.3 to 12.1)	22997 (17965 to 28035)	7.7 (6.0 to 9.4)	15662 (11690 to 19042)	5.0 (3.8 to 6.1)	-42.2 (-55.3 to -20.0)	-47.7 (-59.5 to -27.6)
Global	Lower respiratory infections	5 to 9	Male	27559 (21187 to 33265)	9.4 (7.2 to 11.3)	23809 (18448 to 28576)	7.5 (5.8 to 9.0)	17213 (12815 to 20934)	5.2 (3.9 to 6.3)	-37.5 (-47.9 to -20.2)	-44.6 (-53.8 to -29.2)
Global	Lower respiratory infections	10 to 14	Both	24008 (20005 to 27049)	4.5 (3.8 to 5.1)	24186 (20193 to 27421)	3.9 (3.2 to 4.4)	18865 (15328 to 21360)	3.1 (2.5 to 3.5)	-21.4 (-28.8 to -10.3)	-32.1 (-38.5 to -22.4)
Global	Lower respiratory infections	10 to 14	Female	11637 (9261 to 13691)	4.5 (3.6 to 5.3)	11469 (9122 to 13350)	3.8 (3.0 to 4.4)	8635 (6791 to 10342)	2.9 (2.3 to 3.5)	-25.8 (-35.5 to -5.1)	-35.1 (-43.5 to -16.9)
Global	Lower respiratory infections	10 to 14	Male	12371 (9857 to 14434)	4.6 (3.6 to 5.3)	12717 (10141 to 15056)	4.0 (3.2 to 4.7)	10230 (8030 to 12164)	3.2 (2.5 to 3.8)	-17.3 (-25.5 to -6.6)	-29.3 (-36.3 to -20.2)
Global	Upper respiratory infections	5 to 9	Both	75 (50 to 109)	0.0 (0.0 to 0.0)	59 (39 to 87)	0.0 (0.0 to 0.0)	29 (18 to 43)	0.0 (0.0 to 0.0)	-61.6 (-76.0 to -38.2)	-65.6 (-78.5 to -44.6)
Global	Upper respiratory infections	5 to 9	Female	35 (21 to 57)	0.0 (0.0 to 0.0)	27 (16 to 44)	0.0 (0.0 to 0.0)	12 (7 to 20)	0.0 (0.0 to 0.0)	-64.9 (-82.0 to -29.0)	-68.2 (-83.7 to -35.7)
Global	Upper respiratory infections	5 to 9	Male	39 (24 to 61)	0.0 (0.0 to 0.0)	32 (19 to 51)	0.0 (0.0 to 0.0)	16 (9 to 26)	0.0 (0.0 to 0.0)	-58.6 (-77.1 to -24.3)	-63.3 (-79.7 to -32.9)
Global	Upper respiratory infections	10 to 14	Both	47 (33 to 68)	0.0 (0.0 to 0.0)	43 (30 to 61)	0.0 (0.0 to 0.0)	23 (15 to 33)	0.0 (0.0 to 0.0)	-51.0 (-67.4 to -28.8)	-57.6 (-71.8 to -38.5)
Global	Upper respiratory infections	10 to 14	Female	22 (13 to 34)	0.0 (0.0 to 0.0)	19 (12 to 30)	0.0 (0.0 to 0.0)	10 (6 to 16)	0.0 (0.0 to 0.0)	-54.4 (-73.5 to -15.8)	-60.1 (-76.9 to -26.3)
Global	Upper respiratory infections	10 to 14	Male	26 (16 to 40)	0.0 (0.0 to 0.0)	24 (16 to 36)	0.0 (0.0 to 0.0)	13 (8 to 21)	0.0 (0.0 to 0.0)	-48.1 (-69.4 to -13.3)	-55.6 (-73.9 to -25.9)
Global	Otitis media	5 to 9	Both	489 (311 to 812)	0.1 (0.1 to 0.1)	302 (175 to 556)	0.0 (0.0 to 0.1)	72 (42 to 124)	0.0 (0.0 to 0.0)	-85.3 (-92.5 to -69.4)	-86.8 (-93.3 to -72.6)
Global	Otitis media	5 to 9	Female	298 (159 to 600)	0.1 (0.1 to 0.2)	194 (87 to 442)	0.1 (0.0 to 0.1)	33 (15 to 75)	0.0 (0.0 to 0.0)	-89.0 (-96.1 to -67.6)	-90.0 (-96.5 to -70.7)
Global	Otitis media	5 to 9	Male	191 (85 to 307)	0.1 (0.0 to 0.1)	109 (51 to 183)	0.0 (0.0 to 0.1)	39 (22 to 75)	0.0 (0.0 to 0.0)	-79.5 (-89.8 to -51.1)	-81.8 (-90.9 to -56.6)
Global	Otitis media	10 to 14	Both	399 (277 to 576)	0.1 (0.1 to 0.1)	267 (171 to 420)	0.0 (0.0 to 0.1)	76 (48 to 130)	0.0 (0.0 to 0.0)	-80.9 (-89.0 to -64.4)	-83.5 (-90.5 to -69.2)
Global	Otitis media	10 to 14	Female	207 (132 to 330)	0.1 (0.1 to 0.1)	141 (77 to 269)	0.0 (0.0 to 0.1)	28 (15 to 60)	0.0 (0.0 to 0.0)	-86.3 (-93.6 to -69.5)	-88.0 (-94.4 to -73.3)
Global	Otitis media	10 to 14	Male	193 (96 to 321)	0.1 (0.0 to 0.1)	126 (61 to 216)	0.0 (0.0 to 0.1)	48 (27 to 100)	0.0 (0.0 to 0.0)	-75.0 (-87.3 to -34.7)	-78.7 (-89.2 to -44.2)
Global	Meningitis	5 to 9	Both	24671 (17709 to 31310)	4.3 (3.1 to 5.4)	22384 (16609 to 28221)	3.6 (2.7 to 4.6)	17025 (12207 to 23726)	2.7 (1.9 to 3.7)	-31.0 (-48.8 to 0.0)	-38.2 (-54.1 to -10.4)
Global	Meningitis	5 to 9	Female	12973 (9067 to 15888)	4.6 (3.2 to 5.7)	11207 (8520 to 14001)	3.8 (2.9 to 4.7)	6739 (5103 to 9136)	2.2 (1.6 to 2.9)	-48.0 (-59.0 to -23.8)	-53.0 (-62.9 to -31.1)
Global	Meningitis	5 to 9	Male	11698 (5553 to 16872)	4.0 (1.9 to 5.7)	11177 (6235 to 16376)	3.5 (2.0 to 5.2)	10286 (5963 to 17020)	3.1 (1.8 to 5.1)	-12.1 (-40.9 to 61.5)	-22.0 (-47.6 to 43.2)
Global	Meningitis	10 to 14	Both	15125 (11371 to 18519)	2.9 (2.2 to 3.5)	15663 (12088 to 19810)	2.5 (1.9 to 3.2)	13105 (9806 to 18101)	2.1 (1.6 to 3.0)	-13.4 (-30.0 to 16.1)	-25.1 (-39.5 to 0.4)
Global	Meningitis	10 to 14	Female	8113 (6097 to 9660)	3.1 (2.4 to 3.7)	7998 (6248 to 9748)	2.6 (2.0 to 3.2)	5521 (4404 to 7236)	1.9 (1.5 to 2.5)	-31.9 (-41.9 to -13.0)	-40.4 (-49.2 to -23.9)

eTable 8. All-cause and cause-specific mortality (counts and rates) at the global level and by country in the WHO European Region for ages 5 to 9 and 10 to 14 for 1990, 2000, and 2016

Global	Meningitis	10 to 14	Male	7012 (3702 to 9932)	2.6 (1.4 to 3.7)	7665 (4540 to 11440)	2.4 (1.4 to 3.6)	7584 (4594 to 12493)	2.4 (1.5 to 4.0)	8.2 (-20.5 to 73.6)	-7.6 (-32.1 to 48.3)
Global	Encephalitis	5 to 9	Both	8733 (5309 to 12327)	1.5 (0.9 to 2.1)	8278 (5863 to 10916)	1.3 (1.0 to 1.8)	5142 (3688 to 7727)	0.8 (0.6 to 1.2)	-41.1 (-65.5 to 14.6)	-47.3 (-69.1 to 2.7)
Global	Encephalitis	5 to 9	Female	4503 (2404 to 7523)	1.6 (0.9 to 2.7)	4220 (2655 to 6471)	1.4 (0.9 to 2.2)	2396 (1561 to 3997)	0.8 (0.5 to 1.3)	-46.8 (-70.1 to 14.2)	-51.8 (-73.0 to 3.3)
Global	Encephalitis	5 to 9	Male	4231 (2451 to 6116)	1.4 (0.8 to 2.1)	4058 (2701 to 5347)	1.3 (0.9 to 1.7)	2746 (2003 to 4139)	0.8 (0.6 to 1.2)	-35.1 (-63.1 to 19.8)	-42.4 (-67.3 to 6.3)
Global	Encephalitis	10 to 14	Both	5666 (3742 to 7776)	1.1 (0.7 to 1.5)	6153 (4614 to 8101)	1.0 (0.7 to 1.3)	4249 (3119 to 6167)	0.7 (0.5 to 1.0)	-25.0 (-51.7 to 28.0)	-35.2 (-58.2 to 10.6)
Global	Encephalitis	10 to 14	Female	2716 (1596 to 4436)	1.1 (0.6 to 1.7)	2905 (1961 to 4444)	1.0 (0.6 to 1.5)	1894 (1266 to 3206)	0.6 (0.4 to 1.1)	-30.3 (-57.5 to 31.5)	-39.0 (-62.8 to 15.1)
Global	Encephalitis	10 to 14	Male	2950 (1868 to 3961)	1.1 (0.7 to 1.5)	3248 (2298 to 4140)	1.0 (0.7 to 1.3)	2355 (1780 to 3589)	0.7 (0.6 to 1.1)	-20.2 (-48.7 to 30.1)	-31.8 (-56.1 to 11.2)
Global	Diphtheria	5 to 9	Both	849 (535 to 1231)	0.1 (0.1 to 0.2)	571 (359 to 877)	0.1 (0.1 to 0.1)	96 (57 to 166)	0.0 (0.0 to 0.0)	-88.7 (-93.4 to -78.7)	-89.9 (-94.1 to -80.9)
Global	Diphtheria	5 to 9	Female	417 (242 to 665)	0.1 (0.1 to 0.2)	276 (155 to 474)	0.1 (0.1 to 0.2)	40 (20 to 76)	0.0 (0.0 to 0.0)	-90.4 (-95.5 to -78.2)	-91.3 (-95.9 to -80.2)
Global	Diphtheria	5 to 9	Male	432 (256 to 659)	0.1 (0.1 to 0.2)	295 (157 to 512)	0.1 (0.0 to 0.2)	56 (29 to 112)	0.0 (0.0 to 0.0)	-87.0 (-93.9 to -71.5)	-88.5 (-94.6 to -74.7)
Global	Diphtheria	10 to 14	Both	355 (242 to 491)	0.1 (0.0 to 0.1)	260 (165 to 398)	0.0 (0.0 to 0.1)	48 (31 to 72)	0.0 (0.0 to 0.0)	-86.6 (-91.3 to -79.0)	-88.4 (-92.5 to -81.9)
Global	Diphtheria	10 to 14	Female	170 (107 to 261)	0.1 (0.0 to 0.1)	123 (67 to 210)	0.0 (0.0 to 0.1)	20 (11 to 32)	0.0 (0.0 to 0.0)	-88.4 (-93.6 to -78.8)	-89.9 (-94.4 to -81.5)
Global	Diphtheria	10 to 14	Male	185 (120 to 280)	0.1 (0.0 to 0.1)	138 (78 to 231)	0.0 (0.0 to 0.1)	28 (17 to 46)	0.0 (0.0 to 0.0)	-84.8 (-91.7 to -72.9)	-87.0 (-92.9 to -76.8)
Global	Whooping cough	5 to 9	Both	8523 (3709 to 17305)	1.5 (0.6 to 3.0)	7217 (3265 to 14527)	1.2 (0.5 to 2.4)	3357 (1698 to 6021)	0.5 (0.3 to 0.9)	-60.6 (-80.1 to -20.0)	-64.7 (-82.1 to -28.3)
Global	Whooping cough	5 to 9	Female	4961 (2125 to 10024)	1.8 (0.8 to 3.6)	4145 (1866 to 8419)	1.4 (0.6 to 2.8)	1880 (947 to 3344)	0.6 (0.3 to 1.1)	-62.1 (-80.9 to -21.3)	-65.7 (-82.8 to -28.8)
Global	Whooping cough	5 to 9	Male	3562 (1570 to 7085)	1.2 (0.5 to 2.4)	3072 (1407 to 6098)	1.0 (0.4 to 1.9)	1476 (744 to 2669)	0.4 (0.2 to 0.8)	-58.6 (-78.7 to -16.9)	-63.2 (-81.1 to -26.3)
Global	Whooping cough	10 to 14	Both	1657 (703 to 3452)	0.3 (0.1 to 0.7)	1502 (671 to 3058)	0.2 (0.1 to 0.5)	690 (347 to 1248)	0.1 (0.1 to 0.2)	-58.4 (-79.0 to -14.9)	-64.0 (-81.8 to -26.5)
Global	Whooping cough	10 to 14	Female	911 (388 to 1896)	0.4 (0.2 to 0.7)	818 (363 to 1673)	0.3 (0.1 to 0.5)	370 (184 to 673)	0.1 (0.1 to 0.2)	-59.4 (-79.5 to -15.4)	-64.5 (-82.0 to -26.0)
Global	Whooping cough	10 to 14	Male	746 (315 to 1561)	0.3 (0.1 to 0.6)	684 (302 to 1396)	0.2 (0.1 to 0.4)	320 (164 to 580)	0.1 (0.1 to 0.2)	-57.1 (-78.2 to -12.9)	-63.4 (-81.4 to -25.6)
Global	Tetanus	5 to 9	Both	5510 (2581 to 8687)	1.0 (0.4 to 1.5)	2910 (1386 to 4821)	0.5 (0.2 to 0.8)	727 (344 to 1278)	0.1 (0.1 to 0.2)	-86.8 (-91.7 to -75.3)	-88.2 (-92.6 to -77.9)
Global	Tetanus	5 to 9	Female	1770 (704 to 3738)	0.6 (0.3 to 1.3)	895 (387 to 2035)	0.3 (0.1 to 0.7)	211 (96 to 495)	0.1 (0.0 to 0.2)	-88.1 (-93.6 to -76.1)	-89.2 (-94.2 to -78.4)
Global	Tetanus	5 to 9	Male	3740 (1376 to 6044)	1.3 (0.5 to 2.1)	2015 (786 to 3466)	0.6 (0.2 to 1.1)	516 (210 to 927)	0.2 (0.1 to 0.3)	-86.2 (-92.5 to -65.3)	-87.8 (-93.4 to -69.3)
Global	Tetanus	10 to 14	Both	4768 (2199 to 8566)	0.9 (0.4 to 1.6)	2885 (1353 to 4851)	0.5 (0.2 to 0.8)	838 (411 to 1460)	0.1 (0.1 to 0.2)	-82.4 (-89.8 to -67.0)	-84.8 (-91.2 to -71.4)
Global	Tetanus	10 to 14	Female	2711 (749 to 5977)	1.1 (0.3 to 2.3)	1609 (511 to 3331)	0.5 (0.2 to 1.1)	412 (142 to 897)	0.1 (0.0 to 0.3)	-84.8 (-93.9 to -60.8)	-86.7 (-94.7 to -65.7)
Global	Tetanus	10 to 14	Male	2057 (906 to 3127)	0.8 (0.3 to 1.2)	1277 (591 to 2040)	0.4 (0.2 to 0.6)	426 (211 to 767)	0.1 (0.1 to 0.2)	-79.3 (-87.9 to -53.9)	-82.3 (-89.7 to -60.6)
Global	Measles	5 to 9	Both	88331 (36321 to 175462)	15.4 (6.3 to 30.5)	59600 (23875 to 119403)	9.7 (3.9 to 19.4)	6030 (2124 to 13703)	0.9 (0.3 to 2.1)	-93.2 (-95.1 to -91.1)	-93.9 (-95.6 to -92.1)
Global	Measles	5 to 9	Female	45531 (19085 to 89754)	16.2 (6.8 to 32.0)	30468 (12321 to 61147)	10.2 (4.1 to 20.5)	2979 (1054 to 6566)	1.0 (0.3 to 2.1)	-93.5 (-95.3 to -91.3)	-94.1 (-95.8 to -92.1)
Global	Measles	5 to 9	Male	42799 (17257 to 84746)	14.5 (5.9 to 28.8)	29133 (11205 to 59520)	9.2 (3.5 to 18.8)	3051 (1087 to 6873)	0.9 (0.3 to 2.1)	-92.9 (-94.8 to -90.7)	-93.7 (-95.4 to -91.8)
Global	Measles	10 to 14	Both	23812 (8687 to 51831)	4.5 (1.6 to 9.8)	16906 (6155 to 36813)	2.7 (1.0 to 5.9)	1718 (605 to 3938)	0.3 (0.1 to 0.6)	-92.8 (-94.4 to -90.8)	-93.8 (-95.2 to -92.1)
Global	Measles	10 to 14	Female	12441 (4542 to 26752)	4.8 (1.8 to 10.4)	8776 (3145 to 19220)	2.9 (1.0 to 6.3)	871 (305 to 2008)	0.3 (0.1 to 0.7)	-93.0 (-94.7 to -91.0)	-93.9 (-95.3 to -92.1)
Global	Measles	10 to 14	Male	11371 (4071 to 24887)	4.2 (1.5 to 9.2)	8130 (2853 to 17672)	2.5 (0.9 to 5.5)	846 (299 to 1932)	0.3 (0.1 to 0.6)	-92.6 (-94.3 to -90.5)	-93.6 (-95.1 to -91.9)
Global	Varicella and herpes zoster	5 to 9	Both	1943 (1534 to 2423)	0.3 (0.3 to 0.4)	1617 (1286 to 1993)	0.3 (0.2 to 0.3)	975 (783 to 1226)	0.2 (0.1 to 0.2)	-49.8 (-62.1 to -32.8)	-55.0 (-66.1 to -39.8)

eTable 8. All-cause and cause-specific mortality (counts and rates) at the global level and by country in the WHO European Region for ages 5 to 9 and 10 to 14 for 1990, 2000, and 2016

Global	Varicella and herpes zoster	5 to 9	Female	989 (733 to 1314)	0.4 (0.3 to 0.5)	807 (592 to 1066)	0.3 (0.2 to 0.4)	473 (346 to 628)	0.2 (0.1 to 0.2)	-52.2 (-67.1 to -29.1)	-56.7 (-70.3 to -35.8)
Global	Varicella and herpes zoster	5 to 9	Male	955 (723 to 1244)	0.3 (0.2 to 0.4)	810 (613 to 1054)	0.3 (0.2 to 0.3)	503 (385 to 663)	0.2 (0.1 to 0.2)	-47.4 (-63.4 to -25.4)	-53.3 (-67.5 to -33.8)
Global	Varicella and herpes zoster	10 to 14	Both	606 (504 to 722)	0.1 (0.1 to 0.1)	549 (459 to 665)	0.1 (0.1 to 0.1)	330 (273 to 410)	0.1 (0.0 to 0.1)	-45.5 (-56.2 to -30.5)	-52.9 (-62.1 to -39.9)
Global	Varicella and herpes zoster	10 to 14	Female	302 (234 to 389)	0.1 (0.1 to 0.2)	270 (209 to 352)	0.1 (0.1 to 0.1)	158 (122 to 210)	0.1 (0.0 to 0.1)	-47.6 (-62.1 to -26.9)	-54.2 (-66.9 to -36.0)
Global	Varicella and herpes zoster	10 to 14	Male	304 (245 to 382)	0.1 (0.1 to 0.1)	279 (219 to 351)	0.1 (0.1 to 0.1)	172 (134 to 227)	0.1 (0.0 to 0.1)	-43.4 (-58.4 to -22.2)	-51.7 (-64.4 to -33.5)
Global	Malaria	5 to 9	Both	46618 (30962 to 63599)	8.1 (5.4 to 11.1)	55886 (40404 to 72517)	9.1 (6.6 to 11.8)	44180 (33566 to 57135)	6.9 (5.2 to 8.9)	-5.2 (-35.0 to 48.2)	-15.1 (-41.8 to 32.7)
Global	Malaria	5 to 9	Female	22999 (13032 to 34843)	8.2 (4.6 to 12.4)	28266 (18188 to 39899)	9.5 (6.1 to 13.4)	22269 (14930 to 31530)	7.2 (4.8 to 10.2)	-3.2 (-43.5 to 70.9)	-12.4 (-48.9 to 54.7)
Global	Malaria	5 to 9	Male	23619 (13708 to 33394)	8.0 (4.7 to 11.3)	27620 (17647 to 39470)	8.7 (5.6 to 12.5)	21910 (14542 to 31305)	6.6 (4.4 to 9.4)	-7.2 (-45.1 to 77.1)	-17.7 (-51.4 to 57.1)
Global	Malaria	10 to 14	Both	22135 (15357 to 29169)	4.2 (2.9 to 5.5)	29144 (21269 to 37402)	4.6 (3.4 to 6.0)	22721 (16916 to 29458)	3.7 (2.8 to 4.8)	2.6 (-28.9 to 52.6)	-11.3 (-38.6 to 31.9)
Global	Malaria	10 to 14	Female	10293 (6259 to 14850)	4.0 (2.4 to 5.8)	13848 (9123 to 19462)	4.5 (3.0 to 6.4)	10764 (7181 to 15476)	3.6 (2.4 to 5.2)	4.6 (-37.2 to 76.2)	-8.5 (-45.0 to 54.2)
Global	Malaria	10 to 14	Male	11841 (6838 to 17207)	4.4 (2.5 to 6.4)	15297 (10019 to 21776)	4.8 (3.1 to 6.8)	11957 (7938 to 17042)	3.8 (2.5 to 5.4)	1.0 (-39.1 to 87.5)	-13.7 (-48.0 to 60.2)
Global	Leishmaniasis	5 to 9	Both	3064 (1557 to 5708)	0.5 (0.3 to 1.0)	996 (522 to 1833)	0.2 (0.1 to 0.3)	660 (343 to 1211)	0.1 (0.1 to 0.2)	-78.5 (-83.4 to -71.5)	-80.7 (-85.1 to -74.5)
Global	Leishmaniasis	5 to 9	Female	1559 (711 to 3087)	0.6 (0.3 to 1.1)	499 (235 to 1024)	0.2 (0.1 to 0.3)	302 (137 to 608)	0.1 (0.0 to 0.2)	-80.6 (-86.4 to -73.0)	-82.5 (-87.7 to -75.6)
Global	Leishmaniasis	5 to 9	Male	1505 (821 to 2575)	0.5 (0.3 to 0.9)	497 (275 to 849)	0.2 (0.1 to 0.3)	358 (197 to 626)	0.1 (0.1 to 0.2)	-76.2 (-81.7 to -68.5)	-78.9 (-83.7 to -72.0)
Global	Leishmaniasis	10 to 14	Both	1942 (1014 to 3402)	0.4 (0.2 to 0.6)	669 (347 to 1189)	0.1 (0.1 to 0.2)	461 (241 to 814)	0.1 (0.0 to 0.1)	-76.3 (-80.4 to -71.7)	-79.5 (-83.0 to -75.6)
Global	Leishmaniasis	10 to 14	Female	958 (454 to 1832)	0.4 (0.2 to 0.7)	330 (151 to 641)	0.1 (0.0 to 0.2)	215 (96 to 420)	0.1 (0.0 to 0.1)	-77.5 (-82.4 to -71.2)	-80.3 (-84.6 to -74.8)
Global	Leishmaniasis	10 to 14	Male	984 (546 to 1642)	0.4 (0.2 to 0.6)	339 (186 to 576)	0.1 (0.1 to 0.2)	245 (131 to 412)	0.1 (0.0 to 0.1)	-75.1 (-80.2 to -69.2)	-78.7 (-83.1 to -73.7)
Global	African trypanosomiasis	5 to 9	Both	1407 (434 to 3238)	0.2 (0.1 to 0.6)	1604 (513 to 3814)	0.3 (0.1 to 0.6)	183 (43 to 576)	0.0 (0.0 to 0.1)	-87.0 (-97.2 to -44.8)	-88.3 (-97.5 to -50.6)
Global	African trypanosomiasis	5 to 9	Female	697 (151 to 1923)	0.2 (0.1 to 0.7)	786 (168 to 2438)	0.3 (0.1 to 0.8)	93 (15 to 363)	0.0 (0.0 to 0.1)	-86.6 (-98.2 to -10.3)	-87.9 (-98.4 to -18.8)
Global	African trypanosomiasis	5 to 9	Male	710 (164 to 2008)	0.2 (0.1 to 0.7)	818 (178 to 2438)	0.3 (0.1 to 0.8)	90 (15 to 360)	0.0 (0.0 to 0.1)	-87.4 (-98.3 to -26.0)	-88.8 (-98.5 to -34.4)
Global	African trypanosomiasis	10 to 14	Both	1565 (545 to 3330)	0.3 (0.1 to 0.6)	1816 (657 to 3843)	0.3 (0.1 to 0.6)	223 (55 to 607)	0.0 (0.0 to 0.1)	-85.7 (-96.3 to -46.8)	-87.7 (-96.8 to -54.0)
Global	African trypanosomiasis	10 to 14	Female	768 (190 to 1940)	0.3 (0.1 to 0.8)	877 (229 to 2331)	0.3 (0.1 to 0.8)	105 (18 to 380)	0.0 (0.0 to 0.1)	-86.3 (-97.6 to -37.1)	-88.0 (-97.9 to -45.0)
Global	African trypanosomiasis	10 to 14	Male	797 (196 to 1953)	0.3 (0.1 to 0.7)	938 (230 to 2527)	0.3 (0.1 to 0.8)	118 (21 to 447)	0.0 (0.0 to 0.1)	-85.2 (-97.7 to -12.4)	-87.4 (-98.1 to -25.1)
Global	Schistosomiasis	5 to 9	Both	375 (253 to 548)	0.1 (0.0 to 0.1)	297 (202 to 425)	0.0 (0.0 to 0.1)	174 (115 to 264)	0.0 (0.0 to 0.0)	-53.5 (-72.1 to -19.4)	-58.3 (-75.0 to -27.8)
Global	Schistosomiasis	5 to 9	Female	168 (103 to 265)	0.1 (0.0 to 0.1)	134 (82 to 206)	0.0 (0.0 to 0.1)	76 (45 to 122)	0.0 (0.0 to 0.0)	-55.0 (-75.7 to -15.5)	-59.2 (-78.0 to -23.5)
Global	Schistosomiasis	5 to 9	Male	207 (128 to 331)	0.1 (0.0 to 0.1)	164 (102 to 257)	0.1 (0.0 to 0.1)	99 (56 to 172)	0.0 (0.0 to 0.1)	-52.2 (-76.3 to -2.8)	-57.6 (-79.0 to -13.8)
Global	Schistosomiasis	10 to 14	Both	468 (339 to 626)	0.1 (0.1 to 0.1)	414 (300 to 558)	0.1 (0.0 to 0.1)	265 (184 to 374)	0.0 (0.0 to 0.1)	-43.4 (-61.7 to -14.8)	-51.1 (-66.9 to -26.3)
Global	Schistosomiasis	10 to 14	Female	212 (139 to 311)	0.1 (0.1 to 0.1)	186 (119 to 278)	0.1 (0.0 to 0.1)	112 (71 to 177)	0.0 (0.0 to 0.1)	-47.2 (-68.9 to -12.1)	-53.8 (-72.8 to -23.1)
Global	Schistosomiasis	10 to 14	Male	256 (172 to 363)	0.1 (0.1 to 0.1)	229 (153 to 335)	0.1 (0.0 to 0.1)	153 (97 to 234)	0.0 (0.0 to 0.1)	-40.3 (-65.2 to 2.6)	-48.9 (-70.3 to -12.3)

eTable 8. All-cause and cause-specific mortality (counts and rates) at the global level and by country in the WHO European Region for ages 5 to 9 and 10 to 14 for 1990, 2000, and 2016

Global	Cysticercosis	5 to 9	Both	102<br>(76 to 137)	0.0<br>(0.0 to 0.0)	84<br>(63 to 116)	0.0<br>(0.0 to 0.0)	47<br>(35 to 66)	0.0<br>(0.0 to 0.0)	-53.8<br>(-61.8 to -44.4)	-58.6<br>(-65.8 to -50.2)
Global	Cysticercosis	5 to 9	Female	56<br>(41 to 75)	0.0<br>(0.0 to 0.0)	46<br>(33 to 64)	0.0<br>(0.0 to 0.0)	25<br>(18 to 35)	0.0<br>(0.0 to 0.0)	-55.3<br>(-64.1 to -44.5)	-59.5<br>(-67.5 to -49.8)
Global	Cysticercosis	5 to 9	Male	47<br>(34 to 62)	0.0<br>(0.0 to 0.0)	39<br>(28 to 53)	0.0<br>(0.0 to 0.0)	22<br>(16 to 31)	0.0<br>(0.0 to 0.0)	-52.0<br>(-60.7 to -41.8)	-57.4<br>(-65.1 to -48.4)
Global	Cysticercosis	10 to 14	Both	73<br>(58 to 90)	0.0<br>(0.0 to 0.0)	67<br>(52 to 85)	0.0<br>(0.0 to 0.0)	41<br>(32 to 52)	0.0<br>(0.0 to 0.0)	-43.7<br>(-50.5 to -36.2)	-51.4<br>(-57.2 to -44.8)
Global	Cysticercosis	10 to 14	Female	38<br>(29 to 47)	0.0<br>(0.0 to 0.0)	35<br>(27 to 44)	0.0<br>(0.0 to 0.0)	21<br>(16 to 27)	0.0<br>(0.0 to 0.0)	-44.3<br>(-51.6 to -35.7)	-51.2<br>(-57.7 to -43.7)
Global	Cysticercosis	10 to 14	Male	36<br>(28 to 44)	0.0<br>(0.0 to 0.0)	32<br>(25 to 41)	0.0<br>(0.0 to 0.0)	20<br>(15 to 26)	0.0<br>(0.0 to 0.0)	-43.2<br>(-51.1 to -34.9)	-51.4<br>(-58.3 to -44.3)
Global	Yellow fever	5 to 9	Both	2020<br>(406 to 6113)	0.4<br>(0.1 to 1.1)	1253<br>(257 to 3767)	0.2<br>(0.0 to 0.6)	920<br>(180 to 2725)	0.1<br>(0.0 to 0.4)	-54.5<br>(-72.9 to -25.1)	-59.2<br>(-75.7 to -32.9)
Global	Yellow fever	5 to 9	Female	554<br>(103 to 1724)	0.2<br>(0.0 to 0.6)	357<br>(71 to 1072)	0.1<br>(0.0 to 0.4)	266<br>(50 to 783)	0.1<br>(0.0 to 0.3)	-51.9<br>(-74.3 to -12.0)	-56.5<br>(-76.7 to -20.4)
Global	Yellow fever	5 to 9	Male	1466<br>(291 to 4540)	0.5<br>(0.1 to 1.5)	896<br>(175 to 2747)	0.3<br>(0.1 to 0.9)	653<br>(126 to 1962)	0.2<br>(0.0 to 0.6)	-55.4<br>(-75.5 to -19.0)	-60.5<br>(-78.3 to -28.2)
Global	Yellow fever	10 to 14	Both	1731<br>(385 to 5203)	0.3<br>(0.1 to 1.0)	1139<br>(242 to 3393)	0.2<br>(0.0 to 0.5)	833<br>(176 to 2388)	0.1<br>(0.0 to 0.4)	-51.9<br>(-70.1 to -19.2)	-58.4<br>(-74.2 to -30.2)
Global	Yellow fever	10 to 14	Female	489<br>(96 to 1468)	0.2<br>(0.0 to 0.6)	329<br>(60 to 963)	0.1<br>(0.0 to 0.3)	243<br>(49 to 727)	0.1<br>(0.0 to 0.2)	-50.2<br>(-71.4 to -9.7)	-56.4<br>(-75.0 to -21.0)
Global	Yellow fever	10 to 14	Male	1242<br>(257 to 3767)	0.5<br>(0.1 to 1.4)	811<br>(167 to 2542)	0.3<br>(0.1 to 0.8)	589<br>(120 to 1702)	0.2<br>(0.0 to 0.5)	-52.5<br>(-73.7 to -9.9)	-59.4<br>(-77.5 to -23.0)
Global	Rabies	5 to 9	Both	10129<br>(5143 to 15168)	1.8<br>(0.9 to 2.6)	7319<br>(3470 to 11282)	1.2<br>(0.6 to 1.8)	2130<br>(824 to 3926)	0.3<br>(0.1 to 0.6)	-79.0<br>(-88.5 to -57.9)	-81.2<br>(-89.7 to -62.3)
Global	Rabies	5 to 9	Female	4748<br>(1781 to 8891)	1.7<br>(0.6 to 3.2)	2927<br>(997 to 5877)	1.0<br>(0.3 to 2.0)	636<br>(177 to 1439)	0.2<br>(0.1 to 0.5)	-86.6<br>(-94.4 to -66.6)	-87.9<br>(-94.9 to -69.8)
Global	Rabies	5 to 9	Male	5381<br>(2161 to 8428)	1.8<br>(0.7 to 2.9)	4391<br>(1809 to 7060)	1.4<br>(0.6 to 2.2)	1494<br>(467 to 2981)	0.5<br>(0.1 to 0.9)	-72.2<br>(-86.1 to -37.0)	-75.4<br>(-87.7 to -44.1)
Global	Rabies	10 to 14	Both	5221<br>(2483 to 7281)	1.0<br>(0.5 to 1.4)	4011<br>(1859 to 5507)	0.6<br>(0.3 to 0.9)	1155<br>(508 to 1831)	0.2<br>(0.1 to 0.3)	-77.9<br>(-85.8 to -64.2)	-80.9<br>(-87.7 to -69.0)
Global	Rabies	10 to 14	Female	2310<br>(655 to 3829)	0.9<br>(0.3 to 1.5)	1433<br>(420 to 2414)	0.5<br>(0.1 to 0.8)	344<br>(91 to 687)	0.1<br>(0.0 to 0.2)	-85.1<br>(-92.8 to -68.3)	-87.0<br>(-93.7 to -72.3)
Global	Rabies	10 to 14	Male	2911<br>(1129 to 4353)	1.1<br>(0.4 to 1.6)	2578<br>(968 to 3718)	0.8<br>(0.3 to 1.2)	811<br>(300 to 1332)	0.3<br>(0.1 to 0.4)	-72.1<br>(-83.3 to -51.4)	-76.2<br>(-85.8 to -58.5)
Global	Intestinal nematode infections	5 to 9	Both	1550<br>(1212 to 1948)	0.3<br>(0.2 to 0.3)	1174<br>(913 to 1449)	0.2<br>(0.1 to 0.2)	535<br>(421 to 663)	0.1<br>(0.1 to 0.1)	-65.5<br>(-72.8 to -56.0)	-69.1<br>(-75.7 to -60.6)
Global	Intestinal nematode infections	5 to 9	Female	877<br>(664 to 1142)	0.3<br>(0.2 to 0.4)	660<br>(500 to 848)	0.2<br>(0.2 to 0.3)	282<br>(213 to 368)	0.1<br>(0.1 to 0.1)	-67.8<br>(-76.2 to -56.7)	-70.9<br>(-78.4 to -60.8)
Global	Intestinal nematode infections	5 to 9	Male	674<br>(527 to 847)	0.2<br>(0.2 to 0.3)	514<br>(391 to 646)	0.2<br>(0.1 to 0.2)	253<br>(193 to 332)	0.1<br>(0.1 to 0.1)	-62.5<br>(-71.9 to -50.6)	-66.7<br>(-75.1 to -56.2)
Global	Intestinal nematode infections	10 to 14	Both	290<br>(233 to 359)	0.1<br>(0.0 to 0.1)	243<br>(196 to 302)	0.0<br>(0.0 to 0.0)	121<br>(98 to 150)	0.0<br>(0.0 to 0.0)	-58.2<br>(-65.6 to -49.6)	-63.9<br>(-70.3 to -56.4)
Global	Intestinal nematode infections	10 to 14	Female	160<br>(124 to 208)	0.1<br>(0.0 to 0.1)	132<br>(103 to 169)	0.0<br>(0.0 to 0.1)	63<br>(48 to 80)	0.0<br>(0.0 to 0.0)	-60.9<br>(-69.9 to -49.5)	-65.8<br>(-73.6 to -55.9)
Global	Intestinal nematode infections	10 to 14	Male	130<br>(104 to 160)	0.0<br>(0.0 to 0.1)	111<br>(87 to 140)	0.0<br>(0.0 to 0.0)	59<br>(45 to 76)	0.0<br>(0.0 to 0.0)	-54.9<br>(-64.8 to -41.4)	-61.5<br>(-69.9 to -49.9)
Global	Ebola	5 to 9	Both	0<br>(0 to 0)	0.0<br>(0.0 to 0.0)	11<br>(8 to 14)	0.0<br>(0.0 to 0.0)	0<br>(0 to 0)	0.0<br>(0.0 to 0.0)	0.0<br>(0.0 to 0.0)	0.0<br>(0.0 to 0.0)
Global	Ebola	5 to 9	Female	0<br>(0 to 0)	0.0<br>(0.0 to 0.0)	5<br>(4 to 7)	0.0<br>(0.0 to 0.0)	0<br>(0 to 0)	0.0<br>(0.0 to 0.0)	0.0<br>(0.0 to 0.0)	0.0<br>(0.0 to 0.0)
Global	Ebola	5 to 9	Male	0<br>(0 to 0)	0.0<br>(0.0 to 0.0)	6<br>(4 to 7)	0.0<br>(0.0 to 0.0)	0<br>(0 to 0)	0.0<br>(0.0 to 0.0)	0.0<br>(0.0 to 0.0)	0.0<br>(0.0 to 0.0)
Global	Ebola	10 to 14	Both	0<br>(0 to 0)	0.0<br>(0.0 to 0.0)	10<br>(7 to 13)	0.0<br>(0.0 to 0.0)	0<br>(0 to 0)	0.0<br>(0.0 to 0.0)	0.0<br>(0.0 to 0.0)	0.0<br>(0.0 to 0.0)
Global	Ebola	10 to 14	Female	0<br>(0 to 0)	0.0<br>(0.0 to 0.0)	5<br>(4 to 6)	0.0<br>(0.0 to 0.0)	0<br>(0 to 0)	0.0<br>(0.0 to 0.0)	0.0<br>(0.0 to 0.0)	0.0<br>(0.0 to 0.0)

eTable 8. All-cause and cause-specific mortality (counts and rates) at the global level and by country in the WHO European Region for ages 5 to 9 and 10 to 14 for 1990, 2000, and 2016

Global	Ebola	10 to 14	Male	0<br>(0 to 0)	0.0<br>(0.0 to 0.0)	5<br>(4 to 6)	0.0<br>(0.0 to 0.0)	0<br>(0 to 0)	0.0<br>(0.0 to 0.0)	0.0<br>(0.0 to 0.0)	0.0<br>(0.0 to 0.0)
Global	Zika virus	5 to 9	Both	0<br>(0 to 0)	0.0<br>(0.0 to 0.0)	0<br>(0 to 0)	0.0<br>(0.0 to 0.0)	1<br>(0 to 4)	0.0<br>(0.0 to 0.0)	0.0<br>(0.0 to 0.0)	0.0<br>(0.0 to 0.0)
Global	Zika virus	5 to 9	Female	0<br>(0 to 0)	0.0<br>(0.0 to 0.0)	0<br>(0 to 0)	0.0<br>(0.0 to 0.0)	1<br>(0 to 3)	0.0<br>(0.0 to 0.0)	0.0<br>(0.0 to 0.0)	0.0<br>(0.0 to 0.0)
Global	Zika virus	5 to 9	Male	0<br>(0 to 0)	0.0<br>(0.0 to 0.0)	0<br>(0 to 0)	0.0<br>(0.0 to 0.0)	0<br>(0 to 2)	0.0<br>(0.0 to 0.0)	0.0<br>(0.0 to 0.0)	0.0<br>(0.0 to 0.0)
Global	Zika virus	10 to 14	Both	0<br>(0 to 0)	0.0<br>(0.0 to 0.0)	0<br>(0 to 0)	0.0<br>(0.0 to 0.0)	1<br>(0 to 5)	0.0<br>(0.0 to 0.0)	0.0<br>(0.0 to 0.0)	0.0<br>(0.0 to 0.0)
Global	Zika virus	10 to 14	Female	0<br>(0 to 0)	0.0<br>(0.0 to 0.0)	0<br>(0 to 0)	0.0<br>(0.0 to 0.0)	1<br>(0 to 4)	0.0<br>(0.0 to 0.0)	0.0<br>(0.0 to 0.0)	0.0<br>(0.0 to 0.0)
Global	Zika virus	10 to 14	Male	0<br>(0 to 0)	0.0<br>(0.0 to 0.0)	0<br>(0 to 0)	0.0<br>(0.0 to 0.0)	0<br>(0 to 3)	0.0<br>(0.0 to 0.0)	0.0<br>(0.0 to 0.0)	0.0<br>(0.0 to 0.0)
Global	Other neglected tropical diseases	5 to 9	Both	1952<br>(1171 to 3160)	0.3<br>(0.2 to 0.5)	1787<br>(1079 to 2860)	0.3<br>(0.2 to 0.5)	1854<br>(1043 to 3073)	0.3<br>(0.2 to 0.5)	-5.0<br>(-49.8 to 80.6)	-14.9<br>(-55.0 to 61.8)
Global	Other neglected tropical diseases	5 to 9	Female	892<br>(350 to 1522)	0.3<br>(0.1 to 0.5)	769<br>(351 to 1394)	0.3<br>(0.1 to 0.5)	638<br>(279 to 1342)	0.2<br>(0.1 to 0.4)	-28.5<br>(-67.5 to 63.1)	-35.2<br>(-70.6 to 47.6)
Global	Other neglected tropical diseases	5 to 9	Male	1060<br>(486 to 2001)	0.4<br>(0.2 to 0.7)	1018<br>(484 to 1899)	0.3<br>(0.2 to 0.6)	1216<br>(586 to 2307)	0.4<br>(0.2 to 0.7)	14.7<br>(-52.2 to 149.9)	1.7<br>(-57.6 to 121.6)
Global	Other neglected tropical diseases	10 to 14	Both	758<br>(491 to 1099)	0.1<br>(0.1 to 0.2)	769<br>(513 to 1110)	0.1<br>(0.1 to 0.2)	809<br>(512 to 1253)	0.1<br>(0.1 to 0.2)	6.8<br>(-38.6 to 77.6)	-7.7<br>(-46.9 to 53.5)
Global	Other neglected tropical diseases	10 to 14	Female	388<br>(181 to 645)	0.2<br>(0.1 to 0.2)	390<br>(191 to 674)	0.1<br>(0.1 to 0.2)	368<br>(168 to 726)	0.1<br>(0.1 to 0.2)	-5.3<br>(-52.2 to 96.5)	-17.1<br>(-58.2 to 71.9)
Global	Other neglected tropical diseases	10 to 14	Male	369<br>(229 to 561)	0.1<br>(0.1 to 0.2)	379<br>(236 to 626)	0.1<br>(0.1 to 0.2)	441<br>(267 to 742)	0.1<br>(0.1 to 0.2)	19.4<br>(-39.3 to 124.7)	2.1<br>(-48.2 to 92.1)
Global	Maternal hemorrhage	10 to 14	Both	372<br>(253 to 536)	0.1<br>(0.0 to 0.1)	313<br>(209 to 446)	0.0<br>(0.0 to 0.1)	202<br>(126 to 295)	0.0<br>(0.0 to 0.0)	-45.8<br>(-59.3 to -27.4)	-53.2<br>(-64.8 to -37.2)
Global	Maternal hemorrhage	10 to 14	Female	372<br>(253 to 536)	0.1<br>(0.1 to 0.2)	313<br>(209 to 446)	0.1<br>(0.1 to 0.1)	202<br>(126 to 295)	0.1<br>(0.0 to 0.1)	-45.8<br>(-59.3 to -27.4)	-52.6<br>(-64.4 to -36.5)
Global	Maternal sepsis and other maternal infections	10 to 14	Both	212<br>(139 to 302)	0.0<br>(0.0 to 0.1)	160<br>(101 to 230)	0.0<br>(0.0 to 0.0)	94<br>(57 to 146)	0.0<br>(0.0 to 0.0)	-55.6<br>(-67.8 to -39.6)	-61.7<br>(-72.1 to -47.8)
Global	Maternal sepsis and other maternal infections	10 to 14	Female	212<br>(139 to 302)	0.1<br>(0.1 to 0.1)	160<br>(101 to 230)	0.1<br>(0.0 to 0.1)	94<br>(57 to 146)	0.0<br>(0.0 to 0.0)	-55.6<br>(-67.8 to -39.6)	-61.2<br>(-71.8 to -47.2)
Global	Maternal hypertensive disorders	10 to 14	Both	293<br>(181 to 418)	0.1<br>(0.0 to 0.1)	285<br>(172 to 414)	0.0<br>(0.0 to 0.1)	233<br>(141 to 345)	0.0<br>(0.0 to 0.1)	-20.3<br>(-38.6 to 7.2)	-31.1<br>(-46.9 to -7.3)
Global	Maternal hypertensive disorders	10 to 14	Female	293<br>(181 to 418)	0.1<br>(0.1 to 0.2)	285<br>(172 to 414)	0.1<br>(0.1 to 0.1)	233<br>(141 to 345)	0.1<br>(0.0 to 0.1)	-20.3<br>(-38.6 to 7.2)	-30.3<br>(-46.3 to -6.2)
Global	Maternal obstructed labor and uterine rupture	10 to 14	Both	60<br>(31 to 105)	0.0<br>(0.0 to 0.0)	56<br>(28 to 100)	0.0<br>(0.0 to 0.0)	40<br>(20 to 70)	0.0<br>(0.0 to 0.0)	-32.2<br>(-49.9 to -6.6)	-41.4<br>(-56.7 to -19.3)

eTable 8. All-cause and cause-specific mortality (counts and rates) at the global level and by country in the WHO European Region for ages 5 to 9 and 10 to 14 for 1990, 2000, and 2016

Global	Maternal obstructed labor and uterine rupture	10 to 14	Female	60<br>(31 to 105)	0.0<br>(0.0 to 0.0)	56<br>(28 to 100)	0.0<br>(0.0 to 0.0)	40<br>(20 to 70)	0.0<br>(0.0 to 0.0)	-32.2<br>(-49.9 to -6.6)	-40.6<br>(-56.2 to -18.3)
Global	Maternal abortion, miscarriage, and ectopic pregnancy	10 to 14	Both	162<br>(98 to 242)	0.0<br>(0.0 to 0.0)	113<br>(67 to 186)	0.0<br>(0.0 to 0.0)	95<br>(53 to 153)	0.0<br>(0.0 to 0.0)	-41.4<br>(-57.2 to -20.1)	-49.4<br>(-63.0 to -30.9)
Global	Maternal abortion, miscarriage, and ectopic pregnancy	10 to 14	Female	162<br>(98 to 242)	0.1<br>(0.0 to 0.1)	113<br>(67 to 186)	0.0<br>(0.0 to 0.1)	95<br>(53 to 153)	0.0<br>(0.0 to 0.1)	-41.4<br>(-57.2 to -20.1)	-48.8<br>(-62.6 to -30.0)
Global	Indirect maternal deaths	10 to 14	Both	251<br>(158 to 368)	0.0<br>(0.0 to 0.1)	237<br>(152 to 343)	0.0<br>(0.0 to 0.1)	184<br>(109 to 278)	0.0<br>(0.0 to 0.0)	-26.7<br>(-44.0 to -1.0)	-36.7<br>(-51.5 to -14.4)
Global	Indirect maternal deaths	10 to 14	Female	251<br>(158 to 368)	0.1<br>(0.1 to 0.1)	237<br>(152 to 343)	0.1<br>(0.0 to 0.1)	184<br>(109 to 278)	0.1<br>(0.0 to 0.1)	-26.7<br>(-44.0 to -1.0)	-35.9<br>(-51.0 to -13.3)
Global	Late maternal deaths	10 to 14	Both	42<br>(21 to 76)	0.0<br>(0.0 to 0.0)	36<br>(16 to 73)	0.0<br>(0.0 to 0.0)	23<br>(10 to 49)	0.0<br>(0.0 to 0.0)	-44.0<br>(-63.5 to -20.9)	-51.6<br>(-68.5 to -31.6)
Global	Late maternal deaths	10 to 14	Female	42<br>(21 to 76)	0.0<br>(0.0 to 0.0)	36<br>(16 to 73)	0.0<br>(0.0 to 0.0)	23<br>(10 to 49)	0.0<br>(0.0 to 0.0)	-44.0<br>(-63.5 to -20.9)	-51.0<br>(-68.1 to -30.8)
Global	Maternal deaths aggravated by HIV/AIDS	10 to 14	Both	0<br>(0 to 0)	0.0<br>(0.0 to 0.0)	1<br>(0 to 1)	0.0<br>(0.0 to 0.0)	2<br>(1 to 3)	0.0<br>(0.0 to 0.0)	19210.7<br>(10729.3 to 39259.6)	16593.6<br>(9261.7 to 33925.5)
Global	Maternal deaths aggravated by HIV/AIDS	10 to 14	Female	0<br>(0 to 0)	0.0<br>(0.0 to 0.0)	1<br>(0 to 1)	0.0<br>(0.0 to 0.0)	2<br>(1 to 3)	0.0<br>(0.0 to 0.0)	19210.7<br>(10729.3 to 39259.6)	16798.1<br>(9376.3 to 34342.2)
Global	Other maternal disorders	10 to 14	Both	403<br>(282 to 574)	0.1<br>(0.1 to 0.1)	319<br>(213 to 468)	0.1<br>(0.0 to 0.1)	186<br>(120 to 282)	0.0<br>(0.0 to 0.0)	-53.8<br>(-65.0 to -39.4)	-60.1<br>(-69.8 to -47.6)
Global	Other maternal disorders	10 to 14	Female	403<br>(282 to 574)	0.2<br>(0.1 to 0.2)	319<br>(213 to 468)	0.1<br>(0.1 to 0.2)	186<br>(120 to 282)	0.1<br>(0.0 to 0.1)	-53.8<br>(-65.0 to -39.4)	-59.6<br>(-69.4 to -47.0)
Global	Protein-energy malnutrition	5 to 9	Both	12948<br>(10379 to 15359)	2.3<br>(1.8 to 2.7)	12694<br>(10136 to 15435)	2.1<br>(1.6 to 2.5)	7845<br>(6387 to 9976)	1.2<br>(1.0 to 1.6)	-39.4<br>(-52.8 to -17.8)	-45.7<br>(-57.8 to -26.4)
Global	Protein-energy malnutrition	5 to 9	Female	6040<br>(4712 to 7445)	2.2<br>(1.7 to 2.7)	5867<br>(4532 to 7297)	2.0<br>(1.5 to 2.5)	3348<br>(2642 to 4112)	1.1<br>(0.9 to 1.3)	-44.6<br>(-57.6 to -25.8)	-49.8<br>(-61.6 to -32.8)
Global	Protein-energy malnutrition	5 to 9	Male	6908<br>(5367 to 8699)	2.3<br>(1.8 to 3.0)	6827<br>(5348 to 8619)	2.2<br>(1.7 to 2.7)	4497<br>(3293 to 6640)	1.4<br>(1.0 to 2.0)	-34.9<br>(-54.1 to 7.5)	-42.3<br>(-59.3 to -4.7)
Global	Protein-energy malnutrition	10 to 14	Both	4616<br>(3949 to 5363)	0.9<br>(0.7 to 1.0)	5025<br>(4108 to 6067)	0.8<br>(0.7 to 1.0)	3353<br>(2804 to 4355)	0.5<br>(0.5 to 0.7)	-27.4<br>(-40.3 to -7.5)	-37.2<br>(-48.4 to -20.1)
Global	Protein-energy malnutrition	10 to 14	Female	2351<br>(1937 to 2758)	0.9<br>(0.8 to 1.1)	2519<br>(2020 to 3051)	0.8<br>(0.7 to 1.0)	1568<br>(1284 to 1895)	0.5<br>(0.4 to 0.6)	-33.3<br>(-47.4 to -15.7)	-41.6<br>(-54.0 to -26.2)
Global	Protein-energy malnutrition	10 to 14	Male	2265<br>(1852 to 2760)	0.8<br>(0.7 to 1.0)	2506<br>(1971 to 3225)	0.8<br>(0.6 to 1.0)	1785<br>(1335 to 2781)	0.6<br>(0.4 to 0.9)	-21.2<br>(-42.4 to 21.8)	-32.6<br>(-50.7 to 4.1)
Global	Iodine deficiency	5 to 9	Both	151<br>(52 to 351)	0.0<br>(0.0 to 0.1)	118<br>(35 to 287)	0.0<br>(0.0 to 0.0)	71<br>(21 to 188)	0.0<br>(0.0 to 0.0)	-53.1<br>(-85.6 to 59.6)	-58.0<br>(-87.1 to 42.9)
Global	Iodine deficiency	5 to 9	Female	92<br>(26 to 257)	0.0<br>(0.0 to 0.1)	68<br>(15 to 193)	0.0<br>(0.0 to 0.1)	37<br>(8 to 113)	0.0<br>(0.0 to 0.0)	-60.4<br>(-92.1 to 84.1)	-64.1<br>(-92.8 to 66.7)
Global	Iodine deficiency	5 to 9	Male	59<br>(17 to 175)	0.0<br>(0.0 to 0.1)	50<br>(11 to 157)	0.0<br>(0.0 to 0.0)	34<br>(7 to 116)	0.0<br>(0.0 to 0.0)	-41.7<br>(-89.5 to 161.1)	-48.3<br>(-90.7 to 131.5)
Global	Iodine deficiency	10 to 14	Both	34<br>(9 to 85)	0.0<br>(0.0 to 0.0)	33<br>(9 to 90)	0.0<br>(0.0 to 0.0)	22<br>(6 to 62)	0.0<br>(0.0 to 0.0)	-34.3<br>(-79.1 to 95.0)	-43.2<br>(-81.9 to 68.6)

eTable 8. All-cause and cause-specific mortality (counts and rates) at the global level and by country in the WHO European Region for ages 5 to 9 and 10 to 14 for 1990, 2000, and 2016											
Global	Iodine deficiency	10 to 14	Female	20 (4 to 56)	0.0 (0.0 to 0.0)	19 (4 to 60)	0.0 (0.0 to 0.0)	12 (2 to 42)	0.0 (0.0 to 0.0)	-39.4 (-87.8 to 149.5)	-47.0 (-89.3 to 118.4)
Global	Iodine deficiency	10 to 14	Male	14 (3 to 36)	0.0 (0.0 to 0.0)	14 (3 to 41)	0.0 (0.0 to 0.0)	10 (2 to 28)	0.0 (0.0 to 0.0)	-26.6 (-83.3 to 213.8)	-37.3 (-85.7 to 168.2)
Global	Iron-deficiency anemia	5 to 9	Both	197 (80 to 299)	0.0 (0.0 to 0.1)	153 (79 to 220)	0.0 (0.0 to 0.0)	118 (76 to 184)	0.0 (0.0 to 0.0)	-40.1 (-62.4 to 2.1)	-46.4 (-66.3 to -8.6)
Global	Iron-deficiency anemia	5 to 9	Female	121 (41 to 213)	0.0 (0.0 to 0.1)	83 (39 to 139)	0.0 (0.0 to 0.0)	59 (35 to 126)	0.0 (0.0 to 0.0)	-51.1 (-77.0 to 13.2)	-55.7 (-79.2 to 2.5)
Global	Iron-deficiency anemia	5 to 9	Male	76 (38 to 116)	0.0 (0.0 to 0.0)	70 (38 to 103)	0.0 (0.0 to 0.0)	59 (36 to 82)	0.0 (0.0 to 0.0)	-22.6 (-50.0 to 21.4)	-31.4 (-55.7 to 7.6)
Global	Iron-deficiency anemia	10 to 14	Both	161 (81 to 250)	0.0 (0.0 to 0.0)	135 (85 to 196)	0.0 (0.0 to 0.0)	106 (72 to 151)	0.0 (0.0 to 0.0)	-33.9 (-56.8 to 0.8)	-42.9 (-62.7 to -12.9)
Global	Iron-deficiency anemia	10 to 14	Female	99 (43 to 180)	0.0 (0.0 to 0.1)	71 (43 to 123)	0.0 (0.0 to 0.0)	54 (36 to 95)	0.0 (0.0 to 0.0)	-45.4 (-73.6 to 4.7)	-52.2 (-76.9 to -8.4)
Global	Iron-deficiency anemia	10 to 14	Male	62 (35 to 90)	0.0 (0.0 to 0.0)	65 (39 to 89)	0.0 (0.0 to 0.0)	52 (32 to 69)	0.0 (0.0 to 0.0)	-15.5 (-43.2 to 24.0)	-27.8 (-51.4 to 6.0)
Global	Other nutritional deficiencies	5 to 9	Both	2030 (1482 to 2696)	0.4 (0.3 to 0.5)	1680 (1323 to 2135)	0.3 (0.2 to 0.3)	852 (667 to 1102)	0.1 (0.1 to 0.2)	-58.0 (-69.2 to -37.1)	-62.4 (-72.4 to -43.7)
Global	Other nutritional deficiencies	5 to 9	Female	1217 (853 to 1729)	0.4 (0.3 to 0.6)	995 (722 to 1334)	0.3 (0.2 to 0.4)	398 (290 to 548)	0.1 (0.1 to 0.2)	-67.3 (-78.7 to -50.6)	-70.4 (-80.7 to -55.3)
Global	Other nutritional deficiencies	5 to 9	Male	813 (539 to 1174)	0.3 (0.2 to 0.4)	685 (499 to 947)	0.2 (0.2 to 0.3)	454 (312 to 680)	0.1 (0.1 to 0.2)	-44.1 (-64.8 to 9.4)	-50.5 (-68.8 to -3.0)
Global	Other nutritional deficiencies	10 to 14	Both	982 (793 to 1242)	0.2 (0.2 to 0.2)	955 (797 to 1162)	0.2 (0.1 to 0.2)	598 (488 to 729)	0.1 (0.1 to 0.1)	-39.0 (-52.6 to -19.6)	-47.3 (-59.1 to -30.5)
Global	Other nutritional deficiencies	10 to 14	Female	631 (476 to 830)	0.2 (0.2 to 0.3)	603 (469 to 773)	0.2 (0.2 to 0.3)	331 (254 to 428)	0.1 (0.1 to 0.1)	-47.6 (-62.2 to -29.3)	-54.1 (-66.9 to -38.1)
Global	Other nutritional deficiencies	10 to 14	Male	350 (263 to 481)	0.1 (0.1 to 0.2)	352 (274 to 455)	0.1 (0.1 to 0.1)	268 (200 to 368)	0.1 (0.1 to 0.1)	-23.6 (-49.1 to 19.1)	-34.7 (-56.5 to 1.7)
Global	Sexually transmitted diseases excluding HIV	5 to 9	Both	10564 (5742 to 17637)	1.8 (1.0 to 3.1)	9669 (5346 to 15888)	1.6 (0.9 to 2.6)	6943 (3956 to 10792)	1.1 (0.6 to 1.7)	-34.3 (-47.2 to -16.5)	-41.1 (-52.7 to -25.2)
Global	Sexually transmitted diseases excluding HIV	5 to 9	Female	6793 (3721 to 11488)	2.4 (1.3 to 4.1)	6238 (3438 to 10271)	2.1 (1.2 to 3.4)	4381 (2453 to 6869)	1.4 (0.8 to 2.2)	-35.5 (-48.7 to -15.9)	-41.6 (-53.6 to -23.9)
Global	Sexually transmitted diseases excluding HIV	5 to 9	Male	3771 (2006 to 6337)	1.3 (0.7 to 2.2)	3430 (1848 to 5661)	1.1 (0.6 to 1.8)	2563 (1410 to 4112)	0.8 (0.4 to 1.2)	-32.0 (-46.0 to -13.5)	-39.7 (-52.1 to -23.3)
Global	Sexually transmitted diseases excluding HIV	10 to 14	Both	213 (129 to 300)	0.0 (0.0 to 0.1)	193 (140 to 254)	0.0 (0.0 to 0.0)	140 (113 to 167)	0.0 (0.0 to 0.0)	-34.2 (-51.3 to -4.6)	-43.2 (-57.9 to -17.5)
Global	Sexually transmitted diseases excluding HIV	10 to 14	Female	108 (59 to 151)	0.0 (0.0 to 0.1)	103 (69 to 136)	0.0 (0.0 to 0.0)	78 (61 to 101)	0.0 (0.0 to 0.0)	-27.6 (-49.1 to 11.5)	-36.7 (-55.5 to -2.4)

eTable 8. All-cause and cause-specific mortality (counts and rates) at the global level and by country in the WHO European Region for ages 5 to 9 and 10 to 14 for 1990, 2000, and 2016

Global	Sexually transmitted diseases excluding HIV	10 to 14	Male	106 (60 to 177)	0.0 (0.0 to 0.1)	90 (62 to 143)	0.0 (0.0 to 0.0)	62 (44 to 80)	0.0 (0.0 to 0.0)	-41.0 (-63.6 to -10.2)	-49.6 (-68.9 to -23.2)
Global	Hepatitis	5 to 9	Both	7448 (6074 to 8887)	1.3 (1.1 to 1.5)	6063 (5037 to 7196)	1.0 (0.8 to 1.2)	2635 (2203 to 3174)	0.4 (0.3 to 0.5)	-64.6 (-72.4 to -56.0)	-68.3 (-75.3 to -60.6)
Global	Hepatitis	5 to 9	Female	3826 (3017 to 4687)	1.4 (1.1 to 1.7)	3184 (2512 to 3899)	1.1 (0.8 to 1.3)	1271 (1022 to 1586)	0.4 (0.3 to 0.5)	-66.8 (-75.0 to -56.8)	-69.9 (-77.4 to -60.9)
Global	Hepatitis	5 to 9	Male	3622 (2881 to 4423)	1.2 (1.0 to 1.5)	2879 (2300 to 3516)	0.9 (0.7 to 1.1)	1363 (1097 to 1703)	0.4 (0.3 to 0.5)	-62.4 (-71.5 to -50.9)	-66.6 (-74.7 to -56.5)
Global	Hepatitis	10 to 14	Both	5783 (4962 to 6705)	1.1 (0.9 to 1.3)	5236 (4540 to 5905)	0.8 (0.7 to 0.9)	2883 (2503 to 3328)	0.5 (0.4 to 0.5)	-50.1 (-58.3 to -41.5)	-56.9 (-64.0 to -49.5)
Global	Hepatitis	10 to 14	Female	2896 (2386 to 3439)	1.1 (0.9 to 1.3)	2624 (2137 to 3028)	0.9 (0.7 to 1.0)	1378 (1133 to 1667)	0.5 (0.4 to 0.6)	-52.4 (-62.8 to -41.5)	-58.4 (-67.4 to -48.8)
Global	Hepatitis	10 to 14	Male	2887 (2372 to 3508)	1.1 (0.9 to 1.3)	2612 (2209 to 3059)	0.8 (0.7 to 1.0)	1505 (1257 to 1790)	0.5 (0.4 to 0.6)	-47.9 (-58.8 to -35.0)	-55.5 (-64.8 to -44.4)
Global	Other infectious diseases	5 to 9	Both	3662 (2732 to 4579)	0.6 (0.5 to 0.8)	3032 (2326 to 3719)	0.5 (0.4 to 0.6)	2052 (1504 to 2595)	0.3 (0.2 to 0.4)	-44.0 (-57.2 to -21.9)	-49.8 (-61.7 to -30.1)
Global	Other infectious diseases	5 to 9	Female	1851 (1237 to 2502)	0.7 (0.4 to 0.9)	1523 (1106 to 1948)	0.5 (0.4 to 0.7)	871 (637 to 1183)	0.3 (0.2 to 0.4)	-52.9 (-67.3 to -23.8)	-57.4 (-70.4 to -31.0)
Global	Other infectious diseases	5 to 9	Male	1812 (1289 to 2365)	0.6 (0.4 to 0.8)	1509 (1094 to 1983)	0.5 (0.3 to 0.6)	1181 (781 to 1620)	0.4 (0.2 to 0.5)	-34.8 (-53.0 to -5.5)	-42.2 (-58.4 to -16.2)
Global	Other infectious diseases	10 to 14	Both	2223 (1727 to 2685)	0.4 (0.3 to 0.5)	2043 (1638 to 2414)	0.3 (0.3 to 0.4)	1521 (1146 to 1856)	0.2 (0.2 to 0.3)	-31.6 (-43.6 to -11.7)	-40.8 (-51.3 to -23.6)
Global	Other infectious diseases	10 to 14	Female	987 (694 to 1270)	0.4 (0.3 to 0.5)	881 (657 to 1075)	0.3 (0.2 to 0.4)	566 (429 to 734)	0.2 (0.1 to 0.2)	-42.7 (-56.6 to -11.3)	-49.9 (-62.0 to -22.4)
Global	Other infectious diseases	10 to 14	Male	1236 (950 to 1597)	0.5 (0.4 to 0.6)	1161 (894 to 1464)	0.4 (0.3 to 0.5)	956 (678 to 1245)	0.3 (0.2 to 0.4)	-22.6 (-40.5 to 2.1)	-33.9 (-49.2 to -12.7)
Global	Nasopharynx cancer	5 to 9	Both	231 (197 to 267)	0.0 (0.0 to 0.0)	185 (163 to 209)	0.0 (0.0 to 0.0)	116 (105 to 129)	0.0 (0.0 to 0.0)	-49.7 (-57.6 to -38.7)	-55.0 (-62.1 to -45.1)
Global	Nasopharynx cancer	5 to 9	Female	74 (62 to 90)	0.0 (0.0 to 0.0)	54 (48 to 62)	0.0 (0.0 to 0.0)	36 (32 to 41)	0.0 (0.0 to 0.0)	-51.0 (-60.7 to -40.7)	-55.6 (-64.4 to -46.3)
Global	Nasopharynx cancer	5 to 9	Male	157 (125 to 190)	0.1 (0.0 to 0.1)	132 (112 to 153)	0.0 (0.0 to 0.0)	80 (70 to 91)	0.0 (0.0 to 0.0)	-49.1 (-59.8 to -33.7)	-54.9 (-64.4 to -41.2)
Global	Nasopharynx cancer	10 to 14	Both	388 (337 to 431)	0.1 (0.1 to 0.1)	368 (329 to 402)	0.1 (0.1 to 0.1)	242 (216 to 271)	0.0 (0.0 to 0.0)	-37.7 (-46.0 to -25.8)	-46.1 (-53.3 to -35.8)
Global	Nasopharynx cancer	10 to 14	Female	130 (106 to 169)	0.1 (0.0 to 0.1)	107 (92 to 129)	0.0 (0.0 to 0.0)	59 (52 to 67)	0.0 (0.0 to 0.0)	-54.5 (-65.3 to -42.6)	-60.2 (-69.6 to -49.7)
Global	Nasopharynx cancer	10 to 14	Male	258 (215 to 301)	0.1 (0.1 to 0.1)	261 (226 to 297)	0.1 (0.1 to 0.1)	183 (159 to 209)	0.1 (0.1 to 0.1)	-29.2 (-42.8 to -10.2)	-39.5 (-51.1 to -23.3)
Global	Liver cancer	5 to 9	Both	656 (571 to 774)	0.1 (0.1 to 0.1)	634 (575 to 720)	0.1 (0.1 to 0.1)	440 (388 to 490)	0.1 (0.1 to 0.1)	-32.9 (-44.9 to -19.5)	-39.9 (-50.7 to -27.9)
Global	Liver cancer	5 to 9	Female	244 (162 to 328)	0.1 (0.1 to 0.1)	213 (163 to 257)	0.1 (0.1 to 0.1)	144 (124 to 168)	0.0 (0.0 to 0.1)	-41.1 (-59.3 to -11.1)	-46.7 (-63.1 to -19.5)
Global	Liver cancer	5 to 9	Male	412 (362 to 541)	0.1 (0.1 to 0.2)	421 (377 to 508)	0.1 (0.1 to 0.2)	297 (253 to 340)	0.1 (0.1 to 0.1)	-28.0 (-46.9 to -13.7)	-36.2 (-52.9 to -23.4)
Global	Liver cancer	10 to 14	Both	880 (787 to 988)	0.2 (0.1 to 0.2)	1018 (945 to 1119)	0.2 (0.2 to 0.2)	699 (628 to 775)	0.1 (0.1 to 0.1)	-20.6 (-32.2 to -7.9)	-31.4 (-41.4 to -20.3)
Global	Liver cancer	10 to 14	Female	316 (203 to 402)	0.1 (0.1 to 0.2)	324 (244 to 372)	0.1 (0.1 to 0.1)	201 (172 to 231)	0.1 (0.1 to 0.1)	-36.2 (-53.6 to -5.1)	-44.2 (-59.4 to -17.0)
Global	Liver cancer	10 to 14	Male	565 (506 to 697)	0.2 (0.2 to 0.3)	694 (634 to 811)	0.2 (0.2 to 0.3)	498 (432 to 569)	0.2 (0.1 to 0.2)	-11.9 (-29.0 to 4.4)	-24.7 (-39.3 to -10.8)
Global	Kidney cancer	5 to 9	Both	656 (579 to 754)	0.1 (0.1 to 0.1)	612 (558 to 676)	0.1 (0.1 to 0.1)	617 (551 to 686)	0.1 (0.1 to 0.1)	-6.0 (-23.4 to 12.9)	-15.8 (-31.4 to 1.2)
Global	Kidney cancer	5 to 9	Female	346 (304 to 412)	0.1 (0.1 to 0.1)	307 (275 to 346)	0.1 (0.1 to 0.1)	299 (265 to 333)	0.1 (0.1 to 0.1)	-13.6 (-33.0 to 4.0)	-21.8 (-39.4 to -5.9)
Global	Kidney cancer	5 to 9	Male	310 (268 to 360)	0.1 (0.1 to 0.1)	305 (273 to 342)	0.1 (0.1 to 0.1)	318 (274 to 366)	0.1 (0.1 to 0.1)	2.5 (-17.9 to 26.3)	-9.1 (-27.2 to 12.0)

eTable 8. All-cause and cause-specific mortality (counts and rates) at the global level and by country in the WHO European Region for ages 5 to 9 and 10 to 14 for 1990, 2000, and 2016

Global	Kidney cancer	10 to 14	Both	270 (248 to 295)	0.1 (0.0 to 0.1)	302 (283 to 326)	0.0 (0.0 to 0.1)	322 (300 to 344)	0.1 (0.0 to 0.1)	19.4 (5.8 to 33.8)	3.2 (-8.5 to 15.7)
Global	Kidney cancer	10 to 14	Female	151 (138 to 171)	0.1 (0.1 to 0.1)	160 (148 to 174)	0.1 (0.0 to 0.1)	162 (150 to 175)	0.1 (0.1 to 0.1)	7.5 (-8.5 to 21.8)	-6.0 (-20.0 to 6.6)
Global	Kidney cancer	10 to 14	Male	119 (107 to 132)	0.0 (0.0 to 0.0)	142 (131 to 157)	0.0 (0.0 to 0.0)	160 (144 to 174)	0.1 (0.0 to 0.1)	34.5 (18.4 to 52.6)	15.0 (1.2 to 30.4)
Global	Brain and nervous system cancer	5 to 9	Both	6891 (5439 to 7711)	1.2 (0.9 to 1.3)	6911 (5585 to 7607)	1.1 (0.9 to 1.2)	5584 (4960 to 6277)	0.9 (0.8 to 1.0)	-19.0 (-29.5 to -3.1)	-27.4 (-36.9 to -13.2)
Global	Brain and nervous system cancer	5 to 9	Female	3095 (2234 to 3577)	1.1 (0.8 to 1.3)	2965 (2302 to 3343)	1.0 (0.8 to 1.1)	2430 (2024 to 2820)	0.8 (0.7 to 0.9)	-21.5 (-33.4 to -2.2)	-28.9 (-39.7 to -11.5)
Global	Brain and nervous system cancer	5 to 9	Male	3795 (2846 to 4347)	1.3 (1.0 to 1.5)	3946 (2975 to 4483)	1.2 (0.9 to 1.4)	3154 (2708 to 3726)	1.0 (0.8 to 1.1)	-16.9 (-29.7 to 8.5)	-26.3 (-37.7 to -3.8)
Global	Brain and nervous system cancer	10 to 14	Both	5504 (4467 to 6086)	1.0 (0.8 to 1.2)	6593 (5419 to 7168)	1.1 (0.9 to 1.1)	5097 (4557 to 5708)	0.8 (0.7 to 0.9)	-7.4 (-19.1 to 7.7)	-19.9 (-30.1 to -6.9)
Global	Brain and nervous system cancer	10 to 14	Female	2395 (1807 to 2718)	0.9 (0.7 to 1.1)	2731 (2181 to 3037)	0.9 (0.7 to 1.0)	2139 (1796 to 2488)	0.7 (0.6 to 0.8)	-10.7 (-23.6 to 4.9)	-21.9 (-33.2 to -8.2)
Global	Brain and nervous system cancer	10 to 14	Male	3109 (2367 to 3446)	1.2 (0.9 to 1.3)	3862 (2894 to 4284)	1.2 (0.9 to 1.3)	2959 (2487 to 3474)	0.9 (0.8 to 1.1)	-4.8 (-17.4 to 14.5)	-18.7 (-29.4 to -2.2)
Global	Thyroid cancer	10 to 14	Both	154 (124 to 183)	0.0 (0.0 to 0.0)	153 (127 to 176)	0.0 (0.0 to 0.0)	121 (102 to 141)	0.0 (0.0 to 0.0)	-21.1 (-35.2 to -0.0)	-31.8 (-44.0 to -13.6)
Global	Thyroid cancer	10 to 14	Female	87 (69 to 106)	0.0 (0.0 to 0.0)	81 (67 to 97)	0.0 (0.0 to 0.0)	57 (47 to 69)	0.0 (0.0 to 0.0)	-34.9 (-50.6 to -9.7)	-43.0 (-56.7 to -21.0)
Global	Thyroid cancer	10 to 14	Male	66 (46 to 83)	0.0 (0.0 to 0.0)	72 (53 to 86)	0.0 (0.0 to 0.0)	65 (49 to 80)	0.0 (0.0 to 0.0)	-2.9 (-27.4 to 25.8)	-17.0 (-38.0 to 7.5)
Global	Hodgkin lymphoma	5 to 9	Both	1893 (1034 to 2502)	0.3 (0.2 to 0.4)	1471 (900 to 1902)	0.2 (0.1 to 0.3)	764 (555 to 1071)	0.1 (0.1 to 0.2)	-59.6 (-71.8 to -33.5)	-63.8 (-74.8 to -40.4)
Global	Hodgkin lymphoma	5 to 9	Female	390 (175 to 592)	0.1 (0.1 to 0.2)	286 (144 to 406)	0.1 (0.0 to 0.1)	132 (79 to 194)	0.0 (0.0 to 0.1)	-66.2 (-79.3 to -44.1)	-69.4 (-81.3 to -49.4)
Global	Hodgkin lymphoma	5 to 9	Male	1503 (786 to 2020)	0.5 (0.3 to 0.7)	1186 (689 to 1574)	0.4 (0.2 to 0.5)	632 (442 to 928)	0.2 (0.1 to 0.3)	-57.9 (-72.1 to -30.9)	-62.7 (-75.2 to -38.7)
Global	Hodgkin lymphoma	10 to 14	Both	1823 (1176 to 2160)	0.3 (0.2 to 0.4)	1714 (1160 to 2042)	0.3 (0.2 to 0.3)	1063 (789 to 1356)	0.2 (0.1 to 0.2)	-41.7 (-51.3 to -24.7)	-49.6 (-57.9 to -34.9)
Global	Hodgkin lymphoma	10 to 14	Female	520 (261 to 689)	0.2 (0.1 to 0.3)	472 (246 to 616)	0.2 (0.1 to 0.2)	275 (153 to 376)	0.1 (0.1 to 0.1)	-47.2 (-59.8 to -29.5)	-53.8 (-64.9 to -38.3)
Global	Hodgkin lymphoma	10 to 14	Male	1303 (797 to 1587)	0.5 (0.3 to 0.6)	1242 (794 to 1524)	0.4 (0.2 to 0.5)	789 (568 to 1069)	0.2 (0.2 to 0.3)	-39.5 (-51.1 to -21.9)	-48.3 (-58.2 to -33.3)
Global	Non-Hodgkin lymphoma	5 to 9	Both	3142 (2551 to 3711)	0.5 (0.4 to 0.6)	3428 (2805 to 3940)	0.6 (0.5 to 0.6)	4074 (3250 to 4848)	0.6 (0.5 to 0.8)	29.6 (-2.4 to 64.6)	16.1 (-12.6 to 47.5)
Global	Non-Hodgkin lymphoma	5 to 9	Female	1096 (819 to 1382)	0.4 (0.3 to 0.5)	1216 (940 to 1459)	0.4 (0.3 to 0.5)	1475 (1116 to 1802)	0.5 (0.4 to 0.6)	34.6 (-12.7 to 79.4)	21.8 (-21.0 to 62.4)
Global	Non-Hodgkin lymphoma	5 to 9	Male	2046 (1537 to 2479)	0.7 (0.5 to 0.8)	2212 (1725 to 2577)	0.7 (0.5 to 0.8)	2599 (1964 to 3207)	0.8 (0.6 to 1.0)	27.0 (-7.2 to 67.3)	12.6 (-17.7 to 48.4)
Global	Non-Hodgkin lymphoma	10 to 14	Both	2754 (2333 to 3104)	0.5 (0.4 to 0.6)	3396 (2936 to 3770)	0.5 (0.5 to 0.6)	3853 (3181 to 4407)	0.6 (0.5 to 0.7)	39.9 (18.2 to 64.9)	20.9 (2.2 to 42.6)
Global	Non-Hodgkin lymphoma	10 to 14	Female	979 (796 to 1164)	0.4 (0.3 to 0.5)	1207 (969 to 1397)	0.4 (0.3 to 0.5)	1356 (1096 to 1595)	0.5 (0.4 to 0.5)	38.5 (5.2 to 69.5)	21.2 (-7.9 to 48.3)
Global	Non-Hodgkin lymphoma	10 to 14	Male	1775 (1433 to 2036)	0.7 (0.5 to 0.8)	2188 (1751 to 2481)	0.7 (0.5 to 0.8)	2497 (1957 to 2940)	0.8 (0.6 to 0.9)	40.7 (17.8 to 70.7)	20.2 (0.6 to 45.9)
Global	Leukemia	5 to 9	Both	14577 (12607 to 18079)	2.5 (2.2 to 3.1)	13152 (11791 to 15671)	2.1 (1.9 to 2.6)	8691 (7673 to 9559)	1.4 (1.2 to 1.5)	-40.4 (-50.5 to -29.1)	-46.6 (-55.7 to -36.5)
Global	Leukemia	5 to 9	Female	6675 (4951 to 8951)	2.4 (1.8 to 3.2)	5705 (4414 to 6906)	1.9 (1.5 to 2.3)	3469 (2953 to 4248)	1.1 (1.0 to 1.4)	-48.0 (-58.8 to -29.1)	-53.0 (-62.8 to -35.9)
Global	Leukemia	5 to 9	Male	7902 (6966 to 9704)	2.7 (2.4 to 3.3)	7448 (6628 to 9167)	2.4 (2.1 to 2.9)	5222 (4384 to 5913)	1.6 (1.3 to 1.8)	-33.9 (-45.2 to -22.5)	-41.4 (-51.4 to -31.3)
Global	Leukemia	10 to 14	Both	13553 (12107 to 16125)	2.6 (2.3 to 3.1)	14654 (13403 to 16553)	2.3 (2.1 to 2.6)	9141 (8385 to 10194)	1.5 (1.4 to 1.7)	-32.6 (-40.1 to -24.9)	-41.7 (-48.2 to -35.1)
Global	Leukemia	10 to 14	Female	6328 (4944 to 8550)	2.5 (1.9 to 3.3)	6409 (5240 to 7773)	2.1 (1.7 to 2.5)	3752 (3223 to 4422)	1.3 (1.1 to 1.5)	-40.7 (-51.3 to -26.3)	-48.1 (-57.4 to -35.5)

eTable 8. All-cause and cause-specific mortality (counts and rates) at the global level and by country in the WHO European Region for ages 5 to 9 and 10 to 14 for 1990, 2000, and 2016

Global	Leukemia	10 to 14	Male	7225 (6555 to 8345)	2.7 (2.4 to 3.1)	8245 (7444 to 9497)	2.6 (2.3 to 3.0)	5388 (4819 to 6143)	1.7 (1.5 to 1.9)	-25.4 (-33.5 to -17.5)	-36.3 (-43.2 to -29.5)
Global	Other neoplasms	5 to 9	Both	4836 (4296 to 5654)	0.8 (0.7 to 1.0)	4893 (4438 to 5568)	0.8 (0.7 to 0.9)	4734 (4262 to 5167)	0.7 (0.7 to 0.8)	-2.1 (-19.5 to 12.2)	-12.3 (-27.9 to 0.5)
Global	Other neoplasms	5 to 9	Female	2232 (1936 to 2673)	0.8 (0.7 to 1.0)	2213 (1952 to 2556)	0.7 (0.7 to 0.9)	2145 (1920 to 2395)	0.7 (0.6 to 0.8)	-3.9 (-22.1 to 13.8)	-13.0 (-29.5 to 3.0)
Global	Other neoplasms	5 to 9	Male	2604 (2301 to 3056)	0.9 (0.8 to 1.0)	2680 (2380 to 3107)	0.8 (0.8 to 1.0)	2588 (2276 to 2869)	0.8 (0.7 to 0.9)	-0.6 (-19.7 to 15.8)	-11.9 (-28.8 to 2.7)
Global	Other neoplasms	10 to 14	Both	5508 (5062 to 6069)	1.0 (1.0 to 1.1)	6592 (6120 to 7198)	1.1 (1.0 to 1.1)	6461 (5818 to 7022)	1.1 (1.0 to 1.1)	17.3 (4.4 to 29.4)	1.4 (-9.8 to 11.9)
Global	Other neoplasms	10 to 14	Female	2559 (2274 to 2876)	1.0 (0.9 to 1.1)	3027 (2740 to 3358)	1.0 (0.9 to 1.1)	3013 (2665 to 3326)	1.0 (0.9 to 1.1)	17.8 (2.4 to 35.3)	3.1 (-10.4 to 18.4)
Global	Other neoplasms	10 to 14	Male	2949 (2700 to 3256)	1.1 (1.0 to 1.2)	3564 (3238 to 3950)	1.1 (1.0 to 1.2)	3448 (3017 to 3799)	1.1 (1.0 to 1.2)	16.9 (2.8 to 31.4)	-0.1 (-12.2 to 12.3)
Global	Rheumatic heart disease	5 to 9	Both	4804 (4012 to 5692)	0.8 (0.7 to 1.0)	3642 (3137 to 4192)	0.6 (0.5 to 0.7)	1587 (1363 to 1839)	0.2 (0.2 to 0.3)	-67.0 (-73.0 to -60.2)	-70.4 (-75.8 to -64.4)
Global	Rheumatic heart disease	5 to 9	Female	2500 (1963 to 3081)	0.9 (0.7 to 1.1)	1836 (1482 to 2233)	0.6 (0.5 to 0.8)	724 (591 to 878)	0.2 (0.2 to 0.3)	-71.1 (-76.8 to -63.3)	-73.8 (-79.0 to -66.8)
Global	Rheumatic heart disease	5 to 9	Male	2304 (1880 to 2803)	0.8 (0.6 to 1.0)	1806 (1542 to 2097)	0.6 (0.5 to 0.7)	864 (744 to 1000)	0.3 (0.2 to 0.3)	-62.5 (-69.9 to -54.6)	-66.8 (-73.3 to -59.7)
Global	Rheumatic heart disease	10 to 14	Both	6886 (6063 to 7644)	1.3 (1.1 to 1.4)	6177 (5499 to 6851)	1.0 (0.9 to 1.1)	3297 (2928 to 3723)	0.5 (0.5 to 0.6)	-52.1 (-57.8 to -45.9)	-58.6 (-63.5 to -53.2)
Global	Rheumatic heart disease	10 to 14	Female	3678 (3058 to 4207)	1.4 (1.2 to 1.6)	3213 (2740 to 3691)	1.1 (0.9 to 1.2)	1623 (1379 to 1938)	0.6 (0.5 to 0.7)	-55.9 (-62.2 to -47.2)	-61.4 (-66.9 to -53.8)
Global	Rheumatic heart disease	10 to 14	Male	3209 (2812 to 3636)	1.2 (1.0 to 1.3)	2964 (2620 to 3296)	0.9 (0.8 to 1.0)	1674 (1494 to 1874)	0.5 (0.5 to 0.6)	-47.8 (-54.3 to -40.9)	-55.4 (-60.9 to -49.5)
Global	Ischemic heart disease	5 to 9	Both	1223 (1025 to 1524)	0.2 (0.2 to 0.3)	945 (825 to 1117)	0.2 (0.1 to 0.2)	545 (459 to 646)	0.1 (0.1 to 0.1)	-55.4 (-67.2 to -43.3)	-60.1 (-70.6 to -49.2)
Global	Ischemic heart disease	5 to 9	Female	645 (504 to 872)	0.2 (0.2 to 0.3)	478 (390 to 609)	0.2 (0.1 to 0.2)	249 (207 to 307)	0.1 (0.1 to 0.1)	-61.4 (-74.1 to -45.4)	-65.1 (-76.6 to -50.6)
Global	Ischemic heart disease	5 to 9	Male	578 (482 to 693)	0.2 (0.2 to 0.2)	468 (405 to 550)	0.1 (0.1 to 0.2)	296 (244 to 357)	0.1 (0.1 to 0.1)	-48.8 (-60.6 to -35.6)	-54.6 (-65.1 to -42.9)
Global	Ischemic heart disease	10 to 14	Both	1229 (1089 to 1429)	0.2 (0.2 to 0.3)	1142 (1039 to 1280)	0.2 (0.2 to 0.2)	684 (606 to 772)	0.1 (0.1 to 0.1)	-44.3 (-54.1 to -34.8)	-51.9 (-60.3 to -43.6)
Global	Ischemic heart disease	10 to 14	Female	651 (539 to 817)	0.3 (0.2 to 0.3)	580 (497 to 698)	0.2 (0.2 to 0.2)	346 (297 to 402)	0.1 (0.1 to 0.1)	-46.9 (-59.2 to -34.1)	-53.5 (-64.3 to -42.3)
Global	Ischemic heart disease	10 to 14	Male	578 (508 to 661)	0.2 (0.2 to 0.2)	562 (502 to 626)	0.2 (0.2 to 0.2)	338 (293 to 391)	0.1 (0.1 to 0.1)	-41.4 (-51.4 to -31.0)	-50.0 (-58.5 to -41.0)
Global	Cerebrovascular disease	5 to 9	Both	5860 (5087 to 6792)	1.0 (0.9 to 1.2)	4443 (3989 to 5011)	0.7 (0.6 to 0.8)	2415 (2203 to 2683)	0.4 (0.3 to 0.4)	-58.8 (-65.6 to -51.9)	-63.1 (-69.2 to -57.0)
Global	Cerebrovascular disease	5 to 9	Female	2690 (2237 to 3334)	1.0 (0.8 to 1.2)	1892 (1647 to 2232)	0.6 (0.6 to 0.7)	1025 (913 to 1176)	0.3 (0.3 to 0.4)	-61.9 (-69.7 to -53.7)	-65.5 (-72.6 to -58.1)
Global	Cerebrovascular disease	5 to 9	Male	3170 (2760 to 3731)	1.1 (0.9 to 1.3)	2551 (2236 to 2938)	0.8 (0.7 to 0.9)	1390 (1222 to 1587)	0.4 (0.4 to 0.5)	-56.2 (-63.4 to -48.9)	-61.1 (-67.6 to -54.7)
Global	Cerebrovascular disease	10 to 14	Both	5620 (5134 to 6265)	1.1 (1.0 to 1.2)	5033 (4672 to 5467)	0.8 (0.7 to 0.9)	3073 (2863 to 3356)	0.5 (0.5 to 0.5)	-45.3 (-51.4 to -39.4)	-52.7 (-58.0 to -47.7)
Global	Cerebrovascular disease	10 to 14	Female	2605 (2268 to 3079)	1.0 (0.9 to 1.2)	2181 (1948 to 2483)	0.7 (0.6 to 0.8)	1307 (1189 to 1451)	0.4 (0.4 to 0.5)	-49.8 (-57.4 to -41.7)	-56.1 (-62.7 to -49.0)
Global	Cerebrovascular disease	10 to 14	Male	3014 (2741 to 3372)	1.1 (1.0 to 1.2)	2852 (2605 to 3132)	0.9 (0.8 to 1.0)	1766 (1610 to 1964)	0.6 (0.5 to 0.6)	-41.4 (-48.8 to -34.6)	-49.9 (-56.2 to -44.1)
Global	Hypertensive heart disease	5 to 9	Both	263 (170 to 329)	0.0 (0.0 to 0.1)	196 (148 to 231)	0.0 (0.0 to 0.0)	122 (102 to 142)	0.0 (0.0 to 0.0)	-53.7 (-63.3 to -32.2)	-58.6 (-67.2 to -39.3)
Global	Hypertensive heart disease	5 to 9	Female	144 (62 to 205)	0.1 (0.0 to 0.1)	99 (62 to 130)	0.0 (0.0 to 0.0)	55 (42 to 67)	0.0 (0.0 to 0.0)	-61.9 (-74.7 to -0.8)	-65.5 (-77.1 to -10.2)
Global	Hypertensive heart disease	5 to 9	Male	119 (63 to 153)	0.0 (0.0 to 0.1)	97 (66 to 121)	0.0 (0.0 to 0.0)	67 (54 to 84)	0.0 (0.0 to 0.0)	-43.8 (-57.8 to 6.0)	-50.2 (-62.6 to -6.0)
Global	Hypertensive heart disease	10 to 14	Both	258 (162 to 324)	0.0 (0.0 to 0.1)	245 (176 to 301)	0.0 (0.0 to 0.0)	182 (140 to 222)	0.0 (0.0 to 0.0)	-29.3 (-43.6 to -2.0)	-38.9 (-51.2 to -15.3)
Global	Hypertensive heart disease	10 to 14	Female	123 (59 to 173)	0.0 (0.0 to 0.1)	111 (70 to 150)	0.0 (0.0 to 0.0)	79 (60 to 98)	0.0 (0.0 to 0.0)	-35.7 (-53.4 to 33.3)	-43.7 (-59.3 to 16.6)
Global	Hypertensive heart disease	10 to 14	Male	136 (65 to 182)	0.1 (0.0 to 0.1)	134 (82 to 176)	0.0 (0.0 to 0.1)	104 (70 to 138)	0.0 (0.0 to 0.0)	-23.6 (-46.5 to 32.6)	-34.7 (-54.3 to 13.3)
Global	Cardiomyopathy and myocarditis	5 to 9	Both	1538 (1248 to 1973)	0.3 (0.2 to 0.3)	1566 (1306 to 1777)	0.3 (0.2 to 0.3)	1127 (1000 to 1273)	0.2 (0.2 to 0.2)	-26.7 (-41.5 to -8.9)	-34.3 (-47.6 to -18.4)

eTable 8. All-cause and cause-specific mortality (counts and rates) at the global level and by country in the WHO European Region for ages 5 to 9 and 10 to 14 for 1990, 2000, and 2016

Global	Cardiomyopathy and myocarditis	5 to 9	Female	765 (582 to 1107)	0.3 (0.2 to 0.4)	745 (572 to 889)	0.3 (0.2 to 0.3)	517 (448 to 598)	0.2 (0.1 to 0.2)	-32.4 (-53.1 to -11.3)	-38.8 (-57.5 to -19.7)
Global	Cardiomyopathy and myocarditis	5 to 9	Male	773 (591 to 1027)	0.3 (0.2 to 0.3)	822 (616 to 987)	0.3 (0.2 to 0.3)	610 (501 to 721)	0.2 (0.2 to 0.2)	-21.0 (-38.2 to -1.6)	-30.0 (-45.2 to -12.8)
Global	Cardiomyopathy and myocarditis	10 to 14	Both	1525 (1325 to 1934)	0.3 (0.3 to 0.4)	1821 (1644 to 2099)	0.3 (0.3 to 0.3)	1357 (1195 to 1491)	0.2 (0.2 to 0.2)	-11.0 (-29.3 to 3.7)	-23.1 (-38.9 to -10.3)
Global	Cardiomyopathy and myocarditis	10 to 14	Female	742 (606 to 1027)	0.3 (0.2 to 0.4)	846 (714 to 1001)	0.3 (0.2 to 0.3)	627 (538 to 705)	0.2 (0.2 to 0.2)	-15.4 (-39.1 to 3.8)	-26.0 (-46.7 to -9.2)
Global	Cardiomyopathy and myocarditis	10 to 14	Male	784 (662 to 1042)	0.3 (0.2 to 0.4)	975 (828 to 1174)	0.3 (0.3 to 0.4)	730 (616 to 838)	0.2 (0.2 to 0.3)	-6.9 (-29.0 to 10.9)	-20.5 (-39.3 to -5.2)
Global	Atrial fibrillation and flutter	5 to 9	Both	0 (0 to 0)	0.0 (0.0 to 0.0)	0 (0 to 0)	0.0 (0.0 to 0.0)	0 (0 to 0)	0.0 (0.0 to 0.0)	0.0 (0.0 to 0.0)	0.0 (0.0 to 0.0)
Global	Atrial fibrillation and flutter	5 to 9	Female	0 (0 to 0)	0.0 (0.0 to 0.0)	0 (0 to 0)	0.0 (0.0 to 0.0)	0 (0 to 0)	0.0 (0.0 to 0.0)	0.0 (0.0 to 0.0)	0.0 (0.0 to 0.0)
Global	Atrial fibrillation and flutter	5 to 9	Male	0 (0 to 0)	0.0 (0.0 to 0.0)	0 (0 to 0)	0.0 (0.0 to 0.0)	0 (0 to 0)	0.0 (0.0 to 0.0)	0.0 (0.0 to 0.0)	0.0 (0.0 to 0.0)
Global	Atrial fibrillation and flutter	10 to 14	Both	0 (0 to 0)	0.0 (0.0 to 0.0)	0 (0 to 0)	0.0 (0.0 to 0.0)	0 (0 to 0)	0.0 (0.0 to 0.0)	0.0 (0.0 to 0.0)	0.0 (0.0 to 0.0)
Global	Atrial fibrillation and flutter	10 to 14	Female	0 (0 to 0)	0.0 (0.0 to 0.0)	0 (0 to 0)	0.0 (0.0 to 0.0)	0 (0 to 0)	0.0 (0.0 to 0.0)	0.0 (0.0 to 0.0)	0.0 (0.0 to 0.0)
Global	Atrial fibrillation and flutter	10 to 14	Male	0 (0 to 0)	0.0 (0.0 to 0.0)	0 (0 to 0)	0.0 (0.0 to 0.0)	0 (0 to 0)	0.0 (0.0 to 0.0)	0.0 (0.0 to 0.0)	0.0 (0.0 to 0.0)
Global	Endocarditis	5 to 9	Both	393 (295 to 490)	0.1 (0.1 to 0.1)	403 (316 to 481)	0.1 (0.1 to 0.1)	378 (323 to 455)	0.1 (0.1 to 0.1)	-3.8 (-27.5 to 26.7)	-13.8 (-35.0 to 13.5)
Global	Endocarditis	5 to 9	Female	237 (168 to 331)	0.1 (0.1 to 0.1)	234 (179 to 310)	0.1 (0.1 to 0.1)	210 (173 to 265)	0.1 (0.1 to 0.1)	-11.6 (-35.4 to 21.1)	-19.9 (-41.6 to 9.6)
Global	Endocarditis	5 to 9	Male	155 (105 to 190)	0.1 (0.0 to 0.1)	169 (116 to 203)	0.1 (0.0 to 0.1)	168 (128 to 222)	0.1 (0.0 to 0.1)	8.0 (-18.7 to 38.6)	-4.2 (-27.9 to 22.9)
Global	Endocarditis	10 to 14	Both	462 (382 to 549)	0.1 (0.1 to 0.1)	539 (448 to 633)	0.1 (0.1 to 0.1)	561 (486 to 680)	0.1 (0.1 to 0.1)	21.5 (-0.3 to 42.8)	5.0 (-13.8 to 23.4)
Global	Endocarditis	10 to 14	Female	245 (192 to 308)	0.1 (0.1 to 0.1)	273 (225 to 331)	0.1 (0.1 to 0.1)	267 (227 to 319)	0.1 (0.1 to 0.1)	9.0 (-14.9 to 31.8)	-4.6 (-25.5 to 15.3)
Global	Endocarditis	10 to 14	Male	217 (156 to 273)	0.1 (0.1 to 0.1)	266 (194 to 334)	0.1 (0.1 to 0.1)	294 (230 to 388)	0.1 (0.1 to 0.1)	35.6 (12.6 to 60.1)	15.9 (-3.8 to 36.8)
Global	Other cardiovascular and circulatory diseases	5 to 9	Both	2841 (2144 to 3417)	0.5 (0.4 to 0.6)	2248 (1830 to 2747)	0.4 (0.3 to 0.4)	1476 (1267 to 1908)	0.2 (0.2 to 0.3)	-48.0 (-56.9 to -30.5)	-53.5 (-61.4 to -37.8)
Global	Other cardiovascular and circulatory diseases	5 to 9	Female	1246 (757 to 1551)	0.4 (0.3 to 0.6)	965 (720 to 1188)	0.3 (0.2 to 0.4)	675 (565 to 955)	0.2 (0.2 to 0.3)	-45.8 (-57.8 to -9.5)	-50.9 (-61.8 to -18.1)
Global	Other cardiovascular and circulatory diseases	5 to 9	Male	1595 (1054 to 2002)	0.5 (0.4 to 0.7)	1283 (945 to 1682)	0.4 (0.3 to 0.5)	801 (654 to 1103)	0.2 (0.2 to 0.3)	-49.8 (-59.7 to -32.8)	-55.5 (-64.3 to -40.4)
Global	Other cardiovascular and circulatory diseases	10 to 14	Both	2484 (1888 to 2865)	0.5 (0.4 to 0.5)	2362 (1951 to 2844)	0.4 (0.3 to 0.5)	1664 (1467 to 2145)	0.3 (0.2 to 0.4)	-33.0 (-42.8 to -11.2)	-42.1 (-50.5 to -23.3)

eTable 8. All-cause and cause-specific mortality (counts and rates) at the global level and by country in the WHO European Region for ages 5 to 9 and 10 to 14 for 1990, 2000, and 2016

Global	Other cardiovascular and circulatory diseases	10 to 14	Female	1148 (726 to 1374)	0.4 (0.3 to 0.5)	1064 (789 to 1293)	0.3 (0.3 to 0.4)	761 (641 to 1087)	0.3 (0.2 to 0.4)	-33.7 (-46.3 to 6.7)	-42.0 (-53.0 to -6.6)
Global	Other cardiovascular and circulatory diseases	10 to 14	Male	1336 (912 to 1602)	0.5 (0.3 to 0.6)	1298 (976 to 1687)	0.4 (0.3 to 0.5)	903 (753 to 1238)	0.3 (0.2 to 0.4)	-32.4 (-44.5 to -10.5)	-42.2 (-52.5 to -23.5)
Global	Chronic obstructive pulmonary disease	5 to 9	Both	2214 (1501 to 3133)	0.4 (0.3 to 0.5)	1442 (1105 to 1990)	0.2 (0.2 to 0.3)	528 (436 to 689)	0.1 (0.1 to 0.1)	-76.1 (-82.5 to -63.5)	-78.6 (-84.3 to -67.3)
Global	Chronic obstructive pulmonary disease	5 to 9	Female	1070 (649 to 1473)	0.4 (0.2 to 0.5)	697 (503 to 965)	0.2 (0.2 to 0.3)	249 (187 to 384)	0.1 (0.1 to 0.1)	-76.8 (-83.4 to -57.7)	-79.0 (-85.0 to -61.7)
Global	Chronic obstructive pulmonary disease	5 to 9	Male	1143 (780 to 1787)	0.4 (0.3 to 0.6)	745 (531 to 1101)	0.2 (0.2 to 0.3)	280 (224 to 363)	0.1 (0.1 to 0.1)	-75.5 (-83.9 to -66.4)	-78.3 (-85.7 to -70.2)
Global	Chronic obstructive pulmonary disease	10 to 14	Both	1596 (1138 to 2072)	0.3 (0.2 to 0.4)	1191 (958 to 1529)	0.2 (0.2 to 0.2)	522 (443 to 648)	0.1 (0.1 to 0.1)	-67.3 (-74.1 to -51.4)	-71.7 (-77.6 to -58.0)
Global	Chronic obstructive pulmonary disease	10 to 14	Female	810 (488 to 1031)	0.3 (0.2 to 0.4)	582 (427 to 738)	0.2 (0.1 to 0.2)	266 (209 to 373)	0.1 (0.1 to 0.1)	-67.2 (-75.7 to -44.0)	-71.3 (-78.7 to -51.0)
Global	Chronic obstructive pulmonary disease	10 to 14	Male	786 (566 to 1104)	0.3 (0.2 to 0.4)	609 (457 to 832)	0.2 (0.1 to 0.3)	256 (201 to 329)	0.1 (0.1 to 0.1)	-67.4 (-76.1 to -56.4)	-72.2 (-79.6 to -62.8)
Global	Asthma	5 to 9	Both	5536 (4167 to 6710)	1.0 (0.7 to 1.2)	4018 (3191 to 4915)	0.7 (0.5 to 0.8)	1772 (1488 to 2242)	0.3 (0.2 to 0.3)	-68.0 (-73.6 to -58.5)	-71.3 (-76.3 to -62.8)
Global	Asthma	5 to 9	Female	2664 (1896 to 3368)	0.9 (0.7 to 1.2)	1950 (1506 to 2537)	0.7 (0.5 to 0.9)	864 (687 to 1268)	0.3 (0.2 to 0.4)	-67.6 (-75.5 to -51.3)	-70.6 (-77.8 to -55.9)
Global	Asthma	5 to 9	Male	2872 (2097 to 3696)	1.0 (0.7 to 1.3)	2069 (1554 to 2606)	0.7 (0.5 to 0.8)	907 (731 to 1109)	0.3 (0.2 to 0.3)	-68.4 (-74.5 to -59.5)	-72.0 (-77.4 to -64.1)
Global	Asthma	10 to 14	Both	5389 (4532 to 6238)	1.0 (0.9 to 1.2)	4687 (4015 to 5435)	0.7 (0.6 to 0.9)	2540 (2212 to 3190)	0.4 (0.4 to 0.5)	-52.9 (-59.0 to -43.4)	-59.3 (-64.6 to -51.1)
Global	Asthma	10 to 14	Female	2411 (1885 to 2913)	0.9 (0.7 to 1.1)	2093 (1727 to 2636)	0.7 (0.6 to 0.9)	1193 (978 to 1771)	0.4 (0.3 to 0.6)	-50.5 (-60.3 to -33.0)	-56.7 (-65.3 to -41.4)
Global	Asthma	10 to 14	Male	2978 (2408 to 3564)	1.1 (0.9 to 1.3)	2594 (2132 to 3045)	0.8 (0.7 to 0.9)	1347 (1147 to 1564)	0.4 (0.4 to 0.5)	-54.8 (-60.8 to -46.7)	-61.3 (-66.5 to -54.5)
Global	Interstitial lung disease and pulmonary sarcoidosis	5 to 9	Both	214 (106 to 468)	0.0 (0.0 to 0.1)	196 (106 to 365)	0.0 (0.0 to 0.1)	127 (92 to 172)	0.0 (0.0 to 0.0)	-40.7 (-66.9 to -2.6)	-46.9 (-70.3 to -12.7)
Global	Interstitial lung disease and pulmonary sarcoidosis	5 to 9	Female	138 (60 to 344)	0.0 (0.0 to 0.1)	125 (62 to 256)	0.0 (0.0 to 0.1)	77 (52 to 117)	0.0 (0.0 to 0.0)	-44.2 (-73.5 to 22.7)	-49.5 (-76.0 to 11.1)
Global	Interstitial lung disease and pulmonary sarcoidosis	5 to 9	Male	76 (37 to 167)	0.0 (0.0 to 0.1)	70 (38 to 133)	0.0 (0.0 to 0.0)	50 (35 to 71)	0.0 (0.0 to 0.0)	-34.3 (-63.3 to 13.3)	-41.8 (-67.5 to 0.5)

eTable 8. All-cause and cause-specific mortality (counts and rates) at the global level and by country in the WHO European Region for ages 5 to 9 and 10 to 14 for 1990, 2000, and 2016

Global	Interstitial lung disease and pulmonary sarcoidosis	10 to 14	Both	118 (71 to 215)	0.0 (0.0 to 0.0)	126 (81 to 198)	0.0 (0.0 to 0.0)	98 (75 to 123)	0.0 (0.0 to 0.0)	-16.6 (-45.8 to 17.2)	-27.9 (-53.2 to 1.3)
Global	Interstitial lung disease and pulmonary sarcoidosis	10 to 14	Female	75 (42 to 154)	0.0 (0.0 to 0.1)	79 (49 to 138)	0.0 (0.0 to 0.0)	60 (42 to 83)	0.0 (0.0 to 0.0)	-20.7 (-54.2 to 28.9)	-30.6 (-59.9 to 12.8)
Global	Interstitial lung disease and pulmonary sarcoidosis	10 to 14	Male	43 (22 to 82)	0.0 (0.0 to 0.0)	47 (27 to 78)	0.0 (0.0 to 0.0)	39 (27 to 51)	0.0 (0.0 to 0.0)	-9.5 (-47.5 to 50.7)	-22.6 (-55.1 to 28.8)
Global	Other chronic respiratory diseases	5 to 9	Both	1136 (681 to 1643)	0.2 (0.1 to 0.3)	1079 (718 to 1461)	0.2 (0.1 to 0.2)	857 (657 to 1083)	0.1 (0.1 to 0.2)	-24.5 (-42.6 to 5.6)	-32.4 (-48.6 to -5.4)
Global	Other chronic respiratory diseases	5 to 9	Female	400 (255 to 603)	0.1 (0.1 to 0.2)	403 (262 to 565)	0.1 (0.1 to 0.2)	350 (243 to 477)	0.1 (0.1 to 0.2)	-12.7 (-36.1 to 18.1)	-20.9 (-42.2 to 6.9)
Global	Other chronic respiratory diseases	5 to 9	Male	735 (349 to 1191)	0.2 (0.1 to 0.4)	676 (371 to 1040)	0.2 (0.1 to 0.3)	508 (366 to 682)	0.2 (0.1 to 0.2)	-31.0 (-51.2 to 14.4)	-38.8 (-56.7 to 1.5)
Global	Other chronic respiratory diseases	10 to 14	Both	784 (482 to 1110)	0.1 (0.1 to 0.2)	827 (541 to 1119)	0.1 (0.1 to 0.2)	770 (544 to 959)	0.1 (0.1 to 0.2)	-1.7 (-23.5 to 26.2)	-15.0 (-33.8 to 9.1)
Global	Other chronic respiratory diseases	10 to 14	Female	291 (193 to 409)	0.1 (0.1 to 0.2)	320 (211 to 440)	0.1 (0.1 to 0.1)	329 (214 to 448)	0.1 (0.1 to 0.2)	13.2 (-11.1 to 48.1)	-1.0 (-22.2 to 29.6)
Global	Other chronic respiratory diseases	10 to 14	Male	493 (234 to 817)	0.2 (0.1 to 0.3)	508 (275 to 787)	0.2 (0.1 to 0.2)	441 (291 to 598)	0.1 (0.1 to 0.2)	-10.5 (-36.4 to 32.5)	-23.5 (-45.6 to 13.2)
Global	Cirrhosis and other chronic liver diseases due to hepatitis B	5 to 9	Both	104 (73 to 149)	0.0 (0.0 to 0.0)	97 (68 to 135)	0.0 (0.0 to 0.0)	76 (52 to 112)	0.0 (0.0 to 0.0)	-27.7 (-42.0 to -5.6)	-35.3 (-48.0 to -15.5)
Global	Cirrhosis and other chronic liver diseases due to hepatitis B	5 to 9	Female	25 (18 to 34)	0.0 (0.0 to 0.0)	21 (15 to 28)	0.0 (0.0 to 0.0)	12 (9 to 16)	0.0 (0.0 to 0.0)	-51.5 (-64.1 to -36.9)	-56.1 (-67.5 to -42.9)
Global	Cirrhosis and other chronic liver diseases due to hepatitis B	5 to 9	Male	79 (55 to 115)	0.0 (0.0 to 0.0)	76 (52 to 113)	0.0 (0.0 to 0.0)	63 (43 to 95)	0.0 (0.0 to 0.0)	-20.2 (-37.0 to 6.6)	-29.3 (-44.1 to -5.5)
Global	Cirrhosis and other chronic liver diseases due to hepatitis B	10 to 14	Both	185 (133 to 247)	0.0 (0.0 to 0.0)	197 (144 to 263)	0.0 (0.0 to 0.0)	148 (104 to 209)	0.0 (0.0 to 0.0)	-20.2 (-32.8 to 0.9)	-31.0 (-41.9 to -12.8)
Global	Cirrhosis and other chronic liver diseases due to hepatitis B	10 to 14	Female	49 (35 to 66)	0.0 (0.0 to 0.0)	47 (34 to 63)	0.0 (0.0 to 0.0)	32 (22 to 42)	0.0 (0.0 to 0.0)	-35.8 (-49.6 to -21.2)	-43.9 (-55.9 to -31.0)
Global	Cirrhosis and other chronic liver diseases due to hepatitis B	10 to 14	Male	136 (96 to 186)	0.1 (0.0 to 0.1)	150 (109 to 207)	0.0 (0.0 to 0.1)	116 (81 to 168)	0.0 (0.0 to 0.1)	-14.6 (-30.1 to 10.7)	-27.0 (-40.3 to -5.4)

eTable 8. All-cause and cause-specific mortality (counts and rates) at the global level and by country in the WHO European Region for ages 5 to 9 and 10 to 14 for 1990, 2000, and 2016

Global	Cirrhosis and other chronic liver diseases due to hepatitis C	5 to 9	Both	81 (55 to 116)	0.0 (0.0 to 0.0)	73 (50 to 105)	0.0 (0.0 to 0.0)	54 (35 to 84)	0.0 (0.0 to 0.0)	-33.0 (-46.8 to -13.0)	-40.0 (-52.3 to -22.1)
Global	Cirrhosis and other chronic liver diseases due to hepatitis C	5 to 9	Female	20 (14 to 28)	0.0 (0.0 to 0.0)	18 (12 to 25)	0.0 (0.0 to 0.0)	11 (7 to 16)	0.0 (0.0 to 0.0)	-43.8 (-62.9 to -23.8)	-49.1 (-66.4 to -31.0)
Global	Cirrhosis and other chronic liver diseases due to hepatitis C	5 to 9	Male	60 (41 to 89)	0.0 (0.0 to 0.0)	55 (37 to 83)	0.0 (0.0 to 0.0)	43 (27 to 69)	0.0 (0.0 to 0.0)	-29.4 (-45.4 to -3.7)	-37.4 (-51.6 to -14.6)
Global	Cirrhosis and other chronic liver diseases due to hepatitis C	10 to 14	Both	145 (101 to 198)	0.0 (0.0 to 0.0)	153 (106 to 219)	0.0 (0.0 to 0.0)	118 (80 to 175)	0.0 (0.0 to 0.0)	-18.6 (-32.2 to 1.8)	-29.7 (-41.4 to -12.0)
Global	Cirrhosis and other chronic liver diseases due to hepatitis C	10 to 14	Female	38 (26 to 53)	0.0 (0.0 to 0.0)	39 (27 to 54)	0.0 (0.0 to 0.0)	28 (18 to 40)	0.0 (0.0 to 0.0)	-25.7 (-43.5 to -6.5)	-35.0 (-50.6 to -18.2)
Global	Cirrhosis and other chronic liver diseases due to hepatitis C	10 to 14	Male	107 (74 to 150)	0.0 (0.0 to 0.1)	115 (78 to 167)	0.0 (0.0 to 0.1)	90 (60 to 134)	0.0 (0.0 to 0.0)	-16.1 (-32.5 to 9.3)	-28.3 (-42.3 to -6.6)
Global	Cirrhosis and other chronic liver diseases due to other causes	5 to 9	Both	5150 (4309 to 5999)	0.9 (0.7 to 1.0)	4512 (3880 to 5315)	0.7 (0.6 to 0.9)	2865 (2462 to 3813)	0.4 (0.4 to 0.6)	-44.4 (-54.3 to -29.5)	-50.2 (-59.0 to -36.9)
Global	Cirrhosis and other chronic liver diseases due to other causes	5 to 9	Female	2611 (2137 to 3208)	0.9 (0.8 to 1.1)	2161 (1808 to 2529)	0.7 (0.6 to 0.8)	1173 (979 to 1399)	0.4 (0.3 to 0.5)	-55.1 (-66.9 to -42.8)	-59.3 (-70.1 to -48.2)
Global	Cirrhosis and other chronic liver diseases due to other causes	5 to 9	Male	2538 (2003 to 3342)	0.9 (0.7 to 1.1)	2351 (1922 to 3133)	0.7 (0.6 to 1.0)	1691 (1415 to 2514)	0.5 (0.4 to 0.8)	-33.4 (-46.0 to -13.1)	-40.9 (-52.2 to -22.9)
Global	Cirrhosis and other chronic liver diseases due to other causes	10 to 14	Both	4822 (4262 to 5405)	0.9 (0.8 to 1.0)	4954 (4457 to 5662)	0.8 (0.7 to 0.9)	3318 (2930 to 4246)	0.5 (0.5 to 0.7)	-31.2 (-40.4 to -15.2)	-40.5 (-48.5 to -26.7)
Global	Cirrhosis and other chronic liver diseases due to other causes	10 to 14	Female	2481 (2129 to 2840)	1.0 (0.8 to 1.1)	2418 (2115 to 2686)	0.8 (0.7 to 0.9)	1523 (1303 to 1756)	0.5 (0.4 to 0.6)	-38.6 (-50.2 to -26.8)	-46.3 (-56.5 to -35.9)
Global	Cirrhosis and other chronic liver diseases due to other causes	10 to 14	Male	2341 (1990 to 2774)	0.9 (0.7 to 1.0)	2536 (2191 to 3108)	0.8 (0.7 to 1.0)	1794 (1536 to 2561)	0.6 (0.5 to 0.8)	-23.3 (-35.4 to -0.3)	-34.5 (-44.8 to -14.8)
Global	Peptic ulcer disease	5 to 9	Both	3193 (2428 to 4092)	0.6 (0.4 to 0.7)	1748 (1376 to 2255)	0.3 (0.2 to 0.4)	629 (510 to 815)	0.1 (0.1 to 0.1)	-80.3 (-84.6 to -74.4)	-82.4 (-86.2 to -77.0)
Global	Peptic ulcer disease	5 to 9	Female	1621 (1132 to 2207)	0.6 (0.4 to 0.8)	866 (640 to 1227)	0.3 (0.2 to 0.4)	317 (243 to 445)	0.1 (0.1 to 0.1)	-80.5 (-85.6 to -72.2)	-82.3 (-87.0 to -74.8)

eTable 8. All-cause and cause-specific mortality (counts and rates) at the global level and by country in the WHO European Region for ages 5 to 9 and 10 to 14 for 1990, 2000, and 2016

Global	Peptic ulcer disease	5 to 9	Male	1572 (1178 to 2027)	0.5 (0.4 to 0.7)	882 (676 to 1137)	0.3 (0.2 to 0.4)	312 (240 to 404)	0.1 (0.1 to 0.1)	-80.1 (-85.0 to -72.9)	-82.4 (-86.7 to -76.0)
Global	Peptic ulcer disease	10 to 14	Both	3137 (2656 to 3706)	0.6 (0.5 to 0.7)	2127 (1802 to 2579)	0.3 (0.3 to 0.4)	950 (804 to 1196)	0.2 (0.1 to 0.2)	-69.7 (-74.0 to -64.1)	-73.8 (-77.5 to -69.0)
Global	Peptic ulcer disease	10 to 14	Female	1622 (1279 to 2055)	0.6 (0.5 to 0.8)	1055 (827 to 1374)	0.3 (0.3 to 0.5)	479 (383 to 626)	0.2 (0.1 to 0.2)	-70.5 (-76.0 to -62.7)	-74.2 (-79.0 to -67.4)
Global	Peptic ulcer disease	10 to 14	Male	1515 (1222 to 1814)	0.6 (0.5 to 0.7)	1072 (861 to 1338)	0.3 (0.3 to 0.4)	471 (379 to 599)	0.1 (0.1 to 0.2)	-68.9 (-73.9 to -61.6)	-73.4 (-77.7 to -67.2)
Global	Gastritis and duodenitis	5 to 9	Both	350 (218 to 534)	0.1 (0.0 to 0.1)	274 (191 to 411)	0.0 (0.0 to 0.1)	140 (109 to 182)	0.0 (0.0 to 0.0)	-60.1 (-74.7 to -28.6)	-64.3 (-77.3 to -36.0)
Global	Gastritis and duodenitis	5 to 9	Female	188 (100 to 296)	0.1 (0.0 to 0.1)	143 (89 to 221)	0.0 (0.0 to 0.1)	65 (47 to 94)	0.0 (0.0 to 0.0)	-65.5 (-79.6 to -26.4)	-68.8 (-81.5 to -33.4)
Global	Gastritis and duodenitis	5 to 9	Male	162 (97 to 267)	0.1 (0.0 to 0.1)	132 (87 to 217)	0.0 (0.0 to 0.1)	75 (55 to 105)	0.0 (0.0 to 0.0)	-53.9 (-73.4 to -14.7)	-59.1 (-76.4 to -24.4)
Global	Gastritis and duodenitis	10 to 14	Both	226 (166 to 301)	0.0 (0.0 to 0.1)	220 (170 to 296)	0.0 (0.0 to 0.0)	135 (112 to 170)	0.0 (0.0 to 0.0)	-40.2 (-54.9 to -15.7)	-48.3 (-61.0 to -27.1)
Global	Gastritis and duodenitis	10 to 14	Female	128 (80 to 181)	0.0 (0.0 to 0.1)	117 (82 to 166)	0.0 (0.0 to 0.1)	63 (48 to 86)	0.0 (0.0 to 0.0)	-50.7 (-66.2 to -17.6)	-56.9 (-70.5 to -27.9)
Global	Gastritis and duodenitis	10 to 14	Male	98 (70 to 140)	0.0 (0.0 to 0.1)	103 (78 to 146)	0.0 (0.0 to 0.0)	72 (58 to 92)	0.0 (0.0 to 0.0)	-26.3 (-46.2 to 2.0)	-37.0 (-54.0 to -12.8)
Global	Appendicitis	5 to 9	Both	2562 (1869 to 3168)	0.4 (0.3 to 0.6)	2218 (1700 to 2759)	0.4 (0.3 to 0.4)	1238 (1040 to 1539)	0.2 (0.2 to 0.2)	-51.7 (-61.4 to -34.8)	-56.7 (-65.4 to -41.6)
Global	Appendicitis	5 to 9	Female	1177 (737 to 1525)	0.4 (0.3 to 0.5)	1081 (742 to 1440)	0.4 (0.2 to 0.5)	580 (462 to 786)	0.2 (0.1 to 0.3)	-50.7 (-63.1 to -21.7)	-55.4 (-66.6 to -29.1)
Global	Appendicitis	5 to 9	Male	1385 (1009 to 1779)	0.5 (0.3 to 0.6)	1137 (894 to 1446)	0.4 (0.3 to 0.5)	658 (518 to 839)	0.2 (0.2 to 0.3)	-52.5 (-63.2 to -34.9)	-57.9 (-67.4 to -42.3)
Global	Appendicitis	10 to 14	Both	2083 (1652 to 2417)	0.4 (0.3 to 0.5)	2279 (1873 to 2695)	0.4 (0.3 to 0.4)	1573 (1364 to 1898)	0.3 (0.2 to 0.3)	-24.5 (-35.7 to -7.2)	-34.7 (-44.4 to -19.7)
Global	Appendicitis	10 to 14	Female	1023 (743 to 1247)	0.4 (0.3 to 0.5)	1137 (869 to 1443)	0.4 (0.3 to 0.5)	711 (572 to 921)	0.2 (0.2 to 0.3)	-30.5 (-43.8 to -2.8)	-39.2 (-50.8 to -15.0)
Global	Appendicitis	10 to 14	Male	1060 (803 to 1282)	0.4 (0.3 to 0.5)	1143 (911 to 1403)	0.4 (0.3 to 0.4)	862 (707 to 1093)	0.3 (0.2 to 0.3)	-18.7 (-32.6 to 1.1)	-30.5 (-42.4 to -13.6)
Global	Paralytic ileus and intestinal obstruction	5 to 9	Both	2575 (1594 to 3226)	0.4 (0.3 to 0.6)	2729 (1873 to 3294)	0.4 (0.3 to 0.5)	2262 (1775 to 2722)	0.4 (0.3 to 0.4)	-12.2 (-30.2 to 29.2)	-21.3 (-37.5 to 15.7)
Global	Paralytic ileus and intestinal obstruction	5 to 9	Female	990 (532 to 1321)	0.4 (0.2 to 0.5)	1097 (655 to 1424)	0.4 (0.2 to 0.5)	793 (565 to 1017)	0.3 (0.2 to 0.3)	-19.9 (-40.9 to 30.3)	-27.5 (-46.5 to 17.9)
Global	Paralytic ileus and intestinal obstruction	5 to 9	Male	1585 (933 to 2037)	0.5 (0.3 to 0.7)	1632 (1079 to 1982)	0.5 (0.3 to 0.6)	1469 (1126 to 1834)	0.4 (0.3 to 0.6)	-7.3 (-28.3 to 33.3)	-17.8 (-36.4 to 18.2)
Global	Paralytic ileus and intestinal obstruction	10 to 14	Both	1873 (1253 to 2233)	0.4 (0.2 to 0.4)	2362 (1701 to 2748)	0.4 (0.3 to 0.4)	2245 (1821 to 2612)	0.4 (0.3 to 0.4)	19.9 (1.9 to 57.7)	3.6 (-11.9 to 36.3)
Global	Paralytic ileus and intestinal obstruction	10 to 14	Female	861 (523 to 1078)	0.3 (0.2 to 0.4)	1130 (714 to 1396)	0.4 (0.2 to 0.5)	992 (720 to 1212)	0.3 (0.2 to 0.4)	15.2 (-7.7 to 56.2)	0.8 (-19.2 to 36.7)
Global	Paralytic ileus and intestinal obstruction	10 to 14	Male	1012 (628 to 1243)	0.4 (0.2 to 0.5)	1232 (832 to 1491)	0.4 (0.3 to 0.5)	1253 (960 to 1573)	0.4 (0.3 to 0.5)	23.8 (1.8 to 65.3)	5.8 (-13.0 to 41.3)
Global	Inguinal, femoral, and abdominal hernia	5 to 9	Both	443 (273 to 588)	0.1 (0.0 to 0.1)	407 (267 to 530)	0.1 (0.0 to 0.1)	214 (142 to 279)	0.0 (0.0 to 0.0)	-51.8 (-65.6 to -28.5)	-56.8 (-69.2 to -36.0)
Global	Inguinal, femoral, and abdominal hernia	5 to 9	Female	128 (72 to 183)	0.0 (0.0 to 0.1)	129 (76 to 188)	0.0 (0.0 to 0.1)	64 (43 to 95)	0.0 (0.0 to 0.0)	-50.1 (-69.5 to -19.9)	-54.8 (-72.4 to -27.5)
Global	Inguinal, femoral, and abdominal hernia	5 to 9	Male	316 (173 to 434)	0.1 (0.1 to 0.1)	278 (158 to 371)	0.1 (0.1 to 0.1)	150 (86 to 205)	0.0 (0.0 to 0.1)	-52.5 (-66.6 to -23.6)	-57.9 (-70.4 to -32.2)

eTable 8. All-cause and cause-specific mortality (counts and rates) at the global level and by country in the WHO European Region for ages 5 to 9 and 10 to 14 for 1990, 2000, and 2016

Global	Inguinal, femoral, and abdominal hernia	10 to 14	Both	350 (199 to 464)	0.1 (0.0 to 0.1)	376 (224 to 485)	0.1 (0.0 to 0.1)	260 (154 to 338)	0.0 (0.0 to 0.1)	-25.6 (-43.0 to 0.5)	-35.7 (-50.7 to -13.1)
Global	Inguinal, femoral, and abdominal hernia	10 to 14	Female	80 (52 to 114)	0.0 (0.0 to 0.0)	92 (62 to 136)	0.0 (0.0 to 0.0)	60 (44 to 94)	0.0 (0.0 to 0.0)	-25.8 (-43.2 to -1.7)	-35.1 (-50.3 to -14.0)
Global	Inguinal, femoral, and abdominal hernia	10 to 14	Male	270 (128 to 380)	0.1 (0.0 to 0.1)	284 (139 to 396)	0.1 (0.0 to 0.1)	201 (93 to 280)	0.1 (0.0 to 0.1)	-25.6 (-45.4 to 6.5)	-36.4 (-53.3 to -8.9)
Global	Inflammatory bowel disease	5 to 9	Both	342 (180 to 654)	0.1 (0.0 to 0.1)	325 (195 to 568)	0.1 (0.0 to 0.1)	221 (165 to 292)	0.0 (0.0 to 0.0)	-35.5 (-60.1 to -3.3)	-42.2 (-64.2 to -13.4)
Global	Inflammatory bowel disease	5 to 9	Female	165 (82 to 330)	0.1 (0.0 to 0.1)	152 (86 to 292)	0.1 (0.0 to 0.1)	101 (72 to 152)	0.0 (0.0 to 0.0)	-38.7 (-62.5 to -3.4)	-44.5 (-66.1 to -12.5)
Global	Inflammatory bowel disease	5 to 9	Male	178 (87 to 335)	0.1 (0.0 to 0.1)	172 (92 to 298)	0.1 (0.0 to 0.1)	120 (79 to 156)	0.0 (0.0 to 0.0)	-32.5 (-59.6 to 6.1)	-40.2 (-64.2 to -5.9)
Global	Inflammatory bowel disease	10 to 14	Both	229 (143 to 375)	0.0 (0.0 to 0.1)	261 (178 to 392)	0.0 (0.0 to 0.1)	201 (154 to 255)	0.0 (0.0 to 0.0)	-12.4 (-36.4 to 13.4)	-24.2 (-45.0 to -2.0)
Global	Inflammatory bowel disease	10 to 14	Female	103 (60 to 178)	0.0 (0.0 to 0.1)	114 (72 to 183)	0.0 (0.0 to 0.1)	82 (59 to 117)	0.0 (0.0 to 0.0)	-19.6 (-43.8 to 9.2)	-29.6 (-50.8 to -4.5)
Global	Inflammatory bowel disease	10 to 14	Male	126 (75 to 207)	0.0 (0.0 to 0.1)	147 (93 to 223)	0.0 (0.0 to 0.1)	118 (82 to 153)	0.0 (0.0 to 0.0)	-6.5 (-33.5 to 26.8)	-20.1 (-43.2 to 8.3)
Global	Vascular intestinal disorders	5 to 9	Both	87 (54 to 121)	0.0 (0.0 to 0.0)	80 (51 to 112)	0.0 (0.0 to 0.0)	58 (42 to 80)	0.0 (0.0 to 0.0)	-32.7 (-52.4 to -5.3)	-39.8 (-57.3 to -15.1)
Global	Vascular intestinal disorders	5 to 9	Female	27 (16 to 35)	0.0 (0.0 to 0.0)	22 (14 to 30)	0.0 (0.0 to 0.0)	13 (11 to 17)	0.0 (0.0 to 0.0)	-51.8 (-62.5 to -25.8)	-56.4 (-66.1 to -32.9)
Global	Vascular intestinal disorders	5 to 9	Male	60 (27 to 92)	0.0 (0.0 to 0.0)	58 (30 to 89)	0.0 (0.0 to 0.0)	46 (29 to 68)	0.0 (0.0 to 0.0)	-24.3 (-50.8 to 25.8)	-32.9 (-56.3 to 11.5)
Global	Vascular intestinal disorders	10 to 14	Both	54 (42 to 64)	0.0 (0.0 to 0.0)	58 (46 to 68)	0.0 (0.0 to 0.0)	44 (38 to 54)	0.0 (0.0 to 0.0)	-18.4 (-29.4 to -2.2)	-29.5 (-39.0 to -15.5)
Global	Vascular intestinal disorders	10 to 14	Female	23 (15 to 29)	0.0 (0.0 to 0.0)	24 (17 to 30)	0.0 (0.0 to 0.0)	16 (13 to 20)	0.0 (0.0 to 0.0)	-30.5 (-42.4 to -6.0)	-39.2 (-49.6 to -17.7)
Global	Vascular intestinal disorders	10 to 14	Male	32 (23 to 38)	0.0 (0.0 to 0.0)	34 (25 to 41)	0.0 (0.0 to 0.0)	29 (23 to 36)	0.0 (0.0 to 0.0)	-9.7 (-25.6 to 17.6)	-22.8 (-36.4 to 0.5)
Global	Gallbladder and biliary diseases	5 to 9	Both	364 (259 to 465)	0.1 (0.0 to 0.1)	303 (231 to 417)	0.0 (0.0 to 0.1)	195 (153 to 271)	0.0 (0.0 to 0.0)	-46.6 (-61.5 to -14.2)	-52.2 (-65.6 to -23.2)
Global	Gallbladder and biliary diseases	5 to 9	Female	230 (152 to 311)	0.1 (0.1 to 0.1)	193 (137 to 278)	0.1 (0.0 to 0.1)	125 (92 to 192)	0.0 (0.0 to 0.1)	-45.5 (-63.3 to -10.6)	-50.7 (-66.8 to -19.1)
Global	Gallbladder and biliary diseases	5 to 9	Male	135 (88 to 175)	0.0 (0.0 to 0.1)	111 (86 to 156)	0.0 (0.0 to 0.0)	69 (53 to 101)	0.0 (0.0 to 0.0)	-48.5 (-63.5 to -15.6)	-54.4 (-67.6 to -25.2)
Global	Gallbladder and biliary diseases	10 to 14	Both	302 (236 to 359)	0.1 (0.0 to 0.1)	344 (285 to 418)	0.1 (0.0 to 0.1)	272 (229 to 329)	0.0 (0.0 to 0.1)	-9.9 (-25.2 to 13.0)	-22.1 (-35.3 to -2.3)
Global	Gallbladder and biliary diseases	10 to 14	Female	175 (132 to 216)	0.1 (0.1 to 0.1)	182 (145 to 256)	0.1 (0.0 to 0.1)	116 (93 to 167)	0.0 (0.0 to 0.1)	-33.5 (-48.3 to -1.6)	-41.8 (-54.7 to -13.9)
Global	Gallbladder and biliary diseases	10 to 14	Male	127 (83 to 158)	0.0 (0.0 to 0.1)	162 (109 to 207)	0.1 (0.0 to 0.1)	156 (107 to 200)	0.0 (0.0 to 0.1)	22.4 (-1.2 to 53.8)	4.6 (-15.6 to 31.5)
Global	Pancreatitis	5 to 9	Both	224 (148 to 306)	0.0 (0.0 to 0.1)	189 (136 to 270)	0.0 (0.0 to 0.0)	120 (91 to 152)	0.0 (0.0 to 0.0)	-46.2 (-60.6 to -12.6)	-51.8 (-64.7 to -21.7)
Global	Pancreatitis	5 to 9	Female	133 (63 to 210)	0.0 (0.0 to 0.1)	106 (56 to 180)	0.0 (0.0 to 0.1)	60 (40 to 90)	0.0 (0.0 to 0.0)	-54.4 (-68.0 to -15.8)	-58.7 (-71.0 to -23.8)

eTable 8. All-cause and cause-specific mortality (counts and rates) at the global level and by country in the WHO European Region for ages 5 to 9 and 10 to 14 for 1990, 2000, and 2016

Global	Pancreatitis	5 to 9	Male	91 (59 to 120)	0.0 (0.0 to 0.0)	83 (60 to 111)	0.0 (0.0 to 0.0)	60 (41 to 80)	0.0 (0.0 to 0.0)	-34.3 (-55.1 to 7.0)	-41.8 (-60.2 to -5.2)
Global	Pancreatitis	10 to 14	Both	221 (164 to 287)	0.0 (0.0 to 0.1)	245 (190 to 326)	0.0 (0.0 to 0.1)	185 (142 to 221)	0.0 (0.0 to 0.0)	-16.5 (-33.2 to 17.2)	-27.8 (-42.2 to 1.3)
Global	Pancreatitis	10 to 14	Female	127 (69 to 200)	0.0 (0.0 to 0.1)	135 (85 to 221)	0.0 (0.0 to 0.1)	94 (64 to 127)	0.0 (0.0 to 0.0)	-26.1 (-44.2 to 18.8)	-35.3 (-51.1 to 4.0)
Global	Pancreatitis	10 to 14	Male	95 (67 to 116)	0.0 (0.0 to 0.0)	110 (85 to 139)	0.0 (0.0 to 0.0)	91 (64 to 116)	0.0 (0.0 to 0.0)	-3.8 (-24.1 to 29.3)	-17.8 (-35.2 to 10.5)
Global	Other digestive diseases	5 to 9	Both	1739 (1233 to 2368)	0.3 (0.2 to 0.4)	1144 (792 to 1630)	0.2 (0.1 to 0.3)	723 (570 to 960)	0.1 (0.1 to 0.1)	-58.4 (-66.8 to -48.5)	-62.8 (-70.3 to -53.8)
Global	Other digestive diseases	5 to 9	Female	794 (554 to 1071)	0.3 (0.2 to 0.4)	485 (339 to 673)	0.2 (0.1 to 0.2)	311 (251 to 405)	0.1 (0.1 to 0.1)	-60.8 (-69.0 to -48.8)	-64.5 (-72.0 to -53.6)
Global	Other digestive diseases	5 to 9	Male	945 (630 to 1382)	0.3 (0.2 to 0.5)	659 (418 to 1037)	0.2 (0.1 to 0.3)	412 (295 to 601)	0.1 (0.1 to 0.2)	-56.4 (-67.9 to -43.4)	-61.4 (-71.5 to -49.8)
Global	Other digestive diseases	10 to 14	Both	1052 (815 to 1378)	0.2 (0.2 to 0.3)	817 (628 to 1099)	0.1 (0.1 to 0.2)	560 (474 to 722)	0.1 (0.1 to 0.1)	-46.8 (-56.8 to -35.4)	-54.0 (-62.7 to -44.2)
Global	Other digestive diseases	10 to 14	Female	465 (362 to 599)	0.2 (0.1 to 0.2)	320 (254 to 422)	0.1 (0.1 to 0.1)	198 (166 to 251)	0.1 (0.1 to 0.1)	-57.4 (-64.4 to -46.9)	-62.7 (-68.8 to -53.5)
Global	Other digestive diseases	10 to 14	Male	587 (414 to 843)	0.2 (0.2 to 0.3)	497 (349 to 722)	0.2 (0.1 to 0.2)	361 (289 to 494)	0.1 (0.1 to 0.2)	-38.4 (-54.0 to -20.3)	-47.4 (-60.6 to -31.9)
Global	Alzheimer disease and other dementias	5 to 9	Both	0 (0 to 0)	0.0 (0.0 to 0.0)	0 (0 to 0)	0.0 (0.0 to 0.0)	0 (0 to 0)	0.0 (0.0 to 0.0)	0.0 (0.0 to 0.0)	0.0 (0.0 to 0.0)
Global	Alzheimer disease and other dementias	5 to 9	Female	0 (0 to 0)	0.0 (0.0 to 0.0)	0 (0 to 0)	0.0 (0.0 to 0.0)	0 (0 to 0)	0.0 (0.0 to 0.0)	0.0 (0.0 to 0.0)	0.0 (0.0 to 0.0)
Global	Alzheimer disease and other dementias	5 to 9	Male	0 (0 to 0)	0.0 (0.0 to 0.0)	0 (0 to 0)	0.0 (0.0 to 0.0)	0 (0 to 0)	0.0 (0.0 to 0.0)	0.0 (0.0 to 0.0)	0.0 (0.0 to 0.0)
Global	Alzheimer disease and other dementias	10 to 14	Both	0 (0 to 0)	0.0 (0.0 to 0.0)	0 (0 to 0)	0.0 (0.0 to 0.0)	0 (0 to 0)	0.0 (0.0 to 0.0)	0.0 (0.0 to 0.0)	0.0 (0.0 to 0.0)
Global	Alzheimer disease and other dementias	10 to 14	Female	0 (0 to 0)	0.0 (0.0 to 0.0)	0 (0 to 0)	0.0 (0.0 to 0.0)	0 (0 to 0)	0.0 (0.0 to 0.0)	0.0 (0.0 to 0.0)	0.0 (0.0 to 0.0)
Global	Alzheimer disease and other dementias	10 to 14	Male	0 (0 to 0)	0.0 (0.0 to 0.0)	0 (0 to 0)	0.0 (0.0 to 0.0)	0 (0 to 0)	0.0 (0.0 to 0.0)	0.0 (0.0 to 0.0)	0.0 (0.0 to 0.0)
Global	Parkinson disease	5 to 9	Both	0 (0 to 0)	0.0 (0.0 to 0.0)	0 (0 to 0)	0.0 (0.0 to 0.0)	0 (0 to 0)	0.0 (0.0 to 0.0)	0.0 (0.0 to 0.0)	0.0 (0.0 to 0.0)
Global	Parkinson disease	5 to 9	Female	0 (0 to 0)	0.0 (0.0 to 0.0)	0 (0 to 0)	0.0 (0.0 to 0.0)	0 (0 to 0)	0.0 (0.0 to 0.0)	0.0 (0.0 to 0.0)	0.0 (0.0 to 0.0)
Global	Parkinson disease	5 to 9	Male	0 (0 to 0)	0.0 (0.0 to 0.0)	0 (0 to 0)	0.0 (0.0 to 0.0)	0 (0 to 0)	0.0 (0.0 to 0.0)	0.0 (0.0 to 0.0)	0.0 (0.0 to 0.0)
Global	Parkinson disease	10 to 14	Both	0 (0 to 0)	0.0 (0.0 to 0.0)	0 (0 to 0)	0.0 (0.0 to 0.0)	0 (0 to 0)	0.0 (0.0 to 0.0)	0.0 (0.0 to 0.0)	0.0 (0.0 to 0.0)
Global	Parkinson disease	10 to 14	Female	0 (0 to 0)	0.0 (0.0 to 0.0)	0 (0 to 0)	0.0 (0.0 to 0.0)	0 (0 to 0)	0.0 (0.0 to 0.0)	0.0 (0.0 to 0.0)	0.0 (0.0 to 0.0)
Global	Parkinson disease	10 to 14	Male	0 (0 to 0)	0.0 (0.0 to 0.0)	0 (0 to 0)	0.0 (0.0 to 0.0)	0 (0 to 0)	0.0 (0.0 to 0.0)	0.0 (0.0 to 0.0)	0.0 (0.0 to 0.0)
Global	Epilepsy	5 to 9	Both	5876 (4638 to 7142)	1.0 (0.8 to 1.2)	5339 (4503 to 6212)	0.9 (0.7 to 1.0)	3395 (2964 to 3896)	0.5 (0.5 to 0.6)	-42.2 (-54.2 to -20.8)	-48.3 (-59.0 to -29.0)

eTable 8. All-cause and cause-specific mortality (counts and rates) at the global level and by country in the WHO European Region for ages 5 to 9 and 10 to 14 for 1990, 2000, and 2016

Global	Epilepsy	5 to 9	Female	2668 (1750 to 3472)	1.0 (0.6 to 1.2)	2427 (1732 to 3019)	0.8 (0.6 to 1.0)	1503 (1257 to 1779)	0.5 (0.4 to 0.6)	-43.7 (-57.8 to -9.1)	-49.0 (-61.8 to -17.7)
Global	Epilepsy	5 to 9	Male	3208 (2576 to 3805)	1.1 (0.9 to 1.3)	2912 (2521 to 3319)	0.9 (0.8 to 1.0)	1893 (1628 to 2209)	0.6 (0.5 to 0.7)	-41.0 (-52.8 to -18.4)	-47.7 (-58.2 to -27.7)
Global	Epilepsy	10 to 14	Both	7449 (6282 to 8566)	1.4 (1.2 to 1.6)	7670 (6744 to 8542)	1.2 (1.1 to 1.4)	4969 (4412 to 5560)	0.8 (0.7 to 0.9)	-33.3 (-44.3 to -16.8)	-42.3 (-51.9 to -28.0)
Global	Epilepsy	10 to 14	Female	3365 (2213 to 4128)	1.3 (0.9 to 1.6)	3350 (2506 to 3949)	1.1 (0.8 to 1.3)	2085 (1770 to 2471)	0.7 (0.6 to 0.8)	-38.0 (-51.0 to -5.2)	-45.8 (-57.1 to -17.1)
Global	Epilepsy	10 to 14	Male	4084 (3492 to 4671)	1.5 (1.3 to 1.7)	4320 (3857 to 4827)	1.3 (1.2 to 1.5)	2884 (2527 to 3301)	0.9 (0.8 to 1.0)	-29.4 (-40.8 to -8.7)	-39.6 (-49.4 to -21.9)
Global	Motor neuron disease	5 to 9	Both	51 (43 to 61)	0.0 (0.0 to 0.0)	47 (39 to 55)	0.0 (0.0 to 0.0)	36 (33 to 40)	0.0 (0.0 to 0.0)	-29.1 (-39.6 to -17.9)	-36.5 (-45.9 to -26.4)
Global	Motor neuron disease	5 to 9	Female	26 (21 to 34)	0.0 (0.0 to 0.0)	23 (19 to 29)	0.0 (0.0 to 0.0)	19 (16 to 22)	0.0 (0.0 to 0.0)	-25.3 (-39.9 to -14.0)	-32.4 (-45.6 to -22.2)
Global	Motor neuron disease	5 to 9	Male	26 (21 to 30)	0.0 (0.0 to 0.0)	24 (20 to 27)	0.0 (0.0 to 0.0)	17 (16 to 19)	0.0 (0.0 to 0.0)	-32.9 (-43.8 to -18.1)	-40.5 (-50.2 to -27.3)
Global	Motor neuron disease	10 to 14	Both	77 (66 to 85)	0.0 (0.0 to 0.0)	84 (74 to 94)	0.0 (0.0 to 0.0)	63 (59 to 67)	0.0 (0.0 to 0.0)	-17.9 (-28.5 to -7.3)	-29.1 (-38.2 to -19.9)
Global	Motor neuron disease	10 to 14	Female	25 (21 to 31)	0.0 (0.0 to 0.0)	26 (23 to 31)	0.0 (0.0 to 0.0)	21 (19 to 23)	0.0 (0.0 to 0.0)	-16.4 (-30.6 to -3.8)	-26.8 (-39.3 to -15.8)
Global	Motor neuron disease	10 to 14	Male	51 (44 to 57)	0.0 (0.0 to 0.0)	58 (50 to 65)	0.0 (0.0 to 0.0)	42 (38 to 45)	0.0 (0.0 to 0.0)	-18.7 (-29.9 to -6.0)	-30.5 (-40.1 to -19.7)
Global	Other neurological disorders	5 to 9	Both	1002 (786 to 1323)	0.2 (0.1 to 0.2)	972 (787 to 1244)	0.2 (0.1 to 0.2)	880 (744 to 1058)	0.1 (0.1 to 0.2)	-12.1 (-27.0 to 7.4)	-21.3 (-34.6 to -3.8)
Global	Other neurological disorders	5 to 9	Female	514 (370 to 652)	0.2 (0.1 to 0.2)	492 (368 to 618)	0.2 (0.1 to 0.2)	413 (342 to 487)	0.1 (0.1 to 0.2)	-19.7 (-32.5 to 3.4)	-27.3 (-38.9 to -6.4)
Global	Other neurological disorders	5 to 9	Male	487 (402 to 695)	0.2 (0.1 to 0.2)	480 (405 to 653)	0.2 (0.1 to 0.2)	467 (395 to 588)	0.1 (0.1 to 0.2)	-4.1 (-24.6 to 14.9)	-15.0 (-33.1 to 1.9)
Global	Other neurological disorders	10 to 14	Both	1403 (1193 to 1677)	0.3 (0.2 to 0.3)	1640 (1414 to 1923)	0.3 (0.2 to 0.3)	1605 (1377 to 1834)	0.3 (0.2 to 0.3)	14.4 (-0.1 to 28.4)	-1.1 (-13.7 to 11.0)
Global	Other neurological disorders	10 to 14	Female	567 (415 to 718)	0.2 (0.2 to 0.3)	664 (495 to 845)	0.2 (0.2 to 0.3)	687 (509 to 894)	0.2 (0.2 to 0.3)	21.1 (4.4 to 41.5)	5.9 (-8.7 to 23.8)
Global	Other neurological disorders	10 to 14	Male	836 (751 to 993)	0.3 (0.3 to 0.4)	976 (887 to 1133)	0.3 (0.3 to 0.4)	918 (836 to 995)	0.3 (0.3 to 0.3)	9.8 (-9.2 to 26.1)	-6.1 (-22.4 to 7.8)
Global	Drug use disorders	5 to 9	Both	0 (0 to 0)	0.0 (0.0 to 0.0)	0 (0 to 0)	0.0 (0.0 to 0.0)	0 (0 to 0)	0.0 (0.0 to 0.0)	0.0 (0.0 to 0.0)	0.0 (0.0 to 0.0)
Global	Drug use disorders	5 to 9	Female	0 (0 to 0)	0.0 (0.0 to 0.0)	0 (0 to 0)	0.0 (0.0 to 0.0)	0 (0 to 0)	0.0 (0.0 to 0.0)	0.0 (0.0 to 0.0)	0.0 (0.0 to 0.0)
Global	Drug use disorders	5 to 9	Male	0 (0 to 0)	0.0 (0.0 to 0.0)	0 (0 to 0)	0.0 (0.0 to 0.0)	0 (0 to 0)	0.0 (0.0 to 0.0)	0.0 (0.0 to 0.0)	0.0 (0.0 to 0.0)
Global	Drug use disorders	10 to 14	Both	0 (0 to 0)	0.0 (0.0 to 0.0)	0 (0 to 0)	0.0 (0.0 to 0.0)	0 (0 to 0)	0.0 (0.0 to 0.0)	0.0 (0.0 to 0.0)	0.0 (0.0 to 0.0)
Global	Drug use disorders	10 to 14	Female	0 (0 to 0)	0.0 (0.0 to 0.0)	0 (0 to 0)	0.0 (0.0 to 0.0)	0 (0 to 0)	0.0 (0.0 to 0.0)	0.0 (0.0 to 0.0)	0.0 (0.0 to 0.0)
Global	Drug use disorders	10 to 14	Male	0 (0 to 0)	0.0 (0.0 to 0.0)	0 (0 to 0)	0.0 (0.0 to 0.0)	0 (0 to 0)	0.0 (0.0 to 0.0)	0.0 (0.0 to 0.0)	0.0 (0.0 to 0.0)
Global	Eating disorders	5 to 9	Both	16 (11 to 21)	0.0 (0.0 to 0.0)	18 (13 to 21)	0.0 (0.0 to 0.0)	14 (11 to 16)	0.0 (0.0 to 0.0)	-14.4 (-30.9 to 16.0)	-23.4 (-38.1 to 3.8)
Global	Eating disorders	5 to 9	Female	7 (5 to 11)	0.0 (0.0 to 0.0)	8 (6 to 10)	0.0 (0.0 to 0.0)	8 (6 to 10)	0.0 (0.0 to 0.0)	9.0 (-19.9 to 59.7)	-1.3 (-27.5 to 44.6)
Global	Eating disorders	5 to 9	Male	9 (5 to 11)	0.0 (0.0 to 0.0)	10 (6 to 12)	0.0 (0.0 to 0.0)	6 (5 to 7)	0.0 (0.0 to 0.0)	-34.7 (-47.4 to 4.6)	-42.1 (-53.4 to -7.2)
Global	Eating disorders	10 to 14	Both	14 (10 to 17)	0.0 (0.0 to 0.0)	18 (14 to 21)	0.0 (0.0 to 0.0)	15 (12 to 17)	0.0 (0.0 to 0.0)	8.9 (-9.6 to 37.1)	-5.9 (-21.9 to 18.5)
Global	Eating disorders	10 to 14	Female	8 (5 to 11)	0.0 (0.0 to 0.0)	11 (7 to 13)	0.0 (0.0 to 0.0)	10 (8 to 13)	0.0 (0.0 to 0.0)	23.3 (-2.8 to 64.4)	7.9 (-15.0 to 43.8)
Global	Eating disorders	10 to 14	Male	5 (3 to 7)	0.0 (0.0 to 0.0)	7 (5 to 8)	0.0 (0.0 to 0.0)	4 (4 to 5)	0.0 (0.0 to 0.0)	-14.5 (-30.3 to 27.3)	-26.9 (-40.4 to 8.8)

eTable 8. All-cause and cause-specific mortality (counts and rates) at the global level and by country in the WHO European Region for ages 5 to 9 and 10 to 14 for 1990, 2000, and 2016

Global	Diabetes mellitus	5 to 9	Both	1207 (1039 to 1382)	0.2 (0.2 to 0.2)	1123 (996 to 1249)	0.2 (0.2 to 0.2)	763 (677 to 871)	0.1 (0.1 to 0.1)	-36.8 (-48.0 to -25.7)	-43.4 (-53.4 to -33.4)
Global	Diabetes mellitus	5 to 9	Female	641 (547 to 740)	0.2 (0.2 to 0.3)	576 (501 to 654)	0.2 (0.2 to 0.2)	332 (288 to 390)	0.1 (0.1 to 0.1)	-48.2 (-57.7 to -38.2)	-53.1 (-61.7 to -44.1)
Global	Diabetes mellitus	5 to 9	Male	566 (483 to 651)	0.2 (0.2 to 0.2)	547 (475 to 614)	0.2 (0.1 to 0.2)	431 (374 to 496)	0.1 (0.1 to 0.1)	-23.9 (-37.5 to -8.0)	-32.5 (-44.6 to -18.4)
Global	Diabetes mellitus	10 to 14	Both	1604 (1457 to 1750)	0.3 (0.3 to 0.3)	1791 (1641 to 1938)	0.3 (0.3 to 0.3)	1410 (1291 to 1550)	0.2 (0.2 to 0.3)	-12.1 (-20.9 to -3.3)	-24.0 (-31.6 to -16.4)
Global	Diabetes mellitus	10 to 14	Female	984 (870 to 1092)	0.4 (0.3 to 0.4)	1076 (967 to 1179)	0.4 (0.3 to 0.4)	801 (720 to 892)	0.3 (0.2 to 0.3)	-18.6 (-27.9 to -7.9)	-28.8 (-36.9 to -19.4)
Global	Diabetes mellitus	10 to 14	Male	621 (559 to 686)	0.2 (0.2 to 0.3)	715 (650 to 782)	0.2 (0.2 to 0.2)	609 (553 to 677)	0.2 (0.2 to 0.2)	-1.8 (-12.7 to 9.1)	-16.1 (-25.4 to -6.8)
Global	Acute glomerulonephritis	5 to 9	Both	453 (379 to 548)	0.1 (0.1 to 0.1)	321 (281 to 377)	0.1 (0.0 to 0.1)	153 (132 to 181)	0.0 (0.0 to 0.0)	-66.3 (-72.9 to -57.7)	-69.8 (-75.7 to -62.1)
Global	Acute glomerulonephritis	5 to 9	Female	214 (165 to 280)	0.1 (0.1 to 0.1)	142 (117 to 176)	0.0 (0.0 to 0.1)	76 (62 to 99)	0.0 (0.0 to 0.0)	-64.6 (-74.4 to -50.0)	-67.9 (-76.8 to -54.8)
Global	Acute glomerulonephritis	5 to 9	Male	239 (191 to 307)	0.1 (0.1 to 0.1)	179 (152 to 216)	0.1 (0.0 to 0.1)	77 (64 to 96)	0.0 (0.0 to 0.0)	-67.9 (-75.9 to -56.4)	-71.5 (-78.6 to -61.4)
Global	Acute glomerulonephritis	10 to 14	Both	443 (362 to 550)	0.1 (0.1 to 0.1)	363 (316 to 418)	0.1 (0.1 to 0.1)	181 (152 to 219)	0.0 (0.0 to 0.0)	-59.2 (-68.8 to -45.7)	-64.7 (-73.0 to -53.1)
Global	Acute glomerulonephritis	10 to 14	Female	219 (163 to 296)	0.1 (0.1 to 0.1)	167 (136 to 208)	0.1 (0.0 to 0.1)	91 (71 to 123)	0.0 (0.0 to 0.0)	-58.3 (-71.7 to -35.8)	-63.5 (-75.2 to -43.8)
Global	Acute glomerulonephritis	10 to 14	Male	224 (174 to 291)	0.1 (0.1 to 0.1)	196 (164 to 235)	0.1 (0.1 to 0.1)	89 (74 to 111)	0.0 (0.0 to 0.0)	-60.1 (-71.1 to -44.6)	-65.9 (-75.3 to -52.7)
Global	Chronic kidney disease	5 to 9	Both	5201 (4421 to 5857)	0.9 (0.8 to 1.0)	4515 (3937 to 5009)	0.7 (0.6 to 0.8)	3031 (2694 to 3442)	0.5 (0.4 to 0.5)	-41.7 (-49.8 to -31.7)	-47.8 (-55.1 to -38.9)
Global	Chronic kidney disease	5 to 9	Female	2262 (1924 to 2620)	0.8 (0.7 to 0.9)	1898 (1653 to 2153)	0.6 (0.6 to 0.7)	1221 (1070 to 1414)	0.4 (0.3 to 0.5)	-46.0 (-55.0 to -35.5)	-51.1 (-59.3 to -41.6)
Global	Chronic kidney disease	5 to 9	Male	2939 (2269 to 3370)	1.0 (0.8 to 1.1)	2617 (2084 to 2945)	0.8 (0.7 to 0.9)	1809 (1536 to 2105)	0.5 (0.5 to 0.6)	-38.4 (-48.2 to -26.3)	-45.4 (-54.1 to -34.7)
Global	Chronic kidney disease	10 to 14	Both	5188 (4604 to 5736)	1.0 (0.9 to 1.1)	5314 (4826 to 5835)	0.8 (0.8 to 0.9)	3778 (3461 to 4317)	0.6 (0.6 to 0.7)	-27.2 (-34.3 to -17.9)	-37.1 (-43.2 to -29.0)
Global	Chronic kidney disease	10 to 14	Female	2618 (2313 to 2942)	1.0 (0.9 to 1.1)	2551 (2283 to 2808)	0.8 (0.7 to 0.9)	1712 (1541 to 1934)	0.6 (0.5 to 0.7)	-34.6 (-43.4 to -25.2)	-42.8 (-50.5 to -34.6)
Global	Chronic kidney disease	10 to 14	Male	2570 (2137 to 2877)	1.0 (0.8 to 1.1)	2763 (2371 to 3126)	0.9 (0.7 to 1.0)	2066 (1819 to 2424)	0.7 (0.6 to 0.8)	-19.6 (-28.2 to -8.9)	-31.3 (-38.6 to -22.2)
Global	Urinary diseases and male infertility	5 to 9	Both	1540 (1271 to 1863)	0.3 (0.2 to 0.3)	1473 (1261 to 1713)	0.2 (0.2 to 0.3)	1021 (886 to 1173)	0.2 (0.1 to 0.2)	-33.7 (-48.0 to -17.0)	-40.6 (-53.4 to -25.7)
Global	Urinary diseases and male infertility	5 to 9	Female	660 (543 to 839)	0.2 (0.2 to 0.3)	638 (536 to 769)	0.2 (0.2 to 0.3)	437 (369 to 520)	0.1 (0.1 to 0.2)	-33.7 (-51.3 to -15.9)	-40.0 (-55.9 to -23.9)
Global	Urinary diseases and male infertility	5 to 9	Male	881 (705 to 1080)	0.3 (0.2 to 0.4)	835 (699 to 994)	0.3 (0.2 to 0.3)	584 (489 to 702)	0.2 (0.1 to 0.2)	-33.6 (-47.7 to -14.3)	-41.2 (-53.6 to -24.0)
Global	Urinary diseases and male infertility	10 to 14	Both	1145 (1013 to 1320)	0.2 (0.2 to 0.2)	1297 (1173 to 1435)	0.2 (0.2 to 0.2)	974 (879 to 1088)	0.2 (0.1 to 0.2)	-14.9 (-31.0 to -1.7)	-26.5 (-40.3 to -15.0)
Global	Urinary diseases and male infertility	10 to 14	Female	528 (448 to 657)	0.2 (0.2 to 0.3)	599 (518 to 692)	0.2 (0.2 to 0.2)	430 (368 to 507)	0.1 (0.1 to 0.2)	-18.7 (-41.5 to -2.4)	-28.8 (-48.8 to -14.6)
Global	Urinary diseases and male infertility	10 to 14	Male	617 (538 to 687)	0.2 (0.2 to 0.3)	698 (628 to 771)	0.2 (0.2 to 0.2)	545 (492 to 604)	0.2 (0.2 to 0.2)	-11.7 (-22.7 to 1.0)	-24.5 (-33.9 to -13.7)
Global	Hemoglobinopathies and hemolytic anemias	5 to 9	Both	6681 (4745 to 8491)	1.2 (0.8 to 1.5)	5785 (4394 to 7492)	0.9 (0.7 to 1.2)	4146 (3396 to 5582)	0.6 (0.5 to 0.9)	-37.9 (-50.0 to -20.0)	-44.4 (-55.2 to -28.3)

eTable 8. All-cause and cause-specific mortality (counts and rates) at the global level and by country in the WHO European Region for ages 5 to 9 and 10 to 14 for 1990, 2000, and 2016

Global	Hemoglobinopathies and hemolytic anemias	5 to 9	Female	3239<br>(1720 to 4543)	1.2<br>(0.6 to 1.6)	2752<br>(1694 to 3816)	0.9<br>(0.6 to 1.3)	2004<br>(1422 to 2922)	0.6<br>(0.5 to 0.9)	-38.1<br>(-52.2 to -4.5)	-44.0<br>(-56.7 to -13.6)
Global	Hemoglobinopathies and hemolytic anemias	5 to 9	Male	3442<br>(2486 to 4367)	1.2<br>(0.8 to 1.5)	3034<br>(2350 to 4051)	1.0<br>(0.7 to 1.3)	2142<br>(1705 to 3325)	0.6<br>(0.5 to 1.0)	-37.8<br>(-51.2 to -15.9)	-44.8<br>(-56.7 to -25.4)
Global	Hemoglobinopathies and hemolytic anemias	10 to 14	Both	7495<br>(5561 to 8965)	1.4<br>(1.1 to 1.7)	7797<br>(5906 to 9254)	1.2<br>(0.9 to 1.5)	6728<br>(5344 to 8110)	1.1<br>(0.9 to 1.3)	-10.2<br>(-26.6 to 9.3)	-22.4<br>(-36.6 to -5.5)
Global	Hemoglobinopathies and hemolytic anemias	10 to 14	Female	4129<br>(2766 to 5156)	1.6<br>(1.1 to 2.0)	4341<br>(2954 to 5423)	1.4<br>(1.0 to 1.8)	3872<br>(2689 to 4913)	1.3<br>(0.9 to 1.7)	-6.2<br>(-25.6 to 20.6)	-17.9<br>(-34.9 to 5.5)
Global	Hemoglobinopathies and hemolytic anemias	10 to 14	Male	3366<br>(2216 to 4225)	1.2<br>(0.8 to 1.6)	3456<br>(2403 to 4354)	1.1<br>(0.7 to 1.4)	2856<br>(2143 to 3941)	0.9<br>(0.7 to 1.2)	-15.2<br>(-38.0 to 9.8)	-27.5<br>(-47.0 to -6.2)
Global	Endocrine, metabolic, blood, and immune disorders	5 to 9	Both	2526<br>(2161 to 2998)	0.4<br>(0.4 to 0.5)	2267<br>(2023 to 2823)	0.4<br>(0.3 to 0.5)	1862<br>(1668 to 2356)	0.3<br>(0.3 to 0.4)	-26.3<br>(-36.7 to -13.1)	-34.0<br>(-43.3 to -22.1)
Global	Endocrine, metabolic, blood, and immune disorders	5 to 9	Female	1212<br>(865 to 1474)	0.4<br>(0.3 to 0.5)	1035<br>(852 to 1278)	0.3<br>(0.3 to 0.4)	838<br>(733 to 1063)	0.3<br>(0.2 to 0.3)	-30.8<br>(-44.8 to 0.8)	-37.4<br>(-50.1 to -8.8)
Global	Endocrine, metabolic, blood, and immune disorders	5 to 9	Male	1314<br>(1064 to 1767)	0.4<br>(0.4 to 0.6)	1232<br>(1038 to 1658)	0.4<br>(0.3 to 0.5)	1023<br>(877 to 1408)	0.3<br>(0.3 to 0.4)	-22.1<br>(-35.2 to -0.8)	-30.9<br>(-42.5 to -12.0)
Global	Endocrine, metabolic, blood, and immune disorders	10 to 14	Both	1982<br>(1725 to 2281)	0.4<br>(0.3 to 0.4)	2079<br>(1895 to 2528)	0.3<br>(0.3 to 0.4)	1773<br>(1608 to 2190)	0.3<br>(0.3 to 0.4)	-10.6<br>(-19.9 to 0.5)	-22.7<br>(-30.7 to -13.1)
Global	Endocrine, metabolic, blood, and immune disorders	10 to 14	Female	927<br>(700 to 1077)	0.4<br>(0.3 to 0.4)	906<br>(753 to 1088)	0.3<br>(0.2 to 0.4)	760<br>(654 to 926)	0.3<br>(0.2 to 0.3)	-18.1<br>(-30.5 to 2.9)	-28.3<br>(-39.2 to -9.9)
Global	Endocrine, metabolic, blood, and immune disorders	10 to 14	Male	1055<br>(854 to 1347)	0.4<br>(0.3 to 0.5)	1173<br>(1001 to 1553)	0.4<br>(0.3 to 0.5)	1013<br>(877 to 1365)	0.3<br>(0.3 to 0.4)	-4.0<br>(-15.2 to 11.4)	-17.9<br>(-27.5 to -4.8)
Global	Rheumatoid arthritis	5 to 9	Both	206<br>(107 to 363)	0.0<br>(0.0 to 0.1)	166<br>(105 to 276)	0.0<br>(0.0 to 0.0)	96<br>(80 to 134)	0.0<br>(0.0 to 0.0)	-53.5<br>(-67.6 to -13.4)	-58.3<br>(-71.0 to -22.4)
Global	Rheumatoid arthritis	5 to 9	Female	101<br>(49 to 200)	0.0<br>(0.0 to 0.1)	77<br>(47 to 145)	0.0<br>(0.0 to 0.0)	45<br>(33 to 74)	0.0<br>(0.0 to 0.0)	-54.9<br>(-70.2 to -15.0)	-59.2<br>(-73.0 to -23.0)
Global	Rheumatoid arthritis	5 to 9	Male	106<br>(55 to 186)	0.0<br>(0.0 to 0.1)	90<br>(54 to 147)	0.0<br>(0.0 to 0.0)	51<br>(39 to 68)	0.0<br>(0.0 to 0.0)	-52.1<br>(-68.6 to -8.9)	-57.5<br>(-72.1 to -19.2)
Global	Rheumatoid arthritis	10 to 14	Both	204<br>(126 to 330)	0.0<br>(0.0 to 0.1)	185<br>(135 to 280)	0.0<br>(0.0 to 0.0)	107<br>(88 to 143)	0.0<br>(0.0 to 0.0)	-47.8<br>(-61.9 to -18.7)	-54.9<br>(-67.1 to -29.7)
Global	Rheumatoid arthritis	10 to 14	Female	103<br>(58 to 202)	0.0<br>(0.0 to 0.1)	87<br>(60 to 157)	0.0<br>(0.0 to 0.1)	49<br>(36 to 78)	0.0<br>(0.0 to 0.0)	-53.0<br>(-67.3 to -24.4)	-58.9<br>(-71.4 to -33.9)
Global	Rheumatoid arthritis	10 to 14	Male	101<br>(62 to 157)	0.0<br>(0.0 to 0.1)	98<br>(67 to 142)	0.0<br>(0.0 to 0.0)	58<br>(43 to 77)	0.0<br>(0.0 to 0.0)	-42.6<br>(-58.6 to -7.8)	-50.9<br>(-64.6 to -21.2)
Global	Other musculoskeletal disorders	5 to 9	Both	637<br>(451 to 893)	0.1<br>(0.1 to 0.2)	621<br>(481 to 809)	0.1<br>(0.1 to 0.1)	505<br>(435 to 638)	0.1<br>(0.1 to 0.1)	-20.7<br>(-39.0 to 3.9)	-29.0<br>(-45.4 to -6.9)

eTable 8. All-cause and cause-specific mortality (counts and rates) at the global level and by country in the WHO European Region for ages 5 to 9 and 10 to 14 for 1990, 2000, and 2016

Global	Other musculoskeletal disorders	5 to 9	Female	328 (207 to 507)	0.1 (0.1 to 0.2)	328 (234 to 454)	0.1 (0.1 to 0.2)	283 (217 to 363)	0.1 (0.1 to 0.1)	-13.8 (-39.3 to 20.4)	-22.0 (-45.0 to 9.0)
Global	Other musculoskeletal disorders	5 to 9	Male	309 (222 to 433)	0.1 (0.1 to 0.1)	293 (225 to 388)	0.1 (0.1 to 0.1)	223 (190 to 302)	0.1 (0.1 to 0.1)	-27.9 (-43.9 to -2.9)	-36.1 (-50.3 to -13.9)
Global	Other musculoskeletal disorders	10 to 14	Both	1027 (790 to 1371)	0.2 (0.1 to 0.3)	1163 (957 to 1433)	0.2 (0.2 to 0.2)	929 (810 to 1116)	0.2 (0.1 to 0.2)	-9.6 (-27.0 to 10.6)	-21.8 (-36.9 to -4.4)
Global	Other musculoskeletal disorders	10 to 14	Female	677 (480 to 952)	0.3 (0.2 to 0.4)	772 (597 to 979)	0.3 (0.2 to 0.3)	611 (493 to 759)	0.2 (0.2 to 0.3)	-9.8 (-32.2 to 16.0)	-21.0 (-40.7 to 1.5)
Global	Other musculoskeletal disorders	10 to 14	Male	350 (285 to 446)	0.1 (0.1 to 0.2)	391 (331 to 490)	0.1 (0.1 to 0.2)	318 (279 to 395)	0.1 (0.1 to 0.1)	-9.1 (-25.2 to 9.9)	-22.4 (-36.1 to -6.1)
Global	Congenital birth defects	5 to 9	Both	20773 (16202 to 26352)	3.6 (2.8 to 4.6)	20127 (16736 to 24218)	3.3 (2.7 to 3.9)	15548 (13952 to 18205)	2.4 (2.2 to 2.8)	-25.2 (-39.2 to -0.7)	-33.0 (-45.6 to -11.1)
Global	Congenital birth defects	5 to 9	Female	10240 (7657 to 14558)	3.6 (2.7 to 5.2)	9400 (7629 to 12309)	3.2 (2.6 to 4.1)	7421 (6404 to 8689)	2.4 (2.1 to 2.8)	-27.5 (-47.8 to -1.3)	-34.4 (-52.8 to -10.6)
Global	Congenital birth defects	5 to 9	Male	10533 (7310 to 14741)	3.6 (2.5 to 5.0)	10728 (8452 to 13499)	3.4 (2.7 to 4.3)	8127 (7030 to 10040)	2.4 (2.1 to 3.0)	-22.8 (-43.8 to 10.3)	-31.6 (-50.2 to -2.2)
Global	Congenital birth defects	10 to 14	Both	13357 (10737 to 16227)	2.5 (2.0 to 3.1)	15292 (13114 to 17555)	2.4 (2.1 to 2.8)	11872 (10847 to 13687)	1.9 (1.8 to 2.2)	-11.1 (-27.3 to 12.5)	-23.2 (-37.2 to -2.8)
Global	Congenital birth defects	10 to 14	Female	6854 (5430 to 9018)	2.7 (2.1 to 3.5)	7460 (6331 to 9094)	2.4 (2.1 to 3.0)	5876 (5121 to 6767)	2.0 (1.7 to 2.3)	-14.3 (-35.8 to 9.1)	-25.0 (-43.8 to -4.6)
Global	Congenital birth defects	10 to 14	Male	6503 (4655 to 8697)	2.4 (1.7 to 3.2)	7832 (6132 to 9641)	2.4 (1.9 to 3.0)	5996 (5139 to 7271)	1.9 (1.6 to 2.3)	-7.8 (-30.3 to 23.8)	-21.2 (-40.4 to 5.8)
Global	Skin and subcutaneous diseases	5 to 9	Both	515 (245 to 745)	0.1 (0.0 to 0.1)	579 (280 to 800)	0.1 (0.0 to 0.1)	532 (315 to 713)	0.1 (0.0 to 0.1)	3.3 (-26.0 to 48.4)	-7.5 (-33.7 to 32.9)
Global	Skin and subcutaneous diseases	5 to 9	Female	285 (106 to 447)	0.1 (0.0 to 0.2)	317 (117 to 478)	0.1 (0.0 to 0.2)	286 (125 to 429)	0.1 (0.0 to 0.1)	0.5 (-30.1 to 39.8)	-9.0 (-36.7 to 26.5)
Global	Skin and subcutaneous diseases	5 to 9	Male	230 (99 to 333)	0.1 (0.0 to 0.1)	262 (118 to 353)	0.1 (0.0 to 0.1)	246 (136 to 365)	0.1 (0.0 to 0.1)	6.8 (-28.8 to 67.8)	-5.3 (-36.9 to 48.8)
Global	Skin and subcutaneous diseases	10 to 14	Both	506 (263 to 676)	0.1 (0.0 to 0.1)	653 (329 to 850)	0.1 (0.1 to 0.1)	651 (389 to 860)	0.1 (0.1 to 0.1)	28.7 (-1.1 to 66.6)	11.3 (-14.5 to 44.1)
Global	Skin and subcutaneous diseases	10 to 14	Female	272 (112 to 381)	0.1 (0.0 to 0.1)	345 (139 to 489)	0.1 (0.0 to 0.2)	349 (161 to 530)	0.1 (0.1 to 0.2)	28.3 (-0.0 to 59.7)	12.2 (-12.5 to 39.7)
Global	Skin and subcutaneous diseases	10 to 14	Male	234 (107 to 331)	0.1 (0.0 to 0.1)	308 (144 to 411)	0.1 (0.0 to 0.1)	302 (163 to 438)	0.1 (0.1 to 0.1)	29.3 (-8.6 to 84.9)	10.5 (-21.9 to 58.0)
Global	Road injuries	5 to 9	Both	61025 (55365 to 66282)	10.6 (9.6 to 11.5)	52217 (48197 to 56057)	8.5 (7.8 to 9.1)	31937 (29201 to 35218)	5.0 (4.5 to 5.5)	-47.7 (-53.4 to -41.7)	-53.1 (-58.2 to -47.8)
Global	Road injuries	5 to 9	Female	22778 (19869 to 25696)	8.1 (7.1 to 9.2)	19167 (17141 to 21253)	6.4 (5.8 to 7.1)	11787 (10595 to 13341)	3.8 (3.4 to 4.3)	-48.3 (-55.9 to -40.4)	-53.2 (-60.1 to -46.0)
Global	Road injuries	5 to 9	Male	38247 (34416 to 41642)	13.0 (11.7 to 14.1)	33049 (30340 to 35732)	10.4 (9.6 to 11.3)	20150 (18112 to 22539)	6.1 (5.5 to 6.8)	-47.3 (-53.8 to -40.4)	-53.3 (-59.1 to -47.1)
Global	Road injuries	10 to 14	Both	44497 (41733 to 47220)	8.4 (7.9 to 8.9)	45568 (43222 to 48416)	7.3 (6.9 to 7.7)	30172 (28270 to 32756)	4.9 (4.6 to 5.4)	-32.2 (-36.9 to -27.3)	-41.4 (-45.4 to -37.1)
Global	Road injuries	10 to 14	Female	15071 (13765 to 16405)	5.8 (5.3 to 6.4)	14934 (13740 to 16158)	4.9 (4.5 to 5.3)	9577 (8764 to 10565)	3.2 (3.0 to 3.6)	-36.5 (-43.0 to -29.7)	-44.4 (-50.1 to -38.5)
Global	Road injuries	10 to 14	Male	29426 (27565 to 31477)	10.9 (10.2 to 11.7)	30634 (28894 to 32799)	9.5 (9.0 to 10.2)	20595 (19069 to 22461)	6.5 (6.0 to 7.1)	-30.0 (-35.9 to -24.2)	-40.2 (-45.2 to -35.2)
Global	Other transport injuries	5 to 9	Both	2660 (2025 to 3367)	0.5 (0.4 to 0.6)	2420 (1999 to 2927)	0.4 (0.3 to 0.5)	1558 (1369 to 1827)	0.2 (0.2 to 0.3)	-41.4 (-55.2 to -21.2)	-47.5 (-59.9 to -29.4)
Global	Other transport injuries	5 to 9	Female	874 (596 to 1311)	0.3 (0.2 to 0.5)	763 (589 to 997)	0.3 (0.2 to 0.3)	449 (374 to 559)	0.1 (0.1 to 0.2)	-48.7 (-68.3 to -22.6)	-53.5 (-71.3 to -30.0)

eTable 8. All-cause and cause-specific mortality (counts and rates) at the global level and by country in the WHO European Region for ages 5 to 9 and 10 to 14 for 1990, 2000, and 2016

Global	Other transport injuries	5 to 9	Male	1786 (1312 to 2270)	0.6 (0.4 to 0.8)	1657 (1317 to 2025)	0.5 (0.4 to 0.6)	1109 (941 to 1361)	0.3 (0.3 to 0.4)	-37.9 (-51.8 to -13.9)	-44.9 (-57.3 to -23.7)
Global	Other transport injuries	10 to 14	Both	2705 (2200 to 3221)	0.5 (0.4 to 0.6)	2982 (2561 to 3390)	0.5 (0.4 to 0.5)	2190 (1931 to 2519)	0.4 (0.3 to 0.4)	-19.1 (-34.5 to 3.8)	-30.0 (-43.4 to -10.2)
Global	Other transport injuries	10 to 14	Female	842 (620 to 1126)	0.3 (0.2 to 0.4)	899 (723 to 1072)	0.3 (0.2 to 0.4)	581 (490 to 704)	0.2 (0.2 to 0.2)	-31.0 (-52.8 to -3.3)	-39.6 (-58.7 to -15.4)
Global	Other transport injuries	10 to 14	Male	1863 (1522 to 2261)	0.7 (0.6 to 0.8)	2082 (1753 to 2425)	0.6 (0.5 to 0.8)	1608 (1382 to 1922)	0.5 (0.4 to 0.6)	-13.7 (-31.7 to 10.5)	-26.2 (-41.7 to -5.6)
Global	Falls	5 to 9	Both	10740 (8603 to 12982)	1.9 (1.5 to 2.3)	9291 (7837 to 10828)	1.5 (1.3 to 1.8)	6399 (5606 to 7253)	1.0 (0.9 to 1.1)	-40.4 (-52.0 to -22.3)	-46.6 (-57.0 to -30.4)
Global	Falls	5 to 9	Female	4189 (3250 to 5243)	1.5 (1.2 to 1.9)	3400 (2750 to 4076)	1.1 (0.9 to 1.4)	2172 (1790 to 2530)	0.7 (0.6 to 0.8)	-48.2 (-61.0 to -27.5)	-53.1 (-64.7 to -34.3)
Global	Falls	5 to 9	Male	6551 (4928 to 8059)	2.2 (1.7 to 2.7)	5891 (4748 to 7047)	1.9 (1.5 to 2.2)	4226 (3557 to 4904)	1.3 (1.1 to 1.5)	-35.5 (-49.9 to -11.6)	-42.8 (-55.6 to -21.6)
Global	Falls	10 to 14	Both	8840 (7363 to 10202)	1.7 (1.4 to 1.9)	8972 (7878 to 10121)	1.4 (1.3 to 1.6)	6926 (6065 to 7702)	1.1 (1.0 to 1.3)	-21.7 (-34.3 to -7.2)	-32.3 (-43.2 to -19.8)
Global	Falls	10 to 14	Female	2596 (2105 to 3091)	1.0 (0.8 to 1.2)	2480 (2069 to 2836)	0.8 (0.7 to 0.9)	1688 (1412 to 1941)	0.6 (0.5 to 0.7)	-35.0 (-48.3 to -21.3)	-43.1 (-54.7 to -31.1)
Global	Falls	10 to 14	Male	6245 (4899 to 7283)	2.3 (1.8 to 2.7)	6492 (5461 to 7540)	2.0 (1.7 to 2.3)	5237 (4415 to 5958)	1.7 (1.4 to 1.9)	-16.1 (-31.3 to 2.1)	-28.3 (-41.3 to -12.8)
Global	Drowning	5 to 9	Both	72196 (61510 to 79755)	12.6 (10.7 to 13.9)	58320 (50376 to 63220)	9.5 (8.2 to 10.3)	25790 (23093 to 28665)	4.0 (3.6 to 4.5)	-64.3 (-67.6 to -58.3)	-68.0 (-71.0 to -62.7)
Global	Drowning	5 to 9	Female	22729 (16000 to 26540)	8.1 (5.7 to 9.5)	16915 (11936 to 19581)	5.7 (4.0 to 6.6)	7665 (5890 to 9378)	2.5 (1.9 to 3.0)	-66.3 (-71.9 to -49.6)	-69.5 (-74.6 to -54.4)
Global	Drowning	5 to 9	Male	49467 (44301 to 54362)	16.8 (15.0 to 18.5)	41405 (36873 to 44753)	13.1 (11.6 to 14.1)	18125 (16164 to 20123)	5.5 (4.9 to 6.1)	-63.4 (-66.9 to -59.4)	-67.5 (-70.6 to -64.0)
Global	Drowning	10 to 14	Both	48783 (44771 to 52095)	9.2 (8.5 to 9.9)	48963 (43909 to 51645)	7.8 (7.0 to 8.2)	23333 (21132 to 25344)	3.8 (3.5 to 4.1)	-52.2 (-55.4 to -47.0)	-58.7 (-61.4 to -54.2)
Global	Drowning	10 to 14	Female	13961 (10267 to 15718)	5.4 (4.0 to 6.1)	12400 (9395 to 13733)	4.1 (3.1 to 4.5)	6097 (4774 to 7305)	2.1 (1.6 to 2.5)	-56.3 (-62.3 to -38.8)	-61.8 (-67.1 to -46.5)
Global	Drowning	10 to 14	Male	34821 (32258 to 37639)	12.9 (11.9 to 13.9)	36563 (33681 to 38673)	11.4 (10.5 to 12.0)	17236 (15758 to 18707)	5.5 (5.0 to 5.9)	-50.5 (-54.2 to -46.4)	-57.7 (-60.9 to -54.2)
Global	Fire, heat, and hot substances	5 to 9	Both	8075 (5888 to 9396)	1.4 (1.0 to 1.6)	6406 (4881 to 7363)	1.0 (0.8 to 1.2)	4115 (3432 to 4634)	0.6 (0.5 to 0.7)	-49.0 (-56.4 to -35.7)	-54.4 (-61.0 to -42.4)
Global	Fire, heat, and hot substances	5 to 9	Female	3984 (2749 to 4862)	1.4 (1.0 to 1.7)	3052 (2230 to 3691)	1.0 (0.7 to 1.2)	1851 (1429 to 2183)	0.6 (0.5 to 0.7)	-53.5 (-62.3 to -39.5)	-58.0 (-65.9 to -45.2)
Global	Fire, heat, and hot substances	5 to 9	Male	4090 (2971 to 4814)	1.4 (1.0 to 1.6)	3355 (2520 to 3975)	1.1 (0.8 to 1.3)	2264 (1781 to 2673)	0.7 (0.5 to 0.8)	-44.6 (-53.3 to -19.0)	-50.9 (-58.6 to -28.2)
Global	Fire, heat, and hot substances	10 to 14	Both	5093 (3882 to 5728)	1.0 (0.7 to 1.1)	4731 (3683 to 5249)	0.8 (0.6 to 0.8)	3047 (2435 to 3414)	0.5 (0.4 to 0.6)	-40.2 (-46.1 to -30.1)	-48.3 (-53.4 to -39.5)
Global	Fire, heat, and hot substances	10 to 14	Female	2770 (1933 to 3257)	1.1 (0.7 to 1.3)	2494 (1788 to 2893)	0.8 (0.6 to 0.9)	1507 (1104 to 1757)	0.5 (0.4 to 0.6)	-45.6 (-52.9 to -35.8)	-52.4 (-58.7 to -43.9)
Global	Fire, heat, and hot substances	10 to 14	Male	2324 (1790 to 2667)	0.9 (0.7 to 1.0)	2237 (1741 to 2581)	0.7 (0.5 to 0.8)	1540 (1202 to 1803)	0.5 (0.4 to 0.6)	-33.7 (-40.7 to -12.6)	-43.4 (-49.3 to -25.3)
Global	Poisonings	5 to 9	Both	5049 (3710 to 6360)	0.9 (0.6 to 1.1)	3611 (2845 to 4322)	0.6 (0.5 to 0.7)	1860 (1425 to 2143)	0.3 (0.2 to 0.3)	-63.2 (-72.5 to -51.1)	-67.0 (-75.3 to -56.2)
Global	Poisonings	5 to 9	Female	2088 (1587 to 2852)	0.7 (0.6 to 1.0)	1471 (1187 to 1865)	0.5 (0.4 to 0.6)	789 (604 to 936)	0.3 (0.2 to 0.3)	-62.2 (-72.1 to -52.0)	-65.8 (-74.8 to -56.6)
Global	Poisonings	5 to 9	Male	2961 (1944 to 3878)	1.0 (0.7 to 1.3)	2140 (1494 to 2735)	0.7 (0.5 to 0.9)	1070 (718 to 1288)	0.3 (0.2 to 0.4)	-63.9 (-73.3 to -46.7)	-68.0 (-76.3 to -52.7)
Global	Poisonings	10 to 14	Both	4985 (3806 to 6462)	0.9 (0.7 to 1.2)	4347 (3636 to 5262)	0.7 (0.6 to 0.8)	2061 (1607 to 2339)	0.3 (0.3 to 0.4)	-58.7 (-69.0 to -45.6)	-64.3 (-73.2 to -53.0)
Global	Poisonings	10 to 14	Female	2178 (1813 to 3037)	0.8 (0.7 to 1.2)	1901 (1609 to 2394)	0.6 (0.5 to 0.8)	893 (694 to 1054)	0.3 (0.2 to 0.4)	-59.0 (-69.7 to -49.4)	-64.1 (-73.5 to -55.7)
Global	Poisonings	10 to 14	Male	2807 (1851 to 3846)	1.0 (0.7 to 1.4)	2447 (1787 to 3124)	0.8 (0.6 to 1.0)	1167 (771 to 1358)	0.4 (0.2 to 0.4)	-58.4 (-69.7 to -34.6)	-64.5 (-74.1 to -44.1)
Global	Exposure to mechanical forces	5 to 9	Both	8732 (7342 to 10722)	1.5 (1.3 to 1.9)	7268 (6267 to 8624)	1.2 (1.0 to 1.4)	5097 (4554 to 5747)	0.8 (0.7 to 0.9)	-41.6 (-51.7 to -31.1)	-47.7 (-56.7 to -38.3)

eTable 8. All-cause and cause-specific mortality (counts and rates) at the global level and by country in the WHO European Region for ages 5 to 9 and 10 to 14 for 1990, 2000, and 2016

Global	Exposure to mechanical forces	5 to 9	Female	3026 (2366 to 3814)	1.1 (0.8 to 1.4)	2522 (2054 to 3018)	0.8 (0.7 to 1.0)	1705 (1427 to 1934)	0.5 (0.5 to 0.6)	-43.6 (-55.8 to -27.3)	-49.0 (-60.0 to -34.2)
Global	Exposure to mechanical forces	5 to 9	Male	5705 (4793 to 7082)	1.9 (1.6 to 2.4)	4746 (4020 to 5818)	1.5 (1.3 to 1.8)	3391 (2946 to 3964)	1.0 (0.9 to 1.2)	-40.6 (-51.7 to -29.2)	-47.3 (-57.2 to -37.3)
Global	Exposure to mechanical forces	10 to 14	Both	6409 (5861 to 7675)	1.2 (1.1 to 1.5)	6120 (5604 to 7300)	1.0 (0.9 to 1.2)	4299 (3896 to 4794)	0.7 (0.6 to 0.8)	-32.9 (-42.1 to -26.4)	-42.0 (-50.0 to -36.4)
Global	Exposure to mechanical forces	10 to 14	Female	1737 (1474 to 2153)	0.7 (0.6 to 0.8)	1665 (1439 to 2000)	0.5 (0.5 to 0.7)	1214 (1022 to 1361)	0.4 (0.3 to 0.5)	-30.1 (-42.7 to -19.2)	-38.8 (-49.9 to -29.3)
Global	Exposure to mechanical forces	10 to 14	Male	4672 (4237 to 5832)	1.7 (1.6 to 2.2)	4455 (4048 to 5475)	1.4 (1.3 to 1.7)	3085 (2721 to 3575)	1.0 (0.9 to 1.1)	-34.0 (-43.6 to -26.3)	-43.6 (-51.8 to -37.0)
Global	Adverse effects of medical treatment	5 to 9	Both	3419 (2148 to 4304)	0.6 (0.4 to 0.7)	3012 (2054 to 3713)	0.5 (0.3 to 0.6)	2193 (1791 to 2650)	0.3 (0.3 to 0.4)	-35.9 (-46.7 to -11.6)	-42.6 (-52.3 to -20.8)
Global	Adverse effects of medical treatment	5 to 9	Female	1634 (991 to 2127)	0.6 (0.4 to 0.8)	1357 (891 to 1740)	0.5 (0.3 to 0.6)	888 (706 to 1144)	0.3 (0.2 to 0.4)	-45.6 (-57.2 to -22.8)	-50.8 (-61.3 to -30.2)
Global	Adverse effects of medical treatment	5 to 9	Male	1784 (1104 to 2283)	0.6 (0.4 to 0.8)	1656 (1105 to 2093)	0.5 (0.3 to 0.7)	1305 (1013 to 1660)	0.4 (0.3 to 0.5)	-26.9 (-39.9 to -0.5)	-35.2 (-46.7 to -11.8)
Global	Adverse effects of medical treatment	10 to 14	Both	2391 (1746 to 2738)	0.5 (0.3 to 0.5)	2402 (1857 to 2747)	0.4 (0.3 to 0.4)	1860 (1587 to 2181)	0.3 (0.3 to 0.4)	-22.2 (-32.1 to -5.3)	-32.7 (-41.3 to -18.1)
Global	Adverse effects of medical treatment	10 to 14	Female	1090 (767 to 1314)	0.4 (0.3 to 0.5)	1046 (779 to 1255)	0.3 (0.3 to 0.4)	733 (589 to 917)	0.2 (0.2 to 0.3)	-32.8 (-42.7 to -16.5)	-41.2 (-49.8 to -26.9)
Global	Adverse effects of medical treatment	10 to 14	Male	1301 (922 to 1505)	0.5 (0.3 to 0.6)	1356 (999 to 1586)	0.4 (0.3 to 0.5)	1128 (894 to 1392)	0.4 (0.3 to 0.4)	-13.3 (-25.3 to 4.3)	-25.9 (-36.1 to -10.9)
Global	Animal contact	5 to 9	Both	8103 (5339 to 10218)	1.4 (0.9 to 1.8)	7418 (5090 to 9171)	1.2 (0.8 to 1.5)	4962 (3509 to 5941)	0.8 (0.5 to 0.9)	-38.8 (-52.2 to -17.2)	-45.2 (-57.2 to -25.9)
Global	Animal contact	5 to 9	Female	3452 (1274 to 5312)	1.2 (0.5 to 1.9)	3252 (1253 to 4743)	1.1 (0.4 to 1.6)	2204 (1026 to 2940)	0.7 (0.3 to 0.9)	-36.2 (-59.4 to 2.5)	-42.2 (-63.3 to -7.2)
Global	Animal contact	5 to 9	Male	4651 (3360 to 6134)	1.6 (1.1 to 2.1)	4166 (3278 to 5115)	1.3 (1.0 to 1.6)	2758 (2259 to 3288)	0.8 (0.7 to 1.0)	-40.7 (-56.6 to -15.6)	-47.4 (-61.5 to -25.2)
Global	Animal contact	10 to 14	Both	6865 (4637 to 8450)	1.3 (0.9 to 1.6)	7144 (4922 to 8671)	1.1 (0.8 to 1.4)	5186 (3689 to 6186)	0.8 (0.6 to 1.0)	-24.5 (-37.6 to -1.9)	-34.7 (-46.1 to -15.2)
Global	Animal contact	10 to 14	Female	2650 (923 to 3719)	1.0 (0.4 to 1.4)	2857 (1019 to 3928)	0.9 (0.3 to 1.3)	2095 (847 to 2819)	0.7 (0.3 to 1.0)	-20.9 (-42.1 to 13.4)	-30.8 (-49.4 to -0.8)
Global	Animal contact	10 to 14	Male	4215 (3130 to 5123)	1.6 (1.2 to 1.9)	4287 (3399 to 5121)	1.3 (1.1 to 1.6)	3091 (2576 to 3689)	1.0 (0.8 to 1.2)	-26.7 (-41.2 to 2.4)	-37.3 (-49.7 to -12.5)
Global	Foreign body	5 to 9	Both	3842 (2622 to 5024)	0.7 (0.5 to 0.9)	3464 (2583 to 4291)	0.6 (0.4 to 0.7)	3001 (2546 to 3702)	0.5 (0.4 to 0.6)	-21.9 (-37.8 to 4.4)	-30.1 (-44.3 to -6.5)
Global	Foreign body	5 to 9	Female	1399 (879 to 1946)	0.5 (0.3 to 0.7)	1261 (891 to 1685)	0.4 (0.3 to 0.6)	1125 (877 to 1455)	0.4 (0.3 to 0.5)	-19.6 (-36.4 to 14.4)	-27.2 (-42.4 to 3.5)
Global	Foreign body	5 to 9	Male	2443 (1616 to 3255)	0.8 (0.5 to 1.1)	2204 (1610 to 2789)	0.7 (0.5 to 0.9)	1875 (1512 to 2444)	0.6 (0.5 to 0.7)	-23.2 (-41.0 to 1.9)	-31.9 (-47.7 to -9.7)
Global	Foreign body	10 to 14	Both	2618 (1952 to 3037)	0.5 (0.4 to 0.6)	2617 (2093 to 2979)	0.4 (0.3 to 0.5)	2327 (2019 to 2744)	0.4 (0.3 to 0.4)	-11.1 (-24.0 to 10.7)	-23.2 (-34.3 to -4.3)
Global	Foreign body	10 to 14	Female	826 (556 to 1017)	0.3 (0.2 to 0.4)	837 (617 to 1006)	0.3 (0.2 to 0.3)	750 (610 to 923)	0.3 (0.2 to 0.3)	-9.1 (-22.9 to 18.2)	-20.5 (-32.5 to 3.5)
Global	Foreign body	10 to 14	Male	1793 (1326 to 2100)	0.7 (0.5 to 0.8)	1780 (1405 to 2071)	0.6 (0.4 to 0.6)	1577 (1325 to 1903)	0.5 (0.4 to 0.6)	-12.0 (-26.8 to 9.3)	-24.8 (-37.5 to -6.6)

eTable 8. All-cause and cause-specific mortality (counts and rates) at the global level and by country in the WHO European Region for ages 5 to 9 and 10 to 14 for 1990, 2000, and 2016

Global	Environmental heat and cold exposure	5 to 9	Both	1093 (695 to 1323)	0.2 (0.1 to 0.2)	915 (573 to 1112)	0.1 (0.1 to 0.2)	601 (370 to 724)	0.1 (0.1 to 0.1)	-45.0 (-53.7 to -34.2)	-50.7 (-58.6 to -41.1)
Global	Environmental heat and cold exposure	5 to 9	Female	433 (212 to 572)	0.2 (0.1 to 0.2)	351 (163 to 464)	0.1 (0.1 to 0.2)	218 (109 to 284)	0.1 (0.0 to 0.1)	-49.7 (-60.9 to -34.8)	-54.4 (-64.6 to -41.0)
Global	Environmental heat and cold exposure	5 to 9	Male	660 (387 to 808)	0.2 (0.1 to 0.3)	564 (328 to 690)	0.2 (0.1 to 0.2)	383 (210 to 473)	0.1 (0.1 to 0.1)	-42.0 (-54.5 to -28.6)	-48.5 (-59.6 to -36.7)
Global	Environmental heat and cold exposure	10 to 14	Both	754 (494 to 873)	0.1 (0.1 to 0.2)	725 (456 to 838)	0.1 (0.1 to 0.1)	487 (291 to 575)	0.1 (0.0 to 0.1)	-35.5 (-45.0 to -26.2)	-44.2 (-52.4 to -36.2)
Global	Environmental heat and cold exposure	10 to 14	Female	216 (124 to 269)	0.1 (0.0 to 0.1)	185 (109 to 233)	0.1 (0.0 to 0.1)	123 (71 to 149)	0.0 (0.0 to 0.1)	-43.0 (-51.2 to -34.5)	-50.2 (-57.3 to -42.7)
Global	Environmental heat and cold exposure	10 to 14	Male	539 (310 to 636)	0.2 (0.1 to 0.2)	540 (300 to 637)	0.2 (0.1 to 0.2)	364 (192 to 442)	0.1 (0.1 to 0.1)	-32.4 (-44.0 to -20.4)	-42.2 (-52.1 to -32.0)
Global	Other unintentional injuries	5 to 9	Both	5140 (4325 to 6040)	0.9 (0.8 to 1.1)	4224 (3662 to 4790)	0.7 (0.6 to 0.8)	2474 (2215 to 2757)	0.4 (0.3 to 0.4)	-51.9 (-60.4 to -43.4)	-56.9 (-64.5 to -49.3)
Global	Other unintentional injuries	5 to 9	Female	1741 (1318 to 2179)	0.6 (0.5 to 0.8)	1334 (1073 to 1602)	0.4 (0.4 to 0.5)	704 (572 to 814)	0.2 (0.2 to 0.3)	-59.6 (-68.1 to -45.7)	-63.4 (-71.1 to -50.8)
Global	Other unintentional injuries	5 to 9	Male	3399 (2871 to 4094)	1.2 (1.0 to 1.4)	2890 (2517 to 3332)	0.9 (0.8 to 1.1)	1770 (1543 to 2033)	0.5 (0.5 to 0.6)	-47.9 (-57.8 to -38.0)	-53.8 (-62.5 to -45.0)
Global	Other unintentional injuries	10 to 14	Both	5305 (4699 to 6017)	1.0 (0.9 to 1.1)	5164 (4698 to 5692)	0.8 (0.7 to 0.9)	3106 (2782 to 3391)	0.5 (0.5 to 0.6)	-41.4 (-49.8 to -33.5)	-49.4 (-56.6 to -42.5)
Global	Other unintentional injuries	10 to 14	Female	1635 (1254 to 1952)	0.6 (0.5 to 0.8)	1475 (1187 to 1686)	0.5 (0.4 to 0.6)	824 (619 to 959)	0.3 (0.2 to 0.3)	-49.6 (-58.2 to -35.4)	-55.9 (-63.4 to -43.5)
Global	Other unintentional injuries	10 to 14	Male	3670 (3210 to 4226)	1.4 (1.2 to 1.6)	3688 (3340 to 4083)	1.1 (1.0 to 1.3)	2283 (2053 to 2523)	0.7 (0.6 to 0.8)	-37.8 (-47.3 to -29.1)	-46.9 (-55.0 to -39.4)
Global	Self-harm	10 to 14	Both	13094 (11199 to 14922)	2.5 (2.1 to 2.8)	13146 (11475 to 14273)	2.1 (1.8 to 2.3)	7975 (7140 to 8943)	1.3 (1.2 to 1.5)	-39.1 (-48.3 to -25.4)	-47.3 (-55.3 to -35.5)
Global	Self-harm	10 to 14	Female	6774 (5419 to 8151)	2.6 (2.1 to 3.2)	6154 (5323 to 7048)	2.0 (1.7 to 2.3)	3443 (2935 to 3973)	1.2 (1.0 to 1.3)	-49.2 (-60.5 to -32.1)	-55.5 (-65.4 to -40.6)
Global	Self-harm	10 to 14	Male	6320 (4479 to 7495)	2.3 (1.7 to 2.8)	6992 (5146 to 7912)	2.2 (1.6 to 2.5)	4533 (3759 to 5226)	1.4 (1.2 to 1.7)	-28.3 (-42.8 to -1.5)	-38.7 (-51.1 to -15.8)
Global	Interpersonal violence	5 to 9	Both	7451 (5908 to 8973)	1.3 (1.0 to 1.6)	6865 (5290 to 7820)	1.1 (0.9 to 1.3)	4238 (3582 to 5039)	0.7 (0.6 to 0.8)	-43.1 (-53.1 to -26.5)	-49.1 (-58.0 to -34.2)
Global	Interpersonal violence	5 to 9	Female	3453 (2033 to 4598)	1.2 (0.7 to 1.6)	3065 (2056 to 3678)	1.0 (0.7 to 1.2)	1904 (1420 to 2492)	0.6 (0.5 to 0.8)	-44.9 (-58.7 to 2.6)	-50.1 (-62.7 to -7.2)
Global	Interpersonal violence	5 to 9	Male	3998 (2842 to 4950)	1.4 (1.0 to 1.7)	3800 (2652 to 4401)	1.2 (0.8 to 1.4)	2335 (1793 to 2838)	0.7 (0.5 to 0.9)	-41.6 (-53.1 to -20.6)	-48.2 (-58.4 to -29.6)
Global	Interpersonal violence	10 to 14	Both	8730 (7079 to 10254)	1.7 (1.3 to 1.9)	9564 (7378 to 10613)	1.5 (1.2 to 1.7)	7015 (5745 to 7963)	1.1 (0.9 to 1.3)	-19.6 (-29.7 to -7.0)	-30.5 (-39.2 to -19.6)
Global	Interpersonal violence	10 to 14	Female	3095 (2092 to 3976)	1.2 (0.8 to 1.5)	3274 (2308 to 3857)	1.1 (0.8 to 1.3)	2349 (1793 to 2907)	0.8 (0.6 to 1.0)	-24.1 (-38.5 to 13.6)	-33.6 (-46.2 to -0.6)
Global	Interpersonal violence	10 to 14	Male	5635 (4103 to 6953)	2.1 (1.5 to 2.6)	6290 (4274 to 7214)	2.0 (1.3 to 2.2)	4666 (3612 to 5438)	1.5 (1.1 to 1.7)	-17.2 (-27.9 to -2.5)	-29.2 (-38.4 to -16.7)
Global	Exposure to forces of nature	5 to 9	Both	5568 (1935 to 9174)	1.0 (0.3 to 1.6)	701 (464 to 942)	0.1 (0.1 to 0.2)	429 (275 to 593)	0.1 (0.0 to 0.1)	-92.3 (-96.2 to -78.6)	-93.1 (-96.6 to -80.8)
Global	Exposure to forces of nature	5 to 9	Female	2581 (897 to 4253)	0.9 (0.3 to 1.5)	324 (216 to 435)	0.1 (0.1 to 0.1)	197 (127 to 273)	0.1 (0.0 to 0.1)	-92.4 (-96.2 to -78.7)	-93.1 (-96.6 to -80.8)
Global	Exposure to forces of nature	5 to 9	Male	2986 (1038 to 4921)	1.0 (0.4 to 1.7)	376 (249 to 507)	0.1 (0.1 to 0.2)	231 (148 to 320)	0.1 (0.0 to 0.1)	-92.3 (-96.1 to -78.5)	-93.1 (-96.6 to -80.9)
Global	Exposure to forces of nature	10 to 14	Both	5856 (2096 to 9573)	1.1 (0.4 to 1.8)	870 (580 to 1165)	0.1 (0.1 to 0.2)	528 (337 to 732)	0.1 (0.1 to 0.1)	-91.0 (-95.5 to -75.7)	-92.2 (-96.1 to -79.0)

eTable 8. All-cause and cause-specific mortality (counts and rates) at the global level and by country in the WHO European Region for ages 5 to 9 and 10 to 14 for 1990, 2000, and 2016

Global	Exposure to forces of nature	10 to 14	Female	2330 (835 to 3809)	0.9 (0.3 to 1.5)	348 (232 to 466)	0.1 (0.1 to 0.2)	209 (134 to 289)	0.1 (0.0 to 0.1)	-91.0 (-95.5 to -75.9)	-92.1 (-96.1 to -78.9)
Global	Exposure to forces of nature	10 to 14	Male	3525 (1260 to 5764)	1.3 (0.5 to 2.1)	522 (347 to 700)	0.2 (0.1 to 0.2)	319 (203 to 442)	0.1 (0.1 to 0.1)	-91.0 (-95.5 to -75.6)	-92.3 (-96.1 to -79.2)
Global	Conflict and terrorism	5 to 9	Both	6860 (5879 to 7788)	1.2 (1.0 to 1.4)	6596 (5766 to 7352)	1.1 (0.9 to 1.2)	9402 (6373 to 12653)	1.5 (1.0 to 2.0)	37.1 (-7.8 to 87.0)	22.7 (-17.4 to 67.5)
Global	Conflict and terrorism	5 to 9	Female	3599 (3085 to 4084)	1.3 (1.1 to 1.5)	3455 (3020 to 3850)	1.2 (1.0 to 1.3)	4868 (3304 to 6551)	1.6 (1.1 to 2.1)	35.3 (-8.8 to 84.5)	22.4 (-17.5 to 67.0)
Global	Conflict and terrorism	5 to 9	Male	3261 (2794 to 3703)	1.1 (0.9 to 1.3)	3142 (2747 to 3501)	1.0 (0.9 to 1.1)	4534 (3071 to 6102)	1.4 (0.9 to 1.8)	39.0 (-6.6 to 89.8)	23.3 (-17.2 to 68.3)
Global	Conflict and terrorism	10 to 14	Both	5897 (5040 to 6708)	1.1 (1.0 to 1.3)	5895 (5151 to 6578)	0.9 (0.8 to 1.0)	8954 (5997 to 12117)	1.5 (1.0 to 2.0)	51.9 (0.3 to 109.2)	31.3 (-13.3 to 80.9)
Global	Conflict and terrorism	10 to 14	Female	2847 (2434 to 3238)	1.1 (0.9 to 1.3)	2831 (2474 to 3160)	0.9 (0.8 to 1.0)	4248 (2849 to 5744)	1.4 (1.0 to 1.9)	49.2 (-1.3 to 105.4)	30.6 (-13.6 to 79.8)
Global	Conflict and terrorism	10 to 14	Male	3050 (2605 to 3470)	1.1 (1.0 to 1.3)	3063 (2677 to 3418)	1.0 (0.8 to 1.1)	4706 (3148 to 6373)	1.5 (1.0 to 2.0)	54.3 (1.9 to 112.7)	31.9 (-12.9 to 81.8)
Global	Executions and police conflict	5 to 9	Both	119 (93 to 143)	0.0 (0.0 to 0.0)	91 (78 to 105)	0.0 (0.0 to 0.0)	74 (65 to 88)	0.0 (0.0 to 0.0)	-38.0 (-50.5 to -17.5)	-44.5 (-55.7 to -26.2)
Global	Executions and police conflict	5 to 9	Female	39 (30 to 55)	0.0 (0.0 to 0.0)	37 (30 to 48)	0.0 (0.0 to 0.0)	37 (31 to 49)	0.0 (0.0 to 0.0)	-6.2 (-34.1 to 32.1)	-15.1 (-40.4 to 19.6)
Global	Executions and police conflict	5 to 9	Male	80 (59 to 101)	0.0 (0.0 to 0.0)	54 (46 to 62)	0.0 (0.0 to 0.0)	37 (32 to 43)	0.0 (0.0 to 0.0)	-53.6 (-64.3 to -33.8)	-58.8 (-68.3 to -41.3)
Global	Executions and police conflict	10 to 14	Both	115 (90 to 143)	0.0 (0.0 to 0.0)	108 (92 to 126)	0.0 (0.0 to 0.0)	91 (75 to 114)	0.0 (0.0 to 0.0)	-20.4 (-37.4 to 3.4)	-31.2 (-45.9 to -10.6)
Global	Executions and police conflict	10 to 14	Female	43 (33 to 63)	0.0 (0.0 to 0.0)	47 (37 to 64)	0.0 (0.0 to 0.0)	42 (34 to 62)	0.0 (0.0 to 0.0)	-1.6 (-33.9 to 49.6)	-13.9 (-42.2 to 30.9)
Global	Executions and police conflict	10 to 14	Male	72 (53 to 90)	0.0 (0.0 to 0.0)	61 (52 to 70)	0.0 (0.0 to 0.0)	49 (39 to 62)	0.0 (0.0 to 0.0)	-31.7 (-48.3 to -6.4)	-41.6 (-55.8 to -20.0)
European Region	All causes	5 to 9	Both	29994 (29239 to 30788)	46.3 (45.1 to 47.5)	22286 (21570 to 23063)	39.5 (38.3 to 40.9)	10740 (9970 to 11542)	19.5 (18.1 to 20.9)	-64.2 (-66.8 to -61.4)	-58.0 (-61.1 to -54.7)
European Region	All causes	5 to 9	Female	11726 (11462 to 12018)	36.9 (36.1 to 37.8)	9038 (8760 to 9324)	32.8 (31.8 to 33.9)	4408 (4105 to 4731)	16.4 (15.3 to 17.6)	-62.4 (-65.0 to -59.6)	-55.6 (-58.7 to -52.2)
European Region	All causes	5 to 9	Male	18268 (17759 to 18783)	55.3 (53.7 to 56.8)	13248 (12804 to 13764)	45.9 (44.4 to 47.7)	6332 (5864 to 6838)	22.4 (20.7 to 24.2)	-65.3 (-68.0 to -62.5)	-59.5 (-62.7 to -56.3)
European Region	All causes	10 to 14	Both	24133 (23722 to 24562)	37.9 (37.3 to 38.6)	23588 (23094 to 24084)	36.6 (35.8 to 37.4)	10279 (9652 to 10897)	20.1 (18.8 to 21.3)	-57.4 (-60.1 to -54.7)	-47.1 (-50.4 to -43.8)
European Region	All causes	10 to 14	Female	8826 (8662 to 9010)	28.3 (27.7 to 28.9)	8811 (8617 to 8998)	27.9 (27.3 to 28.5)	3937 (3685 to 4220)	15.8 (14.8 to 16.9)	-55.4 (-58.4 to -52.0)	-44.2 (-48.0 to -40.0)
European Region	All causes	10 to 14	Male	15307 (15000 to 15647)	47.2 (46.3 to 48.3)	14778 (14386 to 15164)	44.9 (43.7 to 46.1)	6342 (5902 to 6791)	24.1 (22.5 to 25.9)	-58.6 (-61.4 to -55.4)	-48.9 (-52.4 to -45.0)
European Region	Tuberculosis	5 to 9	Both	223 (152 to 309)	0.3 (0.2 to 0.5)	128 (107 to 156)	0.2 (0.2 to 0.3)	26 (22 to 31)	0.0 (0.0 to 0.1)	-87.9 (-92.0 to -82.0)	-85.9 (-90.7 to -78.8)
European Region	Tuberculosis	5 to 9	Female	62 (40 to 98)	0.2 (0.1 to 0.3)	51 (40 to 67)	0.2 (0.1 to 0.2)	13 (10 to 16)	0.0 (0.0 to 0.1)	-78.0 (-88.1 to -65.1)	-74.1 (-85.9 to -58.8)
European Region	Tuberculosis	5 to 9	Male	160 (101 to 239)	0.5 (0.3 to 0.7)	78 (62 to 100)	0.3 (0.2 to 0.3)	13 (11 to 16)	0.0 (0.0 to 0.1)	-91.5 (-94.9 to -86.4)	-90.0 (-94.0 to -84.1)
European Region	Tuberculosis	10 to 14	Both	122 (87 to 168)	0.2 (0.1 to 0.3)	119 (104 to 137)	0.2 (0.2 to 0.2)	27 (23 to 31)	0.1 (0.0 to 0.1)	-77.6 (-84.9 to -66.6)	-72.1 (-81.2 to -58.5)
European Region	Tuberculosis	10 to 14	Female	60 (40 to 93)	0.2 (0.1 to 0.3)	63 (53 to 76)	0.2 (0.2 to 0.2)	15 (12 to 18)	0.1 (0.0 to 0.1)	-73.3 (-84.4 to -58.7)	-66.7 (-80.5 to -48.4)
European Region	Tuberculosis	10 to 14	Male	62 (41 to 92)	0.2 (0.1 to 0.3)	56 (45 to 69)	0.2 (0.1 to 0.2)	11 (9 to 14)	0.0 (0.0 to 0.1)	-80.8 (-88.6 to -68.7)	-76.3 (-85.9 to -61.4)
European Region	HIV/AIDS	5 to 9	Both	69 (65 to 73)	0.1 (0.1 to 0.1)	90 (83 to 98)	0.2 (0.1 to 0.2)	46 (41 to 52)	0.1 (0.1 to 0.1)	-32.9 (-40.5 to -23.6)	-21.2 (-30.1 to -10.3)
European Region	HIV/AIDS	5 to 9	Female	24 (21 to 26)	0.1 (0.1 to 0.1)	41 (37 to 46)	0.1 (0.1 to 0.2)	25 (21 to 30)	0.1 (0.1 to 0.1)	7.1 (-13.1 to 32.0)	26.5 (2.7 to 55.9)

eTable 8. All-cause and cause-specific mortality (counts and rates) at the global level and by country in the WHO European Region for ages 5 to 9 and 10 to 14 for 1990, 2000, and 2016

European Region	HIV/AIDS	5 to 9	Male	45 (42 to 48)	0.1 (0.1 to 0.1)	49 (44 to 54)	0.2 (0.2 to 0.2)	21 (18 to 24)	0.1 (0.1 to 0.1)	-53.6 (-59.8 to -46.7)	-45.8 (-53.0 to -37.8)
European Region	HIV/AIDS	10 to 14	Both	47 (45 to 50)	0.1 (0.1 to 0.1)	302 (274 to 331)	0.5 (0.4 to 0.5)	35 (31 to 39)	0.1 (0.1 to 0.1)	-24.8 (-34.1 to -15.2)	-6.6 (-18.1 to 5.4)
European Region	HIV/AIDS	10 to 14	Female	16 (15 to 18)	0.1 (0.0 to 0.1)	129 (114 to 146)	0.4 (0.4 to 0.5)	19 (16 to 23)	0.1 (0.1 to 0.1)	21.9 (-1.3 to 49.7)	52.4 (23.4 to 87.2)
European Region	HIV/AIDS	10 to 14	Male	31 (29 to 33)	0.1 (0.1 to 0.1)	173 (150 to 200)	0.5 (0.5 to 0.6)	16 (14 to 18)	0.1 (0.1 to 0.1)	-48.8 (-55.7 to -41.2)	-36.8 (-45.3 to -27.4)
European Region	Diarrheal diseases	5 to 9	Both	236 (171 to 329)	0.4 (0.3 to 0.5)	205 (136 to 292)	0.4 (0.2 to 0.5)	75 (50 to 114)	0.1 (0.1 to 0.2)	-67.8 (-77.8 to -54.4)	-62.2 (-73.9 to -46.5)
European Region	Diarrheal diseases	5 to 9	Female	117 (78 to 183)	0.4 (0.2 to 0.6)	102 (61 to 160)	0.4 (0.2 to 0.6)	35 (20 to 61)	0.1 (0.1 to 0.2)	-69.9 (-81.2 to -51.9)	-64.4 (-77.8 to -43.2)
European Region	Diarrheal diseases	5 to 9	Male	119 (82 to 172)	0.4 (0.2 to 0.5)	103 (63 to 154)	0.4 (0.2 to 0.5)	40 (24 to 69)	0.1 (0.1 to 0.2)	-65.6 (-78.1 to -46.3)	-59.8 (-74.5 to -37.3)
European Region	Diarrheal diseases	10 to 14	Both	104 (75 to 141)	0.2 (0.1 to 0.2)	105 (72 to 144)	0.2 (0.1 to 0.2)	37 (27 to 53)	0.1 (0.1 to 0.1)	-63.5 (-73.5 to -51.4)	-54.7 (-67.1 to -39.7)
European Region	Diarrheal diseases	10 to 14	Female	47 (31 to 74)	0.2 (0.1 to 0.2)	50 (32 to 76)	0.2 (0.1 to 0.2)	17 (11 to 28)	0.1 (0.0 to 0.1)	-64.4 (-77.1 to -46.7)	-55.5 (-71.3 to -33.3)
European Region	Diarrheal diseases	10 to 14	Male	56 (39 to 79)	0.2 (0.1 to 0.2)	55 (35 to 81)	0.2 (0.1 to 0.2)	21 (13 to 33)	0.1 (0.0 to 0.1)	-62.5 (-74.3 to -45.8)	-53.7 (-68.2 to -33.1)
European Region	Intestinal infectious diseases	5 to 9	Both	134 (61 to 262)	0.2 (0.1 to 0.4)	96 (44 to 181)	0.2 (0.1 to 0.3)	62 (28 to 119)	0.1 (0.1 to 0.2)	-53.3 (-68.4 to -36.0)	-45.2 (-62.9 to -24.9)
European Region	Intestinal infectious diseases	5 to 9	Female	61 (27 to 118)	0.2 (0.1 to 0.4)	43 (20 to 86)	0.2 (0.1 to 0.3)	28 (13 to 54)	0.1 (0.0 to 0.2)	-52.4 (-68.1 to -32.6)	-43.7 (-62.4 to -20.4)
European Region	Intestinal infectious diseases	5 to 9	Male	74 (33 to 141)	0.2 (0.1 to 0.4)	53 (24 to 100)	0.2 (0.1 to 0.3)	34 (15 to 64)	0.1 (0.1 to 0.2)	-53.8 (-70.5 to -33.1)	-46.1 (-65.6 to -21.9)
European Region	Intestinal infectious diseases	10 to 14	Both	145 (68 to 271)	0.2 (0.1 to 0.4)	104 (48 to 196)	0.2 (0.1 to 0.3)	64 (28 to 122)	0.1 (0.1 to 0.2)	-56.1 (-68.1 to -45.6)	-45.4 (-60.4 to -32.4)
European Region	Intestinal infectious diseases	10 to 14	Female	69 (31 to 126)	0.2 (0.1 to 0.4)	48 (22 to 92)	0.2 (0.1 to 0.3)	29 (13 to 54)	0.1 (0.1 to 0.2)	-57.6 (-69.4 to -43.9)	-47.0 (-61.7 to -29.9)
European Region	Intestinal infectious diseases	10 to 14	Male	77 (35 to 143)	0.2 (0.1 to 0.4)	55 (25 to 104)	0.2 (0.1 to 0.3)	35 (15 to 67)	0.1 (0.1 to 0.3)	-54.4 (-69.3 to -39.7)	-43.7 (-62.1 to -25.5)
European Region	Lower respiratory infections	5 to 9	Both	2523 (2210 to 2782)	3.9 (3.4 to 4.3)	2512 (2224 to 2752)	4.5 (3.9 to 4.9)	1041 (894 to 1196)	1.9 (1.6 to 2.2)	-58.6 (-65.1 to -50.8)	-51.4 (-59.0 to -42.2)
European Region	Lower respiratory infections	5 to 9	Female	1198 (1053 to 1333)	3.8 (3.3 to 4.2)	1171 (1035 to 1296)	4.3 (3.8 to 4.7)	482 (411 to 560)	1.8 (1.5 to 2.1)	-59.6 (-66.9 to -51.4)	-52.3 (-60.9 to -42.6)
European Region	Lower respiratory infections	5 to 9	Male	1325 (1113 to 1519)	4.0 (3.4 to 4.6)	1341 (1142 to 1511)	4.6 (4.0 to 5.2)	559 (468 to 687)	2.0 (1.7 to 2.4)	-57.6 (-66.2 to -46.2)	-50.5 (-60.5 to -37.2)
European Region	Lower respiratory infections	10 to 14	Both	1368 (1238 to 1487)	2.1 (1.9 to 2.3)	1644 (1515 to 1775)	2.6 (2.4 to 2.8)	662 (589 to 744)	1.3 (1.2 to 1.5)	-51.5 (-58.1 to -44.6)	-39.8 (-48.0 to -31.2)
European Region	Lower respiratory infections	10 to 14	Female	634 (565 to 709)	2.0 (1.8 to 2.3)	760 (685 to 836)	2.4 (2.2 to 2.6)	297 (256 to 341)	1.2 (1.0 to 1.4)	-53.1 (-60.6 to -44.9)	-41.3 (-50.7 to -31.1)
European Region	Lower respiratory infections	10 to 14	Male	734 (645 to 823)	2.3 (2.0 to 2.5)	883 (782 to 986)	2.7 (2.4 to 3.0)	366 (314 to 431)	1.4 (1.2 to 1.6)	-50.1 (-58.5 to -40.0)	-38.4 (-48.7 to -26.0)
European Region	Upper respiratory infections	5 to 9	Both	1 (1 to 2)	0.0 (0.0 to 0.0)	1 (1 to 2)	0.0 (0.0 to 0.0)	0 (0 to 1)	0.0 (0.0 to 0.0)	-64.7 (-87.4 to -19.3)	-58.6 (-85.2 to -5.2)
European Region	Upper respiratory infections	5 to 9	Female	0 (0 to 1)	0.0 (0.0 to 0.0)	1 (0 to 1)	0.0 (0.0 to 0.0)	0 (0 to 0)	0.0 (0.0 to 0.0)	-60.9 (-91.7 to 6.2)	-53.8 (-90.2 to 25.4)
European Region	Upper respiratory infections	5 to 9	Male	1 (0 to 2)	0.0 (0.0 to 0.0)	1 (0 to 2)	0.0 (0.0 to 0.0)	0 (0 to 1)	0.0 (0.0 to 0.0)	-62.2 (-90.8 to 1.5)	-55.9 (-89.3 to 18.5)

eTable 8. All-cause and cause-specific mortality (counts and rates) at the global level and by country in the WHO European Region for ages 5 to 9 and 10 to 14 for 1990, 2000, and 2016

European Region	Upper respiratory infections	10 to 14	Both	1 (0 to 2)	0.0 (0.0 to 0.0)	1 (1 to 2)	0.0 (0.0 to 0.0)	0 (0 to 1)	0.0 (0.0 to 0.0)	-59.3 (-84.8 to -9.0)	-49.5 (-81.1 to 13.0)
European Region	Upper respiratory infections	10 to 14	Female	0 (0 to 1)	0.0 (0.0 to 0.0)	0 (0 to 1)	0.0 (0.0 to 0.0)	0 (0 to 0)	0.0 (0.0 to 0.0)	-54.4 (-88.7 to 20.0)	-43.0 (-85.8 to 50.0)
European Region	Upper respiratory infections	10 to 14	Male	1 (0 to 1)	0.0 (0.0 to 0.0)	1 (0 to 2)	0.0 (0.0 to 0.0)	0 (0 to 1)	0.0 (0.0 to 0.0)	-57.2 (-89.3 to 12.0)	-47.2 (-86.8 to 38.3)
European Region	Otitis media	5 to 9	Both	8 (3 to 28)	0.0 (0.0 to 0.0)	1 (1 to 3)	0.0 (0.0 to 0.0)	0 (0 to 0)	0.0 (0.0 to 0.0)	-96.9 (-99.4 to -93.8)	-96.3 (-99.2 to -92.8)
European Region	Otitis media	5 to 9	Female	5 (1 to 23)	0.0 (0.0 to 0.1)	1 (0 to 2)	0.0 (0.0 to 0.0)	0 (0 to 0)	0.0 (0.0 to 0.0)	-97.1 (-99.7 to -92.1)	-96.6 (-99.7 to -90.7)
European Region	Otitis media	5 to 9	Male	4 (2 to 12)	0.0 (0.0 to 0.0)	1 (0 to 2)	0.0 (0.0 to 0.0)	0 (0 to 0)	0.0 (0.0 to 0.0)	-95.8 (-99.1 to -92.2)	-95.1 (-98.9 to -90.9)
European Region	Otitis media	10 to 14	Both	6 (3 to 14)	0.0 (0.0 to 0.0)	1 (1 to 2)	0.0 (0.0 to 0.0)	0 (0 to 0)	0.0 (0.0 to 0.0)	-96.9 (-99.0 to -94.8)	-96.2 (-98.8 to -93.5)
European Region	Otitis media	10 to 14	Female	3 (1 to 10)	0.0 (0.0 to 0.0)	1 (0 to 1)	0.0 (0.0 to 0.0)	0 (0 to 0)	0.0 (0.0 to 0.0)	-97.4 (-99.4 to -94.5)	-96.7 (-99.3 to -93.1)
European Region	Otitis media	10 to 14	Male	2 (1 to 6)	0.0 (0.0 to 0.0)	0 (0 to 1)	0.0 (0.0 to 0.0)	0 (0 to 0)	0.0 (0.0 to 0.0)	-95.8 (-98.6 to -93.2)	-94.8 (-98.2 to -91.6)
European Region	Meningitis	5 to 9	Both	819 (498 to 1075)	1.3 (0.8 to 1.7)	628 (391 to 803)	1.1 (0.7 to 1.4)	170 (141 to 211)	0.3 (0.3 to 0.4)	-78.4 (-85.2 to -67.0)	-74.6 (-82.6 to -61.3)
European Region	Meningitis	5 to 9	Female	474 (232 to 653)	1.5 (0.7 to 2.1)	351 (169 to 464)	1.3 (0.6 to 1.7)	71 (59 to 87)	0.3 (0.2 to 0.3)	-83.8 (-89.7 to -70.0)	-80.8 (-87.8 to -64.5)
European Region	Meningitis	5 to 9	Male	345 (232 to 484)	1.0 (0.7 to 1.5)	278 (176 to 375)	1.0 (0.6 to 1.3)	99 (74 to 136)	0.3 (0.3 to 0.5)	-70.2 (-81.0 to -57.5)	-65.2 (-77.9 to -50.4)
European Region	Meningitis	10 to 14	Both	471 (347 to 585)	0.7 (0.5 to 0.9)	446 (348 to 537)	0.7 (0.5 to 0.8)	131 (111 to 163)	0.3 (0.2 to 0.3)	-71.6 (-78.8 to -60.9)	-64.7 (-73.6 to -51.4)
European Region	Meningitis	10 to 14	Female	237 (169 to 299)	0.8 (0.5 to 1.0)	212 (154 to 255)	0.7 (0.5 to 0.8)	55 (47 to 65)	0.2 (0.2 to 0.3)	-76.2 (-82.6 to -66.5)	-70.2 (-78.2 to -58.1)
European Region	Meningitis	10 to 14	Male	234 (162 to 318)	0.7 (0.5 to 1.0)	234 (157 to 304)	0.7 (0.5 to 0.9)	76 (57 to 104)	0.3 (0.2 to 0.4)	-66.5 (-77.8 to -51.5)	-58.6 (-72.6 to -40.2)
European Region	Encephalitis	5 to 9	Both	343 (242 to 423)	0.5 (0.4 to 0.7)	327 (231 to 416)	0.6 (0.4 to 0.7)	229 (179 to 348)	0.4 (0.3 to 0.6)	-32.4 (-50.4 to -7.3)	-20.7 (-41.8 to 8.8)
European Region	Encephalitis	5 to 9	Female	140 (86 to 176)	0.4 (0.3 to 0.6)	135 (81 to 172)	0.5 (0.3 to 0.6)	88 (65 to 119)	0.3 (0.2 to 0.4)	-35.5 (-52.4 to -11.1)	-23.9 (-43.8 to 5.0)
European Region	Encephalitis	5 to 9	Male	203 (128 to 296)	0.6 (0.4 to 0.9)	192 (128 to 280)	0.7 (0.4 to 1.0)	141 (99 to 251)	0.5 (0.3 to 0.9)	-30.3 (-52.8 to -4.1)	-18.6 (-44.9 to 12.0)
European Region	Encephalitis	10 to 14	Both	210 (141 to 252)	0.3 (0.2 to 0.4)	235 (165 to 294)	0.4 (0.3 to 0.5)	138 (111 to 193)	0.3 (0.2 to 0.4)	-33.6 (-49.1 to -14.0)	-17.5 (-36.8 to 6.8)
European Region	Encephalitis	10 to 14	Female	90 (54 to 114)	0.3 (0.2 to 0.4)	101 (62 to 127)	0.3 (0.2 to 0.4)	55 (40 to 71)	0.2 (0.2 to 0.3)	-37.3 (-53.1 to -17.8)	-21.5 (-41.3 to 2.8)
European Region	Encephalitis	10 to 14	Male	120 (77 to 169)	0.4 (0.2 to 0.5)	134 (93 to 199)	0.4 (0.3 to 0.6)	82 (61 to 135)	0.3 (0.2 to 0.5)	-30.7 (-49.7 to -10.1)	-14.5 (-37.9 to 10.9)
European Region	Diphtheria	5 to 9	Both	11 (6 to 20)	0.0 (0.0 to 0.0)	6 (2 to 12)	0.0 (0.0 to 0.0)	1 (1 to 1)	0.0 (0.0 to 0.0)	-91.8 (-96.5 to -83.0)	-90.4 (-95.9 to -80.0)
European Region	Diphtheria	5 to 9	Female	4 (2 to 9)	0.0 (0.0 to 0.0)	2 (1 to 6)	0.0 (0.0 to 0.0)	0 (0 to 1)	0.0 (0.0 to 0.0)	-90.9 (-97.2 to -76.8)	-89.2 (-96.7 to -72.6)
European Region	Diphtheria	5 to 9	Male	7 (3 to 14)	0.0 (0.0 to 0.0)	3 (1 to 9)	0.0 (0.0 to 0.0)	1 (0 to 1)	0.0 (0.0 to 0.0)	-91.2 (-97.3 to -78.7)	-89.7 (-96.8 to -75.1)
European Region	Diphtheria	10 to 14	Both	6 (3 to 10)	0.0 (0.0 to 0.0)	3 (2 to 6)	0.0 (0.0 to 0.0)	1 (0 to 1)	0.0 (0.0 to 0.0)	-89.7 (-95.5 to -79.2)	-87.2 (-94.5 to -74.2)
European Region	Diphtheria	10 to 14	Female	2 (1 to 4)	0.0 (0.0 to 0.0)	1 (0 to 3)	0.0 (0.0 to 0.0)	0 (0 to 0)	0.0 (0.0 to 0.0)	-88.5 (-96.5 to -73.0)	-85.7 (-95.6 to -66.2)
European Region	Diphtheria	10 to 14	Male	4 (1 to 7)	0.0 (0.0 to 0.0)	2 (1 to 4)	0.0 (0.0 to 0.0)	0 (0 to 1)	0.0 (0.0 to 0.0)	-89.1 (-96.6 to -73.4)	-86.5 (-95.8 to -67.2)
European Region	Whooping cough	5 to 9	Both	86 (24 to 206)	0.1 (0.0 to 0.3)	75 (23 to 165)	0.1 (0.0 to 0.3)	16 (4 to 38)	0.0 (0.0 to 0.1)	-76.2 (-96.2 to -20.1)	-72.1 (-95.5 to -6.2)
European Region	Whooping cough	5 to 9	Female	48 (14 to 114)	0.2 (0.0 to 0.4)	42 (13 to 95)	0.2 (0.0 to 0.3)	9 (2 to 22)	0.0 (0.0 to 0.1)	-76.3 (-96.2 to -20.5)	-72.0 (-95.5 to -6.2)
European Region	Whooping cough	5 to 9	Male	38 (10 to 92)	0.1 (0.0 to 0.3)	32 (10 to 71)	0.1 (0.0 to 0.2)	7 (2 to 17)	0.0 (0.0 to 0.1)	-76.0 (-96.1 to -22.1)	-72.0 (-95.4 to -9.0)
European Region	Whooping cough	10 to 14	Both	17 (5 to 43)	0.0 (0.0 to 0.1)	16 (5 to 35)	0.0 (0.0 to 0.1)	3 (1 to 8)	0.0 (0.0 to 0.0)	-74.1 (-95.6 to -14.3)	-67.8 (-94.5 to 6.5)

eTable 8. All-cause and cause-specific mortality (counts and rates) at the global level and by country in the WHO European Region for ages 5 to 9 and 10 to 14 for 1990, 2000, and 2016

European Region	Whooping cough	10 to 14	Female	9 (3 to 24)	0.0 (0.0 to 0.1)	9 (3 to 19)	0.0 (0.0 to 0.1)	2 (1 to 4)	0.0 (0.0 to 0.0)	-75.5 (-95.7 to -17.0)	-69.3 (-94.7 to 3.8)
European Region	Whooping cough	10 to 14	Male	8 (2 to 19)	0.0 (0.0 to 0.1)	7 (2 to 16)	0.0 (0.0 to 0.0)	2 (1 to 4)	0.0 (0.0 to 0.0)	-72.3 (-95.2 to -11.8)	-65.8 (-94.0 to 8.9)
European Region	Tetanus	5 to 9	Both	5 (3 to 9)	0.0 (0.0 to 0.0)	2 (1 to 5)	0.0 (0.0 to 0.0)	1 (1 to 1)	0.0 (0.0 to 0.0)	-74.3 (-88.3 to -58.3)	-69.9 (-86.3 to -51.0)
European Region	Tetanus	5 to 9	Female	2 (1 to 4)	0.0 (0.0 to 0.0)	1 (1 to 2)	0.0 (0.0 to 0.0)	1 (0 to 1)	0.0 (0.0 to 0.0)	-68.8 (-88.1 to -48.7)	-63.2 (-86.0 to -39.4)
European Region	Tetanus	5 to 9	Male	3 (1 to 6)	0.0 (0.0 to 0.0)	1 (1 to 4)	0.0 (0.0 to 0.0)	1 (0 to 1)	0.0 (0.0 to 0.0)	-75.3 (-91.1 to -43.6)	-71.2 (-89.6 to -34.2)
European Region	Tetanus	10 to 14	Both	2 (2 to 3)	0.0 (0.0 to 0.0)	2 (1 to 2)	0.0 (0.0 to 0.0)	1 (1 to 1)	0.0 (0.0 to 0.0)	-53.8 (-68.6 to -31.7)	-42.6 (-61.0 to -15.1)
European Region	Tetanus	10 to 14	Female	1 (1 to 1)	0.0 (0.0 to 0.0)	1 (1 to 1)	0.0 (0.0 to 0.0)	0 (0 to 1)	0.0 (0.0 to 0.0)	-42.8 (-63.4 to -25.6)	-28.4 (-54.2 to -7.0)
European Region	Tetanus	10 to 14	Male	1 (1 to 2)	0.0 (0.0 to 0.0)	1 (1 to 1)	0.0 (0.0 to 0.0)	1 (0 to 1)	0.0 (0.0 to 0.0)	-59.2 (-73.6 to -24.6)	-49.6 (-67.4 to -7.0)
European Region	Measles	5 to 9	Both	614 (264 to 1266)	0.9 (0.4 to 2.0)	56 (21 to 125)	0.1 (0.0 to 0.2)	4 (2 to 9)	0.0 (0.0 to 0.0)	-99.3 (-99.6 to -98.8)	-99.2 (-99.5 to -98.6)
European Region	Measles	5 to 9	Female	303 (130 to 627)	1.0 (0.4 to 2.0)	28 (10 to 62)	0.1 (0.0 to 0.2)	2 (1 to 5)	0.0 (0.0 to 0.0)	-99.3 (-99.6 to -98.8)	-99.1 (-99.5 to -98.6)
European Region	Measles	5 to 9	Male	312 (132 to 640)	0.9 (0.4 to 1.9)	28 (11 to 62)	0.1 (0.0 to 0.2)	2 (1 to 5)	0.0 (0.0 to 0.0)	-99.3 (-99.6 to -98.8)	-99.2 (-99.5 to -98.7)
European Region	Measles	10 to 14	Both	176 (76 to 366)	0.3 (0.1 to 0.6)	17 (7 to 36)	0.0 (0.0 to 0.1)	2 (1 to 3)	0.0 (0.0 to 0.0)	-99.0 (-99.4 to -98.3)	-98.7 (-99.3 to -97.9)
European Region	Measles	10 to 14	Female	92 (39 to 194)	0.3 (0.1 to 0.6)	9 (3 to 19)	0.0 (0.0 to 0.1)	1 (0 to 1)	0.0 (0.0 to 0.0)	-99.1 (-99.5 to -98.5)	-98.8 (-99.3 to -98.1)
European Region	Measles	10 to 14	Male	84 (36 to 178)	0.3 (0.1 to 0.6)	8 (4 to 17)	0.0 (0.0 to 0.1)	1 (1 to 2)	0.0 (0.0 to 0.0)	-98.8 (-99.3 to -98.0)	-98.5 (-99.2 to -97.5)
European Region	Varicella and herpes zoster	5 to 9	Both	28 (17 to 47)	0.0 (0.0 to 0.1)	17 (11 to 25)	0.0 (0.0 to 0.0)	8 (5 to 11)	0.0 (0.0 to 0.0)	-69.8 (-84.6 to -50.5)	-64.6 (-82.0 to -41.9)
European Region	Varicella and herpes zoster	5 to 9	Female	13 (6 to 26)	0.0 (0.0 to 0.1)	8 (4 to 14)	0.0 (0.0 to 0.0)	3 (2 to 6)	0.0 (0.0 to 0.0)	-71.1 (-89.0 to -44.4)	-65.9 (-87.1 to -34.4)
European Region	Varicella and herpes zoster	5 to 9	Male	15 (7 to 28)	0.0 (0.0 to 0.1)	9 (5 to 15)	0.0 (0.0 to 0.1)	5 (2 to 7)	0.0 (0.0 to 0.0)	-66.5 (-85.9 to -40.7)	-60.9 (-83.6 to -30.8)
European Region	Varicella and herpes zoster	10 to 14	Both	9 (5 to 15)	0.0 (0.0 to 0.0)	7 (4 to 10)	0.0 (0.0 to 0.0)	3 (1 to 4)	0.0 (0.0 to 0.0)	-67.9 (-83.9 to -49.0)	-60.1 (-80.0 to -36.6)
European Region	Varicella and herpes zoster	10 to 14	Female	5 (2 to 9)	0.0 (0.0 to 0.0)	4 (2 to 6)	0.0 (0.0 to 0.0)	2 (1 to 3)	0.0 (0.0 to 0.0)	-65.0 (-86.7 to -35.6)	-56.2 (-83.4 to -19.4)
European Region	Varicella and herpes zoster	10 to 14	Male	4 (2 to 9)	0.0 (0.0 to 0.0)	3 (2 to 5)	0.0 (0.0 to 0.0)	1 (1 to 2)	0.0 (0.0 to 0.0)	-68.1 (-87.8 to -39.8)	-60.6 (-85.0 to -25.7)
European Region	Malaria	5 to 9	Both	3 (1 to 9)	0.0 (0.0 to 0.0)	3 (1 to 7)	0.0 (0.0 to 0.0)	2 (0 to 11)	0.0 (0.0 to 0.0)	-8.1 (-94.3 to 456.9)	7.8 (-93.4 to 553.7)
European Region	Malaria	5 to 9	Female	2 (0 to 8)	0.0 (0.0 to 0.0)	2 (0 to 5)	0.0 (0.0 to 0.0)	1 (0 to 10)	0.0 (0.0 to 0.0)	41.1 (-98.2 to 888.1)	66.7 (-97.9 to 1066.9)
European Region	Malaria	5 to 9	Male	1 (1 to 2)	0.0 (0.0 to 0.0)	2 (0 to 4)	0.0 (0.0 to 0.0)	1 (0 to 2)	0.0 (0.0 to 0.0)	-41.2 (-92.3 to 161.4)	-31.4 (-91.0 to 205.0)
European Region	Malaria	10 to 14	Both	1 (1 to 3)	0.0 (0.0 to 0.0)	3 (1 to 5)	0.0 (0.0 to 0.0)	1 (0 to 3)	0.0 (0.0 to 0.0)	-40.6 (-89.5 to 116.8)	-26.2 (-87.0 to 169.4)
European Region	Malaria	10 to 14	Female	1 (0 to 2)	0.0 (0.0 to 0.0)	1 (0 to 3)	0.0 (0.0 to 0.0)	0 (0 to 2)	0.0 (0.0 to 0.0)	-23.0 (-94.7 to 231.2)	-3.7 (-93.4 to 314.2)
European Region	Malaria	10 to 14	Male	1 (0 to 1)	0.0 (0.0 to 0.0)	1 (0 to 3)	0.0 (0.0 to 0.0)	0 (0 to 1)	0.0 (0.0 to 0.0)	-52.2 (-91.2 to 88.1)	-41.0 (-89.1 to 132.2)
European Region	Leishmaniasis	5 to 9	Both	1 (0 to 2)	0.0 (0.0 to 0.0)	0 (0 to 1)	0.0 (0.0 to 0.0)	0 (0 to 1)	0.0 (0.0 to 0.0)	-	-
European Region	Leishmaniasis	5 to 9	Female	0 (0 to 1)	0.0 (0.0 to 0.0)	0 (0 to 1)	0.0 (0.0 to 0.0)	0 (0 to 1)	0.0 (0.0 to 0.0)	-	-
European Region	Leishmaniasis	5 to 9	Male	0 (0 to 1)	0.0 (0.0 to 0.0)	0 (0 to 1)	0.0 (0.0 to 0.0)	0 (0 to 1)	0.0 (0.0 to 0.0)	-	-
European Region	Leishmaniasis	10 to 14	Both	1 (0 to 2)	0.0 (0.0 to 0.0)	0 (0 to 1)	0.0 (0.0 to 0.0)	0 (0 to 1)	0.0 (0.0 to 0.0)	-	-
European Region	Leishmaniasis	10 to 14	Female	0 (0 to 1)	0.0 (0.0 to 0.0)	0 (0 to 0)	0.0 (0.0 to 0.0)	0 (0 to 0)	0.0 (0.0 to 0.0)	-	-
European Region	Leishmaniasis	10 to 14	Male	0 (0 to 1)	0.0 (0.0 to 0.0)	0 (0 to 0)	0.0 (0.0 to 0.0)	0 (0 to 1)	0.0 (0.0 to 0.0)	-	-

eTable 8. All-cause and cause-specific mortality (counts and rates) at the global level and by country in the WHO European Region for ages 5 to 9 and 10 to 14 for 1990, 2000, and 2016

European Region	African trypanosomiasis	5 to 9	Both	0 (0 to 0)	0.0 (0.0 to 0.0)	0 (0 to 0)	0.0 (0.0 to 0.0)	0 (0 to 0)	0.0 (0.0 to 0.0)	-	-
European Region	African trypanosomiasis	5 to 9	Female	0 (0 to 0)	0.0 (0.0 to 0.0)	0 (0 to 0)	0.0 (0.0 to 0.0)	0 (0 to 0)	0.0 (0.0 to 0.0)	-	-
European Region	African trypanosomiasis	5 to 9	Male	0 (0 to 0)	0.0 (0.0 to 0.0)	0 (0 to 0)	0.0 (0.0 to 0.0)	0 (0 to 0)	0.0 (0.0 to 0.0)	-	-
European Region	African trypanosomiasis	10 to 14	Both	0 (0 to 0)	0.0 (0.0 to 0.0)	0 (0 to 0)	0.0 (0.0 to 0.0)	0 (0 to 0)	0.0 (0.0 to 0.0)	-	-
European Region	African trypanosomiasis	10 to 14	Female	0 (0 to 0)	0.0 (0.0 to 0.0)	0 (0 to 0)	0.0 (0.0 to 0.0)	0 (0 to 0)	0.0 (0.0 to 0.0)	-	-
European Region	African trypanosomiasis	10 to 14	Male	0 (0 to 0)	0.0 (0.0 to 0.0)	0 (0 to 0)	0.0 (0.0 to 0.0)	0 (0 to 0)	0.0 (0.0 to 0.0)	-	-
European Region	Schistosomiasis	5 to 9	Both	0 (0 to 0)	0.0 (0.0 to 0.0)	0 (0 to 0)	0.0 (0.0 to 0.0)	0 (0 to 0)	0.0 (0.0 to 0.0)	-	-
European Region	Schistosomiasis	5 to 9	Female	0 (0 to 0)	0.0 (0.0 to 0.0)	0 (0 to 0)	0.0 (0.0 to 0.0)	0 (0 to 0)	0.0 (0.0 to 0.0)	-	-
European Region	Schistosomiasis	5 to 9	Male	0 (0 to 0)	0.0 (0.0 to 0.0)	0 (0 to 0)	0.0 (0.0 to 0.0)	0 (0 to 0)	0.0 (0.0 to 0.0)	-	-
European Region	Schistosomiasis	10 to 14	Both	0 (0 to 0)	0.0 (0.0 to 0.0)	0 (0 to 0)	0.0 (0.0 to 0.0)	0 (0 to 0)	0.0 (0.0 to 0.0)	-	-
European Region	Schistosomiasis	10 to 14	Female	0 (0 to 0)	0.0 (0.0 to 0.0)	0 (0 to 0)	0.0 (0.0 to 0.0)	0 (0 to 0)	0.0 (0.0 to 0.0)	-	-
European Region	Schistosomiasis	10 to 14	Male	0 (0 to 0)	0.0 (0.0 to 0.0)	0 (0 to 0)	0.0 (0.0 to 0.0)	0 (0 to 0)	0.0 (0.0 to 0.0)	-	-
European Region	Cysticercosis	5 to 9	Both	1 (1 to 1)	0.0 (0.0 to 0.0)	0 (0 to 1)	0.0 (0.0 to 0.0)	0 (0 to 0)	0.0 (0.0 to 0.0)	-87.8 (-89.7 to -85.8)	-85.6 (-87.9 to -83.4)
European Region	Cysticercosis	5 to 9	Female	0 (0 to 1)	0.0 (0.0 to 0.0)	0 (0 to 0)	0.0 (0.0 to 0.0)	0 (0 to 0)	0.0 (0.0 to 0.0)	-87.3 (-89.4 to -85.1)	-85.0 (-87.5 to -82.4)
European Region	Cysticercosis	5 to 9	Male	0 (0 to 1)	0.0 (0.0 to 0.0)	0 (0 to 0)	0.0 (0.0 to 0.0)	0 (0 to 0)	0.0 (0.0 to 0.0)	-88.1 (-90.1 to -86.2)	-86.2 (-88.4 to -83.9)
European Region	Cysticercosis	10 to 14	Both	1 (1 to 1)	0.0 (0.0 to 0.0)	0 (0 to 1)	0.0 (0.0 to 0.0)	0 (0 to 0)	0.0 (0.0 to 0.0)	-85.6 (-87.4 to -84.1)	-82.1 (-84.4 to -80.3)
European Region	Cysticercosis	10 to 14	Female	0 (0 to 0)	0.0 (0.0 to 0.0)	0 (0 to 0)	0.0 (0.0 to 0.0)	0 (0 to 0)	0.0 (0.0 to 0.0)	-85.2 (-87.1 to -83.4)	-81.5 (-83.9 to -79.3)
European Region	Cysticercosis	10 to 14	Male	0 (0 to 0)	0.0 (0.0 to 0.0)	0 (0 to 0)	0.0 (0.0 to 0.0)	0 (0 to 0)	0.0 (0.0 to 0.0)	-85.9 (-87.8 to -84.1)	-82.6 (-85.0 to -80.4)
European Region	Yellow fever	5 to 9	Both	0 (0 to 0)	0.0 (0.0 to 0.0)	0 (0 to 0)	0.0 (0.0 to 0.0)	0 (0 to 0)	0.0 (0.0 to 0.0)	-	-
European Region	Yellow fever	5 to 9	Female	0 (0 to 0)	0.0 (0.0 to 0.0)	0 (0 to 0)	0.0 (0.0 to 0.0)	0 (0 to 0)	0.0 (0.0 to 0.0)	-	-
European Region	Yellow fever	5 to 9	Male	0 (0 to 0)	0.0 (0.0 to 0.0)	0 (0 to 0)	0.0 (0.0 to 0.0)	0 (0 to 0)	0.0 (0.0 to 0.0)	-	-
European Region	Yellow fever	10 to 14	Both	0 (0 to 0)	0.0 (0.0 to 0.0)	0 (0 to 0)	0.0 (0.0 to 0.0)	0 (0 to 0)	0.0 (0.0 to 0.0)	-	-
European Region	Yellow fever	10 to 14	Female	0 (0 to 0)	0.0 (0.0 to 0.0)	0 (0 to 0)	0.0 (0.0 to 0.0)	0 (0 to 0)	0.0 (0.0 to 0.0)	-	-
European Region	Yellow fever	10 to 14	Male	0 (0 to 0)	0.0 (0.0 to 0.0)	0 (0 to 0)	0.0 (0.0 to 0.0)	0 (0 to 0)	0.0 (0.0 to 0.0)	-	-
European Region	Rabies	5 to 9	Both	6 (3 to 11)	0.0 (0.0 to 0.0)	7 (3 to 15)	0.0 (0.0 to 0.0)	1 (1 to 3)	0.0 (0.0 to 0.0)	-70.3 (-90.0 to -27.6)	-65.1 (-88.3 to -15.0)
European Region	Rabies	5 to 9	Female	0 (0 to 1)	0.0 (0.0 to 0.0)	1 (0 to 1)	0.0 (0.0 to 0.0)	0 (0 to 0)	0.0 (0.0 to 0.0)	-52.6 (-87.8 to 14.1)	-44.0 (-85.6 to 34.7)
European Region	Rabies	5 to 9	Male	5 (2 to 10)	0.0 (0.0 to 0.0)	7 (3 to 14)	0.0 (0.0 to 0.0)	1 (1 to 3)	0.0 (0.0 to 0.0)	-70.9 (-91.3 to -27.4)	-66.0 (-89.8 to -15.2)
European Region	Rabies	10 to 14	Both	3 (1 to 7)	0.0 (0.0 to 0.0)	5 (2 to 12)	0.0 (0.0 to 0.0)	1 (0 to 1)	0.0 (0.0 to 0.0)	-69.8 (-90.3 to -36.0)	-62.5 (-87.9 to -20.4)
European Region	Rabies	10 to 14	Female	0 (0 to 0)	0.0 (0.0 to 0.0)	0 (0 to 1)	0.0 (0.0 to 0.0)	0 (0 to 0)	0.0 (0.0 to 0.0)	-15.1 (-83.6 to 150.2)	6.2 (-79.4 to 213.0)

eTable 8. All-cause and cause-specific mortality (counts and rates) at the global level and by country in the WHO European Region for ages 5 to 9 and 10 to 14 for 1990, 2000, and 2016

European Region	Rabies	10 to 14	Male	3 (1 to 6)	0.0 (0.0 to 0.0)	5 (2 to 11)	0.0 (0.0 to 0.0)	1 (0 to 1)	0.0 (0.0 to 0.0)	-71.2 (-91.2 to -36.5)	-64.5 (-89.1 to -21.6)
European Region	Intestinal nematode infections	5 to 9	Both	5 (2 to 8)	0.0 (0.0 to 0.0)	3 (1 to 6)	0.0 (0.0 to 0.0)	0 (0 to 1)	0.0 (0.0 to 0.0)	-89.0 (-97.7 to -71.7)	-87.1 (-97.3 to -66.7)
European Region	Intestinal nematode infections	5 to 9	Female	3 (1 to 5)	0.0 (0.0 to 0.0)	2 (0 to 4)	0.0 (0.0 to 0.0)	0 (0 to 1)	0.0 (0.0 to 0.0)	-88.7 (-99.1 to -60.8)	-86.7 (-98.9 to -53.7)
European Region	Intestinal nematode infections	5 to 9	Male	2 (1 to 4)	0.0 (0.0 to 0.0)	1 (0 to 3)	0.0 (0.0 to 0.0)	0 (0 to 1)	0.0 (0.0 to 0.0)	-86.2 (-98.8 to -52.1)	-83.9 (-98.6 to -44.1)
European Region	Intestinal nematode infections	10 to 14	Both	1 (0 to 2)	0.0 (0.0 to 0.0)	1 (0 to 2)	0.0 (0.0 to 0.0)	0 (0 to 0)	0.0 (0.0 to 0.0)	-80.1 (-98.0 to -10.7)	-75.2 (-97.5 to 11.0)
European Region	Intestinal nematode infections	10 to 14	Female	1 (0 to 2)	0.0 (0.0 to 0.0)	0 (0 to 1)	0.0 (0.0 to 0.0)	0 (0 to 0)	0.0 (0.0 to 0.0)	-70.2 (-99.1 to 77.9)	-62.8 (-98.9 to 122.5)
European Region	Intestinal nematode infections	10 to 14	Male	0 (0 to 1)	0.0 (0.0 to 0.0)	0 (0 to 1)	0.0 (0.0 to 0.0)	0 (0 to 0)	0.0 (0.0 to 0.0)	-65.2 (-99.0 to 72.9)	-57.1 (-98.7 to 113.4)
European Region	Ebola	5 to 9	Both	0 (0 to 0)	0.0 (0.0 to 0.0)	0 (0 to 0)	0.0 (0.0 to 0.0)	0 (0 to 0)	0.0 (0.0 to 0.0)	-	-
European Region	Ebola	5 to 9	Female	0 (0 to 0)	0.0 (0.0 to 0.0)	0 (0 to 0)	0.0 (0.0 to 0.0)	0 (0 to 0)	0.0 (0.0 to 0.0)	-	-
European Region	Ebola	5 to 9	Male	0 (0 to 0)	0.0 (0.0 to 0.0)	0 (0 to 0)	0.0 (0.0 to 0.0)	0 (0 to 0)	0.0 (0.0 to 0.0)	-	-
European Region	Ebola	10 to 14	Both	0 (0 to 0)	0.0 (0.0 to 0.0)	0 (0 to 0)	0.0 (0.0 to 0.0)	0 (0 to 0)	0.0 (0.0 to 0.0)	-	-
European Region	Ebola	10 to 14	Female	0 (0 to 0)	0.0 (0.0 to 0.0)	0 (0 to 0)	0.0 (0.0 to 0.0)	0 (0 to 0)	0.0 (0.0 to 0.0)	-	-
European Region	Ebola	10 to 14	Male	0 (0 to 0)	0.0 (0.0 to 0.0)	0 (0 to 0)	0.0 (0.0 to 0.0)	0 (0 to 0)	0.0 (0.0 to 0.0)	-	-
European Region	Zika virus	5 to 9	Both	0 (0 to 0)	0.0 (0.0 to 0.0)	0 (0 to 0)	0.0 (0.0 to 0.0)	0 (0 to 0)	0.0 (0.0 to 0.0)	-	-
European Region	Zika virus	5 to 9	Female	0 (0 to 0)	0.0 (0.0 to 0.0)	0 (0 to 0)	0.0 (0.0 to 0.0)	0 (0 to 0)	0.0 (0.0 to 0.0)	-	-
European Region	Zika virus	5 to 9	Male	0 (0 to 0)	0.0 (0.0 to 0.0)	0 (0 to 0)	0.0 (0.0 to 0.0)	0 (0 to 0)	0.0 (0.0 to 0.0)	-	-
European Region	Zika virus	10 to 14	Both	0 (0 to 0)	0.0 (0.0 to 0.0)	0 (0 to 0)	0.0 (0.0 to 0.0)	0 (0 to 0)	0.0 (0.0 to 0.0)	-	-
European Region	Zika virus	10 to 14	Female	0 (0 to 0)	0.0 (0.0 to 0.0)	0 (0 to 0)	0.0 (0.0 to 0.0)	0 (0 to 0)	0.0 (0.0 to 0.0)	-	-
European Region	Zika virus	10 to 14	Male	0 (0 to 0)	0.0 (0.0 to 0.0)	0 (0 to 0)	0.0 (0.0 to 0.0)	0 (0 to 0)	0.0 (0.0 to 0.0)	-	-
European Region	Other neglected tropical diseases	5 to 9	Both	17 (10 to 32)	0.0 (0.0 to 0.0)	18 (10 to 40)	0.0 (0.0 to 0.1)	15 (8 to 34)	0.0 (0.0 to 0.1)	-3.1 (-63.7 to 128.6)	13.8 (-57.4 to 168.4)
European Region	Other neglected tropical diseases	5 to 9	Female	6 (4 to 16)	0.0 (0.0 to 0.0)	7 (3 to 15)	0.0 (0.0 to 0.1)	5 (3 to 12)	0.0 (0.0 to 0.0)	-7.6 (-67.7 to 150.5)	9.1 (-61.9 to 195.8)
European Region	Other neglected tropical diseases	5 to 9	Male	10 (5 to 24)	0.0 (0.0 to 0.1)	12 (5 to 32)	0.0 (0.0 to 0.1)	10 (4 to 27)	0.0 (0.0 to 0.1)	9.4 (-73.7 to 203.4)	27.6 (-69.4 to 254.1)
European Region	Other neglected tropical diseases	10 to 14	Both	14 (9 to 24)	0.0 (0.0 to 0.0)	18 (11 to 36)	0.0 (0.0 to 0.1)	13 (7 to 27)	0.0 (0.0 to 0.1)	-5.0 (-57.6 to 118.0)	18.0 (-47.3 to 170.8)
European Region	Other neglected tropical diseases	10 to 14	Female	6 (3 to 13)	0.0 (0.0 to 0.0)	7 (4 to 14)	0.0 (0.0 to 0.0)	5 (3 to 11)	0.0 (0.0 to 0.0)	-10.2 (-63.8 to 133.3)	12.3 (-54.7 to 191.7)

eTable 8. All-cause and cause-specific mortality (counts and rates) at the global level and by country in the WHO European Region for ages 5 to 9 and 10 to 14 for 1990, 2000, and 2016

European Region	Other neglected tropical diseases	10 to 14	Male	9 (5 to 16)	0.0 (0.0 to 0.0)	12 (6 to 28)	0.0 (0.0 to 0.1)	8 (4 to 21)	0.0 (0.0 to 0.1)	4.4 (-63.3 to 178.0)	28.9 (-54.8 to 243.1)
European Region	Maternal hemorrhage	10 to 14	Both	2 (1 to 3)	0.0 (0.0 to 0.0)	1 (1 to 1)	0.0 (0.0 to 0.0)	0 (0 to 1)	0.0 (0.0 to 0.0)	-73.1 (-84.7 to -61.2)	-66.5 (-81.0 to -51.8)
European Region	Maternal hemorrhage	10 to 14	Female	2 (1 to 3)	0.0 (0.0 to 0.0)	1 (1 to 1)	0.0 (0.0 to 0.0)	0 (0 to 1)	0.0 (0.0 to 0.0)	-73.1 (-84.7 to -61.2)	-66.3 (-80.9 to -51.5)
European Region	Maternal sepsis and other maternal infections	10 to 14	Both	2 (1 to 4)	0.0 (0.0 to 0.0)	1 (1 to 2)	0.0 (0.0 to 0.0)	0 (0 to 0)	0.0 (0.0 to 0.0)	-85.5 (-93.6 to -72.8)	-81.9 (-92.1 to -66.2)
European Region	Maternal sepsis and other maternal infections	10 to 14	Female	2 (1 to 4)	0.0 (0.0 to 0.0)	1 (1 to 2)	0.0 (0.0 to 0.0)	0 (0 to 0)	0.0 (0.0 to 0.0)	-85.5 (-93.6 to -72.8)	-81.8 (-92.0 to -65.9)
European Region	Maternal hypertensive disorders	10 to 14	Both	3 (2 to 6)	0.0 (0.0 to 0.0)	2 (1 to 3)	0.0 (0.0 to 0.0)	1 (0 to 1)	0.0 (0.0 to 0.0)	-80.4 (-90.8 to -66.7)	-75.7 (-88.5 to -58.6)
European Region	Maternal hypertensive disorders	10 to 14	Female	3 (2 to 6)	0.0 (0.0 to 0.0)	2 (1 to 3)	0.0 (0.0 to 0.0)	1 (0 to 1)	0.0 (0.0 to 0.0)	-80.4 (-90.8 to -66.7)	-75.5 (-88.5 to -58.3)
European Region	Maternal obstructed labor and uterine rupture	10 to 14	Both	0 (0 to 1)	0.0 (0.0 to 0.0)	0 (0 to 0)	0.0 (0.0 to 0.0)	0 (0 to 0)	0.0 (0.0 to 0.0)	-69.6 (-81.3 to -58.3)	-62.3 (-76.8 to -48.2)
European Region	Maternal obstructed labor and uterine rupture	10 to 14	Female	0 (0 to 1)	0.0 (0.0 to 0.0)	0 (0 to 0)	0.0 (0.0 to 0.0)	0 (0 to 0)	0.0 (0.0 to 0.0)	-69.6 (-81.3 to -58.3)	-62.0 (-76.6 to -47.9)
European Region	Maternal abortion, miscarriage, and ectopic pregnancy	10 to 14	Both	5 (2 to 9)	0.0 (0.0 to 0.0)	2 (1 to 4)	0.0 (0.0 to 0.0)	1 (0 to 1)	0.0 (0.0 to 0.0)	-83.2 (-92.4 to -70.3)	-79.1 (-90.6 to -63.2)
European Region	Maternal abortion, miscarriage, and ectopic pregnancy	10 to 14	Female	5 (2 to 9)	0.0 (0.0 to 0.0)	2 (1 to 4)	0.0 (0.0 to 0.0)	1 (0 to 1)	0.0 (0.0 to 0.0)	-83.2 (-92.4 to -70.3)	-79.0 (-90.5 to -62.9)
European Region	Indirect maternal deaths	10 to 14	Both	3 (2 to 6)	0.0 (0.0 to 0.0)	2 (1 to 3)	0.0 (0.0 to 0.0)	1 (0 to 1)	0.0 (0.0 to 0.0)	-80.1 (-90.6 to -64.6)	-75.2 (-88.3 to -56.0)
European Region	Indirect maternal deaths	10 to 14	Female	3 (2 to 6)	0.0 (0.0 to 0.0)	2 (1 to 3)	0.0 (0.0 to 0.0)	1 (0 to 1)	0.0 (0.0 to 0.0)	-80.1 (-90.6 to -64.6)	-75.1 (-88.2 to -55.7)
European Region	Late maternal deaths	10 to 14	Both	0 (0 to 1)	0.0 (0.0 to 0.0)	0 (0 to 0)	0.0 (0.0 to 0.0)	0 (0 to 0)	0.0 (0.0 to 0.0)	-69.9 (-87.5 to -47.2)	-62.6 (-84.5 to -34.4)
European Region	Late maternal deaths	10 to 14	Female	0 (0 to 1)	0.0 (0.0 to 0.0)	0 (0 to 0)	0.0 (0.0 to 0.0)	0 (0 to 0)	0.0 (0.0 to 0.0)	-69.9 (-87.5 to -47.2)	-62.3 (-84.4 to -33.9)
European Region	Maternal deaths aggravated by HIV/AIDS	10 to 14	Both	0 (0 to 0)	0.0 (0.0 to 0.0)	0 (0 to 0)	0.0 (0.0 to 0.0)	0 (0 to 0)	0.0 (0.0 to 0.0)	3476.4 (1892.7 to 6916.8)	4343.0 (2375.5 to 8617.0)
European Region	Maternal deaths aggravated by HIV/AIDS	10 to 14	Female	0 (0 to 0)	0.0 (0.0 to 0.0)	0 (0 to 0)	0.0 (0.0 to 0.0)	0 (0 to 0)	0.0 (0.0 to 0.0)	3476.4 (1892.7 to 6916.8)	4373.0 (2392.2 to 8675.9)
European Region	Other maternal disorders	10 to 14	Both	5 (3 to 8)	0.0 (0.0 to 0.0)	3 (2 to 4)	0.0 (0.0 to 0.0)	1 (1 to 1)	0.0 (0.0 to 0.0)	-76.0 (-86.2 to -64.4)	-70.2 (-82.8 to -55.8)

eTable 8. All-cause and cause-specific mortality (counts and rates) at the global level and by country in the WHO European Region for ages 5 to 9 and 10 to 14 for 1990, 2000, and 2016

European Region	Other maternal disorders	10 to 14	Female	5 (3 to 8)	0.0 (0.0 to 0.0)	3 (2 to 4)	0.0 (0.0 to 0.0)	1 (1 to 1)	0.0 (0.0 to 0.0)	-76.0 (-86.2 to -64.4)	-70.0 (-82.7 to -55.5)
European Region	Protein-energy malnutrition	5 to 9	Both	46 (32 to 67)	0.1 (0.0 to 0.1)	37 (26 to 50)	0.1 (0.0 to 0.1)	13 (9 to 19)	0.0 (0.0 to 0.0)	-70.6 (-83.8 to -51.6)	-65.4 (-81.0 to -43.1)
European Region	Protein-energy malnutrition	5 to 9	Female	21 (13 to 35)	0.1 (0.0 to 0.1)	16 (9 to 24)	0.1 (0.0 to 0.1)	5 (3 to 8)	0.0 (0.0 to 0.0)	-73.3 (-86.8 to -53.4)	-68.5 (-84.4 to -44.9)
European Region	Protein-energy malnutrition	5 to 9	Male	25 (15 to 43)	0.1 (0.0 to 0.1)	21 (13 to 32)	0.1 (0.0 to 0.1)	8 (5 to 12)	0.0 (0.0 to 0.0)	-66.4 (-85.1 to -34.7)	-60.8 (-82.6 to -23.8)
European Region	Protein-energy malnutrition	10 to 14	Both	35 (25 to 49)	0.1 (0.0 to 0.1)	31 (23 to 41)	0.0 (0.0 to 0.1)	10 (7 to 13)	0.0 (0.0 to 0.0)	-70.3 (-81.5 to -56.5)	-63.1 (-77.0 to -45.9)
European Region	Protein-energy malnutrition	10 to 14	Female	19 (11 to 31)	0.1 (0.0 to 0.1)	15 (10 to 23)	0.0 (0.0 to 0.1)	5 (3 to 7)	0.0 (0.0 to 0.0)	-73.1 (-86.4 to -51.8)	-66.3 (-83.0 to -39.7)
European Region	Protein-energy malnutrition	10 to 14	Male	16 (10 to 23)	0.0 (0.0 to 0.1)	16 (11 to 22)	0.0 (0.0 to 0.1)	5 (4 to 7)	0.0 (0.0 to 0.0)	-65.3 (-81.4 to -45.2)	-57.2 (-77.1 to -32.4)
European Region	Iodine deficiency	5 to 9	Both	4 (1 to 14)	0.0 (0.0 to 0.0)	4 (0 to 12)	0.0 (0.0 to 0.0)	1 (0 to 4)	0.0 (0.0 to 0.0)	-52.2 (-97.0 to 122.3)	-43.9 (-96.5 to 161.0)
European Region	Iodine deficiency	5 to 9	Female	2 (0 to 8)	0.0 (0.0 to 0.0)	2 (0 to 7)	0.0 (0.0 to 0.0)	0 (0 to 2)	0.0 (0.0 to 0.0)	-22.3 (-99.0 to 387.8)	-8.2 (-98.8 to 476.1)
European Region	Iodine deficiency	5 to 9	Male	2 (0 to 10)	0.0 (0.0 to 0.0)	2 (0 to 8)	0.0 (0.0 to 0.0)	1 (0 to 3)	0.0 (0.0 to 0.0)	-5.4 (-99.0 to 548.4)	10.5 (-98.9 to 656.7)
European Region	Iodine deficiency	10 to 14	Both	2 (0 to 5)	0.0 (0.0 to 0.0)	2 (0 to 6)	0.0 (0.0 to 0.0)	0 (0 to 2)	0.0 (0.0 to 0.0)	-37.8 (-97.6 to 179.7)	-22.8 (-97.0 to 247.5)
European Region	Iodine deficiency	10 to 14	Female	1 (0 to 3)	0.0 (0.0 to 0.0)	1 (0 to 3)	0.0 (0.0 to 0.0)	0 (0 to 1)	0.0 (0.0 to 0.0)	2.9 (-98.7 to 588.7)	28.7 (-98.4 to 761.4)
European Region	Iodine deficiency	10 to 14	Male	1 (0 to 4)	0.0 (0.0 to 0.0)	1 (0 to 4)	0.0 (0.0 to 0.0)	0 (0 to 1)	0.0 (0.0 to 0.0)	-7.7 (-99.0 to 537.4)	13.9 (-98.8 to 686.8)
European Region	Iron-deficiency anemia	5 to 9	Both	12 (9 to 15)	0.0 (0.0 to 0.0)	8 (6 to 12)	0.0 (0.0 to 0.0)	5 (4 to 6)	0.0 (0.0 to 0.0)	-60.5 (-70.9 to -45.2)	-53.6 (-65.8 to -35.6)
European Region	Iron-deficiency anemia	5 to 9	Female	6 (4 to 8)	0.0 (0.0 to 0.0)	4 (3 to 6)	0.0 (0.0 to 0.0)	2 (2 to 3)	0.0 (0.0 to 0.0)	-65.9 (-77.8 to -50.3)	-59.7 (-73.8 to -41.3)
European Region	Iron-deficiency anemia	5 to 9	Male	6 (4 to 8)	0.0 (0.0 to 0.0)	4 (3 to 7)	0.0 (0.0 to 0.0)	3 (2 to 4)	0.0 (0.0 to 0.0)	-53.4 (-70.8 to -25.8)	-45.6 (-65.9 to -13.4)
European Region	Iron-deficiency anemia	10 to 14	Both	10 (8 to 12)	0.0 (0.0 to 0.0)	9 (7 to 13)	0.0 (0.0 to 0.0)	4 (3 to 5)	0.0 (0.0 to 0.0)	-58.9 (-68.5 to -46.3)	-49.0 (-60.9 to -33.3)
European Region	Iron-deficiency anemia	10 to 14	Female	4 (3 to 6)	0.0 (0.0 to 0.0)	4 (3 to 5)	0.0 (0.0 to 0.0)	2 (1 to 2)	0.0 (0.0 to 0.0)	-60.4 (-72.7 to -44.9)	-50.5 (-65.9 to -31.1)
European Region	Iron-deficiency anemia	10 to 14	Male	5 (4 to 7)	0.0 (0.0 to 0.0)	5 (4 to 8)	0.0 (0.0 to 0.0)	2 (2 to 3)	0.0 (0.0 to 0.0)	-57.1 (-70.5 to -37.8)	-47.0 (-63.5 to -23.2)
European Region	Other nutritional deficiencies	5 to 9	Both	28 (21 to 40)	0.0 (0.0 to 0.1)	27 (21 to 37)	0.0 (0.0 to 0.1)	10 (8 to 13)	0.0 (0.0 to 0.0)	-62.0 (-74.6 to -47.3)	-55.4 (-70.2 to -38.1)
European Region	Other nutritional deficiencies	5 to 9	Female	14 (10 to 21)	0.0 (0.0 to 0.1)	13 (9 to 17)	0.0 (0.0 to 0.1)	4 (3 to 6)	0.0 (0.0 to 0.0)	-68.1 (-80.2 to -53.3)	-62.3 (-76.7 to -44.8)
European Region	Other nutritional deficiencies	5 to 9	Male	14 (10 to 21)	0.0 (0.0 to 0.1)	14 (9 to 20)	0.0 (0.0 to 0.1)	6 (4 to 8)	0.0 (0.0 to 0.0)	-54.6 (-76.1 to -28.6)	-47.1 (-72.1 to -16.7)
European Region	Other nutritional deficiencies	10 to 14	Both	20 (16 to 29)	0.0 (0.0 to 0.0)	24 (19 to 32)	0.0 (0.0 to 0.0)	9 (7 to 11)	0.0 (0.0 to 0.0)	-54.5 (-68.5 to -39.1)	-43.4 (-60.9 to -24.3)
European Region	Other nutritional deficiencies	10 to 14	Female	10 (7 to 16)	0.0 (0.0 to 0.0)	11 (9 to 15)	0.0 (0.0 to 0.0)	4 (3 to 6)	0.0 (0.0 to 0.0)	-57.5 (-73.7 to -37.2)	-46.9 (-67.1 to -21.5)

eTable 8. All-cause and cause-specific mortality (counts and rates) at the global level and by country in the WHO European Region for ages 5 to 9 and 10 to 14 for 1990, 2000, and 2016

European Region	Other nutritional deficiencies	10 to 14	Male	10 (8 to 15)	0.0 (0.0 to 0.0)	13 (9 to 18)	0.0 (0.0 to 0.1)	5 (4 to 6)	0.0 (0.0 to 0.0)	-50.6 (-70.2 to -30.8)	-39.0 (-63.3 to -14.6)
European Region	Sexually transmitted diseases excluding HIV	5 to 9	Both	60 (31 to 106)	0.1 (0.0 to 0.2)	70 (35 to 126)	0.1 (0.1 to 0.2)	11 (5 to 19)	0.0 (0.0 to 0.0)	-81.9 (-88.8 to -73.0)	-78.7 (-86.9 to -68.3)
European Region	Sexually transmitted diseases excluding HIV	5 to 9	Female	38 (19 to 66)	0.1 (0.1 to 0.2)	45 (22 to 80)	0.2 (0.1 to 0.3)	7 (3 to 12)	0.0 (0.0 to 0.0)	-81.7 (-88.7 to -72.6)	-78.4 (-86.7 to -67.6)
European Region	Sexually transmitted diseases excluding HIV	5 to 9	Male	22 (11 to 39)	0.1 (0.0 to 0.1)	25 (13 to 46)	0.1 (0.0 to 0.2)	4 (2 to 7)	0.0 (0.0 to 0.0)	-82.1 (-89.0 to -73.4)	-79.1 (-87.2 to -68.9)
European Region	Sexually transmitted diseases excluding HIV	10 to 14	Both	10 (7 to 11)	0.0 (0.0 to 0.0)	11 (8 to 14)	0.0 (0.0 to 0.0)	4 (3 to 5)	0.0 (0.0 to 0.0)	-59.6 (-68.2 to -39.5)	-49.8 (-60.5 to -24.9)
European Region	Sexually transmitted diseases excluding HIV	10 to 14	Female	5 (5 to 6)	0.0 (0.0 to 0.0)	6 (5 to 8)	0.0 (0.0 to 0.0)	3 (3 to 4)	0.0 (0.0 to 0.0)	-45.0 (-56.5 to -27.6)	-31.2 (-45.6 to -9.5)
European Region	Sexually transmitted diseases excluding HIV	10 to 14	Male	4 (2 to 6)	0.0 (0.0 to 0.0)	5 (3 to 7)	0.0 (0.0 to 0.0)	1 (1 to 2)	0.0 (0.0 to 0.0)	-77.0 (-86.0 to -52.5)	-71.6 (-82.8 to -41.3)
European Region	Hepatitis	5 to 9	Both	204 (176 to 242)	0.3 (0.3 to 0.4)	133 (114 to 156)	0.2 (0.2 to 0.3)	45 (35 to 56)	0.1 (0.1 to 0.1)	-78.0 (-83.9 to -71.0)	-74.1 (-81.1 to -66.0)
European Region	Hepatitis	5 to 9	Female	95 (77 to 119)	0.3 (0.2 to 0.4)	62 (50 to 76)	0.2 (0.2 to 0.3)	20 (15 to 27)	0.1 (0.1 to 0.1)	-78.7 (-85.4 to -69.7)	-74.8 (-82.8 to -64.2)
European Region	Hepatitis	5 to 9	Male	110 (89 to 135)	0.3 (0.3 to 0.4)	71 (58 to 89)	0.2 (0.2 to 0.3)	25 (18 to 33)	0.1 (0.1 to 0.1)	-77.1 (-84.6 to -67.4)	-73.3 (-82.1 to -61.9)
European Region	Hepatitis	10 to 14	Both	146 (125 to 172)	0.2 (0.2 to 0.3)	122 (104 to 145)	0.2 (0.2 to 0.2)	39 (31 to 50)	0.1 (0.1 to 0.1)	-73.2 (-80.2 to -64.4)	-66.8 (-75.4 to -55.7)
European Region	Hepatitis	10 to 14	Female	65 (53 to 80)	0.2 (0.2 to 0.3)	51 (41 to 62)	0.2 (0.1 to 0.2)	16 (12 to 23)	0.1 (0.0 to 0.1)	-74.5 (-82.4 to -63.5)	-68.1 (-78.0 to -54.3)
European Region	Hepatitis	10 to 14	Male	81 (64 to 103)	0.2 (0.2 to 0.3)	72 (56 to 92)	0.2 (0.2 to 0.3)	22 (17 to 31)	0.1 (0.1 to 0.1)	-71.9 (-81.6 to -59.4)	-65.4 (-77.3 to -49.9)
European Region	Other infectious diseases	5 to 9	Both	103 (80 to 128)	0.2 (0.1 to 0.2)	79 (61 to 100)	0.1 (0.1 to 0.2)	50 (39 to 62)	0.1 (0.1 to 0.1)	-50.5 (-63.1 to -36.5)	-41.9 (-56.6 to -25.5)
European Region	Other infectious diseases	5 to 9	Female	45 (23 to 63)	0.1 (0.1 to 0.2)	34 (19 to 48)	0.1 (0.1 to 0.2)	21 (12 to 29)	0.1 (0.0 to 0.1)	-50.5 (-68.0 to -29.7)	-41.6 (-62.1 to -17.0)
European Region	Other infectious diseases	5 to 9	Male	58 (49 to 75)	0.2 (0.1 to 0.2)	45 (37 to 60)	0.2 (0.1 to 0.2)	29 (24 to 37)	0.1 (0.1 to 0.1)	-49.5 (-63.6 to -31.1)	-41.1 (-57.5 to -19.6)
European Region	Other infectious diseases	10 to 14	Both	100 (67 to 133)	0.2 (0.1 to 0.2)	91 (57 to 126)	0.1 (0.1 to 0.2)	57 (31 to 82)	0.1 (0.1 to 0.2)	-42.6 (-60.8 to -15.7)	-28.7 (-51.3 to 4.7)
European Region	Other infectious diseases	10 to 14	Female	39 (20 to 59)	0.1 (0.1 to 0.2)	35 (18 to 52)	0.1 (0.1 to 0.2)	20 (10 to 29)	0.1 (0.0 to 0.1)	-47.1 (-64.6 to -21.8)	-33.9 (-55.8 to -2.2)
European Region	Other infectious diseases	10 to 14	Male	61 (45 to 91)	0.2 (0.1 to 0.3)	56 (36 to 86)	0.2 (0.1 to 0.3)	37 (20 to 60)	0.1 (0.1 to 0.2)	-38.4 (-63.1 to 5.2)	-23.9 (-54.5 to 29.9)
European Region	Nasopharynx cancer	5 to 9	Both	22 (18 to 28)	0.0 (0.0 to 0.0)	18 (15 to 22)	0.0 (0.0 to 0.0)	13 (11 to 15)	0.0 (0.0 to 0.0)	-42.9 (-57.4 to -27.5)	-33.0 (-50.0 to -14.9)

eTable 8. All-cause and cause-specific mortality (counts and rates) at the global level and by country in the WHO European Region for ages 5 to 9 and 10 to 14 for 1990, 2000, and 2016

European Region	Nasopharynx cancer	5 to 9	Female	6 (5 to 7)	0.0 (0.0 to 0.0)	5 (4 to 6)	0.0 (0.0 to 0.0)	4 (3 to 4)	0.0 (0.0 to 0.0)	-35.4 (-46.2 to -22.4)	-23.7 (-36.4 to -8.4)
European Region	Nasopharynx cancer	5 to 9	Male	17 (13 to 22)	0.0 (0.0 to 0.1)	13 (10 to 17)	0.0 (0.0 to 0.1)	9 (7 to 11)	0.0 (0.0 to 0.0)	-45.1 (-63.7 to -23.2)	-36.0 (-57.7 to -10.3)
European Region	Nasopharynx cancer	10 to 14	Both	32 (26 to 40)	0.0 (0.0 to 0.1)	32 (27 to 39)	0.1 (0.0 to 0.1)	19 (17 to 23)	0.0 (0.0 to 0.0)	-38.7 (-54.0 to -20.1)	-23.8 (-42.9 to -0.7)
European Region	Nasopharynx cancer	10 to 14	Female	6 (6 to 7)	0.0 (0.0 to 0.0)	7 (6 to 8)	0.0 (0.0 to 0.0)	4 (4 to 5)	0.0 (0.0 to 0.0)	-34.8 (-44.0 to -24.6)	-18.4 (-29.9 to -5.7)
European Region	Nasopharynx cancer	10 to 14	Male	25 (20 to 33)	0.1 (0.1 to 0.1)	25 (21 to 32)	0.1 (0.1 to 0.1)	15 (13 to 19)	0.1 (0.0 to 0.1)	-39.4 (-57.6 to -15.1)	-25.2 (-47.6 to 4.8)
European Region	Liver cancer	5 to 9	Both	54 (47 to 63)	0.1 (0.1 to 0.1)	39 (35 to 45)	0.1 (0.1 to 0.1)	28 (24 to 32)	0.1 (0.0 to 0.1)	-48.1 (-59.5 to -35.6)	-39.0 (-52.5 to -24.4)
European Region	Liver cancer	5 to 9	Female	20 (16 to 25)	0.1 (0.1 to 0.1)	16 (13 to 20)	0.1 (0.0 to 0.1)	10 (9 to 13)	0.0 (0.0 to 0.0)	-46.2 (-61.1 to -27.6)	-36.4 (-54.0 to -14.5)
European Region	Liver cancer	5 to 9	Male	34 (28 to 43)	0.1 (0.1 to 0.1)	23 (20 to 27)	0.1 (0.1 to 0.1)	17 (14 to 21)	0.1 (0.1 to 0.1)	-48.5 (-63.7 to -32.9)	-39.9 (-57.7 to -21.7)
European Region	Liver cancer	10 to 14	Both	49 (44 to 55)	0.1 (0.1 to 0.1)	49 (45 to 55)	0.1 (0.1 to 0.1)	31 (27 to 36)	0.1 (0.1 to 0.1)	-36.5 (-47.2 to -24.7)	-21.1 (-34.3 to -6.4)
European Region	Liver cancer	10 to 14	Female	17 (14 to 21)	0.1 (0.0 to 0.1)	18 (15 to 22)	0.1 (0.0 to 0.1)	11 (9 to 13)	0.0 (0.0 to 0.1)	-37.5 (-53.1 to -19.6)	-21.8 (-41.4 to 0.5)
European Region	Liver cancer	10 to 14	Male	32 (28 to 38)	0.1 (0.1 to 0.1)	31 (27 to 36)	0.1 (0.1 to 0.1)	21 (17 to 24)	0.1 (0.1 to 0.1)	-35.6 (-49.7 to -20.6)	-20.5 (-37.9 to -2.0)
European Region	Kidney cancer	5 to 9	Both	133 (115 to 161)	0.2 (0.2 to 0.2)	108 (94 to 126)	0.2 (0.2 to 0.2)	84 (72 to 97)	0.2 (0.1 to 0.2)	-35.9 (-52.8 to -21.0)	-24.7 (-44.6 to -7.2)
European Region	Kidney cancer	5 to 9	Female	67 (55 to 83)	0.2 (0.2 to 0.3)	53 (45 to 64)	0.2 (0.2 to 0.2)	41 (35 to 49)	0.2 (0.1 to 0.2)	-37.1 (-54.7 to -18.9)	-25.7 (-46.5 to -4.2)
European Region	Kidney cancer	5 to 9	Male	66 (55 to 82)	0.2 (0.2 to 0.2)	55 (46 to 67)	0.2 (0.2 to 0.2)	43 (34 to 53)	0.2 (0.1 to 0.2)	-34.3 (-53.9 to -13.4)	-23.3 (-46.2 to 1.1)
European Region	Kidney cancer	10 to 14	Both	46 (41 to 53)	0.1 (0.1 to 0.1)	47 (42 to 53)	0.1 (0.1 to 0.1)	34 (30 to 38)	0.1 (0.1 to 0.1)	-26.3 (-39.8 to -12.3)	-8.4 (-25.3 to 9.0)
European Region	Kidney cancer	10 to 14	Female	25 (22 to 29)	0.1 (0.1 to 0.1)	25 (22 to 28)	0.1 (0.1 to 0.1)	18 (15 to 21)	0.1 (0.1 to 0.1)	-28.4 (-43.4 to -10.2)	-10.5 (-29.2 to 12.3)
European Region	Kidney cancer	10 to 14	Male	21 (18 to 25)	0.1 (0.1 to 0.1)	22 (19 to 26)	0.1 (0.1 to 0.1)	16 (13 to 19)	0.1 (0.1 to 0.1)	-23.3 (-39.8 to -5.9)	-5.4 (-25.6 to 16.1)
European Region	Brain and nervous system cancer	5 to 9	Both	1117 (966 to 1260)	1.7 (1.5 to 1.9)	909 (829 to 1010)	1.6 (1.5 to 1.8)	697 (574 to 808)	1.3 (1.0 to 1.5)	-37.3 (-52.3 to -22.2)	-26.3 (-44.0 to -8.7)
European Region	Brain and nervous system cancer	5 to 9	Female	492 (429 to 571)	1.5 (1.4 to 1.8)	411 (357 to 478)	1.5 (1.3 to 1.7)	318 (253 to 379)	1.2 (0.9 to 1.4)	-34.9 (-55.3 to -22.0)	-23.2 (-47.2 to -7.9)
European Region	Brain and nervous system cancer	5 to 9	Male	624 (510 to 724)	1.9 (1.5 to 2.2)	498 (434 to 576)	1.7 (1.5 to 2.0)	378 (284 to 456)	1.3 (1.0 to 1.6)	-38.8 (-57.7 to -15.6)	-28.5 (-50.6 to -1.5)
European Region	Brain and nervous system cancer	10 to 14	Both	836 (761 to 924)	1.3 (1.2 to 1.5)	837 (768 to 922)	1.3 (1.2 to 1.4)	538 (458 to 619)	1.0 (0.9 to 1.2)	-35.5 (-46.7 to -23.9)	-19.9 (-33.8 to -5.5)
European Region	Brain and nervous system cancer	10 to 14	Female	369 (326 to 423)	1.2 (1.0 to 1.4)	374 (331 to 423)	1.2 (1.1 to 1.3)	239 (197 to 283)	1.0 (0.8 to 1.1)	-34.9 (-51.3 to -22.8)	-18.6 (-39.1 to -3.5)
European Region	Brain and nervous system cancer	10 to 14	Male	467 (400 to 531)	1.4 (1.2 to 1.6)	463 (408 to 526)	1.4 (1.2 to 1.6)	298 (245 to 355)	1.1 (0.9 to 1.4)	-35.7 (-51.8 to -18.1)	-20.7 (-40.5 to 1.1)
European Region	Thyroid cancer	10 to 14	Both	9 (8 to 10)	0.0 (0.0 to 0.0)	9 (8 to 10)	0.0 (0.0 to 0.0)	6 (5 to 7)	0.0 (0.0 to 0.0)	-33.7 (-40.8 to -26.0)	-17.7 (-26.5 to -8.1)
European Region	Thyroid cancer	10 to 14	Female	4 (4 to 4)	0.0 (0.0 to 0.0)	4 (4 to 4)	0.0 (0.0 to 0.0)	3 (3 to 3)	0.0 (0.0 to 0.0)	-29.4 (-37.5 to -20.6)	-11.6 (-21.8 to -0.7)
European Region	Thyroid cancer	10 to 14	Male	5 (5 to 6)	0.0 (0.0 to 0.0)	5 (5 to 6)	0.0 (0.0 to 0.0)	3 (3 to 4)	0.0 (0.0 to 0.0)	-36.9 (-46.2 to -25.5)	-22.1 (-33.6 to -8.1)
European Region	Hodgkin lymphoma	5 to 9	Both	69 (45 to 88)	0.1 (0.1 to 0.1)	45 (34 to 59)	0.1 (0.1 to 0.1)	19 (14 to 24)	0.0 (0.0 to 0.0)	-72.0 (-79.9 to -62.3)	-67.1 (-76.5 to -55.8)
European Region	Hodgkin lymphoma	5 to 9	Female	13 (9 to 18)	0.0 (0.0 to 0.1)	10 (7 to 14)	0.0 (0.0 to 0.1)	5 (4 to 7)	0.0 (0.0 to 0.0)	-60.0 (-73.3 to -41.8)	-52.7 (-68.4 to -31.3)
European Region	Hodgkin lymphoma	5 to 9	Male	56 (34 to 74)	0.2 (0.1 to 0.2)	36 (26 to 48)	0.1 (0.1 to 0.2)	14 (10 to 19)	0.0 (0.0 to 0.1)	-74.5 (-83.0 to -63.1)	-70.3 (-80.2 to -56.9)

eTable 8. All-cause and cause-specific mortality (counts and rates) at the global level and by country in the WHO European Region for ages 5 to 9 and 10 to 14 for 1990, 2000, and 2016

European Region	Hodgkin lymphoma	10 to 14	Both	102 (84 to 120)	0.2 (0.1 to 0.2)	84 (72 to 98)	0.1 (0.1 to 0.2)	30 (26 to 35)	0.1 (0.0 to 0.1)	-70.3 (-76.1 to -64.1)	-63.1 (-70.3 to -55.4)
European Region	Hodgkin lymphoma	10 to 14	Female	34 (24 to 45)	0.1 (0.1 to 0.1)	26 (20 to 35)	0.1 (0.1 to 0.1)	9 (7 to 12)	0.0 (0.0 to 0.0)	-71.8 (-80.8 to -60.6)	-64.7 (-76.0 to -50.7)
European Region	Hodgkin lymphoma	10 to 14	Male	68 (55 to 81)	0.2 (0.2 to 0.2)	57 (49 to 69)	0.2 (0.1 to 0.2)	21 (17 to 25)	0.1 (0.1 to 0.1)	-69.3 (-76.0 to -61.9)	-62.1 (-70.4 to -52.9)
European Region	Non-Hodgkin lymphoma	5 to 9	Both	350 (253 to 406)	0.5 (0.4 to 0.6)	237 (203 to 270)	0.4 (0.4 to 0.5)	160 (132 to 189)	0.3 (0.2 to 0.3)	-53.5 (-64.6 to -32.5)	-45.5 (-58.5 to -20.8)
European Region	Non-Hodgkin lymphoma	5 to 9	Female	96 (79 to 117)	0.3 (0.2 to 0.4)	68 (56 to 83)	0.2 (0.2 to 0.3)	51 (40 to 62)	0.2 (0.2 to 0.2)	-46.8 (-61.0 to -28.0)	-37.2 (-54.0 to -14.9)
European Region	Non-Hodgkin lymphoma	5 to 9	Male	253 (156 to 303)	0.8 (0.5 to 0.9)	169 (134 to 200)	0.6 (0.5 to 0.7)	110 (87 to 137)	0.4 (0.3 to 0.5)	-55.4 (-67.7 to -22.2)	-48.0 (-62.4 to -9.2)
European Region	Non-Hodgkin lymphoma	10 to 14	Both	302 (228 to 342)	0.5 (0.4 to 0.5)	270 (233 to 304)	0.4 (0.4 to 0.5)	156 (133 to 181)	0.3 (0.3 to 0.4)	-47.9 (-57.7 to -31.5)	-35.3 (-47.4 to -14.9)
European Region	Non-Hodgkin lymphoma	10 to 14	Female	89 (72 to 106)	0.3 (0.2 to 0.3)	87 (72 to 103)	0.3 (0.2 to 0.3)	53 (42 to 64)	0.2 (0.2 to 0.3)	-39.7 (-53.0 to -23.3)	-24.6 (-41.2 to -4.0)
European Region	Non-Hodgkin lymphoma	10 to 14	Male	213 (142 to 252)	0.7 (0.4 to 0.8)	184 (148 to 213)	0.6 (0.4 to 0.6)	103 (83 to 127)	0.4 (0.3 to 0.5)	-51.0 (-62.5 to -25.7)	-39.5 (-53.7 to -8.3)
European Region	Leukemia	5 to 9	Both	1983 (1814 to 2203)	3.1 (2.8 to 3.4)	1356 (1236 to 1510)	2.4 (2.2 to 2.7)	743 (612 to 866)	1.3 (1.1 to 1.6)	-62.4 (-69.2 to -54.9)	-55.9 (-63.9 to -47.0)
European Region	Leukemia	5 to 9	Female	842 (733 to 972)	2.6 (2.3 to 3.1)	582 (488 to 674)	2.1 (1.8 to 2.4)	291 (242 to 351)	1.1 (0.9 to 1.3)	-65.2 (-72.0 to -55.8)	-58.9 (-66.9 to -47.8)
European Region	Leukemia	5 to 9	Male	1141 (1015 to 1320)	3.5 (3.1 to 4.0)	773 (682 to 898)	2.7 (2.4 to 3.1)	452 (352 to 545)	1.6 (1.2 to 1.9)	-60.1 (-70.1 to -49.8)	-53.5 (-65.1 to -41.4)
European Region	Leukemia	10 to 14	Both	1597 (1468 to 1745)	2.5 (2.3 to 2.7)	1392 (1283 to 1519)	2.2 (2.0 to 2.4)	678 (584 to 765)	1.3 (1.1 to 1.5)	-57.4 (-63.6 to -50.6)	-47.1 (-54.7 to -38.6)
European Region	Leukemia	10 to 14	Female	686 (597 to 786)	2.2 (1.9 to 2.5)	592 (523 to 667)	1.9 (1.7 to 2.1)	261 (222 to 302)	1.0 (0.9 to 1.2)	-61.7 (-68.7 to -53.5)	-52.2 (-60.9 to -41.9)
European Region	Leukemia	10 to 14	Male	911 (821 to 1033)	2.8 (2.5 to 3.2)	800 (717 to 916)	2.4 (2.2 to 2.8)	417 (347 to 489)	1.6 (1.3 to 1.9)	-54.0 (-63.1 to -43.9)	-43.3 (-54.4 to -30.8)
European Region	Other neoplasms	5 to 9	Both	664 (592 to 803)	1.0 (0.9 to 1.2)	548 (493 to 656)	1.0 (0.9 to 1.2)	417 (349 to 475)	0.8 (0.6 to 0.9)	-36.7 (-53.9 to -23.7)	-25.7 (-45.8 to -10.4)
European Region	Other neoplasms	5 to 9	Female	298 (258 to 373)	0.9 (0.8 to 1.2)	245 (215 to 298)	0.9 (0.8 to 1.1)	187 (156 to 220)	0.7 (0.6 to 0.8)	-36.5 (-54.6 to -20.8)	-25.0 (-46.4 to -6.5)
European Region	Other neoplasms	5 to 9	Male	366 (315 to 453)	1.1 (1.0 to 1.4)	303 (266 to 377)	1.1 (0.9 to 1.3)	229 (183 to 272)	0.8 (0.6 to 1.0)	-36.6 (-56.0 to -19.5)	-26.0 (-48.7 to -6.0)
European Region	Other neoplasms	10 to 14	Both	725 (655 to 832)	1.1 (1.0 to 1.3)	714 (644 to 830)	1.1 (1.0 to 1.3)	451 (392 to 504)	0.9 (0.8 to 1.0)	-37.6 (-49.8 to -27.3)	-22.5 (-37.7 to -9.6)
European Region	Other neoplasms	10 to 14	Female	343 (300 to 404)	1.1 (1.0 to 1.3)	338 (297 to 398)	1.1 (0.9 to 1.3)	214 (182 to 249)	0.9 (0.7 to 1.0)	-37.1 (-51.3 to -23.0)	-21.4 (-39.1 to -3.7)
European Region	Other neoplasms	10 to 14	Male	382 (335 to 449)	1.2 (1.0 to 1.4)	376 (331 to 448)	1.1 (1.0 to 1.4)	236 (199 to 274)	0.9 (0.8 to 1.0)	-37.8 (-51.5 to -24.9)	-23.2 (-40.2 to -7.3)
European Region	Rheumatic heart disease	5 to 9	Both	94 (83 to 109)	0.1 (0.1 to 0.2)	90 (80 to 105)	0.2 (0.1 to 0.2)	31 (26 to 38)	0.1 (0.0 to 0.1)	-66.4 (-73.9 to -59.0)	-60.6 (-69.3 to -51.9)
European Region	Rheumatic heart disease	5 to 9	Female	42 (37 to 48)	0.1 (0.1 to 0.2)	41 (35 to 48)	0.1 (0.1 to 0.2)	15 (12 to 19)	0.1 (0.0 to 0.1)	-63.6 (-70.9 to -54.4)	-57.0 (-65.6 to -46.1)
European Region	Rheumatic heart disease	5 to 9	Male	52 (43 to 65)	0.2 (0.1 to 0.2)	50 (42 to 61)	0.2 (0.1 to 0.2)	16 (13 to 21)	0.1 (0.0 to 0.1)	-68.5 (-77.9 to -59.0)	-63.3 (-74.2 to -52.1)
European Region	Rheumatic heart disease	10 to 14	Both	163 (148 to 179)	0.3 (0.2 to 0.3)	174 (159 to 190)	0.3 (0.2 to 0.3)	59 (52 to 67)	0.1 (0.1 to 0.1)	-63.7 (-69.4 to -57.3)	-54.9 (-61.9 to -47.0)
European Region	Rheumatic heart disease	10 to 14	Female	81 (72 to 91)	0.3 (0.2 to 0.3)	87 (78 to 98)	0.3 (0.2 to 0.3)	31 (27 to 36)	0.1 (0.1 to 0.1)	-61.1 (-67.8 to -53.2)	-51.4 (-59.7 to -41.5)
European Region	Rheumatic heart disease	10 to 14	Male	82 (71 to 96)	0.3 (0.2 to 0.3)	87 (77 to 98)	0.3 (0.2 to 0.3)	28 (23 to 33)	0.1 (0.1 to 0.1)	-66.1 (-73.6 to -58.4)	-58.2 (-67.5 to -48.6)
European Region	Ischemic heart disease	5 to 9	Both	21 (19 to 24)	0.0 (0.0 to 0.0)	25 (21 to 30)	0.0 (0.0 to 0.1)	13 (10 to 15)	0.0 (0.0 to 0.0)	-40.9 (-53.3 to -26.2)	-30.6 (-45.2 to -13.4)
European Region	Ischemic heart disease	5 to 9	Female	10 (9 to 11)	0.0 (0.0 to 0.0)	11 (9 to 14)	0.0 (0.0 to 0.1)	6 (5 to 7)	0.0 (0.0 to 0.0)	-42.0 (-54.8 to -26.5)	-31.5 (-46.6 to -13.2)
European Region	Ischemic heart disease	5 to 9	Male	12 (10 to 14)	0.0 (0.0 to 0.0)	14 (11 to 18)	0.0 (0.0 to 0.1)	7 (5 to 9)	0.0 (0.0 to 0.0)	-39.6 (-55.9 to -17.5)	-29.5 (-48.6 to -3.7)
European Region	Ischemic heart disease	10 to 14	Both	34 (30 to 39)	0.1 (0.0 to 0.1)	36 (32 to 41)	0.1 (0.0 to 0.1)	19 (16 to 23)	0.0 (0.0 to 0.0)	-43.4 (-55.1 to -30.4)	-29.7 (-44.2 to -13.5)
European Region	Ischemic heart disease	10 to 14	Female	13 (11 to 16)	0.0 (0.0 to 0.1)	15 (13 to 18)	0.0 (0.0 to 0.1)	8 (7 to 10)	0.0 (0.0 to 0.0)	-37.8 (-51.7 to -22.4)	-22.2 (-39.7 to -3.0)

eTable 8. All-cause and cause-specific mortality (counts and rates) at the global level and by country in the WHO European Region for ages 5 to 9 and 10 to 14 for 1990, 2000, and 2016

European Region	Ischemic heart disease	10 to 14	Male	20 (17 to 25)	0.1 (0.1 to 0.1)	20 (17 to 25)	0.1 (0.1 to 0.1)	11 (8 to 14)	0.0 (0.0 to 0.1)	-46.8 (-62.5 to -27.7)	-34.4 (-53.7 to -10.8)
European Region	Cerebrovascular disease	5 to 9	Both	259 (226 to 300)	0.4 (0.3 to 0.5)	188 (166 to 217)	0.3 (0.3 to 0.4)	86 (70 to 109)	0.2 (0.1 to 0.2)	-66.4 (-75.1 to -56.7)	-60.6 (-70.7 to -49.2)
European Region	Cerebrovascular disease	5 to 9	Female	126 (109 to 149)	0.4 (0.3 to 0.5)	89 (76 to 104)	0.3 (0.3 to 0.4)	42 (34 to 54)	0.2 (0.1 to 0.2)	-66.4 (-75.1 to -56.3)	-60.3 (-70.6 to -48.4)
European Region	Cerebrovascular disease	5 to 9	Male	133 (110 to 165)	0.4 (0.3 to 0.5)	100 (83 to 123)	0.3 (0.3 to 0.4)	44 (34 to 60)	0.2 (0.1 to 0.2)	-66.3 (-76.9 to -52.6)	-60.7 (-73.1 to -44.7)
European Region	Cerebrovascular disease	10 to 14	Both	372 (336 to 421)	0.6 (0.5 to 0.7)	284 (257 to 320)	0.4 (0.4 to 0.5)	121 (103 to 141)	0.2 (0.2 to 0.3)	-67.3 (-73.4 to -61.1)	-59.4 (-66.9 to -51.6)
European Region	Cerebrovascular disease	10 to 14	Female	176 (154 to 204)	0.6 (0.5 to 0.7)	129 (113 to 148)	0.4 (0.4 to 0.5)	53 (45 to 62)	0.2 (0.2 to 0.2)	-69.8 (-75.8 to -62.9)	-62.3 (-69.7 to -53.6)
European Region	Cerebrovascular disease	10 to 14	Male	196 (173 to 227)	0.6 (0.5 to 0.7)	155 (135 to 184)	0.5 (0.4 to 0.6)	68 (56 to 84)	0.3 (0.2 to 0.3)	-64.9 (-73.3 to -55.2)	-56.7 (-67.0 to -44.7)
European Region	Hypertensive heart disease	5 to 9	Both	6 (5 to 7)	0.0 (0.0 to 0.0)	6 (5 to 6)	0.0 (0.0 to 0.0)	5 (4 to 6)	0.0 (0.0 to 0.0)	-16.0 (-32.3 to 1.0)	-1.4 (-20.5 to 18.5)
European Region	Hypertensive heart disease	5 to 9	Female	3 (2 to 4)	0.0 (0.0 to 0.0)	3 (2 to 3)	0.0 (0.0 to 0.0)	3 (2 to 3)	0.0 (0.0 to 0.0)	-15.1 (-34.2 to 9.9)	0.3 (-22.3 to 29.8)
European Region	Hypertensive heart disease	5 to 9	Male	3 (3 to 3)	0.0 (0.0 to 0.0)	3 (3 to 3)	0.0 (0.0 to 0.0)	3 (2 to 3)	0.0 (0.0 to 0.0)	-16.8 (-34.6 to 0.2)	-2.9 (-23.6 to 17.0)
European Region	Hypertensive heart disease	10 to 14	Both	7 (6 to 7)	0.0 (0.0 to 0.0)	7 (6 to 8)	0.0 (0.0 to 0.0)	5 (4 to 6)	0.0 (0.0 to 0.0)	-21.2 (-33.1 to -9.0)	-2.1 (-16.9 to 13.1)
European Region	Hypertensive heart disease	10 to 14	Female	3 (3 to 4)	0.0 (0.0 to 0.0)	3 (3 to 4)	0.0 (0.0 to 0.0)	3 (2 to 3)	0.0 (0.0 to 0.0)	-24.0 (-37.8 to -8.2)	-5.0 (-22.3 to 14.9)
European Region	Hypertensive heart disease	10 to 14	Male	3 (3 to 4)	0.0 (0.0 to 0.0)	3 (3 to 4)	0.0 (0.0 to 0.0)	3 (2 to 3)	0.0 (0.0 to 0.0)	-17.8 (-33.4 to 0.2)	1.4 (-17.8 to 23.7)
European Region	Cardiomyopathy and myocarditis	5 to 9	Both	157 (132 to 189)	0.2 (0.2 to 0.3)	144 (126 to 162)	0.3 (0.2 to 0.3)	86 (74 to 103)	0.2 (0.1 to 0.2)	-44.5 (-55.6 to -28.7)	-34.9 (-47.9 to -16.3)
European Region	Cardiomyopathy and myocarditis	5 to 9	Female	75 (62 to 95)	0.2 (0.2 to 0.3)	67 (58 to 79)	0.2 (0.2 to 0.3)	38 (32 to 50)	0.1 (0.1 to 0.2)	-48.6 (-59.1 to -31.3)	-39.3 (-51.7 to -18.9)
European Region	Cardiomyopathy and myocarditis	5 to 9	Male	82 (65 to 101)	0.2 (0.2 to 0.3)	78 (61 to 91)	0.3 (0.2 to 0.3)	48 (40 to 59)	0.2 (0.1 to 0.2)	-40.6 (-55.9 to -19.5)	-30.6 (-48.5 to -6.1)
European Region	Cardiomyopathy and myocarditis	10 to 14	Both	209 (180 to 252)	0.3 (0.3 to 0.4)	219 (193 to 253)	0.3 (0.3 to 0.4)	127 (111 to 149)	0.2 (0.2 to 0.3)	-38.9 (-49.6 to -26.3)	-24.2 (-37.4 to -8.5)
European Region	Cardiomyopathy and myocarditis	10 to 14	Female	89 (75 to 112)	0.3 (0.2 to 0.4)	89 (77 to 111)	0.3 (0.2 to 0.4)	50 (41 to 66)	0.2 (0.2 to 0.3)	-43.4 (-54.4 to -28.9)	-29.3 (-42.9 to -11.1)
European Region	Cardiomyopathy and myocarditis	10 to 14	Male	120 (100 to 147)	0.4 (0.3 to 0.5)	129 (108 to 154)	0.4 (0.3 to 0.5)	77 (64 to 92)	0.3 (0.2 to 0.4)	-35.5 (-50.4 to -20.4)	-20.3 (-38.8 to -1.7)
European Region	Atrial fibrillation and flutter	5 to 9	Both	0 (0 to 0)	0.0 (0.0 to 0.0)	0 (0 to 0)	0.0 (0.0 to 0.0)	0 (0 to 0)	0.0 (0.0 to 0.0)	-	-
European Region	Atrial fibrillation and flutter	5 to 9	Female	0 (0 to 0)	0.0 (0.0 to 0.0)	0 (0 to 0)	0.0 (0.0 to 0.0)	0 (0 to 0)	0.0 (0.0 to 0.0)	-	-
European Region	Atrial fibrillation and flutter	5 to 9	Male	0 (0 to 0)	0.0 (0.0 to 0.0)	0 (0 to 0)	0.0 (0.0 to 0.0)	0 (0 to 0)	0.0 (0.0 to 0.0)	-	-
European Region	Atrial fibrillation and flutter	10 to 14	Both	0 (0 to 0)	0.0 (0.0 to 0.0)	0 (0 to 0)	0.0 (0.0 to 0.0)	0 (0 to 0)	0.0 (0.0 to 0.0)	-	-
European Region	Atrial fibrillation and flutter	10 to 14	Female	0 (0 to 0)	0.0 (0.0 to 0.0)	0 (0 to 0)	0.0 (0.0 to 0.0)	0 (0 to 0)	0.0 (0.0 to 0.0)	-	-
European Region	Atrial fibrillation and flutter	10 to 14	Male	0 (0 to 0)	0.0 (0.0 to 0.0)	0 (0 to 0)	0.0 (0.0 to 0.0)	0 (0 to 0)	0.0 (0.0 to 0.0)	-	-
European Region	Endocarditis	5 to 9	Both	19 (14 to 24)	0.0 (0.0 to 0.0)	18 (13 to 22)	0.0 (0.0 to 0.0)	17 (13 to 22)	0.0 (0.0 to 0.0)	-10.4 (-37.6 to 18.8)	5.1 (-26.8 to 39.4)

eTable 8. All-cause and cause-specific mortality (counts and rates) at the global level and by country in the WHO European Region for ages 5 to 9 and 10 to 14 for 1990, 2000, and 2016

European Region	Endocarditis	5 to 9	Female	9 (6 to 13)	0.0 (0.0 to 0.0)	8 (6 to 12)	0.0 (0.0 to 0.0)	8 (5 to 11)	0.0 (0.0 to 0.0)	-17.3 (-44.2 to 20.1)	-2.3 (-34.1 to 41.9)
European Region	Endocarditis	5 to 9	Male	10 (6 to 13)	0.0 (0.0 to 0.0)	9 (6 to 13)	0.0 (0.0 to 0.0)	9 (6 to 13)	0.0 (0.0 to 0.0)	-2.5 (-37.6 to 40.9)	13.7 (-27.2 to 64.5)
European Region	Endocarditis	10 to 14	Both	22 (17 to 27)	0.0 (0.0 to 0.0)	23 (19 to 30)	0.0 (0.0 to 0.0)	19 (15 to 25)	0.0 (0.0 to 0.0)	-13.7 (-34.3 to 8.3)	7.2 (-18.4 to 34.5)
European Region	Endocarditis	10 to 14	Female	10 (7 to 15)	0.0 (0.0 to 0.0)	10 (7 to 15)	0.0 (0.0 to 0.0)	8 (6 to 12)	0.0 (0.0 to 0.0)	-21.6 (-45.3 to 5.5)	-2.0 (-31.6 to 31.9)
European Region	Endocarditis	10 to 14	Male	12 (8 to 15)	0.0 (0.0 to 0.0)	13 (9 to 17)	0.0 (0.0 to 0.1)	11 (8 to 15)	0.0 (0.0 to 0.1)	-5.7 (-31.9 to 26.4)	16.3 (-16.0 to 56.0)
European Region	Other cardiovascular and circulatory diseases	5 to 9	Both	81 (67 to 102)	0.1 (0.1 to 0.2)	76 (65 to 90)	0.1 (0.1 to 0.2)	55 (44 to 69)	0.1 (0.1 to 0.1)	-31.6 (-48.9 to -11.1)	-19.7 (-40.0 to 4.4)
European Region	Other cardiovascular and circulatory diseases	5 to 9	Female	33 (26 to 44)	0.1 (0.1 to 0.1)	31 (25 to 39)	0.1 (0.1 to 0.1)	26 (19 to 34)	0.1 (0.1 to 0.1)	-22.3 (-47.3 to 8.1)	-8.3 (-37.8 to 27.7)
European Region	Other cardiovascular and circulatory diseases	5 to 9	Male	48 (37 to 65)	0.1 (0.1 to 0.2)	45 (35 to 58)	0.2 (0.1 to 0.2)	30 (22 to 39)	0.1 (0.1 to 0.1)	-37.0 (-58.0 to -10.7)	-26.5 (-51.0 to 4.2)
European Region	Other cardiovascular and circulatory diseases	10 to 14	Both	102 (84 to 124)	0.2 (0.1 to 0.2)	108 (92 to 125)	0.2 (0.1 to 0.2)	67 (56 to 80)	0.1 (0.1 to 0.2)	-33.2 (-47.9 to -14.9)	-17.0 (-35.3 to 5.7)
European Region	Other cardiovascular and circulatory diseases	10 to 14	Female	44 (34 to 60)	0.1 (0.1 to 0.2)	46 (37 to 57)	0.1 (0.1 to 0.2)	31 (24 to 39)	0.1 (0.1 to 0.2)	-28.9 (-51.1 to -2.9)	-11.1 (-38.8 to 21.5)
European Region	Other cardiovascular and circulatory diseases	10 to 14	Male	58 (45 to 75)	0.2 (0.1 to 0.2)	62 (49 to 77)	0.2 (0.2 to 0.2)	37 (28 to 46)	0.1 (0.1 to 0.2)	-35.5 (-53.5 to -12.7)	-20.4 (-42.6 to 7.7)
European Region	Chronic obstructive pulmonary disease	5 to 9	Both	73 (60 to 96)	0.1 (0.1 to 0.1)	59 (48 to 75)	0.1 (0.1 to 0.1)	19 (16 to 24)	0.0 (0.0 to 0.0)	-73.5 (-79.9 to -67.0)	-68.9 (-76.4 to -61.3)
European Region	Chronic obstructive pulmonary disease	5 to 9	Female	31 (24 to 41)	0.1 (0.1 to 0.1)	25 (19 to 32)	0.1 (0.1 to 0.1)	9 (7 to 11)	0.0 (0.0 to 0.0)	-70.5 (-79.1 to -61.5)	-65.2 (-75.3 to -54.6)
European Region	Chronic obstructive pulmonary disease	5 to 9	Male	42 (31 to 61)	0.1 (0.1 to 0.2)	35 (26 to 48)	0.1 (0.1 to 0.2)	10 (8 to 14)	0.0 (0.0 to 0.0)	-75.2 (-84.1 to -65.8)	-71.1 (-81.4 to -60.1)
European Region	Chronic obstructive pulmonary disease	10 to 14	Both	79 (66 to 104)	0.1 (0.1 to 0.2)	76 (65 to 93)	0.1 (0.1 to 0.1)	28 (24 to 34)	0.1 (0.0 to 0.1)	-64.3 (-72.5 to -56.1)	-55.7 (-65.8 to -45.5)
European Region	Chronic obstructive pulmonary disease	10 to 14	Female	34 (27 to 44)	0.1 (0.1 to 0.1)	32 (26 to 39)	0.1 (0.1 to 0.1)	13 (11 to 16)	0.1 (0.0 to 0.1)	-61.4 (-70.7 to -51.1)	-51.8 (-63.4 to -38.8)
European Region	Chronic obstructive pulmonary disease	10 to 14	Male	46 (35 to 67)	0.1 (0.1 to 0.2)	45 (36 to 61)	0.1 (0.1 to 0.2)	15 (12 to 19)	0.1 (0.0 to 0.1)	-66.0 (-77.7 to -54.3)	-58.0 (-72.5 to -43.6)

eTable 8. All-cause and cause-specific mortality (counts and rates) at the global level and by country in the WHO European Region for ages 5 to 9 and 10 to 14 for 1990, 2000, and 2016

European Region	Asthma	5 to 9	Both	114 (82 to 158)	0.2 (0.1 to 0.2)	71 (53 to 96)	0.1 (0.1 to 0.2)	21 (17 to 27)	0.0 (0.0 to 0.0)	-80.9 (-86.5 to -73.7)	-77.6 (-84.2 to -69.1)
European Region	Asthma	5 to 9	Female	47 (32 to 65)	0.1 (0.1 to 0.2)	32 (23 to 44)	0.1 (0.1 to 0.2)	11 (8 to 14)	0.0 (0.0 to 0.1)	-77.1 (-84.1 to -66.0)	-72.9 (-81.3 to -59.9)
European Region	Asthma	5 to 9	Male	67 (43 to 104)	0.2 (0.1 to 0.3)	39 (26 to 58)	0.1 (0.1 to 0.2)	11 (8 to 15)	0.0 (0.0 to 0.1)	-83.1 (-89.9 to -73.3)	-80.3 (-88.3 to -68.9)
European Region	Asthma	10 to 14	Both	180 (153 to 214)	0.3 (0.2 to 0.3)	130 (112 to 150)	0.2 (0.2 to 0.2)	38 (33 to 45)	0.1 (0.1 to 0.1)	-79.0 (-83.1 to -73.8)	-73.9 (-79.0 to -67.5)
European Region	Asthma	10 to 14	Female	64 (54 to 76)	0.2 (0.2 to 0.2)	49 (41 to 57)	0.2 (0.1 to 0.2)	16 (13 to 19)	0.1 (0.1 to 0.1)	-75.2 (-79.9 to -69.9)	-69.0 (-74.9 to -62.4)
European Region	Asthma	10 to 14	Male	116 (93 to 146)	0.4 (0.3 to 0.4)	82 (67 to 100)	0.2 (0.2 to 0.3)	22 (18 to 29)	0.1 (0.1 to 0.1)	-81.0 (-86.1 to -73.7)	-76.6 (-82.8 to -67.5)
European Region	Interstitial lung disease and pulmonary sarcoidosis	5 to 9	Both	21 (16 to 28)	0.0 (0.0 to 0.0)	18 (13 to 24)	0.0 (0.0 to 0.0)	9 (6 to 10)	0.0 (0.0 to 0.0)	-58.0 (-73.0 to -43.5)	-50.8 (-68.3 to -33.6)
European Region	Interstitial lung disease and pulmonary sarcoidosis	5 to 9	Female	11 (8 to 16)	0.0 (0.0 to 0.1)	11 (7 to 15)	0.0 (0.0 to 0.1)	5 (3 to 6)	0.0 (0.0 to 0.0)	-54.6 (-72.5 to -35.0)	-46.3 (-67.5 to -23.2)
European Region	Interstitial lung disease and pulmonary sarcoidosis	5 to 9	Male	10 (6 to 15)	0.0 (0.0 to 0.0)	7 (5 to 9)	0.0 (0.0 to 0.0)	4 (2 to 5)	0.0 (0.0 to 0.0)	-60.5 (-81.5 to -39.4)	-53.9 (-78.4 to -29.3)
European Region	Interstitial lung disease and pulmonary sarcoidosis	10 to 14	Both	16 (11 to 20)	0.0 (0.0 to 0.0)	15 (10 to 19)	0.0 (0.0 to 0.0)	9 (5 to 11)	0.0 (0.0 to 0.0)	-44.1 (-65.9 to -26.4)	-30.5 (-57.7 to -8.6)
European Region	Interstitial lung disease and pulmonary sarcoidosis	10 to 14	Female	9 (6 to 12)	0.0 (0.0 to 0.0)	9 (6 to 11)	0.0 (0.0 to 0.0)	5 (3 to 6)	0.0 (0.0 to 0.0)	-42.7 (-67.1 to -19.5)	-28.4 (-58.8 to 0.7)
European Region	Interstitial lung disease and pulmonary sarcoidosis	10 to 14	Male	7 (3 to 10)	0.0 (0.0 to 0.0)	7 (4 to 9)	0.0 (0.0 to 0.0)	4 (2 to 5)	0.0 (0.0 to 0.0)	-42.8 (-71.8 to -1.0)	-29.4 (-65.2 to 22.2)
European Region	Other chronic respiratory diseases	5 to 9	Both	88 (50 to 133)	0.1 (0.1 to 0.2)	74 (46 to 101)	0.1 (0.1 to 0.2)	33 (26 to 43)	0.1 (0.0 to 0.1)	-59.6 (-76.2 to -34.9)	-52.6 (-72.0 to -23.5)
European Region	Other chronic respiratory diseases	5 to 9	Female	31 (17 to 49)	0.1 (0.1 to 0.2)	26 (15 to 42)	0.1 (0.1 to 0.2)	14 (9 to 20)	0.1 (0.0 to 0.1)	-51.9 (-72.6 to -27.3)	-43.2 (-67.7 to -14.1)
European Region	Other chronic respiratory diseases	5 to 9	Male	57 (27 to 94)	0.2 (0.1 to 0.3)	47 (27 to 70)	0.2 (0.1 to 0.2)	19 (14 to 27)	0.1 (0.0 to 0.1)	-62.4 (-82.3 to -26.9)	-56.1 (-79.4 to -14.7)
European Region	Other chronic respiratory diseases	10 to 14	Both	61 (37 to 89)	0.1 (0.1 to 0.1)	54 (34 to 74)	0.1 (0.1 to 0.1)	28 (21 to 38)	0.1 (0.0 to 0.1)	-51.2 (-70.0 to -27.0)	-39.4 (-62.7 to -9.3)
European Region	Other chronic respiratory diseases	10 to 14	Female	24 (14 to 36)	0.1 (0.0 to 0.1)	20 (13 to 30)	0.1 (0.0 to 0.1)	11 (8 to 17)	0.0 (0.0 to 0.1)	-51.2 (-71.5 to -27.9)	-39.0 (-64.3 to -9.8)
European Region	Other chronic respiratory diseases	10 to 14	Male	37 (18 to 62)	0.1 (0.1 to 0.2)	33 (19 to 51)	0.1 (0.1 to 0.2)	17 (11 to 24)	0.1 (0.0 to 0.1)	-49.2 (-74.7 to -4.7)	-37.3 (-68.7 to 17.6)
European Region	Cirrhosis and other chronic liver diseases due to hepatitis B	5 to 9	Both	4 (3 to 5)	0.0 (0.0 to 0.0)	5 (4 to 6)	0.0 (0.0 to 0.0)	2 (2 to 3)	0.0 (0.0 to 0.0)	-32.3 (-46.2 to -16.4)	-20.5 (-36.8 to -1.9)

eTable 8. All-cause and cause-specific mortality (counts and rates) at the global level and by country in the WHO European Region for ages 5 to 9 and 10 to 14 for 1990, 2000, and 2016

European Region	Cirrhosis and other chronic liver diseases due to hepatitis B	5 to 9	Female	1 (1 to 1)	0.0 (0.0 to 0.0)	1 (1 to 1)	0.0 (0.0 to 0.0)	0 (0 to 1)	0.0 (0.0 to 0.0)	-34.5 (-50.1 to -17.2)	-22.7 (-41.0 to -2.2)
European Region	Cirrhosis and other chronic liver diseases due to hepatitis B	5 to 9	Male	3 (2 to 4)	0.0 (0.0 to 0.0)	4 (3 to 5)	0.0 (0.0 to 0.0)	2 (2 to 3)	0.0 (0.0 to 0.0)	-31.6 (-47.4 to -12.7)	-20.1 (-38.6 to 1.9)
European Region	Cirrhosis and other chronic liver diseases due to hepatitis B	10 to 14	Both	8 (6 to 10)	0.0 (0.0 to 0.0)	15 (12 to 19)	0.0 (0.0 to 0.0)	7 (5 to 9)	0.0 (0.0 to 0.0)	-15.2 (-30.2 to 1.0)	5.3 (-13.3 to 25.5)
European Region	Cirrhosis and other chronic liver diseases due to hepatitis B	10 to 14	Female	2 (1 to 2)	0.0 (0.0 to 0.0)	3 (2 to 4)	0.0 (0.0 to 0.0)	1 (1 to 2)	0.0 (0.0 to 0.0)	-23.5 (-39.2 to -6.2)	-4.3 (-23.9 to 17.3)
European Region	Cirrhosis and other chronic liver diseases due to hepatitis B	10 to 14	Male	6 (5 to 8)	0.0 (0.0 to 0.0)	12 (9 to 15)	0.0 (0.0 to 0.0)	5 (4 to 7)	0.0 (0.0 to 0.0)	-12.7 (-30.1 to 6.9)	7.7 (-13.7 to 31.9)
European Region	Cirrhosis and other chronic liver diseases due to hepatitis C	5 to 9	Both	3 (2 to 4)	0.0 (0.0 to 0.0)	3 (2 to 4)	0.0 (0.0 to 0.0)	2 (1 to 2)	0.0 (0.0 to 0.0)	-44.0 (-53.2 to -33.5)	-34.3 (-45.1 to -21.9)
European Region	Cirrhosis and other chronic liver diseases due to hepatitis C	5 to 9	Female	1 (1 to 1)	0.0 (0.0 to 0.0)	1 (1 to 1)	0.0 (0.0 to 0.0)	0 (0 to 1)	0.0 (0.0 to 0.0)	-46.3 (-57.1 to -34.8)	-36.5 (-49.3 to -23.0)
European Region	Cirrhosis and other chronic liver diseases due to hepatitis C	5 to 9	Male	2 (2 to 3)	0.0 (0.0 to 0.0)	2 (2 to 3)	0.0 (0.0 to 0.0)	1 (1 to 2)	0.0 (0.0 to 0.0)	-43.3 (-54.1 to -31.1)	-33.8 (-46.5 to -19.5)
European Region	Cirrhosis and other chronic liver diseases due to hepatitis C	10 to 14	Both	7 (5 to 9)	0.0 (0.0 to 0.0)	10 (7 to 12)	0.0 (0.0 to 0.0)	4 (3 to 6)	0.0 (0.0 to 0.0)	-33.7 (-44.0 to -21.8)	-17.6 (-30.5 to -2.8)
European Region	Cirrhosis and other chronic liver diseases due to hepatitis C	10 to 14	Female	2 (1 to 2)	0.0 (0.0 to 0.0)	2 (2 to 3)	0.0 (0.0 to 0.0)	1 (1 to 1)	0.0 (0.0 to 0.0)	-40.0 (-50.7 to -29.2)	-25.0 (-38.3 to -11.4)
European Region	Cirrhosis and other chronic liver diseases due to hepatitis C	10 to 14	Male	5 (3 to 6)	0.0 (0.0 to 0.0)	7 (5 to 10)	0.0 (0.0 to 0.0)	3 (2 to 4)	0.0 (0.0 to 0.0)	-31.4 (-43.6 to -17.5)	-15.3 (-30.3 to 1.8)
European Region	Cirrhosis and other chronic liver diseases due to other causes	5 to 9	Both	216 (189 to 243)	0.3 (0.3 to 0.4)	230 (204 to 257)	0.4 (0.4 to 0.5)	121 (105 to 140)	0.2 (0.2 to 0.3)	-43.6 (-53.9 to -32.4)	-33.8 (-45.9 to -20.6)
European Region	Cirrhosis and other chronic liver diseases due to other causes	5 to 9	Female	92 (80 to 108)	0.3 (0.3 to 0.3)	99 (88 to 113)	0.4 (0.3 to 0.4)	51 (43 to 60)	0.2 (0.2 to 0.2)	-44.4 (-57.0 to -30.2)	-34.3 (-49.2 to -17.5)

eTable 8. All-cause and cause-specific mortality (counts and rates) at the global level and by country in the WHO European Region for ages 5 to 9 and 10 to 14 for 1990, 2000, and 2016

European Region	Cirrhosis and other chronic liver diseases due to other causes	5 to 9	Male	123 (99 to 147)	0.4 (0.3 to 0.4)	130 (108 to 152)	0.5 (0.4 to 0.5)	70 (58 to 84)	0.2 (0.2 to 0.3)	-42.6 (-55.1 to -27.6)	-33.1 (-47.6 to -15.5)
European Region	Cirrhosis and other chronic liver diseases due to other causes	10 to 14	Both	230 (208 to 257)	0.4 (0.3 to 0.4)	350 (319 to 382)	0.5 (0.5 to 0.6)	155 (135 to 174)	0.3 (0.3 to 0.3)	-32.3 (-43.0 to -21.6)	-15.9 (-29.2 to -2.6)
European Region	Cirrhosis and other chronic liver diseases due to other causes	10 to 14	Female	110 (95 to 126)	0.4 (0.3 to 0.4)	156 (138 to 175)	0.5 (0.4 to 0.6)	69 (59 to 80)	0.3 (0.2 to 0.3)	-36.8 (-49.9 to -23.1)	-20.9 (-37.3 to -3.8)
European Region	Cirrhosis and other chronic liver diseases due to other causes	10 to 14	Male	121 (102 to 142)	0.4 (0.3 to 0.4)	194 (170 to 222)	0.6 (0.5 to 0.7)	86 (72 to 101)	0.3 (0.3 to 0.4)	-27.9 (-42.2 to -11.6)	-11.0 (-28.7 to 9.1)
European Region	Peptic ulcer disease	5 to 9	Both	42 (31 to 62)	0.1 (0.0 to 0.1)	30 (24 to 38)	0.1 (0.0 to 0.1)	11 (9 to 14)	0.0 (0.0 to 0.0)	-72.5 (-82.3 to -60.9)	-67.7 (-79.3 to -54.1)
European Region	Peptic ulcer disease	5 to 9	Female	19 (13 to 32)	0.1 (0.0 to 0.1)	14 (11 to 19)	0.1 (0.0 to 0.1)	5 (4 to 7)	0.0 (0.0 to 0.0)	-71.4 (-83.9 to -55.3)	-66.2 (-81.0 to -47.2)
European Region	Peptic ulcer disease	5 to 9	Male	22 (15 to 35)	0.1 (0.0 to 0.1)	16 (12 to 23)	0.1 (0.0 to 0.1)	6 (4 to 9)	0.0 (0.0 to 0.0)	-72.3 (-84.3 to -53.2)	-67.7 (-81.7 to -45.3)
European Region	Peptic ulcer disease	10 to 14	Both	36 (29 to 49)	0.1 (0.0 to 0.1)	37 (31 to 44)	0.1 (0.0 to 0.1)	12 (10 to 15)	0.0 (0.0 to 0.0)	-65.7 (-76.6 to -54.9)	-57.4 (-71.0 to -44.0)
European Region	Peptic ulcer disease	10 to 14	Female	17 (13 to 25)	0.1 (0.0 to 0.1)	18 (14 to 22)	0.1 (0.0 to 0.1)	6 (5 to 7)	0.0 (0.0 to 0.0)	-65.2 (-78.0 to -49.2)	-56.5 (-72.5 to -36.4)
European Region	Peptic ulcer disease	10 to 14	Male	18 (14 to 26)	0.1 (0.0 to 0.1)	19 (15 to 24)	0.1 (0.0 to 0.1)	6 (5 to 9)	0.0 (0.0 to 0.0)	-65.4 (-78.0 to -45.4)	-57.3 (-72.9 to -32.6)
European Region	Gastritis and duodenitis	5 to 9	Both	3 (2 to 5)	0.0 (0.0 to 0.0)	3 (2 to 4)	0.0 (0.0 to 0.0)	2 (1 to 2)	0.0 (0.0 to 0.0)	-51.0 (-72.3 to -26.3)	-42.5 (-67.4 to -13.5)
European Region	Gastritis and duodenitis	5 to 9	Female	2 (1 to 3)	0.0 (0.0 to 0.0)	2 (1 to 3)	0.0 (0.0 to 0.0)	1 (1 to 1)	0.0 (0.0 to 0.0)	-55.9 (-76.3 to -27.9)	-47.9 (-72.0 to -14.8)
European Region	Gastritis and duodenitis	5 to 9	Male	1 (1 to 2)	0.0 (0.0 to 0.0)	1 (1 to 2)	0.0 (0.0 to 0.0)	1 (0 to 1)	0.0 (0.0 to 0.0)	-40.0 (-72.5 to 9.1)	-30.0 (-68.0 to 27.3)
European Region	Gastritis and duodenitis	10 to 14	Both	2 (2 to 3)	0.0 (0.0 to 0.0)	2 (2 to 2)	0.0 (0.0 to 0.0)	1 (1 to 2)	0.0 (0.0 to 0.0)	-48.3 (-62.7 to -32.2)	-35.7 (-53.6 to -15.8)
European Region	Gastritis and duodenitis	10 to 14	Female	1 (1 to 2)	0.0 (0.0 to 0.0)	1 (1 to 1)	0.0 (0.0 to 0.0)	1 (0 to 1)	0.0 (0.0 to 0.0)	-48.1 (-67.7 to -20.6)	-35.0 (-59.7 to -0.7)
European Region	Gastritis and duodenitis	10 to 14	Male	1 (1 to 1)	0.0 (0.0 to 0.0)	1 (1 to 1)	0.0 (0.0 to 0.0)	1 (0 to 1)	0.0 (0.0 to 0.0)	-47.7 (-62.2 to -29.9)	-35.4 (-53.3 to -13.5)
European Region	Appendicitis	5 to 9	Both	95 (66 to 111)	0.1 (0.1 to 0.2)	56 (48 to 74)	0.1 (0.1 to 0.1)	23 (17 to 38)	0.0 (0.0 to 0.1)	-74.8 (-82.3 to -47.4)	-70.4 (-79.2 to -38.3)
European Region	Appendicitis	5 to 9	Female	45 (27 to 56)	0.1 (0.1 to 0.2)	27 (21 to 37)	0.1 (0.1 to 0.1)	11 (8 to 17)	0.0 (0.0 to 0.1)	-73.6 (-82.8 to -55.4)	-68.8 (-79.6 to -47.4)
European Region	Appendicitis	5 to 9	Male	50 (34 to 61)	0.2 (0.1 to 0.2)	30 (24 to 42)	0.1 (0.1 to 0.1)	12 (8 to 22)	0.0 (0.0 to 0.1)	-75.4 (-84.4 to -33.4)	-71.3 (-81.8 to -22.3)
European Region	Appendicitis	10 to 14	Both	92 (65 to 106)	0.1 (0.1 to 0.2)	68 (59 to 88)	0.1 (0.1 to 0.1)	26 (21 to 38)	0.1 (0.0 to 0.1)	-70.9 (-78.1 to -45.1)	-63.8 (-72.9 to -31.7)
European Region	Appendicitis	10 to 14	Female	43 (27 to 54)	0.1 (0.1 to 0.2)	33 (26 to 43)	0.1 (0.1 to 0.1)	13 (10 to 18)	0.1 (0.0 to 0.1)	-70.3 (-78.9 to -52.9)	-62.8 (-73.6 to -41.1)
European Region	Appendicitis	10 to 14	Male	48 (31 to 57)	0.1 (0.1 to 0.2)	35 (29 to 50)	0.1 (0.1 to 0.2)	14 (10 to 23)	0.1 (0.0 to 0.1)	-71.1 (-80.0 to -30.9)	-64.3 (-75.3 to -14.8)
European Region	Paralytic ileus and intestinal obstruction	5 to 9	Both	76 (57 to 93)	0.1 (0.1 to 0.1)	64 (49 to 75)	0.1 (0.1 to 0.1)	39 (31 to 52)	0.1 (0.1 to 0.1)	-47.7 (-59.9 to -24.2)	-38.6 (-52.9 to -11.0)
European Region	Paralytic ileus and intestinal obstruction	5 to 9	Female	31 (22 to 40)	0.1 (0.1 to 0.1)	23 (17 to 29)	0.1 (0.1 to 0.1)	14 (10 to 18)	0.1 (0.0 to 0.1)	-54.1 (-65.9 to -39.6)	-45.8 (-59.7 to -28.7)
European Region	Paralytic ileus and intestinal obstruction	5 to 9	Male	45 (30 to 58)	0.1 (0.1 to 0.2)	40 (29 to 51)	0.1 (0.1 to 0.2)	25 (19 to 38)	0.1 (0.1 to 0.1)	-42.6 (-60.8 to 5.8)	-33.0 (-54.2 to 23.4)

eTable 8. All-cause and cause-specific mortality (counts and rates) at the global level and by country in the WHO European Region for ages 5 to 9 and 10 to 14 for 1990, 2000, and 2016

European Region	Paralytic ileus and intestinal obstruction	10 to 14	Both	60 (49 to 75)	0.1 (0.1 to 0.1)	59 (49 to 73)	0.1 (0.1 to 0.1)	32 (26 to 46)	0.1 (0.1 to 0.1)	-46.7 (-56.8 to -28.5)	-33.8 (-46.4 to -11.2)
European Region	Paralytic ileus and intestinal obstruction	10 to 14	Female	25 (20 to 34)	0.1 (0.1 to 0.1)	24 (19 to 32)	0.1 (0.1 to 0.1)	13 (10 to 17)	0.1 (0.0 to 0.1)	-49.9 (-61.8 to -33.9)	-37.4 (-52.2 to -17.3)
European Region	Paralytic ileus and intestinal obstruction	10 to 14	Male	34 (24 to 44)	0.1 (0.1 to 0.1)	34 (26 to 46)	0.1 (0.1 to 0.1)	19 (15 to 32)	0.1 (0.1 to 0.1)	-43.5 (-58.8 to -1.5)	-30.2 (-49.1 to 21.6)
European Region	Inguinal, femoral, and abdominal hernia	5 to 9	Both	14 (9 to 19)	0.0 (0.0 to 0.0)	10 (7 to 14)	0.0 (0.0 to 0.0)	4 (3 to 6)	0.0 (0.0 to 0.0)	-68.5 (-79.6 to -53.4)	-63.0 (-76.1 to -45.2)
European Region	Inguinal, femoral, and abdominal hernia	5 to 9	Female	5 (3 to 8)	0.0 (0.0 to 0.0)	3 (2 to 5)	0.0 (0.0 to 0.0)	1 (1 to 2)	0.0 (0.0 to 0.0)	-71.9 (-83.6 to -53.0)	-66.8 (-80.6 to -44.5)
European Region	Inguinal, femoral, and abdominal hernia	5 to 9	Male	8 (5 to 14)	0.0 (0.0 to 0.0)	7 (4 to 10)	0.0 (0.0 to 0.0)	3 (1 to 5)	0.0 (0.0 to 0.0)	-65.7 (-81.2 to -41.4)	-60.0 (-78.1 to -31.6)
European Region	Inguinal, femoral, and abdominal hernia	10 to 14	Both	12 (9 to 17)	0.0 (0.0 to 0.0)	12 (8 to 16)	0.0 (0.0 to 0.0)	5 (3 to 7)	0.0 (0.0 to 0.0)	-60.6 (-72.8 to -43.8)	-51.1 (-66.3 to -30.2)
European Region	Inguinal, femoral, and abdominal hernia	10 to 14	Female	5 (4 to 9)	0.0 (0.0 to 0.0)	5 (3 to 8)	0.0 (0.0 to 0.0)	2 (1 to 3)	0.0 (0.0 to 0.0)	-66.8 (-75.8 to -57.1)	-58.5 (-69.7 to -46.4)
European Region	Inguinal, femoral, and abdominal hernia	10 to 14	Male	7 (4 to 12)	0.0 (0.0 to 0.0)	7 (3 to 11)	0.0 (0.0 to 0.0)	3 (1 to 5)	0.0 (0.0 to 0.0)	-55.4 (-74.6 to -28.9)	-44.9 (-68.7 to -12.2)
European Region	Inflammatory bowel disease	5 to 9	Both	33 (23 to 49)	0.1 (0.0 to 0.1)	27 (19 to 38)	0.0 (0.0 to 0.1)	14 (10 to 18)	0.0 (0.0 to 0.0)	-55.9 (-74.4 to -33.3)	-48.2 (-69.9 to -21.7)
European Region	Inflammatory bowel disease	5 to 9	Female	17 (12 to 26)	0.1 (0.0 to 0.1)	14 (10 to 19)	0.0 (0.0 to 0.1)	7 (5 to 9)	0.0 (0.0 to 0.0)	-60.0 (-75.5 to -42.9)	-52.7 (-71.0 to -32.5)
European Region	Inflammatory bowel disease	5 to 9	Male	16 (8 to 26)	0.0 (0.0 to 0.1)	14 (8 to 22)	0.0 (0.0 to 0.1)	7 (4 to 11)	0.0 (0.0 to 0.0)	-49.5 (-77.2 to -7.7)	-41.1 (-73.4 to 7.7)
European Region	Inflammatory bowel disease	10 to 14	Both	26 (19 to 39)	0.0 (0.0 to 0.1)	25 (20 to 34)	0.0 (0.0 to 0.1)	13 (10 to 16)	0.0 (0.0 to 0.0)	-47.8 (-68.3 to -24.1)	-35.1 (-60.6 to -5.7)
European Region	Inflammatory bowel disease	10 to 14	Female	11 (8 to 16)	0.0 (0.0 to 0.1)	10 (8 to 14)	0.0 (0.0 to 0.0)	5 (4 to 7)	0.0 (0.0 to 0.0)	-49.0 (-68.8 to -24.5)	-36.2 (-60.9 to -5.6)
European Region	Inflammatory bowel disease	10 to 14	Male	15 (10 to 24)	0.0 (0.0 to 0.1)	15 (11 to 22)	0.0 (0.0 to 0.1)	8 (6 to 11)	0.0 (0.0 to 0.0)	-45.7 (-70.9 to -10.5)	-33.0 (-64.1 to 10.5)
European Region	Vascular intestinal disorders	5 to 9	Both	5 (3 to 7)	0.0 (0.0 to 0.0)	4 (2 to 5)	0.0 (0.0 to 0.0)	2 (1 to 3)	0.0 (0.0 to 0.0)	-56.1 (-74.8 to -24.4)	-48.4 (-70.5 to -11.3)
European Region	Vascular intestinal disorders	5 to 9	Female	2 (1 to 3)	0.0 (0.0 to 0.0)	1 (1 to 1)	0.0 (0.0 to 0.0)	1 (0 to 1)	0.0 (0.0 to 0.0)	-59.6 (-80.6 to -22.8)	-52.3 (-77.1 to -8.8)
European Region	Vascular intestinal disorders	5 to 9	Male	3 (1 to 5)	0.0 (0.0 to 0.0)	3 (1 to 4)	0.0 (0.0 to 0.0)	2 (1 to 3)	0.0 (0.0 to 0.0)	-52.5 (-75.7 to -5.1)	-44.6 (-71.6 to 10.8)
European Region	Vascular intestinal disorders	10 to 14	Both	6 (5 to 8)	0.0 (0.0 to 0.0)	5 (4 to 6)	0.0 (0.0 to 0.0)	3 (2 to 4)	0.0 (0.0 to 0.0)	-54.9 (-68.4 to -37.4)	-44.0 (-60.8 to -22.3)
European Region	Vascular intestinal disorders	10 to 14	Female	2 (2 to 3)	0.0 (0.0 to 0.0)	2 (1 to 2)	0.0 (0.0 to 0.0)	1 (1 to 1)	0.0 (0.0 to 0.0)	-58.2 (-74.9 to -32.9)	-47.7 (-68.6 to -16.1)
European Region	Vascular intestinal disorders	10 to 14	Male	4 (2 to 5)	0.0 (0.0 to 0.0)	4 (2 to 4)	0.0 (0.0 to 0.0)	2 (1 to 3)	0.0 (0.0 to 0.0)	-52.5 (-69.6 to -32.2)	-41.4 (-62.5 to -16.3)

eTable 8. All-cause and cause-specific mortality (counts and rates) at the global level and by country in the WHO European Region for ages 5 to 9 and 10 to 14 for 1990, 2000, and 2016

European Region	Gallbladder and biliary diseases	5 to 9	Both	8 (6 to 10)	0.0 (0.0 to 0.0)	6 (4 to 7)	0.0 (0.0 to 0.0)	2 (2 to 3)	0.0 (0.0 to 0.0)	-69.0 (-78.3 to -57.4)	-63.6 (-74.6 to -50.0)
European Region	Gallbladder and biliary diseases	5 to 9	Female	3 (2 to 4)	0.0 (0.0 to 0.0)	2 (2 to 3)	0.0 (0.0 to 0.0)	1 (1 to 1)	0.0 (0.0 to 0.0)	-76.7 (-83.2 to -66.6)	-72.5 (-80.1 to -60.6)
European Region	Gallbladder and biliary diseases	5 to 9	Male	4 (3 to 6)	0.0 (0.0 to 0.0)	4 (3 to 5)	0.0 (0.0 to 0.0)	2 (1 to 2)	0.0 (0.0 to 0.0)	-63.0 (-77.6 to -43.0)	-56.8 (-73.8 to -33.4)
European Region	Gallbladder and biliary diseases	10 to 14	Both	7 (6 to 10)	0.0 (0.0 to 0.0)	6 (5 to 8)	0.0 (0.0 to 0.0)	2 (2 to 4)	0.0 (0.0 to 0.0)	-64.4 (-71.9 to -55.9)	-55.8 (-65.0 to -45.3)
European Region	Gallbladder and biliary diseases	10 to 14	Female	3 (2 to 5)	0.0 (0.0 to 0.0)	2 (2 to 4)	0.0 (0.0 to 0.0)	1 (1 to 1)	0.0 (0.0 to 0.0)	-69.6 (-77.1 to -61.0)	-62.0 (-71.4 to -51.2)
European Region	Gallbladder and biliary diseases	10 to 14	Male	4 (3 to 5)	0.0 (0.0 to 0.0)	3 (3 to 5)	0.0 (0.0 to 0.0)	2 (1 to 3)	0.0 (0.0 to 0.0)	-59.7 (-72.5 to -42.1)	-50.3 (-66.1 to -28.6)
European Region	Pancreatitis	5 to 9	Both	7 (5 to 9)	0.0 (0.0 to 0.0)	5 (4 to 7)	0.0 (0.0 to 0.0)	3 (1 to 4)	0.0 (0.0 to 0.0)	-62.1 (-75.1 to -44.2)	-55.5 (-70.8 to -34.5)
European Region	Pancreatitis	5 to 9	Female	4 (3 to 6)	0.0 (0.0 to 0.0)	3 (2 to 4)	0.0 (0.0 to 0.0)	1 (1 to 2)	0.0 (0.0 to 0.0)	-69.5 (-81.2 to -50.8)	-63.9 (-77.8 to -41.9)
European Region	Pancreatitis	5 to 9	Male	3 (1 to 4)	0.0 (0.0 to 0.0)	3 (1 to 4)	0.0 (0.0 to 0.0)	1 (1 to 2)	0.0 (0.0 to 0.0)	-51.0 (-70.8 to -16.7)	-42.8 (-65.9 to -2.7)
European Region	Pancreatitis	10 to 14	Both	11 (7 to 15)	0.0 (0.0 to 0.0)	10 (6 to 13)	0.0 (0.0 to 0.0)	5 (2 to 7)	0.0 (0.0 to 0.0)	-57.1 (-70.1 to -35.8)	-46.7 (-62.9 to -20.3)
European Region	Pancreatitis	10 to 14	Female	6 (4 to 10)	0.0 (0.0 to 0.0)	5 (3 to 8)	0.0 (0.0 to 0.0)	2 (1 to 4)	0.0 (0.0 to 0.0)	-61.9 (-76.8 to -35.9)	-52.3 (-71.0 to -19.8)
European Region	Pancreatitis	10 to 14	Male	4 (2 to 6)	0.0 (0.0 to 0.0)	4 (2 to 6)	0.0 (0.0 to 0.0)	2 (1 to 4)	0.0 (0.0 to 0.0)	-47.6 (-66.3 to -19.2)	-35.3 (-58.4 to -0.3)
European Region	Other digestive diseases	5 to 9	Both	57 (40 to 76)	0.1 (0.1 to 0.1)	45 (32 to 62)	0.1 (0.1 to 0.1)	20 (16 to 26)	0.0 (0.0 to 0.0)	-63.1 (-74.9 to -45.0)	-56.7 (-70.6 to -35.5)
European Region	Other digestive diseases	5 to 9	Female	33 (21 to 48)	0.1 (0.1 to 0.2)	25 (17 to 34)	0.1 (0.1 to 0.1)	11 (9 to 14)	0.0 (0.0 to 0.1)	-64.7 (-78.0 to -43.9)	-58.3 (-74.0 to -33.7)
European Region	Other digestive diseases	5 to 9	Male	24 (16 to 38)	0.1 (0.0 to 0.1)	20 (14 to 31)	0.1 (0.0 to 0.1)	9 (7 to 13)	0.0 (0.0 to 0.0)	-59.1 (-77.6 to -29.7)	-52.2 (-73.8 to -17.9)
European Region	Other digestive diseases	10 to 14	Both	37 (29 to 50)	0.1 (0.0 to 0.1)	37 (29 to 50)	0.1 (0.0 to 0.1)	17 (13 to 20)	0.0 (0.0 to 0.0)	-55.0 (-68.5 to -40.1)	-44.2 (-60.8 to -25.6)
European Region	Other digestive diseases	10 to 14	Female	17 (12 to 23)	0.1 (0.0 to 0.1)	16 (12 to 21)	0.1 (0.0 to 0.1)	7 (6 to 10)	0.0 (0.0 to 0.0)	-54.2 (-70.0 to -32.5)	-42.7 (-62.4 to -15.5)
European Region	Other digestive diseases	10 to 14	Male	21 (16 to 31)	0.1 (0.0 to 0.1)	21 (16 to 31)	0.1 (0.0 to 0.1)	9 (7 to 13)	0.0 (0.0 to 0.0)	-55.0 (-70.7 to -37.4)	-44.5 (-63.8 to -22.7)
European Region	Alzheimer disease and other dementias	5 to 9	Both	0 (0 to 0)	0.0 (0.0 to 0.0)	0 (0 to 0)	0.0 (0.0 to 0.0)	0 (0 to 0)	0.0 (0.0 to 0.0)	-	-
European Region	Alzheimer disease and other dementias	5 to 9	Female	0 (0 to 0)	0.0 (0.0 to 0.0)	0 (0 to 0)	0.0 (0.0 to 0.0)	0 (0 to 0)	0.0 (0.0 to 0.0)	-	-
European Region	Alzheimer disease and other dementias	5 to 9	Male	0 (0 to 0)	0.0 (0.0 to 0.0)	0 (0 to 0)	0.0 (0.0 to 0.0)	0 (0 to 0)	0.0 (0.0 to 0.0)	-	-
European Region	Alzheimer disease and other dementias	10 to 14	Both	0 (0 to 0)	0.0 (0.0 to 0.0)	0 (0 to 0)	0.0 (0.0 to 0.0)	0 (0 to 0)	0.0 (0.0 to 0.0)	-	-

eTable 8. All-cause and cause-specific mortality (counts and rates) at the global level and by country in the WHO European Region for ages 5 to 9 and 10 to 14 for 1990, 2000, and 2016

European Region	Alzheimer disease and other dementias	10 to 14	Female	0 (0 to 0)	0.0 (0.0 to 0.0)	0 (0 to 0)	0.0 (0.0 to 0.0)	0 (0 to 0)	0.0 (0.0 to 0.0)	-	-
European Region	Alzheimer disease and other dementias	10 to 14	Male	0 (0 to 0)	0.0 (0.0 to 0.0)	0 (0 to 0)	0.0 (0.0 to 0.0)	0 (0 to 0)	0.0 (0.0 to 0.0)	-	-
European Region	Parkinson disease	5 to 9	Both	0 (0 to 0)	0.0 (0.0 to 0.0)	0 (0 to 0)	0.0 (0.0 to 0.0)	0 (0 to 0)	0.0 (0.0 to 0.0)	-	-
European Region	Parkinson disease	5 to 9	Female	0 (0 to 0)	0.0 (0.0 to 0.0)	0 (0 to 0)	0.0 (0.0 to 0.0)	0 (0 to 0)	0.0 (0.0 to 0.0)	-	-
European Region	Parkinson disease	5 to 9	Male	0 (0 to 0)	0.0 (0.0 to 0.0)	0 (0 to 0)	0.0 (0.0 to 0.0)	0 (0 to 0)	0.0 (0.0 to 0.0)	-	-
European Region	Parkinson disease	10 to 14	Both	0 (0 to 0)	0.0 (0.0 to 0.0)	0 (0 to 0)	0.0 (0.0 to 0.0)	0 (0 to 0)	0.0 (0.0 to 0.0)	-	-
European Region	Parkinson disease	10 to 14	Female	0 (0 to 0)	0.0 (0.0 to 0.0)	0 (0 to 0)	0.0 (0.0 to 0.0)	0 (0 to 0)	0.0 (0.0 to 0.0)	-	-
European Region	Parkinson disease	10 to 14	Male	0 (0 to 0)	0.0 (0.0 to 0.0)	0 (0 to 0)	0.0 (0.0 to 0.0)	0 (0 to 0)	0.0 (0.0 to 0.0)	-	-
European Region	Epilepsy	5 to 9	Both	529 (444 to 636)	0.8 (0.7 to 1.0)	503 (431 to 581)	0.9 (0.8 to 1.0)	297 (253 to 361)	0.5 (0.5 to 0.7)	-43.1 (-56.4 to -20.5)	-33.2 (-48.8 to -6.7)
European Region	Epilepsy	5 to 9	Female	222 (187 to 284)	0.7 (0.6 to 0.9)	214 (174 to 256)	0.8 (0.6 to 0.9)	130 (106 to 162)	0.5 (0.4 to 0.6)	-41.0 (-56.9 to -19.3)	-30.4 (-49.1 to -4.7)
European Region	Epilepsy	5 to 9	Male	307 (242 to 387)	0.9 (0.7 to 1.2)	289 (239 to 347)	1.0 (0.8 to 1.2)	168 (140 to 210)	0.6 (0.5 to 0.7)	-44.1 (-60.7 to -18.0)	-34.8 (-54.1 to -4.3)
European Region	Epilepsy	10 to 14	Both	546 (467 to 639)	0.9 (0.7 to 1.0)	627 (549 to 707)	1.0 (0.9 to 1.1)	319 (280 to 371)	0.6 (0.5 to 0.7)	-41.0 (-52.2 to -24.5)	-26.8 (-40.6 to -6.2)
European Region	Epilepsy	10 to 14	Female	230 (193 to 286)	0.7 (0.6 to 0.9)	261 (223 to 301)	0.8 (0.7 to 1.0)	135 (115 to 161)	0.5 (0.5 to 0.6)	-40.7 (-54.3 to -23.7)	-25.9 (-42.8 to -4.6)
European Region	Epilepsy	10 to 14	Male	316 (259 to 384)	1.0 (0.8 to 1.2)	365 (309 to 427)	1.1 (0.9 to 1.3)	184 (157 to 220)	0.7 (0.6 to 0.8)	-41.0 (-54.5 to -20.3)	-27.1 (-43.9 to -1.7)
European Region	Motor neuron disease	5 to 9	Both	13 (11 to 17)	0.0 (0.0 to 0.0)	11 (9 to 14)	0.0 (0.0 to 0.0)	8 (6 to 10)	0.0 (0.0 to 0.0)	-40.4 (-55.0 to -22.2)	-30.0 (-47.2 to -8.6)
European Region	Motor neuron disease	5 to 9	Female	9 (7 to 12)	0.0 (0.0 to 0.0)	7 (6 to 10)	0.0 (0.0 to 0.0)	6 (4 to 7)	0.0 (0.0 to 0.0)	-34.5 (-53.5 to -10.6)	-22.6 (-45.1 to 5.6)
European Region	Motor neuron disease	5 to 9	Male	5 (4 to 6)	0.0 (0.0 to 0.0)	4 (3 to 5)	0.0 (0.0 to 0.0)	2 (2 to 3)	0.0 (0.0 to 0.0)	-50.3 (-67.5 to -26.5)	-42.1 (-62.1 to -14.2)
European Region	Motor neuron disease	10 to 14	Both	25 (22 to 29)	0.0 (0.0 to 0.0)	22 (19 to 26)	0.0 (0.0 to 0.0)	17 (14 to 19)	0.0 (0.0 to 0.0)	-32.8 (-45.7 to -19.9)	-16.5 (-32.6 to -0.4)
European Region	Motor neuron disease	10 to 14	Female	10 (8 to 12)	0.0 (0.0 to 0.0)	9 (7 to 11)	0.0 (0.0 to 0.0)	7 (6 to 8)	0.0 (0.0 to 0.0)	-27.1 (-44.1 to -8.5)	-8.9 (-30.1 to 14.4)
European Region	Motor neuron disease	10 to 14	Male	15 (13 to 18)	0.0 (0.0 to 0.1)	13 (11 to 16)	0.0 (0.0 to 0.0)	10 (8 to 12)	0.0 (0.0 to 0.0)	-36.0 (-51.3 to -19.1)	-21.0 (-39.9 to -0.2)
European Region	Other neurological disorders	5 to 9	Both	194 (164 to 248)	0.3 (0.3 to 0.4)	155 (134 to 186)	0.3 (0.2 to 0.3)	120 (104 to 139)	0.2 (0.2 to 0.3)	-37.0 (-56.0 to -20.8)	-26.1 (-48.4 to -7.1)
European Region	Other neurological disorders	5 to 9	Female	93 (76 to 115)	0.3 (0.2 to 0.4)	75 (62 to 90)	0.3 (0.2 to 0.3)	56 (48 to 66)	0.2 (0.2 to 0.2)	-38.4 (-55.1 to -19.4)	-27.3 (-47.0 to -4.8)
European Region	Other neurological disorders	5 to 9	Male	101 (82 to 139)	0.3 (0.2 to 0.4)	80 (68 to 100)	0.3 (0.2 to 0.3)	64 (52 to 78)	0.2 (0.2 to 0.3)	-35.1 (-60.2 to -13.8)	-24.3 (-53.6 to 0.6)
European Region	Other neurological disorders	10 to 14	Both	262 (229 to 317)	0.4 (0.4 to 0.5)	253 (224 to 293)	0.4 (0.3 to 0.5)	170 (150 to 192)	0.3 (0.3 to 0.4)	-34.4 (-49.5 to -21.5)	-18.5 (-37.3 to -2.5)
European Region	Other neurological disorders	10 to 14	Female	83 (72 to 97)	0.3 (0.2 to 0.3)	82 (71 to 97)	0.3 (0.2 to 0.3)	55 (47 to 63)	0.2 (0.2 to 0.3)	-33.9 (-46.9 to -18.0)	-17.4 (-33.6 to 2.5)
European Region	Other neurological disorders	10 to 14	Male	178 (153 to 226)	0.6 (0.5 to 0.7)	171 (147 to 205)	0.5 (0.4 to 0.6)	116 (99 to 134)	0.4 (0.4 to 0.5)	-34.3 (-52.5 to -18.2)	-18.9 (-41.3 to 1.0)
European Region	Drug use disorders	5 to 9	Both	0 (0 to 0)	0.0 (0.0 to 0.0)	0 (0 to 0)	0.0 (0.0 to 0.0)	0 (0 to 0)	0.0 (0.0 to 0.0)	-	-

eTable 8. All-cause and cause-specific mortality (counts and rates) at the global level and by country in the WHO European Region for ages 5 to 9 and 10 to 14 for 1990, 2000, and 2016

European Region	Drug use disorders	5 to 9	Female	0 (0 to 0)	0.0 (0.0 to 0.0)	0 (0 to 0)	0.0 (0.0 to 0.0)	0 (0 to 0)	0.0 (0.0 to 0.0)	-	-
European Region	Drug use disorders	5 to 9	Male	0 (0 to 0)	0.0 (0.0 to 0.0)	0 (0 to 0)	0.0 (0.0 to 0.0)	0 (0 to 0)	0.0 (0.0 to 0.0)	-	-
European Region	Drug use disorders	10 to 14	Both	0 (0 to 0)	0.0 (0.0 to 0.0)	0 (0 to 0)	0.0 (0.0 to 0.0)	0 (0 to 0)	0.0 (0.0 to 0.0)	-	-
European Region	Drug use disorders	10 to 14	Female	0 (0 to 0)	0.0 (0.0 to 0.0)	0 (0 to 0)	0.0 (0.0 to 0.0)	0 (0 to 0)	0.0 (0.0 to 0.0)	-	-
European Region	Drug use disorders	10 to 14	Male	0 (0 to 0)	0.0 (0.0 to 0.0)	0 (0 to 0)	0.0 (0.0 to 0.0)	0 (0 to 0)	0.0 (0.0 to 0.0)	-	-
European Region	Eating disorders	5 to 9	Both	1 (0 to 1)	0.0 (0.0 to 0.0)	1 (0 to 1)	0.0 (0.0 to 0.0)	0 (0 to 1)	0.0 (0.0 to 0.0)	-35.4 (-63.8 to 10.6)	-24.2 (-57.5 to 29.8)
European Region	Eating disorders	5 to 9	Female	0 (0 to 1)	0.0 (0.0 to 0.0)	0 (0 to 1)	0.0 (0.0 to 0.0)	0 (0 to 0)	0.0 (0.0 to 0.0)	-20.0 (-65.3 to 46.9)	-5.5 (-59.0 to 73.5)
European Region	Eating disorders	5 to 9	Male	0 (0 to 1)	0.0 (0.0 to 0.0)	0 (0 to 1)	0.0 (0.0 to 0.0)	0 (0 to 0)	0.0 (0.0 to 0.0)	-43.1 (-74.1 to 10.2)	-33.6 (-69.7 to 28.6)
European Region	Eating disorders	10 to 14	Both	1 (1 to 1)	0.0 (0.0 to 0.0)	1 (1 to 1)	0.0 (0.0 to 0.0)	1 (1 to 1)	0.0 (0.0 to 0.0)	-17.9 (-44.1 to 13.3)	2.0 (-30.6 to 40.7)
European Region	Eating disorders	10 to 14	Female	1 (0 to 1)	0.0 (0.0 to 0.0)	1 (1 to 1)	0.0 (0.0 to 0.0)	1 (0 to 1)	0.0 (0.0 to 0.0)	-8.3 (-38.9 to 28.3)	14.7 (-23.6 to 60.5)
European Region	Eating disorders	10 to 14	Male	0 (0 to 1)	0.0 (0.0 to 0.0)	0 (0 to 1)	0.0 (0.0 to 0.0)	0 (0 to 0)	0.0 (0.0 to 0.0)	-34.6 (-68.4 to 19.3)	-19.2 (-61.0 to 47.2)
European Region	Diabetes mellitus	5 to 9	Both	89 (76 to 107)	0.1 (0.1 to 0.2)	81 (70 to 93)	0.1 (0.1 to 0.2)	36 (31 to 43)	0.1 (0.1 to 0.1)	-58.9 (-67.4 to -49.4)	-51.7 (-61.8 to -40.6)
European Region	Diabetes mellitus	5 to 9	Female	50 (40 to 62)	0.2 (0.1 to 0.2)	43 (37 to 51)	0.2 (0.1 to 0.2)	17 (14 to 20)	0.1 (0.1 to 0.1)	-65.5 (-74.3 to -56.2)	-59.2 (-69.6 to -48.3)
European Region	Diabetes mellitus	5 to 9	Male	39 (32 to 50)	0.1 (0.1 to 0.2)	38 (31 to 47)	0.1 (0.1 to 0.2)	19 (16 to 24)	0.1 (0.1 to 0.1)	-50.0 (-63.8 to -33.6)	-41.7 (-57.7 to -22.5)
European Region	Diabetes mellitus	10 to 14	Both	115 (102 to 131)	0.2 (0.2 to 0.2)	143 (130 to 158)	0.2 (0.2 to 0.2)	63 (55 to 71)	0.1 (0.1 to 0.1)	-45.2 (-54.2 to -34.9)	-31.9 (-43.1 to -19.1)
European Region	Diabetes mellitus	10 to 14	Female	70 (60 to 85)	0.2 (0.2 to 0.3)	88 (78 to 101)	0.3 (0.2 to 0.3)	35 (30 to 41)	0.1 (0.1 to 0.2)	-49.5 (-60.7 to -36.7)	-36.8 (-50.8 to -20.9)
European Region	Diabetes mellitus	10 to 14	Male	45 (39 to 53)	0.1 (0.1 to 0.2)	55 (48 to 63)	0.2 (0.1 to 0.2)	28 (24 to 32)	0.1 (0.1 to 0.1)	-38.1 (-51.1 to -23.0)	-23.6 (-39.6 to -5.0)
European Region	Acute glomerulonephritis	5 to 9	Both	28 (18 to 49)	0.0 (0.0 to 0.1)	29 (18 to 49)	0.1 (0.0 to 0.1)	11 (8 to 17)	0.0 (0.0 to 0.0)	-57.4 (-79.2 to -25.8)	-50.0 (-75.5 to -12.9)
European Region	Acute glomerulonephritis	5 to 9	Female	12 (6 to 26)	0.0 (0.0 to 0.1)	13 (7 to 27)	0.0 (0.0 to 0.1)	5 (3 to 10)	0.0 (0.0 to 0.0)	-51.0 (-81.4 to 5.2)	-42.1 (-78.0 to 24.2)
European Region	Acute glomerulonephritis	5 to 9	Male	16 (8 to 33)	0.0 (0.0 to 0.1)	16 (9 to 34)	0.1 (0.0 to 0.1)	6 (4 to 10)	0.0 (0.0 to 0.0)	-58.6 (-84.8 to -15.7)	-51.7 (-82.3 to -1.6)
European Region	Acute glomerulonephritis	10 to 14	Both	27 (17 to 44)	0.0 (0.0 to 0.1)	24 (17 to 37)	0.0 (0.0 to 0.1)	10 (8 to 15)	0.0 (0.0 to 0.0)	-58.8 (-79.3 to -30.7)	-48.8 (-74.3 to -14.0)
European Region	Acute glomerulonephritis	10 to 14	Female	15 (8 to 28)	0.0 (0.0 to 0.1)	13 (8 to 22)	0.0 (0.0 to 0.1)	5 (4 to 9)	0.0 (0.0 to 0.0)	-58.2 (-82.5 to -16.3)	-47.7 (-78.1 to 4.7)
European Region	Acute glomerulonephritis	10 to 14	Male	12 (7 to 22)	0.0 (0.0 to 0.1)	12 (7 to 20)	0.0 (0.0 to 0.1)	5 (3 to 8)	0.0 (0.0 to 0.0)	-55.6 (-79.8 to -20.3)	-45.2 (-75.0 to -1.6)
European Region	Chronic kidney disease	5 to 9	Both	252 (222 to 297)	0.4 (0.3 to 0.5)	248 (219 to 281)	0.4 (0.4 to 0.5)	127 (109 to 147)	0.2 (0.2 to 0.3)	-49.4 (-60.0 to -38.8)	-40.6 (-53.0 to -28.2)
European Region	Chronic kidney disease	5 to 9	Female	117 (98 to 145)	0.4 (0.3 to 0.5)	118 (100 to 138)	0.4 (0.4 to 0.5)	61 (51 to 72)	0.2 (0.2 to 0.3)	-47.8 (-60.8 to -34.5)	-38.4 (-53.7 to -22.7)
European Region	Chronic kidney disease	5 to 9	Male	135 (114 to 165)	0.4 (0.3 to 0.5)	131 (108 to 152)	0.5 (0.4 to 0.5)	66 (55 to 79)	0.2 (0.2 to 0.3)	-50.5 (-61.9 to -38.3)	-42.2 (-55.5 to -28.0)
European Region	Chronic kidney disease	10 to 14	Both	251 (228 to 280)	0.4 (0.4 to 0.4)	293 (270 to 320)	0.5 (0.4 to 0.5)	154 (137 to 173)	0.3 (0.3 to 0.3)	-38.6 (-47.7 to -29.6)	-23.7 (-35.0 to -12.5)
European Region	Chronic kidney disease	10 to 14	Female	122 (107 to 141)	0.4 (0.3 to 0.5)	146 (130 to 165)	0.5 (0.4 to 0.5)	77 (67 to 88)	0.3 (0.3 to 0.4)	-36.6 (-48.1 to -24.6)	-20.7 (-35.0 to -5.7)
European Region	Chronic kidney disease	10 to 14	Male	129 (113 to 150)	0.4 (0.3 to 0.5)	147 (130 to 165)	0.4 (0.4 to 0.5)	77 (66 to 89)	0.3 (0.3 to 0.3)	-40.2 (-51.4 to -28.4)	-26.2 (-40.0 to -11.6)

eTable 8. All-cause and cause-specific mortality (counts and rates) at the global level and by country in the WHO European Region for ages 5 to 9 and 10 to 14 for 1990, 2000, and 2016

European Region	Urinary diseases and male infertility	5 to 9	Both	78 (68 to 90)	0.1 (0.1 to 0.1)	77 (67 to 89)	0.1 (0.1 to 0.2)	41 (34 to 49)	0.1 (0.1 to 0.1)	-47.5 (-60.1 to -36.0)	-38.4 (-53.2 to -24.8)
European Region	Urinary diseases and male infertility	5 to 9	Female	38 (33 to 45)	0.1 (0.1 to 0.1)	36 (31 to 42)	0.1 (0.1 to 0.2)	20 (17 to 24)	0.1 (0.1 to 0.1)	-46.9 (-58.9 to -33.9)	-37.3 (-51.4 to -22.0)
European Region	Urinary diseases and male infertility	5 to 9	Male	40 (33 to 50)	0.1 (0.1 to 0.2)	41 (34 to 51)	0.1 (0.1 to 0.2)	21 (16 to 26)	0.1 (0.1 to 0.1)	-47.7 (-64.2 to -29.6)	-39.0 (-58.2 to -17.9)
European Region	Urinary diseases and male infertility	10 to 14	Both	82 (75 to 89)	0.1 (0.1 to 0.1)	99 (90 to 109)	0.2 (0.1 to 0.2)	50 (43 to 57)	0.1 (0.1 to 0.1)	-38.9 (-48.4 to -28.8)	-24.0 (-35.9 to -11.6)
European Region	Urinary diseases and male infertility	10 to 14	Female	40 (36 to 45)	0.1 (0.1 to 0.1)	49 (43 to 55)	0.2 (0.1 to 0.2)	24 (20 to 28)	0.1 (0.1 to 0.1)	-40.6 (-52.1 to -28.4)	-25.7 (-40.1 to -10.4)
European Region	Urinary diseases and male infertility	10 to 14	Male	41 (37 to 47)	0.1 (0.1 to 0.1)	50 (45 to 58)	0.2 (0.1 to 0.2)	26 (22 to 31)	0.1 (0.1 to 0.1)	-36.9 (-49.3 to -23.8)	-22.2 (-37.4 to -5.9)
European Region	Hemoglobinopathies and hemolytic anemias	5 to 9	Both	255 (203 to 360)	0.4 (0.3 to 0.6)	221 (175 to 309)	0.4 (0.3 to 0.5)	92 (74 to 128)	0.2 (0.1 to 0.2)	-63.5 (-71.8 to -52.6)	-57.1 (-66.9 to -44.3)
European Region	Hemoglobinopathies and hemolytic anemias	5 to 9	Female	120 (80 to 187)	0.4 (0.3 to 0.6)	103 (71 to 158)	0.4 (0.3 to 0.6)	44 (33 to 71)	0.2 (0.1 to 0.3)	-62.5 (-73.7 to -42.8)	-55.8 (-68.9 to -32.5)
European Region	Hemoglobinopathies and hemolytic anemias	5 to 9	Male	135 (107 to 187)	0.4 (0.3 to 0.6)	118 (94 to 169)	0.4 (0.3 to 0.6)	48 (38 to 67)	0.2 (0.1 to 0.2)	-63.8 (-73.2 to -53.2)	-57.7 (-68.7 to -45.4)
European Region	Hemoglobinopathies and hemolytic anemias	10 to 14	Both	191 (160 to 233)	0.3 (0.3 to 0.4)	181 (150 to 223)	0.3 (0.2 to 0.3)	84 (70 to 102)	0.2 (0.1 to 0.2)	-56.1 (-63.7 to -47.5)	-45.5 (-54.9 to -34.8)
European Region	Hemoglobinopathies and hemolytic anemias	10 to 14	Female	84 (61 to 110)	0.3 (0.2 to 0.4)	79 (57 to 104)	0.3 (0.2 to 0.3)	39 (31 to 55)	0.2 (0.1 to 0.2)	-53.7 (-65.9 to -42.1)	-42.0 (-57.3 to -27.6)
European Region	Hemoglobinopathies and hemolytic anemias	10 to 14	Male	107 (86 to 135)	0.3 (0.3 to 0.4)	102 (79 to 131)	0.3 (0.2 to 0.4)	45 (34 to 57)	0.2 (0.1 to 0.2)	-57.8 (-66.9 to -43.9)	-47.9 (-59.1 to -30.7)
European Region	Endocrine, metabolic, blood, and immune disorders	5 to 9	Both	412 (348 to 535)	0.6 (0.5 to 0.8)	365 (305 to 431)	0.6 (0.5 to 0.8)	261 (203 to 313)	0.5 (0.4 to 0.6)	-36.0 (-53.6 to -18.4)	-24.9 (-45.6 to -4.3)
European Region	Endocrine, metabolic, blood, and immune disorders	5 to 9	Female	208 (152 to 280)	0.7 (0.5 to 0.9)	180 (125 to 237)	0.7 (0.5 to 0.9)	122 (89 to 153)	0.5 (0.3 to 0.6)	-40.1 (-58.9 to -18.1)	-29.3 (-51.4 to -3.3)
European Region	Endocrine, metabolic, blood, and immune disorders	5 to 9	Male	204 (170 to 288)	0.6 (0.5 to 0.9)	185 (150 to 232)	0.6 (0.5 to 0.8)	139 (103 to 182)	0.5 (0.4 to 0.6)	-30.8 (-54.8 to -9.3)	-19.3 (-47.3 to 5.9)
European Region	Endocrine, metabolic, blood, and immune disorders	10 to 14	Both	371 (322 to 472)	0.6 (0.5 to 0.7)	353 (306 to 433)	0.5 (0.5 to 0.7)	236 (198 to 282)	0.5 (0.4 to 0.6)	-36.1 (-47.9 to -24.6)	-20.6 (-35.3 to -6.4)

eTable 8. All-cause and cause-specific mortality (counts and rates) at the global level and by country in the WHO European Region for ages 5 to 9 and 10 to 14 for 1990, 2000, and 2016

European Region	Endocrine, metabolic, blood, and immune disorders	10 to 14	Female	161 (127 to 214)	0.5 (0.4 to 0.7)	150 (121 to 186)	0.5 (0.4 to 0.6)	99 (78 to 122)	0.4 (0.3 to 0.5)	-37.4 (-52.6 to -19.4)	-21.7 (-40.8 to 0.8)
European Region	Endocrine, metabolic, blood, and immune disorders	10 to 14	Male	210 (170 to 279)	0.6 (0.5 to 0.9)	203 (167 to 265)	0.6 (0.5 to 0.8)	137 (107 to 176)	0.5 (0.4 to 0.7)	-34.6 (-48.5 to -19.4)	-19.3 (-36.4 to -0.5)
European Region	Rheumatoid arthritis	5 to 9	Both	16 (12 to 21)	0.0 (0.0 to 0.0)	9 (7 to 14)	0.0 (0.0 to 0.0)	5 (4 to 8)	0.0 (0.0 to 0.0)	-65.5 (-75.1 to -50.7)	-59.5 (-70.8 to -42.1)
European Region	Rheumatoid arthritis	5 to 9	Female	9 (6 to 13)	0.0 (0.0 to 0.0)	5 (4 to 9)	0.0 (0.0 to 0.0)	3 (2 to 4)	0.0 (0.0 to 0.0)	-68.4 (-78.4 to -53.4)	-62.7 (-74.5 to -45.0)
European Region	Rheumatoid arthritis	5 to 9	Male	7 (5 to 10)	0.0 (0.0 to 0.0)	4 (3 to 7)	0.0 (0.0 to 0.0)	3 (2 to 4)	0.0 (0.0 to 0.0)	-61.1 (-75.6 to -37.5)	-54.7 (-71.5 to -27.0)
European Region	Rheumatoid arthritis	10 to 14	Both	18 (13 to 23)	0.0 (0.0 to 0.0)	11 (9 to 17)	0.0 (0.0 to 0.0)	5 (4 to 8)	0.0 (0.0 to 0.0)	-69.9 (-77.8 to -53.8)	-62.6 (-72.5 to -42.6)
European Region	Rheumatoid arthritis	10 to 14	Female	11 (7 to 15)	0.0 (0.0 to 0.0)	6 (5 to 11)	0.0 (0.0 to 0.0)	3 (2 to 5)	0.0 (0.0 to 0.0)	-73.1 (-81.9 to -54.0)	-66.4 (-77.3 to -42.5)
European Region	Rheumatoid arthritis	10 to 14	Male	7 (5 to 10)	0.0 (0.0 to 0.0)	5 (3 to 8)	0.0 (0.0 to 0.0)	2 (2 to 4)	0.0 (0.0 to 0.0)	-64.1 (-76.7 to -41.8)	-55.7 (-71.3 to -28.2)
European Region	Other musculoskeletal disorders	5 to 9	Both	49 (34 to 91)	0.1 (0.1 to 0.1)	43 (32 to 79)	0.1 (0.1 to 0.1)	25 (20 to 45)	0.0 (0.0 to 0.1)	-46.4 (-61.2 to -26.2)	-37.1 (-54.4 to -13.4)
European Region	Other musculoskeletal disorders	5 to 9	Female	22 (16 to 40)	0.1 (0.0 to 0.1)	21 (16 to 37)	0.1 (0.1 to 0.1)	13 (10 to 22)	0.0 (0.0 to 0.1)	-39.0 (-56.7 to -18.0)	-28.0 (-48.9 to -3.2)
European Region	Other musculoskeletal disorders	5 to 9	Male	27 (17 to 54)	0.1 (0.1 to 0.2)	22 (15 to 45)	0.1 (0.1 to 0.2)	12 (9 to 25)	0.0 (0.0 to 0.1)	-52.0 (-68.7 to -27.8)	-44.0 (-63.5 to -15.7)
European Region	Other musculoskeletal disorders	10 to 14	Both	69 (52 to 115)	0.1 (0.1 to 0.2)	75 (59 to 114)	0.1 (0.1 to 0.2)	42 (34 to 65)	0.1 (0.1 to 0.1)	-37.7 (-51.7 to -19.2)	-22.6 (-40.0 to 0.4)
European Region	Other musculoskeletal disorders	10 to 14	Female	40 (30 to 67)	0.1 (0.1 to 0.2)	44 (35 to 68)	0.1 (0.1 to 0.2)	26 (21 to 39)	0.1 (0.1 to 0.2)	-33.8 (-51.7 to -12.0)	-17.1 (-39.5 to 10.0)
European Region	Other musculoskeletal disorders	10 to 14	Male	29 (20 to 50)	0.1 (0.1 to 0.2)	30 (22 to 49)	0.1 (0.1 to 0.1)	17 (12 to 28)	0.1 (0.0 to 0.1)	-42.2 (-60.2 to -19.5)	-28.6 (-50.8 to -0.6)
European Region	Congenital birth defects	5 to 9	Both	1989 (1592 to 2213)	3.1 (2.5 to 3.4)	1482 (1302 to 1657)	2.6 (2.3 to 2.9)	923 (778 to 1112)	1.7 (1.4 to 2.0)	-53.3 (-61.5 to -37.2)	-45.2 (-54.8 to -26.3)
European Region	Congenital birth defects	5 to 9	Female	931 (699 to 1094)	2.9 (2.2 to 3.4)	692 (597 to 839)	2.5 (2.2 to 3.0)	416 (348 to 516)	1.5 (1.3 to 1.9)	-55.0 (-63.7 to -38.2)	-46.8 (-57.1 to -27.0)
European Region	Congenital birth defects	5 to 9	Male	1058 (762 to 1232)	3.2 (2.3 to 3.7)	789 (658 to 913)	2.7 (2.3 to 3.2)	507 (408 to 637)	1.8 (1.4 to 2.3)	-51.5 (-63.4 to -25.3)	-43.3 (-57.3 to -12.8)
European Region	Congenital birth defects	10 to 14	Both	1303 (1047 to 1447)	2.0 (1.6 to 2.3)	1222 (1079 to 1395)	1.9 (1.7 to 2.2)	658 (578 to 785)	1.3 (1.1 to 1.5)	-49.3 (-56.4 to -35.4)	-37.0 (-45.9 to -19.7)
European Region	Congenital birth defects	10 to 14	Female	615 (469 to 715)	2.0 (1.5 to 2.3)	572 (473 to 697)	1.8 (1.5 to 2.2)	297 (254 to 380)	1.2 (1.0 to 1.5)	-51.4 (-59.8 to -36.6)	-39.2 (-49.7 to -20.8)
European Region	Congenital birth defects	10 to 14	Male	689 (513 to 815)	2.1 (1.6 to 2.5)	650 (555 to 761)	2.0 (1.7 to 2.3)	361 (302 to 437)	1.4 (1.1 to 1.7)	-47.0 (-58.0 to -26.7)	-34.6 (-48.1 to -9.5)
European Region	Skin and subcutaneous diseases	5 to 9	Both	12 (9 to 16)	0.0 (0.0 to 0.0)	11 (8 to 14)	0.0 (0.0 to 0.0)	10 (7 to 13)	0.0 (0.0 to 0.0)	-12.8 (-31.0 to 4.4)	2.3 (-19.0 to 22.5)
European Region	Skin and subcutaneous diseases	5 to 9	Female	6 (4 to 9)	0.0 (0.0 to 0.0)	6 (3 to 8)	0.0 (0.0 to 0.0)	5 (3 to 7)	0.0 (0.0 to 0.0)	-13.0 (-31.8 to 10.6)	2.7 (-19.4 to 30.6)
European Region	Skin and subcutaneous diseases	5 to 9	Male	6 (4 to 9)	0.0 (0.0 to 0.0)	6 (4 to 8)	0.0 (0.0 to 0.0)	5 (4 to 8)	0.0 (0.0 to 0.0)	-12.3 (-37.9 to 13.5)	2.4 (-27.6 to 32.5)
European Region	Skin and subcutaneous diseases	10 to 14	Both	12 (9 to 16)	0.0 (0.0 to 0.0)	14 (9 to 17)	0.0 (0.0 to 0.0)	10 (7 to 13)	0.0 (0.0 to 0.0)	-10.3 (-26.7 to 9.8)	11.5 (-9.0 to 36.4)

eTable 8. All-cause and cause-specific mortality (counts and rates) at the global level and by country in the WHO European Region for ages 5 to 9 and 10 to 14 for 1990, 2000, and 2016

European Region	Skin and subcutaneous diseases	10 to 14	Female	6 (4 to 9)	0.0 (0.0 to 0.0)	7 (4 to 10)	0.0 (0.0 to 0.0)	5 (3 to 8)	0.0 (0.0 to 0.0)	-12.3 (-28.3 to 15.8)	9.7 (-10.4 to 44.9)
European Region	Skin and subcutaneous diseases	10 to 14	Male	6 (4 to 8)	0.0 (0.0 to 0.0)	7 (4 to 9)	0.0 (0.0 to 0.0)	5 (3 to 7)	0.0 (0.0 to 0.0)	-7.9 (-28.9 to 15.0)	13.6 (-12.2 to 42.0)
European Region	Road injuries	5 to 9	Both	5463 (5112 to 5899)	8.4 (7.9 to 9.1)	3259 (3033 to 3538)	5.8 (5.4 to 6.3)	1500 (1330 to 1696)	2.7 (2.4 to 3.1)	-72.5 (-76.4 to -68.4)	-67.7 (-72.3 to -62.9)
European Region	Road injuries	5 to 9	Female	1904 (1733 to 2107)	6.0 (5.5 to 6.6)	1184 (1061 to 1329)	4.3 (3.9 to 4.8)	576 (500 to 664)	2.1 (1.9 to 2.5)	-69.7 (-74.7 to -63.8)	-64.2 (-70.1 to -57.3)
European Region	Road injuries	5 to 9	Male	3559 (3279 to 3901)	10.8 (9.9 to 11.8)	2075 (1906 to 2275)	7.2 (6.6 to 7.9)	925 (792 to 1081)	3.3 (2.8 to 3.8)	-74.0 (-78.2 to -69.3)	-69.6 (-74.6 to -64.1)
European Region	Road injuries	10 to 14	Both	4216 (3995 to 4463)	6.6 (6.3 to 7.0)	3322 (3111 to 3570)	5.2 (4.8 to 5.5)	1371 (1223 to 1516)	2.7 (2.4 to 3.0)	-67.5 (-71.5 to -63.7)	-59.6 (-64.6 to -54.9)
European Region	Road injuries	10 to 14	Female	1367 (1264 to 1481)	4.4 (4.0 to 4.7)	1124 (1030 to 1229)	3.6 (3.3 to 3.9)	470 (403 to 535)	1.9 (1.6 to 2.1)	-65.6 (-70.7 to -60.2)	-56.9 (-63.4 to -50.2)
European Region	Road injuries	10 to 14	Male	2849 (2651 to 3069)	8.8 (8.2 to 9.5)	2199 (2017 to 2403)	6.7 (6.1 to 7.3)	901 (786 to 1025)	3.4 (3.0 to 3.9)	-68.4 (-73.1 to -63.6)	-60.9 (-66.8 to -55.1)
European Region	Other transport injuries	5 to 9	Both	229 (189 to 278)	0.4 (0.3 to 0.4)	183 (157 to 214)	0.3 (0.3 to 0.4)	88 (72 to 113)	0.2 (0.1 to 0.2)	-61.2 (-70.5 to -47.5)	-54.4 (-65.4 to -38.4)
European Region	Other transport injuries	5 to 9	Female	73 (60 to 91)	0.2 (0.2 to 0.3)	60 (50 to 74)	0.2 (0.2 to 0.3)	30 (23 to 40)	0.1 (0.1 to 0.1)	-57.8 (-71.6 to -43.4)	-50.2 (-66.5 to -33.1)
European Region	Other transport injuries	5 to 9	Male	156 (118 to 202)	0.5 (0.4 to 0.6)	123 (100 to 151)	0.4 (0.3 to 0.5)	58 (44 to 81)	0.2 (0.2 to 0.3)	-62.2 (-74.9 to -43.4)	-55.9 (-70.7 to -33.9)
European Region	Other transport injuries	10 to 14	Both	289 (250 to 335)	0.5 (0.4 to 0.5)	296 (255 to 345)	0.5 (0.4 to 0.5)	125 (105 to 150)	0.2 (0.2 to 0.3)	-56.6 (-65.7 to -45.6)	-46.1 (-57.3 to -32.4)
European Region	Other transport injuries	10 to 14	Female	78 (65 to 96)	0.3 (0.2 to 0.3)	83 (70 to 101)	0.3 (0.2 to 0.3)	37 (29 to 47)	0.1 (0.1 to 0.2)	-52.0 (-65.8 to -36.6)	-39.9 (-57.3 to -20.7)
European Region	Other transport injuries	10 to 14	Male	210 (175 to 256)	0.6 (0.5 to 0.8)	213 (175 to 257)	0.6 (0.5 to 0.8)	87 (70 to 111)	0.3 (0.3 to 0.4)	-58.1 (-69.2 to -43.9)	-48.3 (-62.0 to -30.8)
European Region	Falls	5 to 9	Both	907 (582 to 1103)	1.4 (0.9 to 1.7)	639 (444 to 757)	1.1 (0.8 to 1.3)	225 (189 to 295)	0.4 (0.3 to 0.5)	-74.3 (-81.0 to -56.3)	-69.9 (-77.7 to -48.7)
European Region	Falls	5 to 9	Female	293 (201 to 370)	0.9 (0.6 to 1.2)	217 (158 to 274)	0.8 (0.6 to 1.0)	77 (63 to 97)	0.3 (0.2 to 0.4)	-73.2 (-80.6 to -61.5)	-68.4 (-77.1 to -54.5)
European Region	Falls	5 to 9	Male	615 (327 to 776)	1.9 (1.0 to 2.3)	421 (263 to 523)	1.5 (0.9 to 1.8)	149 (121 to 204)	0.5 (0.4 to 0.7)	-74.4 (-82.7 to -42.9)	-70.1 (-79.8 to -33.4)
European Region	Falls	10 to 14	Both	706 (499 to 819)	1.1 (0.8 to 1.3)	639 (493 to 726)	1.0 (0.8 to 1.1)	223 (193 to 286)	0.4 (0.4 to 0.6)	-67.7 (-74.8 to -48.2)	-59.9 (-68.7 to -35.7)
European Region	Falls	10 to 14	Female	156 (111 to 188)	0.5 (0.4 to 0.6)	146 (109 to 174)	0.5 (0.3 to 0.6)	56 (43 to 69)	0.2 (0.2 to 0.3)	-63.7 (-71.6 to -54.5)	-54.6 (-64.4 to -43.1)
European Region	Falls	10 to 14	Male	550 (365 to 657)	1.7 (1.1 to 2.0)	493 (365 to 575)	1.5 (1.1 to 1.7)	167 (140 to 229)	0.6 (0.5 to 0.9)	-68.6 (-76.9 to -42.8)	-61.3 (-71.4 to -29.4)
European Region	Drowning	5 to 9	Both	4208 (3926 to 4507)	6.5 (6.1 to 7.0)	2899 (2699 to 3110)	5.1 (4.8 to 5.5)	995 (887 to 1120)	1.8 (1.6 to 2.0)	-76.3 (-79.3 to -72.8)	-72.2 (-75.7 to -68.0)
European Region	Drowning	5 to 9	Female	969 (873 to 1073)	3.1 (2.7 to 3.4)	741 (666 to 819)	2.7 (2.4 to 3.0)	256 (217 to 303)	1.0 (0.8 to 1.1)	-73.5 (-78.2 to -67.2)	-68.7 (-74.2 to -61.3)
European Region	Drowning	5 to 9	Male	3239 (2991 to 3514)	9.8 (9.1 to 10.6)	2158 (1995 to 2354)	7.5 (6.9 to 8.2)	739 (644 to 843)	2.6 (2.3 to 3.0)	-77.1 (-80.6 to -73.3)	-73.3 (-77.4 to -68.9)
European Region	Drowning	10 to 14	Both	2857 (2658 to 3060)	4.5 (4.2 to 4.8)	2730 (2538 to 2942)	4.2 (3.9 to 4.6)	817 (737 to 905)	1.6 (1.4 to 1.8)	-71.4 (-74.9 to -67.1)	-64.4 (-68.8 to -59.1)
European Region	Drowning	10 to 14	Female	745 (676 to 815)	2.4 (2.2 to 2.6)	780 (702 to 857)	2.5 (2.2 to 2.7)	232 (199 to 276)	0.9 (0.8 to 1.1)	-68.8 (-73.9 to -61.9)	-60.9 (-67.3 to -52.3)
European Region	Drowning	10 to 14	Male	2111 (1926 to 2302)	6.5 (5.9 to 7.1)	1950 (1780 to 2134)	5.9 (5.4 to 6.5)	585 (515 to 665)	2.2 (2.0 to 2.5)	-72.2 (-76.4 to -67.2)	-65.7 (-70.9 to -59.5)
European Region	Fire, heat, and hot substances	5 to 9	Both	746 (669 to 898)	1.2 (1.0 to 1.4)	580 (515 to 662)	1.0 (0.9 to 1.2)	235 (204 to 287)	0.4 (0.4 to 0.5)	-68.4 (-73.1 to -62.5)	-63.0 (-68.4 to -56.0)
European Region	Fire, heat, and hot substances	5 to 9	Female	341 (292 to 427)	1.1 (0.9 to 1.3)	262 (224 to 326)	1.0 (0.8 to 1.2)	107 (86 to 158)	0.4 (0.3 to 0.6)	-68.5 (-74.3 to -56.5)	-62.8 (-69.7 to -48.6)

eTable 8. All-cause and cause-specific mortality (counts and rates) at the global level and by country in the WHO European Region for ages 5 to 9 and 10 to 14 for 1990, 2000, and 2016

European Region	Fire, heat, and hot substances	5 to 9	Male	405 (352 to 498)	1.2 (1.1 to 1.5)	318 (272 to 360)	1.1 (0.9 to 1.2)	127 (107 to 155)	0.5 (0.4 to 0.5)	-68.4 (-74.3 to -60.7)	-63.1 (-70.0 to -54.1)
European Region	Fire, heat, and hot substances	10 to 14	Both	400 (358 to 450)	0.6 (0.6 to 0.7)	413 (356 to 459)	0.6 (0.6 to 0.7)	141 (121 to 163)	0.3 (0.2 to 0.3)	-64.8 (-69.7 to -59.3)	-56.3 (-62.3 to -49.4)
European Region	Fire, heat, and hot substances	10 to 14	Female	135 (115 to 166)	0.4 (0.4 to 0.5)	139 (110 to 164)	0.4 (0.3 to 0.5)	51 (40 to 65)	0.2 (0.2 to 0.3)	-62.2 (-68.8 to -53.6)	-52.8 (-61.0 to -42.0)
European Region	Fire, heat, and hot substances	10 to 14	Male	266 (227 to 305)	0.8 (0.7 to 0.9)	274 (229 to 310)	0.8 (0.7 to 0.9)	90 (75 to 109)	0.3 (0.3 to 0.4)	-66.1 (-72.2 to -58.1)	-58.2 (-65.7 to -48.2)
European Region	Poisonings	5 to 9	Both	485 (326 to 568)	0.7 (0.5 to 0.9)	348 (247 to 407)	0.6 (0.4 to 0.7)	146 (106 to 183)	0.3 (0.2 to 0.3)	-69.6 (-76.0 to -60.1)	-64.3 (-71.9 to -53.2)
European Region	Poisonings	5 to 9	Female	195 (116 to 238)	0.6 (0.4 to 0.7)	143 (88 to 174)	0.5 (0.3 to 0.6)	63 (40 to 83)	0.2 (0.1 to 0.3)	-67.6 (-75.4 to -56.1)	-61.7 (-71.0 to -48.1)
European Region	Poisonings	5 to 9	Male	290 (181 to 355)	0.9 (0.5 to 1.1)	205 (143 to 252)	0.7 (0.5 to 0.9)	84 (56 to 113)	0.3 (0.2 to 0.4)	-70.6 (-79.1 to -56.9)	-65.7 (-75.6 to -49.8)
European Region	Poisonings	10 to 14	Both	361 (258 to 421)	0.6 (0.4 to 0.7)	336 (250 to 389)	0.5 (0.4 to 0.6)	124 (87 to 152)	0.2 (0.2 to 0.3)	-65.6 (-72.5 to -55.8)	-57.2 (-65.8 to -45.1)
European Region	Poisonings	10 to 14	Female	129 (83 to 155)	0.4 (0.3 to 0.5)	127 (78 to 156)	0.4 (0.2 to 0.5)	47 (29 to 63)	0.2 (0.1 to 0.3)	-63.0 (-71.8 to -51.6)	-53.7 (-64.7 to -39.4)
European Region	Poisonings	10 to 14	Male	232 (155 to 280)	0.7 (0.5 to 0.9)	210 (156 to 255)	0.6 (0.5 to 0.8)	76 (51 to 101)	0.3 (0.2 to 0.4)	-66.8 (-75.6 to -52.8)	-59.0 (-69.8 to -41.7)
European Region	Exposure to mechanical forces	5 to 9	Both	741 (626 to 1040)	1.1 (1.0 to 1.6)	533 (453 to 703)	0.9 (0.8 to 1.2)	242 (207 to 310)	0.4 (0.4 to 0.6)	-67.1 (-73.7 to -59.2)	-61.4 (-69.1 to -52.1)
European Region	Exposure to mechanical forces	5 to 9	Female	213 (175 to 284)	0.7 (0.5 to 0.9)	172 (142 to 218)	0.6 (0.5 to 0.8)	81 (66 to 117)	0.3 (0.2 to 0.4)	-61.8 (-69.5 to -45.5)	-54.9 (-64.0 to -35.7)
European Region	Exposure to mechanical forces	5 to 9	Male	527 (431 to 767)	1.6 (1.3 to 2.3)	362 (295 to 495)	1.3 (1.0 to 1.7)	161 (134 to 212)	0.6 (0.5 to 0.7)	-69.1 (-76.5 to -60.1)	-64.0 (-72.6 to -53.5)
European Region	Exposure to mechanical forces	10 to 14	Both	689 (597 to 925)	1.1 (0.9 to 1.5)	642 (549 to 852)	1.0 (0.9 to 1.3)	267 (228 to 337)	0.5 (0.4 to 0.7)	-60.9 (-68.6 to -53.4)	-51.5 (-61.0 to -42.2)
European Region	Exposure to mechanical forces	10 to 14	Female	134 (116 to 176)	0.4 (0.4 to 0.6)	141 (118 to 185)	0.4 (0.4 to 0.6)	65 (53 to 86)	0.3 (0.2 to 0.3)	-51.2 (-61.6 to -38.3)	-39.0 (-52.0 to -22.8)
European Region	Exposure to mechanical forces	10 to 14	Male	555 (472 to 767)	1.7 (1.5 to 2.4)	501 (421 to 688)	1.5 (1.3 to 2.1)	202 (167 to 263)	0.8 (0.6 to 1.0)	-63.2 (-71.5 to -55.1)	-54.6 (-64.8 to -44.6)
European Region	Adverse effects of medical treatment	5 to 9	Both	178 (131 to 209)	0.3 (0.2 to 0.3)	138 (103 to 168)	0.2 (0.2 to 0.3)	77 (60 to 100)	0.1 (0.1 to 0.2)	-56.0 (-64.2 to -39.7)	-48.3 (-58.0 to -29.2)
European Region	Adverse effects of medical treatment	5 to 9	Female	73 (54 to 93)	0.2 (0.2 to 0.3)	54 (40 to 73)	0.2 (0.1 to 0.3)	28 (22 to 37)	0.1 (0.1 to 0.1)	-61.4 (-68.6 to -54.7)	-54.4 (-62.9 to -46.5)
European Region	Adverse effects of medical treatment	5 to 9	Male	105 (69 to 128)	0.3 (0.2 to 0.4)	84 (58 to 104)	0.3 (0.2 to 0.4)	49 (34 to 70)	0.2 (0.1 to 0.2)	-51.9 (-63.7 to -26.6)	-43.8 (-57.7 to -14.3)
European Region	Adverse effects of medical treatment	10 to 14	Both	136 (108 to 163)	0.2 (0.2 to 0.3)	127 (102 to 159)	0.2 (0.2 to 0.2)	66 (54 to 87)	0.1 (0.1 to 0.2)	-51.2 (-58.7 to -38.7)	-39.4 (-48.7 to -23.9)
European Region	Adverse effects of medical treatment	10 to 14	Female	49 (38 to 65)	0.2 (0.1 to 0.2)	43 (35 to 61)	0.1 (0.1 to 0.2)	22 (17 to 30)	0.1 (0.1 to 0.1)	-55.4 (-62.0 to -49.3)	-44.2 (-52.4 to -36.6)
European Region	Adverse effects of medical treatment	10 to 14	Male	87 (62 to 108)	0.3 (0.2 to 0.3)	84 (61 to 106)	0.3 (0.2 to 0.3)	44 (33 to 63)	0.2 (0.1 to 0.2)	-48.8 (-58.9 to -30.4)	-36.7 (-49.3 to -14.1)
European Region	Animal contact	5 to 9	Both	78 (56 to 101)	0.1 (0.1 to 0.2)	62 (46 to 76)	0.1 (0.1 to 0.1)	32 (26 to 42)	0.1 (0.0 to 0.1)	-58.5 (-70.5 to -40.9)	-51.3 (-65.4 to -30.6)

eTable 8. All-cause and cause-specific mortality (counts and rates) at the global level and by country in the WHO European Region for ages 5 to 9 and 10 to 14 for 1990, 2000, and 2016

European Region	Animal contact	5 to 9	Female	30 (20 to 45)	0.1 (0.1 to 0.1)	23 (15 to 31)	0.1 (0.1 to 0.1)	11 (9 to 13)	0.0 (0.0 to 0.0)	-62.0 (-76.0 to -44.0)	-55.1 (-71.6 to -33.9)
European Region	Animal contact	5 to 9	Male	48 (31 to 67)	0.1 (0.1 to 0.2)	39 (27 to 50)	0.1 (0.1 to 0.2)	21 (16 to 30)	0.1 (0.1 to 0.1)	-55.3 (-71.0 to -27.0)	-47.9 (-66.2 to -14.8)
European Region	Animal contact	10 to 14	Both	49 (33 to 62)	0.1 (0.1 to 0.1)	47 (33 to 58)	0.1 (0.1 to 0.1)	20 (16 to 25)	0.0 (0.0 to 0.0)	-58.9 (-69.2 to -42.4)	-48.9 (-61.7 to -28.4)
European Region	Animal contact	10 to 14	Female	20 (11 to 30)	0.1 (0.0 to 0.1)	18 (11 to 24)	0.1 (0.0 to 0.1)	7 (6 to 9)	0.0 (0.0 to 0.0)	-62.4 (-77.1 to -41.8)	-53.0 (-71.3 to -27.2)
European Region	Animal contact	10 to 14	Male	29 (19 to 38)	0.1 (0.1 to 0.1)	29 (19 to 38)	0.1 (0.1 to 0.1)	13 (9 to 18)	0.0 (0.0 to 0.1)	-55.3 (-66.9 to -33.4)	-44.8 (-59.2 to -17.8)
European Region	Foreign body	5 to 9	Both	360 (282 to 482)	0.6 (0.4 to 0.7)	298 (244 to 391)	0.5 (0.4 to 0.7)	192 (163 to 249)	0.3 (0.3 to 0.5)	-45.6 (-60.3 to -23.9)	-36.2 (-53.4 to -10.7)
European Region	Foreign body	5 to 9	Female	140 (105 to 185)	0.4 (0.3 to 0.6)	124 (96 to 162)	0.4 (0.3 to 0.6)	80 (67 to 104)	0.3 (0.2 to 0.4)	-41.8 (-55.1 to -21.6)	-31.2 (-47.0 to -7.4)
European Region	Foreign body	5 to 9	Male	220 (165 to 310)	0.7 (0.5 to 0.9)	175 (135 to 236)	0.6 (0.5 to 0.8)	112 (90 to 162)	0.4 (0.3 to 0.6)	-47.9 (-64.8 to -22.6)	-39.2 (-58.9 to -9.6)
European Region	Foreign body	10 to 14	Both	288 (243 to 381)	0.5 (0.4 to 0.6)	279 (233 to 368)	0.4 (0.4 to 0.6)	155 (129 to 214)	0.3 (0.3 to 0.4)	-45.9 (-56.8 to -26.4)	-32.8 (-46.3 to -8.6)
European Region	Foreign body	10 to 14	Female	91 (72 to 112)	0.3 (0.2 to 0.4)	95 (78 to 124)	0.3 (0.2 to 0.4)	55 (46 to 71)	0.2 (0.2 to 0.3)	-39.3 (-51.0 to -23.0)	-24.0 (-38.7 to -3.7)
European Region	Foreign body	10 to 14	Male	197 (157 to 278)	0.6 (0.5 to 0.9)	184 (145 to 265)	0.6 (0.4 to 0.8)	100 (78 to 153)	0.4 (0.3 to 0.6)	-48.9 (-61.6 to -26.6)	-36.9 (-52.6 to -9.3)
European Region	Environmental heat and cold exposure	5 to 9	Both	93 (59 to 119)	0.1 (0.1 to 0.2)	70 (45 to 87)	0.1 (0.1 to 0.2)	29 (19 to 39)	0.1 (0.0 to 0.1)	-68.7 (-75.7 to -58.3)	-63.3 (-71.5 to -51.0)
European Region	Environmental heat and cold exposure	5 to 9	Female	40 (22 to 57)	0.1 (0.1 to 0.2)	27 (16 to 39)	0.1 (0.1 to 0.1)	10 (7 to 16)	0.0 (0.0 to 0.1)	-74.5 (-81.9 to -64.5)	-69.9 (-78.6 to -58.1)
European Region	Environmental heat and cold exposure	5 to 9	Male	53 (27 to 74)	0.2 (0.1 to 0.2)	43 (23 to 58)	0.1 (0.1 to 0.2)	19 (11 to 26)	0.1 (0.0 to 0.1)	-63.9 (-73.9 to -48.7)	-57.9 (-69.6 to -40.2)
European Region	Environmental heat and cold exposure	10 to 14	Both	82 (47 to 107)	0.1 (0.1 to 0.2)	85 (48 to 109)	0.1 (0.1 to 0.2)	28 (18 to 37)	0.1 (0.0 to 0.1)	-65.1 (-72.4 to -56.6)	-56.6 (-65.7 to -46.1)
European Region	Environmental heat and cold exposure	10 to 14	Female	27 (14 to 37)	0.1 (0.0 to 0.1)	25 (14 to 35)	0.1 (0.0 to 0.1)	8 (5 to 12)	0.0 (0.0 to 0.0)	-68.7 (-77.1 to -59.3)	-60.9 (-71.4 to -49.1)
European Region	Environmental heat and cold exposure	10 to 14	Male	56 (26 to 77)	0.2 (0.1 to 0.2)	60 (28 to 83)	0.2 (0.1 to 0.3)	20 (11 to 27)	0.1 (0.0 to 0.1)	-63.0 (-72.7 to -51.2)	-54.4 (-66.3 to -39.7)
European Region	Other unintentional injuries	5 to 9	Both	582 (469 to 721)	0.9 (0.7 to 1.1)	372 (305 to 436)	0.7 (0.5 to 0.8)	98 (84 to 115)	0.2 (0.2 to 0.2)	-82.9 (-87.0 to -77.9)	-79.9 (-84.7 to -74.1)
European Region	Other unintentional injuries	5 to 9	Female	183 (126 to 264)	0.6 (0.4 to 0.8)	113 (82 to 151)	0.4 (0.3 to 0.5)	27 (23 to 33)	0.1 (0.1 to 0.1)	-84.5 (-90.2 to -77.3)	-81.7 (-88.4 to -73.2)
European Region	Other unintentional injuries	5 to 9	Male	399 (311 to 511)	1.2 (0.9 to 1.5)	259 (213 to 309)	0.9 (0.7 to 1.1)	71 (59 to 86)	0.3 (0.2 to 0.3)	-82.0 (-87.2 to -75.7)	-79.0 (-85.1 to -71.6)
European Region	Other unintentional injuries	10 to 14	Both	631 (533 to 739)	1.0 (0.8 to 1.2)	568 (495 to 648)	0.9 (0.8 to 1.0)	145 (124 to 170)	0.3 (0.2 to 0.3)	-76.8 (-81.5 to -71.2)	-71.2 (-77.0 to -64.2)
European Region	Other unintentional injuries	10 to 14	Female	146 (108 to 200)	0.5 (0.3 to 0.6)	120 (94 to 148)	0.4 (0.3 to 0.5)	28 (23 to 35)	0.1 (0.1 to 0.1)	-80.1 (-87.0 to -72.3)	-75.1 (-83.7 to -65.3)
European Region	Other unintentional injuries	10 to 14	Male	485 (400 to 586)	1.5 (1.2 to 1.8)	449 (378 to 523)	1.4 (1.1 to 1.6)	117 (98 to 141)	0.4 (0.4 to 0.5)	-75.7 (-81.2 to -69.1)	-70.0 (-76.8 to -61.9)
European Region	Self-harm	10 to 14	Both	1023 (829 to 1145)	1.6 (1.3 to 1.8)	1287 (935 to 1449)	2.0 (1.5 to 2.2)	610 (479 to 726)	1.2 (0.9 to 1.4)	-40.3 (-51.2 to -28.9)	-25.8 (-39.4 to -11.7)
European Region	Self-harm	10 to 14	Female	233 (211 to 261)	0.7 (0.7 to 0.8)	295 (259 to 335)	0.9 (0.8 to 1.1)	178 (148 to 213)	0.7 (0.6 to 0.9)	-23.3 (-37.0 to -6.6)	-4.1 (-21.2 to 16.8)
European Region	Self-harm	10 to 14	Male	790 (594 to 908)	2.4 (1.8 to 2.8)	992 (646 to 1149)	3.0 (2.0 to 3.5)	432 (301 to 532)	1.6 (1.1 to 2.0)	-45.2 (-58.4 to -31.6)	-32.4 (-48.6 to -15.6)

eTable 8. All-cause and cause-specific mortality (counts and rates) at the global level and by country in the WHO European Region for ages 5 to 9 and 10 to 14 for 1990, 2000, and 2016

European Region	Interpersonal violence	5 to 9	Both	456 (375 to 659)	0.7 (0.6 to 1.0)	400 (288 to 478)	0.7 (0.5 to 0.8)	198 (151 to 270)	0.4 (0.3 to 0.5)	-56.0 (-67.4 to -44.1)	-48.3 (-61.8 to -34.4)
European Region	Interpersonal violence	5 to 9	Female	207 (154 to 308)	0.7 (0.5 to 1.0)	186 (124 to 223)	0.7 (0.5 to 0.8)	96 (66 to 147)	0.4 (0.2 to 0.5)	-52.8 (-68.7 to -23.3)	-44.2 (-63.0 to -9.4)
European Region	Interpersonal violence	5 to 9	Male	249 (171 to 422)	0.8 (0.5 to 1.3)	214 (123 to 284)	0.7 (0.4 to 1.0)	102 (61 to 150)	0.4 (0.2 to 0.5)	-58.3 (-69.1 to -45.9)	-51.3 (-63.9 to -36.9)
European Region	Interpersonal violence	10 to 14	Both	535 (429 to 747)	0.8 (0.7 to 1.2)	669 (436 to 814)	1.0 (0.7 to 1.3)	269 (198 to 346)	0.5 (0.4 to 0.7)	-49.4 (-59.8 to -37.1)	-37.1 (-50.0 to -21.9)
European Region	Interpersonal violence	10 to 14	Female	194 (154 to 287)	0.6 (0.5 to 0.9)	260 (162 to 317)	0.8 (0.5 to 1.0)	107 (75 to 155)	0.4 (0.3 to 0.6)	-44.1 (-60.4 to -24.4)	-30.1 (-50.5 to -5.4)
European Region	Interpersonal violence	10 to 14	Male	341 (226 to 541)	1.1 (0.7 to 1.7)	408 (217 to 531)	1.2 (0.7 to 1.6)	161 (99 to 229)	0.6 (0.4 to 0.9)	-52.1 (-62.4 to -36.3)	-40.9 (-53.5 to -21.3)
European Region	Exposure to forces of nature	5 to 9	Both	25 (12 to 37)	0.0 (0.0 to 0.1)	38 (17 to 58)	0.1 (0.0 to 0.1)	7 (2 to 13)	0.0 (0.0 to 0.0)	-68.3 (-90.8 to -32.8)	-62.8 (-89.2 to -21.1)
European Region	Exposure to forces of nature	5 to 9	Female	12 (6 to 17)	0.0 (0.0 to 0.1)	17 (8 to 27)	0.1 (0.0 to 0.1)	3 (1 to 6)	0.0 (0.0 to 0.0)	-68.6 (-90.9 to -33.5)	-62.9 (-89.2 to -21.4)
European Region	Exposure to forces of nature	5 to 9	Male	13 (7 to 19)	0.0 (0.0 to 0.1)	20 (9 to 31)	0.1 (0.0 to 0.1)	4 (1 to 7)	0.0 (0.0 to 0.0)	-68.0 (-90.7 to -32.2)	-62.7 (-89.1 to -20.9)
European Region	Exposure to forces of nature	10 to 14	Both	29 (15 to 43)	0.0 (0.0 to 0.1)	66 (28 to 103)	0.1 (0.0 to 0.2)	9 (3 to 15)	0.0 (0.0 to 0.0)	-68.6 (-90.9 to -33.4)	-61.0 (-88.6 to -17.3)
European Region	Exposure to forces of nature	10 to 14	Female	12 (6 to 17)	0.0 (0.0 to 0.1)	27 (11 to 41)	0.1 (0.0 to 0.1)	3 (1 to 6)	0.0 (0.0 to 0.0)	-69.2 (-91.0 to -34.6)	-61.4 (-88.8 to -18.2)
European Region	Exposure to forces of nature	10 to 14	Male	17 (9 to 26)	0.1 (0.0 to 0.1)	40 (17 to 62)	0.1 (0.1 to 0.2)	5 (2 to 9)	0.0 (0.0 to 0.0)	-68.2 (-90.7 to -32.7)	-60.8 (-88.6 to -16.9)
European Region	Conflict and terrorism	5 to 9	Both	37 (24 to 51)	0.1 (0.0 to 0.1)	163 (87 to 238)	0.3 (0.2 to 0.4)	91 (21 to 163)	0.2 (0.0 to 0.3)	158.4 (-42.4 to 429.4)	203.3 (-32.4 to 521.5)
European Region	Conflict and terrorism	5 to 9	Female	19 (13 to 27)	0.1 (0.0 to 0.1)	85 (45 to 123)	0.3 (0.2 to 0.4)	47 (11 to 84)	0.2 (0.0 to 0.3)	157.0 (-42.7 to 426.5)	203.5 (-32.3 to 521.8)
European Region	Conflict and terrorism	5 to 9	Male	18 (12 to 24)	0.1 (0.0 to 0.1)	79 (42 to 115)	0.3 (0.1 to 0.4)	44 (10 to 78)	0.2 (0.0 to 0.3)	160.0 (-42.0 to 432.7)	203.4 (-32.3 to 521.6)
European Region	Conflict and terrorism	10 to 14	Both	35 (23 to 49)	0.1 (0.0 to 0.1)	226 (114 to 337)	0.4 (0.2 to 0.5)	94 (21 to 167)	0.2 (0.0 to 0.3)	176.0 (-38.8 to 464.6)	242.8 (-23.9 to 601.4)
European Region	Conflict and terrorism	10 to 14	Female	17 (11 to 23)	0.1 (0.0 to 0.1)	108 (54 to 161)	0.3 (0.2 to 0.5)	45 (10 to 80)	0.2 (0.0 to 0.3)	173.6 (-39.3 to 459.8)	242.2 (-24.1 to 600.1)
European Region	Conflict and terrorism	10 to 14	Male	18 (12 to 25)	0.1 (0.0 to 0.1)	118 (60 to 177)	0.4 (0.2 to 0.5)	49 (11 to 87)	0.2 (0.0 to 0.3)	178.2 (-38.3 to 469.0)	243.3 (-23.8 to 602.3)
European Region	Executions and police conflict	5 to 9	Both	8 (7 to 11)	0.0 (0.0 to 0.0)	7 (6 to 10)	0.0 (0.0 to 0.0)	6 (5 to 7)	0.0 (0.0 to 0.0)	-23.6 (-44.1 to -9.9)	-10.3 (-34.4 to 5.8)
European Region	Executions and police conflict	5 to 9	Female	3 (3 to 5)	0.0 (0.0 to 0.0)	3 (3 to 4)	0.0 (0.0 to 0.0)	3 (2 to 3)	0.0 (0.0 to 0.0)	-24.3 (-44.8 to -14.2)	-10.6 (-34.8 to 1.3)
European Region	Executions and police conflict	5 to 9	Male	4 (3 to 8)	0.0 (0.0 to 0.0)	4 (3 to 6)	0.0 (0.0 to 0.0)	3 (3 to 4)	0.0 (0.0 to 0.0)	-22.1 (-49.0 to -0.2)	-9.1 (-40.5 to 16.4)
European Region	Executions and police conflict	10 to 14	Both	9 (7 to 12)	0.0 (0.0 to 0.0)	8 (7 to 11)	0.0 (0.0 to 0.0)	6 (5 to 6)	0.0 (0.0 to 0.0)	-33.6 (-51.6 to -16.5)	-17.5 (-39.9 to 3.7)
European Region	Executions and police conflict	10 to 14	Female	4 (3 to 6)	0.0 (0.0 to 0.0)	4 (3 to 6)	0.0 (0.0 to 0.0)	2 (2 to 3)	0.0 (0.0 to 0.0)	-31.3 (-58.0 to -20.3)	-14.0 (-47.4 to -0.3)
European Region	Executions and police conflict	10 to 14	Male	5 (4 to 7)	0.0 (0.0 to 0.0)	5 (4 to 7)	0.0 (0.0 to 0.0)	3 (3 to 4)	0.0 (0.0 to 0.0)	-34.2 (-55.6 to -8.9)	-18.7 (-45.2 to 12.5)
Albania	All causes	5 to 9	Both	242 (210 to 277)	68.4 (59.6 to 78.5)	207 (178 to 240)	63.3 (54.4 to 73.1)	50 (36 to 68)	32.4 (23.6 to 43.8)	-79.3 (-85.2 to -71.3)	-52.7 (-66.2 to -34.1)
Albania	All causes	5 to 9	Female	98 (86 to 111)	57.2 (50.5 to 64.9)	78 (66 to 90)	49.1 (41.8 to 56.4)	20 (15 to 26)	26.7 (20.7 to 34.5)	-79.8 (-84.9 to -73.0)	-53.4 (-65.1 to -37.8)

eTable 8. All-cause and cause-specific mortality (counts and rates) at the global level and by country in the WHO European Region for ages 5 to 9 and 10 to 14 for 1990, 2000, and 2016

Albania	All causes	5 to 9	Male	144 (125 to 167)	79.0 (68.5 to 91.7)	129 (111 to 151)	76.6 (66.1 to 89.5)	30 (21 to 42)	37.7 (26.3 to 52.6)	-79.1 (-85.7 to -69.9)	-52.3 (-67.3 to -31.3)
Albania	All causes	10 to 14	Both	162 (151 to 175)	49.0 (45.5 to 52.9)	155 (143 to 168)	45.9 (42.3 to 49.7)	55 (41 to 70)	29.9 (22.3 to 38.2)	-66.2 (-75.2 to -56.1)	-39.0 (-55.3 to -20.7)
Albania	All causes	10 to 14	Female	60 (56 to 64)	37.3 (35.1 to 39.9)	53 (49 to 58)	32.1 (29.6 to 34.5)	19 (15 to 24)	21.8 (17.1 to 27.7)	-67.8 (-75.2 to -59.6)	-41.4 (-54.9 to -26.5)
Albania	All causes	10 to 14	Male	103 (95 to 111)	60.1 (55.4 to 65.2)	102 (94 to 111)	59.2 (54.5 to 64.5)	36 (26 to 46)	37.3 (26.9 to 48.5)	-65.3 (-75.3 to -53.9)	-37.9 (-55.8 to -17.3)
Albania	Tuberculosis	5 to 9	Both	0 (0 to 0)	0.1 (0.1 to 0.1)	0 (0 to 0)	0.1 (0.0 to 0.1)	0 (0 to 0)	0.0 (0.0 to 0.0)	-92.6 (-95.7 to -86.6)	-83.0 (-90.1 to -69.3)
Albania	Tuberculosis	5 to 9	Female	0 (0 to 0)	0.0 (0.0 to 0.1)	0 (0 to 0)	0.0 (0.0 to 0.0)	0 (0 to 0)	0.0 (0.0 to 0.0)	-82.5 (-90.5 to -63.1)	-59.7 (-78.0 to -14.9)
Albania	Tuberculosis	5 to 9	Male	0 (0 to 0)	0.1 (0.1 to 0.2)	0 (0 to 0)	0.1 (0.1 to 0.1)	0 (0 to 0)	0.0 (0.0 to 0.0)	-94.8 (-97.3 to -89.6)	-88.1 (-93.9 to -76.2)
Albania	Tuberculosis	10 to 14	Both	0 (0 to 0)	0.1 (0.1 to 0.1)	0 (0 to 0)	0.0 (0.0 to 0.1)	0 (0 to 0)	0.0 (0.0 to 0.0)	-89.8 (-94.5 to -82.0)	-81.6 (-90.1 to -67.5)
Albania	Tuberculosis	10 to 14	Female	0 (0 to 0)	0.0 (0.0 to 0.0)	0 (0 to 0)	0.0 (0.0 to 0.0)	0 (0 to 0)	0.0 (0.0 to 0.0)	-77.2 (-86.8 to -58.6)	-58.5 (-76.1 to -24.8)
Albania	Tuberculosis	10 to 14	Male	0 (0 to 0)	0.1 (0.1 to 0.2)	0 (0 to 0)	0.1 (0.0 to 0.1)	0 (0 to 0)	0.0 (0.0 to 0.0)	-92.4 (-96.5 to -84.1)	-86.4 (-93.7 to -71.6)
Albania	HIV/AIDS	5 to 9	Both	0 (0 to 0)	0.0 (0.0 to 0.0)	0 (0 to 0)	0.0 (0.0 to 0.0)	0 (0 to 0)	0.0 (0.0 to 0.0)	-60.8 (-76.0 to -39.4)	-10.1 (-45.0 to 38.9)
Albania	HIV/AIDS	5 to 9	Female	0 (0 to 0)	0.0 (0.0 to 0.0)	0 (0 to 0)	0.0 (0.0 to 0.0)	0 (0 to 0)	0.0 (0.0 to 0.0)	-60.7 (-76.1 to -39.2)	-9.4 (-45.0 to 40.1)
Albania	HIV/AIDS	5 to 9	Male	0 (0 to 0)	0.0 (0.0 to 0.0)	0 (0 to 0)	0.0 (0.0 to 0.0)	0 (0 to 0)	0.0 (0.0 to 0.0)	-60.8 (-75.9 to -39.5)	-10.8 (-45.1 to 37.8)
Albania	HIV/AIDS	10 to 14	Both	0 (0 to 0)	0.0 (0.0 to 0.0)	0 (0 to 0)	0.0 (0.0 to 0.0)	0 (0 to 0)	0.0 (0.0 to 0.0)	473.5 (259.7 to 737.5)	934.8 (549.1 to 1411.2)
Albania	HIV/AIDS	10 to 14	Female	0 (0 to 0)	0.0 (0.0 to 0.0)	0 (0 to 0)	0.0 (0.0 to 0.0)	0 (0 to 0)	0.0 (0.0 to 0.0)	468.6 (256.8 to 731.5)	934.1 (548.9 to 1412.1)
Albania	HIV/AIDS	10 to 14	Male	0 (0 to 0)	0.0 (0.0 to 0.0)	0 (0 to 0)	0.0 (0.0 to 0.0)	0 (0 to 0)	0.0 (0.0 to 0.0)	478.0 (260.1 to 742.7)	935.4 (545.0 to 1409.5)
Albania	Diarrheal diseases	5 to 9	Both	1 (0 to 1)	0.2 (0.0 to 0.4)	0 (0 to 1)	0.1 (0.0 to 0.3)	0 (0 to 0)	0.1 (0.0 to 0.1)	-84.5 (-94.4 to -58.3)	-64.5 (-87.1 to -4.4)
Albania	Diarrheal diseases	5 to 9	Female	0 (0 to 1)	0.1 (0.0 to 0.3)	0 (0 to 0)	0.1 (0.0 to 0.2)	0 (0 to 0)	0.0 (0.0 to 0.1)	-86.4 (-96.1 to -60.1)	-68.6 (-91.1 to -8.1)
Albania	Diarrheal diseases	5 to 9	Male	0 (0 to 1)	0.2 (0.1 to 0.5)	0 (0 to 1)	0.2 (0.0 to 0.4)	0 (0 to 0)	0.1 (0.0 to 0.2)	-83.5 (-95.4 to -46.0)	-62.4 (-89.6 to 23.1)
Albania	Diarrheal diseases	10 to 14	Both	0 (0 to 0)	0.1 (0.0 to 0.1)	0 (0 to 0)	0.0 (0.0 to 0.1)	0 (0 to 0)	0.0 (0.0 to 0.1)	-78.1 (-91.5 to -51.0)	-60.5 (-84.7 to -11.6)
Albania	Diarrheal diseases	10 to 14	Female	0 (0 to 0)	0.1 (0.0 to 0.2)	0 (0 to 0)	0.0 (0.0 to 0.1)	0 (0 to 0)	0.0 (0.0 to 0.0)	-83.1 (-94.6 to -49.3)	-69.2 (-90.1 to -7.7)
Albania	Diarrheal diseases	10 to 14	Male	0 (0 to 0)	0.1 (0.0 to 0.2)	0 (0 to 0)	0.1 (0.0 to 0.1)	0 (0 to 0)	0.0 (0.0 to 0.1)	-74.4 (-90.7 to -36.6)	-54.2 (-83.3 to 13.6)
Albania	Intestinal infectious diseases	5 to 9	Both	0 (0 to 0)	0.0 (0.0 to 0.0)	0 (0 to 0)	0.0 (0.0 to 0.0)	0 (0 to 0)	0.0 (0.0 to 0.0)	-49.9 (-74.6 to -10.7)	14.8 (-41.8 to 104.5)
Albania	Intestinal infectious diseases	5 to 9	Female	0 (0 to 0)	0.0 (0.0 to 0.0)	0 (0 to 0)	0.0 (0.0 to 0.0)	0 (0 to 0)	0.0 (0.0 to 0.0)	-52.0 (-80.4 to -0.1)	10.6 (-54.8 to 130.3)
Albania	Intestinal infectious diseases	5 to 9	Male	0 (0 to 0)	0.0 (0.0 to 0.0)	0 (0 to 0)	0.0 (0.0 to 0.0)	0 (0 to 0)	0.0 (0.0 to 0.0)	-48.0 (-75.4 to 4.3)	18.6 (-43.9 to 137.7)
Albania	Intestinal infectious diseases	10 to 14	Both	0 (0 to 0)	0.0 (0.0 to 0.0)	0 (0 to 0)	0.0 (0.0 to 0.0)	0 (0 to 0)	0.0 (0.0 to 0.0)	-35.7 (-63.5 to 1.9)	16.0 (-34.2 to 83.9)
Albania	Intestinal infectious diseases	10 to 14	Female	0 (0 to 0)	0.0 (0.0 to 0.0)	0 (0 to 0)	0.0 (0.0 to 0.0)	0 (0 to 0)	0.0 (0.0 to 0.0)	-40.8 (-70.9 to 13.5)	7.6 (-47.2 to 106.5)
Albania	Intestinal infectious diseases	10 to 14	Male	0 (0 to 0)	0.0 (0.0 to 0.0)	0 (0 to 0)	0.0 (0.0 to 0.0)	0 (0 to 0)	0.0 (0.0 to 0.0)	-30.8 (-64.5 to 20.2)	23.9 (-36.4 to 115.3)
Albania	Lower respiratory infections	5 to 9	Both	27 (12 to 39)	7.6 (3.3 to 10.9)	21 (12 to 29)	6.4 (3.8 to 8.8)	4 (2 to 6)	2.5 (1.5 to 3.7)	-85.9 (-92.0 to -70.7)	-67.7 (-81.7 to -32.9)

eTable 8. All-cause and cause-specific mortality (counts and rates) at the global level and by country in the WHO European Region for ages 5 to 9 and 10 to 14 for 1990, 2000, and 2016

Albania	Lower respiratory infections	5 to 9	Female	14 (5 to 21)	8.1 (3.1 to 12.3)	10 (5 to 14)	6.1 (3.3 to 9.0)	2 (1 to 3)	2.4 (1.4 to 3.9)	-87.2 (-93.5 to -71.8)	-70.5 (-85.1 to -35.1)
Albania	Lower respiratory infections	5 to 9	Male	13 (6 to 20)	7.2 (3.2 to 11.0)	11 (6 to 16)	6.7 (3.8 to 9.7)	2 (1 to 3)	2.5 (1.4 to 4.3)	-84.5 (-92.4 to -67.1)	-64.8 (-82.7 to -25.1)
Albania	Lower respiratory infections	10 to 14	Both	14 (7 to 20)	4.4 (2.2 to 6.0)	11 (7 to 14)	3.1 (2.2 to 4.1)	3 (2 to 4)	1.5 (1.0 to 2.3)	-80.4 (-89.5 to -58.2)	-64.6 (-81.1 to -24.6)
Albania	Lower respiratory infections	10 to 14	Female	7 (3 to 10)	4.3 (2.0 to 6.4)	5 (3 to 7)	2.9 (1.9 to 4.0)	1 (1 to 2)	1.3 (0.7 to 2.1)	-83.1 (-92.5 to -61.9)	-69.2 (-86.3 to -30.8)
Albania	Lower respiratory infections	10 to 14	Male	7 (4 to 11)	4.4 (2.1 to 6.5)	6 (4 to 8)	3.4 (2.2 to 4.8)	2 (1 to 3)	1.7 (1.0 to 2.7)	-77.9 (-89.7 to -54.0)	-60.3 (-81.5 to -17.7)
Albania	Upper respiratory infections	5 to 9	Both	0 (0 to 0)	0.0 (0.0 to 0.0)	0 (0 to 0)	0.0 (0.0 to 0.0)	0 (0 to 0)	0.0 (0.0 to 0.0)	-82.0 (-99.7 to 683.2)	-58.7 (-99.2 to 1694.7)
Albania	Upper respiratory infections	5 to 9	Female	0 (0 to 0)	0.0 (0.0 to 0.0)	0 (0 to 0)	0.0 (0.0 to 0.0)	0 (0 to 0)	0.0 (0.0 to 0.0)	-80.2 (-99.9 to 28702.1)	-54.4 (-99.9 to 66286.5)
Albania	Upper respiratory infections	5 to 9	Male	0 (0 to 0)	0.0 (0.0 to 0.0)	0 (0 to 0)	0.0 (0.0 to 0.0)	0 (0 to 0)	0.0 (0.0 to 0.0)	-83.1 (-100.0 to 18550.7)	-61.5 (-100.0 to 42403.7)
Albania	Upper respiratory infections	10 to 14	Both	0 (0 to 0)	0.0 (0.0 to 0.0)	0 (0 to 0)	0.0 (0.0 to 0.0)	0 (0 to 0)	0.0 (0.0 to 0.0)	-73.2 (-99.6 to 999.3)	-51.6 (-99.2 to 1883.6)
Albania	Upper respiratory infections	10 to 14	Female	0 (0 to 0)	0.0 (0.0 to 0.0)	0 (0 to 0)	0.0 (0.0 to 0.0)	0 (0 to 0)	0.0 (0.0 to 0.0)	-75.0 (-100.0 to 14121.0)	-54.6 (-99.9 to 25763.0)
Albania	Upper respiratory infections	10 to 14	Male	0 (0 to 0)	0.0 (0.0 to 0.0)	0 (0 to 0)	0.0 (0.0 to 0.0)	0 (0 to 0)	0.0 (0.0 to 0.0)	-72.1 (-100.0 to 10130.4)	-50.0 (-100.0 to 18226.3)
Albania	Otitis media	5 to 9	Both	0 (0 to 0)	0.0 (0.0 to 0.0)	0 (0 to 0)	0.0 (0.0 to 0.0)	0 (0 to 0)	0.0 (0.0 to 0.0)	-80.9 (-97.3 to 18.5)	-56.3 (-93.9 to 171.5)
Albania	Otitis media	5 to 9	Female	0 (0 to 0)	0.0 (0.0 to 0.0)	0 (0 to 0)	0.0 (0.0 to 0.0)	0 (0 to 0)	0.0 (0.0 to 0.0)	-78.5 (-97.7 to 90.8)	-50.5 (-94.7 to 339.9)
Albania	Otitis media	5 to 9	Male	0 (0 to 0)	0.0 (0.0 to 0.0)	0 (0 to 0)	0.0 (0.0 to 0.0)	0 (0 to 0)	0.0 (0.0 to 0.0)	-81.1 (-97.7 to 28.2)	-56.9 (-94.7 to 192.2)
Albania	Otitis media	10 to 14	Both	0 (0 to 0)	0.0 (0.0 to 0.0)	0 (0 to 0)	0.0 (0.0 to 0.0)	0 (0 to 0)	0.0 (0.0 to 0.0)	-76.6 (-95.4 to 34.0)	-57.7 (-91.7 to 141.9)
Albania	Otitis media	10 to 14	Female	0 (0 to 0)	0.0 (0.0 to 0.0)	0 (0 to 0)	0.0 (0.0 to 0.0)	0 (0 to 0)	0.0 (0.0 to 0.0)	-63.6 (-97.0 to 169.4)	-33.8 (-94.6 to 389.9)
Albania	Otitis media	10 to 14	Male	0 (0 to 0)	0.0 (0.0 to 0.0)	0 (0 to 0)	0.0 (0.0 to 0.0)	0 (0 to 0)	0.0 (0.0 to 0.0)	-85.3 (-98.1 to 14.7)	-73.6 (-96.5 to 105.5)
Albania	Meningitis	5 to 9	Both	5 (2 to 7)	1.3 (0.7 to 2.1)	4 (2 to 7)	1.2 (0.7 to 2.1)	1 (0 to 2)	0.6 (0.3 to 1.1)	-80.4 (-90.0 to -60.0)	-55.2 (-77.0 to -8.5)
Albania	Meningitis	5 to 9	Female	2 (1 to 3)	1.2 (0.7 to 2.0)	2 (1 to 3)	1.1 (0.6 to 1.9)	0 (0 to 1)	0.4 (0.2 to 0.8)	-86.2 (-93.5 to -68.3)	-68.2 (-85.0 to -27.0)
Albania	Meningitis	5 to 9	Male	3 (1 to 5)	1.4 (0.3 to 2.7)	2 (1 to 5)	1.4 (0.4 to 2.8)	1 (0 to 1)	0.8 (0.4 to 1.6)	-75.8 (-90.0 to -24.6)	-44.9 (-77.1 to 71.8)
Albania	Meningitis	10 to 14	Both	3 (2 to 5)	1.0 (0.6 to 1.5)	3 (2 to 5)	0.8 (0.5 to 1.3)	1 (1 to 2)	0.5 (0.3 to 0.9)	-73.1 (-86.5 to -51.8)	-51.5 (-75.6 to -13.0)
Albania	Meningitis	10 to 14	Female	2 (1 to 2)	1.0 (0.6 to 1.4)	1 (1 to 2)	0.7 (0.4 to 1.1)	0 (0 to 0)	0.3 (0.2 to 0.5)	-83.4 (-92.3 to -63.9)	-69.9 (-86.0 to -34.3)
Albania	Meningitis	10 to 14	Male	2 (1 to 3)	1.1 (0.3 to 1.8)	2 (1 to 3)	0.9 (0.4 to 1.8)	1 (0 to 1)	0.7 (0.3 to 1.3)	-64.3 (-85.2 to -13.1)	-36.0 (-73.6 to 55.7)
Albania	Encephalitis	5 to 9	Both	5 (2 to 7)	1.3 (0.6 to 2.1)	5 (3 to 8)	1.6 (0.8 to 2.4)	1 (1 to 2)	0.9 (0.5 to 1.4)	-70.7 (-86.1 to -33.5)	-33.0 (-68.1 to 52.3)
Albania	Encephalitis	5 to 9	Female	2 (1 to 4)	1.2 (0.5 to 2.2)	2 (1 to 4)	1.3 (0.7 to 2.4)	1 (0 to 1)	0.8 (0.4 to 1.3)	-72.6 (-87.6 to -31.6)	-37.0 (-71.3 to 57.5)
Albania	Encephalitis	5 to 9	Male	2 (1 to 5)	1.3 (0.6 to 2.5)	3 (1 to 5)	1.8 (0.7 to 3.1)	1 (0 to 1)	0.9 (0.4 to 1.7)	-69.1 (-88.8 to -26.0)	-29.5 (-74.5 to 68.6)
Albania	Encephalitis	10 to 14	Both	3 (2 to 5)	0.9 (0.6 to 1.5)	3 (2 to 5)	1.0 (0.7 to 1.4)	1 (1 to 2)	0.7 (0.4 to 1.1)	-58.8 (-77.4 to -26.5)	-25.6 (-59.2 to 32.7)

eTable 8. All-cause and cause-specific mortality (counts and rates) at the global level and by country in the WHO European Region for ages 5 to 9 and 10 to 14 for 1990, 2000, and 2016

Albania	Encephalitis	10 to 14	Female	1 (1 to 2)	0.9 (0.5 to 1.5)	1 (1 to 2)	0.9 (0.5 to 1.4)	1 (0 to 1)	0.6 (0.3 to 1.0)	-63.7 (-82.2 to -28.1)	-34.0 (-67.6 to 30.7)
Albania	Encephalitis	10 to 14	Male	2 (1 to 3)	1.0 (0.6 to 1.7)	2 (1 to 3)	1.1 (0.6 to 1.8)	1 (0 to 1)	0.8 (0.4 to 1.3)	-54.5 (-78.4 to -14.9)	-18.5 (-61.4 to 52.4)
Albania	Diphtheria	5 to 9	Both	0 (0 to 0)	0.0 (0.0 to 0.0)	0 (0 to 0)	0.0 (0.0 to 0.0)	0 (0 to 0)	0.0 (0.0 to 0.0)	-77.7 (-97.4 to 102.1)	-48.8 (-94.1 to 363.2)
Albania	Diphtheria	5 to 9	Female	0 (0 to 0)	0.0 (0.0 to 0.0)	0 (0 to 0)	0.0 (0.0 to 0.0)	0 (0 to 0)	0.0 (0.0 to 0.0)	-78.2 (-98.7 to 338.8)	-49.8 (-96.9 to 911.4)
Albania	Diphtheria	5 to 9	Male	0 (0 to 0)	0.0 (0.0 to 0.0)	0 (0 to 0)	0.0 (0.0 to 0.0)	0 (0 to 0)	0.0 (0.0 to 0.0)	-77.3 (-99.1 to 588.5)	-48.2 (-97.9 to 1469.0)
Albania	Diphtheria	10 to 14	Both	0 (0 to 0)	0.0 (0.0 to 0.0)	0 (0 to 0)	0.0 (0.0 to 0.0)	0 (0 to 0)	0.0 (0.0 to 0.0)	-69.5 (-95.7 to 75.7)	-45.0 (-92.3 to 217.0)
Albania	Diphtheria	10 to 14	Female	0 (0 to 0)	0.0 (0.0 to 0.0)	0 (0 to 0)	0.0 (0.0 to 0.0)	0 (0 to 0)	0.0 (0.0 to 0.0)	-71.6 (-98.7 to 743.8)	-48.4 (-97.6 to 1434.5)
Albania	Diphtheria	10 to 14	Male	0 (0 to 0)	0.0 (0.0 to 0.0)	0 (0 to 0)	0.0 (0.0 to 0.0)	0 (0 to 0)	0.0 (0.0 to 0.0)	-68.4 (-98.3 to 436.3)	-43.3 (-97.0 to 860.8)
Albania	Whooping cough	5 to 9	Both	1 (0 to 2)	0.2 (0.0 to 0.6)	0 (0 to 1)	0.1 (0.0 to 0.4)	0 (0 to 0)	0.0 (0.0 to 0.0)	-97.2 (-99.8 to -66.3)	-93.5 (-99.6 to -22.8)
Albania	Whooping cough	5 to 9	Female	0 (0 to 1)	0.2 (0.0 to 0.7)	0 (0 to 1)	0.1 (0.0 to 0.4)	0 (0 to 0)	0.0 (0.0 to 0.0)	-97.1 (-99.8 to -65.2)	-93.4 (-99.6 to -19.9)
Albania	Whooping cough	5 to 9	Male	0 (0 to 1)	0.2 (0.0 to 0.5)	0 (0 to 1)	0.1 (0.0 to 0.3)	0 (0 to 0)	0.0 (0.0 to 0.0)	-97.2 (-99.8 to -66.1)	-93.6 (-99.6 to -22.9)
Albania	Whooping cough	10 to 14	Both	0 (0 to 0)	0.0 (0.0 to 0.1)	0 (0 to 0)	0.0 (0.0 to 0.1)	0 (0 to 0)	0.0 (0.0 to 0.0)	-96.8 (-99.8 to -61.7)	-94.3 (-99.7 to -30.9)
Albania	Whooping cough	10 to 14	Female	0 (0 to 0)	0.0 (0.0 to 0.1)	0 (0 to 0)	0.0 (0.0 to 0.1)	0 (0 to 0)	0.0 (0.0 to 0.0)	-97.0 (-99.8 to -61.6)	-94.5 (-99.7 to -30.1)
Albania	Whooping cough	10 to 14	Male	0 (0 to 0)	0.0 (0.0 to 0.1)	0 (0 to 0)	0.0 (0.0 to 0.1)	0 (0 to 0)	0.0 (0.0 to 0.0)	-96.7 (-99.8 to -60.1)	-94.0 (-99.6 to -28.6)
Albania	Tetanus	5 to 9	Both	0 (0 to 0)	0.0 (0.0 to 0.0)	0 (0 to 0)	0.0 (0.0 to 0.0)	0 (0 to 0)	0.0 (0.0 to 0.0)	-44.4 (-63.4 to -18.7)	27.5 (-16.2 to 86.3)
Albania	Tetanus	5 to 9	Female	0 (0 to 0)	0.0 (0.0 to 0.0)	0 (0 to 0)	0.0 (0.0 to 0.0)	0 (0 to 0)	0.0 (0.0 to 0.0)	-43.4 (-61.6 to -20.1)	30.4 (-11.6 to 84.2)
Albania	Tetanus	5 to 9	Male	0 (0 to 0)	0.0 (0.0 to 0.0)	0 (0 to 0)	0.0 (0.0 to 0.0)	0 (0 to 0)	0.0 (0.0 to 0.0)	-45.2 (-65.8 to -17.0)	24.8 (-22.0 to 89.2)
Albania	Tetanus	10 to 14	Both	0 (0 to 0)	0.0 (0.0 to 0.0)	0 (0 to 0)	0.0 (0.0 to 0.0)	0 (0 to 0)	0.0 (0.0 to 0.0)	-36.5 (-55.8 to -13.8)	14.5 (-20.2 to 55.6)
Albania	Tetanus	10 to 14	Female	0 (0 to 0)	0.0 (0.0 to 0.0)	0 (0 to 0)	0.0 (0.0 to 0.0)	0 (0 to 0)	0.0 (0.0 to 0.0)	-39.6 (-56.9 to -18.5)	9.9 (-21.6 to 48.3)
Albania	Tetanus	10 to 14	Male	0 (0 to 0)	0.0 (0.0 to 0.0)	0 (0 to 0)	0.0 (0.0 to 0.0)	0 (0 to 0)	0.0 (0.0 to 0.0)	-33.7 (-55.9 to -7.1)	18.7 (-20.9 to 66.5)
Albania	Measles	5 to 9	Both	43 (28 to 60)	12.1 (7.9 to 17.1)	0 (0 to 0)	0.1 (0.0 to 0.1)	0 (0 to 0)	0.0 (0.0 to 0.0)	-100.0 (-100.0 to -99.9)	-99.9 (-99.9 to -99.9)
Albania	Measles	5 to 9	Female	21 (14 to 30)	12.3 (8.1 to 17.3)	0 (0 to 0)	0.1 (0.0 to 0.1)	0 (0 to 0)	0.0 (0.0 to 0.0)	-100.0 (-100.0 to -99.9)	-99.9 (-99.9 to -99.9)
Albania	Measles	5 to 9	Male	22 (14 to 31)	11.9 (7.6 to 17.0)	0 (0 to 0)	0.1 (0.0 to 0.1)	0 (0 to 0)	0.0 (0.0 to 0.0)	-100.0 (-100.0 to -99.9)	-99.9 (-99.9 to -99.8)
Albania	Measles	10 to 14	Both	12 (7 to 17)	3.5 (2.3 to 5.1)	0 (0 to 0)	0.0 (0.0 to 0.0)	0 (0 to 0)	0.0 (0.0 to 0.0)	-100.0 (-100.0 to -99.9)	-99.9 (-100.0 to -99.9)
Albania	Measles	10 to 14	Female	6 (4 to 9)	3.7 (2.4 to 5.5)	0 (0 to 0)	0.0 (0.0 to 0.0)	0 (0 to 0)	0.0 (0.0 to 0.0)	-100.0 (-100.0 to -99.9)	-99.9 (-100.0 to -99.9)
Albania	Measles	10 to 14	Male	6 (4 to 8)	3.3 (2.1 to 5.0)	0 (0 to 0)	0.0 (0.0 to 0.0)	0 (0 to 0)	0.0 (0.0 to 0.0)	-100.0 (-100.0 to -99.9)	-99.9 (-99.9 to -99.9)
Albania	Varicella and herpes zoster	5 to 9	Both	0 (0 to 1)	0.1 (0.0 to 0.2)	0 (0 to 0)	0.1 (0.0 to 0.1)	0 (0 to 0)	0.0 (0.0 to 0.1)	-85.8 (-97.4 to -24.0)	-67.4 (-94.0 to 74.1)
Albania	Varicella and herpes zoster	5 to 9	Female	0 (0 to 0)	0.1 (0.0 to 0.2)	0 (0 to 0)	0.1 (0.0 to 0.1)	0 (0 to 0)	0.0 (0.0 to 0.1)	-85.9 (-99.0 to 93.8)	-67.4 (-97.6 to 346.7)
Albania	Varicella and herpes zoster	5 to 9	Male	0 (0 to 0)	0.1 (0.0 to 0.2)	0 (0 to 0)	0.1 (0.0 to 0.2)	0 (0 to 0)	0.0 (0.0 to 0.1)	-85.7 (-98.6 to 75.3)	-67.4 (-96.9 to 299.5)
Albania	Varicella and herpes zoster	10 to 14	Both	0 (0 to 0)	0.0 (0.0 to 0.1)	0 (0 to 0)	0.0 (0.0 to 0.0)	0 (0 to 0)	0.0 (0.0 to 0.0)	-83.3 (-97.0 to -5.0)	-69.9 (-94.6 to 71.5)
Albania	Varicella and herpes zoster	10 to 14	Female	0 (0 to 0)	0.0 (0.0 to 0.1)	0 (0 to 0)	0.0 (0.0 to 0.0)	0 (0 to 0)	0.0 (0.0 to 0.0)	-84.8 (-99.1 to 156.2)	-72.3 (-98.3 to 365.9)
Albania	Varicella and herpes zoster	10 to 14	Male	0 (0 to 0)	0.0 (0.0 to 0.1)	0 (0 to 0)	0.0 (0.0 to 0.0)	0 (0 to 0)	0.0 (0.0 to 0.0)	-81.9 (-99.0 to 132.0)	-67.6 (-98.2 to 315.6)

eTable 8. All-cause and cause-specific mortality (counts and rates) at the global level and by country in the WHO European Region for ages 5 to 9 and 10 to 14 for 1990, 2000, and 2016

Albania	Malaria	5 to 9	Both	0<br>(0 to 0)	0.0<br>(0.0 to 0.0)	0<br>(0 to 0)	0.0<br>(0.0 to 0.0)	0<br>(0 to 0)	0.0<br>(0.0 to 0.0)	0.0<br>(0.0 to 0.0)	0.0<br>(0.0 to 0.0)
Albania	Malaria	5 to 9	Female	0<br>(0 to 0)	0.0<br>(0.0 to 0.0)	0<br>(0 to 0)	0.0<br>(0.0 to 0.0)	0<br>(0 to 0)	0.0<br>(0.0 to 0.0)	0.0<br>(0.0 to 0.0)	0.0<br>(0.0 to 0.0)
Albania	Malaria	5 to 9	Male	0<br>(0 to 0)	0.0<br>(0.0 to 0.0)	0<br>(0 to 0)	0.0<br>(0.0 to 0.0)	0<br>(0 to 0)	0.0<br>(0.0 to 0.0)	0.0<br>(0.0 to 0.0)	0.0<br>(0.0 to 0.0)
Albania	Malaria	10 to 14	Both	0<br>(0 to 0)	0.0<br>(0.0 to 0.0)	0<br>(0 to 0)	0.0<br>(0.0 to 0.0)	0<br>(0 to 0)	0.0<br>(0.0 to 0.0)	0.0<br>(0.0 to 0.0)	0.0<br>(0.0 to 0.0)
Albania	Malaria	10 to 14	Female	0<br>(0 to 0)	0.0<br>(0.0 to 0.0)	0<br>(0 to 0)	0.0<br>(0.0 to 0.0)	0<br>(0 to 0)	0.0<br>(0.0 to 0.0)	0.0<br>(0.0 to 0.0)	0.0<br>(0.0 to 0.0)
Albania	Malaria	10 to 14	Male	0<br>(0 to 0)	0.0<br>(0.0 to 0.0)	0<br>(0 to 0)	0.0<br>(0.0 to 0.0)	0<br>(0 to 0)	0.0<br>(0.0 to 0.0)	0.0<br>(0.0 to 0.0)	0.0<br>(0.0 to 0.0)
Albania	Leishmaniasis	5 to 9	Both	0<br>(0 to 0)	0.0<br>(0.0 to 0.0)	0<br>(0 to 0)	0.0<br>(0.0 to 0.0)	0<br>(0 to 0)	0.0<br>(0.0 to 0.0)	-13.3<br>(-100.0 to 738.7)	98.7<br>(-100.0 to 1821.7)
Albania	Leishmaniasis	5 to 9	Female	0<br>(0 to 0)	0.0<br>(0.0 to 0.0)	0<br>(0 to 0)	0.0<br>(0.0 to 0.0)	0<br>(0 to 0)	0.0<br>(0.0 to 0.0)	-8.6<br>(-100.0 to 361.8)	110.6<br>(-100.0 to 964.4)
Albania	Leishmaniasis	5 to 9	Male	0<br>(0 to 0)	0.0<br>(0.0 to 0.0)	0<br>(0 to 0)	0.0<br>(0.0 to 0.0)	0<br>(0 to 0)	0.0<br>(0.0 to 0.0)	-17.3<br>(-100.0 to 287.1)	88.5<br>(-100.0 to 782.2)
Albania	Leishmaniasis	10 to 14	Both	0<br>(0 to 0)	0.0<br>(0.0 to 0.0)	0<br>(0 to 0)	0.0<br>(0.0 to 0.0)	0<br>(0 to 0)	0.0<br>(0.0 to 0.0)	-22.5<br>(-100.0 to 768.3)	39.8<br>(-100.0 to 1466.8)
Albania	Leishmaniasis	10 to 14	Female	0<br>(0 to 0)	0.0<br>(0.0 to 0.0)	0<br>(0 to 0)	0.0<br>(0.0 to 0.0)	0<br>(0 to 0)	0.0<br>(0.0 to 0.0)	-23.2<br>(-100.0 to 263.6)	39.7<br>(-100.0 to 561.2)
Albania	Leishmaniasis	10 to 14	Male	0<br>(0 to 0)	0.0<br>(0.0 to 0.0)	0<br>(0 to 0)	0.0<br>(0.0 to 0.0)	0<br>(0 to 0)	0.0<br>(0.0 to 0.0)	-21.9<br>(-100.0 to 261.5)	39.9<br>(-100.0 to 547.5)
Albania	African trypanosomiasis	5 to 9	Both	0<br>(0 to 0)	0.0<br>(0.0 to 0.0)	0<br>(0 to 0)	0.0<br>(0.0 to 0.0)	0<br>(0 to 0)	0.0<br>(0.0 to 0.0)	0.0<br>(0.0 to 0.0)	0.0<br>(0.0 to 0.0)
Albania	African trypanosomiasis	5 to 9	Female	0<br>(0 to 0)	0.0<br>(0.0 to 0.0)	0<br>(0 to 0)	0.0<br>(0.0 to 0.0)	0<br>(0 to 0)	0.0<br>(0.0 to 0.0)	0.0<br>(0.0 to 0.0)	0.0<br>(0.0 to 0.0)
Albania	African trypanosomiasis	5 to 9	Male	0<br>(0 to 0)	0.0<br>(0.0 to 0.0)	0<br>(0 to 0)	0.0<br>(0.0 to 0.0)	0<br>(0 to 0)	0.0<br>(0.0 to 0.0)	0.0<br>(0.0 to 0.0)	0.0<br>(0.0 to 0.0)
Albania	African trypanosomiasis	10 to 14	Both	0<br>(0 to 0)	0.0<br>(0.0 to 0.0)	0<br>(0 to 0)	0.0<br>(0.0 to 0.0)	0<br>(0 to 0)	0.0<br>(0.0 to 0.0)	0.0<br>(0.0 to 0.0)	0.0<br>(0.0 to 0.0)
Albania	African trypanosomiasis	10 to 14	Female	0<br>(0 to 0)	0.0<br>(0.0 to 0.0)	0<br>(0 to 0)	0.0<br>(0.0 to 0.0)	0<br>(0 to 0)	0.0<br>(0.0 to 0.0)	0.0<br>(0.0 to 0.0)	0.0<br>(0.0 to 0.0)
Albania	African trypanosomiasis	10 to 14	Male	0<br>(0 to 0)	0.0<br>(0.0 to 0.0)	0<br>(0 to 0)	0.0<br>(0.0 to 0.0)	0<br>(0 to 0)	0.0<br>(0.0 to 0.0)	0.0<br>(0.0 to 0.0)	0.0<br>(0.0 to 0.0)
Albania	Schistosomiasis	5 to 9	Both	0<br>(0 to 0)	0.0<br>(0.0 to 0.0)	0<br>(0 to 0)	0.0<br>(0.0 to 0.0)	0<br>(0 to 0)	0.0<br>(0.0 to 0.0)	0.0<br>(0.0 to 0.0)	0.0<br>(0.0 to 0.0)
Albania	Schistosomiasis	5 to 9	Female	0<br>(0 to 0)	0.0<br>(0.0 to 0.0)	0<br>(0 to 0)	0.0<br>(0.0 to 0.0)	0<br>(0 to 0)	0.0<br>(0.0 to 0.0)	0.0<br>(0.0 to 0.0)	0.0<br>(0.0 to 0.0)
Albania	Schistosomiasis	5 to 9	Male	0<br>(0 to 0)	0.0<br>(0.0 to 0.0)	0<br>(0 to 0)	0.0<br>(0.0 to 0.0)	0<br>(0 to 0)	0.0<br>(0.0 to 0.0)	0.0<br>(0.0 to 0.0)	0.0<br>(0.0 to 0.0)
Albania	Schistosomiasis	10 to 14	Both	0<br>(0 to 0)	0.0<br>(0.0 to 0.0)	0<br>(0 to 0)	0.0<br>(0.0 to 0.0)	0<br>(0 to 0)	0.0<br>(0.0 to 0.0)	0.0<br>(0.0 to 0.0)	0.0<br>(0.0 to 0.0)
Albania	Schistosomiasis	10 to 14	Female	0<br>(0 to 0)	0.0<br>(0.0 to 0.0)	0<br>(0 to 0)	0.0<br>(0.0 to 0.0)	0<br>(0 to 0)	0.0<br>(0.0 to 0.0)	0.0<br>(0.0 to 0.0)	0.0<br>(0.0 to 0.0)
Albania	Schistosomiasis	10 to 14	Male	0<br>(0 to 0)	0.0<br>(0.0 to 0.0)	0<br>(0 to 0)	0.0<br>(0.0 to 0.0)	0<br>(0 to 0)	0.0<br>(0.0 to 0.0)	0.0<br>(0.0 to 0.0)	0.0<br>(0.0 to 0.0)
Albania	Cysticercosis	5 to 9	Both	0<br>(0 to 0)	0.0<br>(0.0 to 0.0)	0<br>(0 to 0)	0.0<br>(0.0 to 0.0)	0<br>(0 to 0)	0.0<br>(0.0 to 0.0)	-89.8<br>(-93.2 to -85.2)	-76.6<br>(-84.5 to -66.1)
Albania	Cysticercosis	5 to 9	Female	0<br>(0 to 0)	0.0<br>(0.0 to 0.0)	0<br>(0 to 0)	0.0<br>(0.0 to 0.0)	0<br>(0 to 0)	0.0<br>(0.0 to 0.0)	-89.8<br>(-93.2 to -85.6)	-76.5<br>(-84.3 to -66.8)
Albania	Cysticercosis	5 to 9	Male	0<br>(0 to 0)	0.0<br>(0.0 to 0.0)	0<br>(0 to 0)	0.0<br>(0.0 to 0.0)	0<br>(0 to 0)	0.0<br>(0.0 to 0.0)	-89.7<br>(-93.5 to -84.6)	-76.6<br>(-85.2 to -64.8)
Albania	Cysticercosis	10 to 14	Both	0<br>(0 to 0)	0.0<br>(0.0 to 0.0)	0<br>(0 to 0)	0.0<br>(0.0 to 0.0)	0<br>(0 to 0)	0.0<br>(0.0 to 0.0)	-85.1<br>(-89.6 to -79.9)	-73.2<br>(-81.2 to -63.8)
Albania	Cysticercosis	10 to 14	Female	0<br>(0 to 0)	0.0<br>(0.0 to 0.0)	0<br>(0 to 0)	0.0<br>(0.0 to 0.0)	0<br>(0 to 0)	0.0<br>(0.0 to 0.0)	-86.3<br>(-90.3 to -81.6)	-75.2<br>(-82.3 to -66.6)

eTable 8. All-cause and cause-specific mortality (counts and rates) at the global level and by country in the WHO European Region for ages 5 to 9 and 10 to 14 for 1990, 2000, and 2016

Albania	Cysticercosis	10 to 14	Male	0 (0 to 0)	0.0 (0.0 to 0.0)	0 (0 to 0)	0.0 (0.0 to 0.0)	0 (0 to 0)	0.0 (0.0 to 0.0)	-84.1 (-89.4 to -77.8)	-71.6 (-81.0 to -60.3)
Albania	Yellow fever	5 to 9	Both	0 (0 to 0)	0.0 (0.0 to 0.0)	0 (0 to 0)	0.0 (0.0 to 0.0)	0 (0 to 0)	0.0 (0.0 to 0.0)	0.0 (0.0 to 0.0)	0.0 (0.0 to 0.0)
Albania	Yellow fever	5 to 9	Female	0 (0 to 0)	0.0 (0.0 to 0.0)	0 (0 to 0)	0.0 (0.0 to 0.0)	0 (0 to 0)	0.0 (0.0 to 0.0)	0.0 (0.0 to 0.0)	0.0 (0.0 to 0.0)
Albania	Yellow fever	5 to 9	Male	0 (0 to 0)	0.0 (0.0 to 0.0)	0 (0 to 0)	0.0 (0.0 to 0.0)	0 (0 to 0)	0.0 (0.0 to 0.0)	0.0 (0.0 to 0.0)	0.0 (0.0 to 0.0)
Albania	Yellow fever	10 to 14	Both	0 (0 to 0)	0.0 (0.0 to 0.0)	0 (0 to 0)	0.0 (0.0 to 0.0)	0 (0 to 0)	0.0 (0.0 to 0.0)	0.0 (0.0 to 0.0)	0.0 (0.0 to 0.0)
Albania	Yellow fever	10 to 14	Female	0 (0 to 0)	0.0 (0.0 to 0.0)	0 (0 to 0)	0.0 (0.0 to 0.0)	0 (0 to 0)	0.0 (0.0 to 0.0)	0.0 (0.0 to 0.0)	0.0 (0.0 to 0.0)
Albania	Yellow fever	10 to 14	Male	0 (0 to 0)	0.0 (0.0 to 0.0)	0 (0 to 0)	0.0 (0.0 to 0.0)	0 (0 to 0)	0.0 (0.0 to 0.0)	0.0 (0.0 to 0.0)	0.0 (0.0 to 0.0)
Albania	Rabies	5 to 9	Both	0 (0 to 0)	0.0 (0.0 to 0.0)	0 (0 to 0)	0.0 (0.0 to 0.0)	0 (0 to 0)	0.0 (0.0 to 0.0)	-97.1 (-99.6 to -85.3)	-93.4 (-99.1 to -66.4)
Albania	Rabies	5 to 9	Female	0 (0 to 0)	0.0 (0.0 to 0.0)	0 (0 to 0)	0.0 (0.0 to 0.0)	0 (0 to 0)	0.0 (0.0 to 0.0)	-98.3 (-99.9 to -87.8)	-96.0 (-99.7 to -71.9)
Albania	Rabies	5 to 9	Male	0 (0 to 0)	0.0 (0.0 to 0.0)	0 (0 to 0)	0.0 (0.0 to 0.0)	0 (0 to 0)	0.0 (0.0 to 0.0)	-94.8 (-99.6 to -62.2)	-88.2 (-99.1 to -13.9)
Albania	Rabies	10 to 14	Both	0 (0 to 0)	0.0 (0.0 to 0.0)	0 (0 to 0)	0.0 (0.0 to 0.0)	0 (0 to 0)	0.0 (0.0 to 0.0)	-86.3 (-97.4 to 24.0)	-75.2 (-95.4 to 123.8)
Albania	Rabies	10 to 14	Female	0 (0 to 0)	0.0 (0.0 to 0.0)	0 (0 to 0)	0.0 (0.0 to 0.0)	0 (0 to 0)	0.0 (0.0 to 0.0)	-70.9 (-95.6 to 727.0)	-47.0 (-92.0 to 1404.1)
Albania	Rabies	10 to 14	Male	0 (0 to 0)	0.0 (0.0 to 0.0)	0 (0 to 0)	0.0 (0.0 to 0.0)	0 (0 to 0)	0.0 (0.0 to 0.0)	-91.4 (-99.2 to -35.6)	-84.7 (-98.5 to 15.3)
Albania	Intestinal nematode infections	5 to 9	Both	0 (0 to 0)	0.0 (0.0 to 0.0)	0 (0 to 0)	0.0 (0.0 to 0.0)	0 (0 to 0)	0.0 (0.0 to 0.0)	-91.5 (-100.0 to 17.8)	-80.6 (-100.0 to 169.8)
Albania	Intestinal nematode infections	5 to 9	Female	0 (0 to 0)	0.0 (0.0 to 0.1)	0 (0 to 0)	0.0 (0.0 to 0.0)	0 (0 to 0)	0.0 (0.0 to 0.0)	-91.5 (-100.0 to 0.0)	-80.4 (-100.0 to 11.4)
Albania	Intestinal nematode infections	5 to 9	Male	0 (0 to 0)	0.0 (0.0 to 0.0)	0 (0 to 0)	0.0 (0.0 to 0.0)	0 (0 to 0)	0.0 (0.0 to 0.0)	-91.6 (-100.0 to 0.0)	-80.8 (-100.0 to 34.3)
Albania	Intestinal nematode infections	10 to 14	Both	0 (0 to 0)	0.0 (0.0 to 0.0)	0 (0 to 0)	0.0 (0.0 to 0.0)	0 (0 to 0)	0.0 (0.0 to 0.0)	-87.6 (-100.0 to 45.2)	-77.6 (-100.0 to 162.0)
Albania	Intestinal nematode infections	10 to 14	Female	0 (0 to 0)	0.0 (0.0 to 0.0)	0 (0 to 0)	0.0 (0.0 to 0.0)	0 (0 to 0)	0.0 (0.0 to 0.0)	-88.3 (-100.0 to 0.0)	-78.8 (-100.0 to 0.9)
Albania	Intestinal nematode infections	10 to 14	Male	0 (0 to 0)	0.0 (0.0 to 0.0)	0 (0 to 0)	0.0 (0.0 to 0.0)	0 (0 to 0)	0.0 (0.0 to 0.0)	-86.9 (-100.0 to 0.0)	-76.5 (-100.0 to 57.1)
Albania	Ebola	5 to 9	Both	0 (0 to 0)	0.0 (0.0 to 0.0)	0 (0 to 0)	0.0 (0.0 to 0.0)	0 (0 to 0)	0.0 (0.0 to 0.0)	0.0 (0.0 to 0.0)	0.0 (0.0 to 0.0)
Albania	Ebola	5 to 9	Female	0 (0 to 0)	0.0 (0.0 to 0.0)	0 (0 to 0)	0.0 (0.0 to 0.0)	0 (0 to 0)	0.0 (0.0 to 0.0)	0.0 (0.0 to 0.0)	0.0 (0.0 to 0.0)
Albania	Ebola	5 to 9	Male	0 (0 to 0)	0.0 (0.0 to 0.0)	0 (0 to 0)	0.0 (0.0 to 0.0)	0 (0 to 0)	0.0 (0.0 to 0.0)	0.0 (0.0 to 0.0)	0.0 (0.0 to 0.0)
Albania	Ebola	10 to 14	Both	0 (0 to 0)	0.0 (0.0 to 0.0)	0 (0 to 0)	0.0 (0.0 to 0.0)	0 (0 to 0)	0.0 (0.0 to 0.0)	0.0 (0.0 to 0.0)	0.0 (0.0 to 0.0)
Albania	Ebola	10 to 14	Female	0 (0 to 0)	0.0 (0.0 to 0.0)	0 (0 to 0)	0.0 (0.0 to 0.0)	0 (0 to 0)	0.0 (0.0 to 0.0)	0.0 (0.0 to 0.0)	0.0 (0.0 to 0.0)
Albania	Ebola	10 to 14	Male	0 (0 to 0)	0.0 (0.0 to 0.0)	0 (0 to 0)	0.0 (0.0 to 0.0)	0 (0 to 0)	0.0 (0.0 to 0.0)	0.0 (0.0 to 0.0)	0.0 (0.0 to 0.0)
Albania	Zika virus	5 to 9	Both	0 (0 to 0)	0.0 (0.0 to 0.0)	0 (0 to 0)	0.0 (0.0 to 0.0)	0 (0 to 0)	0.0 (0.0 to 0.0)	0.0 (0.0 to 0.0)	0.0 (0.0 to 0.0)
Albania	Zika virus	5 to 9	Female	0 (0 to 0)	0.0 (0.0 to 0.0)	0 (0 to 0)	0.0 (0.0 to 0.0)	0 (0 to 0)	0.0 (0.0 to 0.0)	0.0 (0.0 to 0.0)	0.0 (0.0 to 0.0)
Albania	Zika virus	5 to 9	Male	0 (0 to 0)	0.0 (0.0 to 0.0)	0 (0 to 0)	0.0 (0.0 to 0.0)	0 (0 to 0)	0.0 (0.0 to 0.0)	0.0 (0.0 to 0.0)	0.0 (0.0 to 0.0)
Albania	Zika virus	10 to 14	Both	0 (0 to 0)	0.0 (0.0 to 0.0)	0 (0 to 0)	0.0 (0.0 to 0.0)	0 (0 to 0)	0.0 (0.0 to 0.0)	0.0 (0.0 to 0.0)	0.0 (0.0 to 0.0)

eTable 8. All-cause and cause-specific mortality (counts and rates) at the global level and by country in the WHO European Region for ages 5 to 9 and 10 to 14 for 1990, 2000, and 2016

Albania	Zika virus	10 to 14	Female	0 (0 to 0)	0.0 (0.0 to 0.0)	0 (0 to 0)	0.0 (0.0 to 0.0)	0 (0 to 0)	0.0 (0.0 to 0.0)	0.0 (0.0 to 0.0)	0.0 (0.0 to 0.0)
Albania	Zika virus	10 to 14	Male	0 (0 to 0)	0.0 (0.0 to 0.0)	0 (0 to 0)	0.0 (0.0 to 0.0)	0 (0 to 0)	0.0 (0.0 to 0.0)	0.0 (0.0 to 0.0)	0.0 (0.0 to 0.0)
Albania	Other neglected tropical diseases	5 to 9	Both	0 (0 to 0)	0.1 (0.0 to 0.1)	0 (0 to 0)	0.0 (0.0 to 0.1)	0 (0 to 0)	0.0 (0.0 to 0.1)	-80.3 (-94.2 to -26.1)	-54.9 (-86.7 to 69.2)
Albania	Other neglected tropical diseases	5 to 9	Female	0 (0 to 0)	0.0 (0.0 to 0.0)	0 (0 to 0)	0.0 (0.0 to 0.0)	0 (0 to 0)	0.0 (0.0 to 0.0)	-44.2 (-64.9 to -20.1)	28.5 (-19.1 to 84.2)
Albania	Other neglected tropical diseases	5 to 9	Male	0 (0 to 0)	0.1 (0.0 to 0.3)	0 (0 to 0)	0.1 (0.0 to 0.1)	0 (0 to 0)	0.0 (0.0 to 0.1)	-83.8 (-96.5 to -23.6)	-63.0 (-92.0 to 74.0)
Albania	Other neglected tropical diseases	10 to 14	Both	0 (0 to 0)	0.0 (0.0 to 0.0)	0 (0 to 0)	0.0 (0.0 to 0.0)	0 (0 to 0)	0.0 (0.0 to 0.0)	-38.5 (-64.8 to -12.6)	10.9 (-36.5 to 57.6)
Albania	Other neglected tropical diseases	10 to 14	Female	0 (0 to 0)	0.0 (0.0 to 0.0)	0 (0 to 0)	0.0 (0.0 to 0.0)	0 (0 to 0)	0.0 (0.0 to 0.0)	-39.7 (-57.5 to -18.5)	9.6 (-22.6 to 48.3)
Albania	Other neglected tropical diseases	10 to 14	Male	0 (0 to 0)	0.0 (0.0 to 0.0)	0 (0 to 0)	0.0 (0.0 to 0.0)	0 (0 to 0)	0.0 (0.0 to 0.0)	-37.5 (-68.4 to -3.3)	11.9 (-43.4 to 73.2)
Albania	Maternal hemorrhage	10 to 14	Both	0 (0 to 0)	0.0 (0.0 to 0.0)	0 (0 to 0)	0.0 (0.0 to 0.0)	0 (0 to 0)	0.0 (0.0 to 0.0)	-92.5 (-96.7 to -80.6)	-86.5 (-94.1 to -65.0)
Albania	Maternal hemorrhage	10 to 14	Female	0 (0 to 0)	0.0 (0.0 to 0.0)	0 (0 to 0)	0.0 (0.0 to 0.0)	0 (0 to 0)	0.0 (0.0 to 0.0)	-92.5 (-96.7 to -80.6)	-86.3 (-94.0 to -64.8)
Albania	Maternal sepsis and other maternal infections	10 to 14	Both	0 (0 to 0)	0.0 (0.0 to 0.0)	0 (0 to 0)	0.0 (0.0 to 0.0)	0 (0 to 0)	0.0 (0.0 to 0.0)	-92.9 (-96.7 to -82.2)	-87.3 (-94.1 to -67.9)
Albania	Maternal sepsis and other maternal infections	10 to 14	Female	0 (0 to 0)	0.0 (0.0 to 0.0)	0 (0 to 0)	0.0 (0.0 to 0.0)	0 (0 to 0)	0.0 (0.0 to 0.0)	-92.9 (-96.7 to -82.2)	-87.2 (-94.0 to -67.7)
Albania	Maternal hypertensive disorders	10 to 14	Both	0 (0 to 0)	0.0 (0.0 to 0.0)	0 (0 to 0)	0.0 (0.0 to 0.0)	0 (0 to 0)	0.0 (0.0 to 0.0)	-92.8 (-96.7 to -81.6)	-86.9 (-94.1 to -66.7)
Albania	Maternal hypertensive disorders	10 to 14	Female	0 (0 to 0)	0.0 (0.0 to 0.0)	0 (0 to 0)	0.0 (0.0 to 0.0)	0 (0 to 0)	0.0 (0.0 to 0.0)	-92.8 (-96.7 to -81.6)	-86.8 (-94.1 to -66.5)
Albania	Maternal obstructed labor and uterine rupture	10 to 14	Both	0 (0 to 0)	0.0 (0.0 to 0.0)	0 (0 to 0)	0.0 (0.0 to 0.0)	0 (0 to 0)	0.0 (0.0 to 0.0)	-93.0 (-97.2 to -80.2)	-87.3 (-94.9 to -64.4)
Albania	Maternal obstructed labor and uterine rupture	10 to 14	Female	0 (0 to 0)	0.0 (0.0 to 0.0)	0 (0 to 0)	0.0 (0.0 to 0.0)	0 (0 to 0)	0.0 (0.0 to 0.0)	-93.0 (-97.2 to -80.2)	-87.2 (-94.9 to -64.1)
Albania	Maternal abortion, miscarriage, and ectopic pregnancy	10 to 14	Both	0 (0 to 0)	0.0 (0.0 to 0.0)	0 (0 to 0)	0.0 (0.0 to 0.0)	0 (0 to 0)	0.0 (0.0 to 0.0)	12.8 (-52.5 to 225.2)	103.5 (-14.3 to 486.9)

eTable 8. All-cause and cause-specific mortality (counts and rates) at the global level and by country in the WHO European Region for ages 5 to 9 and 10 to 14 for 1990, 2000, and 2016

Albania	Maternal abortion, miscarriage, and ectopic pregnancy	10 to 14	Female	0 (0 to 0)	0.0 (0.0 to 0.0)	0 (0 to 0)	0.0 (0.0 to 0.0)	0 (0 to 0)	0.0 (0.0 to 0.0)	12.8 (-52.5 to 225.2)	105.1 (-13.7 to 491.5)
Albania	Indirect maternal deaths	10 to 14	Both	0 (0 to 0)	0.0 (0.0 to 0.0)	0 (0 to 0)	0.0 (0.0 to 0.0)	0 (0 to 0)	0.0 (0.0 to 0.0)	-93.0 (-96.8 to -81.9)	-87.3 (-94.2 to -67.4)
Albania	Indirect maternal deaths	10 to 14	Female	0 (0 to 0)	0.0 (0.0 to 0.0)	0 (0 to 0)	0.0 (0.0 to 0.0)	0 (0 to 0)	0.0 (0.0 to 0.0)	-93.0 (-96.8 to -81.9)	-87.2 (-94.2 to -67.2)
Albania	Late maternal deaths	10 to 14	Both	0 (0 to 0)	0.0 (0.0 to 0.0)	0 (0 to 0)	0.0 (0.0 to 0.0)	0 (0 to 0)	0.0 (0.0 to 0.0)	-93.4 (-97.1 to -83.4)	-88.1 (-94.7 to -70.1)
Albania	Late maternal deaths	10 to 14	Female	0 (0 to 0)	0.0 (0.0 to 0.0)	0 (0 to 0)	0.0 (0.0 to 0.0)	0 (0 to 0)	0.0 (0.0 to 0.0)	-93.4 (-97.1 to -83.4)	-88.0 (-94.7 to -69.8)
Albania	Maternal deaths aggravated by HIV/AIDS	10 to 14	Both	0 (0 to 0)	0.0 (0.0 to 0.0)	0 (0 to 0)	0.0 (0.0 to 0.0)	0 (0 to 0)	0.0 (0.0 to 0.0)	48.5 (-41.1 to 303.7)	168.0 (6.2 to 628.5)
Albania	Maternal deaths aggravated by HIV/AIDS	10 to 14	Female	0 (0 to 0)	0.0 (0.0 to 0.0)	0 (0 to 0)	0.0 (0.0 to 0.0)	0 (0 to 0)	0.0 (0.0 to 0.0)	48.5 (-41.1 to 303.7)	170.1 (7.1 to 634.2)
Albania	Other maternal disorders	10 to 14	Both	0 (0 to 0)	0.0 (0.0 to 0.0)	0 (0 to 0)	0.0 (0.0 to 0.0)	0 (0 to 0)	0.0 (0.0 to 0.0)	-93.1 (-96.9 to -83.4)	-87.6 (-94.5 to -70.0)
Albania	Other maternal disorders	10 to 14	Female	0 (0 to 0)	0.0 (0.0 to 0.1)	0 (0 to 0)	0.0 (0.0 to 0.0)	0 (0 to 0)	0.0 (0.0 to 0.0)	-93.1 (-96.9 to -83.4)	-87.5 (-94.4 to -69.8)
Albania	Protein-energy malnutrition	5 to 9	Both	0 (0 to 1)	0.1 (0.1 to 0.2)	0 (0 to 1)	0.1 (0.1 to 0.2)	0 (0 to 0)	0.0 (0.0 to 0.1)	-89.0 (-95.8 to -73.4)	-74.7 (-90.4 to -39.1)
Albania	Protein-energy malnutrition	5 to 9	Female	0 (0 to 0)	0.1 (0.0 to 0.2)	0 (0 to 0)	0.1 (0.0 to 0.3)	0 (0 to 0)	0.0 (0.0 to 0.1)	-86.7 (-96.4 to -55.9)	-69.3 (-91.7 to 1.6)
Albania	Protein-energy malnutrition	5 to 9	Male	0 (0 to 0)	0.1 (0.0 to 0.2)	0 (0 to 0)	0.1 (0.0 to 0.2)	0 (0 to 0)	0.0 (0.0 to 0.0)	-91.9 (-98.2 to -67.7)	-81.6 (-95.8 to -26.3)
Albania	Protein-energy malnutrition	10 to 14	Both	0 (0 to 0)	0.1 (0.0 to 0.1)	0 (0 to 0)	0.1 (0.0 to 0.1)	0 (0 to 0)	0.0 (0.0 to 0.0)	-89.0 (-95.4 to -74.5)	-80.1 (-91.8 to -54.0)
Albania	Protein-energy malnutrition	10 to 14	Female	0 (0 to 0)	0.1 (0.0 to 0.1)	0 (0 to 0)	0.1 (0.0 to 0.1)	0 (0 to 0)	0.0 (0.0 to 0.0)	-86.4 (-95.5 to -59.7)	-75.2 (-91.8 to -26.7)
Albania	Protein-energy malnutrition	10 to 14	Male	0 (0 to 0)	0.1 (0.0 to 0.1)	0 (0 to 0)	0.1 (0.1 to 0.2)	0 (0 to 0)	0.0 (0.0 to 0.0)	-90.7 (-97.3 to -72.6)	-83.3 (-95.1 to -50.8)
Albania	Iodine deficiency	5 to 9	Both	0 (0 to 0)	0.0 (0.0 to 0.1)	0 (0 to 0)	0.0 (0.0 to 0.1)	0 (0 to 0)	0.0 (0.0 to 0.0)	-79.0 (-100.0 to 6.7e+18)	-51.9 (-100.0 to 1.5e+19)
Albania	Iodine deficiency	5 to 9	Female	0 (0 to 0)	0.0 (0.0 to 0.1)	0 (0 to 0)	0.0 (0.0 to 0.1)	0 (0 to 0)	0.0 (0.0 to 0.0)	-81.2 (-100.0 to 1.8e+26)	-56.7 (-100.0 to 4.3e+26)
Albania	Iodine deficiency	5 to 9	Male	0 (0 to 0)	0.0 (0.0 to 0.1)	0 (0 to 0)	0.0 (0.0 to 0.1)	0 (0 to 0)	0.0 (0.0 to 0.0)	-76.6 (-100.0 to 2.5e+25)	-46.6 (-100.0 to 5.8e+25)
Albania	Iodine deficiency	10 to 14	Both	0 (0 to 0)	0.0 (0.0 to 0.0)	0 (0 to 0)	0.0 (0.0 to 0.0)	0 (0 to 0)	0.0 (0.0 to 0.0)	-80.9 (-100.0 to 5.0e+16)	-65.6 (-100.0 to 8.9e+16)
Albania	Iodine deficiency	10 to 14	Female	0 (0 to 0)	0.0 (0.0 to 0.0)	0 (0 to 0)	0.0 (0.0 to 0.0)	0 (0 to 0)	0.0 (0.0 to 0.0)	-85.9 (-100.0 to 4.7e+24)	-74.4 (-100.0 to 8.5e+24)
Albania	Iodine deficiency	10 to 14	Male	0 (0 to 0)	0.0 (0.0 to 0.0)	0 (0 to 0)	0.0 (0.0 to 0.0)	0 (0 to 0)	0.0 (0.0 to 0.0)	-76.3 (-100.0 to 1.6e+26)	-57.5 (-100.0 to 2.8e+26)
Albania	Iron-deficiency anemia	5 to 9	Both	0 (0 to 0)	0.0 (0.0 to 0.0)	0 (0 to 0)	0.0 (0.0 to 0.0)	0 (0 to 0)	0.0 (0.0 to 0.0)	-66.5 (-87.7 to -9.1)	-23.3 (-71.9 to 108.4)
Albania	Iron-deficiency anemia	5 to 9	Female	0 (0 to 0)	0.0 (0.0 to 0.0)	0 (0 to 0)	0.0 (0.0 to 0.0)	0 (0 to 0)	0.0 (0.0 to 0.0)	-71.0 (-92.7 to 4.7)	-33.1 (-83.2 to 141.4)

eTable 8. All-cause and cause-specific mortality (counts and rates) at the global level and by country in the WHO European Region for ages 5 to 9 and 10 to 14 for 1990, 2000, and 2016

Albania	Iron-deficiency anemia	5 to 9	Male	0 (0 to 0)	0.0 (0.0 to 0.0)	0 (0 to 0)	0.0 (0.0 to 0.0)	0 (0 to 0)	0.0 (0.0 to 0.0)	-62.7 (-91.6 to 57.5)	-15.1 (-80.9 to 258.9)
Albania	Iron-deficiency anemia	10 to 14	Both	0 (0 to 0)	0.0 (0.0 to 0.0)	0 (0 to 0)	0.0 (0.0 to 0.0)	0 (0 to 0)	0.0 (0.0 to 0.0)	-59.3 (-82.3 to -6.3)	-26.6 (-68.1 to 69.0)
Albania	Iron-deficiency anemia	10 to 14	Female	0 (0 to 0)	0.0 (0.0 to 0.0)	0 (0 to 0)	0.0 (0.0 to 0.0)	0 (0 to 0)	0.0 (0.0 to 0.0)	-71.5 (-90.5 to -11.5)	-48.1 (-82.7 to 61.0)
Albania	Iron-deficiency anemia	10 to 14	Male	0 (0 to 0)	0.0 (0.0 to 0.0)	0 (0 to 0)	0.0 (0.0 to 0.0)	0 (0 to 0)	0.0 (0.0 to 0.0)	-45.0 (-82.4 to 72.1)	-1.5 (-68.4 to 208.2)
Albania	Other nutritional deficiencies	5 to 9	Both	0 (0 to 0)	0.0 (0.0 to 0.0)	0 (0 to 0)	0.0 (0.0 to 0.0)	0 (0 to 0)	0.0 (0.0 to 0.0)	-67.5 (-88.5 to -8.5)	-25.6 (-73.7 to 109.7)
Albania	Other nutritional deficiencies	5 to 9	Female	0 (0 to 0)	0.0 (0.0 to 0.0)	0 (0 to 0)	0.0 (0.0 to 0.0)	0 (0 to 0)	0.0 (0.0 to 0.0)	-71.0 (-92.7 to 4.7)	-33.2 (-83.2 to 141.4)
Albania	Other nutritional deficiencies	5 to 9	Male	0 (0 to 0)	0.0 (0.0 to 0.0)	0 (0 to 0)	0.0 (0.0 to 0.1)	0 (0 to 0)	0.0 (0.0 to 0.0)	-64.7 (-92.4 to 48.4)	-19.6 (-82.7 to 238.1)
Albania	Other nutritional deficiencies	10 to 14	Both	0 (0 to 0)	0.0 (0.0 to 0.0)	0 (0 to 0)	0.0 (0.0 to 0.0)	0 (0 to 0)	0.0 (0.0 to 0.0)	-59.3 (-82.3 to -6.3)	-26.6 (-68.1 to 69.0)
Albania	Other nutritional deficiencies	10 to 14	Female	0 (0 to 0)	0.0 (0.0 to 0.0)	0 (0 to 0)	0.0 (0.0 to 0.0)	0 (0 to 0)	0.0 (0.0 to 0.0)	-71.5 (-90.5 to -11.5)	-48.1 (-82.7 to 61.0)
Albania	Other nutritional deficiencies	10 to 14	Male	0 (0 to 0)	0.0 (0.0 to 0.0)	0 (0 to 0)	0.0 (0.0 to 0.0)	0 (0 to 0)	0.0 (0.0 to 0.0)	-45.0 (-82.4 to 72.1)	-1.5 (-68.4 to 208.2)
Albania	Sexually transmitted diseases excluding HIV	5 to 9	Both	0 (0 to 0)	0.0 (0.0 to 0.1)	0 (0 to 0)	0.0 (0.0 to 0.0)	0 (0 to 0)	0.0 (0.0 to 0.0)	-97.0 (-98.8 to -92.5)	-93.1 (-97.3 to -82.8)
Albania	Sexually transmitted diseases excluding HIV	5 to 9	Female	0 (0 to 0)	0.1 (0.0 to 0.1)	0 (0 to 0)	0.0 (0.0 to 0.0)	0 (0 to 0)	0.0 (0.0 to 0.0)	-97.0 (-98.8 to -92.6)	-93.0 (-97.3 to -82.9)
Albania	Sexually transmitted diseases excluding HIV	5 to 9	Male	0 (0 to 0)	0.0 (0.0 to 0.1)	0 (0 to 0)	0.0 (0.0 to 0.0)	0 (0 to 0)	0.0 (0.0 to 0.0)	-97.0 (-98.8 to -92.4)	-93.3 (-97.3 to -82.6)
Albania	Sexually transmitted diseases excluding HIV	10 to 14	Both	0 (0 to 0)	0.0 (0.0 to 0.0)	0 (0 to 0)	0.0 (0.0 to 0.0)	0 (0 to 0)	0.0 (0.0 to 0.0)	-64.7 (-83.3 to -29.3)	-36.3 (-69.9 to 27.5)
Albania	Sexually transmitted diseases excluding HIV	10 to 14	Female	0 (0 to 0)	0.0 (0.0 to 0.1)	0 (0 to 0)	0.0 (0.0 to 0.0)	0 (0 to 0)	0.0 (0.0 to 0.0)	-76.8 (-90.4 to -37.2)	-57.8 (-82.5 to 14.2)
Albania	Sexually transmitted diseases excluding HIV	10 to 14	Male	0 (0 to 0)	0.0 (0.0 to 0.0)	0 (0 to 0)	0.0 (0.0 to 0.0)	0 (0 to 0)	0.0 (0.0 to 0.0)	-33.8 (-55.9 to -7.1)	18.5 (-21.0 to 66.5)
Albania	Hepatitis	5 to 9	Both	2 (1 to 3)	0.5 (0.3 to 0.8)	0 (0 to 1)	0.1 (0.1 to 0.2)	0 (0 to 0)	0.0 (0.0 to 0.0)	-98.1 (-99.2 to -95.5)	-95.6 (-98.2 to -89.8)
Albania	Hepatitis	5 to 9	Female	1 (0 to 1)	0.4 (0.2 to 0.7)	0 (0 to 0)	0.1 (0.0 to 0.2)	0 (0 to 0)	0.0 (0.0 to 0.0)	-98.4 (-99.4 to -95.9)	-96.4 (-98.7 to -90.6)
Albania	Hepatitis	5 to 9	Male	1 (1 to 2)	0.6 (0.3 to 1.2)	0 (0 to 1)	0.2 (0.1 to 0.3)	0 (0 to 0)	0.0 (0.0 to 0.1)	-97.9 (-99.4 to -93.7)	-95.2 (-98.7 to -85.6)

eTable 8. All-cause and cause-specific mortality (counts and rates) at the global level and by country in the WHO European Region for ages 5 to 9 and 10 to 14 for 1990, 2000, and 2016

Albania	Hepatitis	10 to 14	Both	1 (0 to 1)	0.2 (0.1 to 0.3)	0 (0 to 0)	0.0 (0.0 to 0.1)	0 (0 to 0)	0.0 (0.0 to 0.0)	-95.8 (-98.3 to -90.4)	-92.4 (-96.9 to -82.6)
Albania	Hepatitis	10 to 14	Female	0 (0 to 1)	0.2 (0.1 to 0.4)	0 (0 to 0)	0.0 (0.0 to 0.1)	0 (0 to 0)	0.0 (0.0 to 0.0)	-97.3 (-99.1 to -92.8)	-95.1 (-98.4 to -86.8)
Albania	Hepatitis	10 to 14	Male	0 (0 to 1)	0.2 (0.1 to 0.3)	0 (0 to 0)	0.0 (0.0 to 0.1)	0 (0 to 0)	0.0 (0.0 to 0.0)	-94.0 (-98.3 to -82.6)	-89.2 (-96.9 to -68.8)
Albania	Other infectious diseases	5 to 9	Both	1 (1 to 2)	0.4 (0.3 to 0.7)	1 (1 to 2)	0.5 (0.3 to 0.7)	0 (0 to 1)	0.2 (0.1 to 0.4)	-74.0 (-86.2 to -49.1)	-40.4 (-68.5 to 16.7)
Albania	Other infectious diseases	5 to 9	Female	1 (0 to 1)	0.4 (0.2 to 0.7)	1 (0 to 1)	0.4 (0.2 to 0.8)	0 (0 to 0)	0.2 (0.1 to 0.4)	-77.3 (-91.7 to -41.4)	-47.7 (-81.0 to 35.1)
Albania	Other infectious diseases	5 to 9	Male	1 (1 to 1)	0.5 (0.3 to 0.8)	1 (1 to 1)	0.5 (0.3 to 0.8)	0 (0 to 0)	0.3 (0.2 to 0.5)	-71.4 (-86.3 to -43.0)	-34.8 (-68.8 to 29.8)
Albania	Other infectious diseases	10 to 14	Both	1 (1 to 2)	0.4 (0.3 to 0.6)	1 (1 to 2)	0.4 (0.3 to 0.5)	0 (0 to 1)	0.2 (0.1 to 0.3)	-69.7 (-83.4 to -47.7)	-45.3 (-70.1 to -5.6)
Albania	Other infectious diseases	10 to 14	Female	1 (0 to 1)	0.4 (0.2 to 0.6)	1 (0 to 1)	0.4 (0.2 to 0.6)	0 (0 to 0)	0.2 (0.1 to 0.3)	-74.2 (-89.6 to -43.0)	-53.1 (-81.1 to 3.6)
Albania	Other infectious diseases	10 to 14	Male	1 (0 to 1)	0.4 (0.3 to 0.7)	1 (0 to 1)	0.4 (0.2 to 0.6)	0 (0 to 0)	0.3 (0.1 to 0.4)	-65.8 (-83.5 to -33.7)	-38.8 (-70.4 to 18.7)
Albania	Nasopharynx cancer	5 to 9	Both	0 (0 to 0)	0.1 (0.0 to 0.1)	0 (0 to 0)	0.1 (0.0 to 0.1)	0 (0 to 0)	0.0 (0.0 to 0.1)	-73.7 (-89.2 to -37.0)	-39.7 (-75.2 to 44.2)
Albania	Nasopharynx cancer	5 to 9	Female	0 (0 to 0)	0.1 (0.0 to 0.1)	0 (0 to 0)	0.1 (0.0 to 0.1)	0 (0 to 0)	0.0 (0.0 to 0.1)	-81.6 (-93.6 to -46.7)	-57.6 (-85.4 to 22.8)
Albania	Nasopharynx cancer	5 to 9	Male	0 (0 to 0)	0.1 (0.0 to 0.2)	0 (0 to 0)	0.1 (0.0 to 0.1)	0 (0 to 0)	0.1 (0.0 to 0.2)	-69.1 (-89.9 to -9.0)	-29.6 (-77.0 to 107.3)
Albania	Nasopharynx cancer	10 to 14	Both	0 (0 to 1)	0.1 (0.1 to 0.2)	0 (0 to 0)	0.1 (0.1 to 0.1)	0 (0 to 0)	0.1 (0.0 to 0.1)	-62.4 (-83.1 to -13.2)	-32.2 (-69.6 to 56.6)
Albania	Nasopharynx cancer	10 to 14	Female	0 (0 to 0)	0.0 (0.0 to 0.0)	0 (0 to 0)	0.0 (0.0 to 0.0)	0 (0 to 0)	0.0 (0.0 to 0.0)	-39.6 (-56.9 to -18.5)	9.9 (-21.6 to 48.3)
Albania	Nasopharynx cancer	10 to 14	Male	0 (0 to 1)	0.2 (0.1 to 0.3)	0 (0 to 0)	0.2 (0.1 to 0.3)	0 (0 to 0)	0.1 (0.1 to 0.3)	-63.5 (-84.4 to -11.9)	-34.7 (-72.1 to 57.9)
Albania	Liver cancer	5 to 9	Both	1 (0 to 1)	0.2 (0.1 to 0.3)	1 (1 to 1)	0.3 (0.2 to 0.5)	0 (0 to 0)	0.2 (0.1 to 0.3)	-66.1 (-82.8 to -31.2)	-22.2 (-60.7 to 57.8)
Albania	Liver cancer	5 to 9	Female	0 (0 to 1)	0.2 (0.1 to 0.4)	0 (0 to 1)	0.3 (0.1 to 0.5)	0 (0 to 0)	0.1 (0.1 to 0.2)	-77.6 (-91.3 to -42.4)	-48.4 (-80.1 to 32.8)
Albania	Liver cancer	5 to 9	Male	0 (0 to 1)	0.2 (0.1 to 0.4)	1 (0 to 1)	0.3 (0.2 to 0.5)	0 (0 to 0)	0.2 (0.1 to 0.4)	-55.8 (-83.8 to 7.4)	0.7 (-63.0 to 144.8)
Albania	Liver cancer	10 to 14	Both	0 (0 to 1)	0.1 (0.1 to 0.2)	1 (0 to 1)	0.2 (0.1 to 0.2)	0 (0 to 0)	0.1 (0.1 to 0.2)	-52.7 (-76.1 to -7.0)	-14.7 (-56.9 to 67.9)
Albania	Liver cancer	10 to 14	Female	0 (0 to 0)	0.1 (0.1 to 0.2)	0 (0 to 0)	0.1 (0.1 to 0.2)	0 (0 to 0)	0.1 (0.0 to 0.1)	-67.0 (-87.5 to -21.5)	-40.0 (-77.3 to 42.8)
Albania	Liver cancer	10 to 14	Male	0 (0 to 0)	0.2 (0.1 to 0.3)	0 (0 to 1)	0.2 (0.1 to 0.3)	0 (0 to 0)	0.2 (0.1 to 0.3)	-43.3 (-77.2 to 30.7)	1.6 (-59.1 to 134.1)
Albania	Kidney cancer	5 to 9	Both	1 (1 to 1)	0.2 (0.1 to 0.3)	1 (1 to 1)	0.3 (0.2 to 0.5)	1 (0 to 1)	0.4 (0.2 to 0.6)	-30.5 (-66.4 to 33.6)	59.2 (-23.0 to 206.2)
Albania	Kidney cancer	5 to 9	Female	0 (0 to 1)	0.2 (0.1 to 0.4)	1 (0 to 1)	0.4 (0.2 to 0.6)	0 (0 to 1)	0.4 (0.2 to 0.7)	-25.8 (-67.9 to 67.1)	70.9 (-26.0 to 285.1)
Albania	Kidney cancer	5 to 9	Male	0 (0 to 1)	0.2 (0.1 to 0.3)	0 (0 to 1)	0.3 (0.2 to 0.5)	0 (0 to 0)	0.3 (0.1 to 0.6)	-35.4 (-73.0 to 47.3)	47.1 (-38.6 to 235.6)
Albania	Kidney cancer	10 to 14	Both	0 (0 to 0)	0.1 (0.0 to 0.1)	0 (0 to 0)	0.1 (0.1 to 0.1)	0 (0 to 0)	0.1 (0.1 to 0.2)	-9.8 (-54.2 to 72.5)	62.8 (-17.3 to 211.2)
Albania	Kidney cancer	10 to 14	Female	0 (0 to 0)	0.1 (0.1 to 0.1)	0 (0 to 0)	0.1 (0.1 to 0.2)	0 (0 to 0)	0.1 (0.1 to 0.3)	-11.4 (-60.9 to 94.7)	61.1 (-28.9 to 254.2)
Albania	Kidney cancer	10 to 14	Male	0 (0 to 0)	0.0 (0.0 to 0.1)	0 (0 to 0)	0.0 (0.0 to 0.1)	0 (0 to 0)	0.1 (0.0 to 0.1)	-6.2 (-54.9 to 101.1)	68.1 (-19.1 to 260.2)
Albania	Brain and nervous system cancer	5 to 9	Both	7 (4 to 17)	2.1 (1.2 to 4.9)	9 (6 to 20)	2.9 (1.8 to 6.1)	4 (3 to 7)	2.8 (1.7 to 4.4)	-42.3 (-70.5 to 17.9)	32.3 (-32.4 to 170.2)

eTable 8. All-cause and cause-specific mortality (counts and rates) at the global level and by country in the WHO European Region for ages 5 to 9 and 10 to 14 for 1990, 2000, and 2016

Albania	Brain and nervous system cancer	5 to 9	Female	3 (1 to 8)	1.8 (0.7 to 4.6)	4 (2 to 9)	2.4 (1.2 to 5.7)	2 (1 to 3)	2.2 (1.0 to 4.0)	-46.7 (-71.1 to 22.2)	22.8 (-33.3 to 181.6)
Albania	Brain and nervous system cancer	5 to 9	Male	4 (2 to 10)	2.5 (1.4 to 5.6)	6 (3 to 12)	3.3 (2.0 to 6.8)	3 (1 to 5)	3.4 (1.9 to 5.8)	-39.3 (-71.3 to 35.4)	38.4 (-34.5 to 208.5)
Albania	Brain and nervous system cancer	10 to 14	Both	5 (3 to 10)	1.5 (0.9 to 3.0)	6 (4 to 11)	1.8 (1.1 to 3.4)	4 (2 to 6)	2.0 (1.2 to 3.0)	-26.1 (-56.6 to 38.3)	33.4 (-21.6 to 149.6)
Albania	Brain and nervous system cancer	10 to 14	Female	2 (1 to 5)	1.4 (0.7 to 2.9)	3 (1 to 5)	1.6 (0.8 to 3.1)	1 (1 to 2)	1.6 (0.9 to 2.7)	-34.1 (-60.6 to 44.3)	19.9 (-28.3 to 162.4)
Albania	Brain and nervous system cancer	10 to 14	Male	3 (2 to 6)	1.6 (0.9 to 3.4)	3 (2 to 7)	1.9 (1.1 to 4.0)	2 (1 to 4)	2.3 (1.3 to 3.9)	-19.8 (-57.7 to 71.0)	43.7 (-24.3 to 206.3)
Albania	Thyroid cancer	10 to 14	Both	0 (0 to 0)	0.0 (0.0 to 0.0)	0 (0 to 0)	0.0 (0.0 to 0.0)	0 (0 to 0)	0.0 (0.0 to 0.0)	-37.0 (-56.8 to -13.8)	13.7 (-22.1 to 55.6)
Albania	Thyroid cancer	10 to 14	Female	0 (0 to 0)	0.0 (0.0 to 0.0)	0 (0 to 0)	0.0 (0.0 to 0.0)	0 (0 to 0)	0.0 (0.0 to 0.0)	-39.6 (-57.1 to -18.5)	9.9 (-22.0 to 48.3)
Albania	Thyroid cancer	10 to 14	Male	0 (0 to 0)	0.0 (0.0 to 0.0)	0 (0 to 0)	0.0 (0.0 to 0.0)	0 (0 to 0)	0.0 (0.0 to 0.0)	-34.7 (-57.8 to -7.1)	17.0 (-24.5 to 66.5)
Albania	Hodgkin lymphoma	5 to 9	Both	1 (0 to 1)	0.2 (0.1 to 0.3)	1 (0 to 1)	0.2 (0.1 to 0.4)	0 (0 to 0)	0.1 (0.0 to 0.2)	-82.4 (-93.6 to -60.5)	-59.7 (-85.3 to -9.5)
Albania	Hodgkin lymphoma	5 to 9	Female	0 (0 to 1)	0.2 (0.0 to 0.4)	0 (0 to 1)	0.2 (0.0 to 0.4)	0 (0 to 0)	0.0 (0.0 to 0.1)	-88.1 (-97.5 to -59.6)	-72.6 (-94.2 to -6.8)
Albania	Hodgkin lymphoma	5 to 9	Male	0 (0 to 1)	0.2 (0.1 to 0.5)	0 (0 to 1)	0.3 (0.1 to 0.5)	0 (0 to 0)	0.1 (0.0 to 0.3)	-78.6 (-93.5 to -40.2)	-51.3 (-85.3 to 36.3)
Albania	Hodgkin lymphoma	10 to 14	Both	1 (0 to 1)	0.2 (0.1 to 0.4)	1 (0 to 1)	0.2 (0.1 to 0.4)	0 (0 to 0)	0.1 (0.0 to 0.2)	-71.5 (-85.0 to -45.6)	-48.5 (-73.0 to -1.9)
Albania	Hodgkin lymphoma	10 to 14	Female	0 (0 to 0)	0.1 (0.1 to 0.3)	0 (0 to 0)	0.1 (0.0 to 0.2)	0 (0 to 0)	0.1 (0.0 to 0.1)	-74.5 (-91.8 to -30.4)	-53.6 (-85.1 to 26.6)
Albania	Hodgkin lymphoma	10 to 14	Male	0 (0 to 1)	0.3 (0.1 to 0.6)	0 (0 to 1)	0.3 (0.2 to 0.6)	0 (0 to 0)	0.1 (0.1 to 0.3)	-70.0 (-86.4 to -38.7)	-46.2 (-75.6 to 9.8)
Albania	Non-Hodgkin lymphoma	5 to 9	Both	2 (1 to 2)	0.5 (0.3 to 0.7)	1 (1 to 2)	0.4 (0.3 to 0.7)	1 (0 to 1)	0.3 (0.2 to 0.6)	-69.3 (-84.5 to -44.0)	-29.7 (-64.6 to 28.4)
Albania	Non-Hodgkin lymphoma	5 to 9	Female	1 (0 to 1)	0.4 (0.1 to 0.6)	0 (0 to 1)	0.3 (0.2 to 0.5)	0 (0 to 0)	0.2 (0.1 to 0.3)	-78.7 (-91.4 to -47.0)	-51.0 (-80.1 to 22.2)
Albania	Non-Hodgkin lymphoma	5 to 9	Male	1 (1 to 2)	0.6 (0.3 to 1.0)	1 (1 to 2)	0.6 (0.3 to 1.2)	0 (0 to 1)	0.5 (0.2 to 0.9)	-63.6 (-83.9 to -26.9)	-17.2 (-63.3 to 66.7)
Albania	Non-Hodgkin lymphoma	10 to 14	Both	1 (1 to 2)	0.4 (0.3 to 0.5)	1 (1 to 2)	0.3 (0.2 to 0.5)	1 (0 to 1)	0.3 (0.2 to 0.4)	-58.4 (-78.2 to -28.5)	-24.9 (-60.7 to 29.0)
Albania	Non-Hodgkin lymphoma	10 to 14	Female	0 (0 to 1)	0.3 (0.2 to 0.5)	0 (0 to 1)	0.2 (0.1 to 0.3)	0 (0 to 0)	0.2 (0.1 to 0.3)	-71.2 (-87.9 to -34.8)	-47.6 (-78.0 to 18.5)
Albania	Non-Hodgkin lymphoma	10 to 14	Male	1 (0 to 1)	0.4 (0.3 to 0.7)	1 (0 to 1)	0.4 (0.2 to 0.8)	0 (0 to 1)	0.4 (0.2 to 0.7)	-50.0 (-77.8 to -1.3)	-10.4 (-60.2 to 76.8)
Albania	Leukemia	5 to 9	Both	10 (7 to 16)	2.9 (2.0 to 4.4)	12 (9 to 17)	3.7 (2.6 to 5.2)	4 (2 to 6)	2.6 (1.6 to 3.9)	-61.8 (-78.8 to -31.5)	-12.4 (-51.4 to 57.1)
Albania	Leukemia	5 to 9	Female	4 (2 to 7)	2.5 (1.4 to 4.3)	5 (3 to 8)	3.3 (1.8 to 4.9)	2 (1 to 3)	2.1 (1.2 to 3.5)	-64.1 (-81.9 to -24.2)	-17.2 (-58.3 to 74.7)
Albania	Leukemia	5 to 9	Male	6 (4 to 10)	3.4 (2.0 to 5.4)	7 (4 to 11)	4.2 (2.5 to 6.5)	2 (1 to 4)	3.0 (1.5 to 5.1)	-60.2 (-80.4 to -11.5)	-9.2 (-55.2 to 101.7)
Albania	Leukemia	10 to 14	Both	8 (5 to 11)	2.3 (1.6 to 3.2)	8 (6 to 11)	2.5 (1.8 to 3.4)	4 (2 to 6)	2.1 (1.3 to 3.1)	-49.6 (-70.9 to -14.7)	-9.1 (-47.5 to 53.9)
Albania	Leukemia	10 to 14	Female	3 (2 to 5)	1.9 (1.2 to 2.9)	3 (2 to 5)	2.0 (1.3 to 2.9)	1 (1 to 2)	1.6 (0.9 to 2.7)	-51.9 (-74.5 to -3.6)	-12.5 (-53.6 to 75.4)
Albania	Leukemia	10 to 14	Male	5 (3 to 7)	2.7 (1.7 to 4.0)	5 (3 to 8)	3.0 (1.9 to 4.4)	2 (1 to 4)	2.5 (1.3 to 4.1)	-48.2 (-73.0 to -0.3)	-7.1 (-51.7 to 78.5)
Albania	Other neoplasms	5 to 9	Both	7 (4 to 9)	1.9 (1.2 to 2.7)	6 (4 to 8)	1.8 (1.2 to 2.6)	3 (1 to 4)	1.7 (0.9 to 2.8)	-60.6 (-79.7 to -27.3)	-9.7 (-53.5 to 66.5)
Albania	Other neoplasms	5 to 9	Female	3 (2 to 4)	1.5 (0.9 to 2.5)	2 (1 to 4)	1.4 (0.8 to 2.2)	1 (0 to 2)	1.3 (0.7 to 2.2)	-63.7 (-84.7 to -20.4)	-16.4 (-64.6 to 83.5)
Albania	Other neoplasms	5 to 9	Male	4 (2 to 6)	2.2 (1.3 to 3.4)	4 (2 to 6)	2.2 (1.3 to 3.4)	2 (1 to 3)	2.0 (1.0 to 3.9)	-58.5 (-80.8 to -10.2)	-5.4 (-56.1 to 104.8)
Albania	Other neoplasms	10 to 14	Both	5 (4 to 7)	1.6 (1.2 to 2.3)	5 (3 to 6)	1.4 (1.0 to 1.8)	3 (2 to 4)	1.5 (0.9 to 2.3)	-49.2 (-70.9 to -15.1)	-8.4 (-47.6 to 53.1)

eTable 8. All-cause and cause-specific mortality (counts and rates) at the global level and by country in the WHO European Region for ages 5 to 9 and 10 to 14 for 1990, 2000, and 2016

Albania	Other neoplasms	10 to 14	Female	2 (1 to 4)	1.5 (0.9 to 2.2)	2 (1 to 3)	1.2 (0.8 to 1.8)	1 (1 to 2)	1.3 (0.7 to 2.2)	-51.8 (-77.2 to 2.0)	-12.3 (-58.6 to 85.5)
Albania	Other neoplasms	10 to 14	Male	3 (2 to 5)	1.8 (1.2 to 2.7)	3 (2 to 4)	1.5 (1.0 to 2.2)	2 (1 to 3)	1.7 (1.0 to 2.8)	-47.3 (-73.7 to -3.9)	-5.6 (-52.9 to 72.2)
Albania	Rheumatic heart disease	5 to 9	Both	2 (1 to 3)	0.5 (0.3 to 0.8)	2 (1 to 3)	0.6 (0.4 to 0.9)	0 (0 to 1)	0.2 (0.1 to 0.4)	-81.5 (-90.8 to -64.8)	-57.6 (-79.0 to -19.3)
Albania	Rheumatic heart disease	5 to 9	Female	1 (1 to 1)	0.5 (0.3 to 0.8)	1 (0 to 1)	0.5 (0.3 to 0.7)	0 (0 to 0)	0.1 (0.1 to 0.2)	-86.9 (-93.7 to -71.7)	-69.7 (-85.4 to -34.8)
Albania	Rheumatic heart disease	5 to 9	Male	1 (1 to 2)	0.6 (0.3 to 0.9)	1 (1 to 2)	0.8 (0.4 to 1.2)	0 (0 to 0)	0.3 (0.1 to 0.6)	-77.1 (-90.5 to -48.6)	-47.9 (-78.4 to 17.1)
Albania	Rheumatic heart disease	10 to 14	Both	2 (1 to 3)	0.6 (0.4 to 0.8)	2 (1 to 3)	0.6 (0.4 to 0.8)	1 (0 to 1)	0.3 (0.2 to 0.5)	-70.7 (-83.7 to -49.3)	-47.1 (-70.6 to -8.5)
Albania	Rheumatic heart disease	10 to 14	Female	1 (1 to 1)	0.5 (0.3 to 0.8)	1 (0 to 1)	0.4 (0.2 to 0.6)	0 (0 to 0)	0.2 (0.1 to 0.3)	-82.6 (-91.3 to -66.6)	-68.3 (-84.3 to -39.3)
Albania	Rheumatic heart disease	10 to 14	Male	1 (1 to 2)	0.7 (0.4 to 1.0)	1 (1 to 2)	0.8 (0.5 to 1.1)	0 (0 to 1)	0.5 (0.2 to 0.7)	-61.7 (-81.5 to -25.8)	-31.3 (-66.8 to 33.0)
Albania	Ischemic heart disease	5 to 9	Both	2 (1 to 3)	0.5 (0.3 to 0.8)	4 (3 to 6)	1.2 (0.8 to 1.8)	1 (1 to 2)	0.7 (0.4 to 1.3)	-38.0 (-69.1 to 22.5)	42.0 (-29.2 to 180.7)
Albania	Ischemic heart disease	5 to 9	Female	1 (0 to 1)	0.5 (0.3 to 0.8)	2 (1 to 2)	1.0 (0.6 to 1.5)	0 (0 to 1)	0.6 (0.3 to 1.1)	-42.4 (-75.0 to 27.8)	32.8 (-42.3 to 194.5)
Albania	Ischemic heart disease	5 to 9	Male	1 (0 to 2)	0.6 (0.3 to 1.0)	3 (1 to 4)	1.5 (0.8 to 2.5)	1 (0 to 1)	0.8 (0.4 to 1.7)	-34.5 (-75.9 to 71.9)	49.2 (-45.1 to 291.8)
Albania	Ischemic heart disease	10 to 14	Both	1 (1 to 2)	0.4 (0.3 to 0.6)	2 (2 to 3)	0.7 (0.5 to 1.0)	1 (1 to 2)	0.6 (0.3 to 0.9)	-28.5 (-64.3 to 42.5)	29.0 (-35.5 to 157.2)
Albania	Ischemic heart disease	10 to 14	Female	1 (0 to 1)	0.4 (0.2 to 0.6)	1 (0 to 1)	0.5 (0.3 to 0.7)	0 (0 to 1)	0.4 (0.2 to 0.6)	-44.2 (-75.1 to 15.7)	1.5 (-54.8 to 110.5)
Albania	Ischemic heart disease	10 to 14	Male	1 (0 to 1)	0.5 (0.3 to 0.8)	2 (1 to 2)	0.9 (0.5 to 1.4)	1 (0 to 1)	0.7 (0.3 to 1.3)	-18.4 (-68.7 to 95.2)	46.2 (-43.9 to 249.8)
Albania	Cerebrovascular disease	5 to 9	Both	6 (5 to 9)	1.8 (1.3 to 2.5)	12 (9 to 17)	3.7 (2.7 to 5.1)	3 (2 to 4)	1.9 (1.1 to 2.9)	-54.4 (-74.6 to -21.4)	4.4 (-41.8 to 80.0)
Albania	Cerebrovascular disease	5 to 9	Female	3 (2 to 5)	1.9 (1.2 to 2.8)	5 (4 to 8)	3.4 (2.4 to 4.8)	1 (1 to 2)	1.7 (1.0 to 2.6)	-60.3 (-78.4 to -26.5)	-8.6 (-50.3 to 69.4)
Albania	Cerebrovascular disease	5 to 9	Male	3 (2 to 5)	1.7 (1.0 to 2.7)	7 (4 to 10)	4.0 (2.6 to 6.0)	2 (1 to 3)	2.0 (1.0 to 3.4)	-48.2 (-76.6 to 9.5)	18.0 (-46.6 to 149.6)
Albania	Cerebrovascular disease	10 to 14	Both	6 (4 to 7)	1.8 (1.3 to 2.3)	9 (7 to 11)	2.7 (2.1 to 3.3)	4 (3 to 6)	2.2 (1.5 to 3.2)	-30.0 (-57.8 to 9.0)	26.3 (-23.8 to 96.7)
Albania	Cerebrovascular disease	10 to 14	Female	3 (2 to 4)	1.7 (1.2 to 2.5)	4 (3 to 5)	2.3 (1.6 to 3.0)	2 (1 to 2)	1.7 (1.1 to 2.6)	-45.5 (-69.5 to -5.1)	-0.9 (-44.5 to 72.5)
Albania	Cerebrovascular disease	10 to 14	Male	3 (2 to 4)	1.8 (1.2 to 2.5)	5 (4 to 7)	3.1 (2.2 to 4.1)	3 (2 to 4)	2.7 (1.6 to 4.1)	-15.7 (-54.0 to 48.1)	51.0 (-17.6 to 165.4)
Albania	Hypertensive heart disease	5 to 9	Both	0 (0 to 0)	0.0 (0.0 to 0.0)	0 (0 to 0)	0.0 (0.0 to 0.0)	0 (0 to 0)	0.0 (0.0 to 0.0)	-54.1 (-79.5 to 6.7)	5.1 (-52.9 to 144.6)
Albania	Hypertensive heart disease	5 to 9	Female	0 (0 to 0)	0.0 (0.0 to 0.1)	0 (0 to 0)	0.0 (0.0 to 0.1)	0 (0 to 0)	0.0 (0.0 to 0.0)	-66.0 (-88.3 to 6.5)	-21.7 (-73.0 to 145.4)
Albania	Hypertensive heart disease	5 to 9	Male	0 (0 to 0)	0.0 (0.0 to 0.0)	0 (0 to 0)	0.0 (0.0 to 0.0)	0 (0 to 0)	0.0 (0.0 to 0.0)	-23.5 (-62.3 to 52.8)	74.4 (-14.1 to 248.3)
Albania	Hypertensive heart disease	10 to 14	Both	0 (0 to 0)	0.0 (0.0 to 0.0)	0 (0 to 0)	0.0 (0.0 to 0.0)	0 (0 to 0)	0.0 (0.0 to 0.0)	-44.6 (-74.7 to 17.4)	0.0 (-54.4 to 111.9)
Albania	Hypertensive heart disease	10 to 14	Female	0 (0 to 0)	0.0 (0.0 to 0.1)	0 (0 to 0)	0.0 (0.0 to 0.1)	0 (0 to 0)	0.0 (0.0 to 0.0)	-60.6 (-86.1 to 13.9)	-28.4 (-74.6 to 107.2)
Albania	Hypertensive heart disease	10 to 14	Male	0 (0 to 0)	0.0 (0.0 to 0.0)	0 (0 to 0)	0.0 (0.0 to 0.0)	0 (0 to 0)	0.0 (0.0 to 0.0)	-0.9 (-44.3 to 72.4)	77.6 (-0.2 to 208.9)
Albania	Cardiomyopathy and myocarditis	5 to 9	Both	3 (2 to 4)	0.8 (0.4 to 1.2)	4 (2 to 5)	1.2 (0.6 to 1.7)	1 (1 to 2)	0.8 (0.4 to 1.3)	-57.4 (-77.5 to -17.3)	-2.3 (-48.4 to 89.5)
Albania	Cardiomyopathy and myocarditis	5 to 9	Female	1 (1 to 2)	0.9 (0.4 to 1.4)	2 (1 to 3)	1.1 (0.6 to 1.7)	1 (0 to 1)	0.7 (0.4 to 1.3)	-62.4 (-82.9 to -17.7)	-13.3 (-60.7 to 89.6)
Albania	Cardiomyopathy and myocarditis	5 to 9	Male	1 (0 to 2)	0.8 (0.3 to 1.3)	2 (1 to 3)	1.2 (0.4 to 2.0)	1 (0 to 1)	0.8 (0.4 to 1.6)	-52.1 (-80.6 to 17.9)	9.1 (-55.7 to 168.7)
Albania	Cardiomyopathy and myocarditis	10 to 14	Both	3 (2 to 4)	0.8 (0.6 to 1.1)	3 (2 to 4)	0.9 (0.6 to 1.3)	2 (1 to 3)	0.9 (0.6 to 1.4)	-35.9 (-61.8 to 10.3)	15.7 (-31.1 to 99.0)

eTable 8. All-cause and cause-specific mortality (counts and rates) at the global level and by country in the WHO European Region for ages 5 to 9 and 10 to 14 for 1990, 2000, and 2016

Albania	Cardiomyopathy and myocarditis	10 to 14	Female	1 (1 to 2)	0.8 (0.5 to 1.2)	1 (1 to 2)	0.8 (0.5 to 1.2)	1 (0 to 1)	0.8 (0.4 to 1.2)	-48.8 (-73.9 to 0.5)	-6.9 (-52.6 to 82.8)
Albania	Cardiomyopathy and myocarditis	10 to 14	Male	1 (1 to 2)	0.8 (0.4 to 1.3)	2 (1 to 3)	1.0 (0.5 to 1.6)	1 (1 to 2)	1.1 (0.6 to 1.9)	-23.6 (-64.3 to 57.4)	36.9 (-36.1 to 182.0)
Albania	Atrial fibrillation and flutter	5 to 9	Both	0 (0 to 0)	0.0 (0.0 to 0.0)	0 (0 to 0)	0.0 (0.0 to 0.0)	0 (0 to 0)	0.0 (0.0 to 0.0)	0.0 (0.0 to 0.0)	0.0 (0.0 to 0.0)
Albania	Atrial fibrillation and flutter	5 to 9	Female	0 (0 to 0)	0.0 (0.0 to 0.0)	0 (0 to 0)	0.0 (0.0 to 0.0)	0 (0 to 0)	0.0 (0.0 to 0.0)	0.0 (0.0 to 0.0)	0.0 (0.0 to 0.0)
Albania	Atrial fibrillation and flutter	5 to 9	Male	0 (0 to 0)	0.0 (0.0 to 0.0)	0 (0 to 0)	0.0 (0.0 to 0.0)	0 (0 to 0)	0.0 (0.0 to 0.0)	0.0 (0.0 to 0.0)	0.0 (0.0 to 0.0)
Albania	Atrial fibrillation and flutter	10 to 14	Both	0 (0 to 0)	0.0 (0.0 to 0.0)	0 (0 to 0)	0.0 (0.0 to 0.0)	0 (0 to 0)	0.0 (0.0 to 0.0)	0.0 (0.0 to 0.0)	0.0 (0.0 to 0.0)
Albania	Atrial fibrillation and flutter	10 to 14	Female	0 (0 to 0)	0.0 (0.0 to 0.0)	0 (0 to 0)	0.0 (0.0 to 0.0)	0 (0 to 0)	0.0 (0.0 to 0.0)	0.0 (0.0 to 0.0)	0.0 (0.0 to 0.0)
Albania	Atrial fibrillation and flutter	10 to 14	Male	0 (0 to 0)	0.0 (0.0 to 0.0)	0 (0 to 0)	0.0 (0.0 to 0.0)	0 (0 to 0)	0.0 (0.0 to 0.0)	0.0 (0.0 to 0.0)	0.0 (0.0 to 0.0)
Albania	Endocarditis	5 to 9	Both	0 (0 to 1)	0.1 (0.0 to 0.2)	0 (0 to 1)	0.1 (0.1 to 0.2)	0 (0 to 0)	0.1 (0.0 to 0.2)	-50.3 (-79.2 to 21.5)	13.8 (-52.4 to 178.3)
Albania	Endocarditis	5 to 9	Female	0 (0 to 1)	0.2 (0.0 to 0.3)	0 (0 to 0)	0.2 (0.1 to 0.3)	0 (0 to 0)	0.2 (0.0 to 0.4)	-54.5 (-85.1 to 35.6)	4.9 (-65.7 to 212.5)
Albania	Endocarditis	5 to 9	Male	0 (0 to 0)	0.0 (0.0 to 0.1)	0 (0 to 0)	0.0 (0.0 to 0.1)	0 (0 to 0)	0.1 (0.0 to 0.1)	-35.4 (-78.3 to 68.5)	47.1 (-50.5 to 284.0)
Albania	Endocarditis	10 to 14	Both	0 (0 to 0)	0.1 (0.0 to 0.1)	0 (0 to 0)	0.1 (0.1 to 0.1)	0 (0 to 0)	0.1 (0.1 to 0.2)	-36.9 (-69.2 to 23.1)	13.9 (-44.4 to 122.2)
Albania	Endocarditis	10 to 14	Female	0 (0 to 0)	0.1 (0.1 to 0.2)	0 (0 to 0)	0.1 (0.1 to 0.2)	0 (0 to 0)	0.1 (0.1 to 0.3)	-44.1 (-77.8 to 29.1)	1.7 (-59.7 to 134.7)
Albania	Endocarditis	10 to 14	Male	0 (0 to 0)	0.1 (0.0 to 0.1)	0 (0 to 0)	0.1 (0.0 to 0.1)	0 (0 to 0)	0.1 (0.0 to 0.1)	-18.8 (-65.6 to 68.9)	45.4 (-38.4 to 202.5)
Albania	Other cardiovascular and circulatory diseases	5 to 9	Both	2 (1 to 4)	0.7 (0.4 to 1.0)	4 (2 to 6)	1.2 (0.6 to 1.7)	1 (0 to 2)	0.8 (0.3 to 1.5)	-45.5 (-77.4 to 5.4)	25.0 (-48.2 to 141.4)
Albania	Other cardiovascular and circulatory diseases	5 to 9	Female	1 (0 to 2)	0.6 (0.2 to 1.0)	2 (1 to 2)	1.0 (0.3 to 1.6)	1 (0 to 1)	0.8 (0.2 to 1.5)	-42.9 (-78.1 to 27.7)	31.6 (-49.4 to 194.4)
Albania	Other cardiovascular and circulatory diseases	5 to 9	Male	1 (1 to 2)	0.7 (0.4 to 1.2)	2 (1 to 4)	1.4 (0.5 to 2.2)	1 (0 to 1)	0.9 (0.3 to 1.6)	-47.4 (-80.3 to 25.2)	19.9 (-55.2 to 185.2)
Albania	Other cardiovascular and circulatory diseases	10 to 14	Both	2 (1 to 3)	0.6 (0.4 to 0.8)	3 (2 to 4)	0.8 (0.5 to 1.1)	1 (1 to 2)	0.8 (0.4 to 1.3)	-26.3 (-63.9 to 37.9)	33.0 (-34.9 to 148.9)
Albania	Other cardiovascular and circulatory diseases	10 to 14	Female	1 (0 to 1)	0.5 (0.3 to 0.8)	1 (1 to 2)	0.6 (0.3 to 1.0)	1 (0 to 1)	0.6 (0.2 to 1.1)	-31.9 (-73.1 to 41.9)	23.9 (-51.1 to 158.2)

eTable 8. All-cause and cause-specific mortality (counts and rates) at the global level and by country in the WHO European Region for ages 5 to 9 and 10 to 14 for 1990, 2000, and 2016

Albania	Other cardiovascular and circulatory diseases	10 to 14	Male	1 (1 to 2)	0.6 (0.4 to 1.0)	2 (1 to 2)	0.9 (0.5 to 1.4)	1 (0 to 2)	0.9 (0.4 to 1.6)	-22.1 (-64.7 to 69.3)	39.5 (-36.7 to 203.3)
Albania	Chronic obstructive pulmonary disease	5 to 9	Both	1 (1 to 2)	0.3 (0.2 to 0.4)	1 (1 to 1)	0.3 (0.2 to 0.4)	0 (0 to 0)	0.1 (0.0 to 0.2)	-83.4 (-91.2 to -67.3)	-61.9 (-79.8 to -25.2)
Albania	Chronic obstructive pulmonary disease	5 to 9	Female	1 (0 to 1)	0.3 (0.2 to 0.5)	1 (0 to 1)	0.3 (0.2 to 0.5)	0 (0 to 0)	0.1 (0.1 to 0.2)	-81.7 (-91.4 to -58.5)	-57.8 (-80.3 to -4.4)
Albania	Chronic obstructive pulmonary disease	5 to 9	Male	0 (0 to 1)	0.3 (0.1 to 0.5)	0 (0 to 1)	0.3 (0.1 to 0.5)	0 (0 to 0)	0.1 (0.0 to 0.2)	-85.1 (-94.5 to -61.6)	-66.1 (-87.5 to -12.5)
Albania	Chronic obstructive pulmonary disease	10 to 14	Both	1 (0 to 1)	0.3 (0.1 to 0.4)	1 (0 to 1)	0.2 (0.1 to 0.3)	0 (0 to 0)	0.1 (0.0 to 0.2)	-76.9 (-88.6 to -55.6)	-58.4 (-79.4 to -19.8)
Albania	Chronic obstructive pulmonary disease	10 to 14	Female	0 (0 to 1)	0.2 (0.1 to 0.4)	0 (0 to 0)	0.2 (0.1 to 0.3)	0 (0 to 0)	0.1 (0.0 to 0.2)	-77.0 (-90.1 to -45.8)	-58.2 (-82.0 to -1.5)
Albania	Chronic obstructive pulmonary disease	10 to 14	Male	1 (0 to 1)	0.3 (0.1 to 0.5)	0 (0 to 1)	0.3 (0.1 to 0.5)	0 (0 to 0)	0.1 (0.0 to 0.2)	-76.9 (-90.6 to -47.4)	-58.7 (-83.2 to -5.8)
Albania	Asthma	5 to 9	Both	1 (0 to 1)	0.1 (0.1 to 0.2)	1 (0 to 1)	0.2 (0.1 to 0.3)	0 (0 to 0)	0.0 (0.0 to 0.1)	-86.0 (-94.0 to -67.1)	-67.8 (-86.2 to -24.7)
Albania	Asthma	5 to 9	Female	0 (0 to 0)	0.2 (0.1 to 0.3)	0 (0 to 1)	0.2 (0.1 to 0.3)	0 (0 to 0)	0.1 (0.0 to 0.1)	-84.8 (-94.5 to -56.2)	-64.9 (-87.4 to 0.9)
Albania	Asthma	5 to 9	Male	0 (0 to 0)	0.1 (0.1 to 0.3)	0 (0 to 1)	0.2 (0.1 to 0.3)	0 (0 to 0)	0.0 (0.0 to 0.1)	-87.3 (-95.9 to -64.0)	-71.0 (-90.6 to -17.9)
Albania	Asthma	10 to 14	Both	1 (0 to 1)	0.2 (0.1 to 0.3)	1 (0 to 1)	0.2 (0.1 to 0.3)	0 (0 to 0)	0.1 (0.0 to 0.1)	-78.2 (-89.7 to -55.1)	-60.7 (-81.4 to -19.1)
Albania	Asthma	10 to 14	Female	0 (0 to 1)	0.3 (0.1 to 0.4)	0 (0 to 1)	0.3 (0.1 to 0.4)	0 (0 to 0)	0.1 (0.0 to 0.2)	-78.2 (-90.8 to -43.6)	-60.4 (-83.2 to 2.6)
Albania	Asthma	10 to 14	Male	0 (0 to 1)	0.1 (0.1 to 0.3)	0 (0 to 0)	0.1 (0.0 to 0.2)	0 (0 to 0)	0.1 (0.0 to 0.1)	-78.2 (-92.8 to -34.3)	-60.9 (-87.2 to 17.8)
Albania	Interstitial lung disease and pulmonary sarcoidosis	5 to 9	Both	0 (0 to 0)	0.0 (0.0 to 0.1)	0 (0 to 0)	0.0 (0.0 to 0.1)	0 (0 to 0)	0.0 (0.0 to 0.1)	-69.3 (-88.7 to -23.0)	-29.6 (-74.0 to 76.5)
Albania	Interstitial lung disease and pulmonary sarcoidosis	5 to 9	Female	0 (0 to 0)	0.1 (0.0 to 0.1)	0 (0 to 0)	0.1 (0.0 to 0.2)	0 (0 to 0)	0.0 (0.0 to 0.1)	-71.3 (-90.8 to -14.1)	-33.9 (-78.8 to 98.0)
Albania	Interstitial lung disease and pulmonary sarcoidosis	5 to 9	Male	0 (0 to 0)	0.0 (0.0 to 0.1)	0 (0 to 0)	0.0 (0.0 to 0.1)	0 (0 to 0)	0.0 (0.0 to 0.0)	-62.6 (-89.2 to 9.3)	-14.8 (-75.3 to 149.0)
Albania	Interstitial lung disease and pulmonary sarcoidosis	10 to 14	Both	0 (0 to 0)	0.0 (0.0 to 0.1)	0 (0 to 0)	0.0 (0.0 to 0.1)	0 (0 to 0)	0.0 (0.0 to 0.1)	-53.4 (-80.2 to -5.6)	-15.8 (-64.3 to 70.4)

eTable 8. All-cause and cause-specific mortality (counts and rates) at the global level and by country in the WHO European Region for ages 5 to 9 and 10 to 14 for 1990, 2000, and 2016

Albania	Interstitial lung disease and pulmonary sarcoidosis	10 to 14	Female	0 (0 to 0)	0.0 (0.0 to 0.1)	0 (0 to 0)	0.0 (0.0 to 0.1)	0 (0 to 0)	0.0 (0.0 to 0.1)	-48.7 (-79.6 to 34.7)	-6.7 (-62.8 to 145.0)
Albania	Interstitial lung disease and pulmonary sarcoidosis	10 to 14	Male	0 (0 to 0)	0.0 (0.0 to 0.1)	0 (0 to 0)	0.0 (0.0 to 0.1)	0 (0 to 0)	0.0 (0.0 to 0.1)	-58.8 (-87.4 to 19.1)	-26.2 (-77.4 to 113.3)
Albania	Other chronic respiratory diseases	5 to 9	Both	1 (0 to 1)	0.2 (0.1 to 0.4)	1 (0 to 1)	0.2 (0.1 to 0.3)	0 (0 to 0)	0.1 (0.1 to 0.2)	-76.5 (-89.5 to -42.7)	-46.0 (-76.0 to 31.2)
Albania	Other chronic respiratory diseases	5 to 9	Female	0 (0 to 0)	0.1 (0.0 to 0.3)	0 (0 to 0)	0.1 (0.1 to 0.3)	0 (0 to 0)	0.1 (0.0 to 0.2)	-68.1 (-90.6 to -22.6)	-26.4 (-78.3 to 78.3)
Albania	Other chronic respiratory diseases	5 to 9	Male	1 (0 to 1)	0.3 (0.1 to 0.6)	1 (0 to 1)	0.3 (0.1 to 0.6)	0 (0 to 0)	0.1 (0.1 to 0.3)	-79.8 (-92.5 to -41.7)	-54.1 (-82.9 to 32.8)
Albania	Other chronic respiratory diseases	10 to 14	Both	1 (0 to 1)	0.2 (0.1 to 0.4)	1 (0 to 1)	0.2 (0.1 to 0.3)	0 (0 to 0)	0.1 (0.1 to 0.3)	-65.8 (-85.0 to -29.1)	-38.4 (-73.0 to 27.9)
Albania	Other chronic respiratory diseases	10 to 14	Female	0 (0 to 0)	0.1 (0.0 to 0.2)	0 (0 to 0)	0.1 (0.0 to 0.2)	0 (0 to 0)	0.1 (0.0 to 0.2)	-60.1 (-89.3 to 10.2)	-27.4 (-80.6 to 100.5)
Albania	Other chronic respiratory diseases	10 to 14	Male	1 (0 to 1)	0.4 (0.2 to 0.6)	1 (0 to 1)	0.3 (0.2 to 0.6)	0 (0 to 0)	0.2 (0.1 to 0.4)	-67.5 (-87.5 to -22.3)	-41.8 (-77.6 to 39.2)
Albania	Cirrhosis and other chronic liver diseases due to hepatitis B	5 to 9	Both	0 (0 to 0)	0.0 (0.0 to 0.0)	0 (0 to 0)	0.0 (0.0 to 0.0)	0 (0 to 0)	0.0 (0.0 to 0.0)	-72.3 (-85.0 to -50.7)	-36.4 (-65.6 to 12.9)
Albania	Cirrhosis and other chronic liver diseases due to hepatitis B	5 to 9	Female	0 (0 to 0)	0.0 (0.0 to 0.0)	0 (0 to 0)	0.0 (0.0 to 0.0)	0 (0 to 0)	0.0 (0.0 to 0.0)	-77.1 (-87.3 to -59.5)	-47.3 (-70.7 to -6.6)
Albania	Cirrhosis and other chronic liver diseases due to hepatitis B	5 to 9	Male	0 (0 to 0)	0.0 (0.0 to 0.0)	0 (0 to 0)	0.0 (0.0 to 0.0)	0 (0 to 0)	0.0 (0.0 to 0.0)	-70.8 (-85.7 to -42.9)	-33.6 (-67.3 to 30.1)
Albania	Cirrhosis and other chronic liver diseases due to hepatitis B	10 to 14	Both	0 (0 to 0)	0.0 (0.0 to 0.0)	0 (0 to 0)	0.0 (0.0 to 0.0)	0 (0 to 0)	0.0 (0.0 to 0.0)	-59.3 (-77.8 to -23.1)	-26.6 (-60.0 to 38.7)
Albania	Cirrhosis and other chronic liver diseases due to hepatitis B	10 to 14	Female	0 (0 to 0)	0.0 (0.0 to 0.0)	0 (0 to 0)	0.0 (0.0 to 0.0)	0 (0 to 0)	0.0 (0.0 to 0.0)	-70.1 (-85.1 to -45.0)	-45.7 (-72.9 to -0.0)
Albania	Cirrhosis and other chronic liver diseases due to hepatitis B	10 to 14	Male	0 (0 to 0)	0.0 (0.0 to 0.0)	0 (0 to 0)	0.0 (0.0 to 0.0)	0 (0 to 0)	0.0 (0.0 to 0.0)	-57.1 (-78.3 to -15.1)	-23.1 (-61.2 to 52.1)
Albania	Cirrhosis and other chronic liver diseases due to hepatitis C	5 to 9	Both	0 (0 to 0)	0.0 (0.0 to 0.0)	0 (0 to 0)	0.0 (0.0 to 0.0)	0 (0 to 0)	0.0 (0.0 to 0.0)	-73.9 (-86.1 to -54.4)	-40.3 (-68.1 to 4.6)

eTable 8. All-cause and cause-specific mortality (counts and rates) at the global level and by country in the WHO European Region for ages 5 to 9 and 10 to 14 for 1990, 2000, and 2016

Albania	Cirrhosis and other chronic liver diseases due to hepatitis C	5 to 9	Female	0<br>(0 to 0)	0.0<br>(0.0 to 0.0)	0<br>(0 to 0)	0.0<br>(0.0 to 0.0)	0<br>(0 to 0)	0.0<br>(0.0 to 0.0)	-78.3<br>(-87.8 to -62.0)	-50.0<br>(-71.8 to -12.4)
Albania	Cirrhosis and other chronic liver diseases due to hepatitis C	5 to 9	Male	0<br>(0 to 0)	0.0<br>(0.0 to 0.0)	0<br>(0 to 0)	0.0<br>(0.0 to 0.0)	0<br>(0 to 0)	0.0<br>(0.0 to 0.0)	-72.5<br>(-86.6 to -46.2)	-37.3<br>(-69.5 to 22.6)
Albania	Cirrhosis and other chronic liver diseases due to hepatitis C	10 to 14	Both	0<br>(0 to 0)	0.0<br>(0.0 to 0.0)	0<br>(0 to 0)	0.0<br>(0.0 to 0.0)	0<br>(0 to 0)	0.0<br>(0.0 to 0.0)	-61.9<br>(-79.2 to -28.9)	-31.2<br>(-62.4 to 28.4)
Albania	Cirrhosis and other chronic liver diseases due to hepatitis C	10 to 14	Female	0<br>(0 to 0)	0.0<br>(0.0 to 0.0)	0<br>(0 to 0)	0.0<br>(0.0 to 0.0)	0<br>(0 to 0)	0.0<br>(0.0 to 0.0)	-71.5<br>(-85.7 to -47.8)	-48.2<br>(-74.0 to -5.1)
Albania	Cirrhosis and other chronic liver diseases due to hepatitis C	10 to 14	Male	0<br>(0 to 0)	0.0<br>(0.0 to 0.0)	0<br>(0 to 0)	0.0<br>(0.0 to 0.0)	0<br>(0 to 0)	0.0<br>(0.0 to 0.0)	-59.5<br>(-79.5 to -18.5)	-27.5<br>(-63.3 to 45.9)
Albania	Cirrhosis and other chronic liver diseases due to other causes	5 to 9	Both	1<br>(1 to 2)	0.4<br>(0.3 to 0.5)	1<br>(1 to 2)	0.4<br>(0.3 to 0.5)	0<br>(0 to 1)	0.2<br>(0.1 to 0.3)	-73.8<br>(-84.7 to -57.4)	-39.9<br>(-64.9 to -2.3)
Albania	Cirrhosis and other chronic liver diseases due to other causes	5 to 9	Female	1<br>(0 to 1)	0.4<br>(0.3 to 0.5)	1<br>(0 to 1)	0.4<br>(0.3 to 0.5)	0<br>(0 to 0)	0.2<br>(0.1 to 0.3)	-77.3<br>(-87.4 to -59.6)	-47.7<br>(-70.9 to -6.9)
Albania	Cirrhosis and other chronic liver diseases due to other causes	5 to 9	Male	1<br>(0 to 1)	0.4<br>(0.2 to 0.5)	1<br>(0 to 1)	0.4<br>(0.3 to 0.6)	0<br>(0 to 0)	0.2<br>(0.1 to 0.4)	-70.4<br>(-85.5 to -42.2)	-32.6<br>(-67.0 to 31.7)
Albania	Cirrhosis and other chronic liver diseases due to other causes	10 to 14	Both	1<br>(1 to 1)	0.3<br>(0.2 to 0.5)	1<br>(1 to 1)	0.3<br>(0.2 to 0.4)	0<br>(0 to 1)	0.2<br>(0.1 to 0.4)	-62.2<br>(-77.9 to -34.8)	-31.9<br>(-60.1 to 17.7)
Albania	Cirrhosis and other chronic liver diseases due to other causes	10 to 14	Female	0<br>(0 to 1)	0.3<br>(0.2 to 0.4)	0<br>(0 to 1)	0.2<br>(0.2 to 0.3)	0<br>(0 to 0)	0.2<br>(0.1 to 0.2)	-70.3<br>(-85.0 to -46.0)	-46.0<br>(-72.8 to -1.9)
Albania	Cirrhosis and other chronic liver diseases due to other causes	10 to 14	Male	1<br>(0 to 1)	0.4<br>(0.2 to 0.6)	1<br>(0 to 1)	0.4<br>(0.2 to 0.5)	0<br>(0 to 0)	0.3<br>(0.1 to 0.5)	-56.6<br>(-78.0 to -12.9)	-22.3<br>(-60.6 to 56.1)
Albania	Peptic ulcer disease	5 to 9	Both	0<br>(0 to 1)	0.1<br>(0.0 to 0.2)	0<br>(0 to 0)	0.1<br>(0.0 to 0.1)	0<br>(0 to 0)	0.0<br>(0.0 to 0.0)	-88.3<br>(-94.4 to -74.2)	-73.2<br>(-87.1 to -40.9)
Albania	Peptic ulcer disease	5 to 9	Female	0<br>(0 to 0)	0.1<br>(0.0 to 0.2)	0<br>(0 to 0)	0.1<br>(0.0 to 0.1)	0<br>(0 to 0)	0.0<br>(0.0 to 0.1)	-86.4<br>(-94.3 to -60.7)	-68.5<br>(-86.9 to -9.4)
Albania	Peptic ulcer disease	5 to 9	Male	0<br>(0 to 0)	0.1<br>(0.0 to 0.2)	0<br>(0 to 0)	0.1<br>(0.0 to 0.2)	0<br>(0 to 0)	0.0<br>(0.0 to 0.0)	-90.0<br>(-96.1 to -70.4)	-77.3<br>(-91.1 to -32.5)
Albania	Peptic ulcer disease	10 to 14	Both	0<br>(0 to 0)	0.0<br>(0.0 to 0.1)	0<br>(0 to 0)	0.0<br>(0.0 to 0.0)	0<br>(0 to 0)	0.0<br>(0.0 to 0.0)	-64.2<br>(-80.0 to -32.3)	-35.4<br>(-63.9 to 22.1)
Albania	Peptic ulcer disease	10 to 14	Female	0<br>(0 to 0)	0.0<br>(0.0 to 0.1)	0<br>(0 to 0)	0.0<br>(0.0 to 0.0)	0<br>(0 to 0)	0.0<br>(0.0 to 0.0)	-53.0<br>(-76.2 to 5.9)	-14.6<br>(-56.8 to 92.5)

eTable 8. All-cause and cause-specific mortality (counts and rates) at the global level and by country in the WHO European Region for ages 5 to 9 and 10 to 14 for 1990, 2000, and 2016

Albania	Peptic ulcer disease	10 to 14	Male	0 (0 to 0)	0.0 (0.0 to 0.1)	0 (0 to 0)	0.0 (0.0 to 0.0)	0 (0 to 0)	0.0 (0.0 to 0.0)	-72.9 (-88.1 to -38.5)	-51.4 (-78.7 to 10.3)
Albania	Gastritis and duodenitis	5 to 9	Both	0 (0 to 0)	0.0 (0.0 to 0.0)	0 (0 to 0)	0.0 (0.0 to 0.0)	0 (0 to 0)	0.0 (0.0 to 0.0)	-43.2 (-74.0 to 22.6)	30.1 (-40.3 to 180.9)
Albania	Gastritis and duodenitis	5 to 9	Female	0 (0 to 0)	0.0 (0.0 to 0.0)	0 (0 to 0)	0.0 (0.0 to 0.0)	0 (0 to 0)	0.0 (0.0 to 0.0)	-39.6 (-73.8 to 33.8)	39.2 (-39.6 to 208.3)
Albania	Gastritis and duodenitis	5 to 9	Male	0 (0 to 0)	0.0 (0.0 to 0.0)	0 (0 to 0)	0.0 (0.0 to 0.0)	0 (0 to 0)	0.0 (0.0 to 0.0)	-46.2 (-81.5 to 74.5)	22.7 (-57.8 to 297.7)
Albania	Gastritis and duodenitis	10 to 14	Both	0 (0 to 0)	0.0 (0.0 to 0.0)	0 (0 to 0)	0.0 (0.0 to 0.0)	0 (0 to 0)	0.0 (0.0 to 0.0)	-41.7 (-67.5 to 4.0)	5.2 (-41.3 to 87.7)
Albania	Gastritis and duodenitis	10 to 14	Female	0 (0 to 0)	0.0 (0.0 to 0.0)	0 (0 to 0)	0.0 (0.0 to 0.0)	0 (0 to 0)	0.0 (0.0 to 0.0)	-45.6 (-72.7 to 16.9)	-1.1 (-50.3 to 112.6)
Albania	Gastritis and duodenitis	10 to 14	Male	0 (0 to 0)	0.0 (0.0 to 0.0)	0 (0 to 0)	0.0 (0.0 to 0.0)	0 (0 to 0)	0.0 (0.0 to 0.0)	-35.4 (-69.2 to 34.5)	15.8 (-44.8 to 141.0)
Albania	Appendicitis	5 to 9	Both	0 (0 to 1)	0.1 (0.1 to 0.2)	0 (0 to 1)	0.1 (0.1 to 0.2)	0 (0 to 0)	0.1 (0.0 to 0.1)	-80.3 (-90.6 to -58.0)	-54.8 (-78.5 to -3.7)
Albania	Appendicitis	5 to 9	Female	0 (0 to 0)	0.1 (0.0 to 0.2)	0 (0 to 0)	0.1 (0.0 to 0.1)	0 (0 to 0)	0.0 (0.0 to 0.1)	-80.6 (-92.4 to -51.7)	-55.3 (-82.4 to 11.3)
Albania	Appendicitis	5 to 9	Male	0 (0 to 1)	0.1 (0.1 to 0.4)	0 (0 to 0)	0.1 (0.1 to 0.3)	0 (0 to 0)	0.1 (0.0 to 0.1)	-80.0 (-92.3 to -46.5)	-54.5 (-82.5 to 21.9)
Albania	Appendicitis	10 to 14	Both	0 (0 to 1)	0.1 (0.1 to 0.2)	0 (0 to 0)	0.1 (0.1 to 0.1)	0 (0 to 0)	0.1 (0.0 to 0.1)	-74.3 (-86.8 to -49.6)	-53.7 (-76.2 to -9.1)
Albania	Appendicitis	10 to 14	Female	0 (0 to 0)	0.1 (0.1 to 0.3)	0 (0 to 0)	0.1 (0.0 to 0.1)	0 (0 to 0)	0.0 (0.0 to 0.1)	-80.7 (-91.9 to -57.4)	-65.0 (-85.3 to -22.5)
Albania	Appendicitis	10 to 14	Male	0 (0 to 0)	0.1 (0.0 to 0.2)	0 (0 to 0)	0.1 (0.1 to 0.2)	0 (0 to 0)	0.1 (0.0 to 0.1)	-68.2 (-85.9 to -28.6)	-43.0 (-74.8 to 27.9)
Albania	Paralytic ileus and intestinal obstruction	5 to 9	Both	1 (0 to 1)	0.2 (0.0 to 0.4)	0 (0 to 1)	0.1 (0.1 to 0.2)	0 (0 to 0)	0.1 (0.0 to 0.2)	-78.7 (-90.6 to -39.3)	-51.2 (-78.4 to 39.1)
Albania	Paralytic ileus and intestinal obstruction	5 to 9	Female	0 (0 to 1)	0.2 (0.0 to 0.3)	0 (0 to 0)	0.1 (0.1 to 0.2)	0 (0 to 0)	0.1 (0.0 to 0.2)	-73.7 (-90.5 to -30.1)	-39.4 (-78.0 to 61.1)
Albania	Paralytic ileus and intestinal obstruction	5 to 9	Male	0 (0 to 1)	0.2 (0.0 to 0.4)	0 (0 to 1)	0.2 (0.0 to 0.3)	0 (0 to 0)	0.1 (0.0 to 0.2)	-82.2 (-94.6 to -33.2)	-59.4 (-87.8 to 52.1)
Albania	Paralytic ileus and intestinal obstruction	10 to 14	Both	0 (0 to 1)	0.1 (0.0 to 0.2)	0 (0 to 0)	0.1 (0.0 to 0.1)	0 (0 to 0)	0.1 (0.0 to 0.1)	-75.7 (-87.9 to -45.5)	-56.1 (-78.1 to -1.7)
Albania	Paralytic ileus and intestinal obstruction	10 to 14	Female	0 (0 to 1)	0.2 (0.1 to 0.4)	0 (0 to 0)	0.1 (0.1 to 0.2)	0 (0 to 0)	0.1 (0.0 to 0.2)	-77.6 (-89.6 to -51.2)	-59.2 (-81.1 to -11.2)
Albania	Paralytic ileus and intestinal obstruction	10 to 14	Male	0 (0 to 0)	0.1 (0.0 to 0.1)	0 (0 to 0)	0.1 (0.0 to 0.1)	0 (0 to 0)	0.0 (0.0 to 0.1)	-70.7 (-89.5 to -15.8)	-47.5 (-81.3 to 50.9)
Albania	Inguinal, femoral, and abdominal hernia	5 to 9	Both	0 (0 to 1)	0.1 (0.0 to 0.2)	0 (0 to 0)	0.1 (0.0 to 0.1)	0 (0 to 0)	0.0 (0.0 to 0.1)	-78.5 (-92.4 to -27.4)	-50.8 (-82.5 to 66.4)
Albania	Inguinal, femoral, and abdominal hernia	5 to 9	Female	0 (0 to 0)	0.0 (0.0 to 0.1)	0 (0 to 0)	0.0 (0.0 to 0.1)	0 (0 to 0)	0.0 (0.0 to 0.0)	-67.7 (-89.5 to 4.7)	-25.6 (-75.8 to 141.3)
Albania	Inguinal, femoral, and abdominal hernia	5 to 9	Male	0 (0 to 1)	0.1 (0.0 to 0.3)	0 (0 to 0)	0.1 (0.0 to 0.2)	0 (0 to 0)	0.1 (0.0 to 0.1)	-81.1 (-94.8 to -19.1)	-57.0 (-88.1 to 84.3)
Albania	Inguinal, femoral, and abdominal hernia	10 to 14	Both	0 (0 to 1)	0.1 (0.0 to 0.2)	0 (0 to 0)	0.1 (0.0 to 0.1)	0 (0 to 0)	0.0 (0.0 to 0.1)	-80.3 (-92.6 to -35.2)	-64.4 (-86.6 to 16.9)
Albania	Inguinal, femoral, and abdominal hernia	10 to 14	Female	0 (0 to 0)	0.1 (0.0 to 0.3)	0 (0 to 0)	0.1 (0.0 to 0.2)	0 (0 to 0)	0.0 (0.0 to 0.1)	-79.6 (-93.6 to -26.4)	-63.0 (-88.3 to 33.9)

eTable 8. All-cause and cause-specific mortality (counts and rates) at the global level and by country in the WHO European Region for ages 5 to 9 and 10 to 14 for 1990, 2000, and 2016

Albania	Inguinal, femoral, and abdominal hernia	10 to 14	Male	0<br>(0 to 0)	0.1<br>(0.0 to 0.3)	0<br>(0 to 0)	0.1<br>(0.0 to 0.1)	0<br>(0 to 0)	0.0<br>(0.0 to 0.1)	-80.9<br>(-95.3 to -11.3)	-65.8<br>(-91.5 to 58.9)
Albania	Inflammatory bowel disease	5 to 9	Both	1<br>(0 to 2)	0.4<br>(0.0 to 0.7)	1<br>(0 to 1)	0.3<br>(0.0 to 0.5)	0<br>(0 to 0)	0.1<br>(0.0 to 0.3)	-84.7<br>(-92.6 to -36.7)	-64.9<br>(-83.0 to 45.1)
Albania	Inflammatory bowel disease	5 to 9	Female	1<br>(0 to 1)	0.3<br>(0.0 to 0.7)	0<br>(0 to 1)	0.2<br>(0.0 to 0.4)	0<br>(0 to 0)	0.1<br>(0.0 to 0.3)	-83.6<br>(-93.2 to -41.0)	-62.1<br>(-84.4 to 36.0)
Albania	Inflammatory bowel disease	5 to 9	Male	1<br>(0 to 2)	0.4<br>(0.0 to 0.9)	0<br>(0 to 1)	0.3<br>(0.1 to 0.6)	0<br>(0 to 0)	0.1<br>(0.0 to 0.3)	-85.5<br>(-94.6 to -32.8)	-66.9<br>(-87.8 to 53.2)
Albania	Inflammatory bowel disease	10 to 14	Both	0<br>(0 to 1)	0.1<br>(0.0 to 0.2)	0<br>(0 to 0)	0.1<br>(0.0 to 0.1)	0<br>(0 to 0)	0.0<br>(0.0 to 0.1)	-76.3<br>(-89.7 to -42.4)	-57.3<br>(-81.3 to 4.0)
Albania	Inflammatory bowel disease	10 to 14	Female	0<br>(0 to 0)	0.0<br>(0.0 to 0.1)	0<br>(0 to 0)	0.0<br>(0.0 to 0.1)	0<br>(0 to 0)	0.0<br>(0.0 to 0.0)	-59.5<br>(-83.6 to 19.2)	-26.4<br>(-70.2 to 116.8)
Albania	Inflammatory bowel disease	10 to 14	Male	0<br>(0 to 1)	0.2<br>(0.0 to 0.3)	0<br>(0 to 0)	0.1<br>(0.0 to 0.2)	0<br>(0 to 0)	0.1<br>(0.0 to 0.1)	-79.2<br>(-92.1 to -9.1)	-62.7<br>(-85.8 to 62.8)
Albania	Vascular intestinal disorders	5 to 9	Both	0<br>(0 to 0)	0.0<br>(0.0 to 0.0)	0<br>(0 to 0)	0.0<br>(0.0 to 0.0)	0<br>(0 to 0)	0.0<br>(0.0 to 0.0)	-72.0<br>(-90.7 to -20.8)	-35.8<br>(-78.7 to 81.5)
Albania	Vascular intestinal disorders	5 to 9	Female	0<br>(0 to 0)	0.0<br>(0.0 to 0.0)	0<br>(0 to 0)	0.0<br>(0.0 to 0.0)	0<br>(0 to 0)	0.0<br>(0.0 to 0.0)	-68.4<br>(-90.3 to 1.2)	-27.1<br>(-77.7 to 133.3)
Albania	Vascular intestinal disorders	5 to 9	Male	0<br>(0 to 0)	0.0<br>(0.0 to 0.0)	0<br>(0 to 0)	0.0<br>(0.0 to 0.0)	0<br>(0 to 0)	0.0<br>(0.0 to 0.0)	-72.7<br>(-92.6 to -1.8)	-37.7<br>(-83.1 to 123.7)
Albania	Vascular intestinal disorders	10 to 14	Both	0<br>(0 to 0)	0.0<br>(0.0 to 0.0)	0<br>(0 to 0)	0.0<br>(0.0 to 0.0)	0<br>(0 to 0)	0.0<br>(0.0 to 0.0)	-71.9<br>(-88.4 to -28.8)	-49.3<br>(-79.1 to 28.5)
Albania	Vascular intestinal disorders	10 to 14	Female	0<br>(0 to 0)	0.0<br>(0.0 to 0.0)	0<br>(0 to 0)	0.0<br>(0.0 to 0.0)	0<br>(0 to 0)	0.0<br>(0.0 to 0.0)	-40.5<br>(-79.1 to 93.7)	8.1<br>(-62.0 to 252.4)
Albania	Vascular intestinal disorders	10 to 14	Male	0<br>(0 to 0)	0.0<br>(0.0 to 0.0)	0<br>(0 to 0)	0.0<br>(0.0 to 0.0)	0<br>(0 to 0)	0.0<br>(0.0 to 0.0)	-75.3<br>(-90.0 to -20.6)	-55.7<br>(-82.1 to 42.2)
Albania	Gallbladder and biliary diseases	5 to 9	Both	0<br>(0 to 0)	0.0<br>(0.0 to 0.0)	0<br>(0 to 0)	0.0<br>(0.0 to 0.0)	0<br>(0 to 0)	0.0<br>(0.0 to 0.0)	-76.8<br>(-90.1 to -46.7)	-46.9<br>(-77.4 to 22.1)
Albania	Gallbladder and biliary diseases	5 to 9	Female	0<br>(0 to 0)	0.0<br>(0.0 to 0.0)	0<br>(0 to 0)	0.0<br>(0.0 to 0.0)	0<br>(0 to 0)	0.0<br>(0.0 to 0.0)	-77.3<br>(-91.9 to -38.4)	-47.8<br>(-81.4 to 42.0)
Albania	Gallbladder and biliary diseases	5 to 9	Male	0<br>(0 to 0)	0.0<br>(0.0 to 0.0)	0<br>(0 to 0)	0.0<br>(0.0 to 0.0)	0<br>(0 to 0)	0.0<br>(0.0 to 0.0)	-75.5<br>(-92.4 to -26.3)	-44.1<br>(-82.6 to 68.0)
Albania	Gallbladder and biliary diseases	10 to 14	Both	0<br>(0 to 0)	0.0<br>(0.0 to 0.0)	0<br>(0 to 0)	0.0<br>(0.0 to 0.0)	0<br>(0 to 0)	0.0<br>(0.0 to 0.0)	-63.6<br>(-84.0 to -13.6)	-34.2<br>(-71.0 to 56.0)
Albania	Gallbladder and biliary diseases	10 to 14	Female	0<br>(0 to 0)	0.0<br>(0.0 to 0.0)	0<br>(0 to 0)	0.0<br>(0.0 to 0.0)	0<br>(0 to 0)	0.0<br>(0.0 to 0.0)	-59.4<br>(-82.1 to 26.2)	-26.3<br>(-67.5 to 129.6)
Albania	Gallbladder and biliary diseases	10 to 14	Male	0<br>(0 to 0)	0.0<br>(0.0 to 0.0)	0<br>(0 to 0)	0.0<br>(0.0 to 0.0)	0<br>(0 to 0)	0.0<br>(0.0 to 0.0)	-65.3<br>(-88.8 to -6.3)	-37.8<br>(-80.0 to 67.8)
Albania	Pancreatitis	5 to 9	Both	0<br>(0 to 0)	0.0<br>(0.0 to 0.1)	0<br>(0 to 0)	0.0<br>(0.0 to 0.1)	0<br>(0 to 0)	0.0<br>(0.0 to 0.0)	-68.6<br>(-86.4 to -28.9)	-28.0<br>(-68.9 to 63.0)
Albania	Pancreatitis	5 to 9	Female	0<br>(0 to 0)	0.0<br>(0.0 to 0.1)	0<br>(0 to 0)	0.0<br>(0.0 to 0.1)	0<br>(0 to 0)	0.0<br>(0.0 to 0.1)	-71.1<br>(-90.0 to -6.6)	-33.3<br>(-77.0 to 115.3)
Albania	Pancreatitis	5 to 9	Male	0<br>(0 to 0)	0.0<br>(0.0 to 0.1)	0<br>(0 to 0)	0.0<br>(0.0 to 0.1)	0<br>(0 to 0)	0.0<br>(0.0 to 0.1)	-65.8<br>(-88.4 to 1.9)	-22.1<br>(-73.7 to 132.2)
Albania	Pancreatitis	10 to 14	Both	0<br>(0 to 0)	0.0<br>(0.0 to 0.0)	0<br>(0 to 0)	0.0<br>(0.0 to 0.1)	0<br>(0 to 0)	0.0<br>(0.0 to 0.0)	-56.2<br>(-80.3 to -4.3)	-21.0<br>(-64.5 to 72.6)
Albania	Pancreatitis	10 to 14	Female	0<br>(0 to 0)	0.0<br>(0.0 to 0.1)	0<br>(0 to 0)	0.0<br>(0.0 to 0.1)	0<br>(0 to 0)	0.0<br>(0.0 to 0.0)	-63.8<br>(-86.0 to 2.9)	-34.2<br>(-74.5 to 87.2)
Albania	Pancreatitis	10 to 14	Male	0<br>(0 to 0)	0.0<br>(0.0 to 0.1)	0<br>(0 to 0)	0.0<br>(0.0 to 0.1)	0<br>(0 to 0)	0.0<br>(0.0 to 0.1)	-48.1<br>(-80.1 to 36.8)	-7.0<br>(-64.3 to 145.0)

eTable 8. All-cause and cause-specific mortality (counts and rates) at the global level and by country in the WHO European Region for ages 5 to 9 and 10 to 14 for 1990, 2000, and 2016

Albania	Other digestive diseases	5 to 9	Both	1 (1 to 2)	0.3 (0.2 to 0.5)	1 (0 to 1)	0.2 (0.1 to 0.3)	0 (0 to 0)	0.1 (0.0 to 0.2)	-85.7 (-93.2 to -70.5)	-67.3 (-84.4 to -32.3)
Albania	Other digestive diseases	5 to 9	Female	0 (0 to 1)	0.3 (0.1 to 0.5)	0 (0 to 1)	0.2 (0.1 to 0.4)	0 (0 to 0)	0.1 (0.0 to 0.2)	-85.0 (-93.8 to -65.9)	-65.4 (-85.7 to -21.4)
Albania	Other digestive diseases	5 to 9	Male	1 (0 to 1)	0.3 (0.1 to 0.7)	0 (0 to 1)	0.2 (0.1 to 0.4)	0 (0 to 0)	0.1 (0.0 to 0.2)	-86.4 (-95.5 to -58.4)	-69.1 (-89.9 to -5.2)
Albania	Other digestive diseases	10 to 14	Both	1 (0 to 1)	0.2 (0.1 to 0.3)	0 (0 to 1)	0.1 (0.1 to 0.2)	0 (0 to 0)	0.1 (0.0 to 0.1)	-79.3 (-90.2 to -56.6)	-62.6 (-82.3 to -21.6)
Albania	Other digestive diseases	10 to 14	Female	0 (0 to 0)	0.0 (0.0 to 0.1)	0 (0 to 0)	0.0 (0.0 to 0.1)	0 (0 to 0)	0.0 (0.0 to 0.0)	-67.6 (-84.9 to -19.0)	-41.0 (-72.6 to 47.2)
Albania	Other digestive diseases	10 to 14	Male	1 (0 to 1)	0.3 (0.1 to 0.5)	0 (0 to 1)	0.2 (0.1 to 0.3)	0 (0 to 0)	0.1 (0.0 to 0.2)	-80.8 (-91.9 to -55.3)	-65.7 (-85.5 to -20.0)
Albania	Alzheimer disease and other dementias	5 to 9	Both	0 (0 to 0)	0.0 (0.0 to 0.0)	0 (0 to 0)	0.0 (0.0 to 0.0)	0 (0 to 0)	0.0 (0.0 to 0.0)	0.0 (0.0 to 0.0)	0.0 (0.0 to 0.0)
Albania	Alzheimer disease and other dementias	5 to 9	Female	0 (0 to 0)	0.0 (0.0 to 0.0)	0 (0 to 0)	0.0 (0.0 to 0.0)	0 (0 to 0)	0.0 (0.0 to 0.0)	0.0 (0.0 to 0.0)	0.0 (0.0 to 0.0)
Albania	Alzheimer disease and other dementias	5 to 9	Male	0 (0 to 0)	0.0 (0.0 to 0.0)	0 (0 to 0)	0.0 (0.0 to 0.0)	0 (0 to 0)	0.0 (0.0 to 0.0)	0.0 (0.0 to 0.0)	0.0 (0.0 to 0.0)
Albania	Alzheimer disease and other dementias	10 to 14	Both	0 (0 to 0)	0.0 (0.0 to 0.0)	0 (0 to 0)	0.0 (0.0 to 0.0)	0 (0 to 0)	0.0 (0.0 to 0.0)	0.0 (0.0 to 0.0)	0.0 (0.0 to 0.0)
Albania	Alzheimer disease and other dementias	10 to 14	Female	0 (0 to 0)	0.0 (0.0 to 0.0)	0 (0 to 0)	0.0 (0.0 to 0.0)	0 (0 to 0)	0.0 (0.0 to 0.0)	0.0 (0.0 to 0.0)	0.0 (0.0 to 0.0)
Albania	Alzheimer disease and other dementias	10 to 14	Male	0 (0 to 0)	0.0 (0.0 to 0.0)	0 (0 to 0)	0.0 (0.0 to 0.0)	0 (0 to 0)	0.0 (0.0 to 0.0)	0.0 (0.0 to 0.0)	0.0 (0.0 to 0.0)
Albania	Parkinson disease	5 to 9	Both	0 (0 to 0)	0.0 (0.0 to 0.0)	0 (0 to 0)	0.0 (0.0 to 0.0)	0 (0 to 0)	0.0 (0.0 to 0.0)	0.0 (0.0 to 0.0)	0.0 (0.0 to 0.0)
Albania	Parkinson disease	5 to 9	Female	0 (0 to 0)	0.0 (0.0 to 0.0)	0 (0 to 0)	0.0 (0.0 to 0.0)	0 (0 to 0)	0.0 (0.0 to 0.0)	0.0 (0.0 to 0.0)	0.0 (0.0 to 0.0)
Albania	Parkinson disease	5 to 9	Male	0 (0 to 0)	0.0 (0.0 to 0.0)	0 (0 to 0)	0.0 (0.0 to 0.0)	0 (0 to 0)	0.0 (0.0 to 0.0)	0.0 (0.0 to 0.0)	0.0 (0.0 to 0.0)
Albania	Parkinson disease	10 to 14	Both	0 (0 to 0)	0.0 (0.0 to 0.0)	0 (0 to 0)	0.0 (0.0 to 0.0)	0 (0 to 0)	0.0 (0.0 to 0.0)	0.0 (0.0 to 0.0)	0.0 (0.0 to 0.0)
Albania	Parkinson disease	10 to 14	Female	0 (0 to 0)	0.0 (0.0 to 0.0)	0 (0 to 0)	0.0 (0.0 to 0.0)	0 (0 to 0)	0.0 (0.0 to 0.0)	0.0 (0.0 to 0.0)	0.0 (0.0 to 0.0)
Albania	Parkinson disease	10 to 14	Male	0 (0 to 0)	0.0 (0.0 to 0.0)	0 (0 to 0)	0.0 (0.0 to 0.0)	0 (0 to 0)	0.0 (0.0 to 0.0)	0.0 (0.0 to 0.0)	0.0 (0.0 to 0.0)
Albania	Epilepsy	5 to 9	Both	5 (3 to 7)	1.4 (0.9 to 2.0)	8 (6 to 11)	2.5 (1.8 to 3.5)	2 (1 to 3)	1.4 (0.9 to 2.2)	-55.1 (-76.8 to -16.3)	2.9 (-46.8 to 91.7)
Albania	Epilepsy	5 to 9	Female	2 (1 to 4)	1.3 (0.8 to 2.6)	4 (2 to 5)	2.3 (1.2 to 3.4)	1 (1 to 2)	1.3 (0.8 to 2.2)	-57.8 (-79.4 to -0.4)	-2.8 (-52.6 to 129.6)
Albania	Epilepsy	5 to 9	Male	3 (2 to 4)	1.4 (1.0 to 2.1)	5 (3 to 7)	2.8 (1.8 to 4.1)	1 (1 to 2)	1.6 (0.8 to 2.7)	-52.7 (-77.2 to -6.0)	7.8 (-48.0 to 114.2)
Albania	Epilepsy	10 to 14	Both	5 (4 to 7)	1.5 (1.1 to 2.0)	7 (5 to 8)	1.9 (1.4 to 2.5)	3 (2 to 4)	1.4 (0.9 to 2.1)	-49.3 (-69.9 to -13.9)	-8.5 (-45.6 to 55.4)
Albania	Epilepsy	10 to 14	Female	2 (1 to 4)	1.4 (0.8 to 2.2)	3 (2 to 4)	1.6 (1.0 to 2.3)	1 (1 to 2)	1.1 (0.6 to 1.7)	-56.7 (-76.8 to -11.2)	-21.2 (-57.7 to 61.5)

eTable 8. All-cause and cause-specific mortality (counts and rates) at the global level and by country in the WHO European Region for ages 5 to 9 and 10 to 14 for 1990, 2000, and 2016

Albania	Epilepsy	10 to 14	Male	3 (2 to 4)	1.6 (1.1 to 2.3)	4 (3 to 5)	2.3 (1.5 to 3.2)	2 (1 to 3)	1.6 (0.9 to 2.7)	-43.5 (-70.0 to 8.2)	1.2 (-46.2 to 93.9)
Albania	Motor neuron disease	5 to 9	Both	0 (0 to 0)	0.0 (0.0 to 0.0)	0 (0 to 0)	0.0 (0.0 to 0.0)	0 (0 to 0)	0.0 (0.0 to 0.0)	-82.6 (-92.4 to -63.0)	-60.2 (-82.7 to -15.3)
Albania	Motor neuron disease	5 to 9	Female	0 (0 to 0)	0.0 (0.0 to 0.0)	0 (0 to 0)	0.0 (0.0 to 0.0)	0 (0 to 0)	0.0 (0.0 to 0.0)	-82.9 (-93.8 to -59.3)	-60.7 (-85.7 to -6.3)
Albania	Motor neuron disease	5 to 9	Male	0 (0 to 0)	0.0 (0.0 to 0.0)	0 (0 to 0)	0.0 (0.0 to 0.0)	0 (0 to 0)	0.0 (0.0 to 0.0)	-82.2 (-95.2 to -43.4)	-59.3 (-89.1 to 29.0)
Albania	Motor neuron disease	10 to 14	Both	0 (0 to 0)	0.0 (0.0 to 0.0)	0 (0 to 0)	0.0 (0.0 to 0.0)	0 (0 to 0)	0.0 (0.0 to 0.0)	-75.9 (-88.6 to -52.5)	-56.6 (-79.4 to -14.4)
Albania	Motor neuron disease	10 to 14	Female	0 (0 to 0)	0.0 (0.0 to 0.0)	0 (0 to 0)	0.0 (0.0 to 0.0)	0 (0 to 0)	0.0 (0.0 to 0.0)	-75.4 (-89.2 to -50.0)	-55.2 (-80.4 to -9.1)
Albania	Motor neuron disease	10 to 14	Male	0 (0 to 0)	0.0 (0.0 to 0.0)	0 (0 to 0)	0.0 (0.0 to 0.0)	0 (0 to 0)	0.0 (0.0 to 0.0)	-76.0 (-89.8 to -46.3)	-57.1 (-81.7 to -3.9)
Albania	Other neurological disorders	5 to 9	Both	2 (2 to 3)	0.7 (0.4 to 0.9)	3 (2 to 5)	1.0 (0.7 to 1.4)	1 (1 to 2)	0.9 (0.5 to 1.5)	-41.5 (-70.1 to 9.0)	34.0 (-31.5 to 149.7)
Albania	Other neurological disorders	5 to 9	Female	1 (1 to 1)	0.6 (0.4 to 0.8)	2 (1 to 2)	1.0 (0.6 to 1.4)	1 (0 to 1)	0.8 (0.5 to 1.4)	-35.5 (-67.3 to 37.8)	48.7 (-24.6 to 217.5)
Albania	Other neurological disorders	5 to 9	Male	1 (1 to 2)	0.7 (0.5 to 1.2)	2 (1 to 3)	1.1 (0.7 to 1.8)	1 (0 to 1)	0.9 (0.4 to 1.8)	-45.8 (-78.6 to 20.7)	23.5 (-51.2 to 175.1)
Albania	Other neurological disorders	10 to 14	Both	2 (1 to 3)	0.6 (0.4 to 0.8)	3 (2 to 4)	0.8 (0.6 to 1.1)	2 (1 to 3)	0.9 (0.5 to 1.4)	-17.8 (-53.7 to 42.2)	48.3 (-16.5 to 156.6)
Albania	Other neurological disorders	10 to 14	Female	1 (1 to 1)	0.5 (0.3 to 0.7)	1 (1 to 2)	0.7 (0.5 to 1.1)	1 (0 to 1)	0.7 (0.4 to 1.3)	-17.3 (-60.4 to 78.5)	50.4 (-28.0 to 224.7)
Albania	Other neurological disorders	10 to 14	Male	1 (1 to 2)	0.7 (0.5 to 1.1)	2 (1 to 2)	1.0 (0.6 to 1.3)	1 (1 to 2)	1.1 (0.6 to 1.9)	-18.1 (-61.6 to 66.7)	46.7 (-31.2 to 198.6)
Albania	Drug use disorders	5 to 9	Both	0 (0 to 0)	0.0 (0.0 to 0.0)	0 (0 to 0)	0.0 (0.0 to 0.0)	0 (0 to 0)	0.0 (0.0 to 0.0)	0.0 (0.0 to 0.0)	0.0 (0.0 to 0.0)
Albania	Drug use disorders	5 to 9	Female	0 (0 to 0)	0.0 (0.0 to 0.0)	0 (0 to 0)	0.0 (0.0 to 0.0)	0 (0 to 0)	0.0 (0.0 to 0.0)	0.0 (0.0 to 0.0)	0.0 (0.0 to 0.0)
Albania	Drug use disorders	5 to 9	Male	0 (0 to 0)	0.0 (0.0 to 0.0)	0 (0 to 0)	0.0 (0.0 to 0.0)	0 (0 to 0)	0.0 (0.0 to 0.0)	0.0 (0.0 to 0.0)	0.0 (0.0 to 0.0)
Albania	Drug use disorders	10 to 14	Both	0 (0 to 0)	0.0 (0.0 to 0.0)	0 (0 to 0)	0.0 (0.0 to 0.0)	0 (0 to 0)	0.0 (0.0 to 0.0)	0.0 (0.0 to 0.0)	0.0 (0.0 to 0.0)
Albania	Drug use disorders	10 to 14	Female	0 (0 to 0)	0.0 (0.0 to 0.0)	0 (0 to 0)	0.0 (0.0 to 0.0)	0 (0 to 0)	0.0 (0.0 to 0.0)	0.0 (0.0 to 0.0)	0.0 (0.0 to 0.0)
Albania	Drug use disorders	10 to 14	Male	0 (0 to 0)	0.0 (0.0 to 0.0)	0 (0 to 0)	0.0 (0.0 to 0.0)	0 (0 to 0)	0.0 (0.0 to 0.0)	0.0 (0.0 to 0.0)	0.0 (0.0 to 0.0)
Albania	Eating disorders	5 to 9	Both	0 (0 to 0)	0.0 (0.0 to 0.0)	0 (0 to 0)	0.0 (0.0 to 0.0)	0 (0 to 0)	0.0 (0.0 to 0.0)	136.1 (-60.9 to 597.2)	441.0 (-10.4 to 1497.6)
Albania	Eating disorders	5 to 9	Female	0 (0 to 0)	0.0 (0.0 to 0.0)	0 (0 to 0)	0.0 (0.0 to 0.0)	0 (0 to 0)	0.0 (0.0 to 0.0)	96.9 (-69.4 to 724.0)	353.9 (-29.5 to 1799.2)
Albania	Eating disorders	5 to 9	Male	0 (0 to 0)	0.0 (0.0 to 0.0)	0 (0 to 0)	0.0 (0.0 to 0.0)	0 (0 to 0)	0.0 (0.0 to 0.0)	215.5 (-54.2 to 867.8)	619.1 (4.4 to 2105.5)
Albania	Eating disorders	10 to 14	Both	0 (0 to 0)	0.0 (0.0 to 0.0)	0 (0 to 0)	0.0 (0.0 to 0.0)	0 (0 to 0)	0.0 (0.0 to 0.0)	97.7 (-47.4 to 460.9)	256.8 (-5.0 to 912.1)
Albania	Eating disorders	10 to 14	Female	0 (0 to 0)	0.0 (0.0 to 0.0)	0 (0 to 0)	0.0 (0.0 to 0.0)	0 (0 to 0)	0.0 (0.0 to 0.0)	68.2 (-58.6 to 478.9)	206.0 (-24.6 to 952.8)
Albania	Eating disorders	10 to 14	Male	0 (0 to 0)	0.0 (0.0 to 0.0)	0 (0 to 0)	0.0 (0.0 to 0.0)	0 (0 to 0)	0.0 (0.0 to 0.0)	300.6 (-47.8 to 1180.9)	617.6 (-6.5 to 2194.5)
Albania	Diabetes mellitus	5 to 9	Both	1 (0 to 1)	0.1 (0.1 to 0.2)	1 (0 to 1)	0.2 (0.2 to 0.3)	0 (0 to 0)	0.1 (0.0 to 0.1)	-80.7 (-89.4 to -66.0)	-55.8 (-75.7 to -22.2)
Albania	Diabetes mellitus	5 to 9	Female	0 (0 to 0)	0.2 (0.1 to 0.2)	0 (0 to 0)	0.2 (0.1 to 0.3)	0 (0 to 0)	0.1 (0.0 to 0.1)	-81.1 (-89.6 to -65.2)	-56.4 (-76.1 to -19.9)
Albania	Diabetes mellitus	5 to 9	Male	0 (0 to 0)	0.1 (0.1 to 0.2)	0 (0 to 1)	0.2 (0.1 to 0.3)	0 (0 to 0)	0.1 (0.0 to 0.1)	-80.2 (-91.7 to -56.0)	-55.0 (-81.2 to 0.3)
Albania	Diabetes mellitus	10 to 14	Both	0 (0 to 1)	0.1 (0.1 to 0.2)	0 (0 to 1)	0.1 (0.1 to 0.2)	0 (0 to 0)	0.1 (0.0 to 0.1)	-68.6 (-82.6 to -48.3)	-43.3 (-68.7 to -6.6)

eTable 8. All-cause and cause-specific mortality (counts and rates) at the global level and by country in the WHO European Region for ages 5 to 9 and 10 to 14 for 1990, 2000, and 2016

Albania	Diabetes mellitus	10 to 14	Female	0 (0 to 0)	0.2 (0.1 to 0.2)	0 (0 to 0)	0.2 (0.1 to 0.3)	0 (0 to 0)	0.1 (0.0 to 0.1)	-73.9 (-87.0 to -50.6)	-52.6 (-76.3 to -10.2)
Albania	Diabetes mellitus	10 to 14	Male	0 (0 to 0)	0.1 (0.0 to 0.1)	0 (0 to 0)	0.1 (0.1 to 0.1)	0 (0 to 0)	0.1 (0.0 to 0.1)	-56.7 (-80.0 to -15.1)	-22.5 (-64.2 to 52.0)
Albania	Acute glomerulonephritis	5 to 9	Both	1 (0 to 2)	0.2 (0.1 to 0.5)	0 (0 to 1)	0.1 (0.1 to 0.2)	0 (0 to 0)	0.0 (0.0 to 0.0)	-97.7 (-99.0 to -94.2)	-94.8 (-97.7 to -86.8)
Albania	Acute glomerulonephritis	5 to 9	Female	0 (0 to 1)	0.2 (0.1 to 0.5)	0 (0 to 0)	0.1 (0.0 to 0.2)	0 (0 to 0)	0.0 (0.0 to 0.0)	-96.9 (-98.9 to -88.4)	-92.9 (-97.4 to -73.3)
Albania	Acute glomerulonephritis	5 to 9	Male	1 (0 to 1)	0.3 (0.1 to 0.7)	0 (0 to 1)	0.2 (0.1 to 0.4)	0 (0 to 0)	0.0 (0.0 to 0.0)	-98.2 (-99.3 to -93.9)	-95.8 (-98.4 to -86.2)
Albania	Acute glomerulonephritis	10 to 14	Both	1 (0 to 1)	0.2 (0.1 to 0.4)	0 (0 to 1)	0.1 (0.0 to 0.2)	0 (0 to 0)	0.0 (0.0 to 0.0)	-96.0 (-98.4 to -86.8)	-92.8 (-97.2 to -76.1)
Albania	Acute glomerulonephritis	10 to 14	Female	0 (0 to 0)	0.0 (0.0 to 0.0)	0 (0 to 0)	0.0 (0.0 to 0.0)	0 (0 to 0)	0.0 (0.0 to 0.0)	-46.4 (-72.6 to -18.9)	-2.4 (-50.2 to 47.5)
Albania	Acute glomerulonephritis	10 to 14	Male	1 (0 to 1)	0.3 (0.1 to 0.8)	0 (0 to 1)	0.2 (0.1 to 0.3)	0 (0 to 0)	0.0 (0.0 to 0.0)	-97.7 (-99.1 to -92.0)	-95.9 (-98.4 to -85.7)
Albania	Chronic kidney disease	5 to 9	Both	3 (2 to 4)	0.8 (0.6 to 1.1)	3 (2 to 4)	1.0 (0.7 to 1.4)	1 (1 to 1)	0.5 (0.3 to 0.8)	-71.9 (-83.5 to -53.3)	-35.6 (-62.2 to 6.9)
Albania	Chronic kidney disease	5 to 9	Female	1 (1 to 2)	0.7 (0.4 to 0.9)	1 (1 to 2)	0.7 (0.5 to 1.1)	0 (0 to 0)	0.4 (0.2 to 0.6)	-72.3 (-85.1 to -50.0)	-36.2 (-65.7 to 15.3)
Albania	Chronic kidney disease	5 to 9	Male	2 (1 to 3)	1.0 (0.6 to 1.5)	2 (1 to 3)	1.3 (0.8 to 1.8)	1 (0 to 1)	0.7 (0.4 to 1.1)	-71.6 (-84.8 to -47.8)	-35.3 (-65.4 to 19.0)
Albania	Chronic kidney disease	10 to 14	Both	4 (3 to 5)	1.2 (0.9 to 1.6)	4 (3 to 5)	1.2 (0.8 to 1.5)	2 (1 to 2)	0.9 (0.5 to 1.2)	-61.3 (-75.1 to -42.7)	-30.2 (-55.1 to 3.4)
Albania	Chronic kidney disease	10 to 14	Female	2 (1 to 3)	1.2 (0.8 to 1.7)	2 (1 to 3)	1.1 (0.8 to 1.5)	1 (0 to 1)	0.8 (0.5 to 1.2)	-64.4 (-79.0 to -39.8)	-35.2 (-61.8 to 9.5)
Albania	Chronic kidney disease	10 to 14	Male	2 (1 to 3)	1.3 (0.7 to 1.9)	2 (1 to 3)	1.4 (0.8 to 1.9)	1 (0 to 1)	1.0 (0.5 to 1.5)	-58.7 (-76.7 to -31.8)	-25.9 (-58.2 to 22.1)
Albania	Urinary diseases and male infertility	5 to 9	Both	1 (0 to 1)	0.2 (0.1 to 0.3)	0 (0 to 1)	0.1 (0.1 to 0.2)	0 (0 to 0)	0.0 (0.0 to 0.1)	-88.1 (-94.0 to -77.3)	-72.8 (-86.4 to -47.9)
Albania	Urinary diseases and male infertility	5 to 9	Female	0 (0 to 0)	0.1 (0.1 to 0.2)	0 (0 to 0)	0.1 (0.1 to 0.1)	0 (0 to 0)	0.0 (0.0 to 0.0)	-89.0 (-94.5 to -79.0)	-74.6 (-87.3 to -51.6)
Albania	Urinary diseases and male infertility	5 to 9	Male	0 (0 to 1)	0.2 (0.1 to 0.4)	0 (0 to 0)	0.2 (0.1 to 0.3)	0 (0 to 0)	0.1 (0.0 to 0.1)	-87.8 (-95.1 to -71.9)	-72.1 (-88.9 to -36.1)
Albania	Urinary diseases and male infertility	10 to 14	Both	1 (0 to 1)	0.2 (0.1 to 0.2)	0 (0 to 0)	0.1 (0.1 to 0.1)	0 (0 to 0)	0.1 (0.0 to 0.1)	-81.8 (-89.5 to -69.8)	-67.1 (-81.0 to -45.5)
Albania	Urinary diseases and male infertility	10 to 14	Female	0 (0 to 0)	0.1 (0.1 to 0.2)	0 (0 to 0)	0.1 (0.0 to 0.1)	0 (0 to 0)	0.0 (0.0 to 0.0)	-86.1 (-92.9 to -73.8)	-74.7 (-87.1 to -52.4)
Albania	Urinary diseases and male infertility	10 to 14	Male	0 (0 to 1)	0.2 (0.1 to 0.3)	0 (0 to 0)	0.1 (0.1 to 0.2)	0 (0 to 0)	0.1 (0.0 to 0.1)	-79.6 (-89.9 to -62.6)	-63.4 (-82.0 to -33.0)
Albania	Hemoglobinopathies and hemolytic anemias	5 to 9	Both	4 (3 to 6)	1.1 (0.8 to 1.8)	4 (2 to 7)	1.1 (0.7 to 2.0)	1 (0 to 2)	0.6 (0.3 to 1.1)	-77.9 (-87.9 to -63.0)	-49.3 (-72.2 to -15.3)
Albania	Hemoglobinopathies and hemolytic anemias	5 to 9	Female	2 (1 to 3)	1.0 (0.6 to 1.5)	1 (1 to 3)	0.9 (0.5 to 1.6)	0 (0 to 1)	0.5 (0.2 to 0.9)	-78.8 (-90.5 to -60.1)	-51.1 (-78.1 to -8.1)
Albania	Hemoglobinopathies and hemolytic anemias	5 to 9	Male	2 (1 to 4)	1.3 (0.8 to 2.3)	2 (1 to 5)	1.4 (0.8 to 2.7)	1 (0 to 1)	0.7 (0.3 to 1.3)	-77.3 (-88.6 to -59.8)	-48.2 (-74.0 to -8.3)

eTable 8. All-cause and cause-specific mortality (counts and rates) at the global level and by country in the WHO European Region for ages 5 to 9 and 10 to 14 for 1990, 2000, and 2016

Albania	Hemoglobinopathies and hemolytic anemias	10 to 14	Both	3 (2 to 4)	0.9 (0.6 to 1.3)	3 (2 to 4)	0.8 (0.6 to 1.1)	1 (1 to 2)	0.6 (0.3 to 0.8)	-67.2 (-80.0 to -47.1)	-40.9 (-64.0 to -4.5)
Albania	Hemoglobinopathies and hemolytic anemias	10 to 14	Female	1 (0 to 2)	0.8 (0.3 to 1.1)	1 (0 to 1)	0.6 (0.3 to 0.9)	0 (0 to 1)	0.3 (0.2 to 0.6)	-74.5 (-86.9 to -52.7)	-53.6 (-76.2 to -14.0)
Albania	Hemoglobinopathies and hemolytic anemias	10 to 14	Male	2 (1 to 3)	1.1 (0.7 to 1.6)	2 (1 to 3)	1.1 (0.7 to 1.6)	1 (0 to 1)	0.7 (0.4 to 1.2)	-62.6 (-79.3 to -34.7)	-33.0 (-63.0 to 17.0)
Albania	Endocrine, metabolic, blood, and immune disorders	5 to 9	Both	4 (2 to 7)	1.1 (0.7 to 2.1)	3 (2 to 8)	1.0 (0.5 to 2.5)	1 (1 to 3)	0.7 (0.3 to 1.7)	-71.1 (-85.2 to -50.1)	-33.8 (-66.2 to 14.3)
Albania	Endocrine, metabolic, blood, and immune disorders	5 to 9	Female	2 (1 to 3)	1.0 (0.6 to 1.9)	1 (1 to 4)	0.8 (0.4 to 2.2)	0 (0 to 1)	0.6 (0.3 to 1.4)	-73.8 (-88.2 to -46.0)	-39.7 (-72.9 to 24.5)
Albania	Endocrine, metabolic, blood, and immune disorders	5 to 9	Male	2 (1 to 5)	1.2 (0.6 to 2.6)	2 (1 to 6)	1.1 (0.5 to 3.4)	1 (0 to 2)	0.8 (0.3 to 2.2)	-69.0 (-86.6 to -36.6)	-29.3 (-69.4 to 44.5)
Albania	Endocrine, metabolic, blood, and immune disorders	10 to 14	Both	3 (2 to 5)	0.8 (0.5 to 1.4)	2 (1 to 5)	0.6 (0.4 to 1.6)	1 (1 to 3)	0.6 (0.3 to 1.4)	-57.3 (-76.9 to -31.8)	-22.9 (-58.2 to 23.1)
Albania	Endocrine, metabolic, blood, and immune disorders	10 to 14	Female	1 (1 to 2)	0.6 (0.4 to 1.0)	1 (0 to 2)	0.4 (0.2 to 1.0)	0 (0 to 1)	0.4 (0.2 to 0.8)	-66.7 (-83.6 to -36.9)	-39.5 (-70.1 to 14.8)
Albania	Endocrine, metabolic, blood, and immune disorders	10 to 14	Male	2 (1 to 3)	1.0 (0.6 to 2.0)	1 (1 to 4)	0.8 (0.4 to 2.4)	1 (0 to 2)	0.8 (0.4 to 2.0)	-51.8 (-76.6 to -16.6)	-13.6 (-58.1 to 49.4)
Albania	Rheumatoid arthritis	5 to 9	Both	0 (0 to 0)	0.0 (0.0 to 0.0)	0 (0 to 0)	0.0 (0.0 to 0.0)	0 (0 to 0)	0.0 (0.0 to 0.0)	-49.0 (-75.0 to 2.0)	16.8 (-42.6 to 133.7)
Albania	Rheumatoid arthritis	5 to 9	Female	0 (0 to 0)	0.0 (0.0 to 0.0)	0 (0 to 0)	0.0 (0.0 to 0.0)	0 (0 to 0)	0.0 (0.0 to 0.0)	-49.7 (-75.0 to 10.0)	15.9 (-42.4 to 153.6)
Albania	Rheumatoid arthritis	5 to 9	Male	0 (0 to 0)	0.0 (0.0 to 0.0)	0 (0 to 0)	0.0 (0.0 to 0.0)	0 (0 to 0)	0.0 (0.0 to 0.0)	-48.5 (-77.7 to 30.0)	17.3 (-49.1 to 196.2)
Albania	Rheumatoid arthritis	10 to 14	Both	0 (0 to 0)	0.0 (0.0 to 0.0)	0 (0 to 0)	0.0 (0.0 to 0.0)	0 (0 to 0)	0.0 (0.0 to 0.0)	-32.9 (-67.3 to 22.7)	21.1 (-41.0 to 121.5)
Albania	Rheumatoid arthritis	10 to 14	Female	0 (0 to 0)	0.0 (0.0 to 0.0)	0 (0 to 0)	0.0 (0.0 to 0.0)	0 (0 to 0)	0.0 (0.0 to 0.0)	-24.9 (-69.3 to 60.5)	36.6 (-44.2 to 191.9)
Albania	Rheumatoid arthritis	10 to 14	Male	0 (0 to 0)	0.0 (0.0 to 0.0)	0 (0 to 0)	0.0 (0.0 to 0.0)	0 (0 to 0)	0.0 (0.0 to 0.0)	-42.8 (-70.4 to 21.4)	2.4 (-47.0 to 117.5)
Albania	Other musculoskeletal disorders	5 to 9	Both	0 (0 to 0)	0.0 (0.0 to 0.1)	0 (0 to 0)	0.0 (0.0 to 0.1)	0 (0 to 0)	0.0 (0.0 to 0.0)	-68.2 (-83.0 to -36.0)	-27.2 (-61.0 to 46.8)
Albania	Other musculoskeletal disorders	5 to 9	Female	0 (0 to 0)	0.0 (0.0 to 0.1)	0 (0 to 0)	0.0 (0.0 to 0.1)	0 (0 to 0)	0.0 (0.0 to 0.1)	-55.2 (-75.7 to 24.8)	3.2 (-44.0 to 187.6)
Albania	Other musculoskeletal disorders	5 to 9	Male	0 (0 to 0)	0.0 (0.0 to 0.1)	0 (0 to 0)	0.0 (0.0 to 0.1)	0 (0 to 0)	0.0 (0.0 to 0.1)	-73.6 (-88.0 to -39.0)	-39.8 (-72.6 to 39.0)

eTable 8. All-cause and cause-specific mortality (counts and rates) at the global level and by country in the WHO European Region for ages 5 to 9 and 10 to 14 for 1990, 2000, and 2016

Albania	Other musculoskeletal disorders	10 to 14	Both	0 (0 to 0)	0.0 (0.0 to 0.1)	0 (0 to 0)	0.0 (0.0 to 0.1)	0 (0 to 0)	0.0 (0.0 to 0.1)	-48.0 (-68.4 to -3.9)	-6.2 (-43.0 to 73.3)
Albania	Other musculoskeletal disorders	10 to 14	Female	0 (0 to 0)	0.0 (0.0 to 0.1)	0 (0 to 0)	0.0 (0.0 to 0.1)	0 (0 to 0)	0.0 (0.0 to 0.1)	-44.1 (-71.3 to 33.8)	1.7 (-47.8 to 143.3)
Albania	Other musculoskeletal disorders	10 to 14	Male	0 (0 to 0)	0.0 (0.0 to 0.1)	0 (0 to 0)	0.0 (0.0 to 0.1)	0 (0 to 0)	0.0 (0.0 to 0.1)	-52.8 (-74.0 to -1.9)	-15.4 (-53.4 to 75.7)
Albania	Congenital birth defects	5 to 9	Both	7 (5 to 12)	2.0 (1.4 to 3.4)	12 (9 to 17)	3.8 (2.7 to 5.1)	5 (3 to 7)	3.0 (1.9 to 4.7)	-34.2 (-68.9 to 18.7)	50.7 (-28.8 to 172.0)
Albania	Congenital birth defects	5 to 9	Female	3 (2 to 5)	1.6 (1.0 to 2.7)	5 (3 to 7)	3.1 (1.8 to 4.6)	2 (1 to 3)	2.6 (1.5 to 4.2)	-28.9 (-67.0 to 47.6)	63.8 (-24.0 to 240.1)
Albania	Congenital birth defects	5 to 9	Male	4 (3 to 8)	2.4 (1.5 to 4.4)	7 (5 to 11)	4.4 (2.8 to 6.6)	3 (2 to 5)	3.5 (1.9 to 5.8)	-37.5 (-73.7 to 32.7)	42.4 (-40.0 to 202.5)
Albania	Congenital birth defects	10 to 14	Both	4 (3 to 7)	1.3 (1.0 to 2.0)	7 (5 to 9)	2.0 (1.5 to 2.6)	4 (2 to 6)	2.1 (1.3 to 3.1)	-11.4 (-54.6 to 53.8)	59.8 (-18.1 to 177.5)
Albania	Congenital birth defects	10 to 14	Female	2 (1 to 3)	1.0 (0.7 to 1.6)	3 (2 to 3)	1.5 (1.0 to 2.1)	1 (1 to 2)	1.6 (0.9 to 2.5)	-13.2 (-57.1 to 59.9)	57.9 (-22.1 to 190.7)
Albania	Congenital birth defects	10 to 14	Male	3 (2 to 4)	1.6 (1.0 to 2.6)	4 (3 to 6)	2.5 (1.7 to 3.5)	2 (1 to 4)	2.5 (1.4 to 4.0)	-10.3 (-61.3 to 76.6)	60.6 (-30.6 to 216.3)
Albania	Skin and subcutaneous diseases	5 to 9	Both	0 (0 to 0)	0.0 (0.0 to 0.1)	0 (0 to 0)	0.0 (0.0 to 0.0)	0 (0 to 0)	0.0 (0.0 to 0.0)	-80.9 (-92.7 to -19.6)	-56.2 (-83.4 to 84.1)
Albania	Skin and subcutaneous diseases	5 to 9	Female	0 (0 to 0)	0.0 (0.0 to 0.1)	0 (0 to 0)	0.0 (0.0 to 0.0)	0 (0 to 0)	0.0 (0.0 to 0.0)	-76.5 (-92.2 to -23.3)	-45.9 (-82.1 to 76.7)
Albania	Skin and subcutaneous diseases	5 to 9	Male	0 (0 to 0)	0.1 (0.0 to 0.1)	0 (0 to 0)	0.0 (0.0 to 0.1)	0 (0 to 0)	0.0 (0.0 to 0.0)	-83.3 (-95.0 to -9.6)	-61.9 (-88.7 to 106.0)
Albania	Skin and subcutaneous diseases	10 to 14	Both	0 (0 to 0)	0.0 (0.0 to 0.1)	0 (0 to 0)	0.0 (0.0 to 0.0)	0 (0 to 0)	0.0 (0.0 to 0.0)	-69.1 (-86.7 to -14.7)	-44.2 (-76.0 to 53.9)
Albania	Skin and subcutaneous diseases	10 to 14	Female	0 (0 to 0)	0.0 (0.0 to 0.0)	0 (0 to 0)	0.0 (0.0 to 0.0)	0 (0 to 0)	0.0 (0.0 to 0.0)	-42.8 (-63.9 to -18.4)	4.1 (-34.3 to 48.4)
Albania	Skin and subcutaneous diseases	10 to 14	Male	0 (0 to 0)	0.0 (0.0 to 0.1)	0 (0 to 0)	0.0 (0.0 to 0.0)	0 (0 to 0)	0.0 (0.0 to 0.0)	-76.0 (-91.7 to -2.3)	-57.1 (-85.2 to 75.0)
Albania	Road injuries	5 to 9	Both	26 (20 to 34)	7.4 (5.5 to 9.7)	16 (12 to 21)	4.9 (3.6 to 6.5)	4 (2 to 6)	2.4 (1.5 to 3.7)	-85.9 (-91.9 to -76.6)	-67.8 (-81.4 to -46.4)
Albania	Road injuries	5 to 9	Female	8 (6 to 12)	4.9 (3.3 to 7.0)	5 (4 to 8)	3.4 (2.2 to 5.3)	1 (1 to 2)	1.9 (1.1 to 3.1)	-83.4 (-91.4 to -68.2)	-61.8 (-80.2 to -26.8)
Albania	Road injuries	5 to 9	Male	18 (13 to 24)	9.8 (6.9 to 13.3)	11 (7 to 15)	6.3 (4.4 to 9.0)	2 (1 to 4)	2.9 (1.6 to 4.6)	-87.1 (-93.3 to -77.6)	-70.6 (-84.7 to -48.9)
Albania	Road injuries	10 to 14	Both	15 (11 to 19)	4.5 (3.4 to 5.8)	10 (8 to 13)	3.0 (2.3 to 3.8)	4 (2 to 5)	2.0 (1.3 to 2.9)	-74.9 (-84.6 to -59.7)	-54.7 (-72.2 to -27.4)
Albania	Road injuries	10 to 14	Female	4 (3 to 6)	2.5 (1.7 to 3.7)	3 (2 to 4)	1.7 (1.1 to 2.6)	1 (1 to 2)	1.2 (0.7 to 1.9)	-72.8 (-85.6 to -50.9)	-50.5 (-73.9 to -10.7)
Albania	Road injuries	10 to 14	Male	11 (8 to 15)	6.4 (4.5 to 8.6)	7 (5 to 10)	4.2 (3.0 to 5.7)	3 (2 to 4)	2.8 (1.6 to 4.3)	-75.6 (-86.7 to -57.6)	-56.4 (-76.2 to -24.1)
Albania	Other transport injuries	5 to 9	Both	1 (1 to 2)	0.4 (0.2 to 0.6)	3 (1 to 4)	0.9 (0.4 to 1.3)	1 (0 to 1)	0.3 (0.1 to 0.6)	-62.5 (-83.6 to -19.9)	-14.0 (-62.3 to 83.6)
Albania	Other transport injuries	5 to 9	Female	0 (0 to 1)	0.2 (0.1 to 0.5)	1 (0 to 2)	0.6 (0.2 to 1.1)	0 (0 to 0)	0.3 (0.1 to 0.5)	-54.5 (-85.9 to 30.7)	4.9 (-67.4 to 201.2)
Albania	Other transport injuries	5 to 9	Male	1 (0 to 2)	0.5 (0.3 to 0.9)	2 (1 to 3)	1.1 (0.4 to 1.9)	0 (0 to 1)	0.4 (0.1 to 0.8)	-65.9 (-87.3 to -14.7)	-22.3 (-71.1 to 94.3)
Albania	Other transport injuries	10 to 14	Both	1 (1 to 2)	0.3 (0.2 to 0.5)	2 (1 to 3)	0.5 (0.2 to 0.8)	1 (0 to 1)	0.3 (0.1 to 0.5)	-43.1 (-73.5 to 16.0)	2.7 (-52.2 to 109.4)

eTable 8. All-cause and cause-specific mortality (counts and rates) at the global level and by country in the WHO European Region for ages 5 to 9 and 10 to 14 for 1990, 2000, and 2016

Albania	Other transport injuries	10 to 14	Female	0 (0 to 0)	0.1 (0.1 to 0.2)	0 (0 to 1)	0.2 (0.1 to 0.4)	0 (0 to 0)	0.2 (0.0 to 0.3)	-29.7 (-74.7 to 76.4)	27.8 (-54.0 to 220.7)
Albania	Other transport injuries	10 to 14	Male	1 (0 to 1)	0.4 (0.2 to 0.8)	1 (1 to 2)	0.8 (0.3 to 1.2)	0 (0 to 1)	0.4 (0.1 to 0.8)	-46.4 (-78.4 to 21.2)	-3.9 (-61.3 to 117.2)
Albania	Falls	5 to 9	Both	3 (1 to 4)	0.9 (0.4 to 1.3)	3 (1 to 5)	0.9 (0.4 to 1.4)	1 (0 to 1)	0.4 (0.2 to 0.6)	-81.8 (-90.1 to -66.2)	-58.3 (-77.4 to -22.5)
Albania	Falls	5 to 9	Female	1 (0 to 1)	0.5 (0.3 to 0.7)	1 (0 to 1)	0.5 (0.3 to 0.8)	0 (0 to 0)	0.2 (0.1 to 0.3)	-81.8 (-91.3 to -62.1)	-58.0 (-79.9 to -12.7)
Albania	Falls	5 to 9	Male	2 (1 to 3)	1.2 (0.4 to 1.9)	2 (1 to 4)	1.3 (0.4 to 2.1)	0 (0 to 1)	0.5 (0.3 to 0.9)	-81.8 (-91.4 to -57.2)	-58.6 (-80.3 to -2.5)
Albania	Falls	10 to 14	Both	2 (1 to 3)	0.7 (0.4 to 0.9)	2 (1 to 3)	0.6 (0.3 to 0.9)	1 (0 to 1)	0.3 (0.2 to 0.6)	-70.6 (-84.1 to -49.6)	-46.9 (-71.3 to -9.0)
Albania	Falls	10 to 14	Female	1 (0 to 1)	0.3 (0.2 to 0.5)	0 (0 to 1)	0.3 (0.1 to 0.4)	0 (0 to 0)	0.1 (0.1 to 0.2)	-76.3 (-88.4 to -53.1)	-56.8 (-78.8 to -14.8)
Albania	Falls	10 to 14	Male	2 (1 to 2)	1.0 (0.4 to 1.5)	2 (1 to 2)	0.9 (0.4 to 1.4)	1 (0 to 1)	0.5 (0.3 to 0.9)	-68.7 (-84.7 to -38.9)	-43.9 (-72.6 to 9.5)
Albania	Drowning	5 to 9	Both	24 (15 to 33)	6.9 (4.3 to 9.4)	17 (12 to 23)	5.2 (3.7 to 7.0)	2 (1 to 4)	1.4 (0.9 to 2.3)	-90.9 (-95.2 to -80.1)	-79.1 (-89.0 to -54.5)
Albania	Drowning	5 to 9	Female	5 (3 to 9)	3.2 (1.6 to 5.0)	3 (2 to 5)	2.1 (1.3 to 3.1)	1 (0 to 1)	0.7 (0.4 to 1.3)	-90.3 (-95.9 to -72.2)	-77.7 (-90.4 to -36.0)
Albania	Drowning	5 to 9	Male	19 (12 to 27)	10.4 (6.6 to 14.7)	14 (9 to 19)	8.1 (5.5 to 11.4)	2 (1 to 3)	2.1 (1.1 to 3.7)	-91.1 (-95.6 to -79.8)	-79.6 (-90.0 to -53.9)
Albania	Drowning	10 to 14	Both	19 (12 to 25)	5.8 (3.5 to 7.5)	14 (10 to 18)	4.0 (2.9 to 5.2)	3 (2 to 4)	1.4 (0.8 to 2.3)	-86.3 (-92.5 to -70.2)	-75.3 (-86.5 to -46.3)
Albania	Drowning	10 to 14	Female	4 (2 to 6)	2.5 (1.2 to 3.7)	3 (2 to 4)	1.6 (1.0 to 2.2)	1 (0 to 1)	0.6 (0.3 to 1.1)	-86.6 (-93.8 to -66.2)	-75.7 (-88.8 to -38.5)
Albania	Drowning	10 to 14	Male	15 (9 to 21)	8.9 (5.5 to 12.0)	11 (8 to 15)	6.4 (4.5 to 8.8)	2 (1 to 4)	2.2 (1.2 to 3.7)	-86.2 (-93.2 to -69.0)	-75.4 (-87.8 to -44.5)
Albania	Fire, heat, and hot substances	5 to 9	Both	2 (1 to 2)	0.5 (0.2 to 0.6)	1 (1 to 2)	0.4 (0.2 to 0.6)	0 (0 to 1)	0.2 (0.1 to 0.3)	-82.8 (-91.9 to -62.4)	-60.6 (-81.4 to -13.8)
Albania	Fire, heat, and hot substances	5 to 9	Female	1 (0 to 1)	0.4 (0.2 to 0.7)	0 (0 to 1)	0.3 (0.2 to 0.6)	0 (0 to 0)	0.2 (0.1 to 0.3)	-83.8 (-92.9 to -61.7)	-62.6 (-83.6 to -11.8)
Albania	Fire, heat, and hot substances	5 to 9	Male	1 (0 to 1)	0.5 (0.2 to 0.8)	1 (0 to 1)	0.5 (0.2 to 0.8)	0 (0 to 0)	0.2 (0.1 to 0.4)	-82.0 (-92.8 to -59.6)	-58.9 (-83.7 to -8.0)
Albania	Fire, heat, and hot substances	10 to 14	Both	1 (1 to 1)	0.3 (0.2 to 0.4)	1 (0 to 1)	0.2 (0.1 to 0.3)	0 (0 to 0)	0.1 (0.1 to 0.2)	-77.7 (-88.6 to -55.0)	-59.8 (-79.4 to -18.8)
Albania	Fire, heat, and hot substances	10 to 14	Female	1 (0 to 1)	0.3 (0.1 to 0.6)	0 (0 to 1)	0.2 (0.1 to 0.3)	0 (0 to 0)	0.1 (0.1 to 0.2)	-82.3 (-91.9 to -60.2)	-67.8 (-85.3 to -27.7)
Albania	Fire, heat, and hot substances	10 to 14	Male	1 (0 to 1)	0.3 (0.2 to 0.4)	0 (0 to 1)	0.2 (0.1 to 0.4)	0 (0 to 0)	0.1 (0.1 to 0.3)	-73.2 (-88.5 to -48.8)	-52.0 (-79.3 to -8.3)
Albania	Poisonings	5 to 9	Both	1 (1 to 2)	0.3 (0.2 to 0.7)	2 (1 to 4)	0.7 (0.3 to 1.1)	0 (0 to 0)	0.2 (0.1 to 0.3)	-78.8 (-89.3 to -56.0)	-51.5 (-75.5 to 0.9)
Albania	Poisonings	5 to 9	Female	1 (0 to 1)	0.3 (0.2 to 0.8)	1 (0 to 2)	0.6 (0.3 to 1.0)	0 (0 to 0)	0.2 (0.1 to 0.3)	-79.2 (-90.7 to -51.7)	-52.2 (-78.6 to 11.3)
Albania	Poisonings	5 to 9	Male	1 (0 to 1)	0.4 (0.2 to 0.7)	1 (0 to 2)	0.8 (0.2 to 1.5)	0 (0 to 0)	0.2 (0.1 to 0.4)	-78.4 (-91.0 to -41.4)	-50.9 (-79.6 to 33.5)
Albania	Poisonings	10 to 14	Both	1 (1 to 2)	0.3 (0.2 to 0.6)	2 (1 to 3)	0.6 (0.3 to 0.9)	0 (0 to 1)	0.2 (0.1 to 0.3)	-69.3 (-83.7 to -40.5)	-44.7 (-70.5 to 7.3)
Albania	Poisonings	10 to 14	Female	1 (0 to 1)	0.4 (0.2 to 0.6)	1 (0 to 2)	0.6 (0.2 to 0.9)	0 (0 to 0)	0.2 (0.1 to 0.3)	-71.5 (-84.9 to -37.7)	-48.2 (-72.6 to 13.3)
Albania	Poisonings	10 to 14	Male	1 (0 to 1)	0.3 (0.2 to 0.6)	1 (0 to 2)	0.7 (0.2 to 1.1)	0 (0 to 0)	0.2 (0.1 to 0.4)	-67.2 (-85.5 to -24.5)	-41.3 (-74.0 to 35.2)
Albania	Exposure to mechanical forces	5 to 9	Both	10 (7 to 14)	2.7 (1.9 to 4.1)	13 (9 to 17)	3.9 (2.9 to 5.3)	1 (1 to 3)	1.0 (0.5 to 1.9)	-84.7 (-92.2 to -73.5)	-64.9 (-82.2 to -39.3)
Albania	Exposure to mechanical forces	5 to 9	Female	3 (2 to 4)	1.6 (1.0 to 2.4)	3 (2 to 5)	2.0 (1.1 to 2.9)	0 (0 to 1)	0.6 (0.3 to 1.2)	-83.0 (-92.1 to -66.0)	-60.9 (-81.7 to -21.7)
Albania	Exposure to mechanical forces	5 to 9	Male	7 (5 to 11)	3.8 (2.5 to 6.0)	9 (6 to 14)	5.6 (3.8 to 8.1)	1 (0 to 2)	1.3 (0.6 to 2.7)	-85.3 (-93.1 to -72.5)	-66.5 (-84.3 to -37.4)

eTable 8. All-cause and cause-specific mortality (counts and rates) at the global level and by country in the WHO European Region for ages 5 to 9 and 10 to 14 for 1990, 2000, and 2016

Albania	Exposure to mechanical forces	10 to 14	Both	8 (6 to 11)	2.5 (1.9 to 3.4)	10 (8 to 13)	2.9 (2.3 to 3.7)	2 (1 to 3)	1.0 (0.6 to 1.8)	-77.8 (-87.6 to -63.7)	-60.0 (-77.6 to -34.6)
Albania	Exposure to mechanical forces	10 to 14	Female	2 (1 to 2)	1.0 (0.7 to 1.4)	2 (1 to 3)	1.1 (0.6 to 1.6)	0 (0 to 1)	0.4 (0.2 to 0.7)	-79.9 (-90.3 to -65.0)	-63.5 (-82.3 to -36.3)
Albania	Exposure to mechanical forces	10 to 14	Male	7 (5 to 9)	4.0 (2.8 to 5.4)	8 (6 to 11)	4.8 (3.5 to 6.4)	2 (1 to 3)	1.6 (0.9 to 3.0)	-77.3 (-88.0 to -61.5)	-59.3 (-78.5 to -31.0)
Albania	Adverse effects of medical treatment	5 to 9	Both	1 (1 to 2)	0.3 (0.2 to 0.5)	2 (1 to 2)	0.5 (0.2 to 0.8)	0 (0 to 1)	0.3 (0.1 to 0.4)	-62.0 (-78.9 to -30.1)	-13.0 (-51.6 to 60.1)
Albania	Adverse effects of medical treatment	5 to 9	Female	0 (0 to 1)	0.2 (0.1 to 0.4)	1 (0 to 1)	0.4 (0.1 to 0.6)	0 (0 to 0)	0.2 (0.1 to 0.3)	-68.4 (-82.7 to -33.4)	-27.2 (-60.2 to 53.4)
Albania	Adverse effects of medical treatment	5 to 9	Male	1 (0 to 1)	0.4 (0.2 to 0.6)	1 (0 to 2)	0.6 (0.2 to 1.0)	0 (0 to 0)	0.3 (0.1 to 0.6)	-57.9 (-78.6 to -15.9)	-4.1 (-51.2 to 91.7)
Albania	Adverse effects of medical treatment	10 to 14	Both	1 (1 to 1)	0.2 (0.2 to 0.3)	1 (0 to 1)	0.3 (0.1 to 0.4)	0 (0 to 1)	0.2 (0.1 to 0.3)	-49.9 (-69.6 to -13.3)	-9.6 (-45.2 to 56.5)
Albania	Adverse effects of medical treatment	10 to 14	Female	0 (0 to 0)	0.2 (0.1 to 0.3)	0 (0 to 1)	0.2 (0.1 to 0.3)	0 (0 to 0)	0.1 (0.1 to 0.2)	-62.5 (-78.0 to -29.7)	-31.7 (-59.9 to 27.8)
Albania	Adverse effects of medical treatment	10 to 14	Male	0 (0 to 1)	0.3 (0.2 to 0.4)	1 (0 to 1)	0.4 (0.2 to 0.6)	0 (0 to 0)	0.3 (0.1 to 0.5)	-41.9 (-67.3 to 11.1)	4.0 (-41.4 to 99.1)
Albania	Animal contact	5 to 9	Both	0 (0 to 0)	0.1 (0.0 to 0.1)	0 (0 to 1)	0.1 (0.1 to 0.2)	0 (0 to 0)	0.0 (0.0 to 0.1)	-82.5 (-91.0 to -65.8)	-59.8 (-79.4 to -21.7)
Albania	Animal contact	5 to 9	Female	0 (0 to 0)	0.1 (0.0 to 0.1)	0 (0 to 0)	0.1 (0.0 to 0.2)	0 (0 to 0)	0.0 (0.0 to 0.1)	-72.0 (-86.6 to -38.4)	-35.5 (-69.2 to 41.9)
Albania	Animal contact	5 to 9	Male	0 (0 to 0)	0.1 (0.0 to 0.2)	0 (0 to 0)	0.1 (0.1 to 0.2)	0 (0 to 0)	0.0 (0.0 to 0.1)	-88.1 (-95.3 to -70.9)	-72.9 (-89.4 to -33.7)
Albania	Animal contact	10 to 14	Both	0 (0 to 0)	0.1 (0.0 to 0.1)	0 (0 to 0)	0.1 (0.0 to 0.1)	0 (0 to 0)	0.0 (0.0 to 0.0)	-82.4 (-90.9 to -65.3)	-68.3 (-83.5 to -37.4)
Albania	Animal contact	10 to 14	Female	0 (0 to 0)	0.0 (0.0 to 0.0)	0 (0 to 0)	0.0 (0.0 to 0.1)	0 (0 to 0)	0.0 (0.0 to 0.0)	-58.6 (-78.3 to -11.0)	-24.8 (-60.5 to 61.9)
Albania	Animal contact	10 to 14	Male	0 (0 to 0)	0.1 (0.1 to 0.2)	0 (0 to 0)	0.1 (0.1 to 0.2)	0 (0 to 0)	0.0 (0.0 to 0.0)	-86.5 (-94.2 to -68.6)	-75.8 (-89.7 to -43.8)
Albania	Foreign body	5 to 9	Both	1 (0 to 1)	0.2 (0.1 to 0.3)	1 (0 to 1)	0.2 (0.1 to 0.3)	0 (0 to 1)	0.2 (0.1 to 0.3)	-54.8 (-75.3 to -16.0)	3.7 (-43.3 to 92.5)
Albania	Foreign body	5 to 9	Female	0 (0 to 0)	0.1 (0.1 to 0.3)	0 (0 to 0)	0.2 (0.1 to 0.3)	0 (0 to 0)	0.2 (0.1 to 0.3)	-52.6 (-76.5 to -8.3)	9.4 (-45.9 to 111.3)
Albania	Foreign body	5 to 9	Male	0 (0 to 1)	0.2 (0.1 to 0.4)	0 (0 to 1)	0.2 (0.1 to 0.4)	0 (0 to 0)	0.2 (0.1 to 0.4)	-56.2 (-78.5 to -6.6)	-0.1 (-51.0 to 113.0)
Albania	Foreign body	10 to 14	Both	0 (0 to 1)	0.1 (0.1 to 0.2)	0 (0 to 1)	0.1 (0.1 to 0.2)	0 (0 to 0)	0.2 (0.1 to 0.2)	-40.9 (-66.2 to -3.0)	6.7 (-38.9 to 75.1)
Albania	Foreign body	10 to 14	Female	0 (0 to 0)	0.1 (0.1 to 0.2)	0 (0 to 0)	0.1 (0.1 to 0.2)	0 (0 to 0)	0.1 (0.1 to 0.3)	-45.5 (-71.1 to 1.1)	-1.0 (-47.4 to 83.8)
Albania	Foreign body	10 to 14	Male	0 (0 to 0)	0.1 (0.1 to 0.2)	0 (0 to 0)	0.1 (0.1 to 0.2)	0 (0 to 0)	0.2 (0.1 to 0.3)	-36.5 (-67.2 to 20.0)	13.8 (-41.2 to 114.9)
Albania	Environmental heat and cold exposure	5 to 9	Both	0 (0 to 0)	0.0 (0.0 to 0.0)	0 (0 to 0)	0.0 (0.0 to 0.1)	0 (0 to 0)	0.0 (0.0 to 0.0)	-67.4 (-82.6 to -40.4)	-25.2 (-60.2 to 36.7)
Albania	Environmental heat and cold exposure	5 to 9	Female	0 (0 to 0)	0.0 (0.0 to 0.0)	0 (0 to 0)	0.0 (0.0 to 0.0)	0 (0 to 0)	0.0 (0.0 to 0.0)	-49.3 (-81.4 to 1.3)	16.8 (-57.2 to 133.4)

eTable 8. All-cause and cause-specific mortality (counts and rates) at the global level and by country in the WHO European Region for ages 5 to 9 and 10 to 14 for 1990, 2000, and 2016

Albania	Environmental heat and cold exposure	5 to 9	Male	0 (0 to 0)	0.0 (0.0 to 0.1)	0 (0 to 0)	0.1 (0.0 to 0.1)	0 (0 to 0)	0.0 (0.0 to 0.0)	-73.6 (-86.4 to -46.4)	-39.8 (-68.9 to 22.1)
Albania	Environmental heat and cold exposure	10 to 14	Both	0 (0 to 0)	0.0 (0.0 to 0.0)	0 (0 to 0)	0.1 (0.0 to 0.1)	0 (0 to 0)	0.0 (0.0 to 0.0)	-58.4 (-76.7 to -26.3)	-24.9 (-57.9 to 32.9)
Albania	Environmental heat and cold exposure	10 to 14	Female	0 (0 to 0)	0.0 (0.0 to 0.0)	0 (0 to 0)	0.0 (0.0 to 0.1)	0 (0 to 0)	0.0 (0.0 to 0.0)	-46.6 (-75.6 to 12.5)	-2.9 (-55.6 to 104.6)
Albania	Environmental heat and cold exposure	10 to 14	Male	0 (0 to 0)	0.0 (0.0 to 0.1)	0 (0 to 0)	0.1 (0.0 to 0.1)	0 (0 to 0)	0.0 (0.0 to 0.0)	-62.4 (-81.7 to -27.7)	-32.7 (-67.2 to 29.6)
Albania	Other unintentional injuries	5 to 9	Both	5 (3 to 7)	1.5 (1.0 to 2.1)	10 (5 to 14)	3.1 (1.4 to 4.3)	0 (0 to 1)	0.2 (0.1 to 0.5)	-93.0 (-96.9 to -85.0)	-84.0 (-92.8 to -65.7)
Albania	Other unintentional injuries	5 to 9	Female	1 (1 to 2)	0.8 (0.4 to 1.2)	3 (1 to 5)	1.9 (0.5 to 3.1)	0 (0 to 0)	0.1 (0.0 to 0.3)	-93.5 (-97.5 to -82.3)	-85.0 (-94.2 to -59.2)
Albania	Other unintentional injuries	5 to 9	Male	4 (2 to 6)	2.1 (1.3 to 3.2)	7 (3 to 10)	4.3 (2.0 to 6.2)	0 (0 to 1)	0.3 (0.2 to 0.8)	-92.8 (-97.0 to -83.9)	-83.7 (-93.2 to -63.2)
Albania	Other unintentional injuries	10 to 14	Both	7 (5 to 9)	2.0 (1.4 to 2.7)	11 (5 to 15)	3.4 (1.5 to 4.5)	1 (0 to 1)	0.4 (0.2 to 0.8)	-89.4 (-94.7 to -77.1)	-80.8 (-90.5 to -58.7)
Albania	Other unintentional injuries	10 to 14	Female	1 (1 to 2)	0.7 (0.4 to 1.1)	2 (1 to 4)	1.4 (0.3 to 2.1)	0 (0 to 0)	0.1 (0.0 to 0.2)	-92.0 (-96.9 to -77.0)	-85.4 (-94.3 to -58.2)
Albania	Other unintentional injuries	10 to 14	Male	5 (4 to 8)	3.2 (2.1 to 4.5)	9 (4 to 13)	5.3 (2.5 to 7.5)	1 (0 to 1)	0.6 (0.3 to 1.2)	-88.8 (-94.8 to -75.3)	-80.0 (-90.7 to -55.7)
Albania	Self-harm	10 to 14	Both	3 (2 to 5)	1.0 (0.7 to 1.4)	5 (3 to 7)	1.4 (0.9 to 1.9)	2 (1 to 4)	1.3 (0.6 to 2.3)	-24.9 (-62.8 to 40.0)	35.4 (-32.8 to 152.7)
Albania	Self-harm	10 to 14	Female	1 (1 to 2)	0.8 (0.5 to 1.3)	2 (1 to 3)	1.1 (0.7 to 1.7)	1 (0 to 2)	1.1 (0.6 to 2.0)	-28.4 (-68.4 to 64.1)	30.2 (-42.6 to 198.5)
Albania	Self-harm	10 to 14	Male	2 (1 to 3)	1.1 (0.6 to 1.7)	3 (1 to 4)	1.6 (0.6 to 2.5)	1 (0 to 3)	1.5 (0.5 to 2.9)	-22.5 (-67.5 to 74.5)	38.9 (-41.7 to 212.6)
Albania	Interpersonal violence	5 to 9	Both	2 (1 to 4)	0.7 (0.4 to 1.2)	3 (2 to 5)	1.0 (0.7 to 1.5)	1 (0 to 1)	0.4 (0.2 to 0.8)	-75.2 (-87.9 to -49.7)	-43.2 (-72.3 to 15.3)
Albania	Interpersonal violence	5 to 9	Female	1 (0 to 2)	0.5 (0.2 to 1.1)	1 (1 to 2)	0.7 (0.4 to 1.2)	0 (0 to 1)	0.3 (0.1 to 0.7)	-69.9 (-86.6 to -26.7)	-30.6 (-69.1 to 68.9)
Albania	Interpersonal violence	5 to 9	Male	1 (1 to 3)	0.8 (0.4 to 1.6)	2 (1 to 4)	1.3 (0.7 to 2.2)	0 (0 to 1)	0.4 (0.2 to 1.0)	-78.3 (-92.1 to -39.2)	-50.5 (-82.0 to 38.6)
Albania	Interpersonal violence	10 to 14	Both	3 (2 to 5)	1.0 (0.7 to 1.5)	5 (2 to 7)	1.4 (0.7 to 2.0)	1 (1 to 2)	0.5 (0.3 to 0.9)	-73.6 (-86.4 to -47.1)	-52.4 (-75.4 to -4.5)
Albania	Interpersonal violence	10 to 14	Female	1 (1 to 2)	0.6 (0.3 to 1.0)	1 (1 to 2)	0.8 (0.4 to 1.3)	0 (0 to 1)	0.4 (0.2 to 0.6)	-68.1 (-86.3 to -29.9)	-41.9 (-75.1 to 27.5)
Albania	Interpersonal violence	10 to 14	Male	2 (1 to 4)	1.4 (0.8 to 2.3)	3 (1 to 5)	2.0 (0.9 to 3.1)	1 (0 to 1)	0.6 (0.3 to 1.3)	-75.9 (-90.4 to -39.5)	-56.8 (-82.7 to 8.4)
Albania	Exposure to forces of nature	5 to 9	Both	0 (0 to 0)	0.0 (0.0 to 0.0)	0 (0 to 0)	0.0 (0.0 to 0.0)	0 (0 to 0)	0.0 (0.0 to 0.0)	0.0 (0.0 to 0.0)	0.0 (0.0 to 0.0)
Albania	Exposure to forces of nature	5 to 9	Female	0 (0 to 0)	0.0 (0.0 to 0.0)	0 (0 to 0)	0.0 (0.0 to 0.0)	0 (0 to 0)	0.0 (0.0 to 0.0)	0.0 (0.0 to 0.0)	0.0 (0.0 to 0.0)
Albania	Exposure to forces of nature	5 to 9	Male	0 (0 to 0)	0.0 (0.0 to 0.0)	0 (0 to 0)	0.0 (0.0 to 0.0)	0 (0 to 0)	0.0 (0.0 to 0.0)	0.0 (0.0 to 0.0)	0.0 (0.0 to 0.0)
Albania	Exposure to forces of nature	10 to 14	Both	0 (0 to 0)	0.0 (0.0 to 0.0)	0 (0 to 0)	0.0 (0.0 to 0.0)	0 (0 to 0)	0.0 (0.0 to 0.0)	0.0 (0.0 to 0.0)	0.0 (0.0 to 0.0)
Albania	Exposure to forces of nature	10 to 14	Female	0 (0 to 0)	0.0 (0.0 to 0.0)	0 (0 to 0)	0.0 (0.0 to 0.0)	0 (0 to 0)	0.0 (0.0 to 0.0)	0.0 (0.0 to 0.0)	0.0 (0.0 to 0.0)
Albania	Exposure to forces of nature	10 to 14	Male	0 (0 to 0)	0.0 (0.0 to 0.0)	0 (0 to 0)	0.0 (0.0 to 0.0)	0 (0 to 0)	0.0 (0.0 to 0.0)	0.0 (0.0 to 0.0)	0.0 (0.0 to 0.0)

eTable 8. All-cause and cause-specific mortality (counts and rates) at the global level and by country in the WHO European Region for ages 5 to 9 and 10 to 14 for 1990, 2000, and 2016

Albania	Conflict and terrorism	5 to 9	Both	0<br>(0 to 0)	0.0<br>(0.0 to 0.0)	0<br>(0 to 0)	0.0<br>(0.0 to 0.0)	0<br>(0 to 0)	0.0<br>(0.0 to 0.0)	0.0<br>(0.0 to 0.0)	0.0<br>(0.0 to 0.0)
Albania	Conflict and terrorism	5 to 9	Female	0<br>(0 to 0)	0.0<br>(0.0 to 0.0)	0<br>(0 to 0)	0.0<br>(0.0 to 0.0)	0<br>(0 to 0)	0.0<br>(0.0 to 0.0)	0.0<br>(0.0 to 0.0)	0.0<br>(0.0 to 0.0)
Albania	Conflict and terrorism	5 to 9	Male	0<br>(0 to 0)	0.0<br>(0.0 to 0.0)	0<br>(0 to 0)	0.0<br>(0.0 to 0.0)	0<br>(0 to 0)	0.0<br>(0.0 to 0.0)	0.0<br>(0.0 to 0.0)	0.0<br>(0.0 to 0.0)
Albania	Conflict and terrorism	10 to 14	Both	0<br>(0 to 0)	0.0<br>(0.0 to 0.0)	0<br>(0 to 0)	0.0<br>(0.0 to 0.0)	0<br>(0 to 0)	0.0<br>(0.0 to 0.0)	0.0<br>(0.0 to 0.0)	0.0<br>(0.0 to 0.0)
Albania	Conflict and terrorism	10 to 14	Female	0<br>(0 to 0)	0.0<br>(0.0 to 0.0)	0<br>(0 to 0)	0.0<br>(0.0 to 0.0)	0<br>(0 to 0)	0.0<br>(0.0 to 0.0)	0.0<br>(0.0 to 0.0)	0.0<br>(0.0 to 0.0)
Albania	Conflict and terrorism	10 to 14	Male	0<br>(0 to 0)	0.0<br>(0.0 to 0.0)	0<br>(0 to 0)	0.0<br>(0.0 to 0.0)	0<br>(0 to 0)	0.0<br>(0.0 to 0.0)	0.0<br>(0.0 to 0.0)	0.0<br>(0.0 to 0.0)
Albania	Executions and police conflict	5 to 9	Both	0<br>(0 to 0)	0.0<br>(0.0 to 0.0)	0<br>(0 to 0)	0.0<br>(0.0 to 0.0)	0<br>(0 to 0)	0.0<br>(0.0 to 0.0)	-40.2<br>(-62.3 to -4.0)	37.0<br>(-13.6 to 120.0)
Albania	Executions and police conflict	5 to 9	Female	0<br>(0 to 0)	0.0<br>(0.0 to 0.0)	0<br>(0 to 0)	0.0<br>(0.0 to 0.0)	0<br>(0 to 0)	0.0<br>(0.0 to 0.0)	-36.4<br>(-61.5 to 38.1)	46.5<br>(-11.3 to 218.4)
Albania	Executions and police conflict	5 to 9	Male	0<br>(0 to 0)	0.0<br>(0.0 to 0.0)	0<br>(0 to 0)	0.0<br>(0.0 to 0.0)	0<br>(0 to 0)	0.0<br>(0.0 to 0.0)	-43.6<br>(-64.4 to -13.6)	28.5<br>(-18.8 to 97.0)
Albania	Executions and police conflict	10 to 14	Both	0<br>(0 to 0)	0.0<br>(0.0 to 0.0)	0<br>(0 to 0)	0.0<br>(0.0 to 0.0)	0<br>(0 to 0)	0.0<br>(0.0 to 0.0)	-30.7<br>(-55.6 to 27.5)	25.0<br>(-19.9 to 130.2)
Albania	Executions and police conflict	10 to 14	Female	0<br>(0 to 0)	0.0<br>(0.0 to 0.0)	0<br>(0 to 0)	0.0<br>(0.0 to 0.0)	0<br>(0 to 0)	0.0<br>(0.0 to 0.0)	-27.5<br>(-56.7 to 81.3)	31.9<br>(-21.3 to 229.8)
Albania	Executions and police conflict	10 to 14	Male	0<br>(0 to 0)	0.0<br>(0.0 to 0.0)	0<br>(0 to 0)	0.0<br>(0.0 to 0.0)	0<br>(0 to 0)	0.0<br>(0.0 to 0.0)	-33.7<br>(-55.9 to -7.1)	18.7<br>(-20.9 to 66.5)
Andorra	All causes	5 to 9	Both	0<br>(0 to 1)	14.6<br>(11.9 to 17.1)	0<br>(0 to 0)	8.8<br>(7.6 to 10.3)	0<br>(0 to 0)	8.5<br>(7.1 to 10.1)	-21.2<br>(-38.7 to 0.8)	-41.6<br>(-54.6 to -25.3)
Andorra	All causes	5 to 9	Female	0<br>(0 to 0)	12.6<br>(9.9 to 15.2)	0<br>(0 to 0)	7.5<br>(6.6 to 8.7)	0<br>(0 to 0)	7.6<br>(6.5 to 8.9)	-18.6<br>(-34.2 to 3.7)	-39.7<br>(-51.3 to -23.2)
Andorra	All causes	5 to 9	Male	0<br>(0 to 0)	16.5<br>(13.1 to 19.8)	0<br>(0 to 0)	10.1<br>(7.9 to 12.1)	0<br>(0 to 0)	9.4<br>(7.1 to 11.8)	-23.1<br>(-44.7 to 4.2)	-43.0<br>(-59.0 to -22.8)
Andorra	All causes	10 to 14	Both	0<br>(0 to 1)	14.1<br>(11.8 to 16.7)	0<br>(0 to 0)	9.5<br>(8.3 to 10.9)	0<br>(0 to 0)	9.0<br>(7.6 to 10.4)	-18.6<br>(-35.0 to 2.3)	-36.3<br>(-49.1 to -19.9)
Andorra	All causes	10 to 14	Female	0<br>(0 to 0)	10.9<br>(8.7 to 13.2)	0<br>(0 to 0)	7.5<br>(6.1 to 9.1)	0<br>(0 to 0)	7.1<br>(5.8 to 8.6)	-17.1<br>(-36.4 to 11.2)	-35.1<br>(-50.2 to -12.9)
Andorra	All causes	10 to 14	Male	0<br>(0 to 0)	17.2<br>(13.7 to 21.4)	0<br>(0 to 0)	11.5<br>(9.7 to 13.2)	0<br>(0 to 0)	10.9<br>(8.5 to 12.9)	-19.5<br>(-41.4 to 6.4)	-37.0<br>(-54.1 to -16.7)
Andorra	Tuberculosis	5 to 9	Both	0<br>(0 to 0)	0.0<br>(0.0 to 0.0)	0<br>(0 to 0)	0.0<br>(0.0 to 0.0)	0<br>(0 to 0)	0.0<br>(0.0 to 0.0)	-13.8<br>(-54.5 to 55.3)	-36.2<br>(-66.3 to 15.0)
Andorra	Tuberculosis	5 to 9	Female	0<br>(0 to 0)	0.0<br>(0.0 to 0.0)	0<br>(0 to 0)	0.0<br>(0.0 to 0.0)	0<br>(0 to 0)	0.0<br>(0.0 to 0.0)	-24.0<br>(-67.0 to 97.6)	-43.7<br>(-75.6 to 46.4)
Andorra	Tuberculosis	5 to 9	Male	0<br>(0 to 0)	0.0<br>(0.0 to 0.0)	0<br>(0 to 0)	0.0<br>(0.0 to 0.0)	0<br>(0 to 0)	0.0<br>(0.0 to 0.0)	1.6<br>(-44.3 to 69.7)	-24.7<br>(-58.7 to 25.7)
Andorra	Tuberculosis	10 to 14	Both	0<br>(0 to 0)	0.0<br>(0.0 to 0.0)	0<br>(0 to 0)	0.0<br>(0.0 to 0.0)	0<br>(0 to 0)	0.0<br>(0.0 to 0.0)	-0.3<br>(-45.3 to 73.0)	-22.0<br>(-57.1 to 35.4)
Andorra	Tuberculosis	10 to 14	Female	0<br>(0 to 0)	0.0<br>(0.0 to 0.0)	0<br>(0 to 0)	0.0<br>(0.0 to 0.0)	0<br>(0 to 0)	0.0<br>(0.0 to 0.0)	-6.6<br>(-56.0 to 101.1)	-26.9<br>(-65.6 to 57.4)
Andorra	Tuberculosis	10 to 14	Male	0<br>(0 to 0)	0.0<br>(0.0 to 0.0)	0<br>(0 to 0)	0.0<br>(0.0 to 0.0)	0<br>(0 to 0)	0.0<br>(0.0 to 0.0)	6.8<br>(-43.7 to 81.2)	-16.3<br>(-55.9 to 41.8)
Andorra	HIV/AIDS	5 to 9	Both	0<br>(0 to 0)	0.0<br>(0.0 to 0.2)	0<br>(0 to 0)	0.0<br>(0.0 to 0.2)	0<br>(0 to 0)	0.0<br>(0.0 to 0.0)	-86.6<br>(-100.0 to -34.2)	-90.1<br>(-100.0 to -51.3)
Andorra	HIV/AIDS	5 to 9	Female	0<br>(0 to 0)	0.0<br>(0.0 to 0.2)	0<br>(0 to 0)	0.0<br>(0.0 to 0.2)	0<br>(0 to 0)	0.0<br>(0.0 to 0.0)	-86.6<br>(-100.0 to -34.7)	-90.1<br>(-100.0 to -51.6)
Andorra	HIV/AIDS	5 to 9	Male	0<br>(0 to 0)	0.0<br>(0.0 to 0.2)	0<br>(0 to 0)	0.0<br>(0.0 to 0.2)	0<br>(0 to 0)	0.0<br>(0.0 to 0.0)	-86.6<br>(-100.0 to -33.8)	-90.1<br>(-100.0 to -51.0)
Andorra	HIV/AIDS	10 to 14	Both	0<br>(0 to 0)	0.0<br>(0.0 to 0.0)	0<br>(0 to 0)	0.1<br>(0.0 to 0.4)	0<br>(0 to 0)	0.0<br>(0.0 to 0.0)	-67.1<br>(-100.0 to 66.3)	-74.3<br>(-100.0 to 30.2)
Andorra	HIV/AIDS	10 to 14	Female	0<br>(0 to 0)	0.0<br>(0.0 to 0.0)	0<br>(0 to 0)	0.1<br>(0.0 to 0.4)	0<br>(0 to 0)	0.0<br>(0.0 to 0.0)	-67.1<br>(-100.0 to 68.0)	-74.2<br>(-100.0 to 31.5)

eTable 8. All-cause and cause-specific mortality (counts and rates) at the global level and by country in the WHO European Region for ages 5 to 9 and 10 to 14 for 1990, 2000, and 2016

Andorra	HIV/AIDS	10 to 14	Male	0 (0 to 0)	0.0 (0.0 to 0.0)	0 (0 to 0)	0.1 (0.0 to 0.4)	0 (0 to 0)	0.0 (0.0 to 0.0)	-67.1 (-100.0 to 64.6)	-74.3 (-100.0 to 28.9)
Andorra	Diarrheal diseases	5 to 9	Both	0 (0 to 0)	0.0 (0.0 to 0.1)	0 (0 to 0)	0.0 (0.0 to 0.0)	0 (0 to 0)	0.0 (0.0 to 0.1)	36.5 (-32.6 to 263.7)	1.1 (-50.1 to 169.4)
Andorra	Diarrheal diseases	5 to 9	Female	0 (0 to 0)	0.0 (0.0 to 0.1)	0 (0 to 0)	0.0 (0.0 to 0.1)	0 (0 to 0)	0.0 (0.0 to 0.1)	34.9 (-33.8 to 361.2)	-0.1 (-51.0 to 241.6)
Andorra	Diarrheal diseases	5 to 9	Male	0 (0 to 0)	0.0 (0.0 to 0.1)	0 (0 to 0)	0.0 (0.0 to 0.1)	0 (0 to 0)	0.0 (0.0 to 0.1)	37.9 (-42.1 to 394.0)	2.1 (-57.1 to 265.9)
Andorra	Diarrheal diseases	10 to 14	Both	0 (0 to 0)	0.0 (0.0 to 0.0)	0 (0 to 0)	0.0 (0.0 to 0.0)	0 (0 to 0)	0.0 (0.0 to 0.0)	34.0 (-19.2 to 187.8)	4.9 (-36.7 to 125.3)
Andorra	Diarrheal diseases	10 to 14	Female	0 (0 to 0)	0.0 (0.0 to 0.0)	0 (0 to 0)	0.0 (0.0 to 0.0)	0 (0 to 0)	0.0 (0.0 to 0.0)	30.9 (-34.4 to 267.7)	2.5 (-48.6 to 187.9)
Andorra	Diarrheal diseases	10 to 14	Male	0 (0 to 0)	0.0 (0.0 to 0.0)	0 (0 to 0)	0.0 (0.0 to 0.0)	0 (0 to 0)	0.0 (0.0 to 0.0)	39.3 (-13.3 to 185.8)	9.1 (-32.1 to 123.7)
Andorra	Intestinal infectious diseases	5 to 9	Both	0 (0 to 0)	0.0 (0.0 to 0.0)	0 (0 to 0)	0.0 (0.0 to 0.0)	0 (0 to 0)	0.0 (0.0 to 0.0)	13.6 (-42.2 to 95.2)	-15.8 (-57.2 to 44.6)
Andorra	Intestinal infectious diseases	5 to 9	Female	0 (0 to 0)	0.0 (0.0 to 0.0)	0 (0 to 0)	0.0 (0.0 to 0.0)	0 (0 to 0)	0.0 (0.0 to 0.0)	14.7 (-52.4 to 175.2)	-15.1 (-64.8 to 103.8)
Andorra	Intestinal infectious diseases	5 to 9	Male	0 (0 to 0)	0.0 (0.0 to 0.0)	0 (0 to 0)	0.0 (0.0 to 0.0)	0 (0 to 0)	0.0 (0.0 to 0.0)	12.6 (-53.9 to 146.0)	-16.6 (-65.8 to 82.2)
Andorra	Intestinal infectious diseases	10 to 14	Both	0 (0 to 0)	0.0 (0.0 to 0.0)	0 (0 to 0)	0.0 (0.0 to 0.0)	0 (0 to 0)	0.0 (0.0 to 0.0)	16.1 (-42.0 to 134.1)	-9.1 (-54.6 to 83.2)
Andorra	Intestinal infectious diseases	10 to 14	Female	0 (0 to 0)	0.0 (0.0 to 0.0)	0 (0 to 0)	0.0 (0.0 to 0.0)	0 (0 to 0)	0.0 (0.0 to 0.0)	19.6 (-52.4 to 177.8)	-6.3 (-62.7 to 117.5)
Andorra	Intestinal infectious diseases	10 to 14	Male	0 (0 to 0)	0.0 (0.0 to 0.0)	0 (0 to 0)	0.0 (0.0 to 0.0)	0 (0 to 0)	0.0 (0.0 to 0.0)	12.8 (-59.2 to 183.3)	-11.7 (-68.0 to 121.8)
Andorra	Lower respiratory infections	5 to 9	Both	0 (0 to 0)	0.5 (0.3 to 0.8)	0 (0 to 0)	0.3 (0.2 to 0.5)	0 (0 to 0)	0.3 (0.2 to 0.4)	-26.1 (-53.8 to 17.1)	-45.3 (-65.8 to -13.3)
Andorra	Lower respiratory infections	5 to 9	Female	0 (0 to 0)	0.6 (0.3 to 1.0)	0 (0 to 0)	0.3 (0.2 to 0.6)	0 (0 to 0)	0.3 (0.2 to 0.5)	-24.6 (-58.8 to 44.1)	-44.2 (-69.5 to 6.7)
Andorra	Lower respiratory infections	5 to 9	Male	0 (0 to 0)	0.5 (0.2 to 0.8)	0 (0 to 0)	0.3 (0.1 to 0.5)	0 (0 to 0)	0.3 (0.1 to 0.4)	-27.8 (-62.2 to 33.1)	-46.6 (-72.0 to -1.4)
Andorra	Lower respiratory infections	10 to 14	Both	0 (0 to 0)	0.4 (0.2 to 0.6)	0 (0 to 0)	0.3 (0.1 to 0.4)	0 (0 to 0)	0.2 (0.1 to 0.3)	-24.7 (-54.1 to 19.7)	-41.0 (-64.1 to -6.3)
Andorra	Lower respiratory infections	10 to 14	Female	0 (0 to 0)	0.4 (0.2 to 0.7)	0 (0 to 0)	0.3 (0.1 to 0.5)	0 (0 to 0)	0.2 (0.1 to 0.4)	-26.3 (-64.4 to 45.9)	-42.3 (-72.1 to 14.3)
Andorra	Lower respiratory infections	10 to 14	Male	0 (0 to 0)	0.4 (0.2 to 0.6)	0 (0 to 0)	0.2 (0.1 to 0.4)	0 (0 to 0)	0.2 (0.1 to 0.4)	-23.0 (-60.4 to 44.2)	-39.7 (-69.0 to 12.9)
Andorra	Upper respiratory infections	5 to 9	Both	0 (0 to 0)	0.0 (0.0 to 0.0)	0 (0 to 0)	0.0 (0.0 to 0.0)	0 (0 to 0)	0.0 (0.0 to 0.0)	-27.9 (-98.4 to 2715.5)	-46.6 (-98.8 to 1985.3)
Andorra	Upper respiratory infections	5 to 9	Female	0 (0 to 0)	0.0 (0.0 to 0.0)	0 (0 to 0)	0.0 (0.0 to 0.0)	0 (0 to 0)	0.0 (0.0 to 0.0)	-29.3 (-100.0 to 76564.7)	-47.6 (-100.0 to 56673.9)
Andorra	Upper respiratory infections	5 to 9	Male	0 (0 to 0)	0.0 (0.0 to 0.0)	0 (0 to 0)	0.0 (0.0 to 0.0)	0 (0 to 0)	0.0 (0.0 to 0.0)	-26.8 (-99.9 to 78711.8)	-45.8 (-99.9 to 58280.3)
Andorra	Upper respiratory infections	10 to 14	Both	0 (0 to 0)	0.0 (0.0 to 0.0)	0 (0 to 0)	0.0 (0.0 to 0.0)	0 (0 to 0)	0.0 (0.0 to 0.0)	-25.3 (-98.8 to 5306.4)	-41.6 (-99.1 to 4132.7)
Andorra	Upper respiratory infections	10 to 14	Female	0 (0 to 0)	0.0 (0.0 to 0.0)	0 (0 to 0)	0.0 (0.0 to 0.0)	0 (0 to 0)	0.0 (0.0 to 0.0)	-28.5 (-99.9 to 113620.5)	-44.0 (-99.9 to 88930.9)

eTable 8. All-cause and cause-specific mortality (counts and rates) at the global level and by country in the WHO European Region for ages 5 to 9 and 10 to 14 for 1990, 2000, and 2016

Andorra	Upper respiratory infections	10 to 14	Male	0<br>(0 to 0)	0.0<br>(0.0 to 0.0)	0<br>(0 to 0)	0.0<br>(0.0 to 0.0)	0<br>(0 to 0)	0.0<br>(0.0 to 0.0)	-23.3<br>(-99.9 to 70689.1)	-40.0<br>(-100.0 to 55322.4)
Andorra	Otitis media	5 to 9	Both	0<br>(0 to 0)	0.0<br>(0.0 to 0.0)	0<br>(0 to 0)	0.0<br>(0.0 to 0.0)	0<br>(0 to 0)	0.0<br>(0.0 to 0.0)	-83.3<br>(-98.2 to 42.7)	-87.7<br>(-98.7 to 5.7)
Andorra	Otitis media	5 to 9	Female	0<br>(0 to 0)	0.0<br>(0.0 to 0.0)	0<br>(0 to 0)	0.0<br>(0.0 to 0.0)	0<br>(0 to 0)	0.0<br>(0.0 to 0.0)	-69.7<br>(-96.6 to 439.9)	-77.6<br>(-97.4 to 299.8)
Andorra	Otitis media	5 to 9	Male	0<br>(0 to 0)	0.0<br>(0.0 to 0.0)	0<br>(0 to 0)	0.0<br>(0.0 to 0.0)	0<br>(0 to 0)	0.0<br>(0.0 to 0.0)	-84.1<br>(-98.6 to 70.7)	-88.2<br>(-98.9 to 26.5)
Andorra	Otitis media	10 to 14	Both	0<br>(0 to 0)	0.0<br>(0.0 to 0.0)	0<br>(0 to 0)	0.0<br>(0.0 to 0.0)	0<br>(0 to 0)	0.0<br>(0.0 to 0.0)	-77.7<br>(-98.2 to 118.4)	-82.6<br>(-98.6 to 71.0)
Andorra	Otitis media	10 to 14	Female	0<br>(0 to 0)	0.0<br>(0.0 to 0.0)	0<br>(0 to 0)	0.0<br>(0.0 to 0.0)	0<br>(0 to 0)	0.0<br>(0.0 to 0.0)	-71.0<br>(-96.0 to 272.7)	-77.3<br>(-96.9 to 191.8)
Andorra	Otitis media	10 to 14	Male	0<br>(0 to 0)	0.0<br>(0.0 to 0.0)	0<br>(0 to 0)	0.0<br>(0.0 to 0.0)	0<br>(0 to 0)	0.0<br>(0.0 to 0.0)	-78.1<br>(-98.5 to 146.0)	-82.9<br>(-98.8 to 92.6)
Andorra	Meningitis	5 to 9	Both	0<br>(0 to 0)	0.4<br>(0.3 to 0.6)	0<br>(0 to 0)	0.2<br>(0.1 to 0.3)	0<br>(0 to 0)	0.2<br>(0.1 to 0.3)	-34.0<br>(-61.7 to 9.0)	-51.1<br>(-71.7 to -19.3)
Andorra	Meningitis	5 to 9	Female	0<br>(0 to 0)	0.4<br>(0.2 to 0.6)	0<br>(0 to 0)	0.2<br>(0.1 to 0.3)	0<br>(0 to 0)	0.2<br>(0.1 to 0.3)	-45.9<br>(-73.4 to 4.8)	-60.0<br>(-80.3 to -22.4)
Andorra	Meningitis	5 to 9	Male	0<br>(0 to 0)	0.4<br>(0.2 to 0.7)	0<br>(0 to 0)	0.2<br>(0.1 to 0.5)	0<br>(0 to 0)	0.2<br>(0.1 to 0.5)	-22.0<br>(-65.3 to 55.6)	-42.2<br>(-74.3 to 15.2)
Andorra	Meningitis	10 to 14	Both	0<br>(0 to 0)	0.3<br>(0.2 to 0.4)	0<br>(0 to 0)	0.2<br>(0.1 to 0.3)	0<br>(0 to 0)	0.2<br>(0.1 to 0.3)	-27.9<br>(-60.7 to 23.7)	-43.6<br>(-69.2 to -3.1)
Andorra	Meningitis	10 to 14	Female	0<br>(0 to 0)	0.3<br>(0.2 to 0.4)	0<br>(0 to 0)	0.2<br>(0.1 to 0.3)	0<br>(0 to 0)	0.1<br>(0.1 to 0.2)	-42.5<br>(-73.7 to 15.5)	-55.0<br>(-79.4 to -9.6)
Andorra	Meningitis	10 to 14	Male	0<br>(0 to 0)	0.3<br>(0.2 to 0.5)	0<br>(0 to 0)	0.2<br>(0.1 to 0.4)	0<br>(0 to 0)	0.2<br>(0.1 to 0.4)	-15.6<br>(-62.5 to 62.8)	-33.9<br>(-70.6 to 27.5)
Andorra	Encephalitis	5 to 9	Both	0<br>(0 to 0)	0.1<br>(0.0 to 0.1)	0<br>(0 to 0)	0.0<br>(0.0 to 0.1)	0<br>(0 to 0)	0.0<br>(0.0 to 0.1)	18.5<br>(-34.9 to 145.1)	-12.3<br>(-51.8 to 81.6)
Andorra	Encephalitis	5 to 9	Female	0<br>(0 to 0)	0.0<br>(0.0 to 0.1)	0<br>(0 to 0)	0.0<br>(0.0 to 0.1)	0<br>(0 to 0)	0.0<br>(0.0 to 0.1)	16.1<br>(-38.1 to 171.0)	-14.0<br>(-54.2 to 100.7)
Andorra	Encephalitis	5 to 9	Male	0<br>(0 to 0)	0.1<br>(0.0 to 0.1)	0<br>(0 to 0)	0.1<br>(0.0 to 0.1)	0<br>(0 to 0)	0.1<br>(0.0 to 0.1)	20.1<br>(-53.1 to 225.5)	-11.1<br>(-65.2 to 141.1)
Andorra	Encephalitis	10 to 14	Both	0<br>(0 to 0)	0.0<br>(0.0 to 0.1)	0<br>(0 to 0)	0.0<br>(0.0 to 0.1)	0<br>(0 to 0)	0.0<br>(0.0 to 0.1)	20.2<br>(-28.6 to 114.5)	-5.9<br>(-44.1 to 67.9)
Andorra	Encephalitis	10 to 14	Female	0<br>(0 to 0)	0.0<br>(0.0 to 0.1)	0<br>(0 to 0)	0.0<br>(0.0 to 0.1)	0<br>(0 to 0)	0.0<br>(0.0 to 0.1)	16.9<br>(-37.0 to 154.5)	-8.5<br>(-50.7 to 99.2)
Andorra	Encephalitis	10 to 14	Male	0<br>(0 to 0)	0.0<br>(0.0 to 0.1)	0<br>(0 to 0)	0.0<br>(0.0 to 0.1)	0<br>(0 to 0)	0.0<br>(0.0 to 0.1)	23.1<br>(-40.3 to 195.4)	-3.7<br>(-53.3 to 131.2)
Andorra	Diphtheria	5 to 9	Both	0<br>(0 to 0)	0.0<br>(0.0 to 0.0)	0<br>(0 to 0)	0.0<br>(0.0 to 0.0)	0<br>(0 to 0)	0.0<br>(0.0 to 0.0)	-66.7<br>(-96.0 to 159.0)	-75.3<br>(-97.1 to 91.8)
Andorra	Diphtheria	5 to 9	Female	0<br>(0 to 0)	0.0<br>(0.0 to 0.0)	0<br>(0 to 0)	0.0<br>(0.0 to 0.0)	0<br>(0 to 0)	0.0<br>(0.0 to 0.0)	-65.3<br>(-98.8 to 638.2)	-74.3<br>(-99.1 to 446.7)
Andorra	Diphtheria	5 to 9	Male	0<br>(0 to 0)	0.0<br>(0.0 to 0.0)	0<br>(0 to 0)	0.0<br>(0.0 to 0.0)	0<br>(0 to 0)	0.0<br>(0.0 to 0.0)	-67.7<br>(-98.9 to 463.1)	-76.1<br>(-99.2 to 317.1)
Andorra	Diphtheria	10 to 14	Both	0<br>(0 to 0)	0.0<br>(0.0 to 0.0)	0<br>(0 to 0)	0.0<br>(0.0 to 0.0)	0<br>(0 to 0)	0.0<br>(0.0 to 0.0)	-63.0<br>(-94.6 to 169.0)	-71.1<br>(-95.8 to 110.6)
Andorra	Diphtheria	10 to 14	Female	0<br>(0 to 0)	0.0<br>(0.0 to 0.0)	0<br>(0 to 0)	0.0<br>(0.0 to 0.0)	0<br>(0 to 0)	0.0<br>(0.0 to 0.0)	-63.2<br>(-97.4 to 715.8)	-71.2<br>(-98.0 to 538.7)
Andorra	Diphtheria	10 to 14	Male	0<br>(0 to 0)	0.0<br>(0.0 to 0.0)	0<br>(0 to 0)	0.0<br>(0.0 to 0.0)	0<br>(0 to 0)	0.0<br>(0.0 to 0.0)	-62.9<br>(-98.0 to 738.2)	-71.0<br>(-98.4 to 556.2)
Andorra	Whooping cough	5 to 9	Both	0<br>(0 to 0)	0.1<br>(0.0 to 0.4)	0<br>(0 to 0)	0.0<br>(0.0 to 0.1)	0<br>(0 to 0)	0.0<br>(0.0 to 0.0)	-81.7<br>(-98.5 to 123.1)	-86.4<br>(-98.9 to 65.2)
Andorra	Whooping cough	5 to 9	Female	0<br>(0 to 0)	0.1<br>(0.0 to 0.4)	0<br>(0 to 0)	0.1<br>(0.0 to 0.2)	0<br>(0 to 0)	0.0<br>(0.0 to 0.1)	-81.7<br>(-98.5 to 134.1)	-86.5<br>(-98.9 to 73.4)
Andorra	Whooping cough	5 to 9	Male	0<br>(0 to 0)	0.1<br>(0.0 to 0.3)	0<br>(0 to 0)	0.0<br>(0.0 to 0.1)	0<br>(0 to 0)	0.0<br>(0.0 to 0.0)	-81.7<br>(-98.4 to 140.0)	-86.4<br>(-98.8 to 77.8)
Andorra	Whooping cough	10 to 14	Both	0<br>(0 to 0)	0.0<br>(0.0 to 0.1)	0<br>(0 to 0)	0.0<br>(0.0 to 0.0)	0<br>(0 to 0)	0.0<br>(0.0 to 0.0)	-82.2<br>(-98.3 to 129.1)	-86.1<br>(-98.6 to 79.4)
Andorra	Whooping cough	10 to 14	Female	0<br>(0 to 0)	0.0<br>(0.0 to 0.1)	0<br>(0 to 0)	0.0<br>(0.0 to 0.0)	0<br>(0 to 0)	0.0<br>(0.0 to 0.0)	-82.6<br>(-98.4 to 134.3)	-86.4<br>(-98.7 to 83.4)
Andorra	Whooping cough	10 to 14	Male	0<br>(0 to 0)	0.0<br>(0.0 to 0.1)	0<br>(0 to 0)	0.0<br>(0.0 to 0.0)	0<br>(0 to 0)	0.0<br>(0.0 to 0.0)	-81.7<br>(-98.3 to 135.9)	-85.7<br>(-98.7 to 84.7)
Andorra	Tetanus	5 to 9	Both	0<br>(0 to 0)	0.0<br>(0.0 to 0.0)	0<br>(0 to 0)	0.0<br>(0.0 to 0.0)	0<br>(0 to 0)	0.0<br>(0.0 to 0.0)	25.3<br>(-3.4 to 64.3)	-7.2<br>(-28.4 to 21.7)

eTable 8. All-cause and cause-specific mortality (counts and rates) at the global level and by country in the WHO European Region for ages 5 to 9 and 10 to 14 for 1990, 2000, and 2016

Andorra	Tetanus	5 to 9	Female	0 (0 to 0)	0.0 (0.0 to 0.0)	0 (0 to 0)	0.0 (0.0 to 0.0)	0 (0 to 0)	0.0 (0.0 to 0.0)	25.0 (-4.7 to 65.9)	-7.5 (-29.4 to 22.9)
Andorra	Tetanus	5 to 9	Male	0 (0 to 0)	0.0 (0.0 to 0.0)	0 (0 to 0)	0.0 (0.0 to 0.0)	0 (0 to 0)	0.0 (0.0 to 0.0)	25.7 (-11.3 to 79.4)	-6.9 (-34.3 to 32.9)
Andorra	Tetanus	10 to 14	Both	0 (0 to 0)	0.0 (0.0 to 0.0)	0 (0 to 0)	0.0 (0.0 to 0.0)	0 (0 to 0)	0.0 (0.0 to 0.0)	23.0 (-4.6 to 55.8)	-3.7 (-25.3 to 22.0)
Andorra	Tetanus	10 to 14	Female	0 (0 to 0)	0.0 (0.0 to 0.0)	0 (0 to 0)	0.0 (0.0 to 0.0)	0 (0 to 0)	0.0 (0.0 to 0.0)	20.4 (-13.3 to 68.9)	-5.7 (-32.1 to 32.2)
Andorra	Tetanus	10 to 14	Male	0 (0 to 0)	0.0 (0.0 to 0.0)	0 (0 to 0)	0.0 (0.0 to 0.0)	0 (0 to 0)	0.0 (0.0 to 0.0)	25.5 (-13.9 to 72.2)	-1.7 (-32.6 to 34.9)
Andorra	Measles	5 to 9	Both	0 (0 to 0)	0.0 (0.0 to 0.1)	0 (0 to 0)	0.0 (0.0 to 0.0)	0 (0 to 0)	0.0 (0.0 to 0.0)	-99.4 (-100.0 to -97.7)	-99.6 (-100.0 to -98.3)
Andorra	Measles	5 to 9	Female	0 (0 to 0)	0.0 (0.0 to 0.1)	0 (0 to 0)	0.0 (0.0 to 0.0)	0 (0 to 0)	0.0 (0.0 to 0.0)	-99.4 (-100.0 to -97.8)	-99.6 (-100.0 to -98.3)
Andorra	Measles	5 to 9	Male	0 (0 to 0)	0.0 (0.0 to 0.1)	0 (0 to 0)	0.0 (0.0 to 0.0)	0 (0 to 0)	0.0 (0.0 to 0.0)	-99.4 (-100.0 to -97.5)	-99.6 (-100.0 to -98.2)
Andorra	Measles	10 to 14	Both	0 (0 to 0)	0.0 (0.0 to 0.0)	0 (0 to 0)	0.0 (0.0 to 0.0)	0 (0 to 0)	0.0 (0.0 to 0.0)	-99.4 (-100.0 to -97.7)	-99.5 (-100.0 to -98.2)
Andorra	Measles	10 to 14	Female	0 (0 to 0)	0.0 (0.0 to 0.0)	0 (0 to 0)	0.0 (0.0 to 0.0)	0 (0 to 0)	0.0 (0.0 to 0.0)	-99.4 (-100.0 to -97.7)	-99.6 (-100.0 to -98.2)
Andorra	Measles	10 to 14	Male	0 (0 to 0)	0.0 (0.0 to 0.0)	0 (0 to 0)	0.0 (0.0 to 0.0)	0 (0 to 0)	0.0 (0.0 to 0.0)	-99.4 (-100.0 to -97.6)	-99.5 (-100.0 to -98.1)
Andorra	Varicella and herpes zoster	5 to 9	Both	0 (0 to 0)	0.0 (0.0 to 0.0)	0 (0 to 0)	0.0 (0.0 to 0.0)	0 (0 to 0)	0.0 (0.0 to 0.0)	-43.8 (-89.4 to 183.0)	-58.4 (-92.2 to 109.6)
Andorra	Varicella and herpes zoster	5 to 9	Female	0 (0 to 0)	0.0 (0.0 to 0.0)	0 (0 to 0)	0.0 (0.0 to 0.0)	0 (0 to 0)	0.0 (0.0 to 0.0)	-43.5 (-95.9 to 810.0)	-58.2 (-97.0 to 573.9)
Andorra	Varicella and herpes zoster	5 to 9	Male	0 (0 to 0)	0.0 (0.0 to 0.0)	0 (0 to 0)	0.0 (0.0 to 0.0)	0 (0 to 0)	0.0 (0.0 to 0.0)	-44.0 (-95.9 to 500.0)	-58.5 (-97.0 to 344.5)
Andorra	Varicella and herpes zoster	10 to 14	Both	0 (0 to 0)	0.0 (0.0 to 0.0)	0 (0 to 0)	0.0 (0.0 to 0.0)	0 (0 to 0)	0.0 (0.0 to 0.0)	-46.4 (-89.0 to 179.7)	-58.1 (-91.4 to 119.0)
Andorra	Varicella and herpes zoster	10 to 14	Female	0 (0 to 0)	0.0 (0.0 to 0.0)	0 (0 to 0)	0.0 (0.0 to 0.0)	0 (0 to 0)	0.0 (0.0 to 0.0)	-48.0 (-95.7 to 593.2)	-59.3 (-96.6 to 442.7)
Andorra	Varicella and herpes zoster	10 to 14	Male	0 (0 to 0)	0.0 (0.0 to 0.0)	0 (0 to 0)	0.0 (0.0 to 0.0)	0 (0 to 0)	0.0 (0.0 to 0.0)	-44.9 (-95.6 to 615.2)	-56.8 (-96.6 to 459.9)
Andorra	Malaria	5 to 9	Both	0 (0 to 0)	0.0 (0.0 to 0.0)	0 (0 to 0)	0.0 (0.0 to 0.0)	0 (0 to 0)	0.0 (0.0 to 0.0)	0.0 (0.0 to 0.0)	0.0 (0.0 to 0.0)
Andorra	Malaria	5 to 9	Female	0 (0 to 0)	0.0 (0.0 to 0.0)	0 (0 to 0)	0.0 (0.0 to 0.0)	0 (0 to 0)	0.0 (0.0 to 0.0)	0.0 (0.0 to 0.0)	0.0 (0.0 to 0.0)
Andorra	Malaria	5 to 9	Male	0 (0 to 0)	0.0 (0.0 to 0.0)	0 (0 to 0)	0.0 (0.0 to 0.0)	0 (0 to 0)	0.0 (0.0 to 0.0)	0.0 (0.0 to 0.0)	0.0 (0.0 to 0.0)
Andorra	Malaria	10 to 14	Both	0 (0 to 0)	0.0 (0.0 to 0.0)	0 (0 to 0)	0.0 (0.0 to 0.0)	0 (0 to 0)	0.0 (0.0 to 0.0)	0.0 (0.0 to 0.0)	0.0 (0.0 to 0.0)
Andorra	Malaria	10 to 14	Female	0 (0 to 0)	0.0 (0.0 to 0.0)	0 (0 to 0)	0.0 (0.0 to 0.0)	0 (0 to 0)	0.0 (0.0 to 0.0)	0.0 (0.0 to 0.0)	0.0 (0.0 to 0.0)
Andorra	Malaria	10 to 14	Male	0 (0 to 0)	0.0 (0.0 to 0.0)	0 (0 to 0)	0.0 (0.0 to 0.0)	0 (0 to 0)	0.0 (0.0 to 0.0)	0.0 (0.0 to 0.0)	0.0 (0.0 to 0.0)
Andorra	Leishmaniasis	5 to 9	Both	0 (0 to 0)	0.0 (0.0 to 0.0)	0 (0 to 0)	0.0 (0.0 to 0.0)	0 (0 to 0)	0.0 (0.0 to 0.0)	0.0 (0.0 to 0.0)	0.0 (0.0 to 0.0)
Andorra	Leishmaniasis	5 to 9	Female	0 (0 to 0)	0.0 (0.0 to 0.0)	0 (0 to 0)	0.0 (0.0 to 0.0)	0 (0 to 0)	0.0 (0.0 to 0.0)	0.0 (0.0 to 0.0)	0.0 (0.0 to 0.0)
Andorra	Leishmaniasis	5 to 9	Male	0 (0 to 0)	0.0 (0.0 to 0.0)	0 (0 to 0)	0.0 (0.0 to 0.0)	0 (0 to 0)	0.0 (0.0 to 0.0)	0.0 (0.0 to 0.0)	0.0 (0.0 to 0.0)
Andorra	Leishmaniasis	10 to 14	Both	0 (0 to 0)	0.0 (0.0 to 0.0)	0 (0 to 0)	0.0 (0.0 to 0.0)	0 (0 to 0)	0.0 (0.0 to 0.0)	0.0 (0.0 to 0.0)	0.0 (0.0 to 0.0)
Andorra	Leishmaniasis	10 to 14	Female	0 (0 to 0)	0.0 (0.0 to 0.0)	0 (0 to 0)	0.0 (0.0 to 0.0)	0 (0 to 0)	0.0 (0.0 to 0.0)	0.0 (0.0 to 0.0)	0.0 (0.0 to 0.0)
Andorra	Leishmaniasis	10 to 14	Male	0 (0 to 0)	0.0 (0.0 to 0.0)	0 (0 to 0)	0.0 (0.0 to 0.0)	0 (0 to 0)	0.0 (0.0 to 0.0)	0.0 (0.0 to 0.0)	0.0 (0.0 to 0.0)
Andorra	African trypanosomiasis	5 to 9	Both	0 (0 to 0)	0.0 (0.0 to 0.0)	0 (0 to 0)	0.0 (0.0 to 0.0)	0 (0 to 0)	0.0 (0.0 to 0.0)	0.0 (0.0 to 0.0)	0.0 (0.0 to 0.0)
Andorra	African trypanosomiasis	5 to 9	Female	0 (0 to 0)	0.0 (0.0 to 0.0)	0 (0 to 0)	0.0 (0.0 to 0.0)	0 (0 to 0)	0.0 (0.0 to 0.0)	0.0 (0.0 to 0.0)	0.0 (0.0 to 0.0)

eTable 8. All-cause and cause-specific mortality (counts and rates) at the global level and by country in the WHO European Region for ages 5 to 9 and 10 to 14 for 1990, 2000, and 2016

Andorra	African trypanosomiasis	5 to 9	Male	0 (0 to 0)	0.0 (0.0 to 0.0)	0 (0 to 0)	0.0 (0.0 to 0.0)	0 (0 to 0)	0.0 (0.0 to 0.0)	0.0 (0.0 to 0.0)	0.0 (0.0 to 0.0)
Andorra	African trypanosomiasis	10 to 14	Both	0 (0 to 0)	0.0 (0.0 to 0.0)	0 (0 to 0)	0.0 (0.0 to 0.0)	0 (0 to 0)	0.0 (0.0 to 0.0)	0.0 (0.0 to 0.0)	0.0 (0.0 to 0.0)
Andorra	African trypanosomiasis	10 to 14	Female	0 (0 to 0)	0.0 (0.0 to 0.0)	0 (0 to 0)	0.0 (0.0 to 0.0)	0 (0 to 0)	0.0 (0.0 to 0.0)	0.0 (0.0 to 0.0)	0.0 (0.0 to 0.0)
Andorra	African trypanosomiasis	10 to 14	Male	0 (0 to 0)	0.0 (0.0 to 0.0)	0 (0 to 0)	0.0 (0.0 to 0.0)	0 (0 to 0)	0.0 (0.0 to 0.0)	0.0 (0.0 to 0.0)	0.0 (0.0 to 0.0)
Andorra	Schistosomiasis	5 to 9	Both	0 (0 to 0)	0.0 (0.0 to 0.0)	0 (0 to 0)	0.0 (0.0 to 0.0)	0 (0 to 0)	0.0 (0.0 to 0.0)	0.0 (0.0 to 0.0)	0.0 (0.0 to 0.0)
Andorra	Schistosomiasis	5 to 9	Female	0 (0 to 0)	0.0 (0.0 to 0.0)	0 (0 to 0)	0.0 (0.0 to 0.0)	0 (0 to 0)	0.0 (0.0 to 0.0)	0.0 (0.0 to 0.0)	0.0 (0.0 to 0.0)
Andorra	Schistosomiasis	5 to 9	Male	0 (0 to 0)	0.0 (0.0 to 0.0)	0 (0 to 0)	0.0 (0.0 to 0.0)	0 (0 to 0)	0.0 (0.0 to 0.0)	0.0 (0.0 to 0.0)	0.0 (0.0 to 0.0)
Andorra	Schistosomiasis	10 to 14	Both	0 (0 to 0)	0.0 (0.0 to 0.0)	0 (0 to 0)	0.0 (0.0 to 0.0)	0 (0 to 0)	0.0 (0.0 to 0.0)	0.0 (0.0 to 0.0)	0.0 (0.0 to 0.0)
Andorra	Schistosomiasis	10 to 14	Female	0 (0 to 0)	0.0 (0.0 to 0.0)	0 (0 to 0)	0.0 (0.0 to 0.0)	0 (0 to 0)	0.0 (0.0 to 0.0)	0.0 (0.0 to 0.0)	0.0 (0.0 to 0.0)
Andorra	Schistosomiasis	10 to 14	Male	0 (0 to 0)	0.0 (0.0 to 0.0)	0 (0 to 0)	0.0 (0.0 to 0.0)	0 (0 to 0)	0.0 (0.0 to 0.0)	0.0 (0.0 to 0.0)	0.0 (0.0 to 0.0)
Andorra	Cysticercosis	5 to 9	Both	0 (0 to 0)	0.0 (0.0 to 0.0)	0 (0 to 0)	0.0 (0.0 to 0.0)	0 (0 to 0)	0.0 (0.0 to 0.0)	-68.8 (-76.1 to -59.2)	-76.9 (-82.3 to -69.8)
Andorra	Cysticercosis	5 to 9	Female	0 (0 to 0)	0.0 (0.0 to 0.0)	0 (0 to 0)	0.0 (0.0 to 0.0)	0 (0 to 0)	0.0 (0.0 to 0.0)	-68.0 (-75.8 to -57.3)	-76.3 (-82.1 to -68.4)
Andorra	Cysticercosis	5 to 9	Male	0 (0 to 0)	0.0 (0.0 to 0.0)	0 (0 to 0)	0.0 (0.0 to 0.0)	0 (0 to 0)	0.0 (0.0 to 0.0)	-69.6 (-78.9 to -56.4)	-77.5 (-84.4 to -67.7)
Andorra	Cysticercosis	10 to 14	Both	0 (0 to 0)	0.0 (0.0 to 0.0)	0 (0 to 0)	0.0 (0.0 to 0.0)	0 (0 to 0)	0.0 (0.0 to 0.0)	-66.7 (-74.3 to -57.8)	-73.9 (-79.8 to -67.0)
Andorra	Cysticercosis	10 to 14	Female	0 (0 to 0)	0.0 (0.0 to 0.0)	0 (0 to 0)	0.0 (0.0 to 0.0)	0 (0 to 0)	0.0 (0.0 to 0.0)	-66.9 (-76.1 to -53.2)	-74.1 (-81.3 to -63.4)
Andorra	Cysticercosis	10 to 14	Male	0 (0 to 0)	0.0 (0.0 to 0.0)	0 (0 to 0)	0.0 (0.0 to 0.0)	0 (0 to 0)	0.0 (0.0 to 0.0)	-66.5 (-77.2 to -54.1)	-73.8 (-82.2 to -64.0)
Andorra	Yellow fever	5 to 9	Both	0 (0 to 0)	0.0 (0.0 to 0.0)	0 (0 to 0)	0.0 (0.0 to 0.0)	0 (0 to 0)	0.0 (0.0 to 0.0)	0.0 (0.0 to 0.0)	0.0 (0.0 to 0.0)
Andorra	Yellow fever	5 to 9	Female	0 (0 to 0)	0.0 (0.0 to 0.0)	0 (0 to 0)	0.0 (0.0 to 0.0)	0 (0 to 0)	0.0 (0.0 to 0.0)	0.0 (0.0 to 0.0)	0.0 (0.0 to 0.0)
Andorra	Yellow fever	5 to 9	Male	0 (0 to 0)	0.0 (0.0 to 0.0)	0 (0 to 0)	0.0 (0.0 to 0.0)	0 (0 to 0)	0.0 (0.0 to 0.0)	0.0 (0.0 to 0.0)	0.0 (0.0 to 0.0)
Andorra	Yellow fever	10 to 14	Both	0 (0 to 0)	0.0 (0.0 to 0.0)	0 (0 to 0)	0.0 (0.0 to 0.0)	0 (0 to 0)	0.0 (0.0 to 0.0)	0.0 (0.0 to 0.0)	0.0 (0.0 to 0.0)
Andorra	Yellow fever	10 to 14	Female	0 (0 to 0)	0.0 (0.0 to 0.0)	0 (0 to 0)	0.0 (0.0 to 0.0)	0 (0 to 0)	0.0 (0.0 to 0.0)	0.0 (0.0 to 0.0)	0.0 (0.0 to 0.0)
Andorra	Yellow fever	10 to 14	Male	0 (0 to 0)	0.0 (0.0 to 0.0)	0 (0 to 0)	0.0 (0.0 to 0.0)	0 (0 to 0)	0.0 (0.0 to 0.0)	0.0 (0.0 to 0.0)	0.0 (0.0 to 0.0)
Andorra	Rabies	5 to 9	Both	0 (0 to 0)	0.0 (0.0 to 0.0)	0 (0 to 0)	0.0 (0.0 to 0.0)	0 (0 to 0)	0.0 (0.0 to 0.0)	-1.6 (-85.6 to 718.5)	-27.1 (-89.3 to 506.2)
Andorra	Rabies	5 to 9	Female	0 (0 to 0)	0.0 (0.0 to 0.0)	0 (0 to 0)	0.0 (0.0 to 0.0)	0 (0 to 0)	0.0 (0.0 to 0.0)	27.5 (-89.8 to 1501.4)	-5.5 (-92.4 to 1085.9)
Andorra	Rabies	5 to 9	Male	0 (0 to 0)	0.0 (0.0 to 0.0)	0 (0 to 0)	0.0 (0.0 to 0.0)	0 (0 to 0)	0.0 (0.0 to 0.0)	-20.7 (-92.0 to 1009.9)	-41.3 (-94.1 to 722.1)
Andorra	Rabies	10 to 14	Both	0 (0 to 0)	0.0 (0.0 to 0.0)	0 (0 to 0)	0.0 (0.0 to 0.0)	0 (0 to 0)	0.0 (0.0 to 0.0)	20.0 (-82.6 to 799.7)	-6.0 (-86.4 to 604.4)
Andorra	Rabies	10 to 14	Female	0 (0 to 0)	0.0 (0.0 to 0.0)	0 (0 to 0)	0.0 (0.0 to 0.0)	0 (0 to 0)	0.0 (0.0 to 0.0)	52.2 (-87.7 to 1794.3)	19.2 (-90.3 to 1383.0)
Andorra	Rabies	10 to 14	Male	0 (0 to 0)	0.0 (0.0 to 0.0)	0 (0 to 0)	0.0 (0.0 to 0.0)	0 (0 to 0)	0.0 (0.0 to 0.0)	-16.9 (-88.1 to 626.8)	-35.0 (-90.7 to 469.1)
Andorra	Intestinal nematode infections	5 to 9	Both	-	-	-	-	-	-	-	-

eTable 8. All-cause and cause-specific mortality (counts and rates) at the global level and by country in the WHO European Region for ages 5 to 9 and 10 to 14 for 1990, 2000, and 2016

Andorra	Intestinal nematode infections	5 to 9	Female	-	-	-	-	-	-	-	-
Andorra	Intestinal nematode infections	5 to 9	Male	-	-	-	-	-	-	-	-
Andorra	Intestinal nematode infections	10 to 14	Both	-	-	-	-	-	-	-	-
Andorra	Intestinal nematode infections	10 to 14	Female	-	-	-	-	-	-	-	-
Andorra	Intestinal nematode infections	10 to 14	Male	-	-	-	-	-	-	-	-
Andorra	Ebola	5 to 9	Both	0 (0 to 0)	0.0 (0.0 to 0.0)	0 (0 to 0)	0.0 (0.0 to 0.0)	0 (0 to 0)	0.0 (0.0 to 0.0)	0.0 (0.0 to 0.0)	0.0 (0.0 to 0.0)
Andorra	Ebola	5 to 9	Female	0 (0 to 0)	0.0 (0.0 to 0.0)	0 (0 to 0)	0.0 (0.0 to 0.0)	0 (0 to 0)	0.0 (0.0 to 0.0)	0.0 (0.0 to 0.0)	0.0 (0.0 to 0.0)
Andorra	Ebola	5 to 9	Male	0 (0 to 0)	0.0 (0.0 to 0.0)	0 (0 to 0)	0.0 (0.0 to 0.0)	0 (0 to 0)	0.0 (0.0 to 0.0)	0.0 (0.0 to 0.0)	0.0 (0.0 to 0.0)
Andorra	Ebola	10 to 14	Both	0 (0 to 0)	0.0 (0.0 to 0.0)	0 (0 to 0)	0.0 (0.0 to 0.0)	0 (0 to 0)	0.0 (0.0 to 0.0)	0.0 (0.0 to 0.0)	0.0 (0.0 to 0.0)
Andorra	Ebola	10 to 14	Female	0 (0 to 0)	0.0 (0.0 to 0.0)	0 (0 to 0)	0.0 (0.0 to 0.0)	0 (0 to 0)	0.0 (0.0 to 0.0)	0.0 (0.0 to 0.0)	0.0 (0.0 to 0.0)
Andorra	Ebola	10 to 14	Male	0 (0 to 0)	0.0 (0.0 to 0.0)	0 (0 to 0)	0.0 (0.0 to 0.0)	0 (0 to 0)	0.0 (0.0 to 0.0)	0.0 (0.0 to 0.0)	0.0 (0.0 to 0.0)
Andorra	Zika virus	5 to 9	Both	0 (0 to 0)	0.0 (0.0 to 0.0)	0 (0 to 0)	0.0 (0.0 to 0.0)	0 (0 to 0)	0.0 (0.0 to 0.0)	0.0 (0.0 to 0.0)	0.0 (0.0 to 0.0)
Andorra	Zika virus	5 to 9	Female	0 (0 to 0)	0.0 (0.0 to 0.0)	0 (0 to 0)	0.0 (0.0 to 0.0)	0 (0 to 0)	0.0 (0.0 to 0.0)	0.0 (0.0 to 0.0)	0.0 (0.0 to 0.0)
Andorra	Zika virus	5 to 9	Male	0 (0 to 0)	0.0 (0.0 to 0.0)	0 (0 to 0)	0.0 (0.0 to 0.0)	0 (0 to 0)	0.0 (0.0 to 0.0)	0.0 (0.0 to 0.0)	0.0 (0.0 to 0.0)
Andorra	Zika virus	10 to 14	Both	0 (0 to 0)	0.0 (0.0 to 0.0)	0 (0 to 0)	0.0 (0.0 to 0.0)	0 (0 to 0)	0.0 (0.0 to 0.0)	0.0 (0.0 to 0.0)	0.0 (0.0 to 0.0)
Andorra	Zika virus	10 to 14	Female	0 (0 to 0)	0.0 (0.0 to 0.0)	0 (0 to 0)	0.0 (0.0 to 0.0)	0 (0 to 0)	0.0 (0.0 to 0.0)	0.0 (0.0 to 0.0)	0.0 (0.0 to 0.0)
Andorra	Zika virus	10 to 14	Male	0 (0 to 0)	0.0 (0.0 to 0.0)	0 (0 to 0)	0.0 (0.0 to 0.0)	0 (0 to 0)	0.0 (0.0 to 0.0)	0.0 (0.0 to 0.0)	0.0 (0.0 to 0.0)
Andorra	Other neglected tropical diseases	5 to 9	Both	0 (0 to 0)	0.0 (0.0 to 0.0)	0 (0 to 0)	0.0 (0.0 to 0.0)	0 (0 to 0)	0.0 (0.0 to 0.0)	25.7 (-3.4 to 65.8)	-6.9 (-28.4 to 22.8)
Andorra	Other neglected tropical diseases	5 to 9	Female	0 (0 to 0)	0.0 (0.0 to 0.0)	0 (0 to 0)	0.0 (0.0 to 0.0)	0 (0 to 0)	0.0 (0.0 to 0.0)	25.5 (-4.7 to 67.3)	-7.1 (-29.4 to 23.9)
Andorra	Other neglected tropical diseases	5 to 9	Male	0 (0 to 0)	0.0 (0.0 to 0.0)	0 (0 to 0)	0.0 (0.0 to 0.0)	0 (0 to 0)	0.0 (0.0 to 0.0)	25.8 (-11.3 to 79.5)	-6.8 (-34.3 to 32.9)
Andorra	Other neglected tropical diseases	10 to 14	Both	0 (0 to 0)	0.0 (0.0 to 0.0)	0 (0 to 0)	0.0 (0.0 to 0.0)	0 (0 to 0)	0.0 (0.0 to 0.0)	23.0 (-4.6 to 55.8)	-3.7 (-25.3 to 22.0)
Andorra	Other neglected tropical diseases	10 to 14	Female	0 (0 to 0)	0.0 (0.0 to 0.0)	0 (0 to 0)	0.0 (0.0 to 0.0)	0 (0 to 0)	0.0 (0.0 to 0.0)	20.4 (-13.3 to 68.9)	-5.7 (-32.1 to 32.2)
Andorra	Other neglected tropical diseases	10 to 14	Male	0 (0 to 0)	0.0 (0.0 to 0.0)	0 (0 to 0)	0.0 (0.0 to 0.0)	0 (0 to 0)	0.0 (0.0 to 0.0)	25.5 (-13.9 to 72.2)	-1.7 (-32.6 to 34.9)
Andorra	Maternal hemorrhage	10 to 14	Both	0 (0 to 0)	0.0 (0.0 to 0.0)	0 (0 to 0)	0.0 (0.0 to 0.0)	0 (0 to 0)	0.0 (0.0 to 0.0)	14.0 (-50.7 to 178.2)	-10.8 (-61.4 to 117.8)

eTable 8. All-cause and cause-specific mortality (counts and rates) at the global level and by country in the WHO European Region for ages 5 to 9 and 10 to 14 for 1990, 2000, and 2016

Andorra	Maternal hemorrhage	10 to 14	Female	0<br>(0 to 0)	0.0<br>(0.0 to 0.0)	0<br>(0 to 0)	0.0<br>(0.0 to 0.0)	0<br>(0 to 0)	0.0<br>(0.0 to 0.0)	14.0<br>(-50.7 to 178.2)	-10.8<br>(-61.4 to 117.8)
Andorra	Maternal sepsis and other maternal infections	10 to 14	Both	0<br>(0 to 0)	0.0<br>(0.0 to 0.0)	0<br>(0 to 0)	0.0<br>(0.0 to 0.0)	0<br>(0 to 0)	0.0<br>(0.0 to 0.0)	9.2<br>(-55.7 to 180.7)	-14.5<br>(-65.3 to 119.8)
Andorra	Maternal sepsis and other maternal infections	10 to 14	Female	0<br>(0 to 0)	0.0<br>(0.0 to 0.0)	0<br>(0 to 0)	0.0<br>(0.0 to 0.0)	0<br>(0 to 0)	0.0<br>(0.0 to 0.0)	9.2<br>(-55.7 to 180.7)	-14.5<br>(-65.3 to 119.8)
Andorra	Maternal hypertensive disorders	10 to 14	Both	0<br>(0 to 0)	0.0<br>(0.0 to 0.0)	0<br>(0 to 0)	0.0<br>(0.0 to 0.0)	0<br>(0 to 0)	0.0<br>(0.0 to 0.0)	16.1<br>(-50.9 to 172.2)	-9.1<br>(-61.6 to 113.1)
Andorra	Maternal hypertensive disorders	10 to 14	Female	0<br>(0 to 0)	0.0<br>(0.0 to 0.0)	0<br>(0 to 0)	0.0<br>(0.0 to 0.0)	0<br>(0 to 0)	0.0<br>(0.0 to 0.0)	16.1<br>(-50.9 to 172.2)	-9.1<br>(-61.6 to 113.1)
Andorra	Maternal obstructed labor and uterine rupture	10 to 14	Both	0<br>(0 to 0)	0.0<br>(0.0 to 0.0)	0<br>(0 to 0)	0.0<br>(0.0 to 0.0)	0<br>(0 to 0)	0.0<br>(0.0 to 0.0)	13.9<br>(-52.0 to 190.7)	-10.8<br>(-62.4 to 127.6)
Andorra	Maternal obstructed labor and uterine rupture	10 to 14	Female	0<br>(0 to 0)	0.0<br>(0.0 to 0.0)	0<br>(0 to 0)	0.0<br>(0.0 to 0.0)	0<br>(0 to 0)	0.0<br>(0.0 to 0.0)	13.9<br>(-52.0 to 190.7)	-10.8<br>(-62.4 to 127.6)
Andorra	Maternal abortion, miscarriage, and ectopic pregnancy	10 to 14	Both	0<br>(0 to 0)	0.0<br>(0.0 to 0.0)	0<br>(0 to 0)	0.0<br>(0.0 to 0.0)	0<br>(0 to 0)	0.0<br>(0.0 to 0.0)	5.5<br>(-57.1 to 149.5)	-17.4<br>(-66.4 to 95.4)
Andorra	Maternal abortion, miscarriage, and ectopic pregnancy	10 to 14	Female	0<br>(0 to 0)	0.0<br>(0.0 to 0.0)	0<br>(0 to 0)	0.0<br>(0.0 to 0.0)	0<br>(0 to 0)	0.0<br>(0.0 to 0.0)	5.5<br>(-57.1 to 149.5)	-17.4<br>(-66.4 to 95.3)
Andorra	Indirect maternal deaths	10 to 14	Both	0<br>(0 to 0)	0.0<br>(0.0 to 0.0)	0<br>(0 to 0)	0.0<br>(0.0 to 0.0)	0<br>(0 to 0)	0.0<br>(0.0 to 0.0)	7.9<br>(-53.4 to 157.9)	-15.6<br>(-63.5 to 101.9)
Andorra	Indirect maternal deaths	10 to 14	Female	0<br>(0 to 0)	0.0<br>(0.0 to 0.0)	0<br>(0 to 0)	0.0<br>(0.0 to 0.0)	0<br>(0 to 0)	0.0<br>(0.0 to 0.0)	7.9<br>(-53.4 to 157.9)	-15.6<br>(-63.5 to 101.9)
Andorra	Late maternal deaths	10 to 14	Both	0<br>(0 to 0)	0.0<br>(0.0 to 0.0)	0<br>(0 to 0)	0.0<br>(0.0 to 0.0)	0<br>(0 to 0)	0.0<br>(0.0 to 0.0)	6.8<br>(-55.1 to 157.2)	-16.4<br>(-64.8 to 101.3)
Andorra	Late maternal deaths	10 to 14	Female	0<br>(0 to 0)	0.0<br>(0.0 to 0.0)	0<br>(0 to 0)	0.0<br>(0.0 to 0.0)	0<br>(0 to 0)	0.0<br>(0.0 to 0.0)	6.8<br>(-55.1 to 157.2)	-16.4<br>(-64.8 to 101.3)
Andorra	Maternal deaths aggravated by HIV/AIDS	10 to 14	Both	0<br>(0 to 0)	0.0<br>(0.0 to 0.0)	0<br>(0 to 0)	0.0<br>(0.0 to 0.0)	0<br>(0 to 0)	0.0<br>(0.0 to 0.0)	259.5<br>(-74.2 to 1542.3)	181.5<br>(-79.8 to 1185.8)
Andorra	Maternal deaths aggravated by HIV/AIDS	10 to 14	Female	0<br>(0 to 0)	0.0<br>(0.0 to 0.0)	0<br>(0 to 0)	0.0<br>(0.0 to 0.0)	0<br>(0 to 0)	0.0<br>(0.0 to 0.0)	259.5<br>(-74.2 to 1542.3)	181.4<br>(-79.8 to 1185.7)
Andorra	Other maternal disorders	10 to 14	Both	0<br>(0 to 0)	0.0<br>(0.0 to 0.0)	0<br>(0 to 0)	0.0<br>(0.0 to 0.0)	0<br>(0 to 0)	0.0<br>(0.0 to 0.0)	5.9<br>(-53.2 to 152.3)	-17.1<br>(-63.4 to 97.5)
Andorra	Other maternal disorders	10 to 14	Female	0<br>(0 to 0)	0.0<br>(0.0 to 0.0)	0<br>(0 to 0)	0.0<br>(0.0 to 0.0)	0<br>(0 to 0)	0.0<br>(0.0 to 0.0)	5.9<br>(-53.2 to 152.3)	-17.1<br>(-63.4 to 97.5)
Andorra	Protein-energy malnutrition	5 to 9	Both	0<br>(0 to 0)	0.0<br>(0.0 to 0.0)	0<br>(0 to 0)	0.0<br>(0.0 to 0.0)	0<br>(0 to 0)	0.0<br>(0.0 to 0.0)	26.8<br>(-14.8 to 108.6)	-6.1<br>(-36.9 to 54.5)

eTable 8. All-cause and cause-specific mortality (counts and rates) at the global level and by country in the WHO European Region for ages 5 to 9 and 10 to 14 for 1990, 2000, and 2016

Andorra	Protein-energy malnutrition	5 to 9	Female	0 (0 to 0)	0.0 (0.0 to 0.0)	0 (0 to 0)	0.0 (0.0 to 0.0)	0 (0 to 0)	0.0 (0.0 to 0.0)	25.8 (-32.7 to 183.7)	-6.9 (-50.2 to 110.1)
Andorra	Protein-energy malnutrition	5 to 9	Male	0 (0 to 0)	0.0 (0.0 to 0.0)	0 (0 to 0)	0.0 (0.0 to 0.0)	0 (0 to 0)	0.0 (0.0 to 0.0)	27.9 (-34.4 to 163.9)	-5.3 (-51.4 to 95.5)
Andorra	Protein-energy malnutrition	10 to 14	Both	0 (0 to 0)	0.0 (0.0 to 0.0)	0 (0 to 0)	0.0 (0.0 to 0.0)	0 (0 to 0)	0.0 (0.0 to 0.0)	24.0 (-7.0 to 72.3)	-2.9 (-27.2 to 34.9)
Andorra	Protein-energy malnutrition	10 to 14	Female	0 (0 to 0)	0.0 (0.0 to 0.0)	0 (0 to 0)	0.0 (0.0 to 0.0)	0 (0 to 0)	0.0 (0.0 to 0.0)	20.9 (-20.0 to 89.5)	-5.3 (-37.3 to 48.4)
Andorra	Protein-energy malnutrition	10 to 14	Male	0 (0 to 0)	0.0 (0.0 to 0.0)	0 (0 to 0)	0.0 (0.0 to 0.0)	0 (0 to 0)	0.0 (0.0 to 0.0)	27.0 (-16.0 to 103.9)	-0.6 (-34.2 to 59.7)
Andorra	Iodine deficiency	5 to 9	Both	0 (0 to 0)	0.0 (0.0 to 0.0)	0 (0 to 0)	0.0 (0.0 to 0.0)	0 (0 to 0)	0.0 (0.0 to 0.0)	7.6 (-100.0 to 1.0e+18)	-20.3 (-100.0 to 7.7e+17)
Andorra	Iodine deficiency	5 to 9	Female	0 (0 to 0)	0.0 (0.0 to 0.0)	0 (0 to 0)	0.0 (0.0 to 0.0)	0 (0 to 0)	0.0 (0.0 to 0.0)	8.3 (-100.0 to 4.1e+24)	-19.8 (-100.0 to 3.0e+24)
Andorra	Iodine deficiency	5 to 9	Male	0 (0 to 0)	0.0 (0.0 to 0.0)	0 (0 to 0)	0.0 (0.0 to 0.0)	0 (0 to 0)	0.0 (0.0 to 0.0)	6.9 (-100.0 to 7.3e+22)	-20.8 (-100.0 to 5.4e+22)
Andorra	Iodine deficiency	10 to 14	Both	0 (0 to 0)	0.0 (0.0 to 0.0)	0 (0 to 0)	0.0 (0.0 to 0.0)	0 (0 to 0)	0.0 (0.0 to 0.0)	-10.8 (-100.0 to 7.6e+17)	-30.1 (-100.0 to 5.9e+17)
Andorra	Iodine deficiency	10 to 14	Female	0 (0 to 0)	0.0 (0.0 to 0.0)	0 (0 to 0)	0.0 (0.0 to 0.0)	0 (0 to 0)	0.0 (0.0 to 0.0)	-16.1 (-100.0 to 1.5e+27)	-34.3 (-100.0 to 1.2e+27)
Andorra	Iodine deficiency	10 to 14	Male	0 (0 to 0)	0.0 (0.0 to 0.0)	0 (0 to 0)	0.0 (0.0 to 0.0)	0 (0 to 0)	0.0 (0.0 to 0.0)	-4.2 (-100.0 to 1.3e+25)	-25.0 (-100.0 to 1.0e+25)
Andorra	Iron-deficiency anemia	5 to 9	Both	0 (0 to 0)	0.0 (0.0 to 0.0)	0 (0 to 0)	0.0 (0.0 to 0.0)	0 (0 to 0)	0.0 (0.0 to 0.0)	26.8 (-14.8 to 108.6)	-6.1 (-36.9 to 54.5)
Andorra	Iron-deficiency anemia	5 to 9	Female	0 (0 to 0)	0.0 (0.0 to 0.0)	0 (0 to 0)	0.0 (0.0 to 0.0)	0 (0 to 0)	0.0 (0.0 to 0.0)	25.7 (-32.7 to 183.7)	-6.9 (-50.2 to 110.1)
Andorra	Iron-deficiency anemia	5 to 9	Male	0 (0 to 0)	0.0 (0.0 to 0.0)	0 (0 to 0)	0.0 (0.0 to 0.0)	0 (0 to 0)	0.0 (0.0 to 0.0)	27.9 (-34.4 to 163.9)	-5.3 (-51.4 to 95.5)
Andorra	Iron-deficiency anemia	10 to 14	Both	0 (0 to 0)	0.0 (0.0 to 0.0)	0 (0 to 0)	0.0 (0.0 to 0.0)	0 (0 to 0)	0.0 (0.0 to 0.0)	24.0 (-7.0 to 72.3)	-2.9 (-27.2 to 34.9)
Andorra	Iron-deficiency anemia	10 to 14	Female	0 (0 to 0)	0.0 (0.0 to 0.0)	0 (0 to 0)	0.0 (0.0 to 0.0)	0 (0 to 0)	0.0 (0.0 to 0.0)	20.9 (-20.0 to 89.5)	-5.3 (-37.3 to 48.4)
Andorra	Iron-deficiency anemia	10 to 14	Male	0 (0 to 0)	0.0 (0.0 to 0.0)	0 (0 to 0)	0.0 (0.0 to 0.0)	0 (0 to 0)	0.0 (0.0 to 0.0)	27.0 (-16.0 to 103.9)	-0.6 (-34.2 to 59.7)
Andorra	Other nutritional deficiencies	5 to 9	Both	0 (0 to 0)	0.0 (0.0 to 0.0)	0 (0 to 0)	0.0 (0.0 to 0.0)	0 (0 to 0)	0.0 (0.0 to 0.0)	26.7 (-14.8 to 108.6)	-6.1 (-36.9 to 54.5)
Andorra	Other nutritional deficiencies	5 to 9	Female	0 (0 to 0)	0.0 (0.0 to 0.0)	0 (0 to 0)	0.0 (0.0 to 0.0)	0 (0 to 0)	0.0 (0.0 to 0.0)	25.6 (-32.7 to 183.7)	-7.0 (-50.2 to 110.1)
Andorra	Other nutritional deficiencies	5 to 9	Male	0 (0 to 0)	0.0 (0.0 to 0.0)	0 (0 to 0)	0.0 (0.0 to 0.0)	0 (0 to 0)	0.0 (0.0 to 0.0)	27.9 (-34.4 to 163.9)	-5.3 (-51.4 to 95.5)
Andorra	Other nutritional deficiencies	10 to 14	Both	0 (0 to 0)	0.0 (0.0 to 0.0)	0 (0 to 0)	0.0 (0.0 to 0.0)	0 (0 to 0)	0.0 (0.0 to 0.0)	23.8 (-7.2 to 72.3)	-3.1 (-27.3 to 34.9)
Andorra	Other nutritional deficiencies	10 to 14	Female	0 (0 to 0)	0.0 (0.0 to 0.0)	0 (0 to 0)	0.0 (0.0 to 0.0)	0 (0 to 0)	0.0 (0.0 to 0.0)	20.4 (-20.0 to 89.5)	-5.7 (-37.4 to 48.4)
Andorra	Other nutritional deficiencies	10 to 14	Male	0 (0 to 0)	0.0 (0.0 to 0.0)	0 (0 to 0)	0.0 (0.0 to 0.0)	0 (0 to 0)	0.0 (0.0 to 0.0)	27.0 (-16.0 to 103.9)	-0.6 (-34.2 to 59.7)

eTable 8. All-cause and cause-specific mortality (counts and rates) at the global level and by country in the WHO European Region for ages 5 to 9 and 10 to 14 for 1990, 2000, and 2016

Andorra	Sexually transmitted diseases excluding HIV	5 to 9	Both	0 (0 to 0)	0.0 (0.0 to 0.0)	0 (0 to 0)	0.0 (0.0 to 0.0)	0 (0 to 0)	0.0 (0.0 to 0.0)	-67.5 (-84.0 to -40.8)	-76.0 (-88.1 to -56.2)
Andorra	Sexually transmitted diseases excluding HIV	5 to 9	Female	0 (0 to 0)	0.0 (0.0 to 0.0)	0 (0 to 0)	0.0 (0.0 to 0.0)	0 (0 to 0)	0.0 (0.0 to 0.0)	-67.6 (-84.4 to -40.6)	-76.0 (-88.5 to -56.0)
Andorra	Sexually transmitted diseases excluding HIV	5 to 9	Male	0 (0 to 0)	0.0 (0.0 to 0.0)	0 (0 to 0)	0.0 (0.0 to 0.0)	0 (0 to 0)	0.0 (0.0 to 0.0)	-67.4 (-83.9 to -37.5)	-75.9 (-88.1 to -53.7)
Andorra	Sexually transmitted diseases excluding HIV	10 to 14	Both	0 (0 to 0)	0.0 (0.0 to 0.0)	0 (0 to 0)	0.0 (0.0 to 0.0)	0 (0 to 0)	0.0 (0.0 to 0.0)	23.1 (-4.6 to 56.6)	-3.7 (-25.3 to 22.6)
Andorra	Sexually transmitted diseases excluding HIV	10 to 14	Female	0 (0 to 0)	0.0 (0.0 to 0.0)	0 (0 to 0)	0.0 (0.0 to 0.0)	0 (0 to 0)	0.0 (0.0 to 0.0)	20.5 (-13.3 to 69.5)	-5.6 (-32.1 to 32.7)
Andorra	Sexually transmitted diseases excluding HIV	10 to 14	Male	0 (0 to 0)	0.0 (0.0 to 0.0)	0 (0 to 0)	0.0 (0.0 to 0.0)	0 (0 to 0)	0.0 (0.0 to 0.0)	25.5 (-13.9 to 72.2)	-1.7 (-32.6 to 34.9)
Andorra	Hepatitis	5 to 9	Both	0 (0 to 0)	0.0 (0.0 to 0.0)	0 (0 to 0)	0.0 (0.0 to 0.0)	0 (0 to 0)	0.0 (0.0 to 0.0)	-37.0 (-69.5 to 25.9)	-53.3 (-77.4 to -6.8)
Andorra	Hepatitis	5 to 9	Female	0 (0 to 0)	0.0 (0.0 to 0.0)	0 (0 to 0)	0.0 (0.0 to 0.0)	0 (0 to 0)	0.0 (0.0 to 0.0)	-41.3 (-72.0 to 26.4)	-56.5 (-79.2 to -6.4)
Andorra	Hepatitis	5 to 9	Male	0 (0 to 0)	0.0 (0.0 to 0.0)	0 (0 to 0)	0.0 (0.0 to 0.0)	0 (0 to 0)	0.0 (0.0 to 0.0)	-30.5 (-81.3 to 137.4)	-48.5 (-86.2 to 75.9)
Andorra	Hepatitis	10 to 14	Both	0 (0 to 0)	0.0 (0.0 to 0.0)	0 (0 to 0)	0.0 (0.0 to 0.0)	0 (0 to 0)	0.0 (0.0 to 0.0)	-36.0 (-67.5 to 25.2)	-49.9 (-74.5 to -1.9)
Andorra	Hepatitis	10 to 14	Female	0 (0 to 0)	0.0 (0.0 to 0.0)	0 (0 to 0)	0.0 (0.0 to 0.0)	0 (0 to 0)	0.0 (0.0 to 0.0)	-41.0 (-72.9 to 30.0)	-53.8 (-78.8 to 1.8)
Andorra	Hepatitis	10 to 14	Male	0 (0 to 0)	0.0 (0.0 to 0.0)	0 (0 to 0)	0.0 (0.0 to 0.0)	0 (0 to 0)	0.0 (0.0 to 0.0)	-21.8 (-77.1 to 168.7)	-38.8 (-82.0 to 110.4)
Andorra	Other infectious diseases	5 to 9	Both	0 (0 to 0)	0.1 (0.0 to 0.2)	0 (0 to 0)	0.1 (0.0 to 0.2)	0 (0 to 0)	0.1 (0.0 to 0.2)	19.5 (-60.4 to 241.9)	-11.5 (-70.6 to 153.2)
Andorra	Other infectious diseases	5 to 9	Female	0 (0 to 0)	0.0 (0.0 to 0.0)	0 (0 to 0)	0.0 (0.0 to 0.0)	0 (0 to 0)	0.0 (0.0 to 0.0)	28.9 (-53.8 to 262.8)	-4.6 (-65.8 to 168.7)
Andorra	Other infectious diseases	5 to 9	Male	0 (0 to 0)	0.1 (0.1 to 0.3)	0 (0 to 0)	0.1 (0.0 to 0.3)	0 (0 to 0)	0.1 (0.0 to 0.3)	17.9 (-68.2 to 311.7)	-12.7 (-76.4 to 205.0)
Andorra	Other infectious diseases	10 to 14	Both	0 (0 to 0)	0.1 (0.0 to 0.1)	0 (0 to 0)	0.1 (0.0 to 0.1)	0 (0 to 0)	0.1 (0.0 to 0.1)	18.1 (-50.5 to 174.8)	-7.6 (-61.2 to 115.2)
Andorra	Other infectious diseases	10 to 14	Female	0 (0 to 0)	0.0 (0.0 to 0.0)	0 (0 to 0)	0.0 (0.0 to 0.0)	0 (0 to 0)	0.0 (0.0 to 0.1)	29.0 (-49.6 to 296.7)	1.0 (-60.5 to 210.6)
Andorra	Other infectious diseases	10 to 14	Male	0 (0 to 0)	0.1 (0.0 to 0.2)	0 (0 to 0)	0.1 (0.0 to 0.2)	0 (0 to 0)	0.1 (0.0 to 0.2)	15.7 (-59.4 to 223.1)	-9.4 (-68.2 to 153.0)
Andorra	Nasopharynx cancer	5 to 9	Both	0 (0 to 0)	0.0 (0.0 to 0.0)	0 (0 to 0)	0.0 (0.0 to 0.0)	0 (0 to 0)	0.0 (0.0 to 0.0)	5.5 (-36.5 to 63.2)	-21.9 (-52.9 to 20.9)
Andorra	Nasopharynx cancer	5 to 9	Female	0 (0 to 0)	0.0 (0.0 to 0.0)	0 (0 to 0)	0.0 (0.0 to 0.0)	0 (0 to 0)	0.0 (0.0 to 0.0)	-4.6 (-52.8 to 77.8)	-29.3 (-65.0 to 31.7)

eTable 8. All-cause and cause-specific mortality (counts and rates) at the global level and by country in the WHO European Region for ages 5 to 9 and 10 to 14 for 1990, 2000, and 2016

Andorra	Nasopharynx cancer	5 to 9	Male	0 (0 to 0)	0.0 (0.0 to 0.0)	0 (0 to 0)	0.0 (0.0 to 0.0)	0 (0 to 0)	0.0 (0.0 to 0.0)	17.8 (-33.3 to 83.1)	-12.8 (-50.6 to 35.6)
Andorra	Nasopharynx cancer	10 to 14	Both	0 (0 to 0)	0.0 (0.0 to 0.1)	0 (0 to 0)	0.0 (0.0 to 0.0)	0 (0 to 0)	0.0 (0.0 to 0.0)	-13.9 (-60.7 to 97.1)	-32.6 (-69.3 to 54.3)
Andorra	Nasopharynx cancer	10 to 14	Female	0 (0 to 0)	0.0 (0.0 to 0.0)	0 (0 to 0)	0.0 (0.0 to 0.0)	0 (0 to 0)	0.0 (0.0 to 0.0)	-2.2 (-48.5 to 75.8)	-23.4 (-59.7 to 37.6)
Andorra	Nasopharynx cancer	10 to 14	Male	0 (0 to 0)	0.1 (0.0 to 0.1)	0 (0 to 0)	0.0 (0.0 to 0.1)	0 (0 to 0)	0.0 (0.0 to 0.1)	-16.9 (-69.6 to 123.8)	-34.9 (-76.2 to 75.2)
Andorra	Liver cancer	5 to 9	Both	0 (0 to 0)	0.0 (0.0 to 0.0)	0 (0 to 0)	0.0 (0.0 to 0.0)	0 (0 to 0)	0.0 (0.0 to 0.0)	20.6 (-36.2 to 139.1)	-10.7 (-52.7 to 77.1)
Andorra	Liver cancer	5 to 9	Female	0 (0 to 0)	0.0 (0.0 to 0.0)	0 (0 to 0)	0.0 (0.0 to 0.0)	0 (0 to 0)	0.0 (0.0 to 0.0)	30.6 (-40.7 to 228.4)	-3.3 (-56.1 to 143.2)
Andorra	Liver cancer	5 to 9	Male	0 (0 to 0)	0.0 (0.0 to 0.0)	0 (0 to 0)	0.0 (0.0 to 0.0)	0 (0 to 0)	0.0 (0.0 to 0.0)	11.6 (-53.5 to 184.6)	-17.3 (-65.5 to 110.8)
Andorra	Liver cancer	10 to 14	Both	0 (0 to 0)	0.0 (0.0 to 0.1)	0 (0 to 0)	0.0 (0.0 to 0.1)	0 (0 to 0)	0.0 (0.0 to 0.1)	25.3 (-33.6 to 138.8)	-1.9 (-48.0 to 86.9)
Andorra	Liver cancer	10 to 14	Female	0 (0 to 0)	0.0 (0.0 to 0.1)	0 (0 to 0)	0.0 (0.0 to 0.0)	0 (0 to 0)	0.0 (0.0 to 0.1)	33.2 (-44.8 to 245.7)	4.3 (-56.8 to 170.7)
Andorra	Liver cancer	10 to 14	Male	0 (0 to 0)	0.0 (0.0 to 0.1)	0 (0 to 0)	0.0 (0.0 to 0.1)	0 (0 to 0)	0.0 (0.0 to 0.1)	20.9 (-47.2 to 185.4)	-5.3 (-58.7 to 123.4)
Andorra	Kidney cancer	5 to 9	Both	0 (0 to 0)	0.2 (0.1 to 0.2)	0 (0 to 0)	0.1 (0.1 to 0.2)	0 (0 to 0)	0.1 (0.1 to 0.2)	6.6 (-43.9 to 117.9)	-21.0 (-58.5 to 61.4)
Andorra	Kidney cancer	5 to 9	Female	0 (0 to 0)	0.2 (0.1 to 0.3)	0 (0 to 0)	0.1 (0.1 to 0.2)	0 (0 to 0)	0.2 (0.1 to 0.3)	11.6 (-50.0 to 189.0)	-17.4 (-63.0 to 114.0)
Andorra	Kidney cancer	5 to 9	Male	0 (0 to 0)	0.1 (0.1 to 0.2)	0 (0 to 0)	0.1 (0.0 to 0.2)	0 (0 to 0)	0.1 (0.0 to 0.2)	-0.2 (-56.0 to 148.4)	-26.0 (-67.4 to 84.0)
Andorra	Kidney cancer	10 to 14	Both	0 (0 to 0)	0.1 (0.0 to 0.1)	0 (0 to 0)	0.0 (0.0 to 0.1)	0 (0 to 0)	0.1 (0.0 to 0.1)	12.5 (-36.7 to 112.1)	-11.9 (-50.4 to 66.0)
Andorra	Kidney cancer	10 to 14	Female	0 (0 to 0)	0.1 (0.0 to 0.1)	0 (0 to 0)	0.1 (0.0 to 0.1)	0 (0 to 0)	0.1 (0.0 to 0.1)	12.9 (-51.9 to 175.0)	-11.6 (-62.3 to 115.3)
Andorra	Kidney cancer	10 to 14	Male	0 (0 to 0)	0.0 (0.0 to 0.1)	0 (0 to 0)	0.0 (0.0 to 0.1)	0 (0 to 0)	0.0 (0.0 to 0.1)	11.9 (-47.2 to 140.2)	-12.4 (-58.7 to 88.1)
Andorra	Brain and nervous system cancer	5 to 9	Both	0 (0 to 0)	1.4 (0.9 to 1.9)	0 (0 to 0)	1.1 (0.7 to 1.4)	0 (0 to 0)	1.2 (0.8 to 1.6)	14.0 (-26.9 to 83.2)	-15.5 (-45.9 to 35.7)
Andorra	Brain and nervous system cancer	5 to 9	Female	0 (0 to 0)	1.3 (0.8 to 1.9)	0 (0 to 0)	0.9 (0.6 to 1.4)	0 (0 to 0)	1.1 (0.6 to 1.5)	12.1 (-38.9 to 98.2)	-17.0 (-54.7 to 46.8)
Andorra	Brain and nervous system cancer	5 to 9	Male	0 (0 to 0)	1.5 (0.9 to 2.2)	0 (0 to 0)	1.2 (0.7 to 1.7)	0 (0 to 0)	1.3 (0.8 to 1.9)	15.7 (-38.2 to 113.1)	-14.3 (-54.2 to 57.8)
Andorra	Brain and nervous system cancer	10 to 14	Both	0 (0 to 0)	1.0 (0.6 to 1.3)	0 (0 to 0)	0.8 (0.5 to 1.1)	0 (0 to 0)	0.9 (0.6 to 1.2)	16.8 (-25.3 to 86.6)	-8.6 (-41.5 to 46.1)
Andorra	Brain and nervous system cancer	10 to 14	Female	0 (0 to 0)	0.9 (0.5 to 1.3)	0 (0 to 0)	0.7 (0.4 to 1.1)	0 (0 to 0)	0.8 (0.5 to 1.2)	12.8 (-35.3 to 120.5)	-11.7 (-49.4 to 72.6)
Andorra	Brain and nervous system cancer	10 to 14	Male	0 (0 to 0)	1.1 (0.6 to 1.6)	0 (0 to 0)	0.9 (0.5 to 1.3)	0 (0 to 0)	1.0 (0.6 to 1.5)	19.9 (-36.2 to 136.3)	-6.1 (-50.1 to 85.0)
Andorra	Thyroid cancer	10 to 14	Both	0 (0 to 0)	0.0 (0.0 to 0.0)	0 (0 to 0)	0.0 (0.0 to 0.0)	0 (0 to 0)	0.0 (0.0 to 0.0)	21.3 (-11.3 to 61.1)	-5.0 (-30.6 to 26.1)
Andorra	Thyroid cancer	10 to 14	Female	0 (0 to 0)	0.0 (0.0 to 0.0)	0 (0 to 0)	0.0 (0.0 to 0.0)	0 (0 to 0)	0.0 (0.0 to 0.0)	18.0 (-24.6 to 82.4)	-7.6 (-41.0 to 42.8)
Andorra	Thyroid cancer	10 to 14	Male	0 (0 to 0)	0.0 (0.0 to 0.0)	0 (0 to 0)	0.0 (0.0 to 0.0)	0 (0 to 0)	0.0 (0.0 to 0.0)	24.6 (-15.5 to 72.0)	-2.5 (-33.8 to 34.7)
Andorra	Hodgkin lymphoma	5 to 9	Both	0 (0 to 0)	0.0 (0.0 to 0.0)	0 (0 to 0)	0.0 (0.0 to 0.0)	0 (0 to 0)	0.0 (0.0 to 0.0)	4.0 (-33.9 to 68.6)	-23.0 (-51.0 to 24.9)
Andorra	Hodgkin lymphoma	5 to 9	Female	0 (0 to 0)	0.0 (0.0 to 0.0)	0 (0 to 0)	0.0 (0.0 to 0.0)	0 (0 to 0)	0.0 (0.0 to 0.0)	23.5 (-11.2 to 67.5)	-8.5 (-34.2 to 24.0)
Andorra	Hodgkin lymphoma	5 to 9	Male	0 (0 to 0)	0.0 (0.0 to 0.0)	0 (0 to 0)	0.0 (0.0 to 0.0)	0 (0 to 0)	0.0 (0.0 to 0.0)	-30.1 (-79.1 to 174.6)	-48.2 (-84.5 to 103.4)
Andorra	Hodgkin lymphoma	10 to 14	Both	0 (0 to 0)	0.0 (0.0 to 0.0)	0 (0 to 0)	0.0 (0.0 to 0.0)	0 (0 to 0)	0.0 (0.0 to 0.0)	-20.5 (-54.0 to 41.9)	-37.7 (-64.0 to 11.1)

eTable 8. All-cause and cause-specific mortality (counts and rates) at the global level and by country in the WHO European Region for ages 5 to 9 and 10 to 14 for 1990, 2000, and 2016

Andorra	Hodgkin lymphoma	10 to 14	Female	0 (0 to 0)	0.0 (0.0 to 0.0)	0 (0 to 0)	0.0 (0.0 to 0.0)	0 (0 to 0)	0.0 (0.0 to 0.0)	5.4 (-38.1 to 73.0)	-17.5 (-51.5 to 35.5)
Andorra	Hodgkin lymphoma	10 to 14	Male	0 (0 to 0)	0.0 (0.0 to 0.1)	0 (0 to 0)	0.0 (0.0 to 0.0)	0 (0 to 0)	0.0 (0.0 to 0.0)	-28.7 (-68.2 to 59.6)	-44.2 (-75.1 to 24.9)
Andorra	Non-Hodgkin lymphoma	5 to 9	Both	0 (0 to 0)	0.2 (0.1 to 0.4)	0 (0 to 0)	0.2 (0.1 to 0.3)	0 (0 to 0)	0.2 (0.1 to 0.3)	-3.4 (-51.1 to 79.0)	-28.5 (-63.8 to 32.5)
Andorra	Non-Hodgkin lymphoma	5 to 9	Female	0 (0 to 0)	0.1 (0.0 to 0.2)	0 (0 to 0)	0.1 (0.0 to 0.1)	0 (0 to 0)	0.1 (0.0 to 0.1)	18.4 (-48.4 to 185.4)	-12.3 (-61.8 to 111.3)
Andorra	Non-Hodgkin lymphoma	5 to 9	Male	0 (0 to 0)	0.4 (0.2 to 0.6)	0 (0 to 0)	0.3 (0.2 to 0.5)	0 (0 to 0)	0.3 (0.1 to 0.4)	-8.3 (-59.7 to 86.0)	-32.1 (-70.2 to 37.8)
Andorra	Non-Hodgkin lymphoma	10 to 14	Both	0 (0 to 0)	0.2 (0.1 to 0.4)	0 (0 to 0)	0.2 (0.1 to 0.3)	0 (0 to 0)	0.2 (0.1 to 0.3)	-4.3 (-50.0 to 72.4)	-25.1 (-60.8 to 35.0)
Andorra	Non-Hodgkin lymphoma	10 to 14	Female	0 (0 to 0)	0.1 (0.1 to 0.2)	0 (0 to 0)	0.1 (0.0 to 0.2)	0 (0 to 0)	0.1 (0.0 to 0.2)	5.8 (-55.8 to 155.4)	-17.2 (-65.4 to 99.9)
Andorra	Non-Hodgkin lymphoma	10 to 14	Male	0 (0 to 0)	0.4 (0.2 to 0.6)	0 (0 to 0)	0.3 (0.1 to 0.5)	0 (0 to 0)	0.3 (0.1 to 0.4)	-7.3 (-60.3 to 95.7)	-27.4 (-68.9 to 53.2)
Andorra	Leukemia	5 to 9	Both	0 (0 to 0)	1.3 (0.9 to 1.9)	0 (0 to 0)	0.9 (0.6 to 1.3)	0 (0 to 0)	0.8 (0.5 to 1.2)	-18.8 (-52.6 to 37.1)	-39.8 (-64.9 to 1.5)
Andorra	Leukemia	5 to 9	Female	0 (0 to 0)	1.0 (0.6 to 1.7)	0 (0 to 0)	0.6 (0.3 to 1.1)	0 (0 to 0)	0.6 (0.3 to 1.0)	-18.9 (-59.8 to 52.9)	-39.9 (-70.2 to 13.2)
Andorra	Leukemia	5 to 9	Male	0 (0 to 0)	1.6 (0.9 to 2.6)	0 (0 to 0)	1.1 (0.6 to 1.7)	0 (0 to 0)	1.0 (0.6 to 1.6)	-18.7 (-59.2 to 69.0)	-39.8 (-69.7 to 25.2)
Andorra	Leukemia	10 to 14	Both	0 (0 to 0)	1.2 (0.8 to 1.7)	0 (0 to 0)	0.8 (0.6 to 1.2)	0 (0 to 0)	0.8 (0.5 to 1.1)	-15.6 (-48.1 to 30.9)	-33.9 (-59.3 to 2.5)
Andorra	Leukemia	10 to 14	Female	0 (0 to 0)	0.9 (0.5 to 1.5)	0 (0 to 0)	0.6 (0.3 to 1.1)	0 (0 to 0)	0.6 (0.3 to 1.0)	-18.4 (-59.9 to 56.2)	-36.1 (-68.6 to 22.3)
Andorra	Leukemia	10 to 14	Male	0 (0 to 0)	1.4 (0.9 to 2.2)	0 (0 to 0)	1.0 (0.6 to 1.7)	0 (0 to 0)	1.0 (0.6 to 1.5)	-14.0 (-54.6 to 60.0)	-32.7 (-64.4 to 25.3)
Andorra	Other neoplasms	5 to 9	Both	0 (0 to 0)	0.9 (0.6 to 1.3)	0 (0 to 0)	0.7 (0.5 to 1.0)	0 (0 to 0)	0.7 (0.4 to 1.0)	4.1 (-41.4 to 81.3)	-22.9 (-56.6 to 34.3)
Andorra	Other neoplasms	5 to 9	Female	0 (0 to 0)	0.8 (0.4 to 1.4)	0 (0 to 0)	0.6 (0.3 to 1.0)	0 (0 to 0)	0.6 (0.4 to 1.0)	3.6 (-51.1 to 117.6)	-23.2 (-63.8 to 61.1)
Andorra	Other neoplasms	5 to 9	Male	0 (0 to 0)	0.9 (0.6 to 1.5)	0 (0 to 0)	0.7 (0.4 to 1.2)	0 (0 to 0)	0.7 (0.4 to 1.2)	4.6 (-49.2 to 120.5)	-22.5 (-62.4 to 63.4)
Andorra	Other neoplasms	10 to 14	Both	0 (0 to 0)	0.9 (0.6 to 1.2)	0 (0 to 0)	0.7 (0.5 to 1.0)	0 (0 to 0)	0.7 (0.5 to 1.0)	7.6 (-36.1 to 81.2)	-15.7 (-50.0 to 41.8)
Andorra	Other neoplasms	10 to 14	Female	0 (0 to 0)	0.9 (0.5 to 1.4)	0 (0 to 0)	0.7 (0.4 to 1.2)	0 (0 to 0)	0.7 (0.4 to 1.1)	6.1 (-46.7 to 133.2)	-16.9 (-58.3 to 82.6)
Andorra	Other neoplasms	10 to 14	Male	0 (0 to 0)	0.9 (0.5 to 1.4)	0 (0 to 0)	0.7 (0.4 to 1.1)	0 (0 to 0)	0.8 (0.4 to 1.2)	9.1 (-43.6 to 118.5)	-14.6 (-55.9 to 71.1)
Andorra	Rheumatic heart disease	5 to 9	Both	0 (0 to 0)	0.0 (0.0 to 0.0)	0 (0 to 0)	0.0 (0.0 to 0.0)	0 (0 to 0)	0.0 (0.0 to 0.0)	-45.4 (-68.8 to -1.8)	-59.6 (-76.9 to -27.2)
Andorra	Rheumatic heart disease	5 to 9	Female	0 (0 to 0)	0.0 (0.0 to 0.1)	0 (0 to 0)	0.0 (0.0 to 0.0)	0 (0 to 0)	0.0 (0.0 to 0.0)	-49.5 (-75.6 to 1.8)	-62.6 (-82.0 to -24.6)
Andorra	Rheumatic heart disease	5 to 9	Male	0 (0 to 0)	0.0 (0.0 to 0.1)	0 (0 to 0)	0.0 (0.0 to 0.0)	0 (0 to 0)	0.0 (0.0 to 0.0)	-40.7 (-75.1 to 45.6)	-56.0 (-81.5 to 7.9)
Andorra	Rheumatic heart disease	10 to 14	Both	0 (0 to 0)	0.0 (0.0 to 0.0)	0 (0 to 0)	0.0 (0.0 to 0.0)	0 (0 to 0)	0.0 (0.0 to 0.0)	-40.7 (-64.2 to -2.8)	-53.6 (-71.9 to -23.9)
Andorra	Rheumatic heart disease	10 to 14	Female	0 (0 to 0)	0.0 (0.0 to 0.0)	0 (0 to 0)	0.0 (0.0 to 0.0)	0 (0 to 0)	0.0 (0.0 to 0.0)	-45.8 (-72.8 to 7.0)	-57.6 (-78.7 to -16.2)
Andorra	Rheumatic heart disease	10 to 14	Male	0 (0 to 0)	0.0 (0.0 to 0.0)	0 (0 to 0)	0.0 (0.0 to 0.0)	0 (0 to 0)	0.0 (0.0 to 0.0)	-34.6 (-66.9 to 26.5)	-48.8 (-74.1 to -1.0)
Andorra	Ischemic heart disease	5 to 9	Both	0 (0 to 0)	0.0 (0.0 to 0.0)	0 (0 to 0)	0.0 (0.0 to 0.0)	0 (0 to 0)	0.0 (0.0 to 0.0)	7.3 (-35.7 to 87.3)	-20.5 (-52.4 to 38.7)
Andorra	Ischemic heart disease	5 to 9	Female	0 (0 to 0)	0.0 (0.0 to 0.0)	0 (0 to 0)	0.0 (0.0 to 0.0)	0 (0 to 0)	0.0 (0.0 to 0.0)	11.9 (-40.4 to 112.9)	-17.1 (-55.9 to 57.7)
Andorra	Ischemic heart disease	5 to 9	Male	0 (0 to 0)	0.0 (0.0 to 0.0)	0 (0 to 0)	0.0 (0.0 to 0.0)	0 (0 to 0)	0.0 (0.0 to 0.0)	3.3 (-51.9 to 114.8)	-23.5 (-64.4 to 59.1)
Andorra	Ischemic heart disease	10 to 14	Both	0 (0 to 0)	0.0 (0.0 to 0.0)	0 (0 to 0)	0.0 (0.0 to 0.0)	0 (0 to 0)	0.0 (0.0 to 0.0)	-21.2 (-56.7 to 46.6)	-38.3 (-66.1 to 14.7)
Andorra	Ischemic heart disease	10 to 14	Female	0 (0 to 0)	0.0 (0.0 to 0.0)	0 (0 to 0)	0.0 (0.0 to 0.0)	0 (0 to 0)	0.0 (0.0 to 0.0)	8.1 (-42.4 to 86.5)	-15.4 (-54.9 to 46.0)
Andorra	Ischemic heart disease	10 to 14	Male	0 (0 to 0)	0.0 (0.0 to 0.0)	0 (0 to 0)	0.0 (0.0 to 0.0)	0 (0 to 0)	0.0 (0.0 to 0.0)	-35.6 (-71.9 to 62.9)	-49.6 (-78.0 to 27.5)

eTable 8. All-cause and cause-specific mortality (counts and rates) at the global level and by country in the WHO European Region for ages 5 to 9 and 10 to 14 for 1990, 2000, and 2016

Andorra	Cerebrovascular disease	5 to 9	Both	0<br>(0 to 0)	0.1<br>(0.1 to 0.2)	0<br>(0 to 0)	0.1<br>(0.0 to 0.1)	0<br>(0 to 0)	0.1<br>(0.0 to 0.1)	-42.9<br>(-67.6 to 0.8)	-57.7<br>(-76.0 to -25.3)
Andorra	Cerebrovascular disease	5 to 9	Female	0<br>(0 to 0)	0.2<br>(0.1 to 0.3)	0<br>(0 to 0)	0.1<br>(0.0 to 0.1)	0<br>(0 to 0)	0.1<br>(0.0 to 0.1)	-41.4<br>(-70.1 to 14.5)	-56.6<br>(-77.8 to -15.2)
Andorra	Cerebrovascular disease	5 to 9	Male	0<br>(0 to 0)	0.1<br>(0.0 to 0.1)	0<br>(0 to 0)	0.0<br>(0.0 to 0.1)	0<br>(0 to 0)	0.0<br>(0.0 to 0.0)	-46.7<br>(-79.1 to 33.9)	-60.5<br>(-84.5 to -0.8)
Andorra	Cerebrovascular disease	10 to 14	Both	0<br>(0 to 0)	0.2<br>(0.1 to 0.3)	0<br>(0 to 0)	0.1<br>(0.1 to 0.1)	0<br>(0 to 0)	0.1<br>(0.1 to 0.1)	-39.0<br>(-61.5 to 0.0)	-52.3<br>(-69.9 to -21.7)
Andorra	Cerebrovascular disease	10 to 14	Female	0<br>(0 to 0)	0.2<br>(0.1 to 0.3)	0<br>(0 to 0)	0.1<br>(0.1 to 0.2)	0<br>(0 to 0)	0.1<br>(0.0 to 0.2)	-40.8<br>(-69.9 to 15.5)	-53.7<br>(-76.5 to -9.6)
Andorra	Cerebrovascular disease	10 to 14	Male	0<br>(0 to 0)	0.2<br>(0.1 to 0.3)	0<br>(0 to 0)	0.1<br>(0.1 to 0.2)	0<br>(0 to 0)	0.1<br>(0.1 to 0.2)	-37.3<br>(-68.3 to 27.4)	-50.9<br>(-75.2 to -0.3)
Andorra	Hypertensive heart disease	5 to 9	Both	0<br>(0 to 0)	0.0<br>(0.0 to 0.0)	0<br>(0 to 0)	0.0<br>(0.0 to 0.0)	0<br>(0 to 0)	0.0<br>(0.0 to 0.0)	9.4<br>(-34.4 to 87.3)	-19.0<br>(-51.4 to 38.7)
Andorra	Hypertensive heart disease	5 to 9	Female	0<br>(0 to 0)	0.0<br>(0.0 to 0.0)	0<br>(0 to 0)	0.0<br>(0.0 to 0.0)	0<br>(0 to 0)	0.0<br>(0.0 to 0.0)	12.1<br>(-39.3 to 112.9)	-17.0<br>(-55.1 to 57.7)
Andorra	Hypertensive heart disease	5 to 9	Male	0<br>(0 to 0)	0.0<br>(0.0 to 0.0)	0<br>(0 to 0)	0.0<br>(0.0 to 0.0)	0<br>(0 to 0)	0.0<br>(0.0 to 0.0)	6.9<br>(-51.2 to 117.8)	-20.8<br>(-63.9 to 61.3)
Andorra	Hypertensive heart disease	10 to 14	Both	0<br>(0 to 0)	0.0<br>(0.0 to 0.0)	0<br>(0 to 0)	0.0<br>(0.0 to 0.0)	0<br>(0 to 0)	0.0<br>(0.0 to 0.0)	14.0<br>(-25.5 to 75.7)	-10.7<br>(-41.6 to 37.5)
Andorra	Hypertensive heart disease	10 to 14	Female	0<br>(0 to 0)	0.0<br>(0.0 to 0.0)	0<br>(0 to 0)	0.0<br>(0.0 to 0.0)	0<br>(0 to 0)	0.0<br>(0.0 to 0.0)	10.4<br>(-40.1 to 87.0)	-13.5<br>(-53.1 to 46.4)
Andorra	Hypertensive heart disease	10 to 14	Male	0<br>(0 to 0)	0.0<br>(0.0 to 0.0)	0<br>(0 to 0)	0.0<br>(0.0 to 0.0)	0<br>(0 to 0)	0.0<br>(0.0 to 0.0)	17.8<br>(-36.4 to 114.4)	-7.8<br>(-50.2 to 67.9)
Andorra	Cardiomyopathy and myocarditis	5 to 9	Both	0<br>(0 to 0)	0.2<br>(0.1 to 0.3)	0<br>(0 to 0)	0.1<br>(0.1 to 0.2)	0<br>(0 to 0)	0.1<br>(0.1 to 0.2)	-19.5<br>(-50.3 to 34.5)	-40.4<br>(-63.2 to -0.4)
Andorra	Cardiomyopathy and myocarditis	5 to 9	Female	0<br>(0 to 0)	0.2<br>(0.1 to 0.3)	0<br>(0 to 0)	0.1<br>(0.1 to 0.2)	0<br>(0 to 0)	0.1<br>(0.1 to 0.2)	-16.6<br>(-57.5 to 65.4)	-38.3<br>(-68.5 to 22.5)
Andorra	Cardiomyopathy and myocarditis	5 to 9	Male	0<br>(0 to 0)	0.2<br>(0.1 to 0.3)	0<br>(0 to 0)	0.1<br>(0.1 to 0.2)	0<br>(0 to 0)	0.1<br>(0.1 to 0.2)	-22.3<br>(-61.2 to 65.6)	-42.5<br>(-71.2 to 22.6)
Andorra	Cardiomyopathy and myocarditis	10 to 14	Both	0<br>(0 to 0)	0.2<br>(0.2 to 0.4)	0<br>(0 to 0)	0.2<br>(0.1 to 0.2)	0<br>(0 to 0)	0.2<br>(0.1 to 0.2)	-14.0<br>(-47.5 to 37.1)	-32.7<br>(-58.9 to 7.3)
Andorra	Cardiomyopathy and myocarditis	10 to 14	Female	0<br>(0 to 0)	0.2<br>(0.1 to 0.3)	0<br>(0 to 0)	0.1<br>(0.1 to 0.2)	0<br>(0 to 0)	0.1<br>(0.1 to 0.2)	-19.6<br>(-60.9 to 69.2)	-37.0<br>(-69.4 to 32.5)
Andorra	Cardiomyopathy and myocarditis	10 to 14	Male	0<br>(0 to 0)	0.3<br>(0.2 to 0.5)	0<br>(0 to 0)	0.2<br>(0.1 to 0.3)	0<br>(0 to 0)	0.2<br>(0.1 to 0.3)	-11.1<br>(-53.1 to 60.5)	-30.4<br>(-63.3 to 25.7)
Andorra	Atrial fibrillation and flutter	5 to 9	Both	0<br>(0 to 0)	0.0<br>(0.0 to 0.0)	0<br>(0 to 0)	0.0<br>(0.0 to 0.0)	0<br>(0 to 0)	0.0<br>(0.0 to 0.0)	0.0<br>(0.0 to 0.0)	0.0<br>(0.0 to 0.0)
Andorra	Atrial fibrillation and flutter	5 to 9	Female	0<br>(0 to 0)	0.0<br>(0.0 to 0.0)	0<br>(0 to 0)	0.0<br>(0.0 to 0.0)	0<br>(0 to 0)	0.0<br>(0.0 to 0.0)	0.0<br>(0.0 to 0.0)	0.0<br>(0.0 to 0.0)
Andorra	Atrial fibrillation and flutter	5 to 9	Male	0<br>(0 to 0)	0.0<br>(0.0 to 0.0)	0<br>(0 to 0)	0.0<br>(0.0 to 0.0)	0<br>(0 to 0)	0.0<br>(0.0 to 0.0)	0.0<br>(0.0 to 0.0)	0.0<br>(0.0 to 0.0)
Andorra	Atrial fibrillation and flutter	10 to 14	Both	0<br>(0 to 0)	0.0<br>(0.0 to 0.0)	0<br>(0 to 0)	0.0<br>(0.0 to 0.0)	0<br>(0 to 0)	0.0<br>(0.0 to 0.0)	0.0<br>(0.0 to 0.0)	0.0<br>(0.0 to 0.0)
Andorra	Atrial fibrillation and flutter	10 to 14	Female	0<br>(0 to 0)	0.0<br>(0.0 to 0.0)	0<br>(0 to 0)	0.0<br>(0.0 to 0.0)	0<br>(0 to 0)	0.0<br>(0.0 to 0.0)	0.0<br>(0.0 to 0.0)	0.0<br>(0.0 to 0.0)
Andorra	Atrial fibrillation and flutter	10 to 14	Male	0<br>(0 to 0)	0.0<br>(0.0 to 0.0)	0<br>(0 to 0)	0.0<br>(0.0 to 0.0)	0<br>(0 to 0)	0.0<br>(0.0 to 0.0)	0.0<br>(0.0 to 0.0)	0.0<br>(0.0 to 0.0)
Andorra	Endocarditis	5 to 9	Both	0<br>(0 to 0)	0.0<br>(0.0 to 0.0)	0<br>(0 to 0)	0.0<br>(0.0 to 0.0)	0<br>(0 to 0)	0.0<br>(0.0 to 0.0)	4.1<br>(-51.4 to 144.9)	-22.9<br>(-64.0 to 81.4)
Andorra	Endocarditis	5 to 9	Female	0<br>(0 to 0)	0.0<br>(0.0 to 0.1)	0<br>(0 to 0)	0.0<br>(0.0 to 0.0)	0<br>(0 to 0)	0.0<br>(0.0 to 0.0)	-2.3<br>(-67.7 to 209.7)	-27.6<br>(-76.1 to 129.4)

eTable 8. All-cause and cause-specific mortality (counts and rates) at the global level and by country in the WHO European Region for ages 5 to 9 and 10 to 14 for 1990, 2000, and 2016

Andorra	Endocarditis	5 to 9	Male	0<br>(0 to 0)	0.0<br>(0.0 to 0.0)	0<br>(0 to 0)	0.0<br>(0.0 to 0.0)	0<br>(0 to 0)	0.0<br>(0.0 to 0.0)	11.8<br>(-58.3 to 196.8)	-17.2<br>(-69.1 to 119.8)
Andorra	Endocarditis	10 to 14	Both	0<br>(0 to 0)	0.0<br>(0.0 to 0.0)	0<br>(0 to 0)	0.0<br>(0.0 to 0.0)	0<br>(0 to 0)	0.0<br>(0.0 to 0.0)	14.4<br>(-38.5 to 115.4)	-10.5<br>(-51.9 to 68.6)
Andorra	Endocarditis	10 to 14	Female	0<br>(0 to 0)	0.0<br>(0.0 to 0.0)	0<br>(0 to 0)	0.0<br>(0.0 to 0.0)	0<br>(0 to 0)	0.0<br>(0.0 to 0.0)	1.8<br>(-56.0 to 142.2)	-20.3<br>(-65.5 to 89.6)
Andorra	Endocarditis	10 to 14	Male	0<br>(0 to 0)	0.0<br>(0.0 to 0.0)	0<br>(0 to 0)	0.0<br>(0.0 to 0.0)	0<br>(0 to 0)	0.0<br>(0.0 to 0.0)	26.5<br>(-48.2 to 215.2)	-1.0<br>(-59.4 to 146.8)
Andorra	Other cardiovascular and circulatory diseases	5 to 9	Both	0<br>(0 to 0)	0.1<br>(0.1 to 0.2)	0<br>(0 to 0)	0.1<br>(0.0 to 0.1)	0<br>(0 to 0)	0.1<br>(0.0 to 0.1)	-29.5<br>(-62.1 to 27.7)	-47.8<br>(-71.9 to -5.4)
Andorra	Other cardiovascular and circulatory diseases	5 to 9	Female	0<br>(0 to 0)	0.1<br>(0.0 to 0.2)	0<br>(0 to 0)	0.0<br>(0.0 to 0.1)	0<br>(0 to 0)	0.0<br>(0.0 to 0.1)	-23.3<br>(-67.5 to 62.3)	-43.2<br>(-75.9 to 20.2)
Andorra	Other cardiovascular and circulatory diseases	5 to 9	Male	0<br>(0 to 0)	0.1<br>(0.1 to 0.3)	0<br>(0 to 0)	0.1<br>(0.0 to 0.1)	0<br>(0 to 0)	0.1<br>(0.0 to 0.1)	-33.2<br>(-69.8 to 47.3)	-50.5<br>(-77.6 to 9.1)
Andorra	Other cardiovascular and circulatory diseases	10 to 14	Both	0<br>(0 to 0)	0.1<br>(0.1 to 0.1)	0<br>(0 to 0)	0.1<br>(0.0 to 0.1)	0<br>(0 to 0)	0.1<br>(0.0 to 0.1)	-20.1<br>(-55.5 to 37.7)	-37.4<br>(-65.2 to 7.8)
Andorra	Other cardiovascular and circulatory diseases	10 to 14	Female	0<br>(0 to 0)	0.1<br>(0.0 to 0.2)	0<br>(0 to 0)	0.1<br>(0.0 to 0.1)	0<br>(0 to 0)	0.1<br>(0.0 to 0.1)	-22.2<br>(-63.0 to 62.4)	-39.1<br>(-71.1 to 27.1)
Andorra	Other cardiovascular and circulatory diseases	10 to 14	Male	0<br>(0 to 0)	0.1<br>(0.0 to 0.2)	0<br>(0 to 0)	0.1<br>(0.0 to 0.1)	0<br>(0 to 0)	0.1<br>(0.0 to 0.1)	-18.3<br>(-63.6 to 80.4)	-36.0<br>(-71.5 to 41.2)
Andorra	Chronic obstructive pulmonary disease	5 to 9	Both	0<br>(0 to 0)	0.1<br>(0.0 to 0.1)	0<br>(0 to 0)	0.0<br>(0.0 to 0.1)	0<br>(0 to 0)	0.0<br>(0.0 to 0.1)	-15.3<br>(-59.6 to 68.3)	-37.3<br>(-70.1 to 24.7)
Andorra	Chronic obstructive pulmonary disease	5 to 9	Female	0<br>(0 to 0)	0.1<br>(0.0 to 0.1)	0<br>(0 to 0)	0.0<br>(0.0 to 0.1)	0<br>(0 to 0)	0.0<br>(0.0 to 0.1)	-10.2<br>(-62.3 to 128.2)	-33.5<br>(-72.1 to 69.0)
Andorra	Chronic obstructive pulmonary disease	5 to 9	Male	0<br>(0 to 0)	0.1<br>(0.0 to 0.1)	0<br>(0 to 0)	0.0<br>(0.0 to 0.1)	0<br>(0 to 0)	0.0<br>(0.0 to 0.1)	-20.0<br>(-73.9 to 110.8)	-40.8<br>(-80.7 to 56.1)
Andorra	Chronic obstructive pulmonary disease	10 to 14	Both	0<br>(0 to 0)	0.1<br>(0.0 to 0.1)	0<br>(0 to 0)	0.0<br>(0.0 to 0.1)	0<br>(0 to 0)	0.0<br>(0.0 to 0.1)	-5.1<br>(-54.4 to 102.7)	-25.7<br>(-64.3 to 58.7)
Andorra	Chronic obstructive pulmonary disease	10 to 14	Female	0<br>(0 to 0)	0.1<br>(0.0 to 0.1)	0<br>(0 to 0)	0.0<br>(0.0 to 0.1)	0<br>(0 to 0)	0.0<br>(0.0 to 0.1)	-1.5<br>(-64.9 to 212.5)	-22.9<br>(-72.5 to 144.6)
Andorra	Chronic obstructive pulmonary disease	10 to 14	Male	0<br>(0 to 0)	0.0<br>(0.0 to 0.1)	0<br>(0 to 0)	0.0<br>(0.0 to 0.1)	0<br>(0 to 0)	0.0<br>(0.0 to 0.1)	-8.7<br>(-68.2 to 160.3)	-28.5<br>(-75.1 to 103.8)
Andorra	Asthma	5 to 9	Both	0<br>(0 to 0)	0.2<br>(0.1 to 0.3)	0<br>(0 to 0)	0.1<br>(0.1 to 0.1)	0<br>(0 to 0)	0.1<br>(0.0 to 0.1)	-34.1<br>(-65.0 to 21.7)	-51.2<br>(-74.1 to -9.9)

eTable 8. All-cause and cause-specific mortality (counts and rates) at the global level and by country in the WHO European Region for ages 5 to 9 and 10 to 14 for 1990, 2000, and 2016

Andorra	Asthma	5 to 9	Female	0 (0 to 0)	0.2 (0.1 to 0.3)	0 (0 to 0)	0.1 (0.0 to 0.2)	0 (0 to 0)	0.1 (0.0 to 0.2)	-27.1 (-71.0 to 73.8)	-46.0 (-78.5 to 28.7)
Andorra	Asthma	5 to 9	Male	0 (0 to 0)	0.2 (0.1 to 0.4)	0 (0 to 0)	0.1 (0.0 to 0.2)	0 (0 to 0)	0.1 (0.0 to 0.2)	-39.6 (-76.1 to 37.8)	-55.2 (-82.3 to 2.1)
Andorra	Asthma	10 to 14	Both	0 (0 to 0)	0.3 (0.2 to 0.5)	0 (0 to 0)	0.2 (0.1 to 0.3)	0 (0 to 0)	0.2 (0.1 to 0.3)	-33.3 (-63.0 to 18.6)	-47.8 (-71.0 to -7.2)
Andorra	Asthma	10 to 14	Female	0 (0 to 0)	0.3 (0.1 to 0.5)	0 (0 to 0)	0.2 (0.1 to 0.3)	0 (0 to 0)	0.2 (0.1 to 0.3)	-28.6 (-68.8 to 69.8)	-44.1 (-75.6 to 33.0)
Andorra	Asthma	10 to 14	Male	0 (0 to 0)	0.4 (0.2 to 0.7)	0 (0 to 0)	0.2 (0.1 to 0.3)	0 (0 to 0)	0.2 (0.1 to 0.3)	-36.6 (-73.0 to 32.9)	-50.3 (-78.9 to 4.0)
Andorra	Interstitial lung disease and pulmonary sarcoidosis	5 to 9	Both	0 (0 to 0)	0.0 (0.0 to 0.0)	0 (0 to 0)	0.0 (0.0 to 0.0)	0 (0 to 0)	0.0 (0.0 to 0.0)	21.8 (-41.9 to 156.7)	-9.8 (-57.0 to 90.1)
Andorra	Interstitial lung disease and pulmonary sarcoidosis	5 to 9	Female	0 (0 to 0)	0.0 (0.0 to 0.0)	0 (0 to 0)	0.0 (0.0 to 0.0)	0 (0 to 0)	0.0 (0.0 to 0.0)	18.2 (-58.1 to 222.8)	-12.5 (-68.9 to 139.1)
Andorra	Interstitial lung disease and pulmonary sarcoidosis	5 to 9	Male	0 (0 to 0)	0.0 (0.0 to 0.0)	0 (0 to 0)	0.0 (0.0 to 0.0)	0 (0 to 0)	0.0 (0.0 to 0.0)	26.4 (-53.9 to 225.0)	-6.4 (-65.9 to 140.8)
Andorra	Interstitial lung disease and pulmonary sarcoidosis	10 to 14	Both	0 (0 to 0)	0.0 (0.0 to 0.0)	0 (0 to 0)	0.0 (0.0 to 0.0)	0 (0 to 0)	0.0 (0.0 to 0.0)	38.5 (-30.1 to 200.5)	8.4 (-45.3 to 135.3)
Andorra	Interstitial lung disease and pulmonary sarcoidosis	10 to 14	Female	0 (0 to 0)	0.0 (0.0 to 0.0)	0 (0 to 0)	0.0 (0.0 to 0.0)	0 (0 to 0)	0.0 (0.0 to 0.0)	33.6 (-45.9 to 285.3)	4.6 (-57.6 to 201.6)
Andorra	Interstitial lung disease and pulmonary sarcoidosis	10 to 14	Male	0 (0 to 0)	0.0 (0.0 to 0.0)	0 (0 to 0)	0.0 (0.0 to 0.0)	0 (0 to 0)	0.0 (0.0 to 0.0)	45.0 (-51.9 to 350.3)	13.5 (-62.3 to 252.5)
Andorra	Other chronic respiratory diseases	5 to 9	Both	0 (0 to 0)	0.1 (0.0 to 0.2)	0 (0 to 0)	0.1 (0.0 to 0.1)	0 (0 to 0)	0.1 (0.0 to 0.1)	18.4 (-43.1 to 155.0)	-12.3 (-57.9 to 88.8)
Andorra	Other chronic respiratory diseases	5 to 9	Female	0 (0 to 0)	0.1 (0.0 to 0.2)	0 (0 to 0)	0.1 (0.0 to 0.2)	0 (0 to 0)	0.1 (0.0 to 0.2)	18.7 (-57.0 to 262.8)	-12.1 (-68.2 to 168.7)
Andorra	Other chronic respiratory diseases	5 to 9	Male	0 (0 to 0)	0.1 (0.0 to 0.2)	0 (0 to 0)	0.1 (0.0 to 0.1)	0 (0 to 0)	0.1 (0.0 to 0.1)	18.0 (-59.6 to 262.1)	-12.6 (-70.1 to 168.3)
Andorra	Other chronic respiratory diseases	10 to 14	Both	0 (0 to 0)	0.1 (0.0 to 0.2)	0 (0 to 0)	0.1 (0.0 to 0.1)	0 (0 to 0)	0.1 (0.0 to 0.1)	28.0 (-44.3 to 204.3)	0.2 (-56.4 to 138.2)
Andorra	Other chronic respiratory diseases	10 to 14	Female	0 (0 to 0)	0.1 (0.0 to 0.2)	0 (0 to 0)	0.1 (0.0 to 0.2)	0 (0 to 0)	0.1 (0.0 to 0.2)	24.1 (-63.6 to 293.3)	-2.9 (-71.5 to 207.9)
Andorra	Other chronic respiratory diseases	10 to 14	Male	0 (0 to 0)	0.1 (0.0 to 0.1)	0 (0 to 0)	0.1 (0.0 to 0.1)	0 (0 to 0)	0.1 (0.0 to 0.1)	33.3 (-57.4 to 345.9)	4.4 (-66.6 to 249.1)
Andorra	Cirrhosis and other chronic liver diseases due to hepatitis B	5 to 9	Both	0 (0 to 0)	0.0 (0.0 to 0.0)	0 (0 to 0)	0.0 (0.0 to 0.0)	0 (0 to 0)	0.0 (0.0 to 0.0)	12.6 (-34.8 to 93.8)	-16.6 (-51.7 to 43.6)

eTable 8. All-cause and cause-specific mortality (counts and rates) at the global level and by country in the WHO European Region for ages 5 to 9 and 10 to 14 for 1990, 2000, and 2016

Andorra	Cirrhosis and other chronic liver diseases due to hepatitis B	5 to 9	Female	0 (0 to 0)	0.0 (0.0 to 0.0)	0 (0 to 0)	0.0 (0.0 to 0.0)	0 (0 to 0)	0.0 (0.0 to 0.0)	-0.5 (-42.2 to 71.9)	-26.3 (-57.2 to 27.3)
Andorra	Cirrhosis and other chronic liver diseases due to hepatitis B	5 to 9	Male	0 (0 to 0)	0.0 (0.0 to 0.0)	0 (0 to 0)	0.0 (0.0 to 0.0)	0 (0 to 0)	0.0 (0.0 to 0.0)	15.2 (-38.6 to 120.5)	-14.7 (-54.5 to 63.3)
Andorra	Cirrhosis and other chronic liver diseases due to hepatitis B	10 to 14	Both	0 (0 to 0)	0.0 (0.0 to 0.0)	0 (0 to 0)	0.0 (0.0 to 0.0)	0 (0 to 0)	0.0 (0.0 to 0.0)	5.7 (-34.9 to 85.1)	-17.3 (-49.1 to 44.9)
Andorra	Cirrhosis and other chronic liver diseases due to hepatitis B	10 to 14	Female	0 (0 to 0)	0.0 (0.0 to 0.0)	0 (0 to 0)	0.0 (0.0 to 0.0)	0 (0 to 0)	0.0 (0.0 to 0.0)	-4.2 (-48.8 to 83.7)	-25.0 (-59.9 to 43.8)
Andorra	Cirrhosis and other chronic liver diseases due to hepatitis B	10 to 14	Male	0 (0 to 0)	0.0 (0.0 to 0.0)	0 (0 to 0)	0.0 (0.0 to 0.0)	0 (0 to 0)	0.0 (0.0 to 0.0)	10.4 (-41.2 to 132.1)	-13.6 (-54.0 to 81.7)
Andorra	Cirrhosis and other chronic liver diseases due to hepatitis C	5 to 9	Both	0 (0 to 0)	0.0 (0.0 to 0.0)	0 (0 to 0)	0.0 (0.0 to 0.0)	0 (0 to 0)	0.0 (0.0 to 0.0)	15.7 (-31.1 to 99.7)	-14.3 (-48.9 to 47.9)
Andorra	Cirrhosis and other chronic liver diseases due to hepatitis C	5 to 9	Female	0 (0 to 0)	0.0 (0.0 to 0.0)	0 (0 to 0)	0.0 (0.0 to 0.0)	0 (0 to 0)	0.0 (0.0 to 0.0)	5.1 (-38.4 to 82.2)	-22.2 (-54.4 to 34.9)
Andorra	Cirrhosis and other chronic liver diseases due to hepatitis C	5 to 9	Male	0 (0 to 0)	0.0 (0.0 to 0.0)	0 (0 to 0)	0.0 (0.0 to 0.0)	0 (0 to 0)	0.0 (0.0 to 0.0)	18.0 (-37.2 to 127.8)	-12.6 (-53.5 to 68.7)
Andorra	Cirrhosis and other chronic liver diseases due to hepatitis C	10 to 14	Both	0 (0 to 0)	0.0 (0.0 to 0.0)	0 (0 to 0)	0.0 (0.0 to 0.0)	0 (0 to 0)	0.0 (0.0 to 0.0)	8.9 (-32.7 to 91.1)	-14.7 (-47.3 to 49.6)
Andorra	Cirrhosis and other chronic liver diseases due to hepatitis C	10 to 14	Female	0 (0 to 0)	0.0 (0.0 to 0.0)	0 (0 to 0)	0.0 (0.0 to 0.0)	0 (0 to 0)	0.0 (0.0 to 0.0)	1.7 (-45.8 to 94.6)	-20.4 (-57.6 to 52.3)
Andorra	Cirrhosis and other chronic liver diseases due to hepatitis C	10 to 14	Male	0 (0 to 0)	0.0 (0.0 to 0.0)	0 (0 to 0)	0.0 (0.0 to 0.0)	0 (0 to 0)	0.0 (0.0 to 0.0)	12.7 (-40.2 to 147.2)	-11.7 (-53.2 to 93.6)
Andorra	Cirrhosis and other chronic liver diseases due to other causes	5 to 9	Both	0 (0 to 0)	0.0 (0.0 to 0.1)	0 (0 to 0)	0.0 (0.0 to 0.0)	0 (0 to 0)	0.0 (0.0 to 0.0)	2.3 (-35.9 to 60.8)	-24.2 (-52.5 to 19.1)
Andorra	Cirrhosis and other chronic liver diseases due to other causes	5 to 9	Female	0 (0 to 0)	0.0 (0.0 to 0.0)	0 (0 to 0)	0.0 (0.0 to 0.0)	0 (0 to 0)	0.0 (0.0 to 0.0)	-6.7 (-45.4 to 61.7)	-30.9 (-59.6 to 19.8)

eTable 8. All-cause and cause-specific mortality (counts and rates) at the global level and by country in the WHO European Region for ages 5 to 9 and 10 to 14 for 1990, 2000, and 2016

Andorra	Cirrhosis and other chronic liver diseases due to other causes	5 to 9	Male	0 (0 to 0)	0.0 (0.0 to 0.1)	0 (0 to 0)	0.0 (0.0 to 0.1)	0 (0 to 0)	0.0 (0.0 to 0.1)	7.4 (-42.6 to 106.5)	-20.4 (-57.5 to 53.0)
Andorra	Cirrhosis and other chronic liver diseases due to other causes	10 to 14	Both	0 (0 to 0)	0.0 (0.0 to 0.0)	0 (0 to 0)	0.0 (0.0 to 0.0)	0 (0 to 0)	0.0 (0.0 to 0.0)	-4.2 (-40.5 to 57.9)	-25.0 (-53.4 to 23.6)
Andorra	Cirrhosis and other chronic liver diseases due to other causes	10 to 14	Female	0 (0 to 0)	0.0 (0.0 to 0.0)	0 (0 to 0)	0.0 (0.0 to 0.0)	0 (0 to 0)	0.0 (0.0 to 0.0)	-9.2 (-50.7 to 71.7)	-28.9 (-61.4 to 34.4)
Andorra	Cirrhosis and other chronic liver diseases due to other causes	10 to 14	Male	0 (0 to 0)	0.0 (0.0 to 0.0)	0 (0 to 0)	0.0 (0.0 to 0.0)	0 (0 to 0)	0.0 (0.0 to 0.0)	2.5 (-45.7 to 115.4)	-19.8 (-57.5 to 68.6)
Andorra	Peptic ulcer disease	5 to 9	Both	0 (0 to 0)	0.0 (0.0 to 0.0)	0 (0 to 0)	0.0 (0.0 to 0.0)	0 (0 to 0)	0.0 (0.0 to 0.0)	5.8 (-43.0 to 92.7)	-21.6 (-57.8 to 42.7)
Andorra	Peptic ulcer disease	5 to 9	Female	0 (0 to 0)	0.0 (0.0 to 0.0)	0 (0 to 0)	0.0 (0.0 to 0.0)	0 (0 to 0)	0.0 (0.0 to 0.0)	4.2 (-53.7 to 130.3)	-22.8 (-65.7 to 70.5)
Andorra	Peptic ulcer disease	5 to 9	Male	0 (0 to 0)	0.0 (0.0 to 0.0)	0 (0 to 0)	0.0 (0.0 to 0.0)	0 (0 to 0)	0.0 (0.0 to 0.0)	7.4 (-51.1 to 142.7)	-20.5 (-63.8 to 79.8)
Andorra	Peptic ulcer disease	10 to 14	Both	0 (0 to 0)	0.0 (0.0 to 0.0)	0 (0 to 0)	0.0 (0.0 to 0.0)	0 (0 to 0)	0.0 (0.0 to 0.0)	7.1 (-34.5 to 75.5)	-16.2 (-48.7 to 37.4)
Andorra	Peptic ulcer disease	10 to 14	Female	0 (0 to 0)	0.0 (0.0 to 0.0)	0 (0 to 0)	0.0 (0.0 to 0.0)	0 (0 to 0)	0.0 (0.0 to 0.0)	2.0 (-44.8 to 101.9)	-20.2 (-56.8 to 58.1)
Andorra	Peptic ulcer disease	10 to 14	Male	0 (0 to 0)	0.0 (0.0 to 0.0)	0 (0 to 0)	0.0 (0.0 to 0.0)	0 (0 to 0)	0.0 (0.0 to 0.0)	12.6 (-44.7 to 128.7)	-11.9 (-56.7 to 79.1)
Andorra	Gastritis and duodenitis	5 to 9	Both	0 (0 to 0)	0.0 (0.0 to 0.0)	0 (0 to 0)	0.0 (0.0 to 0.0)	0 (0 to 0)	0.0 (0.0 to 0.0)	4.5 (-49.4 to 95.6)	-22.6 (-62.6 to 44.9)
Andorra	Gastritis and duodenitis	5 to 9	Female	0 (0 to 0)	0.0 (0.0 to 0.0)	0 (0 to 0)	0.0 (0.0 to 0.0)	0 (0 to 0)	0.0 (0.0 to 0.0)	7.1 (-49.9 to 139.9)	-20.7 (-62.9 to 77.7)
Andorra	Gastritis and duodenitis	5 to 9	Male	0 (0 to 0)	0.0 (0.0 to 0.0)	0 (0 to 0)	0.0 (0.0 to 0.0)	0 (0 to 0)	0.0 (0.0 to 0.0)	2.4 (-60.5 to 157.7)	-24.2 (-70.7 to 90.9)
Andorra	Gastritis and duodenitis	10 to 14	Both	0 (0 to 0)	0.0 (0.0 to 0.0)	0 (0 to 0)	0.0 (0.0 to 0.0)	0 (0 to 0)	0.0 (0.0 to 0.0)	-18.2 (-63.3 to 95.5)	-35.9 (-71.3 to 53.1)
Andorra	Gastritis and duodenitis	10 to 14	Female	0 (0 to 0)	0.0 (0.0 to 0.0)	0 (0 to 0)	0.0 (0.0 to 0.0)	0 (0 to 0)	0.0 (0.0 to 0.0)	3.7 (-46.4 to 109.3)	-18.8 (-58.1 to 63.8)
Andorra	Gastritis and duodenitis	10 to 14	Male	0 (0 to 0)	0.0 (0.0 to 0.0)	0 (0 to 0)	0.0 (0.0 to 0.0)	0 (0 to 0)	0.0 (0.0 to 0.0)	-24.0 (-73.0 to 143.3)	-40.5 (-78.8 to 90.5)
Andorra	Appendicitis	5 to 9	Both	0 (0 to 0)	0.0 (0.0 to 0.1)	0 (0 to 0)	0.0 (0.0 to 0.0)	0 (0 to 0)	0.0 (0.0 to 0.0)	-27.6 (-62.6 to 43.6)	-46.4 (-72.3 to 6.3)
Andorra	Appendicitis	5 to 9	Female	0 (0 to 0)	0.0 (0.0 to 0.1)	0 (0 to 0)	0.0 (0.0 to 0.0)	0 (0 to 0)	0.0 (0.0 to 0.0)	-25.2 (-69.9 to 90.5)	-44.6 (-77.7 to 41.1)
Andorra	Appendicitis	5 to 9	Male	0 (0 to 0)	0.0 (0.0 to 0.1)	0 (0 to 0)	0.0 (0.0 to 0.0)	0 (0 to 0)	0.0 (0.0 to 0.0)	-29.8 (-71.8 to 85.2)	-48.0 (-79.1 to 37.2)
Andorra	Appendicitis	10 to 14	Both	0 (0 to 0)	0.0 (0.0 to 0.1)	0 (0 to 0)	0.0 (0.0 to 0.0)	0 (0 to 0)	0.0 (0.0 to 0.0)	-22.6 (-58.7 to 44.5)	-39.4 (-67.7 to 13.1)
Andorra	Appendicitis	10 to 14	Female	0 (0 to 0)	0.0 (0.0 to 0.1)	0 (0 to 0)	0.0 (0.0 to 0.0)	0 (0 to 0)	0.0 (0.0 to 0.0)	-23.6 (-69.0 to 88.2)	-40.2 (-75.7 to 47.3)
Andorra	Appendicitis	10 to 14	Male	0 (0 to 0)	0.0 (0.0 to 0.1)	0 (0 to 0)	0.0 (0.0 to 0.1)	0 (0 to 0)	0.0 (0.0 to 0.1)	-21.8 (-67.0 to 80.2)	-38.8 (-74.1 to 41.1)
Andorra	Paralytic ileus and intestinal obstruction	5 to 9	Both	0 (0 to 0)	0.1 (0.0 to 0.1)	0 (0 to 0)	0.0 (0.0 to 0.1)	0 (0 to 0)	0.0 (0.0 to 0.1)	-2.5 (-49.3 to 82.7)	-27.8 (-62.5 to 35.3)
Andorra	Paralytic ileus and intestinal obstruction	5 to 9	Female	0 (0 to 0)	0.0 (0.0 to 0.1)	0 (0 to 0)	0.0 (0.0 to 0.1)	0 (0 to 0)	0.0 (0.0 to 0.1)	-7.1 (-64.2 to 136.7)	-31.2 (-73.5 to 75.3)
Andorra	Paralytic ileus and intestinal obstruction	5 to 9	Male	0 (0 to 0)	0.1 (0.0 to 0.1)	0 (0 to 0)	0.0 (0.0 to 0.1)	0 (0 to 0)	0.0 (0.0 to 0.1)	1.1 (-56.8 to 141.3)	-25.1 (-68.0 to 78.8)

eTable 8. All-cause and cause-specific mortality (counts and rates) at the global level and by country in the WHO European Region for ages 5 to 9 and 10 to 14 for 1990, 2000, and 2016

Andorra	Paralytic ileus and intestinal obstruction	10 to 14	Both	0 (0 to 0)	0.0 (0.0 to 0.1)	0 (0 to 0)	0.0 (0.0 to 0.1)	0 (0 to 0)	0.0 (0.0 to 0.1)	0.5 (-44.5 to 84.9)	-21.3 (-56.5 to 44.7)
Andorra	Paralytic ileus and intestinal obstruction	10 to 14	Female	0 (0 to 0)	0.1 (0.0 to 0.1)	0 (0 to 0)	0.0 (0.0 to 0.1)	0 (0 to 0)	0.0 (0.0 to 0.1)	-5.9 (-56.4 to 110.5)	-26.3 (-65.9 to 64.8)
Andorra	Paralytic ileus and intestinal obstruction	10 to 14	Male	0 (0 to 0)	0.0 (0.0 to 0.1)	0 (0 to 0)	0.0 (0.0 to 0.1)	0 (0 to 0)	0.0 (0.0 to 0.1)	7.8 (-53.2 to 161.4)	-15.6 (-63.4 to 104.7)
Andorra	Inguinal, femoral, and abdominal hernia	5 to 9	Both	0 (0 to 0)	0.0 (0.0 to 0.0)	0 (0 to 0)	0.0 (0.0 to 0.0)	0 (0 to 0)	0.0 (0.0 to 0.0)	7.4 (-40.2 to 92.7)	-20.5 (-55.7 to 42.7)
Andorra	Inguinal, femoral, and abdominal hernia	5 to 9	Female	0 (0 to 0)	0.0 (0.0 to 0.0)	0 (0 to 0)	0.0 (0.0 to 0.0)	0 (0 to 0)	0.0 (0.0 to 0.0)	6.6 (-49.9 to 132.8)	-21.1 (-62.9 to 72.4)
Andorra	Inguinal, femoral, and abdominal hernia	5 to 9	Male	0 (0 to 0)	0.0 (0.0 to 0.0)	0 (0 to 0)	0.0 (0.0 to 0.0)	0 (0 to 0)	0.0 (0.0 to 0.0)	8.1 (-49.0 to 142.8)	-19.9 (-62.2 to 79.8)
Andorra	Inguinal, femoral, and abdominal hernia	10 to 14	Both	0 (0 to 0)	0.0 (0.0 to 0.0)	0 (0 to 0)	0.0 (0.0 to 0.0)	0 (0 to 0)	0.0 (0.0 to 0.0)	8.9 (-33.7 to 79.2)	-14.8 (-48.1 to 40.3)
Andorra	Inguinal, femoral, and abdominal hernia	10 to 14	Female	0 (0 to 0)	0.0 (0.0 to 0.0)	0 (0 to 0)	0.0 (0.0 to 0.0)	0 (0 to 0)	0.0 (0.0 to 0.0)	4.4 (-44.1 to 105.5)	-18.3 (-56.2 to 60.9)
Andorra	Inguinal, femoral, and abdominal hernia	10 to 14	Male	0 (0 to 0)	0.0 (0.0 to 0.0)	0 (0 to 0)	0.0 (0.0 to 0.0)	0 (0 to 0)	0.0 (0.0 to 0.0)	13.6 (-44.6 to 130.1)	-11.0 (-56.6 to 80.1)
Andorra	Inflammatory bowel disease	5 to 9	Both	0 (0 to 0)	0.0 (0.0 to 0.0)	0 (0 to 0)	0.0 (0.0 to 0.0)	0 (0 to 0)	0.0 (0.0 to 0.0)	-11.1 (-61.4 to 105.6)	-34.2 (-71.4 to 52.3)
Andorra	Inflammatory bowel disease	5 to 9	Female	0 (0 to 0)	0.0 (0.0 to 0.1)	0 (0 to 0)	0.0 (0.0 to 0.1)	0 (0 to 0)	0.0 (0.0 to 0.1)	-11.1 (-65.4 to 127.4)	-34.2 (-74.4 to 68.4)
Andorra	Inflammatory bowel disease	5 to 9	Male	0 (0 to 0)	0.0 (0.0 to 0.0)	0 (0 to 0)	0.0 (0.0 to 0.0)	0 (0 to 0)	0.0 (0.0 to 0.0)	-11.2 (-73.2 to 212.4)	-34.2 (-80.1 to 131.4)
Andorra	Inflammatory bowel disease	10 to 14	Both	0 (0 to 0)	0.0 (0.0 to 0.0)	0 (0 to 0)	0.0 (0.0 to 0.0)	0 (0 to 0)	0.0 (0.0 to 0.0)	-3.7 (-48.3 to 82.8)	-24.6 (-59.5 to 43.1)
Andorra	Inflammatory bowel disease	10 to 14	Female	0 (0 to 0)	0.0 (0.0 to 0.1)	0 (0 to 0)	0.0 (0.0 to 0.0)	0 (0 to 0)	0.0 (0.0 to 0.0)	-7.9 (-60.1 to 114.7)	-27.9 (-68.8 to 68.1)
Andorra	Inflammatory bowel disease	10 to 14	Male	0 (0 to 0)	0.0 (0.0 to 0.0)	0 (0 to 0)	0.0 (0.0 to 0.0)	0 (0 to 0)	0.0 (0.0 to 0.0)	1.1 (-61.1 to 149.2)	-20.9 (-69.5 to 95.1)
Andorra	Vascular intestinal disorders	5 to 9	Both	0 (0 to 0)	0.0 (0.0 to 0.0)	0 (0 to 0)	0.0 (0.0 to 0.0)	0 (0 to 0)	0.0 (0.0 to 0.0)	-10.8 (-68.4 to 168.4)	-33.9 (-76.6 to 98.8)
Andorra	Vascular intestinal disorders	5 to 9	Female	0 (0 to 0)	0.0 (0.0 to 0.0)	0 (0 to 0)	0.0 (0.0 to 0.0)	0 (0 to 0)	0.0 (0.0 to 0.0)	-10.4 (-72.3 to 250.6)	-33.7 (-79.5 to 159.6)
Andorra	Vascular intestinal disorders	5 to 9	Male	0 (0 to 0)	0.0 (0.0 to 0.0)	0 (0 to 0)	0.0 (0.0 to 0.0)	0 (0 to 0)	0.0 (0.0 to 0.0)	-10.9 (-77.2 to 251.4)	-34.0 (-83.1 to 160.3)
Andorra	Vascular intestinal disorders	10 to 14	Both	0 (0 to 0)	0.0 (0.0 to 0.0)	0 (0 to 0)	0.0 (0.0 to 0.0)	0 (0 to 0)	0.0 (0.0 to 0.0)	-4.9 (-58.3 to 118.5)	-25.6 (-67.3 to 71.1)
Andorra	Vascular intestinal disorders	10 to 14	Female	0 (0 to 0)	0.0 (0.0 to 0.0)	0 (0 to 0)	0.0 (0.0 to 0.0)	0 (0 to 0)	0.0 (0.0 to 0.0)	-4.0 (-71.5 to 236.9)	-24.8 (-77.7 to 163.7)
Andorra	Vascular intestinal disorders	10 to 14	Male	0 (0 to 0)	0.0 (0.0 to 0.0)	0 (0 to 0)	0.0 (0.0 to 0.0)	0 (0 to 0)	0.0 (0.0 to 0.0)	-5.0 (-60.7 to 122.4)	-25.6 (-69.2 to 74.1)

eTable 8. All-cause and cause-specific mortality (counts and rates) at the global level and by country in the WHO European Region for ages 5 to 9 and 10 to 14 for 1990, 2000, and 2016

Andorra	Gallbladder and biliary diseases	5 to 9	Both	0 (0 to 0)	0.0 (0.0 to 0.0)	0 (0 to 0)	0.0 (0.0 to 0.0)	0 (0 to 0)	0.0 (0.0 to 0.0)	-21.7 (-64.1 to 89.5)	-42.0 (-73.4 to 40.3)
Andorra	Gallbladder and biliary diseases	5 to 9	Female	0 (0 to 0)	0.0 (0.0 to 0.0)	0 (0 to 0)	0.0 (0.0 to 0.0)	0 (0 to 0)	0.0 (0.0 to 0.0)	-23.6 (-71.6 to 133.3)	-43.4 (-79.0 to 72.8)
Andorra	Gallbladder and biliary diseases	5 to 9	Male	0 (0 to 0)	0.0 (0.0 to 0.0)	0 (0 to 0)	0.0 (0.0 to 0.0)	0 (0 to 0)	0.0 (0.0 to 0.0)	-19.2 (-72.1 to 133.9)	-40.2 (-79.3 to 73.3)
Andorra	Gallbladder and biliary diseases	10 to 14	Both	0 (0 to 0)	0.0 (0.0 to 0.0)	0 (0 to 0)	0.0 (0.0 to 0.0)	0 (0 to 0)	0.0 (0.0 to 0.0)	-11.2 (-55.5 to 94.9)	-30.5 (-65.2 to 52.6)
Andorra	Gallbladder and biliary diseases	10 to 14	Female	0 (0 to 0)	0.0 (0.0 to 0.0)	0 (0 to 0)	0.0 (0.0 to 0.0)	0 (0 to 0)	0.0 (0.0 to 0.0)	-19.6 (-66.2 to 119.3)	-37.0 (-73.5 to 71.7)
Andorra	Gallbladder and biliary diseases	10 to 14	Male	0 (0 to 0)	0.0 (0.0 to 0.0)	0 (0 to 0)	0.0 (0.0 to 0.0)	0 (0 to 0)	0.0 (0.0 to 0.0)	-5.1 (-66.1 to 180.2)	-25.7 (-73.5 to 119.4)
Andorra	Pancreatitis	5 to 9	Both	0 (0 to 0)	0.0 (0.0 to 0.0)	0 (0 to 0)	0.0 (0.0 to 0.0)	0 (0 to 0)	0.0 (0.0 to 0.0)	-38.4 (-73.0 to 31.0)	-54.3 (-80.0 to -3.0)
Andorra	Pancreatitis	5 to 9	Female	0 (0 to 0)	0.0 (0.0 to 0.0)	0 (0 to 0)	0.0 (0.0 to 0.0)	0 (0 to 0)	0.0 (0.0 to 0.0)	-31.3 (-77.8 to 113.4)	-49.1 (-83.6 to 58.0)
Andorra	Pancreatitis	5 to 9	Male	0 (0 to 0)	0.0 (0.0 to 0.0)	0 (0 to 0)	0.0 (0.0 to 0.0)	0 (0 to 0)	0.0 (0.0 to 0.0)	-43.3 (-81.8 to 60.9)	-58.0 (-86.5 to 19.2)
Andorra	Pancreatitis	10 to 14	Both	0 (0 to 0)	0.0 (0.0 to 0.0)	0 (0 to 0)	0.0 (0.0 to 0.0)	0 (0 to 0)	0.0 (0.0 to 0.0)	-33.1 (-70.2 to 41.4)	-47.7 (-76.6 to 10.7)
Andorra	Pancreatitis	10 to 14	Female	0 (0 to 0)	0.0 (0.0 to 0.0)	0 (0 to 0)	0.0 (0.0 to 0.0)	0 (0 to 0)	0.0 (0.0 to 0.0)	-33.3 (-75.9 to 84.7)	-47.8 (-81.1 to 44.6)
Andorra	Pancreatitis	10 to 14	Male	0 (0 to 0)	0.0 (0.0 to 0.0)	0 (0 to 0)	0.0 (0.0 to 0.0)	0 (0 to 0)	0.0 (0.0 to 0.0)	-33.0 (-78.4 to 80.6)	-47.5 (-83.1 to 41.4)
Andorra	Other digestive diseases	5 to 9	Both	0 (0 to 0)	0.0 (0.0 to 0.1)	0 (0 to 0)	0.0 (0.0 to 0.0)	0 (0 to 0)	0.0 (0.0 to 0.0)	-15.0 (-56.1 to 75.5)	-37.1 (-67.5 to 30.0)
Andorra	Other digestive diseases	5 to 9	Female	0 (0 to 0)	0.0 (0.0 to 0.1)	0 (0 to 0)	0.0 (0.0 to 0.0)	0 (0 to 0)	0.0 (0.0 to 0.1)	-13.3 (-63.8 to 131.2)	-35.8 (-73.2 to 71.2)
Andorra	Other digestive diseases	5 to 9	Male	0 (0 to 0)	0.0 (0.0 to 0.1)	0 (0 to 0)	0.0 (0.0 to 0.1)	0 (0 to 0)	0.0 (0.0 to 0.1)	-16.7 (-67.9 to 137.1)	-38.3 (-76.2 to 75.6)
Andorra	Other digestive diseases	10 to 14	Both	0 (0 to 0)	0.0 (0.0 to 0.0)	0 (0 to 0)	0.0 (0.0 to 0.0)	0 (0 to 0)	0.0 (0.0 to 0.0)	-16.3 (-57.4 to 73.4)	-34.4 (-66.6 to 35.8)
Andorra	Other digestive diseases	10 to 14	Female	0 (0 to 0)	0.0 (0.0 to 0.1)	0 (0 to 0)	0.0 (0.0 to 0.0)	0 (0 to 0)	0.0 (0.0 to 0.0)	-24.1 (-69.7 to 114.8)	-40.5 (-76.3 to 68.2)
Andorra	Other digestive diseases	10 to 14	Male	0 (0 to 0)	0.0 (0.0 to 0.0)	0 (0 to 0)	0.0 (0.0 to 0.0)	0 (0 to 0)	0.0 (0.0 to 0.0)	-7.7 (-61.8 to 136.6)	-27.7 (-70.1 to 85.3)
Andorra	Alzheimer disease and other dementias	5 to 9	Both	0 (0 to 0)	0.0 (0.0 to 0.0)	0 (0 to 0)	0.0 (0.0 to 0.0)	0 (0 to 0)	0.0 (0.0 to 0.0)	0.0 (0.0 to 0.0)	0.0 (0.0 to 0.0)
Andorra	Alzheimer disease and other dementias	5 to 9	Female	0 (0 to 0)	0.0 (0.0 to 0.0)	0 (0 to 0)	0.0 (0.0 to 0.0)	0 (0 to 0)	0.0 (0.0 to 0.0)	0.0 (0.0 to 0.0)	0.0 (0.0 to 0.0)
Andorra	Alzheimer disease and other dementias	5 to 9	Male	0 (0 to 0)	0.0 (0.0 to 0.0)	0 (0 to 0)	0.0 (0.0 to 0.0)	0 (0 to 0)	0.0 (0.0 to 0.0)	0.0 (0.0 to 0.0)	0.0 (0.0 to 0.0)
Andorra	Alzheimer disease and other dementias	10 to 14	Both	0 (0 to 0)	0.0 (0.0 to 0.0)	0 (0 to 0)	0.0 (0.0 to 0.0)	0 (0 to 0)	0.0 (0.0 to 0.0)	0.0 (0.0 to 0.0)	0.0 (0.0 to 0.0)

eTable 8. All-cause and cause-specific mortality (counts and rates) at the global level and by country in the WHO European Region for ages 5 to 9 and 10 to 14 for 1990, 2000, and 2016

Andorra	Alzheimer disease and other dementias	10 to 14	Female	0 (0 to 0)	0.0 (0.0 to 0.0)	0 (0 to 0)	0.0 (0.0 to 0.0)	0 (0 to 0)	0.0 (0.0 to 0.0)	0.0 (0.0 to 0.0)	0.0 (0.0 to 0.0)
Andorra	Alzheimer disease and other dementias	10 to 14	Male	0 (0 to 0)	0.0 (0.0 to 0.0)	0 (0 to 0)	0.0 (0.0 to 0.0)	0 (0 to 0)	0.0 (0.0 to 0.0)	0.0 (0.0 to 0.0)	0.0 (0.0 to 0.0)
Andorra	Parkinson disease	5 to 9	Both	0 (0 to 0)	0.0 (0.0 to 0.0)	0 (0 to 0)	0.0 (0.0 to 0.0)	0 (0 to 0)	0.0 (0.0 to 0.0)	0.0 (0.0 to 0.0)	0.0 (0.0 to 0.0)
Andorra	Parkinson disease	5 to 9	Female	0 (0 to 0)	0.0 (0.0 to 0.0)	0 (0 to 0)	0.0 (0.0 to 0.0)	0 (0 to 0)	0.0 (0.0 to 0.0)	0.0 (0.0 to 0.0)	0.0 (0.0 to 0.0)
Andorra	Parkinson disease	5 to 9	Male	0 (0 to 0)	0.0 (0.0 to 0.0)	0 (0 to 0)	0.0 (0.0 to 0.0)	0 (0 to 0)	0.0 (0.0 to 0.0)	0.0 (0.0 to 0.0)	0.0 (0.0 to 0.0)
Andorra	Parkinson disease	10 to 14	Both	0 (0 to 0)	0.0 (0.0 to 0.0)	0 (0 to 0)	0.0 (0.0 to 0.0)	0 (0 to 0)	0.0 (0.0 to 0.0)	0.0 (0.0 to 0.0)	0.0 (0.0 to 0.0)
Andorra	Parkinson disease	10 to 14	Female	0 (0 to 0)	0.0 (0.0 to 0.0)	0 (0 to 0)	0.0 (0.0 to 0.0)	0 (0 to 0)	0.0 (0.0 to 0.0)	0.0 (0.0 to 0.0)	0.0 (0.0 to 0.0)
Andorra	Parkinson disease	10 to 14	Male	0 (0 to 0)	0.0 (0.0 to 0.0)	0 (0 to 0)	0.0 (0.0 to 0.0)	0 (0 to 0)	0.0 (0.0 to 0.0)	0.0 (0.0 to 0.0)	0.0 (0.0 to 0.0)
Andorra	Epilepsy	5 to 9	Both	0 (0 to 0)	0.3 (0.2 to 0.4)	0 (0 to 0)	0.2 (0.1 to 0.3)	0 (0 to 0)	0.2 (0.1 to 0.3)	5.0 (-37.5 to 75.7)	-22.2 (-53.7 to 30.2)
Andorra	Epilepsy	5 to 9	Female	0 (0 to 0)	0.3 (0.1 to 0.4)	0 (0 to 0)	0.2 (0.1 to 0.3)	0 (0 to 0)	0.2 (0.1 to 0.4)	6.9 (-47.8 to 126.1)	-20.8 (-61.4 to 67.4)
Andorra	Epilepsy	5 to 9	Male	0 (0 to 0)	0.3 (0.2 to 0.5)	0 (0 to 0)	0.2 (0.1 to 0.4)	0 (0 to 0)	0.2 (0.1 to 0.4)	3.6 (-46.5 to 104.4)	-23.3 (-60.4 to 51.4)
Andorra	Epilepsy	10 to 14	Both	0 (0 to 0)	0.3 (0.2 to 0.4)	0 (0 to 0)	0.2 (0.1 to 0.3)	0 (0 to 0)	0.2 (0.1 to 0.4)	9.9 (-34.5 to 85.7)	-13.9 (-48.7 to 45.4)
Andorra	Epilepsy	10 to 14	Female	0 (0 to 0)	0.2 (0.1 to 0.4)	0 (0 to 0)	0.2 (0.1 to 0.3)	0 (0 to 0)	0.2 (0.1 to 0.3)	7.9 (-47.0 to 116.4)	-15.5 (-58.5 to 69.4)
Andorra	Epilepsy	10 to 14	Male	0 (0 to 0)	0.3 (0.2 to 0.5)	0 (0 to 0)	0.2 (0.1 to 0.4)	0 (0 to 0)	0.3 (0.1 to 0.5)	11.4 (-45.7 to 126.1)	-12.8 (-57.5 to 77.0)
Andorra	Motor neuron disease	5 to 9	Both	0 (0 to 0)	0.0 (0.0 to 0.0)	0 (0 to 0)	0.0 (0.0 to 0.0)	0 (0 to 0)	0.0 (0.0 to 0.0)	-13.1 (-65.1 to 103.9)	-35.6 (-74.2 to 51.0)
Andorra	Motor neuron disease	5 to 9	Female	0 (0 to 0)	0.0 (0.0 to 0.1)	0 (0 to 0)	0.0 (0.0 to 0.0)	0 (0 to 0)	0.0 (0.0 to 0.0)	-11.3 (-65.4 to 118.7)	-34.3 (-74.4 to 61.9)
Andorra	Motor neuron disease	5 to 9	Male	0 (0 to 0)	0.0 (0.0 to 0.0)	0 (0 to 0)	0.0 (0.0 to 0.0)	0 (0 to 0)	0.0 (0.0 to 0.0)	-55.6 (-87.6 to 62.5)	-67.1 (-90.8 to 20.4)
Andorra	Motor neuron disease	10 to 14	Both	0 (0 to 0)	0.0 (0.0 to 0.1)	0 (0 to 0)	0.0 (0.0 to 0.0)	0 (0 to 0)	0.0 (0.0 to 0.0)	-10.5 (-49.7 to 58.9)	-29.9 (-60.6 to 24.4)
Andorra	Motor neuron disease	10 to 14	Female	0 (0 to 0)	0.0 (0.0 to 0.1)	0 (0 to 0)	0.0 (0.0 to 0.1)	0 (0 to 0)	0.0 (0.0 to 0.1)	-7.4 (-56.5 to 100.7)	-27.5 (-66.0 to 57.2)
Andorra	Motor neuron disease	10 to 14	Male	0 (0 to 0)	0.0 (0.0 to 0.1)	0 (0 to 0)	0.0 (0.0 to 0.1)	0 (0 to 0)	0.0 (0.0 to 0.1)	-13.6 (-63.0 to 103.2)	-32.4 (-71.1 to 59.1)
Andorra	Other neurological disorders	5 to 9	Both	0 (0 to 0)	0.4 (0.2 to 0.6)	0 (0 to 0)	0.2 (0.2 to 0.4)	0 (0 to 0)	0.2 (0.2 to 0.3)	-19.3 (-52.1 to 37.8)	-40.2 (-64.5 to 2.1)
Andorra	Other neurological disorders	5 to 9	Female	0 (0 to 0)	0.4 (0.2 to 0.6)	0 (0 to 0)	0.2 (0.1 to 0.4)	0 (0 to 0)	0.2 (0.1 to 0.4)	-16.2 (-55.4 to 68.7)	-37.9 (-67.0 to 24.9)
Andorra	Other neurological disorders	5 to 9	Male	0 (0 to 0)	0.4 (0.2 to 0.7)	0 (0 to 0)	0.2 (0.1 to 0.5)	0 (0 to 0)	0.2 (0.1 to 0.4)	-22.3 (-66.1 to 82.3)	-42.5 (-74.9 to 35.0)
Andorra	Other neurological disorders	10 to 14	Both	0 (0 to 0)	0.5 (0.3 to 0.8)	0 (0 to 0)	0.3 (0.2 to 0.5)	0 (0 to 0)	0.3 (0.2 to 0.5)	-15.0 (-49.6 to 49.5)	-33.5 (-60.6 to 17.0)
Andorra	Other neurological disorders	10 to 14	Female	0 (0 to 0)	0.3 (0.2 to 0.4)	0 (0 to 0)	0.2 (0.1 to 0.3)	0 (0 to 0)	0.2 (0.1 to 0.3)	-7.6 (-54.2 to 96.9)	-27.7 (-64.1 to 54.2)
Andorra	Other neurological disorders	10 to 14	Male	0 (0 to 0)	0.7 (0.4 to 1.2)	0 (0 to 0)	0.5 (0.3 to 0.8)	0 (0 to 0)	0.5 (0.3 to 0.8)	-17.8 (-58.9 to 67.1)	-35.6 (-67.8 to 30.8)
Andorra	Drug use disorders	5 to 9	Both	0 (0 to 0)	0.0 (0.0 to 0.0)	0 (0 to 0)	0.0 (0.0 to 0.0)	0 (0 to 0)	0.0 (0.0 to 0.0)	0.0 (0.0 to 0.0)	0.0 (0.0 to 0.0)

eTable 8. All-cause and cause-specific mortality (counts and rates) at the global level and by country in the WHO European Region for ages 5 to 9 and 10 to 14 for 1990, 2000, and 2016

Andorra	Drug use disorders	5 to 9	Female	0 (0 to 0)	0.0 (0.0 to 0.0)	0 (0 to 0)	0.0 (0.0 to 0.0)	0 (0 to 0)	0.0 (0.0 to 0.0)	0.0 (0.0 to 0.0)	0.0 (0.0 to 0.0)
Andorra	Drug use disorders	5 to 9	Male	0 (0 to 0)	0.0 (0.0 to 0.0)	0 (0 to 0)	0.0 (0.0 to 0.0)	0 (0 to 0)	0.0 (0.0 to 0.0)	0.0 (0.0 to 0.0)	0.0 (0.0 to 0.0)
Andorra	Drug use disorders	10 to 14	Both	0 (0 to 0)	0.0 (0.0 to 0.0)	0 (0 to 0)	0.0 (0.0 to 0.0)	0 (0 to 0)	0.0 (0.0 to 0.0)	0.0 (0.0 to 0.0)	0.0 (0.0 to 0.0)
Andorra	Drug use disorders	10 to 14	Female	0 (0 to 0)	0.0 (0.0 to 0.0)	0 (0 to 0)	0.0 (0.0 to 0.0)	0 (0 to 0)	0.0 (0.0 to 0.0)	0.0 (0.0 to 0.0)	0.0 (0.0 to 0.0)
Andorra	Drug use disorders	10 to 14	Male	0 (0 to 0)	0.0 (0.0 to 0.0)	0 (0 to 0)	0.0 (0.0 to 0.0)	0 (0 to 0)	0.0 (0.0 to 0.0)	0.0 (0.0 to 0.0)	0.0 (0.0 to 0.0)
Andorra	Eating disorders	5 to 9	Both	0 (0 to 0)	0.0 (0.0 to 0.0)	0 (0 to 0)	0.0 (0.0 to 0.0)	0 (0 to 0)	0.0 (0.0 to 0.0)	36.0 (-37.6 to 234.0)	0.7 (-53.8 to 147.4)
Andorra	Eating disorders	5 to 9	Female	0 (0 to 0)	0.0 (0.0 to 0.0)	0 (0 to 0)	0.0 (0.0 to 0.0)	0 (0 to 0)	0.0 (0.0 to 0.0)	41.1 (-45.1 to 371.2)	4.5 (-59.3 to 248.9)
Andorra	Eating disorders	5 to 9	Male	0 (0 to 0)	0.0 (0.0 to 0.0)	0 (0 to 0)	0.0 (0.0 to 0.0)	0 (0 to 0)	0.0 (0.0 to 0.0)	20.8 (-64.9 to 275.0)	-10.5 (-74.0 to 177.8)
Andorra	Eating disorders	10 to 14	Both	0 (0 to 0)	0.0 (0.0 to 0.0)	0 (0 to 0)	0.0 (0.0 to 0.0)	0 (0 to 0)	0.0 (0.0 to 0.0)	17.1 (-51.7 to 234.7)	-8.3 (-62.2 to 162.0)
Andorra	Eating disorders	10 to 14	Female	0 (0 to 0)	0.0 (0.0 to 0.0)	0 (0 to 0)	0.0 (0.0 to 0.0)	0 (0 to 0)	0.0 (0.0 to 0.0)	16.1 (-56.4 to 285.3)	-9.1 (-65.9 to 201.6)
Andorra	Eating disorders	10 to 14	Male	0 (0 to 0)	0.0 (0.0 to 0.0)	0 (0 to 0)	0.0 (0.0 to 0.0)	0 (0 to 0)	0.0 (0.0 to 0.0)	25.5 (-66.4 to 372.9)	-1.7 (-73.7 to 270.2)
Andorra	Diabetes mellitus	5 to 9	Both	0 (0 to 0)	0.0 (0.0 to 0.1)	0 (0 to 0)	0.0 (0.0 to 0.0)	0 (0 to 0)	0.0 (0.0 to 0.0)	-26.0 (-52.1 to 14.9)	-45.2 (-64.5 to -14.9)
Andorra	Diabetes mellitus	5 to 9	Female	0 (0 to 0)	0.1 (0.0 to 0.1)	0 (0 to 0)	0.0 (0.0 to 0.0)	0 (0 to 0)	0.0 (0.0 to 0.0)	-34.7 (-62.6 to 13.9)	-51.6 (-72.3 to -15.6)
Andorra	Diabetes mellitus	5 to 9	Male	0 (0 to 0)	0.0 (0.0 to 0.0)	0 (0 to 0)	0.0 (0.0 to 0.0)	0 (0 to 0)	0.0 (0.0 to 0.0)	10.6 (-42.5 to 112.7)	-18.1 (-57.4 to 57.5)
Andorra	Diabetes mellitus	10 to 14	Both	0 (0 to 0)	0.0 (0.0 to 0.1)	0 (0 to 0)	0.0 (0.0 to 0.0)	0 (0 to 0)	0.0 (0.0 to 0.0)	-23.4 (-56.5 to 35.6)	-40.0 (-65.9 to 6.1)
Andorra	Diabetes mellitus	10 to 14	Female	0 (0 to 0)	0.1 (0.0 to 0.1)	0 (0 to 0)	0.0 (0.0 to 0.1)	0 (0 to 0)	0.0 (0.0 to 0.1)	-29.6 (-65.1 to 43.9)	-44.9 (-72.7 to 12.7)
Andorra	Diabetes mellitus	10 to 14	Male	0 (0 to 0)	0.0 (0.0 to 0.0)	0 (0 to 0)	0.0 (0.0 to 0.0)	0 (0 to 0)	0.0 (0.0 to 0.0)	-1.3 (-50.4 to 98.4)	-22.7 (-61.1 to 55.4)
Andorra	Acute glomerulonephritis	5 to 9	Both	0 (0 to 0)	0.0 (0.0 to 0.0)	0 (0 to 0)	0.0 (0.0 to 0.0)	0 (0 to 0)	0.0 (0.0 to 0.0)	25.3 (-3.4 to 64.3)	-7.2 (-28.4 to 21.7)
Andorra	Acute glomerulonephritis	5 to 9	Female	0 (0 to 0)	0.0 (0.0 to 0.0)	0 (0 to 0)	0.0 (0.0 to 0.0)	0 (0 to 0)	0.0 (0.0 to 0.0)	25.0 (-4.7 to 65.9)	-7.5 (-29.4 to 22.9)
Andorra	Acute glomerulonephritis	5 to 9	Male	0 (0 to 0)	0.0 (0.0 to 0.0)	0 (0 to 0)	0.0 (0.0 to 0.0)	0 (0 to 0)	0.0 (0.0 to 0.0)	25.7 (-11.3 to 79.4)	-6.9 (-34.3 to 32.9)
Andorra	Acute glomerulonephritis	10 to 14	Both	0 (0 to 0)	0.0 (0.0 to 0.0)	0 (0 to 0)	0.0 (0.0 to 0.0)	0 (0 to 0)	0.0 (0.0 to 0.0)	23.0 (-4.6 to 55.8)	-3.7 (-25.3 to 22.0)
Andorra	Acute glomerulonephritis	10 to 14	Female	0 (0 to 0)	0.0 (0.0 to 0.0)	0 (0 to 0)	0.0 (0.0 to 0.0)	0 (0 to 0)	0.0 (0.0 to 0.0)	20.4 (-13.3 to 68.9)	-5.7 (-32.1 to 32.2)
Andorra	Acute glomerulonephritis	10 to 14	Male	0 (0 to 0)	0.0 (0.0 to 0.0)	0 (0 to 0)	0.0 (0.0 to 0.0)	0 (0 to 0)	0.0 (0.0 to 0.0)	25.5 (-13.9 to 72.2)	-1.7 (-32.6 to 34.9)
Andorra	Chronic kidney disease	5 to 9	Both	0 (0 to 0)	0.1 (0.0 to 0.1)	0 (0 to 0)	0.0 (0.0 to 0.1)	0 (0 to 0)	0.0 (0.0 to 0.1)	-19.8 (-47.0 to 23.1)	-40.6 (-60.7 to -8.9)
Andorra	Chronic kidney disease	5 to 9	Female	0 (0 to 0)	0.1 (0.0 to 0.1)	0 (0 to 0)	0.0 (0.0 to 0.0)	0 (0 to 0)	0.0 (0.0 to 0.1)	-13.4 (-50.6 to 52.9)	-35.9 (-63.4 to 13.2)
Andorra	Chronic kidney disease	5 to 9	Male	0 (0 to 0)	0.1 (0.0 to 0.1)	0 (0 to 0)	0.0 (0.0 to 0.1)	0 (0 to 0)	0.0 (0.0 to 0.1)	-24.7 (-57.9 to 31.7)	-44.2 (-68.8 to -2.5)
Andorra	Chronic kidney disease	10 to 14	Both	0 (0 to 0)	0.1 (0.0 to 0.1)	0 (0 to 0)	0.0 (0.0 to 0.0)	0 (0 to 0)	0.0 (0.0 to 0.0)	-17.3 (-44.2 to 24.0)	-35.2 (-56.3 to -2.9)
Andorra	Chronic kidney disease	10 to 14	Female	0 (0 to 0)	0.1 (0.0 to 0.1)	0 (0 to 0)	0.0 (0.0 to 0.1)	0 (0 to 0)	0.0 (0.0 to 0.1)	-17.0 (-52.8 to 45.3)	-35.0 (-63.0 to 13.8)
Andorra	Chronic kidney disease	10 to 14	Male	0 (0 to 0)	0.1 (0.0 to 0.1)	0 (0 to 0)	0.0 (0.0 to 0.0)	0 (0 to 0)	0.0 (0.0 to 0.1)	-17.6 (-50.9 to 39.5)	-35.5 (-61.5 to 9.2)

eTable 8. All-cause and cause-specific mortality (counts and rates) at the global level and by country in the WHO European Region for ages 5 to 9 and 10 to 14 for 1990, 2000, and 2016

Andorra	Urinary diseases and male infertility	5 to 9	Both	0 (0 to 0)	0.0 (0.0 to 0.0)	0 (0 to 0)	0.0 (0.0 to 0.0)	0 (0 to 0)	0.0 (0.0 to 0.0)	7.7 (-33.4 to 77.7)	-20.3 (-50.6 to 31.6)
Andorra	Urinary diseases and male infertility	5 to 9	Female	0 (0 to 0)	0.0 (0.0 to 0.1)	0 (0 to 0)	0.0 (0.0 to 0.0)	0 (0 to 0)	0.0 (0.0 to 0.0)	-0.7 (-48.2 to 83.7)	-26.4 (-61.6 to 36.1)
Andorra	Urinary diseases and male infertility	5 to 9	Male	0 (0 to 0)	0.0 (0.0 to 0.0)	0 (0 to 0)	0.0 (0.0 to 0.0)	0 (0 to 0)	0.0 (0.0 to 0.0)	29.5 (-23.3 to 127.6)	-4.1 (-43.2 to 68.6)
Andorra	Urinary diseases and male infertility	10 to 14	Both	0 (0 to 0)	0.0 (0.0 to 0.0)	0 (0 to 0)	0.0 (0.0 to 0.0)	0 (0 to 0)	0.0 (0.0 to 0.0)	-0.9 (-37.7 to 58.7)	-22.4 (-51.2 to 24.3)
Andorra	Urinary diseases and male infertility	10 to 14	Female	0 (0 to 0)	0.0 (0.0 to 0.0)	0 (0 to 0)	0.0 (0.0 to 0.0)	0 (0 to 0)	0.0 (0.0 to 0.0)	3.1 (-46.8 to 99.3)	-19.3 (-58.3 to 56.1)
Andorra	Urinary diseases and male infertility	10 to 14	Male	0 (0 to 0)	0.0 (0.0 to 0.0)	0 (0 to 0)	0.0 (0.0 to 0.0)	0 (0 to 0)	0.0 (0.0 to 0.0)	-6.4 (-49.6 to 73.0)	-26.8 (-60.6 to 35.4)
Andorra	Hemoglobinopathies and hemolytic anemias	5 to 9	Both	0 (0 to 0)	0.1 (0.1 to 0.2)	0 (0 to 0)	0.1 (0.0 to 0.1)	0 (0 to 0)	0.1 (0.0 to 0.1)	-37.0 (-62.4 to -0.9)	-53.3 (-72.1 to -26.6)
Andorra	Hemoglobinopathies and hemolytic anemias	5 to 9	Female	0 (0 to 0)	0.1 (0.1 to 0.2)	0 (0 to 0)	0.1 (0.0 to 0.1)	0 (0 to 0)	0.1 (0.0 to 0.1)	-35.6 (-69.5 to 18.3)	-52.3 (-77.4 to -12.4)
Andorra	Hemoglobinopathies and hemolytic anemias	5 to 9	Male	0 (0 to 0)	0.2 (0.1 to 0.3)	0 (0 to 0)	0.1 (0.0 to 0.2)	0 (0 to 0)	0.1 (0.0 to 0.1)	-38.0 (-68.3 to 11.2)	-54.1 (-76.5 to -17.6)
Andorra	Hemoglobinopathies and hemolytic anemias	10 to 14	Both	0 (0 to 0)	0.1 (0.1 to 0.2)	0 (0 to 0)	0.1 (0.0 to 0.1)	0 (0 to 0)	0.1 (0.0 to 0.1)	-29.9 (-56.8 to 6.0)	-45.1 (-66.2 to -17.0)
Andorra	Hemoglobinopathies and hemolytic anemias	10 to 14	Female	0 (0 to 0)	0.1 (0.1 to 0.2)	0 (0 to 0)	0.1 (0.0 to 0.1)	0 (0 to 0)	0.1 (0.0 to 0.1)	-31.1 (-64.5 to 25.7)	-46.0 (-72.2 to -1.6)
Andorra	Hemoglobinopathies and hemolytic anemias	10 to 14	Male	0 (0 to 0)	0.1 (0.1 to 0.2)	0 (0 to 0)	0.1 (0.0 to 0.1)	0 (0 to 0)	0.1 (0.0 to 0.1)	-29.1 (-63.7 to 25.4)	-44.5 (-71.6 to -1.8)
Andorra	Endocrine, metabolic, blood, and immune disorders	5 to 9	Both	0 (0 to 0)	0.6 (0.3 to 0.8)	0 (0 to 0)	0.3 (0.2 to 0.5)	0 (0 to 0)	0.4 (0.2 to 0.6)	-6.8 (-43.6 to 58.2)	-31.0 (-58.2 to 17.2)
Andorra	Endocrine, metabolic, blood, and immune disorders	5 to 9	Female	0 (0 to 0)	0.5 (0.3 to 0.9)	0 (0 to 0)	0.3 (0.2 to 0.6)	0 (0 to 0)	0.4 (0.2 to 0.6)	-5.7 (-55.6 to 94.6)	-30.2 (-67.1 to 44.1)
Andorra	Endocrine, metabolic, blood, and immune disorders	5 to 9	Male	0 (0 to 0)	0.6 (0.3 to 1.0)	0 (0 to 0)	0.4 (0.2 to 0.6)	0 (0 to 0)	0.4 (0.2 to 0.7)	-7.7 (-54.6 to 87.9)	-31.7 (-66.3 to 39.2)
Andorra	Endocrine, metabolic, blood, and immune disorders	10 to 14	Both	0 (0 to 0)	0.6 (0.4 to 0.8)	0 (0 to 0)	0.4 (0.3 to 0.6)	0 (0 to 0)	0.4 (0.3 to 0.6)	-7.3 (-42.1 to 46.0)	-27.4 (-54.7 to 14.3)

eTable 8. All-cause and cause-specific mortality (counts and rates) at the global level and by country in the WHO European Region for ages 5 to 9 and 10 to 14 for 1990, 2000, and 2016

Andorra	Endocrine, metabolic, blood, and immune disorders	10 to 14	Female	0 (0 to 0)	0.5 (0.3 to 0.9)	0 (0 to 0)	0.3 (0.2 to 0.6)	0 (0 to 0)	0.3 (0.2 to 0.6)	-10.8 (-56.0 to 75.3)	-30.2 (-65.5 to 37.2)
Andorra	Endocrine, metabolic, blood, and immune disorders	10 to 14	Male	0 (0 to 0)	0.6 (0.4 to 1.1)	0 (0 to 0)	0.4 (0.2 to 0.8)	0 (0 to 0)	0.5 (0.3 to 0.8)	-4.8 (-48.9 to 75.1)	-25.4 (-60.0 to 37.1)
Andorra	Rheumatoid arthritis	5 to 9	Both	0 (0 to 0)	0.0 (0.0 to 0.0)	0 (0 to 0)	0.0 (0.0 to 0.0)	0 (0 to 0)	0.0 (0.0 to 0.0)	3.1 (-41.2 to 81.9)	-23.6 (-56.4 to 34.7)
Andorra	Rheumatoid arthritis	5 to 9	Female	0 (0 to 0)	0.0 (0.0 to 0.0)	0 (0 to 0)	0.0 (0.0 to 0.0)	0 (0 to 0)	0.0 (0.0 to 0.0)	-10.1 (-56.8 to 87.6)	-33.4 (-68.0 to 38.9)
Andorra	Rheumatoid arthritis	5 to 9	Male	0 (0 to 0)	0.0 (0.0 to 0.0)	0 (0 to 0)	0.0 (0.0 to 0.0)	0 (0 to 0)	0.0 (0.0 to 0.0)	18.7 (-45.4 to 167.3)	-12.1 (-59.6 to 98.0)
Andorra	Rheumatoid arthritis	10 to 14	Both	0 (0 to 0)	0.0 (0.0 to 0.0)	0 (0 to 0)	0.0 (0.0 to 0.0)	0 (0 to 0)	0.0 (0.0 to 0.0)	3.9 (-41.2 to 89.3)	-18.6 (-53.9 to 48.2)
Andorra	Rheumatoid arthritis	10 to 14	Female	0 (0 to 0)	0.0 (0.0 to 0.0)	0 (0 to 0)	0.0 (0.0 to 0.0)	0 (0 to 0)	0.0 (0.0 to 0.0)	-9.7 (-60.0 to 106.0)	-29.3 (-68.7 to 61.2)
Andorra	Rheumatoid arthritis	10 to 14	Male	0 (0 to 0)	0.0 (0.0 to 0.0)	0 (0 to 0)	0.0 (0.0 to 0.0)	0 (0 to 0)	0.0 (0.0 to 0.0)	17.5 (-43.2 to 160.0)	-8.0 (-55.6 to 103.6)
Andorra	Other musculoskeletal disorders	5 to 9	Both	0 (0 to 0)	0.0 (0.0 to 0.1)	0 (0 to 0)	0.0 (0.0 to 0.0)	0 (0 to 0)	0.0 (0.0 to 0.0)	-0.8 (-39.7 to 60.6)	-26.5 (-55.4 to 18.9)
Andorra	Other musculoskeletal disorders	5 to 9	Female	0 (0 to 0)	0.1 (0.0 to 0.1)	0 (0 to 0)	0.0 (0.0 to 0.1)	0 (0 to 0)	0.0 (0.0 to 0.1)	-2.5 (-47.9 to 85.9)	-27.8 (-61.4 to 37.7)
Andorra	Other musculoskeletal disorders	5 to 9	Male	0 (0 to 0)	0.0 (0.0 to 0.0)	0 (0 to 0)	0.0 (0.0 to 0.0)	0 (0 to 0)	0.0 (0.0 to 0.0)	2.0 (-49.3 to 115.9)	-24.4 (-62.4 to 59.9)
Andorra	Other musculoskeletal disorders	10 to 14	Both	0 (0 to 0)	0.1 (0.0 to 0.1)	0 (0 to 0)	0.0 (0.0 to 0.1)	0 (0 to 0)	0.0 (0.0 to 0.1)	0.7 (-36.6 to 59.0)	-21.1 (-50.4 to 24.5)
Andorra	Other musculoskeletal disorders	10 to 14	Female	0 (0 to 0)	0.1 (0.0 to 0.1)	0 (0 to 0)	0.1 (0.0 to 0.1)	0 (0 to 0)	0.1 (0.0 to 0.1)	-3.5 (-49.8 to 72.1)	-24.5 (-60.7 to 34.7)
Andorra	Other musculoskeletal disorders	10 to 14	Male	0 (0 to 0)	0.0 (0.0 to 0.1)	0 (0 to 0)	0.0 (0.0 to 0.0)	0 (0 to 0)	0.0 (0.0 to 0.1)	9.4 (-43.0 to 118.5)	-14.3 (-55.4 to 71.1)
Andorra	Congenital birth defects	5 to 9	Both	0 (0 to 0)	1.5 (1.1 to 2.1)	0 (0 to 0)	0.9 (0.6 to 1.2)	0 (0 to 0)	0.9 (0.6 to 1.2)	-20.7 (-47.9 to 20.2)	-41.3 (-61.4 to -11.0)
Andorra	Congenital birth defects	5 to 9	Female	0 (0 to 0)	1.4 (0.9 to 2.2)	0 (0 to 0)	0.8 (0.5 to 1.3)	0 (0 to 0)	0.9 (0.6 to 1.3)	-19.3 (-54.6 to 44.3)	-40.2 (-66.4 to 6.9)
Andorra	Congenital birth defects	5 to 9	Male	0 (0 to 0)	1.5 (0.9 to 2.3)	0 (0 to 0)	0.9 (0.5 to 1.3)	0 (0 to 0)	0.9 (0.5 to 1.3)	-22.0 (-57.2 to 41.0)	-42.2 (-68.3 to 4.4)
Andorra	Congenital birth defects	10 to 14	Both	0 (0 to 0)	1.1 (0.8 to 1.5)	0 (0 to 0)	0.7 (0.5 to 1.0)	0 (0 to 0)	0.7 (0.5 to 1.0)	-15.4 (-43.8 to 26.2)	-33.7 (-56.0 to -1.2)
Andorra	Congenital birth defects	10 to 14	Female	0 (0 to 0)	1.1 (0.7 to 1.6)	0 (0 to 0)	0.7 (0.4 to 1.2)	0 (0 to 0)	0.7 (0.4 to 1.1)	-17.8 (-54.0 to 44.7)	-35.7 (-64.0 to 13.3)
Andorra	Congenital birth defects	10 to 14	Male	0 (0 to 0)	1.1 (0.7 to 1.7)	0 (0 to 0)	0.8 (0.5 to 1.1)	0 (0 to 0)	0.8 (0.5 to 1.1)	-13.2 (-50.4 to 49.1)	-32.0 (-61.2 to 16.7)
Andorra	Skin and subcutaneous diseases	5 to 9	Both	0 (0 to 0)	0.0 (0.0 to 0.0)	0 (0 to 0)	0.0 (0.0 to 0.0)	0 (0 to 0)	0.0 (0.0 to 0.0)	30.8 (-29.8 to 139.6)	-3.1 (-48.0 to 77.5)
Andorra	Skin and subcutaneous diseases	5 to 9	Female	0 (0 to 0)	0.0 (0.0 to 0.0)	0 (0 to 0)	0.0 (0.0 to 0.0)	0 (0 to 0)	0.0 (0.0 to 0.0)	29.3 (-37.8 to 173.9)	-4.3 (-54.0 to 102.8)
Andorra	Skin and subcutaneous diseases	5 to 9	Male	0 (0 to 0)	0.0 (0.0 to 0.0)	0 (0 to 0)	0.0 (0.0 to 0.0)	0 (0 to 0)	0.0 (0.0 to 0.0)	32.1 (-44.2 to 198.6)	-2.1 (-58.7 to 121.2)
Andorra	Skin and subcutaneous diseases	10 to 14	Both	0 (0 to 0)	0.0 (0.0 to 0.0)	0 (0 to 0)	0.0 (0.0 to 0.0)	0 (0 to 0)	0.0 (0.0 to 0.0)	36.3 (-19.3 to 146.6)	6.7 (-36.9 to 93.1)

eTable 8. All-cause and cause-specific mortality (counts and rates) at the global level and by country in the WHO European Region for ages 5 to 9 and 10 to 14 for 1990, 2000, and 2016

Andorra	Skin and subcutaneous diseases	10 to 14	Female	0 (0 to 0)	0.0 (0.0 to 0.0)	0 (0 to 0)	0.0 (0.0 to 0.0)	0 (0 to 0)	0.0 (0.0 to 0.0)	27.9 (-36.7 to 167.0)	0.2 (-50.4 to 109.1)
Andorra	Skin and subcutaneous diseases	10 to 14	Male	0 (0 to 0)	0.0 (0.0 to 0.0)	0 (0 to 0)	0.0 (0.0 to 0.0)	0 (0 to 0)	0.0 (0.0 to 0.0)	44.2 (-32.6 to 218.8)	12.9 (-47.2 to 149.6)
Andorra	Road injuries	5 to 9	Both	0 (0 to 0)	3.0 (2.2 to 3.9)	0 (0 to 0)	1.4 (1.0 to 2.0)	0 (0 to 0)	1.1 (0.8 to 1.5)	-49.0 (-67.1 to -21.1)	-62.2 (-75.6 to -41.6)
Andorra	Road injuries	5 to 9	Female	0 (0 to 0)	2.0 (1.2 to 2.9)	0 (0 to 0)	1.0 (0.6 to 1.5)	0 (0 to 0)	0.8 (0.5 to 1.2)	-45.4 (-68.5 to -6.6)	-59.6 (-76.7 to -30.8)
Andorra	Road injuries	5 to 9	Male	0 (0 to 0)	3.9 (2.7 to 5.5)	0 (0 to 0)	1.9 (1.2 to 2.6)	0 (0 to 0)	1.4 (0.9 to 2.1)	-50.7 (-72.8 to -12.5)	-63.5 (-79.9 to -35.2)
Andorra	Road injuries	10 to 14	Both	0 (0 to 0)	3.2 (2.4 to 4.1)	0 (0 to 0)	1.8 (1.3 to 2.3)	0 (0 to 0)	1.4 (1.0 to 1.8)	-45.3 (-63.0 to -19.2)	-57.1 (-71.0 to -36.7)
Andorra	Road injuries	10 to 14	Female	0 (0 to 0)	2.0 (1.3 to 2.8)	0 (0 to 0)	1.2 (0.7 to 1.8)	0 (0 to 0)	0.9 (0.5 to 1.3)	-42.7 (-68.3 to -2.7)	-55.2 (-75.2 to -23.8)
Andorra	Road injuries	10 to 14	Male	0 (0 to 0)	4.4 (3.0 to 6.0)	0 (0 to 0)	2.4 (1.6 to 3.2)	0 (0 to 0)	1.8 (1.2 to 2.6)	-46.4 (-68.2 to -13.6)	-58.0 (-75.1 to -32.3)
Andorra	Other transport injuries	5 to 9	Both	0 (0 to 0)	0.1 (0.0 to 0.1)	0 (0 to 0)	0.0 (0.0 to 0.1)	0 (0 to 0)	0.0 (0.0 to 0.1)	-4.9 (-58.1 to 117.3)	-29.5 (-68.9 to 61.0)
Andorra	Other transport injuries	5 to 9	Female	0 (0 to 0)	0.1 (0.0 to 0.1)	0 (0 to 0)	0.0 (0.0 to 0.1)	0 (0 to 0)	0.0 (0.0 to 0.1)	-8.2 (-70.0 to 187.5)	-32.0 (-77.8 to 112.9)
Andorra	Other transport injuries	5 to 9	Male	0 (0 to 0)	0.1 (0.0 to 0.1)	0 (0 to 0)	0.0 (0.0 to 0.1)	0 (0 to 0)	0.0 (0.0 to 0.1)	-0.9 (-62.0 to 163.6)	-26.6 (-71.9 to 95.3)
Andorra	Other transport injuries	10 to 14	Both	0 (0 to 0)	0.1 (0.1 to 0.2)	0 (0 to 0)	0.1 (0.1 to 0.2)	0 (0 to 0)	0.1 (0.1 to 0.2)	-4.9 (-53.0 to 81.3)	-25.6 (-63.2 to 41.9)
Andorra	Other transport injuries	10 to 14	Female	0 (0 to 0)	0.1 (0.0 to 0.1)	0 (0 to 0)	0.1 (0.0 to 0.1)	0 (0 to 0)	0.1 (0.0 to 0.1)	1.1 (-62.8 to 187.3)	-20.9 (-70.9 to 124.9)
Andorra	Other transport injuries	10 to 14	Male	0 (0 to 0)	0.2 (0.1 to 0.3)	0 (0 to 0)	0.1 (0.1 to 0.3)	0 (0 to 0)	0.1 (0.1 to 0.2)	-7.2 (-59.6 to 113.8)	-27.4 (-68.4 to 67.4)
Andorra	Falls	5 to 9	Both	0 (0 to 0)	0.2 (0.1 to 0.4)	0 (0 to 0)	0.1 (0.1 to 0.2)	0 (0 to 0)	0.1 (0.1 to 0.2)	-26.2 (-57.3 to 24.6)	-45.4 (-68.4 to -7.7)
Andorra	Falls	5 to 9	Female	0 (0 to 0)	0.1 (0.1 to 0.2)	0 (0 to 0)	0.1 (0.0 to 0.1)	0 (0 to 0)	0.1 (0.0 to 0.1)	-20.8 (-60.1 to 57.5)	-41.4 (-70.4 to 16.6)
Andorra	Falls	5 to 9	Male	0 (0 to 0)	0.3 (0.2 to 0.5)	0 (0 to 0)	0.2 (0.1 to 0.3)	0 (0 to 0)	0.2 (0.1 to 0.3)	-28.5 (-64.9 to 39.2)	-47.0 (-74.0 to 3.1)
Andorra	Falls	10 to 14	Both	0 (0 to 0)	0.3 (0.2 to 0.5)	0 (0 to 0)	0.2 (0.1 to 0.3)	0 (0 to 0)	0.2 (0.1 to 0.3)	-22.4 (-54.0 to 30.5)	-39.3 (-64.0 to 2.2)
Andorra	Falls	10 to 14	Female	0 (0 to 0)	0.1 (0.1 to 0.2)	0 (0 to 0)	0.1 (0.0 to 0.1)	0 (0 to 0)	0.1 (0.0 to 0.1)	-20.0 (-58.8 to 49.8)	-37.4 (-67.7 to 17.2)
Andorra	Falls	10 to 14	Male	0 (0 to 0)	0.5 (0.3 to 0.8)	0 (0 to 0)	0.3 (0.2 to 0.5)	0 (0 to 0)	0.3 (0.2 to 0.5)	-23.0 (-61.5 to 39.6)	-39.8 (-69.9 to 9.3)
Andorra	Drowning	5 to 9	Both	0 (0 to 0)	0.4 (0.3 to 0.7)	0 (0 to 0)	0.2 (0.1 to 0.4)	0 (0 to 0)	0.2 (0.1 to 0.4)	-32.3 (-63.2 to 20.7)	-49.9 (-72.7 to -10.6)
Andorra	Drowning	5 to 9	Female	0 (0 to 0)	0.2 (0.1 to 0.4)	0 (0 to 0)	0.1 (0.1 to 0.2)	0 (0 to 0)	0.1 (0.1 to 0.2)	-27.0 (-64.2 to 49.6)	-46.0 (-73.5 to 10.8)
Andorra	Drowning	5 to 9	Male	0 (0 to 0)	0.7 (0.4 to 1.1)	0 (0 to 0)	0.3 (0.2 to 0.6)	0 (0 to 0)	0.3 (0.2 to 0.6)	-33.9 (-68.4 to 30.8)	-51.0 (-76.6 to -3.1)
Andorra	Drowning	10 to 14	Both	0 (0 to 0)	0.3 (0.2 to 0.6)	0 (0 to 0)	0.2 (0.1 to 0.3)	0 (0 to 0)	0.2 (0.1 to 0.3)	-27.0 (-59.9 to 28.9)	-42.8 (-68.6 to 0.9)
Andorra	Drowning	10 to 14	Female	0 (0 to 0)	0.1 (0.1 to 0.3)	0 (0 to 0)	0.1 (0.0 to 0.2)	0 (0 to 0)	0.1 (0.0 to 0.2)	-25.0 (-64.6 to 58.9)	-41.2 (-72.3 to 24.4)
Andorra	Drowning	10 to 14	Male	0 (0 to 0)	0.5 (0.3 to 0.9)	0 (0 to 0)	0.3 (0.2 to 0.5)	0 (0 to 0)	0.3 (0.2 to 0.5)	-27.5 (-65.0 to 46.9)	-43.3 (-72.6 to 15.0)
Andorra	Fire, heat, and hot substances	5 to 9	Both	0 (0 to 0)	0.4 (0.2 to 0.6)	0 (0 to 0)	0.2 (0.1 to 0.3)	0 (0 to 0)	0.2 (0.1 to 0.2)	-42.4 (-67.4 to -4.9)	-57.3 (-75.9 to -29.6)
Andorra	Fire, heat, and hot substances	5 to 9	Female	0 (0 to 0)	0.3 (0.2 to 0.6)	0 (0 to 0)	0.2 (0.1 to 0.3)	0 (0 to 0)	0.1 (0.1 to 0.2)	-41.5 (-71.4 to 2.2)	-56.7 (-78.9 to -24.3)

eTable 8. All-cause and cause-specific mortality (counts and rates) at the global level and by country in the WHO European Region for ages 5 to 9 and 10 to 14 for 1990, 2000, and 2016

Andorra	Fire, heat, and hot substances	5 to 9	Male	0 (0 to 0)	0.4 (0.2 to 0.7)	0 (0 to 0)	0.2 (0.1 to 0.4)	0 (0 to 0)	0.2 (0.1 to 0.3)	-43.1 (-74.5 to 11.2)	-57.8 (-81.1 to -17.6)
Andorra	Fire, heat, and hot substances	10 to 14	Both	0 (0 to 0)	0.2 (0.1 to 0.3)	0 (0 to 0)	0.1 (0.1 to 0.2)	0 (0 to 0)	0.1 (0.1 to 0.1)	-39.2 (-65.4 to 0.7)	-52.4 (-72.9 to -21.2)
Andorra	Fire, heat, and hot substances	10 to 14	Female	0 (0 to 0)	0.2 (0.1 to 0.3)	0 (0 to 0)	0.1 (0.1 to 0.2)	0 (0 to 0)	0.1 (0.0 to 0.1)	-40.5 (-73.1 to 16.7)	-53.4 (-78.9 to -8.6)
Andorra	Fire, heat, and hot substances	10 to 14	Male	0 (0 to 0)	0.2 (0.1 to 0.3)	0 (0 to 0)	0.1 (0.1 to 0.2)	0 (0 to 0)	0.1 (0.1 to 0.2)	-38.2 (-72.3 to 21.3)	-51.6 (-78.3 to -5.0)
Andorra	Poisonings	5 to 9	Both	0 (0 to 0)	0.0 (0.0 to 0.1)	0 (0 to 0)	0.0 (0.0 to 0.0)	0 (0 to 0)	0.0 (0.0 to 0.0)	-40.5 (-68.6 to 16.8)	-55.9 (-76.7 to -13.5)
Andorra	Poisonings	5 to 9	Female	0 (0 to 0)	0.0 (0.0 to 0.1)	0 (0 to 0)	0.0 (0.0 to 0.0)	0 (0 to 0)	0.0 (0.0 to 0.0)	-31.9 (-71.5 to 55.2)	-49.6 (-78.9 to 14.9)
Andorra	Poisonings	5 to 9	Male	0 (0 to 0)	0.0 (0.0 to 0.1)	0 (0 to 0)	0.0 (0.0 to 0.0)	0 (0 to 0)	0.0 (0.0 to 0.0)	-47.8 (-79.5 to 24.9)	-61.4 (-84.8 to -7.5)
Andorra	Poisonings	10 to 14	Both	0 (0 to 0)	0.1 (0.1 to 0.2)	0 (0 to 0)	0.1 (0.0 to 0.1)	0 (0 to 0)	0.1 (0.0 to 0.1)	-39.6 (-66.1 to 3.6)	-52.7 (-73.5 to -18.9)
Andorra	Poisonings	10 to 14	Female	0 (0 to 0)	0.1 (0.1 to 0.2)	0 (0 to 0)	0.1 (0.0 to 0.1)	0 (0 to 0)	0.1 (0.0 to 0.1)	-30.7 (-68.1 to 43.6)	-45.8 (-75.1 to 12.4)
Andorra	Poisonings	10 to 14	Male	0 (0 to 0)	0.1 (0.1 to 0.2)	0 (0 to 0)	0.1 (0.0 to 0.1)	0 (0 to 0)	0.1 (0.0 to 0.1)	-45.5 (-75.6 to 5.9)	-57.3 (-80.9 to -17.1)
Andorra	Exposure to mechanical forces	5 to 9	Both	0 (0 to 0)	0.3 (0.2 to 0.4)	0 (0 to 0)	0.1 (0.1 to 0.2)	0 (0 to 0)	0.1 (0.1 to 0.2)	-26.4 (-56.5 to 24.1)	-45.5 (-67.8 to -8.1)
Andorra	Exposure to mechanical forces	5 to 9	Female	0 (0 to 0)	0.2 (0.1 to 0.3)	0 (0 to 0)	0.1 (0.1 to 0.2)	0 (0 to 0)	0.1 (0.1 to 0.2)	-11.7 (-50.5 to 56.7)	-34.6 (-63.3 to 16.0)
Andorra	Exposure to mechanical forces	5 to 9	Male	0 (0 to 0)	0.3 (0.2 to 0.6)	0 (0 to 0)	0.2 (0.1 to 0.3)	0 (0 to 0)	0.2 (0.1 to 0.3)	-33.7 (-69.7 to 33.4)	-50.9 (-77.6 to -1.2)
Andorra	Exposure to mechanical forces	10 to 14	Both	0 (0 to 0)	0.4 (0.2 to 0.5)	0 (0 to 0)	0.2 (0.1 to 0.3)	0 (0 to 0)	0.2 (0.1 to 0.3)	-23.9 (-51.8 to 22.5)	-40.4 (-62.3 to -4.1)
Andorra	Exposure to mechanical forces	10 to 14	Female	0 (0 to 0)	0.1 (0.1 to 0.2)	0 (0 to 0)	0.1 (0.1 to 0.1)	0 (0 to 0)	0.1 (0.1 to 0.1)	-7.6 (-46.2 to 64.6)	-27.7 (-57.9 to 28.9)
Andorra	Exposure to mechanical forces	10 to 14	Male	0 (0 to 0)	0.6 (0.4 to 0.9)	0 (0 to 0)	0.3 (0.2 to 0.5)	0 (0 to 0)	0.3 (0.2 to 0.5)	-27.1 (-58.9 to 28.5)	-42.9 (-67.9 to 0.6)
Andorra	Adverse effects of medical treatment	5 to 9	Both	0 (0 to 0)	0.1 (0.1 to 0.1)	0 (0 to 0)	0.1 (0.0 to 0.1)	0 (0 to 0)	0.1 (0.0 to 0.1)	-6.4 (-39.5 to 52.3)	-30.7 (-55.2 to 12.8)
Andorra	Adverse effects of medical treatment	5 to 9	Female	0 (0 to 0)	0.1 (0.0 to 0.1)	0 (0 to 0)	0.0 (0.0 to 0.1)	0 (0 to 0)	0.1 (0.0 to 0.1)	-14.9 (-53.2 to 64.4)	-37.0 (-65.3 to 21.8)
Andorra	Adverse effects of medical treatment	5 to 9	Male	0 (0 to 0)	0.1 (0.0 to 0.1)	0 (0 to 0)	0.1 (0.0 to 0.1)	0 (0 to 0)	0.1 (0.0 to 0.1)	1.1 (-42.1 to 102.7)	-25.1 (-57.1 to 50.2)
Andorra	Adverse effects of medical treatment	10 to 14	Both	0 (0 to 0)	0.1 (0.1 to 0.1)	0 (0 to 0)	0.1 (0.0 to 0.1)	0 (0 to 0)	0.1 (0.0 to 0.1)	-1.9 (-35.2 to 62.9)	-23.2 (-49.2 to 27.5)
Andorra	Adverse effects of medical treatment	10 to 14	Female	0 (0 to 0)	0.1 (0.0 to 0.1)	0 (0 to 0)	0.0 (0.0 to 0.1)	0 (0 to 0)	0.0 (0.0 to 0.1)	-12.8 (-51.7 to 71.5)	-31.7 (-62.2 to 34.3)
Andorra	Adverse effects of medical treatment	10 to 14	Male	0 (0 to 0)	0.1 (0.0 to 0.1)	0 (0 to 0)	0.1 (0.0 to 0.1)	0 (0 to 0)	0.1 (0.0 to 0.1)	6.7 (-39.1 to 101.7)	-16.5 (-52.3 to 57.9)
Andorra	Animal contact	5 to 9	Both	0 (0 to 0)	0.0 (0.0 to 0.0)	0 (0 to 0)	0.0 (0.0 to 0.0)	0 (0 to 0)	0.0 (0.0 to 0.0)	22.9 (-21.1 to 83.2)	-9.0 (-41.5 to 35.7)

eTable 8. All-cause and cause-specific mortality (counts and rates) at the global level and by country in the WHO European Region for ages 5 to 9 and 10 to 14 for 1990, 2000, and 2016

Andorra	Animal contact	5 to 9	Female	0<br>(0 to 0)	0.0<br>(0.0 to 0.0)	0<br>(0 to 0)	0.0<br>(0.0 to 0.0)	0<br>(0 to 0)	0.0<br>(0.0 to 0.0)	24.5<br>(-13.8 to 75.7)	-7.8<br>(-36.2 to 30.1)
Andorra	Animal contact	5 to 9	Male	0<br>(0 to 0)	0.0<br>(0.0 to 0.0)	0<br>(0 to 0)	0.0<br>(0.0 to 0.0)	0<br>(0 to 0)	0.0<br>(0.0 to 0.0)	21.5<br>(-35.1 to 118.0)	-10.0<br>(-51.9 to 61.5)
Andorra	Animal contact	10 to 14	Both	0<br>(0 to 0)	0.0<br>(0.0 to 0.0)	0<br>(0 to 0)	0.0<br>(0.0 to 0.0)	0<br>(0 to 0)	0.0<br>(0.0 to 0.0)	16.1<br>(-25.3 to 86.9)	-9.1<br>(-41.5 to 46.3)
Andorra	Animal contact	10 to 14	Female	0<br>(0 to 0)	0.0<br>(0.0 to 0.0)	0<br>(0 to 0)	0.0<br>(0.0 to 0.0)	0<br>(0 to 0)	0.0<br>(0.0 to 0.0)	21.6<br>(-18.5 to 83.9)	-4.8<br>(-36.2 to 44.0)
Andorra	Animal contact	10 to 14	Male	0<br>(0 to 0)	0.0<br>(0.0 to 0.0)	0<br>(0 to 0)	0.0<br>(0.0 to 0.0)	0<br>(0 to 0)	0.0<br>(0.0 to 0.0)	12.3<br>(-42.9 to 131.3)	-12.1<br>(-55.3 to 81.1)
Andorra	Foreign body	5 to 9	Both	0<br>(0 to 0)	0.2<br>(0.1 to 0.3)	0<br>(0 to 0)	0.1<br>(0.1 to 0.2)	0<br>(0 to 0)	0.1<br>(0.1 to 0.2)	-10.8<br>(-52.5 to 54.1)	-34.0<br>(-64.8 to 14.1)
Andorra	Foreign body	5 to 9	Female	0<br>(0 to 0)	0.2<br>(0.1 to 0.3)	0<br>(0 to 0)	0.1<br>(0.1 to 0.2)	0<br>(0 to 0)	0.1<br>(0.1 to 0.2)	-7.9<br>(-56.1 to 83.4)	-31.8<br>(-67.5 to 35.8)
Andorra	Foreign body	5 to 9	Male	0<br>(0 to 0)	0.2<br>(0.1 to 0.3)	0<br>(0 to 0)	0.1<br>(0.1 to 0.2)	0<br>(0 to 0)	0.1<br>(0.1 to 0.2)	-13.3<br>(-64.1 to 78.4)	-35.8<br>(-73.4 to 32.1)
Andorra	Foreign body	10 to 14	Both	0<br>(0 to 0)	0.3<br>(0.2 to 0.4)	0<br>(0 to 0)	0.2<br>(0.1 to 0.3)	0<br>(0 to 0)	0.2<br>(0.1 to 0.3)	-15.2<br>(-55.6 to 41.9)	-33.6<br>(-65.3 to 11.1)
Andorra	Foreign body	10 to 14	Female	0<br>(0 to 0)	0.2<br>(0.1 to 0.2)	0<br>(0 to 0)	0.1<br>(0.1 to 0.2)	0<br>(0 to 0)	0.1<br>(0.1 to 0.2)	-5.2<br>(-56.7 to 77.0)	-25.7<br>(-66.1 to 38.5)
Andorra	Foreign body	10 to 14	Male	0<br>(0 to 0)	0.4<br>(0.2 to 0.7)	0<br>(0 to 0)	0.3<br>(0.2 to 0.5)	0<br>(0 to 0)	0.3<br>(0.1 to 0.5)	-18.7<br>(-63.3 to 54.4)	-36.3<br>(-71.2 to 20.9)
Andorra	Environmental heat and cold exposure	5 to 9	Both	0<br>(0 to 0)	0.0<br>(0.0 to 0.0)	0<br>(0 to 0)	0.0<br>(0.0 to 0.0)	0<br>(0 to 0)	0.0<br>(0.0 to 0.0)	6.8<br>(-30.2 to 71.8)	-20.9<br>(-48.3 to 27.3)
Andorra	Environmental heat and cold exposure	5 to 9	Female	0<br>(0 to 0)	0.0<br>(0.0 to 0.0)	0<br>(0 to 0)	0.0<br>(0.0 to 0.0)	0<br>(0 to 0)	0.0<br>(0.0 to 0.0)	-3.4<br>(-43.5 to 83.6)	-28.5<br>(-58.2 to 35.9)
Andorra	Environmental heat and cold exposure	5 to 9	Male	0<br>(0 to 0)	0.0<br>(0.0 to 0.0)	0<br>(0 to 0)	0.0<br>(0.0 to 0.0)	0<br>(0 to 0)	0.0<br>(0.0 to 0.0)	19.6<br>(-28.7 to 91.5)	-11.4<br>(-47.2 to 41.8)
Andorra	Environmental heat and cold exposure	10 to 14	Both	0<br>(0 to 0)	0.0<br>(0.0 to 0.0)	0<br>(0 to 0)	0.0<br>(0.0 to 0.0)	0<br>(0 to 0)	0.0<br>(0.0 to 0.0)	-8.2<br>(-40.2 to 53.7)	-28.1<br>(-53.2 to 20.3)
Andorra	Environmental heat and cold exposure	10 to 14	Female	0<br>(0 to 0)	0.0<br>(0.0 to 0.0)	0<br>(0 to 0)	0.0<br>(0.0 to 0.0)	0<br>(0 to 0)	0.0<br>(0.0 to 0.0)	6.0<br>(-41.5 to 86.2)	-17.0<br>(-54.2 to 45.8)
Andorra	Environmental heat and cold exposure	10 to 14	Male	0<br>(0 to 0)	0.0<br>(0.0 to 0.0)	0<br>(0 to 0)	0.0<br>(0.0 to 0.0)	0<br>(0 to 0)	0.0<br>(0.0 to 0.0)	-17.2<br>(-52.1 to 67.1)	-35.1<br>(-62.5 to 30.8)
Andorra	Other unintentional injuries	5 to 9	Both	0<br>(0 to 0)	0.0<br>(0.0 to 0.1)	0<br>(0 to 0)	0.0<br>(0.0 to 0.0)	0<br>(0 to 0)	0.0<br>(0.0 to 0.0)	-58.3<br>(-75.2 to -24.2)	-69.1<br>(-81.6 to -43.8)
Andorra	Other unintentional injuries	5 to 9	Female	0<br>(0 to 0)	0.0<br>(0.0 to 0.1)	0<br>(0 to 0)	0.0<br>(0.0 to 0.0)	0<br>(0 to 0)	0.0<br>(0.0 to 0.0)	-54.5<br>(-76.1 to 2.4)	-66.3<br>(-82.3 to -24.2)
Andorra	Other unintentional injuries	5 to 9	Male	0<br>(0 to 0)	0.0<br>(0.0 to 0.1)	0<br>(0 to 0)	0.0<br>(0.0 to 0.0)	0<br>(0 to 0)	0.0<br>(0.0 to 0.0)	-61.0<br>(-81.1 to -13.7)	-71.1<br>(-86.0 to -36.0)
Andorra	Other unintentional injuries	10 to 14	Both	0<br>(0 to 0)	0.1<br>(0.0 to 0.1)	0<br>(0 to 0)	0.0<br>(0.0 to 0.1)	0<br>(0 to 0)	0.0<br>(0.0 to 0.0)	-63.2<br>(-80.7 to -28.7)	-71.2<br>(-84.9 to -44.2)
Andorra	Other unintentional injuries	10 to 14	Female	0<br>(0 to 0)	0.0<br>(0.0 to 0.1)	0<br>(0 to 0)	0.0<br>(0.0 to 0.0)	0<br>(0 to 0)	0.0<br>(0.0 to 0.0)	-54.7<br>(-78.0 to -0.6)	-64.6<br>(-82.8 to -22.2)
Andorra	Other unintentional injuries	10 to 14	Male	0<br>(0 to 0)	0.1<br>(0.1 to 0.2)	0<br>(0 to 0)	0.0<br>(0.0 to 0.1)	0<br>(0 to 0)	0.0<br>(0.0 to 0.1)	-65.0<br>(-85.0 to -22.6)	-72.6<br>(-88.2 to -39.4)
Andorra	Self-harm	10 to 14	Both	0<br>(0 to 0)	0.3<br>(0.2 to 0.5)	0<br>(0 to 0)	0.3<br>(0.2 to 0.4)	0<br>(0 to 0)	0.3<br>(0.2 to 0.4)	0.6<br>(-43.5 to 86.8)	-21.2<br>(-55.8 to 46.3)
Andorra	Self-harm	10 to 14	Female	0<br>(0 to 0)	0.3<br>(0.1 to 0.5)	0<br>(0 to 0)	0.2<br>(0.1 to 0.4)	0<br>(0 to 0)	0.2<br>(0.1 to 0.3)	-15.3<br>(-62.5 to 89.1)	-33.7<br>(-70.6 to 48.1)
Andorra	Self-harm	10 to 14	Male	0<br>(0 to 0)	0.4<br>(0.2 to 0.7)	0<br>(0 to 0)	0.3<br>(0.2 to 0.5)	0<br>(0 to 0)	0.3<br>(0.2 to 0.6)	11.9<br>(-49.6 to 156.6)	-12.4<br>(-60.6 to 100.9)

eTable 8. All-cause and cause-specific mortality (counts and rates) at the global level and by country in the WHO European Region for ages 5 to 9 and 10 to 14 for 1990, 2000, and 2016

Andorra	Interpersonal violence	5 to 9	Both	0 (0 to 0)	0.3 (0.2 to 0.5)	0 (0 to 0)	0.2 (0.1 to 0.3)	0 (0 to 0)	0.2 (0.1 to 0.3)	-17.0 (-55.7 to 45.1)	-38.5 (-67.2 to 7.4)
Andorra	Interpersonal violence	5 to 9	Female	0 (0 to 0)	0.4 (0.2 to 0.6)	0 (0 to 0)	0.2 (0.1 to 0.4)	0 (0 to 0)	0.2 (0.1 to 0.4)	-18.8 (-64.5 to 66.6)	-39.9 (-73.7 to 23.4)
Andorra	Interpersonal violence	5 to 9	Male	0 (0 to 0)	0.3 (0.1 to 0.5)	0 (0 to 0)	0.2 (0.1 to 0.4)	0 (0 to 0)	0.2 (0.1 to 0.4)	-14.7 (-63.4 to 108.1)	-36.8 (-72.9 to 54.1)
Andorra	Interpersonal violence	10 to 14	Both	0 (0 to 0)	0.3 (0.2 to 0.5)	0 (0 to 0)	0.2 (0.1 to 0.4)	0 (0 to 0)	0.2 (0.1 to 0.4)	-10.8 (-50.3 to 65.1)	-30.2 (-61.1 to 29.3)
Andorra	Interpersonal violence	10 to 14	Female	0 (0 to 0)	0.3 (0.1 to 0.5)	0 (0 to 0)	0.2 (0.1 to 0.4)	0 (0 to 0)	0.2 (0.1 to 0.4)	-19.0 (-65.3 to 70.6)	-36.5 (-72.9 to 33.5)
Andorra	Interpersonal violence	10 to 14	Male	0 (0 to 0)	0.3 (0.1 to 0.6)	0 (0 to 0)	0.2 (0.1 to 0.5)	0 (0 to 0)	0.2 (0.1 to 0.5)	-3.6 (-56.1 to 121.6)	-24.5 (-65.7 to 73.5)
Andorra	Exposure to forces of nature	5 to 9	Both	0 (0 to 0)	0.0 (0.0 to 0.0)	0 (0 to 0)	0.0 (0.0 to 0.0)	0 (0 to 0)	0.0 (0.0 to 0.0)	0.0 (0.0 to 0.0)	0.0 (0.0 to 0.0)
Andorra	Exposure to forces of nature	5 to 9	Female	0 (0 to 0)	0.0 (0.0 to 0.0)	0 (0 to 0)	0.0 (0.0 to 0.0)	0 (0 to 0)	0.0 (0.0 to 0.0)	0.0 (0.0 to 0.0)	0.0 (0.0 to 0.0)
Andorra	Exposure to forces of nature	5 to 9	Male	0 (0 to 0)	0.0 (0.0 to 0.0)	0 (0 to 0)	0.0 (0.0 to 0.0)	0 (0 to 0)	0.0 (0.0 to 0.0)	0.0 (0.0 to 0.0)	0.0 (0.0 to 0.0)
Andorra	Exposure to forces of nature	10 to 14	Both	0 (0 to 0)	0.0 (0.0 to 0.0)	0 (0 to 0)	0.0 (0.0 to 0.0)	0 (0 to 0)	0.0 (0.0 to 0.0)	0.0 (0.0 to 0.0)	0.0 (0.0 to 0.0)
Andorra	Exposure to forces of nature	10 to 14	Female	0 (0 to 0)	0.0 (0.0 to 0.0)	0 (0 to 0)	0.0 (0.0 to 0.0)	0 (0 to 0)	0.0 (0.0 to 0.0)	0.0 (0.0 to 0.0)	0.0 (0.0 to 0.0)
Andorra	Exposure to forces of nature	10 to 14	Male	0 (0 to 0)	0.0 (0.0 to 0.0)	0 (0 to 0)	0.0 (0.0 to 0.0)	0 (0 to 0)	0.0 (0.0 to 0.0)	0.0 (0.0 to 0.0)	0.0 (0.0 to 0.0)
Andorra	Conflict and terrorism	5 to 9	Both	0 (0 to 0)	0.0 (0.0 to 0.0)	0 (0 to 0)	0.0 (0.0 to 0.0)	0 (0 to 0)	0.0 (0.0 to 0.0)	0.0 (0.0 to 0.0)	0.0 (0.0 to 0.0)
Andorra	Conflict and terrorism	5 to 9	Female	0 (0 to 0)	0.0 (0.0 to 0.0)	0 (0 to 0)	0.0 (0.0 to 0.0)	0 (0 to 0)	0.0 (0.0 to 0.0)	0.0 (0.0 to 0.0)	0.0 (0.0 to 0.0)
Andorra	Conflict and terrorism	5 to 9	Male	0 (0 to 0)	0.0 (0.0 to 0.0)	0 (0 to 0)	0.0 (0.0 to 0.0)	0 (0 to 0)	0.0 (0.0 to 0.0)	0.0 (0.0 to 0.0)	0.0 (0.0 to 0.0)
Andorra	Conflict and terrorism	10 to 14	Both	0 (0 to 0)	0.0 (0.0 to 0.0)	0 (0 to 0)	0.0 (0.0 to 0.0)	0 (0 to 0)	0.0 (0.0 to 0.0)	0.0 (0.0 to 0.0)	0.0 (0.0 to 0.0)
Andorra	Conflict and terrorism	10 to 14	Female	0 (0 to 0)	0.0 (0.0 to 0.0)	0 (0 to 0)	0.0 (0.0 to 0.0)	0 (0 to 0)	0.0 (0.0 to 0.0)	0.0 (0.0 to 0.0)	0.0 (0.0 to 0.0)
Andorra	Conflict and terrorism	10 to 14	Male	0 (0 to 0)	0.0 (0.0 to 0.0)	0 (0 to 0)	0.0 (0.0 to 0.0)	0 (0 to 0)	0.0 (0.0 to 0.0)	0.0 (0.0 to 0.0)	0.0 (0.0 to 0.0)
Andorra	Executions and police conflict	5 to 9	Both	0 (0 to 0)	0.0 (0.0 to 0.0)	0 (0 to 0)	0.0 (0.0 to 0.0)	0 (0 to 0)	0.0 (0.0 to 0.0)	25.3 (-3.4 to 64.3)	-7.2 (-28.4 to 21.7)
Andorra	Executions and police conflict	5 to 9	Female	0 (0 to 0)	0.0 (0.0 to 0.0)	0 (0 to 0)	0.0 (0.0 to 0.0)	0 (0 to 0)	0.0 (0.0 to 0.0)	25.0 (-4.7 to 65.9)	-7.5 (-29.4 to 22.9)
Andorra	Executions and police conflict	5 to 9	Male	0 (0 to 0)	0.0 (0.0 to 0.0)	0 (0 to 0)	0.0 (0.0 to 0.0)	0 (0 to 0)	0.0 (0.0 to 0.0)	25.7 (-11.3 to 79.4)	-6.9 (-34.3 to 32.9)
Andorra	Executions and police conflict	10 to 14	Both	0 (0 to 0)	0.0 (0.0 to 0.0)	0 (0 to 0)	0.0 (0.0 to 0.0)	0 (0 to 0)	0.0 (0.0 to 0.0)	23.0 (-4.6 to 55.8)	-3.7 (-25.3 to 22.0)
Andorra	Executions and police conflict	10 to 14	Female	0 (0 to 0)	0.0 (0.0 to 0.0)	0 (0 to 0)	0.0 (0.0 to 0.0)	0 (0 to 0)	0.0 (0.0 to 0.0)	20.4 (-13.3 to 68.9)	-5.7 (-32.1 to 32.2)
Andorra	Executions and police conflict	10 to 14	Male	0 (0 to 0)	0.0 (0.0 to 0.0)	0 (0 to 0)	0.0 (0.0 to 0.0)	0 (0 to 0)	0.0 (0.0 to 0.0)	25.5 (-13.9 to 72.2)	-1.7 (-32.6 to 34.9)
Armenia	All causes	5 to 9	Both	233 (202 to 266)	63.0 (54.6 to 72.0)	104 (89 to 123)	38.2 (32.8 to 45.3)	41 (32 to 52)	21.2 (16.3 to 26.9)	-82.2 (-86.8 to -76.5)	-66.3 (-75.0 to -55.5)
Armenia	All causes	5 to 9	Female	94 (81 to 106)	51.6 (44.4 to 58.1)	42 (36 to 50)	31.8 (27.0 to 38.2)	18 (14 to 23)	19.1 (14.7 to 24.1)	-80.6 (-85.7 to -74.0)	-63.1 (-72.8 to -50.5)

eTable 8. All-cause and cause-specific mortality (counts and rates) at the global level and by country in the WHO European Region for ages 5 to 9 and 10 to 14 for 1990, 2000, and 2016

Armenia	All causes	5 to 9	Male	139 (121 to 160)	74.0 (64.4 to 85.6)	62 (54 to 73)	44.3 (38.3 to 51.9)	23 (18 to 30)	23.3 (17.9 to 29.7)	-83.3 (-87.4 to -78.0)	-68.5 (-76.3 to -58.7)
Armenia	All causes	10 to 14	Both	142 (130 to 155)	44.7 (40.9 to 48.7)	105 (96 to 117)	32.2 (29.4 to 35.9)	35 (30 to 42)	21.7 (18.3 to 25.7)	-75.4 (-79.6 to -70.4)	-51.5 (-59.8 to -41.6)
Armenia	All causes	10 to 14	Female	47 (43 to 51)	30.2 (27.6 to 32.8)	39 (35 to 43)	23.9 (21.8 to 26.5)	13 (11 to 15)	16.5 (14.0 to 19.8)	-73.1 (-78.0 to -67.0)	-45.4 (-55.4 to -33.1)
Armenia	All causes	10 to 14	Male	95 (87 to 104)	58.8 (53.7 to 64.5)	67 (60 to 75)	40.4 (36.5 to 45.4)	22 (19 to 27)	26.5 (22.2 to 31.7)	-76.5 (-80.7 to -71.7)	-55.0 (-63.0 to -45.7)
Armenia	Tuberculosis	5 to 9	Both	0 (0 to 1)	0.1 (0.1 to 0.1)	0 (0 to 0)	0.1 (0.1 to 0.1)	0 (0 to 0)	0.0 (0.0 to 0.1)	-80.1 (-87.9 to -67.3)	-62.4 (-77.1 to -38.2)
Armenia	Tuberculosis	5 to 9	Female	0 (0 to 0)	0.1 (0.1 to 0.2)	0 (0 to 0)	0.1 (0.1 to 0.2)	0 (0 to 0)	0.1 (0.0 to 0.1)	-77.7 (-87.6 to -59.7)	-57.6 (-76.4 to -23.4)
Armenia	Tuberculosis	5 to 9	Male	0 (0 to 0)	0.1 (0.1 to 0.1)	0 (0 to 0)	0.1 (0.0 to 0.1)	0 (0 to 0)	0.0 (0.0 to 0.0)	-84.7 (-91.2 to -73.1)	-71.2 (-83.5 to -49.3)
Armenia	Tuberculosis	10 to 14	Both	0 (0 to 0)	0.0 (0.0 to 0.1)	0 (0 to 0)	0.1 (0.0 to 0.1)	0 (0 to 0)	0.0 (0.0 to 0.0)	-65.9 (-77.5 to -48.5)	-32.9 (-55.6 to 1.5)
Armenia	Tuberculosis	10 to 14	Female	0 (0 to 0)	0.1 (0.1 to 0.1)	0 (0 to 0)	0.1 (0.1 to 0.1)	0 (0 to 0)	0.1 (0.0 to 0.1)	-68.7 (-80.8 to -50.3)	-36.6 (-61.0 to 0.9)
Armenia	Tuberculosis	10 to 14	Male	0 (0 to 0)	0.0 (0.0 to 0.0)	0 (0 to 0)	0.0 (0.0 to 0.0)	0 (0 to 0)	0.0 (0.0 to 0.0)	-43.3 (-54.5 to -29.1)	8.8 (-12.6 to 36.0)
Armenia	HIV/AIDS	5 to 9	Both	0 (0 to 0)	0.0 (0.0 to 0.0)	0 (0 to 0)	0.0 (0.0 to 0.0)	0 (0 to 0)	0.1 (0.1 to 0.1)	1033.4 (652.1 to 1598.8)	2044.1 (1322.9 to 3113.7)
Armenia	HIV/AIDS	5 to 9	Female	0 (0 to 0)	0.0 (0.0 to 0.0)	0 (0 to 0)	0.0 (0.0 to 0.0)	0 (0 to 0)	0.2 (0.1 to 0.2)	1680.1 (871.4 to 2938.3)	3285.5 (1747.5 to 5678.2)
Armenia	HIV/AIDS	5 to 9	Male	0 (0 to 0)	0.0 (0.0 to 0.0)	0 (0 to 0)	0.0 (0.0 to 0.0)	0 (0 to 0)	0.1 (0.0 to 0.1)	503.2 (259.5 to 885.9)	1035.2 (576.6 to 1755.5)
Armenia	HIV/AIDS	10 to 14	Both	0 (0 to 0)	0.0 (0.0 to 0.0)	0 (0 to 0)	0.0 (0.0 to 0.0)	0 (0 to 0)	0.0 (0.0 to 0.0)	52.3 (1.7 to 124.8)	200.0 (100.3 to 343.1)
Armenia	HIV/AIDS	10 to 14	Female	0 (0 to 0)	0.0 (0.0 to 0.0)	0 (0 to 0)	0.0 (0.0 to 0.0)	0 (0 to 0)	0.0 (0.0 to 0.0)	1.3 (-45.2 to 80.8)	105.4 (11.1 to 266.4)
Armenia	HIV/AIDS	10 to 14	Male	0 (0 to 0)	0.0 (0.0 to 0.0)	0 (0 to 0)	0.0 (0.0 to 0.0)	0 (0 to 0)	0.0 (0.0 to 0.0)	96.0 (18.0 to 237.6)	276.0 (126.4 to 547.6)
Armenia	Diarrheal diseases	5 to 9	Both	8 (5 to 10)	2.1 (1.5 to 2.8)	3 (2 to 4)	1.0 (0.7 to 1.5)	0 (0 to 1)	0.2 (0.1 to 0.3)	-95.5 (-97.4 to -92.4)	-91.4 (-95.1 to -85.6)
Armenia	Diarrheal diseases	5 to 9	Female	4 (2 to 6)	2.1 (1.4 to 3.0)	1 (1 to 2)	1.1 (0.7 to 1.6)	0 (0 to 0)	0.2 (0.1 to 0.3)	-95.5 (-97.7 to -91.5)	-91.4 (-95.7 to -83.8)
Armenia	Diarrheal diseases	5 to 9	Male	4 (2 to 6)	2.0 (1.3 to 3.0)	1 (1 to 2)	1.0 (0.6 to 1.5)	0 (0 to 0)	0.2 (0.1 to 0.3)	-95.4 (-97.7 to -90.7)	-91.4 (-95.8 to -82.4)
Armenia	Diarrheal diseases	10 to 14	Both	3 (2 to 4)	1.0 (0.7 to 1.3)	2 (1 to 2)	0.6 (0.4 to 0.8)	0 (0 to 0)	0.1 (0.1 to 0.2)	-94.4 (-96.6 to -91.0)	-89.0 (-93.2 to -82.2)
Armenia	Diarrheal diseases	10 to 14	Female	1 (1 to 2)	0.7 (0.5 to 1.0)	1 (0 to 1)	0.4 (0.3 to 0.7)	0 (0 to 0)	0.1 (0.1 to 0.1)	-94.2 (-96.9 to -89.1)	-88.3 (-93.7 to -77.8)
Armenia	Diarrheal diseases	10 to 14	Male	2 (1 to 3)	1.3 (0.8 to 1.8)	1 (1 to 2)	0.7 (0.4 to 1.0)	0 (0 to 0)	0.1 (0.1 to 0.2)	-94.5 (-97.1 to -89.6)	-89.5 (-94.4 to -80.1)
Armenia	Intestinal infectious diseases	5 to 9	Both	0 (0 to 0)	0.0 (0.0 to 0.0)	0 (0 to 0)	0.0 (0.0 to 0.0)	0 (0 to 0)	0.0 (0.0 to 0.0)	-65.9 (-80.7 to -40.4)	-35.5 (-63.6 to 12.8)
Armenia	Intestinal infectious diseases	5 to 9	Female	0 (0 to 0)	0.0 (0.0 to 0.0)	0 (0 to 0)	0.0 (0.0 to 0.0)	0 (0 to 0)	0.0 (0.0 to 0.0)	-65.7 (-82.4 to -34.2)	-34.8 (-66.6 to 25.1)
Armenia	Intestinal infectious diseases	5 to 9	Male	0 (0 to 0)	0.0 (0.0 to 0.0)	0 (0 to 0)	0.0 (0.0 to 0.0)	0 (0 to 0)	0.0 (0.0 to 0.0)	-66.2 (-82.3 to -32.1)	-36.3 (-66.7 to 27.7)
Armenia	Intestinal infectious diseases	10 to 14	Both	0 (0 to 0)	0.0 (0.0 to 0.0)	0 (0 to 0)	0.0 (0.0 to 0.0)	0 (0 to 0)	0.0 (0.0 to 0.0)	-68.6 (-83.1 to -46.5)	-38.2 (-66.7 to 5.5)
Armenia	Intestinal infectious diseases	10 to 14	Female	0 (0 to 0)	0.0 (0.0 to 0.0)	0 (0 to 0)	0.0 (0.0 to 0.0)	0 (0 to 0)	0.0 (0.0 to 0.0)	-70.3 (-84.7 to -46.1)	-39.8 (-69.0 to 9.3)
Armenia	Intestinal infectious diseases	10 to 14	Male	0 (0 to 0)	0.0 (0.0 to 0.0)	0 (0 to 0)	0.0 (0.0 to 0.0)	0 (0 to 0)	0.0 (0.0 to 0.0)	-66.9 (-83.3 to -43.7)	-36.6 (-67.9 to 8.0)
Armenia	Lower respiratory infections	5 to 9	Both	31 (24 to 38)	8.3 (6.4 to 10.3)	12 (9 to 16)	4.5 (3.4 to 5.9)	5 (3 to 6)	2.4 (1.7 to 3.3)	-84.7 (-90.1 to -77.2)	-71.0 (-81.3 to -57.0)

eTable 8. All-cause and cause-specific mortality (counts and rates) at the global level and by country in the WHO European Region for ages 5 to 9 and 10 to 14 for 1990, 2000, and 2016

Armenia	Lower respiratory infections	5 to 9	Female	14 (10 to 20)	7.9 (5.8 to 10.7)	5 (4 to 7)	3.9 (2.8 to 5.4)	2 (1 to 3)	2.1 (1.3 to 3.1)	-85.7 (-91.9 to -77.1)	-72.9 (-84.6 to -56.5)
Armenia	Lower respiratory infections	5 to 9	Male	16 (12 to 22)	8.7 (6.3 to 11.8)	7 (5 to 10)	5.0 (3.6 to 6.9)	3 (2 to 4)	2.7 (1.7 to 4.0)	-83.7 (-90.3 to -73.8)	-69.4 (-81.8 to -50.6)
Armenia	Lower respiratory infections	10 to 14	Both	12 (9 to 15)	3.7 (2.9 to 4.6)	7 (6 to 10)	2.3 (1.8 to 2.9)	3 (2 to 4)	1.7 (1.3 to 2.3)	-76.5 (-84.1 to -66.1)	-53.7 (-68.6 to -33.3)
Armenia	Lower respiratory infections	10 to 14	Female	4 (3 to 6)	2.8 (2.0 to 3.9)	3 (2 to 4)	2.0 (1.4 to 2.7)	1 (1 to 2)	1.5 (1.0 to 2.2)	-73.3 (-84.6 to -55.6)	-45.8 (-68.7 to -10.0)
Armenia	Lower respiratory infections	10 to 14	Male	7 (5 to 10)	4.5 (3.2 to 6.0)	4 (3 to 6)	2.6 (1.8 to 3.6)	2 (1 to 2)	1.9 (1.2 to 2.7)	-78.5 (-86.7 to -64.4)	-58.7 (-74.6 to -31.7)
Armenia	Upper respiratory infections	5 to 9	Both	0 (0 to 0)	0.0 (0.0 to 0.0)	0 (0 to 0)	0.0 (0.0 to 0.0)	0 (0 to 0)	0.0 (0.0 to 0.0)	-86.9 (-99.8 to 568.2)	-75.2 (-99.6 to 1164.0)
Armenia	Upper respiratory infections	5 to 9	Female	0 (0 to 0)	0.0 (0.0 to 0.0)	0 (0 to 0)	0.0 (0.0 to 0.0)	0 (0 to 0)	0.0 (0.0 to 0.0)	-85.9 (-100.0 to 14076.1)	-73.1 (-100.0 to 26860.1)
Armenia	Upper respiratory infections	5 to 9	Male	0 (0 to 0)	0.0 (0.0 to 0.0)	0 (0 to 0)	0.0 (0.0 to 0.0)	0 (0 to 0)	0.0 (0.0 to 0.0)	-87.6 (-100.0 to 18955.0)	-76.6 (-100.0 to 35762.8)
Armenia	Upper respiratory infections	10 to 14	Both	0 (0 to 0)	0.0 (0.0 to 0.0)	0 (0 to 0)	0.0 (0.0 to 0.0)	0 (0 to 0)	0.0 (0.0 to 0.0)	-80.2 (-99.6 to 1065.5)	-61.0 (-99.2 to 2196.6)
Armenia	Upper respiratory infections	10 to 14	Female	0 (0 to 0)	0.0 (0.0 to 0.0)	0 (0 to 0)	0.0 (0.0 to 0.0)	0 (0 to 0)	0.0 (0.0 to 0.0)	-79.8 (-100.0 to 41324.4)	-59.1 (-100.0 to 83881.4)
Armenia	Upper respiratory infections	10 to 14	Male	0 (0 to 0)	0.0 (0.0 to 0.0)	0 (0 to 0)	0.0 (0.0 to 0.0)	0 (0 to 0)	0.0 (0.0 to 0.0)	-80.4 (-100.0 to 10639.9)	-62.4 (-100.0 to 20500.0)
Armenia	Otitis media	5 to 9	Both	0 (0 to 0)	0.0 (0.0 to 0.0)	0 (0 to 0)	0.0 (0.0 to 0.0)	0 (0 to 0)	0.0 (0.0 to 0.0)	-80.2 (-94.2 to -9.7)	-62.6 (-89.1 to 70.8)
Armenia	Otitis media	5 to 9	Female	0 (0 to 0)	0.0 (0.0 to 0.0)	0 (0 to 0)	0.0 (0.0 to 0.0)	0 (0 to 0)	0.0 (0.0 to 0.0)	-96.0 (-99.1 to -73.2)	-92.4 (-98.3 to -49.0)
Armenia	Otitis media	5 to 9	Male	0 (0 to 0)	0.0 (0.0 to 0.0)	0 (0 to 0)	0.0 (0.0 to 0.0)	0 (0 to 0)	0.0 (0.0 to 0.0)	-39.9 (-87.0 to 278.0)	13.2 (-75.5 to 611.4)
Armenia	Otitis media	10 to 14	Both	0 (0 to 0)	0.0 (0.0 to 0.0)	0 (0 to 0)	0.0 (0.0 to 0.0)	0 (0 to 0)	0.0 (0.0 to 0.0)	-87.3 (-96.1 to -50.2)	-75.0 (-92.3 to -1.8)
Armenia	Otitis media	10 to 14	Female	0 (0 to 0)	0.0 (0.0 to 0.0)	0 (0 to 0)	0.0 (0.0 to 0.0)	0 (0 to 0)	0.0 (0.0 to 0.0)	-96.0 (-99.0 to -77.9)	-91.9 (-98.1 to -55.1)
Armenia	Otitis media	10 to 14	Male	0 (0 to 0)	0.0 (0.0 to 0.0)	0 (0 to 0)	0.0 (0.0 to 0.0)	0 (0 to 0)	0.0 (0.0 to 0.0)	-60.7 (-91.2 to 148.3)	-24.5 (-83.2 to 376.4)
Armenia	Meningitis	5 to 9	Both	3 (2 to 4)	0.8 (0.6 to 1.1)	2 (1 to 3)	0.7 (0.5 to 0.9)	0 (0 to 1)	0.2 (0.1 to 0.3)	-86.8 (-91.8 to -79.0)	-75.0 (-84.6 to -60.3)
Armenia	Meningitis	5 to 9	Female	1 (1 to 2)	0.8 (0.5 to 1.1)	1 (0 to 1)	0.6 (0.4 to 0.8)	0 (0 to 0)	0.2 (0.1 to 0.2)	-89.0 (-93.9 to -80.7)	-79.0 (-88.4 to -63.2)
Armenia	Meningitis	5 to 9	Male	2 (1 to 2)	0.9 (0.6 to 1.3)	1 (1 to 2)	0.8 (0.4 to 1.2)	0 (0 to 0)	0.2 (0.1 to 0.4)	-84.9 (-91.9 to -73.1)	-71.7 (-84.7 to -49.4)
Armenia	Meningitis	10 to 14	Both	2 (1 to 2)	0.6 (0.4 to 0.7)	2 (1 to 2)	0.5 (0.4 to 0.7)	0 (0 to 0)	0.2 (0.1 to 0.3)	-81.9 (-88.0 to -73.7)	-64.4 (-76.3 to -48.2)
Armenia	Meningitis	10 to 14	Female	1 (1 to 1)	0.5 (0.4 to 0.7)	1 (1 to 1)	0.5 (0.3 to 0.7)	0 (0 to 0)	0.2 (0.1 to 0.2)	-84.7 (-91.3 to -73.9)	-69.0 (-82.4 to -47.0)
Armenia	Meningitis	10 to 14	Male	1 (1 to 1)	0.6 (0.4 to 0.9)	1 (1 to 1)	0.6 (0.4 to 0.8)	0 (0 to 0)	0.2 (0.2 to 0.4)	-79.6 (-88.3 to -67.1)	-60.9 (-77.5 to -36.9)
Armenia	Encephalitis	5 to 9	Both	1 (1 to 2)	0.3 (0.1 to 0.5)	1 (0 to 1)	0.3 (0.2 to 0.5)	1 (0 to 1)	0.3 (0.2 to 0.5)	-38.8 (-58.9 to 8.8)	15.8 (-22.2 to 105.7)
Armenia	Encephalitis	5 to 9	Female	0 (0 to 1)	0.3 (0.1 to 0.5)	0 (0 to 1)	0.3 (0.2 to 0.5)	0 (0 to 0)	0.3 (0.2 to 0.5)	-44.6 (-65.8 to -1.8)	5.4 (-35.0 to 86.7)
Armenia	Encephalitis	5 to 9	Male	1 (0 to 1)	0.3 (0.1 to 0.7)	0 (0 to 1)	0.3 (0.1 to 0.7)	0 (0 to 1)	0.4 (0.2 to 0.7)	-33.7 (-58.5 to 52.8)	24.9 (-21.8 to 187.7)
Armenia	Encephalitis	10 to 14	Both	1 (0 to 1)	0.2 (0.1 to 0.3)	1 (0 to 1)	0.2 (0.1 to 0.3)	0 (0 to 1)	0.2 (0.2 to 0.4)	-35.3 (-57.1 to 14.1)	27.6 (-15.5 to 124.9)

eTable 8. All-cause and cause-specific mortality (counts and rates) at the global level and by country in the WHO European Region for ages 5 to 9 and 10 to 14 for 1990, 2000, and 2016

Armenia	Encephalitis	10 to 14	Female	0<br>(0 to 1)	0.2<br>(0.1 to 0.3)	0<br>(0 to 1)	0.2<br>(0.1 to 0.3)	0<br>(0 to 0)	0.2<br>(0.1 to 0.3)	-46.9<br>(-65.7 to -0.1)	7.6<br>(-30.4 to 102.5)
Armenia	Encephalitis	10 to 14	Male	0<br>(0 to 1)	0.2<br>(0.1 to 0.4)	0<br>(0 to 1)	0.2<br>(0.1 to 0.4)	0<br>(0 to 0)	0.3<br>(0.2 to 0.4)	-24.8<br>(-55.2 to 65.9)	44.2<br>(-14.0 to 218.2)
Armenia	Diphtheria	5 to 9	Both	0<br>(0 to 0)	0.0<br>(0.0 to 0.0)	0<br>(0 to 0)	0.0<br>(0.0 to 0.0)	0<br>(0 to 0)	0.0<br>(0.0 to 0.0)	-90.6<br>(-98.6 to -41.5)	-82.1<br>(-97.4 to 10.7)
Armenia	Diphtheria	5 to 9	Female	0<br>(0 to 0)	0.0<br>(0.0 to 0.0)	0<br>(0 to 0)	0.0<br>(0.0 to 0.0)	0<br>(0 to 0)	0.0<br>(0.0 to 0.0)	-90.2<br>(-99.5 to 135.4)	-81.4<br>(-99.1 to 347.6)
Armenia	Diphtheria	5 to 9	Male	0<br>(0 to 0)	0.0<br>(0.0 to 0.0)	0<br>(0 to 0)	0.0<br>(0.0 to 0.0)	0<br>(0 to 0)	0.0<br>(0.0 to 0.0)	-90.8<br>(-99.5 to 54.9)	-82.7<br>(-99.1 to 191.6)
Armenia	Diphtheria	10 to 14	Both	0<br>(0 to 0)	0.0<br>(0.0 to 0.0)	0<br>(0 to 0)	0.0<br>(0.0 to 0.0)	0<br>(0 to 0)	0.0<br>(0.0 to 0.0)	-85.5<br>(-98.0 to 13.1)	-71.4<br>(-96.0 to 122.9)
Armenia	Diphtheria	10 to 14	Female	0<br>(0 to 0)	0.0<br>(0.0 to 0.0)	0<br>(0 to 0)	0.0<br>(0.0 to 0.0)	0<br>(0 to 0)	0.0<br>(0.0 to 0.0)	-85.5<br>(-99.5 to 208.5)	-70.6<br>(-98.9 to 525.5)
Armenia	Diphtheria	10 to 14	Male	0<br>(0 to 0)	0.0<br>(0.0 to 0.0)	0<br>(0 to 0)	0.0<br>(0.0 to 0.0)	0<br>(0 to 0)	0.0<br>(0.0 to 0.0)	-85.5<br>(-99.4 to 281.9)	-72.2<br>(-98.8 to 632.6)
Armenia	Whooping cough	5 to 9	Both	0<br>(0 to 0)	0.0<br>(0.0 to 0.0)	0<br>(0 to 0)	0.0<br>(0.0 to 0.0)	0<br>(0 to 0)	0.0<br>(0.0 to 0.0)	61.8<br>(-57.2 to 367.1)	206.0<br>(-19.1 to 783.6)
Armenia	Whooping cough	5 to 9	Female	0<br>(0 to 0)	0.0<br>(0.0 to 0.0)	0<br>(0 to 0)	0.0<br>(0.0 to 0.0)	0<br>(0 to 0)	0.0<br>(0.0 to 0.0)	18.4<br>(-60.6 to 284.3)	125.3<br>(-25.0 to 630.9)
Armenia	Whooping cough	5 to 9	Male	0<br>(0 to 0)	0.0<br>(0.0 to 0.0)	0<br>(0 to 0)	0.0<br>(0.0 to 0.0)	0<br>(0 to 0)	0.0<br>(0.0 to 0.0)	106.5<br>(-57.4 to 610.5)	288.6<br>(-19.8 to 1237.2)
Armenia	Whooping cough	10 to 14	Both	0<br>(0 to 0)	0.0<br>(0.0 to 0.0)	0<br>(0 to 0)	0.0<br>(0.0 to 0.0)	0<br>(0 to 0)	0.0<br>(0.0 to 0.0)	-34.0<br>(-55.8 to 22.0)	30.1<br>(-12.9 to 140.4)
Armenia	Whooping cough	10 to 14	Female	0<br>(0 to 0)	0.0<br>(0.0 to 0.0)	0<br>(0 to 0)	0.0<br>(0.0 to 0.0)	0<br>(0 to 0)	0.0<br>(0.0 to 0.0)	-47.4<br>(-62.0 to 2.5)	6.6<br>(-22.9 to 107.9)
Armenia	Whooping cough	10 to 14	Male	0<br>(0 to 0)	0.0<br>(0.0 to 0.0)	0<br>(0 to 0)	0.0<br>(0.0 to 0.0)	0<br>(0 to 0)	0.0<br>(0.0 to 0.0)	-20.5<br>(-53.5 to 88.1)	52.6<br>(-10.9 to 260.7)
Armenia	Tetanus	5 to 9	Both	0<br>(0 to 0)	0.0<br>(0.0 to 0.0)	0<br>(0 to 0)	0.0<br>(0.0 to 0.0)	0<br>(0 to 0)	0.0<br>(0.0 to 0.0)	-96.9<br>(-98.9 to -91.5)	-94.1<br>(-97.9 to -83.9)
Armenia	Tetanus	5 to 9	Female	0<br>(0 to 0)	0.0<br>(0.0 to 0.0)	0<br>(0 to 0)	0.0<br>(0.0 to 0.0)	0<br>(0 to 0)	0.0<br>(0.0 to 0.0)	-98.0<br>(-99.5 to -93.2)	-96.3<br>(-99.0 to -87.0)
Armenia	Tetanus	5 to 9	Male	0<br>(0 to 0)	0.0<br>(0.0 to 0.0)	0<br>(0 to 0)	0.0<br>(0.0 to 0.0)	0<br>(0 to 0)	0.0<br>(0.0 to 0.0)	-90.1<br>(-97.3 to -72.0)	-81.3<br>(-94.8 to -47.3)
Armenia	Tetanus	10 to 14	Both	0<br>(0 to 0)	0.0<br>(0.0 to 0.0)	0<br>(0 to 0)	0.0<br>(0.0 to 0.0)	0<br>(0 to 0)	0.0<br>(0.0 to 0.0)	-91.9<br>(-97.1 to -77.9)	-84.0<br>(-94.2 to -56.4)
Armenia	Tetanus	10 to 14	Female	0<br>(0 to 0)	0.0<br>(0.0 to 0.0)	0<br>(0 to 0)	0.0<br>(0.0 to 0.0)	0<br>(0 to 0)	0.0<br>(0.0 to 0.0)	-27.5<br>(-83.1 to 138.3)	47.1<br>(-65.8 to 383.1)
Armenia	Tetanus	10 to 14	Male	0<br>(0 to 0)	0.0<br>(0.0 to 0.0)	0<br>(0 to 0)	0.0<br>(0.0 to 0.0)	0<br>(0 to 0)	0.0<br>(0.0 to 0.0)	-93.1<br>(-97.8 to -79.5)	-86.8<br>(-95.8 to -60.6)
Armenia	Measles	5 to 9	Both	0<br>(0 to 0)	0.0<br>(0.0 to 0.0)	0<br>(0 to 0)	0.0<br>(0.0 to 0.0)	0<br>(0 to 0)	0.0<br>(0.0 to 0.0)	-56.5<br>(-83.5 to 2.8)	-17.7<br>(-68.8 to 94.6)
Armenia	Measles	5 to 9	Female	0<br>(0 to 0)	0.0<br>(0.0 to 0.0)	0<br>(0 to 0)	0.0<br>(0.0 to 0.0)	0<br>(0 to 0)	0.0<br>(0.0 to 0.0)	-14.6<br>(-73.9 to 184.5)	62.4<br>(-50.4 to 441.1)
Armenia	Measles	5 to 9	Male	0<br>(0 to 0)	0.0<br>(0.0 to 0.0)	0<br>(0 to 0)	0.0<br>(0.0 to 0.0)	0<br>(0 to 0)	0.0<br>(0.0 to 0.0)	-70.7<br>(-91.9 to -8.5)	-44.9<br>(-84.8 to 72.3)
Armenia	Measles	10 to 14	Both	0<br>(0 to 0)	0.0<br>(0.0 to 0.0)	0<br>(0 to 0)	0.0<br>(0.0 to 0.0)	0<br>(0 to 0)	0.0<br>(0.0 to 0.0)	-26.1<br>(-67.4 to 63.7)	45.7<br>(-35.8 to 222.6)
Armenia	Measles	10 to 14	Female	0<br>(0 to 0)	0.0<br>(0.0 to 0.0)	0<br>(0 to 0)	0.0<br>(0.0 to 0.0)	0<br>(0 to 0)	0.0<br>(0.0 to 0.0)	-44.2<br>(-80.7 to 53.1)	13.2<br>(-60.8 to 210.3)
Armenia	Measles	10 to 14	Male	0<br>(0 to 0)	0.0<br>(0.0 to 0.0)	0<br>(0 to 0)	0.0<br>(0.0 to 0.0)	0<br>(0 to 0)	0.0<br>(0.0 to 0.0)	87.4<br>(-30.4 to 330.9)	259.4<br>(33.5 to 726.5)
Armenia	Varicella and herpes zoster	5 to 9	Both	0<br>(0 to 0)	0.0<br>(0.0 to 0.0)	0<br>(0 to 0)	0.0<br>(0.0 to 0.0)	0<br>(0 to 0)	0.0<br>(0.0 to 0.0)	-80.3<br>(-90.9 to -57.9)	-62.8<br>(-82.7 to -20.4)
Armenia	Varicella and herpes zoster	5 to 9	Female	0<br>(0 to 0)	0.0<br>(0.0 to 0.1)	0<br>(0 to 0)	0.0<br>(0.0 to 0.1)	0<br>(0 to 0)	0.0<br>(0.0 to 0.0)	-81.9<br>(-91.9 to -57.3)	-65.6<br>(-84.5 to -18.8)
Armenia	Varicella and herpes zoster	5 to 9	Male	0<br>(0 to 0)	0.0<br>(0.0 to 0.0)	0<br>(0 to 0)	0.0<br>(0.0 to 0.0)	0<br>(0 to 0)	0.0<br>(0.0 to 0.0)	-72.8<br>(-89.5 to -18.0)	-48.8<br>(-80.3 to 54.3)
Armenia	Varicella and herpes zoster	10 to 14	Both	0<br>(0 to 0)	0.0<br>(0.0 to 0.0)	0<br>(0 to 0)	0.0<br>(0.0 to 0.0)	0<br>(0 to 0)	0.0<br>(0.0 to 0.0)	-81.0<br>(-91.6 to -62.7)	-62.5<br>(-83.4 to -26.5)
Armenia	Varicella and herpes zoster	10 to 14	Female	0<br>(0 to 0)	0.0<br>(0.0 to 0.0)	0<br>(0 to 0)	0.0<br>(0.0 to 0.0)	0<br>(0 to 0)	0.0<br>(0.0 to 0.0)	-89.1<br>(-97.9 to -72.8)	-77.8<br>(-95.7 to -44.8)
Armenia	Varicella and herpes zoster	10 to 14	Male	0<br>(0 to 0)	0.0<br>(0.0 to 0.0)	0<br>(0 to 0)	0.0<br>(0.0 to 0.0)	0<br>(0 to 0)	0.0<br>(0.0 to 0.0)	-54.4<br>(-79.4 to 40.7)	-12.5<br>(-60.5 to 170.0)

eTable 8. All-cause and cause-specific mortality (counts and rates) at the global level and by country in the WHO European Region for ages 5 to 9 and 10 to 14 for 1990, 2000, and 2016

Armenia	Malaria	5 to 9	Both	0<br>(0 to 0)	0.0<br>(0.0 to 0.0)	0<br>(0 to 0)	0.0<br>(0.0 to 0.0)	0<br>(0 to 0)	0.0<br>(0.0 to 0.0)	-100.0<br>(-100.0 to -100.0)	-100.0<br>(-100.0 to -100.0)
Armenia	Malaria	5 to 9	Female	0<br>(0 to 0)	0.0<br>(0.0 to 0.0)	0<br>(0 to 0)	0.0<br>(0.0 to 0.0)	0<br>(0 to 0)	0.0<br>(0.0 to 0.0)	-100.0<br>(-100.0 to -100.0)	-100.0<br>(-100.0 to -100.0)
Armenia	Malaria	5 to 9	Male	0<br>(0 to 0)	0.0<br>(0.0 to 0.0)	0<br>(0 to 0)	0.0<br>(0.0 to 0.0)	0<br>(0 to 0)	0.0<br>(0.0 to 0.0)	-100.0<br>(-100.0 to -100.0)	-100.0<br>(-100.0 to -100.0)
Armenia	Malaria	10 to 14	Both	0<br>(0 to 0)	0.0<br>(0.0 to 0.0)	0<br>(0 to 0)	0.0<br>(0.0 to 0.0)	0<br>(0 to 0)	0.0<br>(0.0 to 0.0)	-100.0<br>(-100.0 to -100.0)	-100.0<br>(-100.0 to -100.0)
Armenia	Malaria	10 to 14	Female	0<br>(0 to 0)	0.0<br>(0.0 to 0.0)	0<br>(0 to 0)	0.0<br>(0.0 to 0.0)	0<br>(0 to 0)	0.0<br>(0.0 to 0.0)	-100.0<br>(-100.0 to -100.0)	-100.0<br>(-100.0 to -100.0)
Armenia	Malaria	10 to 14	Male	0<br>(0 to 0)	0.0<br>(0.0 to 0.0)	0<br>(0 to 0)	0.0<br>(0.0 to 0.0)	0<br>(0 to 0)	0.0<br>(0.0 to 0.0)	-100.0<br>(-100.0 to -100.0)	-100.0<br>(-100.0 to -100.0)
Armenia	Leishmaniasis	5 to 9	Both	0<br>(0 to 0)	0.0<br>(0.0 to 0.0)	0<br>(0 to 0)	0.0<br>(0.0 to 0.0)	0<br>(0 to 0)	0.0<br>(0.0 to 0.0)	507.7<br>(-100.0 to 5764.1)	1049.7<br>(-100.0 to 10993.5)
Armenia	Leishmaniasis	5 to 9	Female	0<br>(0 to 0)	0.0<br>(0.0 to 0.0)	0<br>(0 to 0)	0.0<br>(0.0 to 0.0)	0<br>(0 to 0)	0.0<br>(0.0 to 0.0)	515.0<br>(-100.0 to 3480.8)	1069.7<br>(-100.0 to 6709.9)
Armenia	Leishmaniasis	5 to 9	Male	0<br>(0 to 0)	0.0<br>(0.0 to 0.0)	0<br>(0 to 0)	0.0<br>(0.0 to 0.0)	0<br>(0 to 0)	0.0<br>(0.0 to 0.0)	500.0<br>(-100.0 to 2493.4)	1029.3<br>(-100.0 to 4780.9)
Armenia	Leishmaniasis	10 to 14	Both	0<br>(0 to 0)	0.0<br>(0.0 to 0.0)	0<br>(0 to 0)	0.0<br>(0.0 to 0.0)	0<br>(0 to 0)	0.0<br>(0.0 to 0.0)	512.1<br>(-100.0 to 7225.1)	1106.2<br>(-100.0 to 14333.9)
Armenia	Leishmaniasis	10 to 14	Female	0<br>(0 to 0)	0.0<br>(0.0 to 0.0)	0<br>(0 to 0)	0.0<br>(0.0 to 0.0)	0<br>(0 to 0)	0.0<br>(0.0 to 0.0)	505.9<br>(-100.0 to 3246.3)	1128.3<br>(-100.0 to 6684.0)
Armenia	Leishmaniasis	10 to 14	Male	0<br>(0 to 0)	0.0<br>(0.0 to 0.0)	0<br>(0 to 0)	0.0<br>(0.0 to 0.0)	0<br>(0 to 0)	0.0<br>(0.0 to 0.0)	518.5<br>(-100.0 to 2657.5)	1086.3<br>(-100.0 to 5189.1)
Armenia	African trypanosomiasis	5 to 9	Both	0<br>(0 to 0)	0.0<br>(0.0 to 0.0)	0<br>(0 to 0)	0.0<br>(0.0 to 0.0)	0<br>(0 to 0)	0.0<br>(0.0 to 0.0)	0.0<br>(0.0 to 0.0)	0.0<br>(0.0 to 0.0)
Armenia	African trypanosomiasis	5 to 9	Female	0<br>(0 to 0)	0.0<br>(0.0 to 0.0)	0<br>(0 to 0)	0.0<br>(0.0 to 0.0)	0<br>(0 to 0)	0.0<br>(0.0 to 0.0)	0.0<br>(0.0 to 0.0)	0.0<br>(0.0 to 0.0)
Armenia	African trypanosomiasis	5 to 9	Male	0<br>(0 to 0)	0.0<br>(0.0 to 0.0)	0<br>(0 to 0)	0.0<br>(0.0 to 0.0)	0<br>(0 to 0)	0.0<br>(0.0 to 0.0)	0.0<br>(0.0 to 0.0)	0.0<br>(0.0 to 0.0)
Armenia	African trypanosomiasis	10 to 14	Both	0<br>(0 to 0)	0.0<br>(0.0 to 0.0)	0<br>(0 to 0)	0.0<br>(0.0 to 0.0)	0<br>(0 to 0)	0.0<br>(0.0 to 0.0)	0.0<br>(0.0 to 0.0)	0.0<br>(0.0 to 0.0)
Armenia	African trypanosomiasis	10 to 14	Female	0<br>(0 to 0)	0.0<br>(0.0 to 0.0)	0<br>(0 to 0)	0.0<br>(0.0 to 0.0)	0<br>(0 to 0)	0.0<br>(0.0 to 0.0)	0.0<br>(0.0 to 0.0)	0.0<br>(0.0 to 0.0)
Armenia	African trypanosomiasis	10 to 14	Male	0<br>(0 to 0)	0.0<br>(0.0 to 0.0)	0<br>(0 to 0)	0.0<br>(0.0 to 0.0)	0<br>(0 to 0)	0.0<br>(0.0 to 0.0)	0.0<br>(0.0 to 0.0)	0.0<br>(0.0 to 0.0)
Armenia	Schistosomiasis	5 to 9	Both	0<br>(0 to 0)	0.0<br>(0.0 to 0.0)	0<br>(0 to 0)	0.0<br>(0.0 to 0.0)	0<br>(0 to 0)	0.0<br>(0.0 to 0.0)	0.0<br>(0.0 to 0.0)	0.0<br>(0.0 to 0.0)
Armenia	Schistosomiasis	5 to 9	Female	0<br>(0 to 0)	0.0<br>(0.0 to 0.0)	0<br>(0 to 0)	0.0<br>(0.0 to 0.0)	0<br>(0 to 0)	0.0<br>(0.0 to 0.0)	0.0<br>(0.0 to 0.0)	0.0<br>(0.0 to 0.0)
Armenia	Schistosomiasis	5 to 9	Male	0<br>(0 to 0)	0.0<br>(0.0 to 0.0)	0<br>(0 to 0)	0.0<br>(0.0 to 0.0)	0<br>(0 to 0)	0.0<br>(0.0 to 0.0)	0.0<br>(0.0 to 0.0)	0.0<br>(0.0 to 0.0)
Armenia	Schistosomiasis	10 to 14	Both	0<br>(0 to 0)	0.0<br>(0.0 to 0.0)	0<br>(0 to 0)	0.0<br>(0.0 to 0.0)	0<br>(0 to 0)	0.0<br>(0.0 to 0.0)	0.0<br>(0.0 to 0.0)	0.0<br>(0.0 to 0.0)
Armenia	Schistosomiasis	10 to 14	Female	0<br>(0 to 0)	0.0<br>(0.0 to 0.0)	0<br>(0 to 0)	0.0<br>(0.0 to 0.0)	0<br>(0 to 0)	0.0<br>(0.0 to 0.0)	0.0<br>(0.0 to 0.0)	0.0<br>(0.0 to 0.0)
Armenia	Schistosomiasis	10 to 14	Male	0<br>(0 to 0)	0.0<br>(0.0 to 0.0)	0<br>(0 to 0)	0.0<br>(0.0 to 0.0)	0<br>(0 to 0)	0.0<br>(0.0 to 0.0)	0.0<br>(0.0 to 0.0)	0.0<br>(0.0 to 0.0)
Armenia	Cysticercosis	5 to 9	Both	0<br>(0 to 0)	0.0<br>(0.0 to 0.0)	0<br>(0 to 0)	0.0<br>(0.0 to 0.0)	0<br>(0 to 0)	0.0<br>(0.0 to 0.0)	-92.4<br>(-94.5 to -89.6)	-85.6<br>(-89.7 to -80.3)
Armenia	Cysticercosis	5 to 9	Female	0<br>(0 to 0)	0.0<br>(0.0 to 0.0)	0<br>(0 to 0)	0.0<br>(0.0 to 0.0)	0<br>(0 to 0)	0.0<br>(0.0 to 0.0)	-92.1<br>(-94.4 to -89.1)	-85.0<br>(-89.4 to -79.3)
Armenia	Cysticercosis	5 to 9	Male	0<br>(0 to 0)	0.0<br>(0.0 to 0.0)	0<br>(0 to 0)	0.0<br>(0.0 to 0.0)	0<br>(0 to 0)	0.0<br>(0.0 to 0.0)	-92.7<br>(-94.8 to -89.9)	-86.2<br>(-90.3 to -81.0)
Armenia	Cysticercosis	10 to 14	Both	0<br>(0 to 0)	0.0<br>(0.0 to 0.0)	0<br>(0 to 0)	0.0<br>(0.0 to 0.0)	0<br>(0 to 0)	0.0<br>(0.0 to 0.0)	-89.3<br>(-91.3 to -86.9)	-78.9<br>(-82.9 to -74.1)
Armenia	Cysticercosis	10 to 14	Female	0<br>(0 to 0)	0.0<br>(0.0 to 0.0)	0<br>(0 to 0)	0.0<br>(0.0 to 0.0)	0<br>(0 to 0)	0.0<br>(0.0 to 0.0)	-89.2<br>(-91.5 to -86.4)	-78.1<br>(-82.8 to -72.3)

eTable 8. All-cause and cause-specific mortality (counts and rates) at the global level and by country in the WHO European Region for ages 5 to 9 and 10 to 14 for 1990, 2000, and 2016

Armenia	Cysticercosis	10 to 14	Male	0 (0 to 0)	0.0 (0.0 to 0.0)	0 (0 to 0)	0.0 (0.0 to 0.0)	0 (0 to 0)	0.0 (0.0 to 0.0)	-89.4 (-91.6 to -86.6)	-79.6 (-83.8 to -74.2)
Armenia	Yellow fever	5 to 9	Both	0 (0 to 0)	0.0 (0.0 to 0.0)	0 (0 to 0)	0.0 (0.0 to 0.0)	0 (0 to 0)	0.0 (0.0 to 0.0)	0.0 (0.0 to 0.0)	0.0 (0.0 to 0.0)
Armenia	Yellow fever	5 to 9	Female	0 (0 to 0)	0.0 (0.0 to 0.0)	0 (0 to 0)	0.0 (0.0 to 0.0)	0 (0 to 0)	0.0 (0.0 to 0.0)	0.0 (0.0 to 0.0)	0.0 (0.0 to 0.0)
Armenia	Yellow fever	5 to 9	Male	0 (0 to 0)	0.0 (0.0 to 0.0)	0 (0 to 0)	0.0 (0.0 to 0.0)	0 (0 to 0)	0.0 (0.0 to 0.0)	0.0 (0.0 to 0.0)	0.0 (0.0 to 0.0)
Armenia	Yellow fever	10 to 14	Both	0 (0 to 0)	0.0 (0.0 to 0.0)	0 (0 to 0)	0.0 (0.0 to 0.0)	0 (0 to 0)	0.0 (0.0 to 0.0)	0.0 (0.0 to 0.0)	0.0 (0.0 to 0.0)
Armenia	Yellow fever	10 to 14	Female	0 (0 to 0)	0.0 (0.0 to 0.0)	0 (0 to 0)	0.0 (0.0 to 0.0)	0 (0 to 0)	0.0 (0.0 to 0.0)	0.0 (0.0 to 0.0)	0.0 (0.0 to 0.0)
Armenia	Yellow fever	10 to 14	Male	0 (0 to 0)	0.0 (0.0 to 0.0)	0 (0 to 0)	0.0 (0.0 to 0.0)	0 (0 to 0)	0.0 (0.0 to 0.0)	0.0 (0.0 to 0.0)	0.0 (0.0 to 0.0)
Armenia	Rabies	5 to 9	Both	0 (0 to 0)	0.0 (0.0 to 0.0)	0 (0 to 0)	0.0 (0.0 to 0.0)	0 (0 to 0)	0.0 (0.0 to 0.0)	-85.2 (-96.8 to -12.5)	-72.0 (-94.0 to 65.6)
Armenia	Rabies	5 to 9	Female	0 (0 to 0)	0.0 (0.0 to 0.0)	0 (0 to 0)	0.0 (0.0 to 0.0)	0 (0 to 0)	0.0 (0.0 to 0.0)	-87.4 (-98.5 to 61.4)	-75.9 (-97.2 to 207.0)
Armenia	Rabies	5 to 9	Male	0 (0 to 0)	0.0 (0.0 to 0.0)	0 (0 to 0)	0.0 (0.0 to 0.0)	0 (0 to 0)	0.0 (0.0 to 0.0)	-81.6 (-97.4 to 85.9)	-65.4 (-95.1 to 249.8)
Armenia	Rabies	10 to 14	Both	0 (0 to 0)	0.0 (0.0 to 0.0)	0 (0 to 0)	0.0 (0.0 to 0.0)	0 (0 to 0)	0.0 (0.0 to 0.0)	-96.0 (-99.2 to -73.0)	-92.2 (-98.4 to -46.8)
Armenia	Rabies	10 to 14	Female	0 (0 to 0)	0.0 (0.0 to 0.0)	0 (0 to 0)	0.0 (0.0 to 0.0)	0 (0 to 0)	0.0 (0.0 to 0.0)	-94.0 (-99.3 to -9.2)	-87.9 (-98.7 to 84.1)
Armenia	Rabies	10 to 14	Male	0 (0 to 0)	0.0 (0.0 to 0.0)	0 (0 to 0)	0.0 (0.0 to 0.0)	0 (0 to 0)	0.0 (0.0 to 0.0)	-96.1 (-99.3 to -71.6)	-92.5 (-98.6 to -45.5)
Armenia	Intestinal nematode infections	5 to 9	Both	0 (0 to 0)	0.0 (0.0 to 0.0)	0 (0 to 0)	0.0 (0.0 to 0.0)	0 (0 to 0)	0.0 (0.0 to 0.0)	-92.6 (-100.0 to 0.0)	-85.9 (-100.0 to 39.1)
Armenia	Intestinal nematode infections	5 to 9	Female	0 (0 to 0)	0.0 (0.0 to 0.0)	0 (0 to 0)	0.0 (0.0 to 0.0)	0 (0 to 0)	0.0 (0.0 to 0.0)	-92.0 (-100.0 to 0.0)	-84.7 (-100.0 to 0.0)
Armenia	Intestinal nematode infections	5 to 9	Male	0 (0 to 0)	0.0 (0.0 to 0.0)	0 (0 to 0)	0.0 (0.0 to 0.0)	0 (0 to 0)	0.0 (0.0 to 0.0)	-93.2 (-100.0 to 0.0)	-87.2 (-100.0 to 0.0)
Armenia	Intestinal nematode infections	10 to 14	Both	0 (0 to 0)	0.0 (0.0 to 0.0)	0 (0 to 0)	0.0 (0.0 to 0.0)	0 (0 to 0)	0.0 (0.0 to 0.0)	-89.6 (-100.0 to 21.4)	-79.5 (-100.0 to 139.3)
Armenia	Intestinal nematode infections	10 to 14	Female	0 (0 to 0)	0.0 (0.0 to 0.0)	0 (0 to 0)	0.0 (0.0 to 0.0)	0 (0 to 0)	0.0 (0.0 to 0.0)	-88.9 (-100.0 to 0.0)	-77.6 (-100.0 to 33.9)
Armenia	Intestinal nematode infections	10 to 14	Male	0 (0 to 0)	0.0 (0.0 to 0.0)	0 (0 to 0)	0.0 (0.0 to 0.0)	0 (0 to 0)	0.0 (0.0 to 0.0)	-90.1 (-100.0 to 0.0)	-81.1 (-100.0 to 45.5)
Armenia	Ebola	5 to 9	Both	0 (0 to 0)	0.0 (0.0 to 0.0)	0 (0 to 0)	0.0 (0.0 to 0.0)	0 (0 to 0)	0.0 (0.0 to 0.0)	0.0 (0.0 to 0.0)	0.0 (0.0 to 0.0)
Armenia	Ebola	5 to 9	Female	0 (0 to 0)	0.0 (0.0 to 0.0)	0 (0 to 0)	0.0 (0.0 to 0.0)	0 (0 to 0)	0.0 (0.0 to 0.0)	0.0 (0.0 to 0.0)	0.0 (0.0 to 0.0)
Armenia	Ebola	5 to 9	Male	0 (0 to 0)	0.0 (0.0 to 0.0)	0 (0 to 0)	0.0 (0.0 to 0.0)	0 (0 to 0)	0.0 (0.0 to 0.0)	0.0 (0.0 to 0.0)	0.0 (0.0 to 0.0)
Armenia	Ebola	10 to 14	Both	0 (0 to 0)	0.0 (0.0 to 0.0)	0 (0 to 0)	0.0 (0.0 to 0.0)	0 (0 to 0)	0.0 (0.0 to 0.0)	0.0 (0.0 to 0.0)	0.0 (0.0 to 0.0)
Armenia	Ebola	10 to 14	Female	0 (0 to 0)	0.0 (0.0 to 0.0)	0 (0 to 0)	0.0 (0.0 to 0.0)	0 (0 to 0)	0.0 (0.0 to 0.0)	0.0 (0.0 to 0.0)	0.0 (0.0 to 0.0)
Armenia	Ebola	10 to 14	Male	0 (0 to 0)	0.0 (0.0 to 0.0)	0 (0 to 0)	0.0 (0.0 to 0.0)	0 (0 to 0)	0.0 (0.0 to 0.0)	0.0 (0.0 to 0.0)	0.0 (0.0 to 0.0)
Armenia	Zika virus	5 to 9	Both	0 (0 to 0)	0.0 (0.0 to 0.0)	0 (0 to 0)	0.0 (0.0 to 0.0)	0 (0 to 0)	0.0 (0.0 to 0.0)	0.0 (0.0 to 0.0)	0.0 (0.0 to 0.0)
Armenia	Zika virus	5 to 9	Female	0 (0 to 0)	0.0 (0.0 to 0.0)	0 (0 to 0)	0.0 (0.0 to 0.0)	0 (0 to 0)	0.0 (0.0 to 0.0)	0.0 (0.0 to 0.0)	0.0 (0.0 to 0.0)
Armenia	Zika virus	5 to 9	Male	0 (0 to 0)	0.0 (0.0 to 0.0)	0 (0 to 0)	0.0 (0.0 to 0.0)	0 (0 to 0)	0.0 (0.0 to 0.0)	0.0 (0.0 to 0.0)	0.0 (0.0 to 0.0)
Armenia	Zika virus	10 to 14	Both	0 (0 to 0)	0.0 (0.0 to 0.0)	0 (0 to 0)	0.0 (0.0 to 0.0)	0 (0 to 0)	0.0 (0.0 to 0.0)	0.0 (0.0 to 0.0)	0.0 (0.0 to 0.0)

eTable 8. All-cause and cause-specific mortality (counts and rates) at the global level and by country in the WHO European Region for ages 5 to 9 and 10 to 14 for 1990, 2000, and 2016

Armenia	Zika virus	10 to 14	Female	0 (0 to 0)	0.0 (0.0 to 0.0)	0 (0 to 0)	0.0 (0.0 to 0.0)	0 (0 to 0)	0.0 (0.0 to 0.0)	0.0 (0.0 to 0.0)	0.0 (0.0 to 0.0)
Armenia	Zika virus	10 to 14	Male	0 (0 to 0)	0.0 (0.0 to 0.0)	0 (0 to 0)	0.0 (0.0 to 0.0)	0 (0 to 0)	0.0 (0.0 to 0.0)	0.0 (0.0 to 0.0)	0.0 (0.0 to 0.0)
Armenia	Other neglected tropical diseases	5 to 9	Both	0 (0 to 0)	0.0 (0.0 to 0.0)	0 (0 to 0)	0.0 (0.0 to 0.0)	0 (0 to 0)	0.0 (0.0 to 0.0)	-52.6 (-84.6 to 27.2)	-10.3 (-70.8 to 140.7)
Armenia	Other neglected tropical diseases	5 to 9	Female	0 (0 to 0)	0.0 (0.0 to 0.1)	0 (0 to 0)	0.0 (0.0 to 0.1)	0 (0 to 0)	0.0 (0.0 to 0.1)	-54.6 (-90.4 to 97.4)	-13.7 (-81.7 to 275.4)
Armenia	Other neglected tropical diseases	5 to 9	Male	0 (0 to 0)	0.0 (0.0 to 0.0)	0 (0 to 0)	0.0 (0.0 to 0.0)	0 (0 to 0)	0.0 (0.0 to 0.0)	-48.9 (-81.5 to 16.7)	-3.7 (-65.1 to 119.6)
Armenia	Other neglected tropical diseases	10 to 14	Both	0 (0 to 0)	0.0 (0.0 to 0.0)	0 (0 to 0)	0.0 (0.0 to 0.0)	0 (0 to 0)	0.0 (0.0 to 0.0)	-51.7 (-70.5 to -36.8)	-4.9 (-42.0 to 24.6)
Armenia	Other neglected tropical diseases	10 to 14	Female	0 (0 to 0)	0.0 (0.0 to 0.0)	0 (0 to 0)	0.0 (0.0 to 0.0)	0 (0 to 0)	0.0 (0.0 to 0.0)	-59.0 (-81.7 to -40.8)	-17.0 (-62.9 to 20.0)
Armenia	Other neglected tropical diseases	10 to 14	Male	0 (0 to 0)	0.0 (0.0 to 0.0)	0 (0 to 0)	0.0 (0.0 to 0.0)	0 (0 to 0)	0.0 (0.0 to 0.0)	-43.2 (-54.5 to -28.4)	8.9 (-12.6 to 37.4)
Armenia	Maternal hemorrhage	10 to 14	Both	0 (0 to 0)	0.0 (0.0 to 0.0)	0 (0 to 0)	0.0 (0.0 to 0.0)	0 (0 to 0)	0.0 (0.0 to 0.0)	-74.0 (-82.0 to -62.2)	-48.7 (-64.6 to -25.6)
Armenia	Maternal hemorrhage	10 to 14	Female	0 (0 to 0)	0.0 (0.0 to 0.0)	0 (0 to 0)	0.0 (0.0 to 0.0)	0 (0 to 0)	0.0 (0.0 to 0.0)	-74.0 (-82.0 to -62.2)	-47.2 (-63.5 to -23.4)
Armenia	Maternal sepsis and other maternal infections	10 to 14	Both	0 (0 to 0)	0.0 (0.0 to 0.0)	0 (0 to 0)	0.0 (0.0 to 0.0)	0 (0 to 0)	0.0 (0.0 to 0.0)	-74.9 (-83.3 to -60.1)	-50.4 (-67.1 to -21.3)
Armenia	Maternal sepsis and other maternal infections	10 to 14	Female	0 (0 to 0)	0.0 (0.0 to 0.0)	0 (0 to 0)	0.0 (0.0 to 0.0)	0 (0 to 0)	0.0 (0.0 to 0.0)	-74.9 (-83.3 to -60.1)	-49.0 (-66.1 to -19.0)
Armenia	Maternal hypertensive disorders	10 to 14	Both	0 (0 to 0)	0.0 (0.0 to 0.0)	0 (0 to 0)	0.0 (0.0 to 0.0)	0 (0 to 0)	0.0 (0.0 to 0.0)	-73.5 (-81.6 to -62.9)	-47.7 (-63.7 to -27.0)
Armenia	Maternal hypertensive disorders	10 to 14	Female	0 (0 to 0)	0.0 (0.0 to 0.0)	0 (0 to 0)	0.0 (0.0 to 0.0)	0 (0 to 0)	0.0 (0.0 to 0.0)	-73.5 (-81.6 to -62.9)	-46.2 (-62.7 to -24.9)
Armenia	Maternal obstructed labor and uterine rupture	10 to 14	Both	0 (0 to 0)	0.0 (0.0 to 0.0)	0 (0 to 0)	0.0 (0.0 to 0.0)	0 (0 to 0)	0.0 (0.0 to 0.0)	-78.3 (-86.9 to -65.5)	-57.2 (-74.2 to -32.0)
Armenia	Maternal obstructed labor and uterine rupture	10 to 14	Female	0 (0 to 0)	0.0 (0.0 to 0.0)	0 (0 to 0)	0.0 (0.0 to 0.0)	0 (0 to 0)	0.0 (0.0 to 0.0)	-78.3 (-86.9 to -65.5)	-56.0 (-73.5 to -30.0)
Armenia	Maternal abortion, miscarriage, and ectopic pregnancy	10 to 14	Both	0 (0 to 0)	0.0 (0.0 to 0.0)	0 (0 to 0)	0.0 (0.0 to 0.0)	0 (0 to 0)	0.0 (0.0 to 0.0)	-79.3 (-85.7 to -71.0)	-59.2 (-71.7 to -42.8)

eTable 8. All-cause and cause-specific mortality (counts and rates) at the global level and by country in the WHO European Region for ages 5 to 9 and 10 to 14 for 1990, 2000, and 2016											
Armenia	Maternal abortion, miscarriage, and ectopic pregnancy	10 to 14	Female	0<br>(0 to 0)	0.0<br>(0.0 to 0.0)	0<br>(0 to 0)	0.0<br>(0.0 to 0.0)	0<br>(0 to 0)	0.0<br>(0.0 to 0.0)	-79.3<br>(-85.7 to -71.0)	-58.0<br>(-70.9 to -41.1)
Armenia	Indirect maternal deaths	10 to 14	Both	0<br>(0 to 0)	0.0<br>(0.0 to 0.0)	0<br>(0 to 0)	0.0<br>(0.0 to 0.0)	0<br>(0 to 0)	0.0<br>(0.0 to 0.0)	-73.8<br>(-82.2 to -62.2)	-48.4<br>(-64.9 to -25.6)
Armenia	Indirect maternal deaths	10 to 14	Female	0<br>(0 to 0)	0.0<br>(0.0 to 0.0)	0<br>(0 to 0)	0.0<br>(0.0 to 0.0)	0<br>(0 to 0)	0.0<br>(0.0 to 0.0)	-73.8<br>(-82.2 to -62.2)	-46.9<br>(-63.9 to -23.5)
Armenia	Late maternal deaths	10 to 14	Both	0<br>(0 to 0)	0.0<br>(0.0 to 0.0)	0<br>(0 to 0)	0.0<br>(0.0 to 0.0)	0<br>(0 to 0)	0.0<br>(0.0 to 0.0)	-76.9<br>(-94.2 to -22.1)	-54.5<br>(-88.6 to 53.6)
Armenia	Late maternal deaths	10 to 14	Female	0<br>(0 to 0)	0.0<br>(0.0 to 0.0)	0<br>(0 to 0)	0.0<br>(0.0 to 0.0)	0<br>(0 to 0)	0.0<br>(0.0 to 0.0)	-76.9<br>(-94.2 to -22.1)	-53.2<br>(-88.3 to 58.0)
Armenia	Maternal deaths aggravated by HIV/AIDS	10 to 14	Both	0<br>(0 to 0)	0.0<br>(0.0 to 0.0)	0<br>(0 to 0)	0.0<br>(0.0 to 0.0)	0<br>(0 to 0)	0.0<br>(0.0 to 0.0)	268876.2<br>(0.0 to 0.0)	529907.7<br>(0.0 to 0.0)
Armenia	Maternal deaths aggravated by HIV/AIDS	10 to 14	Female	0<br>(0 to 0)	0.0<br>(0.0 to 0.0)	0<br>(0 to 0)	0.0<br>(0.0 to 0.0)	0<br>(0 to 0)	0.0<br>(0.0 to 0.0)	268876.2<br>(0.0 to 0.0)	545205.9<br>(0.0 to 0.0)
Armenia	Other maternal disorders	10 to 14	Both	0<br>(0 to 0)	0.0<br>(0.0 to 0.0)	0<br>(0 to 0)	0.0<br>(0.0 to 0.0)	0<br>(0 to 0)	0.0<br>(0.0 to 0.0)	-76.4<br>(-83.6 to -65.3)	-53.5<br>(-67.8 to -31.7)
Armenia	Other maternal disorders	10 to 14	Female	0<br>(0 to 0)	0.0<br>(0.0 to 0.0)	0<br>(0 to 0)	0.0<br>(0.0 to 0.0)	0<br>(0 to 0)	0.0<br>(0.0 to 0.0)	-76.4<br>(-83.6 to -65.3)	-52.2<br>(-66.8 to -29.7)
Armenia	Protein-energy malnutrition	5 to 9	Both	0<br>(0 to 1)	0.1<br>(0.1 to 0.2)	0<br>(0 to 0)	0.1<br>(0.0 to 0.1)	0<br>(0 to 0)	0.0<br>(0.0 to 0.0)	-98.3<br>(-99.1 to -96.3)	-96.9<br>(-98.3 to -92.9)
Armenia	Protein-energy malnutrition	5 to 9	Female	0<br>(0 to 0)	0.1<br>(0.0 to 0.2)	0<br>(0 to 0)	0.1<br>(0.0 to 0.1)	0<br>(0 to 0)	0.0<br>(0.0 to 0.0)	-98.3<br>(-99.3 to -90.2)	-96.8<br>(-98.8 to -81.4)
Armenia	Protein-energy malnutrition	5 to 9	Male	0<br>(0 to 0)	0.1<br>(0.0 to 0.2)	0<br>(0 to 0)	0.1<br>(0.0 to 0.1)	0<br>(0 to 0)	0.0<br>(0.0 to 0.0)	-98.4<br>(-99.4 to -93.6)	-96.9<br>(-98.9 to -87.9)
Armenia	Protein-energy malnutrition	10 to 14	Both	1<br>(0 to 1)	0.2<br>(0.1 to 0.2)	0<br>(0 to 1)	0.1<br>(0.1 to 0.2)	0<br>(0 to 0)	0.0<br>(0.0 to 0.0)	-97.9<br>(-98.9 to -96.1)	-96.0<br>(-97.8 to -92.3)
Armenia	Protein-energy malnutrition	10 to 14	Female	0<br>(0 to 0)	0.1<br>(0.1 to 0.2)	0<br>(0 to 0)	0.1<br>(0.1 to 0.1)	0<br>(0 to 0)	0.0<br>(0.0 to 0.0)	-97.7<br>(-99.0 to -94.6)	-95.3<br>(-98.0 to -89.0)
Armenia	Protein-energy malnutrition	10 to 14	Male	0<br>(0 to 1)	0.2<br>(0.1 to 0.3)	0<br>(0 to 0)	0.1<br>(0.1 to 0.2)	0<br>(0 to 0)	0.0<br>(0.0 to 0.0)	-98.1<br>(-99.2 to -95.5)	-96.3<br>(-98.4 to -91.4)
Armenia	Iodine deficiency	5 to 9	Both	0<br>(0 to 1)	0.0<br>(0.0 to 0.2)	0<br>(0 to 0)	0.0<br>(0.0 to 0.1)	0<br>(0 to 0)	0.0<br>(0.0 to 0.0)	-98.9<br>(-100.0 to 1.4e+17)	-97.9<br>(-100.0 to 2.6e+17)
Armenia	Iodine deficiency	5 to 9	Female	0<br>(0 to 1)	0.0<br>(0.0 to 0.3)	0<br>(0 to 0)	0.0<br>(0.0 to 0.2)	0<br>(0 to 0)	0.0<br>(0.0 to 0.0)	-99.0<br>(-100.0 to 1.3e+26)	-98.1<br>(-100.0 to 2.5e+26)
Armenia	Iodine deficiency	5 to 9	Male	0<br>(0 to 1)	0.0<br>(0.0 to 0.3)	0<br>(0 to 0)	0.0<br>(0.0 to 0.2)	0<br>(0 to 0)	0.0<br>(0.0 to 0.0)	-98.8<br>(-100.0 to 3.0e+25)	-97.8<br>(-100.0 to 5.6e+25)
Armenia	Iodine deficiency	10 to 14	Both	0<br>(0 to 1)	0.0<br>(0.0 to 0.2)	0<br>(0 to 0)	0.0<br>(0.0 to 0.1)	0<br>(0 to 0)	0.0<br>(0.0 to 0.0)	-98.7<br>(-100.0 to 2.5e+17)	-97.4<br>(-100.0 to 4.8e+17)
Armenia	Iodine deficiency	10 to 14	Female	0<br>(0 to 0)	0.0<br>(0.0 to 0.2)	0<br>(0 to 0)	0.0<br>(0.0 to 0.1)	0<br>(0 to 0)	0.0<br>(0.0 to 0.0)	-98.8<br>(-100.0 to 5.2e+24)	-97.5<br>(-100.0 to 1.1e+25)
Armenia	Iodine deficiency	10 to 14	Male	0<br>(0 to 0)	0.0<br>(0.0 to 0.3)	0<br>(0 to 0)	0.0<br>(0.0 to 0.2)	0<br>(0 to 0)	0.0<br>(0.0 to 0.0)	-98.6<br>(-100.0 to 1.8e+24)	-97.4<br>(-100.0 to 3.5e+24)
Armenia	Iron-deficiency anemia	5 to 9	Both	0<br>(0 to 1)	0.1<br>(0.1 to 0.2)	0<br>(0 to 0)	0.1<br>(0.0 to 0.1)	0<br>(0 to 0)	0.0<br>(0.0 to 0.0)	-98.3<br>(-99.1 to -96.3)	-96.9<br>(-98.3 to -92.9)
Armenia	Iron-deficiency anemia	5 to 9	Female	0<br>(0 to 0)	0.1<br>(0.0 to 0.2)	0<br>(0 to 0)	0.1<br>(0.0 to 0.1)	0<br>(0 to 0)	0.0<br>(0.0 to 0.0)	-98.3<br>(-99.3 to -90.2)	-96.8<br>(-98.8 to -81.4)

eTable 8. All-cause and cause-specific mortality (counts and rates) at the global level and by country in the WHO European Region for ages 5 to 9 and 10 to 14 for 1990, 2000, and 2016

Armenia	Iron-deficiency anemia	5 to 9	Male	0 (0 to 0)	0.1 (0.0 to 0.2)	0 (0 to 0)	0.1 (0.0 to 0.1)	0 (0 to 0)	0.0 (0.0 to 0.0)	-98.4 (-99.4 to -93.6)	-96.9 (-98.9 to -87.9)
Armenia	Iron-deficiency anemia	10 to 14	Both	1 (0 to 1)	0.2 (0.1 to 0.2)	0 (0 to 1)	0.1 (0.1 to 0.2)	0 (0 to 0)	0.0 (0.0 to 0.0)	-97.9 (-98.9 to -96.1)	-96.0 (-97.8 to -92.3)
Armenia	Iron-deficiency anemia	10 to 14	Female	0 (0 to 0)	0.1 (0.1 to 0.2)	0 (0 to 0)	0.1 (0.1 to 0.1)	0 (0 to 0)	0.0 (0.0 to 0.0)	-97.7 (-99.0 to -94.6)	-95.3 (-98.0 to -89.0)
Armenia	Iron-deficiency anemia	10 to 14	Male	0 (0 to 1)	0.2 (0.1 to 0.3)	0 (0 to 0)	0.1 (0.1 to 0.2)	0 (0 to 0)	0.0 (0.0 to 0.0)	-98.1 (-99.2 to -95.5)	-96.3 (-98.4 to -91.4)
Armenia	Other nutritional deficiencies	5 to 9	Both	1 (0 to 1)	0.1 (0.1 to 0.2)	0 (0 to 0)	0.1 (0.0 to 0.1)	0 (0 to 0)	0.0 (0.0 to 0.0)	-98.6 (-99.3 to -96.7)	-97.3 (-98.6 to -93.8)
Armenia	Other nutritional deficiencies	5 to 9	Female	0 (0 to 0)	0.1 (0.0 to 0.2)	0 (0 to 0)	0.1 (0.0 to 0.1)	0 (0 to 0)	0.0 (0.0 to 0.0)	-98.3 (-99.3 to -90.2)	-96.8 (-98.8 to -81.4)
Armenia	Other nutritional deficiencies	5 to 9	Male	0 (0 to 1)	0.2 (0.0 to 0.3)	0 (0 to 0)	0.1 (0.0 to 0.1)	0 (0 to 0)	0.0 (0.0 to 0.0)	-98.8 (-99.6 to -94.5)	-97.7 (-99.2 to -89.7)
Armenia	Other nutritional deficiencies	10 to 14	Both	1 (0 to 1)	0.2 (0.1 to 0.2)	0 (0 to 1)	0.1 (0.1 to 0.2)	0 (0 to 0)	0.0 (0.0 to 0.0)	-98.0 (-98.9 to -96.1)	-96.0 (-97.8 to -92.4)
Armenia	Other nutritional deficiencies	10 to 14	Female	0 (0 to 0)	0.1 (0.1 to 0.2)	0 (0 to 0)	0.1 (0.1 to 0.1)	0 (0 to 0)	0.0 (0.0 to 0.0)	-97.7 (-99.0 to -94.6)	-95.3 (-98.0 to -89.0)
Armenia	Other nutritional deficiencies	10 to 14	Male	0 (0 to 1)	0.2 (0.1 to 0.3)	0 (0 to 0)	0.1 (0.1 to 0.2)	0 (0 to 0)	0.0 (0.0 to 0.0)	-98.1 (-99.2 to -95.5)	-96.4 (-98.5 to -91.4)
Armenia	Sexually transmitted diseases excluding HIV	5 to 9	Both	0 (0 to 0)	0.0 (0.0 to 0.0)	0 (0 to 0)	0.0 (0.0 to 0.0)	0 (0 to 0)	0.0 (0.0 to 0.0)	-95.9 (-99.7 to -86.2)	-92.3 (-99.4 to -73.8)
Armenia	Sexually transmitted diseases excluding HIV	5 to 9	Female	0 (0 to 0)	0.0 (0.0 to 0.1)	0 (0 to 0)	0.0 (0.0 to 0.0)	0 (0 to 0)	0.0 (0.0 to 0.0)	-96.0 (-99.7 to -86.5)	-92.5 (-99.4 to -74.3)
Armenia	Sexually transmitted diseases excluding HIV	5 to 9	Male	0 (0 to 0)	0.0 (0.0 to 0.0)	0 (0 to 0)	0.0 (0.0 to 0.0)	0 (0 to 0)	0.0 (0.0 to 0.0)	-95.7 (-99.7 to -85.6)	-92.0 (-99.4 to -72.9)
Armenia	Sexually transmitted diseases excluding HIV	10 to 14	Both	0 (0 to 0)	0.0 (0.0 to 0.1)	0 (0 to 0)	0.0 (0.0 to 0.1)	0 (0 to 0)	0.0 (0.0 to 0.0)	-94.7 (-97.1 to -83.3)	-89.5 (-94.2 to -67.0)
Armenia	Sexually transmitted diseases excluding HIV	10 to 14	Female	0 (0 to 0)	0.0 (0.0 to 0.0)	0 (0 to 0)	0.0 (0.0 to 0.0)	0 (0 to 0)	0.0 (0.0 to 0.0)	-52.7 (-62.9 to -40.0)	-4.1 (-24.9 to 21.7)
Armenia	Sexually transmitted diseases excluding HIV	10 to 14	Male	0 (0 to 0)	0.1 (0.0 to 0.1)	0 (0 to 0)	0.1 (0.0 to 0.1)	0 (0 to 0)	0.0 (0.0 to 0.0)	-99.5 (-99.9 to -94.4)	-99.0 (-99.8 to -89.2)
Armenia	Hepatitis	5 to 9	Both	1 (0 to 1)	0.2 (0.1 to 0.2)	0 (0 to 0)	0.1 (0.1 to 0.1)	0 (0 to 0)	0.1 (0.0 to 0.1)	-78.2 (-89.4 to -56.8)	-58.8 (-79.9 to -18.2)
Armenia	Hepatitis	5 to 9	Female	0 (0 to 1)	0.2 (0.1 to 0.3)	0 (0 to 0)	0.1 (0.0 to 0.1)	0 (0 to 0)	0.1 (0.0 to 0.1)	-79.5 (-92.1 to -49.5)	-61.0 (-84.9 to -3.9)
Armenia	Hepatitis	5 to 9	Male	0 (0 to 0)	0.1 (0.1 to 0.2)	0 (0 to 0)	0.1 (0.0 to 0.2)	0 (0 to 0)	0.1 (0.0 to 0.1)	-76.4 (-91.0 to -41.6)	-55.6 (-83.1 to 9.9)

Armenia	Hepatitis	10 to 14	Both	0 (0 to 0)	0.1 (0.0 to 0.1)	0 (0 to 0)	0.1 (0.0 to 0.1)	0 (0 to 0)	0.0 (0.0 to 0.1)	-66.2 (-84.2 to -26.0)	-33.3 (-68.9 to 45.8)
Armenia	Hepatitis	10 to 14	Female	0 (0 to 0)	0.1 (0.0 to 0.1)	0 (0 to 0)	0.0 (0.0 to 0.1)	0 (0 to 0)	0.1 (0.0 to 0.1)	-68.3 (-87.8 to -19.0)	-35.8 (-75.2 to 64.2)
Armenia	Hepatitis	10 to 14	Male	0 (0 to 0)	0.1 (0.0 to 0.1)	0 (0 to 0)	0.1 (0.0 to 0.1)	0 (0 to 0)	0.0 (0.0 to 0.1)	-63.8 (-88.4 to 3.0)	-30.6 (-77.7 to 97.5)
Armenia	Other infectious diseases	5 to 9	Both	0 (0 to 0)	0.0 (0.0 to 0.1)	0 (0 to 0)	0.0 (0.0 to 0.1)	0 (0 to 0)	0.0 (0.0 to 0.1)	-54.4 (-81.1 to 35.0)	-13.7 (-64.3 to 155.4)
Armenia	Other infectious diseases	5 to 9	Female	0 (0 to 0)	0.1 (0.0 to 0.1)	0 (0 to 0)	0.0 (0.0 to 0.1)	0 (0 to 0)	0.1 (0.0 to 0.1)	-49.0 (-83.6 to 95.9)	-2.9 (-68.8 to 272.6)
Armenia	Other infectious diseases	5 to 9	Male	0 (0 to 0)	0.0 (0.0 to 0.1)	0 (0 to 0)	0.0 (0.0 to 0.1)	0 (0 to 0)	0.0 (0.0 to 0.1)	-61.1 (-87.3 to 51.6)	-26.8 (-76.0 to 185.3)
Armenia	Other infectious diseases	10 to 14	Both	0 (0 to 0)	0.0 (0.0 to 0.1)	0 (0 to 0)	0.0 (0.0 to 0.0)	0 (0 to 0)	0.0 (0.0 to 0.0)	-59.1 (-77.2 to -14.0)	-19.4 (-55.0 to 69.4)
Armenia	Other infectious diseases	10 to 14	Female	0 (0 to 0)	0.0 (0.0 to 0.1)	0 (0 to 0)	0.0 (0.0 to 0.1)	0 (0 to 0)	0.0 (0.0 to 0.0)	-59.5 (-82.8 to 3.5)	-17.8 (-65.1 to 109.9)
Armenia	Other infectious diseases	10 to 14	Male	0 (0 to 0)	0.0 (0.0 to 0.1)	0 (0 to 0)	0.0 (0.0 to 0.0)	0 (0 to 0)	0.0 (0.0 to 0.0)	-58.7 (-83.5 to 16.3)	-20.7 (-68.4 to 123.2)
Armenia	Nasopharynx cancer	5 to 9	Both	0 (0 to 0)	0.0 (0.0 to 0.1)	0 (0 to 0)	0.0 (0.0 to 0.0)	0 (0 to 0)	0.0 (0.0 to 0.0)	-66.4 (-82.0 to -37.0)	-36.5 (-65.9 to 19.1)
Armenia	Nasopharynx cancer	5 to 9	Female	0 (0 to 0)	0.1 (0.0 to 0.1)	0 (0 to 0)	0.0 (0.0 to 0.1)	0 (0 to 0)	0.0 (0.0 to 0.1)	-68.1 (-84.7 to -31.6)	-39.4 (-70.9 to 30.1)
Armenia	Nasopharynx cancer	5 to 9	Male	0 (0 to 0)	0.0 (0.0 to 0.0)	0 (0 to 0)	0.0 (0.0 to 0.0)	0 (0 to 0)	0.0 (0.0 to 0.0)	-58.6 (-78.7 to -28.4)	-22.0 (-59.8 to 34.7)
Armenia	Nasopharynx cancer	10 to 14	Both	0 (0 to 0)	0.0 (0.0 to 0.1)	0 (0 to 0)	0.0 (0.0 to 0.0)	0 (0 to 0)	0.0 (0.0 to 0.0)	-68.8 (-82.8 to -33.9)	-38.5 (-66.1 to 30.2)
Armenia	Nasopharynx cancer	10 to 14	Female	0 (0 to 0)	0.0 (0.0 to 0.0)	0 (0 to 0)	0.0 (0.0 to 0.0)	0 (0 to 0)	0.0 (0.0 to 0.0)	-52.3 (-62.4 to -39.9)	-3.3 (-23.7 to 21.8)
Armenia	Nasopharynx cancer	10 to 14	Male	0 (0 to 0)	0.1 (0.0 to 0.1)	0 (0 to 0)	0.1 (0.0 to 0.1)	0 (0 to 0)	0.0 (0.0 to 0.1)	-70.9 (-85.0 to -28.7)	-44.1 (-71.3 to 36.8)
Armenia	Liver cancer	5 to 9	Both	0 (0 to 1)	0.1 (0.0 to 0.2)	0 (0 to 0)	0.1 (0.0 to 0.1)	0 (0 to 0)	0.1 (0.1 to 0.1)	-46.4 (-79.6 to 15.5)	1.4 (-61.4 to 118.5)
Armenia	Liver cancer	5 to 9	Female	0 (0 to 0)	0.0 (0.0 to 0.0)	0 (0 to 0)	0.0 (0.0 to 0.0)	0 (0 to 0)	0.0 (0.0 to 0.0)	-62.7 (-80.7 to -32.9)	-29.0 (-63.2 to 27.6)
Armenia	Liver cancer	5 to 9	Male	0 (0 to 1)	0.2 (0.1 to 0.3)	0 (0 to 0)	0.1 (0.1 to 0.2)	0 (0 to 0)	0.2 (0.1 to 0.3)	-45.0 (-80.3 to 25.3)	3.5 (-62.8 to 135.8)
Armenia	Liver cancer	10 to 14	Both	0 (0 to 0)	0.1 (0.0 to 0.1)	0 (0 to 0)	0.1 (0.0 to 0.1)	0 (0 to 0)	0.1 (0.0 to 0.1)	-41.3 (-73.2 to 20.3)	15.6 (-47.1 to 137.0)
Armenia	Liver cancer	10 to 14	Female	0 (0 to 0)	0.0 (0.0 to 0.0)	0 (0 to 0)	0.0 (0.0 to 0.0)	0 (0 to 0)	0.0 (0.0 to 0.0)	-63.5 (-81.3 to -21.9)	-26.1 (-62.1 to 58.4)
Armenia	Liver cancer	10 to 14	Male	0 (0 to 0)	0.1 (0.1 to 0.2)	0 (0 to 0)	0.1 (0.1 to 0.2)	0 (0 to 0)	0.1 (0.1 to 0.2)	-38.7 (-74.0 to 36.7)	17.5 (-50.1 to 162.1)
Armenia	Kidney cancer	5 to 9	Both	0 (0 to 0)	0.1 (0.0 to 0.1)	0 (0 to 0)	0.1 (0.0 to 0.1)	0 (0 to 0)	0.1 (0.1 to 0.2)	15.3 (-54.0 to 155.3)	118.0 (-12.9 to 382.9)
Armenia	Kidney cancer	5 to 9	Female	0 (0 to 0)	0.1 (0.0 to 0.1)	0 (0 to 0)	0.1 (0.0 to 0.1)	0 (0 to 0)	0.0 (0.0 to 0.1)	-59.2 (-82.4 to 3.8)	-22.3 (-66.6 to 97.4)
Armenia	Kidney cancer	5 to 9	Male	0 (0 to 0)	0.1 (0.0 to 0.1)	0 (0 to 0)	0.1 (0.0 to 0.1)	0 (0 to 0)	0.2 (0.1 to 0.4)	76.9 (-35.8 to 363.1)	232.9 (20.8 to 771.6)
Armenia	Kidney cancer	10 to 14	Both	0 (0 to 0)	0.0 (0.0 to 0.0)	0 (0 to 0)	0.0 (0.0 to 0.0)	0 (0 to 0)	0.0 (0.0 to 0.1)	-22.6 (-54.6 to 36.2)	52.5 (-10.5 to 168.4)
Armenia	Kidney cancer	10 to 14	Female	0 (0 to 0)	0.0 (0.0 to 0.1)	0 (0 to 0)	0.0 (0.0 to 0.0)	0 (0 to 0)	0.0 (0.0 to 0.0)	-51.2 (-74.7 to -1.0)	-1.0 (-48.7 to 100.8)
Armenia	Kidney cancer	10 to 14	Male	0 (0 to 0)	0.0 (0.0 to 0.0)	0 (0 to 0)	0.0 (0.0 to 0.0)	0 (0 to 0)	0.0 (0.0 to 0.1)	26.9 (-37.1 to 145.2)	143.5 (20.7 to 370.3)
Armenia	Brain and nervous system cancer	5 to 9	Both	9 (5 to 17)	2.6 (1.5 to 4.7)	6 (4 to 9)	2.2 (1.5 to 3.2)	4 (3 to 6)	2.0 (1.3 to 2.9)	-59.0 (-81.0 to -18.9)	-22.3 (-64.0 to 53.5)

eTable 8. All-cause and cause-specific mortality (counts and rates) at the global level and by country in the WHO European Region for ages 5 to 9 and 10 to 14 for 1990, 2000, and 2016

Armenia	Brain and nervous system cancer	5 to 9	Female	5<br>(3 to 10)	2.6<br>(1.5 to 5.5)	3<br>(2 to 5)	2.5<br>(1.5 to 3.9)	2<br>(1 to 3)	2.1<br>(1.3 to 3.2)	-57.6<br>(-83.5 to -12.1)	-19.5<br>(-68.6 to 67.2)
Armenia	Brain and nervous system cancer	5 to 9	Male	5<br>(2 to 9)	2.5<br>(1.2 to 4.9)	3<br>(2 to 4)	2.0<br>(1.2 to 3.1)	2<br>(1 to 3)	1.9<br>(1.1 to 2.8)	-60.3<br>(-83.5 to 1.4)	-25.3<br>(-69.0 to 90.8)
Armenia	Brain and nervous system cancer	10 to 14	Both	5<br>(4 to 8)	1.7<br>(1.2 to 2.5)	5<br>(4 to 7)	1.6<br>(1.2 to 2.2)	3<br>(2 to 4)	1.6<br>(1.2 to 2.2)	-51.7<br>(-72.4 to -18.7)	-4.8<br>(-45.6 to 60.2)
Armenia	Brain and nervous system cancer	10 to 14	Female	3<br>(2 to 4)	1.8<br>(1.1 to 2.8)	3<br>(2 to 4)	1.8<br>(1.2 to 2.7)	1<br>(1 to 2)	1.7<br>(1.1 to 2.4)	-53.6<br>(-75.8 to -9.0)	-6.0<br>(-51.0 to 84.5)
Armenia	Brain and nervous system cancer	10 to 14	Male	3<br>(1 to 5)	1.6<br>(0.9 to 2.9)	2<br>(1 to 3)	1.4<br>(0.9 to 2.1)	1<br>(1 to 2)	1.6<br>(1.0 to 2.3)	-49.7<br>(-76.1 to 3.5)	-3.5<br>(-54.2 to 98.6)
Armenia	Thyroid cancer	10 to 14	Both	0<br>(0 to 0)	0.0<br>(0.0 to 0.0)	0<br>(0 to 0)	0.0<br>(0.0 to 0.0)	0<br>(0 to 0)	0.0<br>(0.0 to 0.0)	-20.0<br>(-48.9 to 20.7)	57.7<br>(0.8 to 137.8)
Armenia	Thyroid cancer	10 to 14	Female	0<br>(0 to 0)	0.0<br>(0.0 to 0.0)	0<br>(0 to 0)	0.0<br>(0.0 to 0.0)	0<br>(0 to 0)	0.0<br>(0.0 to 0.0)	-51.9<br>(-62.3 to -38.5)	-2.6<br>(-23.5 to 24.6)
Armenia	Thyroid cancer	10 to 14	Male	0<br>(0 to 0)	0.0<br>(0.0 to 0.0)	0<br>(0 to 0)	0.0<br>(0.0 to 0.0)	0<br>(0 to 0)	0.0<br>(0.0 to 0.0)	11.7<br>(-41.9 to 89.0)	114.2<br>(11.4 to 262.4)
Armenia	Hodgkin lymphoma	5 to 9	Both	0<br>(0 to 0)	0.0<br>(0.0 to 0.0)	0<br>(0 to 0)	0.0<br>(0.0 to 0.0)	0<br>(0 to 0)	0.0<br>(0.0 to 0.0)	-54.2<br>(-79.4 to -24.8)	-13.4<br>(-61.1 to 42.3)
Armenia	Hodgkin lymphoma	5 to 9	Female	0<br>(0 to 0)	0.0<br>(0.0 to 0.0)	0<br>(0 to 0)	0.0<br>(0.0 to 0.0)	0<br>(0 to 0)	0.0<br>(0.0 to 0.0)	-49.2<br>(-65.4 to -29.2)	-3.3<br>(-34.1 to 34.6)
Armenia	Hodgkin lymphoma	5 to 9	Male	0<br>(0 to 0)	0.0<br>(0.0 to 0.1)	0<br>(0 to 0)	0.0<br>(0.0 to 0.0)	0<br>(0 to 0)	0.0<br>(0.0 to 0.0)	-57.9<br>(-84.5 to -13.7)	-20.7<br>(-70.8 to 62.5)
Armenia	Hodgkin lymphoma	10 to 14	Both	0<br>(0 to 0)	0.0<br>(0.0 to 0.1)	0<br>(0 to 0)	0.0<br>(0.0 to 0.0)	0<br>(0 to 0)	0.0<br>(0.0 to 0.1)	-8.9<br>(-76.4 to 106.3)	79.6<br>(-53.4 to 306.5)
Armenia	Hodgkin lymphoma	10 to 14	Female	0<br>(0 to 0)	0.0<br>(0.0 to 0.0)	0<br>(0 to 0)	0.0<br>(0.0 to 0.0)	0<br>(0 to 0)	0.0<br>(0.0 to 0.0)	-52.8<br>(-63.2 to -40.0)	-4.4<br>(-25.5 to 21.7)
Armenia	Hodgkin lymphoma	10 to 14	Male	0<br>(0 to 0)	0.0<br>(0.0 to 0.1)	0<br>(0 to 0)	0.0<br>(0.0 to 0.1)	0<br>(0 to 0)	0.1<br>(0.0 to 0.1)	3.7<br>(-78.5 to 197.5)	99.0<br>(-58.8 to 470.5)
Armenia	Non-Hodgkin lymphoma	5 to 9	Both	1<br>(0 to 1)	0.2<br>(0.1 to 0.4)	0<br>(0 to 1)	0.2<br>(0.1 to 0.3)	0<br>(0 to 1)	0.1<br>(0.1 to 0.3)	-65.5<br>(-85.5 to -31.1)	-34.7<br>(-72.5 to 30.4)
Armenia	Non-Hodgkin lymphoma	5 to 9	Female	0<br>(0 to 0)	0.1<br>(0.0 to 0.2)	0<br>(0 to 0)	0.1<br>(0.0 to 0.2)	0<br>(0 to 0)	0.1<br>(0.0 to 0.1)	-56.9<br>(-80.0 to -1.1)	-18.1<br>(-62.0 to 88.1)
Armenia	Non-Hodgkin lymphoma	5 to 9	Male	1<br>(0 to 1)	0.3<br>(0.1 to 0.6)	0<br>(0 to 1)	0.3<br>(0.1 to 0.5)	0<br>(0 to 0)	0.2<br>(0.1 to 0.4)	-67.9<br>(-88.4 to -25.3)	-39.7<br>(-78.1 to 40.6)
Armenia	Non-Hodgkin lymphoma	10 to 14	Both	1<br>(0 to 1)	0.2<br>(0.1 to 0.3)	1<br>(0 to 1)	0.2<br>(0.1 to 0.3)	0<br>(0 to 0)	0.2<br>(0.1 to 0.3)	-61.0<br>(-80.5 to -29.0)	-23.2<br>(-61.5 to 39.8)
Armenia	Non-Hodgkin lymphoma	10 to 14	Female	0<br>(0 to 0)	0.1<br>(0.0 to 0.2)	0<br>(0 to 0)	0.1<br>(0.0 to 0.2)	0<br>(0 to 0)	0.1<br>(0.0 to 0.2)	-56.4<br>(-80.0 to -3.5)	-11.6<br>(-59.5 to 95.6)
Armenia	Non-Hodgkin lymphoma	10 to 14	Male	0<br>(0 to 1)	0.3<br>(0.2 to 0.5)	0<br>(0 to 1)	0.3<br>(0.2 to 0.5)	0<br>(0 to 0)	0.2<br>(0.1 to 0.4)	-62.4<br>(-83.1 to -24.7)	-27.8<br>(-67.5 to 44.5)
Armenia	Leukemia	5 to 9	Both	16<br>(12 to 20)	4.2<br>(3.2 to 5.5)	9<br>(7 to 12)	3.3<br>(2.5 to 4.3)	4<br>(2 to 5)	1.8<br>(1.2 to 2.6)	-77.0<br>(-85.7 to -64.6)	-56.4<br>(-72.9 to -33.0)
Armenia	Leukemia	5 to 9	Female	7<br>(5 to 10)	3.8<br>(2.6 to 5.3)	4<br>(3 to 6)	3.0<br>(2.0 to 4.2)	2<br>(1 to 3)	1.8<br>(1.1 to 2.7)	-75.6<br>(-86.1 to -55.5)	-53.5<br>(-73.6 to -15.4)
Armenia	Leukemia	5 to 9	Male	9<br>(6 to 12)	4.7<br>(3.1 to 6.6)	5<br>(3 to 7)	3.6<br>(2.5 to 5.1)	2<br>(1 to 3)	1.9<br>(1.2 to 3.0)	-78.1<br>(-88.1 to -60.0)	-58.7<br>(-77.6 to -24.7)
Armenia	Leukemia	10 to 14	Both	9<br>(7 to 12)	2.8<br>(2.2 to 3.6)	8<br>(7 to 11)	2.6<br>(2.0 to 3.3)	3<br>(2 to 4)	1.8<br>(1.3 to 2.5)	-66.9<br>(-77.9 to -50.7)	-34.8<br>(-56.5 to -2.9)
Armenia	Leukemia	10 to 14	Female	4<br>(3 to 5)	2.3<br>(1.6 to 3.2)	3<br>(2 to 5)	2.1<br>(1.5 to 2.8)	1<br>(1 to 2)	1.4<br>(0.9 to 2.1)	-69.0<br>(-82.0 to -47.4)	-37.1<br>(-63.6 to 6.7)
Armenia	Leukemia	10 to 14	Male	5<br>(4 to 7)	3.3<br>(2.4 to 4.6)	5<br>(4 to 7)	3.1<br>(2.2 to 4.2)	2<br>(1 to 3)	2.2<br>(1.5 to 3.3)	-65.5<br>(-80.0 to -42.5)	-33.9<br>(-61.7 to 10.4)
Armenia	Other neoplasms	5 to 9	Both	4<br>(2 to 7)	1.0<br>(0.6 to 1.9)	2<br>(2 to 4)	0.9<br>(0.6 to 1.3)	1<br>(1 to 2)	0.6<br>(0.4 to 0.9)	-67.2<br>(-85.1 to -38.5)	-38.0<br>(-71.9 to 16.3)
Armenia	Other neoplasms	5 to 9	Female	2<br>(1 to 3)	1.0<br>(0.5 to 1.8)	1<br>(1 to 2)	0.8<br>(0.5 to 1.3)	1<br>(0 to 1)	0.6<br>(0.3 to 0.9)	-67.9<br>(-85.6 to -30.5)	-38.9<br>(-72.6 to 32.1)
Armenia	Other neoplasms	5 to 9	Male	2<br>(1 to 4)	1.1<br>(0.6 to 2.3)	1<br>(1 to 2)	1.0<br>(0.5 to 1.5)	1<br>(0 to 1)	0.7<br>(0.4 to 1.2)	-66.7<br>(-87.2 to -23.5)	-37.3<br>(-75.9 to 44.0)
Armenia	Other neoplasms	10 to 14	Both	4<br>(3 to 6)	1.2<br>(0.8 to 1.8)	4<br>(3 to 5)	1.1<br>(0.8 to 1.5)	1<br>(1 to 2)	0.9<br>(0.7 to 1.3)	-61.1<br>(-77.5 to -31.8)	-23.4<br>(-55.6 to 34.3)

eTable 8. All-cause and cause-specific mortality (counts and rates) at the global level and by country in the WHO European Region for ages 5 to 9 and 10 to 14 for 1990, 2000, and 2016

Armenia	Other neoplasms	10 to 14	Female	1 (1 to 2)	0.8 (0.5 to 1.2)	1 (1 to 2)	0.7 (0.5 to 1.1)	0 (0 to 1)	0.5 (0.3 to 0.8)	-65.0 (-81.0 to -33.6)	-29.1 (-61.5 to 34.7)
Armenia	Other neoplasms	10 to 14	Male	3 (2 to 4)	1.6 (1.0 to 2.7)	2 (2 to 4)	1.5 (1.0 to 2.1)	1 (1 to 2)	1.3 (0.8 to 1.9)	-59.4 (-78.9 to -19.2)	-22.1 (-59.5 to 55.0)
Armenia	Rheumatic heart disease	5 to 9	Both	1 (1 to 1)	0.3 (0.2 to 0.4)	0 (0 to 1)	0.1 (0.1 to 0.2)	0 (0 to 0)	0.1 (0.0 to 0.1)	-88.7 (-93.1 to -81.3)	-78.6 (-86.9 to -64.6)
Armenia	Rheumatic heart disease	5 to 9	Female	0 (0 to 1)	0.3 (0.2 to 0.4)	0 (0 to 0)	0.1 (0.1 to 0.2)	0 (0 to 0)	0.1 (0.0 to 0.1)	-85.9 (-92.0 to -75.2)	-73.2 (-84.7 to -52.8)
Armenia	Rheumatic heart disease	5 to 9	Male	1 (0 to 1)	0.3 (0.2 to 0.5)	0 (0 to 0)	0.1 (0.1 to 0.2)	0 (0 to 0)	0.1 (0.0 to 0.1)	-91.0 (-95.6 to -81.6)	-83.1 (-91.7 to -65.3)
Armenia	Rheumatic heart disease	10 to 14	Both	2 (2 to 3)	0.6 (0.5 to 0.8)	1 (1 to 2)	0.4 (0.3 to 0.5)	0 (0 to 0)	0.2 (0.1 to 0.3)	-84.3 (-89.3 to -77.6)	-69.1 (-79.0 to -55.9)
Armenia	Rheumatic heart disease	10 to 14	Female	1 (1 to 2)	0.8 (0.6 to 1.1)	1 (1 to 1)	0.6 (0.4 to 0.8)	0 (0 to 0)	0.3 (0.2 to 0.4)	-82.7 (-88.6 to -73.0)	-65.0 (-77.0 to -45.3)
Armenia	Rheumatic heart disease	10 to 14	Male	1 (1 to 1)	0.5 (0.3 to 0.6)	0 (0 to 1)	0.2 (0.2 to 0.3)	0 (0 to 0)	0.1 (0.1 to 0.2)	-87.0 (-92.2 to -79.2)	-75.1 (-85.1 to -60.2)
Armenia	Ischemic heart disease	5 to 9	Both	1 (1 to 2)	0.4 (0.3 to 0.5)	1 (1 to 1)	0.3 (0.2 to 0.4)	0 (0 to 0)	0.1 (0.1 to 0.2)	-84.9 (-91.2 to -73.7)	-71.5 (-83.4 to -50.2)
Armenia	Ischemic heart disease	5 to 9	Female	1 (0 to 1)	0.3 (0.2 to 0.5)	0 (0 to 0)	0.2 (0.2 to 0.4)	0 (0 to 0)	0.1 (0.0 to 0.1)	-86.6 (-93.2 to -74.3)	-74.6 (-87.0 to -51.1)
Armenia	Ischemic heart disease	5 to 9	Male	1 (0 to 1)	0.4 (0.3 to 0.7)	0 (0 to 1)	0.3 (0.2 to 0.5)	0 (0 to 0)	0.1 (0.1 to 0.2)	-83.7 (-91.7 to -65.7)	-69.2 (-84.4 to -35.5)
Armenia	Ischemic heart disease	10 to 14	Both	1 (1 to 1)	0.3 (0.2 to 0.3)	1 (0 to 1)	0.2 (0.2 to 0.3)	0 (0 to 0)	0.1 (0.1 to 0.2)	-78.3 (-86.6 to -65.4)	-57.2 (-73.6 to -31.9)
Armenia	Ischemic heart disease	10 to 14	Female	0 (0 to 0)	0.2 (0.1 to 0.3)	0 (0 to 0)	0.2 (0.1 to 0.3)	0 (0 to 0)	0.1 (0.0 to 0.1)	-83.0 (-90.3 to -68.7)	-65.6 (-80.3 to -36.6)
Armenia	Ischemic heart disease	10 to 14	Male	0 (0 to 1)	0.3 (0.2 to 0.4)	0 (0 to 1)	0.2 (0.1 to 0.3)	0 (0 to 0)	0.1 (0.1 to 0.2)	-75.1 (-86.7 to -54.2)	-52.2 (-74.5 to -12.2)
Armenia	Cerebrovascular disease	5 to 9	Both	2 (1 to 3)	0.5 (0.4 to 0.7)	1 (1 to 1)	0.4 (0.3 to 0.5)	0 (0 to 0)	0.1 (0.1 to 0.2)	-87.7 (-92.6 to -80.2)	-76.8 (-85.9 to -62.5)
Armenia	Cerebrovascular disease	5 to 9	Female	1 (1 to 2)	0.7 (0.5 to 0.9)	1 (0 to 1)	0.5 (0.3 to 0.7)	0 (0 to 0)	0.1 (0.1 to 0.2)	-88.5 (-93.6 to -81.0)	-78.1 (-87.8 to -63.9)
Armenia	Cerebrovascular disease	5 to 9	Male	1 (0 to 1)	0.4 (0.3 to 0.6)	0 (0 to 1)	0.3 (0.2 to 0.4)	0 (0 to 0)	0.1 (0.1 to 0.2)	-86.5 (-93.6 to -72.4)	-74.6 (-87.9 to -48.1)
Armenia	Cerebrovascular disease	10 to 14	Both	1 (1 to 2)	0.4 (0.3 to 0.5)	1 (1 to 1)	0.3 (0.2 to 0.4)	0 (0 to 0)	0.1 (0.1 to 0.2)	-82.4 (-88.8 to -74.0)	-65.3 (-77.8 to -48.8)
Armenia	Cerebrovascular disease	10 to 14	Female	1 (0 to 1)	0.3 (0.2 to 0.4)	1 (0 to 1)	0.3 (0.2 to 0.4)	0 (0 to 0)	0.1 (0.1 to 0.1)	-85.1 (-91.9 to -74.3)	-69.9 (-83.5 to -47.9)
Armenia	Cerebrovascular disease	10 to 14	Male	1 (0 to 1)	0.4 (0.3 to 0.6)	1 (0 to 1)	0.3 (0.2 to 0.4)	0 (0 to 0)	0.2 (0.1 to 0.2)	-80.4 (-88.7 to -67.7)	-62.3 (-78.3 to -38.1)
Armenia	Hypertensive heart disease	5 to 9	Both	0 (0 to 0)	0.0 (0.0 to 0.0)	0 (0 to 0)	0.0 (0.0 to 0.0)	0 (0 to 0)	0.0 (0.0 to 0.0)	-42.0 (-63.5 to -7.8)	9.7 (-31.0 to 74.4)
Armenia	Hypertensive heart disease	5 to 9	Female	0 (0 to 0)	0.0 (0.0 to 0.0)	0 (0 to 0)	0.0 (0.0 to 0.0)	0 (0 to 0)	0.0 (0.0 to 0.0)	-49.7 (-70.3 to -16.1)	-4.3 (-43.5 to 59.6)
Armenia	Hypertensive heart disease	5 to 9	Male	0 (0 to 0)	0.0 (0.0 to 0.0)	0 (0 to 0)	0.0 (0.0 to 0.0)	0 (0 to 0)	0.0 (0.0 to 0.0)	-34.6 (-64.1 to 22.6)	23.0 (-32.5 to 130.7)
Armenia	Hypertensive heart disease	10 to 14	Both	0 (0 to 0)	0.0 (0.0 to 0.0)	0 (0 to 0)	0.0 (0.0 to 0.0)	0 (0 to 0)	0.0 (0.0 to 0.1)	-3.7 (-59.4 to 143.7)	89.7 (-20.0 to 380.2)
Armenia	Hypertensive heart disease	10 to 14	Female	0 (0 to 0)	0.0 (0.0 to 0.0)	0 (0 to 0)	0.0 (0.0 to 0.0)	0 (0 to 0)	0.0 (0.0 to 0.0)	-58.0 (-73.3 to -35.7)	-14.8 (-45.8 to 30.4)
Armenia	Hypertensive heart disease	10 to 14	Male	0 (0 to 0)	0.0 (0.0 to 0.1)	0 (0 to 0)	0.0 (0.0 to 0.1)	0 (0 to 0)	0.1 (0.0 to 0.1)	18.6 (-58.3 to 297.7)	127.4 (-20.0 to 662.8)
Armenia	Cardiomyopathy and myocarditis	5 to 9	Both	1 (0 to 1)	0.2 (0.1 to 0.3)	0 (0 to 1)	0.1 (0.1 to 0.2)	0 (0 to 0)	0.1 (0.1 to 0.2)	-74.7 (-86.6 to -42.0)	-52.1 (-74.7 to 9.8)
Armenia	Cardiomyopathy and myocarditis	5 to 9	Female	1 (0 to 1)	0.3 (0.1 to 0.5)	0 (0 to 0)	0.2 (0.1 to 0.4)	0 (0 to 0)	0.1 (0.1 to 0.2)	-76.6 (-88.4 to -37.9)	-55.5 (-77.9 to 18.0)
Armenia	Cardiomyopathy and myocarditis	5 to 9	Male	0 (0 to 0)	0.1 (0.1 to 0.2)	0 (0 to 0)	0.1 (0.0 to 0.2)	0 (0 to 0)	0.1 (0.0 to 0.1)	-70.7 (-88.1 to -19.7)	-44.9 (-77.6 to 51.1)
Armenia	Cardiomyopathy and myocarditis	10 to 14	Both	0 (0 to 1)	0.1 (0.1 to 0.2)	0 (0 to 1)	0.1 (0.1 to 0.2)	0 (0 to 0)	0.1 (0.1 to 0.1)	-65.1 (-80.5 to -37.0)	-31.2 (-61.6 to 24.2)

eTable 8. All-cause and cause-specific mortality (counts and rates) at the global level and by country in the WHO European Region for ages 5 to 9 and 10 to 14 for 1990, 2000, and 2016

Armenia	Cardiomyopathy and myocarditis	10 to 14	Female	0 (0 to 0)	0.1 (0.1 to 0.2)	0 (0 to 0)	0.1 (0.1 to 0.2)	0 (0 to 0)	0.1 (0.0 to 0.1)	-73.8 (-87.2 to -48.9)	-46.9 (-74.1 to 3.5)
Armenia	Cardiomyopathy and myocarditis	10 to 14	Male	0 (0 to 0)	0.2 (0.1 to 0.3)	0 (0 to 0)	0.1 (0.1 to 0.2)	0 (0 to 0)	0.1 (0.1 to 0.2)	-58.8 (-79.3 to -11.6)	-20.9 (-60.3 to 69.6)
Armenia	Atrial fibrillation and flutter	5 to 9	Both	0 (0 to 0)	0.0 (0.0 to 0.0)	0 (0 to 0)	0.0 (0.0 to 0.0)	0 (0 to 0)	0.0 (0.0 to 0.0)	0.0 (0.0 to 0.0)	0.0 (0.0 to 0.0)
Armenia	Atrial fibrillation and flutter	5 to 9	Female	0 (0 to 0)	0.0 (0.0 to 0.0)	0 (0 to 0)	0.0 (0.0 to 0.0)	0 (0 to 0)	0.0 (0.0 to 0.0)	0.0 (0.0 to 0.0)	0.0 (0.0 to 0.0)
Armenia	Atrial fibrillation and flutter	5 to 9	Male	0 (0 to 0)	0.0 (0.0 to 0.0)	0 (0 to 0)	0.0 (0.0 to 0.0)	0 (0 to 0)	0.0 (0.0 to 0.0)	0.0 (0.0 to 0.0)	0.0 (0.0 to 0.0)
Armenia	Atrial fibrillation and flutter	10 to 14	Both	0 (0 to 0)	0.0 (0.0 to 0.0)	0 (0 to 0)	0.0 (0.0 to 0.0)	0 (0 to 0)	0.0 (0.0 to 0.0)	0.0 (0.0 to 0.0)	0.0 (0.0 to 0.0)
Armenia	Atrial fibrillation and flutter	10 to 14	Female	0 (0 to 0)	0.0 (0.0 to 0.0)	0 (0 to 0)	0.0 (0.0 to 0.0)	0 (0 to 0)	0.0 (0.0 to 0.0)	0.0 (0.0 to 0.0)	0.0 (0.0 to 0.0)
Armenia	Atrial fibrillation and flutter	10 to 14	Male	0 (0 to 0)	0.0 (0.0 to 0.0)	0 (0 to 0)	0.0 (0.0 to 0.0)	0 (0 to 0)	0.0 (0.0 to 0.0)	0.0 (0.0 to 0.0)	0.0 (0.0 to 0.0)
Armenia	Endocarditis	5 to 9	Both	0 (0 to 0)	0.0 (0.0 to 0.0)	0 (0 to 0)	0.0 (0.0 to 0.0)	0 (0 to 0)	0.0 (0.0 to 0.0)	-42.2 (-64.6 to -8.0)	9.4 (-33.0 to 74.1)
Armenia	Endocarditis	5 to 9	Female	0 (0 to 0)	0.0 (0.0 to 0.0)	0 (0 to 0)	0.0 (0.0 to 0.0)	0 (0 to 0)	0.0 (0.0 to 0.0)	-51.4 (-73.3 to -16.9)	-7.5 (-49.2 to 58.0)
Armenia	Endocarditis	5 to 9	Male	0 (0 to 0)	0.0 (0.0 to 0.0)	0 (0 to 0)	0.0 (0.0 to 0.0)	0 (0 to 0)	0.0 (0.0 to 0.0)	-32.3 (-63.5 to 28.2)	27.4 (-31.2 to 141.2)
Armenia	Endocarditis	10 to 14	Both	0 (0 to 0)	0.0 (0.0 to 0.0)	0 (0 to 0)	0.0 (0.0 to 0.0)	0 (0 to 0)	0.0 (0.0 to 0.0)	-45.6 (-63.8 to -21.6)	7.1 (-28.7 to 54.5)
Armenia	Endocarditis	10 to 14	Female	0 (0 to 0)	0.0 (0.0 to 0.0)	0 (0 to 0)	0.0 (0.0 to 0.0)	0 (0 to 0)	0.0 (0.0 to 0.0)	-58.4 (-73.4 to -35.7)	-15.7 (-46.1 to 30.4)
Armenia	Endocarditis	10 to 14	Male	0 (0 to 0)	0.0 (0.0 to 0.0)	0 (0 to 0)	0.0 (0.0 to 0.0)	0 (0 to 0)	0.0 (0.0 to 0.0)	-31.3 (-59.2 to 12.0)	31.9 (-21.8 to 114.7)
Armenia	Other cardiovascular and circulatory diseases	5 to 9	Both	0 (0 to 0)	0.0 (0.0 to 0.1)	0 (0 to 0)	0.0 (0.0 to 0.0)	0 (0 to 0)	0.1 (0.0 to 0.1)	-21.8 (-54.1 to 37.9)	48.0 (-13.1 to 161.0)
Armenia	Other cardiovascular and circulatory diseases	5 to 9	Female	0 (0 to 0)	0.1 (0.0 to 0.1)	0 (0 to 0)	0.1 (0.0 to 0.1)	0 (0 to 0)	0.1 (0.1 to 0.1)	-18.8 (-55.2 to 54.9)	54.4 (-14.8 to 194.6)
Armenia	Other cardiovascular and circulatory diseases	5 to 9	Male	0 (0 to 0)	0.0 (0.0 to 0.0)	0 (0 to 0)	0.0 (0.0 to 0.0)	0 (0 to 0)	0.0 (0.0 to 0.0)	-39.1 (-68.1 to 21.4)	14.6 (-39.9 to 128.4)
Armenia	Other cardiovascular and circulatory diseases	10 to 14	Both	0 (0 to 0)	0.1 (0.0 to 0.1)	0 (0 to 0)	0.1 (0.0 to 0.1)	0 (0 to 0)	0.1 (0.1 to 0.1)	-1.5 (-38.1 to 55.9)	94.2 (21.9 to 207.2)
Armenia	Other cardiovascular and circulatory diseases	10 to 14	Female	0 (0 to 0)	0.1 (0.0 to 0.1)	0 (0 to 0)	0.1 (0.1 to 0.1)	0 (0 to 0)	0.2 (0.1 to 0.2)	9.6 (-38.2 to 92.4)	122.2 (25.2 to 290.1)

eTable 8. All-cause and cause-specific mortality (counts and rates) at the global level and by country in the WHO European Region for ages 5 to 9 and 10 to 14 for 1990, 2000, and 2016

Armenia	Other cardiovascular and circulatory diseases	10 to 14	Male	0<br>(0 to 0)	0.0<br>(0.0 to 0.0)	0<br>(0 to 0)	0.0<br>(0.0 to 0.0)	0<br>(0 to 0)	0.0<br>(0.0 to 0.1)	-26.0<br>(-61.6 to 45.2)	41.9<br>(-26.4 to 178.5)
Armenia	Chronic obstructive pulmonary disease	5 to 9	Both	1<br>(1 to 2)	0.3<br>(0.2 to 0.4)	0<br>(0 to 1)	0.1<br>(0.1 to 0.2)	0<br>(0 to 0)	0.0<br>(0.0 to 0.1)	-92.0<br>(-95.4 to -86.9)	-84.8<br>(-91.2 to -75.2)
Armenia	Chronic obstructive pulmonary disease	5 to 9	Female	0<br>(0 to 1)	0.3<br>(0.2 to 0.4)	0<br>(0 to 0)	0.1<br>(0.1 to 0.2)	0<br>(0 to 0)	0.0<br>(0.0 to 0.1)	-89.7<br>(-94.7 to -79.5)	-80.5<br>(-90.0 to -61.0)
Armenia	Chronic obstructive pulmonary disease	5 to 9	Male	1<br>(0 to 1)	0.3<br>(0.2 to 0.5)	0<br>(0 to 0)	0.2<br>(0.1 to 0.2)	0<br>(0 to 0)	0.0<br>(0.0 to 0.1)	-93.6<br>(-96.9 to -87.8)	-88.0<br>(-94.1 to -77.0)
Armenia	Chronic obstructive pulmonary disease	10 to 14	Both	1<br>(1 to 1)	0.2<br>(0.2 to 0.3)	0<br>(0 to 1)	0.1<br>(0.1 to 0.2)	0<br>(0 to 0)	0.1<br>(0.1 to 0.1)	-81.4<br>(-87.6 to -73.5)	-63.3<br>(-75.5 to -47.7)
Armenia	Chronic obstructive pulmonary disease	10 to 14	Female	0<br>(0 to 0)	0.2<br>(0.2 to 0.3)	0<br>(0 to 0)	0.1<br>(0.1 to 0.2)	0<br>(0 to 0)	0.1<br>(0.1 to 0.1)	-78.6<br>(-87.0 to -64.8)	-56.7<br>(-73.7 to -28.7)
Armenia	Chronic obstructive pulmonary disease	10 to 14	Male	0<br>(0 to 0)	0.2<br>(0.1 to 0.3)	0<br>(0 to 0)	0.1<br>(0.1 to 0.2)	0<br>(0 to 0)	0.1<br>(0.0 to 0.1)	-84.3<br>(-90.3 to -75.4)	-69.9<br>(-81.4 to -52.9)
Armenia	Asthma	5 to 9	Both	0<br>(0 to 0)	0.1<br>(0.0 to 0.1)	0<br>(0 to 0)	0.0<br>(0.0 to 0.1)	0<br>(0 to 0)	0.0<br>(0.0 to 0.0)	-76.9<br>(-86.9 to -60.4)	-56.3<br>(-75.3 to -25.2)
Armenia	Asthma	5 to 9	Female	0<br>(0 to 0)	0.1<br>(0.0 to 0.1)	0<br>(0 to 0)	0.0<br>(0.0 to 0.1)	0<br>(0 to 0)	0.0<br>(0.0 to 0.0)	-73.5<br>(-86.6 to -44.2)	-49.6<br>(-74.5 to 6.1)
Armenia	Asthma	5 to 9	Male	0<br>(0 to 0)	0.1<br>(0.0 to 0.1)	0<br>(0 to 0)	0.0<br>(0.0 to 0.1)	0<br>(0 to 0)	0.0<br>(0.0 to 0.0)	-79.9<br>(-90.3 to -56.4)	-62.1<br>(-81.8 to -17.9)
Armenia	Asthma	10 to 14	Both	0<br>(0 to 0)	0.0<br>(0.0 to 0.0)	0<br>(0 to 0)	0.0<br>(0.0 to 0.0)	0<br>(0 to 0)	0.0<br>(0.0 to 0.0)	-85.0<br>(-92.7 to -66.7)	-70.5<br>(-85.6 to -34.4)
Armenia	Asthma	10 to 14	Female	0<br>(0 to 0)	0.0<br>(0.0 to 0.0)	0<br>(0 to 0)	0.0<br>(0.0 to 0.0)	0<br>(0 to 0)	0.0<br>(0.0 to 0.0)	-84.8<br>(-93.8 to -61.8)	-69.3<br>(-87.5 to -22.5)
Armenia	Asthma	10 to 14	Male	0<br>(0 to 0)	0.0<br>(0.0 to 0.0)	0<br>(0 to 0)	0.0<br>(0.0 to 0.0)	0<br>(0 to 0)	0.0<br>(0.0 to 0.0)	-85.3<br>(-94.5 to -51.1)	-71.9<br>(-89.5 to -6.1)
Armenia	Interstitial lung disease and pulmonary sarcoidosis	5 to 9	Both	0<br>(0 to 0)	0.1<br>(0.0 to 0.1)	0<br>(0 to 0)	0.0<br>(0.0 to 0.1)	0<br>(0 to 0)	0.0<br>(0.0 to 0.0)	-83.8<br>(-92.6 to -61.3)	-69.4<br>(-85.9 to -26.9)
Armenia	Interstitial lung disease and pulmonary sarcoidosis	5 to 9	Female	0<br>(0 to 0)	0.1<br>(0.0 to 0.2)	0<br>(0 to 0)	0.0<br>(0.0 to 0.1)	0<br>(0 to 0)	0.0<br>(0.0 to 0.0)	-82.3<br>(-93.7 to -45.9)	-66.3<br>(-88.0 to 2.8)
Armenia	Interstitial lung disease and pulmonary sarcoidosis	5 to 9	Male	0<br>(0 to 0)	0.1<br>(0.0 to 0.2)	0<br>(0 to 0)	0.0<br>(0.0 to 0.1)	0<br>(0 to 0)	0.0<br>(0.0 to 0.0)	-85.3<br>(-95.1 to -50.0)	-72.3<br>(-90.8 to -6.0)
Armenia	Interstitial lung disease and pulmonary sarcoidosis	10 to 14	Both	0<br>(0 to 0)	0.0<br>(0.0 to 0.0)	0<br>(0 to 0)	0.0<br>(0.0 to 0.0)	0<br>(0 to 0)	0.0<br>(0.0 to 0.0)	-72.0<br>(-87.6 to -36.5)	-44.7<br>(-75.6 to 25.2)

eTable 8. All-cause and cause-specific mortality (counts and rates) at the global level and by country in the WHO European Region for ages 5 to 9 and 10 to 14 for 1990, 2000, and 2016

Armenia	Interstitial lung disease and pulmonary sarcoidosis	10 to 14	Female	0 (0 to 0)	0.0 (0.0 to 0.0)	0 (0 to 0)	0.0 (0.0 to 0.0)	0 (0 to 0)	0.0 (0.0 to 0.0)	-72.4 (-90.6 to -16.1)	-44.1 (-81.0 to 70.0)
Armenia	Interstitial lung disease and pulmonary sarcoidosis	10 to 14	Male	0 (0 to 0)	0.0 (0.0 to 0.0)	0 (0 to 0)	0.0 (0.0 to 0.0)	0 (0 to 0)	0.0 (0.0 to 0.0)	-70.7 (-89.6 to -17.3)	-43.8 (-80.1 to 58.6)
Armenia	Other chronic respiratory diseases	5 to 9	Both	0 (0 to 0)	0.1 (0.0 to 0.1)	0 (0 to 0)	0.0 (0.0 to 0.1)	0 (0 to 0)	0.0 (0.0 to 0.0)	-75.1 (-85.3 to -59.2)	-52.9 (-72.2 to -22.8)
Armenia	Other chronic respiratory diseases	5 to 9	Female	0 (0 to 0)	0.1 (0.0 to 0.1)	0 (0 to 0)	0.0 (0.0 to 0.1)	0 (0 to 0)	0.0 (0.0 to 0.0)	-73.9 (-86.6 to -46.4)	-50.3 (-74.6 to 1.9)
Armenia	Other chronic respiratory diseases	5 to 9	Male	0 (0 to 0)	0.1 (0.0 to 0.1)	0 (0 to 0)	0.0 (0.0 to 0.1)	0 (0 to 0)	0.0 (0.0 to 0.0)	-76.4 (-88.3 to -50.9)	-55.5 (-78.0 to -7.5)
Armenia	Other chronic respiratory diseases	10 to 14	Both	0 (0 to 0)	0.0 (0.0 to 0.0)	0 (0 to 0)	0.0 (0.0 to 0.0)	0 (0 to 0)	0.0 (0.0 to 0.0)	-67.2 (-82.1 to -44.0)	-35.3 (-64.7 to 10.3)
Armenia	Other chronic respiratory diseases	10 to 14	Female	0 (0 to 0)	0.0 (0.0 to 0.0)	0 (0 to 0)	0.0 (0.0 to 0.0)	0 (0 to 0)	0.0 (0.0 to 0.0)	-67.9 (-85.8 to -34.0)	-35.0 (-71.2 to 33.8)
Armenia	Other chronic respiratory diseases	10 to 14	Male	0 (0 to 0)	0.0 (0.0 to 0.0)	0 (0 to 0)	0.0 (0.0 to 0.0)	0 (0 to 0)	0.0 (0.0 to 0.0)	-65.8 (-82.1 to -34.1)	-34.3 (-65.6 to 26.5)
Armenia	Cirrhosis and other chronic liver diseases due to hepatitis B	5 to 9	Both	0 (0 to 0)	0.0 (0.0 to 0.0)	0 (0 to 0)	0.0 (0.0 to 0.0)	0 (0 to 0)	0.0 (0.0 to 0.0)	-60.6 (-75.7 to -33.2)	-25.4 (-54.1 to 26.3)
Armenia	Cirrhosis and other chronic liver diseases due to hepatitis B	5 to 9	Female	0 (0 to 0)	0.0 (0.0 to 0.0)	0 (0 to 0)	0.0 (0.0 to 0.0)	0 (0 to 0)	0.0 (0.0 to 0.0)	-55.9 (-73.8 to -28.1)	-16.1 (-50.1 to 36.7)
Armenia	Cirrhosis and other chronic liver diseases due to hepatitis B	5 to 9	Male	0 (0 to 0)	0.0 (0.0 to 0.0)	0 (0 to 0)	0.0 (0.0 to 0.0)	0 (0 to 0)	0.0 (0.0 to 0.0)	-61.7 (-77.9 to -30.0)	-28.0 (-58.4 to 31.8)
Armenia	Cirrhosis and other chronic liver diseases due to hepatitis B	10 to 14	Both	0 (0 to 0)	0.0 (0.0 to 0.0)	0 (0 to 0)	0.0 (0.0 to 0.0)	0 (0 to 0)	0.0 (0.0 to 0.0)	-46.4 (-65.3 to -18.1)	5.7 (-31.7 to 61.4)
Armenia	Cirrhosis and other chronic liver diseases due to hepatitis B	10 to 14	Female	0 (0 to 0)	0.0 (0.0 to 0.0)	0 (0 to 0)	0.0 (0.0 to 0.0)	0 (0 to 0)	0.0 (0.0 to 0.0)	-41.6 (-66.2 to -1.0)	18.5 (-31.4 to 100.8)
Armenia	Cirrhosis and other chronic liver diseases due to hepatitis B	10 to 14	Male	0 (0 to 0)	0.0 (0.0 to 0.0)	0 (0 to 0)	0.0 (0.0 to 0.0)	0 (0 to 0)	0.0 (0.0 to 0.1)	-47.7 (-69.6 to -12.2)	0.4 (-41.6 to 68.3)
Armenia	Cirrhosis and other chronic liver diseases due to hepatitis C	5 to 9	Both	0 (0 to 0)	0.0 (0.0 to 0.0)	0 (0 to 0)	0.0 (0.0 to 0.0)	0 (0 to 0)	0.0 (0.0 to 0.0)	-61.8 (-76.6 to -35.9)	-27.8 (-55.7 to 21.3)

eTable 8. All-cause and cause-specific mortality (counts and rates) at the global level and by country in the WHO European Region for ages 5 to 9 and 10 to 14 for 1990, 2000, and 2016

Armenia	Cirrhosis and other chronic liver diseases due to hepatitis C	5 to 9	Female	0 (0 to 0)	0.0 (0.0 to 0.0)	0 (0 to 0)	0.0 (0.0 to 0.0)	0 (0 to 0)	0.0 (0.0 to 0.0)	-55.9 (-74.1 to -28.4)	-16.1 (-50.7 to 36.1)
Armenia	Cirrhosis and other chronic liver diseases due to hepatitis C	5 to 9	Male	0 (0 to 0)	0.0 (0.0 to 0.0)	0 (0 to 0)	0.0 (0.0 to 0.0)	0 (0 to 0)	0.0 (0.0 to 0.0)	-63.4 (-79.1 to -33.4)	-31.1 (-60.7 to 25.4)
Armenia	Cirrhosis and other chronic liver diseases due to hepatitis C	10 to 14	Both	0 (0 to 0)	0.0 (0.0 to 0.0)	0 (0 to 0)	0.0 (0.0 to 0.0)	0 (0 to 0)	0.0 (0.0 to 0.0)	-48.3 (-66.4 to -21.4)	1.8 (-33.7 to 54.9)
Armenia	Cirrhosis and other chronic liver diseases due to hepatitis C	10 to 14	Female	0 (0 to 0)	0.0 (0.0 to 0.0)	0 (0 to 0)	0.0 (0.0 to 0.0)	0 (0 to 0)	0.0 (0.0 to 0.0)	-41.6 (-66.1 to -1.5)	18.5 (-31.2 to 99.7)
Armenia	Cirrhosis and other chronic liver diseases due to hepatitis C	10 to 14	Male	0 (0 to 0)	0.0 (0.0 to 0.0)	0 (0 to 0)	0.0 (0.0 to 0.0)	0 (0 to 0)	0.0 (0.0 to 0.0)	-50.2 (-70.8 to -16.1)	-4.4 (-44.1 to 60.9)
Armenia	Cirrhosis and other chronic liver diseases due to other causes	5 to 9	Both	1 (1 to 2)	0.4 (0.3 to 0.5)	1 (1 to 1)	0.3 (0.2 to 0.4)	1 (0 to 1)	0.3 (0.2 to 0.4)	-60.2 (-74.8 to -36.8)	-24.6 (-52.4 to 19.5)
Armenia	Cirrhosis and other chronic liver diseases due to other causes	5 to 9	Female	1 (0 to 1)	0.3 (0.2 to 0.4)	0 (0 to 0)	0.2 (0.2 to 0.3)	0 (0 to 0)	0.3 (0.2 to 0.4)	-56.7 (-74.5 to -29.6)	-17.6 (-51.5 to 33.8)
Armenia	Cirrhosis and other chronic liver diseases due to other causes	5 to 9	Male	1 (1 to 1)	0.4 (0.3 to 0.6)	0 (0 to 1)	0.3 (0.2 to 0.4)	0 (0 to 0)	0.3 (0.2 to 0.4)	-62.8 (-78.4 to -31.9)	-29.9 (-59.4 to 28.2)
Armenia	Cirrhosis and other chronic liver diseases due to other causes	10 to 14	Both	1 (1 to 2)	0.4 (0.3 to 0.5)	1 (1 to 2)	0.4 (0.3 to 0.5)	1 (1 to 1)	0.4 (0.3 to 0.6)	-46.1 (-63.8 to -21.4)	6.1 (-28.6 to 54.8)
Armenia	Cirrhosis and other chronic liver diseases due to other causes	10 to 14	Female	1 (0 to 1)	0.4 (0.3 to 0.5)	1 (0 to 1)	0.4 (0.3 to 0.5)	0 (0 to 1)	0.4 (0.3 to 0.6)	-42.5 (-66.7 to -3.0)	16.5 (-32.5 to 96.7)
Armenia	Cirrhosis and other chronic liver diseases due to other causes	10 to 14	Male	1 (0 to 1)	0.4 (0.3 to 0.6)	1 (0 to 1)	0.3 (0.2 to 0.5)	0 (0 to 1)	0.4 (0.3 to 0.6)	-49.1 (-70.3 to -14.6)	-2.4 (-43.0 to 63.7)
Armenia	Peptic ulcer disease	5 to 9	Both	0 (0 to 0)	0.0 (0.0 to 0.1)	0 (0 to 0)	0.0 (0.0 to 0.1)	0 (0 to 0)	0.0 (0.0 to 0.1)	-58.8 (-80.1 to -14.0)	-22.0 (-62.4 to 62.7)
Armenia	Peptic ulcer disease	5 to 9	Female	0 (0 to 0)	0.1 (0.0 to 0.1)	0 (0 to 0)	0.0 (0.0 to 0.1)	0 (0 to 0)	0.1 (0.0 to 0.1)	-58.2 (-83.1 to 11.7)	-20.5 (-68.0 to 112.4)
Armenia	Peptic ulcer disease	5 to 9	Male	0 (0 to 0)	0.0 (0.0 to 0.1)	0 (0 to 0)	0.0 (0.0 to 0.0)	0 (0 to 0)	0.0 (0.0 to 0.0)	-60.0 (-83.9 to -8.3)	-24.7 (-69.7 to 72.5)
Armenia	Peptic ulcer disease	10 to 14	Both	0 (0 to 0)	0.1 (0.0 to 0.1)	0 (0 to 0)	0.0 (0.0 to 0.1)	0 (0 to 0)	0.0 (0.0 to 0.1)	-76.1 (-88.6 to -51.5)	-52.9 (-77.5 to -4.3)
Armenia	Peptic ulcer disease	10 to 14	Female	0 (0 to 0)	0.0 (0.0 to 0.0)	0 (0 to 0)	0.0 (0.0 to 0.0)	0 (0 to 0)	0.0 (0.0 to 0.0)	-22.8 (-59.6 to 66.5)	56.4 (-18.1 to 237.5)

eTable 8. All-cause and cause-specific mortality (counts and rates) at the global level and by country in the WHO European Region for ages 5 to 9 and 10 to 14 for 1990, 2000, and 2016

Armenia	Peptic ulcer disease	10 to 14	Male	0 (0 to 0)	0.1 (0.1 to 0.2)	0 (0 to 0)	0.1 (0.0 to 0.1)	0 (0 to 0)	0.0 (0.0 to 0.1)	-80.2 (-91.8 to -53.8)	-62.0 (-84.3 to -11.4)
Armenia	Gastritis and duodenitis	5 to 9	Both	0 (0 to 0)	0.0 (0.0 to 0.0)	0 (0 to 0)	0.0 (0.0 to 0.0)	0 (0 to 0)	0.0 (0.0 to 0.0)	-34.1 (-74.1 to 38.2)	24.7 (-51.0 to 161.5)
Armenia	Gastritis and duodenitis	5 to 9	Female	0 (0 to 0)	0.0 (0.0 to 0.0)	0 (0 to 0)	0.0 (0.0 to 0.0)	0 (0 to 0)	0.0 (0.0 to 0.0)	-20.6 (-60.6 to 77.8)	50.9 (-25.0 to 238.2)
Armenia	Gastritis and duodenitis	5 to 9	Male	0 (0 to 0)	0.0 (0.0 to 0.0)	0 (0 to 0)	0.0 (0.0 to 0.0)	0 (0 to 0)	0.0 (0.0 to 0.0)	-45.9 (-83.7 to 46.0)	1.8 (-69.4 to 174.8)
Armenia	Gastritis and duodenitis	10 to 14	Both	0 (0 to 0)	0.0 (0.0 to 0.0)	0 (0 to 0)	0.0 (0.0 to 0.0)	0 (0 to 0)	0.0 (0.0 to 0.0)	-37.5 (-61.1 to 4.3)	23.2 (-23.3 to 105.4)
Armenia	Gastritis and duodenitis	10 to 14	Female	0 (0 to 0)	0.0 (0.0 to 0.0)	0 (0 to 0)	0.0 (0.0 to 0.0)	0 (0 to 0)	0.0 (0.0 to 0.0)	-22.5 (-59.1 to 61.9)	57.0 (-17.1 to 228.2)
Armenia	Gastritis and duodenitis	10 to 14	Male	0 (0 to 0)	0.0 (0.0 to 0.0)	0 (0 to 0)	0.0 (0.0 to 0.0)	0 (0 to 0)	0.0 (0.0 to 0.0)	-44.2 (-70.1 to 2.7)	6.9 (-42.7 to 96.9)
Armenia	Appendicitis	5 to 9	Both	1 (1 to 2)	0.4 (0.2 to 0.6)	0 (0 to 1)	0.2 (0.1 to 0.3)	0 (0 to 0)	0.1 (0.1 to 0.2)	-81.9 (-90.7 to -56.2)	-65.8 (-82.3 to -17.0)
Armenia	Appendicitis	5 to 9	Female	1 (0 to 1)	0.4 (0.2 to 0.6)	0 (0 to 0)	0.2 (0.1 to 0.3)	0 (0 to 0)	0.2 (0.1 to 0.3)	-74.0 (-88.7 to -38.4)	-50.6 (-78.5 to 17.1)
Armenia	Appendicitis	5 to 9	Male	1 (0 to 1)	0.4 (0.2 to 0.7)	0 (0 to 0)	0.2 (0.1 to 0.3)	0 (0 to 0)	0.1 (0.0 to 0.2)	-88.8 (-96.1 to -55.1)	-78.9 (-92.7 to -15.6)
Armenia	Appendicitis	10 to 14	Both	1 (1 to 2)	0.4 (0.3 to 0.5)	1 (0 to 1)	0.2 (0.1 to 0.3)	0 (0 to 0)	0.2 (0.1 to 0.3)	-76.2 (-86.2 to -53.8)	-53.2 (-72.8 to -9.0)
Armenia	Appendicitis	10 to 14	Female	0 (0 to 1)	0.3 (0.2 to 0.5)	0 (0 to 1)	0.2 (0.1 to 0.3)	0 (0 to 0)	0.2 (0.1 to 0.3)	-64.6 (-81.2 to -37.2)	-28.2 (-61.9 to 27.4)
Armenia	Appendicitis	10 to 14	Male	1 (0 to 1)	0.5 (0.3 to 0.7)	0 (0 to 1)	0.3 (0.1 to 0.4)	0 (0 to 0)	0.2 (0.1 to 0.4)	-83.5 (-92.3 to -55.1)	-68.3 (-85.2 to -13.8)
Armenia	Paralytic ileus and intestinal obstruction	5 to 9	Both	1 (0 to 1)	0.2 (0.1 to 0.3)	0 (0 to 1)	0.2 (0.1 to 0.3)	0 (0 to 1)	0.1 (0.1 to 0.3)	-57.7 (-77.7 to -19.1)	-19.9 (-57.8 to 53.0)
Armenia	Paralytic ileus and intestinal obstruction	5 to 9	Female	0 (0 to 0)	0.1 (0.0 to 0.2)	0 (0 to 0)	0.1 (0.0 to 0.2)	0 (0 to 0)	0.1 (0.1 to 0.2)	-47.7 (-75.8 to 10.3)	-0.5 (-54.0 to 109.7)
Armenia	Paralytic ileus and intestinal obstruction	5 to 9	Male	0 (0 to 1)	0.3 (0.1 to 0.5)	0 (0 to 1)	0.2 (0.1 to 0.4)	0 (0 to 0)	0.2 (0.1 to 0.5)	-62.1 (-83.2 to -9.4)	-28.7 (-68.3 to 70.5)
Armenia	Paralytic ileus and intestinal obstruction	10 to 14	Both	0 (0 to 1)	0.1 (0.1 to 0.2)	0 (0 to 1)	0.1 (0.1 to 0.2)	0 (0 to 0)	0.1 (0.1 to 0.3)	-48.2 (-68.4 to -12.4)	2.0 (-37.7 to 72.7)
Armenia	Paralytic ileus and intestinal obstruction	10 to 14	Female	0 (0 to 0)	0.1 (0.0 to 0.2)	0 (0 to 0)	0.1 (0.0 to 0.2)	0 (0 to 0)	0.1 (0.0 to 0.2)	-39.5 (-69.5 to 20.1)	22.6 (-38.2 to 143.5)
Armenia	Paralytic ileus and intestinal obstruction	10 to 14	Male	0 (0 to 1)	0.2 (0.1 to 0.4)	0 (0 to 1)	0.2 (0.1 to 0.3)	0 (0 to 0)	0.2 (0.1 to 0.4)	-52.5 (-77.4 to -9.2)	-8.9 (-56.6 to 74.2)
Armenia	Inguinal, femoral, and abdominal hernia	5 to 9	Both	0 (0 to 0)	0.0 (0.0 to 0.1)	0 (0 to 0)	0.0 (0.0 to 0.0)	0 (0 to 0)	0.0 (0.0 to 0.0)	-80.1 (-91.6 to -31.1)	-62.3 (-84.0 to 30.4)
Armenia	Inguinal, femoral, and abdominal hernia	5 to 9	Female	0 (0 to 0)	0.0 (0.0 to 0.0)	0 (0 to 0)	0.0 (0.0 to 0.0)	0 (0 to 0)	0.0 (0.0 to 0.0)	-58.6 (-83.4 to 70.5)	-21.3 (-68.5 to 224.2)
Armenia	Inguinal, femoral, and abdominal hernia	5 to 9	Male	0 (0 to 0)	0.0 (0.0 to 0.1)	0 (0 to 0)	0.0 (0.0 to 0.1)	0 (0 to 0)	0.0 (0.0 to 0.0)	-81.7 (-92.7 to -33.1)	-65.6 (-86.3 to 26.0)
Armenia	Inguinal, femoral, and abdominal hernia	10 to 14	Both	0 (0 to 0)	0.0 (0.0 to 0.1)	0 (0 to 0)	0.0 (0.0 to 0.0)	0 (0 to 0)	0.0 (0.0 to 0.0)	-70.0 (-82.5 to -37.8)	-40.9 (-65.6 to 22.6)
Armenia	Inguinal, femoral, and abdominal hernia	10 to 14	Female	0 (0 to 0)	0.0 (0.0 to 0.0)	0 (0 to 0)	0.0 (0.0 to 0.0)	0 (0 to 0)	0.0 (0.0 to 0.0)	-70.3 (-91.4 to -42.3)	-39.8 (-82.6 to 17.0)

eTable 8. All-cause and cause-specific mortality (counts and rates) at the global level and by country in the WHO European Region for ages 5 to 9 and 10 to 14 for 1990, 2000, and 2016

Armenia	Inguinal, femoral, and abdominal hernia	10 to 14	Male	0 (0 to 0)	0.0 (0.0 to 0.1)	0 (0 to 0)	0.0 (0.0 to 0.1)	0 (0 to 0)	0.0 (0.0 to 0.0)	-69.8 (-84.5 to 25.0)	-42.0 (-70.4 to 139.8)
Armenia	Inflammatory bowel disease	5 to 9	Both	0 (0 to 0)	0.0 (0.0 to 0.1)	0 (0 to 0)	0.0 (0.0 to 0.1)	0 (0 to 0)	0.0 (0.0 to 0.1)	-67.7 (-87.0 to 15.9)	-38.9 (-75.4 to 119.3)
Armenia	Inflammatory bowel disease	5 to 9	Female	0 (0 to 0)	0.0 (0.0 to 0.1)	0 (0 to 0)	0.0 (0.0 to 0.0)	0 (0 to 0)	0.0 (0.0 to 0.0)	-52.9 (-78.6 to 91.7)	-10.5 (-59.3 to 264.6)
Armenia	Inflammatory bowel disease	5 to 9	Male	0 (0 to 0)	0.0 (0.0 to 0.2)	0 (0 to 0)	0.0 (0.0 to 0.1)	0 (0 to 0)	0.0 (0.0 to 0.1)	-72.5 (-90.9 to 16.0)	-48.2 (-82.8 to 118.3)
Armenia	Inflammatory bowel disease	10 to 14	Both	0 (0 to 0)	0.0 (0.0 to 0.1)	0 (0 to 0)	0.0 (0.0 to 0.0)	0 (0 to 0)	0.0 (0.0 to 0.0)	-55.9 (-77.5 to 3.9)	-13.0 (-55.7 to 104.7)
Armenia	Inflammatory bowel disease	10 to 14	Female	0 (0 to 0)	0.0 (0.0 to 0.0)	0 (0 to 0)	0.0 (0.0 to 0.0)	0 (0 to 0)	0.0 (0.0 to 0.0)	-55.9 (-80.3 to 3.0)	-10.5 (-60.1 to 108.9)
Armenia	Inflammatory bowel disease	10 to 14	Male	0 (0 to 0)	0.0 (0.0 to 0.1)	0 (0 to 0)	0.0 (0.0 to 0.1)	0 (0 to 0)	0.0 (0.0 to 0.1)	-55.9 (-78.9 to 25.7)	-15.3 (-59.6 to 141.1)
Armenia	Vascular intestinal disorders	5 to 9	Both	0 (0 to 0)	0.0 (0.0 to 0.0)	0 (0 to 0)	0.0 (0.0 to 0.0)	0 (0 to 0)	0.0 (0.0 to 0.0)	-75.3 (-91.3 to -11.1)	-53.2 (-83.5 to 68.2)
Armenia	Vascular intestinal disorders	5 to 9	Female	0 (0 to 0)	0.0 (0.0 to 0.0)	0 (0 to 0)	0.0 (0.0 to 0.0)	0 (0 to 0)	0.0 (0.0 to 0.0)	-34.9 (-75.8 to 120.1)	23.9 (-54.0 to 318.5)
Armenia	Vascular intestinal disorders	5 to 9	Male	0 (0 to 0)	0.0 (0.0 to 0.1)	0 (0 to 0)	0.0 (0.0 to 0.0)	0 (0 to 0)	0.0 (0.0 to 0.0)	-76.3 (-92.5 to -10.5)	-55.5 (-86.0 to 68.5)
Armenia	Vascular intestinal disorders	10 to 14	Both	0 (0 to 0)	0.1 (0.0 to 0.2)	0 (0 to 0)	0.1 (0.0 to 0.1)	0 (0 to 0)	0.1 (0.0 to 0.1)	-60.0 (-81.1 to -1.8)	-21.3 (-62.8 to 93.5)
Armenia	Vascular intestinal disorders	10 to 14	Female	0 (0 to 0)	0.0 (0.0 to 0.0)	0 (0 to 0)	0.0 (0.0 to 0.0)	0 (0 to 0)	0.0 (0.0 to 0.0)	-49.4 (-76.6 to 39.8)	2.5 (-52.5 to 183.5)
Armenia	Vascular intestinal disorders	10 to 14	Male	0 (0 to 0)	0.2 (0.0 to 0.3)	0 (0 to 0)	0.1 (0.0 to 0.2)	0 (0 to 0)	0.1 (0.0 to 0.3)	-60.1 (-81.2 to 1.2)	-23.4 (-63.9 to 94.1)
Armenia	Gallbladder and biliary diseases	5 to 9	Both	0 (0 to 0)	0.1 (0.0 to 0.1)	0 (0 to 0)	0.0 (0.0 to 0.0)	0 (0 to 0)	0.0 (0.0 to 0.1)	-78.4 (-90.4 to -49.0)	-59.1 (-81.8 to -3.4)
Armenia	Gallbladder and biliary diseases	5 to 9	Female	0 (0 to 0)	0.0 (0.0 to 0.0)	0 (0 to 0)	0.0 (0.0 to 0.0)	0 (0 to 0)	0.0 (0.0 to 0.0)	-56.0 (-82.2 to 13.2)	-16.4 (-66.1 to 115.2)
Armenia	Gallbladder and biliary diseases	5 to 9	Male	0 (0 to 0)	0.1 (0.1 to 0.2)	0 (0 to 0)	0.1 (0.0 to 0.1)	0 (0 to 0)	0.0 (0.0 to 0.1)	-79.2 (-91.5 to -49.6)	-60.8 (-83.9 to -5.2)
Armenia	Gallbladder and biliary diseases	10 to 14	Both	0 (0 to 0)	0.0 (0.0 to 0.0)	0 (0 to 0)	0.0 (0.0 to 0.0)	0 (0 to 0)	0.0 (0.0 to 0.0)	-57.0 (-78.9 to -6.0)	-15.3 (-58.4 to 85.2)
Armenia	Gallbladder and biliary diseases	10 to 14	Female	0 (0 to 0)	0.0 (0.0 to 0.0)	0 (0 to 0)	0.0 (0.0 to 0.0)	0 (0 to 0)	0.0 (0.0 to 0.0)	-30.9 (-67.7 to 65.7)	40.1 (-34.4 to 235.9)
Armenia	Gallbladder and biliary diseases	10 to 14	Male	0 (0 to 0)	0.0 (0.0 to 0.1)	0 (0 to 0)	0.0 (0.0 to 0.0)	0 (0 to 0)	0.0 (0.0 to 0.0)	-59.7 (-82.5 to -1.5)	-22.7 (-66.4 to 88.9)
Armenia	Pancreatitis	5 to 9	Both	0 (0 to 0)	0.0 (0.0 to 0.0)	0 (0 to 0)	0.0 (0.0 to 0.0)	0 (0 to 0)	0.0 (0.0 to 0.0)	-65.0 (-86.7 to 15.9)	-33.8 (-74.8 to 119.3)
Armenia	Pancreatitis	5 to 9	Female	0 (0 to 0)	0.0 (0.0 to 0.0)	0 (0 to 0)	0.0 (0.0 to 0.0)	0 (0 to 0)	0.0 (0.0 to 0.0)	-45.6 (-79.6 to 116.0)	3.4 (-61.1 to 310.9)
Armenia	Pancreatitis	5 to 9	Male	0 (0 to 0)	0.0 (0.0 to 0.0)	0 (0 to 0)	0.0 (0.0 to 0.0)	0 (0 to 0)	0.0 (0.0 to 0.0)	-67.9 (-90.2 to 14.8)	-39.6 (-81.6 to 116.1)
Armenia	Pancreatitis	10 to 14	Both	0 (0 to 0)	0.0 (0.0 to 0.0)	0 (0 to 0)	0.0 (0.0 to 0.0)	0 (0 to 0)	0.0 (0.0 to 0.0)	-48.1 (-76.1 to 21.3)	2.3 (-53.0 to 138.9)
Armenia	Pancreatitis	10 to 14	Female	0 (0 to 0)	0.0 (0.0 to 0.0)	0 (0 to 0)	0.0 (0.0 to 0.0)	0 (0 to 0)	0.0 (0.0 to 0.0)	-62.5 (-84.2 to -4.7)	-23.9 (-68.0 to 93.1)
Armenia	Pancreatitis	10 to 14	Male	0 (0 to 0)	0.0 (0.0 to 0.0)	0 (0 to 0)	0.0 (0.0 to 0.0)	0 (0 to 0)	0.0 (0.0 to 0.0)	-45.6 (-77.0 to 40.2)	4.3 (-55.9 to 168.9)

eTable 8. All-cause and cause-specific mortality (counts and rates) at the global level and by country in the WHO European Region for ages 5 to 9 and 10 to 14 for 1990, 2000, and 2016

Armenia	Other digestive diseases	5 to 9	Both	0 (0 to 1)	0.1 (0.1 to 0.3)	0 (0 to 0)	0.1 (0.1 to 0.2)	0 (0 to 0)	0.1 (0.0 to 0.1)	-70.6 (-88.1 to -29.5)	-44.5 (-77.5 to 33.4)
Armenia	Other digestive diseases	5 to 9	Female	0 (0 to 1)	0.2 (0.1 to 0.4)	0 (0 to 0)	0.2 (0.1 to 0.3)	0 (0 to 0)	0.1 (0.1 to 0.2)	-67.8 (-89.1 to 0.6)	-38.7 (-79.3 to 91.3)
Armenia	Other digestive diseases	5 to 9	Male	0 (0 to 0)	0.1 (0.0 to 0.1)	0 (0 to 0)	0.0 (0.0 to 0.1)	0 (0 to 0)	0.0 (0.0 to 0.1)	-79.0 (-91.6 to -37.8)	-60.4 (-84.3 to 17.1)
Armenia	Other digestive diseases	10 to 14	Both	0 (0 to 0)	0.0 (0.0 to 0.1)	0 (0 to 0)	0.0 (0.0 to 0.1)	0 (0 to 0)	0.0 (0.0 to 0.0)	-64.2 (-80.6 to -31.2)	-29.5 (-61.7 to 35.6)
Armenia	Other digestive diseases	10 to 14	Female	0 (0 to 0)	0.0 (0.0 to 0.1)	0 (0 to 0)	0.0 (0.0 to 0.1)	0 (0 to 0)	0.0 (0.0 to 0.0)	-66.4 (-85.8 to -8.5)	-31.8 (-71.2 to 85.5)
Armenia	Other digestive diseases	10 to 14	Male	0 (0 to 0)	0.0 (0.0 to 0.1)	0 (0 to 0)	0.0 (0.0 to 0.1)	0 (0 to 0)	0.0 (0.0 to 0.1)	-62.8 (-80.2 to -20.7)	-28.6 (-62.1 to 52.2)
Armenia	Alzheimer disease and other dementias	5 to 9	Both	0 (0 to 0)	0.0 (0.0 to 0.0)	0 (0 to 0)	0.0 (0.0 to 0.0)	0 (0 to 0)	0.0 (0.0 to 0.0)	0.0 (0.0 to 0.0)	0.0 (0.0 to 0.0)
Armenia	Alzheimer disease and other dementias	5 to 9	Female	0 (0 to 0)	0.0 (0.0 to 0.0)	0 (0 to 0)	0.0 (0.0 to 0.0)	0 (0 to 0)	0.0 (0.0 to 0.0)	0.0 (0.0 to 0.0)	0.0 (0.0 to 0.0)
Armenia	Alzheimer disease and other dementias	5 to 9	Male	0 (0 to 0)	0.0 (0.0 to 0.0)	0 (0 to 0)	0.0 (0.0 to 0.0)	0 (0 to 0)	0.0 (0.0 to 0.0)	0.0 (0.0 to 0.0)	0.0 (0.0 to 0.0)
Armenia	Alzheimer disease and other dementias	10 to 14	Both	0 (0 to 0)	0.0 (0.0 to 0.0)	0 (0 to 0)	0.0 (0.0 to 0.0)	0 (0 to 0)	0.0 (0.0 to 0.0)	0.0 (0.0 to 0.0)	0.0 (0.0 to 0.0)
Armenia	Alzheimer disease and other dementias	10 to 14	Female	0 (0 to 0)	0.0 (0.0 to 0.0)	0 (0 to 0)	0.0 (0.0 to 0.0)	0 (0 to 0)	0.0 (0.0 to 0.0)	0.0 (0.0 to 0.0)	0.0 (0.0 to 0.0)
Armenia	Alzheimer disease and other dementias	10 to 14	Male	0 (0 to 0)	0.0 (0.0 to 0.0)	0 (0 to 0)	0.0 (0.0 to 0.0)	0 (0 to 0)	0.0 (0.0 to 0.0)	0.0 (0.0 to 0.0)	0.0 (0.0 to 0.0)
Armenia	Parkinson disease	5 to 9	Both	0 (0 to 0)	0.0 (0.0 to 0.0)	0 (0 to 0)	0.0 (0.0 to 0.0)	0 (0 to 0)	0.0 (0.0 to 0.0)	0.0 (0.0 to 0.0)	0.0 (0.0 to 0.0)
Armenia	Parkinson disease	5 to 9	Female	0 (0 to 0)	0.0 (0.0 to 0.0)	0 (0 to 0)	0.0 (0.0 to 0.0)	0 (0 to 0)	0.0 (0.0 to 0.0)	0.0 (0.0 to 0.0)	0.0 (0.0 to 0.0)
Armenia	Parkinson disease	5 to 9	Male	0 (0 to 0)	0.0 (0.0 to 0.0)	0 (0 to 0)	0.0 (0.0 to 0.0)	0 (0 to 0)	0.0 (0.0 to 0.0)	0.0 (0.0 to 0.0)	0.0 (0.0 to 0.0)
Armenia	Parkinson disease	10 to 14	Both	0 (0 to 0)	0.0 (0.0 to 0.0)	0 (0 to 0)	0.0 (0.0 to 0.0)	0 (0 to 0)	0.0 (0.0 to 0.0)	0.0 (0.0 to 0.0)	0.0 (0.0 to 0.0)
Armenia	Parkinson disease	10 to 14	Female	0 (0 to 0)	0.0 (0.0 to 0.0)	0 (0 to 0)	0.0 (0.0 to 0.0)	0 (0 to 0)	0.0 (0.0 to 0.0)	0.0 (0.0 to 0.0)	0.0 (0.0 to 0.0)
Armenia	Parkinson disease	10 to 14	Male	0 (0 to 0)	0.0 (0.0 to 0.0)	0 (0 to 0)	0.0 (0.0 to 0.0)	0 (0 to 0)	0.0 (0.0 to 0.0)	0.0 (0.0 to 0.0)	0.0 (0.0 to 0.0)
Armenia	Epilepsy	5 to 9	Both	5 (4 to 7)	1.4 (1.1 to 1.9)	4 (3 to 5)	1.4 (1.0 to 1.9)	1 (0 to 1)	0.3 (0.2 to 0.4)	-88.8 (-93.0 to -81.6)	-78.8 (-86.8 to -65.2)
Armenia	Epilepsy	5 to 9	Female	2 (1 to 3)	1.2 (0.8 to 1.7)	2 (1 to 2)	1.3 (0.8 to 1.9)	0 (0 to 0)	0.2 (0.1 to 0.4)	-89.5 (-94.7 to -80.8)	-80.1 (-90.0 to -63.5)
Armenia	Epilepsy	5 to 9	Male	3 (2 to 4)	1.7 (1.2 to 2.3)	2 (1 to 3)	1.5 (1.0 to 2.1)	0 (0 to 1)	0.4 (0.2 to 0.6)	-88.3 (-93.2 to -79.2)	-77.9 (-87.3 to -60.9)
Armenia	Epilepsy	10 to 14	Both	4 (3 to 5)	1.2 (0.9 to 1.6)	5 (4 to 6)	1.4 (1.1 to 1.8)	1 (0 to 1)	0.3 (0.2 to 0.5)	-85.4 (-90.5 to -78.2)	-71.3 (-81.3 to -57.1)
Armenia	Epilepsy	10 to 14	Female	2 (1 to 3)	1.2 (0.8 to 1.7)	3 (2 to 4)	1.6 (1.1 to 2.3)	0 (0 to 0)	0.3 (0.2 to 0.5)	-86.6 (-92.8 to -76.5)	-72.9 (-85.3 to -52.4)

eTable 8. All-cause and cause-specific mortality (counts and rates) at the global level and by country in the WHO European Region for ages 5 to 9 and 10 to 14 for 1990, 2000, and 2016

Armenia	Epilepsy	10 to 14	Male	2 (1 to 3)	1.3 (0.9 to 1.7)	2 (1 to 3)	1.3 (0.9 to 1.8)	0 (0 to 0)	0.4 (0.2 to 0.6)	-84.4 (-90.6 to -73.7)	-70.0 (-82.0 to -49.5)
Armenia	Motor neuron disease	5 to 9	Both	0 (0 to 0)	0.0 (0.0 to 0.0)	0 (0 to 0)	0.0 (0.0 to 0.0)	0 (0 to 0)	0.0 (0.0 to 0.0)	-70.3 (-82.9 to -44.8)	-43.8 (-67.6 to 4.4)
Armenia	Motor neuron disease	5 to 9	Female	0 (0 to 0)	0.0 (0.0 to 0.0)	0 (0 to 0)	0.0 (0.0 to 0.0)	0 (0 to 0)	0.0 (0.0 to 0.0)	-50.0 (-72.7 to -0.2)	-4.9 (-48.2 to 89.8)
Armenia	Motor neuron disease	5 to 9	Male	0 (0 to 0)	0.0 (0.0 to 0.0)	0 (0 to 0)	0.0 (0.0 to 0.0)	0 (0 to 0)	0.0 (0.0 to 0.0)	-74.9 (-86.5 to -49.0)	-52.7 (-74.6 to -3.9)
Armenia	Motor neuron disease	10 to 14	Both	0 (0 to 0)	0.0 (0.0 to 0.0)	0 (0 to 0)	0.0 (0.0 to 0.0)	0 (0 to 0)	0.0 (0.0 to 0.0)	-68.9 (-82.0 to -47.2)	-38.8 (-64.5 to 4.0)
Armenia	Motor neuron disease	10 to 14	Female	0 (0 to 0)	0.0 (0.0 to 0.0)	0 (0 to 0)	0.0 (0.0 to 0.0)	0 (0 to 0)	0.0 (0.0 to 0.0)	-77.9 (-86.3 to -64.3)	-55.2 (-72.2 to -27.6)
Armenia	Motor neuron disease	10 to 14	Male	0 (0 to 0)	0.0 (0.0 to 0.0)	0 (0 to 0)	0.0 (0.0 to 0.0)	0 (0 to 0)	0.0 (0.0 to 0.0)	-67.3 (-82.3 to -37.1)	-37.3 (-66.1 to 20.6)
Armenia	Other neurological disorders	5 to 9	Both	1 (0 to 1)	0.2 (0.1 to 0.3)	0 (0 to 1)	0.1 (0.1 to 0.2)	0 (0 to 0)	0.1 (0.1 to 0.2)	-65.7 (-82.8 to -24.8)	-35.2 (-67.4 to 42.2)
Armenia	Other neurological disorders	5 to 9	Female	1 (0 to 1)	0.3 (0.1 to 0.5)	0 (0 to 0)	0.2 (0.1 to 0.4)	0 (0 to 0)	0.2 (0.1 to 0.3)	-68.4 (-85.2 to -26.8)	-39.9 (-71.9 to 39.2)
Armenia	Other neurological disorders	5 to 9	Male	0 (0 to 0)	0.1 (0.0 to 0.1)	0 (0 to 0)	0.1 (0.0 to 0.1)	0 (0 to 0)	0.1 (0.0 to 0.1)	-53.3 (-83.6 to 17.8)	-12.0 (-69.2 to 121.6)
Armenia	Other neurological disorders	10 to 14	Both	1 (1 to 1)	0.3 (0.2 to 0.4)	1 (1 to 1)	0.3 (0.2 to 0.4)	0 (0 to 1)	0.3 (0.2 to 0.4)	-47.9 (-67.5 to -11.4)	2.6 (-35.9 to 74.5)
Armenia	Other neurological disorders	10 to 14	Female	0 (0 to 1)	0.3 (0.2 to 0.5)	0 (0 to 1)	0.3 (0.2 to 0.4)	0 (0 to 0)	0.3 (0.2 to 0.4)	-57.0 (-79.0 to -9.9)	-12.9 (-57.5 to 82.6)
Armenia	Other neurological disorders	10 to 14	Male	0 (0 to 1)	0.3 (0.2 to 0.5)	0 (0 to 1)	0.3 (0.2 to 0.4)	0 (0 to 0)	0.3 (0.2 to 0.5)	-38.6 (-67.9 to 19.4)	17.8 (-38.3 to 129.1)
Armenia	Drug use disorders	5 to 9	Both	0 (0 to 0)	0.0 (0.0 to 0.0)	0 (0 to 0)	0.0 (0.0 to 0.0)	0 (0 to 0)	0.0 (0.0 to 0.0)	0.0 (0.0 to 0.0)	0.0 (0.0 to 0.0)
Armenia	Drug use disorders	5 to 9	Female	0 (0 to 0)	0.0 (0.0 to 0.0)	0 (0 to 0)	0.0 (0.0 to 0.0)	0 (0 to 0)	0.0 (0.0 to 0.0)	0.0 (0.0 to 0.0)	0.0 (0.0 to 0.0)
Armenia	Drug use disorders	5 to 9	Male	0 (0 to 0)	0.0 (0.0 to 0.0)	0 (0 to 0)	0.0 (0.0 to 0.0)	0 (0 to 0)	0.0 (0.0 to 0.0)	0.0 (0.0 to 0.0)	0.0 (0.0 to 0.0)
Armenia	Drug use disorders	10 to 14	Both	0 (0 to 0)	0.0 (0.0 to 0.0)	0 (0 to 0)	0.0 (0.0 to 0.0)	0 (0 to 0)	0.0 (0.0 to 0.0)	0.0 (0.0 to 0.0)	0.0 (0.0 to 0.0)
Armenia	Drug use disorders	10 to 14	Female	0 (0 to 0)	0.0 (0.0 to 0.0)	0 (0 to 0)	0.0 (0.0 to 0.0)	0 (0 to 0)	0.0 (0.0 to 0.0)	0.0 (0.0 to 0.0)	0.0 (0.0 to 0.0)
Armenia	Drug use disorders	10 to 14	Male	0 (0 to 0)	0.0 (0.0 to 0.0)	0 (0 to 0)	0.0 (0.0 to 0.0)	0 (0 to 0)	0.0 (0.0 to 0.0)	0.0 (0.0 to 0.0)	0.0 (0.0 to 0.0)
Armenia	Eating disorders	5 to 9	Both	0 (0 to 0)	0.0 (0.0 to 0.0)	0 (0 to 0)	0.0 (0.0 to 0.0)	0 (0 to 0)	0.0 (0.0 to 0.0)	-48.9 (-78.8 to 76.3)	-3.4 (-59.9 to 233.6)
Armenia	Eating disorders	5 to 9	Female	0 (0 to 0)	0.0 (0.0 to 0.0)	0 (0 to 0)	0.0 (0.0 to 0.0)	0 (0 to 0)	0.0 (0.0 to 0.0)	-10.9 (-65.0 to 187.5)	69.4 (-33.5 to 446.7)
Armenia	Eating disorders	5 to 9	Male	0 (0 to 0)	0.0 (0.0 to 0.0)	0 (0 to 0)	0.0 (0.0 to 0.0)	0 (0 to 0)	0.0 (0.0 to 0.0)	-52.8 (-82.3 to 79.3)	-11.1 (-66.7 to 237.4)
Armenia	Eating disorders	10 to 14	Both	0 (0 to 0)	0.0 (0.0 to 0.0)	0 (0 to 0)	0.0 (0.0 to 0.0)	0 (0 to 0)	0.0 (0.0 to 0.0)	-33.1 (-68.3 to 52.8)	31.9 (-37.5 to 201.0)
Armenia	Eating disorders	10 to 14	Female	0 (0 to 0)	0.0 (0.0 to 0.0)	0 (0 to 0)	0.0 (0.0 to 0.0)	0 (0 to 0)	0.0 (0.0 to 0.0)	-87.6 (-94.9 to -55.6)	-74.8 (-89.7 to -10.0)
Armenia	Eating disorders	10 to 14	Male	0 (0 to 0)	0.0 (0.0 to 0.0)	0 (0 to 0)	0.0 (0.0 to 0.0)	0 (0 to 0)	0.0 (0.0 to 0.0)	52.7 (-43.2 to 341.8)	192.9 (8.9 to 747.5)
Armenia	Diabetes mellitus	5 to 9	Both	1 (1 to 1)	0.2 (0.2 to 0.3)	1 (0 to 1)	0.3 (0.2 to 0.4)	0 (0 to 0)	0.1 (0.1 to 0.2)	-63.8 (-79.9 to -35.7)	-31.5 (-61.9 to 21.6)
Armenia	Diabetes mellitus	5 to 9	Female	0 (0 to 0)	0.0 (0.0 to 0.0)	0 (0 to 0)	0.0 (0.0 to 0.0)	0 (0 to 0)	0.0 (0.0 to 0.0)	-63.7 (-78.0 to -43.0)	-30.9 (-58.2 to 8.4)
Armenia	Diabetes mellitus	5 to 9	Male	1 (1 to 1)	0.4 (0.3 to 0.6)	1 (0 to 1)	0.5 (0.3 to 0.7)	0 (0 to 0)	0.3 (0.2 to 0.4)	-63.8 (-80.4 to -33.0)	-31.8 (-63.2 to 26.1)
Armenia	Diabetes mellitus	10 to 14	Both	1 (1 to 1)	0.3 (0.3 to 0.4)	2 (1 to 2)	0.5 (0.4 to 0.6)	1 (0 to 1)	0.4 (0.3 to 0.5)	-48.9 (-65.7 to -23.9)	0.7 (-32.4 to 50.0)

eTable 8. All-cause and cause-specific mortality (counts and rates) at the global level and by country in the WHO European Region for ages 5 to 9 and 10 to 14 for 1990, 2000, and 2016

Armenia	Diabetes mellitus	10 to 14	Female	1 (0 to 1)	0.4 (0.3 to 0.5)	1 (1 to 1)	0.6 (0.4 to 0.8)	0 (0 to 0)	0.4 (0.2 to 0.5)	-51.9 (-71.3 to -17.9)	-2.4 (-41.8 to 66.4)
Armenia	Diabetes mellitus	10 to 14	Male	1 (0 to 1)	0.3 (0.2 to 0.5)	1 (0 to 1)	0.4 (0.3 to 0.6)	0 (0 to 0)	0.3 (0.2 to 0.5)	-45.5 (-69.3 to -7.1)	4.6 (-41.0 to 78.2)
Armenia	Acute glomerulonephritis	5 to 9	Both	0 (0 to 0)	0.0 (0.0 to 0.0)	0 (0 to 0)	0.0 (0.0 to 0.1)	0 (0 to 0)	0.0 (0.0 to 0.0)	-66.0 (-87.3 to -31.4)	-35.7 (-76.0 to 29.8)
Armenia	Acute glomerulonephritis	5 to 9	Female	0 (0 to 0)	0.0 (0.0 to 0.0)	0 (0 to 0)	0.0 (0.0 to 0.0)	0 (0 to 0)	0.0 (0.0 to 0.0)	-48.8 (-64.1 to -29.0)	-2.6 (-31.8 to 35.0)
Armenia	Acute glomerulonephritis	5 to 9	Male	0 (0 to 0)	0.0 (0.0 to 0.1)	0 (0 to 0)	0.0 (0.0 to 0.1)	0 (0 to 0)	0.0 (0.0 to 0.0)	-74.2 (-92.3 to -28.3)	-51.5 (-85.6 to 35.0)
Armenia	Acute glomerulonephritis	10 to 14	Both	0 (0 to 0)	0.0 (0.0 to 0.1)	0 (0 to 0)	0.0 (0.0 to 0.1)	0 (0 to 0)	0.0 (0.0 to 0.0)	-88.5 (-96.6 to -50.2)	-77.4 (-93.2 to -1.9)
Armenia	Acute glomerulonephritis	10 to 14	Female	0 (0 to 0)	0.0 (0.0 to 0.1)	0 (0 to 0)	0.0 (0.0 to 0.1)	0 (0 to 0)	0.0 (0.0 to 0.0)	-82.8 (-96.1 to -43.4)	-65.2 (-92.0 to 14.8)
Armenia	Acute glomerulonephritis	10 to 14	Male	0 (0 to 0)	0.1 (0.0 to 0.3)	0 (0 to 0)	0.1 (0.0 to 0.2)	0 (0 to 0)	0.0 (0.0 to 0.0)	-90.8 (-98.0 to -38.7)	-82.4 (-96.1 to 17.5)
Armenia	Chronic kidney disease	5 to 9	Both	2 (2 to 3)	0.6 (0.5 to 0.8)	1 (1 to 1)	0.4 (0.3 to 0.5)	1 (1 to 1)	0.4 (0.3 to 0.5)	-66.6 (-78.3 to -49.6)	-36.8 (-58.9 to -4.6)
Armenia	Chronic kidney disease	5 to 9	Female	1 (1 to 2)	0.7 (0.5 to 0.9)	1 (0 to 1)	0.4 (0.3 to 0.6)	0 (0 to 1)	0.5 (0.3 to 0.7)	-64.5 (-78.4 to -40.9)	-32.4 (-59.0 to 12.4)
Armenia	Chronic kidney disease	5 to 9	Male	1 (1 to 1)	0.5 (0.4 to 0.7)	0 (0 to 1)	0.3 (0.2 to 0.4)	0 (0 to 0)	0.3 (0.2 to 0.4)	-69.3 (-82.5 to -49.9)	-42.2 (-67.0 to -5.7)
Armenia	Chronic kidney disease	10 to 14	Both	2 (2 to 2)	0.6 (0.5 to 0.8)	2 (1 to 2)	0.5 (0.4 to 0.6)	1 (1 to 1)	0.6 (0.4 to 0.7)	-54.8 (-67.9 to -36.5)	-10.9 (-36.8 to 25.1)
Armenia	Chronic kidney disease	10 to 14	Female	1 (1 to 1)	0.6 (0.4 to 0.8)	1 (1 to 1)	0.5 (0.3 to 0.6)	0 (0 to 1)	0.5 (0.4 to 0.8)	-52.4 (-69.8 to -22.9)	-3.6 (-38.8 to 56.4)
Armenia	Chronic kidney disease	10 to 14	Male	1 (1 to 1)	0.7 (0.5 to 0.9)	1 (1 to 1)	0.5 (0.4 to 0.6)	0 (0 to 1)	0.6 (0.4 to 0.8)	-56.7 (-71.8 to -34.7)	-16.9 (-45.9 to 25.2)
Armenia	Urinary diseases and male infertility	5 to 9	Both	1 (1 to 1)	0.2 (0.1 to 0.3)	0 (0 to 0)	0.1 (0.1 to 0.2)	0 (0 to 1)	0.2 (0.1 to 0.3)	-39.7 (-64.0 to -1.8)	14.0 (-32.0 to 85.8)
Armenia	Urinary diseases and male infertility	5 to 9	Female	0 (0 to 1)	0.2 (0.2 to 0.3)	0 (0 to 0)	0.1 (0.1 to 0.2)	0 (0 to 0)	0.3 (0.2 to 0.4)	-35.8 (-63.9 to 9.7)	22.1 (-31.3 to 108.6)
Armenia	Urinary diseases and male infertility	5 to 9	Male	0 (0 to 0)	0.1 (0.1 to 0.2)	0 (0 to 0)	0.1 (0.1 to 0.1)	0 (0 to 0)	0.1 (0.1 to 0.2)	-46.1 (-74.9 to 9.8)	1.4 (-52.8 to 106.6)
Armenia	Urinary diseases and male infertility	10 to 14	Both	1 (1 to 1)	0.2 (0.2 to 0.3)	1 (0 to 1)	0.2 (0.1 to 0.2)	1 (0 to 1)	0.3 (0.2 to 0.4)	-20.2 (-46.5 to 15.1)	57.2 (5.5 to 126.8)
Armenia	Urinary diseases and male infertility	10 to 14	Female	0 (0 to 0)	0.2 (0.2 to 0.3)	0 (0 to 0)	0.2 (0.1 to 0.2)	0 (0 to 0)	0.4 (0.3 to 0.5)	-12.7 (-47.4 to 46.8)	77.0 (6.6 to 197.6)
Armenia	Urinary diseases and male infertility	10 to 14	Male	0 (0 to 0)	0.2 (0.1 to 0.3)	0 (0 to 0)	0.1 (0.1 to 0.2)	0 (0 to 0)	0.3 (0.2 to 0.4)	-27.8 (-56.2 to 16.0)	38.4 (-16.0 to 122.6)
Armenia	Hemoglobinopathies and hemolytic anemias	5 to 9	Both	1 (0 to 2)	0.4 (0.1 to 0.6)	1 (0 to 1)	0.3 (0.1 to 0.4)	0 (0 to 0)	0.1 (0.0 to 0.2)	-84.9 (-91.6 to -72.5)	-71.5 (-84.2 to -48.0)
Armenia	Hemoglobinopathies and hemolytic anemias	5 to 9	Female	0 (0 to 0)	0.1 (0.0 to 0.2)	0 (0 to 0)	0.1 (0.0 to 0.1)	0 (0 to 0)	0.0 (0.0 to 0.0)	-89.4 (-95.7 to -69.3)	-79.9 (-91.9 to -41.6)
Armenia	Hemoglobinopathies and hemolytic anemias	5 to 9	Male	1 (0 to 2)	0.6 (0.2 to 1.1)	1 (0 to 1)	0.4 (0.1 to 0.8)	0 (0 to 0)	0.2 (0.1 to 0.3)	-84.0 (-91.4 to -69.1)	-69.9 (-83.8 to -41.9)

eTable 8. All-cause and cause-specific mortality (counts and rates) at the global level and by country in the WHO European Region for ages 5 to 9 and 10 to 14 for 1990, 2000, and 2016

Armenia	Hemoglobinopathies and hemolytic anemias	10 to 14	Both	1 (0 to 1)	0.2 (0.1 to 0.3)	1 (0 to 1)	0.2 (0.1 to 0.2)	0 (0 to 0)	0.1 (0.1 to 0.1)	-76.6 (-84.4 to -65.1)	-53.9 (-69.2 to -31.3)
Armenia	Hemoglobinopathies and hemolytic anemias	10 to 14	Female	0 (0 to 1)	0.2 (0.1 to 0.3)	0 (0 to 0)	0.2 (0.1 to 0.3)	0 (0 to 0)	0.1 (0.0 to 0.2)	-77.1 (-87.6 to -57.8)	-53.6 (-74.8 to -14.4)
Armenia	Hemoglobinopathies and hemolytic anemias	10 to 14	Male	0 (0 to 1)	0.2 (0.1 to 0.3)	0 (0 to 0)	0.1 (0.1 to 0.2)	0 (0 to 0)	0.1 (0.1 to 0.2)	-76.1 (-86.1 to -57.5)	-54.2 (-73.4 to -18.6)
Armenia	Endocrine, metabolic, blood, and immune disorders	5 to 9	Both	1 (1 to 1)	0.3 (0.2 to 0.4)	1 (0 to 1)	0.2 (0.1 to 0.3)	1 (0 to 1)	0.3 (0.2 to 0.5)	-44.3 (-69.0 to -10.8)	5.4 (-41.3 to 68.7)
Armenia	Endocrine, metabolic, blood, and immune disorders	5 to 9	Female	1 (0 to 1)	0.3 (0.2 to 0.5)	0 (0 to 0)	0.2 (0.1 to 0.3)	0 (0 to 0)	0.3 (0.1 to 0.5)	-49.6 (-73.3 to -7.5)	-4.1 (-49.3 to 75.9)
Armenia	Endocrine, metabolic, blood, and immune disorders	5 to 9	Male	1 (0 to 1)	0.3 (0.2 to 0.4)	0 (0 to 0)	0.2 (0.1 to 0.3)	0 (0 to 1)	0.3 (0.2 to 0.5)	-38.2 (-70.6 to 16.9)	16.4 (-44.7 to 120.0)
Armenia	Endocrine, metabolic, blood, and immune disorders	10 to 14	Both	1 (1 to 1)	0.2 (0.2 to 0.3)	1 (0 to 1)	0.2 (0.1 to 0.2)	1 (0 to 1)	0.3 (0.2 to 0.5)	-25.6 (-55.7 to 12.3)	46.6 (-12.7 to 121.3)
Armenia	Endocrine, metabolic, blood, and immune disorders	10 to 14	Female	0 (0 to 1)	0.2 (0.1 to 0.3)	0 (0 to 0)	0.2 (0.1 to 0.3)	0 (0 to 0)	0.3 (0.1 to 0.5)	-34.1 (-62.3 to 15.4)	33.5 (-23.6 to 134.0)
Armenia	Endocrine, metabolic, blood, and immune disorders	10 to 14	Male	0 (0 to 1)	0.2 (0.1 to 0.3)	0 (0 to 0)	0.2 (0.1 to 0.3)	0 (0 to 0)	0.3 (0.2 to 0.5)	-16.4 (-58.4 to 41.0)	60.3 (-20.2 to 170.5)
Armenia	Rheumatoid arthritis	5 to 9	Both	0 (0 to 0)	0.0 (0.0 to 0.0)	0 (0 to 0)	0.0 (0.0 to 0.0)	0 (0 to 0)	0.0 (0.0 to 0.0)	11.4 (-63.0 to 190.5)	110.8 (-29.9 to 449.5)
Armenia	Rheumatoid arthritis	5 to 9	Female	0 (0 to 0)	0.0 (0.0 to 0.0)	0 (0 to 0)	0.0 (0.0 to 0.0)	0 (0 to 0)	0.0 (0.0 to 0.0)	2.0 (-71.9 to 247.6)	94.0 (-46.5 to 561.0)
Armenia	Rheumatoid arthritis	5 to 9	Male	0 (0 to 0)	0.0 (0.0 to 0.0)	0 (0 to 0)	0.0 (0.0 to 0.0)	0 (0 to 0)	0.0 (0.0 to 0.0)	27.3 (-62.2 to 301.3)	139.5 (-28.9 to 655.3)
Armenia	Rheumatoid arthritis	10 to 14	Both	0 (0 to 0)	0.0 (0.0 to 0.0)	0 (0 to 0)	0.0 (0.0 to 0.0)	0 (0 to 0)	0.0 (0.0 to 0.0)	-17.0 (-66.1 to 87.8)	63.6 (-33.3 to 270.0)
Armenia	Rheumatoid arthritis	10 to 14	Female	0 (0 to 0)	0.0 (0.0 to 0.0)	0 (0 to 0)	0.0 (0.0 to 0.0)	0 (0 to 0)	0.0 (0.0 to 0.0)	-18.8 (-75.5 to 130.8)	64.6 (-50.3 to 368.0)
Armenia	Rheumatoid arthritis	10 to 14	Male	0 (0 to 0)	0.0 (0.0 to 0.0)	0 (0 to 0)	0.0 (0.0 to 0.0)	0 (0 to 0)	0.0 (0.0 to 0.0)	-14.1 (-68.0 to 140.2)	64.8 (-38.7 to 360.7)
Armenia	Other musculoskeletal disorders	5 to 9	Both	0 (0 to 0)	0.0 (0.0 to 0.0)	0 (0 to 0)	0.0 (0.0 to 0.0)	0 (0 to 0)	0.0 (0.0 to 0.0)	6.7 (-74.1 to 210.4)	101.9 (-50.9 to 487.3)
Armenia	Other musculoskeletal disorders	5 to 9	Female	0 (0 to 0)	0.0 (0.0 to 0.1)	0 (0 to 0)	0.0 (0.0 to 0.0)	0 (0 to 0)	0.0 (0.0 to 0.1)	-8.9 (-75.7 to 193.3)	73.2 (-53.8 to 457.8)
Armenia	Other musculoskeletal disorders	5 to 9	Male	0 (0 to 0)	0.0 (0.0 to 0.0)	0 (0 to 0)	0.0 (0.0 to 0.0)	0 (0 to 0)	0.0 (0.0 to 0.0)	28.7 (-72.5 to 299.8)	142.3 (-48.3 to 652.5)

eTable 8. All-cause and cause-specific mortality (counts and rates) at the global level and by country in the WHO European Region for ages 5 to 9 and 10 to 14 for 1990, 2000, and 2016

Armenia	Other musculoskeletal disorders	10 to 14	Both	0 (0 to 0)	0.0 (0.0 to 0.0)	0 (0 to 0)	0.0 (0.0 to 0.0)	0 (0 to 0)	0.0 (0.0 to 0.1)	0.6 (-62.5 to 127.5)	98.2 (-26.2 to 348.2)
Armenia	Other musculoskeletal disorders	10 to 14	Female	0 (0 to 0)	0.0 (0.0 to 0.1)	0 (0 to 0)	0.0 (0.0 to 0.1)	0 (0 to 0)	0.0 (0.0 to 0.1)	5.6 (-64.7 to 164.3)	114.0 (-28.5 to 435.9)
Armenia	Other musculoskeletal disorders	10 to 14	Male	0 (0 to 0)	0.0 (0.0 to 0.0)	0 (0 to 0)	0.0 (0.0 to 0.0)	0 (0 to 0)	0.0 (0.0 to 0.0)	-4.1 (-60.2 to 167.4)	83.9 (-23.8 to 413.0)
Armenia	Congenital birth defects	5 to 9	Both	11 (8 to 18)	3.0 (2.1 to 5.0)	9 (7 to 12)	3.4 (2.5 to 4.4)	5 (4 to 7)	2.7 (1.8 to 3.8)	-52.6 (-77.1 to -23.8)	-10.4 (-56.6 to 44.2)
Armenia	Congenital birth defects	5 to 9	Female	6 (4 to 10)	3.2 (2.1 to 5.7)	5 (3 to 7)	3.8 (2.6 to 5.2)	3 (2 to 4)	2.8 (1.8 to 4.1)	-53.9 (-78.7 to -18.1)	-12.4 (-59.5 to 55.8)
Armenia	Congenital birth defects	5 to 9	Male	5 (3 to 9)	2.7 (1.8 to 4.8)	4 (3 to 6)	2.9 (2.0 to 4.1)	3 (2 to 4)	2.5 (1.5 to 4.3)	-51.1 (-77.8 to -9.6)	-8.0 (-58.2 to 70.1)
Armenia	Congenital birth defects	10 to 14	Both	6 (5 to 9)	1.9 (1.5 to 3.0)	7 (6 to 9)	2.2 (1.8 to 2.8)	3 (2 to 5)	2.0 (1.5 to 2.9)	-46.6 (-68.3 to -21.4)	5.2 (-37.5 to 54.9)
Armenia	Congenital birth defects	10 to 14	Female	3 (2 to 4)	1.7 (1.2 to 2.8)	3 (2 to 5)	2.2 (1.5 to 2.9)	1 (1 to 2)	1.8 (1.2 to 2.5)	-48.3 (-70.6 to -18.4)	4.9 (-40.4 to 65.3)
Armenia	Congenital birth defects	10 to 14	Male	4 (2 to 5)	2.2 (1.5 to 3.4)	4 (3 to 5)	2.3 (1.6 to 3.2)	2 (1 to 3)	2.3 (1.5 to 3.7)	-45.4 (-70.7 to -11.5)	4.8 (-43.8 to 69.8)
Armenia	Skin and subcutaneous diseases	5 to 9	Both	0 (0 to 0)	0.0 (0.0 to 0.0)	0 (0 to 0)	0.0 (0.0 to 0.0)	0 (0 to 0)	0.0 (0.0 to 0.0)	-35.9 (-56.9 to -6.5)	21.2 (-18.5 to 76.9)
Armenia	Skin and subcutaneous diseases	5 to 9	Female	0 (0 to 0)	0.0 (0.0 to 0.0)	0 (0 to 0)	0.0 (0.0 to 0.0)	0 (0 to 0)	0.0 (0.0 to 0.0)	-46.4 (-62.6 to -22.7)	1.9 (-28.8 to 47.0)
Armenia	Skin and subcutaneous diseases	5 to 9	Male	0 (0 to 0)	0.0 (0.0 to 0.0)	0 (0 to 0)	0.0 (0.0 to 0.0)	0 (0 to 0)	0.0 (0.0 to 0.0)	-25.1 (-55.8 to 17.6)	41.0 (-16.8 to 121.3)
Armenia	Skin and subcutaneous diseases	10 to 14	Both	0 (0 to 0)	0.0 (0.0 to 0.0)	0 (0 to 0)	0.0 (0.0 to 0.0)	0 (0 to 0)	0.0 (0.0 to 0.0)	-42.3 (-54.6 to -21.8)	13.7 (-10.6 to 54.1)
Armenia	Skin and subcutaneous diseases	10 to 14	Female	0 (0 to 0)	0.0 (0.0 to 0.0)	0 (0 to 0)	0.0 (0.0 to 0.0)	0 (0 to 0)	0.0 (0.0 to 0.0)	-49.0 (-61.1 to -29.5)	3.4 (-21.1 to 42.9)
Armenia	Skin and subcutaneous diseases	10 to 14	Male	0 (0 to 0)	0.0 (0.0 to 0.0)	0 (0 to 0)	0.0 (0.0 to 0.0)	0 (0 to 0)	0.0 (0.0 to 0.0)	-35.6 (-52.8 to -4.9)	23.5 (-9.4 to 82.3)
Armenia	Road injuries	5 to 9	Both	37 (29 to 47)	9.9 (7.8 to 12.7)	12 (9 to 15)	4.3 (3.3 to 5.7)	4 (3 to 6)	2.3 (1.6 to 3.2)	-87.8 (-92.3 to -81.3)	-76.8 (-85.4 to -64.7)
Armenia	Road injuries	5 to 9	Female	12 (8 to 17)	6.7 (4.6 to 9.3)	4 (2 to 5)	2.7 (1.7 to 3.8)	1 (1 to 2)	1.5 (1.0 to 2.5)	-87.9 (-93.6 to -77.1)	-76.9 (-87.8 to -56.5)
Armenia	Road injuries	5 to 9	Male	24 (18 to 32)	13.1 (9.8 to 17.1)	8 (6 to 11)	5.8 (4.2 to 8.0)	3 (2 to 4)	3.0 (1.9 to 4.4)	-87.7 (-92.7 to -80.6)	-76.8 (-86.4 to -63.5)
Armenia	Road injuries	10 to 14	Both	19 (15 to 24)	6.0 (4.8 to 7.4)	10 (8 to 12)	3.0 (2.4 to 3.8)	3 (2 to 5)	2.1 (1.5 to 2.8)	-82.1 (-87.4 to -74.3)	-64.8 (-75.1 to -49.4)
Armenia	Road injuries	10 to 14	Female	5 (4 to 7)	3.3 (2.4 to 4.5)	3 (2 to 4)	1.8 (1.3 to 2.4)	1 (1 to 1)	1.1 (0.7 to 1.6)	-83.9 (-90.1 to -73.7)	-67.3 (-80.0 to -46.7)
Armenia	Road injuries	10 to 14	Male	14 (11 to 18)	8.6 (6.6 to 11.0)	7 (5 to 9)	4.3 (3.2 to 5.6)	3 (2 to 4)	3.1 (2.1 to 4.2)	-81.5 (-88.1 to -71.6)	-64.5 (-77.1 to -45.5)
Armenia	Other transport injuries	5 to 9	Both	1 (1 to 2)	0.3 (0.1 to 0.4)	1 (0 to 1)	0.2 (0.1 to 0.3)	0 (0 to 0)	0.1 (0.1 to 0.2)	-77.5 (-89.0 to -48.0)	-57.5 (-79.1 to -1.6)
Armenia	Other transport injuries	5 to 9	Female	0 (0 to 1)	0.3 (0.1 to 0.5)	0 (0 to 0)	0.2 (0.1 to 0.3)	0 (0 to 0)	0.1 (0.0 to 0.2)	-81.4 (-93.2 to -47.8)	-64.7 (-87.0 to -0.7)
Armenia	Other transport injuries	5 to 9	Male	1 (0 to 1)	0.3 (0.1 to 0.6)	0 (0 to 1)	0.2 (0.1 to 0.4)	0 (0 to 0)	0.1 (0.1 to 0.3)	-74.2 (-90.8 to -7.7)	-51.4 (-82.7 to 73.7)
Armenia	Other transport injuries	10 to 14	Both	1 (0 to 1)	0.2 (0.1 to 0.3)	1 (0 to 1)	0.2 (0.1 to 0.2)	0 (0 to 0)	0.1 (0.1 to 0.2)	-68.8 (-82.5 to -39.6)	-38.5 (-65.4 to 19.1)

eTable 8. All-cause and cause-specific mortality (counts and rates) at the global level and by country in the WHO European Region for ages 5 to 9 and 10 to 14 for 1990, 2000, and 2016

Armenia	Other transport injuries	10 to 14	Female	0 (0 to 0)	0.2 (0.1 to 0.3)	0 (0 to 0)	0.1 (0.1 to 0.3)	0 (0 to 0)	0.1 (0.0 to 0.2)	-75.5 (-90.2 to -41.1)	-50.2 (-80.1 to 19.3)
Armenia	Other transport injuries	10 to 14	Male	0 (0 to 1)	0.2 (0.1 to 0.4)	0 (0 to 1)	0.2 (0.1 to 0.3)	0 (0 to 0)	0.2 (0.1 to 0.3)	-63.7 (-84.5 to -6.2)	-30.4 (-70.2 to 79.9)
Armenia	Falls	5 to 9	Both	9 (6 to 12)	2.4 (1.5 to 3.2)	3 (2 to 5)	0.9 (0.6 to 1.7)	1 (1 to 2)	0.5 (0.3 to 1.2)	-89.3 (-94.5 to -62.1)	-79.7 (-89.6 to -28.3)
Armenia	Falls	5 to 9	Female	3 (2 to 5)	1.9 (1.1 to 2.6)	1 (1 to 2)	0.6 (0.4 to 1.3)	0 (0 to 1)	0.4 (0.3 to 0.8)	-87.5 (-93.5 to -68.8)	-76.3 (-87.6 to -40.7)
Armenia	Falls	5 to 9	Male	5 (3 to 8)	2.9 (1.7 to 4.1)	2 (1 to 3)	1.2 (0.8 to 2.0)	1 (0 to 2)	0.5 (0.2 to 1.7)	-90.3 (-96.0 to -55.7)	-81.8 (-92.5 to -16.7)
Armenia	Falls	10 to 14	Both	6 (4 to 9)	2.0 (1.3 to 2.7)	3 (2 to 5)	1.0 (0.7 to 1.5)	1 (1 to 2)	0.6 (0.3 to 1.3)	-86.1 (-92.6 to -58.2)	-72.7 (-85.5 to -17.6)
Armenia	Falls	10 to 14	Female	1 (1 to 2)	0.9 (0.5 to 1.4)	1 (0 to 1)	0.4 (0.2 to 0.7)	0 (0 to 0)	0.3 (0.2 to 0.5)	-83.9 (-91.2 to -67.2)	-67.4 (-82.1 to -33.6)
Armenia	Falls	10 to 14	Male	5 (3 to 7)	3.1 (2.0 to 4.3)	3 (2 to 4)	1.6 (1.1 to 2.4)	1 (0 to 2)	0.8 (0.4 to 2.0)	-86.8 (-93.8 to -55.3)	-74.7 (-88.1 to -14.2)
Armenia	Drowning	5 to 9	Both	37 (29 to 45)	10.0 (7.9 to 12.3)	12 (10 to 16)	4.6 (3.6 to 5.9)	2 (2 to 3)	1.3 (0.9 to 1.8)	-93.3 (-95.6 to -90.0)	-87.3 (-91.6 to -81.1)
Armenia	Drowning	5 to 9	Female	11 (8 to 14)	5.8 (4.2 to 7.6)	3 (2 to 4)	2.1 (1.5 to 2.9)	1 (0 to 1)	0.8 (0.5 to 1.1)	-93.1 (-95.8 to -88.2)	-86.9 (-92.1 to -77.6)
Armenia	Drowning	5 to 9	Male	26 (20 to 33)	14.0 (10.6 to 17.8)	10 (7 to 13)	6.9 (5.2 to 9.0)	2 (1 to 2)	1.7 (1.1 to 2.5)	-93.4 (-95.9 to -89.4)	-87.6 (-92.3 to -80.1)
Armenia	Drowning	10 to 14	Both	26 (21 to 31)	8.0 (6.6 to 9.7)	16 (13 to 19)	4.9 (4.0 to 6.0)	3 (2 to 3)	1.6 (1.2 to 2.2)	-90.1 (-92.9 to -85.8)	-80.4 (-86.1 to -72.1)
Armenia	Drowning	10 to 14	Female	6 (4 to 8)	3.8 (2.8 to 4.8)	3 (2 to 4)	1.8 (1.3 to 2.4)	1 (0 to 1)	0.7 (0.5 to 1.1)	-90.3 (-93.8 to -84.3)	-80.3 (-87.5 to -68.1)
Armenia	Drowning	10 to 14	Male	20 (16 to 24)	12.2 (9.7 to 15.1)	13 (10 to 16)	8.0 (6.3 to 9.9)	2 (1 to 3)	2.3 (1.6 to 3.3)	-90.0 (-93.5 to -85.2)	-80.8 (-87.4 to -71.6)
Armenia	Fire, heat, and hot substances	5 to 9	Both	11 (6 to 14)	2.9 (1.7 to 3.9)	4 (3 to 5)	1.3 (1.0 to 1.8)	1 (1 to 1)	0.4 (0.3 to 0.7)	-92.2 (-95.5 to -82.2)	-85.3 (-91.4 to -66.3)
Armenia	Fire, heat, and hot substances	5 to 9	Female	5 (2 to 8)	3.0 (1.3 to 4.5)	2 (1 to 3)	1.3 (0.9 to 2.0)	0 (0 to 1)	0.5 (0.3 to 0.9)	-90.8 (-95.5 to -70.1)	-82.6 (-91.4 to -43.2)
Armenia	Fire, heat, and hot substances	5 to 9	Male	5 (3 to 7)	2.8 (1.5 to 4.0)	2 (1 to 3)	1.4 (1.0 to 1.9)	0 (0 to 1)	0.3 (0.2 to 0.6)	-93.6 (-96.5 to -84.3)	-88.0 (-93.5 to -70.4)
Armenia	Fire, heat, and hot substances	10 to 14	Both	4 (3 to 6)	1.4 (0.9 to 1.8)	2 (2 to 3)	0.7 (0.6 to 1.0)	0 (0 to 1)	0.3 (0.2 to 0.4)	-90.3 (-94.1 to -79.7)	-81.0 (-88.4 to -59.9)
Armenia	Fire, heat, and hot substances	10 to 14	Female	1 (1 to 1)	0.7 (0.4 to 0.9)	1 (0 to 1)	0.4 (0.2 to 0.7)	0 (0 to 0)	0.2 (0.1 to 0.4)	-87.9 (-94.2 to -70.5)	-75.4 (-88.2 to -40.3)
Armenia	Fire, heat, and hot substances	10 to 14	Male	3 (2 to 5)	2.1 (1.1 to 2.8)	2 (1 to 2)	1.1 (0.8 to 1.5)	0 (0 to 0)	0.3 (0.2 to 0.6)	-91.1 (-94.7 to -79.9)	-83.0 (-89.9 to -61.4)
Armenia	Poisonings	5 to 9	Both	4 (3 to 6)	1.1 (0.8 to 1.5)	1 (1 to 2)	0.6 (0.4 to 0.9)	1 (0 to 1)	0.3 (0.2 to 0.5)	-86.7 (-92.6 to -74.2)	-74.9 (-85.9 to -51.3)
Armenia	Poisonings	5 to 9	Female	2 (1 to 3)	1.0 (0.6 to 1.6)	1 (0 to 1)	0.5 (0.3 to 0.9)	0 (0 to 1)	0.3 (0.2 to 0.6)	-83.8 (-92.0 to -68.0)	-69.3 (-84.8 to -39.2)
Armenia	Poisonings	5 to 9	Male	2 (1 to 3)	1.2 (0.7 to 1.8)	1 (0 to 1)	0.6 (0.3 to 1.1)	0 (0 to 1)	0.2 (0.1 to 0.6)	-89.3 (-95.1 to -68.3)	-79.8 (-90.8 to -40.3)
Armenia	Poisonings	10 to 14	Both	2 (1 to 3)	0.6 (0.5 to 0.9)	1 (1 to 2)	0.4 (0.2 to 0.6)	0 (0 to 1)	0.2 (0.1 to 0.4)	-82.7 (-89.2 to -70.1)	-65.9 (-78.7 to -41.2)
Armenia	Poisonings	10 to 14	Female	1 (1 to 1)	0.6 (0.3 to 0.8)	1 (0 to 1)	0.3 (0.2 to 0.6)	0 (0 to 0)	0.2 (0.1 to 0.4)	-79.9 (-89.5 to -65.1)	-59.3 (-78.7 to -29.1)
Armenia	Poisonings	10 to 14	Male	1 (1 to 2)	0.7 (0.5 to 1.1)	1 (0 to 1)	0.4 (0.2 to 0.7)	0 (0 to 0)	0.2 (0.1 to 0.4)	-84.8 (-92.3 to -68.0)	-70.9 (-85.3 to -38.6)
Armenia	Exposure to mechanical forces	5 to 9	Both	5 (2 to 6)	1.3 (0.6 to 1.7)	2 (2 to 3)	0.9 (0.6 to 1.2)	1 (1 to 1)	0.5 (0.3 to 0.7)	-79.8 (-87.0 to -56.5)	-61.8 (-75.5 to -17.8)
Armenia	Exposure to mechanical forces	5 to 9	Female	1 (1 to 2)	0.8 (0.4 to 1.2)	1 (0 to 1)	0.6 (0.4 to 0.9)	0 (0 to 1)	0.4 (0.2 to 0.5)	-75.0 (-85.9 to -51.5)	-52.4 (-73.1 to -7.7)
Armenia	Exposure to mechanical forces	5 to 9	Male	3 (1 to 5)	1.8 (0.8 to 2.4)	2 (1 to 2)	1.1 (0.7 to 1.6)	1 (0 to 1)	0.6 (0.4 to 0.9)	-81.8 (-89.0 to -55.1)	-65.8 (-79.4 to -15.6)

eTable 8. All-cause and cause-specific mortality (counts and rates) at the global level and by country in the WHO European Region for ages 5 to 9 and 10 to 14 for 1990, 2000, and 2016

Armenia	Exposure to mechanical forces	10 to 14	Both	4 (3 to 5)	1.3 (0.9 to 1.7)	3 (2 to 4)	1.0 (0.7 to 1.3)	1 (1 to 2)	0.7 (0.5 to 0.9)	-74.3 (-81.6 to -60.9)	-49.4 (-63.8 to -23.0)
Armenia	Exposure to mechanical forces	10 to 14	Female	1 (1 to 2)	0.7 (0.4 to 1.1)	1 (1 to 2)	0.7 (0.3 to 0.9)	0 (0 to 0)	0.4 (0.2 to 0.6)	-71.8 (-81.8 to -55.2)	-42.9 (-63.0 to -9.1)
Armenia	Exposure to mechanical forces	10 to 14	Male	3 (2 to 4)	1.9 (1.1 to 2.6)	2 (1 to 3)	1.3 (0.9 to 1.9)	1 (1 to 1)	0.9 (0.6 to 1.4)	-75.3 (-83.6 to -57.8)	-52.7 (-68.5 to -19.0)
Armenia	Adverse effects of medical treatment	5 to 9	Both	3 (1 to 5)	0.8 (0.4 to 1.3)	1 (1 to 2)	0.5 (0.3 to 0.8)	0 (0 to 1)	0.3 (0.2 to 0.4)	-82.4 (-91.7 to -55.6)	-66.8 (-84.3 to -16.1)
Armenia	Adverse effects of medical treatment	5 to 9	Female	1 (1 to 2)	0.7 (0.3 to 1.2)	1 (0 to 1)	0.5 (0.2 to 0.8)	0 (0 to 0)	0.2 (0.1 to 0.3)	-87.4 (-93.7 to -72.5)	-76.1 (-88.1 to -47.7)
Armenia	Adverse effects of medical treatment	5 to 9	Male	2 (1 to 3)	0.8 (0.4 to 1.5)	1 (0 to 1)	0.6 (0.3 to 0.9)	0 (0 to 1)	0.3 (0.2 to 0.6)	-78.1 (-91.4 to -41.6)	-58.7 (-83.9 to 9.8)
Armenia	Adverse effects of medical treatment	10 to 14	Both	1 (1 to 2)	0.4 (0.3 to 0.7)	1 (1 to 2)	0.4 (0.2 to 0.5)	0 (0 to 1)	0.2 (0.1 to 0.4)	-74.4 (-87.6 to -49.0)	-49.5 (-75.5 to 0.5)
Armenia	Adverse effects of medical treatment	10 to 14	Female	0 (0 to 1)	0.2 (0.1 to 0.3)	0 (0 to 0)	0.2 (0.1 to 0.3)	0 (0 to 0)	0.1 (0.0 to 0.1)	-81.9 (-91.3 to -64.7)	-63.3 (-82.3 to -28.5)
Armenia	Adverse effects of medical treatment	10 to 14	Male	1 (1 to 2)	0.7 (0.4 to 1.1)	1 (0 to 1)	0.5 (0.3 to 0.8)	0 (0 to 1)	0.4 (0.2 to 0.6)	-72.0 (-87.4 to -42.8)	-46.2 (-75.9 to 9.6)
Armenia	Animal contact	5 to 9	Both	2 (1 to 3)	0.4 (0.3 to 0.7)	1 (1 to 1)	0.4 (0.2 to 0.5)	1 (0 to 1)	0.3 (0.2 to 0.4)	-69.1 (-83.8 to -41.1)	-41.6 (-69.4 to 11.4)
Armenia	Animal contact	5 to 9	Female	1 (0 to 1)	0.4 (0.2 to 0.7)	0 (0 to 1)	0.3 (0.1 to 0.5)	0 (0 to 0)	0.2 (0.1 to 0.4)	-68.6 (-86.3 to -32.0)	-40.4 (-74.0 to 29.3)
Armenia	Animal contact	5 to 9	Male	1 (0 to 2)	0.5 (0.2 to 1.1)	1 (0 to 1)	0.4 (0.2 to 0.7)	0 (0 to 1)	0.3 (0.2 to 0.6)	-69.4 (-89.1 to -31.4)	-42.5 (-79.5 to 29.2)
Armenia	Animal contact	10 to 14	Both	1 (0 to 1)	0.2 (0.1 to 0.4)	1 (0 to 1)	0.2 (0.1 to 0.3)	0 (0 to 0)	0.2 (0.1 to 0.2)	-64.4 (-80.6 to -38.3)	-29.8 (-61.9 to 21.5)
Armenia	Animal contact	10 to 14	Female	0 (0 to 0)	0.1 (0.1 to 0.2)	0 (0 to 0)	0.1 (0.1 to 0.1)	0 (0 to 0)	0.1 (0.0 to 0.1)	-70.3 (-84.8 to -42.9)	-39.8 (-69.2 to 15.7)
Armenia	Animal contact	10 to 14	Male	1 (0 to 1)	0.4 (0.2 to 0.7)	1 (0 to 1)	0.3 (0.2 to 0.5)	0 (0 to 0)	0.3 (0.2 to 0.4)	-62.9 (-81.6 to -32.2)	-28.8 (-64.7 to 30.1)
Armenia	Foreign body	5 to 9	Both	4 (2 to 6)	1.0 (0.5 to 1.7)	2 (1 to 3)	0.7 (0.5 to 1.2)	1 (1 to 2)	0.5 (0.3 to 0.9)	-69.9 (-82.3 to -48.7)	-43.0 (-66.6 to -3.0)
Armenia	Foreign body	5 to 9	Female	2 (1 to 4)	0.9 (0.4 to 1.9)	1 (0 to 2)	0.7 (0.4 to 1.3)	0 (0 to 1)	0.5 (0.3 to 0.9)	-73.6 (-85.4 to -44.1)	-49.8 (-72.3 to 6.3)
Armenia	Foreign body	5 to 9	Male	2 (1 to 3)	1.0 (0.5 to 1.8)	1 (1 to 2)	0.8 (0.5 to 1.2)	1 (0 to 1)	0.6 (0.4 to 1.0)	-66.6 (-83.5 to -29.8)	-37.2 (-69.0 to 32.1)
Armenia	Foreign body	10 to 14	Both	2 (2 to 3)	0.7 (0.5 to 1.0)	2 (1 to 3)	0.6 (0.5 to 0.8)	1 (1 to 1)	0.5 (0.4 to 0.7)	-65.4 (-76.4 to -50.0)	-31.8 (-53.4 to -1.5)
Armenia	Foreign body	10 to 14	Female	1 (1 to 2)	0.8 (0.4 to 1.2)	1 (1 to 2)	0.7 (0.3 to 1.0)	0 (0 to 1)	0.5 (0.3 to 0.7)	-69.0 (-79.9 to -49.8)	-37.1 (-59.2 to 1.8)
Armenia	Foreign body	10 to 14	Male	1 (1 to 2)	0.7 (0.4 to 1.1)	1 (1 to 1)	0.6 (0.4 to 0.9)	0 (0 to 1)	0.5 (0.3 to 0.8)	-61.5 (-75.7 to -36.7)	-26.1 (-53.3 to 21.4)
Armenia	Environmental heat and cold exposure	5 to 9	Both	1 (1 to 2)	0.3 (0.2 to 0.5)	1 (0 to 1)	0.2 (0.1 to 0.4)	0 (0 to 0)	0.1 (0.1 to 0.2)	-85.1 (-92.2 to -69.0)	-71.9 (-85.3 to -41.4)
Armenia	Environmental heat and cold exposure	5 to 9	Female	0 (0 to 1)	0.2 (0.1 to 0.4)	0 (0 to 0)	0.1 (0.1 to 0.2)	0 (0 to 0)	0.0 (0.0 to 0.1)	-88.1 (-94.1 to -71.3)	-77.3 (-88.7 to -45.4)

eTable 8. All-cause and cause-specific mortality (counts and rates) at the global level and by country in the WHO European Region for ages 5 to 9 and 10 to 14 for 1990, 2000, and 2016

Armenia	Environmental heat and cold exposure	5 to 9	Male	1 (0 to 2)	0.4 (0.2 to 0.8)	0 (0 to 1)	0.3 (0.2 to 0.6)	0 (0 to 0)	0.1 (0.1 to 0.3)	-83.8 (-92.3 to -62.8)	-69.5 (-85.5 to -30.0)
Armenia	Environmental heat and cold exposure	10 to 14	Both	1 (0 to 1)	0.3 (0.2 to 0.4)	1 (0 to 1)	0.2 (0.1 to 0.3)	0 (0 to 0)	0.1 (0.1 to 0.2)	-80.1 (-88.6 to -67.8)	-60.9 (-77.5 to -36.6)
Armenia	Environmental heat and cold exposure	10 to 14	Female	0 (0 to 0)	0.1 (0.1 to 0.2)	0 (0 to 0)	0.1 (0.0 to 0.1)	0 (0 to 0)	0.0 (0.0 to 0.1)	-84.3 (-91.1 to -73.3)	-68.2 (-82.0 to -46.0)
Armenia	Environmental heat and cold exposure	10 to 14	Male	1 (0 to 1)	0.4 (0.2 to 0.7)	0 (0 to 1)	0.3 (0.2 to 0.5)	0 (0 to 0)	0.2 (0.1 to 0.4)	-79.0 (-88.6 to -62.9)	-59.6 (-78.1 to -28.8)
Armenia	Other unintentional injuries	5 to 9	Both	8 (5 to 11)	2.1 (1.5 to 2.9)	3 (2 to 4)	1.2 (0.8 to 1.6)	1 (1 to 1)	0.5 (0.4 to 0.7)	-87.1 (-92.3 to -78.9)	-75.6 (-85.3 to -60.0)
Armenia	Other unintentional injuries	5 to 9	Female	2 (1 to 2)	0.8 (0.6 to 1.2)	1 (0 to 1)	0.4 (0.3 to 0.7)	0 (0 to 0)	0.3 (0.2 to 0.4)	-83.2 (-90.8 to -69.9)	-68.1 (-82.6 to -42.7)
Armenia	Other unintentional injuries	5 to 9	Male	6 (4 to 9)	3.4 (2.2 to 4.7)	3 (2 to 4)	1.9 (1.2 to 2.6)	1 (0 to 1)	0.8 (0.5 to 1.2)	-88.0 (-93.2 to -79.0)	-77.5 (-87.2 to -60.5)
Armenia	Other unintentional injuries	10 to 14	Both	6 (4 to 7)	1.8 (1.3 to 2.3)	4 (3 to 5)	1.1 (0.8 to 1.4)	1 (1 to 1)	0.6 (0.4 to 0.8)	-82.3 (-88.3 to -73.4)	-65.0 (-76.9 to -47.5)
Armenia	Other unintentional injuries	10 to 14	Female	1 (1 to 2)	0.7 (0.4 to 1.0)	1 (0 to 1)	0.4 (0.3 to 0.7)	0 (0 to 0)	0.3 (0.2 to 0.5)	-76.5 (-86.9 to -56.7)	-52.3 (-73.4 to -12.3)
Armenia	Other unintentional injuries	10 to 14	Male	5 (3 to 6)	2.9 (2.0 to 3.8)	3 (2 to 4)	1.7 (1.2 to 2.4)	1 (1 to 1)	0.9 (0.6 to 1.3)	-83.6 (-89.9 to -72.6)	-68.5 (-80.6 to -47.5)
Armenia	Self-harm	10 to 14	Both	2 (1 to 4)	0.6 (0.4 to 1.1)	2 (1 to 3)	0.5 (0.4 to 0.9)	1 (1 to 2)	0.8 (0.5 to 1.1)	-32.2 (-67.4 to 14.4)	33.6 (-35.7 to 125.3)
Armenia	Self-harm	10 to 14	Female	0 (0 to 1)	0.3 (0.2 to 0.4)	0 (0 to 1)	0.3 (0.2 to 0.4)	0 (0 to 0)	0.4 (0.2 to 0.6)	-40.2 (-66.6 to 4.7)	21.2 (-32.2 to 112.2)
Armenia	Self-harm	10 to 14	Male	1 (1 to 3)	0.8 (0.5 to 1.8)	1 (1 to 3)	0.8 (0.5 to 1.6)	1 (1 to 1)	1.1 (0.7 to 1.8)	-29.3 (-71.9 to 36.8)	35.6 (-46.1 to 162.4)
Armenia	Interpersonal violence	5 to 9	Both	2 (1 to 3)	0.5 (0.3 to 0.7)	1 (1 to 2)	0.4 (0.2 to 0.6)	0 (0 to 1)	0.2 (0.1 to 0.4)	-73.4 (-85.6 to -51.9)	-49.7 (-72.7 to -8.9)
Armenia	Interpersonal violence	5 to 9	Female	1 (0 to 1)	0.3 (0.2 to 0.6)	0 (0 to 1)	0.3 (0.2 to 0.5)	0 (0 to 0)	0.2 (0.1 to 0.3)	-70.5 (-86.3 to -35.4)	-43.8 (-73.9 to 22.8)
Armenia	Interpersonal violence	5 to 9	Male	1 (1 to 2)	0.6 (0.3 to 1.0)	1 (0 to 1)	0.5 (0.2 to 0.8)	0 (0 to 0)	0.3 (0.1 to 0.5)	-75.1 (-87.8 to -53.4)	-53.2 (-77.0 to -12.2)
Armenia	Interpersonal violence	10 to 14	Both	2 (1 to 3)	0.7 (0.4 to 1.0)	2 (1 to 3)	0.6 (0.3 to 0.9)	1 (0 to 1)	0.5 (0.3 to 0.7)	-64.5 (-76.8 to -42.7)	-30.0 (-54.3 to 12.8)
Armenia	Interpersonal violence	10 to 14	Female	1 (0 to 1)	0.4 (0.2 to 0.6)	1 (0 to 1)	0.4 (0.2 to 0.7)	0 (0 to 0)	0.3 (0.2 to 0.5)	-60.6 (-78.7 to -24.6)	-20.2 (-56.9 to 52.9)
Armenia	Interpersonal violence	10 to 14	Male	2 (1 to 3)	0.9 (0.5 to 1.6)	1 (1 to 2)	0.8 (0.3 to 1.2)	1 (0 to 1)	0.6 (0.3 to 1.0)	-66.0 (-80.9 to -38.2)	-34.7 (-63.4 to 18.5)
Armenia	Exposure to forces of nature	5 to 9	Both	0 (0 to 0)	0.0 (0.0 to 0.0)	0 (0 to 0)	0.0 (0.0 to 0.0)	0 (0 to 0)	0.0 (0.0 to 0.0)	0.0 (0.0 to 0.0)	0.0 (0.0 to 0.0)
Armenia	Exposure to forces of nature	5 to 9	Female	0 (0 to 0)	0.0 (0.0 to 0.0)	0 (0 to 0)	0.0 (0.0 to 0.0)	0 (0 to 0)	0.0 (0.0 to 0.0)	0.0 (0.0 to 0.0)	0.0 (0.0 to 0.0)
Armenia	Exposure to forces of nature	5 to 9	Male	0 (0 to 0)	0.0 (0.0 to 0.0)	0 (0 to 0)	0.0 (0.0 to 0.0)	0 (0 to 0)	0.0 (0.0 to 0.0)	0.0 (0.0 to 0.0)	0.0 (0.0 to 0.0)
Armenia	Exposure to forces of nature	10 to 14	Both	0 (0 to 0)	0.0 (0.0 to 0.0)	0 (0 to 0)	0.0 (0.0 to 0.0)	0 (0 to 0)	0.0 (0.0 to 0.0)	0.0 (0.0 to 0.0)	0.0 (0.0 to 0.0)
Armenia	Exposure to forces of nature	10 to 14	Female	0 (0 to 0)	0.0 (0.0 to 0.0)	0 (0 to 0)	0.0 (0.0 to 0.0)	0 (0 to 0)	0.0 (0.0 to 0.0)	0.0 (0.0 to 0.0)	0.0 (0.0 to 0.0)
Armenia	Exposure to forces of nature	10 to 14	Male	0 (0 to 0)	0.0 (0.0 to 0.0)	0 (0 to 0)	0.0 (0.0 to 0.0)	0 (0 to 0)	0.0 (0.0 to 0.0)	0.0 (0.0 to 0.0)	0.0 (0.0 to 0.0)

eTable 8. All-cause and cause-specific mortality (counts and rates) at the global level and by country in the WHO European Region for ages 5 to 9 and 10 to 14 for 1990, 2000, and 2016

Armenia	Conflict and terrorism	5 to 9	Both	0<br>(0 to 0)	0.0<br>(0.0 to 0.0)	0<br>(0 to 0)	0.0<br>(0.0 to 0.0)	0<br>(0 to 0)	0.0<br>(0.0 to 0.0)	0.0<br>(0.0 to 0.0)	0.0<br>(0.0 to 0.0)
Armenia	Conflict and terrorism	5 to 9	Female	0<br>(0 to 0)	0.0<br>(0.0 to 0.0)	0<br>(0 to 0)	0.0<br>(0.0 to 0.0)	0<br>(0 to 0)	0.0<br>(0.0 to 0.0)	0.0<br>(0.0 to 0.0)	0.0<br>(0.0 to 0.0)
Armenia	Conflict and terrorism	5 to 9	Male	0<br>(0 to 0)	0.0<br>(0.0 to 0.0)	0<br>(0 to 0)	0.0<br>(0.0 to 0.0)	0<br>(0 to 0)	0.0<br>(0.0 to 0.0)	0.0<br>(0.0 to 0.0)	0.0<br>(0.0 to 0.0)
Armenia	Conflict and terrorism	10 to 14	Both	0<br>(0 to 0)	0.0<br>(0.0 to 0.0)	0<br>(0 to 0)	0.0<br>(0.0 to 0.0)	0<br>(0 to 0)	0.0<br>(0.0 to 0.0)	0.0<br>(0.0 to 0.0)	0.0<br>(0.0 to 0.0)
Armenia	Conflict and terrorism	10 to 14	Female	0<br>(0 to 0)	0.0<br>(0.0 to 0.0)	0<br>(0 to 0)	0.0<br>(0.0 to 0.0)	0<br>(0 to 0)	0.0<br>(0.0 to 0.0)	0.0<br>(0.0 to 0.0)	0.0<br>(0.0 to 0.0)
Armenia	Conflict and terrorism	10 to 14	Male	0<br>(0 to 0)	0.0<br>(0.0 to 0.0)	0<br>(0 to 0)	0.0<br>(0.0 to 0.0)	0<br>(0 to 0)	0.0<br>(0.0 to 0.0)	0.0<br>(0.0 to 0.0)	0.0<br>(0.0 to 0.0)
Armenia	Executions and police conflict	5 to 9	Both	0<br>(0 to 0)	0.0<br>(0.0 to 0.0)	0<br>(0 to 0)	0.0<br>(0.0 to 0.0)	0<br>(0 to 0)	0.0<br>(0.0 to 0.0)	-67.5<br>(-87.1 to -35.4)	-38.5<br>(-75.5 to 22.2)
Armenia	Executions and police conflict	5 to 9	Female	0<br>(0 to 0)	0.0<br>(0.0 to 0.0)	0<br>(0 to 0)	0.0<br>(0.0 to 0.0)	0<br>(0 to 0)	0.0<br>(0.0 to 0.0)	-56.4<br>(-82.9 to -29.9)	-17.1<br>(-67.4 to 33.4)
Armenia	Executions and police conflict	5 to 9	Male	0<br>(0 to 0)	0.0<br>(0.0 to 0.1)	0<br>(0 to 0)	0.0<br>(0.0 to 0.1)	0<br>(0 to 0)	0.0<br>(0.0 to 0.0)	-73.9<br>(-91.8 to -31.1)	-50.9<br>(-84.6 to 29.6)
Armenia	Executions and police conflict	10 to 14	Both	0<br>(0 to 0)	0.0<br>(0.0 to 0.1)	0<br>(0 to 0)	0.0<br>(0.0 to 0.1)	0<br>(0 to 0)	0.0<br>(0.0 to 0.0)	-87.5<br>(-95.5 to -55.3)	-75.3<br>(-91.2 to -11.9)
Armenia	Executions and police conflict	10 to 14	Female	0<br>(0 to 0)	0.0<br>(0.0 to 0.0)	0<br>(0 to 0)	0.0<br>(0.0 to 0.0)	0<br>(0 to 0)	0.0<br>(0.0 to 0.0)	-62.4<br>(-86.4 to -41.6)	-23.7<br>(-72.4 to 18.5)
Armenia	Executions and police conflict	10 to 14	Male	0<br>(0 to 0)	0.1<br>(0.0 to 0.2)	0<br>(0 to 0)	0.1<br>(0.0 to 0.2)	0<br>(0 to 0)	0.0<br>(0.0 to 0.0)	-92.0<br>(-97.4 to -51.4)	-84.6<br>(-95.0 to -6.8)
Austria	All causes	5 to 9	Both	87<br>(84 to 91)	18.8<br>(18.1 to 19.6)	58<br>(55 to 61)	12.3<br>(11.6 to 12.9)	32<br>(28 to 37)	7.9<br>(6.8 to 9.1)	-63.5<br>(-68.6 to -57.3)	-58.2<br>(-64.0 to -51.1)
Austria	All causes	5 to 9	Female	32<br>(31 to 34)	14.2<br>(13.7 to 14.9)	25<br>(24 to 26)	10.8<br>(10.2 to 11.4)	16<br>(14 to 18)	8.0<br>(7.0 to 9.0)	-51.1<br>(-57.1 to -44.1)	-44.2<br>(-51.0 to -36.1)
Austria	All causes	5 to 9	Male	55<br>(53 to 57)	23.2<br>(22.3 to 24.3)	33<br>(32 to 35)	13.7<br>(12.9 to 14.5)	16<br>(14 to 19)	7.8<br>(6.6 to 9.2)	-70.8<br>(-75.5 to -65.3)	-66.5<br>(-71.9 to -60.2)
Austria	All causes	10 to 14	Both	73<br>(71 to 75)	16.7<br>(16.4 to 17.2)	68<br>(66 to 70)	14.4<br>(14.0 to 14.8)	39<br>(36 to 42)	9.4<br>(8.7 to 10.2)	-46.9<br>(-51.4 to -41.9)	-43.8<br>(-48.6 to -38.5)
Austria	All causes	10 to 14	Female	27<br>(26 to 27)	12.5<br>(12.2 to 12.9)	30<br>(29 to 31)	13.0<br>(12.6 to 13.4)	16<br>(15 to 17)	8.0<br>(7.4 to 8.7)	-39.3<br>(-44.2 to -34.4)	-36.0<br>(-41.1 to -30.7)
Austria	All causes	10 to 14	Male	46<br>(45 to 48)	20.7<br>(20.2 to 21.3)	38<br>(37 to 39)	15.7<br>(15.2 to 16.2)	23<br>(21 to 25)	10.7<br>(9.7 to 11.9)	-51.2<br>(-56.2 to -45.6)	-48.2<br>(-53.6 to -42.3)
Austria	Tuberculosis	5 to 9	Both	0<br>(0 to 0)	0.0<br>(0.0 to 0.0)	0<br>(0 to 0)	0.0<br>(0.0 to 0.0)	0<br>(0 to 0)	0.0<br>(0.0 to 0.0)	-59.5<br>(-70.5 to -43.9)	-53.6<br>(-66.2 to -35.8)
Austria	Tuberculosis	5 to 9	Female	0<br>(0 to 0)	0.0<br>(0.0 to 0.0)	0<br>(0 to 0)	0.0<br>(0.0 to 0.0)	0<br>(0 to 0)	0.0<br>(0.0 to 0.0)	-54.5<br>(-68.3 to -33.0)	-48.0<br>(-63.8 to -23.5)
Austria	Tuberculosis	5 to 9	Male	0<br>(0 to 0)	0.0<br>(0.0 to 0.0)	0<br>(0 to 0)	0.0<br>(0.0 to 0.0)	0<br>(0 to 0)	0.0<br>(0.0 to 0.0)	-63.4<br>(-75.2 to -44.0)	-58.1<br>(-71.5 to -35.8)
Austria	Tuberculosis	10 to 14	Both	0<br>(0 to 0)	0.0<br>(0.0 to 0.0)	0<br>(0 to 0)	0.0<br>(0.0 to 0.0)	0<br>(0 to 0)	0.0<br>(0.0 to 0.0)	-53.0<br>(-65.3 to -35.4)	-50.3<br>(-63.3 to -31.6)
Austria	Tuberculosis	10 to 14	Female	0<br>(0 to 0)	0.0<br>(0.0 to 0.0)	0<br>(0 to 0)	0.0<br>(0.0 to 0.0)	0<br>(0 to 0)	0.0<br>(0.0 to 0.0)	-33.2<br>(-51.9 to -8.1)	-29.5<br>(-49.2 to -2.9)
Austria	Tuberculosis	10 to 14	Male	0<br>(0 to 0)	0.0<br>(0.0 to 0.0)	0<br>(0 to 0)	0.0<br>(0.0 to 0.0)	0<br>(0 to 0)	0.0<br>(0.0 to 0.0)	-63.5<br>(-74.8 to -41.6)	-61.3<br>(-73.3 to -38.1)
Austria	HIV/AIDS	5 to 9	Both	0<br>(0 to 0)	0.1<br>(0.1 to 0.1)	0<br>(0 to 0)	0.0<br>(0.0 to 0.0)	0<br>(0 to 0)	0.0<br>(0.0 to 0.0)	-60.4<br>(-68.1 to -51.4)	-54.7<br>(-63.4 to -44.3)
Austria	HIV/AIDS	5 to 9	Female	0<br>(0 to 0)	0.0<br>(0.0 to 0.0)	0<br>(0 to 0)	0.0<br>(0.0 to 0.0)	0<br>(0 to 0)	0.0<br>(0.0 to 0.0)	-28.0<br>(-48.8 to 0.3)	-17.7<br>(-41.5 to 14.6)
Austria	HIV/AIDS	5 to 9	Male	0<br>(0 to 0)	0.1<br>(0.1 to 0.1)	0<br>(0 to 0)	0.0<br>(0.0 to 0.0)	0<br>(0 to 0)	0.0<br>(0.0 to 0.0)	-73.2<br>(-79.4 to -66.2)	-69.3<br>(-76.3 to -61.2)
Austria	HIV/AIDS	10 to 14	Both	0<br>(0 to 0)	0.0<br>(0.0 to 0.1)	0<br>(0 to 0)	0.0<br>(0.0 to 0.0)	0<br>(0 to 0)	0.0<br>(0.0 to 0.0)	-58.3<br>(-67.2 to -47.2)	-55.8<br>(-65.3 to -44.1)
Austria	HIV/AIDS	10 to 14	Female	0<br>(0 to 0)	0.0<br>(0.0 to 0.0)	0<br>(0 to 0)	0.0<br>(0.0 to 0.0)	0<br>(0 to 0)	0.0<br>(0.0 to 0.0)	-42.8<br>(-59.7 to -17.9)	-39.6<br>(-57.5 to -13.4)

eTable 8. All-cause and cause-specific mortality (counts and rates) at the global level and by country in the WHO European Region for ages 5 to 9 and 10 to 14 for 1990, 2000, and 2016

Austria	HIV/AIDS	10 to 14	Male	0 (0 to 0)	0.1 (0.1 to 0.1)	0 (0 to 0)	0.0 (0.0 to 0.1)	0 (0 to 0)	0.0 (0.0 to 0.0)	-59.7 (-69.3 to -47.7)	-57.3 (-67.4 to -44.5)
Austria	Diarrheal diseases	5 to 9	Both	0 (0 to 0)	0.0 (0.0 to 0.0)	0 (0 to 0)	0.0 (0.0 to 0.0)	0 (0 to 0)	0.0 (0.0 to 0.0)	88.6 (25.8 to 179.0)	115.9 (44.0 to 219.4)
Austria	Diarrheal diseases	5 to 9	Female	0 (0 to 0)	0.0 (0.0 to 0.0)	0 (0 to 0)	0.0 (0.0 to 0.0)	0 (0 to 0)	0.0 (0.0 to 0.1)	215.4 (86.5 to 405.8)	260.3 (113.0 to 477.8)
Austria	Diarrheal diseases	5 to 9	Male	0 (0 to 0)	0.0 (0.0 to 0.0)	0 (0 to 0)	0.0 (0.0 to 0.0)	0 (0 to 0)	0.0 (0.0 to 0.0)	-32.6 (-47.6 to -13.0)	-22.7 (-39.9 to -0.3)
Austria	Diarrheal diseases	10 to 14	Both	0 (0 to 0)	0.0 (0.0 to 0.0)	0 (0 to 0)	0.0 (0.0 to 0.0)	0 (0 to 0)	0.0 (0.0 to 0.0)	-5.4 (-23.2 to 26.6)	0.0 (-18.8 to 33.9)
Austria	Diarrheal diseases	10 to 14	Female	0 (0 to 0)	0.0 (0.0 to 0.0)	0 (0 to 0)	0.0 (0.0 to 0.0)	0 (0 to 0)	0.0 (0.0 to 0.0)	6.4 (-23.6 to 67.2)	12.3 (-19.3 to 76.6)
Austria	Diarrheal diseases	10 to 14	Male	0 (0 to 0)	0.0 (0.0 to 0.0)	0 (0 to 0)	0.0 (0.0 to 0.0)	0 (0 to 0)	0.0 (0.0 to 0.0)	-16.5 (-30.8 to -0.5)	-11.5 (-26.7 to 5.5)
Austria	Intestinal infectious diseases	5 to 9	Both	0 (0 to 0)	0.0 (0.0 to 0.0)	0 (0 to 0)	0.0 (0.0 to 0.0)	0 (0 to 0)	0.0 (0.0 to 0.0)	-54.8 (-80.3 to -17.2)	-48.2 (-77.5 to -5.2)
Austria	Intestinal infectious diseases	5 to 9	Female	0 (0 to 0)	0.0 (0.0 to 0.0)	0 (0 to 0)	0.0 (0.0 to 0.0)	0 (0 to 0)	0.0 (0.0 to 0.0)	-50.8 (-82.4 to 16.2)	-43.8 (-79.9 to 32.7)
Austria	Intestinal infectious diseases	5 to 9	Male	0 (0 to 0)	0.0 (0.0 to 0.0)	0 (0 to 0)	0.0 (0.0 to 0.0)	0 (0 to 0)	0.0 (0.0 to 0.0)	-58.1 (-86.1 to -3.6)	-51.9 (-84.0 to 10.6)
Austria	Intestinal infectious diseases	10 to 14	Both	0 (0 to 0)	0.0 (0.0 to 0.0)	0 (0 to 0)	0.0 (0.0 to 0.0)	0 (0 to 0)	0.0 (0.0 to 0.0)	-44.0 (-71.7 to -4.5)	-40.8 (-70.1 to 1.0)
Austria	Intestinal infectious diseases	10 to 14	Female	0 (0 to 0)	0.0 (0.0 to 0.0)	0 (0 to 0)	0.0 (0.0 to 0.0)	0 (0 to 0)	0.0 (0.0 to 0.0)	-43.1 (-74.7 to 14.4)	-39.9 (-73.3 to 20.8)
Austria	Intestinal infectious diseases	10 to 14	Male	0 (0 to 0)	0.0 (0.0 to 0.0)	0 (0 to 0)	0.0 (0.0 to 0.0)	0 (0 to 0)	0.0 (0.0 to 0.0)	-45.1 (-78.3 to 9.7)	-41.9 (-77.0 to 16.3)
Austria	Lower respiratory infections	5 to 9	Both	2 (1 to 2)	0.4 (0.3 to 0.5)	1 (1 to 1)	0.2 (0.2 to 0.3)	0 (0 to 1)	0.1 (0.1 to 0.2)	-76.4 (-84.4 to -65.0)	-72.9 (-82.2 to -59.9)
Austria	Lower respiratory infections	5 to 9	Female	1 (1 to 1)	0.4 (0.3 to 0.6)	1 (0 to 1)	0.3 (0.2 to 0.4)	0 (0 to 0)	0.1 (0.1 to 0.2)	-70.8 (-84.2 to -49.2)	-66.7 (-82.0 to -42.0)
Austria	Lower respiratory infections	5 to 9	Male	1 (1 to 1)	0.4 (0.3 to 0.5)	0 (0 to 1)	0.2 (0.1 to 0.3)	0 (0 to 0)	0.1 (0.0 to 0.1)	-82.6 (-90.2 to -68.7)	-80.1 (-88.7 to -64.1)
Austria	Lower respiratory infections	10 to 14	Both	1 (1 to 2)	0.3 (0.2 to 0.3)	1 (1 to 1)	0.2 (0.2 to 0.3)	0 (0 to 1)	0.1 (0.1 to 0.1)	-64.1 (-75.5 to -45.0)	-62.0 (-74.0 to -41.8)
Austria	Lower respiratory infections	10 to 14	Female	1 (0 to 1)	0.3 (0.2 to 0.4)	1 (0 to 1)	0.3 (0.2 to 0.4)	0 (0 to 0)	0.1 (0.1 to 0.2)	-60.7 (-77.7 to -32.7)	-58.5 (-76.5 to -28.9)
Austria	Lower respiratory infections	10 to 14	Male	1 (0 to 1)	0.2 (0.2 to 0.3)	0 (0 to 1)	0.2 (0.1 to 0.2)	0 (0 to 0)	0.1 (0.1 to 0.1)	-68.0 (-80.7 to -47.2)	-66.0 (-79.6 to -44.1)
Austria	Upper respiratory infections	5 to 9	Both	0 (0 to 0)	0.0 (0.0 to 0.0)	0 (0 to 0)	0.0 (0.0 to 0.0)	0 (0 to 0)	0.0 (0.0 to 0.0)	-78.9 (-99.5 to 1009.6)	-75.9 (-99.4 to 1170.4)
Austria	Upper respiratory infections	5 to 9	Female	0 (0 to 0)	0.0 (0.0 to 0.0)	0 (0 to 0)	0.0 (0.0 to 0.0)	0 (0 to 0)	0.0 (0.0 to 0.0)	-70.6 (-100.0 to 54481.3)	-66.4 (-100.0 to 62255.3)
Austria	Upper respiratory infections	5 to 9	Male	0 (0 to 0)	0.0 (0.0 to 0.0)	0 (0 to 0)	0.0 (0.0 to 0.0)	0 (0 to 0)	0.0 (0.0 to 0.0)	-84.0 (-100.0 to 16932.0)	-81.7 (-100.0 to 19437.9)
Austria	Upper respiratory infections	10 to 14	Both	0 (0 to 0)	0.0 (0.0 to 0.0)	0 (0 to 0)	0.0 (0.0 to 0.0)	0 (0 to 0)	0.0 (0.0 to 0.0)	-62.5 (-99.3 to 1519.1)	-60.3 (-99.2 to 1612.9)
Austria	Upper respiratory infections	10 to 14	Female	0 (0 to 0)	0.0 (0.0 to 0.0)	0 (0 to 0)	0.0 (0.0 to 0.0)	0 (0 to 0)	0.0 (0.0 to 0.0)	-57.0 (-100.0 to 49449.9)	-54.6 (-100.0 to 52217.6)

Austria	Upper respiratory infections	10 to 14	Male	0 (0 to 0)	0.0 (0.0 to 0.0)	0 (0 to 0)	0.0 (0.0 to 0.0)	0 (0 to 0)	0.0 (0.0 to 0.0)	-65.6 (-100.0 to 25407.8)	-63.5 (-100.0 to 26933.6)
Austria	Otitis media	5 to 9	Both	0 (0 to 0)	0.0 (0.0 to 0.0)	0 (0 to 0)	0.0 (0.0 to 0.0)	0 (0 to 0)	0.0 (0.0 to 0.0)	-99.5 (-99.8 to -98.7)	-99.5 (-99.8 to -98.5)
Austria	Otitis media	5 to 9	Female	0 (0 to 0)	0.0 (0.0 to 0.0)	0 (0 to 0)	0.0 (0.0 to 0.0)	0 (0 to 0)	0.0 (0.0 to 0.0)	-92.7 (-98.5 to -71.8)	-91.7 (-98.3 to -67.8)
Austria	Otitis media	5 to 9	Male	0 (0 to 0)	0.0 (0.0 to 0.0)	0 (0 to 0)	0.0 (0.0 to 0.0)	0 (0 to 0)	0.0 (0.0 to 0.0)	-99.8 (-99.9 to -99.4)	-99.8 (-99.9 to -99.4)
Austria	Otitis media	10 to 14	Both	0 (0 to 0)	0.0 (0.0 to 0.0)	0 (0 to 0)	0.0 (0.0 to 0.0)	0 (0 to 0)	0.0 (0.0 to 0.0)	-94.7 (-98.1 to -85.8)	-94.4 (-98.0 to -84.9)
Austria	Otitis media	10 to 14	Female	0 (0 to 0)	0.0 (0.0 to 0.0)	0 (0 to 0)	0.0 (0.0 to 0.0)	0 (0 to 0)	0.0 (0.0 to 0.0)	-90.1 (-97.8 to -61.7)	-89.5 (-97.7 to -59.5)
Austria	Otitis media	10 to 14	Male	0 (0 to 0)	0.0 (0.0 to 0.0)	0 (0 to 0)	0.0 (0.0 to 0.0)	0 (0 to 0)	0.0 (0.0 to 0.0)	-97.6 (-99.2 to -92.5)	-97.4 (-99.2 to -92.0)
Austria	Meningitis	5 to 9	Both	1 (1 to 2)	0.3 (0.2 to 0.4)	1 (1 to 1)	0.2 (0.1 to 0.2)	0 (0 to 0)	0.1 (0.1 to 0.1)	-75.4 (-84.2 to -63.9)	-71.9 (-81.9 to -58.7)
Austria	Meningitis	5 to 9	Female	0 (0 to 1)	0.2 (0.1 to 0.2)	0 (0 to 0)	0.1 (0.1 to 0.2)	0 (0 to 0)	0.1 (0.0 to 0.1)	-62.5 (-76.9 to -38.9)	-57.1 (-73.6 to -30.2)
Austria	Meningitis	5 to 9	Male	1 (1 to 1)	0.4 (0.3 to 0.6)	0 (0 to 1)	0.2 (0.1 to 0.3)	0 (0 to 0)	0.1 (0.1 to 0.1)	-80.9 (-89.1 to -69.2)	-78.1 (-87.5 to -64.6)
Austria	Meningitis	10 to 14	Both	1 (1 to 1)	0.2 (0.1 to 0.3)	1 (1 to 1)	0.2 (0.1 to 0.3)	0 (0 to 1)	0.1 (0.1 to 0.1)	-57.7 (-70.8 to -38.1)	-55.3 (-69.1 to -34.6)
Austria	Meningitis	10 to 14	Female	0 (0 to 1)	0.2 (0.1 to 0.3)	1 (0 to 1)	0.2 (0.2 to 0.3)	0 (0 to 0)	0.1 (0.1 to 0.2)	-51.4 (-72.8 to -18.1)	-48.7 (-71.2 to -13.5)
Austria	Meningitis	10 to 14	Male	0 (0 to 1)	0.2 (0.1 to 0.4)	0 (0 to 1)	0.1 (0.1 to 0.3)	0 (0 to 0)	0.1 (0.0 to 0.1)	-64.6 (-78.5 to -43.1)	-62.5 (-77.3 to -39.7)
Austria	Encephalitis	5 to 9	Both	0 (0 to 1)	0.1 (0.1 to 0.2)	0 (0 to 1)	0.1 (0.1 to 0.1)	0 (0 to 0)	0.1 (0.0 to 0.1)	-44.2 (-69.3 to -14.1)	-36.2 (-64.8 to -1.7)
Austria	Encephalitis	5 to 9	Female	0 (0 to 0)	0.1 (0.0 to 0.1)	0 (0 to 0)	0.1 (0.0 to 0.1)	0 (0 to 0)	0.1 (0.0 to 0.1)	-31.8 (-62.2 to 7.4)	-22.0 (-56.8 to 22.7)
Austria	Encephalitis	5 to 9	Male	0 (0 to 1)	0.1 (0.1 to 0.2)	0 (0 to 0)	0.1 (0.1 to 0.2)	0 (0 to 0)	0.1 (0.0 to 0.1)	-49.6 (-76.8 to -13.7)	-42.2 (-73.4 to -1.0)
Austria	Encephalitis	10 to 14	Both	0 (0 to 0)	0.1 (0.0 to 0.1)	0 (0 to 0)	0.1 (0.0 to 0.1)	0 (0 to 0)	0.1 (0.0 to 0.1)	-24.8 (-52.6 to 3.5)	-20.4 (-49.9 to 9.5)
Austria	Encephalitis	10 to 14	Female	0 (0 to 0)	0.0 (0.0 to 0.1)	0 (0 to 0)	0.1 (0.0 to 0.1)	0 (0 to 0)	0.0 (0.0 to 0.1)	-12.9 (-45.0 to 42.0)	-8.1 (-41.9 to 50.0)
Austria	Encephalitis	10 to 14	Male	0 (0 to 0)	0.1 (0.0 to 0.1)	0 (0 to 0)	0.1 (0.0 to 0.1)	0 (0 to 0)	0.1 (0.0 to 0.1)	-31.1 (-62.4 to 1.1)	-27.0 (-60.2 to 7.2)
Austria	Diphtheria	5 to 9	Both	0 (0 to 0)	0.0 (0.0 to 0.0)	0 (0 to 0)	0.0 (0.0 to 0.0)	0 (0 to 0)	0.0 (0.0 to 0.0)	-77.5 (-96.6 to 59.3)	-74.3 (-96.1 to 82.4)
Austria	Diphtheria	5 to 9	Female	0 (0 to 0)	0.0 (0.0 to 0.0)	0 (0 to 0)	0.0 (0.0 to 0.0)	0 (0 to 0)	0.0 (0.0 to 0.0)	-68.7 (-98.7 to 536.9)	-64.2 (-98.6 to 627.7)
Austria	Diphtheria	5 to 9	Male	0 (0 to 0)	0.0 (0.0 to 0.0)	0 (0 to 0)	0.0 (0.0 to 0.0)	0 (0 to 0)	0.0 (0.0 to 0.0)	-82.8 (-99.1 to 224.0)	-80.2 (-99.0 to 271.6)
Austria	Diphtheria	10 to 14	Both	0 (0 to 0)	0.0 (0.0 to 0.0)	0 (0 to 0)	0.0 (0.0 to 0.0)	0 (0 to 0)	0.0 (0.0 to 0.0)	-62.2 (-94.0 to 201.7)	-60.0 (-93.6 to 219.2)
Austria	Diphtheria	10 to 14	Female	0 (0 to 0)	0.0 (0.0 to 0.0)	0 (0 to 0)	0.0 (0.0 to 0.0)	0 (0 to 0)	0.0 (0.0 to 0.0)	-55.1 (-97.3 to 989.3)	-52.6 (-97.2 to 1050.1)
Austria	Diphtheria	10 to 14	Male	0 (0 to 0)	0.0 (0.0 to 0.0)	0 (0 to 0)	0.0 (0.0 to 0.0)	0 (0 to 0)	0.0 (0.0 to 0.0)	-66.3 (-98.0 to 647.5)	-64.3 (-97.9 to 692.2)
Austria	Whooping cough	5 to 9	Both	0 (0 to 0)	0.0 (0.0 to 0.0)	0 (0 to 0)	0.0 (0.0 to 0.0)	0 (0 to 0)	0.0 (0.0 to 0.0)	74.0 (-38.6 to 490.5)	99.2 (-29.7 to 576.0)
Austria	Whooping cough	5 to 9	Female	0 (0 to 0)	0.0 (0.0 to 0.0)	0 (0 to 0)	0.0 (0.0 to 0.0)	0 (0 to 0)	0.0 (0.0 to 0.0)	180.0 (-39.1 to 1036.5)	219.9 (-30.5 to 1198.4)
Austria	Whooping cough	5 to 9	Male	0 (0 to 0)	0.0 (0.0 to 0.0)	0 (0 to 0)	0.0 (0.0 to 0.0)	0 (0 to 0)	0.0 (0.0 to 0.0)	-25.0 (-46.6 to 38.6)	-14.0 (-38.8 to 59.0)
Austria	Whooping cough	10 to 14	Both	0 (0 to 0)	0.0 (0.0 to 0.0)	0 (0 to 0)	0.0 (0.0 to 0.0)	0 (0 to 0)	0.0 (0.0 to 0.0)	-14.7 (-38.3 to 18.8)	-9.8 (-34.7 to 25.7)
Austria	Whooping cough	10 to 14	Female	0 (0 to 0)	0.0 (0.0 to 0.0)	0 (0 to 0)	0.0 (0.0 to 0.0)	0 (0 to 0)	0.0 (0.0 to 0.0)	-7.2 (-26.6 to 68.4)	-2.1 (-22.5 to 77.8)
Austria	Whooping cough	10 to 14	Male	0 (0 to 0)	0.0 (0.0 to 0.0)	0 (0 to 0)	0.0 (0.0 to 0.0)	0 (0 to 0)	0.0 (0.0 to 0.0)	-21.3 (-52.9 to 1.9)	-16.5 (-50.1 to 8.0)
Austria	Tetanus	5 to 9	Both	0 (0 to 0)	0.0 (0.0 to 0.0)	0 (0 to 0)	0.0 (0.0 to 0.0)	0 (0 to 0)	0.0 (0.0 to 0.0)	-89.9 (-96.6 to -74.0)	-88.4 (-96.1 to -70.3)

eTable 8. All-cause and cause-specific mortality (counts and rates) at the global level and by country in the WHO European Region for ages 5 to 9 and 10 to 14 for 1990, 2000, and 2016

Austria	Tetanus	5 to 9	Female	0 (0 to 0)	0.0 (0.0 to 0.0)	0 (0 to 0)	0.0 (0.0 to 0.0)	0 (0 to 0)	0.0 (0.0 to 0.0)	-88.9 (-97.3 to -63.8)	-87.3 (-96.9 to -58.7)
Austria	Tetanus	5 to 9	Male	0 (0 to 0)	0.0 (0.0 to 0.0)	0 (0 to 0)	0.0 (0.0 to 0.0)	0 (0 to 0)	0.0 (0.0 to 0.0)	-92.9 (-96.2 to -82.4)	-91.8 (-95.6 to -79.9)
Austria	Tetanus	10 to 14	Both	0 (0 to 0)	0.0 (0.0 to 0.0)	0 (0 to 0)	0.0 (0.0 to 0.0)	0 (0 to 0)	0.0 (0.0 to 0.0)	-87.9 (-93.5 to -70.4)	-87.2 (-93.1 to -68.7)
Austria	Tetanus	10 to 14	Female	0 (0 to 0)	0.0 (0.0 to 0.0)	0 (0 to 0)	0.0 (0.0 to 0.0)	0 (0 to 0)	0.0 (0.0 to 0.0)	-82.1 (-92.6 to -49.4)	-81.1 (-92.2 to -46.6)
Austria	Tetanus	10 to 14	Male	0 (0 to 0)	0.0 (0.0 to 0.0)	0 (0 to 0)	0.0 (0.0 to 0.0)	0 (0 to 0)	0.0 (0.0 to 0.0)	-91.2 (-95.7 to -66.0)	-90.7 (-95.4 to -64.0)
Austria	Measles	5 to 9	Both	0 (0 to 0)	0.0 (0.0 to 0.0)	0 (0 to 0)	0.0 (0.0 to 0.0)	0 (0 to 0)	0.0 (0.0 to 0.0)	-96.0 (-98.5 to -89.3)	-95.4 (-98.3 to -87.8)
Austria	Measles	5 to 9	Female	0 (0 to 0)	0.0 (0.0 to 0.0)	0 (0 to 0)	0.0 (0.0 to 0.0)	0 (0 to 0)	0.0 (0.0 to 0.0)	-98.3 (-99.4 to -94.7)	-98.1 (-99.3 to -93.9)
Austria	Measles	5 to 9	Male	0 (0 to 0)	0.0 (0.0 to 0.0)	0 (0 to 0)	0.0 (0.0 to 0.0)	0 (0 to 0)	0.0 (0.0 to 0.0)	-62.1 (-88.8 to 11.4)	-56.5 (-87.1 to 27.8)
Austria	Measles	10 to 14	Both	0 (0 to 0)	0.0 (0.0 to 0.0)	0 (0 to 0)	0.0 (0.0 to 0.0)	0 (0 to 0)	0.0 (0.0 to 0.0)	-94.1 (-97.6 to -85.6)	-93.8 (-97.5 to -84.7)
Austria	Measles	10 to 14	Female	0 (0 to 0)	0.0 (0.0 to 0.0)	0 (0 to 0)	0.0 (0.0 to 0.0)	0 (0 to 0)	0.0 (0.0 to 0.0)	-98.1 (-99.1 to -94.7)	-98.0 (-99.1 to -94.5)
Austria	Measles	10 to 14	Male	0 (0 to 0)	0.0 (0.0 to 0.0)	0 (0 to 0)	0.0 (0.0 to 0.0)	0 (0 to 0)	0.0 (0.0 to 0.0)	-55.2 (-85.6 to 30.5)	-52.5 (-84.7 to 38.3)
Austria	Varicella and herpes zoster	5 to 9	Both	0 (0 to 0)	0.0 (0.0 to 0.0)	0 (0 to 0)	0.0 (0.0 to 0.0)	0 (0 to 0)	0.0 (0.0 to 0.0)	-55.7 (-77.9 to -13.0)	-49.3 (-74.7 to -0.4)
Austria	Varicella and herpes zoster	5 to 9	Female	0 (0 to 0)	0.0 (0.0 to 0.0)	0 (0 to 0)	0.0 (0.0 to 0.0)	0 (0 to 0)	0.0 (0.0 to 0.0)	-28.0 (-70.4 to 93.6)	-17.8 (-66.1 to 121.1)
Austria	Varicella and herpes zoster	5 to 9	Male	0 (0 to 0)	0.0 (0.0 to 0.0)	0 (0 to 0)	0.0 (0.0 to 0.0)	0 (0 to 0)	0.0 (0.0 to 0.0)	-66.3 (-84.2 to -27.8)	-61.3 (-81.8 to -17.2)
Austria	Varicella and herpes zoster	10 to 14	Both	0 (0 to 0)	0.0 (0.0 to 0.0)	0 (0 to 0)	0.0 (0.0 to 0.0)	0 (0 to 0)	0.0 (0.0 to 0.0)	-42.0 (-60.3 to 15.6)	-38.7 (-58.0 to 22.3)
Austria	Varicella and herpes zoster	10 to 14	Female	0 (0 to 0)	0.0 (0.0 to 0.0)	0 (0 to 0)	0.0 (0.0 to 0.0)	0 (0 to 0)	0.0 (0.0 to 0.0)	-40.4 (-60.1 to 55.9)	-37.1 (-57.9 to 64.6)
Austria	Varicella and herpes zoster	10 to 14	Male	0 (0 to 0)	0.0 (0.0 to 0.0)	0 (0 to 0)	0.0 (0.0 to 0.0)	0 (0 to 0)	0.0 (0.0 to 0.0)	-44.2 (-72.5 to 32.8)	-40.8 (-70.8 to 40.7)
Austria	Malaria	5 to 9	Both	0 (0 to 0)	0.0 (0.0 to 0.0)	0 (0 to 0)	0.0 (0.0 to 0.0)	0 (0 to 0)	0.0 (0.0 to 0.0)	0.0 (0.0 to 0.0)	0.0 (0.0 to 0.0)
Austria	Malaria	5 to 9	Female	0 (0 to 0)	0.0 (0.0 to 0.0)	0 (0 to 0)	0.0 (0.0 to 0.0)	0 (0 to 0)	0.0 (0.0 to 0.0)	0.0 (0.0 to 0.0)	0.0 (0.0 to 0.0)
Austria	Malaria	5 to 9	Male	0 (0 to 0)	0.0 (0.0 to 0.0)	0 (0 to 0)	0.0 (0.0 to 0.0)	0 (0 to 0)	0.0 (0.0 to 0.0)	0.0 (0.0 to 0.0)	0.0 (0.0 to 0.0)
Austria	Malaria	10 to 14	Both	0 (0 to 0)	0.0 (0.0 to 0.0)	0 (0 to 0)	0.0 (0.0 to 0.0)	0 (0 to 0)	0.0 (0.0 to 0.0)	0.0 (0.0 to 0.0)	0.0 (0.0 to 0.0)
Austria	Malaria	10 to 14	Female	0 (0 to 0)	0.0 (0.0 to 0.0)	0 (0 to 0)	0.0 (0.0 to 0.0)	0 (0 to 0)	0.0 (0.0 to 0.0)	0.0 (0.0 to 0.0)	0.0 (0.0 to 0.0)
Austria	Malaria	10 to 14	Male	0 (0 to 0)	0.0 (0.0 to 0.0)	0 (0 to 0)	0.0 (0.0 to 0.0)	0 (0 to 0)	0.0 (0.0 to 0.0)	0.0 (0.0 to 0.0)	0.0 (0.0 to 0.0)
Austria	Leishmaniasis	5 to 9	Both	0 (0 to 0)	0.0 (0.0 to 0.0)	0 (0 to 0)	0.0 (0.0 to 0.0)	0 (0 to 0)	0.0 (0.0 to 0.0)	0.0 (0.0 to 0.0)	0.0 (0.0 to 0.0)
Austria	Leishmaniasis	5 to 9	Female	0 (0 to 0)	0.0 (0.0 to 0.0)	0 (0 to 0)	0.0 (0.0 to 0.0)	0 (0 to 0)	0.0 (0.0 to 0.0)	0.0 (0.0 to 0.0)	0.0 (0.0 to 0.0)
Austria	Leishmaniasis	5 to 9	Male	0 (0 to 0)	0.0 (0.0 to 0.0)	0 (0 to 0)	0.0 (0.0 to 0.0)	0 (0 to 0)	0.0 (0.0 to 0.0)	0.0 (0.0 to 0.0)	0.0 (0.0 to 0.0)
Austria	Leishmaniasis	10 to 14	Both	0 (0 to 0)	0.0 (0.0 to 0.0)	0 (0 to 0)	0.0 (0.0 to 0.0)	0 (0 to 0)	0.0 (0.0 to 0.0)	0.0 (0.0 to 0.0)	0.0 (0.0 to 0.0)
Austria	Leishmaniasis	10 to 14	Female	0 (0 to 0)	0.0 (0.0 to 0.0)	0 (0 to 0)	0.0 (0.0 to 0.0)	0 (0 to 0)	0.0 (0.0 to 0.0)	0.0 (0.0 to 0.0)	0.0 (0.0 to 0.0)
Austria	Leishmaniasis	10 to 14	Male	0 (0 to 0)	0.0 (0.0 to 0.0)	0 (0 to 0)	0.0 (0.0 to 0.0)	0 (0 to 0)	0.0 (0.0 to 0.0)	0.0 (0.0 to 0.0)	0.0 (0.0 to 0.0)
Austria	African trypanosomiasis	5 to 9	Both	0 (0 to 0)	0.0 (0.0 to 0.0)	0 (0 to 0)	0.0 (0.0 to 0.0)	0 (0 to 0)	0.0 (0.0 to 0.0)	0.0 (0.0 to 0.0)	0.0 (0.0 to 0.0)
Austria	African trypanosomiasis	5 to 9	Female	0 (0 to 0)	0.0 (0.0 to 0.0)	0 (0 to 0)	0.0 (0.0 to 0.0)	0 (0 to 0)	0.0 (0.0 to 0.0)	0.0 (0.0 to 0.0)	0.0 (0.0 to 0.0)

eTable 8. All-cause and cause-specific mortality (counts and rates) at the global level and by country in the WHO European Region for ages 5 to 9 and 10 to 14 for 1990, 2000, and 2016

Austria	African trypanosomiasis	5 to 9	Male	0 (0 to 0)	0.0 (0.0 to 0.0)	0 (0 to 0)	0.0 (0.0 to 0.0)	0 (0 to 0)	0.0 (0.0 to 0.0)	0.0 (0.0 to 0.0)	0.0 (0.0 to 0.0)
Austria	African trypanosomiasis	10 to 14	Both	0 (0 to 0)	0.0 (0.0 to 0.0)	0 (0 to 0)	0.0 (0.0 to 0.0)	0 (0 to 0)	0.0 (0.0 to 0.0)	0.0 (0.0 to 0.0)	0.0 (0.0 to 0.0)
Austria	African trypanosomiasis	10 to 14	Female	0 (0 to 0)	0.0 (0.0 to 0.0)	0 (0 to 0)	0.0 (0.0 to 0.0)	0 (0 to 0)	0.0 (0.0 to 0.0)	0.0 (0.0 to 0.0)	0.0 (0.0 to 0.0)
Austria	African trypanosomiasis	10 to 14	Male	0 (0 to 0)	0.0 (0.0 to 0.0)	0 (0 to 0)	0.0 (0.0 to 0.0)	0 (0 to 0)	0.0 (0.0 to 0.0)	0.0 (0.0 to 0.0)	0.0 (0.0 to 0.0)
Austria	Schistosomiasis	5 to 9	Both	0 (0 to 0)	0.0 (0.0 to 0.0)	0 (0 to 0)	0.0 (0.0 to 0.0)	0 (0 to 0)	0.0 (0.0 to 0.0)	0.0 (0.0 to 0.0)	0.0 (0.0 to 0.0)
Austria	Schistosomiasis	5 to 9	Female	0 (0 to 0)	0.0 (0.0 to 0.0)	0 (0 to 0)	0.0 (0.0 to 0.0)	0 (0 to 0)	0.0 (0.0 to 0.0)	0.0 (0.0 to 0.0)	0.0 (0.0 to 0.0)
Austria	Schistosomiasis	5 to 9	Male	0 (0 to 0)	0.0 (0.0 to 0.0)	0 (0 to 0)	0.0 (0.0 to 0.0)	0 (0 to 0)	0.0 (0.0 to 0.0)	0.0 (0.0 to 0.0)	0.0 (0.0 to 0.0)
Austria	Schistosomiasis	10 to 14	Both	0 (0 to 0)	0.0 (0.0 to 0.0)	0 (0 to 0)	0.0 (0.0 to 0.0)	0 (0 to 0)	0.0 (0.0 to 0.0)	0.0 (0.0 to 0.0)	0.0 (0.0 to 0.0)
Austria	Schistosomiasis	10 to 14	Female	0 (0 to 0)	0.0 (0.0 to 0.0)	0 (0 to 0)	0.0 (0.0 to 0.0)	0 (0 to 0)	0.0 (0.0 to 0.0)	0.0 (0.0 to 0.0)	0.0 (0.0 to 0.0)
Austria	Schistosomiasis	10 to 14	Male	0 (0 to 0)	0.0 (0.0 to 0.0)	0 (0 to 0)	0.0 (0.0 to 0.0)	0 (0 to 0)	0.0 (0.0 to 0.0)	0.0 (0.0 to 0.0)	0.0 (0.0 to 0.0)
Austria	Cysticercosis	5 to 9	Both	0 (0 to 0)	0.0 (0.0 to 0.0)	0 (0 to 0)	0.0 (0.0 to 0.0)	0 (0 to 0)	0.0 (0.0 to 0.0)	-87.7 (-90.0 to -84.6)	-85.9 (-88.6 to -82.4)
Austria	Cysticercosis	5 to 9	Female	0 (0 to 0)	0.0 (0.0 to 0.0)	0 (0 to 0)	0.0 (0.0 to 0.0)	0 (0 to 0)	0.0 (0.0 to 0.0)	-83.8 (-87.0 to -79.7)	-81.5 (-85.2 to -76.8)
Austria	Cysticercosis	5 to 9	Male	0 (0 to 0)	0.0 (0.0 to 0.0)	0 (0 to 0)	0.0 (0.0 to 0.0)	0 (0 to 0)	0.0 (0.0 to 0.0)	-90.9 (-92.9 to -87.9)	-89.5 (-91.8 to -86.1)
Austria	Cysticercosis	10 to 14	Both	0 (0 to 0)	0.0 (0.0 to 0.0)	0 (0 to 0)	0.0 (0.0 to 0.0)	0 (0 to 0)	0.0 (0.0 to 0.0)	-80.4 (-83.1 to -77.3)	-79.3 (-82.1 to -75.9)
Austria	Cysticercosis	10 to 14	Female	0 (0 to 0)	0.0 (0.0 to 0.0)	0 (0 to 0)	0.0 (0.0 to 0.0)	0 (0 to 0)	0.0 (0.0 to 0.0)	-77.8 (-81.4 to -73.3)	-76.5 (-80.3 to -71.8)
Austria	Cysticercosis	10 to 14	Male	0 (0 to 0)	0.0 (0.0 to 0.0)	0 (0 to 0)	0.0 (0.0 to 0.0)	0 (0 to 0)	0.0 (0.0 to 0.0)	-82.5 (-85.5 to -79.1)	-81.5 (-84.6 to -77.8)
Austria	Yellow fever	5 to 9	Both	0 (0 to 0)	0.0 (0.0 to 0.0)	0 (0 to 0)	0.0 (0.0 to 0.0)	0 (0 to 0)	0.0 (0.0 to 0.0)	0.0 (0.0 to 0.0)	0.0 (0.0 to 0.0)
Austria	Yellow fever	5 to 9	Female	0 (0 to 0)	0.0 (0.0 to 0.0)	0 (0 to 0)	0.0 (0.0 to 0.0)	0 (0 to 0)	0.0 (0.0 to 0.0)	0.0 (0.0 to 0.0)	0.0 (0.0 to 0.0)
Austria	Yellow fever	5 to 9	Male	0 (0 to 0)	0.0 (0.0 to 0.0)	0 (0 to 0)	0.0 (0.0 to 0.0)	0 (0 to 0)	0.0 (0.0 to 0.0)	0.0 (0.0 to 0.0)	0.0 (0.0 to 0.0)
Austria	Yellow fever	10 to 14	Both	0 (0 to 0)	0.0 (0.0 to 0.0)	0 (0 to 0)	0.0 (0.0 to 0.0)	0 (0 to 0)	0.0 (0.0 to 0.0)	0.0 (0.0 to 0.0)	0.0 (0.0 to 0.0)
Austria	Yellow fever	10 to 14	Female	0 (0 to 0)	0.0 (0.0 to 0.0)	0 (0 to 0)	0.0 (0.0 to 0.0)	0 (0 to 0)	0.0 (0.0 to 0.0)	0.0 (0.0 to 0.0)	0.0 (0.0 to 0.0)
Austria	Yellow fever	10 to 14	Male	0 (0 to 0)	0.0 (0.0 to 0.0)	0 (0 to 0)	0.0 (0.0 to 0.0)	0 (0 to 0)	0.0 (0.0 to 0.0)	0.0 (0.0 to 0.0)	0.0 (0.0 to 0.0)
Austria	Rabies	5 to 9	Both	0 (0 to 0)	0.0 (0.0 to 0.0)	0 (0 to 0)	0.0 (0.0 to 0.0)	0 (0 to 0)	0.0 (0.0 to 0.0)	-82.8 (-96.0 to -33.4)	-80.3 (-95.4 to -23.7)
Austria	Rabies	5 to 9	Female	0 (0 to 0)	0.0 (0.0 to 0.0)	0 (0 to 0)	0.0 (0.0 to 0.0)	0 (0 to 0)	0.0 (0.0 to 0.0)	-8.4 (-84.3 to 359.6)	4.6 (-82.1 to 425.1)
Austria	Rabies	5 to 9	Male	0 (0 to 0)	0.0 (0.0 to 0.0)	0 (0 to 0)	0.0 (0.0 to 0.0)	0 (0 to 0)	0.0 (0.0 to 0.0)	-96.5 (-99.4 to -84.5)	-96.0 (-99.3 to -82.2)
Austria	Rabies	10 to 14	Both	0 (0 to 0)	0.0 (0.0 to 0.0)	0 (0 to 0)	0.0 (0.0 to 0.0)	0 (0 to 0)	0.0 (0.0 to 0.0)	-78.4 (-94.0 to -29.6)	-77.1 (-93.6 to -25.5)
Austria	Rabies	10 to 14	Female	0 (0 to 0)	0.0 (0.0 to 0.0)	0 (0 to 0)	0.0 (0.0 to 0.0)	0 (0 to 0)	0.0 (0.0 to 0.0)	166.4 (-47.4 to 1021.0)	181.3 (-44.4 to 1083.6)
Austria	Rabies	10 to 14	Male	0 (0 to 0)	0.0 (0.0 to 0.0)	0 (0 to 0)	0.0 (0.0 to 0.0)	0 (0 to 0)	0.0 (0.0 to 0.0)	-95.3 (-98.9 to -83.5)	-95.0 (-98.8 to -82.5)
Austria	Intestinal nematode infections	5 to 9	Both	-	-	-	-	-	-	-	-

eTable 8. All-cause and cause-specific mortality (counts and rates) at the global level and by country in the WHO European Region for ages 5 to 9 and 10 to 14 for 1990, 2000, and 2016

Austria	Intestinal nematode infections	5 to 9	Female	-	-	-	-	-	-	-	-
Austria	Intestinal nematode infections	5 to 9	Male	-	-	-	-	-	-	-	-
Austria	Intestinal nematode infections	10 to 14	Both	-	-	-	-	-	-	-	-
Austria	Intestinal nematode infections	10 to 14	Female	-	-	-	-	-	-	-	-
Austria	Intestinal nematode infections	10 to 14	Male	-	-	-	-	-	-	-	-
Austria	Ebola	5 to 9	Both	0 (0 to 0)	0.0 (0.0 to 0.0)	0 (0 to 0)	0.0 (0.0 to 0.0)	0 (0 to 0)	0.0 (0.0 to 0.0)	0.0 (0.0 to 0.0)	0.0 (0.0 to 0.0)
Austria	Ebola	5 to 9	Female	0 (0 to 0)	0.0 (0.0 to 0.0)	0 (0 to 0)	0.0 (0.0 to 0.0)	0 (0 to 0)	0.0 (0.0 to 0.0)	0.0 (0.0 to 0.0)	0.0 (0.0 to 0.0)
Austria	Ebola	5 to 9	Male	0 (0 to 0)	0.0 (0.0 to 0.0)	0 (0 to 0)	0.0 (0.0 to 0.0)	0 (0 to 0)	0.0 (0.0 to 0.0)	0.0 (0.0 to 0.0)	0.0 (0.0 to 0.0)
Austria	Ebola	10 to 14	Both	0 (0 to 0)	0.0 (0.0 to 0.0)	0 (0 to 0)	0.0 (0.0 to 0.0)	0 (0 to 0)	0.0 (0.0 to 0.0)	0.0 (0.0 to 0.0)	0.0 (0.0 to 0.0)
Austria	Ebola	10 to 14	Female	0 (0 to 0)	0.0 (0.0 to 0.0)	0 (0 to 0)	0.0 (0.0 to 0.0)	0 (0 to 0)	0.0 (0.0 to 0.0)	0.0 (0.0 to 0.0)	0.0 (0.0 to 0.0)
Austria	Ebola	10 to 14	Male	0 (0 to 0)	0.0 (0.0 to 0.0)	0 (0 to 0)	0.0 (0.0 to 0.0)	0 (0 to 0)	0.0 (0.0 to 0.0)	0.0 (0.0 to 0.0)	0.0 (0.0 to 0.0)
Austria	Zika virus	5 to 9	Both	0 (0 to 0)	0.0 (0.0 to 0.0)	0 (0 to 0)	0.0 (0.0 to 0.0)	0 (0 to 0)	0.0 (0.0 to 0.0)	0.0 (0.0 to 0.0)	0.0 (0.0 to 0.0)
Austria	Zika virus	5 to 9	Female	0 (0 to 0)	0.0 (0.0 to 0.0)	0 (0 to 0)	0.0 (0.0 to 0.0)	0 (0 to 0)	0.0 (0.0 to 0.0)	0.0 (0.0 to 0.0)	0.0 (0.0 to 0.0)
Austria	Zika virus	5 to 9	Male	0 (0 to 0)	0.0 (0.0 to 0.0)	0 (0 to 0)	0.0 (0.0 to 0.0)	0 (0 to 0)	0.0 (0.0 to 0.0)	0.0 (0.0 to 0.0)	0.0 (0.0 to 0.0)
Austria	Zika virus	10 to 14	Both	0 (0 to 0)	0.0 (0.0 to 0.0)	0 (0 to 0)	0.0 (0.0 to 0.0)	0 (0 to 0)	0.0 (0.0 to 0.0)	0.0 (0.0 to 0.0)	0.0 (0.0 to 0.0)
Austria	Zika virus	10 to 14	Female	0 (0 to 0)	0.0 (0.0 to 0.0)	0 (0 to 0)	0.0 (0.0 to 0.0)	0 (0 to 0)	0.0 (0.0 to 0.0)	0.0 (0.0 to 0.0)	0.0 (0.0 to 0.0)
Austria	Zika virus	10 to 14	Male	0 (0 to 0)	0.0 (0.0 to 0.0)	0 (0 to 0)	0.0 (0.0 to 0.0)	0 (0 to 0)	0.0 (0.0 to 0.0)	0.0 (0.0 to 0.0)	0.0 (0.0 to 0.0)
Austria	Other neglected tropical diseases	5 to 9	Both	0 (0 to 0)	0.0 (0.0 to 0.0)	0 (0 to 0)	0.0 (0.0 to 0.0)	0 (0 to 0)	0.0 (0.0 to 0.0)	-30.5 (-43.2 to -13.9)	-20.5 (-34.9 to -1.5)
Austria	Other neglected tropical diseases	5 to 9	Female	0 (0 to 0)	0.0 (0.0 to 0.0)	0 (0 to 0)	0.0 (0.0 to 0.0)	0 (0 to 0)	0.0 (0.0 to 0.0)	-28.3 (-41.9 to -10.6)	-18.0 (-33.6 to 2.1)
Austria	Other neglected tropical diseases	5 to 9	Male	0 (0 to 0)	0.0 (0.0 to 0.0)	0 (0 to 0)	0.0 (0.0 to 0.0)	0 (0 to 0)	0.0 (0.0 to 0.0)	-32.6 (-47.6 to -13.0)	-22.7 (-39.9 to -0.3)
Austria	Other neglected tropical diseases	10 to 14	Both	0 (0 to 0)	0.0 (0.0 to 0.0)	0 (0 to 0)	0.0 (0.0 to 0.0)	0 (0 to 0)	0.0 (0.0 to 0.0)	-8.9 (-26.6 to 32.1)	-3.6 (-22.3 to 39.7)
Austria	Other neglected tropical diseases	10 to 14	Female	0 (0 to 0)	0.0 (0.0 to 0.0)	0 (0 to 0)	0.0 (0.0 to 0.0)	0 (0 to 0)	0.0 (0.0 to 0.0)	-14.3 (-27.5 to 2.8)	-9.5 (-23.5 to 8.5)
Austria	Other neglected tropical diseases	10 to 14	Male	0 (0 to 0)	0.0 (0.0 to 0.0)	0 (0 to 0)	0.0 (0.0 to 0.0)	0 (0 to 0)	0.0 (0.0 to 0.0)	-3.7 (-29.9 to 76.7)	2.0 (-25.7 to 87.2)
Austria	Maternal hemorrhage	10 to 14	Both	0 (0 to 0)	0.0 (0.0 to 0.0)	0 (0 to 0)	0.0 (0.0 to 0.0)	0 (0 to 0)	0.0 (0.0 to 0.0)	-35.8 (-53.9 to -9.7)	-32.0 (-51.3 to -4.5)

eTable 8. All-cause and cause-specific mortality (counts and rates) at the global level and by country in the WHO European Region for ages 5 to 9 and 10 to 14 for 1990, 2000, and 2016

Austria	Maternal hemorrhage	10 to 14	Female	0 (0 to 0)	0.0 (0.0 to 0.0)	0 (0 to 0)	0.0 (0.0 to 0.0)	0 (0 to 0)	0.0 (0.0 to 0.0)	-35.8 (-53.9 to -9.7)	-32.2 (-51.4 to -4.7)
Austria	Maternal sepsis and other maternal infections	10 to 14	Both	0 (0 to 0)	0.0 (0.0 to 0.0)	0 (0 to 0)	0.0 (0.0 to 0.0)	0 (0 to 0)	0.0 (0.0 to 0.0)	-41.4 (-58.6 to -16.0)	-38.0 (-56.2 to -11.1)
Austria	Maternal sepsis and other maternal infections	10 to 14	Female	0 (0 to 0)	0.0 (0.0 to 0.0)	0 (0 to 0)	0.0 (0.0 to 0.0)	0 (0 to 0)	0.0 (0.0 to 0.0)	-41.4 (-58.6 to -16.0)	-38.1 (-56.3 to -11.3)
Austria	Maternal hypertensive disorders	10 to 14	Both	0 (0 to 0)	0.0 (0.0 to 0.0)	0 (0 to 0)	0.0 (0.0 to 0.0)	0 (0 to 0)	0.0 (0.0 to 0.0)	-35.9 (-54.0 to -9.0)	-32.1 (-51.3 to -3.8)
Austria	Maternal hypertensive disorders	10 to 14	Female	0 (0 to 0)	0.0 (0.0 to 0.0)	0 (0 to 0)	0.0 (0.0 to 0.0)	0 (0 to 0)	0.0 (0.0 to 0.0)	-35.9 (-54.0 to -9.0)	-32.3 (-51.4 to -3.9)
Austria	Maternal obstructed labor and uterine rupture	10 to 14	Both	0 (0 to 0)	0.0 (0.0 to 0.0)	0 (0 to 0)	0.0 (0.0 to 0.0)	0 (0 to 0)	0.0 (0.0 to 0.0)	-41.3 (-59.2 to -12.4)	-37.9 (-56.8 to -7.4)
Austria	Maternal obstructed labor and uterine rupture	10 to 14	Female	0 (0 to 0)	0.0 (0.0 to 0.0)	0 (0 to 0)	0.0 (0.0 to 0.0)	0 (0 to 0)	0.0 (0.0 to 0.0)	-41.3 (-59.2 to -12.4)	-38.1 (-56.9 to -7.5)
Austria	Maternal abortion, miscarriage, and ectopic pregnancy	10 to 14	Both	0 (0 to 0)	0.0 (0.0 to 0.0)	0 (0 to 0)	0.0 (0.0 to 0.0)	0 (0 to 0)	0.0 (0.0 to 0.0)	-41.0 (-58.5 to -14.9)	-37.6 (-56.1 to -10.0)
Austria	Maternal abortion, miscarriage, and ectopic pregnancy	10 to 14	Female	0 (0 to 0)	0.0 (0.0 to 0.0)	0 (0 to 0)	0.0 (0.0 to 0.0)	0 (0 to 0)	0.0 (0.0 to 0.0)	-41.0 (-58.5 to -14.9)	-37.7 (-56.2 to -10.2)
Austria	Indirect maternal deaths	10 to 14	Both	0 (0 to 0)	0.0 (0.0 to 0.0)	0 (0 to 0)	0.0 (0.0 to 0.0)	0 (0 to 0)	0.0 (0.0 to 0.0)	-37.9 (-54.1 to -15.8)	-34.3 (-51.4 to -11.0)
Austria	Indirect maternal deaths	10 to 14	Female	0 (0 to 0)	0.0 (0.0 to 0.0)	0 (0 to 0)	0.0 (0.0 to 0.0)	0 (0 to 0)	0.0 (0.0 to 0.0)	-37.9 (-54.1 to -15.8)	-34.4 (-51.5 to -11.1)
Austria	Late maternal deaths	10 to 14	Both	0 (0 to 0)	0.0 (0.0 to 0.0)	0 (0 to 0)	0.0 (0.0 to 0.0)	0 (0 to 0)	0.0 (0.0 to 0.0)	-45.0 (-82.7 to 30.4)	-41.9 (-81.7 to 38.0)
Austria	Late maternal deaths	10 to 14	Female	0 (0 to 0)	0.0 (0.0 to 0.0)	0 (0 to 0)	0.0 (0.0 to 0.0)	0 (0 to 0)	0.0 (0.0 to 0.0)	-45.0 (-82.7 to 30.4)	-42.0 (-81.7 to 37.7)
Austria	Maternal deaths aggravated by HIV/AIDS	10 to 14	Both	0 (0 to 0)	0.0 (0.0 to 0.0)	0 (0 to 0)	0.0 (0.0 to 0.0)	0 (0 to 0)	0.0 (0.0 to 0.0)	4714.2 (0.0 to 45076.2)	4992.8 (0.0 to 47691.3)
Austria	Maternal deaths aggravated by HIV/AIDS	10 to 14	Female	0 (0 to 0)	0.0 (0.0 to 0.0)	0 (0 to 0)	0.0 (0.0 to 0.0)	0 (0 to 0)	0.0 (0.0 to 0.0)	4714.2 (0.0 to 45076.2)	4983.1 (0.0 to 47599.6)
Austria	Other maternal disorders	10 to 14	Both	0 (0 to 0)	0.0 (0.0 to 0.0)	0 (0 to 0)	0.0 (0.0 to 0.0)	0 (0 to 0)	0.0 (0.0 to 0.0)	-39.5 (-54.0 to -17.7)	-36.0 (-51.3 to -13.0)
Austria	Other maternal disorders	10 to 14	Female	0 (0 to 0)	0.0 (0.0 to 0.0)	0 (0 to 0)	0.0 (0.0 to 0.0)	0 (0 to 0)	0.0 (0.0 to 0.0)	-39.5 (-54.0 to -17.7)	-36.1 (-51.4 to -13.2)
Austria	Protein-energy malnutrition	5 to 9	Both	0 (0 to 0)	0.0 (0.0 to 0.0)	0 (0 to 0)	0.0 (0.0 to 0.0)	0 (0 to 0)	0.0 (0.0 to 0.0)	-29.4 (-52.5 to 13.7)	-19.2 (-45.7 to 30.2)

eTable 8. All-cause and cause-specific mortality (counts and rates) at the global level and by country in the WHO European Region for ages 5 to 9 and 10 to 14 for 1990, 2000, and 2016

Austria	Protein-energy malnutrition	5 to 9	Female	0 (0 to 0)	0.0 (0.0 to 0.0)	0 (0 to 0)	0.0 (0.0 to 0.0)	0 (0 to 0)	0.0 (0.0 to 0.0)	-27.6 (-65.5 to 82.5)	-17.3 (-60.6 to 108.5)
Austria	Protein-energy malnutrition	5 to 9	Male	0 (0 to 0)	0.0 (0.0 to 0.0)	0 (0 to 0)	0.0 (0.0 to 0.0)	0 (0 to 0)	0.0 (0.0 to 0.0)	-31.1 (-60.5 to 80.8)	-21.0 (-54.6 to 107.4)
Austria	Protein-energy malnutrition	10 to 14	Both	0 (0 to 0)	0.0 (0.0 to 0.0)	0 (0 to 0)	0.0 (0.0 to 0.0)	0 (0 to 0)	0.0 (0.0 to 0.0)	-14.8 (-34.0 to 13.3)	-9.9 (-30.2 to 19.8)
Austria	Protein-energy malnutrition	10 to 14	Female	0 (0 to 0)	0.0 (0.0 to 0.0)	0 (0 to 0)	0.0 (0.0 to 0.0)	0 (0 to 0)	0.0 (0.0 to 0.0)	-13.7 (-43.1 to 36.2)	-8.8 (-39.9 to 43.8)
Austria	Protein-energy malnutrition	10 to 14	Male	0 (0 to 0)	0.0 (0.0 to 0.0)	0 (0 to 0)	0.0 (0.0 to 0.0)	0 (0 to 0)	0.0 (0.0 to 0.0)	-15.9 (-37.4 to 30.2)	-10.9 (-33.7 to 38.0)
Austria	Iodine deficiency	5 to 9	Both	0 (0 to 0)	0.0 (0.0 to 0.0)	0 (0 to 0)	0.0 (0.0 to 0.0)	0 (0 to 0)	0.0 (0.0 to 0.0)	-50.8 (-100.0 to 4.5e+17)	-43.7 (-100.0 to 5.2e+17)
Austria	Iodine deficiency	5 to 9	Female	0 (0 to 0)	0.0 (0.0 to 0.0)	0 (0 to 0)	0.0 (0.0 to 0.0)	0 (0 to 0)	0.0 (0.0 to 0.0)	-39.9 (-100.0 to 4.8e+24)	-31.3 (-100.0 to 5.5e+24)
Austria	Iodine deficiency	5 to 9	Male	0 (0 to 0)	0.0 (0.0 to 0.0)	0 (0 to 0)	0.0 (0.0 to 0.0)	0 (0 to 0)	0.0 (0.0 to 0.0)	-61.6 (-100.0 to 2.5e+26)	-55.9 (-100.0 to 2.9e+26)
Austria	Iodine deficiency	10 to 14	Both	0 (0 to 0)	0.0 (0.0 to 0.0)	0 (0 to 0)	0.0 (0.0 to 0.0)	0 (0 to 0)	0.0 (0.0 to 0.0)	-38.7 (-100.0 to 2.7e+19)	-35.1 (-100.0 to 2.9e+19)
Austria	Iodine deficiency	10 to 14	Female	0 (0 to 0)	0.0 (0.0 to 0.0)	0 (0 to 0)	0.0 (0.0 to 0.0)	0 (0 to 0)	0.0 (0.0 to 0.0)	-33.4 (-100.0 to 4.5e+24)	-29.7 (-100.0 to 4.8e+24)
Austria	Iodine deficiency	10 to 14	Male	0 (0 to 0)	0.0 (0.0 to 0.0)	0 (0 to 0)	0.0 (0.0 to 0.0)	0 (0 to 0)	0.0 (0.0 to 0.0)	-43.9 (-100.0 to 1.7e+26)	-40.5 (-100.0 to 1.8e+26)
Austria	Iron-deficiency anemia	5 to 9	Both	0 (0 to 0)	0.0 (0.0 to 0.0)	0 (0 to 0)	0.0 (0.0 to 0.0)	0 (0 to 0)	0.0 (0.0 to 0.0)	-29.4 (-52.5 to 13.7)	-19.2 (-45.7 to 30.2)
Austria	Iron-deficiency anemia	5 to 9	Female	0 (0 to 0)	0.0 (0.0 to 0.0)	0 (0 to 0)	0.0 (0.0 to 0.0)	0 (0 to 0)	0.0 (0.0 to 0.0)	-27.6 (-65.5 to 82.5)	-17.3 (-60.6 to 108.5)
Austria	Iron-deficiency anemia	5 to 9	Male	0 (0 to 0)	0.0 (0.0 to 0.0)	0 (0 to 0)	0.0 (0.0 to 0.0)	0 (0 to 0)	0.0 (0.0 to 0.0)	-31.1 (-60.5 to 80.8)	-21.0 (-54.6 to 107.4)
Austria	Iron-deficiency anemia	10 to 14	Both	0 (0 to 0)	0.0 (0.0 to 0.0)	0 (0 to 0)	0.0 (0.0 to 0.0)	0 (0 to 0)	0.0 (0.0 to 0.0)	-14.8 (-34.0 to 13.3)	-9.9 (-30.2 to 19.8)
Austria	Iron-deficiency anemia	10 to 14	Female	0 (0 to 0)	0.0 (0.0 to 0.0)	0 (0 to 0)	0.0 (0.0 to 0.0)	0 (0 to 0)	0.0 (0.0 to 0.0)	-13.7 (-43.1 to 36.2)	-8.8 (-39.9 to 43.8)
Austria	Iron-deficiency anemia	10 to 14	Male	0 (0 to 0)	0.0 (0.0 to 0.0)	0 (0 to 0)	0.0 (0.0 to 0.0)	0 (0 to 0)	0.0 (0.0 to 0.0)	-15.9 (-37.4 to 30.2)	-10.9 (-33.7 to 38.0)
Austria	Other nutritional deficiencies	5 to 9	Both	0 (0 to 0)	0.0 (0.0 to 0.0)	0 (0 to 0)	0.0 (0.0 to 0.0)	0 (0 to 0)	0.0 (0.0 to 0.0)	-29.5 (-52.5 to 13.1)	-19.3 (-45.7 to 29.5)
Austria	Other nutritional deficiencies	5 to 9	Female	0 (0 to 0)	0.0 (0.0 to 0.0)	0 (0 to 0)	0.0 (0.0 to 0.0)	0 (0 to 0)	0.0 (0.0 to 0.0)	-27.8 (-65.5 to 82.5)	-17.5 (-60.6 to 108.5)
Austria	Other nutritional deficiencies	5 to 9	Male	0 (0 to 0)	0.0 (0.0 to 0.0)	0 (0 to 0)	0.0 (0.0 to 0.0)	0 (0 to 0)	0.0 (0.0 to 0.0)	-31.1 (-60.5 to 80.8)	-21.0 (-54.6 to 107.4)
Austria	Other nutritional deficiencies	10 to 14	Both	0 (0 to 0)	0.0 (0.0 to 0.0)	0 (0 to 0)	0.0 (0.0 to 0.0)	0 (0 to 0)	0.0 (0.0 to 0.0)	-14.9 (-34.0 to 12.8)	-10.0 (-30.2 to 19.3)
Austria	Other nutritional deficiencies	10 to 14	Female	0 (0 to 0)	0.0 (0.0 to 0.0)	0 (0 to 0)	0.0 (0.0 to 0.0)	0 (0 to 0)	0.0 (0.0 to 0.0)	-13.9 (-43.1 to 36.2)	-9.1 (-39.9 to 43.8)
Austria	Other nutritional deficiencies	10 to 14	Male	0 (0 to 0)	0.0 (0.0 to 0.0)	0 (0 to 0)	0.0 (0.0 to 0.0)	0 (0 to 0)	0.0 (0.0 to 0.0)	-15.9 (-37.4 to 30.2)	-10.9 (-33.7 to 38.0)

eTable 8. All-cause and cause-specific mortality (counts and rates) at the global level and by country in the WHO European Region for ages 5 to 9 and 10 to 14 for 1990, 2000, and 2016

Austria	Sexually transmitted diseases excluding HIV	5 to 9	Both	0<br>(0 to 0)	0.0<br>(0.0 to 0.0)	0<br>(0 to 0)	0.0<br>(0.0 to 0.0)	0<br>(0 to 0)	0.0<br>(0.0 to 0.0)	-86.8<br>(-93.1 to -74.6)	-84.9<br>(-92.1 to -70.9)
Austria	Sexually transmitted diseases excluding HIV	5 to 9	Female	0<br>(0 to 0)	0.0<br>(0.0 to 0.0)	0<br>(0 to 0)	0.0<br>(0.0 to 0.0)	0<br>(0 to 0)	0.0<br>(0.0 to 0.0)	-86.5<br>(-93.2 to -73.8)	-84.6<br>(-92.2 to -70.0)
Austria	Sexually transmitted diseases excluding HIV	5 to 9	Male	0<br>(0 to 0)	0.0<br>(0.0 to 0.0)	0<br>(0 to 0)	0.0<br>(0.0 to 0.0)	0<br>(0 to 0)	0.0<br>(0.0 to 0.0)	-87.3<br>(-93.5 to -75.7)	-85.4<br>(-92.5 to -72.1)
Austria	Sexually transmitted diseases excluding HIV	10 to 14	Both	0<br>(0 to 0)	0.0<br>(0.0 to 0.0)	0<br>(0 to 0)	0.0<br>(0.0 to 0.0)	0<br>(0 to 0)	0.0<br>(0.0 to 0.0)	-29.2<br>(-42.8 to -10.8)	-25.1<br>(-39.5 to -5.6)
Austria	Sexually transmitted diseases excluding HIV	10 to 14	Female	0<br>(0 to 0)	0.0<br>(0.0 to 0.0)	0<br>(0 to 0)	0.0<br>(0.0 to 0.0)	0<br>(0 to 0)	0.0<br>(0.0 to 0.0)	-14.3<br>(-27.5 to 2.8)	-9.5<br>(-23.5 to 8.5)
Austria	Sexually transmitted diseases excluding HIV	10 to 14	Male	0<br>(0 to 0)	0.0<br>(0.0 to 0.0)	0<br>(0 to 0)	0.0<br>(0.0 to 0.0)	0<br>(0 to 0)	0.0<br>(0.0 to 0.0)	-89.4<br>(-95.6 to -73.3)	-88.8<br>(-95.4 to -71.7)
Austria	Hepatitis	5 to 9	Both	0<br>(0 to 0)	0.0<br>(0.0 to 0.0)	0<br>(0 to 0)	0.0<br>(0.0 to 0.0)	0<br>(0 to 0)	0.0<br>(0.0 to 0.0)	-33.6<br>(-49.6 to -15.1)	-24.0<br>(-42.3 to -2.8)
Austria	Hepatitis	5 to 9	Female	0<br>(0 to 0)	0.0<br>(0.0 to 0.0)	0<br>(0 to 0)	0.0<br>(0.0 to 0.0)	0<br>(0 to 0)	0.0<br>(0.0 to 0.0)	-34.5<br>(-56.1 to -12.4)	-25.2<br>(-49.9 to 0.1)
Austria	Hepatitis	5 to 9	Male	0<br>(0 to 0)	0.0<br>(0.0 to 0.0)	0<br>(0 to 0)	0.0<br>(0.0 to 0.0)	0<br>(0 to 0)	0.0<br>(0.0 to 0.0)	-32.6<br>(-47.6 to -13.0)	-22.7<br>(-39.9 to -0.3)
Austria	Hepatitis	10 to 14	Both	0<br>(0 to 0)	0.0<br>(0.0 to 0.0)	0<br>(0 to 0)	0.0<br>(0.0 to 0.0)	0<br>(0 to 0)	0.0<br>(0.0 to 0.0)	-16.3<br>(-28.1 to -3.1)	-11.5<br>(-24.0 to 2.5)
Austria	Hepatitis	10 to 14	Female	0<br>(0 to 0)	0.0<br>(0.0 to 0.0)	0<br>(0 to 0)	0.0<br>(0.0 to 0.0)	0<br>(0 to 0)	0.0<br>(0.0 to 0.0)	-16.0<br>(-30.9 to 1.8)	-11.3<br>(-27.1 to 7.5)
Austria	Hepatitis	10 to 14	Male	0<br>(0 to 0)	0.0<br>(0.0 to 0.0)	0<br>(0 to 0)	0.0<br>(0.0 to 0.0)	0<br>(0 to 0)	0.0<br>(0.0 to 0.0)	-16.6<br>(-30.8 to -0.5)	-11.7<br>(-26.7 to 5.4)
Austria	Other infectious diseases	5 to 9	Both	0<br>(0 to 0)	0.1<br>(0.1 to 0.1)	0<br>(0 to 1)	0.1<br>(0.0 to 0.1)	0<br>(0 to 1)	0.1<br>(0.0 to 0.1)	13.3<br>(-38.1 to 79.3)	29.7<br>(-29.1 to 105.2)
Austria	Other infectious diseases	5 to 9	Female	0<br>(0 to 0)	0.0<br>(0.0 to 0.1)	0<br>(0 to 0)	0.1<br>(0.0 to 0.1)	0<br>(0 to 0)	0.1<br>(0.0 to 0.2)	94.3<br>(-56.2 to 281.6)	122.0<br>(-50.0 to 335.9)
Austria	Other infectious diseases	5 to 9	Male	0<br>(0 to 0)	0.1<br>(0.1 to 0.1)	0<br>(0 to 0)	0.1<br>(0.1 to 0.1)	0<br>(0 to 0)	0.1<br>(0.1 to 0.2)	-16.3<br>(-50.7 to 37.7)	-4.0<br>(-43.4 to 57.9)
Austria	Other infectious diseases	10 to 14	Both	0<br>(0 to 0)	0.1<br>(0.0 to 0.1)	0<br>(0 to 0)	0.1<br>(0.0 to 0.1)	0<br>(0 to 1)	0.1<br>(0.1 to 0.1)	67.4<br>(-5.4 to 163.3)	77.0<br>(0.1 to 178.6)
Austria	Other infectious diseases	10 to 14	Female	0<br>(0 to 0)	0.0<br>(0.0 to 0.0)	0<br>(0 to 0)	0.1<br>(0.0 to 0.1)	0<br>(0 to 0)	0.1<br>(0.0 to 0.1)	150.2<br>(-42.7 to 443.2)	164.1<br>(-39.5 to 473.6)
Austria	Other infectious diseases	10 to 14	Male	0<br>(0 to 0)	0.1<br>(0.1 to 0.1)	0<br>(0 to 0)	0.1<br>(0.1 to 0.1)	0<br>(0 to 0)	0.1<br>(0.1 to 0.2)	47.0<br>(-17.6 to 143.0)	55.8<br>(-12.6 to 157.5)
Austria	Nasopharynx cancer	5 to 9	Both	0<br>(0 to 0)	0.0<br>(0.0 to 0.0)	0<br>(0 to 0)	0.0<br>(0.0 to 0.0)	0<br>(0 to 0)	0.0<br>(0.0 to 0.0)	-22.4<br>(-50.9 to 25.4)	-11.2<br>(-43.8 to 43.6)
Austria	Nasopharynx cancer	5 to 9	Female	0<br>(0 to 0)	0.0<br>(0.0 to 0.0)	0<br>(0 to 0)	0.0<br>(0.0 to 0.0)	0<br>(0 to 0)	0.0<br>(0.0 to 0.0)	-28.3<br>(-41.9 to -10.6)	-18.0<br>(-33.6 to 2.1)

eTable 8. All-cause and cause-specific mortality (counts and rates) at the global level and by country in the WHO European Region for ages 5 to 9 and 10 to 14 for 1990, 2000, and 2016

Austria	Nasopharynx cancer	5 to 9	Male	0 (0 to 0)	0.0 (0.0 to 0.0)	0 (0 to 0)	0.0 (0.0 to 0.0)	0 (0 to 0)	0.0 (0.0 to 0.0)	-19.3 (-59.2 to 56.3)	-7.4 (-53.2 to 79.3)
Austria	Nasopharynx cancer	10 to 14	Both	0 (0 to 0)	0.0 (0.0 to 0.0)	0 (0 to 0)	0.0 (0.0 to 0.0)	0 (0 to 0)	0.0 (0.0 to 0.0)	14.6 (-20.5 to 73.6)	21.3 (-15.9 to 83.6)
Austria	Nasopharynx cancer	10 to 14	Female	0 (0 to 0)	0.0 (0.0 to 0.0)	0 (0 to 0)	0.0 (0.0 to 0.0)	0 (0 to 0)	0.0 (0.0 to 0.0)	-14.3 (-27.5 to 2.8)	-9.5 (-23.5 to 8.5)
Austria	Nasopharynx cancer	10 to 14	Male	0 (0 to 0)	0.0 (0.0 to 0.0)	0 (0 to 0)	0.0 (0.0 to 0.0)	0 (0 to 0)	0.0 (0.0 to 0.0)	27.3 (-23.0 to 120.6)	34.9 (-18.4 to 133.8)
Austria	Liver cancer	5 to 9	Both	0 (0 to 0)	0.0 (0.0 to 0.1)	0 (0 to 0)	0.0 (0.0 to 0.1)	0 (0 to 0)	0.0 (0.0 to 0.0)	-49.8 (-68.6 to -21.9)	-42.6 (-64.1 to -10.6)
Austria	Liver cancer	5 to 9	Female	0 (0 to 0)	0.0 (0.0 to 0.1)	0 (0 to 0)	0.0 (0.0 to 0.1)	0 (0 to 0)	0.0 (0.0 to 0.0)	-38.2 (-66.8 to 12.2)	-29.4 (-62.1 to 28.1)
Austria	Liver cancer	5 to 9	Male	0 (0 to 0)	0.1 (0.0 to 0.1)	0 (0 to 0)	0.0 (0.0 to 0.1)	0 (0 to 0)	0.0 (0.0 to 0.0)	-56.7 (-78.5 to -21.4)	-50.3 (-75.3 to -9.8)
Austria	Liver cancer	10 to 14	Both	0 (0 to 0)	0.0 (0.0 to 0.1)	0 (0 to 0)	0.0 (0.0 to 0.1)	0 (0 to 0)	0.0 (0.0 to 0.0)	-29.2 (-57.5 to 16.0)	-25.1 (-55.0 to 22.7)
Austria	Liver cancer	10 to 14	Female	0 (0 to 0)	0.0 (0.0 to 0.0)	0 (0 to 0)	0.0 (0.0 to 0.0)	0 (0 to 0)	0.0 (0.0 to 0.0)	-28.9 (-62.0 to 31.8)	-24.9 (-59.8 to 39.2)
Austria	Liver cancer	10 to 14	Male	0 (0 to 0)	0.1 (0.0 to 0.1)	0 (0 to 0)	0.1 (0.0 to 0.1)	0 (0 to 0)	0.1 (0.0 to 0.1)	-29.3 (-62.8 to 26.5)	-25.1 (-60.6 to 34.1)
Austria	Kidney cancer	5 to 9	Both	1 (1 to 1)	0.2 (0.2 to 0.3)	1 (1 to 1)	0.2 (0.1 to 0.2)	0 (0 to 1)	0.1 (0.1 to 0.1)	-60.9 (-75.6 to -37.0)	-55.3 (-72.1 to -27.9)
Austria	Kidney cancer	5 to 9	Female	1 (0 to 1)	0.2 (0.1 to 0.3)	0 (0 to 1)	0.2 (0.1 to 0.2)	0 (0 to 0)	0.1 (0.1 to 0.2)	-52.3 (-75.2 to -12.5)	-45.5 (-71.6 to 0.0)
Austria	Kidney cancer	5 to 9	Male	1 (0 to 1)	0.2 (0.1 to 0.4)	0 (0 to 0)	0.1 (0.1 to 0.2)	0 (0 to 0)	0.1 (0.1 to 0.1)	-68.9 (-84.2 to -34.5)	-64.3 (-81.8 to -24.8)
Austria	Kidney cancer	10 to 14	Both	0 (0 to 1)	0.1 (0.1 to 0.1)	0 (0 to 1)	0.1 (0.1 to 0.1)	0 (0 to 0)	0.1 (0.0 to 0.1)	-45.2 (-63.6 to -18.1)	-42.1 (-61.5 to -13.4)
Austria	Kidney cancer	10 to 14	Female	0 (0 to 0)	0.1 (0.1 to 0.1)	0 (0 to 0)	0.1 (0.1 to 0.1)	0 (0 to 0)	0.1 (0.0 to 0.1)	-37.3 (-65.7 to 12.7)	-33.8 (-63.8 to 19.0)
Austria	Kidney cancer	10 to 14	Male	0 (0 to 0)	0.1 (0.1 to 0.2)	0 (0 to 0)	0.1 (0.1 to 0.1)	0 (0 to 0)	0.1 (0.0 to 0.1)	-52.2 (-71.1 to -19.0)	-49.4 (-69.3 to -14.2)
Austria	Brain and nervous system cancer	5 to 9	Both	6 (5 to 8)	1.4 (1.1 to 1.7)	6 (5 to 7)	1.2 (1.0 to 1.5)	4 (2 to 5)	0.9 (0.6 to 1.3)	-43.5 (-62.8 to -15.5)	-35.4 (-57.4 to -3.2)
Austria	Brain and nervous system cancer	5 to 9	Female	2 (2 to 3)	1.1 (0.8 to 1.4)	2 (2 to 3)	1.0 (0.7 to 1.4)	2 (1 to 3)	1.0 (0.6 to 1.4)	-20.1 (-54.6 to 27.4)	-8.8 (-48.1 to 45.6)
Austria	Brain and nervous system cancer	5 to 9	Male	4 (3 to 5)	1.6 (1.2 to 2.2)	3 (2 to 4)	1.3 (1.0 to 1.8)	2 (1 to 3)	0.8 (0.5 to 1.3)	-58.0 (-75.5 to -24.6)	-51.9 (-71.9 to -13.5)
Austria	Brain and nervous system cancer	10 to 14	Both	4 (3 to 5)	0.9 (0.7 to 1.2)	5 (4 to 6)	1.1 (0.8 to 1.3)	3 (2 to 4)	0.8 (0.6 to 1.0)	-19.5 (-44.7 to 15.7)	-14.8 (-41.5 to 22.4)
Austria	Brain and nervous system cancer	10 to 14	Female	2 (1 to 2)	0.8 (0.5 to 1.0)	2 (2 to 3)	0.9 (0.7 to 1.3)	2 (1 to 2)	0.8 (0.5 to 1.1)	-5.4 (-44.3 to 59.5)	-0.1 (-41.2 to 68.4)
Austria	Brain and nervous system cancer	10 to 14	Male	2 (2 to 3)	1.1 (0.8 to 1.5)	3 (2 to 4)	1.2 (0.8 to 1.6)	2 (1 to 3)	0.8 (0.5 to 1.2)	-28.7 (-55.9 to 11.4)	-24.4 (-53.3 to 18.1)
Austria	Thyroid cancer	10 to 14	Both	0 (0 to 0)	0.0 (0.0 to 0.0)	0 (0 to 0)	0.0 (0.0 to 0.0)	0 (0 to 0)	0.0 (0.0 to 0.0)	-15.5 (-26.9 to -2.6)	-10.6 (-22.7 to 3.0)
Austria	Thyroid cancer	10 to 14	Female	0 (0 to 0)	0.0 (0.0 to 0.0)	0 (0 to 0)	0.0 (0.0 to 0.0)	0 (0 to 0)	0.0 (0.0 to 0.0)	-14.3 (-27.5 to 2.8)	-9.5 (-23.5 to 8.5)
Austria	Thyroid cancer	10 to 14	Male	0 (0 to 0)	0.0 (0.0 to 0.0)	0 (0 to 0)	0.0 (0.0 to 0.0)	0 (0 to 0)	0.0 (0.0 to 0.0)	-16.6 (-30.8 to -0.5)	-11.7 (-26.7 to 5.4)
Austria	Hodgkin lymphoma	5 to 9	Both	0 (0 to 0)	0.1 (0.0 to 0.1)	0 (0 to 0)	0.0 (0.0 to 0.0)	0 (0 to 0)	0.0 (0.0 to 0.0)	-81.8 (-91.0 to -20.5)	-79.2 (-89.7 to -9.0)
Austria	Hodgkin lymphoma	5 to 9	Female	0 (0 to 0)	0.0 (0.0 to 0.0)	0 (0 to 0)	0.0 (0.0 to 0.0)	0 (0 to 0)	0.0 (0.0 to 0.0)	-61.1 (-77.7 to -19.0)	-55.6 (-74.5 to -7.5)
Austria	Hodgkin lymphoma	5 to 9	Male	0 (0 to 0)	0.1 (0.0 to 0.2)	0 (0 to 0)	0.0 (0.0 to 0.1)	0 (0 to 0)	0.0 (0.0 to 0.0)	-86.2 (-94.4 to -15.6)	-84.2 (-93.6 to -3.2)
Austria	Hodgkin lymphoma	10 to 14	Both	0 (0 to 0)	0.1 (0.0 to 0.1)	0 (0 to 0)	0.0 (0.0 to 0.0)	0 (0 to 0)	0.0 (0.0 to 0.0)	-79.3 (-87.4 to -56.8)	-78.1 (-86.6 to -54.3)

eTable 8. All-cause and cause-specific mortality (counts and rates) at the global level and by country in the WHO European Region for ages 5 to 9 and 10 to 14 for 1990, 2000, and 2016

Austria	Hodgkin lymphoma	10 to 14	Female	0 (0 to 0)	0.0 (0.0 to 0.0)	0 (0 to 0)	0.0 (0.0 to 0.0)	0 (0 to 0)	0.0 (0.0 to 0.0)	-66.4 (-78.9 to -41.7)	-64.6 (-77.8 to -38.4)
Austria	Hodgkin lymphoma	10 to 14	Male	0 (0 to 0)	0.1 (0.0 to 0.2)	0 (0 to 0)	0.1 (0.0 to 0.1)	0 (0 to 0)	0.0 (0.0 to 0.0)	-82.2 (-90.6 to -58.5)	-81.2 (-90.1 to -56.1)
Austria	Non-Hodgkin lymphoma	5 to 9	Both	1 (1 to 2)	0.3 (0.2 to 0.4)	1 (1 to 2)	0.3 (0.2 to 0.3)	1 (0 to 1)	0.1 (0.1 to 0.2)	-59.7 (-75.4 to -27.9)	-53.8 (-71.9 to -17.4)
Austria	Non-Hodgkin lymphoma	5 to 9	Female	0 (0 to 0)	0.1 (0.1 to 0.2)	0 (0 to 0)	0.1 (0.1 to 0.2)	0 (0 to 0)	0.1 (0.0 to 0.1)	-51.5 (-74.7 to -12.6)	-44.6 (-71.1 to -0.2)
Austria	Non-Hodgkin lymphoma	5 to 9	Male	1 (1 to 1)	0.4 (0.3 to 0.6)	1 (1 to 1)	0.4 (0.2 to 0.5)	0 (0 to 1)	0.2 (0.1 to 0.3)	-62.2 (-79.8 to -20.5)	-56.6 (-76.8 to -8.8)
Austria	Non-Hodgkin lymphoma	10 to 14	Both	1 (1 to 1)	0.3 (0.2 to 0.3)	1 (1 to 2)	0.3 (0.2 to 0.3)	1 (0 to 1)	0.1 (0.1 to 0.2)	-44.9 (-66.7 to -11.4)	-41.7 (-64.8 to -6.3)
Austria	Non-Hodgkin lymphoma	10 to 14	Female	0 (0 to 0)	0.1 (0.1 to 0.2)	0 (0 to 1)	0.2 (0.1 to 0.2)	0 (0 to 0)	0.1 (0.0 to 0.1)	-41.5 (-69.5 to 5.7)	-38.3 (-67.8 to 11.6)
Austria	Non-Hodgkin lymphoma	10 to 14	Male	1 (1 to 1)	0.4 (0.2 to 0.5)	1 (1 to 1)	0.4 (0.2 to 0.5)	0 (0 to 1)	0.2 (0.1 to 0.3)	-46.1 (-71.5 to -5.4)	-42.9 (-69.8 to 0.2)
Austria	Leukemia	5 to 9	Both	5 (4 to 7)	1.1 (0.9 to 1.4)	4 (3 to 5)	0.8 (0.7 to 1.0)	2 (2 to 3)	0.6 (0.4 to 0.8)	-55.5 (-69.7 to -35.4)	-49.1 (-65.3 to -26.0)
Austria	Leukemia	5 to 9	Female	2 (2 to 3)	0.9 (0.7 to 1.2)	2 (1 to 2)	0.8 (0.6 to 1.0)	1 (1 to 2)	0.6 (0.4 to 0.9)	-44.7 (-66.4 to -8.2)	-36.8 (-61.6 to 4.8)
Austria	Leukemia	5 to 9	Male	3 (2 to 4)	1.3 (0.9 to 1.8)	2 (2 to 3)	0.9 (0.7 to 1.2)	1 (1 to 2)	0.6 (0.3 to 0.8)	-62.9 (-78.2 to -39.2)	-57.4 (-75.0 to -30.2)
Austria	Leukemia	10 to 14	Both	5 (4 to 6)	1.0 (0.8 to 1.3)	5 (4 to 6)	1.0 (0.8 to 1.2)	3 (2 to 4)	0.7 (0.5 to 0.9)	-40.5 (-57.6 to -15.4)	-37.1 (-55.1 to -10.6)
Austria	Leukemia	10 to 14	Female	2 (1 to 2)	0.8 (0.6 to 1.2)	2 (2 to 3)	0.9 (0.7 to 1.2)	1 (1 to 2)	0.6 (0.4 to 0.8)	-35.9 (-61.1 to 5.6)	-32.3 (-58.9 to 11.5)
Austria	Leukemia	10 to 14	Male	3 (2 to 4)	1.2 (0.9 to 1.6)	2 (2 to 3)	1.0 (0.7 to 1.3)	2 (1 to 2)	0.7 (0.5 to 1.0)	-43.5 (-63.7 to -11.1)	-40.1 (-61.6 to -5.7)
Austria	Other neoplasms	5 to 9	Both	4 (3 to 5)	0.8 (0.6 to 1.0)	3 (2 to 3)	0.6 (0.4 to 0.7)	2 (1 to 3)	0.5 (0.3 to 0.7)	-48.7 (-65.4 to -23.3)	-41.3 (-60.3 to -12.2)
Austria	Other neoplasms	5 to 9	Female	2 (1 to 2)	0.7 (0.5 to 1.0)	1 (1 to 2)	0.5 (0.4 to 0.7)	1 (1 to 1)	0.5 (0.3 to 0.8)	-39.5 (-64.1 to 3.6)	-30.9 (-59.0 to 18.4)
Austria	Other neoplasms	5 to 9	Male	2 (1 to 3)	0.9 (0.6 to 1.2)	1 (1 to 2)	0.6 (0.4 to 0.9)	1 (1 to 1)	0.4 (0.3 to 0.7)	-55.8 (-75.4 to -26.1)	-49.3 (-71.8 to -15.3)
Austria	Other neoplasms	10 to 14	Both	3 (3 to 4)	0.8 (0.6 to 1.0)	3 (3 to 4)	0.7 (0.6 to 0.9)	2 (2 to 3)	0.5 (0.4 to 0.7)	-37.1 (-55.2 to -10.1)	-33.5 (-52.6 to -4.9)
Austria	Other neoplasms	10 to 14	Female	2 (1 to 2)	0.7 (0.5 to 1.0)	2 (1 to 2)	0.7 (0.5 to 1.0)	1 (1 to 2)	0.5 (0.4 to 0.8)	-30.3 (-58.3 to 11.6)	-26.4 (-55.9 to 17.8)
Austria	Other neoplasms	10 to 14	Male	2 (1 to 3)	0.9 (0.6 to 1.2)	2 (1 to 2)	0.7 (0.5 to 1.0)	1 (1 to 2)	0.5 (0.3 to 0.8)	-42.6 (-64.2 to -10.5)	-39.1 (-62.0 to -5.2)
Austria	Rheumatic heart disease	5 to 9	Both	0 (0 to 0)	0.0 (0.0 to 0.1)	0 (0 to 0)	0.0 (0.0 to 0.0)	0 (0 to 0)	0.0 (0.0 to 0.0)	-78.8 (-88.6 to -64.9)	-75.7 (-86.9 to -59.9)
Austria	Rheumatic heart disease	5 to 9	Female	0 (0 to 0)	0.0 (0.0 to 0.0)	0 (0 to 0)	0.0 (0.0 to 0.0)	0 (0 to 0)	0.0 (0.0 to 0.0)	-64.8 (-83.2 to -39.0)	-59.7 (-80.8 to -30.3)
Austria	Rheumatic heart disease	5 to 9	Male	0 (0 to 0)	0.1 (0.0 to 0.1)	0 (0 to 0)	0.0 (0.0 to 0.1)	0 (0 to 0)	0.0 (0.0 to 0.0)	-84.1 (-92.5 to -68.2)	-81.8 (-91.4 to -63.6)
Austria	Rheumatic heart disease	10 to 14	Both	0 (0 to 0)	0.0 (0.0 to 0.0)	0 (0 to 0)	0.0 (0.0 to 0.0)	0 (0 to 0)	0.0 (0.0 to 0.0)	-59.9 (-78.0 to -34.2)	-57.5 (-76.7 to -30.4)
Austria	Rheumatic heart disease	10 to 14	Female	0 (0 to 0)	0.0 (0.0 to 0.0)	0 (0 to 0)	0.0 (0.0 to 0.0)	0 (0 to 0)	0.0 (0.0 to 0.0)	-43.7 (-70.3 to 0.1)	-40.6 (-68.7 to 5.7)
Austria	Rheumatic heart disease	10 to 14	Male	0 (0 to 0)	0.0 (0.0 to 0.1)	0 (0 to 0)	0.0 (0.0 to 0.0)	0 (0 to 0)	0.0 (0.0 to 0.0)	-67.0 (-84.8 to -39.8)	-65.0 (-83.9 to -36.2)
Austria	Ischemic heart disease	5 to 9	Both	0 (0 to 0)	0.0 (0.0 to 0.0)	0 (0 to 0)	0.0 (0.0 to 0.0)	0 (0 to 0)	0.0 (0.0 to 0.0)	-44.6 (-68.4 to -11.5)	-36.6 (-63.9 to 1.3)
Austria	Ischemic heart disease	5 to 9	Female	0 (0 to 0)	0.0 (0.0 to 0.0)	0 (0 to 0)	0.0 (0.0 to 0.0)	0 (0 to 0)	0.0 (0.0 to 0.0)	-50.3 (-76.0 to -6.3)	-43.3 (-72.6 to 7.0)
Austria	Ischemic heart disease	5 to 9	Male	0 (0 to 0)	0.0 (0.0 to 0.0)	0 (0 to 0)	0.0 (0.0 to 0.0)	0 (0 to 0)	0.0 (0.0 to 0.0)	-36.1 (-67.1 to 7.6)	-26.7 (-62.3 to 23.4)
Austria	Ischemic heart disease	10 to 14	Both	0 (0 to 0)	0.0 (0.0 to 0.1)	0 (0 to 0)	0.0 (0.0 to 0.1)	0 (0 to 0)	0.0 (0.0 to 0.0)	-52.4 (-74.6 to -21.0)	-49.7 (-73.2 to -16.4)
Austria	Ischemic heart disease	10 to 14	Female	0 (0 to 0)	0.0 (0.0 to 0.1)	0 (0 to 0)	0.0 (0.0 to 0.1)	0 (0 to 0)	0.0 (0.0 to 0.0)	-37.6 (-69.6 to 17.0)	-34.1 (-67.9 to 23.6)
Austria	Ischemic heart disease	10 to 14	Male	0 (0 to 0)	0.0 (0.0 to 0.1)	0 (0 to 0)	0.0 (0.0 to 0.1)	0 (0 to 0)	0.0 (0.0 to 0.0)	-65.4 (-85.4 to -26.6)	-63.3 (-84.5 to -22.2)

eTable 8. All-cause and cause-specific mortality (counts and rates) at the global level and by country in the WHO European Region for ages 5 to 9 and 10 to 14 for 1990, 2000, and 2016

Austria	Cerebrovascular disease	5 to 9	Both	1<br>(1 to 2)	0.3<br>(0.2 to 0.4)	1<br>(0 to 1)	0.1<br>(0.1 to 0.2)	0<br>(0 to 0)	0.0<br>(0.0 to 0.1)	-84.5<br>(-91.5 to -76.5)	-82.3<br>(-90.2 to -73.1)
Austria	Cerebrovascular disease	5 to 9	Female	1<br>(0 to 1)	0.3<br>(0.2 to 0.3)	0<br>(0 to 1)	0.2<br>(0.1 to 0.2)	0<br>(0 to 0)	0.1<br>(0.0 to 0.1)	-77.8<br>(-88.5 to -66.1)	-74.7<br>(-86.9 to -61.3)
Austria	Cerebrovascular disease	5 to 9	Male	1<br>(0 to 1)	0.3<br>(0.2 to 0.4)	0<br>(0 to 0)	0.1<br>(0.1 to 0.2)	0<br>(0 to 0)	0.0<br>(0.0 to 0.1)	-90.0<br>(-95.2 to -80.9)	-88.5<br>(-94.5 to -78.1)
Austria	Cerebrovascular disease	10 to 14	Both	2<br>(1 to 2)	0.4<br>(0.3 to 0.5)	2<br>(1 to 2)	0.3<br>(0.2 to 0.4)	0<br>(0 to 1)	0.1<br>(0.1 to 0.2)	-72.1<br>(-83.9 to -60.2)	-70.5<br>(-83.0 to -57.9)
Austria	Cerebrovascular disease	10 to 14	Female	1<br>(0 to 1)	0.3<br>(0.2 to 0.4)	1<br>(0 to 1)	0.3<br>(0.2 to 0.4)	0<br>(0 to 0)	0.1<br>(0.1 to 0.2)	-68.0<br>(-82.5 to -47.7)	-66.2<br>(-81.5 to -44.8)
Austria	Cerebrovascular disease	10 to 14	Male	1<br>(1 to 1)	0.5<br>(0.3 to 0.6)	1<br>(0 to 1)	0.3<br>(0.2 to 0.5)	0<br>(0 to 0)	0.1<br>(0.1 to 0.2)	-74.5<br>(-87.3 to -58.5)	-73.0<br>(-86.5 to -56.0)
Austria	Hypertensive heart disease	5 to 9	Both	0<br>(0 to 0)	0.0<br>(0.0 to 0.0)	0<br>(0 to 0)	0.0<br>(0.0 to 0.0)	0<br>(0 to 0)	0.0<br>(0.0 to 0.0)	-28.7<br>(-59.1 to 4.6)	-18.4<br>(-53.2 to 19.8)
Austria	Hypertensive heart disease	5 to 9	Female	0<br>(0 to 0)	0.0<br>(0.0 to 0.0)	0<br>(0 to 0)	0.0<br>(0.0 to 0.0)	0<br>(0 to 0)	0.0<br>(0.0 to 0.0)	-19.2<br>(-57.0 to 29.0)	-7.7<br>(-50.9 to 47.3)
Austria	Hypertensive heart disease	5 to 9	Male	0<br>(0 to 0)	0.0<br>(0.0 to 0.0)	0<br>(0 to 0)	0.0<br>(0.0 to 0.0)	0<br>(0 to 0)	0.0<br>(0.0 to 0.0)	-36.1<br>(-67.1 to 7.6)	-26.7<br>(-62.3 to 23.4)
Austria	Hypertensive heart disease	10 to 14	Both	0<br>(0 to 0)	0.0<br>(0.0 to 0.0)	0<br>(0 to 0)	0.0<br>(0.0 to 0.0)	0<br>(0 to 0)	0.0<br>(0.0 to 0.0)	1.6<br>(-40.0 to 46.9)	7.5<br>(-36.5 to 55.4)
Austria	Hypertensive heart disease	10 to 14	Female	0<br>(0 to 0)	0.0<br>(0.0 to 0.0)	0<br>(0 to 0)	0.0<br>(0.0 to 0.0)	0<br>(0 to 0)	0.0<br>(0.0 to 0.0)	2.2<br>(-41.2 to 62.5)	7.9<br>(-37.9 to 71.6)
Austria	Hypertensive heart disease	10 to 14	Male	0<br>(0 to 0)	0.0<br>(0.0 to 0.0)	0<br>(0 to 0)	0.0<br>(0.0 to 0.0)	0<br>(0 to 0)	0.0<br>(0.0 to 0.0)	1.1<br>(-47.9 to 61.4)	7.1<br>(-44.8 to 71.1)
Austria	Cardiomyopathy and myocarditis	5 to 9	Both	1<br>(1 to 1)	0.2<br>(0.1 to 0.3)	0<br>(0 to 1)	0.1<br>(0.1 to 0.2)	0<br>(0 to 1)	0.1<br>(0.0 to 0.1)	-75.2<br>(-88.5 to -53.5)	-71.6<br>(-86.8 to -46.7)
Austria	Cardiomyopathy and myocarditis	5 to 9	Female	0<br>(0 to 1)	0.2<br>(0.1 to 0.3)	0<br>(0 to 1)	0.1<br>(0.0 to 0.2)	0<br>(0 to 0)	0.1<br>(0.0 to 0.2)	-66.2<br>(-85.7 to -40.5)	-61.3<br>(-83.7 to -32.1)
Austria	Cardiomyopathy and myocarditis	5 to 9	Male	0<br>(0 to 1)	0.2<br>(0.1 to 0.4)	0<br>(0 to 1)	0.1<br>(0.0 to 0.2)	0<br>(0 to 0)	0.0<br>(0.0 to 0.1)	-81.6<br>(-92.8 to -54.9)	-78.9<br>(-91.8 to -48.2)
Austria	Cardiomyopathy and myocarditis	10 to 14	Both	1<br>(1 to 2)	0.2<br>(0.1 to 0.4)	1<br>(0 to 2)	0.2<br>(0.1 to 0.4)	0<br>(0 to 1)	0.1<br>(0.0 to 0.2)	-60.6<br>(-79.6 to -33.0)	-58.4<br>(-78.4 to -29.1)
Austria	Cardiomyopathy and myocarditis	10 to 14	Female	0<br>(0 to 1)	0.2<br>(0.1 to 0.3)	0<br>(0 to 1)	0.2<br>(0.1 to 0.3)	0<br>(0 to 0)	0.1<br>(0.0 to 0.2)	-59.6<br>(-83.7 to -18.8)	-57.3<br>(-82.7 to -14.2)
Austria	Cardiomyopathy and myocarditis	10 to 14	Male	1<br>(0 to 1)	0.3<br>(0.1 to 0.5)	0<br>(0 to 1)	0.2<br>(0.1 to 0.4)	0<br>(0 to 1)	0.1<br>(0.0 to 0.3)	-61.4<br>(-82.7 to -33.9)	-59.1<br>(-81.6 to -29.9)
Austria	Atrial fibrillation and flutter	5 to 9	Both	0<br>(0 to 0)	0.0<br>(0.0 to 0.0)	0<br>(0 to 0)	0.0<br>(0.0 to 0.0)	0<br>(0 to 0)	0.0<br>(0.0 to 0.0)	0.0<br>(0.0 to 0.0)	0.0<br>(0.0 to 0.0)
Austria	Atrial fibrillation and flutter	5 to 9	Female	0<br>(0 to 0)	0.0<br>(0.0 to 0.0)	0<br>(0 to 0)	0.0<br>(0.0 to 0.0)	0<br>(0 to 0)	0.0<br>(0.0 to 0.0)	0.0<br>(0.0 to 0.0)	0.0<br>(0.0 to 0.0)
Austria	Atrial fibrillation and flutter	5 to 9	Male	0<br>(0 to 0)	0.0<br>(0.0 to 0.0)	0<br>(0 to 0)	0.0<br>(0.0 to 0.0)	0<br>(0 to 0)	0.0<br>(0.0 to 0.0)	0.0<br>(0.0 to 0.0)	0.0<br>(0.0 to 0.0)
Austria	Atrial fibrillation and flutter	10 to 14	Both	0<br>(0 to 0)	0.0<br>(0.0 to 0.0)	0<br>(0 to 0)	0.0<br>(0.0 to 0.0)	0<br>(0 to 0)	0.0<br>(0.0 to 0.0)	0.0<br>(0.0 to 0.0)	0.0<br>(0.0 to 0.0)
Austria	Atrial fibrillation and flutter	10 to 14	Female	0<br>(0 to 0)	0.0<br>(0.0 to 0.0)	0<br>(0 to 0)	0.0<br>(0.0 to 0.0)	0<br>(0 to 0)	0.0<br>(0.0 to 0.0)	0.0<br>(0.0 to 0.0)	0.0<br>(0.0 to 0.0)
Austria	Atrial fibrillation and flutter	10 to 14	Male	0<br>(0 to 0)	0.0<br>(0.0 to 0.0)	0<br>(0 to 0)	0.0<br>(0.0 to 0.0)	0<br>(0 to 0)	0.0<br>(0.0 to 0.0)	0.0<br>(0.0 to 0.0)	0.0<br>(0.0 to 0.0)
Austria	Endocarditis	5 to 9	Both	0<br>(0 to 0)	0.0<br>(0.0 to 0.1)	0<br>(0 to 0)	0.0<br>(0.0 to 0.0)	0<br>(0 to 0)	0.0<br>(0.0 to 0.0)	-62.4<br>(-82.6 to -15.4)	-57.0<br>(-80.0 to -3.2)
Austria	Endocarditis	5 to 9	Female	0<br>(0 to 0)	0.0<br>(0.0 to 0.0)	0<br>(0 to 0)	0.0<br>(0.0 to 0.0)	0<br>(0 to 0)	0.0<br>(0.0 to 0.0)	-45.8<br>(-76.8 to 3.3)	-38.0<br>(-73.5 to 18.0)

eTable 8. All-cause and cause-specific mortality (counts and rates) at the global level and by country in the WHO European Region for ages 5 to 9 and 10 to 14 for 1990, 2000, and 2016

Austria	Endocarditis	5 to 9	Male	0 (0 to 0)	0.0 (0.0 to 0.1)	0 (0 to 0)	0.0 (0.0 to 0.0)	0 (0 to 0)	0.0 (0.0 to 0.0)	-72.0 (-88.5 to -7.3)	-67.9 (-86.8 to 6.3)
Austria	Endocarditis	10 to 14	Both	0 (0 to 0)	0.0 (0.0 to 0.0)	0 (0 to 0)	0.0 (0.0 to 0.0)	0 (0 to 0)	0.0 (0.0 to 0.0)	-41.0 (-68.8 to 9.9)	-37.6 (-67.0 to 16.2)
Austria	Endocarditis	10 to 14	Female	0 (0 to 0)	0.0 (0.0 to 0.0)	0 (0 to 0)	0.0 (0.0 to 0.0)	0 (0 to 0)	0.0 (0.0 to 0.0)	-33.1 (-71.9 to 37.9)	-29.3 (-70.3 to 45.6)
Austria	Endocarditis	10 to 14	Male	0 (0 to 0)	0.0 (0.0 to 0.1)	0 (0 to 0)	0.0 (0.0 to 0.0)	0 (0 to 0)	0.0 (0.0 to 0.0)	-47.2 (-75.1 to 19.8)	-44.0 (-73.6 to 27.0)
Austria	Other cardiovascular and circulatory diseases	5 to 9	Both	1 (0 to 1)	0.1 (0.1 to 0.2)	0 (0 to 0)	0.1 (0.0 to 0.1)	0 (0 to 0)	0.0 (0.0 to 0.1)	-69.6 (-85.0 to -48.9)	-65.2 (-82.8 to -41.5)
Austria	Other cardiovascular and circulatory diseases	5 to 9	Female	0 (0 to 0)	0.1 (0.0 to 0.1)	0 (0 to 0)	0.1 (0.0 to 0.1)	0 (0 to 0)	0.0 (0.0 to 0.1)	-52.0 (-76.5 to -15.5)	-45.2 (-73.2 to -3.5)
Austria	Other cardiovascular and circulatory diseases	5 to 9	Male	0 (0 to 1)	0.1 (0.1 to 0.2)	0 (0 to 0)	0.1 (0.1 to 0.1)	0 (0 to 0)	0.0 (0.0 to 0.1)	-77.7 (-90.6 to -56.8)	-74.4 (-89.2 to -50.5)
Austria	Other cardiovascular and circulatory diseases	10 to 14	Both	1 (0 to 1)	0.1 (0.1 to 0.2)	1 (0 to 1)	0.1 (0.1 to 0.2)	0 (0 to 0)	0.1 (0.0 to 0.1)	-45.6 (-69.0 to -17.9)	-42.5 (-67.2 to -13.2)
Austria	Other cardiovascular and circulatory diseases	10 to 14	Female	0 (0 to 0)	0.1 (0.1 to 0.2)	0 (0 to 1)	0.2 (0.1 to 0.2)	0 (0 to 0)	0.1 (0.0 to 0.1)	-36.4 (-67.0 to 9.5)	-32.8 (-65.1 to 15.6)
Austria	Other cardiovascular and circulatory diseases	10 to 14	Male	0 (0 to 0)	0.1 (0.1 to 0.2)	0 (0 to 0)	0.1 (0.1 to 0.2)	0 (0 to 0)	0.1 (0.0 to 0.1)	-54.0 (-78.2 to -15.1)	-51.3 (-76.9 to -10.0)
Austria	Chronic obstructive pulmonary disease	5 to 9	Both	0 (0 to 1)	0.1 (0.1 to 0.1)	0 (0 to 0)	0.1 (0.0 to 0.1)	0 (0 to 0)	0.0 (0.0 to 0.1)	-55.8 (-71.4 to -30.2)	-49.4 (-67.3 to -20.1)
Austria	Chronic obstructive pulmonary disease	5 to 9	Female	0 (0 to 0)	0.1 (0.0 to 0.1)	0 (0 to 0)	0.1 (0.0 to 0.1)	0 (0 to 0)	0.1 (0.0 to 0.1)	-27.3 (-60.4 to 29.1)	-17.0 (-54.8 to 47.5)
Austria	Chronic obstructive pulmonary disease	5 to 9	Male	0 (0 to 0)	0.1 (0.1 to 0.1)	0 (0 to 0)	0.1 (0.0 to 0.1)	0 (0 to 0)	0.0 (0.0 to 0.0)	-72.7 (-85.8 to -52.3)	-68.7 (-83.8 to -45.3)
Austria	Chronic obstructive pulmonary disease	10 to 14	Both	0 (0 to 0)	0.0 (0.0 to 0.1)	0 (0 to 1)	0.1 (0.1 to 0.1)	0 (0 to 0)	0.1 (0.0 to 0.1)	8.1 (-33.3 to 73.3)	14.3 (-29.5 to 83.3)
Austria	Chronic obstructive pulmonary disease	10 to 14	Female	0 (0 to 0)	0.1 (0.0 to 0.1)	0 (0 to 0)	0.1 (0.1 to 0.2)	0 (0 to 0)	0.1 (0.0 to 0.1)	22.8 (-32.4 to 129.9)	29.7 (-28.7 to 142.8)
Austria	Chronic obstructive pulmonary disease	10 to 14	Male	0 (0 to 0)	0.0 (0.0 to 0.1)	0 (0 to 0)	0.1 (0.0 to 0.1)	0 (0 to 0)	0.0 (0.0 to 0.1)	-10.9 (-64.2 to 74.7)	-5.5 (-62.1 to 85.1)
Austria	Asthma	5 to 9	Both	0 (0 to 1)	0.1 (0.1 to 0.1)	0 (0 to 0)	0.0 (0.0 to 0.1)	0 (0 to 0)	0.0 (0.0 to 0.0)	-86.1 (-92.4 to -73.2)	-84.1 (-91.3 to -69.3)

eTable 8. All-cause and cause-specific mortality (counts and rates) at the global level and by country in the WHO European Region for ages 5 to 9 and 10 to 14 for 1990, 2000, and 2016

Austria	Asthma	5 to 9	Female	0 (0 to 0)	0.1 (0.0 to 0.1)	0 (0 to 0)	0.0 (0.0 to 0.1)	0 (0 to 0)	0.0 (0.0 to 0.0)	-79.4 (-90.2 to -59.5)	-76.4 (-88.8 to -53.7)
Austria	Asthma	5 to 9	Male	0 (0 to 0)	0.1 (0.1 to 0.2)	0 (0 to 0)	0.0 (0.0 to 0.1)	0 (0 to 0)	0.0 (0.0 to 0.0)	-90.1 (-95.6 to -72.1)	-88.7 (-94.9 to -67.9)
Austria	Asthma	10 to 14	Both	1 (1 to 1)	0.2 (0.1 to 0.2)	1 (0 to 1)	0.1 (0.1 to 0.2)	0 (0 to 0)	0.0 (0.0 to 0.1)	-76.4 (-86.6 to -52.5)	-75.1 (-85.8 to -49.8)
Austria	Asthma	10 to 14	Female	0 (0 to 0)	0.1 (0.1 to 0.2)	0 (0 to 0)	0.1 (0.1 to 0.2)	0 (0 to 0)	0.0 (0.0 to 0.1)	-65.9 (-82.4 to -38.7)	-64.0 (-81.4 to -35.3)
Austria	Asthma	10 to 14	Male	0 (0 to 1)	0.2 (0.2 to 0.3)	0 (0 to 1)	0.1 (0.1 to 0.2)	0 (0 to 0)	0.0 (0.0 to 0.1)	-82.3 (-92.3 to -49.1)	-81.2 (-91.8 to -46.0)
Austria	Interstitial lung disease and pulmonary sarcoidosis	5 to 9	Both	0 (0 to 0)	0.0 (0.0 to 0.0)	0 (0 to 0)	0.0 (0.0 to 0.0)	0 (0 to 0)	0.0 (0.0 to 0.0)	-33.1 (-62.9 to 17.1)	-23.4 (-57.5 to 34.1)
Austria	Interstitial lung disease and pulmonary sarcoidosis	5 to 9	Female	0 (0 to 0)	0.0 (0.0 to 0.0)	0 (0 to 0)	0.0 (0.0 to 0.0)	0 (0 to 0)	0.0 (0.0 to 0.0)	-11.9 (-57.7 to 81.4)	0.6 (-51.7 to 107.2)
Austria	Interstitial lung disease and pulmonary sarcoidosis	5 to 9	Male	0 (0 to 0)	0.0 (0.0 to 0.0)	0 (0 to 0)	0.0 (0.0 to 0.0)	0 (0 to 0)	0.0 (0.0 to 0.0)	-60.5 (-83.9 to 11.4)	-54.7 (-81.5 to 27.7)
Austria	Interstitial lung disease and pulmonary sarcoidosis	10 to 14	Both	0 (0 to 0)	0.0 (0.0 to 0.0)	0 (0 to 0)	0.0 (0.0 to 0.0)	0 (0 to 0)	0.0 (0.0 to 0.0)	35.6 (-28.9 to 130.8)	43.4 (-24.8 to 144.1)
Austria	Interstitial lung disease and pulmonary sarcoidosis	10 to 14	Female	0 (0 to 0)	0.0 (0.0 to 0.0)	0 (0 to 0)	0.0 (0.0 to 0.0)	0 (0 to 0)	0.0 (0.0 to 0.0)	51.4 (-27.7 to 208.4)	59.8 (-23.6 to 225.6)
Austria	Interstitial lung disease and pulmonary sarcoidosis	10 to 14	Male	0 (0 to 0)	0.0 (0.0 to 0.0)	0 (0 to 0)	0.0 (0.0 to 0.0)	0 (0 to 0)	0.0 (0.0 to 0.0)	20.9 (-55.7 to 196.8)	28.1 (-53.1 to 214.6)
Austria	Other chronic respiratory diseases	5 to 9	Both	0 (0 to 0)	0.0 (0.0 to 0.0)	0 (0 to 0)	0.0 (0.0 to 0.0)	0 (0 to 0)	0.0 (0.0 to 0.0)	-33.3 (-61.9 to 9.6)	-23.6 (-56.4 to 25.5)
Austria	Other chronic respiratory diseases	5 to 9	Female	0 (0 to 0)	0.0 (0.0 to 0.1)	0 (0 to 0)	0.0 (0.0 to 0.1)	0 (0 to 0)	0.0 (0.0 to 0.0)	-26.0 (-64.6 to 48.3)	-15.4 (-59.6 to 69.4)
Austria	Other chronic respiratory diseases	5 to 9	Male	0 (0 to 0)	0.0 (0.0 to 0.1)	0 (0 to 0)	0.0 (0.0 to 0.1)	0 (0 to 0)	0.0 (0.0 to 0.0)	-38.7 (-71.3 to 16.2)	-29.7 (-67.0 to 33.4)
Austria	Other chronic respiratory diseases	10 to 14	Both	0 (0 to 0)	0.0 (0.0 to 0.1)	0 (0 to 0)	0.0 (0.0 to 0.1)	0 (0 to 0)	0.0 (0.0 to 0.1)	29.1 (-22.7 to 122.7)	36.6 (-18.2 to 135.5)
Austria	Other chronic respiratory diseases	10 to 14	Female	0 (0 to 0)	0.0 (0.0 to 0.1)	0 (0 to 0)	0.0 (0.0 to 0.1)	0 (0 to 0)	0.0 (0.0 to 0.1)	23.3 (-40.1 to 157.7)	30.2 (-36.8 to 172.1)
Austria	Other chronic respiratory diseases	10 to 14	Male	0 (0 to 0)	0.0 (0.0 to 0.1)	0 (0 to 0)	0.0 (0.0 to 0.1)	0 (0 to 0)	0.1 (0.0 to 0.1)	32.0 (-42.1 to 159.8)	39.9 (-38.6 to 175.3)
Austria	Cirrhosis and other chronic liver diseases due to hepatitis B	5 to 9	Both	0 (0 to 0)	0.0 (0.0 to 0.0)	0 (0 to 0)	0.0 (0.0 to 0.0)	0 (0 to 0)	0.0 (0.0 to 0.0)	-68.8 (-80.1 to -51.8)	-64.3 (-77.2 to -44.8)

eTable 8. All-cause and cause-specific mortality (counts and rates) at the global level and by country in the WHO European Region for ages 5 to 9 and 10 to 14 for 1990, 2000, and 2016

Austria	Cirrhosis and other chronic liver diseases due to hepatitis B	5 to 9	Female	0 (0 to 0)	0.0 (0.0 to 0.0)	0 (0 to 0)	0.0 (0.0 to 0.0)	0 (0 to 0)	0.0 (0.0 to 0.0)	-51.7 (-70.2 to -18.9)	-44.9 (-66.0 to -7.3)
Austria	Cirrhosis and other chronic liver diseases due to hepatitis B	5 to 9	Male	0 (0 to 0)	0.0 (0.0 to 0.0)	0 (0 to 0)	0.0 (0.0 to 0.0)	0 (0 to 0)	0.0 (0.0 to 0.0)	-74.2 (-85.5 to -56.5)	-70.4 (-83.3 to -50.2)
Austria	Cirrhosis and other chronic liver diseases due to hepatitis B	10 to 14	Both	0 (0 to 0)	0.0 (0.0 to 0.0)	0 (0 to 0)	0.0 (0.0 to 0.0)	0 (0 to 0)	0.0 (0.0 to 0.0)	-46.4 (-63.8 to -18.5)	-43.2 (-61.7 to -13.8)
Austria	Cirrhosis and other chronic liver diseases due to hepatitis B	10 to 14	Female	0 (0 to 0)	0.0 (0.0 to 0.0)	0 (0 to 0)	0.0 (0.0 to 0.0)	0 (0 to 0)	0.0 (0.0 to 0.0)	-36.8 (-62.2 to 8.3)	-33.3 (-60.1 to 14.4)
Austria	Cirrhosis and other chronic liver diseases due to hepatitis B	10 to 14	Male	0 (0 to 0)	0.0 (0.0 to 0.0)	0 (0 to 0)	0.0 (0.0 to 0.0)	0 (0 to 0)	0.0 (0.0 to 0.0)	-53.9 (-71.9 to -19.7)	-51.1 (-70.2 to -14.9)
Austria	Cirrhosis and other chronic liver diseases due to hepatitis C	5 to 9	Both	0 (0 to 0)	0.0 (0.0 to 0.0)	0 (0 to 0)	0.0 (0.0 to 0.0)	0 (0 to 0)	0.0 (0.0 to 0.0)	-68.4 (-79.5 to -52.2)	-63.8 (-76.6 to -45.3)
Austria	Cirrhosis and other chronic liver diseases due to hepatitis C	5 to 9	Female	0 (0 to 0)	0.0 (0.0 to 0.0)	0 (0 to 0)	0.0 (0.0 to 0.0)	0 (0 to 0)	0.0 (0.0 to 0.0)	-52.0 (-70.3 to -20.2)	-45.2 (-66.0 to -8.8)
Austria	Cirrhosis and other chronic liver diseases due to hepatitis C	5 to 9	Male	0 (0 to 0)	0.0 (0.0 to 0.0)	0 (0 to 0)	0.0 (0.0 to 0.0)	0 (0 to 0)	0.0 (0.0 to 0.0)	-74.2 (-85.5 to -56.3)	-70.4 (-83.3 to -49.9)
Austria	Cirrhosis and other chronic liver diseases due to hepatitis C	10 to 14	Both	0 (0 to 0)	0.0 (0.0 to 0.0)	0 (0 to 0)	0.0 (0.0 to 0.0)	0 (0 to 0)	0.0 (0.0 to 0.0)	-45.9 (-63.2 to -18.3)	-42.8 (-61.0 to -13.6)
Austria	Cirrhosis and other chronic liver diseases due to hepatitis C	10 to 14	Female	0 (0 to 0)	0.0 (0.0 to 0.0)	0 (0 to 0)	0.0 (0.0 to 0.0)	0 (0 to 0)	0.0 (0.0 to 0.0)	-37.0 (-62.7 to 7.0)	-33.5 (-60.7 to 13.0)
Austria	Cirrhosis and other chronic liver diseases due to hepatitis C	10 to 14	Male	0 (0 to 0)	0.0 (0.0 to 0.0)	0 (0 to 0)	0.0 (0.0 to 0.0)	0 (0 to 0)	0.0 (0.0 to 0.0)	-53.8 (-71.6 to -18.8)	-51.0 (-69.9 to -13.9)
Austria	Cirrhosis and other chronic liver diseases due to other causes	5 to 9	Both	0 (0 to 0)	0.1 (0.0 to 0.1)	0 (0 to 0)	0.0 (0.0 to 0.0)	0 (0 to 0)	0.0 (0.0 to 0.0)	-65.5 (-76.7 to -50.7)	-60.5 (-73.3 to -43.5)
Austria	Cirrhosis and other chronic liver diseases due to other causes	5 to 9	Female	0 (0 to 0)	0.1 (0.0 to 0.1)	0 (0 to 0)	0.0 (0.0 to 0.1)	0 (0 to 0)	0.0 (0.0 to 0.0)	-54.1 (-71.7 to -23.4)	-47.6 (-67.6 to -12.5)

eTable 8. All-cause and cause-specific mortality (counts and rates) at the global level and by country in the WHO European Region for ages 5 to 9 and 10 to 14 for 1990, 2000, and 2016

Austria	Cirrhosis and other chronic liver diseases due to other causes	5 to 9	Male	0 (0 to 0)	0.1 (0.0 to 0.1)	0 (0 to 0)	0.0 (0.0 to 0.0)	0 (0 to 0)	0.0 (0.0 to 0.0)	-75.7 (-86.4 to -59.2)	-72.1 (-84.4 to -53.3)
Austria	Cirrhosis and other chronic liver diseases due to other causes	10 to 14	Both	0 (0 to 0)	0.0 (0.0 to 0.0)	0 (0 to 0)	0.0 (0.0 to 0.1)	0 (0 to 0)	0.0 (0.0 to 0.0)	-45.4 (-64.1 to -17.5)	-42.3 (-62.0 to -12.7)
Austria	Cirrhosis and other chronic liver diseases due to other causes	10 to 14	Female	0 (0 to 0)	0.1 (0.0 to 0.1)	0 (0 to 0)	0.1 (0.0 to 0.1)	0 (0 to 0)	0.0 (0.0 to 0.1)	-40.5 (-64.1 to 2.2)	-37.2 (-62.1 to 7.9)
Austria	Cirrhosis and other chronic liver diseases due to other causes	10 to 14	Male	0 (0 to 0)	0.0 (0.0 to 0.0)	0 (0 to 0)	0.0 (0.0 to 0.0)	0 (0 to 0)	0.0 (0.0 to 0.0)	-56.3 (-73.3 to -25.1)	-53.7 (-71.7 to -20.6)
Austria	Peptic ulcer disease	5 to 9	Both	0 (0 to 0)	0.0 (0.0 to 0.0)	0 (0 to 0)	0.0 (0.0 to 0.0)	0 (0 to 0)	0.0 (0.0 to 0.0)	-56.9 (-75.7 to -23.0)	-50.6 (-72.2 to -11.8)
Austria	Peptic ulcer disease	5 to 9	Female	0 (0 to 0)	0.0 (0.0 to 0.0)	0 (0 to 0)	0.0 (0.0 to 0.0)	0 (0 to 0)	0.0 (0.0 to 0.0)	-58.2 (-80.3 to -18.0)	-52.3 (-77.5 to -6.4)
Austria	Peptic ulcer disease	5 to 9	Male	0 (0 to 0)	0.0 (0.0 to 0.0)	0 (0 to 0)	0.0 (0.0 to 0.0)	0 (0 to 0)	0.0 (0.0 to 0.0)	-55.6 (-80.4 to 2.2)	-49.1 (-77.6 to 17.3)
Austria	Peptic ulcer disease	10 to 14	Both	0 (0 to 0)	0.0 (0.0 to 0.0)	0 (0 to 0)	0.0 (0.0 to 0.0)	0 (0 to 0)	0.0 (0.0 to 0.0)	-50.8 (-70.4 to -17.0)	-48.0 (-68.7 to -12.2)
Austria	Peptic ulcer disease	10 to 14	Female	0 (0 to 0)	0.0 (0.0 to 0.0)	0 (0 to 0)	0.0 (0.0 to 0.0)	0 (0 to 0)	0.0 (0.0 to 0.0)	-54.1 (-75.2 to -13.5)	-51.5 (-73.8 to -8.7)
Austria	Peptic ulcer disease	10 to 14	Male	0 (0 to 0)	0.0 (0.0 to 0.0)	0 (0 to 0)	0.0 (0.0 to 0.0)	0 (0 to 0)	0.0 (0.0 to 0.0)	-47.0 (-74.5 to 9.3)	-43.9 (-72.9 to 15.8)
Austria	Gastritis and duodenitis	5 to 9	Both	0 (0 to 0)	0.0 (0.0 to 0.0)	0 (0 to 0)	0.0 (0.0 to 0.0)	0 (0 to 0)	0.0 (0.0 to 0.0)	-49.0 (-72.2 to -2.8)	-41.6 (-68.2 to 11.2)
Austria	Gastritis and duodenitis	5 to 9	Female	0 (0 to 0)	0.0 (0.0 to 0.0)	0 (0 to 0)	0.0 (0.0 to 0.0)	0 (0 to 0)	0.0 (0.0 to 0.0)	-40.4 (-71.8 to 24.7)	-31.9 (-67.7 to 42.5)
Austria	Gastritis and duodenitis	5 to 9	Male	0 (0 to 0)	0.0 (0.0 to 0.0)	0 (0 to 0)	0.0 (0.0 to 0.0)	0 (0 to 0)	0.0 (0.0 to 0.0)	-55.6 (-80.4 to 2.2)	-49.1 (-77.6 to 17.3)
Austria	Gastritis and duodenitis	10 to 14	Both	0 (0 to 0)	0.0 (0.0 to 0.0)	0 (0 to 0)	0.0 (0.0 to 0.0)	0 (0 to 0)	0.0 (0.0 to 0.0)	-63.2 (-82.5 to -20.3)	-61.0 (-81.5 to -15.7)
Austria	Gastritis and duodenitis	10 to 14	Female	0 (0 to 0)	0.0 (0.0 to 0.0)	0 (0 to 0)	0.0 (0.0 to 0.0)	0 (0 to 0)	0.0 (0.0 to 0.0)	-57.4 (-85.4 to 17.1)	-55.0 (-84.6 to 23.6)
Austria	Gastritis and duodenitis	10 to 14	Male	0 (0 to 0)	0.0 (0.0 to 0.0)	0 (0 to 0)	0.0 (0.0 to 0.0)	0 (0 to 0)	0.0 (0.0 to 0.0)	-69.2 (-87.5 to -24.6)	-67.4 (-86.7 to -20.1)
Austria	Appendicitis	5 to 9	Both	0 (0 to 0)	0.0 (0.0 to 0.1)	0 (0 to 0)	0.0 (0.0 to 0.0)	0 (0 to 0)	0.0 (0.0 to 0.0)	-82.2 (-92.5 to -46.7)	-79.6 (-91.4 to -39.0)
Austria	Appendicitis	5 to 9	Female	0 (0 to 0)	0.0 (0.0 to 0.1)	0 (0 to 0)	0.0 (0.0 to 0.0)	0 (0 to 0)	0.0 (0.0 to 0.0)	-77.6 (-91.8 to -36.4)	-74.5 (-90.7 to -27.3)
Austria	Appendicitis	5 to 9	Male	0 (0 to 0)	0.0 (0.0 to 0.1)	0 (0 to 0)	0.0 (0.0 to 0.0)	0 (0 to 0)	0.0 (0.0 to 0.0)	-87.2 (-96.8 to -40.3)	-85.3 (-96.4 to -31.6)
Austria	Appendicitis	10 to 14	Both	0 (0 to 0)	0.0 (0.0 to 0.1)	0 (0 to 0)	0.0 (0.0 to 0.0)	0 (0 to 0)	0.0 (0.0 to 0.0)	-74.8 (-89.3 to -26.0)	-73.3 (-88.6 to -21.7)
Austria	Appendicitis	10 to 14	Female	0 (0 to 0)	0.0 (0.0 to 0.0)	0 (0 to 0)	0.0 (0.0 to 0.0)	0 (0 to 0)	0.0 (0.0 to 0.0)	-63.4 (-89.0 to -23.4)	-61.4 (-88.4 to -19.2)
Austria	Appendicitis	10 to 14	Male	0 (0 to 0)	0.1 (0.0 to 0.1)	0 (0 to 0)	0.0 (0.0 to 0.1)	0 (0 to 0)	0.0 (0.0 to 0.0)	-78.4 (-91.4 to -22.7)	-77.1 (-90.9 to -18.0)
Austria	Paralytic ileus and intestinal obstruction	5 to 9	Both	0 (0 to 0)	0.0 (0.0 to 0.1)	0 (0 to 0)	0.0 (0.0 to 0.1)	0 (0 to 0)	0.0 (0.0 to 0.0)	-61.4 (-79.3 to -23.0)	-55.8 (-76.3 to -11.8)
Austria	Paralytic ileus and intestinal obstruction	5 to 9	Female	0 (0 to 0)	0.0 (0.0 to 0.1)	0 (0 to 0)	0.0 (0.0 to 0.0)	0 (0 to 0)	0.0 (0.0 to 0.0)	-50.7 (-74.1 to 5.9)	-43.7 (-70.4 to 21.0)
Austria	Paralytic ileus and intestinal obstruction	5 to 9	Male	0 (0 to 0)	0.1 (0.0 to 0.1)	0 (0 to 0)	0.0 (0.0 to 0.1)	0 (0 to 0)	0.0 (0.0 to 0.1)	-65.6 (-85.1 to -2.7)	-60.6 (-82.9 to 11.6)

eTable 8. All-cause and cause-specific mortality (counts and rates) at the global level and by country in the WHO European Region for ages 5 to 9 and 10 to 14 for 1990, 2000, and 2016											
Austria	Paralytic ileus and intestinal obstruction	10 to 14	Both	0<br>(0 to 0)	0.0<br>(0.0 to 0.0)	0<br>(0 to 0)	0.0<br>(0.0 to 0.0)	0<br>(0 to 0)	0.0<br>(0.0 to 0.0)	-36.5<br>(-62.3 to 5.7)	-32.8<br>(-60.1 to 11.8)
Austria	Paralytic ileus and intestinal obstruction	10 to 14	Female	0<br>(0 to 0)	0.0<br>(0.0 to 0.1)	0<br>(0 to 0)	0.0<br>(0.0 to 0.1)	0<br>(0 to 0)	0.0<br>(0.0 to 0.0)	-38.8<br>(-65.3 to 10.2)	-35.4<br>(-63.4 to 16.3)
Austria	Paralytic ileus and intestinal obstruction	10 to 14	Male	0<br>(0 to 0)	0.0<br>(0.0 to 0.0)	0<br>(0 to 0)	0.0<br>(0.0 to 0.1)	0<br>(0 to 0)	0.0<br>(0.0 to 0.1)	-32.2<br>(-65.5 to 39.4)	-28.1<br>(-63.4 to 47.8)
Austria	Inguinal, femoral, and abdominal hernia	5 to 9	Both	0<br>(0 to 0)	0.0<br>(0.0 to 0.0)	0<br>(0 to 0)	0.0<br>(0.0 to 0.0)	0<br>(0 to 0)	0.0<br>(0.0 to 0.0)	-90.6<br>(-96.1 to -69.7)	-89.2<br>(-95.5 to -65.3)
Austria	Inguinal, femoral, and abdominal hernia	5 to 9	Female	0<br>(0 to 0)	0.0<br>(0.0 to 0.0)	0<br>(0 to 0)	0.0<br>(0.0 to 0.0)	0<br>(0 to 0)	0.0<br>(0.0 to 0.0)	-74.7<br>(-89.1 to -42.9)	-71.1<br>(-87.6 to -34.8)
Austria	Inguinal, femoral, and abdominal hernia	5 to 9	Male	0<br>(0 to 0)	0.0<br>(0.0 to 0.0)	0<br>(0 to 0)	0.0<br>(0.0 to 0.0)	0<br>(0 to 0)	0.0<br>(0.0 to 0.0)	-91.6<br>(-96.7 to -72.3)	-90.4<br>(-96.2 to -68.2)
Austria	Inguinal, femoral, and abdominal hernia	10 to 14	Both	0<br>(0 to 0)	0.0<br>(0.0 to 0.0)	0<br>(0 to 0)	0.0<br>(0.0 to 0.0)	0<br>(0 to 0)	0.0<br>(0.0 to 0.0)	-79.1<br>(-90.0 to -47.8)	-77.9<br>(-89.4 to -44.8)
Austria	Inguinal, femoral, and abdominal hernia	10 to 14	Female	0<br>(0 to 0)	0.0<br>(0.0 to 0.0)	0<br>(0 to 0)	0.0<br>(0.0 to 0.0)	0<br>(0 to 0)	0.0<br>(0.0 to 0.0)	-71.0<br>(-87.8 to -32.7)	-69.4<br>(-87.2 to -29.0)
Austria	Inguinal, femoral, and abdominal hernia	10 to 14	Male	0<br>(0 to 0)	0.0<br>(0.0 to 0.0)	0<br>(0 to 0)	0.0<br>(0.0 to 0.0)	0<br>(0 to 0)	0.0<br>(0.0 to 0.0)	-81.5<br>(-92.2 to -46.4)	-80.4<br>(-91.7 to -43.2)
Austria	Inflammatory bowel disease	5 to 9	Both	0<br>(0 to 0)	0.0<br>(0.0 to 0.1)	0<br>(0 to 0)	0.0<br>(0.0 to 0.0)	0<br>(0 to 0)	0.0<br>(0.0 to 0.0)	-72.9<br>(-85.1 to -47.8)	-68.9<br>(-83.0 to -40.3)
Austria	Inflammatory bowel disease	5 to 9	Female	0<br>(0 to 0)	0.0<br>(0.0 to 0.1)	0<br>(0 to 0)	0.0<br>(0.0 to 0.0)	0<br>(0 to 0)	0.0<br>(0.0 to 0.0)	-60.2<br>(-80.1 to -23.8)	-54.6<br>(-77.2 to -12.9)
Austria	Inflammatory bowel disease	5 to 9	Male	0<br>(0 to 0)	0.0<br>(0.0 to 0.1)	0<br>(0 to 0)	0.0<br>(0.0 to 0.0)	0<br>(0 to 0)	0.0<br>(0.0 to 0.0)	-83.9<br>(-93.7 to -55.4)	-81.5<br>(-92.8 to -48.9)
Austria	Inflammatory bowel disease	10 to 14	Both	0<br>(0 to 0)	0.0<br>(0.0 to 0.0)	0<br>(0 to 0)	0.0<br>(0.0 to 0.0)	0<br>(0 to 0)	0.0<br>(0.0 to 0.0)	-52.6<br>(-72.4 to -17.9)	-49.8<br>(-70.8 to -13.2)
Austria	Inflammatory bowel disease	10 to 14	Female	0<br>(0 to 0)	0.0<br>(0.0 to 0.0)	0<br>(0 to 0)	0.0<br>(0.0 to 0.0)	0<br>(0 to 0)	0.0<br>(0.0 to 0.0)	-44.2<br>(-71.3 to 6.1)	-41.1<br>(-69.7 to 12.1)
Austria	Inflammatory bowel disease	10 to 14	Male	0<br>(0 to 0)	0.0<br>(0.0 to 0.1)	0<br>(0 to 0)	0.0<br>(0.0 to 0.0)	0<br>(0 to 0)	0.0<br>(0.0 to 0.0)	-57.1<br>(-79.7 to -13.7)	-54.5<br>(-78.5 to -8.6)
Austria	Vascular intestinal disorders	5 to 9	Both	0<br>(0 to 0)	0.0<br>(0.0 to 0.0)	0<br>(0 to 0)	0.0<br>(0.0 to 0.0)	0<br>(0 to 0)	0.0<br>(0.0 to 0.0)	-85.9<br>(-94.6 to -43.8)	-83.9<br>(-93.9 to -35.6)
Austria	Vascular intestinal disorders	5 to 9	Female	0<br>(0 to 0)	0.0<br>(0.0 to 0.0)	0<br>(0 to 0)	0.0<br>(0.0 to 0.0)	0<br>(0 to 0)	0.0<br>(0.0 to 0.0)	-28.5<br>(-71.9 to 82.6)	-18.3<br>(-67.9 to 108.6)
Austria	Vascular intestinal disorders	5 to 9	Male	0<br>(0 to 0)	0.0<br>(0.0 to 0.0)	0<br>(0 to 0)	0.0<br>(0.0 to 0.0)	0<br>(0 to 0)	0.0<br>(0.0 to 0.0)	-87.5<br>(-95.5 to -53.5)	-85.7<br>(-94.8 to -46.7)
Austria	Vascular intestinal disorders	10 to 14	Both	0<br>(0 to 0)	0.0<br>(0.0 to 0.0)	0<br>(0 to 0)	0.0<br>(0.0 to 0.0)	0<br>(0 to 0)	0.0<br>(0.0 to 0.0)	-63.8<br>(-79.6 to -20.9)	-61.7<br>(-78.4 to -16.3)
Austria	Vascular intestinal disorders	10 to 14	Female	0<br>(0 to 0)	0.0<br>(0.0 to 0.0)	0<br>(0 to 0)	0.0<br>(0.0 to 0.0)	0<br>(0 to 0)	0.0<br>(0.0 to 0.0)	-60.6<br>(-84.7 to -8.6)	-58.4<br>(-83.8 to -3.5)
Austria	Vascular intestinal disorders	10 to 14	Male	0<br>(0 to 0)	0.0<br>(0.0 to 0.1)	0<br>(0 to 0)	0.0<br>(0.0 to 0.0)	0<br>(0 to 0)	0.0<br>(0.0 to 0.0)	-64.6<br>(-81.7 to -11.1)	-62.4<br>(-80.6 to -5.7)

eTable 8. All-cause and cause-specific mortality (counts and rates) at the global level and by country in the WHO European Region for ages 5 to 9 and 10 to 14 for 1990, 2000, and 2016

Austria	Gallbladder and biliary diseases	5 to 9	Both	0 (0 to 0)	0.0 (0.0 to 0.0)	0 (0 to 0)	0.0 (0.0 to 0.0)	0 (0 to 0)	0.0 (0.0 to 0.0)	-83.6 (-92.2 to -67.1)	-81.2 (-91.1 to -62.3)
Austria	Gallbladder and biliary diseases	5 to 9	Female	0 (0 to 0)	0.0 (0.0 to 0.0)	0 (0 to 0)	0.0 (0.0 to 0.0)	0 (0 to 0)	0.0 (0.0 to 0.0)	-76.4 (-90.0 to -41.3)	-73.0 (-88.6 to -33.0)
Austria	Gallbladder and biliary diseases	5 to 9	Male	0 (0 to 0)	0.0 (0.0 to 0.0)	0 (0 to 0)	0.0 (0.0 to 0.0)	0 (0 to 0)	0.0 (0.0 to 0.0)	-86.6 (-94.8 to -65.3)	-84.6 (-94.0 to -60.2)
Austria	Gallbladder and biliary diseases	10 to 14	Both	0 (0 to 0)	0.0 (0.0 to 0.0)	0 (0 to 0)	0.0 (0.0 to 0.0)	0 (0 to 0)	0.0 (0.0 to 0.0)	-68.9 (-83.9 to -41.6)	-67.1 (-83.0 to -38.2)
Austria	Gallbladder and biliary diseases	10 to 14	Female	0 (0 to 0)	0.0 (0.0 to 0.0)	0 (0 to 0)	0.0 (0.0 to 0.0)	0 (0 to 0)	0.0 (0.0 to 0.0)	-67.2 (-83.8 to -36.5)	-65.4 (-82.9 to -32.9)
Austria	Gallbladder and biliary diseases	10 to 14	Male	0 (0 to 0)	0.0 (0.0 to 0.0)	0 (0 to 0)	0.0 (0.0 to 0.0)	0 (0 to 0)	0.0 (0.0 to 0.0)	-69.8 (-87.8 to -28.8)	-68.0 (-87.0 to -24.5)
Austria	Pancreatitis	5 to 9	Both	0 (0 to 0)	0.0 (0.0 to 0.0)	0 (0 to 0)	0.0 (0.0 to 0.0)	0 (0 to 0)	0.0 (0.0 to 0.0)	-87.7 (-94.6 to -45.3)	-85.9 (-93.9 to -37.3)
Austria	Pancreatitis	5 to 9	Female	0 (0 to 0)	0.0 (0.0 to 0.0)	0 (0 to 0)	0.0 (0.0 to 0.0)	0 (0 to 0)	0.0 (0.0 to 0.0)	-73.1 (-89.9 to -37.0)	-69.3 (-88.5 to -28.0)
Austria	Pancreatitis	5 to 9	Male	0 (0 to 0)	0.0 (0.0 to 0.0)	0 (0 to 0)	0.0 (0.0 to 0.0)	0 (0 to 0)	0.0 (0.0 to 0.0)	-88.5 (-95.1 to -36.9)	-86.8 (-94.4 to -27.6)
Austria	Pancreatitis	10 to 14	Both	0 (0 to 0)	0.0 (0.0 to 0.0)	0 (0 to 0)	0.0 (0.0 to 0.0)	0 (0 to 0)	0.0 (0.0 to 0.0)	-75.7 (-86.8 to -29.4)	-74.3 (-86.0 to -25.3)
Austria	Pancreatitis	10 to 14	Female	0 (0 to 0)	0.0 (0.0 to 0.0)	0 (0 to 0)	0.0 (0.0 to 0.0)	0 (0 to 0)	0.0 (0.0 to 0.0)	-73.9 (-89.0 to -29.7)	-72.5 (-88.4 to -25.7)
Austria	Pancreatitis	10 to 14	Male	0 (0 to 0)	0.0 (0.0 to 0.0)	0 (0 to 0)	0.0 (0.0 to 0.0)	0 (0 to 0)	0.0 (0.0 to 0.0)	-77.3 (-90.4 to -13.5)	-75.9 (-89.8 to -8.4)
Austria	Other digestive diseases	5 to 9	Both	0 (0 to 0)	0.0 (0.0 to 0.1)	0 (0 to 0)	0.0 (0.0 to 0.0)	0 (0 to 0)	0.0 (0.0 to 0.0)	-67.5 (-82.8 to -41.6)	-62.8 (-80.3 to -33.1)
Austria	Other digestive diseases	5 to 9	Female	0 (0 to 0)	0.0 (0.0 to 0.1)	0 (0 to 0)	0.0 (0.0 to 0.0)	0 (0 to 0)	0.0 (0.0 to 0.0)	-58.9 (-79.3 to -18.5)	-53.0 (-76.4 to -6.9)
Austria	Other digestive diseases	5 to 9	Male	0 (0 to 0)	0.0 (0.0 to 0.1)	0 (0 to 0)	0.0 (0.0 to 0.0)	0 (0 to 0)	0.0 (0.0 to 0.0)	-76.7 (-90.2 to -47.6)	-73.3 (-88.8 to -39.9)
Austria	Other digestive diseases	10 to 14	Both	0 (0 to 0)	0.0 (0.0 to 0.0)	0 (0 to 0)	0.0 (0.0 to 0.0)	0 (0 to 0)	0.0 (0.0 to 0.0)	-46.6 (-67.2 to -14.7)	-43.5 (-65.3 to -9.8)
Austria	Other digestive diseases	10 to 14	Female	0 (0 to 0)	0.0 (0.0 to 0.0)	0 (0 to 0)	0.0 (0.0 to 0.0)	0 (0 to 0)	0.0 (0.0 to 0.0)	-42.7 (-68.7 to 8.8)	-39.5 (-67.0 to 14.9)
Austria	Other digestive diseases	10 to 14	Male	0 (0 to 0)	0.0 (0.0 to 0.0)	0 (0 to 0)	0.0 (0.0 to 0.0)	0 (0 to 0)	0.0 (0.0 to 0.0)	-50.2 (-75.2 to -7.7)	-47.2 (-73.7 to -2.1)
Austria	Alzheimer disease and other dementias	5 to 9	Both	0 (0 to 0)	0.0 (0.0 to 0.0)	0 (0 to 0)	0.0 (0.0 to 0.0)	0 (0 to 0)	0.0 (0.0 to 0.0)	0.0 (0.0 to 0.0)	0.0 (0.0 to 0.0)
Austria	Alzheimer disease and other dementias	5 to 9	Female	0 (0 to 0)	0.0 (0.0 to 0.0)	0 (0 to 0)	0.0 (0.0 to 0.0)	0 (0 to 0)	0.0 (0.0 to 0.0)	0.0 (0.0 to 0.0)	0.0 (0.0 to 0.0)
Austria	Alzheimer disease and other dementias	5 to 9	Male	0 (0 to 0)	0.0 (0.0 to 0.0)	0 (0 to 0)	0.0 (0.0 to 0.0)	0 (0 to 0)	0.0 (0.0 to 0.0)	0.0 (0.0 to 0.0)	0.0 (0.0 to 0.0)
Austria	Alzheimer disease and other dementias	10 to 14	Both	0 (0 to 0)	0.0 (0.0 to 0.0)	0 (0 to 0)	0.0 (0.0 to 0.0)	0 (0 to 0)	0.0 (0.0 to 0.0)	0.0 (0.0 to 0.0)	0.0 (0.0 to 0.0)

eTable 8. All-cause and cause-specific mortality (counts and rates) at the global level and by country in the WHO European Region for ages 5 to 9 and 10 to 14 for 1990, 2000, and 2016

Austria	Alzheimer disease and other dementias	10 to 14	Female	0 (0 to 0)	0.0 (0.0 to 0.0)	0 (0 to 0)	0.0 (0.0 to 0.0)	0 (0 to 0)	0.0 (0.0 to 0.0)	0.0 (0.0 to 0.0)	0.0 (0.0 to 0.0)
Austria	Alzheimer disease and other dementias	10 to 14	Male	0 (0 to 0)	0.0 (0.0 to 0.0)	0 (0 to 0)	0.0 (0.0 to 0.0)	0 (0 to 0)	0.0 (0.0 to 0.0)	0.0 (0.0 to 0.0)	0.0 (0.0 to 0.0)
Austria	Parkinson disease	5 to 9	Both	0 (0 to 0)	0.0 (0.0 to 0.0)	0 (0 to 0)	0.0 (0.0 to 0.0)	0 (0 to 0)	0.0 (0.0 to 0.0)	0.0 (0.0 to 0.0)	0.0 (0.0 to 0.0)
Austria	Parkinson disease	5 to 9	Female	0 (0 to 0)	0.0 (0.0 to 0.0)	0 (0 to 0)	0.0 (0.0 to 0.0)	0 (0 to 0)	0.0 (0.0 to 0.0)	0.0 (0.0 to 0.0)	0.0 (0.0 to 0.0)
Austria	Parkinson disease	5 to 9	Male	0 (0 to 0)	0.0 (0.0 to 0.0)	0 (0 to 0)	0.0 (0.0 to 0.0)	0 (0 to 0)	0.0 (0.0 to 0.0)	0.0 (0.0 to 0.0)	0.0 (0.0 to 0.0)
Austria	Parkinson disease	10 to 14	Both	0 (0 to 0)	0.0 (0.0 to 0.0)	0 (0 to 0)	0.0 (0.0 to 0.0)	0 (0 to 0)	0.0 (0.0 to 0.0)	0.0 (0.0 to 0.0)	0.0 (0.0 to 0.0)
Austria	Parkinson disease	10 to 14	Female	0 (0 to 0)	0.0 (0.0 to 0.0)	0 (0 to 0)	0.0 (0.0 to 0.0)	0 (0 to 0)	0.0 (0.0 to 0.0)	0.0 (0.0 to 0.0)	0.0 (0.0 to 0.0)
Austria	Parkinson disease	10 to 14	Male	0 (0 to 0)	0.0 (0.0 to 0.0)	0 (0 to 0)	0.0 (0.0 to 0.0)	0 (0 to 0)	0.0 (0.0 to 0.0)	0.0 (0.0 to 0.0)	0.0 (0.0 to 0.0)
Austria	Epilepsy	5 to 9	Both	1 (1 to 2)	0.3 (0.2 to 0.3)	2 (1 to 2)	0.3 (0.2 to 0.4)	1 (1 to 1)	0.2 (0.1 to 0.3)	-34.5 (-56.3 to -1.7)	-25.0 (-49.9 to 12.5)
Austria	Epilepsy	5 to 9	Female	1 (0 to 1)	0.2 (0.2 to 0.3)	1 (1 to 1)	0.4 (0.3 to 0.5)	1 (0 to 1)	0.3 (0.2 to 0.4)	8.9 (-39.3 to 93.6)	24.4 (-30.7 to 121.2)
Austria	Epilepsy	5 to 9	Male	1 (1 to 1)	0.3 (0.2 to 0.4)	1 (0 to 1)	0.3 (0.2 to 0.4)	0 (0 to 0)	0.1 (0.1 to 0.2)	-64.5 (-78.8 to -37.3)	-59.3 (-75.7 to -28.1)
Austria	Epilepsy	10 to 14	Both	1 (1 to 1)	0.2 (0.1 to 0.3)	1 (1 to 2)	0.3 (0.2 to 0.4)	1 (1 to 1)	0.2 (0.1 to 0.3)	-5.5 (-37.3 to 38.1)	0.0 (-33.6 to 46.1)
Austria	Epilepsy	10 to 14	Female	0 (0 to 0)	0.1 (0.1 to 0.2)	1 (0 to 1)	0.3 (0.2 to 0.5)	0 (0 to 1)	0.2 (0.1 to 0.3)	51.1 (-13.4 to 162.0)	59.5 (-8.6 to 176.6)
Austria	Epilepsy	10 to 14	Male	1 (0 to 1)	0.2 (0.2 to 0.3)	1 (0 to 1)	0.3 (0.2 to 0.4)	0 (0 to 1)	0.2 (0.1 to 0.2)	-36.2 (-61.2 to 3.7)	-32.4 (-58.8 to 9.9)
Austria	Motor neuron disease	5 to 9	Both	0 (0 to 0)	0.0 (0.0 to 0.1)	0 (0 to 0)	0.0 (0.0 to 0.1)	0 (0 to 0)	0.0 (0.0 to 0.1)	-20.2 (-53.9 to 40.0)	-8.6 (-47.3 to 60.3)
Austria	Motor neuron disease	5 to 9	Female	0 (0 to 0)	0.1 (0.0 to 0.1)	0 (0 to 0)	0.1 (0.0 to 0.1)	0 (0 to 0)	0.1 (0.0 to 0.1)	1.5 (-47.8 to 99.1)	16.0 (-40.4 to 127.5)
Austria	Motor neuron disease	5 to 9	Male	0 (0 to 0)	0.0 (0.0 to 0.1)	0 (0 to 0)	0.0 (0.0 to 0.0)	0 (0 to 0)	0.0 (0.0 to 0.0)	-57.0 (-82.9 to -2.6)	-50.7 (-80.3 to 11.8)
Austria	Motor neuron disease	10 to 14	Both	0 (0 to 0)	0.1 (0.0 to 0.1)	0 (0 to 1)	0.1 (0.1 to 0.1)	0 (0 to 1)	0.1 (0.1 to 0.1)	26.2 (-27.1 to 107.8)	33.5 (-22.8 to 119.9)
Austria	Motor neuron disease	10 to 14	Female	0 (0 to 0)	0.0 (0.0 to 0.0)	0 (0 to 0)	0.1 (0.0 to 0.1)	0 (0 to 0)	0.1 (0.0 to 0.1)	45.4 (-13.6 to 149.5)	53.5 (-8.8 to 163.4)
Austria	Motor neuron disease	10 to 14	Male	0 (0 to 0)	0.1 (0.1 to 0.2)	0 (0 to 0)	0.1 (0.1 to 0.2)	0 (0 to 0)	0.1 (0.1 to 0.2)	20.5 (-40.6 to 125.0)	27.7 (-37.0 to 138.5)
Austria	Other neurological disorders	5 to 9	Both	1 (1 to 2)	0.3 (0.2 to 0.4)	1 (1 to 1)	0.2 (0.2 to 0.3)	1 (1 to 1)	0.2 (0.2 to 0.3)	-31.1 (-55.3 to 3.7)	-21.1 (-48.8 to 18.7)
Austria	Other neurological disorders	5 to 9	Female	1 (0 to 1)	0.3 (0.2 to 0.4)	1 (0 to 1)	0.3 (0.2 to 0.4)	1 (0 to 1)	0.3 (0.2 to 0.4)	-14.5 (-50.7 to 41.7)	-2.3 (-43.6 to 61.9)
Austria	Other neurological disorders	5 to 9	Male	1 (0 to 1)	0.3 (0.2 to 0.5)	1 (0 to 1)	0.2 (0.1 to 0.3)	0 (0 to 1)	0.2 (0.1 to 0.3)	-46.5 (-72.8 to -2.5)	-38.6 (-68.8 to 11.8)
Austria	Other neurological disorders	10 to 14	Both	2 (1 to 2)	0.4 (0.3 to 0.5)	2 (1 to 2)	0.3 (0.3 to 0.4)	1 (1 to 2)	0.3 (0.2 to 0.4)	-16.2 (-43.6 to 22.3)	-11.3 (-40.3 to 29.4)
Austria	Other neurological disorders	10 to 14	Female	0 (0 to 1)	0.2 (0.1 to 0.3)	1 (0 to 1)	0.3 (0.2 to 0.4)	1 (0 to 1)	0.3 (0.2 to 0.4)	8.0 (-40.0 to 94.8)	14.1 (-36.7 to 105.7)
Austria	Other neurological disorders	10 to 14	Male	1 (1 to 2)	0.5 (0.4 to 0.7)	1 (1 to 1)	0.4 (0.3 to 0.6)	1 (1 to 1)	0.4 (0.3 to 0.6)	-26.1 (-56.7 to 24.1)	-21.7 (-54.1 to 31.5)
Austria	Drug use disorders	5 to 9	Both	0 (0 to 0)	0.0 (0.0 to 0.0)	0 (0 to 0)	0.0 (0.0 to 0.0)	0 (0 to 0)	0.0 (0.0 to 0.0)	0.0 (0.0 to 0.0)	0.0 (0.0 to 0.0)

eTable 8. All-cause and cause-specific mortality (counts and rates) at the global level and by country in the WHO European Region for ages 5 to 9 and 10 to 14 for 1990, 2000, and 2016

Austria	Drug use disorders	5 to 9	Female	0 (0 to 0)	0.0 (0.0 to 0.0)	0 (0 to 0)	0.0 (0.0 to 0.0)	0 (0 to 0)	0.0 (0.0 to 0.0)	0.0 (0.0 to 0.0)	0.0 (0.0 to 0.0)
Austria	Drug use disorders	5 to 9	Male	0 (0 to 0)	0.0 (0.0 to 0.0)	0 (0 to 0)	0.0 (0.0 to 0.0)	0 (0 to 0)	0.0 (0.0 to 0.0)	0.0 (0.0 to 0.0)	0.0 (0.0 to 0.0)
Austria	Drug use disorders	10 to 14	Both	0 (0 to 0)	0.0 (0.0 to 0.0)	0 (0 to 0)	0.0 (0.0 to 0.0)	0 (0 to 0)	0.0 (0.0 to 0.0)	0.0 (0.0 to 0.0)	0.0 (0.0 to 0.0)
Austria	Drug use disorders	10 to 14	Female	0 (0 to 0)	0.0 (0.0 to 0.0)	0 (0 to 0)	0.0 (0.0 to 0.0)	0 (0 to 0)	0.0 (0.0 to 0.0)	0.0 (0.0 to 0.0)	0.0 (0.0 to 0.0)
Austria	Drug use disorders	10 to 14	Male	0 (0 to 0)	0.0 (0.0 to 0.0)	0 (0 to 0)	0.0 (0.0 to 0.0)	0 (0 to 0)	0.0 (0.0 to 0.0)	0.0 (0.0 to 0.0)	0.0 (0.0 to 0.0)
Austria	Eating disorders	5 to 9	Both	0 (0 to 0)	0.0 (0.0 to 0.0)	0 (0 to 0)	0.0 (0.0 to 0.0)	0 (0 to 0)	0.0 (0.0 to 0.0)	130.7 (23.2 to 345.3)	164.1 (41.1 to 409.8)
Austria	Eating disorders	5 to 9	Female	0 (0 to 0)	0.0 (0.0 to 0.0)	0 (0 to 0)	0.0 (0.0 to 0.0)	0 (0 to 0)	0.0 (0.0 to 0.0)	146.8 (14.4 to 563.5)	182.0 (30.7 to 658.0)
Austria	Eating disorders	5 to 9	Male	0 (0 to 0)	0.0 (0.0 to 0.0)	0 (0 to 0)	0.0 (0.0 to 0.0)	0 (0 to 0)	0.0 (0.0 to 0.0)	121.7 (-6.1 to 382.5)	154.4 (7.7 to 453.5)
Austria	Eating disorders	10 to 14	Both	0 (0 to 0)	0.0 (0.0 to 0.0)	0 (0 to 0)	0.0 (0.0 to 0.0)	0 (0 to 0)	0.0 (0.0 to 0.0)	201.1 (10.2 to 527.6)	218.5 (16.6 to 564.0)
Austria	Eating disorders	10 to 14	Female	0 (0 to 0)	0.0 (0.0 to 0.0)	0 (0 to 0)	0.0 (0.0 to 0.0)	0 (0 to 0)	0.0 (0.0 to 0.0)	205.6 (11.6 to 542.8)	222.7 (17.8 to 578.7)
Austria	Eating disorders	10 to 14	Male	0 (0 to 0)	0.0 (0.0 to 0.0)	0 (0 to 0)	0.0 (0.0 to 0.0)	0 (0 to 0)	0.0 (0.0 to 0.0)	-14.2 (-60.7 to 81.7)	-9.0 (-58.4 to 92.5)
Austria	Diabetes mellitus	5 to 9	Both	0 (0 to 0)	0.0 (0.0 to 0.0)	0 (0 to 0)	0.0 (0.0 to 0.0)	0 (0 to 0)	0.0 (0.0 to 0.0)	-50.7 (-66.0 to -29.0)	-43.5 (-61.1 to -18.7)
Austria	Diabetes mellitus	5 to 9	Female	0 (0 to 0)	0.0 (0.0 to 0.1)	0 (0 to 0)	0.0 (0.0 to 0.0)	0 (0 to 0)	0.0 (0.0 to 0.0)	-51.7 (-69.0 to -25.3)	-44.8 (-64.6 to -14.6)
Austria	Diabetes mellitus	5 to 9	Male	0 (0 to 0)	0.0 (0.0 to 0.0)	0 (0 to 0)	0.0 (0.0 to 0.0)	0 (0 to 0)	0.0 (0.0 to 0.0)	-48.1 (-70.7 to -8.0)	-40.5 (-66.3 to 5.5)
Austria	Diabetes mellitus	10 to 14	Both	0 (0 to 0)	0.0 (0.0 to 0.1)	0 (0 to 0)	0.0 (0.0 to 0.1)	0 (0 to 0)	0.0 (0.0 to 0.0)	-31.2 (-54.1 to 4.4)	-27.2 (-51.5 to 10.5)
Austria	Diabetes mellitus	10 to 14	Female	0 (0 to 0)	0.1 (0.0 to 0.1)	0 (0 to 0)	0.1 (0.0 to 0.1)	0 (0 to 0)	0.0 (0.0 to 0.1)	-32.5 (-59.7 to 16.2)	-28.8 (-57.4 to 22.7)
Austria	Diabetes mellitus	10 to 14	Male	0 (0 to 0)	0.0 (0.0 to 0.0)	0 (0 to 0)	0.0 (0.0 to 0.0)	0 (0 to 0)	0.0 (0.0 to 0.0)	-27.8 (-57.4 to 25.4)	-23.5 (-54.8 to 32.9)
Austria	Acute glomerulonephritis	5 to 9	Both	0 (0 to 0)	0.0 (0.0 to 0.0)	0 (0 to 0)	0.0 (0.0 to 0.0)	0 (0 to 0)	0.0 (0.0 to 0.0)	-30.5 (-43.2 to -13.9)	-20.5 (-34.9 to -1.5)
Austria	Acute glomerulonephritis	5 to 9	Female	0 (0 to 0)	0.0 (0.0 to 0.0)	0 (0 to 0)	0.0 (0.0 to 0.0)	0 (0 to 0)	0.0 (0.0 to 0.0)	-28.3 (-41.9 to -10.6)	-18.0 (-33.6 to 2.1)
Austria	Acute glomerulonephritis	5 to 9	Male	0 (0 to 0)	0.0 (0.0 to 0.0)	0 (0 to 0)	0.0 (0.0 to 0.0)	0 (0 to 0)	0.0 (0.0 to 0.0)	-32.6 (-47.6 to -13.0)	-22.7 (-39.9 to -0.3)
Austria	Acute glomerulonephritis	10 to 14	Both	0 (0 to 0)	0.0 (0.0 to 0.0)	0 (0 to 0)	0.0 (0.0 to 0.0)	0 (0 to 0)	0.0 (0.0 to 0.0)	-15.5 (-26.9 to -2.6)	-10.6 (-22.7 to 3.0)
Austria	Acute glomerulonephritis	10 to 14	Female	0 (0 to 0)	0.0 (0.0 to 0.0)	0 (0 to 0)	0.0 (0.0 to 0.0)	0 (0 to 0)	0.0 (0.0 to 0.0)	-14.3 (-27.5 to 2.8)	-9.5 (-23.5 to 8.5)
Austria	Acute glomerulonephritis	10 to 14	Male	0 (0 to 0)	0.0 (0.0 to 0.0)	0 (0 to 0)	0.0 (0.0 to 0.0)	0 (0 to 0)	0.0 (0.0 to 0.0)	-16.6 (-30.8 to -0.5)	-11.7 (-26.7 to 5.4)
Austria	Chronic kidney disease	5 to 9	Both	0 (0 to 0)	0.0 (0.0 to 0.0)	0 (0 to 0)	0.0 (0.0 to 0.0)	0 (0 to 0)	0.0 (0.0 to 0.1)	-7.1 (-36.5 to 31.9)	6.3 (-27.3 to 51.0)
Austria	Chronic kidney disease	5 to 9	Female	0 (0 to 0)	0.0 (0.0 to 0.1)	0 (0 to 0)	0.0 (0.0 to 0.1)	0 (0 to 0)	0.1 (0.0 to 0.1)	14.5 (-27.3 to 75.3)	30.8 (-16.9 to 100.2)
Austria	Chronic kidney disease	5 to 9	Male	0 (0 to 0)	0.0 (0.0 to 0.0)	0 (0 to 0)	0.0 (0.0 to 0.0)	0 (0 to 0)	0.0 (0.0 to 0.0)	-39.3 (-62.9 to -1.5)	-30.4 (-57.4 to 13.0)
Austria	Chronic kidney disease	10 to 14	Both	0 (0 to 0)	0.0 (0.0 to 0.1)	0 (0 to 0)	0.1 (0.0 to 0.1)	0 (0 to 0)	0.1 (0.0 to 0.1)	14.3 (-15.4 to 53.2)	21.0 (-10.5 to 62.0)
Austria	Chronic kidney disease	10 to 14	Female	0 (0 to 0)	0.0 (0.0 to 0.0)	0 (0 to 0)	0.0 (0.0 to 0.1)	0 (0 to 0)	0.0 (0.0 to 0.1)	22.1 (-20.2 to 83.8)	28.9 (-15.7 to 94.0)
Austria	Chronic kidney disease	10 to 14	Male	0 (0 to 0)	0.1 (0.0 to 0.1)	0 (0 to 0)	0.1 (0.0 to 0.1)	0 (0 to 0)	0.1 (0.0 to 0.1)	9.4 (-25.6 to 61.1)	16.0 (-21.1 to 70.7)

eTable 8. All-cause and cause-specific mortality (counts and rates) at the global level and by country in the WHO European Region for ages 5 to 9 and 10 to 14 for 1990, 2000, and 2016

Austria	Urinary diseases and male infertility	5 to 9	Both	0 (0 to 0)	0.0 (0.0 to 0.0)	0 (0 to 0)	0.0 (0.0 to 0.0)	0 (0 to 0)	0.0 (0.0 to 0.0)	-57.2 (-69.2 to -39.7)	-51.0 (-64.7 to -30.9)
Austria	Urinary diseases and male infertility	5 to 9	Female	0 (0 to 0)	0.0 (0.0 to 0.0)	0 (0 to 0)	0.0 (0.0 to 0.0)	0 (0 to 0)	0.0 (0.0 to 0.0)	-67.7 (-78.7 to -46.8)	-63.1 (-75.7 to -39.2)
Austria	Urinary diseases and male infertility	5 to 9	Male	0 (0 to 0)	0.0 (0.0 to 0.0)	0 (0 to 0)	0.0 (0.0 to 0.0)	0 (0 to 0)	0.0 (0.0 to 0.0)	-32.6 (-47.6 to -13.0)	-22.7 (-39.9 to -0.3)
Austria	Urinary diseases and male infertility	10 to 14	Both	0 (0 to 0)	0.0 (0.0 to 0.0)	0 (0 to 0)	0.0 (0.0 to 0.0)	0 (0 to 0)	0.0 (0.0 to 0.0)	-30.0 (-45.0 to -12.7)	-26.0 (-41.8 to -7.6)
Austria	Urinary diseases and male infertility	10 to 14	Female	0 (0 to 0)	0.0 (0.0 to 0.0)	0 (0 to 0)	0.0 (0.0 to 0.0)	0 (0 to 0)	0.0 (0.0 to 0.0)	-36.4 (-54.6 to -11.2)	-32.9 (-52.1 to -6.2)
Austria	Urinary diseases and male infertility	10 to 14	Male	0 (0 to 0)	0.0 (0.0 to 0.0)	0 (0 to 0)	0.0 (0.0 to 0.0)	0 (0 to 0)	0.0 (0.0 to 0.0)	-22.4 (-42.6 to -3.3)	-17.8 (-39.2 to 2.5)
Austria	Hemoglobinopathies and hemolytic anemias	5 to 9	Both	1 (1 to 1)	0.2 (0.1 to 0.2)	1 (0 to 1)	0.1 (0.1 to 0.1)	0 (0 to 0)	0.0 (0.0 to 0.1)	-78.1 (-85.2 to -67.8)	-74.9 (-83.1 to -63.1)
Austria	Hemoglobinopathies and hemolytic anemias	5 to 9	Female	0 (0 to 0)	0.1 (0.1 to 0.2)	0 (0 to 0)	0.1 (0.1 to 0.1)	0 (0 to 0)	0.0 (0.0 to 0.1)	-67.1 (-80.4 to -48.5)	-62.5 (-77.6 to -41.2)
Austria	Hemoglobinopathies and hemolytic anemias	5 to 9	Male	0 (0 to 1)	0.2 (0.1 to 0.3)	0 (0 to 0)	0.1 (0.1 to 0.2)	0 (0 to 0)	0.0 (0.0 to 0.1)	-83.6 (-89.9 to -72.7)	-81.2 (-88.5 to -68.6)
Austria	Hemoglobinopathies and hemolytic anemias	10 to 14	Both	0 (0 to 1)	0.1 (0.1 to 0.2)	0 (0 to 1)	0.1 (0.1 to 0.1)	0 (0 to 0)	0.0 (0.0 to 0.1)	-53.6 (-72.7 to -26.4)	-50.9 (-71.1 to -22.1)
Austria	Hemoglobinopathies and hemolytic anemias	10 to 14	Female	0 (0 to 0)	0.1 (0.1 to 0.2)	0 (0 to 0)	0.1 (0.1 to 0.2)	0 (0 to 0)	0.1 (0.0 to 0.2)	-35.1 (-67.3 to -1.3)	-31.5 (-65.5 to 4.2)
Austria	Hemoglobinopathies and hemolytic anemias	10 to 14	Male	0 (0 to 0)	0.1 (0.1 to 0.2)	0 (0 to 0)	0.1 (0.1 to 0.1)	0 (0 to 0)	0.0 (0.0 to 0.1)	-68.2 (-81.2 to -47.4)	-66.3 (-80.1 to -44.3)
Austria	Endocrine, metabolic, blood, and immune disorders	5 to 9	Both	2 (1 to 3)	0.4 (0.3 to 0.7)	2 (1 to 3)	0.3 (0.2 to 0.6)	3 (1 to 4)	0.7 (0.3 to 1.0)	58.4 (-55.1 to 168.2)	81.3 (-48.6 to 207.1)
Austria	Endocrine, metabolic, blood, and immune disorders	5 to 9	Female	1 (1 to 2)	0.4 (0.2 to 0.7)	1 (0 to 1)	0.3 (0.2 to 0.6)	1 (1 to 2)	0.7 (0.3 to 1.1)	79.7 (-43.3 to 244.7)	105.3 (-35.2 to 293.9)
Austria	Endocrine, metabolic, blood, and immune disorders	5 to 9	Male	1 (1 to 2)	0.4 (0.3 to 0.8)	1 (1 to 2)	0.4 (0.2 to 0.8)	1 (1 to 2)	0.7 (0.3 to 1.0)	40.6 (-64.7 to 179.2)	61.3 (-59.5 to 220.2)
Austria	Endocrine, metabolic, blood, and immune disorders	10 to 14	Both	1 (1 to 3)	0.3 (0.2 to 0.7)	1 (1 to 4)	0.3 (0.2 to 0.8)	3 (2 to 4)	0.7 (0.4 to 0.9)	136.2 (-32.5 to 302.8)	149.9 (-28.6 to 326.1)

eTable 8. All-cause and cause-specific mortality (counts and rates) at the global level and by country in the WHO European Region for ages 5 to 9 and 10 to 14 for 1990, 2000, and 2016

Austria	Endocrine, metabolic, blood, and immune disorders	10 to 14	Female	0<br>(0 to 1)	0.2<br>(0.1 to 0.6)	1<br>(0 to 2)	0.3<br>(0.2 to 0.7)	1<br>(1 to 2)	0.6<br>(0.3 to 0.9)	157.3<br>(-25.3 to 414.5)	171.6<br>(-21.1 to 443.3)
Austria	Endocrine, metabolic, blood, and immune disorders	10 to 14	Male	1<br>(0 to 2)	0.3<br>(0.2 to 0.8)	1<br>(1 to 2)	0.4<br>(0.2 to 0.9)	2<br>(1 to 2)	0.8<br>(0.5 to 1.1)	122.3<br>(-38.8 to 323.2)	135.6<br>(-35.1 to 348.5)
Austria	Rheumatoid arthritis	5 to 9	Both	0<br>(0 to 0)	0.0<br>(0.0 to 0.0)	0<br>(0 to 0)	0.0<br>(0.0 to 0.0)	0<br>(0 to 0)	0.0<br>(0.0 to 0.0)	-66.6<br>(-80.4 to -40.8)	-61.8<br>(-77.5 to -32.2)
Austria	Rheumatoid arthritis	5 to 9	Female	0<br>(0 to 0)	0.0<br>(0.0 to 0.0)	0<br>(0 to 0)	0.0<br>(0.0 to 0.0)	0<br>(0 to 0)	0.0<br>(0.0 to 0.0)	-66.0<br>(-81.7 to -32.7)	-61.2<br>(-79.1 to -23.2)
Austria	Rheumatoid arthritis	5 to 9	Male	0<br>(0 to 0)	0.0<br>(0.0 to 0.0)	0<br>(0 to 0)	0.0<br>(0.0 to 0.0)	0<br>(0 to 0)	0.0<br>(0.0 to 0.0)	-67.4<br>(-85.4 to -23.4)	-62.7<br>(-83.2 to -12.1)
Austria	Rheumatoid arthritis	10 to 14	Both	0<br>(0 to 0)	0.0<br>(0.0 to 0.0)	0<br>(0 to 0)	0.0<br>(0.0 to 0.0)	0<br>(0 to 0)	0.0<br>(0.0 to 0.0)	-57.5<br>(-76.0 to -24.6)	-55.0<br>(-74.6 to -20.2)
Austria	Rheumatoid arthritis	10 to 14	Female	0<br>(0 to 0)	0.0<br>(0.0 to 0.0)	0<br>(0 to 0)	0.0<br>(0.0 to 0.0)	0<br>(0 to 0)	0.0<br>(0.0 to 0.0)	-65.2<br>(-83.8 to -28.3)	-63.2<br>(-82.9 to -24.3)
Austria	Rheumatoid arthritis	10 to 14	Male	0<br>(0 to 0)	0.0<br>(0.0 to 0.0)	0<br>(0 to 0)	0.0<br>(0.0 to 0.0)	0<br>(0 to 0)	0.0<br>(0.0 to 0.0)	-33.4<br>(-64.9 to 17.0)	-29.4<br>(-62.8 to 24.0)
Austria	Other musculoskeletal disorders	5 to 9	Both	0<br>(0 to 0)	0.0<br>(0.0 to 0.0)	0<br>(0 to 0)	0.0<br>(0.0 to 0.0)	0<br>(0 to 0)	0.0<br>(0.0 to 0.0)	-58.2<br>(-73.6 to -31.6)	-52.2<br>(-69.8 to -21.7)
Austria	Other musculoskeletal disorders	5 to 9	Female	0<br>(0 to 0)	0.0<br>(0.0 to 0.1)	0<br>(0 to 0)	0.0<br>(0.0 to 0.0)	0<br>(0 to 0)	0.0<br>(0.0 to 0.0)	-47.2<br>(-69.6 to -7.8)	-39.6<br>(-65.2 to 5.3)
Austria	Other musculoskeletal disorders	5 to 9	Male	0<br>(0 to 0)	0.0<br>(0.0 to 0.0)	0<br>(0 to 0)	0.0<br>(0.0 to 0.0)	0<br>(0 to 0)	0.0<br>(0.0 to 0.0)	-76.1<br>(-86.9 to -45.2)	-72.6<br>(-84.9 to -37.2)
Austria	Other musculoskeletal disorders	10 to 14	Both	0<br>(0 to 0)	0.0<br>(0.0 to 0.1)	0<br>(0 to 0)	0.0<br>(0.0 to 0.1)	0<br>(0 to 0)	0.0<br>(0.0 to 0.0)	-34.2<br>(-57.1 to 0.5)	-30.3<br>(-54.6 to 6.3)
Austria	Other musculoskeletal disorders	10 to 14	Female	0<br>(0 to 0)	0.1<br>(0.0 to 0.1)	0<br>(0 to 0)	0.1<br>(0.0 to 0.1)	0<br>(0 to 0)	0.1<br>(0.0 to 0.1)	-29.6<br>(-57.3 to 15.3)	-25.7<br>(-54.9 to 21.8)
Austria	Other musculoskeletal disorders	10 to 14	Male	0<br>(0 to 0)	0.0<br>(0.0 to 0.0)	0<br>(0 to 0)	0.0<br>(0.0 to 0.0)	0<br>(0 to 0)	0.0<br>(0.0 to 0.0)	-53.6<br>(-73.1 to -21.9)	-50.9<br>(-71.5 to -17.3)
Austria	Congenital birth defects	5 to 9	Both	10<br>(7 to 12)	2.1<br>(1.4 to 2.6)	7<br>(5 to 9)	1.5<br>(1.0 to 1.9)	5<br>(3 to 6)	1.1<br>(0.7 to 1.5)	-52.5<br>(-65.5 to -32.8)	-45.7<br>(-60.5 to -23.1)
Austria	Congenital birth defects	5 to 9	Female	4<br>(3 to 5)	1.7<br>(1.2 to 2.2)	3<br>(2 to 4)	1.5<br>(0.9 to 1.9)	2<br>(1 to 3)	1.1<br>(0.7 to 1.5)	-42.4<br>(-60.1 to -14.7)	-34.2<br>(-54.4 to -2.6)
Austria	Congenital birth defects	5 to 9	Male	6<br>(3 to 8)	2.5<br>(1.5 to 3.3)	4<br>(2 to 5)	1.6<br>(0.9 to 2.2)	2<br>(1 to 4)	1.2<br>(0.6 to 1.7)	-59.2<br>(-74.2 to -35.0)	-53.2<br>(-70.4 to -25.5)
Austria	Congenital birth defects	10 to 14	Both	5<br>(4 to 6)	1.1<br>(0.9 to 1.4)	6<br>(4 to 7)	1.2<br>(0.9 to 1.5)	4<br>(3 to 5)	1.0<br>(0.6 to 1.2)	-20.2<br>(-44.8 to 10.7)	-15.5<br>(-41.6 to 17.1)
Austria	Congenital birth defects	10 to 14	Female	2<br>(2 to 3)	1.0<br>(0.8 to 1.4)	3<br>(2 to 4)	1.4<br>(0.8 to 1.8)	2<br>(1 to 3)	1.0<br>(0.6 to 1.3)	-13.0<br>(-42.5 to 29.5)	-8.2<br>(-39.3 to 36.8)
Austria	Congenital birth defects	10 to 14	Male	3<br>(2 to 4)	1.2<br>(0.9 to 1.7)	3<br>(2 to 4)	1.1<br>(0.7 to 1.5)	2<br>(1 to 3)	1.0<br>(0.6 to 1.4)	-25.7<br>(-53.1 to 16.4)	-21.3<br>(-50.3 to 23.4)
Austria	Skin and subcutaneous diseases	5 to 9	Both	0<br>(0 to 0)	0.0<br>(0.0 to 0.0)	0<br>(0 to 0)	0.0<br>(0.0 to 0.0)	0<br>(0 to 0)	0.0<br>(0.0 to 0.0)	-30.4<br>(-43.2 to -13.8)	-20.3<br>(-34.9 to -1.3)
Austria	Skin and subcutaneous diseases	5 to 9	Female	0<br>(0 to 0)	0.0<br>(0.0 to 0.0)	0<br>(0 to 0)	0.0<br>(0.0 to 0.0)	0<br>(0 to 0)	0.0<br>(0.0 to 0.0)	-28.2<br>(-41.9 to -10.4)	-17.9<br>(-33.6 to 2.4)
Austria	Skin and subcutaneous diseases	5 to 9	Male	0<br>(0 to 0)	0.0<br>(0.0 to 0.0)	0<br>(0 to 0)	0.0<br>(0.0 to 0.0)	0<br>(0 to 0)	0.0<br>(0.0 to 0.0)	-32.5<br>(-47.6 to -12.1)	-22.6<br>(-39.9 to 0.8)
Austria	Skin and subcutaneous diseases	10 to 14	Both	0<br>(0 to 0)	0.0<br>(0.0 to 0.0)	0<br>(0 to 0)	0.0<br>(0.0 to 0.0)	0<br>(0 to 0)	0.0<br>(0.0 to 0.0)	-15.4<br>(-26.9 to -2.5)	-10.5<br>(-22.7 to 3.2)

eTable 8. All-cause and cause-specific mortality (counts and rates) at the global level and by country in the WHO European Region for ages 5 to 9 and 10 to 14 for 1990, 2000, and 2016

Austria	Skin and subcutaneous diseases	10 to 14	Female	0 (0 to 0)	0.0 (0.0 to 0.0)	0 (0 to 0)	0.0 (0.0 to 0.0)	0 (0 to 0)	0.0 (0.0 to 0.0)	-14.2 (-27.5 to 3.1)	-9.4 (-23.5 to 8.9)
Austria	Skin and subcutaneous diseases	10 to 14	Male	0 (0 to 0)	0.0 (0.0 to 0.0)	0 (0 to 0)	0.0 (0.0 to 0.0)	0 (0 to 0)	0.0 (0.0 to 0.0)	-16.5 (-30.8 to -0.3)	-11.5 (-26.7 to 5.6)
Austria	Road injuries	5 to 9	Both	23 (19 to 27)	5.0 (4.2 to 5.8)	10 (8 to 12)	2.1 (1.7 to 2.6)	3 (2 to 5)	0.8 (0.6 to 1.1)	-85.9 (-90.1 to -78.8)	-83.9 (-88.7 to -75.8)
Austria	Road injuries	5 to 9	Female	8 (6 to 10)	3.6 (2.9 to 4.6)	4 (3 to 5)	1.7 (1.2 to 2.2)	1 (1 to 2)	0.6 (0.4 to 1.0)	-84.6 (-90.9 to -74.4)	-82.4 (-89.6 to -70.8)
Austria	Road injuries	5 to 9	Male	15 (12 to 18)	6.2 (5.0 to 7.5)	6 (5 to 8)	2.5 (2.0 to 3.2)	2 (1 to 3)	1.0 (0.6 to 1.4)	-86.7 (-91.4 to -78.7)	-84.7 (-90.1 to -75.6)
Austria	Road injuries	10 to 14	Both	20 (17 to 23)	4.6 (4.0 to 5.2)	14 (11 to 16)	2.9 (2.4 to 3.4)	5 (4 to 6)	1.2 (0.9 to 1.5)	-75.3 (-81.7 to -67.8)	-73.9 (-80.6 to -65.9)
Austria	Road injuries	10 to 14	Female	7 (6 to 9)	3.5 (2.8 to 4.4)	6 (5 to 8)	2.6 (2.0 to 3.3)	2 (1 to 3)	1.0 (0.7 to 1.4)	-73.1 (-82.6 to -58.9)	-71.6 (-81.6 to -56.6)
Austria	Road injuries	10 to 14	Male	13 (10 to 15)	5.6 (4.6 to 6.6)	8 (6 to 9)	3.1 (2.5 to 3.8)	3 (2 to 4)	1.4 (1.0 to 1.9)	-76.7 (-83.5 to -67.3)	-75.3 (-82.5 to -65.3)
Austria	Other transport injuries	5 to 9	Both	1 (1 to 2)	0.3 (0.2 to 0.4)	1 (1 to 1)	0.2 (0.1 to 0.2)	0 (0 to 1)	0.1 (0.1 to 0.2)	-60.9 (-77.6 to -31.2)	-55.2 (-74.3 to -21.3)
Austria	Other transport injuries	5 to 9	Female	1 (0 to 1)	0.2 (0.1 to 0.3)	0 (0 to 1)	0.1 (0.1 to 0.2)	0 (0 to 0)	0.1 (0.1 to 0.2)	-59.6 (-82.4 to -14.4)	-53.9 (-79.9 to -2.3)
Austria	Other transport injuries	5 to 9	Male	1 (0 to 1)	0.3 (0.1 to 0.5)	0 (0 to 1)	0.2 (0.1 to 0.3)	0 (0 to 0)	0.1 (0.1 to 0.2)	-61.8 (-82.7 to -17.2)	-56.2 (-80.1 to -5.0)
Austria	Other transport injuries	10 to 14	Both	2 (1 to 2)	0.4 (0.3 to 0.5)	1 (1 to 2)	0.3 (0.2 to 0.4)	1 (1 to 1)	0.2 (0.2 to 0.4)	-38.5 (-62.1 to 0.1)	-35.0 (-60.0 to 5.9)
Austria	Other transport injuries	10 to 14	Female	1 (0 to 1)	0.3 (0.2 to 0.4)	1 (0 to 1)	0.2 (0.2 to 0.4)	0 (0 to 1)	0.2 (0.1 to 0.3)	-47.6 (-74.8 to 5.3)	-44.7 (-73.4 to 11.2)
Austria	Other transport injuries	10 to 14	Male	1 (1 to 2)	0.5 (0.3 to 0.7)	1 (1 to 1)	0.3 (0.2 to 0.5)	1 (0 to 1)	0.3 (0.2 to 0.5)	-33.4 (-66.0 to 27.0)	-29.4 (-63.9 to 34.6)
Austria	Falls	5 to 9	Both	2 (2 to 3)	0.5 (0.4 to 0.7)	1 (1 to 2)	0.3 (0.2 to 0.4)	1 (1 to 1)	0.2 (0.1 to 0.3)	-70.0 (-80.2 to -43.7)	-65.7 (-77.4 to -35.5)
Austria	Falls	5 to 9	Female	1 (0 to 1)	0.3 (0.2 to 0.4)	0 (0 to 1)	0.2 (0.1 to 0.2)	0 (0 to 0)	0.1 (0.1 to 0.2)	-64.6 (-78.9 to -43.2)	-59.6 (-75.9 to -35.1)
Austria	Falls	5 to 9	Male	2 (1 to 2)	0.8 (0.5 to 1.0)	1 (1 to 1)	0.4 (0.3 to 0.6)	1 (0 to 1)	0.2 (0.2 to 0.4)	-71.9 (-83.4 to -42.9)	-67.7 (-80.9 to -34.5)
Austria	Falls	10 to 14	Both	3 (2 to 4)	0.7 (0.5 to 0.9)	2 (2 to 3)	0.5 (0.4 to 0.7)	1 (1 to 2)	0.3 (0.2 to 0.5)	-55.2 (-70.3 to -32.2)	-52.6 (-68.6 to -28.2)
Austria	Falls	10 to 14	Female	1 (0 to 1)	0.4 (0.2 to 0.6)	1 (0 to 1)	0.3 (0.2 to 0.4)	0 (0 to 0)	0.2 (0.1 to 0.2)	-55.2 (-72.5 to -27.8)	-52.7 (-71.0 to -23.8)
Austria	Falls	10 to 14	Male	2 (2 to 3)	1.1 (0.8 to 1.5)	2 (1 to 3)	0.8 (0.6 to 1.1)	1 (1 to 2)	0.5 (0.3 to 0.7)	-55.2 (-72.2 to -28.9)	-52.5 (-70.5 to -24.6)
Austria	Drowning	5 to 9	Both	6 (5 to 7)	1.3 (1.0 to 1.6)	3 (2 to 4)	0.6 (0.5 to 0.8)	1 (1 to 2)	0.3 (0.2 to 0.4)	-77.5 (-84.4 to -67.6)	-74.2 (-82.1 to -62.9)
Austria	Drowning	5 to 9	Female	1 (1 to 2)	0.5 (0.4 to 0.7)	1 (1 to 1)	0.4 (0.3 to 0.6)	0 (0 to 1)	0.2 (0.2 to 0.3)	-60.2 (-75.5 to -38.4)	-54.6 (-72.0 to -29.6)
Austria	Drowning	5 to 9	Male	5 (4 to 6)	1.9 (1.5 to 2.5)	2 (2 to 3)	0.9 (0.6 to 1.1)	1 (1 to 1)	0.4 (0.3 to 0.6)	-82.0 (-88.9 to -72.0)	-79.4 (-87.3 to -67.8)
Austria	Drowning	10 to 14	Both	2 (2 to 3)	0.5 (0.4 to 0.6)	2 (1 to 2)	0.4 (0.3 to 0.5)	1 (1 to 1)	0.2 (0.2 to 0.3)	-55.3 (-70.2 to -36.1)	-52.7 (-68.5 to -32.4)
Austria	Drowning	10 to 14	Female	0 (0 to 1)	0.2 (0.1 to 0.3)	1 (0 to 1)	0.2 (0.2 to 0.3)	0 (0 to 0)	0.1 (0.1 to 0.2)	-39.7 (-63.5 to 0.7)	-36.3 (-61.5 to 6.3)
Austria	Drowning	10 to 14	Male	2 (1 to 2)	0.7 (0.5 to 1.0)	1 (1 to 2)	0.5 (0.4 to 0.7)	1 (0 to 1)	0.3 (0.2 to 0.4)	-59.6 (-75.2 to -37.2)	-57.2 (-73.7 to -33.4)
Austria	Fire, heat, and hot substances	5 to 9	Both	1 (1 to 2)	0.3 (0.2 to 0.4)	3 (2 to 4)	0.6 (0.4 to 0.9)	0 (0 to 1)	0.1 (0.1 to 0.2)	-61.2 (-74.8 to -40.4)	-55.6 (-71.1 to -31.7)
Austria	Fire, heat, and hot substances	5 to 9	Female	0 (0 to 1)	0.2 (0.1 to 0.4)	1 (1 to 2)	0.6 (0.3 to 0.9)	0 (0 to 0)	0.1 (0.1 to 0.2)	-49.3 (-70.7 to -11.9)	-42.0 (-66.5 to 0.7)

eTable 8. All-cause and cause-specific mortality (counts and rates) at the global level and by country in the WHO European Region for ages 5 to 9 and 10 to 14 for 1990, 2000, and 2016

Austria	Fire, heat, and hot substances	5 to 9	Male	1<br>(0 to 1)	0.3<br>(0.2 to 0.5)	2<br>(1 to 2)	0.7<br>(0.4 to 1.0)	0<br>(0 to 0)	0.1<br>(0.1 to 0.2)	-69.0<br>(-81.0 to -45.4)	-64.4<br>(-78.2 to -37.3)
Austria	Fire, heat, and hot substances	10 to 14	Both	1<br>(0 to 1)	0.1<br>(0.1 to 0.2)	2<br>(1 to 3)	0.4<br>(0.2 to 0.6)	0<br>(0 to 1)	0.1<br>(0.1 to 0.1)	-41.0<br>(-61.6 to -12.1)	-37.6<br>(-59.4 to -7.1)
Austria	Fire, heat, and hot substances	10 to 14	Female	0<br>(0 to 0)	0.1<br>(0.1 to 0.2)	1<br>(0 to 1)	0.3<br>(0.2 to 0.5)	0<br>(0 to 0)	0.1<br>(0.0 to 0.1)	-32.6<br>(-66.9 to 28.7)	-28.8<br>(-65.0 to 35.9)
Austria	Fire, heat, and hot substances	10 to 14	Male	0<br>(0 to 1)	0.2<br>(0.1 to 0.3)	1<br>(1 to 2)	0.4<br>(0.3 to 0.6)	0<br>(0 to 0)	0.1<br>(0.1 to 0.2)	-45.6<br>(-66.0 to -15.7)	-42.3<br>(-63.9 to -10.7)
Austria	Poisonings	5 to 9	Both	1<br>(0 to 1)	0.1<br>(0.0 to 0.2)	0<br>(0 to 0)	0.0<br>(0.0 to 0.1)	0<br>(0 to 0)	0.0<br>(0.0 to 0.0)	-78.5<br>(-87.6 to -55.1)	-75.4<br>(-85.8 to -48.6)
Austria	Poisonings	5 to 9	Female	0<br>(0 to 0)	0.1<br>(0.0 to 0.1)	0<br>(0 to 0)	0.0<br>(0.0 to 0.1)	0<br>(0 to 0)	0.0<br>(0.0 to 0.0)	-73.4<br>(-87.2 to -47.7)	-69.6<br>(-85.4 to -40.2)
Austria	Poisonings	5 to 9	Male	0<br>(0 to 1)	0.1<br>(0.1 to 0.2)	0<br>(0 to 0)	0.1<br>(0.0 to 0.1)	0<br>(0 to 0)	0.0<br>(0.0 to 0.1)	-80.9<br>(-90.5 to -51.3)	-78.1<br>(-89.2 to -44.2)
Austria	Poisonings	10 to 14	Both	1<br>(1 to 1)	0.2<br>(0.1 to 0.3)	0<br>(0 to 1)	0.1<br>(0.1 to 0.2)	0<br>(0 to 0)	0.1<br>(0.0 to 0.1)	-62.3<br>(-77.2 to -38.3)	-60.2<br>(-75.9 to -34.7)
Austria	Poisonings	10 to 14	Female	0<br>(0 to 0)	0.1<br>(0.1 to 0.2)	0<br>(0 to 0)	0.1<br>(0.1 to 0.2)	0<br>(0 to 0)	0.1<br>(0.0 to 0.1)	-62.6<br>(-81.8 to -34.7)	-60.5<br>(-80.8 to -31.1)
Austria	Poisonings	10 to 14	Male	0<br>(0 to 1)	0.2<br>(0.1 to 0.3)	0<br>(0 to 0)	0.1<br>(0.1 to 0.2)	0<br>(0 to 0)	0.1<br>(0.1 to 0.1)	-62.2<br>(-79.3 to -30.8)	-59.9<br>(-78.1 to -26.7)
Austria	Exposure to mechanical forces	5 to 9	Both	3<br>(2 to 5)	0.7<br>(0.4 to 1.0)	2<br>(1 to 3)	0.4<br>(0.3 to 0.6)	1<br>(1 to 1)	0.2<br>(0.1 to 0.3)	-71.3<br>(-80.3 to -56.8)	-67.2<br>(-77.5 to -50.6)
Austria	Exposure to mechanical forces	5 to 9	Female	1<br>(0 to 1)	0.4<br>(0.2 to 0.6)	1<br>(0 to 1)	0.3<br>(0.2 to 0.4)	0<br>(0 to 1)	0.2<br>(0.1 to 0.4)	-47.4<br>(-66.9 to -16.4)	-39.9<br>(-62.2 to -4.5)
Austria	Exposure to mechanical forces	5 to 9	Male	3<br>(1 to 3)	1.1<br>(0.6 to 1.5)	1<br>(1 to 2)	0.6<br>(0.3 to 0.8)	1<br>(0 to 1)	0.2<br>(0.1 to 0.4)	-79.8<br>(-87.0 to -62.6)	-76.8<br>(-85.1 to -57.1)
Austria	Exposure to mechanical forces	10 to 14	Both	2<br>(2 to 3)	0.5<br>(0.4 to 0.7)	2<br>(1 to 2)	0.4<br>(0.3 to 0.5)	1<br>(1 to 1)	0.2<br>(0.2 to 0.3)	-56.5<br>(-67.7 to -40.2)	-54.0<br>(-65.8 to -36.7)
Austria	Exposure to mechanical forces	10 to 14	Female	0<br>(0 to 1)	0.2<br>(0.1 to 0.3)	0<br>(0 to 1)	0.2<br>(0.1 to 0.3)	0<br>(0 to 0)	0.2<br>(0.1 to 0.2)	-31.1<br>(-52.9 to 4.4)	-27.2<br>(-50.3 to 10.2)
Austria	Exposure to mechanical forces	10 to 14	Male	2<br>(1 to 2)	0.8<br>(0.6 to 1.1)	1<br>(1 to 2)	0.6<br>(0.4 to 0.8)	1<br>(1 to 1)	0.3<br>(0.2 to 0.5)	-62.6<br>(-74.8 to -43.2)	-60.3<br>(-73.3 to -39.8)
Austria	Adverse effects of medical treatment	5 to 9	Both	1<br>(0 to 1)	0.1<br>(0.1 to 0.2)	1<br>(0 to 1)	0.1<br>(0.1 to 0.2)	0<br>(0 to 1)	0.1<br>(0.0 to 0.2)	-36.2<br>(-69.0 to 6.6)	-27.0<br>(-64.6 to 22.1)
Austria	Adverse effects of medical treatment	5 to 9	Female	0<br>(0 to 0)	0.1<br>(0.1 to 0.1)	0<br>(0 to 0)	0.1<br>(0.1 to 0.1)	0<br>(0 to 0)	0.1<br>(0.0 to 0.2)	-16.3<br>(-59.3 to 62.0)	-4.4<br>(-53.5 to 85.1)
Austria	Adverse effects of medical treatment	5 to 9	Male	0<br>(0 to 1)	0.2<br>(0.1 to 0.3)	0<br>(0 to 1)	0.2<br>(0.1 to 0.3)	0<br>(0 to 0)	0.1<br>(0.0 to 0.2)	-46.9<br>(-78.1 to -3.3)	-39.1<br>(-74.9 to 10.9)
Austria	Adverse effects of medical treatment	10 to 14	Both	0<br>(0 to 1)	0.1<br>(0.1 to 0.2)	1<br>(0 to 1)	0.1<br>(0.1 to 0.2)	0<br>(0 to 1)	0.1<br>(0.1 to 0.2)	-0.2<br>(-48.9 to 67.4)	5.6<br>(-45.9 to 77.1)
Austria	Adverse effects of medical treatment	10 to 14	Female	0<br>(0 to 0)	0.1<br>(0.1 to 0.1)	0<br>(0 to 0)	0.1<br>(0.1 to 0.2)	0<br>(0 to 0)	0.1<br>(0.0 to 0.2)	17.9<br>(-43.3 to 121.4)	24.5<br>(-40.1 to 133.8)
Austria	Adverse effects of medical treatment	10 to 14	Male	0<br>(0 to 0)	0.1<br>(0.1 to 0.2)	0<br>(0 to 0)	0.1<br>(0.1 to 0.2)	0<br>(0 to 0)	0.1<br>(0.1 to 0.2)	-13.1<br>(-56.3 to 61.2)	-7.9<br>(-53.6 to 70.9)
Austria	Animal contact	5 to 9	Both	0<br>(0 to 0)	0.0<br>(0.0 to 0.0)	0<br>(0 to 0)	0.0<br>(0.0 to 0.0)	0<br>(0 to 0)	0.0<br>(0.0 to 0.0)	-54.4<br>(-75.0 to -21.6)	-47.8<br>(-71.4 to -10.3)

eTable 8. All-cause and cause-specific mortality (counts and rates) at the global level and by country in the WHO European Region for ages 5 to 9 and 10 to 14 for 1990, 2000, and 2016

Austria	Animal contact	5 to 9	Female	0 (0 to 0)	0.0 (0.0 to 0.0)	0 (0 to 0)	0.0 (0.0 to 0.0)	0 (0 to 0)	0.0 (0.0 to 0.0)	-27.6 (-41.9 to -7.7)	-17.3 (-33.6 to 5.5)
Austria	Animal contact	5 to 9	Male	0 (0 to 0)	0.0 (0.0 to 0.1)	0 (0 to 0)	0.0 (0.0 to 0.0)	0 (0 to 0)	0.0 (0.0 to 0.0)	-61.8 (-82.4 to -19.6)	-56.1 (-79.8 to -7.8)
Austria	Animal contact	10 to 14	Both	0 (0 to 0)	0.0 (0.0 to 0.0)	0 (0 to 0)	0.0 (0.0 to 0.0)	0 (0 to 0)	0.0 (0.0 to 0.0)	-14.5 (-40.7 to 35.7)	-9.6 (-37.3 to 43.5)
Austria	Animal contact	10 to 14	Female	0 (0 to 0)	0.0 (0.0 to 0.0)	0 (0 to 0)	0.0 (0.0 to 0.0)	0 (0 to 0)	0.0 (0.0 to 0.0)	-0.4 (-47.3 to 87.4)	5.2 (-44.3 to 97.8)
Austria	Animal contact	10 to 14	Male	0 (0 to 0)	0.0 (0.0 to 0.0)	0 (0 to 0)	0.0 (0.0 to 0.0)	0 (0 to 0)	0.0 (0.0 to 0.0)	-26.6 (-50.7 to -1.9)	-22.2 (-47.7 to 3.9)
Austria	Foreign body	5 to 9	Both	1 (1 to 1)	0.2 (0.2 to 0.3)	1 (1 to 1)	0.2 (0.1 to 0.2)	1 (0 to 1)	0.1 (0.1 to 0.2)	-44.8 (-63.8 to -14.7)	-36.8 (-58.5 to -2.3)
Austria	Foreign body	5 to 9	Female	0 (0 to 0)	0.1 (0.1 to 0.2)	0 (0 to 0)	0.1 (0.1 to 0.2)	0 (0 to 0)	0.1 (0.1 to 0.2)	-21.3 (-50.5 to 36.8)	-10.1 (-43.4 to 56.3)
Austria	Foreign body	5 to 9	Male	1 (0 to 1)	0.3 (0.2 to 0.4)	0 (0 to 1)	0.2 (0.1 to 0.3)	0 (0 to 1)	0.2 (0.1 to 0.3)	-55.0 (-75.1 to -19.4)	-48.4 (-71.4 to -7.6)
Austria	Foreign body	10 to 14	Both	1 (1 to 2)	0.3 (0.2 to 0.4)	1 (1 to 2)	0.2 (0.1 to 0.3)	1 (1 to 1)	0.2 (0.1 to 0.3)	-27.1 (-53.3 to 25.1)	-22.9 (-50.6 to 32.4)
Austria	Foreign body	10 to 14	Female	0 (0 to 0)	0.1 (0.1 to 0.2)	0 (0 to 0)	0.1 (0.1 to 0.2)	0 (0 to 0)	0.1 (0.1 to 0.1)	4.3 (-31.2 to 90.7)	10.1 (-27.3 to 101.3)
Austria	Foreign body	10 to 14	Male	1 (1 to 1)	0.4 (0.3 to 0.7)	1 (0 to 1)	0.3 (0.2 to 0.5)	1 (0 to 1)	0.3 (0.2 to 0.5)	-33.5 (-62.7 to 22.5)	-29.5 (-60.4 to 29.9)
Austria	Environmental heat and cold exposure	5 to 9	Both	0 (0 to 0)	0.0 (0.0 to 0.0)	0 (0 to 0)	0.0 (0.0 to 0.0)	0 (0 to 0)	0.0 (0.0 to 0.0)	-35.2 (-66.7 to 11.1)	-25.9 (-61.8 to 27.2)
Austria	Environmental heat and cold exposure	5 to 9	Female	0 (0 to 0)	0.0 (0.0 to 0.0)	0 (0 to 0)	0.0 (0.0 to 0.0)	0 (0 to 0)	0.0 (0.0 to 0.0)	-22.4 (-64.2 to 30.2)	-11.4 (-59.1 to 48.8)
Austria	Environmental heat and cold exposure	5 to 9	Male	0 (0 to 0)	0.0 (0.0 to 0.0)	0 (0 to 0)	0.0 (0.0 to 0.0)	0 (0 to 0)	0.0 (0.0 to 0.0)	-42.0 (-73.6 to 16.8)	-33.4 (-69.7 to 34.0)
Austria	Environmental heat and cold exposure	10 to 14	Both	0 (0 to 0)	0.0 (0.0 to 0.0)	0 (0 to 0)	0.0 (0.0 to 0.0)	0 (0 to 0)	0.0 (0.0 to 0.0)	-24.2 (-59.6 to 29.2)	-19.9 (-57.2 to 36.7)
Austria	Environmental heat and cold exposure	10 to 14	Female	0 (0 to 0)	0.0 (0.0 to 0.0)	0 (0 to 0)	0.0 (0.0 to 0.0)	0 (0 to 0)	0.0 (0.0 to 0.0)	15.6 (-52.9 to 93.7)	22.1 (-50.3 to 104.6)
Austria	Environmental heat and cold exposure	10 to 14	Male	0 (0 to 0)	0.0 (0.0 to 0.1)	0 (0 to 0)	0.0 (0.0 to 0.0)	0 (0 to 0)	0.0 (0.0 to 0.0)	-41.1 (-65.7 to 14.0)	-37.6 (-63.6 to 20.8)
Austria	Other unintentional injuries	5 to 9	Both	1 (0 to 1)	0.1 (0.1 to 0.2)	0 (0 to 0)	0.0 (0.0 to 0.1)	0 (0 to 0)	0.0 (0.0 to 0.0)	-84.8 (-91.3 to -72.7)	-82.6 (-90.0 to -68.8)
Austria	Other unintentional injuries	5 to 9	Female	0 (0 to 0)	0.0 (0.0 to 0.0)	0 (0 to 0)	0.0 (0.0 to 0.0)	0 (0 to 0)	0.0 (0.0 to 0.0)	-59.8 (-74.1 to -28.8)	-54.1 (-70.4 to -18.6)
Austria	Other unintentional injuries	5 to 9	Male	0 (0 to 1)	0.2 (0.1 to 0.3)	0 (0 to 0)	0.1 (0.1 to 0.1)	0 (0 to 0)	0.0 (0.0 to 0.1)	-87.2 (-93.6 to -75.2)	-85.4 (-92.7 to -71.6)
Austria	Other unintentional injuries	10 to 14	Both	1 (0 to 1)	0.2 (0.1 to 0.2)	0 (0 to 1)	0.1 (0.1 to 0.1)	0 (0 to 0)	0.0 (0.0 to 0.1)	-72.1 (-82.1 to -56.1)	-70.5 (-81.0 to -53.5)
Austria	Other unintentional injuries	10 to 14	Female	0 (0 to 0)	0.0 (0.0 to 0.0)	0 (0 to 0)	0.0 (0.0 to 0.0)	0 (0 to 0)	0.0 (0.0 to 0.0)	-54.3 (-74.2 to -16.5)	-51.8 (-72.8 to -11.8)
Austria	Other unintentional injuries	10 to 14	Male	1 (0 to 1)	0.3 (0.2 to 0.4)	0 (0 to 1)	0.2 (0.1 to 0.2)	0 (0 to 0)	0.1 (0.1 to 0.1)	-73.8 (-84.1 to -56.7)	-72.2 (-83.1 to -54.1)
Austria	Self-harm	10 to 14	Both	4 (3 to 6)	1.0 (0.7 to 1.3)	4 (3 to 6)	1.0 (0.6 to 1.2)	2 (2 to 3)	0.6 (0.4 to 0.8)	-47.1 (-63.8 to -24.7)	-44.1 (-61.7 to -20.4)
Austria	Self-harm	10 to 14	Female	1 (1 to 2)	0.7 (0.5 to 0.9)	2 (1 to 2)	0.7 (0.5 to 1.0)	1 (0 to 1)	0.4 (0.2 to 0.5)	-48.9 (-69.0 to -14.9)	-46.1 (-67.3 to -10.1)
Austria	Self-harm	10 to 14	Male	3 (2 to 4)	1.4 (0.7 to 1.9)	3 (1 to 4)	1.2 (0.6 to 1.6)	2 (1 to 2)	0.8 (0.4 to 1.1)	-46.3 (-67.0 to -16.5)	-43.1 (-65.0 to -11.5)

eTable 8. All-cause and cause-specific mortality (counts and rates) at the global level and by country in the WHO European Region for ages 5 to 9 and 10 to 14 for 1990, 2000, and 2016

Austria	Interpersonal violence	5 to 9	Both	3 (2 to 4)	0.6 (0.4 to 0.8)	2 (1 to 3)	0.4 (0.3 to 0.5)	1 (1 to 1)	0.2 (0.2 to 0.3)	-67.1 (-78.5 to -49.2)	-62.3 (-75.4 to -41.9)
Austria	Interpersonal violence	5 to 9	Female	2 (1 to 2)	0.7 (0.4 to 1.0)	1 (1 to 2)	0.5 (0.3 to 0.7)	1 (0 to 1)	0.3 (0.2 to 0.5)	-60.1 (-77.5 to -29.1)	-54.5 (-74.3 to -19.0)
Austria	Interpersonal violence	5 to 9	Male	1 (1 to 2)	0.6 (0.3 to 0.9)	1 (0 to 1)	0.3 (0.2 to 0.5)	0 (0 to 1)	0.2 (0.1 to 0.3)	-74.6 (-84.7 to -63.0)	-70.9 (-82.5 to -57.6)
Austria	Interpersonal violence	10 to 14	Both	2 (1 to 3)	0.4 (0.3 to 0.7)	2 (1 to 3)	0.4 (0.3 to 0.6)	1 (1 to 2)	0.2 (0.2 to 0.4)	-50.8 (-66.9 to -28.1)	-48.0 (-65.0 to -23.9)
Austria	Interpersonal violence	10 to 14	Female	1 (1 to 1)	0.4 (0.3 to 0.6)	1 (1 to 1)	0.4 (0.3 to 0.6)	0 (0 to 1)	0.2 (0.1 to 0.5)	-42.9 (-68.1 to -13.0)	-39.8 (-66.4 to -8.1)
Austria	Interpersonal violence	10 to 14	Male	1 (1 to 2)	0.5 (0.3 to 0.9)	1 (0 to 2)	0.3 (0.2 to 0.6)	0 (0 to 1)	0.2 (0.1 to 0.4)	-56.7 (-75.2 to -27.6)	-54.1 (-73.7 to -23.3)
Austria	Exposure to forces of nature	5 to 9	Both	0 (0 to 0)	0.0 (0.0 to 0.0)	0 (0 to 0)	0.0 (0.0 to 0.0)	0 (0 to 0)	0.0 (0.0 to 0.0)	0.0 (0.0 to 0.0)	0.0 (0.0 to 0.0)
Austria	Exposure to forces of nature	5 to 9	Female	0 (0 to 0)	0.0 (0.0 to 0.0)	0 (0 to 0)	0.0 (0.0 to 0.0)	0 (0 to 0)	0.0 (0.0 to 0.0)	0.0 (0.0 to 0.0)	0.0 (0.0 to 0.0)
Austria	Exposure to forces of nature	5 to 9	Male	0 (0 to 0)	0.0 (0.0 to 0.0)	0 (0 to 0)	0.0 (0.0 to 0.0)	0 (0 to 0)	0.0 (0.0 to 0.0)	0.0 (0.0 to 0.0)	0.0 (0.0 to 0.0)
Austria	Exposure to forces of nature	10 to 14	Both	0 (0 to 0)	0.0 (0.0 to 0.0)	0 (0 to 0)	0.0 (0.0 to 0.0)	0 (0 to 0)	0.0 (0.0 to 0.0)	0.0 (0.0 to 0.0)	0.0 (0.0 to 0.0)
Austria	Exposure to forces of nature	10 to 14	Female	0 (0 to 0)	0.0 (0.0 to 0.0)	0 (0 to 0)	0.0 (0.0 to 0.0)	0 (0 to 0)	0.0 (0.0 to 0.0)	0.0 (0.0 to 0.0)	0.0 (0.0 to 0.0)
Austria	Exposure to forces of nature	10 to 14	Male	0 (0 to 0)	0.0 (0.0 to 0.0)	0 (0 to 0)	0.0 (0.0 to 0.0)	0 (0 to 0)	0.0 (0.0 to 0.0)	0.0 (0.0 to 0.0)	0.0 (0.0 to 0.0)
Austria	Conflict and terrorism	5 to 9	Both	0 (0 to 0)	0.0 (0.0 to 0.0)	0 (0 to 0)	0.0 (0.0 to 0.0)	0 (0 to 0)	0.0 (0.0 to 0.0)	0.0 (0.0 to 0.0)	0.0 (0.0 to 0.0)
Austria	Conflict and terrorism	5 to 9	Female	0 (0 to 0)	0.0 (0.0 to 0.0)	0 (0 to 0)	0.0 (0.0 to 0.0)	0 (0 to 0)	0.0 (0.0 to 0.0)	0.0 (0.0 to 0.0)	0.0 (0.0 to 0.0)
Austria	Conflict and terrorism	5 to 9	Male	0 (0 to 0)	0.0 (0.0 to 0.0)	0 (0 to 0)	0.0 (0.0 to 0.0)	0 (0 to 0)	0.0 (0.0 to 0.0)	0.0 (0.0 to 0.0)	0.0 (0.0 to 0.0)
Austria	Conflict and terrorism	10 to 14	Both	0 (0 to 0)	0.0 (0.0 to 0.0)	0 (0 to 0)	0.0 (0.0 to 0.0)	0 (0 to 0)	0.0 (0.0 to 0.0)	0.0 (0.0 to 0.0)	0.0 (0.0 to 0.0)
Austria	Conflict and terrorism	10 to 14	Female	0 (0 to 0)	0.0 (0.0 to 0.0)	0 (0 to 0)	0.0 (0.0 to 0.0)	0 (0 to 0)	0.0 (0.0 to 0.0)	0.0 (0.0 to 0.0)	0.0 (0.0 to 0.0)
Austria	Conflict and terrorism	10 to 14	Male	0 (0 to 0)	0.0 (0.0 to 0.0)	0 (0 to 0)	0.0 (0.0 to 0.0)	0 (0 to 0)	0.0 (0.0 to 0.0)	0.0 (0.0 to 0.0)	0.0 (0.0 to 0.0)
Austria	Executions and police conflict	5 to 9	Both	0 (0 to 0)	0.0 (0.0 to 0.0)	0 (0 to 0)	0.0 (0.0 to 0.0)	0 (0 to 0)	0.0 (0.0 to 0.0)	-30.5 (-43.2 to -13.9)	-20.5 (-34.9 to -1.5)
Austria	Executions and police conflict	5 to 9	Female	0 (0 to 0)	0.0 (0.0 to 0.0)	0 (0 to 0)	0.0 (0.0 to 0.0)	0 (0 to 0)	0.0 (0.0 to 0.0)	-28.3 (-41.9 to -10.6)	-18.0 (-33.6 to 2.1)
Austria	Executions and police conflict	5 to 9	Male	0 (0 to 0)	0.0 (0.0 to 0.0)	0 (0 to 0)	0.0 (0.0 to 0.0)	0 (0 to 0)	0.0 (0.0 to 0.0)	-32.6 (-47.6 to -13.0)	-22.7 (-39.9 to -0.3)
Austria	Executions and police conflict	10 to 14	Both	0 (0 to 0)	0.0 (0.0 to 0.0)	0 (0 to 0)	0.0 (0.0 to 0.0)	0 (0 to 0)	0.0 (0.0 to 0.0)	-15.5 (-26.9 to -2.6)	-10.6 (-22.7 to 3.0)
Austria	Executions and police conflict	10 to 14	Female	0 (0 to 0)	0.0 (0.0 to 0.0)	0 (0 to 0)	0.0 (0.0 to 0.0)	0 (0 to 0)	0.0 (0.0 to 0.0)	-14.3 (-27.5 to 2.8)	-9.5 (-23.5 to 8.5)
Austria	Executions and police conflict	10 to 14	Male	0 (0 to 0)	0.0 (0.0 to 0.0)	0 (0 to 0)	0.0 (0.0 to 0.0)	0 (0 to 0)	0.0 (0.0 to 0.0)	-16.6 (-30.8 to -0.5)	-11.7 (-26.7 to 5.4)
Azerbaijan	All causes	5 to 9	Both	923 (802 to 1049)	112.8 (98.0 to 128.2)	1427 (1257 to 1644)	160.8 (141.6 to 185.2)	419 (325 to 535)	64.0 (49.6 to 81.7)	-54.6 (-66.2 to -38.7)	-43.2 (-57.8 to -23.5)
Azerbaijan	All causes	5 to 9	Female	380 (332 to 430)	95.3 (83.4 to 107.9)	596 (524 to 678)	137.6 (121.1 to 156.7)	164 (133 to 203)	53.4 (43.3 to 66.2)	-56.9 (-67.0 to -44.1)	-43.9 (-57.0 to -27.3)

eTable 8. All-cause and cause-specific mortality (counts and rates) at the global level and by country in the WHO European Region for ages 5 to 9 and 10 to 14 for 1990, 2000, and 2016

Azerbaijan	All causes	5 to 9	Male	543 (469 to 623)	129.3 (111.8 to 148.4)	832 (724 to 966)	182.7 (159.0 to 212.3)	256 (192 to 333)	73.3 (55.2 to 95.4)	-52.9 (-65.9 to -36.2)	-43.3 (-58.9 to -23.2)
Azerbaijan	All causes	10 to 14	Both	551 (503 to 597)	77.8 (71.1 to 84.3)	928 (856 to 1010)	99.1 (91.4 to 108.0)	328 (273 to 391)	56.8 (47.3 to 67.8)	-40.5 (-51.4 to -27.7)	-27.0 (-40.4 to -11.3)
Azerbaijan	All causes	10 to 14	Female	202 (185 to 217)	58.2 (53.5 to 62.7)	358 (330 to 388)	78.2 (72.1 to 84.9)	118 (99 to 137)	43.2 (36.2 to 50.3)	-41.7 (-51.8 to -30.6)	-25.9 (-38.8 to -11.8)
Azerbaijan	All causes	10 to 14	Male	349 (313 to 383)	96.5 (86.6 to 105.7)	570 (522 to 630)	119.2 (109.1 to 131.7)	210 (170 to 257)	69.0 (55.8 to 84.5)	-39.8 (-51.6 to -23.3)	-28.5 (-42.5 to -8.8)
Azerbaijan	Tuberculosis	5 to 9	Both	6 (4 to 8)	0.7 (0.5 to 1.0)	17 (12 to 24)	1.9 (1.4 to 2.7)	2 (1 to 3)	0.3 (0.2 to 0.4)	-68.4 (-82.2 to -46.5)	-60.5 (-77.8 to -33.1)
Azerbaijan	Tuberculosis	5 to 9	Female	3 (2 to 5)	0.7 (0.4 to 1.1)	8 (5 to 12)	1.9 (1.2 to 2.8)	1 (0 to 1)	0.3 (0.1 to 0.5)	-73.2 (-87.6 to -45.8)	-65.2 (-83.8 to -29.5)
Azerbaijan	Tuberculosis	5 to 9	Male	3 (2 to 4)	0.7 (0.4 to 1.0)	9 (6 to 13)	2.0 (1.3 to 2.9)	1 (1 to 2)	0.3 (0.2 to 0.5)	-63.6 (-80.8 to -28.9)	-56.2 (-76.9 to -14.4)
Azerbaijan	Tuberculosis	10 to 14	Both	6 (4 to 8)	0.8 (0.5 to 1.1)	18 (13 to 25)	1.9 (1.4 to 2.6)	2 (1 to 3)	0.4 (0.2 to 0.6)	-62.7 (-78.2 to -34.9)	-54.2 (-73.3 to -20.2)
Azerbaijan	Tuberculosis	10 to 14	Female	3 (2 to 4)	0.8 (0.5 to 1.2)	9 (6 to 13)	2.0 (1.3 to 2.9)	1 (1 to 2)	0.4 (0.2 to 0.7)	-61.4 (-80.2 to -27.7)	-50.9 (-74.8 to -8.1)
Azerbaijan	Tuberculosis	10 to 14	Male	3 (2 to 4)	0.7 (0.4 to 1.1)	9 (6 to 13)	1.9 (1.2 to 2.8)	1 (0 to 2)	0.3 (0.2 to 0.6)	-64.2 (-83.8 to -22.8)	-57.4 (-80.7 to -8.3)
Azerbaijan	HIV/AIDS	5 to 9	Both	0 (0 to 0)	0.0 (0.0 to 0.0)	0 (0 to 0)	0.0 (0.0 to 0.0)	0 (0 to 0)	0.0 (0.0 to 0.0)	-37.3 (-100.0 to 13294.8)	-21.7 (-100.0 to 16637.8)
Azerbaijan	HIV/AIDS	5 to 9	Female	0 (0 to 0)	0.0 (0.0 to 0.0)	0 (0 to 0)	0.0 (0.0 to 0.0)	0 (0 to 0)	0.0 (0.0 to 0.0)	-39.7 (-100.0 to 12980.1)	-21.5 (-100.0 to 16932.0)
Azerbaijan	HIV/AIDS	5 to 9	Male	0 (0 to 0)	0.0 (0.0 to 0.0)	0 (0 to 0)	0.0 (0.0 to 0.0)	0 (0 to 0)	0.0 (0.0 to 0.0)	-35.1 (-100.0 to 13846.2)	-21.9 (-100.0 to 16683.5)
Azerbaijan	HIV/AIDS	10 to 14	Both	0 (0 to 0)	0.0 (0.0 to 0.0)	0 (0 to 0)	0.0 (0.0 to 0.0)	0 (0 to 0)	0.0 (0.0 to 0.0)	21.7 (-100.0 to 2960.7)	49.3 (-100.0 to 3655.8)
Azerbaijan	HIV/AIDS	10 to 14	Female	0 (0 to 0)	0.0 (0.0 to 0.0)	0 (0 to 0)	0.0 (0.0 to 0.0)	0 (0 to 0)	0.0 (0.0 to 0.0)	17.2 (-100.0 to 2896.6)	49.0 (-100.0 to 3708.0)
Azerbaijan	HIV/AIDS	10 to 14	Male	0 (0 to 0)	0.0 (0.0 to 0.0)	0 (0 to 0)	0.0 (0.0 to 0.0)	0 (0 to 0)	0.0 (0.0 to 0.0)	26.0 (-100.0 to 3025.8)	49.6 (-100.0 to 3613.7)
Azerbaijan	Diarrheal diseases	5 to 9	Both	13 (6 to 23)	1.6 (0.7 to 2.8)	18 (6 to 36)	2.0 (0.7 to 4.0)	2 (1 to 5)	0.4 (0.2 to 0.7)	-81.3 (-90.2 to -68.2)	-76.6 (-87.8 to -60.2)
Azerbaijan	Diarrheal diseases	5 to 9	Female	6 (2 to 14)	1.6 (0.5 to 3.4)	9 (2 to 22)	2.1 (0.5 to 5.2)	1 (0 to 3)	0.4 (0.1 to 0.9)	-81.1 (-92.0 to -64.1)	-75.4 (-89.6 to -53.2)
Azerbaijan	Diarrheal diseases	5 to 9	Male	7 (2 to 13)	1.6 (0.5 to 3.1)	9 (3 to 19)	1.9 (0.6 to 4.2)	1 (0 to 3)	0.4 (0.1 to 0.8)	-81.5 (-92.4 to -64.8)	-77.8 (-90.8 to -57.6)
Azerbaijan	Diarrheal diseases	10 to 14	Both	5 (2 to 9)	0.7 (0.3 to 1.3)	8 (3 to 14)	0.8 (0.3 to 1.5)	1 (1 to 2)	0.2 (0.1 to 0.4)	-79.0 (-88.4 to -62.8)	-74.3 (-85.8 to -54.3)
Azerbaijan	Diarrheal diseases	10 to 14	Female	2 (1 to 4)	0.6 (0.2 to 1.2)	3 (1 to 7)	0.7 (0.2 to 1.5)	0 (0 to 1)	0.2 (0.1 to 0.4)	-77.8 (-90.0 to -52.0)	-71.8 (-87.3 to -39.0)
Azerbaijan	Diarrheal diseases	10 to 14	Male	3 (1 to 6)	0.9 (0.3 to 1.7)	5 (1 to 10)	1.0 (0.3 to 2.0)	1 (0 to 1)	0.2 (0.1 to 0.5)	-79.8 (-90.9 to -58.8)	-76.0 (-89.2 to -51.1)
Azerbaijan	Intestinal infectious diseases	5 to 9	Both	0 (0 to 0)	0.0 (0.0 to 0.0)	0 (0 to 0)	0.0 (0.0 to 0.0)	0 (0 to 0)	0.0 (0.0 to 0.0)	-72.0 (-87.5 to -46.2)	-65.1 (-84.4 to -32.7)
Azerbaijan	Intestinal infectious diseases	5 to 9	Female	0 (0 to 0)	0.0 (0.0 to 0.0)	0 (0 to 0)	0.0 (0.0 to 0.0)	0 (0 to 0)	0.0 (0.0 to 0.0)	-72.8 (-89.2 to -42.7)	-64.6 (-85.9 to -25.4)
Azerbaijan	Intestinal infectious diseases	5 to 9	Male	0 (0 to 0)	0.0 (0.0 to 0.0)	0 (0 to 0)	0.0 (0.0 to 0.0)	0 (0 to 0)	0.0 (0.0 to 0.0)	-71.3 (-87.5 to -38.9)	-65.4 (-85.0 to -26.5)
Azerbaijan	Intestinal infectious diseases	10 to 14	Both	0 (0 to 0)	0.0 (0.0 to 0.0)	0 (0 to 0)	0.0 (0.0 to 0.0)	0 (0 to 0)	0.0 (0.0 to 0.0)	-70.6 (-85.7 to -43.8)	-63.9 (-82.5 to -31.1)
Azerbaijan	Intestinal infectious diseases	10 to 14	Female	0 (0 to 0)	0.0 (0.0 to 0.0)	0 (0 to 0)	0.0 (0.0 to 0.0)	0 (0 to 0)	0.0 (0.0 to 0.0)	-71.6 (-87.3 to -41.7)	-63.9 (-83.8 to -25.9)
Azerbaijan	Intestinal infectious diseases	10 to 14	Male	0 (0 to 0)	0.0 (0.0 to 0.0)	0 (0 to 0)	0.0 (0.0 to 0.0)	0 (0 to 0)	0.0 (0.0 to 0.0)	-69.6 (-86.1 to -35.4)	-63.9 (-83.5 to -23.3)
Azerbaijan	Lower respiratory infections	5 to 9	Both	225 (156 to 285)	27.4 (19.0 to 34.8)	359 (259 to 453)	40.5 (29.1 to 51.0)	88 (60 to 128)	13.4 (9.1 to 19.5)	-60.9 (-74.8 to -41.0)	-51.1 (-68.6 to -26.3)

eTable 8. All-cause and cause-specific mortality (counts and rates) at the global level and by country in the WHO European Region for ages 5 to 9 and 10 to 14 for 1990, 2000, and 2016											
Azerbaijan	Lower respiratory infections	5 to 9	Female	108 (73 to 140)	27.0 (18.3 to 35.2)	163 (115 to 213)	37.7 (26.5 to 49.3)	41 (25 to 64)	13.4 (8.3 to 20.8)	-61.9 (-77.6 to -38.6)	-50.4 (-70.8 to -20.0)
Azerbaijan	Lower respiratory infections	5 to 9	Male	117 (75 to 156)	27.8 (17.9 to 37.1)	196 (128 to 267)	43.2 (28.1 to 58.6)	47 (28 to 75)	13.4 (8.1 to 21.4)	-59.9 (-77.6 to -33.8)	-51.7 (-73.0 to -20.4)
Azerbaijan	Lower respiratory infections	10 to 14	Both	90 (61 to 114)	12.7 (8.5 to 16.1)	153 (112 to 191)	16.3 (12.0 to 20.4)	43 (29 to 58)	7.4 (5.0 to 10.0)	-52.3 (-69.9 to -30.5)	-41.5 (-63.1 to -14.8)
Azerbaijan	Lower respiratory infections	10 to 14	Female	41 (27 to 55)	11.8 (7.8 to 16.0)	68 (49 to 88)	14.8 (10.6 to 19.3)	18 (11 to 28)	6.7 (3.9 to 10.2)	-55.1 (-76.9 to -30.4)	-42.9 (-70.6 to -11.5)
Azerbaijan	Lower respiratory infections	10 to 14	Male	49 (31 to 66)	13.5 (8.6 to 18.1)	85 (55 to 114)	17.8 (11.4 to 23.9)	24 (15 to 35)	8.0 (4.9 to 11.6)	-50.0 (-71.9 to -23.4)	-40.6 (-66.6 to -9.0)
Azerbaijan	Upper respiratory infections	5 to 9	Both	0 (0 to 0)	0.0 (0.0 to 0.0)	0 (0 to 0)	0.0 (0.0 to 0.1)	0 (0 to 0)	0.0 (0.0 to 0.0)	-64.1 (-99.3 to 2489.3)	-55.2 (-99.1 to 3135.5)
Azerbaijan	Upper respiratory infections	5 to 9	Female	0 (0 to 0)	0.0 (0.0 to 0.0)	0 (0 to 0)	0.0 (0.0 to 0.1)	0 (0 to 0)	0.0 (0.0 to 0.0)	-67.0 (-100.0 to 51740.0)	-57.1 (-100.0 to 67402.7)
Azerbaijan	Upper respiratory infections	5 to 9	Male	0 (0 to 0)	0.0 (0.0 to 0.0)	0 (0 to 0)	0.0 (0.0 to 0.1)	0 (0 to 0)	0.0 (0.0 to 0.0)	-62.1 (-100.0 to 30630.2)	-54.4 (-100.0 to 36882.1)
Azerbaijan	Upper respiratory infections	10 to 14	Both	0 (0 to 0)	0.0 (0.0 to 0.0)	0 (0 to 0)	0.0 (0.0 to 0.0)	0 (0 to 0)	0.0 (0.0 to 0.0)	-56.4 (-99.3 to 1673.6)	-46.5 (-99.1 to 2076.4)
Azerbaijan	Upper respiratory infections	10 to 14	Female	0 (0 to 0)	0.0 (0.0 to 0.0)	0 (0 to 0)	0.0 (0.0 to 0.0)	0 (0 to 0)	0.0 (0.0 to 0.0)	-57.1 (-100.0 to 66703.7)	-45.5 (-100.0 to 84791.9)
Azerbaijan	Upper respiratory infections	10 to 14	Male	0 (0 to 0)	0.0 (0.0 to 0.0)	0 (0 to 0)	0.0 (0.0 to 0.0)	0 (0 to 0)	0.0 (0.0 to 0.0)	-56.0 (-99.9 to 56820.3)	-47.7 (-99.9 to 67524.5)
Azerbaijan	Otitis media	5 to 9	Both	0 (0 to 0)	0.0 (0.0 to 0.0)	0 (0 to 0)	0.0 (0.0 to 0.0)	0 (0 to 0)	0.0 (0.0 to 0.0)	-62.6 (-96.2 to 351.6)	-53.3 (-95.3 to 464.3)
Azerbaijan	Otitis media	5 to 9	Female	0 (0 to 0)	0.0 (0.0 to 0.0)	0 (0 to 0)	0.0 (0.0 to 0.0)	0 (0 to 0)	0.0 (0.0 to 0.0)	-79.3 (-99.3 to 587.8)	-73.0 (-99.1 to 795.7)
Azerbaijan	Otitis media	5 to 9	Male	0 (0 to 0)	0.0 (0.0 to 0.0)	0 (0 to 0)	0.0 (0.0 to 0.0)	0 (0 to 0)	0.0 (0.0 to 0.0)	-39.9 (-95.7 to 972.8)	-27.6 (-94.8 to 1191.1)
Azerbaijan	Otitis media	10 to 14	Both	0 (0 to 0)	0.0 (0.0 to 0.0)	0 (0 to 0)	0.0 (0.0 to 0.0)	0 (0 to 0)	0.0 (0.0 to 0.0)	-60.5 (-94.8 to 252.5)	-51.5 (-93.6 to 332.5)
Azerbaijan	Otitis media	10 to 14	Female	0 (0 to 0)	0.0 (0.0 to 0.0)	0 (0 to 0)	0.0 (0.0 to 0.0)	0 (0 to 0)	0.0 (0.0 to 0.0)	-78.7 (-98.9 to 223.8)	-72.9 (-98.6 to 311.5)
Azerbaijan	Otitis media	10 to 14	Male	0 (0 to 0)	0.0 (0.0 to 0.0)	0 (0 to 0)	0.0 (0.0 to 0.0)	0 (0 to 0)	0.0 (0.0 to 0.0)	-24.8 (-95.3 to 1000.3)	-10.7 (-94.4 to 1207.2)
Azerbaijan	Meningitis	5 to 9	Both	15 (9 to 26)	1.8 (1.1 to 3.2)	39 (21 to 59)	4.4 (2.4 to 6.7)	10 (5 to 18)	1.5 (0.8 to 2.7)	-33.3 (-65.9 to 49.8)	-16.7 (-57.5 to 87.2)
Azerbaijan	Meningitis	5 to 9	Female	6 (3 to 10)	1.5 (0.9 to 2.4)	18 (7 to 27)	4.1 (1.7 to 6.3)	3 (1 to 5)	0.9 (0.5 to 1.5)	-54.2 (-76.9 to -9.0)	-40.4 (-70.0 to 18.5)
Azerbaijan	Meningitis	5 to 9	Male	9 (4 to 18)	2.1 (1.1 to 4.3)	22 (6 to 37)	4.8 (1.3 to 8.2)	7 (3 to 15)	2.1 (1.0 to 4.2)	-19.8 (-62.8 to 122.1)	-3.4 (-55.3 to 167.2)
Azerbaijan	Meningitis	10 to 14	Both	10 (7 to 16)	1.4 (0.9 to 2.3)	26 (15 to 37)	2.8 (1.7 to 4.0)	7 (4 to 12)	1.3 (0.8 to 2.0)	-27.5 (-55.4 to 36.5)	-11.0 (-45.3 to 67.6)
Azerbaijan	Meningitis	10 to 14	Female	4 (3 to 6)	1.2 (0.8 to 1.8)	13 (6 to 19)	2.8 (1.2 to 4.2)	2 (1 to 4)	0.8 (0.4 to 1.4)	-47.0 (-73.5 to 6.7)	-32.6 (-66.3 to 35.5)
Azerbaijan	Meningitis	10 to 14	Male	6 (3 to 11)	1.6 (0.9 to 3.0)	14 (5 to 22)	2.9 (1.0 to 4.7)	5 (3 to 9)	1.7 (0.8 to 3.1)	-13.6 (-51.1 to 95.6)	2.6 (-41.9 to 132.4)
Azerbaijan	Encephalitis	5 to 9	Both	15 (7 to 26)	1.8 (0.8 to 3.1)	23 (8 to 39)	2.6 (0.9 to 4.4)	10 (5 to 19)	1.5 (0.8 to 2.9)	-33.6 (-67.1 to 34.9)	-17.0 (-58.9 to 68.6)
Azerbaijan	Encephalitis	5 to 9	Female	7 (3 to 12)	1.7 (0.7 to 2.9)	11 (3 to 19)	2.5 (0.8 to 4.5)	4 (2 to 9)	1.4 (0.7 to 2.8)	-38.8 (-69.6 to 36.8)	-20.3 (-60.5 to 78.1)
Azerbaijan	Encephalitis	5 to 9	Male	8 (3 to 17)	1.9 (0.7 to 4.1)	12 (4 to 25)	2.7 (0.9 to 5.4)	6 (2 to 13)	1.6 (0.7 to 3.7)	-29.1 (-75.6 to 87.0)	-14.6 (-70.6 to 125.1)
Azerbaijan	Encephalitis	10 to 14	Both	6 (4 to 9)	0.8 (0.5 to 1.2)	10 (5 to 14)	1.0 (0.6 to 1.5)	5 (3 to 8)	0.8 (0.5 to 1.4)	-17.4 (-50.6 to 62.4)	1.4 (-39.4 to 99.3)

eTable 8. All-cause and cause-specific mortality (counts and rates) at the global level and by country in the WHO European Region for ages 5 to 9 and 10 to 14 for 1990, 2000, and 2016

Azerbaijan	Encephalitis	10 to 14	Female	2 (1 to 4)	0.7 (0.4 to 1.2)	4 (2 to 7)	0.9 (0.5 to 1.6)	2 (1 to 3)	0.6 (0.4 to 1.0)	-32.9 (-63.0 to 40.0)	-14.7 (-52.9 to 78.0)
Azerbaijan	Encephalitis	10 to 14	Male	3 (2 to 6)	0.9 (0.4 to 1.7)	6 (2 to 10)	1.2 (0.5 to 2.1)	3 (1 to 6)	1.0 (0.4 to 2.1)	-6.0 (-52.8 to 120.6)	11.7 (-43.9 to 162.1)
Azerbaijan	Diphtheria	5 to 9	Both	3 (1 to 9)	0.4 (0.1 to 1.0)	3 (1 to 9)	0.4 (0.1 to 1.0)	0 (0 to 0)	0.0 (0.0 to 0.0)	-98.6 (-99.8 to -91.2)	-98.3 (-99.8 to -88.9)
Azerbaijan	Diphtheria	5 to 9	Female	1 (0 to 5)	0.3 (0.0 to 1.2)	1 (0 to 5)	0.3 (0.0 to 1.1)	0 (0 to 0)	0.0 (0.0 to 0.0)	-98.8 (-99.9 to -79.4)	-98.5 (-99.9 to -73.2)
Azerbaijan	Diphtheria	5 to 9	Male	2 (0 to 6)	0.4 (0.0 to 1.5)	2 (0 to 7)	0.4 (0.0 to 1.5)	0 (0 to 0)	0.0 (0.0 to 0.0)	-98.5 (-99.9 to -69.1)	-98.2 (-99.9 to -62.8)
Azerbaijan	Diphtheria	10 to 14	Both	1 (0 to 3)	0.2 (0.0 to 0.5)	1 (0 to 4)	0.2 (0.0 to 0.4)	0 (0 to 0)	0.0 (0.0 to 0.0)	-98.2 (-99.7 to -87.6)	-97.8 (-99.7 to -84.8)
Azerbaijan	Diphtheria	10 to 14	Female	0 (0 to 2)	0.1 (0.0 to 0.4)	1 (0 to 2)	0.1 (0.0 to 0.4)	0 (0 to 0)	0.0 (0.0 to 0.0)	-98.3 (-99.9 to -67.4)	-97.8 (-99.9 to -58.6)
Azerbaijan	Diphtheria	10 to 14	Male	1 (0 to 3)	0.2 (0.0 to 0.7)	1 (0 to 3)	0.2 (0.0 to 0.6)	0 (0 to 0)	0.0 (0.0 to 0.0)	-98.1 (-99.9 to -65.5)	-97.8 (-99.9 to -59.0)
Azerbaijan	Whooping cough	5 to 9	Both	11 (1 to 34)	1.4 (0.1 to 4.2)	22 (2 to 72)	2.5 (0.2 to 8.1)	1 (0 to 5)	0.2 (0.0 to 0.7)	-87.0 (-99.1 to 70.4)	-83.8 (-98.8 to 112.9)
Azerbaijan	Whooping cough	5 to 9	Female	6 (1 to 19)	1.6 (0.2 to 4.9)	13 (1 to 40)	2.9 (0.2 to 9.3)	1 (0 to 3)	0.3 (0.0 to 0.8)	-87.8 (-99.2 to 51.3)	-84.1 (-99.0 to 97.0)
Azerbaijan	Whooping cough	5 to 9	Male	5 (0 to 15)	1.2 (0.1 to 3.7)	10 (1 to 32)	2.1 (0.2 to 6.9)	1 (0 to 2)	0.2 (0.0 to 0.7)	-86.1 (-99.0 to 97.2)	-83.2 (-98.8 to 137.3)
Azerbaijan	Whooping cough	10 to 14	Both	2 (0 to 6)	0.3 (0.0 to 0.9)	5 (0 to 14)	0.5 (0.0 to 1.5)	0 (0 to 1)	0.0 (0.0 to 0.1)	-86.6 (-99.1 to 75.0)	-83.6 (-98.8 to 114.7)
Azerbaijan	Whooping cough	10 to 14	Female	1 (0 to 3)	0.3 (0.0 to 1.0)	2 (0 to 8)	0.5 (0.0 to 1.7)	0 (0 to 0)	0.1 (0.0 to 0.2)	-87.4 (-99.1 to 59.2)	-84.0 (-98.9 to 102.3)
Azerbaijan	Whooping cough	10 to 14	Male	1 (0 to 3)	0.3 (0.0 to 0.8)	2 (0 to 7)	0.5 (0.0 to 1.4)	0 (0 to 0)	0.0 (0.0 to 0.1)	-85.7 (-99.0 to 93.1)	-83.0 (-98.8 to 129.4)
Azerbaijan	Tetanus	5 to 9	Both	0 (0 to 0)	0.0 (0.0 to 0.0)	0 (0 to 0)	0.0 (0.0 to 0.0)	0 (0 to 0)	0.0 (0.0 to 0.0)	-21.5 (-60.1 to 71.5)	-1.9 (-50.1 to 114.3)
Azerbaijan	Tetanus	5 to 9	Female	0 (0 to 0)	0.0 (0.0 to 0.0)	0 (0 to 0)	0.0 (0.0 to 0.0)	0 (0 to 0)	0.0 (0.0 to 0.0)	-23.3 (-46.2 to 9.1)	-0.1 (-30.0 to 42.1)
Azerbaijan	Tetanus	5 to 9	Male	0 (0 to 0)	0.0 (0.0 to 0.0)	0 (0 to 0)	0.0 (0.0 to 0.0)	0 (0 to 0)	0.0 (0.0 to 0.0)	-20.4 (-70.2 to 139.7)	-4.2 (-64.2 to 188.4)
Azerbaijan	Tetanus	10 to 14	Both	0 (0 to 0)	0.0 (0.0 to 0.0)	0 (0 to 0)	0.0 (0.0 to 0.0)	0 (0 to 0)	0.0 (0.0 to 0.0)	-25.8 (-70.6 to 133.3)	-8.9 (-63.9 to 186.3)
Azerbaijan	Tetanus	10 to 14	Female	0 (0 to 0)	0.0 (0.0 to 0.0)	0 (0 to 0)	0.0 (0.0 to 0.0)	0 (0 to 0)	0.0 (0.0 to 0.0)	-20.0 (-37.5 to 3.2)	1.7 (-20.6 to 31.2)
Azerbaijan	Tetanus	10 to 14	Male	0 (0 to 0)	0.0 (0.0 to 0.1)	0 (0 to 0)	0.0 (0.0 to 0.0)	0 (0 to 0)	0.0 (0.0 to 0.1)	-28.4 (-81.2 to 220.0)	-14.9 (-77.6 to 280.1)
Azerbaijan	Measles	5 to 9	Both	1 (1 to 2)	0.2 (0.1 to 0.2)	0 (0 to 0)	0.0 (0.0 to 0.0)	0 (0 to 0)	0.0 (0.0 to 0.0)	-99.7 (-100.0 to -98.2)	-99.6 (-100.0 to -97.8)
Azerbaijan	Measles	5 to 9	Female	1 (0 to 1)	0.2 (0.1 to 0.2)	0 (0 to 0)	0.0 (0.0 to 0.0)	0 (0 to 0)	0.0 (0.0 to 0.0)	-99.7 (-100.0 to -98.3)	-99.6 (-100.0 to -97.8)
Azerbaijan	Measles	5 to 9	Male	1 (0 to 1)	0.2 (0.1 to 0.2)	0 (0 to 0)	0.0 (0.0 to 0.0)	0 (0 to 0)	0.0 (0.0 to 0.0)	-99.7 (-100.0 to -98.1)	-99.6 (-100.0 to -97.7)
Azerbaijan	Measles	10 to 14	Both	0 (0 to 0)	0.0 (0.0 to 0.1)	0 (0 to 0)	0.0 (0.0 to 0.0)	0 (0 to 0)	0.0 (0.0 to 0.0)	-99.7 (-100.0 to -98.2)	-99.6 (-100.0 to -97.8)
Azerbaijan	Measles	10 to 14	Female	0 (0 to 0)	0.0 (0.0 to 0.1)	0 (0 to 0)	0.0 (0.0 to 0.0)	0 (0 to 0)	0.0 (0.0 to 0.0)	-99.7 (-100.0 to -98.4)	-99.6 (-100.0 to -97.9)
Azerbaijan	Measles	10 to 14	Male	0 (0 to 0)	0.0 (0.0 to 0.1)	0 (0 to 0)	0.0 (0.0 to 0.0)	0 (0 to 0)	0.0 (0.0 to 0.0)	-99.7 (-100.0 to -98.1)	-99.6 (-100.0 to -97.7)
Azerbaijan	Varicella and herpes zoster	5 to 9	Both	1 (0 to 3)	0.1 (0.0 to 0.3)	1 (0 to 3)	0.2 (0.0 to 0.4)	0 (0 to 1)	0.1 (0.0 to 0.1)	-69.0 (-93.4 to 61.9)	-61.3 (-91.8 to 102.4)
Azerbaijan	Varicella and herpes zoster	5 to 9	Female	1 (0 to 2)	0.1 (0.0 to 0.4)	1 (0 to 2)	0.2 (0.0 to 0.5)	0 (0 to 0)	0.1 (0.0 to 0.2)	-71.9 (-97.2 to 253.2)	-63.5 (-96.4 to 360.0)
Azerbaijan	Varicella and herpes zoster	5 to 9	Male	1 (0 to 2)	0.1 (0.0 to 0.4)	1 (0 to 2)	0.2 (0.0 to 0.5)	0 (0 to 1)	0.1 (0.0 to 0.2)	-66.2 (-98.1 to 370.2)	-59.3 (-97.7 to 465.9)
Azerbaijan	Varicella and herpes zoster	10 to 14	Both	0 (0 to 1)	0.0 (0.0 to 0.1)	0 (0 to 1)	0.1 (0.0 to 0.1)	0 (0 to 0)	0.0 (0.0 to 0.0)	-69.5 (-93.7 to 59.0)	-62.6 (-92.3 to 95.1)
Azerbaijan	Varicella and herpes zoster	10 to 14	Female	0 (0 to 1)	0.0 (0.0 to 0.1)	0 (0 to 1)	0.0 (0.0 to 0.1)	0 (0 to 0)	0.0 (0.0 to 0.1)	-72.5 (-97.9 to 196.6)	-65.1 (-97.3 to 276.9)
Azerbaijan	Varicella and herpes zoster	10 to 14	Male	0 (0 to 1)	0.0 (0.0 to 0.1)	0 (0 to 1)	0.1 (0.0 to 0.2)	0 (0 to 0)	0.0 (0.0 to 0.1)	-66.7 (-97.5 to 346.3)	-60.5 (-97.0 to 430.2)

eTable 8. All-cause and cause-specific mortality (counts and rates) at the global level and by country in the WHO European Region for ages 5 to 9 and 10 to 14 for 1990, 2000, and 2016

Azerbaijan	Malaria	5 to 9	Both	0 (0 to 0)	0.0 (0.0 to 0.0)	0 (0 to 0)	0.0 (0.0 to 0.0)	0 (0 to 0)	0.0 (0.0 to 0.0)	-90.3 (-93.3 to -86.4)	-87.8 (-91.6 to -83.0)
Azerbaijan	Malaria	5 to 9	Female	0 (0 to 0)	0.0 (0.0 to 0.0)	0 (0 to 0)	0.0 (0.0 to 0.0)	0 (0 to 0)	0.0 (0.0 to 0.0)	-90.9 (-93.8 to -87.1)	-88.2 (-92.0 to -83.2)
Azerbaijan	Malaria	5 to 9	Male	0 (0 to 0)	0.0 (0.0 to 0.0)	0 (0 to 0)	0.0 (0.0 to 0.0)	0 (0 to 0)	0.0 (0.0 to 0.0)	-89.8 (-93.1 to -85.3)	-87.7 (-91.7 to -82.3)
Azerbaijan	Malaria	10 to 14	Both	0 (0 to 0)	0.0 (0.0 to 0.0)	0 (0 to 0)	0.0 (0.0 to 0.0)	0 (0 to 0)	0.0 (0.0 to 0.0)	-86.7 (-89.7 to -82.9)	-83.7 (-87.4 to -79.0)
Azerbaijan	Malaria	10 to 14	Female	0 (0 to 0)	0.0 (0.0 to 0.0)	0 (0 to 0)	0.0 (0.0 to 0.0)	0 (0 to 0)	0.0 (0.0 to 0.0)	-87.7 (-90.5 to -83.7)	-84.3 (-87.9 to -79.3)
Azerbaijan	Malaria	10 to 14	Male	0 (0 to 0)	0.0 (0.0 to 0.0)	0 (0 to 0)	0.0 (0.0 to 0.0)	0 (0 to 0)	0.0 (0.0 to 0.0)	-86.2 (-89.9 to -81.6)	-83.6 (-87.9 to -78.2)
Azerbaijan	Leishmaniasis	5 to 9	Both	0 (0 to 0)	0.0 (0.0 to 0.0)	0 (0 to 0)	0.0 (0.0 to 0.0)	0 (0 to 0)	0.0 (0.0 to 0.0)	87.3 (-100.0 to 1799.6)	134.0 (-100.0 to 2273.7)
Azerbaijan	Leishmaniasis	5 to 9	Female	0 (0 to 0)	0.0 (0.0 to 0.0)	0 (0 to 0)	0.0 (0.0 to 0.0)	0 (0 to 0)	0.0 (0.0 to 0.0)	80.2 (-100.0 to 854.1)	134.7 (-100.0 to 1142.3)
Azerbaijan	Leishmaniasis	5 to 9	Male	0 (0 to 0)	0.0 (0.0 to 0.0)	0 (0 to 0)	0.0 (0.0 to 0.0)	0 (0 to 0)	0.0 (0.0 to 0.0)	94.1 (-100.0 to 768.7)	133.6 (-100.0 to 945.5)
Azerbaijan	Leishmaniasis	10 to 14	Both	0 (0 to 0)	0.0 (0.0 to 0.0)	0 (0 to 0)	0.0 (0.0 to 0.0)	0 (0 to 0)	0.0 (0.0 to 0.0)	90.5 (-100.0 to 2118.8)	133.8 (-100.0 to 2622.7)
Azerbaijan	Leishmaniasis	10 to 14	Female	0 (0 to 0)	0.0 (0.0 to 0.0)	0 (0 to 0)	0.0 (0.0 to 0.0)	0 (0 to 0)	0.0 (0.0 to 0.0)	81.0 (-100.0 to 768.4)	130.0 (-100.0 to 1003.5)
Azerbaijan	Leishmaniasis	10 to 14	Male	0 (0 to 0)	0.0 (0.0 to 0.0)	0 (0 to 0)	0.0 (0.0 to 0.0)	0 (0 to 0)	0.0 (0.0 to 0.0)	99.9 (-100.0 to 810.7)	137.5 (-100.0 to 981.9)
Azerbaijan	African trypanosomiasis	5 to 9	Both	0 (0 to 0)	0.0 (0.0 to 0.0)	0 (0 to 0)	0.0 (0.0 to 0.0)	0 (0 to 0)	0.0 (0.0 to 0.0)	0.0 (0.0 to 0.0)	0.0 (0.0 to 0.0)
Azerbaijan	African trypanosomiasis	5 to 9	Female	0 (0 to 0)	0.0 (0.0 to 0.0)	0 (0 to 0)	0.0 (0.0 to 0.0)	0 (0 to 0)	0.0 (0.0 to 0.0)	0.0 (0.0 to 0.0)	0.0 (0.0 to 0.0)
Azerbaijan	African trypanosomiasis	5 to 9	Male	0 (0 to 0)	0.0 (0.0 to 0.0)	0 (0 to 0)	0.0 (0.0 to 0.0)	0 (0 to 0)	0.0 (0.0 to 0.0)	0.0 (0.0 to 0.0)	0.0 (0.0 to 0.0)
Azerbaijan	African trypanosomiasis	10 to 14	Both	0 (0 to 0)	0.0 (0.0 to 0.0)	0 (0 to 0)	0.0 (0.0 to 0.0)	0 (0 to 0)	0.0 (0.0 to 0.0)	0.0 (0.0 to 0.0)	0.0 (0.0 to 0.0)
Azerbaijan	African trypanosomiasis	10 to 14	Female	0 (0 to 0)	0.0 (0.0 to 0.0)	0 (0 to 0)	0.0 (0.0 to 0.0)	0 (0 to 0)	0.0 (0.0 to 0.0)	0.0 (0.0 to 0.0)	0.0 (0.0 to 0.0)
Azerbaijan	African trypanosomiasis	10 to 14	Male	0 (0 to 0)	0.0 (0.0 to 0.0)	0 (0 to 0)	0.0 (0.0 to 0.0)	0 (0 to 0)	0.0 (0.0 to 0.0)	0.0 (0.0 to 0.0)	0.0 (0.0 to 0.0)
Azerbaijan	Schistosomiasis	5 to 9	Both	0 (0 to 0)	0.0 (0.0 to 0.0)	0 (0 to 0)	0.0 (0.0 to 0.0)	0 (0 to 0)	0.0 (0.0 to 0.0)	0.0 (0.0 to 0.0)	0.0 (0.0 to 0.0)
Azerbaijan	Schistosomiasis	5 to 9	Female	0 (0 to 0)	0.0 (0.0 to 0.0)	0 (0 to 0)	0.0 (0.0 to 0.0)	0 (0 to 0)	0.0 (0.0 to 0.0)	0.0 (0.0 to 0.0)	0.0 (0.0 to 0.0)
Azerbaijan	Schistosomiasis	5 to 9	Male	0 (0 to 0)	0.0 (0.0 to 0.0)	0 (0 to 0)	0.0 (0.0 to 0.0)	0 (0 to 0)	0.0 (0.0 to 0.0)	0.0 (0.0 to 0.0)	0.0 (0.0 to 0.0)
Azerbaijan	Schistosomiasis	10 to 14	Both	0 (0 to 0)	0.0 (0.0 to 0.0)	0 (0 to 0)	0.0 (0.0 to 0.0)	0 (0 to 0)	0.0 (0.0 to 0.0)	0.0 (0.0 to 0.0)	0.0 (0.0 to 0.0)
Azerbaijan	Schistosomiasis	10 to 14	Female	0 (0 to 0)	0.0 (0.0 to 0.0)	0 (0 to 0)	0.0 (0.0 to 0.0)	0 (0 to 0)	0.0 (0.0 to 0.0)	0.0 (0.0 to 0.0)	0.0 (0.0 to 0.0)
Azerbaijan	Schistosomiasis	10 to 14	Male	0 (0 to 0)	0.0 (0.0 to 0.0)	0 (0 to 0)	0.0 (0.0 to 0.0)	0 (0 to 0)	0.0 (0.0 to 0.0)	0.0 (0.0 to 0.0)	0.0 (0.0 to 0.0)
Azerbaijan	Cysticercosis	5 to 9	Both	0 (0 to 0)	0.0 (0.0 to 0.0)	0 (0 to 0)	0.0 (0.0 to 0.0)	0 (0 to 0)	0.0 (0.0 to 0.0)	-83.2 (-88.3 to -76.7)	-79.0 (-85.3 to -70.9)
Azerbaijan	Cysticercosis	5 to 9	Female	0 (0 to 0)	0.0 (0.0 to 0.0)	0 (0 to 0)	0.0 (0.0 to 0.0)	0 (0 to 0)	0.0 (0.0 to 0.0)	-84.5 (-89.4 to -78.1)	-79.8 (-86.2 to -71.5)
Azerbaijan	Cysticercosis	5 to 9	Male	0 (0 to 0)	0.0 (0.0 to 0.0)	0 (0 to 0)	0.0 (0.0 to 0.0)	0 (0 to 0)	0.0 (0.0 to 0.0)	-82.0 (-87.8 to -74.2)	-78.3 (-85.3 to -69.0)
Azerbaijan	Cysticercosis	10 to 14	Both	0 (0 to 0)	0.0 (0.0 to 0.0)	0 (0 to 0)	0.0 (0.0 to 0.0)	0 (0 to 0)	0.0 (0.0 to 0.0)	-77.5 (-82.3 to -71.5)	-72.4 (-78.3 to -65.0)
Azerbaijan	Cysticercosis	10 to 14	Female	0 (0 to 0)	0.0 (0.0 to 0.0)	0 (0 to 0)	0.0 (0.0 to 0.0)	0 (0 to 0)	0.0 (0.0 to 0.0)	-78.6 (-83.6 to -72.1)	-72.9 (-79.1 to -64.5)

eTable 8. All-cause and cause-specific mortality (counts and rates) at the global level and by country in the WHO European Region for ages 5 to 9 and 10 to 14 for 1990, 2000, and 2016

Azerbaijan	Cysticercosis	10 to 14	Male	0 (0 to 0)	0.0 (0.0 to 0.0)	0 (0 to 0)	0.0 (0.0 to 0.0)	0 (0 to 0)	0.0 (0.0 to 0.0)	-76.5 (-82.3 to -69.3)	-72.1 (-79.0 to -63.6)
Azerbaijan	Yellow fever	5 to 9	Both	0 (0 to 0)	0.0 (0.0 to 0.0)	0 (0 to 0)	0.0 (0.0 to 0.0)	0 (0 to 0)	0.0 (0.0 to 0.0)	0.0 (0.0 to 0.0)	0.0 (0.0 to 0.0)
Azerbaijan	Yellow fever	5 to 9	Female	0 (0 to 0)	0.0 (0.0 to 0.0)	0 (0 to 0)	0.0 (0.0 to 0.0)	0 (0 to 0)	0.0 (0.0 to 0.0)	0.0 (0.0 to 0.0)	0.0 (0.0 to 0.0)
Azerbaijan	Yellow fever	5 to 9	Male	0 (0 to 0)	0.0 (0.0 to 0.0)	0 (0 to 0)	0.0 (0.0 to 0.0)	0 (0 to 0)	0.0 (0.0 to 0.0)	0.0 (0.0 to 0.0)	0.0 (0.0 to 0.0)
Azerbaijan	Yellow fever	10 to 14	Both	0 (0 to 0)	0.0 (0.0 to 0.0)	0 (0 to 0)	0.0 (0.0 to 0.0)	0 (0 to 0)	0.0 (0.0 to 0.0)	0.0 (0.0 to 0.0)	0.0 (0.0 to 0.0)
Azerbaijan	Yellow fever	10 to 14	Female	0 (0 to 0)	0.0 (0.0 to 0.0)	0 (0 to 0)	0.0 (0.0 to 0.0)	0 (0 to 0)	0.0 (0.0 to 0.0)	0.0 (0.0 to 0.0)	0.0 (0.0 to 0.0)
Azerbaijan	Yellow fever	10 to 14	Male	0 (0 to 0)	0.0 (0.0 to 0.0)	0 (0 to 0)	0.0 (0.0 to 0.0)	0 (0 to 0)	0.0 (0.0 to 0.0)	0.0 (0.0 to 0.0)	0.0 (0.0 to 0.0)
Azerbaijan	Rabies	5 to 9	Both	1 (0 to 5)	0.2 (0.0 to 0.7)	3 (0 to 11)	0.4 (0.1 to 1.2)	0 (0 to 1)	0.0 (0.0 to 0.1)	-85.8 (-99.2 to 80.7)	-82.3 (-99.0 to 125.8)
Azerbaijan	Rabies	5 to 9	Female	0 (0 to 0)	0.0 (0.0 to 0.0)	0 (0 to 0)	0.0 (0.0 to 0.0)	0 (0 to 0)	0.0 (0.0 to 0.0)	-94.3 (-99.7 to 11.9)	-92.6 (-99.6 to 45.7)
Azerbaijan	Rabies	5 to 9	Male	1 (0 to 5)	0.3 (0.0 to 1.3)	3 (0 to 11)	0.7 (0.1 to 2.4)	0 (0 to 1)	0.1 (0.0 to 0.2)	-85.8 (-99.2 to 89.8)	-82.9 (-99.1 to 128.4)
Azerbaijan	Rabies	10 to 14	Both	1 (0 to 4)	0.2 (0.0 to 0.6)	3 (0 to 9)	0.3 (0.0 to 1.0)	0 (0 to 1)	0.0 (0.0 to 0.1)	-85.0 (-98.6 to 43.4)	-81.6 (-98.2 to 75.9)
Azerbaijan	Rabies	10 to 14	Female	0 (0 to 0)	0.0 (0.0 to 0.0)	0 (0 to 0)	0.0 (0.0 to 0.0)	0 (0 to 0)	0.0 (0.0 to 0.0)	-81.9 (-99.0 to 297.7)	-77.0 (-98.7 to 405.3)
Azerbaijan	Rabies	10 to 14	Male	1 (0 to 4)	0.3 (0.0 to 1.2)	3 (0 to 9)	0.6 (0.1 to 1.9)	0 (0 to 1)	0.1 (0.0 to 0.2)	-85.0 (-98.6 to 43.3)	-82.2 (-98.3 to 70.3)
Azerbaijan	Intestinal nematode infections	5 to 9	Both	0 (0 to 1)	0.0 (0.0 to 0.1)	0 (0 to 1)	0.0 (0.0 to 0.1)	0 (0 to 0)	0.0 (0.0 to 0.0)	-83.4 (-100.0 to 263.3)	-79.3 (-100.0 to 354.0)
Azerbaijan	Intestinal nematode infections	5 to 9	Female	0 (0 to 0)	0.0 (0.0 to 0.1)	0 (0 to 1)	0.0 (0.0 to 0.2)	0 (0 to 0)	0.0 (0.0 to 0.0)	-84.8 (-100.0 to 0.1)	-80.2 (-100.0 to 26.7)
Azerbaijan	Intestinal nematode infections	5 to 9	Male	0 (0 to 0)	0.0 (0.0 to 0.1)	0 (0 to 1)	0.0 (0.0 to 0.2)	0 (0 to 0)	0.0 (0.0 to 0.0)	-81.9 (-100.0 to 24.2)	-78.2 (-100.0 to 49.5)
Azerbaijan	Intestinal nematode infections	10 to 14	Both	0 (0 to 0)	0.0 (0.0 to 0.0)	0 (0 to 0)	0.0 (0.0 to 0.0)	0 (0 to 0)	0.0 (0.0 to 0.0)	-78.0 (-100.0 to 113.7)	-73.0 (-100.0 to 162.2)
Azerbaijan	Intestinal nematode infections	10 to 14	Female	0 (0 to 0)	0.0 (0.0 to 0.0)	0 (0 to 0)	0.0 (0.0 to 0.0)	0 (0 to 0)	0.0 (0.0 to 0.0)	-79.0 (-100.0 to 30.7)	-73.3 (-100.0 to 66.1)
Azerbaijan	Intestinal nematode infections	10 to 14	Male	0 (0 to 0)	0.0 (0.0 to 0.0)	0 (0 to 0)	0.0 (0.0 to 0.0)	0 (0 to 0)	0.0 (0.0 to 0.0)	-77.0 (-100.0 to 33.1)	-72.7 (-100.0 to 58.1)
Azerbaijan	Ebola	5 to 9	Both	0 (0 to 0)	0.0 (0.0 to 0.0)	0 (0 to 0)	0.0 (0.0 to 0.0)	0 (0 to 0)	0.0 (0.0 to 0.0)	0.0 (0.0 to 0.0)	0.0 (0.0 to 0.0)
Azerbaijan	Ebola	5 to 9	Female	0 (0 to 0)	0.0 (0.0 to 0.0)	0 (0 to 0)	0.0 (0.0 to 0.0)	0 (0 to 0)	0.0 (0.0 to 0.0)	0.0 (0.0 to 0.0)	0.0 (0.0 to 0.0)
Azerbaijan	Ebola	5 to 9	Male	0 (0 to 0)	0.0 (0.0 to 0.0)	0 (0 to 0)	0.0 (0.0 to 0.0)	0 (0 to 0)	0.0 (0.0 to 0.0)	0.0 (0.0 to 0.0)	0.0 (0.0 to 0.0)
Azerbaijan	Ebola	10 to 14	Both	0 (0 to 0)	0.0 (0.0 to 0.0)	0 (0 to 0)	0.0 (0.0 to 0.0)	0 (0 to 0)	0.0 (0.0 to 0.0)	0.0 (0.0 to 0.0)	0.0 (0.0 to 0.0)
Azerbaijan	Ebola	10 to 14	Female	0 (0 to 0)	0.0 (0.0 to 0.0)	0 (0 to 0)	0.0 (0.0 to 0.0)	0 (0 to 0)	0.0 (0.0 to 0.0)	0.0 (0.0 to 0.0)	0.0 (0.0 to 0.0)
Azerbaijan	Ebola	10 to 14	Male	0 (0 to 0)	0.0 (0.0 to 0.0)	0 (0 to 0)	0.0 (0.0 to 0.0)	0 (0 to 0)	0.0 (0.0 to 0.0)	0.0 (0.0 to 0.0)	0.0 (0.0 to 0.0)
Azerbaijan	Zika virus	5 to 9	Both	0 (0 to 0)	0.0 (0.0 to 0.0)	0 (0 to 0)	0.0 (0.0 to 0.0)	0 (0 to 0)	0.0 (0.0 to 0.0)	0.0 (0.0 to 0.0)	0.0 (0.0 to 0.0)
Azerbaijan	Zika virus	5 to 9	Female	0 (0 to 0)	0.0 (0.0 to 0.0)	0 (0 to 0)	0.0 (0.0 to 0.0)	0 (0 to 0)	0.0 (0.0 to 0.0)	0.0 (0.0 to 0.0)	0.0 (0.0 to 0.0)
Azerbaijan	Zika virus	5 to 9	Male	0 (0 to 0)	0.0 (0.0 to 0.0)	0 (0 to 0)	0.0 (0.0 to 0.0)	0 (0 to 0)	0.0 (0.0 to 0.0)	0.0 (0.0 to 0.0)	0.0 (0.0 to 0.0)
Azerbaijan	Zika virus	10 to 14	Both	0 (0 to 0)	0.0 (0.0 to 0.0)	0 (0 to 0)	0.0 (0.0 to 0.0)	0 (0 to 0)	0.0 (0.0 to 0.0)	0.0 (0.0 to 0.0)	0.0 (0.0 to 0.0)

eTable 8. All-cause and cause-specific mortality (counts and rates) at the global level and by country in the WHO European Region for ages 5 to 9 and 10 to 14 for 1990, 2000, and 2016

Azerbaijan	Zika virus	10 to 14	Female	0 (0 to 0)	0.0 (0.0 to 0.0)	0 (0 to 0)	0.0 (0.0 to 0.0)	0 (0 to 0)	0.0 (0.0 to 0.0)	0.0 (0.0 to 0.0)	0.0 (0.0 to 0.0)
Azerbaijan	Zika virus	10 to 14	Male	0 (0 to 0)	0.0 (0.0 to 0.0)	0 (0 to 0)	0.0 (0.0 to 0.0)	0 (0 to 0)	0.0 (0.0 to 0.0)	0.0 (0.0 to 0.0)	0.0 (0.0 to 0.0)
Azerbaijan	Other neglected tropical diseases	5 to 9	Both	0 (0 to 0)	0.0 (0.0 to 0.0)	0 (0 to 0)	0.0 (0.0 to 0.0)	0 (0 to 0)	0.0 (0.0 to 0.0)	-18.9 (-51.2 to 25.0)	1.4 (-39.0 to 56.2)
Azerbaijan	Other neglected tropical diseases	5 to 9	Female	0 (0 to 0)	0.0 (0.0 to 0.0)	0 (0 to 0)	0.0 (0.0 to 0.0)	0 (0 to 0)	0.0 (0.0 to 0.0)	-25.5 (-64.8 to 33.7)	-2.9 (-54.2 to 74.1)
Azerbaijan	Other neglected tropical diseases	5 to 9	Male	0 (0 to 0)	0.0 (0.0 to 0.0)	0 (0 to 0)	0.0 (0.0 to 0.0)	0 (0 to 0)	0.0 (0.0 to 0.0)	-12.2 (-41.6 to 29.7)	5.7 (-29.7 to 56.0)
Azerbaijan	Other neglected tropical diseases	10 to 14	Both	0 (0 to 0)	0.0 (0.0 to 0.0)	0 (0 to 0)	0.0 (0.0 to 0.0)	0 (0 to 0)	0.0 (0.0 to 0.0)	-14.8 (-37.2 to 10.5)	4.5 (-23.0 to 35.6)
Azerbaijan	Other neglected tropical diseases	10 to 14	Female	0 (0 to 0)	0.0 (0.0 to 0.0)	0 (0 to 0)	0.0 (0.0 to 0.0)	0 (0 to 0)	0.0 (0.0 to 0.0)	-19.8 (-38.7 to 6.5)	2.0 (-22.1 to 35.4)
Azerbaijan	Other neglected tropical diseases	10 to 14	Male	0 (0 to 0)	0.0 (0.0 to 0.0)	0 (0 to 0)	0.0 (0.0 to 0.0)	0 (0 to 0)	0.0 (0.0 to 0.0)	-10.2 (-35.9 to 21.8)	6.7 (-23.8 to 44.7)
Azerbaijan	Maternal hemorrhage	10 to 14	Both	0 (0 to 0)	0.0 (0.0 to 0.0)	0 (0 to 0)	0.0 (0.0 to 0.0)	0 (0 to 0)	0.0 (0.0 to 0.0)	-72.9 (-84.9 to -48.4)	-66.7 (-81.5 to -36.6)
Azerbaijan	Maternal hemorrhage	10 to 14	Female	0 (0 to 0)	0.0 (0.0 to 0.0)	0 (0 to 0)	0.0 (0.0 to 0.0)	0 (0 to 0)	0.0 (0.0 to 0.0)	-72.9 (-84.9 to -48.4)	-65.5 (-80.9 to -34.4)
Azerbaijan	Maternal sepsis and other maternal infections	10 to 14	Both	0 (0 to 0)	0.0 (0.0 to 0.0)	0 (0 to 0)	0.0 (0.0 to 0.0)	0 (0 to 0)	0.0 (0.0 to 0.0)	-74.4 (-86.1 to -52.1)	-68.6 (-83.0 to -41.2)
Azerbaijan	Maternal sepsis and other maternal infections	10 to 14	Female	0 (0 to 0)	0.0 (0.0 to 0.0)	0 (0 to 0)	0.0 (0.0 to 0.0)	0 (0 to 0)	0.0 (0.0 to 0.0)	-74.4 (-86.1 to -52.1)	-67.5 (-82.4 to -39.1)
Azerbaijan	Maternal hypertensive disorders	10 to 14	Both	0 (0 to 0)	0.0 (0.0 to 0.0)	0 (0 to 0)	0.0 (0.0 to 0.0)	0 (0 to 0)	0.0 (0.0 to 0.0)	-72.1 (-84.7 to -48.2)	-65.7 (-81.2 to -36.5)
Azerbaijan	Maternal hypertensive disorders	10 to 14	Female	0 (0 to 0)	0.0 (0.0 to 0.0)	0 (0 to 0)	0.0 (0.0 to 0.0)	0 (0 to 0)	0.0 (0.0 to 0.0)	-72.1 (-84.7 to -48.2)	-64.5 (-80.5 to -34.2)
Azerbaijan	Maternal obstructed labor and uterine rupture	10 to 14	Both	0 (0 to 0)	0.0 (0.0 to 0.0)	0 (0 to 0)	0.0 (0.0 to 0.0)	0 (0 to 0)	0.0 (0.0 to 0.0)	-74.8 (-86.5 to -50.4)	-69.1 (-83.4 to -39.2)
Azerbaijan	Maternal obstructed labor and uterine rupture	10 to 14	Female	0 (0 to 0)	0.0 (0.0 to 0.0)	0 (0 to 0)	0.0 (0.0 to 0.0)	0 (0 to 0)	0.0 (0.0 to 0.0)	-74.8 (-86.5 to -50.4)	-68.0 (-82.8 to -37.0)
Azerbaijan	Maternal abortion, miscarriage, and ectopic pregnancy	10 to 14	Both	0 (0 to 0)	0.0 (0.0 to 0.0)	0 (0 to 0)	0.0 (0.0 to 0.0)	0 (0 to 0)	0.0 (0.0 to 0.0)	-75.5 (-87.1 to -53.5)	-69.9 (-84.2 to -43.0)

eTable 8. All-cause and cause-specific mortality (counts and rates) at the global level and by country in the WHO European Region for ages 5 to 9 and 10 to 14 for 1990, 2000, and 2016

Azerbaijan	Maternal abortion, miscarriage, and ectopic pregnancy	10 to 14	Female	0 (0 to 0)	0.0 (0.0 to 0.0)	0 (0 to 0)	0.0 (0.0 to 0.0)	0 (0 to 0)	0.0 (0.0 to 0.0)	-75.5 (-87.1 to -53.5)	-68.8 (-83.6 to -41.0)
Azerbaijan	Indirect maternal deaths	10 to 14	Both	0 (0 to 0)	0.0 (0.0 to 0.0)	0 (0 to 0)	0.0 (0.0 to 0.0)	0 (0 to 0)	0.0 (0.0 to 0.0)	-74.5 (-85.8 to -52.5)	-68.7 (-82.5 to -41.7)
Azerbaijan	Indirect maternal deaths	10 to 14	Female	0 (0 to 0)	0.0 (0.0 to 0.0)	0 (0 to 0)	0.0 (0.0 to 0.0)	0 (0 to 0)	0.0 (0.0 to 0.0)	-74.5 (-85.8 to -52.5)	-67.6 (-81.9 to -39.6)
Azerbaijan	Late maternal deaths	10 to 14	Both	0 (0 to 0)	0.0 (0.0 to 0.0)	0 (0 to 0)	0.0 (0.0 to 0.0)	0 (0 to 0)	0.0 (0.0 to 0.0)	-75.8 (-88.1 to -53.5)	-70.4 (-85.3 to -43.0)
Azerbaijan	Late maternal deaths	10 to 14	Female	0 (0 to 0)	0.0 (0.0 to 0.0)	0 (0 to 0)	0.0 (0.0 to 0.0)	0 (0 to 0)	0.0 (0.0 to 0.0)	-75.8 (-88.1 to -53.5)	-69.3 (-84.8 to -41.0)
Azerbaijan	Maternal deaths aggravated by HIV/AIDS	10 to 14	Both	0 (0 to 0)	0.0 (0.0 to 0.0)	0 (0 to 0)	0.0 (0.0 to 0.0)	0 (0 to 0)	0.0 (0.0 to 0.0)	389.9 (-12.0 to 10699.5)	501.1 (0.0 to 13152.3)
Azerbaijan	Maternal deaths aggravated by HIV/AIDS	10 to 14	Female	0 (0 to 0)	0.0 (0.0 to 0.0)	0 (0 to 0)	0.0 (0.0 to 0.0)	0 (0 to 0)	0.0 (0.0 to 0.0)	389.9 (-12.0 to 10699.5)	522.5 (0.0 to 13623.7)
Azerbaijan	Other maternal disorders	10 to 14	Both	0 (0 to 0)	0.0 (0.0 to 0.0)	0 (0 to 0)	0.0 (0.0 to 0.0)	0 (0 to 0)	0.0 (0.0 to 0.0)	-74.0 (-86.3 to -50.8)	-68.1 (-83.2 to -39.6)
Azerbaijan	Other maternal disorders	10 to 14	Female	0 (0 to 0)	0.0 (0.0 to 0.0)	0 (0 to 0)	0.0 (0.0 to 0.0)	0 (0 to 0)	0.0 (0.0 to 0.0)	-74.0 (-86.3 to -50.8)	-66.9 (-82.7 to -37.4)
Azerbaijan	Protein-energy malnutrition	5 to 9	Both	2 (1 to 3)	0.2 (0.1 to 0.4)	3 (1 to 5)	0.3 (0.1 to 0.5)	1 (0 to 1)	0.1 (0.0 to 0.2)	-70.7 (-90.6 to -7.0)	-63.4 (-88.3 to 16.3)
Azerbaijan	Protein-energy malnutrition	5 to 9	Female	1 (0 to 1)	0.1 (0.0 to 0.3)	1 (0 to 1)	0.2 (0.0 to 0.3)	0 (0 to 0)	0.0 (0.0 to 0.1)	-73.0 (-97.5 to 152.1)	-64.8 (-96.8 to 228.3)
Azerbaijan	Protein-energy malnutrition	5 to 9	Male	1 (0 to 3)	0.3 (0.1 to 0.7)	2 (0 to 4)	0.4 (0.1 to 0.9)	0 (0 to 1)	0.1 (0.0 to 0.3)	-69.8 (-94.6 to 99.1)	-63.7 (-93.5 to 139.6)
Azerbaijan	Protein-energy malnutrition	10 to 14	Both	1 (0 to 1)	0.1 (0.0 to 0.1)	1 (0 to 1)	0.1 (0.0 to 0.1)	0 (0 to 0)	0.0 (0.0 to 0.1)	-60.3 (-83.4 to -0.1)	-51.3 (-79.6 to 22.6)
Azerbaijan	Protein-energy malnutrition	10 to 14	Female	0 (0 to 1)	0.1 (0.0 to 0.2)	0 (0 to 1)	0.1 (0.0 to 0.2)	0 (0 to 0)	0.0 (0.0 to 0.1)	-65.7 (-92.2 to 62.9)	-56.4 (-90.1 to 107.0)
Azerbaijan	Protein-energy malnutrition	10 to 14	Male	0 (0 to 1)	0.1 (0.0 to 0.2)	0 (0 to 1)	0.1 (0.0 to 0.2)	0 (0 to 0)	0.0 (0.0 to 0.1)	-55.7 (-88.0 to 101.6)	-47.4 (-85.8 to 139.5)
Azerbaijan	Iodine deficiency	5 to 9	Both	0 (0 to 2)	0.0 (0.0 to 0.3)	1 (0 to 4)	0.1 (0.0 to 0.4)	0 (0 to 1)	0.0 (0.0 to 0.1)	-73.1 (-100.0 to 2.5e+20)	-66.4 (-100.0 to 3.2e+20)
Azerbaijan	Iodine deficiency	5 to 9	Female	0 (0 to 2)	0.0 (0.0 to 0.5)	0 (0 to 3)	0.1 (0.0 to 0.6)	0 (0 to 0)	0.0 (0.0 to 0.2)	-71.7 (-100.0 to 6.8e+28)	-63.2 (-100.0 to 8.8e+28)
Azerbaijan	Iodine deficiency	5 to 9	Male	0 (0 to 2)	0.0 (0.0 to 0.5)	0 (0 to 3)	0.1 (0.0 to 0.6)	0 (0 to 1)	0.0 (0.0 to 0.1)	-74.7 (-100.0 to 5.2e+25)	-69.6 (-100.0 to 6.2e+25)
Azerbaijan	Iodine deficiency	10 to 14	Both	0 (0 to 1)	0.0 (0.0 to 0.1)	0 (0 to 1)	0.0 (0.0 to 0.1)	0 (0 to 0)	0.0 (0.0 to 0.1)	-72.8 (-100.0 to 3.1e+19)	-66.6 (-100.0 to 3.8e+19)
Azerbaijan	Iodine deficiency	10 to 14	Female	0 (0 to 1)	0.0 (0.0 to 0.2)	0 (0 to 1)	0.0 (0.0 to 0.2)	0 (0 to 0)	0.0 (0.0 to 0.1)	-68.1 (-100.0 to 2.2e+26)	-59.4 (-100.0 to 2.8e+26)
Azerbaijan	Iodine deficiency	10 to 14	Male	0 (0 to 1)	0.0 (0.0 to 0.3)	0 (0 to 1)	0.0 (0.0 to 0.3)	0 (0 to 0)	0.0 (0.0 to 0.1)	-75.4 (-100.0 to 1.3e+27)	-70.8 (-100.0 to 1.6e+27)
Azerbaijan	Iron-deficiency anemia	5 to 9	Both	1 (0 to 1)	0.1 (0.0 to 0.2)	1 (0 to 2)	0.1 (0.0 to 0.2)	0 (0 to 1)	0.0 (0.0 to 0.1)	-66.1 (-90.1 to 14.6)	-57.6 (-87.6 to 43.2)
Azerbaijan	Iron-deficiency anemia	5 to 9	Female	0 (0 to 1)	0.1 (0.0 to 0.3)	1 (0 to 1)	0.1 (0.0 to 0.3)	0 (0 to 0)	0.0 (0.0 to 0.1)	-68.2 (-97.1 to 186.0)	-58.5 (-96.3 to 272.4)

eTable 8. All-cause and cause-specific mortality (counts and rates) at the global level and by country in the WHO European Region for ages 5 to 9 and 10 to 14 for 1990, 2000, and 2016

Azerbaijan	Iron-deficiency anemia	5 to 9	Male	0 (0 to 1)	0.1 (0.0 to 0.2)	0 (0 to 1)	0.1 (0.0 to 0.2)	0 (0 to 0)	0.0 (0.0 to 0.1)	-63.5 (-93.4 to 159.4)	-56.1 (-92.1 to 212.2)
Azerbaijan	Iron-deficiency anemia	10 to 14	Both	1 (0 to 1)	0.1 (0.0 to 0.1)	1 (0 to 1)	0.1 (0.0 to 0.1)	0 (0 to 0)	0.0 (0.0 to 0.1)	-56.9 (-84.0 to 21.0)	-47.1 (-80.3 to 48.5)
Azerbaijan	Iron-deficiency anemia	10 to 14	Female	0 (0 to 0)	0.1 (0.0 to 0.1)	0 (0 to 1)	0.1 (0.0 to 0.1)	0 (0 to 0)	0.0 (0.0 to 0.1)	-58.3 (-90.6 to 81.5)	-47.0 (-88.1 to 130.7)
Azerbaijan	Iron-deficiency anemia	10 to 14	Male	0 (0 to 1)	0.1 (0.0 to 0.2)	0 (0 to 1)	0.1 (0.0 to 0.2)	0 (0 to 0)	0.0 (0.0 to 0.1)	-56.1 (-88.0 to 101.6)	-47.8 (-85.8 to 139.5)
Azerbaijan	Other nutritional deficiencies	5 to 9	Both	1 (0 to 3)	0.2 (0.1 to 0.3)	2 (1 to 4)	0.2 (0.1 to 0.4)	0 (0 to 1)	0.0 (0.0 to 0.1)	-80.0 (-94.1 to -28.0)	-75.0 (-92.6 to -10.0)
Azerbaijan	Other nutritional deficiencies	5 to 9	Female	1 (0 to 2)	0.2 (0.0 to 0.5)	1 (0 to 3)	0.3 (0.0 to 0.7)	0 (0 to 0)	0.1 (0.0 to 0.1)	-83.7 (-98.5 to 60.4)	-78.7 (-98.1 to 108.9)
Azerbaijan	Other nutritional deficiencies	5 to 9	Male	0 (0 to 1)	0.1 (0.0 to 0.3)	1 (0 to 2)	0.1 (0.0 to 0.3)	0 (0 to 0)	0.0 (0.0 to 0.1)	-72.5 (-94.9 to 114.3)	-66.9 (-93.9 to 157.9)
Azerbaijan	Other nutritional deficiencies	10 to 14	Both	1 (0 to 2)	0.1 (0.1 to 0.2)	2 (1 to 3)	0.2 (0.1 to 0.3)	0 (0 to 1)	0.0 (0.0 to 0.1)	-69.3 (-87.3 to -23.5)	-62.4 (-84.4 to -6.2)
Azerbaijan	Other nutritional deficiencies	10 to 14	Female	1 (0 to 1)	0.2 (0.0 to 0.3)	1 (0 to 2)	0.2 (0.1 to 0.4)	0 (0 to 0)	0.1 (0.0 to 0.1)	-74.0 (-94.1 to 6.9)	-67.0 (-92.5 to 35.8)
Azerbaijan	Other nutritional deficiencies	10 to 14	Male	0 (0 to 1)	0.1 (0.0 to 0.2)	1 (0 to 1)	0.1 (0.0 to 0.3)	0 (0 to 0)	0.0 (0.0 to 0.1)	-62.9 (-90.1 to 64.6)	-56.0 (-88.3 to 95.5)
Azerbaijan	Sexually transmitted diseases excluding HIV	5 to 9	Both	1 (0 to 2)	0.1 (0.0 to 0.3)	1 (0 to 1)	0.1 (0.0 to 0.1)	0 (0 to 0)	0.0 (0.0 to 0.1)	-82.5 (-99.6 to -36.9)	-78.1 (-99.5 to -21.1)
Azerbaijan	Sexually transmitted diseases excluding HIV	5 to 9	Female	1 (0 to 1)	0.2 (0.0 to 0.3)	0 (0 to 1)	0.1 (0.0 to 0.1)	0 (0 to 0)	0.0 (0.0 to 0.1)	-82.8 (-99.6 to -39.8)	-77.7 (-99.5 to -21.6)
Azerbaijan	Sexually transmitted diseases excluding HIV	5 to 9	Male	0 (0 to 1)	0.1 (0.0 to 0.2)	0 (0 to 0)	0.0 (0.0 to 0.1)	0 (0 to 0)	0.0 (0.0 to 0.1)	-81.8 (-99.6 to -34.2)	-78.1 (-99.5 to -20.8)
Azerbaijan	Sexually transmitted diseases excluding HIV	10 to 14	Both	1 (0 to 1)	0.1 (0.0 to 0.1)	0 (0 to 1)	0.0 (0.0 to 0.1)	0 (0 to 0)	0.0 (0.0 to 0.0)	-85.4 (-93.7 to -55.9)	-82.0 (-92.3 to -45.8)
Azerbaijan	Sexually transmitted diseases excluding HIV	10 to 14	Female	0 (0 to 1)	0.1 (0.0 to 0.2)	0 (0 to 1)	0.1 (0.0 to 0.2)	0 (0 to 0)	0.0 (0.0 to 0.0)	-86.8 (-96.0 to -39.7)	-83.2 (-94.9 to -23.3)
Azerbaijan	Sexually transmitted diseases excluding HIV	10 to 14	Male	0 (0 to 0)	0.1 (0.0 to 0.1)	0 (0 to 0)	0.0 (0.0 to 0.1)	0 (0 to 0)	0.0 (0.0 to 0.0)	-82.8 (-91.9 to -56.8)	-79.6 (-90.4 to -48.7)
Azerbaijan	Hepatitis	5 to 9	Both	6 (4 to 9)	0.7 (0.4 to 1.1)	8 (5 to 12)	0.9 (0.5 to 1.4)	2 (1 to 4)	0.3 (0.2 to 0.7)	-61.9 (-84.0 to -13.9)	-52.4 (-80.0 to 7.6)
Azerbaijan	Hepatitis	5 to 9	Female	2 (1 to 3)	0.5 (0.3 to 0.8)	2 (1 to 4)	0.6 (0.3 to 0.9)	1 (0 to 1)	0.2 (0.1 to 0.5)	-64.8 (-88.2 to -16.6)	-54.2 (-84.7 to 8.5)
Azerbaijan	Hepatitis	5 to 9	Male	4 (2 to 7)	0.9 (0.5 to 1.7)	5 (3 to 10)	1.2 (0.6 to 2.2)	2 (1 to 4)	0.4 (0.1 to 1.1)	-60.4 (-88.2 to 17.3)	-52.4 (-85.8 to 41.1)

Azerbaijan	Hepatitis	10 to 14	Both	4 (3 to 6)	0.6 (0.4 to 0.9)	6 (4 to 9)	0.6 (0.4 to 1.0)	2 (1 to 4)	0.4 (0.2 to 0.7)	-51.1 (-78.6 to 5.0)	-40.0 (-73.8 to 28.8)
Azerbaijan	Hepatitis	10 to 14	Female	1 (1 to 2)	0.4 (0.2 to 0.7)	2 (1 to 3)	0.3 (0.2 to 0.6)	1 (0 to 1)	0.2 (0.1 to 0.4)	-56.5 (-85.6 to 15.1)	-44.7 (-81.7 to 46.3)
Azerbaijan	Hepatitis	10 to 14	Male	3 (2 to 5)	0.8 (0.4 to 1.3)	4 (2 to 7)	0.9 (0.5 to 1.5)	1 (1 to 3)	0.5 (0.2 to 1.1)	-48.6 (-82.5 to 37.5)	-38.9 (-79.2 to 63.4)
Azerbaijan	Other infectious diseases	5 to 9	Both	3 (2 to 6)	0.4 (0.2 to 0.7)	5 (2 to 8)	0.5 (0.2 to 0.9)	1 (1 to 3)	0.2 (0.1 to 0.4)	-56.3 (-81.1 to 1.7)	-45.4 (-76.3 to 27.1)
Azerbaijan	Other infectious diseases	5 to 9	Female	1 (1 to 3)	0.4 (0.2 to 0.8)	2 (1 to 4)	0.5 (0.2 to 0.9)	1 (0 to 1)	0.2 (0.1 to 0.5)	-54.2 (-83.9 to 20.8)	-40.3 (-79.0 to 57.3)
Azerbaijan	Other infectious diseases	5 to 9	Male	2 (1 to 4)	0.4 (0.2 to 0.9)	3 (1 to 5)	0.6 (0.2 to 1.2)	1 (0 to 2)	0.2 (0.1 to 0.4)	-58.2 (-87.7 to 35.4)	-49.7 (-85.2 to 62.9)
Azerbaijan	Other infectious diseases	10 to 14	Both	1 (1 to 2)	0.2 (0.1 to 0.3)	2 (1 to 3)	0.2 (0.1 to 0.3)	1 (0 to 1)	0.1 (0.1 to 0.1)	-52.2 (-77.9 to -7.1)	-41.4 (-72.9 to 14.0)
Azerbaijan	Other infectious diseases	10 to 14	Female	1 (0 to 1)	0.2 (0.1 to 0.4)	1 (1 to 2)	0.2 (0.1 to 0.4)	0 (0 to 1)	0.1 (0.1 to 0.2)	-53.3 (-84.0 to 24.8)	-40.6 (-79.6 to 58.6)
Azerbaijan	Other infectious diseases	10 to 14	Male	0 (0 to 1)	0.1 (0.1 to 0.2)	1 (0 to 1)	0.1 (0.1 to 0.2)	0 (0 to 0)	0.1 (0.0 to 0.1)	-50.7 (-83.2 to 32.8)	-41.4 (-80.1 to 57.8)
Azerbaijan	Nasopharynx cancer	5 to 9	Both	1 (0 to 1)	0.1 (0.0 to 0.2)	1 (1 to 2)	0.1 (0.1 to 0.2)	1 (0 to 1)	0.1 (0.0 to 0.2)	-1.7 (-63.8 to 164.9)	22.9 (-54.8 to 231.1)
Azerbaijan	Nasopharynx cancer	5 to 9	Female	0 (0 to 0)	0.0 (0.0 to 0.0)	0 (0 to 0)	0.0 (0.0 to 0.1)	0 (0 to 0)	0.0 (0.0 to 0.0)	-33.8 (-79.3 to 112.1)	-13.8 (-73.1 to 176.2)
Azerbaijan	Nasopharynx cancer	5 to 9	Male	1 (0 to 1)	0.1 (0.1 to 0.3)	1 (0 to 2)	0.2 (0.1 to 0.4)	1 (0 to 1)	0.2 (0.1 to 0.4)	3.0 (-66.2 to 213.5)	24.0 (-59.3 to 277.3)
Azerbaijan	Nasopharynx cancer	10 to 14	Both	0 (0 to 1)	0.1 (0.0 to 0.1)	1 (0 to 1)	0.1 (0.0 to 0.1)	1 (0 to 1)	0.1 (0.0 to 0.2)	19.2 (-50.9 to 181.0)	46.3 (-39.8 to 244.8)
Azerbaijan	Nasopharynx cancer	10 to 14	Female	0 (0 to 0)	0.0 (0.0 to 0.0)	0 (0 to 0)	0.0 (0.0 to 0.0)	0 (0 to 0)	0.0 (0.0 to 0.0)	-20.0 (-37.5 to 3.2)	1.7 (-20.6 to 31.2)
Azerbaijan	Nasopharynx cancer	10 to 14	Male	0 (0 to 1)	0.1 (0.1 to 0.2)	1 (0 to 1)	0.1 (0.1 to 0.2)	1 (0 to 1)	0.2 (0.1 to 0.3)	22.4 (-52.5 to 211.1)	45.4 (-43.6 to 269.6)
Azerbaijan	Liver cancer	5 to 9	Both	2 (1 to 3)	0.2 (0.1 to 0.3)	2 (1 to 3)	0.3 (0.2 to 0.4)	1 (1 to 2)	0.2 (0.1 to 0.3)	-27.2 (-67.5 to 51.2)	-9.1 (-59.4 to 89.0)
Azerbaijan	Liver cancer	5 to 9	Female	1 (0 to 1)	0.2 (0.1 to 0.3)	1 (0 to 2)	0.2 (0.1 to 0.4)	0 (0 to 1)	0.1 (0.1 to 0.3)	-41.6 (-77.5 to 48.6)	-24.0 (-70.7 to 93.5)
Azerbaijan	Liver cancer	5 to 9	Male	1 (0 to 2)	0.2 (0.1 to 0.4)	1 (1 to 2)	0.3 (0.1 to 0.5)	1 (0 to 2)	0.2 (0.1 to 0.5)	-17.2 (-71.9 to 120.7)	-0.3 (-66.1 to 165.6)
Azerbaijan	Liver cancer	10 to 14	Both	2 (1 to 3)	0.2 (0.1 to 0.4)	3 (2 to 4)	0.3 (0.2 to 0.4)	1 (1 to 2)	0.3 (0.1 to 0.4)	-16.6 (-60.6 to 76.4)	2.3 (-51.7 to 116.4)
Azerbaijan	Liver cancer	10 to 14	Female	1 (0 to 1)	0.2 (0.1 to 0.3)	1 (0 to 2)	0.2 (0.1 to 0.4)	0 (0 to 1)	0.1 (0.1 to 0.3)	-29.5 (-72.9 to 80.6)	-10.4 (-65.5 to 129.4)
Azerbaijan	Liver cancer	10 to 14	Male	1 (1 to 2)	0.3 (0.2 to 0.6)	2 (1 to 3)	0.4 (0.2 to 0.6)	1 (1 to 2)	0.4 (0.2 to 0.6)	-11.1 (-63.5 to 116.3)	5.6 (-56.6 to 156.9)
Azerbaijan	Kidney cancer	5 to 9	Both	4 (3 to 7)	0.5 (0.3 to 0.9)	7 (4 to 11)	0.8 (0.4 to 1.3)	3 (2 to 5)	0.5 (0.3 to 0.8)	-29.9 (-63.0 to 44.2)	-12.5 (-53.8 to 80.2)
Azerbaijan	Kidney cancer	5 to 9	Female	2 (1 to 3)	0.4 (0.2 to 0.8)	3 (1 to 5)	0.6 (0.3 to 1.1)	1 (0 to 2)	0.3 (0.2 to 0.6)	-42.1 (-75.7 to 37.0)	-24.6 (-68.3 to 78.5)
Azerbaijan	Kidney cancer	5 to 9	Male	3 (1 to 5)	0.6 (0.3 to 1.1)	4 (1 to 8)	0.9 (0.3 to 1.7)	2 (1 to 4)	0.6 (0.3 to 1.2)	-21.6 (-66.5 to 97.4)	-5.7 (-59.7 to 137.6)
Azerbaijan	Kidney cancer	10 to 14	Both	2 (1 to 2)	0.2 (0.1 to 0.3)	3 (2 to 4)	0.3 (0.2 to 0.4)	1 (1 to 2)	0.2 (0.1 to 0.3)	-20.6 (-54.5 to 43.6)	-2.6 (-44.1 to 76.2)
Azerbaijan	Kidney cancer	10 to 14	Female	1 (0 to 1)	0.2 (0.1 to 0.4)	1 (1 to 2)	0.3 (0.1 to 0.4)	1 (0 to 1)	0.2 (0.1 to 0.3)	-31.9 (-70.5 to 52.1)	-13.4 (-62.5 to 93.3)
Azerbaijan	Kidney cancer	10 to 14	Male	1 (0 to 1)	0.2 (0.1 to 0.4)	1 (1 to 2)	0.3 (0.1 to 0.5)	1 (0 to 1)	0.3 (0.1 to 0.4)	-10.4 (-58.7 to 85.7)	6.4 (-50.9 to 120.7)
Azerbaijan	Brain and nervous system cancer	5 to 9	Both	21 (13 to 31)	2.5 (1.6 to 3.8)	31 (16 to 50)	3.5 (1.8 to 5.7)	15 (9 to 22)	2.2 (1.4 to 3.4)	-29.6 (-59.6 to 21.8)	-12.0 (-49.5 to 52.2)

eTable 8. All-cause and cause-specific mortality (counts and rates) at the global level and by country in the WHO European Region for ages 5 to 9 and 10 to 14 for 1990, 2000, and 2016

Azerbaijan	Brain and nervous system cancer	5 to 9	Female	10 (6 to 17)	2.5 (1.4 to 4.2)	15 (7 to 28)	3.5 (1.6 to 6.4)	6 (3 to 10)	1.9 (1.0 to 3.4)	-40.4 (-72.9 to 9.8)	-22.3 (-64.8 to 42.9)
Azerbaijan	Brain and nervous system cancer	5 to 9	Male	11 (6 to 18)	2.6 (1.4 to 4.2)	16 (8 to 28)	3.5 (1.7 to 6.2)	9 (5 to 15)	2.5 (1.3 to 4.2)	-19.6 (-62.2 to 71.4)	-3.3 (-54.6 to 106.3)
Azerbaijan	Brain and nervous system cancer	10 to 14	Both	16 (10 to 22)	2.2 (1.5 to 3.1)	26 (15 to 37)	2.7 (1.6 to 4.0)	12 (8 to 17)	2.1 (1.4 to 3.0)	-21.0 (-52.3 to 22.4)	-3.1 (-41.5 to 50.1)
Azerbaijan	Brain and nervous system cancer	10 to 14	Female	8 (5 to 12)	2.2 (1.3 to 3.5)	13 (7 to 20)	2.8 (1.4 to 4.5)	6 (3 to 8)	2.0 (1.3 to 3.1)	-27.7 (-63.0 to 29.9)	-8.1 (-52.9 to 65.1)
Azerbaijan	Brain and nervous system cancer	10 to 14	Male	8 (5 to 13)	2.2 (1.3 to 3.6)	13 (7 to 21)	2.7 (1.4 to 4.3)	7 (4 to 11)	2.2 (1.3 to 3.7)	-14.7 (-55.3 to 64.4)	1.3 (-46.9 to 95.4)
Azerbaijan	Thyroid cancer	10 to 14	Both	0 (0 to 1)	0.0 (0.0 to 0.1)	1 (0 to 1)	0.1 (0.0 to 0.1)	0 (0 to 0)	0.0 (0.0 to 0.1)	-28.9 (-70.6 to 64.8)	-12.7 (-63.9 to 102.2)
Azerbaijan	Thyroid cancer	10 to 14	Female	0 (0 to 0)	0.0 (0.0 to 0.1)	0 (0 to 0)	0.0 (0.0 to 0.1)	0 (0 to 0)	0.0 (0.0 to 0.1)	-17.6 (-79.2 to 215.4)	4.7 (-73.5 to 300.9)
Azerbaijan	Thyroid cancer	10 to 14	Male	0 (0 to 0)	0.1 (0.0 to 0.1)	0 (0 to 1)	0.1 (0.0 to 0.1)	0 (0 to 0)	0.0 (0.0 to 0.1)	-35.7 (-76.3 to 70.1)	-23.6 (-71.9 to 102.1)
Azerbaijan	Hodgkin lymphoma	5 to 9	Both	2 (1 to 3)	0.2 (0.1 to 0.4)	3 (1 to 5)	0.3 (0.1 to 0.6)	1 (0 to 2)	0.1 (0.0 to 0.3)	-45.7 (-81.9 to 89.5)	-32.1 (-77.4 to 136.8)
Azerbaijan	Hodgkin lymphoma	5 to 9	Female	0 (0 to 1)	0.1 (0.0 to 0.2)	0 (0 to 1)	0.1 (0.0 to 0.2)	0 (0 to 0)	0.0 (0.0 to 0.1)	-58.7 (-91.6 to 132.9)	-46.3 (-89.1 to 203.3)
Azerbaijan	Hodgkin lymphoma	5 to 9	Male	1 (0 to 3)	0.3 (0.1 to 0.8)	2 (1 to 5)	0.5 (0.2 to 1.0)	1 (0 to 2)	0.2 (0.1 to 0.6)	-43.7 (-83.3 to 138.5)	-32.2 (-79.9 to 187.0)
Azerbaijan	Hodgkin lymphoma	10 to 14	Both	2 (1 to 3)	0.3 (0.2 to 0.5)	4 (2 to 6)	0.4 (0.2 to 0.6)	1 (1 to 2)	0.2 (0.1 to 0.4)	-32.5 (-65.5 to 52.4)	-17.2 (-57.7 to 87.0)
Azerbaijan	Hodgkin lymphoma	10 to 14	Female	0 (0 to 0)	0.1 (0.0 to 0.1)	0 (0 to 1)	0.1 (0.0 to 0.2)	0 (0 to 0)	0.0 (0.0 to 0.1)	-46.6 (-83.3 to 89.2)	-32.1 (-78.8 to 140.5)
Azerbaijan	Hodgkin lymphoma	10 to 14	Male	2 (1 to 3)	0.5 (0.3 to 0.8)	3 (2 to 5)	0.7 (0.4 to 1.1)	1 (1 to 2)	0.4 (0.2 to 0.7)	-31.0 (-65.7 to 61.0)	-18.0 (-59.2 to 91.3)
Azerbaijan	Non-Hodgkin lymphoma	5 to 9	Both	4 (3 to 7)	0.5 (0.3 to 0.8)	6 (3 to 9)	0.6 (0.4 to 1.0)	4 (2 to 7)	0.7 (0.4 to 1.1)	2.7 (-46.2 to 107.6)	28.4 (-32.8 to 159.4)
Azerbaijan	Non-Hodgkin lymphoma	5 to 9	Female	1 (1 to 3)	0.4 (0.2 to 0.6)	2 (1 to 3)	0.4 (0.2 to 0.8)	1 (1 to 2)	0.4 (0.2 to 0.8)	-8.0 (-61.1 to 125.8)	19.9 (-49.3 to 194.0)
Azerbaijan	Non-Hodgkin lymphoma	5 to 9	Male	3 (2 to 5)	0.7 (0.4 to 1.2)	4 (2 to 7)	0.8 (0.4 to 1.6)	3 (2 to 6)	0.9 (0.4 to 1.6)	8.0 (-49.8 to 161.8)	30.0 (-39.5 to 215.1)
Azerbaijan	Non-Hodgkin lymphoma	10 to 14	Both	3 (2 to 5)	0.5 (0.3 to 0.7)	5 (3 to 7)	0.5 (0.3 to 0.8)	4 (2 to 6)	0.6 (0.4 to 1.0)	12.3 (-36.0 to 116.6)	37.7 (-21.5 to 165.8)
Azerbaijan	Non-Hodgkin lymphoma	10 to 14	Female	1 (1 to 2)	0.4 (0.2 to 0.7)	2 (1 to 3)	0.4 (0.2 to 0.8)	1 (1 to 3)	0.5 (0.3 to 0.9)	5.9 (-54.4 to 151.7)	34.6 (-42.0 to 219.8)
Azerbaijan	Non-Hodgkin lymphoma	10 to 14	Male	2 (1 to 3)	0.5 (0.3 to 0.9)	3 (2 to 5)	0.6 (0.3 to 1.1)	2 (1 to 4)	0.7 (0.4 to 1.3)	16.7 (-42.0 to 184.1)	38.6 (-31.1 to 237.5)
Azerbaijan	Leukemia	5 to 9	Both	44 (31 to 62)	5.4 (3.8 to 7.6)	107 (68 to 148)	12.1 (7.6 to 16.7)	40 (23 to 63)	6.1 (3.5 to 9.6)	-9.6 (-54.5 to 64.3)	13.0 (-43.1 to 105.3)
Azerbaijan	Leukemia	5 to 9	Female	18 (12 to 28)	4.6 (3.0 to 7.0)	44 (18 to 67)	10.1 (4.1 to 15.5)	13 (7 to 22)	4.1 (2.2 to 7.2)	-31.1 (-63.9 to 49.1)	-10.3 (-53.0 to 94.1)
Azerbaijan	Leukemia	5 to 9	Male	26 (16 to 40)	6.1 (3.8 to 9.5)	63 (38 to 96)	13.9 (8.4 to 21.0)	27 (15 to 48)	7.8 (4.2 to 13.8)	5.7 (-51.1 to 118.3)	27.3 (-41.2 to 162.7)
Azerbaijan	Leukemia	10 to 14	Both	31 (24 to 42)	4.4 (3.3 to 5.9)	85 (52 to 114)	9.1 (5.6 to 12.2)	33 (21 to 50)	5.7 (3.6 to 8.7)	5.0 (-39.0 to 75.4)	28.9 (-25.2 to 115.3)
Azerbaijan	Leukemia	10 to 14	Female	13 (9 to 18)	3.7 (2.5 to 5.2)	33 (15 to 50)	7.2 (3.4 to 10.8)	10 (6 to 17)	3.8 (2.1 to 6.3)	-19.3 (-53.1 to 54.7)	2.5 (-40.5 to 96.6)
Azerbaijan	Leukemia	10 to 14	Male	19 (12 to 26)	5.2 (3.4 to 7.3)	52 (31 to 73)	10.9 (6.6 to 15.2)	23 (13 to 37)	7.5 (4.3 to 12.2)	21.8 (-35.4 to 132.1)	44.7 (-23.2 to 175.7)
Azerbaijan	Other neoplasms	5 to 9	Both	19 (10 to 29)	2.4 (1.3 to 3.6)	28 (13 to 47)	3.2 (1.4 to 5.2)	13 (7 to 20)	2.0 (1.1 to 3.1)	-32.8 (-62.8 to 24.4)	-16.0 (-53.5 to 55.4)
Azerbaijan	Other neoplasms	5 to 9	Female	8 (4 to 13)	2.0 (1.1 to 3.4)	12 (6 to 20)	2.7 (1.3 to 4.5)	5 (3 to 9)	1.7 (0.9 to 2.9)	-36.2 (-71.6 to 37.9)	-16.9 (-63.1 to 79.5)
Azerbaijan	Other neoplasms	5 to 9	Male	11 (5 to 19)	2.7 (1.1 to 4.5)	17 (5 to 30)	3.7 (1.2 to 6.7)	8 (4 to 14)	2.2 (1.0 to 4.0)	-30.3 (-68.0 to 58.8)	-16.2 (-61.5 to 91.1)
Azerbaijan	Other neoplasms	10 to 14	Both	19 (10 to 29)	2.7 (1.5 to 4.0)	31 (16 to 45)	3.3 (1.7 to 4.9)	15 (8 to 22)	2.6 (1.3 to 3.8)	-23.6 (-54.9 to 24.3)	-6.3 (-44.6 to 52.5)

Azerbaijan	Other neoplasms	10 to 14	Female	8 (4 to 12)	2.2 (1.2 to 3.6)	12 (6 to 20)	2.7 (1.4 to 4.4)	6 (3 to 9)	2.1 (1.1 to 3.5)	-27.5 (-66.4 to 60.4)	-7.8 (-57.2 to 103.8)
Azerbaijan	Other neoplasms	10 to 14	Male	12 (5 to 19)	3.2 (1.4 to 5.2)	19 (7 to 30)	3.9 (1.5 to 6.3)	9 (4 to 15)	3.0 (1.4 to 4.9)	-21.0 (-60.2 to 58.4)	-6.2 (-52.7 to 88.2)
Azerbaijan	Rheumatic heart disease	5 to 9	Both	5 (3 to 9)	0.6 (0.4 to 1.0)	15 (9 to 25)	1.7 (1.0 to 2.8)	3 (2 to 6)	0.5 (0.3 to 0.8)	-35.5 (-64.7 to 11.1)	-19.4 (-55.9 to 38.8)
Azerbaijan	Rheumatic heart disease	5 to 9	Female	2 (1 to 5)	0.6 (0.3 to 1.1)	7 (4 to 12)	1.6 (0.9 to 2.8)	1 (1 to 2)	0.5 (0.2 to 0.8)	-41.6 (-69.6 to 16.5)	-24.0 (-60.5 to 51.7)
Azerbaijan	Rheumatic heart disease	5 to 9	Male	3 (1 to 5)	0.6 (0.3 to 1.2)	8 (4 to 15)	1.9 (1.0 to 3.3)	2 (1 to 4)	0.5 (0.2 to 1.0)	-30.0 (-70.9 to 58.4)	-15.8 (-65.0 to 90.7)
Azerbaijan	Rheumatic heart disease	10 to 14	Both	8 (6 to 12)	1.2 (0.8 to 1.8)	20 (14 to 29)	2.1 (1.5 to 3.1)	6 (4 to 8)	1.0 (0.6 to 1.4)	-33.2 (-55.7 to 4.0)	-18.0 (-45.6 to 27.6)
Azerbaijan	Rheumatic heart disease	10 to 14	Female	4 (3 to 7)	1.3 (0.8 to 2.1)	10 (6 to 15)	2.1 (1.4 to 3.4)	3 (2 to 4)	1.0 (0.6 to 1.6)	-37.6 (-62.6 to 13.8)	-20.7 (-52.5 to 44.6)
Azerbaijan	Rheumatic heart disease	10 to 14	Male	4 (2 to 6)	1.1 (0.7 to 1.7)	10 (6 to 15)	2.0 (1.3 to 3.1)	3 (2 to 4)	0.9 (0.5 to 1.4)	-28.3 (-58.9 to 33.3)	-14.8 (-51.2 to 58.4)
Azerbaijan	Ischemic heart disease	5 to 9	Both	2 (1 to 4)	0.2 (0.1 to 0.5)	5 (3 to 9)	0.6 (0.3 to 1.0)	1 (0 to 2)	0.2 (0.1 to 0.3)	-50.4 (-76.4 to 2.3)	-38.0 (-70.5 to 27.9)
Azerbaijan	Ischemic heart disease	5 to 9	Female	1 (1 to 2)	0.3 (0.1 to 0.5)	3 (1 to 5)	0.6 (0.3 to 1.1)	0 (0 to 1)	0.1 (0.1 to 0.3)	-56.5 (-80.9 to -0.1)	-43.3 (-75.1 to 30.1)
Azerbaijan	Ischemic heart disease	5 to 9	Male	1 (0 to 2)	0.2 (0.1 to 0.5)	3 (1 to 5)	0.6 (0.2 to 1.2)	1 (0 to 1)	0.2 (0.1 to 0.3)	-43.5 (-81.0 to 57.3)	-32.0 (-77.2 to 89.3)
Azerbaijan	Ischemic heart disease	10 to 14	Both	2 (1 to 3)	0.3 (0.2 to 0.5)	5 (3 to 8)	0.6 (0.4 to 0.9)	2 (1 to 3)	0.3 (0.2 to 0.5)	-25.2 (-59.0 to 28.6)	-8.2 (-49.6 to 57.8)
Azerbaijan	Ischemic heart disease	10 to 14	Female	1 (1 to 2)	0.3 (0.2 to 0.6)	3 (1 to 4)	0.6 (0.3 to 0.9)	1 (0 to 1)	0.3 (0.1 to 0.5)	-36.0 (-69.4 to 34.5)	-18.7 (-61.2 to 71.0)
Azerbaijan	Ischemic heart disease	10 to 14	Male	1 (1 to 2)	0.3 (0.1 to 0.5)	3 (1 to 5)	0.6 (0.3 to 1.0)	1 (0 to 2)	0.3 (0.1 to 0.5)	-13.3 (-64.1 to 101.2)	3.0 (-57.3 to 139.0)
Azerbaijan	Cerebrovascular disease	5 to 9	Both	4 (2 to 7)	0.5 (0.3 to 0.8)	8 (5 to 14)	0.9 (0.6 to 1.5)	2 (1 to 4)	0.3 (0.2 to 0.6)	-44.4 (-69.5 to 1.5)	-30.5 (-61.9 to 26.8)
Azerbaijan	Cerebrovascular disease	5 to 9	Female	2 (1 to 4)	0.5 (0.3 to 1.0)	4 (2 to 7)	1.0 (0.5 to 1.7)	1 (0 to 2)	0.3 (0.1 to 0.6)	-51.9 (-76.4 to -2.5)	-37.3 (-69.3 to 26.9)
Azerbaijan	Cerebrovascular disease	5 to 9	Male	2 (1 to 3)	0.4 (0.2 to 0.8)	4 (2 to 8)	0.9 (0.4 to 1.7)	1 (0 to 2)	0.3 (0.1 to 0.6)	-35.5 (-73.5 to 54.0)	-22.3 (-68.1 to 85.3)
Azerbaijan	Cerebrovascular disease	10 to 14	Both	4 (3 to 6)	0.5 (0.4 to 0.8)	7 (5 to 11)	0.8 (0.5 to 1.2)	2 (2 to 4)	0.4 (0.3 to 0.6)	-36.3 (-61.1 to 4.6)	-21.8 (-52.3 to 28.3)
Azerbaijan	Cerebrovascular disease	10 to 14	Female	2 (1 to 3)	0.6 (0.3 to 1.0)	4 (2 to 6)	0.8 (0.5 to 1.3)	1 (1 to 2)	0.4 (0.2 to 0.7)	-45.2 (-71.9 to 5.8)	-30.3 (-64.3 to 34.5)
Azerbaijan	Cerebrovascular disease	10 to 14	Male	2 (1 to 3)	0.5 (0.3 to 0.8)	4 (2 to 6)	0.7 (0.4 to 1.2)	1 (1 to 2)	0.4 (0.2 to 0.7)	-26.5 (-62.3 to 42.4)	-12.7 (-55.2 to 69.2)
Azerbaijan	Hypertensive heart disease	5 to 9	Both	0 (0 to 0)	0.0 (0.0 to 0.0)	0 (0 to 0)	0.0 (0.0 to 0.0)	0 (0 to 0)	0.0 (0.0 to 0.0)	14.8 (-36.7 to 95.4)	43.5 (-20.9 to 144.2)
Azerbaijan	Hypertensive heart disease	5 to 9	Female	0 (0 to 0)	0.0 (0.0 to 0.0)	0 (0 to 0)	0.0 (0.0 to 0.0)	0 (0 to 0)	0.0 (0.0 to 0.0)	-2.6 (-49.6 to 83.4)	26.8 (-34.4 to 138.9)
Azerbaijan	Hypertensive heart disease	5 to 9	Male	0 (0 to 0)	0.0 (0.0 to 0.0)	0 (0 to 0)	0.0 (0.0 to 0.0)	0 (0 to 0)	0.0 (0.0 to 0.0)	31.4 (-39.9 to 168.2)	58.2 (-27.6 to 222.7)
Azerbaijan	Hypertensive heart disease	10 to 14	Both	0 (0 to 0)	0.0 (0.0 to 0.0)	0 (0 to 0)	0.0 (0.0 to 0.0)	0 (0 to 0)	0.0 (0.0 to 0.0)	11.6 (-24.3 to 62.1)	37.0 (-7.1 to 98.9)
Azerbaijan	Hypertensive heart disease	10 to 14	Female	0 (0 to 0)	0.0 (0.0 to 0.0)	0 (0 to 0)	0.0 (0.0 to 0.0)	0 (0 to 0)	0.0 (0.0 to 0.0)	-3.3 (-40.4 to 65.3)	22.9 (-24.2 to 110.0)
Azerbaijan	Hypertensive heart disease	10 to 14	Male	0 (0 to 0)	0.0 (0.0 to 0.0)	0 (0 to 0)	0.0 (0.0 to 0.0)	0 (0 to 0)	0.0 (0.0 to 0.0)	27.7 (-23.4 to 121.5)	51.8 (-9.0 to 163.2)
Azerbaijan	Cardiomyopathy and myocarditis	5 to 9	Both	10 (5 to 14)	1.2 (0.6 to 1.7)	22 (11 to 33)	2.5 (1.2 to 3.8)	7 (4 to 10)	1.0 (0.6 to 1.5)	-30.7 (-59.6 to 21.9)	-13.5 (-49.5 to 52.3)
Azerbaijan	Cardiomyopathy and myocarditis	5 to 9	Female	5 (2 to 7)	1.1 (0.5 to 1.8)	10 (3 to 16)	2.4 (0.7 to 3.8)	3 (1 to 4)	0.8 (0.4 to 1.3)	-45.0 (-71.8 to 4.4)	-28.3 (-63.3 to 35.9)
Azerbaijan	Cardiomyopathy and myocarditis	5 to 9	Male	5 (2 to 8)	1.2 (0.5 to 1.9)	12 (4 to 20)	2.7 (0.8 to 4.5)	4 (2 to 7)	1.2 (0.6 to 2.0)	-17.7 (-60.6 to 66.5)	-1.0 (-52.6 to 100.4)
Azerbaijan	Cardiomyopathy and myocarditis	10 to 14	Both	8 (5 to 11)	1.1 (0.7 to 1.6)	16 (9 to 23)	1.8 (1.0 to 2.4)	7 (4 to 10)	1.1 (0.8 to 1.7)	-17.2 (-47.3 to 28.4)	1.6 (-35.3 to 57.5)

eTable 8. All-cause and cause-specific mortality (counts and rates) at the global level and by country in the WHO European Region for ages 5 to 9 and 10 to 14 for 1990, 2000, and 2016

Azerbaijan	Cardiomyopathy and myocarditis	10 to 14	Female	4 (2 to 6)	1.1 (0.6 to 1.7)	8 (4 to 12)	1.7 (0.8 to 2.6)	3 (1 to 4)	0.9 (0.5 to 1.5)	-32.7 (-65.1 to 19.4)	-14.5 (-55.7 to 51.8)
Azerbaijan	Cardiomyopathy and myocarditis	10 to 14	Male	4 (2 to 7)	1.2 (0.6 to 1.8)	9 (4 to 13)	1.9 (0.8 to 2.8)	4 (2 to 6)	1.3 (0.8 to 2.1)	-3.5 (-48.5 to 72.1)	14.6 (-38.8 to 104.5)
Azerbaijan	Atrial fibrillation and flutter	5 to 9	Both	0 (0 to 0)	0.0 (0.0 to 0.0)	0 (0 to 0)	0.0 (0.0 to 0.0)	0 (0 to 0)	0.0 (0.0 to 0.0)	0.0 (0.0 to 0.0)	0.0 (0.0 to 0.0)
Azerbaijan	Atrial fibrillation and flutter	5 to 9	Female	0 (0 to 0)	0.0 (0.0 to 0.0)	0 (0 to 0)	0.0 (0.0 to 0.0)	0 (0 to 0)	0.0 (0.0 to 0.0)	0.0 (0.0 to 0.0)	0.0 (0.0 to 0.0)
Azerbaijan	Atrial fibrillation and flutter	5 to 9	Male	0 (0 to 0)	0.0 (0.0 to 0.0)	0 (0 to 0)	0.0 (0.0 to 0.0)	0 (0 to 0)	0.0 (0.0 to 0.0)	0.0 (0.0 to 0.0)	0.0 (0.0 to 0.0)
Azerbaijan	Atrial fibrillation and flutter	10 to 14	Both	0 (0 to 0)	0.0 (0.0 to 0.0)	0 (0 to 0)	0.0 (0.0 to 0.0)	0 (0 to 0)	0.0 (0.0 to 0.0)	0.0 (0.0 to 0.0)	0.0 (0.0 to 0.0)
Azerbaijan	Atrial fibrillation and flutter	10 to 14	Female	0 (0 to 0)	0.0 (0.0 to 0.0)	0 (0 to 0)	0.0 (0.0 to 0.0)	0 (0 to 0)	0.0 (0.0 to 0.0)	0.0 (0.0 to 0.0)	0.0 (0.0 to 0.0)
Azerbaijan	Atrial fibrillation and flutter	10 to 14	Male	0 (0 to 0)	0.0 (0.0 to 0.0)	0 (0 to 0)	0.0 (0.0 to 0.0)	0 (0 to 0)	0.0 (0.0 to 0.0)	0.0 (0.0 to 0.0)	0.0 (0.0 to 0.0)
Azerbaijan	Endocarditis	5 to 9	Both	1 (0 to 2)	0.1 (0.0 to 0.2)	2 (1 to 3)	0.2 (0.1 to 0.4)	1 (0 to 1)	0.1 (0.1 to 0.2)	-7.7 (-63.3 to 119.4)	15.4 (-54.2 to 174.1)
Azerbaijan	Endocarditis	5 to 9	Female	0 (0 to 1)	0.1 (0.0 to 0.3)	1 (0 to 2)	0.2 (0.1 to 0.5)	0 (0 to 1)	0.1 (0.0 to 0.2)	-30.0 (-78.5 to 145.9)	-8.8 (-72.0 to 220.2)
Azerbaijan	Endocarditis	5 to 9	Male	0 (0 to 1)	0.1 (0.0 to 0.2)	1 (0 to 1)	0.1 (0.1 to 0.3)	0 (0 to 1)	0.1 (0.0 to 0.3)	22.0 (-59.9 to 231.2)	46.8 (-51.7 to 298.6)
Azerbaijan	Endocarditis	10 to 14	Both	1 (0 to 1)	0.1 (0.1 to 0.2)	1 (1 to 2)	0.2 (0.1 to 0.2)	1 (0 to 1)	0.1 (0.1 to 0.2)	-4.1 (-50.3 to 86.9)	17.7 (-39.0 to 129.3)
Azerbaijan	Endocarditis	10 to 14	Female	0 (0 to 1)	0.1 (0.1 to 0.2)	1 (0 to 1)	0.2 (0.1 to 0.3)	0 (0 to 1)	0.1 (0.1 to 0.2)	-23.1 (-68.0 to 96.2)	-2.3 (-59.3 to 149.3)
Azerbaijan	Endocarditis	10 to 14	Male	0 (0 to 1)	0.1 (0.0 to 0.2)	1 (0 to 1)	0.2 (0.1 to 0.3)	0 (0 to 1)	0.1 (0.1 to 0.3)	17.3 (-45.9 to 164.8)	39.3 (-35.8 to 214.6)
Azerbaijan	Other cardiovascular and circulatory diseases	5 to 9	Both	1 (0 to 1)	0.1 (0.0 to 0.2)	1 (1 to 3)	0.2 (0.1 to 0.3)	1 (0 to 1)	0.1 (0.1 to 0.2)	29.6 (-47.3 to 204.0)	61.9 (-34.2 to 279.9)
Azerbaijan	Other cardiovascular and circulatory diseases	5 to 9	Female	0 (0 to 1)	0.1 (0.0 to 0.2)	1 (0 to 1)	0.2 (0.1 to 0.3)	0 (0 to 1)	0.1 (0.1 to 0.3)	19.9 (-56.4 to 213.1)	56.1 (-43.2 to 307.7)
Azerbaijan	Other cardiovascular and circulatory diseases	5 to 9	Male	0 (0 to 1)	0.1 (0.0 to 0.2)	1 (0 to 2)	0.2 (0.1 to 0.4)	0 (0 to 1)	0.1 (0.1 to 0.2)	41.0 (-49.0 to 332.7)	69.7 (-38.7 to 420.7)
Azerbaijan	Other cardiovascular and circulatory diseases	10 to 14	Both	1 (0 to 1)	0.1 (0.1 to 0.2)	2 (1 to 3)	0.2 (0.1 to 0.3)	1 (1 to 2)	0.2 (0.1 to 0.3)	47.8 (-35.1 to 222.5)	81.3 (-20.3 to 295.7)
Azerbaijan	Other cardiovascular and circulatory diseases	10 to 14	Female	0 (0 to 1)	0.1 (0.1 to 0.3)	1 (0 to 2)	0.2 (0.1 to 0.4)	1 (0 to 1)	0.2 (0.1 to 0.4)	39.8 (-48.8 to 228.1)	77.7 (-34.9 to 317.0)

eTable 8. All-cause and cause-specific mortality (counts and rates) at the global level and by country in the WHO European Region for ages 5 to 9 and 10 to 14 for 1990, 2000, and 2016

Azerbaijan	Other cardiovascular and circulatory diseases	10 to 14	Male	0 (0 to 1)	0.1 (0.1 to 0.2)	1 (0 to 2)	0.2 (0.1 to 0.4)	1 (0 to 1)	0.2 (0.1 to 0.4)	55.4 (-35.4 to 286.5)	84.6 (-23.2 to 359.2)
Azerbaijan	Chronic obstructive pulmonary disease	5 to 9	Both	4 (2 to 7)	0.5 (0.2 to 0.9)	9 (4 to 15)	1.0 (0.5 to 1.7)	1 (1 to 2)	0.2 (0.1 to 0.3)	-71.5 (-87.2 to -34.3)	-64.4 (-84.0 to -17.9)
Azerbaijan	Chronic obstructive pulmonary disease	5 to 9	Female	1 (1 to 3)	0.4 (0.2 to 0.6)	3 (1 to 5)	0.7 (0.3 to 1.2)	0 (0 to 1)	0.1 (0.0 to 0.2)	-74.0 (-91.2 to -22.9)	-66.1 (-88.5 to 0.4)
Azerbaijan	Chronic obstructive pulmonary disease	5 to 9	Male	3 (1 to 5)	0.6 (0.2 to 1.3)	6 (2 to 12)	1.2 (0.4 to 2.6)	1 (0 to 2)	0.2 (0.1 to 0.5)	-70.2 (-90.9 to -15.8)	-64.1 (-89.0 to 1.3)
Azerbaijan	Chronic obstructive pulmonary disease	10 to 14	Both	5 (3 to 7)	0.7 (0.4 to 1.0)	9 (6 to 13)	1.0 (0.6 to 1.3)	2 (1 to 3)	0.4 (0.2 to 0.6)	-56.1 (-74.2 to -24.8)	-46.2 (-68.3 to -7.7)
Azerbaijan	Chronic obstructive pulmonary disease	10 to 14	Female	2 (1 to 3)	0.5 (0.2 to 0.8)	3 (2 to 5)	0.6 (0.3 to 1.0)	1 (0 to 1)	0.2 (0.1 to 0.4)	-56.9 (-81.6 to -2.0)	-45.2 (-76.6 to 24.5)
Azerbaijan	Chronic obstructive pulmonary disease	10 to 14	Male	3 (2 to 5)	0.9 (0.5 to 1.4)	6 (3 to 9)	1.3 (0.7 to 1.9)	1 (1 to 2)	0.5 (0.2 to 0.8)	-55.8 (-78.3 to -12.9)	-47.5 (-74.2 to 3.5)
Azerbaijan	Asthma	5 to 9	Both	1 (0 to 1)	0.1 (0.0 to 0.2)	1 (0 to 2)	0.1 (0.1 to 0.2)	0 (0 to 1)	0.1 (0.0 to 0.1)	-55.4 (-81.3 to 2.7)	-44.3 (-76.7 to 28.3)
Azerbaijan	Asthma	5 to 9	Female	1 (0 to 1)	0.2 (0.1 to 0.3)	1 (0 to 2)	0.2 (0.1 to 0.4)	0 (0 to 1)	0.1 (0.0 to 0.2)	-56.2 (-82.7 to 8.3)	-43.0 (-77.5 to 41.0)
Azerbaijan	Asthma	5 to 9	Male	0 (0 to 1)	0.0 (0.0 to 0.1)	0 (0 to 1)	0.0 (0.0 to 0.2)	0 (0 to 0)	0.0 (0.0 to 0.0)	-51.4 (-84.5 to 75.0)	-41.5 (-81.3 to 110.6)
Azerbaijan	Asthma	10 to 14	Both	1 (0 to 2)	0.1 (0.1 to 0.3)	2 (1 to 3)	0.2 (0.1 to 0.4)	0 (0 to 1)	0.1 (0.0 to 0.1)	-66.9 (-84.6 to -31.2)	-59.4 (-81.1 to -15.6)
Azerbaijan	Asthma	10 to 14	Female	1 (0 to 1)	0.2 (0.1 to 0.3)	1 (0 to 2)	0.2 (0.1 to 0.5)	0 (0 to 0)	0.1 (0.0 to 0.2)	-65.8 (-87.3 to -12.5)	-56.6 (-83.9 to 11.2)
Azerbaijan	Asthma	10 to 14	Male	0 (0 to 1)	0.1 (0.0 to 0.4)	0 (0 to 2)	0.1 (0.0 to 0.5)	0 (0 to 0)	0.0 (0.0 to 0.1)	-68.9 (-89.7 to 2.0)	-63.1 (-87.7 to 21.2)
Azerbaijan	Interstitial lung disease and pulmonary sarcoidosis	5 to 9	Both	2 (1 to 3)	0.2 (0.1 to 0.4)	3 (1 to 6)	0.3 (0.1 to 0.6)	1 (0 to 1)	0.1 (0.0 to 0.2)	-56.9 (-85.1 to 18.2)	-46.1 (-81.4 to 47.8)
Azerbaijan	Interstitial lung disease and pulmonary sarcoidosis	5 to 9	Female	2 (1 to 3)	0.4 (0.2 to 0.8)	3 (1 to 5)	0.7 (0.3 to 1.3)	1 (0 to 1)	0.2 (0.1 to 0.4)	-57.4 (-85.8 to 25.9)	-44.6 (-81.5 to 63.9)
Azerbaijan	Interstitial lung disease and pulmonary sarcoidosis	5 to 9	Male	0 (0 to 0)	0.0 (0.0 to 0.0)	0 (0 to 0)	0.0 (0.0 to 0.0)	0 (0 to 0)	0.0 (0.0 to 0.0)	-40.0 (-84.6 to 109.9)	-27.8 (-81.5 to 152.6)
Azerbaijan	Interstitial lung disease and pulmonary sarcoidosis	10 to 14	Both	1 (0 to 1)	0.1 (0.0 to 0.2)	1 (0 to 2)	0.1 (0.0 to 0.2)	0 (0 to 1)	0.1 (0.0 to 0.1)	-32.1 (-75.4 to 54.8)	-16.7 (-69.8 to 89.9)

eTable 8. All-cause and cause-specific mortality (counts and rates) at the global level and by country in the WHO European Region for ages 5 to 9 and 10 to 14 for 1990, 2000, and 2016

Azerbaijan	Interstitial lung disease and pulmonary sarcoidosis	10 to 14	Female	1<br>(0 to 1)	0.2<br>(0.1 to 0.3)	1<br>(0 to 2)	0.2<br>(0.1 to 0.4)	0<br>(0 to 1)	0.1<br>(0.0 to 0.3)	-32.7<br>(-76.8 to 62.2)	-14.4<br>(-70.5 to 106.1)
Azerbaijan	Interstitial lung disease and pulmonary sarcoidosis	10 to 14	Male	0<br>(0 to 0)	0.0<br>(0.0 to 0.0)	0<br>(0 to 0)	0.0<br>(0.0 to 0.0)	0<br>(0 to 0)	0.0<br>(0.0 to 0.0)	-21.9<br>(-74.7 to 98.9)	-7.2<br>(-70.0 to 136.4)
Azerbaijan	Other chronic respiratory diseases	5 to 9	Both	6<br>(3 to 9)	0.7<br>(0.4 to 1.1)	10<br>(5 to 16)	1.1<br>(0.5 to 1.8)	3<br>(1 to 4)	0.4<br>(0.2 to 0.7)	-55.8<br>(-77.6 to -14.6)	-44.8<br>(-72.1 to 6.7)
Azerbaijan	Other chronic respiratory diseases	5 to 9	Female	2<br>(1 to 4)	0.5<br>(0.1 to 0.9)	3<br>(1 to 6)	0.7<br>(0.1 to 1.5)	1<br>(0 to 2)	0.3<br>(0.1 to 0.6)	-54.9<br>(-84.1 to 26.0)	-41.3<br>(-79.2 to 64.1)
Azerbaijan	Other chronic respiratory diseases	5 to 9	Male	4<br>(2 to 7)	0.9<br>(0.4 to 1.6)	7<br>(2 to 12)	1.6<br>(0.5 to 2.7)	2<br>(1 to 3)	0.5<br>(0.2 to 0.9)	-56.2<br>(-80.5 to -3.8)	-47.3<br>(-76.5 to 15.8)
Azerbaijan	Other chronic respiratory diseases	10 to 14	Both	2<br>(1 to 4)	0.3<br>(0.1 to 0.5)	3<br>(1 to 6)	0.3<br>(0.1 to 0.6)	1<br>(1 to 2)	0.2<br>(0.1 to 0.4)	-41.9<br>(-72.5 to 22.2)	-28.8<br>(-66.3 to 49.9)
Azerbaijan	Other chronic respiratory diseases	10 to 14	Female	1<br>(0 to 2)	0.2<br>(0.0 to 0.5)	1<br>(0 to 3)	0.2<br>(0.0 to 0.6)	0<br>(0 to 1)	0.2<br>(0.0 to 0.4)	-37.7<br>(-77.5 to 60.9)	-20.8<br>(-71.5 to 104.5)
Azerbaijan	Other chronic respiratory diseases	10 to 14	Male	1<br>(1 to 3)	0.4<br>(0.1 to 0.7)	2<br>(1 to 4)	0.5<br>(0.1 to 0.9)	1<br>(0 to 1)	0.2<br>(0.1 to 0.5)	-44.1<br>(-80.1 to 50.6)	-33.6<br>(-76.4 to 78.9)
Azerbaijan	Cirrhosis and other chronic liver diseases due to hepatitis B	5 to 9	Both	0<br>(0 to 0)	0.0<br>(0.0 to 0.0)	0<br>(0 to 1)	0.0<br>(0.0 to 0.1)	0<br>(0 to 0)	0.0<br>(0.0 to 0.0)	-36.3<br>(-64.4 to 13.6)	-20.4<br>(-55.5 to 42.0)
Azerbaijan	Cirrhosis and other chronic liver diseases due to hepatitis B	5 to 9	Female	0<br>(0 to 0)	0.0<br>(0.0 to 0.0)	0<br>(0 to 0)	0.0<br>(0.0 to 0.0)	0<br>(0 to 0)	0.0<br>(0.0 to 0.0)	-46.2<br>(-71.7 to -1.5)	-30.0<br>(-63.1 to 28.2)
Azerbaijan	Cirrhosis and other chronic liver diseases due to hepatitis B	5 to 9	Male	0<br>(0 to 0)	0.0<br>(0.0 to 0.1)	0<br>(0 to 0)	0.1<br>(0.0 to 0.1)	0<br>(0 to 0)	0.0<br>(0.0 to 0.1)	-33.9<br>(-67.1 to 29.2)	-20.4<br>(-60.4 to 55.5)
Azerbaijan	Cirrhosis and other chronic liver diseases due to hepatitis B	10 to 14	Both	0<br>(0 to 1)	0.1<br>(0.0 to 0.1)	1<br>(1 to 1)	0.1<br>(0.1 to 0.1)	0<br>(0 to 1)	0.1<br>(0.0 to 0.1)	-14.1<br>(-52.7 to 54.0)	5.5<br>(-41.9 to 89.0)
Azerbaijan	Cirrhosis and other chronic liver diseases due to hepatitis B	10 to 14	Female	0<br>(0 to 0)	0.0<br>(0.0 to 0.0)	0<br>(0 to 0)	0.0<br>(0.0 to 0.1)	0<br>(0 to 0)	0.0<br>(0.0 to 0.0)	-26.4<br>(-59.0 to 35.3)	-6.4<br>(-47.9 to 71.9)
Azerbaijan	Cirrhosis and other chronic liver diseases due to hepatitis B	10 to 14	Male	0<br>(0 to 1)	0.1<br>(0.1 to 0.2)	1<br>(0 to 1)	0.1<br>(0.1 to 0.2)	0<br>(0 to 1)	0.1<br>(0.1 to 0.2)	-10.8<br>(-58.1 to 84.5)	5.9<br>(-50.2 to 119.2)
Azerbaijan	Cirrhosis and other chronic liver diseases due to hepatitis C	5 to 9	Both	0<br>(0 to 0)	0.0<br>(0.0 to 0.0)	0<br>(0 to 0)	0.0<br>(0.0 to 0.0)	0<br>(0 to 0)	0.0<br>(0.0 to 0.0)	-39.0<br>(-65.8 to 8.8)	-23.7<br>(-57.2 to 35.9)

eTable 8. All-cause and cause-specific mortality (counts and rates) at the global level and by country in the WHO European Region for ages 5 to 9 and 10 to 14 for 1990, 2000, and 2016

Azerbaijan	Cirrhosis and other chronic liver diseases due to hepatitis C	5 to 9	Female	0 (0 to 0)	0.0 (0.0 to 0.0)	0 (0 to 0)	0.0 (0.0 to 0.0)	0 (0 to 0)	0.0 (0.0 to 0.0)	-46.5 (-71.3 to -1.8)	-30.4 (-62.7 to 27.9)
Azerbaijan	Cirrhosis and other chronic liver diseases due to hepatitis C	5 to 9	Male	0 (0 to 0)	0.0 (0.0 to 0.0)	0 (0 to 0)	0.0 (0.0 to 0.1)	0 (0 to 0)	0.0 (0.0 to 0.0)	-37.1 (-68.7 to 22.8)	-24.2 (-62.3 to 47.8)
Azerbaijan	Cirrhosis and other chronic liver diseases due to hepatitis C	10 to 14	Both	0 (0 to 0)	0.0 (0.0 to 0.1)	0 (0 to 1)	0.0 (0.0 to 0.1)	0 (0 to 0)	0.0 (0.0 to 0.1)	-17.4 (-54.4 to 49.2)	1.4 (-44.0 to 83.1)
Azerbaijan	Cirrhosis and other chronic liver diseases due to hepatitis C	10 to 14	Female	0 (0 to 0)	0.0 (0.0 to 0.0)	0 (0 to 0)	0.0 (0.0 to 0.0)	0 (0 to 0)	0.0 (0.0 to 0.0)	-26.4 (-59.4 to 33.7)	-6.5 (-48.4 to 69.9)
Azerbaijan	Cirrhosis and other chronic liver diseases due to hepatitis C	10 to 14	Male	0 (0 to 0)	0.0 (0.0 to 0.1)	0 (0 to 1)	0.1 (0.0 to 0.1)	0 (0 to 0)	0.0 (0.0 to 0.1)	-14.9 (-60.0 to 76.4)	1.1 (-52.5 to 109.5)
Azerbaijan	Cirrhosis and other chronic liver diseases due to other causes	5 to 9	Both	8 (6 to 12)	1.0 (0.7 to 1.5)	13 (9 to 19)	1.5 (1.1 to 2.2)	5 (3 to 8)	0.8 (0.5 to 1.2)	-40.2 (-63.6 to 0.5)	-25.3 (-54.5 to 25.6)
Azerbaijan	Cirrhosis and other chronic liver diseases due to other causes	5 to 9	Female	4 (2 to 5)	0.9 (0.6 to 1.3)	6 (4 to 9)	1.4 (0.9 to 2.1)	2 (1 to 3)	0.6 (0.3 to 1.0)	-46.9 (-71.6 to -2.8)	-30.9 (-63.0 to 26.6)
Azerbaijan	Cirrhosis and other chronic liver diseases due to other causes	5 to 9	Male	5 (3 to 8)	1.1 (0.7 to 1.9)	7 (4 to 12)	1.6 (1.0 to 2.7)	3 (2 to 6)	0.9 (0.5 to 1.6)	-35.2 (-67.8 to 26.0)	-22.1 (-61.2 to 51.6)
Azerbaijan	Cirrhosis and other chronic liver diseases due to other causes	10 to 14	Both	8 (6 to 12)	1.2 (0.8 to 1.7)	15 (11 to 22)	1.6 (1.2 to 2.3)	7 (4 to 10)	1.2 (0.8 to 1.8)	-19.2 (-50.7 to 33.2)	-0.9 (-39.5 to 63.4)
Azerbaijan	Cirrhosis and other chronic liver diseases due to other causes	10 to 14	Female	4 (2 to 6)	1.1 (0.7 to 1.7)	7 (5 to 10)	1.6 (1.0 to 2.3)	3 (2 to 4)	1.0 (0.6 to 1.6)	-27.2 (-60.0 to 32.1)	-7.5 (-49.2 to 67.9)
Azerbaijan	Cirrhosis and other chronic liver diseases due to other causes	10 to 14	Male	5 (3 to 8)	1.3 (0.8 to 2.2)	8 (5 to 13)	1.7 (1.0 to 2.8)	4 (2 to 7)	1.3 (0.7 to 2.2)	-12.8 (-58.9 to 80.7)	3.6 (-51.2 to 114.7)
Azerbaijan	Peptic ulcer disease	5 to 9	Both	2 (1 to 3)	0.2 (0.1 to 0.4)	3 (2 to 6)	0.4 (0.2 to 0.7)	0 (0 to 1)	0.1 (0.0 to 0.1)	-74.3 (-89.4 to -46.8)	-67.8 (-86.7 to -33.6)
Azerbaijan	Peptic ulcer disease	5 to 9	Female	1 (0 to 1)	0.2 (0.1 to 0.3)	1 (1 to 3)	0.3 (0.2 to 0.7)	0 (0 to 0)	0.1 (0.0 to 0.1)	-73.2 (-91.0 to -39.8)	-65.1 (-88.2 to -21.6)
Azerbaijan	Peptic ulcer disease	5 to 9	Male	1 (0 to 2)	0.3 (0.1 to 0.5)	2 (1 to 4)	0.4 (0.2 to 0.8)	0 (0 to 1)	0.1 (0.0 to 0.2)	-74.9 (-92.9 to -27.1)	-69.8 (-91.4 to -12.2)
Azerbaijan	Peptic ulcer disease	10 to 14	Both	2 (1 to 3)	0.3 (0.2 to 0.4)	4 (2 to 6)	0.4 (0.3 to 0.6)	1 (0 to 1)	0.1 (0.1 to 0.2)	-66.5 (-83.7 to -36.7)	-58.9 (-80.1 to -22.3)
Azerbaijan	Peptic ulcer disease	10 to 14	Female	1 (0 to 1)	0.2 (0.1 to 0.4)	2 (1 to 3)	0.4 (0.2 to 0.6)	0 (0 to 0)	0.1 (0.0 to 0.2)	-69.6 (-88.4 to -27.1)	-61.4 (-85.3 to -7.3)

eTable 8. All-cause and cause-specific mortality (counts and rates) at the global level and by country in the WHO European Region for ages 5 to 9 and 10 to 14 for 1990, 2000, and 2016											
Azerbaijan	Peptic ulcer disease	10 to 14	Male	1<br>(1 to 2)	0.3<br>(0.2 to 0.5)	2<br>(1 to 3)	0.4<br>(0.2 to 0.7)	0<br>(0 to 1)	0.1<br>(0.1 to 0.3)	-64.4<br>(-86.8 to -18.9)	-57.7<br>(-84.4 to -3.7)
Azerbaijan	Gastritis and duodenitis	5 to 9	Both	0<br>(0 to 0)	0.0<br>(0.0 to 0.0)	0<br>(0 to 1)	0.0<br>(0.0 to 0.1)	0<br>(0 to 0)	0.0<br>(0.0 to 0.0)	-66.8<br>(-93.2 to 69.1)	-58.5<br>(-91.5 to 111.4)
Azerbaijan	Gastritis and duodenitis	5 to 9	Female	0<br>(0 to 0)	0.0<br>(0.0 to 0.1)	0<br>(0 to 0)	0.0<br>(0.0 to 0.1)	0<br>(0 to 0)	0.0<br>(0.0 to 0.0)	-68.3<br>(-94.9 to 96.3)	-58.7<br>(-93.4 to 155.6)
Azerbaijan	Gastritis and duodenitis	5 to 9	Male	0<br>(0 to 0)	0.0<br>(0.0 to 0.0)	0<br>(0 to 0)	0.0<br>(0.0 to 0.0)	0<br>(0 to 0)	0.0<br>(0.0 to 0.0)	-50.4<br>(-87.5 to 102.9)	-40.4<br>(-85.0 to 144.2)
Azerbaijan	Gastritis and duodenitis	10 to 14	Both	0<br>(0 to 0)	0.0<br>(0.0 to 0.0)	0<br>(0 to 0)	0.0<br>(0.0 to 0.0)	0<br>(0 to 0)	0.0<br>(0.0 to 0.0)	-24.0<br>(-67.0 to 71.9)	-6.8<br>(-59.5 to 111.0)
Azerbaijan	Gastritis and duodenitis	10 to 14	Female	0<br>(0 to 0)	0.0<br>(0.0 to 0.0)	0<br>(0 to 0)	0.0<br>(0.0 to 0.0)	0<br>(0 to 0)	0.0<br>(0.0 to 0.0)	-23.9<br>(-77.7 to 134.5)	-3.3<br>(-71.7 to 198.0)
Azerbaijan	Gastritis and duodenitis	10 to 14	Male	0<br>(0 to 0)	0.0<br>(0.0 to 0.0)	0<br>(0 to 0)	0.0<br>(0.0 to 0.0)	0<br>(0 to 0)	0.0<br>(0.0 to 0.0)	-24.2<br>(-67.7 to 73.6)	-10.0<br>(-61.7 to 106.3)
Azerbaijan	Appendicitis	5 to 9	Both	5<br>(4 to 8)	0.7<br>(0.4 to 1.0)	5<br>(3 to 10)	0.6<br>(0.3 to 1.1)	1<br>(1 to 2)	0.2<br>(0.1 to 0.3)	-80.2<br>(-90.6 to -59.0)	-75.3<br>(-88.2 to -48.7)
Azerbaijan	Appendicitis	5 to 9	Female	2<br>(1 to 3)	0.5<br>(0.3 to 0.9)	2<br>(1 to 5)	0.5<br>(0.2 to 1.1)	0<br>(0 to 1)	0.2<br>(0.1 to 0.3)	-79.0<br>(-91.7 to -57.8)	-72.7<br>(-89.2 to -45.0)
Azerbaijan	Appendicitis	5 to 9	Male	3<br>(2 to 5)	0.8<br>(0.4 to 1.3)	3<br>(2 to 6)	0.7<br>(0.4 to 1.3)	1<br>(0 to 1)	0.2<br>(0.1 to 0.4)	-81.0<br>(-93.3 to -52.6)	-77.1<br>(-91.9 to -43.0)
Azerbaijan	Appendicitis	10 to 14	Both	4<br>(3 to 6)	0.6<br>(0.4 to 0.9)	4<br>(2 to 8)	0.4<br>(0.3 to 0.8)	1<br>(1 to 2)	0.2<br>(0.1 to 0.4)	-70.2<br>(-84.5 to -42.4)	-63.5<br>(-81.0 to -29.3)
Azerbaijan	Appendicitis	10 to 14	Female	2<br>(1 to 3)	0.5<br>(0.3 to 0.7)	2<br>(1 to 4)	0.4<br>(0.2 to 0.8)	1<br>(0 to 1)	0.2<br>(0.1 to 0.4)	-66.0<br>(-84.7 to -36.4)	-56.8<br>(-80.6 to -19.2)
Azerbaijan	Appendicitis	10 to 14	Male	3<br>(1 to 4)	0.7<br>(0.4 to 1.2)	2<br>(1 to 4)	0.5<br>(0.3 to 0.9)	1<br>(0 to 1)	0.2<br>(0.1 to 0.4)	-72.9<br>(-89.2 to -35.7)	-67.8<br>(-87.2 to -23.6)
Azerbaijan	Paralytic ileus and intestinal obstruction	5 to 9	Both	3<br>(2 to 6)	0.4<br>(0.2 to 0.7)	4<br>(2 to 7)	0.5<br>(0.2 to 0.8)	2<br>(1 to 4)	0.3<br>(0.2 to 0.5)	-40.1<br>(-71.3 to 17.5)	-25.2<br>(-64.1 to 46.8)
Azerbaijan	Paralytic ileus and intestinal obstruction	5 to 9	Female	1<br>(0 to 2)	0.2<br>(0.1 to 0.4)	1<br>(0 to 2)	0.3<br>(0.1 to 0.5)	0<br>(0 to 1)	0.2<br>(0.1 to 0.3)	-40.0<br>(-76.0 to 46.5)	-21.9<br>(-68.7 to 90.7)
Azerbaijan	Paralytic ileus and intestinal obstruction	5 to 9	Male	3<br>(1 to 5)	0.6<br>(0.3 to 1.1)	3<br>(1 to 6)	0.7<br>(0.3 to 1.3)	2<br>(1 to 3)	0.4<br>(0.2 to 0.8)	-40.1<br>(-75.5 to 39.4)	-28.0<br>(-70.5 to 67.8)
Azerbaijan	Paralytic ileus and intestinal obstruction	10 to 14	Both	2<br>(1 to 3)	0.2<br>(0.1 to 0.4)	2<br>(1 to 3)	0.2<br>(0.1 to 0.4)	1<br>(1 to 2)	0.2<br>(0.1 to 0.3)	-26.2<br>(-61.8 to 33.5)	-9.4<br>(-53.2 to 63.9)
Azerbaijan	Paralytic ileus and intestinal obstruction	10 to 14	Female	0<br>(0 to 1)	0.1<br>(0.1 to 0.2)	1<br>(0 to 1)	0.1<br>(0.1 to 0.3)	0<br>(0 to 1)	0.1<br>(0.1 to 0.2)	-27.7<br>(-69.4 to 60.0)	-8.1<br>(-61.2 to 103.3)
Azerbaijan	Paralytic ileus and intestinal obstruction	10 to 14	Male	1<br>(1 to 2)	0.3<br>(0.1 to 0.5)	2<br>(1 to 3)	0.3<br>(0.2 to 0.6)	1<br>(0 to 2)	0.3<br>(0.1 to 0.5)	-25.5<br>(-67.4 to 59.4)	-11.5<br>(-61.3 to 89.4)
Azerbaijan	Inguinal, femoral, and abdominal hernia	5 to 9	Both	0<br>(0 to 1)	0.0<br>(0.0 to 0.1)	0<br>(0 to 1)	0.1<br>(0.0 to 0.1)	0<br>(0 to 0)	0.0<br>(0.0 to 0.0)	-64.1<br>(-89.5 to 5.8)	-55.1<br>(-86.8 to 32.2)
Azerbaijan	Inguinal, femoral, and abdominal hernia	5 to 9	Female	0<br>(0 to 0)	0.0<br>(0.0 to 0.0)	0<br>(0 to 0)	0.0<br>(0.0 to 0.0)	0<br>(0 to 0)	0.0<br>(0.0 to 0.0)	-32.9<br>(-68.9 to 27.0)	-12.6<br>(-59.5 to 65.3)
Azerbaijan	Inguinal, femoral, and abdominal hernia	5 to 9	Male	0<br>(0 to 1)	0.1<br>(0.0 to 0.2)	0<br>(0 to 1)	0.1<br>(0.0 to 0.2)	0<br>(0 to 0)	0.0<br>(0.0 to 0.1)	-70.6<br>(-93.8 to 20.0)	-64.7<br>(-92.6 to 44.4)
Azerbaijan	Inguinal, femoral, and abdominal hernia	10 to 14	Both	1<br>(0 to 1)	0.1<br>(0.0 to 0.2)	1<br>(0 to 3)	0.1<br>(0.0 to 0.3)	0<br>(0 to 1)	0.0<br>(0.0 to 0.1)	-50.5<br>(-87.2 to 97.1)	-39.3<br>(-84.3 to 141.9)
Azerbaijan	Inguinal, femoral, and abdominal hernia	10 to 14	Female	0<br>(0 to 0)	0.0<br>(0.0 to 0.0)	0<br>(0 to 0)	0.0<br>(0.0 to 0.0)	0<br>(0 to 0)	0.0<br>(0.0 to 0.0)	-27.6<br>(-61.8 to 41.1)	-8.0<br>(-51.5 to 79.3)

eTable 8. All-cause and cause-specific mortality (counts and rates) at the global level and by country in the WHO European Region for ages 5 to 9 and 10 to 14 for 1990, 2000, and 2016

Azerbaijan	Inguinal, femoral, and abdominal hernia	10 to 14	Male	0 (0 to 1)	0.1 (0.0 to 0.4)	1 (0 to 3)	0.2 (0.0 to 0.5)	0 (0 to 1)	0.1 (0.0 to 0.2)	-53.4 (-90.6 to 131.7)	-44.7 (-88.9 to 175.3)
Azerbaijan	Inflammatory bowel disease	5 to 9	Both	0 (0 to 1)	0.0 (0.0 to 0.1)	0 (0 to 1)	0.1 (0.0 to 0.1)	0 (0 to 0)	0.0 (0.0 to 0.1)	-45.0 (-78.7 to 34.9)	-31.2 (-73.4 to 68.5)
Azerbaijan	Inflammatory bowel disease	5 to 9	Female	0 (0 to 0)	0.0 (0.0 to 0.1)	0 (0 to 0)	0.0 (0.0 to 0.1)	0 (0 to 0)	0.0 (0.0 to 0.0)	-44.4 (-79.3 to 47.9)	-27.6 (-73.0 to 92.6)
Azerbaijan	Inflammatory bowel disease	5 to 9	Male	0 (0 to 0)	0.0 (0.0 to 0.1)	0 (0 to 1)	0.1 (0.0 to 0.2)	0 (0 to 0)	0.0 (0.0 to 0.1)	-45.2 (-85.0 to 83.4)	-34.1 (-82.0 to 120.7)
Azerbaijan	Inflammatory bowel disease	10 to 14	Both	0 (0 to 0)	0.0 (0.0 to 0.0)	0 (0 to 0)	0.0 (0.0 to 0.0)	0 (0 to 0)	0.0 (0.0 to 0.0)	-30.9 (-67.5 to 35.8)	-15.2 (-60.1 to 66.6)
Azerbaijan	Inflammatory bowel disease	10 to 14	Female	0 (0 to 0)	0.0 (0.0 to 0.0)	0 (0 to 0)	0.0 (0.0 to 0.0)	0 (0 to 0)	0.0 (0.0 to 0.0)	-31.7 (-72.8 to 72.6)	-13.2 (-65.4 to 119.3)
Azerbaijan	Inflammatory bowel disease	10 to 14	Male	0 (0 to 0)	0.0 (0.0 to 0.0)	0 (0 to 0)	0.0 (0.0 to 0.1)	0 (0 to 0)	0.0 (0.0 to 0.0)	-30.3 (-74.9 to 60.6)	-17.2 (-70.2 to 90.8)
Azerbaijan	Vascular intestinal disorders	5 to 9	Both	0 (0 to 0)	0.0 (0.0 to 0.0)	0 (0 to 0)	0.0 (0.0 to 0.0)	0 (0 to 0)	0.0 (0.0 to 0.0)	-50.4 (-81.9 to 23.7)	-38.1 (-77.4 to 54.6)
Azerbaijan	Vascular intestinal disorders	5 to 9	Female	0 (0 to 0)	0.0 (0.0 to 0.0)	0 (0 to 0)	0.0 (0.0 to 0.0)	0 (0 to 0)	0.0 (0.0 to 0.0)	-47.9 (-84.5 to 65.6)	-32.1 (-79.9 to 115.6)
Azerbaijan	Vascular intestinal disorders	5 to 9	Male	0 (0 to 0)	0.0 (0.0 to 0.0)	0 (0 to 0)	0.0 (0.0 to 0.0)	0 (0 to 0)	0.0 (0.0 to 0.0)	-51.7 (-88.7 to 91.7)	-41.9 (-86.4 to 130.7)
Azerbaijan	Vascular intestinal disorders	10 to 14	Both	0 (0 to 0)	0.0 (0.0 to 0.0)	0 (0 to 0)	0.0 (0.0 to 0.0)	0 (0 to 0)	0.0 (0.0 to 0.0)	-30.6 (-68.1 to 34.8)	-14.8 (-60.9 to 65.5)
Azerbaijan	Vascular intestinal disorders	10 to 14	Female	0 (0 to 0)	0.0 (0.0 to 0.0)	0 (0 to 0)	0.0 (0.0 to 0.0)	0 (0 to 0)	0.0 (0.0 to 0.0)	-30.8 (-78.7 to 127.9)	-12.1 (-72.9 to 189.6)
Azerbaijan	Vascular intestinal disorders	10 to 14	Male	0 (0 to 0)	0.0 (0.0 to 0.0)	0 (0 to 0)	0.0 (0.0 to 0.0)	0 (0 to 0)	0.0 (0.0 to 0.0)	-30.4 (-72.3 to 61.3)	-17.3 (-67.1 to 91.7)
Azerbaijan	Gallbladder and biliary diseases	5 to 9	Both	0 (0 to 1)	0.1 (0.0 to 0.1)	1 (0 to 1)	0.1 (0.0 to 0.1)	0 (0 to 0)	0.0 (0.0 to 0.0)	-71.2 (-88.8 to -30.7)	-64.0 (-86.0 to -13.4)
Azerbaijan	Gallbladder and biliary diseases	5 to 9	Female	0 (0 to 0)	0.0 (0.0 to 0.1)	0 (0 to 0)	0.0 (0.0 to 0.1)	0 (0 to 0)	0.0 (0.0 to 0.0)	-68.2 (-89.9 to -22.0)	-58.6 (-86.8 to 1.5)
Azerbaijan	Gallbladder and biliary diseases	5 to 9	Male	0 (0 to 1)	0.1 (0.0 to 0.1)	0 (0 to 1)	0.1 (0.0 to 0.2)	0 (0 to 0)	0.0 (0.0 to 0.1)	-72.3 (-92.2 to -21.5)	-66.7 (-90.6 to -5.5)
Azerbaijan	Gallbladder and biliary diseases	10 to 14	Both	0 (0 to 0)	0.0 (0.0 to 0.1)	0 (0 to 1)	0.0 (0.0 to 0.1)	0 (0 to 0)	0.0 (0.0 to 0.0)	-58.6 (-80.7 to -17.1)	-49.2 (-76.3 to 1.7)
Azerbaijan	Gallbladder and biliary diseases	10 to 14	Female	0 (0 to 0)	0.0 (0.0 to 0.1)	0 (0 to 0)	0.1 (0.0 to 0.1)	0 (0 to 0)	0.0 (0.0 to 0.0)	-56.4 (-82.9 to 0.2)	-44.6 (-78.3 to 27.3)
Azerbaijan	Gallbladder and biliary diseases	10 to 14	Male	0 (0 to 0)	0.0 (0.0 to 0.1)	0 (0 to 0)	0.0 (0.0 to 0.1)	0 (0 to 0)	0.0 (0.0 to 0.0)	-61.3 (-87.8 to 1.7)	-54.0 (-85.5 to 20.8)
Azerbaijan	Pancreatitis	5 to 9	Both	0 (0 to 0)	0.0 (0.0 to 0.0)	0 (0 to 1)	0.0 (0.0 to 0.1)	0 (0 to 0)	0.0 (0.0 to 0.0)	-52.0 (-79.4 to 12.8)	-40.1 (-74.3 to 41.0)
Azerbaijan	Pancreatitis	5 to 9	Female	0 (0 to 0)	0.0 (0.0 to 0.1)	0 (0 to 1)	0.0 (0.0 to 0.1)	0 (0 to 0)	0.0 (0.0 to 0.0)	-53.9 (-82.8 to 39.0)	-39.9 (-77.6 to 81.0)
Azerbaijan	Pancreatitis	5 to 9	Male	0 (0 to 0)	0.0 (0.0 to 0.0)	0 (0 to 0)	0.0 (0.0 to 0.1)	0 (0 to 0)	0.0 (0.0 to 0.0)	-49.5 (-83.6 to 46.9)	-39.2 (-80.3 to 76.8)
Azerbaijan	Pancreatitis	10 to 14	Both	0 (0 to 0)	0.0 (0.0 to 0.0)	0 (0 to 0)	0.0 (0.0 to 0.0)	0 (0 to 0)	0.0 (0.0 to 0.0)	-32.8 (-70.4 to 49.8)	-17.6 (-63.6 to 83.9)
Azerbaijan	Pancreatitis	10 to 14	Female	0 (0 to 0)	0.0 (0.0 to 0.0)	0 (0 to 0)	0.0 (0.0 to 0.0)	0 (0 to 0)	0.0 (0.0 to 0.0)	-35.1 (-75.7 to 65.6)	-17.6 (-69.2 to 110.5)
Azerbaijan	Pancreatitis	10 to 14	Male	0 (0 to 0)	0.0 (0.0 to 0.0)	0 (0 to 0)	0.0 (0.0 to 0.0)	0 (0 to 0)	0.0 (0.0 to 0.0)	-31.7 (-75.7 to 91.1)	-18.9 (-71.1 to 127.0)

eTable 8. All-cause and cause-specific mortality (counts and rates) at the global level and by country in the WHO European Region for ages 5 to 9 and 10 to 14 for 1990, 2000, and 2016

Azerbaijan	Other digestive diseases	5 to 9	Both	3 (2 to 6)	0.4 (0.2 to 0.7)	5 (2 to 10)	0.6 (0.2 to 1.2)	1 (1 to 2)	0.2 (0.1 to 0.3)	-67.1 (-84.8 to -31.5)	-58.9 (-81.0 to -14.4)
Azerbaijan	Other digestive diseases	5 to 9	Female	1 (1 to 3)	0.4 (0.2 to 0.7)	2 (1 to 5)	0.6 (0.2 to 1.2)	1 (0 to 1)	0.2 (0.1 to 0.3)	-63.2 (-85.1 to -14.6)	-52.1 (-80.6 to 11.2)
Azerbaijan	Other digestive diseases	5 to 9	Male	2 (1 to 4)	0.4 (0.1 to 0.9)	3 (1 to 6)	0.6 (0.2 to 1.3)	0 (0 to 1)	0.1 (0.0 to 0.3)	-70.3 (-89.5 to 0.1)	-64.3 (-87.4 to 20.5)
Azerbaijan	Other digestive diseases	10 to 14	Both	2 (1 to 4)	0.3 (0.2 to 0.5)	4 (2 to 6)	0.4 (0.2 to 0.7)	1 (1 to 2)	0.2 (0.1 to 0.3)	-55.3 (-78.5 to -3.7)	-45.1 (-73.7 to 18.1)
Azerbaijan	Other digestive diseases	10 to 14	Female	0 (0 to 1)	0.1 (0.0 to 0.2)	1 (0 to 1)	0.1 (0.1 to 0.3)	0 (0 to 0)	0.1 (0.0 to 0.1)	-50.5 (-82.8 to 28.7)	-37.1 (-78.1 to 63.6)
Azerbaijan	Other digestive diseases	10 to 14	Male	2 (1 to 3)	0.5 (0.2 to 0.9)	3 (1 to 5)	0.6 (0.3 to 1.1)	1 (0 to 1)	0.3 (0.1 to 0.5)	-56.3 (-81.4 to 3.0)	-48.1 (-77.9 to 22.4)
Azerbaijan	Alzheimer disease and other dementias	5 to 9	Both	0 (0 to 0)	0.0 (0.0 to 0.0)	0 (0 to 0)	0.0 (0.0 to 0.0)	0 (0 to 0)	0.0 (0.0 to 0.0)	0.0 (0.0 to 0.0)	0.0 (0.0 to 0.0)
Azerbaijan	Alzheimer disease and other dementias	5 to 9	Female	0 (0 to 0)	0.0 (0.0 to 0.0)	0 (0 to 0)	0.0 (0.0 to 0.0)	0 (0 to 0)	0.0 (0.0 to 0.0)	0.0 (0.0 to 0.0)	0.0 (0.0 to 0.0)
Azerbaijan	Alzheimer disease and other dementias	5 to 9	Male	0 (0 to 0)	0.0 (0.0 to 0.0)	0 (0 to 0)	0.0 (0.0 to 0.0)	0 (0 to 0)	0.0 (0.0 to 0.0)	0.0 (0.0 to 0.0)	0.0 (0.0 to 0.0)
Azerbaijan	Alzheimer disease and other dementias	10 to 14	Both	0 (0 to 0)	0.0 (0.0 to 0.0)	0 (0 to 0)	0.0 (0.0 to 0.0)	0 (0 to 0)	0.0 (0.0 to 0.0)	0.0 (0.0 to 0.0)	0.0 (0.0 to 0.0)
Azerbaijan	Alzheimer disease and other dementias	10 to 14	Female	0 (0 to 0)	0.0 (0.0 to 0.0)	0 (0 to 0)	0.0 (0.0 to 0.0)	0 (0 to 0)	0.0 (0.0 to 0.0)	0.0 (0.0 to 0.0)	0.0 (0.0 to 0.0)
Azerbaijan	Alzheimer disease and other dementias	10 to 14	Male	0 (0 to 0)	0.0 (0.0 to 0.0)	0 (0 to 0)	0.0 (0.0 to 0.0)	0 (0 to 0)	0.0 (0.0 to 0.0)	0.0 (0.0 to 0.0)	0.0 (0.0 to 0.0)
Azerbaijan	Parkinson disease	5 to 9	Both	0 (0 to 0)	0.0 (0.0 to 0.0)	0 (0 to 0)	0.0 (0.0 to 0.0)	0 (0 to 0)	0.0 (0.0 to 0.0)	0.0 (0.0 to 0.0)	0.0 (0.0 to 0.0)
Azerbaijan	Parkinson disease	5 to 9	Female	0 (0 to 0)	0.0 (0.0 to 0.0)	0 (0 to 0)	0.0 (0.0 to 0.0)	0 (0 to 0)	0.0 (0.0 to 0.0)	0.0 (0.0 to 0.0)	0.0 (0.0 to 0.0)
Azerbaijan	Parkinson disease	5 to 9	Male	0 (0 to 0)	0.0 (0.0 to 0.0)	0 (0 to 0)	0.0 (0.0 to 0.0)	0 (0 to 0)	0.0 (0.0 to 0.0)	0.0 (0.0 to 0.0)	0.0 (0.0 to 0.0)
Azerbaijan	Parkinson disease	10 to 14	Both	0 (0 to 0)	0.0 (0.0 to 0.0)	0 (0 to 0)	0.0 (0.0 to 0.0)	0 (0 to 0)	0.0 (0.0 to 0.0)	0.0 (0.0 to 0.0)	0.0 (0.0 to 0.0)
Azerbaijan	Parkinson disease	10 to 14	Female	0 (0 to 0)	0.0 (0.0 to 0.0)	0 (0 to 0)	0.0 (0.0 to 0.0)	0 (0 to 0)	0.0 (0.0 to 0.0)	0.0 (0.0 to 0.0)	0.0 (0.0 to 0.0)
Azerbaijan	Parkinson disease	10 to 14	Male	0 (0 to 0)	0.0 (0.0 to 0.0)	0 (0 to 0)	0.0 (0.0 to 0.0)	0 (0 to 0)	0.0 (0.0 to 0.0)	0.0 (0.0 to 0.0)	0.0 (0.0 to 0.0)
Azerbaijan	Epilepsy	5 to 9	Both	23 (17 to 30)	2.8 (2.0 to 3.7)	56 (37 to 78)	6.3 (4.2 to 8.8)	18 (11 to 29)	2.8 (1.7 to 4.5)	-19.8 (-55.2 to 47.5)	0.2 (-44.0 to 84.4)
Azerbaijan	Epilepsy	5 to 9	Female	10 (6 to 15)	2.5 (1.5 to 3.8)	23 (8 to 38)	5.4 (1.8 to 8.7)	7 (3 to 13)	2.3 (1.1 to 4.3)	-28.2 (-64.6 to 57.6)	-6.5 (-53.8 to 105.3)
Azerbaijan	Epilepsy	5 to 9	Male	13 (9 to 19)	3.0 (2.0 to 4.5)	32 (20 to 49)	7.1 (4.4 to 10.7)	11 (6 to 19)	3.2 (1.7 to 5.3)	-13.3 (-55.6 to 71.4)	4.4 (-46.5 to 106.2)
Azerbaijan	Epilepsy	10 to 14	Both	17 (13 to 23)	2.5 (1.8 to 3.3)	44 (31 to 60)	4.7 (3.3 to 6.4)	17 (11 to 25)	2.9 (1.9 to 4.3)	-3.5 (-39.9 to 60.8)	18.4 (-26.3 to 97.4)
Azerbaijan	Epilepsy	10 to 14	Female	8 (5 to 11)	2.3 (1.5 to 3.3)	18 (9 to 28)	4.0 (2.0 to 6.2)	7 (4 to 11)	2.4 (1.4 to 4.1)	-16.3 (-54.7 to 68.8)	6.3 (-42.4 to 114.5)

eTable 8. All-cause and cause-specific mortality (counts and rates) at the global level and by country in the WHO European Region for ages 5 to 9 and 10 to 14 for 1990, 2000, and 2016

Azerbaijan	Epilepsy	10 to 14	Male	10 (6 to 14)	2.7 (1.8 to 3.8)	26 (16 to 39)	5.4 (3.4 to 8.1)	10 (6 to 17)	3.4 (2.0 to 5.6)	6.9 (-41.4 to 99.6)	27.0 (-30.4 to 137.1)
Azerbaijan	Motor neuron disease	5 to 9	Both	0 (0 to 0)	0.0 (0.0 to 0.0)	0 (0 to 0)	0.0 (0.0 to 0.0)	0 (0 to 0)	0.0 (0.0 to 0.0)	-19.9 (-68.4 to 110.3)	0.1 (-60.5 to 162.8)
Azerbaijan	Motor neuron disease	5 to 9	Female	0 (0 to 0)	0.0 (0.0 to 0.0)	0 (0 to 0)	0.0 (0.0 to 0.0)	0 (0 to 0)	0.0 (0.0 to 0.0)	-29.6 (-74.9 to 90.2)	-8.4 (-67.3 to 147.6)
Azerbaijan	Motor neuron disease	5 to 9	Male	0 (0 to 0)	0.0 (0.0 to 0.0)	0 (0 to 0)	0.0 (0.0 to 0.0)	0 (0 to 0)	0.0 (0.0 to 0.0)	-12.1 (-78.5 to 300.2)	5.8 (-74.1 to 381.7)
Azerbaijan	Motor neuron disease	10 to 14	Both	0 (0 to 0)	0.0 (0.0 to 0.0)	0 (0 to 0)	0.0 (0.0 to 0.0)	0 (0 to 0)	0.0 (0.0 to 0.0)	8.8 (-50.1 to 149.0)	33.5 (-38.8 to 205.5)
Azerbaijan	Motor neuron disease	10 to 14	Female	0 (0 to 0)	0.0 (0.0 to 0.0)	0 (0 to 0)	0.0 (0.0 to 0.0)	0 (0 to 0)	0.0 (0.0 to 0.0)	-6.6 (-59.8 to 99.6)	18.6 (-48.9 to 153.6)
Azerbaijan	Motor neuron disease	10 to 14	Male	0 (0 to 0)	0.0 (0.0 to 0.0)	0 (0 to 0)	0.0 (0.0 to 0.0)	0 (0 to 0)	0.0 (0.0 to 0.0)	10.8 (-53.7 to 194.9)	31.6 (-45.0 to 250.4)
Azerbaijan	Other neurological disorders	5 to 9	Both	1 (0 to 2)	0.1 (0.1 to 0.3)	2 (1 to 4)	0.2 (0.1 to 0.5)	1 (0 to 2)	0.2 (0.1 to 0.3)	-2.7 (-47.6 to 76.9)	21.6 (-34.5 to 121.1)
Azerbaijan	Other neurological disorders	5 to 9	Female	1 (0 to 1)	0.2 (0.1 to 0.3)	1 (0 to 2)	0.3 (0.1 to 0.6)	1 (0 to 1)	0.2 (0.1 to 0.4)	-6.6 (-53.6 to 101.5)	21.6 (-39.6 to 162.4)
Azerbaijan	Other neurological disorders	5 to 9	Male	1 (0 to 1)	0.1 (0.0 to 0.3)	1 (0 to 2)	0.2 (0.1 to 0.5)	1 (0 to 1)	0.1 (0.1 to 0.4)	2.1 (-56.6 to 140.9)	22.9 (-47.7 to 189.9)
Azerbaijan	Other neurological disorders	10 to 14	Both	2 (1 to 3)	0.3 (0.1 to 0.4)	3 (2 to 6)	0.3 (0.2 to 0.6)	2 (1 to 4)	0.4 (0.2 to 0.7)	22.1 (-26.4 to 104.6)	49.9 (-9.7 to 151.0)
Azerbaijan	Other neurological disorders	10 to 14	Female	1 (0 to 1)	0.2 (0.1 to 0.4)	1 (0 to 3)	0.3 (0.1 to 0.6)	1 (0 to 2)	0.3 (0.1 to 0.6)	18.0 (-44.5 to 135.5)	50.0 (-29.5 to 199.3)
Azerbaijan	Other neurological disorders	10 to 14	Male	1 (1 to 2)	0.3 (0.2 to 0.6)	2 (1 to 4)	0.4 (0.2 to 0.9)	1 (1 to 3)	0.5 (0.2 to 0.9)	24.6 (-37.0 to 137.3)	48.0 (-25.1 to 181.9)
Azerbaijan	Drug use disorders	5 to 9	Both	0 (0 to 0)	0.0 (0.0 to 0.0)	0 (0 to 0)	0.0 (0.0 to 0.0)	0 (0 to 0)	0.0 (0.0 to 0.0)	0.0 (0.0 to 0.0)	0.0 (0.0 to 0.0)
Azerbaijan	Drug use disorders	5 to 9	Female	0 (0 to 0)	0.0 (0.0 to 0.0)	0 (0 to 0)	0.0 (0.0 to 0.0)	0 (0 to 0)	0.0 (0.0 to 0.0)	0.0 (0.0 to 0.0)	0.0 (0.0 to 0.0)
Azerbaijan	Drug use disorders	5 to 9	Male	0 (0 to 0)	0.0 (0.0 to 0.0)	0 (0 to 0)	0.0 (0.0 to 0.0)	0 (0 to 0)	0.0 (0.0 to 0.0)	0.0 (0.0 to 0.0)	0.0 (0.0 to 0.0)
Azerbaijan	Drug use disorders	10 to 14	Both	0 (0 to 0)	0.0 (0.0 to 0.0)	0 (0 to 0)	0.0 (0.0 to 0.0)	0 (0 to 0)	0.0 (0.0 to 0.0)	0.0 (0.0 to 0.0)	0.0 (0.0 to 0.0)
Azerbaijan	Drug use disorders	10 to 14	Female	0 (0 to 0)	0.0 (0.0 to 0.0)	0 (0 to 0)	0.0 (0.0 to 0.0)	0 (0 to 0)	0.0 (0.0 to 0.0)	0.0 (0.0 to 0.0)	0.0 (0.0 to 0.0)
Azerbaijan	Drug use disorders	10 to 14	Male	0 (0 to 0)	0.0 (0.0 to 0.0)	0 (0 to 0)	0.0 (0.0 to 0.0)	0 (0 to 0)	0.0 (0.0 to 0.0)	0.0 (0.0 to 0.0)	0.0 (0.0 to 0.0)
Azerbaijan	Eating disorders	5 to 9	Both	0 (0 to 0)	0.0 (0.0 to 0.0)	0 (0 to 0)	0.0 (0.0 to 0.0)	0 (0 to 0)	0.0 (0.0 to 0.0)	-31.2 (-73.4 to 96.7)	-14.0 (-66.7 to 145.8)
Azerbaijan	Eating disorders	5 to 9	Female	0 (0 to 0)	0.0 (0.0 to 0.0)	0 (0 to 0)	0.0 (0.0 to 0.0)	0 (0 to 0)	0.0 (0.0 to 0.0)	-32.3 (-78.1 to 127.9)	-11.9 (-71.5 to 196.8)
Azerbaijan	Eating disorders	5 to 9	Male	0 (0 to 0)	0.0 (0.0 to 0.0)	0 (0 to 0)	0.0 (0.0 to 0.0)	0 (0 to 0)	0.0 (0.0 to 0.0)	-30.8 (-78.2 to 155.6)	-16.8 (-73.8 to 207.6)
Azerbaijan	Eating disorders	10 to 14	Both	0 (0 to 0)	0.0 (0.0 to 0.0)	0 (0 to 0)	0.0 (0.0 to 0.0)	0 (0 to 0)	0.0 (0.0 to 0.0)	-9.8 (-66.7 to 165.6)	10.6 (-59.1 to 225.9)
Azerbaijan	Eating disorders	10 to 14	Female	0 (0 to 0)	0.0 (0.0 to 0.0)	0 (0 to 0)	0.0 (0.0 to 0.0)	0 (0 to 0)	0.0 (0.0 to 0.0)	-10.2 (-72.4 to 166.3)	14.1 (-65.0 to 238.4)
Azerbaijan	Eating disorders	10 to 14	Male	0 (0 to 0)	0.0 (0.0 to 0.0)	0 (0 to 0)	0.0 (0.0 to 0.0)	0 (0 to 0)	0.0 (0.0 to 0.0)	-9.7 (-75.9 to 290.8)	7.3 (-71.4 to 364.3)
Azerbaijan	Diabetes mellitus	5 to 9	Both	3 (2 to 4)	0.4 (0.3 to 0.5)	8 (5 to 11)	0.9 (0.6 to 1.2)	2 (1 to 3)	0.3 (0.2 to 0.5)	-32.1 (-61.3 to 13.2)	-15.2 (-51.6 to 41.4)
Azerbaijan	Diabetes mellitus	5 to 9	Female	1 (1 to 2)	0.3 (0.2 to 0.5)	3 (2 to 5)	0.8 (0.5 to 1.1)	1 (0 to 1)	0.3 (0.1 to 0.4)	-38.4 (-67.1 to 9.2)	-19.8 (-57.2 to 42.2)
Azerbaijan	Diabetes mellitus	5 to 9	Male	2 (1 to 3)	0.4 (0.3 to 0.6)	4 (3 to 7)	0.9 (0.6 to 1.5)	1 (1 to 2)	0.4 (0.2 to 0.6)	-27.4 (-66.9 to 55.2)	-12.6 (-60.2 to 86.7)
Azerbaijan	Diabetes mellitus	10 to 14	Both	5 (4 to 7)	0.7 (0.5 to 0.9)	14 (10 to 18)	1.4 (1.1 to 2.0)	5 (3 to 7)	0.8 (0.6 to 1.2)	-2.6 (-38.5 to 52.2)	19.5 (-24.6 to 86.8)

eTable 8. All-cause and cause-specific mortality (counts and rates) at the global level and by country in the WHO European Region for ages 5 to 9 and 10 to 14 for 1990, 2000, and 2016

Azerbaijan	Diabetes mellitus	10 to 14	Female	3 (2 to 4)	0.8 (0.5 to 1.2)	8 (5 to 12)	1.8 (1.2 to 2.6)	3 (2 to 4)	1.0 (0.6 to 1.6)	-0.1 (-45.4 to 82.3)	27.0 (-30.6 to 131.6)
Azerbaijan	Diabetes mellitus	10 to 14	Male	2 (1 to 3)	0.6 (0.4 to 0.9)	5 (3 to 8)	1.1 (0.7 to 1.6)	2 (1 to 3)	0.7 (0.4 to 1.1)	-5.7 (-49.5 to 79.4)	12.1 (-40.0 to 113.1)
Azerbaijan	Acute glomerulonephritis	5 to 9	Both	9 (2 to 29)	1.1 (0.2 to 3.5)	16 (6 to 37)	1.8 (0.7 to 4.2)	2 (0 to 7)	0.4 (0.1 to 1.0)	-74.0 (-96.4 to 92.8)	-67.5 (-95.4 to 140.9)
Azerbaijan	Acute glomerulonephritis	5 to 9	Female	4 (0 to 15)	0.9 (0.1 to 3.8)	7 (2 to 21)	1.6 (0.4 to 4.8)	1 (0 to 5)	0.4 (0.0 to 1.6)	-69.5 (-97.9 to 377.8)	-60.3 (-97.2 to 522.1)
Azerbaijan	Acute glomerulonephritis	5 to 9	Male	5 (1 to 20)	1.3 (0.1 to 4.8)	9 (2 to 27)	2.1 (0.5 to 5.9)	1 (0 to 4)	0.4 (0.0 to 1.3)	-77.0 (-97.9 to 209.4)	-72.3 (-97.5 to 272.3)
Azerbaijan	Acute glomerulonephritis	10 to 14	Both	4 (1 to 15)	0.6 (0.1 to 2.2)	6 (2 to 15)	0.7 (0.2 to 1.6)	1 (0 to 5)	0.2 (0.0 to 0.9)	-68.0 (-96.1 to 150.1)	-60.7 (-95.2 to 206.9)
Azerbaijan	Acute glomerulonephritis	10 to 14	Female	2 (0 to 11)	0.6 (0.0 to 3.1)	3 (0 to 10)	0.6 (0.1 to 2.1)	1 (0 to 4)	0.3 (0.0 to 1.3)	-65.7 (-98.5 to 708.4)	-56.4 (-98.1 to 927.3)
Azerbaijan	Acute glomerulonephritis	10 to 14	Male	2 (0 to 10)	0.7 (0.1 to 2.7)	3 (1 to 9)	0.7 (0.1 to 1.9)	1 (0 to 3)	0.2 (0.0 to 0.9)	-70.0 (-97.9 to 251.7)	-64.3 (-97.5 to 317.9)
Azerbaijan	Chronic kidney disease	5 to 9	Both	15 (11 to 20)	1.8 (1.4 to 2.4)	35 (25 to 46)	3.9 (2.8 to 5.2)	11 (7 to 17)	1.7 (1.1 to 2.6)	-23.4 (-50.2 to 21.1)	-4.3 (-37.7 to 51.3)
Azerbaijan	Chronic kidney disease	5 to 9	Female	7 (5 to 10)	1.8 (1.3 to 2.6)	17 (11 to 24)	3.9 (2.5 to 5.6)	5 (3 to 8)	1.7 (1.0 to 2.7)	-27.0 (-57.0 to 26.1)	-4.9 (-44.0 to 64.2)
Azerbaijan	Chronic kidney disease	5 to 9	Male	8 (5 to 11)	1.8 (1.3 to 2.6)	18 (11 to 25)	4.0 (2.5 to 5.6)	6 (3 to 10)	1.7 (1.0 to 2.8)	-20.1 (-55.7 to 39.5)	-3.8 (-46.7 to 67.8)
Azerbaijan	Chronic kidney disease	10 to 14	Both	11 (9 to 15)	1.6 (1.2 to 2.1)	27 (21 to 34)	2.9 (2.2 to 3.6)	12 (8 to 16)	2.0 (1.4 to 2.7)	0.5 (-33.7 to 48.6)	23.3 (-18.7 to 82.4)
Azerbaijan	Chronic kidney disease	10 to 14	Female	5 (4 to 7)	1.6 (1.1 to 2.1)	13 (9 to 17)	2.7 (1.9 to 3.8)	5 (3 to 7)	1.8 (1.2 to 2.7)	-7.7 (-43.1 to 51.4)	17.3 (-27.7 to 92.4)
Azerbaijan	Chronic kidney disease	10 to 14	Male	6 (4 to 8)	1.7 (1.2 to 2.3)	14 (10 to 20)	3.0 (2.1 to 4.1)	6 (4 to 10)	2.1 (1.4 to 3.1)	8.0 (-38.4 to 82.5)	28.3 (-26.8 to 116.8)
Azerbaijan	Urinary diseases and male infertility	5 to 9	Both	3 (2 to 4)	0.3 (0.2 to 0.5)	10 (7 to 14)	1.1 (0.8 to 1.6)	3 (2 to 5)	0.4 (0.3 to 0.7)	10.7 (-40.4 to 101.0)	38.4 (-25.5 to 151.2)
Azerbaijan	Urinary diseases and male infertility	5 to 9	Female	1 (1 to 2)	0.3 (0.2 to 0.4)	4 (3 to 6)	1.0 (0.6 to 1.4)	1 (1 to 2)	0.4 (0.2 to 0.7)	19.9 (-37.8 to 111.5)	56.1 (-19.0 to 175.4)
Azerbaijan	Urinary diseases and male infertility	5 to 9	Male	2 (1 to 3)	0.4 (0.2 to 0.6)	6 (3 to 9)	1.2 (0.7 to 2.0)	2 (1 to 3)	0.5 (0.2 to 0.9)	4.0 (-59.4 to 154.1)	25.2 (-51.1 to 205.8)
Azerbaijan	Urinary diseases and male infertility	10 to 14	Both	2 (2 to 3)	0.3 (0.2 to 0.4)	8 (6 to 11)	0.9 (0.7 to 1.2)	3 (2 to 5)	0.6 (0.4 to 0.8)	47.4 (-7.2 to 124.0)	80.9 (13.9 to 174.9)
Azerbaijan	Urinary diseases and male infertility	10 to 14	Female	1 (1 to 2)	0.3 (0.2 to 0.4)	4 (3 to 6)	0.8 (0.6 to 1.2)	2 (1 to 3)	0.6 (0.4 to 0.9)	57.3 (-16.6 to 180.5)	99.8 (6.0 to 256.5)
Azerbaijan	Urinary diseases and male infertility	10 to 14	Male	1 (1 to 2)	0.3 (0.2 to 0.5)	4 (3 to 6)	0.9 (0.6 to 1.3)	2 (1 to 3)	0.6 (0.3 to 0.9)	39.1 (-25.3 to 141.8)	65.2 (-11.3 to 187.3)
Azerbaijan	Hemoglobinopathies and hemolytic anemias	5 to 9	Both	23 (14 to 38)	2.8 (1.8 to 4.7)	40 (25 to 65)	4.5 (2.9 to 7.3)	13 (8 to 20)	1.9 (1.2 to 3.0)	-44.7 (-67.2 to -4.0)	-30.9 (-59.0 to 20.0)
Azerbaijan	Hemoglobinopathies and hemolytic anemias	5 to 9	Female	8 (5 to 15)	2.1 (1.2 to 3.7)	14 (7 to 23)	3.3 (1.7 to 5.4)	4 (2 to 8)	1.4 (0.8 to 2.5)	-48.1 (-71.1 to 14.9)	-32.5 (-62.3 to 49.6)
Azerbaijan	Hemoglobinopathies and hemolytic anemias	5 to 9	Male	15 (8 to 26)	3.5 (2.0 to 6.2)	25 (15 to 45)	5.6 (3.2 to 9.8)	8 (5 to 14)	2.4 (1.4 to 4.0)	-42.7 (-69.2 to 11.6)	-31.1 (-63.0 to 34.3)

eTable 8. All-cause and cause-specific mortality (counts and rates) at the global level and by country in the WHO European Region for ages 5 to 9 and 10 to 14 for 1990, 2000, and 2016

Azerbaijan	Hemoglobinopathies and hemolytic anemias	10 to 14	Both	8 (6 to 10)	1.1 (0.8 to 1.5)	15 (10 to 20)	1.6 (1.1 to 2.2)	6 (3 to 9)	1.0 (0.6 to 1.5)	-26.7 (-53.5 to 23.0)	-10.0 (-42.9 to 50.9)
Azerbaijan	Hemoglobinopathies and hemolytic anemias	10 to 14	Female	3 (2 to 5)	0.9 (0.5 to 1.4)	6 (3 to 10)	1.4 (0.6 to 2.2)	2 (1 to 4)	0.8 (0.3 to 1.4)	-34.3 (-63.2 to 27.7)	-16.5 (-53.2 to 62.3)
Azerbaijan	Hemoglobinopathies and hemolytic anemias	10 to 14	Male	5 (3 to 7)	1.3 (0.8 to 1.9)	9 (5 to 13)	1.8 (1.1 to 2.8)	4 (2 to 6)	1.2 (0.6 to 1.9)	-21.2 (-52.9 to 57.9)	-6.4 (-44.1 to 87.7)
Azerbaijan	Endocrine, metabolic, blood, and immune disorders	5 to 9	Both	16 (7 to 23)	1.9 (0.9 to 2.8)	39 (11 to 59)	4.4 (1.2 to 6.7)	11 (5 to 18)	1.7 (0.8 to 2.8)	-26.5 (-55.9 to 20.2)	-8.2 (-44.9 to 50.2)
Azerbaijan	Endocrine, metabolic, blood, and immune disorders	5 to 9	Female	7 (3 to 11)	1.7 (0.7 to 2.7)	18 (4 to 30)	4.2 (0.8 to 7.0)	5 (2 to 10)	1.7 (0.6 to 3.1)	-22.6 (-60.2 to 46.2)	0.8 (-48.1 to 90.3)
Azerbaijan	Endocrine, metabolic, blood, and immune disorders	5 to 9	Male	9 (3 to 15)	2.1 (0.8 to 3.5)	21 (4 to 34)	4.6 (0.9 to 7.6)	6 (3 to 11)	1.8 (0.7 to 3.2)	-29.5 (-63.9 to 24.0)	-15.2 (-56.5 to 49.3)
Azerbaijan	Endocrine, metabolic, blood, and immune disorders	10 to 14	Both	6 (4 to 8)	0.9 (0.6 to 1.2)	17 (8 to 24)	1.8 (0.8 to 2.6)	7 (4 to 11)	1.2 (0.7 to 1.9)	13.5 (-25.3 to 76.1)	39.3 (-8.3 to 116.1)
Azerbaijan	Endocrine, metabolic, blood, and immune disorders	10 to 14	Female	2 (2 to 4)	0.7 (0.4 to 1.1)	6 (3 to 10)	1.4 (0.6 to 2.2)	3 (1 to 5)	1.1 (0.5 to 1.7)	17.1 (-35.8 to 127.4)	48.8 (-18.5 to 189.0)
Azerbaijan	Endocrine, metabolic, blood, and immune disorders	10 to 14	Male	4 (2 to 6)	1.1 (0.6 to 1.6)	10 (3 to 16)	2.2 (0.7 to 3.4)	4 (2 to 7)	1.4 (0.7 to 2.3)	11.3 (-37.5 to 94.5)	32.3 (-25.7 to 131.0)
Azerbaijan	Rheumatoid arthritis	5 to 9	Both	0 (0 to 0)	0.0 (0.0 to 0.0)	0 (0 to 1)	0.0 (0.0 to 0.1)	0 (0 to 0)	0.0 (0.0 to 0.0)	-3.3 (-59.1 to 104.5)	20.9 (-48.9 to 155.6)
Azerbaijan	Rheumatoid arthritis	5 to 9	Female	0 (0 to 0)	0.0 (0.0 to 0.1)	0 (0 to 0)	0.1 (0.0 to 0.1)	0 (0 to 0)	0.0 (0.0 to 0.1)	2.1 (-62.9 to 139.5)	33.0 (-51.7 to 211.9)
Azerbaijan	Rheumatoid arthritis	5 to 9	Male	0 (0 to 0)	0.0 (0.0 to 0.0)	0 (0 to 0)	0.0 (0.0 to 0.0)	0 (0 to 0)	0.0 (0.0 to 0.0)	-17.1 (-74.8 to 204.4)	-0.3 (-69.6 to 266.4)
Azerbaijan	Rheumatoid arthritis	10 to 14	Both	0 (0 to 0)	0.0 (0.0 to 0.0)	0 (0 to 0)	0.0 (0.0 to 0.0)	0 (0 to 0)	0.0 (0.0 to 0.0)	-0.3 (-58.2 to 150.7)	22.3 (-48.7 to 207.7)
Azerbaijan	Rheumatoid arthritis	10 to 14	Female	0 (0 to 0)	0.0 (0.0 to 0.0)	0 (0 to 0)	0.0 (0.0 to 0.0)	0 (0 to 0)	0.0 (0.0 to 0.0)	8.7 (-66.9 to 258.9)	38.1 (-57.9 to 356.1)
Azerbaijan	Rheumatoid arthritis	10 to 14	Male	0 (0 to 0)	0.0 (0.0 to 0.0)	0 (0 to 0)	0.0 (0.0 to 0.0)	0 (0 to 0)	0.0 (0.0 to 0.0)	-6.4 (-69.2 to 183.4)	11.2 (-63.4 to 236.7)
Azerbaijan	Other musculoskeletal disorders	5 to 9	Both	0 (0 to 0)	0.0 (0.0 to 0.0)	0 (0 to 1)	0.0 (0.0 to 0.1)	0 (0 to 0)	0.0 (0.0 to 0.1)	-11.8 (-55.0 to 65.1)	10.2 (-43.8 to 106.3)
Azerbaijan	Other musculoskeletal disorders	5 to 9	Female	0 (0 to 0)	0.0 (0.0 to 0.0)	0 (0 to 0)	0.0 (0.0 to 0.1)	0 (0 to 0)	0.0 (0.0 to 0.1)	9.7 (-59.7 to 184.7)	42.9 (-47.5 to 270.8)
Azerbaijan	Other musculoskeletal disorders	5 to 9	Male	0 (0 to 0)	0.0 (0.0 to 0.1)	0 (0 to 0)	0.1 (0.0 to 0.1)	0 (0 to 0)	0.0 (0.0 to 0.1)	-22.9 (-62.2 to 74.6)	-7.2 (-54.5 to 110.1)

eTable 8. All-cause and cause-specific mortality (counts and rates) at the global level and by country in the WHO European Region for ages 5 to 9 and 10 to 14 for 1990, 2000, and 2016

Azerbaijan	Other musculoskeletal disorders	10 to 14	Both	0 (0 to 0)	0.0 (0.0 to 0.0)	0 (0 to 1)	0.0 (0.0 to 0.1)	0 (0 to 0)	0.0 (0.0 to 0.1)	5.6 (-38.1 to 82.7)	29.6 (-24.0 to 124.2)
Azerbaijan	Other musculoskeletal disorders	10 to 14	Female	0 (0 to 0)	0.0 (0.0 to 0.0)	0 (0 to 0)	0.0 (0.0 to 0.1)	0 (0 to 0)	0.0 (0.0 to 0.1)	30.0 (-36.3 to 176.4)	65.2 (-19.1 to 251.2)
Azerbaijan	Other musculoskeletal disorders	10 to 14	Male	0 (0 to 0)	0.0 (0.0 to 0.1)	0 (0 to 0)	0.0 (0.0 to 0.1)	0 (0 to 0)	0.0 (0.0 to 0.1)	-8.5 (-52.0 to 82.6)	8.7 (-43.0 to 117.0)
Azerbaijan	Congenital birth defects	5 to 9	Both	34 (24 to 45)	4.2 (2.9 to 5.5)	40 (29 to 59)	4.5 (3.2 to 6.7)	20 (10 to 36)	3.1 (1.6 to 5.4)	-40.7 (-70.9 to 12.3)	-25.9 (-63.6 to 40.4)
Azerbaijan	Congenital birth defects	5 to 9	Female	16 (9 to 23)	3.9 (2.4 to 5.9)	18 (11 to 28)	4.2 (2.4 to 6.4)	9 (4 to 16)	2.8 (1.3 to 5.1)	-45.2 (-75.1 to 9.8)	-28.6 (-67.6 to 43.0)
Azerbaijan	Congenital birth defects	5 to 9	Male	18 (11 to 27)	4.4 (2.6 to 6.3)	22 (14 to 35)	4.9 (3.0 to 7.7)	12 (5 to 24)	3.3 (1.4 to 6.8)	-36.9 (-75.0 to 54.1)	-24.1 (-69.9 to 85.5)
Azerbaijan	Congenital birth defects	10 to 14	Both	19 (13 to 24)	2.6 (1.9 to 3.4)	24 (18 to 34)	2.6 (1.9 to 3.7)	14 (7 to 24)	2.4 (1.3 to 4.2)	-26.6 (-62.8 to 42.0)	-10.0 (-54.3 to 74.2)
Azerbaijan	Congenital birth defects	10 to 14	Female	8 (5 to 12)	2.4 (1.5 to 3.5)	11 (7 to 16)	2.4 (1.5 to 3.4)	6 (3 to 10)	2.0 (1.0 to 3.7)	-33.8 (-68.3 to 33.3)	-15.8 (-59.7 to 69.4)
Azerbaijan	Congenital birth defects	10 to 14	Male	10 (7 to 14)	2.8 (1.8 to 4.0)	13 (9 to 21)	2.7 (1.9 to 4.4)	8 (3 to 17)	2.7 (1.1 to 5.5)	-20.8 (-67.7 to 98.5)	-5.9 (-61.7 to 135.8)
Azerbaijan	Skin and subcutaneous diseases	5 to 9	Both	0 (0 to 0)	0.0 (0.0 to 0.0)	0 (0 to 0)	0.0 (0.0 to 0.0)	0 (0 to 0)	0.0 (0.0 to 0.0)	-14.4 (-39.9 to 21.1)	7.0 (-24.9 to 51.4)
Azerbaijan	Skin and subcutaneous diseases	5 to 9	Female	0 (0 to 0)	0.0 (0.0 to 0.0)	0 (0 to 0)	0.0 (0.0 to 0.0)	0 (0 to 0)	0.0 (0.0 to 0.0)	-22.6 (-46.2 to 10.7)	0.8 (-30.0 to 44.1)
Azerbaijan	Skin and subcutaneous diseases	5 to 9	Male	0 (0 to 0)	0.0 (0.0 to 0.0)	0 (0 to 0)	0.0 (0.0 to 0.0)	0 (0 to 0)	0.0 (0.0 to 0.0)	-6.5 (-37.8 to 43.1)	12.5 (-25.2 to 72.2)
Azerbaijan	Skin and subcutaneous diseases	10 to 14	Both	0 (0 to 0)	0.0 (0.0 to 0.0)	0 (0 to 0)	0.0 (0.0 to 0.0)	0 (0 to 0)	0.0 (0.0 to 0.0)	-11.1 (-30.6 to 20.7)	9.1 (-14.8 to 48.2)
Azerbaijan	Skin and subcutaneous diseases	10 to 14	Female	0 (0 to 0)	0.0 (0.0 to 0.0)	0 (0 to 0)	0.0 (0.0 to 0.0)	0 (0 to 0)	0.0 (0.0 to 0.0)	-19.4 (-37.5 to 6.0)	2.4 (-20.5 to 34.7)
Azerbaijan	Skin and subcutaneous diseases	10 to 14	Male	0 (0 to 0)	0.0 (0.0 to 0.0)	0 (0 to 0)	0.0 (0.0 to 0.0)	0 (0 to 0)	0.0 (0.0 to 0.0)	-3.3 (-29.9 to 44.4)	14.9 (-16.7 to 71.5)
Azerbaijan	Road injuries	5 to 9	Both	97 (73 to 128)	11.9 (8.9 to 15.7)	86 (62 to 118)	9.7 (6.9 to 13.3)	26 (15 to 40)	3.9 (2.3 to 6.0)	-73.7 (-84.8 to -57.9)	-67.1 (-81.1 to -47.3)
Azerbaijan	Road injuries	5 to 9	Female	30 (20 to 44)	7.6 (5.1 to 10.9)	28 (18 to 44)	6.5 (4.1 to 10.2)	8 (4 to 14)	2.7 (1.4 to 4.5)	-72.0 (-86.2 to -48.9)	-63.6 (-82.0 to -33.5)
Azerbaijan	Road injuries	5 to 9	Male	67 (46 to 93)	16.0 (11.0 to 22.1)	58 (39 to 84)	12.7 (8.5 to 18.5)	17 (9 to 29)	4.9 (2.7 to 8.2)	-74.4 (-87.3 to -53.1)	-69.2 (-84.7 to -43.5)
Azerbaijan	Road injuries	10 to 14	Both	57 (43 to 73)	8.0 (6.1 to 10.4)	53 (40 to 69)	5.7 (4.3 to 7.4)	19 (13 to 28)	3.4 (2.3 to 4.9)	-65.7 (-78.0 to -47.6)	-57.9 (-73.0 to -35.7)
Azerbaijan	Road injuries	10 to 14	Female	14 (9 to 19)	3.9 (2.7 to 5.6)	14 (9 to 20)	3.1 (2.0 to 4.5)	5 (3 to 8)	1.9 (1.1 to 3.1)	-61.2 (-79.5 to -33.2)	-50.6 (-74.0 to -15.1)
Azerbaijan	Road injuries	10 to 14	Male	43 (31 to 58)	11.9 (8.6 to 16.1)	39 (27 to 53)	8.2 (5.6 to 11.1)	14 (9 to 22)	4.7 (2.9 to 7.2)	-67.1 (-81.1 to -46.6)	-60.9 (-77.5 to -36.5)
Azerbaijan	Other transport injuries	5 to 9	Both	2 (1 to 4)	0.3 (0.2 to 0.5)	4 (2 to 7)	0.5 (0.2 to 0.8)	1 (1 to 2)	0.2 (0.1 to 0.4)	-45.5 (-75.8 to 12.7)	-32.0 (-69.7 to 40.8)
Azerbaijan	Other transport injuries	5 to 9	Female	1 (0 to 2)	0.3 (0.1 to 0.5)	2 (1 to 4)	0.4 (0.2 to 0.9)	1 (0 to 1)	0.2 (0.1 to 0.4)	-49.6 (-84.9 to 56.2)	-34.3 (-80.3 to 103.4)
Azerbaijan	Other transport injuries	5 to 9	Male	1 (1 to 2)	0.3 (0.2 to 0.6)	2 (1 to 4)	0.5 (0.2 to 0.9)	1 (0 to 2)	0.2 (0.1 to 0.5)	-42.4 (-80.7 to 53.3)	-30.7 (-76.7 to 84.5)
Azerbaijan	Other transport injuries	10 to 14	Both	1 (1 to 2)	0.2 (0.1 to 0.3)	2 (1 to 4)	0.2 (0.1 to 0.4)	1 (1 to 2)	0.2 (0.1 to 0.3)	-28.2 (-66.8 to 49.4)	-11.9 (-59.2 to 83.3)

eTable 8. All-cause and cause-specific mortality (counts and rates) at the global level and by country in the WHO European Region for ages 5 to 9 and 10 to 14 for 1990, 2000, and 2016

Azerbaijan	Other transport injuries	10 to 14	Female	1 (0 to 1)	0.1 (0.1 to 0.3)	1 (0 to 2)	0.2 (0.1 to 0.4)	0 (0 to 1)	0.1 (0.1 to 0.3)	-30.9 (-78.1 to 90.9)	-12.2 (-72.1 to 142.6)
Azerbaijan	Other transport injuries	10 to 14	Male	1 (0 to 1)	0.2 (0.1 to 0.4)	1 (1 to 2)	0.3 (0.1 to 0.5)	1 (0 to 1)	0.2 (0.1 to 0.4)	-26.4 (-72.2 to 86.0)	-12.6 (-66.9 to 121.0)
Azerbaijan	Falls	5 to 9	Both	17 (12 to 23)	2.1 (1.5 to 2.8)	17 (12 to 31)	2.0 (1.3 to 3.5)	6 (3 to 11)	0.9 (0.5 to 1.7)	-64.3 (-81.3 to -36.6)	-55.4 (-76.6 to -20.8)
Azerbaijan	Falls	5 to 9	Female	6 (4 to 9)	1.6 (1.1 to 2.3)	7 (4 to 11)	1.5 (0.9 to 2.6)	2 (1 to 4)	0.6 (0.3 to 1.2)	-69.0 (-84.6 to -41.6)	-59.6 (-79.9 to -23.9)
Azerbaijan	Falls	5 to 9	Male	11 (7 to 15)	2.5 (1.6 to 3.7)	11 (6 to 22)	2.4 (1.4 to 4.7)	4 (2 to 8)	1.2 (0.6 to 2.4)	-61.5 (-82.4 to -21.5)	-53.7 (-78.8 to -5.5)
Azerbaijan	Falls	10 to 14	Both	14 (10 to 19)	2.0 (1.5 to 2.7)	15 (10 to 25)	1.6 (1.1 to 2.7)	6 (4 to 11)	1.1 (0.6 to 1.8)	-56.2 (-77.6 to -23.6)	-46.3 (-72.5 to -6.2)
Azerbaijan	Falls	10 to 14	Female	3 (2 to 4)	0.9 (0.6 to 1.2)	3 (2 to 5)	0.8 (0.5 to 1.2)	1 (1 to 2)	0.5 (0.3 to 0.8)	-56.9 (-77.8 to -23.0)	-45.3 (-71.8 to -2.2)
Azerbaijan	Falls	10 to 14	Male	11 (7 to 16)	3.1 (2.1 to 4.4)	12 (7 to 20)	2.4 (1.5 to 4.2)	5 (3 to 9)	1.6 (0.8 to 2.9)	-56.0 (-79.9 to -16.7)	-47.8 (-76.1 to -1.1)
Azerbaijan	Drowning	5 to 9	Both	103 (79 to 135)	12.6 (9.7 to 16.4)	103 (72 to 165)	11.5 (8.2 to 18.6)	27 (16 to 50)	4.1 (2.5 to 7.6)	-73.6 (-84.3 to -54.1)	-67.1 (-80.4 to -42.7)
Azerbaijan	Drowning	5 to 9	Female	26 (18 to 37)	6.5 (4.5 to 9.4)	27 (16 to 51)	6.3 (3.7 to 11.9)	7 (4 to 14)	2.4 (1.2 to 4.6)	-72.4 (-86.2 to -45.4)	-64.1 (-82.0 to -28.9)
Azerbaijan	Drowning	5 to 9	Male	77 (57 to 102)	18.3 (13.6 to 24.3)	75 (51 to 120)	16.6 (11.3 to 26.4)	20 (11 to 36)	5.7 (3.1 to 10.5)	-74.1 (-85.9 to -51.9)	-68.8 (-83.0 to -42.1)
Azerbaijan	Drowning	10 to 14	Both	72 (56 to 91)	10.2 (7.9 to 12.9)	86 (65 to 115)	9.2 (7.0 to 12.3)	25 (18 to 38)	4.4 (3.1 to 6.7)	-65.0 (-77.0 to -43.3)	-57.0 (-71.7 to -30.4)
Azerbaijan	Drowning	10 to 14	Female	15 (10 to 21)	4.4 (2.9 to 6.1)	20 (13 to 34)	4.4 (2.8 to 7.4)	7 (4 to 11)	2.4 (1.4 to 4.1)	-56.2 (-74.7 to -24.5)	-44.3 (-67.8 to -4.1)
Azerbaijan	Drowning	10 to 14	Male	57 (42 to 75)	15.9 (11.7 to 20.7)	66 (48 to 89)	13.9 (10.1 to 18.6)	19 (12 to 29)	6.2 (4.0 to 9.5)	-67.3 (-80.4 to -41.2)	-61.1 (-76.7 to -30.1)
Azerbaijan	Fire, heat, and hot substances	5 to 9	Both	44 (24 to 59)	5.4 (3.0 to 7.2)	59 (28 to 81)	6.6 (3.2 to 9.1)	14 (9 to 21)	2.2 (1.3 to 3.3)	-67.8 (-81.6 to -42.0)	-59.8 (-77.1 to -27.5)
Azerbaijan	Fire, heat, and hot substances	5 to 9	Female	23 (12 to 33)	5.8 (3.1 to 8.2)	27 (14 to 44)	6.1 (3.3 to 10.1)	6 (3 to 11)	2.1 (1.1 to 3.7)	-71.9 (-86.8 to -46.5)	-63.4 (-82.8 to -30.3)
Azerbaijan	Fire, heat, and hot substances	5 to 9	Male	21 (12 to 30)	5.0 (2.8 to 7.2)	32 (13 to 47)	7.1 (2.8 to 10.4)	8 (4 to 12)	2.2 (1.3 to 3.6)	-63.3 (-81.1 to -31.1)	-55.9 (-77.2 to -17.1)
Azerbaijan	Fire, heat, and hot substances	10 to 14	Both	19 (13 to 24)	2.6 (1.8 to 3.4)	29 (18 to 38)	3.1 (2.0 to 4.1)	9 (6 to 12)	1.5 (1.1 to 2.1)	-52.6 (-69.1 to -32.5)	-41.8 (-62.1 to -17.2)
Azerbaijan	Fire, heat, and hot substances	10 to 14	Female	8 (5 to 11)	2.2 (1.4 to 3.1)	10 (6 to 16)	2.2 (1.4 to 3.5)	3 (2 to 5)	1.1 (0.6 to 1.7)	-60.9 (-79.9 to -35.4)	-50.3 (-74.5 to -17.9)
Azerbaijan	Fire, heat, and hot substances	10 to 14	Male	11 (7 to 15)	3.1 (2.0 to 4.3)	19 (11 to 26)	4.0 (2.3 to 5.4)	6 (4 to 9)	1.9 (1.2 to 3.0)	-47.0 (-68.1 to -14.0)	-37.0 (-62.2 to 2.2)
Azerbaijan	Poisonings	5 to 9	Both	11 (7 to 14)	1.3 (0.8 to 1.7)	13 (8 to 20)	1.4 (0.9 to 2.3)	3 (2 to 6)	0.5 (0.3 to 0.9)	-68.6 (-84.3 to -40.0)	-60.8 (-80.3 to -25.0)
Azerbaijan	Poisonings	5 to 9	Female	4 (3 to 7)	1.1 (0.6 to 1.7)	6 (3 to 9)	1.3 (0.7 to 2.2)	1 (1 to 3)	0.5 (0.2 to 0.9)	-65.8 (-84.8 to -28.4)	-55.5 (-80.2 to -6.8)
Azerbaijan	Poisonings	5 to 9	Male	6 (3 to 10)	1.5 (0.8 to 2.3)	7 (4 to 13)	1.6 (0.8 to 2.9)	2 (1 to 4)	0.5 (0.2 to 1.1)	-70.5 (-88.9 to -28.8)	-64.5 (-86.7 to -14.3)
Azerbaijan	Poisonings	10 to 14	Both	5 (4 to 8)	0.8 (0.5 to 1.1)	7 (5 to 11)	0.8 (0.5 to 1.1)	2 (1 to 4)	0.4 (0.2 to 0.6)	-60.6 (-79.2 to -32.0)	-51.7 (-74.4 to -16.5)
Azerbaijan	Poisonings	10 to 14	Female	2 (1 to 3)	0.6 (0.4 to 1.0)	3 (2 to 5)	0.7 (0.4 to 1.1)	1 (0 to 2)	0.4 (0.2 to 0.6)	-55.1 (-80.1 to -4.7)	-42.9 (-74.7 to 21.1)
Azerbaijan	Poisonings	10 to 14	Male	3 (2 to 5)	0.9 (0.5 to 1.4)	4 (2 to 6)	0.9 (0.5 to 1.4)	1 (1 to 2)	0.4 (0.2 to 0.8)	-64.2 (-85.0 to -22.2)	-57.5 (-82.2 to -7.5)
Azerbaijan	Exposure to mechanical forces	5 to 9	Both	21 (14 to 31)	2.6 (1.7 to 3.8)	33 (19 to 51)	3.7 (2.1 to 5.7)	10 (6 to 16)	1.5 (0.8 to 2.5)	-52.4 (-72.3 to -16.9)	-40.5 (-65.4 to 3.9)
Azerbaijan	Exposure to mechanical forces	5 to 9	Female	7 (4 to 10)	1.7 (1.1 to 2.5)	10 (6 to 16)	2.3 (1.3 to 3.6)	3 (2 to 5)	1.1 (0.7 to 1.6)	-51.4 (-71.7 to -15.0)	-36.7 (-63.1 to 10.7)
Azerbaijan	Exposure to mechanical forces	5 to 9	Male	14 (8 to 24)	3.4 (1.9 to 5.7)	23 (11 to 39)	5.0 (2.5 to 8.5)	7 (3 to 12)	1.9 (0.8 to 3.5)	-52.8 (-77.5 to -4.4)	-43.2 (-72.9 to 15.1)

eTable 8. All-cause and cause-specific mortality (counts and rates) at the global level and by country in the WHO European Region for ages 5 to 9 and 10 to 14 for 1990, 2000, and 2016

Azerbaijan	Exposure to mechanical forces	10 to 14	Both	14 (9 to 19)	2.0 (1.3 to 2.8)	23 (15 to 33)	2.5 (1.6 to 3.6)	8 (5 to 12)	1.4 (0.8 to 2.1)	-43.2 (-64.4 to -6.1)	-30.3 (-56.3 to 15.2)
Azerbaijan	Exposure to mechanical forces	10 to 14	Female	3 (2 to 5)	1.0 (0.6 to 1.4)	5 (3 to 8)	1.2 (0.7 to 1.8)	2 (1 to 3)	0.7 (0.5 to 1.1)	-39.4 (-66.4 to 5.3)	-22.9 (-57.2 to 33.8)
Azerbaijan	Exposure to mechanical forces	10 to 14	Male	11 (7 to 16)	2.9 (1.8 to 4.4)	18 (11 to 28)	3.7 (2.2 to 5.8)	6 (3 to 10)	1.9 (1.1 to 3.1)	-44.4 (-69.2 to 1.5)	-33.9 (-63.4 to 20.5)
Azerbaijan	Adverse effects of medical treatment	5 to 9	Both	8 (4 to 11)	0.9 (0.5 to 1.4)	12 (5 to 18)	1.3 (0.5 to 2.1)	4 (2 to 7)	0.7 (0.4 to 1.0)	-41.6 (-63.9 to -1.1)	-27.0 (-54.9 to 23.6)
Azerbaijan	Adverse effects of medical treatment	5 to 9	Female	3 (1 to 5)	0.7 (0.3 to 1.1)	4 (2 to 7)	1.0 (0.4 to 1.7)	1 (1 to 2)	0.4 (0.2 to 0.7)	-54.8 (-77.4 to -14.3)	-41.2 (-70.6 to 11.5)
Azerbaijan	Adverse effects of medical treatment	5 to 9	Male	5 (3 to 8)	1.1 (0.6 to 1.8)	7 (3 to 13)	1.6 (0.7 to 2.8)	3 (2 to 5)	0.9 (0.5 to 1.5)	-33.5 (-63.4 to 31.1)	-20.0 (-56.0 to 57.7)
Azerbaijan	Adverse effects of medical treatment	10 to 14	Both	4 (3 to 6)	0.6 (0.4 to 0.8)	7 (4 to 10)	0.7 (0.4 to 1.1)	3 (2 to 5)	0.5 (0.3 to 0.8)	-23.9 (-51.7 to 25.4)	-6.6 (-40.7 to 53.8)
Azerbaijan	Adverse effects of medical treatment	10 to 14	Female	1 (1 to 1)	0.3 (0.2 to 0.4)	2 (1 to 3)	0.4 (0.2 to 0.6)	1 (0 to 1)	0.2 (0.1 to 0.3)	-43.9 (-70.0 to 0.2)	-28.8 (-61.9 to 27.4)
Azerbaijan	Adverse effects of medical treatment	10 to 14	Male	3 (2 to 5)	0.8 (0.5 to 1.3)	5 (3 to 8)	1.0 (0.6 to 1.7)	2 (1 to 4)	0.8 (0.5 to 1.3)	-17.3 (-50.2 to 51.8)	-1.8 (-40.8 to 80.4)
Azerbaijan	Animal contact	5 to 9	Both	3 (2 to 5)	0.4 (0.2 to 0.6)	5 (3 to 7)	0.5 (0.3 to 0.8)	2 (1 to 3)	0.3 (0.2 to 0.5)	-43.2 (-72.6 to 11.3)	-29.0 (-65.8 to 39.1)
Azerbaijan	Animal contact	5 to 9	Female	1 (0 to 2)	0.2 (0.1 to 0.4)	1 (0 to 3)	0.3 (0.1 to 0.6)	1 (0 to 1)	0.2 (0.1 to 0.3)	-40.5 (-72.0 to 42.0)	-22.5 (-63.5 to 84.9)
Azerbaijan	Animal contact	5 to 9	Male	2 (1 to 4)	0.5 (0.2 to 1.0)	3 (2 to 6)	0.7 (0.3 to 1.3)	1 (1 to 2)	0.4 (0.2 to 0.7)	-44.3 (-77.7 to 33.5)	-32.9 (-73.2 to 60.7)
Azerbaijan	Animal contact	10 to 14	Both	1 (1 to 2)	0.2 (0.1 to 0.3)	2 (1 to 3)	0.2 (0.1 to 0.4)	1 (1 to 1)	0.2 (0.1 to 0.3)	-35.8 (-65.7 to 31.2)	-21.2 (-58.0 to 61.0)
Azerbaijan	Animal contact	10 to 14	Female	0 (0 to 1)	0.1 (0.0 to 0.2)	1 (0 to 1)	0.1 (0.1 to 0.2)	0 (0 to 0)	0.1 (0.0 to 0.2)	-28.9 (-63.4 to 82.3)	-9.7 (-53.5 to 131.7)
Azerbaijan	Animal contact	10 to 14	Male	1 (1 to 2)	0.3 (0.2 to 0.5)	2 (1 to 3)	0.4 (0.2 to 0.6)	1 (0 to 1)	0.2 (0.1 to 0.4)	-37.8 (-71.8 to 50.3)	-26.1 (-66.5 to 78.5)
Azerbaijan	Foreign body	5 to 9	Both	12 (5 to 21)	1.5 (0.6 to 2.6)	17 (5 to 31)	1.9 (0.6 to 3.5)	8 (4 to 13)	1.2 (0.5 to 2.0)	-36.0 (-61.9 to 11.6)	-20.1 (-52.4 to 39.4)
Azerbaijan	Foreign body	5 to 9	Female	7 (3 to 13)	1.8 (0.7 to 3.2)	9 (3 to 19)	2.2 (0.7 to 4.5)	4 (2 to 7)	1.3 (0.6 to 2.2)	-43.4 (-70.8 to 12.7)	-26.2 (-62.0 to 46.7)
Azerbaijan	Foreign body	5 to 9	Male	5 (2 to 11)	1.3 (0.4 to 2.5)	7 (2 to 15)	1.6 (0.4 to 3.3)	4 (1 to 8)	1.2 (0.4 to 2.2)	-26.7 (-64.7 to 53.0)	-11.8 (-57.5 to 84.1)
Azerbaijan	Foreign body	10 to 14	Both	7 (3 to 10)	1.0 (0.5 to 1.5)	10 (4 to 16)	1.1 (0.4 to 1.8)	5 (2 to 8)	0.9 (0.4 to 1.4)	-24.4 (-50.1 to 22.6)	-7.3 (-38.8 to 50.5)
Azerbaijan	Foreign body	10 to 14	Female	4 (2 to 6)	1.1 (0.5 to 1.7)	5 (2 to 9)	1.2 (0.5 to 2.1)	3 (1 to 4)	0.9 (0.4 to 1.6)	-29.6 (-60.5 to 31.9)	-10.5 (-49.8 to 67.6)
Azerbaijan	Foreign body	10 to 14	Male	3 (1 to 6)	0.9 (0.3 to 1.6)	5 (2 to 9)	1.0 (0.3 to 1.9)	3 (1 to 5)	0.8 (0.3 to 1.6)	-18.4 (-56.8 to 55.6)	-3.1 (-48.7 to 84.9)
Azerbaijan	Environmental heat and cold exposure	5 to 9	Both	2 (1 to 3)	0.2 (0.1 to 0.4)	3 (1 to 5)	0.4 (0.2 to 0.6)	1 (0 to 1)	0.1 (0.1 to 0.2)	-61.0 (-79.6 to -15.8)	-51.3 (-74.5 to 5.2)
Azerbaijan	Environmental heat and cold exposure	5 to 9	Female	0 (0 to 1)	0.1 (0.0 to 0.2)	1 (0 to 1)	0.1 (0.0 to 0.3)	0 (0 to 0)	0.1 (0.0 to 0.1)	-57.4 (-81.9 to 11.5)	-44.5 (-76.4 to 45.2)

eTable 8. All-cause and cause-specific mortality (counts and rates) at the global level and by country in the WHO European Region for ages 5 to 9 and 10 to 14 for 1990, 2000, and 2016

Azerbaijan	Environmental heat and cold exposure	5 to 9	Male	2 (1 to 3)	0.4 (0.2 to 0.6)	3 (1 to 4)	0.6 (0.2 to 1.0)	1 (0 to 1)	0.2 (0.1 to 0.3)	-61.9 (-81.7 to -2.5)	-54.2 (-77.9 to 17.3)
Azerbaijan	Environmental heat and cold exposure	10 to 14	Both	1 (1 to 2)	0.2 (0.1 to 0.3)	2 (1 to 4)	0.3 (0.1 to 0.4)	1 (0 to 1)	0.1 (0.1 to 0.2)	-50.6 (-74.5 to 8.6)	-39.4 (-68.7 to 33.2)
Azerbaijan	Environmental heat and cold exposure	10 to 14	Female	0 (0 to 0)	0.1 (0.0 to 0.1)	0 (0 to 1)	0.1 (0.0 to 0.1)	0 (0 to 0)	0.0 (0.0 to 0.1)	-47.9 (-80.7 to 34.9)	-33.8 (-75.5 to 71.4)
Azerbaijan	Environmental heat and cold exposure	10 to 14	Male	1 (1 to 2)	0.3 (0.2 to 0.6)	2 (1 to 3)	0.4 (0.2 to 0.7)	1 (0 to 1)	0.2 (0.1 to 0.4)	-51.1 (-76.7 to 19.9)	-41.9 (-72.4 to 42.5)
Azerbaijan	Other unintentional injuries	5 to 9	Both	7 (4 to 12)	0.9 (0.5 to 1.5)	11 (7 to 17)	1.3 (0.8 to 1.9)	4 (2 to 7)	0.6 (0.4 to 1.0)	-44.3 (-72.8 to 18.1)	-30.5 (-65.9 to 47.6)
Azerbaijan	Other unintentional injuries	5 to 9	Female	2 (1 to 3)	0.4 (0.2 to 0.8)	3 (1 to 5)	0.6 (0.3 to 1.1)	1 (0 to 2)	0.3 (0.1 to 0.5)	-52.3 (-80.1 to 19.8)	-37.9 (-74.1 to 56.0)
Azerbaijan	Other unintentional injuries	5 to 9	Male	6 (3 to 10)	1.3 (0.7 to 2.3)	8 (5 to 14)	1.9 (1.0 to 3.1)	3 (2 to 6)	0.9 (0.5 to 1.6)	-41.8 (-74.9 to 41.0)	-30.0 (-69.8 to 69.7)
Azerbaijan	Other unintentional injuries	10 to 14	Both	6 (4 to 10)	0.9 (0.6 to 1.4)	10 (7 to 16)	1.1 (0.7 to 1.7)	5 (3 to 8)	0.8 (0.5 to 1.3)	-25.8 (-61.9 to 44.3)	-8.9 (-53.2 to 77.1)
Azerbaijan	Other unintentional injuries	10 to 14	Female	1 (1 to 2)	0.4 (0.2 to 0.6)	2 (1 to 4)	0.5 (0.2 to 0.8)	1 (0 to 1)	0.3 (0.1 to 0.5)	-37.2 (-73.7 to 60.5)	-20.2 (-66.6 to 104.0)
Azerbaijan	Other unintentional injuries	10 to 14	Male	5 (3 to 8)	1.4 (0.8 to 2.3)	8 (5 to 14)	1.7 (1.1 to 2.9)	4 (2 to 7)	1.3 (0.7 to 2.2)	-23.0 (-64.0 to 67.3)	-8.5 (-57.3 to 98.7)
Azerbaijan	Self-harm	10 to 14	Both	4 (3 to 7)	0.6 (0.4 to 1.0)	7 (5 to 13)	0.8 (0.5 to 1.4)	5 (3 to 7)	0.8 (0.5 to 1.3)	8.3 (-38.3 to 95.9)	32.9 (-24.3 to 140.4)
Azerbaijan	Self-harm	10 to 14	Female	1 (1 to 2)	0.3 (0.2 to 0.5)	1 (1 to 2)	0.3 (0.2 to 0.5)	1 (0 to 1)	0.3 (0.2 to 0.5)	-25.7 (-67.9 to 74.0)	-5.6 (-59.2 to 121.1)
Azerbaijan	Self-harm	10 to 14	Male	3 (2 to 6)	0.9 (0.6 to 1.7)	6 (4 to 11)	1.2 (0.8 to 2.3)	4 (2 to 6)	1.3 (0.7 to 2.1)	20.2 (-37.6 to 132.5)	42.8 (-25.8 to 176.3)
Azerbaijan	Interpersonal violence	5 to 9	Both	7 (5 to 10)	0.9 (0.6 to 1.3)	11 (7 to 17)	1.3 (0.8 to 1.9)	3 (2 to 5)	0.5 (0.3 to 0.8)	-56.7 (-78.2 to -19.9)	-45.9 (-72.7 to 0.1)
Azerbaijan	Interpersonal violence	5 to 9	Female	3 (2 to 5)	0.7 (0.4 to 1.2)	4 (2 to 7)	1.0 (0.4 to 1.7)	1 (1 to 2)	0.4 (0.2 to 0.7)	-59.6 (-84.7 to -8.0)	-47.4 (-80.1 to 19.8)
Azerbaijan	Interpersonal violence	5 to 9	Male	4 (2 to 7)	1.0 (0.6 to 1.6)	7 (4 to 11)	1.5 (0.8 to 2.5)	2 (1 to 4)	0.5 (0.2 to 1.0)	-54.7 (-82.3 to -5.2)	-45.5 (-78.7 to 14.1)
Azerbaijan	Interpersonal violence	10 to 14	Both	10 (6 to 14)	1.4 (0.9 to 2.0)	17 (11 to 25)	1.9 (1.2 to 2.7)	5 (3 to 9)	0.9 (0.5 to 1.5)	-46.4 (-70.7 to -8.2)	-34.3 (-64.0 to 12.6)
Azerbaijan	Interpersonal violence	10 to 14	Female	3 (2 to 4)	0.8 (0.5 to 1.2)	5 (2 to 7)	1.0 (0.5 to 1.6)	1 (1 to 3)	0.5 (0.3 to 1.0)	-44.7 (-76.5 to 12.5)	-29.7 (-70.1 to 42.9)
Azerbaijan	Interpersonal violence	10 to 14	Male	7 (4 to 11)	2.0 (1.1 to 3.1)	13 (6 to 21)	2.7 (1.3 to 4.3)	4 (2 to 7)	1.3 (0.6 to 2.2)	-47.1 (-75.5 to 5.4)	-37.1 (-70.9 to 25.2)
Azerbaijan	Exposure to forces of nature	5 to 9	Both	0 (0 to 0)	0.0 (0.0 to 0.0)	3 (1 to 5)	0.4 (0.2 to 0.6)	0 (0 to 0)	0.0 (0.0 to 0.0)	0.0 (0.0 to 0.0)	0.0 (0.0 to 0.0)
Azerbaijan	Exposure to forces of nature	5 to 9	Female	0 (0 to 0)	0.0 (0.0 to 0.0)	1 (1 to 2)	0.3 (0.1 to 0.5)	0 (0 to 0)	0.0 (0.0 to 0.0)	0.0 (0.0 to 0.0)	0.0 (0.0 to 0.0)
Azerbaijan	Exposure to forces of nature	5 to 9	Male	0 (0 to 0)	0.0 (0.0 to 0.0)	2 (1 to 3)	0.4 (0.2 to 0.6)	0 (0 to 0)	0.0 (0.0 to 0.0)	0.0 (0.0 to 0.0)	0.0 (0.0 to 0.0)
Azerbaijan	Exposure to forces of nature	10 to 14	Both	0 (0 to 0)	0.0 (0.0 to 0.0)	4 (2 to 7)	0.5 (0.2 to 0.7)	0 (0 to 0)	0.0 (0.0 to 0.0)	0.0 (0.0 to 0.0)	0.0 (0.0 to 0.0)
Azerbaijan	Exposure to forces of nature	10 to 14	Female	0 (0 to 0)	0.0 (0.0 to 0.0)	2 (1 to 3)	0.4 (0.2 to 0.6)	0 (0 to 0)	0.0 (0.0 to 0.0)	0.0 (0.0 to 0.0)	0.0 (0.0 to 0.0)
Azerbaijan	Exposure to forces of nature	10 to 14	Male	0 (0 to 0)	0.0 (0.0 to 0.0)	3 (1 to 4)	0.5 (0.2 to 0.9)	0 (0 to 0)	0.0 (0.0 to 0.0)	0.0 (0.0 to 0.0)	0.0 (0.0 to 0.0)

eTable 8. All-cause and cause-specific mortality (counts and rates) at the global level and by country in the WHO European Region for ages 5 to 9 and 10 to 14 for 1990, 2000, and 2016

Azerbaijan	Conflict and terrorism	5 to 9	Both	0 (0 to 0)	0.0 (0.0 to 0.0)	0 (0 to 0)	0.0 (0.0 to 0.0)	0 (0 to 0)	0.0 (0.0 to 0.0)	0.0 (0.0 to 0.0)	0.0 (0.0 to 0.0)
Azerbaijan	Conflict and terrorism	5 to 9	Female	0 (0 to 0)	0.0 (0.0 to 0.0)	0 (0 to 0)	0.0 (0.0 to 0.0)	0 (0 to 0)	0.0 (0.0 to 0.0)	0.0 (0.0 to 0.0)	0.0 (0.0 to 0.0)
Azerbaijan	Conflict and terrorism	5 to 9	Male	0 (0 to 0)	0.0 (0.0 to 0.0)	0 (0 to 0)	0.0 (0.0 to 0.0)	0 (0 to 0)	0.0 (0.0 to 0.0)	0.0 (0.0 to 0.0)	0.0 (0.0 to 0.0)
Azerbaijan	Conflict and terrorism	10 to 14	Both	0 (0 to 0)	0.0 (0.0 to 0.0)	0 (0 to 0)	0.0 (0.0 to 0.0)	0 (0 to 0)	0.0 (0.0 to 0.0)	0.0 (0.0 to 0.0)	0.0 (0.0 to 0.0)
Azerbaijan	Conflict and terrorism	10 to 14	Female	0 (0 to 0)	0.0 (0.0 to 0.0)	0 (0 to 0)	0.0 (0.0 to 0.0)	0 (0 to 0)	0.0 (0.0 to 0.0)	0.0 (0.0 to 0.0)	0.0 (0.0 to 0.0)
Azerbaijan	Conflict and terrorism	10 to 14	Male	0 (0 to 0)	0.0 (0.0 to 0.0)	0 (0 to 0)	0.0 (0.0 to 0.0)	0 (0 to 0)	0.0 (0.0 to 0.0)	0.0 (0.0 to 0.0)	0.0 (0.0 to 0.0)
Azerbaijan	Executions and police conflict	5 to 9	Both	0 (0 to 0)	0.0 (0.0 to 0.0)	0 (0 to 0)	0.0 (0.0 to 0.0)	0 (0 to 0)	0.0 (0.0 to 0.0)	-32.4 (-73.9 to 43.1)	-15.5 (-67.3 to 78.8)
Azerbaijan	Executions and police conflict	5 to 9	Female	0 (0 to 0)	0.0 (0.0 to 0.0)	0 (0 to 0)	0.0 (0.0 to 0.0)	0 (0 to 0)	0.0 (0.0 to 0.0)	-25.1 (-79.5 to 122.1)	-2.5 (-73.3 to 189.2)
Azerbaijan	Executions and police conflict	5 to 9	Male	0 (0 to 0)	0.0 (0.0 to 0.0)	0 (0 to 0)	0.0 (0.0 to 0.0)	0 (0 to 0)	0.0 (0.0 to 0.0)	-38.3 (-78.2 to 20.5)	-25.7 (-73.7 to 45.0)
Azerbaijan	Executions and police conflict	10 to 14	Both	0 (0 to 1)	0.0 (0.0 to 0.1)	0 (0 to 1)	0.0 (0.0 to 0.1)	0 (0 to 0)	0.0 (0.0 to 0.0)	-65.3 (-89.5 to 10.9)	-57.5 (-87.1 to 36.0)
Azerbaijan	Executions and police conflict	10 to 14	Female	0 (0 to 0)	0.0 (0.0 to 0.0)	0 (0 to 0)	0.0 (0.0 to 0.0)	0 (0 to 0)	0.0 (0.0 to 0.0)	-24.3 (-72.5 to 78.4)	-3.8 (-65.1 to 126.7)
Azerbaijan	Executions and police conflict	10 to 14	Male	0 (0 to 1)	0.0 (0.0 to 0.2)	0 (0 to 1)	0.0 (0.0 to 0.1)	0 (0 to 0)	0.0 (0.0 to 0.0)	-75.4 (-93.8 to 20.5)	-70.7 (-92.7 to 43.1)
Belarus	All causes	5 to 9	Both	430 (369 to 500)	53.1 (45.6 to 61.8)	207 (176 to 239)	34.6 (29.4 to 40.0)	108 (85 to 139)	20.7 (16.2 to 26.5)	-74.9 (-80.7 to -66.1)	-61.0 (-70.2 to -47.5)
Belarus	All causes	5 to 9	Female	153 (133 to 177)	38.5 (33.4 to 44.5)	76 (65 to 87)	26.1 (22.3 to 29.9)	41 (33 to 52)	16.3 (13.1 to 20.6)	-72.9 (-79.0 to -64.1)	-57.6 (-67.0 to -43.8)
Belarus	All causes	5 to 9	Male	277 (236 to 324)	67.1 (57.3 to 78.5)	131 (111 to 152)	42.7 (36.2 to 49.6)	66 (52 to 86)	24.8 (19.2 to 32.1)	-76.0 (-81.7 to -67.1)	-63.1 (-72.0 to -49.6)
Belarus	All causes	10 to 14	Both	313 (287 to 341)	42.3 (38.8 to 46.1)	262 (239 to 284)	32.6 (29.8 to 35.3)	81 (70 to 93)	18.3 (15.8 to 21.2)	-74.2 (-78.0 to -69.7)	-56.8 (-63.1 to -49.2)
Belarus	All causes	10 to 14	Female	111 (102 to 120)	30.4 (28.1 to 32.9)	90 (83 to 97)	23.0 (21.2 to 24.7)	31 (27 to 37)	14.7 (12.5 to 17.2)	-71.5 (-76.2 to -66.1)	-51.8 (-59.7 to -42.6)
Belarus	All causes	10 to 14	Male	203 (185 to 223)	53.9 (49.1 to 59.4)	172 (155 to 189)	41.8 (37.8 to 46.0)	49 (41 to 58)	21.8 (18.4 to 25.9)	-75.7 (-79.8 to -70.6)	-59.6 (-66.4 to -51.0)
Belarus	Tuberculosis	5 to 9	Both	0 (0 to 0)	0.0 (0.0 to 0.0)	0 (0 to 0)	0.0 (0.0 to 0.0)	0 (0 to 0)	0.0 (0.0 to 0.0)	-76.1 (-85.3 to -61.2)	-62.9 (-77.3 to -39.8)
Belarus	Tuberculosis	5 to 9	Female	0 (0 to 0)	0.0 (0.0 to 0.0)	0 (0 to 0)	0.0 (0.0 to 0.0)	0 (0 to 0)	0.0 (0.0 to 0.0)	-77.0 (-86.3 to -60.6)	-63.9 (-78.5 to -38.3)
Belarus	Tuberculosis	5 to 9	Male	0 (0 to 0)	0.0 (0.0 to 0.1)	0 (0 to 0)	0.0 (0.0 to 0.0)	0 (0 to 0)	0.0 (0.0 to 0.0)	-75.4 (-86.1 to -54.9)	-62.2 (-78.7 to -30.7)
Belarus	Tuberculosis	10 to 14	Both	0 (0 to 0)	0.0 (0.0 to 0.0)	0 (0 to 0)	0.0 (0.0 to 0.0)	0 (0 to 0)	0.0 (0.0 to 0.0)	-69.4 (-78.9 to -54.9)	-48.6 (-64.6 to -24.3)
Belarus	Tuberculosis	10 to 14	Female	0 (0 to 0)	0.0 (0.0 to 0.0)	0 (0 to 0)	0.0 (0.0 to 0.0)	0 (0 to 0)	0.0 (0.0 to 0.0)	-75.8 (-84.8 to -60.3)	-59.1 (-74.3 to -32.9)
Belarus	Tuberculosis	10 to 14	Male	0 (0 to 0)	0.0 (0.0 to 0.0)	0 (0 to 0)	0.0 (0.0 to 0.0)	0 (0 to 0)	0.0 (0.0 to 0.0)	-55.3 (-74.3 to -15.8)	-25.6 (-57.2 to 40.2)
Belarus	HIV/AIDS	5 to 9	Both	0 (0 to 0)	0.0 (0.0 to 0.1)	0 (0 to 0)	0.0 (0.0 to 0.0)	0 (0 to 0)	0.1 (0.1 to 0.1)	16.2 (-20.9 to 69.8)	80.1 (22.5 to 163.1)
Belarus	HIV/AIDS	5 to 9	Female	0 (0 to 0)	0.0 (0.0 to 0.1)	0 (0 to 0)	0.0 (0.0 to 0.0)	0 (0 to 0)	0.1 (0.1 to 0.1)	43.5 (-21.7 to 151.4)	124.7 (22.6 to 293.5)
Belarus	HIV/AIDS	5 to 9	Male	0 (0 to 0)	0.0 (0.0 to 0.1)	0 (0 to 0)	0.0 (0.0 to 0.1)	0 (0 to 0)	0.1 (0.0 to 0.1)	-4.6 (-43.7 to 55.3)	46.5 (-13.6 to 138.3)
Belarus	HIV/AIDS	10 to 14	Both	0 (0 to 0)	0.0 (0.0 to 0.0)	0 (0 to 0)	0.0 (0.0 to 0.0)	0 (0 to 0)	0.0 (0.0 to 0.0)	14.3 (-24.0 to 65.4)	91.8 (27.6 to 177.7)
Belarus	HIV/AIDS	10 to 14	Female	0 (0 to 0)	0.0 (0.0 to 0.0)	0 (0 to 0)	0.0 (0.0 to 0.0)	0 (0 to 0)	0.0 (0.0 to 0.0)	28.1 (-29.6 to 127.3)	116.8 (19.1 to 284.8)

eTable 8. All-cause and cause-specific mortality (counts and rates) at the global level and by country in the WHO European Region for ages 5 to 9 and 10 to 14 for 1990, 2000, and 2016

Belarus	HIV/AIDS	10 to 14	Male	0 (0 to 0)	0.0 (0.0 to 0.0)	0 (0 to 0)	0.0 (0.0 to 0.0)	0 (0 to 0)	0.0 (0.0 to 0.1)	7.7 (-35.6 to 80.4)	79.3 (7.3 to 200.4)
Belarus	Diarrheal diseases	5 to 9	Both	1 (1 to 2)	0.1 (0.1 to 0.2)	0 (0 to 1)	0.1 (0.1 to 0.1)	0 (0 to 0)	0.0 (0.0 to 0.0)	-94.4 (-96.7 to -90.6)	-91.3 (-95.0 to -85.4)
Belarus	Diarrheal diseases	5 to 9	Female	0 (0 to 1)	0.1 (0.1 to 0.2)	0 (0 to 0)	0.0 (0.0 to 0.1)	0 (0 to 0)	0.0 (0.0 to 0.0)	-94.5 (-96.7 to -89.9)	-91.3 (-94.8 to -84.3)
Belarus	Diarrheal diseases	5 to 9	Male	1 (0 to 1)	0.2 (0.1 to 0.3)	0 (0 to 0)	0.1 (0.1 to 0.2)	0 (0 to 0)	0.0 (0.0 to 0.0)	-94.4 (-97.2 to -88.8)	-91.3 (-95.7 to -82.8)
Belarus	Diarrheal diseases	10 to 14	Both	0 (0 to 1)	0.1 (0.0 to 0.1)	0 (0 to 0)	0.0 (0.0 to 0.0)	0 (0 to 0)	0.0 (0.0 to 0.0)	-90.2 (-93.1 to -86.1)	-83.6 (-88.5 to -76.7)
Belarus	Diarrheal diseases	10 to 14	Female	0 (0 to 0)	0.1 (0.0 to 0.1)	0 (0 to 0)	0.0 (0.0 to 0.0)	0 (0 to 0)	0.0 (0.0 to 0.0)	-90.7 (-94.3 to -85.0)	-84.2 (-90.3 to -74.6)
Belarus	Diarrheal diseases	10 to 14	Male	0 (0 to 0)	0.1 (0.0 to 0.1)	0 (0 to 0)	0.0 (0.0 to 0.1)	0 (0 to 0)	0.0 (0.0 to 0.0)	-89.8 (-93.7 to -82.6)	-83.0 (-89.5 to -71.1)
Belarus	Intestinal infectious diseases	5 to 9	Both	0 (0 to 0)	0.0 (0.0 to 0.0)	0 (0 to 0)	0.0 (0.0 to 0.0)	0 (0 to 0)	0.0 (0.0 to 0.0)	-44.9 (-69.3 to -7.6)	-14.5 (-52.4 to 43.3)
Belarus	Intestinal infectious diseases	5 to 9	Female	0 (0 to 0)	0.0 (0.0 to 0.0)	0 (0 to 0)	0.0 (0.0 to 0.0)	0 (0 to 0)	0.0 (0.0 to 0.0)	-45.3 (-74.5 to 16.8)	-14.4 (-60.0 to 82.8)
Belarus	Intestinal infectious diseases	5 to 9	Male	0 (0 to 0)	0.0 (0.0 to 0.0)	0 (0 to 0)	0.0 (0.0 to 0.0)	0 (0 to 0)	0.0 (0.0 to 0.0)	-44.3 (-73.8 to 15.7)	-14.4 (-59.8 to 77.5)
Belarus	Intestinal infectious diseases	10 to 14	Both	0 (0 to 0)	0.0 (0.0 to 0.0)	0 (0 to 0)	0.0 (0.0 to 0.0)	0 (0 to 0)	0.0 (0.0 to 0.0)	-49.2 (-72.1 to -10.5)	-14.7 (-53.2 to 50.2)
Belarus	Intestinal infectious diseases	10 to 14	Female	0 (0 to 0)	0.0 (0.0 to 0.0)	0 (0 to 0)	0.0 (0.0 to 0.0)	0 (0 to 0)	0.0 (0.0 to 0.0)	-49.7 (-77.2 to 30.1)	-14.8 (-61.4 to 120.2)
Belarus	Intestinal infectious diseases	10 to 14	Male	0 (0 to 0)	0.0 (0.0 to 0.0)	0 (0 to 0)	0.0 (0.0 to 0.0)	0 (0 to 0)	0.0 (0.0 to 0.0)	-48.4 (-78.7 to 1.4)	-14.1 (-64.6 to 68.8)
Belarus	Lower respiratory infections	5 to 9	Both	16 (11 to 20)	1.9 (1.4 to 2.5)	6 (4 to 8)	1.0 (0.7 to 1.3)	3 (2 to 4)	0.5 (0.3 to 0.7)	-82.9 (-89.5 to -72.6)	-73.5 (-83.7 to -57.5)
Belarus	Lower respiratory infections	5 to 9	Female	8 (6 to 12)	2.1 (1.4 to 2.9)	3 (2 to 4)	0.9 (0.6 to 1.3)	1 (1 to 2)	0.4 (0.3 to 0.7)	-86.4 (-92.9 to -74.7)	-78.7 (-88.9 to -60.4)
Belarus	Lower respiratory infections	5 to 9	Male	7 (5 to 10)	1.8 (1.2 to 2.5)	3 (2 to 4)	1.0 (0.6 to 1.4)	2 (1 to 2)	0.6 (0.4 to 0.9)	-78.8 (-88.4 to -59.6)	-67.5 (-82.2 to -38.0)
Belarus	Lower respiratory infections	10 to 14	Both	8 (6 to 10)	1.0 (0.8 to 1.3)	5 (4 to 6)	0.6 (0.5 to 0.8)	1 (1 to 2)	0.3 (0.2 to 0.5)	-80.8 (-87.0 to -71.2)	-67.8 (-78.2 to -51.6)
Belarus	Lower respiratory infections	10 to 14	Female	4 (3 to 6)	1.2 (0.8 to 1.7)	2 (2 to 3)	0.6 (0.4 to 0.9)	1 (0 to 1)	0.3 (0.2 to 0.5)	-84.5 (-91.3 to -72.5)	-73.8 (-85.2 to -53.4)
Belarus	Lower respiratory infections	10 to 14	Male	3 (2 to 5)	0.9 (0.6 to 1.2)	3 (2 to 4)	0.7 (0.5 to 0.9)	1 (1 to 1)	0.4 (0.2 to 0.5)	-75.8 (-85.8 to -59.2)	-59.8 (-76.4 to -32.0)
Belarus	Upper respiratory infections	5 to 9	Both	0 (0 to 0)	0.0 (0.0 to 0.0)	0 (0 to 0)	0.0 (0.0 to 0.0)	0 (0 to 0)	0.0 (0.0 to 0.0)	-80.6 (-99.6 to 850.6)	-69.9 (-99.4 to 1373.3)
Belarus	Upper respiratory infections	5 to 9	Female	0 (0 to 0)	0.0 (0.0 to 0.0)	0 (0 to 0)	0.0 (0.0 to 0.0)	0 (0 to 0)	0.0 (0.0 to 0.0)	-80.8 (-100.0 to 13818.3)	-70.0 (-100.0 to 21689.4)
Belarus	Upper respiratory infections	5 to 9	Male	0 (0 to 0)	0.0 (0.0 to 0.0)	0 (0 to 0)	0.0 (0.0 to 0.0)	0 (0 to 0)	0.0 (0.0 to 0.0)	-80.4 (-100.0 to 25905.1)	-70.0 (-100.0 to 39818.1)
Belarus	Upper respiratory infections	10 to 14	Both	0 (0 to 0)	0.0 (0.0 to 0.0)	0 (0 to 0)	0.0 (0.0 to 0.0)	0 (0 to 0)	0.0 (0.0 to 0.0)	-80.7 (-99.6 to 995.7)	-67.7 (-99.3 to 1739.4)
Belarus	Upper respiratory infections	10 to 14	Female	0 (0 to 0)	0.0 (0.0 to 0.0)	0 (0 to 0)	0.0 (0.0 to 0.0)	0 (0 to 0)	0.0 (0.0 to 0.0)	-80.2 (-100.0 to 29040.7)	-66.6 (-100.0 to 49228.8)

eTable 8. All-cause and cause-specific mortality (counts and rates) at the global level and by country in the WHO European Region for ages 5 to 9 and 10 to 14 for 1990, 2000, and 2016

Belarus	Upper respiratory infections	10 to 14	Male	0 (0 to 0)	0.0 (0.0 to 0.0)	0 (0 to 0)	0.0 (0.0 to 0.0)	0 (0 to 0)	0.0 (0.0 to 0.0)	-81.0 (-100.0 to 42665.3)	-68.4 (-100.0 to 71122.0)
Belarus	Otitis media	5 to 9	Both	0 (0 to 0)	0.0 (0.0 to 0.0)	0 (0 to 0)	0.0 (0.0 to 0.0)	0 (0 to 0)	0.0 (0.0 to 0.0)	-87.5 (-96.1 to -53.3)	-80.7 (-93.9 to -27.7)
Belarus	Otitis media	5 to 9	Female	0 (0 to 0)	0.0 (0.0 to 0.0)	0 (0 to 0)	0.0 (0.0 to 0.0)	0 (0 to 0)	0.0 (0.0 to 0.0)	-81.4 (-95.7 to -17.3)	-70.9 (-93.3 to 29.5)
Belarus	Otitis media	5 to 9	Male	0 (0 to 0)	0.0 (0.0 to 0.0)	0 (0 to 0)	0.0 (0.0 to 0.0)	0 (0 to 0)	0.0 (0.0 to 0.0)	-91.0 (-98.0 to -48.5)	-86.1 (-97.0 to -21.0)
Belarus	Otitis media	10 to 14	Both	0 (0 to 0)	0.0 (0.0 to 0.0)	0 (0 to 0)	0.0 (0.0 to 0.0)	0 (0 to 0)	0.0 (0.0 to 0.0)	-88.2 (-96.4 to -56.4)	-80.2 (-93.9 to -26.8)
Belarus	Otitis media	10 to 14	Female	0 (0 to 0)	0.0 (0.0 to 0.0)	0 (0 to 0)	0.0 (0.0 to 0.0)	0 (0 to 0)	0.0 (0.0 to 0.0)	-85.5 (-96.8 to -20.7)	-75.4 (-94.7 to 34.2)
Belarus	Otitis media	10 to 14	Male	0 (0 to 0)	0.0 (0.0 to 0.0)	0 (0 to 0)	0.0 (0.0 to 0.0)	0 (0 to 0)	0.0 (0.0 to 0.0)	-90.1 (-97.9 to -44.6)	-83.5 (-96.6 to -7.8)
Belarus	Meningitis	5 to 9	Both	4 (3 to 5)	0.5 (0.3 to 0.6)	2 (1 to 3)	0.3 (0.2 to 0.4)	1 (0 to 1)	0.1 (0.1 to 0.2)	-83.0 (-89.4 to -72.3)	-73.7 (-83.5 to -57.1)
Belarus	Meningitis	5 to 9	Female	2 (1 to 2)	0.4 (0.3 to 0.6)	1 (0 to 1)	0.2 (0.2 to 0.3)	0 (0 to 0)	0.1 (0.1 to 0.2)	-83.9 (-91.2 to -71.4)	-74.9 (-86.2 to -55.2)
Belarus	Meningitis	5 to 9	Male	2 (1 to 3)	0.5 (0.4 to 0.8)	1 (1 to 2)	0.4 (0.2 to 0.6)	0 (0 to 1)	0.1 (0.1 to 0.2)	-82.4 (-90.4 to -68.3)	-73.0 (-85.2 to -51.4)
Belarus	Meningitis	10 to 14	Both	3 (2 to 4)	0.4 (0.3 to 0.5)	2 (2 to 3)	0.3 (0.2 to 0.4)	0 (0 to 1)	0.1 (0.1 to 0.2)	-83.9 (-89.6 to -75.0)	-73.0 (-82.6 to -58.0)
Belarus	Meningitis	10 to 14	Female	2 (1 to 2)	0.4 (0.3 to 0.6)	1 (1 to 2)	0.3 (0.2 to 0.4)	0 (0 to 0)	0.1 (0.1 to 0.2)	-83.6 (-90.8 to -71.8)	-72.3 (-84.4 to -52.3)
Belarus	Meningitis	10 to 14	Male	2 (1 to 2)	0.4 (0.2 to 0.6)	1 (1 to 2)	0.3 (0.2 to 0.5)	0 (0 to 0)	0.1 (0.1 to 0.2)	-84.2 (-91.4 to -72.9)	-73.6 (-85.6 to -54.9)
Belarus	Encephalitis	5 to 9	Both	5 (2 to 7)	0.6 (0.3 to 0.9)	3 (2 to 5)	0.6 (0.3 to 0.9)	3 (2 to 5)	0.6 (0.3 to 0.9)	-37.1 (-59.3 to -3.4)	-2.4 (-36.9 to 49.6)
Belarus	Encephalitis	5 to 9	Female	2 (1 to 3)	0.4 (0.2 to 0.7)	1 (1 to 2)	0.4 (0.2 to 0.7)	1 (0 to 2)	0.4 (0.2 to 0.7)	-39.4 (-62.8 to 2.6)	-5.1 (-41.8 to 60.6)
Belarus	Encephalitis	5 to 9	Male	3 (1 to 5)	0.8 (0.3 to 1.2)	2 (1 to 4)	0.7 (0.3 to 1.1)	2 (1 to 3)	0.7 (0.3 to 1.2)	-35.7 (-63.0 to 6.4)	-1.3 (-43.2 to 63.3)
Belarus	Encephalitis	10 to 14	Both	2 (1 to 4)	0.3 (0.2 to 0.5)	2 (1 to 4)	0.3 (0.2 to 0.5)	1 (1 to 2)	0.3 (0.2 to 0.5)	-38.9 (-57.5 to -8.3)	2.6 (-28.7 to 54.0)
Belarus	Encephalitis	10 to 14	Female	1 (0 to 1)	0.3 (0.1 to 0.4)	1 (0 to 2)	0.2 (0.1 to 0.4)	1 (0 to 1)	0.3 (0.1 to 0.5)	-37.1 (-63.3 to 10.0)	6.5 (-37.9 to 86.3)
Belarus	Encephalitis	10 to 14	Male	1 (1 to 2)	0.4 (0.2 to 0.6)	2 (1 to 2)	0.4 (0.2 to 0.6)	1 (0 to 1)	0.4 (0.2 to 0.6)	-40.0 (-64.1 to -0.9)	-0.1 (-40.2 to 65.1)
Belarus	Diphtheria	5 to 9	Both	0 (0 to 0)	0.0 (0.0 to 0.0)	0 (0 to 0)	0.0 (0.0 to 0.0)	0 (0 to 0)	0.0 (0.0 to 0.0)	-83.2 (-98.0 to 19.9)	-74.0 (-96.9 to 85.8)
Belarus	Diphtheria	5 to 9	Female	0 (0 to 0)	0.0 (0.0 to 0.0)	0 (0 to 0)	0.0 (0.0 to 0.0)	0 (0 to 0)	0.0 (0.0 to 0.0)	-82.8 (-99.2 to 281.9)	-73.1 (-98.8 to 497.9)
Belarus	Diphtheria	5 to 9	Male	0 (0 to 0)	0.0 (0.0 to 0.0)	0 (0 to 0)	0.0 (0.0 to 0.0)	0 (0 to 0)	0.0 (0.0 to 0.0)	-83.4 (-99.4 to 257.1)	-74.5 (-99.1 to 448.1)
Belarus	Diphtheria	10 to 14	Both	0 (0 to 0)	0.0 (0.0 to 0.0)	0 (0 to 0)	0.0 (0.0 to 0.0)	0 (0 to 0)	0.0 (0.0 to 0.0)	-83.5 (-97.5 to 19.4)	-72.2 (-95.8 to 100.5)
Belarus	Diphtheria	10 to 14	Female	0 (0 to 0)	0.0 (0.0 to 0.0)	0 (0 to 0)	0.0 (0.0 to 0.0)	0 (0 to 0)	0.0 (0.0 to 0.0)	-81.5 (-99.1 to 210.9)	-68.7 (-98.4 to 426.3)
Belarus	Diphtheria	10 to 14	Male	0 (0 to 0)	0.0 (0.0 to 0.0)	0 (0 to 0)	0.0 (0.0 to 0.0)	0 (0 to 0)	0.0 (0.0 to 0.0)	-84.5 (-99.1 to 266.2)	-74.2 (-98.5 to 510.0)
Belarus	Whooping cough	5 to 9	Both	0 (0 to 0)	0.0 (0.0 to 0.0)	0 (0 to 0)	0.0 (0.0 to 0.0)	0 (0 to 0)	0.0 (0.0 to 0.0)	-33.8 (-53.6 to 5.5)	2.6 (-28.1 to 63.4)
Belarus	Whooping cough	5 to 9	Female	0 (0 to 0)	0.0 (0.0 to 0.0)	0 (0 to 0)	0.0 (0.0 to 0.0)	0 (0 to 0)	0.0 (0.0 to 0.0)	-35.9 (-57.7 to 32.0)	0.4 (-33.8 to 106.6)
Belarus	Whooping cough	5 to 9	Male	0 (0 to 0)	0.0 (0.0 to 0.0)	0 (0 to 0)	0.0 (0.0 to 0.0)	0 (0 to 0)	0.0 (0.0 to 0.0)	-31.8 (-54.5 to 10.7)	4.7 (-30.1 to 69.9)
Belarus	Whooping cough	10 to 14	Both	0 (0 to 0)	0.0 (0.0 to 0.0)	0 (0 to 0)	0.0 (0.0 to 0.0)	0 (0 to 0)	0.0 (0.0 to 0.0)	-41.9 (-54.4 to -23.5)	-2.4 (-23.4 to 28.4)
Belarus	Whooping cough	10 to 14	Female	0 (0 to 0)	0.0 (0.0 to 0.0)	0 (0 to 0)	0.0 (0.0 to 0.0)	0 (0 to 0)	0.0 (0.0 to 0.0)	-43.2 (-57.1 to -23.7)	-3.8 (-27.3 to 29.1)
Belarus	Whooping cough	10 to 14	Male	0 (0 to 0)	0.0 (0.0 to 0.0)	0 (0 to 0)	0.0 (0.0 to 0.0)	0 (0 to 0)	0.0 (0.0 to 0.0)	-40.6 (-55.9 to -15.3)	-1.0 (-26.6 to 41.1)
Belarus	Tetanus	5 to 9	Both	0 (0 to 0)	0.0 (0.0 to 0.0)	0 (0 to 0)	0.0 (0.0 to 0.0)	0 (0 to 0)	0.0 (0.0 to 0.0)	-80.8 (-93.8 to -49.5)	-70.2 (-90.4 to -21.8)

eTable 8. All-cause and cause-specific mortality (counts and rates) at the global level and by country in the WHO European Region for ages 5 to 9 and 10 to 14 for 1990, 2000, and 2016

Belarus	Tetanus	5 to 9	Female	0 (0 to 0)	0.0 (0.0 to 0.0)	0 (0 to 0)	0.0 (0.0 to 0.0)	0 (0 to 0)	0.0 (0.0 to 0.0)	-58.2 (-84.5 to 60.3)	-34.6 (-75.7 to 151.0)
Belarus	Tetanus	5 to 9	Male	0 (0 to 0)	0.0 (0.0 to 0.0)	0 (0 to 0)	0.0 (0.0 to 0.0)	0 (0 to 0)	0.0 (0.0 to 0.0)	-87.5 (-95.5 to -64.8)	-80.8 (-93.0 to -46.0)
Belarus	Tetanus	10 to 14	Both	0 (0 to 0)	0.0 (0.0 to 0.0)	0 (0 to 0)	0.0 (0.0 to 0.0)	0 (0 to 0)	0.0 (0.0 to 0.0)	-26.5 (-86.3 to 94.3)	23.5 (-77.0 to 226.1)
Belarus	Tetanus	10 to 14	Female	0 (0 to 0)	0.0 (0.0 to 0.0)	0 (0 to 0)	0.0 (0.0 to 0.0)	0 (0 to 0)	0.0 (0.0 to 0.0)	21.7 (-62.4 to 239.7)	106.1 (-36.3 to 475.0)
Belarus	Tetanus	10 to 14	Male	0 (0 to 0)	0.0 (0.0 to 0.0)	0 (0 to 0)	0.0 (0.0 to 0.0)	0 (0 to 0)	0.0 (0.0 to 0.0)	-46.4 (-93.0 to 104.8)	-10.7 (-88.3 to 241.1)
Belarus	Measles	5 to 9	Both	0 (0 to 0)	0.0 (0.0 to 0.0)	0 (0 to 0)	0.0 (0.0 to 0.0)	0 (0 to 0)	0.0 (0.0 to 0.0)	-82.3 (-92.6 to -56.9)	-72.6 (-88.6 to -33.2)
Belarus	Measles	5 to 9	Female	0 (0 to 0)	0.0 (0.0 to 0.0)	0 (0 to 0)	0.0 (0.0 to 0.0)	0 (0 to 0)	0.0 (0.0 to 0.0)	-87.7 (-96.0 to -61.5)	-80.7 (-93.7 to -39.7)
Belarus	Measles	5 to 9	Male	0 (0 to 0)	0.0 (0.0 to 0.0)	0 (0 to 0)	0.0 (0.0 to 0.0)	0 (0 to 0)	0.0 (0.0 to 0.0)	-48.9 (-76.6 to 22.1)	-21.6 (-64.1 to 87.5)
Belarus	Measles	10 to 14	Both	0 (0 to 0)	0.0 (0.0 to 0.0)	0 (0 to 0)	0.0 (0.0 to 0.0)	0 (0 to 0)	0.0 (0.0 to 0.0)	-33.8 (-56.5 to 7.0)	11.1 (-27.0 to 79.6)
Belarus	Measles	10 to 14	Female	0 (0 to 0)	0.0 (0.0 to 0.0)	0 (0 to 0)	0.0 (0.0 to 0.0)	0 (0 to 0)	0.0 (0.0 to 0.0)	-34.3 (-62.9 to 31.5)	11.3 (-37.2 to 122.6)
Belarus	Measles	10 to 14	Male	0 (0 to 0)	0.0 (0.0 to 0.0)	0 (0 to 0)	0.0 (0.0 to 0.0)	0 (0 to 0)	0.0 (0.0 to 0.0)	-33.3 (-55.5 to 29.9)	11.1 (-25.9 to 116.3)
Belarus	Varicella and herpes zoster	5 to 9	Both	0 (0 to 0)	0.0 (0.0 to 0.0)	0 (0 to 0)	0.0 (0.0 to 0.0)	0 (0 to 0)	0.0 (0.0 to 0.0)	-74.4 (-87.0 to -49.6)	-60.3 (-79.8 to -21.8)
Belarus	Varicella and herpes zoster	5 to 9	Female	0 (0 to 0)	0.0 (0.0 to 0.0)	0 (0 to 0)	0.0 (0.0 to 0.0)	0 (0 to 0)	0.0 (0.0 to 0.0)	-77.1 (-90.8 to -44.6)	-64.2 (-85.6 to -13.3)
Belarus	Varicella and herpes zoster	5 to 9	Male	0 (0 to 0)	0.0 (0.0 to 0.0)	0 (0 to 0)	0.0 (0.0 to 0.0)	0 (0 to 0)	0.0 (0.0 to 0.0)	-69.9 (-88.9 to -18.9)	-53.8 (-82.9 to 24.4)
Belarus	Varicella and herpes zoster	10 to 14	Both	0 (0 to 0)	0.0 (0.0 to 0.0)	0 (0 to 0)	0.0 (0.0 to 0.0)	0 (0 to 0)	0.0 (0.0 to 0.0)	-73.1 (-85.7 to -48.0)	-54.9 (-76.1 to -12.7)
Belarus	Varicella and herpes zoster	10 to 14	Female	0 (0 to 0)	0.0 (0.0 to 0.0)	0 (0 to 0)	0.0 (0.0 to 0.0)	0 (0 to 0)	0.0 (0.0 to 0.0)	-76.0 (-88.6 to -46.0)	-59.3 (-80.8 to -8.6)
Belarus	Varicella and herpes zoster	10 to 14	Male	0 (0 to 0)	0.0 (0.0 to 0.0)	0 (0 to 0)	0.0 (0.0 to 0.0)	0 (0 to 0)	0.0 (0.0 to 0.0)	-69.1 (-87.6 to -21.7)	-48.5 (-79.3 to 30.5)
Belarus	Malaria	5 to 9	Both	0 (0 to 0)	0.0 (0.0 to 0.0)	0 (0 to 0)	0.0 (0.0 to 0.0)	0 (0 to 0)	0.0 (0.0 to 0.0)	0.0 (0.0 to 0.0)	0.0 (0.0 to 0.0)
Belarus	Malaria	5 to 9	Female	0 (0 to 0)	0.0 (0.0 to 0.0)	0 (0 to 0)	0.0 (0.0 to 0.0)	0 (0 to 0)	0.0 (0.0 to 0.0)	0.0 (0.0 to 0.0)	0.0 (0.0 to 0.0)
Belarus	Malaria	5 to 9	Male	0 (0 to 0)	0.0 (0.0 to 0.0)	0 (0 to 0)	0.0 (0.0 to 0.0)	0 (0 to 0)	0.0 (0.0 to 0.0)	0.0 (0.0 to 0.0)	0.0 (0.0 to 0.0)
Belarus	Malaria	10 to 14	Both	0 (0 to 0)	0.0 (0.0 to 0.0)	0 (0 to 0)	0.0 (0.0 to 0.0)	0 (0 to 0)	0.0 (0.0 to 0.0)	0.0 (0.0 to 0.0)	0.0 (0.0 to 0.0)
Belarus	Malaria	10 to 14	Female	0 (0 to 0)	0.0 (0.0 to 0.0)	0 (0 to 0)	0.0 (0.0 to 0.0)	0 (0 to 0)	0.0 (0.0 to 0.0)	0.0 (0.0 to 0.0)	0.0 (0.0 to 0.0)
Belarus	Malaria	10 to 14	Male	0 (0 to 0)	0.0 (0.0 to 0.0)	0 (0 to 0)	0.0 (0.0 to 0.0)	0 (0 to 0)	0.0 (0.0 to 0.0)	0.0 (0.0 to 0.0)	0.0 (0.0 to 0.0)
Belarus	Leishmaniasis	5 to 9	Both	0 (0 to 0)	0.0 (0.0 to 0.0)	0 (0 to 0)	0.0 (0.0 to 0.0)	0 (0 to 0)	0.0 (0.0 to 0.0)	0.0 (0.0 to 0.0)	0.0 (0.0 to 0.0)
Belarus	Leishmaniasis	5 to 9	Female	0 (0 to 0)	0.0 (0.0 to 0.0)	0 (0 to 0)	0.0 (0.0 to 0.0)	0 (0 to 0)	0.0 (0.0 to 0.0)	0.0 (0.0 to 0.0)	0.0 (0.0 to 0.0)
Belarus	Leishmaniasis	5 to 9	Male	0 (0 to 0)	0.0 (0.0 to 0.0)	0 (0 to 0)	0.0 (0.0 to 0.0)	0 (0 to 0)	0.0 (0.0 to 0.0)	0.0 (0.0 to 0.0)	0.0 (0.0 to 0.0)
Belarus	Leishmaniasis	10 to 14	Both	0 (0 to 0)	0.0 (0.0 to 0.0)	0 (0 to 0)	0.0 (0.0 to 0.0)	0 (0 to 0)	0.0 (0.0 to 0.0)	0.0 (0.0 to 0.0)	0.0 (0.0 to 0.0)
Belarus	Leishmaniasis	10 to 14	Female	0 (0 to 0)	0.0 (0.0 to 0.0)	0 (0 to 0)	0.0 (0.0 to 0.0)	0 (0 to 0)	0.0 (0.0 to 0.0)	0.0 (0.0 to 0.0)	0.0 (0.0 to 0.0)
Belarus	Leishmaniasis	10 to 14	Male	0 (0 to 0)	0.0 (0.0 to 0.0)	0 (0 to 0)	0.0 (0.0 to 0.0)	0 (0 to 0)	0.0 (0.0 to 0.0)	0.0 (0.0 to 0.0)	0.0 (0.0 to 0.0)
Belarus	African trypanosomiasis	5 to 9	Both	0 (0 to 0)	0.0 (0.0 to 0.0)	0 (0 to 0)	0.0 (0.0 to 0.0)	0 (0 to 0)	0.0 (0.0 to 0.0)	0.0 (0.0 to 0.0)	0.0 (0.0 to 0.0)
Belarus	African trypanosomiasis	5 to 9	Female	0 (0 to 0)	0.0 (0.0 to 0.0)	0 (0 to 0)	0.0 (0.0 to 0.0)	0 (0 to 0)	0.0 (0.0 to 0.0)	0.0 (0.0 to 0.0)	0.0 (0.0 to 0.0)

eTable 8. All-cause and cause-specific mortality (counts and rates) at the global level and by country in the WHO European Region for ages 5 to 9 and 10 to 14 for 1990, 2000, and 2016

Belarus	African trypanosomiasis	5 to 9	Male	0 (0 to 0)	0.0 (0.0 to 0.0)	0 (0 to 0)	0.0 (0.0 to 0.0)	0 (0 to 0)	0.0 (0.0 to 0.0)	0.0 (0.0 to 0.0)	0.0 (0.0 to 0.0)
Belarus	African trypanosomiasis	10 to 14	Both	0 (0 to 0)	0.0 (0.0 to 0.0)	0 (0 to 0)	0.0 (0.0 to 0.0)	0 (0 to 0)	0.0 (0.0 to 0.0)	0.0 (0.0 to 0.0)	0.0 (0.0 to 0.0)
Belarus	African trypanosomiasis	10 to 14	Female	0 (0 to 0)	0.0 (0.0 to 0.0)	0 (0 to 0)	0.0 (0.0 to 0.0)	0 (0 to 0)	0.0 (0.0 to 0.0)	0.0 (0.0 to 0.0)	0.0 (0.0 to 0.0)
Belarus	African trypanosomiasis	10 to 14	Male	0 (0 to 0)	0.0 (0.0 to 0.0)	0 (0 to 0)	0.0 (0.0 to 0.0)	0 (0 to 0)	0.0 (0.0 to 0.0)	0.0 (0.0 to 0.0)	0.0 (0.0 to 0.0)
Belarus	Schistosomiasis	5 to 9	Both	0 (0 to 0)	0.0 (0.0 to 0.0)	0 (0 to 0)	0.0 (0.0 to 0.0)	0 (0 to 0)	0.0 (0.0 to 0.0)	0.0 (0.0 to 0.0)	0.0 (0.0 to 0.0)
Belarus	Schistosomiasis	5 to 9	Female	0 (0 to 0)	0.0 (0.0 to 0.0)	0 (0 to 0)	0.0 (0.0 to 0.0)	0 (0 to 0)	0.0 (0.0 to 0.0)	0.0 (0.0 to 0.0)	0.0 (0.0 to 0.0)
Belarus	Schistosomiasis	5 to 9	Male	0 (0 to 0)	0.0 (0.0 to 0.0)	0 (0 to 0)	0.0 (0.0 to 0.0)	0 (0 to 0)	0.0 (0.0 to 0.0)	0.0 (0.0 to 0.0)	0.0 (0.0 to 0.0)
Belarus	Schistosomiasis	10 to 14	Both	0 (0 to 0)	0.0 (0.0 to 0.0)	0 (0 to 0)	0.0 (0.0 to 0.0)	0 (0 to 0)	0.0 (0.0 to 0.0)	0.0 (0.0 to 0.0)	0.0 (0.0 to 0.0)
Belarus	Schistosomiasis	10 to 14	Female	0 (0 to 0)	0.0 (0.0 to 0.0)	0 (0 to 0)	0.0 (0.0 to 0.0)	0 (0 to 0)	0.0 (0.0 to 0.0)	0.0 (0.0 to 0.0)	0.0 (0.0 to 0.0)
Belarus	Schistosomiasis	10 to 14	Male	0 (0 to 0)	0.0 (0.0 to 0.0)	0 (0 to 0)	0.0 (0.0 to 0.0)	0 (0 to 0)	0.0 (0.0 to 0.0)	0.0 (0.0 to 0.0)	0.0 (0.0 to 0.0)
Belarus	Cysticercosis	5 to 9	Both	0 (0 to 0)	0.0 (0.0 to 0.0)	0 (0 to 0)	0.0 (0.0 to 0.0)	0 (0 to 0)	0.0 (0.0 to 0.0)	-90.5 (-93.3 to -86.7)	-85.3 (-89.6 to -79.4)
Belarus	Cysticercosis	5 to 9	Female	0 (0 to 0)	0.0 (0.0 to 0.0)	0 (0 to 0)	0.0 (0.0 to 0.0)	0 (0 to 0)	0.0 (0.0 to 0.0)	-90.4 (-93.3 to -86.6)	-84.9 (-89.5 to -79.0)
Belarus	Cysticercosis	5 to 9	Male	0 (0 to 0)	0.0 (0.0 to 0.0)	0 (0 to 0)	0.0 (0.0 to 0.0)	0 (0 to 0)	0.0 (0.0 to 0.0)	-90.6 (-93.5 to -86.5)	-85.6 (-90.0 to -79.3)
Belarus	Cysticercosis	10 to 14	Both	0 (0 to 0)	0.0 (0.0 to 0.0)	0 (0 to 0)	0.0 (0.0 to 0.0)	0 (0 to 0)	0.0 (0.0 to 0.0)	-90.3 (-92.3 to -88.0)	-83.7 (-87.1 to -79.8)
Belarus	Cysticercosis	10 to 14	Female	0 (0 to 0)	0.0 (0.0 to 0.0)	0 (0 to 0)	0.0 (0.0 to 0.0)	0 (0 to 0)	0.0 (0.0 to 0.0)	-89.6 (-92.1 to -86.7)	-82.4 (-86.6 to -77.4)
Belarus	Cysticercosis	10 to 14	Male	0 (0 to 0)	0.0 (0.0 to 0.0)	0 (0 to 0)	0.0 (0.0 to 0.0)	0 (0 to 0)	0.0 (0.0 to 0.0)	-90.9 (-93.1 to -88.0)	-84.8 (-88.5 to -80.1)
Belarus	Yellow fever	5 to 9	Both	0 (0 to 0)	0.0 (0.0 to 0.0)	0 (0 to 0)	0.0 (0.0 to 0.0)	0 (0 to 0)	0.0 (0.0 to 0.0)	0.0 (0.0 to 0.0)	0.0 (0.0 to 0.0)
Belarus	Yellow fever	5 to 9	Female	0 (0 to 0)	0.0 (0.0 to 0.0)	0 (0 to 0)	0.0 (0.0 to 0.0)	0 (0 to 0)	0.0 (0.0 to 0.0)	0.0 (0.0 to 0.0)	0.0 (0.0 to 0.0)
Belarus	Yellow fever	5 to 9	Male	0 (0 to 0)	0.0 (0.0 to 0.0)	0 (0 to 0)	0.0 (0.0 to 0.0)	0 (0 to 0)	0.0 (0.0 to 0.0)	0.0 (0.0 to 0.0)	0.0 (0.0 to 0.0)
Belarus	Yellow fever	10 to 14	Both	0 (0 to 0)	0.0 (0.0 to 0.0)	0 (0 to 0)	0.0 (0.0 to 0.0)	0 (0 to 0)	0.0 (0.0 to 0.0)	0.0 (0.0 to 0.0)	0.0 (0.0 to 0.0)
Belarus	Yellow fever	10 to 14	Female	0 (0 to 0)	0.0 (0.0 to 0.0)	0 (0 to 0)	0.0 (0.0 to 0.0)	0 (0 to 0)	0.0 (0.0 to 0.0)	0.0 (0.0 to 0.0)	0.0 (0.0 to 0.0)
Belarus	Yellow fever	10 to 14	Male	0 (0 to 0)	0.0 (0.0 to 0.0)	0 (0 to 0)	0.0 (0.0 to 0.0)	0 (0 to 0)	0.0 (0.0 to 0.0)	0.0 (0.0 to 0.0)	0.0 (0.0 to 0.0)
Belarus	Rabies	5 to 9	Both	0 (0 to 0)	0.0 (0.0 to 0.0)	0 (0 to 0)	0.0 (0.0 to 0.0)	0 (0 to 0)	0.0 (0.0 to 0.0)	-54.0 (-92.2 to 228.1)	-28.7 (-87.9 to 408.5)
Belarus	Rabies	5 to 9	Female	0 (0 to 0)	0.0 (0.0 to 0.0)	0 (0 to 0)	0.0 (0.0 to 0.0)	0 (0 to 0)	0.0 (0.0 to 0.0)	-60.2 (-95.7 to 341.7)	-37.7 (-93.3 to 591.4)
Belarus	Rabies	5 to 9	Male	0 (0 to 0)	0.0 (0.0 to 0.0)	0 (0 to 0)	0.0 (0.0 to 0.0)	0 (0 to 0)	0.0 (0.0 to 0.0)	-24.9 (-90.4 to 637.1)	15.3 (-85.3 to 1031.4)
Belarus	Rabies	10 to 14	Both	0 (0 to 0)	0.0 (0.0 to 0.0)	0 (0 to 0)	0.0 (0.0 to 0.0)	0 (0 to 0)	0.0 (0.0 to 0.0)	2.7 (-80.8 to 506.9)	72.5 (-67.7 to 918.9)
Belarus	Rabies	10 to 14	Female	0 (0 to 0)	0.0 (0.0 to 0.0)	0 (0 to 0)	0.0 (0.0 to 0.0)	0 (0 to 0)	0.0 (0.0 to 0.0)	-29.4 (-92.7 to 777.5)	19.5 (-87.7 to 1385.3)
Belarus	Rabies	10 to 14	Male	0 (0 to 0)	0.0 (0.0 to 0.0)	0 (0 to 0)	0.0 (0.0 to 0.0)	0 (0 to 0)	0.0 (0.0 to 0.0)	118.6 (-61.9 to 1253.6)	264.1 (-36.6 to 2154.3)
Belarus	Intestinal nematode infections	5 to 9	Both	-	-	-	-	-	-	-	-

eTable 8. All-cause and cause-specific mortality (counts and rates) at the global level and by country in the WHO European Region for ages 5 to 9 and 10 to 14 for 1990, 2000, and 2016

Belarus	Intestinal nematode infections	5 to 9	Female	-	-	-	-	-	-	-	-
Belarus	Intestinal nematode infections	5 to 9	Male	-	-	-	-	-	-	-	-
Belarus	Intestinal nematode infections	10 to 14	Both	-	-	-	-	-	-	-	-
Belarus	Intestinal nematode infections	10 to 14	Female	-	-	-	-	-	-	-	-
Belarus	Intestinal nematode infections	10 to 14	Male	-	-	-	-	-	-	-	-
Belarus	Ebola	5 to 9	Both	0 (0 to 0)	0.0 (0.0 to 0.0)	0 (0 to 0)	0.0 (0.0 to 0.0)	0 (0 to 0)	0.0 (0.0 to 0.0)	0.0 (0.0 to 0.0)	0.0 (0.0 to 0.0)
Belarus	Ebola	5 to 9	Female	0 (0 to 0)	0.0 (0.0 to 0.0)	0 (0 to 0)	0.0 (0.0 to 0.0)	0 (0 to 0)	0.0 (0.0 to 0.0)	0.0 (0.0 to 0.0)	0.0 (0.0 to 0.0)
Belarus	Ebola	5 to 9	Male	0 (0 to 0)	0.0 (0.0 to 0.0)	0 (0 to 0)	0.0 (0.0 to 0.0)	0 (0 to 0)	0.0 (0.0 to 0.0)	0.0 (0.0 to 0.0)	0.0 (0.0 to 0.0)
Belarus	Ebola	10 to 14	Both	0 (0 to 0)	0.0 (0.0 to 0.0)	0 (0 to 0)	0.0 (0.0 to 0.0)	0 (0 to 0)	0.0 (0.0 to 0.0)	0.0 (0.0 to 0.0)	0.0 (0.0 to 0.0)
Belarus	Ebola	10 to 14	Female	0 (0 to 0)	0.0 (0.0 to 0.0)	0 (0 to 0)	0.0 (0.0 to 0.0)	0 (0 to 0)	0.0 (0.0 to 0.0)	0.0 (0.0 to 0.0)	0.0 (0.0 to 0.0)
Belarus	Ebola	10 to 14	Male	0 (0 to 0)	0.0 (0.0 to 0.0)	0 (0 to 0)	0.0 (0.0 to 0.0)	0 (0 to 0)	0.0 (0.0 to 0.0)	0.0 (0.0 to 0.0)	0.0 (0.0 to 0.0)
Belarus	Zika virus	5 to 9	Both	0 (0 to 0)	0.0 (0.0 to 0.0)	0 (0 to 0)	0.0 (0.0 to 0.0)	0 (0 to 0)	0.0 (0.0 to 0.0)	0.0 (0.0 to 0.0)	0.0 (0.0 to 0.0)
Belarus	Zika virus	5 to 9	Female	0 (0 to 0)	0.0 (0.0 to 0.0)	0 (0 to 0)	0.0 (0.0 to 0.0)	0 (0 to 0)	0.0 (0.0 to 0.0)	0.0 (0.0 to 0.0)	0.0 (0.0 to 0.0)
Belarus	Zika virus	5 to 9	Male	0 (0 to 0)	0.0 (0.0 to 0.0)	0 (0 to 0)	0.0 (0.0 to 0.0)	0 (0 to 0)	0.0 (0.0 to 0.0)	0.0 (0.0 to 0.0)	0.0 (0.0 to 0.0)
Belarus	Zika virus	10 to 14	Both	0 (0 to 0)	0.0 (0.0 to 0.0)	0 (0 to 0)	0.0 (0.0 to 0.0)	0 (0 to 0)	0.0 (0.0 to 0.0)	0.0 (0.0 to 0.0)	0.0 (0.0 to 0.0)
Belarus	Zika virus	10 to 14	Female	0 (0 to 0)	0.0 (0.0 to 0.0)	0 (0 to 0)	0.0 (0.0 to 0.0)	0 (0 to 0)	0.0 (0.0 to 0.0)	0.0 (0.0 to 0.0)	0.0 (0.0 to 0.0)
Belarus	Zika virus	10 to 14	Male	0 (0 to 0)	0.0 (0.0 to 0.0)	0 (0 to 0)	0.0 (0.0 to 0.0)	0 (0 to 0)	0.0 (0.0 to 0.0)	0.0 (0.0 to 0.0)	0.0 (0.0 to 0.0)
Belarus	Other neglected tropical diseases	5 to 9	Both	0 (0 to 0)	0.0 (0.0 to 0.0)	0 (0 to 0)	0.0 (0.0 to 0.0)	0 (0 to 0)	0.0 (0.0 to 0.0)	-38.0 (-58.0 to -4.9)	-3.9 (-35.0 to 47.4)
Belarus	Other neglected tropical diseases	5 to 9	Female	0 (0 to 0)	0.0 (0.0 to 0.0)	0 (0 to 0)	0.0 (0.0 to 0.0)	0 (0 to 0)	0.0 (0.0 to 0.0)	-41.3 (-62.0 to -8.9)	-8.1 (-40.4 to 42.7)
Belarus	Other neglected tropical diseases	5 to 9	Male	0 (0 to 0)	0.0 (0.0 to 0.0)	0 (0 to 0)	0.0 (0.0 to 0.0)	0 (0 to 0)	0.0 (0.0 to 0.0)	-34.6 (-56.2 to -2.3)	0.3 (-32.8 to 49.9)
Belarus	Other neglected tropical diseases	10 to 14	Both	0 (0 to 0)	0.0 (0.0 to 0.0)	0 (0 to 0)	0.0 (0.0 to 0.0)	0 (0 to 0)	0.0 (0.0 to 0.0)	-43.3 (-54.7 to -28.8)	-4.7 (-23.9 to 19.6)
Belarus	Other neglected tropical diseases	10 to 14	Female	0 (0 to 0)	0.0 (0.0 to 0.0)	0 (0 to 0)	0.0 (0.0 to 0.0)	0 (0 to 0)	0.0 (0.0 to 0.0)	-44.4 (-57.9 to -28.6)	-5.9 (-28.8 to 20.8)
Belarus	Other neglected tropical diseases	10 to 14	Male	0 (0 to 0)	0.0 (0.0 to 0.0)	0 (0 to 0)	0.0 (0.0 to 0.0)	0 (0 to 0)	0.0 (0.0 to 0.0)	-42.1 (-56.0 to -23.3)	-3.6 (-26.7 to 27.7)
Belarus	Maternal hemorrhage	10 to 14	Both	0 (0 to 0)	0.0 (0.0 to 0.0)	0 (0 to 0)	0.0 (0.0 to 0.0)	0 (0 to 0)	0.0 (0.0 to 0.0)	-40.5 (-58.3 to -16.8)	-0.1 (-30.1 to 39.7)

eTable 8. All-cause and cause-specific mortality (counts and rates) at the global level and by country in the WHO European Region for ages 5 to 9 and 10 to 14 for 1990, 2000, and 2016

Belarus	Maternal hemorrhage	10 to 14	Female	0 (0 to 0)	0.0 (0.0 to 0.0)	0 (0 to 0)	0.0 (0.0 to 0.0)	0 (0 to 0)	0.0 (0.0 to 0.0)	-40.5 (-58.3 to -16.8)	0.7 (-29.5 to 40.9)
Belarus	Maternal sepsis and other maternal infections	10 to 14	Both	0 (0 to 0)	0.0 (0.0 to 0.0)	0 (0 to 0)	0.0 (0.0 to 0.0)	0 (0 to 0)	0.0 (0.0 to 0.0)	-47.0 (-64.5 to -21.8)	-11.0 (-40.4 to 31.2)
Belarus	Maternal sepsis and other maternal infections	10 to 14	Female	0 (0 to 0)	0.0 (0.0 to 0.0)	0 (0 to 0)	0.0 (0.0 to 0.0)	0 (0 to 0)	0.0 (0.0 to 0.0)	-47.0 (-64.5 to -21.8)	-10.3 (-39.9 to 32.3)
Belarus	Maternal hypertensive disorders	10 to 14	Both	0 (0 to 0)	0.0 (0.0 to 0.0)	0 (0 to 0)	0.0 (0.0 to 0.0)	0 (0 to 0)	0.0 (0.0 to 0.0)	-45.5 (-61.1 to -25.6)	-8.4 (-34.7 to 24.9)
Belarus	Maternal hypertensive disorders	10 to 14	Female	0 (0 to 0)	0.0 (0.0 to 0.0)	0 (0 to 0)	0.0 (0.0 to 0.0)	0 (0 to 0)	0.0 (0.0 to 0.0)	-45.5 (-61.1 to -25.6)	-7.7 (-34.2 to 25.9)
Belarus	Maternal obstructed labor and uterine rupture	10 to 14	Both	0 (0 to 0)	0.0 (0.0 to 0.0)	0 (0 to 0)	0.0 (0.0 to 0.0)	0 (0 to 0)	0.0 (0.0 to 0.0)	-45.0 (-66.5 to -14.2)	-7.7 (-43.8 to 44.1)
Belarus	Maternal obstructed labor and uterine rupture	10 to 14	Female	0 (0 to 0)	0.0 (0.0 to 0.0)	0 (0 to 0)	0.0 (0.0 to 0.0)	0 (0 to 0)	0.0 (0.0 to 0.0)	-45.0 (-66.5 to -14.2)	-6.9 (-43.3 to 45.3)
Belarus	Maternal abortion, miscarriage, and ectopic pregnancy	10 to 14	Both	0 (0 to 0)	0.0 (0.0 to 0.0)	0 (0 to 0)	0.0 (0.0 to 0.0)	0 (0 to 0)	0.0 (0.0 to 0.0)	-45.3 (-60.8 to -24.0)	-8.1 (-34.3 to 27.6)
Belarus	Maternal abortion, miscarriage, and ectopic pregnancy	10 to 14	Female	0 (0 to 0)	0.0 (0.0 to 0.0)	0 (0 to 0)	0.0 (0.0 to 0.0)	0 (0 to 0)	0.0 (0.0 to 0.0)	-45.3 (-60.8 to -24.0)	-7.3 (-33.7 to 28.6)
Belarus	Indirect maternal deaths	10 to 14	Both	0 (0 to 0)	0.0 (0.0 to 0.0)	0 (0 to 0)	0.0 (0.0 to 0.0)	0 (0 to 0)	0.0 (0.0 to 0.0)	-44.9 (-60.2 to -25.3)	-7.5 (-33.1 to 25.3)
Belarus	Indirect maternal deaths	10 to 14	Female	0 (0 to 0)	0.0 (0.0 to 0.0)	0 (0 to 0)	0.0 (0.0 to 0.0)	0 (0 to 0)	0.0 (0.0 to 0.0)	-44.9 (-60.2 to -25.3)	-6.8 (-32.6 to 26.4)
Belarus	Late maternal deaths	10 to 14	Both	0 (0 to 0)	0.0 (0.0 to 0.0)	0 (0 to 0)	0.0 (0.0 to 0.0)	0 (0 to 0)	0.0 (0.0 to 0.0)	-46.4 (-63.4 to -16.8)	-9.9 (-38.5 to 39.8)
Belarus	Late maternal deaths	10 to 14	Female	0 (0 to 0)	0.0 (0.0 to 0.0)	0 (0 to 0)	0.0 (0.0 to 0.0)	0 (0 to 0)	0.0 (0.0 to 0.0)	-46.4 (-63.4 to -16.8)	-9.2 (-38.0 to 40.9)
Belarus	Maternal deaths aggravated by HIV/AIDS	10 to 14	Both	0 (0 to 0)	0.0 (0.0 to 0.0)	0 (0 to 0)	0.0 (0.0 to 0.0)	0 (0 to 0)	0.0 (0.0 to 0.0)	0.0 (0.0 to 0.0)	0.0 (0.0 to 0.0)
Belarus	Maternal deaths aggravated by HIV/AIDS	10 to 14	Female	0 (0 to 0)	0.0 (0.0 to 0.0)	0 (0 to 0)	0.0 (0.0 to 0.0)	0 (0 to 0)	0.0 (0.0 to 0.0)	0.0 (0.0 to 0.0)	0.0 (0.0 to 0.0)
Belarus	Other maternal disorders	10 to 14	Both	0 (0 to 0)	0.0 (0.0 to 0.0)	0 (0 to 0)	0.0 (0.0 to 0.0)	0 (0 to 0)	0.0 (0.0 to 0.0)	-44.7 (-61.2 to -24.2)	-7.2 (-34.8 to 27.3)
Belarus	Other maternal disorders	10 to 14	Female	0 (0 to 0)	0.0 (0.0 to 0.0)	0 (0 to 0)	0.0 (0.0 to 0.0)	0 (0 to 0)	0.0 (0.0 to 0.0)	-44.7 (-61.2 to -24.2)	-6.4 (-34.3 to 28.4)
Belarus	Protein-energy malnutrition	5 to 9	Both	1 (0 to 1)	0.1 (0.1 to 0.2)	0 (0 to 1)	0.1 (0.0 to 0.1)	0 (0 to 0)	0.0 (0.0 to 0.0)	-92.5 (-96.6 to -82.9)	-88.3 (-94.7 to -73.5)

eTable 8. All-cause and cause-specific mortality (counts and rates) at the global level and by country in the WHO European Region for ages 5 to 9 and 10 to 14 for 1990, 2000, and 2016

Belarus	Protein-energy malnutrition	5 to 9	Female	0 (0 to 1)	0.1 (0.0 to 0.2)	0 (0 to 0)	0.0 (0.0 to 0.1)	0 (0 to 0)	0.0 (0.0 to 0.0)	-92.9 (-97.8 to -65.1)	-88.9 (-96.5 to -45.4)
Belarus	Protein-energy malnutrition	5 to 9	Male	0 (0 to 1)	0.1 (0.0 to 0.2)	0 (0 to 1)	0.1 (0.0 to 0.2)	0 (0 to 0)	0.0 (0.0 to 0.0)	-92.1 (-97.7 to -52.4)	-87.9 (-96.4 to -26.9)
Belarus	Protein-energy malnutrition	10 to 14	Both	1 (0 to 1)	0.1 (0.1 to 0.1)	0 (0 to 1)	0.1 (0.0 to 0.1)	0 (0 to 0)	0.0 (0.0 to 0.0)	-91.9 (-95.8 to -84.9)	-86.3 (-92.9 to -74.6)
Belarus	Protein-energy malnutrition	10 to 14	Female	0 (0 to 1)	0.1 (0.0 to 0.1)	0 (0 to 0)	0.0 (0.0 to 0.1)	0 (0 to 0)	0.0 (0.0 to 0.0)	-92.8 (-97.0 to -80.3)	-87.8 (-94.9 to -66.6)
Belarus	Protein-energy malnutrition	10 to 14	Male	0 (0 to 1)	0.1 (0.0 to 0.2)	0 (0 to 0)	0.1 (0.0 to 0.1)	0 (0 to 0)	0.0 (0.0 to 0.0)	-91.1 (-96.6 to -74.3)	-85.1 (-94.3 to -57.3)
Belarus	Iodine deficiency	5 to 9	Both	0 (0 to 1)	0.0 (0.0 to 0.2)	0 (0 to 1)	0.0 (0.0 to 0.1)	0 (0 to 0)	0.0 (0.0 to 0.0)	-94.7 (-100.0 to 6.4e+16)	-91.8 (-100.0 to 1.0e+17)
Belarus	Iodine deficiency	5 to 9	Female	0 (0 to 1)	0.0 (0.0 to 0.3)	0 (0 to 0)	0.0 (0.0 to 0.1)	0 (0 to 0)	0.0 (0.0 to 0.0)	-95.3 (-100.0 to 4.8e+26)	-92.7 (-100.0 to 7.6e+26)
Belarus	Iodine deficiency	5 to 9	Male	0 (0 to 1)	0.0 (0.0 to 0.3)	0 (0 to 1)	0.0 (0.0 to 0.2)	0 (0 to 0)	0.0 (0.0 to 0.0)	-94.2 (-100.0 to 3.5e+25)	-91.0 (-100.0 to 5.4e+25)
Belarus	Iodine deficiency	10 to 14	Both	0 (0 to 1)	0.0 (0.0 to 0.1)	0 (0 to 0)	0.0 (0.0 to 0.1)	0 (0 to 0)	0.0 (0.0 to 0.0)	-95.9 (-100.0 to 3.9e+17)	-93.1 (-100.0 to 6.5e+17)
Belarus	Iodine deficiency	10 to 14	Female	0 (0 to 1)	0.0 (0.0 to 0.1)	0 (0 to 0)	0.0 (0.0 to 0.1)	0 (0 to 0)	0.0 (0.0 to 0.0)	-95.3 (-100.0 to 2.7e+25)	-92.1 (-100.0 to 4.6e+25)
Belarus	Iodine deficiency	10 to 14	Male	0 (0 to 1)	0.0 (0.0 to 0.2)	0 (0 to 0)	0.0 (0.0 to 0.1)	0 (0 to 0)	0.0 (0.0 to 0.0)	-96.3 (-100.0 to 1.5e+22)	-93.9 (-100.0 to 2.5e+22)
Belarus	Iron-deficiency anemia	5 to 9	Both	1 (0 to 1)	0.1 (0.0 to 0.1)	0 (0 to 0)	0.0 (0.0 to 0.1)	0 (0 to 0)	0.0 (0.0 to 0.0)	-91.0 (-96.0 to -79.5)	-86.1 (-93.8 to -68.2)
Belarus	Iron-deficiency anemia	5 to 9	Female	0 (0 to 1)	0.1 (0.0 to 0.2)	0 (0 to 0)	0.0 (0.0 to 0.1)	0 (0 to 0)	0.0 (0.0 to 0.0)	-92.9 (-97.8 to -64.2)	-88.9 (-96.5 to -43.9)
Belarus	Iron-deficiency anemia	5 to 9	Male	0 (0 to 1)	0.1 (0.0 to 0.2)	0 (0 to 0)	0.1 (0.0 to 0.1)	0 (0 to 0)	0.0 (0.0 to 0.0)	-88.7 (-96.6 to -29.6)	-82.6 (-94.8 to 8.1)
Belarus	Iron-deficiency anemia	10 to 14	Both	1 (0 to 1)	0.1 (0.1 to 0.1)	0 (0 to 1)	0.1 (0.0 to 0.1)	0 (0 to 0)	0.0 (0.0 to 0.0)	-91.8 (-95.7 to -84.9)	-86.3 (-92.8 to -74.6)
Belarus	Iron-deficiency anemia	10 to 14	Female	0 (0 to 1)	0.1 (0.0 to 0.1)	0 (0 to 0)	0.0 (0.0 to 0.1)	0 (0 to 0)	0.0 (0.0 to 0.0)	-92.8 (-97.0 to -80.3)	-87.8 (-94.9 to -66.6)
Belarus	Iron-deficiency anemia	10 to 14	Male	0 (0 to 1)	0.1 (0.0 to 0.2)	0 (0 to 0)	0.1 (0.0 to 0.1)	0 (0 to 0)	0.0 (0.0 to 0.0)	-91.0 (-96.5 to -74.3)	-85.0 (-94.2 to -57.3)
Belarus	Other nutritional deficiencies	5 to 9	Both	1 (0 to 1)	0.1 (0.0 to 0.1)	0 (0 to 0)	0.0 (0.0 to 0.1)	0 (0 to 0)	0.0 (0.0 to 0.0)	-91.0 (-96.0 to -79.5)	-86.1 (-93.8 to -68.2)
Belarus	Other nutritional deficiencies	5 to 9	Female	0 (0 to 1)	0.1 (0.0 to 0.2)	0 (0 to 0)	0.0 (0.0 to 0.1)	0 (0 to 0)	0.0 (0.0 to 0.0)	-92.9 (-97.8 to -64.2)	-88.9 (-96.5 to -43.9)
Belarus	Other nutritional deficiencies	5 to 9	Male	0 (0 to 1)	0.1 (0.0 to 0.2)	0 (0 to 0)	0.1 (0.0 to 0.1)	0 (0 to 0)	0.0 (0.0 to 0.0)	-88.7 (-96.6 to -29.6)	-82.6 (-94.8 to 8.1)
Belarus	Other nutritional deficiencies	10 to 14	Both	1 (0 to 1)	0.1 (0.1 to 0.1)	0 (0 to 1)	0.1 (0.0 to 0.1)	0 (0 to 0)	0.0 (0.0 to 0.0)	-91.8 (-95.7 to -84.9)	-86.3 (-92.8 to -74.6)
Belarus	Other nutritional deficiencies	10 to 14	Female	0 (0 to 1)	0.1 (0.0 to 0.1)	0 (0 to 0)	0.0 (0.0 to 0.1)	0 (0 to 0)	0.0 (0.0 to 0.0)	-92.8 (-97.0 to -80.3)	-87.8 (-94.9 to -66.6)
Belarus	Other nutritional deficiencies	10 to 14	Male	0 (0 to 1)	0.1 (0.0 to 0.2)	0 (0 to 0)	0.1 (0.0 to 0.1)	0 (0 to 0)	0.0 (0.0 to 0.0)	-91.0 (-96.5 to -74.3)	-85.0 (-94.2 to -57.3)

eTable 8. All-cause and cause-specific mortality (counts and rates) at the global level and by country in the WHO European Region for ages 5 to 9 and 10 to 14 for 1990, 2000, and 2016

Belarus	Sexually transmitted diseases excluding HIV	5 to 9	Both	0 (0 to 0)	0.0 (0.0 to 0.1)	0 (0 to 1)	0.0 (0.0 to 0.1)	0 (0 to 0)	0.0 (0.0 to 0.0)	-88.9 (-97.1 to -40.9)	-82.7 (-95.5 to -8.4)
Belarus	Sexually transmitted diseases excluding HIV	5 to 9	Female	0 (0 to 0)	0.0 (0.0 to 0.1)	0 (0 to 0)	0.1 (0.0 to 0.1)	0 (0 to 0)	0.0 (0.0 to 0.0)	-89.2 (-97.1 to -43.2)	-83.1 (-95.5 to -11.1)
Belarus	Sexually transmitted diseases excluding HIV	5 to 9	Male	0 (0 to 0)	0.0 (0.0 to 0.0)	0 (0 to 0)	0.0 (0.0 to 0.1)	0 (0 to 0)	0.0 (0.0 to 0.0)	-88.2 (-97.0 to -38.8)	-81.9 (-95.3 to -6.0)
Belarus	Sexually transmitted diseases excluding HIV	10 to 14	Both	0 (0 to 0)	0.0 (0.0 to 0.0)	0 (0 to 0)	0.0 (0.0 to 0.0)	0 (0 to 0)	0.0 (0.0 to 0.0)	-83.3 (-89.4 to -61.2)	-71.9 (-82.2 to -34.8)
Belarus	Sexually transmitted diseases excluding HIV	10 to 14	Female	0 (0 to 0)	0.0 (0.0 to 0.0)	0 (0 to 0)	0.0 (0.0 to 0.0)	0 (0 to 0)	0.0 (0.0 to 0.0)	-71.5 (-83.9 to -39.9)	-51.7 (-72.8 to 1.8)
Belarus	Sexually transmitted diseases excluding HIV	10 to 14	Male	0 (0 to 0)	0.0 (0.0 to 0.0)	0 (0 to 0)	0.0 (0.0 to 0.0)	0 (0 to 0)	0.0 (0.0 to 0.0)	-98.3 (-99.4 to -93.5)	-97.1 (-99.0 to -89.2)
Belarus	Hepatitis	5 to 9	Both	1 (1 to 2)	0.2 (0.1 to 0.2)	1 (0 to 1)	0.1 (0.1 to 0.2)	0 (0 to 1)	0.1 (0.0 to 0.1)	-71.6 (-86.4 to -35.7)	-56.0 (-78.9 to -0.4)
Belarus	Hepatitis	5 to 9	Female	1 (0 to 1)	0.1 (0.1 to 0.2)	0 (0 to 0)	0.1 (0.0 to 0.1)	0 (0 to 0)	0.0 (0.0 to 0.1)	-83.1 (-93.5 to -59.3)	-73.5 (-89.8 to -36.3)
Belarus	Hepatitis	5 to 9	Male	1 (0 to 1)	0.2 (0.1 to 0.3)	0 (0 to 1)	0.1 (0.1 to 0.2)	0 (0 to 1)	0.1 (0.0 to 0.2)	-64.0 (-87.2 to -2.6)	-44.8 (-80.4 to 49.5)
Belarus	Hepatitis	10 to 14	Both	1 (0 to 1)	0.1 (0.1 to 0.1)	1 (0 to 1)	0.1 (0.0 to 0.1)	0 (0 to 0)	0.0 (0.0 to 0.1)	-72.8 (-87.3 to -42.9)	-54.4 (-78.7 to -4.2)
Belarus	Hepatitis	10 to 14	Female	0 (0 to 1)	0.1 (0.1 to 0.2)	0 (0 to 1)	0.1 (0.0 to 0.2)	0 (0 to 0)	0.0 (0.0 to 0.1)	-81.9 (-93.6 to -56.7)	-69.4 (-89.2 to -26.7)
Belarus	Hepatitis	10 to 14	Male	0 (0 to 0)	0.1 (0.0 to 0.1)	0 (0 to 1)	0.1 (0.0 to 0.1)	0 (0 to 0)	0.0 (0.0 to 0.1)	-56.6 (-86.3 to 39.7)	-27.7 (-77.1 to 132.7)
Belarus	Other infectious diseases	5 to 9	Both	2 (1 to 4)	0.3 (0.1 to 0.5)	1 (1 to 2)	0.2 (0.1 to 0.4)	1 (0 to 2)	0.2 (0.1 to 0.3)	-58.2 (-82.7 to 4.6)	-35.2 (-73.2 to 62.1)
Belarus	Other infectious diseases	5 to 9	Female	1 (0 to 2)	0.2 (0.1 to 0.4)	0 (0 to 1)	0.2 (0.1 to 0.3)	0 (0 to 0)	0.1 (0.1 to 0.2)	-64.5 (-86.2 to -14.8)	-44.5 (-78.4 to 33.3)
Belarus	Other infectious diseases	5 to 9	Male	1 (0 to 3)	0.3 (0.1 to 0.7)	1 (0 to 2)	0.3 (0.1 to 0.6)	1 (0 to 1)	0.2 (0.1 to 0.5)	-54.1 (-86.5 to 66.0)	-29.5 (-79.3 to 154.8)
Belarus	Other infectious diseases	10 to 14	Both	1 (1 to 2)	0.2 (0.1 to 0.3)	1 (1 to 2)	0.1 (0.1 to 0.2)	0 (0 to 1)	0.1 (0.1 to 0.2)	-59.3 (-80.2 to -18.0)	-31.6 (-66.8 to 37.6)
Belarus	Other infectious diseases	10 to 14	Female	1 (0 to 1)	0.2 (0.1 to 0.3)	1 (0 to 1)	0.1 (0.1 to 0.3)	0 (0 to 0)	0.1 (0.0 to 0.2)	-61.5 (-83.6 to -9.1)	-34.8 (-72.3 to 53.8)
Belarus	Other infectious diseases	10 to 14	Male	1 (0 to 1)	0.2 (0.1 to 0.3)	1 (0 to 1)	0.1 (0.1 to 0.3)	0 (0 to 0)	0.1 (0.0 to 0.2)	-57.1 (-84.8 to 26.6)	-28.5 (-74.7 to 110.9)
Belarus	Nasopharynx cancer	5 to 9	Both	0 (0 to 0)	0.0 (0.0 to 0.0)	0 (0 to 0)	0.0 (0.0 to 0.0)	0 (0 to 0)	0.0 (0.0 to 0.0)	-43.8 (-67.5 to -7.5)	-12.9 (-49.6 to 43.3)
Belarus	Nasopharynx cancer	5 to 9	Female	0 (0 to 0)	0.0 (0.0 to 0.0)	0 (0 to 0)	0.0 (0.0 to 0.0)	0 (0 to 0)	0.0 (0.0 to 0.0)	-47.7 (-76.3 to 8.0)	-18.1 (-62.9 to 69.1)

eTable 8. All-cause and cause-specific mortality (counts and rates) at the global level and by country in the WHO European Region for ages 5 to 9 and 10 to 14 for 1990, 2000, and 2016

Belarus	Nasopharynx cancer	5 to 9	Male	0 (0 to 0)	0.0 (0.0 to 0.0)	0 (0 to 0)	0.0 (0.0 to 0.0)	0 (0 to 0)	0.0 (0.0 to 0.0)	-37.5 (-58.9 to -8.4)	-4.0 (-36.9 to 40.6)
Belarus	Nasopharynx cancer	10 to 14	Both	0 (0 to 0)	0.0 (0.0 to 0.0)	0 (0 to 0)	0.0 (0.0 to 0.0)	0 (0 to 0)	0.0 (0.0 to 0.0)	-50.3 (-61.7 to -35.2)	-16.6 (-35.7 to 8.7)
Belarus	Nasopharynx cancer	10 to 14	Female	0 (0 to 0)	0.0 (0.0 to 0.0)	0 (0 to 0)	0.0 (0.0 to 0.0)	0 (0 to 0)	0.0 (0.0 to 0.0)	-44.7 (-57.7 to -29.6)	-6.4 (-28.4 to 19.2)
Belarus	Nasopharynx cancer	10 to 14	Male	0 (0 to 0)	0.0 (0.0 to 0.0)	0 (0 to 0)	0.0 (0.0 to 0.0)	0 (0 to 0)	0.0 (0.0 to 0.0)	-54.7 (-70.1 to -31.2)	-24.6 (-50.2 to 14.6)
Belarus	Liver cancer	5 to 9	Both	0 (0 to 1)	0.1 (0.0 to 0.1)	0 (0 to 0)	0.0 (0.0 to 0.1)	0 (0 to 0)	0.0 (0.0 to 0.1)	-59.8 (-79.3 to -23.5)	-37.7 (-68.0 to 18.5)
Belarus	Liver cancer	5 to 9	Female	0 (0 to 1)	0.1 (0.0 to 0.2)	0 (0 to 0)	0.1 (0.0 to 0.1)	0 (0 to 0)	0.1 (0.0 to 0.1)	-60.4 (-81.9 to -13.0)	-38.0 (-71.6 to 36.2)
Belarus	Liver cancer	5 to 9	Male	0 (0 to 0)	0.0 (0.0 to 0.0)	0 (0 to 0)	0.0 (0.0 to 0.0)	0 (0 to 0)	0.0 (0.0 to 0.0)	-57.5 (-80.4 to -4.6)	-34.7 (-70.0 to 46.5)
Belarus	Liver cancer	10 to 14	Both	1 (0 to 1)	0.1 (0.1 to 0.2)	1 (0 to 1)	0.1 (0.1 to 0.1)	0 (0 to 1)	0.1 (0.1 to 0.1)	-48.5 (-70.9 to -9.7)	-13.5 (-51.1 to 51.7)
Belarus	Liver cancer	10 to 14	Female	0 (0 to 1)	0.1 (0.0 to 0.1)	0 (0 to 0)	0.1 (0.0 to 0.1)	0 (0 to 0)	0.1 (0.0 to 0.1)	-54.5 (-78.8 to 2.6)	-23.0 (-64.0 to 73.7)
Belarus	Liver cancer	10 to 14	Male	0 (0 to 1)	0.1 (0.1 to 0.2)	0 (0 to 1)	0.1 (0.1 to 0.2)	0 (0 to 0)	0.1 (0.1 to 0.2)	-43.4 (-74.6 to 20.9)	-5.8 (-57.7 to 101.3)
Belarus	Kidney cancer	5 to 9	Both	1 (1 to 2)	0.2 (0.1 to 0.3)	1 (1 to 1)	0.1 (0.1 to 0.2)	1 (0 to 1)	0.1 (0.1 to 0.2)	-46.1 (-71.2 to 4.3)	-16.5 (-55.4 to 61.7)
Belarus	Kidney cancer	5 to 9	Female	1 (0 to 1)	0.2 (0.1 to 0.3)	0 (0 to 1)	0.2 (0.1 to 0.2)	0 (0 to 1)	0.2 (0.1 to 0.3)	-45.4 (-74.8 to 23.0)	-14.6 (-60.5 to 92.5)
Belarus	Kidney cancer	5 to 9	Male	1 (0 to 1)	0.2 (0.1 to 0.3)	0 (0 to 1)	0.1 (0.1 to 0.2)	0 (0 to 1)	0.1 (0.1 to 0.2)	-46.9 (-77.4 to 22.2)	-18.6 (-65.3 to 87.5)
Belarus	Kidney cancer	10 to 14	Both	0 (0 to 1)	0.1 (0.0 to 0.1)	0 (0 to 1)	0.1 (0.0 to 0.1)	0 (0 to 0)	0.1 (0.0 to 0.1)	-47.4 (-69.4 to -8.8)	-11.7 (-48.7 to 53.1)
Belarus	Kidney cancer	10 to 14	Female	0 (0 to 0)	0.1 (0.0 to 0.1)	0 (0 to 0)	0.1 (0.0 to 0.1)	0 (0 to 0)	0.1 (0.0 to 0.1)	-44.8 (-72.7 to 11.8)	-6.5 (-53.8 to 89.3)
Belarus	Kidney cancer	10 to 14	Male	0 (0 to 0)	0.1 (0.0 to 0.1)	0 (0 to 0)	0.1 (0.0 to 0.1)	0 (0 to 0)	0.0 (0.0 to 0.1)	-50.2 (-75.3 to -1.9)	-17.0 (-58.8 to 63.4)
Belarus	Brain and nervous system cancer	5 to 9	Both	12 (8 to 18)	1.5 (1.0 to 2.3)	7 (5 to 10)	1.2 (0.9 to 1.6)	7 (4 to 9)	1.2 (0.8 to 1.8)	-47.3 (-72.9 to -6.1)	-18.3 (-57.9 to 45.5)
Belarus	Brain and nervous system cancer	5 to 9	Female	5 (3 to 9)	1.4 (0.8 to 2.2)	3 (2 to 5)	1.1 (0.7 to 1.6)	3 (2 to 4)	1.1 (0.7 to 1.7)	-49.7 (-76.4 to 0.1)	-21.3 (-63.1 to 56.8)
Belarus	Brain and nervous system cancer	5 to 9	Male	7 (4 to 11)	1.7 (1.0 to 2.8)	4 (3 to 6)	1.3 (0.9 to 2.0)	4 (2 to 6)	1.4 (0.8 to 2.2)	-45.4 (-74.5 to 20.5)	-16.1 (-60.9 to 85.0)
Belarus	Brain and nervous system cancer	10 to 14	Both	11 (8 to 16)	1.4 (1.0 to 2.1)	10 (8 to 13)	1.3 (1.0 to 1.7)	5 (3 to 7)	1.1 (0.8 to 1.6)	-52.6 (-72.9 to -24.7)	-20.5 (-54.5 to 26.3)
Belarus	Brain and nervous system cancer	10 to 14	Female	5 (3 to 8)	1.3 (0.8 to 2.1)	5 (3 to 7)	1.2 (0.8 to 1.7)	3 (2 to 4)	1.2 (0.7 to 1.8)	-47.5 (-73.3 to -3.5)	-11.1 (-54.9 to 63.3)
Belarus	Brain and nervous system cancer	10 to 14	Male	6 (4 to 9)	1.6 (1.0 to 2.4)	6 (4 to 8)	1.4 (0.9 to 1.9)	3 (2 to 4)	1.1 (0.7 to 1.7)	-56.9 (-77.7 to -20.4)	-28.3 (-62.9 to 32.5)
Belarus	Thyroid cancer	10 to 14	Both	0 (0 to 0)	0.0 (0.0 to 0.0)	0 (0 to 0)	0.0 (0.0 to 0.0)	0 (0 to 0)	0.0 (0.0 to 0.0)	-62.6 (-79.0 to -33.3)	-37.2 (-64.7 to 12.0)
Belarus	Thyroid cancer	10 to 14	Female	0 (0 to 0)	0.0 (0.0 to 0.0)	0 (0 to 0)	0.0 (0.0 to 0.0)	0 (0 to 0)	0.0 (0.0 to 0.0)	-56.3 (-77.3 to -17.0)	-26.1 (-61.6 to 40.6)
Belarus	Thyroid cancer	10 to 14	Male	0 (0 to 0)	0.0 (0.0 to 0.0)	0 (0 to 0)	0.0 (0.0 to 0.0)	0 (0 to 0)	0.0 (0.0 to 0.0)	-65.9 (-84.8 to -24.6)	-43.3 (-74.7 to 25.6)
Belarus	Hodgkin lymphoma	5 to 9	Both	1 (0 to 1)	0.1 (0.0 to 0.2)	0 (0 to 1)	0.1 (0.0 to 0.1)	0 (0 to 0)	0.0 (0.0 to 0.0)	-83.1 (-93.6 to -56.5)	-73.8 (-90.1 to -32.6)
Belarus	Hodgkin lymphoma	5 to 9	Female	0 (0 to 0)	0.0 (0.0 to 0.1)	0 (0 to 0)	0.0 (0.0 to 0.1)	0 (0 to 0)	0.0 (0.0 to 0.0)	-77.9 (-92.5 to -21.5)	-65.3 (-88.2 to 22.9)
Belarus	Hodgkin lymphoma	5 to 9	Male	1 (0 to 1)	0.1 (0.1 to 0.3)	0 (0 to 0)	0.1 (0.0 to 0.1)	0 (0 to 0)	0.0 (0.0 to 0.1)	-84.6 (-95.4 to -50.0)	-76.3 (-92.9 to -23.2)
Belarus	Hodgkin lymphoma	10 to 14	Both	1 (1 to 1)	0.1 (0.1 to 0.2)	1 (0 to 1)	0.1 (0.1 to 0.1)	0 (0 to 0)	0.0 (0.0 to 0.1)	-77.5 (-87.4 to -60.6)	-62.3 (-78.8 to -33.9)

eTable 8. All-cause and cause-specific mortality (counts and rates) at the global level and by country in the WHO European Region for ages 5 to 9 and 10 to 14 for 1990, 2000, and 2016

Belarus	Hodgkin lymphoma	10 to 14	Female	0 (0 to 0)	0.1 (0.0 to 0.1)	0 (0 to 0)	0.0 (0.0 to 0.1)	0 (0 to 0)	0.0 (0.0 to 0.0)	-77.2 (-90.6 to -43.4)	-61.4 (-84.2 to -4.2)
Belarus	Hodgkin lymphoma	10 to 14	Male	1 (0 to 1)	0.1 (0.1 to 0.2)	0 (0 to 1)	0.1 (0.1 to 0.2)	0 (0 to 0)	0.1 (0.0 to 0.1)	-77.7 (-89.3 to -55.6)	-62.8 (-82.1 to -26.0)
Belarus	Non-Hodgkin lymphoma	5 to 9	Both	3 (2 to 4)	0.3 (0.2 to 0.5)	2 (1 to 2)	0.3 (0.2 to 0.4)	1 (1 to 2)	0.3 (0.2 to 0.4)	-48.1 (-72.3 to -0.8)	-19.6 (-57.0 to 53.8)
Belarus	Non-Hodgkin lymphoma	5 to 9	Female	1 (0 to 1)	0.1 (0.1 to 0.2)	0 (0 to 1)	0.1 (0.1 to 0.2)	0 (0 to 0)	0.1 (0.1 to 0.2)	-45.8 (-75.1 to 21.7)	-15.2 (-61.0 to 90.5)
Belarus	Non-Hodgkin lymphoma	5 to 9	Male	2 (1 to 4)	0.5 (0.3 to 0.9)	1 (1 to 2)	0.5 (0.3 to 0.7)	1 (1 to 2)	0.4 (0.2 to 0.7)	-48.7 (-75.3 to 5.7)	-21.2 (-62.0 to 62.3)
Belarus	Non-Hodgkin lymphoma	10 to 14	Both	3 (2 to 4)	0.3 (0.2 to 0.5)	2 (2 to 4)	0.3 (0.2 to 0.4)	1 (1 to 2)	0.3 (0.2 to 0.4)	-51.1 (-73.9 to -6.1)	-17.9 (-56.1 to 57.7)
Belarus	Non-Hodgkin lymphoma	10 to 14	Female	0 (0 to 1)	0.1 (0.1 to 0.2)	0 (0 to 1)	0.1 (0.1 to 0.2)	0 (0 to 0)	0.1 (0.1 to 0.2)	-51.5 (-77.5 to 5.3)	-17.8 (-62.0 to 78.3)
Belarus	Non-Hodgkin lymphoma	10 to 14	Male	2 (1 to 3)	0.5 (0.3 to 0.8)	2 (1 to 3)	0.5 (0.3 to 0.8)	1 (1 to 2)	0.4 (0.3 to 0.7)	-51.0 (-76.2 to 10.1)	-18.4 (-60.4 to 83.3)
Belarus	Leukemia	5 to 9	Both	40 (31 to 51)	4.9 (3.8 to 6.3)	13 (9 to 16)	2.1 (1.6 to 2.7)	7 (5 to 10)	1.3 (0.9 to 1.8)	-82.9 (-89.2 to -74.2)	-73.6 (-83.3 to -60.0)
Belarus	Leukemia	5 to 9	Female	18 (13 to 24)	4.5 (3.2 to 5.9)	5 (3 to 7)	1.7 (1.2 to 2.5)	3 (2 to 5)	1.3 (0.8 to 2.0)	-81.6 (-89.4 to -68.7)	-71.1 (-83.4 to -51.0)
Belarus	Leukemia	5 to 9	Male	22 (16 to 30)	5.4 (3.9 to 7.3)	7 (5 to 10)	2.4 (1.7 to 3.4)	4 (2 to 6)	1.3 (0.8 to 2.1)	-84.1 (-91.4 to -71.8)	-75.5 (-86.8 to -56.7)
Belarus	Leukemia	10 to 14	Both	26 (21 to 32)	3.5 (2.8 to 4.3)	16 (13 to 20)	2.0 (1.6 to 2.5)	5 (3 to 6)	1.1 (0.7 to 1.4)	-82.2 (-88.2 to -74.1)	-70.1 (-80.2 to -56.5)
Belarus	Leukemia	10 to 14	Female	12 (9 to 16)	3.4 (2.5 to 4.4)	7 (5 to 10)	1.8 (1.3 to 2.5)	2 (1 to 3)	1.0 (0.6 to 1.4)	-82.7 (-90.1 to -71.4)	-70.7 (-83.2 to -51.5)
Belarus	Leukemia	10 to 14	Male	14 (10 to 19)	3.7 (2.7 to 5.0)	9 (6 to 12)	2.2 (1.6 to 3.0)	3 (2 to 4)	1.1 (0.7 to 1.8)	-81.8 (-90.0 to -68.6)	-69.7 (-83.4 to -47.7)
Belarus	Other neoplasms	5 to 9	Both	7 (4 to 10)	0.8 (0.5 to 1.2)	4 (3 to 6)	0.7 (0.5 to 0.9)	3 (2 to 5)	0.7 (0.4 to 0.9)	-48.2 (-71.3 to -7.1)	-19.7 (-55.6 to 44.0)
Belarus	Other neoplasms	5 to 9	Female	4 (2 to 6)	0.9 (0.5 to 1.4)	2 (1 to 3)	0.7 (0.5 to 1.1)	2 (1 to 3)	0.7 (0.4 to 1.1)	-50.6 (-74.1 to -0.6)	-22.7 (-59.5 to 55.5)
Belarus	Other neoplasms	5 to 9	Male	3 (2 to 5)	0.7 (0.4 to 1.2)	2 (1 to 3)	0.6 (0.4 to 0.9)	2 (1 to 3)	0.6 (0.4 to 0.9)	-45.3 (-73.8 to 20.0)	-16.0 (-59.7 to 84.2)
Belarus	Other neoplasms	10 to 14	Both	7 (5 to 10)	1.0 (0.7 to 1.3)	7 (5 to 9)	0.9 (0.6 to 1.1)	3 (2 to 4)	0.7 (0.5 to 0.9)	-57.5 (-74.5 to -28.9)	-28.7 (-57.2 to 19.3)
Belarus	Other neoplasms	10 to 14	Female	3 (2 to 5)	0.9 (0.6 to 1.4)	3 (2 to 5)	0.8 (0.5 to 1.2)	1 (1 to 2)	0.7 (0.4 to 1.0)	-57.4 (-77.7 to -17.2)	-28.0 (-62.2 to 40.2)
Belarus	Other neoplasms	10 to 14	Male	4 (2 to 5)	1.0 (0.6 to 1.5)	4 (2 to 5)	0.9 (0.6 to 1.3)	2 (1 to 2)	0.7 (0.4 to 1.1)	-57.6 (-78.0 to -18.1)	-29.4 (-63.3 to 36.4)
Belarus	Rheumatic heart disease	5 to 9	Both	1 (0 to 1)	0.1 (0.0 to 0.1)	0 (0 to 0)	0.0 (0.0 to 0.0)	0 (0 to 0)	0.0 (0.0 to 0.0)	-90.1 (-94.5 to -83.5)	-84.7 (-91.5 to -74.4)
Belarus	Rheumatic heart disease	5 to 9	Female	0 (0 to 0)	0.0 (0.0 to 0.1)	0 (0 to 0)	0.0 (0.0 to 0.0)	0 (0 to 0)	0.0 (0.0 to 0.0)	-88.3 (-94.0 to -79.6)	-81.7 (-90.7 to -68.1)
Belarus	Rheumatic heart disease	5 to 9	Male	0 (0 to 1)	0.1 (0.1 to 0.1)	0 (0 to 0)	0.0 (0.0 to 0.1)	0 (0 to 0)	0.0 (0.0 to 0.0)	-91.1 (-95.7 to -82.1)	-86.3 (-93.4 to -72.5)
Belarus	Rheumatic heart disease	10 to 14	Both	1 (1 to 1)	0.1 (0.1 to 0.2)	1 (0 to 1)	0.1 (0.1 to 0.1)	0 (0 to 0)	0.0 (0.0 to 0.0)	-89.2 (-93.2 to -83.8)	-81.9 (-88.7 to -72.9)
Belarus	Rheumatic heart disease	10 to 14	Female	0 (0 to 1)	0.1 (0.1 to 0.2)	0 (0 to 0)	0.1 (0.0 to 0.1)	0 (0 to 0)	0.0 (0.0 to 0.0)	-86.9 (-92.7 to -76.8)	-77.8 (-87.6 to -60.6)
Belarus	Rheumatic heart disease	10 to 14	Male	1 (0 to 1)	0.2 (0.1 to 0.2)	0 (0 to 0)	0.1 (0.1 to 0.1)	0 (0 to 0)	0.0 (0.0 to 0.0)	-90.8 (-94.9 to -84.0)	-84.7 (-91.6 to -73.3)
Belarus	Ischemic heart disease	5 to 9	Both	0 (0 to 0)	0.0 (0.0 to 0.0)	0 (0 to 0)	0.0 (0.0 to 0.0)	0 (0 to 0)	0.0 (0.0 to 0.0)	-49.6 (-71.9 to -12.0)	-21.9 (-56.5 to 36.4)
Belarus	Ischemic heart disease	5 to 9	Female	0 (0 to 0)	0.0 (0.0 to 0.0)	0 (0 to 0)	0.0 (0.0 to 0.0)	0 (0 to 0)	0.0 (0.0 to 0.0)	-62.7 (-81.7 to -27.4)	-41.6 (-71.4 to 13.7)
Belarus	Ischemic heart disease	5 to 9	Male	0 (0 to 0)	0.0 (0.0 to 0.0)	0 (0 to 0)	0.0 (0.0 to 0.0)	0 (0 to 0)	0.0 (0.0 to 0.0)	-24.6 (-61.1 to 39.1)	15.8 (-40.2 to 113.5)
Belarus	Ischemic heart disease	10 to 14	Both	0 (0 to 0)	0.0 (0.0 to 0.0)	0 (0 to 0)	0.0 (0.0 to 0.0)	0 (0 to 0)	0.0 (0.0 to 0.0)	-61.9 (-78.6 to -34.8)	-36.1 (-64.0 to 9.4)
Belarus	Ischemic heart disease	10 to 14	Female	0 (0 to 0)	0.0 (0.0 to 0.0)	0 (0 to 0)	0.0 (0.0 to 0.0)	0 (0 to 0)	0.0 (0.0 to 0.0)	-60.0 (-79.6 to -24.2)	-32.4 (-65.5 to 28.4)
Belarus	Ischemic heart disease	10 to 14	Male	0 (0 to 0)	0.0 (0.0 to 0.0)	0 (0 to 0)	0.0 (0.0 to 0.0)	0 (0 to 0)	0.0 (0.0 to 0.0)	-64.2 (-82.3 to -25.4)	-40.4 (-70.6 to 24.2)

eTable 8. All-cause and cause-specific mortality (counts and rates) at the global level and by country in the WHO European Region for ages 5 to 9 and 10 to 14 for 1990, 2000, and 2016

Belarus	Cerebrovascular disease	5 to 9	Both	1 (1 to 2)	0.1 (0.1 to 0.2)	1 (0 to 1)	0.1 (0.1 to 0.1)	0 (0 to 0)	0.0 (0.0 to 0.1)	-77.6 (-87.6 to -62.7)	-65.3 (-80.7 to -42.3)
Belarus	Cerebrovascular disease	5 to 9	Female	1 (0 to 1)	0.1 (0.1 to 0.2)	0 (0 to 0)	0.1 (0.1 to 0.1)	0 (0 to 0)	0.0 (0.0 to 0.1)	-77.4 (-87.7 to -60.7)	-64.6 (-80.7 to -38.4)
Belarus	Cerebrovascular disease	5 to 9	Male	1 (0 to 1)	0.1 (0.1 to 0.2)	0 (0 to 0)	0.1 (0.1 to 0.1)	0 (0 to 0)	0.1 (0.0 to 0.1)	-77.7 (-89.3 to -54.2)	-65.8 (-83.5 to -29.7)
Belarus	Cerebrovascular disease	10 to 14	Both	2 (1 to 2)	0.2 (0.2 to 0.3)	1 (1 to 2)	0.2 (0.1 to 0.2)	0 (0 to 1)	0.1 (0.1 to 0.1)	-76.6 (-85.0 to -64.1)	-60.7 (-74.8 to -39.7)
Belarus	Cerebrovascular disease	10 to 14	Female	1 (1 to 1)	0.3 (0.2 to 0.4)	1 (1 to 1)	0.2 (0.1 to 0.3)	0 (0 to 0)	0.1 (0.1 to 0.2)	-76.0 (-86.3 to -59.5)	-59.3 (-76.7 to -31.5)
Belarus	Cerebrovascular disease	10 to 14	Male	1 (0 to 1)	0.2 (0.1 to 0.2)	1 (0 to 1)	0.1 (0.1 to 0.2)	0 (0 to 0)	0.1 (0.0 to 0.1)	-77.5 (-87.4 to -61.1)	-62.6 (-79.0 to -35.3)
Belarus	Hypertensive heart disease	5 to 9	Both	0 (0 to 0)	0.0 (0.0 to 0.0)	0 (0 to 0)	0.0 (0.0 to 0.0)	0 (0 to 0)	0.0 (0.0 to 0.0)	-29.6 (-59.4 to 14.0)	9.0 (-37.0 to 76.7)
Belarus	Hypertensive heart disease	5 to 9	Female	0 (0 to 0)	0.0 (0.0 to 0.0)	0 (0 to 0)	0.0 (0.0 to 0.0)	0 (0 to 0)	0.0 (0.0 to 0.0)	-35.2 (-64.9 to 8.0)	1.5 (-45.1 to 69.1)
Belarus	Hypertensive heart disease	5 to 9	Male	0 (0 to 0)	0.0 (0.0 to 0.0)	0 (0 to 0)	0.0 (0.0 to 0.0)	0 (0 to 0)	0.0 (0.0 to 0.0)	-24.6 (-61.1 to 39.1)	15.8 (-40.2 to 113.5)
Belarus	Hypertensive heart disease	10 to 14	Both	0 (0 to 0)	0.0 (0.0 to 0.0)	0 (0 to 0)	0.0 (0.0 to 0.0)	0 (0 to 0)	0.0 (0.0 to 0.0)	-34.5 (-57.8 to -4.7)	10.0 (-29.1 to 60.0)
Belarus	Hypertensive heart disease	10 to 14	Female	0 (0 to 0)	0.0 (0.0 to 0.0)	0 (0 to 0)	0.0 (0.0 to 0.0)	0 (0 to 0)	0.0 (0.0 to 0.0)	-37.2 (-62.6 to 3.1)	6.3 (-36.7 to 74.6)
Belarus	Hypertensive heart disease	10 to 14	Male	0 (0 to 0)	0.0 (0.0 to 0.0)	0 (0 to 0)	0.0 (0.0 to 0.0)	0 (0 to 0)	0.0 (0.0 to 0.0)	-31.4 (-60.6 to 11.4)	14.2 (-34.4 to 85.5)
Belarus	Cardiomyopathy and myocarditis	5 to 9	Both	1 (0 to 1)	0.1 (0.1 to 0.2)	0 (0 to 1)	0.1 (0.0 to 0.1)	0 (0 to 0)	0.1 (0.0 to 0.1)	-66.3 (-80.2 to -29.7)	-47.7 (-69.4 to 9.0)
Belarus	Cardiomyopathy and myocarditis	5 to 9	Female	0 (0 to 1)	0.1 (0.0 to 0.2)	0 (0 to 0)	0.1 (0.0 to 0.1)	0 (0 to 0)	0.1 (0.0 to 0.1)	-70.0 (-84.3 to -29.5)	-53.0 (-75.5 to 10.3)
Belarus	Cardiomyopathy and myocarditis	5 to 9	Male	0 (0 to 1)	0.1 (0.0 to 0.2)	0 (0 to 0)	0.1 (0.0 to 0.1)	0 (0 to 0)	0.1 (0.0 to 0.1)	-62.5 (-82.0 to -12.0)	-42.4 (-72.4 to 35.1)
Belarus	Cardiomyopathy and myocarditis	10 to 14	Both	1 (1 to 1)	0.1 (0.1 to 0.2)	1 (1 to 1)	0.1 (0.1 to 0.2)	0 (0 to 1)	0.1 (0.1 to 0.1)	-64.7 (-77.7 to -37.3)	-40.7 (-62.6 to 5.2)
Belarus	Cardiomyopathy and myocarditis	10 to 14	Female	0 (0 to 1)	0.1 (0.1 to 0.2)	0 (0 to 1)	0.1 (0.1 to 0.2)	0 (0 to 0)	0.1 (0.0 to 0.1)	-67.6 (-82.6 to -29.1)	-45.1 (-70.5 to 20.1)
Belarus	Cardiomyopathy and myocarditis	10 to 14	Male	1 (0 to 1)	0.1 (0.1 to 0.2)	1 (0 to 1)	0.1 (0.1 to 0.2)	0 (0 to 0)	0.1 (0.1 to 0.1)	-62.1 (-78.2 to -27.4)	-36.9 (-63.7 to 20.9)
Belarus	Atrial fibrillation and flutter	5 to 9	Both	0 (0 to 0)	0.0 (0.0 to 0.0)	0 (0 to 0)	0.0 (0.0 to 0.0)	0 (0 to 0)	0.0 (0.0 to 0.0)	0.0 (0.0 to 0.0)	0.0 (0.0 to 0.0)
Belarus	Atrial fibrillation and flutter	5 to 9	Female	0 (0 to 0)	0.0 (0.0 to 0.0)	0 (0 to 0)	0.0 (0.0 to 0.0)	0 (0 to 0)	0.0 (0.0 to 0.0)	0.0 (0.0 to 0.0)	0.0 (0.0 to 0.0)
Belarus	Atrial fibrillation and flutter	5 to 9	Male	0 (0 to 0)	0.0 (0.0 to 0.0)	0 (0 to 0)	0.0 (0.0 to 0.0)	0 (0 to 0)	0.0 (0.0 to 0.0)	0.0 (0.0 to 0.0)	0.0 (0.0 to 0.0)
Belarus	Atrial fibrillation and flutter	10 to 14	Both	0 (0 to 0)	0.0 (0.0 to 0.0)	0 (0 to 0)	0.0 (0.0 to 0.0)	0 (0 to 0)	0.0 (0.0 to 0.0)	0.0 (0.0 to 0.0)	0.0 (0.0 to 0.0)
Belarus	Atrial fibrillation and flutter	10 to 14	Female	0 (0 to 0)	0.0 (0.0 to 0.0)	0 (0 to 0)	0.0 (0.0 to 0.0)	0 (0 to 0)	0.0 (0.0 to 0.0)	0.0 (0.0 to 0.0)	0.0 (0.0 to 0.0)
Belarus	Atrial fibrillation and flutter	10 to 14	Male	0 (0 to 0)	0.0 (0.0 to 0.0)	0 (0 to 0)	0.0 (0.0 to 0.0)	0 (0 to 0)	0.0 (0.0 to 0.0)	0.0 (0.0 to 0.0)	0.0 (0.0 to 0.0)
Belarus	Endocarditis	5 to 9	Both	0 (0 to 0)	0.0 (0.0 to 0.0)	0 (0 to 0)	0.0 (0.0 to 0.0)	0 (0 to 0)	0.0 (0.0 to 0.0)	-29.4 (-66.0 to 29.4)	9.4 (-47.4 to 100.5)
Belarus	Endocarditis	5 to 9	Female	0 (0 to 0)	0.0 (0.0 to 0.0)	0 (0 to 0)	0.0 (0.0 to 0.0)	0 (0 to 0)	0.0 (0.0 to 0.0)	-37.5 (-69.2 to 9.1)	-2.2 (-51.8 to 70.8)

eTable 8. All-cause and cause-specific mortality (counts and rates) at the global level and by country in the WHO European Region for ages 5 to 9 and 10 to 14 for 1990, 2000, and 2016

Belarus	Endocarditis	5 to 9	Male	0<br>(0 to 0)	0.0<br>(0.0 to 0.0)	0<br>(0 to 0)	0.0<br>(0.0 to 0.0)	0<br>(0 to 0)	0.0<br>(0.0 to 0.0)	-24.2<br>(-70.3 to 71.5)	16.4<br>(-54.4 to 163.3)
Belarus	Endocarditis	10 to 14	Both	0<br>(0 to 0)	0.0<br>(0.0 to 0.0)	0<br>(0 to 0)	0.0<br>(0.0 to 0.0)	0<br>(0 to 0)	0.0<br>(0.0 to 0.0)	-32.2<br>(-62.8 to 14.9)	13.9<br>(-37.5 to 92.8)
Belarus	Endocarditis	10 to 14	Female	0<br>(0 to 0)	0.0<br>(0.0 to 0.0)	0<br>(0 to 0)	0.0<br>(0.0 to 0.0)	0<br>(0 to 0)	0.0<br>(0.0 to 0.0)	-38.7<br>(-69.1 to 11.9)	3.8<br>(-47.7 to 89.4)
Belarus	Endocarditis	10 to 14	Male	0<br>(0 to 0)	0.0<br>(0.0 to 0.0)	0<br>(0 to 0)	0.0<br>(0.0 to 0.0)	0<br>(0 to 0)	0.0<br>(0.0 to 0.1)	-28.9<br>(-65.5 to 39.4)	18.4<br>(-42.6 to 132.1)
Belarus	Other cardiovascular and circulatory diseases	5 to 9	Both	0<br>(0 to 0)	0.0<br>(0.0 to 0.0)	0<br>(0 to 0)	0.0<br>(0.0 to 0.0)	0<br>(0 to 0)	0.0<br>(0.0 to 0.0)	-33.1<br>(-61.3 to 13.3)	3.7<br>(-39.9 to 75.5)
Belarus	Other cardiovascular and circulatory diseases	5 to 9	Female	0<br>(0 to 0)	0.0<br>(0.0 to 0.0)	0<br>(0 to 0)	0.0<br>(0.0 to 0.0)	0<br>(0 to 0)	0.0<br>(0.0 to 0.0)	-35.7<br>(-65.9 to 8.8)	0.6<br>(-46.6 to 70.3)
Belarus	Other cardiovascular and circulatory diseases	5 to 9	Male	0<br>(0 to 0)	0.0<br>(0.0 to 0.0)	0<br>(0 to 0)	0.0<br>(0.0 to 0.0)	0<br>(0 to 0)	0.0<br>(0.0 to 0.0)	-30.9<br>(-64.6 to 36.3)	6.0<br>(-45.6 to 109.2)
Belarus	Other cardiovascular and circulatory diseases	10 to 14	Both	0<br>(0 to 0)	0.0<br>(0.0 to 0.0)	0<br>(0 to 0)	0.0<br>(0.0 to 0.0)	0<br>(0 to 0)	0.0<br>(0.0 to 0.0)	-51.2<br>(-70.7 to -13.8)	-18.1<br>(-50.8 to 44.7)
Belarus	Other cardiovascular and circulatory diseases	10 to 14	Female	0<br>(0 to 0)	0.0<br>(0.0 to 0.0)	0<br>(0 to 0)	0.0<br>(0.0 to 0.0)	0<br>(0 to 0)	0.0<br>(0.0 to 0.0)	-48.9<br>(-73.3 to 6.4)	-13.5<br>(-54.9 to 80.2)
Belarus	Other cardiovascular and circulatory diseases	10 to 14	Male	0<br>(0 to 0)	0.0<br>(0.0 to 0.0)	0<br>(0 to 0)	0.0<br>(0.0 to 0.0)	0<br>(0 to 0)	0.0<br>(0.0 to 0.0)	-53.4<br>(-76.0 to -7.1)	-22.4<br>(-60.0 to 54.7)
Belarus	Chronic obstructive pulmonary disease	5 to 9	Both	1<br>(0 to 1)	0.1<br>(0.1 to 0.1)	0<br>(0 to 0)	0.0<br>(0.0 to 0.0)	0<br>(0 to 0)	0.0<br>(0.0 to 0.0)	-94.6<br>(-97.2 to -89.4)	-91.6<br>(-95.7 to -83.5)
Belarus	Chronic obstructive pulmonary disease	5 to 9	Female	0<br>(0 to 0)	0.1<br>(0.0 to 0.1)	0<br>(0 to 0)	0.0<br>(0.0 to 0.0)	0<br>(0 to 0)	0.0<br>(0.0 to 0.0)	-94.9<br>(-97.5 to -89.3)	-92.1<br>(-96.1 to -83.3)
Belarus	Chronic obstructive pulmonary disease	5 to 9	Male	0<br>(0 to 1)	0.1<br>(0.1 to 0.2)	0<br>(0 to 0)	0.0<br>(0.0 to 0.1)	0<br>(0 to 0)	0.0<br>(0.0 to 0.0)	-94.4<br>(-97.6 to -86.9)	-91.4<br>(-96.3 to -79.8)
Belarus	Chronic obstructive pulmonary disease	10 to 14	Both	1<br>(0 to 1)	0.1<br>(0.1 to 0.1)	0<br>(0 to 1)	0.1<br>(0.0 to 0.1)	0<br>(0 to 0)	0.0<br>(0.0 to 0.0)	-91.8<br>(-95.5 to -85.4)	-86.2<br>(-92.5 to -75.5)
Belarus	Chronic obstructive pulmonary disease	10 to 14	Female	0<br>(0 to 1)	0.1<br>(0.1 to 0.2)	0<br>(0 to 0)	0.1<br>(0.0 to 0.1)	0<br>(0 to 0)	0.0<br>(0.0 to 0.0)	-92.7<br>(-96.5 to -84.5)	-87.7<br>(-94.1 to -73.8)
Belarus	Chronic obstructive pulmonary disease	10 to 14	Male	0<br>(0 to 0)	0.1<br>(0.0 to 0.1)	0<br>(0 to 0)	0.0<br>(0.0 to 0.1)	0<br>(0 to 0)	0.0<br>(0.0 to 0.0)	-90.3<br>(-96.4 to -79.0)	-83.9<br>(-93.9 to -65.1)
Belarus	Asthma	5 to 9	Both	0<br>(0 to 1)	0.0<br>(0.0 to 0.1)	0<br>(0 to 0)	0.0<br>(0.0 to 0.0)	0<br>(0 to 0)	0.0<br>(0.0 to 0.0)	-94.2<br>(-96.8 to -89.5)	-91.1<br>(-95.0 to -83.8)

eTable 8. All-cause and cause-specific mortality (counts and rates) at the global level and by country in the WHO European Region for ages 5 to 9 and 10 to 14 for 1990, 2000, and 2016

Belarus	Asthma	5 to 9	Female	0 (0 to 0)	0.1 (0.0 to 0.1)	0 (0 to 0)	0.0 (0.0 to 0.0)	0 (0 to 0)	0.0 (0.0 to 0.0)	-94.8 (-97.4 to -88.8)	-91.9 (-95.9 to -82.5)
Belarus	Asthma	5 to 9	Male	0 (0 to 0)	0.0 (0.0 to 0.1)	0 (0 to 0)	0.0 (0.0 to 0.0)	0 (0 to 0)	0.0 (0.0 to 0.0)	-93.4 (-97.0 to -83.4)	-89.9 (-95.3 to -74.6)
Belarus	Asthma	10 to 14	Both	1 (1 to 2)	0.2 (0.1 to 0.3)	1 (1 to 1)	0.1 (0.1 to 0.1)	0 (0 to 0)	0.0 (0.0 to 0.0)	-95.1 (-97.0 to -92.0)	-91.8 (-95.0 to -86.6)
Belarus	Asthma	10 to 14	Female	1 (0 to 1)	0.2 (0.1 to 0.2)	0 (0 to 0)	0.1 (0.0 to 0.1)	0 (0 to 0)	0.0 (0.0 to 0.0)	-96.5 (-98.3 to -93.3)	-94.1 (-97.1 to -88.7)
Belarus	Asthma	10 to 14	Male	1 (1 to 1)	0.2 (0.2 to 0.3)	0 (0 to 1)	0.1 (0.1 to 0.2)	0 (0 to 0)	0.0 (0.0 to 0.0)	-94.2 (-96.8 to -89.0)	-90.3 (-94.6 to -81.6)
Belarus	Interstitial lung disease and pulmonary sarcoidosis	5 to 9	Both	0 (0 to 1)	0.1 (0.0 to 0.1)	0 (0 to 0)	0.0 (0.0 to 0.0)	0 (0 to 0)	0.0 (0.0 to 0.0)	-89.5 (-94.4 to -80.2)	-83.7 (-91.3 to -69.3)
Belarus	Interstitial lung disease and pulmonary sarcoidosis	5 to 9	Female	0 (0 to 0)	0.0 (0.0 to 0.1)	0 (0 to 0)	0.0 (0.0 to 0.0)	0 (0 to 0)	0.0 (0.0 to 0.0)	-92.0 (-96.7 to -82.0)	-87.5 (-94.8 to -71.8)
Belarus	Interstitial lung disease and pulmonary sarcoidosis	5 to 9	Male	0 (0 to 0)	0.1 (0.0 to 0.1)	0 (0 to 0)	0.0 (0.0 to 0.1)	0 (0 to 0)	0.0 (0.0 to 0.0)	-88.0 (-94.8 to -73.3)	-81.5 (-92.0 to -59.0)
Belarus	Interstitial lung disease and pulmonary sarcoidosis	10 to 14	Both	0 (0 to 0)	0.0 (0.0 to 0.0)	0 (0 to 0)	0.0 (0.0 to 0.0)	0 (0 to 0)	0.0 (0.0 to 0.0)	-84.5 (-91.7 to -70.1)	-74.0 (-86.1 to -49.8)
Belarus	Interstitial lung disease and pulmonary sarcoidosis	10 to 14	Female	0 (0 to 0)	0.0 (0.0 to 0.1)	0 (0 to 0)	0.0 (0.0 to 0.0)	0 (0 to 0)	0.0 (0.0 to 0.0)	-88.0 (-94.6 to -72.2)	-79.7 (-90.9 to -53.0)
Belarus	Interstitial lung disease and pulmonary sarcoidosis	10 to 14	Male	0 (0 to 0)	0.0 (0.0 to 0.0)	0 (0 to 0)	0.0 (0.0 to 0.0)	0 (0 to 0)	0.0 (0.0 to 0.0)	-78.7 (-92.1 to -41.4)	-64.5 (-86.8 to -2.3)
Belarus	Other chronic respiratory diseases	5 to 9	Both	0 (0 to 0)	0.0 (0.0 to 0.0)	0 (0 to 0)	0.0 (0.0 to 0.0)	0 (0 to 0)	0.0 (0.0 to 0.0)	-88.6 (-93.9 to -79.9)	-82.3 (-90.6 to -68.8)
Belarus	Other chronic respiratory diseases	5 to 9	Female	0 (0 to 0)	0.0 (0.0 to 0.0)	0 (0 to 0)	0.0 (0.0 to 0.0)	0 (0 to 0)	0.0 (0.0 to 0.0)	-90.3 (-94.9 to -80.2)	-84.8 (-92.0 to -69.1)
Belarus	Other chronic respiratory diseases	5 to 9	Male	0 (0 to 0)	0.0 (0.0 to 0.0)	0 (0 to 0)	0.0 (0.0 to 0.0)	0 (0 to 0)	0.0 (0.0 to 0.0)	-85.9 (-94.0 to -68.5)	-78.3 (-90.9 to -51.6)
Belarus	Other chronic respiratory diseases	10 to 14	Both	0 (0 to 0)	0.0 (0.0 to 0.0)	0 (0 to 0)	0.0 (0.0 to 0.0)	0 (0 to 0)	0.0 (0.0 to 0.0)	-85.0 (-92.2 to -72.9)	-74.8 (-86.9 to -54.6)
Belarus	Other chronic respiratory diseases	10 to 14	Female	0 (0 to 0)	0.0 (0.0 to 0.1)	0 (0 to 0)	0.0 (0.0 to 0.0)	0 (0 to 0)	0.0 (0.0 to 0.0)	-89.1 (-94.4 to -77.8)	-81.5 (-90.5 to -62.5)
Belarus	Other chronic respiratory diseases	10 to 14	Male	0 (0 to 0)	0.0 (0.0 to 0.0)	0 (0 to 0)	0.0 (0.0 to 0.0)	0 (0 to 0)	0.0 (0.0 to 0.0)	-77.6 (-91.9 to -50.0)	-62.8 (-86.5 to -16.7)
Belarus	Cirrhosis and other chronic liver diseases due to hepatitis B	5 to 9	Both	0 (0 to 0)	0.0 (0.0 to 0.0)	0 (0 to 0)	0.0 (0.0 to 0.0)	0 (0 to 0)	0.0 (0.0 to 0.0)	-33.9 (-60.3 to 7.2)	2.5 (-38.5 to 66.1)

eTable 8. All-cause and cause-specific mortality (counts and rates) at the global level and by country in the WHO European Region for ages 5 to 9 and 10 to 14 for 1990, 2000, and 2016

Belarus	Cirrhosis and other chronic liver diseases due to hepatitis B	5 to 9	Female	0 (0 to 0)	0.0 (0.0 to 0.0)	0 (0 to 0)	0.0 (0.0 to 0.0)	0 (0 to 0)	0.0 (0.0 to 0.0)	-27.8 (-58.5 to 19.6)	13.1 (-35.1 to 87.3)
Belarus	Cirrhosis and other chronic liver diseases due to hepatitis B	5 to 9	Male	0 (0 to 0)	0.0 (0.0 to 0.0)	0 (0 to 0)	0.0 (0.0 to 0.0)	0 (0 to 0)	0.0 (0.0 to 0.0)	-35.8 (-65.3 to 14.4)	-1.4 (-46.8 to 75.5)
Belarus	Cirrhosis and other chronic liver diseases due to hepatitis B	10 to 14	Both	0 (0 to 0)	0.0 (0.0 to 0.0)	0 (0 to 0)	0.0 (0.0 to 0.0)	0 (0 to 0)	0.0 (0.0 to 0.0)	-33.5 (-58.3 to 2.0)	11.7 (-29.9 to 71.2)
Belarus	Cirrhosis and other chronic liver diseases due to hepatitis B	10 to 14	Female	0 (0 to 0)	0.0 (0.0 to 0.0)	0 (0 to 0)	0.0 (0.0 to 0.0)	0 (0 to 0)	0.0 (0.0 to 0.0)	-26.4 (-59.5 to 26.4)	24.5 (-31.5 to 114.0)
Belarus	Cirrhosis and other chronic liver diseases due to hepatitis B	10 to 14	Male	0 (0 to 0)	0.0 (0.0 to 0.0)	0 (0 to 0)	0.0 (0.0 to 0.0)	0 (0 to 0)	0.0 (0.0 to 0.0)	-36.6 (-64.3 to 12.0)	5.6 (-40.5 to 86.6)
Belarus	Cirrhosis and other chronic liver diseases due to hepatitis C	5 to 9	Both	0 (0 to 0)	0.0 (0.0 to 0.0)	0 (0 to 0)	0.0 (0.0 to 0.0)	0 (0 to 0)	0.0 (0.0 to 0.0)	-33.2 (-60.0 to 6.8)	3.6 (-38.0 to 65.5)
Belarus	Cirrhosis and other chronic liver diseases due to hepatitis C	5 to 9	Female	0 (0 to 0)	0.0 (0.0 to 0.0)	0 (0 to 0)	0.0 (0.0 to 0.0)	0 (0 to 0)	0.0 (0.0 to 0.0)	-29.9 (-59.5 to 16.5)	9.7 (-36.5 to 82.4)
Belarus	Cirrhosis and other chronic liver diseases due to hepatitis C	5 to 9	Male	0 (0 to 0)	0.0 (0.0 to 0.0)	0 (0 to 0)	0.0 (0.0 to 0.0)	0 (0 to 0)	0.0 (0.0 to 0.0)	-34.3 (-64.6 to 16.8)	0.8 (-45.7 to 79.3)
Belarus	Cirrhosis and other chronic liver diseases due to hepatitis C	10 to 14	Both	0 (0 to 0)	0.0 (0.0 to 0.0)	0 (0 to 0)	0.0 (0.0 to 0.0)	0 (0 to 0)	0.0 (0.0 to 0.0)	-32.9 (-57.7 to 1.6)	12.7 (-28.9 to 70.6)
Belarus	Cirrhosis and other chronic liver diseases due to hepatitis C	10 to 14	Female	0 (0 to 0)	0.0 (0.0 to 0.0)	0 (0 to 0)	0.0 (0.0 to 0.0)	0 (0 to 0)	0.0 (0.0 to 0.0)	-28.6 (-60.6 to 22.7)	20.9 (-33.3 to 107.8)
Belarus	Cirrhosis and other chronic liver diseases due to hepatitis C	10 to 14	Male	0 (0 to 0)	0.0 (0.0 to 0.0)	0 (0 to 0)	0.0 (0.0 to 0.0)	0 (0 to 0)	0.0 (0.0 to 0.0)	-35.0 (-63.7 to 14.5)	8.2 (-39.6 to 90.6)
Belarus	Cirrhosis and other chronic liver diseases due to other causes	5 to 9	Both	1 (1 to 2)	0.1 (0.1 to 0.2)	1 (1 to 1)	0.1 (0.1 to 0.2)	1 (1 to 1)	0.2 (0.1 to 0.2)	-31.2 (-57.6 to 7.6)	6.6 (-34.4 to 66.8)
Belarus	Cirrhosis and other chronic liver diseases due to other causes	5 to 9	Female	1 (0 to 1)	0.1 (0.1 to 0.2)	0 (0 to 1)	0.1 (0.1 to 0.2)	0 (0 to 1)	0.2 (0.1 to 0.2)	-27.0 (-58.3 to 21.3)	14.2 (-34.7 to 89.9)

eTable 8. All-cause and cause-specific mortality (counts and rates) at the global level and by country in the WHO European Region for ages 5 to 9 and 10 to 14 for 1990, 2000, and 2016

Belarus	Cirrhosis and other chronic liver diseases due to other causes	5 to 9	Male	1 (0 to 1)	0.1 (0.1 to 0.2)	0 (0 to 1)	0.1 (0.1 to 0.2)	0 (0 to 1)	0.1 (0.1 to 0.2)	-35.4 (-65.2 to 14.1)	-0.8 (-46.6 to 75.1)
Belarus	Cirrhosis and other chronic liver diseases due to other causes	10 to 14	Both	1 (1 to 2)	0.2 (0.1 to 0.2)	1 (1 to 2)	0.2 (0.1 to 0.2)	1 (1 to 1)	0.2 (0.1 to 0.3)	-30.3 (-55.8 to 3.7)	17.0 (-25.8 to 74.1)
Belarus	Cirrhosis and other chronic liver diseases due to other causes	10 to 14	Female	1 (0 to 1)	0.2 (0.1 to 0.3)	1 (1 to 1)	0.2 (0.1 to 0.3)	1 (0 to 1)	0.2 (0.2 to 0.4)	-25.9 (-59.0 to 27.6)	25.4 (-30.7 to 116.0)
Belarus	Cirrhosis and other chronic liver diseases due to other causes	10 to 14	Male	0 (0 to 1)	0.1 (0.1 to 0.2)	1 (0 to 1)	0.1 (0.1 to 0.2)	0 (0 to 0)	0.1 (0.1 to 0.2)	-36.5 (-64.3 to 12.4)	5.7 (-40.5 to 87.1)
Belarus	Peptic ulcer disease	5 to 9	Both	0 (0 to 0)	0.0 (0.0 to 0.0)	0 (0 to 0)	0.0 (0.0 to 0.0)	0 (0 to 0)	0.0 (0.0 to 0.0)	-51.7 (-75.3 to -10.9)	-25.1 (-61.7 to 38.0)
Belarus	Peptic ulcer disease	5 to 9	Female	0 (0 to 0)	0.0 (0.0 to 0.1)	0 (0 to 0)	0.0 (0.0 to 0.0)	0 (0 to 0)	0.0 (0.0 to 0.0)	-57.9 (-83.8 to 5.2)	-34.1 (-74.6 to 64.7)
Belarus	Peptic ulcer disease	5 to 9	Male	0 (0 to 0)	0.0 (0.0 to 0.0)	0 (0 to 0)	0.0 (0.0 to 0.0)	0 (0 to 0)	0.0 (0.0 to 0.0)	-41.8 (-69.9 to 30.5)	-10.7 (-53.8 to 100.3)
Belarus	Peptic ulcer disease	10 to 14	Both	0 (0 to 0)	0.0 (0.0 to 0.1)	0 (0 to 0)	0.0 (0.0 to 0.0)	0 (0 to 0)	0.0 (0.0 to 0.0)	-63.8 (-82.4 to -30.7)	-39.3 (-70.4 to 16.4)
Belarus	Peptic ulcer disease	10 to 14	Female	0 (0 to 0)	0.1 (0.0 to 0.1)	0 (0 to 0)	0.0 (0.0 to 0.1)	0 (0 to 0)	0.0 (0.0 to 0.1)	-66.2 (-86.0 to -24.1)	-42.8 (-76.2 to 28.5)
Belarus	Peptic ulcer disease	10 to 14	Male	0 (0 to 0)	0.0 (0.0 to 0.0)	0 (0 to 0)	0.0 (0.0 to 0.0)	0 (0 to 0)	0.0 (0.0 to 0.0)	-54.3 (-75.9 to -5.6)	-23.8 (-59.9 to 57.1)
Belarus	Gastritis and duodenitis	5 to 9	Both	0 (0 to 0)	0.0 (0.0 to 0.0)	0 (0 to 0)	0.0 (0.0 to 0.0)	0 (0 to 0)	0.0 (0.0 to 0.0)	-45.3 (-69.6 to 4.1)	-15.2 (-53.0 to 61.4)
Belarus	Gastritis and duodenitis	5 to 9	Female	0 (0 to 0)	0.0 (0.0 to 0.0)	0 (0 to 0)	0.0 (0.0 to 0.0)	0 (0 to 0)	0.0 (0.0 to 0.0)	-46.7 (-76.8 to 31.6)	-16.5 (-63.6 to 106.0)
Belarus	Gastritis and duodenitis	5 to 9	Male	0 (0 to 0)	0.0 (0.0 to 0.0)	0 (0 to 0)	0.0 (0.0 to 0.0)	0 (0 to 0)	0.0 (0.0 to 0.0)	-44.3 (-73.1 to 25.1)	-14.5 (-58.7 to 92.0)
Belarus	Gastritis and duodenitis	10 to 14	Both	0 (0 to 0)	0.0 (0.0 to 0.0)	0 (0 to 0)	0.0 (0.0 to 0.0)	0 (0 to 0)	0.0 (0.0 to 0.0)	-50.3 (-68.5 to -19.5)	-16.6 (-47.2 to 35.1)
Belarus	Gastritis and duodenitis	10 to 14	Female	0 (0 to 0)	0.0 (0.0 to 0.0)	0 (0 to 0)	0.0 (0.0 to 0.0)	0 (0 to 0)	0.0 (0.0 to 0.0)	-46.8 (-71.2 to -0.2)	-9.9 (-51.3 to 68.9)
Belarus	Gastritis and duodenitis	10 to 14	Male	0 (0 to 0)	0.0 (0.0 to 0.0)	0 (0 to 0)	0.0 (0.0 to 0.0)	0 (0 to 0)	0.0 (0.0 to 0.0)	-54.3 (-75.9 to -5.6)	-23.8 (-59.9 to 57.1)
Belarus	Appendicitis	5 to 9	Both	2 (1 to 2)	0.2 (0.1 to 0.3)	0 (0 to 1)	0.1 (0.0 to 0.1)	0 (0 to 0)	0.1 (0.0 to 0.1)	-81.5 (-89.6 to -48.9)	-71.3 (-83.8 to -20.7)
Belarus	Appendicitis	5 to 9	Female	1 (0 to 1)	0.2 (0.0 to 0.3)	0 (0 to 0)	0.1 (0.0 to 0.1)	0 (0 to 0)	0.1 (0.0 to 0.1)	-80.6 (-90.9 to -50.3)	-69.7 (-85.8 to -22.3)
Belarus	Appendicitis	5 to 9	Male	1 (0 to 1)	0.2 (0.1 to 0.3)	0 (0 to 0)	0.1 (0.0 to 0.1)	0 (0 to 0)	0.1 (0.0 to 0.1)	-82.1 (-92.1 to -37.8)	-72.4 (-87.9 to -4.4)
Belarus	Appendicitis	10 to 14	Both	1 (0 to 1)	0.1 (0.1 to 0.2)	0 (0 to 1)	0.0 (0.0 to 0.1)	0 (0 to 0)	0.0 (0.0 to 0.1)	-81.4 (-89.7 to -56.9)	-68.8 (-82.7 to -27.7)
Belarus	Appendicitis	10 to 14	Female	1 (0 to 1)	0.2 (0.1 to 0.3)	0 (0 to 0)	0.1 (0.0 to 0.1)	0 (0 to 0)	0.1 (0.0 to 0.1)	-78.4 (-90.0 to -53.5)	-63.4 (-83.1 to -21.2)
Belarus	Appendicitis	10 to 14	Male	0 (0 to 1)	0.1 (0.1 to 0.2)	0 (0 to 0)	0.0 (0.0 to 0.1)	0 (0 to 0)	0.0 (0.0 to 0.1)	-85.1 (-93.4 to -47.7)	-75.2 (-89.0 to -12.8)
Belarus	Paralytic ileus and intestinal obstruction	5 to 9	Both	0 (0 to 1)	0.1 (0.0 to 0.1)	0 (0 to 0)	0.1 (0.0 to 0.1)	0 (0 to 0)	0.0 (0.0 to 0.1)	-45.2 (-69.1 to 4.8)	-15.1 (-52.1 to 62.4)
Belarus	Paralytic ileus and intestinal obstruction	5 to 9	Female	0 (0 to 1)	0.1 (0.0 to 0.1)	0 (0 to 0)	0.1 (0.0 to 0.1)	0 (0 to 0)	0.1 (0.0 to 0.1)	-45.9 (-74.8 to 44.0)	-15.4 (-60.5 to 125.5)
Belarus	Paralytic ileus and intestinal obstruction	5 to 9	Male	0 (0 to 0)	0.0 (0.0 to 0.1)	0 (0 to 0)	0.1 (0.0 to 0.1)	0 (0 to 0)	0.0 (0.0 to 0.1)	-44.3 (-74.0 to 32.9)	-14.5 (-60.0 to 104.0)

eTable 8. All-cause and cause-specific mortality (counts and rates) at the global level and by country in the WHO European Region for ages 5 to 9 and 10 to 14 for 1990, 2000, and 2016

Belarus	Paralytic ileus and intestinal obstruction	10 to 14	Both	1<br>(0 to 1)	0.1<br>(0.0 to 0.1)	1<br>(0 to 1)	0.1<br>(0.0 to 0.1)	0<br>(0 to 0)	0.1<br>(0.0 to 0.1)	-52.0<br>(-70.9 to -6.8)	-19.4<br>(-51.1 to 56.4)
Belarus	Paralytic ileus and intestinal obstruction	10 to 14	Female	0<br>(0 to 1)	0.1<br>(0.0 to 0.2)	0<br>(0 to 1)	0.1<br>(0.0 to 0.2)	0<br>(0 to 0)	0.1<br>(0.0 to 0.1)	-49.0<br>(-73.5 to 25.2)	-13.7<br>(-55.1 to 111.9)
Belarus	Paralytic ileus and intestinal obstruction	10 to 14	Male	0<br>(0 to 0)	0.1<br>(0.0 to 0.1)	0<br>(0 to 0)	0.1<br>(0.0 to 0.1)	0<br>(0 to 0)	0.0<br>(0.0 to 0.1)	-56.4<br>(-78.2 to -9.6)	-27.4<br>(-63.6 to 50.5)
Belarus	Inguinal, femoral, and abdominal hernia	5 to 9	Both	0<br>(0 to 0)	0.0<br>(0.0 to 0.0)	0<br>(0 to 0)	0.0<br>(0.0 to 0.0)	0<br>(0 to 0)	0.0<br>(0.0 to 0.0)	-79.3<br>(-88.7 to -62.8)	-68.0<br>(-82.5 to -42.4)
Belarus	Inguinal, femoral, and abdominal hernia	5 to 9	Female	0<br>(0 to 0)	0.0<br>(0.0 to 0.0)	0<br>(0 to 0)	0.0<br>(0.0 to 0.0)	0<br>(0 to 0)	0.0<br>(0.0 to 0.0)	-77.5<br>(-89.3 to -49.7)	-64.7<br>(-83.3 to -21.2)
Belarus	Inguinal, femoral, and abdominal hernia	5 to 9	Male	0<br>(0 to 0)	0.0<br>(0.0 to 0.0)	0<br>(0 to 0)	0.0<br>(0.0 to 0.0)	0<br>(0 to 0)	0.0<br>(0.0 to 0.0)	-81.8<br>(-92.3 to -53.7)	-72.0<br>(-88.1 to -29.0)
Belarus	Inguinal, femoral, and abdominal hernia	10 to 14	Both	0<br>(0 to 0)	0.0<br>(0.0 to 0.0)	0<br>(0 to 0)	0.0<br>(0.0 to 0.0)	0<br>(0 to 0)	0.0<br>(0.0 to 0.0)	-79.5<br>(-88.8 to -65.0)	-65.7<br>(-81.3 to -41.2)
Belarus	Inguinal, femoral, and abdominal hernia	10 to 14	Female	0<br>(0 to 0)	0.0<br>(0.0 to 0.0)	0<br>(0 to 0)	0.0<br>(0.0 to 0.0)	0<br>(0 to 0)	0.0<br>(0.0 to 0.0)	-77.3<br>(-89.0 to -56.1)	-61.6<br>(-81.4 to -25.7)
Belarus	Inguinal, femoral, and abdominal hernia	10 to 14	Male	0<br>(0 to 0)	0.0<br>(0.0 to 0.0)	0<br>(0 to 0)	0.0<br>(0.0 to 0.0)	0<br>(0 to 0)	0.0<br>(0.0 to 0.0)	-84.3<br>(-93.2 to -66.8)	-73.8<br>(-88.7 to -44.6)
Belarus	Inflammatory bowel disease	5 to 9	Both	0<br>(0 to 0)	0.0<br>(0.0 to 0.0)	0<br>(0 to 0)	0.0<br>(0.0 to 0.0)	0<br>(0 to 0)	0.0<br>(0.0 to 0.0)	-68.9<br>(-84.9 to -30.9)	-51.8<br>(-76.6 to 7.1)
Belarus	Inflammatory bowel disease	5 to 9	Female	0<br>(0 to 0)	0.0<br>(0.0 to 0.1)	0<br>(0 to 0)	0.0<br>(0.0 to 0.0)	0<br>(0 to 0)	0.0<br>(0.0 to 0.0)	-66.4<br>(-85.6 to -19.3)	-47.3<br>(-77.4 to 26.3)
Belarus	Inflammatory bowel disease	5 to 9	Male	0<br>(0 to 0)	0.0<br>(0.0 to 0.1)	0<br>(0 to 0)	0.0<br>(0.0 to 0.0)	0<br>(0 to 0)	0.0<br>(0.0 to 0.0)	-71.3<br>(-90.3 to -6.0)	-56.0<br>(-85.2 to 44.2)
Belarus	Inflammatory bowel disease	10 to 14	Both	0<br>(0 to 0)	0.0<br>(0.0 to 0.0)	0<br>(0 to 0)	0.0<br>(0.0 to 0.0)	0<br>(0 to 0)	0.0<br>(0.0 to 0.0)	-69.3<br>(-85.1 to -33.9)	-48.4<br>(-74.9 to 11.0)
Belarus	Inflammatory bowel disease	10 to 14	Female	0<br>(0 to 0)	0.0<br>(0.0 to 0.0)	0<br>(0 to 0)	0.0<br>(0.0 to 0.0)	0<br>(0 to 0)	0.0<br>(0.0 to 0.0)	-62.7<br>(-83.9 to -7.2)	-36.8<br>(-72.8 to 57.0)
Belarus	Inflammatory bowel disease	10 to 14	Male	0<br>(0 to 0)	0.0<br>(0.0 to 0.1)	0<br>(0 to 0)	0.0<br>(0.0 to 0.0)	0<br>(0 to 0)	0.0<br>(0.0 to 0.0)	-72.4<br>(-89.5 to -28.3)	-54.0<br>(-82.5 to 19.5)
Belarus	Vascular intestinal disorders	5 to 9	Both	0<br>(0 to 0)	0.0<br>(0.0 to 0.0)	0<br>(0 to 0)	0.0<br>(0.0 to 0.0)	0<br>(0 to 0)	0.0<br>(0.0 to 0.0)	-54.1<br>(-78.9 to 14.4)	-28.9<br>(-67.2 to 77.3)
Belarus	Vascular intestinal disorders	5 to 9	Female	0<br>(0 to 0)	0.0<br>(0.0 to 0.0)	0<br>(0 to 0)	0.0<br>(0.0 to 0.0)	0<br>(0 to 0)	0.0<br>(0.0 to 0.0)	-58.6<br>(-84.7 to 17.4)	-35.2<br>(-76.0 to 83.8)
Belarus	Vascular intestinal disorders	5 to 9	Male	0<br>(0 to 0)	0.0<br>(0.0 to 0.0)	0<br>(0 to 0)	0.0<br>(0.0 to 0.0)	0<br>(0 to 0)	0.0<br>(0.0 to 0.0)	-52.6<br>(-81.1 to 43.3)	-27.2<br>(-70.9 to 120.0)
Belarus	Vascular intestinal disorders	10 to 14	Both	0<br>(0 to 0)	0.0<br>(0.0 to 0.0)	0<br>(0 to 0)	0.0<br>(0.0 to 0.0)	0<br>(0 to 0)	0.0<br>(0.0 to 0.0)	-60.0<br>(-80.8 to -7.8)	-32.8<br>(-67.7 to 54.8)
Belarus	Vascular intestinal disorders	10 to 14	Female	0<br>(0 to 0)	0.0<br>(0.0 to 0.0)	0<br>(0 to 0)	0.0<br>(0.0 to 0.0)	0<br>(0 to 0)	0.0<br>(0.0 to 0.0)	-59.7<br>(-83.4 to 11.4)	-31.7<br>(-71.8 to 88.5)
Belarus	Vascular intestinal disorders	10 to 14	Male	0<br>(0 to 0)	0.0<br>(0.0 to 0.0)	0<br>(0 to 0)	0.0<br>(0.0 to 0.0)	0<br>(0 to 0)	0.0<br>(0.0 to 0.0)	-60.7<br>(-83.1 to 0.8)	-34.6<br>(-71.8 to 67.8)

eTable 8. All-cause and cause-specific mortality (counts and rates) at the global level and by country in the WHO European Region for ages 5 to 9 and 10 to 14 for 1990, 2000, and 2016

Belarus	Gallbladder and biliary diseases	5 to 9	Both	0 (0 to 0)	0.0 (0.0 to 0.0)	0 (0 to 0)	0.0 (0.0 to 0.0)	0 (0 to 0)	0.0 (0.0 to 0.0)	-85.8 (-93.6 to -66.3)	-78.0 (-90.2 to -47.7)
Belarus	Gallbladder and biliary diseases	5 to 9	Female	0 (0 to 0)	0.0 (0.0 to 0.0)	0 (0 to 0)	0.0 (0.0 to 0.0)	0 (0 to 0)	0.0 (0.0 to 0.0)	-79.9 (-92.7 to -46.9)	-68.5 (-88.6 to -16.8)
Belarus	Gallbladder and biliary diseases	5 to 9	Male	0 (0 to 0)	0.0 (0.0 to 0.1)	0 (0 to 0)	0.0 (0.0 to 0.0)	0 (0 to 0)	0.0 (0.0 to 0.0)	-86.2 (-94.2 to -64.7)	-78.8 (-91.1 to -45.8)
Belarus	Gallbladder and biliary diseases	10 to 14	Both	0 (0 to 0)	0.0 (0.0 to 0.0)	0 (0 to 0)	0.0 (0.0 to 0.0)	0 (0 to 0)	0.0 (0.0 to 0.0)	-80.7 (-90.0 to -64.8)	-67.6 (-83.2 to -40.9)
Belarus	Gallbladder and biliary diseases	10 to 14	Female	0 (0 to 0)	0.0 (0.0 to 0.0)	0 (0 to 0)	0.0 (0.0 to 0.0)	0 (0 to 0)	0.0 (0.0 to 0.0)	-80.3 (-91.1 to -57.9)	-66.6 (-84.9 to -28.7)
Belarus	Gallbladder and biliary diseases	10 to 14	Male	0 (0 to 0)	0.0 (0.0 to 0.0)	0 (0 to 0)	0.0 (0.0 to 0.0)	0 (0 to 0)	0.0 (0.0 to 0.0)	-81.1 (-92.7 to -51.7)	-68.5 (-87.8 to -19.6)
Belarus	Pancreatitis	5 to 9	Both	0 (0 to 0)	0.0 (0.0 to 0.0)	0 (0 to 0)	0.0 (0.0 to 0.0)	0 (0 to 0)	0.0 (0.0 to 0.0)	-64.5 (-84.5 to -12.7)	-45.0 (-76.0 to 35.3)
Belarus	Pancreatitis	5 to 9	Female	0 (0 to 0)	0.0 (0.0 to 0.0)	0 (0 to 0)	0.0 (0.0 to 0.0)	0 (0 to 0)	0.0 (0.0 to 0.0)	-67.4 (-85.6 to -9.3)	-49.0 (-77.5 to 42.0)
Belarus	Pancreatitis	5 to 9	Male	0 (0 to 0)	0.0 (0.0 to 0.0)	0 (0 to 0)	0.0 (0.0 to 0.0)	0 (0 to 0)	0.0 (0.0 to 0.0)	-63.9 (-86.3 to 4.4)	-44.6 (-79.0 to 60.3)
Belarus	Pancreatitis	10 to 14	Both	0 (0 to 0)	0.0 (0.0 to 0.0)	0 (0 to 0)	0.0 (0.0 to 0.0)	0 (0 to 0)	0.0 (0.0 to 0.0)	-67.8 (-83.8 to -36.3)	-46.0 (-72.8 to 6.9)
Belarus	Pancreatitis	10 to 14	Female	0 (0 to 0)	0.0 (0.0 to 0.0)	0 (0 to 0)	0.0 (0.0 to 0.0)	0 (0 to 0)	0.0 (0.0 to 0.0)	-67.2 (-86.9 to -19.0)	-44.4 (-77.8 to 37.1)
Belarus	Pancreatitis	10 to 14	Male	0 (0 to 0)	0.0 (0.0 to 0.0)	0 (0 to 0)	0.0 (0.0 to 0.0)	0 (0 to 0)	0.0 (0.0 to 0.0)	-68.5 (-87.8 to -24.9)	-47.6 (-79.7 to 25.1)
Belarus	Other digestive diseases	5 to 9	Both	0 (0 to 1)	0.1 (0.0 to 0.1)	0 (0 to 0)	0.0 (0.0 to 0.1)	0 (0 to 0)	0.0 (0.0 to 0.1)	-61.4 (-80.9 to -22.4)	-40.2 (-70.5 to 20.3)
Belarus	Other digestive diseases	5 to 9	Female	0 (0 to 0)	0.0 (0.0 to 0.1)	0 (0 to 0)	0.0 (0.0 to 0.1)	0 (0 to 0)	0.0 (0.0 to 0.1)	-53.4 (-82.8 to 29.5)	-27.0 (-73.1 to 102.8)
Belarus	Other digestive diseases	5 to 9	Male	0 (0 to 0)	0.1 (0.0 to 0.1)	0 (0 to 0)	0.1 (0.0 to 0.1)	0 (0 to 0)	0.0 (0.0 to 0.1)	-66.8 (-85.1 to -22.3)	-49.0 (-77.2 to 19.3)
Belarus	Other digestive diseases	10 to 14	Both	0 (0 to 0)	0.0 (0.0 to 0.1)	0 (0 to 0)	0.0 (0.0 to 0.1)	0 (0 to 0)	0.0 (0.0 to 0.0)	-65.5 (-82.7 to -34.6)	-42.1 (-71.0 to 9.8)
Belarus	Other digestive diseases	10 to 14	Female	0 (0 to 0)	0.0 (0.0 to 0.0)	0 (0 to 0)	0.0 (0.0 to 0.0)	0 (0 to 0)	0.0 (0.0 to 0.0)	-47.1 (-77.9 to 16.3)	-10.4 (-62.6 to 96.9)
Belarus	Other digestive diseases	10 to 14	Male	0 (0 to 0)	0.1 (0.0 to 0.1)	0 (0 to 0)	0.1 (0.0 to 0.1)	0 (0 to 0)	0.0 (0.0 to 0.1)	-71.1 (-87.2 to -38.9)	-51.9 (-78.6 to 1.7)
Belarus	Alzheimer disease and other dementias	5 to 9	Both	0 (0 to 0)	0.0 (0.0 to 0.0)	0 (0 to 0)	0.0 (0.0 to 0.0)	0 (0 to 0)	0.0 (0.0 to 0.0)	0.0 (0.0 to 0.0)	0.0 (0.0 to 0.0)
Belarus	Alzheimer disease and other dementias	5 to 9	Female	0 (0 to 0)	0.0 (0.0 to 0.0)	0 (0 to 0)	0.0 (0.0 to 0.0)	0 (0 to 0)	0.0 (0.0 to 0.0)	0.0 (0.0 to 0.0)	0.0 (0.0 to 0.0)
Belarus	Alzheimer disease and other dementias	5 to 9	Male	0 (0 to 0)	0.0 (0.0 to 0.0)	0 (0 to 0)	0.0 (0.0 to 0.0)	0 (0 to 0)	0.0 (0.0 to 0.0)	0.0 (0.0 to 0.0)	0.0 (0.0 to 0.0)
Belarus	Alzheimer disease and other dementias	10 to 14	Both	0 (0 to 0)	0.0 (0.0 to 0.0)	0 (0 to 0)	0.0 (0.0 to 0.0)	0 (0 to 0)	0.0 (0.0 to 0.0)	0.0 (0.0 to 0.0)	0.0 (0.0 to 0.0)

eTable 8. All-cause and cause-specific mortality (counts and rates) at the global level and by country in the WHO European Region for ages 5 to 9 and 10 to 14 for 1990, 2000, and 2016

Belarus	Alzheimer disease and other dementias	10 to 14	Female	0 (0 to 0)	0.0 (0.0 to 0.0)	0 (0 to 0)	0.0 (0.0 to 0.0)	0 (0 to 0)	0.0 (0.0 to 0.0)	0.0 (0.0 to 0.0)	0.0 (0.0 to 0.0)
Belarus	Alzheimer disease and other dementias	10 to 14	Male	0 (0 to 0)	0.0 (0.0 to 0.0)	0 (0 to 0)	0.0 (0.0 to 0.0)	0 (0 to 0)	0.0 (0.0 to 0.0)	0.0 (0.0 to 0.0)	0.0 (0.0 to 0.0)
Belarus	Parkinson disease	5 to 9	Both	0 (0 to 0)	0.0 (0.0 to 0.0)	0 (0 to 0)	0.0 (0.0 to 0.0)	0 (0 to 0)	0.0 (0.0 to 0.0)	0.0 (0.0 to 0.0)	0.0 (0.0 to 0.0)
Belarus	Parkinson disease	5 to 9	Female	0 (0 to 0)	0.0 (0.0 to 0.0)	0 (0 to 0)	0.0 (0.0 to 0.0)	0 (0 to 0)	0.0 (0.0 to 0.0)	0.0 (0.0 to 0.0)	0.0 (0.0 to 0.0)
Belarus	Parkinson disease	5 to 9	Male	0 (0 to 0)	0.0 (0.0 to 0.0)	0 (0 to 0)	0.0 (0.0 to 0.0)	0 (0 to 0)	0.0 (0.0 to 0.0)	0.0 (0.0 to 0.0)	0.0 (0.0 to 0.0)
Belarus	Parkinson disease	10 to 14	Both	0 (0 to 0)	0.0 (0.0 to 0.0)	0 (0 to 0)	0.0 (0.0 to 0.0)	0 (0 to 0)	0.0 (0.0 to 0.0)	0.0 (0.0 to 0.0)	0.0 (0.0 to 0.0)
Belarus	Parkinson disease	10 to 14	Female	0 (0 to 0)	0.0 (0.0 to 0.0)	0 (0 to 0)	0.0 (0.0 to 0.0)	0 (0 to 0)	0.0 (0.0 to 0.0)	0.0 (0.0 to 0.0)	0.0 (0.0 to 0.0)
Belarus	Parkinson disease	10 to 14	Male	0 (0 to 0)	0.0 (0.0 to 0.0)	0 (0 to 0)	0.0 (0.0 to 0.0)	0 (0 to 0)	0.0 (0.0 to 0.0)	0.0 (0.0 to 0.0)	0.0 (0.0 to 0.0)
Belarus	Epilepsy	5 to 9	Both	6 (4 to 8)	0.7 (0.5 to 1.0)	3 (2 to 4)	0.5 (0.3 to 0.6)	2 (1 to 3)	0.3 (0.2 to 0.6)	-71.7 (-84.0 to -46.8)	-56.2 (-75.2 to -17.5)
Belarus	Epilepsy	5 to 9	Female	3 (2 to 4)	0.6 (0.4 to 0.9)	1 (1 to 2)	0.4 (0.2 to 0.6)	1 (0 to 1)	0.3 (0.2 to 0.6)	-68.9 (-84.8 to -35.5)	-51.4 (-76.2 to 1.0)
Belarus	Epilepsy	5 to 9	Male	4 (2 to 5)	0.9 (0.6 to 1.2)	2 (1 to 2)	0.5 (0.3 to 0.7)	1 (1 to 2)	0.3 (0.2 to 0.6)	-73.7 (-87.1 to -42.8)	-59.7 (-80.2 to -12.2)
Belarus	Epilepsy	10 to 14	Both	7 (5 to 9)	0.9 (0.7 to 1.2)	5 (4 to 6)	0.6 (0.4 to 0.8)	2 (1 to 3)	0.4 (0.2 to 0.6)	-74.7 (-85.3 to -56.8)	-57.4 (-75.3 to -27.4)
Belarus	Epilepsy	10 to 14	Female	3 (2 to 5)	0.9 (0.7 to 1.3)	2 (2 to 3)	0.6 (0.4 to 0.8)	1 (1 to 2)	0.4 (0.2 to 0.8)	-72.2 (-85.3 to -47.8)	-52.9 (-75.2 to -11.7)
Belarus	Epilepsy	10 to 14	Male	3 (2 to 5)	0.9 (0.6 to 1.2)	2 (2 to 3)	0.6 (0.4 to 0.8)	1 (0 to 1)	0.3 (0.2 to 0.6)	-77.1 (-88.2 to -54.8)	-61.9 (-80.4 to -24.8)
Belarus	Motor neuron disease	5 to 9	Both	0 (0 to 0)	0.0 (0.0 to 0.0)	0 (0 to 0)	0.0 (0.0 to 0.0)	0 (0 to 0)	0.0 (0.0 to 0.0)	-70.4 (-82.5 to -49.8)	-54.1 (-72.9 to -22.2)
Belarus	Motor neuron disease	5 to 9	Female	0 (0 to 0)	0.0 (0.0 to 0.0)	0 (0 to 0)	0.0 (0.0 to 0.0)	0 (0 to 0)	0.0 (0.0 to 0.0)	-70.8 (-84.9 to -44.5)	-54.3 (-76.3 to -13.1)
Belarus	Motor neuron disease	5 to 9	Male	0 (0 to 0)	0.0 (0.0 to 0.0)	0 (0 to 0)	0.0 (0.0 to 0.0)	0 (0 to 0)	0.0 (0.0 to 0.0)	-69.8 (-84.6 to -41.4)	-53.6 (-76.4 to -10.1)
Belarus	Motor neuron disease	10 to 14	Both	0 (0 to 0)	0.0 (0.0 to 0.0)	0 (0 to 0)	0.0 (0.0 to 0.0)	0 (0 to 0)	0.0 (0.0 to 0.0)	-65.9 (-79.9 to -40.6)	-42.8 (-66.3 to -0.3)
Belarus	Motor neuron disease	10 to 14	Female	0 (0 to 0)	0.0 (0.0 to 0.0)	0 (0 to 0)	0.0 (0.0 to 0.0)	0 (0 to 0)	0.0 (0.0 to 0.0)	-66.3 (-79.1 to -45.6)	-42.9 (-64.7 to -7.9)
Belarus	Motor neuron disease	10 to 14	Male	0 (0 to 0)	0.0 (0.0 to 0.0)	0 (0 to 0)	0.0 (0.0 to 0.0)	0 (0 to 0)	0.0 (0.0 to 0.0)	-65.8 (-81.9 to -34.3)	-43.1 (-69.9 to 9.3)
Belarus	Other neurological disorders	5 to 9	Both	2 (1 to 4)	0.3 (0.2 to 0.4)	1 (1 to 2)	0.2 (0.1 to 0.3)	1 (1 to 1)	0.2 (0.1 to 0.3)	-53.7 (-74.9 to -14.4)	-28.2 (-61.2 to 32.6)
Belarus	Other neurological disorders	5 to 9	Female	1 (1 to 2)	0.3 (0.2 to 0.4)	1 (0 to 1)	0.2 (0.1 to 0.3)	0 (0 to 1)	0.2 (0.1 to 0.3)	-57.8 (-77.8 to -15.1)	-33.9 (-65.2 to 32.9)
Belarus	Other neurological disorders	5 to 9	Male	1 (1 to 2)	0.3 (0.1 to 0.6)	1 (0 to 1)	0.2 (0.1 to 0.4)	1 (0 to 1)	0.2 (0.1 to 0.3)	-49.8 (-79.1 to 19.3)	-23.0 (-67.9 to 83.1)
Belarus	Other neurological disorders	10 to 14	Both	3 (2 to 4)	0.4 (0.2 to 0.5)	3 (2 to 4)	0.3 (0.2 to 0.5)	1 (1 to 2)	0.3 (0.2 to 0.4)	-52.6 (-74.8 to -16.1)	-20.3 (-57.7 to 40.8)
Belarus	Other neurological disorders	10 to 14	Female	1 (0 to 1)	0.2 (0.1 to 0.3)	1 (0 to 1)	0.2 (0.1 to 0.3)	0 (0 to 1)	0.2 (0.1 to 0.3)	-54.6 (-76.9 to -8.0)	-23.2 (-61.0 to 55.7)
Belarus	Other neurological disorders	10 to 14	Male	2 (1 to 3)	0.5 (0.3 to 0.9)	2 (1 to 3)	0.5 (0.3 to 0.7)	1 (1 to 1)	0.4 (0.3 to 0.6)	-51.8 (-76.9 to 0.7)	-19.7 (-61.5 to 67.8)
Belarus	Drug use disorders	5 to 9	Both	0 (0 to 0)	0.0 (0.0 to 0.0)	0 (0 to 0)	0.0 (0.0 to 0.0)	0 (0 to 0)	0.0 (0.0 to 0.0)	0.0 (0.0 to 0.0)	0.0 (0.0 to 0.0)

eTable 8. All-cause and cause-specific mortality (counts and rates) at the global level and by country in the WHO European Region for ages 5 to 9 and 10 to 14 for 1990, 2000, and 2016

Belarus	Drug use disorders	5 to 9	Female	0<br>(0 to 0)	0.0<br>(0.0 to 0.0)	0<br>(0 to 0)	0.0<br>(0.0 to 0.0)	0<br>(0 to 0)	0.0<br>(0.0 to 0.0)	0.0<br>(0.0 to 0.0)	0.0<br>(0.0 to 0.0)
Belarus	Drug use disorders	5 to 9	Male	0<br>(0 to 0)	0.0<br>(0.0 to 0.0)	0<br>(0 to 0)	0.0<br>(0.0 to 0.0)	0<br>(0 to 0)	0.0<br>(0.0 to 0.0)	0.0<br>(0.0 to 0.0)	0.0<br>(0.0 to 0.0)
Belarus	Drug use disorders	10 to 14	Both	0<br>(0 to 0)	0.0<br>(0.0 to 0.0)	0<br>(0 to 0)	0.0<br>(0.0 to 0.0)	0<br>(0 to 0)	0.0<br>(0.0 to 0.0)	0.0<br>(0.0 to 0.0)	0.0<br>(0.0 to 0.0)
Belarus	Drug use disorders	10 to 14	Female	0<br>(0 to 0)	0.0<br>(0.0 to 0.0)	0<br>(0 to 0)	0.0<br>(0.0 to 0.0)	0<br>(0 to 0)	0.0<br>(0.0 to 0.0)	0.0<br>(0.0 to 0.0)	0.0<br>(0.0 to 0.0)
Belarus	Drug use disorders	10 to 14	Male	0<br>(0 to 0)	0.0<br>(0.0 to 0.0)	0<br>(0 to 0)	0.0<br>(0.0 to 0.0)	0<br>(0 to 0)	0.0<br>(0.0 to 0.0)	0.0<br>(0.0 to 0.0)	0.0<br>(0.0 to 0.0)
Belarus	Eating disorders	5 to 9	Both	0<br>(0 to 0)	0.0<br>(0.0 to 0.0)	0<br>(0 to 0)	0.0<br>(0.0 to 0.0)	0<br>(0 to 0)	0.0<br>(0.0 to 0.0)	-16.5<br>(-59.7 to 77.9)	29.4<br>(-37.6 to 175.7)
Belarus	Eating disorders	5 to 9	Female	0<br>(0 to 0)	0.0<br>(0.0 to 0.0)	0<br>(0 to 0)	0.0<br>(0.0 to 0.0)	0<br>(0 to 0)	0.0<br>(0.0 to 0.0)	-10.7<br>(-63.1 to 118.9)	39.8<br>(-42.2 to 242.6)
Belarus	Eating disorders	5 to 9	Male	0<br>(0 to 0)	0.0<br>(0.0 to 0.0)	0<br>(0 to 0)	0.0<br>(0.0 to 0.0)	0<br>(0 to 0)	0.0<br>(0.0 to 0.0)	-20.1<br>(-70.5 to 122.7)	22.6<br>(-54.7 to 241.8)
Belarus	Eating disorders	10 to 14	Both	0<br>(0 to 0)	0.0<br>(0.0 to 0.0)	0<br>(0 to 0)	0.0<br>(0.0 to 0.0)	0<br>(0 to 0)	0.0<br>(0.0 to 0.0)	-33.2<br>(-65.6 to 38.3)	12.1<br>(-42.3 to 132.1)
Belarus	Eating disorders	10 to 14	Female	0<br>(0 to 0)	0.0<br>(0.0 to 0.0)	0<br>(0 to 0)	0.0<br>(0.0 to 0.0)	0<br>(0 to 0)	0.0<br>(0.0 to 0.0)	-34.8<br>(-72.2 to 63.4)	10.3<br>(-52.9 to 176.6)
Belarus	Eating disorders	10 to 14	Male	0<br>(0 to 0)	0.0<br>(0.0 to 0.0)	0<br>(0 to 0)	0.0<br>(0.0 to 0.0)	0<br>(0 to 0)	0.0<br>(0.0 to 0.0)	-29.1<br>(-74.3 to 87.9)	18.0<br>(-57.2 to 212.9)
Belarus	Diabetes mellitus	5 to 9	Both	1<br>(1 to 1)	0.1<br>(0.1 to 0.1)	0<br>(0 to 0)	0.0<br>(0.0 to 0.1)	0<br>(0 to 0)	0.0<br>(0.0 to 0.0)	-86.9<br>(-91.8 to -79.8)	-79.7<br>(-87.3 to -68.7)
Belarus	Diabetes mellitus	5 to 9	Female	0<br>(0 to 0)	0.1<br>(0.1 to 0.1)	0<br>(0 to 0)	0.0<br>(0.0 to 0.1)	0<br>(0 to 0)	0.0<br>(0.0 to 0.0)	-87.8<br>(-92.8 to -80.8)	-80.9<br>(-88.7 to -69.9)
Belarus	Diabetes mellitus	5 to 9	Male	0<br>(0 to 1)	0.1<br>(0.1 to 0.1)	0<br>(0 to 0)	0.1<br>(0.0 to 0.1)	0<br>(0 to 0)	0.0<br>(0.0 to 0.0)	-86.2<br>(-92.5 to -74.8)	-78.8<br>(-88.4 to -61.2)
Belarus	Diabetes mellitus	10 to 14	Both	1<br>(1 to 1)	0.1<br>(0.1 to 0.2)	1<br>(1 to 1)	0.1<br>(0.1 to 0.1)	0<br>(0 to 0)	0.0<br>(0.0 to 0.0)	-87.1<br>(-91.7 to -80.9)	-78.3<br>(-86.0 to -68.0)
Belarus	Diabetes mellitus	10 to 14	Female	1<br>(1 to 1)	0.2<br>(0.1 to 0.3)	1<br>(0 to 1)	0.1<br>(0.1 to 0.2)	0<br>(0 to 0)	0.0<br>(0.0 to 0.1)	-87.2<br>(-92.6 to -78.6)	-78.3<br>(-87.6 to -63.7)
Belarus	Diabetes mellitus	10 to 14	Male	0<br>(0 to 0)	0.1<br>(0.1 to 0.1)	0<br>(0 to 0)	0.1<br>(0.0 to 0.1)	0<br>(0 to 0)	0.0<br>(0.0 to 0.0)	-86.9<br>(-92.8 to -78.2)	-78.2<br>(-88.0 to -63.6)
Belarus	Acute glomerulonephritis	5 to 9	Both	0<br>(0 to 0)	0.0<br>(0.0 to 0.0)	0<br>(0 to 0)	0.0<br>(0.0 to 0.0)	0<br>(0 to 0)	0.0<br>(0.0 to 0.0)	-38.2<br>(-56.3 to -14.2)	-4.2<br>(-32.3 to 33.0)
Belarus	Acute glomerulonephritis	5 to 9	Female	0<br>(0 to 0)	0.0<br>(0.0 to 0.0)	0<br>(0 to 0)	0.0<br>(0.0 to 0.0)	0<br>(0 to 0)	0.0<br>(0.0 to 0.0)	-41.7<br>(-58.9 to -19.4)	-8.7<br>(-35.6 to 26.2)
Belarus	Acute glomerulonephritis	5 to 9	Male	0<br>(0 to 0)	0.0<br>(0.0 to 0.0)	0<br>(0 to 0)	0.0<br>(0.0 to 0.0)	0<br>(0 to 0)	0.0<br>(0.0 to 0.0)	-34.8<br>(-54.9 to -6.8)	0.1<br>(-30.7 to 43.1)
Belarus	Acute glomerulonephritis	10 to 14	Both	0<br>(0 to 0)	0.0<br>(0.0 to 0.0)	0<br>(0 to 0)	0.0<br>(0.0 to 0.0)	0<br>(0 to 0)	0.0<br>(0.0 to 0.0)	-58.1<br>(-84.9 to -25.7)	-29.7<br>(-74.7 to 24.7)
Belarus	Acute glomerulonephritis	10 to 14	Female	0<br>(0 to 0)	0.0<br>(0.0 to 0.1)	0<br>(0 to 0)	0.0<br>(0.0 to 0.1)	0<br>(0 to 0)	0.0<br>(0.0 to 0.0)	-67.1<br>(-91.0 to -18.7)	-44.4<br>(-84.7 to 37.6)
Belarus	Acute glomerulonephritis	10 to 14	Male	0<br>(0 to 0)	0.0<br>(0.0 to 0.0)	0<br>(0 to 0)	0.0<br>(0.0 to 0.0)	0<br>(0 to 0)	0.0<br>(0.0 to 0.0)	-42.2<br>(-55.9 to -24.8)	-3.8<br>(-26.6 to 25.3)
Belarus	Chronic kidney disease	5 to 9	Both	1<br>(1 to 2)	0.2<br>(0.1 to 0.2)	1<br>(0 to 1)	0.1<br>(0.1 to 0.1)	0<br>(0 to 0)	0.1<br>(0.0 to 0.1)	-74.8<br>(-84.0 to -60.5)	-60.9<br>(-75.2 to -38.7)
Belarus	Chronic kidney disease	5 to 9	Female	1<br>(0 to 1)	0.2<br>(0.1 to 0.2)	0<br>(0 to 0)	0.1<br>(0.1 to 0.1)	0<br>(0 to 0)	0.1<br>(0.0 to 0.1)	-73.7<br>(-84.8 to -56.8)	-58.9<br>(-76.1 to -32.4)
Belarus	Chronic kidney disease	5 to 9	Male	1<br>(0 to 1)	0.2<br>(0.1 to 0.2)	0<br>(0 to 0)	0.1<br>(0.1 to 0.1)	0<br>(0 to 0)	0.1<br>(0.0 to 0.1)	-75.8<br>(-86.3 to -58.5)	-62.9<br>(-78.9 to -36.3)
Belarus	Chronic kidney disease	10 to 14	Both	2<br>(2 to 2)	0.3<br>(0.2 to 0.3)	1<br>(1 to 2)	0.2<br>(0.1 to 0.2)	1<br>(0 to 1)	0.1<br>(0.1 to 0.1)	-74.5<br>(-82.2 to -63.2)	-57.2<br>(-70.1 to -38.3)
Belarus	Chronic kidney disease	10 to 14	Female	1<br>(1 to 1)	0.3<br>(0.2 to 0.4)	1<br>(0 to 1)	0.2<br>(0.1 to 0.2)	0<br>(0 to 0)	0.1<br>(0.1 to 0.2)	-73.2<br>(-83.4 to -57.3)	-54.7<br>(-72.0 to -27.8)
Belarus	Chronic kidney disease	10 to 14	Male	1<br>(1 to 1)	0.2<br>(0.2 to 0.3)	1<br>(0 to 1)	0.1<br>(0.1 to 0.2)	0<br>(0 to 0)	0.1<br>(0.1 to 0.1)	-76.0<br>(-84.7 to -61.7)	-60.0<br>(-74.5 to -36.1)

eTable 8. All-cause and cause-specific mortality (counts and rates) at the global level and by country in the WHO European Region for ages 5 to 9 and 10 to 14 for 1990, 2000, and 2016

Belarus	Urinary diseases and male infertility	5 to 9	Both	1 (1 to 2)	0.2 (0.1 to 0.2)	1 (0 to 1)	0.1 (0.1 to 0.1)	0 (0 to 0)	0.0 (0.0 to 0.1)	-83.0 (-90.3 to -70.5)	-73.6 (-84.9 to -54.3)
Belarus	Urinary diseases and male infertility	5 to 9	Female	1 (0 to 1)	0.1 (0.1 to 0.2)	0 (0 to 0)	0.1 (0.1 to 0.1)	0 (0 to 0)	0.0 (0.0 to 0.1)	-81.1 (-89.2 to -65.3)	-70.3 (-83.1 to -45.7)
Belarus	Urinary diseases and male infertility	5 to 9	Male	1 (0 to 1)	0.2 (0.1 to 0.3)	0 (0 to 0)	0.1 (0.1 to 0.2)	0 (0 to 0)	0.0 (0.0 to 0.1)	-84.4 (-93.1 to -66.0)	-76.0 (-89.3 to -47.9)
Belarus	Urinary diseases and male infertility	10 to 14	Both	1 (1 to 2)	0.2 (0.1 to 0.2)	1 (1 to 1)	0.1 (0.1 to 0.2)	0 (0 to 0)	0.1 (0.0 to 0.1)	-82.0 (-87.8 to -73.9)	-69.7 (-79.5 to -56.2)
Belarus	Urinary diseases and male infertility	10 to 14	Female	1 (0 to 1)	0.2 (0.1 to 0.3)	1 (0 to 1)	0.1 (0.1 to 0.2)	0 (0 to 0)	0.1 (0.0 to 0.1)	-80.1 (-88.2 to -66.4)	-66.3 (-80.1 to -43.2)
Belarus	Urinary diseases and male infertility	10 to 14	Male	1 (0 to 1)	0.2 (0.1 to 0.2)	1 (0 to 1)	0.1 (0.1 to 0.2)	0 (0 to 0)	0.0 (0.0 to 0.1)	-84.0 (-90.5 to -73.2)	-73.3 (-84.2 to -55.4)
Belarus	Hemoglobinopathies and hemolytic anemias	5 to 9	Both	3 (2 to 5)	0.4 (0.3 to 0.6)	2 (1 to 2)	0.3 (0.2 to 0.4)	1 (1 to 1)	0.1 (0.1 to 0.2)	-75.8 (-85.3 to -60.1)	-62.5 (-77.2 to -38.2)
Belarus	Hemoglobinopathies and hemolytic anemias	5 to 9	Female	2 (1 to 3)	0.4 (0.2 to 0.7)	1 (0 to 1)	0.3 (0.2 to 0.5)	0 (0 to 1)	0.2 (0.1 to 0.3)	-75.2 (-87.1 to -48.9)	-61.2 (-79.8 to -20.1)
Belarus	Hemoglobinopathies and hemolytic anemias	5 to 9	Male	2 (1 to 2)	0.4 (0.2 to 0.6)	1 (0 to 1)	0.3 (0.2 to 0.4)	0 (0 to 1)	0.1 (0.1 to 0.2)	-76.4 (-87.4 to -57.8)	-63.8 (-80.7 to -35.3)
Belarus	Hemoglobinopathies and hemolytic anemias	10 to 14	Both	2 (1 to 2)	0.2 (0.2 to 0.3)	1 (1 to 2)	0.2 (0.1 to 0.3)	0 (0 to 1)	0.1 (0.1 to 0.1)	-76.1 (-84.6 to -62.7)	-59.9 (-74.1 to -37.4)
Belarus	Hemoglobinopathies and hemolytic anemias	10 to 14	Female	1 (0 to 1)	0.1 (0.1 to 0.2)	0 (0 to 1)	0.1 (0.1 to 0.2)	0 (0 to 0)	0.1 (0.0 to 0.1)	-73.9 (-85.8 to -51.8)	-55.9 (-76.0 to -18.4)
Belarus	Hemoglobinopathies and hemolytic anemias	10 to 14	Male	1 (1 to 2)	0.3 (0.2 to 0.5)	1 (1 to 2)	0.3 (0.2 to 0.4)	0 (0 to 0)	0.1 (0.1 to 0.2)	-77.0 (-86.8 to -59.1)	-61.7 (-78.0 to -32.0)
Belarus	Endocrine, metabolic, blood, and immune disorders	5 to 9	Both	1 (1 to 2)	0.2 (0.1 to 0.2)	1 (1 to 1)	0.1 (0.1 to 0.2)	1 (1 to 2)	0.2 (0.1 to 0.3)	-10.9 (-63.2 to 57.5)	38.1 (-43.0 to 144.1)
Belarus	Endocrine, metabolic, blood, and immune disorders	5 to 9	Female	1 (0 to 1)	0.1 (0.1 to 0.2)	0 (0 to 0)	0.1 (0.1 to 0.2)	0 (0 to 1)	0.2 (0.1 to 0.3)	-27.3 (-68.0 to 37.7)	13.8 (-49.9 to 115.6)
Belarus	Endocrine, metabolic, blood, and immune disorders	5 to 9	Male	1 (0 to 1)	0.2 (0.1 to 0.3)	0 (0 to 1)	0.1 (0.1 to 0.3)	1 (0 to 1)	0.3 (0.1 to 0.5)	3.1 (-62.8 to 119.1)	58.3 (-42.9 to 236.2)
Belarus	Endocrine, metabolic, blood, and immune disorders	10 to 14	Both	1 (1 to 2)	0.2 (0.1 to 0.2)	1 (1 to 2)	0.1 (0.1 to 0.2)	1 (1 to 1)	0.2 (0.1 to 0.3)	-16.4 (-63.6 to 30.3)	40.4 (-38.9 to 118.7)

eTable 8. All-cause and cause-specific mortality (counts and rates) at the global level and by country in the WHO European Region for ages 5 to 9 and 10 to 14 for 1990, 2000, and 2016

Belarus	Endocrine, metabolic, blood, and immune disorders	10 to 14	Female	1 (0 to 1)	0.1 (0.1 to 0.2)	0 (0 to 1)	0.1 (0.1 to 0.2)	0 (0 to 1)	0.2 (0.1 to 0.3)	-28.2 (-66.1 to 26.3)	21.5 (-42.6 to 113.8)
Belarus	Endocrine, metabolic, blood, and immune disorders	10 to 14	Male	1 (0 to 1)	0.2 (0.1 to 0.3)	1 (0 to 1)	0.2 (0.1 to 0.3)	1 (0 to 1)	0.3 (0.2 to 0.5)	-7.4 (-64.1 to 69.6)	54.3 (-40.2 to 182.4)
Belarus	Rheumatoid arthritis	5 to 9	Both	0 (0 to 0)	0.0 (0.0 to 0.0)	0 (0 to 0)	0.0 (0.0 to 0.0)	0 (0 to 0)	0.0 (0.0 to 0.0)	-63.7 (-80.0 to -29.0)	-43.8 (-69.0 to 10.0)
Belarus	Rheumatoid arthritis	5 to 9	Female	0 (0 to 0)	0.0 (0.0 to 0.0)	0 (0 to 0)	0.0 (0.0 to 0.0)	0 (0 to 0)	0.0 (0.0 to 0.0)	-70.8 (-86.1 to -29.3)	-54.3 (-78.2 to 10.6)
Belarus	Rheumatoid arthritis	5 to 9	Male	0 (0 to 0)	0.0 (0.0 to 0.0)	0 (0 to 0)	0.0 (0.0 to 0.0)	0 (0 to 0)	0.0 (0.0 to 0.0)	-51.2 (-78.7 to 9.0)	-25.0 (-67.3 to 67.3)
Belarus	Rheumatoid arthritis	10 to 14	Both	0 (0 to 0)	0.0 (0.0 to 0.0)	0 (0 to 0)	0.0 (0.0 to 0.0)	0 (0 to 0)	0.0 (0.0 to 0.0)	-68.5 (-84.6 to -33.4)	-47.0 (-74.1 to 11.8)
Belarus	Rheumatoid arthritis	10 to 14	Female	0 (0 to 0)	0.0 (0.0 to 0.0)	0 (0 to 0)	0.0 (0.0 to 0.0)	0 (0 to 0)	0.0 (0.0 to 0.0)	-77.2 (-91.7 to -36.2)	-61.4 (-86.0 to 8.0)
Belarus	Rheumatoid arthritis	10 to 14	Male	0 (0 to 0)	0.0 (0.0 to 0.0)	0 (0 to 0)	0.0 (0.0 to 0.0)	0 (0 to 0)	0.0 (0.0 to 0.0)	-57.0 (-82.0 to -3.9)	-28.4 (-70.0 to 60.0)
Belarus	Other musculoskeletal disorders	5 to 9	Both	0 (0 to 0)	0.0 (0.0 to 0.0)	0 (0 to 0)	0.0 (0.0 to 0.0)	0 (0 to 0)	0.0 (0.0 to 0.0)	-50.4 (-67.8 to -18.0)	-23.2 (-50.0 to 27.1)
Belarus	Other musculoskeletal disorders	5 to 9	Female	0 (0 to 0)	0.0 (0.0 to 0.0)	0 (0 to 0)	0.0 (0.0 to 0.0)	0 (0 to 0)	0.0 (0.0 to 0.0)	-46.7 (-68.6 to -8.7)	-16.5 (-50.8 to 42.9)
Belarus	Other musculoskeletal disorders	5 to 9	Male	0 (0 to 0)	0.0 (0.0 to 0.0)	0 (0 to 0)	0.0 (0.0 to 0.0)	0 (0 to 0)	0.0 (0.0 to 0.0)	-53.4 (-71.6 to -18.6)	-28.4 (-56.4 to 25.0)
Belarus	Other musculoskeletal disorders	10 to 14	Both	0 (0 to 0)	0.0 (0.0 to 0.0)	0 (0 to 0)	0.0 (0.0 to 0.0)	0 (0 to 0)	0.0 (0.0 to 0.0)	-59.2 (-74.8 to -28.6)	-31.6 (-57.8 to 19.9)
Belarus	Other musculoskeletal disorders	10 to 14	Female	0 (0 to 0)	0.0 (0.0 to 0.0)	0 (0 to 0)	0.0 (0.0 to 0.0)	0 (0 to 0)	0.0 (0.0 to 0.0)	-58.2 (-77.8 to -17.5)	-29.3 (-62.5 to 39.7)
Belarus	Other musculoskeletal disorders	10 to 14	Male	0 (0 to 0)	0.0 (0.0 to 0.0)	0 (0 to 0)	0.0 (0.0 to 0.0)	0 (0 to 0)	0.0 (0.0 to 0.0)	-60.6 (-76.5 to -22.6)	-34.4 (-60.9 to 28.9)
Belarus	Congenital birth defects	5 to 9	Both	40 (30 to 52)	4.9 (3.7 to 6.4)	21 (16 to 28)	3.6 (2.7 to 4.6)	13 (9 to 19)	2.4 (1.6 to 3.7)	-68.5 (-79.4 to -45.5)	-51.2 (-68.1 to -15.5)
Belarus	Congenital birth defects	5 to 9	Female	18 (13 to 25)	4.6 (3.3 to 6.3)	9 (7 to 13)	3.2 (2.3 to 4.4)	6 (4 to 8)	2.2 (1.4 to 3.2)	-69.7 (-81.3 to -48.2)	-52.6 (-70.7 to -18.9)
Belarus	Congenital birth defects	5 to 9	Male	22 (15 to 30)	5.2 (3.6 to 7.4)	12 (8 to 17)	3.9 (2.8 to 5.4)	7 (4 to 12)	2.6 (1.5 to 4.6)	-67.5 (-81.9 to -35.5)	-50.1 (-72.2 to -1.0)
Belarus	Congenital birth defects	10 to 14	Both	22 (17 to 27)	2.9 (2.3 to 3.7)	19 (15 to 24)	2.4 (1.9 to 3.0)	7 (5 to 10)	1.6 (1.1 to 2.3)	-68.2 (-77.5 to -50.3)	-46.6 (-62.2 to -16.5)
Belarus	Congenital birth defects	10 to 14	Female	11 (8 to 14)	2.9 (2.2 to 3.8)	9 (7 to 12)	2.3 (1.7 to 3.1)	3 (2 to 5)	1.5 (1.1 to 2.3)	-69.0 (-79.3 to -49.5)	-47.6 (-65.0 to -14.6)
Belarus	Congenital birth defects	10 to 14	Male	11 (8 to 15)	3.0 (2.1 to 4.0)	10 (7 to 14)	2.5 (1.8 to 3.4)	4 (2 to 6)	1.6 (1.0 to 2.7)	-67.4 (-79.9 to -44.2)	-45.6 (-66.5 to -7.1)
Belarus	Skin and subcutaneous diseases	5 to 9	Both	0 (0 to 0)	0.0 (0.0 to 0.0)	0 (0 to 0)	0.0 (0.0 to 0.0)	0 (0 to 0)	0.0 (0.0 to 0.0)	-38.2 (-56.3 to -14.2)	-4.2 (-32.3 to 33.0)
Belarus	Skin and subcutaneous diseases	5 to 9	Female	0 (0 to 0)	0.0 (0.0 to 0.0)	0 (0 to 0)	0.0 (0.0 to 0.0)	0 (0 to 0)	0.0 (0.0 to 0.0)	-41.7 (-58.9 to -19.4)	-8.7 (-35.6 to 26.2)
Belarus	Skin and subcutaneous diseases	5 to 9	Male	0 (0 to 0)	0.0 (0.0 to 0.0)	0 (0 to 0)	0.0 (0.0 to 0.0)	0 (0 to 0)	0.0 (0.0 to 0.0)	-34.8 (-54.9 to -6.8)	0.1 (-30.7 to 43.1)
Belarus	Skin and subcutaneous diseases	10 to 14	Both	0 (0 to 0)	0.0 (0.0 to 0.0)	0 (0 to 0)	0.0 (0.0 to 0.0)	0 (0 to 0)	0.0 (0.0 to 0.0)	-43.4 (-54.7 to -29.9)	-5.1 (-23.9 to 17.6)

eTable 8. All-cause and cause-specific mortality (counts and rates) at the global level and by country in the WHO European Region for ages 5 to 9 and 10 to 14 for 1990, 2000, and 2016

Belarus	Skin and subcutaneous diseases	10 to 14	Female	0<br>(0 to 0)	0.0<br>(0.0 to 0.0)	0<br>(0 to 0)	0.0<br>(0.0 to 0.0)	0<br>(0 to 0)	0.0<br>(0.0 to 0.0)	-44.7<br>(-57.7 to -29.5)	-6.3<br>(-28.4 to 19.3)
Belarus	Skin and subcutaneous diseases	10 to 14	Male	0<br>(0 to 0)	0.0<br>(0.0 to 0.0)	0<br>(0 to 0)	0.0<br>(0.0 to 0.0)	0<br>(0 to 0)	0.0<br>(0.0 to 0.0)	-42.2<br>(-55.9 to -24.6)	-3.7<br>(-26.6 to 25.5)
Belarus	Road injuries	5 to 9	Both	85<br>(67 to 106)	10.5<br>(8.3 to 13.1)	35<br>(27 to 45)	5.9<br>(4.6 to 7.5)	17<br>(12 to 23)	3.2<br>(2.3 to 4.3)	-80.2<br>(-86.5 to -71.2)	-69.4<br>(-79.0 to -55.4)
Belarus	Road injuries	5 to 9	Female	29<br>(22 to 39)	7.4<br>(5.5 to 9.8)	15<br>(11 to 20)	5.1<br>(3.7 to 6.8)	7<br>(5 to 11)	2.9<br>(2.0 to 4.2)	-75.0<br>(-84.2 to -60.5)	-60.9<br>(-75.3 to -38.1)
Belarus	Road injuries	5 to 9	Male	56<br>(42 to 72)	13.5<br>(10.2 to 17.4)	20<br>(15 to 27)	6.5<br>(4.8 to 8.8)	9<br>(6 to 13)	3.5<br>(2.4 to 4.9)	-83.0<br>(-89.6 to -73.2)	-73.9<br>(-84.1 to -58.9)
Belarus	Road injuries	10 to 14	Both	53<br>(44 to 63)	7.2<br>(6.0 to 8.6)	38<br>(31 to 45)	4.7<br>(3.8 to 5.6)	11<br>(8 to 14)	2.4<br>(1.8 to 3.1)	-79.7<br>(-85.5 to -72.0)	-65.9<br>(-75.6 to -53.0)
Belarus	Road injuries	10 to 14	Female	17<br>(13 to 22)	4.7<br>(3.6 to 6.0)	13<br>(10 to 17)	3.3<br>(2.5 to 4.4)	5<br>(3 to 6)	2.1<br>(1.4 to 2.9)	-73.3<br>(-83.3 to -59.1)	-54.8<br>(-71.7 to -30.7)
Belarus	Road injuries	10 to 14	Male	36<br>(29 to 45)	9.6<br>(7.6 to 12.0)	25<br>(20 to 32)	6.0<br>(4.8 to 7.7)	6<br>(4 to 9)	2.8<br>(1.9 to 3.8)	-82.7<br>(-88.6 to -74.3)	-71.2<br>(-81.1 to -57.2)
Belarus	Other transport injuries	5 to 9	Both	3<br>(2 to 5)	0.4<br>(0.2 to 0.6)	2<br>(1 to 3)	0.3<br>(0.2 to 0.5)	1<br>(1 to 2)	0.2<br>(0.1 to 0.3)	-69.4<br>(-84.4 to -37.5)	-52.7<br>(-75.8 to -3.1)
Belarus	Other transport injuries	5 to 9	Female	1<br>(1 to 2)	0.3<br>(0.1 to 0.4)	1<br>(0 to 1)	0.2<br>(0.1 to 0.3)	0<br>(0 to 0)	0.1<br>(0.1 to 0.2)	-71.4<br>(-88.9 to -36.6)	-55.2<br>(-82.6 to -0.8)
Belarus	Other transport injuries	5 to 9	Male	2<br>(1 to 4)	0.5<br>(0.3 to 1.0)	1<br>(1 to 2)	0.4<br>(0.2 to 0.7)	1<br>(0 to 1)	0.3<br>(0.1 to 0.5)	-68.6<br>(-87.2 to -22.3)	-51.7<br>(-80.4 to 19.3)
Belarus	Other transport injuries	10 to 14	Both	4<br>(2 to 5)	0.5<br>(0.3 to 0.7)	3<br>(2 to 5)	0.4<br>(0.3 to 0.6)	1<br>(1 to 2)	0.2<br>(0.2 to 0.4)	-69.5<br>(-83.6 to -43.5)	-48.8<br>(-72.5 to -5.2)
Belarus	Other transport injuries	10 to 14	Female	1<br>(0 to 1)	0.2<br>(0.1 to 0.4)	1<br>(0 to 1)	0.2<br>(0.1 to 0.3)	0<br>(0 to 0)	0.1<br>(0.1 to 0.2)	-67.0<br>(-87.6 to -20.5)	-44.1<br>(-79.1 to 34.6)
Belarus	Other transport injuries	10 to 14	Male	3<br>(2 to 4)	0.7<br>(0.4 to 1.1)	3<br>(2 to 4)	0.6<br>(0.4 to 1.0)	1<br>(0 to 1)	0.4<br>(0.2 to 0.6)	-70.3<br>(-86.2 to -35.2)	-50.5<br>(-77.0 to 7.9)
Belarus	Falls	5 to 9	Both	11<br>(7 to 15)	1.4<br>(0.8 to 1.9)	7<br>(4 to 9)	1.1<br>(0.7 to 1.6)	4<br>(2 to 6)	0.8<br>(0.4 to 1.2)	-63.2<br>(-77.4 to -37.2)	-43.0<br>(-65.0 to -2.6)
Belarus	Falls	5 to 9	Female	4<br>(2 to 5)	0.9<br>(0.5 to 1.3)	2<br>(1 to 3)	0.7<br>(0.4 to 1.1)	1<br>(1 to 2)	0.6<br>(0.2 to 0.9)	-58.0<br>(-76.1 to -29.4)	-34.3<br>(-62.5 to 10.5)
Belarus	Falls	5 to 9	Male	8<br>(4 to 11)	1.8<br>(1.1 to 2.7)	5<br>(3 to 7)	1.5<br>(0.9 to 2.2)	3<br>(1 to 4)	1.0<br>(0.6 to 1.6)	-65.6<br>(-81.0 to -34.9)	-47.2<br>(-70.8 to -0.0)
Belarus	Falls	10 to 14	Both	9<br>(7 to 13)	1.3<br>(0.9 to 1.7)	9<br>(6 to 11)	1.1<br>(0.8 to 1.4)	3<br>(2 to 4)	0.7<br>(0.4 to 1.0)	-67.6<br>(-78.5 to -50.3)	-45.6<br>(-63.9 to -16.5)
Belarus	Falls	10 to 14	Female	2<br>(1 to 3)	0.5<br>(0.4 to 0.7)	2<br>(1 to 3)	0.5<br>(0.3 to 0.7)	1<br>(0 to 1)	0.4<br>(0.2 to 0.6)	-51.6<br>(-72.9 to -17.6)	-18.1<br>(-54.1 to 39.4)
Belarus	Falls	10 to 14	Male	7<br>(5 to 11)	2.0<br>(1.3 to 2.8)	7<br>(5 to 10)	1.6<br>(1.1 to 2.3)	2<br>(1 to 3)	0.9<br>(0.5 to 1.4)	-71.8<br>(-83.5 to -49.9)	-53.1<br>(-72.5 to -16.5)
Belarus	Drowning	5 to 9	Both	110<br>(90 to 132)	13.6<br>(11.1 to 16.3)	54<br>(44 to 66)	9.1<br>(7.4 to 11.1)	20<br>(14 to 27)	3.9<br>(2.8 to 5.2)	-81.6<br>(-87.5 to -73.1)	-71.5<br>(-80.6 to -58.3)
Belarus	Drowning	5 to 9	Female	24<br>(18 to 30)	6.0<br>(4.4 to 7.6)	13<br>(9 to 17)	4.4<br>(3.3 to 5.7)	5<br>(3 to 6)	1.8<br>(1.2 to 2.5)	-80.9<br>(-87.7 to -68.7)	-70.1<br>(-80.8 to -51.0)
Belarus	Drowning	5 to 9	Male	86<br>(70 to 106)	20.9<br>(16.9 to 25.6)	41<br>(33 to 51)	13.5<br>(10.7 to 16.7)	16<br>(11 to 22)	5.8<br>(4.0 to 8.3)	-81.8<br>(-88.2 to -72.1)	-72.1<br>(-81.8 to -57.2)
Belarus	Drowning	10 to 14	Both	75<br>(64 to 86)	10.1<br>(8.6 to 11.7)	62<br>(52 to 73)	7.7<br>(6.5 to 9.1)	14<br>(10 to 17)	3.1<br>(2.4 to 3.8)	-81.9<br>(-86.3 to -76.1)	-69.6<br>(-77.1 to -59.9)
Belarus	Drowning	10 to 14	Female	20<br>(16 to 25)	5.4<br>(4.3 to 6.7)	17<br>(14 to 21)	4.4<br>(3.5 to 5.5)	5<br>(3 to 6)	2.1<br>(1.5 to 2.9)	-76.9<br>(-84.6 to -65.3)	-60.8<br>(-73.9 to -41.2)
Belarus	Drowning	10 to 14	Male	55<br>(45 to 65)	14.6<br>(12.0 to 17.4)	45<br>(37 to 54)	10.9<br>(9.0 to 13.1)	9<br>(7 to 12)	4.0<br>(2.9 to 5.3)	-83.7<br>(-88.5 to -77.1)	-72.9<br>(-80.8 to -61.9)
Belarus	Fire, heat, and hot substances	5 to 9	Both	16<br>(12 to 21)	1.9<br>(1.4 to 2.6)	10<br>(7 to 14)	1.8<br>(1.2 to 2.3)	5<br>(3 to 7)	0.9<br>(0.6 to 1.3)	-69.2<br>(-79.8 to -51.6)	-52.2<br>(-68.6 to -25.0)
Belarus	Fire, heat, and hot substances	5 to 9	Female	7<br>(5 to 10)	1.7<br>(1.1 to 2.4)	5<br>(3 to 6)	1.6<br>(1.0 to 2.2)	2<br>(1 to 3)	0.8<br>(0.5 to 1.3)	-68.8<br>(-81.3 to -44.6)	-51.2<br>(-70.8 to -13.3)

eTable 8. All-cause and cause-specific mortality (counts and rates) at the global level and by country in the WHO European Region for ages 5 to 9 and 10 to 14 for 1990, 2000, and 2016

Belarus	Fire, heat, and hot substances	5 to 9	Male	9 (6 to 13)	2.2 (1.5 to 3.0)	6 (4 to 8)	1.9 (1.3 to 2.7)	3 (2 to 4)	1.0 (0.7 to 1.5)	-69.4 (-81.8 to -46.7)	-53.1 (-72.1 to -18.1)
Belarus	Fire, heat, and hot substances	10 to 14	Both	7 (5 to 8)	0.9 (0.7 to 1.1)	7 (5 to 8)	0.8 (0.6 to 1.1)	2 (1 to 3)	0.4 (0.3 to 0.6)	-71.2 (-80.5 to -56.8)	-51.7 (-67.2 to -27.6)
Belarus	Fire, heat, and hot substances	10 to 14	Female	2 (1 to 2)	0.5 (0.3 to 0.7)	2 (1 to 3)	0.5 (0.3 to 0.7)	1 (0 to 1)	0.3 (0.2 to 0.4)	-64.4 (-79.5 to -33.8)	-39.7 (-65.4 to 12.1)
Belarus	Fire, heat, and hot substances	10 to 14	Male	5 (4 to 6)	1.3 (0.9 to 1.7)	5 (3 to 6)	1.1 (0.8 to 1.5)	1 (1 to 2)	0.6 (0.4 to 0.8)	-73.6 (-83.5 to -58.3)	-56.1 (-72.5 to -30.6)
Belarus	Poisonings	5 to 9	Both	12 (8 to 16)	1.4 (1.0 to 2.0)	7 (5 to 10)	1.2 (0.8 to 1.7)	4 (2 to 6)	0.7 (0.4 to 1.1)	-66.8 (-80.5 to -46.2)	-48.5 (-69.7 to -16.7)
Belarus	Poisonings	5 to 9	Female	4 (3 to 6)	1.1 (0.7 to 1.6)	3 (2 to 4)	1.0 (0.5 to 1.5)	1 (1 to 2)	0.6 (0.3 to 0.9)	-66.5 (-81.0 to -37.7)	-47.6 (-70.3 to -2.4)
Belarus	Poisonings	5 to 9	Male	7 (4 to 11)	1.7 (1.0 to 2.6)	4 (3 to 7)	1.4 (0.8 to 2.3)	2 (1 to 4)	0.9 (0.4 to 1.5)	-66.9 (-83.1 to -38.1)	-49.2 (-74.1 to -4.9)
Belarus	Poisonings	10 to 14	Both	6 (4 to 8)	0.8 (0.5 to 1.1)	6 (4 to 8)	0.7 (0.5 to 1.0)	2 (1 to 3)	0.5 (0.3 to 0.7)	-64.4 (-79.8 to -41.2)	-40.3 (-66.0 to -1.3)
Belarus	Poisonings	10 to 14	Female	2 (1 to 3)	0.5 (0.3 to 0.8)	2 (1 to 3)	0.5 (0.3 to 0.7)	1 (0 to 1)	0.3 (0.2 to 0.5)	-60.2 (-78.9 to -16.7)	-32.7 (-64.2 to 41.1)
Belarus	Poisonings	10 to 14	Male	4 (2 to 6)	1.0 (0.7 to 1.5)	4 (3 to 6)	1.0 (0.6 to 1.4)	1 (1 to 2)	0.6 (0.3 to 0.9)	-66.4 (-82.7 to -40.4)	-44.0 (-71.2 to -0.8)
Belarus	Exposure to mechanical forces	5 to 9	Both	12 (9 to 16)	1.5 (1.1 to 2.0)	6 (4 to 8)	1.0 (0.7 to 1.3)	3 (2 to 4)	0.6 (0.4 to 0.8)	-76.0 (-84.2 to -60.4)	-62.8 (-75.5 to -38.6)
Belarus	Exposure to mechanical forces	5 to 9	Female	3 (2 to 5)	0.8 (0.5 to 1.1)	2 (1 to 2)	0.5 (0.4 to 0.8)	1 (1 to 2)	0.4 (0.2 to 0.6)	-69.9 (-82.3 to -45.1)	-52.8 (-72.3 to -14.1)
Belarus	Exposure to mechanical forces	5 to 9	Male	9 (6 to 12)	2.2 (1.5 to 3.0)	4 (3 to 6)	1.4 (0.9 to 1.8)	2 (1 to 3)	0.7 (0.5 to 1.1)	-78.2 (-87.1 to -60.3)	-66.5 (-80.1 to -39.1)
Belarus	Exposure to mechanical forces	10 to 14	Both	8 (7 to 10)	1.1 (0.9 to 1.4)	7 (5 to 9)	0.8 (0.7 to 1.1)	2 (1 to 3)	0.4 (0.3 to 0.6)	-76.0 (-82.8 to -65.5)	-59.6 (-71.1 to -42.0)
Belarus	Exposure to mechanical forces	10 to 14	Female	2 (1 to 3)	0.5 (0.4 to 0.7)	2 (1 to 2)	0.4 (0.3 to 0.5)	1 (0 to 1)	0.3 (0.2 to 0.4)	-68.9 (-79.8 to -50.7)	-47.3 (-65.8 to -16.6)
Belarus	Exposure to mechanical forces	10 to 14	Male	6 (5 to 8)	1.7 (1.3 to 2.2)	5 (4 to 7)	1.2 (0.9 to 1.7)	1 (1 to 2)	0.6 (0.4 to 0.9)	-78.1 (-85.6 to -65.6)	-63.6 (-76.1 to -42.7)
Belarus	Adverse effects of medical treatment	5 to 9	Both	2 (1 to 4)	0.3 (0.1 to 0.5)	1 (1 to 2)	0.2 (0.1 to 0.4)	1 (0 to 1)	0.2 (0.1 to 0.3)	-64.1 (-78.2 to -39.1)	-44.3 (-66.2 to -5.6)
Belarus	Adverse effects of medical treatment	5 to 9	Female	1 (0 to 2)	0.2 (0.1 to 0.5)	0 (0 to 1)	0.2 (0.1 to 0.3)	0 (0 to 1)	0.1 (0.0 to 0.2)	-70.3 (-81.5 to -52.7)	-53.6 (-71.1 to -26.0)
Belarus	Adverse effects of medical treatment	5 to 9	Male	1 (1 to 2)	0.3 (0.2 to 0.6)	1 (0 to 1)	0.2 (0.1 to 0.4)	1 (0 to 1)	0.2 (0.1 to 0.4)	-59.6 (-79.5 to -16.4)	-38.0 (-68.5 to 28.3)
Belarus	Adverse effects of medical treatment	10 to 14	Both	2 (1 to 3)	0.2 (0.1 to 0.4)	1 (1 to 3)	0.2 (0.1 to 0.3)	1 (0 to 1)	0.1 (0.1 to 0.3)	-64.3 (-76.3 to -46.4)	-40.0 (-60.1 to -10.0)
Belarus	Adverse effects of medical treatment	10 to 14	Female	1 (0 to 1)	0.2 (0.1 to 0.4)	1 (0 to 1)	0.1 (0.1 to 0.3)	0 (0 to 0)	0.1 (0.0 to 0.2)	-68.0 (-78.5 to -51.7)	-45.9 (-63.6 to -18.2)
Belarus	Adverse effects of medical treatment	10 to 14	Male	1 (1 to 2)	0.3 (0.2 to 0.5)	1 (1 to 2)	0.2 (0.1 to 0.4)	0 (0 to 1)	0.2 (0.1 to 0.4)	-61.9 (-78.7 to -35.0)	-36.6 (-64.5 to 8.2)
Belarus	Animal contact	5 to 9	Both	1 (1 to 2)	0.2 (0.1 to 0.3)	1 (1 to 1)	0.1 (0.1 to 0.2)	1 (0 to 1)	0.1 (0.1 to 0.2)	-58.3 (-78.9 to -15.8)	-35.4 (-67.3 to 30.5)

eTable 8. All-cause and cause-specific mortality (counts and rates) at the global level and by country in the WHO European Region for ages 5 to 9 and 10 to 14 for 1990, 2000, and 2016

Belarus	Animal contact	5 to 9	Female	0 (0 to 1)	0.1 (0.0 to 0.1)	0 (0 to 0)	0.1 (0.0 to 0.1)	0 (0 to 0)	0.0 (0.0 to 0.1)	-61.6 (-82.4 to -20.2)	-39.8 (-72.5 to 25.0)
Belarus	Animal contact	5 to 9	Male	1 (1 to 2)	0.3 (0.1 to 0.5)	1 (0 to 1)	0.2 (0.1 to 0.4)	0 (0 to 1)	0.2 (0.1 to 0.3)	-57.3 (-81.4 to -5.2)	-34.5 (-71.4 to 45.6)
Belarus	Animal contact	10 to 14	Both	1 (0 to 1)	0.1 (0.1 to 0.1)	1 (0 to 1)	0.1 (0.1 to 0.1)	0 (0 to 0)	0.1 (0.0 to 0.1)	-60.1 (-76.6 to -32.0)	-33.0 (-60.8 to 14.2)
Belarus	Animal contact	10 to 14	Female	0 (0 to 0)	0.0 (0.0 to 0.1)	0 (0 to 0)	0.0 (0.0 to 0.1)	0 (0 to 0)	0.0 (0.0 to 0.1)	-59.8 (-79.7 to -22.1)	-32.0 (-65.6 to 31.9)
Belarus	Animal contact	10 to 14	Male	1 (0 to 1)	0.1 (0.1 to 0.2)	1 (0 to 1)	0.1 (0.1 to 0.2)	0 (0 to 0)	0.1 (0.1 to 0.2)	-60.2 (-79.4 to -23.7)	-33.7 (-65.7 to 27.0)
Belarus	Foreign body	5 to 9	Both	4 (3 to 6)	0.5 (0.3 to 0.7)	2 (2 to 3)	0.4 (0.3 to 0.5)	2 (1 to 2)	0.3 (0.2 to 0.4)	-59.5 (-76.8 to -27.7)	-37.2 (-64.1 to 12.1)
Belarus	Foreign body	5 to 9	Female	1 (1 to 2)	0.3 (0.2 to 0.5)	1 (0 to 1)	0.2 (0.2 to 0.4)	1 (0 to 1)	0.2 (0.1 to 0.3)	-56.6 (-77.0 to -25.4)	-32.1 (-63.9 to 16.8)
Belarus	Foreign body	5 to 9	Male	3 (1 to 4)	0.7 (0.4 to 1.0)	2 (1 to 2)	0.5 (0.3 to 0.8)	1 (1 to 2)	0.4 (0.2 to 0.6)	-60.9 (-80.2 to -22.5)	-39.9 (-69.6 to 19.0)
Belarus	Foreign body	10 to 14	Both	3 (2 to 4)	0.4 (0.3 to 0.6)	3 (2 to 4)	0.4 (0.3 to 0.5)	1 (1 to 2)	0.3 (0.2 to 0.4)	-61.5 (-77.5 to -37.2)	-35.4 (-62.2 to 5.3)
Belarus	Foreign body	10 to 14	Female	1 (1 to 1)	0.2 (0.1 to 0.3)	1 (0 to 1)	0.2 (0.1 to 0.2)	0 (0 to 0)	0.1 (0.1 to 0.2)	-55.7 (-74.4 to -29.5)	-25.0 (-56.6 to 19.3)
Belarus	Foreign body	10 to 14	Male	2 (2 to 3)	0.6 (0.4 to 0.9)	2 (2 to 3)	0.5 (0.4 to 0.8)	1 (1 to 1)	0.4 (0.2 to 0.6)	-63.2 (-80.6 to -35.2)	-38.8 (-67.7 to 7.9)
Belarus	Environmental heat and cold exposure	5 to 9	Both	0 (0 to 1)	0.0 (0.0 to 0.1)	0 (0 to 0)	0.0 (0.0 to 0.1)	0 (0 to 0)	0.0 (0.0 to 0.0)	-72.3 (-82.6 to -51.3)	-57.1 (-73.0 to -24.5)
Belarus	Environmental heat and cold exposure	5 to 9	Female	0 (0 to 0)	0.1 (0.0 to 0.1)	0 (0 to 0)	0.0 (0.0 to 0.1)	0 (0 to 0)	0.0 (0.0 to 0.0)	-74.5 (-85.0 to -50.4)	-60.2 (-76.5 to -22.4)
Belarus	Environmental heat and cold exposure	5 to 9	Male	0 (0 to 0)	0.0 (0.0 to 0.1)	0 (0 to 0)	0.0 (0.0 to 0.0)	0 (0 to 0)	0.0 (0.0 to 0.0)	-69.1 (-83.0 to -35.5)	-52.5 (-73.9 to -1.0)
Belarus	Environmental heat and cold exposure	10 to 14	Both	0 (0 to 0)	0.0 (0.0 to 0.1)	0 (0 to 0)	0.0 (0.0 to 0.0)	0 (0 to 0)	0.0 (0.0 to 0.0)	-71.7 (-81.3 to -55.6)	-52.4 (-68.7 to -25.4)
Belarus	Environmental heat and cold exposure	10 to 14	Female	0 (0 to 0)	0.0 (0.0 to 0.1)	0 (0 to 0)	0.0 (0.0 to 0.1)	0 (0 to 0)	0.0 (0.0 to 0.0)	-72.7 (-83.1 to -52.2)	-53.8 (-71.3 to -19.2)
Belarus	Environmental heat and cold exposure	10 to 14	Male	0 (0 to 0)	0.0 (0.0 to 0.1)	0 (0 to 0)	0.0 (0.0 to 0.1)	0 (0 to 0)	0.0 (0.0 to 0.0)	-70.7 (-82.6 to -46.6)	-51.2 (-71.1 to -11.1)
Belarus	Other unintentional injuries	5 to 9	Both	5 (3 to 6)	0.6 (0.4 to 0.8)	2 (1 to 2)	0.3 (0.2 to 0.4)	1 (1 to 1)	0.2 (0.1 to 0.2)	-80.5 (-87.7 to -68.1)	-69.7 (-81.0 to -50.5)
Belarus	Other unintentional injuries	5 to 9	Female	1 (1 to 1)	0.3 (0.2 to 0.4)	0 (0 to 1)	0.1 (0.1 to 0.2)	0 (0 to 0)	0.1 (0.0 to 0.1)	-82.9 (-90.4 to -70.7)	-73.2 (-85.0 to -54.2)
Belarus	Other unintentional injuries	5 to 9	Male	4 (2 to 5)	0.9 (0.6 to 1.2)	1 (1 to 2)	0.5 (0.3 to 0.6)	1 (0 to 1)	0.3 (0.2 to 0.4)	-79.8 (-88.1 to -63.5)	-68.9 (-81.8 to -44.0)
Belarus	Other unintentional injuries	10 to 14	Both	4 (3 to 6)	0.6 (0.4 to 0.8)	3 (2 to 4)	0.4 (0.3 to 0.5)	1 (1 to 1)	0.2 (0.1 to 0.3)	-80.4 (-87.1 to -69.6)	-67.0 (-78.3 to -49.0)
Belarus	Other unintentional injuries	10 to 14	Female	1 (1 to 1)	0.2 (0.2 to 0.4)	1 (0 to 1)	0.1 (0.1 to 0.2)	0 (0 to 0)	0.1 (0.0 to 0.1)	-81.2 (-89.8 to -64.4)	-68.2 (-82.7 to -39.7)
Belarus	Other unintentional injuries	10 to 14	Male	3 (2 to 5)	0.9 (0.6 to 1.3)	2 (2 to 3)	0.6 (0.4 to 0.8)	1 (0 to 1)	0.3 (0.2 to 0.4)	-80.1 (-88.1 to -67.0)	-66.9 (-80.2 to -45.1)
Belarus	Self-harm	10 to 14	Both	19 (14 to 24)	2.6 (1.9 to 3.3)	23 (15 to 30)	2.9 (1.9 to 3.7)	7 (5 to 10)	1.5 (1.1 to 2.2)	-65.1 (-77.4 to -48.0)	-41.4 (-62.1 to -12.7)
Belarus	Self-harm	10 to 14	Female	5 (3 to 6)	1.3 (0.9 to 1.8)	4 (3 to 6)	1.1 (0.8 to 1.5)	1 (1 to 2)	0.7 (0.4 to 1.0)	-68.0 (-80.6 to -44.6)	-45.8 (-67.2 to -6.1)
Belarus	Self-harm	10 to 14	Male	15 (10 to 19)	3.9 (2.7 to 5.2)	19 (11 to 25)	4.5 (2.7 to 6.0)	5 (3 to 8)	2.3 (1.5 to 3.6)	-64.2 (-78.5 to -41.4)	-40.4 (-64.3 to -2.3)

eTable 8. All-cause and cause-specific mortality (counts and rates) at the global level and by country in the WHO European Region for ages 5 to 9 and 10 to 14 for 1990, 2000, and 2016

Belarus	Interpersonal violence	5 to 9	Both	6 (4 to 9)	0.8 (0.5 to 1.1)	4 (2 to 5)	0.6 (0.4 to 0.9)	2 (1 to 3)	0.4 (0.2 to 0.6)	-70.4 (-81.5 to -48.6)	-54.2 (-71.4 to -20.4)
Belarus	Interpersonal violence	5 to 9	Female	3 (2 to 5)	0.9 (0.5 to 1.3)	2 (1 to 3)	0.7 (0.4 to 1.1)	1 (1 to 2)	0.4 (0.2 to 0.7)	-73.7 (-86.7 to -34.3)	-58.9 (-79.1 to 2.8)
Belarus	Interpersonal violence	5 to 9	Male	3 (2 to 5)	0.7 (0.4 to 1.2)	2 (1 to 3)	0.6 (0.3 to 0.9)	1 (1 to 2)	0.4 (0.2 to 0.7)	-66.4 (-79.9 to -42.8)	-48.4 (-69.2 to -12.2)
Belarus	Interpersonal violence	10 to 14	Both	7 (5 to 9)	0.9 (0.6 to 1.2)	7 (4 to 10)	0.9 (0.5 to 1.3)	2 (1 to 3)	0.5 (0.3 to 0.7)	-70.9 (-81.0 to -53.8)	-51.2 (-68.2 to -22.4)
Belarus	Interpersonal violence	10 to 14	Female	3 (2 to 5)	0.9 (0.5 to 1.3)	4 (2 to 6)	1.1 (0.5 to 1.6)	1 (1 to 2)	0.5 (0.3 to 0.8)	-71.3 (-84.1 to -37.7)	-51.4 (-73.1 to 5.4)
Belarus	Interpersonal violence	10 to 14	Male	3 (2 to 6)	0.9 (0.5 to 1.5)	3 (1 to 5)	0.8 (0.4 to 1.2)	1 (1 to 2)	0.5 (0.2 to 0.8)	-70.6 (-82.1 to -53.7)	-51.0 (-70.3 to -22.8)
Belarus	Exposure to forces of nature	5 to 9	Both	0 (0 to 0)	0.0 (0.0 to 0.0)	0 (0 to 0)	0.0 (0.0 to 0.0)	0 (0 to 0)	0.0 (0.0 to 0.0)	0.0 (0.0 to 0.0)	0.0 (0.0 to 0.0)
Belarus	Exposure to forces of nature	5 to 9	Female	0 (0 to 0)	0.0 (0.0 to 0.0)	0 (0 to 0)	0.0 (0.0 to 0.0)	0 (0 to 0)	0.0 (0.0 to 0.0)	0.0 (0.0 to 0.0)	0.0 (0.0 to 0.0)
Belarus	Exposure to forces of nature	5 to 9	Male	0 (0 to 0)	0.0 (0.0 to 0.0)	0 (0 to 0)	0.0 (0.0 to 0.0)	0 (0 to 0)	0.0 (0.0 to 0.0)	0.0 (0.0 to 0.0)	0.0 (0.0 to 0.0)
Belarus	Exposure to forces of nature	10 to 14	Both	0 (0 to 0)	0.0 (0.0 to 0.0)	0 (0 to 0)	0.0 (0.0 to 0.0)	0 (0 to 0)	0.0 (0.0 to 0.0)	0.0 (0.0 to 0.0)	0.0 (0.0 to 0.0)
Belarus	Exposure to forces of nature	10 to 14	Female	0 (0 to 0)	0.0 (0.0 to 0.0)	0 (0 to 0)	0.0 (0.0 to 0.0)	0 (0 to 0)	0.0 (0.0 to 0.0)	0.0 (0.0 to 0.0)	0.0 (0.0 to 0.0)
Belarus	Exposure to forces of nature	10 to 14	Male	0 (0 to 0)	0.0 (0.0 to 0.0)	0 (0 to 0)	0.0 (0.0 to 0.0)	0 (0 to 0)	0.0 (0.0 to 0.0)	0.0 (0.0 to 0.0)	0.0 (0.0 to 0.0)
Belarus	Conflict and terrorism	5 to 9	Both	0 (0 to 0)	0.0 (0.0 to 0.0)	0 (0 to 0)	0.0 (0.0 to 0.0)	0 (0 to 0)	0.0 (0.0 to 0.0)	0.0 (0.0 to 0.0)	0.0 (0.0 to 0.0)
Belarus	Conflict and terrorism	5 to 9	Female	0 (0 to 0)	0.0 (0.0 to 0.0)	0 (0 to 0)	0.0 (0.0 to 0.0)	0 (0 to 0)	0.0 (0.0 to 0.0)	0.0 (0.0 to 0.0)	0.0 (0.0 to 0.0)
Belarus	Conflict and terrorism	5 to 9	Male	0 (0 to 0)	0.0 (0.0 to 0.0)	0 (0 to 0)	0.0 (0.0 to 0.0)	0 (0 to 0)	0.0 (0.0 to 0.0)	0.0 (0.0 to 0.0)	0.0 (0.0 to 0.0)
Belarus	Conflict and terrorism	10 to 14	Both	0 (0 to 0)	0.0 (0.0 to 0.0)	0 (0 to 0)	0.0 (0.0 to 0.0)	0 (0 to 0)	0.0 (0.0 to 0.0)	0.0 (0.0 to 0.0)	0.0 (0.0 to 0.0)
Belarus	Conflict and terrorism	10 to 14	Female	0 (0 to 0)	0.0 (0.0 to 0.0)	0 (0 to 0)	0.0 (0.0 to 0.0)	0 (0 to 0)	0.0 (0.0 to 0.0)	0.0 (0.0 to 0.0)	0.0 (0.0 to 0.0)
Belarus	Conflict and terrorism	10 to 14	Male	0 (0 to 0)	0.0 (0.0 to 0.0)	0 (0 to 0)	0.0 (0.0 to 0.0)	0 (0 to 0)	0.0 (0.0 to 0.0)	0.0 (0.0 to 0.0)	0.0 (0.0 to 0.0)
Belarus	Executions and police conflict	5 to 9	Both	0 (0 to 0)	0.0 (0.0 to 0.0)	0 (0 to 0)	0.0 (0.0 to 0.0)	0 (0 to 0)	0.0 (0.0 to 0.0)	-46.8 (-72.7 to -15.2)	-17.5 (-57.7 to 31.5)
Belarus	Executions and police conflict	5 to 9	Female	0 (0 to 0)	0.0 (0.0 to 0.0)	0 (0 to 0)	0.0 (0.0 to 0.0)	0 (0 to 0)	0.0 (0.0 to 0.0)	-42.3 (-59.9 to -19.4)	-9.7 (-37.2 to 26.2)
Belarus	Executions and police conflict	5 to 9	Male	0 (0 to 0)	0.0 (0.0 to 0.1)	0 (0 to 0)	0.0 (0.0 to 0.0)	0 (0 to 0)	0.0 (0.0 to 0.0)	-49.9 (-78.3 to -7.1)	-23.1 (-66.6 to 42.6)
Belarus	Executions and police conflict	10 to 14	Both	0 (0 to 0)	0.0 (0.0 to 0.1)	0 (0 to 0)	0.0 (0.0 to 0.0)	0 (0 to 0)	0.0 (0.0 to 0.0)	-56.5 (-85.5 to 37.8)	-26.9 (-75.6 to 131.3)
Belarus	Executions and police conflict	10 to 14	Female	0 (0 to 0)	0.0 (0.0 to 0.0)	0 (0 to 0)	0.0 (0.0 to 0.0)	0 (0 to 0)	0.0 (0.0 to 0.0)	-46.1 (-60.2 to -30.2)	-8.7 (-32.7 to 18.1)
Belarus	Executions and police conflict	10 to 14	Male	0 (0 to 0)	0.0 (0.0 to 0.1)	0 (0 to 0)	0.0 (0.0 to 0.1)	0 (0 to 0)	0.0 (0.0 to 0.0)	-59.9 (-90.2 to 102.8)	-33.3 (-83.7 to 237.7)
Belgium	All causes	5 to 9	Both	126 (124 to 128)	21.0 (20.6 to 21.4)	79 (77 to 81)	12.8 (12.5 to 13.1)	57 (53 to 61)	8.5 (7.9 to 9.2)	-54.7 (-57.9 to -51.2)	-59.5 (-62.3 to -56.3)
Belgium	All causes	5 to 9	Female	55 (54 to 56)	18.8 (18.5 to 19.1)	33 (33 to 34)	11.1 (10.8 to 11.3)	26 (24 to 28)	7.9 (7.4 to 8.4)	-53.3 (-56.2 to -50.0)	-58.2 (-60.9 to -55.3)

eTable 8. All-cause and cause-specific mortality (counts and rates) at the global level and by country in the WHO European Region for ages 5 to 9 and 10 to 14 for 1990, 2000, and 2016

Belgium	All causes	5 to 9	Male	71 (69 to 72)	23.0 (22.6 to 23.6)	46 (44 to 47)	14.5 (14.1 to 14.9)	31 (29 to 34)	9.1 (8.4 to 9.9)	-55.9 (-59.2 to -52.1)	-60.5 (-63.4 to -57.1)
Belgium	All causes	10 to 14	Both	124 (122 to 127)	20.4 (20.0 to 20.8)	98 (96 to 100)	16.1 (15.7 to 16.4)	61 (57 to 64)	9.8 (9.2 to 10.4)	-50.9 (-54.2 to -48.1)	-52.0 (-55.2 to -49.2)
Belgium	All causes	10 to 14	Female	51 (50 to 53)	17.3 (16.8 to 17.8)	44 (43 to 45)	14.7 (14.3 to 15.2)	27 (25 to 29)	8.9 (8.2 to 9.6)	-47.7 (-52.3 to -43.0)	-48.8 (-53.3 to -44.1)
Belgium	All causes	10 to 14	Male	73 (71 to 75)	23.4 (22.7 to 24.0)	54 (53 to 56)	17.3 (16.9 to 17.7)	34 (31 to 36)	10.7 (9.9 to 11.5)	-53.2 (-56.8 to -49.7)	-54.3 (-57.8 to -50.8)
Belgium	Tuberculosis	5 to 9	Both	0 (0 to 0)	0.0 (0.0 to 0.0)	0 (0 to 0)	0.0 (0.0 to 0.0)	0 (0 to 0)	0.0 (0.0 to 0.0)	-63.0 (-72.3 to -49.1)	-66.9 (-75.2 to -54.5)
Belgium	Tuberculosis	5 to 9	Female	0 (0 to 0)	0.0 (0.0 to 0.1)	0 (0 to 0)	0.0 (0.0 to 0.0)	0 (0 to 0)	0.0 (0.0 to 0.0)	-68.3 (-78.5 to -49.4)	-71.7 (-80.8 to -54.8)
Belgium	Tuberculosis	5 to 9	Male	0 (0 to 0)	0.0 (0.0 to 0.0)	0 (0 to 0)	0.0 (0.0 to 0.0)	0 (0 to 0)	0.0 (0.0 to 0.0)	-54.4 (-68.1 to -34.2)	-59.1 (-71.4 to -41.1)
Belgium	Tuberculosis	10 to 14	Both	0 (0 to 0)	0.0 (0.0 to 0.0)	0 (0 to 0)	0.0 (0.0 to 0.0)	0 (0 to 0)	0.0 (0.0 to 0.0)	-42.7 (-56.8 to -23.3)	-43.9 (-57.7 to -24.9)
Belgium	Tuberculosis	10 to 14	Female	0 (0 to 0)	0.0 (0.0 to 0.0)	0 (0 to 0)	0.0 (0.0 to 0.0)	0 (0 to 0)	0.0 (0.0 to 0.0)	-40.9 (-56.7 to -16.6)	-42.1 (-57.6 to -18.3)
Belgium	Tuberculosis	10 to 14	Male	0 (0 to 0)	0.0 (0.0 to 0.0)	0 (0 to 0)	0.0 (0.0 to 0.0)	0 (0 to 0)	0.0 (0.0 to 0.0)	-44.5 (-63.4 to -11.0)	-45.7 (-64.2 to -12.9)
Belgium	HIV/AIDS	5 to 9	Both	1 (1 to 2)	0.2 (0.2 to 0.3)	1 (0 to 1)	0.1 (0.1 to 0.1)	0 (0 to 0)	0.1 (0.1 to 0.1)	-68.0 (-74.4 to -60.3)	-71.4 (-77.1 to -64.5)
Belgium	HIV/AIDS	5 to 9	Female	0 (0 to 1)	0.2 (0.1 to 0.2)	0 (0 to 0)	0.1 (0.1 to 0.1)	0 (0 to 0)	0.1 (0.1 to 0.1)	-57.0 (-69.3 to -40.4)	-61.6 (-72.6 to -46.8)
Belgium	HIV/AIDS	5 to 9	Male	1 (1 to 1)	0.3 (0.2 to 0.3)	0 (0 to 0)	0.1 (0.1 to 0.1)	0 (0 to 0)	0.1 (0.1 to 0.1)	-74.6 (-80.2 to -66.5)	-77.2 (-82.3 to -70.0)
Belgium	HIV/AIDS	10 to 14	Both	1 (1 to 1)	0.2 (0.1 to 0.2)	0 (0 to 1)	0.1 (0.1 to 0.1)	0 (0 to 0)	0.1 (0.0 to 0.1)	-67.9 (-74.2 to -59.7)	-68.6 (-74.8 to -60.5)
Belgium	HIV/AIDS	10 to 14	Female	0 (0 to 1)	0.1 (0.1 to 0.2)	0 (0 to 0)	0.1 (0.1 to 0.1)	0 (0 to 0)	0.1 (0.0 to 0.1)	-59.7 (-71.4 to -42.8)	-60.6 (-72.0 to -44.0)
Belgium	HIV/AIDS	10 to 14	Male	1 (1 to 1)	0.2 (0.2 to 0.2)	0 (0 to 0)	0.1 (0.1 to 0.1)	0 (0 to 0)	0.1 (0.0 to 0.1)	-73.5 (-79.4 to -65.7)	-74.1 (-79.8 to -66.4)
Belgium	Diarrheal diseases	5 to 9	Both	0 (0 to 0)	0.0 (0.0 to 0.0)	0 (0 to 0)	0.0 (0.0 to 0.0)	0 (0 to 0)	0.0 (0.0 to 0.1)	128.3 (43.4 to 250.7)	104.2 (28.3 to 213.8)
Belgium	Diarrheal diseases	5 to 9	Female	0 (0 to 0)	0.0 (0.0 to 0.0)	0 (0 to 0)	0.0 (0.0 to 0.1)	0 (0 to 0)	0.1 (0.0 to 0.1)	125.6 (19.7 to 312.9)	101.5 (6.9 to 268.9)
Belgium	Diarrheal diseases	5 to 9	Male	0 (0 to 0)	0.0 (0.0 to 0.0)	0 (0 to 0)	0.0 (0.0 to 0.0)	0 (0 to 0)	0.0 (0.0 to 0.0)	133.3 (26.0 to 313.2)	109.0 (12.9 to 270.1)
Belgium	Diarrheal diseases	10 to 14	Both	0 (0 to 0)	0.0 (0.0 to 0.0)	0 (0 to 0)	0.0 (0.0 to 0.0)	0 (0 to 0)	0.0 (0.0 to 0.0)	152.5 (67.7 to 265.7)	147.1 (64.1 to 257.9)
Belgium	Diarrheal diseases	10 to 14	Female	0 (0 to 0)	0.0 (0.0 to 0.0)	0 (0 to 0)	0.0 (0.0 to 0.0)	0 (0 to 0)	0.0 (0.0 to 0.0)	179.4 (67.0 to 353.5)	173.7 (63.5 to 344.2)
Belgium	Diarrheal diseases	10 to 14	Male	0 (0 to 0)	0.0 (0.0 to 0.0)	0 (0 to 0)	0.0 (0.0 to 0.0)	0 (0 to 0)	0.0 (0.0 to 0.0)	125.4 (19.8 to 283.3)	120.4 (17.2 to 274.9)
Belgium	Intestinal infectious diseases	5 to 9	Both	0 (0 to 0)	0.0 (0.0 to 0.0)	0 (0 to 0)	0.0 (0.0 to 0.0)	0 (0 to 0)	0.0 (0.0 to 0.0)	-29.8 (-65.9 to 18.9)	-37.2 (-69.5 to 6.4)
Belgium	Intestinal infectious diseases	5 to 9	Female	0 (0 to 0)	0.0 (0.0 to 0.0)	0 (0 to 0)	0.0 (0.0 to 0.0)	0 (0 to 0)	0.0 (0.0 to 0.0)	-25.4 (-68.9 to 44.8)	-33.4 (-72.2 to 29.4)
Belgium	Intestinal infectious diseases	5 to 9	Male	0 (0 to 0)	0.0 (0.0 to 0.0)	0 (0 to 0)	0.0 (0.0 to 0.0)	0 (0 to 0)	0.0 (0.0 to 0.0)	-38.5 (-77.7 to 41.3)	-44.9 (-80.0 to 26.6)
Belgium	Intestinal infectious diseases	10 to 14	Both	0 (0 to 0)	0.0 (0.0 to 0.0)	0 (0 to 0)	0.0 (0.0 to 0.0)	0 (0 to 0)	0.0 (0.0 to 0.0)	-22.5 (-60.7 to 21.2)	-24.1 (-61.5 to 18.6)
Belgium	Intestinal infectious diseases	10 to 14	Female	0 (0 to 0)	0.0 (0.0 to 0.0)	0 (0 to 0)	0.0 (0.0 to 0.0)	0 (0 to 0)	0.0 (0.0 to 0.0)	-14.4 (-58.7 to 49.6)	-16.2 (-59.6 to 46.5)
Belgium	Intestinal infectious diseases	10 to 14	Male	0 (0 to 0)	0.0 (0.0 to 0.0)	0 (0 to 0)	0.0 (0.0 to 0.0)	0 (0 to 0)	0.0 (0.0 to 0.0)	-39.8 (-75.6 to 27.6)	-41.2 (-76.1 to 24.8)
Belgium	Lower respiratory infections	5 to 9	Both	3 (2 to 3)	0.5 (0.3 to 0.6)	2 (2 to 3)	0.3 (0.3 to 0.4)	1 (1 to 2)	0.2 (0.2 to 0.3)	-48.1 (-65.1 to -23.6)	-53.6 (-68.8 to -31.7)

eTable 8. All-cause and cause-specific mortality (counts and rates) at the global level and by country in the WHO European Region for ages 5 to 9 and 10 to 14 for 1990, 2000, and 2016

Belgium	Lower respiratory infections	5 to 9	Female	1 (1 to 2)	0.4 (0.3 to 0.6)	1 (1 to 1)	0.3 (0.2 to 0.4)	1 (0 to 1)	0.2 (0.1 to 0.3)	-43.8 (-68.8 to 0.4)	-49.8 (-72.1 to -10.3)
Belgium	Lower respiratory infections	5 to 9	Male	2 (1 to 2)	0.5 (0.4 to 0.7)	1 (1 to 2)	0.4 (0.3 to 0.5)	1 (0 to 1)	0.2 (0.1 to 0.3)	-51.4 (-71.3 to -18.6)	-56.4 (-74.3 to -27.1)
Belgium	Lower respiratory infections	10 to 14	Both	2 (1 to 2)	0.3 (0.2 to 0.4)	2 (1 to 2)	0.3 (0.2 to 0.4)	1 (1 to 1)	0.2 (0.1 to 0.2)	-38.2 (-58.3 to -7.3)	-39.5 (-59.2 to -9.3)
Belgium	Lower respiratory infections	10 to 14	Female	1 (1 to 1)	0.3 (0.2 to 0.5)	1 (1 to 2)	0.4 (0.2 to 0.5)	1 (0 to 1)	0.2 (0.1 to 0.3)	-33.8 (-61.7 to 15.4)	-35.2 (-62.4 to 13.0)
Belgium	Lower respiratory infections	10 to 14	Male	1 (1 to 1)	0.3 (0.2 to 0.4)	1 (1 to 1)	0.2 (0.2 to 0.3)	0 (0 to 1)	0.1 (0.1 to 0.2)	-43.5 (-66.8 to -2.3)	-44.7 (-67.6 to -4.5)
Belgium	Upper respiratory infections	5 to 9	Both	0 (0 to 0)	0.0 (0.0 to 0.0)	0 (0 to 0)	0.0 (0.0 to 0.0)	0 (0 to 0)	0.0 (0.0 to 0.0)	-68.9 (-99.5 to 1432.4)	-72.2 (-99.6 to 1270.9)
Belgium	Upper respiratory infections	5 to 9	Female	0 (0 to 0)	0.0 (0.0 to 0.0)	0 (0 to 0)	0.0 (0.0 to 0.0)	0 (0 to 0)	0.0 (0.0 to 0.0)	-67.4 (-100.0 to 40090.1)	-70.9 (-100.0 to 35807.2)
Belgium	Upper respiratory infections	5 to 9	Male	0 (0 to 0)	0.0 (0.0 to 0.0)	0 (0 to 0)	0.0 (0.0 to 0.0)	0 (0 to 0)	0.0 (0.0 to 0.0)	-70.0 (-100.0 to 30764.5)	-73.1 (-100.0 to 27548.3)
Belgium	Upper respiratory infections	10 to 14	Both	0 (0 to 0)	0.0 (0.0 to 0.0)	0 (0 to 0)	0.0 (0.0 to 0.0)	0 (0 to 0)	0.0 (0.0 to 0.0)	-65.3 (-99.4 to 1817.4)	-66.0 (-99.5 to 1776.6)
Belgium	Upper respiratory infections	10 to 14	Female	0 (0 to 0)	0.0 (0.0 to 0.0)	0 (0 to 0)	0.0 (0.0 to 0.0)	0 (0 to 0)	0.0 (0.0 to 0.0)	-59.2 (-100.0 to 34732.5)	-60.1 (-100.0 to 34016.1)
Belgium	Upper respiratory infections	10 to 14	Male	0 (0 to 0)	0.0 (0.0 to 0.0)	0 (0 to 0)	0.0 (0.0 to 0.0)	0 (0 to 0)	0.0 (0.0 to 0.0)	-69.2 (-100.0 to 47252.6)	-69.8 (-100.0 to 46211.9)
Belgium	Otitis media	5 to 9	Both	0 (0 to 0)	0.0 (0.0 to 0.0)	0 (0 to 0)	0.0 (0.0 to 0.0)	0 (0 to 0)	0.0 (0.0 to 0.0)	-95.1 (-98.1 to -88.2)	-95.6 (-98.3 to -89.4)
Belgium	Otitis media	5 to 9	Female	0 (0 to 0)	0.0 (0.0 to 0.0)	0 (0 to 0)	0.0 (0.0 to 0.0)	0 (0 to 0)	0.0 (0.0 to 0.0)	-97.1 (-99.3 to -89.7)	-97.4 (-99.4 to -90.8)
Belgium	Otitis media	5 to 9	Male	0 (0 to 0)	0.0 (0.0 to 0.0)	0 (0 to 0)	0.0 (0.0 to 0.0)	0 (0 to 0)	0.0 (0.0 to 0.0)	-91.1 (-97.2 to -76.1)	-92.0 (-97.5 to -78.6)
Belgium	Otitis media	10 to 14	Both	0 (0 to 0)	0.0 (0.0 to 0.0)	0 (0 to 0)	0.0 (0.0 to 0.0)	0 (0 to 0)	0.0 (0.0 to 0.0)	-98.0 (-99.3 to -94.7)	-98.0 (-99.3 to -94.8)
Belgium	Otitis media	10 to 14	Female	0 (0 to 0)	0.0 (0.0 to 0.0)	0 (0 to 0)	0.0 (0.0 to 0.0)	0 (0 to 0)	0.0 (0.0 to 0.0)	-83.1 (-96.3 to -36.1)	-83.5 (-96.3 to -37.4)
Belgium	Otitis media	10 to 14	Male	0 (0 to 0)	0.0 (0.0 to 0.0)	0 (0 to 0)	0.0 (0.0 to 0.0)	0 (0 to 0)	0.0 (0.0 to 0.0)	-98.6 (-99.6 to -95.7)	-98.6 (-99.6 to -95.8)
Belgium	Meningitis	5 to 9	Both	2 (1 to 2)	0.3 (0.2 to 0.4)	1 (1 to 2)	0.2 (0.2 to 0.3)	1 (1 to 1)	0.1 (0.1 to 0.1)	-59.1 (-72.5 to -37.9)	-63.4 (-75.4 to -44.5)
Belgium	Meningitis	5 to 9	Female	1 (0 to 1)	0.2 (0.2 to 0.3)	1 (0 to 1)	0.2 (0.1 to 0.3)	0 (0 to 0)	0.1 (0.1 to 0.1)	-56.7 (-74.0 to -28.8)	-61.3 (-76.8 to -36.3)
Belgium	Meningitis	5 to 9	Male	1 (1 to 2)	0.3 (0.2 to 0.5)	1 (1 to 1)	0.2 (0.2 to 0.3)	0 (0 to 1)	0.1 (0.1 to 0.2)	-60.8 (-77.4 to -29.0)	-64.9 (-79.8 to -36.4)
Belgium	Meningitis	10 to 14	Both	1 (1 to 2)	0.2 (0.1 to 0.3)	1 (1 to 1)	0.2 (0.1 to 0.2)	0 (0 to 1)	0.1 (0.1 to 0.1)	-53.9 (-68.2 to -29.8)	-54.9 (-68.9 to -31.3)
Belgium	Meningitis	10 to 14	Female	0 (0 to 1)	0.1 (0.1 to 0.2)	0 (0 to 1)	0.2 (0.1 to 0.2)	0 (0 to 0)	0.1 (0.0 to 0.1)	-47.1 (-71.2 to -7.1)	-48.2 (-71.8 to -9.1)
Belgium	Meningitis	10 to 14	Male	1 (0 to 1)	0.2 (0.1 to 0.4)	1 (0 to 1)	0.2 (0.1 to 0.3)	0 (0 to 0)	0.1 (0.1 to 0.1)	-58.2 (-74.1 to -26.9)	-59.1 (-74.7 to -28.5)
Belgium	Encephalitis	5 to 9	Both	1 (0 to 1)	0.1 (0.1 to 0.1)	1 (0 to 1)	0.1 (0.1 to 0.1)	1 (0 to 1)	0.1 (0.1 to 0.1)	-0.7 (-31.3 to 39.9)	-11.2 (-38.5 to 25.2)
Belgium	Encephalitis	5 to 9	Female	0 (0 to 0)	0.1 (0.0 to 0.1)	0 (0 to 0)	0.1 (0.0 to 0.1)	0 (0 to 0)	0.1 (0.0 to 0.1)	9.1 (-34.7 to 80.6)	-2.6 (-41.7 to 61.4)
Belgium	Encephalitis	5 to 9	Male	0 (0 to 0)	0.1 (0.1 to 0.2)	0 (0 to 1)	0.1 (0.1 to 0.2)	0 (0 to 0)	0.1 (0.0 to 0.1)	-8.1 (-47.3 to 37.0)	-17.7 (-52.8 to 22.7)
Belgium	Encephalitis	10 to 14	Both	0 (0 to 1)	0.1 (0.0 to 0.1)	0 (0 to 1)	0.1 (0.0 to 0.1)	0 (0 to 1)	0.1 (0.0 to 0.1)	0.9 (-28.7 to 47.3)	-1.3 (-30.2 to 44.2)

eTable 8. All-cause and cause-specific mortality (counts and rates) at the global level and by country in the WHO European Region for ages 5 to 9 and 10 to 14 for 1990, 2000, and 2016

Belgium	Encephalitis	10 to 14	Female	0 (0 to 0)	0.1 (0.0 to 0.1)	0 (0 to 0)	0.1 (0.0 to 0.1)	0 (0 to 0)	0.1 (0.0 to 0.1)	6.0 (-31.4 to 80.8)	3.9 (-32.8 to 77.1)
Belgium	Encephalitis	10 to 14	Male	0 (0 to 0)	0.1 (0.0 to 0.1)	0 (0 to 0)	0.1 (0.0 to 0.1)	0 (0 to 0)	0.1 (0.0 to 0.1)	-5.5 (-43.7 to 50.6)	-7.5 (-44.9 to 47.3)
Belgium	Diphtheria	5 to 9	Both	0 (0 to 0)	0.0 (0.0 to 0.0)	0 (0 to 0)	0.0 (0.0 to 0.0)	0 (0 to 0)	0.0 (0.0 to 0.0)	-73.5 (-96.0 to 97.9)	-76.3 (-96.4 to 77.1)
Belgium	Diphtheria	5 to 9	Female	0 (0 to 0)	0.0 (0.0 to 0.0)	0 (0 to 0)	0.0 (0.0 to 0.0)	0 (0 to 0)	0.0 (0.0 to 0.0)	-72.7 (-98.9 to 325.3)	-75.6 (-99.0 to 280.0)
Belgium	Diphtheria	5 to 9	Male	0 (0 to 0)	0.0 (0.0 to 0.0)	0 (0 to 0)	0.0 (0.0 to 0.0)	0 (0 to 0)	0.0 (0.0 to 0.0)	-74.2 (-98.7 to 415.8)	-76.9 (-98.8 to 362.0)
Belgium	Diphtheria	10 to 14	Both	0 (0 to 0)	0.0 (0.0 to 0.0)	0 (0 to 0)	0.0 (0.0 to 0.0)	0 (0 to 0)	0.0 (0.0 to 0.0)	-70.9 (-95.6 to 115.2)	-71.5 (-95.7 to 110.6)
Belgium	Diphtheria	10 to 14	Female	0 (0 to 0)	0.0 (0.0 to 0.0)	0 (0 to 0)	0.0 (0.0 to 0.0)	0 (0 to 0)	0.0 (0.0 to 0.0)	-69.5 (-98.5 to 607.0)	-70.1 (-98.6 to 592.5)
Belgium	Diphtheria	10 to 14	Male	0 (0 to 0)	0.0 (0.0 to 0.0)	0 (0 to 0)	0.0 (0.0 to 0.0)	0 (0 to 0)	0.0 (0.0 to 0.0)	-72.0 (-98.9 to 439.3)	-72.6 (-98.9 to 427.4)
Belgium	Whooping cough	5 to 9	Both	0 (0 to 0)	0.0 (0.0 to 0.0)	0 (0 to 0)	0.0 (0.0 to 0.0)	0 (0 to 0)	0.0 (0.0 to 0.0)	637.1 (-44.8 to 2988.6)	559.5 (-50.7 to 2663.2)
Belgium	Whooping cough	5 to 9	Female	0 (0 to 0)	0.0 (0.0 to 0.0)	0 (0 to 0)	0.0 (0.0 to 0.0)	0 (0 to 0)	0.0 (0.0 to 0.0)	1041.9 (-59.8 to 5774.8)	920.2 (-64.1 to 5148.8)
Belgium	Whooping cough	5 to 9	Male	0 (0 to 0)	0.0 (0.0 to 0.0)	0 (0 to 0)	0.0 (0.0 to 0.0)	0 (0 to 0)	0.0 (0.0 to 0.0)	267.5 (-16.2 to 1416.9)	229.2 (-25.0 to 1258.8)
Belgium	Whooping cough	10 to 14	Both	0 (0 to 0)	0.0 (0.0 to 0.0)	0 (0 to 0)	0.0 (0.0 to 0.0)	0 (0 to 0)	0.0 (0.0 to 0.0)	342.5 (-32.0 to 1359.2)	333.1 (-33.4 to 1328.1)
Belgium	Whooping cough	10 to 14	Female	0 (0 to 0)	0.0 (0.0 to 0.0)	0 (0 to 0)	0.0 (0.0 to 0.0)	0 (0 to 0)	0.0 (0.0 to 0.0)	200.5 (-47.9 to 1023.2)	194.3 (-48.9 to 1000.1)
Belgium	Whooping cough	10 to 14	Male	0 (0 to 0)	0.0 (0.0 to 0.0)	0 (0 to 0)	0.0 (0.0 to 0.0)	0 (0 to 0)	0.0 (0.0 to 0.0)	449.4 (-20.0 to 2077.7)	437.3 (-21.8 to 2029.8)
Belgium	Tetanus	5 to 9	Both	0 (0 to 0)	0.0 (0.0 to 0.0)	0 (0 to 0)	0.0 (0.0 to 0.0)	0 (0 to 0)	0.0 (0.0 to 0.0)	-77.2 (-88.3 to -56.1)	-79.6 (-89.5 to -60.7)
Belgium	Tetanus	5 to 9	Female	0 (0 to 0)	0.0 (0.0 to 0.0)	0 (0 to 0)	0.0 (0.0 to 0.0)	0 (0 to 0)	0.0 (0.0 to 0.0)	-57.7 (-85.6 to 9.8)	-62.2 (-87.1 to -1.9)
Belgium	Tetanus	5 to 9	Male	0 (0 to 0)	0.0 (0.0 to 0.0)	0 (0 to 0)	0.0 (0.0 to 0.0)	0 (0 to 0)	0.0 (0.0 to 0.0)	-83.7 (-92.2 to -60.6)	-85.4 (-93.1 to -64.7)
Belgium	Tetanus	10 to 14	Both	0 (0 to 0)	0.0 (0.0 to 0.0)	0 (0 to 0)	0.0 (0.0 to 0.0)	0 (0 to 0)	0.0 (0.0 to 0.0)	-61.5 (-83.1 to -7.0)	-62.3 (-83.5 to -9.0)
Belgium	Tetanus	10 to 14	Female	0 (0 to 0)	0.0 (0.0 to 0.0)	0 (0 to 0)	0.0 (0.0 to 0.0)	0 (0 to 0)	0.0 (0.0 to 0.0)	-25.7 (-67.1 to 56.7)	-27.2 (-67.8 to 53.5)
Belgium	Tetanus	10 to 14	Male	0 (0 to 0)	0.0 (0.0 to 0.0)	0 (0 to 0)	0.0 (0.0 to 0.0)	0 (0 to 0)	0.0 (0.0 to 0.0)	-68.2 (-90.1 to -0.3)	-68.9 (-90.3 to -2.5)
Belgium	Measles	5 to 9	Both	0 (0 to 0)	0.0 (0.0 to 0.0)	0 (0 to 0)	0.0 (0.0 to 0.0)	0 (0 to 0)	0.0 (0.0 to 0.0)	-11.0 (-62.1 to 106.8)	-20.4 (-66.1 to 85.0)
Belgium	Measles	5 to 9	Female	0 (0 to 0)	0.0 (0.0 to 0.0)	0 (0 to 0)	0.0 (0.0 to 0.0)	0 (0 to 0)	0.0 (0.0 to 0.0)	-35.2 (-81.6 to 97.0)	-42.1 (-83.6 to 76.1)
Belgium	Measles	5 to 9	Male	0 (0 to 0)	0.0 (0.0 to 0.0)	0 (0 to 0)	0.0 (0.0 to 0.0)	0 (0 to 0)	0.0 (0.0 to 0.0)	37.1 (-55.8 to 295.0)	22.8 (-60.5 to 253.8)
Belgium	Measles	10 to 14	Both	0 (0 to 0)	0.0 (0.0 to 0.0)	0 (0 to 0)	0.0 (0.0 to 0.0)	0 (0 to 0)	0.0 (0.0 to 0.0)	-5.9 (-58.4 to 111.2)	-7.9 (-59.3 to 106.7)
Belgium	Measles	10 to 14	Female	0 (0 to 0)	0.0 (0.0 to 0.0)	0 (0 to 0)	0.0 (0.0 to 0.0)	0 (0 to 0)	0.0 (0.0 to 0.0)	-23.0 (-73.6 to 111.3)	-24.6 (-74.2 to 106.9)
Belgium	Measles	10 to 14	Male	0 (0 to 0)	0.0 (0.0 to 0.0)	0 (0 to 0)	0.0 (0.0 to 0.0)	0 (0 to 0)	0.0 (0.0 to 0.0)	30.7 (-58.6 to 272.4)	27.9 (-59.5 to 264.2)
Belgium	Varicella and herpes zoster	5 to 9	Both	0 (0 to 1)	0.1 (0.0 to 0.1)	0 (0 to 0)	0.0 (0.0 to 0.1)	0 (0 to 0)	0.0 (0.0 to 0.1)	-32.4 (-66.0 to 26.6)	-39.5 (-69.6 to 13.3)
Belgium	Varicella and herpes zoster	5 to 9	Female	0 (0 to 0)	0.0 (0.0 to 0.1)	0 (0 to 0)	0.0 (0.0 to 0.0)	0 (0 to 0)	0.0 (0.0 to 0.0)	-32.4 (-68.7 to 67.0)	-39.6 (-72.0 to 49.2)
Belgium	Varicella and herpes zoster	5 to 9	Male	0 (0 to 0)	0.1 (0.0 to 0.1)	0 (0 to 0)	0.1 (0.0 to 0.1)	0 (0 to 0)	0.0 (0.0 to 0.1)	-32.3 (-67.7 to 41.1)	-39.4 (-71.0 to 26.4)
Belgium	Varicella and herpes zoster	10 to 14	Both	0 (0 to 0)	0.0 (0.0 to 0.0)	0 (0 to 0)	0.0 (0.0 to 0.0)	0 (0 to 0)	0.0 (0.0 to 0.0)	-32.5 (-62.9 to 42.7)	-34.0 (-63.7 to 39.7)
Belgium	Varicella and herpes zoster	10 to 14	Female	0 (0 to 0)	0.0 (0.0 to 0.0)	0 (0 to 0)	0.0 (0.0 to 0.0)	0 (0 to 0)	0.0 (0.0 to 0.0)	-34.0 (-62.7 to 82.7)	-35.3 (-63.5 to 78.9)
Belgium	Varicella and herpes zoster	10 to 14	Male	0 (0 to 0)	0.0 (0.0 to 0.0)	0 (0 to 0)	0.0 (0.0 to 0.0)	0 (0 to 0)	0.0 (0.0 to 0.0)	-31.7 (-71.3 to 64.6)	-33.2 (-71.9 to 61.0)

eTable 8. All-cause and cause-specific mortality (counts and rates) at the global level and by country in the WHO European Region for ages 5 to 9 and 10 to 14 for 1990, 2000, and 2016

Belgium	Malaria	5 to 9	Both	0 (0 to 0)	0.0 (0.0 to 0.0)	0 (0 to 0)	0.0 (0.0 to 0.0)	0 (0 to 0)	0.0 (0.0 to 0.0)	0.0 (0.0 to 0.0)	0.0 (0.0 to 0.0)
Belgium	Malaria	5 to 9	Female	0 (0 to 0)	0.0 (0.0 to 0.0)	0 (0 to 0)	0.0 (0.0 to 0.0)	0 (0 to 0)	0.0 (0.0 to 0.0)	0.0 (0.0 to 0.0)	0.0 (0.0 to 0.0)
Belgium	Malaria	5 to 9	Male	0 (0 to 0)	0.0 (0.0 to 0.0)	0 (0 to 0)	0.0 (0.0 to 0.0)	0 (0 to 0)	0.0 (0.0 to 0.0)	0.0 (0.0 to 0.0)	0.0 (0.0 to 0.0)
Belgium	Malaria	10 to 14	Both	0 (0 to 0)	0.0 (0.0 to 0.0)	0 (0 to 0)	0.0 (0.0 to 0.0)	0 (0 to 0)	0.0 (0.0 to 0.0)	0.0 (0.0 to 0.0)	0.0 (0.0 to 0.0)
Belgium	Malaria	10 to 14	Female	0 (0 to 0)	0.0 (0.0 to 0.0)	0 (0 to 0)	0.0 (0.0 to 0.0)	0 (0 to 0)	0.0 (0.0 to 0.0)	0.0 (0.0 to 0.0)	0.0 (0.0 to 0.0)
Belgium	Malaria	10 to 14	Male	0 (0 to 0)	0.0 (0.0 to 0.0)	0 (0 to 0)	0.0 (0.0 to 0.0)	0 (0 to 0)	0.0 (0.0 to 0.0)	0.0 (0.0 to 0.0)	0.0 (0.0 to 0.0)
Belgium	Leishmaniasis	5 to 9	Both	0 (0 to 0)	0.0 (0.0 to 0.0)	0 (0 to 0)	0.0 (0.0 to 0.0)	0 (0 to 0)	0.0 (0.0 to 0.0)	0.0 (0.0 to 0.0)	0.0 (0.0 to 0.0)
Belgium	Leishmaniasis	5 to 9	Female	0 (0 to 0)	0.0 (0.0 to 0.0)	0 (0 to 0)	0.0 (0.0 to 0.0)	0 (0 to 0)	0.0 (0.0 to 0.0)	0.0 (0.0 to 0.0)	0.0 (0.0 to 0.0)
Belgium	Leishmaniasis	5 to 9	Male	0 (0 to 0)	0.0 (0.0 to 0.0)	0 (0 to 0)	0.0 (0.0 to 0.0)	0 (0 to 0)	0.0 (0.0 to 0.0)	0.0 (0.0 to 0.0)	0.0 (0.0 to 0.0)
Belgium	Leishmaniasis	10 to 14	Both	0 (0 to 0)	0.0 (0.0 to 0.0)	0 (0 to 0)	0.0 (0.0 to 0.0)	0 (0 to 0)	0.0 (0.0 to 0.0)	0.0 (0.0 to 0.0)	0.0 (0.0 to 0.0)
Belgium	Leishmaniasis	10 to 14	Female	0 (0 to 0)	0.0 (0.0 to 0.0)	0 (0 to 0)	0.0 (0.0 to 0.0)	0 (0 to 0)	0.0 (0.0 to 0.0)	0.0 (0.0 to 0.0)	0.0 (0.0 to 0.0)
Belgium	Leishmaniasis	10 to 14	Male	0 (0 to 0)	0.0 (0.0 to 0.0)	0 (0 to 0)	0.0 (0.0 to 0.0)	0 (0 to 0)	0.0 (0.0 to 0.0)	0.0 (0.0 to 0.0)	0.0 (0.0 to 0.0)
Belgium	African trypanosomiasis	5 to 9	Both	0 (0 to 0)	0.0 (0.0 to 0.0)	0 (0 to 0)	0.0 (0.0 to 0.0)	0 (0 to 0)	0.0 (0.0 to 0.0)	0.0 (0.0 to 0.0)	0.0 (0.0 to 0.0)
Belgium	African trypanosomiasis	5 to 9	Female	0 (0 to 0)	0.0 (0.0 to 0.0)	0 (0 to 0)	0.0 (0.0 to 0.0)	0 (0 to 0)	0.0 (0.0 to 0.0)	0.0 (0.0 to 0.0)	0.0 (0.0 to 0.0)
Belgium	African trypanosomiasis	5 to 9	Male	0 (0 to 0)	0.0 (0.0 to 0.0)	0 (0 to 0)	0.0 (0.0 to 0.0)	0 (0 to 0)	0.0 (0.0 to 0.0)	0.0 (0.0 to 0.0)	0.0 (0.0 to 0.0)
Belgium	African trypanosomiasis	10 to 14	Both	0 (0 to 0)	0.0 (0.0 to 0.0)	0 (0 to 0)	0.0 (0.0 to 0.0)	0 (0 to 0)	0.0 (0.0 to 0.0)	0.0 (0.0 to 0.0)	0.0 (0.0 to 0.0)
Belgium	African trypanosomiasis	10 to 14	Female	0 (0 to 0)	0.0 (0.0 to 0.0)	0 (0 to 0)	0.0 (0.0 to 0.0)	0 (0 to 0)	0.0 (0.0 to 0.0)	0.0 (0.0 to 0.0)	0.0 (0.0 to 0.0)
Belgium	African trypanosomiasis	10 to 14	Male	0 (0 to 0)	0.0 (0.0 to 0.0)	0 (0 to 0)	0.0 (0.0 to 0.0)	0 (0 to 0)	0.0 (0.0 to 0.0)	0.0 (0.0 to 0.0)	0.0 (0.0 to 0.0)
Belgium	Schistosomiasis	5 to 9	Both	0 (0 to 0)	0.0 (0.0 to 0.0)	0 (0 to 0)	0.0 (0.0 to 0.0)	0 (0 to 0)	0.0 (0.0 to 0.0)	0.0 (0.0 to 0.0)	0.0 (0.0 to 0.0)
Belgium	Schistosomiasis	5 to 9	Female	0 (0 to 0)	0.0 (0.0 to 0.0)	0 (0 to 0)	0.0 (0.0 to 0.0)	0 (0 to 0)	0.0 (0.0 to 0.0)	0.0 (0.0 to 0.0)	0.0 (0.0 to 0.0)
Belgium	Schistosomiasis	5 to 9	Male	0 (0 to 0)	0.0 (0.0 to 0.0)	0 (0 to 0)	0.0 (0.0 to 0.0)	0 (0 to 0)	0.0 (0.0 to 0.0)	0.0 (0.0 to 0.0)	0.0 (0.0 to 0.0)
Belgium	Schistosomiasis	10 to 14	Both	0 (0 to 0)	0.0 (0.0 to 0.0)	0 (0 to 0)	0.0 (0.0 to 0.0)	0 (0 to 0)	0.0 (0.0 to 0.0)	0.0 (0.0 to 0.0)	0.0 (0.0 to 0.0)
Belgium	Schistosomiasis	10 to 14	Female	0 (0 to 0)	0.0 (0.0 to 0.0)	0 (0 to 0)	0.0 (0.0 to 0.0)	0 (0 to 0)	0.0 (0.0 to 0.0)	0.0 (0.0 to 0.0)	0.0 (0.0 to 0.0)
Belgium	Schistosomiasis	10 to 14	Male	0 (0 to 0)	0.0 (0.0 to 0.0)	0 (0 to 0)	0.0 (0.0 to 0.0)	0 (0 to 0)	0.0 (0.0 to 0.0)	0.0 (0.0 to 0.0)	0.0 (0.0 to 0.0)
Belgium	Cysticercosis	5 to 9	Both	0 (0 to 0)	0.0 (0.0 to 0.0)	0 (0 to 0)	0.0 (0.0 to 0.0)	0 (0 to 0)	0.0 (0.0 to 0.0)	-83.4 (-85.9 to -80.7)	-85.2 (-87.3 to -82.7)
Belgium	Cysticercosis	5 to 9	Female	0 (0 to 0)	0.0 (0.0 to 0.0)	0 (0 to 0)	0.0 (0.0 to 0.0)	0 (0 to 0)	0.0 (0.0 to 0.0)	-82.9 (-85.9 to -79.5)	-84.7 (-87.4 to -81.7)
Belgium	Cysticercosis	5 to 9	Male	0 (0 to 0)	0.0 (0.0 to 0.0)	0 (0 to 0)	0.0 (0.0 to 0.0)	0 (0 to 0)	0.0 (0.0 to 0.0)	-84.0 (-86.8 to -80.7)	-85.7 (-88.2 to -82.7)
Belgium	Cysticercosis	10 to 14	Both	0 (0 to 0)	0.0 (0.0 to 0.0)	0 (0 to 0)	0.0 (0.0 to 0.0)	0 (0 to 0)	0.0 (0.0 to 0.0)	-80.8 (-83.3 to -78.2)	-81.2 (-83.7 to -78.6)
Belgium	Cysticercosis	10 to 14	Female	0 (0 to 0)	0.0 (0.0 to 0.0)	0 (0 to 0)	0.0 (0.0 to 0.0)	0 (0 to 0)	0.0 (0.0 to 0.0)	-79.3 (-82.8 to -75.2)	-79.7 (-83.1 to -75.7)

eTable 8. All-cause and cause-specific mortality (counts and rates) at the global level and by country in the WHO European Region for ages 5 to 9 and 10 to 14 for 1990, 2000, and 2016

Belgium	Cysticercosis	10 to 14	Male	0 (0 to 0)	0.0 (0.0 to 0.0)	0 (0 to 0)	0.0 (0.0 to 0.0)	0 (0 to 0)	0.0 (0.0 to 0.0)	-82.5 (-85.3 to -79.1)	-82.9 (-85.7 to -79.6)
Belgium	Yellow fever	5 to 9	Both	0 (0 to 0)	0.0 (0.0 to 0.0)	0 (0 to 0)	0.0 (0.0 to 0.0)	0 (0 to 0)	0.0 (0.0 to 0.0)	0.0 (0.0 to 0.0)	0.0 (0.0 to 0.0)
Belgium	Yellow fever	5 to 9	Female	0 (0 to 0)	0.0 (0.0 to 0.0)	0 (0 to 0)	0.0 (0.0 to 0.0)	0 (0 to 0)	0.0 (0.0 to 0.0)	0.0 (0.0 to 0.0)	0.0 (0.0 to 0.0)
Belgium	Yellow fever	5 to 9	Male	0 (0 to 0)	0.0 (0.0 to 0.0)	0 (0 to 0)	0.0 (0.0 to 0.0)	0 (0 to 0)	0.0 (0.0 to 0.0)	0.0 (0.0 to 0.0)	0.0 (0.0 to 0.0)
Belgium	Yellow fever	10 to 14	Both	0 (0 to 0)	0.0 (0.0 to 0.0)	0 (0 to 0)	0.0 (0.0 to 0.0)	0 (0 to 0)	0.0 (0.0 to 0.0)	0.0 (0.0 to 0.0)	0.0 (0.0 to 0.0)
Belgium	Yellow fever	10 to 14	Female	0 (0 to 0)	0.0 (0.0 to 0.0)	0 (0 to 0)	0.0 (0.0 to 0.0)	0 (0 to 0)	0.0 (0.0 to 0.0)	0.0 (0.0 to 0.0)	0.0 (0.0 to 0.0)
Belgium	Yellow fever	10 to 14	Male	0 (0 to 0)	0.0 (0.0 to 0.0)	0 (0 to 0)	0.0 (0.0 to 0.0)	0 (0 to 0)	0.0 (0.0 to 0.0)	0.0 (0.0 to 0.0)	0.0 (0.0 to 0.0)
Belgium	Rabies	5 to 9	Both	0 (0 to 0)	0.0 (0.0 to 0.0)	0 (0 to 0)	0.0 (0.0 to 0.0)	0 (0 to 0)	0.0 (0.0 to 0.0)	2.6 (-77.8 to 406.5)	-8.2 (-80.1 to 353.1)
Belgium	Rabies	5 to 9	Female	0 (0 to 0)	0.0 (0.0 to 0.0)	0 (0 to 0)	0.0 (0.0 to 0.0)	0 (0 to 0)	0.0 (0.0 to 0.0)	933.3 (69.8 to 3311.8)	823.2 (51.7 to 2948.2)
Belgium	Rabies	5 to 9	Male	0 (0 to 0)	0.0 (0.0 to 0.0)	0 (0 to 0)	0.0 (0.0 to 0.0)	0 (0 to 0)	0.0 (0.0 to 0.0)	-12.9 (-83.4 to 367.1)	-22.0 (-85.2 to 318.4)
Belgium	Rabies	10 to 14	Both	0 (0 to 0)	0.0 (0.0 to 0.0)	0 (0 to 0)	0.0 (0.0 to 0.0)	0 (0 to 0)	0.0 (0.0 to 0.0)	63.1 (-60.4 to 562.8)	59.6 (-61.3 to 548.7)
Belgium	Rabies	10 to 14	Female	0 (0 to 0)	0.0 (0.0 to 0.0)	0 (0 to 0)	0.0 (0.0 to 0.0)	0 (0 to 0)	0.0 (0.0 to 0.0)	2220.3 (321.6 to 7121.3)	2172.6 (313.0 to 6972.8)
Belgium	Rabies	10 to 14	Male	0 (0 to 0)	0.0 (0.0 to 0.0)	0 (0 to 0)	0.0 (0.0 to 0.0)	0 (0 to 0)	0.0 (0.0 to 0.0)	-41.2 (-87.5 to 143.3)	-42.5 (-87.7 to 138.0)
Belgium	Intestinal nematode infections	5 to 9	Both	-	-	-	-	-	-	-	-
Belgium	Intestinal nematode infections	5 to 9	Female	-	-	-	-	-	-	-	-
Belgium	Intestinal nematode infections	5 to 9	Male	-	-	-	-	-	-	-	-
Belgium	Intestinal nematode infections	10 to 14	Both	-	-	-	-	-	-	-	-
Belgium	Intestinal nematode infections	10 to 14	Female	-	-	-	-	-	-	-	-
Belgium	Intestinal nematode infections	10 to 14	Male	-	-	-	-	-	-	-	-
Belgium	Ebola	5 to 9	Both	0 (0 to 0)	0.0 (0.0 to 0.0)	0 (0 to 0)	0.0 (0.0 to 0.0)	0 (0 to 0)	0.0 (0.0 to 0.0)	0.0 (0.0 to 0.0)	0.0 (0.0 to 0.0)
Belgium	Ebola	5 to 9	Female	0 (0 to 0)	0.0 (0.0 to 0.0)	0 (0 to 0)	0.0 (0.0 to 0.0)	0 (0 to 0)	0.0 (0.0 to 0.0)	0.0 (0.0 to 0.0)	0.0 (0.0 to 0.0)
Belgium	Ebola	5 to 9	Male	0 (0 to 0)	0.0 (0.0 to 0.0)	0 (0 to 0)	0.0 (0.0 to 0.0)	0 (0 to 0)	0.0 (0.0 to 0.0)	0.0 (0.0 to 0.0)	0.0 (0.0 to 0.0)
Belgium	Ebola	10 to 14	Both	0 (0 to 0)	0.0 (0.0 to 0.0)	0 (0 to 0)	0.0 (0.0 to 0.0)	0 (0 to 0)	0.0 (0.0 to 0.0)	0.0 (0.0 to 0.0)	0.0 (0.0 to 0.0)
Belgium	Ebola	10 to 14	Female	0 (0 to 0)	0.0 (0.0 to 0.0)	0 (0 to 0)	0.0 (0.0 to 0.0)	0 (0 to 0)	0.0 (0.0 to 0.0)	0.0 (0.0 to 0.0)	0.0 (0.0 to 0.0)
Belgium	Ebola	10 to 14	Male	0 (0 to 0)	0.0 (0.0 to 0.0)	0 (0 to 0)	0.0 (0.0 to 0.0)	0 (0 to 0)	0.0 (0.0 to 0.0)	0.0 (0.0 to 0.0)	0.0 (0.0 to 0.0)
Belgium	Zika virus	5 to 9	Both	0 (0 to 0)	0.0 (0.0 to 0.0)	0 (0 to 0)	0.0 (0.0 to 0.0)	0 (0 to 0)	0.0 (0.0 to 0.0)	0.0 (0.0 to 0.0)	0.0 (0.0 to 0.0)
Belgium	Zika virus	5 to 9	Female	0 (0 to 0)	0.0 (0.0 to 0.0)	0 (0 to 0)	0.0 (0.0 to 0.0)	0 (0 to 0)	0.0 (0.0 to 0.0)	0.0 (0.0 to 0.0)	0.0 (0.0 to 0.0)
Belgium	Zika virus	5 to 9	Male	0 (0 to 0)	0.0 (0.0 to 0.0)	0 (0 to 0)	0.0 (0.0 to 0.0)	0 (0 to 0)	0.0 (0.0 to 0.0)	0.0 (0.0 to 0.0)	0.0 (0.0 to 0.0)
Belgium	Zika virus	10 to 14	Both	0 (0 to 0)	0.0 (0.0 to 0.0)	0 (0 to 0)	0.0 (0.0 to 0.0)	0 (0 to 0)	0.0 (0.0 to 0.0)	0.0 (0.0 to 0.0)	0.0 (0.0 to 0.0)

eTable 8. All-cause and cause-specific mortality (counts and rates) at the global level and by country in the WHO European Region for ages 5 to 9 and 10 to 14 for 1990, 2000, and 2016

Belgium	Zika virus	10 to 14	Female	0<br>(0 to 0)	0.0<br>(0.0 to 0.0)	0<br>(0 to 0)	0.0<br>(0.0 to 0.0)	0<br>(0 to 0)	0.0<br>(0.0 to 0.0)	0.0<br>(0.0 to 0.0)	0.0<br>(0.0 to 0.0)
Belgium	Zika virus	10 to 14	Male	0<br>(0 to 0)	0.0<br>(0.0 to 0.0)	0<br>(0 to 0)	0.0<br>(0.0 to 0.0)	0<br>(0 to 0)	0.0<br>(0.0 to 0.0)	0.0<br>(0.0 to 0.0)	0.0<br>(0.0 to 0.0)
Belgium	Other neglected tropical diseases	5 to 9	Both	0<br>(0 to 0)	0.0<br>(0.0 to 0.0)	0<br>(0 to 0)	0.0<br>(0.0 to 0.0)	0<br>(0 to 0)	0.0<br>(0.0 to 0.0)	-1.0<br>(-14.9 to 15.1)	-11.4<br>(-23.8 to 3.0)
Belgium	Other neglected tropical diseases	5 to 9	Female	0<br>(0 to 0)	0.0<br>(0.0 to 0.0)	0<br>(0 to 0)	0.0<br>(0.0 to 0.0)	0<br>(0 to 0)	0.0<br>(0.0 to 0.0)	0.1<br>(-17.1 to 19.8)	-10.6<br>(-26.0 to 7.1)
Belgium	Other neglected tropical diseases	5 to 9	Male	0<br>(0 to 0)	0.0<br>(0.0 to 0.0)	0<br>(0 to 0)	0.0<br>(0.0 to 0.0)	0<br>(0 to 0)	0.0<br>(0.0 to 0.0)	-2.0<br>(-18.3 to 16.8)	-12.2<br>(-26.8 to 4.6)
Belgium	Other neglected tropical diseases	10 to 14	Both	0<br>(0 to 0)	0.0<br>(0.0 to 0.0)	0<br>(0 to 0)	0.0<br>(0.0 to 0.0)	0<br>(0 to 0)	0.0<br>(0.0 to 0.0)	-4.3<br>(-16.1 to 7.7)	-6.4<br>(-17.9 to 5.4)
Belgium	Other neglected tropical diseases	10 to 14	Female	0<br>(0 to 0)	0.0<br>(0.0 to 0.0)	0<br>(0 to 0)	0.0<br>(0.0 to 0.0)	0<br>(0 to 0)	0.0<br>(0.0 to 0.0)	-1.5<br>(-17.2 to 18.0)	-3.6<br>(-18.9 to 15.6)
Belgium	Other neglected tropical diseases	10 to 14	Male	0<br>(0 to 0)	0.0<br>(0.0 to 0.0)	0<br>(0 to 0)	0.0<br>(0.0 to 0.0)	0<br>(0 to 0)	0.0<br>(0.0 to 0.0)	-7.1<br>(-21.6 to 10.3)	-9.1<br>(-23.3 to 7.9)
Belgium	Maternal hemorrhage	10 to 14	Both	0<br>(0 to 0)	0.0<br>(0.0 to 0.0)	0<br>(0 to 0)	0.0<br>(0.0 to 0.0)	0<br>(0 to 0)	0.0<br>(0.0 to 0.0)	-53.5<br>(-69.4 to -30.4)	-54.4<br>(-70.0 to -31.9)
Belgium	Maternal hemorrhage	10 to 14	Female	0<br>(0 to 0)	0.0<br>(0.0 to 0.0)	0<br>(0 to 0)	0.0<br>(0.0 to 0.0)	0<br>(0 to 0)	0.0<br>(0.0 to 0.0)	-53.5<br>(-69.4 to -30.4)	-54.4<br>(-70.0 to -31.8)
Belgium	Maternal sepsis and other maternal infections	10 to 14	Both	0<br>(0 to 0)	0.0<br>(0.0 to 0.0)	0<br>(0 to 0)	0.0<br>(0.0 to 0.0)	0<br>(0 to 0)	0.0<br>(0.0 to 0.0)	-58.2<br>(-71.7 to -35.9)	-59.1<br>(-72.3 to -37.3)
Belgium	Maternal sepsis and other maternal infections	10 to 14	Female	0<br>(0 to 0)	0.0<br>(0.0 to 0.0)	0<br>(0 to 0)	0.0<br>(0.0 to 0.0)	0<br>(0 to 0)	0.0<br>(0.0 to 0.0)	-58.2<br>(-71.7 to -35.9)	-59.0<br>(-72.3 to -37.2)
Belgium	Maternal hypertensive disorders	10 to 14	Both	0<br>(0 to 0)	0.0<br>(0.0 to 0.0)	0<br>(0 to 0)	0.0<br>(0.0 to 0.0)	0<br>(0 to 0)	0.0<br>(0.0 to 0.0)	-54.1<br>(-69.0 to -32.9)	-55.1<br>(-69.6 to -34.3)
Belgium	Maternal hypertensive disorders	10 to 14	Female	0<br>(0 to 0)	0.0<br>(0.0 to 0.0)	0<br>(0 to 0)	0.0<br>(0.0 to 0.0)	0<br>(0 to 0)	0.0<br>(0.0 to 0.0)	-54.1<br>(-69.0 to -32.9)	-55.1<br>(-69.6 to -34.3)
Belgium	Maternal obstructed labor and uterine rupture	10 to 14	Both	0<br>(0 to 0)	0.0<br>(0.0 to 0.0)	0<br>(0 to 0)	0.0<br>(0.0 to 0.0)	0<br>(0 to 0)	0.0<br>(0.0 to 0.0)	-53.9<br>(-70.1 to -27.5)	-54.9<br>(-70.7 to -29.0)
Belgium	Maternal obstructed labor and uterine rupture	10 to 14	Female	0<br>(0 to 0)	0.0<br>(0.0 to 0.0)	0<br>(0 to 0)	0.0<br>(0.0 to 0.0)	0<br>(0 to 0)	0.0<br>(0.0 to 0.0)	-53.9<br>(-70.1 to -27.5)	-54.9<br>(-70.7 to -28.9)
Belgium	Maternal abortion, miscarriage, and ectopic pregnancy	10 to 14	Both	0<br>(0 to 0)	0.0<br>(0.0 to 0.0)	0<br>(0 to 0)	0.0<br>(0.0 to 0.0)	0<br>(0 to 0)	0.0<br>(0.0 to 0.0)	-60.5<br>(-74.3 to -39.7)	-61.3<br>(-74.9 to -40.9)

eTable 8. All-cause and cause-specific mortality (counts and rates) at the global level and by country in the WHO European Region for ages 5 to 9 and 10 to 14 for 1990, 2000, and 2016

Belgium	Maternal abortion, miscarriage, and ectopic pregnancy	10 to 14	Female	0 (0 to 0)	0.0 (0.0 to 0.0)	0 (0 to 0)	0.0 (0.0 to 0.0)	0 (0 to 0)	0.0 (0.0 to 0.0)	-60.5 (-74.3 to -39.7)	-61.3 (-74.9 to -40.9)
Belgium	Indirect maternal deaths	10 to 14	Both	0 (0 to 0)	0.0 (0.0 to 0.0)	0 (0 to 0)	0.0 (0.0 to 0.0)	0 (0 to 0)	0.0 (0.0 to 0.0)	-57.2 (-71.1 to -38.3)	-58.1 (-71.7 to -39.6)
Belgium	Indirect maternal deaths	10 to 14	Female	0 (0 to 0)	0.0 (0.0 to 0.0)	0 (0 to 0)	0.0 (0.0 to 0.0)	0 (0 to 0)	0.0 (0.0 to 0.0)	-57.2 (-71.1 to -38.3)	-58.1 (-71.7 to -39.6)
Belgium	Late maternal deaths	10 to 14	Both	0 (0 to 0)	0.0 (0.0 to 0.0)	0 (0 to 0)	0.0 (0.0 to 0.0)	0 (0 to 0)	0.0 (0.0 to 0.0)	-61.7 (-82.4 to -21.1)	-62.5 (-82.8 to -22.8)
Belgium	Late maternal deaths	10 to 14	Female	0 (0 to 0)	0.0 (0.0 to 0.0)	0 (0 to 0)	0.0 (0.0 to 0.0)	0 (0 to 0)	0.0 (0.0 to 0.0)	-61.7 (-82.4 to -21.1)	-62.5 (-82.8 to -22.7)
Belgium	Maternal deaths aggravated by HIV/AIDS	10 to 14	Both	0 (0 to 0)	0.0 (0.0 to 0.0)	0 (0 to 0)	0.0 (0.0 to 0.0)	0 (0 to 0)	0.0 (0.0 to 0.0)	4648.9 (834.1 to 25372.5)	4547.8 (814.2 to 24830.2)
Belgium	Maternal deaths aggravated by HIV/AIDS	10 to 14	Female	0 (0 to 0)	0.0 (0.0 to 0.0)	0 (0 to 0)	0.0 (0.0 to 0.0)	0 (0 to 0)	0.0 (0.0 to 0.0)	4648.9 (834.1 to 25372.5)	4551.2 (814.9 to 24848.7)
Belgium	Other maternal disorders	10 to 14	Both	0 (0 to 0)	0.0 (0.0 to 0.0)	0 (0 to 0)	0.0 (0.0 to 0.0)	0 (0 to 0)	0.0 (0.0 to 0.0)	-54.4 (-68.5 to -34.2)	-55.4 (-69.2 to -35.6)
Belgium	Other maternal disorders	10 to 14	Female	0 (0 to 0)	0.0 (0.0 to 0.0)	0 (0 to 0)	0.0 (0.0 to 0.0)	0 (0 to 0)	0.0 (0.0 to 0.0)	-54.4 (-68.5 to -34.2)	-55.4 (-69.2 to -35.6)
Belgium	Protein-energy malnutrition	5 to 9	Both	0 (0 to 0)	0.0 (0.0 to 0.0)	0 (0 to 0)	0.0 (0.0 to 0.0)	0 (0 to 0)	0.0 (0.0 to 0.0)	58.2 (-10.0 to 214.2)	41.5 (-19.5 to 181.1)
Belgium	Protein-energy malnutrition	5 to 9	Female	0 (0 to 0)	0.0 (0.0 to 0.0)	0 (0 to 0)	0.0 (0.0 to 0.0)	0 (0 to 0)	0.0 (0.0 to 0.0)	19.3 (-48.6 to 292.1)	6.6 (-54.1 to 250.3)
Belgium	Protein-energy malnutrition	5 to 9	Male	0 (0 to 0)	0.0 (0.0 to 0.0)	0 (0 to 0)	0.0 (0.0 to 0.0)	0 (0 to 0)	0.0 (0.0 to 0.0)	92.8 (-20.0 to 462.0)	72.7 (-28.4 to 403.4)
Belgium	Protein-energy malnutrition	10 to 14	Both	0 (0 to 0)	0.0 (0.0 to 0.0)	0 (0 to 0)	0.0 (0.0 to 0.0)	0 (0 to 0)	0.0 (0.0 to 0.0)	80.9 (-2.1 to 245.2)	77.1 (-4.2 to 237.8)
Belgium	Protein-energy malnutrition	10 to 14	Female	0 (0 to 0)	0.0 (0.0 to 0.0)	0 (0 to 0)	0.0 (0.0 to 0.0)	0 (0 to 0)	0.0 (0.0 to 0.0)	56.9 (-37.5 to 282.0)	53.7 (-38.8 to 274.1)
Belgium	Protein-energy malnutrition	10 to 14	Male	0 (0 to 0)	0.0 (0.0 to 0.0)	0 (0 to 0)	0.0 (0.0 to 0.0)	0 (0 to 0)	0.0 (0.0 to 0.0)	100.2 (-15.6 to 411.0)	95.8 (-17.4 to 399.8)
Belgium	Iodine deficiency	5 to 9	Both	0 (0 to 0)	0.0 (0.0 to 0.0)	0 (0 to 0)	0.0 (0.0 to 0.0)	0 (0 to 0)	0.0 (0.0 to 0.0)	-12.2 (-100.0 to 3.9e+20)	-21.5 (-100.0 to 3.5e+20)
Belgium	Iodine deficiency	5 to 9	Female	0 (0 to 0)	0.0 (0.0 to 0.0)	0 (0 to 0)	0.0 (0.0 to 0.0)	0 (0 to 0)	0.0 (0.0 to 0.0)	-21.6 (-100.0 to 1.2e+27)	-30.0 (-100.0 to 1.0e+27)
Belgium	Iodine deficiency	5 to 9	Male	0 (0 to 0)	0.0 (0.0 to 0.0)	0 (0 to 0)	0.0 (0.0 to 0.0)	0 (0 to 0)	0.0 (0.0 to 0.0)	-1.9 (-100.0 to 3.5e+26)	-12.1 (-100.0 to 3.2e+26)
Belgium	Iodine deficiency	10 to 14	Both	0 (0 to 0)	0.0 (0.0 to 0.0)	0 (0 to 0)	0.0 (0.0 to 0.0)	0 (0 to 0)	0.0 (0.0 to 0.0)	-7.0 (-100.0 to 6.3e+15)	-9.0 (-100.0 to 6.1e+15)
Belgium	Iodine deficiency	10 to 14	Female	0 (0 to 0)	0.0 (0.0 to 0.0)	0 (0 to 0)	0.0 (0.0 to 0.0)	0 (0 to 0)	0.0 (0.0 to 0.0)	-9.1 (-100.0 to 8.7e+25)	-10.9 (-100.0 to 8.6e+25)
Belgium	Iodine deficiency	10 to 14	Male	0 (0 to 0)	0.0 (0.0 to 0.0)	0 (0 to 0)	0.0 (0.0 to 0.0)	0 (0 to 0)	0.0 (0.0 to 0.0)	-5.0 (-100.0 to 1.3e+25)	-7.1 (-100.0 to 1.3e+25)
Belgium	Iron-deficiency anemia	5 to 9	Both	0 (0 to 0)	0.0 (0.0 to 0.0)	0 (0 to 0)	0.0 (0.0 to 0.0)	0 (0 to 0)	0.0 (0.0 to 0.0)	31.3 (-24.6 to 143.7)	17.4 (-32.6 to 118.1)
Belgium	Iron-deficiency anemia	5 to 9	Female	0 (0 to 0)	0.0 (0.0 to 0.0)	0 (0 to 0)	0.0 (0.0 to 0.0)	0 (0 to 0)	0.0 (0.0 to 0.0)	19.3 (-48.6 to 292.2)	6.6 (-54.1 to 250.4)

eTable 8. All-cause and cause-specific mortality (counts and rates) at the global level and by country in the WHO European Region for ages 5 to 9 and 10 to 14 for 1990, 2000, and 2016

Belgium	Iron-deficiency anemia	5 to 9	Male	0<br>(0 to 0)	0.0<br>(0.0 to 0.0)	0<br>(0 to 0)	0.0<br>(0.0 to 0.0)	0<br>(0 to 0)	0.0<br>(0.0 to 0.0)	41.9<br>(-42.3 to 291.2)	27.1<br>(-48.3 to 250.5)
Belgium	Iron-deficiency anemia	10 to 14	Both	0<br>(0 to 0)	0.0<br>(0.0 to 0.0)	0<br>(0 to 0)	0.0<br>(0.0 to 0.0)	0<br>(0 to 0)	0.0<br>(0.0 to 0.0)	65.2<br>(-9.1 to 200.8)	61.7<br>(-11.1 to 194.4)
Belgium	Iron-deficiency anemia	10 to 14	Female	0<br>(0 to 0)	0.0<br>(0.0 to 0.0)	0<br>(0 to 0)	0.0<br>(0.0 to 0.0)	0<br>(0 to 0)	0.0<br>(0.0 to 0.0)	56.3<br>(-37.5 to 282.0)	53.1<br>(-38.7 to 274.2)
Belgium	Iron-deficiency anemia	10 to 14	Male	0<br>(0 to 0)	0.0<br>(0.0 to 0.0)	0<br>(0 to 0)	0.0<br>(0.0 to 0.0)	0<br>(0 to 0)	0.0<br>(0.0 to 0.0)	72.3<br>(-27.8 to 344.8)	68.5<br>(-29.4 to 335.0)
Belgium	Other nutritional deficiencies	5 to 9	Both	0<br>(0 to 0)	0.0<br>(0.0 to 0.0)	0<br>(0 to 0)	0.0<br>(0.0 to 0.0)	0<br>(0 to 0)	0.0<br>(0.0 to 0.0)	24.3<br>(-28.2 to 138.2)	11.2<br>(-35.8 to 113.1)
Belgium	Other nutritional deficiencies	5 to 9	Female	0<br>(0 to 0)	0.0<br>(0.0 to 0.0)	0<br>(0 to 0)	0.0<br>(0.0 to 0.0)	0<br>(0 to 0)	0.0<br>(0.0 to 0.0)	6.7<br>(-55.1 to 292.5)	-4.6<br>(-59.9 to 250.6)
Belgium	Other nutritional deficiencies	5 to 9	Male	0<br>(0 to 0)	0.0<br>(0.0 to 0.0)	0<br>(0 to 0)	0.0<br>(0.0 to 0.0)	0<br>(0 to 0)	0.0<br>(0.0 to 0.0)	41.9<br>(-42.3 to 291.3)	27.1<br>(-48.3 to 250.5)
Belgium	Other nutritional deficiencies	10 to 14	Both	0<br>(0 to 0)	0.0<br>(0.0 to 0.0)	0<br>(0 to 0)	0.0<br>(0.0 to 0.0)	0<br>(0 to 0)	0.0<br>(0.0 to 0.0)	23.9<br>(-34.2 to 131.8)	21.3<br>(-35.6 to 126.8)
Belgium	Other nutritional deficiencies	10 to 14	Female	0<br>(0 to 0)	0.0<br>(0.0 to 0.0)	0<br>(0 to 0)	0.0<br>(0.0 to 0.0)	0<br>(0 to 0)	0.0<br>(0.0 to 0.0)	-0.8<br>(-58.8 to 157.1)	-2.9<br>(-59.7 to 151.9)
Belgium	Other nutritional deficiencies	10 to 14	Male	0<br>(0 to 0)	0.0<br>(0.0 to 0.0)	0<br>(0 to 0)	0.0<br>(0.0 to 0.0)	0<br>(0 to 0)	0.0<br>(0.0 to 0.0)	56.3<br>(-36.5 to 300.1)	52.9<br>(-37.9 to 291.3)
Belgium	Sexually transmitted diseases excluding HIV	5 to 9	Both	0<br>(0 to 0)	0.0<br>(0.0 to 0.0)	0<br>(0 to 0)	0.0<br>(0.0 to 0.0)	0<br>(0 to 0)	0.0<br>(0.0 to 0.0)	-82.7<br>(-91.2 to -69.1)	-84.6<br>(-92.1 to -72.4)
Belgium	Sexually transmitted diseases excluding HIV	5 to 9	Female	0<br>(0 to 0)	0.0<br>(0.0 to 0.0)	0<br>(0 to 0)	0.0<br>(0.0 to 0.0)	0<br>(0 to 0)	0.0<br>(0.0 to 0.0)	-82.6<br>(-91.1 to -69.3)	-84.5<br>(-92.1 to -72.5)
Belgium	Sexually transmitted diseases excluding HIV	5 to 9	Male	0<br>(0 to 0)	0.0<br>(0.0 to 0.0)	0<br>(0 to 0)	0.0<br>(0.0 to 0.0)	0<br>(0 to 0)	0.0<br>(0.0 to 0.0)	-82.9<br>(-91.3 to -68.4)	-84.7<br>(-92.2 to -71.7)
Belgium	Sexually transmitted diseases excluding HIV	10 to 14	Both	0<br>(0 to 0)	0.0<br>(0.0 to 0.0)	0<br>(0 to 0)	0.0<br>(0.0 to 0.0)	0<br>(0 to 0)	0.0<br>(0.0 to 0.0)	-47.5<br>(-63.6 to -7.5)	-48.6<br>(-64.4 to -9.5)
Belgium	Sexually transmitted diseases excluding HIV	10 to 14	Female	0<br>(0 to 0)	0.0<br>(0.0 to 0.0)	0<br>(0 to 0)	0.0<br>(0.0 to 0.0)	0<br>(0 to 0)	0.0<br>(0.0 to 0.0)	-2.1<br>(-19.0 to 18.0)	-4.1<br>(-20.7 to 15.6)
Belgium	Sexually transmitted diseases excluding HIV	10 to 14	Male	0<br>(0 to 0)	0.0<br>(0.0 to 0.0)	0<br>(0 to 0)	0.0<br>(0.0 to 0.0)	0<br>(0 to 0)	0.0<br>(0.0 to 0.0)	-98.3<br>(-99.3 to -85.2)	-98.3<br>(-99.3 to -85.5)
Belgium	Hepatitis	5 to 9	Both	0<br>(0 to 0)	0.0<br>(0.0 to 0.0)	0<br>(0 to 0)	0.0<br>(0.0 to 0.0)	0<br>(0 to 0)	0.0<br>(0.0 to 0.0)	-17.7<br>(-42.2 to 13.6)	-26.4<br>(-48.3 to 1.6)
Belgium	Hepatitis	5 to 9	Female	0<br>(0 to 0)	0.0<br>(0.0 to 0.0)	0<br>(0 to 0)	0.0<br>(0.0 to 0.0)	0<br>(0 to 0)	0.0<br>(0.0 to 0.0)	-29.2<br>(-59.5 to 24.9)	-36.7<br>(-63.9 to 11.6)
Belgium	Hepatitis	5 to 9	Male	0<br>(0 to 0)	0.0<br>(0.0 to 0.0)	0<br>(0 to 0)	0.0<br>(0.0 to 0.0)	0<br>(0 to 0)	0.0<br>(0.0 to 0.0)	-2.0<br>(-18.3 to 16.8)	-12.2<br>(-26.8 to 4.6)

eTable 8. All-cause and cause-specific mortality (counts and rates) at the global level and by country in the WHO European Region for ages 5 to 9 and 10 to 14 for 1990, 2000, and 2016

Belgium	Hepatitis	10 to 14	Both	0 (0 to 0)	0.0 (0.0 to 0.0)	0 (0 to 0)	0.0 (0.0 to 0.0)	0 (0 to 0)	0.0 (0.0 to 0.0)	-25.2 (-52.9 to 22.1)	-26.8 (-53.9 to 19.5)
Belgium	Hepatitis	10 to 14	Female	0 (0 to 0)	0.0 (0.0 to 0.0)	0 (0 to 0)	0.0 (0.0 to 0.0)	0 (0 to 0)	0.0 (0.0 to 0.0)	-30.7 (-64.5 to 46.4)	-32.1 (-65.2 to 43.4)
Belgium	Hepatitis	10 to 14	Male	0 (0 to 0)	0.0 (0.0 to 0.0)	0 (0 to 0)	0.0 (0.0 to 0.0)	0 (0 to 0)	0.0 (0.0 to 0.0)	-16.2 (-50.8 to 17.8)	-18.1 (-51.9 to 15.2)
Belgium	Other infectious diseases	5 to 9	Both	0 (0 to 1)	0.1 (0.1 to 0.1)	1 (0 to 1)	0.1 (0.1 to 0.1)	1 (0 to 1)	0.1 (0.0 to 0.1)	16.0 (-29.6 to 79.7)	3.7 (-37.0 to 60.7)
Belgium	Other infectious diseases	5 to 9	Female	0 (0 to 0)	0.1 (0.0 to 0.1)	0 (0 to 0)	0.1 (0.0 to 0.1)	0 (0 to 0)	0.1 (0.0 to 0.1)	29.7 (-58.2 to 180.3)	15.9 (-62.6 to 150.4)
Belgium	Other infectious diseases	5 to 9	Male	0 (0 to 0)	0.1 (0.1 to 0.1)	0 (0 to 1)	0.1 (0.1 to 0.2)	0 (0 to 0)	0.1 (0.1 to 0.1)	7.9 (-35.4 to 72.4)	-3.4 (-42.2 to 54.4)
Belgium	Other infectious diseases	10 to 14	Both	0 (0 to 0)	0.1 (0.0 to 0.1)	1 (0 to 1)	0.1 (0.1 to 0.1)	0 (0 to 1)	0.1 (0.0 to 0.1)	22.5 (-25.8 to 94.1)	19.9 (-27.4 to 90.0)
Belgium	Other infectious diseases	10 to 14	Female	0 (0 to 0)	0.0 (0.0 to 0.1)	0 (0 to 0)	0.1 (0.0 to 0.1)	0 (0 to 0)	0.0 (0.0 to 0.1)	36.6 (-53.3 to 213.0)	33.7 (-54.2 to 206.6)
Belgium	Other infectious diseases	10 to 14	Male	0 (0 to 0)	0.1 (0.0 to 0.1)	0 (0 to 0)	0.1 (0.1 to 0.2)	0 (0 to 0)	0.1 (0.0 to 0.1)	16.0 (-35.8 to 92.7)	13.4 (-37.2 to 88.5)
Belgium	Nasopharynx cancer	5 to 9	Both	0 (0 to 0)	0.0 (0.0 to 0.0)	0 (0 to 0)	0.0 (0.0 to 0.0)	0 (0 to 0)	0.0 (0.0 to 0.0)	-54.0 (-71.9 to -22.9)	-58.9 (-74.8 to -31.0)
Belgium	Nasopharynx cancer	5 to 9	Female	0 (0 to 0)	0.0 (0.0 to 0.0)	0 (0 to 0)	0.0 (0.0 to 0.0)	0 (0 to 0)	0.0 (0.0 to 0.0)	0.1 (-17.1 to 19.8)	-10.6 (-26.0 to 7.1)
Belgium	Nasopharynx cancer	5 to 9	Male	0 (0 to 0)	0.0 (0.0 to 0.1)	0 (0 to 0)	0.0 (0.0 to 0.0)	0 (0 to 0)	0.0 (0.0 to 0.0)	-65.7 (-82.7 to -31.1)	-69.3 (-84.5 to -38.3)
Belgium	Nasopharynx cancer	10 to 14	Both	0 (0 to 0)	0.0 (0.0 to 0.0)	0 (0 to 0)	0.0 (0.0 to 0.0)	0 (0 to 0)	0.0 (0.0 to 0.0)	-53.4 (-68.2 to -30.2)	-54.4 (-68.9 to -31.7)
Belgium	Nasopharynx cancer	10 to 14	Female	0 (0 to 0)	0.0 (0.0 to 0.0)	0 (0 to 0)	0.0 (0.0 to 0.0)	0 (0 to 0)	0.0 (0.0 to 0.0)	-1.5 (-17.2 to 18.0)	-3.6 (-18.9 to 15.6)
Belgium	Nasopharynx cancer	10 to 14	Male	0 (0 to 0)	0.1 (0.0 to 0.1)	0 (0 to 0)	0.0 (0.0 to 0.0)	0 (0 to 0)	0.0 (0.0 to 0.0)	-62.9 (-78.1 to -36.6)	-63.7 (-78.6 to -38.0)
Belgium	Liver cancer	5 to 9	Both	0 (0 to 0)	0.0 (0.0 to 0.1)	0 (0 to 0)	0.0 (0.0 to 0.0)	0 (0 to 0)	0.0 (0.0 to 0.0)	-21.6 (-51.4 to 28.6)	-29.8 (-56.6 to 15.0)
Belgium	Liver cancer	5 to 9	Female	0 (0 to 0)	0.0 (0.0 to 0.0)	0 (0 to 0)	0.0 (0.0 to 0.0)	0 (0 to 0)	0.0 (0.0 to 0.0)	-25.4 (-59.8 to 46.0)	-33.4 (-64.1 to 30.4)
Belgium	Liver cancer	5 to 9	Male	0 (0 to 0)	0.1 (0.0 to 0.1)	0 (0 to 0)	0.0 (0.0 to 0.1)	0 (0 to 0)	0.0 (0.0 to 0.1)	-19.8 (-58.2 to 47.2)	-28.2 (-62.5 to 31.8)
Belgium	Liver cancer	10 to 14	Both	0 (0 to 0)	0.0 (0.0 to 0.1)	0 (0 to 0)	0.0 (0.0 to 0.0)	0 (0 to 0)	0.0 (0.0 to 0.1)	-7.8 (-45.2 to 51.2)	-9.8 (-46.4 to 48.0)
Belgium	Liver cancer	10 to 14	Female	0 (0 to 0)	0.0 (0.0 to 0.0)	0 (0 to 0)	0.0 (0.0 to 0.0)	0 (0 to 0)	0.0 (0.0 to 0.0)	-33.0 (-66.9 to 32.5)	-34.4 (-67.6 to 29.8)
Belgium	Liver cancer	10 to 14	Male	0 (0 to 0)	0.0 (0.0 to 0.1)	0 (0 to 0)	0.0 (0.0 to 0.1)	0 (0 to 0)	0.1 (0.0 to 0.1)	7.7 (-43.8 to 110.3)	5.4 (-45.0 to 105.7)
Belgium	Kidney cancer	5 to 9	Both	1 (1 to 1)	0.2 (0.1 to 0.2)	1 (0 to 1)	0.1 (0.1 to 0.1)	1 (0 to 1)	0.1 (0.1 to 0.1)	-42.5 (-63.9 to -9.2)	-48.5 (-67.7 to -18.8)
Belgium	Kidney cancer	5 to 9	Female	1 (0 to 1)	0.2 (0.1 to 0.3)	0 (0 to 0)	0.1 (0.1 to 0.1)	0 (0 to 0)	0.1 (0.1 to 0.1)	-47.8 (-73.0 to -4.9)	-53.4 (-75.9 to -15.0)
Belgium	Kidney cancer	5 to 9	Male	0 (0 to 1)	0.1 (0.1 to 0.2)	0 (0 to 0)	0.1 (0.1 to 0.1)	0 (0 to 0)	0.1 (0.0 to 0.1)	-34.4 (-65.9 to 24.3)	-41.2 (-69.4 to 11.3)
Belgium	Kidney cancer	10 to 14	Both	0 (0 to 1)	0.1 (0.0 to 0.1)	0 (0 to 0)	0.0 (0.0 to 0.1)	0 (0 to 0)	0.0 (0.0 to 0.1)	-37.4 (-59.9 to -2.4)	-38.8 (-60.7 to -4.4)
Belgium	Kidney cancer	10 to 14	Female	0 (0 to 0)	0.1 (0.1 to 0.1)	0 (0 to 0)	0.1 (0.0 to 0.1)	0 (0 to 0)	0.1 (0.0 to 0.1)	-38.3 (-65.2 to 14.0)	-39.5 (-65.9 to 11.7)
Belgium	Kidney cancer	10 to 14	Male	0 (0 to 0)	0.0 (0.0 to 0.1)	0 (0 to 0)	0.0 (0.0 to 0.0)	0 (0 to 0)	0.0 (0.0 to 0.0)	-35.7 (-62.7 to 12.9)	-37.1 (-63.5 to 10.5)
Belgium	Brain and nervous system cancer	5 to 9	Both	10 (8 to 12)	1.6 (1.3 to 2.0)	6 (5 to 9)	1.0 (0.7 to 1.4)	6 (4 to 9)	0.9 (0.7 to 1.3)	-36.2 (-58.2 to 0.8)	-42.9 (-62.6 to -9.9)

eTable 8. All-cause and cause-specific mortality (counts and rates) at the global level and by country in the WHO European Region for ages 5 to 9 and 10 to 14 for 1990, 2000, and 2016

Belgium	Brain and nervous system cancer	5 to 9	Female	4 (3 to 6)	1.5 (1.0 to 2.1)	3 (2 to 4)	0.9 (0.6 to 1.4)	3 (2 to 4)	0.9 (0.5 to 1.3)	-34.6 (-63.7 to 10.5)	-41.6 (-67.6 to -1.2)
Belgium	Brain and nervous system cancer	5 to 9	Male	5 (4 to 7)	1.8 (1.3 to 2.4)	3 (2 to 5)	1.1 (0.7 to 1.6)	3 (2 to 5)	1.0 (0.6 to 1.5)	-37.4 (-62.1 to 4.5)	-43.9 (-66.1 to -6.4)
Belgium	Brain and nervous system cancer	10 to 14	Both	7 (5 to 8)	1.1 (0.9 to 1.4)	6 (4 to 7)	0.9 (0.7 to 1.2)	5 (3 to 6)	0.7 (0.5 to 1.0)	-32.4 (-55.2 to -1.9)	-33.9 (-56.2 to -4.0)
Belgium	Brain and nervous system cancer	10 to 14	Female	3 (2 to 4)	1.1 (0.8 to 1.5)	3 (2 to 4)	1.0 (0.7 to 1.3)	2 (1 to 3)	0.7 (0.5 to 1.1)	-30.2 (-59.3 to 13.6)	-31.6 (-60.1 to 11.2)
Belgium	Brain and nervous system cancer	10 to 14	Male	3 (3 to 5)	1.1 (0.8 to 1.5)	3 (2 to 4)	0.9 (0.6 to 1.3)	2 (1 to 4)	0.7 (0.5 to 1.1)	-34.5 (-60.4 to 6.4)	-36.0 (-61.3 to 4.0)
Belgium	Thyroid cancer	10 to 14	Both	0 (0 to 0)	0.0 (0.0 to 0.0)	0 (0 to 0)	0.0 (0.0 to 0.0)	0 (0 to 0)	0.0 (0.0 to 0.0)	-22.8 (-47.8 to 16.0)	-24.4 (-48.9 to 13.5)
Belgium	Thyroid cancer	10 to 14	Female	0 (0 to 0)	0.0 (0.0 to 0.0)	0 (0 to 0)	0.0 (0.0 to 0.0)	0 (0 to 0)	0.0 (0.0 to 0.0)	-12.0 (-40.6 to 13.7)	-13.8 (-41.8 to 11.4)
Belgium	Thyroid cancer	10 to 14	Male	0 (0 to 0)	0.0 (0.0 to 0.0)	0 (0 to 0)	0.0 (0.0 to 0.0)	0 (0 to 0)	0.0 (0.0 to 0.0)	-30.2 (-61.7 to 31.1)	-31.8 (-62.6 to 28.2)
Belgium	Hodgkin lymphoma	5 to 9	Both	0 (0 to 0)	0.0 (0.0 to 0.0)	0 (0 to 0)	0.0 (0.0 to 0.0)	0 (0 to 0)	0.0 (0.0 to 0.0)	-24.2 (-50.8 to 12.7)	-32.2 (-56.0 to 0.9)
Belgium	Hodgkin lymphoma	5 to 9	Female	0 (0 to 0)	0.0 (0.0 to 0.0)	0 (0 to 0)	0.0 (0.0 to 0.0)	0 (0 to 0)	0.0 (0.0 to 0.0)	0.1 (-17.1 to 19.8)	-10.6 (-26.0 to 7.1)
Belgium	Hodgkin lymphoma	5 to 9	Male	0 (0 to 0)	0.0 (0.0 to 0.0)	0 (0 to 0)	0.0 (0.0 to 0.0)	0 (0 to 0)	0.0 (0.0 to 0.0)	-38.2 (-67.2 to 15.4)	-44.7 (-70.7 to 3.4)
Belgium	Hodgkin lymphoma	10 to 14	Both	0 (0 to 0)	0.0 (0.0 to 0.1)	0 (0 to 0)	0.0 (0.0 to 0.0)	0 (0 to 0)	0.0 (0.0 to 0.0)	-64.3 (-78.2 to -43.3)	-65.1 (-78.7 to -44.5)
Belgium	Hodgkin lymphoma	10 to 14	Female	0 (0 to 0)	0.0 (0.0 to 0.1)	0 (0 to 0)	0.0 (0.0 to 0.0)	0 (0 to 0)	0.0 (0.0 to 0.0)	-62.2 (-80.1 to -20.9)	-63.0 (-80.5 to -22.6)
Belgium	Hodgkin lymphoma	10 to 14	Male	0 (0 to 0)	0.1 (0.0 to 0.1)	0 (0 to 0)	0.0 (0.0 to 0.1)	0 (0 to 0)	0.0 (0.0 to 0.0)	-65.7 (-81.4 to -37.6)	-66.4 (-81.8 to -39.0)
Belgium	Non-Hodgkin lymphoma	5 to 9	Both	2 (1 to 2)	0.3 (0.2 to 0.3)	1 (1 to 2)	0.2 (0.2 to 0.3)	1 (1 to 2)	0.2 (0.1 to 0.2)	-29.3 (-53.2 to 9.9)	-36.8 (-58.1 to -1.7)
Belgium	Non-Hodgkin lymphoma	5 to 9	Female	0 (0 to 1)	0.2 (0.1 to 0.2)	0 (0 to 1)	0.1 (0.1 to 0.2)	0 (0 to 1)	0.1 (0.1 to 0.2)	-30.4 (-63.3 to 30.3)	-37.9 (-67.2 to 16.4)
Belgium	Non-Hodgkin lymphoma	5 to 9	Male	1 (1 to 2)	0.4 (0.2 to 0.5)	1 (1 to 1)	0.3 (0.2 to 0.4)	1 (0 to 1)	0.2 (0.1 to 0.3)	-28.8 (-58.7 to 21.7)	-36.3 (-63.0 to 9.0)
Belgium	Non-Hodgkin lymphoma	10 to 14	Both	1 (1 to 2)	0.2 (0.2 to 0.3)	1 (1 to 2)	0.2 (0.2 to 0.3)	1 (1 to 1)	0.2 (0.1 to 0.2)	-20.9 (-48.6 to 22.1)	-22.6 (-49.7 to 19.5)
Belgium	Non-Hodgkin lymphoma	10 to 14	Female	0 (0 to 1)	0.1 (0.1 to 0.2)	0 (0 to 1)	0.1 (0.1 to 0.2)	0 (0 to 0)	0.1 (0.1 to 0.2)	-23.2 (-61.6 to 48.6)	-24.8 (-62.4 to 45.6)
Belgium	Non-Hodgkin lymphoma	10 to 14	Male	1 (1 to 1)	0.3 (0.2 to 0.4)	1 (1 to 1)	0.3 (0.2 to 0.4)	1 (0 to 1)	0.2 (0.1 to 0.3)	-19.9 (-54.0 to 38.4)	-21.6 (-55.0 to 35.4)
Belgium	Leukemia	5 to 9	Both	9 (7 to 11)	1.5 (1.1 to 1.8)	5 (4 to 6)	0.8 (0.7 to 1.0)	4 (3 to 6)	0.6 (0.5 to 0.9)	-50.3 (-65.8 to -27.0)	-55.5 (-69.4 to -34.7)
Belgium	Leukemia	5 to 9	Female	4 (3 to 5)	1.2 (0.9 to 1.7)	2 (1 to 3)	0.6 (0.5 to 0.9)	2 (1 to 3)	0.6 (0.4 to 0.8)	-50.1 (-70.5 to -16.5)	-55.4 (-73.7 to -25.4)
Belgium	Leukemia	5 to 9	Male	5 (4 to 7)	1.7 (1.2 to 2.3)	3 (2 to 4)	1.0 (0.7 to 1.3)	3 (2 to 4)	0.7 (0.5 to 1.1)	-50.5 (-71.2 to -15.1)	-55.6 (-74.2 to -23.9)
Belgium	Leukemia	10 to 14	Both	8 (7 to 10)	1.4 (1.1 to 1.7)	6 (5 to 8)	1.0 (0.8 to 1.3)	5 (3 to 6)	0.7 (0.5 to 1.0)	-43.9 (-61.0 to -21.4)	-45.1 (-61.9 to -23.1)
Belgium	Leukemia	10 to 14	Female	3 (2 to 5)	1.1 (0.8 to 1.5)	3 (2 to 4)	0.9 (0.7 to 1.3)	2 (1 to 3)	0.7 (0.4 to 0.9)	-39.6 (-64.2 to -2.5)	-40.8 (-65.0 to -4.5)
Belgium	Leukemia	10 to 14	Male	5 (4 to 7)	1.6 (1.1 to 2.1)	4 (3 to 5)	1.2 (0.8 to 1.5)	3 (2 to 4)	0.8 (0.5 to 1.2)	-46.9 (-67.9 to -15.9)	-48.1 (-68.6 to -17.7)
Belgium	Other neoplasms	5 to 9	Both	5 (4 to 6)	0.8 (0.6 to 1.0)	3 (2 to 4)	0.5 (0.4 to 0.7)	3 (2 to 4)	0.4 (0.3 to 0.6)	-36.9 (-57.1 to -7.1)	-43.5 (-61.6 to -16.9)
Belgium	Other neoplasms	5 to 9	Female	2 (1 to 3)	0.7 (0.5 to 1.0)	1 (1 to 2)	0.5 (0.3 to 0.6)	1 (1 to 2)	0.4 (0.3 to 0.6)	-36.5 (-63.3 to 10.8)	-43.2 (-67.2 to -1.0)
Belgium	Other neoplasms	5 to 9	Male	3 (2 to 3)	0.8 (0.6 to 1.1)	2 (1 to 3)	0.6 (0.4 to 0.8)	2 (1 to 2)	0.5 (0.3 to 0.7)	-37.2 (-63.2 to 5.9)	-43.8 (-67.1 to -5.1)
Belgium	Other neoplasms	10 to 14	Both	5 (4 to 6)	0.8 (0.6 to 1.0)	4 (3 to 5)	0.6 (0.5 to 0.8)	3 (2 to 4)	0.5 (0.4 to 0.7)	-29.3 (-50.4 to 3.5)	-30.8 (-51.4 to 1.3)

eTable 8. All-cause and cause-specific mortality (counts and rates) at the global level and by country in the WHO European Region for ages 5 to 9 and 10 to 14 for 1990, 2000, and 2016

Belgium	Other neoplasms	10 to 14	Female	2 (2 to 3)	0.7 (0.5 to 1.0)	2 (1 to 3)	0.7 (0.5 to 0.9)	2 (1 to 2)	0.5 (0.3 to 0.7)	-30.4 (-57.8 to 21.6)	-31.8 (-58.7 to 19.1)
Belgium	Other neoplasms	10 to 14	Male	2 (2 to 3)	0.8 (0.6 to 1.1)	2 (1 to 3)	0.6 (0.5 to 0.9)	2 (1 to 3)	0.6 (0.4 to 0.8)	-28.4 (-57.3 to 14.5)	-30.0 (-58.2 to 12.0)
Belgium	Rheumatic heart disease	5 to 9	Both	0 (0 to 0)	0.0 (0.0 to 0.0)	0 (0 to 0)	0.0 (0.0 to 0.0)	0 (0 to 0)	0.0 (0.0 to 0.0)	-50.4 (-67.2 to -24.6)	-55.6 (-70.7 to -32.6)
Belgium	Rheumatic heart disease	5 to 9	Female	0 (0 to 0)	0.0 (0.0 to 0.0)	0 (0 to 0)	0.0 (0.0 to 0.0)	0 (0 to 0)	0.0 (0.0 to 0.0)	-35.5 (-62.0 to 6.6)	-42.4 (-66.0 to -4.7)
Belgium	Rheumatic heart disease	5 to 9	Male	0 (0 to 0)	0.0 (0.0 to 0.0)	0 (0 to 0)	0.0 (0.0 to 0.0)	0 (0 to 0)	0.0 (0.0 to 0.0)	-63.2 (-81.0 to -31.9)	-67.1 (-82.9 to -39.0)
Belgium	Rheumatic heart disease	10 to 14	Both	0 (0 to 0)	0.0 (0.0 to 0.0)	0 (0 to 0)	0.0 (0.0 to 0.0)	0 (0 to 0)	0.0 (0.0 to 0.0)	-40.5 (-61.4 to -12.0)	-41.7 (-62.2 to -13.8)
Belgium	Rheumatic heart disease	10 to 14	Female	0 (0 to 0)	0.0 (0.0 to 0.0)	0 (0 to 0)	0.0 (0.0 to 0.0)	0 (0 to 0)	0.0 (0.0 to 0.0)	-18.5 (-54.1 to 42.1)	-20.1 (-55.1 to 39.2)
Belgium	Rheumatic heart disease	10 to 14	Male	0 (0 to 0)	0.0 (0.0 to 0.0)	0 (0 to 0)	0.0 (0.0 to 0.0)	0 (0 to 0)	0.0 (0.0 to 0.0)	-59.8 (-75.5 to -33.0)	-60.6 (-76.0 to -34.5)
Belgium	Ischemic heart disease	5 to 9	Both	0 (0 to 0)	0.0 (0.0 to 0.0)	0 (0 to 0)	0.0 (0.0 to 0.0)	0 (0 to 0)	0.0 (0.0 to 0.0)	-68.2 (-79.2 to -49.5)	-71.5 (-81.4 to -54.8)
Belgium	Ischemic heart disease	5 to 9	Female	0 (0 to 0)	0.0 (0.0 to 0.1)	0 (0 to 0)	0.0 (0.0 to 0.0)	0 (0 to 0)	0.0 (0.0 to 0.0)	-72.3 (-84.8 to -49.4)	-75.3 (-86.4 to -54.8)
Belgium	Ischemic heart disease	5 to 9	Male	0 (0 to 0)	0.0 (0.0 to 0.0)	0 (0 to 0)	0.0 (0.0 to 0.0)	0 (0 to 0)	0.0 (0.0 to 0.0)	-59.7 (-78.9 to -24.1)	-63.9 (-81.1 to -32.0)
Belgium	Ischemic heart disease	10 to 14	Both	0 (0 to 0)	0.0 (0.0 to 0.0)	0 (0 to 0)	0.0 (0.0 to 0.0)	0 (0 to 0)	0.0 (0.0 to 0.0)	-61.5 (-75.3 to -41.7)	-62.3 (-75.8 to -42.9)
Belgium	Ischemic heart disease	10 to 14	Female	0 (0 to 0)	0.0 (0.0 to 0.0)	0 (0 to 0)	0.0 (0.0 to 0.0)	0 (0 to 0)	0.0 (0.0 to 0.0)	-63.0 (-79.6 to -35.2)	-63.8 (-80.0 to -36.5)
Belgium	Ischemic heart disease	10 to 14	Male	0 (0 to 0)	0.0 (0.0 to 0.0)	0 (0 to 0)	0.0 (0.0 to 0.0)	0 (0 to 0)	0.0 (0.0 to 0.0)	-59.4 (-77.1 to -27.8)	-60.3 (-77.6 to -29.4)
Belgium	Cerebrovascular disease	5 to 9	Both	3 (2 to 4)	0.5 (0.4 to 0.6)	1 (1 to 1)	0.2 (0.1 to 0.2)	1 (0 to 1)	0.1 (0.1 to 0.1)	-80.5 (-86.4 to -72.3)	-82.5 (-87.8 to -75.2)
Belgium	Cerebrovascular disease	5 to 9	Female	2 (1 to 3)	0.7 (0.5 to 0.9)	1 (1 to 1)	0.2 (0.2 to 0.3)	0 (0 to 1)	0.1 (0.1 to 0.2)	-79.2 (-86.8 to -68.3)	-81.4 (-88.2 to -71.7)
Belgium	Cerebrovascular disease	5 to 9	Male	1 (1 to 1)	0.3 (0.2 to 0.5)	0 (0 to 1)	0.1 (0.1 to 0.2)	0 (0 to 0)	0.1 (0.0 to 0.1)	-82.9 (-90.7 to -70.5)	-84.7 (-91.6 to -73.6)
Belgium	Cerebrovascular disease	10 to 14	Both	4 (3 to 5)	0.6 (0.5 to 0.8)	2 (2 to 2)	0.3 (0.2 to 0.4)	1 (1 to 1)	0.2 (0.1 to 0.2)	-75.2 (-82.1 to -66.6)	-75.7 (-82.5 to -67.3)
Belgium	Cerebrovascular disease	10 to 14	Female	2 (1 to 2)	0.6 (0.5 to 0.8)	1 (1 to 1)	0.3 (0.2 to 0.4)	1 (0 to 1)	0.2 (0.1 to 0.2)	-72.2 (-82.3 to -57.0)	-72.8 (-82.7 to -57.8)
Belgium	Cerebrovascular disease	10 to 14	Male	2 (1 to 3)	0.6 (0.5 to 0.8)	1 (1 to 1)	0.3 (0.2 to 0.4)	0 (0 to 1)	0.1 (0.1 to 0.2)	-78.1 (-86.1 to -65.7)	-78.6 (-86.4 to -66.5)
Belgium	Hypertensive heart disease	5 to 9	Both	0 (0 to 0)	0.0 (0.0 to 0.0)	0 (0 to 0)	0.0 (0.0 to 0.0)	0 (0 to 0)	0.0 (0.0 to 0.0)	-26.9 (-48.0 to 2.1)	-34.6 (-53.5 to -8.6)
Belgium	Hypertensive heart disease	5 to 9	Female	0 (0 to 0)	0.0 (0.0 to 0.0)	0 (0 to 0)	0.0 (0.0 to 0.0)	0 (0 to 0)	0.0 (0.0 to 0.0)	-20.0 (-47.2 to 21.3)	-28.5 (-52.9 to 8.4)
Belgium	Hypertensive heart disease	5 to 9	Male	0 (0 to 0)	0.0 (0.0 to 0.0)	0 (0 to 0)	0.0 (0.0 to 0.0)	0 (0 to 0)	0.0 (0.0 to 0.0)	-32.7 (-60.1 to 10.8)	-39.7 (-64.3 to -0.7)
Belgium	Hypertensive heart disease	10 to 14	Both	0 (0 to 0)	0.0 (0.0 to 0.0)	0 (0 to 0)	0.0 (0.0 to 0.0)	0 (0 to 0)	0.0 (0.0 to 0.0)	-26.8 (-51.0 to 3.4)	-28.4 (-52.0 to 1.2)
Belgium	Hypertensive heart disease	10 to 14	Female	0 (0 to 0)	0.0 (0.0 to 0.0)	0 (0 to 0)	0.0 (0.0 to 0.0)	0 (0 to 0)	0.0 (0.0 to 0.0)	-16.0 (-48.1 to 28.3)	-17.7 (-49.2 to 25.6)
Belgium	Hypertensive heart disease	10 to 14	Male	0 (0 to 0)	0.0 (0.0 to 0.0)	0 (0 to 0)	0.0 (0.0 to 0.0)	0 (0 to 0)	0.0 (0.0 to 0.0)	-36.6 (-66.7 to 8.6)	-38.0 (-67.4 to 6.2)
Belgium	Cardiomyopathy and myocarditis	5 to 9	Both	1 (1 to 2)	0.2 (0.2 to 0.3)	1 (1 to 1)	0.1 (0.1 to 0.2)	0 (0 to 1)	0.1 (0.0 to 0.1)	-66.6 (-79.3 to -37.8)	-70.1 (-81.5 to -44.4)
Belgium	Cardiomyopathy and myocarditis	5 to 9	Female	1 (0 to 1)	0.3 (0.2 to 0.4)	0 (0 to 1)	0.1 (0.1 to 0.2)	0 (0 to 0)	0.1 (0.1 to 0.1)	-65.3 (-81.7 to -23.9)	-69.0 (-83.6 to -32.0)
Belgium	Cardiomyopathy and myocarditis	5 to 9	Male	1 (0 to 1)	0.2 (0.1 to 0.3)	0 (0 to 0)	0.1 (0.1 to 0.2)	0 (0 to 0)	0.1 (0.0 to 0.1)	-68.3 (-84.6 to -31.6)	-71.6 (-86.2 to -38.8)
Belgium	Cardiomyopathy and myocarditis	10 to 14	Both	1 (1 to 2)	0.2 (0.2 to 0.3)	1 (1 to 1)	0.2 (0.1 to 0.2)	1 (0 to 1)	0.1 (0.1 to 0.1)	-60.6 (-75.0 to -36.4)	-61.5 (-75.6 to -37.8)

eTable 8. All-cause and cause-specific mortality (counts and rates) at the global level and by country in the WHO European Region for ages 5 to 9 and 10 to 14 for 1990, 2000, and 2016

Belgium	Cardiomyopathy and myocarditis	10 to 14	Female	1 (0 to 1)	0.2 (0.2 to 0.4)	0 (0 to 1)	0.2 (0.1 to 0.2)	0 (0 to 0)	0.1 (0.1 to 0.2)	-59.1 (-77.4 to -20.0)	-59.9 (-77.8 to -21.6)
Belgium	Cardiomyopathy and myocarditis	10 to 14	Male	1 (0 to 1)	0.2 (0.1 to 0.4)	0 (0 to 1)	0.2 (0.1 to 0.2)	0 (0 to 1)	0.1 (0.1 to 0.2)	-62.2 (-78.9 to -29.8)	-63.0 (-79.3 to -31.3)
Belgium	Atrial fibrillation and flutter	5 to 9	Both	0 (0 to 0)	0.0 (0.0 to 0.0)	0 (0 to 0)	0.0 (0.0 to 0.0)	0 (0 to 0)	0.0 (0.0 to 0.0)	0.0 (0.0 to 0.0)	0.0 (0.0 to 0.0)
Belgium	Atrial fibrillation and flutter	5 to 9	Female	0 (0 to 0)	0.0 (0.0 to 0.0)	0 (0 to 0)	0.0 (0.0 to 0.0)	0 (0 to 0)	0.0 (0.0 to 0.0)	0.0 (0.0 to 0.0)	0.0 (0.0 to 0.0)
Belgium	Atrial fibrillation and flutter	5 to 9	Male	0 (0 to 0)	0.0 (0.0 to 0.0)	0 (0 to 0)	0.0 (0.0 to 0.0)	0 (0 to 0)	0.0 (0.0 to 0.0)	0.0 (0.0 to 0.0)	0.0 (0.0 to 0.0)
Belgium	Atrial fibrillation and flutter	10 to 14	Both	0 (0 to 0)	0.0 (0.0 to 0.0)	0 (0 to 0)	0.0 (0.0 to 0.0)	0 (0 to 0)	0.0 (0.0 to 0.0)	0.0 (0.0 to 0.0)	0.0 (0.0 to 0.0)
Belgium	Atrial fibrillation and flutter	10 to 14	Female	0 (0 to 0)	0.0 (0.0 to 0.0)	0 (0 to 0)	0.0 (0.0 to 0.0)	0 (0 to 0)	0.0 (0.0 to 0.0)	0.0 (0.0 to 0.0)	0.0 (0.0 to 0.0)
Belgium	Atrial fibrillation and flutter	10 to 14	Male	0 (0 to 0)	0.0 (0.0 to 0.0)	0 (0 to 0)	0.0 (0.0 to 0.0)	0 (0 to 0)	0.0 (0.0 to 0.0)	0.0 (0.0 to 0.0)	0.0 (0.0 to 0.0)
Belgium	Endocarditis	5 to 9	Both	0 (0 to 0)	0.0 (0.0 to 0.0)	0 (0 to 0)	0.0 (0.0 to 0.0)	0 (0 to 0)	0.0 (0.0 to 0.0)	-23.1 (-49.7 to 15.0)	-31.2 (-55.0 to 2.9)
Belgium	Endocarditis	5 to 9	Female	0 (0 to 0)	0.0 (0.0 to 0.1)	0 (0 to 0)	0.0 (0.0 to 0.0)	0 (0 to 0)	0.0 (0.0 to 0.0)	-22.6 (-56.5 to 34.0)	-30.8 (-61.1 to 19.7)
Belgium	Endocarditis	5 to 9	Male	0 (0 to 0)	0.0 (0.0 to 0.0)	0 (0 to 0)	0.0 (0.0 to 0.0)	0 (0 to 0)	0.0 (0.0 to 0.0)	-23.9 (-60.4 to 43.5)	-31.8 (-64.5 to 28.5)
Belgium	Endocarditis	10 to 14	Both	0 (0 to 0)	0.0 (0.0 to 0.1)	0 (0 to 0)	0.0 (0.0 to 0.1)	0 (0 to 0)	0.0 (0.0 to 0.0)	-19.5 (-48.4 to 22.5)	-21.2 (-49.5 to 19.9)
Belgium	Endocarditis	10 to 14	Female	0 (0 to 0)	0.0 (0.0 to 0.1)	0 (0 to 0)	0.0 (0.0 to 0.1)	0 (0 to 0)	0.0 (0.0 to 0.1)	-20.6 (-53.9 to 37.6)	-22.2 (-54.9 to 34.7)
Belgium	Endocarditis	10 to 14	Male	0 (0 to 0)	0.0 (0.0 to 0.0)	0 (0 to 0)	0.0 (0.0 to 0.0)	0 (0 to 0)	0.0 (0.0 to 0.0)	-17.9 (-55.5 to 48.4)	-19.7 (-56.5 to 45.1)
Belgium	Other cardiovascular and circulatory diseases	5 to 9	Both	1 (0 to 1)	0.1 (0.1 to 0.1)	0 (0 to 1)	0.1 (0.1 to 0.1)	0 (0 to 0)	0.0 (0.0 to 0.1)	-46.6 (-65.8 to -14.5)	-52.2 (-69.4 to -23.5)
Belgium	Other cardiovascular and circulatory diseases	5 to 9	Female	0 (0 to 0)	0.1 (0.1 to 0.2)	0 (0 to 0)	0.1 (0.1 to 0.1)	0 (0 to 0)	0.1 (0.0 to 0.1)	-44.1 (-68.5 to 2.3)	-50.1 (-71.9 to -8.6)
Belgium	Other cardiovascular and circulatory diseases	5 to 9	Male	0 (0 to 0)	0.1 (0.0 to 0.1)	0 (0 to 0)	0.1 (0.0 to 0.1)	0 (0 to 0)	0.0 (0.0 to 0.1)	-50.0 (-73.2 to -5.6)	-55.2 (-76.0 to -15.5)
Belgium	Other cardiovascular and circulatory diseases	10 to 14	Both	1 (0 to 1)	0.1 (0.1 to 0.1)	0 (0 to 1)	0.1 (0.1 to 0.1)	0 (0 to 0)	0.0 (0.0 to 0.1)	-38.4 (-60.0 to -4.8)	-39.7 (-60.8 to -6.8)
Belgium	Other cardiovascular and circulatory diseases	10 to 14	Female	0 (0 to 0)	0.1 (0.1 to 0.1)	0 (0 to 0)	0.1 (0.1 to 0.1)	0 (0 to 0)	0.1 (0.0 to 0.1)	-31.6 (-62.2 to 23.1)	-33.1 (-63.0 to 20.5)

eTable 8. All-cause and cause-specific mortality (counts and rates) at the global level and by country in the WHO European Region for ages 5 to 9 and 10 to 14 for 1990, 2000, and 2016

Belgium	Other cardiovascular and circulatory diseases	10 to 14	Male	0 (0 to 0)	0.1 (0.1 to 0.1)	0 (0 to 0)	0.1 (0.1 to 0.1)	0 (0 to 0)	0.0 (0.0 to 0.1)	-44.0 (-69.0 to -0.3)	-45.3 (-69.7 to -2.5)
Belgium	Chronic obstructive pulmonary disease	5 to 9	Both	0 (0 to 1)	0.1 (0.0 to 0.1)	0 (0 to 0)	0.0 (0.0 to 0.1)	0 (0 to 0)	0.0 (0.0 to 0.0)	-57.6 (-74.5 to -31.5)	-62.1 (-77.2 to -38.7)
Belgium	Chronic obstructive pulmonary disease	5 to 9	Female	0 (0 to 0)	0.1 (0.0 to 0.1)	0 (0 to 0)	0.0 (0.0 to 0.1)	0 (0 to 0)	0.0 (0.0 to 0.1)	-38.9 (-68.0 to 15.8)	-45.4 (-71.4 to 3.4)
Belgium	Chronic obstructive pulmonary disease	5 to 9	Male	0 (0 to 0)	0.1 (0.0 to 0.1)	0 (0 to 0)	0.1 (0.0 to 0.1)	0 (0 to 0)	0.0 (0.0 to 0.0)	-72.1 (-86.4 to -49.1)	-75.0 (-87.8 to -54.4)
Belgium	Chronic obstructive pulmonary disease	10 to 14	Both	0 (0 to 0)	0.1 (0.0 to 0.1)	0 (0 to 0)	0.1 (0.0 to 0.1)	0 (0 to 0)	0.0 (0.0 to 0.0)	-34.4 (-61.8 to 8.2)	-35.7 (-62.6 to 5.9)
Belgium	Chronic obstructive pulmonary disease	10 to 14	Female	0 (0 to 0)	0.1 (0.0 to 0.1)	0 (0 to 0)	0.1 (0.0 to 0.1)	0 (0 to 0)	0.0 (0.0 to 0.1)	-16.0 (-59.3 to 72.4)	-17.8 (-60.2 to 68.9)
Belgium	Chronic obstructive pulmonary disease	10 to 14	Male	0 (0 to 0)	0.0 (0.0 to 0.1)	0 (0 to 0)	0.0 (0.0 to 0.1)	0 (0 to 0)	0.0 (0.0 to 0.0)	-53.7 (-80.0 to -11.0)	-54.7 (-80.4 to -13.0)
Belgium	Asthma	5 to 9	Both	1 (1 to 1)	0.1 (0.1 to 0.2)	0 (0 to 0)	0.1 (0.0 to 0.1)	0 (0 to 0)	0.0 (0.0 to 0.0)	-82.7 (-90.5 to -68.6)	-84.5 (-91.5 to -71.9)
Belgium	Asthma	5 to 9	Female	0 (0 to 0)	0.1 (0.0 to 0.1)	0 (0 to 0)	0.0 (0.0 to 0.1)	0 (0 to 0)	0.0 (0.0 to 0.0)	-74.7 (-87.9 to -51.3)	-77.4 (-89.1 to -56.5)
Belgium	Asthma	5 to 9	Male	0 (0 to 1)	0.2 (0.1 to 0.2)	0 (0 to 0)	0.1 (0.0 to 0.1)	0 (0 to 0)	0.0 (0.0 to 0.1)	-86.6 (-94.1 to -66.4)	-88.0 (-94.8 to -69.9)
Belgium	Asthma	10 to 14	Both	2 (1 to 2)	0.3 (0.2 to 0.4)	1 (1 to 2)	0.2 (0.1 to 0.3)	0 (0 to 1)	0.1 (0.0 to 0.1)	-76.7 (-85.5 to -61.9)	-77.2 (-85.8 to -62.7)
Belgium	Asthma	10 to 14	Female	1 (0 to 1)	0.2 (0.1 to 0.3)	0 (0 to 1)	0.1 (0.1 to 0.2)	0 (0 to 0)	0.1 (0.0 to 0.1)	-67.7 (-83.3 to -42.0)	-68.4 (-83.7 to -43.2)
Belgium	Asthma	10 to 14	Male	1 (1 to 2)	0.4 (0.3 to 0.6)	1 (1 to 1)	0.2 (0.2 to 0.3)	0 (0 to 0)	0.1 (0.0 to 0.1)	-80.5 (-89.7 to -61.4)	-80.9 (-89.9 to -62.3)
Belgium	Interstitial lung disease and pulmonary sarcoidosis	5 to 9	Both	0 (0 to 0)	0.0 (0.0 to 0.0)	0 (0 to 0)	0.0 (0.0 to 0.0)	0 (0 to 0)	0.0 (0.0 to 0.0)	-22.9 (-62.8 to 32.2)	-31.0 (-66.8 to 18.3)
Belgium	Interstitial lung disease and pulmonary sarcoidosis	5 to 9	Female	0 (0 to 0)	0.0 (0.0 to 0.0)	0 (0 to 0)	0.0 (0.0 to 0.0)	0 (0 to 0)	0.0 (0.0 to 0.0)	-22.2 (-62.1 to 58.0)	-30.4 (-66.1 to 41.1)
Belgium	Interstitial lung disease and pulmonary sarcoidosis	5 to 9	Male	0 (0 to 0)	0.0 (0.0 to 0.0)	0 (0 to 0)	0.0 (0.0 to 0.0)	0 (0 to 0)	0.0 (0.0 to 0.0)	-23.6 (-77.0 to 62.3)	-31.6 (-79.4 to 45.4)
Belgium	Interstitial lung disease and pulmonary sarcoidosis	10 to 14	Both	0 (0 to 0)	0.0 (0.0 to 0.0)	0 (0 to 0)	0.0 (0.0 to 0.0)	0 (0 to 0)	0.0 (0.0 to 0.0)	28.0 (-48.4 to 128.8)	25.2 (-49.5 to 124.0)

eTable 8. All-cause and cause-specific mortality (counts and rates) at the global level and by country in the WHO European Region for ages 5 to 9 and 10 to 14 for 1990, 2000, and 2016

Belgium	Interstitial lung disease and pulmonary sarcoidosis	10 to 14	Female	0<br>(0 to 0)	0.0<br>(0.0 to 0.0)	0<br>(0 to 0)	0.0<br>(0.0 to 0.1)	0<br>(0 to 0)	0.0<br>(0.0 to 0.0)	28.7<br>(-46.7 to 183.7)	26.0<br>(-47.8 to 177.8)
Belgium	Interstitial lung disease and pulmonary sarcoidosis	10 to 14	Male	0<br>(0 to 0)	0.0<br>(0.0 to 0.0)	0<br>(0 to 0)	0.0<br>(0.0 to 0.0)	0<br>(0 to 0)	0.0<br>(0.0 to 0.0)	26.9<br>(-68.3 to 165.8)	24.1<br>(-69.0 to 160.0)
Belgium	Other chronic respiratory diseases	5 to 9	Both	0<br>(0 to 1)	0.1<br>(0.0 to 0.1)	0<br>(0 to 1)	0.1<br>(0.0 to 0.1)	0<br>(0 to 0)	0.0<br>(0.0 to 0.1)	-34.0<br>(-62.9 to 11.5)	-41.0<br>(-66.8 to -0.3)
Belgium	Other chronic respiratory diseases	5 to 9	Female	0<br>(0 to 0)	0.0<br>(0.0 to 0.1)	0<br>(0 to 0)	0.0<br>(0.0 to 0.1)	0<br>(0 to 0)	0.0<br>(0.0 to 0.1)	-9.5<br>(-51.0 to 92.2)	-19.1<br>(-56.2 to 71.7)
Belgium	Other chronic respiratory diseases	5 to 9	Male	0<br>(0 to 1)	0.1<br>(0.1 to 0.2)	0<br>(0 to 0)	0.1<br>(0.0 to 0.1)	0<br>(0 to 0)	0.1<br>(0.0 to 0.1)	-39.7<br>(-69.3 to 15.8)	-46.0<br>(-72.5 to 3.8)
Belgium	Other chronic respiratory diseases	10 to 14	Both	0<br>(0 to 1)	0.1<br>(0.0 to 0.1)	0<br>(0 to 1)	0.1<br>(0.0 to 0.1)	0<br>(0 to 1)	0.1<br>(0.0 to 0.1)	-8.4<br>(-46.4 to 50.0)	-10.3<br>(-47.5 to 46.8)
Belgium	Other chronic respiratory diseases	10 to 14	Female	0<br>(0 to 0)	0.1<br>(0.0 to 0.1)	0<br>(0 to 0)	0.1<br>(0.0 to 0.1)	0<br>(0 to 0)	0.0<br>(0.0 to 0.1)	-12.9<br>(-61.2 to 80.6)	-14.7<br>(-62.0 to 76.9)
Belgium	Other chronic respiratory diseases	10 to 14	Male	0<br>(0 to 0)	0.1<br>(0.0 to 0.1)	0<br>(0 to 0)	0.1<br>(0.0 to 0.1)	0<br>(0 to 0)	0.1<br>(0.0 to 0.1)	-4.9<br>(-56.1 to 90.1)	-7.0<br>(-57.1 to 85.9)
Belgium	Cirrhosis and other chronic liver diseases due to hepatitis B	5 to 9	Both	0<br>(0 to 0)	0.0<br>(0.0 to 0.0)	0<br>(0 to 0)	0.0<br>(0.0 to 0.0)	0<br>(0 to 0)	0.0<br>(0.0 to 0.0)	-49.9<br>(-67.6 to -25.9)	-55.1<br>(-71.0 to -33.7)
Belgium	Cirrhosis and other chronic liver diseases due to hepatitis B	5 to 9	Female	0<br>(0 to 0)	0.0<br>(0.0 to 0.0)	0<br>(0 to 0)	0.0<br>(0.0 to 0.0)	0<br>(0 to 0)	0.0<br>(0.0 to 0.0)	-52.1<br>(-69.5 to -25.8)	-57.2<br>(-72.8 to -33.7)
Belgium	Cirrhosis and other chronic liver diseases due to hepatitis B	5 to 9	Male	0<br>(0 to 0)	0.0<br>(0.0 to 0.0)	0<br>(0 to 0)	0.0<br>(0.0 to 0.0)	0<br>(0 to 0)	0.0<br>(0.0 to 0.0)	-49.3<br>(-70.3 to -18.4)	-54.6<br>(-73.4 to -26.9)
Belgium	Cirrhosis and other chronic liver diseases due to hepatitis B	10 to 14	Both	0<br>(0 to 0)	0.0<br>(0.0 to 0.0)	0<br>(0 to 0)	0.0<br>(0.0 to 0.0)	0<br>(0 to 0)	0.0<br>(0.0 to 0.0)	-45.3<br>(-62.7 to -20.5)	-46.5<br>(-63.4 to -22.2)
Belgium	Cirrhosis and other chronic liver diseases due to hepatitis B	10 to 14	Female	0<br>(0 to 0)	0.0<br>(0.0 to 0.0)	0<br>(0 to 0)	0.0<br>(0.0 to 0.0)	0<br>(0 to 0)	0.0<br>(0.0 to 0.0)	-45.3<br>(-67.3 to -7.5)	-46.4<br>(-68.0 to -9.4)
Belgium	Cirrhosis and other chronic liver diseases due to hepatitis B	10 to 14	Male	0<br>(0 to 0)	0.0<br>(0.0 to 0.0)	0<br>(0 to 0)	0.0<br>(0.0 to 0.0)	0<br>(0 to 0)	0.0<br>(0.0 to 0.0)	-45.4<br>(-66.9 to -7.8)	-46.6<br>(-67.6 to -9.8)
Belgium	Cirrhosis and other chronic liver diseases due to hepatitis C	5 to 9	Both	0<br>(0 to 0)	0.0<br>(0.0 to 0.0)	0<br>(0 to 0)	0.0<br>(0.0 to 0.0)	0<br>(0 to 0)	0.0<br>(0.0 to 0.0)	-49.9<br>(-67.7 to -25.8)	-55.2<br>(-71.1 to -33.6)

eTable 8. All-cause and cause-specific mortality (counts and rates) at the global level and by country in the WHO European Region for ages 5 to 9 and 10 to 14 for 1990, 2000, and 2016

Belgium	Cirrhosis and other chronic liver diseases due to hepatitis C	5 to 9	Female	0 (0 to 0)	0.0 (0.0 to 0.0)	0 (0 to 0)	0.0 (0.0 to 0.0)	0 (0 to 0)	0.0 (0.0 to 0.0)	-53.4 (-70.4 to -28.0)	-58.3 (-73.6 to -35.7)
Belgium	Cirrhosis and other chronic liver diseases due to hepatitis C	5 to 9	Male	0 (0 to 0)	0.0 (0.0 to 0.0)	0 (0 to 0)	0.0 (0.0 to 0.0)	0 (0 to 0)	0.0 (0.0 to 0.0)	-49.1 (-70.2 to -18.0)	-54.4 (-73.3 to -26.6)
Belgium	Cirrhosis and other chronic liver diseases due to hepatitis C	10 to 14	Both	0 (0 to 0)	0.0 (0.0 to 0.0)	0 (0 to 0)	0.0 (0.0 to 0.0)	0 (0 to 0)	0.0 (0.0 to 0.0)	-45.8 (-63.0 to -20.9)	-47.0 (-63.8 to -22.6)
Belgium	Cirrhosis and other chronic liver diseases due to hepatitis C	10 to 14	Female	0 (0 to 0)	0.0 (0.0 to 0.0)	0 (0 to 0)	0.0 (0.0 to 0.0)	0 (0 to 0)	0.0 (0.0 to 0.0)	-46.7 (-68.3 to -9.5)	-47.8 (-68.9 to -11.3)
Belgium	Cirrhosis and other chronic liver diseases due to hepatitis C	10 to 14	Male	0 (0 to 0)	0.0 (0.0 to 0.0)	0 (0 to 0)	0.0 (0.0 to 0.0)	0 (0 to 0)	0.0 (0.0 to 0.0)	-45.2 (-66.5 to -7.3)	-46.4 (-67.3 to -9.3)
Belgium	Cirrhosis and other chronic liver diseases due to other causes	5 to 9	Both	1 (1 to 1)	0.1 (0.1 to 0.1)	0 (0 to 1)	0.1 (0.1 to 0.1)	0 (0 to 0)	0.0 (0.0 to 0.1)	-51.3 (-66.2 to -30.7)	-56.5 (-69.8 to -38.0)
Belgium	Cirrhosis and other chronic liver diseases due to other causes	5 to 9	Female	0 (0 to 0)	0.1 (0.1 to 0.1)	0 (0 to 0)	0.1 (0.0 to 0.1)	0 (0 to 0)	0.0 (0.0 to 0.1)	-53.2 (-70.3 to -27.4)	-58.2 (-73.5 to -35.1)
Belgium	Cirrhosis and other chronic liver diseases due to other causes	5 to 9	Male	0 (0 to 1)	0.1 (0.1 to 0.2)	0 (0 to 0)	0.1 (0.1 to 0.1)	0 (0 to 0)	0.1 (0.0 to 0.1)	-50.0 (-71.0 to -19.4)	-55.3 (-74.0 to -27.8)
Belgium	Cirrhosis and other chronic liver diseases due to other causes	10 to 14	Both	0 (0 to 0)	0.1 (0.0 to 0.1)	0 (0 to 0)	0.1 (0.0 to 0.1)	0 (0 to 0)	0.0 (0.0 to 0.0)	-46.4 (-63.9 to -21.1)	-47.5 (-64.7 to -22.8)
Belgium	Cirrhosis and other chronic liver diseases due to other causes	10 to 14	Female	0 (0 to 0)	0.1 (0.1 to 0.1)	0 (0 to 0)	0.1 (0.0 to 0.1)	0 (0 to 0)	0.0 (0.0 to 0.1)	-46.4 (-68.1 to -9.2)	-47.5 (-68.7 to -11.1)
Belgium	Cirrhosis and other chronic liver diseases due to other causes	10 to 14	Male	0 (0 to 0)	0.0 (0.0 to 0.1)	0 (0 to 0)	0.0 (0.0 to 0.0)	0 (0 to 0)	0.0 (0.0 to 0.0)	-46.4 (-67.5 to -9.3)	-47.5 (-68.2 to -11.3)
Belgium	Peptic ulcer disease	5 to 9	Both	0 (0 to 0)	0.0 (0.0 to 0.0)	0 (0 to 0)	0.0 (0.0 to 0.0)	0 (0 to 0)	0.0 (0.0 to 0.0)	-50.2 (-71.7 to -11.5)	-55.5 (-74.7 to -20.9)
Belgium	Peptic ulcer disease	5 to 9	Female	0 (0 to 0)	0.0 (0.0 to 0.1)	0 (0 to 0)	0.0 (0.0 to 0.0)	0 (0 to 0)	0.0 (0.0 to 0.0)	-61.5 (-82.2 to -22.3)	-65.6 (-84.1 to -30.6)
Belgium	Peptic ulcer disease	5 to 9	Male	0 (0 to 0)	0.0 (0.0 to 0.0)	0 (0 to 0)	0.0 (0.0 to 0.0)	0 (0 to 0)	0.0 (0.0 to 0.0)	-27.6 (-64.4 to 49.5)	-35.2 (-68.1 to 33.9)
Belgium	Peptic ulcer disease	10 to 14	Both	0 (0 to 0)	0.0 (0.0 to 0.0)	0 (0 to 0)	0.0 (0.0 to 0.0)	0 (0 to 0)	0.0 (0.0 to 0.0)	-45.7 (-67.2 to -9.7)	-46.9 (-67.9 to -11.6)
Belgium	Peptic ulcer disease	10 to 14	Female	0 (0 to 0)	0.0 (0.0 to 0.0)	0 (0 to 0)	0.0 (0.0 to 0.0)	0 (0 to 0)	0.0 (0.0 to 0.0)	-17.8 (-54.0 to 44.9)	-19.5 (-54.9 to 41.9)

eTable 8. All-cause and cause-specific mortality (counts and rates) at the global level and by country in the WHO European Region for ages 5 to 9 and 10 to 14 for 1990, 2000, and 2016

Belgium	Peptic ulcer disease	10 to 14	Male	0 (0 to 0)	0.0 (0.0 to 0.0)	0 (0 to 0)	0.0 (0.0 to 0.0)	0 (0 to 0)	0.0 (0.0 to 0.0)	-60.0 (-79.3 to -20.9)	-60.9 (-79.7 to -22.7)
Belgium	Gastritis and duodenitis	5 to 9	Both	0 (0 to 0)	0.0 (0.0 to 0.0)	0 (0 to 0)	0.0 (0.0 to 0.0)	0 (0 to 0)	0.0 (0.0 to 0.0)	-56.0 (-86.9 to 24.6)	-60.7 (-88.2 to 11.4)
Belgium	Gastritis and duodenitis	5 to 9	Female	0 (0 to 0)	0.0 (0.0 to 0.0)	0 (0 to 0)	0.0 (0.0 to 0.0)	0 (0 to 0)	0.0 (0.0 to 0.0)	10.7 (-55.1 to 183.5)	-1.1 (-59.9 to 153.3)
Belgium	Gastritis and duodenitis	5 to 9	Male	0 (0 to 0)	0.0 (0.0 to 0.0)	0 (0 to 0)	0.0 (0.0 to 0.0)	0 (0 to 0)	0.0 (0.0 to 0.0)	-60.9 (-89.8 to 24.5)	-65.0 (-90.9 to 11.5)
Belgium	Gastritis and duodenitis	10 to 14	Both	0 (0 to 0)	0.0 (0.0 to 0.0)	0 (0 to 0)	0.0 (0.0 to 0.0)	0 (0 to 0)	0.0 (0.0 to 0.0)	-12.1 (-45.2 to 41.3)	-14.0 (-46.4 to 38.3)
Belgium	Gastritis and duodenitis	10 to 14	Female	0 (0 to 0)	0.0 (0.0 to 0.0)	0 (0 to 0)	0.0 (0.0 to 0.0)	0 (0 to 0)	0.0 (0.0 to 0.0)	-15.8 (-54.0 to 60.1)	-17.5 (-55.0 to 56.8)
Belgium	Gastritis and duodenitis	10 to 14	Male	0 (0 to 0)	0.0 (0.0 to 0.0)	0 (0 to 0)	0.0 (0.0 to 0.0)	0 (0 to 0)	0.0 (0.0 to 0.0)	-7.8 (-49.2 to 60.0)	-9.8 (-50.3 to 56.4)
Belgium	Appendicitis	5 to 9	Both	0 (0 to 0)	0.0 (0.0 to 0.0)	0 (0 to 0)	0.0 (0.0 to 0.0)	0 (0 to 0)	0.0 (0.0 to 0.0)	-60.5 (-80.6 to -20.3)	-64.7 (-82.7 to -28.7)
Belgium	Appendicitis	5 to 9	Female	0 (0 to 0)	0.0 (0.0 to 0.1)	0 (0 to 0)	0.0 (0.0 to 0.0)	0 (0 to 0)	0.0 (0.0 to 0.0)	-56.2 (-80.7 to -1.6)	-60.8 (-82.7 to -12.1)
Belgium	Appendicitis	5 to 9	Male	0 (0 to 0)	0.0 (0.0 to 0.1)	0 (0 to 0)	0.0 (0.0 to 0.0)	0 (0 to 0)	0.0 (0.0 to 0.0)	-69.5 (-88.7 to -13.7)	-72.6 (-89.9 to -22.7)
Belgium	Appendicitis	10 to 14	Both	0 (0 to 0)	0.0 (0.0 to 0.1)	0 (0 to 0)	0.0 (0.0 to 0.0)	0 (0 to 0)	0.0 (0.0 to 0.0)	-62.4 (-79.6 to -24.1)	-63.2 (-80.1 to -25.7)
Belgium	Appendicitis	10 to 14	Female	0 (0 to 0)	0.1 (0.0 to 0.1)	0 (0 to 0)	0.0 (0.0 to 0.1)	0 (0 to 0)	0.0 (0.0 to 0.0)	-61.5 (-81.9 to -20.0)	-62.3 (-82.3 to -21.7)
Belgium	Appendicitis	10 to 14	Male	0 (0 to 0)	0.0 (0.0 to 0.1)	0 (0 to 0)	0.0 (0.0 to 0.1)	0 (0 to 0)	0.0 (0.0 to 0.0)	-63.8 (-84.7 to -10.1)	-64.6 (-85.0 to -12.1)
Belgium	Paralytic ileus and intestinal obstruction	5 to 9	Both	0 (0 to 0)	0.1 (0.0 to 0.1)	0 (0 to 0)	0.0 (0.0 to 0.1)	0 (0 to 0)	0.0 (0.0 to 0.1)	-33.8 (-63.3 to 13.1)	-40.8 (-67.2 to 1.2)
Belgium	Paralytic ileus and intestinal obstruction	5 to 9	Female	0 (0 to 0)	0.0 (0.0 to 0.1)	0 (0 to 0)	0.0 (0.0 to 0.0)	0 (0 to 0)	0.0 (0.0 to 0.0)	-32.4 (-64.7 to 54.4)	-39.6 (-68.4 to 38.0)
Belgium	Paralytic ileus and intestinal obstruction	5 to 9	Male	0 (0 to 0)	0.1 (0.0 to 0.1)	0 (0 to 0)	0.1 (0.0 to 0.1)	0 (0 to 0)	0.0 (0.0 to 0.1)	-34.3 (-67.1 to 34.5)	-41.2 (-70.5 to 20.5)
Belgium	Paralytic ileus and intestinal obstruction	10 to 14	Both	0 (0 to 0)	0.0 (0.0 to 0.1)	0 (0 to 0)	0.0 (0.0 to 0.1)	0 (0 to 0)	0.0 (0.0 to 0.1)	-30.8 (-58.6 to 5.7)	-32.3 (-59.5 to 3.4)
Belgium	Paralytic ileus and intestinal obstruction	10 to 14	Female	0 (0 to 0)	0.0 (0.0 to 0.1)	0 (0 to 0)	0.0 (0.0 to 0.1)	0 (0 to 0)	0.0 (0.0 to 0.1)	-42.7 (-70.6 to 7.6)	-43.9 (-71.2 to 5.4)
Belgium	Paralytic ileus and intestinal obstruction	10 to 14	Male	0 (0 to 0)	0.0 (0.0 to 0.1)	0 (0 to 0)	0.0 (0.0 to 0.1)	0 (0 to 0)	0.0 (0.0 to 0.1)	-15.7 (-56.1 to 50.8)	-17.6 (-57.1 to 47.5)
Belgium	Inguinal, femoral, and abdominal hernia	5 to 9	Both	0 (0 to 0)	0.0 (0.0 to 0.0)	0 (0 to 0)	0.0 (0.0 to 0.0)	0 (0 to 0)	0.0 (0.0 to 0.0)	-76.9 (-88.2 to -53.4)	-79.4 (-89.4 to -58.3)
Belgium	Inguinal, femoral, and abdominal hernia	5 to 9	Female	0 (0 to 0)	0.0 (0.0 to 0.0)	0 (0 to 0)	0.0 (0.0 to 0.0)	0 (0 to 0)	0.0 (0.0 to 0.0)	-72.6 (-89.1 to -29.3)	-75.5 (-90.3 to -36.9)
Belgium	Inguinal, femoral, and abdominal hernia	5 to 9	Male	0 (0 to 0)	0.0 (0.0 to 0.0)	0 (0 to 0)	0.0 (0.0 to 0.0)	0 (0 to 0)	0.0 (0.0 to 0.0)	-80.0 (-91.4 to -50.7)	-82.1 (-92.3 to -55.8)
Belgium	Inguinal, femoral, and abdominal hernia	10 to 14	Both	0 (0 to 0)	0.0 (0.0 to 0.0)	0 (0 to 0)	0.0 (0.0 to 0.0)	0 (0 to 0)	0.0 (0.0 to 0.0)	-70.8 (-84.4 to -46.1)	-71.4 (-84.8 to -47.3)
Belgium	Inguinal, femoral, and abdominal hernia	10 to 14	Female	0 (0 to 0)	0.0 (0.0 to 0.0)	0 (0 to 0)	0.0 (0.0 to 0.0)	0 (0 to 0)	0.0 (0.0 to 0.0)	-71.0 (-87.1 to -36.9)	-71.6 (-87.4 to -38.2)

eTable 8. All-cause and cause-specific mortality (counts and rates) at the global level and by country in the WHO European Region for ages 5 to 9 and 10 to 14 for 1990, 2000, and 2016

Belgium	Inguinal, femoral, and abdominal hernia	10 to 14	Male	0 (0 to 0)	0.0 (0.0 to 0.0)	0 (0 to 0)	0.0 (0.0 to 0.0)	0 (0 to 0)	0.0 (0.0 to 0.0)	-70.7 (-87.4 to -36.2)	-71.3 (-87.7 to -37.6)
Belgium	Inflammatory bowel disease	5 to 9	Both	0 (0 to 0)	0.0 (0.0 to 0.0)	0 (0 to 0)	0.0 (0.0 to 0.0)	0 (0 to 0)	0.0 (0.0 to 0.0)	-36.4 (-70.5 to 49.7)	-43.1 (-73.6 to 33.9)
Belgium	Inflammatory bowel disease	5 to 9	Female	0 (0 to 0)	0.0 (0.0 to 0.0)	0 (0 to 0)	0.0 (0.0 to 0.0)	0 (0 to 0)	0.0 (0.0 to 0.0)	-30.8 (-72.0 to 107.2)	-38.2 (-75.0 to 85.1)
Belgium	Inflammatory bowel disease	5 to 9	Male	0 (0 to 0)	0.0 (0.0 to 0.0)	0 (0 to 0)	0.0 (0.0 to 0.0)	0 (0 to 0)	0.0 (0.0 to 0.0)	-51.9 (-83.3 to 26.1)	-56.9 (-85.1 to 13.0)
Belgium	Inflammatory bowel disease	10 to 14	Both	0 (0 to 0)	0.0 (0.0 to 0.0)	0 (0 to 0)	0.0 (0.0 to 0.0)	0 (0 to 0)	0.0 (0.0 to 0.0)	-21.9 (-63.5 to 62.3)	-23.6 (-64.3 to 58.8)
Belgium	Inflammatory bowel disease	10 to 14	Female	0 (0 to 0)	0.0 (0.0 to 0.0)	0 (0 to 0)	0.0 (0.0 to 0.1)	0 (0 to 0)	0.0 (0.0 to 0.0)	-16.9 (-69.4 to 85.9)	-18.6 (-70.1 to 82.1)
Belgium	Inflammatory bowel disease	10 to 14	Male	0 (0 to 0)	0.0 (0.0 to 0.0)	0 (0 to 0)	0.0 (0.0 to 0.0)	0 (0 to 0)	0.0 (0.0 to 0.0)	-35.6 (-71.0 to 41.3)	-37.0 (-71.6 to 38.2)
Belgium	Vascular intestinal disorders	5 to 9	Both	0 (0 to 0)	0.0 (0.0 to 0.0)	0 (0 to 0)	0.0 (0.0 to 0.0)	0 (0 to 0)	0.0 (0.0 to 0.0)	-57.7 (-84.8 to 15.6)	-62.2 (-86.4 to 3.4)
Belgium	Vascular intestinal disorders	5 to 9	Female	0 (0 to 0)	0.0 (0.0 to 0.0)	0 (0 to 0)	0.0 (0.0 to 0.0)	0 (0 to 0)	0.0 (0.0 to 0.0)	-26.9 (-76.4 to 118.5)	-34.7 (-78.9 to 95.2)
Belgium	Vascular intestinal disorders	5 to 9	Male	0 (0 to 0)	0.0 (0.0 to 0.0)	0 (0 to 0)	0.0 (0.0 to 0.0)	0 (0 to 0)	0.0 (0.0 to 0.0)	-59.0 (-85.9 to 22.6)	-63.3 (-87.4 to 9.8)
Belgium	Vascular intestinal disorders	10 to 14	Both	0 (0 to 0)	0.0 (0.0 to 0.0)	0 (0 to 0)	0.0 (0.0 to 0.0)	0 (0 to 0)	0.0 (0.0 to 0.0)	-33.8 (-67.1 to 36.2)	-35.2 (-67.8 to 33.3)
Belgium	Vascular intestinal disorders	10 to 14	Female	0 (0 to 0)	0.0 (0.0 to 0.0)	0 (0 to 0)	0.0 (0.0 to 0.0)	0 (0 to 0)	0.0 (0.0 to 0.0)	-19.7 (-68.1 to 98.4)	-21.4 (-68.7 to 94.3)
Belgium	Vascular intestinal disorders	10 to 14	Male	0 (0 to 0)	0.0 (0.0 to 0.0)	0 (0 to 0)	0.0 (0.0 to 0.0)	0 (0 to 0)	0.0 (0.0 to 0.0)	-34.3 (-67.5 to 38.9)	-35.8 (-68.2 to 35.9)
Belgium	Gallbladder and biliary diseases	5 to 9	Both	0 (0 to 0)	0.0 (0.0 to 0.0)	0 (0 to 0)	0.0 (0.0 to 0.0)	0 (0 to 0)	0.0 (0.0 to 0.0)	-71.8 (-86.8 to -38.2)	-74.8 (-88.2 to -44.8)
Belgium	Gallbladder and biliary diseases	5 to 9	Female	0 (0 to 0)	0.0 (0.0 to 0.0)	0 (0 to 0)	0.0 (0.0 to 0.0)	0 (0 to 0)	0.0 (0.0 to 0.0)	-72.4 (-88.7 to -30.3)	-75.3 (-89.9 to -37.8)
Belgium	Gallbladder and biliary diseases	5 to 9	Male	0 (0 to 0)	0.0 (0.0 to 0.0)	0 (0 to 0)	0.0 (0.0 to 0.0)	0 (0 to 0)	0.0 (0.0 to 0.0)	-68.6 (-86.2 to -28.1)	-71.9 (-87.6 to -35.6)
Belgium	Gallbladder and biliary diseases	10 to 14	Both	0 (0 to 0)	0.0 (0.0 to 0.0)	0 (0 to 0)	0.0 (0.0 to 0.0)	0 (0 to 0)	0.0 (0.0 to 0.0)	-64.6 (-81.2 to -34.6)	-65.3 (-81.6 to -36.0)
Belgium	Gallbladder and biliary diseases	10 to 14	Female	0 (0 to 0)	0.0 (0.0 to 0.0)	0 (0 to 0)	0.0 (0.0 to 0.0)	0 (0 to 0)	0.0 (0.0 to 0.0)	-67.8 (-85.3 to -28.1)	-68.5 (-85.6 to -29.6)
Belgium	Gallbladder and biliary diseases	10 to 14	Male	0 (0 to 0)	0.0 (0.0 to 0.0)	0 (0 to 0)	0.0 (0.0 to 0.0)	0 (0 to 0)	0.0 (0.0 to 0.0)	-63.1 (-83.9 to -22.7)	-63.9 (-84.2 to -24.4)
Belgium	Pancreatitis	5 to 9	Both	0 (0 to 0)	0.0 (0.0 to 0.0)	0 (0 to 0)	0.0 (0.0 to 0.0)	0 (0 to 0)	0.0 (0.0 to 0.0)	-69.1 (-86.2 to -20.8)	-72.4 (-87.6 to -29.1)
Belgium	Pancreatitis	5 to 9	Female	0 (0 to 0)	0.0 (0.0 to 0.0)	0 (0 to 0)	0.0 (0.0 to 0.0)	0 (0 to 0)	0.0 (0.0 to 0.0)	-69.5 (-89.3 to -18.6)	-72.8 (-90.4 to -27.3)
Belgium	Pancreatitis	5 to 9	Male	0 (0 to 0)	0.0 (0.0 to 0.0)	0 (0 to 0)	0.0 (0.0 to 0.0)	0 (0 to 0)	0.0 (0.0 to 0.0)	-69.1 (-87.1 to 2.4)	-72.3 (-88.5 to -8.2)
Belgium	Pancreatitis	10 to 14	Both	0 (0 to 0)	0.0 (0.0 to 0.0)	0 (0 to 0)	0.0 (0.0 to 0.0)	0 (0 to 0)	0.0 (0.0 to 0.0)	-71.0 (-86.3 to -27.5)	-71.6 (-86.6 to -29.0)
Belgium	Pancreatitis	10 to 14	Female	0 (0 to 0)	0.0 (0.0 to 0.0)	0 (0 to 0)	0.0 (0.0 to 0.0)	0 (0 to 0)	0.0 (0.0 to 0.0)	-72.3 (-87.6 to -34.9)	-72.8 (-87.8 to -36.2)
Belgium	Pancreatitis	10 to 14	Male	0 (0 to 0)	0.0 (0.0 to 0.0)	0 (0 to 0)	0.0 (0.0 to 0.0)	0 (0 to 0)	0.0 (0.0 to 0.0)	-58.8 (-84.4 to 12.5)	-59.7 (-84.8 to 10.0)

eTable 8. All-cause and cause-specific mortality (counts and rates) at the global level and by country in the WHO European Region for ages 5 to 9 and 10 to 14 for 1990, 2000, and 2016

Belgium	Other digestive diseases	5 to 9	Both	0 (0 to 0)	0.0 (0.0 to 0.0)	0 (0 to 0)	0.0 (0.0 to 0.0)	0 (0 to 0)	0.0 (0.0 to 0.0)	-44.6 (-72.7 to 1.1)	-50.5 (-75.6 to -9.5)
Belgium	Other digestive diseases	5 to 9	Female	0 (0 to 0)	0.0 (0.0 to 0.0)	0 (0 to 0)	0.0 (0.0 to 0.0)	0 (0 to 0)	0.0 (0.0 to 0.0)	-33.9 (-72.2 to 44.8)	-41.0 (-75.2 to 29.4)
Belgium	Other digestive diseases	5 to 9	Male	0 (0 to 0)	0.0 (0.0 to 0.1)	0 (0 to 0)	0.0 (0.0 to 0.1)	0 (0 to 0)	0.0 (0.0 to 0.0)	-51.2 (-78.3 to 8.6)	-56.3 (-80.6 to -2.7)
Belgium	Other digestive diseases	10 to 14	Both	0 (0 to 0)	0.0 (0.0 to 0.0)	0 (0 to 0)	0.0 (0.0 to 0.0)	0 (0 to 0)	0.0 (0.0 to 0.0)	-30.7 (-61.7 to 12.9)	-32.2 (-62.5 to 10.5)
Belgium	Other digestive diseases	10 to 14	Female	0 (0 to 0)	0.0 (0.0 to 0.0)	0 (0 to 0)	0.0 (0.0 to 0.0)	0 (0 to 0)	0.0 (0.0 to 0.0)	-22.5 (-57.7 to 41.2)	-24.1 (-58.6 to 38.3)
Belgium	Other digestive diseases	10 to 14	Male	0 (0 to 0)	0.0 (0.0 to 0.1)	0 (0 to 0)	0.0 (0.0 to 0.1)	0 (0 to 0)	0.0 (0.0 to 0.0)	-34.3 (-69.3 to 19.8)	-35.8 (-70.0 to 17.2)
Belgium	Alzheimer disease and other dementias	5 to 9	Both	0 (0 to 0)	0.0 (0.0 to 0.0)	0 (0 to 0)	0.0 (0.0 to 0.0)	0 (0 to 0)	0.0 (0.0 to 0.0)	0.0 (0.0 to 0.0)	0.0 (0.0 to 0.0)
Belgium	Alzheimer disease and other dementias	5 to 9	Female	0 (0 to 0)	0.0 (0.0 to 0.0)	0 (0 to 0)	0.0 (0.0 to 0.0)	0 (0 to 0)	0.0 (0.0 to 0.0)	0.0 (0.0 to 0.0)	0.0 (0.0 to 0.0)
Belgium	Alzheimer disease and other dementias	5 to 9	Male	0 (0 to 0)	0.0 (0.0 to 0.0)	0 (0 to 0)	0.0 (0.0 to 0.0)	0 (0 to 0)	0.0 (0.0 to 0.0)	0.0 (0.0 to 0.0)	0.0 (0.0 to 0.0)
Belgium	Alzheimer disease and other dementias	10 to 14	Both	0 (0 to 0)	0.0 (0.0 to 0.0)	0 (0 to 0)	0.0 (0.0 to 0.0)	0 (0 to 0)	0.0 (0.0 to 0.0)	0.0 (0.0 to 0.0)	0.0 (0.0 to 0.0)
Belgium	Alzheimer disease and other dementias	10 to 14	Female	0 (0 to 0)	0.0 (0.0 to 0.0)	0 (0 to 0)	0.0 (0.0 to 0.0)	0 (0 to 0)	0.0 (0.0 to 0.0)	0.0 (0.0 to 0.0)	0.0 (0.0 to 0.0)
Belgium	Alzheimer disease and other dementias	10 to 14	Male	0 (0 to 0)	0.0 (0.0 to 0.0)	0 (0 to 0)	0.0 (0.0 to 0.0)	0 (0 to 0)	0.0 (0.0 to 0.0)	0.0 (0.0 to 0.0)	0.0 (0.0 to 0.0)
Belgium	Parkinson disease	5 to 9	Both	0 (0 to 0)	0.0 (0.0 to 0.0)	0 (0 to 0)	0.0 (0.0 to 0.0)	0 (0 to 0)	0.0 (0.0 to 0.0)	0.0 (0.0 to 0.0)	0.0 (0.0 to 0.0)
Belgium	Parkinson disease	5 to 9	Female	0 (0 to 0)	0.0 (0.0 to 0.0)	0 (0 to 0)	0.0 (0.0 to 0.0)	0 (0 to 0)	0.0 (0.0 to 0.0)	0.0 (0.0 to 0.0)	0.0 (0.0 to 0.0)
Belgium	Parkinson disease	5 to 9	Male	0 (0 to 0)	0.0 (0.0 to 0.0)	0 (0 to 0)	0.0 (0.0 to 0.0)	0 (0 to 0)	0.0 (0.0 to 0.0)	0.0 (0.0 to 0.0)	0.0 (0.0 to 0.0)
Belgium	Parkinson disease	10 to 14	Both	0 (0 to 0)	0.0 (0.0 to 0.0)	0 (0 to 0)	0.0 (0.0 to 0.0)	0 (0 to 0)	0.0 (0.0 to 0.0)	0.0 (0.0 to 0.0)	0.0 (0.0 to 0.0)
Belgium	Parkinson disease	10 to 14	Female	0 (0 to 0)	0.0 (0.0 to 0.0)	0 (0 to 0)	0.0 (0.0 to 0.0)	0 (0 to 0)	0.0 (0.0 to 0.0)	0.0 (0.0 to 0.0)	0.0 (0.0 to 0.0)
Belgium	Parkinson disease	10 to 14	Male	0 (0 to 0)	0.0 (0.0 to 0.0)	0 (0 to 0)	0.0 (0.0 to 0.0)	0 (0 to 0)	0.0 (0.0 to 0.0)	0.0 (0.0 to 0.0)	0.0 (0.0 to 0.0)
Belgium	Epilepsy	5 to 9	Both	2 (1 to 2)	0.3 (0.2 to 0.4)	2 (1 to 2)	0.3 (0.2 to 0.4)	2 (1 to 2)	0.3 (0.2 to 0.3)	1.8 (-30.0 to 50.9)	-8.9 (-37.4 to 35.0)
Belgium	Epilepsy	5 to 9	Female	1 (1 to 1)	0.3 (0.2 to 0.4)	1 (1 to 1)	0.3 (0.2 to 0.4)	1 (1 to 1)	0.3 (0.2 to 0.4)	16.3 (-32.0 to 98.6)	3.9 (-39.3 to 77.4)
Belgium	Epilepsy	5 to 9	Male	1 (1 to 1)	0.3 (0.2 to 0.4)	1 (1 to 1)	0.3 (0.2 to 0.4)	1 (1 to 1)	0.2 (0.2 to 0.3)	-12.7 (-44.1 to 39.8)	-21.8 (-50.0 to 25.2)
Belgium	Epilepsy	10 to 14	Both	1 (1 to 2)	0.2 (0.2 to 0.3)	2 (1 to 2)	0.3 (0.2 to 0.4)	1 (1 to 2)	0.2 (0.2 to 0.3)	9.2 (-24.4 to 60.1)	6.9 (-26.0 to 56.7)
Belgium	Epilepsy	10 to 14	Female	1 (0 to 1)	0.2 (0.1 to 0.3)	1 (1 to 1)	0.3 (0.2 to 0.4)	1 (0 to 1)	0.2 (0.1 to 0.3)	33.4 (-23.9 to 127.8)	30.6 (-25.5 to 123.1)

eTable 8. All-cause and cause-specific mortality (counts and rates) at the global level and by country in the WHO European Region for ages 5 to 9 and 10 to 14 for 1990, 2000, and 2016

Belgium	Epilepsy	10 to 14	Male	1 (1 to 1)	0.2 (0.2 to 0.3)	1 (1 to 1)	0.3 (0.2 to 0.4)	1 (0 to 1)	0.2 (0.1 to 0.3)	-7.6 (-44.7 to 50.7)	-9.6 (-46.0 to 47.4)
Belgium	Motor neuron disease	5 to 9	Both	0 (0 to 0)	0.0 (0.0 to 0.1)	0 (0 to 0)	0.0 (0.0 to 0.0)	0 (0 to 0)	0.0 (0.0 to 0.1)	-19.7 (-52.5 to 38.5)	-28.1 (-57.5 to 23.9)
Belgium	Motor neuron disease	5 to 9	Female	0 (0 to 0)	0.1 (0.0 to 0.1)	0 (0 to 0)	0.0 (0.0 to 0.0)	0 (0 to 0)	0.1 (0.0 to 0.1)	-15.1 (-54.9 to 64.4)	-24.2 (-59.7 to 46.9)
Belgium	Motor neuron disease	5 to 9	Male	0 (0 to 0)	0.0 (0.0 to 0.0)	0 (0 to 0)	0.0 (0.0 to 0.0)	0 (0 to 0)	0.0 (0.0 to 0.0)	-32.3 (-72.9 to 68.8)	-39.3 (-75.7 to 51.2)
Belgium	Motor neuron disease	10 to 14	Both	0 (0 to 1)	0.1 (0.1 to 0.1)	0 (0 to 0)	0.0 (0.0 to 0.1)	0 (0 to 1)	0.1 (0.0 to 0.1)	-3.3 (-43.5 to 59.6)	-5.3 (-44.7 to 56.2)
Belgium	Motor neuron disease	10 to 14	Female	0 (0 to 0)	0.0 (0.0 to 0.1)	0 (0 to 0)	0.0 (0.0 to 0.0)	0 (0 to 0)	0.0 (0.0 to 0.1)	6.1 (-35.3 to 74.5)	4.0 (-36.7 to 70.9)
Belgium	Motor neuron disease	10 to 14	Male	0 (0 to 1)	0.1 (0.1 to 0.2)	0 (0 to 0)	0.1 (0.0 to 0.1)	0 (0 to 1)	0.1 (0.1 to 0.2)	-7.1 (-54.7 to 88.3)	-9.1 (-55.7 to 84.2)
Belgium	Other neurological disorders	5 to 9	Both	2 (2 to 3)	0.4 (0.3 to 0.5)	1 (1 to 2)	0.2 (0.2 to 0.3)	1 (1 to 2)	0.2 (0.1 to 0.3)	-40.0 (-61.5 to -5.5)	-46.3 (-65.6 to -15.4)
Belgium	Other neurological disorders	5 to 9	Female	1 (1 to 1)	0.3 (0.2 to 0.5)	1 (0 to 1)	0.2 (0.2 to 0.3)	1 (0 to 1)	0.2 (0.1 to 0.2)	-42.7 (-66.3 to 4.8)	-48.8 (-69.9 to -6.4)
Belgium	Other neurological disorders	5 to 9	Male	1 (1 to 2)	0.4 (0.2 to 0.5)	1 (1 to 1)	0.3 (0.2 to 0.4)	1 (0 to 1)	0.2 (0.1 to 0.3)	-37.8 (-68.4 to 14.7)	-44.2 (-71.6 to 2.8)
Belgium	Other neurological disorders	10 to 14	Both	3 (2 to 3)	0.4 (0.3 to 0.6)	2 (2 to 3)	0.3 (0.3 to 0.4)	2 (1 to 2)	0.3 (0.2 to 0.4)	-36.6 (-57.6 to -4.2)	-38.0 (-58.5 to -6.3)
Belgium	Other neurological disorders	10 to 14	Female	1 (0 to 1)	0.2 (0.2 to 0.4)	1 (0 to 1)	0.2 (0.1 to 0.3)	0 (0 to 1)	0.2 (0.1 to 0.2)	-36.9 (-65.8 to 22.8)	-38.2 (-66.5 to 20.2)
Belgium	Other neurological disorders	10 to 14	Male	2 (1 to 3)	0.6 (0.4 to 0.8)	1 (1 to 2)	0.5 (0.3 to 0.6)	1 (1 to 2)	0.4 (0.2 to 0.5)	-36.5 (-60.0 to 3.6)	-37.9 (-60.9 to 1.4)
Belgium	Drug use disorders	5 to 9	Both	0 (0 to 0)	0.0 (0.0 to 0.0)	0 (0 to 0)	0.0 (0.0 to 0.0)	0 (0 to 0)	0.0 (0.0 to 0.0)	0.0 (0.0 to 0.0)	0.0 (0.0 to 0.0)
Belgium	Drug use disorders	5 to 9	Female	0 (0 to 0)	0.0 (0.0 to 0.0)	0 (0 to 0)	0.0 (0.0 to 0.0)	0 (0 to 0)	0.0 (0.0 to 0.0)	0.0 (0.0 to 0.0)	0.0 (0.0 to 0.0)
Belgium	Drug use disorders	5 to 9	Male	0 (0 to 0)	0.0 (0.0 to 0.0)	0 (0 to 0)	0.0 (0.0 to 0.0)	0 (0 to 0)	0.0 (0.0 to 0.0)	0.0 (0.0 to 0.0)	0.0 (0.0 to 0.0)
Belgium	Drug use disorders	10 to 14	Both	0 (0 to 0)	0.0 (0.0 to 0.0)	0 (0 to 0)	0.0 (0.0 to 0.0)	0 (0 to 0)	0.0 (0.0 to 0.0)	0.0 (0.0 to 0.0)	0.0 (0.0 to 0.0)
Belgium	Drug use disorders	10 to 14	Female	0 (0 to 0)	0.0 (0.0 to 0.0)	0 (0 to 0)	0.0 (0.0 to 0.0)	0 (0 to 0)	0.0 (0.0 to 0.0)	0.0 (0.0 to 0.0)	0.0 (0.0 to 0.0)
Belgium	Drug use disorders	10 to 14	Male	0 (0 to 0)	0.0 (0.0 to 0.0)	0 (0 to 0)	0.0 (0.0 to 0.0)	0 (0 to 0)	0.0 (0.0 to 0.0)	0.0 (0.0 to 0.0)	0.0 (0.0 to 0.0)
Belgium	Eating disorders	5 to 9	Both	0 (0 to 0)	0.0 (0.0 to 0.0)	0 (0 to 0)	0.0 (0.0 to 0.0)	0 (0 to 0)	0.0 (0.0 to 0.0)	70.0 (-7.0 to 220.7)	52.1 (-16.8 to 186.9)
Belgium	Eating disorders	5 to 9	Female	0 (0 to 0)	0.0 (0.0 to 0.0)	0 (0 to 0)	0.0 (0.0 to 0.0)	0 (0 to 0)	0.0 (0.0 to 0.0)	-13.8 (-60.3 to 82.3)	-23.0 (-64.5 to 62.9)
Belgium	Eating disorders	5 to 9	Male	0 (0 to 0)	0.0 (0.0 to 0.0)	0 (0 to 0)	0.0 (0.0 to 0.0)	0 (0 to 0)	0.0 (0.0 to 0.0)	316.3 (92.8 to 782.9)	272.9 (72.7 to 690.9)
Belgium	Eating disorders	10 to 14	Both	0 (0 to 0)	0.0 (0.0 to 0.0)	0 (0 to 0)	0.0 (0.0 to 0.0)	0 (0 to 0)	0.0 (0.0 to 0.0)	4.8 (-45.8 to 109.3)	2.6 (-47.0 to 104.9)
Belgium	Eating disorders	10 to 14	Female	0 (0 to 0)	0.0 (0.0 to 0.0)	0 (0 to 0)	0.0 (0.0 to 0.0)	0 (0 to 0)	0.0 (0.0 to 0.0)	-6.3 (-54.4 to 98.7)	-8.2 (-55.3 to 94.6)
Belgium	Eating disorders	10 to 14	Male	0 (0 to 0)	0.0 (0.0 to 0.0)	0 (0 to 0)	0.0 (0.0 to 0.0)	0 (0 to 0)	0.0 (0.0 to 0.0)	342.2 (85.6 to 909.5)	332.5 (81.5 to 887.3)
Belgium	Diabetes mellitus	5 to 9	Both	0 (0 to 0)	0.1 (0.0 to 0.1)	0 (0 to 0)	0.0 (0.0 to 0.0)	0 (0 to 0)	0.0 (0.0 to 0.0)	-64.4 (-75.0 to -48.8)	-68.1 (-77.6 to -54.2)
Belgium	Diabetes mellitus	5 to 9	Female	0 (0 to 0)	0.1 (0.1 to 0.1)	0 (0 to 0)	0.0 (0.0 to 0.0)	0 (0 to 0)	0.0 (0.0 to 0.0)	-72.9 (-82.2 to -58.4)	-75.8 (-84.1 to -62.8)
Belgium	Diabetes mellitus	5 to 9	Male	0 (0 to 0)	0.0 (0.0 to 0.1)	0 (0 to 0)	0.0 (0.0 to 0.0)	0 (0 to 0)	0.0 (0.0 to 0.0)	-51.2 (-71.8 to -12.7)	-56.3 (-74.8 to -21.8)
Belgium	Diabetes mellitus	10 to 14	Both	0 (0 to 0)	0.1 (0.0 to 0.1)	0 (0 to 0)	0.0 (0.0 to 0.1)	0 (0 to 0)	0.0 (0.0 to 0.0)	-62.2 (-74.3 to -45.8)	-63.0 (-74.8 to -47.0)

eTable 8. All-cause and cause-specific mortality (counts and rates) at the global level and by country in the WHO European Region for ages 5 to 9 and 10 to 14 for 1990, 2000, and 2016

Belgium	Diabetes mellitus	10 to 14	Female	0 (0 to 0)	0.1 (0.0 to 0.1)	0 (0 to 0)	0.0 (0.0 to 0.1)	0 (0 to 0)	0.0 (0.0 to 0.0)	-66.3 (-79.1 to -43.2)	-67.0 (-79.6 to -44.4)
Belgium	Diabetes mellitus	10 to 14	Male	0 (0 to 0)	0.0 (0.0 to 0.1)	0 (0 to 0)	0.0 (0.0 to 0.0)	0 (0 to 0)	0.0 (0.0 to 0.0)	-56.4 (-75.4 to -26.4)	-57.4 (-75.9 to -28.0)
Belgium	Acute glomerulonephritis	5 to 9	Both	0 (0 to 0)	0.0 (0.0 to 0.0)	0 (0 to 0)	0.0 (0.0 to 0.0)	0 (0 to 0)	0.0 (0.0 to 0.0)	-1.0 (-14.9 to 15.1)	-11.4 (-23.8 to 3.0)
Belgium	Acute glomerulonephritis	5 to 9	Female	0 (0 to 0)	0.0 (0.0 to 0.0)	0 (0 to 0)	0.0 (0.0 to 0.0)	0 (0 to 0)	0.0 (0.0 to 0.0)	0.1 (-17.1 to 19.8)	-10.6 (-26.0 to 7.1)
Belgium	Acute glomerulonephritis	5 to 9	Male	0 (0 to 0)	0.0 (0.0 to 0.0)	0 (0 to 0)	0.0 (0.0 to 0.0)	0 (0 to 0)	0.0 (0.0 to 0.0)	-2.0 (-18.3 to 16.8)	-12.2 (-26.8 to 4.6)
Belgium	Acute glomerulonephritis	10 to 14	Both	0 (0 to 0)	0.0 (0.0 to 0.0)	0 (0 to 0)	0.0 (0.0 to 0.0)	0 (0 to 0)	0.0 (0.0 to 0.0)	-4.3 (-16.1 to 7.7)	-6.4 (-17.9 to 5.4)
Belgium	Acute glomerulonephritis	10 to 14	Female	0 (0 to 0)	0.0 (0.0 to 0.0)	0 (0 to 0)	0.0 (0.0 to 0.0)	0 (0 to 0)	0.0 (0.0 to 0.0)	-1.5 (-17.2 to 18.0)	-3.6 (-18.9 to 15.6)
Belgium	Acute glomerulonephritis	10 to 14	Male	0 (0 to 0)	0.0 (0.0 to 0.0)	0 (0 to 0)	0.0 (0.0 to 0.0)	0 (0 to 0)	0.0 (0.0 to 0.0)	-7.1 (-21.6 to 10.3)	-9.1 (-23.3 to 7.9)
Belgium	Chronic kidney disease	5 to 9	Both	0 (0 to 1)	0.1 (0.1 to 0.1)	0 (0 to 0)	0.0 (0.0 to 0.0)	0 (0 to 0)	0.0 (0.0 to 0.0)	-56.1 (-68.5 to -38.6)	-60.8 (-71.8 to -45.1)
Belgium	Chronic kidney disease	5 to 9	Female	0 (0 to 0)	0.1 (0.0 to 0.1)	0 (0 to 0)	0.0 (0.0 to 0.0)	0 (0 to 0)	0.0 (0.0 to 0.0)	-54.6 (-71.9 to -26.7)	-59.5 (-74.9 to -34.5)
Belgium	Chronic kidney disease	5 to 9	Male	0 (0 to 0)	0.1 (0.1 to 0.1)	0 (0 to 0)	0.0 (0.0 to 0.0)	0 (0 to 0)	0.0 (0.0 to 0.0)	-57.6 (-73.3 to -34.9)	-62.0 (-76.1 to -41.7)
Belgium	Chronic kidney disease	10 to 14	Both	1 (0 to 1)	0.1 (0.1 to 0.1)	0 (0 to 0)	0.1 (0.0 to 0.1)	0 (0 to 0)	0.0 (0.0 to 0.0)	-51.7 (-64.5 to -34.0)	-52.7 (-65.3 to -35.4)
Belgium	Chronic kidney disease	10 to 14	Female	0 (0 to 0)	0.1 (0.1 to 0.1)	0 (0 to 0)	0.1 (0.1 to 0.1)	0 (0 to 0)	0.1 (0.0 to 0.1)	-48.7 (-66.6 to -21.7)	-49.8 (-67.2 to -23.3)
Belgium	Chronic kidney disease	10 to 14	Male	0 (0 to 0)	0.1 (0.1 to 0.1)	0 (0 to 0)	0.0 (0.0 to 0.1)	0 (0 to 0)	0.0 (0.0 to 0.0)	-55.8 (-71.3 to -35.0)	-56.8 (-71.9 to -36.4)
Belgium	Urinary diseases and male infertility	5 to 9	Both	0 (0 to 0)	0.0 (0.0 to 0.0)	0 (0 to 0)	0.0 (0.0 to 0.0)	0 (0 to 0)	0.0 (0.0 to 0.0)	9.5 (-12.0 to 39.6)	-2.0 (-21.3 to 24.9)
Belgium	Urinary diseases and male infertility	5 to 9	Female	0 (0 to 0)	0.0 (0.0 to 0.0)	0 (0 to 0)	0.0 (0.0 to 0.0)	0 (0 to 0)	0.0 (0.0 to 0.0)	18.4 (-14.0 to 66.2)	5.8 (-23.2 to 48.5)
Belgium	Urinary diseases and male infertility	5 to 9	Male	0 (0 to 0)	0.0 (0.0 to 0.0)	0 (0 to 0)	0.0 (0.0 to 0.0)	0 (0 to 0)	0.0 (0.0 to 0.0)	0.9 (-17.4 to 30.0)	-9.6 (-26.0 to 16.4)
Belgium	Urinary diseases and male infertility	10 to 14	Both	0 (0 to 0)	0.0 (0.0 to 0.0)	0 (0 to 0)	0.0 (0.0 to 0.0)	0 (0 to 0)	0.0 (0.0 to 0.0)	46.9 (4.2 to 102.7)	43.8 (2.0 to 98.4)
Belgium	Urinary diseases and male infertility	10 to 14	Female	0 (0 to 0)	0.0 (0.0 to 0.0)	0 (0 to 0)	0.0 (0.0 to 0.0)	0 (0 to 0)	0.0 (0.0 to 0.0)	65.9 (2.7 to 167.3)	62.5 (0.6 to 161.8)
Belgium	Urinary diseases and male infertility	10 to 14	Male	0 (0 to 0)	0.0 (0.0 to 0.0)	0 (0 to 0)	0.0 (0.0 to 0.0)	0 (0 to 0)	0.0 (0.0 to 0.0)	22.9 (-21.6 to 91.0)	20.2 (-23.3 to 86.8)
Belgium	Hemoglobinopathies and hemolytic anemias	5 to 9	Both	1 (1 to 1)	0.2 (0.1 to 0.2)	1 (0 to 1)	0.1 (0.1 to 0.1)	0 (0 to 0)	0.0 (0.0 to 0.1)	-70.7 (-79.6 to -58.5)	-73.8 (-81.8 to -62.9)
Belgium	Hemoglobinopathies and hemolytic anemias	5 to 9	Female	0 (0 to 1)	0.2 (0.1 to 0.2)	0 (0 to 0)	0.1 (0.1 to 0.1)	0 (0 to 0)	0.0 (0.0 to 0.1)	-69.8 (-80.5 to -51.9)	-73.0 (-82.5 to -57.0)
Belgium	Hemoglobinopathies and hemolytic anemias	5 to 9	Male	1 (0 to 1)	0.2 (0.1 to 0.3)	0 (0 to 0)	0.1 (0.1 to 0.1)	0 (0 to 0)	0.0 (0.0 to 0.1)	-71.5 (-82.4 to -52.6)	-74.5 (-84.2 to -57.5)

eTable 8. All-cause and cause-specific mortality (counts and rates) at the global level and by country in the WHO European Region for ages 5 to 9 and 10 to 14 for 1990, 2000, and 2016

Belgium	Hemoglobinopathies and hemolytic anemias	10 to 14	Both	1 (1 to 1)	0.1 (0.1 to 0.2)	1 (0 to 1)	0.1 (0.1 to 0.2)	0 (0 to 1)	0.1 (0.0 to 0.1)	-53.9 (-73.1 to -21.6)	-54.9 (-73.7 to -23.2)
Belgium	Hemoglobinopathies and hemolytic anemias	10 to 14	Female	0 (0 to 1)	0.1 (0.1 to 0.2)	0 (0 to 1)	0.1 (0.1 to 0.2)	0 (0 to 1)	0.1 (0.0 to 0.2)	-39.1 (-72.4 to 3.4)	-40.3 (-73.0 to 1.3)
Belgium	Hemoglobinopathies and hemolytic anemias	10 to 14	Male	0 (0 to 1)	0.1 (0.1 to 0.2)	0 (0 to 0)	0.1 (0.1 to 0.1)	0 (0 to 0)	0.0 (0.0 to 0.1)	-67.3 (-80.1 to -46.2)	-68.0 (-80.5 to -47.3)
Belgium	Endocrine, metabolic, blood, and immune disorders	5 to 9	Both	5 (3 to 6)	0.8 (0.6 to 1.0)	4 (3 to 5)	0.6 (0.5 to 0.9)	3 (2 to 5)	0.5 (0.4 to 0.7)	-27.8 (-49.7 to 7.9)	-35.4 (-55.0 to -3.4)
Belgium	Endocrine, metabolic, blood, and immune disorders	5 to 9	Female	2 (2 to 3)	0.8 (0.5 to 1.2)	2 (1 to 3)	0.6 (0.4 to 0.9)	2 (1 to 2)	0.5 (0.3 to 0.8)	-31.1 (-56.6 to 20.4)	-38.5 (-61.2 to 7.6)
Belgium	Endocrine, metabolic, blood, and immune disorders	5 to 9	Male	2 (1 to 3)	0.7 (0.5 to 1.0)	2 (1 to 3)	0.6 (0.4 to 1.0)	2 (1 to 3)	0.5 (0.3 to 0.8)	-24.1 (-55.1 to 39.9)	-32.1 (-59.8 to 25.3)
Belgium	Endocrine, metabolic, blood, and immune disorders	10 to 14	Both	5 (4 to 6)	0.8 (0.6 to 1.0)	5 (3 to 7)	0.8 (0.6 to 1.1)	3 (3 to 5)	0.5 (0.4 to 0.8)	-27.1 (-48.3 to 7.8)	-28.7 (-49.4 to 5.5)
Belgium	Endocrine, metabolic, blood, and immune disorders	10 to 14	Female	2 (2 to 3)	0.8 (0.6 to 1.2)	2 (2 to 3)	0.8 (0.5 to 1.1)	2 (1 to 3)	0.5 (0.4 to 0.8)	-31.9 (-56.6 to 12.1)	-33.3 (-57.5 to 9.8)
Belgium	Endocrine, metabolic, blood, and immune disorders	10 to 14	Male	2 (2 to 3)	0.7 (0.5 to 1.1)	2 (2 to 4)	0.7 (0.5 to 1.2)	2 (1 to 3)	0.5 (0.3 to 1.0)	-21.9 (-51.2 to 38.0)	-23.6 (-52.2 to 35.0)
Belgium	Rheumatoid arthritis	5 to 9	Both	0 (0 to 0)	0.0 (0.0 to 0.0)	0 (0 to 0)	0.0 (0.0 to 0.0)	0 (0 to 0)	0.0 (0.0 to 0.0)	-57.2 (-73.1 to -15.3)	-61.7 (-76.0 to -24.2)
Belgium	Rheumatoid arthritis	5 to 9	Female	0 (0 to 0)	0.0 (0.0 to 0.0)	0 (0 to 0)	0.0 (0.0 to 0.0)	0 (0 to 0)	0.0 (0.0 to 0.0)	-65.0 (-80.8 to -18.5)	-68.8 (-82.9 to -27.2)
Belgium	Rheumatoid arthritis	5 to 9	Male	0 (0 to 0)	0.0 (0.0 to 0.0)	0 (0 to 0)	0.0 (0.0 to 0.0)	0 (0 to 0)	0.0 (0.0 to 0.0)	-33.8 (-64.4 to 37.6)	-40.7 (-68.1 to 23.3)
Belgium	Rheumatoid arthritis	10 to 14	Both	0 (0 to 0)	0.0 (0.0 to 0.0)	0 (0 to 0)	0.0 (0.0 to 0.0)	0 (0 to 0)	0.0 (0.0 to 0.0)	-43.1 (-65.5 to 9.8)	-44.3 (-66.2 to 7.4)
Belgium	Rheumatoid arthritis	10 to 14	Female	0 (0 to 0)	0.0 (0.0 to 0.0)	0 (0 to 0)	0.0 (0.0 to 0.0)	0 (0 to 0)	0.0 (0.0 to 0.0)	-37.4 (-67.6 to 29.6)	-38.7 (-68.3 to 27.0)
Belgium	Rheumatoid arthritis	10 to 14	Male	0 (0 to 0)	0.0 (0.0 to 0.0)	0 (0 to 0)	0.0 (0.0 to 0.0)	0 (0 to 0)	0.0 (0.0 to 0.0)	-47.7 (-73.6 to 18.5)	-48.8 (-74.2 to 15.9)
Belgium	Other musculoskeletal disorders	5 to 9	Both	0 (0 to 0)	0.0 (0.0 to 0.0)	0 (0 to 0)	0.0 (0.0 to 0.0)	0 (0 to 0)	0.0 (0.0 to 0.0)	-19.6 (-50.4 to 26.9)	-28.1 (-55.6 to 13.6)
Belgium	Other musculoskeletal disorders	5 to 9	Female	0 (0 to 0)	0.0 (0.0 to 0.1)	0 (0 to 0)	0.0 (0.0 to 0.1)	0 (0 to 0)	0.0 (0.0 to 0.0)	-14.2 (-50.0 to 53.8)	-23.4 (-55.3 to 37.4)
Belgium	Other musculoskeletal disorders	5 to 9	Male	0 (0 to 0)	0.0 (0.0 to 0.0)	0 (0 to 0)	0.0 (0.0 to 0.0)	0 (0 to 0)	0.0 (0.0 to 0.0)	-30.8 (-60.1 to 32.3)	-38.0 (-64.2 to 18.5)

eTable 8. All-cause and cause-specific mortality (counts and rates) at the global level and by country in the WHO European Region for ages 5 to 9 and 10 to 14 for 1990, 2000, and 2016

Belgium	Other musculoskeletal disorders	10 to 14	Both	0 (0 to 0)	0.0 (0.0 to 0.0)	0 (0 to 0)	0.1 (0.0 to 0.1)	0 (0 to 0)	0.0 (0.0 to 0.0)	-20.1 (-46.2 to 16.7)	-21.8 (-47.3 to 14.2)
Belgium	Other musculoskeletal disorders	10 to 14	Female	0 (0 to 0)	0.0 (0.0 to 0.1)	0 (0 to 0)	0.1 (0.0 to 0.1)	0 (0 to 0)	0.0 (0.0 to 0.0)	-20.9 (-52.5 to 29.6)	-22.5 (-53.5 to 26.9)
Belgium	Other musculoskeletal disorders	10 to 14	Male	0 (0 to 0)	0.0 (0.0 to 0.0)	0 (0 to 0)	0.0 (0.0 to 0.1)	0 (0 to 0)	0.0 (0.0 to 0.0)	-19.2 (-51.3 to 41.4)	-20.9 (-52.4 to 38.3)
Belgium	Congenital birth defects	5 to 9	Both	8 (6 to 11)	1.4 (1.1 to 1.8)	5 (4 to 7)	0.9 (0.7 to 1.1)	4 (3 to 5)	0.6 (0.5 to 0.8)	-50.2 (-63.7 to -27.9)	-55.5 (-67.5 to -35.5)
Belgium	Congenital birth defects	5 to 9	Female	4 (3 to 6)	1.5 (1.0 to 1.9)	3 (2 to 3)	0.8 (0.6 to 1.1)	2 (1 to 3)	0.6 (0.4 to 0.8)	-52.6 (-68.8 to -22.6)	-57.6 (-72.1 to -30.8)
Belgium	Congenital birth defects	5 to 9	Male	4 (3 to 6)	1.4 (0.9 to 1.9)	3 (2 to 4)	0.9 (0.7 to 1.2)	2 (1 to 3)	0.6 (0.4 to 0.9)	-47.9 (-66.6 to -20.2)	-53.3 (-70.1 to -28.5)
Belgium	Congenital birth defects	10 to 14	Both	6 (5 to 8)	1.0 (0.8 to 1.3)	5 (4 to 6)	0.8 (0.6 to 1.0)	4 (3 to 5)	0.6 (0.5 to 0.8)	-38.6 (-55.0 to -17.0)	-39.9 (-56.0 to -18.8)
Belgium	Congenital birth defects	10 to 14	Female	3 (2 to 4)	1.0 (0.7 to 1.4)	3 (2 to 4)	0.9 (0.6 to 1.2)	2 (1 to 3)	0.6 (0.4 to 0.8)	-37.6 (-58.0 to -4.2)	-38.9 (-58.9 to -6.2)
Belgium	Congenital birth defects	10 to 14	Male	3 (2 to 4)	1.0 (0.7 to 1.4)	2 (2 to 3)	0.8 (0.5 to 1.1)	2 (1 to 3)	0.6 (0.4 to 0.9)	-39.5 (-60.1 to -8.9)	-40.9 (-61.0 to -10.9)
Belgium	Skin and subcutaneous diseases	5 to 9	Both	0 (0 to 0)	0.0 (0.0 to 0.0)	0 (0 to 0)	0.0 (0.0 to 0.0)	0 (0 to 0)	0.0 (0.0 to 0.0)	14.6 (-10.5 to 49.1)	2.5 (-19.9 to 33.4)
Belgium	Skin and subcutaneous diseases	5 to 9	Female	0 (0 to 0)	0.0 (0.0 to 0.0)	0 (0 to 0)	0.0 (0.0 to 0.0)	0 (0 to 0)	0.0 (0.0 to 0.0)	3.8 (-17.0 to 26.6)	-7.3 (-25.9 to 13.1)
Belgium	Skin and subcutaneous diseases	5 to 9	Male	0 (0 to 0)	0.0 (0.0 to 0.0)	0 (0 to 0)	0.0 (0.0 to 0.0)	0 (0 to 0)	0.0 (0.0 to 0.0)	24.9 (-22.9 to 98.9)	11.9 (-30.9 to 78.2)
Belgium	Skin and subcutaneous diseases	10 to 14	Both	0 (0 to 0)	0.0 (0.0 to 0.0)	0 (0 to 0)	0.0 (0.0 to 0.0)	0 (0 to 0)	0.0 (0.0 to 0.0)	10.5 (-12.2 to 39.5)	8.1 (-14.1 to 36.5)
Belgium	Skin and subcutaneous diseases	10 to 14	Female	0 (0 to 0)	0.0 (0.0 to 0.0)	0 (0 to 0)	0.0 (0.0 to 0.0)	0 (0 to 0)	0.0 (0.0 to 0.0)	1.3 (-16.6 to 21.2)	-0.8 (-18.3 to 18.7)
Belgium	Skin and subcutaneous diseases	10 to 14	Male	0 (0 to 0)	0.0 (0.0 to 0.0)	0 (0 to 0)	0.0 (0.0 to 0.0)	0 (0 to 0)	0.0 (0.0 to 0.0)	19.6 (-17.9 to 79.5)	17.0 (-19.7 to 75.5)
Belgium	Road injuries	5 to 9	Both	37 (32 to 42)	6.1 (5.3 to 6.9)	20 (17 to 23)	3.2 (2.7 to 3.8)	9 (7 to 12)	1.3 (1.1 to 1.7)	-75.5 (-81.4 to -67.6)	-78.0 (-83.4 to -71.0)
Belgium	Road injuries	5 to 9	Female	15 (12 to 19)	5.2 (4.1 to 6.4)	8 (6 to 10)	2.6 (2.0 to 3.3)	4 (3 to 5)	1.1 (0.8 to 1.6)	-76.3 (-84.3 to -63.2)	-78.8 (-86.0 to -67.1)
Belgium	Road injuries	5 to 9	Male	21 (18 to 25)	7.0 (5.8 to 8.2)	12 (10 to 14)	3.8 (3.1 to 4.6)	5 (4 to 7)	1.6 (1.1 to 2.1)	-74.9 (-82.5 to -64.3)	-77.5 (-84.4 to -68.0)
Belgium	Road injuries	10 to 14	Both	39 (34 to 45)	6.5 (5.6 to 7.3)	28 (25 to 32)	4.6 (4.1 to 5.3)	11 (9 to 13)	1.7 (1.4 to 2.1)	-72.4 (-78.2 to -64.6)	-73.0 (-78.7 to -65.3)
Belgium	Road injuries	10 to 14	Female	15 (12 to 19)	5.2 (4.2 to 6.4)	13 (10 to 16)	4.3 (3.4 to 5.2)	5 (3 to 6)	1.5 (1.1 to 2.1)	-69.7 (-79.1 to -54.7)	-70.3 (-79.5 to -55.6)
Belgium	Road injuries	10 to 14	Male	24 (21 to 27)	7.7 (6.6 to 8.8)	15 (13 to 18)	4.9 (4.1 to 5.7)	6 (5 to 8)	1.9 (1.5 to 2.5)	-74.1 (-80.7 to -65.7)	-74.7 (-81.1 to -66.4)
Belgium	Other transport injuries	5 to 9	Both	1 (1 to 2)	0.2 (0.1 to 0.3)	1 (1 to 1)	0.2 (0.1 to 0.2)	1 (0 to 1)	0.1 (0.1 to 0.1)	-46.8 (-70.6 to -4.8)	-52.4 (-73.7 to -14.8)
Belgium	Other transport injuries	5 to 9	Female	1 (0 to 1)	0.2 (0.1 to 0.3)	0 (0 to 1)	0.1 (0.1 to 0.2)	0 (0 to 1)	0.1 (0.1 to 0.2)	-44.4 (-74.0 to 14.8)	-50.3 (-76.8 to 2.6)
Belgium	Other transport injuries	5 to 9	Male	1 (0 to 1)	0.2 (0.1 to 0.4)	1 (0 to 1)	0.2 (0.1 to 0.3)	0 (0 to 1)	0.1 (0.1 to 0.2)	-48.9 (-78.6 to 22.6)	-54.2 (-80.8 to 9.8)
Belgium	Other transport injuries	10 to 14	Both	2 (1 to 3)	0.4 (0.2 to 0.5)	2 (1 to 3)	0.3 (0.2 to 0.4)	1 (1 to 2)	0.2 (0.1 to 0.3)	-47.0 (-68.4 to -7.4)	-48.2 (-69.0 to -9.3)

eTable 8. All-cause and cause-specific mortality (counts and rates) at the global level and by country in the WHO European Region for ages 5 to 9 and 10 to 14 for 1990, 2000, and 2016

Belgium	Other transport injuries	10 to 14	Female	1 (0 to 1)	0.2 (0.1 to 0.4)	1 (0 to 1)	0.2 (0.1 to 0.3)	0 (0 to 1)	0.1 (0.1 to 0.2)	-39.6 (-72.8 to 20.2)	-40.8 (-73.4 to 17.8)
Belgium	Other transport injuries	10 to 14	Male	1 (1 to 2)	0.5 (0.3 to 0.7)	1 (1 to 2)	0.4 (0.2 to 0.6)	1 (0 to 1)	0.2 (0.1 to 0.4)	-50.5 (-74.6 to 2.1)	-51.6 (-75.2 to -0.2)
Belgium	Falls	5 to 9	Both	2 (2 to 3)	0.3 (0.3 to 0.4)	2 (1 to 2)	0.2 (0.2 to 0.3)	1 (1 to 2)	0.2 (0.1 to 0.2)	-38.9 (-61.3 to -7.9)	-45.3 (-65.4 to -17.6)
Belgium	Falls	5 to 9	Female	1 (1 to 1)	0.3 (0.2 to 0.4)	1 (0 to 1)	0.2 (0.1 to 0.3)	1 (0 to 1)	0.2 (0.1 to 0.2)	-40.3 (-61.4 to -2.9)	-46.6 (-65.5 to -13.2)
Belgium	Falls	5 to 9	Male	1 (1 to 2)	0.4 (0.3 to 0.5)	1 (1 to 1)	0.3 (0.2 to 0.4)	1 (0 to 1)	0.2 (0.1 to 0.3)	-37.9 (-64.6 to 6.7)	-44.4 (-68.3 to -4.5)
Belgium	Falls	10 to 14	Both	2 (1 to 3)	0.3 (0.2 to 0.5)	2 (1 to 3)	0.3 (0.2 to 0.5)	1 (1 to 2)	0.2 (0.2 to 0.3)	-30.9 (-56.1 to 4.4)	-32.4 (-57.0 to 2.2)
Belgium	Falls	10 to 14	Female	1 (1 to 1)	0.2 (0.2 to 0.4)	1 (0 to 1)	0.2 (0.2 to 0.3)	1 (0 to 1)	0.2 (0.1 to 0.2)	-29.9 (-56.4 to 19.8)	-31.4 (-57.3 to 17.3)
Belgium	Falls	10 to 14	Male	1 (1 to 2)	0.4 (0.3 to 0.7)	1 (1 to 2)	0.4 (0.3 to 0.6)	1 (1 to 2)	0.3 (0.2 to 0.5)	-31.5 (-58.7 to 16.3)	-33.0 (-59.6 to 13.8)
Belgium	Drowning	5 to 9	Both	6 (5 to 8)	1.1 (0.8 to 1.3)	3 (3 to 4)	0.5 (0.4 to 0.7)	2 (2 to 3)	0.3 (0.3 to 0.4)	-65.0 (-74.9 to -51.4)	-68.7 (-77.5 to -56.5)
Belgium	Drowning	5 to 9	Female	2 (1 to 2)	0.6 (0.4 to 0.8)	1 (1 to 1)	0.3 (0.2 to 0.4)	1 (0 to 1)	0.2 (0.1 to 0.3)	-64.0 (-77.2 to -43.8)	-67.8 (-79.6 to -49.8)
Belgium	Drowning	5 to 9	Male	5 (3 to 6)	1.5 (1.1 to 2.0)	2 (2 to 3)	0.8 (0.6 to 1.0)	2 (1 to 2)	0.5 (0.3 to 0.6)	-65.3 (-77.8 to -47.8)	-69.0 (-80.2 to -53.2)
Belgium	Drowning	10 to 14	Both	4 (3 to 5)	0.6 (0.5 to 0.8)	2 (2 to 3)	0.4 (0.3 to 0.5)	1 (1 to 2)	0.2 (0.2 to 0.3)	-60.3 (-71.8 to -45.7)	-61.2 (-72.4 to -46.8)
Belgium	Drowning	10 to 14	Female	1 (1 to 2)	0.4 (0.3 to 0.6)	1 (1 to 1)	0.3 (0.2 to 0.4)	1 (0 to 1)	0.2 (0.1 to 0.3)	-56.2 (-72.8 to -31.0)	-57.1 (-73.3 to -32.5)
Belgium	Drowning	10 to 14	Male	2 (2 to 3)	0.7 (0.6 to 1.0)	1 (1 to 2)	0.5 (0.3 to 0.6)	1 (1 to 1)	0.3 (0.2 to 0.4)	-62.6 (-76.1 to -42.2)	-63.5 (-76.7 to -43.5)
Belgium	Fire, heat, and hot substances	5 to 9	Both	5 (4 to 6)	0.8 (0.6 to 0.9)	3 (2 to 4)	0.5 (0.4 to 0.6)	2 (1 to 2)	0.3 (0.2 to 0.4)	-59.1 (-70.7 to -41.7)	-63.4 (-73.8 to -47.9)
Belgium	Fire, heat, and hot substances	5 to 9	Female	2 (1 to 3)	0.7 (0.5 to 0.9)	1 (1 to 2)	0.4 (0.3 to 0.6)	1 (1 to 1)	0.3 (0.2 to 0.4)	-54.1 (-71.5 to -22.0)	-58.9 (-74.5 to -30.3)
Belgium	Fire, heat, and hot substances	5 to 9	Male	3 (2 to 3)	0.8 (0.6 to 1.1)	2 (1 to 2)	0.5 (0.4 to 0.7)	1 (1 to 1)	0.3 (0.2 to 0.4)	-62.8 (-76.0 to -38.7)	-66.7 (-78.5 to -45.1)
Belgium	Fire, heat, and hot substances	10 to 14	Both	2 (1 to 3)	0.3 (0.2 to 0.4)	1 (1 to 2)	0.2 (0.2 to 0.3)	1 (1 to 1)	0.1 (0.1 to 0.2)	-54.9 (-67.5 to -35.6)	-55.8 (-68.2 to -36.9)
Belgium	Fire, heat, and hot substances	10 to 14	Female	1 (0 to 1)	0.3 (0.2 to 0.4)	1 (0 to 1)	0.2 (0.1 to 0.3)	0 (0 to 1)	0.1 (0.1 to 0.2)	-50.1 (-70.1 to -13.9)	-51.1 (-70.7 to -15.7)
Belgium	Fire, heat, and hot substances	10 to 14	Male	1 (1 to 2)	0.4 (0.3 to 0.5)	1 (1 to 1)	0.3 (0.2 to 0.4)	0 (0 to 1)	0.1 (0.1 to 0.2)	-58.0 (-72.2 to -34.2)	-58.9 (-72.8 to -35.7)
Belgium	Poisonings	5 to 9	Both	2 (1 to 3)	0.3 (0.1 to 0.4)	0 (0 to 1)	0.1 (0.1 to 0.1)	0 (0 to 0)	0.0 (0.0 to 0.1)	-85.8 (-91.6 to -48.3)	-87.3 (-92.5 to -53.7)
Belgium	Poisonings	5 to 9	Female	1 (0 to 1)	0.3 (0.1 to 0.4)	0 (0 to 0)	0.1 (0.0 to 0.1)	0 (0 to 0)	0.0 (0.0 to 0.1)	-84.0 (-92.1 to -36.3)	-85.7 (-93.0 to -43.0)
Belgium	Poisonings	5 to 9	Male	1 (0 to 2)	0.3 (0.1 to 0.5)	0 (0 to 0)	0.1 (0.0 to 0.1)	0 (0 to 0)	0.0 (0.0 to 0.1)	-87.2 (-93.7 to -34.9)	-88.6 (-94.3 to -41.7)
Belgium	Poisonings	10 to 14	Both	3 (2 to 5)	0.6 (0.2 to 0.8)	1 (1 to 2)	0.2 (0.1 to 0.3)	1 (0 to 1)	0.1 (0.1 to 0.2)	-82.7 (-90.1 to -44.7)	-83.1 (-90.3 to -45.9)
Belgium	Poisonings	10 to 14	Female	2 (1 to 3)	0.6 (0.2 to 1.0)	1 (0 to 1)	0.2 (0.1 to 0.3)	0 (0 to 1)	0.1 (0.1 to 0.2)	-81.6 (-91.5 to -38.3)	-81.9 (-91.6 to -39.6)
Belgium	Poisonings	10 to 14	Male	2 (1 to 2)	0.5 (0.3 to 0.8)	1 (0 to 1)	0.2 (0.1 to 0.3)	0 (0 to 1)	0.1 (0.0 to 0.2)	-84.1 (-92.0 to -37.5)	-84.4 (-92.1 to -38.9)
Belgium	Exposure to mechanical forces	5 to 9	Both	2 (2 to 3)	0.4 (0.3 to 0.5)	2 (1 to 2)	0.3 (0.2 to 0.4)	1 (1 to 2)	0.2 (0.1 to 0.3)	-48.5 (-64.4 to -26.0)	-53.9 (-68.2 to -33.8)
Belgium	Exposure to mechanical forces	5 to 9	Female	1 (1 to 1)	0.3 (0.2 to 0.4)	1 (1 to 1)	0.2 (0.2 to 0.3)	1 (0 to 1)	0.2 (0.1 to 0.2)	-42.1 (-61.4 to -9.8)	-48.3 (-65.5 to -19.4)
Belgium	Exposure to mechanical forces	5 to 9	Male	2 (1 to 2)	0.5 (0.4 to 0.7)	1 (1 to 1)	0.4 (0.2 to 0.5)	1 (0 to 1)	0.2 (0.1 to 0.3)	-52.2 (-70.3 to -25.5)	-57.2 (-73.4 to -33.3)

eTable 8. All-cause and cause-specific mortality (counts and rates) at the global level and by country in the WHO European Region for ages 5 to 9 and 10 to 14 for 1990, 2000, and 2016

Belgium	Exposure to mechanical forces	10 to 14	Both	2 (2 to 4)	0.4 (0.3 to 0.7)	2 (2 to 3)	0.3 (0.3 to 0.5)	1 (1 to 2)	0.2 (0.2 to 0.3)	-45.0 (-60.4 to -24.0)	-46.2 (-61.2 to -25.6)
Belgium	Exposure to mechanical forces	10 to 14	Female	1 (0 to 1)	0.2 (0.1 to 0.4)	1 (0 to 1)	0.2 (0.1 to 0.3)	0 (0 to 1)	0.1 (0.1 to 0.2)	-34.5 (-55.7 to -0.3)	-35.8 (-56.6 to -2.3)
Belgium	Exposure to mechanical forces	10 to 14	Male	2 (1 to 3)	0.6 (0.4 to 1.0)	2 (1 to 2)	0.5 (0.4 to 0.8)	1 (1 to 2)	0.3 (0.2 to 0.5)	-48.0 (-63.8 to -24.1)	-49.2 (-64.6 to -25.8)
Belgium	Adverse effects of medical treatment	5 to 9	Both	1 (1 to 2)	0.2 (0.2 to 0.3)	1 (0 to 1)	0.1 (0.1 to 0.2)	1 (0 to 1)	0.1 (0.1 to 0.1)	-60.3 (-77.7 to -19.7)	-64.4 (-80.0 to -28.1)
Belgium	Adverse effects of medical treatment	5 to 9	Female	1 (0 to 1)	0.2 (0.1 to 0.3)	0 (0 to 1)	0.1 (0.1 to 0.2)	0 (0 to 0)	0.1 (0.1 to 0.1)	-62.2 (-78.7 to -42.6)	-66.2 (-81.0 to -48.7)
Belgium	Adverse effects of medical treatment	5 to 9	Male	1 (0 to 1)	0.2 (0.1 to 0.4)	0 (0 to 1)	0.1 (0.1 to 0.2)	0 (0 to 1)	0.1 (0.0 to 0.2)	-58.5 (-81.5 to 8.6)	-62.8 (-83.4 to -2.7)
Belgium	Adverse effects of medical treatment	10 to 14	Both	1 (1 to 2)	0.2 (0.1 to 0.3)	1 (1 to 1)	0.1 (0.1 to 0.2)	1 (0 to 1)	0.1 (0.1 to 0.2)	-54.4 (-73.4 to -18.6)	-55.4 (-74.0 to -20.3)
Belgium	Adverse effects of medical treatment	10 to 14	Female	1 (0 to 1)	0.2 (0.1 to 0.3)	0 (0 to 1)	0.1 (0.1 to 0.2)	0 (0 to 0)	0.1 (0.1 to 0.1)	-53.9 (-73.0 to -34.2)	-54.8 (-73.5 to -35.6)
Belgium	Adverse effects of medical treatment	10 to 14	Male	1 (0 to 1)	0.2 (0.1 to 0.3)	0 (0 to 1)	0.1 (0.1 to 0.2)	0 (0 to 1)	0.1 (0.0 to 0.2)	-54.8 (-79.2 to 1.1)	-55.8 (-79.6 to -1.1)
Belgium	Animal contact	5 to 9	Both	0 (0 to 0)	0.0 (0.0 to 0.0)	0 (0 to 0)	0.0 (0.0 to 0.0)	0 (0 to 0)	0.0 (0.0 to 0.0)	9.0 (-40.9 to 99.9)	-2.5 (-47.1 to 78.8)
Belgium	Animal contact	5 to 9	Female	0 (0 to 0)	0.0 (0.0 to 0.0)	0 (0 to 0)	0.0 (0.0 to 0.0)	0 (0 to 0)	0.0 (0.0 to 0.0)	14.5 (-54.9 to 174.6)	2.3 (-59.7 to 145.3)
Belgium	Animal contact	5 to 9	Male	0 (0 to 0)	0.0 (0.0 to 0.0)	0 (0 to 0)	0.0 (0.0 to 0.0)	0 (0 to 0)	0.0 (0.0 to 0.0)	2.9 (-53.5 to 116.7)	-7.8 (-58.3 to 94.1)
Belgium	Animal contact	10 to 14	Both	0 (0 to 0)	0.0 (0.0 to 0.0)	0 (0 to 0)	0.0 (0.0 to 0.0)	0 (0 to 0)	0.0 (0.0 to 0.0)	3.6 (-43.7 to 92.5)	1.3 (-44.9 to 88.4)
Belgium	Animal contact	10 to 14	Female	0 (0 to 0)	0.0 (0.0 to 0.1)	0 (0 to 0)	0.0 (0.0 to 0.1)	0 (0 to 0)	0.0 (0.0 to 0.1)	3.4 (-57.1 to 129.6)	1.2 (-58.0 to 124.9)
Belgium	Animal contact	10 to 14	Male	0 (0 to 0)	0.0 (0.0 to 0.0)	0 (0 to 0)	0.0 (0.0 to 0.0)	0 (0 to 0)	0.0 (0.0 to 0.0)	4.0 (-49.9 to 106.7)	1.7 (-51.0 to 102.2)
Belgium	Foreign body	5 to 9	Both	1 (1 to 2)	0.2 (0.1 to 0.4)	1 (1 to 2)	0.2 (0.1 to 0.3)	1 (1 to 2)	0.2 (0.1 to 0.3)	-4.6 (-50.2 to 58.4)	-14.7 (-55.4 to 41.7)
Belgium	Foreign body	5 to 9	Female	1 (0 to 1)	0.2 (0.1 to 0.4)	0 (0 to 1)	0.2 (0.1 to 0.3)	1 (0 to 1)	0.2 (0.1 to 0.3)	4.5 (-47.3 to 104.0)	-6.6 (-52.9 to 82.3)
Belgium	Foreign body	5 to 9	Male	1 (0 to 1)	0.2 (0.1 to 0.4)	1 (0 to 1)	0.2 (0.1 to 0.3)	1 (0 to 1)	0.2 (0.1 to 0.3)	-11.2 (-57.1 to 69.4)	-20.5 (-61.6 to 51.7)
Belgium	Foreign body	10 to 14	Both	2 (1 to 3)	0.3 (0.1 to 0.5)	2 (1 to 3)	0.3 (0.2 to 0.5)	2 (1 to 3)	0.2 (0.2 to 0.4)	-5.9 (-47.0 to 81.5)	-7.9 (-48.1 to 77.6)
Belgium	Foreign body	10 to 14	Female	0 (0 to 1)	0.1 (0.1 to 0.3)	0 (0 to 1)	0.2 (0.1 to 0.3)	0 (0 to 1)	0.1 (0.1 to 0.3)	4.9 (-41.6 to 109.3)	2.7 (-42.8 to 105.0)
Belgium	Foreign body	10 to 14	Male	1 (1 to 2)	0.4 (0.2 to 0.8)	1 (1 to 2)	0.4 (0.2 to 0.7)	1 (1 to 2)	0.3 (0.2 to 0.6)	-9.8 (-52.6 to 101.9)	-11.8 (-53.7 to 97.4)
Belgium	Environmental heat and cold exposure	5 to 9	Both	0 (0 to 0)	0.0 (0.0 to 0.0)	0 (0 to 0)	0.0 (0.0 to 0.0)	0 (0 to 0)	0.0 (0.0 to 0.0)	-31.5 (-60.2 to 10.6)	-38.7 (-64.4 to -1.1)
Belgium	Environmental heat and cold exposure	5 to 9	Female	0 (0 to 0)	0.0 (0.0 to 0.0)	0 (0 to 0)	0.0 (0.0 to 0.0)	0 (0 to 0)	0.0 (0.0 to 0.0)	-37.5 (-64.6 to 2.9)	-44.1 (-68.4 to -8.1)

eTable 8. All-cause and cause-specific mortality (counts and rates) at the global level and by country in the WHO European Region for ages 5 to 9 and 10 to 14 for 1990, 2000, and 2016

Belgium	Environmental heat and cold exposure	5 to 9	Male	0 (0 to 0)	0.0 (0.0 to 0.0)	0 (0 to 0)	0.0 (0.0 to 0.0)	0 (0 to 0)	0.0 (0.0 to 0.0)	-27.3 (-63.0 to 33.1)	-34.8 (-66.9 to 19.3)
Belgium	Environmental heat and cold exposure	10 to 14	Both	0 (0 to 0)	0.0 (0.0 to 0.0)	0 (0 to 0)	0.0 (0.0 to 0.0)	0 (0 to 0)	0.0 (0.0 to 0.0)	-42.2 (-61.8 to -15.6)	-43.5 (-62.6 to -17.4)
Belgium	Environmental heat and cold exposure	10 to 14	Female	0 (0 to 0)	0.0 (0.0 to 0.0)	0 (0 to 0)	0.0 (0.0 to 0.0)	0 (0 to 0)	0.0 (0.0 to 0.0)	-31.6 (-60.9 to 6.4)	-33.0 (-61.7 to 4.2)
Belgium	Environmental heat and cold exposure	10 to 14	Male	0 (0 to 0)	0.0 (0.0 to 0.1)	0 (0 to 0)	0.0 (0.0 to 0.0)	0 (0 to 0)	0.0 (0.0 to 0.0)	-48.5 (-66.0 to -13.6)	-49.6 (-66.8 to -15.5)
Belgium	Other unintentional injuries	5 to 9	Both	1 (0 to 1)	0.1 (0.1 to 0.2)	0 (0 to 0)	0.1 (0.0 to 0.1)	0 (0 to 0)	0.0 (0.0 to 0.0)	-66.6 (-79.6 to -44.0)	-70.1 (-81.8 to -49.9)
Belgium	Other unintentional injuries	5 to 9	Female	0 (0 to 0)	0.1 (0.0 to 0.1)	0 (0 to 0)	0.0 (0.0 to 0.0)	0 (0 to 0)	0.0 (0.0 to 0.0)	-57.0 (-76.9 to -18.9)	-61.5 (-79.4 to -27.5)
Belgium	Other unintentional injuries	5 to 9	Male	0 (0 to 1)	0.2 (0.1 to 0.2)	0 (0 to 0)	0.1 (0.1 to 0.1)	0 (0 to 0)	0.0 (0.0 to 0.1)	-70.1 (-85.0 to -41.2)	-73.3 (-86.6 to -47.4)
Belgium	Other unintentional injuries	10 to 14	Both	1 (1 to 1)	0.1 (0.1 to 0.2)	1 (0 to 1)	0.1 (0.1 to 0.1)	0 (0 to 0)	0.1 (0.0 to 0.1)	-62.3 (-74.6 to -45.4)	-63.1 (-75.1 to -46.6)
Belgium	Other unintentional injuries	10 to 14	Female	0 (0 to 0)	0.1 (0.1 to 0.1)	0 (0 to 0)	0.1 (0.0 to 0.1)	0 (0 to 0)	0.0 (0.0 to 0.0)	-56.1 (-76.0 to -24.3)	-57.0 (-76.5 to -25.8)
Belgium	Other unintentional injuries	10 to 14	Male	1 (0 to 1)	0.2 (0.2 to 0.3)	0 (0 to 1)	0.1 (0.1 to 0.2)	0 (0 to 0)	0.1 (0.1 to 0.1)	-64.4 (-77.8 to -42.6)	-65.2 (-78.3 to -43.8)
Belgium	Self-harm	10 to 14	Both	6 (4 to 7)	0.9 (0.6 to 1.2)	6 (4 to 8)	1.0 (0.6 to 1.3)	4 (2 to 5)	0.6 (0.4 to 0.9)	-31.0 (-52.0 to -1.5)	-32.5 (-53.0 to -3.6)
Belgium	Self-harm	10 to 14	Female	2 (1 to 2)	0.6 (0.4 to 0.8)	2 (1 to 3)	0.6 (0.4 to 0.9)	1 (1 to 2)	0.4 (0.3 to 0.6)	-25.5 (-56.1 to 29.4)	-27.1 (-57.0 to 26.7)
Belgium	Self-harm	10 to 14	Male	4 (2 to 6)	1.3 (0.7 to 1.9)	4 (2 to 6)	1.3 (0.6 to 1.8)	3 (1 to 4)	0.9 (0.4 to 1.3)	-33.3 (-59.5 to 3.9)	-34.7 (-60.4 to 1.6)
Belgium	Interpersonal violence	5 to 9	Both	4 (3 to 6)	0.7 (0.5 to 1.0)	4 (2 to 5)	0.6 (0.4 to 0.8)	2 (2 to 3)	0.4 (0.2 to 0.5)	-43.8 (-60.6 to -17.4)	-49.7 (-64.8 to -26.1)
Belgium	Interpersonal violence	5 to 9	Female	2 (1 to 3)	0.7 (0.5 to 1.1)	2 (1 to 2)	0.5 (0.4 to 0.8)	1 (1 to 2)	0.4 (0.2 to 0.6)	-41.8 (-64.9 to 10.3)	-48.0 (-68.7 to -1.5)
Belgium	Interpersonal violence	5 to 9	Male	2 (1 to 3)	0.7 (0.4 to 1.1)	2 (1 to 3)	0.6 (0.2 to 0.9)	1 (0 to 2)	0.3 (0.1 to 0.5)	-45.8 (-61.7 to -11.6)	-51.5 (-65.7 to -20.8)
Belgium	Interpersonal violence	10 to 14	Both	4 (3 to 6)	0.6 (0.4 to 1.0)	4 (3 to 5)	0.6 (0.4 to 0.8)	2 (1 to 3)	0.3 (0.2 to 0.5)	-43.0 (-58.0 to -16.2)	-44.2 (-58.8 to -17.9)
Belgium	Interpersonal violence	10 to 14	Female	2 (1 to 3)	0.6 (0.4 to 1.0)	2 (1 to 3)	0.6 (0.4 to 0.9)	1 (1 to 2)	0.4 (0.2 to 0.6)	-37.6 (-61.1 to 3.5)	-38.9 (-61.9 to 1.4)
Belgium	Interpersonal violence	10 to 14	Male	2 (1 to 4)	0.6 (0.4 to 1.3)	2 (1 to 3)	0.6 (0.3 to 0.9)	1 (1 to 2)	0.3 (0.2 to 0.6)	-47.5 (-63.1 to -4.3)	-48.7 (-63.9 to -6.4)
Belgium	Exposure to forces of nature	5 to 9	Both	0 (0 to 0)	0.0 (0.0 to 0.0)	0 (0 to 0)	0.0 (0.0 to 0.0)	0 (0 to 0)	0.0 (0.0 to 0.0)	0.0 (0.0 to 0.0)	0.0 (0.0 to 0.0)
Belgium	Exposure to forces of nature	5 to 9	Female	0 (0 to 0)	0.0 (0.0 to 0.0)	0 (0 to 0)	0.0 (0.0 to 0.0)	0 (0 to 0)	0.0 (0.0 to 0.0)	0.0 (0.0 to 0.0)	0.0 (0.0 to 0.0)
Belgium	Exposure to forces of nature	5 to 9	Male	0 (0 to 0)	0.0 (0.0 to 0.0)	0 (0 to 0)	0.0 (0.0 to 0.0)	0 (0 to 0)	0.0 (0.0 to 0.0)	0.0 (0.0 to 0.0)	0.0 (0.0 to 0.0)
Belgium	Exposure to forces of nature	10 to 14	Both	0 (0 to 0)	0.0 (0.0 to 0.0)	0 (0 to 0)	0.0 (0.0 to 0.0)	0 (0 to 0)	0.0 (0.0 to 0.0)	0.0 (0.0 to 0.0)	0.0 (0.0 to 0.0)
Belgium	Exposure to forces of nature	10 to 14	Female	0 (0 to 0)	0.0 (0.0 to 0.0)	0 (0 to 0)	0.0 (0.0 to 0.0)	0 (0 to 0)	0.0 (0.0 to 0.0)	0.0 (0.0 to 0.0)	0.0 (0.0 to 0.0)
Belgium	Exposure to forces of nature	10 to 14	Male	0 (0 to 0)	0.0 (0.0 to 0.0)	0 (0 to 0)	0.0 (0.0 to 0.0)	0 (0 to 0)	0.0 (0.0 to 0.0)	0.0 (0.0 to 0.0)	0.0 (0.0 to 0.0)

eTable 8. All-cause and cause-specific mortality (counts and rates) at the global level and by country in the WHO European Region for ages 5 to 9 and 10 to 14 for 1990, 2000, and 2016

Belgium	Conflict and terrorism	5 to 9	Both	1 (0 to 1)	0.1 (0.0 to 0.1)	0 (0 to 0)	0.0 (0.0 to 0.0)	1 (0 to 1)	0.1 (0.1 to 0.2)	68.7 (66.2 to 77.5)	51.0 (48.7 to 58.8)
Belgium	Conflict and terrorism	5 to 9	Female	0 (0 to 0)	0.1 (0.0 to 0.1)	0 (0 to 0)	0.0 (0.0 to 0.0)	0 (0 to 1)	0.1 (0.1 to 0.2)	69.0 (66.5 to 77.7)	51.0 (48.7 to 58.8)
Belgium	Conflict and terrorism	5 to 9	Male	0 (0 to 0)	0.1 (0.0 to 0.1)	0 (0 to 0)	0.0 (0.0 to 0.0)	0 (0 to 1)	0.1 (0.1 to 0.2)	68.5 (66.0 to 77.2)	50.9 (48.7 to 58.7)
Belgium	Conflict and terrorism	10 to 14	Both	1 (0 to 1)	0.1 (0.0 to 0.1)	0 (0 to 0)	0.0 (0.0 to 0.0)	1 (0 to 1)	0.1 (0.1 to 0.2)	65.6 (63.1 to 74.1)	62.0 (59.7 to 70.4)
Belgium	Conflict and terrorism	10 to 14	Female	0 (0 to 0)	0.1 (0.0 to 0.1)	0 (0 to 0)	0.0 (0.0 to 0.0)	0 (0 to 1)	0.1 (0.1 to 0.2)	66.8 (64.4 to 75.5)	63.4 (61.0 to 71.9)
Belgium	Conflict and terrorism	10 to 14	Male	0 (0 to 0)	0.1 (0.0 to 0.1)	0 (0 to 0)	0.0 (0.0 to 0.0)	0 (0 to 1)	0.1 (0.1 to 0.2)	64.4 (62.0 to 72.9)	60.8 (58.4 to 69.1)
Belgium	Executions and police conflict	5 to 9	Both	0 (0 to 0)	0.0 (0.0 to 0.0)	0 (0 to 0)	0.0 (0.0 to 0.0)	0 (0 to 0)	0.0 (0.0 to 0.0)	-1.0 (-14.9 to 15.1)	-11.4 (-23.8 to 3.0)
Belgium	Executions and police conflict	5 to 9	Female	0 (0 to 0)	0.0 (0.0 to 0.0)	0 (0 to 0)	0.0 (0.0 to 0.0)	0 (0 to 0)	0.0 (0.0 to 0.0)	0.1 (-17.1 to 19.8)	-10.6 (-26.0 to 7.1)
Belgium	Executions and police conflict	5 to 9	Male	0 (0 to 0)	0.0 (0.0 to 0.0)	0 (0 to 0)	0.0 (0.0 to 0.0)	0 (0 to 0)	0.0 (0.0 to 0.0)	-2.0 (-18.3 to 16.8)	-12.2 (-26.8 to 4.6)
Belgium	Executions and police conflict	10 to 14	Both	0 (0 to 0)	0.0 (0.0 to 0.0)	0 (0 to 0)	0.0 (0.0 to 0.0)	0 (0 to 0)	0.0 (0.0 to 0.0)	-4.3 (-16.1 to 7.7)	-6.4 (-17.9 to 5.4)
Belgium	Executions and police conflict	10 to 14	Female	0 (0 to 0)	0.0 (0.0 to 0.0)	0 (0 to 0)	0.0 (0.0 to 0.0)	0 (0 to 0)	0.0 (0.0 to 0.0)	-1.5 (-17.2 to 18.0)	-3.6 (-18.9 to 15.6)
Belgium	Executions and police conflict	10 to 14	Male	0 (0 to 0)	0.0 (0.0 to 0.0)	0 (0 to 0)	0.0 (0.0 to 0.0)	0 (0 to 0)	0.0 (0.0 to 0.0)	-7.1 (-21.6 to 10.3)	-9.1 (-23.3 to 7.9)
Bosnia and Herzegovina	All causes	5 to 9	Both	105 (100 to 110)	27.4 (26.1 to 28.8)	43 (41 to 45)	19.1 (18.2 to 20.0)	19 (17 to 22)	10.7 (9.6 to 12.1)	-81.8 (-83.9 to -79.3)	-60.9 (-65.5 to -55.6)
Bosnia and Herzegovina	All causes	5 to 9	Female	39 (37 to 40)	20.7 (19.8 to 21.6)	17 (16 to 18)	15.8 (15.1 to 16.6)	8 (7 to 9)	9.1 (8.1 to 10.2)	-79.5 (-81.9 to -76.8)	-55.9 (-61.0 to -50.1)
Bosnia and Herzegovina	All causes	5 to 9	Male	66 (63 to 70)	33.8 (32.1 to 35.6)	26 (25 to 27)	22.1 (21.1 to 23.2)	11 (10 to 13)	12.2 (10.9 to 13.8)	-83.1 (-85.1 to -80.7)	-63.8 (-68.1 to -58.7)
Bosnia and Herzegovina	All causes	10 to 14	Both	102 (94 to 109)	28.4 (26.4 to 30.5)	58 (54 to 61)	20.2 (19.0 to 21.6)	22 (20 to 24)	13.4 (12.1 to 14.8)	-78.5 (-81.0 to -75.8)	-52.9 (-58.3 to -47.1)
Bosnia and Herzegovina	All causes	10 to 14	Female	38 (35 to 42)	21.9 (19.9 to 24.1)	22 (19 to 24)	15.7 (14.1 to 17.4)	9 (8 to 10)	11.1 (9.7 to 12.5)	-76.8 (-80.4 to -73.4)	-49.2 (-56.9 to -41.7)
Bosnia and Herzegovina	All causes	10 to 14	Male	64 (58 to 69)	34.6 (31.5 to 37.7)	36 (33 to 39)	24.5 (22.8 to 26.8)	13 (12 to 15)	15.5 (13.8 to 17.6)	-79.5 (-82.5 to -76.3)	-55.1 (-61.7 to -48.0)
Bosnia and Herzegovina	Tuberculosis	5 to 9	Both	0 (0 to 1)	0.1 (0.1 to 0.2)	0 (0 to 0)	0.0 (0.0 to 0.1)	0 (0 to 0)	0.0 (0.0 to 0.0)	-91.9 (-95.8 to -84.8)	-82.7 (-91.1 to -67.3)
Bosnia and Herzegovina	Tuberculosis	5 to 9	Female	0 (0 to 0)	0.1 (0.1 to 0.2)	0 (0 to 0)	0.0 (0.0 to 0.1)	0 (0 to 0)	0.0 (0.0 to 0.0)	-93.0 (-97.2 to -82.3)	-85.0 (-94.1 to -61.9)
Bosnia and Herzegovina	Tuberculosis	5 to 9	Male	0 (0 to 0)	0.1 (0.1 to 0.1)	0 (0 to 0)	0.0 (0.0 to 0.1)	0 (0 to 0)	0.0 (0.0 to 0.0)	-90.3 (-95.6 to -81.1)	-79.3 (-90.5 to -59.5)
Bosnia and Herzegovina	Tuberculosis	10 to 14	Both	1 (0 to 1)	0.1 (0.1 to 0.2)	0 (0 to 0)	0.1 (0.0 to 0.1)	0 (0 to 0)	0.0 (0.0 to 0.0)	-91.4 (-95.4 to -84.5)	-81.2 (-89.9 to -66.1)
Bosnia and Herzegovina	Tuberculosis	10 to 14	Female	0 (0 to 0)	0.1 (0.1 to 0.2)	0 (0 to 0)	0.0 (0.0 to 0.1)	0 (0 to 0)	0.0 (0.0 to 0.0)	-93.4 (-96.8 to -85.4)	-85.4 (-93.1 to -68.1)
Bosnia and Herzegovina	Tuberculosis	10 to 14	Male	0 (0 to 1)	0.2 (0.1 to 0.3)	0 (0 to 0)	0.1 (0.0 to 0.1)	0 (0 to 0)	0.0 (0.0 to 0.1)	-90.3 (-95.6 to -79.0)	-78.6 (-90.4 to -54.0)
Bosnia and Herzegovina	HIV/AIDS	5 to 9	Both	0 (0 to 0)	0.0 (0.0 to 0.0)	0 (0 to 0)	0.0 (0.0 to 0.0)	0 (0 to 0)	0.0 (0.0 to 0.0)	-60.3 (-76.7 to -38.1)	-14.9 (-50.1 to 32.9)
Bosnia and Herzegovina	HIV/AIDS	5 to 9	Female	0 (0 to 0)	0.0 (0.0 to 0.0)	0 (0 to 0)	0.0 (0.0 to 0.0)	0 (0 to 0)	0.0 (0.0 to 0.0)	-60.4 (-76.7 to -38.1)	-14.9 (-49.9 to 33.2)
Bosnia and Herzegovina	HIV/AIDS	5 to 9	Male	0 (0 to 0)	0.0 (0.0 to 0.0)	0 (0 to 0)	0.0 (0.0 to 0.0)	0 (0 to 0)	0.0 (0.0 to 0.0)	-60.2 (-76.8 to -37.9)	-15.0 (-50.4 to 32.8)
Bosnia and Herzegovina	HIV/AIDS	10 to 14	Both	0 (0 to 0)	0.0 (0.0 to 0.0)	0 (0 to 0)	0.0 (0.0 to 0.0)	0 (0 to 0)	0.0 (0.0 to 0.0)	337.6 (176.3 to 546.4)	859.2 (505.8 to 1317.0)
Bosnia and Herzegovina	HIV/AIDS	10 to 14	Female	0 (0 to 0)	0.0 (0.0 to 0.0)	0 (0 to 0)	0.0 (0.0 to 0.0)	0 (0 to 0)	0.0 (0.0 to 0.0)	337.7 (177.6 to 552.6)	860.1 (509.0 to 1331.5)

eTable 8. All-cause and cause-specific mortality (counts and rates) at the global level and by country in the WHO European Region for ages 5 to 9 and 10 to 14 for 1990, 2000, and 2016

Bosnia and Herzegovina	HIV/AIDS	10 to 14	Male	0 (0 to 0)	0.0 (0.0 to 0.0)	0 (0 to 0)	0.0 (0.0 to 0.0)	0 (0 to 0)	0.0 (0.0 to 0.0)	337.5 (176.6 to 551.0)	858.4 (506.0 to 1326.3)
Bosnia and Herzegovina	Diarrheal diseases	5 to 9	Both	0 (0 to 1)	0.1 (0.0 to 0.1)	0 (0 to 0)	0.0 (0.0 to 0.1)	0 (0 to 0)	0.0 (0.0 to 0.0)	-85.7 (-92.6 to -72.3)	-69.2 (-84.2 to -40.5)
Bosnia and Herzegovina	Diarrheal diseases	5 to 9	Female	0 (0 to 0)	0.1 (0.0 to 0.1)	0 (0 to 0)	0.0 (0.0 to 0.1)	0 (0 to 0)	0.0 (0.0 to 0.1)	-84.6 (-93.0 to -60.3)	-66.8 (-84.8 to -14.4)
Bosnia and Herzegovina	Diarrheal diseases	5 to 9	Male	0 (0 to 0)	0.1 (0.0 to 0.2)	0 (0 to 0)	0.0 (0.0 to 0.1)	0 (0 to 0)	0.0 (0.0 to 0.1)	-86.5 (-94.6 to -63.8)	-71.2 (-88.6 to -22.5)
Bosnia and Herzegovina	Diarrheal diseases	10 to 14	Both	0 (0 to 0)	0.0 (0.0 to 0.1)	0 (0 to 0)	0.0 (0.0 to 0.0)	0 (0 to 0)	0.0 (0.0 to 0.0)	-79.7 (-89.1 to -57.5)	-55.5 (-76.1 to -6.9)
Bosnia and Herzegovina	Diarrheal diseases	10 to 14	Female	0 (0 to 0)	0.0 (0.0 to 0.1)	0 (0 to 0)	0.0 (0.0 to 0.0)	0 (0 to 0)	0.0 (0.0 to 0.0)	-81.6 (-91.3 to -57.8)	-59.6 (-80.9 to -7.4)
Bosnia and Herzegovina	Diarrheal diseases	10 to 14	Male	0 (0 to 0)	0.0 (0.0 to 0.1)	0 (0 to 0)	0.0 (0.0 to 0.0)	0 (0 to 0)	0.0 (0.0 to 0.0)	-76.9 (-89.8 to -38.9)	-49.5 (-77.7 to 34.0)
Bosnia and Herzegovina	Intestinal infectious diseases	5 to 9	Both	0 (0 to 0)	0.0 (0.0 to 0.0)	0 (0 to 0)	0.0 (0.0 to 0.0)	0 (0 to 0)	0.0 (0.0 to 0.0)	-67.0 (-82.3 to -48.9)	-29.3 (-62.0 to 9.7)
Bosnia and Herzegovina	Intestinal infectious diseases	5 to 9	Female	0 (0 to 0)	0.0 (0.0 to 0.0)	0 (0 to 0)	0.0 (0.0 to 0.0)	0 (0 to 0)	0.0 (0.0 to 0.0)	-65.1 (-85.2 to -29.4)	-24.8 (-68.2 to 52.1)
Bosnia and Herzegovina	Intestinal infectious diseases	5 to 9	Male	0 (0 to 0)	0.0 (0.0 to 0.0)	0 (0 to 0)	0.0 (0.0 to 0.0)	0 (0 to 0)	0.0 (0.0 to 0.0)	-68.6 (-84.1 to -47.0)	-32.9 (-66.1 to 13.3)
Bosnia and Herzegovina	Intestinal infectious diseases	10 to 14	Both	0 (0 to 0)	0.0 (0.0 to 0.0)	0 (0 to 0)	0.0 (0.0 to 0.0)	0 (0 to 0)	0.0 (0.0 to 0.0)	-62.9 (-80.5 to -39.1)	-18.7 (-57.4 to 33.4)
Bosnia and Herzegovina	Intestinal infectious diseases	10 to 14	Female	0 (0 to 0)	0.0 (0.0 to 0.0)	0 (0 to 0)	0.0 (0.0 to 0.0)	0 (0 to 0)	0.0 (0.0 to 0.0)	-65.3 (-85.4 to -17.4)	-24.0 (-68.1 to 81.2)
Bosnia and Herzegovina	Intestinal infectious diseases	10 to 14	Male	0 (0 to 0)	0.0 (0.0 to 0.0)	0 (0 to 0)	0.0 (0.0 to 0.0)	0 (0 to 0)	0.0 (0.0 to 0.0)	-60.3 (-78.2 to -34.5)	-13.1 (-52.3 to 43.5)
Bosnia and Herzegovina	Lower respiratory infections	5 to 9	Both	7 (4 to 10)	1.9 (1.1 to 2.6)	3 (2 to 4)	1.2 (0.8 to 1.8)	1 (1 to 1)	0.5 (0.3 to 0.7)	-87.8 (-92.3 to -81.6)	-73.8 (-83.6 to -60.5)
Bosnia and Herzegovina	Lower respiratory infections	5 to 9	Female	3 (2 to 4)	1.6 (1.0 to 2.4)	1 (1 to 2)	1.1 (0.7 to 1.8)	0 (0 to 1)	0.5 (0.3 to 0.8)	-85.5 (-92.6 to -74.6)	-68.7 (-84.1 to -45.2)
Bosnia and Herzegovina	Lower respiratory infections	5 to 9	Male	4 (2 to 6)	2.1 (1.1 to 3.3)	1 (1 to 2)	1.3 (0.7 to 2.1)	0 (0 to 1)	0.5 (0.3 to 0.8)	-89.4 (-94.0 to -82.9)	-77.4 (-87.2 to -63.5)
Bosnia and Herzegovina	Lower respiratory infections	10 to 14	Both	6 (4 to 8)	1.7 (1.0 to 2.3)	3 (2 to 4)	1.1 (0.7 to 1.6)	1 (1 to 1)	0.5 (0.4 to 0.7)	-85.6 (-91.1 to -78.1)	-68.4 (-80.6 to -52.0)
Bosnia and Herzegovina	Lower respiratory infections	10 to 14	Female	3 (2 to 5)	1.8 (1.0 to 2.9)	2 (1 to 2)	1.1 (0.7 to 1.8)	0 (0 to 1)	0.6 (0.3 to 0.8)	-86.1 (-92.8 to -76.1)	-69.4 (-84.3 to -47.5)
Bosnia and Herzegovina	Lower respiratory infections	10 to 14	Male	3 (1 to 4)	1.5 (0.8 to 2.4)	2 (1 to 2)	1.1 (0.6 to 1.7)	0 (0 to 1)	0.5 (0.3 to 0.8)	-85.0 (-91.9 to -75.3)	-67.2 (-82.2 to -45.8)
Bosnia and Herzegovina	Upper respiratory infections	5 to 9	Both	0 (0 to 0)	0.0 (0.0 to 0.0)	0 (0 to 0)	0.0 (0.0 to 0.0)	0 (0 to 0)	0.0 (0.0 to 0.0)	-93.3 (-99.9 to 234.8)	-85.7 (-99.8 to 618.2)
Bosnia and Herzegovina	Upper respiratory infections	5 to 9	Female	0 (0 to 0)	0.0 (0.0 to 0.0)	0 (0 to 0)	0.0 (0.0 to 0.0)	0 (0 to 0)	0.0 (0.0 to 0.0)	-92.0 (-100.0 to 8521.5)	-82.8 (-100.0 to 18458.7)
Bosnia and Herzegovina	Upper respiratory infections	5 to 9	Male	0 (0 to 0)	0.0 (0.0 to 0.0)	0 (0 to 0)	0.0 (0.0 to 0.0)	0 (0 to 0)	0.0 (0.0 to 0.0)	-94.0 (-100.0 to 7815.3)	-87.2 (-100.0 to 16823.5)
Bosnia and Herzegovina	Upper respiratory infections	10 to 14	Both	0 (0 to 0)	0.0 (0.0 to 0.0)	0 (0 to 0)	0.0 (0.0 to 0.0)	0 (0 to 0)	0.0 (0.0 to 0.0)	-90.2 (-99.8 to 326.1)	-78.4 (-99.5 to 834.0)
Bosnia and Herzegovina	Upper respiratory infections	10 to 14	Female	0 (0 to 0)	0.0 (0.0 to 0.0)	0 (0 to 0)	0.0 (0.0 to 0.0)	0 (0 to 0)	0.0 (0.0 to 0.0)	-89.9 (-100.0 to 24569.4)	-78.0 (-100.0 to 54011.1)

Bosnia and Herzegovina	Upper respiratory infections	10 to 14	Male	0 (0 to 0)	0.0 (0.0 to 0.0)	0 (0 to 0)	0.0 (0.0 to 0.0)	0 (0 to 0)	0.0 (0.0 to 0.0)	-90.3 (-100.0 to 9028.8)	-78.7 (-100.0 to 19899.9)
Bosnia and Herzegovina	Otitis media	5 to 9	Both	0 (0 to 0)	0.0 (0.0 to 0.0)	0 (0 to 0)	0.0 (0.0 to 0.0)	0 (0 to 0)	0.0 (0.0 to 0.0)	-84.7 (-97.4 to -25.8)	-67.2 (-94.4 to 59.1)
Bosnia and Herzegovina	Otitis media	5 to 9	Female	0 (0 to 0)	0.0 (0.0 to 0.0)	0 (0 to 0)	0.0 (0.0 to 0.0)	0 (0 to 0)	0.0 (0.0 to 0.0)	-95.7 (-99.0 to -72.3)	-90.8 (-97.8 to -40.3)
Bosnia and Herzegovina	Otitis media	5 to 9	Male	0 (0 to 0)	0.0 (0.0 to 0.0)	0 (0 to 0)	0.0 (0.0 to 0.0)	0 (0 to 0)	0.0 (0.0 to 0.0)	-78.6 (-97.5 to 54.7)	-54.2 (-94.6 to 230.8)
Bosnia and Herzegovina	Otitis media	10 to 14	Both	0 (0 to 0)	0.0 (0.0 to 0.1)	0 (0 to 0)	0.0 (0.0 to 0.0)	0 (0 to 0)	0.0 (0.0 to 0.0)	-99.9 (-100.0 to -99.7)	-99.9 (-100.0 to -99.3)
Bosnia and Herzegovina	Otitis media	10 to 14	Female	0 (0 to 0)	0.1 (0.0 to 0.1)	0 (0 to 0)	0.0 (0.0 to 0.0)	0 (0 to 0)	0.0 (0.0 to 0.0)	-100.0 (-100.0 to -99.9)	-99.9 (-100.0 to -99.7)
Bosnia and Herzegovina	Otitis media	10 to 14	Male	0 (0 to 0)	0.0 (0.0 to 0.0)	0 (0 to 0)	0.0 (0.0 to 0.0)	0 (0 to 0)	0.0 (0.0 to 0.0)	-93.1 (-98.6 to -62.0)	-84.9 (-96.9 to -16.8)
Bosnia and Herzegovina	Meningitis	5 to 9	Both	1 (1 to 2)	0.3 (0.2 to 0.4)	0 (0 to 1)	0.2 (0.1 to 0.3)	0 (0 to 0)	0.1 (0.1 to 0.2)	-83.0 (-89.9 to -67.1)	-63.4 (-78.3 to -29.5)
Bosnia and Herzegovina	Meningitis	5 to 9	Female	1 (0 to 1)	0.3 (0.2 to 0.5)	0 (0 to 0)	0.2 (0.1 to 0.3)	0 (0 to 0)	0.1 (0.1 to 0.2)	-83.7 (-91.0 to -69.8)	-65.0 (-80.7 to -34.9)
Bosnia and Herzegovina	Meningitis	5 to 9	Male	0 (0 to 1)	0.2 (0.1 to 0.4)	0 (0 to 0)	0.2 (0.1 to 0.3)	0 (0 to 0)	0.1 (0.0 to 0.3)	-81.9 (-93.1 to -53.2)	-61.2 (-85.3 to 0.0)
Bosnia and Herzegovina	Meningitis	10 to 14	Both	1 (1 to 1)	0.2 (0.1 to 0.4)	0 (0 to 1)	0.2 (0.1 to 0.3)	0 (0 to 0)	0.1 (0.1 to 0.2)	-80.4 (-89.2 to -60.4)	-57.1 (-76.3 to -13.2)
Bosnia and Herzegovina	Meningitis	10 to 14	Female	0 (0 to 1)	0.2 (0.1 to 0.3)	0 (0 to 0)	0.1 (0.1 to 0.2)	0 (0 to 0)	0.1 (0.1 to 0.1)	-80.7 (-89.7 to -64.3)	-57.6 (-77.5 to -21.6)
Bosnia and Herzegovina	Meningitis	10 to 14	Male	0 (0 to 1)	0.3 (0.1 to 0.5)	0 (0 to 1)	0.2 (0.1 to 0.4)	0 (0 to 0)	0.1 (0.1 to 0.2)	-80.3 (-91.6 to -44.1)	-56.7 (-81.7 to 22.4)
Bosnia and Herzegovina	Encephalitis	5 to 9	Both	1 (1 to 2)	0.3 (0.1 to 0.5)	1 (0 to 1)	0.2 (0.1 to 0.4)	0 (0 to 1)	0.1 (0.0 to 0.3)	-79.7 (-93.1 to -60.8)	-56.5 (-85.1 to -15.8)
Bosnia and Herzegovina	Encephalitis	5 to 9	Female	0 (0 to 1)	0.2 (0.1 to 0.5)	0 (0 to 0)	0.2 (0.1 to 0.4)	0 (0 to 0)	0.1 (0.0 to 0.4)	-73.9 (-90.2 to -55.0)	-43.7 (-79.0 to -3.1)
Bosnia and Herzegovina	Encephalitis	5 to 9	Male	1 (0 to 1)	0.3 (0.2 to 0.5)	0 (0 to 1)	0.3 (0.1 to 0.4)	0 (0 to 0)	0.1 (0.0 to 0.3)	-83.0 (-95.3 to -61.5)	-63.7 (-89.9 to -17.7)
Bosnia and Herzegovina	Encephalitis	10 to 14	Both	1 (0 to 2)	0.2 (0.1 to 0.4)	1 (0 to 1)	0.2 (0.1 to 0.3)	0 (0 to 0)	0.1 (0.0 to 0.3)	-76.3 (-90.2 to -58.4)	-47.9 (-78.6 to -8.9)
Bosnia and Herzegovina	Encephalitis	10 to 14	Female	0 (0 to 1)	0.2 (0.1 to 0.5)	0 (0 to 1)	0.2 (0.1 to 0.4)	0 (0 to 0)	0.1 (0.0 to 0.3)	-75.6 (-91.3 to -58.3)	-46.5 (-80.9 to -8.5)
Bosnia and Herzegovina	Encephalitis	10 to 14	Male	0 (0 to 1)	0.2 (0.1 to 0.5)	0 (0 to 1)	0.2 (0.1 to 0.4)	0 (0 to 0)	0.1 (0.0 to 0.3)	-76.8 (-92.5 to -54.8)	-49.2 (-83.5 to -1.0)
Bosnia and Herzegovina	Diphtheria	5 to 9	Both	0 (0 to 0)	0.0 (0.0 to 0.0)	0 (0 to 0)	0.0 (0.0 to 0.0)	0 (0 to 0)	0.0 (0.0 to 0.0)	-95.0 (-99.3 to -66.7)	-89.3 (-98.5 to -28.6)
Bosnia and Herzegovina	Diphtheria	5 to 9	Female	0 (0 to 0)	0.0 (0.0 to 0.0)	0 (0 to 0)	0.0 (0.0 to 0.0)	0 (0 to 0)	0.0 (0.0 to 0.0)	-94.2 (-99.7 to 17.2)	-87.6 (-99.3 to 152.3)
Bosnia and Herzegovina	Diphtheria	5 to 9	Male	0 (0 to 0)	0.0 (0.0 to 0.0)	0 (0 to 0)	0.0 (0.0 to 0.0)	0 (0 to 0)	0.0 (0.0 to 0.0)	-95.4 (-99.8 to 7.8)	-90.3 (-99.6 to 130.4)
Bosnia and Herzegovina	Diphtheria	10 to 14	Both	0 (0 to 0)	0.0 (0.0 to 0.0)	0 (0 to 0)	0.0 (0.0 to 0.0)	0 (0 to 0)	0.0 (0.0 to 0.0)	-93.4 (-99.1 to -53.1)	-85.5 (-98.1 to 2.7)
Bosnia and Herzegovina	Diphtheria	10 to 14	Female	0 (0 to 0)	0.0 (0.0 to 0.0)	0 (0 to 0)	0.0 (0.0 to 0.0)	0 (0 to 0)	0.0 (0.0 to 0.0)	-93.5 (-99.6 to 21.9)	-85.6 (-99.2 to 167.3)
Bosnia and Herzegovina	Diphtheria	10 to 14	Male	0 (0 to 0)	0.0 (0.0 to 0.0)	0 (0 to 0)	0.0 (0.0 to 0.0)	0 (0 to 0)	0.0 (0.0 to 0.0)	-93.3 (-99.8 to 42.9)	-85.4 (-99.5 to 213.0)
Bosnia and Herzegovina	Whooping cough	5 to 9	Both	7 (1 to 20)	1.7 (0.2 to 5.2)	1 (0 to 3)	0.5 (0.0 to 1.5)	0 (0 to 1)	0.1 (0.0 to 0.3)	-97.1 (-99.8 to -73.2)	-93.8 (-99.5 to -42.5)
Bosnia and Herzegovina	Whooping cough	5 to 9	Female	4 (0 to 11)	2.0 (0.2 to 5.7)	1 (0 to 2)	0.5 (0.1 to 1.7)	0 (0 to 0)	0.1 (0.0 to 0.4)	-97.0 (-99.8 to -72.0)	-93.5 (-99.5 to -39.7)
Bosnia and Herzegovina	Whooping cough	5 to 9	Male	3 (0 to 10)	1.5 (0.2 to 4.9)	0 (0 to 1)	0.4 (0.0 to 1.2)	0 (0 to 0)	0.1 (0.0 to 0.3)	-97.3 (-99.8 to -73.4)	-94.2 (-99.6 to -43.2)
Bosnia and Herzegovina	Whooping cough	10 to 14	Both	1 (0 to 4)	0.3 (0.0 to 1.2)	0 (0 to 1)	0.1 (0.0 to 0.3)	0 (0 to 0)	0.0 (0.0 to 0.1)	-97.0 (-99.8 to -68.8)	-93.5 (-99.5 to -31.5)
Bosnia and Herzegovina	Whooping cough	10 to 14	Female	1 (0 to 2)	0.4 (0.0 to 1.4)	0 (0 to 0)	0.1 (0.0 to 0.3)	0 (0 to 0)	0.0 (0.0 to 0.1)	-97.2 (-99.8 to -70.5)	-93.8 (-99.5 to -35.3)
Bosnia and Herzegovina	Whooping cough	10 to 14	Male	1 (0 to 2)	0.3 (0.0 to 1.0)	0 (0 to 0)	0.1 (0.0 to 0.3)	0 (0 to 0)	0.0 (0.0 to 0.1)	-96.9 (-99.8 to -66.6)	-93.1 (-99.5 to -26.8)
Bosnia and Herzegovina	Tetanus	5 to 9	Both	0 (0 to 0)	0.0 (0.0 to 0.0)	0 (0 to 0)	0.0 (0.0 to 0.0)	0 (0 to 0)	0.0 (0.0 to 0.0)	-58.9 (-67.1 to -48.6)	-11.9 (-29.5 to 10.2)

eTable 8. All-cause and cause-specific mortality (counts and rates) at the global level and by country in the WHO European Region for ages 5 to 9 and 10 to 14 for 1990, 2000, and 2016

Bosnia and Herzegovina	Tetanus	5 to 9	Female	0 (0 to 0)	0.0 (0.0 to 0.0)	0 (0 to 0)	0.0 (0.0 to 0.0)	0 (0 to 0)	0.0 (0.0 to 0.0)	-57.5 (-66.8 to -43.8)	-8.5 (-28.6 to 21.0)
Bosnia and Herzegovina	Tetanus	5 to 9	Male	0 (0 to 0)	0.0 (0.0 to 0.0)	0 (0 to 0)	0.0 (0.0 to 0.0)	0 (0 to 0)	0.0 (0.0 to 0.0)	-60.3 (-69.6 to -48.9)	-15.2 (-35.0 to 9.2)
Bosnia and Herzegovina	Tetanus	10 to 14	Both	0 (0 to 0)	0.0 (0.0 to 0.0)	0 (0 to 0)	0.0 (0.0 to 0.0)	0 (0 to 0)	0.0 (0.0 to 0.0)	-56.0 (-64.6 to -46.6)	-3.4 (-22.3 to 17.1)
Bosnia and Herzegovina	Tetanus	10 to 14	Female	0 (0 to 0)	0.0 (0.0 to 0.0)	0 (0 to 0)	0.0 (0.0 to 0.0)	0 (0 to 0)	0.0 (0.0 to 0.0)	-58.2 (-66.5 to -48.2)	-8.4 (-26.5 to 13.7)
Bosnia and Herzegovina	Tetanus	10 to 14	Male	0 (0 to 0)	0.0 (0.0 to 0.0)	0 (0 to 0)	0.0 (0.0 to 0.0)	0 (0 to 0)	0.0 (0.0 to 0.0)	-53.5 (-66.0 to -39.6)	1.9 (-25.4 to 32.4)
Bosnia and Herzegovina	Measles	5 to 9	Both	0 (0 to 0)	0.0 (0.0 to 0.0)	0 (0 to 0)	0.0 (0.0 to 0.0)	0 (0 to 0)	0.0 (0.0 to 0.0)	-44.7 (-61.5 to -23.6)	18.6 (-17.5 to 64.0)
Bosnia and Herzegovina	Measles	5 to 9	Female	0 (0 to 0)	0.0 (0.0 to 0.0)	0 (0 to 0)	0.0 (0.0 to 0.0)	0 (0 to 0)	0.0 (0.0 to 0.1)	-42.8 (-60.9 to -18.4)	23.2 (-15.8 to 75.7)
Bosnia and Herzegovina	Measles	5 to 9	Male	0 (0 to 0)	0.0 (0.0 to 0.0)	0 (0 to 0)	0.0 (0.0 to 0.0)	0 (0 to 0)	0.0 (0.0 to 0.0)	-46.7 (-64.6 to -24.4)	14.0 (-24.4 to 61.6)
Bosnia and Herzegovina	Measles	10 to 14	Both	0 (0 to 0)	0.0 (0.0 to 0.0)	0 (0 to 0)	0.0 (0.0 to 0.0)	0 (0 to 0)	0.0 (0.0 to 0.0)	-40.9 (-59.5 to -18.4)	29.5 (-11.2 to 78.8)
Bosnia and Herzegovina	Measles	10 to 14	Female	0 (0 to 0)	0.0 (0.0 to 0.0)	0 (0 to 0)	0.0 (0.0 to 0.0)	0 (0 to 0)	0.0 (0.0 to 0.0)	-43.9 (-61.3 to -20.7)	23.1 (-15.2 to 73.9)
Bosnia and Herzegovina	Measles	10 to 14	Male	0 (0 to 0)	0.0 (0.0 to 0.0)	0 (0 to 0)	0.0 (0.0 to 0.0)	0 (0 to 0)	0.0 (0.0 to 0.0)	-37.3 (-59.9 to -10.0)	37.3 (-12.2 to 97.1)
Bosnia and Herzegovina	Varicella and herpes zoster	5 to 9	Both	0 (0 to 1)	0.1 (0.0 to 0.2)	0 (0 to 0)	0.0 (0.0 to 0.1)	0 (0 to 0)	0.0 (0.0 to 0.0)	-90.4 (-98.0 to -47.4)	-79.3 (-95.8 to 12.8)
Bosnia and Herzegovina	Varicella and herpes zoster	5 to 9	Female	0 (0 to 1)	0.1 (0.0 to 0.3)	0 (0 to 0)	0.0 (0.0 to 0.1)	0 (0 to 0)	0.0 (0.0 to 0.1)	-90.4 (-99.2 to 24.3)	-79.4 (-98.4 to 167.7)
Bosnia and Herzegovina	Varicella and herpes zoster	5 to 9	Male	0 (0 to 1)	0.1 (0.0 to 0.3)	0 (0 to 0)	0.0 (0.0 to 0.1)	0 (0 to 0)	0.0 (0.0 to 0.1)	-90.3 (-99.1 to 31.5)	-79.2 (-98.1 to 181.1)
Bosnia and Herzegovina	Varicella and herpes zoster	10 to 14	Both	0 (0 to 0)	0.0 (0.0 to 0.1)	0 (0 to 0)	0.0 (0.0 to 0.0)	0 (0 to 0)	0.0 (0.0 to 0.0)	-89.9 (-97.9 to -44.0)	-77.8 (-95.4 to 22.7)
Bosnia and Herzegovina	Varicella and herpes zoster	10 to 14	Female	0 (0 to 0)	0.0 (0.0 to 0.1)	0 (0 to 0)	0.0 (0.0 to 0.0)	0 (0 to 0)	0.0 (0.0 to 0.0)	-90.6 (-99.3 to -1.0)	-79.4 (-98.5 to 117.3)
Bosnia and Herzegovina	Varicella and herpes zoster	10 to 14	Male	0 (0 to 0)	0.0 (0.0 to 0.1)	0 (0 to 0)	0.0 (0.0 to 0.0)	0 (0 to 0)	0.0 (0.0 to 0.0)	-89.0 (-99.1 to 29.6)	-75.8 (-98.0 to 184.0)
Bosnia and Herzegovina	Malaria	5 to 9	Both	0 (0 to 0)	0.0 (0.0 to 0.0)	0 (0 to 0)	0.0 (0.0 to 0.0)	0 (0 to 0)	0.0 (0.0 to 0.0)	0.0 (0.0 to 0.0)	0.0 (0.0 to 0.0)
Bosnia and Herzegovina	Malaria	5 to 9	Female	0 (0 to 0)	0.0 (0.0 to 0.0)	0 (0 to 0)	0.0 (0.0 to 0.0)	0 (0 to 0)	0.0 (0.0 to 0.0)	0.0 (0.0 to 0.0)	0.0 (0.0 to 0.0)
Bosnia and Herzegovina	Malaria	5 to 9	Male	0 (0 to 0)	0.0 (0.0 to 0.0)	0 (0 to 0)	0.0 (0.0 to 0.0)	0 (0 to 0)	0.0 (0.0 to 0.0)	0.0 (0.0 to 0.0)	0.0 (0.0 to 0.0)
Bosnia and Herzegovina	Malaria	10 to 14	Both	0 (0 to 0)	0.0 (0.0 to 0.0)	0 (0 to 0)	0.0 (0.0 to 0.0)	0 (0 to 0)	0.0 (0.0 to 0.0)	0.0 (0.0 to 0.0)	0.0 (0.0 to 0.0)
Bosnia and Herzegovina	Malaria	10 to 14	Female	0 (0 to 0)	0.0 (0.0 to 0.0)	0 (0 to 0)	0.0 (0.0 to 0.0)	0 (0 to 0)	0.0 (0.0 to 0.0)	0.0 (0.0 to 0.0)	0.0 (0.0 to 0.0)
Bosnia and Herzegovina	Malaria	10 to 14	Male	0 (0 to 0)	0.0 (0.0 to 0.0)	0 (0 to 0)	0.0 (0.0 to 0.0)	0 (0 to 0)	0.0 (0.0 to 0.0)	0.0 (0.0 to 0.0)	0.0 (0.0 to 0.0)
Bosnia and Herzegovina	Leishmaniasis	5 to 9	Both	0 (0 to 0)	0.0 (0.0 to 0.0)	0 (0 to 0)	0.0 (0.0 to 0.0)	0 (0 to 0)	0.0 (0.0 to 0.0)	-87.0 (-100.0 to 15.8)	-72.1 (-100.0 to 148.4)
Bosnia and Herzegovina	Leishmaniasis	5 to 9	Female	0 (0 to 0)	0.0 (0.0 to 0.0)	0 (0 to 0)	0.0 (0.0 to 0.0)	0 (0 to 0)	0.0 (0.0 to 0.0)	-86.4 (-100.0 to 0.0)	-70.8 (-100.0 to 76.4)
Bosnia and Herzegovina	Leishmaniasis	5 to 9	Male	0 (0 to 0)	0.0 (0.0 to 0.0)	0 (0 to 0)	0.0 (0.0 to 0.0)	0 (0 to 0)	0.0 (0.0 to 0.0)	-87.5 (-100.0 to 0.0)	-73.2 (-100.0 to 22.8)
Bosnia and Herzegovina	Leishmaniasis	10 to 14	Both	0 (0 to 0)	0.0 (0.0 to 0.0)	0 (0 to 0)	0.0 (0.0 to 0.0)	0 (0 to 0)	0.0 (0.0 to 0.0)	-85.7 (-100.0 to 82.8)	-68.7 (-100.0 to 300.6)
Bosnia and Herzegovina	Leishmaniasis	10 to 14	Female	0 (0 to 0)	0.0 (0.0 to 0.0)	0 (0 to 0)	0.0 (0.0 to 0.0)	0 (0 to 0)	0.0 (0.0 to 0.0)	-86.5 (-100.0 to 0.0)	-70.4 (-100.0 to 50.1)
Bosnia and Herzegovina	Leishmaniasis	10 to 14	Male	0 (0 to 0)	0.0 (0.0 to 0.0)	0 (0 to 0)	0.0 (0.0 to 0.0)	0 (0 to 0)	0.0 (0.0 to 0.0)	-84.9 (-100.0 to 0.0)	-67.0 (-100.0 to 43.6)
Bosnia and Herzegovina	African trypanosomiasis	5 to 9	Both	0 (0 to 0)	0.0 (0.0 to 0.0)	0 (0 to 0)	0.0 (0.0 to 0.0)	0 (0 to 0)	0.0 (0.0 to 0.0)	0.0 (0.0 to 0.0)	0.0 (0.0 to 0.0)
Bosnia and Herzegovina	African trypanosomiasis	5 to 9	Female	0 (0 to 0)	0.0 (0.0 to 0.0)	0 (0 to 0)	0.0 (0.0 to 0.0)	0 (0 to 0)	0.0 (0.0 to 0.0)	0.0 (0.0 to 0.0)	0.0 (0.0 to 0.0)

eTable 8. All-cause and cause-specific mortality (counts and rates) at the global level and by country in the WHO European Region for ages 5 to 9 and 10 to 14 for 1990, 2000, and 2016

Bosnia and Herzegovina	African trypanosomiasis	5 to 9	Male	0 (0 to 0)	0.0 (0.0 to 0.0)	0 (0 to 0)	0.0 (0.0 to 0.0)	0 (0 to 0)	0.0 (0.0 to 0.0)	0.0 (0.0 to 0.0)	0.0 (0.0 to 0.0)
Bosnia and Herzegovina	African trypanosomiasis	10 to 14	Both	0 (0 to 0)	0.0 (0.0 to 0.0)	0 (0 to 0)	0.0 (0.0 to 0.0)	0 (0 to 0)	0.0 (0.0 to 0.0)	0.0 (0.0 to 0.0)	0.0 (0.0 to 0.0)
Bosnia and Herzegovina	African trypanosomiasis	10 to 14	Female	0 (0 to 0)	0.0 (0.0 to 0.0)	0 (0 to 0)	0.0 (0.0 to 0.0)	0 (0 to 0)	0.0 (0.0 to 0.0)	0.0 (0.0 to 0.0)	0.0 (0.0 to 0.0)
Bosnia and Herzegovina	African trypanosomiasis	10 to 14	Male	0 (0 to 0)	0.0 (0.0 to 0.0)	0 (0 to 0)	0.0 (0.0 to 0.0)	0 (0 to 0)	0.0 (0.0 to 0.0)	0.0 (0.0 to 0.0)	0.0 (0.0 to 0.0)
Bosnia and Herzegovina	Schistosomiasis	5 to 9	Both	0 (0 to 0)	0.0 (0.0 to 0.0)	0 (0 to 0)	0.0 (0.0 to 0.0)	0 (0 to 0)	0.0 (0.0 to 0.0)	0.0 (0.0 to 0.0)	0.0 (0.0 to 0.0)
Bosnia and Herzegovina	Schistosomiasis	5 to 9	Female	0 (0 to 0)	0.0 (0.0 to 0.0)	0 (0 to 0)	0.0 (0.0 to 0.0)	0 (0 to 0)	0.0 (0.0 to 0.0)	0.0 (0.0 to 0.0)	0.0 (0.0 to 0.0)
Bosnia and Herzegovina	Schistosomiasis	5 to 9	Male	0 (0 to 0)	0.0 (0.0 to 0.0)	0 (0 to 0)	0.0 (0.0 to 0.0)	0 (0 to 0)	0.0 (0.0 to 0.0)	0.0 (0.0 to 0.0)	0.0 (0.0 to 0.0)
Bosnia and Herzegovina	Schistosomiasis	10 to 14	Both	0 (0 to 0)	0.0 (0.0 to 0.0)	0 (0 to 0)	0.0 (0.0 to 0.0)	0 (0 to 0)	0.0 (0.0 to 0.0)	0.0 (0.0 to 0.0)	0.0 (0.0 to 0.0)
Bosnia and Herzegovina	Schistosomiasis	10 to 14	Female	0 (0 to 0)	0.0 (0.0 to 0.0)	0 (0 to 0)	0.0 (0.0 to 0.0)	0 (0 to 0)	0.0 (0.0 to 0.0)	0.0 (0.0 to 0.0)	0.0 (0.0 to 0.0)
Bosnia and Herzegovina	Schistosomiasis	10 to 14	Male	0 (0 to 0)	0.0 (0.0 to 0.0)	0 (0 to 0)	0.0 (0.0 to 0.0)	0 (0 to 0)	0.0 (0.0 to 0.0)	0.0 (0.0 to 0.0)	0.0 (0.0 to 0.0)
Bosnia and Herzegovina	Cysticercosis	5 to 9	Both	0 (0 to 0)	0.0 (0.0 to 0.0)	0 (0 to 0)	0.0 (0.0 to 0.0)	0 (0 to 0)	0.0 (0.0 to 0.0)	-92.9 (-94.4 to -91.0)	-84.9 (-88.0 to -80.8)
Bosnia and Herzegovina	Cysticercosis	5 to 9	Female	0 (0 to 0)	0.0 (0.0 to 0.0)	0 (0 to 0)	0.0 (0.0 to 0.0)	0 (0 to 0)	0.0 (0.0 to 0.0)	-92.0 (-93.7 to -89.3)	-82.7 (-86.5 to -77.1)
Bosnia and Herzegovina	Cysticercosis	5 to 9	Male	0 (0 to 0)	0.0 (0.0 to 0.0)	0 (0 to 0)	0.0 (0.0 to 0.0)	0 (0 to 0)	0.0 (0.0 to 0.0)	-93.7 (-95.2 to -91.9)	-86.6 (-89.8 to -82.8)
Bosnia and Herzegovina	Cysticercosis	10 to 14	Both	0 (0 to 0)	0.0 (0.0 to 0.0)	0 (0 to 0)	0.0 (0.0 to 0.0)	0 (0 to 0)	0.0 (0.0 to 0.0)	-90.8 (-92.8 to -88.9)	-79.9 (-84.1 to -75.6)
Bosnia and Herzegovina	Cysticercosis	10 to 14	Female	0 (0 to 0)	0.0 (0.0 to 0.0)	0 (0 to 0)	0.0 (0.0 to 0.0)	0 (0 to 0)	0.0 (0.0 to 0.0)	-91.0 (-92.8 to -88.8)	-80.3 (-84.2 to -75.3)
Bosnia and Herzegovina	Cysticercosis	10 to 14	Male	0 (0 to 0)	0.0 (0.0 to 0.0)	0 (0 to 0)	0.0 (0.0 to 0.0)	0 (0 to 0)	0.0 (0.0 to 0.0)	-90.7 (-93.2 to -87.8)	-79.6 (-85.1 to -73.3)
Bosnia and Herzegovina	Yellow fever	5 to 9	Both	0 (0 to 0)	0.0 (0.0 to 0.0)	0 (0 to 0)	0.0 (0.0 to 0.0)	0 (0 to 0)	0.0 (0.0 to 0.0)	0.0 (0.0 to 0.0)	0.0 (0.0 to 0.0)
Bosnia and Herzegovina	Yellow fever	5 to 9	Female	0 (0 to 0)	0.0 (0.0 to 0.0)	0 (0 to 0)	0.0 (0.0 to 0.0)	0 (0 to 0)	0.0 (0.0 to 0.0)	0.0 (0.0 to 0.0)	0.0 (0.0 to 0.0)
Bosnia and Herzegovina	Yellow fever	5 to 9	Male	0 (0 to 0)	0.0 (0.0 to 0.0)	0 (0 to 0)	0.0 (0.0 to 0.0)	0 (0 to 0)	0.0 (0.0 to 0.0)	0.0 (0.0 to 0.0)	0.0 (0.0 to 0.0)
Bosnia and Herzegovina	Yellow fever	10 to 14	Both	0 (0 to 0)	0.0 (0.0 to 0.0)	0 (0 to 0)	0.0 (0.0 to 0.0)	0 (0 to 0)	0.0 (0.0 to 0.0)	0.0 (0.0 to 0.0)	0.0 (0.0 to 0.0)
Bosnia and Herzegovina	Yellow fever	10 to 14	Female	0 (0 to 0)	0.0 (0.0 to 0.0)	0 (0 to 0)	0.0 (0.0 to 0.0)	0 (0 to 0)	0.0 (0.0 to 0.0)	0.0 (0.0 to 0.0)	0.0 (0.0 to 0.0)
Bosnia and Herzegovina	Yellow fever	10 to 14	Male	0 (0 to 0)	0.0 (0.0 to 0.0)	0 (0 to 0)	0.0 (0.0 to 0.0)	0 (0 to 0)	0.0 (0.0 to 0.0)	0.0 (0.0 to 0.0)	0.0 (0.0 to 0.0)
Bosnia and Herzegovina	Rabies	5 to 9	Both	0 (0 to 0)	0.0 (0.0 to 0.0)	0 (0 to 0)	0.0 (0.0 to 0.0)	0 (0 to 0)	0.0 (0.0 to 0.0)	-98.3 (-99.8 to -83.9)	-96.3 (-99.5 to -65.5)
Bosnia and Herzegovina	Rabies	5 to 9	Female	0 (0 to 0)	0.0 (0.0 to 0.0)	0 (0 to 0)	0.0 (0.0 to 0.0)	0 (0 to 0)	0.0 (0.0 to 0.0)	-29.2 (-95.6 to 1295.6)	52.3 (-90.6 to 2904.2)
Bosnia and Herzegovina	Rabies	5 to 9	Male	0 (0 to 0)	0.0 (0.0 to 0.0)	0 (0 to 0)	0.0 (0.0 to 0.0)	0 (0 to 0)	0.0 (0.0 to 0.0)	-98.6 (-99.8 to -84.1)	-96.9 (-99.6 to -65.9)
Bosnia and Herzegovina	Rabies	10 to 14	Both	0 (0 to 0)	0.0 (0.0 to 0.0)	0 (0 to 0)	0.0 (0.0 to 0.0)	0 (0 to 0)	0.0 (0.0 to 0.0)	-92.7 (-98.8 to -30.0)	-84.1 (-97.5 to 53.4)
Bosnia and Herzegovina	Rabies	10 to 14	Female	0 (0 to 0)	0.0 (0.0 to 0.0)	0 (0 to 0)	0.0 (0.0 to 0.0)	0 (0 to 0)	0.0 (0.0 to 0.0)	11.4 (-95.6 to 1979.9)	144.4 (-90.2 to 4462.1)
Bosnia and Herzegovina	Rabies	10 to 14	Male	0 (0 to 0)	0.0 (0.0 to 0.0)	0 (0 to 0)	0.0 (0.0 to 0.0)	0 (0 to 0)	0.0 (0.0 to 0.0)	-97.0 (-99.7 to -66.5)	-93.5 (-99.3 to -26.5)
Bosnia and Herzegovina	Intestinal nematode infections	5 to 9	Both	-	-	-	-	-	-	-	-

eTable 8. All-cause and cause-specific mortality (counts and rates) at the global level and by country in the WHO European Region for ages 5 to 9 and 10 to 14 for 1990, 2000, and 2016

Bosnia and Herzegovina	Intestinal nematode infections	5 to 9	Female	-	-	-	-	-	-	-	-
Bosnia and Herzegovina	Intestinal nematode infections	5 to 9	Male	-	-	-	-	-	-	-	-
Bosnia and Herzegovina	Intestinal nematode infections	10 to 14	Both	-	-	-	-	-	-	-	-
Bosnia and Herzegovina	Intestinal nematode infections	10 to 14	Female	-	-	-	-	-	-	-	-
Bosnia and Herzegovina	Intestinal nematode infections	10 to 14	Male	-	-	-	-	-	-	-	-
Bosnia and Herzegovina	Ebola	5 to 9	Both	0 (0 to 0)	0.0 (0.0 to 0.0)	0 (0 to 0)	0.0 (0.0 to 0.0)	0 (0 to 0)	0.0 (0.0 to 0.0)	0.0 (0.0 to 0.0)	0.0 (0.0 to 0.0)
Bosnia and Herzegovina	Ebola	5 to 9	Female	0 (0 to 0)	0.0 (0.0 to 0.0)	0 (0 to 0)	0.0 (0.0 to 0.0)	0 (0 to 0)	0.0 (0.0 to 0.0)	0.0 (0.0 to 0.0)	0.0 (0.0 to 0.0)
Bosnia and Herzegovina	Ebola	5 to 9	Male	0 (0 to 0)	0.0 (0.0 to 0.0)	0 (0 to 0)	0.0 (0.0 to 0.0)	0 (0 to 0)	0.0 (0.0 to 0.0)	0.0 (0.0 to 0.0)	0.0 (0.0 to 0.0)
Bosnia and Herzegovina	Ebola	10 to 14	Both	0 (0 to 0)	0.0 (0.0 to 0.0)	0 (0 to 0)	0.0 (0.0 to 0.0)	0 (0 to 0)	0.0 (0.0 to 0.0)	0.0 (0.0 to 0.0)	0.0 (0.0 to 0.0)
Bosnia and Herzegovina	Ebola	10 to 14	Female	0 (0 to 0)	0.0 (0.0 to 0.0)	0 (0 to 0)	0.0 (0.0 to 0.0)	0 (0 to 0)	0.0 (0.0 to 0.0)	0.0 (0.0 to 0.0)	0.0 (0.0 to 0.0)
Bosnia and Herzegovina	Ebola	10 to 14	Male	0 (0 to 0)	0.0 (0.0 to 0.0)	0 (0 to 0)	0.0 (0.0 to 0.0)	0 (0 to 0)	0.0 (0.0 to 0.0)	0.0 (0.0 to 0.0)	0.0 (0.0 to 0.0)
Bosnia and Herzegovina	Zika virus	5 to 9	Both	0 (0 to 0)	0.0 (0.0 to 0.0)	0 (0 to 0)	0.0 (0.0 to 0.0)	0 (0 to 0)	0.0 (0.0 to 0.0)	0.0 (0.0 to 0.0)	0.0 (0.0 to 0.0)
Bosnia and Herzegovina	Zika virus	5 to 9	Female	0 (0 to 0)	0.0 (0.0 to 0.0)	0 (0 to 0)	0.0 (0.0 to 0.0)	0 (0 to 0)	0.0 (0.0 to 0.0)	0.0 (0.0 to 0.0)	0.0 (0.0 to 0.0)
Bosnia and Herzegovina	Zika virus	5 to 9	Male	0 (0 to 0)	0.0 (0.0 to 0.0)	0 (0 to 0)	0.0 (0.0 to 0.0)	0 (0 to 0)	0.0 (0.0 to 0.0)	0.0 (0.0 to 0.0)	0.0 (0.0 to 0.0)
Bosnia and Herzegovina	Zika virus	10 to 14	Both	0 (0 to 0)	0.0 (0.0 to 0.0)	0 (0 to 0)	0.0 (0.0 to 0.0)	0 (0 to 0)	0.0 (0.0 to 0.0)	0.0 (0.0 to 0.0)	0.0 (0.0 to 0.0)
Bosnia and Herzegovina	Zika virus	10 to 14	Female	0 (0 to 0)	0.0 (0.0 to 0.0)	0 (0 to 0)	0.0 (0.0 to 0.0)	0 (0 to 0)	0.0 (0.0 to 0.0)	0.0 (0.0 to 0.0)	0.0 (0.0 to 0.0)
Bosnia and Herzegovina	Zika virus	10 to 14	Male	0 (0 to 0)	0.0 (0.0 to 0.0)	0 (0 to 0)	0.0 (0.0 to 0.0)	0 (0 to 0)	0.0 (0.0 to 0.0)	0.0 (0.0 to 0.0)	0.0 (0.0 to 0.0)
Bosnia and Herzegovina	Other neglected tropical diseases	5 to 9	Both	0 (0 to 0)	0.0 (0.0 to 0.0)	0 (0 to 0)	0.0 (0.0 to 0.0)	0 (0 to 0)	0.0 (0.0 to 0.0)	-58.9 (-67.1 to -48.5)	-11.7 (-29.4 to 10.4)
Bosnia and Herzegovina	Other neglected tropical diseases	5 to 9	Female	0 (0 to 0)	0.0 (0.0 to 0.0)	0 (0 to 0)	0.0 (0.0 to 0.0)	0 (0 to 0)	0.0 (0.0 to 0.0)	-57.5 (-66.8 to -43.8)	-8.5 (-28.6 to 21.0)
Bosnia and Herzegovina	Other neglected tropical diseases	5 to 9	Male	0 (0 to 0)	0.0 (0.0 to 0.0)	0 (0 to 0)	0.0 (0.0 to 0.0)	0 (0 to 0)	0.0 (0.0 to 0.0)	-60.1 (-69.6 to -48.7)	-14.8 (-35.0 to 9.6)
Bosnia and Herzegovina	Other neglected tropical diseases	10 to 14	Both	0 (0 to 0)	0.0 (0.0 to 0.0)	0 (0 to 0)	0.0 (0.0 to 0.0)	0 (0 to 0)	0.0 (0.0 to 0.0)	-56.0 (-64.8 to -46.6)	-3.5 (-22.8 to 17.1)
Bosnia and Herzegovina	Other neglected tropical diseases	10 to 14	Female	0 (0 to 0)	0.0 (0.0 to 0.0)	0 (0 to 0)	0.0 (0.0 to 0.0)	0 (0 to 0)	0.0 (0.0 to 0.0)	-58.2 (-66.5 to -48.2)	-8.4 (-26.5 to 13.7)
Bosnia and Herzegovina	Other neglected tropical diseases	10 to 14	Male	0 (0 to 0)	0.0 (0.0 to 0.0)	0 (0 to 0)	0.0 (0.0 to 0.0)	0 (0 to 0)	0.0 (0.0 to 0.0)	-53.6 (-66.3 to -39.5)	1.7 (-26.2 to 32.6)
Bosnia and Herzegovina	Maternal hemorrhage	10 to 14	Both	0 (0 to 0)	0.0 (0.0 to 0.0)	0 (0 to 0)	0.0 (0.0 to 0.0)	0 (0 to 0)	0.0 (0.0 to 0.0)	-60.2 (-71.8 to -42.4)	-12.8 (-38.3 to 26.3)

eTable 8. All-cause and cause-specific mortality (counts and rates) at the global level and by country in the WHO European Region for ages 5 to 9 and 10 to 14 for 1990, 2000, and 2016

Bosnia and Herzegovina	Maternal hemorrhage	10 to 14	Female	0 (0 to 0)	0.0 (0.0 to 0.0)	0 (0 to 0)	0.0 (0.0 to 0.0)	0 (0 to 0)	0.0 (0.0 to 0.0)	-60.2 (-71.8 to -42.4)	-12.7 (-38.2 to 26.3)
Bosnia and Herzegovina	Maternal sepsis and other maternal infections	10 to 14	Both	0 (0 to 0)	0.0 (0.0 to 0.0)	0 (0 to 0)	0.0 (0.0 to 0.0)	0 (0 to 0)	0.0 (0.0 to 0.0)	-66.9 (-79.5 to -46.8)	-27.5 (-55.1 to 16.7)
Bosnia and Herzegovina	Maternal sepsis and other maternal infections	10 to 14	Female	0 (0 to 0)	0.0 (0.0 to 0.0)	0 (0 to 0)	0.0 (0.0 to 0.0)	0 (0 to 0)	0.0 (0.0 to 0.0)	-66.9 (-79.5 to -46.8)	-27.4 (-55.1 to 16.8)
Bosnia and Herzegovina	Maternal hypertensive disorders	10 to 14	Both	0 (0 to 0)	0.0 (0.0 to 0.0)	0 (0 to 0)	0.0 (0.0 to 0.0)	0 (0 to 0)	0.0 (0.0 to 0.0)	-57.5 (-68.0 to -42.3)	-6.8 (-30.0 to 26.6)
Bosnia and Herzegovina	Maternal hypertensive disorders	10 to 14	Female	0 (0 to 0)	0.0 (0.0 to 0.0)	0 (0 to 0)	0.0 (0.0 to 0.0)	0 (0 to 0)	0.0 (0.0 to 0.0)	-57.5 (-68.0 to -42.3)	-6.7 (-29.9 to 26.6)
Bosnia and Herzegovina	Maternal obstructed labor and uterine rupture	10 to 14	Both	0 (0 to 0)	0.0 (0.0 to 0.0)	0 (0 to 0)	0.0 (0.0 to 0.0)	0 (0 to 0)	0.0 (0.0 to 0.0)	-62.3 (-75.9 to -30.2)	-17.3 (-47.2 to 53.1)
Bosnia and Herzegovina	Maternal obstructed labor and uterine rupture	10 to 14	Female	0 (0 to 0)	0.0 (0.0 to 0.0)	0 (0 to 0)	0.0 (0.0 to 0.0)	0 (0 to 0)	0.0 (0.0 to 0.0)	-62.3 (-75.9 to -30.2)	-17.2 (-47.1 to 53.1)
Bosnia and Herzegovina	Maternal abortion, miscarriage, and ectopic pregnancy	10 to 14	Both	0 (0 to 0)	0.0 (0.0 to 0.0)	0 (0 to 0)	0.0 (0.0 to 0.0)	0 (0 to 0)	0.0 (0.0 to 0.0)	-53.1 (-67.0 to -31.5)	2.9 (-27.6 to 50.1)
Bosnia and Herzegovina	Maternal abortion, miscarriage, and ectopic pregnancy	10 to 14	Female	0 (0 to 0)	0.0 (0.0 to 0.0)	0 (0 to 0)	0.0 (0.0 to 0.0)	0 (0 to 0)	0.0 (0.0 to 0.0)	-53.1 (-67.0 to -31.5)	3.0 (-27.6 to 50.2)
Bosnia and Herzegovina	Indirect maternal deaths	10 to 14	Both	0 (0 to 0)	0.0 (0.0 to 0.0)	0 (0 to 0)	0.0 (0.0 to 0.0)	0 (0 to 0)	0.0 (0.0 to 0.0)	-58.8 (-69.6 to -43.7)	-9.7 (-33.3 to 23.4)
Bosnia and Herzegovina	Indirect maternal deaths	10 to 14	Female	0 (0 to 0)	0.0 (0.0 to 0.0)	0 (0 to 0)	0.0 (0.0 to 0.0)	0 (0 to 0)	0.0 (0.0 to 0.0)	-58.8 (-69.6 to -43.7)	-9.7 (-33.2 to 23.5)
Bosnia and Herzegovina	Late maternal deaths	10 to 14	Both	0 (0 to 0)	0.0 (0.0 to 0.0)	0 (0 to 0)	0.0 (0.0 to 0.0)	0 (0 to 0)	0.0 (0.0 to 0.0)	-59.3 (-91.9 to 45.6)	-10.8 (-82.3 to 219.1)
Bosnia and Herzegovina	Late maternal deaths	10 to 14	Female	0 (0 to 0)	0.0 (0.0 to 0.0)	0 (0 to 0)	0.0 (0.0 to 0.0)	0 (0 to 0)	0.0 (0.0 to 0.0)	-59.3 (-91.9 to 45.6)	-10.7 (-82.3 to 219.3)
Bosnia and Herzegovina	Maternal deaths aggravated by HIV/AIDS	10 to 14	Both	0 (0 to 0)	0.0 (0.0 to 0.0)	0 (0 to 0)	0.0 (0.0 to 0.0)	0 (0 to 0)	0.0 (0.0 to 0.0)	494.5 (244.8 to 835.0)	1203.1 (655.8 to 1949.6)
Bosnia and Herzegovina	Maternal deaths aggravated by HIV/AIDS	10 to 14	Female	0 (0 to 0)	0.0 (0.0 to 0.0)	0 (0 to 0)	0.0 (0.0 to 0.0)	0 (0 to 0)	0.0 (0.0 to 0.0)	494.5 (244.8 to 835.0)	1203.9 (656.3 to 1950.8)
Bosnia and Herzegovina	Other maternal disorders	10 to 14	Both	0 (0 to 0)	0.0 (0.0 to 0.0)	0 (0 to 0)	0.0 (0.0 to 0.0)	0 (0 to 0)	0.0 (0.0 to 0.0)	-58.8 (-67.5 to -47.7)	-9.6 (-28.8 to 14.6)
Bosnia and Herzegovina	Other maternal disorders	10 to 14	Female	0 (0 to 0)	0.0 (0.0 to 0.0)	0 (0 to 0)	0.0 (0.0 to 0.0)	0 (0 to 0)	0.0 (0.0 to 0.0)	-58.8 (-67.5 to -47.7)	-9.6 (-28.8 to 14.7)
Bosnia and Herzegovina	Protein-energy malnutrition	5 to 9	Both	0 (0 to 0)	0.0 (0.0 to 0.0)	0 (0 to 0)	0.0 (0.0 to 0.0)	0 (0 to 0)	0.0 (0.0 to 0.0)	-58.0 (-72.1 to -28.1)	-9.9 (-40.1 to 54.1)

eTable 8. All-cause and cause-specific mortality (counts and rates) at the global level and by country in the WHO European Region for ages 5 to 9 and 10 to 14 for 1990, 2000, and 2016

Bosnia and Herzegovina	Protein-energy malnutrition	5 to 9	Female	0 (0 to 0)	0.0 (0.0 to 0.0)	0 (0 to 0)	0.0 (0.0 to 0.0)	0 (0 to 0)	0.0 (0.0 to 0.0)	-56.6 (-78.0 to 41.8)	-6.6 (-52.7 to 205.3)
Bosnia and Herzegovina	Protein-energy malnutrition	5 to 9	Male	0 (0 to 0)	0.0 (0.0 to 0.0)	0 (0 to 0)	0.0 (0.0 to 0.0)	0 (0 to 0)	0.0 (0.0 to 0.0)	-59.3 (-78.9 to 33.2)	-13.0 (-54.9 to 184.8)
Bosnia and Herzegovina	Protein-energy malnutrition	10 to 14	Both	0 (0 to 0)	0.0 (0.0 to 0.0)	0 (0 to 0)	0.0 (0.0 to 0.0)	0 (0 to 0)	0.0 (0.0 to 0.0)	-55.2 (-66.2 to -38.4)	-1.8 (-25.8 to 35.0)
Bosnia and Herzegovina	Protein-energy malnutrition	10 to 14	Female	0 (0 to 0)	0.0 (0.0 to 0.0)	0 (0 to 0)	0.0 (0.0 to 0.0)	0 (0 to 0)	0.0 (0.0 to 0.0)	-57.7 (-71.9 to -27.4)	-7.1 (-38.3 to 59.3)
Bosnia and Herzegovina	Protein-energy malnutrition	10 to 14	Male	0 (0 to 0)	0.0 (0.0 to 0.0)	0 (0 to 0)	0.0 (0.0 to 0.0)	0 (0 to 0)	0.0 (0.0 to 0.0)	-52.6 (-67.5 to -21.4)	3.9 (-28.9 to 72.2)
Bosnia and Herzegovina	Iodine deficiency	5 to 9	Both	0 (0 to 0)	0.0 (0.0 to 0.0)	0 (0 to 0)	0.0 (0.0 to 0.0)	0 (0 to 0)	0.0 (0.0 to 0.0)	-74.2 (-100.0 to 5.1e+18)	-44.7 (-100.0 to 1.1e+19)
Bosnia and Herzegovina	Iodine deficiency	5 to 9	Female	0 (0 to 0)	0.0 (0.0 to 0.0)	0 (0 to 0)	0.0 (0.0 to 0.0)	0 (0 to 0)	0.0 (0.0 to 0.0)	-71.4 (-100.0 to 2.6e+25)	-38.4 (-100.0 to 5.6e+25)
Bosnia and Herzegovina	Iodine deficiency	5 to 9	Male	0 (0 to 0)	0.0 (0.0 to 0.0)	0 (0 to 0)	0.0 (0.0 to 0.0)	0 (0 to 0)	0.0 (0.0 to 0.0)	-76.9 (-100.0 to 4.7e+26)	-50.6 (-100.0 to 1.0e+27)
Bosnia and Herzegovina	Iodine deficiency	10 to 14	Both	0 (0 to 0)	0.0 (0.0 to 0.0)	0 (0 to 0)	0.0 (0.0 to 0.0)	0 (0 to 0)	0.0 (0.0 to 0.0)	-74.6 (-100.0 to 2.4e+18)	-44.3 (-100.0 to 5.3e+18)
Bosnia and Herzegovina	Iodine deficiency	10 to 14	Female	0 (0 to 0)	0.0 (0.0 to 0.0)	0 (0 to 0)	0.0 (0.0 to 0.0)	0 (0 to 0)	0.0 (0.0 to 0.0)	-73.2 (-100.0 to 8.6e+26)	-41.2 (-100.0 to 1.9e+27)
Bosnia and Herzegovina	Iodine deficiency	10 to 14	Male	0 (0 to 0)	0.0 (0.0 to 0.0)	0 (0 to 0)	0.0 (0.0 to 0.0)	0 (0 to 0)	0.0 (0.0 to 0.0)	-76.2 (-100.0 to 9.8e+24)	-47.9 (-100.0 to 2.1e+25)
Bosnia and Herzegovina	Iron-deficiency anemia	5 to 9	Both	0 (0 to 0)	0.0 (0.0 to 0.0)	0 (0 to 0)	0.0 (0.0 to 0.0)	0 (0 to 0)	0.0 (0.0 to 0.0)	-58.0 (-72.1 to -29.9)	-10.0 (-40.1 to 50.5)
Bosnia and Herzegovina	Iron-deficiency anemia	5 to 9	Female	0 (0 to 0)	0.0 (0.0 to 0.0)	0 (0 to 0)	0.0 (0.0 to 0.0)	0 (0 to 0)	0.0 (0.0 to 0.0)	-56.7 (-79.0 to 41.8)	-6.7 (-54.7 to 205.3)
Bosnia and Herzegovina	Iron-deficiency anemia	5 to 9	Male	0 (0 to 0)	0.0 (0.0 to 0.0)	0 (0 to 0)	0.0 (0.0 to 0.0)	0 (0 to 0)	0.0 (0.0 to 0.0)	-59.3 (-78.9 to 33.2)	-13.0 (-54.9 to 184.8)
Bosnia and Herzegovina	Iron-deficiency anemia	10 to 14	Both	0 (0 to 0)	0.0 (0.0 to 0.0)	0 (0 to 0)	0.0 (0.0 to 0.0)	0 (0 to 0)	0.0 (0.0 to 0.0)	-55.3 (-66.3 to -39.2)	-2.1 (-26.2 to 33.4)
Bosnia and Herzegovina	Iron-deficiency anemia	10 to 14	Female	0 (0 to 0)	0.0 (0.0 to 0.0)	0 (0 to 0)	0.0 (0.0 to 0.0)	0 (0 to 0)	0.0 (0.0 to 0.0)	-57.9 (-72.7 to -30.5)	-7.6 (-40.1 to 52.5)
Bosnia and Herzegovina	Iron-deficiency anemia	10 to 14	Male	0 (0 to 0)	0.0 (0.0 to 0.0)	0 (0 to 0)	0.0 (0.0 to 0.0)	0 (0 to 0)	0.0 (0.0 to 0.0)	-52.6 (-67.5 to -21.4)	3.9 (-28.9 to 72.2)
Bosnia and Herzegovina	Other nutritional deficiencies	5 to 9	Both	0 (0 to 0)	0.0 (0.0 to 0.0)	0 (0 to 0)	0.0 (0.0 to 0.0)	0 (0 to 0)	0.0 (0.0 to 0.0)	-58.0 (-72.1 to -28.1)	-9.9 (-40.1 to 54.1)
Bosnia and Herzegovina	Other nutritional deficiencies	5 to 9	Female	0 (0 to 0)	0.0 (0.0 to 0.0)	0 (0 to 0)	0.0 (0.0 to 0.0)	0 (0 to 0)	0.0 (0.0 to 0.0)	-56.6 (-78.0 to 41.8)	-6.6 (-52.7 to 205.3)
Bosnia and Herzegovina	Other nutritional deficiencies	5 to 9	Male	0 (0 to 0)	0.0 (0.0 to 0.0)	0 (0 to 0)	0.0 (0.0 to 0.0)	0 (0 to 0)	0.0 (0.0 to 0.0)	-59.3 (-78.9 to 33.2)	-13.0 (-54.9 to 184.8)
Bosnia and Herzegovina	Other nutritional deficiencies	10 to 14	Both	0 (0 to 0)	0.0 (0.0 to 0.0)	0 (0 to 0)	0.0 (0.0 to 0.0)	0 (0 to 0)	0.0 (0.0 to 0.0)	-55.2 (-66.2 to -38.4)	-1.8 (-25.8 to 35.0)
Bosnia and Herzegovina	Other nutritional deficiencies	10 to 14	Female	0 (0 to 0)	0.0 (0.0 to 0.0)	0 (0 to 0)	0.0 (0.0 to 0.0)	0 (0 to 0)	0.0 (0.0 to 0.0)	-57.7 (-71.9 to -27.4)	-7.1 (-38.3 to 59.3)
Bosnia and Herzegovina	Other nutritional deficiencies	10 to 14	Male	0 (0 to 0)	0.0 (0.0 to 0.0)	0 (0 to 0)	0.0 (0.0 to 0.0)	0 (0 to 0)	0.0 (0.0 to 0.0)	-52.6 (-67.5 to -21.4)	3.9 (-28.9 to 72.2)

eTable 8. All-cause and cause-specific mortality (counts and rates) at the global level and by country in the WHO European Region for ages 5 to 9 and 10 to 14 for 1990, 2000, and 2016

Bosnia and Herzegovina	Sexually transmitted diseases excluding HIV	5 to 9	Both	0<br>(0 to 1)	0.1<br>(0.0 to 0.2)	0<br>(0 to 0)	0.0<br>(0.0 to 0.1)	0<br>(0 to 0)	0.0<br>(0.0 to 0.0)	-98.9<br>(-99.6 to -96.2)	-97.6<br>(-99.1 to -91.9)
Bosnia and Herzegovina	Sexually transmitted diseases excluding HIV	5 to 9	Female	0<br>(0 to 1)	0.1<br>(0.0 to 0.3)	0<br>(0 to 0)	0.0<br>(0.0 to 0.1)	0<br>(0 to 0)	0.0<br>(0.0 to 0.0)	-98.9<br>(-99.6 to -96.0)	-97.6<br>(-99.1 to -91.4)
Bosnia and Herzegovina	Sexually transmitted diseases excluding HIV	5 to 9	Male	0<br>(0 to 0)	0.1<br>(0.0 to 0.2)	0<br>(0 to 0)	0.0<br>(0.0 to 0.0)	0<br>(0 to 0)	0.0<br>(0.0 to 0.0)	-98.9<br>(-99.6 to -96.5)	-97.8<br>(-99.1 to -92.5)
Bosnia and Herzegovina	Sexually transmitted diseases excluding HIV	10 to 14	Both	0<br>(0 to 0)	0.0<br>(0.0 to 0.0)	0<br>(0 to 0)	0.0<br>(0.0 to 0.0)	0<br>(0 to 0)	0.0<br>(0.0 to 0.0)	-56.0<br>(-64.6 to -46.6)	-3.4<br>(-22.3 to 17.1)
Bosnia and Herzegovina	Sexually transmitted diseases excluding HIV	10 to 14	Female	0<br>(0 to 0)	0.0<br>(0.0 to 0.0)	0<br>(0 to 0)	0.0<br>(0.0 to 0.0)	0<br>(0 to 0)	0.0<br>(0.0 to 0.0)	-58.2<br>(-66.5 to -48.2)	-8.4<br>(-26.5 to 13.7)
Bosnia and Herzegovina	Sexually transmitted diseases excluding HIV	10 to 14	Male	0<br>(0 to 0)	0.0<br>(0.0 to 0.0)	0<br>(0 to 0)	0.0<br>(0.0 to 0.0)	0<br>(0 to 0)	0.0<br>(0.0 to 0.0)	-53.5<br>(-66.0 to -39.6)	1.9<br>(-25.4 to 32.4)
Bosnia and Herzegovina	Hepatitis	5 to 9	Both	0<br>(0 to 0)	0.1<br>(0.0 to 0.1)	0<br>(0 to 0)	0.0<br>(0.0 to 0.1)	0<br>(0 to 0)	0.0<br>(0.0 to 0.0)	-80.6<br>(-90.9 to -59.4)	-58.4<br>(-80.5 to -12.9)
Bosnia and Herzegovina	Hepatitis	5 to 9	Female	0<br>(0 to 0)	0.0<br>(0.0 to 0.1)	0<br>(0 to 0)	0.0<br>(0.0 to 0.1)	0<br>(0 to 0)	0.0<br>(0.0 to 0.0)	-80.2<br>(-90.2 to -59.6)	-57.4<br>(-78.9 to -13.0)
Bosnia and Herzegovina	Hepatitis	5 to 9	Male	0<br>(0 to 0)	0.1<br>(0.0 to 0.1)	0<br>(0 to 0)	0.1<br>(0.0 to 0.1)	0<br>(0 to 0)	0.0<br>(0.0 to 0.1)	-80.8<br>(-93.7 to -46.9)	-59.0<br>(-86.5 to 13.6)
Bosnia and Herzegovina	Hepatitis	10 to 14	Both	0<br>(0 to 0)	0.1<br>(0.0 to 0.1)	0<br>(0 to 0)	0.1<br>(0.0 to 0.1)	0<br>(0 to 0)	0.0<br>(0.0 to 0.1)	-73.8<br>(-87.2 to -47.1)	-42.6<br>(-71.9 to 16.1)
Bosnia and Herzegovina	Hepatitis	10 to 14	Female	0<br>(0 to 0)	0.1<br>(0.0 to 0.1)	0<br>(0 to 0)	0.0<br>(0.0 to 0.1)	0<br>(0 to 0)	0.0<br>(0.0 to 0.0)	-77.5<br>(-89.0 to -53.1)	-50.6<br>(-75.8 to 2.9)
Bosnia and Herzegovina	Hepatitis	10 to 14	Male	0<br>(0 to 0)	0.1<br>(0.0 to 0.1)	0<br>(0 to 0)	0.1<br>(0.0 to 0.1)	0<br>(0 to 0)	0.0<br>(0.0 to 0.1)	-71.5<br>(-90.6 to -19.5)	-37.6<br>(-79.3 to 76.4)
Bosnia and Herzegovina	Other infectious diseases	5 to 9	Both	0<br>(0 to 0)	0.1<br>(0.0 to 0.1)	0<br>(0 to 0)	0.0<br>(0.0 to 0.1)	0<br>(0 to 0)	0.0<br>(0.0 to 0.1)	-73.0<br>(-86.9 to -48.5)	-42.0<br>(-72.0 to 10.4)
Bosnia and Herzegovina	Other infectious diseases	5 to 9	Female	0<br>(0 to 0)	0.1<br>(0.0 to 0.1)	0<br>(0 to 0)	0.1<br>(0.0 to 0.1)	0<br>(0 to 0)	0.0<br>(0.0 to 0.1)	-76.7<br>(-91.5 to -50.5)	-49.8<br>(-81.8 to 6.5)
Bosnia and Herzegovina	Other infectious diseases	5 to 9	Male	0<br>(0 to 0)	0.0<br>(0.0 to 0.1)	0<br>(0 to 0)	0.0<br>(0.0 to 0.1)	0<br>(0 to 0)	0.0<br>(0.0 to 0.1)	-67.0<br>(-87.3 to 9.8)	-29.4<br>(-72.9 to 134.7)
Bosnia and Herzegovina	Other infectious diseases	10 to 14	Both	0<br>(0 to 0)	0.1<br>(0.0 to 0.1)	0<br>(0 to 0)	0.1<br>(0.0 to 0.1)	0<br>(0 to 0)	0.0<br>(0.0 to 0.1)	-63.4<br>(-81.6 to -19.1)	-19.7<br>(-59.6 to 77.3)
Bosnia and Herzegovina	Other infectious diseases	10 to 14	Female	0<br>(0 to 0)	0.0<br>(0.0 to 0.1)	0<br>(0 to 0)	0.0<br>(0.0 to 0.1)	0<br>(0 to 0)	0.0<br>(0.0 to 0.1)	-65.2<br>(-83.8 to -4.8)	-23.6<br>(-64.5 to 108.8)
Bosnia and Herzegovina	Other infectious diseases	10 to 14	Male	0<br>(0 to 0)	0.1<br>(0.0 to 0.1)	0<br>(0 to 0)	0.1<br>(0.0 to 0.1)	0<br>(0 to 0)	0.1<br>(0.0 to 0.1)	-62.2<br>(-84.2 to -5.2)	-17.3<br>(-65.5 to 107.7)
Bosnia and Herzegovina	Nasopharynx cancer	5 to 9	Both	0<br>(0 to 0)	0.0<br>(0.0 to 0.0)	0<br>(0 to 0)	0.0<br>(0.0 to 0.0)	0<br>(0 to 0)	0.0<br>(0.0 to 0.0)	-59.0<br>(-67.3 to -48.7)	-12.1<br>(-29.8 to 10.1)
Bosnia and Herzegovina	Nasopharynx cancer	5 to 9	Female	0<br>(0 to 0)	0.0<br>(0.0 to 0.0)	0<br>(0 to 0)	0.0<br>(0.0 to 0.0)	0<br>(0 to 0)	0.0<br>(0.0 to 0.0)	-57.5<br>(-66.8 to -43.8)	-8.5<br>(-28.6 to 21.0)

eTable 8. All-cause and cause-specific mortality (counts and rates) at the global level and by country in the WHO European Region for ages 5 to 9 and 10 to 14 for 1990, 2000, and 2016

Bosnia and Herzegovina	Nasopharynx cancer	5 to 9	Male	0 (0 to 0)	0.0 (0.0 to 0.0)	0 (0 to 0)	0.0 (0.0 to 0.0)	0 (0 to 0)	0.0 (0.0 to 0.0)	-60.5 (-69.9 to -48.9)	-15.4 (-35.7 to 9.2)
Bosnia and Herzegovina	Nasopharynx cancer	10 to 14	Both	0 (0 to 0)	0.0 (0.0 to 0.0)	0 (0 to 0)	0.0 (0.0 to 0.0)	0 (0 to 0)	0.0 (0.0 to 0.0)	-52.8 (-67.9 to -27.8)	3.4 (-29.6 to 58.3)
Bosnia and Herzegovina	Nasopharynx cancer	10 to 14	Female	0 (0 to 0)	0.0 (0.0 to 0.0)	0 (0 to 0)	0.0 (0.0 to 0.0)	0 (0 to 0)	0.0 (0.0 to 0.0)	-58.2 (-66.5 to -48.2)	-8.4 (-26.5 to 13.7)
Bosnia and Herzegovina	Nasopharynx cancer	10 to 14	Male	0 (0 to 0)	0.0 (0.0 to 0.0)	0 (0 to 0)	0.0 (0.0 to 0.0)	0 (0 to 0)	0.0 (0.0 to 0.0)	-47.7 (-72.4 to 4.4)	14.5 (-39.5 to 128.8)
Bosnia and Herzegovina	Liver cancer	5 to 9	Both	0 (0 to 0)	0.0 (0.0 to 0.1)	0 (0 to 0)	0.0 (0.0 to 0.1)	0 (0 to 0)	0.0 (0.0 to 0.0)	-73.4 (-87.2 to -45.6)	-42.9 (-72.6 to 16.6)
Bosnia and Herzegovina	Liver cancer	5 to 9	Female	0 (0 to 0)	0.0 (0.0 to 0.1)	0 (0 to 0)	0.0 (0.0 to 0.1)	0 (0 to 0)	0.0 (0.0 to 0.0)	-62.2 (-77.9 to -33.7)	-18.7 (-52.4 to 42.8)
Bosnia and Herzegovina	Liver cancer	5 to 9	Male	0 (0 to 0)	0.1 (0.0 to 0.1)	0 (0 to 0)	0.1 (0.0 to 0.1)	0 (0 to 0)	0.0 (0.0 to 0.1)	-75.8 (-90.2 to -43.0)	-48.3 (-79.0 to 21.8)
Bosnia and Herzegovina	Liver cancer	10 to 14	Both	0 (0 to 0)	0.0 (0.0 to 0.1)	0 (0 to 0)	0.0 (0.0 to 0.1)	0 (0 to 0)	0.0 (0.0 to 0.1)	-62.5 (-80.2 to -31.1)	-17.7 (-56.6 to 51.0)
Bosnia and Herzegovina	Liver cancer	10 to 14	Female	0 (0 to 0)	0.0 (0.0 to 0.1)	0 (0 to 0)	0.0 (0.0 to 0.1)	0 (0 to 0)	0.0 (0.0 to 0.0)	-57.8 (-79.5 to -4.0)	-7.5 (-55.0 to 110.5)
Bosnia and Herzegovina	Liver cancer	10 to 14	Male	0 (0 to 0)	0.1 (0.0 to 0.1)	0 (0 to 0)	0.1 (0.0 to 0.1)	0 (0 to 0)	0.0 (0.0 to 0.1)	-64.1 (-84.4 to -21.2)	-21.3 (-65.9 to 72.7)
Bosnia and Herzegovina	Kidney cancer	5 to 9	Both	1 (1 to 1)	0.3 (0.1 to 0.4)	1 (0 to 1)	0.3 (0.1 to 0.4)	0 (0 to 1)	0.2 (0.1 to 0.3)	-63.3 (-79.7 to -33.1)	-21.2 (-56.5 to 43.6)
Bosnia and Herzegovina	Kidney cancer	5 to 9	Female	0 (0 to 0)	0.1 (0.1 to 0.2)	0 (0 to 0)	0.1 (0.1 to 0.2)	0 (0 to 0)	0.1 (0.0 to 0.2)	-65.6 (-84.7 to -25.7)	-26.0 (-67.1 to 60.0)
Bosnia and Herzegovina	Kidney cancer	5 to 9	Male	1 (0 to 1)	0.4 (0.2 to 0.6)	0 (0 to 1)	0.4 (0.2 to 0.7)	0 (0 to 0)	0.3 (0.1 to 0.5)	-62.6 (-82.0 to -23.7)	-20.0 (-61.5 to 63.1)
Bosnia and Herzegovina	Kidney cancer	10 to 14	Both	0 (0 to 0)	0.1 (0.0 to 0.1)	0 (0 to 0)	0.1 (0.0 to 0.1)	0 (0 to 0)	0.1 (0.0 to 0.1)	-61.0 (-76.5 to -34.8)	-14.6 (-48.5 to 42.9)
Bosnia and Herzegovina	Kidney cancer	10 to 14	Female	0 (0 to 0)	0.1 (0.1 to 0.1)	0 (0 to 0)	0.1 (0.0 to 0.1)	0 (0 to 0)	0.1 (0.0 to 0.1)	-67.2 (-83.8 to -32.4)	-28.1 (-64.5 to 48.3)
Bosnia and Herzegovina	Kidney cancer	10 to 14	Male	0 (0 to 0)	0.0 (0.0 to 0.1)	0 (0 to 0)	0.0 (0.0 to 0.1)	0 (0 to 0)	0.0 (0.0 to 0.1)	-45.3 (-70.9 to 13.0)	19.9 (-36.3 to 147.5)
Bosnia and Herzegovina	Brain and nervous system cancer	5 to 9	Both	8 (5 to 10)	2.0 (1.4 to 2.7)	5 (3 to 6)	2.0 (1.4 to 2.7)	3 (2 to 4)	1.4 (1.0 to 2.0)	-65.5 (-77.3 to -50.4)	-26.0 (-51.3 to 6.4)
Bosnia and Herzegovina	Brain and nervous system cancer	5 to 9	Female	3 (2 to 4)	1.6 (0.9 to 2.3)	2 (1 to 3)	1.8 (1.1 to 2.5)	1 (1 to 2)	1.4 (0.9 to 2.0)	-57.3 (-74.4 to -26.4)	-8.1 (-44.9 to 58.5)
Bosnia and Herzegovina	Brain and nervous system cancer	5 to 9	Male	5 (3 to 7)	2.3 (1.5 to 3.4)	3 (2 to 4)	2.3 (1.4 to 3.3)	1 (1 to 2)	1.5 (0.8 to 2.4)	-70.6 (-84.9 to -53.2)	-37.2 (-67.6 to 0.1)
Bosnia and Herzegovina	Brain and nervous system cancer	10 to 14	Both	5 (4 to 7)	1.5 (1.0 to 2.1)	4 (3 to 6)	1.6 (1.0 to 2.2)	2 (2 to 3)	1.4 (0.9 to 1.9)	-56.9 (-70.3 to -36.4)	-5.6 (-34.9 to 39.3)
Bosnia and Herzegovina	Brain and nervous system cancer	10 to 14	Female	2 (1 to 3)	1.4 (0.8 to 1.9)	2 (1 to 3)	1.4 (0.8 to 2.1)	1 (1 to 2)	1.4 (0.8 to 1.9)	-54.8 (-72.4 to -21.5)	-0.9 (-39.4 to 72.2)
Bosnia and Herzegovina	Brain and nervous system cancer	10 to 14	Male	3 (2 to 4)	1.5 (0.9 to 2.4)	2 (1 to 4)	1.7 (0.9 to 2.7)	1 (1 to 2)	1.4 (0.8 to 2.2)	-58.7 (-76.5 to -30.1)	-9.6 (-48.5 to 53.0)
Bosnia and Herzegovina	Thyroid cancer	10 to 14	Both	0 (0 to 0)	0.0 (0.0 to 0.0)	0 (0 to 0)	0.0 (0.0 to 0.0)	0 (0 to 0)	0.0 (0.0 to 0.0)	-56.0 (-64.6 to -46.6)	-3.4 (-22.3 to 17.1)
Bosnia and Herzegovina	Thyroid cancer	10 to 14	Female	0 (0 to 0)	0.0 (0.0 to 0.0)	0 (0 to 0)	0.0 (0.0 to 0.0)	0 (0 to 0)	0.0 (0.0 to 0.0)	-58.2 (-66.5 to -48.2)	-8.4 (-26.5 to 13.7)
Bosnia and Herzegovina	Thyroid cancer	10 to 14	Male	0 (0 to 0)	0.0 (0.0 to 0.0)	0 (0 to 0)	0.0 (0.0 to 0.0)	0 (0 to 0)	0.0 (0.0 to 0.0)	-53.5 (-66.0 to -39.6)	1.9 (-25.4 to 32.4)
Bosnia and Herzegovina	Hodgkin lymphoma	5 to 9	Both	0 (0 to 0)	0.0 (0.0 to 0.1)	0 (0 to 0)	0.0 (0.0 to 0.0)	0 (0 to 0)	0.0 (0.0 to 0.0)	-76.9 (-88.5 to -22.9)	-50.5 (-75.4 to 65.4)
Bosnia and Herzegovina	Hodgkin lymphoma	5 to 9	Female	0 (0 to 0)	0.0 (0.0 to 0.0)	0 (0 to 0)	0.0 (0.0 to 0.0)	0 (0 to 0)	0.0 (0.0 to 0.0)	-67.2 (-85.0 to -44.4)	-29.4 (-67.7 to 19.6)
Bosnia and Herzegovina	Hodgkin lymphoma	5 to 9	Male	0 (0 to 0)	0.0 (0.0 to 0.1)	0 (0 to 0)	0.0 (0.0 to 0.1)	0 (0 to 0)	0.0 (0.0 to 0.0)	-81.2 (-90.3 to 45.0)	-59.8 (-79.2 to 210.1)
Bosnia and Herzegovina	Hodgkin lymphoma	10 to 14	Both	0 (0 to 1)	0.1 (0.1 to 0.2)	0 (0 to 0)	0.1 (0.0 to 0.1)	0 (0 to 0)	0.0 (0.0 to 0.1)	-81.2 (-90.2 to -61.7)	-58.8 (-78.5 to -16.0)

eTable 8. All-cause and cause-specific mortality (counts and rates) at the global level and by country in the WHO European Region for ages 5 to 9 and 10 to 14 for 1990, 2000, and 2016

Bosnia and Herzegovina	Hodgkin lymphoma	10 to 14	Female	0 (0 to 0)	0.1 (0.0 to 0.2)	0 (0 to 0)	0.1 (0.0 to 0.1)	0 (0 to 0)	0.0 (0.0 to 0.1)	-82.6 (-94.0 to -54.7)	-61.7 (-86.9 to -0.6)
Bosnia and Herzegovina	Hodgkin lymphoma	10 to 14	Male	0 (0 to 0)	0.1 (0.0 to 0.2)	0 (0 to 0)	0.1 (0.0 to 0.1)	0 (0 to 0)	0.0 (0.0 to 0.1)	-79.4 (-87.9 to -53.8)	-54.8 (-73.5 to 1.1)
Bosnia and Herzegovina	Non-Hodgkin lymphoma	5 to 9	Both	2 (1 to 3)	0.5 (0.4 to 0.8)	1 (1 to 2)	0.5 (0.4 to 0.9)	1 (0 to 1)	0.4 (0.3 to 0.7)	-61.8 (-77.2 to -32.3)	-18.1 (-51.0 to 45.2)
Bosnia and Herzegovina	Non-Hodgkin lymphoma	5 to 9	Female	0 (0 to 1)	0.2 (0.1 to 0.4)	0 (0 to 0)	0.3 (0.1 to 0.4)	0 (0 to 0)	0.2 (0.1 to 0.4)	-50.3 (-76.6 to 1.2)	7.0 (-49.7 to 117.9)
Bosnia and Herzegovina	Non-Hodgkin lymphoma	5 to 9	Male	2 (1 to 2)	0.8 (0.5 to 1.3)	1 (1 to 2)	0.8 (0.5 to 1.4)	1 (0 to 1)	0.6 (0.4 to 1.0)	-64.9 (-81.9 to -29.3)	-24.9 (-61.2 to 51.1)
Bosnia and Herzegovina	Non-Hodgkin lymphoma	10 to 14	Both	1 (1 to 2)	0.4 (0.2 to 0.5)	1 (1 to 2)	0.4 (0.2 to 0.6)	1 (0 to 1)	0.4 (0.3 to 0.6)	-49.8 (-69.3 to -6.4)	10.0 (-32.7 to 105.2)
Bosnia and Herzegovina	Non-Hodgkin lymphoma	10 to 14	Female	0 (0 to 0)	0.2 (0.1 to 0.3)	0 (0 to 0)	0.2 (0.1 to 0.3)	0 (0 to 0)	0.2 (0.1 to 0.4)	-44.1 (-74.0 to 27.9)	22.6 (-42.9 to 180.5)
Bosnia and Herzegovina	Non-Hodgkin lymphoma	10 to 14	Male	1 (1 to 1)	0.5 (0.3 to 0.8)	1 (0 to 1)	0.6 (0.3 to 0.9)	0 (0 to 1)	0.6 (0.4 to 0.9)	-51.6 (-72.3 to 6.7)	6.1 (-39.2 to 133.7)
Bosnia and Herzegovina	Leukemia	5 to 9	Both	8 (6 to 10)	2.0 (1.4 to 2.7)	4 (3 to 6)	1.8 (1.2 to 2.5)	2 (1 to 3)	1.2 (0.7 to 1.7)	-72.0 (-82.3 to -54.2)	-40.0 (-62.0 to -1.7)
Bosnia and Herzegovina	Leukemia	5 to 9	Female	3 (2 to 4)	1.4 (0.8 to 2.1)	1 (1 to 2)	1.3 (0.7 to 2.1)	1 (0 to 1)	1.0 (0.5 to 1.5)	-67.6 (-82.9 to -35.0)	-30.2 (-63.2 to 40.0)
Bosnia and Herzegovina	Leukemia	5 to 9	Male	5 (3 to 7)	2.6 (1.7 to 3.8)	3 (2 to 4)	2.2 (1.4 to 3.5)	1 (1 to 2)	1.4 (0.8 to 2.3)	-74.3 (-85.8 to -50.3)	-45.1 (-69.6 to 6.2)
Bosnia and Herzegovina	Leukemia	10 to 14	Both	7 (5 to 9)	1.9 (1.4 to 2.5)	5 (3 to 6)	1.6 (1.2 to 2.2)	2 (1 to 3)	1.4 (0.9 to 2.0)	-66.0 (-77.6 to -46.2)	-25.4 (-51.0 to 18.0)
Bosnia and Herzegovina	Leukemia	10 to 14	Female	3 (2 to 4)	1.6 (1.0 to 2.4)	2 (1 to 3)	1.3 (0.7 to 2.0)	1 (0 to 1)	1.2 (0.5 to 1.9)	-67.0 (-80.8 to -37.2)	-27.7 (-57.9 to 37.8)
Bosnia and Herzegovina	Leukemia	10 to 14	Male	4 (3 to 6)	2.2 (1.4 to 3.1)	3 (2 to 4)	1.9 (1.2 to 2.8)	1 (1 to 2)	1.6 (1.0 to 2.5)	-65.2 (-79.9 to -34.8)	-23.8 (-56.1 to 42.9)
Bosnia and Herzegovina	Other neoplasms	5 to 9	Both	3 (2 to 4)	0.7 (0.5 to 1.0)	1 (1 to 2)	0.7 (0.4 to 0.9)	1 (0 to 1)	0.4 (0.3 to 0.7)	-73.1 (-84.2 to -57.5)	-42.3 (-66.0 to -8.8)
Bosnia and Herzegovina	Other neoplasms	5 to 9	Female	1 (1 to 2)	0.5 (0.3 to 0.8)	1 (0 to 1)	0.5 (0.3 to 0.8)	0 (0 to 1)	0.4 (0.2 to 0.7)	-66.8 (-83.9 to -31.9)	-28.4 (-65.4 to 46.5)
Bosnia and Herzegovina	Other neoplasms	5 to 9	Male	2 (1 to 3)	0.9 (0.6 to 1.4)	1 (1 to 1)	0.8 (0.4 to 1.2)	0 (0 to 1)	0.5 (0.3 to 0.8)	-76.2 (-88.1 to -54.4)	-49.2 (-74.6 to -2.5)
Bosnia and Herzegovina	Other neoplasms	10 to 14	Both	3 (2 to 4)	0.8 (0.6 to 1.2)	2 (2 to 3)	0.8 (0.5 to 1.1)	1 (1 to 1)	0.6 (0.4 to 0.9)	-67.3 (-79.8 to -48.0)	-28.3 (-55.6 to 14.0)
Bosnia and Herzegovina	Other neoplasms	10 to 14	Female	2 (1 to 3)	0.9 (0.6 to 1.4)	1 (1 to 2)	0.9 (0.5 to 1.4)	1 (0 to 1)	0.7 (0.4 to 1.1)	-67.5 (-83.7 to -32.4)	-28.7 (-64.3 to 48.3)
Bosnia and Herzegovina	Other neoplasms	10 to 14	Male	1 (1 to 2)	0.7 (0.5 to 1.2)	1 (1 to 2)	0.7 (0.4 to 1.1)	0 (0 to 1)	0.5 (0.3 to 0.9)	-67.1 (-82.4 to -38.0)	-27.8 (-61.4 to 35.9)
Bosnia and Herzegovina	Rheumatic heart disease	5 to 9	Both	0 (0 to 0)	0.1 (0.0 to 0.1)	0 (0 to 0)	0.0 (0.0 to 0.1)	0 (0 to 0)	0.0 (0.0 to 0.0)	-86.6 (-93.3 to -74.3)	-71.2 (-85.6 to -44.8)
Bosnia and Herzegovina	Rheumatic heart disease	5 to 9	Female	0 (0 to 0)	0.0 (0.0 to 0.0)	0 (0 to 0)	0.0 (0.0 to 0.0)	0 (0 to 0)	0.0 (0.0 to 0.0)	-83.6 (-91.6 to -67.6)	-64.7 (-81.9 to -30.3)
Bosnia and Herzegovina	Rheumatic heart disease	5 to 9	Male	0 (0 to 0)	0.1 (0.0 to 0.1)	0 (0 to 0)	0.0 (0.0 to 0.1)	0 (0 to 0)	0.0 (0.0 to 0.0)	-87.4 (-94.9 to -71.9)	-73.0 (-89.0 to -40.0)
Bosnia and Herzegovina	Rheumatic heart disease	10 to 14	Both	1 (0 to 1)	0.2 (0.1 to 0.2)	0 (0 to 0)	0.1 (0.1 to 0.1)	0 (0 to 0)	0.1 (0.0 to 0.1)	-82.1 (-89.6 to -70.2)	-60.8 (-77.1 to -34.6)
Bosnia and Herzegovina	Rheumatic heart disease	10 to 14	Female	0 (0 to 0)	0.1 (0.1 to 0.2)	0 (0 to 0)	0.1 (0.0 to 0.1)	0 (0 to 0)	0.0 (0.0 to 0.1)	-84.7 (-92.3 to -69.6)	-66.5 (-83.2 to -33.4)
Bosnia and Herzegovina	Rheumatic heart disease	10 to 14	Male	0 (0 to 0)	0.2 (0.1 to 0.3)	0 (0 to 0)	0.1 (0.1 to 0.2)	0 (0 to 0)	0.1 (0.0 to 0.1)	-80.2 (-90.9 to -62.2)	-56.7 (-80.0 to -17.1)
Bosnia and Herzegovina	Ischemic heart disease	5 to 9	Both	0 (0 to 0)	0.0 (0.0 to 0.0)	0 (0 to 0)	0.0 (0.0 to 0.0)	0 (0 to 0)	0.0 (0.0 to 0.0)	-64.4 (-78.1 to -43.0)	-23.6 (-53.0 to 22.3)
Bosnia and Herzegovina	Ischemic heart disease	5 to 9	Female	0 (0 to 0)	0.0 (0.0 to 0.0)	0 (0 to 0)	0.0 (0.0 to 0.0)	0 (0 to 0)	0.0 (0.0 to 0.0)	-60.1 (-78.2 to -26.8)	-14.1 (-53.0 to 57.7)
Bosnia and Herzegovina	Ischemic heart disease	5 to 9	Male	0 (0 to 0)	0.0 (0.0 to 0.0)	0 (0 to 0)	0.0 (0.0 to 0.0)	0 (0 to 0)	0.0 (0.0 to 0.0)	-67.8 (-83.9 to -38.2)	-31.3 (-65.6 to 32.1)
Bosnia and Herzegovina	Ischemic heart disease	10 to 14	Both	0 (0 to 0)	0.0 (0.0 to 0.0)	0 (0 to 0)	0.0 (0.0 to 0.0)	0 (0 to 0)	0.0 (0.0 to 0.0)	-69.6 (-82.1 to -50.1)	-33.4 (-60.7 to 9.3)
Bosnia and Herzegovina	Ischemic heart disease	10 to 14	Female	0 (0 to 0)	0.0 (0.0 to 0.0)	0 (0 to 0)	0.0 (0.0 to 0.0)	0 (0 to 0)	0.0 (0.0 to 0.0)	-71.4 (-85.5 to -41.5)	-37.2 (-68.3 to 28.3)
Bosnia and Herzegovina	Ischemic heart disease	10 to 14	Male	0 (0 to 0)	0.0 (0.0 to 0.0)	0 (0 to 0)	0.0 (0.0 to 0.0)	0 (0 to 0)	0.0 (0.0 to 0.0)	-67.4 (-84.2 to -36.2)	-28.6 (-65.4 to 39.8)

eTable 8. All-cause and cause-specific mortality (counts and rates) at the global level and by country in the WHO European Region for ages 5 to 9 and 10 to 14 for 1990, 2000, and 2016

Bosnia and Herzegovina	Cerebrovascular disease	5 to 9	Both	3 (3 to 4)	0.9 (0.7 to 1.1)	1 (1 to 2)	0.6 (0.5 to 0.8)	0 (0 to 1)	0.3 (0.2 to 0.4)	-85.1 (-90.6 to -77.6)	-68.1 (-79.8 to -52.0)
Bosnia and Herzegovina	Cerebrovascular disease	5 to 9	Female	1 (1 to 2)	0.7 (0.5 to 1.0)	1 (0 to 1)	0.6 (0.4 to 0.8)	0 (0 to 0)	0.3 (0.2 to 0.4)	-82.2 (-89.7 to -69.9)	-61.7 (-77.7 to -35.3)
Bosnia and Herzegovina	Cerebrovascular disease	5 to 9	Male	2 (1 to 3)	1.0 (0.7 to 1.4)	1 (1 to 1)	0.7 (0.5 to 1.0)	0 (0 to 0)	0.3 (0.2 to 0.4)	-87.1 (-93.1 to -77.2)	-72.5 (-85.2 to -51.3)
Bosnia and Herzegovina	Cerebrovascular disease	10 to 14	Both	5 (3 to 6)	1.3 (1.0 to 1.6)	3 (2 to 3)	1.0 (0.7 to 1.2)	1 (1 to 1)	0.5 (0.4 to 0.7)	-80.4 (-86.8 to -71.2)	-57.0 (-71.2 to -36.8)
Bosnia and Herzegovina	Cerebrovascular disease	10 to 14	Female	2 (1 to 3)	1.2 (0.9 to 1.7)	1 (1 to 2)	0.8 (0.6 to 1.1)	0 (0 to 1)	0.5 (0.4 to 0.8)	-80.1 (-87.9 to -67.6)	-56.3 (-73.4 to -28.9)
Bosnia and Herzegovina	Cerebrovascular disease	10 to 14	Male	2 (2 to 4)	1.3 (0.9 to 2.0)	2 (1 to 2)	1.1 (0.7 to 1.5)	0 (0 to 1)	0.6 (0.4 to 0.8)	-80.6 (-88.7 to -68.1)	-57.6 (-75.2 to -30.1)
Bosnia and Herzegovina	Hypertensive heart disease	5 to 9	Both	0 (0 to 0)	0.0 (0.0 to 0.0)	0 (0 to 0)	0.0 (0.0 to 0.0)	0 (0 to 0)	0.0 (0.0 to 0.0)	-64.3 (-78.0 to -43.0)	-23.4 (-52.8 to 22.3)
Bosnia and Herzegovina	Hypertensive heart disease	5 to 9	Female	0 (0 to 0)	0.0 (0.0 to 0.0)	0 (0 to 0)	0.0 (0.0 to 0.0)	0 (0 to 0)	0.0 (0.0 to 0.0)	-60.1 (-78.2 to -26.8)	-14.1 (-53.0 to 57.7)
Bosnia and Herzegovina	Hypertensive heart disease	5 to 9	Male	0 (0 to 0)	0.0 (0.0 to 0.0)	0 (0 to 0)	0.0 (0.0 to 0.0)	0 (0 to 0)	0.0 (0.0 to 0.0)	-67.7 (-83.8 to -38.2)	-31.0 (-65.4 to 32.1)
Bosnia and Herzegovina	Hypertensive heart disease	10 to 14	Both	0 (0 to 0)	0.0 (0.0 to 0.0)	0 (0 to 0)	0.0 (0.0 to 0.0)	0 (0 to 0)	0.0 (0.0 to 0.0)	-59.9 (-73.8 to -40.0)	-12.2 (-42.6 to 31.6)
Bosnia and Herzegovina	Hypertensive heart disease	10 to 14	Female	0 (0 to 0)	0.0 (0.0 to 0.0)	0 (0 to 0)	0.0 (0.0 to 0.0)	0 (0 to 0)	0.0 (0.0 to 0.0)	-59.6 (-76.3 to -28.8)	-11.3 (-48.0 to 56.3)
Bosnia and Herzegovina	Hypertensive heart disease	10 to 14	Male	0 (0 to 0)	0.0 (0.0 to 0.0)	0 (0 to 0)	0.0 (0.0 to 0.0)	0 (0 to 0)	0.0 (0.0 to 0.0)	-60.3 (-78.2 to -29.9)	-13.1 (-52.2 to 53.6)
Bosnia and Herzegovina	Cardiomyopathy and myocarditis	5 to 9	Both	1 (0 to 1)	0.2 (0.1 to 0.3)	0 (0 to 1)	0.2 (0.1 to 0.3)	0 (0 to 0)	0.1 (0.1 to 0.2)	-77.8 (-87.5 to -58.9)	-52.3 (-73.3 to -11.8)
Bosnia and Herzegovina	Cardiomyopathy and myocarditis	5 to 9	Female	0 (0 to 1)	0.2 (0.1 to 0.4)	0 (0 to 0)	0.2 (0.1 to 0.3)	0 (0 to 0)	0.1 (0.0 to 0.2)	-76.9 (-89.6 to -48.5)	-50.2 (-77.7 to 10.8)
Bosnia and Herzegovina	Cardiomyopathy and myocarditis	5 to 9	Male	0 (0 to 1)	0.2 (0.1 to 0.4)	0 (0 to 0)	0.2 (0.1 to 0.3)	0 (0 to 0)	0.1 (0.0 to 0.2)	-78.7 (-91.1 to -49.5)	-54.4 (-81.0 to 7.9)
Bosnia and Herzegovina	Cardiomyopathy and myocarditis	10 to 14	Both	1 (1 to 2)	0.3 (0.2 to 0.5)	1 (0 to 1)	0.3 (0.2 to 0.4)	0 (0 to 0)	0.2 (0.1 to 0.3)	-71.7 (-83.0 to -51.4)	-37.9 (-62.8 to 6.5)
Bosnia and Herzegovina	Cardiomyopathy and myocarditis	10 to 14	Female	1 (0 to 1)	0.4 (0.2 to 0.6)	0 (0 to 1)	0.3 (0.2 to 0.5)	0 (0 to 0)	0.2 (0.1 to 0.3)	-74.1 (-86.9 to -43.8)	-43.1 (-71.2 to 23.2)
Bosnia and Herzegovina	Cardiomyopathy and myocarditis	10 to 14	Male	0 (0 to 1)	0.3 (0.1 to 0.5)	0 (0 to 1)	0.3 (0.1 to 0.5)	0 (0 to 0)	0.2 (0.1 to 0.3)	-68.6 (-85.4 to -32.6)	-31.3 (-68.0 to 47.6)
Bosnia and Herzegovina	Atrial fibrillation and flutter	5 to 9	Both	0 (0 to 0)	0.0 (0.0 to 0.0)	0 (0 to 0)	0.0 (0.0 to 0.0)	0 (0 to 0)	0.0 (0.0 to 0.0)	0.0 (0.0 to 0.0)	0.0 (0.0 to 0.0)
Bosnia and Herzegovina	Atrial fibrillation and flutter	5 to 9	Female	0 (0 to 0)	0.0 (0.0 to 0.0)	0 (0 to 0)	0.0 (0.0 to 0.0)	0 (0 to 0)	0.0 (0.0 to 0.0)	0.0 (0.0 to 0.0)	0.0 (0.0 to 0.0)
Bosnia and Herzegovina	Atrial fibrillation and flutter	5 to 9	Male	0 (0 to 0)	0.0 (0.0 to 0.0)	0 (0 to 0)	0.0 (0.0 to 0.0)	0 (0 to 0)	0.0 (0.0 to 0.0)	0.0 (0.0 to 0.0)	0.0 (0.0 to 0.0)
Bosnia and Herzegovina	Atrial fibrillation and flutter	10 to 14	Both	0 (0 to 0)	0.0 (0.0 to 0.0)	0 (0 to 0)	0.0 (0.0 to 0.0)	0 (0 to 0)	0.0 (0.0 to 0.0)	0.0 (0.0 to 0.0)	0.0 (0.0 to 0.0)
Bosnia and Herzegovina	Atrial fibrillation and flutter	10 to 14	Female	0 (0 to 0)	0.0 (0.0 to 0.0)	0 (0 to 0)	0.0 (0.0 to 0.0)	0 (0 to 0)	0.0 (0.0 to 0.0)	0.0 (0.0 to 0.0)	0.0 (0.0 to 0.0)
Bosnia and Herzegovina	Atrial fibrillation and flutter	10 to 14	Male	0 (0 to 0)	0.0 (0.0 to 0.0)	0 (0 to 0)	0.0 (0.0 to 0.0)	0 (0 to 0)	0.0 (0.0 to 0.0)	0.0 (0.0 to 0.0)	0.0 (0.0 to 0.0)
Bosnia and Herzegovina	Endocarditis	5 to 9	Both	0 (0 to 0)	0.0 (0.0 to 0.0)	0 (0 to 0)	0.0 (0.0 to 0.0)	0 (0 to 0)	0.0 (0.0 to 0.0)	-67.4 (-82.8 to -30.4)	-30.1 (-63.1 to 49.3)
Bosnia and Herzegovina	Endocarditis	5 to 9	Female	0 (0 to 0)	0.0 (0.0 to 0.0)	0 (0 to 0)	0.0 (0.0 to 0.0)	0 (0 to 0)	0.0 (0.0 to 0.0)	-62.8 (-82.4 to -15.5)	-20.0 (-62.2 to 81.9)

eTable 8. All-cause and cause-specific mortality (counts and rates) at the global level and by country in the WHO European Region for ages 5 to 9 and 10 to 14 for 1990, 2000, and 2016

Bosnia and Herzegovina	Endocarditis	5 to 9	Male	0 (0 to 0)	0.0 (0.0 to 0.0)	0 (0 to 0)	0.0 (0.0 to 0.0)	0 (0 to 0)	0.0 (0.0 to 0.0)	-70.1 (-87.4 to -22.3)	-36.0 (-73.1 to 66.1)
Bosnia and Herzegovina	Endocarditis	10 to 14	Both	0 (0 to 0)	0.0 (0.0 to 0.0)	0 (0 to 0)	0.0 (0.0 to 0.0)	0 (0 to 0)	0.0 (0.0 to 0.0)	-55.2 (-75.9 to -10.4)	-1.8 (-47.2 to 96.4)
Bosnia and Herzegovina	Endocarditis	10 to 14	Female	0 (0 to 0)	0.0 (0.0 to 0.0)	0 (0 to 0)	0.0 (0.0 to 0.0)	0 (0 to 0)	0.0 (0.0 to 0.0)	-54.8 (-80.9 to 8.6)	-0.8 (-58.2 to 138.1)
Bosnia and Herzegovina	Endocarditis	10 to 14	Male	0 (0 to 0)	0.0 (0.0 to 0.0)	0 (0 to 0)	0.0 (0.0 to 0.0)	0 (0 to 0)	0.0 (0.0 to 0.0)	-55.7 (-79.3 to 12.7)	-2.8 (-54.7 to 147.0)
Bosnia and Herzegovina	Other cardiovascular and circulatory diseases	5 to 9	Both	0 (0 to 1)	0.1 (0.1 to 0.2)	0 (0 to 0)	0.1 (0.0 to 0.2)	0 (0 to 0)	0.0 (0.0 to 0.1)	-89.8 (-95.4 to -78.8)	-78.1 (-90.1 to -54.5)
Bosnia and Herzegovina	Other cardiovascular and circulatory diseases	5 to 9	Female	0 (0 to 0)	0.1 (0.0 to 0.2)	0 (0 to 0)	0.1 (0.0 to 0.2)	0 (0 to 0)	0.0 (0.0 to 0.1)	-87.6 (-95.9 to -71.2)	-73.2 (-91.1 to -38.1)
Bosnia and Herzegovina	Other cardiovascular and circulatory diseases	5 to 9	Male	0 (0 to 1)	0.2 (0.1 to 0.3)	0 (0 to 0)	0.1 (0.0 to 0.2)	0 (0 to 0)	0.0 (0.0 to 0.1)	-90.9 (-96.3 to -78.4)	-80.4 (-92.1 to -53.8)
Bosnia and Herzegovina	Other cardiovascular and circulatory diseases	10 to 14	Both	1 (0 to 1)	0.2 (0.1 to 0.3)	0 (0 to 1)	0.2 (0.1 to 0.2)	0 (0 to 0)	0.1 (0.0 to 0.1)	-87.2 (-93.6 to -76.0)	-72.0 (-86.0 to -47.5)
Bosnia and Herzegovina	Other cardiovascular and circulatory diseases	10 to 14	Female	0 (0 to 1)	0.2 (0.1 to 0.3)	0 (0 to 0)	0.1 (0.1 to 0.2)	0 (0 to 0)	0.1 (0.0 to 0.2)	-88.4 (-95.5 to -67.4)	-74.6 (-90.1 to -28.5)
Bosnia and Herzegovina	Other cardiovascular and circulatory diseases	10 to 14	Male	0 (0 to 1)	0.2 (0.1 to 0.4)	0 (0 to 0)	0.2 (0.1 to 0.3)	0 (0 to 0)	0.1 (0.0 to 0.1)	-86.2 (-93.9 to -70.1)	-69.7 (-86.7 to -34.5)
Bosnia and Herzegovina	Chronic obstructive pulmonary disease	5 to 9	Both	0 (0 to 1)	0.1 (0.1 to 0.1)	0 (0 to 0)	0.0 (0.0 to 0.1)	0 (0 to 0)	0.0 (0.0 to 0.0)	-91.9 (-95.5 to -86.6)	-82.6 (-90.3 to -71.2)
Bosnia and Herzegovina	Chronic obstructive pulmonary disease	5 to 9	Female	0 (0 to 0)	0.1 (0.0 to 0.1)	0 (0 to 0)	0.0 (0.0 to 0.1)	0 (0 to 0)	0.0 (0.0 to 0.0)	-90.2 (-95.3 to -81.5)	-78.8 (-89.8 to -60.2)
Bosnia and Herzegovina	Chronic obstructive pulmonary disease	5 to 9	Male	0 (0 to 0)	0.1 (0.1 to 0.2)	0 (0 to 0)	0.1 (0.0 to 0.1)	0 (0 to 0)	0.0 (0.0 to 0.0)	-92.9 (-96.5 to -86.2)	-84.7 (-92.5 to -70.5)
Bosnia and Herzegovina	Chronic obstructive pulmonary disease	10 to 14	Both	0 (0 to 1)	0.1 (0.1 to 0.2)	0 (0 to 0)	0.1 (0.1 to 0.1)	0 (0 to 0)	0.0 (0.0 to 0.1)	-84.9 (-91.1 to -74.3)	-66.9 (-80.5 to -43.6)
Bosnia and Herzegovina	Chronic obstructive pulmonary disease	10 to 14	Female	0 (0 to 0)	0.1 (0.1 to 0.2)	0 (0 to 0)	0.1 (0.0 to 0.1)	0 (0 to 0)	0.0 (0.0 to 0.1)	-86.5 (-94.2 to -71.9)	-70.3 (-87.3 to -38.4)
Bosnia and Herzegovina	Chronic obstructive pulmonary disease	10 to 14	Male	0 (0 to 0)	0.2 (0.1 to 0.2)	0 (0 to 0)	0.1 (0.1 to 0.2)	0 (0 to 0)	0.1 (0.0 to 0.1)	-83.9 (-92.4 to -67.8)	-64.7 (-83.4 to -29.6)
Bosnia and Herzegovina	Asthma	5 to 9	Both	0 (0 to 0)	0.0 (0.0 to 0.0)	0 (0 to 0)	0.0 (0.0 to 0.0)	0 (0 to 0)	0.0 (0.0 to 0.0)	-80.5 (-88.7 to -67.0)	-58.1 (-75.9 to -29.2)

eTable 8. All-cause and cause-specific mortality (counts and rates) at the global level and by country in the WHO European Region for ages 5 to 9 and 10 to 14 for 1990, 2000, and 2016

Bosnia and Herzegovina	Asthma	5 to 9	Female	0 (0 to 0)	0.0 (0.0 to 0.0)	0 (0 to 0)	0.0 (0.0 to 0.0)	0 (0 to 0)	0.0 (0.0 to 0.0)	-80.3 (-90.0 to -62.4)	-57.6 (-78.5 to -19.1)
Bosnia and Herzegovina	Asthma	5 to 9	Male	0 (0 to 0)	0.0 (0.0 to 0.1)	0 (0 to 0)	0.0 (0.0 to 0.0)	0 (0 to 0)	0.0 (0.0 to 0.0)	-80.6 (-90.6 to -59.8)	-58.5 (-80.0 to -14.1)
Bosnia and Herzegovina	Asthma	10 to 14	Both	0 (0 to 0)	0.1 (0.0 to 0.1)	0 (0 to 0)	0.0 (0.0 to 0.0)	0 (0 to 0)	0.0 (0.0 to 0.0)	-85.7 (-92.1 to -72.5)	-68.7 (-82.6 to -39.8)
Bosnia and Herzegovina	Asthma	10 to 14	Female	0 (0 to 0)	0.1 (0.0 to 0.1)	0 (0 to 0)	0.0 (0.0 to 0.1)	0 (0 to 0)	0.0 (0.0 to 0.0)	-88.2 (-94.8 to -71.5)	-74.1 (-88.7 to -37.4)
Bosnia and Herzegovina	Asthma	10 to 14	Male	0 (0 to 0)	0.1 (0.0 to 0.1)	0 (0 to 0)	0.0 (0.0 to 0.1)	0 (0 to 0)	0.0 (0.0 to 0.0)	-83.1 (-93.1 to -51.0)	-63.0 (-84.9 to 7.4)
Bosnia and Herzegovina	Interstitial lung disease and pulmonary sarcoidosis	5 to 9	Both	0 (0 to 0)	0.0 (0.0 to 0.1)	0 (0 to 0)	0.0 (0.0 to 0.0)	0 (0 to 0)	0.0 (0.0 to 0.0)	-80.6 (-90.9 to -60.5)	-58.4 (-80.4 to -15.2)
Bosnia and Herzegovina	Interstitial lung disease and pulmonary sarcoidosis	5 to 9	Female	0 (0 to 0)	0.0 (0.0 to 0.1)	0 (0 to 0)	0.0 (0.0 to 0.1)	0 (0 to 0)	0.0 (0.0 to 0.0)	-80.3 (-90.5 to -59.2)	-57.7 (-79.6 to -12.1)
Bosnia and Herzegovina	Interstitial lung disease and pulmonary sarcoidosis	5 to 9	Male	0 (0 to 0)	0.0 (0.0 to 0.1)	0 (0 to 0)	0.0 (0.0 to 0.0)	0 (0 to 0)	0.0 (0.0 to 0.0)	-81.0 (-94.4 to -36.1)	-59.3 (-88.0 to 36.6)
Bosnia and Herzegovina	Interstitial lung disease and pulmonary sarcoidosis	10 to 14	Both	0 (0 to 0)	0.0 (0.0 to 0.1)	0 (0 to 0)	0.0 (0.0 to 0.0)	0 (0 to 0)	0.0 (0.0 to 0.0)	-70.7 (-87.2 to -39.6)	-35.9 (-71.9 to 32.4)
Bosnia and Herzegovina	Interstitial lung disease and pulmonary sarcoidosis	10 to 14	Female	0 (0 to 0)	0.0 (0.0 to 0.1)	0 (0 to 0)	0.0 (0.0 to 0.1)	0 (0 to 0)	0.0 (0.0 to 0.1)	-75.6 (-91.0 to -41.0)	-46.5 (-80.3 to 29.3)
Bosnia and Herzegovina	Interstitial lung disease and pulmonary sarcoidosis	10 to 14	Male	0 (0 to 0)	0.0 (0.0 to 0.0)	0 (0 to 0)	0.0 (0.0 to 0.0)	0 (0 to 0)	0.0 (0.0 to 0.0)	-57.2 (-87.3 to 44.6)	-6.2 (-72.1 to 216.8)
Bosnia and Herzegovina	Other chronic respiratory diseases	5 to 9	Both	0 (0 to 0)	0.0 (0.0 to 0.0)	0 (0 to 0)	0.0 (0.0 to 0.0)	0 (0 to 0)	0.0 (0.0 to 0.0)	-80.4 (-88.7 to -66.8)	-58.0 (-75.8 to -28.8)
Bosnia and Herzegovina	Other chronic respiratory diseases	5 to 9	Female	0 (0 to 0)	0.0 (0.0 to 0.0)	0 (0 to 0)	0.0 (0.0 to 0.0)	0 (0 to 0)	0.0 (0.0 to 0.0)	-80.3 (-90.0 to -62.4)	-57.5 (-78.5 to -19.1)
Bosnia and Herzegovina	Other chronic respiratory diseases	5 to 9	Male	0 (0 to 0)	0.0 (0.0 to 0.1)	0 (0 to 0)	0.0 (0.0 to 0.0)	0 (0 to 0)	0.0 (0.0 to 0.0)	-80.5 (-90.5 to -59.2)	-58.3 (-79.6 to -12.7)
Bosnia and Herzegovina	Other chronic respiratory diseases	10 to 14	Both	0 (0 to 0)	0.0 (0.0 to 0.0)	0 (0 to 0)	0.0 (0.0 to 0.0)	0 (0 to 0)	0.0 (0.0 to 0.0)	-69.3 (-82.7 to -46.7)	-32.8 (-62.0 to 16.9)
Bosnia and Herzegovina	Other chronic respiratory diseases	10 to 14	Female	0 (0 to 0)	0.0 (0.0 to 0.0)	0 (0 to 0)	0.0 (0.0 to 0.0)	0 (0 to 0)	0.0 (0.0 to 0.0)	-75.5 (-88.4 to -50.2)	-46.3 (-74.6 to 9.3)
Bosnia and Herzegovina	Other chronic respiratory diseases	10 to 14	Male	0 (0 to 0)	0.0 (0.0 to 0.0)	0 (0 to 0)	0.0 (0.0 to 0.0)	0 (0 to 0)	0.0 (0.0 to 0.0)	-62.5 (-83.5 to -16.0)	-17.9 (-63.8 to 84.0)
Bosnia and Herzegovina	Cirrhosis and other chronic liver diseases due to hepatitis B	5 to 9	Both	0 (0 to 0)	0.0 (0.0 to 0.0)	0 (0 to 0)	0.0 (0.0 to 0.0)	0 (0 to 0)	0.0 (0.0 to 0.0)	-85.4 (-91.0 to -76.6)	-68.7 (-80.7 to -49.9)

eTable 8. All-cause and cause-specific mortality (counts and rates) at the global level and by country in the WHO European Region for ages 5 to 9 and 10 to 14 for 1990, 2000, and 2016

Bosnia and Herzegovina	Cirrhosis and other chronic liver diseases due to hepatitis B	5 to 9	Female	0<br>(0 to 0)	0.0<br>(0.0 to 0.0)	0<br>(0 to 0)	0.0<br>(0.0 to 0.0)	0<br>(0 to 0)	0.0<br>(0.0 to 0.0)	-83.9<br>(-90.5 to -71.9)	-65.3<br>(-79.6 to -39.5)
Bosnia and Herzegovina	Cirrhosis and other chronic liver diseases due to hepatitis B	5 to 9	Male	0<br>(0 to 0)	0.0<br>(0.0 to 0.0)	0<br>(0 to 0)	0.0<br>(0.0 to 0.0)	0<br>(0 to 0)	0.0<br>(0.0 to 0.0)	-85.9<br>(-92.3 to -73.9)	-69.8<br>(-83.5 to -44.2)
Bosnia and Herzegovina	Cirrhosis and other chronic liver diseases due to hepatitis B	10 to 14	Both	0<br>(0 to 0)	0.0<br>(0.0 to 0.0)	0<br>(0 to 0)	0.0<br>(0.0 to 0.0)	0<br>(0 to 0)	0.0<br>(0.0 to 0.0)	-80.9<br>(-87.6 to -71.3)	-58.1<br>(-72.8 to -37.0)
Bosnia and Herzegovina	Cirrhosis and other chronic liver diseases due to hepatitis B	10 to 14	Female	0<br>(0 to 0)	0.0<br>(0.0 to 0.0)	0<br>(0 to 0)	0.0<br>(0.0 to 0.0)	0<br>(0 to 0)	0.0<br>(0.0 to 0.0)	-83.4<br>(-90.7 to -70.7)	-63.7<br>(-79.5 to -35.7)
Bosnia and Herzegovina	Cirrhosis and other chronic liver diseases due to hepatitis B	10 to 14	Male	0<br>(0 to 0)	0.0<br>(0.0 to 0.0)	0<br>(0 to 0)	0.0<br>(0.0 to 0.0)	0<br>(0 to 0)	0.0<br>(0.0 to 0.0)	-79.5<br>(-88.4 to -63.8)	-55.1<br>(-74.7 to -20.6)
Bosnia and Herzegovina	Cirrhosis and other chronic liver diseases due to hepatitis C	5 to 9	Both	0<br>(0 to 0)	0.0<br>(0.0 to 0.0)	0<br>(0 to 0)	0.0<br>(0.0 to 0.0)	0<br>(0 to 0)	0.0<br>(0.0 to 0.0)	-85.5<br>(-91.0 to -77.1)	-69.0<br>(-80.7 to -51.0)
Bosnia and Herzegovina	Cirrhosis and other chronic liver diseases due to hepatitis C	5 to 9	Female	0<br>(0 to 0)	0.0<br>(0.0 to 0.0)	0<br>(0 to 0)	0.0<br>(0.0 to 0.0)	0<br>(0 to 0)	0.0<br>(0.0 to 0.0)	-83.8<br>(-90.5 to -71.7)	-65.2<br>(-79.7 to -39.2)
Bosnia and Herzegovina	Cirrhosis and other chronic liver diseases due to hepatitis C	5 to 9	Male	0<br>(0 to 0)	0.0<br>(0.0 to 0.0)	0<br>(0 to 0)	0.0<br>(0.0 to 0.0)	0<br>(0 to 0)	0.0<br>(0.0 to 0.0)	-86.1<br>(-92.4 to -74.4)	-70.4<br>(-83.8 to -45.2)
Bosnia and Herzegovina	Cirrhosis and other chronic liver diseases due to hepatitis C	10 to 14	Both	0<br>(0 to 0)	0.0<br>(0.0 to 0.0)	0<br>(0 to 0)	0.0<br>(0.0 to 0.0)	0<br>(0 to 0)	0.0<br>(0.0 to 0.0)	-81.3<br>(-87.8 to -71.9)	-58.9<br>(-73.3 to -38.5)
Bosnia and Herzegovina	Cirrhosis and other chronic liver diseases due to hepatitis C	10 to 14	Female	0<br>(0 to 0)	0.0<br>(0.0 to 0.0)	0<br>(0 to 0)	0.0<br>(0.0 to 0.0)	0<br>(0 to 0)	0.0<br>(0.0 to 0.0)	-83.4<br>(-90.7 to -70.4)	-63.5<br>(-79.6 to -35.1)
Bosnia and Herzegovina	Cirrhosis and other chronic liver diseases due to hepatitis C	10 to 14	Male	0<br>(0 to 0)	0.0<br>(0.0 to 0.0)	0<br>(0 to 0)	0.0<br>(0.0 to 0.0)	0<br>(0 to 0)	0.0<br>(0.0 to 0.0)	-79.9<br>(-88.7 to -64.3)	-55.9<br>(-75.2 to -21.8)
Bosnia and Herzegovina	Cirrhosis and other chronic liver diseases due to other causes	5 to 9	Both	1<br>(1 to 1)	0.2<br>(0.2 to 0.3)	0<br>(0 to 1)	0.2<br>(0.1 to 0.2)	0<br>(0 to 0)	0.1<br>(0.1 to 0.1)	-85.3<br>(-90.4 to -78.0)	-68.4<br>(-79.5 to -52.9)
Bosnia and Herzegovina	Cirrhosis and other chronic liver diseases due to other causes	5 to 9	Female	0<br>(0 to 1)	0.2<br>(0.2 to 0.3)	0<br>(0 to 0)	0.2<br>(0.1 to 0.3)	0<br>(0 to 0)	0.1<br>(0.1 to 0.1)	-84.6<br>(-90.9 to -73.1)	-66.8<br>(-80.5 to -42.2)

eTable 8. All-cause and cause-specific mortality (counts and rates) at the global level and by country in the WHO European Region for ages 5 to 9 and 10 to 14 for 1990, 2000, and 2016

Bosnia and Herzegovina	Cirrhosis and other chronic liver diseases due to other causes	5 to 9	Male	0<br>(0 to 1)	0.2<br>(0.1 to 0.3)	0<br>(0 to 0)	0.2<br>(0.1 to 0.3)	0<br>(0 to 0)	0.1<br>(0.0 to 0.1)	-85.9<br>(-92.3 to -74.3)	-70.0<br>(-83.5 to -45.0)
Bosnia and Herzegovina	Cirrhosis and other chronic liver diseases due to other causes	10 to 14	Both	1<br>(1 to 2)	0.3<br>(0.3 to 0.5)	1<br>(1 to 1)	0.2<br>(0.2 to 0.3)	0<br>(0 to 0)	0.1<br>(0.1 to 0.2)	-82.6<br>(-88.4 to -73.9)	-61.8<br>(-74.6 to -42.8)
Bosnia and Herzegovina	Cirrhosis and other chronic liver diseases due to other causes	10 to 14	Female	1<br>(1 to 1)	0.5<br>(0.3 to 0.7)	0<br>(0 to 1)	0.3<br>(0.2 to 0.5)	0<br>(0 to 0)	0.2<br>(0.1 to 0.2)	-84.1<br>(-91.0 to -71.5)	-65.0<br>(-80.2 to -37.5)
Bosnia and Herzegovina	Cirrhosis and other chronic liver diseases due to other causes	10 to 14	Male	0<br>(0 to 1)	0.2<br>(0.1 to 0.4)	0<br>(0 to 0)	0.2<br>(0.1 to 0.3)	0<br>(0 to 0)	0.1<br>(0.1 to 0.2)	-79.8<br>(-88.6 to -64.1)	-55.6<br>(-74.9 to -21.4)
Bosnia and Herzegovina	Peptic ulcer disease	5 to 9	Both	0<br>(0 to 1)	0.1<br>(0.1 to 0.2)	0<br>(0 to 0)	0.0<br>(0.0 to 0.1)	0<br>(0 to 0)	0.0<br>(0.0 to 0.0)	-93.8<br>(-97.0 to -86.6)	-86.7<br>(-93.5 to -71.2)
Bosnia and Herzegovina	Peptic ulcer disease	5 to 9	Female	0<br>(0 to 0)	0.1<br>(0.0 to 0.1)	0<br>(0 to 0)	0.0<br>(0.0 to 0.1)	0<br>(0 to 0)	0.0<br>(0.0 to 0.0)	-91.9<br>(-97.0 to -79.5)	-82.5<br>(-93.5 to -55.9)
Bosnia and Herzegovina	Peptic ulcer disease	5 to 9	Male	0<br>(0 to 0)	0.1<br>(0.1 to 0.2)	0<br>(0 to 0)	0.1<br>(0.0 to 0.1)	0<br>(0 to 0)	0.0<br>(0.0 to 0.0)	-94.6<br>(-97.9 to -85.2)	-88.5<br>(-95.4 to -68.3)
Bosnia and Herzegovina	Peptic ulcer disease	10 to 14	Both	0<br>(0 to 0)	0.0<br>(0.0 to 0.0)	0<br>(0 to 0)	0.0<br>(0.0 to 0.0)	0<br>(0 to 0)	0.0<br>(0.0 to 0.0)	-79.2<br>(-87.6 to -62.2)	-54.4<br>(-72.8 to -17.2)
Bosnia and Herzegovina	Peptic ulcer disease	10 to 14	Female	0<br>(0 to 0)	0.0<br>(0.0 to 0.1)	0<br>(0 to 0)	0.0<br>(0.0 to 0.0)	0<br>(0 to 0)	0.0<br>(0.0 to 0.0)	-81.0<br>(-91.4 to -57.9)	-58.3<br>(-81.1 to -7.7)
Bosnia and Herzegovina	Peptic ulcer disease	10 to 14	Male	0<br>(0 to 0)	0.0<br>(0.0 to 0.1)	0<br>(0 to 0)	0.0<br>(0.0 to 0.0)	0<br>(0 to 0)	0.0<br>(0.0 to 0.0)	-77.3<br>(-88.6 to -45.7)	-50.2<br>(-75.1 to 19.0)
Bosnia and Herzegovina	Gastritis and duodenitis	5 to 9	Both	0<br>(0 to 0)	0.0<br>(0.0 to 0.0)	0<br>(0 to 0)	0.0<br>(0.0 to 0.0)	0<br>(0 to 0)	0.0<br>(0.0 to 0.0)	-68.8<br>(-81.9 to -44.6)	-33.2<br>(-61.1 to 18.8)
Bosnia and Herzegovina	Gastritis and duodenitis	5 to 9	Female	0<br>(0 to 0)	0.0<br>(0.0 to 0.0)	0<br>(0 to 0)	0.0<br>(0.0 to 0.0)	0<br>(0 to 0)	0.0<br>(0.0 to 0.0)	-72.0<br>(-87.8 to -41.9)	-39.8<br>(-73.7 to 25.1)
Bosnia and Herzegovina	Gastritis and duodenitis	5 to 9	Male	0<br>(0 to 0)	0.0<br>(0.0 to 0.0)	0<br>(0 to 0)	0.0<br>(0.0 to 0.0)	0<br>(0 to 0)	0.0<br>(0.0 to 0.0)	-66.3<br>(-83.4 to -28.9)	-27.9<br>(-64.5 to 52.1)
Bosnia and Herzegovina	Gastritis and duodenitis	10 to 14	Both	0<br>(0 to 0)	0.0<br>(0.0 to 0.0)	0<br>(0 to 0)	0.0<br>(0.0 to 0.0)	0<br>(0 to 0)	0.0<br>(0.0 to 0.0)	-66.6<br>(-79.0 to -45.9)	-26.8<br>(-53.9 to 18.7)
Bosnia and Herzegovina	Gastritis and duodenitis	10 to 14	Female	0<br>(0 to 0)	0.0<br>(0.0 to 0.0)	0<br>(0 to 0)	0.0<br>(0.0 to 0.0)	0<br>(0 to 0)	0.0<br>(0.0 to 0.0)	-70.6<br>(-85.7 to -42.9)	-35.6<br>(-68.5 to 25.2)
Bosnia and Herzegovina	Gastritis and duodenitis	10 to 14	Male	0<br>(0 to 0)	0.0<br>(0.0 to 0.0)	0<br>(0 to 0)	0.0<br>(0.0 to 0.0)	0<br>(0 to 0)	0.0<br>(0.0 to 0.0)	-62.0<br>(-79.0 to -25.3)	-16.8<br>(-54.0 to 63.8)
Bosnia and Herzegovina	Appendicitis	5 to 9	Both	1<br>(0 to 2)	0.3<br>(0.1 to 0.5)	0<br>(0 to 1)	0.2<br>(0.1 to 0.3)	0<br>(0 to 0)	0.0<br>(0.0 to 0.1)	-92.6<br>(-96.0 to -86.2)	-84.2<br>(-91.4 to -70.4)
Bosnia and Herzegovina	Appendicitis	5 to 9	Female	0<br>(0 to 0)	0.1<br>(0.1 to 0.2)	0<br>(0 to 0)	0.1<br>(0.0 to 0.1)	0<br>(0 to 0)	0.0<br>(0.0 to 0.0)	-94.0<br>(-97.9 to -85.3)	-87.0<br>(-95.5 to -68.4)
Bosnia and Herzegovina	Appendicitis	5 to 9	Male	1<br>(0 to 1)	0.4<br>(0.1 to 0.8)	0<br>(0 to 1)	0.2<br>(0.1 to 0.4)	0<br>(0 to 0)	0.1<br>(0.0 to 0.1)	-92.3<br>(-96.2 to -84.8)	-83.6<br>(-91.8 to -67.5)
Bosnia and Herzegovina	Appendicitis	10 to 14	Both	1<br>(0 to 1)	0.2<br>(0.1 to 0.3)	0<br>(0 to 1)	0.1<br>(0.1 to 0.2)	0<br>(0 to 0)	0.0<br>(0.0 to 0.1)	-90.7<br>(-94.5 to -84.6)	-79.7<br>(-87.9 to -66.2)
Bosnia and Herzegovina	Appendicitis	10 to 14	Female	0<br>(0 to 1)	0.2<br>(0.1 to 0.3)	0<br>(0 to 0)	0.1<br>(0.0 to 0.2)	0<br>(0 to 0)	0.0<br>(0.0 to 0.1)	-93.7<br>(-97.5 to -84.5)	-86.1<br>(-94.5 to -65.9)
Bosnia and Herzegovina	Appendicitis	10 to 14	Male	0<br>(0 to 1)	0.3<br>(0.1 to 0.4)	0<br>(0 to 0)	0.2<br>(0.1 to 0.3)	0<br>(0 to 0)	0.1<br>(0.0 to 0.1)	-88.6<br>(-93.8 to -78.4)	-75.1<br>(-86.4 to -52.7)
Bosnia and Herzegovina	Paralytic ileus and intestinal obstruction	5 to 9	Both	1<br>(0 to 1)	0.2<br>(0.1 to 0.3)	0<br>(0 to 1)	0.2<br>(0.1 to 0.2)	0<br>(0 to 0)	0.1<br>(0.1 to 0.1)	-77.3<br>(-87.5 to -60.4)	-51.4<br>(-73.2 to -15.2)
Bosnia and Herzegovina	Paralytic ileus and intestinal obstruction	5 to 9	Female	0<br>(0 to 1)	0.2<br>(0.1 to 0.4)	0<br>(0 to 0)	0.2<br>(0.1 to 0.3)	0<br>(0 to 0)	0.1<br>(0.1 to 0.2)	-74.9<br>(-87.6 to -50.4)	-46.0<br>(-73.3 to 6.7)
Bosnia and Herzegovina	Paralytic ileus and intestinal obstruction	5 to 9	Male	0<br>(0 to 0)	0.1<br>(0.1 to 0.3)	0<br>(0 to 0)	0.1<br>(0.0 to 0.2)	0<br>(0 to 0)	0.1<br>(0.0 to 0.1)	-80.8<br>(-92.0 to -56.1)	-58.9<br>(-83.0 to -6.1)

eTable 8. All-cause and cause-specific mortality (counts and rates) at the global level and by country in the WHO European Region for ages 5 to 9 and 10 to 14 for 1990, 2000, and 2016

Bosnia and Herzegovina	Paralytic ileus and intestinal obstruction	10 to 14	Both	0 (0 to 1)	0.1 (0.1 to 0.2)	0 (0 to 0)	0.1 (0.1 to 0.1)	0 (0 to 0)	0.1 (0.0 to 0.1)	-70.8 (-83.2 to -48.2)	-36.0 (-63.3 to 13.6)
Bosnia and Herzegovina	Paralytic ileus and intestinal obstruction	10 to 14	Female	0 (0 to 1)	0.2 (0.1 to 0.3)	0 (0 to 0)	0.1 (0.1 to 0.3)	0 (0 to 0)	0.1 (0.1 to 0.2)	-71.3 (-84.9 to -45.2)	-37.1 (-66.9 to 20.2)
Bosnia and Herzegovina	Paralytic ileus and intestinal obstruction	10 to 14	Male	0 (0 to 0)	0.0 (0.0 to 0.1)	0 (0 to 0)	0.0 (0.0 to 0.1)	0 (0 to 0)	0.0 (0.0 to 0.1)	-69.0 (-83.8 to -10.0)	-32.1 (-64.5 to 97.2)
Bosnia and Herzegovina	Inguinal, femoral, and abdominal hernia	5 to 9	Both	0 (0 to 0)	0.0 (0.0 to 0.0)	0 (0 to 0)	0.0 (0.0 to 0.0)	0 (0 to 0)	0.0 (0.0 to 0.0)	-76.2 (-88.6 to -52.6)	-49.0 (-75.6 to 1.7)
Bosnia and Herzegovina	Inguinal, femoral, and abdominal hernia	5 to 9	Female	0 (0 to 0)	0.0 (0.0 to 0.0)	0 (0 to 0)	0.0 (0.0 to 0.0)	0 (0 to 0)	0.0 (0.0 to 0.0)	-72.4 (-88.3 to -40.8)	-40.5 (-74.9 to 27.4)
Bosnia and Herzegovina	Inguinal, femoral, and abdominal hernia	5 to 9	Male	0 (0 to 0)	0.0 (0.0 to 0.1)	0 (0 to 0)	0.0 (0.0 to 0.1)	0 (0 to 0)	0.0 (0.0 to 0.0)	-78.3 (-91.3 to -47.2)	-53.6 (-81.5 to 12.8)
Bosnia and Herzegovina	Inguinal, femoral, and abdominal hernia	10 to 14	Both	0 (0 to 0)	0.0 (0.0 to 0.0)	0 (0 to 0)	0.0 (0.0 to 0.0)	0 (0 to 0)	0.0 (0.0 to 0.0)	-69.9 (-82.8 to -49.0)	-33.9 (-62.3 to 11.9)
Bosnia and Herzegovina	Inguinal, femoral, and abdominal hernia	10 to 14	Female	0 (0 to 0)	0.0 (0.0 to 0.0)	0 (0 to 0)	0.0 (0.0 to 0.0)	0 (0 to 0)	0.0 (0.0 to 0.0)	-71.2 (-86.0 to -43.4)	-36.9 (-69.4 to 24.2)
Bosnia and Herzegovina	Inguinal, femoral, and abdominal hernia	10 to 14	Male	0 (0 to 0)	0.0 (0.0 to 0.0)	0 (0 to 0)	0.0 (0.0 to 0.1)	0 (0 to 0)	0.0 (0.0 to 0.0)	-68.5 (-85.4 to -29.0)	-31.1 (-68.0 to 55.6)
Bosnia and Herzegovina	Inflammatory bowel disease	5 to 9	Both	0 (0 to 0)	0.0 (0.0 to 0.0)	0 (0 to 0)	0.0 (0.0 to 0.0)	0 (0 to 0)	0.0 (0.0 to 0.0)	-70.1 (-89.1 to 9.6)	-35.9 (-76.5 to 135.1)
Bosnia and Herzegovina	Inflammatory bowel disease	5 to 9	Female	0 (0 to 0)	0.0 (0.0 to 0.0)	0 (0 to 0)	0.0 (0.0 to 0.0)	0 (0 to 0)	0.0 (0.0 to 0.0)	-78.9 (-93.1 to -39.8)	-54.6 (-85.1 to 29.6)
Bosnia and Herzegovina	Inflammatory bowel disease	5 to 9	Male	0 (0 to 0)	0.0 (0.0 to 0.0)	0 (0 to 0)	0.0 (0.0 to 0.0)	0 (0 to 0)	0.0 (0.0 to 0.0)	-67.1 (-89.9 to 69.3)	-29.7 (-78.5 to 262.0)
Bosnia and Herzegovina	Inflammatory bowel disease	10 to 14	Both	0 (0 to 0)	0.0 (0.0 to 0.0)	0 (0 to 0)	0.0 (0.0 to 0.0)	0 (0 to 0)	0.0 (0.0 to 0.0)	-71.5 (-86.3 to -28.8)	-37.5 (-69.9 to 56.2)
Bosnia and Herzegovina	Inflammatory bowel disease	10 to 14	Female	0 (0 to 0)	0.0 (0.0 to 0.0)	0 (0 to 0)	0.0 (0.0 to 0.0)	0 (0 to 0)	0.0 (0.0 to 0.0)	-77.2 (-91.4 to -44.9)	-50.1 (-81.1 to 20.9)
Bosnia and Herzegovina	Inflammatory bowel disease	10 to 14	Male	0 (0 to 0)	0.0 (0.0 to 0.0)	0 (0 to 0)	0.0 (0.0 to 0.0)	0 (0 to 0)	0.0 (0.0 to 0.0)	-69.1 (-87.2 to -5.5)	-32.3 (-71.9 to 107.1)
Bosnia and Herzegovina	Vascular intestinal disorders	5 to 9	Both	0 (0 to 0)	0.0 (0.0 to 0.0)	0 (0 to 0)	0.0 (0.0 to 0.0)	0 (0 to 0)	0.0 (0.0 to 0.0)	-78.7 (-93.2 to -17.5)	-54.2 (-85.3 to 76.9)
Bosnia and Herzegovina	Vascular intestinal disorders	5 to 9	Female	0 (0 to 0)	0.0 (0.0 to 0.0)	0 (0 to 0)	0.0 (0.0 to 0.0)	0 (0 to 0)	0.0 (0.0 to 0.0)	-81.9 (-95.0 to -38.3)	-61.0 (-89.2 to 32.7)
Bosnia and Herzegovina	Vascular intestinal disorders	5 to 9	Male	0 (0 to 0)	0.0 (0.0 to 0.0)	0 (0 to 0)	0.0 (0.0 to 0.0)	0 (0 to 0)	0.0 (0.0 to 0.0)	-78.0 (-94.0 to 9.9)	-52.9 (-87.2 to 135.0)
Bosnia and Herzegovina	Vascular intestinal disorders	10 to 14	Both	0 (0 to 0)	0.0 (0.0 to 0.0)	0 (0 to 0)	0.0 (0.0 to 0.0)	0 (0 to 0)	0.0 (0.0 to 0.0)	-76.6 (-87.8 to -46.2)	-48.6 (-73.2 to 18.0)
Bosnia and Herzegovina	Vascular intestinal disorders	10 to 14	Female	0 (0 to 0)	0.0 (0.0 to 0.0)	0 (0 to 0)	0.0 (0.0 to 0.0)	0 (0 to 0)	0.0 (0.0 to 0.0)	-80.0 (-93.8 to -28.4)	-56.2 (-86.5 to 57.0)
Bosnia and Herzegovina	Vascular intestinal disorders	10 to 14	Male	0 (0 to 0)	0.0 (0.0 to 0.0)	0 (0 to 0)	0.0 (0.0 to 0.0)	0 (0 to 0)	0.0 (0.0 to 0.0)	-73.2 (-87.4 to -16.7)	-41.4 (-72.5 to 82.4)

eTable 8. All-cause and cause-specific mortality (counts and rates) at the global level and by country in the WHO European Region for ages 5 to 9 and 10 to 14 for 1990, 2000, and 2016

Bosnia and Herzegovina	Gallbladder and biliary diseases	5 to 9	Both	0 (0 to 0)	0.0 (0.0 to 0.0)	0 (0 to 0)	0.0 (0.0 to 0.0)	0 (0 to 0)	0.0 (0.0 to 0.0)	-66.9 (-88.9 to -4.1)	-28.9 (-76.1 to 105.7)
Bosnia and Herzegovina	Gallbladder and biliary diseases	5 to 9	Female	0 (0 to 0)	0.0 (0.0 to 0.0)	0 (0 to 0)	0.0 (0.0 to 0.0)	0 (0 to 0)	0.0 (0.0 to 0.0)	-85.7 (-95.6 to -57.2)	-69.2 (-90.5 to -7.9)
Bosnia and Herzegovina	Gallbladder and biliary diseases	5 to 9	Male	0 (0 to 0)	0.0 (0.0 to 0.0)	0 (0 to 0)	0.0 (0.0 to 0.0)	0 (0 to 0)	0.0 (0.0 to 0.0)	-45.9 (-89.6 to 112.8)	15.7 (-77.9 to 354.9)
Bosnia and Herzegovina	Gallbladder and biliary diseases	10 to 14	Both	0 (0 to 0)	0.0 (0.0 to 0.0)	0 (0 to 0)	0.0 (0.0 to 0.0)	0 (0 to 0)	0.0 (0.0 to 0.0)	-77.8 (-88.5 to -53.3)	-51.4 (-74.9 to 2.5)
Bosnia and Herzegovina	Gallbladder and biliary diseases	10 to 14	Female	0 (0 to 0)	0.0 (0.0 to 0.0)	0 (0 to 0)	0.0 (0.0 to 0.0)	0 (0 to 0)	0.0 (0.0 to 0.0)	-87.5 (-95.5 to -70.6)	-72.6 (-90.1 to -35.4)
Bosnia and Herzegovina	Gallbladder and biliary diseases	10 to 14	Male	0 (0 to 0)	0.0 (0.0 to 0.0)	0 (0 to 0)	0.0 (0.0 to 0.0)	0 (0 to 0)	0.0 (0.0 to 0.0)	-70.7 (-87.4 to -21.4)	-35.8 (-72.4 to 72.3)
Bosnia and Herzegovina	Pancreatitis	5 to 9	Both	0 (0 to 0)	0.0 (0.0 to 0.0)	0 (0 to 0)	0.0 (0.0 to 0.0)	0 (0 to 0)	0.0 (0.0 to 0.0)	-81.6 (-90.2 to -44.6)	-60.4 (-79.0 to 18.9)
Bosnia and Herzegovina	Pancreatitis	5 to 9	Female	0 (0 to 0)	0.0 (0.0 to 0.0)	0 (0 to 0)	0.0 (0.0 to 0.0)	0 (0 to 0)	0.0 (0.0 to 0.0)	-84.0 (-96.0 to -48.9)	-65.5 (-91.3 to 10.1)
Bosnia and Herzegovina	Pancreatitis	5 to 9	Male	0 (0 to 0)	0.0 (0.0 to 0.0)	0 (0 to 0)	0.0 (0.0 to 0.0)	0 (0 to 0)	0.0 (0.0 to 0.0)	-77.5 (-90.6 to -15.1)	-51.9 (-80.0 to 81.6)
Bosnia and Herzegovina	Pancreatitis	10 to 14	Both	0 (0 to 0)	0.0 (0.0 to 0.0)	0 (0 to 0)	0.0 (0.0 to 0.0)	0 (0 to 0)	0.0 (0.0 to 0.0)	-79.4 (-89.6 to -52.4)	-54.8 (-77.2 to 4.3)
Bosnia and Herzegovina	Pancreatitis	10 to 14	Female	0 (0 to 0)	0.0 (0.0 to 0.0)	0 (0 to 0)	0.0 (0.0 to 0.0)	0 (0 to 0)	0.0 (0.0 to 0.0)	-81.0 (-94.0 to -49.6)	-58.4 (-86.8 to 10.7)
Bosnia and Herzegovina	Pancreatitis	10 to 14	Male	0 (0 to 0)	0.0 (0.0 to 0.0)	0 (0 to 0)	0.0 (0.0 to 0.0)	0 (0 to 0)	0.0 (0.0 to 0.0)	-76.7 (-89.9 to -24.3)	-49.0 (-77.8 to 65.9)
Bosnia and Herzegovina	Other digestive diseases	5 to 9	Both	0 (0 to 0)	0.0 (0.0 to 0.1)	0 (0 to 0)	0.0 (0.0 to 0.0)	0 (0 to 0)	0.0 (0.0 to 0.0)	-79.5 (-90.0 to -54.1)	-56.0 (-78.5 to -1.6)
Bosnia and Herzegovina	Other digestive diseases	5 to 9	Female	0 (0 to 0)	0.0 (0.0 to 0.0)	0 (0 to 0)	0.0 (0.0 to 0.0)	0 (0 to 0)	0.0 (0.0 to 0.0)	-78.9 (-91.8 to -48.9)	-54.6 (-82.4 to 10.0)
Bosnia and Herzegovina	Other digestive diseases	5 to 9	Male	0 (0 to 0)	0.0 (0.0 to 0.1)	0 (0 to 0)	0.0 (0.0 to 0.1)	0 (0 to 0)	0.0 (0.0 to 0.0)	-79.8 (-92.1 to -35.6)	-56.8 (-83.1 to 37.8)
Bosnia and Herzegovina	Other digestive diseases	10 to 14	Both	0 (0 to 0)	0.0 (0.0 to 0.1)	0 (0 to 0)	0.0 (0.0 to 0.0)	0 (0 to 0)	0.0 (0.0 to 0.0)	-77.9 (-87.8 to -55.8)	-51.6 (-73.4 to -3.0)
Bosnia and Herzegovina	Other digestive diseases	10 to 14	Female	0 (0 to 0)	0.0 (0.0 to 0.1)	0 (0 to 0)	0.0 (0.0 to 0.0)	0 (0 to 0)	0.0 (0.0 to 0.0)	-75.7 (-90.0 to -39.9)	-46.7 (-78.2 to 31.8)
Bosnia and Herzegovina	Other digestive diseases	10 to 14	Male	0 (0 to 0)	0.0 (0.0 to 0.1)	0 (0 to 0)	0.0 (0.0 to 0.1)	0 (0 to 0)	0.0 (0.0 to 0.0)	-79.8 (-91.1 to -46.2)	-55.7 (-80.4 to 17.8)
Bosnia and Herzegovina	Alzheimer disease and other dementias	5 to 9	Both	0 (0 to 0)	0.0 (0.0 to 0.0)	0 (0 to 0)	0.0 (0.0 to 0.0)	0 (0 to 0)	0.0 (0.0 to 0.0)	0.0 (0.0 to 0.0)	0.0 (0.0 to 0.0)
Bosnia and Herzegovina	Alzheimer disease and other dementias	5 to 9	Female	0 (0 to 0)	0.0 (0.0 to 0.0)	0 (0 to 0)	0.0 (0.0 to 0.0)	0 (0 to 0)	0.0 (0.0 to 0.0)	0.0 (0.0 to 0.0)	0.0 (0.0 to 0.0)
Bosnia and Herzegovina	Alzheimer disease and other dementias	5 to 9	Male	0 (0 to 0)	0.0 (0.0 to 0.0)	0 (0 to 0)	0.0 (0.0 to 0.0)	0 (0 to 0)	0.0 (0.0 to 0.0)	0.0 (0.0 to 0.0)	0.0 (0.0 to 0.0)
Bosnia and Herzegovina	Alzheimer disease and other dementias	10 to 14	Both	0 (0 to 0)	0.0 (0.0 to 0.0)	0 (0 to 0)	0.0 (0.0 to 0.0)	0 (0 to 0)	0.0 (0.0 to 0.0)	0.0 (0.0 to 0.0)	0.0 (0.0 to 0.0)

eTable 8. All-cause and cause-specific mortality (counts and rates) at the global level and by country in the WHO European Region for ages 5 to 9 and 10 to 14 for 1990, 2000, and 2016

Bosnia and Herzegovina	Alzheimer disease and other dementias	10 to 14	Female	0<br>(0 to 0)	0.0<br>(0.0 to 0.0)	0<br>(0 to 0)	0.0<br>(0.0 to 0.0)	0<br>(0 to 0)	0.0<br>(0.0 to 0.0)	0.0<br>(0.0 to 0.0)	0.0<br>(0.0 to 0.0)
Bosnia and Herzegovina	Alzheimer disease and other dementias	10 to 14	Male	0<br>(0 to 0)	0.0<br>(0.0 to 0.0)	0<br>(0 to 0)	0.0<br>(0.0 to 0.0)	0<br>(0 to 0)	0.0<br>(0.0 to 0.0)	0.0<br>(0.0 to 0.0)	0.0<br>(0.0 to 0.0)
Bosnia and Herzegovina	Parkinson disease	5 to 9	Both	0<br>(0 to 0)	0.0<br>(0.0 to 0.0)	0<br>(0 to 0)	0.0<br>(0.0 to 0.0)	0<br>(0 to 0)	0.0<br>(0.0 to 0.0)	0.0<br>(0.0 to 0.0)	0.0<br>(0.0 to 0.0)
Bosnia and Herzegovina	Parkinson disease	5 to 9	Female	0<br>(0 to 0)	0.0<br>(0.0 to 0.0)	0<br>(0 to 0)	0.0<br>(0.0 to 0.0)	0<br>(0 to 0)	0.0<br>(0.0 to 0.0)	0.0<br>(0.0 to 0.0)	0.0<br>(0.0 to 0.0)
Bosnia and Herzegovina	Parkinson disease	5 to 9	Male	0<br>(0 to 0)	0.0<br>(0.0 to 0.0)	0<br>(0 to 0)	0.0<br>(0.0 to 0.0)	0<br>(0 to 0)	0.0<br>(0.0 to 0.0)	0.0<br>(0.0 to 0.0)	0.0<br>(0.0 to 0.0)
Bosnia and Herzegovina	Parkinson disease	10 to 14	Both	0<br>(0 to 0)	0.0<br>(0.0 to 0.0)	0<br>(0 to 0)	0.0<br>(0.0 to 0.0)	0<br>(0 to 0)	0.0<br>(0.0 to 0.0)	0.0<br>(0.0 to 0.0)	0.0<br>(0.0 to 0.0)
Bosnia and Herzegovina	Parkinson disease	10 to 14	Female	0<br>(0 to 0)	0.0<br>(0.0 to 0.0)	0<br>(0 to 0)	0.0<br>(0.0 to 0.0)	0<br>(0 to 0)	0.0<br>(0.0 to 0.0)	0.0<br>(0.0 to 0.0)	0.0<br>(0.0 to 0.0)
Bosnia and Herzegovina	Parkinson disease	10 to 14	Male	0<br>(0 to 0)	0.0<br>(0.0 to 0.0)	0<br>(0 to 0)	0.0<br>(0.0 to 0.0)	0<br>(0 to 0)	0.0<br>(0.0 to 0.0)	0.0<br>(0.0 to 0.0)	0.0<br>(0.0 to 0.0)
Bosnia and Herzegovina	Epilepsy	5 to 9	Both	7<br>(5 to 10)	1.9<br>(1.4 to 2.5)	3<br>(2 to 5)	1.5<br>(1.0 to 2.1)	1<br>(1 to 2)	0.7<br>(0.5 to 1.0)	-82.8<br>(-88.8 to -73.0)	-63.2<br>(-75.9 to -42.1)
Bosnia and Herzegovina	Epilepsy	5 to 9	Female	3<br>(2 to 5)	1.8<br>(1.0 to 2.9)	2<br>(1 to 3)	1.6<br>(0.8 to 2.5)	1<br>(0 to 1)	0.7<br>(0.4 to 1.2)	-81.1<br>(-89.9 to -64.6)	-59.3<br>(-78.3 to -23.9)
Bosnia and Herzegovina	Epilepsy	5 to 9	Male	4<br>(3 to 6)	1.9<br>(1.3 to 2.8)	2<br>(1 to 3)	1.5<br>(1.0 to 2.2)	1<br>(0 to 1)	0.7<br>(0.4 to 1.0)	-84.4<br>(-91.1 to -72.5)	-66.6<br>(-81.0 to -41.2)
Bosnia and Herzegovina	Epilepsy	10 to 14	Both	6<br>(5 to 8)	1.7<br>(1.3 to 2.3)	4<br>(3 to 6)	1.4<br>(1.1 to 2.0)	1<br>(1 to 2)	0.8<br>(0.6 to 1.1)	-78.4<br>(-85.9 to -68.4)	-52.7<br>(-69.0 to -30.6)
Bosnia and Herzegovina	Epilepsy	10 to 14	Female	3<br>(2 to 4)	1.6<br>(1.0 to 2.4)	2<br>(1 to 3)	1.3<br>(0.8 to 2.1)	1<br>(0 to 1)	0.8<br>(0.5 to 1.2)	-77.7<br>(-88.1 to -60.0)	-51.1<br>(-73.8 to -12.3)
Bosnia and Herzegovina	Epilepsy	10 to 14	Male	3<br>(2 to 5)	1.8<br>(1.2 to 2.7)	2<br>(1 to 3)	1.6<br>(1.0 to 2.3)	1<br>(0 to 1)	0.8<br>(0.5 to 1.3)	-79.0<br>(-88.4 to -62.8)	-54.0<br>(-74.6 to -18.5)
Bosnia and Herzegovina	Motor neuron disease	5 to 9	Both	0<br>(0 to 0)	0.0<br>(0.0 to 0.0)	0<br>(0 to 0)	0.0<br>(0.0 to 0.0)	0<br>(0 to 0)	0.0<br>(0.0 to 0.0)	-52.2<br>(-82.7 to 28.5)	2.5<br>(-62.9 to 175.6)
Bosnia and Herzegovina	Motor neuron disease	5 to 9	Female	0<br>(0 to 0)	0.0<br>(0.0 to 0.0)	0<br>(0 to 0)	0.0<br>(0.0 to 0.1)	0<br>(0 to 0)	0.0<br>(0.0 to 0.0)	-52.3<br>(-82.9 to 29.0)	2.7<br>(-63.2 to 177.7)
Bosnia and Herzegovina	Motor neuron disease	5 to 9	Male	0<br>(0 to 0)	0.0<br>(0.0 to 0.0)	0<br>(0 to 0)	0.0<br>(0.0 to 0.0)	0<br>(0 to 0)	0.0<br>(0.0 to 0.0)	-43.3<br>(-85.5 to 142.9)	21.3<br>(-69.1 to 419.4)
Bosnia and Herzegovina	Motor neuron disease	10 to 14	Both	0<br>(0 to 0)	0.0<br>(0.0 to 0.0)	0<br>(0 to 0)	0.0<br>(0.0 to 0.0)	0<br>(0 to 0)	0.0<br>(0.0 to 0.0)	-50.4<br>(-76.7 to 6.6)	8.7<br>(-48.9 to 133.6)
Bosnia and Herzegovina	Motor neuron disease	10 to 14	Female	0<br>(0 to 0)	0.0<br>(0.0 to 0.1)	0<br>(0 to 0)	0.0<br>(0.0 to 0.1)	0<br>(0 to 0)	0.0<br>(0.0 to 0.1)	-50.4<br>(-77.2 to 11.9)	8.8<br>(-50.1 to 145.4)
Bosnia and Herzegovina	Motor neuron disease	10 to 14	Male	0<br>(0 to 0)	0.0<br>(0.0 to 0.0)	0<br>(0 to 0)	0.0<br>(0.0 to 0.0)	0<br>(0 to 0)	0.0<br>(0.0 to 0.0)	-50.5<br>(-79.4 to 26.2)	8.5<br>(-54.9 to 176.4)
Bosnia and Herzegovina	Other neurological disorders	5 to 9	Both	0<br>(0 to 0)	0.1<br>(0.0 to 0.1)	0<br>(0 to 0)	0.1<br>(0.0 to 0.1)	0<br>(0 to 0)	0.1<br>(0.0 to 0.1)	-60.7<br>(-79.9 to -20.9)	-15.8<br>(-56.8 to 69.6)
Bosnia and Herzegovina	Other neurological disorders	5 to 9	Female	0<br>(0 to 0)	0.0<br>(0.0 to 0.1)	0<br>(0 to 0)	0.1<br>(0.0 to 0.1)	0<br>(0 to 0)	0.0<br>(0.0 to 0.1)	-60.9<br>(-81.0 to -15.3)	-15.9<br>(-59.2 to 82.4)
Bosnia and Herzegovina	Other neurological disorders	5 to 9	Male	0<br>(0 to 0)	0.1<br>(0.0 to 0.1)	0<br>(0 to 0)	0.1<br>(0.0 to 0.1)	0<br>(0 to 0)	0.1<br>(0.0 to 0.1)	-60.7<br>(-83.9 to 3.4)	-15.9<br>(-65.6 to 121.1)
Bosnia and Herzegovina	Other neurological disorders	10 to 14	Both	0<br>(0 to 1)	0.1<br>(0.1 to 0.2)	0<br>(0 to 1)	0.1<br>(0.1 to 0.2)	0<br>(0 to 0)	0.1<br>(0.1 to 0.2)	-50.8<br>(-73.7 to -7.9)	7.9<br>(-42.4 to 101.9)
Bosnia and Herzegovina	Other neurological disorders	10 to 14	Female	0<br>(0 to 0)	0.1<br>(0.0 to 0.1)	0<br>(0 to 0)	0.1<br>(0.0 to 0.1)	0<br>(0 to 0)	0.0<br>(0.0 to 0.1)	-58.9<br>(-81.0 to -10.0)	-9.7<br>(-58.4 to 97.3)
Bosnia and Herzegovina	Other neurological disorders	10 to 14	Male	0<br>(0 to 1)	0.2<br>(0.1 to 0.3)	0<br>(0 to 1)	0.2<br>(0.1 to 0.3)	0<br>(0 to 0)	0.2<br>(0.1 to 0.3)	-48.6<br>(-75.3 to 6.1)	12.6<br>(-45.9 to 132.5)
Bosnia and Herzegovina	Drug use disorders	5 to 9	Both	0<br>(0 to 0)	0.0<br>(0.0 to 0.0)	0<br>(0 to 0)	0.0<br>(0.0 to 0.0)	0<br>(0 to 0)	0.0<br>(0.0 to 0.0)	0.0<br>(0.0 to 0.0)	0.0<br>(0.0 to 0.0)

eTable 8. All-cause and cause-specific mortality (counts and rates) at the global level and by country in the WHO European Region for ages 5 to 9 and 10 to 14 for 1990, 2000, and 2016

Bosnia and Herzegovina	Drug use disorders	5 to 9	Female	0 (0 to 0)	0.0 (0.0 to 0.0)	0 (0 to 0)	0.0 (0.0 to 0.0)	0 (0 to 0)	0.0 (0.0 to 0.0)	0.0 (0.0 to 0.0)	0.0 (0.0 to 0.0)
Bosnia and Herzegovina	Drug use disorders	5 to 9	Male	0 (0 to 0)	0.0 (0.0 to 0.0)	0 (0 to 0)	0.0 (0.0 to 0.0)	0 (0 to 0)	0.0 (0.0 to 0.0)	0.0 (0.0 to 0.0)	0.0 (0.0 to 0.0)
Bosnia and Herzegovina	Drug use disorders	10 to 14	Both	0 (0 to 0)	0.0 (0.0 to 0.0)	0 (0 to 0)	0.0 (0.0 to 0.0)	0 (0 to 0)	0.0 (0.0 to 0.0)	0.0 (0.0 to 0.0)	0.0 (0.0 to 0.0)
Bosnia and Herzegovina	Drug use disorders	10 to 14	Female	0 (0 to 0)	0.0 (0.0 to 0.0)	0 (0 to 0)	0.0 (0.0 to 0.0)	0 (0 to 0)	0.0 (0.0 to 0.0)	0.0 (0.0 to 0.0)	0.0 (0.0 to 0.0)
Bosnia and Herzegovina	Drug use disorders	10 to 14	Male	0 (0 to 0)	0.0 (0.0 to 0.0)	0 (0 to 0)	0.0 (0.0 to 0.0)	0 (0 to 0)	0.0 (0.0 to 0.0)	0.0 (0.0 to 0.0)	0.0 (0.0 to 0.0)
Bosnia and Herzegovina	Eating disorders	5 to 9	Both	0 (0 to 0)	0.0 (0.0 to 0.0)	0 (0 to 0)	0.0 (0.0 to 0.0)	0 (0 to 0)	0.0 (0.0 to 0.0)	-4.2 (-62.7 to 149.8)	105.5 (-20.1 to 435.9)
Bosnia and Herzegovina	Eating disorders	5 to 9	Female	0 (0 to 0)	0.0 (0.0 to 0.0)	0 (0 to 0)	0.0 (0.0 to 0.0)	0 (0 to 0)	0.0 (0.0 to 0.0)	-55.9 (-83.1 to 11.5)	-5.0 (-63.6 to 140.0)
Bosnia and Herzegovina	Eating disorders	5 to 9	Male	0 (0 to 0)	0.0 (0.0 to 0.0)	0 (0 to 0)	0.0 (0.0 to 0.0)	0 (0 to 0)	0.0 (0.0 to 0.0)	24.5 (-64.3 to 306.0)	166.2 (-23.6 to 768.0)
Bosnia and Herzegovina	Eating disorders	10 to 14	Both	0 (0 to 0)	0.0 (0.0 to 0.0)	0 (0 to 0)	0.0 (0.0 to 0.0)	0 (0 to 0)	0.0 (0.0 to 0.0)	-52.5 (-78.6 to 15.5)	4.1 (-53.1 to 153.1)
Bosnia and Herzegovina	Eating disorders	10 to 14	Female	0 (0 to 0)	0.0 (0.0 to 0.0)	0 (0 to 0)	0.0 (0.0 to 0.0)	0 (0 to 0)	0.0 (0.0 to 0.0)	-58.7 (-86.1 to 19.3)	-9.4 (-69.4 to 161.6)
Bosnia and Herzegovina	Eating disorders	10 to 14	Male	0 (0 to 0)	0.0 (0.0 to 0.0)	0 (0 to 0)	0.0 (0.0 to 0.0)	0 (0 to 0)	0.0 (0.0 to 0.0)	-47.1 (-84.2 to 109.2)	15.8 (-65.3 to 358.3)
Bosnia and Herzegovina	Diabetes mellitus	5 to 9	Both	0 (0 to 0)	0.0 (0.0 to 0.1)	0 (0 to 0)	0.0 (0.0 to 0.1)	0 (0 to 0)	0.0 (0.0 to 0.1)	-53.2 (-70.3 to -27.6)	0.4 (-36.4 to 55.2)
Bosnia and Herzegovina	Diabetes mellitus	5 to 9	Female	0 (0 to 0)	0.1 (0.0 to 0.1)	0 (0 to 0)	0.1 (0.0 to 0.1)	0 (0 to 0)	0.1 (0.0 to 0.1)	-55.3 (-73.1 to -24.5)	-3.7 (-42.0 to 62.6)
Bosnia and Herzegovina	Diabetes mellitus	5 to 9	Male	0 (0 to 0)	0.0 (0.0 to 0.0)	0 (0 to 0)	0.0 (0.0 to 0.0)	0 (0 to 0)	0.0 (0.0 to 0.0)	-48.1 (-76.0 to 0.2)	10.9 (-48.7 to 114.3)
Bosnia and Herzegovina	Diabetes mellitus	10 to 14	Both	0 (0 to 1)	0.1 (0.1 to 0.2)	0 (0 to 0)	0.1 (0.1 to 0.1)	0 (0 to 0)	0.1 (0.1 to 0.2)	-39.5 (-63.5 to 0.7)	32.7 (-20.0 to 120.7)
Bosnia and Herzegovina	Diabetes mellitus	10 to 14	Female	0 (0 to 0)	0.2 (0.1 to 0.2)	0 (0 to 0)	0.1 (0.1 to 0.2)	0 (0 to 0)	0.2 (0.1 to 0.3)	-45.1 (-70.9 to 3.6)	20.4 (-36.2 to 127.2)
Bosnia and Herzegovina	Diabetes mellitus	10 to 14	Male	0 (0 to 0)	0.1 (0.0 to 0.1)	0 (0 to 0)	0.1 (0.0 to 0.1)	0 (0 to 0)	0.1 (0.1 to 0.2)	-28.5 (-64.2 to 31.0)	56.7 (-21.5 to 187.0)
Bosnia and Herzegovina	Acute glomerulonephritis	5 to 9	Both	0 (0 to 0)	0.0 (0.0 to 0.0)	0 (0 to 0)	0.0 (0.0 to 0.0)	0 (0 to 0)	0.0 (0.0 to 0.0)	-58.9 (-67.1 to -48.6)	-11.9 (-29.5 to 10.2)
Bosnia and Herzegovina	Acute glomerulonephritis	5 to 9	Female	0 (0 to 0)	0.0 (0.0 to 0.0)	0 (0 to 0)	0.0 (0.0 to 0.0)	0 (0 to 0)	0.0 (0.0 to 0.0)	-57.5 (-66.8 to -43.8)	-8.5 (-28.6 to 21.0)
Bosnia and Herzegovina	Acute glomerulonephritis	5 to 9	Male	0 (0 to 0)	0.0 (0.0 to 0.0)	0 (0 to 0)	0.0 (0.0 to 0.0)	0 (0 to 0)	0.0 (0.0 to 0.0)	-60.3 (-69.6 to -48.9)	-15.2 (-35.0 to 9.2)
Bosnia and Herzegovina	Acute glomerulonephritis	10 to 14	Both	0 (0 to 0)	0.0 (0.0 to 0.0)	0 (0 to 0)	0.0 (0.0 to 0.0)	0 (0 to 0)	0.0 (0.0 to 0.0)	-55.9 (-64.6 to -46.6)	-3.4 (-22.3 to 17.1)
Bosnia and Herzegovina	Acute glomerulonephritis	10 to 14	Female	0 (0 to 0)	0.0 (0.0 to 0.0)	0 (0 to 0)	0.0 (0.0 to 0.0)	0 (0 to 0)	0.0 (0.0 to 0.0)	-58.2 (-66.5 to -48.2)	-8.4 (-26.5 to 13.7)
Bosnia and Herzegovina	Acute glomerulonephritis	10 to 14	Male	0 (0 to 0)	0.0 (0.0 to 0.0)	0 (0 to 0)	0.0 (0.0 to 0.0)	0 (0 to 0)	0.0 (0.0 to 0.0)	-53.5 (-66.0 to -39.6)	1.9 (-25.4 to 32.4)
Bosnia and Herzegovina	Chronic kidney disease	5 to 9	Both	1 (1 to 1)	0.2 (0.1 to 0.3)	0 (0 to 0)	0.2 (0.1 to 0.2)	0 (0 to 0)	0.1 (0.1 to 0.1)	-78.9 (-85.7 to -68.7)	-54.8 (-69.3 to -32.8)
Bosnia and Herzegovina	Chronic kidney disease	5 to 9	Female	0 (0 to 0)	0.2 (0.1 to 0.2)	0 (0 to 0)	0.2 (0.1 to 0.2)	0 (0 to 0)	0.1 (0.1 to 0.1)	-76.7 (-85.9 to -60.0)	-49.8 (-69.7 to -13.9)
Bosnia and Herzegovina	Chronic kidney disease	5 to 9	Male	0 (0 to 1)	0.2 (0.2 to 0.3)	0 (0 to 0)	0.2 (0.1 to 0.3)	0 (0 to 0)	0.1 (0.1 to 0.1)	-80.5 (-88.2 to -67.6)	-58.3 (-74.7 to -30.8)
Bosnia and Herzegovina	Chronic kidney disease	10 to 14	Both	1 (1 to 2)	0.4 (0.3 to 0.5)	1 (1 to 1)	0.3 (0.2 to 0.4)	0 (0 to 0)	0.2 (0.2 to 0.3)	-72.2 (-81.0 to -59.0)	-39.1 (-58.4 to -10.1)
Bosnia and Herzegovina	Chronic kidney disease	10 to 14	Female	1 (1 to 1)	0.5 (0.3 to 0.7)	1 (0 to 1)	0.4 (0.3 to 0.6)	0 (0 to 0)	0.3 (0.2 to 0.4)	-73.2 (-83.7 to -55.6)	-41.2 (-64.2 to -2.7)
Bosnia and Herzegovina	Chronic kidney disease	10 to 14	Male	0 (0 to 1)	0.2 (0.2 to 0.4)	0 (0 to 1)	0.2 (0.1 to 0.3)	0 (0 to 0)	0.1 (0.1 to 0.2)	-70.3 (-81.3 to -48.8)	-34.8 (-59.1 to 12.3)

eTable 8. All-cause and cause-specific mortality (counts and rates) at the global level and by country in the WHO European Region for ages 5 to 9 and 10 to 14 for 1990, 2000, and 2016

Bosnia and Herzegovina	Urinary diseases and male infertility	5 to 9	Both	0 (0 to 0)	0.0 (0.0 to 0.0)	0 (0 to 0)	0.0 (0.0 to 0.0)	0 (0 to 0)	0.0 (0.0 to 0.0)	-78.5 (-84.9 to -69.0)	-53.9 (-67.7 to -33.5)
Bosnia and Herzegovina	Urinary diseases and male infertility	5 to 9	Female	0 (0 to 0)	0.0 (0.0 to 0.0)	0 (0 to 0)	0.0 (0.0 to 0.0)	0 (0 to 0)	0.0 (0.0 to 0.0)	-82.8 (-88.5 to -72.3)	-63.1 (-75.3 to -40.4)
Bosnia and Herzegovina	Urinary diseases and male infertility	5 to 9	Male	0 (0 to 0)	0.0 (0.0 to 0.0)	0 (0 to 0)	0.0 (0.0 to 0.0)	0 (0 to 0)	0.0 (0.0 to 0.0)	-71.2 (-83.1 to -53.2)	-38.5 (-63.9 to 0.1)
Bosnia and Herzegovina	Urinary diseases and male infertility	10 to 14	Both	0 (0 to 0)	0.0 (0.0 to 0.1)	0 (0 to 0)	0.0 (0.0 to 0.0)	0 (0 to 0)	0.0 (0.0 to 0.0)	-88.5 (-92.3 to -82.1)	-74.7 (-83.1 to -60.8)
Bosnia and Herzegovina	Urinary diseases and male infertility	10 to 14	Female	0 (0 to 0)	0.1 (0.1 to 0.1)	0 (0 to 0)	0.0 (0.0 to 0.1)	0 (0 to 0)	0.0 (0.0 to 0.0)	-93.0 (-95.7 to -87.7)	-84.6 (-90.6 to -73.1)
Bosnia and Herzegovina	Urinary diseases and male infertility	10 to 14	Male	0 (0 to 0)	0.0 (0.0 to 0.0)	0 (0 to 0)	0.0 (0.0 to 0.0)	0 (0 to 0)	0.0 (0.0 to 0.0)	-58.8 (-73.1 to -42.6)	-9.7 (-41.0 to 25.8)
Bosnia and Herzegovina	Hemoglobinopathies and hemolytic anemias	5 to 9	Both	0 (0 to 1)	0.1 (0.1 to 0.2)	0 (0 to 0)	0.1 (0.1 to 0.1)	0 (0 to 0)	0.0 (0.0 to 0.1)	-83.3 (-89.7 to -69.3)	-64.1 (-77.9 to -34.2)
Bosnia and Herzegovina	Hemoglobinopathies and hemolytic anemias	5 to 9	Female	0 (0 to 1)	0.2 (0.1 to 0.3)	0 (0 to 0)	0.1 (0.1 to 0.2)	0 (0 to 0)	0.1 (0.0 to 0.1)	-82.1 (-90.1 to -59.9)	-61.5 (-78.7 to -13.6)
Bosnia and Herzegovina	Hemoglobinopathies and hemolytic anemias	5 to 9	Male	0 (0 to 0)	0.1 (0.0 to 0.1)	0 (0 to 0)	0.1 (0.0 to 0.1)	0 (0 to 0)	0.0 (0.0 to 0.0)	-85.8 (-91.6 to -72.9)	-69.7 (-82.1 to -42.0)
Bosnia and Herzegovina	Hemoglobinopathies and hemolytic anemias	10 to 14	Both	1 (0 to 1)	0.2 (0.1 to 0.3)	0 (0 to 1)	0.1 (0.1 to 0.2)	0 (0 to 0)	0.1 (0.1 to 0.1)	-80.1 (-85.5 to -67.8)	-56.5 (-68.3 to -29.4)
Bosnia and Herzegovina	Hemoglobinopathies and hemolytic anemias	10 to 14	Female	0 (0 to 1)	0.2 (0.1 to 0.4)	0 (0 to 0)	0.1 (0.1 to 0.2)	0 (0 to 0)	0.1 (0.1 to 0.1)	-80.3 (-88.5 to -61.1)	-56.8 (-74.7 to -14.7)
Bosnia and Herzegovina	Hemoglobinopathies and hemolytic anemias	10 to 14	Male	0 (0 to 1)	0.2 (0.1 to 0.3)	0 (0 to 0)	0.1 (0.1 to 0.2)	0 (0 to 0)	0.1 (0.0 to 0.1)	-79.9 (-87.9 to -62.3)	-56.0 (-73.5 to -17.4)
Bosnia and Herzegovina	Endocrine, metabolic, blood, and immune disorders	5 to 9	Both	1 (1 to 2)	0.2 (0.1 to 0.5)	1 (0 to 1)	0.2 (0.1 to 0.5)	0 (0 to 1)	0.1 (0.1 to 0.3)	-71.4 (-82.9 to -56.5)	-38.6 (-63.4 to -6.8)
Bosnia and Herzegovina	Endocrine, metabolic, blood, and immune disorders	5 to 9	Female	0 (0 to 1)	0.2 (0.1 to 0.5)	0 (0 to 1)	0.2 (0.1 to 0.5)	0 (0 to 0)	0.1 (0.1 to 0.4)	-71.6 (-86.2 to -48.7)	-38.8 (-70.3 to 10.4)
Bosnia and Herzegovina	Endocrine, metabolic, blood, and immune disorders	5 to 9	Male	0 (0 to 1)	0.2 (0.1 to 0.5)	0 (0 to 1)	0.2 (0.1 to 0.5)	0 (0 to 0)	0.2 (0.1 to 0.4)	-71.3 (-85.1 to -47.6)	-38.6 (-68.1 to 12.0)
Bosnia and Herzegovina	Endocrine, metabolic, blood, and immune disorders	10 to 14	Both	1 (0 to 1)	0.2 (0.1 to 0.4)	1 (0 to 1)	0.2 (0.1 to 0.4)	0 (0 to 1)	0.2 (0.1 to 0.3)	-64.4 (-76.5 to -45.0)	-22.1 (-48.6 to 20.5)

eTable 8. All-cause and cause-specific mortality (counts and rates) at the global level and by country in the WHO European Region for ages 5 to 9 and 10 to 14 for 1990, 2000, and 2016

Bosnia and Herzegovina	Endocrine, metabolic, blood, and immune disorders	10 to 14	Female	0 (0 to 1)	0.2 (0.1 to 0.4)	0 (0 to 0)	0.2 (0.1 to 0.4)	0 (0 to 0)	0.1 (0.1 to 0.3)	-69.7 (-84.9 to -46.4)	-33.6 (-66.8 to 17.6)
Bosnia and Herzegovina	Endocrine, metabolic, blood, and immune disorders	10 to 14	Male	0 (0 to 1)	0.2 (0.1 to 0.5)	0 (0 to 1)	0.3 (0.1 to 0.5)	0 (0 to 0)	0.2 (0.1 to 0.4)	-60.6 (-77.6 to -29.4)	-13.6 (-50.9 to 54.6)
Bosnia and Herzegovina	Rheumatoid arthritis	5 to 9	Both	0 (0 to 0)	0.0 (0.0 to 0.0)	0 (0 to 0)	0.0 (0.0 to 0.0)	0 (0 to 0)	0.0 (0.0 to 0.0)	-67.4 (-80.3 to -41.3)	-30.0 (-57.7 to 25.9)
Bosnia and Herzegovina	Rheumatoid arthritis	5 to 9	Female	0 (0 to 0)	0.0 (0.0 to 0.0)	0 (0 to 0)	0.0 (0.0 to 0.0)	0 (0 to 0)	0.0 (0.0 to 0.0)	-69.4 (-83.3 to -30.0)	-34.2 (-64.0 to 50.8)
Bosnia and Herzegovina	Rheumatoid arthritis	5 to 9	Male	0 (0 to 0)	0.0 (0.0 to 0.0)	0 (0 to 0)	0.0 (0.0 to 0.0)	0 (0 to 0)	0.0 (0.0 to 0.0)	-65.5 (-81.6 to -30.7)	-26.2 (-60.6 to 48.1)
Bosnia and Herzegovina	Rheumatoid arthritis	10 to 14	Both	0 (0 to 0)	0.0 (0.0 to 0.0)	0 (0 to 0)	0.0 (0.0 to 0.0)	0 (0 to 0)	0.0 (0.0 to 0.0)	-69.1 (-81.5 to -30.9)	-32.2 (-59.4 to 51.6)
Bosnia and Herzegovina	Rheumatoid arthritis	10 to 14	Female	0 (0 to 0)	0.0 (0.0 to 0.0)	0 (0 to 0)	0.0 (0.0 to 0.0)	0 (0 to 0)	0.0 (0.0 to 0.0)	-74.9 (-88.6 to -23.3)	-44.9 (-75.1 to 68.2)
Bosnia and Herzegovina	Rheumatoid arthritis	10 to 14	Male	0 (0 to 0)	0.0 (0.0 to 0.0)	0 (0 to 0)	0.0 (0.0 to 0.0)	0 (0 to 0)	0.0 (0.0 to 0.0)	-60.6 (-80.5 to -20.4)	-13.7 (-57.2 to 74.5)
Bosnia and Herzegovina	Other musculoskeletal disorders	5 to 9	Both	0 (0 to 0)	0.0 (0.0 to 0.0)	0 (0 to 0)	0.0 (0.0 to 0.0)	0 (0 to 0)	0.0 (0.0 to 0.0)	-32.3 (-79.7 to 51.3)	45.2 (-56.4 to 224.5)
Bosnia and Herzegovina	Other musculoskeletal disorders	5 to 9	Female	0 (0 to 0)	0.0 (0.0 to 0.0)	0 (0 to 0)	0.0 (0.0 to 0.0)	0 (0 to 0)	0.0 (0.0 to 0.0)	1.7 (-75.7 to 165.2)	119.0 (-47.7 to 471.0)
Bosnia and Herzegovina	Other musculoskeletal disorders	5 to 9	Male	0 (0 to 0)	0.0 (0.0 to 0.0)	0 (0 to 0)	0.0 (0.0 to 0.0)	0 (0 to 0)	0.0 (0.0 to 0.0)	-60.4 (-84.1 to -7.9)	-15.4 (-66.1 to 97.0)
Bosnia and Herzegovina	Other musculoskeletal disorders	10 to 14	Both	0 (0 to 0)	0.0 (0.0 to 0.0)	0 (0 to 0)	0.0 (0.0 to 0.0)	0 (0 to 0)	0.0 (0.0 to 0.1)	-7.3 (-73.4 to 122.9)	103.1 (-41.6 to 388.6)
Bosnia and Herzegovina	Other musculoskeletal disorders	10 to 14	Female	0 (0 to 0)	0.0 (0.0 to 0.1)	0 (0 to 0)	0.0 (0.0 to 0.1)	0 (0 to 0)	0.1 (0.0 to 0.1)	18.4 (-71.6 to 232.3)	159.7 (-37.7 to 628.9)
Bosnia and Herzegovina	Other musculoskeletal disorders	10 to 14	Male	0 (0 to 0)	0.0 (0.0 to 0.0)	0 (0 to 0)	0.0 (0.0 to 0.0)	0 (0 to 0)	0.0 (0.0 to 0.0)	-51.4 (-77.4 to 15.2)	6.4 (-50.6 to 152.5)
Bosnia and Herzegovina	Congenital birth defects	5 to 9	Both	7 (5 to 10)	1.7 (1.2 to 2.5)	3 (3 to 5)	1.5 (1.1 to 2.1)	2 (1 to 2)	1.1 (0.8 to 1.4)	-71.1 (-82.3 to -54.2)	-37.9 (-62.0 to -1.7)
Bosnia and Herzegovina	Congenital birth defects	5 to 9	Female	3 (2 to 4)	1.6 (1.0 to 2.3)	2 (1 to 2)	1.6 (1.0 to 2.3)	1 (1 to 2)	1.2 (0.8 to 1.8)	-64.7 (-79.8 to -37.5)	-24.1 (-56.4 to 34.5)
Bosnia and Herzegovina	Congenital birth defects	5 to 9	Male	4 (2 to 6)	1.8 (1.1 to 3.2)	2 (1 to 3)	1.5 (1.0 to 2.3)	1 (1 to 1)	0.9 (0.6 to 1.4)	-76.2 (-86.2 to -58.2)	-49.1 (-70.5 to -10.7)
Bosnia and Herzegovina	Congenital birth defects	10 to 14	Both	5 (4 to 7)	1.4 (1.1 to 2.0)	4 (3 to 5)	1.3 (1.0 to 1.7)	2 (1 to 2)	1.1 (0.8 to 1.4)	-65.6 (-78.2 to -48.9)	-24.7 (-52.1 to 11.9)
Bosnia and Herzegovina	Congenital birth defects	10 to 14	Female	3 (2 to 4)	1.5 (1.0 to 2.1)	2 (1 to 3)	1.3 (0.9 to 1.9)	1 (1 to 1)	1.2 (0.8 to 1.7)	-63.7 (-77.8 to -40.5)	-20.4 (-51.3 to 30.5)
Bosnia and Herzegovina	Congenital birth defects	10 to 14	Male	3 (2 to 4)	1.4 (0.9 to 2.3)	2 (1 to 3)	1.3 (0.9 to 1.8)	1 (1 to 1)	1.0 (0.7 to 1.4)	-67.6 (-81.2 to -44.7)	-29.1 (-58.7 to 21.2)
Bosnia and Herzegovina	Skin and subcutaneous diseases	5 to 9	Both	0 (0 to 0)	0.0 (0.0 to 0.0)	0 (0 to 0)	0.0 (0.0 to 0.0)	0 (0 to 0)	0.0 (0.0 to 0.0)	-56.7 (-66.7 to -39.9)	-7.1 (-28.6 to 28.9)
Bosnia and Herzegovina	Skin and subcutaneous diseases	5 to 9	Female	0 (0 to 0)	0.0 (0.0 to 0.0)	0 (0 to 0)	0.0 (0.0 to 0.0)	0 (0 to 0)	0.0 (0.0 to 0.0)	-55.7 (-66.8 to -30.6)	-4.6 (-28.5 to 49.5)
Bosnia and Herzegovina	Skin and subcutaneous diseases	5 to 9	Male	0 (0 to 0)	0.0 (0.0 to 0.0)	0 (0 to 0)	0.0 (0.0 to 0.0)	0 (0 to 0)	0.0 (0.0 to 0.0)	-57.6 (-69.0 to -37.7)	-9.4 (-33.7 to 33.3)
Bosnia and Herzegovina	Skin and subcutaneous diseases	10 to 14	Both	0 (0 to 0)	0.0 (0.0 to 0.0)	0 (0 to 0)	0.0 (0.0 to 0.0)	0 (0 to 0)	0.0 (0.0 to 0.0)	-51.4 (-64.1 to -27.0)	6.5 (-21.3 to 60.0)

eTable 8. All-cause and cause-specific mortality (counts and rates) at the global level and by country in the WHO European Region for ages 5 to 9 and 10 to 14 for 1990, 2000, and 2016

Bosnia and Herzegovina	Skin and subcutaneous diseases	10 to 14	Female	0 (0 to 0)	0.0 (0.0 to 0.0)	0 (0 to 0)	0.0 (0.0 to 0.0)	0 (0 to 0)	0.0 (0.0 to 0.0)	-51.2 (-66.1 to -8.7)	7.1 (-25.6 to 100.2)
Bosnia and Herzegovina	Skin and subcutaneous diseases	10 to 14	Male	0 (0 to 0)	0.0 (0.0 to 0.0)	0 (0 to 0)	0.0 (0.0 to 0.0)	0 (0 to 0)	0.0 (0.0 to 0.0)	-51.7 (-65.3 to -34.1)	5.9 (-24.0 to 44.4)
Bosnia and Herzegovina	Road injuries	5 to 9	Both	9 (7 to 13)	2.4 (1.7 to 3.4)	4 (3 to 6)	1.9 (1.4 to 2.6)	2 (2 to 3)	1.2 (0.9 to 1.7)	-76.6 (-84.5 to -63.4)	-49.8 (-66.7 to -21.5)
Bosnia and Herzegovina	Road injuries	5 to 9	Female	3 (2 to 5)	1.7 (1.1 to 2.5)	2 (1 to 2)	1.5 (0.9 to 2.2)	1 (0 to 1)	0.7 (0.5 to 1.1)	-79.6 (-88.4 to -64.2)	-56.1 (-75.1 to -22.9)
Bosnia and Herzegovina	Road injuries	5 to 9	Male	6 (4 to 9)	3.2 (2.1 to 4.7)	3 (2 to 4)	2.4 (1.6 to 3.4)	2 (1 to 2)	1.7 (1.2 to 2.4)	-75.1 (-85.1 to -56.2)	-46.8 (-68.1 to -6.2)
Bosnia and Herzegovina	Road injuries	10 to 14	Both	7 (5 to 10)	1.9 (1.4 to 2.8)	4 (3 to 6)	1.6 (1.1 to 2.1)	2 (1 to 3)	1.2 (0.9 to 1.6)	-72.5 (-81.6 to -58.1)	-39.7 (-59.7 to -8.1)
Bosnia and Herzegovina	Road injuries	10 to 14	Female	2 (2 to 3)	1.3 (0.9 to 2.0)	1 (1 to 2)	1.0 (0.7 to 1.6)	1 (0 to 1)	0.7 (0.5 to 1.1)	-75.8 (-86.2 to -57.9)	-46.9 (-69.8 to -7.7)
Bosnia and Herzegovina	Road injuries	10 to 14	Male	5 (3 to 7)	2.5 (1.6 to 3.9)	3 (2 to 4)	2.1 (1.3 to 3.1)	1 (1 to 2)	1.6 (1.1 to 2.3)	-70.9 (-82.7 to -50.4)	-36.2 (-62.0 to 8.7)
Bosnia and Herzegovina	Other transport injuries	5 to 9	Both	1 (1 to 2)	0.3 (0.1 to 0.4)	0 (0 to 1)	0.2 (0.1 to 0.3)	0 (0 to 0)	0.1 (0.1 to 0.2)	-83.6 (-91.9 to -66.1)	-64.9 (-82.7 to -27.4)
Bosnia and Herzegovina	Other transport injuries	5 to 9	Female	0 (0 to 1)	0.2 (0.1 to 0.3)	0 (0 to 0)	0.1 (0.1 to 0.3)	0 (0 to 0)	0.0 (0.0 to 0.1)	-87.8 (-95.7 to -68.8)	-73.8 (-90.8 to -32.8)
Bosnia and Herzegovina	Other transport injuries	5 to 9	Male	1 (0 to 1)	0.3 (0.2 to 0.6)	0 (0 to 0)	0.2 (0.1 to 0.4)	0 (0 to 0)	0.1 (0.1 to 0.3)	-81.5 (-92.3 to -54.3)	-60.5 (-83.6 to -2.4)
Bosnia and Herzegovina	Other transport injuries	10 to 14	Both	1 (1 to 2)	0.3 (0.2 to 0.5)	1 (0 to 1)	0.2 (0.1 to 0.3)	0 (0 to 0)	0.1 (0.1 to 0.2)	-78.3 (-88.6 to -59.9)	-52.5 (-75.1 to -12.0)
Bosnia and Herzegovina	Other transport injuries	10 to 14	Female	1 (0 to 1)	0.3 (0.2 to 0.6)	0 (0 to 1)	0.2 (0.1 to 0.4)	0 (0 to 0)	0.1 (0.0 to 0.2)	-86.7 (-94.4 to -66.4)	-70.7 (-87.8 to -26.3)
Bosnia and Herzegovina	Other transport injuries	10 to 14	Male	1 (0 to 1)	0.3 (0.2 to 0.5)	0 (0 to 1)	0.2 (0.1 to 0.4)	0 (0 to 0)	0.2 (0.1 to 0.3)	-69.2 (-86.3 to -29.8)	-32.5 (-70.0 to 53.9)
Bosnia and Herzegovina	Falls	5 to 9	Both	1 (1 to 1)	0.2 (0.1 to 0.4)	0 (0 to 1)	0.2 (0.1 to 0.3)	0 (0 to 0)	0.1 (0.1 to 0.1)	-82.4 (-89.9 to -65.6)	-62.3 (-78.3 to -26.1)
Bosnia and Herzegovina	Falls	5 to 9	Female	0 (0 to 0)	0.1 (0.0 to 0.1)	0 (0 to 0)	0.1 (0.0 to 0.1)	0 (0 to 0)	0.0 (0.0 to 0.1)	-78.4 (-89.6 to -52.0)	-53.6 (-77.7 to 3.4)
Bosnia and Herzegovina	Falls	5 to 9	Male	1 (0 to 1)	0.4 (0.2 to 0.6)	0 (0 to 1)	0.3 (0.2 to 0.4)	0 (0 to 0)	0.1 (0.1 to 0.2)	-83.2 (-91.2 to -63.0)	-64.0 (-81.1 to -20.8)
Bosnia and Herzegovina	Falls	10 to 14	Both	1 (1 to 2)	0.3 (0.2 to 0.4)	1 (0 to 1)	0.2 (0.1 to 0.4)	0 (0 to 0)	0.1 (0.1 to 0.2)	-76.6 (-87.3 to -53.9)	-48.8 (-72.2 to 1.1)
Bosnia and Herzegovina	Falls	10 to 14	Female	0 (0 to 0)	0.1 (0.0 to 0.1)	0 (0 to 0)	0.0 (0.0 to 0.1)	0 (0 to 0)	0.0 (0.0 to 0.0)	-77.0 (-88.3 to -54.4)	-49.5 (-74.4 to 0.1)
Bosnia and Herzegovina	Falls	10 to 14	Male	1 (0 to 1)	0.5 (0.3 to 0.8)	1 (0 to 1)	0.4 (0.2 to 0.7)	0 (0 to 0)	0.2 (0.2 to 0.4)	-76.6 (-88.0 to -48.9)	-48.7 (-73.8 to 11.9)
Bosnia and Herzegovina	Drowning	5 to 9	Both	3 (2 to 7)	0.8 (0.5 to 1.7)	1 (1 to 2)	0.6 (0.4 to 1.1)	1 (0 to 1)	0.3 (0.2 to 0.6)	-80.1 (-88.2 to -62.2)	-57.4 (-74.7 to -18.8)
Bosnia and Herzegovina	Drowning	5 to 9	Female	0 (0 to 1)	0.3 (0.1 to 0.7)	0 (0 to 1)	0.2 (0.1 to 0.5)	0 (0 to 0)	0.1 (0.1 to 0.3)	-75.6 (-87.6 to -40.0)	-47.5 (-73.2 to 29.1)
Bosnia and Herzegovina	Drowning	5 to 9	Male	3 (1 to 5)	1.3 (0.8 to 2.8)	1 (1 to 2)	1.0 (0.6 to 1.8)	1 (0 to 1)	0.5 (0.3 to 0.9)	-81.0 (-90.0 to -61.7)	-59.3 (-78.7 to -18.1)
Bosnia and Herzegovina	Drowning	10 to 14	Both	3 (2 to 6)	0.7 (0.4 to 1.7)	2 (1 to 3)	0.6 (0.4 to 1.2)	1 (0 to 1)	0.4 (0.3 to 0.7)	-72.6 (-84.7 to -48.4)	-40.0 (-66.6 to 13.1)
Bosnia and Herzegovina	Drowning	10 to 14	Female	0 (0 to 1)	0.2 (0.1 to 0.7)	0 (0 to 1)	0.2 (0.1 to 0.5)	0 (0 to 0)	0.1 (0.1 to 0.3)	-75.6 (-87.3 to -41.1)	-46.5 (-72.1 to 29.3)
Bosnia and Herzegovina	Drowning	10 to 14	Male	2 (1 to 5)	1.2 (0.6 to 2.8)	2 (1 to 3)	1.1 (0.6 to 1.9)	1 (0 to 1)	0.7 (0.4 to 1.2)	-72.0 (-86.1 to -43.4)	-38.8 (-69.6 to 24.1)
Bosnia and Herzegovina	Fire, heat, and hot substances	5 to 9	Both	1 (1 to 2)	0.4 (0.2 to 0.6)	1 (0 to 1)	0.3 (0.2 to 0.4)	0 (0 to 0)	0.1 (0.1 to 0.2)	-84.6 (-91.0 to -72.8)	-66.9 (-80.7 to -41.6)
Bosnia and Herzegovina	Fire, heat, and hot substances	5 to 9	Female	1 (0 to 1)	0.3 (0.2 to 0.4)	0 (0 to 0)	0.2 (0.1 to 0.3)	0 (0 to 0)	0.1 (0.1 to 0.2)	-82.7 (-91.2 to -70.0)	-62.8 (-81.0 to -35.4)

eTable 8. All-cause and cause-specific mortality (counts and rates) at the global level and by country in the WHO European Region for ages 5 to 9 and 10 to 14 for 1990, 2000, and 2016

Bosnia and Herzegovina	Fire, heat, and hot substances	5 to 9	Male	1 (0 to 2)	0.5 (0.2 to 0.8)	0 (0 to 1)	0.3 (0.2 to 0.5)	0 (0 to 0)	0.1 (0.1 to 0.2)	-85.7 (-92.8 to -68.0)	-69.3 (-84.7 to -31.5)
Bosnia and Herzegovina	Fire, heat, and hot substances	10 to 14	Both	1 (1 to 2)	0.3 (0.2 to 0.5)	1 (0 to 1)	0.3 (0.2 to 0.4)	0 (0 to 0)	0.1 (0.1 to 0.2)	-80.0 (-88.3 to -66.9)	-56.1 (-74.3 to -27.4)
Bosnia and Herzegovina	Fire, heat, and hot substances	10 to 14	Female	0 (0 to 1)	0.2 (0.1 to 0.4)	0 (0 to 0)	0.2 (0.1 to 0.2)	0 (0 to 0)	0.1 (0.1 to 0.1)	-82.4 (-91.8 to -68.8)	-61.5 (-81.9 to -31.5)
Bosnia and Herzegovina	Fire, heat, and hot substances	10 to 14	Male	1 (0 to 1)	0.4 (0.2 to 0.8)	1 (0 to 1)	0.4 (0.2 to 0.6)	0 (0 to 0)	0.2 (0.1 to 0.3)	-78.7 (-88.6 to -58.1)	-53.4 (-75.0 to -8.1)
Bosnia and Herzegovina	Poisonings	5 to 9	Both	8 (2 to 13)	2.0 (0.6 to 3.4)	1 (1 to 2)	0.4 (0.3 to 0.7)	0 (0 to 0)	0.1 (0.1 to 0.2)	-97.3 (-98.9 to -86.0)	-94.3 (-97.6 to -70.0)
Bosnia and Herzegovina	Poisonings	5 to 9	Female	2 (1 to 4)	1.3 (0.4 to 2.3)	1 (0 to 1)	0.5 (0.3 to 0.8)	0 (0 to 0)	0.1 (0.1 to 0.3)	-95.0 (-98.1 to -83.3)	-89.2 (-95.8 to -64.1)
Bosnia and Herzegovina	Poisonings	5 to 9	Male	5 (1 to 10)	2.6 (0.5 to 4.9)	0 (0 to 1)	0.4 (0.2 to 0.7)	0 (0 to 0)	0.1 (0.0 to 0.2)	-98.4 (-99.5 to -86.0)	-96.7 (-99.0 to -70.2)
Bosnia and Herzegovina	Poisonings	10 to 14	Both	8 (2 to 12)	2.2 (0.6 to 3.5)	1 (1 to 2)	0.4 (0.3 to 0.6)	0 (0 to 0)	0.1 (0.1 to 0.3)	-97.0 (-98.6 to -84.6)	-93.3 (-96.9 to -66.2)
Bosnia and Herzegovina	Poisonings	10 to 14	Female	2 (1 to 3)	1.1 (0.5 to 1.8)	1 (0 to 1)	0.4 (0.3 to 0.7)	0 (0 to 0)	0.2 (0.1 to 0.3)	-92.9 (-97.0 to -83.3)	-84.5 (-93.3 to -63.3)
Bosnia and Herzegovina	Poisonings	10 to 14	Male	6 (1 to 10)	3.2 (0.5 to 5.6)	1 (0 to 1)	0.5 (0.3 to 0.8)	0 (0 to 0)	0.1 (0.1 to 0.3)	-98.2 (-99.4 to -82.5)	-96.2 (-98.7 to -61.7)
Bosnia and Herzegovina	Exposure to mechanical forces	5 to 9	Both	8 (4 to 13)	2.1 (1.0 to 3.3)	1 (1 to 2)	0.6 (0.3 to 0.9)	1 (0 to 1)	0.3 (0.1 to 0.5)	-93.8 (-97.2 to -84.7)	-86.8 (-94.0 to -67.1)
Bosnia and Herzegovina	Exposure to mechanical forces	5 to 9	Female	2 (1 to 3)	1.0 (0.4 to 1.7)	0 (0 to 0)	0.2 (0.1 to 0.3)	0 (0 to 0)	0.1 (0.1 to 0.2)	-94.9 (-97.7 to -87.2)	-89.1 (-95.1 to -72.5)
Bosnia and Herzegovina	Exposure to mechanical forces	5 to 9	Male	6 (3 to 10)	3.2 (1.5 to 4.8)	1 (1 to 2)	0.9 (0.4 to 1.5)	0 (0 to 1)	0.4 (0.2 to 0.8)	-93.5 (-97.1 to -84.4)	-86.1 (-93.7 to -66.7)
Bosnia and Herzegovina	Exposure to mechanical forces	10 to 14	Both	8 (4 to 13)	2.3 (1.0 to 3.7)	1 (1 to 2)	0.5 (0.3 to 0.7)	0 (0 to 1)	0.3 (0.2 to 0.4)	-94.5 (-97.3 to -86.4)	-87.8 (-94.0 to -70.2)
Bosnia and Herzegovina	Exposure to mechanical forces	10 to 14	Female	1 (1 to 2)	0.8 (0.3 to 1.3)	0 (0 to 0)	0.1 (0.1 to 0.2)	0 (0 to 0)	0.1 (0.1 to 0.1)	-94.7 (-97.3 to -86.0)	-88.3 (-94.0 to -69.3)
Bosnia and Herzegovina	Exposure to mechanical forces	10 to 14	Male	7 (3 to 11)	3.7 (1.7 to 6.0)	1 (1 to 2)	0.8 (0.4 to 1.2)	0 (0 to 1)	0.5 (0.2 to 0.7)	-94.4 (-97.4 to -86.2)	-87.8 (-94.2 to -69.8)
Bosnia and Herzegovina	Adverse effects of medical treatment	5 to 9	Both	3 (2 to 4)	0.7 (0.5 to 1.0)	1 (1 to 2)	0.5 (0.4 to 0.8)	0 (0 to 1)	0.2 (0.1 to 0.3)	-88.0 (-94.0 to -72.8)	-74.3 (-87.1 to -41.6)
Bosnia and Herzegovina	Adverse effects of medical treatment	5 to 9	Female	1 (0 to 1)	0.5 (0.2 to 0.8)	0 (0 to 1)	0.4 (0.2 to 0.6)	0 (0 to 0)	0.1 (0.1 to 0.2)	-86.9 (-93.8 to -73.3)	-71.7 (-86.6 to -42.5)
Bosnia and Herzegovina	Adverse effects of medical treatment	5 to 9	Male	2 (1 to 3)	0.9 (0.6 to 1.4)	1 (1 to 1)	0.7 (0.5 to 1.1)	0 (0 to 0)	0.2 (0.1 to 0.5)	-88.6 (-95.6 to -71.0)	-75.6 (-90.5 to -38.1)
Bosnia and Herzegovina	Adverse effects of medical treatment	10 to 14	Both	3 (1 to 4)	0.7 (0.4 to 1.0)	2 (1 to 2)	0.6 (0.3 to 0.9)	0 (0 to 1)	0.2 (0.1 to 0.3)	-85.9 (-92.6 to -69.3)	-69.1 (-83.7 to -32.6)
Bosnia and Herzegovina	Adverse effects of medical treatment	10 to 14	Female	1 (0 to 1)	0.4 (0.2 to 0.7)	0 (0 to 1)	0.3 (0.2 to 0.5)	0 (0 to 0)	0.1 (0.1 to 0.2)	-85.5 (-92.9 to -73.4)	-68.1 (-84.4 to -41.7)
Bosnia and Herzegovina	Adverse effects of medical treatment	10 to 14	Male	2 (1 to 3)	1.0 (0.5 to 1.5)	1 (1 to 2)	0.9 (0.4 to 1.4)	0 (0 to 0)	0.3 (0.2 to 0.5)	-86.0 (-93.8 to -65.9)	-69.4 (-86.5 to -25.2)
Bosnia and Herzegovina	Animal contact	5 to 9	Both	1 (0 to 1)	0.2 (0.1 to 0.4)	0 (0 to 0)	0.1 (0.1 to 0.2)	0 (0 to 0)	0.0 (0.0 to 0.1)	-91.3 (-95.6 to -82.5)	-81.3 (-90.6 to -62.4)

eTable 8. All-cause and cause-specific mortality (counts and rates) at the global level and by country in the WHO European Region for ages 5 to 9 and 10 to 14 for 1990, 2000, and 2016

Bosnia and Herzegovina	Animal contact	5 to 9	Female	0 (0 to 0)	0.1 (0.1 to 0.2)	0 (0 to 0)	0.1 (0.0 to 0.1)	0 (0 to 0)	0.0 (0.0 to 0.1)	-82.6 (-92.9 to -67.0)	-62.5 (-84.7 to -29.0)
Bosnia and Herzegovina	Animal contact	5 to 9	Male	1 (0 to 1)	0.3 (0.1 to 0.6)	0 (0 to 0)	0.2 (0.1 to 0.3)	0 (0 to 0)	0.0 (0.0 to 0.1)	-94.0 (-97.6 to -83.9)	-87.3 (-94.8 to -65.6)
Bosnia and Herzegovina	Animal contact	10 to 14	Both	1 (0 to 1)	0.2 (0.1 to 0.4)	0 (0 to 1)	0.1 (0.1 to 0.2)	0 (0 to 0)	0.0 (0.0 to 0.1)	-91.7 (-95.8 to -81.5)	-81.8 (-90.9 to -59.4)
Bosnia and Herzegovina	Animal contact	10 to 14	Female	0 (0 to 0)	0.1 (0.0 to 0.2)	0 (0 to 0)	0.1 (0.0 to 0.1)	0 (0 to 0)	0.0 (0.0 to 0.0)	-86.7 (-94.6 to -73.6)	-70.9 (-88.1 to -42.0)
Bosnia and Herzegovina	Animal contact	10 to 14	Male	1 (0 to 1)	0.4 (0.2 to 0.7)	0 (0 to 1)	0.2 (0.1 to 0.3)	0 (0 to 0)	0.1 (0.0 to 0.1)	-92.8 (-96.9 to -81.2)	-84.2 (-93.1 to -58.9)
Bosnia and Herzegovina	Foreign body	5 to 9	Both	1 (1 to 2)	0.3 (0.1 to 0.5)	1 (0 to 1)	0.3 (0.1 to 0.4)	0 (0 to 1)	0.2 (0.1 to 0.3)	-71.8 (-83.9 to -50.6)	-39.5 (-65.4 to 6.0)
Bosnia and Herzegovina	Foreign body	5 to 9	Female	0 (0 to 1)	0.2 (0.1 to 0.3)	0 (0 to 0)	0.2 (0.1 to 0.3)	0 (0 to 0)	0.1 (0.1 to 0.2)	-71.6 (-85.0 to -49.0)	-38.9 (-67.7 to 9.7)
Bosnia and Herzegovina	Foreign body	5 to 9	Male	1 (0 to 2)	0.4 (0.2 to 0.8)	0 (0 to 1)	0.3 (0.1 to 0.6)	0 (0 to 0)	0.3 (0.1 to 0.5)	-71.9 (-86.4 to -42.0)	-39.9 (-71.0 to 24.0)
Bosnia and Herzegovina	Foreign body	10 to 14	Both	1 (0 to 2)	0.3 (0.1 to 0.6)	1 (0 to 1)	0.3 (0.1 to 0.4)	0 (0 to 0)	0.2 (0.1 to 0.3)	-73.7 (-85.5 to -55.5)	-42.4 (-68.3 to -2.5)
Bosnia and Herzegovina	Foreign body	10 to 14	Female	0 (0 to 1)	0.2 (0.1 to 0.3)	0 (0 to 0)	0.2 (0.1 to 0.3)	0 (0 to 0)	0.1 (0.1 to 0.2)	-75.4 (-87.6 to -55.5)	-46.1 (-72.8 to -2.5)
Bosnia and Herzegovina	Foreign body	10 to 14	Male	1 (0 to 2)	0.4 (0.1 to 0.9)	0 (0 to 1)	0.3 (0.1 to 0.6)	0 (0 to 0)	0.2 (0.1 to 0.4)	-72.9 (-87.0 to -44.7)	-40.7 (-71.6 to 21.1)
Bosnia and Herzegovina	Environmental heat and cold exposure	5 to 9	Both	0 (0 to 0)	0.0 (0.0 to 0.0)	0 (0 to 0)	0.0 (0.0 to 0.0)	0 (0 to 0)	0.0 (0.0 to 0.0)	-58.8 (-67.1 to -48.3)	-11.7 (-29.5 to 10.9)
Bosnia and Herzegovina	Environmental heat and cold exposure	5 to 9	Female	0 (0 to 0)	0.0 (0.0 to 0.0)	0 (0 to 0)	0.0 (0.0 to 0.0)	0 (0 to 0)	0.0 (0.0 to 0.0)	-57.4 (-66.8 to -43.6)	-8.4 (-28.5 to 21.4)
Bosnia and Herzegovina	Environmental heat and cold exposure	5 to 9	Male	0 (0 to 0)	0.0 (0.0 to 0.0)	0 (0 to 0)	0.0 (0.0 to 0.0)	0 (0 to 0)	0.0 (0.0 to 0.0)	-60.1 (-69.6 to -48.3)	-14.7 (-35.0 to 10.5)
Bosnia and Herzegovina	Environmental heat and cold exposure	10 to 14	Both	0 (0 to 0)	0.0 (0.0 to 0.0)	0 (0 to 0)	0.0 (0.0 to 0.0)	0 (0 to 0)	0.0 (0.0 to 0.0)	-56.0 (-64.7 to -46.6)	-3.5 (-22.5 to 17.1)
Bosnia and Herzegovina	Environmental heat and cold exposure	10 to 14	Female	0 (0 to 0)	0.0 (0.0 to 0.0)	0 (0 to 0)	0.0 (0.0 to 0.0)	0 (0 to 0)	0.0 (0.0 to 0.0)	-58.2 (-66.5 to -48.2)	-8.4 (-26.5 to 13.7)
Bosnia and Herzegovina	Environmental heat and cold exposure	10 to 14	Male	0 (0 to 0)	0.0 (0.0 to 0.0)	0 (0 to 0)	0.0 (0.0 to 0.0)	0 (0 to 0)	0.0 (0.0 to 0.0)	-53.6 (-66.2 to -39.7)	1.7 (-26.0 to 32.1)
Bosnia and Herzegovina	Other unintentional injuries	5 to 9	Both	1 (0 to 2)	0.2 (0.1 to 0.4)	0 (0 to 1)	0.1 (0.1 to 0.2)	0 (0 to 0)	0.1 (0.0 to 0.1)	-87.4 (-93.1 to -75.8)	-72.9 (-85.2 to -48.0)
Bosnia and Herzegovina	Other unintentional injuries	5 to 9	Female	0 (0 to 1)	0.1 (0.1 to 0.4)	0 (0 to 0)	0.1 (0.0 to 0.2)	0 (0 to 0)	0.0 (0.0 to 0.1)	-89.3 (-95.5 to -74.8)	-77.1 (-90.2 to -45.7)
Bosnia and Herzegovina	Other unintentional injuries	5 to 9	Male	1 (0 to 1)	0.3 (0.1 to 0.5)	0 (0 to 0)	0.2 (0.1 to 0.3)	0 (0 to 0)	0.1 (0.0 to 0.1)	-86.4 (-93.7 to -69.5)	-70.9 (-86.5 to -34.7)
Bosnia and Herzegovina	Other unintentional injuries	10 to 14	Both	1 (1 to 2)	0.3 (0.2 to 0.6)	1 (0 to 1)	0.2 (0.1 to 0.4)	0 (0 to 0)	0.1 (0.1 to 0.2)	-83.4 (-90.4 to -67.9)	-63.5 (-79.0 to -29.6)
Bosnia and Herzegovina	Other unintentional injuries	10 to 14	Female	0 (0 to 1)	0.2 (0.1 to 0.4)	0 (0 to 0)	0.1 (0.1 to 0.2)	0 (0 to 0)	0.0 (0.0 to 0.1)	-89.4 (-95.8 to -75.0)	-76.7 (-90.8 to -45.1)
Bosnia and Herzegovina	Other unintentional injuries	10 to 14	Male	1 (0 to 1)	0.4 (0.2 to 0.8)	1 (0 to 1)	0.4 (0.2 to 0.6)	0 (0 to 0)	0.2 (0.1 to 0.3)	-80.7 (-90.0 to -57.0)	-57.8 (-78.2 to -5.9)
Bosnia and Herzegovina	Self-harm	10 to 14	Both	10 (6 to 14)	2.9 (1.6 to 4.0)	6 (3 to 9)	2.1 (1.1 to 3.1)	2 (1 to 2)	1.0 (0.7 to 1.4)	-84.0 (-90.1 to -72.5)	-64.9 (-78.3 to -39.8)
Bosnia and Herzegovina	Self-harm	10 to 14	Female	3 (2 to 4)	1.5 (0.9 to 2.2)	1 (1 to 2)	0.9 (0.5 to 1.6)	0 (0 to 1)	0.6 (0.3 to 1.0)	-81.7 (-90.9 to -61.6)	-59.8 (-80.1 to -15.8)
Bosnia and Herzegovina	Self-harm	10 to 14	Male	8 (3 to 11)	4.2 (1.7 to 6.2)	5 (2 to 7)	3.1 (1.4 to 4.9)	1 (1 to 2)	1.4 (0.9 to 2.1)	-84.8 (-91.6 to -68.5)	-66.7 (-81.7 to -31.0)

eTable 8. All-cause and cause-specific mortality (counts and rates) at the global level and by country in the WHO European Region for ages 5 to 9 and 10 to 14 for 1990, 2000, and 2016

Bosnia and Herzegovina	Interpersonal violence	5 to 9	Both	3 (1 to 5)	0.8 (0.4 to 1.4)	1 (1 to 2)	0.6 (0.3 to 1.0)	1 (0 to 1)	0.3 (0.2 to 0.5)	-82.9 (-91.8 to -61.0)	-63.2 (-82.4 to -16.4)
Bosnia and Herzegovina	Interpersonal violence	5 to 9	Female	1 (0 to 1)	0.4 (0.2 to 0.8)	0 (0 to 1)	0.4 (0.2 to 0.7)	0 (0 to 0)	0.2 (0.1 to 0.4)	-76.9 (-90.2 to -40.6)	-50.3 (-79.0 to 27.9)
Bosnia and Herzegovina	Interpersonal violence	5 to 9	Male	2 (1 to 4)	1.1 (0.4 to 2.1)	1 (0 to 2)	0.8 (0.3 to 1.5)	0 (0 to 1)	0.4 (0.2 to 0.6)	-85.1 (-94.2 to -58.8)	-68.0 (-87.5 to -11.9)
Bosnia and Herzegovina	Interpersonal violence	10 to 14	Both	3 (2 to 5)	0.9 (0.4 to 1.4)	2 (1 to 3)	0.7 (0.4 to 1.2)	1 (0 to 1)	0.4 (0.3 to 0.6)	-78.8 (-88.8 to -57.1)	-53.6 (-75.5 to -5.9)
Bosnia and Herzegovina	Interpersonal violence	10 to 14	Female	1 (0 to 1)	0.4 (0.2 to 0.7)	0 (0 to 1)	0.3 (0.2 to 0.5)	0 (0 to 0)	0.2 (0.1 to 0.3)	-78.1 (-91.3 to -46.8)	-51.9 (-80.8 to 16.8)
Bosnia and Herzegovina	Interpersonal violence	10 to 14	Male	2 (1 to 4)	1.3 (0.5 to 2.3)	2 (1 to 3)	1.1 (0.5 to 1.9)	0 (0 to 1)	0.6 (0.3 to 1.0)	-79.0 (-90.3 to -50.8)	-54.1 (-78.8 to 7.9)
Bosnia and Herzegovina	Exposure to forces of nature	5 to 9	Both	0 (0 to 0)	0.0 (0.0 to 0.0)	0 (0 to 0)	0.0 (0.0 to 0.0)	0 (0 to 0)	0.0 (0.0 to 0.0)	0.0 (0.0 to 0.0)	0.0 (0.0 to 0.0)
Bosnia and Herzegovina	Exposure to forces of nature	5 to 9	Female	0 (0 to 0)	0.0 (0.0 to 0.0)	0 (0 to 0)	0.0 (0.0 to 0.0)	0 (0 to 0)	0.0 (0.0 to 0.0)	0.0 (0.0 to 0.0)	0.0 (0.0 to 0.0)
Bosnia and Herzegovina	Exposure to forces of nature	5 to 9	Male	0 (0 to 0)	0.0 (0.0 to 0.0)	0 (0 to 0)	0.0 (0.0 to 0.0)	0 (0 to 0)	0.0 (0.0 to 0.0)	0.0 (0.0 to 0.0)	0.0 (0.0 to 0.0)
Bosnia and Herzegovina	Exposure to forces of nature	10 to 14	Both	0 (0 to 0)	0.0 (0.0 to 0.0)	0 (0 to 0)	0.0 (0.0 to 0.0)	0 (0 to 0)	0.0 (0.0 to 0.0)	0.0 (0.0 to 0.0)	0.0 (0.0 to 0.0)
Bosnia and Herzegovina	Exposure to forces of nature	10 to 14	Female	0 (0 to 0)	0.0 (0.0 to 0.0)	0 (0 to 0)	0.0 (0.0 to 0.0)	0 (0 to 0)	0.0 (0.0 to 0.0)	0.0 (0.0 to 0.0)	0.0 (0.0 to 0.0)
Bosnia and Herzegovina	Exposure to forces of nature	10 to 14	Male	0 (0 to 0)	0.0 (0.0 to 0.0)	0 (0 to 0)	0.0 (0.0 to 0.0)	0 (0 to 0)	0.0 (0.0 to 0.0)	0.0 (0.0 to 0.0)	0.0 (0.0 to 0.0)
Bosnia and Herzegovina	Conflict and terrorism	5 to 9	Both	0 (0 to 0)	0.0 (0.0 to 0.0)	0 (0 to 0)	0.0 (0.0 to 0.0)	0 (0 to 0)	0.0 (0.0 to 0.0)	0.0 (0.0 to 0.0)	0.0 (0.0 to 0.0)
Bosnia and Herzegovina	Conflict and terrorism	5 to 9	Female	0 (0 to 0)	0.0 (0.0 to 0.0)	0 (0 to 0)	0.0 (0.0 to 0.0)	0 (0 to 0)	0.0 (0.0 to 0.0)	0.0 (0.0 to 0.0)	0.0 (0.0 to 0.0)
Bosnia and Herzegovina	Conflict and terrorism	5 to 9	Male	0 (0 to 0)	0.0 (0.0 to 0.0)	0 (0 to 0)	0.0 (0.0 to 0.0)	0 (0 to 0)	0.0 (0.0 to 0.0)	0.0 (0.0 to 0.0)	0.0 (0.0 to 0.0)
Bosnia and Herzegovina	Conflict and terrorism	10 to 14	Both	0 (0 to 0)	0.0 (0.0 to 0.0)	0 (0 to 0)	0.0 (0.0 to 0.0)	0 (0 to 0)	0.0 (0.0 to 0.0)	0.0 (0.0 to 0.0)	0.0 (0.0 to 0.0)
Bosnia and Herzegovina	Conflict and terrorism	10 to 14	Female	0 (0 to 0)	0.0 (0.0 to 0.0)	0 (0 to 0)	0.0 (0.0 to 0.0)	0 (0 to 0)	0.0 (0.0 to 0.0)	0.0 (0.0 to 0.0)	0.0 (0.0 to 0.0)
Bosnia and Herzegovina	Conflict and terrorism	10 to 14	Male	0 (0 to 0)	0.0 (0.0 to 0.0)	0 (0 to 0)	0.0 (0.0 to 0.0)	0 (0 to 0)	0.0 (0.0 to 0.0)	0.0 (0.0 to 0.0)	0.0 (0.0 to 0.0)
Bosnia and Herzegovina	Executions and police conflict	5 to 9	Both	0 (0 to 0)	0.0 (0.0 to 0.0)	0 (0 to 0)	0.0 (0.0 to 0.0)	0 (0 to 0)	0.0 (0.0 to 0.0)	-58.9 (-67.1 to -48.6)	-11.9 (-29.5 to 10.2)
Bosnia and Herzegovina	Executions and police conflict	5 to 9	Female	0 (0 to 0)	0.0 (0.0 to 0.0)	0 (0 to 0)	0.0 (0.0 to 0.0)	0 (0 to 0)	0.0 (0.0 to 0.0)	-57.5 (-66.8 to -43.8)	-8.5 (-28.6 to 21.0)
Bosnia and Herzegovina	Executions and police conflict	5 to 9	Male	0 (0 to 0)	0.0 (0.0 to 0.0)	0 (0 to 0)	0.0 (0.0 to 0.0)	0 (0 to 0)	0.0 (0.0 to 0.0)	-60.3 (-69.6 to -48.9)	-15.2 (-35.0 to 9.2)
Bosnia and Herzegovina	Executions and police conflict	10 to 14	Both	0 (0 to 0)	0.0 (0.0 to 0.0)	0 (0 to 0)	0.0 (0.0 to 0.0)	0 (0 to 0)	0.0 (0.0 to 0.0)	-56.0 (-64.6 to -46.6)	-3.4 (-22.3 to 17.1)
Bosnia and Herzegovina	Executions and police conflict	10 to 14	Female	0 (0 to 0)	0.0 (0.0 to 0.0)	0 (0 to 0)	0.0 (0.0 to 0.0)	0 (0 to 0)	0.0 (0.0 to 0.0)	-58.2 (-66.5 to -48.2)	-8.4 (-26.5 to 13.7)
Bosnia and Herzegovina	Executions and police conflict	10 to 14	Male	0 (0 to 0)	0.0 (0.0 to 0.0)	0 (0 to 0)	0.0 (0.0 to 0.0)	0 (0 to 0)	0.0 (0.0 to 0.0)	-53.5 (-66.0 to -39.6)	1.9 (-25.4 to 32.4)
Bulgaria	All causes	5 to 9	Both	242 (235 to 250)	41.3 (40.1 to 42.7)	132 (127 to 137)	32.5 (31.1 to 33.8)	65 (48 to 88)	20.4 (15.0 to 27.6)	-73.3 (-80.3 to -64.0)	-50.7 (-63.7 to -33.5)
Bulgaria	All causes	5 to 9	Female	101 (97 to 104)	35.3 (34.2 to 36.5)	52 (50 to 54)	26.1 (25.0 to 27.1)	28 (21 to 37)	18.3 (13.8 to 24.4)	-72.1 (-79.0 to -62.8)	-48.1 (-61.0 to -30.7)

eTable 8. All-cause and cause-specific mortality (counts and rates) at the global level and by country in the WHO European Region for ages 5 to 9 and 10 to 14 for 1990, 2000, and 2016

Bulgaria	All causes	5 to 9	Male	141 (137 to 146)	47.0 (45.5 to 48.6)	81 (77 to 84)	38.5 (36.9 to 40.2)	37 (26 to 50)	22.3 (16.2 to 30.6)	-74.2 (-81.3 to -64.6)	-52.6 (-65.6 to -35.1)
Bulgaria	All causes	10 to 14	Both	252 (247 to 258)	38.8 (38.0 to 39.7)	149 (145 to 154)	28.9 (28.1 to 29.8)	64 (53 to 77)	22.3 (18.5 to 26.8)	-74.5 (-78.9 to -69.5)	-42.5 (-52.4 to -31.2)
Bulgaria	All causes	10 to 14	Female	91 (88 to 93)	28.7 (27.9 to 29.4)	56 (55 to 58)	22.4 (21.7 to 23.1)	24 (19 to 28)	17.6 (14.6 to 21.3)	-74.1 (-78.6 to -68.8)	-38.5 (-49.2 to -26.0)
Bulgaria	All causes	10 to 14	Male	162 (158 to 166)	48.4 (47.2 to 49.8)	93 (90 to 96)	35.1 (33.9 to 36.2)	41 (33 to 50)	26.3 (21.5 to 32.2)	-74.8 (-79.3 to -69.0)	-45.7 (-55.5 to -33.2)
Bulgaria	Tuberculosis	5 to 9	Both	0 (0 to 0)	0.0 (0.0 to 0.0)	0 (0 to 0)	0.0 (0.0 to 0.0)	0 (0 to 0)	0.0 (0.0 to 0.0)	-72.3 (-81.8 to -57.1)	-48.8 (-66.3 to -20.8)
Bulgaria	Tuberculosis	5 to 9	Female	0 (0 to 0)	0.0 (0.0 to 0.0)	0 (0 to 0)	0.0 (0.0 to 0.0)	0 (0 to 0)	0.0 (0.0 to 0.0)	-53.6 (-69.1 to -33.7)	-13.6 (-42.6 to 23.2)
Bulgaria	Tuberculosis	5 to 9	Male	0 (0 to 0)	0.0 (0.0 to 0.0)	0 (0 to 0)	0.0 (0.0 to 0.0)	0 (0 to 0)	0.0 (0.0 to 0.0)	-79.1 (-87.3 to -63.6)	-61.6 (-76.6 to -33.3)
Bulgaria	Tuberculosis	10 to 14	Both	0 (0 to 0)	0.0 (0.0 to 0.0)	0 (0 to 0)	0.0 (0.0 to 0.0)	0 (0 to 0)	0.0 (0.0 to 0.0)	-72.9 (-80.7 to -62.2)	-38.9 (-56.5 to -14.7)
Bulgaria	Tuberculosis	10 to 14	Female	0 (0 to 0)	0.0 (0.0 to 0.0)	0 (0 to 0)	0.0 (0.0 to 0.0)	0 (0 to 0)	0.0 (0.0 to 0.0)	-62.1 (-72.6 to -50.3)	-10.1 (-35.1 to 18.1)
Bulgaria	Tuberculosis	10 to 14	Male	0 (0 to 0)	0.0 (0.0 to 0.0)	0 (0 to 0)	0.0 (0.0 to 0.0)	0 (0 to 0)	0.0 (0.0 to 0.0)	-78.1 (-86.3 to -65.1)	-52.9 (-70.6 to -24.9)
Bulgaria	HIV/AIDS	5 to 9	Both	0 (0 to 0)	0.0 (0.0 to 0.1)	0 (0 to 1)	0.1 (0.1 to 0.1)	0 (0 to 0)	0.1 (0.0 to 0.1)	-27.2 (-52.8 to 12.1)	34.4 (-12.8 to 107.1)
Bulgaria	HIV/AIDS	5 to 9	Female	0 (0 to 0)	0.0 (0.0 to 0.1)	0 (0 to 0)	0.1 (0.1 to 0.1)	0 (0 to 0)	0.1 (0.0 to 0.1)	-3.4 (-48.4 to 82.6)	79.6 (-4.0 to 239.7)
Bulgaria	HIV/AIDS	5 to 9	Male	0 (0 to 0)	0.1 (0.0 to 0.1)	0 (0 to 0)	0.1 (0.1 to 0.1)	0 (0 to 0)	0.1 (0.0 to 0.1)	-41.1 (-67.6 to 1.1)	8.0 (-40.6 to 85.5)
Bulgaria	HIV/AIDS	10 to 14	Both	0 (0 to 0)	0.0 (0.0 to 0.1)	0 (0 to 1)	0.1 (0.1 to 0.1)	0 (0 to 0)	0.1 (0.0 to 0.1)	-43.5 (-63.4 to -12.7)	27.5 (-17.4 to 97.0)
Bulgaria	HIV/AIDS	10 to 14	Female	0 (0 to 0)	0.0 (0.0 to 0.1)	0 (0 to 0)	0.1 (0.1 to 0.1)	0 (0 to 0)	0.1 (0.0 to 0.1)	-36.4 (-67.4 to 23.9)	51.0 (-22.5 to 194.3)
Bulgaria	HIV/AIDS	10 to 14	Male	0 (0 to 0)	0.0 (0.0 to 0.1)	0 (0 to 0)	0.1 (0.0 to 0.1)	0 (0 to 0)	0.1 (0.0 to 0.1)	-48.9 (-70.9 to -13.3)	10.0 (-37.2 to 86.7)
Bulgaria	Diarrheal diseases	5 to 9	Both	1 (1 to 2)	0.2 (0.1 to 0.3)	1 (0 to 1)	0.1 (0.1 to 0.2)	0 (0 to 1)	0.1 (0.1 to 0.2)	-71.6 (-84.1 to -51.9)	-47.6 (-70.7 to -11.2)
Bulgaria	Diarrheal diseases	5 to 9	Female	1 (0 to 1)	0.2 (0.2 to 0.4)	0 (0 to 0)	0.2 (0.1 to 0.2)	0 (0 to 0)	0.1 (0.1 to 0.2)	-72.4 (-86.7 to -46.6)	-48.7 (-75.2 to -0.8)
Bulgaria	Diarrheal diseases	5 to 9	Male	0 (0 to 1)	0.2 (0.1 to 0.2)	0 (0 to 0)	0.1 (0.1 to 0.2)	0 (0 to 0)	0.1 (0.0 to 0.1)	-70.4 (-85.6 to -39.7)	-45.8 (-73.6 to 10.5)
Bulgaria	Diarrheal diseases	10 to 14	Both	1 (0 to 1)	0.1 (0.1 to 0.1)	0 (0 to 0)	0.1 (0.0 to 0.1)	0 (0 to 0)	0.1 (0.0 to 0.1)	-72.2 (-83.4 to -55.1)	-37.4 (-62.5 to 1.2)
Bulgaria	Diarrheal diseases	10 to 14	Female	0 (0 to 0)	0.1 (0.0 to 0.1)	0 (0 to 0)	0.1 (0.0 to 0.1)	0 (0 to 0)	0.0 (0.0 to 0.1)	-71.5 (-84.5 to -45.8)	-32.3 (-63.2 to 28.7)
Bulgaria	Diarrheal diseases	10 to 14	Male	0 (0 to 1)	0.1 (0.1 to 0.2)	0 (0 to 0)	0.1 (0.0 to 0.1)	0 (0 to 0)	0.1 (0.0 to 0.1)	-72.7 (-86.8 to -48.2)	-41.3 (-71.5 to 11.5)
Bulgaria	Intestinal infectious diseases	5 to 9	Both	0 (0 to 0)	0.0 (0.0 to 0.0)	0 (0 to 0)	0.0 (0.0 to 0.0)	0 (0 to 0)	0.0 (0.0 to 0.0)	-49.7 (-68.0 to -22.6)	-7.2 (-40.9 to 42.9)
Bulgaria	Intestinal infectious diseases	5 to 9	Female	0 (0 to 0)	0.0 (0.0 to 0.0)	0 (0 to 0)	0.0 (0.0 to 0.0)	0 (0 to 0)	0.0 (0.0 to 0.0)	-55.2 (-76.9 to -17.0)	-16.6 (-57.1 to 54.3)
Bulgaria	Intestinal infectious diseases	5 to 9	Male	0 (0 to 0)	0.0 (0.0 to 0.0)	0 (0 to 0)	0.0 (0.0 to 0.0)	0 (0 to 0)	0.0 (0.0 to 0.0)	-45.2 (-65.7 to -12.1)	0.5 (-37.1 to 61.2)
Bulgaria	Intestinal infectious diseases	10 to 14	Both	0 (0 to 0)	0.0 (0.0 to 0.0)	0 (0 to 0)	0.0 (0.0 to 0.0)	0 (0 to 0)	0.0 (0.0 to 0.0)	-53.2 (-66.2 to -33.0)	5.5 (-23.8 to 51.1)
Bulgaria	Intestinal infectious diseases	10 to 14	Female	0 (0 to 0)	0.0 (0.0 to 0.0)	0 (0 to 0)	0.0 (0.0 to 0.0)	0 (0 to 0)	0.0 (0.0 to 0.0)	-59.6 (-74.6 to -20.4)	-4.2 (-39.8 to 89.0)
Bulgaria	Intestinal infectious diseases	10 to 14	Male	0 (0 to 0)	0.0 (0.0 to 0.0)	0 (0 to 0)	0.0 (0.0 to 0.0)	0 (0 to 0)	0.0 (0.0 to 0.0)	-47.7 (-68.1 to -14.0)	12.7 (-31.4 to 85.2)
Bulgaria	Lower respiratory infections	5 to 9	Both	36 (29 to 43)	6.1 (5.0 to 7.3)	15 (12 to 18)	3.6 (2.9 to 4.4)	6 (4 to 9)	1.8 (1.2 to 2.8)	-84.1 (-89.9 to -74.8)	-70.6 (-81.3 to -53.4)

eTable 8. All-cause and cause-specific mortality (counts and rates) at the global level and by country in the WHO European Region for ages 5 to 9 and 10 to 14 for 1990, 2000, and 2016

Bulgaria	Lower respiratory infections	5 to 9	Female	18 (14 to 23)	6.4 (4.8 to 7.9)	7 (5 to 9)	3.4 (2.5 to 4.4)	3 (2 to 4)	1.8 (1.0 to 2.8)	-85.1 (-91.5 to -75.6)	-72.3 (-84.3 to -54.6)
Bulgaria	Lower respiratory infections	5 to 9	Male	18 (13 to 23)	5.9 (4.4 to 7.6)	8 (6 to 10)	3.8 (2.8 to 5.0)	3 (2 to 5)	1.8 (1.1 to 3.0)	-83.0 (-90.3 to -69.8)	-68.9 (-82.2 to -44.6)
Bulgaria	Lower respiratory infections	10 to 14	Both	28 (23 to 33)	4.3 (3.5 to 5.1)	11 (9 to 14)	2.2 (1.7 to 2.6)	4 (3 to 6)	1.4 (1.0 to 1.9)	-85.3 (-90.0 to -78.5)	-66.8 (-77.5 to -51.6)
Bulgaria	Lower respiratory infections	10 to 14	Female	13 (10 to 17)	4.2 (3.3 to 5.4)	5 (4 to 7)	2.0 (1.5 to 2.6)	2 (1 to 3)	1.3 (0.8 to 1.9)	-87.4 (-92.5 to -79.9)	-70.1 (-82.3 to -52.2)
Bulgaria	Lower respiratory infections	10 to 14	Male	14 (11 to 19)	4.3 (3.3 to 5.6)	6 (4 to 8)	2.3 (1.7 to 3.1)	2 (2 to 3)	1.6 (1.0 to 2.3)	-83.3 (-89.8 to -73.4)	-64.1 (-78.1 to -42.6)
Bulgaria	Upper respiratory infections	5 to 9	Both	0 (0 to 0)	0.0 (0.0 to 0.0)	0 (0 to 0)	0.0 (0.0 to 0.0)	0 (0 to 0)	0.0 (0.0 to 0.0)	-78.8 (-99.7 to 1140.7)	-60.9 (-99.5 to 2191.0)
Bulgaria	Upper respiratory infections	5 to 9	Female	0 (0 to 0)	0.0 (0.0 to 0.0)	0 (0 to 0)	0.0 (0.0 to 0.0)	0 (0 to 0)	0.0 (0.0 to 0.0)	-79.5 (-100.0 to 26738.9)	-61.9 (-100.0 to 49816.2)
Bulgaria	Upper respiratory infections	5 to 9	Male	0 (0 to 0)	0.0 (0.0 to 0.0)	0 (0 to 0)	0.0 (0.0 to 0.0)	0 (0 to 0)	0.0 (0.0 to 0.0)	-78.3 (-100.0 to 18142.0)	-60.2 (-100.0 to 33358.2)
Bulgaria	Upper respiratory infections	10 to 14	Both	0 (0 to 0)	0.0 (0.0 to 0.0)	0 (0 to 0)	0.0 (0.0 to 0.0)	0 (0 to 0)	0.0 (0.0 to 0.0)	-80.0 (-99.6 to 728.3)	-54.9 (-99.1 to 1768.3)
Bulgaria	Upper respiratory infections	10 to 14	Female	0 (0 to 0)	0.0 (0.0 to 0.0)	0 (0 to 0)	0.0 (0.0 to 0.0)	0 (0 to 0)	0.0 (0.0 to 0.0)	-81.6 (-100.0 to 17385.8)	-56.4 (-100.0 to 41415.1)
Bulgaria	Upper respiratory infections	10 to 14	Male	0 (0 to 0)	0.0 (0.0 to 0.0)	0 (0 to 0)	0.0 (0.0 to 0.0)	0 (0 to 0)	0.0 (0.0 to 0.0)	-79.0 (-100.0 to 31450.1)	-54.8 (-100.0 to 67835.8)
Bulgaria	Otitis media	5 to 9	Both	0 (0 to 0)	0.0 (0.0 to 0.0)	0 (0 to 0)	0.0 (0.0 to 0.0)	0 (0 to 0)	0.0 (0.0 to 0.0)	-94.9 (-98.4 to -86.2)	-90.6 (-97.0 to -74.6)
Bulgaria	Otitis media	5 to 9	Female	0 (0 to 0)	0.0 (0.0 to 0.0)	0 (0 to 0)	0.0 (0.0 to 0.0)	0 (0 to 0)	0.0 (0.0 to 0.0)	-91.7 (-98.5 to -67.1)	-84.6 (-97.2 to -38.8)
Bulgaria	Otitis media	5 to 9	Male	0 (0 to 0)	0.0 (0.0 to 0.0)	0 (0 to 0)	0.0 (0.0 to 0.0)	0 (0 to 0)	0.0 (0.0 to 0.0)	-97.0 (-99.1 to -91.4)	-94.4 (-98.4 to -84.2)
Bulgaria	Otitis media	10 to 14	Both	0 (0 to 0)	0.0 (0.0 to 0.0)	0 (0 to 0)	0.0 (0.0 to 0.0)	0 (0 to 0)	0.0 (0.0 to 0.0)	-91.7 (-98.1 to -71.7)	-81.3 (-95.7 to -36.1)
Bulgaria	Otitis media	10 to 14	Female	0 (0 to 0)	0.0 (0.0 to 0.0)	0 (0 to 0)	0.0 (0.0 to 0.0)	0 (0 to 0)	0.0 (0.0 to 0.0)	-90.6 (-98.3 to -60.5)	-77.8 (-95.9 to -6.3)
Bulgaria	Otitis media	10 to 14	Male	0 (0 to 0)	0.0 (0.0 to 0.0)	0 (0 to 0)	0.0 (0.0 to 0.0)	0 (0 to 0)	0.0 (0.0 to 0.0)	-96.6 (-99.1 to -90.0)	-92.7 (-98.0 to -78.4)
Bulgaria	Meningitis	5 to 9	Both	3 (2 to 4)	0.5 (0.4 to 0.7)	2 (1 to 2)	0.4 (0.3 to 0.5)	1 (0 to 1)	0.2 (0.1 to 0.3)	-78.3 (-86.7 to -65.7)	-59.9 (-75.5 to -36.6)
Bulgaria	Meningitis	5 to 9	Female	1 (1 to 2)	0.5 (0.3 to 0.6)	1 (0 to 1)	0.3 (0.2 to 0.5)	0 (0 to 0)	0.2 (0.1 to 0.3)	-77.7 (-88.1 to -60.5)	-58.5 (-77.8 to -26.6)
Bulgaria	Meningitis	5 to 9	Male	2 (1 to 2)	0.6 (0.4 to 0.8)	1 (1 to 1)	0.5 (0.3 to 0.6)	0 (0 to 1)	0.2 (0.1 to 0.4)	-78.8 (-88.0 to -63.2)	-61.1 (-78.0 to -32.4)
Bulgaria	Meningitis	10 to 14	Both	3 (2 to 4)	0.4 (0.3 to 0.6)	2 (1 to 2)	0.3 (0.2 to 0.4)	1 (0 to 1)	0.2 (0.1 to 0.3)	-79.1 (-86.0 to -69.4)	-53.0 (-68.4 to -31.0)
Bulgaria	Meningitis	10 to 14	Female	1 (1 to 2)	0.4 (0.3 to 0.6)	1 (1 to 1)	0.3 (0.2 to 0.4)	0 (0 to 0)	0.2 (0.1 to 0.3)	-78.6 (-87.8 to -62.0)	-49.1 (-71.1 to -9.7)
Bulgaria	Meningitis	10 to 14	Male	2 (1 to 2)	0.5 (0.3 to 0.7)	1 (1 to 1)	0.3 (0.2 to 0.4)	0 (0 to 0)	0.2 (0.1 to 0.3)	-79.6 (-87.8 to -64.8)	-56.1 (-73.8 to -24.1)
Bulgaria	Encephalitis	5 to 9	Both	3 (1 to 4)	0.5 (0.2 to 0.7)	2 (1 to 3)	0.5 (0.3 to 0.7)	1 (1 to 2)	0.5 (0.2 to 0.8)	-46.9 (-66.2 to -16.3)	-2.0 (-37.6 to 54.5)
Bulgaria	Encephalitis	5 to 9	Female	1 (1 to 2)	0.5 (0.2 to 0.8)	1 (0 to 1)	0.5 (0.2 to 0.7)	1 (0 to 1)	0.5 (0.2 to 0.9)	-47.4 (-71.0 to -7.0)	-2.2 (-46.1 to 73.1)
Bulgaria	Encephalitis	5 to 9	Male	1 (1 to 2)	0.4 (0.2 to 0.7)	1 (0 to 2)	0.5 (0.2 to 0.8)	1 (0 to 1)	0.4 (0.2 to 0.8)	-46.4 (-69.2 to -5.1)	-1.7 (-43.6 to 74.0)
Bulgaria	Encephalitis	10 to 14	Both	2 (2 to 3)	0.4 (0.2 to 0.5)	2 (1 to 3)	0.4 (0.2 to 0.5)	1 (1 to 2)	0.4 (0.2 to 0.6)	-49.8 (-64.3 to -23.5)	13.1 (-19.5 to 72.6)

eTable 8. All-cause and cause-specific mortality (counts and rates) at the global level and by country in the WHO European Region for ages 5 to 9 and 10 to 14 for 1990, 2000, and 2016

Bulgaria	Encephalitis	10 to 14	Female	1 (1 to 2)	0.4 (0.2 to 0.6)	1 (0 to 1)	0.3 (0.2 to 0.5)	1 (0 to 1)	0.4 (0.2 to 0.7)	-52.5 (-70.3 to -16.8)	12.7 (-29.4 to 97.4)
Bulgaria	Encephalitis	10 to 14	Male	1 (1 to 2)	0.4 (0.2 to 0.6)	1 (1 to 2)	0.4 (0.2 to 0.6)	1 (0 to 1)	0.4 (0.2 to 0.7)	-47.4 (-65.6 to -6.5)	13.3 (-26.0 to 101.4)
Bulgaria	Diphtheria	5 to 9	Both	0 (0 to 0)	0.0 (0.0 to 0.0)	0 (0 to 0)	0.0 (0.0 to 0.0)	0 (0 to 0)	0.0 (0.0 to 0.0)	-67.8 (-95.3 to 115.4)	-40.5 (-91.4 to 297.7)
Bulgaria	Diphtheria	5 to 9	Female	0 (0 to 0)	0.0 (0.0 to 0.0)	0 (0 to 0)	0.0 (0.0 to 0.0)	0 (0 to 0)	0.0 (0.0 to 0.0)	-67.3 (-98.4 to 811.6)	-39.2 (-97.1 to 1595.5)
Bulgaria	Diphtheria	5 to 9	Male	0 (0 to 0)	0.0 (0.0 to 0.0)	0 (0 to 0)	0.0 (0.0 to 0.0)	0 (0 to 0)	0.0 (0.0 to 0.0)	-68.2 (-98.9 to 537.5)	-41.6 (-98.0 to 1069.2)
Bulgaria	Diphtheria	10 to 14	Both	0 (0 to 0)	0.0 (0.0 to 0.0)	0 (0 to 0)	0.0 (0.0 to 0.0)	0 (0 to 0)	0.0 (0.0 to 0.0)	-69.4 (-95.6 to 110.3)	-31.0 (-90.2 to 374.2)
Bulgaria	Diphtheria	10 to 14	Female	0 (0 to 0)	0.0 (0.0 to 0.0)	0 (0 to 0)	0.0 (0.0 to 0.0)	0 (0 to 0)	0.0 (0.0 to 0.0)	-67.6 (-97.9 to 441.0)	-23.0 (-95.0 to 1184.5)
Bulgaria	Diphtheria	10 to 14	Male	0 (0 to 0)	0.0 (0.0 to 0.0)	0 (0 to 0)	0.0 (0.0 to 0.0)	0 (0 to 0)	0.0 (0.0 to 0.0)	-70.4 (-98.4 to 390.8)	-36.2 (-96.7 to 956.7)
Bulgaria	Whooping cough	5 to 9	Both	0 (0 to 0)	0.0 (0.0 to 0.0)	0 (0 to 0)	0.0 (0.0 to 0.0)	0 (0 to 0)	0.0 (0.0 to 0.0)	1317.6 (-64.7 to 8663.7)	2517.7 (-34.9 to 16082.5)
Bulgaria	Whooping cough	5 to 9	Female	0 (0 to 0)	0.0 (0.0 to 0.0)	0 (0 to 0)	0.0 (0.0 to 0.0)	0 (0 to 0)	0.0 (0.0 to 0.0)	907.4 (-75.0 to 7114.6)	1773.6 (-53.5 to 13318.0)
Bulgaria	Whooping cough	5 to 9	Male	0 (0 to 0)	0.0 (0.0 to 0.0)	0 (0 to 0)	0.0 (0.0 to 0.0)	0 (0 to 0)	0.0 (0.0 to 0.0)	1627.9 (-56.6 to 13380.2)	3069.2 (-20.4 to 24624.4)
Bulgaria	Whooping cough	10 to 14	Both	0 (0 to 0)	0.0 (0.0 to 0.0)	0 (0 to 0)	0.0 (0.0 to 0.0)	0 (0 to 0)	0.0 (0.0 to 0.0)	1944.3 (-65.2 to 11605.9)	4511.1 (-21.4 to 26303.1)
Bulgaria	Whooping cough	10 to 14	Female	0 (0 to 0)	0.0 (0.0 to 0.0)	0 (0 to 0)	0.0 (0.0 to 0.0)	0 (0 to 0)	0.0 (0.0 to 0.0)	2152.9 (-74.6 to 11249.5)	5248.8 (-39.6 to 26846.1)
Bulgaria	Whooping cough	10 to 14	Male	0 (0 to 0)	0.0 (0.0 to 0.0)	0 (0 to 0)	0.0 (0.0 to 0.0)	0 (0 to 0)	0.0 (0.0 to 0.0)	1830.9 (-60.1 to 17403.4)	4057.7 (-14.1 to 37589.5)
Bulgaria	Tetanus	5 to 9	Both	0 (0 to 0)	0.0 (0.0 to 0.0)	0 (0 to 0)	0.0 (0.0 to 0.0)	0 (0 to 0)	0.0 (0.0 to 0.0)	-98.2 (-99.4 to -88.0)	-96.6 (-98.9 to -77.8)
Bulgaria	Tetanus	5 to 9	Female	0 (0 to 0)	0.0 (0.0 to 0.0)	0 (0 to 0)	0.0 (0.0 to 0.0)	0 (0 to 0)	0.0 (0.0 to 0.0)	-49.7 (-92.8 to 74.4)	-6.5 (-86.6 to 224.4)
Bulgaria	Tetanus	5 to 9	Male	0 (0 to 0)	0.0 (0.0 to 0.0)	0 (0 to 0)	0.0 (0.0 to 0.0)	0 (0 to 0)	0.0 (0.0 to 0.0)	-98.5 (-99.6 to -93.6)	-97.3 (-99.2 to -88.2)
Bulgaria	Tetanus	10 to 14	Both	0 (0 to 0)	0.0 (0.0 to 0.0)	0 (0 to 0)	0.0 (0.0 to 0.0)	0 (0 to 0)	0.0 (0.0 to 0.0)	-98.6 (-99.6 to -93.0)	-96.9 (-99.0 to -84.1)
Bulgaria	Tetanus	10 to 14	Female	0 (0 to 0)	0.0 (0.0 to 0.0)	0 (0 to 0)	0.0 (0.0 to 0.0)	0 (0 to 0)	0.0 (0.0 to 0.0)	-70.8 (-94.9 to 16.6)	-30.8 (-87.8 to 176.8)
Bulgaria	Tetanus	10 to 14	Male	0 (0 to 0)	0.0 (0.0 to 0.0)	0 (0 to 0)	0.0 (0.0 to 0.0)	0 (0 to 0)	0.0 (0.0 to 0.0)	-98.8 (-99.7 to -93.9)	-97.5 (-99.3 to -86.9)
Bulgaria	Measles	5 to 9	Both	0 (0 to 0)	0.0 (0.0 to 0.0)	0 (0 to 0)	0.0 (0.0 to 0.0)	0 (0 to 0)	0.0 (0.0 to 0.0)	36.9 (-49.9 to 234.1)	152.9 (-7.5 to 517.0)
Bulgaria	Measles	5 to 9	Female	0 (0 to 0)	0.0 (0.0 to 0.0)	0 (0 to 0)	0.0 (0.0 to 0.0)	0 (0 to 0)	0.0 (0.0 to 0.0)	60.6 (-57.3 to 410.3)	198.7 (-20.6 to 849.0)
Bulgaria	Measles	5 to 9	Male	0 (0 to 0)	0.0 (0.0 to 0.0)	0 (0 to 0)	0.0 (0.0 to 0.0)	0 (0 to 0)	0.0 (0.0 to 0.0)	-9.1 (-74.0 to 178.0)	66.6 (-52.3 to 410.0)
Bulgaria	Measles	10 to 14	Both	0 (0 to 0)	0.0 (0.0 to 0.0)	0 (0 to 0)	0.0 (0.0 to 0.0)	0 (0 to 0)	0.0 (0.0 to 0.0)	35.8 (-53.5 to 257.4)	206.3 (4.9 to 706.1)
Bulgaria	Measles	10 to 14	Female	0 (0 to 0)	0.0 (0.0 to 0.0)	0 (0 to 0)	0.0 (0.0 to 0.0)	0 (0 to 0)	0.0 (0.0 to 0.0)	4.1 (-63.9 to 199.4)	147.1 (-14.4 to 610.9)
Bulgaria	Measles	10 to 14	Male	0 (0 to 0)	0.0 (0.0 to 0.0)	0 (0 to 0)	0.0 (0.0 to 0.0)	0 (0 to 0)	0.0 (0.0 to 0.0)	40.7 (-56.8 to 322.8)	202.9 (-6.9 to 810.4)
Bulgaria	Varicella and herpes zoster	5 to 9	Both	0 (0 to 0)	0.0 (0.0 to 0.0)	0 (0 to 0)	0.0 (0.0 to 0.0)	0 (0 to 0)	0.0 (0.0 to 0.0)	-73.9 (-87.3 to -51.3)	-51.9 (-76.6 to -10.1)
Bulgaria	Varicella and herpes zoster	5 to 9	Female	0 (0 to 0)	0.0 (0.0 to 0.0)	0 (0 to 0)	0.0 (0.0 to 0.0)	0 (0 to 0)	0.0 (0.0 to 0.0)	-72.6 (-84.3 to -17.1)	-49.0 (-70.9 to 54.2)
Bulgaria	Varicella and herpes zoster	5 to 9	Male	0 (0 to 0)	0.0 (0.0 to 0.0)	0 (0 to 0)	0.0 (0.0 to 0.0)	0 (0 to 0)	0.0 (0.0 to 0.0)	-74.8 (-90.9 to -47.6)	-53.7 (-83.4 to -3.8)
Bulgaria	Varicella and herpes zoster	10 to 14	Both	0 (0 to 0)	0.0 (0.0 to 0.0)	0 (0 to 0)	0.0 (0.0 to 0.0)	0 (0 to 0)	0.0 (0.0 to 0.0)	-71.8 (-84.8 to -43.2)	-36.5 (-65.8 to 28.0)
Bulgaria	Varicella and herpes zoster	10 to 14	Female	0 (0 to 0)	0.0 (0.0 to 0.0)	0 (0 to 0)	0.0 (0.0 to 0.0)	0 (0 to 0)	0.0 (0.0 to 0.0)	-70.1 (-84.3 to -32.3)	-29.0 (-62.7 to 60.8)
Bulgaria	Varicella and herpes zoster	10 to 14	Male	0 (0 to 0)	0.0 (0.0 to 0.0)	0 (0 to 0)	0.0 (0.0 to 0.0)	0 (0 to 0)	0.0 (0.0 to 0.0)	-76.1 (-91.0 to -43.7)	-48.5 (-80.6 to 21.2)

eTable 8. All-cause and cause-specific mortality (counts and rates) at the global level and by country in the WHO European Region for ages 5 to 9 and 10 to 14 for 1990, 2000, and 2016

Bulgaria	Malaria	5 to 9	Both	0 (0 to 0)	0.0 (0.0 to 0.0)	0 (0 to 0)	0.0 (0.0 to 0.0)	0 (0 to 0)	0.0 (0.0 to 0.0)	0.0 (0.0 to 0.0)	0.0 (0.0 to 0.0)
Bulgaria	Malaria	5 to 9	Female	0 (0 to 0)	0.0 (0.0 to 0.0)	0 (0 to 0)	0.0 (0.0 to 0.0)	0 (0 to 0)	0.0 (0.0 to 0.0)	0.0 (0.0 to 0.0)	0.0 (0.0 to 0.0)
Bulgaria	Malaria	5 to 9	Male	0 (0 to 0)	0.0 (0.0 to 0.0)	0 (0 to 0)	0.0 (0.0 to 0.0)	0 (0 to 0)	0.0 (0.0 to 0.0)	0.0 (0.0 to 0.0)	0.0 (0.0 to 0.0)
Bulgaria	Malaria	10 to 14	Both	0 (0 to 0)	0.0 (0.0 to 0.0)	0 (0 to 0)	0.0 (0.0 to 0.0)	0 (0 to 0)	0.0 (0.0 to 0.0)	0.0 (0.0 to 0.0)	0.0 (0.0 to 0.0)
Bulgaria	Malaria	10 to 14	Female	0 (0 to 0)	0.0 (0.0 to 0.0)	0 (0 to 0)	0.0 (0.0 to 0.0)	0 (0 to 0)	0.0 (0.0 to 0.0)	0.0 (0.0 to 0.0)	0.0 (0.0 to 0.0)
Bulgaria	Malaria	10 to 14	Male	0 (0 to 0)	0.0 (0.0 to 0.0)	0 (0 to 0)	0.0 (0.0 to 0.0)	0 (0 to 0)	0.0 (0.0 to 0.0)	0.0 (0.0 to 0.0)	0.0 (0.0 to 0.0)
Bulgaria	Leishmaniasis	5 to 9	Both	0 (0 to 0)	0.0 (0.0 to 0.0)	0 (0 to 0)	0.0 (0.0 to 0.0)	0 (0 to 0)	0.0 (0.0 to 0.0)	-49.0 (-100.0 to 333.3)	-5.8 (-100.0 to 700.1)
Bulgaria	Leishmaniasis	5 to 9	Female	0 (0 to 0)	0.0 (0.0 to 0.0)	0 (0 to 0)	0.0 (0.0 to 0.0)	0 (0 to 0)	0.0 (0.0 to 0.0)	-50.0 (-100.0 to 215.6)	-7.0 (-100.0 to 486.9)
Bulgaria	Leishmaniasis	5 to 9	Male	0 (0 to 0)	0.0 (0.0 to 0.0)	0 (0 to 0)	0.0 (0.0 to 0.0)	0 (0 to 0)	0.0 (0.0 to 0.0)	-47.9 (-100.0 to 131.3)	-4.5 (-100.0 to 324.2)
Bulgaria	Leishmaniasis	10 to 14	Both	0 (0 to 0)	0.0 (0.0 to 0.0)	0 (0 to 0)	0.0 (0.0 to 0.0)	0 (0 to 0)	0.0 (0.0 to 0.0)	-48.0 (-100.0 to 554.8)	17.2 (-100.0 to 1377.0)
Bulgaria	Leishmaniasis	10 to 14	Female	0 (0 to 0)	0.0 (0.0 to 0.0)	0 (0 to 0)	0.0 (0.0 to 0.0)	0 (0 to 0)	0.0 (0.0 to 0.0)	-47.6 (-100.0 to 186.4)	24.3 (-100.0 to 579.9)
Bulgaria	Leishmaniasis	10 to 14	Male	0 (0 to 0)	0.0 (0.0 to 0.0)	0 (0 to 0)	0.0 (0.0 to 0.0)	0 (0 to 0)	0.0 (0.0 to 0.0)	-48.4 (-100.0 to 136.8)	11.1 (-100.0 to 409.9)
Bulgaria	African trypanosomiasis	5 to 9	Both	0 (0 to 0)	0.0 (0.0 to 0.0)	0 (0 to 0)	0.0 (0.0 to 0.0)	0 (0 to 0)	0.0 (0.0 to 0.0)	0.0 (0.0 to 0.0)	0.0 (0.0 to 0.0)
Bulgaria	African trypanosomiasis	5 to 9	Female	0 (0 to 0)	0.0 (0.0 to 0.0)	0 (0 to 0)	0.0 (0.0 to 0.0)	0 (0 to 0)	0.0 (0.0 to 0.0)	0.0 (0.0 to 0.0)	0.0 (0.0 to 0.0)
Bulgaria	African trypanosomiasis	5 to 9	Male	0 (0 to 0)	0.0 (0.0 to 0.0)	0 (0 to 0)	0.0 (0.0 to 0.0)	0 (0 to 0)	0.0 (0.0 to 0.0)	0.0 (0.0 to 0.0)	0.0 (0.0 to 0.0)
Bulgaria	African trypanosomiasis	10 to 14	Both	0 (0 to 0)	0.0 (0.0 to 0.0)	0 (0 to 0)	0.0 (0.0 to 0.0)	0 (0 to 0)	0.0 (0.0 to 0.0)	0.0 (0.0 to 0.0)	0.0 (0.0 to 0.0)
Bulgaria	African trypanosomiasis	10 to 14	Female	0 (0 to 0)	0.0 (0.0 to 0.0)	0 (0 to 0)	0.0 (0.0 to 0.0)	0 (0 to 0)	0.0 (0.0 to 0.0)	0.0 (0.0 to 0.0)	0.0 (0.0 to 0.0)
Bulgaria	African trypanosomiasis	10 to 14	Male	0 (0 to 0)	0.0 (0.0 to 0.0)	0 (0 to 0)	0.0 (0.0 to 0.0)	0 (0 to 0)	0.0 (0.0 to 0.0)	0.0 (0.0 to 0.0)	0.0 (0.0 to 0.0)
Bulgaria	Schistosomiasis	5 to 9	Both	0 (0 to 0)	0.0 (0.0 to 0.0)	0 (0 to 0)	0.0 (0.0 to 0.0)	0 (0 to 0)	0.0 (0.0 to 0.0)	0.0 (0.0 to 0.0)	0.0 (0.0 to 0.0)
Bulgaria	Schistosomiasis	5 to 9	Female	0 (0 to 0)	0.0 (0.0 to 0.0)	0 (0 to 0)	0.0 (0.0 to 0.0)	0 (0 to 0)	0.0 (0.0 to 0.0)	0.0 (0.0 to 0.0)	0.0 (0.0 to 0.0)
Bulgaria	Schistosomiasis	5 to 9	Male	0 (0 to 0)	0.0 (0.0 to 0.0)	0 (0 to 0)	0.0 (0.0 to 0.0)	0 (0 to 0)	0.0 (0.0 to 0.0)	0.0 (0.0 to 0.0)	0.0 (0.0 to 0.0)
Bulgaria	Schistosomiasis	10 to 14	Both	0 (0 to 0)	0.0 (0.0 to 0.0)	0 (0 to 0)	0.0 (0.0 to 0.0)	0 (0 to 0)	0.0 (0.0 to 0.0)	0.0 (0.0 to 0.0)	0.0 (0.0 to 0.0)
Bulgaria	Schistosomiasis	10 to 14	Female	0 (0 to 0)	0.0 (0.0 to 0.0)	0 (0 to 0)	0.0 (0.0 to 0.0)	0 (0 to 0)	0.0 (0.0 to 0.0)	0.0 (0.0 to 0.0)	0.0 (0.0 to 0.0)
Bulgaria	Schistosomiasis	10 to 14	Male	0 (0 to 0)	0.0 (0.0 to 0.0)	0 (0 to 0)	0.0 (0.0 to 0.0)	0 (0 to 0)	0.0 (0.0 to 0.0)	0.0 (0.0 to 0.0)	0.0 (0.0 to 0.0)
Bulgaria	Cysticercosis	5 to 9	Both	0 (0 to 0)	0.0 (0.0 to 0.0)	0 (0 to 0)	0.0 (0.0 to 0.0)	0 (0 to 0)	0.0 (0.0 to 0.0)	-88.9 (-92.1 to -84.5)	-79.5 (-85.4 to -71.4)
Bulgaria	Cysticercosis	5 to 9	Female	0 (0 to 0)	0.0 (0.0 to 0.0)	0 (0 to 0)	0.0 (0.0 to 0.0)	0 (0 to 0)	0.0 (0.0 to 0.0)	-88.8 (-92.0 to -84.4)	-79.2 (-85.1 to -71.1)
Bulgaria	Cysticercosis	5 to 9	Male	0 (0 to 0)	0.0 (0.0 to 0.0)	0 (0 to 0)	0.0 (0.0 to 0.0)	0 (0 to 0)	0.0 (0.0 to 0.0)	-89.0 (-92.1 to -84.7)	-79.8 (-85.5 to -71.9)
Bulgaria	Cysticercosis	10 to 14	Both	0 (0 to 0)	0.0 (0.0 to 0.0)	0 (0 to 0)	0.0 (0.0 to 0.0)	0 (0 to 0)	0.0 (0.0 to 0.0)	-89.3 (-91.3 to -86.8)	-75.9 (-80.4 to -70.2)
Bulgaria	Cysticercosis	10 to 14	Female	0 (0 to 0)	0.0 (0.0 to 0.0)	0 (0 to 0)	0.0 (0.0 to 0.0)	0 (0 to 0)	0.0 (0.0 to 0.0)	-89.2 (-91.4 to -86.5)	-74.4 (-79.6 to -67.9)

Bulgaria	Cysticercosis	10 to 14	Male	0 (0 to 0)	0.0 (0.0 to 0.0)	0 (0 to 0)	0.0 (0.0 to 0.0)	0 (0 to 0)	0.0 (0.0 to 0.0)	-89.4 (-91.6 to -86.3)	-77.1 (-82.0 to -70.6)
Bulgaria	Yellow fever	5 to 9	Both	0 (0 to 0)	0.0 (0.0 to 0.0)	0 (0 to 0)	0.0 (0.0 to 0.0)	0 (0 to 0)	0.0 (0.0 to 0.0)	0.0 (0.0 to 0.0)	0.0 (0.0 to 0.0)
Bulgaria	Yellow fever	5 to 9	Female	0 (0 to 0)	0.0 (0.0 to 0.0)	0 (0 to 0)	0.0 (0.0 to 0.0)	0 (0 to 0)	0.0 (0.0 to 0.0)	0.0 (0.0 to 0.0)	0.0 (0.0 to 0.0)
Bulgaria	Yellow fever	5 to 9	Male	0 (0 to 0)	0.0 (0.0 to 0.0)	0 (0 to 0)	0.0 (0.0 to 0.0)	0 (0 to 0)	0.0 (0.0 to 0.0)	0.0 (0.0 to 0.0)	0.0 (0.0 to 0.0)
Bulgaria	Yellow fever	10 to 14	Both	0 (0 to 0)	0.0 (0.0 to 0.0)	0 (0 to 0)	0.0 (0.0 to 0.0)	0 (0 to 0)	0.0 (0.0 to 0.0)	0.0 (0.0 to 0.0)	0.0 (0.0 to 0.0)
Bulgaria	Yellow fever	10 to 14	Female	0 (0 to 0)	0.0 (0.0 to 0.0)	0 (0 to 0)	0.0 (0.0 to 0.0)	0 (0 to 0)	0.0 (0.0 to 0.0)	0.0 (0.0 to 0.0)	0.0 (0.0 to 0.0)
Bulgaria	Yellow fever	10 to 14	Male	0 (0 to 0)	0.0 (0.0 to 0.0)	0 (0 to 0)	0.0 (0.0 to 0.0)	0 (0 to 0)	0.0 (0.0 to 0.0)	0.0 (0.0 to 0.0)	0.0 (0.0 to 0.0)
Bulgaria	Rabies	5 to 9	Both	0 (0 to 0)	0.0 (0.0 to 0.0)	0 (0 to 0)	0.0 (0.0 to 0.0)	0 (0 to 0)	0.0 (0.0 to 0.0)	-54.6 (-90.4 to 72.9)	-16.2 (-82.3 to 219.3)
Bulgaria	Rabies	5 to 9	Female	0 (0 to 0)	0.0 (0.0 to 0.0)	0 (0 to 0)	0.0 (0.0 to 0.0)	0 (0 to 0)	0.0 (0.0 to 0.0)	-77.8 (-96.9 to 32.0)	-58.7 (-94.2 to 145.5)
Bulgaria	Rabies	5 to 9	Male	0 (0 to 0)	0.0 (0.0 to 0.0)	0 (0 to 0)	0.0 (0.0 to 0.0)	0 (0 to 0)	0.0 (0.0 to 0.0)	9.5 (-83.3 to 439.7)	100.8 (-69.3 to 890.0)
Bulgaria	Rabies	10 to 14	Both	0 (0 to 0)	0.0 (0.0 to 0.0)	0 (0 to 0)	0.0 (0.0 to 0.0)	0 (0 to 0)	0.0 (0.0 to 0.0)	-67.9 (-91.3 to 7.0)	-27.5 (-80.3 to 141.3)
Bulgaria	Rabies	10 to 14	Female	0 (0 to 0)	0.0 (0.0 to 0.0)	0 (0 to 0)	0.0 (0.0 to 0.0)	0 (0 to 0)	0.0 (0.0 to 0.0)	-69.9 (-96.1 to 70.7)	-28.6 (-90.8 to 305.2)
Bulgaria	Rabies	10 to 14	Male	0 (0 to 0)	0.0 (0.0 to 0.0)	0 (0 to 0)	0.0 (0.0 to 0.0)	0 (0 to 0)	0.0 (0.0 to 0.0)	-66.2 (-93.5 to 62.9)	-27.1 (-85.9 to 250.7)
Bulgaria	Intestinal nematode infections	5 to 9	Both	-	-	-	-	-	-	-	-
Bulgaria	Intestinal nematode infections	5 to 9	Female	-	-	-	-	-	-	-	-
Bulgaria	Intestinal nematode infections	5 to 9	Male	-	-	-	-	-	-	-	-
Bulgaria	Intestinal nematode infections	10 to 14	Both	-	-	-	-	-	-	-	-
Bulgaria	Intestinal nematode infections	10 to 14	Female	-	-	-	-	-	-	-	-
Bulgaria	Intestinal nematode infections	10 to 14	Male	-	-	-	-	-	-	-	-
Bulgaria	Ebola	5 to 9	Both	0 (0 to 0)	0.0 (0.0 to 0.0)	0 (0 to 0)	0.0 (0.0 to 0.0)	0 (0 to 0)	0.0 (0.0 to 0.0)	0.0 (0.0 to 0.0)	0.0 (0.0 to 0.0)
Bulgaria	Ebola	5 to 9	Female	0 (0 to 0)	0.0 (0.0 to 0.0)	0 (0 to 0)	0.0 (0.0 to 0.0)	0 (0 to 0)	0.0 (0.0 to 0.0)	0.0 (0.0 to 0.0)	0.0 (0.0 to 0.0)
Bulgaria	Ebola	5 to 9	Male	0 (0 to 0)	0.0 (0.0 to 0.0)	0 (0 to 0)	0.0 (0.0 to 0.0)	0 (0 to 0)	0.0 (0.0 to 0.0)	0.0 (0.0 to 0.0)	0.0 (0.0 to 0.0)
Bulgaria	Ebola	10 to 14	Both	0 (0 to 0)	0.0 (0.0 to 0.0)	0 (0 to 0)	0.0 (0.0 to 0.0)	0 (0 to 0)	0.0 (0.0 to 0.0)	0.0 (0.0 to 0.0)	0.0 (0.0 to 0.0)
Bulgaria	Ebola	10 to 14	Female	0 (0 to 0)	0.0 (0.0 to 0.0)	0 (0 to 0)	0.0 (0.0 to 0.0)	0 (0 to 0)	0.0 (0.0 to 0.0)	0.0 (0.0 to 0.0)	0.0 (0.0 to 0.0)
Bulgaria	Ebola	10 to 14	Male	0 (0 to 0)	0.0 (0.0 to 0.0)	0 (0 to 0)	0.0 (0.0 to 0.0)	0 (0 to 0)	0.0 (0.0 to 0.0)	0.0 (0.0 to 0.0)	0.0 (0.0 to 0.0)
Bulgaria	Zika virus	5 to 9	Both	0 (0 to 0)	0.0 (0.0 to 0.0)	0 (0 to 0)	0.0 (0.0 to 0.0)	0 (0 to 0)	0.0 (0.0 to 0.0)	0.0 (0.0 to 0.0)	0.0 (0.0 to 0.0)
Bulgaria	Zika virus	5 to 9	Female	0 (0 to 0)	0.0 (0.0 to 0.0)	0 (0 to 0)	0.0 (0.0 to 0.0)	0 (0 to 0)	0.0 (0.0 to 0.0)	0.0 (0.0 to 0.0)	0.0 (0.0 to 0.0)
Bulgaria	Zika virus	5 to 9	Male	0 (0 to 0)	0.0 (0.0 to 0.0)	0 (0 to 0)	0.0 (0.0 to 0.0)	0 (0 to 0)	0.0 (0.0 to 0.0)	0.0 (0.0 to 0.0)	0.0 (0.0 to 0.0)
Bulgaria	Zika virus	10 to 14	Both	0 (0 to 0)	0.0 (0.0 to 0.0)	0 (0 to 0)	0.0 (0.0 to 0.0)	0 (0 to 0)	0.0 (0.0 to 0.0)	0.0 (0.0 to 0.0)	0.0 (0.0 to 0.0)

eTable 8. All-cause and cause-specific mortality (counts and rates) at the global level and by country in the WHO European Region for ages 5 to 9 and 10 to 14 for 1990, 2000, and 2016

Bulgaria	Zika virus	10 to 14	Female	0<br>(0 to 0)	0.0<br>(0.0 to 0.0)	0<br>(0 to 0)	0.0<br>(0.0 to 0.0)	0<br>(0 to 0)	0.0<br>(0.0 to 0.0)	0.0<br>(0.0 to 0.0)	0.0<br>(0.0 to 0.0)
Bulgaria	Zika virus	10 to 14	Male	0<br>(0 to 0)	0.0<br>(0.0 to 0.0)	0<br>(0 to 0)	0.0<br>(0.0 to 0.0)	0<br>(0 to 0)	0.0<br>(0.0 to 0.0)	0.0<br>(0.0 to 0.0)	0.0<br>(0.0 to 0.0)
Bulgaria	Other neglected tropical diseases	5 to 9	Both	0<br>(0 to 0)	0.0<br>(0.0 to 0.0)	0<br>(0 to 0)	0.0<br>(0.0 to 0.1)	0<br>(0 to 0)	0.0<br>(0.0 to 0.0)	-34.7<br>(-69.9 to 61.6)	20.5<br>(-44.5 to 198.4)
Bulgaria	Other neglected tropical diseases	5 to 9	Female	0<br>(0 to 0)	0.0<br>(0.0 to 0.0)	0<br>(0 to 0)	0.0<br>(0.0 to 0.0)	0<br>(0 to 0)	0.0<br>(0.0 to 0.0)	-50.0<br>(-65.1 to -29.1)	-6.9<br>(-35.1 to 31.9)
Bulgaria	Other neglected tropical diseases	5 to 9	Male	0<br>(0 to 0)	0.0<br>(0.0 to 0.0)	0<br>(0 to 0)	0.0<br>(0.0 to 0.1)	0<br>(0 to 0)	0.0<br>(0.0 to 0.1)	-23.7<br>(-75.0 to 159.9)	39.9<br>(-54.2 to 376.7)
Bulgaria	Other neglected tropical diseases	10 to 14	Both	0<br>(0 to 0)	0.0<br>(0.0 to 0.0)	0<br>(0 to 0)	0.0<br>(0.0 to 0.1)	0<br>(0 to 0)	0.0<br>(0.0 to 0.0)	-39.1<br>(-72.8 to 43.6)	37.4<br>(-38.5 to 223.9)
Bulgaria	Other neglected tropical diseases	10 to 14	Female	0<br>(0 to 0)	0.0<br>(0.0 to 0.0)	0<br>(0 to 0)	0.0<br>(0.0 to 0.0)	0<br>(0 to 0)	0.0<br>(0.0 to 0.0)	-58.9<br>(-67.5 to -45.7)	-2.4<br>(-22.9 to 28.9)
Bulgaria	Other neglected tropical diseases	10 to 14	Male	0<br>(0 to 0)	0.0<br>(0.0 to 0.0)	0<br>(0 to 0)	0.1<br>(0.0 to 0.1)	0<br>(0 to 0)	0.0<br>(0.0 to 0.1)	-30.0<br>(-76.8 to 115.4)	50.7<br>(-50.0 to 363.8)
Bulgaria	Maternal hemorrhage	10 to 14	Both	0<br>(0 to 0)	0.0<br>(0.0 to 0.0)	0<br>(0 to 0)	0.0<br>(0.0 to 0.0)	0<br>(0 to 0)	0.0<br>(0.0 to 0.0)	-85.4<br>(-90.7 to -77.9)	-67.2<br>(-78.9 to -50.1)
Bulgaria	Maternal hemorrhage	10 to 14	Female	0<br>(0 to 0)	0.0<br>(0.0 to 0.0)	0<br>(0 to 0)	0.0<br>(0.0 to 0.0)	0<br>(0 to 0)	0.0<br>(0.0 to 0.0)	-85.4<br>(-90.7 to -77.9)	-65.4<br>(-77.8 to -47.4)
Bulgaria	Maternal sepsis and other maternal infections	10 to 14	Both	0<br>(0 to 0)	0.0<br>(0.0 to 0.0)	0<br>(0 to 0)	0.0<br>(0.0 to 0.0)	0<br>(0 to 0)	0.0<br>(0.0 to 0.0)	-84.2<br>(-89.4 to -75.9)	-64.5<br>(-76.0 to -45.7)
Bulgaria	Maternal sepsis and other maternal infections	10 to 14	Female	0<br>(0 to 0)	0.0<br>(0.0 to 0.0)	0<br>(0 to 0)	0.0<br>(0.0 to 0.0)	0<br>(0 to 0)	0.0<br>(0.0 to 0.0)	-84.2<br>(-89.4 to -75.9)	-62.6<br>(-74.8 to -42.8)
Bulgaria	Maternal hypertensive disorders	10 to 14	Both	0<br>(0 to 0)	0.0<br>(0.0 to 0.0)	0<br>(0 to 0)	0.0<br>(0.0 to 0.0)	0<br>(0 to 0)	0.0<br>(0.0 to 0.0)	-83.5<br>(-89.1 to -75.0)	-62.8<br>(-75.4 to -43.7)
Bulgaria	Maternal hypertensive disorders	10 to 14	Female	0<br>(0 to 0)	0.0<br>(0.0 to 0.0)	0<br>(0 to 0)	0.0<br>(0.0 to 0.0)	0<br>(0 to 0)	0.0<br>(0.0 to 0.0)	-83.5<br>(-89.1 to -75.0)	-60.8<br>(-74.1 to -40.7)
Bulgaria	Maternal obstructed labor and uterine rupture	10 to 14	Both	0<br>(0 to 0)	0.0<br>(0.0 to 0.0)	0<br>(0 to 0)	0.0<br>(0.0 to 0.0)	0<br>(0 to 0)	0.0<br>(0.0 to 0.0)	-84.0<br>(-89.6 to -75.8)	-63.9<br>(-76.6 to -45.4)
Bulgaria	Maternal obstructed labor and uterine rupture	10 to 14	Female	0<br>(0 to 0)	0.0<br>(0.0 to 0.0)	0<br>(0 to 0)	0.0<br>(0.0 to 0.0)	0<br>(0 to 0)	0.0<br>(0.0 to 0.0)	-84.0<br>(-89.6 to -75.8)	-62.0<br>(-75.4 to -42.5)
Bulgaria	Maternal abortion, miscarriage, and ectopic pregnancy	10 to 14	Both	0<br>(0 to 0)	0.0<br>(0.0 to 0.0)	0<br>(0 to 0)	0.0<br>(0.0 to 0.0)	0<br>(0 to 0)	0.0<br>(0.0 to 0.0)	-86.5<br>(-91.6 to -79.9)	-69.7<br>(-81.0 to -54.7)

eTable 8. All-cause and cause-specific mortality (counts and rates) at the global level and by country in the WHO European Region for ages 5 to 9 and 10 to 14 for 1990, 2000, and 2016

Bulgaria	Maternal abortion, miscarriage, and ectopic pregnancy	10 to 14	Female	0 (0 to 0)	0.0 (0.0 to 0.0)	0 (0 to 0)	0.0 (0.0 to 0.0)	0 (0 to 0)	0.0 (0.0 to 0.0)	-86.5 (-91.6 to -79.9)	-68.1 (-80.0 to -52.3)
Bulgaria	Indirect maternal deaths	10 to 14	Both	0 (0 to 0)	0.0 (0.0 to 0.0)	0 (0 to 0)	0.0 (0.0 to 0.0)	0 (0 to 0)	0.0 (0.0 to 0.0)	-83.6 (-89.2 to -75.3)	-63.0 (-75.7 to -44.3)
Bulgaria	Indirect maternal deaths	10 to 14	Female	0 (0 to 0)	0.0 (0.0 to 0.0)	0 (0 to 0)	0.0 (0.0 to 0.0)	0 (0 to 0)	0.0 (0.0 to 0.0)	-83.6 (-89.2 to -75.3)	-61.1 (-74.4 to -41.3)
Bulgaria	Late maternal deaths	10 to 14	Both	0 (0 to 0)	0.0 (0.0 to 0.0)	0 (0 to 0)	0.0 (0.0 to 0.0)	0 (0 to 0)	0.0 (0.0 to 0.0)	-83.2 (-96.4 to -41.3)	-62.2 (-91.8 to 32.4)
Bulgaria	Late maternal deaths	10 to 14	Female	0 (0 to 0)	0.0 (0.0 to 0.0)	0 (0 to 0)	0.0 (0.0 to 0.0)	0 (0 to 0)	0.0 (0.0 to 0.0)	-83.2 (-96.4 to -41.3)	-60.2 (-91.4 to 39.4)
Bulgaria	Maternal deaths aggravated by HIV/AIDS	10 to 14	Both	0 (0 to 0)	0.0 (0.0 to 0.0)	0 (0 to 0)	0.0 (0.0 to 0.0)	0 (0 to 0)	0.0 (0.0 to 0.0)	7413.8 (0.0 to 56994.7)	16847.6 (0.0 to 128679.6)
Bulgaria	Maternal deaths aggravated by HIV/AIDS	10 to 14	Female	0 (0 to 0)	0.0 (0.0 to 0.0)	0 (0 to 0)	0.0 (0.0 to 0.0)	0 (0 to 0)	0.0 (0.0 to 0.0)	7413.8 (0.0 to 56994.7)	17739.3 (0.0 to 135455.3)
Bulgaria	Other maternal disorders	10 to 14	Both	0 (0 to 0)	0.0 (0.0 to 0.0)	0 (0 to 0)	0.0 (0.0 to 0.0)	0 (0 to 0)	0.0 (0.0 to 0.0)	-84.0 (-89.3 to -76.0)	-63.8 (-76.0 to -45.8)
Bulgaria	Other maternal disorders	10 to 14	Female	0 (0 to 0)	0.0 (0.0 to 0.0)	0 (0 to 0)	0.0 (0.0 to 0.0)	0 (0 to 0)	0.0 (0.0 to 0.0)	-84.0 (-89.3 to -76.0)	-61.9 (-74.7 to -42.9)
Bulgaria	Protein-energy malnutrition	5 to 9	Both	0 (0 to 0)	0.0 (0.0 to 0.0)	0 (0 to 0)	0.0 (0.0 to 0.0)	0 (0 to 0)	0.0 (0.0 to 0.0)	19.4 (-45.3 to 162.6)	120.4 (1.0 to 385.0)
Bulgaria	Protein-energy malnutrition	5 to 9	Female	0 (0 to 0)	0.0 (0.0 to 0.0)	0 (0 to 0)	0.0 (0.0 to 0.0)	0 (0 to 0)	0.0 (0.0 to 0.0)	-10.0 (-68.8 to 203.9)	67.4 (-42.0 to 465.2)
Bulgaria	Protein-energy malnutrition	5 to 9	Male	0 (0 to 0)	0.0 (0.0 to 0.0)	0 (0 to 0)	0.0 (0.0 to 0.0)	0 (0 to 0)	0.0 (0.0 to 0.0)	50.1 (-53.8 to 388.9)	175.2 (-15.3 to 796.7)
Bulgaria	Protein-energy malnutrition	10 to 14	Both	0 (0 to 0)	0.0 (0.0 to 0.0)	0 (0 to 0)	0.0 (0.0 to 0.0)	0 (0 to 0)	0.0 (0.0 to 0.0)	-21.6 (-55.0 to 39.9)	76.9 (1.5 to 215.5)
Bulgaria	Protein-energy malnutrition	10 to 14	Female	0 (0 to 0)	0.0 (0.0 to 0.0)	0 (0 to 0)	0.0 (0.0 to 0.0)	0 (0 to 0)	0.0 (0.0 to 0.0)	-46.8 (-69.0 to 1.4)	26.3 (-26.3 to 140.7)
Bulgaria	Protein-energy malnutrition	10 to 14	Male	0 (0 to 0)	0.0 (0.0 to 0.0)	0 (0 to 0)	0.0 (0.0 to 0.0)	0 (0 to 0)	0.0 (0.0 to 0.0)	2.7 (-53.2 to 152.4)	121.2 (0.9 to 443.5)
Bulgaria	Iodine deficiency	5 to 9	Both	0 (0 to 0)	0.0 (0.0 to 0.0)	0 (0 to 0)	0.0 (0.0 to 0.0)	0 (0 to 0)	0.0 (0.0 to 0.0)	-18.3 (-100.0 to 3.3e+17)	50.8 (-100.0 to 6.2e+17)
Bulgaria	Iodine deficiency	5 to 9	Female	0 (0 to 0)	0.0 (0.0 to 0.0)	0 (0 to 0)	0.0 (0.0 to 0.0)	0 (0 to 0)	0.0 (0.0 to 0.0)	-43.9 (-100.0 to 2.8e+25)	4.3 (-100.0 to 5.3e+25)
Bulgaria	Iodine deficiency	5 to 9	Male	0 (0 to 0)	0.0 (0.0 to 0.0)	0 (0 to 0)	0.0 (0.0 to 0.0)	0 (0 to 0)	0.0 (0.0 to 0.0)	12.8 (-100.0 to 2.0e+29)	107.0 (-100.0 to 3.7e+29)
Bulgaria	Iodine deficiency	10 to 14	Both	0 (0 to 0)	0.0 (0.0 to 0.0)	0 (0 to 0)	0.0 (0.0 to 0.0)	0 (0 to 0)	0.0 (0.0 to 0.0)	-46.5 (-100.0 to 7.5e+17)	20.6 (-100.0 to 1.7e+18)
Bulgaria	Iodine deficiency	10 to 14	Female	0 (0 to 0)	0.0 (0.0 to 0.0)	0 (0 to 0)	0.0 (0.0 to 0.0)	0 (0 to 0)	0.0 (0.0 to 0.0)	-50.9 (-100.0 to 3.0e+27)	16.5 (-100.0 to 7.2e+27)
Bulgaria	Iodine deficiency	10 to 14	Male	0 (0 to 0)	0.0 (0.0 to 0.0)	0 (0 to 0)	0.0 (0.0 to 0.0)	0 (0 to 0)	0.0 (0.0 to 0.0)	-43.5 (-100.0 to 2.3e+26)	21.6 (-100.0 to 5.0e+26)
Bulgaria	Iron-deficiency anemia	5 to 9	Both	0 (0 to 0)	0.0 (0.0 to 0.0)	0 (0 to 0)	0.0 (0.0 to 0.0)	0 (0 to 0)	0.0 (0.0 to 0.0)	9.2 (-50.0 to 131.3)	101.6 (-7.6 to 327.1)
Bulgaria	Iron-deficiency anemia	5 to 9	Female	0 (0 to 0)	0.0 (0.0 to 0.0)	0 (0 to 0)	0.0 (0.0 to 0.0)	0 (0 to 0)	0.0 (0.0 to 0.0)	-12.6 (-69.7 to 203.9)	62.5 (-43.7 to 465.1)

eTable 8. All-cause and cause-specific mortality (counts and rates) at the global level and by country in the WHO European Region for ages 5 to 9 and 10 to 14 for 1990, 2000, and 2016											
Bulgaria	Iron-deficiency anemia	5 to 9	Male	0 (0 to 0)	0.0 (0.0 to 0.0)	0 (0 to 0)	0.0 (0.0 to 0.0)	0 (0 to 0)	0.0 (0.0 to 0.0)	32.1 (-57.0 to 318.5)	142.2 (-21.1 to 667.7)
Bulgaria	Iron-deficiency anemia	10 to 14	Both	0 (0 to 0)	0.0 (0.0 to 0.0)	0 (0 to 0)	0.0 (0.0 to 0.0)	0 (0 to 0)	0.0 (0.0 to 0.0)	-27.2 (-58.0 to 24.9)	64.3 (-5.4 to 181.6)
Bulgaria	Iron-deficiency anemia	10 to 14	Female	0 (0 to 0)	0.0 (0.0 to 0.0)	0 (0 to 0)	0.0 (0.0 to 0.0)	0 (0 to 0)	0.0 (0.0 to 0.0)	-46.8 (-69.0 to 1.4)	26.3 (-26.3 to 140.7)
Bulgaria	Iron-deficiency anemia	10 to 14	Male	0 (0 to 0)	0.0 (0.0 to 0.0)	0 (0 to 0)	0.0 (0.0 to 0.0)	0 (0 to 0)	0.0 (0.0 to 0.0)	-8.2 (-58.7 to 141.5)	97.7 (-11.1 to 420.0)
Bulgaria	Other nutritional deficiencies	5 to 9	Both	0 (0 to 0)	0.0 (0.0 to 0.0)	0 (0 to 0)	0.0 (0.0 to 0.0)	0 (0 to 0)	0.0 (0.0 to 0.0)	70.6 (-27.2 to 270.2)	215.0 (34.5 to 583.6)
Bulgaria	Other nutritional deficiencies	5 to 9	Female	0 (0 to 0)	0.0 (0.0 to 0.0)	0 (0 to 0)	0.0 (0.0 to 0.0)	0 (0 to 0)	0.0 (0.0 to 0.0)	-12.8 (-69.7 to 203.9)	62.2 (-43.7 to 465.2)
Bulgaria	Other nutritional deficiencies	5 to 9	Male	0 (0 to 0)	0.0 (0.0 to 0.0)	0 (0 to 0)	0.0 (0.0 to 0.0)	0 (0 to 0)	0.0 (0.0 to 0.0)	156.6 (-22.7 to 740.7)	370.6 (41.8 to 1441.9)
Bulgaria	Other nutritional deficiencies	10 to 14	Both	0 (0 to 0)	0.0 (0.0 to 0.0)	0 (0 to 0)	0.0 (0.0 to 0.0)	0 (0 to 0)	0.0 (0.0 to 0.0)	-26.5 (-58.0 to 25.3)	65.7 (-5.2 to 182.7)
Bulgaria	Other nutritional deficiencies	10 to 14	Female	0 (0 to 0)	0.0 (0.0 to 0.0)	0 (0 to 0)	0.0 (0.0 to 0.0)	0 (0 to 0)	0.0 (0.0 to 0.0)	-46.8 (-69.0 to 1.4)	26.4 (-26.3 to 140.8)
Bulgaria	Other nutritional deficiencies	10 to 14	Male	0 (0 to 0)	0.0 (0.0 to 0.0)	0 (0 to 0)	0.0 (0.0 to 0.0)	0 (0 to 0)	0.0 (0.0 to 0.0)	-7.0 (-57.8 to 141.5)	100.2 (-9.2 to 420.0)
Bulgaria	Sexually transmitted diseases excluding HIV	5 to 9	Both	0 (0 to 0)	0.0 (0.0 to 0.1)	0 (0 to 0)	0.0 (0.0 to 0.1)	0 (0 to 0)	0.0 (0.0 to 0.0)	-87.2 (-95.6 to -66.6)	-76.4 (-91.9 to -38.3)
Bulgaria	Sexually transmitted diseases excluding HIV	5 to 9	Female	0 (0 to 0)	0.0 (0.0 to 0.1)	0 (0 to 0)	0.0 (0.0 to 0.1)	0 (0 to 0)	0.0 (0.0 to 0.0)	-87.5 (-95.7 to -67.6)	-76.7 (-91.9 to -39.7)
Bulgaria	Sexually transmitted diseases excluding HIV	5 to 9	Male	0 (0 to 0)	0.0 (0.0 to 0.0)	0 (0 to 0)	0.0 (0.0 to 0.0)	0 (0 to 0)	0.0 (0.0 to 0.0)	-86.7 (-95.5 to -65.0)	-75.7 (-91.8 to -35.9)
Bulgaria	Sexually transmitted diseases excluding HIV	10 to 14	Both	0 (0 to 0)	0.0 (0.0 to 0.0)	0 (0 to 0)	0.0 (0.0 to 0.0)	0 (0 to 0)	0.0 (0.0 to 0.0)	-55.2 (-66.0 to -41.1)	1.1 (-23.4 to 32.8)
Bulgaria	Sexually transmitted diseases excluding HIV	10 to 14	Female	0 (0 to 0)	0.0 (0.0 to 0.0)	0 (0 to 0)	0.0 (0.0 to 0.0)	0 (0 to 0)	0.0 (0.0 to 0.0)	-59.3 (-67.3 to -48.5)	-3.4 (-22.3 to 22.4)
Bulgaria	Sexually transmitted diseases excluding HIV	10 to 14	Male	0 (0 to 0)	0.0 (0.0 to 0.0)	0 (0 to 0)	0.0 (0.0 to 0.0)	0 (0 to 0)	0.0 (0.0 to 0.0)	-20.8 (-94.6 to 122.0)	70.4 (-88.4 to 378.0)
Bulgaria	Hepatitis	5 to 9	Both	1 (1 to 1)	0.2 (0.1 to 0.3)	1 (0 to 1)	0.1 (0.1 to 0.2)	0 (0 to 0)	0.1 (0.0 to 0.1)	-82.7 (-91.5 to -65.6)	-68.1 (-84.4 to -36.4)
Bulgaria	Hepatitis	5 to 9	Female	0 (0 to 1)	0.2 (0.1 to 0.3)	0 (0 to 0)	0.1 (0.1 to 0.2)	0 (0 to 0)	0.1 (0.0 to 0.1)	-83.7 (-93.1 to -64.5)	-69.7 (-87.2 to -34.0)
Bulgaria	Hepatitis	5 to 9	Male	1 (0 to 1)	0.2 (0.1 to 0.3)	0 (0 to 1)	0.1 (0.1 to 0.3)	0 (0 to 0)	0.1 (0.0 to 0.1)	-81.8 (-93.5 to -53.2)	-66.7 (-88.1 to -14.1)

eTable 8. All-cause and cause-specific mortality (counts and rates) at the global level and by country in the WHO European Region for ages 5 to 9 and 10 to 14 for 1990, 2000, and 2016

Bulgaria	Hepatitis	10 to 14	Both	2 (1 to 2)	0.2 (0.1 to 0.4)	1 (1 to 1)	0.2 (0.1 to 0.2)	0 (0 to 0)	0.1 (0.0 to 0.1)	-83.6 (-92.5 to -66.2)	-63.0 (-83.2 to -23.9)
Bulgaria	Hepatitis	10 to 14	Female	1 (0 to 1)	0.2 (0.1 to 0.3)	0 (0 to 1)	0.1 (0.1 to 0.2)	0 (0 to 0)	0.1 (0.0 to 0.1)	-82.9 (-92.7 to -60.4)	-59.3 (-82.7 to -6.0)
Bulgaria	Hepatitis	10 to 14	Male	1 (0 to 2)	0.3 (0.1 to 0.5)	0 (0 to 1)	0.2 (0.1 to 0.3)	0 (0 to 0)	0.1 (0.0 to 0.2)	-84.1 (-95.2 to -54.3)	-65.7 (-89.6 to -1.6)
Bulgaria	Other infectious diseases	5 to 9	Both	1 (1 to 1)	0.2 (0.1 to 0.2)	1 (1 to 1)	0.2 (0.1 to 0.3)	0 (0 to 0)	0.1 (0.0 to 0.1)	-79.2 (-88.7 to -62.3)	-61.7 (-79.1 to -30.3)
Bulgaria	Other infectious diseases	5 to 9	Female	1 (0 to 1)	0.2 (0.1 to 0.3)	0 (0 to 1)	0.2 (0.1 to 0.4)	0 (0 to 0)	0.1 (0.0 to 0.2)	-76.1 (-90.4 to -42.4)	-55.5 (-82.1 to 7.1)
Bulgaria	Other infectious diseases	5 to 9	Male	0 (0 to 1)	0.2 (0.1 to 0.2)	0 (0 to 1)	0.2 (0.2 to 0.3)	0 (0 to 0)	0.1 (0.0 to 0.1)	-82.8 (-91.0 to -68.6)	-68.5 (-83.4 to -42.3)
Bulgaria	Other infectious diseases	10 to 14	Both	1 (1 to 1)	0.1 (0.1 to 0.2)	1 (1 to 1)	0.1 (0.1 to 0.2)	0 (0 to 0)	0.1 (0.0 to 0.1)	-80.4 (-88.7 to -67.2)	-55.7 (-74.5 to -26.0)
Bulgaria	Other infectious diseases	10 to 14	Female	0 (0 to 1)	0.1 (0.1 to 0.2)	0 (0 to 1)	0.1 (0.1 to 0.2)	0 (0 to 0)	0.1 (0.0 to 0.1)	-77.5 (-90.3 to -54.1)	-46.5 (-76.9 to 8.9)
Bulgaria	Other infectious diseases	10 to 14	Male	0 (0 to 1)	0.1 (0.1 to 0.2)	0 (0 to 1)	0.1 (0.1 to 0.2)	0 (0 to 0)	0.0 (0.0 to 0.1)	-83.2 (-90.6 to -69.9)	-63.7 (-79.8 to -35.2)
Bulgaria	Nasopharynx cancer	5 to 9	Both	0 (0 to 0)	0.0 (0.0 to 0.0)	0 (0 to 0)	0.0 (0.0 to 0.0)	0 (0 to 0)	0.0 (0.0 to 0.0)	-41.0 (-69.5 to 6.9)	9.0 (-43.6 to 97.4)
Bulgaria	Nasopharynx cancer	5 to 9	Female	0 (0 to 0)	0.0 (0.0 to 0.0)	0 (0 to 0)	0.0 (0.0 to 0.0)	0 (0 to 0)	0.0 (0.0 to 0.0)	-49.8 (-64.3 to -30.4)	-6.6 (-33.5 to 29.5)
Bulgaria	Nasopharynx cancer	5 to 9	Male	0 (0 to 0)	0.0 (0.0 to 0.1)	0 (0 to 0)	0.0 (0.0 to 0.1)	0 (0 to 0)	0.0 (0.0 to 0.1)	-38.5 (-72.1 to 25.0)	12.9 (-48.8 to 129.2)
Bulgaria	Nasopharynx cancer	10 to 14	Both	0 (0 to 0)	0.0 (0.0 to 0.0)	0 (0 to 0)	0.0 (0.0 to 0.0)	0 (0 to 0)	0.0 (0.0 to 0.0)	-53.9 (-71.4 to -24.1)	3.9 (-35.5 to 71.2)
Bulgaria	Nasopharynx cancer	10 to 14	Female	0 (0 to 0)	0.0 (0.0 to 0.0)	0 (0 to 0)	0.0 (0.0 to 0.0)	0 (0 to 0)	0.0 (0.0 to 0.0)	-69.0 (-81.4 to -48.3)	-26.4 (-55.9 to 22.8)
Bulgaria	Nasopharynx cancer	10 to 14	Male	0 (0 to 0)	0.0 (0.0 to 0.1)	0 (0 to 0)	0.0 (0.0 to 0.1)	0 (0 to 0)	0.0 (0.0 to 0.1)	-48.0 (-70.8 to -4.5)	11.9 (-37.1 to 105.7)
Bulgaria	Liver cancer	5 to 9	Both	1 (0 to 1)	0.1 (0.1 to 0.1)	0 (0 to 0)	0.1 (0.1 to 0.1)	0 (0 to 0)	0.1 (0.0 to 0.1)	-64.2 (-80.1 to -37.0)	-33.9 (-63.2 to 16.3)
Bulgaria	Liver cancer	5 to 9	Female	0 (0 to 0)	0.1 (0.1 to 0.2)	0 (0 to 0)	0.1 (0.0 to 0.1)	0 (0 to 0)	0.1 (0.0 to 0.1)	-67.0 (-84.3 to -30.3)	-38.6 (-70.7 to 29.6)
Bulgaria	Liver cancer	5 to 9	Male	0 (0 to 0)	0.1 (0.1 to 0.2)	0 (0 to 0)	0.1 (0.0 to 0.1)	0 (0 to 0)	0.1 (0.0 to 0.1)	-61.4 (-81.5 to -19.0)	-29.3 (-66.1 to 48.5)
Bulgaria	Liver cancer	10 to 14	Both	1 (0 to 1)	0.1 (0.1 to 0.2)	0 (0 to 1)	0.1 (0.1 to 0.1)	0 (0 to 0)	0.1 (0.0 to 0.1)	-70.6 (-82.7 to -49.7)	-33.7 (-61.0 to 13.4)
Bulgaria	Liver cancer	10 to 14	Female	0 (0 to 1)	0.1 (0.1 to 0.2)	0 (0 to 0)	0.1 (0.1 to 0.1)	0 (0 to 0)	0.1 (0.0 to 0.1)	-72.5 (-86.5 to -42.6)	-34.8 (-67.9 to 36.3)
Bulgaria	Liver cancer	10 to 14	Male	0 (0 to 0)	0.1 (0.1 to 0.1)	0 (0 to 0)	0.1 (0.1 to 0.1)	0 (0 to 0)	0.1 (0.0 to 0.1)	-68.3 (-84.5 to -36.6)	-31.8 (-66.5 to 36.5)
Bulgaria	Kidney cancer	5 to 9	Both	1 (1 to 1)	0.1 (0.1 to 0.2)	0 (0 to 1)	0.1 (0.1 to 0.1)	1 (1 to 1)	0.2 (0.2 to 0.4)	-9.9 (-46.9 to 48.5)	66.4 (-2.0 to 174.2)
Bulgaria	Kidney cancer	5 to 9	Female	0 (0 to 1)	0.1 (0.1 to 0.2)	0 (0 to 0)	0.1 (0.0 to 0.1)	0 (0 to 1)	0.2 (0.1 to 0.4)	-2.1 (-50.8 to 87.7)	82.0 (-8.4 to 249.1)
Bulgaria	Kidney cancer	5 to 9	Male	0 (0 to 1)	0.2 (0.1 to 0.2)	0 (0 to 0)	0.1 (0.1 to 0.2)	0 (0 to 1)	0.3 (0.1 to 0.4)	-15.8 (-58.4 to 64.5)	54.4 (-23.7 to 201.7)
Bulgaria	Kidney cancer	10 to 14	Both	0 (0 to 0)	0.1 (0.0 to 0.1)	0 (0 to 0)	0.0 (0.0 to 0.0)	0 (0 to 0)	0.1 (0.1 to 0.1)	-16.5 (-46.4 to 27.8)	88.3 (21.0 to 188.3)
Bulgaria	Kidney cancer	10 to 14	Female	0 (0 to 0)	0.1 (0.0 to 0.1)	0 (0 to 0)	0.0 (0.0 to 0.0)	0 (0 to 0)	0.1 (0.1 to 0.2)	-12.0 (-51.9 to 61.2)	108.9 (14.2 to 282.7)
Bulgaria	Kidney cancer	10 to 14	Male	0 (0 to 0)	0.1 (0.0 to 0.1)	0 (0 to 0)	0.0 (0.0 to 0.1)	0 (0 to 0)	0.1 (0.1 to 0.1)	-20.3 (-56.4 to 40.2)	71.5 (-6.0 to 202.0)
Bulgaria	Brain and nervous system cancer	5 to 9	Both	12 (9 to 16)	2.1 (1.6 to 2.7)	7 (6 to 9)	1.7 (1.4 to 2.2)	5 (3 to 8)	1.7 (1.1 to 2.4)	-56.5 (-73.8 to -31.0)	-19.8 (-51.6 to 27.3)

eTable 8. All-cause and cause-specific mortality (counts and rates) at the global level and by country in the WHO European Region for ages 5 to 9 and 10 to 14 for 1990, 2000, and 2016

Bulgaria	Brain and nervous system cancer	5 to 9	Female	6<br>(4 to 8)	2.0<br>(1.4 to 2.8)	3<br>(2 to 4)	1.6<br>(1.1 to 2.1)	3<br>(2 to 4)	1.8<br>(1.1 to 2.7)	-53.2<br>(-75.3 to -16.3)	-12.9<br>(-54.1 to 55.7)
Bulgaria	Brain and nervous system cancer	5 to 9	Male	7<br>(5 to 9)	2.2<br>(1.6 to 3.0)	4<br>(3 to 5)	1.9<br>(1.4 to 2.6)	3<br>(2 to 4)	1.6<br>(0.9 to 2.6)	-59.5<br>(-77.9 to -25.2)	-25.7<br>(-59.5 to 37.2)
Bulgaria	Brain and nervous system cancer	10 to 14	Both	10<br>(8 to 13)	1.6<br>(1.2 to 2.0)	7<br>(5 to 8)	1.3<br>(1.0 to 1.5)	4<br>(3 to 6)	1.4<br>(1.0 to 2.0)	-59.4<br>(-73.9 to -39.3)	-8.4<br>(-41.1 to 36.9)
Bulgaria	Brain and nervous system cancer	10 to 14	Female	5<br>(3 to 7)	1.5<br>(1.0 to 2.1)	3<br>(2 to 4)	1.3<br>(0.9 to 1.7)	2<br>(1 to 3)	1.4<br>(0.9 to 2.2)	-59.6<br>(-77.1 to -34.5)	-4.1<br>(-45.7 to 55.4)
Bulgaria	Brain and nervous system cancer	10 to 14	Male	5<br>(4 to 7)	1.6<br>(1.1 to 2.2)	3<br>(3 to 4)	1.3<br>(1.0 to 1.7)	2<br>(1 to 3)	1.4<br>(0.9 to 2.1)	-59.2<br>(-77.0 to -30.7)	-12.1<br>(-50.6 to 49.3)
Bulgaria	Thyroid cancer	10 to 14	Both	0<br>(0 to 0)	0.0<br>(0.0 to 0.0)	0<br>(0 to 0)	0.0<br>(0.0 to 0.0)	0<br>(0 to 0)	0.0<br>(0.0 to 0.0)	-57.7<br>(-66.7 to -47.4)	-4.5<br>(-24.8 to 18.7)
Bulgaria	Thyroid cancer	10 to 14	Female	0<br>(0 to 0)	0.0<br>(0.0 to 0.0)	0<br>(0 to 0)	0.0<br>(0.0 to 0.0)	0<br>(0 to 0)	0.0<br>(0.0 to 0.0)	-60.7<br>(-71.8 to -48.2)	-6.7<br>(-33.1 to 23.1)
Bulgaria	Thyroid cancer	10 to 14	Male	0<br>(0 to 0)	0.0<br>(0.0 to 0.0)	0<br>(0 to 0)	0.0<br>(0.0 to 0.0)	0<br>(0 to 0)	0.0<br>(0.0 to 0.0)	-54.7<br>(-64.4 to -42.9)	-2.4<br>(-23.3 to 22.9)
Bulgaria	Hodgkin lymphoma	5 to 9	Both	1<br>(1 to 2)	0.2<br>(0.1 to 0.3)	1<br>(0 to 1)	0.2<br>(0.1 to 0.3)	0<br>(0 to 0)	0.1<br>(0.0 to 0.2)	-75.5<br>(-91.2 to -44.0)	-54.8<br>(-83.8 to 3.4)
Bulgaria	Hodgkin lymphoma	5 to 9	Female	0<br>(0 to 0)	0.1<br>(0.0 to 0.1)	0<br>(0 to 0)	0.1<br>(0.0 to 0.1)	0<br>(0 to 0)	0.0<br>(0.0 to 0.1)	-78.4<br>(-93.3 to -37.4)	-59.9<br>(-87.6 to 16.5)
Bulgaria	Hodgkin lymphoma	5 to 9	Male	1<br>(0 to 1)	0.3<br>(0.1 to 0.5)	1<br>(0 to 1)	0.3<br>(0.1 to 0.5)	0<br>(0 to 0)	0.1<br>(0.0 to 0.3)	-74.8<br>(-92.3 to -33.6)	-53.8<br>(-85.8 to 21.8)
Bulgaria	Hodgkin lymphoma	10 to 14	Both	1<br>(1 to 2)	0.2<br>(0.2 to 0.3)	1<br>(1 to 1)	0.2<br>(0.1 to 0.2)	0<br>(0 to 1)	0.1<br>(0.1 to 0.2)	-76.9<br>(-87.1 to -61.2)	-47.9<br>(-70.9 to -12.6)
Bulgaria	Hodgkin lymphoma	10 to 14	Female	0<br>(0 to 1)	0.1<br>(0.1 to 0.2)	0<br>(0 to 0)	0.1<br>(0.1 to 0.2)	0<br>(0 to 0)	0.1<br>(0.0 to 0.1)	-77.8<br>(-90.3 to -48.9)	-47.3<br>(-76.9 to 21.4)
Bulgaria	Hodgkin lymphoma	10 to 14	Male	1<br>(1 to 2)	0.3<br>(0.2 to 0.5)	1<br>(0 to 1)	0.2<br>(0.2 to 0.3)	0<br>(0 to 0)	0.2<br>(0.1 to 0.3)	-76.6<br>(-88.4 to -55.8)	-49.6<br>(-75.1 to -4.9)
Bulgaria	Non-Hodgkin lymphoma	5 to 9	Both	3<br>(2 to 4)	0.5<br>(0.4 to 0.7)	2<br>(1 to 2)	0.5<br>(0.3 to 0.6)	2<br>(1 to 3)	0.5<br>(0.3 to 0.8)	-46.3<br>(-67.5 to -12.1)	-0.8<br>(-40.0 to 62.4)
Bulgaria	Non-Hodgkin lymphoma	5 to 9	Female	1<br>(1 to 1)	0.3<br>(0.2 to 0.5)	1<br>(0 to 1)	0.3<br>(0.2 to 0.4)	1<br>(0 to 1)	0.4<br>(0.2 to 0.6)	-39.3<br>(-69.5 to 22.7)	12.9<br>(-43.4 to 128.3)
Bulgaria	Non-Hodgkin lymphoma	5 to 9	Male	2<br>(1 to 3)	0.7<br>(0.4 to 1.0)	1<br>(1 to 2)	0.6<br>(0.4 to 0.9)	1<br>(1 to 2)	0.6<br>(0.3 to 1.1)	-49.4<br>(-73.2 to -7.7)	-7.2<br>(-50.8 to 69.3)
Bulgaria	Non-Hodgkin lymphoma	10 to 14	Both	3<br>(2 to 4)	0.4<br>(0.3 to 0.6)	2<br>(1 to 2)	0.4<br>(0.3 to 0.5)	1<br>(1 to 2)	0.5<br>(0.3 to 0.7)	-54.1<br>(-72.0 to -25.0)	3.6<br>(-36.9 to 69.1)
Bulgaria	Non-Hodgkin lymphoma	10 to 14	Female	1<br>(0 to 1)	0.2<br>(0.1 to 0.3)	1<br>(0 to 1)	0.2<br>(0.1 to 0.3)	0<br>(0 to 1)	0.3<br>(0.2 to 0.5)	-44.2<br>(-71.5 to 12.7)	32.4<br>(-32.3 to 167.5)
Bulgaria	Non-Hodgkin lymphoma	10 to 14	Male	2<br>(1 to 3)	0.7<br>(0.4 to 0.9)	1<br>(1 to 2)	0.5<br>(0.3 to 0.7)	1<br>(1 to 2)	0.6<br>(0.3 to 1.0)	-57.3<br>(-76.2 to -24.3)	-8.0<br>(-48.8 to 63.0)
Bulgaria	Leukemia	5 to 9	Both	15<br>(12 to 19)	2.6<br>(2.1 to 3.3)	9<br>(7 to 11)	2.2<br>(1.7 to 2.7)	5<br>(3 to 8)	1.7<br>(1.1 to 2.5)	-65.7<br>(-79.0 to -45.7)	-36.7<br>(-61.2 to 0.3)
Bulgaria	Leukemia	5 to 9	Female	7<br>(5 to 9)	2.3<br>(1.7 to 3.2)	4<br>(3 to 5)	1.8<br>(1.3 to 2.4)	2<br>(1 to 3)	1.4<br>(0.8 to 2.2)	-68.0<br>(-82.2 to -42.8)	-40.5<br>(-66.9 to 6.4)
Bulgaria	Leukemia	5 to 9	Male	9<br>(6 to 12)	2.9<br>(2.1 to 4.0)	5<br>(4 to 7)	2.5<br>(1.9 to 3.3)	3<br>(2 to 5)	1.9<br>(1.1 to 3.2)	-64.0<br>(-80.4 to -36.1)	-34.1<br>(-64.1 to 17.2)
Bulgaria	Leukemia	10 to 14	Both	16<br>(12 to 19)	2.4<br>(1.9 to 2.9)	10<br>(8 to 12)	1.9<br>(1.5 to 2.3)	5<br>(3 to 6)	1.6<br>(1.2 to 2.2)	-69.9<br>(-79.8 to -55.8)	-32.0<br>(-54.4 to -0.3)
Bulgaria	Leukemia	10 to 14	Female	5<br>(4 to 7)	1.6<br>(1.1 to 2.2)	4<br>(3 to 5)	1.5<br>(1.1 to 2.0)	2<br>(1 to 3)	1.3<br>(0.8 to 1.9)	-66.7<br>(-80.9 to -43.4)	-20.9<br>(-54.6 to 34.3)
Bulgaria	Leukemia	10 to 14	Male	10<br>(8 to 13)	3.1<br>(2.3 to 4.0)	6<br>(4 to 8)	2.3<br>(1.7 to 3.0)	3<br>(2 to 4)	1.9<br>(1.3 to 2.8)	-71.4<br>(-82.9 to -52.6)	-38.5<br>(-63.2 to 2.2)
Bulgaria	Other neoplasms	5 to 9	Both	5<br>(4 to 7)	0.9<br>(0.7 to 1.1)	3<br>(3 to 4)	0.8<br>(0.7 to 1.0)	2<br>(1 to 3)	0.7<br>(0.5 to 1.1)	-56.2<br>(-73.4 to -29.9)	-19.2<br>(-50.8 to 29.5)
Bulgaria	Other neoplasms	5 to 9	Female	2<br>(1 to 3)	0.8<br>(0.5 to 1.1)	1<br>(1 to 2)	0.8<br>(0.5 to 1.0)	1<br>(1 to 2)	0.7<br>(0.4 to 1.2)	-48.3<br>(-72.0 to -3.1)	-3.9<br>(-47.8 to 80.2)
Bulgaria	Other neoplasms	5 to 9	Male	3<br>(2 to 4)	1.0<br>(0.6 to 1.4)	2<br>(1 to 3)	0.9<br>(0.7 to 1.2)	1<br>(1 to 2)	0.7<br>(0.4 to 1.1)	-62.2<br>(-79.7 to -31.1)	-30.7<br>(-62.8 to 26.4)
Bulgaria	Other neoplasms	10 to 14	Both	7<br>(5 to 8)	1.0<br>(0.8 to 1.3)	5<br>(4 to 6)	0.9<br>(0.7 to 1.2)	3<br>(2 to 4)	0.9<br>(0.6 to 1.2)	-61.6<br>(-74.3 to -41.8)	-13.4<br>(-42.0 to 31.2)

eTable 8. All-cause and cause-specific mortality (counts and rates) at the global level and by country in the WHO European Region for ages 5 to 9 and 10 to 14 for 1990, 2000, and 2016

Bulgaria	Other neoplasms	10 to 14	Female	4 (2 to 5)	1.1 (0.8 to 1.5)	3 (2 to 4)	1.1 (0.7 to 1.5)	1 (1 to 2)	1.1 (0.7 to 1.6)	-60.6 (-76.9 to -30.6)	-6.4 (-45.3 to 64.8)
Bulgaria	Other neoplasms	10 to 14	Male	3 (2 to 4)	1.0 (0.7 to 1.3)	2 (2 to 3)	0.8 (0.6 to 1.1)	1 (1 to 2)	0.8 (0.5 to 1.2)	-62.8 (-79.0 to -34.9)	-19.9 (-54.9 to 40.3)
Bulgaria	Rheumatic heart disease	5 to 9	Both	2 (1 to 2)	0.3 (0.2 to 0.4)	1 (0 to 1)	0.2 (0.1 to 0.2)	0 (0 to 0)	0.1 (0.0 to 0.1)	-89.3 (-93.9 to -81.9)	-80.2 (-88.7 to -66.6)
Bulgaria	Rheumatic heart disease	5 to 9	Female	1 (0 to 1)	0.2 (0.2 to 0.3)	0 (0 to 0)	0.1 (0.1 to 0.2)	0 (0 to 0)	0.0 (0.0 to 0.1)	-89.6 (-94.2 to -82.2)	-80.7 (-89.2 to -66.8)
Bulgaria	Rheumatic heart disease	5 to 9	Male	1 (1 to 1)	0.3 (0.2 to 0.5)	0 (0 to 1)	0.2 (0.1 to 0.3)	0 (0 to 0)	0.1 (0.0 to 0.1)	-89.0 (-94.6 to -79.8)	-79.8 (-90.1 to -63.0)
Bulgaria	Rheumatic heart disease	10 to 14	Both	3 (2 to 4)	0.4 (0.3 to 0.5)	1 (1 to 1)	0.2 (0.2 to 0.3)	0 (0 to 0)	0.1 (0.1 to 0.2)	-88.7 (-92.4 to -83.4)	-74.5 (-82.9 to -62.6)
Bulgaria	Rheumatic heart disease	10 to 14	Female	1 (1 to 1)	0.3 (0.2 to 0.4)	0 (0 to 0)	0.1 (0.1 to 0.2)	0 (0 to 0)	0.1 (0.0 to 0.1)	-89.3 (-93.9 to -81.7)	-74.5 (-85.5 to -56.6)
Bulgaria	Rheumatic heart disease	10 to 14	Male	2 (1 to 2)	0.6 (0.4 to 0.7)	1 (1 to 1)	0.3 (0.2 to 0.4)	0 (0 to 0)	0.1 (0.1 to 0.2)	-88.4 (-93.1 to -81.3)	-75.0 (-85.0 to -59.7)
Bulgaria	Ischemic heart disease	5 to 9	Both	0 (0 to 0)	0.0 (0.0 to 0.0)	0 (0 to 0)	0.0 (0.0 to 0.0)	0 (0 to 0)	0.0 (0.0 to 0.0)	-48.4 (-67.2 to -22.4)	-4.7 (-39.4 to 43.2)
Bulgaria	Ischemic heart disease	5 to 9	Female	0 (0 to 0)	0.0 (0.0 to 0.0)	0 (0 to 0)	0.0 (0.0 to 0.0)	0 (0 to 0)	0.0 (0.0 to 0.0)	-52.0 (-70.4 to -22.6)	-10.7 (-44.9 to 43.9)
Bulgaria	Ischemic heart disease	5 to 9	Male	0 (0 to 0)	0.0 (0.0 to 0.0)	0 (0 to 0)	0.0 (0.0 to 0.0)	0 (0 to 0)	0.0 (0.0 to 0.0)	-44.9 (-70.6 to -7.8)	1.0 (-46.0 to 69.1)
Bulgaria	Ischemic heart disease	10 to 14	Both	0 (0 to 0)	0.0 (0.0 to 0.0)	0 (0 to 0)	0.0 (0.0 to 0.0)	0 (0 to 0)	0.0 (0.0 to 0.0)	-58.2 (-71.5 to -38.9)	-5.7 (-35.7 to 37.8)
Bulgaria	Ischemic heart disease	10 to 14	Female	0 (0 to 0)	0.0 (0.0 to 0.0)	0 (0 to 0)	0.0 (0.0 to 0.0)	0 (0 to 0)	0.0 (0.0 to 0.0)	-59.3 (-75.4 to -33.8)	-3.3 (-41.6 to 57.1)
Bulgaria	Ischemic heart disease	10 to 14	Male	0 (0 to 0)	0.0 (0.0 to 0.0)	0 (0 to 0)	0.0 (0.0 to 0.0)	0 (0 to 0)	0.0 (0.0 to 0.0)	-57.3 (-75.4 to -29.8)	-8.0 (-47.1 to 51.1)
Bulgaria	Cerebrovascular disease	5 to 9	Both	6 (5 to 7)	1.0 (0.8 to 1.3)	3 (2 to 4)	0.7 (0.5 to 0.9)	1 (1 to 2)	0.4 (0.3 to 0.6)	-78.5 (-86.6 to -66.0)	-60.3 (-75.3 to -37.3)
Bulgaria	Cerebrovascular disease	5 to 9	Female	3 (2 to 4)	1.0 (0.7 to 1.3)	1 (1 to 2)	0.6 (0.4 to 0.8)	1 (0 to 1)	0.4 (0.2 to 0.6)	-79.8 (-88.4 to -66.6)	-62.5 (-78.5 to -37.8)
Bulgaria	Cerebrovascular disease	5 to 9	Male	3 (2 to 4)	1.0 (0.7 to 1.4)	2 (1 to 2)	0.8 (0.5 to 1.2)	1 (0 to 1)	0.4 (0.2 to 0.7)	-77.2 (-88.1 to -59.3)	-58.2 (-78.2 to -25.4)
Bulgaria	Cerebrovascular disease	10 to 14	Both	9 (8 to 11)	1.4 (1.2 to 1.7)	5 (3 to 6)	0.9 (0.7 to 1.1)	2 (1 to 3)	0.6 (0.5 to 0.9)	-80.0 (-86.4 to -71.5)	-55.0 (-69.3 to -35.8)
Bulgaria	Cerebrovascular disease	10 to 14	Female	4 (3 to 5)	1.3 (1.0 to 1.6)	2 (1 to 2)	0.7 (0.5 to 1.0)	1 (0 to 1)	0.6 (0.4 to 0.8)	-81.2 (-88.6 to -70.4)	-55.3 (-72.8 to -29.8)
Bulgaria	Cerebrovascular disease	10 to 14	Male	5 (4 to 7)	1.6 (1.2 to 2.0)	3 (2 to 4)	1.0 (0.7 to 1.3)	1 (1 to 2)	0.7 (0.5 to 1.0)	-79.2 (-87.0 to -66.9)	-55.1 (-72.1 to -28.8)
Bulgaria	Hypertensive heart disease	5 to 9	Both	0 (0 to 0)	0.0 (0.0 to 0.0)	0 (0 to 0)	0.0 (0.0 to 0.0)	0 (0 to 0)	0.0 (0.0 to 0.0)	-48.4 (-67.2 to -22.4)	-4.7 (-39.4 to 43.2)
Bulgaria	Hypertensive heart disease	5 to 9	Female	0 (0 to 0)	0.0 (0.0 to 0.0)	0 (0 to 0)	0.0 (0.0 to 0.0)	0 (0 to 0)	0.0 (0.0 to 0.0)	-52.0 (-70.4 to -22.6)	-10.7 (-44.9 to 43.9)
Bulgaria	Hypertensive heart disease	5 to 9	Male	0 (0 to 0)	0.0 (0.0 to 0.0)	0 (0 to 0)	0.0 (0.0 to 0.0)	0 (0 to 0)	0.0 (0.0 to 0.0)	-44.9 (-70.3 to -7.8)	1.1 (-45.5 to 69.1)
Bulgaria	Hypertensive heart disease	10 to 14	Both	0 (0 to 0)	0.0 (0.0 to 0.1)	0 (0 to 0)	0.0 (0.0 to 0.1)	0 (0 to 0)	0.0 (0.0 to 0.1)	-48.8 (-74.9 to 2.4)	15.5 (-43.4 to 130.9)
Bulgaria	Hypertensive heart disease	10 to 14	Female	0 (0 to 0)	0.0 (0.0 to 0.1)	0 (0 to 0)	0.0 (0.0 to 0.1)	0 (0 to 0)	0.0 (0.0 to 0.1)	-56.6 (-78.8 to -9.4)	3.1 (-49.7 to 115.2)
Bulgaria	Hypertensive heart disease	10 to 14	Male	0 (0 to 0)	0.0 (0.0 to 0.1)	0 (0 to 0)	0.0 (0.0 to 0.1)	0 (0 to 0)	0.0 (0.0 to 0.1)	-41.5 (-80.2 to 55.7)	26.0 (-57.3 to 235.2)
Bulgaria	Cardiomyopathy and myocarditis	5 to 9	Both	1 (0 to 1)	0.1 (0.1 to 0.2)	1 (0 to 1)	0.2 (0.1 to 0.2)	0 (0 to 1)	0.1 (0.1 to 0.2)	-40.6 (-75.7 to 12.0)	9.6 (-55.2 to 106.8)
Bulgaria	Cardiomyopathy and myocarditis	5 to 9	Female	0 (0 to 1)	0.1 (0.1 to 0.2)	0 (0 to 0)	0.1 (0.1 to 0.2)	0 (0 to 0)	0.1 (0.1 to 0.2)	-53.0 (-79.2 to 1.1)	-12.5 (-61.4 to 88.1)
Bulgaria	Cardiomyopathy and myocarditis	5 to 9	Male	0 (0 to 1)	0.1 (0.1 to 0.2)	0 (0 to 1)	0.2 (0.1 to 0.4)	0 (0 to 1)	0.2 (0.1 to 0.3)	-31.8 (-76.6 to 46.7)	25.0 (-57.2 to 169.1)
Bulgaria	Cardiomyopathy and myocarditis	10 to 14	Both	1 (1 to 2)	0.2 (0.1 to 0.3)	1 (1 to 1)	0.2 (0.1 to 0.3)	1 (0 to 1)	0.2 (0.1 to 0.3)	-41.6 (-74.8 to 11.6)	31.6 (-43.3 to 151.7)

eTable 8. All-cause and cause-specific mortality (counts and rates) at the global level and by country in the WHO European Region for ages 5 to 9 and 10 to 14 for 1990, 2000, and 2016

Bulgaria	Cardiomyopathy and myocarditis	10 to 14	Female	0<br>(0 to 1)	0.1<br>(0.1 to 0.2)	0<br>(0 to 1)	0.1<br>(0.1 to 0.2)	0<br>(0 to 0)	0.2<br>(0.1 to 0.3)	-51.0<br>(-77.9 to 4.8)	16.4<br>(-47.6 to 148.8)
Bulgaria	Cardiomyopathy and myocarditis	10 to 14	Male	1<br>(0 to 1)	0.2<br>(0.1 to 0.3)	1<br>(0 to 1)	0.2<br>(0.1 to 0.4)	0<br>(0 to 1)	0.3<br>(0.1 to 0.5)	-35.2<br>(-76.7 to 41.1)	39.6<br>(-49.9 to 203.9)
Bulgaria	Atrial fibrillation and flutter	5 to 9	Both	0<br>(0 to 0)	0.0<br>(0.0 to 0.0)	0<br>(0 to 0)	0.0<br>(0.0 to 0.0)	0<br>(0 to 0)	0.0<br>(0.0 to 0.0)	0.0<br>(0.0 to 0.0)	0.0<br>(0.0 to 0.0)
Bulgaria	Atrial fibrillation and flutter	5 to 9	Female	0<br>(0 to 0)	0.0<br>(0.0 to 0.0)	0<br>(0 to 0)	0.0<br>(0.0 to 0.0)	0<br>(0 to 0)	0.0<br>(0.0 to 0.0)	0.0<br>(0.0 to 0.0)	0.0<br>(0.0 to 0.0)
Bulgaria	Atrial fibrillation and flutter	5 to 9	Male	0<br>(0 to 0)	0.0<br>(0.0 to 0.0)	0<br>(0 to 0)	0.0<br>(0.0 to 0.0)	0<br>(0 to 0)	0.0<br>(0.0 to 0.0)	0.0<br>(0.0 to 0.0)	0.0<br>(0.0 to 0.0)
Bulgaria	Atrial fibrillation and flutter	10 to 14	Both	0<br>(0 to 0)	0.0<br>(0.0 to 0.0)	0<br>(0 to 0)	0.0<br>(0.0 to 0.0)	0<br>(0 to 0)	0.0<br>(0.0 to 0.0)	0.0<br>(0.0 to 0.0)	0.0<br>(0.0 to 0.0)
Bulgaria	Atrial fibrillation and flutter	10 to 14	Female	0<br>(0 to 0)	0.0<br>(0.0 to 0.0)	0<br>(0 to 0)	0.0<br>(0.0 to 0.0)	0<br>(0 to 0)	0.0<br>(0.0 to 0.0)	0.0<br>(0.0 to 0.0)	0.0<br>(0.0 to 0.0)
Bulgaria	Atrial fibrillation and flutter	10 to 14	Male	0<br>(0 to 0)	0.0<br>(0.0 to 0.0)	0<br>(0 to 0)	0.0<br>(0.0 to 0.0)	0<br>(0 to 0)	0.0<br>(0.0 to 0.0)	0.0<br>(0.0 to 0.0)	0.0<br>(0.0 to 0.0)
Bulgaria	Endocarditis	5 to 9	Both	1<br>(0 to 1)	0.1<br>(0.0 to 0.2)	0<br>(0 to 1)	0.1<br>(0.0 to 0.2)	0<br>(0 to 0)	0.1<br>(0.0 to 0.1)	-54.6<br>(-76.7 to -17.6)	-16.1<br>(-57.0 to 52.2)
Bulgaria	Endocarditis	5 to 9	Female	0<br>(0 to 1)	0.1<br>(0.0 to 0.2)	0<br>(0 to 0)	0.1<br>(0.0 to 0.2)	0<br>(0 to 0)	0.1<br>(0.0 to 0.1)	-56.7<br>(-79.1 to -20.7)	-19.5<br>(-61.1 to 47.6)
Bulgaria	Endocarditis	5 to 9	Male	0<br>(0 to 0)	0.1<br>(0.0 to 0.2)	0<br>(0 to 0)	0.1<br>(0.0 to 0.2)	0<br>(0 to 0)	0.1<br>(0.0 to 0.1)	-52.2<br>(-79.1 to 11.1)	-12.3<br>(-61.6 to 103.8)
Bulgaria	Endocarditis	10 to 14	Both	1<br>(0 to 1)	0.1<br>(0.1 to 0.1)	0<br>(0 to 1)	0.1<br>(0.1 to 0.1)	0<br>(0 to 0)	0.1<br>(0.1 to 0.1)	-54.8<br>(-74.2 to -28.4)	2.0<br>(-41.9 to 61.6)
Bulgaria	Endocarditis	10 to 14	Female	0<br>(0 to 0)	0.1<br>(0.0 to 0.1)	0<br>(0 to 0)	0.1<br>(0.0 to 0.1)	0<br>(0 to 0)	0.1<br>(0.0 to 0.1)	-59.3<br>(-79.3 to -28.8)	-3.4<br>(-50.7 to 69.1)
Bulgaria	Endocarditis	10 to 14	Male	0<br>(0 to 0)	0.1<br>(0.1 to 0.1)	0<br>(0 to 0)	0.1<br>(0.0 to 0.1)	0<br>(0 to 0)	0.1<br>(0.1 to 0.2)	-51.0<br>(-76.4 to -10.1)	5.5<br>(-49.2 to 93.5)
Bulgaria	Other cardiovascular and circulatory diseases	5 to 9	Both	4<br>(3 to 5)	0.7<br>(0.5 to 0.8)	4<br>(3 to 5)	1.0<br>(0.8 to 1.3)	1<br>(1 to 2)	0.4<br>(0.3 to 0.6)	-67.8<br>(-80.0 to -48.9)	-40.6<br>(-63.1 to -5.6)
Bulgaria	Other cardiovascular and circulatory diseases	5 to 9	Female	2<br>(1 to 2)	0.6<br>(0.4 to 0.8)	2<br>(1 to 2)	0.8<br>(0.6 to 1.1)	1<br>(0 to 1)	0.4<br>(0.2 to 0.6)	-68.2<br>(-82.1 to -46.4)	-40.9<br>(-66.7 to -0.4)
Bulgaria	Other cardiovascular and circulatory diseases	5 to 9	Male	2<br>(1 to 3)	0.7<br>(0.5 to 1.0)	2<br>(2 to 3)	1.2<br>(0.8 to 1.6)	1<br>(0 to 1)	0.4<br>(0.2 to 0.7)	-67.5<br>(-82.6 to -40.4)	-40.3<br>(-68.0 to 9.3)
Bulgaria	Other cardiovascular and circulatory diseases	10 to 14	Both	4<br>(3 to 5)	0.7<br>(0.5 to 0.8)	5<br>(4 to 6)	0.9<br>(0.7 to 1.2)	1<br>(1 to 2)	0.5<br>(0.4 to 0.7)	-66.1<br>(-77.5 to -49.1)	-23.5<br>(-49.2 to 14.7)
Bulgaria	Other cardiovascular and circulatory diseases	10 to 14	Female	2<br>(1 to 3)	0.6<br>(0.4 to 0.8)	2<br>(2 to 3)	0.8<br>(0.6 to 1.1)	1<br>(0 to 1)	0.4<br>(0.3 to 0.6)	-68.3<br>(-82.1 to -47.0)	-24.8<br>(-57.6 to 25.7)

eTable 8. All-cause and cause-specific mortality (counts and rates) at the global level and by country in the WHO European Region for ages 5 to 9 and 10 to 14 for 1990, 2000, and 2016

Bulgaria	Other cardiovascular and circulatory diseases	10 to 14	Male	2 (2 to 3)	0.7 (0.5 to 1.0)	3 (2 to 4)	1.0 (0.7 to 1.4)	1 (1 to 1)	0.6 (0.3 to 0.8)	-64.3 (-79.2 to -41.0)	-23.2 (-55.2 to 27.0)
Bulgaria	Chronic obstructive pulmonary disease	5 to 9	Both	1 (0 to 1)	0.1 (0.1 to 0.1)	0 (0 to 0)	0.1 (0.0 to 0.1)	0 (0 to 0)	0.0 (0.0 to 0.1)	-76.0 (-86.0 to -59.4)	-55.7 (-74.2 to -25.0)
Bulgaria	Chronic obstructive pulmonary disease	5 to 9	Female	0 (0 to 0)	0.1 (0.1 to 0.2)	0 (0 to 0)	0.1 (0.0 to 0.1)	0 (0 to 0)	0.1 (0.0 to 0.1)	-71.4 (-85.8 to -46.7)	-46.9 (-73.5 to -0.8)
Bulgaria	Chronic obstructive pulmonary disease	5 to 9	Male	0 (0 to 0)	0.1 (0.1 to 0.2)	0 (0 to 0)	0.1 (0.0 to 0.1)	0 (0 to 0)	0.0 (0.0 to 0.1)	-80.4 (-90.1 to -62.6)	-64.0 (-81.9 to -31.4)
Bulgaria	Chronic obstructive pulmonary disease	10 to 14	Both	1 (1 to 1)	0.1 (0.1 to 0.1)	0 (0 to 0)	0.1 (0.0 to 0.1)	0 (0 to 0)	0.1 (0.0 to 0.1)	-70.3 (-82.0 to -55.3)	-33.1 (-59.5 to 0.8)
Bulgaria	Chronic obstructive pulmonary disease	10 to 14	Female	0 (0 to 0)	0.1 (0.0 to 0.1)	0 (0 to 0)	0.0 (0.0 to 0.1)	0 (0 to 0)	0.1 (0.0 to 0.1)	-64.4 (-80.6 to -35.2)	-15.4 (-53.9 to 53.9)
Bulgaria	Chronic obstructive pulmonary disease	10 to 14	Male	0 (0 to 1)	0.1 (0.1 to 0.2)	0 (0 to 0)	0.1 (0.1 to 0.1)	0 (0 to 0)	0.1 (0.0 to 0.1)	-73.3 (-85.8 to -54.7)	-42.5 (-69.5 to -2.4)
Bulgaria	Asthma	5 to 9	Both	0 (0 to 0)	0.0 (0.0 to 0.0)	0 (0 to 0)	0.0 (0.0 to 0.0)	0 (0 to 0)	0.0 (0.0 to 0.0)	-79.9 (-90.0 to -55.5)	-62.9 (-81.5 to -17.8)
Bulgaria	Asthma	5 to 9	Female	0 (0 to 0)	0.0 (0.0 to 0.0)	0 (0 to 0)	0.0 (0.0 to 0.0)	0 (0 to 0)	0.0 (0.0 to 0.0)	-60.9 (-80.9 to -24.5)	-27.3 (-64.6 to 40.5)
Bulgaria	Asthma	5 to 9	Male	0 (0 to 0)	0.0 (0.0 to 0.1)	0 (0 to 0)	0.0 (0.0 to 0.0)	0 (0 to 0)	0.0 (0.0 to 0.0)	-85.6 (-93.5 to -60.0)	-73.6 (-88.1 to -26.7)
Bulgaria	Asthma	10 to 14	Both	1 (0 to 1)	0.1 (0.0 to 0.1)	0 (0 to 0)	0.0 (0.0 to 0.1)	0 (0 to 0)	0.0 (0.0 to 0.0)	-90.1 (-94.5 to -78.1)	-77.6 (-87.6 to -50.6)
Bulgaria	Asthma	10 to 14	Female	0 (0 to 0)	0.0 (0.0 to 0.0)	0 (0 to 0)	0.0 (0.0 to 0.0)	0 (0 to 0)	0.0 (0.0 to 0.0)	-85.5 (-92.7 to -70.4)	-65.6 (-82.6 to -29.7)
Bulgaria	Asthma	10 to 14	Male	0 (0 to 1)	0.1 (0.1 to 0.2)	0 (0 to 0)	0.1 (0.0 to 0.1)	0 (0 to 0)	0.0 (0.0 to 0.0)	-91.2 (-96.0 to -76.0)	-81.0 (-91.3 to -48.4)
Bulgaria	Interstitial lung disease and pulmonary sarcoidosis	5 to 9	Both	0 (0 to 0)	0.0 (0.0 to 0.0)	0 (0 to 0)	0.0 (0.0 to 0.0)	0 (0 to 0)	0.0 (0.0 to 0.0)	-73.0 (-86.7 to -48.2)	-50.1 (-75.4 to -4.4)
Bulgaria	Interstitial lung disease and pulmonary sarcoidosis	5 to 9	Female	0 (0 to 0)	0.0 (0.0 to 0.0)	0 (0 to 0)	0.0 (0.0 to 0.0)	0 (0 to 0)	0.0 (0.0 to 0.0)	-73.4 (-89.1 to -34.8)	-50.5 (-79.7 to 21.3)
Bulgaria	Interstitial lung disease and pulmonary sarcoidosis	5 to 9	Male	0 (0 to 0)	0.0 (0.0 to 0.0)	0 (0 to 0)	0.0 (0.0 to 0.0)	0 (0 to 0)	0.0 (0.0 to 0.0)	-72.6 (-90.0 to -32.2)	-49.7 (-81.6 to 24.4)
Bulgaria	Interstitial lung disease and pulmonary sarcoidosis	10 to 14	Both	0 (0 to 0)	0.0 (0.0 to 0.0)	0 (0 to 0)	0.0 (0.0 to 0.0)	0 (0 to 0)	0.0 (0.0 to 0.0)	-70.3 (-83.9 to -45.1)	-33.0 (-63.7 to 23.7)

eTable 8. All-cause and cause-specific mortality (counts and rates) at the global level and by country in the WHO European Region for ages 5 to 9 and 10 to 14 for 1990, 2000, and 2016

Bulgaria	Interstitial lung disease and pulmonary sarcoidosis	10 to 14	Female	0<br>(0 to 0)	0.0<br>(0.0 to 0.0)	0<br>(0 to 0)	0.0<br>(0.0 to 0.0)	0<br>(0 to 0)	0.0<br>(0.0 to 0.0)	-72.3<br>(-87.3 to -31.6)	-34.2<br>(-69.9 to 62.5)
Bulgaria	Interstitial lung disease and pulmonary sarcoidosis	10 to 14	Male	0<br>(0 to 0)	0.0<br>(0.0 to 0.0)	0<br>(0 to 0)	0.0<br>(0.0 to 0.0)	0<br>(0 to 0)	0.0<br>(0.0 to 0.0)	-65.9<br>(-85.0 to -19.7)	-26.5<br>(-67.7 to 72.9)
Bulgaria	Other chronic respiratory diseases	5 to 9	Both	0<br>(0 to 0)	0.1<br>(0.0 to 0.1)	0<br>(0 to 0)	0.1<br>(0.0 to 0.1)	0<br>(0 to 0)	0.0<br>(0.0 to 0.1)	-61.0<br>(-77.7 to -33.3)	-28.0<br>(-58.8 to 23.2)
Bulgaria	Other chronic respiratory diseases	5 to 9	Female	0<br>(0 to 0)	0.1<br>(0.0 to 0.1)	0<br>(0 to 0)	0.1<br>(0.0 to 0.1)	0<br>(0 to 0)	0.0<br>(0.0 to 0.1)	-60.0<br>(-81.1 to -19.0)	-25.6<br>(-64.9 to 50.7)
Bulgaria	Other chronic respiratory diseases	5 to 9	Male	0<br>(0 to 0)	0.1<br>(0.0 to 0.1)	0<br>(0 to 0)	0.1<br>(0.0 to 0.1)	0<br>(0 to 0)	0.0<br>(0.0 to 0.1)	-61.8<br>(-81.0 to -24.1)	-29.9<br>(-65.1 to 39.2)
Bulgaria	Other chronic respiratory diseases	10 to 14	Both	0<br>(0 to 0)	0.0<br>(0.0 to 0.1)	0<br>(0 to 0)	0.1<br>(0.0 to 0.1)	0<br>(0 to 0)	0.1<br>(0.0 to 0.1)	-51.5<br>(-72.0 to -15.5)	9.3<br>(-36.9 to 90.7)
Bulgaria	Other chronic respiratory diseases	10 to 14	Female	0<br>(0 to 0)	0.0<br>(0.0 to 0.1)	0<br>(0 to 0)	0.0<br>(0.0 to 0.1)	0<br>(0 to 0)	0.0<br>(0.0 to 0.1)	-53.4<br>(-78.0 to 6.4)	10.5<br>(-47.7 to 152.6)
Bulgaria	Other chronic respiratory diseases	10 to 14	Male	0<br>(0 to 0)	0.1<br>(0.0 to 0.1)	0<br>(0 to 0)	0.1<br>(0.0 to 0.1)	0<br>(0 to 0)	0.1<br>(0.0 to 0.1)	-50.8<br>(-74.8 to -8.1)	5.9<br>(-45.8 to 97.8)
Bulgaria	Cirrhosis and other chronic liver diseases due to hepatitis B	5 to 9	Both	0<br>(0 to 0)	0.0<br>(0.0 to 0.0)	0<br>(0 to 0)	0.0<br>(0.0 to 0.0)	0<br>(0 to 0)	0.0<br>(0.0 to 0.0)	-64.5<br>(-78.5 to -41.7)	-34.5<br>(-60.3 to 7.6)
Bulgaria	Cirrhosis and other chronic liver diseases due to hepatitis B	5 to 9	Female	0<br>(0 to 0)	0.0<br>(0.0 to 0.0)	0<br>(0 to 0)	0.0<br>(0.0 to 0.0)	0<br>(0 to 0)	0.0<br>(0.0 to 0.0)	-65.3<br>(-79.3 to -44.4)	-35.5<br>(-61.5 to 3.4)
Bulgaria	Cirrhosis and other chronic liver diseases due to hepatitis B	5 to 9	Male	0<br>(0 to 0)	0.0<br>(0.0 to 0.0)	0<br>(0 to 0)	0.0<br>(0.0 to 0.0)	0<br>(0 to 0)	0.0<br>(0.0 to 0.0)	-64.3<br>(-79.4 to -38.3)	-34.5<br>(-62.2 to 13.2)
Bulgaria	Cirrhosis and other chronic liver diseases due to hepatitis B	10 to 14	Both	0<br>(0 to 0)	0.0<br>(0.0 to 0.0)	0<br>(0 to 0)	0.0<br>(0.0 to 0.0)	0<br>(0 to 0)	0.0<br>(0.0 to 0.0)	-67.8<br>(-80.3 to -48.7)	-27.4<br>(-55.5 to 15.7)
Bulgaria	Cirrhosis and other chronic liver diseases due to hepatitis B	10 to 14	Female	0<br>(0 to 0)	0.0<br>(0.0 to 0.0)	0<br>(0 to 0)	0.0<br>(0.0 to 0.0)	0<br>(0 to 0)	0.0<br>(0.0 to 0.0)	-66.7<br>(-80.8 to -44.1)	-20.9<br>(-54.5 to 32.8)
Bulgaria	Cirrhosis and other chronic liver diseases due to hepatitis B	10 to 14	Male	0<br>(0 to 0)	0.0<br>(0.0 to 0.0)	0<br>(0 to 0)	0.0<br>(0.0 to 0.0)	0<br>(0 to 0)	0.0<br>(0.0 to 0.0)	-68.0<br>(-81.8 to -46.0)	-31.2<br>(-60.9 to 16.3)
Bulgaria	Cirrhosis and other chronic liver diseases due to hepatitis C	5 to 9	Both	0<br>(0 to 0)	0.0<br>(0.0 to 0.0)	0<br>(0 to 0)	0.0<br>(0.0 to 0.0)	0<br>(0 to 0)	0.0<br>(0.0 to 0.0)	-63.8<br>(-78.0 to -41.6)	-33.1<br>(-59.3 to 7.8)

eTable 8. All-cause and cause-specific mortality (counts and rates) at the global level and by country in the WHO European Region for ages 5 to 9 and 10 to 14 for 1990, 2000, and 2016

Bulgaria	Cirrhosis and other chronic liver diseases due to hepatitis C	5 to 9	Female	0 (0 to 0)	0.0 (0.0 to 0.0)	0 (0 to 0)	0.0 (0.0 to 0.0)	0 (0 to 0)	0.0 (0.0 to 0.0)	-64.7 (-78.8 to -43.5)	-34.3 (-60.5 to 5.1)
Bulgaria	Cirrhosis and other chronic liver diseases due to hepatitis C	5 to 9	Male	0 (0 to 0)	0.0 (0.0 to 0.0)	0 (0 to 0)	0.0 (0.0 to 0.0)	0 (0 to 0)	0.0 (0.0 to 0.0)	-63.5 (-79.0 to -37.0)	-33.1 (-61.4 to 15.6)
Bulgaria	Cirrhosis and other chronic liver diseases due to hepatitis C	10 to 14	Both	0 (0 to 0)	0.0 (0.0 to 0.0)	0 (0 to 0)	0.0 (0.0 to 0.0)	0 (0 to 0)	0.0 (0.0 to 0.0)	-67.1 (-79.7 to -48.8)	-25.9 (-54.2 to 15.5)
Bulgaria	Cirrhosis and other chronic liver diseases due to hepatitis C	10 to 14	Female	0 (0 to 0)	0.0 (0.0 to 0.0)	0 (0 to 0)	0.0 (0.0 to 0.0)	0 (0 to 0)	0.0 (0.0 to 0.0)	-66.2 (-80.7 to -43.0)	-19.9 (-54.2 to 35.3)
Bulgaria	Cirrhosis and other chronic liver diseases due to hepatitis C	10 to 14	Male	0 (0 to 0)	0.0 (0.0 to 0.0)	0 (0 to 0)	0.0 (0.0 to 0.0)	0 (0 to 0)	0.0 (0.0 to 0.0)	-67.3 (-81.5 to -44.6)	-29.6 (-60.1 to 19.3)
Bulgaria	Cirrhosis and other chronic liver diseases due to other causes	5 to 9	Both	2 (2 to 3)	0.4 (0.3 to 0.5)	1 (1 to 2)	0.3 (0.3 to 0.4)	1 (0 to 1)	0.2 (0.2 to 0.3)	-66.6 (-79.2 to -48.3)	-38.4 (-61.6 to -4.5)
Bulgaria	Cirrhosis and other chronic liver diseases due to other causes	5 to 9	Female	1 (1 to 1)	0.4 (0.3 to 0.5)	1 (0 to 1)	0.3 (0.2 to 0.4)	0 (0 to 0)	0.2 (0.1 to 0.3)	-67.7 (-80.7 to -48.1)	-39.9 (-64.0 to -3.5)
Bulgaria	Cirrhosis and other chronic liver diseases due to other causes	5 to 9	Male	1 (1 to 2)	0.4 (0.3 to 0.6)	1 (1 to 1)	0.4 (0.3 to 0.5)	0 (0 to 1)	0.3 (0.2 to 0.4)	-65.8 (-80.3 to -40.7)	-37.3 (-63.9 to 8.8)
Bulgaria	Cirrhosis and other chronic liver diseases due to other causes	10 to 14	Both	2 (2 to 3)	0.4 (0.3 to 0.5)	1 (1 to 2)	0.3 (0.2 to 0.4)	1 (1 to 1)	0.3 (0.2 to 0.4)	-69.2 (-79.7 to -54.0)	-30.6 (-54.3 to 3.7)
Bulgaria	Cirrhosis and other chronic liver diseases due to other causes	10 to 14	Female	1 (1 to 1)	0.3 (0.2 to 0.4)	1 (0 to 1)	0.2 (0.2 to 0.3)	0 (0 to 0)	0.2 (0.1 to 0.3)	-68.8 (-82.3 to -48.1)	-26.0 (-58.0 to 23.3)
Bulgaria	Cirrhosis and other chronic liver diseases due to other causes	10 to 14	Male	1 (1 to 2)	0.4 (0.3 to 0.6)	1 (1 to 1)	0.3 (0.2 to 0.5)	0 (0 to 1)	0.3 (0.2 to 0.4)	-69.5 (-82.6 to -48.2)	-34.3 (-62.6 to 11.5)
Bulgaria	Peptic ulcer disease	5 to 9	Both	0 (0 to 1)	0.1 (0.0 to 0.1)	0 (0 to 0)	0.0 (0.0 to 0.1)	0 (0 to 0)	0.0 (0.0 to 0.0)	-85.7 (-93.0 to -71.4)	-73.6 (-87.1 to -47.2)
Bulgaria	Peptic ulcer disease	5 to 9	Female	0 (0 to 0)	0.1 (0.0 to 0.1)	0 (0 to 0)	0.0 (0.0 to 0.1)	0 (0 to 0)	0.0 (0.0 to 0.0)	-84.8 (-93.7 to -65.3)	-71.7 (-88.4 to -35.4)
Bulgaria	Peptic ulcer disease	5 to 9	Male	0 (0 to 0)	0.1 (0.0 to 0.1)	0 (0 to 0)	0.0 (0.0 to 0.1)	0 (0 to 0)	0.0 (0.0 to 0.0)	-86.7 (-95.2 to -63.2)	-75.5 (-91.1 to -32.5)
Bulgaria	Peptic ulcer disease	10 to 14	Both	0 (0 to 0)	0.1 (0.0 to 0.1)	0 (0 to 0)	0.0 (0.0 to 0.0)	0 (0 to 0)	0.0 (0.0 to 0.0)	-83.7 (-91.1 to -70.2)	-63.3 (-79.9 to -32.9)
Bulgaria	Peptic ulcer disease	10 to 14	Female	0 (0 to 0)	0.1 (0.0 to 0.1)	0 (0 to 0)	0.0 (0.0 to 0.1)	0 (0 to 0)	0.0 (0.0 to 0.0)	-83.2 (-92.7 to -62.0)	-60.0 (-82.6 to -9.8)

eTable 8. All-cause and cause-specific mortality (counts and rates) at the global level and by country in the WHO European Region for ages 5 to 9 and 10 to 14 for 1990, 2000, and 2016

Bulgaria	Peptic ulcer disease	10 to 14	Male	0 (0 to 0)	0.0 (0.0 to 0.1)	0 (0 to 0)	0.0 (0.0 to 0.0)	0 (0 to 0)	0.0 (0.0 to 0.0)	-84.5 (-93.2 to -64.6)	-66.7 (-85.4 to -23.7)
Bulgaria	Gastritis and duodenitis	5 to 9	Both	0 (0 to 0)	0.0 (0.0 to 0.0)	0 (0 to 0)	0.0 (0.0 to 0.0)	0 (0 to 0)	0.0 (0.0 to 0.0)	-38.0 (-78.9 to 98.9)	14.5 (-61.1 to 267.3)
Bulgaria	Gastritis and duodenitis	5 to 9	Female	0 (0 to 0)	0.0 (0.0 to 0.0)	0 (0 to 0)	0.0 (0.0 to 0.0)	0 (0 to 0)	0.0 (0.0 to 0.0)	-38.4 (-83.7 to 197.5)	14.5 (-69.7 to 453.3)
Bulgaria	Gastritis and duodenitis	5 to 9	Male	0 (0 to 0)	0.0 (0.0 to 0.0)	0 (0 to 0)	0.0 (0.0 to 0.0)	0 (0 to 0)	0.0 (0.0 to 0.0)	-36.9 (-82.0 to 103.1)	15.8 (-67.0 to 272.4)
Bulgaria	Gastritis and duodenitis	10 to 14	Both	0 (0 to 0)	0.0 (0.0 to 0.0)	0 (0 to 0)	0.0 (0.0 to 0.0)	0 (0 to 0)	0.0 (0.0 to 0.0)	-42.7 (-81.4 to 69.2)	29.3 (-58.0 to 281.6)
Bulgaria	Gastritis and duodenitis	10 to 14	Female	0 (0 to 0)	0.0 (0.0 to 0.0)	0 (0 to 0)	0.0 (0.0 to 0.0)	0 (0 to 0)	0.0 (0.0 to 0.1)	-41.8 (-82.9 to 82.4)	38.1 (-59.4 to 333.1)
Bulgaria	Gastritis and duodenitis	10 to 14	Male	0 (0 to 0)	0.0 (0.0 to 0.0)	0 (0 to 0)	0.0 (0.0 to 0.0)	0 (0 to 0)	0.0 (0.0 to 0.0)	-53.8 (-76.7 to -2.9)	-0.5 (-49.7 to 109.0)
Bulgaria	Appendicitis	5 to 9	Both	0 (0 to 1)	0.1 (0.0 to 0.1)	0 (0 to 0)	0.0 (0.0 to 0.1)	0 (0 to 0)	0.0 (0.0 to 0.1)	-69.8 (-84.4 to -38.5)	-44.2 (-71.3 to 13.6)
Bulgaria	Appendicitis	5 to 9	Female	0 (0 to 0)	0.1 (0.0 to 0.1)	0 (0 to 0)	0.0 (0.0 to 0.1)	0 (0 to 0)	0.0 (0.0 to 0.1)	-58.3 (-82.6 to -8.5)	-22.5 (-67.6 to 70.3)
Bulgaria	Appendicitis	5 to 9	Male	0 (0 to 0)	0.1 (0.0 to 0.2)	0 (0 to 0)	0.0 (0.0 to 0.1)	0 (0 to 0)	0.0 (0.0 to 0.1)	-77.2 (-90.2 to -34.9)	-58.1 (-82.1 to 19.3)
Bulgaria	Appendicitis	10 to 14	Both	1 (0 to 1)	0.1 (0.0 to 0.1)	0 (0 to 0)	0.0 (0.0 to 0.1)	0 (0 to 0)	0.1 (0.0 to 0.1)	-71.1 (-83.4 to -50.0)	-34.9 (-62.6 to 12.8)
Bulgaria	Appendicitis	10 to 14	Female	0 (0 to 0)	0.1 (0.0 to 0.1)	0 (0 to 0)	0.0 (0.0 to 0.1)	0 (0 to 0)	0.1 (0.0 to 0.1)	-64.2 (-82.6 to -25.4)	-15.0 (-58.6 to 77.2)
Bulgaria	Appendicitis	10 to 14	Male	0 (0 to 1)	0.1 (0.0 to 0.2)	0 (0 to 0)	0.0 (0.0 to 0.1)	0 (0 to 0)	0.1 (0.0 to 0.1)	-75.5 (-88.2 to -46.0)	-47.2 (-74.6 to 16.4)
Bulgaria	Paralytic ileus and intestinal obstruction	5 to 9	Both	1 (1 to 1)	0.1 (0.1 to 0.2)	0 (0 to 1)	0.1 (0.1 to 0.2)	0 (0 to 1)	0.1 (0.1 to 0.2)	-60.2 (-77.4 to -31.5)	-26.5 (-58.4 to 26.6)
Bulgaria	Paralytic ileus and intestinal obstruction	5 to 9	Female	0 (0 to 1)	0.1 (0.1 to 0.2)	0 (0 to 0)	0.1 (0.0 to 0.1)	0 (0 to 0)	0.1 (0.0 to 0.1)	-61.2 (-81.3 to -15.1)	-27.8 (-65.2 to 58.0)
Bulgaria	Paralytic ileus and intestinal obstruction	5 to 9	Male	1 (0 to 1)	0.2 (0.1 to 0.3)	0 (0 to 0)	0.1 (0.1 to 0.2)	0 (0 to 0)	0.1 (0.1 to 0.2)	-59.7 (-80.0 to -9.8)	-26.0 (-63.4 to 65.4)
Bulgaria	Paralytic ileus and intestinal obstruction	10 to 14	Both	1 (0 to 1)	0.1 (0.1 to 0.1)	0 (0 to 1)	0.1 (0.0 to 0.1)	0 (0 to 0)	0.1 (0.0 to 0.1)	-62.6 (-76.9 to -39.9)	-15.5 (-48.0 to 35.6)
Bulgaria	Paralytic ileus and intestinal obstruction	10 to 14	Female	0 (0 to 0)	0.1 (0.0 to 0.1)	0 (0 to 0)	0.0 (0.0 to 0.1)	0 (0 to 0)	0.0 (0.0 to 0.1)	-66.2 (-81.9 to -30.4)	-19.9 (-57.0 to 65.3)
Bulgaria	Paralytic ileus and intestinal obstruction	10 to 14	Male	0 (0 to 1)	0.1 (0.1 to 0.2)	0 (0 to 0)	0.1 (0.1 to 0.1)	0 (0 to 0)	0.1 (0.1 to 0.2)	-61.1 (-78.3 to -27.5)	-16.1 (-53.2 to 56.1)
Bulgaria	Inguinal, femoral, and abdominal hernia	5 to 9	Both	0 (0 to 0)	0.0 (0.0 to 0.0)	0 (0 to 0)	0.0 (0.0 to 0.0)	0 (0 to 0)	0.0 (0.0 to 0.0)	-89.0 (-96.0 to -68.2)	-79.8 (-92.6 to -41.3)
Bulgaria	Inguinal, femoral, and abdominal hernia	5 to 9	Female	0 (0 to 0)	0.0 (0.0 to 0.1)	0 (0 to 0)	0.0 (0.0 to 0.0)	0 (0 to 0)	0.0 (0.0 to 0.0)	-88.3 (-96.6 to -64.1)	-78.2 (-93.6 to -33.2)
Bulgaria	Inguinal, femoral, and abdominal hernia	5 to 9	Male	0 (0 to 0)	0.0 (0.0 to 0.0)	0 (0 to 0)	0.0 (0.0 to 0.0)	0 (0 to 0)	0.0 (0.0 to 0.0)	-90.5 (-97.1 to -61.4)	-82.5 (-94.8 to -29.2)
Bulgaria	Inguinal, femoral, and abdominal hernia	10 to 14	Both	0 (0 to 0)	0.0 (0.0 to 0.0)	0 (0 to 0)	0.0 (0.0 to 0.0)	0 (0 to 0)	0.0 (0.0 to 0.0)	-89.5 (-95.5 to -71.6)	-76.3 (-89.9 to -36.0)
Bulgaria	Inguinal, femoral, and abdominal hernia	10 to 14	Female	0 (0 to 0)	0.0 (0.0 to 0.0)	0 (0 to 0)	0.0 (0.0 to 0.0)	0 (0 to 0)	0.0 (0.0 to 0.0)	-89.1 (-96.1 to -68.8)	-74.0 (-90.7 to -26.0)

eTable 8. All-cause and cause-specific mortality (counts and rates) at the global level and by country in the WHO European Region for ages 5 to 9 and 10 to 14 for 1990, 2000, and 2016

Bulgaria	Inguinal, femoral, and abdominal hernia	10 to 14	Male	0 (0 to 0)	0.0 (0.0 to 0.0)	0 (0 to 0)	0.0 (0.0 to 0.0)	0 (0 to 0)	0.0 (0.0 to 0.0)	-89.7 (-96.7 to -66.7)	-77.9 (-92.8 to -28.3)
Bulgaria	Inflammatory bowel disease	5 to 9	Both	0 (0 to 1)	0.1 (0.0 to 0.1)	0 (0 to 0)	0.0 (0.0 to 0.1)	0 (0 to 0)	0.0 (0.0 to 0.1)	-57.8 (-81.6 to -17.6)	-22.1 (-66.0 to 52.2)
Bulgaria	Inflammatory bowel disease	5 to 9	Female	0 (0 to 0)	0.0 (0.0 to 0.1)	0 (0 to 0)	0.0 (0.0 to 0.1)	0 (0 to 0)	0.0 (0.0 to 0.1)	-52.1 (-82.7 to 0.7)	-10.9 (-67.9 to 87.3)
Bulgaria	Inflammatory bowel disease	5 to 9	Male	0 (0 to 0)	0.1 (0.0 to 0.1)	0 (0 to 0)	0.0 (0.0 to 0.1)	0 (0 to 0)	0.0 (0.0 to 0.1)	-61.9 (-85.8 to -3.2)	-30.2 (-74.0 to 77.5)
Bulgaria	Inflammatory bowel disease	10 to 14	Both	0 (0 to 0)	0.0 (0.0 to 0.0)	0 (0 to 0)	0.0 (0.0 to 0.0)	0 (0 to 0)	0.0 (0.0 to 0.0)	-61.0 (-80.0 to -21.1)	-11.9 (-54.8 to 77.9)
Bulgaria	Inflammatory bowel disease	10 to 14	Female	0 (0 to 0)	0.0 (0.0 to 0.0)	0 (0 to 0)	0.0 (0.0 to 0.0)	0 (0 to 0)	0.0 (0.0 to 0.0)	-57.1 (-81.5 to 6.2)	1.7 (-56.1 to 152.1)
Bulgaria	Inflammatory bowel disease	10 to 14	Male	0 (0 to 0)	0.0 (0.0 to 0.0)	0 (0 to 0)	0.0 (0.0 to 0.0)	0 (0 to 0)	0.0 (0.0 to 0.0)	-66.2 (-84.6 to -18.2)	-27.2 (-66.9 to 76.0)
Bulgaria	Vascular intestinal disorders	5 to 9	Both	0 (0 to 0)	0.0 (0.0 to 0.0)	0 (0 to 0)	0.0 (0.0 to 0.0)	0 (0 to 0)	0.0 (0.0 to 0.0)	-69.4 (-87.6 to -29.7)	-43.6 (-77.1 to 29.9)
Bulgaria	Vascular intestinal disorders	5 to 9	Female	0 (0 to 0)	0.0 (0.0 to 0.0)	0 (0 to 0)	0.0 (0.0 to 0.0)	0 (0 to 0)	0.0 (0.0 to 0.0)	-69.6 (-88.1 to -24.5)	-43.5 (-77.9 to 40.5)
Bulgaria	Vascular intestinal disorders	5 to 9	Male	0 (0 to 0)	0.0 (0.0 to 0.0)	0 (0 to 0)	0.0 (0.0 to 0.0)	0 (0 to 0)	0.0 (0.0 to 0.0)	-67.5 (-89.1 to -5.8)	-40.3 (-80.0 to 72.8)
Bulgaria	Vascular intestinal disorders	10 to 14	Both	0 (0 to 0)	0.0 (0.0 to 0.0)	0 (0 to 0)	0.0 (0.0 to 0.0)	0 (0 to 0)	0.0 (0.0 to 0.0)	-66.5 (-82.2 to -33.9)	-24.5 (-59.9 to 49.1)
Bulgaria	Vascular intestinal disorders	10 to 14	Female	0 (0 to 0)	0.0 (0.0 to 0.0)	0 (0 to 0)	0.0 (0.0 to 0.0)	0 (0 to 0)	0.0 (0.0 to 0.0)	-64.3 (-85.4 to -8.7)	-15.2 (-65.4 to 116.7)
Bulgaria	Vascular intestinal disorders	10 to 14	Male	0 (0 to 0)	0.0 (0.0 to 0.0)	0 (0 to 0)	0.0 (0.0 to 0.0)	0 (0 to 0)	0.0 (0.0 to 0.0)	-69.7 (-85.0 to -36.8)	-34.8 (-67.8 to 36.1)
Bulgaria	Gallbladder and biliary diseases	5 to 9	Both	0 (0 to 0)	0.0 (0.0 to 0.0)	0 (0 to 0)	0.0 (0.0 to 0.0)	0 (0 to 0)	0.0 (0.0 to 0.0)	-85.3 (-92.8 to -72.4)	-72.9 (-86.7 to -49.0)
Bulgaria	Gallbladder and biliary diseases	5 to 9	Female	0 (0 to 0)	0.0 (0.0 to 0.0)	0 (0 to 0)	0.0 (0.0 to 0.0)	0 (0 to 0)	0.0 (0.0 to 0.0)	-85.7 (-94.0 to -68.0)	-73.3 (-88.8 to -40.5)
Bulgaria	Gallbladder and biliary diseases	5 to 9	Male	0 (0 to 0)	0.0 (0.0 to 0.0)	0 (0 to 0)	0.0 (0.0 to 0.0)	0 (0 to 0)	0.0 (0.0 to 0.0)	-84.9 (-94.1 to -61.9)	-72.2 (-89.2 to -30.1)
Bulgaria	Gallbladder and biliary diseases	10 to 14	Both	0 (0 to 0)	0.0 (0.0 to 0.0)	0 (0 to 0)	0.0 (0.0 to 0.0)	0 (0 to 0)	0.0 (0.0 to 0.0)	-86.2 (-92.7 to -75.0)	-68.8 (-83.4 to -43.6)
Bulgaria	Gallbladder and biliary diseases	10 to 14	Female	0 (0 to 0)	0.0 (0.0 to 0.0)	0 (0 to 0)	0.0 (0.0 to 0.0)	0 (0 to 0)	0.0 (0.0 to 0.0)	-85.9 (-93.1 to -72.6)	-66.6 (-83.6 to -35.0)
Bulgaria	Gallbladder and biliary diseases	10 to 14	Male	0 (0 to 0)	0.0 (0.0 to 0.0)	0 (0 to 0)	0.0 (0.0 to 0.0)	0 (0 to 0)	0.0 (0.0 to 0.0)	-86.3 (-94.0 to -69.2)	-70.5 (-87.1 to -33.7)
Bulgaria	Pancreatitis	5 to 9	Both	0 (0 to 0)	0.0 (0.0 to 0.1)	0 (0 to 0)	0.0 (0.0 to 0.1)	0 (0 to 0)	0.0 (0.0 to 0.1)	-66.2 (-84.0 to -32.7)	-37.6 (-70.5 to 24.2)
Bulgaria	Pancreatitis	5 to 9	Female	0 (0 to 0)	0.1 (0.0 to 0.2)	0 (0 to 0)	0.1 (0.0 to 0.1)	0 (0 to 0)	0.1 (0.0 to 0.1)	-66.3 (-85.0 to -26.5)	-37.3 (-72.1 to 36.7)
Bulgaria	Pancreatitis	5 to 9	Male	0 (0 to 0)	0.0 (0.0 to 0.0)	0 (0 to 0)	0.0 (0.0 to 0.0)	0 (0 to 0)	0.0 (0.0 to 0.0)	-66.0 (-86.8 to -10.8)	-37.6 (-75.7 to 63.6)
Bulgaria	Pancreatitis	10 to 14	Both	0 (0 to 0)	0.0 (0.0 to 0.0)	0 (0 to 0)	0.0 (0.0 to 0.0)	0 (0 to 0)	0.0 (0.0 to 0.0)	-68.2 (-84.0 to -34.5)	-28.2 (-63.8 to 47.8)
Bulgaria	Pancreatitis	10 to 14	Female	0 (0 to 0)	0.0 (0.0 to 0.1)	0 (0 to 0)	0.0 (0.0 to 0.1)	0 (0 to 0)	0.0 (0.0 to 0.1)	-68.3 (-85.1 to -30.8)	-24.7 (-64.7 to 64.3)
Bulgaria	Pancreatitis	10 to 14	Male	0 (0 to 0)	0.0 (0.0 to 0.0)	0 (0 to 0)	0.0 (0.0 to 0.0)	0 (0 to 0)	0.0 (0.0 to 0.0)	-66.9 (-85.9 to -17.0)	-28.6 (-69.6 to 78.7)

eTable 8. All-cause and cause-specific mortality (counts and rates) at the global level and by country in the WHO European Region for ages 5 to 9 and 10 to 14 for 1990, 2000, and 2016

Bulgaria	Other digestive diseases	5 to 9	Both	0 (0 to 0)	0.1 (0.0 to 0.1)	0 (0 to 0)	0.0 (0.0 to 0.1)	0 (0 to 0)	0.0 (0.0 to 0.1)	-58.8 (-77.1 to -25.0)	-24.0 (-57.7 to 38.5)
Bulgaria	Other digestive diseases	5 to 9	Female	0 (0 to 0)	0.1 (0.0 to 0.1)	0 (0 to 0)	0.0 (0.0 to 0.1)	0 (0 to 0)	0.1 (0.0 to 0.1)	-53.3 (-76.6 to -4.5)	-13.2 (-56.4 to 77.6)
Bulgaria	Other digestive diseases	5 to 9	Male	0 (0 to 0)	0.0 (0.0 to 0.1)	0 (0 to 0)	0.0 (0.0 to 0.1)	0 (0 to 0)	0.0 (0.0 to 0.0)	-69.5 (-87.2 to -28.2)	-44.0 (-76.4 to 31.8)
Bulgaria	Other digestive diseases	10 to 14	Both	0 (0 to 1)	0.1 (0.0 to 0.1)	0 (0 to 0)	0.0 (0.0 to 0.1)	0 (0 to 0)	0.0 (0.0 to 0.1)	-63.5 (-77.3 to -39.9)	-17.6 (-48.9 to 35.5)
Bulgaria	Other digestive diseases	10 to 14	Female	0 (0 to 0)	0.1 (0.0 to 0.1)	0 (0 to 0)	0.0 (0.0 to 0.1)	0 (0 to 0)	0.1 (0.0 to 0.1)	-56.6 (-77.3 to -17.4)	3.0 (-46.1 to 96.2)
Bulgaria	Other digestive diseases	10 to 14	Male	0 (0 to 0)	0.1 (0.0 to 0.1)	0 (0 to 0)	0.0 (0.0 to 0.1)	0 (0 to 0)	0.0 (0.0 to 0.1)	-68.8 (-86.0 to -38.5)	-32.8 (-69.9 to 32.4)
Bulgaria	Alzheimer disease and other dementias	5 to 9	Both	0 (0 to 0)	0.0 (0.0 to 0.0)	0 (0 to 0)	0.0 (0.0 to 0.0)	0 (0 to 0)	0.0 (0.0 to 0.0)	0.0 (0.0 to 0.0)	0.0 (0.0 to 0.0)
Bulgaria	Alzheimer disease and other dementias	5 to 9	Female	0 (0 to 0)	0.0 (0.0 to 0.0)	0 (0 to 0)	0.0 (0.0 to 0.0)	0 (0 to 0)	0.0 (0.0 to 0.0)	0.0 (0.0 to 0.0)	0.0 (0.0 to 0.0)
Bulgaria	Alzheimer disease and other dementias	5 to 9	Male	0 (0 to 0)	0.0 (0.0 to 0.0)	0 (0 to 0)	0.0 (0.0 to 0.0)	0 (0 to 0)	0.0 (0.0 to 0.0)	0.0 (0.0 to 0.0)	0.0 (0.0 to 0.0)
Bulgaria	Alzheimer disease and other dementias	10 to 14	Both	0 (0 to 0)	0.0 (0.0 to 0.0)	0 (0 to 0)	0.0 (0.0 to 0.0)	0 (0 to 0)	0.0 (0.0 to 0.0)	0.0 (0.0 to 0.0)	0.0 (0.0 to 0.0)
Bulgaria	Alzheimer disease and other dementias	10 to 14	Female	0 (0 to 0)	0.0 (0.0 to 0.0)	0 (0 to 0)	0.0 (0.0 to 0.0)	0 (0 to 0)	0.0 (0.0 to 0.0)	0.0 (0.0 to 0.0)	0.0 (0.0 to 0.0)
Bulgaria	Alzheimer disease and other dementias	10 to 14	Male	0 (0 to 0)	0.0 (0.0 to 0.0)	0 (0 to 0)	0.0 (0.0 to 0.0)	0 (0 to 0)	0.0 (0.0 to 0.0)	0.0 (0.0 to 0.0)	0.0 (0.0 to 0.0)
Bulgaria	Parkinson disease	5 to 9	Both	0 (0 to 0)	0.0 (0.0 to 0.0)	0 (0 to 0)	0.0 (0.0 to 0.0)	0 (0 to 0)	0.0 (0.0 to 0.0)	0.0 (0.0 to 0.0)	0.0 (0.0 to 0.0)
Bulgaria	Parkinson disease	5 to 9	Female	0 (0 to 0)	0.0 (0.0 to 0.0)	0 (0 to 0)	0.0 (0.0 to 0.0)	0 (0 to 0)	0.0 (0.0 to 0.0)	0.0 (0.0 to 0.0)	0.0 (0.0 to 0.0)
Bulgaria	Parkinson disease	5 to 9	Male	0 (0 to 0)	0.0 (0.0 to 0.0)	0 (0 to 0)	0.0 (0.0 to 0.0)	0 (0 to 0)	0.0 (0.0 to 0.0)	0.0 (0.0 to 0.0)	0.0 (0.0 to 0.0)
Bulgaria	Parkinson disease	10 to 14	Both	0 (0 to 0)	0.0 (0.0 to 0.0)	0 (0 to 0)	0.0 (0.0 to 0.0)	0 (0 to 0)	0.0 (0.0 to 0.0)	0.0 (0.0 to 0.0)	0.0 (0.0 to 0.0)
Bulgaria	Parkinson disease	10 to 14	Female	0 (0 to 0)	0.0 (0.0 to 0.0)	0 (0 to 0)	0.0 (0.0 to 0.0)	0 (0 to 0)	0.0 (0.0 to 0.0)	0.0 (0.0 to 0.0)	0.0 (0.0 to 0.0)
Bulgaria	Parkinson disease	10 to 14	Male	0 (0 to 0)	0.0 (0.0 to 0.0)	0 (0 to 0)	0.0 (0.0 to 0.0)	0 (0 to 0)	0.0 (0.0 to 0.0)	0.0 (0.0 to 0.0)	0.0 (0.0 to 0.0)
Bulgaria	Epilepsy	5 to 9	Both	4 (3 to 5)	0.7 (0.5 to 0.8)	2 (2 to 3)	0.5 (0.4 to 0.7)	1 (1 to 2)	0.4 (0.3 to 0.6)	-64.0 (-77.3 to -41.7)	-33.5 (-58.1 to 7.7)
Bulgaria	Epilepsy	5 to 9	Female	2 (1 to 2)	0.6 (0.4 to 0.9)	1 (1 to 1)	0.4 (0.3 to 0.6)	1 (0 to 1)	0.4 (0.2 to 0.7)	-64.0 (-81.2 to -33.4)	-33.1 (-64.9 to 23.9)
Bulgaria	Epilepsy	5 to 9	Male	2 (2 to 3)	0.7 (0.5 to 0.9)	1 (1 to 2)	0.6 (0.4 to 0.8)	1 (0 to 1)	0.5 (0.3 to 0.7)	-63.9 (-79.5 to -37.8)	-33.9 (-62.4 to 14.2)
Bulgaria	Epilepsy	10 to 14	Both	6 (5 to 7)	0.9 (0.7 to 1.1)	3 (2 to 4)	0.6 (0.5 to 0.7)	2 (1 to 2)	0.6 (0.4 to 0.9)	-69.5 (-79.7 to -54.7)	-31.3 (-54.3 to 2.1)
Bulgaria	Epilepsy	10 to 14	Female	2 (1 to 3)	0.7 (0.4 to 0.9)	1 (1 to 2)	0.4 (0.3 to 0.6)	1 (0 to 1)	0.5 (0.3 to 0.8)	-69.4 (-83.3 to -45.1)	-27.4 (-60.2 to 30.3)

eTable 8. All-cause and cause-specific mortality (counts and rates) at the global level and by country in the WHO European Region for ages 5 to 9 and 10 to 14 for 1990, 2000, and 2016

Bulgaria	Epilepsy	10 to 14	Male	4 (3 to 5)	1.1 (0.8 to 1.5)	2 (1 to 3)	0.7 (0.5 to 1.0)	1 (1 to 2)	0.7 (0.5 to 1.1)	-69.6 (-81.4 to -50.1)	-34.5 (-60.0 to 7.5)
Bulgaria	Motor neuron disease	5 to 9	Both	0 (0 to 0)	0.0 (0.0 to 0.0)	0 (0 to 0)	0.0 (0.0 to 0.0)	0 (0 to 0)	0.0 (0.0 to 0.0)	-88.2 (-94.3 to -76.6)	-78.2 (-89.6 to -56.9)
Bulgaria	Motor neuron disease	5 to 9	Female	0 (0 to 0)	0.0 (0.0 to 0.0)	0 (0 to 0)	0.0 (0.0 to 0.0)	0 (0 to 0)	0.0 (0.0 to 0.0)	-89.4 (-95.2 to -77.7)	-80.3 (-91.1 to -58.6)
Bulgaria	Motor neuron disease	5 to 9	Male	0 (0 to 0)	0.0 (0.0 to 0.0)	0 (0 to 0)	0.0 (0.0 to 0.0)	0 (0 to 0)	0.0 (0.0 to 0.0)	-86.8 (-95.9 to -66.0)	-75.8 (-92.5 to -37.7)
Bulgaria	Motor neuron disease	10 to 14	Both	0 (0 to 1)	0.1 (0.0 to 0.1)	0 (0 to 0)	0.0 (0.0 to 0.0)	0 (0 to 0)	0.0 (0.0 to 0.0)	-97.3 (-98.7 to -94.0)	-93.8 (-97.0 to -86.5)
Bulgaria	Motor neuron disease	10 to 14	Female	0 (0 to 0)	0.0 (0.0 to 0.0)	0 (0 to 0)	0.0 (0.0 to 0.0)	0 (0 to 0)	0.0 (0.0 to 0.0)	-91.7 (-95.4 to -85.6)	-80.4 (-89.2 to -65.7)
Bulgaria	Motor neuron disease	10 to 14	Male	0 (0 to 1)	0.1 (0.1 to 0.2)	0 (0 to 0)	0.1 (0.0 to 0.1)	0 (0 to 0)	0.0 (0.0 to 0.0)	-97.4 (-98.8 to -94.0)	-94.3 (-97.4 to -87.1)
Bulgaria	Other neurological disorders	5 to 9	Both	1 (1 to 1)	0.2 (0.1 to 0.2)	1 (1 to 1)	0.2 (0.1 to 0.3)	1 (0 to 1)	0.2 (0.1 to 0.4)	-30.9 (-60.0 to 29.1)	27.6 (-26.1 to 138.4)
Bulgaria	Other neurological disorders	5 to 9	Female	0 (0 to 0)	0.1 (0.1 to 0.2)	0 (0 to 0)	0.1 (0.1 to 0.1)	0 (0 to 0)	0.1 (0.1 to 0.2)	-45.9 (-70.7 to 1.4)	0.6 (-45.6 to 88.6)
Bulgaria	Other neurological disorders	5 to 9	Male	1 (0 to 1)	0.2 (0.1 to 0.3)	1 (0 to 1)	0.3 (0.2 to 0.4)	1 (0 to 1)	0.3 (0.2 to 0.5)	-23.3 (-63.1 to 65.8)	40.6 (-32.4 to 204.1)
Bulgaria	Other neurological disorders	10 to 14	Both	1 (1 to 2)	0.2 (0.1 to 0.3)	1 (1 to 1)	0.2 (0.2 to 0.3)	1 (1 to 1)	0.3 (0.2 to 0.4)	-34.3 (-59.9 to 8.4)	48.2 (-9.5 to 144.6)
Bulgaria	Other neurological disorders	10 to 14	Female	0 (0 to 1)	0.1 (0.1 to 0.2)	0 (0 to 0)	0.1 (0.1 to 0.1)	0 (0 to 0)	0.1 (0.1 to 0.2)	-45.5 (-70.5 to 5.3)	29.4 (-30.1 to 150.1)
Bulgaria	Other neurological disorders	10 to 14	Male	1 (1 to 1)	0.3 (0.2 to 0.4)	1 (1 to 1)	0.3 (0.2 to 0.4)	1 (0 to 1)	0.4 (0.2 to 0.6)	-29.7 (-62.1 to 31.7)	51.4 (-18.5 to 183.6)
Bulgaria	Drug use disorders	5 to 9	Both	0 (0 to 0)	0.0 (0.0 to 0.0)	0 (0 to 0)	0.0 (0.0 to 0.0)	0 (0 to 0)	0.0 (0.0 to 0.0)	0.0 (0.0 to 0.0)	0.0 (0.0 to 0.0)
Bulgaria	Drug use disorders	5 to 9	Female	0 (0 to 0)	0.0 (0.0 to 0.0)	0 (0 to 0)	0.0 (0.0 to 0.0)	0 (0 to 0)	0.0 (0.0 to 0.0)	0.0 (0.0 to 0.0)	0.0 (0.0 to 0.0)
Bulgaria	Drug use disorders	5 to 9	Male	0 (0 to 0)	0.0 (0.0 to 0.0)	0 (0 to 0)	0.0 (0.0 to 0.0)	0 (0 to 0)	0.0 (0.0 to 0.0)	0.0 (0.0 to 0.0)	0.0 (0.0 to 0.0)
Bulgaria	Drug use disorders	10 to 14	Both	0 (0 to 0)	0.0 (0.0 to 0.0)	0 (0 to 0)	0.0 (0.0 to 0.0)	0 (0 to 0)	0.0 (0.0 to 0.0)	0.0 (0.0 to 0.0)	0.0 (0.0 to 0.0)
Bulgaria	Drug use disorders	10 to 14	Female	0 (0 to 0)	0.0 (0.0 to 0.0)	0 (0 to 0)	0.0 (0.0 to 0.0)	0 (0 to 0)	0.0 (0.0 to 0.0)	0.0 (0.0 to 0.0)	0.0 (0.0 to 0.0)
Bulgaria	Drug use disorders	10 to 14	Male	0 (0 to 0)	0.0 (0.0 to 0.0)	0 (0 to 0)	0.0 (0.0 to 0.0)	0 (0 to 0)	0.0 (0.0 to 0.0)	0.0 (0.0 to 0.0)	0.0 (0.0 to 0.0)
Bulgaria	Eating disorders	5 to 9	Both	0 (0 to 0)	0.0 (0.0 to 0.0)	0 (0 to 0)	0.0 (0.0 to 0.0)	0 (0 to 0)	0.0 (0.0 to 0.0)	280.8 (83.8 to 622.7)	603.2 (239.3 to 1234.5)
Bulgaria	Eating disorders	5 to 9	Female	0 (0 to 0)	0.0 (0.0 to 0.0)	0 (0 to 0)	0.0 (0.0 to 0.0)	0 (0 to 0)	0.0 (0.0 to 0.0)	310.3 (48.5 to 813.5)	663.1 (176.1 to 1598.9)
Bulgaria	Eating disorders	5 to 9	Male	0 (0 to 0)	0.0 (0.0 to 0.0)	0 (0 to 0)	0.0 (0.0 to 0.0)	0 (0 to 0)	0.0 (0.0 to 0.0)	248.3 (44.3 to 717.6)	538.9 (164.7 to 1399.6)
Bulgaria	Eating disorders	10 to 14	Both	0 (0 to 0)	0.0 (0.0 to 0.0)	0 (0 to 0)	0.0 (0.0 to 0.0)	0 (0 to 0)	0.0 (0.0 to 0.0)	19.6 (-36.6 to 119.0)	169.7 (43.0 to 394.0)
Bulgaria	Eating disorders	10 to 14	Female	0 (0 to 0)	0.0 (0.0 to 0.0)	0 (0 to 0)	0.0 (0.0 to 0.0)	0 (0 to 0)	0.0 (0.0 to 0.0)	-9.9 (-59.3 to 98.7)	114.0 (-3.3 to 371.8)
Bulgaria	Eating disorders	10 to 14	Male	0 (0 to 0)	0.0 (0.0 to 0.0)	0 (0 to 0)	0.0 (0.0 to 0.0)	0 (0 to 0)	0.0 (0.0 to 0.0)	100.0 (-16.9 to 365.0)	330.6 (78.9 to 901.4)
Bulgaria	Diabetes mellitus	5 to 9	Both	0 (0 to 0)	0.1 (0.0 to 0.1)	0 (0 to 0)	0.1 (0.0 to 0.1)	0 (0 to 0)	0.0 (0.0 to 0.0)	-71.6 (-81.6 to -55.0)	-47.5 (-66.0 to -16.8)
Bulgaria	Diabetes mellitus	5 to 9	Female	0 (0 to 0)	0.1 (0.0 to 0.1)	0 (0 to 0)	0.1 (0.0 to 0.1)	0 (0 to 0)	0.0 (0.0 to 0.1)	-72.4 (-83.7 to -54.2)	-48.6 (-69.6 to -14.7)
Bulgaria	Diabetes mellitus	5 to 9	Male	0 (0 to 0)	0.1 (0.0 to 0.1)	0 (0 to 0)	0.1 (0.0 to 0.1)	0 (0 to 0)	0.0 (0.0 to 0.0)	-70.7 (-84.7 to -44.9)	-46.2 (-71.9 to 1.0)
Bulgaria	Diabetes mellitus	10 to 14	Both	1 (1 to 1)	0.2 (0.1 to 0.2)	1 (1 to 1)	0.2 (0.1 to 0.2)	0 (0 to 0)	0.1 (0.1 to 0.1)	-73.9 (-82.3 to -61.9)	-41.1 (-60.1 to -14.0)

Bulgaria	Diabetes mellitus	10 to 14	Female	1 (0 to 1)	0.2 (0.1 to 0.3)	0 (0 to 1)	0.2 (0.1 to 0.2)	0 (0 to 0)	0.1 (0.1 to 0.2)	-75.6 (-85.7 to -59.7)	-42.0 (-66.0 to -4.2)
Bulgaria	Diabetes mellitus	10 to 14	Male	1 (0 to 1)	0.2 (0.1 to 0.2)	0 (0 to 0)	0.1 (0.1 to 0.2)	0 (0 to 0)	0.1 (0.1 to 0.1)	-71.6 (-83.7 to -52.0)	-38.9 (-65.0 to 3.3)
Bulgaria	Acute glomerulonephritis	5 to 9	Both	0 (0 to 0)	0.0 (0.0 to 0.0)	0 (0 to 0)	0.0 (0.0 to 0.0)	0 (0 to 0)	0.0 (0.0 to 0.0)	-47.9 (-62.0 to -27.7)	-3.7 (-29.9 to 33.6)
Bulgaria	Acute glomerulonephritis	5 to 9	Female	0 (0 to 0)	0.0 (0.0 to 0.0)	0 (0 to 0)	0.0 (0.0 to 0.0)	0 (0 to 0)	0.0 (0.0 to 0.0)	-49.8 (-64.3 to -30.4)	-6.6 (-33.5 to 29.5)
Bulgaria	Acute glomerulonephritis	5 to 9	Male	0 (0 to 0)	0.0 (0.0 to 0.0)	0 (0 to 0)	0.0 (0.0 to 0.0)	0 (0 to 0)	0.0 (0.0 to 0.0)	-46.0 (-61.4 to -25.3)	-1.0 (-29.3 to 37.0)
Bulgaria	Acute glomerulonephritis	10 to 14	Both	0 (0 to 0)	0.0 (0.0 to 0.0)	0 (0 to 0)	0.0 (0.0 to 0.0)	0 (0 to 0)	0.0 (0.0 to 0.0)	-55.2 (-64.3 to -37.7)	1.0 (-19.5 to 40.4)
Bulgaria	Acute glomerulonephritis	10 to 14	Female	0 (0 to 0)	0.0 (0.0 to 0.0)	0 (0 to 0)	0.0 (0.0 to 0.0)	0 (0 to 0)	0.0 (0.0 to 0.0)	-56.1 (-67.1 to -21.0)	4.3 (-21.8 to 87.6)
Bulgaria	Acute glomerulonephritis	10 to 14	Male	0 (0 to 0)	0.0 (0.0 to 0.0)	0 (0 to 0)	0.0 (0.0 to 0.0)	0 (0 to 0)	0.0 (0.0 to 0.0)	-54.4 (-63.9 to -42.6)	-1.9 (-22.3 to 23.6)
Bulgaria	Chronic kidney disease	5 to 9	Both	1 (1 to 2)	0.2 (0.2 to 0.3)	1 (1 to 1)	0.3 (0.2 to 0.3)	1 (0 to 1)	0.2 (0.1 to 0.3)	-53.5 (-69.6 to -27.2)	-14.1 (-43.9 to 34.4)
Bulgaria	Chronic kidney disease	5 to 9	Female	1 (0 to 1)	0.2 (0.1 to 0.3)	0 (0 to 1)	0.2 (0.1 to 0.3)	0 (0 to 0)	0.2 (0.1 to 0.3)	-53.3 (-72.5 to -21.6)	-13.2 (-48.8 to 45.8)
Bulgaria	Chronic kidney disease	5 to 9	Male	1 (1 to 1)	0.3 (0.2 to 0.4)	1 (0 to 1)	0.3 (0.2 to 0.4)	0 (0 to 1)	0.2 (0.1 to 0.4)	-53.6 (-72.9 to -20.5)	-14.9 (-50.4 to 45.8)
Bulgaria	Chronic kidney disease	10 to 14	Both	2 (2 to 3)	0.4 (0.3 to 0.4)	2 (1 to 2)	0.3 (0.3 to 0.4)	1 (1 to 1)	0.3 (0.3 to 0.5)	-56.8 (-68.4 to -39.9)	-2.6 (-28.7 to 35.5)
Bulgaria	Chronic kidney disease	10 to 14	Female	1 (1 to 1)	0.3 (0.2 to 0.4)	1 (1 to 1)	0.3 (0.2 to 0.4)	0 (0 to 1)	0.3 (0.2 to 0.5)	-58.1 (-72.8 to -34.4)	-0.4 (-35.5 to 55.7)
Bulgaria	Chronic kidney disease	10 to 14	Male	1 (1 to 2)	0.4 (0.3 to 0.5)	1 (1 to 1)	0.3 (0.3 to 0.4)	1 (0 to 1)	0.4 (0.3 to 0.5)	-55.8 (-70.6 to -31.8)	-4.8 (-36.8 to 46.9)
Bulgaria	Urinary diseases and male infertility	5 to 9	Both	1 (1 to 2)	0.2 (0.2 to 0.3)	0 (0 to 0)	0.1 (0.0 to 0.1)	0 (0 to 0)	0.0 (0.0 to 0.0)	-93.8 (-96.3 to -89.7)	-88.5 (-93.2 to -81.0)
Bulgaria	Urinary diseases and male infertility	5 to 9	Female	1 (1 to 1)	0.3 (0.2 to 0.3)	0 (0 to 0)	0.1 (0.0 to 0.1)	0 (0 to 0)	0.0 (0.0 to 0.0)	-93.3 (-96.2 to -88.6)	-87.6 (-92.9 to -78.7)
Bulgaria	Urinary diseases and male infertility	5 to 9	Male	1 (0 to 1)	0.2 (0.1 to 0.3)	0 (0 to 0)	0.0 (0.0 to 0.1)	0 (0 to 0)	0.0 (0.0 to 0.0)	-94.5 (-97.5 to -87.5)	-89.9 (-95.4 to -77.1)
Bulgaria	Urinary diseases and male infertility	10 to 14	Both	2 (1 to 2)	0.2 (0.2 to 0.3)	0 (0 to 0)	0.1 (0.0 to 0.1)	0 (0 to 0)	0.0 (0.0 to 0.0)	-93.8 (-95.7 to -91.1)	-86.0 (-90.4 to -79.9)
Bulgaria	Urinary diseases and male infertility	10 to 14	Female	1 (1 to 1)	0.3 (0.2 to 0.3)	0 (0 to 0)	0.1 (0.0 to 0.1)	0 (0 to 0)	0.0 (0.0 to 0.1)	-93.5 (-96.0 to -89.2)	-84.5 (-90.5 to -74.3)
Bulgaria	Urinary diseases and male infertility	10 to 14	Male	1 (1 to 1)	0.2 (0.2 to 0.3)	0 (0 to 0)	0.1 (0.0 to 0.1)	0 (0 to 0)	0.0 (0.0 to 0.0)	-94.1 (-96.6 to -90.4)	-87.3 (-92.6 to -79.4)
Bulgaria	Hemoglobinopathies and hemolytic anemias	5 to 9	Both	1 (1 to 2)	0.2 (0.2 to 0.3)	1 (1 to 2)	0.3 (0.3 to 0.4)	1 (1 to 1)	0.3 (0.2 to 0.4)	-28.4 (-58.2 to 14.1)	32.2 (-22.7 to 110.7)
Bulgaria	Hemoglobinopathies and hemolytic anemias	5 to 9	Female	1 (0 to 1)	0.2 (0.2 to 0.3)	1 (0 to 1)	0.3 (0.2 to 0.4)	0 (0 to 1)	0.3 (0.2 to 0.5)	-27.8 (-62.5 to 27.8)	34.3 (-30.3 to 137.7)
Bulgaria	Hemoglobinopathies and hemolytic anemias	5 to 9	Male	1 (0 to 1)	0.2 (0.1 to 0.3)	1 (0 to 1)	0.3 (0.2 to 0.4)	0 (0 to 1)	0.3 (0.2 to 0.4)	-29.1 (-59.7 to 27.3)	30.1 (-26.1 to 133.5)

eTable 8. All-cause and cause-specific mortality (counts and rates) at the global level and by country in the WHO European Region for ages 5 to 9 and 10 to 14 for 1990, 2000, and 2016

Bulgaria	Hemoglobinopathies and hemolytic anemias	10 to 14	Both	2 (1 to 4)	0.3 (0.2 to 0.6)	2 (2 to 3)	0.4 (0.3 to 0.5)	1 (1 to 2)	0.4 (0.3 to 0.6)	-36.0 (-71.0 to 8.7)	44.4 (-34.7 to 145.2)
Bulgaria	Hemoglobinopathies and hemolytic anemias	10 to 14	Female	1 (1 to 2)	0.3 (0.2 to 0.6)	1 (1 to 1)	0.4 (0.2 to 0.6)	1 (0 to 1)	0.4 (0.3 to 0.7)	-31.7 (-63.9 to 37.6)	62.1 (-14.4 to 226.7)
Bulgaria	Hemoglobinopathies and hemolytic anemias	10 to 14	Male	1 (1 to 2)	0.3 (0.2 to 0.7)	1 (1 to 2)	0.4 (0.3 to 0.6)	1 (0 to 1)	0.4 (0.3 to 0.7)	-39.2 (-77.9 to 13.5)	30.9 (-52.4 to 144.5)
Bulgaria	Endocrine, metabolic, blood, and immune disorders	5 to 9	Both	2 (2 to 4)	0.4 (0.3 to 0.8)	2 (1 to 2)	0.4 (0.3 to 0.6)	1 (1 to 2)	0.3 (0.2 to 0.5)	-54.9 (-76.6 to -22.4)	-16.8 (-56.9 to 43.2)
Bulgaria	Endocrine, metabolic, blood, and immune disorders	5 to 9	Female	1 (1 to 2)	0.4 (0.3 to 0.8)	1 (1 to 1)	0.4 (0.3 to 0.6)	1 (0 to 1)	0.3 (0.2 to 0.5)	-55.9 (-80.5 to -13.1)	-17.9 (-63.8 to 61.6)
Bulgaria	Endocrine, metabolic, blood, and immune disorders	5 to 9	Male	1 (1 to 2)	0.4 (0.3 to 0.8)	1 (1 to 1)	0.5 (0.3 to 0.7)	1 (0 to 1)	0.3 (0.2 to 0.6)	-54.1 (-78.1 to -9.3)	-15.7 (-59.8 to 66.4)
Bulgaria	Endocrine, metabolic, blood, and immune disorders	10 to 14	Both	2 (1 to 4)	0.3 (0.2 to 0.6)	2 (1 to 2)	0.3 (0.2 to 0.4)	1 (1 to 1)	0.3 (0.2 to 0.4)	-57.6 (-76.8 to -31.3)	-4.3 (-47.6 to 55.0)
Bulgaria	Endocrine, metabolic, blood, and immune disorders	10 to 14	Female	1 (1 to 2)	0.3 (0.2 to 0.5)	1 (0 to 1)	0.3 (0.2 to 0.4)	0 (0 to 1)	0.3 (0.2 to 0.4)	-59.7 (-81.3 to -25.3)	-4.3 (-55.7 to 77.3)
Bulgaria	Endocrine, metabolic, blood, and immune disorders	10 to 14	Male	1 (1 to 3)	0.4 (0.2 to 0.8)	1 (1 to 1)	0.4 (0.2 to 0.5)	1 (0 to 1)	0.3 (0.2 to 0.6)	-56.1 (-78.3 to -18.7)	-5.4 (-53.2 to 75.1)
Bulgaria	Rheumatoid arthritis	5 to 9	Both	0 (0 to 0)	0.0 (0.0 to 0.0)	0 (0 to 0)	0.0 (0.0 to 0.0)	0 (0 to 0)	0.0 (0.0 to 0.0)	-59.1 (-76.5 to -24.6)	-24.4 (-56.5 to 39.1)
Bulgaria	Rheumatoid arthritis	5 to 9	Female	0 (0 to 0)	0.0 (0.0 to 0.0)	0 (0 to 0)	0.0 (0.0 to 0.0)	0 (0 to 0)	0.0 (0.0 to 0.0)	-64.9 (-84.5 to -19.4)	-34.6 (-71.2 to 49.8)
Bulgaria	Rheumatoid arthritis	5 to 9	Male	0 (0 to 0)	0.0 (0.0 to 0.0)	0 (0 to 0)	0.0 (0.0 to 0.0)	0 (0 to 0)	0.0 (0.0 to 0.0)	-52.9 (-75.6 to -1.5)	-13.6 (-55.3 to 80.6)
Bulgaria	Rheumatoid arthritis	10 to 14	Both	0 (0 to 0)	0.0 (0.0 to 0.0)	0 (0 to 0)	0.0 (0.0 to 0.0)	0 (0 to 0)	0.0 (0.0 to 0.0)	-65.7 (-80.3 to -34.7)	-22.6 (-55.5 to 47.4)
Bulgaria	Rheumatoid arthritis	10 to 14	Female	0 (0 to 0)	0.0 (0.0 to 0.0)	0 (0 to 0)	0.0 (0.0 to 0.0)	0 (0 to 0)	0.0 (0.0 to 0.0)	-71.6 (-86.7 to -24.5)	-32.7 (-68.4 to 79.3)
Bulgaria	Rheumatoid arthritis	10 to 14	Male	0 (0 to 0)	0.0 (0.0 to 0.0)	0 (0 to 0)	0.0 (0.0 to 0.0)	0 (0 to 0)	0.0 (0.0 to 0.0)	-60.4 (-79.9 to -23.1)	-14.8 (-56.7 to 65.7)
Bulgaria	Other musculoskeletal disorders	5 to 9	Both	0 (0 to 0)	0.1 (0.0 to 0.1)	0 (0 to 0)	0.0 (0.0 to 0.1)	0 (0 to 0)	0.0 (0.0 to 0.1)	-55.8 (-75.7 to -29.3)	-18.4 (-55.2 to 30.6)
Bulgaria	Other musculoskeletal disorders	5 to 9	Female	0 (0 to 0)	0.1 (0.0 to 0.1)	0 (0 to 0)	0.0 (0.0 to 0.1)	0 (0 to 0)	0.0 (0.0 to 0.1)	-48.2 (-75.5 to -7.6)	-3.6 (-54.4 to 71.9)
Bulgaria	Other musculoskeletal disorders	5 to 9	Male	0 (0 to 0)	0.1 (0.0 to 0.1)	0 (0 to 0)	0.0 (0.0 to 0.1)	0 (0 to 0)	0.0 (0.0 to 0.1)	-63.0 (-79.4 to -34.4)	-32.1 (-62.3 to 20.3)

eTable 8. All-cause and cause-specific mortality (counts and rates) at the global level and by country in the WHO European Region for ages 5 to 9 and 10 to 14 for 1990, 2000, and 2016

Bulgaria	Other musculoskeletal disorders	10 to 14	Both	0 (0 to 0)	0.0 (0.0 to 0.1)	0 (0 to 0)	0.0 (0.0 to 0.1)	0 (0 to 0)	0.0 (0.0 to 0.1)	-57.6 (-74.8 to -34.5)	-4.3 (-43.1 to 47.7)
Bulgaria	Other musculoskeletal disorders	10 to 14	Female	0 (0 to 0)	0.0 (0.0 to 0.1)	0 (0 to 0)	0.0 (0.0 to 0.0)	0 (0 to 0)	0.0 (0.0 to 0.1)	-46.9 (-73.6 to -2.9)	26.0 (-37.3 to 130.5)
Bulgaria	Other musculoskeletal disorders	10 to 14	Male	0 (0 to 0)	0.1 (0.0 to 0.1)	0 (0 to 0)	0.0 (0.0 to 0.1)	0 (0 to 0)	0.0 (0.0 to 0.1)	-63.6 (-79.1 to -37.2)	-21.5 (-55.0 to 35.2)
Bulgaria	Congenital birth defects	5 to 9	Both	20 (12 to 24)	3.4 (2.1 to 4.1)	13 (10 to 15)	3.1 (2.4 to 3.8)	8 (5 to 12)	2.6 (1.7 to 3.7)	-58.5 (-73.1 to -34.7)	-23.5 (-50.4 to 20.6)
Bulgaria	Congenital birth defects	5 to 9	Female	10 (6 to 13)	3.6 (2.0 to 4.7)	5 (4 to 7)	2.8 (2.0 to 3.6)	4 (2 to 5)	2.3 (1.4 to 3.5)	-64.9 (-79.1 to -38.9)	-34.6 (-61.1 to 13.6)
Bulgaria	Congenital birth defects	5 to 9	Male	10 (5 to 13)	3.2 (1.8 to 4.2)	7 (5 to 9)	3.4 (2.4 to 4.5)	5 (3 to 7)	2.8 (1.7 to 4.4)	-51.8 (-72.5 to -14.1)	-11.6 (-49.7 to 57.6)
Bulgaria	Congenital birth defects	10 to 14	Both	14 (9 to 18)	2.2 (1.5 to 2.7)	10 (8 to 12)	2.0 (1.5 to 2.4)	6 (4 to 8)	2.0 (1.4 to 2.7)	-59.9 (-72.5 to -39.5)	-9.6 (-37.9 to 36.5)
Bulgaria	Congenital birth defects	10 to 14	Female	6 (4 to 8)	2.0 (1.3 to 2.7)	4 (3 to 6)	1.7 (1.2 to 2.2)	2 (1 to 3)	1.7 (1.1 to 2.4)	-65.4 (-77.8 to -44.0)	-17.8 (-47.3 to 33.0)
Bulgaria	Congenital birth defects	10 to 14	Male	8 (4 to 11)	2.4 (1.3 to 3.3)	6 (4 to 8)	2.2 (1.6 to 3.0)	4 (2 to 5)	2.3 (1.4 to 3.4)	-55.5 (-74.1 to -26.6)	-4.2 (-44.2 to 58.0)
Bulgaria	Skin and subcutaneous diseases	5 to 9	Both	0 (0 to 0)	0.0 (0.0 to 0.1)	0 (0 to 0)	0.0 (0.0 to 0.1)	0 (0 to 0)	0.0 (0.0 to 0.1)	-44.3 (-66.1 to -13.2)	2.9 (-37.4 to 60.4)
Bulgaria	Skin and subcutaneous diseases	5 to 9	Female	0 (0 to 0)	0.0 (0.0 to 0.1)	0 (0 to 0)	0.0 (0.0 to 0.1)	0 (0 to 0)	0.0 (0.0 to 0.1)	-39.1 (-62.9 to -1.3)	13.3 (-31.0 to 83.6)
Bulgaria	Skin and subcutaneous diseases	5 to 9	Male	0 (0 to 0)	0.0 (0.0 to 0.1)	0 (0 to 0)	0.0 (0.0 to 0.1)	0 (0 to 0)	0.0 (0.0 to 0.1)	-49.9 (-75.3 to -11.8)	-8.1 (-54.7 to 61.8)
Bulgaria	Skin and subcutaneous diseases	10 to 14	Both	0 (0 to 0)	0.0 (0.0 to 0.1)	0 (0 to 0)	0.0 (0.0 to 0.1)	0 (0 to 0)	0.0 (0.0 to 0.1)	-51.3 (-68.9 to -30.5)	9.9 (-29.9 to 56.9)
Bulgaria	Skin and subcutaneous diseases	10 to 14	Female	0 (0 to 0)	0.0 (0.0 to 0.1)	0 (0 to 0)	0.0 (0.0 to 0.1)	0 (0 to 0)	0.0 (0.0 to 0.1)	-47.1 (-64.4 to -13.5)	25.6 (-15.4 to 105.3)
Bulgaria	Skin and subcutaneous diseases	10 to 14	Male	0 (0 to 0)	0.0 (0.0 to 0.1)	0 (0 to 0)	0.0 (0.0 to 0.1)	0 (0 to 0)	0.0 (0.0 to 0.1)	-54.6 (-75.9 to -24.7)	-2.2 (-48.2 to 62.1)
Bulgaria	Road injuries	5 to 9	Both	40 (34 to 49)	6.9 (5.7 to 8.4)	19 (15 to 23)	4.6 (3.7 to 5.6)	7 (4 to 10)	2.2 (1.4 to 3.2)	-83.0 (-89.1 to -73.3)	-68.6 (-80.0 to -50.8)
Bulgaria	Road injuries	5 to 9	Female	16 (12 to 22)	5.7 (4.2 to 7.6)	8 (6 to 10)	3.9 (2.8 to 5.1)	3 (2 to 5)	2.3 (1.3 to 3.6)	-78.6 (-88.3 to -63.7)	-60.2 (-78.2 to -32.5)
Bulgaria	Road injuries	5 to 9	Male	24 (19 to 30)	8.1 (6.2 to 10.1)	11 (8 to 14)	5.1 (3.8 to 6.7)	3 (2 to 6)	2.1 (1.3 to 3.4)	-85.9 (-91.9 to -75.2)	-74.1 (-85.2 to -54.5)
Bulgaria	Road injuries	10 to 14	Both	32 (26 to 38)	4.9 (4.0 to 5.9)	18 (15 to 22)	3.5 (2.9 to 4.2)	7 (5 to 9)	2.4 (1.7 to 3.2)	-78.5 (-85.0 to -69.3)	-51.5 (-66.1 to -30.7)
Bulgaria	Road injuries	10 to 14	Female	10 (8 to 13)	3.3 (2.4 to 4.3)	7 (5 to 9)	2.8 (2.1 to 3.7)	2 (2 to 4)	1.8 (1.1 to 2.7)	-76.6 (-86.3 to -61.3)	-44.5 (-67.4 to -8.2)
Bulgaria	Road injuries	10 to 14	Male	21 (17 to 27)	6.4 (4.9 to 8.0)	11 (9 to 14)	4.2 (3.2 to 5.3)	4 (3 to 6)	2.8 (1.9 to 4.0)	-79.4 (-87.0 to -68.4)	-55.7 (-71.9 to -32.0)
Bulgaria	Other transport injuries	5 to 9	Both	2 (2 to 3)	0.4 (0.3 to 0.6)	1 (1 to 2)	0.2 (0.2 to 0.4)	0 (0 to 1)	0.1 (0.1 to 0.3)	-81.1 (-91.1 to -61.3)	-65.0 (-83.6 to -28.5)
Bulgaria	Other transport injuries	5 to 9	Female	1 (1 to 1)	0.3 (0.2 to 0.5)	0 (0 to 1)	0.2 (0.1 to 0.4)	0 (0 to 0)	0.1 (0.1 to 0.2)	-80.5 (-91.7 to -57.2)	-63.7 (-84.6 to -20.5)
Bulgaria	Other transport injuries	5 to 9	Male	2 (1 to 2)	0.5 (0.3 to 0.8)	1 (0 to 1)	0.3 (0.1 to 0.5)	0 (0 to 1)	0.2 (0.1 to 0.3)	-81.4 (-93.2 to -54.0)	-65.9 (-87.6 to -15.6)
Bulgaria	Other transport injuries	10 to 14	Both	3 (2 to 5)	0.5 (0.4 to 0.7)	2 (1 to 3)	0.3 (0.2 to 0.5)	1 (0 to 1)	0.2 (0.1 to 0.4)	-80.1 (-89.1 to -65.4)	-55.2 (-75.3 to -21.9)

eTable 8. All-cause and cause-specific mortality (counts and rates) at the global level and by country in the WHO European Region for ages 5 to 9 and 10 to 14 for 1990, 2000, and 2016

Bulgaria	Other transport injuries	10 to 14	Female	1 (1 to 2)	0.4 (0.2 to 0.6)	1 (0 to 1)	0.3 (0.2 to 0.5)	0 (0 to 0)	0.2 (0.1 to 0.3)	-81.2 (-91.8 to -60.1)	-55.3 (-80.6 to -5.4)
Bulgaria	Other transport injuries	10 to 14	Male	2 (1 to 3)	0.6 (0.4 to 1.0)	1 (1 to 2)	0.3 (0.2 to 0.6)	0 (0 to 1)	0.3 (0.1 to 0.5)	-79.5 (-91.0 to -58.0)	-55.9 (-80.7 to -9.7)
Bulgaria	Falls	5 to 9	Both	8 (3 to 10)	1.3 (0.5 to 1.8)	4 (2 to 5)	0.9 (0.5 to 1.2)	2 (1 to 3)	0.6 (0.3 to 0.9)	-76.9 (-86.1 to -55.4)	-57.3 (-74.3 to -17.6)
Bulgaria	Falls	5 to 9	Female	3 (1 to 4)	0.9 (0.4 to 1.3)	1 (1 to 2)	0.6 (0.3 to 0.9)	1 (0 to 1)	0.5 (0.2 to 0.7)	-72.5 (-84.0 to -53.2)	-48.9 (-70.3 to -13.0)
Bulgaria	Falls	5 to 9	Male	5 (2 to 7)	1.7 (0.5 to 2.5)	2 (1 to 3)	1.1 (0.5 to 1.6)	1 (1 to 2)	0.6 (0.3 to 1.0)	-79.1 (-88.9 to -52.6)	-61.6 (-79.6 to -13.0)
Bulgaria	Falls	10 to 14	Both	7 (3 to 10)	1.1 (0.5 to 1.5)	4 (2 to 5)	0.7 (0.4 to 0.9)	2 (1 to 3)	0.6 (0.3 to 0.9)	-76.0 (-84.2 to -62.1)	-46.0 (-64.4 to -14.6)
Bulgaria	Falls	10 to 14	Female	2 (1 to 2)	0.5 (0.2 to 0.8)	1 (0 to 1)	0.4 (0.2 to 0.5)	0 (0 to 1)	0.3 (0.2 to 0.5)	-73.4 (-83.6 to -56.1)	-36.9 (-61.0 to 4.2)
Bulgaria	Falls	10 to 14	Male	6 (2 to 8)	1.7 (0.7 to 2.4)	3 (2 to 4)	1.0 (0.6 to 1.4)	1 (1 to 2)	0.8 (0.5 to 1.3)	-76.8 (-86.4 to -59.6)	-50.1 (-70.7 to -13.1)
Bulgaria	Drowning	5 to 9	Both	28 (23 to 33)	4.8 (4.0 to 5.7)	14 (12 to 17)	3.5 (2.9 to 4.2)	5 (3 to 8)	1.6 (1.1 to 2.4)	-81.6 (-88.2 to -72.2)	-66.0 (-78.2 to -48.7)
Bulgaria	Drowning	5 to 9	Female	6 (4 to 8)	2.1 (1.5 to 2.7)	3 (2 to 4)	1.5 (1.1 to 2.0)	1 (1 to 2)	0.8 (0.5 to 1.2)	-79.3 (-87.5 to -66.5)	-61.5 (-76.7 to -37.7)
Bulgaria	Drowning	5 to 9	Male	22 (18 to 27)	7.3 (5.9 to 9.1)	11 (9 to 14)	5.4 (4.3 to 6.7)	4 (3 to 6)	2.4 (1.5 to 3.6)	-82.2 (-89.3 to -71.9)	-67.3 (-80.3 to -48.4)
Bulgaria	Drowning	10 to 14	Both	30 (25 to 35)	4.6 (3.8 to 5.4)	15 (13 to 19)	3.0 (2.5 to 3.6)	6 (4 to 8)	2.0 (1.5 to 2.6)	-81.1 (-86.9 to -74.0)	-57.3 (-70.4 to -41.5)
Bulgaria	Drowning	10 to 14	Female	7 (5 to 9)	2.3 (1.7 to 3.0)	4 (3 to 5)	1.6 (1.2 to 2.1)	1 (1 to 2)	1.0 (0.7 to 1.5)	-81.2 (-88.6 to -69.6)	-55.3 (-73.0 to -27.9)
Bulgaria	Drowning	10 to 14	Male	23 (18 to 27)	6.8 (5.4 to 8.2)	11 (9 to 14)	4.3 (3.4 to 5.3)	4 (3 to 6)	2.8 (1.9 to 3.8)	-81.1 (-87.4 to -72.3)	-59.2 (-72.8 to -40.4)
Bulgaria	Fire, heat, and hot substances	5 to 9	Both	4 (3 to 6)	0.7 (0.5 to 1.0)	2 (2 to 3)	0.5 (0.4 to 0.7)	1 (1 to 2)	0.3 (0.2 to 0.5)	-76.6 (-85.1 to -62.5)	-56.8 (-72.5 to -30.7)
Bulgaria	Fire, heat, and hot substances	5 to 9	Female	2 (1 to 3)	0.7 (0.4 to 1.1)	1 (1 to 1)	0.5 (0.3 to 0.7)	0 (0 to 1)	0.3 (0.2 to 0.5)	-78.3 (-88.1 to -61.6)	-59.7 (-77.8 to -28.7)
Bulgaria	Fire, heat, and hot substances	5 to 9	Male	2 (2 to 3)	0.8 (0.5 to 1.0)	1 (1 to 2)	0.6 (0.4 to 0.8)	1 (0 to 1)	0.3 (0.2 to 0.5)	-75.0 (-85.5 to -56.9)	-54.2 (-73.4 to -21.0)
Bulgaria	Fire, heat, and hot substances	10 to 14	Both	2 (2 to 3)	0.4 (0.3 to 0.5)	1 (1 to 2)	0.2 (0.2 to 0.3)	1 (0 to 1)	0.2 (0.1 to 0.3)	-77.9 (-84.6 to -67.6)	-50.0 (-65.2 to -26.8)
Bulgaria	Fire, heat, and hot substances	10 to 14	Female	1 (1 to 1)	0.3 (0.2 to 0.4)	0 (0 to 1)	0.2 (0.1 to 0.2)	0 (0 to 0)	0.1 (0.1 to 0.2)	-78.2 (-87.9 to -63.5)	-48.2 (-71.2 to -13.3)
Bulgaria	Fire, heat, and hot substances	10 to 14	Male	2 (1 to 2)	0.5 (0.3 to 0.6)	1 (1 to 1)	0.3 (0.2 to 0.4)	0 (0 to 1)	0.2 (0.2 to 0.3)	-77.7 (-85.9 to -63.9)	-51.9 (-69.6 to -22.2)
Bulgaria	Poisonings	5 to 9	Both	3 (2 to 4)	0.6 (0.4 to 0.8)	2 (1 to 3)	0.5 (0.3 to 0.6)	1 (0 to 1)	0.2 (0.1 to 0.3)	-85.0 (-91.8 to -69.5)	-72.2 (-84.9 to -43.7)
Bulgaria	Poisonings	5 to 9	Female	2 (1 to 3)	0.6 (0.3 to 0.9)	1 (1 to 2)	0.5 (0.3 to 0.8)	0 (0 to 1)	0.2 (0.1 to 0.3)	-83.4 (-92.4 to -66.5)	-69.0 (-85.8 to -37.7)
Bulgaria	Poisonings	5 to 9	Male	2 (1 to 2)	0.5 (0.3 to 0.8)	1 (1 to 1)	0.4 (0.3 to 0.6)	0 (0 to 0)	0.1 (0.1 to 0.3)	-86.8 (-94.1 to -65.8)	-75.8 (-89.2 to -37.2)
Bulgaria	Poisonings	10 to 14	Both	3 (2 to 4)	0.5 (0.4 to 0.7)	2 (1 to 3)	0.4 (0.3 to 0.5)	0 (0 to 1)	0.2 (0.1 to 0.3)	-86.0 (-91.9 to -73.5)	-68.5 (-81.6 to -40.2)
Bulgaria	Poisonings	10 to 14	Female	1 (1 to 2)	0.4 (0.3 to 0.6)	1 (1 to 1)	0.4 (0.2 to 0.5)	0 (0 to 0)	0.2 (0.1 to 0.3)	-84.5 (-92.8 to -71.4)	-63.2 (-82.9 to -32.1)
Bulgaria	Poisonings	10 to 14	Male	2 (1 to 3)	0.6 (0.4 to 0.9)	1 (1 to 2)	0.4 (0.3 to 0.6)	0 (0 to 1)	0.2 (0.1 to 0.4)	-87.1 (-93.5 to -70.3)	-72.2 (-86.0 to -36.0)
Bulgaria	Exposure to mechanical forces	5 to 9	Both	5 (4 to 9)	0.9 (0.7 to 1.6)	3 (2 to 5)	0.7 (0.5 to 1.3)	2 (1 to 3)	0.6 (0.4 to 0.9)	-66.0 (-78.8 to -46.1)	-37.2 (-60.8 to -0.5)
Bulgaria	Exposure to mechanical forces	5 to 9	Female	1 (1 to 3)	0.5 (0.4 to 0.9)	1 (1 to 1)	0.4 (0.3 to 0.7)	1 (0 to 1)	0.4 (0.3 to 0.6)	-58.0 (-77.7 to -22.0)	-21.8 (-58.6 to 45.1)
Bulgaria	Exposure to mechanical forces	5 to 9	Male	4 (3 to 7)	1.3 (0.9 to 2.3)	2 (1 to 4)	1.0 (0.6 to 1.9)	1 (1 to 2)	0.7 (0.4 to 1.3)	-69.0 (-80.9 to -48.2)	-43.2 (-65.0 to -4.9)

eTable 8. All-cause and cause-specific mortality (counts and rates) at the global level and by country in the WHO European Region for ages 5 to 9 and 10 to 14 for 1990, 2000, and 2016

Bulgaria	Exposure to mechanical forces	10 to 14	Both	7 (5 to 11)	1.1 (0.8 to 1.8)	4 (3 to 7)	0.7 (0.5 to 1.3)	2 (2 to 3)	0.8 (0.6 to 1.2)	-68.5 (-78.4 to -54.5)	-28.9 (-51.2 to 2.6)
Bulgaria	Exposure to mechanical forces	10 to 14	Female	1 (1 to 2)	0.4 (0.3 to 0.5)	1 (1 to 1)	0.3 (0.3 to 0.5)	1 (0 to 1)	0.4 (0.2 to 0.6)	-58.2 (-77.5 to -29.9)	-0.8 (-46.5 to 66.5)
Bulgaria	Exposure to mechanical forces	10 to 14	Male	6 (4 to 10)	1.7 (1.2 to 3.0)	3 (2 to 6)	1.1 (0.7 to 2.1)	2 (1 to 3)	1.1 (0.7 to 1.8)	-70.7 (-79.8 to -56.1)	-37.0 (-56.4 to -5.6)
Bulgaria	Adverse effects of medical treatment	5 to 9	Both	1 (1 to 2)	0.2 (0.1 to 0.3)	1 (0 to 1)	0.2 (0.1 to 0.2)	0 (0 to 1)	0.1 (0.1 to 0.2)	-63.3 (-76.5 to -43.2)	-32.3 (-56.7 to 4.9)
Bulgaria	Adverse effects of medical treatment	5 to 9	Female	1 (0 to 1)	0.2 (0.1 to 0.3)	0 (0 to 0)	0.1 (0.1 to 0.2)	0 (0 to 0)	0.1 (0.1 to 0.2)	-64.5 (-78.0 to -39.3)	-33.9 (-59.0 to 12.8)
Bulgaria	Adverse effects of medical treatment	5 to 9	Male	1 (0 to 1)	0.2 (0.1 to 0.3)	0 (0 to 1)	0.2 (0.1 to 0.3)	0 (0 to 0)	0.2 (0.1 to 0.3)	-62.4 (-77.8 to -33.5)	-31.0 (-59.4 to 22.0)
Bulgaria	Adverse effects of medical treatment	10 to 14	Both	1 (1 to 1)	0.2 (0.1 to 0.2)	1 (0 to 1)	0.1 (0.1 to 0.2)	0 (0 to 1)	0.1 (0.1 to 0.2)	-64.5 (-75.4 to -47.6)	-19.8 (-44.6 to 18.3)
Bulgaria	Adverse effects of medical treatment	10 to 14	Female	0 (0 to 0)	0.1 (0.1 to 0.2)	0 (0 to 0)	0.1 (0.1 to 0.1)	0 (0 to 0)	0.1 (0.0 to 0.1)	-64.8 (-76.6 to -44.4)	-16.5 (-44.4 to 32.0)
Bulgaria	Adverse effects of medical treatment	10 to 14	Male	1 (0 to 1)	0.2 (0.1 to 0.3)	0 (0 to 1)	0.1 (0.1 to 0.2)	0 (0 to 0)	0.2 (0.1 to 0.3)	-64.3 (-77.6 to -41.7)	-23.0 (-51.8 to 25.6)
Bulgaria	Animal contact	5 to 9	Both	0 (0 to 1)	0.1 (0.0 to 0.1)	0 (0 to 0)	0.1 (0.0 to 0.1)	0 (0 to 0)	0.1 (0.0 to 0.1)	-56.6 (-78.3 to -13.6)	-19.8 (-60.0 to 59.6)
Bulgaria	Animal contact	5 to 9	Female	0 (0 to 0)	0.1 (0.0 to 0.1)	0 (0 to 0)	0.0 (0.0 to 0.1)	0 (0 to 0)	0.1 (0.0 to 0.1)	-49.6 (-80.6 to 28.3)	-6.3 (-63.9 to 138.6)
Bulgaria	Animal contact	5 to 9	Male	0 (0 to 0)	0.1 (0.0 to 0.1)	0 (0 to 0)	0.1 (0.0 to 0.1)	0 (0 to 0)	0.0 (0.0 to 0.1)	-63.4 (-84.2 to -20.7)	-32.8 (-70.9 to 45.5)
Bulgaria	Animal contact	10 to 14	Both	0 (0 to 1)	0.1 (0.0 to 0.1)	0 (0 to 0)	0.0 (0.0 to 0.1)	0 (0 to 0)	0.0 (0.0 to 0.1)	-63.0 (-79.6 to -35.1)	-16.6 (-54.0 to 46.4)
Bulgaria	Animal contact	10 to 14	Female	0 (0 to 0)	0.0 (0.0 to 0.1)	0 (0 to 0)	0.0 (0.0 to 0.0)	0 (0 to 0)	0.0 (0.0 to 0.1)	-55.7 (-82.5 to 5.6)	5.2 (-58.5 to 150.6)
Bulgaria	Animal contact	10 to 14	Male	0 (0 to 0)	0.1 (0.0 to 0.1)	0 (0 to 0)	0.1 (0.0 to 0.1)	0 (0 to 0)	0.1 (0.0 to 0.1)	-65.8 (-83.2 to -32.0)	-26.3 (-63.8 to 46.5)
Bulgaria	Foreign body	5 to 9	Both	2 (1 to 3)	0.4 (0.2 to 0.5)	1 (1 to 2)	0.3 (0.2 to 0.4)	1 (0 to 1)	0.2 (0.1 to 0.3)	-67.7 (-81.5 to -43.4)	-40.4 (-65.8 to 4.6)
Bulgaria	Foreign body	5 to 9	Female	1 (0 to 1)	0.2 (0.1 to 0.4)	0 (0 to 1)	0.2 (0.1 to 0.3)	0 (0 to 0)	0.2 (0.1 to 0.3)	-65.4 (-81.6 to -38.9)	-35.6 (-65.8 to 13.6)
Bulgaria	Foreign body	5 to 9	Male	1 (1 to 2)	0.5 (0.3 to 0.7)	1 (1 to 1)	0.4 (0.3 to 0.5)	0 (0 to 1)	0.3 (0.1 to 0.4)	-68.9 (-84.0 to -42.1)	-43.0 (-70.7 to 6.1)
Bulgaria	Foreign body	10 to 14	Both	2 (1 to 2)	0.3 (0.2 to 0.4)	1 (1 to 1)	0.2 (0.2 to 0.3)	0 (0 to 1)	0.2 (0.1 to 0.3)	-72.0 (-85.1 to -53.4)	-36.8 (-66.4 to 5.2)
Bulgaria	Foreign body	10 to 14	Female	1 (0 to 1)	0.2 (0.1 to 0.2)	0 (0 to 1)	0.1 (0.1 to 0.2)	0 (0 to 0)	0.1 (0.1 to 0.2)	-71.1 (-84.5 to -52.7)	-31.5 (-63.3 to 12.3)
Bulgaria	Foreign body	10 to 14	Male	1 (1 to 2)	0.4 (0.2 to 0.6)	1 (0 to 1)	0.3 (0.2 to 0.4)	0 (0 to 1)	0.2 (0.1 to 0.4)	-72.3 (-87.0 to -50.4)	-40.4 (-72.0 to 6.8)
Bulgaria	Environmental heat and cold exposure	5 to 9	Both	1 (0 to 1)	0.1 (0.1 to 0.1)	0 (0 to 1)	0.1 (0.0 to 0.1)	0 (0 to 0)	0.1 (0.0 to 0.1)	-69.6 (-82.6 to -48.5)	-43.9 (-67.8 to -5.0)
Bulgaria	Environmental heat and cold exposure	5 to 9	Female	0 (0 to 0)	0.1 (0.0 to 0.1)	0 (0 to 0)	0.1 (0.0 to 0.1)	0 (0 to 0)	0.0 (0.0 to 0.1)	-70.7 (-83.4 to -46.3)	-45.5 (-69.2 to -0.1)

eTable 8. All-cause and cause-specific mortality (counts and rates) at the global level and by country in the WHO European Region for ages 5 to 9 and 10 to 14 for 1990, 2000, and 2016

Bulgaria	Environmental heat and cold exposure	5 to 9	Male	0<br>(0 to 1)	0.1<br>(0.0 to 0.2)	0<br>(0 to 0)	0.1<br>(0.0 to 0.2)	0<br>(0 to 0)	0.1<br>(0.0 to 0.1)	-69.1<br>(-83.9 to -42.4)	-43.3<br>(-70.4 to 5.7)
Bulgaria	Environmental heat and cold exposure	10 to 14	Both	1<br>(0 to 1)	0.1<br>(0.0 to 0.1)	0<br>(0 to 1)	0.1<br>(0.0 to 0.1)	0<br>(0 to 0)	0.1<br>(0.0 to 0.1)	-71.6<br>(-81.1 to -56.9)	-36.0<br>(-57.3 to -2.8)
Bulgaria	Environmental heat and cold exposure	10 to 14	Female	0<br>(0 to 0)	0.1<br>(0.0 to 0.1)	0<br>(0 to 0)	0.0<br>(0.0 to 0.1)	0<br>(0 to 0)	0.0<br>(0.0 to 0.1)	-71.9<br>(-82.8 to -50.5)	-33.4<br>(-59.1 to 17.5)
Bulgaria	Environmental heat and cold exposure	10 to 14	Male	0<br>(0 to 1)	0.1<br>(0.0 to 0.2)	0<br>(0 to 0)	0.1<br>(0.0 to 0.2)	0<br>(0 to 0)	0.1<br>(0.0 to 0.1)	-71.5<br>(-81.8 to -53.2)	-38.6<br>(-60.8 to 0.8)
Bulgaria	Other unintentional injuries	5 to 9	Both	7<br>(5 to 9)	1.2<br>(0.9 to 1.6)	6<br>(4 to 8)	1.5<br>(1.1 to 1.9)	1<br>(0 to 1)	0.2<br>(0.1 to 0.4)	-89.7<br>(-94.1 to -83.0)	-81.0<br>(-89.1 to -68.6)
Bulgaria	Other unintentional injuries	5 to 9	Female	2<br>(1 to 3)	0.7<br>(0.5 to 1.0)	2<br>(1 to 2)	0.8<br>(0.5 to 1.1)	0<br>(0 to 0)	0.1<br>(0.1 to 0.2)	-90.1<br>(-95.0 to -80.9)	-81.6<br>(-90.7 to -64.5)
Bulgaria	Other unintentional injuries	5 to 9	Male	5<br>(3 to 7)	1.6<br>(1.1 to 2.4)	4<br>(3 to 6)	2.1<br>(1.4 to 2.9)	1<br>(0 to 1)	0.3<br>(0.2 to 0.5)	-89.6<br>(-94.8 to -80.7)	-80.9<br>(-90.5 to -64.6)
Bulgaria	Other unintentional injuries	10 to 14	Both	10<br>(8 to 12)	1.5<br>(1.2 to 1.9)	8<br>(6 to 9)	1.5<br>(1.2 to 1.8)	1<br>(1 to 1)	0.3<br>(0.2 to 0.5)	-89.9<br>(-93.5 to -84.7)	-77.2<br>(-85.2 to -65.6)
Bulgaria	Other unintentional injuries	10 to 14	Female	2<br>(1 to 3)	0.7<br>(0.5 to 0.9)	2<br>(1 to 3)	0.8<br>(0.5 to 1.2)	0<br>(0 to 0)	0.2<br>(0.1 to 0.2)	-90.2<br>(-94.6 to -82.8)	-76.8<br>(-87.2 to -59.2)
Bulgaria	Other unintentional injuries	10 to 14	Male	8<br>(6 to 10)	2.3<br>(1.7 to 3.0)	6<br>(4 to 7)	2.1<br>(1.6 to 2.7)	1<br>(1 to 1)	0.5<br>(0.3 to 0.7)	-89.8<br>(-93.9 to -83.5)	-78.0<br>(-86.8 to -64.4)
Bulgaria	Self-harm	10 to 14	Both	17<br>(11 to 22)	2.7<br>(1.7 to 3.4)	10<br>(7 to 12)	1.9<br>(1.4 to 2.4)	3<br>(2 to 5)	1.2<br>(0.8 to 1.7)	-80.5<br>(-87.8 to -65.0)	-56.0<br>(-72.5 to -20.9)
Bulgaria	Self-harm	10 to 14	Female	5<br>(4 to 7)	1.6<br>(1.2 to 2.2)	3<br>(2 to 4)	1.1<br>(0.8 to 1.6)	1<br>(1 to 1)	0.7<br>(0.4 to 1.0)	-83.1<br>(-90.5 to -72.2)	-59.8<br>(-77.4 to -34.0)
Bulgaria	Self-harm	10 to 14	Male	12<br>(6 to 17)	3.7<br>(1.9 to 5.0)	7<br>(5 to 9)	2.6<br>(1.8 to 3.4)	3<br>(2 to 4)	1.6<br>(1.0 to 2.6)	-79.4<br>(-88.4 to -54.2)	-55.7<br>(-74.9 to -1.3)
Bulgaria	Interpersonal violence	5 to 9	Both	4<br>(3 to 6)	0.7<br>(0.5 to 1.0)	2<br>(2 to 3)	0.6<br>(0.4 to 0.8)	1<br>(1 to 2)	0.3<br>(0.2 to 0.6)	-72.4<br>(-83.8 to -55.9)	-49.0<br>(-70.1 to -18.6)
Bulgaria	Interpersonal violence	5 to 9	Female	2<br>(1 to 3)	0.6<br>(0.4 to 1.0)	1<br>(1 to 2)	0.5<br>(0.3 to 0.8)	1<br>(0 to 1)	0.3<br>(0.2 to 0.6)	-71.9<br>(-85.4 to -42.0)	-47.8<br>(-72.9 to 7.8)
Bulgaria	Interpersonal violence	5 to 9	Male	2<br>(1 to 4)	0.7<br>(0.4 to 1.3)	1<br>(1 to 2)	0.6<br>(0.3 to 1.1)	1<br>(0 to 1)	0.3<br>(0.2 to 0.7)	-72.8<br>(-87.0 to -53.8)	-50.1<br>(-76.1 to -15.2)
Bulgaria	Interpersonal violence	10 to 14	Both	5<br>(3 to 7)	0.7<br>(0.5 to 1.1)	3<br>(2 to 4)	0.6<br>(0.4 to 0.9)	1<br>(1 to 2)	0.4<br>(0.3 to 0.7)	-74.5<br>(-84.4 to -63.5)	-42.6<br>(-64.9 to -17.7)
Bulgaria	Interpersonal violence	10 to 14	Female	2<br>(1 to 3)	0.5<br>(0.3 to 0.8)	1<br>(1 to 2)	0.5<br>(0.3 to 0.7)	0<br>(0 to 1)	0.3<br>(0.2 to 0.6)	-74.1<br>(-85.4 to -54.8)	-38.5<br>(-65.3 to 7.3)
Bulgaria	Interpersonal violence	10 to 14	Male	3<br>(2 to 6)	0.9<br>(0.5 to 1.7)	2<br>(1 to 3)	0.8<br>(0.4 to 1.2)	1<br>(0 to 2)	0.5<br>(0.3 to 1.0)	-74.8<br>(-87.5 to -61.3)	-45.8<br>(-73.1 to -16.6)
Bulgaria	Exposure to forces of nature	5 to 9	Both	0<br>(0 to 0)	0.0<br>(0.0 to 0.0)	1<br>(0 to 1)	0.2<br>(0.0 to 0.3)	0<br>(0 to 0)	0.0<br>(0.0 to 0.0)	0.0<br>(0.0 to 0.0)	0.0<br>(0.0 to 0.0)
Bulgaria	Exposure to forces of nature	5 to 9	Female	0<br>(0 to 0)	0.0<br>(0.0 to 0.0)	0<br>(0 to 1)	0.2<br>(0.0 to 0.3)	0<br>(0 to 0)	0.0<br>(0.0 to 0.0)	0.0<br>(0.0 to 0.0)	0.0<br>(0.0 to 0.0)
Bulgaria	Exposure to forces of nature	5 to 9	Male	0<br>(0 to 0)	0.0<br>(0.0 to 0.0)	0<br>(0 to 1)	0.2<br>(0.0 to 0.4)	0<br>(0 to 0)	0.0<br>(0.0 to 0.0)	0.0<br>(0.0 to 0.0)	0.0<br>(0.0 to 0.0)
Bulgaria	Exposure to forces of nature	10 to 14	Both	0<br>(0 to 0)	0.0<br>(0.0 to 0.0)	1<br>(0 to 2)	0.2<br>(0.0 to 0.4)	0<br>(0 to 0)	0.0<br>(0.0 to 0.0)	0.0<br>(0.0 to 0.0)	0.0<br>(0.0 to 0.0)
Bulgaria	Exposure to forces of nature	10 to 14	Female	0<br>(0 to 0)	0.0<br>(0.0 to 0.0)	0<br>(0 to 1)	0.2<br>(0.0 to 0.3)	0<br>(0 to 0)	0.0<br>(0.0 to 0.0)	0.0<br>(0.0 to 0.0)	0.0<br>(0.0 to 0.0)
Bulgaria	Exposure to forces of nature	10 to 14	Male	0<br>(0 to 0)	0.0<br>(0.0 to 0.0)	1<br>(0 to 1)	0.3<br>(0.1 to 0.5)	0<br>(0 to 0)	0.0<br>(0.0 to 0.0)	0.0<br>(0.0 to 0.0)	0.0<br>(0.0 to 0.0)

eTable 8. All-cause and cause-specific mortality (counts and rates) at the global level and by country in the WHO European Region for ages 5 to 9 and 10 to 14 for 1990, 2000, and 2016

Bulgaria	Conflict and terrorism	5 to 9	Both	0 (0 to 0)	0.0 (0.0 to 0.0)	0 (0 to 0)	0.0 (0.0 to 0.0)	0 (0 to 0)	0.0 (0.0 to 0.0)	0.0 (0.0 to 0.0)	0.0 (0.0 to 0.0)
Bulgaria	Conflict and terrorism	5 to 9	Female	0 (0 to 0)	0.0 (0.0 to 0.0)	0 (0 to 0)	0.0 (0.0 to 0.0)	0 (0 to 0)	0.0 (0.0 to 0.0)	0.0 (0.0 to 0.0)	0.0 (0.0 to 0.0)
Bulgaria	Conflict and terrorism	5 to 9	Male	0 (0 to 0)	0.0 (0.0 to 0.0)	0 (0 to 0)	0.0 (0.0 to 0.0)	0 (0 to 0)	0.0 (0.0 to 0.0)	0.0 (0.0 to 0.0)	0.0 (0.0 to 0.0)
Bulgaria	Conflict and terrorism	10 to 14	Both	0 (0 to 0)	0.0 (0.0 to 0.0)	0 (0 to 0)	0.0 (0.0 to 0.0)	0 (0 to 0)	0.0 (0.0 to 0.0)	0.0 (0.0 to 0.0)	0.0 (0.0 to 0.0)
Bulgaria	Conflict and terrorism	10 to 14	Female	0 (0 to 0)	0.0 (0.0 to 0.0)	0 (0 to 0)	0.0 (0.0 to 0.0)	0 (0 to 0)	0.0 (0.0 to 0.0)	0.0 (0.0 to 0.0)	0.0 (0.0 to 0.0)
Bulgaria	Conflict and terrorism	10 to 14	Male	0 (0 to 0)	0.0 (0.0 to 0.0)	0 (0 to 0)	0.0 (0.0 to 0.0)	0 (0 to 0)	0.0 (0.0 to 0.0)	0.0 (0.0 to 0.0)	0.0 (0.0 to 0.0)
Bulgaria	Executions and police conflict	5 to 9	Both	0 (0 to 0)	0.0 (0.0 to 0.0)	0 (0 to 0)	0.0 (0.0 to 0.0)	0 (0 to 0)	0.0 (0.0 to 0.0)	-48.0 (-62.3 to -27.6)	-4.0 (-30.4 to 33.6)
Bulgaria	Executions and police conflict	5 to 9	Female	0 (0 to 0)	0.0 (0.0 to 0.0)	0 (0 to 0)	0.0 (0.0 to 0.0)	0 (0 to 0)	0.0 (0.0 to 0.0)	-49.7 (-64.3 to -30.3)	-6.5 (-33.5 to 29.7)
Bulgaria	Executions and police conflict	5 to 9	Male	0 (0 to 0)	0.0 (0.0 to 0.0)	0 (0 to 0)	0.0 (0.0 to 0.0)	0 (0 to 0)	0.0 (0.0 to 0.0)	-46.3 (-62.0 to -25.3)	-1.5 (-30.3 to 37.0)
Bulgaria	Executions and police conflict	10 to 14	Both	0 (0 to 0)	0.0 (0.0 to 0.0)	0 (0 to 0)	0.0 (0.0 to 0.0)	0 (0 to 0)	0.0 (0.0 to 0.0)	-59.4 (-72.3 to -47.8)	-8.5 (-37.5 to 17.8)
Bulgaria	Executions and police conflict	10 to 14	Female	0 (0 to 0)	0.0 (0.0 to 0.0)	0 (0 to 0)	0.0 (0.0 to 0.0)	0 (0 to 0)	0.0 (0.0 to 0.0)	-59.3 (-67.3 to -48.5)	-3.4 (-22.3 to 22.4)
Bulgaria	Executions and police conflict	10 to 14	Male	0 (0 to 0)	0.0 (0.0 to 0.0)	0 (0 to 0)	0.0 (0.0 to 0.0)	0 (0 to 0)	0.0 (0.0 to 0.0)	-59.5 (-77.4 to -42.9)	-12.9 (-51.4 to 22.9)
Croatia	All causes	5 to 9	Both	93 (89 to 96)	28.6 (27.6 to 29.7)	45 (43 to 47)	18.6 (17.8 to 19.4)	21 (18 to 23)	9.5 (8.3 to 10.7)	-77.7 (-80.7 to -74.6)	-67.0 (-71.4 to -62.4)
Croatia	All causes	5 to 9	Female	36 (35 to 38)	23.2 (22.2 to 24.1)	17 (16 to 18)	14.7 (14.0 to 15.3)	9 (8 to 10)	8.4 (7.4 to 9.4)	-75.5 (-78.8 to -72.1)	-63.8 (-68.6 to -58.6)
Croatia	All causes	5 to 9	Male	56 (54 to 59)	33.8 (32.5 to 35.2)	28 (26 to 29)	22.3 (21.2 to 23.4)	12 (10 to 13)	10.5 (9.1 to 12.0)	-79.1 (-81.9 to -76.0)	-69.0 (-73.3 to -64.4)
Croatia	All causes	10 to 14	Both	88 (85 to 92)	26.9 (25.8 to 28.1)	52 (50 to 54)	18.4 (17.6 to 19.2)	23 (21 to 25)	11.2 (10.2 to 12.2)	-73.9 (-76.5 to -71.2)	-58.3 (-62.4 to -54.0)
Croatia	All causes	10 to 14	Female	31 (30 to 33)	19.7 (18.5 to 20.8)	18 (17 to 19)	12.7 (12.0 to 13.5)	9 (8 to 10)	8.5 (7.6 to 9.6)	-72.9 (-76.1 to -69.3)	-56.6 (-61.7 to -50.8)
Croatia	All causes	10 to 14	Male	57 (54 to 60)	33.8 (32.0 to 35.7)	35 (33 to 36)	23.9 (22.7 to 25.1)	15 (13 to 16)	13.8 (12.3 to 15.4)	-74.4 (-77.2 to -71.2)	-59.2 (-63.7 to -54.1)
Croatia	Tuberculosis	5 to 9	Both	0 (0 to 0)	0.0 (0.0 to 0.0)	0 (0 to 0)	0.0 (0.0 to 0.0)	0 (0 to 0)	0.0 (0.0 to 0.0)	-76.6 (-83.3 to -67.2)	-65.4 (-75.2 to -51.4)
Croatia	Tuberculosis	5 to 9	Female	0 (0 to 0)	0.0 (0.0 to 0.1)	0 (0 to 0)	0.0 (0.0 to 0.0)	0 (0 to 0)	0.0 (0.0 to 0.0)	-85.9 (-90.3 to -78.9)	-79.1 (-85.6 to -68.7)
Croatia	Tuberculosis	5 to 9	Male	0 (0 to 0)	0.0 (0.0 to 0.0)	0 (0 to 0)	0.0 (0.0 to 0.0)	0 (0 to 0)	0.0 (0.0 to 0.0)	-40.4 (-53.9 to -24.2)	-11.8 (-31.7 to 12.1)
Croatia	Tuberculosis	10 to 14	Both	0 (0 to 0)	0.1 (0.1 to 0.1)	0 (0 to 0)	0.0 (0.0 to 0.0)	0 (0 to 0)	0.0 (0.0 to 0.0)	-91.8 (-94.1 to -88.6)	-87.0 (-90.6 to -81.7)
Croatia	Tuberculosis	10 to 14	Female	0 (0 to 0)	0.1 (0.0 to 0.1)	0 (0 to 0)	0.0 (0.0 to 0.0)	0 (0 to 0)	0.0 (0.0 to 0.0)	-89.1 (-92.2 to -84.3)	-82.5 (-87.5 to -74.8)
Croatia	Tuberculosis	10 to 14	Male	0 (0 to 0)	0.1 (0.1 to 0.1)	0 (0 to 0)	0.0 (0.0 to 0.1)	0 (0 to 0)	0.0 (0.0 to 0.0)	-93.3 (-95.8 to -89.2)	-89.4 (-93.2 to -82.8)
Croatia	HIV/AIDS	5 to 9	Both	0 (0 to 0)	0.0 (0.0 to 0.0)	0 (0 to 0)	0.0 (0.0 to 0.0)	0 (0 to 0)	0.0 (0.0 to 0.0)	-42.1 (-64.4 to -10.8)	-14.4 (-47.3 to 32.1)
Croatia	HIV/AIDS	5 to 9	Female	0 (0 to 0)	0.0 (0.0 to 0.0)	0 (0 to 0)	0.0 (0.0 to 0.0)	0 (0 to 0)	0.0 (0.0 to 0.0)	-19.4 (-56.1 to 54.1)	19.4 (-35.0 to 128.2)
Croatia	HIV/AIDS	5 to 9	Male	0 (0 to 0)	0.0 (0.0 to 0.0)	0 (0 to 0)	0.1 (0.0 to 0.1)	0 (0 to 0)	0.0 (0.0 to 0.0)	-48.1 (-71.5 to -9.3)	-23.2 (-57.8 to 34.2)
Croatia	HIV/AIDS	10 to 14	Both	0 (0 to 0)	0.0 (0.0 to 0.0)	0 (0 to 0)	0.0 (0.0 to 0.0)	0 (0 to 0)	0.0 (0.0 to 0.0)	-43.9 (-62.0 to -14.0)	-10.4 (-39.3 to 37.3)
Croatia	HIV/AIDS	10 to 14	Female	0 (0 to 0)	0.0 (0.0 to 0.0)	0 (0 to 0)	0.0 (0.0 to 0.0)	0 (0 to 0)	0.0 (0.0 to 0.0)	-42.7 (-69.0 to 6.8)	-8.3 (-50.5 to 70.9)

eTable 8. All-cause and cause-specific mortality (counts and rates) at the global level and by country in the WHO European Region for ages 5 to 9 and 10 to 14 for 1990, 2000, and 2016

Croatia	HIV/AIDS	10 to 14	Male	0 (0 to 0)	0.0 (0.0 to 0.0)	0 (0 to 0)	0.0 (0.0 to 0.0)	0 (0 to 0)	0.0 (0.0 to 0.0)	-44.5 (-67.2 to -1.9)	-11.6 (-47.7 to 56.2)
Croatia	Diarrheal diseases	5 to 9	Both	0 (0 to 0)	0.1 (0.0 to 0.1)	0 (0 to 0)	0.0 (0.0 to 0.0)	0 (0 to 0)	0.0 (0.0 to 0.0)	-80.5 (-87.7 to -68.8)	-71.1 (-81.8 to -53.9)
Croatia	Diarrheal diseases	5 to 9	Female	0 (0 to 0)	0.0 (0.0 to 0.1)	0 (0 to 0)	0.0 (0.0 to 0.0)	0 (0 to 0)	0.0 (0.0 to 0.0)	-75.5 (-86.9 to -53.3)	-63.8 (-80.6 to -30.9)
Croatia	Diarrheal diseases	5 to 9	Male	0 (0 to 0)	0.1 (0.1 to 0.1)	0 (0 to 0)	0.0 (0.0 to 0.0)	0 (0 to 0)	0.0 (0.0 to 0.0)	-82.4 (-90.6 to -66.7)	-74.0 (-86.1 to -50.8)
Croatia	Diarrheal diseases	10 to 14	Both	0 (0 to 0)	0.0 (0.0 to 0.1)	0 (0 to 0)	0.0 (0.0 to 0.0)	0 (0 to 0)	0.0 (0.0 to 0.0)	-76.7 (-86.4 to -62.7)	-62.9 (-78.3 to -40.5)
Croatia	Diarrheal diseases	10 to 14	Female	0 (0 to 0)	0.0 (0.0 to 0.0)	0 (0 to 0)	0.0 (0.0 to 0.0)	0 (0 to 0)	0.0 (0.0 to 0.0)	-50.3 (-66.2 to -33.3)	-20.5 (-45.9 to 6.6)
Croatia	Diarrheal diseases	10 to 14	Male	0 (0 to 0)	0.1 (0.0 to 0.1)	0 (0 to 0)	0.0 (0.0 to 0.0)	0 (0 to 0)	0.0 (0.0 to 0.0)	-80.8 (-90.4 to -66.0)	-69.5 (-84.7 to -45.9)
Croatia	Intestinal infectious diseases	5 to 9	Both	0 (0 to 0)	0.0 (0.0 to 0.0)	0 (0 to 0)	0.0 (0.0 to 0.0)	0 (0 to 0)	0.0 (0.0 to 0.0)	-47.5 (-66.4 to -21.1)	-22.3 (-50.2 to 16.7)
Croatia	Intestinal infectious diseases	5 to 9	Female	0 (0 to 0)	0.0 (0.0 to 0.0)	0 (0 to 0)	0.0 (0.0 to 0.0)	0 (0 to 0)	0.0 (0.0 to 0.0)	-40.3 (-67.6 to -3.5)	-11.5 (-52.0 to 42.9)
Croatia	Intestinal infectious diseases	5 to 9	Male	0 (0 to 0)	0.0 (0.0 to 0.0)	0 (0 to 0)	0.0 (0.0 to 0.0)	0 (0 to 0)	0.0 (0.0 to 0.0)	-51.3 (-72.7 to -17.4)	-28.0 (-59.6 to 22.2)
Croatia	Intestinal infectious diseases	10 to 14	Both	0 (0 to 0)	0.0 (0.0 to 0.0)	0 (0 to 0)	0.0 (0.0 to 0.0)	0 (0 to 0)	0.0 (0.0 to 0.0)	-41.6 (-63.3 to -17.4)	-6.7 (-41.4 to 31.8)
Croatia	Intestinal infectious diseases	10 to 14	Female	0 (0 to 0)	0.0 (0.0 to 0.0)	0 (0 to 0)	0.0 (0.0 to 0.0)	0 (0 to 0)	0.0 (0.0 to 0.0)	-35.0 (-60.5 to 1.3)	4.0 (-36.8 to 62.1)
Croatia	Intestinal infectious diseases	10 to 14	Male	0 (0 to 0)	0.0 (0.0 to 0.0)	0 (0 to 0)	0.0 (0.0 to 0.0)	0 (0 to 0)	0.0 (0.0 to 0.0)	-45.5 (-70.5 to -9.0)	-13.1 (-53.1 to 44.9)
Croatia	Lower respiratory infections	5 to 9	Both	3 (2 to 3)	0.8 (0.6 to 1.0)	2 (1 to 2)	0.6 (0.5 to 0.8)	0 (0 to 1)	0.2 (0.1 to 0.2)	-85.6 (-90.6 to -77.7)	-78.7 (-86.0 to -67.0)
Croatia	Lower respiratory infections	5 to 9	Female	1 (1 to 2)	0.8 (0.5 to 1.1)	1 (1 to 1)	0.6 (0.4 to 0.9)	0 (0 to 0)	0.2 (0.1 to 0.3)	-85.6 (-92.5 to -74.1)	-78.7 (-88.9 to -61.7)
Croatia	Lower respiratory infections	5 to 9	Male	1 (1 to 2)	0.9 (0.6 to 1.2)	1 (1 to 1)	0.7 (0.5 to 0.9)	0 (0 to 0)	0.2 (0.1 to 0.3)	-85.6 (-91.5 to -74.7)	-78.6 (-87.4 to -62.6)
Croatia	Lower respiratory infections	10 to 14	Both	3 (2 to 3)	0.8 (0.6 to 1.0)	2 (1 to 2)	0.6 (0.5 to 0.8)	0 (0 to 1)	0.2 (0.2 to 0.3)	-83.4 (-88.7 to -75.3)	-73.4 (-81.9 to -60.5)
Croatia	Lower respiratory infections	10 to 14	Female	1 (1 to 2)	0.8 (0.6 to 1.2)	1 (1 to 1)	0.6 (0.4 to 0.8)	0 (0 to 0)	0.2 (0.1 to 0.3)	-84.2 (-90.7 to -72.7)	-74.7 (-85.1 to -56.3)
Croatia	Lower respiratory infections	10 to 14	Male	1 (1 to 2)	0.7 (0.5 to 1.0)	1 (1 to 1)	0.6 (0.4 to 0.8)	0 (0 to 0)	0.2 (0.1 to 0.3)	-82.5 (-89.9 to -69.8)	-72.1 (-83.9 to -51.9)
Croatia	Upper respiratory infections	5 to 9	Both	0 (0 to 0)	0.0 (0.0 to 0.0)	0 (0 to 0)	0.0 (0.0 to 0.0)	0 (0 to 0)	0.0 (0.0 to 0.0)	-83.7 (-99.7 to 647.1)	-75.8 (-99.5 to 1005.8)
Croatia	Upper respiratory infections	5 to 9	Female	0 (0 to 0)	0.0 (0.0 to 0.0)	0 (0 to 0)	0.0 (0.0 to 0.0)	0 (0 to 0)	0.0 (0.0 to 0.0)	-81.7 (-100.0 to 43492.4)	-72.8 (-100.0 to 64458.0)
Croatia	Upper respiratory infections	5 to 9	Male	0 (0 to 0)	0.0 (0.0 to 0.0)	0 (0 to 0)	0.0 (0.0 to 0.0)	0 (0 to 0)	0.0 (0.0 to 0.0)	-84.9 (-100.0 to 28872.8)	-77.7 (-100.0 to 42764.0)
Croatia	Upper respiratory infections	10 to 14	Both	0 (0 to 0)	0.0 (0.0 to 0.0)	0 (0 to 0)	0.0 (0.0 to 0.0)	0 (0 to 0)	0.0 (0.0 to 0.0)	-80.0 (-99.7 to 1474.4)	-68.1 (-99.5 to 2413.5)
Croatia	Upper respiratory infections	10 to 14	Female	0 (0 to 0)	0.0 (0.0 to 0.0)	0 (0 to 0)	0.0 (0.0 to 0.0)	0 (0 to 0)	0.0 (0.0 to 0.0)	-79.0 (-100.0 to 40174.7)	-66.4 (-100.0 to 64336.7)

eTable 8. All-cause and cause-specific mortality (counts and rates) at the global level and by country in the WHO European Region for ages 5 to 9 and 10 to 14 for 1990, 2000, and 2016

Croatia	Upper respiratory infections	10 to 14	Male	0 (0 to 0)	0.0 (0.0 to 0.0)	0 (0 to 0)	0.0 (0.0 to 0.0)	0 (0 to 0)	0.0 (0.0 to 0.0)	-80.6 (-100.0 to 18012.7)	-69.2 (-100.0 to 28755.9)
Croatia	Otitis media	5 to 9	Both	0 (0 to 0)	0.0 (0.0 to 0.0)	0 (0 to 0)	0.0 (0.0 to 0.0)	0 (0 to 0)	0.0 (0.0 to 0.0)	-95.5 (-98.5 to -87.5)	-93.3 (-97.8 to -81.5)
Croatia	Otitis media	5 to 9	Female	0 (0 to 0)	0.0 (0.0 to 0.0)	0 (0 to 0)	0.0 (0.0 to 0.0)	0 (0 to 0)	0.0 (0.0 to 0.0)	-94.2 (-98.9 to -76.3)	-91.5 (-98.3 to -64.8)
Croatia	Otitis media	5 to 9	Male	0 (0 to 0)	0.0 (0.0 to 0.0)	0 (0 to 0)	0.0 (0.0 to 0.0)	0 (0 to 0)	0.0 (0.0 to 0.0)	-97.2 (-99.0 to -92.5)	-95.9 (-98.6 to -89.0)
Croatia	Otitis media	10 to 14	Both	0 (0 to 0)	0.0 (0.0 to 0.0)	0 (0 to 0)	0.0 (0.0 to 0.0)	0 (0 to 0)	0.0 (0.0 to 0.0)	-77.0 (-91.4 to -43.9)	-63.4 (-86.3 to -10.5)
Croatia	Otitis media	10 to 14	Female	0 (0 to 0)	0.0 (0.0 to 0.0)	0 (0 to 0)	0.0 (0.0 to 0.0)	0 (0 to 0)	0.0 (0.0 to 0.0)	-91.3 (-98.2 to -68.7)	-86.0 (-97.1 to -49.9)
Croatia	Otitis media	10 to 14	Male	0 (0 to 0)	0.0 (0.0 to 0.0)	0 (0 to 0)	0.0 (0.0 to 0.0)	0 (0 to 0)	0.0 (0.0 to 0.0)	-66.0 (-89.9 to 7.3)	-45.8 (-83.9 to 70.9)
Croatia	Meningitis	5 to 9	Both	1 (1 to 2)	0.4 (0.3 to 0.5)	0 (0 to 1)	0.2 (0.2 to 0.3)	0 (0 to 0)	0.1 (0.0 to 0.1)	-90.1 (-93.7 to -84.8)	-85.3 (-90.7 to -77.5)
Croatia	Meningitis	5 to 9	Female	1 (0 to 1)	0.4 (0.3 to 0.6)	0 (0 to 0)	0.2 (0.1 to 0.3)	0 (0 to 0)	0.1 (0.0 to 0.1)	-89.1 (-93.6 to -82.3)	-83.8 (-90.5 to -73.9)
Croatia	Meningitis	5 to 9	Male	1 (0 to 1)	0.4 (0.3 to 0.6)	0 (0 to 0)	0.2 (0.1 to 0.3)	0 (0 to 0)	0.1 (0.0 to 0.1)	-91.0 (-95.3 to -84.3)	-86.7 (-93.0 to -76.7)
Croatia	Meningitis	10 to 14	Both	1 (1 to 1)	0.3 (0.2 to 0.4)	0 (0 to 1)	0.1 (0.1 to 0.2)	0 (0 to 0)	0.1 (0.0 to 0.1)	-87.8 (-91.9 to -81.9)	-80.6 (-87.1 to -71.1)
Croatia	Meningitis	10 to 14	Female	0 (0 to 1)	0.3 (0.2 to 0.4)	0 (0 to 0)	0.1 (0.1 to 0.2)	0 (0 to 0)	0.1 (0.0 to 0.1)	-87.1 (-92.3 to -78.2)	-79.4 (-87.7 to -65.1)
Croatia	Meningitis	10 to 14	Male	1 (0 to 1)	0.3 (0.2 to 0.4)	0 (0 to 0)	0.2 (0.1 to 0.2)	0 (0 to 0)	0.1 (0.0 to 0.1)	-88.4 (-93.4 to -80.8)	-81.6 (-89.6 to -69.4)
Croatia	Encephalitis	5 to 9	Both	0 (0 to 1)	0.1 (0.1 to 0.2)	0 (0 to 1)	0.1 (0.1 to 0.2)	0 (0 to 0)	0.1 (0.0 to 0.2)	-52.4 (-77.9 to -30.3)	-29.6 (-67.3 to 3.2)
Croatia	Encephalitis	5 to 9	Female	0 (0 to 0)	0.1 (0.1 to 0.3)	0 (0 to 0)	0.1 (0.1 to 0.3)	0 (0 to 0)	0.1 (0.0 to 0.2)	-53.4 (-83.0 to -31.5)	-30.9 (-74.8 to 1.4)
Croatia	Encephalitis	5 to 9	Male	0 (0 to 0)	0.1 (0.1 to 0.2)	0 (0 to 0)	0.1 (0.1 to 0.2)	0 (0 to 0)	0.1 (0.0 to 0.2)	-51.6 (-78.4 to -22.9)	-28.4 (-68.0 to 14.1)
Croatia	Encephalitis	10 to 14	Both	0 (0 to 1)	0.1 (0.1 to 0.2)	0 (0 to 1)	0.1 (0.1 to 0.2)	0 (0 to 0)	0.1 (0.1 to 0.2)	-52.8 (-73.1 to -34.6)	-24.7 (-57.0 to 4.4)
Croatia	Encephalitis	10 to 14	Female	0 (0 to 0)	0.1 (0.1 to 0.2)	0 (0 to 0)	0.1 (0.1 to 0.2)	0 (0 to 0)	0.1 (0.0 to 0.2)	-56.7 (-81.5 to -35.1)	-30.6 (-70.5 to 3.9)
Croatia	Encephalitis	10 to 14	Male	0 (0 to 0)	0.2 (0.1 to 0.3)	0 (0 to 0)	0.2 (0.1 to 0.3)	0 (0 to 0)	0.1 (0.1 to 0.2)	-50.3 (-73.6 to -28.4)	-20.9 (-58.0 to 14.1)
Croatia	Diphtheria	5 to 9	Both	0 (0 to 0)	0.0 (0.0 to 0.0)	0 (0 to 0)	0.0 (0.0 to 0.0)	0 (0 to 0)	0.0 (0.0 to 0.0)	-90.9 (-98.6 to -38.8)	-86.5 (-97.9 to -9.5)
Croatia	Diphtheria	5 to 9	Female	0 (0 to 0)	0.0 (0.0 to 0.0)	0 (0 to 0)	0.0 (0.0 to 0.0)	0 (0 to 0)	0.0 (0.0 to 0.0)	-89.5 (-99.6 to 65.8)	-84.4 (-99.5 to 145.6)
Croatia	Diphtheria	5 to 9	Male	0 (0 to 0)	0.0 (0.0 to 0.0)	0 (0 to 0)	0.0 (0.0 to 0.0)	0 (0 to 0)	0.0 (0.0 to 0.0)	-91.7 (-99.6 to 44.7)	-87.8 (-99.4 to 114.1)
Croatia	Diphtheria	10 to 14	Both	0 (0 to 0)	0.0 (0.0 to 0.0)	0 (0 to 0)	0.0 (0.0 to 0.0)	0 (0 to 0)	0.0 (0.0 to 0.0)	-88.7 (-98.5 to -22.8)	-81.9 (-97.7 to 23.3)
Croatia	Diphtheria	10 to 14	Female	0 (0 to 0)	0.0 (0.0 to 0.0)	0 (0 to 0)	0.0 (0.0 to 0.0)	0 (0 to 0)	0.0 (0.0 to 0.0)	-88.2 (-99.4 to 145.5)	-81.2 (-99.1 to 292.8)
Croatia	Diphtheria	10 to 14	Male	0 (0 to 0)	0.0 (0.0 to 0.0)	0 (0 to 0)	0.0 (0.0 to 0.0)	0 (0 to 0)	0.0 (0.0 to 0.0)	-88.9 (-99.4 to 114.2)	-82.4 (-99.1 to 241.2)
Croatia	Whooping cough	5 to 9	Both	0 (0 to 0)	0.0 (0.0 to 0.0)	0 (0 to 0)	0.0 (0.0 to 0.0)	0 (0 to 0)	0.0 (0.0 to 0.0)	-24.8 (-72.4 to 111.8)	11.3 (-59.1 to 213.4)
Croatia	Whooping cough	5 to 9	Female	0 (0 to 0)	0.0 (0.0 to 0.0)	0 (0 to 0)	0.0 (0.0 to 0.0)	0 (0 to 0)	0.0 (0.0 to 0.0)	-36.6 (-80.4 to 120.5)	-6.2 (-71.0 to 226.6)
Croatia	Whooping cough	5 to 9	Male	0 (0 to 0)	0.0 (0.0 to 0.0)	0 (0 to 0)	0.0 (0.0 to 0.0)	0 (0 to 0)	0.0 (0.0 to 0.0)	-12.3 (-56.3 to 168.8)	29.8 (-35.4 to 297.7)
Croatia	Whooping cough	10 to 14	Both	0 (0 to 0)	0.0 (0.0 to 0.0)	0 (0 to 0)	0.0 (0.0 to 0.0)	0 (0 to 0)	0.0 (0.0 to 0.0)	-5.1 (-68.8 to 236.2)	51.5 (-50.2 to 436.7)
Croatia	Whooping cough	10 to 14	Female	0 (0 to 0)	0.0 (0.0 to 0.0)	0 (0 to 0)	0.0 (0.0 to 0.0)	0 (0 to 0)	0.0 (0.0 to 0.0)	-50.9 (-77.4 to -23.8)	-21.5 (-63.8 to 21.9)
Croatia	Whooping cough	10 to 14	Male	0 (0 to 0)	0.0 (0.0 to 0.0)	0 (0 to 0)	0.0 (0.0 to 0.0)	0 (0 to 0)	0.0 (0.0 to 0.0)	33.5 (-57.4 to 469.1)	112.7 (-32.1 to 806.7)
Croatia	Tetanus	5 to 9	Both	0 (0 to 0)	0.0 (0.0 to 0.0)	0 (0 to 0)	0.0 (0.0 to 0.0)	0 (0 to 0)	0.0 (0.0 to 0.0)	-93.3 (-97.0 to -84.9)	-90.0 (-95.5 to -77.6)

eTable 8. All-cause and cause-specific mortality (counts and rates) at the global level and by country in the WHO European Region for ages 5 to 9 and 10 to 14 for 1990, 2000, and 2016

Croatia	Tetanus	5 to 9	Female	0 (0 to 0)	0.0 (0.0 to 0.0)	0 (0 to 0)	0.0 (0.0 to 0.0)	0 (0 to 0)	0.0 (0.0 to 0.0)	-92.0 (-96.8 to -74.7)	-88.2 (-95.3 to -62.5)
Croatia	Tetanus	5 to 9	Male	0 (0 to 0)	0.0 (0.0 to 0.0)	0 (0 to 0)	0.0 (0.0 to 0.0)	0 (0 to 0)	0.0 (0.0 to 0.0)	-94.0 (-97.2 to -85.3)	-91.1 (-95.9 to -78.3)
Croatia	Tetanus	10 to 14	Both	0 (0 to 0)	0.0 (0.0 to 0.0)	0 (0 to 0)	0.0 (0.0 to 0.0)	0 (0 to 0)	0.0 (0.0 to 0.0)	-81.3 (-96.9 to -35.1)	-70.2 (-95.0 to 3.7)
Croatia	Tetanus	10 to 14	Female	0 (0 to 0)	0.0 (0.0 to 0.0)	0 (0 to 0)	0.0 (0.0 to 0.0)	0 (0 to 0)	0.0 (0.0 to 0.0)	-92.4 (-96.7 to -79.3)	-87.8 (-94.7 to -66.8)
Croatia	Tetanus	10 to 14	Male	0 (0 to 0)	0.0 (0.0 to 0.0)	0 (0 to 0)	0.0 (0.0 to 0.0)	0 (0 to 0)	0.0 (0.0 to 0.0)	-77.2 (-96.7 to 0.1)	-63.7 (-94.7 to 59.4)
Croatia	Measles	5 to 9	Both	0 (0 to 0)	0.0 (0.0 to 0.0)	0 (0 to 0)	0.0 (0.0 to 0.0)	0 (0 to 0)	0.0 (0.0 to 0.0)	-93.2 (-97.0 to -84.1)	-89.9 (-95.6 to -76.5)
Croatia	Measles	5 to 9	Female	0 (0 to 0)	0.0 (0.0 to 0.0)	0 (0 to 0)	0.0 (0.0 to 0.0)	0 (0 to 0)	0.0 (0.0 to 0.0)	-94.8 (-98.4 to -85.6)	-92.3 (-97.6 to -78.7)
Croatia	Measles	5 to 9	Male	0 (0 to 0)	0.0 (0.0 to 0.0)	0 (0 to 0)	0.0 (0.0 to 0.0)	0 (0 to 0)	0.0 (0.0 to 0.0)	-74.4 (-87.5 to -40.9)	-62.1 (-81.5 to -12.6)
Croatia	Measles	10 to 14	Both	0 (0 to 0)	0.0 (0.0 to 0.0)	0 (0 to 0)	0.0 (0.0 to 0.0)	0 (0 to 0)	0.0 (0.0 to 0.0)	-95.0 (-97.9 to -88.7)	-92.1 (-96.7 to -82.0)
Croatia	Measles	10 to 14	Female	0 (0 to 0)	0.0 (0.0 to 0.0)	0 (0 to 0)	0.0 (0.0 to 0.0)	0 (0 to 0)	0.0 (0.0 to 0.0)	-98.2 (-99.3 to -95.2)	-97.1 (-98.9 to -92.3)
Croatia	Measles	10 to 14	Male	0 (0 to 0)	0.0 (0.0 to 0.0)	0 (0 to 0)	0.0 (0.0 to 0.0)	0 (0 to 0)	0.0 (0.0 to 0.0)	-78.7 (-93.6 to -36.2)	-66.0 (-89.8 to 1.7)
Croatia	Varicella and herpes zoster	5 to 9	Both	0 (0 to 0)	0.0 (0.0 to 0.0)	0 (0 to 0)	0.0 (0.0 to 0.0)	0 (0 to 0)	0.0 (0.0 to 0.0)	-68.9 (-84.4 to -34.4)	-54.0 (-76.9 to -3.0)
Croatia	Varicella and herpes zoster	5 to 9	Female	0 (0 to 0)	0.0 (0.0 to 0.0)	0 (0 to 0)	0.0 (0.0 to 0.0)	0 (0 to 0)	0.0 (0.0 to 0.0)	-83.8 (-93.5 to -71.9)	-76.0 (-90.4 to -58.4)
Croatia	Varicella and herpes zoster	5 to 9	Male	0 (0 to 0)	0.0 (0.0 to 0.0)	0 (0 to 0)	0.0 (0.0 to 0.0)	0 (0 to 0)	0.0 (0.0 to 0.0)	-59.2 (-84.8 to 1.4)	-39.7 (-77.6 to 50.0)
Croatia	Varicella and herpes zoster	10 to 14	Both	0 (0 to 0)	0.0 (0.0 to 0.0)	0 (0 to 0)	0.0 (0.0 to 0.0)	0 (0 to 0)	0.0 (0.0 to 0.0)	-61.9 (-79.7 to -8.7)	-39.1 (-67.5 to 45.7)
Croatia	Varicella and herpes zoster	10 to 14	Female	0 (0 to 0)	0.0 (0.0 to 0.0)	0 (0 to 0)	0.0 (0.0 to 0.0)	0 (0 to 0)	0.0 (0.0 to 0.0)	-79.8 (-90.8 to -67.0)	-67.6 (-85.3 to -47.2)
Croatia	Varicella and herpes zoster	10 to 14	Male	0 (0 to 0)	0.0 (0.0 to 0.0)	0 (0 to 0)	0.0 (0.0 to 0.0)	0 (0 to 0)	0.0 (0.0 to 0.0)	-49.7 (-79.8 to 61.3)	-19.9 (-67.8 to 157.0)
Croatia	Malaria	5 to 9	Both	0 (0 to 0)	0.0 (0.0 to 0.0)	0 (0 to 0)	0.0 (0.0 to 0.0)	0 (0 to 0)	0.0 (0.0 to 0.0)	0.0 (0.0 to 0.0)	0.0 (0.0 to 0.0)
Croatia	Malaria	5 to 9	Female	0 (0 to 0)	0.0 (0.0 to 0.0)	0 (0 to 0)	0.0 (0.0 to 0.0)	0 (0 to 0)	0.0 (0.0 to 0.0)	0.0 (0.0 to 0.0)	0.0 (0.0 to 0.0)
Croatia	Malaria	5 to 9	Male	0 (0 to 0)	0.0 (0.0 to 0.0)	0 (0 to 0)	0.0 (0.0 to 0.0)	0 (0 to 0)	0.0 (0.0 to 0.0)	0.0 (0.0 to 0.0)	0.0 (0.0 to 0.0)
Croatia	Malaria	10 to 14	Both	0 (0 to 0)	0.0 (0.0 to 0.0)	0 (0 to 0)	0.0 (0.0 to 0.0)	0 (0 to 0)	0.0 (0.0 to 0.0)	0.0 (0.0 to 0.0)	0.0 (0.0 to 0.0)
Croatia	Malaria	10 to 14	Female	0 (0 to 0)	0.0 (0.0 to 0.0)	0 (0 to 0)	0.0 (0.0 to 0.0)	0 (0 to 0)	0.0 (0.0 to 0.0)	0.0 (0.0 to 0.0)	0.0 (0.0 to 0.0)
Croatia	Malaria	10 to 14	Male	0 (0 to 0)	0.0 (0.0 to 0.0)	0 (0 to 0)	0.0 (0.0 to 0.0)	0 (0 to 0)	0.0 (0.0 to 0.0)	0.0 (0.0 to 0.0)	0.0 (0.0 to 0.0)
Croatia	Leishmaniasis	5 to 9	Both	0 (0 to 0)	0.0 (0.0 to 0.0)	0 (0 to 0)	0.0 (0.0 to 0.0)	0 (0 to 0)	0.0 (0.0 to 0.0)	-73.7 (-100.0 to 142.1)	-61.0 (-100.0 to 258.4)
Croatia	Leishmaniasis	5 to 9	Female	0 (0 to 0)	0.0 (0.0 to 0.0)	0 (0 to 0)	0.0 (0.0 to 0.0)	0 (0 to 0)	0.0 (0.0 to 0.0)	-73.6 (-100.0 to 63.3)	-60.9 (-100.0 to 141.8)
Croatia	Leishmaniasis	5 to 9	Male	0 (0 to 0)	0.0 (0.0 to 0.0)	0 (0 to 0)	0.0 (0.0 to 0.0)	0 (0 to 0)	0.0 (0.0 to 0.0)	-73.7 (-100.0 to 14.5)	-61.1 (-100.0 to 69.3)
Croatia	Leishmaniasis	10 to 14	Both	0 (0 to 0)	0.0 (0.0 to 0.0)	0 (0 to 0)	0.0 (0.0 to 0.0)	0 (0 to 0)	0.0 (0.0 to 0.0)	-72.6 (-100.0 to 210.3)	-56.2 (-100.0 to 395.3)
Croatia	Leishmaniasis	10 to 14	Female	0 (0 to 0)	0.0 (0.0 to 0.0)	0 (0 to 0)	0.0 (0.0 to 0.0)	0 (0 to 0)	0.0 (0.0 to 0.0)	-72.7 (-100.0 to 40.9)	-56.3 (-100.0 to 125.4)
Croatia	Leishmaniasis	10 to 14	Male	0 (0 to 0)	0.0 (0.0 to 0.0)	0 (0 to 0)	0.0 (0.0 to 0.0)	0 (0 to 0)	0.0 (0.0 to 0.0)	-72.5 (-100.0 to 20.1)	-56.1 (-100.0 to 91.3)
Croatia	African trypanosomiasis	5 to 9	Both	0 (0 to 0)	0.0 (0.0 to 0.0)	0 (0 to 0)	0.0 (0.0 to 0.0)	0 (0 to 0)	0.0 (0.0 to 0.0)	0.0 (0.0 to 0.0)	0.0 (0.0 to 0.0)
Croatia	African trypanosomiasis	5 to 9	Female	0 (0 to 0)	0.0 (0.0 to 0.0)	0 (0 to 0)	0.0 (0.0 to 0.0)	0 (0 to 0)	0.0 (0.0 to 0.0)	0.0 (0.0 to 0.0)	0.0 (0.0 to 0.0)

eTable 8. All-cause and cause-specific mortality (counts and rates) at the global level and by country in the WHO European Region for ages 5 to 9 and 10 to 14 for 1990, 2000, and 2016

Croatia	African trypanosomiasis	5 to 9	Male	0 (0 to 0)	0.0 (0.0 to 0.0)	0 (0 to 0)	0.0 (0.0 to 0.0)	0 (0 to 0)	0.0 (0.0 to 0.0)	0.0 (0.0 to 0.0)	0.0 (0.0 to 0.0)
Croatia	African trypanosomiasis	10 to 14	Both	0 (0 to 0)	0.0 (0.0 to 0.0)	0 (0 to 0)	0.0 (0.0 to 0.0)	0 (0 to 0)	0.0 (0.0 to 0.0)	0.0 (0.0 to 0.0)	0.0 (0.0 to 0.0)
Croatia	African trypanosomiasis	10 to 14	Female	0 (0 to 0)	0.0 (0.0 to 0.0)	0 (0 to 0)	0.0 (0.0 to 0.0)	0 (0 to 0)	0.0 (0.0 to 0.0)	0.0 (0.0 to 0.0)	0.0 (0.0 to 0.0)
Croatia	African trypanosomiasis	10 to 14	Male	0 (0 to 0)	0.0 (0.0 to 0.0)	0 (0 to 0)	0.0 (0.0 to 0.0)	0 (0 to 0)	0.0 (0.0 to 0.0)	0.0 (0.0 to 0.0)	0.0 (0.0 to 0.0)
Croatia	Schistosomiasis	5 to 9	Both	0 (0 to 0)	0.0 (0.0 to 0.0)	0 (0 to 0)	0.0 (0.0 to 0.0)	0 (0 to 0)	0.0 (0.0 to 0.0)	0.0 (0.0 to 0.0)	0.0 (0.0 to 0.0)
Croatia	Schistosomiasis	5 to 9	Female	0 (0 to 0)	0.0 (0.0 to 0.0)	0 (0 to 0)	0.0 (0.0 to 0.0)	0 (0 to 0)	0.0 (0.0 to 0.0)	0.0 (0.0 to 0.0)	0.0 (0.0 to 0.0)
Croatia	Schistosomiasis	5 to 9	Male	0 (0 to 0)	0.0 (0.0 to 0.0)	0 (0 to 0)	0.0 (0.0 to 0.0)	0 (0 to 0)	0.0 (0.0 to 0.0)	0.0 (0.0 to 0.0)	0.0 (0.0 to 0.0)
Croatia	Schistosomiasis	10 to 14	Both	0 (0 to 0)	0.0 (0.0 to 0.0)	0 (0 to 0)	0.0 (0.0 to 0.0)	0 (0 to 0)	0.0 (0.0 to 0.0)	0.0 (0.0 to 0.0)	0.0 (0.0 to 0.0)
Croatia	Schistosomiasis	10 to 14	Female	0 (0 to 0)	0.0 (0.0 to 0.0)	0 (0 to 0)	0.0 (0.0 to 0.0)	0 (0 to 0)	0.0 (0.0 to 0.0)	0.0 (0.0 to 0.0)	0.0 (0.0 to 0.0)
Croatia	Schistosomiasis	10 to 14	Male	0 (0 to 0)	0.0 (0.0 to 0.0)	0 (0 to 0)	0.0 (0.0 to 0.0)	0 (0 to 0)	0.0 (0.0 to 0.0)	0.0 (0.0 to 0.0)	0.0 (0.0 to 0.0)
Croatia	Cysticercosis	5 to 9	Both	0 (0 to 0)	0.0 (0.0 to 0.0)	0 (0 to 0)	0.0 (0.0 to 0.0)	0 (0 to 0)	0.0 (0.0 to 0.0)	-91.6 (-93.3 to -89.7)	-87.5 (-90.0 to -84.7)
Croatia	Cysticercosis	5 to 9	Female	0 (0 to 0)	0.0 (0.0 to 0.0)	0 (0 to 0)	0.0 (0.0 to 0.0)	0 (0 to 0)	0.0 (0.0 to 0.0)	-90.9 (-92.9 to -88.6)	-86.6 (-89.4 to -83.1)
Croatia	Cysticercosis	5 to 9	Male	0 (0 to 0)	0.0 (0.0 to 0.0)	0 (0 to 0)	0.0 (0.0 to 0.0)	0 (0 to 0)	0.0 (0.0 to 0.0)	-92.2 (-93.9 to -90.0)	-88.4 (-91.0 to -85.3)
Croatia	Cysticercosis	10 to 14	Both	0 (0 to 0)	0.0 (0.0 to 0.0)	0 (0 to 0)	0.0 (0.0 to 0.0)	0 (0 to 0)	0.0 (0.0 to 0.0)	-89.8 (-91.4 to -88.1)	-83.7 (-86.2 to -81.1)
Croatia	Cysticercosis	10 to 14	Female	0 (0 to 0)	0.0 (0.0 to 0.0)	0 (0 to 0)	0.0 (0.0 to 0.0)	0 (0 to 0)	0.0 (0.0 to 0.0)	-89.5 (-91.4 to -87.3)	-83.2 (-86.3 to -79.7)
Croatia	Cysticercosis	10 to 14	Male	0 (0 to 0)	0.0 (0.0 to 0.0)	0 (0 to 0)	0.0 (0.0 to 0.0)	0 (0 to 0)	0.0 (0.0 to 0.0)	-90.0 (-91.9 to -87.8)	-84.1 (-87.1 to -80.6)
Croatia	Yellow fever	5 to 9	Both	0 (0 to 0)	0.0 (0.0 to 0.0)	0 (0 to 0)	0.0 (0.0 to 0.0)	0 (0 to 0)	0.0 (0.0 to 0.0)	0.0 (0.0 to 0.0)	0.0 (0.0 to 0.0)
Croatia	Yellow fever	5 to 9	Female	0 (0 to 0)	0.0 (0.0 to 0.0)	0 (0 to 0)	0.0 (0.0 to 0.0)	0 (0 to 0)	0.0 (0.0 to 0.0)	0.0 (0.0 to 0.0)	0.0 (0.0 to 0.0)
Croatia	Yellow fever	5 to 9	Male	0 (0 to 0)	0.0 (0.0 to 0.0)	0 (0 to 0)	0.0 (0.0 to 0.0)	0 (0 to 0)	0.0 (0.0 to 0.0)	0.0 (0.0 to 0.0)	0.0 (0.0 to 0.0)
Croatia	Yellow fever	10 to 14	Both	0 (0 to 0)	0.0 (0.0 to 0.0)	0 (0 to 0)	0.0 (0.0 to 0.0)	0 (0 to 0)	0.0 (0.0 to 0.0)	0.0 (0.0 to 0.0)	0.0 (0.0 to 0.0)
Croatia	Yellow fever	10 to 14	Female	0 (0 to 0)	0.0 (0.0 to 0.0)	0 (0 to 0)	0.0 (0.0 to 0.0)	0 (0 to 0)	0.0 (0.0 to 0.0)	0.0 (0.0 to 0.0)	0.0 (0.0 to 0.0)
Croatia	Yellow fever	10 to 14	Male	0 (0 to 0)	0.0 (0.0 to 0.0)	0 (0 to 0)	0.0 (0.0 to 0.0)	0 (0 to 0)	0.0 (0.0 to 0.0)	0.0 (0.0 to 0.0)	0.0 (0.0 to 0.0)
Croatia	Rabies	5 to 9	Both	0 (0 to 0)	0.0 (0.0 to 0.0)	0 (0 to 0)	0.0 (0.0 to 0.0)	0 (0 to 0)	0.0 (0.0 to 0.0)	-90.8 (-97.8 to -67.0)	-86.3 (-96.8 to -51.2)
Croatia	Rabies	5 to 9	Female	0 (0 to 0)	0.0 (0.0 to 0.0)	0 (0 to 0)	0.0 (0.0 to 0.0)	0 (0 to 0)	0.0 (0.0 to 0.0)	-80.7 (-96.8 to -11.5)	-71.3 (-95.3 to 31.1)
Croatia	Rabies	5 to 9	Male	0 (0 to 0)	0.0 (0.0 to 0.0)	0 (0 to 0)	0.0 (0.0 to 0.0)	0 (0 to 0)	0.0 (0.0 to 0.0)	-95.0 (-99.2 to -72.8)	-92.6 (-98.8 to -59.8)
Croatia	Rabies	10 to 14	Both	0 (0 to 0)	0.0 (0.0 to 0.0)	0 (0 to 0)	0.0 (0.0 to 0.0)	0 (0 to 0)	0.0 (0.0 to 0.0)	-62.8 (-88.3 to 4.7)	-40.6 (-81.3 to 67.2)
Croatia	Rabies	10 to 14	Female	0 (0 to 0)	0.0 (0.0 to 0.0)	0 (0 to 0)	0.0 (0.0 to 0.0)	0 (0 to 0)	0.0 (0.0 to 0.0)	-69.8 (-94.9 to 38.4)	-51.6 (-91.9 to 121.5)
Croatia	Rabies	10 to 14	Male	0 (0 to 0)	0.0 (0.0 to 0.0)	0 (0 to 0)	0.0 (0.0 to 0.0)	0 (0 to 0)	0.0 (0.0 to 0.0)	-61.0 (-90.2 to 33.0)	-37.9 (-84.4 to 111.9)
Croatia	Intestinal nematode infections	5 to 9	Both	-	-	-	-	-	-	-	-

eTable 8. All-cause and cause-specific mortality (counts and rates) at the global level and by country in the WHO European Region for ages 5 to 9 and 10 to 14 for 1990, 2000, and 2016

Croatia	Intestinal nematode infections	5 to 9	Female	-	-	-	-	-	-	-	-
Croatia	Intestinal nematode infections	5 to 9	Male	-	-	-	-	-	-	-	-
Croatia	Intestinal nematode infections	10 to 14	Both	-	-	-	-	-	-	-	-
Croatia	Intestinal nematode infections	10 to 14	Female	-	-	-	-	-	-	-	-
Croatia	Intestinal nematode infections	10 to 14	Male	-	-	-	-	-	-	-	-
Croatia	Ebola	5 to 9	Both	0 (0 to 0)	0.0 (0.0 to 0.0)	0 (0 to 0)	0.0 (0.0 to 0.0)	0 (0 to 0)	0.0 (0.0 to 0.0)	0.0 (0.0 to 0.0)	0.0 (0.0 to 0.0)
Croatia	Ebola	5 to 9	Female	0 (0 to 0)	0.0 (0.0 to 0.0)	0 (0 to 0)	0.0 (0.0 to 0.0)	0 (0 to 0)	0.0 (0.0 to 0.0)	0.0 (0.0 to 0.0)	0.0 (0.0 to 0.0)
Croatia	Ebola	5 to 9	Male	0 (0 to 0)	0.0 (0.0 to 0.0)	0 (0 to 0)	0.0 (0.0 to 0.0)	0 (0 to 0)	0.0 (0.0 to 0.0)	0.0 (0.0 to 0.0)	0.0 (0.0 to 0.0)
Croatia	Ebola	10 to 14	Both	0 (0 to 0)	0.0 (0.0 to 0.0)	0 (0 to 0)	0.0 (0.0 to 0.0)	0 (0 to 0)	0.0 (0.0 to 0.0)	0.0 (0.0 to 0.0)	0.0 (0.0 to 0.0)
Croatia	Ebola	10 to 14	Female	0 (0 to 0)	0.0 (0.0 to 0.0)	0 (0 to 0)	0.0 (0.0 to 0.0)	0 (0 to 0)	0.0 (0.0 to 0.0)	0.0 (0.0 to 0.0)	0.0 (0.0 to 0.0)
Croatia	Ebola	10 to 14	Male	0 (0 to 0)	0.0 (0.0 to 0.0)	0 (0 to 0)	0.0 (0.0 to 0.0)	0 (0 to 0)	0.0 (0.0 to 0.0)	0.0 (0.0 to 0.0)	0.0 (0.0 to 0.0)
Croatia	Zika virus	5 to 9	Both	0 (0 to 0)	0.0 (0.0 to 0.0)	0 (0 to 0)	0.0 (0.0 to 0.0)	0 (0 to 0)	0.0 (0.0 to 0.0)	0.0 (0.0 to 0.0)	0.0 (0.0 to 0.0)
Croatia	Zika virus	5 to 9	Female	0 (0 to 0)	0.0 (0.0 to 0.0)	0 (0 to 0)	0.0 (0.0 to 0.0)	0 (0 to 0)	0.0 (0.0 to 0.0)	0.0 (0.0 to 0.0)	0.0 (0.0 to 0.0)
Croatia	Zika virus	5 to 9	Male	0 (0 to 0)	0.0 (0.0 to 0.0)	0 (0 to 0)	0.0 (0.0 to 0.0)	0 (0 to 0)	0.0 (0.0 to 0.0)	0.0 (0.0 to 0.0)	0.0 (0.0 to 0.0)
Croatia	Zika virus	10 to 14	Both	0 (0 to 0)	0.0 (0.0 to 0.0)	0 (0 to 0)	0.0 (0.0 to 0.0)	0 (0 to 0)	0.0 (0.0 to 0.0)	0.0 (0.0 to 0.0)	0.0 (0.0 to 0.0)
Croatia	Zika virus	10 to 14	Female	0 (0 to 0)	0.0 (0.0 to 0.0)	0 (0 to 0)	0.0 (0.0 to 0.0)	0 (0 to 0)	0.0 (0.0 to 0.0)	0.0 (0.0 to 0.0)	0.0 (0.0 to 0.0)
Croatia	Zika virus	10 to 14	Male	0 (0 to 0)	0.0 (0.0 to 0.0)	0 (0 to 0)	0.0 (0.0 to 0.0)	0 (0 to 0)	0.0 (0.0 to 0.0)	0.0 (0.0 to 0.0)	0.0 (0.0 to 0.0)
Croatia	Other neglected tropical diseases	5 to 9	Both	0 (0 to 0)	0.0 (0.0 to 0.0)	0 (0 to 0)	0.0 (0.0 to 0.0)	0 (0 to 0)	0.0 (0.0 to 0.0)	-40.2 (-51.4 to -27.0)	-11.5 (-28.0 to 8.0)
Croatia	Other neglected tropical diseases	5 to 9	Female	0 (0 to 0)	0.0 (0.0 to 0.0)	0 (0 to 0)	0.0 (0.0 to 0.0)	0 (0 to 0)	0.0 (0.0 to 0.0)	-40.6 (-52.9 to -25.7)	-12.0 (-30.3 to 10.0)
Croatia	Other neglected tropical diseases	5 to 9	Male	0 (0 to 0)	0.0 (0.0 to 0.0)	0 (0 to 0)	0.0 (0.0 to 0.0)	0 (0 to 0)	0.0 (0.0 to 0.0)	-39.9 (-53.4 to -23.8)	-11.1 (-31.1 to 12.8)
Croatia	Other neglected tropical diseases	10 to 14	Both	0 (0 to 0)	0.0 (0.0 to 0.0)	0 (0 to 0)	0.0 (0.0 to 0.0)	0 (0 to 0)	0.0 (0.0 to 0.0)	-42.2 (-50.6 to -33.7)	-7.8 (-21.1 to 5.9)
Croatia	Other neglected tropical diseases	10 to 14	Female	0 (0 to 0)	0.0 (0.0 to 0.0)	0 (0 to 0)	0.0 (0.0 to 0.0)	0 (0 to 0)	0.0 (0.0 to 0.0)	-42.7 (-52.7 to -31.2)	-8.2 (-24.3 to 10.1)
Croatia	Other neglected tropical diseases	10 to 14	Male	0 (0 to 0)	0.0 (0.0 to 0.0)	0 (0 to 0)	0.0 (0.0 to 0.0)	0 (0 to 0)	0.0 (0.0 to 0.0)	-41.9 (-52.5 to -29.9)	-7.4 (-24.4 to 11.7)
Croatia	Maternal hemorrhage	10 to 14	Both	0 (0 to 0)	0.0 (0.0 to 0.0)	0 (0 to 0)	0.0 (0.0 to 0.0)	0 (0 to 0)	0.0 (0.0 to 0.0)	-72.3 (-81.6 to -58.8)	-55.8 (-70.7 to -34.2)

eTable 8. All-cause and cause-specific mortality (counts and rates) at the global level and by country in the WHO European Region for ages 5 to 9 and 10 to 14 for 1990, 2000, and 2016

Croatia	Maternal hemorrhage	10 to 14	Female	0 (0 to 0)	0.0 (0.0 to 0.0)	0 (0 to 0)	0.0 (0.0 to 0.0)	0 (0 to 0)	0.0 (0.0 to 0.0)	-72.3 (-81.6 to -58.8)	-55.7 (-70.6 to -34.0)
Croatia	Maternal sepsis and other maternal infections	10 to 14	Both	0 (0 to 0)	0.0 (0.0 to 0.0)	0 (0 to 0)	0.0 (0.0 to 0.0)	0 (0 to 0)	0.0 (0.0 to 0.0)	-75.7 (-83.4 to -64.8)	-61.3 (-73.5 to -43.9)
Croatia	Maternal sepsis and other maternal infections	10 to 14	Female	0 (0 to 0)	0.0 (0.0 to 0.0)	0 (0 to 0)	0.0 (0.0 to 0.0)	0 (0 to 0)	0.0 (0.0 to 0.0)	-75.7 (-83.4 to -64.8)	-61.2 (-73.4 to -43.8)
Croatia	Maternal hypertensive disorders	10 to 14	Both	0 (0 to 0)	0.0 (0.0 to 0.0)	0 (0 to 0)	0.0 (0.0 to 0.0)	0 (0 to 0)	0.0 (0.0 to 0.0)	-70.3 (-80.0 to -57.1)	-52.5 (-68.0 to -31.5)
Croatia	Maternal hypertensive disorders	10 to 14	Female	0 (0 to 0)	0.0 (0.0 to 0.0)	0 (0 to 0)	0.0 (0.0 to 0.0)	0 (0 to 0)	0.0 (0.0 to 0.0)	-70.3 (-80.0 to -57.1)	-52.4 (-67.9 to -31.4)
Croatia	Maternal obstructed labor and uterine rupture	10 to 14	Both	0 (0 to 0)	0.0 (0.0 to 0.0)	0 (0 to 0)	0.0 (0.0 to 0.0)	0 (0 to 0)	0.0 (0.0 to 0.0)	-74.2 (-86.8 to -57.4)	-58.9 (-78.9 to -32.0)
Croatia	Maternal obstructed labor and uterine rupture	10 to 14	Female	0 (0 to 0)	0.0 (0.0 to 0.0)	0 (0 to 0)	0.0 (0.0 to 0.0)	0 (0 to 0)	0.0 (0.0 to 0.0)	-74.2 (-86.8 to -57.4)	-58.8 (-78.8 to -31.9)
Croatia	Maternal abortion, miscarriage, and ectopic pregnancy	10 to 14	Both	0 (0 to 0)	0.0 (0.0 to 0.0)	0 (0 to 0)	0.0 (0.0 to 0.0)	0 (0 to 0)	0.0 (0.0 to 0.0)	-74.9 (-83.3 to -63.1)	-59.9 (-73.3 to -41.1)
Croatia	Maternal abortion, miscarriage, and ectopic pregnancy	10 to 14	Female	0 (0 to 0)	0.0 (0.0 to 0.0)	0 (0 to 0)	0.0 (0.0 to 0.0)	0 (0 to 0)	0.0 (0.0 to 0.0)	-74.9 (-83.3 to -63.1)	-59.8 (-73.2 to -41.0)
Croatia	Indirect maternal deaths	10 to 14	Both	0 (0 to 0)	0.0 (0.0 to 0.0)	0 (0 to 0)	0.0 (0.0 to 0.0)	0 (0 to 0)	0.0 (0.0 to 0.0)	-71.5 (-80.5 to -58.6)	-54.5 (-68.9 to -34.0)
Croatia	Indirect maternal deaths	10 to 14	Female	0 (0 to 0)	0.0 (0.0 to 0.0)	0 (0 to 0)	0.0 (0.0 to 0.0)	0 (0 to 0)	0.0 (0.0 to 0.0)	-71.5 (-80.5 to -58.6)	-54.4 (-68.9 to -33.8)
Croatia	Late maternal deaths	10 to 14	Both	0 (0 to 0)	0.0 (0.0 to 0.0)	0 (0 to 0)	0.0 (0.0 to 0.0)	0 (0 to 0)	0.0 (0.0 to 0.0)	-73.4 (-83.0 to -58.1)	-57.5 (-72.9 to -33.1)
Croatia	Late maternal deaths	10 to 14	Female	0 (0 to 0)	0.0 (0.0 to 0.0)	0 (0 to 0)	0.0 (0.0 to 0.0)	0 (0 to 0)	0.0 (0.0 to 0.0)	-73.4 (-83.0 to -58.1)	-57.4 (-72.8 to -32.9)
Croatia	Maternal deaths aggravated by HIV/AIDS	10 to 14	Both	0 (0 to 0)	0.0 (0.0 to 0.0)	0 (0 to 0)	0.0 (0.0 to 0.0)	0 (0 to 0)	0.0 (0.0 to 0.0)	1764.8 (0.0 to 4127.8)	2877.1 (0.0 to 6649.5)
Croatia	Maternal deaths aggravated by HIV/AIDS	10 to 14	Female	0 (0 to 0)	0.0 (0.0 to 0.0)	0 (0 to 0)	0.0 (0.0 to 0.0)	0 (0 to 0)	0.0 (0.0 to 0.0)	1764.8 (0.0 to 4127.8)	2883.6 (0.0 to 6664.3)
Croatia	Other maternal disorders	10 to 14	Both	0 (0 to 0)	0.0 (0.0 to 0.0)	0 (0 to 0)	0.0 (0.0 to 0.0)	0 (0 to 0)	0.0 (0.0 to 0.0)	-72.5 (-81.1 to -60.8)	-56.2 (-69.8 to -37.4)
Croatia	Other maternal disorders	10 to 14	Female	0 (0 to 0)	0.0 (0.0 to 0.0)	0 (0 to 0)	0.0 (0.0 to 0.0)	0 (0 to 0)	0.0 (0.0 to 0.0)	-72.5 (-81.1 to -60.8)	-56.1 (-69.7 to -37.3)
Croatia	Protein-energy malnutrition	5 to 9	Both	0 (0 to 0)	0.0 (0.0 to 0.0)	0 (0 to 0)	0.0 (0.0 to 0.0)	0 (0 to 0)	0.0 (0.0 to 0.0)	-5.3 (-47.0 to 71.1)	40.2 (-21.5 to 153.2)

eTable 8. All-cause and cause-specific mortality (counts and rates) at the global level and by country in the WHO European Region for ages 5 to 9 and 10 to 14 for 1990, 2000, and 2016

Croatia	Protein-energy malnutrition	5 to 9	Female	0<br>(0 to 0)	0.0<br>(0.0 to 0.0)	0<br>(0 to 0)	0.0<br>(0.0 to 0.0)	0<br>(0 to 0)	0.0<br>(0.0 to 0.0)	12.8<br>(-50.5 to 241.0)	67.1<br>(-26.7 to 405.1)
Croatia	Protein-energy malnutrition	5 to 9	Male	0<br>(0 to 0)	0.0<br>(0.0 to 0.0)	0<br>(0 to 0)	0.0<br>(0.0 to 0.0)	0<br>(0 to 0)	0.0<br>(0.0 to 0.0)	-23.0<br>(-64.7 to 113.8)	13.9<br>(-47.7 to 216.3)
Croatia	Protein-energy malnutrition	10 to 14	Both	0<br>(0 to 0)	0.0<br>(0.0 to 0.0)	0<br>(0 to 0)	0.0<br>(0.0 to 0.0)	0<br>(0 to 0)	0.0<br>(0.0 to 0.0)	-8.4<br>(-40.9 to 42.7)	46.2<br>(-5.6 to 127.8)
Croatia	Protein-energy malnutrition	10 to 14	Female	0<br>(0 to 0)	0.0<br>(0.0 to 0.0)	0<br>(0 to 0)	0.0<br>(0.0 to 0.0)	0<br>(0 to 0)	0.0<br>(0.0 to 0.0)	26.2<br>(-35.6 to 139.9)	101.9<br>(3.1 to 283.9)
Croatia	Protein-energy malnutrition	10 to 14	Male	0<br>(0 to 0)	0.0<br>(0.0 to 0.0)	0<br>(0 to 0)	0.0<br>(0.0 to 0.0)	0<br>(0 to 0)	0.0<br>(0.0 to 0.0)	-41.4<br>(-56.3 to -14.7)	-6.6<br>(-30.3 to 36.0)
Croatia	Iodine deficiency	5 to 9	Both	0<br>(0 to 0)	0.0<br>(0.0 to 0.0)	0<br>(0 to 0)	0.0<br>(0.0 to 0.0)	0<br>(0 to 0)	0.0<br>(0.0 to 0.0)	-44.1<br>(-100.0 to 5.5e+18)	-17.3<br>(-100.0 to 8.1e+18)
Croatia	Iodine deficiency	5 to 9	Female	0<br>(0 to 0)	0.0<br>(0.0 to 0.0)	0<br>(0 to 0)	0.0<br>(0.0 to 0.0)	0<br>(0 to 0)	0.0<br>(0.0 to 0.0)	-28.8<br>(-100.0 to 2.5e+27)	5.5<br>(-100.0 to 3.7e+27)
Croatia	Iodine deficiency	5 to 9	Male	0<br>(0 to 0)	0.0<br>(0.0 to 0.0)	0<br>(0 to 0)	0.0<br>(0.0 to 0.0)	0<br>(0 to 0)	0.0<br>(0.0 to 0.0)	-58.2<br>(-100.0 to 2.6e+25)	-38.1<br>(-100.0 to 3.8e+25)
Croatia	Iodine deficiency	10 to 14	Both	0<br>(0 to 0)	0.0<br>(0.0 to 0.0)	0<br>(0 to 0)	0.0<br>(0.0 to 0.0)	0<br>(0 to 0)	0.0<br>(0.0 to 0.0)	-45.6<br>(-100.0 to 1.1e+16)	-13.2<br>(-100.0 to 1.7e+16)
Croatia	Iodine deficiency	10 to 14	Female	0<br>(0 to 0)	0.0<br>(0.0 to 0.0)	0<br>(0 to 0)	0.0<br>(0.0 to 0.0)	0<br>(0 to 0)	0.0<br>(0.0 to 0.0)	-30.7<br>(-100.0 to 2.9e+24)	10.9<br>(-100.0 to 4.7e+24)
Croatia	Iodine deficiency	10 to 14	Male	0<br>(0 to 0)	0.0<br>(0.0 to 0.0)	0<br>(0 to 0)	0.0<br>(0.0 to 0.0)	0<br>(0 to 0)	0.0<br>(0.0 to 0.0)	-61.1<br>(-100.0 to 6.0e+26)	-38.1<br>(-100.0 to 9.6e+26)
Croatia	Iron-deficiency anemia	5 to 9	Both	0<br>(0 to 0)	0.0<br>(0.0 to 0.0)	0<br>(0 to 0)	0.0<br>(0.0 to 0.0)	0<br>(0 to 0)	0.0<br>(0.0 to 0.0)	-5.3<br>(-47.0 to 71.1)	40.2<br>(-21.5 to 153.2)
Croatia	Iron-deficiency anemia	5 to 9	Female	0<br>(0 to 0)	0.0<br>(0.0 to 0.0)	0<br>(0 to 0)	0.0<br>(0.0 to 0.0)	0<br>(0 to 0)	0.0<br>(0.0 to 0.0)	12.8<br>(-50.5 to 241.0)	67.1<br>(-26.7 to 405.1)
Croatia	Iron-deficiency anemia	5 to 9	Male	0<br>(0 to 0)	0.0<br>(0.0 to 0.0)	0<br>(0 to 0)	0.0<br>(0.0 to 0.0)	0<br>(0 to 0)	0.0<br>(0.0 to 0.0)	-23.0<br>(-64.7 to 113.8)	13.9<br>(-47.7 to 216.3)
Croatia	Iron-deficiency anemia	10 to 14	Both	0<br>(0 to 0)	0.0<br>(0.0 to 0.0)	0<br>(0 to 0)	0.0<br>(0.0 to 0.0)	0<br>(0 to 0)	0.0<br>(0.0 to 0.0)	-8.3<br>(-40.9 to 42.7)	46.3<br>(-5.6 to 127.8)
Croatia	Iron-deficiency anemia	10 to 14	Female	0<br>(0 to 0)	0.0<br>(0.0 to 0.0)	0<br>(0 to 0)	0.0<br>(0.0 to 0.0)	0<br>(0 to 0)	0.0<br>(0.0 to 0.0)	26.4<br>(-35.6 to 139.9)	102.2<br>(3.1 to 283.9)
Croatia	Iron-deficiency anemia	10 to 14	Male	0<br>(0 to 0)	0.0<br>(0.0 to 0.0)	0<br>(0 to 0)	0.0<br>(0.0 to 0.0)	0<br>(0 to 0)	0.0<br>(0.0 to 0.0)	-41.4<br>(-56.3 to -14.7)	-6.6<br>(-30.3 to 36.0)
Croatia	Other nutritional deficiencies	5 to 9	Both	0<br>(0 to 0)	0.0<br>(0.0 to 0.0)	0<br>(0 to 0)	0.0<br>(0.0 to 0.0)	0<br>(0 to 0)	0.0<br>(0.0 to 0.0)	46.2<br>(-17.7 to 164.9)	116.5<br>(21.8 to 292.2)
Croatia	Other nutritional deficiencies	5 to 9	Female	0<br>(0 to 0)	0.0<br>(0.0 to 0.0)	0<br>(0 to 0)	0.0<br>(0.0 to 0.0)	0<br>(0 to 0)	0.0<br>(0.0 to 0.0)	74.7<br>(-24.6 to 431.8)	158.7<br>(11.7 to 687.5)
Croatia	Other nutritional deficiencies	5 to 9	Male	0<br>(0 to 0)	0.0<br>(0.0 to 0.0)	0<br>(0 to 0)	0.0<br>(0.0 to 0.0)	0<br>(0 to 0)	0.0<br>(0.0 to 0.0)	18.6<br>(-44.5 to 233.1)	75.4<br>(-17.9 to 392.9)
Croatia	Other nutritional deficiencies	10 to 14	Both	0<br>(0 to 0)	0.0<br>(0.0 to 0.0)	0<br>(0 to 0)	0.0<br>(0.0 to 0.0)	0<br>(0 to 0)	0.0<br>(0.0 to 0.0)	-2.6<br>(-36.7 to 57.8)	55.4<br>(1.1 to 151.9)
Croatia	Other nutritional deficiencies	10 to 14	Female	0<br>(0 to 0)	0.0<br>(0.0 to 0.0)	0<br>(0 to 0)	0.0<br>(0.0 to 0.0)	0<br>(0 to 0)	0.0<br>(0.0 to 0.0)	38.1<br>(-28.8 to 182.5)	121.0<br>(13.9 to 351.9)
Croatia	Other nutritional deficiencies	10 to 14	Male	0<br>(0 to 0)	0.0<br>(0.0 to 0.0)	0<br>(0 to 0)	0.0<br>(0.0 to 0.0)	0<br>(0 to 0)	0.0<br>(0.0 to 0.0)	-41.4<br>(-56.3 to -14.7)	-6.6<br>(-30.3 to 36.0)

eTable 8. All-cause and cause-specific mortality (counts and rates) at the global level and by country in the WHO European Region for ages 5 to 9 and 10 to 14 for 1990, 2000, and 2016

Croatia	Sexually transmitted diseases excluding HIV	5 to 9	Both	0 (0 to 0)	0.0 (0.0 to 0.0)	0 (0 to 0)	0.0 (0.0 to 0.0)	0 (0 to 0)	0.0 (0.0 to 0.0)	-86.3 (-95.9 to -58.1)	-79.7 (-94.0 to -38.0)
Croatia	Sexually transmitted diseases excluding HIV	5 to 9	Female	0 (0 to 0)	0.0 (0.0 to 0.0)	0 (0 to 0)	0.0 (0.0 to 0.0)	0 (0 to 0)	0.0 (0.0 to 0.0)	-86.3 (-95.9 to -58.9)	-79.7 (-93.9 to -39.1)
Croatia	Sexually transmitted diseases excluding HIV	5 to 9	Male	0 (0 to 0)	0.0 (0.0 to 0.0)	0 (0 to 0)	0.0 (0.0 to 0.0)	0 (0 to 0)	0.0 (0.0 to 0.0)	-86.2 (-95.8 to -57.4)	-79.5 (-93.8 to -37.0)
Croatia	Sexually transmitted diseases excluding HIV	10 to 14	Both	0 (0 to 0)	0.0 (0.0 to 0.0)	0 (0 to 0)	0.0 (0.0 to 0.0)	0 (0 to 0)	0.0 (0.0 to 0.0)	-49.1 (-58.1 to -37.7)	-18.7 (-33.1 to -0.6)
Croatia	Sexually transmitted diseases excluding HIV	10 to 14	Female	0 (0 to 0)	0.0 (0.0 to 0.0)	0 (0 to 0)	0.0 (0.0 to 0.0)	0 (0 to 0)	0.0 (0.0 to 0.0)	-42.7 (-52.7 to -31.2)	-8.2 (-24.3 to 10.1)
Croatia	Sexually transmitted diseases excluding HIV	10 to 14	Male	0 (0 to 0)	0.0 (0.0 to 0.0)	0 (0 to 0)	0.0 (0.0 to 0.0)	0 (0 to 0)	0.0 (0.0 to 0.0)	-93.0 (-97.0 to -81.1)	-88.8 (-95.2 to -69.9)
Croatia	Hepatitis	5 to 9	Both	0 (0 to 0)	0.0 (0.0 to 0.0)	0 (0 to 0)	0.0 (0.0 to 0.0)	0 (0 to 0)	0.0 (0.0 to 0.0)	-64.6 (-77.3 to -44.7)	-47.6 (-66.4 to -18.1)
Croatia	Hepatitis	5 to 9	Female	0 (0 to 0)	0.0 (0.0 to 0.0)	0 (0 to 0)	0.0 (0.0 to 0.0)	0 (0 to 0)	0.0 (0.0 to 0.0)	-48.1 (-58.9 to -35.1)	-23.1 (-39.1 to -3.9)
Croatia	Hepatitis	5 to 9	Male	0 (0 to 0)	0.0 (0.0 to 0.0)	0 (0 to 0)	0.0 (0.0 to 0.0)	0 (0 to 0)	0.0 (0.0 to 0.0)	-71.4 (-83.8 to -44.1)	-57.7 (-76.1 to -17.2)
Croatia	Hepatitis	10 to 14	Both	0 (0 to 0)	0.0 (0.0 to 0.0)	0 (0 to 0)	0.0 (0.0 to 0.0)	0 (0 to 0)	0.0 (0.0 to 0.0)	-58.9 (-71.8 to -41.5)	-34.4 (-55.0 to -6.6)
Croatia	Hepatitis	10 to 14	Female	0 (0 to 0)	0.0 (0.0 to 0.0)	0 (0 to 0)	0.0 (0.0 to 0.0)	0 (0 to 0)	0.0 (0.0 to 0.0)	-68.6 (-81.2 to -44.3)	-49.7 (-69.9 to -10.9)
Croatia	Hepatitis	10 to 14	Male	0 (0 to 0)	0.0 (0.0 to 0.0)	0 (0 to 0)	0.0 (0.0 to 0.0)	0 (0 to 0)	0.0 (0.0 to 0.0)	-41.9 (-52.5 to -29.9)	-7.4 (-24.4 to 11.7)
Croatia	Other infectious diseases	5 to 9	Both	0 (0 to 0)	0.1 (0.0 to 0.1)	0 (0 to 0)	0.0 (0.0 to 0.1)	0 (0 to 0)	0.0 (0.0 to 0.0)	-69.2 (-80.2 to -52.0)	-54.4 (-70.6 to -29.0)
Croatia	Other infectious diseases	5 to 9	Female	0 (0 to 0)	0.0 (0.0 to 0.1)	0 (0 to 0)	0.0 (0.0 to 0.1)	0 (0 to 0)	0.0 (0.0 to 0.1)	-60.6 (-80.6 to -20.9)	-41.7 (-71.2 to 17.2)
Croatia	Other infectious diseases	5 to 9	Male	0 (0 to 0)	0.1 (0.0 to 0.1)	0 (0 to 0)	0.1 (0.0 to 0.1)	0 (0 to 0)	0.0 (0.0 to 0.0)	-74.6 (-84.6 to -57.4)	-62.4 (-77.3 to -36.9)
Croatia	Other infectious diseases	10 to 14	Both	0 (0 to 0)	0.1 (0.0 to 0.1)	0 (0 to 0)	0.1 (0.0 to 0.1)	0 (0 to 0)	0.0 (0.0 to 0.0)	-67.2 (-78.8 to -49.2)	-47.7 (-66.2 to -19.0)
Croatia	Other infectious diseases	10 to 14	Female	0 (0 to 0)	0.1 (0.0 to 0.1)	0 (0 to 0)	0.0 (0.0 to 0.1)	0 (0 to 0)	0.0 (0.0 to 0.1)	-62.6 (-81.7 to -25.5)	-40.2 (-70.7 to 19.2)
Croatia	Other infectious diseases	10 to 14	Male	0 (0 to 0)	0.1 (0.1 to 0.1)	0 (0 to 0)	0.1 (0.1 to 0.1)	0 (0 to 0)	0.0 (0.0 to 0.1)	-70.3 (-82.6 to -48.7)	-52.6 (-72.3 to -18.3)
Croatia	Nasopharynx cancer	5 to 9	Both	0 (0 to 0)	0.0 (0.0 to 0.0)	0 (0 to 0)	0.0 (0.0 to 0.0)	0 (0 to 0)	0.0 (0.0 to 0.0)	-73.2 (-82.9 to -58.6)	-60.3 (-74.6 to -38.7)
Croatia	Nasopharynx cancer	5 to 9	Female	0 (0 to 0)	0.0 (0.0 to 0.0)	0 (0 to 0)	0.0 (0.0 to 0.0)	0 (0 to 0)	0.0 (0.0 to 0.0)	-40.6 (-52.9 to -25.7)	-12.0 (-30.3 to 10.0)

eTable 8. All-cause and cause-specific mortality (counts and rates) at the global level and by country in the WHO European Region for ages 5 to 9 and 10 to 14 for 1990, 2000, and 2016

Croatia	Nasopharynx cancer	5 to 9	Male	0 (0 to 0)	0.0 (0.0 to 0.1)	0 (0 to 0)	0.0 (0.0 to 0.0)	0 (0 to 0)	0.0 (0.0 to 0.0)	-81.2 (-89.7 to -66.5)	-72.3 (-84.7 to -50.5)
Croatia	Nasopharynx cancer	10 to 14	Both	0 (0 to 0)	0.0 (0.0 to 0.0)	0 (0 to 0)	0.0 (0.0 to 0.0)	0 (0 to 0)	0.0 (0.0 to 0.0)	-73.1 (-81.0 to -59.1)	-57.0 (-69.7 to -34.7)
Croatia	Nasopharynx cancer	10 to 14	Female	0 (0 to 0)	0.0 (0.0 to 0.0)	0 (0 to 0)	0.0 (0.0 to 0.0)	0 (0 to 0)	0.0 (0.0 to 0.0)	-42.7 (-52.7 to -31.2)	-8.2 (-24.3 to 10.1)
Croatia	Nasopharynx cancer	10 to 14	Male	0 (0 to 0)	0.0 (0.0 to 0.1)	0 (0 to 0)	0.0 (0.0 to 0.0)	0 (0 to 0)	0.0 (0.0 to 0.0)	-79.6 (-87.7 to -63.3)	-67.6 (-80.4 to -41.5)
Croatia	Liver cancer	5 to 9	Both	0 (0 to 0)	0.0 (0.0 to 0.0)	0 (0 to 0)	0.0 (0.0 to 0.1)	0 (0 to 0)	0.0 (0.0 to 0.0)	-31.5 (-61.9 to 17.0)	1.3 (-43.6 to 73.2)
Croatia	Liver cancer	5 to 9	Female	0 (0 to 0)	0.0 (0.0 to 0.0)	0 (0 to 0)	0.0 (0.0 to 0.0)	0 (0 to 0)	0.0 (0.0 to 0.0)	-46.5 (-65.5 to -20.8)	-20.8 (-48.9 to 17.3)
Croatia	Liver cancer	5 to 9	Male	0 (0 to 0)	0.0 (0.0 to 0.1)	0 (0 to 0)	0.1 (0.0 to 0.1)	0 (0 to 0)	0.0 (0.0 to 0.1)	-27.8 (-64.5 to 37.3)	6.9 (-47.4 to 103.2)
Croatia	Liver cancer	10 to 14	Both	0 (0 to 0)	0.0 (0.0 to 0.0)	0 (0 to 0)	0.1 (0.0 to 0.1)	0 (0 to 0)	0.0 (0.0 to 0.1)	-30.4 (-58.5 to 12.3)	11.2 (-33.7 to 79.3)
Croatia	Liver cancer	10 to 14	Female	0 (0 to 0)	0.0 (0.0 to 0.0)	0 (0 to 0)	0.0 (0.0 to 0.1)	0 (0 to 0)	0.0 (0.0 to 0.0)	-46.1 (-73.3 to 5.4)	-13.7 (-57.4 to 68.6)
Croatia	Liver cancer	10 to 14	Male	0 (0 to 0)	0.0 (0.0 to 0.1)	0 (0 to 0)	0.1 (0.0 to 0.1)	0 (0 to 0)	0.0 (0.0 to 0.1)	-19.5 (-60.8 to 46.4)	28.2 (-37.5 to 133.3)
Croatia	Kidney cancer	5 to 9	Both	0 (0 to 0)	0.1 (0.1 to 0.1)	0 (0 to 0)	0.1 (0.1 to 0.1)	0 (0 to 0)	0.1 (0.1 to 0.1)	-17.7 (-50.8 to 30.3)	21.9 (-27.1 to 92.8)
Croatia	Kidney cancer	5 to 9	Female	0 (0 to 0)	0.1 (0.1 to 0.1)	0 (0 to 0)	0.1 (0.1 to 0.1)	0 (0 to 0)	0.1 (0.1 to 0.2)	-14.7 (-55.9 to 57.6)	26.3 (-34.7 to 133.4)
Croatia	Kidney cancer	5 to 9	Male	0 (0 to 0)	0.1 (0.1 to 0.1)	0 (0 to 0)	0.1 (0.1 to 0.1)	0 (0 to 0)	0.1 (0.1 to 0.2)	-20.2 (-62.1 to 52.5)	18.0 (-43.9 to 125.6)
Croatia	Kidney cancer	10 to 14	Both	0 (0 to 0)	0.0 (0.0 to 0.0)	0 (0 to 0)	0.0 (0.0 to 0.1)	0 (0 to 0)	0.0 (0.0 to 0.1)	2.7 (-37.4 to 64.0)	63.9 (-0.0 to 161.8)
Croatia	Kidney cancer	10 to 14	Female	0 (0 to 0)	0.0 (0.0 to 0.0)	0 (0 to 0)	0.0 (0.0 to 0.1)	0 (0 to 0)	0.1 (0.0 to 0.1)	18.1 (-39.6 to 112.9)	88.9 (-3.3 to 240.6)
Croatia	Kidney cancer	10 to 14	Male	0 (0 to 0)	0.0 (0.0 to 0.0)	0 (0 to 0)	0.0 (0.0 to 0.1)	0 (0 to 0)	0.0 (0.0 to 0.1)	-12.0 (-50.9 to 59.7)	40.2 (-21.9 to 154.4)
Croatia	Brain and nervous system cancer	5 to 9	Both	6 (5 to 8)	1.9 (1.5 to 2.5)	4 (4 to 6)	1.9 (1.5 to 2.3)	3 (2 to 4)	1.3 (0.9 to 1.8)	-54.9 (-73.6 to -32.5)	-33.2 (-61.0 to -0.0)
Croatia	Brain and nervous system cancer	5 to 9	Female	3 (2 to 4)	1.7 (1.2 to 2.3)	2 (1 to 2)	1.6 (1.1 to 2.1)	1 (1 to 2)	1.2 (0.7 to 1.8)	-50.4 (-74.9 to -14.4)	-26.5 (-62.9 to 26.8)
Croatia	Brain and nervous system cancer	5 to 9	Male	4 (2 to 5)	2.1 (1.5 to 3.0)	3 (2 to 3)	2.1 (1.5 to 2.8)	1 (1 to 2)	1.3 (0.8 to 2.0)	-58.3 (-77.8 to -30.6)	-38.3 (-67.2 to 2.6)
Croatia	Brain and nervous system cancer	10 to 14	Both	4 (3 to 6)	1.2 (0.9 to 1.8)	4 (3 to 5)	1.3 (1.0 to 1.6)	2 (1 to 3)	1.0 (0.7 to 1.4)	-49.7 (-68.8 to -26.3)	-19.7 (-50.3 to 17.6)
Croatia	Brain and nervous system cancer	10 to 14	Female	2 (1 to 2)	1.0 (0.7 to 1.5)	1 (1 to 2)	1.0 (0.7 to 1.4)	1 (1 to 1)	0.9 (0.6 to 1.3)	-43.7 (-70.7 to -3.4)	-9.9 (-53.2 to 54.6)
Croatia	Brain and nervous system cancer	10 to 14	Male	2 (2 to 4)	1.5 (1.0 to 2.1)	2 (2 to 3)	1.5 (1.1 to 2.0)	1 (1 to 2)	1.1 (0.7 to 1.7)	-53.7 (-74.0 to -24.8)	-26.3 (-58.5 to 19.8)
Croatia	Thyroid cancer	10 to 14	Both	0 (0 to 0)	0.0 (0.0 to 0.0)	0 (0 to 0)	0.0 (0.0 to 0.0)	0 (0 to 0)	0.0 (0.0 to 0.0)	-70.2 (-81.3 to -49.6)	-52.5 (-70.1 to -19.6)
Croatia	Thyroid cancer	10 to 14	Female	0 (0 to 0)	0.0 (0.0 to 0.0)	0 (0 to 0)	0.0 (0.0 to 0.0)	0 (0 to 0)	0.0 (0.0 to 0.0)	-42.7 (-52.7 to -31.2)	-8.3 (-24.3 to 10.1)
Croatia	Thyroid cancer	10 to 14	Male	0 (0 to 0)	0.0 (0.0 to 0.1)	0 (0 to 0)	0.0 (0.0 to 0.0)	0 (0 to 0)	0.0 (0.0 to 0.0)	-77.5 (-88.3 to -53.5)	-64.1 (-81.4 to -25.9)
Croatia	Hodgkin lymphoma	5 to 9	Both	0 (0 to 0)	0.0 (0.0 to 0.1)	0 (0 to 0)	0.0 (0.0 to 0.0)	0 (0 to 0)	0.0 (0.0 to 0.0)	-79.3 (-89.6 to -54.1)	-69.3 (-84.6 to -32.0)
Croatia	Hodgkin lymphoma	5 to 9	Female	0 (0 to 0)	0.0 (0.0 to 0.0)	0 (0 to 0)	0.0 (0.0 to 0.0)	0 (0 to 0)	0.0 (0.0 to 0.0)	-43.3 (-67.9 to -26.6)	-16.1 (-52.5 to 8.7)
Croatia	Hodgkin lymphoma	5 to 9	Male	0 (0 to 0)	0.1 (0.0 to 0.1)	0 (0 to 0)	0.0 (0.0 to 0.1)	0 (0 to 0)	0.0 (0.0 to 0.0)	-84.4 (-94.0 to -57.6)	-76.9 (-91.1 to -37.2)
Croatia	Hodgkin lymphoma	10 to 14	Both	0 (0 to 0)	0.1 (0.1 to 0.1)	0 (0 to 0)	0.1 (0.0 to 0.1)	0 (0 to 0)	0.0 (0.0 to 0.0)	-80.8 (-89.2 to -67.8)	-69.4 (-82.7 to -48.7)

eTable 8. All-cause and cause-specific mortality (counts and rates) at the global level and by country in the WHO European Region for ages 5 to 9 and 10 to 14 for 1990, 2000, and 2016

Croatia	Hodgkin lymphoma	10 to 14	Female	0 (0 to 0)	0.0 (0.0 to 0.1)	0 (0 to 0)	0.0 (0.0 to 0.1)	0 (0 to 0)	0.0 (0.0 to 0.0)	-70.6 (-82.3 to -44.8)	-52.9 (-71.6 to -11.7)
Croatia	Hodgkin lymphoma	10 to 14	Male	0 (0 to 0)	0.1 (0.1 to 0.2)	0 (0 to 0)	0.1 (0.1 to 0.1)	0 (0 to 0)	0.0 (0.0 to 0.1)	-82.4 (-90.9 to -68.1)	-72.0 (-85.5 to -49.1)
Croatia	Non-Hodgkin lymphoma	5 to 9	Both	1 (1 to 2)	0.5 (0.3 to 0.6)	1 (1 to 1)	0.4 (0.3 to 0.6)	1 (0 to 1)	0.3 (0.2 to 0.4)	-54.7 (-73.1 to -24.9)	-32.9 (-60.2 to 11.2)
Croatia	Non-Hodgkin lymphoma	5 to 9	Female	0 (0 to 1)	0.2 (0.1 to 0.3)	0 (0 to 0)	0.2 (0.1 to 0.3)	0 (0 to 0)	0.2 (0.1 to 0.3)	-51.5 (-74.1 to -8.3)	-28.1 (-61.6 to 35.8)
Croatia	Non-Hodgkin lymphoma	5 to 9	Male	1 (1 to 2)	0.7 (0.5 to 1.0)	1 (1 to 1)	0.7 (0.4 to 0.9)	0 (0 to 1)	0.4 (0.3 to 0.7)	-55.8 (-76.6 to -18.5)	-34.5 (-65.4 to 20.5)
Croatia	Non-Hodgkin lymphoma	10 to 14	Both	1 (1 to 2)	0.4 (0.3 to 0.5)	1 (1 to 2)	0.4 (0.3 to 0.6)	1 (0 to 1)	0.3 (0.2 to 0.5)	-46.1 (-67.1 to -13.3)	-14.0 (-47.4 to 38.4)
Croatia	Non-Hodgkin lymphoma	10 to 14	Female	0 (0 to 1)	0.3 (0.2 to 0.4)	0 (0 to 1)	0.3 (0.2 to 0.4)	0 (0 to 0)	0.2 (0.1 to 0.4)	-48.6 (-74.4 to 3.9)	-17.7 (-59.1 to 66.3)
Croatia	Non-Hodgkin lymphoma	10 to 14	Male	1 (0 to 1)	0.4 (0.3 to 0.8)	1 (1 to 1)	0.6 (0.4 to 0.8)	0 (0 to 1)	0.4 (0.2 to 0.6)	-44.7 (-70.9 to 2.0)	-11.9 (-53.7 to 62.5)
Croatia	Leukemia	5 to 9	Both	5 (4 to 7)	1.7 (1.3 to 2.1)	3 (2 to 4)	1.2 (0.9 to 1.5)	2 (1 to 2)	0.7 (0.5 to 1.0)	-71.1 (-80.8 to -56.1)	-57.3 (-71.6 to -35.0)
Croatia	Leukemia	5 to 9	Female	2 (2 to 3)	1.4 (1.0 to 2.0)	1 (1 to 2)	1.1 (0.7 to 1.4)	1 (0 to 1)	0.6 (0.4 to 0.9)	-70.4 (-82.7 to -48.9)	-56.2 (-74.3 to -24.3)
Croatia	Leukemia	5 to 9	Male	3 (2 to 4)	1.9 (1.4 to 2.7)	2 (1 to 2)	1.3 (0.9 to 1.7)	1 (1 to 1)	0.8 (0.5 to 1.2)	-71.7 (-84.2 to -49.4)	-58.1 (-76.6 to -25.2)
Croatia	Leukemia	10 to 14	Both	4 (3 to 5)	1.3 (1.0 to 1.6)	3 (2 to 4)	1.1 (0.8 to 1.3)	2 (1 to 2)	0.8 (0.6 to 1.0)	-62.5 (-75.4 to -43.2)	-40.1 (-60.8 to -9.3)
Croatia	Leukemia	10 to 14	Female	1 (1 to 2)	0.9 (0.6 to 1.3)	1 (1 to 1)	0.8 (0.5 to 1.0)	0 (0 to 1)	0.5 (0.3 to 0.8)	-65.8 (-81.1 to -37.4)	-45.3 (-69.7 to 0.2)
Croatia	Leukemia	10 to 14	Male	3 (2 to 4)	1.6 (1.2 to 2.2)	2 (1 to 3)	1.3 (1.0 to 1.8)	1 (1 to 2)	1.0 (0.7 to 1.5)	-60.7 (-76.4 to -34.8)	-37.4 (-62.4 to 3.9)
Croatia	Other neoplasms	5 to 9	Both	3 (3 to 4)	1.1 (0.8 to 1.4)	2 (1 to 2)	0.8 (0.6 to 1.0)	1 (1 to 2)	0.5 (0.3 to 0.7)	-66.6 (-79.8 to -48.4)	-50.5 (-70.0 to -23.6)
Croatia	Other neoplasms	5 to 9	Female	1 (1 to 2)	0.9 (0.6 to 1.3)	1 (1 to 1)	0.6 (0.4 to 0.9)	1 (0 to 1)	0.5 (0.3 to 0.8)	-65.2 (-80.6 to -38.3)	-48.5 (-71.3 to -8.7)
Croatia	Other neoplasms	5 to 9	Male	2 (1 to 3)	1.2 (0.8 to 1.6)	1 (1 to 2)	0.9 (0.6 to 1.2)	1 (0 to 1)	0.6 (0.3 to 0.8)	-67.6 (-83.0 to -38.6)	-52.1 (-74.8 to -9.2)
Croatia	Other neoplasms	10 to 14	Both	3 (2 to 4)	1.0 (0.7 to 1.2)	2 (2 to 3)	0.8 (0.6 to 1.0)	1 (1 to 2)	0.6 (0.4 to 0.8)	-62.1 (-74.9 to -42.8)	-39.5 (-59.9 to -8.8)
Croatia	Other neoplasms	10 to 14	Female	2 (1 to 2)	1.0 (0.6 to 1.3)	1 (1 to 1)	0.7 (0.5 to 0.9)	1 (0 to 1)	0.6 (0.4 to 0.9)	-62.9 (-78.7 to -35.8)	-40.6 (-65.8 to 2.7)
Croatia	Other neoplasms	10 to 14	Male	2 (1 to 2)	1.0 (0.7 to 1.4)	1 (1 to 2)	0.9 (0.6 to 1.2)	1 (0 to 1)	0.6 (0.4 to 0.9)	-61.4 (-77.0 to -33.9)	-38.6 (-63.4 to 5.2)
Croatia	Rheumatic heart disease	5 to 9	Both	0 (0 to 0)	0.1 (0.1 to 0.1)	0 (0 to 0)	0.0 (0.0 to 0.0)	0 (0 to 0)	0.0 (0.0 to 0.0)	-89.2 (-93.2 to -83.0)	-84.0 (-90.0 to -74.8)
Croatia	Rheumatic heart disease	5 to 9	Female	0 (0 to 0)	0.1 (0.1 to 0.1)	0 (0 to 0)	0.0 (0.0 to 0.0)	0 (0 to 0)	0.0 (0.0 to 0.0)	-91.5 (-95.2 to -85.9)	-87.4 (-92.9 to -79.1)
Croatia	Rheumatic heart disease	5 to 9	Male	0 (0 to 0)	0.0 (0.0 to 0.1)	0 (0 to 0)	0.0 (0.0 to 0.0)	0 (0 to 0)	0.0 (0.0 to 0.0)	-83.1 (-91.3 to -68.8)	-75.0 (-87.1 to -53.9)
Croatia	Rheumatic heart disease	10 to 14	Both	0 (0 to 0)	0.1 (0.1 to 0.1)	0 (0 to 0)	0.0 (0.0 to 0.1)	0 (0 to 0)	0.0 (0.0 to 0.0)	-89.6 (-93.2 to -84.3)	-83.5 (-89.2 to -74.9)
Croatia	Rheumatic heart disease	10 to 14	Female	0 (0 to 0)	0.1 (0.1 to 0.1)	0 (0 to 0)	0.0 (0.0 to 0.0)	0 (0 to 0)	0.0 (0.0 to 0.0)	-89.3 (-93.9 to -81.7)	-82.9 (-90.2 to -70.8)
Croatia	Rheumatic heart disease	10 to 14	Male	0 (0 to 0)	0.1 (0.1 to 0.2)	0 (0 to 0)	0.1 (0.0 to 0.1)	0 (0 to 0)	0.0 (0.0 to 0.0)	-89.9 (-94.3 to -82.6)	-83.9 (-91.0 to -72.3)
Croatia	Ischemic heart disease	5 to 9	Both	0 (0 to 0)	0.0 (0.0 to 0.0)	0 (0 to 0)	0.0 (0.0 to 0.0)	0 (0 to 0)	0.0 (0.0 to 0.0)	-67.9 (-80.9 to -44.8)	-52.5 (-71.8 to -18.3)
Croatia	Ischemic heart disease	5 to 9	Female	0 (0 to 0)	0.0 (0.0 to 0.0)	0 (0 to 0)	0.0 (0.0 to 0.0)	0 (0 to 0)	0.0 (0.0 to 0.0)	-42.9 (-65.0 to -13.0)	-15.5 (-48.1 to 28.8)
Croatia	Ischemic heart disease	5 to 9	Male	0 (0 to 0)	0.0 (0.0 to 0.0)	0 (0 to 0)	0.0 (0.0 to 0.0)	0 (0 to 0)	0.0 (0.0 to 0.0)	-77.8 (-89.5 to -53.5)	-67.2 (-84.5 to -31.2)
Croatia	Ischemic heart disease	10 to 14	Both	0 (0 to 0)	0.0 (0.0 to 0.0)	0 (0 to 0)	0.0 (0.0 to 0.0)	0 (0 to 0)	0.0 (0.0 to 0.0)	-46.3 (-62.4 to -23.5)	-14.3 (-40.0 to 22.2)
Croatia	Ischemic heart disease	10 to 14	Female	0 (0 to 0)	0.0 (0.0 to 0.0)	0 (0 to 0)	0.0 (0.0 to 0.0)	0 (0 to 0)	0.0 (0.0 to 0.0)	-41.8 (-62.8 to -11.7)	-6.8 (-40.5 to 41.3)
Croatia	Ischemic heart disease	10 to 14	Male	0 (0 to 0)	0.0 (0.0 to 0.0)	0 (0 to 0)	0.0 (0.0 to 0.0)	0 (0 to 0)	0.0 (0.0 to 0.0)	-50.1 (-71.2 to -15.7)	-20.5 (-54.1 to 34.4)

eTable 8. All-cause and cause-specific mortality (counts and rates) at the global level and by country in the WHO European Region for ages 5 to 9 and 10 to 14 for 1990, 2000, and 2016

Croatia	Cerebrovascular disease	5 to 9	Both	1 (1 to 2)	0.4 (0.3 to 0.5)	0 (0 to 1)	0.2 (0.1 to 0.3)	0 (0 to 0)	0.1 (0.1 to 0.1)	-85.7 (-90.9 to -78.6)	-78.9 (-86.5 to -68.3)
Croatia	Cerebrovascular disease	5 to 9	Female	1 (0 to 1)	0.4 (0.3 to 0.6)	0 (0 to 0)	0.2 (0.1 to 0.3)	0 (0 to 0)	0.1 (0.1 to 0.1)	-83.9 (-90.4 to -74.8)	-76.1 (-85.7 to -62.6)
Croatia	Cerebrovascular disease	5 to 9	Male	1 (0 to 1)	0.3 (0.2 to 0.5)	0 (0 to 0)	0.2 (0.1 to 0.3)	0 (0 to 0)	0.1 (0.0 to 0.1)	-88.1 (-94.2 to -77.2)	-82.4 (-91.4 to -66.3)
Croatia	Cerebrovascular disease	10 to 14	Both	2 (2 to 2)	0.6 (0.5 to 0.7)	1 (1 to 1)	0.3 (0.2 to 0.5)	0 (0 to 0)	0.2 (0.1 to 0.2)	-81.5 (-86.7 to -74.0)	-70.5 (-78.7 to -58.6)
Croatia	Cerebrovascular disease	10 to 14	Female	1 (1 to 1)	0.6 (0.4 to 0.8)	0 (0 to 1)	0.3 (0.2 to 0.5)	0 (0 to 0)	0.2 (0.1 to 0.3)	-80.5 (-87.5 to -69.8)	-68.8 (-80.1 to -51.6)
Croatia	Cerebrovascular disease	10 to 14	Male	1 (1 to 1)	0.6 (0.4 to 0.8)	1 (0 to 1)	0.4 (0.2 to 0.5)	0 (0 to 0)	0.2 (0.1 to 0.2)	-82.6 (-89.3 to -72.2)	-72.2 (-82.9 to -55.7)
Croatia	Hypertensive heart disease	5 to 9	Both	0 (0 to 0)	0.0 (0.0 to 0.0)	0 (0 to 0)	0.0 (0.0 to 0.0)	0 (0 to 0)	0.0 (0.0 to 0.0)	-46.9 (-65.7 to -20.8)	-21.3 (-49.3 to 17.2)
Croatia	Hypertensive heart disease	5 to 9	Female	0 (0 to 0)	0.0 (0.0 to 0.0)	0 (0 to 0)	0.0 (0.0 to 0.0)	0 (0 to 0)	0.0 (0.0 to 0.0)	-42.9 (-65.0 to -13.0)	-15.5 (-48.1 to 28.8)
Croatia	Hypertensive heart disease	5 to 9	Male	0 (0 to 0)	0.0 (0.0 to 0.0)	0 (0 to 0)	0.0 (0.0 to 0.0)	0 (0 to 0)	0.0 (0.0 to 0.0)	-50.5 (-73.1 to -15.4)	-26.8 (-60.2 to 25.1)
Croatia	Hypertensive heart disease	10 to 14	Both	0 (0 to 0)	0.0 (0.0 to 0.0)	0 (0 to 0)	0.0 (0.0 to 0.0)	0 (0 to 0)	0.0 (0.0 to 0.0)	-55.4 (-77.5 to -22.6)	-28.7 (-64.0 to 23.5)
Croatia	Hypertensive heart disease	10 to 14	Female	0 (0 to 0)	0.0 (0.0 to 0.0)	0 (0 to 0)	0.0 (0.0 to 0.0)	0 (0 to 0)	0.0 (0.0 to 0.0)	-41.8 (-62.8 to -11.7)	-6.8 (-40.5 to 41.3)
Croatia	Hypertensive heart disease	10 to 14	Male	0 (0 to 0)	0.0 (0.0 to 0.1)	0 (0 to 0)	0.0 (0.0 to 0.0)	0 (0 to 0)	0.0 (0.0 to 0.0)	-62.0 (-85.2 to -10.6)	-39.4 (-76.4 to 42.4)
Croatia	Cardiomyopathy and myocarditis	5 to 9	Both	1 (1 to 1)	0.2 (0.2 to 0.3)	1 (0 to 1)	0.3 (0.1 to 0.4)	0 (0 to 0)	0.1 (0.1 to 0.1)	-71.1 (-82.2 to -51.1)	-57.2 (-73.6 to -27.7)
Croatia	Cardiomyopathy and myocarditis	5 to 9	Female	0 (0 to 1)	0.2 (0.1 to 0.3)	0 (0 to 0)	0.3 (0.1 to 0.4)	0 (0 to 0)	0.1 (0.1 to 0.2)	-69.6 (-82.4 to -44.1)	-55.0 (-74.0 to -17.2)
Croatia	Cardiomyopathy and myocarditis	5 to 9	Male	0 (0 to 1)	0.2 (0.1 to 0.3)	0 (0 to 0)	0.3 (0.1 to 0.4)	0 (0 to 0)	0.1 (0.1 to 0.1)	-72.5 (-85.8 to -46.8)	-59.3 (-79.1 to -21.4)
Croatia	Cardiomyopathy and myocarditis	10 to 14	Both	1 (1 to 1)	0.3 (0.2 to 0.4)	1 (1 to 1)	0.4 (0.2 to 0.5)	0 (0 to 0)	0.2 (0.1 to 0.2)	-62.5 (-75.6 to -43.6)	-40.1 (-61.1 to -10.0)
Croatia	Cardiomyopathy and myocarditis	10 to 14	Female	0 (0 to 1)	0.3 (0.2 to 0.4)	0 (0 to 1)	0.4 (0.2 to 0.5)	0 (0 to 0)	0.2 (0.1 to 0.2)	-64.2 (-78.8 to -36.1)	-42.7 (-66.1 to 2.3)
Croatia	Cardiomyopathy and myocarditis	10 to 14	Male	0 (0 to 1)	0.3 (0.2 to 0.4)	1 (0 to 1)	0.4 (0.2 to 0.6)	0 (0 to 0)	0.2 (0.1 to 0.3)	-60.9 (-78.1 to -32.8)	-37.7 (-65.1 to 7.1)
Croatia	Atrial fibrillation and flutter	5 to 9	Both	0 (0 to 0)	0.0 (0.0 to 0.0)	0 (0 to 0)	0.0 (0.0 to 0.0)	0 (0 to 0)	0.0 (0.0 to 0.0)	0.0 (0.0 to 0.0)	0.0 (0.0 to 0.0)
Croatia	Atrial fibrillation and flutter	5 to 9	Female	0 (0 to 0)	0.0 (0.0 to 0.0)	0 (0 to 0)	0.0 (0.0 to 0.0)	0 (0 to 0)	0.0 (0.0 to 0.0)	0.0 (0.0 to 0.0)	0.0 (0.0 to 0.0)
Croatia	Atrial fibrillation and flutter	5 to 9	Male	0 (0 to 0)	0.0 (0.0 to 0.0)	0 (0 to 0)	0.0 (0.0 to 0.0)	0 (0 to 0)	0.0 (0.0 to 0.0)	0.0 (0.0 to 0.0)	0.0 (0.0 to 0.0)
Croatia	Atrial fibrillation and flutter	10 to 14	Both	0 (0 to 0)	0.0 (0.0 to 0.0)	0 (0 to 0)	0.0 (0.0 to 0.0)	0 (0 to 0)	0.0 (0.0 to 0.0)	0.0 (0.0 to 0.0)	0.0 (0.0 to 0.0)
Croatia	Atrial fibrillation and flutter	10 to 14	Female	0 (0 to 0)	0.0 (0.0 to 0.0)	0 (0 to 0)	0.0 (0.0 to 0.0)	0 (0 to 0)	0.0 (0.0 to 0.0)	0.0 (0.0 to 0.0)	0.0 (0.0 to 0.0)
Croatia	Atrial fibrillation and flutter	10 to 14	Male	0 (0 to 0)	0.0 (0.0 to 0.0)	0 (0 to 0)	0.0 (0.0 to 0.0)	0 (0 to 0)	0.0 (0.0 to 0.0)	0.0 (0.0 to 0.0)	0.0 (0.0 to 0.0)
Croatia	Endocarditis	5 to 9	Both	0 (0 to 0)	0.0 (0.0 to 0.1)	0 (0 to 0)	0.0 (0.0 to 0.0)	0 (0 to 0)	0.0 (0.0 to 0.0)	-61.3 (-81.3 to -21.9)	-42.7 (-72.3 to 15.7)
Croatia	Endocarditis	5 to 9	Female	0 (0 to 0)	0.0 (0.0 to 0.1)	0 (0 to 0)	0.0 (0.0 to 0.1)	0 (0 to 0)	0.0 (0.0 to 0.0)	-59.4 (-83.2 to -16.6)	-39.9 (-75.1 to 23.5)

eTable 8. All-cause and cause-specific mortality (counts and rates) at the global level and by country in the WHO European Region for ages 5 to 9 and 10 to 14 for 1990, 2000, and 2016

Croatia	Endocarditis	5 to 9	Male	0<br>(0 to 0)	0.0<br>(0.0 to 0.0)	0<br>(0 to 0)	0.0<br>(0.0 to 0.0)	0<br>(0 to 0)	0.0<br>(0.0 to 0.0)	-64.1<br>(-85.8 to -8.7)	-46.8<br>(-79.0 to 35.0)
Croatia	Endocarditis	10 to 14	Both	0<br>(0 to 0)	0.0<br>(0.0 to 0.0)	0<br>(0 to 0)	0.0<br>(0.0 to 0.0)	0<br>(0 to 0)	0.0<br>(0.0 to 0.0)	-50.2<br>(-73.9 to -20.4)	-20.4<br>(-58.3 to 27.1)
Croatia	Endocarditis	10 to 14	Female	0<br>(0 to 0)	0.0<br>(0.0 to 0.1)	0<br>(0 to 0)	0.0<br>(0.0 to 0.1)	0<br>(0 to 0)	0.0<br>(0.0 to 0.1)	-53.9<br>(-80.0 to -15.9)	-26.3<br>(-68.0 to 34.6)
Croatia	Endocarditis	10 to 14	Male	0<br>(0 to 0)	0.0<br>(0.0 to 0.0)	0<br>(0 to 0)	0.0<br>(0.0 to 0.0)	0<br>(0 to 0)	0.0<br>(0.0 to 0.0)	-43.4<br>(-70.8 to -7.6)	-9.8<br>(-53.4 to 47.2)
Croatia	Other cardiovascular and circulatory diseases	5 to 9	Both	1<br>(1 to 1)	0.2<br>(0.2 to 0.3)	0<br>(0 to 0)	0.1<br>(0.0 to 0.1)	0<br>(0 to 0)	0.0<br>(0.0 to 0.0)	-93.2<br>(-95.9 to -89.2)	-89.9<br>(-93.9 to -83.9)
Croatia	Other cardiovascular and circulatory diseases	5 to 9	Female	0<br>(0 to 1)	0.2<br>(0.2 to 0.3)	0<br>(0 to 0)	0.1<br>(0.0 to 0.1)	0<br>(0 to 0)	0.0<br>(0.0 to 0.0)	-92.7<br>(-96.0 to -87.2)	-89.1<br>(-94.0 to -81.0)
Croatia	Other cardiovascular and circulatory diseases	5 to 9	Male	0<br>(0 to 1)	0.2<br>(0.1 to 0.3)	0<br>(0 to 0)	0.1<br>(0.0 to 0.1)	0<br>(0 to 0)	0.0<br>(0.0 to 0.0)	-93.7<br>(-96.8 to -88.0)	-90.7<br>(-95.3 to -82.3)
Croatia	Other cardiovascular and circulatory diseases	10 to 14	Both	1<br>(1 to 1)	0.3<br>(0.3 to 0.4)	0<br>(0 to 0)	0.1<br>(0.1 to 0.1)	0<br>(0 to 0)	0.0<br>(0.0 to 0.1)	-91.5<br>(-94.3 to -87.0)	-86.4<br>(-91.0 to -79.3)
Croatia	Other cardiovascular and circulatory diseases	10 to 14	Female	1<br>(0 to 1)	0.3<br>(0.2 to 0.5)	0<br>(0 to 0)	0.1<br>(0.0 to 0.1)	0<br>(0 to 0)	0.0<br>(0.0 to 0.1)	-91.6<br>(-95.2 to -85.3)	-86.6<br>(-92.3 to -76.5)
Croatia	Other cardiovascular and circulatory diseases	10 to 14	Male	1<br>(0 to 1)	0.3<br>(0.2 to 0.5)	0<br>(0 to 0)	0.1<br>(0.1 to 0.2)	0<br>(0 to 0)	0.0<br>(0.0 to 0.1)	-91.4<br>(-95.2 to -84.1)	-86.3<br>(-92.4 to -74.7)
Croatia	Chronic obstructive pulmonary disease	5 to 9	Both	0<br>(0 to 0)	0.1<br>(0.0 to 0.1)	0<br>(0 to 0)	0.0<br>(0.0 to 0.1)	0<br>(0 to 0)	0.0<br>(0.0 to 0.0)	-73.9<br>(-83.6 to -58.9)	-61.4<br>(-75.8 to -39.2)
Croatia	Chronic obstructive pulmonary disease	5 to 9	Female	0<br>(0 to 0)	0.1<br>(0.1 to 0.1)	0<br>(0 to 0)	0.1<br>(0.0 to 0.1)	0<br>(0 to 0)	0.0<br>(0.0 to 0.1)	-71.2<br>(-83.6 to -51.6)	-57.3<br>(-75.7 to -28.4)
Croatia	Chronic obstructive pulmonary disease	5 to 9	Male	0<br>(0 to 0)	0.0<br>(0.0 to 0.0)	0<br>(0 to 0)	0.0<br>(0.0 to 0.0)	0<br>(0 to 0)	0.0<br>(0.0 to 0.0)	-82.8<br>(-90.4 to -69.6)	-74.6<br>(-85.8 to -55.0)
Croatia	Chronic obstructive pulmonary disease	10 to 14	Both	0<br>(0 to 0)	0.0<br>(0.0 to 0.0)	0<br>(0 to 0)	0.0<br>(0.0 to 0.0)	0<br>(0 to 0)	0.0<br>(0.0 to 0.0)	-45.8<br>(-69.4 to -15.8)	-13.5<br>(-51.1 to 34.5)
Croatia	Chronic obstructive pulmonary disease	10 to 14	Female	0<br>(0 to 0)	0.0<br>(0.0 to 0.1)	0<br>(0 to 0)	0.0<br>(0.0 to 0.1)	0<br>(0 to 0)	0.0<br>(0.0 to 0.0)	-48.9<br>(-72.7 to -9.5)	-18.2<br>(-56.3 to 44.8)
Croatia	Chronic obstructive pulmonary disease	10 to 14	Male	0<br>(0 to 0)	0.0<br>(0.0 to 0.0)	0<br>(0 to 0)	0.0<br>(0.0 to 0.0)	0<br>(0 to 0)	0.0<br>(0.0 to 0.0)	-42.4<br>(-78.1 to 9.6)	-8.2<br>(-65.1 to 74.7)
Croatia	Asthma	5 to 9	Both	0<br>(0 to 0)	0.0<br>(0.0 to 0.0)	0<br>(0 to 0)	0.0<br>(0.0 to 0.0)	0<br>(0 to 0)	0.0<br>(0.0 to 0.0)	-60.8<br>(-75.8 to -38.6)	-42.0<br>(-64.2 to -9.1)

eTable 8. All-cause and cause-specific mortality (counts and rates) at the global level and by country in the WHO European Region for ages 5 to 9 and 10 to 14 for 1990, 2000, and 2016

Croatia	Asthma	5 to 9	Female	0<br>(0 to 0)	0.0<br>(0.0 to 0.0)	0<br>(0 to 0)	0.0<br>(0.0 to 0.0)	0<br>(0 to 0)	0.0<br>(0.0 to 0.0)	-53.8<br>(-76.0 to -15.2)	-31.6<br>(-64.4 to 25.6)
Croatia	Asthma	5 to 9	Male	0<br>(0 to 0)	0.0<br>(0.0 to 0.0)	0<br>(0 to 0)	0.0<br>(0.0 to 0.0)	0<br>(0 to 0)	0.0<br>(0.0 to 0.0)	-67.1<br>(-81.7 to -39.4)	-51.3<br>(-72.9 to -10.3)
Croatia	Asthma	10 to 14	Both	0<br>(0 to 0)	0.1<br>(0.1 to 0.1)	0<br>(0 to 0)	0.1<br>(0.0 to 0.1)	0<br>(0 to 0)	0.0<br>(0.0 to 0.0)	-86.6<br>(-91.8 to -74.5)	-78.6<br>(-86.9 to -59.4)
Croatia	Asthma	10 to 14	Female	0<br>(0 to 0)	0.0<br>(0.0 to 0.1)	0<br>(0 to 0)	0.0<br>(0.0 to 0.0)	0<br>(0 to 0)	0.0<br>(0.0 to 0.0)	-84.5<br>(-91.5 to -70.6)	-75.1<br>(-86.5 to -52.9)
Croatia	Asthma	10 to 14	Male	0<br>(0 to 0)	0.1<br>(0.1 to 0.2)	0<br>(0 to 0)	0.1<br>(0.1 to 0.1)	0<br>(0 to 0)	0.0<br>(0.0 to 0.0)	-87.3<br>(-93.2 to -71.2)	-79.7<br>(-89.2 to -54.1)
Croatia	Interstitial lung disease and pulmonary sarcoidosis	5 to 9	Both	0<br>(0 to 0)	0.0<br>(0.0 to 0.0)	0<br>(0 to 0)	0.0<br>(0.0 to 0.0)	0<br>(0 to 0)	0.0<br>(0.0 to 0.0)	-73.8<br>(-85.7 to -53.1)	-61.2<br>(-78.8 to -30.6)
Croatia	Interstitial lung disease and pulmonary sarcoidosis	5 to 9	Female	0<br>(0 to 0)	0.0<br>(0.0 to 0.0)	0<br>(0 to 0)	0.0<br>(0.0 to 0.0)	0<br>(0 to 0)	0.0<br>(0.0 to 0.0)	-72.1<br>(-87.4 to -35.9)	-58.7<br>(-81.4 to -5.1)
Croatia	Interstitial lung disease and pulmonary sarcoidosis	5 to 9	Male	0<br>(0 to 0)	0.0<br>(0.0 to 0.0)	0<br>(0 to 0)	0.0<br>(0.0 to 0.0)	0<br>(0 to 0)	0.0<br>(0.0 to 0.0)	-76.3<br>(-90.0 to -36.8)	-64.9<br>(-85.2 to -6.5)
Croatia	Interstitial lung disease and pulmonary sarcoidosis	10 to 14	Both	0<br>(0 to 0)	0.0<br>(0.0 to 0.0)	0<br>(0 to 0)	0.0<br>(0.0 to 0.0)	0<br>(0 to 0)	0.0<br>(0.0 to 0.0)	-52.6<br>(-74.1 to -13.1)	-24.3<br>(-58.7 to 38.7)
Croatia	Interstitial lung disease and pulmonary sarcoidosis	10 to 14	Female	0<br>(0 to 0)	0.0<br>(0.0 to 0.0)	0<br>(0 to 0)	0.0<br>(0.0 to 0.0)	0<br>(0 to 0)	0.0<br>(0.0 to 0.0)	-61.1<br>(-79.9 to -16.9)	-37.7<br>(-67.8 to 32.9)
Croatia	Interstitial lung disease and pulmonary sarcoidosis	10 to 14	Male	0<br>(0 to 0)	0.0<br>(0.0 to 0.0)	0<br>(0 to 0)	0.0<br>(0.0 to 0.0)	0<br>(0 to 0)	0.0<br>(0.0 to 0.0)	-21.4<br>(-75.4 to 102.6)	25.2<br>(-60.8 to 222.8)
Croatia	Other chronic respiratory diseases	5 to 9	Both	0<br>(0 to 0)	0.0<br>(0.0 to 0.0)	0<br>(0 to 0)	0.0<br>(0.0 to 0.0)	0<br>(0 to 0)	0.0<br>(0.0 to 0.0)	-56.1<br>(-71.9 to -31.0)	-35.0<br>(-58.4 to 2.1)
Croatia	Other chronic respiratory diseases	5 to 9	Female	0<br>(0 to 0)	0.0<br>(0.0 to 0.0)	0<br>(0 to 0)	0.0<br>(0.0 to 0.0)	0<br>(0 to 0)	0.0<br>(0.0 to 0.0)	-53.7<br>(-75.8 to -15.2)	-31.5<br>(-64.2 to 25.6)
Croatia	Other chronic respiratory diseases	5 to 9	Male	0<br>(0 to 0)	0.0<br>(0.0 to 0.0)	0<br>(0 to 0)	0.0<br>(0.0 to 0.0)	0<br>(0 to 0)	0.0<br>(0.0 to 0.0)	-58.7<br>(-76.5 to -27.2)	-38.9<br>(-65.2 to 7.7)
Croatia	Other chronic respiratory diseases	10 to 14	Both	0<br>(0 to 0)	0.0<br>(0.0 to 0.0)	0<br>(0 to 0)	0.0<br>(0.0 to 0.0)	0<br>(0 to 0)	0.0<br>(0.0 to 0.0)	-32.9<br>(-63.7 to 26.3)	7.1<br>(-42.1 to 101.7)
Croatia	Other chronic respiratory diseases	10 to 14	Female	0<br>(0 to 0)	0.0<br>(0.0 to 0.0)	0<br>(0 to 0)	0.0<br>(0.0 to 0.0)	0<br>(0 to 0)	0.0<br>(0.0 to 0.0)	-36.0<br>(-63.5 to 10.5)	2.3<br>(-41.6 to 76.8)
Croatia	Other chronic respiratory diseases	10 to 14	Male	0<br>(0 to 0)	0.0<br>(0.0 to 0.0)	0<br>(0 to 0)	0.0<br>(0.0 to 0.0)	0<br>(0 to 0)	0.0<br>(0.0 to 0.0)	-31.1<br>(-70.4 to 80.4)	9.7<br>(-52.8 to 187.4)
Croatia	Cirrhosis and other chronic liver diseases due to hepatitis B	5 to 9	Both	0<br>(0 to 0)	0.0<br>(0.0 to 0.0)	0<br>(0 to 0)	0.0<br>(0.0 to 0.0)	0<br>(0 to 0)	0.0<br>(0.0 to 0.0)	-79.2<br>(-87.5 to -66.5)	-69.2<br>(-81.5 to -50.5)

Croatia	Cirrhosis and other chronic liver diseases due to hepatitis B	5 to 9	Female	0<br>(0 to 0)	0.0<br>(0.0 to 0.0)	0<br>(0 to 0)	0.0<br>(0.0 to 0.0)	0<br>(0 to 0)	0.0<br>(0.0 to 0.0)	-80.1<br>(-87.7 to -69.1)	-70.5<br>(-81.8 to -54.3)
Croatia	Cirrhosis and other chronic liver diseases due to hepatitis B	5 to 9	Male	0<br>(0 to 0)	0.0<br>(0.0 to 0.0)	0<br>(0 to 0)	0.0<br>(0.0 to 0.0)	0<br>(0 to 0)	0.0<br>(0.0 to 0.0)	-79.1<br>(-88.0 to -64.5)	-69.0<br>(-82.3 to -47.5)
Croatia	Cirrhosis and other chronic liver diseases due to hepatitis B	10 to 14	Both	0<br>(0 to 0)	0.0<br>(0.0 to 0.0)	0<br>(0 to 0)	0.0<br>(0.0 to 0.0)	0<br>(0 to 0)	0.0<br>(0.0 to 0.0)	-74.2<br>(-83.4 to -59.8)	-58.8<br>(-73.4 to -35.8)
Croatia	Cirrhosis and other chronic liver diseases due to hepatitis B	10 to 14	Female	0<br>(0 to 0)	0.0<br>(0.0 to 0.0)	0<br>(0 to 0)	0.0<br>(0.0 to 0.0)	0<br>(0 to 0)	0.0<br>(0.0 to 0.0)	-77.4<br>(-87.0 to -61.1)	-63.8<br>(-79.2 to -37.8)
Croatia	Cirrhosis and other chronic liver diseases due to hepatitis B	10 to 14	Male	0<br>(0 to 0)	0.0<br>(0.0 to 0.0)	0<br>(0 to 0)	0.0<br>(0.0 to 0.0)	0<br>(0 to 0)	0.0<br>(0.0 to 0.0)	-72.9<br>(-84.2 to -52.4)	-56.8<br>(-74.9 to -24.2)
Croatia	Cirrhosis and other chronic liver diseases due to hepatitis C	5 to 9	Both	0<br>(0 to 0)	0.0<br>(0.0 to 0.0)	0<br>(0 to 0)	0.0<br>(0.0 to 0.0)	0<br>(0 to 0)	0.0<br>(0.0 to 0.0)	-78.8<br>(-87.1 to -66.2)	-68.5<br>(-80.8 to -50.0)
Croatia	Cirrhosis and other chronic liver diseases due to hepatitis C	5 to 9	Female	0<br>(0 to 0)	0.0<br>(0.0 to 0.0)	0<br>(0 to 0)	0.0<br>(0.0 to 0.0)	0<br>(0 to 0)	0.0<br>(0.0 to 0.0)	-79.9<br>(-87.7 to -69.0)	-70.3<br>(-81.8 to -54.1)
Croatia	Cirrhosis and other chronic liver diseases due to hepatitis C	5 to 9	Male	0<br>(0 to 0)	0.0<br>(0.0 to 0.0)	0<br>(0 to 0)	0.0<br>(0.0 to 0.0)	0<br>(0 to 0)	0.0<br>(0.0 to 0.0)	-78.5<br>(-87.8 to -63.8)	-68.3<br>(-82.0 to -46.4)
Croatia	Cirrhosis and other chronic liver diseases due to hepatitis C	10 to 14	Both	0<br>(0 to 0)	0.0<br>(0.0 to 0.0)	0<br>(0 to 0)	0.0<br>(0.0 to 0.0)	0<br>(0 to 0)	0.0<br>(0.0 to 0.0)	-73.9<br>(-83.1 to -59.6)	-58.4<br>(-73.1 to -35.5)
Croatia	Cirrhosis and other chronic liver diseases due to hepatitis C	10 to 14	Female	0<br>(0 to 0)	0.0<br>(0.0 to 0.0)	0<br>(0 to 0)	0.0<br>(0.0 to 0.0)	0<br>(0 to 0)	0.0<br>(0.0 to 0.0)	-77.3<br>(-86.9 to -61.1)	-63.6<br>(-79.1 to -37.8)
Croatia	Cirrhosis and other chronic liver diseases due to hepatitis C	10 to 14	Male	0<br>(0 to 0)	0.0<br>(0.0 to 0.0)	0<br>(0 to 0)	0.0<br>(0.0 to 0.0)	0<br>(0 to 0)	0.0<br>(0.0 to 0.0)	-72.3<br>(-83.8 to -51.5)	-55.9<br>(-74.3 to -22.7)
Croatia	Cirrhosis and other chronic liver diseases due to other causes	5 to 9	Both	0<br>(0 to 0)	0.1<br>(0.1 to 0.1)	0<br>(0 to 0)	0.1<br>(0.1 to 0.1)	0<br>(0 to 0)	0.0<br>(0.0 to 0.0)	-80.3<br>(-87.4 to -70.7)	-70.8<br>(-81.4 to -56.6)
Croatia	Cirrhosis and other chronic liver diseases due to other causes	5 to 9	Female	0<br>(0 to 0)	0.1<br>(0.1 to 0.1)	0<br>(0 to 0)	0.0<br>(0.0 to 0.1)	0<br>(0 to 0)	0.0<br>(0.0 to 0.0)	-81.4<br>(-88.6 to -71.2)	-72.4<br>(-83.1 to -57.4)

eTable 8. All-cause and cause-specific mortality (counts and rates) at the global level and by country in the WHO European Region for ages 5 to 9 and 10 to 14 for 1990, 2000, and 2016

Croatia	Cirrhosis and other chronic liver diseases due to other causes	5 to 9	Male	0<br>(0 to 0)	0.1<br>(0.1 to 0.2)	0<br>(0 to 0)	0.1<br>(0.1 to 0.1)	0<br>(0 to 0)	0.0<br>(0.0 to 0.1)	-79.7<br>(-88.4 to -65.5)	-70.0<br>(-82.8 to -49.0)
Croatia	Cirrhosis and other chronic liver diseases due to other causes	10 to 14	Both	0<br>(0 to 0)	0.1<br>(0.1 to 0.1)	0<br>(0 to 0)	0.1<br>(0.0 to 0.1)	0<br>(0 to 0)	0.0<br>(0.0 to 0.0)	-76.8<br>(-84.4 to -66.0)	-62.9<br>(-75.2 to -45.7)
Croatia	Cirrhosis and other chronic liver diseases due to other causes	10 to 14	Female	0<br>(0 to 0)	0.1<br>(0.1 to 0.1)	0<br>(0 to 0)	0.1<br>(0.0 to 0.1)	0<br>(0 to 0)	0.0<br>(0.0 to 0.0)	-78.8<br>(-88.0 to -64.0)	-66.2<br>(-80.8 to -42.4)
Croatia	Cirrhosis and other chronic liver diseases due to other causes	10 to 14	Male	0<br>(0 to 0)	0.1<br>(0.0 to 0.1)	0<br>(0 to 0)	0.0<br>(0.0 to 0.1)	0<br>(0 to 0)	0.0<br>(0.0 to 0.0)	-73.9<br>(-84.8 to -54.2)	-58.5<br>(-75.8 to -27.0)
Croatia	Peptic ulcer disease	5 to 9	Both	0<br>(0 to 0)	0.0<br>(0.0 to 0.1)	0<br>(0 to 0)	0.0<br>(0.0 to 0.0)	0<br>(0 to 0)	0.0<br>(0.0 to 0.0)	-79.6<br>(-90.0 to -59.4)	-69.7<br>(-85.2 to -39.9)
Croatia	Peptic ulcer disease	5 to 9	Female	0<br>(0 to 0)	0.0<br>(0.0 to 0.0)	0<br>(0 to 0)	0.0<br>(0.0 to 0.0)	0<br>(0 to 0)	0.0<br>(0.0 to 0.0)	-57.0<br>(-78.3 to -12.9)	-36.2<br>(-67.8 to 29.0)
Croatia	Peptic ulcer disease	5 to 9	Male	0<br>(0 to 0)	0.1<br>(0.0 to 0.1)	0<br>(0 to 0)	0.0<br>(0.0 to 0.1)	0<br>(0 to 0)	0.0<br>(0.0 to 0.0)	-84.2<br>(-94.2 to -60.3)	-76.6<br>(-91.4 to -41.3)
Croatia	Peptic ulcer disease	10 to 14	Both	0<br>(0 to 0)	0.0<br>(0.0 to 0.0)	0<br>(0 to 0)	0.0<br>(0.0 to 0.0)	0<br>(0 to 0)	0.0<br>(0.0 to 0.0)	-74.1<br>(-86.4 to -52.1)	-58.7<br>(-78.3 to -23.5)
Croatia	Peptic ulcer disease	10 to 14	Female	0<br>(0 to 0)	0.0<br>(0.0 to 0.1)	0<br>(0 to 0)	0.0<br>(0.0 to 0.0)	0<br>(0 to 0)	0.0<br>(0.0 to 0.0)	-80.0<br>(-90.5 to -53.0)	-68.0<br>(-84.9 to -24.7)
Croatia	Peptic ulcer disease	10 to 14	Male	0<br>(0 to 0)	0.0<br>(0.0 to 0.0)	0<br>(0 to 0)	0.0<br>(0.0 to 0.0)	0<br>(0 to 0)	0.0<br>(0.0 to 0.0)	-53.1<br>(-74.2 to -18.3)	-25.3<br>(-58.9 to 30.1)
Croatia	Gastritis and duodenitis	5 to 9	Both	0<br>(0 to 0)	0.0<br>(0.0 to 0.0)	0<br>(0 to 0)	0.0<br>(0.0 to 0.0)	0<br>(0 to 0)	0.0<br>(0.0 to 0.0)	-28.4<br>(-70.8 to 69.5)	6.0<br>(-56.7 to 150.9)
Croatia	Gastritis and duodenitis	5 to 9	Female	0<br>(0 to 0)	0.0<br>(0.0 to 0.0)	0<br>(0 to 0)	0.0<br>(0.0 to 0.0)	0<br>(0 to 0)	0.0<br>(0.0 to 0.0)	-42.5<br>(-78.4 to 56.9)	-14.9<br>(-68.0 to 132.4)
Croatia	Gastritis and duodenitis	5 to 9	Male	0<br>(0 to 0)	0.0<br>(0.0 to 0.0)	0<br>(0 to 0)	0.0<br>(0.0 to 0.0)	0<br>(0 to 0)	0.0<br>(0.0 to 0.0)	-10.4<br>(-72.9 to 160.1)	32.5<br>(-59.8 to 284.8)
Croatia	Gastritis and duodenitis	10 to 14	Both	0<br>(0 to 0)	0.0<br>(0.0 to 0.0)	0<br>(0 to 0)	0.0<br>(0.0 to 0.0)	0<br>(0 to 0)	0.0<br>(0.0 to 0.0)	-31.7<br>(-63.9 to 25.6)	9.0<br>(-42.4 to 100.5)
Croatia	Gastritis and duodenitis	10 to 14	Female	0<br>(0 to 0)	0.0<br>(0.0 to 0.0)	0<br>(0 to 0)	0.0<br>(0.0 to 0.0)	0<br>(0 to 0)	0.0<br>(0.0 to 0.0)	-42.3<br>(-72.4 to 40.9)	-7.7<br>(-55.8 to 125.4)
Croatia	Gastritis and duodenitis	10 to 14	Male	0<br>(0 to 0)	0.0<br>(0.0 to 0.0)	0<br>(0 to 0)	0.0<br>(0.0 to 0.0)	0<br>(0 to 0)	0.0<br>(0.0 to 0.0)	-18.0<br>(-64.3 to 58.7)	30.6<br>(-43.1 to 152.9)
Croatia	Appendicitis	5 to 9	Both	0<br>(0 to 0)	0.0<br>(0.0 to 0.1)	0<br>(0 to 0)	0.0<br>(0.0 to 0.0)	0<br>(0 to 0)	0.0<br>(0.0 to 0.0)	-84.4<br>(-93.1 to -57.0)	-76.9<br>(-89.8 to -36.4)
Croatia	Appendicitis	5 to 9	Female	0<br>(0 to 0)	0.1<br>(0.0 to 0.1)	0<br>(0 to 0)	0.0<br>(0.0 to 0.1)	0<br>(0 to 0)	0.0<br>(0.0 to 0.0)	-84.9<br>(-93.7 to -58.5)	-77.6<br>(-90.7 to -38.5)
Croatia	Appendicitis	5 to 9	Male	0<br>(0 to 0)	0.0<br>(0.0 to 0.0)	0<br>(0 to 0)	0.0<br>(0.0 to 0.0)	0<br>(0 to 0)	0.0<br>(0.0 to 0.0)	-82.2<br>(-95.4 to -35.1)	-73.6<br>(-93.3 to -3.9)
Croatia	Appendicitis	10 to 14	Both	0<br>(0 to 0)	0.0<br>(0.0 to 0.0)	0<br>(0 to 0)	0.0<br>(0.0 to 0.0)	0<br>(0 to 0)	0.0<br>(0.0 to 0.0)	-79.0<br>(-91.0 to -54.5)	-66.5<br>(-85.6 to -27.3)
Croatia	Appendicitis	10 to 14	Female	0<br>(0 to 0)	0.0<br>(0.0 to 0.0)	0<br>(0 to 0)	0.0<br>(0.0 to 0.0)	0<br>(0 to 0)	0.0<br>(0.0 to 0.0)	-77.7<br>(-92.7 to -50.5)	-64.3<br>(-88.4 to -20.8)
Croatia	Appendicitis	10 to 14	Male	0<br>(0 to 0)	0.0<br>(0.0 to 0.1)	0<br>(0 to 0)	0.0<br>(0.0 to 0.1)	0<br>(0 to 0)	0.0<br>(0.0 to 0.0)	-80.3<br>(-93.0 to -48.9)	-68.6<br>(-88.9 to -18.7)
Croatia	Paralytic ileus and intestinal obstruction	5 to 9	Both	0<br>(0 to 0)	0.1<br>(0.1 to 0.1)	0<br>(0 to 0)	0.1<br>(0.0 to 0.1)	0<br>(0 to 0)	0.0<br>(0.0 to 0.1)	-69.7<br>(-83.3 to -46.1)	-55.2<br>(-75.3 to -20.3)
Croatia	Paralytic ileus and intestinal obstruction	5 to 9	Female	0<br>(0 to 0)	0.1<br>(0.1 to 0.2)	0<br>(0 to 0)	0.1<br>(0.0 to 0.1)	0<br>(0 to 0)	0.0<br>(0.0 to 0.1)	-71.7<br>(-85.3 to -42.2)	-58.1<br>(-78.2 to -14.4)
Croatia	Paralytic ileus and intestinal obstruction	5 to 9	Male	0<br>(0 to 0)	0.1<br>(0.0 to 0.1)	0<br>(0 to 0)	0.1<br>(0.0 to 0.1)	0<br>(0 to 0)	0.0<br>(0.0 to 0.1)	-66.6<br>(-86.5 to -9.2)	-50.6<br>(-80.1 to 34.3)

eTable 8. All-cause and cause-specific mortality (counts and rates) at the global level and by country in the WHO European Region for ages 5 to 9 and 10 to 14 for 1990, 2000, and 2016

Croatia	Paralytic ileus and intestinal obstruction	10 to 14	Both	0 (0 to 0)	0.0 (0.0 to 0.1)	0 (0 to 0)	0.0 (0.0 to 0.1)	0 (0 to 0)	0.0 (0.0 to 0.1)	-65.4 (-81.6 to -40.8)	-44.8 (-70.7 to -5.5)
Croatia	Paralytic ileus and intestinal obstruction	10 to 14	Female	0 (0 to 0)	0.1 (0.0 to 0.1)	0 (0 to 0)	0.0 (0.0 to 0.1)	0 (0 to 0)	0.0 (0.0 to 0.1)	-70.1 (-84.8 to -43.7)	-52.2 (-75.7 to -9.9)
Croatia	Paralytic ileus and intestinal obstruction	10 to 14	Male	0 (0 to 0)	0.0 (0.0 to 0.1)	0 (0 to 0)	0.0 (0.0 to 0.1)	0 (0 to 0)	0.0 (0.0 to 0.1)	-54.0 (-76.3 to -16.4)	-26.7 (-62.3 to 33.2)
Croatia	Inguinal, femoral, and abdominal hernia	5 to 9	Both	0 (0 to 0)	0.0 (0.0 to 0.0)	0 (0 to 0)	0.0 (0.0 to 0.0)	0 (0 to 0)	0.0 (0.0 to 0.0)	-86.4 (-94.5 to -74.0)	-79.8 (-91.8 to -61.6)
Croatia	Inguinal, femoral, and abdominal hernia	5 to 9	Female	0 (0 to 0)	0.0 (0.0 to 0.0)	0 (0 to 0)	0.0 (0.0 to 0.0)	0 (0 to 0)	0.0 (0.0 to 0.0)	-86.7 (-95.8 to -70.5)	-80.3 (-93.8 to -56.3)
Croatia	Inguinal, femoral, and abdominal hernia	5 to 9	Male	0 (0 to 0)	0.0 (0.0 to 0.0)	0 (0 to 0)	0.0 (0.0 to 0.0)	0 (0 to 0)	0.0 (0.0 to 0.0)	-85.9 (-95.5 to -61.7)	-79.1 (-93.4 to -43.4)
Croatia	Inguinal, femoral, and abdominal hernia	10 to 14	Both	0 (0 to 0)	0.0 (0.0 to 0.0)	0 (0 to 0)	0.0 (0.0 to 0.0)	0 (0 to 0)	0.0 (0.0 to 0.0)	-86.2 (-94.2 to -74.0)	-77.9 (-90.7 to -58.5)
Croatia	Inguinal, femoral, and abdominal hernia	10 to 14	Female	0 (0 to 0)	0.0 (0.0 to 0.0)	0 (0 to 0)	0.0 (0.0 to 0.0)	0 (0 to 0)	0.0 (0.0 to 0.0)	-88.5 (-96.1 to -69.6)	-81.5 (-93.7 to -51.3)
Croatia	Inguinal, femoral, and abdominal hernia	10 to 14	Male	0 (0 to 0)	0.0 (0.0 to 0.0)	0 (0 to 0)	0.0 (0.0 to 0.0)	0 (0 to 0)	0.0 (0.0 to 0.0)	-82.5 (-93.0 to -65.6)	-72.1 (-88.9 to -45.2)
Croatia	Inflammatory bowel disease	5 to 9	Both	0 (0 to 0)	0.0 (0.0 to 0.0)	0 (0 to 0)	0.0 (0.0 to 0.0)	0 (0 to 0)	0.0 (0.0 to 0.0)	-55.2 (-85.9 to 13.1)	-33.8 (-79.2 to 67.5)
Croatia	Inflammatory bowel disease	5 to 9	Female	0 (0 to 0)	0.0 (0.0 to 0.0)	0 (0 to 0)	0.0 (0.0 to 0.0)	0 (0 to 0)	0.0 (0.0 to 0.0)	-51.2 (-88.4 to 23.9)	-27.7 (-82.9 to 83.5)
Croatia	Inflammatory bowel disease	5 to 9	Male	0 (0 to 0)	0.0 (0.0 to 0.0)	0 (0 to 0)	0.0 (0.0 to 0.0)	0 (0 to 0)	0.0 (0.0 to 0.0)	-65.6 (-88.1 to 21.5)	-49.1 (-82.3 to 79.8)
Croatia	Inflammatory bowel disease	10 to 14	Both	0 (0 to 0)	0.0 (0.0 to 0.0)	0 (0 to 0)	0.0 (0.0 to 0.0)	0 (0 to 0)	0.0 (0.0 to 0.0)	-56.7 (-80.9 to 1.0)	-30.9 (-69.5 to 61.3)
Croatia	Inflammatory bowel disease	10 to 14	Female	0 (0 to 0)	0.0 (0.0 to 0.0)	0 (0 to 0)	0.0 (0.0 to 0.0)	0 (0 to 0)	0.0 (0.0 to 0.0)	-55.5 (-83.5 to 29.9)	-28.9 (-73.6 to 107.8)
Croatia	Inflammatory bowel disease	10 to 14	Male	0 (0 to 0)	0.0 (0.0 to 0.0)	0 (0 to 0)	0.0 (0.0 to 0.0)	0 (0 to 0)	0.0 (0.0 to 0.0)	-58.8 (-82.4 to 7.8)	-34.4 (-72.0 to 71.8)
Croatia	Vascular intestinal disorders	5 to 9	Both	0 (0 to 0)	0.0 (0.0 to 0.0)	0 (0 to 0)	0.0 (0.0 to 0.0)	0 (0 to 0)	0.0 (0.0 to 0.0)	-60.3 (-84.3 to 32.6)	-41.2 (-76.7 to 96.2)
Croatia	Vascular intestinal disorders	5 to 9	Female	0 (0 to 0)	0.0 (0.0 to 0.0)	0 (0 to 0)	0.0 (0.0 to 0.0)	0 (0 to 0)	0.0 (0.0 to 0.0)	-48.8 (-82.8 to 56.8)	-24.2 (-74.5 to 132.1)
Croatia	Vascular intestinal disorders	5 to 9	Male	0 (0 to 0)	0.0 (0.0 to 0.0)	0 (0 to 0)	0.0 (0.0 to 0.0)	0 (0 to 0)	0.0 (0.0 to 0.0)	-64.6 (-87.1 to 66.9)	-47.7 (-81.0 to 146.9)
Croatia	Vascular intestinal disorders	10 to 14	Both	0 (0 to 0)	0.0 (0.0 to 0.0)	0 (0 to 0)	0.0 (0.0 to 0.0)	0 (0 to 0)	0.0 (0.0 to 0.0)	-72.7 (-87.7 to -34.1)	-56.5 (-80.3 to 5.2)
Croatia	Vascular intestinal disorders	10 to 14	Female	0 (0 to 0)	0.0 (0.0 to 0.0)	0 (0 to 0)	0.0 (0.0 to 0.0)	0 (0 to 0)	0.0 (0.0 to 0.0)	-73.3 (-88.7 to -33.5)	-57.2 (-81.9 to 6.4)
Croatia	Vascular intestinal disorders	10 to 14	Male	0 (0 to 0)	0.0 (0.0 to 0.0)	0 (0 to 0)	0.0 (0.0 to 0.0)	0 (0 to 0)	0.0 (0.0 to 0.0)	-63.1 (-82.5 to -9.2)	-41.2 (-72.2 to 44.6)

eTable 8. All-cause and cause-specific mortality (counts and rates) at the global level and by country in the WHO European Region for ages 5 to 9 and 10 to 14 for 1990, 2000, and 2016

Croatia	Gallbladder and biliary diseases	5 to 9	Both	0 (0 to 0)	0.0 (0.0 to 0.0)	0 (0 to 0)	0.0 (0.0 to 0.0)	0 (0 to 0)	0.0 (0.0 to 0.0)	-79.8 (-90.3 to -57.7)	-70.1 (-85.6 to -37.4)
Croatia	Gallbladder and biliary diseases	5 to 9	Female	0 (0 to 0)	0.0 (0.0 to 0.0)	0 (0 to 0)	0.0 (0.0 to 0.0)	0 (0 to 0)	0.0 (0.0 to 0.0)	-81.1 (-92.5 to -52.7)	-72.0 (-88.8 to -30.0)
Croatia	Gallbladder and biliary diseases	5 to 9	Male	0 (0 to 0)	0.0 (0.0 to 0.0)	0 (0 to 0)	0.0 (0.0 to 0.0)	0 (0 to 0)	0.0 (0.0 to 0.0)	-78.3 (-92.6 to -40.8)	-67.9 (-89.0 to -12.4)
Croatia	Gallbladder and biliary diseases	10 to 14	Both	0 (0 to 0)	0.0 (0.0 to 0.0)	0 (0 to 0)	0.0 (0.0 to 0.0)	0 (0 to 0)	0.0 (0.0 to 0.0)	-77.2 (-87.6 to -61.1)	-63.6 (-80.1 to -37.9)
Croatia	Gallbladder and biliary diseases	10 to 14	Female	0 (0 to 0)	0.0 (0.0 to 0.0)	0 (0 to 0)	0.0 (0.0 to 0.0)	0 (0 to 0)	0.0 (0.0 to 0.0)	-79.7 (-90.8 to -57.6)	-67.5 (-85.2 to -32.2)
Croatia	Gallbladder and biliary diseases	10 to 14	Male	0 (0 to 0)	0.0 (0.0 to 0.0)	0 (0 to 0)	0.0 (0.0 to 0.0)	0 (0 to 0)	0.0 (0.0 to 0.0)	-72.3 (-87.8 to -40.4)	-55.8 (-80.6 to -5.0)
Croatia	Pancreatitis	5 to 9	Both	0 (0 to 0)	0.0 (0.0 to 0.0)	0 (0 to 0)	0.0 (0.0 to 0.0)	0 (0 to 0)	0.0 (0.0 to 0.0)	-82.6 (-92.7 to -59.4)	-74.2 (-89.1 to -39.9)
Croatia	Pancreatitis	5 to 9	Female	0 (0 to 0)	0.0 (0.0 to 0.1)	0 (0 to 0)	0.0 (0.0 to 0.0)	0 (0 to 0)	0.0 (0.0 to 0.0)	-82.7 (-93.2 to -59.0)	-74.4 (-89.9 to -39.2)
Croatia	Pancreatitis	5 to 9	Male	0 (0 to 0)	0.0 (0.0 to 0.0)	0 (0 to 0)	0.0 (0.0 to 0.0)	0 (0 to 0)	0.0 (0.0 to 0.0)	-79.5 (-95.8 to -36.1)	-69.6 (-93.8 to -5.4)
Croatia	Pancreatitis	10 to 14	Both	0 (0 to 0)	0.0 (0.0 to 0.0)	0 (0 to 0)	0.0 (0.0 to 0.0)	0 (0 to 0)	0.0 (0.0 to 0.0)	-79.9 (-91.3 to -61.9)	-68.0 (-86.1 to -39.2)
Croatia	Pancreatitis	10 to 14	Female	0 (0 to 0)	0.0 (0.0 to 0.0)	0 (0 to 0)	0.0 (0.0 to 0.0)	0 (0 to 0)	0.0 (0.0 to 0.0)	-79.9 (-91.6 to -54.0)	-67.8 (-86.6 to -26.5)
Croatia	Pancreatitis	10 to 14	Male	0 (0 to 0)	0.0 (0.0 to 0.0)	0 (0 to 0)	0.0 (0.0 to 0.0)	0 (0 to 0)	0.0 (0.0 to 0.0)	-80.1 (-95.1 to -45.9)	-68.2 (-92.2 to -13.9)
Croatia	Other digestive diseases	5 to 9	Both	0 (0 to 0)	0.0 (0.0 to 0.0)	0 (0 to 0)	0.0 (0.0 to 0.0)	0 (0 to 0)	0.0 (0.0 to 0.0)	-48.4 (-75.8 to 7.7)	-23.6 (-64.2 to 59.4)
Croatia	Other digestive diseases	5 to 9	Female	0 (0 to 0)	0.0 (0.0 to 0.1)	0 (0 to 0)	0.0 (0.0 to 0.1)	0 (0 to 0)	0.0 (0.0 to 0.1)	-41.9 (-75.2 to 33.8)	-13.9 (-63.3 to 98.1)
Croatia	Other digestive diseases	5 to 9	Male	0 (0 to 0)	0.0 (0.0 to 0.0)	0 (0 to 0)	0.0 (0.0 to 0.0)	0 (0 to 0)	0.0 (0.0 to 0.0)	-61.5 (-86.0 to -1.1)	-43.1 (-79.3 to 46.3)
Croatia	Other digestive diseases	10 to 14	Both	0 (0 to 0)	0.0 (0.0 to 0.0)	0 (0 to 0)	0.0 (0.0 to 0.0)	0 (0 to 0)	0.0 (0.0 to 0.0)	-43.1 (-69.6 to 1.7)	-9.2 (-51.5 to 62.4)
Croatia	Other digestive diseases	10 to 14	Female	0 (0 to 0)	0.0 (0.0 to 0.1)	0 (0 to 0)	0.0 (0.0 to 0.1)	0 (0 to 0)	0.0 (0.0 to 0.1)	-30.7 (-64.2 to 36.4)	10.9 (-42.8 to 118.2)
Croatia	Other digestive diseases	10 to 14	Male	0 (0 to 0)	0.0 (0.0 to 0.0)	0 (0 to 0)	0.0 (0.0 to 0.0)	0 (0 to 0)	0.0 (0.0 to 0.0)	-66.4 (-84.6 to -28.2)	-46.5 (-75.5 to 14.3)
Croatia	Alzheimer disease and other dementias	5 to 9	Both	0 (0 to 0)	0.0 (0.0 to 0.0)	0 (0 to 0)	0.0 (0.0 to 0.0)	0 (0 to 0)	0.0 (0.0 to 0.0)	0.0 (0.0 to 0.0)	0.0 (0.0 to 0.0)
Croatia	Alzheimer disease and other dementias	5 to 9	Female	0 (0 to 0)	0.0 (0.0 to 0.0)	0 (0 to 0)	0.0 (0.0 to 0.0)	0 (0 to 0)	0.0 (0.0 to 0.0)	0.0 (0.0 to 0.0)	0.0 (0.0 to 0.0)
Croatia	Alzheimer disease and other dementias	5 to 9	Male	0 (0 to 0)	0.0 (0.0 to 0.0)	0 (0 to 0)	0.0 (0.0 to 0.0)	0 (0 to 0)	0.0 (0.0 to 0.0)	0.0 (0.0 to 0.0)	0.0 (0.0 to 0.0)
Croatia	Alzheimer disease and other dementias	10 to 14	Both	0 (0 to 0)	0.0 (0.0 to 0.0)	0 (0 to 0)	0.0 (0.0 to 0.0)	0 (0 to 0)	0.0 (0.0 to 0.0)	0.0 (0.0 to 0.0)	0.0 (0.0 to 0.0)

eTable 8. All-cause and cause-specific mortality (counts and rates) at the global level and by country in the WHO European Region for ages 5 to 9 and 10 to 14 for 1990, 2000, and 2016

Croatia	Alzheimer disease and other dementias	10 to 14	Female	0 (0 to 0)	0.0 (0.0 to 0.0)	0 (0 to 0)	0.0 (0.0 to 0.0)	0 (0 to 0)	0.0 (0.0 to 0.0)	0.0 (0.0 to 0.0)	0.0 (0.0 to 0.0)
Croatia	Alzheimer disease and other dementias	10 to 14	Male	0 (0 to 0)	0.0 (0.0 to 0.0)	0 (0 to 0)	0.0 (0.0 to 0.0)	0 (0 to 0)	0.0 (0.0 to 0.0)	0.0 (0.0 to 0.0)	0.0 (0.0 to 0.0)
Croatia	Parkinson disease	5 to 9	Both	0 (0 to 0)	0.0 (0.0 to 0.0)	0 (0 to 0)	0.0 (0.0 to 0.0)	0 (0 to 0)	0.0 (0.0 to 0.0)	0.0 (0.0 to 0.0)	0.0 (0.0 to 0.0)
Croatia	Parkinson disease	5 to 9	Female	0 (0 to 0)	0.0 (0.0 to 0.0)	0 (0 to 0)	0.0 (0.0 to 0.0)	0 (0 to 0)	0.0 (0.0 to 0.0)	0.0 (0.0 to 0.0)	0.0 (0.0 to 0.0)
Croatia	Parkinson disease	5 to 9	Male	0 (0 to 0)	0.0 (0.0 to 0.0)	0 (0 to 0)	0.0 (0.0 to 0.0)	0 (0 to 0)	0.0 (0.0 to 0.0)	0.0 (0.0 to 0.0)	0.0 (0.0 to 0.0)
Croatia	Parkinson disease	10 to 14	Both	0 (0 to 0)	0.0 (0.0 to 0.0)	0 (0 to 0)	0.0 (0.0 to 0.0)	0 (0 to 0)	0.0 (0.0 to 0.0)	0.0 (0.0 to 0.0)	0.0 (0.0 to 0.0)
Croatia	Parkinson disease	10 to 14	Female	0 (0 to 0)	0.0 (0.0 to 0.0)	0 (0 to 0)	0.0 (0.0 to 0.0)	0 (0 to 0)	0.0 (0.0 to 0.0)	0.0 (0.0 to 0.0)	0.0 (0.0 to 0.0)
Croatia	Parkinson disease	10 to 14	Male	0 (0 to 0)	0.0 (0.0 to 0.0)	0 (0 to 0)	0.0 (0.0 to 0.0)	0 (0 to 0)	0.0 (0.0 to 0.0)	0.0 (0.0 to 0.0)	0.0 (0.0 to 0.0)
Croatia	Epilepsy	5 to 9	Both	3 (2 to 3)	0.8 (0.6 to 1.0)	1 (1 to 2)	0.6 (0.4 to 0.7)	1 (1 to 1)	0.5 (0.3 to 0.6)	-61.4 (-74.4 to -42.8)	-42.9 (-62.2 to -15.4)
Croatia	Epilepsy	5 to 9	Female	1 (1 to 2)	0.9 (0.6 to 1.2)	1 (0 to 1)	0.5 (0.3 to 0.7)	0 (0 to 1)	0.5 (0.3 to 0.7)	-64.2 (-79.2 to -37.1)	-47.0 (-69.1 to -6.8)
Croatia	Epilepsy	5 to 9	Male	1 (1 to 2)	0.8 (0.5 to 1.0)	1 (1 to 1)	0.7 (0.5 to 0.9)	1 (0 to 1)	0.5 (0.3 to 0.7)	-58.4 (-74.4 to -33.0)	-38.5 (-62.1 to -0.9)
Croatia	Epilepsy	10 to 14	Both	2 (2 to 3)	0.6 (0.5 to 0.8)	1 (1 to 2)	0.5 (0.4 to 0.6)	1 (1 to 1)	0.5 (0.3 to 0.6)	-53.8 (-68.6 to -31.3)	-26.3 (-49.9 to 9.7)
Croatia	Epilepsy	10 to 14	Female	1 (1 to 1)	0.6 (0.4 to 0.8)	0 (0 to 1)	0.3 (0.2 to 0.5)	0 (0 to 1)	0.4 (0.2 to 0.6)	-57.4 (-75.6 to -25.7)	-31.8 (-61.0 to 18.9)
Croatia	Epilepsy	10 to 14	Male	1 (1 to 2)	0.7 (0.5 to 1.0)	1 (1 to 1)	0.6 (0.4 to 0.8)	1 (0 to 1)	0.6 (0.4 to 0.8)	-51.2 (-70.6 to -16.0)	-22.3 (-53.2 to 33.8)
Croatia	Motor neuron disease	5 to 9	Both	0 (0 to 1)	0.1 (0.1 to 0.2)	0 (0 to 0)	0.1 (0.1 to 0.1)	0 (0 to 0)	0.1 (0.0 to 0.1)	-59.3 (-77.2 to -30.5)	-39.8 (-66.3 to 2.9)
Croatia	Motor neuron disease	5 to 9	Female	0 (0 to 0)	0.2 (0.1 to 0.3)	0 (0 to 0)	0.2 (0.1 to 0.3)	0 (0 to 0)	0.1 (0.1 to 0.2)	-56.0 (-77.1 to -15.4)	-34.8 (-66.0 to 25.3)
Croatia	Motor neuron disease	5 to 9	Male	0 (0 to 0)	0.1 (0.0 to 0.1)	0 (0 to 0)	0.0 (0.0 to 0.1)	0 (0 to 0)	0.0 (0.0 to 0.0)	-71.1 (-87.9 to -31.4)	-57.3 (-82.0 to 1.5)
Croatia	Motor neuron disease	10 to 14	Both	0 (0 to 0)	0.1 (0.1 to 0.1)	0 (0 to 0)	0.1 (0.1 to 0.1)	0 (0 to 0)	0.1 (0.1 to 0.1)	-52.1 (-69.6 to -26.6)	-23.6 (-51.5 to 17.2)
Croatia	Motor neuron disease	10 to 14	Female	0 (0 to 0)	0.1 (0.1 to 0.2)	0 (0 to 0)	0.1 (0.1 to 0.1)	0 (0 to 0)	0.1 (0.1 to 0.1)	-46.0 (-68.6 to -7.5)	-13.5 (-49.8 to 48.0)
Croatia	Motor neuron disease	10 to 14	Male	0 (0 to 0)	0.1 (0.1 to 0.1)	0 (0 to 0)	0.1 (0.0 to 0.1)	0 (0 to 0)	0.1 (0.0 to 0.1)	-59.3 (-80.3 to -25.0)	-35.1 (-68.6 to 19.5)
Croatia	Other neurological disorders	5 to 9	Both	1 (0 to 1)	0.2 (0.1 to 0.2)	0 (0 to 1)	0.2 (0.1 to 0.2)	0 (0 to 1)	0.2 (0.1 to 0.2)	-38.1 (-60.2 to -1.3)	-8.4 (-41.1 to 46.1)
Croatia	Other neurological disorders	5 to 9	Female	0 (0 to 0)	0.2 (0.2 to 0.3)	0 (0 to 0)	0.2 (0.1 to 0.2)	0 (0 to 0)	0.2 (0.1 to 0.3)	-35.3 (-62.2 to 16.2)	-4.2 (-44.0 to 72.1)
Croatia	Other neurological disorders	5 to 9	Male	0 (0 to 0)	0.2 (0.1 to 0.2)	0 (0 to 0)	0.2 (0.1 to 0.2)	0 (0 to 0)	0.1 (0.1 to 0.2)	-41.8 (-71.4 to 9.4)	-13.8 (-57.8 to 61.8)
Croatia	Other neurological disorders	10 to 14	Both	1 (1 to 1)	0.3 (0.2 to 0.4)	1 (1 to 1)	0.3 (0.2 to 0.4)	1 (0 to 1)	0.3 (0.2 to 0.4)	-32.5 (-56.6 to 4.0)	7.7 (-30.7 to 66.1)
Croatia	Other neurological disorders	10 to 14	Female	0 (0 to 0)	0.1 (0.1 to 0.2)	0 (0 to 0)	0.1 (0.1 to 0.1)	0 (0 to 0)	0.1 (0.1 to 0.2)	-33.8 (-64.5 to 24.5)	6.0 (-43.2 to 99.2)
Croatia	Other neurological disorders	10 to 14	Male	1 (1 to 1)	0.4 (0.3 to 0.6)	1 (0 to 1)	0.5 (0.3 to 0.7)	0 (0 to 1)	0.5 (0.3 to 0.7)	-32.1 (-61.1 to 15.6)	8.2 (-38.0 to 84.1)
Croatia	Drug use disorders	5 to 9	Both	0 (0 to 0)	0.0 (0.0 to 0.0)	0 (0 to 0)	0.0 (0.0 to 0.0)	0 (0 to 0)	0.0 (0.0 to 0.0)	0.0 (0.0 to 0.0)	0.0 (0.0 to 0.0)

eTable 8. All-cause and cause-specific mortality (counts and rates) at the global level and by country in the WHO European Region for ages 5 to 9 and 10 to 14 for 1990, 2000, and 2016

Croatia	Drug use disorders	5 to 9	Female	0 (0 to 0)	0.0 (0.0 to 0.0)	0 (0 to 0)	0.0 (0.0 to 0.0)	0 (0 to 0)	0.0 (0.0 to 0.0)	0.0 (0.0 to 0.0)	0.0 (0.0 to 0.0)
Croatia	Drug use disorders	5 to 9	Male	0 (0 to 0)	0.0 (0.0 to 0.0)	0 (0 to 0)	0.0 (0.0 to 0.0)	0 (0 to 0)	0.0 (0.0 to 0.0)	0.0 (0.0 to 0.0)	0.0 (0.0 to 0.0)
Croatia	Drug use disorders	10 to 14	Both	0 (0 to 0)	0.0 (0.0 to 0.0)	0 (0 to 0)	0.0 (0.0 to 0.0)	0 (0 to 0)	0.0 (0.0 to 0.0)	0.0 (0.0 to 0.0)	0.0 (0.0 to 0.0)
Croatia	Drug use disorders	10 to 14	Female	0 (0 to 0)	0.0 (0.0 to 0.0)	0 (0 to 0)	0.0 (0.0 to 0.0)	0 (0 to 0)	0.0 (0.0 to 0.0)	0.0 (0.0 to 0.0)	0.0 (0.0 to 0.0)
Croatia	Drug use disorders	10 to 14	Male	0 (0 to 0)	0.0 (0.0 to 0.0)	0 (0 to 0)	0.0 (0.0 to 0.0)	0 (0 to 0)	0.0 (0.0 to 0.0)	0.0 (0.0 to 0.0)	0.0 (0.0 to 0.0)
Croatia	Eating disorders	5 to 9	Both	0 (0 to 0)	0.0 (0.0 to 0.0)	0 (0 to 0)	0.0 (0.0 to 0.0)	0 (0 to 0)	0.0 (0.0 to 0.0)	334.0 (107.9 to 790.4)	542.4 (207.8 to 1217.9)
Croatia	Eating disorders	5 to 9	Female	0 (0 to 0)	0.0 (0.0 to 0.0)	0 (0 to 0)	0.0 (0.0 to 0.0)	0 (0 to 0)	0.0 (0.0 to 0.0)	243.8 (28.4 to 687.7)	409.1 (90.1 to 1066.5)
Croatia	Eating disorders	5 to 9	Male	0 (0 to 0)	0.0 (0.0 to 0.0)	0 (0 to 0)	0.0 (0.0 to 0.0)	0 (0 to 0)	0.0 (0.0 to 0.0)	367.0 (96.4 to 989.6)	591.0 (190.6 to 1512.1)
Croatia	Eating disorders	10 to 14	Both	0 (0 to 0)	0.0 (0.0 to 0.0)	0 (0 to 0)	0.0 (0.0 to 0.0)	0 (0 to 0)	0.0 (0.0 to 0.0)	1127.1 (432.5 to 2358.4)	1859.0 (750.2 to 3824.7)
Croatia	Eating disorders	10 to 14	Female	0 (0 to 0)	0.0 (0.0 to 0.0)	0 (0 to 0)	0.0 (0.0 to 0.0)	0 (0 to 0)	0.0 (0.0 to 0.0)	1534.6 (476.3 to 3457.2)	2515.3 (822.1 to 5591.2)
Croatia	Eating disorders	10 to 14	Male	0 (0 to 0)	0.0 (0.0 to 0.0)	0 (0 to 0)	0.0 (0.0 to 0.0)	0 (0 to 0)	0.0 (0.0 to 0.0)	221.9 (37.0 to 604.4)	412.8 (118.3 to 1022.2)
Croatia	Diabetes mellitus	5 to 9	Both	0 (0 to 0)	0.0 (0.0 to 0.0)	0 (0 to 0)	0.0 (0.0 to 0.0)	0 (0 to 0)	0.0 (0.0 to 0.0)	-52.0 (-64.3 to -36.9)	-29.0 (-47.2 to -6.6)
Croatia	Diabetes mellitus	5 to 9	Female	0 (0 to 0)	0.0 (0.0 to 0.0)	0 (0 to 0)	0.0 (0.0 to 0.0)	0 (0 to 0)	0.0 (0.0 to 0.0)	-55.0 (-68.5 to -37.3)	-33.4 (-53.3 to -7.1)
Croatia	Diabetes mellitus	5 to 9	Male	0 (0 to 0)	0.0 (0.0 to 0.0)	0 (0 to 0)	0.0 (0.0 to 0.0)	0 (0 to 0)	0.0 (0.0 to 0.0)	-48.9 (-65.8 to -28.1)	-24.4 (-49.4 to 6.4)
Croatia	Diabetes mellitus	10 to 14	Both	0 (0 to 0)	0.0 (0.0 to 0.1)	0 (0 to 0)	0.0 (0.0 to 0.0)	0 (0 to 0)	0.0 (0.0 to 0.0)	-68.9 (-79.2 to -52.9)	-50.3 (-66.7 to -24.8)
Croatia	Diabetes mellitus	10 to 14	Female	0 (0 to 0)	0.0 (0.0 to 0.0)	0 (0 to 0)	0.0 (0.0 to 0.0)	0 (0 to 0)	0.0 (0.0 to 0.0)	-70.3 (-80.1 to -54.0)	-52.4 (-68.1 to -26.4)
Croatia	Diabetes mellitus	10 to 14	Male	0 (0 to 0)	0.1 (0.0 to 0.1)	0 (0 to 0)	0.0 (0.0 to 0.1)	0 (0 to 0)	0.0 (0.0 to 0.0)	-68.4 (-81.7 to -46.6)	-49.6 (-70.8 to -14.9)
Croatia	Acute glomerulonephritis	5 to 9	Both	0 (0 to 0)	0.0 (0.0 to 0.0)	0 (0 to 0)	0.0 (0.0 to 0.0)	0 (0 to 0)	0.0 (0.0 to 0.0)	-40.2 (-51.4 to -27.0)	-11.5 (-28.0 to 8.0)
Croatia	Acute glomerulonephritis	5 to 9	Female	0 (0 to 0)	0.0 (0.0 to 0.0)	0 (0 to 0)	0.0 (0.0 to 0.0)	0 (0 to 0)	0.0 (0.0 to 0.0)	-40.6 (-52.9 to -25.7)	-12.0 (-30.3 to 10.0)
Croatia	Acute glomerulonephritis	5 to 9	Male	0 (0 to 0)	0.0 (0.0 to 0.0)	0 (0 to 0)	0.0 (0.0 to 0.0)	0 (0 to 0)	0.0 (0.0 to 0.0)	-39.9 (-53.4 to -23.8)	-11.1 (-31.1 to 12.8)
Croatia	Acute glomerulonephritis	10 to 14	Both	0 (0 to 0)	0.0 (0.0 to 0.0)	0 (0 to 0)	0.0 (0.0 to 0.0)	0 (0 to 0)	0.0 (0.0 to 0.0)	-42.2 (-50.6 to -33.7)	-7.8 (-21.1 to 5.9)
Croatia	Acute glomerulonephritis	10 to 14	Female	0 (0 to 0)	0.0 (0.0 to 0.0)	0 (0 to 0)	0.0 (0.0 to 0.0)	0 (0 to 0)	0.0 (0.0 to 0.0)	-42.7 (-52.7 to -31.2)	-8.2 (-24.3 to 10.1)
Croatia	Acute glomerulonephritis	10 to 14	Male	0 (0 to 0)	0.0 (0.0 to 0.0)	0 (0 to 0)	0.0 (0.0 to 0.0)	0 (0 to 0)	0.0 (0.0 to 0.0)	-41.9 (-52.5 to -29.9)	-7.4 (-24.4 to 11.7)
Croatia	Chronic kidney disease	5 to 9	Both	0 (0 to 0)	0.1 (0.1 to 0.1)	0 (0 to 0)	0.1 (0.1 to 0.1)	0 (0 to 0)	0.0 (0.0 to 0.1)	-66.5 (-77.2 to -52.8)	-50.4 (-66.2 to -30.2)
Croatia	Chronic kidney disease	5 to 9	Female	0 (0 to 0)	0.1 (0.1 to 0.2)	0 (0 to 0)	0.1 (0.1 to 0.1)	0 (0 to 0)	0.1 (0.0 to 0.1)	-61.8 (-76.3 to -41.8)	-43.5 (-65.0 to -13.9)
Croatia	Chronic kidney disease	5 to 9	Male	0 (0 to 0)	0.1 (0.1 to 0.1)	0 (0 to 0)	0.1 (0.1 to 0.1)	0 (0 to 0)	0.0 (0.0 to 0.0)	-72.7 (-83.0 to -55.1)	-59.6 (-74.8 to -33.5)
Croatia	Chronic kidney disease	10 to 14	Both	0 (0 to 1)	0.1 (0.1 to 0.2)	0 (0 to 0)	0.1 (0.1 to 0.1)	0 (0 to 0)	0.1 (0.1 to 0.1)	-60.9 (-71.1 to -46.5)	-37.6 (-53.9 to -14.6)
Croatia	Chronic kidney disease	10 to 14	Female	0 (0 to 0)	0.2 (0.1 to 0.2)	0 (0 to 0)	0.1 (0.1 to 0.2)	0 (0 to 0)	0.1 (0.1 to 0.1)	-58.2 (-72.7 to -36.9)	-33.1 (-56.4 to 1.0)
Croatia	Chronic kidney disease	10 to 14	Male	0 (0 to 0)	0.1 (0.1 to 0.1)	0 (0 to 0)	0.1 (0.1 to 0.1)	0 (0 to 0)	0.1 (0.0 to 0.1)	-65.3 (-77.5 to -46.7)	-44.7 (-64.2 to -15.1)

eTable 8. All-cause and cause-specific mortality (counts and rates) at the global level and by country in the WHO European Region for ages 5 to 9 and 10 to 14 for 1990, 2000, and 2016

Croatia	Urinary diseases and male infertility	5 to 9	Both	0<br>(0 to 0)	0.0<br>(0.0 to 0.1)	0<br>(0 to 0)	0.0<br>(0.0 to 0.0)	0<br>(0 to 0)	0.0<br>(0.0 to 0.0)	-73.1<br>(-83.3 to -56.9)	-60.2<br>(-75.3 to -36.3)
Croatia	Urinary diseases and male infertility	5 to 9	Female	0<br>(0 to 0)	0.0<br>(0.0 to 0.1)	0<br>(0 to 0)	0.0<br>(0.0 to 0.0)	0<br>(0 to 0)	0.0<br>(0.0 to 0.0)	-69.0<br>(-81.5 to -48.8)	-54.1<br>(-72.5 to -24.2)
Croatia	Urinary diseases and male infertility	5 to 9	Male	0<br>(0 to 0)	0.0<br>(0.0 to 0.1)	0<br>(0 to 0)	0.0<br>(0.0 to 0.0)	0<br>(0 to 0)	0.0<br>(0.0 to 0.0)	-76.4<br>(-88.7 to -49.4)	-65.1<br>(-83.3 to -25.1)
Croatia	Urinary diseases and male infertility	10 to 14	Both	0<br>(0 to 0)	0.1<br>(0.1 to 0.1)	0<br>(0 to 0)	0.0<br>(0.0 to 0.0)	0<br>(0 to 0)	0.0<br>(0.0 to 0.0)	-67.8<br>(-77.3 to -53.8)	-48.7<br>(-63.8 to -26.2)
Croatia	Urinary diseases and male infertility	10 to 14	Female	0<br>(0 to 0)	0.1<br>(0.0 to 0.1)	0<br>(0 to 0)	0.0<br>(0.0 to 0.0)	0<br>(0 to 0)	0.0<br>(0.0 to 0.0)	-62.8<br>(-77.0 to -39.3)	-40.5<br>(-63.2 to -2.9)
Croatia	Urinary diseases and male infertility	10 to 14	Male	0<br>(0 to 0)	0.1<br>(0.1 to 0.1)	0<br>(0 to 0)	0.0<br>(0.0 to 0.1)	0<br>(0 to 0)	0.0<br>(0.0 to 0.0)	-71.5<br>(-82.5 to -55.4)	-54.7<br>(-72.1 to -29.0)
Croatia	Hemoglobinopathies and hemolytic anemias	5 to 9	Both	0<br>(0 to 1)	0.1<br>(0.1 to 0.2)	0<br>(0 to 0)	0.1<br>(0.1 to 0.2)	0<br>(0 to 0)	0.0<br>(0.0 to 0.0)	-84.9<br>(-90.2 to -76.9)	-77.7<br>(-85.5 to -65.8)
Croatia	Hemoglobinopathies and hemolytic anemias	5 to 9	Female	0<br>(0 to 0)	0.1<br>(0.0 to 0.1)	0<br>(0 to 0)	0.0<br>(0.0 to 0.1)	0<br>(0 to 0)	0.0<br>(0.0 to 0.0)	-82.8<br>(-89.7 to -73.1)	-74.5<br>(-84.7 to -60.2)
Croatia	Hemoglobinopathies and hemolytic anemias	5 to 9	Male	0<br>(0 to 0)	0.2<br>(0.2 to 0.3)	0<br>(0 to 0)	0.2<br>(0.1 to 0.3)	0<br>(0 to 0)	0.0<br>(0.0 to 0.1)	-85.4<br>(-91.3 to -75.7)	-78.4<br>(-87.2 to -64.0)
Croatia	Hemoglobinopathies and hemolytic anemias	10 to 14	Both	0<br>(0 to 1)	0.1<br>(0.1 to 0.2)	0<br>(0 to 0)	0.1<br>(0.1 to 0.2)	0<br>(0 to 0)	0.1<br>(0.0 to 0.1)	-75.0<br>(-85.2 to -56.6)	-60.0<br>(-76.4 to -30.7)
Croatia	Hemoglobinopathies and hemolytic anemias	10 to 14	Female	0<br>(0 to 0)	0.1<br>(0.1 to 0.2)	0<br>(0 to 0)	0.1<br>(0.1 to 0.2)	0<br>(0 to 0)	0.1<br>(0.0 to 0.2)	-69.3<br>(-85.9 to -40.3)	-50.8<br>(-77.4 to -4.5)
Croatia	Hemoglobinopathies and hemolytic anemias	10 to 14	Male	0<br>(0 to 0)	0.1<br>(0.1 to 0.2)	0<br>(0 to 0)	0.1<br>(0.1 to 0.2)	0<br>(0 to 0)	0.0<br>(0.0 to 0.1)	-79.9<br>(-88.3 to -66.0)	-68.0<br>(-81.3 to -45.8)
Croatia	Endocrine, metabolic, blood, and immune disorders	5 to 9	Both	2<br>(1 to 3)	0.6<br>(0.3 to 0.8)	1<br>(1 to 1)	0.4<br>(0.3 to 0.5)	1<br>(0 to 1)	0.3<br>(0.2 to 0.4)	-69.3<br>(-81.3 to -35.2)	-54.6<br>(-72.4 to -4.1)
Croatia	Endocrine, metabolic, blood, and immune disorders	5 to 9	Female	1<br>(0 to 1)	0.5<br>(0.3 to 0.7)	0<br>(0 to 0)	0.3<br>(0.2 to 0.4)	0<br>(0 to 0)	0.2<br>(0.1 to 0.3)	-72.8<br>(-85.7 to -40.5)	-59.8<br>(-78.8 to -12.0)
Croatia	Endocrine, metabolic, blood, and immune disorders	5 to 9	Male	1<br>(1 to 2)	0.7<br>(0.4 to 1.0)	1<br>(0 to 1)	0.5<br>(0.3 to 0.8)	0<br>(0 to 1)	0.3<br>(0.2 to 0.6)	-67.0<br>(-82.0 to -11.4)	-51.2<br>(-73.4 to 31.1)
Croatia	Endocrine, metabolic, blood, and immune disorders	10 to 14	Both	2<br>(1 to 2)	0.5<br>(0.3 to 0.7)	1<br>(1 to 1)	0.4<br>(0.3 to 0.5)	1<br>(0 to 1)	0.3<br>(0.2 to 0.4)	-65.2<br>(-77.4 to -31.0)	-44.5<br>(-63.9 to 10.2)

eTable 8. All-cause and cause-specific mortality (counts and rates) at the global level and by country in the WHO European Region for ages 5 to 9 and 10 to 14 for 1990, 2000, and 2016

Croatia	Endocrine, metabolic, blood, and immune disorders	10 to 14	Female	1 (0 to 1)	0.5 (0.2 to 0.7)	0 (0 to 1)	0.3 (0.2 to 0.4)	0 (0 to 0)	0.2 (0.1 to 0.3)	-71.2 (-83.3 to -43.7)	-53.9 (-73.3 to -10.0)
Croatia	Endocrine, metabolic, blood, and immune disorders	10 to 14	Male	1 (1 to 1)	0.6 (0.3 to 0.8)	1 (0 to 1)	0.4 (0.3 to 0.6)	0 (0 to 1)	0.4 (0.2 to 0.6)	-60.5 (-78.0 to -8.5)	-37.1 (-64.9 to 45.7)
Croatia	Rheumatoid arthritis	5 to 9	Both	0 (0 to 0)	0.0 (0.0 to 0.0)	0 (0 to 0)	0.0 (0.0 to 0.0)	0 (0 to 0)	0.0 (0.0 to 0.0)	-23.2 (-55.7 to 33.3)	13.7 (-34.4 to 97.3)
Croatia	Rheumatoid arthritis	5 to 9	Female	0 (0 to 0)	0.0 (0.0 to 0.0)	0 (0 to 0)	0.0 (0.0 to 0.0)	0 (0 to 0)	0.0 (0.0 to 0.0)	-14.1 (-59.5 to 67.6)	27.2 (-40.0 to 148.2)
Croatia	Rheumatoid arthritis	5 to 9	Male	0 (0 to 0)	0.0 (0.0 to 0.0)	0 (0 to 0)	0.0 (0.0 to 0.0)	0 (0 to 0)	0.0 (0.0 to 0.0)	-31.6 (-63.1 to 39.7)	1.1 (-45.4 to 106.7)
Croatia	Rheumatoid arthritis	10 to 14	Both	0 (0 to 0)	0.0 (0.0 to 0.0)	0 (0 to 0)	0.0 (0.0 to 0.0)	0 (0 to 0)	0.0 (0.0 to 0.0)	13.9 (-38.9 to 108.5)	81.8 (-2.5 to 232.8)
Croatia	Rheumatoid arthritis	10 to 14	Female	0 (0 to 0)	0.0 (0.0 to 0.0)	0 (0 to 0)	0.0 (0.0 to 0.0)	0 (0 to 0)	0.0 (0.0 to 0.1)	45.4 (-39.8 to 232.5)	132.6 (-3.7 to 432.0)
Croatia	Rheumatoid arthritis	10 to 14	Male	0 (0 to 0)	0.0 (0.0 to 0.0)	0 (0 to 0)	0.0 (0.0 to 0.0)	0 (0 to 0)	0.0 (0.0 to 0.0)	-27.2 (-62.3 to 50.3)	16.0 (-40.0 to 139.5)
Croatia	Other musculoskeletal disorders	5 to 9	Both	0 (0 to 0)	0.1 (0.0 to 0.1)	0 (0 to 0)	0.1 (0.0 to 0.1)	0 (0 to 0)	0.1 (0.0 to 0.1)	-43.2 (-75.6 to -10.4)	-15.9 (-63.9 to 32.6)
Croatia	Other musculoskeletal disorders	5 to 9	Female	0 (0 to 0)	0.1 (0.1 to 0.1)	0 (0 to 0)	0.1 (0.0 to 0.1)	0 (0 to 0)	0.1 (0.0 to 0.1)	-44.8 (-75.7 to -5.2)	-18.3 (-64.1 to 40.4)
Croatia	Other musculoskeletal disorders	5 to 9	Male	0 (0 to 0)	0.0 (0.0 to 0.1)	0 (0 to 0)	0.1 (0.0 to 0.1)	0 (0 to 0)	0.0 (0.0 to 0.1)	-40.6 (-77.2 to 8.8)	-12.1 (-66.3 to 60.9)
Croatia	Other musculoskeletal disorders	10 to 14	Both	0 (0 to 0)	0.1 (0.1 to 0.1)	0 (0 to 0)	0.1 (0.1 to 0.1)	0 (0 to 0)	0.1 (0.1 to 0.2)	-36.5 (-71.6 to -2.0)	1.4 (-54.6 to 56.5)
Croatia	Other musculoskeletal disorders	10 to 14	Female	0 (0 to 0)	0.2 (0.1 to 0.2)	0 (0 to 0)	0.1 (0.1 to 0.2)	0 (0 to 0)	0.1 (0.1 to 0.2)	-41.4 (-74.8 to 0.7)	-6.2 (-59.7 to 61.1)
Croatia	Other musculoskeletal disorders	10 to 14	Male	0 (0 to 0)	0.1 (0.0 to 0.1)	0 (0 to 0)	0.1 (0.1 to 0.1)	0 (0 to 0)	0.1 (0.0 to 0.1)	-24.4 (-70.4 to 32.4)	20.4 (-52.8 to 110.9)
Croatia	Congenital birth defects	5 to 9	Both	7 (5 to 10)	2.0 (1.5 to 3.0)	4 (3 to 5)	1.6 (1.3 to 2.2)	2 (2 to 3)	1.0 (0.7 to 1.4)	-67.3 (-78.0 to -51.9)	-51.6 (-67.5 to -28.8)
Croatia	Congenital birth defects	5 to 9	Female	3 (2 to 5)	1.8 (1.2 to 3.2)	2 (1 to 3)	1.6 (1.1 to 2.3)	1 (1 to 1)	1.0 (0.7 to 1.4)	-63.9 (-78.1 to -39.8)	-46.5 (-67.6 to -10.8)
Croatia	Congenital birth defects	5 to 9	Male	4 (3 to 5)	2.2 (1.5 to 3.2)	2 (1 to 3)	1.7 (1.2 to 2.5)	1 (1 to 2)	1.0 (0.7 to 1.5)	-69.8 (-81.9 to -49.2)	-55.3 (-73.3 to -24.8)
Croatia	Congenital birth defects	10 to 14	Both	5 (4 to 7)	1.5 (1.2 to 2.2)	3 (3 to 5)	1.2 (0.9 to 1.7)	2 (1 to 2)	0.9 (0.7 to 1.2)	-61.5 (-73.2 to -46.1)	-38.5 (-57.2 to -14.0)
Croatia	Congenital birth defects	10 to 14	Female	2 (2 to 4)	1.5 (1.1 to 2.4)	2 (1 to 2)	1.2 (0.9 to 1.7)	1 (1 to 1)	1.0 (0.7 to 1.3)	-60.7 (-74.9 to -39.0)	-37.1 (-59.8 to -2.4)
Croatia	Congenital birth defects	10 to 14	Male	2 (2 to 4)	1.5 (1.0 to 2.2)	2 (1 to 3)	1.2 (0.8 to 1.9)	1 (1 to 1)	0.9 (0.6 to 1.3)	-62.2 (-75.6 to -40.3)	-39.8 (-61.2 to -5.0)
Croatia	Skin and subcutaneous diseases	5 to 9	Both	0 (0 to 0)	0.0 (0.0 to 0.0)	0 (0 to 0)	0.0 (0.0 to 0.0)	0 (0 to 0)	0.0 (0.0 to 0.0)	-38.7 (-51.8 to -23.8)	-9.3 (-28.6 to 12.8)
Croatia	Skin and subcutaneous diseases	5 to 9	Female	0 (0 to 0)	0.0 (0.0 to 0.0)	0 (0 to 0)	0.0 (0.0 to 0.0)	0 (0 to 0)	0.0 (0.0 to 0.0)	-37.4 (-52.0 to -17.7)	-7.2 (-28.9 to 21.9)
Croatia	Skin and subcutaneous diseases	5 to 9	Male	0 (0 to 0)	0.0 (0.0 to 0.0)	0 (0 to 0)	0.0 (0.0 to 0.0)	0 (0 to 0)	0.0 (0.0 to 0.0)	-40.0 (-55.5 to -22.2)	-11.2 (-34.2 to 15.1)
Croatia	Skin and subcutaneous diseases	10 to 14	Both	0 (0 to 0)	0.0 (0.0 to 0.0)	0 (0 to 0)	0.0 (0.0 to 0.0)	0 (0 to 0)	0.0 (0.0 to 0.0)	-45.6 (-62.0 to -27.7)	-13.1 (-39.3 to 15.5)

eTable 8. All-cause and cause-specific mortality (counts and rates) at the global level and by country in the WHO European Region for ages 5 to 9 and 10 to 14 for 1990, 2000, and 2016

Croatia	Skin and subcutaneous diseases	10 to 14	Female	0 (0 to 0)	0.0 (0.0 to 0.0)	0 (0 to 0)	0.0 (0.0 to 0.0)	0 (0 to 0)	0.0 (0.0 to 0.0)	-40.1 (-52.1 to -25.1)	-4.2 (-23.3 to 19.9)
Croatia	Skin and subcutaneous diseases	10 to 14	Male	0 (0 to 0)	0.0 (0.0 to 0.0)	0 (0 to 0)	0.0 (0.0 to 0.0)	0 (0 to 0)	0.0 (0.0 to 0.0)	-49.1 (-70.8 to -21.6)	-19.0 (-53.4 to 24.9)
Croatia	Road injuries	5 to 9	Both	28 (24 to 32)	8.5 (7.3 to 9.8)	12 (10 to 14)	5.1 (4.4 to 5.9)	4 (3 to 5)	1.9 (1.5 to 2.4)	-84.9 (-88.6 to -79.8)	-77.6 (-83.2 to -70.1)
Croatia	Road injuries	5 to 9	Female	10 (8 to 13)	6.7 (5.0 to 8.3)	4 (3 to 5)	3.7 (2.9 to 4.7)	2 (1 to 2)	1.6 (1.1 to 2.2)	-84.1 (-89.8 to -76.2)	-76.4 (-84.9 to -64.8)
Croatia	Road injuries	5 to 9	Male	17 (14 to 20)	10.3 (8.5 to 12.1)	8 (7 to 9)	6.4 (5.3 to 7.6)	3 (2 to 3)	2.2 (1.6 to 3.0)	-85.4 (-89.6 to -79.2)	-78.3 (-84.6 to -69.3)
Croatia	Road injuries	10 to 14	Both	19 (16 to 23)	5.9 (5.0 to 6.9)	11 (9 to 13)	4.0 (3.3 to 4.6)	4 (3 to 5)	1.9 (1.5 to 2.4)	-79.8 (-84.8 to -73.8)	-67.8 (-75.8 to -58.2)
Croatia	Road injuries	10 to 14	Female	6 (5 to 8)	3.9 (2.9 to 5.0)	3 (2 to 4)	2.3 (1.8 to 3.0)	1 (1 to 2)	1.2 (0.8 to 1.6)	-81.6 (-87.9 to -71.2)	-70.6 (-80.6 to -53.9)
Croatia	Road injuries	10 to 14	Male	13 (11 to 16)	7.8 (6.3 to 9.5)	8 (6 to 10)	5.6 (4.5 to 6.6)	3 (2 to 4)	2.6 (1.9 to 3.4)	-79.0 (-85.0 to -71.1)	-66.5 (-76.1 to -53.9)
Croatia	Other transport injuries	5 to 9	Both	1 (1 to 1)	0.3 (0.2 to 0.4)	1 (1 to 1)	0.3 (0.2 to 0.5)	0 (0 to 1)	0.2 (0.1 to 0.2)	-68.0 (-82.3 to -44.1)	-52.7 (-73.8 to -17.3)
Croatia	Other transport injuries	5 to 9	Female	0 (0 to 1)	0.3 (0.2 to 0.4)	0 (0 to 0)	0.2 (0.1 to 0.4)	0 (0 to 0)	0.1 (0.1 to 0.2)	-74.1 (-87.5 to -47.3)	-61.7 (-81.4 to -22.0)
Croatia	Other transport injuries	5 to 9	Male	1 (0 to 1)	0.3 (0.2 to 0.5)	1 (0 to 1)	0.4 (0.2 to 0.7)	0 (0 to 0)	0.2 (0.1 to 0.3)	-63.2 (-84.1 to -17.9)	-45.6 (-76.5 to 21.5)
Croatia	Other transport injuries	10 to 14	Both	1 (1 to 2)	0.4 (0.2 to 0.5)	1 (1 to 2)	0.4 (0.3 to 0.6)	0 (0 to 1)	0.2 (0.1 to 0.3)	-61.2 (-77.3 to -33.4)	-38.1 (-63.7 to 6.3)
Croatia	Other transport injuries	10 to 14	Female	0 (0 to 1)	0.3 (0.2 to 0.5)	0 (0 to 1)	0.2 (0.1 to 0.4)	0 (0 to 0)	0.1 (0.1 to 0.2)	-72.0 (-87.2 to -41.0)	-55.2 (-79.5 to -5.5)
Croatia	Other transport injuries	10 to 14	Male	1 (0 to 1)	0.4 (0.2 to 0.7)	1 (0 to 1)	0.6 (0.3 to 0.9)	0 (0 to 1)	0.3 (0.2 to 0.5)	-54.0 (-78.4 to -3.8)	-26.8 (-65.5 to 53.2)
Croatia	Falls	5 to 9	Both	1 (1 to 2)	0.3 (0.2 to 0.5)	1 (0 to 1)	0.2 (0.2 to 0.4)	0 (0 to 1)	0.1 (0.1 to 0.2)	-67.9 (-82.5 to -49.9)	-52.4 (-74.1 to -25.8)
Croatia	Falls	5 to 9	Female	1 (0 to 1)	0.4 (0.3 to 0.5)	0 (0 to 0)	0.3 (0.2 to 0.4)	0 (0 to 0)	0.2 (0.1 to 0.3)	-68.0 (-81.1 to -46.6)	-52.6 (-72.1 to -21.0)
Croatia	Falls	5 to 9	Male	0 (0 to 1)	0.2 (0.1 to 0.6)	0 (0 to 1)	0.2 (0.1 to 0.4)	0 (0 to 0)	0.1 (0.1 to 0.3)	-67.7 (-84.7 to -34.7)	-52.2 (-77.4 to -3.4)
Croatia	Falls	10 to 14	Both	1 (1 to 2)	0.3 (0.2 to 0.5)	1 (1 to 1)	0.3 (0.2 to 0.4)	0 (0 to 1)	0.2 (0.1 to 0.3)	-61.7 (-78.0 to -39.7)	-38.9 (-64.8 to -3.8)
Croatia	Falls	10 to 14	Female	0 (0 to 1)	0.2 (0.1 to 0.3)	0 (0 to 0)	0.1 (0.1 to 0.2)	0 (0 to 0)	0.1 (0.1 to 0.2)	-62.9 (-78.2 to -35.1)	-40.7 (-65.2 to 3.8)
Croatia	Falls	10 to 14	Male	1 (0 to 1)	0.5 (0.3 to 0.7)	1 (0 to 1)	0.4 (0.3 to 0.6)	0 (0 to 0)	0.3 (0.2 to 0.4)	-61.2 (-79.0 to -31.0)	-38.2 (-66.5 to 10.0)
Croatia	Drowning	5 to 9	Both	7 (5 to 8)	2.0 (1.6 to 2.5)	4 (3 to 4)	1.5 (1.2 to 1.8)	1 (1 to 2)	0.6 (0.4 to 0.8)	-80.3 (-86.1 to -71.4)	-70.8 (-79.4 to -57.7)
Croatia	Drowning	5 to 9	Female	1 (1 to 2)	0.9 (0.7 to 1.2)	1 (1 to 1)	0.6 (0.4 to 0.8)	0 (0 to 0)	0.3 (0.2 to 0.4)	-79.5 (-87.0 to -67.6)	-69.6 (-80.7 to -52.0)
Croatia	Drowning	5 to 9	Male	5 (4 to 7)	3.1 (2.3 to 4.0)	3 (2 to 4)	2.3 (1.7 to 2.9)	1 (1 to 1)	0.9 (0.6 to 1.3)	-80.5 (-87.4 to -69.7)	-71.1 (-81.3 to -55.1)
Croatia	Drowning	10 to 14	Both	7 (5 to 8)	2.1 (1.7 to 2.6)	4 (3 to 5)	1.5 (1.2 to 1.8)	2 (1 to 2)	0.7 (0.6 to 1.0)	-77.6 (-84.1 to -69.0)	-64.2 (-74.6 to -50.6)
Croatia	Drowning	10 to 14	Female	2 (1 to 2)	1.1 (0.8 to 1.5)	1 (1 to 1)	0.7 (0.5 to 1.0)	0 (0 to 1)	0.4 (0.3 to 0.5)	-77.8 (-86.0 to -65.9)	-64.5 (-77.5 to -45.5)
Croatia	Drowning	10 to 14	Male	5 (4 to 7)	3.0 (2.3 to 3.9)	3 (2 to 4)	2.2 (1.6 to 2.9)	1 (1 to 2)	1.1 (0.8 to 1.5)	-77.5 (-85.6 to -66.6)	-64.2 (-77.1 to -46.8)
Croatia	Fire, heat, and hot substances	5 to 9	Both	1 (1 to 2)	0.4 (0.3 to 0.5)	1 (0 to 1)	0.2 (0.2 to 0.3)	0 (0 to 0)	0.1 (0.1 to 0.1)	-86.5 (-90.9 to -78.7)	-80.0 (-86.5 to -68.5)
Croatia	Fire, heat, and hot substances	5 to 9	Female	1 (0 to 1)	0.4 (0.2 to 0.5)	0 (0 to 0)	0.2 (0.1 to 0.2)	0 (0 to 0)	0.1 (0.0 to 0.1)	-87.7 (-93.4 to -63.9)	-81.8 (-90.3 to -46.6)

eTable 8. All-cause and cause-specific mortality (counts and rates) at the global level and by country in the WHO European Region for ages 5 to 9 and 10 to 14 for 1990, 2000, and 2016

Croatia	Fire, heat, and hot substances	5 to 9	Male	1<br>(1 to 1)	0.5<br>(0.3 to 0.7)	0<br>(0 to 1)	0.3<br>(0.2 to 0.4)	0<br>(0 to 0)	0.1<br>(0.1 to 0.2)	-85.6<br>(-91.3 to -74.2)	-78.7<br>(-87.2 to -61.9)
Croatia	Fire, heat, and hot substances	10 to 14	Both	1<br>(0 to 1)	0.2<br>(0.1 to 0.2)	0<br>(0 to 0)	0.1<br>(0.1 to 0.2)	0<br>(0 to 0)	0.1<br>(0.0 to 0.1)	-80.2<br>(-86.1 to -71.1)	-68.4<br>(-77.8 to -53.8)
Croatia	Fire, heat, and hot substances	10 to 14	Female	0<br>(0 to 0)	0.1<br>(0.1 to 0.2)	0<br>(0 to 0)	0.1<br>(0.0 to 0.1)	0<br>(0 to 0)	0.0<br>(0.0 to 0.1)	-74.6<br>(-85.8 to -54.5)	-59.3<br>(-77.3 to -27.2)
Croatia	Fire, heat, and hot substances	10 to 14	Male	0<br>(0 to 1)	0.3<br>(0.2 to 0.4)	0<br>(0 to 0)	0.2<br>(0.1 to 0.2)	0<br>(0 to 0)	0.1<br>(0.1 to 0.1)	-82.0<br>(-88.5 to -71.9)	-71.4<br>(-81.6 to -55.2)
Croatia	Poisonings	5 to 9	Both	1<br>(0 to 2)	0.4<br>(0.1 to 0.5)	0<br>(0 to 0)	0.1<br>(0.1 to 0.1)	0<br>(0 to 0)	0.0<br>(0.0 to 0.1)	-94.3<br>(-97.1 to -71.7)	-91.6<br>(-95.7 to -58.1)
Croatia	Poisonings	5 to 9	Female	1<br>(0 to 1)	0.3<br>(0.1 to 0.5)	0<br>(0 to 0)	0.1<br>(0.0 to 0.1)	0<br>(0 to 0)	0.0<br>(0.0 to 0.1)	-93.2<br>(-97.2 to -65.1)	-90.0<br>(-95.8 to -48.3)
Croatia	Poisonings	5 to 9	Male	1<br>(0 to 1)	0.4<br>(0.1 to 0.6)	0<br>(0 to 0)	0.1<br>(0.1 to 0.1)	0<br>(0 to 0)	0.0<br>(0.0 to 0.1)	-95.2<br>(-97.9 to -64.8)	-92.9<br>(-96.9 to -47.9)
Croatia	Poisonings	10 to 14	Both	1<br>(0 to 2)	0.4<br>(0.1 to 0.6)	0<br>(0 to 0)	0.1<br>(0.1 to 0.1)	0<br>(0 to 0)	0.0<br>(0.0 to 0.1)	-93.6<br>(-96.4 to -69.6)	-89.9<br>(-94.3 to -51.5)
Croatia	Poisonings	10 to 14	Female	1<br>(0 to 1)	0.4<br>(0.1 to 0.7)	0<br>(0 to 0)	0.1<br>(0.1 to 0.2)	0<br>(0 to 0)	0.0<br>(0.0 to 0.1)	-93.1<br>(-97.0 to -65.0)	-89.0<br>(-95.1 to -43.9)
Croatia	Poisonings	10 to 14	Male	1<br>(0 to 1)	0.4<br>(0.1 to 0.7)	0<br>(0 to 0)	0.1<br>(0.1 to 0.2)	0<br>(0 to 0)	0.0<br>(0.0 to 0.1)	-94.2<br>(-97.3 to -63.9)	-90.7<br>(-95.6 to -42.5)
Croatia	Exposure to mechanical forces	5 to 9	Both	2<br>(2 to 3)	0.8<br>(0.6 to 0.9)	1<br>(1 to 1)	0.5<br>(0.4 to 0.6)	0<br>(0 to 1)	0.2<br>(0.1 to 0.3)	-81.6<br>(-88.3 to -72.2)	-72.8<br>(-82.7 to -58.9)
Croatia	Exposure to mechanical forces	5 to 9	Female	1<br>(0 to 1)	0.4<br>(0.3 to 0.6)	0<br>(0 to 0)	0.2<br>(0.1 to 0.3)	0<br>(0 to 0)	0.1<br>(0.1 to 0.2)	-82.6<br>(-90.8 to -58.9)	-74.2<br>(-86.4 to -39.1)
Croatia	Exposure to mechanical forces	5 to 9	Male	2<br>(1 to 2)	1.1<br>(0.8 to 1.4)	1<br>(1 to 1)	0.8<br>(0.5 to 1.0)	0<br>(0 to 1)	0.3<br>(0.2 to 0.4)	-81.3<br>(-89.2 to -71.2)	-72.3<br>(-84.1 to -57.4)
Croatia	Exposure to mechanical forces	10 to 14	Both	3<br>(2 to 3)	0.8<br>(0.5 to 1.0)	2<br>(1 to 2)	0.6<br>(0.4 to 0.7)	1<br>(0 to 1)	0.3<br>(0.2 to 0.4)	-78.7<br>(-85.6 to -69.8)	-66.0<br>(-77.1 to -51.9)
Croatia	Exposure to mechanical forces	10 to 14	Female	0<br>(0 to 1)	0.3<br>(0.2 to 0.4)	0<br>(0 to 0)	0.1<br>(0.1 to 0.2)	0<br>(0 to 0)	0.1<br>(0.1 to 0.2)	-76.0<br>(-86.8 to -55.3)	-61.6<br>(-78.9 to -28.5)
Croatia	Exposure to mechanical forces	10 to 14	Male	2<br>(2 to 3)	1.3<br>(0.9 to 1.7)	1<br>(1 to 2)	0.9<br>(0.6 to 1.2)	0<br>(0 to 1)	0.4<br>(0.3 to 0.6)	-79.3<br>(-87.2 to -69.5)	-67.0<br>(-79.7 to -51.4)
Croatia	Adverse effects of medical treatment	5 to 9	Both	0<br>(0 to 1)	0.1<br>(0.1 to 0.2)	0<br>(0 to 0)	0.1<br>(0.1 to 0.1)	0<br>(0 to 0)	0.1<br>(0.0 to 0.1)	-73.1<br>(-82.9 to -55.1)	-60.2<br>(-74.6 to -33.5)
Croatia	Adverse effects of medical treatment	5 to 9	Female	0<br>(0 to 0)	0.1<br>(0.1 to 0.2)	0<br>(0 to 0)	0.1<br>(0.1 to 0.1)	0<br>(0 to 0)	0.0<br>(0.0 to 0.1)	-77.4<br>(-86.7 to -68.3)	-66.5<br>(-80.2 to -53.0)
Croatia	Adverse effects of medical treatment	5 to 9	Male	0<br>(0 to 0)	0.1<br>(0.1 to 0.2)	0<br>(0 to 0)	0.1<br>(0.1 to 0.2)	0<br>(0 to 0)	0.1<br>(0.0 to 0.1)	-69.1<br>(-83.6 to -34.2)	-54.2<br>(-75.8 to -2.6)
Croatia	Adverse effects of medical treatment	10 to 14	Both	0<br>(0 to 1)	0.1<br>(0.1 to 0.2)	0<br>(0 to 0)	0.1<br>(0.1 to 0.1)	0<br>(0 to 0)	0.1<br>(0.0 to 0.1)	-70.0<br>(-79.5 to -53.6)	-52.1<br>(-67.3 to -26.0)
Croatia	Adverse effects of medical treatment	10 to 14	Female	0<br>(0 to 0)	0.1<br>(0.1 to 0.2)	0<br>(0 to 0)	0.1<br>(0.1 to 0.1)	0<br>(0 to 0)	0.1<br>(0.0 to 0.1)	-75.1<br>(-84.2 to -64.7)	-60.2<br>(-74.7 to -43.5)
Croatia	Adverse effects of medical treatment	10 to 14	Male	0<br>(0 to 0)	0.1<br>(0.1 to 0.2)	0<br>(0 to 0)	0.1<br>(0.1 to 0.2)	0<br>(0 to 0)	0.1<br>(0.0 to 0.1)	-64.1<br>(-78.8 to -38.2)	-42.8<br>(-66.3 to -1.6)
Croatia	Animal contact	5 to 9	Both	0<br>(0 to 0)	0.0<br>(0.0 to 0.0)	0<br>(0 to 0)	0.0<br>(0.0 to 0.0)	0<br>(0 to 0)	0.0<br>(0.0 to 0.0)	-61.2<br>(-75.3 to -37.0)	-42.5<br>(-63.5 to -6.8)

eTable 8. All-cause and cause-specific mortality (counts and rates) at the global level and by country in the WHO European Region for ages 5 to 9 and 10 to 14 for 1990, 2000, and 2016

Croatia	Animal contact	5 to 9	Female	0<br>(0 to 0)	0.0<br>(0.0 to 0.0)	0<br>(0 to 0)	0.0<br>(0.0 to 0.0)	0<br>(0 to 0)	0.0<br>(0.0 to 0.0)	-63.0<br>(-79.4 to -30.9)	-45.1<br>(-69.5 to 2.3)
Croatia	Animal contact	5 to 9	Male	0<br>(0 to 0)	0.0<br>(0.0 to 0.0)	0<br>(0 to 0)	0.0<br>(0.0 to 0.0)	0<br>(0 to 0)	0.0<br>(0.0 to 0.0)	-59.7<br>(-79.3 to -23.1)	-40.4<br>(-69.3 to 13.8)
Croatia	Animal contact	10 to 14	Both	0<br>(0 to 0)	0.0<br>(0.0 to 0.0)	0<br>(0 to 0)	0.0<br>(0.0 to 0.0)	0<br>(0 to 0)	0.0<br>(0.0 to 0.0)	-59.1<br>(-74.7 to -34.8)	-34.7<br>(-59.7 to 4.0)
Croatia	Animal contact	10 to 14	Female	0<br>(0 to 0)	0.0<br>(0.0 to 0.0)	0<br>(0 to 0)	0.0<br>(0.0 to 0.0)	0<br>(0 to 0)	0.0<br>(0.0 to 0.0)	-49.9<br>(-69.2 to -31.7)	-19.8<br>(-50.7 to 9.3)
Croatia	Animal contact	10 to 14	Male	0<br>(0 to 0)	0.0<br>(0.0 to 0.1)	0<br>(0 to 0)	0.0<br>(0.0 to 0.0)	0<br>(0 to 0)	0.0<br>(0.0 to 0.0)	-61.8<br>(-79.5 to -31.6)	-39.1<br>(-67.3 to 9.0)
Croatia	Foreign body	5 to 9	Both	1<br>(0 to 1)	0.2<br>(0.1 to 0.3)	0<br>(0 to 1)	0.2<br>(0.1 to 0.2)	0<br>(0 to 0)	0.1<br>(0.1 to 0.2)	-67.7<br>(-80.7 to -36.4)	-52.3<br>(-71.5 to -5.9)
Croatia	Foreign body	5 to 9	Female	0<br>(0 to 0)	0.1<br>(0.1 to 0.2)	0<br>(0 to 0)	0.1<br>(0.1 to 0.1)	0<br>(0 to 0)	0.1<br>(0.0 to 0.1)	-60.1<br>(-78.0 to -35.5)	-40.9<br>(-67.4 to -4.5)
Croatia	Foreign body	5 to 9	Male	0<br>(0 to 1)	0.3<br>(0.2 to 0.4)	0<br>(0 to 0)	0.2<br>(0.2 to 0.3)	0<br>(0 to 0)	0.1<br>(0.1 to 0.2)	-70.9<br>(-85.1 to -32.4)	-56.9<br>(-77.9 to -0.0)
Croatia	Foreign body	10 to 14	Both	1<br>(0 to 1)	0.2<br>(0.1 to 0.3)	0<br>(0 to 1)	0.2<br>(0.1 to 0.2)	0<br>(0 to 0)	0.1<br>(0.1 to 0.2)	-67.6<br>(-80.6 to -39.1)	-48.2<br>(-69.0 to -2.8)
Croatia	Foreign body	10 to 14	Female	0<br>(0 to 0)	0.1<br>(0.1 to 0.2)	0<br>(0 to 0)	0.1<br>(0.0 to 0.1)	0<br>(0 to 0)	0.1<br>(0.0 to 0.1)	-63.4<br>(-79.7 to -37.7)	-41.4<br>(-67.6 to -0.4)
Croatia	Foreign body	10 to 14	Male	0<br>(0 to 1)	0.3<br>(0.2 to 0.4)	0<br>(0 to 0)	0.2<br>(0.1 to 0.3)	0<br>(0 to 0)	0.1<br>(0.1 to 0.2)	-69.2<br>(-83.5 to -38.2)	-51.0<br>(-73.7 to -1.5)
Croatia	Environmental heat and cold exposure	5 to 9	Both	0<br>(0 to 0)	0.1<br>(0.0 to 0.1)	0<br>(0 to 0)	0.0<br>(0.0 to 0.0)	0<br>(0 to 0)	0.0<br>(0.0 to 0.0)	-84.4<br>(-90.7 to -71.4)	-77.0<br>(-86.2 to -57.7)
Croatia	Environmental heat and cold exposure	5 to 9	Female	0<br>(0 to 0)	0.0<br>(0.0 to 0.1)	0<br>(0 to 0)	0.0<br>(0.0 to 0.0)	0<br>(0 to 0)	0.0<br>(0.0 to 0.0)	-86.3<br>(-92.0 to -68.6)	-79.7<br>(-88.1 to -53.5)
Croatia	Environmental heat and cold exposure	5 to 9	Male	0<br>(0 to 0)	0.1<br>(0.0 to 0.1)	0<br>(0 to 0)	0.0<br>(0.0 to 0.1)	0<br>(0 to 0)	0.0<br>(0.0 to 0.0)	-82.7<br>(-91.3 to -61.8)	-74.4<br>(-87.2 to -43.5)
Croatia	Environmental heat and cold exposure	10 to 14	Both	0<br>(0 to 0)	0.0<br>(0.0 to 0.1)	0<br>(0 to 0)	0.0<br>(0.0 to 0.0)	0<br>(0 to 0)	0.0<br>(0.0 to 0.0)	-77.9<br>(-83.7 to -69.6)	-64.8<br>(-73.9 to -51.4)
Croatia	Environmental heat and cold exposure	10 to 14	Female	0<br>(0 to 0)	0.0<br>(0.0 to 0.1)	0<br>(0 to 0)	0.0<br>(0.0 to 0.0)	0<br>(0 to 0)	0.0<br>(0.0 to 0.0)	-79.6<br>(-86.1 to -69.9)	-67.4<br>(-77.8 to -51.9)
Croatia	Environmental heat and cold exposure	10 to 14	Male	0<br>(0 to 0)	0.0<br>(0.0 to 0.1)	0<br>(0 to 0)	0.0<br>(0.0 to 0.1)	0<br>(0 to 0)	0.0<br>(0.0 to 0.0)	-76.3<br>(-83.8 to -64.3)	-62.3<br>(-74.2 to -43.1)
Croatia	Other unintentional injuries	5 to 9	Both	8<br>(7 to 11)	2.6<br>(2.0 to 3.3)	0<br>(0 to 0)	0.1<br>(0.1 to 0.2)	0<br>(0 to 0)	0.0<br>(0.0 to 0.1)	-99.0<br>(-99.4 to -98.5)	-98.6<br>(-99.1 to -97.7)
Croatia	Other unintentional injuries	5 to 9	Female	3<br>(2 to 3)	1.6<br>(1.1 to 2.2)	0<br>(0 to 0)	0.1<br>(0.0 to 0.1)	0<br>(0 to 0)	0.0<br>(0.0 to 0.0)	-99.3<br>(-99.6 to -98.7)	-98.9<br>(-99.4 to -98.1)
Croatia	Other unintentional injuries	5 to 9	Male	6<br>(4 to 8)	3.5<br>(2.5 to 4.7)	0<br>(0 to 0)	0.2<br>(0.1 to 0.3)	0<br>(0 to 0)	0.1<br>(0.0 to 0.1)	-98.9<br>(-99.4 to -98.1)	-98.4<br>(-99.1 to -97.2)
Croatia	Other unintentional injuries	10 to 14	Both	9<br>(7 to 11)	2.8<br>(2.2 to 3.4)	1<br>(0 to 1)	0.2<br>(0.1 to 0.2)	0<br>(0 to 0)	0.1<br>(0.0 to 0.1)	-98.6<br>(-99.1 to -98.0)	-97.8<br>(-98.6 to -96.8)
Croatia	Other unintentional injuries	10 to 14	Female	2<br>(2 to 3)	1.4<br>(1.0 to 1.9)	0<br>(0 to 0)	0.0<br>(0.0 to 0.1)	0<br>(0 to 0)	0.0<br>(0.0 to 0.0)	-99.1<br>(-99.5 to -98.4)	-98.6<br>(-99.2 to -97.5)
Croatia	Other unintentional injuries	10 to 14	Male	7<br>(5 to 9)	4.1<br>(3.1 to 5.1)	0<br>(0 to 1)	0.3<br>(0.2 to 0.4)	0<br>(0 to 0)	0.1<br>(0.1 to 0.1)	-98.5<br>(-99.1 to -97.6)	-97.5<br>(-98.5 to -96.1)
Croatia	Self-harm	10 to 14	Both	7<br>(5 to 9)	2.2<br>(1.7 to 2.8)	5<br>(4 to 6)	1.7<br>(1.3 to 2.1)	2<br>(1 to 3)	1.0<br>(0.7 to 1.5)	-72.3<br>(-81.8 to -54.2)	-55.8<br>(-71.0 to -26.8)
Croatia	Self-harm	10 to 14	Female	2<br>(1 to 3)	1.2<br>(0.8 to 1.6)	1<br>(1 to 1)	0.8<br>(0.5 to 1.0)	0<br>(0 to 1)	0.5<br>(0.3 to 0.7)	-75.9<br>(-85.9 to -58.6)	-61.5<br>(-77.4 to -33.8)
Croatia	Self-harm	10 to 14	Male	5<br>(4 to 7)	3.2<br>(2.2 to 4.3)	4<br>(3 to 5)	2.6<br>(1.9 to 3.4)	2<br>(1 to 3)	1.5<br>(1.0 to 2.5)	-71.0<br>(-82.4 to -45.7)	-53.8<br>(-71.9 to -13.4)

eTable 8. All-cause and cause-specific mortality (counts and rates) at the global level and by country in the WHO European Region for ages 5 to 9 and 10 to 14 for 1990, 2000, and 2016

Croatia	Interpersonal violence	5 to 9	Both	2 (1 to 2)	0.5 (0.3 to 0.7)	1 (1 to 1)	0.3 (0.2 to 0.5)	0 (0 to 1)	0.2 (0.1 to 0.3)	-79.5 (-87.5 to -55.7)	-69.6 (-81.6 to -34.4)
Croatia	Interpersonal violence	5 to 9	Female	1 (0 to 1)	0.5 (0.3 to 0.8)	0 (0 to 1)	0.4 (0.2 to 0.5)	0 (0 to 0)	0.2 (0.1 to 0.4)	-75.7 (-86.5 to -38.2)	-64.0 (-80.0 to -8.4)
Croatia	Interpersonal violence	5 to 9	Male	1 (0 to 1)	0.5 (0.2 to 0.7)	0 (0 to 1)	0.3 (0.1 to 0.5)	0 (0 to 0)	0.1 (0.1 to 0.2)	-83.6 (-91.8 to -69.3)	-75.7 (-87.9 to -54.6)
Croatia	Interpersonal violence	10 to 14	Both	2 (1 to 2)	0.5 (0.3 to 0.7)	1 (1 to 1)	0.4 (0.2 to 0.5)	0 (0 to 1)	0.2 (0.1 to 0.3)	-77.4 (-86.0 to -59.2)	-63.9 (-77.7 to -34.8)
Croatia	Interpersonal violence	10 to 14	Female	1 (0 to 1)	0.5 (0.3 to 0.7)	0 (0 to 1)	0.4 (0.2 to 0.5)	0 (0 to 0)	0.2 (0.1 to 0.4)	-73.2 (-85.2 to -40.3)	-57.2 (-76.3 to -4.4)
Croatia	Interpersonal violence	10 to 14	Male	1 (0 to 1)	0.6 (0.3 to 0.9)	1 (0 to 1)	0.4 (0.2 to 0.6)	0 (0 to 0)	0.2 (0.1 to 0.3)	-80.9 (-90.4 to -70.0)	-69.6 (-84.8 to -52.1)
Croatia	Exposure to forces of nature	5 to 9	Both	0 (0 to 0)	0.0 (0.0 to 0.0)	0 (0 to 0)	0.0 (0.0 to 0.0)	0 (0 to 0)	0.0 (0.0 to 0.0)	0.0 (0.0 to 0.0)	0.0 (0.0 to 0.0)
Croatia	Exposure to forces of nature	5 to 9	Female	0 (0 to 0)	0.0 (0.0 to 0.0)	0 (0 to 0)	0.0 (0.0 to 0.0)	0 (0 to 0)	0.0 (0.0 to 0.0)	0.0 (0.0 to 0.0)	0.0 (0.0 to 0.0)
Croatia	Exposure to forces of nature	5 to 9	Male	0 (0 to 0)	0.0 (0.0 to 0.0)	0 (0 to 0)	0.0 (0.0 to 0.0)	0 (0 to 0)	0.0 (0.0 to 0.0)	0.0 (0.0 to 0.0)	0.0 (0.0 to 0.0)
Croatia	Exposure to forces of nature	10 to 14	Both	0 (0 to 0)	0.0 (0.0 to 0.0)	0 (0 to 0)	0.0 (0.0 to 0.0)	0 (0 to 0)	0.0 (0.0 to 0.0)	0.0 (0.0 to 0.0)	0.0 (0.0 to 0.0)
Croatia	Exposure to forces of nature	10 to 14	Female	0 (0 to 0)	0.0 (0.0 to 0.0)	0 (0 to 0)	0.0 (0.0 to 0.0)	0 (0 to 0)	0.0 (0.0 to 0.0)	0.0 (0.0 to 0.0)	0.0 (0.0 to 0.0)
Croatia	Exposure to forces of nature	10 to 14	Male	0 (0 to 0)	0.0 (0.0 to 0.0)	0 (0 to 0)	0.0 (0.0 to 0.0)	0 (0 to 0)	0.0 (0.0 to 0.0)	0.0 (0.0 to 0.0)	0.0 (0.0 to 0.0)
Croatia	Conflict and terrorism	5 to 9	Both	0 (0 to 0)	0.0 (0.0 to 0.0)	0 (0 to 0)	0.1 (0.0 to 0.1)	0 (0 to 0)	0.0 (0.0 to 0.0)	0.0 (0.0 to 0.0)	0.0 (0.0 to 0.0)
Croatia	Conflict and terrorism	5 to 9	Female	0 (0 to 0)	0.0 (0.0 to 0.0)	0 (0 to 0)	0.1 (0.0 to 0.1)	0 (0 to 0)	0.0 (0.0 to 0.0)	0.0 (0.0 to 0.0)	0.0 (0.0 to 0.0)
Croatia	Conflict and terrorism	5 to 9	Male	0 (0 to 0)	0.0 (0.0 to 0.0)	0 (0 to 0)	0.1 (0.0 to 0.1)	0 (0 to 0)	0.0 (0.0 to 0.0)	0.0 (0.0 to 0.0)	0.0 (0.0 to 0.0)
Croatia	Conflict and terrorism	10 to 14	Both	0 (0 to 0)	0.0 (0.0 to 0.0)	0 (0 to 0)	0.1 (0.0 to 0.1)	0 (0 to 0)	0.0 (0.0 to 0.0)	0.0 (0.0 to 0.0)	0.0 (0.0 to 0.0)
Croatia	Conflict and terrorism	10 to 14	Female	0 (0 to 0)	0.0 (0.0 to 0.0)	0 (0 to 0)	0.1 (0.0 to 0.1)	0 (0 to 0)	0.0 (0.0 to 0.0)	0.0 (0.0 to 0.0)	0.0 (0.0 to 0.0)
Croatia	Conflict and terrorism	10 to 14	Male	0 (0 to 0)	0.0 (0.0 to 0.0)	0 (0 to 0)	0.1 (0.0 to 0.1)	0 (0 to 0)	0.0 (0.0 to 0.0)	0.0 (0.0 to 0.0)	0.0 (0.0 to 0.0)
Croatia	Executions and police conflict	5 to 9	Both	0 (0 to 0)	0.0 (0.0 to 0.0)	0 (0 to 0)	0.0 (0.0 to 0.0)	0 (0 to 0)	0.0 (0.0 to 0.0)	-40.2 (-51.4 to -27.0)	-11.5 (-28.0 to 8.0)
Croatia	Executions and police conflict	5 to 9	Female	0 (0 to 0)	0.0 (0.0 to 0.0)	0 (0 to 0)	0.0 (0.0 to 0.0)	0 (0 to 0)	0.0 (0.0 to 0.0)	-40.6 (-52.9 to -25.7)	-12.0 (-30.3 to 10.0)
Croatia	Executions and police conflict	5 to 9	Male	0 (0 to 0)	0.0 (0.0 to 0.0)	0 (0 to 0)	0.0 (0.0 to 0.0)	0 (0 to 0)	0.0 (0.0 to 0.0)	-39.9 (-53.4 to -23.8)	-11.1 (-31.1 to 12.8)
Croatia	Executions and police conflict	10 to 14	Both	0 (0 to 0)	0.0 (0.0 to 0.0)	0 (0 to 0)	0.0 (0.0 to 0.0)	0 (0 to 0)	0.0 (0.0 to 0.0)	-42.1 (-50.6 to -33.3)	-7.5 (-21.1 to 6.5)
Croatia	Executions and police conflict	10 to 14	Female	0 (0 to 0)	0.0 (0.0 to 0.0)	0 (0 to 0)	0.0 (0.0 to 0.0)	0 (0 to 0)	0.0 (0.0 to 0.0)	-42.7 (-52.7 to -31.2)	-8.2 (-24.3 to 10.1)
Croatia	Executions and police conflict	10 to 14	Male	0 (0 to 0)	0.0 (0.0 to 0.0)	0 (0 to 0)	0.0 (0.0 to 0.0)	0 (0 to 0)	0.0 (0.0 to 0.0)	-41.5 (-52.5 to -29.1)	-6.8 (-24.4 to 13.0)
Cyprus	All causes	5 to 9	Both	14 (13 to 14)	24.3 (23.3 to 25.4)	12 (11 to 13)	22.5 (21.4 to 23.6)	5 (4 to 5)	10.6 (9.4 to 12.0)	-65.0 (-69.2 to -60.3)	-56.4 (-61.7 to -50.5)
Cyprus	All causes	5 to 9	Female	6 (5 to 6)	20.2 (19.8 to 20.8)	5 (5 to 6)	20.4 (19.0 to 22.1)	3 (2 to 3)	11.7 (10.3 to 13.3)	-53.5 (-59.0 to -47.3)	-42.0 (-48.9 to -34.3)

eTable 8. All-cause and cause-specific mortality (counts and rates) at the global level and by country in the WHO European Region for ages 5 to 9 and 10 to 14 for 1990, 2000, and 2016

Cyprus	All causes	5 to 9	Male	8 (8 to 9)	28.2 (26.1 to 30.2)	7 (6 to 7)	24.4 (22.4 to 26.1)	2 (2 to 3)	9.6 (8.3 to 11.0)	-72.8 (-77.1 to -68.0)	-66.1 (-71.5 to -60.2)
Cyprus	All causes	10 to 14	Both	9 (9 to 10)	15.9 (15.4 to 16.4)	11 (11 to 12)	20.4 (19.7 to 21.2)	5 (4 to 5)	10.5 (9.8 to 11.3)	-48.8 (-53.0 to -44.1)	-33.9 (-39.4 to -27.8)
Cyprus	All causes	10 to 14	Female	3 (3 to 3)	11.5 (11.1 to 11.9)	4 (4 to 4)	14.7 (14.1 to 15.5)	1 (1 to 2)	6.2 (5.8 to 6.7)	-57.6 (-60.8 to -53.9)	-45.9 (-49.9 to -41.1)
Cyprus	All causes	10 to 14	Male	6 (6 to 6)	20.0 (19.0 to 21.1)	7 (7 to 8)	25.8 (24.5 to 27.1)	3 (3 to 4)	14.6 (13.5 to 15.9)	-44.1 (-49.1 to -37.9)	-27.0 (-33.5 to -19.0)
Cyprus	Tuberculosis	5 to 9	Both	0 (0 to 0)	0.0 (0.0 to 0.0)	0 (0 to 0)	0.0 (0.0 to 0.0)	0 (0 to 0)	0.0 (0.0 to 0.0)	-67.7 (-77.4 to -51.2)	-59.8 (-71.9 to -39.3)
Cyprus	Tuberculosis	5 to 9	Female	0 (0 to 0)	0.0 (0.0 to 0.0)	0 (0 to 0)	0.0 (0.0 to 0.0)	0 (0 to 0)	0.0 (0.0 to 0.0)	-69.7 (-81.6 to -45.2)	-62.2 (-77.0 to -31.7)
Cyprus	Tuberculosis	5 to 9	Male	0 (0 to 0)	0.0 (0.0 to 0.0)	0 (0 to 0)	0.0 (0.0 to 0.0)	0 (0 to 0)	0.0 (0.0 to 0.0)	-65.4 (-78.8 to -42.1)	-56.9 (-73.6 to -28.0)
Cyprus	Tuberculosis	10 to 14	Both	0 (0 to 0)	0.0 (0.0 to 0.0)	0 (0 to 0)	0.0 (0.0 to 0.0)	0 (0 to 0)	0.0 (0.0 to 0.0)	-43.1 (-64.0 to -21.1)	-26.6 (-53.5 to 1.8)
Cyprus	Tuberculosis	10 to 14	Female	0 (0 to 0)	0.0 (0.0 to 0.0)	0 (0 to 0)	0.0 (0.0 to 0.0)	0 (0 to 0)	0.0 (0.0 to 0.0)	-45.0 (-71.5 to -16.8)	-29.7 (-63.6 to 6.2)
Cyprus	Tuberculosis	10 to 14	Male	0 (0 to 0)	0.0 (0.0 to 0.0)	0 (0 to 0)	0.0 (0.0 to 0.0)	0 (0 to 0)	0.0 (0.0 to 0.0)	-41.5 (-67.4 to -11.9)	-23.7 (-57.5 to 15.0)
Cyprus	HIV/AIDS	5 to 9	Both	0 (0 to 0)	0.0 (0.0 to 0.0)	0 (0 to 0)	0.0 (0.0 to 0.0)	0 (0 to 0)	0.0 (0.0 to 0.0)	-54.5 (-100.0 to 67.9)	-43.3 (-100.0 to 109.2)
Cyprus	HIV/AIDS	5 to 9	Female	0 (0 to 0)	0.0 (0.0 to 0.0)	0 (0 to 0)	0.0 (0.0 to 0.0)	0 (0 to 0)	0.0 (0.0 to 0.0)	-54.7 (-100.0 to 66.7)	-43.5 (-100.0 to 108.0)
Cyprus	HIV/AIDS	5 to 9	Male	0 (0 to 0)	0.0 (0.0 to 0.0)	0 (0 to 0)	0.0 (0.0 to 0.0)	0 (0 to 0)	0.0 (0.0 to 0.0)	-54.4 (-100.0 to 68.5)	-43.2 (-100.0 to 109.6)
Cyprus	HIV/AIDS	10 to 14	Both	0 (0 to 0)	0.0 (0.0 to 0.0)	0 (0 to 0)	0.0 (0.0 to 0.0)	0 (0 to 0)	0.0 (0.0 to 0.0)	8.7 (-100.0 to 297.0)	40.4 (-100.0 to 412.5)
Cyprus	HIV/AIDS	10 to 14	Female	0 (0 to 0)	0.0 (0.0 to 0.0)	0 (0 to 0)	0.0 (0.0 to 0.0)	0 (0 to 0)	0.0 (0.0 to 0.0)	8.7 (-100.0 to 297.5)	38.7 (-100.0 to 407.4)
Cyprus	HIV/AIDS	10 to 14	Male	0 (0 to 0)	0.0 (0.0 to 0.0)	0 (0 to 0)	0.0 (0.0 to 0.0)	0 (0 to 0)	0.0 (0.0 to 0.0)	8.8 (-100.0 to 304.8)	42.0 (-100.0 to 428.3)
Cyprus	Diarrheal diseases	5 to 9	Both	0 (0 to 0)	0.1 (0.0 to 0.2)	0 (0 to 0)	0.1 (0.0 to 0.2)	0 (0 to 0)	0.1 (0.0 to 0.1)	-42.1 (-72.2 to 11.8)	-27.9 (-65.3 to 39.2)
Cyprus	Diarrheal diseases	5 to 9	Female	0 (0 to 0)	0.1 (0.0 to 0.2)	0 (0 to 0)	0.1 (0.0 to 0.2)	0 (0 to 0)	0.1 (0.0 to 0.1)	-31.8 (-63.2 to 82.5)	-15.0 (-54.1 to 127.6)
Cyprus	Diarrheal diseases	5 to 9	Male	0 (0 to 0)	0.1 (0.0 to 0.2)	0 (0 to 0)	0.1 (0.0 to 0.2)	0 (0 to 0)	0.1 (0.0 to 0.1)	-49.4 (-81.6 to 1.7)	-37.0 (-77.1 to 26.5)
Cyprus	Diarrheal diseases	10 to 14	Both	0 (0 to 0)	0.0 (0.0 to 0.1)	0 (0 to 0)	0.0 (0.0 to 0.1)	0 (0 to 0)	0.0 (0.0 to 0.1)	-28.7 (-58.6 to 45.0)	-8.0 (-46.6 to 87.2)
Cyprus	Diarrheal diseases	10 to 14	Female	0 (0 to 0)	0.0 (0.0 to 0.1)	0 (0 to 0)	0.1 (0.0 to 0.1)	0 (0 to 0)	0.0 (0.0 to 0.1)	-36.8 (-68.1 to 36.2)	-19.4 (-59.3 to 73.8)
Cyprus	Diarrheal diseases	10 to 14	Male	0 (0 to 0)	0.0 (0.0 to 0.1)	0 (0 to 0)	0.0 (0.0 to 0.1)	0 (0 to 0)	0.0 (0.0 to 0.1)	-17.1 (-52.0 to 117.1)	8.1 (-37.4 to 183.3)
Cyprus	Intestinal infectious diseases	5 to 9	Both	0 (0 to 0)	0.0 (0.0 to 0.0)	0 (0 to 0)	0.0 (0.0 to 0.0)	0 (0 to 0)	0.0 (0.0 to 0.0)	-46.7 (-75.9 to -8.5)	-33.6 (-70.0 to 14.0)
Cyprus	Intestinal infectious diseases	5 to 9	Female	0 (0 to 0)	0.0 (0.0 to 0.0)	0 (0 to 0)	0.0 (0.0 to 0.0)	0 (0 to 0)	0.0 (0.0 to 0.0)	-41.3 (-78.2 to 40.7)	-26.7 (-72.8 to 75.5)
Cyprus	Intestinal infectious diseases	5 to 9	Male	0 (0 to 0)	0.0 (0.0 to 0.0)	0 (0 to 0)	0.0 (0.0 to 0.0)	0 (0 to 0)	0.0 (0.0 to 0.0)	-51.8 (-83.1 to 0.0)	-40.0 (-79.0 to 24.4)
Cyprus	Intestinal infectious diseases	10 to 14	Both	0 (0 to 0)	0.0 (0.0 to 0.0)	0 (0 to 0)	0.0 (0.0 to 0.0)	0 (0 to 0)	0.0 (0.0 to 0.0)	-30.5 (-66.5 to 18.2)	-10.3 (-56.8 to 52.5)
Cyprus	Intestinal infectious diseases	10 to 14	Female	0 (0 to 0)	0.0 (0.0 to 0.0)	0 (0 to 0)	0.0 (0.0 to 0.0)	0 (0 to 0)	0.0 (0.0 to 0.0)	-28.5 (-69.2 to 34.3)	-8.7 (-60.7 to 71.4)
Cyprus	Intestinal infectious diseases	10 to 14	Male	0 (0 to 0)	0.0 (0.0 to 0.0)	0 (0 to 0)	0.0 (0.0 to 0.0)	0 (0 to 0)	0.0 (0.0 to 0.0)	-32.2 (-75.6 to 50.4)	-11.5 (-68.2 to 96.3)
Cyprus	Lower respiratory infections	5 to 9	Both	0 (0 to 0)	0.5 (0.3 to 0.7)	0 (0 to 0)	0.4 (0.3 to 0.6)	0 (0 to 0)	0.2 (0.1 to 0.3)	-68.9 (-81.5 to -49.6)	-61.3 (-76.9 to -37.3)

eTable 8. All-cause and cause-specific mortality (counts and rates) at the global level and by country in the WHO European Region for ages 5 to 9 and 10 to 14 for 1990, 2000, and 2016

Cyprus	Lower respiratory infections	5 to 9	Female	0 (0 to 0)	0.5 (0.3 to 0.9)	0 (0 to 0)	0.5 (0.3 to 0.7)	0 (0 to 0)	0.2 (0.1 to 0.4)	-60.5 (-80.6 to -24.2)	-50.7 (-75.8 to -5.5)
Cyprus	Lower respiratory infections	5 to 9	Male	0 (0 to 0)	0.5 (0.3 to 0.8)	0 (0 to 0)	0.4 (0.2 to 0.6)	0 (0 to 0)	0.1 (0.1 to 0.2)	-77.9 (-87.5 to -57.5)	-72.5 (-84.5 to -47.2)
Cyprus	Lower respiratory infections	10 to 14	Both	0 (0 to 0)	0.2 (0.1 to 0.4)	0 (0 to 0)	0.3 (0.2 to 0.4)	0 (0 to 0)	0.1 (0.1 to 0.2)	-57.6 (-73.6 to -31.0)	-45.3 (-66.0 to -11.0)
Cyprus	Lower respiratory infections	10 to 14	Female	0 (0 to 0)	0.2 (0.1 to 0.5)	0 (0 to 0)	0.3 (0.2 to 0.4)	0 (0 to 0)	0.1 (0.1 to 0.2)	-64.7 (-83.0 to -31.3)	-54.9 (-78.2 to -12.3)
Cyprus	Lower respiratory infections	10 to 14	Male	0 (0 to 0)	0.3 (0.1 to 0.5)	0 (0 to 0)	0.3 (0.2 to 0.5)	0 (0 to 0)	0.2 (0.1 to 0.3)	-51.2 (-73.3 to -11.7)	-36.3 (-65.2 to 15.2)
Cyprus	Upper respiratory infections	5 to 9	Both	0 (0 to 0)	0.0 (0.0 to 0.0)	0 (0 to 0)	0.0 (0.0 to 0.0)	0 (0 to 0)	0.0 (0.0 to 0.0)	-77.8 (-99.7 to 682.2)	-72.4 (-99.6 to 874.3)
Cyprus	Upper respiratory infections	5 to 9	Female	0 (0 to 0)	0.0 (0.0 to 0.0)	0 (0 to 0)	0.0 (0.0 to 0.0)	0 (0 to 0)	0.0 (0.0 to 0.0)	-71.1 (-100.0 to 21388.3)	-63.9 (-100.0 to 26700.2)
Cyprus	Upper respiratory infections	5 to 9	Male	0 (0 to 0)	0.0 (0.0 to 0.0)	0 (0 to 0)	0.0 (0.0 to 0.0)	0 (0 to 0)	0.0 (0.0 to 0.0)	-82.7 (-100.0 to 12846.9)	-78.5 (-100.0 to 16007.0)
Cyprus	Upper respiratory infections	10 to 14	Both	0 (0 to 0)	0.0 (0.0 to 0.0)	0 (0 to 0)	0.0 (0.0 to 0.0)	0 (0 to 0)	0.0 (0.0 to 0.0)	-61.4 (-99.2 to 1901.0)	-50.1 (-99.0 to 2483.2)
Cyprus	Upper respiratory infections	10 to 14	Female	0 (0 to 0)	0.0 (0.0 to 0.0)	0 (0 to 0)	0.0 (0.0 to 0.0)	0 (0 to 0)	0.0 (0.0 to 0.0)	-68.5 (-100.0 to 61167.5)	-59.8 (-100.0 to 78105.9)
Cyprus	Upper respiratory infections	10 to 14	Male	0 (0 to 0)	0.0 (0.0 to 0.0)	0 (0 to 0)	0.0 (0.0 to 0.0)	0 (0 to 0)	0.0 (0.0 to 0.0)	-57.9 (-100.0 to 56342.5)	-45.1 (-100.0 to 73554.3)
Cyprus	Otitis media	5 to 9	Both	0 (0 to 0)	0.0 (0.0 to 0.0)	0 (0 to 0)	0.0 (0.0 to 0.0)	0 (0 to 0)	0.0 (0.0 to 0.0)	-85.0 (-96.2 to -30.5)	-81.3 (-95.3 to -13.4)
Cyprus	Otitis media	5 to 9	Female	0 (0 to 0)	0.0 (0.0 to 0.0)	0 (0 to 0)	0.0 (0.0 to 0.0)	0 (0 to 0)	0.0 (0.0 to 0.0)	-77.7 (-97.4 to 189.9)	-72.2 (-96.8 to 261.6)
Cyprus	Otitis media	5 to 9	Male	0 (0 to 0)	0.0 (0.0 to 0.0)	0 (0 to 0)	0.0 (0.0 to 0.0)	0 (0 to 0)	0.0 (0.0 to 0.0)	-89.6 (-97.9 to -29.2)	-87.0 (-97.4 to -11.9)
Cyprus	Otitis media	10 to 14	Both	0 (0 to 0)	0.0 (0.0 to 0.0)	0 (0 to 0)	0.0 (0.0 to 0.0)	0 (0 to 0)	0.0 (0.0 to 0.0)	-82.7 (-96.1 to -17.2)	-77.6 (-94.9 to 6.9)
Cyprus	Otitis media	10 to 14	Female	0 (0 to 0)	0.0 (0.0 to 0.0)	0 (0 to 0)	0.0 (0.0 to 0.0)	0 (0 to 0)	0.0 (0.0 to 0.0)	-86.5 (-98.4 to 82.2)	-82.8 (-97.9 to 132.6)
Cyprus	Otitis media	10 to 14	Male	0 (0 to 0)	0.0 (0.0 to 0.0)	0 (0 to 0)	0.0 (0.0 to 0.0)	0 (0 to 0)	0.0 (0.0 to 0.0)	-80.7 (-96.5 to 34.1)	-74.8 (-95.4 to 75.0)
Cyprus	Meningitis	5 to 9	Both	0 (0 to 0)	0.3 (0.2 to 0.5)	0 (0 to 0)	0.3 (0.2 to 0.4)	0 (0 to 0)	0.1 (0.1 to 0.2)	-69.0 (-82.5 to -41.5)	-61.4 (-78.2 to -27.1)
Cyprus	Meningitis	5 to 9	Female	0 (0 to 0)	0.3 (0.2 to 0.6)	0 (0 to 0)	0.3 (0.2 to 0.5)	0 (0 to 0)	0.1 (0.1 to 0.2)	-67.7 (-84.1 to -37.9)	-59.7 (-80.1 to -22.6)
Cyprus	Meningitis	5 to 9	Male	0 (0 to 0)	0.3 (0.1 to 0.6)	0 (0 to 0)	0.3 (0.1 to 0.5)	0 (0 to 0)	0.1 (0.1 to 0.2)	-70.5 (-87.9 to -24.0)	-63.4 (-85.0 to -5.4)
Cyprus	Meningitis	10 to 14	Both	0 (0 to 0)	0.2 (0.1 to 0.3)	0 (0 to 0)	0.2 (0.1 to 0.3)	0 (0 to 0)	0.1 (0.1 to 0.2)	-55.3 (-75.1 to -30.7)	-42.3 (-67.8 to -10.5)
Cyprus	Meningitis	10 to 14	Female	0 (0 to 0)	0.1 (0.1 to 0.3)	0 (0 to 0)	0.2 (0.1 to 0.3)	0 (0 to 0)	0.1 (0.0 to 0.1)	-69.9 (-85.7 to -39.7)	-61.6 (-81.8 to -23.0)
Cyprus	Meningitis	10 to 14	Male	0 (0 to 0)	0.2 (0.1 to 0.3)	0 (0 to 0)	0.2 (0.1 to 0.4)	0 (0 to 0)	0.1 (0.1 to 0.2)	-43.0 (-69.3 to -5.1)	-25.6 (-60.0 to 23.9)
Cyprus	Encephalitis	5 to 9	Both	0 (0 to 0)	0.2 (0.1 to 0.3)	0 (0 to 0)	0.1 (0.1 to 0.2)	0 (0 to 0)	0.1 (0.1 to 0.1)	-52.2 (-75.0 to -11.8)	-40.5 (-68.9 to 9.8)
Cyprus	Encephalitis	5 to 9	Female	0 (0 to 0)	0.1 (0.1 to 0.2)	0 (0 to 0)	0.1 (0.1 to 0.2)	0 (0 to 0)	0.1 (0.0 to 0.1)	-40.9 (-66.6 to 23.4)	-26.3 (-58.4 to 53.9)
Cyprus	Encephalitis	5 to 9	Male	0 (0 to 0)	0.2 (0.1 to 0.4)	0 (0 to 0)	0.2 (0.1 to 0.3)	0 (0 to 0)	0.1 (0.1 to 0.2)	-58.4 (-83.3 to -8.6)	-48.3 (-79.2 to 13.8)
Cyprus	Encephalitis	10 to 14	Both	0 (0 to 0)	0.1 (0.1 to 0.1)	0 (0 to 0)	0.1 (0.1 to 0.2)	0 (0 to 0)	0.1 (0.0 to 0.1)	-34.8 (-57.1 to 9.2)	-15.8 (-44.6 to 41.0)

eTable 8. All-cause and cause-specific mortality (counts and rates) at the global level and by country in the WHO European Region for ages 5 to 9 and 10 to 14 for 1990, 2000, and 2016

Cyprus	Encephalitis	10 to 14	Female	0<br>(0 to 0)	0.1<br>(0.0 to 0.1)	0<br>(0 to 0)	0.1<br>(0.0 to 0.1)	0<br>(0 to 0)	0.0<br>(0.0 to 0.1)	-44.9<br>(-71.8 to 5.0)	-29.7<br>(-64.0 to 34.1)
Cyprus	Encephalitis	10 to 14	Male	0<br>(0 to 0)	0.1<br>(0.1 to 0.2)	0<br>(0 to 0)	0.1<br>(0.1 to 0.2)	0<br>(0 to 0)	0.1<br>(0.1 to 0.2)	-29.0<br>(-58.7 to 30.0)	-7.3<br>(-46.1 to 69.6)
Cyprus	Diphtheria	5 to 9	Both	0<br>(0 to 0)	0.0<br>(0.0 to 0.0)	0<br>(0 to 0)	0.0<br>(0.0 to 0.0)	0<br>(0 to 0)	0.0<br>(0.0 to 0.0)	-80.2<br>(-97.3 to 35.6)	-75.3<br>(-96.6 to 68.9)
Cyprus	Diphtheria	5 to 9	Female	0<br>(0 to 0)	0.0<br>(0.0 to 0.0)	0<br>(0 to 0)	0.0<br>(0.0 to 0.0)	0<br>(0 to 0)	0.0<br>(0.0 to 0.0)	-72.4<br>(-98.5 to 553.4)	-65.6<br>(-98.1 to 715.0)
Cyprus	Diphtheria	5 to 9	Male	0<br>(0 to 0)	0.0<br>(0.0 to 0.0)	0<br>(0 to 0)	0.0<br>(0.0 to 0.0)	0<br>(0 to 0)	0.0<br>(0.0 to 0.0)	-85.3<br>(-99.3 to 160.1)	-81.8<br>(-99.1 to 223.6)
Cyprus	Diphtheria	10 to 14	Both	0<br>(0 to 0)	0.0<br>(0.0 to 0.0)	0<br>(0 to 0)	0.0<br>(0.0 to 0.0)	0<br>(0 to 0)	0.0<br>(0.0 to 0.0)	-64.4<br>(-95.0 to 180.1)	-54.0<br>(-93.6 to 261.5)
Cyprus	Diphtheria	10 to 14	Female	0<br>(0 to 0)	0.0<br>(0.0 to 0.0)	0<br>(0 to 0)	0.0<br>(0.0 to 0.0)	0<br>(0 to 0)	0.0<br>(0.0 to 0.0)	-72.0<br>(-98.6 to 553.6)	-64.2<br>(-98.2 to 734.3)
Cyprus	Diphtheria	10 to 14	Male	0<br>(0 to 0)	0.0<br>(0.0 to 0.0)	0<br>(0 to 0)	0.0<br>(0.0 to 0.0)	0<br>(0 to 0)	0.0<br>(0.0 to 0.0)	-60.6<br>(-98.1 to 795.9)	-48.6<br>(-97.6 to 1069.1)
Cyprus	Whooping cough	5 to 9	Both	0<br>(0 to 0)	0.1<br>(0.0 to 0.4)	0<br>(0 to 0)	0.0<br>(0.0 to 0.0)	0<br>(0 to 0)	0.0<br>(0.0 to 0.0)	-98.7<br>(-99.9 to -84.4)	-98.4<br>(-99.9 to -80.6)
Cyprus	Whooping cough	5 to 9	Female	0<br>(0 to 0)	0.1<br>(0.0 to 0.4)	0<br>(0 to 0)	0.0<br>(0.0 to 0.0)	0<br>(0 to 0)	0.0<br>(0.0 to 0.0)	-98.7<br>(-99.9 to -83.1)	-98.3<br>(-99.9 to -78.9)
Cyprus	Whooping cough	5 to 9	Male	0<br>(0 to 0)	0.1<br>(0.0 to 0.3)	0<br>(0 to 0)	0.0<br>(0.0 to 0.0)	0<br>(0 to 0)	0.0<br>(0.0 to 0.0)	-98.8<br>(-99.9 to -86.3)	-98.5<br>(-99.9 to -83.0)
Cyprus	Whooping cough	10 to 14	Both	0<br>(0 to 0)	0.0<br>(0.0 to 0.1)	0<br>(0 to 0)	0.0<br>(0.0 to 0.0)	0<br>(0 to 0)	0.0<br>(0.0 to 0.0)	-98.6<br>(-99.9 to -83.9)	-98.2<br>(-99.9 to -79.3)
Cyprus	Whooping cough	10 to 14	Female	0<br>(0 to 0)	0.0<br>(0.0 to 0.1)	0<br>(0 to 0)	0.0<br>(0.0 to 0.0)	0<br>(0 to 0)	0.0<br>(0.0 to 0.0)	-98.7<br>(-99.9 to -84.1)	-98.3<br>(-99.9 to -79.8)
Cyprus	Whooping cough	10 to 14	Male	0<br>(0 to 0)	0.0<br>(0.0 to 0.1)	0<br>(0 to 0)	0.0<br>(0.0 to 0.0)	0<br>(0 to 0)	0.0<br>(0.0 to 0.0)	-98.6<br>(-99.9 to -83.5)	-98.2<br>(-99.9 to -78.4)
Cyprus	Tetanus	5 to 9	Both	0<br>(0 to 0)	0.0<br>(0.0 to 0.0)	0<br>(0 to 0)	0.0<br>(0.0 to 0.0)	0<br>(0 to 0)	0.0<br>(0.0 to 0.0)	-32.5<br>(-45.0 to -18.0)	-16.0<br>(-31.5 to 2.2)
Cyprus	Tetanus	5 to 9	Female	0<br>(0 to 0)	0.0<br>(0.0 to 0.0)	0<br>(0 to 0)	0.0<br>(0.0 to 0.0)	0<br>(0 to 0)	0.0<br>(0.0 to 0.0)	-28.3<br>(-42.8 to -11.4)	-10.6<br>(-28.7 to 10.5)
Cyprus	Tetanus	5 to 9	Male	0<br>(0 to 0)	0.0<br>(0.0 to 0.0)	0<br>(0 to 0)	0.0<br>(0.0 to 0.0)	0<br>(0 to 0)	0.0<br>(0.0 to 0.0)	-36.4<br>(-52.1 to -16.9)	-20.8<br>(-40.4 to 3.3)
Cyprus	Tetanus	10 to 14	Both	0<br>(0 to 0)	0.0<br>(0.0 to 0.0)	0<br>(0 to 0)	0.0<br>(0.0 to 0.0)	0<br>(0 to 0)	0.0<br>(0.0 to 0.0)	-26.5<br>(-36.8 to -13.7)	-5.1<br>(-18.4 to 11.4)
Cyprus	Tetanus	10 to 14	Female	0<br>(0 to 0)	0.0<br>(0.0 to 0.0)	0<br>(0 to 0)	0.0<br>(0.0 to 0.0)	0<br>(0 to 0)	0.0<br>(0.0 to 0.0)	-28.4<br>(-40.8 to -11.9)	-8.6<br>(-24.4 to 12.5)
Cyprus	Tetanus	10 to 14	Male	0<br>(0 to 0)	0.0<br>(0.0 to 0.0)	0<br>(0 to 0)	0.0<br>(0.0 to 0.0)	0<br>(0 to 0)	0.0<br>(0.0 to 0.0)	-24.8<br>(-37.9 to -8.4)	-1.8<br>(-18.9 to 19.5)
Cyprus	Measles	5 to 9	Both	0<br>(0 to 0)	0.0<br>(0.0 to 0.0)	0<br>(0 to 0)	0.0<br>(0.0 to 0.0)	0<br>(0 to 0)	0.0<br>(0.0 to 0.0)	-98.1<br>(-100.0 to -68.6)	-97.6<br>(-100.0 to -60.8)
Cyprus	Measles	5 to 9	Female	0<br>(0 to 0)	0.0<br>(0.0 to 0.0)	0<br>(0 to 0)	0.0<br>(0.0 to 0.0)	0<br>(0 to 0)	0.0<br>(0.0 to 0.0)	-98.0<br>(-100.0 to -66.3)	-97.5<br>(-100.0 to -58.0)
Cyprus	Measles	5 to 9	Male	0<br>(0 to 0)	0.0<br>(0.0 to 0.0)	0<br>(0 to 0)	0.0<br>(0.0 to 0.0)	0<br>(0 to 0)	0.0<br>(0.0 to 0.0)	-98.2<br>(-100.0 to -68.5)	-97.7<br>(-100.0 to -60.8)
Cyprus	Measles	10 to 14	Both	0<br>(0 to 0)	0.0<br>(0.0 to 0.0)	0<br>(0 to 0)	0.0<br>(0.0 to 0.0)	0<br>(0 to 0)	0.0<br>(0.0 to 0.0)	-97.9<br>(-100.0 to -63.7)	-97.3<br>(-100.0 to -53.2)
Cyprus	Measles	10 to 14	Female	0<br>(0 to 0)	0.0<br>(0.0 to 0.0)	0<br>(0 to 0)	0.0<br>(0.0 to 0.0)	0<br>(0 to 0)	0.0<br>(0.0 to 0.0)	-98.0<br>(-100.0 to -63.6)	-97.4<br>(-100.0 to -53.5)
Cyprus	Measles	10 to 14	Male	0<br>(0 to 0)	0.0<br>(0.0 to 0.0)	0<br>(0 to 0)	0.0<br>(0.0 to 0.0)	0<br>(0 to 0)	0.0<br>(0.0 to 0.0)	-97.9<br>(-100.0 to -66.3)	-97.2<br>(-100.0 to -56.1)
Cyprus	Varicella and herpes zoster	5 to 9	Both	0<br>(0 to 0)	0.0<br>(0.0 to 0.1)	0<br>(0 to 0)	0.0<br>(0.0 to 0.0)	0<br>(0 to 0)	0.0<br>(0.0 to 0.0)	-77.8<br>(-96.2 to 28.3)	-72.3<br>(-95.2 to 59.9)
Cyprus	Varicella and herpes zoster	5 to 9	Female	0<br>(0 to 0)	0.0<br>(0.0 to 0.1)	0<br>(0 to 0)	0.0<br>(0.0 to 0.1)	0<br>(0 to 0)	0.0<br>(0.0 to 0.0)	-76.0<br>(-98.4 to 188.3)	-70.0<br>(-98.0 to 259.6)
Cyprus	Varicella and herpes zoster	5 to 9	Male	0<br>(0 to 0)	0.0<br>(0.0 to 0.1)	0<br>(0 to 0)	0.0<br>(0.0 to 0.1)	0<br>(0 to 0)	0.0<br>(0.0 to 0.0)	-79.3<br>(-98.5 to 191.8)	-74.3<br>(-98.1 to 263.0)
Cyprus	Varicella and herpes zoster	10 to 14	Both	0<br>(0 to 0)	0.0<br>(0.0 to 0.0)	0<br>(0 to 0)	0.0<br>(0.0 to 0.0)	0<br>(0 to 0)	0.0<br>(0.0 to 0.0)	-76.0<br>(-95.8 to 41.9)	-69.0<br>(-94.6 to 83.2)
Cyprus	Varicella and herpes zoster	10 to 14	Female	0<br>(0 to 0)	0.0<br>(0.0 to 0.0)	0<br>(0 to 0)	0.0<br>(0.0 to 0.0)	0<br>(0 to 0)	0.0<br>(0.0 to 0.0)	-77.1<br>(-98.1 to 252.6)	-70.8<br>(-97.5 to 350.1)
Cyprus	Varicella and herpes zoster	10 to 14	Male	0<br>(0 to 0)	0.0<br>(0.0 to 0.0)	0<br>(0 to 0)	0.0<br>(0.0 to 0.0)	0<br>(0 to 0)	0.0<br>(0.0 to 0.0)	-75.0<br>(-98.4 to 329.0)	-67.4<br>(-97.9 to 459.8)

eTable 8. All-cause and cause-specific mortality (counts and rates) at the global level and by country in the WHO European Region for ages 5 to 9 and 10 to 14 for 1990, 2000, and 2016

Cyprus	Malaria	5 to 9	Both	0 (0 to 0)	0.0 (0.0 to 0.0)	0 (0 to 0)	0.0 (0.0 to 0.0)	0 (0 to 0)	0.0 (0.0 to 0.0)	0.0 (0.0 to 0.0)	0.0 (0.0 to 0.0)
Cyprus	Malaria	5 to 9	Female	0 (0 to 0)	0.0 (0.0 to 0.0)	0 (0 to 0)	0.0 (0.0 to 0.0)	0 (0 to 0)	0.0 (0.0 to 0.0)	0.0 (0.0 to 0.0)	0.0 (0.0 to 0.0)
Cyprus	Malaria	5 to 9	Male	0 (0 to 0)	0.0 (0.0 to 0.0)	0 (0 to 0)	0.0 (0.0 to 0.0)	0 (0 to 0)	0.0 (0.0 to 0.0)	0.0 (0.0 to 0.0)	0.0 (0.0 to 0.0)
Cyprus	Malaria	10 to 14	Both	0 (0 to 0)	0.0 (0.0 to 0.0)	0 (0 to 0)	0.0 (0.0 to 0.0)	0 (0 to 0)	0.0 (0.0 to 0.0)	0.0 (0.0 to 0.0)	0.0 (0.0 to 0.0)
Cyprus	Malaria	10 to 14	Female	0 (0 to 0)	0.0 (0.0 to 0.0)	0 (0 to 0)	0.0 (0.0 to 0.0)	0 (0 to 0)	0.0 (0.0 to 0.0)	0.0 (0.0 to 0.0)	0.0 (0.0 to 0.0)
Cyprus	Malaria	10 to 14	Male	0 (0 to 0)	0.0 (0.0 to 0.0)	0 (0 to 0)	0.0 (0.0 to 0.0)	0 (0 to 0)	0.0 (0.0 to 0.0)	0.0 (0.0 to 0.0)	0.0 (0.0 to 0.0)
Cyprus	Leishmaniasis	5 to 9	Both	0 (0 to 0)	0.0 (0.0 to 0.0)	0 (0 to 0)	0.0 (0.0 to 0.0)	0 (0 to 0)	0.0 (0.0 to 0.0)	-64.7 (-100.0 to 212.2)	-56.1 (-100.0 to 288.9)
Cyprus	Leishmaniasis	5 to 9	Female	0 (0 to 0)	0.0 (0.0 to 0.0)	0 (0 to 0)	0.0 (0.0 to 0.0)	0 (0 to 0)	0.0 (0.0 to 0.0)	-61.5 (-100.0 to 133.5)	-52.0 (-100.0 to 191.2)
Cyprus	Leishmaniasis	5 to 9	Male	0 (0 to 0)	0.0 (0.0 to 0.0)	0 (0 to 0)	0.0 (0.0 to 0.0)	0 (0 to 0)	0.0 (0.0 to 0.0)	-67.7 (-100.0 to 43.2)	-59.8 (-100.0 to 78.2)
Cyprus	Leishmaniasis	10 to 14	Both	0 (0 to 0)	0.0 (0.0 to 0.0)	0 (0 to 0)	0.0 (0.0 to 0.0)	0 (0 to 0)	0.0 (0.0 to 0.0)	-60.2 (-100.0 to 367.4)	-48.7 (-100.0 to 503.4)
Cyprus	Leishmaniasis	10 to 14	Female	0 (0 to 0)	0.0 (0.0 to 0.0)	0 (0 to 0)	0.0 (0.0 to 0.0)	0 (0 to 0)	0.0 (0.0 to 0.0)	-60.5 (-100.0 to 89.4)	-49.6 (-100.0 to 141.8)
Cyprus	Leishmaniasis	10 to 14	Male	0 (0 to 0)	0.0 (0.0 to 0.0)	0 (0 to 0)	0.0 (0.0 to 0.0)	0 (0 to 0)	0.0 (0.0 to 0.0)	-60.0 (-100.0 to 87.1)	-47.8 (-100.0 to 144.2)
Cyprus	African trypanosomiasis	5 to 9	Both	0 (0 to 0)	0.0 (0.0 to 0.0)	0 (0 to 0)	0.0 (0.0 to 0.0)	0 (0 to 0)	0.0 (0.0 to 0.0)	0.0 (0.0 to 0.0)	0.0 (0.0 to 0.0)
Cyprus	African trypanosomiasis	5 to 9	Female	0 (0 to 0)	0.0 (0.0 to 0.0)	0 (0 to 0)	0.0 (0.0 to 0.0)	0 (0 to 0)	0.0 (0.0 to 0.0)	0.0 (0.0 to 0.0)	0.0 (0.0 to 0.0)
Cyprus	African trypanosomiasis	5 to 9	Male	0 (0 to 0)	0.0 (0.0 to 0.0)	0 (0 to 0)	0.0 (0.0 to 0.0)	0 (0 to 0)	0.0 (0.0 to 0.0)	0.0 (0.0 to 0.0)	0.0 (0.0 to 0.0)
Cyprus	African trypanosomiasis	10 to 14	Both	0 (0 to 0)	0.0 (0.0 to 0.0)	0 (0 to 0)	0.0 (0.0 to 0.0)	0 (0 to 0)	0.0 (0.0 to 0.0)	0.0 (0.0 to 0.0)	0.0 (0.0 to 0.0)
Cyprus	African trypanosomiasis	10 to 14	Female	0 (0 to 0)	0.0 (0.0 to 0.0)	0 (0 to 0)	0.0 (0.0 to 0.0)	0 (0 to 0)	0.0 (0.0 to 0.0)	0.0 (0.0 to 0.0)	0.0 (0.0 to 0.0)
Cyprus	African trypanosomiasis	10 to 14	Male	0 (0 to 0)	0.0 (0.0 to 0.0)	0 (0 to 0)	0.0 (0.0 to 0.0)	0 (0 to 0)	0.0 (0.0 to 0.0)	0.0 (0.0 to 0.0)	0.0 (0.0 to 0.0)
Cyprus	Schistosomiasis	5 to 9	Both	0 (0 to 0)	0.0 (0.0 to 0.0)	0 (0 to 0)	0.0 (0.0 to 0.0)	0 (0 to 0)	0.0 (0.0 to 0.0)	0.0 (0.0 to 0.0)	0.0 (0.0 to 0.0)
Cyprus	Schistosomiasis	5 to 9	Female	0 (0 to 0)	0.0 (0.0 to 0.0)	0 (0 to 0)	0.0 (0.0 to 0.0)	0 (0 to 0)	0.0 (0.0 to 0.0)	0.0 (0.0 to 0.0)	0.0 (0.0 to 0.0)
Cyprus	Schistosomiasis	5 to 9	Male	0 (0 to 0)	0.0 (0.0 to 0.0)	0 (0 to 0)	0.0 (0.0 to 0.0)	0 (0 to 0)	0.0 (0.0 to 0.0)	0.0 (0.0 to 0.0)	0.0 (0.0 to 0.0)
Cyprus	Schistosomiasis	10 to 14	Both	0 (0 to 0)	0.0 (0.0 to 0.0)	0 (0 to 0)	0.0 (0.0 to 0.0)	0 (0 to 0)	0.0 (0.0 to 0.0)	0.0 (0.0 to 0.0)	0.0 (0.0 to 0.0)
Cyprus	Schistosomiasis	10 to 14	Female	0 (0 to 0)	0.0 (0.0 to 0.0)	0 (0 to 0)	0.0 (0.0 to 0.0)	0 (0 to 0)	0.0 (0.0 to 0.0)	0.0 (0.0 to 0.0)	0.0 (0.0 to 0.0)
Cyprus	Schistosomiasis	10 to 14	Male	0 (0 to 0)	0.0 (0.0 to 0.0)	0 (0 to 0)	0.0 (0.0 to 0.0)	0 (0 to 0)	0.0 (0.0 to 0.0)	0.0 (0.0 to 0.0)	0.0 (0.0 to 0.0)
Cyprus	Cysticercosis	5 to 9	Both	0 (0 to 0)	0.0 (0.0 to 0.0)	0 (0 to 0)	0.0 (0.0 to 0.0)	0 (0 to 0)	0.0 (0.0 to 0.0)	-87.9 (-90.2 to -85.3)	-85.0 (-87.7 to -81.7)
Cyprus	Cysticercosis	5 to 9	Female	0 (0 to 0)	0.0 (0.0 to 0.0)	0 (0 to 0)	0.0 (0.0 to 0.0)	0 (0 to 0)	0.0 (0.0 to 0.0)	-83.9 (-87.3 to -80.1)	-80.0 (-84.1 to -75.2)
Cyprus	Cysticercosis	5 to 9	Male	0 (0 to 0)	0.0 (0.0 to 0.0)	0 (0 to 0)	0.0 (0.0 to 0.0)	0 (0 to 0)	0.0 (0.0 to 0.0)	-91.7 (-93.8 to -89.1)	-89.6 (-92.3 to -86.4)
Cyprus	Cysticercosis	10 to 14	Both	0 (0 to 0)	0.0 (0.0 to 0.0)	0 (0 to 0)	0.0 (0.0 to 0.0)	0 (0 to 0)	0.0 (0.0 to 0.0)	-81.4 (-84.3 to -77.8)	-75.9 (-79.7 to -71.3)
Cyprus	Cysticercosis	10 to 14	Female	0 (0 to 0)	0.0 (0.0 to 0.0)	0 (0 to 0)	0.0 (0.0 to 0.0)	0 (0 to 0)	0.0 (0.0 to 0.0)	-85.0 (-87.6 to -81.6)	-80.9 (-84.1 to -76.5)

eTable 8. All-cause and cause-specific mortality (counts and rates) at the global level and by country in the WHO European Region for ages 5 to 9 and 10 to 14 for 1990, 2000, and 2016

Cyprus	Cysticercosis	10 to 14	Male	0 (0 to 0)	0.0 (0.0 to 0.0)	0 (0 to 0)	0.0 (0.0 to 0.0)	0 (0 to 0)	0.0 (0.0 to 0.0)	-78.7 (-82.6 to -73.7)	-72.3 (-77.3 to -65.7)
Cyprus	Yellow fever	5 to 9	Both	0 (0 to 0)	0.0 (0.0 to 0.0)	0 (0 to 0)	0.0 (0.0 to 0.0)	0 (0 to 0)	0.0 (0.0 to 0.0)	0.0 (0.0 to 0.0)	0.0 (0.0 to 0.0)
Cyprus	Yellow fever	5 to 9	Female	0 (0 to 0)	0.0 (0.0 to 0.0)	0 (0 to 0)	0.0 (0.0 to 0.0)	0 (0 to 0)	0.0 (0.0 to 0.0)	0.0 (0.0 to 0.0)	0.0 (0.0 to 0.0)
Cyprus	Yellow fever	5 to 9	Male	0 (0 to 0)	0.0 (0.0 to 0.0)	0 (0 to 0)	0.0 (0.0 to 0.0)	0 (0 to 0)	0.0 (0.0 to 0.0)	0.0 (0.0 to 0.0)	0.0 (0.0 to 0.0)
Cyprus	Yellow fever	10 to 14	Both	0 (0 to 0)	0.0 (0.0 to 0.0)	0 (0 to 0)	0.0 (0.0 to 0.0)	0 (0 to 0)	0.0 (0.0 to 0.0)	0.0 (0.0 to 0.0)	0.0 (0.0 to 0.0)
Cyprus	Yellow fever	10 to 14	Female	0 (0 to 0)	0.0 (0.0 to 0.0)	0 (0 to 0)	0.0 (0.0 to 0.0)	0 (0 to 0)	0.0 (0.0 to 0.0)	0.0 (0.0 to 0.0)	0.0 (0.0 to 0.0)
Cyprus	Yellow fever	10 to 14	Male	0 (0 to 0)	0.0 (0.0 to 0.0)	0 (0 to 0)	0.0 (0.0 to 0.0)	0 (0 to 0)	0.0 (0.0 to 0.0)	0.0 (0.0 to 0.0)	0.0 (0.0 to 0.0)
Cyprus	Rabies	5 to 9	Both	0 (0 to 0)	0.0 (0.0 to 0.0)	0 (0 to 0)	0.0 (0.0 to 0.0)	0 (0 to 0)	0.0 (0.0 to 0.0)	-86.4 (-98.4 to 75.3)	-83.0 (-98.0 to 118.3)
Cyprus	Rabies	5 to 9	Female	0 (0 to 0)	0.0 (0.0 to 0.0)	0 (0 to 0)	0.0 (0.0 to 0.0)	0 (0 to 0)	0.0 (0.0 to 0.0)	-63.4 (-96.1 to 437.5)	-54.3 (-95.1 to 570.4)
Cyprus	Rabies	5 to 9	Male	0 (0 to 0)	0.0 (0.0 to 0.0)	0 (0 to 0)	0.0 (0.0 to 0.0)	0 (0 to 0)	0.0 (0.0 to 0.0)	-86.8 (-98.6 to 84.8)	-83.5 (-98.3 to 129.9)
Cyprus	Rabies	10 to 14	Both	0 (0 to 0)	0.0 (0.0 to 0.0)	0 (0 to 0)	0.0 (0.0 to 0.0)	0 (0 to 0)	0.0 (0.0 to 0.0)	-57.2 (-94.0 to 255.4)	-44.7 (-92.3 to 358.9)
Cyprus	Rabies	10 to 14	Female	0 (0 to 0)	0.0 (0.0 to 0.0)	0 (0 to 0)	0.0 (0.0 to 0.0)	0 (0 to 0)	0.0 (0.0 to 0.0)	-44.3 (-93.7 to 770.4)	-28.9 (-91.9 to 1011.0)
Cyprus	Rabies	10 to 14	Male	0 (0 to 0)	0.0 (0.0 to 0.0)	0 (0 to 0)	0.0 (0.0 to 0.0)	0 (0 to 0)	0.0 (0.0 to 0.0)	-57.5 (-94.2 to 325.1)	-44.5 (-92.5 to 454.7)
Cyprus	Intestinal nematode infections	5 to 9	Both	-	-	-	-	-	-	-	-
Cyprus	Intestinal nematode infections	5 to 9	Female	-	-	-	-	-	-	-	-
Cyprus	Intestinal nematode infections	5 to 9	Male	-	-	-	-	-	-	-	-
Cyprus	Intestinal nematode infections	10 to 14	Both	-	-	-	-	-	-	-	-
Cyprus	Intestinal nematode infections	10 to 14	Female	-	-	-	-	-	-	-	-
Cyprus	Intestinal nematode infections	10 to 14	Male	-	-	-	-	-	-	-	-
Cyprus	Ebola	5 to 9	Both	0 (0 to 0)	0.0 (0.0 to 0.0)	0 (0 to 0)	0.0 (0.0 to 0.0)	0 (0 to 0)	0.0 (0.0 to 0.0)	0.0 (0.0 to 0.0)	0.0 (0.0 to 0.0)
Cyprus	Ebola	5 to 9	Female	0 (0 to 0)	0.0 (0.0 to 0.0)	0 (0 to 0)	0.0 (0.0 to 0.0)	0 (0 to 0)	0.0 (0.0 to 0.0)	0.0 (0.0 to 0.0)	0.0 (0.0 to 0.0)
Cyprus	Ebola	5 to 9	Male	0 (0 to 0)	0.0 (0.0 to 0.0)	0 (0 to 0)	0.0 (0.0 to 0.0)	0 (0 to 0)	0.0 (0.0 to 0.0)	0.0 (0.0 to 0.0)	0.0 (0.0 to 0.0)
Cyprus	Ebola	10 to 14	Both	0 (0 to 0)	0.0 (0.0 to 0.0)	0 (0 to 0)	0.0 (0.0 to 0.0)	0 (0 to 0)	0.0 (0.0 to 0.0)	0.0 (0.0 to 0.0)	0.0 (0.0 to 0.0)
Cyprus	Ebola	10 to 14	Female	0 (0 to 0)	0.0 (0.0 to 0.0)	0 (0 to 0)	0.0 (0.0 to 0.0)	0 (0 to 0)	0.0 (0.0 to 0.0)	0.0 (0.0 to 0.0)	0.0 (0.0 to 0.0)
Cyprus	Ebola	10 to 14	Male	0 (0 to 0)	0.0 (0.0 to 0.0)	0 (0 to 0)	0.0 (0.0 to 0.0)	0 (0 to 0)	0.0 (0.0 to 0.0)	0.0 (0.0 to 0.0)	0.0 (0.0 to 0.0)
Cyprus	Zika virus	5 to 9	Both	0 (0 to 0)	0.0 (0.0 to 0.0)	0 (0 to 0)	0.0 (0.0 to 0.0)	0 (0 to 0)	0.0 (0.0 to 0.0)	0.0 (0.0 to 0.0)	0.0 (0.0 to 0.0)
Cyprus	Zika virus	5 to 9	Female	0 (0 to 0)	0.0 (0.0 to 0.0)	0 (0 to 0)	0.0 (0.0 to 0.0)	0 (0 to 0)	0.0 (0.0 to 0.0)	0.0 (0.0 to 0.0)	0.0 (0.0 to 0.0)
Cyprus	Zika virus	5 to 9	Male	0 (0 to 0)	0.0 (0.0 to 0.0)	0 (0 to 0)	0.0 (0.0 to 0.0)	0 (0 to 0)	0.0 (0.0 to 0.0)	0.0 (0.0 to 0.0)	0.0 (0.0 to 0.0)
Cyprus	Zika virus	10 to 14	Both	0 (0 to 0)	0.0 (0.0 to 0.0)	0 (0 to 0)	0.0 (0.0 to 0.0)	0 (0 to 0)	0.0 (0.0 to 0.0)	0.0 (0.0 to 0.0)	0.0 (0.0 to 0.0)

eTable 8. All-cause and cause-specific mortality (counts and rates) at the global level and by country in the WHO European Region for ages 5 to 9 and 10 to 14 for 1990, 2000, and 2016

Cyprus	Zika virus	10 to 14	Female	0 (0 to 0)	0.0 (0.0 to 0.0)	0 (0 to 0)	0.0 (0.0 to 0.0)	0 (0 to 0)	0.0 (0.0 to 0.0)	0.0 (0.0 to 0.0)	0.0 (0.0 to 0.0)
Cyprus	Zika virus	10 to 14	Male	0 (0 to 0)	0.0 (0.0 to 0.0)	0 (0 to 0)	0.0 (0.0 to 0.0)	0 (0 to 0)	0.0 (0.0 to 0.0)	0.0 (0.0 to 0.0)	0.0 (0.0 to 0.0)
Cyprus	Other neglected tropical diseases	5 to 9	Both	0 (0 to 0)	0.0 (0.0 to 0.0)	0 (0 to 0)	0.0 (0.0 to 0.0)	0 (0 to 0)	0.0 (0.0 to 0.0)	-42.5 (-76.8 to 28.1)	-28.4 (-71.1 to 59.6)
Cyprus	Other neglected tropical diseases	5 to 9	Female	0 (0 to 0)	0.0 (0.0 to 0.0)	0 (0 to 0)	0.0 (0.0 to 0.0)	0 (0 to 0)	0.0 (0.0 to 0.0)	-39.9 (-84.1 to 103.2)	-25.1 (-80.1 to 153.4)
Cyprus	Other neglected tropical diseases	5 to 9	Male	0 (0 to 0)	0.0 (0.0 to 0.0)	0 (0 to 0)	0.0 (0.0 to 0.0)	0 (0 to 0)	0.0 (0.0 to 0.0)	-45.6 (-76.4 to -14.9)	-32.3 (-70.7 to 5.9)
Cyprus	Other neglected tropical diseases	10 to 14	Both	0 (0 to 0)	0.0 (0.0 to 0.0)	0 (0 to 0)	0.0 (0.0 to 0.0)	0 (0 to 0)	0.0 (0.0 to 0.0)	-35.2 (-66.1 to -2.3)	-16.3 (-56.2 to 26.1)
Cyprus	Other neglected tropical diseases	10 to 14	Female	0 (0 to 0)	0.0 (0.0 to 0.0)	0 (0 to 0)	0.0 (0.0 to 0.0)	0 (0 to 0)	0.0 (0.0 to 0.0)	-41.4 (-79.2 to 9.0)	-25.2 (-73.4 to 39.1)
Cyprus	Other neglected tropical diseases	10 to 14	Male	0 (0 to 0)	0.0 (0.0 to 0.0)	0 (0 to 0)	0.0 (0.0 to 0.0)	0 (0 to 0)	0.0 (0.0 to 0.0)	-28.6 (-57.9 to -6.3)	-6.8 (-45.0 to 22.3)
Cyprus	Maternal hemorrhage	10 to 14	Both	0 (0 to 0)	0.0 (0.0 to 0.0)	0 (0 to 0)	0.0 (0.0 to 0.0)	0 (0 to 0)	0.0 (0.0 to 0.0)	-67.0 (-89.4 to -2.1)	-57.4 (-86.3 to 26.3)
Cyprus	Maternal hemorrhage	10 to 14	Female	0 (0 to 0)	0.0 (0.0 to 0.0)	0 (0 to 0)	0.0 (0.0 to 0.0)	0 (0 to 0)	0.0 (0.0 to 0.0)	-67.0 (-89.4 to -2.1)	-57.9 (-86.4 to 24.9)
Cyprus	Maternal sepsis and other maternal infections	10 to 14	Both	0 (0 to 0)	0.0 (0.0 to 0.0)	0 (0 to 0)	0.0 (0.0 to 0.0)	0 (0 to 0)	0.0 (0.0 to 0.0)	-70.8 (-90.5 to -8.4)	-62.3 (-87.7 to 18.3)
Cyprus	Maternal sepsis and other maternal infections	10 to 14	Female	0 (0 to 0)	0.0 (0.0 to 0.0)	0 (0 to 0)	0.0 (0.0 to 0.0)	0 (0 to 0)	0.0 (0.0 to 0.0)	-70.8 (-90.5 to -8.4)	-62.8 (-87.8 to 17.0)
Cyprus	Maternal hypertensive disorders	10 to 14	Both	0 (0 to 0)	0.0 (0.0 to 0.0)	0 (0 to 0)	0.0 (0.0 to 0.0)	0 (0 to 0)	0.0 (0.0 to 0.0)	-68.1 (-88.9 to -2.9)	-58.8 (-85.7 to 25.3)
Cyprus	Maternal hypertensive disorders	10 to 14	Female	0 (0 to 0)	0.0 (0.0 to 0.0)	0 (0 to 0)	0.0 (0.0 to 0.0)	0 (0 to 0)	0.0 (0.0 to 0.0)	-68.1 (-88.9 to -2.9)	-59.3 (-85.9 to 23.9)
Cyprus	Maternal obstructed labor and uterine rupture	10 to 14	Both	0 (0 to 0)	0.0 (0.0 to 0.0)	0 (0 to 0)	0.0 (0.0 to 0.0)	0 (0 to 0)	0.0 (0.0 to 0.0)	-69.7 (-90.9 to 3.4)	-60.8 (-88.2 to 33.4)
Cyprus	Maternal obstructed labor and uterine rupture	10 to 14	Female	0 (0 to 0)	0.0 (0.0 to 0.0)	0 (0 to 0)	0.0 (0.0 to 0.0)	0 (0 to 0)	0.0 (0.0 to 0.0)	-69.7 (-90.9 to 3.4)	-61.3 (-88.3 to 31.9)
Cyprus	Maternal abortion, miscarriage, and ectopic pregnancy	10 to 14	Both	0 (0 to 0)	0.0 (0.0 to 0.0)	0 (0 to 0)	0.0 (0.0 to 0.0)	0 (0 to 0)	0.0 (0.0 to 0.0)	-71.0 (-90.3 to -9.5)	-62.5 (-87.5 to 16.8)

eTable 8. All-cause and cause-specific mortality (counts and rates) at the global level and by country in the WHO European Region for ages 5 to 9 and 10 to 14 for 1990, 2000, and 2016

Cyprus	Maternal abortion, miscarriage, and ectopic pregnancy	10 to 14	Female	0 (0 to 0)	0.0 (0.0 to 0.0)	0 (0 to 0)	0.0 (0.0 to 0.0)	0 (0 to 0)	0.0 (0.0 to 0.0)	-71.0 (-90.3 to -9.5)	-63.0 (-87.7 to 15.5)
Cyprus	Indirect maternal deaths	10 to 14	Both	0 (0 to 0)	0.0 (0.0 to 0.0)	0 (0 to 0)	0.0 (0.0 to 0.0)	0 (0 to 0)	0.0 (0.0 to 0.0)	-70.0 (-90.1 to -9.3)	-61.3 (-87.2 to 17.1)
Cyprus	Indirect maternal deaths	10 to 14	Female	0 (0 to 0)	0.0 (0.0 to 0.0)	0 (0 to 0)	0.0 (0.0 to 0.0)	0 (0 to 0)	0.0 (0.0 to 0.0)	-70.0 (-90.1 to -9.3)	-61.8 (-87.3 to 15.8)
Cyprus	Late maternal deaths	10 to 14	Both	0 (0 to 0)	0.0 (0.0 to 0.0)	0 (0 to 0)	0.0 (0.0 to 0.0)	0 (0 to 0)	0.0 (0.0 to 0.0)	-68.8 (-92.4 to 44.6)	-59.7 (-90.2 to 86.6)
Cyprus	Late maternal deaths	10 to 14	Female	0 (0 to 0)	0.0 (0.0 to 0.0)	0 (0 to 0)	0.0 (0.0 to 0.0)	0 (0 to 0)	0.0 (0.0 to 0.0)	-68.8 (-92.4 to 44.6)	-60.2 (-90.3 to 84.5)
Cyprus	Maternal deaths aggravated by HIV/AIDS	10 to 14	Both	0 (0 to 0)	0.0 (0.0 to 0.0)	0 (0 to 0)	0.0 (0.0 to 0.0)	0 (0 to 0)	0.0 (0.0 to 0.0)	478.7 (-3.2 to 2316.5)	647.0 (25.0 to 3019.5)
Cyprus	Maternal deaths aggravated by HIV/AIDS	10 to 14	Female	0 (0 to 0)	0.0 (0.0 to 0.0)	0 (0 to 0)	0.0 (0.0 to 0.0)	0 (0 to 0)	0.0 (0.0 to 0.0)	478.7 (-3.2 to 2316.5)	638.6 (23.6 to 2984.5)
Cyprus	Other maternal disorders	10 to 14	Both	0 (0 to 0)	0.0 (0.0 to 0.0)	0 (0 to 0)	0.0 (0.0 to 0.0)	0 (0 to 0)	0.0 (0.0 to 0.0)	-69.2 (-89.7 to -3.2)	-60.2 (-86.7 to 25.0)
Cyprus	Other maternal disorders	10 to 14	Female	0 (0 to 0)	0.0 (0.0 to 0.1)	0 (0 to 0)	0.0 (0.0 to 0.1)	0 (0 to 0)	0.0 (0.0 to 0.0)	-69.2 (-89.7 to -3.2)	-60.6 (-86.8 to 23.6)
Cyprus	Protein-energy malnutrition	5 to 9	Both	0 (0 to 0)	0.0 (0.0 to 0.0)	0 (0 to 0)	0.0 (0.0 to 0.0)	0 (0 to 0)	0.0 (0.0 to 0.0)	2.8 (-65.9 to 250.0)	28.1 (-57.6 to 336.0)
Cyprus	Protein-energy malnutrition	5 to 9	Female	0 (0 to 0)	0.0 (0.0 to 0.0)	0 (0 to 0)	0.0 (0.0 to 0.0)	0 (0 to 0)	0.0 (0.0 to 0.0)	12.3 (-69.8 to 433.7)	40.1 (-62.3 to 565.6)
Cyprus	Protein-energy malnutrition	5 to 9	Male	0 (0 to 0)	0.0 (0.0 to 0.0)	0 (0 to 0)	0.0 (0.0 to 0.0)	0 (0 to 0)	0.0 (0.0 to 0.0)	-42.2 (-80.7 to 109.8)	-28.1 (-76.0 to 161.0)
Cyprus	Protein-energy malnutrition	10 to 14	Both	0 (0 to 0)	0.0 (0.0 to 0.0)	0 (0 to 0)	0.0 (0.0 to 0.0)	0 (0 to 0)	0.0 (0.0 to 0.0)	-35.1 (-68.3 to 40.1)	-16.3 (-59.0 to 80.9)
Cyprus	Protein-energy malnutrition	10 to 14	Female	0 (0 to 0)	0.0 (0.0 to 0.0)	0 (0 to 0)	0.0 (0.0 to 0.0)	0 (0 to 0)	0.0 (0.0 to 0.0)	-38.5 (-74.6 to 74.9)	-21.5 (-67.6 to 123.3)
Cyprus	Protein-energy malnutrition	10 to 14	Male	0 (0 to 0)	0.0 (0.0 to 0.0)	0 (0 to 0)	0.0 (0.0 to 0.0)	0 (0 to 0)	0.0 (0.0 to 0.0)	-32.6 (-76.0 to 92.6)	-12.1 (-68.7 to 151.4)
Cyprus	Iodine deficiency	5 to 9	Both	0 (0 to 0)	0.0 (0.0 to 0.0)	0 (0 to 0)	0.0 (0.0 to 0.0)	0 (0 to 0)	0.0 (0.0 to 0.0)	-47.3 (-100.0 to 2.0e+15)	-34.4 (-100.0 to 2.5e+15)
Cyprus	Iodine deficiency	5 to 9	Female	0 (0 to 0)	0.0 (0.0 to 0.1)	0 (0 to 0)	0.0 (0.0 to 0.1)	0 (0 to 0)	0.0 (0.0 to 0.0)	-41.7 (-100.0 to 4.3e+25)	-27.3 (-100.0 to 5.3e+25)
Cyprus	Iodine deficiency	5 to 9	Male	0 (0 to 0)	0.0 (0.0 to 0.0)	0 (0 to 0)	0.0 (0.0 to 0.0)	0 (0 to 0)	0.0 (0.0 to 0.0)	-76.7 (-100.0 to 5.2e+23)	-71.0 (-100.0 to 6.5e+23)
Cyprus	Iodine deficiency	10 to 14	Both	0 (0 to 0)	0.0 (0.0 to 0.0)	0 (0 to 0)	0.0 (0.0 to 0.0)	0 (0 to 0)	0.0 (0.0 to 0.0)	-58.3 (-100.0 to 2.6e+18)	-46.2 (-100.0 to 3.4e+18)
Cyprus	Iodine deficiency	10 to 14	Female	0 (0 to 0)	0.0 (0.0 to 0.0)	0 (0 to 0)	0.0 (0.0 to 0.0)	0 (0 to 0)	0.0 (0.0 to 0.0)	-75.1 (-100.0 to 3.6e+25)	-68.3 (-100.0 to 4.6e+25)
Cyprus	Iodine deficiency	10 to 14	Male	0 (0 to 0)	0.0 (0.0 to 0.0)	0 (0 to 0)	0.0 (0.0 to 0.0)	0 (0 to 0)	0.0 (0.0 to 0.0)	-43.1 (-100.0 to 6.8e+24)	-25.8 (-100.0 to 8.9e+24)
Cyprus	Iron-deficiency anemia	5 to 9	Both	0 (0 to 0)	0.0 (0.0 to 0.0)	0 (0 to 0)	0.0 (0.0 to 0.0)	0 (0 to 0)	0.0 (0.0 to 0.0)	1.2 (-65.9 to 246.9)	26.1 (-57.6 to 332.1)
Cyprus	Iron-deficiency anemia	5 to 9	Female	0 (0 to 0)	0.0 (0.0 to 0.0)	0 (0 to 0)	0.0 (0.0 to 0.0)	0 (0 to 0)	0.0 (0.0 to 0.0)	12.3 (-69.8 to 433.7)	40.1 (-62.3 to 565.6)

eTable 8. All-cause and cause-specific mortality (counts and rates) at the global level and by country in the WHO European Region for ages 5 to 9 and 10 to 14 for 1990, 2000, and 2016

Cyprus	Iron-deficiency anemia	5 to 9	Male	0 (0 to 0)	0.0 (0.0 to 0.0)	0 (0 to 0)	0.0 (0.0 to 0.0)	0 (0 to 0)	0.0 (0.0 to 0.0)	-46.5 (-83.0 to 97.1)	-33.4 (-78.9 to 145.2)
Cyprus	Iron-deficiency anemia	10 to 14	Both	0 (0 to 0)	0.0 (0.0 to 0.0)	0 (0 to 0)	0.0 (0.0 to 0.0)	0 (0 to 0)	0.0 (0.0 to 0.0)	-35.1 (-68.3 to 40.1)	-16.2 (-59.0 to 80.9)
Cyprus	Iron-deficiency anemia	10 to 14	Female	0 (0 to 0)	0.0 (0.0 to 0.0)	0 (0 to 0)	0.0 (0.0 to 0.0)	0 (0 to 0)	0.0 (0.0 to 0.0)	-38.5 (-74.6 to 74.9)	-21.5 (-67.6 to 123.3)
Cyprus	Iron-deficiency anemia	10 to 14	Male	0 (0 to 0)	0.0 (0.0 to 0.0)	0 (0 to 0)	0.0 (0.0 to 0.0)	0 (0 to 0)	0.0 (0.0 to 0.0)	-32.6 (-75.9 to 92.6)	-12.0 (-68.6 to 151.4)
Cyprus	Other nutritional deficiencies	5 to 9	Both	0 (0 to 0)	0.0 (0.0 to 0.1)	0 (0 to 0)	0.0 (0.0 to 0.1)	0 (0 to 0)	0.0 (0.0 to 0.1)	-15.1 (-68.6 to 160.5)	5.8 (-60.9 to 224.5)
Cyprus	Other nutritional deficiencies	5 to 9	Female	0 (0 to 0)	0.0 (0.0 to 0.1)	0 (0 to 0)	0.1 (0.0 to 0.1)	0 (0 to 0)	0.1 (0.0 to 0.1)	-7.1 (-69.0 to 273.8)	15.9 (-61.3 to 366.1)
Cyprus	Other nutritional deficiencies	5 to 9	Male	0 (0 to 0)	0.0 (0.0 to 0.0)	0 (0 to 0)	0.0 (0.0 to 0.0)	0 (0 to 0)	0.0 (0.0 to 0.0)	-58.5 (-85.7 to 50.3)	-48.3 (-82.3 to 87.0)
Cyprus	Other nutritional deficiencies	10 to 14	Both	0 (0 to 0)	0.0 (0.0 to 0.0)	0 (0 to 0)	0.0 (0.0 to 0.0)	0 (0 to 0)	0.0 (0.0 to 0.0)	-48.2 (-74.6 to 18.6)	-33.1 (-67.2 to 53.1)
Cyprus	Other nutritional deficiencies	10 to 14	Female	0 (0 to 0)	0.0 (0.0 to 0.0)	0 (0 to 0)	0.0 (0.0 to 0.0)	0 (0 to 0)	0.0 (0.0 to 0.0)	-56.9 (-82.1 to 8.0)	-44.9 (-77.1 to 37.9)
Cyprus	Other nutritional deficiencies	10 to 14	Male	0 (0 to 0)	0.0 (0.0 to 0.0)	0 (0 to 0)	0.0 (0.0 to 0.0)	0 (0 to 0)	0.0 (0.0 to 0.0)	-41.6 (-78.3 to 74.6)	-23.8 (-71.7 to 127.9)
Cyprus	Sexually transmitted diseases excluding HIV	5 to 9	Both	0 (0 to 0)	0.0 (0.0 to 0.0)	0 (0 to 0)	0.0 (0.0 to 0.0)	0 (0 to 0)	0.0 (0.0 to 0.0)	-86.9 (-93.1 to -76.3)	-83.7 (-91.5 to -70.5)
Cyprus	Sexually transmitted diseases excluding HIV	5 to 9	Female	0 (0 to 0)	0.0 (0.0 to 0.0)	0 (0 to 0)	0.0 (0.0 to 0.0)	0 (0 to 0)	0.0 (0.0 to 0.0)	-86.4 (-92.9 to -74.9)	-83.0 (-91.1 to -68.7)
Cyprus	Sexually transmitted diseases excluding HIV	5 to 9	Male	0 (0 to 0)	0.0 (0.0 to 0.0)	0 (0 to 0)	0.0 (0.0 to 0.0)	0 (0 to 0)	0.0 (0.0 to 0.0)	-87.9 (-94.0 to -77.8)	-85.0 (-92.6 to -72.4)
Cyprus	Sexually transmitted diseases excluding HIV	10 to 14	Both	0 (0 to 0)	0.0 (0.0 to 0.0)	0 (0 to 0)	0.0 (0.0 to 0.0)	0 (0 to 0)	0.0 (0.0 to 0.0)	-29.7 (-50.6 to -13.7)	-9.2 (-36.2 to 11.4)
Cyprus	Sexually transmitted diseases excluding HIV	10 to 14	Female	0 (0 to 0)	0.0 (0.0 to 0.0)	0 (0 to 0)	0.0 (0.0 to 0.0)	0 (0 to 0)	0.0 (0.0 to 0.0)	-34.3 (-64.1 to -11.8)	-16.1 (-54.2 to 12.6)
Cyprus	Sexually transmitted diseases excluding HIV	10 to 14	Male	0 (0 to 0)	0.0 (0.0 to 0.0)	0 (0 to 0)	0.0 (0.0 to 0.0)	0 (0 to 0)	0.0 (0.0 to 0.0)	-25.4 (-39.2 to -7.9)	-2.6 (-20.7 to 20.1)
Cyprus	Hepatitis	5 to 9	Both	0 (0 to 0)	0.0 (0.0 to 0.0)	0 (0 to 0)	0.0 (0.0 to 0.0)	0 (0 to 0)	0.0 (0.0 to 0.0)	-80.4 (-91.2 to -55.1)	-75.6 (-89.0 to -44.1)
Cyprus	Hepatitis	5 to 9	Female	0 (0 to 0)	0.0 (0.0 to 0.0)	0 (0 to 0)	0.0 (0.0 to 0.0)	0 (0 to 0)	0.0 (0.0 to 0.0)	-72.5 (-90.2 to -23.2)	-65.7 (-87.7 to -4.2)
Cyprus	Hepatitis	5 to 9	Male	0 (0 to 0)	0.0 (0.0 to 0.1)	0 (0 to 0)	0.0 (0.0 to 0.0)	0 (0 to 0)	0.0 (0.0 to 0.0)	-86.3 (-95.9 to -50.1)	-83.0 (-94.9 to -37.9)

eTable 8. All-cause and cause-specific mortality (counts and rates) at the global level and by country in the WHO European Region for ages 5 to 9 and 10 to 14 for 1990, 2000, and 2016

Cyprus	Hepatitis	10 to 14	Both	0 (0 to 0)	0.0 (0.0 to 0.0)	0 (0 to 0)	0.0 (0.0 to 0.0)	0 (0 to 0)	0.0 (0.0 to 0.0)	-64.2 (-82.1 to -25.0)	-53.8 (-76.9 to -3.1)
Cyprus	Hepatitis	10 to 14	Female	0 (0 to 0)	0.0 (0.0 to 0.0)	0 (0 to 0)	0.0 (0.0 to 0.0)	0 (0 to 0)	0.0 (0.0 to 0.0)	-72.4 (-86.6 to -39.9)	-64.8 (-82.9 to -23.2)
Cyprus	Hepatitis	10 to 14	Male	0 (0 to 0)	0.0 (0.0 to 0.0)	0 (0 to 0)	0.0 (0.0 to 0.0)	0 (0 to 0)	0.0 (0.0 to 0.0)	-54.7 (-85.1 to 39.0)	-40.9 (-80.6 to 81.3)
Cyprus	Other infectious diseases	5 to 9	Both	0 (0 to 0)	0.1 (0.0 to 0.2)	0 (0 to 0)	0.1 (0.0 to 0.2)	0 (0 to 0)	0.1 (0.0 to 0.1)	-46.3 (-78.7 to 34.6)	-33.1 (-73.4 to 67.6)
Cyprus	Other infectious diseases	5 to 9	Female	0 (0 to 0)	0.0 (0.0 to 0.1)	0 (0 to 0)	0.1 (0.0 to 0.1)	0 (0 to 0)	0.0 (0.0 to 0.1)	-31.0 (-72.9 to 81.3)	-13.9 (-66.3 to 126.1)
Cyprus	Other infectious diseases	5 to 9	Male	0 (0 to 0)	0.1 (0.0 to 0.3)	0 (0 to 0)	0.1 (0.1 to 0.3)	0 (0 to 0)	0.1 (0.0 to 0.1)	-52.7 (-86.0 to 54.3)	-41.1 (-82.6 to 92.0)
Cyprus	Other infectious diseases	10 to 14	Both	0 (0 to 0)	0.0 (0.0 to 0.1)	0 (0 to 0)	0.1 (0.1 to 0.1)	0 (0 to 0)	0.1 (0.0 to 0.1)	-9.3 (-55.9 to 59.0)	17.1 (-43.1 to 105.2)
Cyprus	Other infectious diseases	10 to 14	Female	0 (0 to 0)	0.0 (0.0 to 0.1)	0 (0 to 0)	0.0 (0.0 to 0.1)	0 (0 to 0)	0.0 (0.0 to 0.1)	-43.5 (-78.2 to 29.5)	-27.9 (-72.1 to 65.3)
Cyprus	Other infectious diseases	10 to 14	Male	0 (0 to 0)	0.1 (0.0 to 0.1)	0 (0 to 0)	0.1 (0.1 to 0.2)	0 (0 to 0)	0.1 (0.0 to 0.1)	10.1 (-55.6 to 123.1)	43.7 (-42.1 to 191.1)
Cyprus	Nasopharynx cancer	5 to 9	Both	0 (0 to 0)	0.0 (0.0 to 0.0)	0 (0 to 0)	0.0 (0.0 to 0.0)	0 (0 to 0)	0.0 (0.0 to 0.0)	-32.8 (-54.8 to -4.0)	-16.3 (-43.7 to 19.6)
Cyprus	Nasopharynx cancer	5 to 9	Female	0 (0 to 0)	0.0 (0.0 to 0.0)	0 (0 to 0)	0.0 (0.0 to 0.0)	0 (0 to 0)	0.0 (0.0 to 0.0)	-37.2 (-65.5 to -13.3)	-21.6 (-56.9 to 8.1)
Cyprus	Nasopharynx cancer	5 to 9	Male	0 (0 to 0)	0.0 (0.0 to 0.0)	0 (0 to 0)	0.0 (0.0 to 0.0)	0 (0 to 0)	0.0 (0.0 to 0.0)	-28.3 (-50.2 to 24.7)	-10.8 (-38.1 to 55.2)
Cyprus	Nasopharynx cancer	10 to 14	Both	0 (0 to 0)	0.0 (0.0 to 0.0)	0 (0 to 0)	0.0 (0.0 to 0.0)	0 (0 to 0)	0.0 (0.0 to 0.0)	2.7 (-41.7 to 85.4)	32.6 (-24.8 to 139.3)
Cyprus	Nasopharynx cancer	10 to 14	Female	0 (0 to 0)	0.0 (0.0 to 0.0)	0 (0 to 0)	0.0 (0.0 to 0.0)	0 (0 to 0)	0.0 (0.0 to 0.0)	-35.3 (-61.1 to -14.8)	-17.4 (-50.4 to 8.8)
Cyprus	Nasopharynx cancer	10 to 14	Male	0 (0 to 0)	0.0 (0.0 to 0.0)	0 (0 to 0)	0.0 (0.0 to 0.0)	0 (0 to 0)	0.0 (0.0 to 0.0)	29.8 (-41.7 to 183.2)	69.4 (-23.9 to 269.6)
Cyprus	Liver cancer	5 to 9	Both	0 (0 to 0)	0.0 (0.0 to 0.0)	0 (0 to 0)	0.0 (0.0 to 0.0)	0 (0 to 0)	0.0 (0.0 to 0.0)	-34.6 (-64.9 to 29.4)	-18.5 (-56.2 to 61.2)
Cyprus	Liver cancer	5 to 9	Female	0 (0 to 0)	0.0 (0.0 to 0.0)	0 (0 to 0)	0.0 (0.0 to 0.0)	0 (0 to 0)	0.0 (0.0 to 0.0)	-29.5 (-67.8 to 68.8)	-12.1 (-59.9 to 110.5)
Cyprus	Liver cancer	5 to 9	Male	0 (0 to 0)	0.0 (0.0 to 0.1)	0 (0 to 0)	0.0 (0.0 to 0.1)	0 (0 to 0)	0.0 (0.0 to 0.0)	-38.8 (-75.2 to 44.4)	-23.8 (-69.1 to 79.6)
Cyprus	Liver cancer	10 to 14	Both	0 (0 to 0)	0.0 (0.0 to 0.0)	0 (0 to 0)	0.0 (0.0 to 0.1)	0 (0 to 0)	0.0 (0.0 to 0.0)	-27.7 (-60.6 to 42.7)	-6.6 (-49.2 to 84.3)
Cyprus	Liver cancer	10 to 14	Female	0 (0 to 0)	0.0 (0.0 to 0.0)	0 (0 to 0)	0.0 (0.0 to 0.0)	0 (0 to 0)	0.0 (0.0 to 0.0)	-34.5 (-72.2 to 50.9)	-16.4 (-64.5 to 92.6)
Cyprus	Liver cancer	10 to 14	Male	0 (0 to 0)	0.0 (0.0 to 0.1)	0 (0 to 0)	0.0 (0.0 to 0.1)	0 (0 to 0)	0.0 (0.0 to 0.1)	-24.8 (-65.3 to 68.5)	-1.9 (-54.7 to 119.9)
Cyprus	Kidney cancer	5 to 9	Both	0 (0 to 0)	0.1 (0.0 to 0.1)	0 (0 to 0)	0.1 (0.0 to 0.1)	0 (0 to 0)	0.1 (0.0 to 0.1)	-12.8 (-52.6 to 59.1)	8.6 (-41.0 to 98.1)
Cyprus	Kidney cancer	5 to 9	Female	0 (0 to 0)	0.1 (0.0 to 0.1)	0 (0 to 0)	0.1 (0.0 to 0.1)	0 (0 to 0)	0.1 (0.0 to 0.1)	6.0 (-52.0 to 154.2)	32.3 (-40.1 to 217.0)
Cyprus	Kidney cancer	5 to 9	Male	0 (0 to 0)	0.1 (0.0 to 0.1)	0 (0 to 0)	0.1 (0.0 to 0.1)	0 (0 to 0)	0.1 (0.0 to 0.1)	-30.7 (-70.2 to 67.5)	-13.7 (-63.0 to 108.4)
Cyprus	Kidney cancer	10 to 14	Both	0 (0 to 0)	0.0 (0.0 to 0.0)	0 (0 to 0)	0.0 (0.0 to 0.0)	0 (0 to 0)	0.0 (0.0 to 0.0)	6.9 (-36.3 to 84.8)	38.0 (-17.8 to 138.6)
Cyprus	Kidney cancer	10 to 14	Female	0 (0 to 0)	0.0 (0.0 to 0.0)	0 (0 to 0)	0.0 (0.0 to 0.0)	0 (0 to 0)	0.0 (0.0 to 0.0)	-13.6 (-59.2 to 90.9)	10.3 (-48.0 to 143.7)
Cyprus	Kidney cancer	10 to 14	Male	0 (0 to 0)	0.0 (0.0 to 0.0)	0 (0 to 0)	0.0 (0.0 to 0.0)	0 (0 to 0)	0.0 (0.0 to 0.0)	29.0 (-34.9 to 160.3)	68.3 (-15.0 to 239.7)
Cyprus	Brain and nervous system cancer	5 to 9	Both	1 (0 to 1)	1.2 (0.9 to 1.7)	1 (0 to 1)	1.3 (0.9 to 1.8)	0 (0 to 1)	0.9 (0.6 to 1.2)	-39.5 (-62.6 to -9.5)	-24.7 (-53.4 to 12.8)

eTable 8. All-cause and cause-specific mortality (counts and rates) at the global level and by country in the WHO European Region for ages 5 to 9 and 10 to 14 for 1990, 2000, and 2016

Cyprus	Brain and nervous system cancer	5 to 9	Female	0 (0 to 0)	1.1 (0.7 to 1.8)	0 (0 to 1)	1.3 (0.8 to 1.9)	0 (0 to 0)	1.0 (0.6 to 1.5)	-28.1 (-64.5 to 23.9)	-10.3 (-55.8 to 54.5)
Cyprus	Brain and nervous system cancer	5 to 9	Male	0 (0 to 1)	1.3 (0.8 to 2.1)	0 (0 to 1)	1.3 (0.9 to 1.9)	0 (0 to 0)	0.8 (0.5 to 1.2)	-48.7 (-73.8 to -9.8)	-36.2 (-67.4 to 12.2)
Cyprus	Brain and nervous system cancer	10 to 14	Both	0 (0 to 1)	0.7 (0.4 to 1.1)	0 (0 to 1)	0.9 (0.7 to 1.2)	0 (0 to 0)	0.7 (0.5 to 0.9)	-28.5 (-57.8 to 16.2)	-7.7 (-45.6 to 50.0)
Cyprus	Brain and nervous system cancer	10 to 14	Female	0 (0 to 0)	0.6 (0.3 to 0.9)	0 (0 to 0)	0.8 (0.5 to 1.1)	0 (0 to 0)	0.5 (0.3 to 0.7)	-35.5 (-66.4 to 12.7)	-17.7 (-57.2 to 43.8)
Cyprus	Brain and nervous system cancer	10 to 14	Male	0 (0 to 0)	0.8 (0.5 to 1.4)	0 (0 to 0)	1.0 (0.7 to 1.6)	0 (0 to 0)	0.8 (0.5 to 1.2)	-23.9 (-60.0 to 53.8)	-0.7 (-47.8 to 100.7)
Cyprus	Thyroid cancer	10 to 14	Both	0 (0 to 0)	0.0 (0.0 to 0.0)	0 (0 to 0)	0.0 (0.0 to 0.0)	0 (0 to 0)	0.0 (0.0 to 0.0)	-51.6 (-73.0 to -13.4)	-37.5 (-65.2 to 11.8)
Cyprus	Thyroid cancer	10 to 14	Female	0 (0 to 0)	0.0 (0.0 to 0.0)	0 (0 to 0)	0.0 (0.0 to 0.0)	0 (0 to 0)	0.0 (0.0 to 0.0)	-55.5 (-80.5 to -17.5)	-43.2 (-75.2 to 5.3)
Cyprus	Thyroid cancer	10 to 14	Male	0 (0 to 0)	0.0 (0.0 to 0.0)	0 (0 to 0)	0.0 (0.0 to 0.0)	0 (0 to 0)	0.0 (0.0 to 0.0)	-48.5 (-77.3 to 17.8)	-32.8 (-70.4 to 53.7)
Cyprus	Hodgkin lymphoma	5 to 9	Both	0 (0 to 0)	0.0 (0.0 to 0.0)	0 (0 to 0)	0.0 (0.0 to 0.0)	0 (0 to 0)	0.0 (0.0 to 0.0)	-62.7 (-84.0 to -10.8)	-53.6 (-80.0 to 11.2)
Cyprus	Hodgkin lymphoma	5 to 9	Female	0 (0 to 0)	0.0 (0.0 to 0.0)	0 (0 to 0)	0.0 (0.0 to 0.0)	0 (0 to 0)	0.0 (0.0 to 0.0)	-35.0 (-78.2 to 74.5)	-18.9 (-72.8 to 117.7)
Cyprus	Hodgkin lymphoma	5 to 9	Male	0 (0 to 0)	0.0 (0.0 to 0.1)	0 (0 to 0)	0.0 (0.0 to 0.1)	0 (0 to 0)	0.0 (0.0 to 0.0)	-77.8 (-93.0 to -30.9)	-72.3 (-91.3 to -14.0)
Cyprus	Hodgkin lymphoma	10 to 14	Both	0 (0 to 0)	0.0 (0.0 to 0.1)	0 (0 to 0)	0.1 (0.0 to 0.1)	0 (0 to 0)	0.0 (0.0 to 0.0)	-62.7 (-81.3 to -33.0)	-51.9 (-75.8 to -13.5)
Cyprus	Hodgkin lymphoma	10 to 14	Female	0 (0 to 0)	0.0 (0.0 to 0.1)	0 (0 to 0)	0.0 (0.0 to 0.1)	0 (0 to 0)	0.0 (0.0 to 0.0)	-66.7 (-88.8 to 2.1)	-57.4 (-85.7 to 30.4)
Cyprus	Hodgkin lymphoma	10 to 14	Male	0 (0 to 0)	0.1 (0.0 to 0.1)	0 (0 to 0)	0.1 (0.0 to 0.1)	0 (0 to 0)	0.0 (0.0 to 0.0)	-61.2 (-82.6 to -21.5)	-49.4 (-77.3 to 2.4)
Cyprus	Non-Hodgkin lymphoma	5 to 9	Both	0 (0 to 0)	0.3 (0.2 to 0.5)	0 (0 to 0)	0.4 (0.2 to 0.5)	0 (0 to 0)	0.2 (0.1 to 0.3)	-49.1 (-73.6 to -8.7)	-36.6 (-67.1 to 13.7)
Cyprus	Non-Hodgkin lymphoma	5 to 9	Female	0 (0 to 0)	0.1 (0.1 to 0.3)	0 (0 to 0)	0.2 (0.1 to 0.3)	0 (0 to 0)	0.1 (0.1 to 0.2)	-23.4 (-63.6 to 61.4)	-4.5 (-54.6 to 101.2)
Cyprus	Non-Hodgkin lymphoma	5 to 9	Male	0 (0 to 0)	0.5 (0.3 to 0.9)	0 (0 to 0)	0.5 (0.3 to 0.9)	0 (0 to 0)	0.3 (0.2 to 0.5)	-55.8 (-80.1 to -8.3)	-45.1 (-75.2 to 14.1)
Cyprus	Non-Hodgkin lymphoma	10 to 14	Both	0 (0 to 0)	0.3 (0.2 to 0.5)	0 (0 to 0)	0.3 (0.2 to 0.5)	0 (0 to 0)	0.2 (0.1 to 0.4)	-37.4 (-66.8 to 14.7)	-19.2 (-57.1 to 48.0)
Cyprus	Non-Hodgkin lymphoma	10 to 14	Female	0 (0 to 0)	0.1 (0.1 to 0.2)	0 (0 to 0)	0.2 (0.1 to 0.3)	0 (0 to 0)	0.1 (0.0 to 0.2)	-38.6 (-71.0 to 27.5)	-21.6 (-62.9 to 62.7)
Cyprus	Non-Hodgkin lymphoma	10 to 14	Male	0 (0 to 0)	0.4 (0.2 to 0.7)	0 (0 to 0)	0.5 (0.3 to 0.8)	0 (0 to 0)	0.3 (0.2 to 0.6)	-37.0 (-71.5 to 32.2)	-17.8 (-62.8 to 72.5)
Cyprus	Leukemia	5 to 9	Both	1 (1 to 1)	1.4 (1.0 to 2.0)	1 (1 to 1)	1.5 (1.1 to 2.0)	0 (0 to 1)	0.9 (0.6 to 1.3)	-47.8 (-68.3 to -10.9)	-35.0 (-60.5 to 11.0)
Cyprus	Leukemia	5 to 9	Female	0 (0 to 1)	1.2 (0.7 to 2.0)	0 (0 to 1)	1.3 (0.7 to 2.0)	0 (0 to 0)	0.9 (0.5 to 1.3)	-43.4 (-69.0 to 12.1)	-29.4 (-61.3 to 39.9)
Cyprus	Leukemia	5 to 9	Male	0 (0 to 1)	1.6 (0.9 to 2.4)	0 (0 to 1)	1.6 (1.0 to 2.4)	0 (0 to 0)	1.0 (0.5 to 1.5)	-51.0 (-75.2 to 5.1)	-39.1 (-69.2 to 30.7)
Cyprus	Leukemia	10 to 14	Both	1 (0 to 1)	1.1 (0.7 to 1.6)	1 (1 to 1)	1.3 (1.0 to 1.7)	0 (0 to 1)	0.9 (0.6 to 1.2)	-36.2 (-59.7 to -0.5)	-17.6 (-48.0 to 28.5)
Cyprus	Leukemia	10 to 14	Female	0 (0 to 0)	0.8 (0.5 to 1.4)	0 (0 to 0)	1.1 (0.7 to 1.6)	0 (0 to 0)	0.5 (0.3 to 0.8)	-52.9 (-74.7 to -13.4)	-39.9 (-67.7 to 10.5)
Cyprus	Leukemia	10 to 14	Male	0 (0 to 1)	1.3 (0.8 to 2.1)	0 (0 to 1)	1.5 (1.0 to 2.1)	0 (0 to 0)	1.2 (0.8 to 1.9)	-25.8 (-58.4 to 33.2)	-3.2 (-45.8 to 73.8)
Cyprus	Other neoplasms	5 to 9	Both	0 (0 to 0)	0.4 (0.3 to 0.6)	0 (0 to 0)	0.5 (0.3 to 0.6)	0 (0 to 0)	0.5 (0.4 to 0.8)	-4.5 (-46.0 to 76.3)	19.0 (-32.8 to 119.6)
Cyprus	Other neoplasms	5 to 9	Female	0 (0 to 0)	0.4 (0.2 to 0.6)	0 (0 to 0)	0.4 (0.2 to 0.6)	0 (0 to 0)	0.6 (0.4 to 0.9)	20.6 (-38.4 to 162.6)	50.4 (-23.2 to 227.5)
Cyprus	Other neoplasms	5 to 9	Male	0 (0 to 0)	0.5 (0.3 to 0.8)	0 (0 to 0)	0.5 (0.3 to 0.8)	0 (0 to 0)	0.5 (0.3 to 0.8)	-22.8 (-65.6 to 62.4)	-4.0 (-57.1 to 102.1)
Cyprus	Other neoplasms	10 to 14	Both	0 (0 to 0)	0.4 (0.2 to 0.6)	0 (0 to 0)	0.5 (0.3 to 0.6)	0 (0 to 0)	0.5 (0.4 to 0.7)	0.8 (-36.4 to 65.9)	30.2 (-17.8 to 114.2)

eTable 8. All-cause and cause-specific mortality (counts and rates) at the global level and by country in the WHO European Region for ages 5 to 9 and 10 to 14 for 1990, 2000, and 2016

Cyprus	Other neoplasms	10 to 14	Female	0 (0 to 0)	0.4 (0.2 to 0.6)	0 (0 to 0)	0.4 (0.2 to 0.6)	0 (0 to 0)	0.4 (0.2 to 0.7)	-8.8 (-49.9 to 91.3)	16.4 (-36.0 to 144.1)
Cyprus	Other neoplasms	10 to 14	Male	0 (0 to 0)	0.5 (0.2 to 0.7)	0 (0 to 0)	0.5 (0.4 to 0.8)	0 (0 to 0)	0.6 (0.4 to 1.0)	8.1 (-39.4 to 120.8)	41.1 (-20.9 to 188.2)
Cyprus	Rheumatic heart disease	5 to 9	Both	0 (0 to 0)	0.1 (0.1 to 0.2)	0 (0 to 0)	0.1 (0.1 to 0.1)	0 (0 to 0)	0.0 (0.0 to 0.0)	-79.9 (-88.4 to -65.7)	-75.0 (-85.5 to -57.3)
Cyprus	Rheumatic heart disease	5 to 9	Female	0 (0 to 0)	0.1 (0.1 to 0.2)	0 (0 to 0)	0.1 (0.1 to 0.1)	0 (0 to 0)	0.0 (0.0 to 0.1)	-68.8 (-83.4 to -39.6)	-61.1 (-79.3 to -24.6)
Cyprus	Rheumatic heart disease	5 to 9	Male	0 (0 to 0)	0.1 (0.1 to 0.2)	0 (0 to 0)	0.1 (0.1 to 0.2)	0 (0 to 0)	0.0 (0.0 to 0.0)	-87.3 (-94.8 to -70.9)	-84.2 (-93.6 to -63.8)
Cyprus	Rheumatic heart disease	10 to 14	Both	0 (0 to 0)	0.1 (0.0 to 0.1)	0 (0 to 0)	0.1 (0.1 to 0.1)	0 (0 to 0)	0.0 (0.0 to 0.0)	-62.6 (-77.5 to -32.4)	-51.8 (-70.9 to -12.7)
Cyprus	Rheumatic heart disease	10 to 14	Female	0 (0 to 0)	0.1 (0.0 to 0.1)	0 (0 to 0)	0.1 (0.0 to 0.1)	0 (0 to 0)	0.0 (0.0 to 0.0)	-71.8 (-86.3 to -42.4)	-64.0 (-82.6 to -26.5)
Cyprus	Rheumatic heart disease	10 to 14	Male	0 (0 to 0)	0.1 (0.0 to 0.1)	0 (0 to 0)	0.1 (0.1 to 0.2)	0 (0 to 0)	0.0 (0.0 to 0.1)	-57.0 (-78.0 to -15.8)	-43.9 (-71.3 to 9.9)
Cyprus	Ischemic heart disease	5 to 9	Both	0 (0 to 0)	0.0 (0.0 to 0.0)	0 (0 to 0)	0.0 (0.0 to 0.0)	0 (0 to 0)	0.0 (0.0 to 0.0)	-61.4 (-80.8 to -19.2)	-51.9 (-76.1 to 0.6)
Cyprus	Ischemic heart disease	5 to 9	Female	0 (0 to 0)	0.0 (0.0 to 0.0)	0 (0 to 0)	0.0 (0.0 to 0.0)	0 (0 to 0)	0.0 (0.0 to 0.0)	-51.9 (-77.6 to 4.8)	-40.0 (-72.1 to 30.7)
Cyprus	Ischemic heart disease	5 to 9	Male	0 (0 to 0)	0.0 (0.0 to 0.1)	0 (0 to 0)	0.0 (0.0 to 0.1)	0 (0 to 0)	0.0 (0.0 to 0.0)	-67.9 (-88.7 to -1.7)	-60.1 (-86.0 to 22.2)
Cyprus	Ischemic heart disease	10 to 14	Both	0 (0 to 0)	0.0 (0.0 to 0.1)	0 (0 to 0)	0.1 (0.0 to 0.1)	0 (0 to 0)	0.0 (0.0 to 0.0)	-61.2 (-81.7 to -21.4)	-49.9 (-76.4 to 1.4)
Cyprus	Ischemic heart disease	10 to 14	Female	0 (0 to 0)	0.0 (0.0 to 0.0)	0 (0 to 0)	0.0 (0.0 to 0.0)	0 (0 to 0)	0.0 (0.0 to 0.0)	-57.5 (-81.5 to 3.1)	-45.8 (-76.3 to 31.6)
Cyprus	Ischemic heart disease	10 to 14	Male	0 (0 to 0)	0.1 (0.0 to 0.1)	0 (0 to 0)	0.1 (0.0 to 0.1)	0 (0 to 0)	0.0 (0.0 to 0.1)	-62.3 (-84.8 to -8.6)	-50.8 (-80.1 to 19.2)
Cyprus	Cerebrovascular disease	5 to 9	Both	0 (0 to 0)	0.4 (0.3 to 0.5)	0 (0 to 0)	0.3 (0.2 to 0.5)	0 (0 to 0)	0.1 (0.1 to 0.1)	-77.7 (-85.7 to -63.9)	-72.2 (-82.2 to -55.0)
Cyprus	Cerebrovascular disease	5 to 9	Female	0 (0 to 0)	0.4 (0.3 to 0.6)	0 (0 to 0)	0.4 (0.3 to 0.7)	0 (0 to 0)	0.2 (0.1 to 0.2)	-71.0 (-83.3 to -49.4)	-63.8 (-79.2 to -36.9)
Cyprus	Cerebrovascular disease	5 to 9	Male	0 (0 to 0)	0.3 (0.2 to 0.6)	0 (0 to 0)	0.3 (0.1 to 0.4)	0 (0 to 0)	0.1 (0.0 to 0.1)	-86.2 (-94.1 to -69.6)	-82.8 (-92.6 to -62.1)
Cyprus	Cerebrovascular disease	10 to 14	Both	0 (0 to 0)	0.3 (0.2 to 0.4)	0 (0 to 0)	0.4 (0.3 to 0.6)	0 (0 to 0)	0.1 (0.1 to 0.2)	-63.0 (-75.6 to -43.1)	-52.3 (-68.5 to -26.5)
Cyprus	Cerebrovascular disease	10 to 14	Female	0 (0 to 0)	0.2 (0.1 to 0.4)	0 (0 to 0)	0.3 (0.2 to 0.5)	0 (0 to 0)	0.1 (0.1 to 0.1)	-72.9 (-84.3 to -51.6)	-65.4 (-79.9 to -38.3)
Cyprus	Cerebrovascular disease	10 to 14	Male	0 (0 to 0)	0.4 (0.2 to 0.5)	0 (0 to 0)	0.5 (0.3 to 0.8)	0 (0 to 0)	0.2 (0.1 to 0.3)	-57.0 (-75.4 to -25.4)	-43.9 (-68.0 to -2.6)
Cyprus	Hypertensive heart disease	5 to 9	Both	0 (0 to 0)	0.0 (0.0 to 0.0)	0 (0 to 0)	0.0 (0.0 to 0.0)	0 (0 to 0)	0.0 (0.0 to 0.0)	-43.7 (-65.8 to -12.4)	-29.9 (-57.3 to 9.1)
Cyprus	Hypertensive heart disease	5 to 9	Female	0 (0 to 0)	0.0 (0.0 to 0.0)	0 (0 to 0)	0.0 (0.0 to 0.0)	0 (0 to 0)	0.0 (0.0 to 0.0)	-35.6 (-64.3 to 18.3)	-19.7 (-55.5 to 47.5)
Cyprus	Hypertensive heart disease	5 to 9	Male	0 (0 to 0)	0.0 (0.0 to 0.0)	0 (0 to 0)	0.0 (0.0 to 0.0)	0 (0 to 0)	0.0 (0.0 to 0.0)	-50.1 (-76.2 to -3.3)	-37.9 (-70.4 to 20.4)
Cyprus	Hypertensive heart disease	10 to 14	Both	0 (0 to 0)	0.0 (0.0 to 0.0)	0 (0 to 0)	0.0 (0.0 to 0.0)	0 (0 to 0)	0.0 (0.0 to 0.0)	-33.3 (-61.8 to 7.8)	-13.8 (-50.7 to 39.1)
Cyprus	Hypertensive heart disease	10 to 14	Female	0 (0 to 0)	0.0 (0.0 to 0.0)	0 (0 to 0)	0.0 (0.0 to 0.0)	0 (0 to 0)	0.0 (0.0 to 0.0)	-39.2 (-67.9 to 7.2)	-22.4 (-59.1 to 36.8)
Cyprus	Hypertensive heart disease	10 to 14	Male	0 (0 to 0)	0.0 (0.0 to 0.0)	0 (0 to 0)	0.0 (0.0 to 0.0)	0 (0 to 0)	0.0 (0.0 to 0.0)	-28.3 (-67.9 to 37.6)	-6.4 (-58.1 to 79.5)
Cyprus	Cardiomyopathy and myocarditis	5 to 9	Both	0 (0 to 0)	0.1 (0.1 to 0.2)	0 (0 to 0)	0.1 (0.1 to 0.2)	0 (0 to 0)	0.1 (0.0 to 0.1)	-68.2 (-82.7 to -41.8)	-60.4 (-78.4 to -27.5)
Cyprus	Cardiomyopathy and myocarditis	5 to 9	Female	0 (0 to 0)	0.1 (0.1 to 0.3)	0 (0 to 0)	0.1 (0.1 to 0.3)	0 (0 to 0)	0.1 (0.0 to 0.1)	-58.6 (-80.9 to -8.5)	-48.3 (-76.2 to 14.2)
Cyprus	Cardiomyopathy and myocarditis	5 to 9	Male	0 (0 to 0)	0.2 (0.1 to 0.3)	0 (0 to 0)	0.1 (0.1 to 0.2)	0 (0 to 0)	0.0 (0.0 to 0.1)	-76.5 (-90.4 to -45.9)	-70.7 (-88.0 to -32.7)
Cyprus	Cardiomyopathy and myocarditis	10 to 14	Both	0 (0 to 0)	0.1 (0.1 to 0.2)	0 (0 to 0)	0.2 (0.1 to 0.2)	0 (0 to 0)	0.1 (0.0 to 0.1)	-45.0 (-68.0 to -8.7)	-29.0 (-58.7 to 17.9)

eTable 8. All-cause and cause-specific mortality (counts and rates) at the global level and by country in the WHO European Region for ages 5 to 9 and 10 to 14 for 1990, 2000, and 2016

Cyprus	Cardiomyopathy and myocarditis	10 to 14	Female	0<br>(0 to 0)	0.1<br>(0.0 to 0.1)	0<br>(0 to 0)	0.1<br>(0.1 to 0.2)	0<br>(0 to 0)	0.0<br>(0.0 to 0.1)	-59.9<br>(-81.6 to -12.9)	-48.8<br>(-76.5 to 11.2)
Cyprus	Cardiomyopathy and myocarditis	10 to 14	Male	0<br>(0 to 0)	0.1<br>(0.1 to 0.2)	0<br>(0 to 0)	0.2<br>(0.1 to 0.3)	0<br>(0 to 0)	0.1<br>(0.1 to 0.2)	-38.0<br>(-67.8 to 16.7)	-19.1<br>(-58.0 to 52.3)
Cyprus	Atrial fibrillation and flutter	5 to 9	Both	0<br>(0 to 0)	0.0<br>(0.0 to 0.0)	0<br>(0 to 0)	0.0<br>(0.0 to 0.0)	0<br>(0 to 0)	0.0<br>(0.0 to 0.0)	0.0<br>(0.0 to 0.0)	0.0<br>(0.0 to 0.0)
Cyprus	Atrial fibrillation and flutter	5 to 9	Female	0<br>(0 to 0)	0.0<br>(0.0 to 0.0)	0<br>(0 to 0)	0.0<br>(0.0 to 0.0)	0<br>(0 to 0)	0.0<br>(0.0 to 0.0)	0.0<br>(0.0 to 0.0)	0.0<br>(0.0 to 0.0)
Cyprus	Atrial fibrillation and flutter	5 to 9	Male	0<br>(0 to 0)	0.0<br>(0.0 to 0.0)	0<br>(0 to 0)	0.0<br>(0.0 to 0.0)	0<br>(0 to 0)	0.0<br>(0.0 to 0.0)	0.0<br>(0.0 to 0.0)	0.0<br>(0.0 to 0.0)
Cyprus	Atrial fibrillation and flutter	10 to 14	Both	0<br>(0 to 0)	0.0<br>(0.0 to 0.0)	0<br>(0 to 0)	0.0<br>(0.0 to 0.0)	0<br>(0 to 0)	0.0<br>(0.0 to 0.0)	0.0<br>(0.0 to 0.0)	0.0<br>(0.0 to 0.0)
Cyprus	Atrial fibrillation and flutter	10 to 14	Female	0<br>(0 to 0)	0.0<br>(0.0 to 0.0)	0<br>(0 to 0)	0.0<br>(0.0 to 0.0)	0<br>(0 to 0)	0.0<br>(0.0 to 0.0)	0.0<br>(0.0 to 0.0)	0.0<br>(0.0 to 0.0)
Cyprus	Atrial fibrillation and flutter	10 to 14	Male	0<br>(0 to 0)	0.0<br>(0.0 to 0.0)	0<br>(0 to 0)	0.0<br>(0.0 to 0.0)	0<br>(0 to 0)	0.0<br>(0.0 to 0.0)	0.0<br>(0.0 to 0.0)	0.0<br>(0.0 to 0.0)
Cyprus	Endocarditis	5 to 9	Both	0<br>(0 to 0)	0.0<br>(0.0 to 0.1)	0<br>(0 to 0)	0.0<br>(0.0 to 0.1)	0<br>(0 to 0)	0.0<br>(0.0 to 0.0)	-52.1<br>(-75.0 to -14.2)	-40.4<br>(-68.8 to 6.8)
Cyprus	Endocarditis	5 to 9	Female	0<br>(0 to 0)	0.0<br>(0.0 to 0.1)	0<br>(0 to 0)	0.0<br>(0.0 to 0.1)	0<br>(0 to 0)	0.0<br>(0.0 to 0.0)	-48.2<br>(-76.8 to 21.8)	-35.4<br>(-71.1 to 51.9)
Cyprus	Endocarditis	5 to 9	Male	0<br>(0 to 0)	0.0<br>(0.0 to 0.1)	0<br>(0 to 0)	0.0<br>(0.0 to 0.1)	0<br>(0 to 0)	0.0<br>(0.0 to 0.0)	-56.3<br>(-82.6 to 1.6)	-45.6<br>(-78.3 to 26.4)
Cyprus	Endocarditis	10 to 14	Both	0<br>(0 to 0)	0.0<br>(0.0 to 0.0)	0<br>(0 to 0)	0.0<br>(0.0 to 0.1)	0<br>(0 to 0)	0.0<br>(0.0 to 0.0)	-38.7<br>(-65.1 to 5.4)	-20.8<br>(-54.9 to 36.1)
Cyprus	Endocarditis	10 to 14	Female	0<br>(0 to 0)	0.0<br>(0.0 to 0.1)	0<br>(0 to 0)	0.0<br>(0.0 to 0.1)	0<br>(0 to 0)	0.0<br>(0.0 to 0.0)	-55.3<br>(-79.1 to -3.2)	-43.0<br>(-73.3 to 23.6)
Cyprus	Endocarditis	10 to 14	Male	0<br>(0 to 0)	0.0<br>(0.0 to 0.1)	0<br>(0 to 0)	0.0<br>(0.0 to 0.1)	0<br>(0 to 0)	0.0<br>(0.0 to 0.1)	-25.6<br>(-63.5 to 51.5)	-3.0<br>(-52.4 to 97.8)
Cyprus	Other cardiovascular and circulatory diseases	5 to 9	Both	0<br>(0 to 0)	0.2<br>(0.1 to 0.2)	0<br>(0 to 0)	0.1<br>(0.1 to 0.2)	0<br>(0 to 0)	0.1<br>(0.0 to 0.1)	-70.0<br>(-82.7 to -47.3)	-62.6<br>(-78.4 to -34.4)
Cyprus	Other cardiovascular and circulatory diseases	5 to 9	Female	0<br>(0 to 0)	0.1<br>(0.1 to 0.2)	0<br>(0 to 0)	0.1<br>(0.1 to 0.2)	0<br>(0 to 0)	0.1<br>(0.0 to 0.1)	-54.9<br>(-78.7 to -0.4)	-43.8<br>(-73.5 to 24.3)
Cyprus	Other cardiovascular and circulatory diseases	5 to 9	Male	0<br>(0 to 0)	0.2<br>(0.1 to 0.3)	0<br>(0 to 0)	0.2<br>(0.1 to 0.3)	0<br>(0 to 0)	0.1<br>(0.0 to 0.1)	-78.9<br>(-90.1 to -51.5)	-73.8<br>(-87.7 to -39.6)
Cyprus	Other cardiovascular and circulatory diseases	10 to 14	Both	0<br>(0 to 0)	0.1<br>(0.1 to 0.1)	0<br>(0 to 0)	0.1<br>(0.1 to 0.2)	0<br>(0 to 0)	0.1<br>(0.0 to 0.1)	-50.3<br>(-70.0 to -17.2)	-35.8<br>(-61.2 to 6.9)
Cyprus	Other cardiovascular and circulatory diseases	10 to 14	Female	0<br>(0 to 0)	0.1<br>(0.0 to 0.2)	0<br>(0 to 0)	0.1<br>(0.1 to 0.2)	0<br>(0 to 0)	0.0<br>(0.0 to 0.1)	-62.2<br>(-82.4 to -17.8)	-51.7<br>(-77.5 to 4.9)

eTable 8. All-cause and cause-specific mortality (counts and rates) at the global level and by country in the WHO European Region for ages 5 to 9 and 10 to 14 for 1990, 2000, and 2016

Cyprus	Other cardiovascular and circulatory diseases	10 to 14	Male	0 (0 to 0)	0.1 (0.1 to 0.2)	0 (0 to 0)	0.1 (0.1 to 0.2)	0 (0 to 0)	0.1 (0.0 to 0.1)	-40.4 (-68.4 to 20.2)	-22.2 (-58.8 to 56.8)
Cyprus	Chronic obstructive pulmonary disease	5 to 9	Both	0 (0 to 0)	0.1 (0.1 to 0.1)	0 (0 to 0)	0.1 (0.1 to 0.1)	0 (0 to 0)	0.0 (0.0 to 0.1)	-68.3 (-83.1 to -41.7)	-60.5 (-78.9 to -27.4)
Cyprus	Chronic obstructive pulmonary disease	5 to 9	Female	0 (0 to 0)	0.1 (0.1 to 0.2)	0 (0 to 0)	0.1 (0.1 to 0.2)	0 (0 to 0)	0.1 (0.0 to 0.1)	-59.6 (-81.3 to -4.1)	-49.6 (-76.7 to 19.6)
Cyprus	Chronic obstructive pulmonary disease	5 to 9	Male	0 (0 to 0)	0.1 (0.0 to 0.1)	0 (0 to 0)	0.1 (0.0 to 0.1)	0 (0 to 0)	0.0 (0.0 to 0.0)	-81.9 (-92.9 to -54.6)	-77.5 (-91.2 to -43.6)
Cyprus	Chronic obstructive pulmonary disease	10 to 14	Both	0 (0 to 0)	0.0 (0.0 to 0.1)	0 (0 to 0)	0.1 (0.0 to 0.1)	0 (0 to 0)	0.0 (0.0 to 0.0)	-51.5 (-72.9 to -3.0)	-37.3 (-65.1 to 25.2)
Cyprus	Chronic obstructive pulmonary disease	10 to 14	Female	0 (0 to 0)	0.1 (0.0 to 0.1)	0 (0 to 0)	0.1 (0.0 to 0.1)	0 (0 to 0)	0.0 (0.0 to 0.1)	-61.3 (-82.8 to -9.6)	-50.6 (-78.1 to 15.4)
Cyprus	Chronic obstructive pulmonary disease	10 to 14	Male	0 (0 to 0)	0.0 (0.0 to 0.1)	0 (0 to 0)	0.0 (0.0 to 0.1)	0 (0 to 0)	0.0 (0.0 to 0.0)	-35.5 (-70.2 to 67.5)	-15.8 (-61.1 to 118.5)
Cyprus	Asthma	5 to 9	Both	0 (0 to 0)	0.2 (0.1 to 0.3)	0 (0 to 0)	0.2 (0.1 to 0.2)	0 (0 to 0)	0.0 (0.0 to 0.1)	-80.2 (-89.8 to -64.8)	-75.4 (-87.2 to -56.1)
Cyprus	Asthma	5 to 9	Female	0 (0 to 0)	0.2 (0.1 to 0.3)	0 (0 to 0)	0.1 (0.1 to 0.3)	0 (0 to 0)	0.1 (0.0 to 0.1)	-69.0 (-86.1 to -24.4)	-61.3 (-82.7 to -5.7)
Cyprus	Asthma	5 to 9	Male	0 (0 to 0)	0.2 (0.1 to 0.4)	0 (0 to 0)	0.2 (0.1 to 0.3)	0 (0 to 0)	0.0 (0.0 to 0.1)	-87.8 (-94.8 to -72.8)	-84.8 (-93.6 to -66.1)
Cyprus	Asthma	10 to 14	Both	0 (0 to 0)	0.2 (0.1 to 0.3)	0 (0 to 0)	0.3 (0.2 to 0.4)	0 (0 to 0)	0.1 (0.1 to 0.1)	-64.2 (-78.3 to -41.6)	-53.7 (-72.0 to -24.5)
Cyprus	Asthma	10 to 14	Female	0 (0 to 0)	0.2 (0.1 to 0.3)	0 (0 to 0)	0.2 (0.1 to 0.4)	0 (0 to 0)	0.1 (0.0 to 0.1)	-69.2 (-85.4 to -27.4)	-60.7 (-81.3 to -7.4)
Cyprus	Asthma	10 to 14	Male	0 (0 to 0)	0.3 (0.2 to 0.4)	0 (0 to 0)	0.3 (0.2 to 0.5)	0 (0 to 0)	0.1 (0.1 to 0.2)	-61.1 (-78.6 to -34.2)	-49.2 (-72.1 to -14.2)
Cyprus	Interstitial lung disease and pulmonary sarcoidosis	5 to 9	Both	0 (0 to 0)	0.0 (0.0 to 0.1)	0 (0 to 0)	0.0 (0.0 to 0.1)	0 (0 to 0)	0.0 (0.0 to 0.0)	-40.2 (-71.2 to 25.9)	-25.6 (-64.1 to 56.9)
Cyprus	Interstitial lung disease and pulmonary sarcoidosis	5 to 9	Female	0 (0 to 0)	0.0 (0.0 to 0.1)	0 (0 to 0)	0.1 (0.0 to 0.1)	0 (0 to 0)	0.0 (0.0 to 0.1)	-33.9 (-71.5 to 73.3)	-17.5 (-64.4 to 116.1)
Cyprus	Interstitial lung disease and pulmonary sarcoidosis	5 to 9	Male	0 (0 to 0)	0.0 (0.0 to 0.1)	0 (0 to 0)	0.0 (0.0 to 0.1)	0 (0 to 0)	0.0 (0.0 to 0.0)	-50.7 (-82.4 to 63.6)	-38.7 (-78.2 to 103.5)
Cyprus	Interstitial lung disease and pulmonary sarcoidosis	10 to 14	Both	0 (0 to 0)	0.0 (0.0 to 0.0)	0 (0 to 0)	0.0 (0.0 to 0.1)	0 (0 to 0)	0.0 (0.0 to 0.0)	-16.8 (-59.5 to 72.0)	7.4 (-47.7 to 122.1)

eTable 8. All-cause and cause-specific mortality (counts and rates) at the global level and by country in the WHO European Region for ages 5 to 9 and 10 to 14 for 1990, 2000, and 2016

Cyprus	Interstitial lung disease and pulmonary sarcoidosis	10 to 14	Female	0<br>(0 to 0)	0.0<br>(0.0 to 0.1)	0<br>(0 to 0)	0.0<br>(0.0 to 0.1)	0<br>(0 to 0)	0.0<br>(0.0 to 0.1)	-34.8<br>(-73.1 to 62.8)	-16.8<br>(-65.7 to 107.8)
Cyprus	Interstitial lung disease and pulmonary sarcoidosis	10 to 14	Male	0<br>(0 to 0)	0.0<br>(0.0 to 0.0)	0<br>(0 to 0)	0.0<br>(0.0 to 0.0)	0<br>(0 to 0)	0.0<br>(0.0 to 0.0)	20.0<br>(-51.5 to 224.1)	56.5<br>(-36.8 to 322.9)
Cyprus	Other chronic respiratory diseases	5 to 9	Both	0<br>(0 to 0)	0.1<br>(0.0 to 0.1)	0<br>(0 to 0)	0.1<br>(0.0 to 0.1)	0<br>(0 to 0)	0.0<br>(0.0 to 0.1)	-49.9<br>(-76.4 to 3.4)	-37.6<br>(-70.6 to 28.8)
Cyprus	Other chronic respiratory diseases	5 to 9	Female	0<br>(0 to 0)	0.1<br>(0.0 to 0.2)	0<br>(0 to 0)	0.1<br>(0.0 to 0.2)	0<br>(0 to 0)	0.1<br>(0.0 to 0.1)	-39.7<br>(-73.9 to 52.0)	-24.8<br>(-67.4 to 89.6)
Cyprus	Other chronic respiratory diseases	5 to 9	Male	0<br>(0 to 0)	0.0<br>(0.0 to 0.1)	0<br>(0 to 0)	0.0<br>(0.0 to 0.1)	0<br>(0 to 0)	0.0<br>(0.0 to 0.0)	-66.8<br>(-89.3 to 0.3)	-58.7<br>(-86.7 to 24.8)
Cyprus	Other chronic respiratory diseases	10 to 14	Both	0<br>(0 to 0)	0.0<br>(0.0 to 0.1)	0<br>(0 to 0)	0.0<br>(0.0 to 0.1)	0<br>(0 to 0)	0.0<br>(0.0 to 0.1)	-36.9<br>(-71.5 to 27.8)	-18.5<br>(-63.2 to 65.0)
Cyprus	Other chronic respiratory diseases	10 to 14	Female	0<br>(0 to 0)	0.1<br>(0.0 to 0.1)	0<br>(0 to 0)	0.1<br>(0.0 to 0.2)	0<br>(0 to 0)	0.0<br>(0.0 to 0.1)	-43.0<br>(-80.0 to 42.6)	-27.3<br>(-74.5 to 82.0)
Cyprus	Other chronic respiratory diseases	10 to 14	Male	0<br>(0 to 0)	0.0<br>(0.0 to 0.1)	0<br>(0 to 0)	0.0<br>(0.0 to 0.1)	0<br>(0 to 0)	0.0<br>(0.0 to 0.1)	-23.4<br>(-70.4 to 102.2)	-0.0<br>(-61.4 to 163.8)
Cyprus	Cirrhosis and other chronic liver diseases due to hepatitis B	5 to 9	Both	0<br>(0 to 0)	0.0<br>(0.0 to 0.0)	0<br>(0 to 0)	0.0<br>(0.0 to 0.0)	0<br>(0 to 0)	0.0<br>(0.0 to 0.0)	-68.6<br>(-81.9 to -41.1)	-60.8<br>(-77.4 to -26.6)
Cyprus	Cirrhosis and other chronic liver diseases due to hepatitis B	5 to 9	Female	0<br>(0 to 0)	0.0<br>(0.0 to 0.0)	0<br>(0 to 0)	0.0<br>(0.0 to 0.0)	0<br>(0 to 0)	0.0<br>(0.0 to 0.0)	-53.6<br>(-74.5 to -17.0)	-42.1<br>(-68.2 to 3.6)
Cyprus	Cirrhosis and other chronic liver diseases due to hepatitis B	5 to 9	Male	0<br>(0 to 0)	0.0<br>(0.0 to 0.0)	0<br>(0 to 0)	0.0<br>(0.0 to 0.0)	0<br>(0 to 0)	0.0<br>(0.0 to 0.0)	-72.4<br>(-86.1 to -37.3)	-65.7<br>(-82.7 to -22.0)
Cyprus	Cirrhosis and other chronic liver diseases due to hepatitis B	10 to 14	Both	0<br>(0 to 0)	0.0<br>(0.0 to 0.0)	0<br>(0 to 0)	0.0<br>(0.0 to 0.0)	0<br>(0 to 0)	0.0<br>(0.0 to 0.0)	-41.1<br>(-66.2 to 7.0)	-24.0<br>(-56.4 to 38.2)
Cyprus	Cirrhosis and other chronic liver diseases due to hepatitis B	10 to 14	Female	0<br>(0 to 0)	0.0<br>(0.0 to 0.0)	0<br>(0 to 0)	0.0<br>(0.0 to 0.0)	0<br>(0 to 0)	0.0<br>(0.0 to 0.0)	-56.3<br>(-76.7 to -18.4)	-44.2<br>(-70.3 to 4.2)
Cyprus	Cirrhosis and other chronic liver diseases due to hepatitis B	10 to 14	Male	0<br>(0 to 0)	0.0<br>(0.0 to 0.0)	0<br>(0 to 0)	0.0<br>(0.0 to 0.0)	0<br>(0 to 0)	0.0<br>(0.0 to 0.0)	-35.4<br>(-68.7 to 39.0)	-15.8<br>(-59.1 to 81.4)
Cyprus	Cirrhosis and other chronic liver diseases due to hepatitis C	5 to 9	Both	0<br>(0 to 0)	0.0<br>(0.0 to 0.0)	0<br>(0 to 0)	0.0<br>(0.0 to 0.0)	0<br>(0 to 0)	0.0<br>(0.0 to 0.0)	-68.2<br>(-81.4 to -40.3)	-60.4<br>(-76.9 to -25.6)

eTable 8. All-cause and cause-specific mortality (counts and rates) at the global level and by country in the WHO European Region for ages 5 to 9 and 10 to 14 for 1990, 2000, and 2016

Cyprus	Cirrhosis and other chronic liver diseases due to hepatitis C	5 to 9	Female	0<br>(0 to 0)	0.0<br>(0.0 to 0.0)	0<br>(0 to 0)	0.0<br>(0.0 to 0.0)	0<br>(0 to 0)	0.0<br>(0.0 to 0.0)	-53.2<br>(-74.5 to -16.9)	-41.6<br>(-68.1 to 3.7)
Cyprus	Cirrhosis and other chronic liver diseases due to hepatitis C	5 to 9	Male	0<br>(0 to 0)	0.0<br>(0.0 to 0.0)	0<br>(0 to 0)	0.0<br>(0.0 to 0.0)	0<br>(0 to 0)	0.0<br>(0.0 to 0.0)	-72.5<br>(-86.3 to -35.6)	-65.8<br>(-82.9 to -19.9)
Cyprus	Cirrhosis and other chronic liver diseases due to hepatitis C	10 to 14	Both	0<br>(0 to 0)	0.0<br>(0.0 to 0.0)	0<br>(0 to 0)	0.0<br>(0.0 to 0.0)	0<br>(0 to 0)	0.0<br>(0.0 to 0.0)	-41.4<br>(-66.4 to 5.8)	-24.3<br>(-56.6 to 36.5)
Cyprus	Cirrhosis and other chronic liver diseases due to hepatitis C	10 to 14	Female	0<br>(0 to 0)	0.0<br>(0.0 to 0.0)	0<br>(0 to 0)	0.0<br>(0.0 to 0.0)	0<br>(0 to 0)	0.0<br>(0.0 to 0.0)	-56.0<br>(-76.5 to -17.0)	-43.9<br>(-70.0 to 5.9)
Cyprus	Cirrhosis and other chronic liver diseases due to hepatitis C	10 to 14	Male	0<br>(0 to 0)	0.0<br>(0.0 to 0.0)	0<br>(0 to 0)	0.0<br>(0.0 to 0.0)	0<br>(0 to 0)	0.0<br>(0.0 to 0.0)	-35.2<br>(-68.9 to 39.9)	-15.4<br>(-59.5 to 82.6)
Cyprus	Cirrhosis and other chronic liver diseases due to other causes	5 to 9	Both	0<br>(0 to 0)	0.1<br>(0.1 to 0.1)	0<br>(0 to 0)	0.1<br>(0.1 to 0.1)	0<br>(0 to 0)	0.0<br>(0.0 to 0.0)	-65.7<br>(-78.6 to -42.9)	-57.3<br>(-73.3 to -28.9)
Cyprus	Cirrhosis and other chronic liver diseases due to other causes	5 to 9	Female	0<br>(0 to 0)	0.1<br>(0.0 to 0.1)	0<br>(0 to 0)	0.1<br>(0.0 to 0.1)	0<br>(0 to 0)	0.0<br>(0.0 to 0.1)	-55.0<br>(-75.5 to -20.7)	-43.9<br>(-69.5 to -1.1)
Cyprus	Cirrhosis and other chronic liver diseases due to other causes	5 to 9	Male	0<br>(0 to 0)	0.1<br>(0.0 to 0.1)	0<br>(0 to 0)	0.1<br>(0.0 to 0.1)	0<br>(0 to 0)	0.0<br>(0.0 to 0.0)	-73.5<br>(-86.8 to -39.7)	-67.0<br>(-83.6 to -25.0)
Cyprus	Cirrhosis and other chronic liver diseases due to other causes	10 to 14	Both	0<br>(0 to 0)	0.0<br>(0.0 to 0.0)	0<br>(0 to 0)	0.0<br>(0.0 to 0.1)	0<br>(0 to 0)	0.0<br>(0.0 to 0.0)	-48.0<br>(-68.1 to -13.8)	-32.9<br>(-58.8 to 11.3)
Cyprus	Cirrhosis and other chronic liver diseases due to other causes	10 to 14	Female	0<br>(0 to 0)	0.0<br>(0.0 to 0.0)	0<br>(0 to 0)	0.0<br>(0.0 to 0.1)	0<br>(0 to 0)	0.0<br>(0.0 to 0.0)	-57.9<br>(-77.8 to -22.0)	-46.2<br>(-71.6 to -0.4)
Cyprus	Cirrhosis and other chronic liver diseases due to other causes	10 to 14	Male	0<br>(0 to 0)	0.0<br>(0.0 to 0.0)	0<br>(0 to 0)	0.0<br>(0.0 to 0.1)	0<br>(0 to 0)	0.0<br>(0.0 to 0.0)	-37.6<br>(-70.3 to 33.6)	-18.5<br>(-61.2 to 74.3)
Cyprus	Peptic ulcer disease	5 to 9	Both	0<br>(0 to 0)	0.0<br>(0.0 to 0.0)	0<br>(0 to 0)	0.0<br>(0.0 to 0.0)	0<br>(0 to 0)	0.0<br>(0.0 to 0.0)	-53.5<br>(-72.7 to -21.2)	-42.1<br>(-66.0 to -1.9)
Cyprus	Peptic ulcer disease	5 to 9	Female	0<br>(0 to 0)	0.0<br>(0.0 to 0.0)	0<br>(0 to 0)	0.0<br>(0.0 to 0.0)	0<br>(0 to 0)	0.0<br>(0.0 to 0.0)	-47.0<br>(-76.4 to 9.6)	-33.9<br>(-70.5 to 36.7)
Cyprus	Peptic ulcer disease	5 to 9	Male	0<br>(0 to 0)	0.0<br>(0.0 to 0.0)	0<br>(0 to 0)	0.0<br>(0.0 to 0.0)	0<br>(0 to 0)	0.0<br>(0.0 to 0.0)	-60.1<br>(-80.1 to -11.3)	-50.4<br>(-75.3 to 10.3)
Cyprus	Peptic ulcer disease	10 to 14	Both	0<br>(0 to 0)	0.0<br>(0.0 to 0.0)	0<br>(0 to 0)	0.0<br>(0.0 to 0.0)	0<br>(0 to 0)	0.0<br>(0.0 to 0.0)	-41.1<br>(-62.3 to -1.1)	-23.9<br>(-51.4 to 27.7)
Cyprus	Peptic ulcer disease	10 to 14	Female	0<br>(0 to 0)	0.0<br>(0.0 to 0.0)	0<br>(0 to 0)	0.0<br>(0.0 to 0.0)	0<br>(0 to 0)	0.0<br>(0.0 to 0.0)	-47.5<br>(-71.0 to 8.4)	-32.9<br>(-63.0 to 38.4)

eTable 8. All-cause and cause-specific mortality (counts and rates) at the global level and by country in the WHO European Region for ages 5 to 9 and 10 to 14 for 1990, 2000, and 2016

Cyprus	Peptic ulcer disease	10 to 14	Male	0<br>(0 to 0)	0.0<br>(0.0 to 0.0)	0<br>(0 to 0)	0.0<br>(0.0 to 0.0)	0<br>(0 to 0)	0.0<br>(0.0 to 0.0)	-34.8<br>(-66.3 to 35.1)	-14.9<br>(-56.0 to 76.3)
Cyprus	Gastritis and duodenitis	5 to 9	Both	0<br>(0 to 0)	0.0<br>(0.0 to 0.0)	0<br>(0 to 0)	0.0<br>(0.0 to 0.0)	0<br>(0 to 0)	0.0<br>(0.0 to 0.0)	-49.7<br>(-84.2 to 42.1)	-37.4<br>(-80.4 to 77.0)
Cyprus	Gastritis and duodenitis	5 to 9	Female	0<br>(0 to 0)	0.0<br>(0.0 to 0.0)	0<br>(0 to 0)	0.0<br>(0.0 to 0.0)	0<br>(0 to 0)	0.0<br>(0.0 to 0.0)	-34.7<br>(-85.0 to 188.2)	-18.5<br>(-81.2 to 259.5)
Cyprus	Gastritis and duodenitis	5 to 9	Male	0<br>(0 to 0)	0.0<br>(0.0 to 0.0)	0<br>(0 to 0)	0.0<br>(0.0 to 0.0)	0<br>(0 to 0)	0.0<br>(0.0 to 0.0)	-66.9<br>(-90.5 to 18.4)	-58.8<br>(-88.1 to 47.4)
Cyprus	Gastritis and duodenitis	10 to 14	Both	0<br>(0 to 0)	0.0<br>(0.0 to 0.0)	0<br>(0 to 0)	0.0<br>(0.0 to 0.0)	0<br>(0 to 0)	0.0<br>(0.0 to 0.0)	-57.3<br>(-82.6 to 8.1)	-44.9<br>(-77.6 to 39.5)
Cyprus	Gastritis and duodenitis	10 to 14	Female	0<br>(0 to 0)	0.0<br>(0.0 to 0.0)	0<br>(0 to 0)	0.0<br>(0.0 to 0.0)	0<br>(0 to 0)	0.0<br>(0.0 to 0.0)	-52.5<br>(-88.1 to 88.1)	-39.4<br>(-84.8 to 140.1)
Cyprus	Gastritis and duodenitis	10 to 14	Male	0<br>(0 to 0)	0.0<br>(0.0 to 0.0)	0<br>(0 to 0)	0.0<br>(0.0 to 0.0)	0<br>(0 to 0)	0.0<br>(0.0 to 0.0)	-60.1<br>(-86.6 to 14.3)	-47.9<br>(-82.5 to 49.1)
Cyprus	Appendicitis	5 to 9	Both	0<br>(0 to 0)	0.0<br>(0.0 to 0.1)	0<br>(0 to 0)	0.0<br>(0.0 to 0.1)	0<br>(0 to 0)	0.0<br>(0.0 to 0.0)	-79.2<br>(-88.9 to -60.4)	-74.1<br>(-86.1 to -50.7)
Cyprus	Appendicitis	5 to 9	Female	0<br>(0 to 0)	0.0<br>(0.0 to 0.1)	0<br>(0 to 0)	0.0<br>(0.0 to 0.1)	0<br>(0 to 0)	0.0<br>(0.0 to 0.0)	-68.9<br>(-86.5 to -32.4)	-61.2<br>(-83.2 to -15.6)
Cyprus	Appendicitis	5 to 9	Male	0<br>(0 to 0)	0.1<br>(0.0 to 0.1)	0<br>(0 to 0)	0.0<br>(0.0 to 0.1)	0<br>(0 to 0)	0.0<br>(0.0 to 0.0)	-86.5<br>(-94.6 to -66.2)	-83.3<br>(-93.3 to -58.0)
Cyprus	Appendicitis	10 to 14	Both	0<br>(0 to 0)	0.0<br>(0.0 to 0.1)	0<br>(0 to 0)	0.0<br>(0.0 to 0.1)	0<br>(0 to 0)	0.0<br>(0.0 to 0.0)	-64.2<br>(-81.9 to -30.5)	-53.8<br>(-76.6 to -10.2)
Cyprus	Appendicitis	10 to 14	Female	0<br>(0 to 0)	0.0<br>(0.0 to 0.0)	0<br>(0 to 0)	0.0<br>(0.0 to 0.1)	0<br>(0 to 0)	0.0<br>(0.0 to 0.0)	-73.2<br>(-88.4 to -34.6)	-65.8<br>(-85.3 to -16.6)
Cyprus	Appendicitis	10 to 14	Male	0<br>(0 to 0)	0.0<br>(0.0 to 0.1)	0<br>(0 to 0)	0.0<br>(0.0 to 0.1)	0<br>(0 to 0)	0.0<br>(0.0 to 0.0)	-59.1<br>(-82.9 to -6.3)	-46.6<br>(-77.7 to 22.3)
Cyprus	Paralytic ileus and intestinal obstruction	5 to 9	Both	0<br>(0 to 0)	0.1<br>(0.0 to 0.1)	0<br>(0 to 0)	0.0<br>(0.0 to 0.1)	0<br>(0 to 0)	0.0<br>(0.0 to 0.0)	-62.5<br>(-80.8 to -32.6)	-53.3<br>(-76.1 to -16.1)
Cyprus	Paralytic ileus and intestinal obstruction	5 to 9	Female	0<br>(0 to 0)	0.0<br>(0.0 to 0.1)	0<br>(0 to 0)	0.0<br>(0.0 to 0.1)	0<br>(0 to 0)	0.0<br>(0.0 to 0.0)	-50.6<br>(-78.7 to 8.2)	-38.4<br>(-73.4 to 34.9)
Cyprus	Paralytic ileus and intestinal obstruction	5 to 9	Male	0<br>(0 to 0)	0.1<br>(0.0 to 0.1)	0<br>(0 to 0)	0.1<br>(0.0 to 0.1)	0<br>(0 to 0)	0.0<br>(0.0 to 0.0)	-69.9<br>(-87.4 to -27.6)	-62.5<br>(-84.3 to -10.0)
Cyprus	Paralytic ileus and intestinal obstruction	10 to 14	Both	0<br>(0 to 0)	0.0<br>(0.0 to 0.1)	0<br>(0 to 0)	0.0<br>(0.0 to 0.1)	0<br>(0 to 0)	0.0<br>(0.0 to 0.0)	-53.2<br>(-75.8 to -15.9)	-39.6<br>(-68.8 to 8.5)
Cyprus	Paralytic ileus and intestinal obstruction	10 to 14	Female	0<br>(0 to 0)	0.0<br>(0.0 to 0.1)	0<br>(0 to 0)	0.0<br>(0.0 to 0.1)	0<br>(0 to 0)	0.0<br>(0.0 to 0.0)	-60.7<br>(-83.9 to -22.2)	-49.9<br>(-79.5 to -0.6)
Cyprus	Paralytic ileus and intestinal obstruction	10 to 14	Male	0<br>(0 to 0)	0.0<br>(0.0 to 0.1)	0<br>(0 to 0)	0.0<br>(0.0 to 0.1)	0<br>(0 to 0)	0.0<br>(0.0 to 0.0)	-47.3<br>(-78.1 to 10.7)	-31.2<br>(-71.5 to 44.5)
Cyprus	Inguinal, femoral, and abdominal hernia	5 to 9	Both	0<br>(0 to 0)	0.0<br>(0.0 to 0.0)	0<br>(0 to 0)	0.0<br>(0.0 to 0.0)	0<br>(0 to 0)	0.0<br>(0.0 to 0.0)	-50.6<br>(-70.6 to -18.6)	-38.5<br>(-63.4 to 1.3)
Cyprus	Inguinal, femoral, and abdominal hernia	5 to 9	Female	0<br>(0 to 0)	0.0<br>(0.0 to 0.0)	0<br>(0 to 0)	0.0<br>(0.0 to 0.0)	0<br>(0 to 0)	0.0<br>(0.0 to 0.0)	-40.8<br>(-69.4 to 19.5)	-26.1<br>(-61.8 to 49.0)
Cyprus	Inguinal, femoral, and abdominal hernia	5 to 9	Male	0<br>(0 to 0)	0.0<br>(0.0 to 0.0)	0<br>(0 to 0)	0.0<br>(0.0 to 0.0)	0<br>(0 to 0)	0.0<br>(0.0 to 0.0)	-59.5<br>(-79.8 to -10.4)	-49.6<br>(-74.9 to 11.5)
Cyprus	Inguinal, femoral, and abdominal hernia	10 to 14	Both	0<br>(0 to 0)	0.0<br>(0.0 to 0.0)	0<br>(0 to 0)	0.0<br>(0.0 to 0.0)	0<br>(0 to 0)	0.0<br>(0.0 to 0.0)	-39.4<br>(-61.0 to -0.8)	-21.7<br>(-49.6 to 28.0)
Cyprus	Inguinal, femoral, and abdominal hernia	10 to 14	Female	0<br>(0 to 0)	0.0<br>(0.0 to 0.0)	0<br>(0 to 0)	0.0<br>(0.0 to 0.0)	0<br>(0 to 0)	0.0<br>(0.0 to 0.0)	-44.4<br>(-68.4 to 9.6)	-29.1<br>(-59.7 to 39.9)

eTable 8. All-cause and cause-specific mortality (counts and rates) at the global level and by country in the WHO European Region for ages 5 to 9 and 10 to 14 for 1990, 2000, and 2016

Cyprus	Inguinal, femoral, and abdominal hernia	10 to 14	Male	0 (0 to 0)	0.0 (0.0 to 0.0)	0 (0 to 0)	0.0 (0.0 to 0.0)	0 (0 to 0)	0.0 (0.0 to 0.0)	-34.7 (-66.7 to 35.1)	-14.7 (-56.5 to 76.3)
Cyprus	Inflammatory bowel disease	5 to 9	Both	0 (0 to 0)	0.0 (0.0 to 0.1)	0 (0 to 0)	0.0 (0.0 to 0.0)	0 (0 to 0)	0.0 (0.0 to 0.0)	-67.5 (-86.0 to -34.0)	-59.6 (-82.6 to -17.8)
Cyprus	Inflammatory bowel disease	5 to 9	Female	0 (0 to 0)	0.0 (0.0 to 0.1)	0 (0 to 0)	0.0 (0.0 to 0.1)	0 (0 to 0)	0.0 (0.0 to 0.0)	-64.0 (-86.1 to -14.1)	-55.1 (-82.7 to 7.2)
Cyprus	Inflammatory bowel disease	5 to 9	Male	0 (0 to 0)	0.0 (0.0 to 0.0)	0 (0 to 0)	0.0 (0.0 to 0.0)	0 (0 to 0)	0.0 (0.0 to 0.0)	-77.8 (-93.9 to -25.0)	-72.4 (-92.5 to -6.7)
Cyprus	Inflammatory bowel disease	10 to 14	Both	0 (0 to 0)	0.0 (0.0 to 0.0)	0 (0 to 0)	0.0 (0.0 to 0.0)	0 (0 to 0)	0.0 (0.0 to 0.0)	-65.2 (-83.4 to -37.4)	-55.1 (-78.6 to -19.2)
Cyprus	Inflammatory bowel disease	10 to 14	Female	0 (0 to 0)	0.0 (0.0 to 0.0)	0 (0 to 0)	0.0 (0.0 to 0.0)	0 (0 to 0)	0.0 (0.0 to 0.0)	-70.1 (-88.6 to -35.1)	-61.8 (-85.5 to -17.2)
Cyprus	Inflammatory bowel disease	10 to 14	Male	0 (0 to 0)	0.0 (0.0 to 0.1)	0 (0 to 0)	0.0 (0.0 to 0.0)	0 (0 to 0)	0.0 (0.0 to 0.0)	-61.4 (-85.7 to -15.8)	-49.6 (-81.4 to 9.9)
Cyprus	Vascular intestinal disorders	5 to 9	Both	0 (0 to 0)	0.0 (0.0 to 0.0)	0 (0 to 0)	0.0 (0.0 to 0.0)	0 (0 to 0)	0.0 (0.0 to 0.0)	-64.7 (-84.8 to -11.0)	-56.0 (-81.1 to 10.9)
Cyprus	Vascular intestinal disorders	5 to 9	Female	0 (0 to 0)	0.0 (0.0 to 0.0)	0 (0 to 0)	0.0 (0.0 to 0.0)	0 (0 to 0)	0.0 (0.0 to 0.0)	-56.2 (-85.1 to 47.0)	-45.4 (-81.5 to 83.4)
Cyprus	Vascular intestinal disorders	5 to 9	Male	0 (0 to 0)	0.0 (0.0 to 0.0)	0 (0 to 0)	0.0 (0.0 to 0.0)	0 (0 to 0)	0.0 (0.0 to 0.0)	-75.8 (-93.0 to -8.7)	-69.9 (-91.3 to 13.5)
Cyprus	Vascular intestinal disorders	10 to 14	Both	0 (0 to 0)	0.0 (0.0 to 0.0)	0 (0 to 0)	0.0 (0.0 to 0.0)	0 (0 to 0)	0.0 (0.0 to 0.0)	-53.9 (-79.2 to -4.0)	-40.4 (-73.2 to 24.0)
Cyprus	Vascular intestinal disorders	10 to 14	Female	0 (0 to 0)	0.0 (0.0 to 0.0)	0 (0 to 0)	0.0 (0.0 to 0.0)	0 (0 to 0)	0.0 (0.0 to 0.0)	-66.2 (-89.6 to 23.6)	-56.8 (-86.8 to 57.7)
Cyprus	Vascular intestinal disorders	10 to 14	Male	0 (0 to 0)	0.0 (0.0 to 0.0)	0 (0 to 0)	0.0 (0.0 to 0.0)	0 (0 to 0)	0.0 (0.0 to 0.0)	-52.0 (-80.4 to 9.1)	-37.4 (-74.4 to 42.3)
Cyprus	Gallbladder and biliary diseases	5 to 9	Both	0 (0 to 0)	0.0 (0.0 to 0.0)	0 (0 to 0)	0.0 (0.0 to 0.0)	0 (0 to 0)	0.0 (0.0 to 0.0)	-67.2 (-83.7 to -24.5)	-59.1 (-79.7 to -6.0)
Cyprus	Gallbladder and biliary diseases	5 to 9	Female	0 (0 to 0)	0.0 (0.0 to 0.0)	0 (0 to 0)	0.0 (0.0 to 0.0)	0 (0 to 0)	0.0 (0.0 to 0.0)	-60.0 (-84.5 to 16.7)	-50.1 (-80.7 to 45.5)
Cyprus	Gallbladder and biliary diseases	5 to 9	Male	0 (0 to 0)	0.0 (0.0 to 0.0)	0 (0 to 0)	0.0 (0.0 to 0.0)	0 (0 to 0)	0.0 (0.0 to 0.0)	-78.7 (-92.1 to -29.3)	-73.6 (-90.2 to -12.0)
Cyprus	Gallbladder and biliary diseases	10 to 14	Both	0 (0 to 0)	0.0 (0.0 to 0.0)	0 (0 to 0)	0.0 (0.0 to 0.0)	0 (0 to 0)	0.0 (0.0 to 0.0)	-50.2 (-76.9 to 11.4)	-35.6 (-70.2 to 43.8)
Cyprus	Gallbladder and biliary diseases	10 to 14	Female	0 (0 to 0)	0.0 (0.0 to 0.0)	0 (0 to 0)	0.0 (0.0 to 0.0)	0 (0 to 0)	0.0 (0.0 to 0.0)	-66.3 (-86.1 to -13.0)	-57.0 (-82.2 to 11.1)
Cyprus	Gallbladder and biliary diseases	10 to 14	Male	0 (0 to 0)	0.0 (0.0 to 0.0)	0 (0 to 0)	0.0 (0.0 to 0.0)	0 (0 to 0)	0.0 (0.0 to 0.0)	-39.4 (-79.2 to 82.6)	-21.0 (-72.9 to 138.2)
Cyprus	Pancreatitis	5 to 9	Both	0 (0 to 0)	0.0 (0.0 to 0.0)	0 (0 to 0)	0.0 (0.0 to 0.0)	0 (0 to 0)	0.0 (0.0 to 0.0)	-71.2 (-87.9 to -35.3)	-64.2 (-84.9 to -19.4)
Cyprus	Pancreatitis	5 to 9	Female	0 (0 to 0)	0.0 (0.0 to 0.0)	0 (0 to 0)	0.0 (0.0 to 0.0)	0 (0 to 0)	0.0 (0.0 to 0.0)	-58.9 (-84.8 to 23.2)	-48.8 (-81.0 to 53.7)
Cyprus	Pancreatitis	5 to 9	Male	0 (0 to 0)	0.0 (0.0 to 0.0)	0 (0 to 0)	0.0 (0.0 to 0.0)	0 (0 to 0)	0.0 (0.0 to 0.0)	-78.5 (-92.3 to -30.2)	-73.3 (-90.5 to -13.2)
Cyprus	Pancreatitis	10 to 14	Both	0 (0 to 0)	0.0 (0.0 to 0.0)	0 (0 to 0)	0.0 (0.0 to 0.0)	0 (0 to 0)	0.0 (0.0 to 0.0)	-52.2 (-77.1 to 9.3)	-38.2 (-70.5 to 41.1)
Cyprus	Pancreatitis	10 to 14	Female	0 (0 to 0)	0.0 (0.0 to 0.0)	0 (0 to 0)	0.0 (0.0 to 0.0)	0 (0 to 0)	0.0 (0.0 to 0.0)	-65.1 (-85.9 to -1.8)	-55.5 (-82.0 to 25.4)
Cyprus	Pancreatitis	10 to 14	Male	0 (0 to 0)	0.0 (0.0 to 0.0)	0 (0 to 0)	0.0 (0.0 to 0.0)	0 (0 to 0)	0.0 (0.0 to 0.0)	-43.8 (-79.5 to 55.4)	-26.7 (-73.3 to 102.8)

eTable 8. All-cause and cause-specific mortality (counts and rates) at the global level and by country in the WHO European Region for ages 5 to 9 and 10 to 14 for 1990, 2000, and 2016

Cyprus	Other digestive diseases	5 to 9	Both	0<br>(0 to 0)	0.0<br>(0.0 to 0.0)	0<br>(0 to 0)	0.0<br>(0.0 to 0.0)	0<br>(0 to 0)	0.0<br>(0.0 to 0.0)	-72.2<br>(-84.7 to -42.9)	-65.3<br>(-80.9 to -28.8)
Cyprus	Other digestive diseases	5 to 9	Female	0<br>(0 to 0)	0.0<br>(0.0 to 0.1)	0<br>(0 to 0)	0.0<br>(0.0 to 0.1)	0<br>(0 to 0)	0.0<br>(0.0 to 0.0)	-64.1<br>(-82.6 to -17.2)	-55.3<br>(-78.3 to 3.2)
Cyprus	Other digestive diseases	5 to 9	Male	0<br>(0 to 0)	0.0<br>(0.0 to 0.0)	0<br>(0 to 0)	0.0<br>(0.0 to 0.0)	0<br>(0 to 0)	0.0<br>(0.0 to 0.0)	-81.2<br>(-91.9 to -40.4)	-76.6<br>(-89.9 to -25.8)
Cyprus	Other digestive diseases	10 to 14	Both	0<br>(0 to 0)	0.0<br>(0.0 to 0.0)	0<br>(0 to 0)	0.0<br>(0.0 to 0.0)	0<br>(0 to 0)	0.0<br>(0.0 to 0.0)	-54.0<br>(-76.3 to -15.8)	-40.6<br>(-69.4 to 8.6)
Cyprus	Other digestive diseases	10 to 14	Female	0<br>(0 to 0)	0.0<br>(0.0 to 0.0)	0<br>(0 to 0)	0.0<br>(0.0 to 0.0)	0<br>(0 to 0)	0.0<br>(0.0 to 0.0)	-59.9<br>(-82.5 to -11.1)	-48.8<br>(-77.7 to 13.5)
Cyprus	Other digestive diseases	10 to 14	Male	0<br>(0 to 0)	0.0<br>(0.0 to 0.0)	0<br>(0 to 0)	0.0<br>(0.0 to 0.0)	0<br>(0 to 0)	0.0<br>(0.0 to 0.0)	-49.1<br>(-79.4 to 12.3)	-33.5<br>(-73.2 to 46.5)
Cyprus	Alzheimer disease and other dementias	5 to 9	Both	0<br>(0 to 0)	0.0<br>(0.0 to 0.0)	0<br>(0 to 0)	0.0<br>(0.0 to 0.0)	0<br>(0 to 0)	0.0<br>(0.0 to 0.0)	0.0<br>(0.0 to 0.0)	0.0<br>(0.0 to 0.0)
Cyprus	Alzheimer disease and other dementias	5 to 9	Female	0<br>(0 to 0)	0.0<br>(0.0 to 0.0)	0<br>(0 to 0)	0.0<br>(0.0 to 0.0)	0<br>(0 to 0)	0.0<br>(0.0 to 0.0)	0.0<br>(0.0 to 0.0)	0.0<br>(0.0 to 0.0)
Cyprus	Alzheimer disease and other dementias	5 to 9	Male	0<br>(0 to 0)	0.0<br>(0.0 to 0.0)	0<br>(0 to 0)	0.0<br>(0.0 to 0.0)	0<br>(0 to 0)	0.0<br>(0.0 to 0.0)	0.0<br>(0.0 to 0.0)	0.0<br>(0.0 to 0.0)
Cyprus	Alzheimer disease and other dementias	10 to 14	Both	0<br>(0 to 0)	0.0<br>(0.0 to 0.0)	0<br>(0 to 0)	0.0<br>(0.0 to 0.0)	0<br>(0 to 0)	0.0<br>(0.0 to 0.0)	0.0<br>(0.0 to 0.0)	0.0<br>(0.0 to 0.0)
Cyprus	Alzheimer disease and other dementias	10 to 14	Female	0<br>(0 to 0)	0.0<br>(0.0 to 0.0)	0<br>(0 to 0)	0.0<br>(0.0 to 0.0)	0<br>(0 to 0)	0.0<br>(0.0 to 0.0)	0.0<br>(0.0 to 0.0)	0.0<br>(0.0 to 0.0)
Cyprus	Alzheimer disease and other dementias	10 to 14	Male	0<br>(0 to 0)	0.0<br>(0.0 to 0.0)	0<br>(0 to 0)	0.0<br>(0.0 to 0.0)	0<br>(0 to 0)	0.0<br>(0.0 to 0.0)	0.0<br>(0.0 to 0.0)	0.0<br>(0.0 to 0.0)
Cyprus	Parkinson disease	5 to 9	Both	0<br>(0 to 0)	0.0<br>(0.0 to 0.0)	0<br>(0 to 0)	0.0<br>(0.0 to 0.0)	0<br>(0 to 0)	0.0<br>(0.0 to 0.0)	0.0<br>(0.0 to 0.0)	0.0<br>(0.0 to 0.0)
Cyprus	Parkinson disease	5 to 9	Female	0<br>(0 to 0)	0.0<br>(0.0 to 0.0)	0<br>(0 to 0)	0.0<br>(0.0 to 0.0)	0<br>(0 to 0)	0.0<br>(0.0 to 0.0)	0.0<br>(0.0 to 0.0)	0.0<br>(0.0 to 0.0)
Cyprus	Parkinson disease	5 to 9	Male	0<br>(0 to 0)	0.0<br>(0.0 to 0.0)	0<br>(0 to 0)	0.0<br>(0.0 to 0.0)	0<br>(0 to 0)	0.0<br>(0.0 to 0.0)	0.0<br>(0.0 to 0.0)	0.0<br>(0.0 to 0.0)
Cyprus	Parkinson disease	10 to 14	Both	0<br>(0 to 0)	0.0<br>(0.0 to 0.0)	0<br>(0 to 0)	0.0<br>(0.0 to 0.0)	0<br>(0 to 0)	0.0<br>(0.0 to 0.0)	0.0<br>(0.0 to 0.0)	0.0<br>(0.0 to 0.0)
Cyprus	Parkinson disease	10 to 14	Female	0<br>(0 to 0)	0.0<br>(0.0 to 0.0)	0<br>(0 to 0)	0.0<br>(0.0 to 0.0)	0<br>(0 to 0)	0.0<br>(0.0 to 0.0)	0.0<br>(0.0 to 0.0)	0.0<br>(0.0 to 0.0)
Cyprus	Parkinson disease	10 to 14	Male	0<br>(0 to 0)	0.0<br>(0.0 to 0.0)	0<br>(0 to 0)	0.0<br>(0.0 to 0.0)	0<br>(0 to 0)	0.0<br>(0.0 to 0.0)	0.0<br>(0.0 to 0.0)	0.0<br>(0.0 to 0.0)
Cyprus	Epilepsy	5 to 9	Both	0<br>(0 to 0)	0.2<br>(0.2 to 0.3)	0<br>(0 to 0)	0.2<br>(0.1 to 0.3)	0<br>(0 to 0)	0.1<br>(0.1 to 0.2)	-58.5<br>(-75.1 to -28.1)	-48.3<br>(-68.9 to -10.4)
Cyprus	Epilepsy	5 to 9	Female	0<br>(0 to 0)	0.2<br>(0.1 to 0.4)	0<br>(0 to 0)	0.2<br>(0.1 to 0.4)	0<br>(0 to 0)	0.2<br>(0.1 to 0.3)	-45.2<br>(-72.1 to 13.3)	-31.7<br>(-65.2 to 41.2)
Cyprus	Epilepsy	5 to 9	Male	0<br>(0 to 0)	0.2<br>(0.1 to 0.4)	0<br>(0 to 0)	0.2<br>(0.1 to 0.3)	0<br>(0 to 0)	0.1<br>(0.1 to 0.1)	-70.4<br>(-85.0 to -37.1)	-63.1<br>(-81.3 to -21.7)
Cyprus	Epilepsy	10 to 14	Both	0<br>(0 to 0)	0.2<br>(0.1 to 0.2)	0<br>(0 to 0)	0.2<br>(0.1 to 0.3)	0<br>(0 to 0)	0.1<br>(0.1 to 0.2)	-45.1<br>(-65.8 to -8.2)	-29.1<br>(-55.8 to 18.4)
Cyprus	Epilepsy	10 to 14	Female	0<br>(0 to 0)	0.1<br>(0.1 to 0.2)	0<br>(0 to 0)	0.2<br>(0.1 to 0.3)	0<br>(0 to 0)	0.1<br>(0.1 to 0.2)	-50.6<br>(-76.0 to -0.6)	-36.9<br>(-69.3 to 26.9)

eTable 8. All-cause and cause-specific mortality (counts and rates) at the global level and by country in the WHO European Region for ages 5 to 9 and 10 to 14 for 1990, 2000, and 2016

Cyprus	Epilepsy	10 to 14	Male	0 (0 to 0)	0.2 (0.1 to 0.3)	0 (0 to 0)	0.2 (0.1 to 0.3)	0 (0 to 0)	0.1 (0.1 to 0.2)	-41.0 (-68.9 to 11.2)	-23.0 (-59.5 to 45.1)
Cyprus	Motor neuron disease	5 to 9	Both	0 (0 to 0)	0.0 (0.0 to 0.0)	0 (0 to 0)	0.0 (0.0 to 0.0)	0 (0 to 0)	0.0 (0.0 to 0.0)	-33.1 (-70.8 to 58.0)	-16.6 (-63.7 to 96.8)
Cyprus	Motor neuron disease	5 to 9	Female	0 (0 to 0)	0.0 (0.0 to 0.0)	0 (0 to 0)	0.0 (0.0 to 0.1)	0 (0 to 0)	0.0 (0.0 to 0.0)	-30.4 (-73.5 to 98.1)	-13.2 (-67.0 to 147.1)
Cyprus	Motor neuron disease	5 to 9	Male	0 (0 to 0)	0.0 (0.0 to 0.0)	0 (0 to 0)	0.0 (0.0 to 0.0)	0 (0 to 0)	0.0 (0.0 to 0.0)	-41.9 (-85.0 to 118.9)	-27.7 (-81.3 to 172.3)
Cyprus	Motor neuron disease	10 to 14	Both	0 (0 to 0)	0.0 (0.0 to 0.1)	0 (0 to 0)	0.1 (0.0 to 0.1)	0 (0 to 0)	0.0 (0.0 to 0.1)	-27.8 (-67.0 to 51.4)	-6.8 (-57.3 to 95.5)
Cyprus	Motor neuron disease	10 to 14	Female	0 (0 to 0)	0.0 (0.0 to 0.0)	0 (0 to 0)	0.0 (0.0 to 0.0)	0 (0 to 0)	0.0 (0.0 to 0.0)	-38.0 (-72.4 to 38.6)	-20.9 (-64.7 to 76.9)
Cyprus	Motor neuron disease	10 to 14	Male	0 (0 to 0)	0.1 (0.0 to 0.1)	0 (0 to 0)	0.1 (0.0 to 0.2)	0 (0 to 0)	0.1 (0.0 to 0.1)	-25.7 (-69.2 to 75.6)	-3.0 (-59.8 to 129.2)
Cyprus	Other neurological disorders	5 to 9	Both	0 (0 to 0)	0.3 (0.2 to 0.4)	0 (0 to 0)	0.3 (0.2 to 0.4)	0 (0 to 0)	0.2 (0.1 to 0.3)	-42.6 (-66.7 to 0.4)	-28.5 (-58.5 to 25.0)
Cyprus	Other neurological disorders	5 to 9	Female	0 (0 to 0)	0.3 (0.1 to 0.4)	0 (0 to 0)	0.3 (0.2 to 0.5)	0 (0 to 0)	0.2 (0.1 to 0.3)	-30.8 (-63.4 to 33.7)	-13.6 (-54.4 to 66.7)
Cyprus	Other neurological disorders	5 to 9	Male	0 (0 to 0)	0.3 (0.2 to 0.5)	0 (0 to 0)	0.3 (0.2 to 0.5)	0 (0 to 0)	0.2 (0.1 to 0.3)	-51.7 (-79.4 to 8.8)	-39.9 (-74.3 to 35.4)
Cyprus	Other neurological disorders	10 to 14	Both	0 (0 to 0)	0.3 (0.2 to 0.4)	0 (0 to 0)	0.4 (0.3 to 0.5)	0 (0 to 0)	0.3 (0.2 to 0.4)	-28.4 (-58.9 to 20.7)	-7.6 (-46.9 to 55.9)
Cyprus	Other neurological disorders	10 to 14	Female	0 (0 to 0)	0.1 (0.1 to 0.2)	0 (0 to 0)	0.2 (0.1 to 0.3)	0 (0 to 0)	0.1 (0.1 to 0.2)	-38.1 (-70.4 to 33.6)	-21.0 (-62.2 to 70.6)
Cyprus	Other neurological disorders	10 to 14	Male	0 (0 to 0)	0.4 (0.3 to 0.7)	0 (0 to 0)	0.5 (0.4 to 0.8)	0 (0 to 0)	0.4 (0.3 to 0.7)	-25.6 (-61.3 to 39.5)	-3.0 (-49.5 to 82.0)
Cyprus	Drug use disorders	5 to 9	Both	0 (0 to 0)	0.0 (0.0 to 0.0)	0 (0 to 0)	0.0 (0.0 to 0.0)	0 (0 to 0)	0.0 (0.0 to 0.0)	0.0 (0.0 to 0.0)	0.0 (0.0 to 0.0)
Cyprus	Drug use disorders	5 to 9	Female	0 (0 to 0)	0.0 (0.0 to 0.0)	0 (0 to 0)	0.0 (0.0 to 0.0)	0 (0 to 0)	0.0 (0.0 to 0.0)	0.0 (0.0 to 0.0)	0.0 (0.0 to 0.0)
Cyprus	Drug use disorders	5 to 9	Male	0 (0 to 0)	0.0 (0.0 to 0.0)	0 (0 to 0)	0.0 (0.0 to 0.0)	0 (0 to 0)	0.0 (0.0 to 0.0)	0.0 (0.0 to 0.0)	0.0 (0.0 to 0.0)
Cyprus	Drug use disorders	10 to 14	Both	0 (0 to 0)	0.0 (0.0 to 0.0)	0 (0 to 0)	0.0 (0.0 to 0.0)	0 (0 to 0)	0.0 (0.0 to 0.0)	0.0 (0.0 to 0.0)	0.0 (0.0 to 0.0)
Cyprus	Drug use disorders	10 to 14	Female	0 (0 to 0)	0.0 (0.0 to 0.0)	0 (0 to 0)	0.0 (0.0 to 0.0)	0 (0 to 0)	0.0 (0.0 to 0.0)	0.0 (0.0 to 0.0)	0.0 (0.0 to 0.0)
Cyprus	Drug use disorders	10 to 14	Male	0 (0 to 0)	0.0 (0.0 to 0.0)	0 (0 to 0)	0.0 (0.0 to 0.0)	0 (0 to 0)	0.0 (0.0 to 0.0)	0.0 (0.0 to 0.0)	0.0 (0.0 to 0.0)
Cyprus	Eating disorders	5 to 9	Both	0 (0 to 0)	0.0 (0.0 to 0.0)	0 (0 to 0)	0.0 (0.0 to 0.0)	0 (0 to 0)	0.0 (0.0 to 0.0)	-33.0 (-71.4 to 55.9)	-16.5 (-64.4 to 94.1)
Cyprus	Eating disorders	5 to 9	Female	0 (0 to 0)	0.0 (0.0 to 0.0)	0 (0 to 0)	0.0 (0.0 to 0.0)	0 (0 to 0)	0.0 (0.0 to 0.0)	-24.6 (-72.0 to 108.0)	-5.9 (-65.1 to 159.4)
Cyprus	Eating disorders	5 to 9	Male	0 (0 to 0)	0.0 (0.0 to 0.0)	0 (0 to 0)	0.0 (0.0 to 0.0)	0 (0 to 0)	0.0 (0.0 to 0.0)	-56.1 (-84.3 to 30.5)	-45.4 (-80.4 to 62.3)
Cyprus	Eating disorders	10 to 14	Both	0 (0 to 0)	0.0 (0.0 to 0.0)	0 (0 to 0)	0.0 (0.0 to 0.0)	0 (0 to 0)	0.0 (0.0 to 0.0)	-26.9 (-71.5 to 83.6)	-5.6 (-63.2 to 137.0)
Cyprus	Eating disorders	10 to 14	Female	0 (0 to 0)	0.0 (0.0 to 0.0)	0 (0 to 0)	0.0 (0.0 to 0.0)	0 (0 to 0)	0.0 (0.0 to 0.0)	-30.3 (-76.5 to 97.3)	-11.0 (-70.0 to 151.8)
Cyprus	Eating disorders	10 to 14	Male	0 (0 to 0)	0.0 (0.0 to 0.0)	0 (0 to 0)	0.0 (0.0 to 0.0)	0 (0 to 0)	0.0 (0.0 to 0.0)	4.5 (-67.8 to 270.9)	36.4 (-58.0 to 383.9)
Cyprus	Diabetes mellitus	5 to 9	Both	0 (0 to 0)	0.2 (0.1 to 0.2)	0 (0 to 0)	0.1 (0.1 to 0.2)	0 (0 to 0)	0.1 (0.0 to 0.1)	-71.4 (-82.1 to -53.6)	-64.3 (-77.8 to -42.2)
Cyprus	Diabetes mellitus	5 to 9	Female	0 (0 to 0)	0.2 (0.1 to 0.3)	0 (0 to 0)	0.2 (0.1 to 0.3)	0 (0 to 0)	0.1 (0.0 to 0.1)	-69.0 (-81.4 to -46.1)	-61.3 (-76.9 to -32.8)
Cyprus	Diabetes mellitus	5 to 9	Male	0 (0 to 0)	0.1 (0.1 to 0.2)	0 (0 to 0)	0.1 (0.1 to 0.2)	0 (0 to 0)	0.0 (0.0 to 0.1)	-74.9 (-88.4 to -48.3)	-68.7 (-85.6 to -35.6)
Cyprus	Diabetes mellitus	10 to 14	Both	0 (0 to 0)	0.1 (0.1 to 0.1)	0 (0 to 0)	0.1 (0.1 to 0.2)	0 (0 to 0)	0.1 (0.0 to 0.1)	-56.6 (-72.6 to -30.4)	-43.9 (-64.6 to -10.1)

Cyprus	Diabetes mellitus	10 to 14	Female	0 (0 to 0)	0.1 (0.1 to 0.2)	0 (0 to 0)	0.1 (0.1 to 0.2)	0 (0 to 0)	0.0 (0.0 to 0.1)	-70.7 (-83.5 to -43.8)	-62.6 (-79.0 to -28.2)
Cyprus	Diabetes mellitus	10 to 14	Male	0 (0 to 0)	0.1 (0.1 to 0.1)	0 (0 to 0)	0.1 (0.1 to 0.2)	0 (0 to 0)	0.1 (0.0 to 0.1)	-38.7 (-67.7 to 17.3)	-20.0 (-57.9 to 53.1)
Cyprus	Acute glomerulonephritis	5 to 9	Both	0 (0 to 0)	0.0 (0.0 to 0.0)	0 (0 to 0)	0.0 (0.0 to 0.0)	0 (0 to 0)	0.0 (0.0 to 0.0)	-32.6 (-45.0 to -18.0)	-16.0 (-31.5 to 2.2)
Cyprus	Acute glomerulonephritis	5 to 9	Female	0 (0 to 0)	0.0 (0.0 to 0.0)	0 (0 to 0)	0.0 (0.0 to 0.0)	0 (0 to 0)	0.0 (0.0 to 0.0)	-28.4 (-43.0 to -11.4)	-10.6 (-28.9 to 10.5)
Cyprus	Acute glomerulonephritis	5 to 9	Male	0 (0 to 0)	0.0 (0.0 to 0.0)	0 (0 to 0)	0.0 (0.0 to 0.0)	0 (0 to 0)	0.0 (0.0 to 0.0)	-36.4 (-52.3 to -16.9)	-20.9 (-40.6 to 3.3)
Cyprus	Acute glomerulonephritis	10 to 14	Both	0 (0 to 0)	0.0 (0.0 to 0.0)	0 (0 to 0)	0.0 (0.0 to 0.0)	0 (0 to 0)	0.0 (0.0 to 0.0)	-27.1 (-37.7 to -13.6)	-5.9 (-19.6 to 11.5)
Cyprus	Acute glomerulonephritis	10 to 14	Female	0 (0 to 0)	0.0 (0.0 to 0.0)	0 (0 to 0)	0.0 (0.0 to 0.0)	0 (0 to 0)	0.0 (0.0 to 0.0)	-29.3 (-41.3 to -11.6)	-9.8 (-25.1 to 12.8)
Cyprus	Acute glomerulonephritis	10 to 14	Male	0 (0 to 0)	0.0 (0.0 to 0.0)	0 (0 to 0)	0.0 (0.0 to 0.0)	0 (0 to 0)	0.0 (0.0 to 0.0)	-25.1 (-38.5 to -8.0)	-2.2 (-19.8 to 20.1)
Cyprus	Chronic kidney disease	5 to 9	Both	0 (0 to 0)	0.2 (0.2 to 0.3)	0 (0 to 0)	0.2 (0.1 to 0.3)	0 (0 to 0)	0.1 (0.1 to 0.1)	-65.0 (-76.3 to -49.5)	-56.4 (-70.5 to -37.1)
Cyprus	Chronic kidney disease	5 to 9	Female	0 (0 to 0)	0.2 (0.1 to 0.2)	0 (0 to 0)	0.2 (0.1 to 0.3)	0 (0 to 0)	0.1 (0.1 to 0.2)	-48.4 (-70.2 to -10.3)	-35.7 (-62.8 to 11.9)
Cyprus	Chronic kidney disease	5 to 9	Male	0 (0 to 0)	0.2 (0.2 to 0.4)	0 (0 to 0)	0.2 (0.1 to 0.3)	0 (0 to 0)	0.1 (0.0 to 0.1)	-75.2 (-85.3 to -59.3)	-69.1 (-81.8 to -49.4)
Cyprus	Chronic kidney disease	10 to 14	Both	0 (0 to 0)	0.1 (0.1 to 0.1)	0 (0 to 0)	0.2 (0.1 to 0.2)	0 (0 to 0)	0.1 (0.1 to 0.1)	-44.1 (-61.6 to -19.0)	-27.8 (-50.5 to 4.5)
Cyprus	Chronic kidney disease	10 to 14	Female	0 (0 to 0)	0.1 (0.1 to 0.1)	0 (0 to 0)	0.1 (0.1 to 0.2)	0 (0 to 0)	0.1 (0.0 to 0.1)	-53.1 (-71.1 to -22.3)	-40.2 (-63.1 to -0.8)
Cyprus	Chronic kidney disease	10 to 14	Male	0 (0 to 0)	0.1 (0.1 to 0.2)	0 (0 to 0)	0.2 (0.1 to 0.2)	0 (0 to 0)	0.1 (0.1 to 0.1)	-37.6 (-62.0 to 5.2)	-18.6 (-50.5 to 37.2)
Cyprus	Urinary diseases and male infertility	5 to 9	Both	0 (0 to 0)	0.0 (0.0 to 0.0)	0 (0 to 0)	0.0 (0.0 to 0.1)	0 (0 to 0)	0.0 (0.0 to 0.0)	-45.8 (-68.1 to -8.5)	-32.5 (-60.2 to 14.0)
Cyprus	Urinary diseases and male infertility	5 to 9	Female	0 (0 to 0)	0.0 (0.0 to 0.1)	0 (0 to 0)	0.1 (0.0 to 0.1)	0 (0 to 0)	0.0 (0.0 to 0.0)	-46.0 (-70.9 to 1.1)	-32.6 (-63.6 to 26.1)
Cyprus	Urinary diseases and male infertility	5 to 9	Male	0 (0 to 0)	0.0 (0.0 to 0.0)	0 (0 to 0)	0.0 (0.0 to 0.0)	0 (0 to 0)	0.0 (0.0 to 0.0)	-45.5 (-78.1 to 33.3)	-32.2 (-72.8 to 65.9)
Cyprus	Urinary diseases and male infertility	10 to 14	Both	0 (0 to 0)	0.0 (0.0 to 0.0)	0 (0 to 0)	0.0 (0.0 to 0.1)	0 (0 to 0)	0.0 (0.0 to 0.0)	-46.1 (-65.2 to -16.5)	-30.4 (-55.1 to 7.8)
Cyprus	Urinary diseases and male infertility	10 to 14	Female	0 (0 to 0)	0.0 (0.0 to 0.0)	0 (0 to 0)	0.0 (0.0 to 0.1)	0 (0 to 0)	0.0 (0.0 to 0.0)	-48.4 (-71.2 to -6.5)	-34.1 (-63.2 to 19.3)
Cyprus	Urinary diseases and male infertility	10 to 14	Male	0 (0 to 0)	0.0 (0.0 to 0.0)	0 (0 to 0)	0.0 (0.0 to 0.1)	0 (0 to 0)	0.0 (0.0 to 0.0)	-44.3 (-70.5 to 0.8)	-27.3 (-61.5 to 31.6)
Cyprus	Hemoglobinopathies and hemolytic anemias	5 to 9	Both	0 (0 to 0)	0.3 (0.2 to 0.4)	0 (0 to 0)	0.2 (0.2 to 0.3)	0 (0 to 0)	0.1 (0.1 to 0.1)	-72.3 (-83.5 to -57.9)	-65.5 (-79.5 to -47.6)
Cyprus	Hemoglobinopathies and hemolytic anemias	5 to 9	Female	0 (0 to 0)	0.2 (0.1 to 0.4)	0 (0 to 0)	0.2 (0.1 to 0.4)	0 (0 to 0)	0.1 (0.1 to 0.2)	-62.5 (-80.7 to -30.1)	-53.2 (-75.9 to -12.9)
Cyprus	Hemoglobinopathies and hemolytic anemias	5 to 9	Male	0 (0 to 0)	0.3 (0.2 to 0.5)	0 (0 to 0)	0.2 (0.1 to 0.4)	0 (0 to 0)	0.1 (0.0 to 0.1)	-79.7 (-89.3 to -65.9)	-74.8 (-86.6 to -57.6)

eTable 8. All-cause and cause-specific mortality (counts and rates) at the global level and by country in the WHO European Region for ages 5 to 9 and 10 to 14 for 1990, 2000, and 2016

Cyprus	Hemoglobinopathies and hemolytic anemias	10 to 14	Both	0 (0 to 0)	0.2 (0.1 to 0.2)	0 (0 to 0)	0.2 (0.2 to 0.3)	0 (0 to 0)	0.1 (0.1 to 0.1)	-55.8 (-69.8 to -32.5)	-42.9 (-61.0 to -12.8)
Cyprus	Hemoglobinopathies and hemolytic anemias	10 to 14	Female	0 (0 to 0)	0.2 (0.1 to 0.2)	0 (0 to 0)	0.2 (0.1 to 0.3)	0 (0 to 0)	0.1 (0.0 to 0.1)	-65.4 (-81.6 to -37.0)	-55.9 (-76.5 to -19.6)
Cyprus	Hemoglobinopathies and hemolytic anemias	10 to 14	Male	0 (0 to 0)	0.2 (0.1 to 0.3)	0 (0 to 0)	0.2 (0.2 to 0.4)	0 (0 to 0)	0.1 (0.1 to 0.2)	-48.1 (-69.3 to -12.6)	-32.3 (-59.9 to 14.1)
Cyprus	Endocrine, metabolic, blood, and immune disorders	5 to 9	Both	1 (0 to 1)	1.3 (0.9 to 2.0)	1 (0 to 1)	1.2 (0.8 to 1.8)	0 (0 to 0)	0.7 (0.5 to 1.0)	-58.3 (-73.6 to -27.3)	-48.0 (-67.1 to -9.4)
Cyprus	Endocrine, metabolic, blood, and immune disorders	5 to 9	Female	0 (0 to 1)	1.3 (0.7 to 2.1)	0 (0 to 1)	1.3 (0.7 to 2.1)	0 (0 to 0)	0.8 (0.5 to 1.3)	-47.6 (-71.2 to 5.0)	-34.6 (-64.1 to 31.0)
Cyprus	Endocrine, metabolic, blood, and immune disorders	5 to 9	Male	0 (0 to 1)	1.3 (0.7 to 2.3)	0 (0 to 1)	1.2 (0.7 to 2.1)	0 (0 to 0)	0.5 (0.3 to 0.9)	-68.2 (-83.3 to -33.5)	-60.5 (-79.3 to -17.3)
Cyprus	Endocrine, metabolic, blood, and immune disorders	10 to 14	Both	1 (0 to 1)	0.8 (0.5 to 1.4)	1 (0 to 1)	1.1 (0.7 to 1.7)	0 (0 to 0)	0.6 (0.4 to 1.0)	-41.8 (-60.7 to -11.4)	-24.9 (-49.2 to 14.4)
Cyprus	Endocrine, metabolic, blood, and immune disorders	10 to 14	Female	0 (0 to 0)	0.7 (0.4 to 1.3)	0 (0 to 0)	0.9 (0.6 to 1.5)	0 (0 to 0)	0.4 (0.3 to 0.8)	-52.6 (-73.1 to -17.3)	-39.4 (-65.7 to 5.6)
Cyprus	Endocrine, metabolic, blood, and immune disorders	10 to 14	Male	0 (0 to 1)	0.9 (0.5 to 1.7)	0 (0 to 1)	1.3 (0.7 to 2.3)	0 (0 to 0)	0.8 (0.5 to 1.4)	-34.0 (-60.6 to 18.2)	-13.8 (-48.6 to 54.2)
Cyprus	Rheumatoid arthritis	5 to 9	Both	0 (0 to 0)	0.0 (0.0 to 0.0)	0 (0 to 0)	0.0 (0.0 to 0.0)	0 (0 to 0)	0.0 (0.0 to 0.0)	-62.3 (-80.7 to -20.5)	-53.0 (-75.9 to -1.0)
Cyprus	Rheumatoid arthritis	5 to 9	Female	0 (0 to 0)	0.0 (0.0 to 0.1)	0 (0 to 0)	0.0 (0.0 to 0.1)	0 (0 to 0)	0.0 (0.0 to 0.0)	-62.7 (-84.0 to -6.8)	-53.5 (-80.1 to 16.2)
Cyprus	Rheumatoid arthritis	5 to 9	Male	0 (0 to 0)	0.0 (0.0 to 0.0)	0 (0 to 0)	0.0 (0.0 to 0.0)	0 (0 to 0)	0.0 (0.0 to 0.0)	-61.6 (-83.3 to 19.2)	-52.2 (-79.3 to 48.3)
Cyprus	Rheumatoid arthritis	10 to 14	Both	0 (0 to 0)	0.0 (0.0 to 0.0)	0 (0 to 0)	0.0 (0.0 to 0.0)	0 (0 to 0)	0.0 (0.0 to 0.0)	-54.0 (-75.9 to -9.2)	-40.7 (-68.9 to 17.2)
Cyprus	Rheumatoid arthritis	10 to 14	Female	0 (0 to 0)	0.0 (0.0 to 0.0)	0 (0 to 0)	0.0 (0.0 to 0.0)	0 (0 to 0)	0.0 (0.0 to 0.0)	-63.1 (-85.6 to -1.7)	-52.9 (-81.7 to 25.4)
Cyprus	Rheumatoid arthritis	10 to 14	Male	0 (0 to 0)	0.0 (0.0 to 0.0)	0 (0 to 0)	0.0 (0.0 to 0.0)	0 (0 to 0)	0.0 (0.0 to 0.0)	-41.4 (-74.8 to 45.7)	-23.5 (-67.2 to 90.1)
Cyprus	Other musculoskeletal disorders	5 to 9	Both	0 (0 to 0)	0.0 (0.0 to 0.1)	0 (0 to 0)	0.0 (0.0 to 0.1)	0 (0 to 0)	0.0 (0.0 to 0.0)	-49.6 (-70.9 to -12.5)	-37.2 (-63.8 to 9.0)
Cyprus	Other musculoskeletal disorders	5 to 9	Female	0 (0 to 0)	0.1 (0.0 to 0.1)	0 (0 to 0)	0.1 (0.0 to 0.1)	0 (0 to 0)	0.0 (0.0 to 0.1)	-38.9 (-68.7 to 18.5)	-23.8 (-60.9 to 47.7)
Cyprus	Other musculoskeletal disorders	5 to 9	Male	0 (0 to 0)	0.0 (0.0 to 0.0)	0 (0 to 0)	0.0 (0.0 to 0.0)	0 (0 to 0)	0.0 (0.0 to 0.0)	-72.1 (-87.4 to -34.0)	-65.2 (-84.3 to -17.8)

Cyprus	Other musculoskeletal disorders	10 to 14	Both	0<br>(0 to 0)	0.0<br>(0.0 to 0.1)	0<br>(0 to 0)	0.1<br>(0.0 to 0.1)	0<br>(0 to 0)	0.0<br>(0.0 to 0.0)	-42.4<br>(-65.3 to -6.6)	-25.7<br>(-55.1 to 20.6)
Cyprus	Other musculoskeletal disorders	10 to 14	Female	0<br>(0 to 0)	0.1<br>(0.0 to 0.1)	0<br>(0 to 0)	0.1<br>(0.1 to 0.2)	0<br>(0 to 0)	0.0<br>(0.0 to 0.1)	-46.9<br>(-71.6 to 3.6)	-32.2<br>(-63.8 to 32.2)
Cyprus	Other musculoskeletal disorders	10 to 14	Male	0<br>(0 to 0)	0.0<br>(0.0 to 0.0)	0<br>(0 to 0)	0.0<br>(0.0 to 0.1)	0<br>(0 to 0)	0.0<br>(0.0 to 0.0)	-30.4<br>(-66.0 to 42.1)	-9.1<br>(-55.6 to 85.4)
Cyprus	Congenital birth defects	5 to 9	Both	1<br>(1 to 1)	1.5<br>(0.9 to 2.0)	1<br>(1 to 1)	1.5<br>(1.0 to 1.9)	0<br>(0 to 0)	0.7<br>(0.5 to 1.0)	-60.0<br>(-73.3 to -34.7)	-50.1<br>(-66.8 to -18.7)
Cyprus	Congenital birth defects	5 to 9	Female	0<br>(0 to 1)	1.5<br>(0.7 to 2.3)	0<br>(0 to 1)	1.6<br>(0.8 to 2.4)	0<br>(0 to 0)	1.0<br>(0.6 to 1.4)	-47.2<br>(-68.9 to -0.1)	-34.1<br>(-61.2 to 24.6)
Cyprus	Congenital birth defects	5 to 9	Male	0<br>(0 to 1)	1.5<br>(0.9 to 2.2)	0<br>(0 to 1)	1.4<br>(0.8 to 2.0)	0<br>(0 to 0)	0.5<br>(0.3 to 0.8)	-71.9<br>(-84.3 to -36.7)	-65.1<br>(-80.5 to -21.3)
Cyprus	Congenital birth defects	10 to 14	Both	0<br>(0 to 1)	0.7<br>(0.5 to 0.9)	1<br>(0 to 1)	1.0<br>(0.7 to 1.3)	0<br>(0 to 0)	0.5<br>(0.4 to 0.7)	-42.0<br>(-58.6 to -17.1)	-25.1<br>(-46.6 to 7.0)
Cyprus	Congenital birth defects	10 to 14	Female	0<br>(0 to 0)	0.7<br>(0.5 to 1.1)	0<br>(0 to 0)	1.0<br>(0.6 to 1.4)	0<br>(0 to 0)	0.4<br>(0.3 to 0.7)	-51.7<br>(-70.4 to -14.1)	-38.4<br>(-62.2 to 9.7)
Cyprus	Congenital birth defects	10 to 14	Male	0<br>(0 to 0)	0.7<br>(0.5 to 1.0)	0<br>(0 to 0)	1.1<br>(0.7 to 1.5)	0<br>(0 to 0)	0.6<br>(0.4 to 0.9)	-33.0<br>(-57.7 to 10.9)	-12.6<br>(-44.8 to 44.7)
Cyprus	Skin and subcutaneous diseases	5 to 9	Both	0<br>(0 to 0)	0.0<br>(0.0 to 0.0)	0<br>(0 to 0)	0.0<br>(0.0 to 0.0)	0<br>(0 to 0)	0.0<br>(0.0 to 0.0)	-36.7<br>(-64.3 to -5.5)	-21.2<br>(-55.5 to 17.8)
Cyprus	Skin and subcutaneous diseases	5 to 9	Female	0<br>(0 to 0)	0.0<br>(0.0 to 0.0)	0<br>(0 to 0)	0.0<br>(0.0 to 0.0)	0<br>(0 to 0)	0.0<br>(0.0 to 0.0)	-34.1<br>(-66.8 to 6.5)	-17.9<br>(-58.6 to 32.8)
Cyprus	Skin and subcutaneous diseases	5 to 9	Male	0<br>(0 to 0)	0.0<br>(0.0 to 0.0)	0<br>(0 to 0)	0.0<br>(0.0 to 0.0)	0<br>(0 to 0)	0.0<br>(0.0 to 0.0)	-39.2<br>(-72.7 to 5.0)	-24.3<br>(-66.0 to 30.6)
Cyprus	Skin and subcutaneous diseases	10 to 14	Both	0<br>(0 to 0)	0.0<br>(0.0 to 0.0)	0<br>(0 to 0)	0.0<br>(0.0 to 0.0)	0<br>(0 to 0)	0.0<br>(0.0 to 0.0)	-30.1<br>(-57.2 to 0.8)	-9.8<br>(-44.8 to 30.2)
Cyprus	Skin and subcutaneous diseases	10 to 14	Female	0<br>(0 to 0)	0.0<br>(0.0 to 0.0)	0<br>(0 to 0)	0.0<br>(0.0 to 0.0)	0<br>(0 to 0)	0.0<br>(0.0 to 0.0)	-39.6<br>(-70.3 to -8.0)	-22.9<br>(-62.0 to 17.4)
Cyprus	Skin and subcutaneous diseases	10 to 14	Male	0<br>(0 to 0)	0.0<br>(0.0 to 0.0)	0<br>(0 to 0)	0.0<br>(0.0 to 0.0)	0<br>(0 to 0)	0.0<br>(0.0 to 0.0)	-22.1<br>(-58.6 to 38.2)	1.7<br>(-46.0 to 80.3)
Cyprus	Road injuries	5 to 9	Both	4<br>(3 to 5)	7.0<br>(5.7 to 8.4)	3<br>(3 to 4)	6.0<br>(5.0 to 7.1)	1<br>(1 to 1)	2.0<br>(1.5 to 2.6)	-77.1<br>(-83.7 to -67.6)	-71.5<br>(-79.7 to -59.6)
Cyprus	Road injuries	5 to 9	Female	1<br>(1 to 2)	5.0<br>(3.6 to 6.4)	1<br>(1 to 2)	4.8<br>(3.4 to 6.2)	0<br>(0 to 1)	2.0<br>(1.4 to 2.7)	-67.4<br>(-79.7 to -50.0)	-59.4<br>(-74.7 to -37.6)
Cyprus	Road injuries	5 to 9	Male	3<br>(2 to 3)	8.9<br>(6.7 to 11.2)	2<br>(2 to 2)	7.2<br>(5.6 to 9.0)	0<br>(0 to 1)	2.0<br>(1.4 to 2.7)	-82.2<br>(-88.5 to -72.6)	-77.8<br>(-85.6 to -65.9)
Cyprus	Road injuries	10 to 14	Both	3<br>(2 to 3)	4.4<br>(3.6 to 5.2)	3<br>(3 to 4)	5.7<br>(4.8 to 6.6)	1<br>(1 to 1)	2.2<br>(1.8 to 2.7)	-60.8<br>(-70.1 to -48.9)	-49.4<br>(-61.4 to -34.0)
Cyprus	Road injuries	10 to 14	Female	1<br>(1 to 1)	2.9<br>(2.1 to 4.0)	1<br>(1 to 1)	3.7<br>(2.8 to 4.7)	0<br>(0 to 0)	1.1<br>(0.8 to 1.5)	-69.8<br>(-79.1 to -55.5)	-61.5<br>(-73.3 to -43.2)
Cyprus	Road injuries	10 to 14	Male	2<br>(1 to 2)	5.8<br>(4.4 to 7.3)	2<br>(2 to 3)	7.6<br>(6.1 to 9.1)	1<br>(1 to 1)	3.3<br>(2.5 to 4.2)	-56.5<br>(-69.0 to -37.9)	-43.3<br>(-59.6 to -19.0)
Cyprus	Other transport injuries	5 to 9	Both	0<br>(0 to 1)	0.8<br>(0.5 to 1.3)	0<br>(0 to 1)	0.9<br>(0.6 to 1.4)	0<br>(0 to 0)	0.4<br>(0.2 to 0.5)	-64.7<br>(-80.3 to -37.7)	-56.0<br>(-75.5 to -22.4)
Cyprus	Other transport injuries	5 to 9	Female	0<br>(0 to 0)	1.0<br>(0.6 to 1.7)	0<br>(0 to 1)	1.2<br>(0.7 to 2.1)	0<br>(0 to 0)	0.5<br>(0.3 to 0.8)	-58.6<br>(-79.5 to -19.0)	-48.4<br>(-74.4 to 1.0)
Cyprus	Other transport injuries	5 to 9	Male	0<br>(0 to 0)	0.6<br>(0.3 to 1.1)	0<br>(0 to 0)	0.6<br>(0.3 to 1.1)	0<br>(0 to 0)	0.2<br>(0.1 to 0.4)	-74.2<br>(-90.4 to -26.9)	-67.9<br>(-88.1 to -9.1)
Cyprus	Other transport injuries	10 to 14	Both	0<br>(0 to 1)	0.8<br>(0.5 to 1.1)	1<br>(0 to 1)	1.2<br>(0.8 to 1.7)	0<br>(0 to 0)	0.5<br>(0.3 to 0.7)	-51.0<br>(-71.7 to -18.4)	-36.8<br>(-63.5 to 5.3)

eTable 8. All-cause and cause-specific mortality (counts and rates) at the global level and by country in the WHO European Region for ages 5 to 9 and 10 to 14 for 1990, 2000, and 2016

Cyprus	Other transport injuries	10 to 14	Female	0 (0 to 0)	0.7 (0.3 to 1.2)	0 (0 to 0)	1.1 (0.7 to 1.7)	0 (0 to 0)	0.3 (0.2 to 0.6)	-60.5 (-80.8 to -21.1)	-49.6 (-75.4 to 0.7)
Cyprus	Other transport injuries	10 to 14	Male	0 (0 to 0)	0.9 (0.5 to 1.5)	0 (0 to 1)	1.3 (0.7 to 2.0)	0 (0 to 0)	0.6 (0.4 to 1.0)	-44.1 (-71.6 to 14.0)	-27.1 (-63.0 to 48.8)
Cyprus	Falls	5 to 9	Both	0 (0 to 0)	0.3 (0.2 to 0.5)	0 (0 to 0)	0.3 (0.2 to 0.4)	0 (0 to 0)	0.1 (0.1 to 0.2)	-66.9 (-79.6 to -40.8)	-58.8 (-74.6 to -26.3)
Cyprus	Falls	5 to 9	Female	0 (0 to 0)	0.2 (0.1 to 0.3)	0 (0 to 0)	0.2 (0.1 to 0.3)	0 (0 to 0)	0.1 (0.1 to 0.2)	-51.5 (-72.5 to -15.4)	-39.5 (-65.7 to 5.5)
Cyprus	Falls	5 to 9	Male	0 (0 to 0)	0.4 (0.2 to 0.7)	0 (0 to 0)	0.4 (0.2 to 0.6)	0 (0 to 0)	0.2 (0.1 to 0.2)	-72.6 (-85.8 to -42.3)	-66.0 (-82.3 to -28.2)
Cyprus	Falls	10 to 14	Both	0 (0 to 0)	0.3 (0.2 to 0.4)	0 (0 to 0)	0.3 (0.2 to 0.5)	0 (0 to 0)	0.2 (0.1 to 0.3)	-42.6 (-64.8 to -7.4)	-25.9 (-54.6 to 19.6)
Cyprus	Falls	10 to 14	Female	0 (0 to 0)	0.1 (0.1 to 0.1)	0 (0 to 0)	0.1 (0.1 to 0.2)	0 (0 to 0)	0.0 (0.0 to 0.1)	-56.2 (-75.5 to -19.7)	-44.1 (-68.7 to 2.5)
Cyprus	Falls	10 to 14	Male	0 (0 to 0)	0.4 (0.3 to 0.6)	0 (0 to 0)	0.5 (0.3 to 0.8)	0 (0 to 0)	0.3 (0.2 to 0.5)	-39.9 (-65.7 to 4.7)	-21.6 (-55.3 to 36.6)
Cyprus	Drowning	5 to 9	Both	2 (1 to 2)	2.7 (2.0 to 3.5)	1 (1 to 2)	2.4 (1.8 to 3.0)	0 (0 to 1)	1.0 (0.7 to 1.3)	-70.4 (-79.5 to -55.3)	-63.1 (-74.5 to -44.3)
Cyprus	Drowning	5 to 9	Female	0 (0 to 1)	1.8 (1.0 to 2.5)	0 (0 to 1)	1.8 (1.0 to 2.6)	0 (0 to 0)	1.0 (0.6 to 1.4)	-55.2 (-73.8 to -21.8)	-44.1 (-67.3 to -2.5)
Cyprus	Drowning	5 to 9	Male	1 (1 to 1)	3.6 (2.5 to 5.0)	1 (1 to 1)	3.0 (2.0 to 4.0)	0 (0 to 0)	1.0 (0.7 to 1.4)	-77.4 (-86.3 to -62.1)	-71.9 (-82.9 to -52.8)
Cyprus	Drowning	10 to 14	Both	1 (1 to 1)	1.2 (0.9 to 1.6)	1 (1 to 1)	1.6 (1.2 to 2.0)	0 (0 to 0)	0.8 (0.6 to 1.0)	-49.7 (-66.4 to -24.2)	-35.1 (-56.6 to -2.1)
Cyprus	Drowning	10 to 14	Female	0 (0 to 0)	0.7 (0.4 to 1.1)	0 (0 to 0)	0.9 (0.6 to 1.3)	0 (0 to 0)	0.3 (0.2 to 0.6)	-60.4 (-76.9 to -29.4)	-49.4 (-70.5 to -9.9)
Cyprus	Drowning	10 to 14	Male	0 (0 to 1)	1.6 (1.1 to 2.3)	1 (0 to 1)	2.2 (1.5 to 3.0)	0 (0 to 0)	1.2 (0.8 to 1.6)	-45.4 (-66.9 to -11.1)	-28.8 (-56.8 to 16.0)
Cyprus	Fire, heat, and hot substances	5 to 9	Both	0 (0 to 1)	0.6 (0.4 to 0.9)	0 (0 to 0)	0.5 (0.3 to 0.8)	0 (0 to 0)	0.2 (0.1 to 0.3)	-73.7 (-83.8 to -58.8)	-67.3 (-79.8 to -48.6)
Cyprus	Fire, heat, and hot substances	5 to 9	Female	0 (0 to 0)	0.5 (0.3 to 0.9)	0 (0 to 0)	0.5 (0.2 to 0.8)	0 (0 to 0)	0.2 (0.1 to 0.4)	-65.2 (-82.0 to -41.2)	-56.6 (-77.5 to -26.7)
Cyprus	Fire, heat, and hot substances	5 to 9	Male	0 (0 to 0)	0.7 (0.4 to 1.1)	0 (0 to 0)	0.6 (0.3 to 0.9)	0 (0 to 0)	0.2 (0.1 to 0.3)	-79.6 (-89.4 to -55.7)	-74.6 (-86.8 to -44.9)
Cyprus	Fire, heat, and hot substances	10 to 14	Both	0 (0 to 0)	0.2 (0.2 to 0.3)	0 (0 to 0)	0.3 (0.2 to 0.4)	0 (0 to 0)	0.1 (0.1 to 0.2)	-60.7 (-74.8 to -42.8)	-49.3 (-67.5 to -26.1)
Cyprus	Fire, heat, and hot substances	10 to 14	Female	0 (0 to 0)	0.2 (0.1 to 0.3)	0 (0 to 0)	0.2 (0.1 to 0.3)	0 (0 to 0)	0.1 (0.0 to 0.1)	-68.9 (-84.5 to -45.0)	-60.4 (-80.2 to -29.7)
Cyprus	Fire, heat, and hot substances	10 to 14	Male	0 (0 to 0)	0.3 (0.2 to 0.4)	0 (0 to 0)	0.3 (0.2 to 0.5)	0 (0 to 0)	0.1 (0.1 to 0.2)	-54.8 (-73.4 to -27.6)	-41.0 (-65.3 to -5.5)
Cyprus	Poisonings	5 to 9	Both	0 (0 to 0)	0.0 (0.0 to 0.1)	0 (0 to 0)	0.0 (0.0 to 0.0)	0 (0 to 0)	0.0 (0.0 to 0.0)	-72.2 (-83.9 to -47.6)	-65.4 (-80.0 to -34.7)
Cyprus	Poisonings	5 to 9	Female	0 (0 to 0)	0.0 (0.0 to 0.1)	0 (0 to 0)	0.0 (0.0 to 0.1)	0 (0 to 0)	0.0 (0.0 to 0.0)	-62.6 (-82.3 to -20.2)	-53.4 (-77.9 to -0.5)
Cyprus	Poisonings	5 to 9	Male	0 (0 to 0)	0.0 (0.0 to 0.1)	0 (0 to 0)	0.0 (0.0 to 0.1)	0 (0 to 0)	0.0 (0.0 to 0.0)	-79.9 (-90.9 to -49.4)	-75.0 (-88.6 to -37.0)
Cyprus	Poisonings	10 to 14	Both	0 (0 to 0)	0.1 (0.0 to 0.1)	0 (0 to 0)	0.1 (0.0 to 0.1)	0 (0 to 0)	0.0 (0.0 to 0.0)	-67.9 (-82.8 to -42.4)	-58.6 (-77.8 to -25.7)
Cyprus	Poisonings	10 to 14	Female	0 (0 to 0)	0.1 (0.0 to 0.1)	0 (0 to 0)	0.1 (0.0 to 0.1)	0 (0 to 0)	0.0 (0.0 to 0.0)	-70.5 (-86.7 to -34.5)	-62.4 (-83.1 to -16.4)
Cyprus	Poisonings	10 to 14	Male	0 (0 to 0)	0.1 (0.0 to 0.2)	0 (0 to 0)	0.1 (0.0 to 0.1)	0 (0 to 0)	0.0 (0.0 to 0.1)	-66.4 (-85.9 to -21.2)	-56.2 (-81.5 to 2.9)
Cyprus	Exposure to mechanical forces	5 to 9	Both	0 (0 to 1)	0.8 (0.6 to 1.0)	0 (0 to 0)	0.7 (0.5 to 0.9)	0 (0 to 0)	0.3 (0.2 to 0.3)	-73.2 (-81.5 to -60.7)	-66.6 (-76.9 to -51.0)
Cyprus	Exposure to mechanical forces	5 to 9	Female	0 (0 to 0)	0.4 (0.3 to 0.6)	0 (0 to 0)	0.4 (0.3 to 0.6)	0 (0 to 0)	0.2 (0.2 to 0.3)	-57.1 (-74.1 to -29.5)	-46.5 (-67.7 to -12.1)
Cyprus	Exposure to mechanical forces	5 to 9	Male	0 (0 to 0)	1.1 (0.8 to 1.6)	0 (0 to 0)	0.9 (0.6 to 1.2)	0 (0 to 0)	0.3 (0.2 to 0.4)	-79.3 (-87.2 to -64.8)	-74.3 (-84.1 to -56.2)

eTable 8. All-cause and cause-specific mortality (counts and rates) at the global level and by country in the WHO European Region for ages 5 to 9 and 10 to 14 for 1990, 2000, and 2016

Cyprus	Exposure to mechanical forces	10 to 14	Both	0<br>(0 to 1)	0.7<br>(0.5 to 1.0)	0<br>(0 to 1)	0.8<br>(0.6 to 1.0)	0<br>(0 to 0)	0.4<br>(0.3 to 0.5)	-58.0<br>(-73.1 to -38.6)	-45.7<br>(-65.2 to -20.8)
Cyprus	Exposure to mechanical forces	10 to 14	Female	0<br>(0 to 0)	0.2<br>(0.1 to 0.3)	0<br>(0 to 0)	0.2<br>(0.2 to 0.3)	0<br>(0 to 0)	0.1<br>(0.1 to 0.2)	-59.7<br>(-74.5 to -39.0)	-48.6<br>(-67.5 to -22.1)
Cyprus	Exposure to mechanical forces	10 to 14	Male	0<br>(0 to 1)	1.1<br>(0.8 to 1.7)	0<br>(0 to 0)	1.3<br>(0.9 to 1.7)	0<br>(0 to 0)	0.6<br>(0.5 to 0.9)	-57.7<br>(-73.9 to -35.2)	-44.8<br>(-65.9 to -15.4)
Cyprus	Adverse effects of medical treatment	5 to 9	Both	0<br>(0 to 0)	0.2<br>(0.1 to 0.2)	0<br>(0 to 0)	0.2<br>(0.1 to 0.2)	0<br>(0 to 0)	0.1<br>(0.1 to 0.1)	-62.0<br>(-75.2 to -45.5)	-52.6<br>(-69.1 to -32.2)
Cyprus	Adverse effects of medical treatment	5 to 9	Female	0<br>(0 to 0)	0.2<br>(0.1 to 0.3)	0<br>(0 to 0)	0.2<br>(0.1 to 0.3)	0<br>(0 to 0)	0.1<br>(0.1 to 0.2)	-56.4<br>(-75.7 to -24.5)	-45.7<br>(-69.6 to -5.8)
Cyprus	Adverse effects of medical treatment	5 to 9	Male	0<br>(0 to 0)	0.2<br>(0.1 to 0.2)	0<br>(0 to 0)	0.1<br>(0.1 to 0.2)	0<br>(0 to 0)	0.1<br>(0.0 to 0.1)	-68.7<br>(-82.7 to -42.1)	-61.0<br>(-78.5 to -28.0)
Cyprus	Adverse effects of medical treatment	10 to 14	Both	0<br>(0 to 0)	0.1<br>(0.1 to 0.2)	0<br>(0 to 0)	0.1<br>(0.1 to 0.2)	0<br>(0 to 0)	0.1<br>(0.1 to 0.1)	-51.0<br>(-71.0 to -25.4)	-36.7<br>(-62.6 to -3.7)
Cyprus	Adverse effects of medical treatment	10 to 14	Female	0<br>(0 to 0)	0.1<br>(0.1 to 0.2)	0<br>(0 to 0)	0.1<br>(0.1 to 0.2)	0<br>(0 to 0)	0.1<br>(0.0 to 0.1)	-61.4<br>(-78.5 to -37.3)	-50.7<br>(-72.6 to -19.9)
Cyprus	Adverse effects of medical treatment	10 to 14	Male	0<br>(0 to 0)	0.1<br>(0.1 to 0.2)	0<br>(0 to 0)	0.1<br>(0.1 to 0.2)	0<br>(0 to 0)	0.1<br>(0.1 to 0.1)	-41.1<br>(-68.7 to 0.9)	-23.1<br>(-59.2 to 31.6)
Cyprus	Animal contact	5 to 9	Both	0<br>(0 to 0)	0.1<br>(0.0 to 0.1)	0<br>(0 to 0)	0.1<br>(0.0 to 0.1)	0<br>(0 to 0)	0.0<br>(0.0 to 0.1)	-57.6<br>(-82.3 to -1.9)	-47.2<br>(-77.9 to 22.2)
Cyprus	Animal contact	5 to 9	Female	0<br>(0 to 0)	0.0<br>(0.0 to 0.1)	0<br>(0 to 0)	0.0<br>(0.0 to 0.1)	0<br>(0 to 0)	0.0<br>(0.0 to 0.1)	-43.5<br>(-83.8 to 108.6)	-29.5<br>(-79.7 to 160.2)
Cyprus	Animal contact	5 to 9	Male	0<br>(0 to 0)	0.1<br>(0.0 to 0.1)	0<br>(0 to 0)	0.1<br>(0.0 to 0.1)	0<br>(0 to 0)	0.0<br>(0.0 to 0.0)	-67.7<br>(-88.9 to -4.4)	-59.8<br>(-86.1 to 18.9)
Cyprus	Animal contact	10 to 14	Both	0<br>(0 to 0)	0.0<br>(0.0 to 0.1)	0<br>(0 to 0)	0.0<br>(0.0 to 0.1)	0<br>(0 to 0)	0.0<br>(0.0 to 0.0)	-58.5<br>(-79.5 to -16.1)	-46.5<br>(-73.5 to 8.3)
Cyprus	Animal contact	10 to 14	Female	0<br>(0 to 0)	0.0<br>(0.0 to 0.1)	0<br>(0 to 0)	0.0<br>(0.0 to 0.1)	0<br>(0 to 0)	0.0<br>(0.0 to 0.0)	-53.7<br>(-84.3 to 51.4)	-40.8<br>(-79.9 to 93.3)
Cyprus	Animal contact	10 to 14	Male	0<br>(0 to 0)	0.1<br>(0.0 to 0.1)	0<br>(0 to 0)	0.1<br>(0.0 to 0.1)	0<br>(0 to 0)	0.0<br>(0.0 to 0.1)	-61.1<br>(-83.1 to -7.7)	-49.2<br>(-77.9 to 20.4)
Cyprus	Foreign body	5 to 9	Both	0<br>(0 to 0)	0.2<br>(0.2 to 0.3)	0<br>(0 to 0)	0.2<br>(0.1 to 0.3)	0<br>(0 to 0)	0.1<br>(0.1 to 0.2)	-52.5<br>(-72.4 to -25.9)	-40.9<br>(-65.6 to -7.6)
Cyprus	Foreign body	5 to 9	Female	0<br>(0 to 0)	0.2<br>(0.1 to 0.3)	0<br>(0 to 0)	0.2<br>(0.1 to 0.3)	0<br>(0 to 0)	0.1<br>(0.1 to 0.2)	-40.3<br>(-69.9 to 5.1)	-25.6<br>(-62.4 to 31.1)
Cyprus	Foreign body	5 to 9	Male	0<br>(0 to 0)	0.3<br>(0.1 to 0.4)	0<br>(0 to 0)	0.2<br>(0.1 to 0.4)	0<br>(0 to 0)	0.1<br>(0.1 to 0.2)	-60.4<br>(-80.0 to -25.7)	-50.7<br>(-75.1 to -7.5)
Cyprus	Foreign body	10 to 14	Both	0<br>(0 to 0)	0.2<br>(0.1 to 0.4)	0<br>(0 to 0)	0.3<br>(0.2 to 0.4)	0<br>(0 to 0)	0.2<br>(0.1 to 0.3)	-42.7<br>(-72.0 to -1.6)	-26.0<br>(-63.9 to 27.1)
Cyprus	Foreign body	10 to 14	Female	0<br>(0 to 0)	0.1<br>(0.1 to 0.2)	0<br>(0 to 0)	0.1<br>(0.1 to 0.2)	0<br>(0 to 0)	0.1<br>(0.0 to 0.2)	-46.7<br>(-74.1 to -12.1)	-31.9<br>(-67.0 to 12.2)
Cyprus	Foreign body	10 to 14	Male	0<br>(0 to 0)	0.4<br>(0.2 to 0.6)	0<br>(0 to 0)	0.4<br>(0.2 to 0.7)	0<br>(0 to 0)	0.3<br>(0.1 to 0.5)	-41.4<br>(-76.0 to 21.1)	-23.6<br>(-68.7 to 58.0)
Cyprus	Environmental heat and cold exposure	5 to 9	Both	0<br>(0 to 0)	0.1<br>(0.0 to 0.1)	0<br>(0 to 0)	0.1<br>(0.0 to 0.1)	0<br>(0 to 0)	0.0<br>(0.0 to 0.0)	-67.9<br>(-81.1 to -46.3)	-60.0<br>(-76.4 to -33.1)
Cyprus	Environmental heat and cold exposure	5 to 9	Female	0<br>(0 to 0)	0.1<br>(0.0 to 0.1)	0<br>(0 to 0)	0.1<br>(0.0 to 0.1)	0<br>(0 to 0)	0.0<br>(0.0 to 0.1)	-66.1<br>(-82.3 to -32.4)	-57.7<br>(-78.0 to -15.7)

Cyprus	Environmental heat and cold exposure	5 to 9	Male	0 (0 to 0)	0.1 (0.0 to 0.1)	0 (0 to 0)	0.0 (0.0 to 0.1)	0 (0 to 0)	0.0 (0.0 to 0.0)	-70.2 (-86.1 to -20.4)	-62.9 (-82.7 to -1.0)
Cyprus	Environmental heat and cold exposure	10 to 14	Both	0 (0 to 0)	0.0 (0.0 to 0.1)	0 (0 to 0)	0.1 (0.0 to 0.1)	0 (0 to 0)	0.0 (0.0 to 0.0)	-57.2 (-74.8 to -29.0)	-44.7 (-67.4 to -8.3)
Cyprus	Environmental heat and cold exposure	10 to 14	Female	0 (0 to 0)	0.0 (0.0 to 0.1)	0 (0 to 0)	0.0 (0.0 to 0.1)	0 (0 to 0)	0.0 (0.0 to 0.0)	-62.7 (-83.1 to -13.4)	-52.4 (-78.5 to 10.5)
Cyprus	Environmental heat and cold exposure	10 to 14	Male	0 (0 to 0)	0.1 (0.0 to 0.1)	0 (0 to 0)	0.1 (0.0 to 0.1)	0 (0 to 0)	0.0 (0.0 to 0.1)	-53.8 (-75.9 to -16.2)	-39.7 (-68.6 to 9.4)
Cyprus	Other unintentional injuries	5 to 9	Both	0 (0 to 0)	0.1 (0.1 to 0.2)	0 (0 to 0)	0.1 (0.0 to 0.1)	0 (0 to 0)	0.0 (0.0 to 0.0)	-81.6 (-89.8 to -64.8)	-77.0 (-87.3 to -56.1)
Cyprus	Other unintentional injuries	5 to 9	Female	0 (0 to 0)	0.1 (0.0 to 0.1)	0 (0 to 0)	0.0 (0.0 to 0.1)	0 (0 to 0)	0.0 (0.0 to 0.0)	-72.6 (-87.8 to -37.7)	-65.9 (-84.8 to -22.3)
Cyprus	Other unintentional injuries	5 to 9	Male	0 (0 to 0)	0.2 (0.1 to 0.3)	0 (0 to 0)	0.1 (0.1 to 0.2)	0 (0 to 0)	0.0 (0.0 to 0.1)	-84.7 (-92.8 to -64.9)	-80.9 (-91.1 to -56.3)
Cyprus	Other unintentional injuries	10 to 14	Both	0 (0 to 0)	0.1 (0.1 to 0.2)	0 (0 to 0)	0.2 (0.1 to 0.2)	0 (0 to 0)	0.1 (0.0 to 0.1)	-67.5 (-82.1 to -40.5)	-58.0 (-76.9 to -23.2)
Cyprus	Other unintentional injuries	10 to 14	Female	0 (0 to 0)	0.0 (0.0 to 0.1)	0 (0 to 0)	0.0 (0.0 to 0.0)	0 (0 to 0)	0.0 (0.0 to 0.0)	-75.7 (-88.9 to -47.5)	-69.0 (-85.9 to -33.0)
Cyprus	Other unintentional injuries	10 to 14	Male	0 (0 to 0)	0.2 (0.2 to 0.4)	0 (0 to 0)	0.3 (0.2 to 0.4)	0 (0 to 0)	0.1 (0.1 to 0.2)	-66.5 (-83.3 to -35.8)	-56.3 (-78.2 to -16.2)
Cyprus	Self-harm	10 to 14	Both	0 (0 to 0)	0.2 (0.1 to 0.3)	0 (0 to 0)	0.2 (0.2 to 0.4)	0 (0 to 0)	0.1 (0.1 to 0.2)	-33.6 (-60.4 to 15.6)	-14.3 (-48.9 to 49.3)
Cyprus	Self-harm	10 to 14	Female	0 (0 to 0)	0.1 (0.1 to 0.2)	0 (0 to 0)	0.1 (0.1 to 0.2)	0 (0 to 0)	0.1 (0.0 to 0.1)	-56.5 (-80.9 to -7.7)	-44.5 (-75.6 to 17.9)
Cyprus	Self-harm	10 to 14	Male	0 (0 to 0)	0.2 (0.1 to 0.4)	0 (0 to 0)	0.3 (0.2 to 0.5)	0 (0 to 0)	0.2 (0.1 to 0.4)	-23.7 (-60.0 to 45.6)	-0.5 (-47.8 to 90.0)
Cyprus	Interpersonal violence	5 to 9	Both	0 (0 to 1)	0.8 (0.6 to 1.2)	0 (0 to 1)	0.8 (0.6 to 1.1)	0 (0 to 0)	0.4 (0.3 to 0.6)	-62.1 (-75.8 to -34.6)	-52.8 (-69.8 to -18.5)
Cyprus	Interpersonal violence	5 to 9	Female	0 (0 to 0)	0.7 (0.3 to 1.1)	0 (0 to 0)	0.7 (0.4 to 1.2)	0 (0 to 0)	0.4 (0.3 to 0.7)	-50.0 (-72.1 to -0.0)	-37.6 (-65.1 to 24.7)
Cyprus	Interpersonal violence	5 to 9	Male	0 (0 to 0)	1.0 (0.5 to 1.5)	0 (0 to 0)	0.9 (0.5 to 1.3)	0 (0 to 0)	0.4 (0.2 to 0.6)	-70.3 (-84.4 to -32.7)	-63.1 (-80.5 to -16.3)
Cyprus	Interpersonal violence	10 to 14	Both	0 (0 to 0)	0.6 (0.4 to 0.8)	0 (0 to 1)	0.7 (0.5 to 1.1)	0 (0 to 0)	0.4 (0.3 to 0.6)	-45.3 (-68.2 to -8.7)	-29.3 (-59.0 to 17.9)
Cyprus	Interpersonal violence	10 to 14	Female	0 (0 to 0)	0.4 (0.2 to 0.5)	0 (0 to 0)	0.5 (0.3 to 0.7)	0 (0 to 0)	0.2 (0.1 to 0.4)	-53.5 (-75.1 to -13.9)	-40.7 (-68.2 to 9.9)
Cyprus	Interpersonal violence	10 to 14	Male	0 (0 to 0)	0.8 (0.4 to 1.2)	0 (0 to 0)	1.0 (0.6 to 1.6)	0 (0 to 0)	0.6 (0.3 to 0.9)	-41.7 (-70.1 to 10.6)	-23.9 (-61.0 to 44.4)
Cyprus	Exposure to forces of nature	5 to 9	Both	0 (0 to 0)	0.0 (0.0 to 0.0)	0 (0 to 0)	0.0 (0.0 to 0.0)	0 (0 to 0)	0.0 (0.0 to 0.0)	0.0 (0.0 to 0.0)	0.0 (0.0 to 0.0)
Cyprus	Exposure to forces of nature	5 to 9	Female	0 (0 to 0)	0.0 (0.0 to 0.0)	0 (0 to 0)	0.0 (0.0 to 0.0)	0 (0 to 0)	0.0 (0.0 to 0.0)	0.0 (0.0 to 0.0)	0.0 (0.0 to 0.0)
Cyprus	Exposure to forces of nature	5 to 9	Male	0 (0 to 0)	0.0 (0.0 to 0.0)	0 (0 to 0)	0.0 (0.0 to 0.0)	0 (0 to 0)	0.0 (0.0 to 0.0)	0.0 (0.0 to 0.0)	0.0 (0.0 to 0.0)
Cyprus	Exposure to forces of nature	10 to 14	Both	0 (0 to 0)	0.0 (0.0 to 0.0)	0 (0 to 0)	0.0 (0.0 to 0.0)	0 (0 to 0)	0.0 (0.0 to 0.0)	0.0 (0.0 to 0.0)	0.0 (0.0 to 0.0)
Cyprus	Exposure to forces of nature	10 to 14	Female	0 (0 to 0)	0.0 (0.0 to 0.0)	0 (0 to 0)	0.0 (0.0 to 0.0)	0 (0 to 0)	0.0 (0.0 to 0.0)	0.0 (0.0 to 0.0)	0.0 (0.0 to 0.0)
Cyprus	Exposure to forces of nature	10 to 14	Male	0 (0 to 0)	0.0 (0.0 to 0.0)	0 (0 to 0)	0.0 (0.0 to 0.0)	0 (0 to 0)	0.0 (0.0 to 0.0)	0.0 (0.0 to 0.0)	0.0 (0.0 to 0.0)

eTable 8. All-cause and cause-specific mortality (counts and rates) at the global level and by country in the WHO European Region for ages 5 to 9 and 10 to 14 for 1990, 2000, and 2016

Cyprus	Conflict and terrorism	5 to 9	Both	0 (0 to 0)	0.0 (0.0 to 0.0)	0 (0 to 0)	0.0 (0.0 to 0.0)	0 (0 to 0)	0.0 (0.0 to 0.0)	0.0 (0.0 to 0.0)	0.0 (0.0 to 0.0)
Cyprus	Conflict and terrorism	5 to 9	Female	0 (0 to 0)	0.0 (0.0 to 0.0)	0 (0 to 0)	0.0 (0.0 to 0.0)	0 (0 to 0)	0.0 (0.0 to 0.0)	0.0 (0.0 to 0.0)	0.0 (0.0 to 0.0)
Cyprus	Conflict and terrorism	5 to 9	Male	0 (0 to 0)	0.0 (0.0 to 0.0)	0 (0 to 0)	0.0 (0.0 to 0.0)	0 (0 to 0)	0.0 (0.0 to 0.0)	0.0 (0.0 to 0.0)	0.0 (0.0 to 0.0)
Cyprus	Conflict and terrorism	10 to 14	Both	0 (0 to 0)	0.0 (0.0 to 0.0)	0 (0 to 0)	0.0 (0.0 to 0.0)	0 (0 to 0)	0.0 (0.0 to 0.0)	0.0 (0.0 to 0.0)	0.0 (0.0 to 0.0)
Cyprus	Conflict and terrorism	10 to 14	Female	0 (0 to 0)	0.0 (0.0 to 0.0)	0 (0 to 0)	0.0 (0.0 to 0.0)	0 (0 to 0)	0.0 (0.0 to 0.0)	0.0 (0.0 to 0.0)	0.0 (0.0 to 0.0)
Cyprus	Conflict and terrorism	10 to 14	Male	0 (0 to 0)	0.0 (0.0 to 0.0)	0 (0 to 0)	0.0 (0.0 to 0.0)	0 (0 to 0)	0.0 (0.0 to 0.0)	0.0 (0.0 to 0.0)	0.0 (0.0 to 0.0)
Cyprus	Executions and police conflict	5 to 9	Both	0 (0 to 0)	0.0 (0.0 to 0.0)	0 (0 to 0)	0.0 (0.0 to 0.0)	0 (0 to 0)	0.0 (0.0 to 0.0)	-36.0 (-69.2 to 11.0)	-20.2 (-61.6 to 38.3)
Cyprus	Executions and police conflict	5 to 9	Female	0 (0 to 0)	0.0 (0.0 to 0.0)	0 (0 to 0)	0.0 (0.0 to 0.0)	0 (0 to 0)	0.0 (0.0 to 0.0)	-28.5 (-46.7 to -9.3)	-10.8 (-33.5 to 13.1)
Cyprus	Executions and police conflict	5 to 9	Male	0 (0 to 0)	0.0 (0.0 to 0.0)	0 (0 to 0)	0.0 (0.0 to 0.0)	0 (0 to 0)	0.0 (0.0 to 0.0)	-41.7 (-79.0 to 35.5)	-27.5 (-73.8 to 68.6)
Cyprus	Executions and police conflict	10 to 14	Both	0 (0 to 0)	0.0 (0.0 to 0.1)	0 (0 to 0)	0.0 (0.0 to 0.0)	0 (0 to 0)	0.0 (0.0 to 0.0)	-29.9 (-88.5 to 266.2)	-9.5 (-85.2 to 372.8)
Cyprus	Executions and police conflict	10 to 14	Female	0 (0 to 0)	0.0 (0.0 to 0.0)	0 (0 to 0)	0.0 (0.0 to 0.0)	0 (0 to 0)	0.0 (0.0 to 0.0)	-29.2 (-41.3 to -12.1)	-9.7 (-25.1 to 12.2)
Cyprus	Executions and police conflict	10 to 14	Male	0 (0 to 0)	0.0 (0.0 to 0.1)	0 (0 to 0)	0.0 (0.0 to 0.1)	0 (0 to 0)	0.0 (0.0 to 0.1)	-30.1 (-93.2 to 514.8)	-8.8 (-91.1 to 702.2)
Czech Republic	All causes	5 to 9	Both	170 (165 to 176)	24.7 (23.8 to 25.5)	98 (93 to 104)	16.8 (15.8 to 17.7)	53 (46 to 61)	8.7 (7.6 to 10.0)	-68.8 (-72.9 to -63.7)	-64.7 (-69.3 to -59.0)
Czech Republic	All causes	5 to 9	Female	65 (63 to 67)	19.2 (18.6 to 19.7)	39 (37 to 41)	13.6 (12.9 to 14.4)	25 (22 to 28)	8.3 (7.3 to 9.4)	-61.8 (-66.3 to -56.3)	-56.9 (-61.9 to -50.6)
Czech Republic	All causes	5 to 9	Male	106 (102 to 110)	29.9 (28.8 to 31.0)	59 (56 to 63)	19.7 (18.6 to 20.9)	29 (25 to 33)	9.1 (7.9 to 10.6)	-73.0 (-76.8 to -68.2)	-69.5 (-73.8 to -64.0)
Czech Republic	All causes	10 to 14	Both	194 (190 to 198)	22.3 (21.9 to 22.8)	117 (113 to 121)	18.0 (17.5 to 18.6)	46 (42 to 49)	9.6 (8.9 to 10.4)	-76.5 (-78.4 to -74.3)	-56.9 (-60.3 to -52.9)
Czech Republic	All causes	10 to 14	Female	71 (69 to 73)	16.7 (16.3 to 17.1)	47 (46 to 49)	14.9 (14.4 to 15.3)	16 (15 to 18)	7.2 (6.7 to 7.7)	-76.8 (-78.4 to -75.0)	-57.1 (-60.0 to -53.8)
Czech Republic	All causes	10 to 14	Male	123 (120 to 126)	27.7 (27.0 to 28.4)	70 (68 to 72)	21.0 (20.3 to 21.8)	29 (27 to 32)	11.9 (10.9 to 13.0)	-76.3 (-78.5 to -74.0)	-56.9 (-60.9 to -52.7)
Czech Republic	Tuberculosis	5 to 9	Both	0 (0 to 0)	0.0 (0.0 to 0.0)	0 (0 to 0)	0.0 (0.0 to 0.0)	0 (0 to 0)	0.0 (0.0 to 0.0)	-47.3 (-60.1 to -30.7)	-40.4 (-54.9 to -21.6)
Czech Republic	Tuberculosis	5 to 9	Female	0 (0 to 0)	0.0 (0.0 to 0.0)	0 (0 to 0)	0.0 (0.0 to 0.0)	0 (0 to 0)	0.0 (0.0 to 0.0)	-56.5 (-70.0 to -36.4)	-50.8 (-66.1 to -28.1)
Czech Republic	Tuberculosis	5 to 9	Male	0 (0 to 0)	0.0 (0.0 to 0.0)	0 (0 to 0)	0.0 (0.0 to 0.0)	0 (0 to 0)	0.0 (0.0 to 0.0)	-34.4 (-53.1 to -11.1)	-25.8 (-47.0 to 0.7)
Czech Republic	Tuberculosis	10 to 14	Both	0 (0 to 0)	0.0 (0.0 to 0.0)	0 (0 to 0)	0.0 (0.0 to 0.0)	0 (0 to 0)	0.0 (0.0 to 0.0)	-80.7 (-85.7 to -74.1)	-64.7 (-73.8 to -52.6)
Czech Republic	Tuberculosis	10 to 14	Female	0 (0 to 0)	0.0 (0.0 to 0.0)	0 (0 to 0)	0.0 (0.0 to 0.0)	0 (0 to 0)	0.0 (0.0 to 0.0)	-80.1 (-85.8 to -71.8)	-63.3 (-73.7 to -47.9)
Czech Republic	Tuberculosis	10 to 14	Male	0 (0 to 0)	0.0 (0.0 to 0.0)	0 (0 to 0)	0.0 (0.0 to 0.0)	0 (0 to 0)	0.0 (0.0 to 0.0)	-81.2 (-87.5 to -70.1)	-65.9 (-77.2 to -45.7)
Czech Republic	HIV/AIDS	5 to 9	Both	0 (0 to 0)	0.0 (0.0 to 0.0)	0 (0 to 0)	0.0 (0.0 to 0.0)	0 (0 to 0)	0.0 (0.0 to 0.0)	-11.3 (-42.4 to 35.1)	0.3 (-34.9 to 52.8)
Czech Republic	HIV/AIDS	5 to 9	Female	0 (0 to 0)	0.0 (0.0 to 0.0)	0 (0 to 0)	0.0 (0.0 to 0.0)	0 (0 to 0)	0.0 (0.0 to 0.0)	55.9 (-16.5 to 195.5)	76.2 (-5.7 to 234.0)
Czech Republic	HIV/AIDS	5 to 9	Male	0 (0 to 0)	0.0 (0.0 to 0.0)	0 (0 to 0)	0.0 (0.0 to 0.0)	0 (0 to 0)	0.0 (0.0 to 0.0)	-41.0 (-66.3 to 5.9)	-33.2 (-61.9 to 19.8)
Czech Republic	HIV/AIDS	10 to 14	Both	0 (0 to 0)	0.0 (0.0 to 0.0)	0 (0 to 0)	0.0 (0.0 to 0.0)	0 (0 to 0)	0.0 (0.0 to 0.0)	18.2 (-21.1 to 80.7)	116.8 (44.7 to 231.5)
Czech Republic	HIV/AIDS	10 to 14	Female	0 (0 to 0)	0.0 (0.0 to 0.0)	0 (0 to 0)	0.0 (0.0 to 0.0)	0 (0 to 0)	0.0 (0.0 to 0.0)	-5.1 (-48.2 to 73.7)	75.5 (-4.3 to 221.3)

eTable 8. All-cause and cause-specific mortality (counts and rates) at the global level and by country in the WHO European Region for ages 5 to 9 and 10 to 14 for 1990, 2000, and 2016

Czech Republic	HIV/AIDS	10 to 14	Male	0<br>(0 to 0)	0.0<br>(0.0 to 0.0)	0<br>(0 to 0)	0.0<br>(0.0 to 0.0)	0<br>(0 to 0)	0.0<br>(0.0 to 0.1)	39.0<br>(-19.9 to 157.6)	153.0<br>(45.7 to 368.8)
Czech Republic	Diarrheal diseases	5 to 9	Both	0<br>(0 to 0)	0.0<br>(0.0 to 0.1)	0<br>(0 to 0)	0.0<br>(0.0 to 0.0)	0<br>(0 to 1)	0.1<br>(0.1 to 0.1)	62.7<br>(-1.0 to 168.3)	84.0<br>(12.0 to 203.4)
Czech Republic	Diarrheal diseases	5 to 9	Female	0<br>(0 to 0)	0.0<br>(0.0 to 0.0)	0<br>(0 to 0)	0.0<br>(0.0 to 0.0)	0<br>(0 to 0)	0.1<br>(0.0 to 0.1)	107.1<br>(9.1 to 283.2)	134.1<br>(23.3 to 333.1)
Czech Republic	Diarrheal diseases	5 to 9	Male	0<br>(0 to 0)	0.1<br>(0.0 to 0.1)	0<br>(0 to 0)	0.0<br>(0.0 to 0.0)	0<br>(0 to 0)	0.1<br>(0.0 to 0.1)	39.7<br>(-25.7 to 165.6)	58.1<br>(-16.0 to 200.6)
Czech Republic	Diarrheal diseases	10 to 14	Both	0<br>(0 to 0)	0.0<br>(0.0 to 0.0)	0<br>(0 to 0)	0.0<br>(0.0 to 0.0)	0<br>(0 to 0)	0.0<br>(0.0 to 0.1)	7.3<br>(-35.6 to 83.7)	96.7<br>(18.2 to 236.9)
Czech Republic	Diarrheal diseases	10 to 14	Female	0<br>(0 to 0)	0.0<br>(0.0 to 0.0)	0<br>(0 to 0)	0.0<br>(0.0 to 0.0)	0<br>(0 to 0)	0.0<br>(0.0 to 0.0)	-19.7<br>(-51.1 to 24.6)	48.6<br>(-9.6 to 130.5)
Czech Republic	Diarrheal diseases	10 to 14	Male	0<br>(0 to 0)	0.0<br>(0.0 to 0.0)	0<br>(0 to 0)	0.0<br>(0.0 to 0.0)	0<br>(0 to 0)	0.1<br>(0.0 to 0.1)	16.9<br>(-38.9 to 129.0)	112.8<br>(11.2 to 316.7)
Czech Republic	Intestinal infectious diseases	5 to 9	Both	0<br>(0 to 0)	0.0<br>(0.0 to 0.0)	0<br>(0 to 0)	0.0<br>(0.0 to 0.0)	0<br>(0 to 0)	0.0<br>(0.0 to 0.0)	-33.7<br>(-56.3 to -2.1)	-25.1<br>(-50.5 to 10.7)
Czech Republic	Intestinal infectious diseases	5 to 9	Female	0<br>(0 to 0)	0.0<br>(0.0 to 0.0)	0<br>(0 to 0)	0.0<br>(0.0 to 0.0)	0<br>(0 to 0)	0.0<br>(0.0 to 0.0)	-32.7<br>(-58.7 to -0.3)	-23.9<br>(-53.3 to 12.7)
Czech Republic	Intestinal infectious diseases	5 to 9	Male	0<br>(0 to 0)	0.0<br>(0.0 to 0.0)	0<br>(0 to 0)	0.0<br>(0.0 to 0.0)	0<br>(0 to 0)	0.0<br>(0.0 to 0.0)	-33.9<br>(-58.9 to 2.9)	-25.1<br>(-53.5 to 16.4)
Czech Republic	Intestinal infectious diseases	10 to 14	Both	0<br>(0 to 0)	0.0<br>(0.0 to 0.0)	0<br>(0 to 0)	0.0<br>(0.0 to 0.0)	0<br>(0 to 0)	0.0<br>(0.0 to 0.0)	-55.0<br>(-67.9 to -31.8)	-17.4<br>(-41.2 to 25.2)
Czech Republic	Intestinal infectious diseases	10 to 14	Female	0<br>(0 to 0)	0.0<br>(0.0 to 0.0)	0<br>(0 to 0)	0.0<br>(0.0 to 0.0)	0<br>(0 to 0)	0.0<br>(0.0 to 0.0)	-61.1<br>(-80.0 to -39.5)	-28.0<br>(-63.0 to 11.9)
Czech Republic	Intestinal infectious diseases	10 to 14	Male	0<br>(0 to 0)	0.0<br>(0.0 to 0.0)	0<br>(0 to 0)	0.0<br>(0.0 to 0.0)	0<br>(0 to 0)	0.0<br>(0.0 to 0.0)	-53.8<br>(-68.2 to -23.2)	-15.9<br>(-42.1 to 39.8)
Czech Republic	Lower respiratory infections	5 to 9	Both	6<br>(5 to 8)	0.9<br>(0.7 to 1.2)	4<br>(3 to 5)	0.7<br>(0.6 to 0.9)	3<br>(2 to 4)	0.4<br>(0.3 to 0.6)	-58.9<br>(-73.1 to -37.0)	-53.5<br>(-69.5 to -28.7)
Czech Republic	Lower respiratory infections	5 to 9	Female	2<br>(2 to 3)	0.7<br>(0.5 to 1.0)	2<br>(1 to 2)	0.6<br>(0.4 to 0.9)	1<br>(1 to 2)	0.4<br>(0.2 to 0.5)	-56.5<br>(-77.5 to -25.6)	-50.9<br>(-74.5 to -16.0)
Czech Republic	Lower respiratory infections	5 to 9	Male	4<br>(3 to 5)	1.1<br>(0.8 to 1.5)	3<br>(2 to 3)	0.9<br>(0.6 to 1.2)	2<br>(1 to 2)	0.5<br>(0.3 to 0.7)	-60.4<br>(-77.1 to -31.5)	-55.1<br>(-74.1 to -22.4)
Czech Republic	Lower respiratory infections	10 to 14	Both	7<br>(6 to 9)	0.8<br>(0.7 to 1.1)	5<br>(4 to 6)	0.8<br>(0.6 to 1.0)	2<br>(1 to 2)	0.4<br>(0.3 to 0.5)	-74.2<br>(-82.3 to -62.0)	-52.7<br>(-67.5 to -30.3)
Czech Republic	Lower respiratory infections	10 to 14	Female	4<br>(3 to 5)	0.8<br>(0.6 to 1.2)	2<br>(2 to 3)	0.8<br>(0.5 to 1.0)	1<br>(0 to 1)	0.3<br>(0.2 to 0.4)	-79.3<br>(-87.6 to -66.7)	-61.8<br>(-77.1 to -38.4)
Czech Republic	Lower respiratory infections	10 to 14	Male	4<br>(3 to 5)	0.8<br>(0.6 to 1.2)	3<br>(2 to 3)	0.8<br>(0.5 to 1.0)	1<br>(1 to 2)	0.5<br>(0.3 to 0.7)	-69.4<br>(-81.4 to -49.2)	-44.4<br>(-66.1 to -7.6)
Czech Republic	Upper respiratory infections	5 to 9	Both	0<br>(0 to 0)	0.0<br>(0.0 to 0.0)	0<br>(0 to 0)	0.0<br>(0.0 to 0.0)	0<br>(0 to 0)	0.0<br>(0.0 to 0.0)	-80.9<br>(-99.6 to 924.2)	-78.4<br>(-99.5 to 1058.4)
Czech Republic	Upper respiratory infections	5 to 9	Female	0<br>(0 to 0)	0.0<br>(0.0 to 0.0)	0<br>(0 to 0)	0.0<br>(0.0 to 0.0)	0<br>(0 to 0)	0.0<br>(0.0 to 0.0)	-77.9<br>(-100.0 to 31890.9)	-75.0<br>(-100.0 to 36054.7)
Czech Republic	Upper respiratory infections	5 to 9	Male	0<br>(0 to 0)	0.0<br>(0.0 to 0.0)	0<br>(0 to 0)	0.0<br>(0.0 to 0.0)	0<br>(0 to 0)	0.0<br>(0.0 to 0.0)	-82.6<br>(-100.0 to 14841.5)	-80.3<br>(-100.0 to 16811.6)
Czech Republic	Upper respiratory infections	10 to 14	Both	0<br>(0 to 0)	0.0<br>(0.0 to 0.0)	0<br>(0 to 0)	0.0<br>(0.0 to 0.0)	0<br>(0 to 0)	0.0<br>(0.0 to 0.0)	-84.9<br>(-99.9 to 881.3)	-72.3<br>(-99.7 to 1699.7)
Czech Republic	Upper respiratory infections	10 to 14	Female	0<br>(0 to 0)	0.0<br>(0.0 to 0.0)	0<br>(0 to 0)	0.0<br>(0.0 to 0.0)	0<br>(0 to 0)	0.0<br>(0.0 to 0.0)	-85.9<br>(-100.0 to 16774.7)	-74.0<br>(-100.0 to 31110.1)

eTable 8. All-cause and cause-specific mortality (counts and rates) at the global level and by country in the WHO European Region for ages 5 to 9 and 10 to 14 for 1990, 2000, and 2016

Czech Republic	Upper respiratory infections	10 to 14	Male	0 (0 to 0)	0.0 (0.0 to 0.0)	0 (0 to 0)	0.0 (0.0 to 0.0)	0 (0 to 0)	0.0 (0.0 to 0.0)	-84.2 (-100.0 to 43799.2)	-71.3 (-100.0 to 79780.4)
Czech Republic	Otitis media	5 to 9	Both	0 (0 to 0)	0.0 (0.0 to 0.0)	0 (0 to 0)	0.0 (0.0 to 0.0)	0 (0 to 0)	0.0 (0.0 to 0.0)	-92.7 (-97.4 to -80.1)	-91.7 (-97.1 to -77.5)
Czech Republic	Otitis media	5 to 9	Female	0 (0 to 0)	0.0 (0.0 to 0.0)	0 (0 to 0)	0.0 (0.0 to 0.0)	0 (0 to 0)	0.0 (0.0 to 0.0)	-96.1 (-99.2 to -84.5)	-95.6 (-99.1 to -82.5)
Czech Republic	Otitis media	5 to 9	Male	0 (0 to 0)	0.0 (0.0 to 0.0)	0 (0 to 0)	0.0 (0.0 to 0.0)	0 (0 to 0)	0.0 (0.0 to 0.0)	-92.3 (-97.4 to -77.2)	-91.3 (-97.1 to -74.2)
Czech Republic	Otitis media	10 to 14	Both	0 (0 to 0)	0.0 (0.0 to 0.0)	0 (0 to 0)	0.0 (0.0 to 0.0)	0 (0 to 0)	0.0 (0.0 to 0.0)	-95.4 (-98.3 to -89.3)	-91.6 (-96.9 to -80.4)
Czech Republic	Otitis media	10 to 14	Female	0 (0 to 0)	0.0 (0.0 to 0.0)	0 (0 to 0)	0.0 (0.0 to 0.0)	0 (0 to 0)	0.0 (0.0 to 0.0)	-97.6 (-99.4 to -91.8)	-95.5 (-98.9 to -84.9)
Czech Republic	Otitis media	10 to 14	Male	0 (0 to 0)	0.0 (0.0 to 0.0)	0 (0 to 0)	0.0 (0.0 to 0.0)	0 (0 to 0)	0.0 (0.0 to 0.0)	-93.0 (-98.0 to -78.1)	-87.3 (-96.4 to -60.1)
Czech Republic	Meningitis	5 to 9	Both	2 (2 to 3)	0.4 (0.3 to 0.5)	1 (1 to 2)	0.2 (0.2 to 0.3)	0 (0 to 1)	0.1 (0.1 to 0.1)	-79.7 (-86.5 to -70.1)	-77.0 (-84.7 to -66.1)
Czech Republic	Meningitis	5 to 9	Female	1 (1 to 1)	0.3 (0.2 to 0.4)	1 (0 to 1)	0.2 (0.2 to 0.3)	0 (0 to 0)	0.1 (0.1 to 0.1)	-77.0 (-86.6 to -61.4)	-74.0 (-84.8 to -56.4)
Czech Republic	Meningitis	5 to 9	Male	1 (1 to 2)	0.4 (0.3 to 0.6)	1 (1 to 1)	0.3 (0.2 to 0.4)	0 (0 to 0)	0.1 (0.0 to 0.1)	-81.8 (-89.7 to -68.4)	-79.3 (-88.4 to -64.3)
Czech Republic	Meningitis	10 to 14	Both	2 (1 to 3)	0.2 (0.2 to 0.3)	1 (1 to 2)	0.2 (0.1 to 0.2)	0 (0 to 0)	0.1 (0.0 to 0.1)	-85.1 (-89.9 to -78.1)	-72.6 (-81.4 to -59.9)
Czech Republic	Meningitis	10 to 14	Female	1 (1 to 1)	0.2 (0.2 to 0.3)	1 (0 to 1)	0.2 (0.2 to 0.3)	0 (0 to 0)	0.1 (0.0 to 0.1)	-86.8 (-92.6 to -77.4)	-75.7 (-86.2 to -58.2)
Czech Republic	Meningitis	10 to 14	Male	1 (1 to 2)	0.2 (0.1 to 0.3)	1 (0 to 1)	0.2 (0.1 to 0.2)	0 (0 to 0)	0.1 (0.0 to 0.1)	-83.3 (-89.5 to -71.8)	-69.6 (-81.0 to -48.8)
Czech Republic	Encephalitis	5 to 9	Both	0 (0 to 1)	0.1 (0.0 to 0.1)	1 (0 to 1)	0.1 (0.1 to 0.1)	0 (0 to 1)	0.1 (0.0 to 0.1)	7.7 (-40.3 to 178.4)	21.8 (-32.4 to 214.8)
Czech Republic	Encephalitis	5 to 9	Female	0 (0 to 1)	0.1 (0.0 to 0.1)	0 (0 to 1)	0.1 (0.1 to 0.2)	0 (0 to 0)	0.1 (0.0 to 0.1)	20.5 (-41.0 to 266.1)	36.2 (-33.3 to 313.7)
Czech Republic	Encephalitis	5 to 9	Male	0 (0 to 1)	0.1 (0.0 to 0.1)	0 (0 to 0)	0.1 (0.0 to 0.1)	0 (0 to 0)	0.1 (0.0 to 0.1)	-5.4 (-40.6 to 168.5)	7.1 (-32.8 to 203.9)
Czech Republic	Encephalitis	10 to 14	Both	0 (0 to 1)	0.1 (0.0 to 0.1)	1 (0 to 1)	0.1 (0.1 to 0.2)	0 (0 to 1)	0.1 (0.1 to 0.1)	-26.3 (-61.1 to 84.6)	35.1 (-28.7 to 238.5)
Czech Republic	Encephalitis	10 to 14	Female	0 (0 to 1)	0.0 (0.0 to 0.1)	0 (0 to 1)	0.1 (0.1 to 0.2)	0 (0 to 0)	0.1 (0.0 to 0.1)	-30.0 (-63.6 to 93.4)	29.4 (-32.7 to 257.7)
Czech Republic	Encephalitis	10 to 14	Male	0 (0 to 1)	0.1 (0.0 to 0.2)	0 (0 to 1)	0.1 (0.1 to 0.2)	0 (0 to 0)	0.1 (0.1 to 0.1)	-23.6 (-60.3 to 135.1)	39.0 (-27.8 to 327.9)
Czech Republic	Diphtheria	5 to 9	Both	0 (0 to 0)	0.0 (0.0 to 0.0)	0 (0 to 0)	0.0 (0.0 to 0.0)	0 (0 to 0)	0.0 (0.0 to 0.0)	-78.7 (-97.0 to 46.3)	-76.0 (-96.6 to 65.5)
Czech Republic	Diphtheria	5 to 9	Female	0 (0 to 0)	0.0 (0.0 to 0.0)	0 (0 to 0)	0.0 (0.0 to 0.0)	0 (0 to 0)	0.0 (0.0 to 0.0)	-75.1 (-98.9 to 461.6)	-71.9 (-98.8 to 534.7)
Czech Republic	Diphtheria	5 to 9	Male	0 (0 to 0)	0.0 (0.0 to 0.0)	0 (0 to 0)	0.0 (0.0 to 0.0)	0 (0 to 0)	0.0 (0.0 to 0.0)	-81.1 (-99.2 to 246.1)	-78.6 (-99.1 to 291.7)
Czech Republic	Diphtheria	10 to 14	Both	0 (0 to 0)	0.0 (0.0 to 0.0)	0 (0 to 0)	0.0 (0.0 to 0.0)	0 (0 to 0)	0.0 (0.0 to 0.0)	-84.5 (-98.0 to 5.2)	-71.6 (-96.3 to 93.0)
Czech Republic	Diphtheria	10 to 14	Female	0 (0 to 0)	0.0 (0.0 to 0.0)	0 (0 to 0)	0.0 (0.0 to 0.0)	0 (0 to 0)	0.0 (0.0 to 0.0)	-85.7 (-99.4 to 227.3)	-73.5 (-98.9 to 505.4)
Czech Republic	Diphtheria	10 to 14	Male	0 (0 to 0)	0.0 (0.0 to 0.0)	0 (0 to 0)	0.0 (0.0 to 0.0)	0 (0 to 0)	0.0 (0.0 to 0.0)	-83.8 (-99.2 to 221.2)	-70.5 (-98.6 to 484.4)
Czech Republic	Whooping cough	5 to 9	Both	0 (0 to 0)	0.0 (0.0 to 0.0)	0 (0 to 0)	0.0 (0.0 to 0.0)	0 (0 to 0)	0.0 (0.0 to 0.0)	73.9 (-65.7 to 829.8)	96.7 (-61.2 to 951.6)
Czech Republic	Whooping cough	5 to 9	Female	0 (0 to 0)	0.0 (0.0 to 0.0)	0 (0 to 0)	0.0 (0.0 to 0.0)	0 (0 to 0)	0.0 (0.0 to 0.0)	-34.1 (-76.4 to 223.7)	-25.5 (-73.3 to 265.9)
Czech Republic	Whooping cough	5 to 9	Male	0 (0 to 0)	0.0 (0.0 to 0.0)	0 (0 to 0)	0.0 (0.0 to 0.0)	0 (0 to 0)	0.0 (0.0 to 0.0)	201.1 (-42.3 to 1657.5)	240.8 (-34.7 to 1889.3)
Czech Republic	Whooping cough	10 to 14	Both	0 (0 to 0)	0.0 (0.0 to 0.0)	0 (0 to 0)	0.0 (0.0 to 0.0)	0 (0 to 0)	0.0 (0.0 to 0.0)	208.4 (-75.9 to 1278.8)	465.6 (-55.8 to 2428.8)
Czech Republic	Whooping cough	10 to 14	Female	0 (0 to 0)	0.0 (0.0 to 0.0)	0 (0 to 0)	0.0 (0.0 to 0.0)	0 (0 to 0)	0.0 (0.0 to 0.0)	39.2 (-84.1 to 551.1)	157.4 (-70.5 to 1104.3)
Czech Republic	Whooping cough	10 to 14	Male	0 (0 to 0)	0.0 (0.0 to 0.0)	0 (0 to 0)	0.0 (0.0 to 0.0)	0 (0 to 0)	0.0 (0.0 to 0.0)	433.6 (-61.5 to 3829.7)	871.0 (-29.9 to 7050.6)
Czech Republic	Tetanus	5 to 9	Both	0 (0 to 0)	0.0 (0.0 to 0.0)	0 (0 to 0)	0.0 (0.0 to 0.0)	0 (0 to 0)	0.0 (0.0 to 0.0)	-92.8 (-97.1 to -82.8)	-91.9 (-96.8 to -80.5)

eTable 8. All-cause and cause-specific mortality (counts and rates) at the global level and by country in the WHO European Region for ages 5 to 9 and 10 to 14 for 1990, 2000, and 2016

Czech Republic	Tetanus	5 to 9	Female	0 (0 to 0)	0.0 (0.0 to 0.0)	0 (0 to 0)	0.0 (0.0 to 0.0)	0 (0 to 0)	0.0 (0.0 to 0.0)	-95.9 (-98.1 to -88.7)	-95.4 (-97.9 to -87.3)
Czech Republic	Tetanus	5 to 9	Male	0 (0 to 0)	0.0 (0.0 to 0.0)	0 (0 to 0)	0.0 (0.0 to 0.0)	0 (0 to 0)	0.0 (0.0 to 0.0)	-90.0 (-97.4 to -68.5)	-88.7 (-97.0 to -64.3)
Czech Republic	Tetanus	10 to 14	Both	0 (0 to 0)	0.0 (0.0 to 0.0)	0 (0 to 0)	0.0 (0.0 to 0.0)	0 (0 to 0)	0.0 (0.0 to 0.0)	-96.4 (-98.5 to -91.0)	-93.4 (-97.3 to -83.5)
Czech Republic	Tetanus	10 to 14	Female	0 (0 to 0)	0.0 (0.0 to 0.0)	0 (0 to 0)	0.0 (0.0 to 0.0)	0 (0 to 0)	0.0 (0.0 to 0.0)	-97.4 (-99.2 to -91.5)	-95.2 (-98.5 to -84.3)
Czech Republic	Tetanus	10 to 14	Male	0 (0 to 0)	0.0 (0.0 to 0.0)	0 (0 to 0)	0.0 (0.0 to 0.0)	0 (0 to 0)	0.0 (0.0 to 0.0)	-94.8 (-98.5 to -82.6)	-90.5 (-97.2 to -68.4)
Czech Republic	Measles	5 to 9	Both	0 (0 to 0)	0.0 (0.0 to 0.0)	0 (0 to 0)	0.0 (0.0 to 0.0)	0 (0 to 0)	0.0 (0.0 to 0.0)	-71.0 (-87.6 to -34.8)	-67.2 (-86.0 to -26.2)
Czech Republic	Measles	5 to 9	Female	0 (0 to 0)	0.0 (0.0 to 0.0)	0 (0 to 0)	0.0 (0.0 to 0.0)	0 (0 to 0)	0.0 (0.0 to 0.0)	-58.4 (-86.9 to 29.3)	-53.0 (-85.2 to 46.1)
Czech Republic	Measles	5 to 9	Male	0 (0 to 0)	0.0 (0.0 to 0.0)	0 (0 to 0)	0.0 (0.0 to 0.0)	0 (0 to 0)	0.0 (0.0 to 0.0)	-77.8 (-92.8 to -33.3)	-74.9 (-91.9 to -24.6)
Czech Republic	Measles	10 to 14	Both	0 (0 to 0)	0.0 (0.0 to 0.0)	0 (0 to 0)	0.0 (0.0 to 0.0)	0 (0 to 0)	0.0 (0.0 to 0.0)	-78.8 (-90.3 to -53.2)	-61.2 (-82.2 to -14.1)
Czech Republic	Measles	10 to 14	Female	0 (0 to 0)	0.0 (0.0 to 0.0)	0 (0 to 0)	0.0 (0.0 to 0.0)	0 (0 to 0)	0.0 (0.0 to 0.0)	-78.4 (-92.3 to -39.5)	-60.0 (-85.8 to 11.8)
Czech Republic	Measles	10 to 14	Male	0 (0 to 0)	0.0 (0.0 to 0.0)	0 (0 to 0)	0.0 (0.0 to 0.0)	0 (0 to 0)	0.0 (0.0 to 0.0)	-79.3 (-93.0 to -37.5)	-62.4 (-87.3 to 13.6)
Czech Republic	Varicella and herpes zoster	5 to 9	Both	0 (0 to 0)	0.0 (0.0 to 0.0)	0 (0 to 0)	0.0 (0.0 to 0.0)	0 (0 to 0)	0.0 (0.0 to 0.0)	-67.0 (-81.6 to -40.3)	-62.7 (-79.2 to -32.5)
Czech Republic	Varicella and herpes zoster	5 to 9	Female	0 (0 to 0)	0.0 (0.0 to 0.0)	0 (0 to 0)	0.0 (0.0 to 0.0)	0 (0 to 0)	0.0 (0.0 to 0.0)	-63.9 (-80.5 to -23.5)	-59.2 (-78.0 to -13.5)
Czech Republic	Varicella and herpes zoster	5 to 9	Male	0 (0 to 0)	0.0 (0.0 to 0.1)	0 (0 to 0)	0.0 (0.0 to 0.0)	0 (0 to 0)	0.0 (0.0 to 0.0)	-69.7 (-85.5 to -34.7)	-65.7 (-83.6 to -26.1)
Czech Republic	Varicella and herpes zoster	10 to 14	Both	0 (0 to 0)	0.0 (0.0 to 0.0)	0 (0 to 0)	0.0 (0.0 to 0.0)	0 (0 to 0)	0.0 (0.0 to 0.0)	-76.7 (-84.5 to -54.7)	-57.3 (-71.7 to -16.9)
Czech Republic	Varicella and herpes zoster	10 to 14	Female	0 (0 to 0)	0.0 (0.0 to 0.0)	0 (0 to 0)	0.0 (0.0 to 0.0)	0 (0 to 0)	0.0 (0.0 to 0.0)	-80.9 (-87.5 to -58.0)	-64.7 (-76.9 to -22.4)
Czech Republic	Varicella and herpes zoster	10 to 14	Male	0 (0 to 0)	0.0 (0.0 to 0.0)	0 (0 to 0)	0.0 (0.0 to 0.0)	0 (0 to 0)	0.0 (0.0 to 0.0)	-73.0 (-86.1 to -30.0)	-50.9 (-74.7 to 27.4)
Czech Republic	Malaria	5 to 9	Both	0 (0 to 0)	0.0 (0.0 to 0.0)	0 (0 to 0)	0.0 (0.0 to 0.0)	0 (0 to 0)	0.0 (0.0 to 0.0)	0.0 (0.0 to 0.0)	0.0 (0.0 to 0.0)
Czech Republic	Malaria	5 to 9	Female	0 (0 to 0)	0.0 (0.0 to 0.0)	0 (0 to 0)	0.0 (0.0 to 0.0)	0 (0 to 0)	0.0 (0.0 to 0.0)	0.0 (0.0 to 0.0)	0.0 (0.0 to 0.0)
Czech Republic	Malaria	5 to 9	Male	0 (0 to 0)	0.0 (0.0 to 0.0)	0 (0 to 0)	0.0 (0.0 to 0.0)	0 (0 to 0)	0.0 (0.0 to 0.0)	0.0 (0.0 to 0.0)	0.0 (0.0 to 0.0)
Czech Republic	Malaria	10 to 14	Both	0 (0 to 0)	0.0 (0.0 to 0.0)	0 (0 to 0)	0.0 (0.0 to 0.0)	0 (0 to 0)	0.0 (0.0 to 0.0)	0.0 (0.0 to 0.0)	0.0 (0.0 to 0.0)
Czech Republic	Malaria	10 to 14	Female	0 (0 to 0)	0.0 (0.0 to 0.0)	0 (0 to 0)	0.0 (0.0 to 0.0)	0 (0 to 0)	0.0 (0.0 to 0.0)	0.0 (0.0 to 0.0)	0.0 (0.0 to 0.0)
Czech Republic	Malaria	10 to 14	Male	0 (0 to 0)	0.0 (0.0 to 0.0)	0 (0 to 0)	0.0 (0.0 to 0.0)	0 (0 to 0)	0.0 (0.0 to 0.0)	0.0 (0.0 to 0.0)	0.0 (0.0 to 0.0)
Czech Republic	Leishmaniasis	5 to 9	Both	0 (0 to 0)	0.0 (0.0 to 0.0)	0 (0 to 0)	0.0 (0.0 to 0.0)	0 (0 to 0)	0.0 (0.0 to 0.0)	0.0 (0.0 to 0.0)	0.0 (0.0 to 0.0)
Czech Republic	Leishmaniasis	5 to 9	Female	0 (0 to 0)	0.0 (0.0 to 0.0)	0 (0 to 0)	0.0 (0.0 to 0.0)	0 (0 to 0)	0.0 (0.0 to 0.0)	0.0 (0.0 to 0.0)	0.0 (0.0 to 0.0)
Czech Republic	Leishmaniasis	5 to 9	Male	0 (0 to 0)	0.0 (0.0 to 0.0)	0 (0 to 0)	0.0 (0.0 to 0.0)	0 (0 to 0)	0.0 (0.0 to 0.0)	0.0 (0.0 to 0.0)	0.0 (0.0 to 0.0)
Czech Republic	Leishmaniasis	10 to 14	Both	0 (0 to 0)	0.0 (0.0 to 0.0)	0 (0 to 0)	0.0 (0.0 to 0.0)	0 (0 to 0)	0.0 (0.0 to 0.0)	0.0 (0.0 to 0.0)	0.0 (0.0 to 0.0)
Czech Republic	Leishmaniasis	10 to 14	Female	0 (0 to 0)	0.0 (0.0 to 0.0)	0 (0 to 0)	0.0 (0.0 to 0.0)	0 (0 to 0)	0.0 (0.0 to 0.0)	0.0 (0.0 to 0.0)	0.0 (0.0 to 0.0)
Czech Republic	Leishmaniasis	10 to 14	Male	0 (0 to 0)	0.0 (0.0 to 0.0)	0 (0 to 0)	0.0 (0.0 to 0.0)	0 (0 to 0)	0.0 (0.0 to 0.0)	0.0 (0.0 to 0.0)	0.0 (0.0 to 0.0)
Czech Republic	African trypanosomiasis	5 to 9	Both	0 (0 to 0)	0.0 (0.0 to 0.0)	0 (0 to 0)	0.0 (0.0 to 0.0)	0 (0 to 0)	0.0 (0.0 to 0.0)	0.0 (0.0 to 0.0)	0.0 (0.0 to 0.0)
Czech Republic	African trypanosomiasis	5 to 9	Female	0 (0 to 0)	0.0 (0.0 to 0.0)	0 (0 to 0)	0.0 (0.0 to 0.0)	0 (0 to 0)	0.0 (0.0 to 0.0)	0.0 (0.0 to 0.0)	0.0 (0.0 to 0.0)

eTable 8. All-cause and cause-specific mortality (counts and rates) at the global level and by country in the WHO European Region for ages 5 to 9 and 10 to 14 for 1990, 2000, and 2016

Czech Republic	African trypanosomiasis	5 to 9	Male	0 (0 to 0)	0.0 (0.0 to 0.0)	0 (0 to 0)	0.0 (0.0 to 0.0)	0 (0 to 0)	0.0 (0.0 to 0.0)	0.0 (0.0 to 0.0)	0.0 (0.0 to 0.0)
Czech Republic	African trypanosomiasis	10 to 14	Both	0 (0 to 0)	0.0 (0.0 to 0.0)	0 (0 to 0)	0.0 (0.0 to 0.0)	0 (0 to 0)	0.0 (0.0 to 0.0)	0.0 (0.0 to 0.0)	0.0 (0.0 to 0.0)
Czech Republic	African trypanosomiasis	10 to 14	Female	0 (0 to 0)	0.0 (0.0 to 0.0)	0 (0 to 0)	0.0 (0.0 to 0.0)	0 (0 to 0)	0.0 (0.0 to 0.0)	0.0 (0.0 to 0.0)	0.0 (0.0 to 0.0)
Czech Republic	African trypanosomiasis	10 to 14	Male	0 (0 to 0)	0.0 (0.0 to 0.0)	0 (0 to 0)	0.0 (0.0 to 0.0)	0 (0 to 0)	0.0 (0.0 to 0.0)	0.0 (0.0 to 0.0)	0.0 (0.0 to 0.0)
Czech Republic	Schistosomiasis	5 to 9	Both	0 (0 to 0)	0.0 (0.0 to 0.0)	0 (0 to 0)	0.0 (0.0 to 0.0)	0 (0 to 0)	0.0 (0.0 to 0.0)	0.0 (0.0 to 0.0)	0.0 (0.0 to 0.0)
Czech Republic	Schistosomiasis	5 to 9	Female	0 (0 to 0)	0.0 (0.0 to 0.0)	0 (0 to 0)	0.0 (0.0 to 0.0)	0 (0 to 0)	0.0 (0.0 to 0.0)	0.0 (0.0 to 0.0)	0.0 (0.0 to 0.0)
Czech Republic	Schistosomiasis	5 to 9	Male	0 (0 to 0)	0.0 (0.0 to 0.0)	0 (0 to 0)	0.0 (0.0 to 0.0)	0 (0 to 0)	0.0 (0.0 to 0.0)	0.0 (0.0 to 0.0)	0.0 (0.0 to 0.0)
Czech Republic	Schistosomiasis	10 to 14	Both	0 (0 to 0)	0.0 (0.0 to 0.0)	0 (0 to 0)	0.0 (0.0 to 0.0)	0 (0 to 0)	0.0 (0.0 to 0.0)	0.0 (0.0 to 0.0)	0.0 (0.0 to 0.0)
Czech Republic	Schistosomiasis	10 to 14	Female	0 (0 to 0)	0.0 (0.0 to 0.0)	0 (0 to 0)	0.0 (0.0 to 0.0)	0 (0 to 0)	0.0 (0.0 to 0.0)	0.0 (0.0 to 0.0)	0.0 (0.0 to 0.0)
Czech Republic	Schistosomiasis	10 to 14	Male	0 (0 to 0)	0.0 (0.0 to 0.0)	0 (0 to 0)	0.0 (0.0 to 0.0)	0 (0 to 0)	0.0 (0.0 to 0.0)	0.0 (0.0 to 0.0)	0.0 (0.0 to 0.0)
Czech Republic	Cysticercosis	5 to 9	Both	0 (0 to 0)	0.0 (0.0 to 0.0)	0 (0 to 0)	0.0 (0.0 to 0.0)	0 (0 to 0)	0.0 (0.0 to 0.0)	-89.1 (-91.1 to -86.6)	-87.7 (-89.9 to -84.8)
Czech Republic	Cysticercosis	5 to 9	Female	0 (0 to 0)	0.0 (0.0 to 0.0)	0 (0 to 0)	0.0 (0.0 to 0.0)	0 (0 to 0)	0.0 (0.0 to 0.0)	-87.4 (-89.7 to -84.5)	-85.7 (-88.4 to -82.5)
Czech Republic	Cysticercosis	5 to 9	Male	0 (0 to 0)	0.0 (0.0 to 0.0)	0 (0 to 0)	0.0 (0.0 to 0.0)	0 (0 to 0)	0.0 (0.0 to 0.0)	-90.6 (-92.6 to -88.1)	-89.4 (-91.7 to -86.5)
Czech Republic	Cysticercosis	10 to 14	Both	0 (0 to 0)	0.0 (0.0 to 0.0)	0 (0 to 0)	0.0 (0.0 to 0.0)	0 (0 to 0)	0.0 (0.0 to 0.0)	-92.2 (-93.3 to -90.9)	-85.6 (-87.6 to -83.2)
Czech Republic	Cysticercosis	10 to 14	Female	0 (0 to 0)	0.0 (0.0 to 0.0)	0 (0 to 0)	0.0 (0.0 to 0.0)	0 (0 to 0)	0.0 (0.0 to 0.0)	-92.8 (-94.0 to -91.3)	-86.6 (-88.8 to -83.9)
Czech Republic	Cysticercosis	10 to 14	Male	0 (0 to 0)	0.0 (0.0 to 0.0)	0 (0 to 0)	0.0 (0.0 to 0.0)	0 (0 to 0)	0.0 (0.0 to 0.0)	-91.6 (-93.0 to -90.0)	-84.7 (-87.2 to -81.8)
Czech Republic	Yellow fever	5 to 9	Both	0 (0 to 0)	0.0 (0.0 to 0.0)	0 (0 to 0)	0.0 (0.0 to 0.0)	0 (0 to 0)	0.0 (0.0 to 0.0)	0.0 (0.0 to 0.0)	0.0 (0.0 to 0.0)
Czech Republic	Yellow fever	5 to 9	Female	0 (0 to 0)	0.0 (0.0 to 0.0)	0 (0 to 0)	0.0 (0.0 to 0.0)	0 (0 to 0)	0.0 (0.0 to 0.0)	0.0 (0.0 to 0.0)	0.0 (0.0 to 0.0)
Czech Republic	Yellow fever	5 to 9	Male	0 (0 to 0)	0.0 (0.0 to 0.0)	0 (0 to 0)	0.0 (0.0 to 0.0)	0 (0 to 0)	0.0 (0.0 to 0.0)	0.0 (0.0 to 0.0)	0.0 (0.0 to 0.0)
Czech Republic	Yellow fever	10 to 14	Both	0 (0 to 0)	0.0 (0.0 to 0.0)	0 (0 to 0)	0.0 (0.0 to 0.0)	0 (0 to 0)	0.0 (0.0 to 0.0)	0.0 (0.0 to 0.0)	0.0 (0.0 to 0.0)
Czech Republic	Yellow fever	10 to 14	Female	0 (0 to 0)	0.0 (0.0 to 0.0)	0 (0 to 0)	0.0 (0.0 to 0.0)	0 (0 to 0)	0.0 (0.0 to 0.0)	0.0 (0.0 to 0.0)	0.0 (0.0 to 0.0)
Czech Republic	Yellow fever	10 to 14	Male	0 (0 to 0)	0.0 (0.0 to 0.0)	0 (0 to 0)	0.0 (0.0 to 0.0)	0 (0 to 0)	0.0 (0.0 to 0.0)	0.0 (0.0 to 0.0)	0.0 (0.0 to 0.0)
Czech Republic	Rabies	5 to 9	Both	0 (0 to 0)	0.0 (0.0 to 0.0)	0 (0 to 0)	0.0 (0.0 to 0.0)	0 (0 to 0)	0.0 (0.0 to 0.0)	-81.5 (-95.5 to -40.2)	-79.1 (-94.9 to -32.4)
Czech Republic	Rabies	5 to 9	Female	0 (0 to 0)	0.0 (0.0 to 0.0)	0 (0 to 0)	0.0 (0.0 to 0.0)	0 (0 to 0)	0.0 (0.0 to 0.0)	-95.6 (-99.3 to -79.3)	-95.1 (-99.2 to -76.6)
Czech Republic	Rabies	5 to 9	Male	0 (0 to 0)	0.0 (0.0 to 0.0)	0 (0 to 0)	0.0 (0.0 to 0.0)	0 (0 to 0)	0.0 (0.0 to 0.0)	-63.7 (-92.5 to 58.3)	-58.9 (-91.5 to 79.2)
Czech Republic	Rabies	10 to 14	Both	0 (0 to 0)	0.0 (0.0 to 0.0)	0 (0 to 0)	0.0 (0.0 to 0.0)	0 (0 to 0)	0.0 (0.0 to 0.0)	-92.6 (-97.6 to -78.9)	-86.5 (-95.6 to -61.3)
Czech Republic	Rabies	10 to 14	Female	0 (0 to 0)	0.0 (0.0 to 0.0)	0 (0 to 0)	0.0 (0.0 to 0.0)	0 (0 to 0)	0.0 (0.0 to 0.0)	-95.6 (-99.2 to -80.1)	-91.9 (-98.5 to -63.1)
Czech Republic	Rabies	10 to 14	Male	0 (0 to 0)	0.0 (0.0 to 0.0)	0 (0 to 0)	0.0 (0.0 to 0.0)	0 (0 to 0)	0.0 (0.0 to 0.0)	-84.6 (-96.2 to -43.1)	-71.9 (-93.1 to 3.6)
Czech Republic	Intestinal nematode infections	5 to 9	Both	-	-	-	-	-	-	-	-

eTable 8. All-cause and cause-specific mortality (counts and rates) at the global level and by country in the WHO European Region for ages 5 to 9 and 10 to 14 for 1990, 2000, and 2016

Czech Republic	Intestinal nematode infections	5 to 9	Female	-	-	-	-	-	-	-	-
Czech Republic	Intestinal nematode infections	5 to 9	Male	-	-	-	-	-	-	-	-
Czech Republic	Intestinal nematode infections	10 to 14	Both	-	-	-	-	-	-	-	-
Czech Republic	Intestinal nematode infections	10 to 14	Female	-	-	-	-	-	-	-	-
Czech Republic	Intestinal nematode infections	10 to 14	Male	-	-	-	-	-	-	-	-
Czech Republic	Ebola	5 to 9	Both	0 (0 to 0)	0.0 (0.0 to 0.0)	0 (0 to 0)	0.0 (0.0 to 0.0)	0 (0 to 0)	0.0 (0.0 to 0.0)	0.0 (0.0 to 0.0)	0.0 (0.0 to 0.0)
Czech Republic	Ebola	5 to 9	Female	0 (0 to 0)	0.0 (0.0 to 0.0)	0 (0 to 0)	0.0 (0.0 to 0.0)	0 (0 to 0)	0.0 (0.0 to 0.0)	0.0 (0.0 to 0.0)	0.0 (0.0 to 0.0)
Czech Republic	Ebola	5 to 9	Male	0 (0 to 0)	0.0 (0.0 to 0.0)	0 (0 to 0)	0.0 (0.0 to 0.0)	0 (0 to 0)	0.0 (0.0 to 0.0)	0.0 (0.0 to 0.0)	0.0 (0.0 to 0.0)
Czech Republic	Ebola	10 to 14	Both	0 (0 to 0)	0.0 (0.0 to 0.0)	0 (0 to 0)	0.0 (0.0 to 0.0)	0 (0 to 0)	0.0 (0.0 to 0.0)	0.0 (0.0 to 0.0)	0.0 (0.0 to 0.0)
Czech Republic	Ebola	10 to 14	Female	0 (0 to 0)	0.0 (0.0 to 0.0)	0 (0 to 0)	0.0 (0.0 to 0.0)	0 (0 to 0)	0.0 (0.0 to 0.0)	0.0 (0.0 to 0.0)	0.0 (0.0 to 0.0)
Czech Republic	Ebola	10 to 14	Male	0 (0 to 0)	0.0 (0.0 to 0.0)	0 (0 to 0)	0.0 (0.0 to 0.0)	0 (0 to 0)	0.0 (0.0 to 0.0)	0.0 (0.0 to 0.0)	0.0 (0.0 to 0.0)
Czech Republic	Zika virus	5 to 9	Both	0 (0 to 0)	0.0 (0.0 to 0.0)	0 (0 to 0)	0.0 (0.0 to 0.0)	0 (0 to 0)	0.0 (0.0 to 0.0)	0.0 (0.0 to 0.0)	0.0 (0.0 to 0.0)
Czech Republic	Zika virus	5 to 9	Female	0 (0 to 0)	0.0 (0.0 to 0.0)	0 (0 to 0)	0.0 (0.0 to 0.0)	0 (0 to 0)	0.0 (0.0 to 0.0)	0.0 (0.0 to 0.0)	0.0 (0.0 to 0.0)
Czech Republic	Zika virus	5 to 9	Male	0 (0 to 0)	0.0 (0.0 to 0.0)	0 (0 to 0)	0.0 (0.0 to 0.0)	0 (0 to 0)	0.0 (0.0 to 0.0)	0.0 (0.0 to 0.0)	0.0 (0.0 to 0.0)
Czech Republic	Zika virus	10 to 14	Both	0 (0 to 0)	0.0 (0.0 to 0.0)	0 (0 to 0)	0.0 (0.0 to 0.0)	0 (0 to 0)	0.0 (0.0 to 0.0)	0.0 (0.0 to 0.0)	0.0 (0.0 to 0.0)
Czech Republic	Zika virus	10 to 14	Female	0 (0 to 0)	0.0 (0.0 to 0.0)	0 (0 to 0)	0.0 (0.0 to 0.0)	0 (0 to 0)	0.0 (0.0 to 0.0)	0.0 (0.0 to 0.0)	0.0 (0.0 to 0.0)
Czech Republic	Zika virus	10 to 14	Male	0 (0 to 0)	0.0 (0.0 to 0.0)	0 (0 to 0)	0.0 (0.0 to 0.0)	0 (0 to 0)	0.0 (0.0 to 0.0)	0.0 (0.0 to 0.0)	0.0 (0.0 to 0.0)
Czech Republic	Other neglected tropical diseases	5 to 9	Both	0 (0 to 0)	0.0 (0.0 to 0.0)	0 (0 to 0)	0.0 (0.0 to 0.0)	0 (0 to 0)	0.0 (0.0 to 0.0)	-29.6 (-42.3 to -12.8)	-20.3 (-34.8 to -1.4)
Czech Republic	Other neglected tropical diseases	5 to 9	Female	0 (0 to 0)	0.0 (0.0 to 0.0)	0 (0 to 0)	0.0 (0.0 to 0.0)	0 (0 to 0)	0.0 (0.0 to 0.0)	-31.8 (-43.8 to -16.8)	-22.9 (-36.5 to -6.0)
Czech Republic	Other neglected tropical diseases	5 to 9	Male	0 (0 to 0)	0.0 (0.0 to 0.0)	0 (0 to 0)	0.0 (0.0 to 0.0)	0 (0 to 0)	0.0 (0.0 to 0.0)	-27.4 (-42.7 to -6.1)	-17.9 (-35.1 to 6.3)
Czech Republic	Other neglected tropical diseases	10 to 14	Both	0 (0 to 0)	0.0 (0.0 to 0.0)	0 (0 to 0)	0.0 (0.0 to 0.0)	0 (0 to 0)	0.0 (0.0 to 0.0)	-57.7 (-63.3 to -51.3)	-22.3 (-32.6 to -10.7)
Czech Republic	Other neglected tropical diseases	10 to 14	Female	0 (0 to 0)	0.0 (0.0 to 0.0)	0 (0 to 0)	0.0 (0.0 to 0.0)	0 (0 to 0)	0.0 (0.0 to 0.0)	-60.6 (-66.7 to -53.6)	-27.1 (-38.5 to -14.1)
Czech Republic	Other neglected tropical diseases	10 to 14	Male	0 (0 to 0)	0.0 (0.0 to 0.0)	0 (0 to 0)	0.0 (0.0 to 0.0)	0 (0 to 0)	0.0 (0.0 to 0.0)	-54.7 (-62.0 to -46.6)	-17.5 (-30.8 to -2.8)
Czech Republic	Maternal hemorrhage	10 to 14	Both	0 (0 to 0)	0.0 (0.0 to 0.0)	0 (0 to 0)	0.0 (0.0 to 0.0)	0 (0 to 0)	0.0 (0.0 to 0.0)	-86.7 (-91.1 to -79.5)	-75.6 (-83.7 to -62.5)

eTable 8. All-cause and cause-specific mortality (counts and rates) at the global level and by country in the WHO European Region for ages 5 to 9 and 10 to 14 for 1990, 2000, and 2016

Czech Republic	Maternal hemorrhage	10 to 14	Female	0 (0 to 0)	0.0 (0.0 to 0.0)	0 (0 to 0)	0.0 (0.0 to 0.0)	0 (0 to 0)	0.0 (0.0 to 0.0)	-86.7 (-91.1 to -79.5)	-75.4 (-83.6 to -62.1)
Czech Republic	Maternal sepsis and other maternal infections	10 to 14	Both	0 (0 to 0)	0.0 (0.0 to 0.0)	0 (0 to 0)	0.0 (0.0 to 0.0)	0 (0 to 0)	0.0 (0.0 to 0.0)	-88.1 (-92.2 to -82.1)	-78.2 (-85.6 to -67.2)
Czech Republic	Maternal sepsis and other maternal infections	10 to 14	Female	0 (0 to 0)	0.0 (0.0 to 0.0)	0 (0 to 0)	0.0 (0.0 to 0.0)	0 (0 to 0)	0.0 (0.0 to 0.0)	-88.1 (-92.2 to -82.1)	-78.1 (-85.5 to -66.9)
Czech Republic	Maternal hypertensive disorders	10 to 14	Both	0 (0 to 0)	0.0 (0.0 to 0.0)	0 (0 to 0)	0.0 (0.0 to 0.0)	0 (0 to 0)	0.0 (0.0 to 0.0)	-87.8 (-91.8 to -82.4)	-77.7 (-85.0 to -67.7)
Czech Republic	Maternal hypertensive disorders	10 to 14	Female	0 (0 to 0)	0.0 (0.0 to 0.0)	0 (0 to 0)	0.0 (0.0 to 0.0)	0 (0 to 0)	0.0 (0.0 to 0.0)	-87.8 (-91.8 to -82.4)	-77.5 (-84.8 to -67.4)
Czech Republic	Maternal obstructed labor and uterine rupture	10 to 14	Both	0 (0 to 0)	0.0 (0.0 to 0.0)	0 (0 to 0)	0.0 (0.0 to 0.0)	0 (0 to 0)	0.0 (0.0 to 0.0)	-86.2 (-91.4 to -78.9)	-74.7 (-84.2 to -61.3)
Czech Republic	Maternal obstructed labor and uterine rupture	10 to 14	Female	0 (0 to 0)	0.0 (0.0 to 0.0)	0 (0 to 0)	0.0 (0.0 to 0.0)	0 (0 to 0)	0.0 (0.0 to 0.0)	-86.2 (-91.4 to -78.9)	-74.5 (-84.1 to -61.0)
Czech Republic	Maternal abortion, miscarriage, and ectopic pregnancy	10 to 14	Both	0 (0 to 0)	0.0 (0.0 to 0.0)	0 (0 to 0)	0.0 (0.0 to 0.0)	0 (0 to 0)	0.0 (0.0 to 0.0)	-88.8 (-92.5 to -83.2)	-79.4 (-86.3 to -69.1)
Czech Republic	Maternal abortion, miscarriage, and ectopic pregnancy	10 to 14	Female	0 (0 to 0)	0.0 (0.0 to 0.0)	0 (0 to 0)	0.0 (0.0 to 0.0)	0 (0 to 0)	0.0 (0.0 to 0.0)	-88.8 (-92.5 to -83.2)	-79.2 (-86.2 to -68.8)
Czech Republic	Indirect maternal deaths	10 to 14	Both	0 (0 to 0)	0.0 (0.0 to 0.0)	0 (0 to 0)	0.0 (0.0 to 0.0)	0 (0 to 0)	0.0 (0.0 to 0.0)	-87.8 (-91.7 to -82.2)	-77.7 (-84.7 to -67.4)
Czech Republic	Indirect maternal deaths	10 to 14	Female	0 (0 to 0)	0.0 (0.0 to 0.0)	0 (0 to 0)	0.0 (0.0 to 0.0)	0 (0 to 0)	0.0 (0.0 to 0.0)	-87.8 (-91.7 to -82.2)	-77.5 (-84.6 to -67.1)
Czech Republic	Late maternal deaths	10 to 14	Both	0 (0 to 0)	0.0 (0.0 to 0.0)	0 (0 to 0)	0.0 (0.0 to 0.0)	0 (0 to 0)	0.0 (0.0 to 0.0)	-88.5 (-92.2 to -83.1)	-79.0 (-85.8 to -69.0)
Czech Republic	Late maternal deaths	10 to 14	Female	0 (0 to 0)	0.0 (0.0 to 0.0)	0 (0 to 0)	0.0 (0.0 to 0.0)	0 (0 to 0)	0.0 (0.0 to 0.0)	-88.5 (-92.2 to -83.1)	-78.8 (-85.6 to -68.8)
Czech Republic	Maternal deaths aggravated by HIV/AIDS	10 to 14	Both	0 (0 to 0)	0.0 (0.0 to 0.0)	0 (0 to 0)	0.0 (0.0 to 0.0)	0 (0 to 0)	0.0 (0.0 to 0.0)	1219.5 (0.0 to 4085.9)	2320.2 (0.0 to 7577.5)
Czech Republic	Maternal deaths aggravated by HIV/AIDS	10 to 14	Female	0 (0 to 0)	0.0 (0.0 to 0.0)	0 (0 to 0)	0.0 (0.0 to 0.0)	0 (0 to 0)	0.0 (0.0 to 0.0)	1219.5 (0.0 to 4085.9)	2340.5 (0.0 to 7642.0)
Czech Republic	Other maternal disorders	10 to 14	Both	0 (0 to 0)	0.0 (0.0 to 0.0)	0 (0 to 0)	0.0 (0.0 to 0.0)	0 (0 to 0)	0.0 (0.0 to 0.0)	-87.4 (-91.2 to -82.1)	-76.9 (-83.8 to -67.1)
Czech Republic	Other maternal disorders	10 to 14	Female	0 (0 to 0)	0.0 (0.0 to 0.1)	0 (0 to 0)	0.0 (0.0 to 0.0)	0 (0 to 0)	0.0 (0.0 to 0.0)	-87.4 (-91.2 to -82.1)	-76.7 (-83.6 to -66.8)
Czech Republic	Protein-energy malnutrition	5 to 9	Both	0 (0 to 0)	0.0 (0.0 to 0.0)	0 (0 to 0)	0.0 (0.0 to 0.0)	0 (0 to 0)	0.0 (0.0 to 0.0)	190.3 (21.3 to 535.5)	228.4 (37.2 to 618.7)

eTable 8. All-cause and cause-specific mortality (counts and rates) at the global level and by country in the WHO European Region for ages 5 to 9 and 10 to 14 for 1990, 2000, and 2016

Czech Republic	Protein-energy malnutrition	5 to 9	Female	0 (0 to 0)	0.0 (0.0 to 0.0)	0 (0 to 0)	0.0 (0.0 to 0.0)	0 (0 to 0)	0.0 (0.0 to 0.1)	227.9 (-2.2 to 861.1)	270.5 (10.6 to 986.1)
Czech Republic	Protein-energy malnutrition	5 to 9	Male	0 (0 to 0)	0.0 (0.0 to 0.0)	0 (0 to 0)	0.0 (0.0 to 0.0)	0 (0 to 0)	0.0 (0.0 to 0.0)	109.7 (-17.4 to 481.0)	137.4 (-6.5 to 557.6)
Czech Republic	Protein-energy malnutrition	10 to 14	Both	0 (0 to 0)	0.0 (0.0 to 0.0)	0 (0 to 0)	0.0 (0.0 to 0.0)	0 (0 to 0)	0.0 (0.0 to 0.0)	51.5 (-35.6 to 207.6)	177.9 (18.2 to 464.1)
Czech Republic	Protein-energy malnutrition	10 to 14	Female	0 (0 to 0)	0.0 (0.0 to 0.0)	0 (0 to 0)	0.0 (0.0 to 0.0)	0 (0 to 0)	0.0 (0.0 to 0.0)	103.9 (-28.2 to 411.7)	277.1 (32.7 to 846.3)
Czech Republic	Protein-energy malnutrition	10 to 14	Male	0 (0 to 0)	0.0 (0.0 to 0.0)	0 (0 to 0)	0.0 (0.0 to 0.0)	0 (0 to 0)	0.0 (0.0 to 0.0)	-50.8 (-63.1 to -22.1)	-10.4 (-32.9 to 41.8)
Czech Republic	Iodine deficiency	5 to 9	Both	0 (0 to 0)	0.0 (0.0 to 0.0)	0 (0 to 0)	0.0 (0.0 to 0.0)	0 (0 to 0)	0.0 (0.0 to 0.0)	-24.4 (-100.0 to 1.1e+18)	-14.5 (-100.0 to 1.3e+18)
Czech Republic	Iodine deficiency	5 to 9	Female	0 (0 to 0)	0.0 (0.0 to 0.0)	0 (0 to 0)	0.0 (0.0 to 0.0)	0 (0 to 0)	0.0 (0.0 to 0.0)	-29.1 (-100.0 to 3.1e+25)	-19.8 (-100.0 to 3.5e+25)
Czech Republic	Iodine deficiency	5 to 9	Male	0 (0 to 0)	0.0 (0.0 to 0.0)	0 (0 to 0)	0.0 (0.0 to 0.0)	0 (0 to 0)	0.0 (0.0 to 0.0)	-16.3 (-100.0 to 1.9e+24)	-5.3 (-100.0 to 2.2e+24)
Czech Republic	Iodine deficiency	10 to 14	Both	0 (0 to 0)	0.0 (0.0 to 0.0)	0 (0 to 0)	0.0 (0.0 to 0.0)	0 (0 to 0)	0.0 (0.0 to 0.0)	-56.8 (-100.0 to 1.1e+17)	-20.7 (-100.0 to 2.1e+17)
Czech Republic	Iodine deficiency	10 to 14	Female	0 (0 to 0)	0.0 (0.0 to 0.0)	0 (0 to 0)	0.0 (0.0 to 0.0)	0 (0 to 0)	0.0 (0.0 to 0.0)	-55.4 (-100.0 to 2.0e+25)	-17.5 (-100.0 to 3.7e+25)
Czech Republic	Iodine deficiency	10 to 14	Male	0 (0 to 0)	0.0 (0.0 to 0.0)	0 (0 to 0)	0.0 (0.0 to 0.0)	0 (0 to 0)	0.0 (0.0 to 0.0)	-60.3 (-100.0 to 7.7e+23)	-27.8 (-100.0 to 1.4e+24)
Czech Republic	Iron-deficiency anemia	5 to 9	Both	0 (0 to 0)	0.0 (0.0 to 0.0)	0 (0 to 0)	0.0 (0.0 to 0.0)	0 (0 to 0)	0.0 (0.0 to 0.0)	40.8 (-35.9 to 209.8)	59.2 (-27.5 to 250.4)
Czech Republic	Iron-deficiency anemia	5 to 9	Female	0 (0 to 0)	0.0 (0.0 to 0.0)	0 (0 to 0)	0.0 (0.0 to 0.0)	0 (0 to 0)	0.0 (0.0 to 0.0)	5.0 (-67.4 to 285.1)	18.7 (-63.1 to 335.2)
Czech Republic	Iron-deficiency anemia	5 to 9	Male	0 (0 to 0)	0.0 (0.0 to 0.0)	0 (0 to 0)	0.0 (0.0 to 0.0)	0 (0 to 0)	0.0 (0.0 to 0.0)	110.7 (-17.4 to 486.1)	138.5 (-6.5 to 563.4)
Czech Republic	Iron-deficiency anemia	10 to 14	Both	0 (0 to 0)	0.0 (0.0 to 0.0)	0 (0 to 0)	0.0 (0.0 to 0.0)	0 (0 to 0)	0.0 (0.0 to 0.0)	-41.0 (-73.6 to 38.1)	8.3 (-51.5 to 153.3)
Czech Republic	Iron-deficiency anemia	10 to 14	Female	0 (0 to 0)	0.0 (0.0 to 0.0)	0 (0 to 0)	0.0 (0.0 to 0.0)	0 (0 to 0)	0.0 (0.0 to 0.0)	-35.8 (-81.6 to 91.1)	18.7 (-66.0 to 253.4)
Czech Republic	Iron-deficiency anemia	10 to 14	Male	0 (0 to 0)	0.0 (0.0 to 0.0)	0 (0 to 0)	0.0 (0.0 to 0.0)	0 (0 to 0)	0.0 (0.0 to 0.0)	-50.4 (-63.1 to -20.6)	-9.8 (-32.9 to 44.4)
Czech Republic	Other nutritional deficiencies	5 to 9	Both	0 (0 to 0)	0.0 (0.0 to 0.0)	0 (0 to 0)	0.0 (0.0 to 0.0)	0 (0 to 0)	0.0 (0.0 to 0.0)	7.1 (-54.1 to 146.0)	21.1 (-48.1 to 178.3)
Czech Republic	Other nutritional deficiencies	5 to 9	Female	0 (0 to 0)	0.0 (0.0 to 0.0)	0 (0 to 0)	0.0 (0.0 to 0.0)	0 (0 to 0)	0.0 (0.0 to 0.0)	-15.7 (-75.7 to 242.0)	-4.7 (-72.6 to 286.5)
Czech Republic	Other nutritional deficiencies	5 to 9	Male	0 (0 to 0)	0.0 (0.0 to 0.0)	0 (0 to 0)	0.0 (0.0 to 0.0)	0 (0 to 0)	0.0 (0.0 to 0.0)	45.2 (-45.7 to 314.5)	64.4 (-38.5 to 369.1)
Czech Republic	Other nutritional deficiencies	10 to 14	Both	0 (0 to 0)	0.0 (0.0 to 0.0)	0 (0 to 0)	0.0 (0.0 to 0.0)	0 (0 to 0)	0.0 (0.0 to 0.0)	-42.7 (-74.5 to 30.3)	5.1 (-53.3 to 139.0)
Czech Republic	Other nutritional deficiencies	10 to 14	Female	0 (0 to 0)	0.0 (0.0 to 0.0)	0 (0 to 0)	0.0 (0.0 to 0.0)	0 (0 to 0)	0.0 (0.0 to 0.0)	-38.7 (-82.2 to 80.6)	13.4 (-67.1 to 234.1)
Czech Republic	Other nutritional deficiencies	10 to 14	Male	0 (0 to 0)	0.0 (0.0 to 0.0)	0 (0 to 0)	0.0 (0.0 to 0.0)	0 (0 to 0)	0.0 (0.0 to 0.0)	-50.4 (-63.4 to -20.6)	-9.8 (-33.3 to 44.4)

eTable 8. All-cause and cause-specific mortality (counts and rates) at the global level and by country in the WHO European Region for ages 5 to 9 and 10 to 14 for 1990, 2000, and 2016

Czech Republic	Sexually transmitted diseases excluding HIV	5 to 9	Both	0 (0 to 0)	0.0 (0.0 to 0.0)	0 (0 to 0)	0.0 (0.0 to 0.0)	0 (0 to 0)	0.0 (0.0 to 0.0)	-93.2 (-97.7 to -81.5)	-92.3 (-97.4 to -79.1)
Czech Republic	Sexually transmitted diseases excluding HIV	5 to 9	Female	0 (0 to 0)	0.0 (0.0 to 0.0)	0 (0 to 0)	0.0 (0.0 to 0.0)	0 (0 to 0)	0.0 (0.0 to 0.0)	-93.4 (-97.7 to -81.4)	-92.5 (-97.4 to -78.9)
Czech Republic	Sexually transmitted diseases excluding HIV	5 to 9	Male	0 (0 to 0)	0.0 (0.0 to 0.0)	0 (0 to 0)	0.0 (0.0 to 0.0)	0 (0 to 0)	0.0 (0.0 to 0.0)	-93.0 (-97.7 to -80.6)	-92.1 (-97.4 to -78.0)
Czech Republic	Sexually transmitted diseases excluding HIV	10 to 14	Both	0 (0 to 0)	0.0 (0.0 to 0.0)	0 (0 to 0)	0.0 (0.0 to 0.0)	0 (0 to 0)	0.0 (0.0 to 0.0)	-71.9 (-79.4 to -57.4)	-48.4 (-62.3 to -22.0)
Czech Republic	Sexually transmitted diseases excluding HIV	10 to 14	Female	0 (0 to 0)	0.0 (0.0 to 0.0)	0 (0 to 0)	0.0 (0.0 to 0.0)	0 (0 to 0)	0.0 (0.0 to 0.0)	-59.6 (-66.9 to -46.7)	-25.3 (-38.8 to -1.3)
Czech Republic	Sexually transmitted diseases excluding HIV	10 to 14	Male	0 (0 to 0)	0.0 (0.0 to 0.0)	0 (0 to 0)	0.0 (0.0 to 0.0)	0 (0 to 0)	0.0 (0.0 to 0.0)	-98.2 (-99.3 to -94.7)	-96.7 (-98.7 to -90.4)
Czech Republic	Hepatitis	5 to 9	Both	0 (0 to 0)	0.0 (0.0 to 0.0)	0 (0 to 0)	0.0 (0.0 to 0.0)	0 (0 to 0)	0.0 (0.0 to 0.0)	-28.5 (-41.3 to -13.3)	-19.1 (-33.6 to -2.0)
Czech Republic	Hepatitis	5 to 9	Female	0 (0 to 0)	0.0 (0.0 to 0.0)	0 (0 to 0)	0.0 (0.0 to 0.0)	0 (0 to 0)	0.0 (0.0 to 0.0)	-28.6 (-41.2 to -13.0)	-19.3 (-33.5 to -1.7)
Czech Republic	Hepatitis	5 to 9	Male	0 (0 to 0)	0.0 (0.0 to 0.0)	0 (0 to 0)	0.0 (0.0 to 0.0)	0 (0 to 0)	0.0 (0.0 to 0.0)	-28.4 (-43.8 to -8.7)	-18.9 (-36.4 to 3.3)
Czech Republic	Hepatitis	10 to 14	Both	0 (0 to 0)	0.0 (0.0 to 0.0)	0 (0 to 0)	0.0 (0.0 to 0.0)	0 (0 to 0)	0.0 (0.0 to 0.0)	-63.2 (-74.5 to -52.1)	-32.5 (-53.3 to -12.1)
Czech Republic	Hepatitis	10 to 14	Female	0 (0 to 0)	0.0 (0.0 to 0.0)	0 (0 to 0)	0.0 (0.0 to 0.0)	0 (0 to 0)	0.0 (0.0 to 0.0)	-60.0 (-66.2 to -52.8)	-26.0 (-37.5 to -12.7)
Czech Republic	Hepatitis	10 to 14	Male	0 (0 to 0)	0.0 (0.0 to 0.0)	0 (0 to 0)	0.0 (0.0 to 0.0)	0 (0 to 0)	0.0 (0.0 to 0.0)	-65.7 (-81.0 to -48.0)	-37.5 (-65.4 to -5.5)
Czech Republic	Other infectious diseases	5 to 9	Both	1 (0 to 1)	0.1 (0.1 to 0.1)	0 (0 to 0)	0.0 (0.0 to 0.1)	0 (0 to 0)	0.0 (0.0 to 0.1)	-51.1 (-69.6 to -21.9)	-44.7 (-65.6 to -11.7)
Czech Republic	Other infectious diseases	5 to 9	Female	0 (0 to 0)	0.1 (0.0 to 0.1)	0 (0 to 0)	0.0 (0.0 to 0.1)	0 (0 to 0)	0.1 (0.0 to 0.1)	-40.7 (-72.5 to 30.2)	-32.9 (-69.0 to 47.1)
Czech Republic	Other infectious diseases	5 to 9	Male	0 (0 to 0)	0.1 (0.1 to 0.1)	0 (0 to 0)	0.0 (0.0 to 0.1)	0 (0 to 0)	0.0 (0.0 to 0.1)	-59.8 (-76.7 to -32.1)	-54.5 (-73.6 to -23.1)
Czech Republic	Other infectious diseases	10 to 14	Both	1 (1 to 1)	0.1 (0.1 to 0.1)	0 (0 to 1)	0.1 (0.0 to 0.1)	0 (0 to 0)	0.1 (0.0 to 0.1)	-65.4 (-78.1 to -47.9)	-36.6 (-59.8 to -4.5)
Czech Republic	Other infectious diseases	10 to 14	Female	0 (0 to 1)	0.1 (0.0 to 0.1)	0 (0 to 0)	0.0 (0.0 to 0.1)	0 (0 to 0)	0.1 (0.0 to 0.1)	-64.8 (-83.3 to -28.5)	-34.9 (-69.1 to 32.2)
Czech Republic	Other infectious diseases	10 to 14	Male	0 (0 to 1)	0.1 (0.1 to 0.1)	0 (0 to 0)	0.1 (0.0 to 0.1)	0 (0 to 0)	0.1 (0.0 to 0.1)	-65.8 (-79.8 to -44.4)	-37.8 (-63.2 to 1.1)
Czech Republic	Nasopharynx cancer	5 to 9	Both	0 (0 to 0)	0.0 (0.0 to 0.0)	0 (0 to 0)	0.0 (0.0 to 0.0)	0 (0 to 0)	0.0 (0.0 to 0.0)	-40.9 (-58.8 to -10.8)	-33.2 (-53.4 to 0.9)
Czech Republic	Nasopharynx cancer	5 to 9	Female	0 (0 to 0)	0.0 (0.0 to 0.0)	0 (0 to 0)	0.0 (0.0 to 0.0)	0 (0 to 0)	0.0 (0.0 to 0.0)	-43.7 (-66.8 to 6.1)	-36.4 (-62.5 to 19.9)

eTable 8. All-cause and cause-specific mortality (counts and rates) at the global level and by country in the WHO European Region for ages 5 to 9 and 10 to 14 for 1990, 2000, and 2016

Czech Republic	Nasopharynx cancer	5 to 9	Male	0 (0 to 0)	0.0 (0.0 to 0.0)	0 (0 to 0)	0.0 (0.0 to 0.0)	0 (0 to 0)	0.0 (0.0 to 0.0)	-37.0 (-58.3 to -12.9)	-28.7 (-52.8 to -1.4)
Czech Republic	Nasopharynx cancer	10 to 14	Both	0 (0 to 0)	0.0 (0.0 to 0.0)	0 (0 to 0)	0.0 (0.0 to 0.0)	0 (0 to 0)	0.0 (0.0 to 0.0)	-68.9 (-76.4 to -59.0)	-42.9 (-56.7 to -24.7)
Czech Republic	Nasopharynx cancer	10 to 14	Female	0 (0 to 0)	0.0 (0.0 to 0.0)	0 (0 to 0)	0.0 (0.0 to 0.0)	0 (0 to 0)	0.0 (0.0 to 0.0)	-71.2 (-80.3 to -56.8)	-46.7 (-63.6 to -20.0)
Czech Republic	Nasopharynx cancer	10 to 14	Male	0 (0 to 0)	0.0 (0.0 to 0.0)	0 (0 to 0)	0.0 (0.0 to 0.0)	0 (0 to 0)	0.0 (0.0 to 0.0)	-66.4 (-76.3 to -51.3)	-38.8 (-56.9 to -11.4)
Czech Republic	Liver cancer	5 to 9	Both	0 (0 to 1)	0.1 (0.1 to 0.1)	0 (0 to 0)	0.1 (0.0 to 0.1)	0 (0 to 0)	0.0 (0.0 to 0.0)	-58.9 (-76.1 to -29.1)	-53.5 (-72.9 to -19.8)
Czech Republic	Liver cancer	5 to 9	Female	0 (0 to 0)	0.0 (0.0 to 0.1)	0 (0 to 0)	0.0 (0.0 to 0.0)	0 (0 to 0)	0.0 (0.0 to 0.0)	-69.7 (-83.8 to -41.8)	-65.8 (-81.7 to -34.3)
Czech Republic	Liver cancer	5 to 9	Male	0 (0 to 0)	0.1 (0.1 to 0.1)	0 (0 to 0)	0.1 (0.1 to 0.1)	0 (0 to 0)	0.1 (0.0 to 0.1)	-55.1 (-76.3 to -13.5)	-49.2 (-73.2 to -2.1)
Czech Republic	Liver cancer	10 to 14	Both	1 (0 to 1)	0.1 (0.0 to 0.1)	0 (0 to 1)	0.1 (0.0 to 0.1)	0 (0 to 0)	0.0 (0.0 to 0.1)	-69.0 (-82.0 to -46.7)	-43.2 (-66.9 to -2.2)
Czech Republic	Liver cancer	10 to 14	Female	0 (0 to 0)	0.0 (0.0 to 0.0)	0 (0 to 0)	0.0 (0.0 to 0.0)	0 (0 to 0)	0.0 (0.0 to 0.0)	-76.9 (-86.9 to -55.7)	-57.4 (-75.7 to -18.0)
Czech Republic	Liver cancer	10 to 14	Male	0 (0 to 1)	0.1 (0.1 to 0.1)	0 (0 to 0)	0.1 (0.1 to 0.1)	0 (0 to 0)	0.1 (0.0 to 0.1)	-66.7 (-82.9 to -36.0)	-39.3 (-68.9 to 16.5)
Czech Republic	Kidney cancer	5 to 9	Both	1 (1 to 1)	0.1 (0.1 to 0.1)	1 (0 to 1)	0.1 (0.1 to 0.1)	1 (0 to 1)	0.1 (0.1 to 0.1)	-31.5 (-56.2 to 6.7)	-22.5 (-50.5 to 20.7)
Czech Republic	Kidney cancer	5 to 9	Female	0 (0 to 1)	0.1 (0.1 to 0.2)	0 (0 to 1)	0.1 (0.1 to 0.2)	0 (0 to 0)	0.1 (0.1 to 0.1)	-25.2 (-57.5 to 32.7)	-15.5 (-52.0 to 49.9)
Czech Republic	Kidney cancer	5 to 9	Male	0 (0 to 0)	0.1 (0.1 to 0.1)	0 (0 to 0)	0.1 (0.1 to 0.1)	0 (0 to 0)	0.1 (0.0 to 0.1)	-38.5 (-67.8 to 13.3)	-30.4 (-63.5 to 28.3)
Czech Republic	Kidney cancer	10 to 14	Both	0 (0 to 0)	0.0 (0.0 to 0.1)	0 (0 to 0)	0.0 (0.0 to 0.1)	0 (0 to 0)	0.0 (0.0 to 0.1)	-51.3 (-69.2 to -24.7)	-10.8 (-43.6 to 38.1)
Czech Republic	Kidney cancer	10 to 14	Female	0 (0 to 0)	0.1 (0.0 to 0.1)	0 (0 to 0)	0.1 (0.0 to 0.1)	0 (0 to 0)	0.1 (0.0 to 0.1)	-47.5 (-70.9 to -7.7)	-2.9 (-46.2 to 70.8)
Czech Republic	Kidney cancer	10 to 14	Male	0 (0 to 0)	0.0 (0.0 to 0.0)	0 (0 to 0)	0.0 (0.0 to 0.0)	0 (0 to 0)	0.0 (0.0 to 0.0)	-59.1 (-74.7 to -32.0)	-25.6 (-54.0 to 23.8)
Czech Republic	Brain and nervous system cancer	5 to 9	Both	13 (10 to 16)	1.9 (1.5 to 2.3)	10 (8 to 12)	1.7 (1.4 to 2.0)	6 (4 to 9)	1.0 (0.7 to 1.4)	-50.8 (-69.5 to -28.2)	-44.3 (-65.5 to -18.7)
Czech Republic	Brain and nervous system cancer	5 to 9	Female	5 (4 to 7)	1.6 (1.1 to 2.1)	4 (3 to 6)	1.5 (1.1 to 2.0)	3 (2 to 4)	1.0 (0.6 to 1.4)	-44.7 (-69.0 to -11.9)	-37.5 (-65.0 to -0.4)
Czech Republic	Brain and nervous system cancer	5 to 9	Male	8 (5 to 10)	2.1 (1.5 to 2.8)	6 (4 to 7)	1.8 (1.4 to 2.4)	3 (2 to 5)	1.1 (0.7 to 1.6)	-55.1 (-76.9 to -23.5)	-49.2 (-73.9 to -13.4)
Czech Republic	Brain and nervous system cancer	10 to 14	Both	12 (10 to 14)	1.3 (1.1 to 1.7)	9 (8 to 12)	1.5 (1.2 to 1.8)	4 (3 to 6)	0.9 (0.6 to 1.2)	-64.3 (-76.9 to -50.4)	-34.6 (-57.6 to -9.1)
Czech Republic	Brain and nervous system cancer	10 to 14	Female	5 (4 to 7)	1.2 (0.8 to 1.6)	4 (3 to 6)	1.4 (1.0 to 1.8)	2 (1 to 2)	0.7 (0.5 to 1.1)	-65.7 (-81.4 to -44.5)	-36.5 (-65.5 to 2.6)
Czech Republic	Brain and nervous system cancer	10 to 14	Male	7 (5 to 9)	1.5 (1.2 to 1.9)	5 (4 to 7)	1.6 (1.1 to 2.0)	2 (2 to 3)	1.0 (0.6 to 1.4)	-63.4 (-78.9 to -45.1)	-33.3 (-61.5 to -0.2)
Czech Republic	Thyroid cancer	10 to 14	Both	0 (0 to 0)	0.0 (0.0 to 0.0)	0 (0 to 0)	0.0 (0.0 to 0.0)	0 (0 to 0)	0.0 (0.0 to 0.0)	-71.8 (-80.4 to -57.2)	-48.2 (-64.1 to -21.4)
Czech Republic	Thyroid cancer	10 to 14	Female	0 (0 to 0)	0.0 (0.0 to 0.0)	0 (0 to 0)	0.0 (0.0 to 0.0)	0 (0 to 0)	0.0 (0.0 to 0.0)	-70.7 (-80.5 to -56.2)	-45.9 (-63.8 to -19.0)
Czech Republic	Thyroid cancer	10 to 14	Male	0 (0 to 0)	0.0 (0.0 to 0.0)	0 (0 to 0)	0.0 (0.0 to 0.0)	0 (0 to 0)	0.0 (0.0 to 0.0)	-72.5 (-83.2 to -50.8)	-50.0 (-69.3 to -10.5)
Czech Republic	Hodgkin lymphoma	5 to 9	Both	0 (0 to 1)	0.1 (0.0 to 0.1)	0 (0 to 0)	0.0 (0.0 to 0.1)	0 (0 to 0)	0.0 (0.0 to 0.0)	-81.0 (-90.4 to -63.2)	-78.5 (-89.1 to -58.4)
Czech Republic	Hodgkin lymphoma	5 to 9	Female	0 (0 to 0)	0.0 (0.0 to 0.1)	0 (0 to 0)	0.0 (0.0 to 0.0)	0 (0 to 0)	0.0 (0.0 to 0.0)	-69.6 (-84.6 to -31.7)	-65.7 (-82.6 to -22.8)
Czech Republic	Hodgkin lymphoma	5 to 9	Male	0 (0 to 1)	0.1 (0.1 to 0.2)	0 (0 to 0)	0.0 (0.0 to 0.1)	0 (0 to 0)	0.0 (0.0 to 0.0)	-84.5 (-93.7 to -65.5)	-82.5 (-92.9 to -60.9)
Czech Republic	Hodgkin lymphoma	10 to 14	Both	1 (1 to 1)	0.1 (0.1 to 0.2)	1 (0 to 1)	0.1 (0.1 to 0.1)	0 (0 to 0)	0.0 (0.0 to 0.0)	-88.2 (-93.0 to -81.4)	-78.4 (-87.2 to -65.9)

eTable 8. All-cause and cause-specific mortality (counts and rates) at the global level and by country in the WHO European Region for ages 5 to 9 and 10 to 14 for 1990, 2000, and 2016

Czech Republic	Hodgkin lymphoma	10 to 14	Female	0 (0 to 1)	0.1 (0.1 to 0.2)	0 (0 to 0)	0.1 (0.0 to 0.1)	0 (0 to 0)	0.0 (0.0 to 0.0)	-90.2 (-95.5 to -80.5)	-81.9 (-91.8 to -63.9)
Czech Republic	Hodgkin lymphoma	10 to 14	Male	1 (0 to 1)	0.2 (0.1 to 0.2)	0 (0 to 0)	0.1 (0.1 to 0.1)	0 (0 to 0)	0.0 (0.0 to 0.1)	-86.9 (-93.1 to -77.9)	-76.1 (-87.4 to -59.7)
Czech Republic	Non-Hodgkin lymphoma	5 to 9	Both	3 (3 to 4)	0.5 (0.4 to 0.6)	2 (2 to 3)	0.4 (0.3 to 0.5)	1 (1 to 2)	0.2 (0.1 to 0.4)	-58.9 (-75.1 to -30.6)	-53.5 (-71.8 to -21.6)
Czech Republic	Non-Hodgkin lymphoma	5 to 9	Female	1 (1 to 1)	0.2 (0.2 to 0.3)	1 (0 to 1)	0.2 (0.1 to 0.3)	0 (0 to 1)	0.1 (0.1 to 0.2)	-45.2 (-70.3 to -2.3)	-38.1 (-66.4 to 10.4)
Czech Republic	Non-Hodgkin lymphoma	5 to 9	Male	3 (2 to 3)	0.7 (0.5 to 1.0)	2 (1 to 2)	0.5 (0.4 to 0.8)	1 (1 to 2)	0.3 (0.2 to 0.6)	-63.0 (-80.9 to -24.5)	-58.2 (-78.4 to -14.5)
Czech Republic	Non-Hodgkin lymphoma	10 to 14	Both	4 (3 to 6)	0.5 (0.4 to 0.7)	3 (2 to 4)	0.4 (0.3 to 0.6)	1 (1 to 2)	0.3 (0.2 to 0.4)	-71.6 (-82.8 to -55.1)	-47.9 (-68.4 to -17.6)
Czech Republic	Non-Hodgkin lymphoma	10 to 14	Female	1 (1 to 1)	0.2 (0.2 to 0.3)	1 (1 to 1)	0.3 (0.2 to 0.4)	0 (0 to 1)	0.1 (0.1 to 0.2)	-67.3 (-82.7 to -39.5)	-39.5 (-67.9 to 11.9)
Czech Republic	Non-Hodgkin lymphoma	10 to 14	Male	3 (2 to 5)	0.7 (0.5 to 1.0)	2 (1 to 3)	0.6 (0.4 to 0.8)	1 (1 to 1)	0.4 (0.2 to 0.6)	-72.9 (-85.3 to -51.5)	-50.7 (-73.2 to -11.7)
Czech Republic	Leukemia	5 to 9	Both	12 (10 to 15)	1.8 (1.4 to 2.2)	7 (5 to 8)	1.1 (0.9 to 1.4)	4 (3 to 6)	0.7 (0.5 to 0.9)	-66.4 (-77.5 to -50.5)	-62.0 (-74.6 to -44.0)
Czech Republic	Leukemia	5 to 9	Female	5 (4 to 6)	1.4 (1.0 to 1.9)	3 (2 to 4)	0.9 (0.7 to 1.3)	2 (1 to 3)	0.6 (0.4 to 0.8)	-64.9 (-78.7 to -41.0)	-60.3 (-75.9 to -33.3)
Czech Republic	Leukemia	5 to 9	Male	7 (6 to 10)	2.1 (1.6 to 2.8)	4 (3 to 5)	1.3 (1.0 to 1.8)	2 (1 to 4)	0.8 (0.5 to 1.2)	-67.4 (-81.6 to -45.5)	-63.1 (-79.2 to -38.3)
Czech Republic	Leukemia	10 to 14	Both	14 (12 to 17)	1.6 (1.4 to 2.0)	8 (7 to 10)	1.2 (1.0 to 1.5)	3 (2 to 4)	0.6 (0.4 to 0.8)	-80.3 (-86.1 to -71.5)	-63.9 (-74.5 to -47.7)
Czech Republic	Leukemia	10 to 14	Female	6 (4 to 7)	1.3 (0.9 to 1.7)	3 (2 to 4)	1.0 (0.7 to 1.4)	1 (1 to 2)	0.4 (0.3 to 0.7)	-81.5 (-88.3 to -70.1)	-65.7 (-78.4 to -44.7)
Czech Republic	Leukemia	10 to 14	Male	9 (7 to 11)	2.0 (1.5 to 2.6)	5 (4 to 6)	1.4 (1.1 to 1.9)	2 (1 to 3)	0.7 (0.5 to 1.1)	-79.6 (-86.9 to -67.4)	-62.8 (-76.2 to -40.7)
Czech Republic	Other neoplasms	5 to 9	Both	4 (3 to 5)	0.6 (0.5 to 0.8)	3 (3 to 4)	0.5 (0.4 to 0.7)	3 (2 to 4)	0.5 (0.3 to 0.6)	-35.4 (-57.2 to -3.7)	-27.0 (-51.6 to 8.9)
Czech Republic	Other neoplasms	5 to 9	Female	2 (1 to 3)	0.6 (0.4 to 0.8)	1 (1 to 2)	0.5 (0.3 to 0.7)	1 (1 to 2)	0.4 (0.3 to 0.6)	-32.1 (-61.5 to 10.5)	-23.3 (-56.5 to 24.9)
Czech Republic	Other neoplasms	5 to 9	Male	2 (2 to 3)	0.7 (0.5 to 0.9)	2 (1 to 2)	0.6 (0.4 to 0.8)	2 (1 to 2)	0.5 (0.3 to 0.7)	-38.0 (-64.9 to 9.4)	-29.9 (-60.3 to 23.9)
Czech Republic	Other neoplasms	10 to 14	Both	6 (5 to 8)	0.7 (0.6 to 0.9)	4 (3 to 5)	0.6 (0.5 to 0.8)	2 (2 to 3)	0.5 (0.4 to 0.7)	-60.7 (-73.1 to -43.3)	-27.8 (-50.7 to 4.0)
Czech Republic	Other neoplasms	10 to 14	Female	3 (2 to 4)	0.7 (0.5 to 0.9)	2 (1 to 3)	0.6 (0.5 to 0.9)	1 (1 to 2)	0.5 (0.3 to 0.7)	-64.2 (-78.7 to -39.4)	-33.8 (-60.6 to 12.1)
Czech Republic	Other neoplasms	10 to 14	Male	3 (2 to 4)	0.7 (0.5 to 1.0)	2 (2 to 3)	0.6 (0.5 to 0.9)	1 (1 to 2)	0.6 (0.4 to 0.8)	-57.4 (-74.6 to -31.1)	-22.4 (-53.8 to 25.3)
Czech Republic	Rheumatic heart disease	5 to 9	Both	0 (0 to 1)	0.1 (0.0 to 0.1)	0 (0 to 0)	0.0 (0.0 to 0.0)	0 (0 to 0)	0.0 (0.0 to 0.0)	-86.1 (-91.2 to -78.6)	-84.3 (-90.0 to -75.8)
Czech Republic	Rheumatic heart disease	5 to 9	Female	0 (0 to 0)	0.1 (0.0 to 0.1)	0 (0 to 0)	0.0 (0.0 to 0.0)	0 (0 to 0)	0.0 (0.0 to 0.0)	-84.5 (-90.9 to -73.5)	-82.5 (-89.7 to -70.1)
Czech Republic	Rheumatic heart disease	5 to 9	Male	0 (0 to 0)	0.1 (0.0 to 0.1)	0 (0 to 0)	0.0 (0.0 to 0.0)	0 (0 to 0)	0.0 (0.0 to 0.0)	-87.6 (-93.7 to -76.5)	-86.0 (-92.9 to -73.4)
Czech Republic	Rheumatic heart disease	10 to 14	Both	1 (1 to 1)	0.1 (0.1 to 0.1)	0 (0 to 0)	0.1 (0.0 to 0.1)	0 (0 to 0)	0.0 (0.0 to 0.0)	-91.9 (-95.1 to -87.9)	-85.1 (-91.0 to -77.7)
Czech Republic	Rheumatic heart disease	10 to 14	Female	0 (0 to 1)	0.1 (0.1 to 0.1)	0 (0 to 0)	0.1 (0.0 to 0.1)	0 (0 to 0)	0.0 (0.0 to 0.0)	-90.5 (-95.0 to -83.5)	-82.5 (-90.7 to -69.4)
Czech Republic	Rheumatic heart disease	10 to 14	Male	0 (0 to 1)	0.1 (0.1 to 0.2)	0 (0 to 0)	0.0 (0.0 to 0.1)	0 (0 to 0)	0.0 (0.0 to 0.0)	-92.9 (-96.1 to -88.1)	-87.0 (-92.9 to -78.3)
Czech Republic	Ischemic heart disease	5 to 9	Both	0 (0 to 0)	0.0 (0.0 to 0.0)	0 (0 to 0)	0.0 (0.0 to 0.0)	0 (0 to 0)	0.0 (0.0 to 0.0)	-41.0 (-60.4 to -12.7)	-33.3 (-55.2 to -1.3)
Czech Republic	Ischemic heart disease	5 to 9	Female	0 (0 to 0)	0.0 (0.0 to 0.0)	0 (0 to 0)	0.0 (0.0 to 0.0)	0 (0 to 0)	0.0 (0.0 to 0.0)	-40.2 (-63.9 to -7.4)	-32.4 (-59.2 to 4.7)
Czech Republic	Ischemic heart disease	5 to 9	Male	0 (0 to 0)	0.0 (0.0 to 0.0)	0 (0 to 0)	0.0 (0.0 to 0.0)	0 (0 to 0)	0.0 (0.0 to 0.0)	-41.9 (-69.0 to 5.1)	-34.2 (-64.9 to 18.9)
Czech Republic	Ischemic heart disease	10 to 14	Both	0 (0 to 0)	0.0 (0.0 to 0.0)	0 (0 to 0)	0.0 (0.0 to 0.0)	0 (0 to 0)	0.0 (0.0 to 0.0)	-69.9 (-80.9 to -53.8)	-44.8 (-65.1 to -15.3)
Czech Republic	Ischemic heart disease	10 to 14	Female	0 (0 to 0)	0.0 (0.0 to 0.0)	0 (0 to 0)	0.0 (0.0 to 0.0)	0 (0 to 0)	0.0 (0.0 to 0.0)	-62.2 (-78.0 to -41.0)	-30.0 (-59.3 to 9.0)
Czech Republic	Ischemic heart disease	10 to 14	Male	0 (0 to 0)	0.0 (0.0 to 0.0)	0 (0 to 0)	0.0 (0.0 to 0.0)	0 (0 to 0)	0.0 (0.0 to 0.0)	-74.7 (-85.9 to -55.1)	-53.9 (-74.4 to -18.4)

eTable 8. All-cause and cause-specific mortality (counts and rates) at the global level and by country in the WHO European Region for ages 5 to 9 and 10 to 14 for 1990, 2000, and 2016

Czech Republic	Cerebrovascular disease	5 to 9	Both	2 (1 to 3)	0.3 (0.2 to 0.4)	1 (1 to 1)	0.2 (0.1 to 0.2)	0 (0 to 0)	0.0 (0.0 to 0.1)	-88.3 (-92.9 to -81.6)	-86.8 (-91.9 to -79.2)
Czech Republic	Cerebrovascular disease	5 to 9	Female	1 (0 to 1)	0.2 (0.1 to 0.3)	0 (0 to 1)	0.2 (0.1 to 0.2)	0 (0 to 0)	0.0 (0.0 to 0.1)	-85.5 (-92.1 to -76.0)	-83.7 (-91.1 to -72.8)
Czech Republic	Cerebrovascular disease	5 to 9	Male	1 (1 to 2)	0.3 (0.2 to 0.5)	1 (0 to 1)	0.2 (0.1 to 0.3)	0 (0 to 0)	0.0 (0.0 to 0.1)	-90.0 (-95.1 to -80.5)	-88.7 (-94.5 to -78.0)
Czech Republic	Cerebrovascular disease	10 to 14	Both	3 (2 to 4)	0.3 (0.2 to 0.4)	2 (1 to 2)	0.3 (0.2 to 0.4)	0 (0 to 0)	0.1 (0.0 to 0.1)	-91.0 (-94.5 to -86.3)	-83.4 (-89.8 to -74.9)
Czech Republic	Cerebrovascular disease	10 to 14	Female	1 (1 to 2)	0.3 (0.2 to 0.4)	1 (1 to 1)	0.3 (0.2 to 0.4)	0 (0 to 0)	0.0 (0.0 to 0.1)	-90.6 (-95.1 to -84.2)	-82.7 (-90.9 to -70.7)
Czech Republic	Cerebrovascular disease	10 to 14	Male	2 (1 to 2)	0.4 (0.3 to 0.5)	1 (1 to 1)	0.3 (0.2 to 0.4)	0 (0 to 0)	0.1 (0.0 to 0.1)	-91.2 (-95.1 to -85.3)	-83.9 (-91.0 to -73.3)
Czech Republic	Hypertensive heart disease	5 to 9	Both	0 (0 to 0)	0.0 (0.0 to 0.0)	0 (0 to 0)	0.0 (0.0 to 0.0)	0 (0 to 0)	0.0 (0.0 to 0.0)	-41.0 (-60.4 to -12.7)	-33.3 (-55.2 to -1.3)
Czech Republic	Hypertensive heart disease	5 to 9	Female	0 (0 to 0)	0.0 (0.0 to 0.0)	0 (0 to 0)	0.0 (0.0 to 0.0)	0 (0 to 0)	0.0 (0.0 to 0.0)	-40.2 (-63.9 to -7.4)	-32.4 (-59.2 to 4.7)
Czech Republic	Hypertensive heart disease	5 to 9	Male	0 (0 to 0)	0.0 (0.0 to 0.0)	0 (0 to 0)	0.0 (0.0 to 0.0)	0 (0 to 0)	0.0 (0.0 to 0.0)	-41.9 (-69.0 to 5.1)	-34.2 (-64.9 to 18.9)
Czech Republic	Hypertensive heart disease	10 to 14	Both	0 (0 to 0)	0.0 (0.0 to 0.0)	0 (0 to 0)	0.0 (0.0 to 0.0)	0 (0 to 0)	0.0 (0.0 to 0.0)	-64.2 (-76.1 to -48.2)	-34.3 (-56.2 to -5.0)
Czech Republic	Hypertensive heart disease	10 to 14	Female	0 (0 to 0)	0.0 (0.0 to 0.0)	0 (0 to 0)	0.0 (0.0 to 0.0)	0 (0 to 0)	0.0 (0.0 to 0.0)	-62.2 (-78.0 to -41.0)	-30.0 (-59.3 to 9.0)
Czech Republic	Hypertensive heart disease	10 to 14	Male	0 (0 to 0)	0.0 (0.0 to 0.0)	0 (0 to 0)	0.0 (0.0 to 0.0)	0 (0 to 0)	0.0 (0.0 to 0.0)	-65.9 (-79.8 to -46.7)	-37.9 (-63.3 to -2.9)
Czech Republic	Cardiomyopathy and myocarditis	5 to 9	Both	2 (1 to 2)	0.2 (0.1 to 0.4)	1 (1 to 1)	0.2 (0.1 to 0.2)	1 (0 to 1)	0.1 (0.0 to 0.1)	-64.6 (-76.7 to -39.5)	-59.9 (-73.6 to -31.6)
Czech Republic	Cardiomyopathy and myocarditis	5 to 9	Female	1 (0 to 1)	0.3 (0.1 to 0.4)	1 (0 to 1)	0.2 (0.1 to 0.3)	0 (0 to 1)	0.1 (0.0 to 0.2)	-63.3 (-77.3 to -33.5)	-58.5 (-74.3 to -24.8)
Czech Republic	Cardiomyopathy and myocarditis	5 to 9	Male	1 (0 to 1)	0.2 (0.1 to 0.3)	0 (0 to 1)	0.1 (0.1 to 0.2)	0 (0 to 0)	0.1 (0.0 to 0.1)	-66.1 (-82.7 to -28.0)	-61.6 (-80.4 to -18.5)
Czech Republic	Cardiomyopathy and myocarditis	10 to 14	Both	3 (1 to 3)	0.3 (0.2 to 0.4)	2 (1 to 2)	0.2 (0.2 to 0.3)	1 (0 to 1)	0.1 (0.1 to 0.2)	-75.1 (-82.9 to -61.6)	-54.3 (-68.7 to -29.6)
Czech Republic	Cardiomyopathy and myocarditis	10 to 14	Female	1 (1 to 1)	0.2 (0.1 to 0.3)	1 (0 to 1)	0.2 (0.1 to 0.3)	0 (0 to 0)	0.1 (0.1 to 0.2)	-75.1 (-84.9 to -56.3)	-54.0 (-72.1 to -19.2)
Czech Republic	Cardiomyopathy and myocarditis	10 to 14	Male	1 (1 to 2)	0.3 (0.2 to 0.5)	1 (0 to 1)	0.3 (0.1 to 0.4)	0 (0 to 1)	0.2 (0.1 to 0.2)	-75.1 (-84.8 to -58.9)	-54.6 (-72.4 to -25.2)
Czech Republic	Atrial fibrillation and flutter	5 to 9	Both	0 (0 to 0)	0.0 (0.0 to 0.0)	0 (0 to 0)	0.0 (0.0 to 0.0)	0 (0 to 0)	0.0 (0.0 to 0.0)	0.0 (0.0 to 0.0)	0.0 (0.0 to 0.0)
Czech Republic	Atrial fibrillation and flutter	5 to 9	Female	0 (0 to 0)	0.0 (0.0 to 0.0)	0 (0 to 0)	0.0 (0.0 to 0.0)	0 (0 to 0)	0.0 (0.0 to 0.0)	0.0 (0.0 to 0.0)	0.0 (0.0 to 0.0)
Czech Republic	Atrial fibrillation and flutter	5 to 9	Male	0 (0 to 0)	0.0 (0.0 to 0.0)	0 (0 to 0)	0.0 (0.0 to 0.0)	0 (0 to 0)	0.0 (0.0 to 0.0)	0.0 (0.0 to 0.0)	0.0 (0.0 to 0.0)
Czech Republic	Atrial fibrillation and flutter	10 to 14	Both	0 (0 to 0)	0.0 (0.0 to 0.0)	0 (0 to 0)	0.0 (0.0 to 0.0)	0 (0 to 0)	0.0 (0.0 to 0.0)	0.0 (0.0 to 0.0)	0.0 (0.0 to 0.0)
Czech Republic	Atrial fibrillation and flutter	10 to 14	Female	0 (0 to 0)	0.0 (0.0 to 0.0)	0 (0 to 0)	0.0 (0.0 to 0.0)	0 (0 to 0)	0.0 (0.0 to 0.0)	0.0 (0.0 to 0.0)	0.0 (0.0 to 0.0)
Czech Republic	Atrial fibrillation and flutter	10 to 14	Male	0 (0 to 0)	0.0 (0.0 to 0.0)	0 (0 to 0)	0.0 (0.0 to 0.0)	0 (0 to 0)	0.0 (0.0 to 0.0)	0.0 (0.0 to 0.0)	0.0 (0.0 to 0.0)
Czech Republic	Endocarditis	5 to 9	Both	0 (0 to 0)	0.0 (0.0 to 0.0)	0 (0 to 0)	0.0 (0.0 to 0.0)	0 (0 to 0)	0.0 (0.0 to 0.0)	-31.7 (-58.4 to 7.1)	-22.7 (-53.0 to 21.1)
Czech Republic	Endocarditis	5 to 9	Female	0 (0 to 0)	0.0 (0.0 to 0.1)	0 (0 to 0)	0.0 (0.0 to 0.1)	0 (0 to 0)	0.0 (0.0 to 0.0)	-32.1 (-61.7 to 28.8)	-23.3 (-56.7 to 45.5)

eTable 8. All-cause and cause-specific mortality (counts and rates) at the global level and by country in the WHO European Region for ages 5 to 9 and 10 to 14 for 1990, 2000, and 2016

Czech Republic	Endocarditis	5 to 9	Male	0 (0 to 0)	0.0 (0.0 to 0.0)	0 (0 to 0)	0.0 (0.0 to 0.0)	0 (0 to 0)	0.0 (0.0 to 0.0)	-31.1 (-69.4 to 32.6)	-22.0 (-65.3 to 50.1)
Czech Republic	Endocarditis	10 to 14	Both	0 (0 to 0)	0.0 (0.0 to 0.0)	0 (0 to 0)	0.0 (0.0 to 0.1)	0 (0 to 0)	0.0 (0.0 to 0.0)	-54.7 (-70.3 to -27.3)	-16.9 (-45.6 to 33.4)
Czech Republic	Endocarditis	10 to 14	Female	0 (0 to 0)	0.0 (0.0 to 0.0)	0 (0 to 0)	0.0 (0.0 to 0.1)	0 (0 to 0)	0.0 (0.0 to 0.0)	-54.3 (-73.9 to -19.1)	-15.5 (-51.7 to 49.5)
[truncated: 673,543 more chars]
